# DNA methylome profiling of circulating tumor cells in lung cancer at single base-pair resolution

**DOI:** 10.1038/s41388-021-01657-0

**Published:** 2021-02-09

**Authors:** Lei Zhao, Xiaohong Wu, Junnian Zheng, Dong Dong

**Affiliations:** 1 Cancer Institute, Xuzhou Medical University, Xuzhou, Jiangsu China; 2 Center of Clinical Oncology, Affiliated Hospital of Xuzhou Medical University, Xuzhou, China; 3 Shanghai Key Laboratory of Regulatory Biology, Institute of Biomedical Sciences, School of Life Sciences, East China Normal University, Shanghai, China; 4 Department of General Surgery, Affiliated Yixing Hospital of Jiangsu University, Yixing, 214200 Jiangsu China

**Keywords:** Cancer genomics, DNA methylation

## Abstract

DNA methylation plays a pivotal role in regulating cellular processes, and altered DNA methylation pattern is a general hallmark of cancer. However, DNA methylome in circulating tumor cells (CTCs) is still a mystery due to the lack of proper analytical techniques. We introduced an efficient workflow, LCM–µWGBS, which can efficiently profile the DNA methylation of microdissected CTC samples. LCM–µWGBS combines the laser capture microdissection (LCM)-based CTC capture method and whole-genome bisulfite sequencing in very small CTC population (µWGBS) to gain insight into the DNA methylation landscape of CTCs. We herein profiled the DNA methylome of CTCs from lung cancer patients. Deriving from a comprehensive analysis of CTC methylome, a unique “CTC DNA methylation signature” that is distinct from primary lung cancer tissues was identified. Further analysis showed that promoter hypermethylation of epithelial genes is a hallmark of stable epithelial–mesenchymal transition process. Moreover, it has been suggested that CTCs are endowed with a stemness-related feature during dissemination and metastasis. This work constitutes a unique DNA methylation analysis of CTCs at single base-pair resolution, which might facilitate to propose noninvasive CTC DNA methylation biomarkers contributing to clinical diagnosis.

## Introduction

Circulating tumor cells (CTCs) are cells that have shed into the bloodstream from primary or metastatic tumors and circulate in the bloodstream of cancer patients. CTCs have the potential to form metastases in distant organs that are ultimately responsible for the vast majority of cancer-related deaths [1]. The development of “liquid biopsies” presents new opportunities for noninvasive monitoring of cancer, with applications ranging from early detection to treatment selection and monitoring response. Given the critical role of CTCs in the metastatic cascade, the number of CTCs in patient peripheral blood has been found to correlate with the diagnosis and clinical outcomes. The enumeration of CTCs enables detecting cancers and monitoring therapeutic response by a noninvasive way. Moreover, CTCs captured as a “liquid biopsy” contain intact genomic and transcriptomic information of cancer cells, which made them suitable for use as clinical biomarkers [2–5]. The rarity of CTCs in circulating blood becomes the primary issue to overcome for CTC assay. Differentiating CTCs from the vast contaminating leukocytes background requires highly sensitive and specific assays. Classical CTC enumeration includes the presence of cell surface epithelial cell adhesion molecule (EpCAM), cytoplasmic epithelial cytokeratins, and the absence of the hematopoietic CD45 marker [6]. However, recent works have documented that CTCs were frequently in an intermediate epithelial-to-mesenchymal (EMT) state, and some epithelial markers become rapidly downregulated when cancer cells enter the bloodstream [7–9]. To date, the primary method for isolating and capturing CTCs is micromanipulation; however, this process is too time consuming and laborious for routine clinical application. Laser capture microdissection (LCM) has been widely used to isolate cells from solid tissue samples, especially in fixed in formalin and embedded in paraffin samples. LCM enables a highly accurate isolation of CTCs from leukocytes, and has been successfully used to isolate CTC single cells. The ability of LCM to capture CTCs without loss of cells and cell integrity is critical in subsequent sequencing analysis [10].

DNA methylation is an epigenetic mechanism used by cells to control gene expression, which can fix genes in the “off” position [11, 12]. Extensive DNA methylation perturbation has been widely explored in human cancer, causing changes in gene regulation that promote oncogenesis. Understanding epigenetic changes shows promise for improving the characterization of malignancy to predict diagnosis and prognosis [13–16]. Some DNA methylation changes are even found in some cases of a specific type of cancer. With the emerging of high-throughput technologies, such as comprehensive DNA methylation microarrays and genome-wide bisulfite sequencing (WGBS), large amount of DNA methylation profiling data have been generated [12, 17]. These data provided us great importance to improve the prediction of cancer diagnosis and prognosis. To date, few works focused on the DNA methylation analysis of CTCs, which is most likely explained by the combined technical challenges of CTC isolation and DNA methylation analyses on extremely rare cells. Some pioneering studies explored the DNA methylation on some specific genes. For example, Chimonidou et al. investigated the methylation status of three tumor-associated genes (*CST6*, *BRMS1*, and *SOX17*) in breast cancer CTCs [18–20], and the results suggested that the expressions of these genes are modulated by DNA methylation.

The epigenetic landscape of CTCs remains a largely unexplored field with great potential. Considering the mounting evidence for the role in epigenetics, especially DNA methylation in several cellular mechanisms, elucidation of DNA methylation profiles of CTCs is essential to understand the molecular mechanism of tumor metastasis [21, 22]. In this study, we sought to better understand the whole-genome DNA methylome of CTCs at single base-pair resolution. LCM platform was employed for CTCs isolation. CTC DNA methylation data were generated by using WGBS method from lung cancer CTCs. Our work comprehensively addressed the epigenetic state of CTCs, and provided an epigenetic picture to elucidate mechanisms during dissemination and metastasis and to develop tumor biomarkers.

## Results

### LCM–µWGBS workflow for DNA methylation profiling

**Fig. 1:**
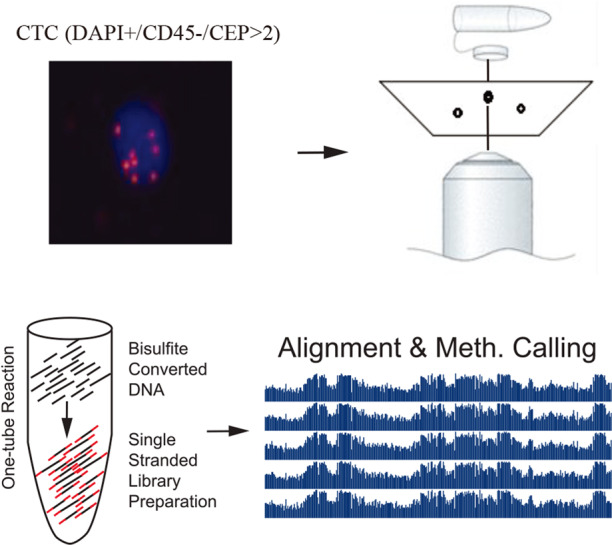
Schematics of LCM–µWGBS for CTC DNA methylation profiling. Subtraction enrichment and immunostaining-fluorescence in situ hybridization (SE-iFISH) was used to detect CTCs. LCM method was then applied to capture CTCs. At last, whole-genome bisulfite sequencing of a small number of cells was employed for DNA methylation profiling.

Subtraction enrichment and immunostaining-fluorescence in situ hybridization (SE-iFISH) was an established method to detect CTCs. In general, CTCs were characterized as nucleated cells with epithelial markers and/or hyperdiploid, with absence of lymphocytic marker CD45. This method is independent of downregulation or loss of EpCAM expression. According to previous works [23, 24], CTCs were defined as DAPI+/CD45−/CEP8 > 2 in our work (Fig. 1). LCM method was then applied to capture CTCs on the polyethylene-napthalate (PEN) membrane-coated slide. The enriched CTC samples were encapsulated in a hydrogel matrix, which made them easily to be isolated by LCM and compatible with downstream analysis. µWGBS is an established DNA methylation analysis method of a small number of cells based on the post-bisulfite adapter tagging (PBAT) assay. With the attempt to robustly and efficiently identify the whole-genome DNA methylation profiles of CTCs, we have developed a LCM–µWGBS workflow by combining LCM-based CTC capture and µWGBS method (Fig. 1).

**Fig. 2:**
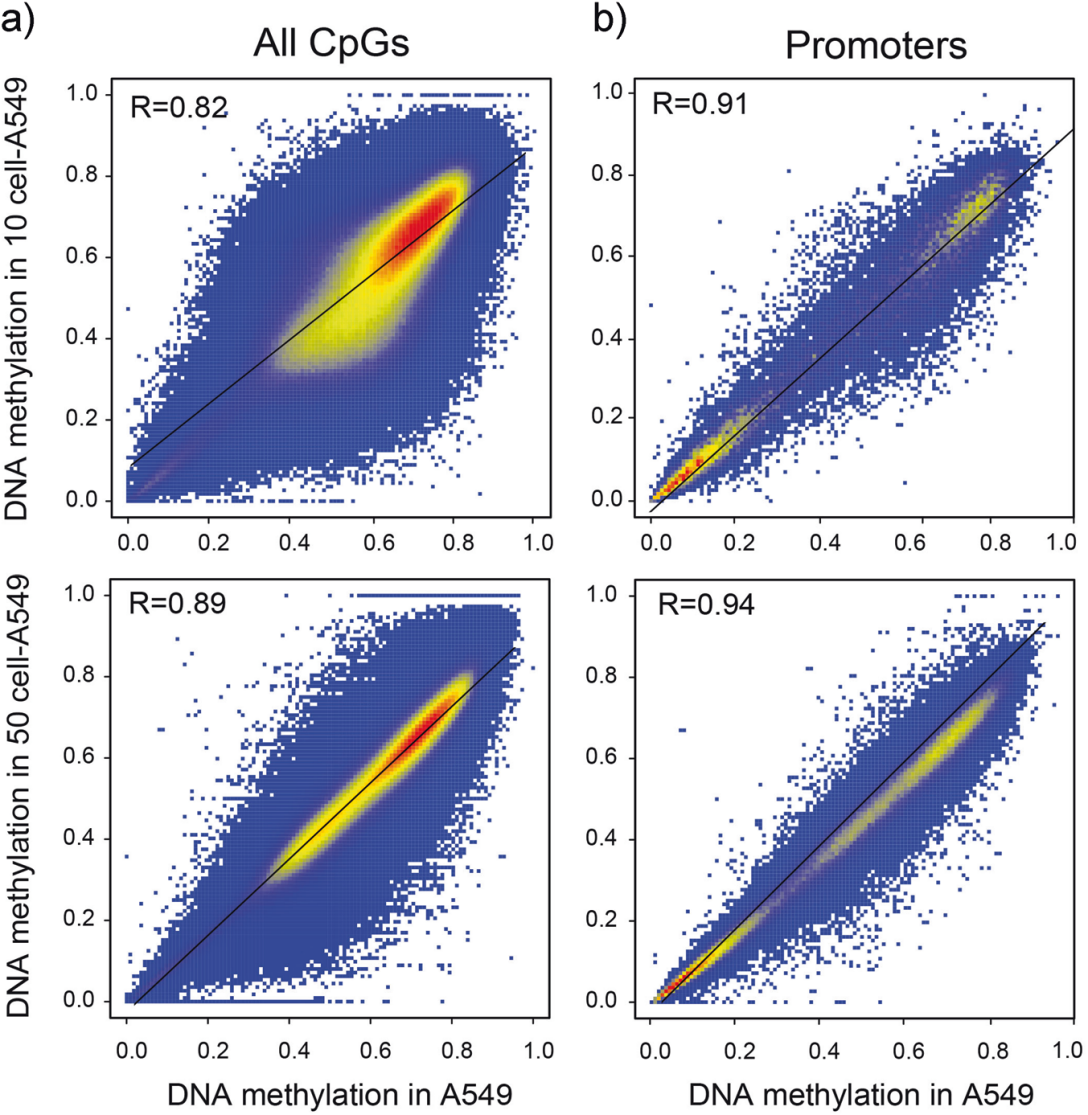
The repeatability and robustness of LCM–µWGBS workflow. Micromanipulated A549 cells were spiked into the whole blood of the healthy donors. 10 cells and 50 cells were captured from the mixed samples, respectively, and the DNA methylomes were profiled subsequently. **a** DNA methylation levels of A549 cell lines measured using LCM–µWGBS and WGBS at the whole-genome-wide level. **b** DNA methylation levels of A549 cell lines measured using LCM–µWGBS and WGBS at the promoter region.

By optimizing multiple steps in the procedure, we can now acquire high-quality DNA methylation data down to LCM dissected cells. To technically validate this strategy, we detected the DNA methylome measured from the introduction of individually micromanipulated cells from lung cancer cell line A549 into 5 ml of blood from a healthy donor, followed by cancer cells subtraction enrichment and LCM–µWGBS processing. LCM procedure requires a dedicated instrument in a confined space to avoid contamination of experimental materials. The parameters for LCM should be set at the lowest energy to ensure the integrity of those dissected cells. Because of the rarity of CTCs, experimental groups of 10 and 50 A549 cells were isolated from the mixed blood cells using LCM method, respectively. Those harvested cells of interests were pooled together for each sample to provide enough starting materials for subsequent µWGBS. The result showed that the conversion rate resulting from bisulfite treatment is more than 98% for the 10-cell and 50-cell A549 DNA methylome, suggesting the high fidelity of this method. An average of 145 million paired-end reads were generated, covering 58% of the human genome (Table 1). We determined the presence of 16,660,129 and 19,437,132 CpG sites for the 10-cell and 50-cell A549 DNA methylome, which account for 58% and 61% of the total CpG sites at the whole-genome-wide level. To evaluate the performance of LCM–µWGBS, we benchmarked them against human A549 cell line WGBS data. The result showed significantly high Pearson correlation coefficients of 0.82 and 0.89 with 10-cell A549 and 50-cell A549, respectively (Fig. 2). As for the gene promoter regions, we observed Pearson correlation coefficients of 0.91 and 0.94 with 10-cell A549 and 50-cell A549 (Fig. 2), respectively. Moreover, these two data display the similar DNA methylation level as the A549 cell line WGBS data (Supplementary Fig. S1). These results suggested that LCM–µWGBS method provides an accurate and efficient way to interrogate CTCs DNA methylation information at the whole-genome-wide level.

**Table 1:** Statistics of µWGBS data of CTCs.

Sample ID	Origins	CTC#	Total clean reads	Percentage of mapped reads	Coverage	Unique CpGs	CpG coverage	Bisulfite conversion rate
Patient1_CTC	No LCLM	16	255320380	38%	0.544	18602441	0.648	0.992
Patient2_CTC	No LCLM	11	131833442	47%	0.189	6262136	0.218	0.991
Patient3_CTC	No LCLM	13	131827972	50%	0.314	10285461	0.358	0.990
Patient4_CTC	No LCLM	10	131916942	49%	0.182	5734767	0.200	0.984
Patient5_CTC	No LCLM	18	131575810	50%	0.108	3532767	0.123	0.992
Patient6_CTC	LCLM	18	263668458	48%	0.512	17650885	0.615	0.991
Patient7_CTC	LCLM	22	176002976	33%	0.639	19175555	0.668	0.995
Patient8_CTC	LCLM	18	163784488	37%	0.572	17405709	0.606	0.995
Patient9_CTC	LCLM	20	141161092	25%	0.545	16200091	0.564	0.994
Patient10_CTC	LCLM	20	141161092	50%	0.641	19896348	0.693	0.995
Patient11_CTC	No LCLM	11	133144320	37%	0.148	4550251	0.159	0.992
Patient12_CTC	No LCLM	16	130581964	47%	0.210	8219373	0.286	0.991
Patient13_CTC	No LCLM	18	131534266	50%	0.151	4884290	0.170	0.992
Patient14_CTC	No LCLM	18	136890898	51%	0.234	7522107	0.262	0.990
Patient15_CTC	No LCLM	16	131982568	48%	0.189	6222565	0.217	0.992

*LCLM* lung cancer liver metastasis.

### DNA methylation signature of lung cancer CTCs

**Fig. 3:**
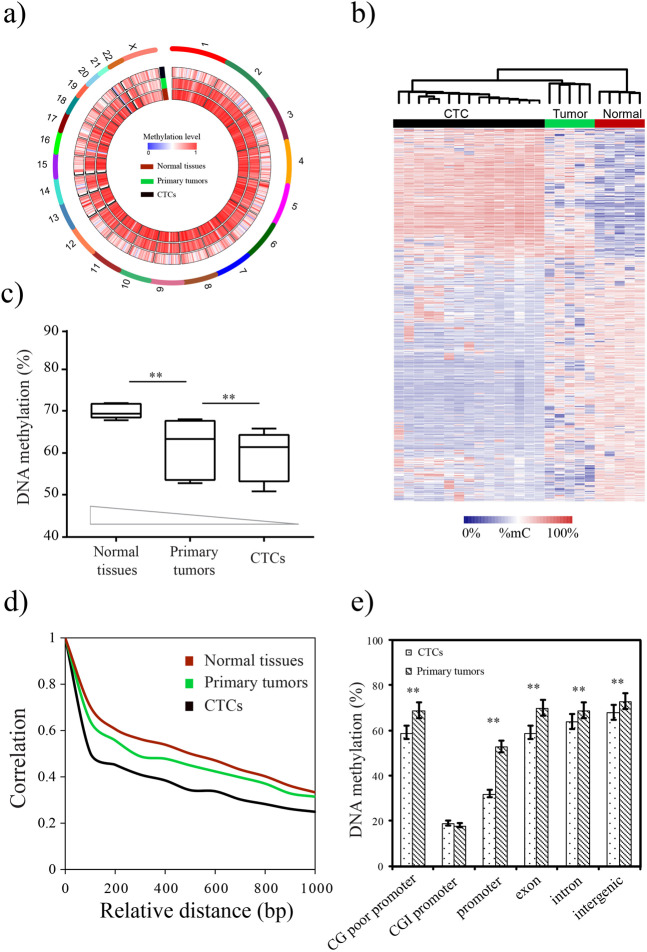
Genome-wide DNA methylation changes in lung cancer CTCs. **a** Whole-genome representation of DNA methylation levels of lung cancer CTCs, primary tumors and normal tissues. **b** Unsupervised hierarchical clustering of DNA methylation data from lung cancer CTCs, primary tumors and normal tissues. **c** DNA methylation level was significantly lower in CTCs compared with normal and cancer samples. **d** Correlation of DNA methylation levels between neighboring CpG sites. **e** DNA methylation levels among different genomic sequences. The error bars represent the 95% confidence intervals.

According to the CTCs collection strategy, CTCs were isolated from a cohort of 15 lung cancer patients in this way, yielding between 10 and 22 CTCs/5 ml of patient blood (Table 1). To gain a comprehensive insight into the DNA methylation changes during CTC dissemination and metastasis, genome-wide DNA methylation profiling of 15 CTC samples, 5 matched primary tumor samples, and 5 matched adjacent normal tissue samples derived from lung cancer patients were performed using LCM–µWGBS method (Fig. 3a, Table 1, Supplementary Table S1). Biopsy specimens contain a mix of tumor and normal tissue cells, tumor infiltrating lymphocytes. The tumor tissues had been stained with H&E before µWGBS. Moreover, we employed a deconvolution method to estimate the tumor purity [25]. The result showed that the tumor purity was >90%. Bisulfite sequencing reads were generated with an average of 150 million reads per sample (7.5× coverage). Two CTC samples (patient 1 and patient 6) were sequenced to 250 million reads per sample (12.5× coverage), and sequencing saturation analysis was performed to determine the sequencing coverage needed for LCM–µWGBS. As sequencing depth increases, more CpG sites were detected. The number of CpG sites reaches saturation at the 6× to 10× genome coverage (Supplementary Fig. S2). This result suggested that the sequencing depth of this work is enough for further analysis. After mapping to the human reference genome (hg38), we identified an average of 13,474,616 CpG sites for CTC samples, which covered on average 46.9% of the total CpG sites at the genome-wide level. We investigated the DNA methylation levels along all chromosomes by assessing with a 300-kb sliding window. To assess the DNA methylation similarities of the CTCs samples, Pearson correlation coefficients were calculated for every two samples. The average correlation coefficient was observed to be 0.68, indicating a relatively higher consistency of CTCs samples. Further unsupervised hierarchical clustering analysis showed that CTCs clustered together as a single group, indicating that CTCs resemble each other more closely than their primary tumor counterparts (Fig. 3b).

Consistent with previous reports [12, 17], normal tissues have higher CpG methylation level (70–80%), whereas dramatic DNA methylation losses occur in cancer samples in this context. Notably, we found that global DNA methylation was significantly lower in CTCs compared with normal and cancer samples (Wilcoxon rank sum test, *P* < 2.2 × 10^−16^, Fig. 3c). This progressive decrease in global DNA methylation from normal tissues to primary tumors and, in turn, to their associated CTCs suggested a successive loss of DNA methylation during tumorigenesis. Further analysis suggested a significant loss of the association between methylation levels of adjacent CpG sites (Fig. 3d), indicating DNA methylation loss may occur randomly rather than at consecutive CpG sites. From a genomic perspective, the decrease of DNA methylation occurred at all genomic compartments, such as promoter, gene body, intron, and intergenic region (Fig. 3e). After we subgrouped the promoters based on their CpG contents, we found that the CTC DNA exhibited more unmethylated CpGs at the CpG-poor promoters (Wilcoxon rank sum test, *P* < 2.2 × 10^−16^) and similar methylated CpGs at CpG island promoters (Wilcoxon rank sum test, *P* = 0.37, Fig. 3e) than the primary tumor samples, suggesting hypomethylated CpG sites in CTCs mainly occurred in the promoter regions lacking CpG islands.

### Identification of differentially methylated regions (DMRs)

**Fig. 4:**
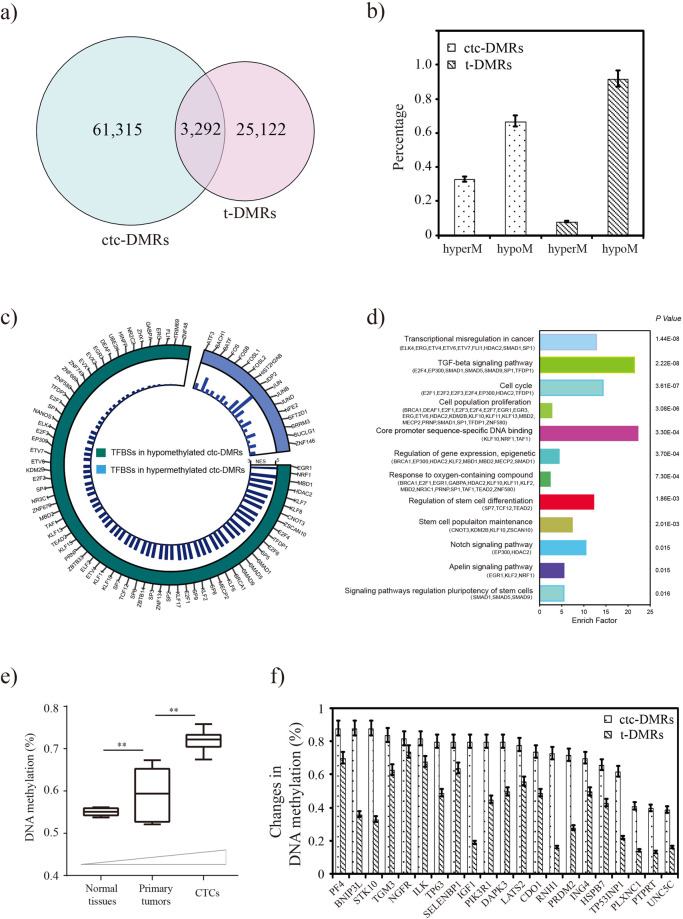
Functional characterization of DMRs. **a** The number of DMRs between CTCs and primary tumors (ctc-DMRs), and the number of DMRs between primary tumors and normal tissues (t-DMRs). A total of 3292 DMRs were classified as overlapping DMRs. **b** More hypomethylated DMRs were observed than hypermethylated DMRs in both ctc-DMRs and t-DMRs. **c** Normalized enrichment score (NES) representing enrichment (NES ≥ 3.4) of transcription factor binding sites (TFBSs) in hypomethylated ctc-DMRs and hypermethylated ctc-DMRs. **d** Integrated pathway analysis of TFBSs in hypomethylated ctc-DMRs. The bars represent the percentage of genes detected per pathway category with *P* value ≤ 0.05. **e** CpG sites in hypermethylated t-DMRs further gained methylation intensities in CTCs. **f** Representative tumor suppressor genes show increased methylation in CTCs and primary tumors. The error bars represent the 95% confidence intervals.

The discrepancies between the CTCs and primary lung cancer DNA methylome prompted us to detect DMRs. DMRs were identified using a smoothing approach, and CpGs with correlated methylation values were grouped together (see “Materials and methods“). We totally identified 64,607 DMRs (ctc-DMRs) between CTCs and primary tumor samples, and 28,414 DMRs (t-DMRs) between primary tumors and normal tissues. A total of 3292 DMRs were classified as overlapping (Fig. 4a). The complete lists of ctc-DMRs and t-DMRs can be found in Supplementary Tables S2 and S3. The ctc-DMRs and t-DMRs represent 2,223,957 CpG sites and 1,281,976 CpG sites in the reference genome that are distributed across all human chromosomes, respectively. To validate those identified DMRs, we performed a pairwise sample comparison, and the result showed that most of the DMRs (90%) were existed in at least three samples (Supplementary Fig. S3).

The DMRs were distributed across the human genome, and provided information on all genomic contexts. DMRs were identified in promoters (16,719 for ctc-DMRs and 6368 for t-DMRs), exonic, (22,901 for ctc-DMRs and 10,345 for t-DMRs), intronic (39,857 for ctc-DMRs and 16,824 for t-DMRs), and intergenic regions (35,140 for ctc-DMRs and 15,826 for t-DMRs). Consistent with previous reports, 1884 (6.6%) of these t-DMRs were hypermethylated, whereas 26,530 (93.4%) were hypomethylated. Global DNA hypomethylation is a common genetic feature of lung cancer tumorigenesis. As for ctc-DMRs, 43,283 (67.0%) of them were hypomethylated, indicating a greater tendency toward decreased DNA methylation at aberrantly methylated loci in CTCs (Fig. 4b).

Hypomethylated regions in CTC samples covered 4.2% of the entire human genome. In all, 42.8% of the hypomethylated regions are located within gene promoters. Previous reports suggested that DNA hypomethylation and transcription factor binding sites (TFBSs) are highly correlated, describing a codependency of these regulatory mechanisms. In this work, we found that CTC DNA hypomethylation and transcription factor occupancy revealed a significant relationship (Fisher’s exact test, *P* < 2.2 × 10^−16^). We then analyzed ctc-DMRs using i-cisTarget method [26], and found a significant enrichment for several TFBSs among DNA hypomethylated regions that are specific to CTCs (Fig. 4c). For example, transcription factor EGR1 shows the most remarkable enrichment in the hypomethylation regions specific to CTCs. EGR1 has been reported to be involved in the regulation of cell growth, differentiation, and apoptosis in several cancer types [27]. Transcription factors E2F1 and TCF12 both regulated target genes mainly involved in cell cycle and/or cell division processes [28, 29]. Further Gene Ontology analysis of global CTC hypomethylated TFBSs demonstrating that stemness-related transcription factors are significantly enriched to coordinately regulate proliferation and pluripotency (Fig. 4d), such as CNOT3, KDM2B, and KLF10. Thus, CTCs are clearly distinguishable from primary tumors based on their DNA methylation status at DMRs, where they feature more hypomethylated TFBSs.

Despite the progressive loss of DNA methylation during tumorigenesis, we found that CpG sites in the hypermethylated t-DMRs further gained methylation intensities in CTCs (Fig. 4e). Several tumor suppressor genes promoters that were hypermethylated in tumors showed a greater tendency toward increased methylation in CTCs with potential cancer-driving effects (Fig. 4f). For example, *PIK3R1* is a suppressor of the mitogenic AKT pathway, and highly frequent promoter methylation and *PIK3R1* has been reported in lung cancer. The promoter hypermethylation of *PIK3R1* is significantly associated with gene expression in lung cancer samples (TCGA, LUAD, *r* = 0.54, *P* < 2.2 × 10^−16^). This focal gain of DNA methylation in tumors and even CTCs highlights the extensive changes in DNA methylation that occur in cancer progression and dissemination. To further gain insight in to the functional significance of DNA methylation changes, we performed KEGG pathway enrichment analysis for those hypo- and hypermethylation related genes (Table 2). This analysis revealed that genes in the hypomethylation regions are involved in the “TGF-beta signaling pathway,” “hippo signaling pathway,” and “hedgehog signaling pathway.” These pathways have been documented to be significantly related to EMT process [30]. On the other extreme, genes in the hypermethylation regions are involved in the “tight junction,” “focal adhesion” functions.

**Table 2:** KEGG pathway enrichment analysis.

	KEGG ID	Description	No. of genes	F.D.R.
Hypomethylation	hsa04392	Hippo signaling pathway	14	0.03793
	hsa04012	ErbB signaling pathway	41	0.00192
	hsa04120	Ubiquitin mediated proteolysis	61	0.00158
	hsa04350	TGF-beta signaling pathway	36	0.02109
	hsa01521	EGFR tyrosine kinase inhibitor resistance	34	0.02774
	hsa04668	TNF signaling pathway	44	0.03742
	hsa04015	Rap1 signaling pathway	79	0.04836
	hsa04152	AMPK signaling pathway	47	0.06318
	hsa04340	Hedgehog signaling pathway	20	0.07179
	hsa00510	N-Glycan biosynthesis	21	0.07652
	hsa04066	HIF-1 signaling pathway	39	0.07821
Hypermethylation	hsa04810	Regulation of actin cytoskeleton	34	0.00002
	hsa04530	Tight junction	16	0.02035
	hsa04974	Protein digestion and absorption	16	0.00087
	hsa04020	Calcium signaling pathway	26	0.00092
	hsa04370	VEGF signaling pathway	12	0.00125
	hsa04510	Focal adhesion	27	0.00192
	hsa04670	Leukocyte transendothelial migration	17	0.00370
	hsa04015	Rap1 signaling pathway	26	0.00746
	hsa04973	Carbohydrate digestion and absorption	8	0.01561
	hsa04520	Adherens junction	11	0.01809
	hsa04014	Ras signaling pathway	26	0.02038
	hsa04964	Proximal tubule bicarbonate reclamation	5	0.02578

Among ten lung cancer patients, only four of them had clinically detectable liver metastasis (LCLM, Table 1). We next sought to detect distinct patterns of liver metastatic CTCs. The result showed that global DNA methylation was nearly similar in CTCs derived from four patients diagnosed with LCLM compared with no LCLM group (Wilcoxon rank sum test, *P* = 0.36, Supplementary Fig. S4). We totally detected 487 genes which showed significantly distinct DNA methylation in their promoter regions. Subsequently pathway analysis revealed higher activation of some known pathways in LCLM patients (Supplementary Table S4), such as Notch signaling pathway, Hippo signaling pathway, etc. The comparison of patient cohort of LCLM and no LCLM revealed the epigenetic signature of liver metastatic CTCs.

### CTC DNA methylation of epithelial, mesenchymal, and stem cell markers

**Fig. 5:**
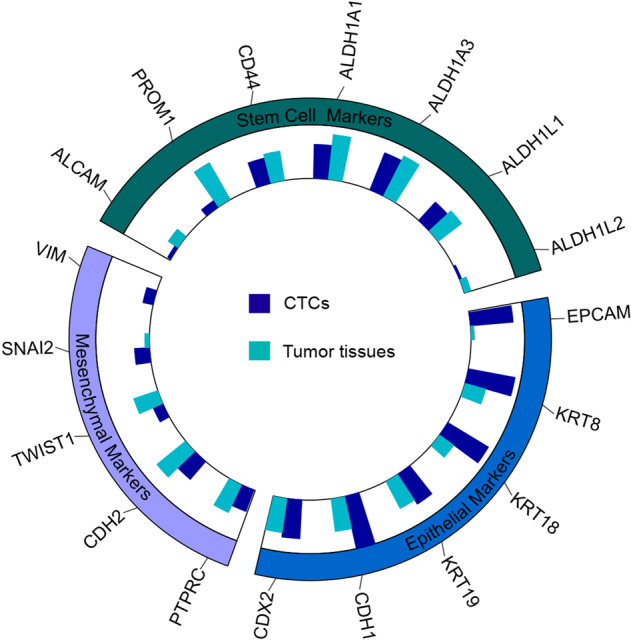
Targeted analysis of DNA methylome data. DNA methylation levels of epithelial, mesenchymal and stem cell markers. DNA methylation levels of epithelial, mesenchymal, and stem cell markers.

Previous works have documented that CTCs were frequently in an intermediate EMT state, and significantly lost the epithelial markers. Since different epithelial and mesenchymal genes are actually known to act in concert during EMT process, we herein comprehensively interrogated the epigenetic status of epithelial and mesenchymal markers on CTCs. As shown in Fig. 5, epithelial gene promoters are hypermethylated in CTC samples. For example, epithelial gene *Cdh1* and *EpCAM*, key features of EMT process, were significantly hypermethylated in CTCs (Fig. 5). This result is consistent with previous findings that CTCs loss some of their epithelial characteristics, and promoter hypermethylation of epithelial genes is hallmarks of stable EMT process. Furthermore, the DNA methylation of mesenchymal gene promoters are mixed, with some showing DNA hypermethylation in CTCs (*Vim* and *Snail2*) and others with DNA hypomethylation status in CTCs (*TWIST1*, *CDH2*, and *PTPRC*). The promoters of putative stem cell genes are hypomethylated in CTCs (such as *ALDH1A1*, *CD44*), which strongly supports the findings proposed with the TFBS enrichment analysis, suggesting that CTCs are endowed with a stemness-related feature, and this program may play a pivotal role in determining the metastasis-seeding ability of CTCs.

## Discussion

The concept of liquid biopsies for noninvasive detecting and monitoring cancer has been embedded in our minds as a promising approach in cancer diagnosis and prognosis. CTCs provide a source of intact genomic and epigenomic information for lineage-based analysis [31]. Efficient isolation of CTCs in the bloodstream, followed by genomic and epigenomic quantification, may provide a highly specific diagnostic assay. Here, we have described a new workflow, LCM–µWGBS, which can accurately and reproducibly profile the DNA methylome based on microdissected CTC samples at the single base-pair resolution. Our approach combines the CTC negative enrichment with LCM method, enabling isolation of CTCs with intact DNA, together with DNA methylation profiling in very small cell population. This work provided a comprehensive genome-wide analysis of the DNA methylation events that characterize CTCs in lung cancer, and compared the methylation changes with matched primary tumors and normal tissues. We herein have reinforced the notion of epigenetic disruption as a signature during dissemination and metastasis.

As expected, the DNA methylation level was significantly lower in primary lung cancer samples compared with normal tissues [12, 14, 17, 32]. We described a clear distinction in DNA methylation patterns between CTCs and primary tumors, mostly in the form of hypomethylation in the former. From a functional genomic standpoint, the DNA methylation decrease was observed in the CTC DNA covered all genomic compartments. Since DNA methylation changes were associated with profound effects on gene expression, it suggested that the progressive decrease in global DNA methylation plays important roles during tumorigenesis and blood-borne dissemination. Interestingly, we found progressive increase in DNA methylation in focal regions, suggesting that DNA methylation hypermethylation of tumor suppressor exists in CTCs. Our results added a new dimension to the dissemination and metastasis. Whether the DNA methylation change in CTCs is of prognostic, predictive, or therapeutic importance has yet to be determined.

EMT plasticity of CTCs reveals that epigenetic landscape is implicated in the dynamic events during dissemination and metastasis. The epithelial genes tend to be hypermethylated in their promoters, such as *Cdh1*, a key feature of EMT transition [33]. Such promoter hypermethylation leads to the loss of the epithelial features, and acquisition of a mesenchymal-like phenotype. As for the mesenchymal genes, the DNA methylation status showed plastic, which suggested that CTCs appear arrested in a biphenotypic state. Another observation suggests that CTCs have several properties that commonly feature stem cell implication. For instance, CTCs result in hypomethylation of TFBS of master stemness and proliferation regulators, such as CNOT3, KDM2B, and KLF10. Moreover, putative stem cell genes are hypomethylated in their promoters in CTCs, such as ALDH1A1 and CD44, suggesting that CTCs are endowed with a stemness-related feature. This finding reinforced the stem cell-like characteristics of CTCs in lung cancer.

Our LCM–µWGBS method has several advantages. First, it is an efficient workflow, which can accurately and reproducibly profile the DNA methylation of microdissected CTC samples, and will facilitate the investigation of CTC DNA methylome. Second, this method is independent of loss of EpCAM expression. Classical CTC staining criteria include the presence of cell surface EpCAM. However, EpCAM is not a perfect marker for CTC selection due to the high variation in its gene expression. Third, we provided the CTC DNA methylome at single base resolution, which can discover a unique “CTC DNA methylation signature” that is distinct from primary tumors. Several limitations of our work should be addressed. First, only few lung cancer patients (15 patients) were involved in this analysis. Second, CTCs are very heterogeneous tumor cells; however, this method is difficult to attain single-cell resolution with the current LCM technology because many harvested cells do not remain intact after laser microdissection. Third, the field of single-cell epigenomics is still in its infancy.

Taken together, we provided a robust and efficient method for CTC DNA methylation analysis. Using this method, we revealed a detailed description of the DNA methylation landscape in lung cancer CTCs at single base-pair resolution. It might facilitate to propose noninvasive CTC DNA methylation biomarkers contributing to clinical diagnosis.

## Materials and methods

### Patient specimens and cell lines

Blood, primary tumors, and paired adjacent normal tissues were collected from lung cancer patients with written consent, which was approval by the Affiliated Hospital of Xuzhou Medical University Agency Ethics Committee. We collected patient peripheral venous blood (7.5 ml) using EDTA tubes and stored them at room temperature for processing within 24 h. Tissue samples were snap-frozen in liquid nitrogen and stored at −80 °C. Human lung cancer cell line A549 was purchased from Cell Bank of Type Culture Collection of the Chinese Academy of Sciences (Shanghai, China) and maintained in a humidified incubator at 37 °C, using minimum essential media, supplemented with fetal bovine serum (10% v/v).

### CTCs enrichment and detection

An EpCAM-independent method was employed for CTCs subtraction enrichment. At first, red blood cells were lysed using RBC lysis buffer (G-bioscience) according to the manufacturer’s protocol. The sample was then centrifuged at 800 × *g* for 10 min at room temperature, and the supernatants above the red blood cells were removed to deplete serum. All the sedimented cells were mixed with 3 ml nonhematopoietic cell separation matrix, followed by centrifugation at 400 × *g* for 8 min. White buffy was collected and subjected to magnetic separation of beads to deplete red blood cells. The resulting pellet containing rare cells was incubated with 150 μl immunomagnetic particles coupled to anti-CD45 monoclonal antibody for 10 min, followed by magnetic separation. The cell pellet was mixed with 100 μl cell fixative solution and applied to the formatted and coated CTC slide. After the drying process, the slides were suitable for iFISH.

CTCs were detected by immunostaining of CD45, 4′,6-diamidino-2-phenylindole, dihydrochloride (DAPI), and FISH of the centromere of chromosome 8 probe (CEP8). CTCs are identified by combining immunofluorescent staining of CD45 and FISH with the CEP8 probe method. In brief, we added hybridization solution containing CEP8 probe to the slides. After hybridization, the antibody of CD45 was added to the slides. Finally, the nuclear dye DAPI (100 mg/ml, Sigma) was added and slides were mounted for microscopic observation. Cells with the DAPI+/CD45−/CEP8 ≥ 3 pattern were considered to be CTCs. Cells with DAPI+/CD45+/CEP8 = 2 pattern were defined as white blood cells and cells with DAPI+/CD45−/CEP8 = 2 pattern were defined as indeterminate cells.

### CTCs extraction using LCM

The target cells in fixed slide were laser microdissected following the manufacturer’s protocol for the PALM Laser MicroBeam System (Zeiss AG, Oberkochen, Germany). To facilitate LCM extraction, a PEN membrane-coated glass slide was used to prepare samples, which made it easily to be cut together with the samples. When we identified the target cell, a circle was drawn around it. Then, the area of the interest was laser microdissected, and catapulted into an adhesive cap of the collection tube. All CTCs harvested from each patient were pooled into one tube for subsequent µWGBS analysis.

### µWGBS analysis

The tumor tissues had been stained with H&E before µWGBS to maximize sample tumor purity and the efficiency of tissue utilization. µWGBS was performed according to the protocol of previous single-cell bisulfite sequencing echnology [7]. The PBAT method was used to avoid high DNA loss from limited starting material. Bisulfite conversion was performed according to the manufacturer’s instructions for EZ DNA Methylation Direct Kit (Zymo Research D5020), and bisulfite-treated DNA was eluted using 9 µl of elution buffer. Here, bisulfite treatment was performed directly on lysed cells by placing cells in 10 µl digestion buffer and 1 µl proteinase K for 20 min at 50 °C. Sequencing library was prepared using the TruSeq DNA Methylation Kit (Illumina, EGMK91396) according to manufacturer’s instructions. For library amplification, 18 PCR cycles were performed. The final library purification was performed twice using Agencourt AMPure XP beads. Library concentration was estimated using Qubit dsDNA HS Assay Kit. Sequencing was performed on Illumina HiSeq X Ten platform.

### Bioinformatics analysis of DNA methylation sequencing data

Several bioinformatics steps were carried out to analyze the bisulfite sequencing reads: (1) adapter trimming, (2) alignment of bisulfite-treated reads to human genome, (3) determination of methylation state at each cytosine, and (4) filtering the contaminating reads. Library adapter was trimmed using trimmomatic [34], and fastqc is used to evaluate clean data. Bisulfite sequencing reads alignment was done using Bismark [35] with default parameters. All analyses were performed based on the human reference genome assembly hg38.

DMRs were determined using DMRseq [36] and bsseq software package [37]. In brief, we processed CpG count matrixes to merge symmetric CpG sites across stands and filtered those CpG sites for at least 1× coverage across the human genome, which accords with the minimum requirements for DMR inference. DMRs were identified by testing for differences between samples. We set the sliding window size and step at 1000 and 100 bp. DMRseq was employed by setting a cutoff of 0.05 and increasing the number of permutations to 500. Background regions were considered as the testable regions, and used as the candidate regions for permutation analysis to determine significant DMRs.

### TFBSs enrichment analysis in DMRs

TFBS enrichment in DMRs was calculated using i-cisTarget software [26] (https://gbiomed.kuleuven.be/apps/lcb/i-cisTarget/) based on the normalized enrichment score (NES). NES corresponds to the enrichment score, which reflects the degree to which the motif set is overrepresented at the top or bottom of a ranked list of motifs. NES is positive if it was enriched in DMRs. NES score threshold > 3.4 was used (passes a FDR threshold of 0.05). Motif search is performed in DMR regions overlapping with predefined candidate regularity regions.

### Functional enrichment analysis

Functional enrichment analysis of KEGG was performed using the using the DAVID software [38] for the methylation related genes. The KEGG categories were corrected using the Benjamini–Hochberg method, and the genes with F.D.R of less than 0.05 were considered to be significantly enriched.

## Supplementary information

Supplementary information file

Supplementary FigureS1

Supplementary FigureS2

Supplementary FigureS3

Supplementary FigureS4

Supplementary TableS1

Supplementary TableS2

Supplementary TableS3

Supplementary TableS4

## Data Availability

The bisulfite sequencing data have been deposited in the NCBI Sequencing Read Archive database (SRA, http://www.ncbi.nlm.nih.gov/sra/) under the accession number PRJNA649023.
