# Supplementary material for: DNA methylome profiling of circulating tumor cells in lung cancer at single base-pair resolution
Source: Oncogene. 2021 Feb 9;40(10):1884–95. doi: 10.1038/s41388-021-01657-0 (PMC7946637; doi:10.1038/s41388-021-01657-0)
Supplement: Supplementary file 1 — Supplementary information file [file 41388_2021_1657_MOESM1_ESM.pdf]

Supplementary figure legends

Figure S1 DNA methylation levels in each sample.

Figure S2 Figure S2 Sequencing saturation analysis.

Figure S3 Pairwise sample comparisons of DMRs between different samples.

Figure S4 DNA methylation levels in primary tumors, CTCs in LCLM and non-LCLM samples.
