# Supplementary figures and images for: DNA methylome profiling of circulating tumor cells in lung cancer at single base-pair resolution
Source: Oncogene. 2021 Feb 9;40(10):1884–95. doi: 10.1038/s41388-021-01657-0 (PMC7946637; doi:10.1038/s41388-021-01657-0)

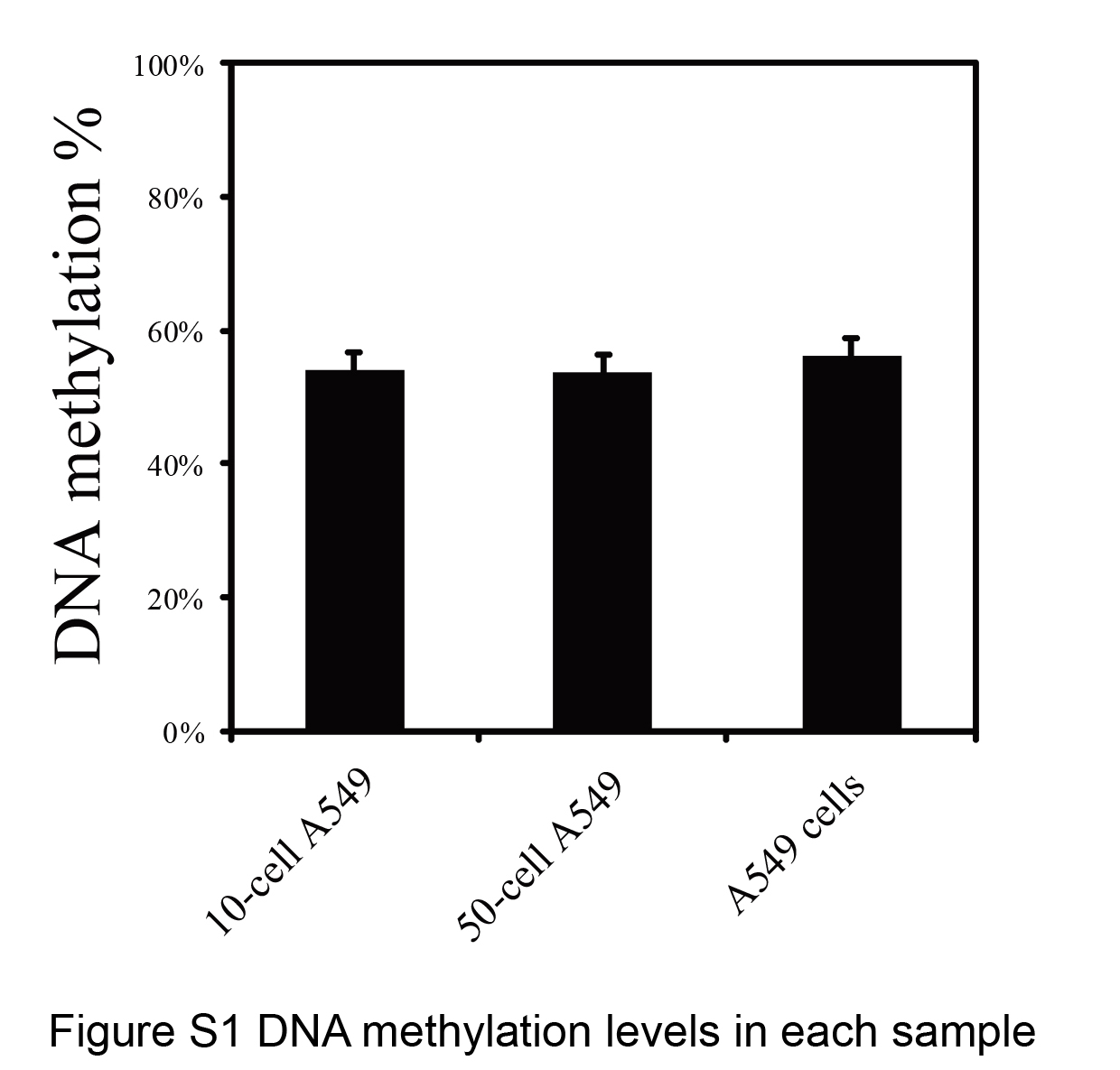

Supplement: Supplementary file 2 — Supplementary FigureS1 [file 41388_2021_1657_MOESM2_ESM.jpg]

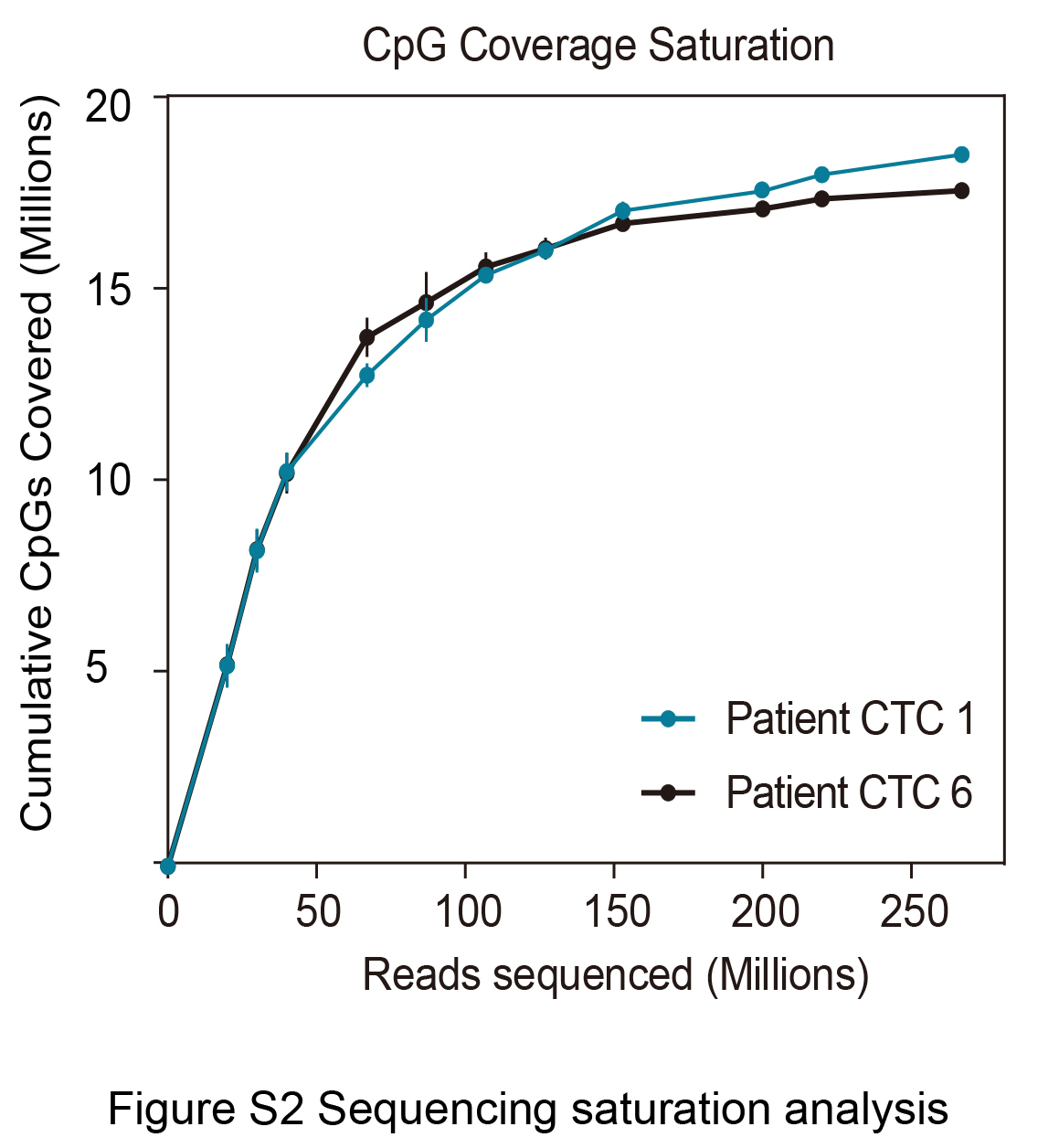

Supplement: Supplementary file 3 — Supplementary FigureS2 [file 41388_2021_1657_MOESM3_ESM.jpg]

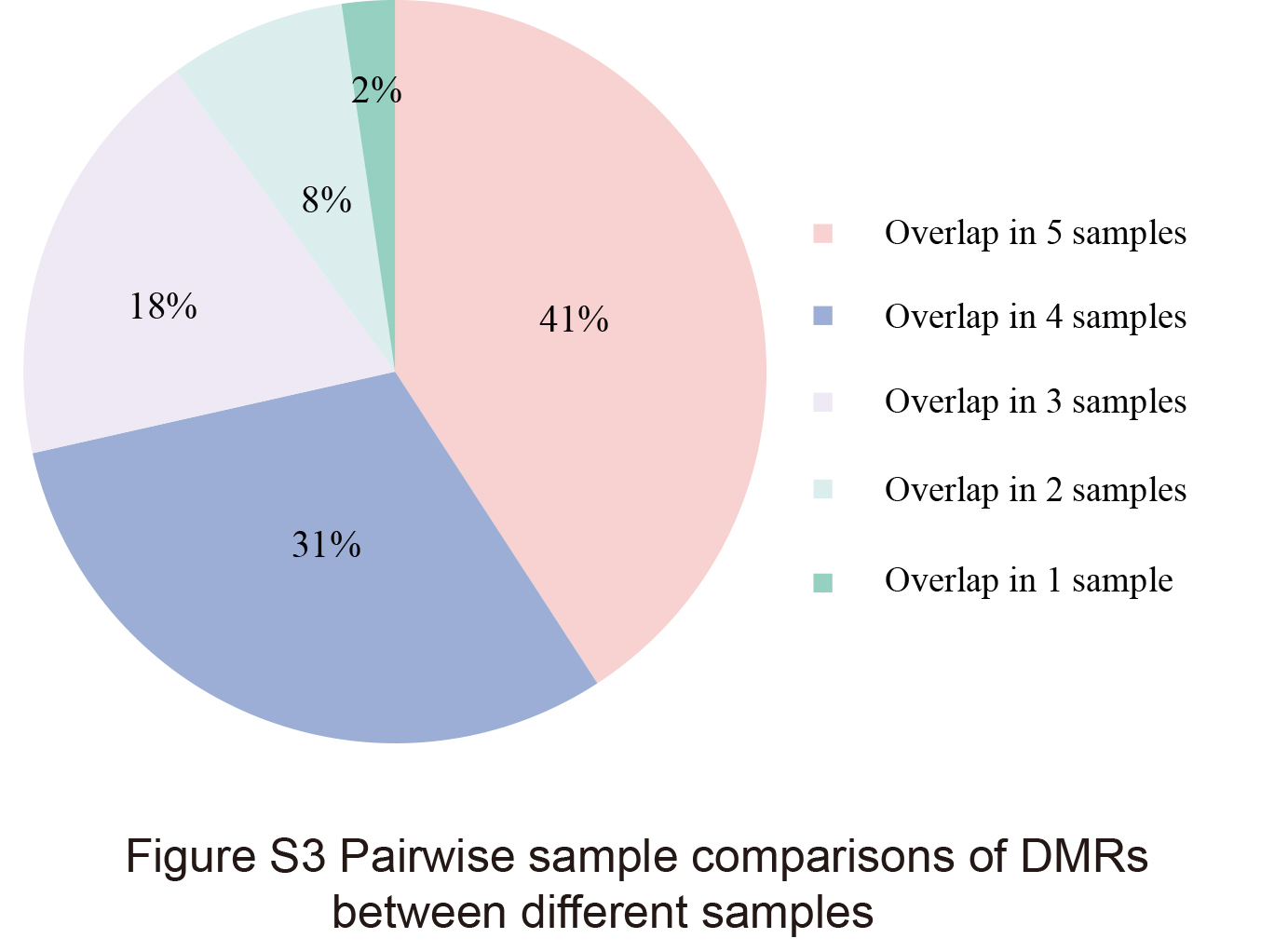

Supplement: Supplementary file 4 — Supplementary FigureS3 [file 41388_2021_1657_MOESM4_ESM.jpg]

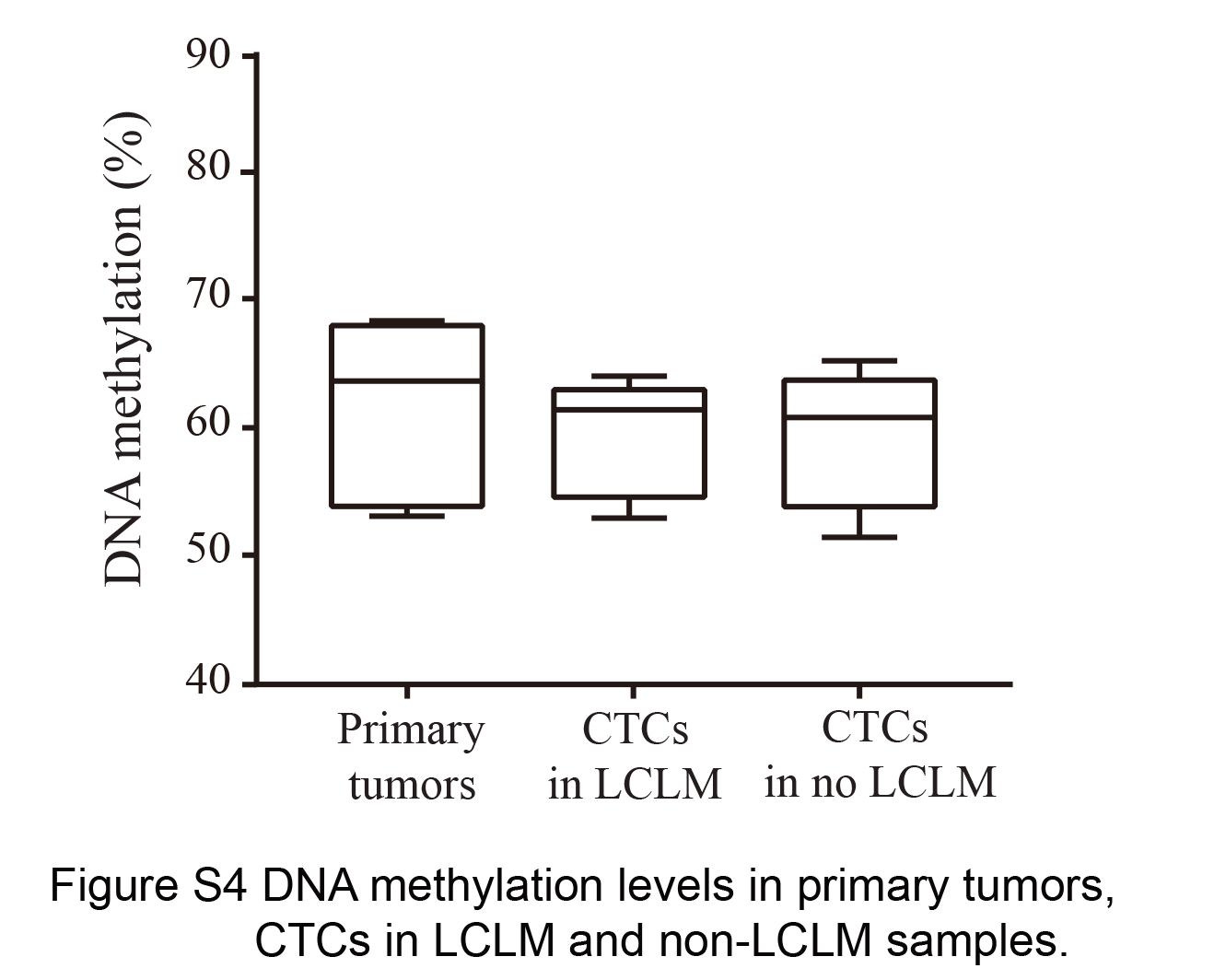

Supplement: Supplementary file 5 — Supplementary FigureS4 [file 41388_2021_1657_MOESM5_ESM.jpg]
