# Supplementary material for: DNA methylome profiling of circulating tumor cells in lung cancer at single base-pair resolution
Source: Oncogene. 2021 Feb 9;40(10):1884–95. doi: 10.1038/s41388-021-01657-0 (PMC7946637; doi:10.1038/s41388-021-01657-0)
Supplement: Supplementary file 8 — Supplementary TableS3 [file 41388_2021_1657_MOESM8_ESM.pdf]

Supplemental Table S3. List of identified DMRs between tumor tissues and normal tissues

chr	start	end	qvalue	direction (Tumor compared to normal)
chr1	936001	941000	8.57E-05	hyper
chr1	937001	942000	8.57E-05	
chr1	938001	943000	4.15E-07	
chr1	1047001	1052000	1.55E-17	
chr1	1048001	1053000	3.84E-23	
chr1	1049001	1054000	1.66E-18	
chr1	1050001	1055000	5.86E-14	
chr1	1306001	1311000	7.44E-06	
chr1	1307001	1312000	7.44E-06	
chr1	1440001	1445000	5.65E-07	
chr1	1441001	1446000	1.24E-07	
chr1	2105001	2110000	8.44E-06	
chr1	2122001	2127000	0.004811	
chr1	2157001	2162000	1.97E-07	
chr1	2413001	2418000	0.006578	
chr1	2477001	2482000	1.86E-07	
chr1	2478001	2483000	8.37E-10	
chr1	2485001	2490000	0.001244	
chr1	2981001	2986000	1.98E-10	
chr1	2982001	2987000	2.89E-07	
chr1	3130001	3135000	9.54E-07	
chr1	3395001	3400000	0.005902	
chr1	6048001	6053000	1.53E-11	
chr1	6261001	6266000	3.02E-06	
chr1	6262001	6267000	2.30E-08	
chr1	6263001	6268000	1.24E-07	
chr1	6505001	6510000	1.18E-05	
chr1	6506001	6511000	3.59E-07	
chr1	6507001	6512000	6.37E-08	
chr1	6610001	6615000	1.33E-08	
chr1	6611001	6616000	1.38E-08	
chr1	6612001	6617000	5.10E-10	
chr1	6613001	6618000	4.21E-05	
chr1	6614001	6619000	0.004312	
chr1	8939001	8944000	7.48E-06	
chr1	8940001	8945000	2.73E-05	
chr1	9194001	9199000	9.57E-05	
chr1	9489001	9494000	6.43E-07	
chr1	15247001	15252000	0.004147	
chr1	15248001	15253000	1.73E-05	
chr1	16008001	16013000	0.003393	
chr1	16025001	16030000	0.000797	
chr1	16082001	16087000	0.027332	
chr1	16373001	16378000	0.002092	
chr1	16528001	16533000	8.43E-06	

chr1	16529001	16534000	1.08E-06
chr1	16530001	16535000	8.98E-05
chr1	17334001	17339000	0.004505
chr1	17335001	17340000	0.001679
chr1	17336001	17341000	1.31E-05
chr1	17697001	17702000	0.015538
chr1	17914001	17919000	0.001669
chr1	19806001	19811000	0.010448
chr1	19970001	19975000	0.000913
chr1	20122001	20127000	2.00E-14
chr1	20123001	20128000	1.34E-12
chr1	20124001	20129000	3.21E-07
chr1	20125001	20130000	2.53E-07
chr1	20126001	20131000	7.62E-07
chr1	20158001	20163000	0.001769
chr1	21502001	21507000	4.39E-11
chr1	21614001	21619000	2.13E-05
chr1	21615001	21620000	3.52E-05
chr1	21616001	21621000	3.10E-06
chr1	23540001	23545000	0.000513
chr1	24066001	24071000	0.0036
chr1	24242001	24247000	1.54E-06
chr1	26484001	26489000	7.40E-09
chr1	26825001	26830000	0.025963
chr1	26826001	26831000	0.000123
chr1	27882001	27887000	0.000163
chr1	27981001	27986000	0.002503
chr1	27982001	27987000	8.83E-05
chr1	27983001	27988000	0.000397
chr1	28238001	28243000	5.06E-08
chr1	28239001	28244000	4.41E-08
chr1	28240001	28245000	2.08E-08
chr1	28844001	28849000	3.23E-05
chr1	28969001	28974000	7.07E-05
chr1	28990001	28995000	4.84E-06
chr1	28991001	28996000	2.91E-06
chr1	30875001	30880000	0.000982
chr1	30876001	30881000	7.02E-05
chr1	31885001	31890000	5.22E-10
chr1	31886001	31891000	1.72E-11
chr1	32042001	32047000	5.92E-05
chr1	32223001	32228000	1.38E-06
chr1	32224001	32229000	3.87E-07
chr1	32225001	32230000	0.00479
chr1	33164001	33169000	2.57E-05
chr1	33165001	33170000	1.78E-05
chr1	33191001	33196000	0.000298
chr1	33192001	33197000	0.000407

chr1	33721001	33726000	0.000525
chr1	33938001	33943000	1.94E-08
chr1	35321001	35326000	1.37E-07
chr1	35541001	35546000	1.02E-05
chr1	36811001	36816000	0.030151
chr1	36915001	36920000	0.000165
chr1	38467001	38472000	0.001061
chr1	38468001	38473000	0.007135
chr1	41326001	41331000	3.97E-06
chr1	41828001	41833000	0.005697
chr1	41830001	41835000	0.000502
chr1	41831001	41836000	0.001788
chr1	43822001	43827000	0.000328
chr1	44781001	44786000	0.0045
chr1	44868001	44873000	1.73E-14
chr1	45201001	45206000	5.95E-22
chr1	45202001	45207000	5.68E-11
chr1	45203001	45208000	4.89E-17
chr1	45204001	45209000	3.16E-17
chr1	45205001	45210000	2.02E-15
chr1	45271001	45276000	1.65E-08
chr1	45273001	45278000	0.000154
chr1	45475001	45480000	4.76E-12
chr1	45477001	45482000	2.37E-07
chr1	45789001	45794000	0.009974
chr1	45961001	45966000	7.41E-06
chr1	45962001	45967000	3.32E-06
chr1	45964001	45969000	9.07E-06
chr1	45965001	45970000	3.24E-05
chr1	46015001	46020000	0.01827
chr1	46016001	46021000	0.002148
chr1	46048001	46053000	3.79E-12
chr1	46712001	46717000	0.000175
chr1	47006001	47011000	9.17E-05
chr1	47078001	47083000	0.001099
chr1	47079001	47084000	0.000146
chr1	47080001	47085000	4.00E-05
chr1	47081001	47086000	0.000644
chr1	47082001	47087000	3.23E-05
chr1	47533001	47538000	0.006862
chr1	47880001	47885000	1.87E-09
chr1	47906001	47911000	3.46E-12
chr1	48186001	48191000	1.75E-06
chr1	48187001	48192000	1.00E-07
chr1	53192001	53197000	3.04E-05
chr1	53791001	53796000	0.000439
chr1	55010001	55015000	2.39E-05
chr1	55013001	55018000	4.43E-06

chr1	63830001	63835000	0.002109
chr1	65613001	65618000	0.000105
chr1	78221001	78226000	1.39E-10
chr1	78222001	78227000	1.27E-10
chr1	78223001	78228000	3.08E-11
chr1	78224001	78229000	4.10E-10
chr1	109641001	109646000	4.92E-13
chr1	109642001	109647000	3.22E-11
chr1	109643001	109648000	5.90E-06
chr1	112294001	112299000	1.50E-09
chr1	113217001	113222000	4.67E-16
chr1	114446001	114451000	9.21E-07
chr1	114447001	114452000	7.24E-08
chr1	115319001	115324000	1.02E-11
chr1	115320001	115325000	1.17E-12
chr1	119544001	119549000	1.26E-15
chr1	119545001	119550000	7.54E-15
chr1	119546001	119551000	3.32E-19
chr1	119547001	119552000	1.32E-09
chr1	145574001	145579000	9.07E-06
chr1	145575001	145580000	4.47E-08
chr1	150266001	150271000	2.29E-07
chr1	150520001	150525000	1.55E-05
chr1	150528001	150533000	8.97E-06
chr1	151255001	151260000	7.63E-08
chr1	153534001	153539000	0.004073
chr1	153646001	153651000	8.45E-06
chr1	153647001	153652000	8.45E-06
chr1	153753001	153758000	2.32E-07
chr1	154167001	154172000	0.00024
chr1	154835001	154840000	3.95E-06
chr1	155162001	155167000	4.65E-15
chr1	155178001	155183000	4.77E-06
chr1	155293001	155298000	0.008596
chr1	155901001	155906000	1.55E-06
chr1	155903001	155908000	7.70E-08
chr1	155904001	155909000	2.93E-09
chr1	156258001	156263000	1.54E-05
chr1	156259001	156264000	2.63E-05
chr1	156752001	156757000	0.025001
chr1	160940001	160945000	0.000477
chr1	161183001	161188000	1.80E-06
chr1	202825001	202830000	2.73E-05
chr1	206223001	206228000	7.86E-09
chr1	211432001	211437000	1.44E-05
chr1	211433001	211438000	1.53E-06
chr1	213027001	213032000	2.72E-10
chr1	213029001	213034000	2.52E-07

chr1	228111001	228116000	0.025542
chr1	247290001	247295000	1.28E-06
chr1	247291001	247296000	4.28E-05
chr2	5832001	5837000	4.25E-05
chr2	7001001	7006000	1.48E-12
chr2	10572001	10577000	0.00108
chr2	25560001	25565000	0.000129
chr2	27349001	27354000	2.27E-06
chr2	27350001	27355000	0.004217
chr2	27528001	27533000	7.39E-06
chr2	27529001	27534000	4.72E-07
chr2	27530001	27535000	3.49E-06
chr2	27715001	27720000	1.33E-05
chr2	27716001	27721000	5.64E-05
chr2	27717001	27722000	3.82E-05
chr2	37546001	37551000	9.44E-13
chr2	45160001	45165000	1.98E-13
chr2	54781001	54786000	3.80E-07
chr2	62418001	62423000	4.13E-07
chr2	62419001	62424000	1.55E-09
chr2	63278001	63283000	7.91E-11
chr2	63279001	63284000	3.17E-17
chr2	63280001	63285000	3.70E-24
chr2	63281001	63286000	1.66E-19
chr2	64868001	64873000	2.88E-05
chr2	64869001	64874000	6.49E-09
chr2	72858001	72863000	0.00099
chr2	73340001	73345000	9.11E-07
chr2	74778001	74783000	4.06E-06
chr2	74779001	74784000	4.68E-05
chr2	74780001	74785000	0.001383
chr2	87288001	87293000	0.004344
chr2	87879001	87884000	1.01E-05
chr2	87889001	87894000	1.22E-05
chr2	87892001	87897000	4.90E-05
chr2	87962001	87967000	8.48E-05
chr2	87963001	87968000	1.88E-05
chr2	87964001	87969000	1.88E-05
chr2	96092001	96097000	0.006736
chr2	97069001	97074000	0.000102
chr2	97071001	97076000	0.002769
chr2	97523001	97528000	0.001013
chr2	97619001	97624000	4.19E-05
chr2	97620001	97625000	1.59E-05
chr2	98419001	98424000	2.36E-19
chr2	98420001	98425000	4.87E-16
chr2	98421001	98426000	3.89E-15
chr2	99549001	99554000	1.38E-11

chr2	99550001	99555000	5.66E-09
chr2	99552001	99557000	0.000187
chr2	112110001	112115000	0.001072
chr2	112111001	112116000	0.001302
chr2	114296001	114301000	3.30E-05
chr2	114297001	114302000	3.06E-06
chr2	114298001	114303000	6.93E-05
chr2	114299001	114304000	6.00E-06
chr2	121621001	121626000	3.06E-19
chr2	121622001	121627000	5.62E-21
chr2	121623001	121628000	2.30E-17
chr2	121624001	121629000	3.78E-16
chr2	121625001	121630000	8.38E-10
chr2	122756001	122761000	0.000133
chr2	131181001	131186000	6.61E-06
chr2	131552001	131557000	4.55E-05
chr2	187215001	187220000	0.042677
chr2	187216001	187221000	0.003041
chr2	198299001	198304000	6.21E-16
chr2	201672001	201677000	4.00E-08
chr2	203495001	203500000	1.69E-13
chr2	203496001	203501000	6.01E-12
chr2	203498001	203503000	1.10E-09
chr2	206787001	206792000	3.47E-06
chr2	206788001	206793000	2.27E-07
chr2	208391001	208396000	9.57E-11
chr2	219571001	219576000	3.91E-10
chr2	219863001	219868000	5.07E-08
chr2	220024001	220029000	4.55E-06
chr2	220383001	220388000	1.53E-07
chr2	220384001	220389000	1.36E-06
chr2	220385001	220390000	4.25E-07
chr2	220386001	220391000	8.17E-09
chr2	220387001	220392000	7.58E-10
chr2	220435001	220440000	5.15E-08
chr2	220436001	220441000	9.20E-09
chr2	230928001	230933000	1.94E-07
chr2	232325001	232330000	1.43E-12
chr2	232328001	232333000	6.66E-08
chr2	232466001	232471000	0.029433
chr2	232550001	232555000	0.000425
chr2	232551001	232556000	0.000282
chr2	232822001	232827000	8.39E-13
chr2	233252001	233257000	0.000877
chr2	233272001	233277000	0.047069
chr2	233346001	233351000	3.91E-06
chr2	233347001	233352000	1.55E-05
chr2	233348001	233353000	1.12E-05

chr2	233349001	233354000	1.44E-05
chr2	234144001	234149000	0.000789
chr2	234158001	234163000	1.67E-06
chr2	234159001	234164000	6.96E-07
chr2	234160001	234165000	1.64E-14
chr2	235332001	235337000	1.58E-08
chr2	235404001	235409000	9.08E-18
chr2	235405001	235410000	4.52E-15
chr2	238596001	238601000	0.00109
chr2	238597001	238602000	1.21E-08
chr2	238598001	238603000	5.01E-07
chr2	238599001	238604000	3.15E-09
chr2	239013001	239018000	0.012875
chr2	241395001	241400000	0.001403
chr2	241756001	241761000	0.010077
chr2	241830001	241835000	0.003708
chr2	241831001	241836000	0.003708
chr2	241832001	241837000	0.003798
chr2	241927001	241932000	0.00012
chr2	242252001	242257000	3.19E-06
chr2	242446001	242451000	1.04E-08
chr2	242447001	242452000	6.73E-08
chr3	10154001	10159000	6.49E-06
chr3	10155001	10160000	0.000104
chr3	10235001	10240000	0.003205
chr3	12836001	12841000	0.024789
chr3	13150001	13155000	2.46E-05
chr3	14442001	14447000	4.63E-06
chr3	15138001	15143000	2.94E-11
chr3	15139001	15144000	1.48E-07
chr3	18483001	18488000	2.28E-10
chr3	32818001	32823000	3.05E-05
chr3	32819001	32824000	0.000584
chr3	37898001	37903000	5.38E-07
chr3	37899001	37904000	0.000324
chr3	39144001	39149000	8.31E-06
chr3	39145001	39150000	4.23E-09
chr3	39146001	39151000	1.10E-09
chr3	39147001	39152000	1.10E-09
chr3	39148001	39153000	0.000241
chr3	40563001	40568000	6.18E-08
chr3	40564001	40569000	2.47E-08
chr3	42051001	42056000	4.99E-06
chr3	42052001	42057000	0.001167
chr3	42054001	42059000	2.04E-05
chr3	42055001	42060000	3.96E-05
chr3	42630001	42635000	3.63E-06
chr3	42631001	42636000	1.69E-06

chr3	42632001	42637000	1.11E-07
chr3	42638001	42643000	6.31E-17
chr3	43427001	43432000	0.000435
chr3	43428001	43433000	0.000628
chr3	43429001	43434000	0.000961
chr3	43728001	43733000	1.16E-05
chr3	45426001	45431000	6.18E-08
chr3	46920001	46925000	1.18E-05
chr3	46921001	46926000	0.000254
chr3	47025001	47030000	0.000749
chr3	47026001	47031000	0.008798
chr3	47502001	47507000	0.020574
chr3	47551001	47556000	0.000118
chr3	48353001	48358000	0.033391
chr3	48538001	48543000	0.010022
chr3	48542001	48547000	0.001445
chr3	48809001	48814000	0.003671
chr3	48810001	48815000	0.002854
chr3	48956001	48961000	3.07E-09
chr3	48957001	48962000	1.40E-05
chr3	49023001	49028000	0.0003
chr3	49024001	49029000	4.67E-05
chr3	49066001	49071000	0.000239
chr3	49131001	49136000	3.84E-07
chr3	49138001	49143000	0.003189
chr3	49936001	49941000	0.017447
chr3	49937001	49942000	1.06E-08
chr3	49938001	49943000	6.29E-08
chr3	49939001	49944000	4.96E-10
chr3	49940001	49945000	7.60E-08
chr3	49941001	49946000	5.68E-05
chr3	50123001	50128000	0.000651
chr3	50124001	50129000	0.00017
chr3	50125001	50130000	3.51E-10
chr3	50126001	50131000	2.76E-11
chr3	50127001	50132000	2.01E-11
chr3	50250001	50255000	0.005544
chr3	50327001	50332000	3.61E-06
chr3	50328001	50333000	1.54E-07
chr3	50329001	50334000	4.95E-06
chr3	50346001	50351000	0.000802
chr3	50394001	50399000	0.003419
chr3	50485001	50490000	0.005572
chr3	50486001	50491000	0.000172
chr3	50602001	50607000	2.30E-05
chr3	50607001	50612000	0.007844
chr3	51530001	51535000	0.00097
chr3	51573001	51578000	4.90E-05

chr3	52476001	52481000	0.008712
chr3	52477001	52482000	0.007367
chr3	53379001	53384000	4.60E-13
chr3	53380001	53385000	2.86E-14
chr3	53381001	53386000	9.40E-07
chr3	57019001	57024000	0.009726
chr3	57258001	57263000	2.10E-09
chr3	57737001	57742000	1.12E-06
chr3	57738001	57743000	6.50E-11
chr3	57739001	57744000	2.97E-06
chr3	58221001	58226000	5.50E-05
chr3	58222001	58227000	0.000183
chr3	58223001	58228000	0.000226
chr3	65534001	65539000	0.003645
chr3	71772001	71777000	3.51E-07
chr3	71773001	71778000	1.45E-06
chr3	98312001	98317000	1.19E-09
chr3	101279001	101284000	8.19E-07
chr3	114377001	114382000	3.99E-08
chr3	122279001	122284000	2.24E-06
chr3	122742001	122747000	0.000245
chr3	125634001	125639000	0.008353
chr3	127791001	127796000	1.61E-05
chr3	129481001	129486000	0.027642
chr3	133746001	133751000	2.37E-11
chr3	133747001	133752000	8.43E-15
chr3	133748001	133753000	8.92E-15
chr3	135965001	135970000	1.22E-06
chr3	138666001	138671000	2.46E-05
chr3	147108001	147113000	2.46E-11
chr3	172309001	172314000	1.68E-06
chr3	181439001	181444000	2.15E-27
chr3	181440001	181445000	5.77E-31
chr4	329001	334000	0.00099
chr4	556001	561000	0.01038
chr4	1003001	1008000	0.000228
chr4	1162001	1167000	5.02E-05
chr4	1718001	1723000	9.88E-14
chr4	2797001	2802000	0.00595
chr4	2798001	2803000	0.00595
chr4	2799001	2804000	0.0021
chr4	2819001	2824000	6.66E-06
chr4	2820001	2825000	2.87E-06
chr4	6916001	6921000	2.83E-08
chr4	7306001	7311000	0.000154
chr4	8894001	8899000	3.32E-10
chr4	8895001	8900000	3.54E-10
chr4	8896001	8901000	2.39E-07

chr4	8897001	8902000	0.000123
chr4	40057001	40062000	2.66E-10
chr4	40058001	40063000	2.48E-10
chr4	41866001	41871000	3.25E-14
chr4	41867001	41872000	4.36E-13
chr4	41868001	41873000	1.19E-14
chr4	41869001	41874000	9.07E-12
chr4	41878001	41883000	6.41E-13
chr4	41879001	41884000	4.58E-15
chr4	56258001	56263000	1.11E-15
chr4	57687001	57692000	1.90E-05
chr4	57845001	57850000	3.70E-15
chr4	78740001	78745000	4.02E-16
chr4	78741001	78746000	1.22E-19
chr4	81109001	81114000	3.01E-10
chr4	81110001	81115000	1.86E-11
chr4	82132001	82137000	1.95E-07
chr4	82133001	82138000	1.14E-09
chr4	82134001	82139000	1.01E-09
chr4	82135001	82140000	4.05E-10
chr4	99845001	99850000	4.15E-12
chr4	99846001	99851000	2.18E-06
chr4	110478001	110483000	1.31E-07
chr4	112818001	112823000	1.64E-05
chr4	119273001	119278000	7.53E-09
chr4	139903001	139908000	0.004911
chr4	140477001	140482000	0.000908
chr4	140478001	140483000	1.80E-08
chr4	166029001	166034000	5.45E-09
chr4	166790001	166795000	8.42E-06
chr4	166791001	166796000	1.67E-08
chr4	166792001	166797000	3.35E-15
chr4	166793001	166798000	1.52E-10
chr4	176922001	176927000	9.04E-06
chr4	183726001	183731000	1.17E-08
chr4	183727001	183732000	1.84E-13
chr4	183728001	183733000	5.85E-05
chr4	185746001	185751000	9.96E-08
chr4	190918001	190923000	0.024742
chr5	1004001	1009000	5.67E-05
chr5	1877001	1882000	1.39E-05
chr5	1878001	1883000	8.02E-06
chr5	1879001	1884000	4.99E-19
chr5	3599001	3604000	1.46E-15
chr5	16791001	16796000	1.29E-07
chr5	55573001	55578000	3.00E-08
chr5	60626001	60631000	0.000599
chr5	63794001	63799000	0.002728

chr5	63795001	63800000	0.000691
chr5	65017001	65022000	1.05E-17
chr5	65018001	65023000	1.73E-16
chr5	68898001	68903000	0.006422
chr5	69933001	69938000	0.029464
chr5	69934001	69939000	0.029464
chr5	69935001	69940000	0.029464
chr5	77657001	77662000	3.17E-08
chr5	78904001	78909000	2.82E-07
chr5	79539001	79544000	0.006279
chr5	79547001	79552000	1.93E-09
chr5	79603001	79608000	0.001719
chr5	79950001	79955000	4.34E-08
chr5	131825001	131830000	6.73E-05
chr5	131828001	131833000	1.19E-07
chr5	131829001	131834000	4.58E-06
chr5	132444001	132449000	6.34E-05
chr5	132445001	132450000	3.94E-06
chr5	132446001	132451000	3.37E-05
chr5	133355001	133360000	0.007781
chr5	133561001	133566000	2.92E-11
chr5	133979001	133984000	8.14E-06
chr5	137364001	137369000	2.29E-05
chr5	137798001	137803000	0.000236
chr5	137799001	137804000	1.32E-11
chr5	139088001	139093000	0.000118
chr5	139127001	139132000	3.08E-05
chr5	139281001	139286000	9.33E-05
chr5	139282001	139287000	3.50E-07
chr5	139283001	139288000	1.11E-06
chr5	139741001	139746000	0.008482
chr5	139926001	139931000	0.004567
chr5	141017001	141022000	0.004348
chr5	145562001	145567000	0.001467
chr5	149155001	149160000	0.001642
chr5	149734001	149739000	1.07E-08
chr5	149788001	149793000	4.03E-08
chr5	149791001	149796000	0.001357
chr5	149855001	149860000	0.000129
chr5	150408001	150413000	7.75E-06
chr5	157094001	157099000	2.38E-12
chr5	157095001	157100000	3.78E-14
chr5	157096001	157101000	5.79E-25
chr5	157097001	157102000	3.77E-25
chr5	157098001	157103000	8.40E-21
chr5	159251001	159256000	0.000129
chr5	167910001	167915000	0.000331
chr5	167911001	167916000	4.89E-08

chr5	167912001	167917000	4.71E-09
chr5	167913001	167918000	2.22E-08
chr5	172386001	172391000	4.58E-06
chr5	172483001	172488000	5.42E-10
chr5	175153001	175158000	7.38E-06
chr5	175154001	175159000	1.04E-05
chr5	175155001	175160000	6.12E-06
chr5	175618001	175623000	1.60E-05
chr5	175788001	175793000	1.14E-08
chr5	175816001	175821000	0.000147
chr5	176439001	176444000	8.69E-05
chr5	176440001	176445000	0.000394
chr5	176560001	176565000	4.54E-05
chr5	176815001	176820000	6.32E-07
chr5	176816001	176821000	0.000105
chr5	176817001	176822000	1.12E-07
chr5	176922001	176927000	2.58E-07
chr5	176923001	176928000	6.78E-06
chr5	176924001	176929000	3.45E-06
chr5	176925001	176930000	1.84E-07
chr5	176977001	176982000	7.42E-05
chr5	177400001	177405000	0.03526
chr5	177444001	177449000	0.024659
chr5	177503001	177508000	0.010538
chr5	177576001	177581000	1.09E-08
chr5	179243001	179248000	0.001454
chr5	180059001	180064000	0.002998
chr5	180258001	180263000	5.04E-08
chr6	2762001	2767000	1.11E-15
chr6	2763001	2768000	3.55E-17
chr6	2764001	2769000	2.54E-13
chr6	3022001	3027000	9.45E-07
chr6	3023001	3028000	5.02E-06
chr6	3062001	3067000	2.32E-06
chr6	3063001	3068000	9.30E-06
chr6	4020001	4025000	9.87E-11
chr6	5000001	5005000	1.29E-29
chr6	5001001	5006000	9.44E-32
chr6	5002001	5007000	8.26E-27
chr6	5003001	5008000	6.38E-27
chr6	5004001	5009000	1.06E-19
chr6	7050001	7055000	6.88E-08
chr6	7104001	7109000	1.00E-06
chr6	7105001	7110000	8.72E-05
chr6	7313001	7318000	5.38E-08
chr6	11090001	11095000	2.13E-11
chr6	13486001	13491000	2.61E-08
chr6	13487001	13492000	6.28E-09

chr6	13768001	13773000	0.001568
chr6	17282001	17287000	1.31E-10
chr6	21662001	21667000	2.01E-08
chr6	21666001	21671000	2.51E-07
chr6	26268001	26273000	1.54E-16
chr6	26610001	26615000	7.03E-06
chr6	27276001	27281000	1.99E-07
chr6	27761001	27766000	8.30E-05
chr6	27763001	27768000	0.000656
chr6	33539001	33544000	0.00493
chr6	33585001	33590000	0.007074
chr6	33586001	33591000	0.001618
chr6	33587001	33592000	8.55E-08
chr6	33588001	33593000	4.34E-08
chr6	33637001	33642000	0.023431
chr6	34189001	34194000	1.07E-05
chr6	34190001	34195000	1.10E-06
chr6	34191001	34196000	6.65E-06
chr6	35419001	35424000	0.000554
chr6	35432001	35437000	3.57E-11
chr6	35433001	35438000	1.98E-14
chr6	35434001	35439000	2.58E-09
chr6	40145001	40150000	0.00014
chr6	41510001	41515000	5.52E-08
chr6	41698001	41703000	0.000218
chr6	41995001	42000000	6.37E-05
chr6	41996001	42001000	8.22E-09
chr6	41997001	42002000	5.83E-07
chr6	41998001	42003000	8.27E-06
chr6	42739001	42744000	0.004229
chr6	42952001	42957000	0.032716
chr6	43240001	43245000	3.97E-05
chr6	43241001	43246000	0.001131
chr6	43242001	43247000	0.001604
chr6	43252001	43257000	1.78E-05
chr6	44118001	44123000	7.16E-05
chr6	44119001	44124000	8.91E-05
chr6	44594001	44599000	0.000128
chr6	45631001	45636000	1.90E-09
chr6	56951001	56956000	2.65E-10
chr6	56952001	56957000	1.05E-12
chr6	74293001	74298000	0.000589
chr6	74294001	74299000	0.000589
chr6	74405001	74410000	1.36E-09
chr6	90121001	90126000	2.98E-05
chr6	90122001	90127000	1.57E-06
chr6	107475001	107480000	0.003814
chr6	107476001	107481000	0.003814

chr6	111276001	111281000	0.000348
chr6	111277001	111282000	0.001139
chr6	114179001	114184000	0.00115
chr6	114659001	114664000	5.60E-12
chr6	116585001	116590000	4.89E-07
chr6	116586001	116591000	1.97E-05
chr6	116587001	116592000	0.000138
chr6	134590001	134595000	0.001908
chr6	137141001	137146000	2.47E-09
chr6	137142001	137147000	6.34E-08
chr6	137242001	137247000	1.36E-06
chr6	138482001	138487000	2.38E-13
chr6	138483001	138488000	4.05E-12
chr6	138722001	138727000	8.55E-11
chr7	499001	504000	0.000563
chr7	699001	704000	0.001056
chr7	700001	705000	0.001093
chr7	701001	706000	0.000121
chr7	1077001	1082000	2.48E-05
chr7	1090001	1095000	2.69E-06
chr7	1091001	1096000	9.69E-05
chr7	1104001	1109000	0.003623
chr7	1311001	1316000	0.020193
chr7	1312001	1317000	0.003787
chr7	1779001	1784000	2.07E-05
chr7	2901001	2906000	9.33E-06
chr7	4872001	4877000	0.044184
chr7	4997001	5002000	1.27E-05
chr7	4998001	5003000	4.05E-09
chr7	26896001	26901000	2.66E-07
chr7	26897001	26902000	4.70E-13
chr7	32533001	32538000	2.13E-11
chr7	32535001	32540000	4.42E-06
chr7	42926001	42931000	4.11E-07
chr7	44181001	44186000	8.35E-09
chr7	44785001	44790000	3.43E-07
chr7	45808001	45813000	1.37E-15
chr7	47621001	47626000	7.34E-09
chr7	61640001	61645000	0.033978
chr7	62893001	62898000	0.031515
chr7	62894001	62899000	0.031515
chr7	62895001	62900000	0.031515
chr7	64462001	64467000	7.93E-05
chr7	64463001	64468000	1.78E-11
chr7	64464001	64469000	7.21E-12
chr7	64465001	64470000	4.96E-05
chr7	66123001	66128000	0.02253
chr7	70155001	70160000	0.003654

chr7	72834001	72839000	0.002716
chr7	72835001	72840000	9.18E-05
chr7	73035001	73040000	9.48E-06
chr7	73036001	73041000	0.000595
chr7	73173001	73178000	0.002756
chr7	73174001	73179000	0.008179
chr7	73175001	73180000	0.00655
chr7	73176001	73181000	0.027985
chr7	73177001	73182000	0.027623
chr7	75068001	75073000	1.13E-14
chr7	75069001	75074000	2.47E-14
chr7	75070001	75075000	1.59E-06
chr7	75507001	75512000	4.36E-06
chr7	75803001	75808000	2.10E-06
chr7	76018001	76023000	0.000448
chr7	76234001	76239000	0.006682
chr7	76235001	76240000	0.011787
chr7	92464001	92469000	1.91E-06
chr7	96647001	96652000	8.21E-21
chr7	99093001	99098000	3.61E-07
chr7	100271001	100276000	1.09E-11
chr7	100424001	100429000	1.23E-05
chr7	100446001	100451000	0.000305
chr7	101053001	101058000	0.001977
chr7	101128001	101133000	4.18E-05
chr7	101129001	101134000	0.000603
chr7	101130001	101135000	0.000251
chr7	101333001	101338000	0.000125
chr7	101382001	101387000	5.09E-16
chr7	101384001	101389000	1.16E-12
chr7	101385001	101390000	1.95E-13
chr7	102308001	102313000	2.38E-12
chr7	128730001	128735000	0.009173
chr7	128850001	128855000	4.30E-05
chr7	129073001	129078000	0.000102
chr7	129737001	129742000	0.006558
chr7	129738001	129743000	0.002348
chr7	139184001	139189000	6.26E-10
chr7	139331001	139336000	5.07E-05
chr7	143517001	143522000	0.02497
chr7	148763001	148768000	6.96E-07
chr7	150449001	150454000	0.000799
chr7	150703001	150708000	4.52E-06
chr7	150704001	150709000	1.24E-06
chr7	151102001	151107000	2.39E-07
chr7	151103001	151108000	1.87E-12
chr7	151104001	151109000	3.89E-13
chr7	151105001	151110000	1.25E-08

chr7	151216001	151221000	8.00E-06
chr7	151217001	151222000	3.42E-08
chr7	152157001	152162000	0.001668
chr7	154994001	154999000	0.000194
chr7	154995001	155000000	8.01E-05
chr7	154997001	155002000	1.49E-06
chr7	156796001	156801000	1.60E-10
chr7	156797001	156802000	2.10E-09
chr7	157214001	157219000	3.40E-05
chr8	6561001	6566000	1.25E-05
chr8	11201001	11206000	1.19E-05
chr8	22012001	22017000	7.01E-07
chr8	22013001	22018000	1.63E-06
chr8	22014001	22019000	4.91E-07
chr8	22015001	22020000	7.47E-08
chr8	22923001	22928000	0.000225
chr8	22924001	22929000	2.27E-05
chr8	22959001	22964000	0.000236
chr8	22960001	22965000	0.000121
chr8	23079001	23084000	5.74E-10
chr8	23585001	23590000	2.82E-05
chr8	30082001	30087000	4.21E-06
chr8	30083001	30088000	9.54E-08
chr8	33422001	33427000	0.000751
chr8	37652001	37657000	0.004177
chr8	38299001	38304000	0.00075
chr8	38300001	38305000	0.001636
chr8	41347001	41352000	1.24E-07
chr8	41348001	41353000	9.11E-08
chr8	144821001	144826000	1.17E-08
chr8	144822001	144827000	7.10E-11
chr9	5624001	5629000	0.0001
chr9	5625001	5630000	6.21E-06
chr9	19123001	19128000	0.000161
chr9	19124001	19129000	1.72E-05
chr9	19462001	19467000	0.000436
chr9	21961001	21966000	7.36E-08
chr9	21969001	21974000	3.08E-06
chr9	21992001	21997000	6.41E-12
chr9	21993001	21998000	3.52E-08
chr9	26888001	26893000	1.04E-09
chr9	26889001	26894000	1.39E-10
chr9	32997001	33002000	8.22E-05
chr9	33457001	33462000	0.005385
chr9	34175001	34180000	3.04E-05
chr9	34178001	34183000	2.09E-08
chr9	34374001	34379000	0.00074
chr9	35071001	35076000	7.64E-07

chr9	35107001	35112000	9.56E-05
chr9	35108001	35113000	2.59E-07
chr9	35109001	35114000	1.93E-07
chr9	35110001	35115000	6.12E-07
chr9	35745001	35750000	0.003312
chr9	35906001	35911000	0.012672
chr9	37420001	37425000	7.51E-05
chr9	37422001	37427000	0.001164
chr9	37922001	37927000	0.00036
chr9	42243001	42248000	0.039552
chr9	42244001	42249000	0.027507
chr9	42245001	42250000	0.036308
chr9	44429001	44434000	0.034995
chr9	45648001	45653000	0.013725
chr9	46207001	46212000	0.004454
chr9	66095001	66100000	0.020258
chr9	67924001	67929000	0.013189
chr9	69384001	69389000	0.010924
chr9	69660001	69665000	0.013745
chr9	70479001	70484000	0.033309
chr9	71681001	71686000	0.013496
chr9	77113001	77118000	2.49E-11
chr9	79182001	79187000	1.19E-05
chr9	94119001	94124000	4.26E-08
chr9	94711001	94716000	5.38E-05
chr9	97429001	97434000	7.74E-07
chr9	103359001	103364000	6.90E-14
chr9	104207001	104212000	4.87E-07
chr9	114655001	114660000	1.07E-08
chr9	114935001	114940000	3.03E-05
chr9	114936001	114941000	4.87E-06
chr9	115141001	115146000	1.39E-10
chr9	115848001	115853000	4.79E-09
chr9	118351001	118356000	1.83E-07
chr9	118352001	118357000	3.49E-07
chr9	126773001	126778000	1.09E-07
chr9	126774001	126779000	9.22E-10
chr9	129438001	129443000	1.70E-05
chr9	129439001	129444000	6.65E-05
chr9	130283001	130288000	0.013244
chr9	130366001	130371000	8.98E-07
chr9	130367001	130372000	9.88E-09
chr9	130368001	130373000	6.11E-07
chr9	130369001	130374000	3.09E-08
chr9	130370001	130375000	7.86E-09
chr9	130492001	130497000	0.009824
chr9	130493001	130498000	0.033391
chr9	130557001	130562000	0.000241

chr9	130558001	130563000	6.16E-05
chr9	130559001	130564000	0.00031
chr9	130586001	130591000	9.67E-06
chr9	130724001	130729000	1.67E-06
chr9	130826001	130831000	3.62E-10
chr9	131868001	131873000	0.003412
chr9	132544001	132549000	0.001889
chr9	133808001	133813000	0.000563
chr9	133809001	133814000	0.000382
chr9	133810001	133815000	2.08E-07
chr9	133811001	133816000	3.30E-10
chr9	134222001	134227000	1.35E-05
chr9	134249001	134254000	1.56E-05
chr9	134613001	134618000	0.010681
chr9	135901001	135906000	2.97E-05
chr9	138984001	138989000	0.000528
chr9	138985001	138990000	0.000159
chr9	138986001	138991000	0.00014
chr9	138987001	138992000	0.00014
chr9	139292001	139297000	1.21E-05
chr9	139328001	139333000	1.55E-07
chr9	139494001	139499000	0.000354
chr9	139563001	139568000	0.000264
chr9	139564001	139569000	0.008004
chr9	139578001	139583000	0.000191
chr9	140020001	140025000	2.93E-13
chr9	140315001	140320000	2.81E-16
chr9	140316001	140321000	5.78E-19
chr9	140509001	140514000	0.001559
chr10	8097001	8102000	8.52E-11
chr10	8103001	8108000	2.09E-05
chr10	27385001	27390000	1.38E-14
chr10	27386001	27391000	1.55E-11
chr10	27387001	27392000	1.90E-13
chr10	27388001	27393000	2.06E-07
chr10	30021001	30026000	2.01E-08
chr10	30022001	30027000	2.83E-07
chr10	30023001	30028000	3.46E-10
chr10	33801001	33806000	0.009501
chr10	35926001	35931000	1.53E-10
chr10	35927001	35932000	2.33E-09
chr10	43777001	43782000	0.000193
chr10	45912001	45917000	0.001993
chr10	45913001	45918000	0.003564
chr10	45914001	45919000	9.34E-06
chr10	46524001	46529000	0.001262
chr10	46525001	46530000	0.001262
chr10	46526001	46531000	0.001262

chr10	46558001	46563000	0.024345
chr10	51614001	51619000	0.005421
chr10	51735001	51740000	0.001396
chr10	64446001	64451000	0.00172
chr10	70746001	70751000	1.60E-09
chr10	73623001	73628000	0.005417
chr10	73624001	73629000	0.031934
chr10	73971001	73976000	2.77E-06
chr10	74006001	74011000	0.035738
chr10	74008001	74013000	0.021999
chr10	74055001	74060000	0.000251
chr10	77152001	77157000	0.000131
chr10	79395001	79400000	2.22E-07
chr10	79396001	79401000	1.16E-14
chr10	79397001	79402000	4.84E-08
chr10	79398001	79403000	4.84E-08
chr10	79399001	79404000	2.33E-05
chr10	79699001	79704000	7.04E-06
chr10	79785001	79790000	1.59E-16
chr10	79786001	79791000	1.46E-11
chr10	79787001	79792000	6.61E-15
chr10	79788001	79793000	2.86E-21
chr10	79789001	79794000	9.96E-12
chr10	80828001	80833000	3.26E-08
chr10	80859001	80864000	0.003425
chr10	80860001	80865000	1.09E-05
chr10	88388001	88393000	2.68E-11
chr10	88389001	88394000	1.31E-09
chr10	89618001	89623000	8.71E-11
chr10	89619001	89624000	5.01E-11
chr10	95214001	95219000	2.27E-06
chr10	95216001	95221000	2.68E-05
chr10	95217001	95222000	0.00013
chr10	96887001	96892000	0.01242
chr10	97800001	97805000	6.97E-08
chr10	97801001	97806000	1.12E-07
chr10	98797001	98802000	3.21E-05
chr10	99471001	99476000	0.001279
chr10	99472001	99477000	0.002224
chr10	99473001	99478000	0.009007
chr10	102490001	102495000	1.98E-08
chr10	102493001	102498000	0.000405
chr10	102494001	102499000	2.10E-05
chr10	102881001	102886000	1.72E-07
chr10	102883001	102888000	1.92E-07
chr10	103577001	103582000	1.63E-08
chr10	103578001	103583000	7.16E-09
chr10	104501001	104506000	1.01E-09

chr10	104502001	104507000	2.46E-11
chr10	105128001	105133000	0.000881
chr10	116171001	116176000	0.00012
chr10	118498001	118503000	1.48E-05
chr10	123859001	123864000	0.000602
chr10	126067001	126072000	1.96E-06
chr10	126134001	126139000	3.46E-06
chr10	126476001	126481000	7.36E-06
chr10	126477001	126482000	9.85E-07
chr10	126478001	126483000	1.73E-05
chr10	126479001	126484000	2.74E-09
chr10	126480001	126485000	4.63E-05
chr10	134172001	134177000	0.019834
chr10	135138001	135143000	0.015837
chr10	135139001	135144000	0.004723
chr10	135189001	135194000	0.000502
chr10	135191001	135196000	2.41E-06
chr11	387001	392000	0.001554
chr11	388001	393000	0.00322
chr11	605001	610000	0.000137
chr11	1142001	1147000	3.50E-05
chr11	1143001	1148000	1.46E-05
chr11	1144001	1149000	0.000294
chr11	1526001	1531000	0.001232
chr11	1708001	1713000	0.015807
chr11	1768001	1773000	3.52E-06
chr11	2287001	2292000	8.78E-09
chr11	2288001	2293000	3.60E-19
chr11	2289001	2294000	2.95E-08
chr11	2290001	2295000	2.39E-07
chr11	2799001	2804000	5.25E-08
chr11	2907001	2912000	0.001842
chr11	2908001	2913000	0.001842
chr11	3184001	3189000	0.000631
chr11	5711001	5716000	1.75E-05
chr11	6620001	6625000	0.000119
chr11	6621001	6626000	9.98E-12
chr11	8929001	8934000	2.18E-05
chr11	8930001	8935000	5.04E-05
chr11	9284001	9289000	9.24E-17
chr11	9285001	9290000	3.56E-14
chr11	10311001	10316000	1.01E-05
chr11	11639001	11644000	1.63E-07
chr11	11642001	11647000	1.27E-05
chr11	16943001	16948000	2.60E-06
chr11	16946001	16951000	5.09E-12
chr11	18034001	18039000	3.62E-05
chr11	18342001	18347000	4.14E-09

chr11	18401001	18406000	0.004968
chr11	18402001	18407000	0.001201
chr11	44630001	44635000	1.67E-06
chr11	44631001	44636000	9.25E-05
chr11	44967001	44972000	7.42E-07
chr11	44968001	44973000	9.65E-09
chr11	44969001	44974000	1.09E-09
chr11	44970001	44975000	1.18E-10
chr11	44971001	44976000	4.21E-09
chr11	45168001	45173000	8.78E-10
chr11	45917001	45922000	0.000264
chr11	45918001	45923000	2.99E-05
chr11	45920001	45925000	0.020877
chr11	46413001	46418000	0.005179
chr11	46722001	46727000	4.99E-05
chr11	47789001	47794000	1.97E-08
chr11	47790001	47795000	4.12E-07
chr11	47963001	47968000	0.000334
chr11	47965001	47970000	1.38E-05
chr11	47966001	47971000	0.00134
chr11	47967001	47972000	0.000271
chr11	47968001	47973000	3.09E-05
chr11	47969001	47974000	2.60E-06
chr11	48190001	48195000	0.000449
chr11	48191001	48196000	2.91E-06
chr11	51141001	51146000	0.042728
chr11	61450001	61455000	3.54E-06
chr11	62429001	62434000	0.001193
chr11	62430001	62435000	0.000785
chr11	62527001	62532000	1.23E-08
chr11	62528001	62533000	2.52E-10
chr11	62529001	62534000	2.36E-05
chr11	63741001	63746000	7.36E-08
chr11	63800001	63805000	1.25E-05
chr11	64004001	64009000	8.14E-05
chr11	64005001	64010000	8.14E-05
chr11	64067001	64072000	2.10E-05
chr11	64413001	64418000	7.79E-05
chr11	64876001	64881000	0.003332
chr11	66025001	66030000	0.000637
chr11	66058001	66063000	0.001457
chr11	66860001	66865000	0.020345
chr11	66861001	66866000	0.004266
chr11	66862001	66867000	0.000667
chr11	66863001	66868000	0.001721
chr11	66864001	66869000	0.002401
chr11	66865001	66870000	0.009858
chr11	67047001	67052000	0.008156

chr11	67207001	67212000	5.44E-06
chr11	67208001	67213000	4.04E-05
chr11	67209001	67214000	0.004753
chr11	67210001	67215000	0.001087
chr11	67380001	67385000	0.000623
chr11	67910001	67915000	0.001734
chr11	71753001	71758000	1.63E-05
chr11	72351001	72356000	3.34E-08
chr11	72352001	72357000	1.47E-10
chr11	72353001	72358000	5.23E-12
chr11	72354001	72359000	2.17E-08
chr11	72926001	72931000	0.000985
chr11	72928001	72933000	0.000385
chr11	72929001	72934000	0.003787
chr11	74459001	74464000	1.50E-10
chr11	74460001	74465000	2.21E-18
chr11	75942001	75947000	1.57E-08
chr11	75943001	75948000	4.89E-11
chr11	89686001	89691000	0.035711
chr11	93156001	93161000	0.00475
chr11	94470001	94475000	7.55E-08
chr11	108464001	108469000	9.53E-09
chr11	111844001	111849000	6.30E-07
chr11	113641001	113646000	3.11E-06
chr11	113642001	113647000	2.78E-07
chr11	116654001	116659000	1.31E-09
chr11	116655001	116660000	2.84E-09
chr11	116656001	116661000	1.62E-07
chr11	117085001	117090000	0.000386
chr11	117198001	117203000	1.58E-07
chr11	119062001	119067000	0.000278
chr11	120170001	120175000	0.016466
chr11	120171001	120176000	0.034937
chr11	124610001	124615000	1.19E-06
chr12	2999001	3004000	1.94E-07
chr12	7187001	7192000	6.06E-05
chr12	12658001	12663000	5.75E-13
chr12	48107001	48112000	5.60E-08
chr12	48208001	48213000	7.42E-05
chr12	48209001	48214000	0.000459
chr12	48210001	48215000	2.07E-05
chr12	48211001	48216000	0.001121
chr12	51734001	51739000	0.000348
chr12	52536001	52541000	0.000115
chr12	54764001	54769000	8.33E-05
chr12	56220001	56225000	5.10E-06
chr12	56221001	56226000	6.44E-06
chr12	56222001	56227000	5.92E-06

chr12	56592001	56597000	0.000389
chr12	56593001	56598000	0.004248
chr12	57040001	57045000	4.55E-05
chr12	57619001	57624000	1.20E-06
chr12	57622001	57627000	6.59E-14
chr12	57623001	57628000	1.48E-14
chr12	58130001	58135000	3.39E-05
chr12	58148001	58153000	6.45E-07
chr12	58234001	58239000	1.17E-08
chr12	64795001	64800000	6.15E-20
chr12	64796001	64801000	3.85E-24
chr12	64797001	64802000	2.72E-21
chr12	64798001	64803000	2.86E-19
chr12	110305001	110310000	0.000143
chr12	112200001	112205000	8.40E-09
chr12	120989001	120994000	0.001203
chr12	120990001	120995000	0.001436
chr12	123920001	123925000	0.000535
chr12	125052001	125057000	0.04357
chr12	132284001	132289000	0.000737
chr12	132647001	132652000	2.18E-06
chr13	19129001	19134000	0.025963
chr13	20432001	20437000	4.64E-06
chr13	21095001	21100000	2.91E-09
chr13	21096001	21101000	1.30E-12
chr13	21097001	21102000	4.23E-15
chr13	21098001	21103000	1.71E-16
chr13	21099001	21104000	1.27E-08
chr13	21517001	21522000	1.07E-05
chr13	21747001	21752000	3.20E-11
chr13	21748001	21753000	3.89E-10
chr13	25316001	25321000	9.02E-06
chr13	31039001	31044000	1.60E-06
chr13	31770001	31775000	1.57E-07
chr13	31772001	31777000	2.56E-13
chr13	31773001	31778000	2.27E-13
chr13	31774001	31779000	9.94E-13
chr13	42621001	42626000	8.94E-16
chr13	42622001	42627000	1.61E-15
chr13	45621001	45626000	1.08E-08
chr13	53418001	53423000	4.35E-08
chr13	95951001	95956000	6.09E-05
chr13	95952001	95957000	0.000121
chr13	110434001	110439000	4.82E-10
chr13	110436001	110441000	1.78E-07
chr13	115045001	115050000	9.75E-08
chr13	115046001	115051000	1.39E-10
chr13	115047001	115052000	3.45E-10

chr14	19934001	19939000	0.00421
chr14	19935001	19940000	0.010287
chr14	20905001	20910000	0.000879
chr14	23067001	23072000	1.10E-07
chr14	24708001	24713000	2.85E-06
chr14	24796001	24801000	0.008867
chr14	24802001	24807000	3.68E-07
chr14	24803001	24808000	1.64E-08
chr14	51293001	51298000	1.30E-12
chr14	57275001	57280000	2.35E-25
chr14	61185001	61190000	3.42E-07
chr14	61186001	61191000	1.29E-08
chr14	64758001	64763000	2.82E-16
chr14	64759001	64764000	8.44E-16
chr14	64802001	64807000	8.63E-08
chr14	65003001	65008000	6.43E-10
chr14	69373001	69378000	0.000161
chr14	69374001	69379000	2.57E-05
chr14	71371001	71376000	6.74E-14
chr14	71372001	71377000	5.34E-12
chr14	72049001	72054000	1.07E-07
chr14	74110001	74115000	4.77E-05
chr14	74111001	74116000	0.00116
chr14	75408001	75413000	2.09E-07
chr14	89824001	89829000	5.63E-05
chr14	91577001	91582000	6.08E-05
chr14	91581001	91586000	0.000155
chr14	93648001	93653000	3.98E-07
chr14	93649001	93654000	2.09E-08
chr14	94451001	94456000	0.018941
chr14	100658001	100663000	0.000252
chr14	101034001	101039000	0.000226
chr14	101035001	101040000	0.000959
chr14	101037001	101042000	0.001078
chr14	103385001	103390000	6.57E-19
chr14	103386001	103391000	1.48E-17
chr14	103387001	103392000	3.94E-15
chr14	103944001	103949000	3.54E-08
chr14	105247001	105252000	3.88E-05
chr14	105248001	105253000	2.06E-06
chr14	105249001	105254000	3.67E-08
chr14	105250001	105255000	1.10E-06
chr14	105251001	105256000	8.69E-07
chr14	105267001	105272000	4.89E-09
chr14	105631001	105636000	3.99E-07
chr14	105632001	105637000	1.51E-05
chr14	105658001	105663000	0.000228
chr14	105659001	105664000	0.000228

chr14	105878001	105883000	0.001363
chr14	106543001	106548000	0.025002
chr15	20893001	20898000	0.001898
chr15	20978001	20983000	0.003921
chr15	20979001	20984000	0.003921
chr15	20980001	20985000	0.003921
chr15	20981001	20986000	0.003921
chr15	20997001	21002000	0.022631
chr15	22889001	22894000	1.94E-11
chr15	22890001	22895000	4.87E-09
chr15	23597001	23602000	0.000259
chr15	28633001	28638000	0.043923
chr15	28634001	28639000	0.043923
chr15	31615001	31620000	4.88E-13
chr15	32488001	32493000	0.024279
chr15	32489001	32494000	0.013236
chr15	32718001	32723000	2.63E-07
chr15	32719001	32724000	1.36E-05
chr15	32720001	32725000	4.14E-06
chr15	40802001	40807000	7.09E-07
chr15	40803001	40808000	1.26E-09
chr15	41293001	41298000	1.27E-05
chr15	41780001	41785000	0.00078
chr15	41781001	41786000	8.50E-09
chr15	41782001	41787000	1.63E-05
chr15	41789001	41794000	2.53E-07
chr15	41790001	41795000	8.08E-10
chr15	41791001	41796000	1.81E-06
chr15	41792001	41797000	1.08E-12
chr15	41793001	41798000	1.05E-14
chr15	45992001	45997000	1.89E-06
chr15	45995001	46000000	3.31E-16
chr15	58762001	58767000	0.000191
chr15	59060001	59065000	7.94E-12
chr15	59061001	59066000	7.44E-12
chr15	64269001	64274000	0.009412
chr15	64270001	64275000	0.004387
chr15	65113001	65118000	1.02E-05
chr15	65114001	65119000	3.15E-06
chr15	65602001	65607000	0.000331
chr15	65710001	65715000	0.000556
chr15	67015001	67020000	0.000448
chr15	67033001	67038000	1.18E-07
chr15	72489001	72494000	0.000529
chr15	73979001	73984000	0.00017
chr15	74425001	74430000	2.99E-09
chr15	74426001	74431000	2.24E-09
chr15	74502001	74507000	0.00011

chr15	74656001	74661000	3.67E-05
chr15	75014001	75019000	0.001564
chr15	75015001	75020000	3.29E-07
chr15	82651001	82656000	0.001161
chr15	84912001	84917000	0.01278
chr15	89496001	89501000	0.015456
chr15	90575001	90580000	4.14E-07
chr15	96909001	96914000	4.07E-10
chr15	98969001	98974000	4.31E-09
chr15	98970001	98975000	2.87E-07
chr16	177001	182000	9.12E-05
chr16	212001	217000	0.014075
chr16	446001	451000	7.58E-07
chr16	690001	695000	4.78E-07
chr16	1291001	1296000	0.012448
chr16	1391001	1396000	0.000762
chr16	1540001	1545000	0.000956
chr16	1558001	1563000	0.019753
chr16	1821001	1826000	0.000146
chr16	1822001	1827000	0.000115
chr16	2182001	2187000	5.29E-06
chr16	2183001	2188000	1.83E-09
chr16	2532001	2537000	1.02E-09
chr16	2653001	2658000	1.02E-09
chr16	2888001	2893000	0.000343
chr16	3091001	3096000	3.72E-09
chr16	3092001	3097000	3.89E-09
chr16	3093001	3098000	2.14E-09
chr16	3094001	3099000	8.90E-12
chr16	3095001	3100000	1.16E-08
chr16	3219001	3224000	9.63E-14
chr16	3986001	3991000	0.004581
chr16	4389001	4394000	0.000195
chr16	4674001	4679000	4.92E-10
chr16	10646001	10651000	5.40E-08
chr16	10647001	10652000	1.22E-06
chr16	11697001	11702000	3.97E-06
chr16	11698001	11703000	0.000105
chr16	11944001	11949000	3.06E-11
chr16	15005001	15010000	0.002427
chr16	16324001	16329000	8.57E-10
chr16	19121001	19126000	1.66E-20
chr16	19122001	19127000	3.31E-21
chr16	19123001	19128000	8.40E-26
chr16	19124001	19129000	1.04E-15
chr16	19125001	19130000	5.61E-13
chr16	19894001	19899000	8.44E-10
chr16	22177001	22182000	0.032809

chr16	22197001	22202000	9.46E-06
chr16	28893001	28898000	0.003091
chr16	28894001	28899000	0.00668
chr16	29532001	29537000	0.006005
chr16	29533001	29538000	0.006005
chr16	30103001	30108000	1.22E-07
chr16	30377001	30382000	1.57E-06
chr16	30378001	30383000	4.86E-08
chr16	30402001	30407000	9.58E-05
chr16	30403001	30408000	7.31E-07
chr16	30405001	30410000	1.08E-05
chr16	30409001	30414000	2.02E-06
chr16	30783001	30788000	1.69E-07
chr16	30784001	30789000	1.03E-12
chr16	30785001	30790000	3.85E-09
chr16	30786001	30791000	1.28E-09
chr16	30787001	30792000	1.77E-08
chr16	30813001	30818000	0.000709
chr16	30882001	30887000	5.15E-05
chr16	30883001	30888000	6.60E-06
chr16	30884001	30889000	0.000272
chr16	31020001	31025000	0.00253
chr16	31128001	31133000	7.48E-07
chr16	46917001	46922000	0.001147
chr16	50279001	50284000	0.001818
chr16	56349001	56354000	6.11E-07
chr16	57503001	57508000	4.87E-06
chr16	57516001	57521000	0.000259
chr16	57517001	57522000	5.83E-05
chr16	57518001	57523000	6.70E-06
chr16	58018001	58023000	4.37E-07
chr16	58663001	58668000	2.38E-08
chr16	58664001	58669000	2.22E-12
chr16	66579001	66584000	3.57E-05
chr16	66580001	66585000	3.03E-05
chr16	66634001	66639000	1.91E-10
chr16	66635001	66640000	2.70E-09
chr16	67429001	67434000	3.47E-05
chr16	67430001	67435000	1.68E-06
chr16	67548001	67553000	0.002707
chr16	67549001	67554000	0.005561
chr16	67593001	67598000	4.24E-10
chr16	67596001	67601000	7.81E-09
chr16	67597001	67602000	1.82E-08
chr16	74706001	74711000	0.012378
chr16	75281001	75286000	6.16E-05
chr16	75287001	75292000	0.000626
chr16	75288001	75293000	0.001719

chr16	75289001	75294000	0.004277
chr16	75295001	75300000	0.000155
chr16	84216001	84221000	1.73E-08
chr16	84217001	84222000	2.10E-10
chr16	85363001	85368000	0.000556
chr16	86543001	86548000	0.000209
chr16	86544001	86549000	1.14E-05
chr16	86545001	86550000	9.34E-06
chr16	86546001	86551000	3.30E-08
chr16	86584001	86589000	2.78E-09
chr16	87346001	87351000	6.69E-11
chr16	88879001	88884000	1.31E-06
chr16	89719001	89724000	5.47E-05
chr16	90175001	90180000	0.000453
chr16	90176001	90181000	0.000453
chr16	90177001	90182000	0.000196
chr17	1161001	1166000	3.41E-07
chr17	1393001	1398000	1.74E-07
chr17	1394001	1399000	0.000103
chr17	1620001	1625000	1.74E-07
chr17	1835001	1840000	0.035501
chr17	1836001	1841000	0.031772
chr17	3866001	3871000	8.00E-06
chr17	4454001	4459000	3.54E-10
chr17	4455001	4460000	2.07E-09
chr17	4456001	4461000	1.23E-10
chr17	4457001	4462000	3.09E-12
chr17	4852001	4857000	5.67E-13
chr17	4889001	4894000	0.000132
chr17	4890001	4895000	0.001728
chr17	6567001	6572000	4.53E-11
chr17	6568001	6573000	3.71E-12
chr17	7280001	7285000	5.30E-07
chr17	7281001	7286000	9.12E-06
chr17	7282001	7287000	8.82E-06
chr17	7380001	7385000	1.06E-06
chr17	7381001	7386000	1.95E-10
chr17	7382001	7387000	3.74E-06
chr17	7383001	7388000	7.04E-07
chr17	7384001	7389000	3.74E-06
chr17	8130001	8135000	2.01E-05
chr17	8196001	8201000	8.11E-06
chr17	8197001	8202000	1.12E-08
chr17	8198001	8203000	9.63E-06
chr17	17585001	17590000	2.23E-07
chr17	17586001	17591000	1.79E-08
chr17	17681001	17686000	4.21E-07
chr17	17682001	17687000	1.23E-10

chr17	17683001	17688000	1.47E-06
chr17	18023001	18028000	0.004217
chr17	18083001	18088000	5.06E-06
chr17	18157001	18162000	0.000114
chr17	18684001	18689000	3.14E-05
chr17	19390001	19395000	1.38E-06
chr17	20743001	20748000	1.24E-05
chr17	20744001	20749000	9.34E-08
chr17	26817001	26822000	0.000456
chr17	26876001	26881000	0.000543
chr17	26877001	26882000	1.68E-06
chr17	26878001	26883000	7.22E-06
chr17	29315001	29320000	3.00E-05
chr17	36878001	36883000	0.00198
chr17	36977001	36982000	2.05E-12
chr17	36978001	36983000	1.64E-08
chr17	36979001	36984000	2.86E-07
chr17	36980001	36985000	7.87E-06
chr17	37772001	37777000	0.000212
chr17	38268001	38273000	6.23E-06
chr17	38497001	38502000	2.37E-07
chr17	39930001	39935000	0.014346
chr17	39931001	39936000	1.71E-05
chr17	39932001	39937000	0.006987
chr17	39933001	39938000	0.029333
chr17	39934001	39939000	0.024104
chr17	40342001	40347000	0.001765
chr17	40930001	40935000	0.013032
chr17	41652001	41657000	0.000522
chr17	42848001	42853000	1.75E-06
chr17	42907001	42912000	1.03E-09
chr17	42972001	42977000	8.85E-19
chr17	43301001	43306000	0.001592
chr17	44064001	44069000	9.87E-06
chr17	44327001	44332000	0.000807
chr17	44844001	44849000	2.21E-05
chr17	44845001	44850000	1.11E-08
chr17	44846001	44851000	2.03E-06
chr17	44847001	44852000	8.05E-08
chr17	44848001	44853000	4.87E-05
chr17	46751001	46756000	0.000139
chr17	47027001	47032000	8.50E-06
chr17	47409001	47414000	0.010255
chr17	47488001	47493000	2.73E-05
chr17	47958001	47963000	5.80E-06
chr17	48204001	48209000	0.001789
chr17	48205001	48210000	0.00701
chr17	48364001	48369000	7.64E-06

chr17	48677001	48682000	0.004023
chr17	55119001	55124000	1.90E-12
chr17	55120001	55125000	1.17E-10
chr17	56009001	56014000	0.011485
chr17	56159001	56164000	4.08E-05
chr17	56160001	56165000	4.91E-06
chr17	56161001	56166000	3.77E-06
chr17	57077001	57082000	0.00011
chr17	57078001	57083000	2.61E-12
chr17	57284001	57289000	1.34E-09
chr17	57285001	57290000	3.75E-09
chr17	57286001	57291000	3.09E-10
chr17	59463001	59468000	0.00111
chr17	60546001	60551000	0.00031
chr17	60549001	60554000	0.010148
chr17	64593001	64598000	0.005474
chr17	64594001	64599000	0.009703
chr17	66191001	66196000	6.36E-08
chr17	71224001	71229000	1.48E-09
chr17	71226001	71231000	5.64E-09
chr17	71305001	71310000	3.72E-10
chr17	71306001	71311000	4.33E-12
chr17	71307001	71312000	2.45E-10
chr17	72461001	72466000	3.22E-08
chr17	72916001	72921000	0.000201
chr17	73288001	73293000	0.000262
chr17	73838001	73843000	0.016013
chr17	73848001	73853000	5.26E-18
chr17	73849001	73854000	8.66E-13
chr17	73850001	73855000	3.25E-13
chr17	73851001	73856000	3.55E-14
chr17	73852001	73857000	3.76E-20
chr17	75378001	75383000	1.53E-10
chr17	75382001	75387000	9.92E-05
chr17	75416001	75421000	6.21E-07
chr17	75417001	75422000	3.69E-05
chr17	77768001	77773000	5.02E-10
chr17	77787001	77792000	5.42E-05
chr17	78039001	78044000	2.25E-10
chr17	78040001	78045000	2.63E-11
chr17	78041001	78046000	1.50E-15
chr17	78447001	78452000	8.58E-07
chr17	78940001	78945000	0.002862
chr17	78941001	78946000	0.000587
chr17	79338001	79343000	0.000123
chr17	79581001	79586000	0.005539
chr17	79582001	79587000	0.007034
chr17	79867001	79872000	1.32E-05

chr17	79883001	79888000	0.000236
chr17	80060001	80065000	8.83E-05
chr17	80176001	80181000	0.039091
chr17	80729001	80734000	1.42E-12
chr18	5626001	5631000	1.90E-07
chr18	12267001	12272000	1.20E-05
chr18	12268001	12273000	4.00E-08
chr18	12284001	12289000	0.004777
chr18	12375001	12380000	2.29E-14
chr18	14455001	14460000	0.000262
chr18	14456001	14461000	0.004001
chr18	19281001	19286000	9.63E-07
chr18	20829001	20834000	2.10E-05
chr18	20830001	20835000	2.62E-05
chr18	20987001	20992000	0.000935
chr18	20988001	20993000	0.000345
chr18	20989001	20994000	0.000382
chr18	20990001	20995000	0.000151
chr18	34853001	34858000	0.000681
chr18	35343001	35348000	0.003234
chr18	45684001	45689000	9.71E-17
chr18	45685001	45690000	1.42E-19
chr18	45686001	45691000	6.77E-07
chr18	46304001	46309000	7.84E-07
chr18	56938001	56943000	2.44E-08
chr18	57072001	57077000	0.005164
chr18	74841001	74846000	3.21E-06
chr19	348001	353000	0.000662
chr19	659001	664000	1.91E-05
chr19	929001	934000	1.65E-05
chr19	1091001	1096000	2.71E-15
chr19	1092001	1097000	5.26E-20
chr19	1093001	1098000	3.86E-20
chr19	1094001	1099000	1.51E-19
chr19	1095001	1100000	1.43E-14
chr19	1439001	1444000	4.32E-07
chr19	1489001	1494000	9.10E-06
chr19	1490001	1495000	6.91E-07
chr19	1761001	1766000	0.001683
chr19	2246001	2251000	0.000266
chr19	2454001	2459000	0.003804
chr19	2456001	2461000	0.005557
chr19	2699001	2704000	0.002997
chr19	2721001	2726000	0.005547
chr19	2941001	2946000	0.000331
chr19	3381001	3386000	9.98E-07
chr19	3382001	3387000	0.001837
chr19	3386001	3391000	0.009347

chr19	3957001	3962000	0.02029
chr19	3971001	3976000	1.34E-14
chr19	4119001	4124000	0.018605
chr19	4145001	4150000	0.000244
chr19	4807001	4812000	4.52E-05
chr19	4808001	4813000	4.50E-05
chr19	4809001	4814000	2.50E-05
chr19	4810001	4815000	0.000798
chr19	4973001	4978000	0.006279
chr19	4975001	4980000	0.001632
chr19	4976001	4981000	0.01055
chr19	4977001	4982000	0.019087
chr19	5433001	5438000	0.00097
chr19	5968001	5973000	2.38E-05
chr19	6067001	6072000	2.29E-05
chr19	6196001	6201000	8.69E-07
chr19	6198001	6203000	5.31E-05
chr19	6389001	6394000	2.11E-05
chr19	6391001	6396000	0.001334
chr19	6392001	6397000	2.29E-05
chr19	6474001	6479000	0.00216
chr19	7419001	7424000	0.047657
chr19	7566001	7571000	0.000183
chr19	7979001	7984000	0.000706
chr19	8429001	8434000	4.41E-05
chr19	8451001	8456000	5.16E-11
chr19	8452001	8457000	2.67E-13
chr19	9167001	9172000	0.000197
chr19	9875001	9880000	1.53E-08
chr19	9876001	9881000	8.60E-07
chr19	9877001	9882000	5.54E-07
chr19	9878001	9883000	2.39E-11
chr19	9879001	9884000	2.35E-10
chr19	9895001	9900000	1.74E-07
chr19	10529001	10534000	4.13E-05
chr19	10530001	10535000	1.87E-05
chr19	10626001	10631000	8.08E-05
chr19	10627001	10632000	0.00376
chr19	10628001	10633000	0.000841
chr19	10672001	10677000	1.17E-07
chr19	10710001	10715000	2.34E-05
chr19	10711001	10716000	2.87E-07
chr19	10712001	10717000	6.20E-07
chr19	10823001	10828000	5.51E-07
chr19	10981001	10986000	6.41E-09
chr19	10982001	10987000	4.64E-07
chr19	10983001	10988000	7.42E-08
chr19	11196001	11201000	1.15E-07

chr19	11197001	11202000	5.51E-07
chr19	11527001	11532000	3.39E-08
chr19	11528001	11533000	5.54E-11
chr19	11955001	11960000	1.85E-10
chr19	11956001	11961000	2.73E-07
chr19	11957001	11962000	5.59E-07
chr19	12987001	12992000	0.000437
chr19	12988001	12993000	0.008575
chr19	12989001	12994000	0.001697
chr19	12991001	12996000	0.005144
chr19	12992001	12997000	0.00125
chr19	12993001	12998000	6.26E-05
chr19	12995001	13000000	0.00019
chr19	12996001	13001000	0.000262
chr19	13049001	13054000	1.50E-05
chr19	13050001	13055000	5.94E-08
chr19	13051001	13056000	0.000427
chr19	13183001	13188000	0.004685
chr19	13184001	13189000	0.008463
chr19	13903001	13908000	2.43E-05
chr19	13904001	13909000	0.002353
chr19	13946001	13951000	0.00033
chr19	13947001	13952000	0.000108
chr19	14180001	14185000	1.35E-05
chr19	14182001	14187000	0.000625
chr19	14227001	14232000	0.010171
chr19	14245001	14250000	1.04E-05
chr19	14325001	14330000	0.002095
chr19	14441001	14446000	2.46E-16
chr19	14540001	14545000	0.00265
chr19	14541001	14546000	9.31E-05
chr19	14542001	14547000	4.42E-08
chr19	14543001	14548000	1.13E-09
chr19	15305001	15310000	0.027555
chr19	16433001	16438000	0.00062
chr19	16579001	16584000	0.000184
chr19	16635001	16640000	0.024598
chr19	16797001	16802000	0.035497
chr19	17186001	17191000	7.92E-08
chr19	17187001	17192000	0.000117
chr19	17209001	17214000	0.016911
chr19	17336001	17341000	1.02E-06
chr19	17370001	17375000	0.001612
chr19	17455001	17460000	8.65E-06
chr19	17493001	17498000	7.30E-05
chr19	17578001	17583000	0.007034
chr19	17595001	17600000	0.001829
chr19	17968001	17973000	2.68E-07

chr19	17969001	17974000	1.34E-07
chr19	17970001	17975000	2.99E-08
chr19	17971001	17976000	2.97E-06
chr19	18074001	18079000	0.002364
chr19	18075001	18080000	0.020138
chr19	18165001	18170000	0.001744
chr19	18166001	18171000	0.000457
chr19	18167001	18172000	0.000292
chr19	18168001	18173000	7.86E-07
chr19	18169001	18174000	8.64E-05
chr19	18258001	18263000	0.000342
chr19	18387001	18392000	2.92E-08
chr19	18388001	18393000	2.92E-08
chr19	18389001	18394000	5.35E-06
chr19	18392001	18397000	0.000558
chr19	18398001	18403000	8.09E-05
chr19	18399001	18404000	0.000896
chr19	18433001	18438000	3.54E-13
chr19	18464001	18469000	0.041778
chr19	18505001	18510000	0.000389
chr19	18545001	18550000	0.000274
chr19	18571001	18576000	0.004417
chr19	18614001	18619000	3.98E-05
chr19	18615001	18620000	1.22E-05
chr19	18616001	18621000	4.80E-07
chr19	18746001	18751000	0.00232
chr19	18747001	18752000	0.000896
chr19	18758001	18763000	1.01E-07
chr19	18759001	18764000	1.64E-05
chr19	18760001	18765000	2.75E-05
chr19	18919001	18924000	7.40E-06
chr19	18920001	18925000	5.59E-06
chr19	19427001	19432000	9.31E-09
chr19	19750001	19755000	7.91E-12
chr19	19751001	19756000	2.30E-09
chr19	19752001	19757000	2.14E-05
chr19	19753001	19758000	3.06E-05
chr19	30333001	30338000	4.36E-08
chr19	34285001	34290000	3.46E-18
chr19	34286001	34291000	2.30E-10
chr19	36248001	36253000	2.55E-10
chr19	36249001	36254000	1.86E-08
chr19	36336001	36341000	7.33E-05
chr19	36390001	36395000	0.000815
chr19	36496001	36501000	0.002711
chr19	36979001	36984000	6.73E-12
chr19	37064001	37069000	1.37E-07
chr19	38356001	38361000	0.003461

chr19	38357001	38362000	0.003153
chr19	38696001	38701000	0.000528
chr19	38697001	38702000	0.001399
chr19	39084001	39089000	0.000254
chr19	39085001	39090000	0.00037
chr19	39086001	39091000	0.001563
chr19	39147001	39152000	3.30E-08
chr19	39170001	39175000	0.005562
chr19	40474001	40479000	4.05E-05
chr19	40475001	40480000	1.20E-06
chr19	40476001	40481000	9.17E-12
chr19	40477001	40482000	0.000122
chr19	40768001	40773000	0.00014
chr19	42385001	42390000	5.23E-06
chr19	42571001	42576000	0.000126
chr19	44121001	44126000	0.000444
chr19	44122001	44127000	0.000313
chr19	44123001	44128000	0.001193
chr19	45394001	45399000	0.001131
chr19	45395001	45400000	0.000906
chr19	45636001	45641000	1.95E-05
chr19	45637001	45642000	7.98E-05
chr19	45638001	45643000	9.15E-05
chr19	45715001	45720000	0.003859
chr19	45846001	45851000	0.002038
chr19	45898001	45903000	6.67E-06
chr19	45966001	45971000	5.80E-05
chr19	45967001	45972000	9.95E-09
chr19	46191001	46196000	0.000245
chr19	46193001	46198000	2.04E-07
chr19	46194001	46199000	8.08E-11
chr19	46195001	46200000	5.17E-12
chr19	46196001	46201000	8.35E-07
chr19	46375001	46380000	1.07E-05
chr19	46376001	46381000	4.65E-07
chr19	46377001	46382000	1.32E-08
chr19	46378001	46383000	2.94E-08
chr19	46802001	46807000	0.00403
chr19	47155001	47160000	1.25E-05
chr19	47547001	47552000	4.17E-07
chr19	47573001	47578000	0.000107
chr19	47726001	47731000	0.000174
chr19	47727001	47732000	0.000116
chr19	47750001	47755000	7.96E-05
chr19	47758001	47763000	0.004539
chr19	47867001	47872000	0.003689
chr19	48012001	48017000	0.000751
chr19	48918001	48923000	0.008867

chr19	49216001	49221000	0.014047
chr19	49217001	49222000	1.31E-05
chr19	49318001	49323000	0.005527
chr19	49727001	49732000	0.013021
chr19	49939001	49944000	0.000832
chr19	51179001	51184000	4.21E-05
chr19	51181001	51186000	1.86E-07
chr19	52692001	52697000	6.74E-11
chr19	52693001	52698000	2.86E-13
chr19	53070001	53075000	1.66E-10
chr19	53071001	53076000	2.35E-10
chr19	53072001	53077000	8.84E-17
chr19	53073001	53078000	3.04E-14
chr19	54614001	54619000	3.73E-09
chr19	54615001	54620000	2.72E-07
chr19	54618001	54623000	1.05E-10
chr19	54619001	54624000	3.39E-15
chr19	58787001	58792000	1.18E-09
chr19	58869001	58874000	1.42E-11
chr19	58870001	58875000	4.01E-13
chr19	58871001	58876000	4.30E-11
chr19	58872001	58877000	1.14E-12
chr19	58919001	58924000	5.27E-11
chr19	58979001	58984000	0.000303
chr19	59007001	59012000	2.99E-06
chr19	59051001	59056000	8.36E-09
chr19	59073001	59078000	3.25E-06
chr20	2821001	2826000	1.97E-07
chr20	3828001	3833000	5.78E-05
chr20	4800001	4805000	2.34E-07
chr20	4801001	4806000	1.97E-08
chr20	4802001	4807000	1.18E-05
chr20	4803001	4808000	2.81E-06
chr20	5668001	5673000	7.69E-06
chr20	25174001	25179000	2.06E-11
chr20	25175001	25180000	4.42E-09
chr20	25176001	25181000	2.02E-09
chr20	25990001	25995000	4.05E-07
chr20	30774001	30779000	0.000472
chr20	30775001	30780000	0.000162
chr20	31346001	31351000	5.29E-07
chr20	32318001	32323000	0.000337
chr20	32699001	32704000	2.67E-07
chr20	33491001	33496000	8.73E-09
chr20	33538001	33543000	2.56E-06
chr20	35547001	35552000	2.66E-05
chr20	35548001	35553000	0.000881
chr20	36748001	36753000	0.004424

chr20	44030001	44035000	5.38E-05
chr20	44033001	44038000	0.000101
chr20	44517001	44522000	0.01353
chr20	47663001	47668000	8.54E-08
chr20	57221001	57226000	2.56E-14
chr20	57222001	57227000	4.02E-09
chr20	57579001	57584000	0.00539
chr20	60370001	60375000	0.000431
chr20	60810001	60815000	0.000785
chr20	61846001	61851000	4.93E-09
chr20	61847001	61852000	1.36E-08
chr20	62072001	62077000	0.005398
chr20	62317001	62322000	2.18E-07
chr20	62608001	62613000	0.000139
chr21	30387001	30392000	2.69E-10
chr21	30388001	30393000	1.73E-10
chr21	32714001	32719000	2.61E-13
chr21	32928001	32933000	1.53E-11
chr21	32929001	32934000	4.76E-15
chr21	32931001	32936000	1.57E-10
chr21	34850001	34855000	3.57E-05
chr21	34851001	34856000	0.00121
chr21	40749001	40754000	1.13E-05
chr21	40750001	40755000	0.000593
chr21	42875001	42880000	9.98E-07
chr21	44392001	44397000	5.38E-05
chr21	44393001	44398000	3.30E-06
chr21	44394001	44399000	9.42E-07
chr21	44395001	44400000	4.66E-07
chr21	44755001	44760000	0.042939
chr21	44780001	44785000	1.14E-05
chr21	45860001	45865000	0.001293
chr21	45861001	45866000	0.002243
chr21	46221001	46226000	1.47E-10
chr21	46289001	46294000	2.82E-09
chr21	46349001	46354000	0.000523
chr21	46350001	46355000	0.000219
chr21	46401001	46406000	1.30E-05
chr21	46402001	46407000	2.09E-05
chr21	47713001	47718000	3.97E-07
chr21	47717001	47722000	0.000463
chr22	20253001	20258000	0.000423
chr22	20254001	20259000	0.002444
chr22	20255001	20260000	0.000549
chr22	20919001	20924000	1.70E-05
chr22	20921001	20926000	0.002489
chr22	21320001	21325000	0.001659
chr22	23860001	23865000	1.67E-05

chr22	24937001	24942000	0.000906
chr22	24938001	24943000	0.000139
chr22	25411001	25416000	0.00114
chr22	29277001	29282000	0.005448
chr22	30589001	30594000	2.97E-05
chr22	30685001	30690000	4.23E-05
chr22	30686001	30691000	6.43E-05
chr22	35739001	35744000	0.012321
chr22	36811001	36816000	3.79E-05
chr22	36812001	36817000	2.55E-05
chr22	37941001	37946000	3.22E-06
chr22	37942001	37947000	0.000108
chr22	37943001	37948000	1.69E-05
chr22	38050001	38055000	3.03E-07
chr22	38051001	38056000	6.51E-07
chr22	41797001	41802000	0.000385
chr22	41936001	41941000	8.16E-05
chr22	42225001	42230000	1.63E-08
chr22	42568001	42573000	0.011509
chr22	43527001	43532000	2.02E-05
chr22	44287001	44292000	3.43E-07
chr22	45089001	45094000	0.000787
chr22	45655001	45660000	0.00841
chr22	50467001	50472000	5.60E-06
chr22	50683001	50688000	1.50E-05
chr22	51121001	51126000	0.046903
chr22	51122001	51127000	0.018297
chr22	51135001	51140000	0.001184
chr22	51220001	51225000	4.07E-05
chr22	51221001	51226000	1.24E-06
chrX	3292001	3297000	0.00039
chrX	9751001	9756000	2.68E-05
chrX	9752001	9757000	3.59E-05
chrX	9799001	9804000	0.000401
chrX	13128001	13133000	0.019333
chrX	18874001	18879000	0.036834
chrX	24258001	24263000	0.001656
chrX	40007001	40012000	2.16E-06
chrX	40328001	40333000	0.000211
chrX	40329001	40334000	6.66E-05
chrX	40806001	40811000	0.016999
chrX	40807001	40812000	0.002002
chrX	40808001	40813000	0.011313
chrX	40865001	40870000	0.000508
chrX	41320001	41325000	0.002582
chrX	44294001	44299000	0.003631
chrX	44391001	44396000	0.002222
chrX	46320001	46325000	0.003084

chrX	46697001	46702000	3.16E-06
chrX	47217001	47222000	2.05E-05
chrX	48376001	48381000	5.72E-06
chrX	48670001	48675000	6.24E-05
chrX	53025001	53030000	8.88E-06
chrX	53110001	53115000	1.02E-08
chrX	53111001	53116000	7.09E-07
chrX	53755001	53760000	0.002708
chrX	54463001	54468000	0.001042
chrX	54469001	54474000	0.000229
chrX	55514001	55519000	9.25E-11
chrX	55515001	55520000	1.42E-09
chrX	56457001	56462000	0.001495
chrX	62202001	62207000	3.21E-07
chrX	62302001	62307000	0.004259
chrX	62303001	62308000	0.000257
chrX	62304001	62309000	9.97E-05
chrX	64314001	64319000	0.01638
chrX	68137001	68142000	0.026431
chrX	68140001	68145000	0.011213
chrX	68141001	68146000	0.012564
chrX	70284001	70289000	0.000676
chrX	71342001	71347000	0.000417
chrX	72849001	72854000	0.04286
chrX	74855001	74860000	4.60E-05
chrX	75205001	75210000	0.00058
chrX	75448001	75453000	0.012924
chrX	75450001	75455000	0.016929
chrX	77496001	77501000	0.04286
chrX	77498001	77503000	0.000889
chrX	87998001	88003000	0.000121
chrX	87999001	88004000	1.00E-06
chrX	100746001	100751000	0.004225
chrX	100747001	100752000	0.010571
chrX	102762001	102767000	0.000399
chrX	102815001	102820000	0.003637
chrX	102919001	102924000	0.000223
chrX	106909001	106914000	0.002786
chrX	106910001	106915000	0.003134
chrX	106911001	106916000	0.000981
chrX	106912001	106917000	0.000149
chrX	108980001	108985000	8.49E-07
chrX	108981001	108986000	2.84E-05
chrX	114260001	114265000	0.000189
chrX	117469001	117474000	0.002512
chrX	129066001	129071000	0.000685
chrX	129067001	129072000	0.006409
chrX	132695001	132700000	0.0132

chrX	132696001	132701000	0.004098	
chrX	145620001	145625000	0.001152	
chrX	148230001	148235000	0.00044	
chrX	148614001	148619000	0.030239	
chrX	150143001	150148000	2.63E-05	
chrX	150144001	150149000	5.73E-06	
chrX	150145001	150150000	1.89E-07	
chrX	152954001	152959000	0.020717	
chrY	4868001	4873000	0.000621	
chrY	4869001	4874000	0.001127	
chrY	8619001	8624000	0.005979	
chrY	13328001	13333000	0.003513	
chrY	13329001	13334000	0.003513	
chrY	13625001	13630000	0.010245	
chrY	13626001	13631000	0.00728	
chr1	574001	579000	0.007706	hypo
chr1	575001	580000	0.045521	
chr1	576001	581000	0.045521	
chr1	577001	582000	0.00134	
chr1	578001	583000	4.56E-05	
chr1	1114001	1119000	0.005413	
chr1	1120001	1125000	1.55E-08	
chr1	1143001	1148000	0.000124	
chr1	1182001	1187000	0.005756	
chr1	1226001	1231000	0.000157	
chr1	1232001	1237000	0.024629	
chr1	1233001	1238000	0.024629	
chr1	1259001	1264000	0.00026	
chr1	1260001	1265000	0.000375	
chr1	1284001	1289000	0.001769	
chr1	1374001	1379000	0.001144	
chr1	1375001	1380000	9.31E-07	
chr1	1376001	1381000	2.22E-06	
chr1	1377001	1382000	0.012609	
chr1	1408001	1413000	4.28E-07	
chr1	1530001	1535000	0.024297	
chr1	1533001	1538000	1.61E-05	
chr1	1534001	1539000	0.005033	
chr1	1535001	1540000	0.00343	
chr1	1537001	1542000	0.003942	
chr1	1538001	1543000	0.001778	
chr1	1539001	1544000	0.000476	
chr1	1593001	1598000	0.000353	
chr1	1594001	1599000	0.00034	
chr1	1605001	1610000	3.41E-08	
chr1	1607001	1612000	5.11E-06	
chr1	1667001	1672000	0.003207	
chr1	1676001	1681000	0.00011	

chr1	1707001	1712000	0.000837
chr1	1711001	1716000	4.91E-06
chr1	1821001	1826000	3.29E-05
chr1	1830001	1835000	1.42E-05
chr1	1831001	1836000	0.000554
chr1	1899001	1904000	0.000799
chr1	1915001	1920000	0.00014
chr1	1932001	1937000	2.52E-07
chr1	1933001	1938000	1.51E-08
chr1	1934001	1939000	1.71E-10
chr1	1935001	1940000	4.94E-07
chr1	2050001	2055000	1.14E-05
chr1	2118001	2123000	2.86E-12
chr1	2119001	2124000	2.19E-06
chr1	2130001	2135000	0.002995
chr1	2282001	2287000	2.65E-06
chr1	2423001	2428000	2.33E-09
chr1	2433001	2438000	6.66E-05
chr1	2435001	2440000	0.00533
chr1	2436001	2441000	7.05E-06
chr1	2437001	2442000	2.92E-05
chr1	2466001	2471000	5.23E-10
chr1	2564001	2569000	0.004472
chr1	2570001	2575000	0.000581
chr1	2571001	2576000	3.36E-07
chr1	2573001	2578000	0.048537
chr1	2574001	2579000	3.73E-05
chr1	2693001	2698000	3.65E-08
chr1	2695001	2700000	3.29E-09
chr1	2696001	2701000	7.50E-09
chr1	2826001	2831000	0.003519
chr1	2868001	2873000	3.12E-05
chr1	2869001	2874000	4.70E-05
chr1	2870001	2875000	8.06E-05
chr1	2871001	2876000	3.51E-05
chr1	2875001	2880000	9.84E-07
chr1	2877001	2882000	7.50E-10
chr1	2878001	2883000	6.03E-11
chr1	2879001	2884000	3.30E-09
chr1	2902001	2907000	5.55E-05
chr1	2914001	2919000	1.68E-07
chr1	2951001	2956000	0.001197
chr1	3365001	3370000	9.38E-05
chr1	3366001	3371000	9.16E-06
chr1	3367001	3372000	2.68E-05
chr1	3368001	3373000	5.03E-08
chr1	3369001	3374000	1.58E-06
chr1	3407001	3412000	0.008556

chr1	3408001	3413000	0.008556
chr1	3451001	3456000	0.000705
chr1	3601001	3606000	0.000153
chr1	3605001	3610000	1.88E-05
chr1	3633001	3638000	3.71E-08
chr1	3634001	3639000	1.07E-07
chr1	3690001	3695000	0.00023
chr1	4096001	4101000	3.18E-05
chr1	4109001	4114000	0.000109
chr1	4135001	4140000	8.60E-06
chr1	4145001	4150000	3.33E-06
chr1	4148001	4153000	0.000657
chr1	4152001	4157000	0.000174
chr1	4165001	4170000	3.17E-06
chr1	4166001	4171000	6.15E-08
chr1	4167001	4172000	5.18E-08
chr1	4168001	4173000	5.71E-08
chr1	4218001	4223000	7.69E-05
chr1	4219001	4224000	7.76E-06
chr1	4237001	4242000	0.001188
chr1	4239001	4244000	8.36E-09
chr1	4430001	4435000	2.71E-05
chr1	4634001	4639000	5.45E-06
chr1	4794001	4799000	5.86E-08
chr1	4795001	4800000	1.42E-10
chr1	4915001	4920000	0.003806
chr1	5308001	5313000	3.21E-06
chr1	5589001	5594000	0.000249
chr1	5590001	5595000	3.14E-06
chr1	5591001	5596000	8.69E-06
chr1	5634001	5639000	4.62E-05
chr1	5684001	5689000	2.28E-08
chr1	5717001	5722000	7.46E-05
chr1	5718001	5723000	0.000267
chr1	5851001	5856000	0.015588
chr1	6077001	6082000	1.57E-05
chr1	6125001	6130000	3.96E-05
chr1	6152001	6157000	3.00E-06
chr1	6234001	6239000	0.000425
chr1	6235001	6240000	5.99E-07
chr1	6236001	6241000	1.95E-06
chr1	6269001	6274000	0.000464
chr1	6450001	6455000	0.000673
chr1	6451001	6456000	4.27E-09
chr1	6452001	6457000	2.33E-12
chr1	6453001	6458000	3.98E-09
chr1	6454001	6459000	5.67E-07
chr1	6472001	6477000	1.30E-06

chr1	6473001	6478000	9.63E-09
chr1	6474001	6479000	8.43E-08
chr1	6475001	6480000	8.43E-08
chr1	6500001	6505000	1.61E-08
chr1	6545001	6550000	0.000345
chr1	6548001	6553000	0.014993
chr1	6551001	6556000	3.54E-06
chr1	6552001	6557000	2.19E-05
chr1	6655001	6660000	1.05E-07
chr1	6656001	6661000	3.24E-06
chr1	6657001	6662000	8.58E-07
chr1	6658001	6663000	5.09E-05
chr1	6668001	6673000	2.76E-14
chr1	6669001	6674000	1.20E-14
chr1	6670001	6675000	2.93E-12
chr1	6757001	6762000	5.91E-12
chr1	6800001	6805000	2.64E-06
chr1	6801001	6806000	4.74E-05
chr1	6802001	6807000	0.002382
chr1	7136001	7141000	2.05E-08
chr1	7137001	7142000	1.37E-07
chr1	7659001	7664000	2.09E-07
chr1	7695001	7700000	1.87E-05
chr1	7729001	7734000	0.016082
chr1	7730001	7735000	0.000409
chr1	7954001	7959000	2.54E-07
chr1	7955001	7960000	2.43E-05
chr1	7956001	7961000	0.001424
chr1	8242001	8247000	1.98E-10
chr1	9095001	9100000	8.40E-05
chr1	9127001	9132000	8.24E-05
chr1	9128001	9133000	9.42E-05
chr1	9290001	9295000	0.001149
chr1	9646001	9651000	0.015088
chr1	9647001	9652000	1.29E-06
chr1	9649001	9654000	1.53E-05
chr1	9684001	9689000	9.30E-05
chr1	9685001	9690000	2.84E-05
chr1	9686001	9691000	1.12E-05
chr1	9701001	9706000	0.000891
chr1	9702001	9707000	0.000133
chr1	9703001	9708000	0.002544
chr1	9743001	9748000	4.04E-07
chr1	9880001	9885000	2.94E-14
chr1	9892001	9897000	6.36E-05
chr1	10006001	10011000	4.29E-06
chr1	10007001	10012000	7.23E-09
chr1	10008001	10013000	2.99E-07

chr1	10009001	10014000	9.03E-05
chr1	10010001	10015000	5.46E-06
chr1	10085001	10090000	0.001513
chr1	10086001	10091000	0.000506
chr1	10534001	10539000	7.38E-10
chr1	10699001	10704000	0.008312
chr1	10957001	10962000	3.33E-06
chr1	11071001	11076000	0.00087
chr1	11072001	11077000	6.62E-05
chr1	11073001	11078000	9.89E-05
chr1	11091001	11096000	5.15E-05
chr1	11092001	11097000	3.31E-05
chr1	11109001	11114000	9.44E-07
chr1	11110001	11115000	6.73E-10
chr1	11111001	11116000	8.15E-08
chr1	11112001	11117000	3.29E-05
chr1	11156001	11161000	1.02E-08
chr1	11374001	11379000	3.86E-05
chr1	11406001	11411000	0.000569
chr1	11407001	11412000	2.32E-05
chr1	11431001	11436000	1.67E-08
chr1	11432001	11437000	8.61E-07
chr1	11433001	11438000	4.70E-07
chr1	11434001	11439000	7.62E-08
chr1	11435001	11440000	2.42E-05
chr1	11449001	11454000	6.04E-09
chr1	11450001	11455000	1.14E-06
chr1	11451001	11456000	6.11E-08
chr1	11453001	11458000	6.96E-05
chr1	11463001	11468000	2.48E-07
chr1	11477001	11482000	2.14E-10
chr1	11478001	11483000	7.58E-11
chr1	11479001	11484000	8.79E-12
chr1	11480001	11485000	1.52E-07
chr1	11570001	11575000	0.00032
chr1	11571001	11576000	0.000306
chr1	11720001	11725000	1.05E-07
chr1	11737001	11742000	0.02993
chr1	11820001	11825000	4.42E-07
chr1	11911001	11916000	1.18E-06
chr1	11950001	11955000	9.78E-05
chr1	11951001	11956000	0.000921
chr1	11952001	11957000	0.00036
chr1	11993001	11998000	0.000147
chr1	12127001	12132000	8.19E-05
chr1	12128001	12133000	6.44E-06
chr1	12129001	12134000	0.000218
chr1	12240001	12245000	0.000144

chr1	12241001	12246000	0.02526
chr1	12286001	12291000	2.82E-07
chr1	12287001	12292000	1.09E-07
chr1	12743001	12748000	1.27E-07
chr1	12744001	12749000	1.18E-07
chr1	12874001	12879000	1.47E-05
chr1	12875001	12880000	7.78E-05
chr1	12975001	12980000	0.000198
chr1	12976001	12981000	9.19E-05
chr1	12988001	12993000	0.036834
chr1	12997001	13002000	0.000143
chr1	12998001	13003000	0.002449
chr1	12999001	13004000	0.002449
chr1	13000001	13005000	0.002449
chr1	13001001	13006000	0.002449
chr1	13147001	13152000	0.006117
chr1	13148001	13153000	0.010287
chr1	13370001	13375000	0.001063
chr1	13371001	13376000	0.001063
chr1	13790001	13795000	1.32E-06
chr1	15361001	15366000	4.96E-06
chr1	15430001	15435000	0.000298
chr1	15748001	15753000	0.000395
chr1	15749001	15754000	2.20E-05
chr1	15750001	15755000	0.000453
chr1	15751001	15756000	0.000453
chr1	15812001	15817000	5.10E-05
chr1	15909001	15914000	0.005667
chr1	16122001	16127000	0.000331
chr1	16125001	16130000	0.023624
chr1	16126001	16131000	0.003298
chr1	16128001	16133000	0.000252
chr1	16161001	16166000	5.81E-08
chr1	16162001	16167000	5.49E-07
chr1	16293001	16298000	2.14E-06
chr1	16379001	16384000	0.0063
chr1	16414001	16419000	0.00479
chr1	16436001	16441000	0.005169
chr1	16468001	16473000	1.66E-06
chr1	16469001	16474000	4.89E-06
chr1	16488001	16493000	6.52E-06
chr1	16516001	16521000	0.001309
chr1	16517001	16522000	6.84E-07
chr1	16518001	16523000	1.98E-06
chr1	17540001	17545000	1.38E-05
chr1	17585001	17590000	0.00925
chr1	17809001	17814000	0.026812
chr1	17818001	17823000	0.001479

chr1	17820001	17825000	0.000331
chr1	17821001	17826000	6.20E-05
chr1	17828001	17833000	3.98E-06
chr1	17829001	17834000	2.17E-05
chr1	18110001	18115000	2.15E-06
chr1	18111001	18116000	1.73E-05
chr1	18431001	18436000	1.46E-07
chr1	18515001	18520000	1.46E-05
chr1	18549001	18554000	5.97E-07
chr1	18660001	18665000	2.59E-06
chr1	18793001	18798000	1.83E-07
chr1	18794001	18799000	6.97E-08
chr1	18795001	18800000	9.32E-07
chr1	18796001	18801000	3.09E-06
chr1	18873001	18878000	0.000379
chr1	18928001	18933000	0.001446
chr1	18929001	18934000	2.72E-05
chr1	18930001	18935000	2.84E-05
chr1	18931001	18936000	6.62E-06
chr1	19024001	19029000	3.08E-05
chr1	19025001	19030000	5.30E-09
chr1	19026001	19031000	1.70E-13
chr1	19027001	19032000	5.65E-09
chr1	19028001	19033000	2.93E-07
chr1	19029001	19034000	1.02E-07
chr1	19041001	19046000	1.76E-07
chr1	19044001	19049000	1.91E-06
chr1	19250001	19255000	4.17E-08
chr1	19916001	19921000	0.005491
chr1	20000001	20005000	9.07E-05
chr1	20001001	20006000	1.24E-10
chr1	20002001	20007000	1.87E-07
chr1	20003001	20008000	3.83E-07
chr1	20360001	20365000	1.87E-05
chr1	20361001	20366000	2.22E-05
chr1	20363001	20368000	5.25E-06
chr1	20726001	20731000	0.001014
chr1	20910001	20915000	2.82E-07
chr1	20911001	20916000	0.000103
chr1	20912001	20917000	0.001213
chr1	20913001	20918000	0.001074
chr1	21035001	21040000	0.000678
chr1	21036001	21041000	3.51E-07
chr1	21037001	21042000	9.80E-06
chr1	21048001	21053000	0.000459
chr1	21049001	21054000	0.002559
chr1	21050001	21055000	0.004697
chr1	21051001	21056000	0.004853

chr1	21759001	21764000	0.005206
chr1	21840001	21845000	1.56E-08
chr1	21961001	21966000	1.37E-05
chr1	21994001	21999000	7.88E-06
chr1	21995001	22000000	7.83E-09
chr1	22197001	22202000	1.91E-05
chr1	22198001	22203000	0.000203
chr1	22199001	22204000	2.94E-05
chr1	22281001	22286000	0.000484
chr1	22282001	22287000	0.000271
chr1	22361001	22366000	0.000102
chr1	22454001	22459000	3.60E-06
chr1	22586001	22591000	1.00E-06
chr1	22877001	22882000	1.97E-05
chr1	22878001	22883000	3.29E-05
chr1	22919001	22924000	8.44E-05
chr1	22920001	22925000	0.000134
chr1	22921001	22926000	1.89E-05
chr1	22923001	22928000	4.17E-05
chr1	23249001	23254000	1.06E-08
chr1	23250001	23255000	8.70E-10
chr1	23251001	23256000	5.29E-08
chr1	23509001	23514000	6.75E-06
chr1	23805001	23810000	2.04E-07
chr1	23806001	23811000	6.17E-07
chr1	23963001	23968000	8.92E-06
chr1	23964001	23969000	6.19E-09
chr1	23991001	23996000	0.000338
chr1	23992001	23997000	0.000178
chr1	23993001	23998000	0.000103
chr1	24149001	24154000	0.002523
chr1	24151001	24156000	5.30E-05
chr1	24212001	24217000	5.85E-05
chr1	24254001	24259000	0.00084
chr1	24917001	24922000	0.000122
chr1	25068001	25073000	4.42E-07
chr1	25357001	25362000	6.86E-07
chr1	25430001	25435000	0.000167
chr1	25431001	25436000	0.002174
chr1	25728001	25733000	6.26E-05
chr1	25729001	25734000	1.04E-05
chr1	25843001	25848000	7.26E-05
chr1	25844001	25849000	0.009011
chr1	25918001	25923000	1.93E-10
chr1	25919001	25924000	4.50E-07
chr1	25935001	25940000	2.68E-08
chr1	25944001	25949000	2.21E-12
chr1	26093001	26098000	0.021511

chr1	26143001	26148000	0.000228
chr1	26353001	26358000	0.001068
chr1	26354001	26359000	0.000852
chr1	26355001	26360000	0.00126
chr1	26512001	26517000	0.000629
chr1	26513001	26518000	3.13E-05
chr1	26514001	26519000	0.008865
chr1	26540001	26545000	0.001548
chr1	26557001	26562000	1.36E-10
chr1	26558001	26563000	4.43E-12
chr1	26559001	26564000	2.74E-09
chr1	26710001	26715000	2.93E-06
chr1	26712001	26717000	4.66E-05
chr1	26722001	26727000	8.52E-06
chr1	26724001	26729000	5.66E-06
chr1	26758001	26763000	1.45E-05
chr1	27018001	27023000	1.27E-09
chr1	27019001	27024000	0.001506
chr1	27215001	27220000	3.97E-09
chr1	27216001	27221000	6.72E-14
chr1	27224001	27229000	2.67E-08
chr1	27244001	27249000	0.000212
chr1	27245001	27250000	2.87E-05
chr1	27409001	27414000	0.00028
chr1	27410001	27415000	5.49E-05
chr1	27411001	27416000	0.000129
chr1	27424001	27429000	0.029309
chr1	27425001	27430000	0.006237
chr1	27461001	27466000	0.002682
chr1	27464001	27469000	1.06E-05
chr1	27467001	27472000	0.002167
chr1	27484001	27489000	4.48E-07
chr1	27485001	27490000	1.43E-07
chr1	27486001	27491000	1.11E-06
chr1	27487001	27492000	7.70E-06
chr1	27666001	27671000	0.000153
chr1	27667001	27672000	0.000147
chr1	27668001	27673000	0.000471
chr1	27670001	27675000	0.000779
chr1	27925001	27930000	0.000312
chr1	28410001	28415000	1.56E-11
chr1	28688001	28693000	0.000512
chr1	28689001	28694000	3.94E-05
chr1	29043001	29048000	3.37E-05
chr1	29149001	29154000	0.004321
chr1	29237001	29242000	9.10E-09
chr1	29238001	29243000	5.95E-05
chr1	29240001	29245000	3.06E-06

chr1	29241001	29246000	1.86E-09
chr1	29556001	29561000	1.96E-08
chr1	29557001	29562000	2.63E-09
chr1	29595001	29600000	0.000248
chr1	29774001	29779000	5.93E-05
chr1	29833001	29838000	8.10E-06
chr1	30227001	30232000	8.48E-05
chr1	30228001	30233000	0.001103
chr1	30529001	30534000	7.40E-07
chr1	30658001	30663000	6.72E-05
chr1	30659001	30664000	1.02E-05
chr1	30660001	30665000	3.51E-06
chr1	31044001	31049000	4.65E-05
chr1	31108001	31113000	0.001141
chr1	31537001	31542000	1.34E-09
chr1	31538001	31543000	1.19E-12
chr1	31576001	31581000	0.009622
chr1	31604001	31609000	0.002001
chr1	31605001	31610000	0.028038
chr1	31625001	31630000	0.008055
chr1	31644001	31649000	6.93E-12
chr1	31645001	31650000	2.81E-14
chr1	31646001	31651000	3.32E-12
chr1	31647001	31652000	9.13E-19
chr1	31648001	31653000	1.88E-21
chr1	31649001	31654000	0.000619
chr1	31674001	31679000	1.79E-09
chr1	31675001	31680000	4.82E-09
chr1	31676001	31681000	1.13E-07
chr1	31685001	31690000	0.000222
chr1	32088001	32093000	0.008439
chr1	32089001	32094000	0.000733
chr1	32533001	32538000	0.002233
chr1	32726001	32731000	0.007017
chr1	32728001	32733000	0.027408
chr1	32931001	32936000	7.80E-10
chr1	32977001	32982000	1.41E-05
chr1	33186001	33191000	0.013938
chr1	33224001	33229000	0.004056
chr1	33351001	33356000	2.00E-06
chr1	33352001	33357000	0.014797
chr1	33353001	33358000	0.00141
chr1	33354001	33359000	0.000656
chr1	33355001	33360000	0.000656
chr1	33356001	33361000	0.00674
chr1	33437001	33442000	7.55E-06
chr1	33438001	33443000	7.30E-07
chr1	33439001	33444000	1.15E-08

chr1	33440001	33445000	3.23E-05
chr1	33759001	33764000	0.000365
chr1	33760001	33765000	0.002658
chr1	35029001	35034000	0.002112
chr1	35057001	35062000	3.12E-05
chr1	35059001	35064000	2.73E-08
chr1	35060001	35065000	1.41E-08
chr1	35063001	35068000	4.22E-08
chr1	35064001	35069000	1.00E-05
chr1	35514001	35519000	0.000701
chr1	35709001	35714000	0.002095
chr1	35716001	35721000	0.000158
chr1	35730001	35735000	0.001209
chr1	36019001	36024000	1.36E-13
chr1	36020001	36025000	8.49E-14
chr1	36021001	36026000	7.32E-14
chr1	36023001	36028000	3.59E-07
chr1	36102001	36107000	3.09E-14
chr1	36103001	36108000	1.68E-11
chr1	36104001	36109000	3.85E-15
chr1	36126001	36131000	3.34E-05
chr1	36158001	36163000	0.00023
chr1	36161001	36166000	8.60E-07
chr1	36162001	36167000	3.54E-07
chr1	36163001	36168000	1.54E-06
chr1	36169001	36174000	3.88E-09
chr1	36170001	36175000	8.17E-10
chr1	36171001	36176000	6.35E-08
chr1	36383001	36388000	0.000161
chr1	36385001	36390000	0.00221
chr1	36548001	36553000	0.000146
chr1	36554001	36559000	1.28E-05
chr1	36555001	36560000	1.53E-09
chr1	36640001	36645000	0.000246
chr1	36851001	36856000	7.55E-06
chr1	36862001	36867000	9.87E-08
chr1	37018001	37023000	1.35E-07
chr1	37019001	37024000	2.17E-06
chr1	37020001	37025000	6.71E-07
chr1	37036001	37041000	0.001048
chr1	37063001	37068000	2.12E-06
chr1	37064001	37069000	2.35E-05
chr1	37065001	37070000	1.80E-05
chr1	37066001	37071000	3.78E-06
chr1	37084001	37089000	6.74E-10
chr1	37087001	37092000	4.88E-06
chr1	37092001	37097000	7.33E-05
chr1	37113001	37118000	6.49E-07

chr1	37174001	37179000	3.13E-08
chr1	37176001	37181000	1.64E-08
chr1	37178001	37183000	9.87E-11
chr1	37214001	37219000	2.75E-05
chr1	37392001	37397000	3.38E-10
chr1	37463001	37468000	4.46E-06
chr1	37464001	37469000	6.39E-07
chr1	37465001	37470000	5.62E-06
chr1	37466001	37471000	7.47E-06
chr1	37784001	37789000	3.68E-06
chr1	37785001	37790000	3.32E-08
chr1	37786001	37791000	2.46E-07
chr1	37919001	37924000	0.001153
chr1	37920001	37925000	0.000226
chr1	37949001	37954000	0.002404
chr1	37983001	37988000	0.000305
chr1	37984001	37989000	0.000338
chr1	38156001	38161000	5.51E-11
chr1	38157001	38162000	1.24E-13
chr1	38158001	38163000	9.88E-15
chr1	38393001	38398000	8.99E-11
chr1	38394001	38399000	1.00E-07
chr1	38395001	38400000	2.75E-09
chr1	38396001	38401000	3.96E-07
chr1	38397001	38402000	3.34E-12
chr1	38418001	38423000	0.012162
chr1	38419001	38424000	0.014879
chr1	38527001	38532000	0.001262
chr1	38806001	38811000	5.60E-09
chr1	38807001	38812000	4.05E-08
chr1	38853001	38858000	0.000196
chr1	39159001	39164000	2.64E-05
chr1	39161001	39166000	8.45E-05
chr1	39454001	39459000	1.23E-07
chr1	39488001	39493000	4.14E-06
chr1	39489001	39494000	1.75E-10
chr1	39490001	39495000	3.41E-14
chr1	39491001	39496000	5.32E-13
chr1	39492001	39497000	9.61E-13
chr1	39545001	39550000	0.000558
chr1	39547001	39552000	1.82E-05
chr1	39733001	39738000	2.49E-05
chr1	39953001	39958000	7.49E-06
chr1	39955001	39960000	8.99E-06
chr1	40133001	40138000	5.33E-06
chr1	40134001	40139000	1.29E-08
chr1	40135001	40140000	7.03E-08
chr1	40136001	40141000	0.000798

chr1	40137001	40142000	1.25E-09
chr1	40138001	40143000	4.94E-05
chr1	40232001	40237000	1.27E-06
chr1	40233001	40238000	1.51E-05
chr1	40261001	40266000	0.003314
chr1	40499001	40504000	9.28E-07
chr1	40500001	40505000	9.68E-11
chr1	40501001	40506000	6.78E-14
chr1	40502001	40507000	3.29E-08
chr1	40503001	40508000	6.32E-13
chr1	40778001	40783000	0.000584
chr1	40779001	40784000	0.000967
chr1	41127001	41132000	9.00E-07
chr1	41128001	41133000	3.36E-06
chr1	41280001	41285000	0.000561
chr1	41281001	41286000	0.000167
chr1	42235001	42240000	2.97E-07
chr1	43339001	43344000	0.003301
chr1	43390001	43395000	6.17E-09
chr1	43824001	43829000	0.000416
chr1	43828001	43833000	3.76E-09
chr1	43834001	43839000	1.95E-08
chr1	44015001	44020000	6.57E-08
chr1	44016001	44021000	1.18E-07
chr1	44027001	44032000	0.002254
chr1	44304001	44309000	7.35E-09
chr1	44431001	44436000	5.89E-13
chr1	44432001	44437000	1.84E-16
chr1	44433001	44438000	1.81E-13
chr1	44434001	44439000	7.05E-11
chr1	44454001	44459000	0.002891
chr1	44455001	44460000	8.63E-05
chr1	44456001	44461000	0.000332
chr1	44604001	44609000	0.000257
chr1	44605001	44610000	0.00024
chr1	44606001	44611000	0.000887
chr1	44764001	44769000	1.56E-06
chr1	45118001	45123000	1.33E-05
chr1	45175001	45180000	1.06E-07
chr1	45255001	45260000	0.009909
chr1	45256001	45261000	0.008716
chr1	45257001	45262000	0.008716
chr1	45276001	45281000	8.26E-06
chr1	45292001	45297000	3.08E-05
chr1	45293001	45298000	0.000971
chr1	45307001	45312000	1.43E-08
chr1	45447001	45452000	1.86E-05
chr1	45450001	45455000	8.43E-07

chr1	45982001	45987000	3.58E-07
chr1	45983001	45988000	4.01E-07
chr1	46088001	46093000	1.13E-06
chr1	46213001	46218000	1.48E-15
chr1	46214001	46219000	1.52E-08
chr1	46215001	46220000	3.97E-07
chr1	46216001	46221000	1.02E-05
chr1	46641001	46646000	0.012547
chr1	46769001	46774000	3.22E-13
chr1	47675001	47680000	9.04E-06
chr1	47696001	47701000	3.22E-11
chr1	48755001	48760000	1.01E-06
chr1	48756001	48761000	5.56E-08
chr1	48757001	48762000	2.52E-06
chr1	48890001	48895000	1.14E-05
chr1	48997001	49002000	5.95E-07
chr1	49388001	49393000	1.23E-06
chr1	49491001	49496000	2.26E-05
chr1	50313001	50318000	4.19E-07
chr1	51068001	51073000	6.72E-08
chr1	51599001	51604000	6.76E-07
chr1	51600001	51605000	3.34E-07
chr1	51601001	51606000	2.14E-07
chr1	51602001	51607000	1.98E-06
chr1	52522001	52527000	0.008725
chr1	53064001	53069000	8.43E-14
chr1	53065001	53070000	2.91E-15
chr1	53066001	53071000	2.17E-19
chr1	53067001	53072000	9.56E-12
chr1	53068001	53073000	7.79E-10
chr1	53156001	53161000	0.000136
chr1	53170001	53175000	0.00026
chr1	53171001	53176000	0.000963
chr1	53407001	53412000	7.60E-07
chr1	54004001	54009000	1.92E-05
chr1	54125001	54130000	0.000587
chr1	54355001	54360000	1.64E-11
chr1	54463001	54468000	0.000253
chr1	54464001	54469000	3.33E-06
chr1	54888001	54893000	0.003323
chr1	54917001	54922000	0.00105
chr1	55032001	55037000	0.00013
chr1	55057001	55062000	0.004635
chr1	55058001	55063000	0.013134
chr1	55178001	55183000	9.42E-06
chr1	55179001	55184000	2.87E-06
chr1	55180001	55185000	7.48E-08
chr1	55181001	55186000	7.49E-08

chr1	58169001	58174000	3.92E-06
chr1	58170001	58175000	8.92E-10
chr1	58171001	58176000	2.27E-09
chr1	58172001	58177000	9.70E-09
chr1	58317001	58322000	2.33E-05
chr1	58388001	58393000	3.50E-07
chr1	58389001	58394000	3.57E-09
chr1	58413001	58418000	0.000116
chr1	58598001	58603000	2.36E-05
chr1	58666001	58671000	2.28E-07
chr1	59082001	59087000	3.80E-11
chr1	60546001	60551000	2.03E-05
chr1	60547001	60552000	0.000102
chr1	61260001	61265000	0.006381
chr1	61261001	61266000	0.000101
chr1	61291001	61296000	1.20E-14
chr1	61293001	61298000	1.20E-15
chr1	62186001	62191000	6.14E-09
chr1	62207001	62212000	0.000649
chr1	62853001	62858000	1.20E-07
chr1	63765001	63770000	3.82E-05
chr1	64633001	64638000	1.43E-08
chr1	64634001	64639000	1.38E-06
chr1	66159001	66164000	2.26E-08
chr1	66255001	66260000	1.75E-05
chr1	66546001	66551000	2.89E-06
chr1	67519001	67524000	1.19E-06
chr1	67573001	67578000	2.02E-06
chr1	67574001	67579000	1.06E-05
chr1	68024001	68029000	2.64E-08
chr1	68224001	68229000	3.45E-10
chr1	68225001	68230000	3.25E-11
chr1	69173001	69178000	0.00039
chr1	69269001	69274000	7.13E-05
chr1	69300001	69305000	7.33E-09
chr1	69301001	69306000	1.54E-09
chr1	69308001	69313000	1.36E-08
chr1	69309001	69314000	1.08E-07
chr1	69321001	69326000	2.56E-05
chr1	69322001	69327000	1.10E-05
chr1	69323001	69328000	4.59E-05
chr1	69324001	69329000	3.28E-05
chr1	69468001	69473000	1.78E-07
chr1	69469001	69474000	6.68E-08
chr1	69691001	69696000	6.21E-05
chr1	70329001	70334000	4.23E-09
chr1	70341001	70346000	1.02E-09
chr1	70355001	70360000	1.19E-06

chr1	70927001	70932000	0.000131
chr1	71027001	71032000	7.15E-06
chr1	71132001	71137000	1.07E-08
chr1	71267001	71272000	0.000305
chr1	72766001	72771000	0.037446
chr1	73616001	73621000	0.000164
chr1	75493001	75498000	3.84E-09
chr1	75494001	75499000	3.15E-07
chr1	75841001	75846000	0.000225
chr1	75912001	75917000	5.94E-06
chr1	76661001	76666000	1.21E-07
chr1	76662001	76667000	7.72E-08
chr1	77795001	77800000	4.44E-08
chr1	77796001	77801000	4.08E-08
chr1	77829001	77834000	1.05E-05
chr1	78378001	78383000	0.000506
chr1	79280001	79285000	3.58E-06
chr1	80401001	80406000	3.25E-07
chr1	80558001	80563000	0.003918
chr1	81101001	81106000	3.92E-08
chr1	81102001	81107000	1.29E-06
chr1	81193001	81198000	3.47E-06
chr1	81194001	81199000	2.30E-07
chr1	81407001	81412000	8.01E-07
chr1	81408001	81413000	1.22E-05
chr1	81409001	81414000	0.000754
chr1	83192001	83197000	3.61E-05
chr1	83193001	83198000	4.57E-05
chr1	85073001	85078000	0.000334
chr1	86396001	86401000	0.001362
chr1	86397001	86402000	0.007415
chr1	86474001	86479000	1.46E-06
chr1	86915001	86920000	1.29E-07
chr1	89674001	89679000	0.000734
chr1	90226001	90231000	1.04E-07
chr1	90227001	90232000	1.36E-12
chr1	90228001	90233000	1.73E-07
chr1	90282001	90287000	9.21E-13
chr1	90283001	90288000	7.27E-11
chr1	91312001	91317000	7.85E-07
chr1	91553001	91558000	1.88E-10
chr1	91705001	91710000	0.000216
chr1	91838001	91843000	1.10E-06
chr1	94507001	94512000	1.95E-05
chr1	95316001	95321000	3.51E-08
chr1	99328001	99333000	7.52E-06
chr1	99329001	99334000	0.000909
chr1	99330001	99335000	0.000476

chr1	100311001	100316000	3.94E-09
chr1	100814001	100819000	2.74E-09
chr1	101910001	101915000	6.78E-08
chr1	102957001	102962000	2.22E-05
chr1	102959001	102964000	4.39E-08
chr1	102960001	102965000	7.83E-08
chr1	103353001	103358000	8.89E-10
chr1	104723001	104728000	6.92E-06
chr1	105312001	105317000	0.000309
chr1	105313001	105318000	0.000302
chr1	105446001	105451000	2.04E-06
chr1	105580001	105585000	1.60E-07
chr1	105581001	105586000	1.77E-06
chr1	105803001	105808000	1.52E-07
chr1	105861001	105866000	9.89E-09
chr1	105862001	105867000	3.42E-10
chr1	105863001	105868000	1.85E-11
chr1	105864001	105869000	3.90E-07
chr1	106103001	106108000	0.003213
chr1	106106001	106111000	4.78E-07
chr1	106121001	106126000	2.56E-14
chr1	106124001	106129000	4.84E-16
chr1	106201001	106206000	0.000835
chr1	106732001	106737000	3.51E-05
chr1	106795001	106800000	1.52E-09
chr1	106796001	106801000	2.23E-08
chr1	106797001	106802000	1.67E-07
chr1	106904001	106909000	8.20E-10
chr1	107423001	107428000	0.002938
chr1	108808001	108813000	0.019039
chr1	108809001	108814000	0.019039
chr1	108813001	108818000	0.002382
chr1	108814001	108819000	0.002745
chr1	109006001	109011000	7.79E-05
chr1	109007001	109012000	0.000481
chr1	109099001	109104000	6.97E-13
chr1	109100001	109105000	6.34E-16
chr1	109101001	109106000	9.29E-21
chr1	109366001	109371000	0.000359
chr1	109592001	109597000	0.000603
chr1	109593001	109598000	0.001883
chr1	109627001	109632000	0.000233
chr1	109629001	109634000	7.67E-07
chr1	109657001	109662000	1.47E-08
chr1	110278001	110283000	1.07E-12
chr1	110281001	110286000	1.21E-06
chr1	110282001	110287000	2.43E-07
chr1	110299001	110304000	0.000447

chr1	110302001	110307000	0.000196
chr1	110450001	110455000	4.68E-06
chr1	112521001	112526000	2.73E-09
chr1	112565001	112570000	1.46E-06
chr1	112695001	112700000	0.001266
chr1	112697001	112702000	0.001429
chr1	112718001	112723000	0.00015
chr1	112727001	112732000	6.72E-06
chr1	112728001	112733000	4.16E-05
chr1	112729001	112734000	7.14E-08
chr1	112730001	112735000	6.23E-09
chr1	112874001	112879000	1.47E-06
chr1	112929001	112934000	2.87E-06
chr1	112930001	112935000	3.83E-06
chr1	113045001	113050000	0.000329
chr1	113046001	113051000	0.007929
chr1	113047001	113052000	0.048793
chr1	113303001	113308000	5.49E-06
chr1	113348001	113353000	1.58E-06
chr1	113495001	113500000	1.70E-08
chr1	114548001	114553000	3.51E-06
chr1	114558001	114563000	1.44E-06
chr1	114691001	114696000	7.60E-05
chr1	114692001	114697000	9.56E-12
chr1	114693001	114698000	0.000312
chr1	117110001	117115000	2.95E-05
chr1	117111001	117116000	0.000205
chr1	120217001	120222000	1.33E-09
chr1	120886001	120891000	0.013237
chr1	120887001	120892000	0.010669
chr1	120921001	120926000	2.13E-11
chr1	120925001	120930000	9.77E-12
chr1	142868001	142873000	0.00562
chr1	142878001	142883000	0.024789
chr1	142879001	142884000	0.024789
chr1	142938001	142943000	0.000511
chr1	142939001	142944000	0.000511
chr1	142940001	142945000	0.000619
chr1	142941001	142946000	0.002567
chr1	143653001	143658000	0.003864
chr1	143770001	143775000	0.006931
chr1	144577001	144582000	0.00626
chr1	144578001	144583000	8.70E-06
chr1	144579001	144584000	1.05E-05
chr1	144580001	144585000	0.000119
chr1	144581001	144586000	1.40E-06
chr1	146311001	146316000	0.032403
chr1	146312001	146317000	0.032403

chr1	146313001	146318000	0.032403
chr1	146314001	146319000	0.026089
chr1	146315001	146320000	0.026089
chr1	146321001	146326000	0.00234
chr1	146322001	146327000	0.001605
chr1	146323001	146328000	0.001605
chr1	146324001	146329000	0.001605
chr1	146325001	146330000	0.000999
chr1	147210001	147215000	2.55E-06
chr1	147211001	147216000	2.51E-08
chr1	147951001	147956000	3.54E-05
chr1	147952001	147957000	3.23E-05
chr1	147953001	147958000	1.39E-05
chr1	148173001	148178000	1.40E-06
chr1	148538001	148543000	7.21E-19
chr1	148539001	148544000	2.45E-17
chr1	148601001	148606000	0.000156
chr1	148645001	148650000	1.76E-07
chr1	148646001	148651000	1.83E-06
chr1	148647001	148652000	8.90E-09
chr1	148810001	148815000	0.036894
chr1	148837001	148842000	0.000252
chr1	149054001	149059000	4.40E-11
chr1	149055001	149060000	1.93E-11
chr1	149056001	149061000	2.94E-14
chr1	149057001	149062000	5.42E-18
chr1	149058001	149063000	2.10E-10
chr1	149077001	149082000	3.64E-07
chr1	149126001	149131000	9.04E-10
chr1	149127001	149132000	2.23E-09
chr1	149128001	149133000	4.17E-06
chr1	149371001	149376000	5.82E-07
chr1	149372001	149377000	2.03E-07
chr1	149426001	149431000	3.47E-05
chr1	150894001	150899000	1.02E-09
chr1	150945001	150950000	0.001256
chr1	151134001	151139000	1.61E-15
chr1	151135001	151140000	2.91E-18
chr1	151136001	151141000	5.73E-15
chr1	151137001	151142000	0.009651
chr1	151138001	151143000	1.67E-08
chr1	151772001	151777000	2.45E-05
chr1	151773001	151778000	1.62E-05
chr1	151774001	151779000	1.46E-06
chr1	152529001	152534000	2.19E-09
chr1	152530001	152535000	7.17E-10
chr1	152543001	152548000	1.09E-06
chr1	153234001	153239000	9.08E-15

chr1	153302001	153307000	2.81E-08
chr1	153303001	153308000	3.54E-08
chr1	153397001	153402000	7.44E-07
chr1	153398001	153403000	1.68E-07
chr1	153399001	153404000	2.24E-06
chr1	153400001	153405000	5.80E-08
chr1	153426001	153431000	5.71E-10
chr1	153586001	153591000	4.95E-05
chr1	153587001	153592000	0.001204
chr1	153622001	153627000	0.001601
chr1	153918001	153923000	0.00025
chr1	153957001	153962000	4.59E-08
chr1	154092001	154097000	0.000297
chr1	154093001	154098000	8.34E-05
chr1	154391001	154396000	7.19E-06
chr1	154536001	154541000	0.000648
chr1	154602001	154607000	4.53E-11
chr1	154605001	154610000	3.40E-08
chr1	154606001	154611000	5.71E-08
chr1	154607001	154612000	0.000387
chr1	154608001	154613000	0.000127
chr1	154901001	154906000	0.000475
chr1	154902001	154907000	5.78E-05
chr1	154904001	154909000	2.67E-14
chr1	154905001	154910000	4.25E-11
chr1	154931001	154936000	0.000784
chr1	154932001	154937000	0.002153
chr1	154933001	154938000	1.37E-06
chr1	155036001	155041000	2.50E-11
chr1	155071001	155076000	3.39E-05
chr1	155072001	155077000	2.96E-07
chr1	155144001	155149000	1.41E-06
chr1	155212001	155217000	1.08E-06
chr1	155213001	155218000	9.42E-08
chr1	155214001	155219000	4.48E-11
chr1	155216001	155221000	0.000207
chr1	155225001	155230000	4.80E-05
chr1	155240001	155245000	4.41E-12
chr1	155241001	155246000	7.10E-09
chr1	155242001	155247000	2.73E-10
chr1	155244001	155249000	0.001355
chr1	155272001	155277000	1.58E-05
chr1	155273001	155278000	0.000199
chr1	155653001	155658000	1.06E-09
chr1	155828001	155833000	4.06E-14
chr1	155829001	155834000	1.01E-17
chr1	156161001	156166000	5.50E-11
chr1	156162001	156167000	1.58E-09

chr1	156163001	156168000	7.24E-11
chr1	156164001	156169000	1.76E-07
chr1	156251001	156256000	4.90E-11
chr1	156252001	156257000	6.10E-09
chr1	156328001	156333000	1.05E-10
chr1	156329001	156334000	8.29E-10
chr1	156601001	156606000	2.01E-05
chr1	156623001	156628000	0.001648
chr1	156706001	156711000	1.53E-08
chr1	156707001	156712000	5.00E-10
chr1	156708001	156713000	2.42E-11
chr1	156709001	156714000	1.02E-06
chr1	156710001	156715000	3.18E-06
chr1	156781001	156786000	2.24E-08
chr1	156782001	156787000	8.89E-06
chr1	156783001	156788000	1.35E-05
chr1	156875001	156880000	8.03E-06
chr1	157036001	157041000	8.67E-07
chr1	157124001	157129000	8.90E-06
chr1	157379001	157384000	1.31E-05
chr1	157380001	157385000	0.000276
chr1	157430001	157435000	1.61E-10
chr1	157431001	157436000	7.22E-08
chr1	157664001	157669000	6.85E-06
chr1	157751001	157756000	0.019182
chr1	157940001	157945000	1.03E-06
chr1	157941001	157946000	1.41E-06
chr1	158192001	158197000	9.18E-05
chr1	158439001	158444000	4.69E-15
chr1	158717001	158722000	1.24E-17
chr1	158718001	158723000	1.39E-17
chr1	158719001	158724000	2.33E-16
chr1	158784001	158789000	1.31E-09
chr1	158785001	158790000	2.29E-09
chr1	158877001	158882000	0.00185
chr1	159149001	159154000	7.37E-08
chr1	159150001	159155000	2.13E-08
chr1	159222001	159227000	1.81E-06
chr1	159223001	159228000	7.37E-07
chr1	159351001	159356000	1.55E-06
chr1	159352001	159357000	5.37E-05
chr1	159353001	159358000	1.21E-05
chr1	159410001	159415000	3.32E-06
chr1	159449001	159454000	4.03E-10
chr1	159451001	159456000	4.92E-12
chr1	159452001	159457000	2.46E-11
chr1	159497001	159502000	9.98E-11
chr1	159608001	159613000	5.36E-08

chr1	160098001	160103000	1.52E-07
chr1	160592001	160597000	6.62E-14
chr1	160679001	160684000	9.15E-07
chr1	160889001	160894000	0.001418
chr1	161011001	161016000	0.005316
chr1	161133001	161138000	3.81E-11
chr1	161134001	161139000	1.55E-08
chr1	161135001	161140000	3.13E-09
chr1	161136001	161141000	1.31E-05
chr1	161169001	161174000	5.43E-05
chr1	161634001	161639000	0.000236
chr1	161635001	161640000	0.006558
chr1	161636001	161641000	0.006815
chr1	161637001	161642000	0.001277
chr1	161667001	161672000	1.46E-09
chr1	161938001	161943000	3.90E-06
chr1	162412001	162417000	0.001135
chr1	162413001	162418000	0.000848
chr1	163615001	163620000	1.67E-10
chr1	164578001	164583000	2.63E-09
chr1	164871001	164876000	4.21E-12
chr1	165945001	165950000	1.27E-05
chr1	165947001	165952000	3.31E-05
chr1	165985001	165990000	2.19E-09
chr1	165986001	165991000	1.19E-09
chr1	165988001	165993000	1.53E-07
chr1	166027001	166032000	1.61E-08
chr1	166029001	166034000	4.03E-09
chr1	166036001	166041000	1.20E-07
chr1	166198001	166203000	9.42E-06
chr1	166232001	166237000	1.97E-08
chr1	166236001	166241000	9.62E-05
chr1	166253001	166258000	3.64E-09
chr1	166361001	166366000	2.12E-08
chr1	166741001	166746000	2.03E-06
chr1	166792001	166797000	2.09E-07
chr1	166794001	166799000	1.23E-09
chr1	166795001	166800000	2.27E-08
chr1	166796001	166801000	5.37E-06
chr1	167006001	167011000	3.01E-10
chr1	168102001	168107000	5.79E-07
chr1	168803001	168808000	3.85E-12
chr1	168804001	168809000	7.73E-14
chr1	170171001	170176000	1.28E-06
chr1	172410001	172415000	4.10E-10
chr1	175073001	175078000	0.000898
chr1	175328001	175333000	4.27E-07
chr1	175331001	175336000	2.91E-07

chr1	175332001	175337000	4.07E-07
chr1	175382001	175387000	1.30E-09
chr1	175388001	175393000	4.18E-12
chr1	175627001	175632000	4.23E-06
chr1	175628001	175633000	7.56E-08
chr1	175629001	175634000	1.62E-07
chr1	175639001	175644000	1.52E-07
chr1	175708001	175713000	2.56E-06
chr1	175745001	175750000	9.46E-09
chr1	175747001	175752000	1.12E-08
chr1	176232001	176237000	1.55E-06
chr1	176258001	176263000	1.12E-08
chr1	176387001	176392000	1.08E-05
chr1	176388001	176393000	1.02E-05
chr1	176389001	176394000	2.81E-07
chr1	176390001	176395000	1.87E-05
chr1	176391001	176396000	8.79E-06
chr1	176760001	176765000	7.31E-08
chr1	176761001	176766000	3.92E-10
chr1	176762001	176767000	9.23E-09
chr1	176763001	176768000	2.14E-07
chr1	176961001	176966000	2.50E-08
chr1	176983001	176988000	3.89E-12
chr1	177088001	177093000	8.75E-10
chr1	177089001	177094000	3.73E-10
chr1	177473001	177478000	0.000299
chr1	177491001	177496000	5.13E-09
chr1	177565001	177570000	1.27E-08
chr1	177649001	177654000	1.52E-09
chr1	177703001	177708000	7.72E-08
chr1	178058001	178063000	4.19E-11
chr1	178624001	178629000	5.28E-09
chr1	179259001	179264000	1.51E-18
chr1	179260001	179265000	1.53E-22
chr1	179724001	179729000	7.44E-07
chr1	180195001	180200000	7.97E-08
chr1	180540001	180545000	7.15E-08
chr1	181001001	181006000	3.69E-07
chr1	181322001	181327000	1.28E-05
chr1	181323001	181328000	1.16E-05
chr1	181324001	181329000	7.21E-06
chr1	181325001	181330000	2.46E-05
chr1	181326001	181331000	5.45E-05
chr1	181479001	181484000	1.18E-09
chr1	181815001	181820000	7.50E-07
chr1	181917001	181922000	1.39E-05
chr1	181928001	181933000	8.32E-08
chr1	181931001	181936000	0.000545

chr1	183440001	183445000	2.67E-17
chr1	183441001	183446000	2.53E-14
chr1	183442001	183447000	7.89E-11
chr1	184274001	184279000	2.74E-09
chr1	184275001	184280000	3.07E-07
chr1	184276001	184281000	7.36E-05
chr1	187274001	187279000	6.03E-14
chr1	190995001	191000000	3.90E-05
chr1	191202001	191207000	1.16E-08
chr1	191203001	191208000	9.03E-10
chr1	191204001	191209000	3.93E-09
chr1	191647001	191652000	4.04E-09
chr1	191785001	191790000	1.72E-08
chr1	192090001	192095000	3.94E-11
chr1	192091001	192096000	1.07E-14
chr1	192092001	192097000	5.87E-13
chr1	193688001	193693000	0.013159
chr1	194415001	194420000	2.93E-10
chr1	194531001	194536000	1.57E-10
chr1	195594001	195599000	1.41E-07
chr1	195788001	195793000	4.10E-05
chr1	197040001	197045000	0.000659
chr1	198762001	198767000	2.72E-07
chr1	198764001	198769000	3.39E-07
chr1	199505001	199510000	1.15E-06
chr1	199864001	199869000	7.63E-06
chr1	199865001	199870000	7.60E-05
chr1	199954001	199959000	9.53E-05
chr1	199955001	199960000	0.000482
chr1	200153001	200158000	1.89E-07
chr1	200327001	200332000	9.54E-05
chr1	200602001	200607000	0.000138
chr1	200603001	200608000	7.20E-05
chr1	201365001	201370000	0.002893
chr1	201434001	201439000	1.95E-09
chr1	201435001	201440000	7.04E-11
chr1	201436001	201441000	1.50E-10
chr1	201437001	201442000	2.56E-10
chr1	201567001	201572000	4.17E-12
chr1	201568001	201573000	1.42E-10
chr1	201569001	201574000	2.19E-12
chr1	201570001	201575000	2.14E-11
chr1	201571001	201576000	5.04E-12
chr1	201572001	201577000	1.83E-10
chr1	201615001	201620000	3.15E-06
chr1	201616001	201621000	0.000163
chr1	201617001	201622000	1.26E-07
chr1	201654001	201659000	1.11E-11

chr1	201655001	201660000	2.93E-11
chr1	201975001	201980000	1.55E-14
chr1	201976001	201981000	9.77E-15
chr1	201977001	201982000	5.07E-16
chr1	201978001	201983000	1.51E-13
chr1	202120001	202125000	8.73E-11
chr1	202307001	202312000	2.65E-08
chr1	202793001	202798000	0.000323
chr1	202932001	202937000	4.58E-12
chr1	202933001	202938000	1.98E-10
chr1	203096001	203101000	2.19E-06
chr1	203097001	203102000	8.16E-08
chr1	203148001	203153000	7.55E-08
chr1	203149001	203154000	1.43E-06
chr1	203297001	203302000	6.10E-06
chr1	203557001	203562000	6.40E-05
chr1	204771001	204776000	1.65E-09
chr1	204772001	204777000	3.60E-13
chr1	204773001	204778000	4.36E-10
chr1	204774001	204779000	3.15E-08
chr1	205309001	205314000	9.15E-26
chr1	205310001	205315000	6.23E-26
chr1	205311001	205316000	3.11E-19
chr1	205312001	205317000	7.89E-14
chr1	205313001	205318000	3.68E-13
chr1	205417001	205422000	1.13E-05
chr1	205469001	205474000	1.18E-06
chr1	205649001	205654000	1.02E-07
chr1	208952001	208957000	4.16E-07
chr1	208953001	208958000	7.64E-10
chr1	209062001	209067000	8.78E-07
chr1	209343001	209348000	0.000287
chr1	209997001	210002000	3.65E-15
chr1	209999001	210004000	2.53E-15
chr1	210000001	210005000	3.65E-12
chr1	211503001	211508000	2.51E-06
chr1	212681001	212686000	1.52E-11
chr1	212682001	212687000	5.76E-13
chr1	212683001	212688000	1.75E-10
chr1	212684001	212689000	3.33E-11
chr1	212685001	212690000	1.70E-13
chr1	212961001	212966000	9.98E-14
chr1	212962001	212967000	1.87E-12
chr1	213149001	213154000	2.79E-07
chr1	213150001	213155000	1.22E-06
chr1	214968001	214973000	4.34E-07
chr1	214969001	214974000	9.63E-05
chr1	214970001	214975000	3.50E-05

chr1	215349001	215354000	1.21E-09
chr1	215350001	215355000	1.43E-06
chr1	215351001	215356000	1.60E-08
chr1	215579001	215584000	7.78E-13
chr1	218179001	218184000	0.000351
chr1	218180001	218185000	0.000943
chr1	219146001	219151000	0.000934
chr1	219794001	219799000	2.13E-08
chr1	220697001	220702000	6.45E-17
chr1	220701001	220706000	4.14E-19
chr1	221074001	221079000	1.43E-11
chr1	221075001	221080000	6.55E-07
chr1	221076001	221081000	6.64E-08
chr1	221129001	221134000	6.71E-08
chr1	221234001	221239000	0.000248
chr1	221236001	221241000	1.65E-08
chr1	221237001	221242000	9.93E-07
chr1	221252001	221257000	7.13E-10
chr1	221291001	221296000	4.78E-13
chr1	221292001	221297000	2.87E-15
chr1	221293001	221298000	3.46E-11
chr1	221389001	221394000	6.18E-10
chr1	221466001	221471000	7.30E-08
chr1	222353001	222358000	3.32E-07
chr1	222354001	222359000	5.89E-07
chr1	222573001	222578000	8.44E-07
chr1	222936001	222941000	1.87E-07
chr1	222938001	222943000	5.06E-06
chr1	223796001	223801000	0.048801
chr1	223797001	223802000	0.00573
chr1	224220001	224225000	0.000371
chr1	226369001	226374000	4.05E-10
chr1	226370001	226375000	1.13E-19
chr1	226371001	226376000	1.37E-13
chr1	226810001	226815000	6.78E-14
chr1	226811001	226816000	1.09E-11
chr1	226887001	226892000	2.72E-11
chr1	227672001	227677000	2.81E-05
chr1	227674001	227679000	3.55E-06
chr1	227728001	227733000	0.006945
chr1	227747001	227752000	2.33E-09
chr1	228072001	228077000	0.001549
chr1	228073001	228078000	0.003196
chr1	228074001	228079000	0.001234
chr1	228075001	228080000	0.000507
chr1	228076001	228081000	8.31E-06
chr1	228134001	228139000	0.022264
chr1	228323001	228328000	3.17E-12

chr1	228324001	228329000	4.31E-12
chr1	228325001	228330000	1.04E-17
chr1	228326001	228331000	2.56E-13
chr1	228553001	228558000	8.28E-07
chr1	228554001	228559000	2.99E-06
chr1	229361001	229366000	1.10E-06
chr1	229365001	229370000	5.95E-13
chr1	229556001	229561000	1.08E-05
chr1	230446001	230451000	1.35E-08
chr1	230447001	230452000	2.39E-05
chr1	231285001	231290000	2.02E-06
chr1	231759001	231764000	2.58E-08
chr1	231760001	231765000	2.01E-11
chr1	232167001	232172000	1.24E-05
chr1	233961001	233966000	3.15E-09
chr1	233963001	233968000	4.58E-06
chr1	233964001	233969000	1.97E-06
chr1	234070001	234075000	1.65E-08
chr1	234071001	234076000	1.12E-07
chr1	234282001	234287000	1.50E-08
chr1	234283001	234288000	1.80E-09
chr1	235026001	235031000	5.99E-07
chr1	235813001	235818000	1.25E-14
chr1	236224001	236229000	1.52E-06
chr1	236250001	236255000	6.47E-05
chr1	237075001	237080000	1.88E-06
chr1	237076001	237081000	4.17E-05
chr1	240122001	240127000	5.91E-16
chr1	240785001	240790000	2.67E-10
chr1	241964001	241969000	3.06E-05
chr1	242114001	242119000	0.006136
chr1	242568001	242573000	8.16E-07
chr1	242721001	242726000	3.51E-15
chr1	242722001	242727000	1.93E-17
chr1	242723001	242728000	1.36E-11
chr1	242744001	242749000	8.57E-17
chr1	242745001	242750000	1.22E-16
chr1	242916001	242921000	2.84E-11
chr1	243046001	243051000	1.98E-07
chr1	243071001	243076000	1.52E-08
chr1	243072001	243077000	2.63E-08
chr1	243097001	243102000	5.07E-06
chr1	243098001	243103000	6.13E-08
chr1	243110001	243115000	1.92E-05
chr1	243169001	243174000	2.19E-13
chr1	243170001	243175000	1.83E-12
chr1	243246001	243251000	1.76E-05
chr1	243247001	243252000	0.000252

chr1	245432001	245437000	6.41E-05
chr1	247491001	247496000	0.000385
chr1	247690001	247695000	1.41E-08
chr1	247691001	247696000	7.91E-08
chr1	247692001	247697000	9.36E-09
chr1	247693001	247698000	8.52E-10
chr1	247694001	247699000	4.89E-08
chr1	247756001	247761000	5.40E-08
chr1	247830001	247835000	8.46E-06
chr1	247831001	247836000	5.46E-07
chr1	247988001	247993000	1.66E-09
chr1	247989001	247994000	8.95E-08
chr1	248117001	248122000	4.24E-07
chr1	248118001	248123000	2.25E-09
chr1	248119001	248124000	2.46E-08
chr1	248120001	248125000	4.68E-09
chr1	248460001	248465000	5.48E-13
chr1	248461001	248466000	3.31E-12
chr1	248556001	248561000	8.80E-09
chr1	248557001	248562000	4.84E-08
chr1	248797001	248802000	0.000633
chr1	248798001	248803000	0.006674
chr1	248799001	248804000	0.00362
chr1	248800001	248805000	0.026145
chr1	249056001	249061000	0.001293
chr1	249150001	249155000	8.89E-11
chr1	249151001	249156000	4.82E-15
chr1	249152001	249157000	4.32E-12
chr1	249153001	249158000	1.49E-14
chr2	180001	185000	0.002682
chr2	285001	290000	1.74E-14
chr2	402001	407000	5.56E-09
chr2	614001	619000	1.30E-07
chr2	615001	620000	1.32E-07
chr2	616001	621000	2.01E-16
chr2	617001	622000	6.34E-16
chr2	618001	623000	2.97E-13
chr2	682001	687000	4.30E-06
chr2	921001	926000	1.72E-10
chr2	974001	979000	9.17E-13
chr2	975001	980000	1.43E-18
chr2	976001	981000	5.71E-19
chr2	977001	982000	1.24E-19
chr2	985001	990000	2.09E-08
chr2	986001	991000	2.17E-07
chr2	1015001	1020000	2.30E-13
chr2	1016001	1021000	1.23E-14
chr2	1017001	1022000	2.06E-16

chr2	1018001	1023000	1.31E-15
chr2	1019001	1024000	6.21E-12
chr2	1348001	1353000	2.07E-06
chr2	1349001	1354000	2.51E-07
chr2	1367001	1372000	2.36E-06
chr2	1528001	1533000	0.00204
chr2	1529001	1534000	0.00204
chr2	1530001	1535000	0.001132
chr2	1531001	1536000	0.004185
chr2	1532001	1537000	0.00531
chr2	1582001	1587000	1.90E-13
chr2	1588001	1593000	9.53E-06
chr2	1590001	1595000	0.000107
chr2	1610001	1615000	2.29E-10
chr2	1611001	1616000	2.67E-11
chr2	1612001	1617000	8.33E-12
chr2	1613001	1618000	1.22E-09
chr2	1663001	1668000	1.24E-07
chr2	1747001	1752000	6.77E-05
chr2	1895001	1900000	9.61E-09
chr2	2598001	2603000	8.37E-09
chr2	2599001	2604000	1.25E-10
chr2	3144001	3149000	4.90E-07
chr2	3145001	3150000	1.16E-05
chr2	3519001	3524000	1.55E-28
chr2	3520001	3525000	9.20E-25
chr2	3521001	3526000	2.81E-22
chr2	3522001	3527000	1.95E-11
chr2	3650001	3655000	0.000308
chr2	3865001	3870000	1.32E-08
chr2	3942001	3947000	3.63E-05
chr2	3943001	3948000	1.08E-05
chr2	4107001	4112000	2.13E-07
chr2	4162001	4167000	1.14E-09
chr2	4214001	4219000	1.42E-10
chr2	4217001	4222000	2.30E-07
chr2	4236001	4241000	8.05E-09
chr2	4422001	4427000	0.000379
chr2	4423001	4428000	0.001254
chr2	4424001	4429000	6.79E-06
chr2	4426001	4431000	3.26E-08
chr2	4860001	4865000	9.36E-09
chr2	5246001	5251000	3.64E-09
chr2	5247001	5252000	3.35E-08
chr2	5431001	5436000	7.33E-10
chr2	5684001	5689000	2.01E-05
chr2	5685001	5690000	2.62E-06
chr2	6294001	6299000	2.85E-06

chr2	6431001	6436000	1.92E-07
chr2	6432001	6437000	1.35E-09
chr2	6433001	6438000	1.38E-09
chr2	6434001	6439000	5.07E-10
chr2	6546001	6551000	5.88E-09
chr2	6547001	6552000	2.19E-08
chr2	7666001	7671000	0.000522
chr2	7667001	7672000	0.000466
chr2	7695001	7700000	2.18E-07
chr2	7697001	7702000	1.02E-05
chr2	7698001	7703000	3.00E-07
chr2	7707001	7712000	1.79E-10
chr2	7844001	7849000	5.69E-08
chr2	7845001	7850000	4.22E-07
chr2	7901001	7906000	1.69E-07
chr2	7939001	7944000	0.000567
chr2	8336001	8341000	7.72E-11
chr2	8337001	8342000	6.75E-11
chr2	9526001	9531000	9.44E-10
chr2	10149001	10154000	6.81E-18
chr2	10150001	10155000	4.93E-21
chr2	10151001	10156000	6.25E-28
chr2	10152001	10157000	4.68E-33
chr2	10153001	10158000	2.54E-28
chr2	10154001	10159000	7.34E-06
chr2	10349001	10354000	1.42E-05
chr2	10439001	10444000	8.63E-07
chr2	10984001	10989000	4.86E-06
chr2	10986001	10991000	2.23E-09
chr2	10990001	10995000	1.09E-09
chr2	11036001	11041000	5.44E-10
chr2	11038001	11043000	4.30E-07
chr2	11039001	11044000	6.36E-05
chr2	11052001	11057000	7.24E-07
chr2	11201001	11206000	7.91E-08
chr2	12145001	12150000	2.16E-16
chr2	12146001	12151000	9.99E-13
chr2	13478001	13483000	9.40E-11
chr2	14949001	14954000	3.40E-07
chr2	15148001	15153000	3.71E-07
chr2	15149001	15154000	9.51E-07
chr2	15983001	15988000	4.03E-07
chr2	16053001	16058000	3.94E-08
chr2	16077001	16082000	8.32E-05
chr2	16078001	16083000	4.74E-07
chr2	16326001	16331000	0.007244
chr2	16532001	16537000	5.01E-09
chr2	16533001	16538000	8.77E-10

chr2	16534001	16539000	1.50E-10
chr2	16893001	16898000	1.51E-12
chr2	18055001	18060000	4.18E-06
chr2	18056001	18061000	9.63E-07
chr2	18094001	18099000	2.42E-05
chr2	18095001	18100000	3.00E-05
chr2	18287001	18292000	2.46E-09
chr2	18326001	18331000	4.47E-08
chr2	18327001	18332000	3.46E-09
chr2	18422001	18427000	3.96E-06
chr2	18814001	18819000	2.84E-05
chr2	19085001	19090000	4.12E-07
chr2	19086001	19091000	4.93E-06
chr2	19543001	19548000	1.89E-10
chr2	19544001	19549000	7.06E-12
chr2	19545001	19550000	2.85E-09
chr2	19939001	19944000	1.63E-05
chr2	20036001	20041000	1.27E-06
chr2	20208001	20213000	6.13E-07
chr2	20421001	20426000	0.000869
chr2	20641001	20646000	1.01E-09
chr2	20685001	20690000	0.000251
chr2	21126001	21131000	2.05E-06
chr2	21175001	21180000	0.010555
chr2	21426001	21431000	1.46E-06
chr2	21592001	21597000	8.01E-10
chr2	21693001	21698000	2.84E-08
chr2	21886001	21891000	4.45E-08
chr2	21963001	21968000	2.72E-10
chr2	21964001	21969000	1.87E-12
chr2	21965001	21970000	9.77E-12
chr2	22216001	22221000	4.80E-13
chr2	23062001	23067000	4.15E-08
chr2	23083001	23088000	1.02E-08
chr2	23372001	23377000	3.28E-06
chr2	23374001	23379000	2.18E-08
chr2	23375001	23380000	3.12E-08
chr2	23376001	23381000	5.32E-07
chr2	23377001	23382000	1.79E-07
chr2	23825001	23830000	2.20E-05
chr2	23855001	23860000	0.003763
chr2	24237001	24242000	4.61E-07
chr2	24369001	24374000	1.31E-07
chr2	24370001	24375000	3.80E-06
chr2	25151001	25156000	0.000919
chr2	25255001	25260000	0.003378
chr2	25438001	25443000	0.004496
chr2	25439001	25444000	0.001898

chr2	25470001	25475000	7.92E-09
chr2	25471001	25476000	2.31E-07
chr2	25472001	25477000	5.68E-17
chr2	25473001	25478000	1.17E-13
chr2	25474001	25479000	7.57E-13
chr2	25838001	25843000	6.39E-11
chr2	26118001	26123000	9.91E-05
chr2	26133001	26138000	0.00021
chr2	26134001	26139000	5.97E-06
chr2	26200001	26205000	0.000585
chr2	26201001	26206000	0.000854
chr2	26202001	26207000	0.000238
chr2	26366001	26371000	2.81E-07
chr2	26367001	26372000	4.04E-07
chr2	26368001	26373000	1.34E-07
chr2	26780001	26785000	5.02E-07
chr2	26835001	26840000	2.64E-05
chr2	26927001	26932000	2.32E-08
chr2	27269001	27274000	6.93E-12
chr2	27341001	27346000	3.96E-05
chr2	27415001	27420000	1.32E-06
chr2	27601001	27606000	1.60E-05
chr2	28786001	28791000	0.001369
chr2	28787001	28792000	2.73E-05
chr2	28788001	28793000	0.000201
chr2	28789001	28794000	2.90E-05
chr2	29514001	29519000	9.15E-09
chr2	30978001	30983000	0.000135
chr2	31927001	31932000	0.000573
chr2	32288001	32293000	2.42E-06
chr2	33862001	33867000	1.80E-06
chr2	33863001	33868000	7.80E-06
chr2	33866001	33871000	2.04E-11
chr2	33871001	33876000	9.90E-07
chr2	34016001	34021000	4.46E-09
chr2	34079001	34084000	6.11E-12
chr2	34080001	34085000	5.20E-13
chr2	34081001	34086000	3.75E-10
chr2	34082001	34087000	3.07E-13
chr2	34103001	34108000	1.77E-09
chr2	34104001	34109000	1.84E-10
chr2	34105001	34110000	2.99E-10
chr2	34121001	34126000	0.003176
chr2	34124001	34129000	1.38E-05
chr2	34125001	34130000	2.33E-08
chr2	34203001	34208000	9.22E-13
chr2	34204001	34209000	4.83E-13
chr2	34205001	34210000	3.75E-14

chr2	34272001	34277000	1.22E-10
chr2	34273001	34278000	3.09E-12
chr2	34524001	34529000	6.81E-06
chr2	34572001	34577000	1.56E-13
chr2	34684001	34689000	0.000175
chr2	34737001	34742000	6.92E-10
chr2	34770001	34775000	4.84E-08
chr2	34773001	34778000	4.64E-06
chr2	34797001	34802000	0.005122
chr2	34798001	34803000	0.000565
chr2	34853001	34858000	6.48E-10
chr2	34856001	34861000	5.84E-12
chr2	34857001	34862000	2.14E-12
chr2	35468001	35473000	2.53E-07
chr2	35469001	35474000	7.43E-10
chr2	35655001	35660000	1.72E-11
chr2	35656001	35661000	9.85E-11
chr2	35657001	35662000	1.45E-12
chr2	35821001	35826000	0.000163
chr2	35822001	35827000	0.000719
chr2	35960001	35965000	9.56E-05
chr2	35977001	35982000	9.86E-08
chr2	35978001	35983000	3.39E-10
chr2	35979001	35984000	2.38E-10
chr2	35980001	35985000	1.29E-09
chr2	35981001	35986000	7.88E-11
chr2	35982001	35987000	1.56E-08
chr2	35983001	35988000	9.81E-06
chr2	36123001	36128000	1.07E-08
chr2	36143001	36148000	1.60E-08
chr2	36216001	36221000	0.000261
chr2	36297001	36302000	5.61E-11
chr2	36368001	36373000	2.79E-06
chr2	38301001	38306000	5.34E-05
chr2	38302001	38307000	6.04E-09
chr2	38303001	38308000	6.57E-11
chr2	38600001	38605000	9.05E-10
chr2	38875001	38880000	2.92E-06
chr2	38876001	38881000	2.10E-07
chr2	38952001	38957000	7.58E-05
chr2	39416001	39421000	4.14E-09
chr2	39418001	39423000	2.00E-08
chr2	40358001	40363000	2.27E-09
chr2	40359001	40364000	4.29E-10
chr2	40485001	40490000	5.69E-11
chr2	40486001	40491000	7.24E-08
chr2	41376001	41381000	1.36E-11
chr2	41622001	41627000	1.51E-05

chr2	41623001	41628000	1.69E-07
chr2	42055001	42060000	1.14E-09
chr2	42300001	42305000	4.91E-05
chr2	42746001	42751000	0.026559
chr2	42749001	42754000	0.000214
chr2	42750001	42755000	2.12E-05
chr2	43033001	43038000	1.67E-15
chr2	43034001	43039000	1.16E-12
chr2	43035001	43040000	1.47E-10
chr2	43036001	43041000	8.05E-16
chr2	43037001	43042000	5.80E-30
chr2	43038001	43043000	3.35E-14
chr2	43358001	43363000	6.65E-10
chr2	43359001	43364000	2.09E-05
chr2	43383001	43388000	0.00055
chr2	44242001	44247000	1.43E-07
chr2	44287001	44292000	9.24E-06
chr2	44288001	44293000	1.46E-07
chr2	45306001	45311000	5.11E-05
chr2	45968001	45973000	1.10E-08
chr2	46427001	46432000	3.78E-06
chr2	47621001	47626000	4.87E-05
chr2	47622001	47627000	0.00021
chr2	47748001	47753000	9.30E-13
chr2	47749001	47754000	1.29E-07
chr2	47797001	47802000	4.35E-07
chr2	47802001	47807000	1.94E-05
chr2	47803001	47808000	5.07E-09
chr2	47804001	47809000	2.29E-08
chr2	47805001	47810000	5.71E-08
chr2	47806001	47811000	6.54E-11
chr2	47807001	47812000	4.27E-10
chr2	48312001	48317000	8.33E-06
chr2	48351001	48356000	6.49E-07
chr2	48664001	48669000	6.60E-17
chr2	48990001	48995000	3.58E-10
chr2	48991001	48996000	7.34E-09
chr2	49120001	49125000	0.002437
chr2	49122001	49127000	4.19E-06
chr2	49498001	49503000	1.50E-09
chr2	49499001	49504000	6.87E-09
chr2	49500001	49505000	1.20E-09
chr2	49501001	49506000	4.71E-08
chr2	49502001	49507000	4.77E-08
chr2	49698001	49703000	3.55E-06
chr2	49723001	49728000	1.14E-07
chr2	49769001	49774000	7.97E-10
chr2	49770001	49775000	1.52E-10

chr2	49787001	49792000	6.64E-07
chr2	49851001	49856000	1.47E-10
chr2	50001001	50006000	4.39E-07
chr2	50002001	50007000	1.11E-08
chr2	50003001	50008000	2.97E-07
chr2	50359001	50364000	1.31E-06
chr2	50643001	50648000	1.11E-07
chr2	51262001	51267000	6.54E-13
chr2	51603001	51608000	1.25E-05
chr2	51604001	51609000	2.11E-05
chr2	51841001	51846000	2.06E-09
chr2	52028001	52033000	2.02E-08
chr2	52180001	52185000	1.25E-09
chr2	52283001	52288000	4.43E-05
chr2	52599001	52604000	2.42E-11
chr2	52600001	52605000	8.78E-19
chr2	52876001	52881000	3.90E-06
chr2	53467001	53472000	2.04E-08
chr2	55426001	55431000	0.000937
chr2	55496001	55501000	1.94E-07
chr2	55505001	55510000	4.80E-12
chr2	55846001	55851000	1.42E-11
chr2	55919001	55924000	1.92E-12
chr2	56411001	56416000	5.52E-10
chr2	56469001	56474000	4.79E-06
chr2	56704001	56709000	8.63E-08
chr2	56870001	56875000	4.21E-06
chr2	56871001	56876000	3.84E-06
chr2	56872001	56877000	2.75E-06
chr2	57153001	57158000	8.09E-05
chr2	57172001	57177000	1.00E-08
chr2	57262001	57267000	4.84E-08
chr2	57416001	57421000	4.07E-06
chr2	57501001	57506000	1.53E-06
chr2	57502001	57507000	3.08E-05
chr2	57840001	57845000	4.75E-06
chr2	60819001	60824000	7.09E-05
chr2	61404001	61409000	2.51E-10
chr2	62079001	62084000	9.28E-06
chr2	64371001	64376000	7.74E-13
chr2	65661001	65666000	2.30E-12
chr2	65662001	65667000	7.71E-09
chr2	65663001	65668000	2.29E-05
chr2	66083001	66088000	0.001469
chr2	67344001	67349000	2.15E-06
chr2	67345001	67350000	7.23E-08
chr2	67346001	67351000	1.64E-06
chr2	67347001	67352000	1.96E-06

chr2	70352001	70357000	2.84E-06
chr2	70516001	70521000	4.34E-20
chr2	70566001	70571000	2.99E-06
chr2	70567001	70572000	8.47E-07
chr2	71013001	71018000	2.19E-06
chr2	71014001	71019000	5.58E-08
chr2	71210001	71215000	2.92E-06
chr2	71392001	71397000	3.16E-07
chr2	71815001	71820000	0.001114
chr2	71817001	71822000	0.000314
chr2	71941001	71946000	1.33E-05
chr2	71942001	71947000	2.93E-05
chr2	72112001	72117000	4.06E-10
chr2	72146001	72151000	0.00028
chr2	72291001	72296000	0.000214
chr2	72305001	72310000	9.18E-08
chr2	72306001	72311000	1.00E-08
chr2	72366001	72371000	1.65E-08
chr2	72367001	72372000	3.10E-06
chr2	73396001	73401000	0.000381
chr2	73439001	73444000	2.58E-08
chr2	73440001	73445000	2.85E-12
chr2	73441001	73446000	6.87E-10
chr2	73543001	73548000	1.04E-05
chr2	73923001	73928000	0.000202
chr2	74010001	74015000	2.27E-06
chr2	74211001	74216000	4.80E-08
chr2	74212001	74217000	1.18E-07
chr2	74429001	74434000	9.74E-08
chr2	74641001	74646000	0.007175
chr2	74644001	74649000	4.85E-08
chr2	74683001	74688000	3.06E-10
chr2	74721001	74726000	5.09E-10
chr2	74722001	74727000	3.10E-07
chr2	74723001	74728000	4.96E-05
chr2	75237001	75242000	1.59E-06
chr2	75322001	75327000	1.69E-05
chr2	75323001	75328000	3.60E-05
chr2	75487001	75492000	5.29E-05
chr2	76035001	76040000	1.96E-08
chr2	76140001	76145000	3.34E-08
chr2	76165001	76170000	2.87E-07
chr2	76217001	76222000	0.000848
chr2	76270001	76275000	9.40E-06
chr2	76296001	76301000	3.23E-08
chr2	76297001	76302000	3.50E-07
chr2	76298001	76303000	1.01E-08
chr2	76335001	76340000	9.35E-07

chr2	76523001	76528000	0.000252
chr2	76524001	76529000	0.000102
chr2	77183001	77188000	1.16E-12
chr2	77433001	77438000	1.69E-08
chr2	77800001	77805000	2.93E-13
chr2	78015001	78020000	1.14E-15
chr2	78116001	78121000	1.54E-09
chr2	78409001	78414000	7.77E-09
chr2	78416001	78421000	2.33E-05
chr2	78867001	78872000	5.77E-11
chr2	78868001	78873000	3.27E-09
chr2	78869001	78874000	2.20E-08
chr2	78870001	78875000	9.55E-10
chr2	78929001	78934000	1.45E-07
chr2	79013001	79018000	2.68E-09
chr2	79018001	79023000	1.42E-12
chr2	79019001	79024000	3.87E-08
chr2	79021001	79026000	1.03E-08
chr2	79022001	79027000	1.15E-07
chr2	79104001	79109000	6.15E-11
chr2	79120001	79125000	6.68E-09
chr2	79121001	79126000	2.44E-11
chr2	79122001	79127000	7.01E-10
chr2	79196001	79201000	5.05E-08
chr2	79243001	79248000	2.64E-13
chr2	79347001	79352000	1.06E-11
chr2	79368001	79373000	1.87E-16
chr2	79579001	79584000	3.98E-08
chr2	79586001	79591000	2.16E-05
chr2	79588001	79593000	7.58E-06
chr2	79589001	79594000	1.31E-06
chr2	80399001	80404000	4.41E-14
chr2	80400001	80405000	4.43E-15
chr2	80401001	80406000	3.86E-10
chr2	80402001	80407000	1.32E-11
chr2	80456001	80461000	8.55E-07
chr2	80509001	80514000	1.00E-05
chr2	80602001	80607000	6.87E-08
chr2	80647001	80652000	1.69E-06
chr2	80661001	80666000	5.28E-11
chr2	80719001	80724000	5.94E-09
chr2	80720001	80725000	2.70E-10
chr2	80883001	80888000	2.35E-11
chr2	80884001	80889000	6.70E-11
chr2	80885001	80890000	9.22E-12
chr2	81144001	81149000	4.47E-07
chr2	81169001	81174000	1.06E-11
chr2	81254001	81259000	2.65E-09

chr2	81255001	81260000	3.24E-09
chr2	81256001	81261000	4.06E-08
chr2	81360001	81365000	0.000126
chr2	82045001	82050000	8.28E-07
chr2	83032001	83037000	2.51E-07
chr2	83176001	83181000	2.55E-06
chr2	83178001	83183000	3.83E-08
chr2	83333001	83338000	6.86E-09
chr2	83392001	83397000	8.65E-12
chr2	83497001	83502000	6.77E-11
chr2	83498001	83503000	1.34E-11
chr2	83499001	83504000	3.12E-10
chr2	83501001	83506000	2.36E-08
chr2	83519001	83524000	5.47E-13
chr2	83520001	83525000	8.55E-15
chr2	83553001	83558000	5.57E-11
chr2	83556001	83561000	1.42E-05
chr2	84232001	84237000	5.46E-06
chr2	84318001	84323000	9.25E-06
chr2	84373001	84378000	1.34E-09
chr2	84374001	84379000	3.34E-10
chr2	84389001	84394000	6.19E-07
chr2	84570001	84575000	5.53E-11
chr2	84571001	84576000	1.12E-07
chr2	84572001	84577000	1.01E-06
chr2	84590001	84595000	5.00E-06
chr2	84591001	84596000	3.65E-06
chr2	84592001	84597000	5.22E-06
chr2	84637001	84642000	6.74E-08
chr2	84638001	84643000	6.63E-08
chr2	84639001	84644000	0.000163
chr2	85133001	85138000	1.35E-09
chr2	85148001	85153000	4.48E-08
chr2	85149001	85154000	4.67E-08
chr2	85150001	85155000	1.21E-09
chr2	85151001	85156000	5.72E-08
chr2	85577001	85582000	5.14E-18
chr2	85578001	85583000	4.03E-23
chr2	85579001	85584000	1.69E-22
chr2	85580001	85585000	1.45E-10
chr2	86026001	86031000	7.88E-07
chr2	86169001	86174000	0.000235
chr2	87314001	87319000	0.000344
chr2	87316001	87321000	0.002881
chr2	87418001	87423000	0.001871
chr2	87473001	87478000	0.000551
chr2	87474001	87479000	0.000666
chr2	87475001	87480000	1.60E-06

chr2	87476001	87481000	8.56E-05
chr2	87617001	87622000	8.30E-05
chr2	87649001	87654000	0.000125
chr2	87650001	87655000	0.000125
chr2	87651001	87656000	1.15E-06
chr2	87652001	87657000	1.82E-05
chr2	87653001	87658000	1.82E-05
chr2	87654001	87659000	0.001479
chr2	87655001	87660000	0.001479
chr2	87801001	87806000	0.002286
chr2	87802001	87807000	0.001693
chr2	87959001	87964000	1.49E-05
chr2	88248001	88253000	1.86E-05
chr2	88249001	88254000	0.000105
chr2	88251001	88256000	4.35E-05
chr2	88252001	88257000	7.03E-05
chr2	89180001	89185000	5.58E-11
chr2	89270001	89275000	7.78E-09
chr2	89281001	89286000	2.83E-06
chr2	89343001	89348000	0.042066
chr2	89345001	89350000	0.004654
chr2	89364001	89369000	4.37E-09
chr2	89365001	89370000	7.71E-13
chr2	89376001	89381000	0.00026
chr2	89378001	89383000	0.003247
chr2	89379001	89384000	0.000379
chr2	89380001	89385000	2.79E-05
chr2	89381001	89386000	2.09E-05
chr2	89386001	89391000	9.42E-06
chr2	89387001	89392000	4.10E-05
chr2	89389001	89394000	9.66E-09
chr2	89390001	89395000	2.61E-10
chr2	89413001	89418000	1.16E-06
chr2	89414001	89419000	8.37E-07
chr2	89415001	89420000	9.98E-11
chr2	89523001	89528000	2.80E-06
chr2	89533001	89538000	0.002296
chr2	89540001	89545000	3.84E-06
chr2	89584001	89589000	0.000524
chr2	89585001	89590000	0.001466
chr2	89586001	89591000	0.000474
chr2	89587001	89592000	0.000764
chr2	89588001	89593000	0.001326
chr2	89589001	89594000	0.003821
chr2	89590001	89595000	0.005128
chr2	89593001	89598000	0.00348
chr2	89618001	89623000	0.024109
chr2	89619001	89624000	0.024109

chr2	89620001	89625000	0.024109
chr2	89910001	89915000	3.70E-10
chr2	89947001	89952000	3.00E-07
chr2	90080001	90085000	3.31E-15
chr2	90081001	90086000	5.05E-16
chr2	90082001	90087000	1.43E-13
chr2	90088001	90093000	2.14E-09
chr2	90089001	90094000	2.93E-10
chr2	90190001	90195000	0.000343
chr2	90191001	90196000	3.28E-05
chr2	90192001	90197000	1.15E-05
chr2	96389001	96394000	2.26E-08
chr2	96439001	96444000	0.022169
chr2	96462001	96467000	0.00035
chr2	96463001	96468000	0.00404
chr2	96464001	96469000	9.62E-05
chr2	96465001	96470000	3.97E-05
chr2	96688001	96693000	0.008706
chr2	96721001	96726000	0.001279
chr2	96781001	96786000	0.001509
chr2	96808001	96813000	9.71E-09
chr2	96810001	96815000	2.47E-05
chr2	96841001	96846000	6.62E-05
chr2	96874001	96879000	5.63E-08
chr2	97084001	97089000	6.27E-06
chr2	97241001	97246000	0.000241
chr2	97302001	97307000	1.48E-13
chr2	97303001	97308000	3.78E-16
chr2	97304001	97309000	1.33E-09
chr2	97480001	97485000	2.59E-10
chr2	97980001	97985000	0.023689
chr2	98088001	98093000	0.004512
chr2	98233001	98238000	0.000718
chr2	98275001	98280000	2.63E-05
chr2	98677001	98682000	0.000125
chr2	98975001	98980000	2.13E-06
chr2	98976001	98981000	1.83E-07
chr2	98977001	98982000	3.68E-08
chr2	99577001	99582000	1.42E-05
chr2	99578001	99583000	0.000231
chr2	99579001	99584000	4.00E-05
chr2	99859001	99864000	2.24E-05
chr2	99860001	99865000	1.38E-06
chr2	100824001	100829000	1.79E-08
chr2	100825001	100830000	8.55E-09
chr2	101095001	101100000	8.02E-07
chr2	101096001	101101000	1.81E-07
chr2	101097001	101102000	3.60E-06

chr2	101177001	101182000	4.61E-06
chr2	101178001	101183000	5.60E-07
chr2	101179001	101184000	2.64E-10
chr2	101208001	101213000	0.009519
chr2	101922001	101927000	1.56E-10
chr2	101923001	101928000	8.05E-10
chr2	103708001	103713000	0.00208
chr2	104345001	104350000	3.74E-07
chr2	104346001	104351000	1.03E-06
chr2	104683001	104688000	9.56E-07
chr2	105257001	105262000	7.73E-09
chr2	105258001	105263000	4.50E-08
chr2	105816001	105821000	6.78E-06
chr2	106552001	106557000	4.90E-05
chr2	106747001	106752000	1.65E-06
chr2	106748001	106753000	1.63E-05
chr2	106749001	106754000	0.000189
chr2	108463001	108468000	0.004317
chr2	108487001	108492000	0.000224
chr2	108489001	108494000	0.001334
chr2	108490001	108495000	4.76E-07
chr2	108491001	108496000	9.46E-06
chr2	109320001	109325000	0.000744
chr2	110791001	110796000	0.002544
chr2	110792001	110797000	0.000843
chr2	110793001	110798000	0.001002
chr2	110794001	110799000	0.001002
chr2	110795001	110800000	0.002215
chr2	111052001	111057000	1.78E-08
chr2	111053001	111058000	3.74E-08
chr2	111054001	111059000	3.74E-08
chr2	111055001	111060000	3.74E-08
chr2	111056001	111061000	3.74E-08
chr2	111136001	111141000	0.018584
chr2	111138001	111143000	0.040071
chr2	111139001	111144000	0.040071
chr2	111187001	111192000	3.09E-05
chr2	111188001	111193000	3.09E-05
chr2	111189001	111194000	3.09E-05
chr2	111190001	111195000	3.09E-05
chr2	112052001	112057000	0.026169
chr2	112136001	112141000	0.002137
chr2	112305001	112310000	7.14E-05
chr2	112306001	112311000	0.000718
chr2	112315001	112320000	6.39E-05
chr2	112316001	112321000	5.65E-05
chr2	112916001	112921000	2.34E-10
chr2	113341001	113346000	0.000277

chr2	113396001	113401000	1.89E-05
chr2	113825001	113830000	4.89E-06
chr2	113826001	113831000	4.91E-05
chr2	113921001	113926000	0.000117
chr2	113922001	113927000	0.000634
chr2	113967001	113972000	2.27E-06
chr2	114634001	114639000	2.08E-07
chr2	114635001	114640000	5.17E-08
chr2	114752001	114757000	0.000293
chr2	115789001	115794000	1.35E-07
chr2	116734001	116739000	6.25E-09
chr2	116735001	116740000	1.76E-09
chr2	116857001	116862000	5.89E-08
chr2	116858001	116863000	3.44E-09
chr2	117360001	117365000	7.65E-07
chr2	117713001	117718000	8.64E-06
chr2	118438001	118443000	2.49E-06
chr2	118439001	118444000	6.26E-06
chr2	118592001	118597000	7.82E-06
chr2	118593001	118598000	4.43E-05
chr2	119845001	119850000	0.001758
chr2	120180001	120185000	0.000122
chr2	120275001	120280000	6.21E-06
chr2	120297001	120302000	2.73E-14
chr2	120298001	120303000	4.45E-15
chr2	121152001	121157000	2.60E-05
chr2	121316001	121321000	4.02E-08
chr2	121542001	121547000	4.43E-05
chr2	121543001	121548000	3.21E-05
chr2	121544001	121549000	2.30E-05
chr2	121587001	121592000	0.018652
chr2	121822001	121827000	0.000691
chr2	121958001	121963000	0.002265
chr2	121959001	121964000	0.011265
chr2	122425001	122430000	0.003236
chr2	122822001	122827000	2.79E-07
chr2	122823001	122828000	5.77E-06
chr2	122824001	122829000	3.65E-07
chr2	122882001	122887000	0.000928
chr2	122883001	122888000	0.002738
chr2	123333001	123338000	3.69E-06
chr2	123334001	123339000	2.26E-06
chr2	123335001	123340000	1.03E-06
chr2	123572001	123577000	1.07E-06
chr2	123573001	123578000	6.40E-09
chr2	123901001	123906000	1.42E-08
chr2	124030001	124035000	3.94E-06
chr2	124031001	124036000	4.57E-08

chr2	124032001	124037000	1.28E-06
chr2	124033001	124038000	5.54E-07
chr2	124034001	124039000	3.82E-07
chr2	124140001	124145000	1.01E-05
chr2	124919001	124924000	1.68E-07
chr2	125844001	125849000	4.29E-10
chr2	125978001	125983000	1.23E-05
chr2	126255001	126260000	1.15E-10
chr2	126256001	126261000	3.02E-11
chr2	126257001	126262000	1.75E-09
chr2	126557001	126562000	1.47E-08
chr2	126832001	126837000	1.19E-05
chr2	126833001	126838000	1.70E-07
chr2	127041001	127046000	7.29E-09
chr2	127062001	127067000	1.52E-10
chr2	127360001	127365000	4.66E-07
chr2	127443001	127448000	0.000568
chr2	127446001	127451000	0.000545
chr2	127481001	127486000	0.000418
chr2	127482001	127487000	5.66E-06
chr2	127483001	127488000	8.71E-07
chr2	127532001	127537000	6.17E-06
chr2	127533001	127538000	2.09E-06
chr2	127534001	127539000	1.44E-10
chr2	127535001	127540000	5.51E-07
chr2	127537001	127542000	8.38E-05
chr2	127580001	127585000	0.001637
chr2	127581001	127586000	0.000747
chr2	127642001	127647000	3.28E-05
chr2	127643001	127648000	0.000114
chr2	127644001	127649000	1.46E-05
chr2	127645001	127650000	2.96E-05
chr2	127734001	127739000	9.15E-07
chr2	127735001	127740000	5.01E-06
chr2	127952001	127957000	4.51E-08
chr2	128164001	128169000	9.74E-06
chr2	128165001	128170000	1.93E-07
chr2	128411001	128416000	0.000371
chr2	128415001	128420000	0.002991
chr2	128780001	128785000	1.60E-13
chr2	128781001	128786000	2.22E-06
chr2	128782001	128787000	1.09E-06
chr2	128784001	128789000	1.59E-11
chr2	128813001	128818000	0.000137
chr2	128837001	128842000	0.006251
chr2	128999001	129004000	8.43E-06
chr2	129056001	129061000	0.004717
chr2	129569001	129574000	1.21E-07

chr2	129640001	129645000	0.000303
chr2	129641001	129646000	0.000238
chr2	129642001	129647000	6.09E-06
chr2	129977001	129982000	2.45E-09
chr2	130137001	130142000	7.79E-08
chr2	130138001	130143000	1.17E-08
chr2	130140001	130145000	6.35E-05
chr2	130155001	130160000	0.000243
chr2	130261001	130266000	3.32E-06
chr2	130262001	130267000	4.21E-06
chr2	130263001	130268000	3.88E-07
chr2	130285001	130290000	5.53E-05
chr2	130448001	130453000	0.000167
chr2	130515001	130520000	3.16E-07
chr2	130516001	130521000	3.61E-09
chr2	130530001	130535000	3.86E-08
chr2	130571001	130576000	0.000458
chr2	130635001	130640000	8.13E-08
chr2	130636001	130641000	2.85E-09
chr2	130645001	130650000	0.001564
chr2	130843001	130848000	0.00499
chr2	131173001	131178000	0.001245
chr2	131241001	131246000	1.86E-05
chr2	131242001	131247000	0.004164
chr2	131439001	131444000	0.002124
chr2	131440001	131445000	0.003347
chr2	131446001	131451000	7.55E-06
chr2	131449001	131454000	5.70E-07
chr2	131450001	131455000	1.07E-09
chr2	131526001	131531000	6.69E-05
chr2	131527001	131532000	1.29E-09
chr2	131792001	131797000	6.15E-08
chr2	131793001	131798000	1.25E-13
chr2	131965001	131970000	1.72E-09
chr2	132039001	132044000	0.006223
chr2	132144001	132149000	0.006436
chr2	132246001	132251000	8.07E-05
chr2	132249001	132254000	2.10E-10
chr2	132250001	132255000	6.96E-12
chr2	134307001	134312000	5.23E-05
chr2	134515001	134520000	7.35E-08
chr2	136553001	136558000	0.000349
chr2	137089001	137094000	0.000159
chr2	137208001	137213000	1.54E-05
chr2	137209001	137214000	9.29E-07
chr2	137210001	137215000	5.85E-05
chr2	137211001	137216000	9.76E-05
chr2	137212001	137217000	6.12E-05

chr2	137326001	137331000	3.01E-06
chr2	137335001	137340000	2.90E-05
chr2	137441001	137446000	2.02E-05
chr2	137564001	137569000	4.00E-07
chr2	140041001	140046000	6.88E-08
chr2	140090001	140095000	0.000828
chr2	140137001	140142000	1.25E-05
chr2	144624001	144629000	0.000172
chr2	146709001	146714000	1.44E-06
chr2	146710001	146715000	4.57E-07
chr2	146711001	146716000	2.69E-07
chr2	149360001	149365000	1.02E-06
chr2	149361001	149366000	7.18E-06
chr2	151839001	151844000	0.00012
chr2	151840001	151845000	0.001466
chr2	152557001	152562000	2.55E-08
chr2	152913001	152918000	0.000113
chr2	153198001	153203000	0.03223
chr2	153777001	153782000	5.22E-06
chr2	153778001	153783000	4.12E-11
chr2	154123001	154128000	0.002003
chr2	154675001	154680000	4.80E-05
chr2	154676001	154681000	4.91E-05
chr2	155162001	155167000	0.00025
chr2	156133001	156138000	3.96E-06
chr2	161437001	161442000	0.000245
chr2	164674001	164679000	1.04E-07
chr2	165118001	165123000	2.86E-05
chr2	167575001	167580000	0.000844
chr2	167834001	167839000	1.51E-09
chr2	167836001	167841000	7.63E-09
chr2	168030001	168035000	3.60E-08
chr2	173004001	173009000	0.000488
chr2	173005001	173010000	0.001932
chr2	173006001	173011000	0.000256
chr2	173007001	173012000	0.000496
chr2	173420001	173425000	1.10E-11
chr2	178186001	178191000	2.71E-05
chr2	179518001	179523000	0.005549
chr2	179939001	179944000	2.68E-05
chr2	181552001	181557000	0.001049
chr2	183359001	183364000	2.15E-11
chr2	183360001	183365000	4.01E-12
chr2	183361001	183366000	5.70E-14
chr2	183362001	183367000	1.36E-13
chr2	184667001	184672000	1.49E-06
chr2	186273001	186278000	1.59E-07
chr2	187810001	187815000	1.12E-07

chr2	188056001	188061000	4.91E-08
chr2	188193001	188198000	0.000497
chr2	188388001	188393000	8.71E-05
chr2	188629001	188634000	2.92E-09
chr2	189695001	189700000	0.000213
chr2	189696001	189701000	0.000124
chr2	189698001	189703000	0.004751
chr2	189724001	189729000	2.22E-05
chr2	190184001	190189000	1.41E-10
chr2	190525001	190530000	3.84E-16
chr2	190526001	190531000	2.89E-19
chr2	193499001	193504000	0.000177
chr2	194693001	194698000	0.000526
chr2	196300001	196305000	6.22E-08
chr2	196301001	196306000	3.95E-08
chr2	196302001	196307000	6.50E-08
chr2	196303001	196308000	1.67E-09
chr2	196304001	196309000	5.32E-08
chr2	199422001	199427000	3.39E-07
chr2	200383001	200388000	0.000958
chr2	200739001	200744000	0.007509
chr2	201168001	201173000	7.53E-13
chr2	201170001	201175000	7.13E-16
chr2	201171001	201176000	1.81E-23
chr2	201172001	201177000	2.49E-18
chr2	201530001	201535000	5.91E-06
chr2	201531001	201536000	1.35E-06
chr2	202178001	202183000	0.002413
chr2	202471001	202476000	1.21E-05
chr2	203201001	203206000	2.02E-09
chr2	203202001	203207000	2.36E-13
chr2	203203001	203208000	2.88E-13
chr2	203236001	203241000	1.45E-12
chr2	203237001	203242000	5.29E-14
chr2	203238001	203243000	5.16E-11
chr2	203733001	203738000	3.77E-20
chr2	207019001	207024000	4.69E-13
chr2	207020001	207025000	1.69E-24
chr2	208572001	208577000	8.20E-07
chr2	208956001	208961000	0.007292
chr2	209222001	209227000	2.58E-08
chr2	209223001	209228000	6.77E-10
chr2	209224001	209229000	3.20E-11
chr2	209771001	209776000	4.09E-07
chr2	209772001	209777000	1.93E-08
chr2	210360001	210365000	0.008247
chr2	211093001	211098000	7.63E-08
chr2	211854001	211859000	0.00888

chr2	212461001	212466000	6.26E-06
chr2	215328001	215333000	8.48E-05
chr2	216634001	216639000	2.95E-07
chr2	216635001	216640000	1.33E-08
chr2	217273001	217278000	4.22E-09
chr2	218783001	218788000	0.022835
chr2	219004001	219009000	0.000253
chr2	219153001	219158000	1.47E-05
chr2	219186001	219191000	0.001029
chr2	219187001	219192000	0.001053
chr2	219293001	219298000	0.001614
chr2	219294001	219299000	0.010523
chr2	219471001	219476000	1.30E-05
chr2	219534001	219539000	7.93E-05
chr2	219711001	219716000	5.30E-07
chr2	219761001	219766000	7.66E-07
chr2	220030001	220035000	4.77E-06
chr2	220068001	220073000	1.31E-09
chr2	220069001	220074000	1.10E-09
chr2	220070001	220075000	9.63E-09
chr2	220071001	220076000	0.000444
chr2	220080001	220085000	0.00048
chr2	220081001	220086000	0.000238
chr2	220082001	220087000	7.19E-05
chr2	220221001	220226000	0.000109
chr2	220308001	220313000	0.004176
chr2	220309001	220314000	0.029436
chr2	220310001	220315000	0.028417
chr2	220323001	220328000	0.000635
chr2	220370001	220375000	0.000304
chr2	220516001	220521000	1.03E-05
chr2	224699001	224704000	3.76E-05
chr2	224702001	224707000	0.000198
chr2	224809001	224814000	7.16E-08
chr2	226014001	226019000	1.38E-08
chr2	228813001	228818000	0.011319
chr2	230585001	230590000	0.000431
chr2	231108001	231113000	6.63E-07
chr2	231203001	231208000	3.22E-12
chr2	231204001	231209000	5.96E-11
chr2	231205001	231210000	1.65E-13
chr2	231206001	231211000	1.77E-11
chr2	231727001	231732000	1.09E-06
chr2	231819001	231824000	5.13E-13
chr2	231820001	231825000	3.91E-11
chr2	231821001	231826000	2.85E-11
chr2	231850001	231855000	7.11E-07
chr2	232053001	232058000	1.91E-06

chr2	232054001	232059000	4.44E-06
chr2	232262001	232267000	8.88E-05
chr2	232273001	232278000	2.13E-11
chr2	232392001	232397000	0.008897
chr2	232393001	232398000	0.00523
chr2	232453001	232458000	0.000233
chr2	232454001	232459000	4.46E-05
chr2	233196001	233201000	7.74E-05
chr2	233197001	233202000	0.0001
chr2	233198001	233203000	0.000134
chr2	233270001	233275000	0.000615
chr2	233385001	233390000	0.000259
chr2	233740001	233745000	2.73E-07
chr2	233844001	233849000	6.48E-05
chr2	234001001	234006000	0.00705
chr2	234094001	234099000	1.55E-06
chr2	234762001	234767000	6.20E-14
chr2	234763001	234768000	5.51E-17
chr2	235914001	235919000	6.83E-07
chr2	236402001	236407000	2.18E-06
chr2	236498001	236503000	3.60E-05
chr2	237140001	237145000	1.22E-05
chr2	237142001	237147000	1.50E-05
chr2	237151001	237156000	0.001005
chr2	237596001	237601000	4.72E-05
chr2	237930001	237935000	0.00205
chr2	238543001	238548000	5.89E-06
chr2	238715001	238720000	4.73E-05
chr2	239039001	239044000	1.33E-09
chr2	239066001	239071000	0.000203
chr2	239067001	239072000	8.30E-06
chr2	239068001	239073000	1.44E-07
chr2	239069001	239074000	4.33E-06
chr2	239070001	239075000	4.62E-09
chr2	239071001	239076000	2.79E-09
chr2	239072001	239077000	2.47E-06
chr2	239126001	239131000	0.000167
chr2	239289001	239294000	3.05E-06
chr2	239415001	239420000	0.001384
chr2	239443001	239448000	0.003925
chr2	239845001	239850000	0.04966
chr2	239953001	239958000	1.94E-06
chr2	239954001	239959000	0.000156
chr2	240423001	240428000	1.20E-07
chr2	240437001	240442000	2.65E-05
chr2	240512001	240517000	0.003889
chr2	240583001	240588000	2.51E-07
chr2	240584001	240589000	3.79E-10

chr2	240585001	240590000	2.48E-09
chr2	240586001	240591000	2.74E-12
chr2	240587001	240592000	1.60E-08
chr2	240588001	240593000	1.58E-06
chr2	240655001	240660000	0.000736
chr2	240676001	240681000	1.05E-09
chr2	240678001	240683000	3.69E-08
chr2	240679001	240684000	3.91E-08
chr2	240738001	240743000	0.000328
chr2	240867001	240872000	0.000365
chr2	240870001	240875000	3.53E-13
chr2	240871001	240876000	7.93E-10
chr2	240872001	240877000	7.86E-12
chr2	240873001	240878000	2.06E-12
chr2	240874001	240879000	4.11E-12
chr2	241146001	241151000	0.004287
chr2	241301001	241306000	0.013557
chr2	241302001	241307000	4.01E-06
chr2	241303001	241308000	9.92E-05
chr2	241304001	241309000	1.50E-06
chr2	241305001	241310000	5.80E-07
chr2	241377001	241382000	8.07E-06
chr2	241493001	241498000	8.07E-11
chr2	241494001	241499000	1.21E-06
chr2	241533001	241538000	7.13E-06
chr2	241534001	241539000	4.92E-07
chr2	241640001	241645000	2.15E-09
chr2	241668001	241673000	0.000846
chr2	241812001	241817000	0.000139
chr2	241855001	241860000	0.007291
chr2	241856001	241861000	0.004343
chr2	241857001	241862000	0.003826
chr2	241913001	241918000	2.24E-07
chr2	242001001	242006000	0.036883
chr2	242145001	242150000	0.007178
chr2	242146001	242151000	0.003158
chr2	242486001	242491000	3.27E-05
chr2	242841001	242846000	2.63E-12
chr2	242842001	242847000	4.41E-12
chr2	242843001	242848000	3.50E-10
chr2	242931001	242936000	1.79E-10
chr3	732001	737000	4.16E-07
chr3	1072001	1077000	5.81E-06
chr3	1089001	1094000	3.11E-05
chr3	2418001	2423000	1.35E-05
chr3	2575001	2580000	7.95E-08
chr3	3164001	3169000	9.88E-10
chr3	3165001	3170000	2.33E-12

chr3	3166001	3171000	5.87E-10
chr3	3167001	3172000	2.82E-08
chr3	3938001	3943000	0.001478
chr3	3939001	3944000	0.000301
chr3	4002001	4007000	1.17E-05
chr3	4190001	4195000	5.80E-06
chr3	4191001	4196000	3.35E-07
chr3	4192001	4197000	0.000234
chr3	5052001	5057000	0.000339
chr3	5777001	5782000	0.000101
chr3	5889001	5894000	0.003754
chr3	6012001	6017000	1.01E-05
chr3	6706001	6711000	0.000464
chr3	6707001	6712000	0.002021
chr3	7922001	7927000	0.001812
chr3	7924001	7929000	0.000292
chr3	8122001	8127000	9.24E-05
chr3	8123001	8128000	0.000127
chr3	8124001	8129000	0.000978
chr3	9538001	9543000	1.79E-06
chr3	9542001	9547000	2.03E-05
chr3	9550001	9555000	0.007125
chr3	9551001	9556000	0.00126
chr3	9812001	9817000	0.000107
chr3	9904001	9909000	0.010782
chr3	9905001	9910000	0.000365
chr3	9928001	9933000	5.23E-05
chr3	10291001	10296000	1.52E-05
chr3	10359001	10364000	4.47E-06
chr3	10360001	10365000	6.10E-05
chr3	10418001	10423000	0.0007
chr3	10419001	10424000	6.53E-05
chr3	10505001	10510000	0.011013
chr3	10546001	10551000	0.002943
chr3	10547001	10552000	0.00021
chr3	10585001	10590000	4.60E-07
chr3	10688001	10693000	3.82E-05
chr3	10990001	10995000	0.00932
chr3	10991001	10996000	0.011258
chr3	11085001	11090000	3.93E-05
chr3	11685001	11690000	3.27E-09
chr3	11773001	11778000	3.68E-05
chr3	12014001	12019000	5.77E-06
chr3	12211001	12216000	7.75E-05
chr3	12487001	12492000	0.000258
chr3	12585001	12590000	8.61E-06
chr3	12586001	12591000	1.41E-06
chr3	12757001	12762000	4.80E-07

chr3	12759001	12764000	2.92E-05
chr3	13174001	13179000	3.51E-05
chr3	13192001	13197000	2.22E-06
chr3	13220001	13225000	0.000729
chr3	13237001	13242000	8.35E-09
chr3	13238001	13243000	1.37E-07
chr3	13349001	13354000	0.009555
chr3	13660001	13665000	0.044802
chr3	13668001	13673000	0.024279
chr3	13686001	13691000	8.91E-05
chr3	13687001	13692000	6.57E-05
chr3	13694001	13699000	0.000207
chr3	13854001	13859000	0.026632
chr3	13940001	13945000	0.00975
chr3	14644001	14649000	0.002197
chr3	14744001	14749000	0.016947
chr3	14745001	14750000	3.12E-05
chr3	14755001	14760000	0.00095
chr3	14892001	14897000	5.70E-07
chr3	14958001	14963000	0.000161
chr3	14960001	14965000	8.69E-06
chr3	14961001	14966000	0.00017
chr3	14962001	14967000	2.65E-06
chr3	14984001	14989000	1.99E-07
chr3	15159001	15164000	1.03E-07
chr3	15160001	15165000	5.43E-08
chr3	15506001	15511000	0.01228
chr3	15664001	15669000	8.55E-05
chr3	15665001	15670000	3.73E-06
chr3	16022001	16027000	0.000191
chr3	17880001	17885000	2.80E-05
chr3	17882001	17887000	1.55E-05
chr3	19890001	19895000	0.011036
chr3	20437001	20442000	0.000281
chr3	20438001	20443000	0.000245
chr3	22229001	22234000	7.11E-08
chr3	22691001	22696000	0.000138
chr3	22692001	22697000	0.000586
chr3	22947001	22952000	8.29E-06
chr3	23817001	23822000	0.001627
chr3	24181001	24186000	9.74E-05
chr3	25852001	25857000	5.45E-07
chr3	25853001	25858000	1.61E-06
chr3	26066001	26071000	0.000165
chr3	26234001	26239000	4.58E-05
chr3	26425001	26430000	0.001634
chr3	26640001	26645000	1.86E-06
chr3	26641001	26646000	1.84E-08

chr3	26642001	26647000	1.38E-05
chr3	26695001	26700000	1.89E-05
chr3	26725001	26730000	7.44E-07
chr3	26726001	26731000	1.42E-05
chr3	26990001	26995000	0.000495
chr3	26991001	26996000	0.000128
chr3	26992001	26997000	4.14E-05
chr3	26993001	26998000	8.21E-05
chr3	27243001	27248000	4.79E-08
chr3	27524001	27529000	3.52E-05
chr3	31503001	31508000	0.00067
chr3	32266001	32271000	4.19E-05
chr3	32267001	32272000	1.88E-05
chr3	32429001	32434000	9.84E-05
chr3	32430001	32435000	9.49E-06
chr3	32431001	32436000	1.07E-05
chr3	32432001	32437000	2.99E-08
chr3	32433001	32438000	8.36E-07
chr3	32629001	32634000	0.000471
chr3	33086001	33091000	2.68E-05
chr3	33209001	33214000	0.010327
chr3	34765001	34770000	0.002542
chr3	34805001	34810000	3.58E-06
chr3	36047001	36052000	0.000564
chr3	36048001	36053000	1.89E-05
chr3	36172001	36177000	0.008197
chr3	36356001	36361000	1.54E-08
chr3	36357001	36362000	1.00E-06
chr3	36578001	36583000	0.000267
chr3	36666001	36671000	0.000757
chr3	36667001	36672000	0.001047
chr3	36801001	36806000	2.54E-11
chr3	36804001	36809000	1.13E-09
chr3	38042001	38047000	4.48E-06
chr3	38043001	38048000	2.70E-06
chr3	38044001	38049000	1.48E-05
chr3	38596001	38601000	0.013334
chr3	38620001	38625000	0.002831
chr3	38621001	38626000	0.005934
chr3	38682001	38687000	0.003179
chr3	39192001	39197000	9.32E-07
chr3	39193001	39198000	7.73E-06
chr3	39957001	39962000	0.000537
chr3	39958001	39963000	0.000109
chr3	39959001	39964000	0.000691
chr3	39960001	39965000	0.005624
chr3	40047001	40052000	0.000176
chr3	40150001	40155000	0.000151

chr3	41468001	41473000	1.52E-05
chr3	41470001	41475000	6.01E-05
chr3	41471001	41476000	5.39E-06
chr3	41472001	41477000	3.67E-07
chr3	42228001	42233000	1.36E-06
chr3	42421001	42426000	4.98E-06
chr3	42422001	42427000	4.02E-05
chr3	42485001	42490000	0.000678
chr3	42486001	42491000	0.001486
chr3	42543001	42548000	0.0254
chr3	42695001	42700000	0.038629
chr3	42788001	42793000	0.000494
chr3	43040001	43045000	2.47E-06
chr3	43158001	43163000	3.49E-05
chr3	43276001	43281000	2.28E-05
chr3	43277001	43282000	0.000158
chr3	43297001	43302000	2.62E-07
chr3	43298001	43303000	8.50E-09
chr3	43300001	43305000	7.50E-08
chr3	43901001	43906000	0.0007
chr3	43902001	43907000	7.65E-05
chr3	44576001	44581000	2.36E-05
chr3	45079001	45084000	2.86E-07
chr3	46597001	46602000	5.53E-06
chr3	46692001	46697000	0.001468
chr3	46713001	46718000	3.82E-05
chr3	46759001	46764000	0.003048
chr3	46950001	46955000	4.39E-09
chr3	47778001	47783000	0.000646
chr3	47828001	47833000	0.003458
chr3	47830001	47835000	0.00119
chr3	48193001	48198000	0.001889
chr3	48231001	48236000	0.000353
chr3	48327001	48332000	0.042145
chr3	48340001	48345000	7.68E-06
chr3	48341001	48346000	1.10E-05
chr3	48503001	48508000	0.0008
chr3	48593001	48598000	4.44E-07
chr3	48666001	48671000	0.000953
chr3	48720001	48725000	0.000772
chr3	48721001	48726000	0.012911
chr3	48754001	48759000	2.43E-05
chr3	48881001	48886000	4.86E-09
chr3	48882001	48887000	1.09E-09
chr3	48883001	48888000	5.57E-13
chr3	48884001	48889000	2.13E-07
chr3	48885001	48890000	9.45E-05
chr3	48947001	48952000	0.003155

chr3	49157001	49162000	0.000722
chr3	49188001	49193000	0.027072
chr3	49207001	49212000	3.24E-07
chr3	49208001	49213000	8.14E-08
chr3	49209001	49214000	8.14E-08
chr3	49276001	49281000	0.00021
chr3	49308001	49313000	5.07E-06
chr3	49309001	49314000	6.77E-06
chr3	49337001	49342000	0.001003
chr3	49338001	49343000	0.001003
chr3	49367001	49372000	0.029692
chr3	49464001	49469000	8.91E-05
chr3	49474001	49479000	0.005253
chr3	49594001	49599000	0.002108
chr3	49597001	49602000	0.005567
chr3	49598001	49603000	0.005567
chr3	49652001	49657000	0.000139
chr3	49701001	49706000	0.004379
chr3	49702001	49707000	0.000278
chr3	49724001	49729000	5.83E-05
chr3	49725001	49730000	6.15E-05
chr3	49726001	49731000	2.50E-11
chr3	49727001	49732000	4.11E-15
chr3	49759001	49764000	3.48E-09
chr3	49760001	49765000	3.34E-07
chr3	49761001	49766000	9.59E-07
chr3	49842001	49847000	0.011544
chr3	49896001	49901000	0.004085
chr3	49949001	49954000	0.001188
chr3	49952001	49957000	0.006596
chr3	49963001	49968000	3.73E-10
chr3	49964001	49969000	1.14E-11
chr3	49965001	49970000	3.46E-15
chr3	49966001	49971000	3.25E-21
chr3	49967001	49972000	4.34E-24
chr3	50241001	50246000	0.016214
chr3	50242001	50247000	0.002711
chr3	50264001	50269000	0.00014
chr3	50270001	50275000	0.009336
chr3	50278001	50283000	0.001229
chr3	50279001	50284000	0.003925
chr3	50280001	50285000	0.003925
chr3	50296001	50301000	6.87E-07
chr3	50297001	50302000	1.75E-07
chr3	50298001	50303000	4.74E-06
chr3	50299001	50304000	0.002002
chr3	50351001	50356000	0.000979
chr3	50353001	50358000	0.017638

chr3	50385001	50390000	0.000678
chr3	50386001	50391000	0.006459
chr3	50406001	50411000	8.44E-05
chr3	50530001	50535000	0.012806
chr3	50652001	50657000	1.79E-12
chr3	50653001	50658000	1.24E-14
chr3	50654001	50659000	1.17E-08
chr3	50681001	50686000	0.001168
chr3	50690001	50695000	0.002342
chr3	50691001	50696000	0.005369
chr3	50705001	50710000	4.68E-06
chr3	50823001	50828000	0.000645
chr3	50824001	50829000	0.000958
chr3	51058001	51063000	2.38E-09
chr3	51059001	51064000	8.52E-07
chr3	51060001	51065000	8.87E-06
chr3	51061001	51066000	2.78E-06
chr3	51212001	51217000	0.000455
chr3	51213001	51218000	0.000347
chr3	51536001	51541000	2.37E-06
chr3	51550001	51555000	0.02097
chr3	51728001	51733000	0.017145
chr3	51736001	51741000	0.000642
chr3	51737001	51742000	0.003094
chr3	51738001	51743000	0.0006
chr3	51739001	51744000	0.000333
chr3	51740001	51745000	0.001891
chr3	51741001	51746000	0.008027
chr3	51749001	51754000	0.00011
chr3	51761001	51766000	1.44E-05
chr3	51897001	51902000	0.000661
chr3	51898001	51903000	4.48E-05
chr3	51907001	51912000	0.000616
chr3	51943001	51948000	0.001135
chr3	52017001	52022000	0.002069
chr3	52020001	52025000	0.004263
chr3	52022001	52027000	0.000374
chr3	52038001	52043000	3.23E-05
chr3	52043001	52048000	0.005847
chr3	52193001	52198000	0.002205
chr3	52194001	52199000	0.002002
chr3	52222001	52227000	0.00165
chr3	52229001	52234000	0.002025
chr3	52249001	52254000	3.11E-05
chr3	52251001	52256000	3.47E-06
chr3	52345001	52350000	0.000384
chr3	52441001	52446000	1.84E-05
chr3	52442001	52447000	4.11E-06

chr3	52735001	52740000	7.52E-07
chr3	52821001	52826000	0.011319
chr3	52822001	52827000	0.00193
chr3	52823001	52828000	0.006534
chr3	52824001	52829000	0.011633
chr3	53004001	53009000	7.99E-05
chr3	53185001	53190000	0.00288
chr3	53238001	53243000	0.006601
chr3	53239001	53244000	0.009168
chr3	53240001	53245000	0.001846
chr3	53241001	53246000	0.003577
chr3	53251001	53256000	0.004859
chr3	53252001	53257000	0.000253
chr3	53253001	53258000	0.000234
chr3	53254001	53259000	0.002316
chr3	53255001	53260000	0.000502
chr3	53263001	53268000	0.02214
chr3	53264001	53269000	0.017679
chr3	53265001	53270000	0.014485
chr3	53266001	53271000	0.014485
chr3	54429001	54434000	0.019895
chr3	54430001	54435000	0.019895
chr3	56603001	56608000	0.000668
chr3	56790001	56795000	0.004844
chr3	57011001	57016000	0.001009
chr3	57526001	57531000	0.002353
chr3	57528001	57533000	3.78E-05
chr3	57904001	57909000	0.000148
chr3	57941001	57946000	1.74E-06
chr3	57987001	57992000	0.007579
chr3	57993001	57998000	0.021492
chr3	58005001	58010000	5.40E-05
chr3	58006001	58011000	4.07E-05
chr3	58007001	58012000	0.000197
chr3	58448001	58453000	0.00016
chr3	58449001	58454000	0.000163
chr3	58450001	58455000	1.50E-05
chr3	58550001	58555000	5.45E-06
chr3	58551001	58556000	5.88E-10
chr3	58552001	58557000	7.91E-07
chr3	58592001	58597000	3.13E-05
chr3	58593001	58598000	5.32E-06
chr3	58594001	58599000	8.35E-05
chr3	58606001	58611000	3.73E-06
chr3	58607001	58612000	4.32E-08
chr3	58608001	58613000	2.15E-07
chr3	58609001	58614000	3.77E-06
chr3	61494001	61499000	8.39E-06

chr3	61539001	61544000	0.000105
chr3	64095001	64100000	0.023972
chr3	64096001	64101000	0.023972
chr3	64384001	64389000	5.75E-06
chr3	67182001	67187000	0.000152
chr3	67183001	67188000	0.000858
chr3	67184001	67189000	0.0001
chr3	68008001	68013000	0.000995
chr3	68244001	68249000	2.73E-07
chr3	68922001	68927000	3.44E-10
chr3	68923001	68928000	1.00E-10
chr3	68924001	68929000	1.13E-12
chr3	69134001	69139000	1.36E-07
chr3	69282001	69287000	5.46E-05
chr3	69729001	69734000	4.03E-06
chr3	69730001	69735000	6.63E-06
chr3	69882001	69887000	3.07E-05
chr3	71240001	71245000	5.99E-05
chr3	71241001	71246000	6.68E-07
chr3	71366001	71371000	4.56E-09
chr3	71949001	71954000	0.000252
chr3	72029001	72034000	1.74E-06
chr3	72030001	72035000	1.05E-05
chr3	72334001	72339000	2.37E-07
chr3	72396001	72401000	0.000934
chr3	72794001	72799000	1.26E-08
chr3	72996001	73001000	0.000106
chr3	73152001	73157000	0.007029
chr3	73153001	73158000	0.005636
chr3	73285001	73290000	0.001294
chr3	74267001	74272000	0.000183
chr3	74774001	74779000	0.000183
chr3	75207001	75212000	2.12E-05
chr3	75208001	75213000	0.000571
chr3	75562001	75567000	0.004751
chr3	75755001	75760000	6.66E-05
chr3	75756001	75761000	3.31E-05
chr3	75757001	75762000	3.31E-05
chr3	75759001	75764000	0.000148
chr3	75843001	75848000	7.36E-07
chr3	75844001	75849000	5.06E-07
chr3	75845001	75850000	3.11E-08
chr3	77247001	77252000	2.83E-06
chr3	88027001	88032000	4.86E-05
chr3	88268001	88273000	2.22E-08
chr3	88418001	88423000	8.45E-07
chr3	88419001	88424000	2.14E-08
chr3	88470001	88475000	1.01E-13

chr3	88840001	88845000	8.14E-06
chr3	88841001	88846000	3.15E-06
chr3	89342001	89347000	5.09E-13
chr3	89343001	89348000	1.32E-07
chr3	89651001	89656000	5.94E-06
chr3	89979001	89984000	0.000673
chr3	89980001	89985000	0.001759
chr3	89997001	90002000	1.22E-06
chr3	90062001	90067000	1.10E-10
chr3	90063001	90068000	2.06E-08
chr3	90114001	90119000	2.08E-09
chr3	90189001	90194000	7.22E-07
chr3	90357001	90362000	9.49E-14
chr3	90358001	90363000	2.62E-12
chr3	90359001	90364000	2.82E-08
chr3	90360001	90365000	1.84E-07
chr3	90361001	90366000	1.34E-06
chr3	93570001	93575000	0.000137
chr3	93885001	93890000	7.97E-10
chr3	93886001	93891000	1.04E-10
chr3	93937001	93942000	8.12E-06
chr3	93939001	93944000	1.06E-05
chr3	94190001	94195000	5.78E-06
chr3	94266001	94271000	0.002169
chr3	94267001	94272000	0.00061
chr3	94268001	94273000	0.000821
chr3	94318001	94323000	0.000218
chr3	95049001	95054000	0.00289
chr3	95139001	95144000	3.01E-08
chr3	95777001	95782000	7.61E-07
chr3	95778001	95783000	2.90E-07
chr3	95812001	95817000	2.29E-08
chr3	96126001	96131000	1.05E-08
chr3	96240001	96245000	2.06E-06
chr3	96241001	96246000	6.74E-08
chr3	96256001	96261000	1.06E-05
chr3	96257001	96262000	3.07E-05
chr3	96397001	96402000	4.36E-08
chr3	96398001	96403000	1.70E-09
chr3	97266001	97271000	0.000146
chr3	97267001	97272000	7.87E-05
chr3	97268001	97273000	0.000235
chr3	97436001	97441000	2.42E-09
chr3	97456001	97461000	7.15E-06
chr3	97457001	97462000	3.66E-08
chr3	97458001	97463000	3.83E-08
chr3	98356001	98361000	1.07E-10
chr3	98357001	98362000	3.93E-14

chr3	98358001	98363000	1.44E-09
chr3	98359001	98364000	2.53E-09
chr3	98360001	98365000	6.55E-07
chr3	98553001	98558000	0.000763
chr3	98897001	98902000	0.01483
chr3	100240001	100245000	7.71E-05
chr3	100241001	100246000	1.89E-07
chr3	100242001	100247000	1.38E-07
chr3	100296001	100301000	2.57E-05
chr3	100725001	100730000	6.27E-09
chr3	100726001	100731000	9.71E-07
chr3	100745001	100750000	2.01E-06
chr3	100746001	100751000	7.77E-06
chr3	100921001	100926000	7.82E-11
chr3	100922001	100927000	2.25E-09
chr3	101001001	101006000	2.00E-07
chr3	101002001	101007000	9.61E-05
chr3	101151001	101156000	1.25E-06
chr3	101152001	101157000	9.77E-05
chr3	101153001	101158000	5.52E-05
chr3	101494001	101499000	5.07E-11
chr3	102321001	102326000	3.54E-08
chr3	102363001	102368000	3.27E-11
chr3	102364001	102369000	6.04E-11
chr3	102412001	102417000	2.37E-07
chr3	102569001	102574000	0.000573
chr3	102690001	102695000	6.14E-07
chr3	102691001	102696000	3.04E-08
chr3	102692001	102697000	9.44E-08
chr3	102804001	102809000	2.14E-07
chr3	102805001	102810000	2.87E-09
chr3	102814001	102819000	1.98E-06
chr3	103217001	103222000	2.56E-07
chr3	103313001	103318000	3.70E-07
chr3	103314001	103319000	8.28E-07
chr3	103315001	103320000	9.05E-06
chr3	103707001	103712000	0.000205
chr3	103708001	103713000	1.15E-05
chr3	103730001	103735000	1.45E-09
chr3	103731001	103736000	3.72E-08
chr3	104352001	104357000	2.93E-08
chr3	104539001	104544000	5.66E-07
chr3	106690001	106695000	2.38E-07
chr3	106691001	106696000	3.25E-08
chr3	106692001	106697000	2.47E-08
chr3	106693001	106698000	1.50E-06
chr3	108165001	108170000	0.0003
chr3	108470001	108475000	0.001391

chr3	108471001	108476000	3.35E-05
chr3	108887001	108892000	1.37E-07
chr3	108888001	108893000	2.40E-08
chr3	108891001	108896000	2.55E-08
chr3	108945001	108950000	6.97E-08
chr3	109015001	109020000	6.48E-05
chr3	109023001	109028000	4.72E-08
chr3	109088001	109093000	0.000246
chr3	109089001	109094000	7.72E-06
chr3	109090001	109095000	0.000102
chr3	109127001	109132000	2.91E-08
chr3	109128001	109133000	5.15E-10
chr3	109215001	109220000	6.34E-06
chr3	109216001	109221000	2.28E-08
chr3	109247001	109252000	4.14E-07
chr3	109249001	109254000	0.001758
chr3	109252001	109257000	3.33E-05
chr3	109300001	109305000	8.46E-06
chr3	109379001	109384000	1.18E-10
chr3	109421001	109426000	3.43E-07
chr3	109497001	109502000	0.000155
chr3	109576001	109581000	3.25E-08
chr3	109577001	109582000	1.61E-08
chr3	109578001	109583000	6.75E-08
chr3	109579001	109584000	4.45E-08
chr3	109580001	109585000	6.37E-06
chr3	109633001	109638000	0.000216
chr3	109699001	109704000	1.76E-10
chr3	109765001	109770000	8.89E-07
chr3	109786001	109791000	1.66E-05
chr3	109950001	109955000	3.48E-09
chr3	109970001	109975000	1.09E-05
chr3	109971001	109976000	1.51E-05
chr3	110666001	110671000	0.001199
chr3	110667001	110672000	0.003347
chr3	110725001	110730000	1.33E-08
chr3	110726001	110731000	2.64E-06
chr3	110727001	110732000	1.59E-05
chr3	110753001	110758000	0.000191
chr3	110754001	110759000	3.30E-05
chr3	110757001	110762000	2.08E-05
chr3	110758001	110763000	5.30E-05
chr3	111083001	111088000	2.34E-09
chr3	111184001	111189000	4.41E-05
chr3	111185001	111190000	3.95E-08
chr3	111345001	111350000	0.000379
chr3	111346001	111351000	0.000314
chr3	111977001	111982000	4.93E-08

chr3	111978001	111983000	2.08E-08
chr3	112404001	112409000	3.23E-06
chr3	112757001	112762000	1.12E-06
chr3	112758001	112763000	3.96E-05
chr3	112759001	112764000	2.01E-05
chr3	112795001	112800000	2.55E-06
chr3	112825001	112830000	5.11E-06
chr3	112826001	112831000	1.28E-06
chr3	112827001	112832000	1.60E-07
chr3	113961001	113966000	0.000131
chr3	113964001	113969000	2.22E-05
chr3	113977001	113982000	5.98E-06
chr3	113978001	113983000	9.66E-06
chr3	113979001	113984000	1.95E-06
chr3	113980001	113985000	6.11E-08
chr3	113981001	113986000	1.59E-08
chr3	113982001	113987000	7.28E-09
chr3	116104001	116109000	6.66E-07
chr3	116105001	116110000	2.96E-05
chr3	116793001	116798000	6.45E-10
chr3	116794001	116799000	2.49E-09
chr3	117895001	117900000	5.30E-12
chr3	118049001	118054000	1.85E-05
chr3	118073001	118078000	1.48E-08
chr3	118170001	118175000	2.23E-06
chr3	118171001	118176000	2.25E-06
chr3	118177001	118182000	2.66E-07
chr3	118179001	118184000	1.17E-07
chr3	118186001	118191000	5.18E-05
chr3	118323001	118328000	1.05E-06
chr3	118324001	118329000	1.61E-06
chr3	118337001	118342000	6.31E-06
chr3	118338001	118343000	1.51E-05
chr3	118339001	118344000	4.41E-09
chr3	118340001	118345000	6.12E-08
chr3	118341001	118346000	1.35E-07
chr3	118342001	118347000	1.15E-06
chr3	118551001	118556000	6.16E-06
chr3	118575001	118580000	8.96E-08
chr3	118576001	118581000	1.45E-07
chr3	119276001	119281000	1.51E-08
chr3	120899001	120904000	3.95E-06
chr3	120900001	120905000	1.49E-05
chr3	120901001	120906000	1.92E-05
chr3	121274001	121279000	0.00458
chr3	121289001	121294000	3.96E-05
chr3	121290001	121295000	2.04E-07
chr3	122630001	122635000	2.65E-05

chr3	122640001	122645000	2.90E-09
chr3	123020001	123025000	0.000364
chr3	123021001	123026000	0.000302
chr3	123057001	123062000	1.67E-06
chr3	123058001	123063000	3.49E-07
chr3	123059001	123064000	1.48E-06
chr3	123060001	123065000	4.46E-06
chr3	123122001	123127000	8.70E-07
chr3	123123001	123128000	5.58E-08
chr3	123166001	123171000	5.94E-08
chr3	123167001	123172000	7.41E-12
chr3	123368001	123373000	1.52E-06
chr3	123369001	123374000	1.26E-09
chr3	123370001	123375000	2.09E-05
chr3	123426001	123431000	1.99E-06
chr3	123427001	123432000	3.66E-06
chr3	123706001	123711000	5.24E-07
chr3	123751001	123756000	0.000135
chr3	123974001	123979000	0.00025
chr3	124094001	124099000	8.69E-07
chr3	124095001	124100000	4.75E-08
chr3	124096001	124101000	1.02E-05
chr3	124211001	124216000	7.90E-06
chr3	124248001	124253000	6.71E-06
chr3	124445001	124450000	1.34E-07
chr3	124446001	124451000	3.41E-08
chr3	124448001	124453000	3.95E-09
chr3	124449001	124454000	1.42E-12
chr3	125569001	125574000	1.82E-05
chr3	125570001	125575000	1.51E-07
chr3	125572001	125577000	0.000201
chr3	126075001	126080000	0.003994
chr3	126117001	126122000	9.15E-07
chr3	126675001	126680000	2.04E-05
chr3	126852001	126857000	7.10E-07
chr3	126854001	126859000	1.16E-06
chr3	126856001	126861000	0.000467
chr3	127042001	127047000	7.07E-08
chr3	127043001	127048000	6.03E-06
chr3	127044001	127049000	0.000373
chr3	127051001	127056000	0.000321
chr3	127053001	127058000	7.92E-07
chr3	127096001	127101000	2.82E-11
chr3	127097001	127102000	5.28E-09
chr3	127098001	127103000	1.19E-08
chr3	127167001	127172000	3.95E-06
chr3	127170001	127175000	0.000162
chr3	127171001	127176000	4.63E-05

chr3	127173001	127178000	9.86E-05
chr3	127174001	127179000	1.65E-07
chr3	127175001	127180000	6.71E-06
chr3	127254001	127259000	2.29E-07
chr3	127264001	127269000	0.001232
chr3	127269001	127274000	6.00E-06
chr3	127342001	127347000	1.73E-07
chr3	127343001	127348000	3.79E-08
chr3	127369001	127374000	8.45E-10
chr3	127387001	127392000	2.71E-06
chr3	127549001	127554000	2.76E-07
chr3	127620001	127625000	4.70E-07
chr3	127621001	127626000	3.74E-07
chr3	127622001	127627000	3.60E-07
chr3	127752001	127757000	1.96E-08
chr3	127755001	127760000	6.68E-07
chr3	128131001	128136000	5.04E-05
chr3	128194001	128199000	9.12E-11
chr3	128195001	128200000	5.70E-09
chr3	128246001	128251000	3.26E-08
chr3	128322001	128327000	2.22E-06
chr3	128323001	128328000	4.84E-08
chr3	128332001	128337000	0.000536
chr3	128333001	128338000	1.04E-06
chr3	128650001	128655000	0.016912
chr3	128651001	128656000	0.001493
chr3	128653001	128658000	2.71E-05
chr3	128746001	128751000	2.15E-07
chr3	129058001	129063000	1.60E-06
chr3	129059001	129064000	1.88E-07
chr3	129165001	129170000	1.46E-06
chr3	129626001	129631000	9.65E-07
chr3	129628001	129633000	2.14E-07
chr3	129874001	129879000	1.98E-05
chr3	129875001	129880000	9.50E-05
chr3	129952001	129957000	1.70E-08
chr3	131765001	131770000	3.73E-05
chr3	131805001	131810000	0.003965
chr3	131806001	131811000	0.000117
chr3	133089001	133094000	0.000539
chr3	133141001	133146000	1.39E-05
chr3	133555001	133560000	0.000674
chr3	133602001	133607000	1.29E-05
chr3	133603001	133608000	9.12E-07
chr3	133796001	133801000	5.92E-06
chr3	134078001	134083000	3.69E-08
chr3	134079001	134084000	3.51E-09
chr3	134080001	134085000	3.86E-09

chr3	134081001	134086000	5.64E-10
chr3	134082001	134087000	2.76E-07
chr3	134114001	134119000	6.70E-12
chr3	134115001	134120000	4.70E-12
chr3	134116001	134121000	5.81E-12
chr3	134431001	134436000	0.000702
chr3	134432001	134437000	9.69E-05
chr3	134518001	134523000	0.000528
chr3	135145001	135150000	8.73E-06
chr3	135245001	135250000	2.25E-09
chr3	135273001	135278000	5.85E-09
chr3	135274001	135279000	3.19E-08
chr3	135280001	135285000	9.15E-09
chr3	135281001	135286000	7.22E-08
chr3	135310001	135315000	1.76E-07
chr3	135356001	135361000	0.00016
chr3	135357001	135362000	4.23E-05
chr3	135365001	135370000	0.003127
chr3	135367001	135372000	0.0001
chr3	135400001	135405000	8.60E-05
chr3	135494001	135499000	0.000185
chr3	135547001	135552000	2.17E-07
chr3	135623001	135628000	4.65E-07
chr3	135624001	135629000	5.46E-10
chr3	136332001	136337000	3.64E-05
chr3	136554001	136559000	0.007171
chr3	137112001	137117000	2.87E-09
chr3	137143001	137148000	1.01E-06
chr3	137377001	137382000	1.81E-09
chr3	137378001	137383000	6.39E-11
chr3	137379001	137384000	5.22E-13
chr3	137424001	137429000	3.73E-08
chr3	138040001	138045000	7.48E-05
chr3	138041001	138046000	4.67E-05
chr3	138324001	138329000	0.000987
chr3	138325001	138330000	2.66E-07
chr3	138326001	138331000	2.85E-06
chr3	138327001	138332000	6.17E-09
chr3	138640001	138645000	0.000147
chr3	139104001	139109000	2.77E-10
chr3	139105001	139110000	4.72E-14
chr3	139106001	139111000	5.92E-09
chr3	139805001	139810000	6.88E-08
chr3	139827001	139832000	2.73E-06
chr3	139843001	139848000	0.000257
chr3	139865001	139870000	9.86E-07
chr3	139944001	139949000	2.15E-05
chr3	139945001	139950000	1.75E-06

chr3	139946001	139951000	1.88E-07
chr3	140098001	140103000	8.72E-06
chr3	140376001	140381000	0.00048
chr3	140434001	140439000	6.07E-07
chr3	140466001	140471000	5.76E-06
chr3	140467001	140472000	5.17E-07
chr3	140809001	140814000	1.80E-06
chr3	140810001	140815000	2.30E-05
chr3	141370001	141375000	0.001713
chr3	141372001	141377000	0.002684
chr3	141373001	141378000	0.000164
chr3	141552001	141557000	0.013073
chr3	142838001	142843000	1.90E-06
chr3	144694001	144699000	2.61E-08
chr3	144695001	144700000	2.54E-09
chr3	144743001	144748000	0.000196
chr3	144744001	144749000	7.53E-06
chr3	144745001	144750000	0.000212
chr3	144757001	144762000	3.60E-10
chr3	144959001	144964000	2.82E-07
chr3	145702001	145707000	1.60E-08
chr3	146346001	146351000	1.62E-09
chr3	146355001	146360000	3.58E-11
chr3	146424001	146429000	6.62E-16
chr3	146425001	146430000	1.33E-16
chr3	146427001	146432000	3.20E-18
chr3	146428001	146433000	1.79E-19
chr3	146435001	146440000	5.02E-16
chr3	146439001	146444000	3.64E-16
chr3	146446001	146451000	4.66E-14
chr3	146447001	146452000	5.65E-13
chr3	146455001	146460000	1.80E-13
chr3	146467001	146472000	4.11E-11
chr3	146468001	146473000	4.71E-17
chr3	146469001	146474000	4.98E-18
chr3	146470001	146475000	4.64E-20
chr3	146471001	146476000	5.70E-18
chr3	146477001	146482000	7.00E-14
chr3	146518001	146523000	1.63E-13
chr3	146577001	146582000	4.58E-10
chr3	146641001	146646000	5.29E-19
chr3	146653001	146658000	1.28E-11
chr3	146654001	146659000	4.34E-11
chr3	146711001	146716000	1.64E-09
chr3	146723001	146728000	3.82E-14
chr3	146724001	146729000	6.70E-13
chr3	146725001	146730000	8.74E-14
chr3	146771001	146776000	4.45E-07

chr3	146772001	146777000	1.02E-07
chr3	146789001	146794000	2.83E-07
chr3	146790001	146795000	2.30E-08
chr3	146791001	146796000	1.67E-08
chr3	146792001	146797000	2.74E-08
chr3	146855001	146860000	1.49E-10
chr3	146862001	146867000	9.18E-11
chr3	146884001	146889000	5.07E-08
chr3	146885001	146890000	3.14E-08
chr3	146902001	146907000	7.93E-12
chr3	146955001	146960000	5.64E-09
chr3	146986001	146991000	3.84E-07
chr3	147003001	147008000	1.32E-05
chr3	147025001	147030000	3.71E-14
chr3	147060001	147065000	7.35E-12
chr3	147061001	147066000	4.83E-15
chr3	147062001	147067000	9.75E-12
chr3	147144001	147149000	5.19E-10
chr3	147153001	147158000	7.71E-18
chr3	147154001	147159000	4.87E-18
chr3	147156001	147161000	1.35E-11
chr3	147160001	147165000	5.00E-12
chr3	147161001	147166000	8.06E-10
chr3	147162001	147167000	4.11E-08
chr3	147163001	147168000	4.68E-11
chr3	147164001	147169000	2.22E-09
chr3	147165001	147170000	3.12E-09
chr3	147174001	147179000	3.25E-10
chr3	147197001	147202000	4.05E-10
chr3	147198001	147203000	2.65E-09
chr3	147200001	147205000	1.42E-12
chr3	147256001	147261000	2.58E-08
chr3	147277001	147282000	5.25E-14
chr3	147278001	147283000	1.27E-12
chr3	147279001	147284000	2.17E-08
chr3	147341001	147346000	8.40E-11
chr3	147349001	147354000	1.28E-07
chr3	147350001	147355000	7.23E-08
chr3	147351001	147356000	2.13E-09
chr3	147352001	147357000	1.11E-11
chr3	147353001	147358000	1.04E-08
chr3	147387001	147392000	3.31E-09
chr3	147388001	147393000	3.57E-10
chr3	147389001	147394000	2.32E-14
chr3	147390001	147395000	5.12E-12
chr3	147391001	147396000	1.60E-11
chr3	147392001	147397000	1.60E-11
chr3	147393001	147398000	3.50E-10

chr3	147575001	147580000	2.34E-09
chr3	147577001	147582000	1.61E-07
chr3	147578001	147583000	1.84E-07
chr3	147579001	147584000	5.61E-06
chr3	147591001	147596000	3.43E-06
chr3	147592001	147597000	4.59E-11
chr3	147593001	147598000	1.66E-07
chr3	147612001	147617000	1.79E-10
chr3	147627001	147632000	1.03E-14
chr3	147628001	147633000	3.29E-13
chr3	147640001	147645000	3.65E-10
chr3	147641001	147646000	3.58E-09
chr3	147642001	147647000	8.50E-11
chr3	147643001	147648000	7.14E-13
chr3	147644001	147649000	1.60E-10
chr3	147694001	147699000	3.28E-11
chr3	147695001	147700000	1.96E-11
chr3	147697001	147702000	2.78E-09
chr3	147731001	147736000	6.28E-06
chr3	147740001	147745000	2.31E-13
chr3	147792001	147797000	8.75E-16
chr3	147952001	147957000	1.73E-09
chr3	147953001	147958000	3.57E-09
chr3	147954001	147959000	1.41E-11
chr3	148070001	148075000	1.18E-15
chr3	148071001	148076000	5.03E-14
chr3	148072001	148077000	1.32E-10
chr3	148073001	148078000	1.42E-11
chr3	148101001	148106000	6.17E-08
chr3	148102001	148107000	3.39E-07
chr3	148103001	148108000	1.22E-05
chr3	148104001	148109000	1.64E-11
chr3	148105001	148110000	2.14E-08
chr3	148106001	148111000	5.72E-11
chr3	148107001	148112000	1.62E-09
chr3	148108001	148113000	4.71E-13
chr3	148125001	148130000	1.27E-08
chr3	148185001	148190000	6.24E-10
chr3	148186001	148191000	1.84E-09
chr3	148195001	148200000	4.66E-12
chr3	148196001	148201000	3.01E-09
chr3	148197001	148202000	4.57E-09
chr3	148198001	148203000	2.84E-07
chr3	148263001	148268000	8.61E-11
chr3	148264001	148269000	5.52E-12
chr3	148265001	148270000	3.78E-13
chr3	148266001	148271000	5.72E-13
chr3	148267001	148272000	9.95E-12

chr3	148285001	148290000	1.55E-11
chr3	148286001	148291000	1.37E-12
chr3	148289001	148294000	5.23E-11
chr3	148320001	148325000	4.80E-09
chr3	148321001	148326000	3.73E-10
chr3	148322001	148327000	8.54E-17
chr3	148400001	148405000	3.60E-09
chr3	148401001	148406000	9.03E-15
chr3	148402001	148407000	1.52E-17
chr3	148409001	148414000	3.82E-12
chr3	148426001	148431000	8.33E-15
chr3	148427001	148432000	7.54E-10
chr3	148464001	148469000	0.000115
chr3	148466001	148471000	1.71E-14
chr3	148467001	148472000	6.73E-18
chr3	148484001	148489000	0.000217
chr3	148485001	148490000	6.20E-06
chr3	148547001	148552000	1.66E-09
chr3	148593001	148598000	3.32E-15
chr3	148594001	148599000	1.11E-10
chr3	148621001	148626000	3.06E-22
chr3	148623001	148628000	2.19E-18
chr3	148648001	148653000	1.17E-12
chr3	148649001	148654000	1.48E-13
chr3	148650001	148655000	1.08E-11
chr3	148651001	148656000	6.19E-10
chr3	148652001	148657000	4.47E-11
chr3	149391001	149396000	5.38E-13
chr3	149392001	149397000	1.39E-13
chr3	149393001	149398000	1.03E-10
chr3	149469001	149474000	3.65E-19
chr3	149470001	149475000	1.61E-18
chr3	149748001	149753000	1.00E-13
chr3	149762001	149767000	4.88E-11
chr3	149805001	149810000	3.82E-16
chr3	149823001	149828000	6.97E-13
chr3	149824001	149829000	8.14E-23
chr3	149847001	149852000	3.40E-13
chr3	149849001	149854000	2.21E-10
chr3	149850001	149855000	1.55E-09
chr3	149914001	149919000	5.64E-09
chr3	150384001	150389000	5.62E-08
chr3	150388001	150393000	1.10E-05
chr3	150389001	150394000	7.36E-06
chr3	150417001	150422000	1.13E-13
chr3	150535001	150540000	0.00136
chr3	150536001	150541000	1.60E-05
chr3	150538001	150543000	1.36E-07

chr3	150539001	150544000	4.86E-07
chr3	150634001	150639000	5.39E-11
chr3	150701001	150706000	1.74E-09
chr3	150702001	150707000	4.16E-09
chr3	150703001	150708000	8.20E-09
chr3	150714001	150719000	4.75E-14
chr3	151981001	151986000	9.07E-09
chr3	152248001	152253000	3.30E-07
chr3	152249001	152254000	2.79E-09
chr3	152250001	152255000	7.03E-10
chr3	152251001	152256000	3.31E-10
chr3	152252001	152257000	2.91E-08
chr3	152402001	152407000	1.07E-06
chr3	152467001	152472000	1.62E-08
chr3	152468001	152473000	5.00E-09
chr3	152470001	152475000	8.80E-11
chr3	152549001	152554000	5.68E-23
chr3	152550001	152555000	9.33E-29
chr3	152563001	152568000	3.31E-11
chr3	152856001	152861000	6.79E-10
chr3	153551001	153556000	4.14E-14
chr3	153552001	153557000	2.88E-18
chr3	153553001	153558000	6.41E-13
chr3	153581001	153586000	7.08E-10
chr3	153621001	153626000	2.19E-12
chr3	153628001	153633000	1.14E-10
chr3	153629001	153634000	4.84E-12
chr3	153630001	153635000	2.08E-09
chr3	153631001	153636000	1.14E-09
chr3	153788001	153793000	2.94E-08
chr3	153918001	153923000	6.77E-07
chr3	153919001	153924000	4.10E-07
chr3	154093001	154098000	3.40E-07
chr3	154290001	154295000	1.10E-05
chr3	154345001	154350000	1.97E-11
chr3	154346001	154351000	7.55E-09
chr3	154347001	154352000	8.15E-11
chr3	154348001	154353000	3.66E-09
chr3	154458001	154463000	2.35E-09
chr3	154569001	154574000	1.12E-13
chr3	154570001	154575000	4.86E-17
chr3	154571001	154576000	3.23E-17
chr3	154572001	154577000	7.44E-19
chr3	154573001	154578000	1.93E-21
chr3	154574001	154579000	2.05E-12
chr3	154575001	154580000	1.22E-12
chr3	154576001	154581000	4.44E-11
chr3	154585001	154590000	3.78E-09

chr3	154607001	154612000	7.06E-08
chr3	154608001	154613000	4.90E-12
chr3	154609001	154614000	7.04E-13
chr3	154610001	154615000	2.80E-13
chr3	154611001	154616000	2.38E-10
chr3	154656001	154661000	1.41E-09
chr3	154728001	154733000	1.33E-11
chr3	154763001	154768000	3.65E-13
chr3	154764001	154769000	2.93E-16
chr3	154765001	154770000	3.79E-13
chr3	154767001	154772000	2.15E-13
chr3	154851001	154856000	3.82E-17
chr3	154853001	154858000	1.67E-13
chr3	154951001	154956000	4.92E-14
chr3	155013001	155018000	0.000232
chr3	155014001	155019000	5.70E-05
chr3	155015001	155020000	2.79E-05
chr3	155064001	155069000	1.38E-08
chr3	155095001	155100000	2.48E-08
chr3	155812001	155817000	2.37E-10
chr3	155813001	155818000	3.52E-10
chr3	155884001	155889000	7.93E-13
chr3	155913001	155918000	3.98E-10
chr3	155914001	155919000	2.39E-11
chr3	155923001	155928000	9.94E-12
chr3	155999001	156004000	3.15E-13
chr3	156000001	156005000	1.22E-14
chr3	156001001	156006000	3.03E-15
chr3	156002001	156007000	1.76E-13
chr3	156065001	156070000	5.19E-05
chr3	156103001	156108000	8.24E-11
chr3	156563001	156568000	1.24E-09
chr3	156565001	156570000	4.54E-06
chr3	156581001	156586000	2.65E-09
chr3	156582001	156587000	8.60E-12
chr3	156583001	156588000	6.95E-13
chr3	156584001	156589000	1.51E-14
chr3	156585001	156590000	2.64E-13
chr3	156662001	156667000	1.67E-08
chr3	156726001	156731000	6.10E-14
chr3	156802001	156807000	6.21E-12
chr3	156928001	156933000	2.39E-15
chr3	156929001	156934000	3.31E-11
chr3	157030001	157035000	1.57E-13
chr3	157031001	157036000	1.39E-14
chr3	157076001	157081000	6.46E-14
chr3	157104001	157109000	8.08E-08
chr3	157118001	157123000	1.11E-08

chr3	157126001	157131000	8.13E-08
chr3	157130001	157135000	5.22E-05
chr3	157131001	157136000	2.98E-06
chr3	157134001	157139000	8.81E-14
chr3	157136001	157141000	9.07E-12
chr3	157137001	157142000	4.82E-11
chr3	157138001	157143000	5.32E-11
chr3	157139001	157144000	3.80E-08
chr3	157169001	157174000	3.28E-07
chr3	157170001	157175000	4.45E-08
chr3	157200001	157205000	3.07E-10
chr3	157201001	157206000	2.05E-10
chr3	157202001	157207000	7.84E-11
chr3	157203001	157208000	1.15E-10
chr3	157204001	157209000	2.63E-12
chr3	157205001	157210000	8.12E-06
chr3	157238001	157243000	6.06E-08
chr3	157252001	157257000	5.73E-13
chr3	157253001	157258000	5.61E-13
chr3	157292001	157297000	1.09E-13
chr3	157366001	157371000	1.10E-08
chr3	157367001	157372000	1.28E-07
chr3	157378001	157383000	1.61E-09
chr3	157395001	157400000	1.74E-12
chr3	157396001	157401000	3.15E-13
chr3	157398001	157403000	5.55E-11
chr3	157428001	157433000	1.37E-14
chr3	157429001	157434000	5.42E-14
chr3	157430001	157435000	1.52E-14
chr3	157458001	157463000	6.28E-09
chr3	157460001	157465000	2.33E-10
chr3	157461001	157466000	2.00E-08
chr3	157463001	157468000	2.48E-05
chr3	157487001	157492000	5.63E-11
chr3	157503001	157508000	3.36E-16
chr3	157524001	157529000	9.15E-16
chr3	157525001	157530000	4.05E-13
chr3	157526001	157531000	5.19E-11
chr3	157541001	157546000	3.86E-18
chr3	157542001	157547000	7.63E-15
chr3	157543001	157548000	1.28E-13
chr3	157575001	157580000	5.06E-11
chr3	157576001	157581000	7.64E-12
chr3	157577001	157582000	6.40E-12
chr3	157578001	157583000	9.70E-11
chr3	157579001	157584000	4.82E-11
chr3	157580001	157585000	4.11E-10
chr3	157617001	157622000	6.90E-09

chr3	157618001	157623000	8.79E-10
chr3	157620001	157625000	8.05E-09
chr3	157628001	157633000	4.05E-14
chr3	157629001	157634000	4.67E-14
chr3	157630001	157635000	4.98E-12
chr3	157701001	157706000	1.47E-12
chr3	157703001	157708000	1.47E-06
chr3	157706001	157711000	3.41E-10
chr3	157712001	157717000	3.34E-13
chr3	157713001	157718000	1.04E-12
chr3	157726001	157731000	3.20E-07
chr3	157727001	157732000	3.47E-17
chr3	157728001	157733000	1.48E-13
chr3	157729001	157734000	1.48E-15
chr3	157730001	157735000	3.04E-14
chr3	157731001	157736000	1.02E-15
chr3	157757001	157762000	8.45E-12
chr3	158348001	158353000	5.22E-09
chr3	158554001	158559000	3.25E-14
chr3	158555001	158560000	4.60E-15
chr3	158556001	158561000	1.19E-13
chr3	158595001	158600000	2.93E-08
chr3	158687001	158692000	5.76E-13
chr3	158707001	158712000	1.34E-10
chr3	158708001	158713000	2.09E-10
chr3	158729001	158734000	7.61E-13
chr3	158732001	158737000	6.85E-15
chr3	159043001	159048000	1.26E-10
chr3	159071001	159076000	3.99E-15
chr3	159116001	159121000	1.68E-08
chr3	159118001	159123000	6.50E-08
chr3	159223001	159228000	7.01E-16
chr3	159250001	159255000	1.64E-08
chr3	159251001	159256000	4.62E-10
chr3	159252001	159257000	2.76E-08
chr3	159263001	159268000	7.36E-11
chr3	159264001	159269000	6.39E-11
chr3	159286001	159291000	1.18E-12
chr3	159287001	159292000	4.82E-17
chr3	159288001	159293000	6.99E-18
chr3	159289001	159294000	9.36E-19
chr3	159290001	159295000	1.21E-15
chr3	159291001	159296000	1.95E-13
chr3	159316001	159321000	1.98E-05
chr3	159703001	159708000	6.69E-10
chr3	159763001	159768000	1.84E-05
chr3	159832001	159837000	6.15E-11
chr3	159833001	159838000	1.37E-13

chr3	159931001	159936000	7.76E-11
chr3	160330001	160335000	3.02E-07
chr3	160522001	160527000	5.87E-11
chr3	160591001	160596000	7.00E-09
chr3	160592001	160597000	3.49E-07
chr3	160839001	160844000	0.000267
chr3	160840001	160845000	7.86E-05
chr3	160841001	160846000	8.13E-05
chr3	160842001	160847000	9.81E-06
chr3	161208001	161213000	4.29E-09
chr3	161212001	161217000	2.34E-10
chr3	161213001	161218000	1.39E-11
chr3	161214001	161219000	1.44E-09
chr3	161215001	161220000	1.82E-08
chr3	161225001	161230000	3.73E-10
chr3	161241001	161246000	1.94E-16
chr3	161318001	161323000	1.71E-10
chr3	161319001	161324000	1.07E-09
chr3	161320001	161325000	1.01E-09
chr3	161321001	161326000	2.49E-09
chr3	161331001	161336000	1.06E-09
chr3	161343001	161348000	6.26E-15
chr3	161344001	161349000	2.41E-14
chr3	161372001	161377000	4.42E-09
chr3	161466001	161471000	5.79E-15
chr3	161467001	161472000	1.51E-13
chr3	161468001	161473000	5.44E-15
chr3	161469001	161474000	2.78E-12
chr3	161610001	161615000	1.50E-07
chr3	161611001	161616000	1.87E-08
chr3	161637001	161642000	2.05E-09
chr3	161638001	161643000	1.36E-09
chr3	161639001	161644000	1.18E-06
chr3	161640001	161645000	1.86E-07
chr3	161641001	161646000	3.01E-09
chr3	161766001	161771000	9.76E-07
chr3	161935001	161940000	7.44E-14
chr3	161996001	162001000	3.43E-11
chr3	162031001	162036000	4.86E-15
chr3	162047001	162052000	2.93E-15
chr3	162126001	162131000	2.50E-08
chr3	162215001	162220000	5.01E-06
chr3	162216001	162221000	3.62E-08
chr3	162217001	162222000	4.08E-07
chr3	162264001	162269000	4.32E-12
chr3	162341001	162346000	4.80E-09
chr3	162352001	162357000	9.34E-07
chr3	162353001	162358000	2.15E-05

chr3	162500001	162505000	1.88E-05
chr3	162511001	162516000	1.42E-05
chr3	162624001	162629000	9.05E-06
chr3	162630001	162635000	3.16E-12
chr3	162726001	162731000	1.41E-10
chr3	162727001	162732000	6.94E-09
chr3	162735001	162740000	1.35E-09
chr3	162755001	162760000	7.52E-14
chr3	162756001	162761000	1.96E-12
chr3	162758001	162763000	7.21E-11
chr3	162766001	162771000	3.77E-06
chr3	162781001	162786000	5.61E-07
chr3	162802001	162807000	7.94E-09
chr3	162803001	162808000	1.19E-12
chr3	162888001	162893000	2.11E-14
chr3	162916001	162921000	1.02E-08
chr3	162922001	162927000	1.22E-16
chr3	162923001	162928000	1.37E-17
chr3	162924001	162929000	9.19E-17
chr3	162925001	162930000	1.02E-16
chr3	162936001	162941000	5.63E-08
chr3	162991001	162996000	4.96E-10
chr3	162993001	162998000	1.82E-10
chr3	163062001	163067000	6.01E-13
chr3	163165001	163170000	1.01E-08
chr3	163166001	163171000	1.30E-09
chr3	163167001	163172000	1.86E-10
chr3	163225001	163230000	2.82E-08
chr3	163226001	163231000	7.44E-08
chr3	163311001	163316000	7.74E-13
chr3	163397001	163402000	1.12E-05
chr3	163398001	163403000	1.05E-06
chr3	163399001	163404000	2.37E-10
chr3	163480001	163485000	3.98E-11
chr3	163505001	163510000	5.72E-07
chr3	163551001	163556000	8.32E-09
chr3	163552001	163557000	2.63E-11
chr3	163666001	163671000	9.81E-09
chr3	163667001	163672000	1.71E-09
chr3	163670001	163675000	1.27E-12
chr3	163673001	163678000	2.20E-12
chr3	163674001	163679000	5.01E-10
chr3	163709001	163714000	2.21E-05
chr3	163710001	163715000	2.81E-08
chr3	163711001	163716000	2.12E-10
chr3	163712001	163717000	5.28E-16
chr3	163714001	163719000	3.44E-12
chr3	163898001	163903000	1.64E-07

chr3	163916001	163921000	1.08E-09
chr3	163925001	163930000	6.58E-09
chr3	163971001	163976000	1.80E-14
chr3	163973001	163978000	1.07E-09
chr3	163989001	163994000	4.74E-06
chr3	164063001	164068000	1.12E-10
chr3	164064001	164069000	6.29E-09
chr3	164246001	164251000	3.62E-11
chr3	164587001	164592000	3.20E-11
chr3	164946001	164951000	2.01E-10
chr3	164950001	164955000	6.24E-15
chr3	164994001	164999000	4.01E-15
chr3	164995001	165000000	9.53E-15
chr3	165095001	165100000	2.15E-13
chr3	165096001	165101000	3.65E-15
chr3	165104001	165109000	4.49E-11
chr3	165183001	165188000	3.07E-12
chr3	165184001	165189000	5.95E-10
chr3	166318001	166323000	7.84E-16
chr3	166320001	166325000	9.99E-16
chr3	166325001	166330000	4.29E-07
chr3	166354001	166359000	1.76E-09
chr3	166715001	166720000	2.39E-05
chr3	166837001	166842000	2.95E-06
chr3	166847001	166852000	1.76E-14
chr3	166848001	166853000	4.29E-12
chr3	166849001	166854000	7.35E-11
chr3	166850001	166855000	5.15E-11
chr3	166851001	166856000	1.24E-07
chr3	167023001	167028000	1.19E-05
chr3	167135001	167140000	2.78E-07
chr3	167136001	167141000	9.76E-09
chr3	167480001	167485000	1.03E-06
chr3	168009001	168014000	2.73E-10
chr3	168112001	168117000	4.28E-09
chr3	169532001	169537000	7.22E-13
chr3	169533001	169538000	1.28E-10
chr3	169564001	169569000	3.59E-12
chr3	170703001	170708000	3.39E-08
chr3	172604001	172609000	1.38E-07
chr3	172605001	172610000	2.28E-07
chr3	172606001	172611000	9.54E-09
chr3	172612001	172617000	1.30E-16
chr3	172613001	172618000	2.39E-14
chr3	172614001	172619000	5.73E-16
chr3	172661001	172666000	4.53E-13
chr3	172662001	172667000	1.09E-11
chr3	172681001	172686000	2.17E-19

chr3	172711001	172716000	5.93E-15
chr3	172727001	172732000	1.41E-14
chr3	172729001	172734000	2.36E-09
chr3	172730001	172735000	1.83E-07
chr3	172731001	172736000	1.22E-08
chr3	172764001	172769000	1.49E-15
chr3	172789001	172794000	6.24E-07
chr3	172790001	172795000	1.54E-11
chr3	172823001	172828000	5.35E-13
chr3	172839001	172844000	2.34E-16
chr3	172864001	172869000	2.38E-09
chr3	172865001	172870000	1.68E-10
chr3	172866001	172871000	4.69E-11
chr3	172867001	172872000	4.21E-11
chr3	172898001	172903000	3.95E-09
chr3	172899001	172904000	8.33E-08
chr3	172909001	172914000	6.88E-09
chr3	172912001	172917000	2.41E-09
chr3	173046001	173051000	1.44E-14
chr3	173065001	173070000	7.24E-09
chr3	173066001	173071000	1.38E-08
chr3	173224001	173229000	6.78E-17
chr3	173225001	173230000	1.66E-16
chr3	173226001	173231000	4.20E-16
chr3	173227001	173232000	3.10E-17
chr3	173228001	173233000	5.33E-13
chr3	173310001	173315000	1.22E-10
chr3	173312001	173317000	4.24E-11
chr3	173341001	173346000	1.57E-13
chr3	173359001	173364000	4.62E-14
chr3	173360001	173365000	4.66E-10
chr3	173362001	173367000	6.05E-07
chr3	173443001	173448000	2.30E-09
chr3	173466001	173471000	9.51E-13
chr3	173467001	173472000	8.15E-15
chr3	173468001	173473000	8.02E-14
chr3	173469001	173474000	7.68E-13
chr3	173470001	173475000	3.59E-13
chr3	173509001	173514000	3.31E-13
chr3	173510001	173515000	1.34E-16
chr3	173511001	173516000	2.53E-19
chr3	173512001	173517000	5.13E-17
chr3	173513001	173518000	6.52E-19
chr3	173514001	173519000	3.60E-15
chr3	173520001	173525000	3.09E-20
chr3	173521001	173526000	3.45E-23
chr3	173522001	173527000	5.61E-23
chr3	173558001	173563000	3.93E-07

chr3	173567001	173572000	8.61E-16
chr3	173568001	173573000	1.36E-14
chr3	173569001	173574000	3.70E-17
chr3	173570001	173575000	1.28E-13
chr3	173611001	173616000	2.71E-10
chr3	173621001	173626000	1.46E-16
chr3	173622001	173627000	1.66E-16
chr3	173646001	173651000	9.95E-11
chr3	173647001	173652000	5.80E-10
chr3	173649001	173654000	3.35E-08
chr3	173691001	173696000	1.36E-12
chr3	173692001	173697000	5.72E-14
chr3	173693001	173698000	1.36E-14
chr3	173694001	173699000	6.34E-14
chr3	173722001	173727000	2.07E-18
chr3	173723001	173728000	3.87E-21
chr3	173724001	173729000	1.15E-15
chr3	173775001	173780000	1.56E-11
chr3	173781001	173786000	3.66E-11
chr3	173823001	173828000	9.79E-16
chr3	173877001	173882000	1.41E-17
chr3	173878001	173883000	2.73E-14
chr3	173897001	173902000	7.36E-09
chr3	174011001	174016000	2.84E-13
chr3	175745001	175750000	2.59E-16
chr3	175746001	175751000	3.74E-14
chr3	175909001	175914000	1.60E-07
chr3	175910001	175915000	1.40E-09
chr3	175911001	175916000	1.40E-09
chr3	175912001	175917000	1.29E-10
chr3	175938001	175943000	8.44E-09
chr3	176107001	176112000	0.000305
chr3	176201001	176206000	1.07E-08
chr3	176239001	176244000	1.98E-13
chr3	176240001	176245000	1.47E-13
chr3	176241001	176246000	1.71E-14
chr3	176242001	176247000	1.60E-11
chr3	176338001	176343000	4.38E-12
chr3	176363001	176368000	3.17E-08
chr3	176364001	176369000	4.59E-11
chr3	176365001	176370000	1.08E-09
chr3	176366001	176371000	5.21E-09
chr3	176367001	176372000	6.45E-06
chr3	176368001	176373000	6.37E-08
chr3	176371001	176376000	6.67E-09
chr3	176393001	176398000	3.39E-08
chr3	176394001	176399000	2.98E-07
chr3	176437001	176442000	1.46E-12

chr3	176481001	176486000	4.41E-09
chr3	176482001	176487000	4.19E-10
chr3	176483001	176488000	2.15E-07
chr3	176568001	176573000	3.49E-08
chr3	176674001	176679000	1.10E-11
chr3	176675001	176680000	3.38E-13
chr3	176676001	176681000	1.32E-13
chr3	176677001	176682000	4.32E-14
chr3	178183001	178188000	5.17E-08
chr3	178184001	178189000	1.10E-08
chr3	178706001	178711000	4.81E-07
chr3	179194001	179199000	8.71E-18
chr3	179195001	179200000	6.08E-19
chr3	179196001	179201000	2.25E-16
chr3	179562001	179567000	2.50E-16
chr3	179628001	179633000	1.64E-08
chr3	179629001	179634000	1.15E-11
chr3	179630001	179635000	3.96E-12
chr3	179714001	179719000	2.01E-10
chr3	179728001	179733000	7.36E-21
chr3	179839001	179844000	2.07E-07
chr3	179874001	179879000	4.79E-09
chr3	179889001	179894000	3.06E-10
chr3	179890001	179895000	8.72E-14
chr3	179891001	179896000	7.77E-13
chr3	179892001	179897000	3.81E-10
chr3	179893001	179898000	2.39E-09
chr3	179909001	179914000	6.04E-16
chr3	180002001	180007000	2.53E-09
chr3	180003001	180008000	8.21E-09
chr3	180004001	180009000	2.09E-07
chr3	180005001	180010000	1.73E-09
chr3	180006001	180011000	2.10E-07
chr3	180083001	180088000	2.36E-10
chr3	180114001	180119000	9.44E-09
chr3	180115001	180120000	6.93E-12
chr3	180116001	180121000	6.12E-11
chr3	180117001	180122000	1.17E-09
chr3	180132001	180137000	5.80E-06
chr3	180133001	180138000	1.57E-07
chr3	180160001	180165000	3.44E-09
chr3	180161001	180166000	1.47E-12
chr3	180191001	180196000	2.07E-10
chr3	180216001	180221000	5.83E-09
chr3	180237001	180242000	8.55E-10
chr3	180238001	180243000	6.40E-11
chr3	180240001	180245000	9.53E-11
chr3	180289001	180294000	2.88E-15

chr3	180290001	180295000	8.81E-19
chr3	180291001	180296000	1.66E-18
chr3	180292001	180297000	5.21E-16
chr3	180418001	180423000	5.40E-11
chr3	180447001	180452000	1.23E-10
chr3	180448001	180453000	2.02E-11
chr3	180483001	180488000	5.58E-08
chr3	180484001	180489000	9.06E-11
chr3	180485001	180490000	5.40E-11
chr3	180555001	180560000	6.73E-09
chr3	180574001	180579000	9.38E-08
chr3	180575001	180580000	2.87E-08
chr3	180576001	180581000	3.80E-10
chr3	180598001	180603000	5.48E-10
chr3	180723001	180728000	7.90E-09
chr3	180736001	180741000	3.42E-10
chr3	180737001	180742000	2.21E-13
chr3	180738001	180743000	2.44E-10
chr3	180739001	180744000	5.78E-12
chr3	180740001	180745000	4.27E-12
chr3	180743001	180748000	3.97E-09
chr3	180744001	180749000	1.77E-10
chr3	180745001	180750000	2.29E-10
chr3	180746001	180751000	5.18E-14
chr3	180747001	180752000	7.34E-13
chr3	180748001	180753000	6.67E-12
chr3	180749001	180754000	1.27E-12
chr3	180756001	180761000	6.79E-09
chr3	180757001	180762000	2.99E-09
chr3	180758001	180763000	1.64E-09
chr3	180815001	180820000	2.13E-10
chr3	180841001	180846000	8.33E-09
chr3	180849001	180854000	4.40E-08
chr3	180972001	180977000	1.60E-06
chr3	181011001	181016000	3.04E-14
chr3	181013001	181018000	2.75E-15
chr3	181152001	181157000	8.43E-17
chr3	181153001	181158000	1.01E-15
chr3	181477001	181482000	1.47E-10
chr3	181478001	181483000	1.08E-10
chr3	183161001	183166000	2.16E-19
chr3	183207001	183212000	5.75E-12
chr3	183692001	183697000	1.43E-06
chr3	183707001	183712000	2.48E-12
chr3	183732001	183737000	2.86E-13
chr3	183733001	183738000	1.94E-18
chr3	183734001	183739000	6.59E-20
chr3	183735001	183740000	6.43E-19

chr3	183872001	183877000	1.26E-06
chr3	184012001	184017000	4.19E-11
chr3	184056001	184061000	1.05E-10
chr3	184057001	184062000	3.54E-09
chr3	184076001	184081000	1.21E-11
chr3	185265001	185270000	0.000753
chr3	186007001	186012000	1.30E-13
chr3	186017001	186022000	3.05E-08
chr3	186018001	186023000	5.10E-11
chr3	186019001	186024000	1.47E-14
chr3	186020001	186025000	1.68E-07
chr3	186040001	186045000	9.64E-15
chr3	186041001	186046000	4.64E-15
chr3	186042001	186047000	4.57E-09
chr3	186043001	186048000	3.15E-09
chr3	186055001	186060000	2.31E-11
chr3	186067001	186072000	1.13E-10
chr3	186098001	186103000	1.38E-09
chr3	186146001	186151000	1.88E-11
chr3	186147001	186152000	1.26E-14
chr3	186148001	186153000	6.35E-19
chr3	186149001	186154000	2.92E-18
chr3	186150001	186155000	2.66E-18
chr3	186526001	186531000	6.27E-05
chr3	186527001	186532000	0.003296
chr3	186547001	186552000	3.75E-08
chr3	187027001	187032000	4.23E-06
chr3	187261001	187266000	1.32E-08
chr3	187268001	187273000	1.20E-09
chr3	187284001	187289000	3.79E-09
chr3	187285001	187290000	1.77E-09
chr3	187286001	187291000	2.29E-08
chr3	187287001	187292000	1.58E-07
chr3	187321001	187326000	8.54E-15
chr3	187324001	187329000	5.56E-08
chr3	187325001	187330000	3.39E-08
chr3	187326001	187331000	1.62E-06
chr3	187327001	187332000	1.39E-08
chr3	187328001	187333000	3.89E-07
chr3	187607001	187612000	5.92E-15
chr3	187608001	187613000	8.47E-12
chr3	187960001	187965000	1.08E-10
chr3	190647001	190652000	1.34E-14
chr3	190667001	190672000	8.95E-07
chr3	190682001	190687000	8.02E-11
chr3	190683001	190688000	1.66E-10
chr3	190878001	190883000	9.91E-09
chr3	190929001	190934000	3.13E-11

chr3	190930001	190935000	8.68E-12
chr3	191249001	191254000	7.42E-08
chr3	191250001	191255000	1.72E-08
chr3	191251001	191256000	2.12E-10
chr3	191257001	191262000	1.06E-13
chr3	191259001	191264000	6.78E-16
chr3	191260001	191265000	4.23E-14
chr3	191261001	191266000	1.89E-09
chr3	191303001	191308000	1.60E-12
chr3	191312001	191317000	1.20E-27
chr3	191313001	191318000	1.25E-26
chr3	191314001	191319000	7.22E-28
chr3	191315001	191320000	6.11E-27
chr3	191316001	191321000	2.18E-17
chr3	191339001	191344000	5.83E-10
chr3	191366001	191371000	1.73E-08
chr3	191367001	191372000	4.33E-08
chr3	191370001	191375000	2.03E-09
chr3	191371001	191376000	3.87E-06
chr3	191416001	191421000	1.10E-09
chr3	191417001	191422000	8.60E-11
chr3	191418001	191423000	1.29E-11
chr3	191431001	191436000	1.28E-15
chr3	191440001	191445000	7.17E-09
chr3	191441001	191446000	1.36E-09
chr3	191442001	191447000	1.09E-09
chr3	191454001	191459000	1.82E-11
chr3	191468001	191473000	7.61E-11
chr3	191521001	191526000	6.38E-07
chr3	191522001	191527000	5.82E-08
chr3	191523001	191528000	6.98E-08
chr3	191524001	191529000	2.08E-07
chr3	191539001	191544000	1.28E-16
chr3	191540001	191545000	3.55E-20
chr3	191541001	191546000	8.21E-14
chr3	191542001	191547000	1.25E-15
chr3	191597001	191602000	9.98E-15
chr3	191598001	191603000	1.40E-17
chr3	191623001	191628000	6.70E-12
chr3	191624001	191629000	4.57E-10
chr3	191650001	191655000	2.23E-16
chr3	191664001	191669000	7.22E-14
chr3	191665001	191670000	3.37E-13
chr3	191674001	191679000	1.89E-13
chr3	191694001	191699000	5.92E-07
chr3	191695001	191700000	4.90E-06
chr3	191736001	191741000	3.81E-06
chr3	191737001	191742000	2.47E-06

chr3	191761001	191766000	6.91E-08
chr3	191866001	191871000	2.80E-05
chr3	191953001	191958000	3.42E-13
chr3	191954001	191959000	3.99E-14
chr3	192090001	192095000	1.23E-18
chr3	192091001	192096000	1.64E-18
chr3	192166001	192171000	3.60E-18
chr3	192236001	192241000	4.80E-19
chr3	192237001	192242000	2.16E-18
chr3	192252001	192257000	7.80E-13
chr3	192785001	192790000	2.77E-14
chr3	192786001	192791000	8.83E-11
chr3	192788001	192793000	2.59E-06
chr3	192789001	192794000	2.34E-06
chr3	192860001	192865000	1.62E-11
chr3	192875001	192880000	6.70E-05
chr3	192881001	192886000	0.019042
chr3	193544001	193549000	1.32E-12
chr3	193545001	193550000	1.19E-15
chr3	193546001	193551000	9.39E-16
chr3	194036001	194041000	3.50E-09
chr3	194239001	194244000	2.00E-06
chr3	194240001	194245000	2.69E-05
chr3	194241001	194246000	3.31E-05
chr3	194303001	194308000	2.26E-10
chr3	194304001	194309000	4.00E-07
chr3	194351001	194356000	1.32E-07
chr3	194352001	194357000	4.82E-14
chr3	194353001	194358000	1.54E-16
chr3	195173001	195178000	4.06E-06
chr3	195600001	195605000	3.51E-05
chr3	195647001	195652000	6.32E-23
chr3	195648001	195653000	2.78E-34
chr3	195649001	195654000	8.24E-14
chr3	195804001	195809000	2.22E-18
chr3	195833001	195838000	0.00062
chr3	195909001	195914000	4.53E-13
chr3	195910001	195915000	1.33E-12
chr3	195913001	195918000	1.46E-08
chr3	196224001	196229000	2.49E-10
chr3	196251001	196256000	3.98E-10
chr3	196725001	196730000	7.63E-13
chr3	196726001	196731000	2.75E-14
chr3	196727001	196732000	1.61E-17
chr3	196728001	196733000	8.99E-15
chr3	196729001	196734000	1.31E-09
chr3	196754001	196759000	0.001975
chr3	197237001	197242000	2.32E-08

chr3	197243001	197248000	6.46E-12
chr3	197244001	197249000	1.17E-13
chr3	197245001	197250000	3.55E-11
chr3	197327001	197332000	2.91E-13
chr3	197461001	197466000	1.12E-19
chr3	197462001	197467000	3.68E-21
chr3	197463001	197468000	1.60E-12
chr3	197634001	197639000	6.52E-16
chr3	197635001	197640000	2.15E-23
chr3	197636001	197641000	5.29E-28
chr3	197637001	197642000	6.58E-23
chr3	197638001	197643000	1.50E-18
chr4	98001	103000	2.94E-06
chr4	113001	118000	2.08E-05
chr4	115001	120000	5.29E-05
chr4	300001	305000	5.51E-10
chr4	301001	306000	4.61E-07
chr4	603001	608000	4.04E-09
chr4	621001	626000	0.000493
chr4	622001	627000	1.16E-05
chr4	624001	629000	6.12E-07
chr4	628001	633000	3.39E-09
chr4	629001	634000	1.02E-06
chr4	654001	659000	7.96E-11
chr4	655001	660000	2.08E-09
chr4	656001	661000	1.77E-07
chr4	657001	662000	2.20E-12
chr4	658001	663000	7.77E-06
chr4	659001	664000	0.018661
chr4	670001	675000	0.002757
chr4	671001	676000	0.002757
chr4	677001	682000	0.002876
chr4	678001	683000	0.00032
chr4	679001	684000	1.90E-07
chr4	697001	702000	6.01E-05
chr4	698001	703000	1.74E-07
chr4	815001	820000	1.61E-05
chr4	816001	821000	4.09E-05
chr4	922001	927000	0.000246
chr4	996001	1001000	7.58E-06
chr4	1033001	1038000	0.004557
chr4	1034001	1039000	4.15E-05
chr4	1035001	1040000	2.50E-05
chr4	1036001	1041000	2.60E-08
chr4	1037001	1042000	2.31E-14
chr4	1038001	1043000	5.21E-23
chr4	1039001	1044000	1.07E-14
chr4	1040001	1045000	6.11E-12

chr4	1050001	1055000	6.87E-08
chr4	1072001	1077000	2.14E-08
chr4	1279001	1284000	7.63E-17
chr4	1280001	1285000	3.49E-43
chr4	1281001	1286000	6.33E-42
chr4	1282001	1287000	3.51E-31
chr4	1283001	1288000	1.36E-06
chr4	1476001	1481000	0.000705
chr4	1477001	1482000	3.48E-06
chr4	1478001	1483000	0.000114
chr4	1479001	1484000	1.32E-05
chr4	1486001	1491000	2.39E-05
chr4	1487001	1492000	2.61E-05
chr4	1488001	1493000	1.20E-06
chr4	1509001	1514000	1.16E-07
chr4	1510001	1515000	7.66E-12
chr4	1511001	1516000	1.72E-12
chr4	1512001	1517000	4.61E-10
chr4	1513001	1518000	5.43E-09
chr4	1540001	1545000	3.05E-07
chr4	1541001	1546000	0.000161
chr4	1544001	1549000	0.003196
chr4	1564001	1569000	0.000671
chr4	1565001	1570000	1.12E-05
chr4	1597001	1602000	0.003044
chr4	1615001	1620000	2.59E-07
chr4	1616001	1621000	5.41E-08
chr4	1617001	1622000	2.98E-09
chr4	1618001	1623000	1.20E-14
chr4	1619001	1624000	6.50E-06
chr4	1667001	1672000	1.53E-06
chr4	1723001	1728000	2.20E-07
chr4	1757001	1762000	0.001943
chr4	1758001	1763000	0.007553
chr4	1759001	1764000	0.003523
chr4	1760001	1765000	0.007666
chr4	1800001	1805000	0.000117
chr4	1804001	1809000	1.10E-05
chr4	2263001	2268000	0.000422
chr4	2318001	2323000	2.88E-09
chr4	2319001	2324000	2.23E-11
chr4	2370001	2375000	3.17E-07
chr4	2400001	2405000	8.55E-05
chr4	2443001	2448000	0.00084
chr4	2761001	2766000	0.000437
chr4	2813001	2818000	0.00044
chr4	2814001	2819000	8.43E-05
chr4	2815001	2820000	8.95E-05

chr4	2932001	2937000	0.000599
chr4	2935001	2940000	1.79E-16
chr4	2936001	2941000	1.18E-16
chr4	3308001	3313000	0.000103
chr4	3309001	3314000	1.23E-06
chr4	3737001	3742000	1.31E-09
chr4	3738001	3743000	5.44E-08
chr4	3817001	3822000	0.000224
chr4	3877001	3882000	7.67E-07
chr4	4338001	4343000	4.93E-09
chr4	4339001	4344000	2.91E-07
chr4	4340001	4345000	8.94E-07
chr4	4341001	4346000	3.53E-09
chr4	4385001	4390000	0.000294
chr4	4393001	4398000	5.02E-09
chr4	4394001	4399000	2.44E-08
chr4	4395001	4400000	6.02E-09
chr4	4396001	4401000	6.36E-09
chr4	4397001	4402000	2.99E-08
chr4	4406001	4411000	1.25E-05
chr4	4407001	4412000	1.83E-06
chr4	4827001	4832000	7.54E-05
chr4	4917001	4922000	0.001166
chr4	4975001	4980000	0.001181
chr4	4976001	4981000	0.000232
chr4	4977001	4982000	3.49E-07
chr4	5025001	5030000	1.41E-05
chr4	5708001	5713000	6.12E-07
chr4	5872001	5877000	1.83E-09
chr4	5945001	5950000	9.05E-06
chr4	6241001	6246000	3.19E-05
chr4	6242001	6247000	2.85E-09
chr4	6243001	6248000	4.74E-10
chr4	6244001	6249000	5.01E-11
chr4	6245001	6250000	6.76E-07
chr4	6325001	6330000	4.08E-07
chr4	6327001	6332000	1.49E-09
chr4	6352001	6357000	9.87E-05
chr4	6353001	6358000	0.001515
chr4	6372001	6377000	8.25E-05
chr4	6575001	6580000	0.004691
chr4	6909001	6914000	7.97E-09
chr4	7118001	7123000	6.85E-06
chr4	7119001	7124000	2.99E-07
chr4	7120001	7125000	3.65E-07
chr4	7121001	7126000	3.41E-06
chr4	7147001	7152000	7.34E-06
chr4	7192001	7197000	0.000112

chr4	7425001	7430000	0.02328
chr4	8164001	8169000	6.25E-06
chr4	8239001	8244000	5.64E-05
chr4	8610001	8615000	0.000404
chr4	8656001	8661000	1.16E-09
chr4	8667001	8672000	1.38E-10
chr4	8668001	8673000	8.90E-09
chr4	8684001	8689000	3.25E-05
chr4	8685001	8690000	1.59E-05
chr4	8686001	8691000	6.14E-08
chr4	8699001	8704000	6.87E-08
chr4	8700001	8705000	1.23E-06
chr4	8823001	8828000	5.13E-07
chr4	8918001	8923000	1.40E-07
chr4	8919001	8924000	6.70E-08
chr4	9066001	9071000	2.20E-05
chr4	9088001	9093000	2.48E-06
chr4	9153001	9158000	5.13E-06
chr4	9154001	9159000	5.09E-07
chr4	9155001	9160000	2.43E-07
chr4	9464001	9469000	3.47E-07
chr4	9465001	9470000	7.90E-08
chr4	9466001	9471000	2.99E-06
chr4	9547001	9552000	1.23E-05
chr4	9557001	9562000	0.000138
chr4	9578001	9583000	0.005641
chr4	9591001	9596000	0.000175
chr4	9592001	9597000	0.000382
chr4	9659001	9664000	7.82E-08
chr4	9777001	9782000	1.75E-10
chr4	10159001	10164000	1.51E-10
chr4	10161001	10166000	1.50E-09
chr4	10225001	10230000	3.75E-05
chr4	10226001	10231000	5.41E-07
chr4	10227001	10232000	1.36E-06
chr4	10228001	10233000	4.07E-08
chr4	10229001	10234000	3.53E-08
chr4	10230001	10235000	1.66E-05
chr4	12024001	12029000	1.55E-13
chr4	12192001	12197000	2.36E-09
chr4	12250001	12255000	3.21E-05
chr4	12251001	12256000	2.63E-05
chr4	12344001	12349000	0.00016
chr4	12488001	12493000	3.72E-08
chr4	12689001	12694000	1.24E-05
chr4	12833001	12838000	0.000209
chr4	12994001	12999000	8.62E-05
chr4	13138001	13143000	9.44E-06

chr4	13768001	13773000	6.56E-07
chr4	13776001	13781000	0.003382
chr4	14553001	14558000	2.69E-09
chr4	14783001	14788000	4.25E-09
chr4	14784001	14789000	1.33E-08
chr4	14836001	14841000	0.00255
chr4	15788001	15793000	0.000374
chr4	15789001	15794000	0.001649
chr4	16452001	16457000	1.86E-07
chr4	16453001	16458000	1.17E-08
chr4	16454001	16459000	5.91E-08
chr4	16455001	16460000	1.42E-07
chr4	16615001	16620000	1.44E-09
chr4	16616001	16621000	4.69E-08
chr4	16632001	16637000	8.74E-10
chr4	16633001	16638000	6.20E-10
chr4	16634001	16639000	1.57E-12
chr4	16635001	16640000	8.24E-12
chr4	16636001	16641000	3.22E-12
chr4	16725001	16730000	5.33E-14
chr4	16726001	16731000	2.29E-16
chr4	16727001	16732000	1.51E-16
chr4	16766001	16771000	1.16E-10
chr4	16767001	16772000	3.85E-10
chr4	16782001	16787000	1.19E-08
chr4	16861001	16866000	4.69E-12
chr4	16905001	16910000	3.99E-09
chr4	16908001	16913000	1.89E-10
chr4	16959001	16964000	8.47E-08
chr4	16960001	16965000	1.72E-07
chr4	16961001	16966000	4.88E-06
chr4	16966001	16971000	2.17E-07
chr4	17018001	17023000	5.23E-09
chr4	17019001	17024000	1.97E-08
chr4	17020001	17025000	1.51E-09
chr4	17021001	17026000	1.66E-09
chr4	17050001	17055000	6.68E-09
chr4	17093001	17098000	9.48E-07
chr4	17094001	17099000	9.40E-11
chr4	17095001	17100000	3.14E-11
chr4	17096001	17101000	6.13E-12
chr4	17097001	17102000	1.21E-11
chr4	17161001	17166000	1.94E-06
chr4	17162001	17167000	1.01E-05
chr4	17179001	17184000	0.000155
chr4	17181001	17186000	1.66E-07
chr4	17182001	17187000	1.41E-06
chr4	17203001	17208000	1.93E-08

chr4	17206001	17211000	8.35E-11
chr4	17208001	17213000	2.16E-10
chr4	17209001	17214000	6.88E-09
chr4	17210001	17215000	2.51E-11
chr4	17211001	17216000	2.35E-08
chr4	17212001	17217000	2.82E-08
chr4	17216001	17221000	0.000887
chr4	17217001	17222000	0.000673
chr4	17218001	17223000	0.000376
chr4	17219001	17224000	3.29E-06
chr4	17220001	17225000	4.59E-06
chr4	17221001	17226000	0.000466
chr4	17386001	17391000	7.57E-05
chr4	17405001	17410000	4.40E-05
chr4	17406001	17411000	4.25E-08
chr4	17407001	17412000	4.54E-07
chr4	17408001	17413000	6.52E-05
chr4	17438001	17443000	1.56E-05
chr4	17439001	17444000	1.96E-05
chr4	17447001	17452000	1.94E-07
chr4	17448001	17453000	1.27E-07
chr4	17449001	17454000	3.50E-08
chr4	17450001	17455000	6.64E-07
chr4	17574001	17579000	1.72E-06
chr4	17577001	17582000	3.14E-08
chr4	17578001	17583000	5.47E-07
chr4	17613001	17618000	6.92E-07
chr4	17646001	17651000	0.002761
chr4	17647001	17652000	0.000922
chr4	17648001	17653000	9.47E-05
chr4	17649001	17654000	7.61E-06
chr4	17650001	17655000	0.000991
chr4	18071001	18076000	1.49E-09
chr4	18072001	18077000	8.61E-11
chr4	18073001	18078000	1.65E-09
chr4	18074001	18079000	7.44E-10
chr4	18227001	18232000	2.01E-05
chr4	18248001	18253000	2.03E-08
chr4	18255001	18260000	4.36E-11
chr4	18262001	18267000	1.43E-05
chr4	18325001	18330000	5.81E-05
chr4	18831001	18836000	3.86E-07
chr4	18832001	18837000	1.18E-06
chr4	19642001	19647000	9.85E-06
chr4	19972001	19977000	9.54E-07
chr4	19973001	19978000	4.74E-05
chr4	21777001	21782000	1.72E-05
chr4	21778001	21783000	2.07E-06

chr4	21779001	21784000	9.75E-05
chr4	21780001	21785000	3.97E-05
chr4	21986001	21991000	7.95E-07
chr4	22138001	22143000	6.13E-07
chr4	22182001	22187000	3.08E-09
chr4	23415001	23420000	1.10E-08
chr4	23512001	23517000	7.73E-13
chr4	23513001	23518000	1.22E-10
chr4	24293001	24298000	5.27E-09
chr4	24294001	24299000	1.98E-07
chr4	24330001	24335000	4.01E-10
chr4	24331001	24336000	7.53E-08
chr4	24343001	24348000	2.81E-07
chr4	24953001	24958000	0.000483
chr4	25556001	25561000	0.000444
chr4	25985001	25990000	2.03E-09
chr4	25986001	25991000	1.42E-09
chr4	25987001	25992000	5.56E-11
chr4	25988001	25993000	4.57E-10
chr4	27196001	27201000	5.66E-05
chr4	27335001	27340000	7.89E-14
chr4	27482001	27487000	1.23E-07
chr4	28202001	28207000	4.08E-05
chr4	28203001	28208000	0.000461
chr4	28205001	28210000	7.32E-05
chr4	28206001	28211000	1.66E-05
chr4	28557001	28562000	2.38E-08
chr4	28764001	28769000	5.09E-05
chr4	29591001	29596000	1.76E-08
chr4	29592001	29597000	5.98E-07
chr4	29593001	29598000	7.23E-07
chr4	30504001	30509000	7.40E-08
chr4	30677001	30682000	6.52E-08
chr4	32839001	32844000	1.13E-05
chr4	33186001	33191000	5.29E-05
chr4	33500001	33505000	2.65E-09
chr4	33502001	33507000	1.80E-08
chr4	33503001	33508000	1.49E-06
chr4	33595001	33600000	2.75E-06
chr4	34323001	34328000	2.15E-07
chr4	34324001	34329000	1.22E-07
chr4	34325001	34330000	3.76E-08
chr4	34326001	34331000	2.35E-06
chr4	34553001	34558000	7.28E-13
chr4	34554001	34559000	3.55E-10
chr4	34555001	34560000	1.07E-12
chr4	34780001	34785000	2.30E-05
chr4	34781001	34786000	9.15E-08

chr4	36705001	36710000	0.000395
chr4	37825001	37830000	3.74E-15
chr4	37826001	37831000	4.54E-18
chr4	37827001	37832000	9.28E-19
chr4	37828001	37833000	1.25E-23
chr4	39538001	39543000	4.00E-07
chr4	39681001	39686000	6.24E-05
chr4	39813001	39818000	4.42E-06
chr4	39814001	39819000	1.35E-05
chr4	40625001	40630000	1.25E-08
chr4	41975001	41980000	1.71E-05
chr4	43174001	43179000	4.99E-05
chr4	43304001	43309000	4.20E-08
chr4	43305001	43310000	7.48E-13
chr4	43447001	43452000	1.09E-08
chr4	43448001	43453000	5.29E-09
chr4	43450001	43455000	3.79E-09
chr4	43553001	43558000	9.10E-06
chr4	43593001	43598000	2.04E-10
chr4	43594001	43599000	4.80E-08
chr4	43595001	43600000	1.70E-11
chr4	43615001	43620000	1.13E-08
chr4	43683001	43688000	1.11E-05
chr4	44041001	44046000	9.39E-06
chr4	44199001	44204000	9.66E-05
chr4	44200001	44205000	3.95E-06
chr4	44464001	44469000	9.17E-08
chr4	44466001	44471000	7.82E-09
chr4	44493001	44498000	2.24E-06
chr4	44587001	44592000	9.16E-06
chr4	44890001	44895000	6.46E-08
chr4	44964001	44969000	0.000199
chr4	45018001	45023000	2.92E-06
chr4	45055001	45060000	3.84E-12
chr4	45126001	45131000	3.67E-11
chr4	45313001	45318000	0.00057
chr4	45314001	45319000	2.82E-06
chr4	45381001	45386000	1.42E-07
chr4	45438001	45443000	4.72E-05
chr4	45882001	45887000	3.50E-07
chr4	45883001	45888000	2.91E-12
chr4	45884001	45889000	7.11E-10
chr4	46012001	46017000	2.87E-08
chr4	46013001	46018000	2.13E-09
chr4	46015001	46020000	1.47E-08
chr4	46148001	46153000	1.55E-10
chr4	46179001	46184000	0.000489
chr4	46455001	46460000	1.99E-10

chr4	46456001	46461000	5.36E-09
chr4	46457001	46462000	4.12E-09
chr4	46458001	46463000	6.53E-07
chr4	46527001	46532000	4.61E-08
chr4	46528001	46533000	4.81E-10
chr4	46621001	46626000	3.32E-11
chr4	46622001	46627000	8.55E-12
chr4	46623001	46628000	4.59E-12
chr4	46624001	46629000	2.01E-13
chr4	46625001	46630000	5.53E-13
chr4	46626001	46631000	1.49E-12
chr4	46631001	46636000	2.99E-13
chr4	46632001	46637000	2.93E-12
chr4	46633001	46638000	9.56E-12
chr4	46641001	46646000	8.98E-06
chr4	46642001	46647000	6.81E-07
chr4	46643001	46648000	4.99E-08
chr4	46644001	46649000	9.18E-07
chr4	46645001	46650000	0.008247
chr4	46674001	46679000	1.96E-08
chr4	46675001	46680000	1.72E-10
chr4	46676001	46681000	6.04E-07
chr4	46714001	46719000	1.29E-07
chr4	47259001	47264000	3.96E-06
chr4	47261001	47266000	2.79E-07
chr4	47279001	47284000	1.66E-06
chr4	47311001	47316000	0.002462
chr4	47342001	47347000	1.52E-07
chr4	47343001	47348000	1.27E-07
chr4	47344001	47349000	3.14E-05
chr4	47364001	47369000	4.85E-07
chr4	47371001	47376000	4.91E-08
chr4	47372001	47377000	6.82E-07
chr4	47373001	47378000	1.23E-07
chr4	47396001	47401000	1.37E-09
chr4	47397001	47402000	8.90E-12
chr4	47398001	47403000	4.90E-09
chr4	47399001	47404000	3.60E-05
chr4	47612001	47617000	1.10E-06
chr4	47613001	47618000	7.43E-06
chr4	47782001	47787000	1.30E-06
chr4	47783001	47788000	1.04E-06
chr4	47784001	47789000	1.65E-08
chr4	48018001	48023000	6.42E-06
chr4	48055001	48060000	0.000251
chr4	48058001	48063000	4.95E-07
chr4	48061001	48066000	0.000116
chr4	48338001	48343000	5.23E-14

chr4	48339001	48344000	1.97E-20
chr4	48904001	48909000	2.79E-09
chr4	48905001	48910000	1.17E-08
chr4	48906001	48911000	4.66E-10
chr4	48907001	48912000	1.01E-08
chr4	48908001	48913000	2.91E-08
chr4	49283001	49288000	1.94E-06
chr4	49284001	49289000	2.96E-18
chr4	49285001	49290000	2.37E-37
chr4	53137001	53142000	8.67E-06
chr4	53138001	53143000	3.96E-07
chr4	53202001	53207000	0.000112
chr4	53203001	53208000	9.86E-05
chr4	53218001	53223000	1.69E-07
chr4	53525001	53530000	5.69E-06
chr4	53526001	53531000	7.63E-05
chr4	54150001	54155000	0.001827
chr4	54454001	54459000	2.10E-08
chr4	54455001	54460000	1.72E-16
chr4	54456001	54461000	2.98E-19
chr4	54457001	54462000	6.03E-22
chr4	55004001	55009000	1.41E-07
chr4	55005001	55010000	1.34E-07
chr4	55006001	55011000	9.20E-08
chr4	55007001	55012000	9.82E-07
chr4	55008001	55013000	1.45E-06
chr4	55099001	55104000	1.76E-08
chr4	55100001	55105000	1.10E-09
chr4	55188001	55193000	6.16E-12
chr4	55190001	55195000	3.32E-10
chr4	55550001	55555000	1.31E-07
chr4	55572001	55577000	2.90E-16
chr4	55625001	55630000	3.27E-12
chr4	55626001	55631000	5.50E-10
chr4	55844001	55849000	0.000293
chr4	56072001	56077000	3.43E-08
chr4	56208001	56213000	4.16E-08
chr4	56498001	56503000	5.80E-10
chr4	56501001	56506000	4.44E-06
chr4	56802001	56807000	5.60E-08
chr4	57249001	57254000	2.81E-19
chr4	57252001	57257000	6.93E-16
chr4	57408001	57413000	4.58E-15
chr4	57409001	57414000	1.46E-15
chr4	57410001	57415000	6.94E-13
chr4	57449001	57454000	5.43E-06
chr4	57463001	57468000	0.001087
chr4	57714001	57719000	4.73E-05

chr4	57839001	57844000	7.53E-05
chr4	58016001	58021000	1.11E-07
chr4	58025001	58030000	1.74E-10
chr4	58201001	58206000	2.80E-06
chr4	58202001	58207000	7.61E-07
chr4	58203001	58208000	4.07E-06
chr4	58360001	58365000	2.47E-07
chr4	58380001	58385000	6.96E-05
chr4	58404001	58409000	4.42E-07
chr4	58557001	58562000	2.34E-06
chr4	58558001	58563000	5.46E-06
chr4	58559001	58564000	2.03E-08
chr4	58560001	58565000	3.12E-08
chr4	58598001	58603000	4.99E-07
chr4	58599001	58604000	8.38E-08
chr4	58600001	58605000	2.30E-07
chr4	58601001	58606000	1.10E-05
chr4	58711001	58716000	4.64E-08
chr4	59110001	59115000	2.57E-09
chr4	59111001	59116000	3.09E-09
chr4	59113001	59118000	5.88E-09
chr4	59262001	59267000	1.52E-06
chr4	59446001	59451000	1.85E-10
chr4	59447001	59452000	5.27E-11
chr4	59448001	59453000	4.90E-12
chr4	60184001	60189000	1.03E-09
chr4	60337001	60342000	9.48E-07
chr4	60338001	60343000	1.07E-10
chr4	60339001	60344000	9.69E-11
chr4	60340001	60345000	3.80E-08
chr4	60860001	60865000	2.39E-07
chr4	61877001	61882000	1.33E-11
chr4	61878001	61883000	2.83E-09
chr4	61879001	61884000	1.34E-08
chr4	62671001	62676000	4.45E-13
chr4	62807001	62812000	0.001339
chr4	63142001	63147000	1.80E-09
chr4	63143001	63148000	1.39E-10
chr4	63167001	63172000	2.68E-06
chr4	63168001	63173000	4.84E-05
chr4	63225001	63230000	3.66E-08
chr4	63226001	63231000	1.54E-10
chr4	63227001	63232000	2.05E-12
chr4	63228001	63233000	6.83E-12
chr4	63235001	63240000	1.92E-10
chr4	63236001	63241000	7.80E-10
chr4	63237001	63242000	2.04E-11
chr4	63238001	63243000	3.45E-12

chr4	63296001	63301000	3.47E-11
chr4	63297001	63302000	7.50E-07
chr4	63942001	63947000	5.35E-07
chr4	63943001	63948000	3.74E-05
chr4	63944001	63949000	1.67E-05
chr4	63945001	63950000	0.000678
chr4	63946001	63951000	0.00204
chr4	64782001	64787000	8.57E-05
chr4	64876001	64881000	1.41E-08
chr4	64877001	64882000	7.58E-08
chr4	65292001	65297000	1.53E-06
chr4	65542001	65547000	1.02E-09
chr4	65543001	65548000	7.53E-13
chr4	65926001	65931000	9.49E-07
chr4	66159001	66164000	1.67E-09
chr4	66162001	66167000	3.92E-11
chr4	66631001	66636000	6.23E-08
chr4	67289001	67294000	0.001448
chr4	67482001	67487000	2.12E-08
chr4	67483001	67488000	2.48E-09
chr4	67484001	67489000	5.16E-09
chr4	67800001	67805000	9.70E-13
chr4	67801001	67806000	1.18E-12
chr4	67802001	67807000	1.00E-11
chr4	67976001	67981000	3.59E-08
chr4	68131001	68136000	0.000186
chr4	68142001	68147000	3.90E-07
chr4	68215001	68220000	2.58E-08
chr4	70343001	70348000	5.05E-08
chr4	70344001	70349000	1.07E-12
chr4	70412001	70417000	1.89E-05
chr4	70414001	70419000	3.61E-05
chr4	70415001	70420000	7.34E-06
chr4	70605001	70610000	1.32E-05
chr4	70694001	70699000	2.35E-10
chr4	70695001	70700000	3.30E-12
chr4	71002001	71007000	8.27E-10
chr4	71003001	71008000	3.17E-09
chr4	71004001	71009000	5.53E-09
chr4	71005001	71010000	4.64E-11
chr4	71255001	71260000	7.89E-07
chr4	71256001	71261000	7.41E-10
chr4	71258001	71263000	9.16E-08
chr4	71291001	71296000	3.20E-07
chr4	71292001	71297000	2.14E-06
chr4	71311001	71316000	1.21E-05
chr4	71312001	71317000	3.94E-06
chr4	71570001	71575000	5.20E-08

chr4	71702001	71707000	3.13E-11
chr4	71704001	71709000	1.91E-09
chr4	72012001	72017000	0.000395
chr4	72661001	72666000	6.70E-08
chr4	72853001	72858000	0.004535
chr4	73075001	73080000	3.03E-08
chr4	73076001	73081000	3.94E-09
chr4	73402001	73407000	1.09E-07
chr4	73620001	73625000	0.001335
chr4	73635001	73640000	5.22E-07
chr4	73637001	73642000	4.01E-08
chr4	73638001	73643000	7.42E-06
chr4	73702001	73707000	8.97E-06
chr4	73704001	73709000	0.000135
chr4	73935001	73940000	1.18E-15
chr4	74281001	74286000	2.92E-09
chr4	74563001	74568000	1.36E-12
chr4	76079001	76084000	0.000522
chr4	76859001	76864000	8.40E-08
chr4	76860001	76865000	3.93E-08
chr4	79469001	79474000	1.23E-16
chr4	80131001	80136000	5.41E-10
chr4	80135001	80140000	0.010112
chr4	80227001	80232000	1.47E-10
chr4	80629001	80634000	0.005936
chr4	80752001	80757000	0.001775
chr4	80753001	80758000	0.004068
chr4	80754001	80759000	0.000104
chr4	81673001	81678000	3.68E-06
chr4	82929001	82934000	0.001049
chr4	82930001	82935000	0.000524
chr4	83932001	83937000	1.85E-12
chr4	83933001	83938000	2.79E-15
chr4	83951001	83956000	1.50E-05
chr4	83952001	83957000	1.28E-18
chr4	83953001	83958000	4.17E-18
chr4	83954001	83959000	7.17E-07
chr4	84452001	84457000	2.03E-07
chr4	84467001	84472000	0.000972
chr4	86654001	86659000	6.10E-06
chr4	87852001	87857000	0.000857
chr4	89067001	89072000	0.002662
chr4	89104001	89109000	0.016509
chr4	89509001	89514000	2.07E-07
chr4	91416001	91421000	3.74E-06
chr4	92490001	92495000	0.0135
chr4	97369001	97374000	0.000289
chr4	98978001	98983000	0.003925

chr4	99060001	99065000	6.90E-08
chr4	99061001	99066000	3.70E-16
chr4	99062001	99067000	1.44E-16
chr4	99063001	99068000	2.36E-15
chr4	99064001	99069000	9.67E-18
chr4	99634001	99639000	0.000276
chr4	100322001	100327000	4.84E-05
chr4	100323001	100328000	1.73E-06
chr4	100683001	100688000	0.000264
chr4	100899001	100904000	5.24E-05
chr4	101163001	101168000	1.57E-06
chr4	101325001	101330000	5.64E-05
chr4	102692001	102697000	0.002714
chr4	104641001	104646000	2.55E-06
chr4	105013001	105018000	0.006974
chr4	105014001	105019000	0.014695
chr4	105015001	105020000	8.77E-05
chr4	105214001	105219000	0.000858
chr4	106930001	106935000	0.000264
chr4	106931001	106936000	6.85E-05
chr4	106932001	106937000	0.002752
chr4	109325001	109330000	0.0207
chr4	109326001	109331000	0.001283
chr4	109644001	109649000	0.000245
chr4	109761001	109766000	0.001384
chr4	109762001	109767000	0.00068
chr4	109937001	109942000	2.05E-07
chr4	110277001	110282000	1.76E-09
chr4	111114001	111119000	5.75E-06
chr4	111115001	111120000	1.76E-11
chr4	111116001	111121000	1.00E-11
chr4	111117001	111122000	0.020157
chr4	111119001	111124000	7.40E-05
chr4	111137001	111142000	0.016236
chr4	113697001	113702000	1.01E-06
chr4	113698001	113703000	6.94E-07
chr4	116840001	116845000	6.70E-07
chr4	117407001	117412000	2.30E-10
chr4	117408001	117413000	1.60E-08
chr4	118055001	118060000	1.45E-08
chr4	118056001	118061000	3.18E-07
chr4	119487001	119492000	6.93E-07
chr4	122518001	122523000	1.15E-06
chr4	122570001	122575000	1.01E-05
chr4	122571001	122576000	1.99E-07
chr4	122938001	122943000	9.24E-06
chr4	125823001	125828000	5.98E-05
chr4	125824001	125829000	5.55E-06

chr4	126238001	126243000	2.19E-17
chr4	126797001	126802000	4.60E-06
chr4	126798001	126803000	4.52E-10
chr4	126799001	126804000	3.57E-09
chr4	126800001	126805000	9.19E-08
chr4	127816001	127821000	7.73E-05
chr4	127817001	127822000	4.13E-06
chr4	128191001	128196000	1.36E-07
chr4	128441001	128446000	5.67E-08
chr4	128442001	128447000	2.91E-07
chr4	128443001	128448000	6.64E-08
chr4	128444001	128449000	2.39E-07
chr4	129148001	129153000	3.33E-05
chr4	129565001	129570000	6.40E-05
chr4	129566001	129571000	3.64E-06
chr4	129597001	129602000	8.71E-06
chr4	129598001	129603000	0.000465
chr4	129674001	129679000	1.20E-07
chr4	129698001	129703000	3.48E-05
chr4	130396001	130401000	2.44E-06
chr4	130544001	130549000	0.000389
chr4	131493001	131498000	1.41E-06
chr4	133311001	133316000	5.46E-07
chr4	133545001	133550000	2.37E-05
chr4	137507001	137512000	6.54E-05
chr4	137508001	137513000	3.05E-05
chr4	137509001	137514000	1.63E-06
chr4	137755001	137760000	3.16E-06
chr4	142905001	142910000	9.65E-07
chr4	142982001	142987000	0.000111
chr4	143111001	143116000	6.24E-05
chr4	143650001	143655000	9.57E-10
chr4	144019001	144024000	0.002184
chr4	144045001	144050000	0.01892
chr4	145823001	145828000	0.000127
chr4	147402001	147407000	0.002866
chr4	147526001	147531000	9.43E-06
chr4	147527001	147532000	9.33E-07
chr4	148648001	148653000	1.09E-08
chr4	149413001	149418000	0.010667
chr4	149414001	149419000	0.007342
chr4	149415001	149420000	2.04E-05
chr4	150175001	150180000	4.10E-05
chr4	150873001	150878000	0.000587
chr4	151579001	151584000	1.33E-06
chr4	154967001	154972000	1.29E-06
chr4	154968001	154973000	1.01E-08
chr4	155114001	155119000	6.06E-07

chr4	155115001	155120000	6.65E-07
chr4	155805001	155810000	0.000173
chr4	155914001	155919000	0.000504
chr4	159502001	159507000	0.000205
chr4	159505001	159510000	5.57E-07
chr4	160368001	160373000	1.38E-05
chr4	160369001	160374000	1.52E-05
chr4	162146001	162151000	2.66E-06
chr4	163595001	163600000	0.008336
chr4	163612001	163617000	2.76E-06
chr4	163613001	163618000	1.45E-05
chr4	164222001	164227000	3.43E-07
chr4	165665001	165670000	0.004231
chr4	165666001	165671000	0.00124
chr4	165667001	165672000	0.002302
chr4	165946001	165951000	4.92E-06
chr4	165947001	165952000	1.95E-06
chr4	166126001	166131000	1.19E-09
chr4	166483001	166488000	2.58E-05
chr4	167369001	167374000	6.63E-07
chr4	167370001	167375000	2.94E-06
chr4	169074001	169079000	1.27E-11
chr4	169075001	169080000	4.37E-09
chr4	170161001	170166000	0.000138
chr4	171125001	171130000	0.007664
chr4	171822001	171827000	5.00E-08
chr4	171824001	171829000	2.97E-11
chr4	172136001	172141000	7.14E-05
chr4	172137001	172142000	3.95E-05
chr4	172138001	172143000	0.000149
chr4	172417001	172422000	7.36E-05
chr4	172418001	172423000	4.39E-05
chr4	172512001	172517000	4.18E-05
chr4	172513001	172518000	0.000116
chr4	172514001	172519000	5.78E-06
chr4	172784001	172789000	2.06E-09
chr4	174288001	174293000	1.94E-07
chr4	174490001	174495000	1.57E-05
chr4	175383001	175388000	0.000206
chr4	176391001	176396000	1.83E-09
chr4	177909001	177914000	0.000674
chr4	179051001	179056000	4.74E-09
chr4	179096001	179101000	1.23E-05
chr4	179519001	179524000	0.003024
chr4	180263001	180268000	3.59E-08
chr4	180569001	180574000	1.01E-08
chr4	181769001	181774000	1.12E-08
chr4	186347001	186352000	7.00E-17

chr4	186390001	186395000	1.38E-11
chr4	186391001	186396000	3.35E-11
chr4	186651001	186656000	5.86E-05
chr4	187022001	187027000	3.79E-17
chr4	187023001	187028000	1.01E-18
chr4	187024001	187029000	7.22E-13
chr4	187026001	187031000	2.23E-09
chr4	187033001	187038000	0.000113
chr4	187035001	187040000	8.48E-08
chr4	187310001	187315000	5.97E-05
chr4	190061001	190066000	0.002604
chr4	190062001	190067000	0.000131
chr4	190445001	190450000	9.61E-06
chr5	391001	396000	5.85E-05
chr5	471001	476000	8.54E-17
chr5	472001	477000	6.87E-11
chr5	474001	479000	2.70E-05
chr5	688001	693000	0.002277
chr5	692001	697000	1.40E-08
chr5	693001	698000	6.77E-10
chr5	744001	749000	4.69E-15
chr5	745001	750000	1.63E-12
chr5	746001	751000	3.46E-10
chr5	747001	752000	3.69E-06
chr5	748001	753000	9.73E-07
chr5	847001	852000	3.40E-06
chr5	848001	853000	0.000166
chr5	1032001	1037000	7.14E-05
chr5	1741001	1746000	6.76E-09
chr5	2410001	2415000	2.49E-08
chr5	2434001	2439000	1.50E-09
chr5	2435001	2440000	5.01E-11
chr5	2436001	2441000	2.82E-09
chr5	3637001	3642000	0.000157
chr5	5046001	5051000	2.25E-14
chr5	5047001	5052000	4.73E-12
chr5	5633001	5638000	3.69E-11
chr5	6132001	6137000	4.68E-05
chr5	6570001	6575000	0.000341
chr5	6629001	6634000	2.37E-14
chr5	6630001	6635000	6.21E-15
chr5	6631001	6636000	1.10E-19
chr5	6632001	6637000	1.21E-24
chr5	6633001	6638000	2.46E-25
chr5	8218001	8223000	6.07E-07
chr5	8397001	8402000	3.72E-06
chr5	8399001	8404000	7.08E-09
chr5	8408001	8413000	1.29E-08

chr5	9024001	9029000	0.000245
chr5	10816001	10821000	1.32E-05
chr5	10817001	10822000	1.29E-05
chr5	11038001	11043000	3.41E-06
chr5	11295001	11300000	1.35E-11
chr5	11448001	11453000	1.77E-16
chr5	11931001	11936000	0.0001
chr5	11981001	11986000	1.65E-10
chr5	11982001	11987000	5.85E-11
chr5	11983001	11988000	3.30E-09
chr5	11984001	11989000	2.52E-08
chr5	12130001	12135000	3.33E-05
chr5	12381001	12386000	3.66E-08
chr5	12544001	12549000	1.12E-09
chr5	12545001	12550000	1.50E-08
chr5	12586001	12591000	4.14E-08
chr5	13199001	13204000	9.50E-11
chr5	13565001	13570000	7.99E-08
chr5	13566001	13571000	2.83E-06
chr5	13567001	13572000	1.42E-06
chr5	13654001	13659000	1.77E-06
chr5	13796001	13801000	6.72E-07
chr5	14262001	14267000	1.01E-17
chr5	15045001	15050000	1.30E-08
chr5	15085001	15090000	1.00E-15
chr5	15086001	15091000	1.77E-11
chr5	15088001	15093000	5.58E-10
chr5	15428001	15433000	3.28E-06
chr5	15885001	15890000	2.23E-09
chr5	16046001	16051000	2.11E-06
chr5	16047001	16052000	1.40E-06
chr5	16072001	16077000	5.47E-09
chr5	16098001	16103000	2.00E-07
chr5	16134001	16139000	9.04E-10
chr5	16135001	16140000	1.84E-09
chr5	16136001	16141000	6.01E-10
chr5	16193001	16198000	3.37E-05
chr5	16194001	16199000	1.06E-05
chr5	16195001	16200000	7.43E-06
chr5	16196001	16201000	4.10E-07
chr5	16203001	16208000	2.53E-10
chr5	16204001	16209000	5.72E-11
chr5	16206001	16211000	7.35E-10
chr5	16207001	16212000	3.27E-11
chr5	16208001	16213000	7.16E-09
chr5	16351001	16356000	9.24E-10
chr5	16359001	16364000	3.92E-07
chr5	18225001	18230000	1.63E-05

chr5	18226001	18231000	3.23E-06
chr5	18327001	18332000	2.94E-09
chr5	19304001	19309000	3.62E-08
chr5	19335001	19340000	1.96E-08
chr5	19370001	19375000	6.99E-07
chr5	19754001	19759000	7.54E-08
chr5	19755001	19760000	3.13E-11
chr5	19756001	19761000	1.31E-19
chr5	19757001	19762000	1.97E-18
chr5	19758001	19763000	8.72E-17
chr5	19759001	19764000	1.88E-13
chr5	19760001	19765000	9.59E-12
chr5	19938001	19943000	6.94E-08
chr5	20334001	20339000	8.22E-09
chr5	20384001	20389000	3.41E-13
chr5	20475001	20480000	1.45E-05
chr5	20477001	20482000	1.64E-06
chr5	20740001	20745000	1.25E-10
chr5	20742001	20747000	5.15E-08
chr5	21934001	21939000	3.40E-05
chr5	21936001	21941000	2.82E-05
chr5	21938001	21943000	4.29E-05
chr5	22057001	22062000	3.36E-09
chr5	22065001	22070000	3.36E-07
chr5	22066001	22071000	5.10E-07
chr5	22292001	22297000	7.09E-12
chr5	22592001	22597000	6.77E-08
chr5	22801001	22806000	2.61E-16
chr5	23249001	23254000	5.30E-06
chr5	23314001	23319000	1.73E-08
chr5	23315001	23320000	2.70E-09
chr5	23361001	23366000	3.48E-11
chr5	23362001	23367000	5.85E-12
chr5	23363001	23368000	1.14E-12
chr5	23455001	23460000	5.66E-11
chr5	23520001	23525000	4.05E-05
chr5	23521001	23526000	4.99E-09
chr5	23564001	23569000	6.22E-10
chr5	23565001	23570000	2.99E-10
chr5	23674001	23679000	0.000265
chr5	24055001	24060000	3.11E-06
chr5	24056001	24061000	4.15E-10
chr5	24057001	24062000	1.67E-10
chr5	24087001	24092000	4.02E-08
chr5	24362001	24367000	1.00E-09
chr5	24363001	24368000	1.53E-11
chr5	24529001	24534000	2.28E-09
chr5	24660001	24665000	1.17E-07

chr5	24682001	24687000	7.43E-07
chr5	24683001	24688000	7.46E-05
chr5	24684001	24689000	6.46E-06
chr5	25911001	25916000	1.56E-06
chr5	26680001	26685000	2.42E-08
chr5	26940001	26945000	2.30E-06
chr5	26941001	26946000	3.91E-09
chr5	28824001	28829000	9.04E-05
chr5	29271001	29276000	4.17E-10
chr5	30009001	30014000	0.000631
chr5	30010001	30015000	1.95E-05
chr5	30011001	30016000	0.000312
chr5	30287001	30292000	1.73E-05
chr5	30288001	30293000	4.90E-07
chr5	30375001	30380000	4.11E-07
chr5	31576001	31581000	2.66E-06
chr5	31639001	31644000	1.86E-06
chr5	31649001	31654000	1.20E-08
chr5	32525001	32530000	1.75E-08
chr5	32526001	32531000	5.66E-07
chr5	32527001	32532000	0.000167
chr5	32528001	32533000	5.61E-05
chr5	32662001	32667000	0.000461
chr5	32720001	32725000	6.57E-10
chr5	32721001	32726000	1.63E-10
chr5	32792001	32797000	1.57E-08
chr5	32793001	32798000	1.35E-08
chr5	32862001	32867000	1.26E-11
chr5	32863001	32868000	3.03E-12
chr5	32864001	32869000	6.90E-08
chr5	32865001	32870000	4.66E-08
chr5	32891001	32896000	5.31E-09
chr5	32892001	32897000	3.14E-13
chr5	32893001	32898000	1.36E-10
chr5	32894001	32899000	7.06E-06
chr5	32901001	32906000	6.63E-06
chr5	32902001	32907000	7.07E-07
chr5	32903001	32908000	1.31E-07
chr5	32968001	32973000	1.18E-11
chr5	33104001	33109000	1.46E-05
chr5	33397001	33402000	0.000191
chr5	33398001	33403000	3.98E-05
chr5	33657001	33662000	5.29E-06
chr5	33846001	33851000	1.24E-09
chr5	33871001	33876000	3.86E-07
chr5	33880001	33885000	8.65E-16
chr5	33896001	33901000	5.33E-10
chr5	33897001	33902000	8.78E-11

chr5	33898001	33903000	4.07E-10
chr5	33918001	33923000	0.000518
chr5	34149001	34154000	0.002823
chr5	34970001	34975000	5.03E-08
chr5	35103001	35108000	2.85E-07
chr5	35555001	35560000	3.65E-08
chr5	35556001	35561000	2.12E-08
chr5	35557001	35562000	5.04E-07
chr5	35558001	35563000	3.21E-06
chr5	35608001	35613000	1.94E-05
chr5	36152001	36157000	7.50E-21
chr5	38291001	38296000	1.57E-08
chr5	39254001	39259000	4.28E-10
chr5	39255001	39260000	1.12E-09
chr5	40459001	40464000	0.00048
chr5	41063001	41068000	1.63E-13
chr5	41064001	41069000	8.96E-14
chr5	41065001	41070000	1.76E-11
chr5	41066001	41071000	3.76E-11
chr5	41107001	41112000	5.30E-07
chr5	41108001	41113000	3.55E-07
chr5	41109001	41114000	3.20E-08
chr5	41471001	41476000	5.22E-07
chr5	41472001	41477000	5.30E-07
chr5	41473001	41478000	1.54E-06
chr5	41995001	42000000	4.89E-07
chr5	42068001	42073000	2.62E-06
chr5	42069001	42074000	2.75E-07
chr5	42178001	42183000	3.31E-08
chr5	42208001	42213000	0.003455
chr5	42224001	42229000	1.83E-10
chr5	42538001	42543000	5.47E-08
chr5	42539001	42544000	2.60E-10
chr5	43101001	43106000	2.85E-09
chr5	43104001	43109000	1.81E-10
chr5	43481001	43486000	1.73E-16
chr5	43482001	43487000	3.01E-16
chr5	43484001	43489000	8.43E-16
chr5	44197001	44202000	3.13E-05
chr5	44626001	44631000	3.62E-09
chr5	44627001	44632000	9.16E-08
chr5	44923001	44928000	1.83E-05
chr5	44989001	44994000	3.40E-06
chr5	45005001	45010000	2.33E-05
chr5	45006001	45011000	5.56E-06
chr5	45007001	45012000	4.66E-07
chr5	45008001	45013000	4.93E-08
chr5	45114001	45119000	5.77E-09

chr5	45115001	45120000	5.07E-10
chr5	45116001	45121000	8.95E-14
chr5	45117001	45122000	1.86E-10
chr5	45401001	45406000	2.56E-08
chr5	45402001	45407000	1.17E-08
chr5	45438001	45443000	8.79E-10
chr5	45527001	45532000	4.63E-05
chr5	45528001	45533000	2.34E-06
chr5	46273001	46278000	1.06E-05
chr5	46274001	46279000	1.67E-05
chr5	46275001	46280000	1.47E-06
chr5	49424001	49429000	0.001798
chr5	49466001	49471000	1.54E-08
chr5	49467001	49472000	4.17E-09
chr5	49712001	49717000	3.35E-08
chr5	50003001	50008000	1.64E-06
chr5	50192001	50197000	7.40E-07
chr5	50431001	50436000	3.13E-07
chr5	51362001	51367000	5.98E-07
chr5	51790001	51795000	0.000112
chr5	51791001	51796000	3.30E-05
chr5	54322001	54327000	2.67E-11
chr5	54422001	54427000	2.04E-06
chr5	54423001	54428000	6.42E-07
chr5	54890001	54895000	0.00025
chr5	54893001	54898000	1.45E-08
chr5	55309001	55314000	8.10E-05
chr5	55310001	55315000	0.00123
chr5	56430001	56435000	1.71E-05
chr5	56685001	56690000	2.54E-07
chr5	57447001	57452000	1.12E-06
chr5	57454001	57459000	8.66E-06
chr5	57456001	57461000	3.90E-08
chr5	57811001	57816000	0.000392
chr5	57812001	57817000	5.21E-05
chr5	57891001	57896000	6.17E-07
chr5	57913001	57918000	2.35E-10
chr5	57914001	57919000	1.96E-11
chr5	57915001	57920000	2.86E-10
chr5	60137001	60142000	1.23E-09
chr5	60138001	60143000	1.83E-07
chr5	60140001	60145000	1.87E-05
chr5	61704001	61709000	2.41E-09
chr5	61708001	61713000	5.75E-12
chr5	63190001	63195000	0.000493
chr5	66509001	66514000	6.46E-07
chr5	66510001	66515000	2.38E-05
chr5	66560001	66565000	2.35E-07

chr5	68516001	68521000	3.22E-08
chr5	68585001	68590000	0.022471
chr5	68624001	68629000	1.96E-16
chr5	68857001	68862000	0.000484
chr5	70081001	70086000	0.002635
chr5	70880001	70885000	8.53E-07
chr5	72009001	72014000	1.43E-06
chr5	73914001	73919000	0.000371
chr5	74530001	74535000	3.68E-09
chr5	75366001	75371000	1.96E-07
chr5	75367001	75372000	3.97E-08
chr5	75843001	75848000	0.000589
chr5	76279001	76284000	0.000484
chr5	76280001	76285000	0.000303
chr5	77961001	77966000	9.10E-06
chr5	77962001	77967000	3.43E-06
chr5	78276001	78281000	2.56E-16
chr5	78277001	78282000	2.23E-18
chr5	78278001	78283000	2.58E-19
chr5	78504001	78509000	0.002041
chr5	79477001	79482000	0.000128
chr5	79595001	79600000	0.014102
chr5	80685001	80690000	0.000228
chr5	81143001	81148000	4.92E-08
chr5	81144001	81149000	2.44E-08
chr5	81145001	81150000	1.37E-12
chr5	81146001	81151000	4.95E-06
chr5	81147001	81152000	2.71E-08
chr5	82746001	82751000	3.02E-05
chr5	82747001	82752000	1.63E-05
chr5	82748001	82753000	1.95E-05
chr5	82749001	82754000	1.51E-05
chr5	82750001	82755000	1.52E-06
chr5	84178001	84183000	0.001406
chr5	86164001	86169000	8.16E-07
chr5	86165001	86170000	8.35E-08
chr5	86235001	86240000	3.29E-05
chr5	86799001	86804000	0.003576
chr5	86800001	86805000	0.004694
chr5	89038001	89043000	1.61E-06
chr5	89039001	89044000	1.42E-06
chr5	89097001	89102000	2.70E-08
chr5	91511001	91516000	2.97E-05
chr5	95292001	95297000	1.52E-10
chr5	95293001	95298000	1.52E-10
chr5	97512001	97517000	0.002777
chr5	97933001	97938000	1.27E-07
chr5	100839001	100844000	1.31E-05

chr5	100840001	100845000	2.02E-05
chr5	101772001	101777000	0.001154
chr5	103315001	103320000	0.002035
chr5	103811001	103816000	3.90E-05
chr5	103812001	103817000	4.16E-05
chr5	104213001	104218000	0.000125
chr5	105689001	105694000	9.84E-06
chr5	106024001	106029000	1.20E-07
chr5	106025001	106030000	9.98E-08
chr5	106065001	106070000	2.29E-05
chr5	106066001	106071000	5.67E-05
chr5	110422001	110427000	4.13E-09
chr5	110423001	110428000	5.39E-11
chr5	110424001	110429000	1.27E-16
chr5	110660001	110665000	1.57E-08
chr5	110661001	110666000	2.31E-07
chr5	110992001	110997000	9.00E-07
chr5	112290001	112295000	0.00015
chr5	112291001	112296000	2.07E-07
chr5	112292001	112297000	1.43E-06
chr5	113374001	113379000	7.29E-07
chr5	113599001	113604000	0.010147
chr5	113977001	113982000	4.40E-07
chr5	113978001	113983000	2.82E-08
chr5	114051001	114056000	6.09E-09
chr5	114429001	114434000	2.01E-05
chr5	114967001	114972000	5.24E-07
chr5	114968001	114973000	5.26E-06
chr5	115051001	115056000	4.08E-05
chr5	117064001	117069000	1.15E-07
chr5	117065001	117070000	1.06E-06
chr5	117164001	117169000	0.000312
chr5	117220001	117225000	0.000269
chr5	117221001	117226000	8.10E-05
chr5	117222001	117227000	0.000277
chr5	118038001	118043000	4.03E-06
chr5	118358001	118363000	0.0001
chr5	119974001	119979000	8.99E-05
chr5	120902001	120907000	4.66E-07
chr5	120903001	120908000	4.66E-07
chr5	120904001	120909000	3.33E-07
chr5	120905001	120910000	1.07E-05
chr5	121408001	121413000	1.98E-10
chr5	121409001	121414000	4.61E-24
chr5	121591001	121596000	0.000154
chr5	125854001	125859000	0.000178
chr5	125855001	125860000	5.54E-05
chr5	126097001	126102000	4.54E-08

chr5	126098001	126103000	4.11E-08
chr5	130444001	130449000	1.05E-06
chr5	130445001	130450000	1.75E-05
chr5	131235001	131240000	0.000957
chr5	131399001	131404000	0.000563
chr5	131400001	131405000	0.032365
chr5	131525001	131530000	6.47E-05
chr5	131527001	131532000	0.000145
chr5	131528001	131533000	0.005316
chr5	131626001	131631000	1.72E-06
chr5	132361001	132366000	3.49E-05
chr5	132917001	132922000	0.001375
chr5	132919001	132924000	0.001241
chr5	132920001	132925000	0.004332
chr5	133335001	133340000	0.002246
chr5	133339001	133344000	2.10E-08
chr5	133889001	133894000	0.00284
chr5	133890001	133895000	0.000133
chr5	133919001	133924000	0.000564
chr5	133920001	133925000	0.001783
chr5	133921001	133926000	9.44E-05
chr5	133922001	133927000	7.70E-06
chr5	134563001	134568000	0.003735
chr5	134564001	134569000	0.001409
chr5	135050001	135055000	2.15E-06
chr5	135610001	135615000	1.53E-07
chr5	136042001	136047000	6.31E-09
chr5	136043001	136048000	1.16E-05
chr5	136044001	136049000	6.38E-05
chr5	136756001	136761000	1.07E-06
chr5	137546001	137551000	2.48E-12
chr5	137547001	137552000	1.16E-07
chr5	137557001	137562000	0.002569
chr5	137558001	137563000	0.0099
chr5	137599001	137604000	0.004581
chr5	137600001	137605000	0.016786
chr5	137601001	137606000	0.005869
chr5	137781001	137786000	0.00329
chr5	137909001	137914000	2.24E-11
chr5	137910001	137915000	1.01E-11
chr5	138089001	138094000	6.12E-05
chr5	138284001	138289000	0.000337
chr5	138285001	138290000	8.16E-06
chr5	138286001	138291000	2.55E-06
chr5	138605001	138610000	2.66E-05
chr5	138725001	138730000	0.000183
chr5	138727001	138732000	0.001131
chr5	138728001	138733000	6.28E-07

chr5	138729001	138734000	2.38E-08
chr5	138749001	138754000	1.10E-07
chr5	138851001	138856000	1.38E-07
chr5	138852001	138857000	1.29E-07
chr5	138853001	138858000	1.09E-09
chr5	138854001	138859000	1.29E-08
chr5	138855001	138860000	0.002132
chr5	138866001	138871000	0.000739
chr5	138888001	138893000	3.07E-06
chr5	138889001	138894000	4.45E-06
chr5	138890001	138895000	3.45E-06
chr5	138891001	138896000	3.68E-05
chr5	139043001	139048000	2.09E-09
chr5	139044001	139049000	1.35E-11
chr5	139045001	139050000	3.14E-14
chr5	139046001	139051000	7.31E-11
chr5	139047001	139052000	6.97E-12
chr5	139145001	139150000	0.004064
chr5	139242001	139247000	0.004219
chr5	139523001	139528000	0.005704
chr5	139778001	139783000	3.16E-09
chr5	139779001	139784000	3.60E-07
chr5	139780001	139785000	6.79E-06
chr5	139940001	139945000	4.04E-06
chr5	140345001	140350000	1.51E-06
chr5	140889001	140894000	2.77E-06
chr5	140890001	140895000	0.002673
chr5	140892001	140897000	1.13E-05
chr5	140893001	140898000	3.11E-05
chr5	141100001	141105000	0.013554
chr5	141117001	141122000	2.70E-05
chr5	141118001	141123000	9.96E-06
chr5	141134001	141139000	5.43E-05
chr5	141149001	141154000	0.00274
chr5	141188001	141193000	1.37E-05
chr5	141217001	141222000	0.00206
chr5	141218001	141223000	0.000686
chr5	141219001	141224000	0.000372
chr5	141229001	141234000	9.79E-08
chr5	141260001	141265000	2.30E-05
chr5	141261001	141266000	4.25E-06
chr5	141262001	141267000	1.89E-05
chr5	141279001	141284000	2.16E-05
chr5	141698001	141703000	0.000306
chr5	141746001	141751000	0.000101
chr5	141747001	141752000	0.000499
chr5	142062001	142067000	1.78E-06
chr5	142064001	142069000	1.95E-05

chr5	142065001	142070000	1.94E-07
chr5	142078001	142083000	4.83E-05
chr5	143051001	143056000	2.87E-06
chr5	143454001	143459000	2.77E-05
chr5	144388001	144393000	3.03E-05
chr5	144459001	144464000	0.000314
chr5	145925001	145930000	7.62E-06
chr5	145926001	145931000	0.000826
chr5	147841001	147846000	3.20E-09
chr5	148479001	148484000	0.006333
chr5	148647001	148652000	1.34E-16
chr5	148648001	148653000	1.75E-16
chr5	148649001	148654000	3.81E-14
chr5	148692001	148697000	5.23E-05
chr5	148693001	148698000	0.000542
chr5	148866001	148871000	2.57E-06
chr5	148929001	148934000	6.19E-12
chr5	148957001	148962000	2.08E-08
chr5	148959001	148964000	5.71E-08
chr5	149497001	149502000	5.72E-09
chr5	149498001	149503000	2.47E-08
chr5	149547001	149552000	2.22E-07
chr5	149548001	149553000	7.54E-07
chr5	149627001	149632000	0.00019
chr5	149628001	149633000	0.001113
chr5	149674001	149679000	0.000278
chr5	150035001	150040000	6.09E-06
chr5	150036001	150041000	0.000255
chr5	150344001	150349000	1.50E-05
chr5	151314001	151319000	0.000251
chr5	151315001	151320000	0.001031
chr5	151540001	151545000	0.000237
chr5	151722001	151727000	0.001681
chr5	152258001	152263000	9.12E-08
chr5	153325001	153330000	3.59E-06
chr5	153896001	153901000	2.77E-05
chr5	153897001	153902000	3.19E-05
chr5	154090001	154095000	0.000423
chr5	155060001	155065000	4.85E-05
chr5	155061001	155066000	3.65E-05
chr5	155220001	155225000	7.95E-05
chr5	156236001	156241000	4.02E-05
chr5	156825001	156830000	0.004249
chr5	156954001	156959000	3.01E-05
chr5	157057001	157062000	5.70E-05
chr5	158762001	158767000	3.93E-05
chr5	159024001	159029000	0.001227
chr5	159396001	159401000	1.65E-06

chr5	159397001	159402000	1.57E-06
chr5	159398001	159403000	1.87E-06
chr5	159399001	159404000	1.74E-05
chr5	159572001	159577000	1.72E-05
chr5	159573001	159578000	0.00014
chr5	159611001	159616000	2.71E-07
chr5	159625001	159630000	0.001362
chr5	160539001	160544000	0.00019
chr5	161633001	161638000	4.81E-06
chr5	162654001	162659000	5.51E-06
chr5	162655001	162660000	6.38E-05
chr5	162656001	162661000	0.006215
chr5	162959001	162964000	7.51E-06
chr5	162960001	162965000	9.81E-07
chr5	164125001	164130000	0.000363
chr5	164202001	164207000	2.69E-09
chr5	164204001	164209000	3.01E-09
chr5	164227001	164232000	1.03E-05
chr5	164522001	164527000	3.22E-06
chr5	165120001	165125000	1.49E-05
chr5	166628001	166633000	6.82E-10
chr5	166705001	166710000	2.42E-06
chr5	166825001	166830000	1.38E-13
chr5	166826001	166831000	6.59E-11
chr5	168773001	168778000	0.00242
chr5	169072001	169077000	2.49E-08
chr5	169073001	169078000	1.18E-10
chr5	169074001	169079000	6.25E-10
chr5	169158001	169163000	1.28E-07
chr5	169361001	169366000	2.02E-08
chr5	169779001	169784000	1.94E-14
chr5	169780001	169785000	2.42E-13
chr5	170810001	170815000	1.57E-09
chr5	170811001	170816000	1.38E-11
chr5	170812001	170817000	1.09E-13
chr5	170993001	170998000	0.003942
chr5	171599001	171604000	4.47E-06
chr5	171600001	171605000	5.95E-06
chr5	171905001	171910000	0.000198
chr5	172591001	172596000	0.002709
chr5	172623001	172628000	0.000202
chr5	173536001	173541000	1.89E-08
chr5	173740001	173745000	1.82E-07
chr5	173741001	173746000	1.85E-09
chr5	173742001	173747000	1.90E-07
chr5	174094001	174099000	1.48E-05
chr5	174095001	174100000	7.86E-06
chr5	174174001	174179000	7.78E-07

chr5	174175001	174180000	4.43E-09
chr5	174176001	174181000	1.27E-12
chr5	174177001	174182000	7.44E-19
chr5	174178001	174183000	4.38E-22
chr5	174180001	174185000	2.34E-06
chr5	174181001	174186000	3.26E-07
chr5	174191001	174196000	1.99E-07
chr5	174192001	174197000	9.72E-07
chr5	174227001	174232000	5.10E-05
chr5	174240001	174245000	2.12E-05
chr5	174342001	174347000	0.00216
chr5	174358001	174363000	0.000383
chr5	175018001	175023000	2.85E-10
chr5	175019001	175024000	5.28E-11
chr5	175020001	175025000	2.15E-14
chr5	175021001	175026000	9.37E-10
chr5	175029001	175034000	3.54E-08
chr5	175030001	175035000	6.77E-07
chr5	175073001	175078000	0.000277
chr5	175218001	175223000	9.02E-06
chr5	175219001	175224000	1.71E-05
chr5	175310001	175315000	0.000121
chr5	175318001	175323000	8.27E-05
chr5	175442001	175447000	0.003677
chr5	175444001	175449000	0.006513
chr5	175520001	175525000	0.000152
chr5	175622001	175627000	0.000769
chr5	175623001	175628000	0.000123
chr5	175632001	175637000	0.002934
chr5	175837001	175842000	2.61E-06
chr5	175838001	175843000	4.66E-06
chr5	175839001	175844000	0.000328
chr5	176003001	176008000	0.001691
chr5	176004001	176009000	0.001939
chr5	176005001	176010000	0.000762
chr5	176022001	176027000	0.000276
chr5	176237001	176242000	2.70E-08
chr5	176238001	176243000	1.07E-05
chr5	176290001	176295000	0.006413
chr5	176448001	176453000	2.98E-06
chr5	176513001	176518000	1.20E-05
chr5	176514001	176519000	5.68E-08
chr5	176793001	176798000	4.57E-05
chr5	176794001	176799000	9.42E-07
chr5	176795001	176800000	2.77E-06
chr5	176910001	176915000	0.001149
chr5	176931001	176936000	6.38E-05
chr5	177019001	177024000	0.000762

chr5	177022001	177027000	0.000231
chr5	177126001	177131000	0.000135
chr5	177127001	177132000	0.000135
chr5	177161001	177166000	6.69E-05
chr5	177351001	177356000	0.002897
chr5	177527001	177532000	0.002816
chr5	177553001	177558000	1.78E-05
chr5	177631001	177636000	2.99E-06
chr5	177632001	177637000	1.90E-05
chr5	177747001	177752000	0.002825
chr5	177861001	177866000	7.98E-06
chr5	177933001	177938000	1.35E-09
chr5	177971001	177976000	0.000207
chr5	177981001	177986000	6.24E-06
chr5	177983001	177988000	6.66E-09
chr5	177984001	177989000	4.14E-09
chr5	177985001	177990000	1.73E-06
chr5	178375001	178380000	1.56E-14
chr5	178376001	178381000	4.73E-16
chr5	178377001	178382000	3.67E-12
chr5	178378001	178383000	5.17E-10
chr5	178393001	178398000	1.33E-08
chr5	178394001	178399000	1.09E-10
chr5	178395001	178400000	1.36E-09
chr5	178396001	178401000	1.99E-09
chr5	178397001	178402000	2.25E-09
chr5	178417001	178422000	5.52E-09
chr5	178536001	178541000	4.93E-11
chr5	178576001	178581000	7.42E-09
chr5	178635001	178640000	0.000528
chr5	178647001	178652000	0.021308
chr5	178648001	178653000	0.013954
chr5	178793001	178798000	1.29E-06
chr5	178794001	178799000	7.43E-08
chr5	178877001	178882000	0.000245
chr5	178879001	178884000	0.00014
chr5	178880001	178885000	0.000105
chr5	178881001	178886000	0.000434
chr5	178890001	178895000	0.000271
chr5	179123001	179128000	3.62E-07
chr5	179233001	179238000	0.000185
chr5	179234001	179239000	6.44E-06
chr5	179235001	179240000	8.15E-10
chr5	179633001	179638000	1.62E-06
chr5	179828001	179833000	2.68E-05
chr5	179917001	179922000	2.00E-08
chr5	180358001	180363000	0.000695
chr5	180505001	180510000	3.10E-05

chr5	180572001	180577000	6.63E-06
chr6	1993001	1998000	1.34E-06
chr6	2531001	2536000	3.67E-07
chr6	2532001	2537000	3.32E-08
chr6	3000001	3005000	2.70E-06
chr6	3185001	3190000	3.72E-06
chr6	3190001	3195000	1.75E-08
chr6	3219001	3224000	2.20E-05
chr6	4983001	4988000	1.43E-08
chr6	5033001	5038000	3.98E-07
chr6	5082001	5087000	1.32E-07
chr6	5083001	5088000	4.66E-23
chr6	5084001	5089000	1.02E-25
chr6	5085001	5090000	2.32E-26
chr6	5086001	5091000	3.18E-17
chr6	5832001	5837000	0.000676
chr6	6009001	6014000	3.41E-12
chr6	6284001	6289000	6.05E-08
chr6	6285001	6290000	1.62E-10
chr6	6286001	6291000	7.09E-10
chr6	6583001	6588000	0.000137
chr6	6818001	6823000	5.60E-08
chr6	7612001	7617000	1.12E-06
chr6	11140001	11145000	7.09E-12
chr6	11141001	11146000	7.79E-10
chr6	11803001	11808000	1.85E-08
chr6	12208001	12213000	3.98E-08
chr6	12209001	12214000	2.26E-06
chr6	12211001	12216000	9.79E-08
chr6	12212001	12217000	1.66E-07
chr6	12836001	12841000	0.000128
chr6	12837001	12842000	1.72E-05
chr6	12838001	12843000	1.88E-05
chr6	13333001	13338000	4.00E-06
chr6	13334001	13339000	5.22E-07
chr6	13763001	13768000	6.02E-06
chr6	18614001	18619000	1.47E-06
chr6	18615001	18620000	8.75E-05
chr6	20703001	20708000	1.16E-06
chr6	20704001	20709000	3.92E-06
chr6	21806001	21811000	1.42E-05
chr6	21807001	21812000	2.07E-08
chr6	21808001	21813000	5.84E-10
chr6	21809001	21814000	9.48E-11
chr6	23741001	23746000	1.03E-06
chr6	24358001	24363000	3.30E-12
chr6	24359001	24364000	1.72E-15
chr6	24360001	24365000	4.39E-08

chr6	24391001	24396000	0.000475
chr6	25995001	26000000	1.39E-09
chr6	26070001	26075000	5.04E-08
chr6	26116001	26121000	1.37E-07
chr6	26153001	26158000	4.70E-18
chr6	27091001	27096000	3.58E-12
chr6	27092001	27097000	4.31E-11
chr6	27093001	27098000	1.95E-13
chr6	27094001	27099000	4.13E-12
chr6	27224001	27229000	6.57E-07
chr6	27395001	27400000	8.52E-06
chr6	27630001	27635000	3.06E-06
chr6	31306001	31311000	0.001648
chr6	31307001	31312000	0.001648
chr6	31308001	31313000	0.001648
chr6	33571001	33576000	0.002221
chr6	33579001	33584000	0.002404
chr6	33582001	33587000	0.000218
chr6	33621001	33626000	0.000699
chr6	33653001	33658000	0.000168
chr6	33705001	33710000	0.002327
chr6	33785001	33790000	0.000109
chr6	33799001	33804000	0.0139
chr6	34110001	34115000	3.15E-07
chr6	34111001	34116000	5.61E-15
chr6	34112001	34117000	4.59E-12
chr6	34113001	34118000	1.03E-10
chr6	34146001	34151000	0.000377
chr6	34204001	34209000	0.018511
chr6	34325001	34330000	3.56E-08
chr6	34424001	34429000	0.00285
chr6	34425001	34430000	0.006796
chr6	34433001	34438000	2.22E-05
chr6	34465001	34470000	0.000147
chr6	34466001	34471000	0.000643
chr6	34489001	34494000	2.68E-05
chr6	34491001	34496000	6.29E-13
chr6	34492001	34497000	4.10E-12
chr6	34493001	34498000	5.49E-12
chr6	34722001	34727000	1.09E-07
chr6	34723001	34728000	8.12E-09
chr6	34725001	34730000	5.40E-08
chr6	35516001	35521000	0.000494
chr6	35517001	35522000	5.20E-06
chr6	35518001	35523000	6.49E-07
chr6	35519001	35524000	1.89E-05
chr6	35671001	35676000	0.000332
chr6	35729001	35734000	1.36E-08

chr6	35730001	35735000	1.28E-12
chr6	35731001	35736000	1.18E-08
chr6	35732001	35737000	1.69E-08
chr6	35782001	35787000	0.013893
chr6	36095001	36100000	9.47E-07
chr6	36096001	36101000	2.22E-05
chr6	36097001	36102000	2.85E-06
chr6	36401001	36406000	0.001555
chr6	36512001	36517000	3.07E-09
chr6	36513001	36518000	1.43E-07
chr6	36514001	36519000	2.77E-10
chr6	36515001	36520000	3.52E-07
chr6	36557001	36562000	0.000191
chr6	36624001	36629000	9.34E-12
chr6	36647001	36652000	3.04E-05
chr6	36729001	36734000	0.000352
chr6	36730001	36735000	0.00196
chr6	36737001	36742000	5.49E-05
chr6	37154001	37159000	0.012396
chr6	37174001	37179000	7.78E-06
chr6	37175001	37180000	0.001567
chr6	37176001	37181000	0.000404
chr6	37177001	37182000	0.000289
chr6	37190001	37195000	1.65E-07
chr6	37204001	37209000	0.008124
chr6	37396001	37401000	7.62E-09
chr6	37397001	37402000	3.38E-08
chr6	37398001	37403000	1.18E-12
chr6	37399001	37404000	2.05E-07
chr6	37400001	37405000	7.52E-09
chr6	37514001	37519000	4.11E-06
chr6	37560001	37565000	9.63E-09
chr6	37561001	37566000	9.27E-09
chr6	39821001	39826000	4.18E-05
chr6	40255001	40260000	0.000159
chr6	40261001	40266000	0.009904
chr6	40454001	40459000	0.000315
chr6	40455001	40460000	0.006246
chr6	40522001	40527000	8.39E-05
chr6	40530001	40535000	3.98E-05
chr6	40531001	40536000	3.33E-05
chr6	40626001	40631000	3.87E-07
chr6	40630001	40635000	4.97E-05
chr6	40674001	40679000	3.11E-05
chr6	40676001	40681000	0.001653
chr6	41089001	41094000	3.89E-05
chr6	41119001	41124000	2.25E-05
chr6	41565001	41570000	0.039229

chr6	41601001	41606000	1.22E-05
chr6	41703001	41708000	1.40E-10
chr6	41744001	41749000	1.09E-06
chr6	41880001	41885000	0.004035
chr6	41959001	41964000	1.97E-06
chr6	42693001	42698000	8.42E-05
chr6	42694001	42699000	0.000734
chr6	42744001	42749000	3.34E-05
chr6	42870001	42875000	1.15E-07
chr6	42927001	42932000	4.46E-08
chr6	42928001	42933000	7.10E-05
chr6	42929001	42934000	0.000544
chr6	42931001	42936000	0.000993
chr6	43031001	43036000	0.007738
chr6	43212001	43217000	2.96E-08
chr6	43213001	43218000	3.48E-07
chr6	43236001	43241000	8.11E-06
chr6	43237001	43242000	1.30E-08
chr6	43287001	43292000	4.23E-05
chr6	43288001	43293000	2.45E-06
chr6	43424001	43429000	1.41E-05
chr6	43596001	43601000	1.05E-07
chr6	43597001	43602000	3.08E-16
chr6	43599001	43604000	4.66E-09
chr6	43689001	43694000	2.68E-10
chr6	43690001	43695000	3.87E-07
chr6	43733001	43738000	0.000256
chr6	43907001	43912000	1.65E-05
chr6	44091001	44096000	5.06E-05
chr6	44092001	44097000	4.23E-06
chr6	44093001	44098000	0.003165
chr6	44094001	44099000	0.00056
chr6	44188001	44193000	0.000255
chr6	44537001	44542000	0.000418
chr6	45903001	45908000	2.05E-07
chr6	47414001	47419000	0.00012
chr6	47415001	47420000	1.89E-06
chr6	47856001	47861000	1.43E-06
chr6	47857001	47862000	8.65E-08
chr6	47858001	47863000	3.12E-10
chr6	47859001	47864000	4.41E-09
chr6	49519001	49524000	5.84E-13
chr6	50228001	50233000	0.000164
chr6	50320001	50325000	5.02E-06
chr6	50420001	50425000	5.26E-06
chr6	51659001	51664000	0.001328
chr6	53223001	53228000	4.75E-13
chr6	53224001	53229000	1.15E-09

chr6	54978001	54983000	5.19E-06
chr6	55234001	55239000	2.94E-09
chr6	55235001	55240000	3.37E-10
chr6	55237001	55242000	1.06E-12
chr6	55238001	55243000	1.70E-12
chr6	55239001	55244000	5.62E-11
chr6	55272001	55277000	5.39E-11
chr6	55517001	55522000	1.93E-11
chr6	55518001	55523000	1.51E-11
chr6	55519001	55524000	2.35E-11
chr6	55544001	55549000	3.74E-09
chr6	55857001	55862000	3.00E-06
chr6	55858001	55863000	3.01E-07
chr6	56136001	56141000	3.94E-05
chr6	56707001	56712000	9.25E-09
chr6	56708001	56713000	4.32E-09
chr6	57659001	57664000	4.53E-11
chr6	57660001	57665000	1.09E-10
chr6	57720001	57725000	0.000302
chr6	57721001	57726000	0.000265
chr6	58024001	58029000	5.31E-06
chr6	58025001	58030000	1.25E-06
chr6	58027001	58032000	0.000165
chr6	58341001	58346000	5.93E-09
chr6	58342001	58347000	3.50E-09
chr6	58352001	58357000	1.36E-06
chr6	58356001	58361000	8.82E-06
chr6	58384001	58389000	2.75E-09
chr6	58385001	58390000	1.55E-11
chr6	58386001	58391000	2.12E-11
chr6	58387001	58392000	1.41E-13
chr6	58388001	58393000	8.39E-15
chr6	61933001	61938000	9.57E-07
chr6	61934001	61939000	1.05E-08
chr6	61935001	61940000	1.76E-09
chr6	61936001	61941000	1.10E-08
chr6	61960001	61965000	1.04E-09
chr6	61961001	61966000	8.87E-12
chr6	61962001	61967000	8.03E-11
chr6	62066001	62071000	3.16E-09
chr6	62069001	62074000	4.20E-07
chr6	62120001	62125000	0.036918
chr6	62121001	62126000	0.036918
chr6	62201001	62206000	1.85E-05
chr6	62202001	62207000	0.000755
chr6	62203001	62208000	0.002152
chr6	62204001	62209000	1.20E-05
chr6	62233001	62238000	0.000239

chr6	62234001	62239000	7.16E-05
chr6	62265001	62270000	1.58E-06
chr6	62274001	62279000	7.44E-06
chr6	62298001	62303000	0.000255
chr6	62460001	62465000	2.30E-09
chr6	62489001	62494000	4.31E-08
chr6	62550001	62555000	4.40E-08
chr6	62577001	62582000	4.78E-09
chr6	62724001	62729000	1.51E-11
chr6	62800001	62805000	4.14E-10
chr6	62808001	62813000	1.72E-07
chr6	62809001	62814000	1.23E-07
chr6	62830001	62835000	1.00E-08
chr6	62831001	62836000	8.38E-08
chr6	63006001	63011000	7.64E-07
chr6	63153001	63158000	4.61E-09
chr6	63359001	63364000	9.37E-05
chr6	63504001	63509000	5.27E-12
chr6	63774001	63779000	2.62E-07
chr6	63783001	63788000	2.44E-06
chr6	63816001	63821000	9.88E-10
chr6	63817001	63822000	8.57E-11
chr6	63923001	63928000	2.58E-08
chr6	64025001	64030000	1.43E-07
chr6	64986001	64991000	1.93E-09
chr6	65067001	65072000	1.20E-09
chr6	65461001	65466000	4.90E-06
chr6	65462001	65467000	5.93E-06
chr6	65463001	65468000	2.48E-08
chr6	65469001	65474000	6.24E-08
chr6	65566001	65571000	1.94E-07
chr6	65568001	65573000	8.51E-10
chr6	65582001	65587000	6.90E-09
chr6	65627001	65632000	4.62E-16
chr6	65628001	65633000	4.96E-12
chr6	65629001	65634000	1.10E-13
chr6	65669001	65674000	8.40E-09
chr6	65740001	65745000	1.62E-06
chr6	65761001	65766000	1.90E-08
chr6	65973001	65978000	1.05E-15
chr6	65974001	65979000	2.97E-14
chr6	65975001	65980000	3.70E-13
chr6	65977001	65982000	2.94E-11
chr6	66188001	66193000	1.46E-14
chr6	66315001	66320000	9.81E-12
chr6	66613001	66618000	3.45E-05
chr6	66853001	66858000	1.73E-07
chr6	66918001	66923000	9.74E-05

chr6	66961001	66966000	7.63E-05
chr6	66969001	66974000	2.13E-08
chr6	67139001	67144000	1.70E-12
chr6	67212001	67217000	6.51E-09
chr6	67302001	67307000	2.83E-09
chr6	67571001	67576000	9.23E-09
chr6	67611001	67616000	3.68E-09
chr6	67612001	67617000	4.09E-09
chr6	67696001	67701000	1.10E-05
chr6	68018001	68023000	8.41E-08
chr6	68845001	68850000	0.000122
chr6	69159001	69164000	4.26E-12
chr6	69160001	69165000	2.29E-09
chr6	69162001	69167000	1.40E-08
chr6	69163001	69168000	2.68E-07
chr6	69399001	69404000	1.03E-12
chr6	69564001	69569000	0.000143
chr6	69699001	69704000	5.81E-12
chr6	69700001	69705000	7.20E-11
chr6	70361001	70366000	5.14E-05
chr6	70364001	70369000	0.001104
chr6	72266001	72271000	1.18E-06
chr6	74170001	74175000	6.59E-06
chr6	74288001	74293000	4.70E-06
chr6	74289001	74294000	8.29E-08
chr6	74290001	74295000	1.46E-07
chr6	74609001	74614000	3.77E-06
chr6	74610001	74615000	4.19E-06
chr6	74950001	74955000	1.40E-05
chr6	75765001	75770000	0.00096
chr6	75766001	75771000	0.011338
chr6	76252001	76257000	0.000145
chr6	76740001	76745000	5.32E-06
chr6	76768001	76773000	4.13E-05
chr6	76769001	76774000	0.000135
chr6	76770001	76775000	0.001965
chr6	76901001	76906000	0.000697
chr6	76949001	76954000	1.57E-07
chr6	77098001	77103000	1.42E-05
chr6	77110001	77115000	6.07E-06
chr6	77120001	77125000	1.42E-07
chr6	77121001	77126000	7.38E-09
chr6	77160001	77165000	0.000432
chr6	77161001	77166000	5.83E-05
chr6	77162001	77167000	8.78E-06
chr6	77163001	77168000	5.82E-09
chr6	77429001	77434000	2.34E-05
chr6	77430001	77435000	4.62E-07

chr6	77674001	77679000	2.93E-05
chr6	77705001	77710000	1.98E-05
chr6	78101001	78106000	1.11E-06
chr6	78102001	78107000	2.24E-07
chr6	78220001	78225000	6.49E-05
chr6	78221001	78226000	7.30E-07
chr6	78446001	78451000	3.11E-07
chr6	78570001	78575000	5.39E-07
chr6	78571001	78576000	5.71E-07
chr6	78595001	78600000	7.02E-06
chr6	78654001	78659000	4.64E-05
chr6	78656001	78661000	1.22E-06
chr6	78753001	78758000	2.76E-08
chr6	79089001	79094000	3.33E-05
chr6	79189001	79194000	3.09E-06
chr6	79290001	79295000	2.57E-05
chr6	79380001	79385000	3.69E-05
chr6	85034001	85039000	3.70E-06
chr6	85181001	85186000	0.004874
chr6	85501001	85506000	5.81E-05
chr6	85502001	85507000	0.000285
chr6	85705001	85710000	0.000165
chr6	85706001	85711000	0.000202
chr6	85707001	85712000	0.000156
chr6	85928001	85933000	0.009827
chr6	85963001	85968000	0.003334
chr6	86521001	86526000	0.01632
chr6	86522001	86527000	0.002509
chr6	86558001	86563000	2.15E-05
chr6	86655001	86660000	3.01E-06
chr6	86878001	86883000	2.53E-06
chr6	86879001	86884000	4.12E-07
chr6	87015001	87020000	7.85E-05
chr6	87016001	87021000	5.36E-05
chr6	87017001	87022000	3.50E-05
chr6	87059001	87064000	2.53E-08
chr6	87060001	87065000	2.93E-07
chr6	87080001	87085000	1.52E-06
chr6	87629001	87634000	9.11E-09
chr6	87630001	87635000	2.69E-09
chr6	87631001	87636000	7.15E-10
chr6	87645001	87650000	9.01E-10
chr6	87646001	87651000	1.12E-12
chr6	87647001	87652000	1.00E-10
chr6	87727001	87732000	2.00E-05
chr6	88038001	88043000	1.84E-08
chr6	88039001	88044000	2.54E-07
chr6	89690001	89695000	8.82E-06

chr6	89693001	89698000	0.000497
chr6	89694001	89699000	0.000112
chr6	90133001	90138000	0.001134
chr6	90187001	90192000	1.86E-06
chr6	91101001	91106000	5.62E-06
chr6	93724001	93729000	2.76E-05
chr6	96319001	96324000	0.000214
chr6	96781001	96786000	5.73E-05
chr6	98681001	98686000	1.93E-08
chr6	98682001	98687000	1.20E-08
chr6	99967001	99972000	1.60E-08
chr6	99968001	99973000	1.33E-07
chr6	103271001	103276000	0.002497
chr6	104447001	104452000	1.36E-05
chr6	104914001	104919000	0.000127
chr6	105422001	105427000	2.91E-06
chr6	107339001	107344000	8.48E-05
chr6	108583001	108588000	8.02E-06
chr6	108875001	108880000	1.85E-08
chr6	110708001	110713000	9.08E-05
chr6	110709001	110714000	2.69E-06
chr6	111266001	111271000	0.000129
chr6	112364001	112369000	9.46E-12
chr6	115069001	115074000	7.89E-07
chr6	116690001	116695000	7.02E-14
chr6	116691001	116696000	8.65E-16
chr6	116934001	116939000	9.91E-08
chr6	116935001	116940000	1.62E-06
chr6	116936001	116941000	9.26E-10
chr6	116937001	116942000	8.34E-11
chr6	117237001	117242000	5.87E-13
chr6	117423001	117428000	0.006314
chr6	117426001	117431000	0.012414
chr6	117427001	117432000	0.006635
chr6	118137001	118142000	7.43E-07
chr6	118138001	118143000	6.16E-08
chr6	118141001	118146000	8.46E-07
chr6	118144001	118149000	9.25E-07
chr6	118161001	118166000	0.000175
chr6	118162001	118167000	2.35E-06
chr6	118163001	118168000	2.55E-06
chr6	118164001	118169000	1.56E-07
chr6	118165001	118170000	5.94E-07
chr6	118201001	118206000	1.48E-05
chr6	119014001	119019000	8.56E-08
chr6	119117001	119122000	1.16E-07
chr6	119119001	119124000	5.12E-09
chr6	119120001	119125000	3.89E-11

chr6	119121001	119126000	2.40E-07
chr6	119403001	119408000	1.51E-08
chr6	119404001	119409000	3.12E-09
chr6	119405001	119410000	6.74E-08
chr6	119521001	119526000	1.75E-11
chr6	120344001	120349000	2.92E-08
chr6	120493001	120498000	5.07E-09
chr6	120669001	120674000	0.00042
chr6	121212001	121217000	4.03E-05
chr6	122844001	122849000	1.02E-06
chr6	123017001	123022000	0.000133
chr6	123409001	123414000	0.000275
chr6	123410001	123415000	0.000123
chr6	123529001	123534000	8.08E-09
chr6	124056001	124061000	7.25E-06
chr6	124058001	124063000	1.12E-10
chr6	124059001	124064000	1.33E-07
chr6	124660001	124665000	3.68E-07
chr6	125737001	125742000	1.68E-08
chr6	125738001	125743000	1.28E-08
chr6	125777001	125782000	1.94E-05
chr6	126185001	126190000	9.55E-07
chr6	126186001	126191000	2.57E-07
chr6	126187001	126192000	4.10E-09
chr6	127381001	127386000	9.47E-08
chr6	129145001	129150000	9.51E-07
chr6	129741001	129746000	1.07E-07
chr6	129743001	129748000	3.44E-10
chr6	130121001	130126000	2.25E-05
chr6	130525001	130530000	1.59E-05
chr6	130598001	130603000	6.95E-06
chr6	130628001	130633000	9.31E-09
chr6	131028001	131033000	2.43E-06
chr6	131029001	131034000	4.72E-11
chr6	131441001	131446000	0.000465
chr6	131854001	131859000	3.80E-06
chr6	131855001	131860000	4.78E-08
chr6	132158001	132163000	5.65E-09
chr6	133977001	133982000	9.50E-05
chr6	134472001	134477000	0.000111
chr6	138189001	138194000	4.02E-09
chr6	138190001	138195000	2.99E-12
chr6	138191001	138196000	3.94E-10
chr6	139013001	139018000	2.60E-09
chr6	139014001	139019000	4.94E-10
chr6	140754001	140759000	3.00E-07
chr6	141510001	141515000	0.000166
chr6	142450001	142455000	1.53E-06

chr6	145479001	145484000	2.17E-07
chr6	145824001	145829000	0.00139
chr6	145825001	145830000	0.001029
chr6	146300001	146305000	4.87E-07
chr6	146969001	146974000	4.14E-09
chr6	146970001	146975000	2.43E-09
chr6	147037001	147042000	1.05E-05
chr6	148409001	148414000	3.39E-05
chr6	148410001	148415000	3.39E-07
chr6	148884001	148889000	1.16E-05
chr6	150282001	150287000	1.22E-10
chr6	150283001	150288000	1.49E-12
chr6	150284001	150289000	1.07E-17
chr6	150285001	150290000	2.17E-15
chr6	150341001	150346000	0.003219
chr6	150342001	150347000	8.15E-06
chr6	150343001	150348000	8.86E-07
chr6	150344001	150349000	1.56E-05
chr6	150345001	150350000	1.10E-06
chr6	150346001	150351000	7.99E-09
chr6	150347001	150352000	0.000222
chr6	151642001	151647000	1.07E-05
chr6	151844001	151849000	6.53E-07
chr6	152150001	152155000	2.30E-07
chr6	152713001	152718000	1.05E-07
chr6	152958001	152963000	2.34E-06
chr6	153672001	153677000	5.94E-10
chr6	153673001	153678000	4.92E-06
chr6	153674001	153679000	1.78E-09
chr6	153766001	153771000	2.82E-06
chr6	155314001	155319000	0.001074
chr6	156195001	156200000	2.08E-07
chr6	156320001	156325000	3.06E-05
chr6	157700001	157705000	2.00E-06
chr6	157701001	157706000	1.01E-07
chr6	158371001	158376000	1.53E-07
chr6	158372001	158377000	1.09E-09
chr6	159113001	159118000	9.82E-07
chr6	159650001	159655000	4.80E-08
chr6	159815001	159820000	1.73E-11
chr6	160182001	160187000	4.54E-06
chr6	160275001	160280000	2.02E-08
chr6	163459001	163464000	5.36E-07
chr6	163460001	163465000	4.00E-08
chr6	165066001	165071000	1.46E-05
chr6	165666001	165671000	1.25E-09
chr6	165667001	165672000	3.86E-07
chr6	165669001	165674000	9.59E-07

chr6	165726001	165731000	0.000191
chr6	165727001	165732000	0.001361
chr6	166140001	166145000	2.32E-08
chr6	166238001	166243000	5.35E-08
chr6	166397001	166402000	2.02E-09
chr6	166398001	166403000	1.00E-07
chr6	167480001	167485000	5.34E-05
chr6	167481001	167486000	1.57E-05
chr6	167482001	167487000	1.50E-05
chr6	167815001	167820000	8.66E-06
chr6	170227001	170232000	4.12E-12
chr6	170278001	170283000	0.003472
chr6	170574001	170579000	6.28E-09
chr6	171002001	171007000	0.010526
chr6	171003001	171008000	0.034995
chr6	171004001	171009000	0.025002
chr6	171042001	171047000	1.43E-05
chr6	171043001	171048000	2.14E-06
chr7	78001	83000	4.62E-06
chr7	493001	498000	0.016133
chr7	495001	500000	0.020983
chr7	517001	522000	0.002662
chr7	540001	545000	2.98E-06
chr7	554001	559000	3.05E-09
chr7	569001	574000	2.27E-09
chr7	591001	596000	0.000374
chr7	848001	853000	2.29E-07
chr7	849001	854000	3.06E-08
chr7	850001	855000	5.76E-05
chr7	851001	856000	4.15E-05
chr7	1318001	1323000	1.48E-07
chr7	1319001	1324000	2.03E-05
chr7	1320001	1325000	8.90E-05
chr7	1321001	1326000	0.000129
chr7	1322001	1327000	0.000691
chr7	1428001	1433000	6.20E-06
chr7	1454001	1459000	3.09E-06
chr7	1455001	1460000	6.27E-07
chr7	1474001	1479000	5.09E-08
chr7	1475001	1480000	6.24E-05
chr7	1489001	1494000	0.006382
chr7	1573001	1578000	2.44E-12
chr7	1574001	1579000	6.59E-13
chr7	1577001	1582000	2.71E-09
chr7	1630001	1635000	0.002126
chr7	1631001	1636000	0.002144
chr7	1685001	1690000	1.59E-06
chr7	1729001	1734000	1.98E-07

chr7	1763001	1768000	2.55E-08
chr7	1764001	1769000	1.74E-07
chr7	1765001	1770000	3.43E-07
chr7	1786001	1791000	1.64E-06
chr7	1787001	1792000	9.88E-11
chr7	1788001	1793000	4.17E-11
chr7	1794001	1799000	3.28E-09
chr7	1952001	1957000	5.93E-05
chr7	2354001	2359000	2.12E-05
chr7	2390001	2395000	8.99E-05
chr7	2531001	2536000	0.000213
chr7	2589001	2594000	1.56E-07
chr7	2590001	2595000	1.63E-06
chr7	2591001	2596000	1.49E-06
chr7	2737001	2742000	6.07E-09
chr7	2738001	2743000	1.77E-06
chr7	2896001	2901000	1.38E-14
chr7	2897001	2902000	1.38E-10
chr7	2898001	2903000	3.03E-10
chr7	2926001	2931000	1.69E-06
chr7	2927001	2932000	0.000128
chr7	3106001	3111000	5.25E-06
chr7	4464001	4469000	4.08E-06
chr7	4465001	4470000	6.96E-06
chr7	4466001	4471000	8.09E-06
chr7	4853001	4858000	3.58E-07
chr7	4854001	4859000	0.000116
chr7	4855001	4860000	0.018405
chr7	4868001	4873000	0.000122
chr7	4973001	4978000	0.000243
chr7	4983001	4988000	0.021029
chr7	5005001	5010000	0.003602
chr7	5118001	5123000	1.67E-09
chr7	5318001	5323000	2.76E-05
chr7	5569001	5574000	3.35E-09
chr7	5570001	5575000	1.79E-08
chr7	5605001	5610000	0.001657
chr7	5606001	5611000	2.14E-05
chr7	5607001	5612000	7.23E-06
chr7	5608001	5613000	0.009732
chr7	5611001	5616000	4.61E-05
chr7	5612001	5617000	4.16E-06
chr7	5643001	5648000	1.32E-06
chr7	5644001	5649000	1.32E-06
chr7	5857001	5862000	0.000294
chr7	5860001	5865000	3.32E-10
chr7	5861001	5866000	5.45E-13
chr7	5862001	5867000	3.42E-07

chr7	5863001	5868000	4.60E-06
chr7	6046001	6051000	1.48E-06
chr7	6049001	6054000	0.002533
chr7	6389001	6394000	0.001237
chr7	6423001	6428000	0.000113
chr7	6520001	6525000	1.56E-14
chr7	6521001	6526000	1.51E-09
chr7	6522001	6527000	3.75E-07
chr7	6566001	6571000	7.25E-07
chr7	6567001	6572000	1.58E-11
chr7	6568001	6573000	2.76E-09
chr7	6574001	6579000	0.000184
chr7	6604001	6609000	8.45E-05
chr7	6605001	6610000	7.10E-06
chr7	6613001	6618000	9.31E-10
chr7	6614001	6619000	2.46E-10
chr7	6615001	6620000	3.21E-10
chr7	6616001	6621000	5.87E-10
chr7	6653001	6658000	2.31E-06
chr7	6654001	6659000	2.81E-06
chr7	6655001	6660000	8.59E-09
chr7	6693001	6698000	0.002136
chr7	6694001	6699000	0.000832
chr7	6695001	6700000	4.43E-05
chr7	6696001	6701000	4.04E-06
chr7	6703001	6708000	4.82E-08
chr7	7052001	7057000	6.17E-07
chr7	7053001	7058000	2.59E-08
chr7	7179001	7184000	5.31E-05
chr7	7715001	7720000	1.79E-05
chr7	9209001	9214000	5.87E-05
chr7	9210001	9215000	1.88E-05
chr7	10031001	10036000	2.12E-05
chr7	10032001	10037000	1.13E-05
chr7	10097001	10102000	1.03E-09
chr7	10393001	10398000	2.08E-06
chr7	10707001	10712000	2.68E-08
chr7	11557001	11562000	6.88E-07
chr7	14750001	14755000	0.000101
chr7	15806001	15811000	1.54E-05
chr7	15926001	15931000	2.08E-06
chr7	15927001	15932000	9.91E-09
chr7	15928001	15933000	1.72E-07
chr7	15929001	15934000	1.15E-06
chr7	20533001	20538000	0.000693
chr7	20534001	20539000	0.00127
chr7	20535001	20540000	0.001956
chr7	23634001	23639000	8.13E-07

chr7	24034001	24039000	0.000464
chr7	24035001	24040000	3.49E-07
chr7	24036001	24041000	4.28E-06
chr7	24037001	24042000	3.03E-07
chr7	24059001	24064000	0.000479
chr7	25165001	25170000	3.71E-10
chr7	25489001	25494000	1.29E-06
chr7	25914001	25919000	1.53E-08
chr7	28133001	28138000	1.16E-07
chr7	28246001	28251000	1.13E-05
chr7	29812001	29817000	2.94E-05
chr7	29813001	29818000	3.32E-07
chr7	30721001	30726000	4.64E-07
chr7	30766001	30771000	0.000393
chr7	30767001	30772000	0.000427
chr7	30773001	30778000	4.44E-06
chr7	30883001	30888000	1.79E-05
chr7	31111001	31116000	4.98E-06
chr7	31524001	31529000	2.47E-07
chr7	31709001	31714000	2.97E-06
chr7	31786001	31791000	5.81E-06
chr7	32008001	32013000	1.17E-07
chr7	32265001	32270000	1.62E-09
chr7	32266001	32271000	1.43E-08
chr7	32272001	32277000	1.89E-07
chr7	32273001	32278000	1.10E-07
chr7	32274001	32279000	1.22E-06
chr7	32275001	32280000	1.18E-06
chr7	32276001	32281000	2.82E-06
chr7	32401001	32406000	0.000599
chr7	32468001	32473000	1.03E-05
chr7	32487001	32492000	3.37E-06
chr7	32488001	32493000	8.75E-06
chr7	33363001	33368000	3.55E-05
chr7	34060001	34065000	6.14E-07
chr7	36536001	36541000	1.97E-06
chr7	37345001	37350000	4.00E-06
chr7	37472001	37477000	4.88E-07
chr7	38300001	38305000	2.07E-07
chr7	39453001	39458000	1.36E-12
chr7	40345001	40350000	1.32E-05
chr7	40351001	40356000	1.54E-08
chr7	40993001	40998000	0.000162
chr7	40999001	41004000	0.002317
chr7	42276001	42281000	1.04E-07
chr7	43794001	43799000	0.000269
chr7	43887001	43892000	0.000599
chr7	43962001	43967000	9.96E-05

chr7	43963001	43968000	1.10E-05
chr7	43964001	43969000	4.56E-05
chr7	43965001	43970000	5.97E-05
chr7	44064001	44069000	2.16E-05
chr7	44065001	44070000	6.59E-06
chr7	44103001	44108000	0.003049
chr7	44104001	44109000	0.001237
chr7	44224001	44229000	0.000128
chr7	44225001	44230000	0.000141
chr7	44397001	44402000	0.001021
chr7	44774001	44779000	5.01E-05
chr7	44822001	44827000	0.037317
chr7	44823001	44828000	0.019125
chr7	45001001	45006000	0.000279
chr7	45002001	45007000	0.001131
chr7	45021001	45026000	8.89E-06
chr7	45035001	45040000	7.96E-08
chr7	45119001	45124000	0.003161
chr7	45120001	45125000	0.001399
chr7	45215001	45220000	0.001164
chr7	45538001	45543000	0.00025
chr7	45901001	45906000	1.44E-06
chr7	46502001	46507000	2.77E-05
chr7	46503001	46508000	0.000248
chr7	46846001	46851000	3.35E-06
chr7	46847001	46852000	1.66E-06
chr7	47492001	47497000	2.80E-05
chr7	47493001	47498000	2.59E-05
chr7	48726001	48731000	3.55E-05
chr7	49260001	49265000	0.000432
chr7	49335001	49340000	5.58E-08
chr7	49643001	49648000	4.73E-06
chr7	49773001	49778000	1.20E-05
chr7	50182001	50187000	8.11E-08
chr7	51799001	51804000	4.35E-06
chr7	51962001	51967000	1.93E-07
chr7	51963001	51968000	6.04E-07
chr7	51973001	51978000	3.03E-07
chr7	51974001	51979000	3.95E-07
chr7	52201001	52206000	8.36E-06
chr7	52202001	52207000	0.00028
chr7	52203001	52208000	9.82E-05
chr7	52204001	52209000	5.18E-05
chr7	53101001	53106000	2.49E-05
chr7	53102001	53107000	2.13E-06
chr7	53126001	53131000	0.000397
chr7	53127001	53132000	3.86E-07
chr7	53762001	53767000	1.15E-06

chr7	53873001	53878000	1.95E-07
chr7	53882001	53887000	0.000275
chr7	53883001	53888000	2.59E-05
chr7	53884001	53889000	9.16E-05
chr7	53885001	53890000	0.000867
chr7	54571001	54576000	2.35E-07
chr7	54572001	54577000	0.000257
chr7	54622001	54627000	4.89E-05
chr7	54664001	54669000	2.90E-10
chr7	54783001	54788000	1.60E-05
chr7	55397001	55402000	9.88E-13
chr7	55398001	55403000	6.92E-10
chr7	55399001	55404000	4.10E-11
chr7	55400001	55405000	7.21E-11
chr7	55401001	55406000	1.64E-12
chr7	55658001	55663000	0.000955
chr7	55659001	55664000	1.75E-06
chr7	55660001	55665000	2.00E-07
chr7	55661001	55666000	7.70E-07
chr7	55817001	55822000	3.17E-10
chr7	55818001	55823000	5.36E-13
chr7	55819001	55824000	5.76E-11
chr7	55820001	55825000	8.31E-10
chr7	56015001	56020000	1.66E-18
chr7	56016001	56021000	9.81E-12
chr7	56114001	56119000	6.73E-12
chr7	56115001	56120000	1.34E-27
chr7	56116001	56121000	2.35E-32
chr7	56117001	56122000	7.54E-42
chr7	56118001	56123000	2.64E-19
chr7	56119001	56124000	1.21E-17
chr7	56227001	56232000	5.39E-13
chr7	56228001	56233000	1.38E-17
chr7	56229001	56234000	5.46E-15
chr7	56230001	56235000	8.22E-14
chr7	56231001	56236000	3.74E-08
chr7	56251001	56256000	1.30E-12
chr7	56252001	56257000	7.23E-13
chr7	56254001	56259000	5.10E-12
chr7	56284001	56289000	4.20E-12
chr7	56285001	56290000	1.06E-15
chr7	56286001	56291000	1.47E-10
chr7	56287001	56292000	2.57E-10
chr7	56305001	56310000	2.44E-09
chr7	56315001	56320000	2.59E-16
chr7	56316001	56321000	1.03E-13
chr7	56317001	56322000	1.32E-10
chr7	56318001	56323000	1.27E-08

chr7	56319001	56324000	5.27E-08
chr7	56327001	56332000	0.000663
chr7	56340001	56345000	7.58E-06
chr7	56342001	56347000	1.56E-13
chr7	56343001	56348000	1.22E-12
chr7	56344001	56349000	6.28E-13
chr7	56345001	56350000	6.10E-12
chr7	56346001	56351000	5.92E-12
chr7	56347001	56352000	4.16E-06
chr7	56411001	56416000	3.12E-06
chr7	56413001	56418000	1.96E-08
chr7	56414001	56419000	1.07E-08
chr7	56415001	56420000	2.17E-10
chr7	56425001	56430000	1.04E-10
chr7	56426001	56431000	1.01E-13
chr7	56427001	56432000	1.07E-07
chr7	56428001	56433000	8.35E-06
chr7	56461001	56466000	8.77E-09
chr7	56462001	56467000	5.74E-11
chr7	56463001	56468000	4.30E-12
chr7	56464001	56469000	1.06E-09
chr7	56725001	56730000	2.95E-07
chr7	56726001	56731000	5.72E-07
chr7	56787001	56792000	2.79E-06
chr7	56788001	56793000	2.17E-06
chr7	56823001	56828000	0.006674
chr7	56824001	56829000	3.45E-05
chr7	56825001	56830000	0.000279
chr7	56865001	56870000	2.20E-06
chr7	56866001	56871000	7.36E-05
chr7	56991001	56996000	0.001417
chr7	56996001	57001000	0.007256
chr7	56998001	57003000	0.001342
chr7	57036001	57041000	0.000629
chr7	57037001	57042000	0.000637
chr7	57075001	57080000	3.86E-07
chr7	57076001	57081000	4.06E-06
chr7	57077001	57082000	6.03E-06
chr7	57081001	57086000	0.001888
chr7	57082001	57087000	0.001888
chr7	57083001	57088000	0.001888
chr7	57084001	57089000	0.003278
chr7	57099001	57104000	0.002673
chr7	57117001	57122000	2.92E-06
chr7	57450001	57455000	2.84E-05
chr7	57462001	57467000	1.71E-06
chr7	57464001	57469000	0.000406
chr7	57507001	57512000	1.31E-05

chr7	57508001	57513000	5.83E-06
chr7	57828001	57833000	2.17E-07
chr7	57829001	57834000	1.16E-05
chr7	57830001	57835000	2.14E-06
chr7	61274001	61279000	0.000924
chr7	61388001	61393000	0.009459
chr7	61389001	61394000	0.009459
chr7	61390001	61395000	0.009459
chr7	61391001	61396000	0.009459
chr7	61392001	61397000	0.009459
chr7	61415001	61420000	0.00952
chr7	61417001	61422000	0.018792
chr7	61455001	61460000	0.003447
chr7	61620001	61625000	0.004598
chr7	61645001	61650000	0.000284
chr7	61646001	61651000	0.01561
chr7	62401001	62406000	3.68E-07
chr7	62499001	62504000	1.92E-07
chr7	62502001	62507000	7.33E-06
chr7	62503001	62508000	1.96E-06
chr7	62504001	62509000	0.000164
chr7	62505001	62510000	1.56E-07
chr7	62506001	62511000	4.73E-05
chr7	62535001	62540000	2.65E-05
chr7	62566001	62571000	3.87E-06
chr7	62648001	62653000	4.11E-09
chr7	62649001	62654000	9.29E-09
chr7	62652001	62657000	2.20E-09
chr7	62743001	62748000	0.000967
chr7	62744001	62749000	0.00012
chr7	62750001	62755000	1.77E-05
chr7	62781001	62786000	0.008182
chr7	62914001	62919000	0.001522
chr7	62915001	62920000	0.001187
chr7	62935001	62940000	4.94E-05
chr7	62952001	62957000	0.037771
chr7	62953001	62958000	0.037771
chr7	62954001	62959000	0.024659
chr7	62969001	62974000	0.001262
chr7	63000001	63005000	0.009793
chr7	63001001	63006000	0.002814
chr7	63002001	63007000	0.002814
chr7	63029001	63034000	2.72E-08
chr7	63160001	63165000	4.33E-05
chr7	63161001	63166000	0.000137
chr7	63162001	63167000	9.28E-05
chr7	63169001	63174000	0.003709
chr7	63348001	63353000	1.04E-05

chr7	63483001	63488000	0.000224
chr7	63484001	63489000	0.000378
chr7	63485001	63490000	7.49E-07
chr7	63486001	63491000	3.28E-06
chr7	63487001	63492000	4.16E-07
chr7	63601001	63606000	2.61E-07
chr7	63602001	63607000	3.06E-07
chr7	63661001	63666000	7.57E-08
chr7	63683001	63688000	0.001359
chr7	63760001	63765000	0.000281
chr7	63761001	63766000	5.69E-06
chr7	63762001	63767000	1.02E-06
chr7	63763001	63768000	3.97E-08
chr7	63764001	63769000	1.35E-07
chr7	63766001	63771000	1.13E-05
chr7	63767001	63772000	2.34E-08
chr7	63936001	63941000	2.33E-06
chr7	63938001	63943000	2.45E-06
chr7	64049001	64054000	1.65E-07
chr7	64050001	64055000	1.67E-06
chr7	64222001	64227000	0.000752
chr7	64622001	64627000	0.00062
chr7	64675001	64680000	6.44E-07
chr7	64700001	64705000	3.20E-11
chr7	64701001	64706000	1.12E-15
chr7	64702001	64707000	4.15E-16
chr7	64703001	64708000	1.18E-10
chr7	64886001	64891000	0.004991
chr7	64919001	64924000	4.80E-07
chr7	64987001	64992000	0.00021
chr7	64988001	64993000	0.004675
chr7	64989001	64994000	0.002004
chr7	65009001	65014000	0.00427
chr7	65025001	65030000	0.023068
chr7	65026001	65031000	0.023068
chr7	65027001	65032000	0.023068
chr7	65028001	65033000	0.023068
chr7	65029001	65034000	0.009155
chr7	65112001	65117000	4.22E-05
chr7	65304001	65309000	0.013482
chr7	65857001	65862000	0.000421
chr7	65878001	65883000	3.43E-05
chr7	65879001	65884000	0.00049
chr7	65958001	65963000	9.74E-07
chr7	65981001	65986000	2.97E-07
chr7	65982001	65987000	3.03E-08
chr7	66074001	66079000	0.000169
chr7	66204001	66209000	6.58E-07

chr7	66205001	66210000	1.04E-11
chr7	66765001	66770000	1.71E-08
chr7	66766001	66771000	6.43E-07
chr7	66767001	66772000	1.05E-11
chr7	66861001	66866000	1.80E-06
chr7	66911001	66916000	0.000101
chr7	66912001	66917000	3.87E-07
chr7	66924001	66929000	7.38E-06
chr7	66925001	66930000	2.98E-05
chr7	66926001	66931000	6.73E-05
chr7	66969001	66974000	0.018771
chr7	66970001	66975000	2.77E-06
chr7	66972001	66977000	1.03E-07
chr7	67031001	67036000	4.83E-11
chr7	67038001	67043000	5.22E-06
chr7	67039001	67044000	4.83E-06
chr7	67048001	67053000	0.000109
chr7	67049001	67054000	0.000181
chr7	67050001	67055000	0.000723
chr7	67092001	67097000	9.76E-09
chr7	67093001	67098000	9.93E-11
chr7	67094001	67099000	7.40E-05
chr7	67153001	67158000	6.99E-10
chr7	67154001	67159000	1.31E-09
chr7	67155001	67160000	3.29E-06
chr7	67239001	67244000	9.36E-07
chr7	67240001	67245000	1.06E-06
chr7	67353001	67358000	1.53E-05
chr7	67354001	67359000	0.000265
chr7	67362001	67367000	4.73E-05
chr7	67604001	67609000	2.65E-05
chr7	67698001	67703000	1.10E-05
chr7	67843001	67848000	6.66E-10
chr7	67844001	67849000	2.20E-10
chr7	68071001	68076000	8.40E-05
chr7	68073001	68078000	0.000136
chr7	68074001	68079000	0.000126
chr7	68155001	68160000	0.000218
chr7	68180001	68185000	2.66E-06
chr7	68181001	68186000	8.01E-06
chr7	68182001	68187000	1.51E-05
chr7	68183001	68188000	8.40E-06
chr7	68184001	68189000	0.000662
chr7	68185001	68190000	0.000517
chr7	68283001	68288000	0.000349
chr7	68284001	68289000	4.67E-05
chr7	68285001	68290000	0.000137
chr7	68520001	68525000	5.50E-05

chr7	68521001	68526000	1.10E-05
chr7	68565001	68570000	1.75E-07
chr7	68566001	68571000	1.48E-08
chr7	68567001	68572000	4.28E-09
chr7	68775001	68780000	0.000125
chr7	68832001	68837000	4.96E-10
chr7	68962001	68967000	9.05E-05
chr7	69064001	69069000	2.02E-12
chr7	70328001	70333000	0.000203
chr7	70329001	70334000	2.77E-05
chr7	70330001	70335000	9.09E-06
chr7	70332001	70337000	0.000475
chr7	70367001	70372000	2.41E-05
chr7	70423001	70428000	0.007637
chr7	70693001	70698000	0.000453
chr7	70725001	70730000	6.08E-05
chr7	70726001	70731000	0.00045
chr7	71057001	71062000	2.30E-07
chr7	71058001	71063000	1.05E-09
chr7	71229001	71234000	0.000389
chr7	71282001	71287000	6.89E-07
chr7	71494001	71499000	0.002042
chr7	71597001	71602000	1.03E-10
chr7	71619001	71624000	0.001391
chr7	71684001	71689000	0.002395
chr7	71695001	71700000	1.31E-05
chr7	71722001	71727000	5.33E-05
chr7	71737001	71742000	0.000105
chr7	71738001	71743000	0.000294
chr7	71739001	71744000	0.000507
chr7	71803001	71808000	6.16E-05
chr7	71804001	71809000	1.39E-06
chr7	71888001	71893000	0.000213
chr7	71889001	71894000	0.000679
chr7	72300001	72305000	5.36E-08
chr7	72724001	72729000	0.024645
chr7	72969001	72974000	7.20E-10
chr7	72970001	72975000	9.50E-08
chr7	72988001	72993000	3.83E-06
chr7	73017001	73022000	4.24E-07
chr7	73018001	73023000	1.08E-06
chr7	73019001	73024000	6.52E-08
chr7	73020001	73025000	1.48E-07
chr7	73021001	73026000	1.22E-10
chr7	73023001	73028000	1.28E-07
chr7	73024001	73029000	1.02E-08
chr7	73025001	73030000	8.73E-09
chr7	73026001	73031000	2.77E-07

chr7	73027001	73032000	8.13E-08
chr7	73119001	73124000	0.003558
chr7	73120001	73125000	0.000143
chr7	73121001	73126000	2.90E-05
chr7	73181001	73186000	8.23E-05
chr7	73182001	73187000	2.01E-05
chr7	73253001	73258000	1.65E-05
chr7	73263001	73268000	0.000108
chr7	73303001	73308000	1.53E-06
chr7	73320001	73325000	0.000294
chr7	73395001	73400000	6.94E-09
chr7	73396001	73401000	6.70E-08
chr7	73412001	73417000	9.00E-05
chr7	73413001	73418000	5.39E-07
chr7	73414001	73419000	1.21E-06
chr7	73508001	73513000	0.000658
chr7	73548001	73553000	0.02104
chr7	73584001	73589000	1.24E-05
chr7	73625001	73630000	0.00134
chr7	73687001	73692000	0.01422
chr7	73688001	73693000	0.02627
chr7	73689001	73694000	0.022106
chr7	73759001	73764000	0.000174
chr7	73760001	73765000	8.76E-06
chr7	73761001	73766000	6.55E-09
chr7	73762001	73767000	5.65E-13
chr7	73827001	73832000	0.000783
chr7	73890001	73895000	0.003031
chr7	73937001	73942000	2.42E-05
chr7	73938001	73943000	3.03E-05
chr7	73939001	73944000	0.000215
chr7	73956001	73961000	0.000322
chr7	73957001	73962000	0.001181
chr7	74002001	74007000	2.10E-05
chr7	74010001	74015000	0.002344
chr7	74011001	74016000	0.005247
chr7	74067001	74072000	2.04E-07
chr7	74068001	74073000	9.35E-15
chr7	74069001	74074000	1.06E-18
chr7	74070001	74075000	1.39E-07
chr7	74208001	74213000	0.000777
chr7	74209001	74214000	0.000142
chr7	74981001	74986000	0.009347
chr7	74982001	74987000	0.009347
chr7	74983001	74988000	0.009347
chr7	75153001	75158000	5.71E-10
chr7	75154001	75159000	7.79E-09
chr7	75155001	75160000	1.20E-06

chr7	75156001	75161000	4.65E-08
chr7	75157001	75162000	2.40E-08
chr7	75464001	75469000	3.24E-05
chr7	75544001	75549000	5.29E-06
chr7	75824001	75829000	0.000763
chr7	75827001	75832000	1.32E-05
chr7	75828001	75833000	1.50E-09
chr7	75829001	75834000	4.69E-06
chr7	75830001	75835000	1.75E-06
chr7	75831001	75836000	2.91E-05
chr7	75834001	75839000	0.001644
chr7	75835001	75840000	0.000301
chr7	75838001	75843000	0.002993
chr7	75861001	75866000	8.53E-06
chr7	75921001	75926000	9.69E-05
chr7	75922001	75927000	0.00042
chr7	75923001	75928000	0.000143
chr7	75927001	75932000	0.000239
chr7	75944001	75949000	0.000362
chr7	75945001	75950000	5.48E-07
chr7	75946001	75951000	1.39E-08
chr7	75947001	75952000	8.57E-10
chr7	75948001	75953000	4.39E-06
chr7	76062001	76067000	0.003514
chr7	76071001	76076000	5.34E-07
chr7	76072001	76077000	3.29E-09
chr7	76073001	76078000	1.56E-05
chr7	76074001	76079000	2.47E-05
chr7	76228001	76233000	1.59E-05
chr7	76585001	76590000	0.00275
chr7	76612001	76617000	0.000754
chr7	77162001	77167000	2.94E-10
chr7	77163001	77168000	2.38E-13
chr7	84228001	84233000	9.29E-08
chr7	84229001	84234000	1.02E-07
chr7	85954001	85959000	2.04E-06
chr7	86240001	86245000	9.57E-06
chr7	86241001	86246000	5.83E-06
chr7	86242001	86247000	1.37E-06
chr7	86243001	86248000	1.22E-06
chr7	88172001	88177000	3.64E-05
chr7	89061001	89066000	1.24E-09
chr7	89306001	89311000	5.26E-05
chr7	90081001	90086000	1.21E-05
chr7	90094001	90099000	1.94E-07
chr7	91324001	91329000	0.000531
chr7	91325001	91330000	0.001211
chr7	92584001	92589000	3.09E-06

chr7	93933001	93938000	1.55E-10
chr7	94782001	94787000	8.37E-07
chr7	94783001	94788000	8.48E-05
chr7	95397001	95402000	1.73E-10
chr7	95398001	95403000	4.71E-10
chr7	96956001	96961000	1.62E-06
chr7	96957001	96962000	1.06E-07
chr7	96958001	96963000	2.34E-06
chr7	96959001	96964000	1.86E-07
chr7	97020001	97025000	3.49E-09
chr7	97021001	97026000	3.98E-14
chr7	97022001	97027000	1.02E-13
chr7	97068001	97073000	1.33E-10
chr7	97070001	97075000	1.61E-09
chr7	97138001	97143000	1.19E-08
chr7	97154001	97159000	2.27E-12
chr7	97187001	97192000	4.10E-06
chr7	97190001	97195000	7.37E-05
chr7	97248001	97253000	1.95E-09
chr7	97249001	97254000	1.02E-10
chr7	97250001	97255000	1.14E-12
chr7	97251001	97256000	1.70E-11
chr7	97268001	97273000	7.53E-13
chr7	97269001	97274000	3.69E-10
chr7	97270001	97275000	9.35E-11
chr7	97271001	97276000	4.27E-10
chr7	97272001	97277000	1.97E-07
chr7	97273001	97278000	5.43E-08
chr7	97274001	97279000	3.21E-11
chr7	97275001	97280000	2.44E-10
chr7	97276001	97281000	3.90E-07
chr7	97304001	97309000	0.000833
chr7	97453001	97458000	3.99E-11
chr7	97602001	97607000	2.57E-07
chr7	97685001	97690000	0.000142
chr7	97839001	97844000	2.80E-09
chr7	97840001	97845000	9.84E-11
chr7	97982001	97987000	2.15E-06
chr7	98150001	98155000	6.08E-05
chr7	98152001	98157000	4.70E-06
chr7	98153001	98158000	1.17E-08
chr7	98154001	98159000	1.26E-08
chr7	98156001	98161000	2.16E-06
chr7	98192001	98197000	6.76E-06
chr7	98195001	98200000	3.02E-11
chr7	98196001	98201000	4.81E-08
chr7	98197001	98202000	2.13E-07
chr7	98198001	98203000	4.20E-09

chr7	98199001	98204000	1.68E-10
chr7	98263001	98268000	1.43E-10
chr7	98281001	98286000	8.33E-06
chr7	98300001	98305000	0.000109
chr7	98301001	98306000	3.71E-06
chr7	98302001	98307000	1.95E-07
chr7	98703001	98708000	1.82E-11
chr7	98864001	98869000	1.28E-16
chr7	98865001	98870000	8.27E-14
chr7	98866001	98871000	5.13E-14
chr7	98867001	98872000	3.34E-08
chr7	98868001	98873000	8.10E-07
chr7	98970001	98975000	2.47E-06
chr7	99406001	99411000	1.57E-08
chr7	99407001	99412000	8.85E-05
chr7	99528001	99533000	5.30E-07
chr7	99552001	99557000	9.20E-08
chr7	99712001	99717000	7.92E-08
chr7	99745001	99750000	3.16E-12
chr7	99748001	99753000	7.53E-06
chr7	99772001	99777000	0.002503
chr7	99773001	99778000	3.67E-08
chr7	99774001	99779000	4.59E-07
chr7	99775001	99780000	2.15E-05
chr7	99838001	99843000	3.52E-06
chr7	99840001	99845000	1.92E-09
chr7	99841001	99846000	1.16E-11
chr7	99842001	99847000	8.63E-10
chr7	99843001	99848000	1.06E-07
chr7	100029001	100034000	3.47E-06
chr7	100030001	100035000	3.47E-06
chr7	100083001	100088000	0.000449
chr7	100084001	100089000	0.00161
chr7	100099001	100104000	1.56E-06
chr7	100103001	100108000	4.79E-08
chr7	100197001	100202000	0.002128
chr7	100198001	100203000	1.97E-05
chr7	100199001	100204000	0.000157
chr7	100200001	100205000	0.002593
chr7	100201001	100206000	0.02713
chr7	100237001	100242000	6.88E-08
chr7	100238001	100243000	9.26E-08
chr7	100252001	100257000	1.47E-05
chr7	100253001	100258000	0.000187
chr7	100268001	100273000	3.14E-09
chr7	100299001	100304000	0.00014
chr7	100390001	100395000	0.000109
chr7	100459001	100464000	0.000166

chr7	100460001	100465000	0.007603
chr7	100699001	100704000	6.43E-08
chr7	100725001	100730000	0.00081
chr7	100726001	100731000	0.000652
chr7	100729001	100734000	9.39E-06
chr7	100947001	100952000	2.94E-07
chr7	100948001	100953000	3.53E-10
chr7	100987001	100992000	0.000263
chr7	100992001	100997000	3.66E-05
chr7	100999001	101004000	0.001254
chr7	101146001	101151000	7.06E-13
chr7	101147001	101152000	2.21E-15
chr7	101148001	101153000	9.86E-16
chr7	101149001	101154000	6.01E-18
chr7	101150001	101155000	1.27E-14
chr7	101153001	101158000	8.87E-06
chr7	101284001	101289000	5.01E-10
chr7	101285001	101290000	5.16E-11
chr7	101286001	101291000	3.61E-13
chr7	101287001	101292000	2.44E-05
chr7	101372001	101377000	1.39E-08
chr7	101742001	101747000	6.21E-07
chr7	101743001	101748000	8.93E-09
chr7	101933001	101938000	0.000191
chr7	101934001	101939000	0.000235
chr7	101935001	101940000	0.000165
chr7	102032001	102037000	2.92E-07
chr7	102115001	102120000	0.001909
chr7	102229001	102234000	1.11E-13
chr7	102230001	102235000	1.11E-13
chr7	102231001	102236000	1.11E-13
chr7	102232001	102237000	9.55E-13
chr7	102265001	102270000	2.97E-09
chr7	102266001	102271000	3.20E-10
chr7	102344001	102349000	0.000512
chr7	102630001	102635000	6.20E-05
chr7	102989001	102994000	1.89E-10
chr7	103055001	103060000	1.02E-05
chr7	103281001	103286000	8.36E-07
chr7	103501001	103506000	1.26E-11
chr7	103502001	103507000	3.97E-11
chr7	103503001	103508000	6.74E-14
chr7	103928001	103933000	4.66E-07
chr7	103929001	103934000	2.27E-08
chr7	104176001	104181000	7.65E-10
chr7	104177001	104182000	1.28E-08
chr7	104178001	104183000	3.13E-08
chr7	104179001	104184000	8.59E-08

chr7	104180001	104185000	4.46E-08
chr7	104416001	104421000	4.48E-06
chr7	104417001	104422000	8.78E-07
chr7	104418001	104423000	4.48E-09
chr7	104514001	104519000	1.66E-05
chr7	104984001	104989000	4.38E-09
chr7	105562001	105567000	7.93E-08
chr7	105770001	105775000	5.76E-09
chr7	106324001	106329000	3.05E-08
chr7	106583001	106588000	1.16E-07
chr7	106596001	106601000	6.69E-05
chr7	106597001	106602000	6.09E-05
chr7	106608001	106613000	1.85E-06
chr7	106609001	106614000	2.40E-07
chr7	106610001	106615000	8.18E-08
chr7	106685001	106690000	1.28E-14
chr7	106686001	106691000	3.53E-13
chr7	107363001	107368000	8.57E-08
chr7	107364001	107369000	1.10E-06
chr7	107365001	107370000	1.28E-07
chr7	107639001	107644000	2.65E-07
chr7	107892001	107897000	1.91E-14
chr7	107893001	107898000	6.32E-13
chr7	107894001	107899000	3.61E-14
chr7	107895001	107900000	5.04E-13
chr7	108393001	108398000	0.000625
chr7	108395001	108400000	5.88E-07
chr7	108396001	108401000	1.55E-06
chr7	108413001	108418000	1.50E-07
chr7	108414001	108419000	1.93E-07
chr7	108461001	108466000	2.47E-10
chr7	108484001	108489000	1.41E-06
chr7	108786001	108791000	8.36E-10
chr7	108787001	108792000	1.43E-08
chr7	108941001	108946000	1.09E-08
chr7	109218001	109223000	2.01E-05
chr7	109263001	109268000	7.27E-11
chr7	109543001	109548000	2.34E-06
chr7	109544001	109549000	5.41E-08
chr7	109545001	109550000	8.25E-07
chr7	109641001	109646000	1.03E-06
chr7	109713001	109718000	9.56E-12
chr7	109787001	109792000	3.23E-08
chr7	109947001	109952000	8.34E-08
chr7	110041001	110046000	3.23E-07
chr7	110247001	110252000	1.55E-08
chr7	110248001	110253000	6.43E-08
chr7	110344001	110349000	1.61E-05

chr7	110668001	110673000	2.23E-07
chr7	112194001	112199000	2.06E-06
chr7	112195001	112200000	1.55E-07
chr7	112685001	112690000	1.11E-07
chr7	114352001	114357000	6.20E-06
chr7	114353001	114358000	2.34E-07
chr7	116267001	116272000	2.44E-11
chr7	116268001	116273000	8.86E-09
chr7	116566001	116571000	1.63E-08
chr7	117912001	117917000	2.25E-06
chr7	118225001	118230000	0.000125
chr7	120372001	120377000	1.04E-11
chr7	120373001	120378000	9.35E-12
chr7	124812001	124817000	9.10E-07
chr7	124814001	124819000	1.06E-07
chr7	125397001	125402000	0.000561
chr7	125398001	125403000	0.000378
chr7	125399001	125404000	0.001906
chr7	126176001	126181000	5.94E-09
chr7	126207001	126212000	6.56E-05
chr7	126208001	126213000	2.69E-05
chr7	126741001	126746000	0.025795
chr7	127194001	127199000	2.42E-05
chr7	127273001	127278000	0.001049
chr7	128113001	128118000	1.53E-12
chr7	128114001	128119000	4.11E-07
chr7	128115001	128120000	7.94E-07
chr7	128116001	128121000	6.62E-07
chr7	128168001	128173000	7.58E-06
chr7	128309001	128314000	5.69E-08
chr7	128310001	128315000	2.74E-08
chr7	128312001	128317000	2.20E-06
chr7	128313001	128318000	4.40E-08
chr7	128546001	128551000	0.01675
chr7	128547001	128552000	0.000918
chr7	128548001	128553000	0.041966
chr7	129093001	129098000	3.92E-05
chr7	129438001	129443000	3.18E-07
chr7	129591001	129596000	0.000253
chr7	129609001	129614000	0.006904
chr7	129613001	129618000	0.008353
chr7	130575001	130580000	8.20E-06
chr7	130583001	130588000	1.66E-12
chr7	130584001	130589000	4.76E-07
chr7	130585001	130590000	4.66E-05
chr7	131272001	131277000	0.00013
chr7	131273001	131278000	6.85E-05
chr7	131672001	131677000	0.017428

chr7	131701001	131706000	1.06E-06
chr7	131702001	131707000	4.80E-06
chr7	131703001	131708000	5.45E-06
chr7	131873001	131878000	0.001145
chr7	131874001	131879000	3.08E-06
chr7	131875001	131880000	8.28E-08
chr7	131876001	131881000	4.79E-10
chr7	131877001	131882000	5.31E-06
chr7	131878001	131883000	4.22E-07
chr7	132175001	132180000	3.26E-05
chr7	132209001	132214000	5.25E-10
chr7	132210001	132215000	0.007666
chr7	132261001	132266000	4.23E-08
chr7	132386001	132391000	5.77E-06
chr7	132387001	132392000	2.00E-05
chr7	134892001	134897000	3.22E-11
chr7	134893001	134898000	9.52E-08
chr7	134894001	134899000	2.72E-11
chr7	134895001	134900000	5.62E-09
chr7	134935001	134940000	0.001443
chr7	135194001	135199000	1.06E-05
chr7	135195001	135200000	1.03E-06
chr7	135398001	135403000	0.00308
chr7	135399001	135404000	0.000351
chr7	135401001	135406000	0.000307
chr7	136432001	136437000	1.67E-05
chr7	136480001	136485000	0.000206
chr7	136550001	136555000	2.39E-13
chr7	137044001	137049000	1.26E-06
chr7	137045001	137050000	0.000104
chr7	138328001	138333000	0.00284
chr7	138330001	138335000	0.001009
chr7	138381001	138386000	0.003157
chr7	138408001	138413000	9.68E-07
chr7	139026001	139031000	4.64E-07
chr7	140004001	140009000	0.000146
chr7	140005001	140010000	0.00013
chr7	140192001	140197000	0.003683
chr7	140193001	140198000	0.002362
chr7	140393001	140398000	3.77E-08
chr7	140394001	140399000	3.29E-06
chr7	140395001	140400000	3.04E-08
chr7	140396001	140401000	3.29E-11
chr7	140723001	140728000	0.000591
chr7	141207001	141212000	1.54E-07
chr7	141208001	141213000	2.06E-08
chr7	141209001	141214000	1.24E-05
chr7	142387001	142392000	1.33E-05

chr7	142388001	142393000	3.77E-05
chr7	142431001	142436000	6.66E-06
chr7	142432001	142437000	2.33E-06
chr7	142433001	142438000	9.05E-06
chr7	142466001	142471000	0.000162
chr7	142780001	142785000	1.19E-05
chr7	142781001	142786000	2.76E-07
chr7	143052001	143057000	9.99E-06
chr7	143133001	143138000	1.09E-05
chr7	143148001	143153000	3.75E-05
chr7	143150001	143155000	1.79E-06
chr7	143241001	143246000	0.000165
chr7	143242001	143247000	9.30E-05
chr7	143243001	143248000	0.001467
chr7	143244001	143249000	0.001467
chr7	143245001	143250000	0.000434
chr7	143629001	143634000	3.12E-06
chr7	143907001	143912000	0.006718
chr7	143994001	143999000	0.001478
chr7	143995001	144000000	1.98E-05
chr7	143996001	144001000	0.000781
chr7	143997001	144002000	0.000781
chr7	144631001	144636000	0.00087
chr7	147223001	147228000	2.63E-07
chr7	148659001	148664000	0.000157
chr7	148976001	148981000	0.001518
chr7	148978001	148983000	6.83E-07
chr7	148979001	148984000	0.039552
chr7	148980001	148985000	0.000252
chr7	149345001	149350000	4.67E-05
chr7	149347001	149352000	4.27E-05
chr7	149421001	149426000	0.010132
chr7	149509001	149514000	0.004778
chr7	149524001	149529000	3.53E-06
chr7	149525001	149530000	4.70E-06
chr7	150076001	150081000	1.99E-06
chr7	150077001	150082000	8.89E-09
chr7	150225001	150230000	5.23E-05
chr7	150479001	150484000	2.21E-07
chr7	150480001	150485000	1.08E-06
chr7	150651001	150656000	4.96E-05
chr7	150747001	150752000	0.000225
chr7	150810001	150815000	8.19E-06
chr7	150811001	150816000	3.81E-07
chr7	151074001	151079000	1.50E-09
chr7	151076001	151081000	5.89E-06
chr7	151077001	151082000	2.86E-13
chr7	151078001	151083000	6.33E-11

chr7	151124001	151129000	0.000299
chr7	151125001	151130000	1.30E-06
chr7	151422001	151427000	0.000219
chr7	151423001	151428000	2.15E-05
chr7	152132001	152137000	9.19E-10
chr7	152133001	152138000	8.92E-06
chr7	152444001	152449000	0.000749
chr7	152707001	152712000	2.07E-09
chr7	152708001	152713000	5.95E-10
chr7	152959001	152964000	7.30E-05
chr7	152960001	152965000	8.87E-05
chr7	152961001	152966000	6.54E-05
chr7	152962001	152967000	2.53E-05
chr7	153175001	153180000	7.83E-07
chr7	153176001	153181000	3.88E-06
chr7	153177001	153182000	1.60E-05
chr7	153178001	153183000	5.60E-07
chr7	153295001	153300000	5.16E-11
chr7	153461001	153466000	1.09E-07
chr7	153489001	153494000	8.25E-09
chr7	153490001	153495000	8.77E-09
chr7	153497001	153502000	3.94E-09
chr7	153544001	153549000	1.82E-07
chr7	153553001	153558000	7.27E-07
chr7	153752001	153757000	2.73E-06
chr7	154266001	154271000	3.26E-10
chr7	154267001	154272000	1.70E-09
chr7	154268001	154273000	2.73E-10
chr7	154269001	154274000	6.55E-08
chr7	154270001	154275000	7.36E-08
chr7	154514001	154519000	0.000104
chr7	154575001	154580000	7.92E-08
chr7	154622001	154627000	4.04E-08
chr7	154674001	154679000	0.000108
chr7	154675001	154680000	3.47E-06
chr7	155042001	155047000	3.69E-06
chr7	155197001	155202000	0.000213
chr7	155433001	155438000	1.50E-09
chr7	155947001	155952000	1.41E-07
chr7	155948001	155953000	1.27E-07
chr7	156742001	156747000	1.28E-16
chr7	156743001	156748000	2.74E-19
chr7	157130001	157135000	1.10E-06
chr7	157287001	157292000	3.87E-06
chr7	157288001	157293000	1.35E-05
chr7	157289001	157294000	0.000275
chr7	157662001	157667000	0.004341
chr7	158282001	158287000	5.42E-05

chr7	158283001	158288000	4.63E-06
chr7	158508001	158513000	2.45E-06
chr7	158509001	158514000	2.17E-05
chr7	158510001	158515000	1.34E-06
chr7	158511001	158516000	7.21E-06
chr8	133001	138000	0.02051
chr8	135001	140000	0.006391
chr8	136001	141000	0.006391
chr8	137001	142000	0.005122
chr8	138001	143000	0.015738
chr8	586001	591000	2.57E-05
chr8	596001	601000	4.74E-05
chr8	1164001	1169000	5.54E-05
chr8	1281001	1286000	4.71E-09
chr8	1553001	1558000	2.02E-11
chr8	1554001	1559000	8.34E-10
chr8	1555001	1560000	1.03E-10
chr8	1778001	1783000	0.00543
chr8	2491001	2496000	9.36E-12
chr8	2492001	2497000	6.94E-12
chr8	3070001	3075000	1.05E-05
chr8	3485001	3490000	0.000381
chr8	6092001	6097000	0.000235
chr8	6631001	6636000	9.46E-05
chr8	6632001	6637000	5.02E-05
chr8	6633001	6638000	8.70E-05
chr8	6843001	6848000	0.005674
chr8	7024001	7029000	0.013938
chr8	7231001	7236000	0.036801
chr8	7297001	7302000	0.000385
chr8	7368001	7373000	0.000256
chr8	7536001	7541000	2.57E-06
chr8	7537001	7542000	3.64E-06
chr8	7642001	7647000	0.003457
chr8	7705001	7710000	0.000596
chr8	7766001	7771000	0.048281
chr8	7952001	7957000	0.040071
chr8	7953001	7958000	0.017193
chr8	7954001	7959000	0.047302
chr8	7955001	7960000	0.002184
chr8	7956001	7961000	0.002184
chr8	8124001	8129000	2.37E-05
chr8	9122001	9127000	0.002332
chr8	9324001	9329000	0.001316
chr8	9377001	9382000	0.000146
chr8	9378001	9383000	3.80E-05
chr8	10039001	10044000	0.000148
chr8	10307001	10312000	9.67E-08

chr8	10458001	10463000	0.001526
chr8	10549001	10554000	0.016141
chr8	10591001	10596000	2.46E-06
chr8	11721001	11726000	0.000582
chr8	11907001	11912000	0.002422
chr8	12170001	12175000	0.009988
chr8	12171001	12176000	0.020717
chr8	12172001	12177000	3.00E-05
chr8	12219001	12224000	0.000191
chr8	12265001	12270000	8.39E-05
chr8	12266001	12271000	6.06E-05
chr8	12267001	12272000	1.05E-05
chr8	12277001	12282000	1.99E-05
chr8	12278001	12283000	6.56E-05
chr8	12289001	12294000	1.10E-09
chr8	12290001	12295000	6.46E-15
chr8	12762001	12767000	0.000497
chr8	12763001	12768000	1.89E-05
chr8	12932001	12937000	5.27E-05
chr8	12933001	12938000	0.010961
chr8	13125001	13130000	0.000752
chr8	14103001	14108000	3.49E-08
chr8	18950001	18955000	5.91E-07
chr8	18951001	18956000	1.13E-05
chr8	18952001	18957000	7.19E-06
chr8	18997001	19002000	2.00E-05
chr8	18998001	19003000	1.00E-05
chr8	19614001	19619000	1.68E-12
chr8	20085001	20090000	4.51E-06
chr8	20086001	20091000	1.68E-07
chr8	20196001	20201000	1.59E-06
chr8	20199001	20204000	1.08E-06
chr8	20202001	20207000	0.001381
chr8	20294001	20299000	0.000744
chr8	20326001	20331000	6.59E-05
chr8	20534001	20539000	2.13E-07
chr8	20859001	20864000	1.12E-05
chr8	20879001	20884000	0.031057
chr8	20895001	20900000	1.62E-05
chr8	20903001	20908000	0.000214
chr8	20921001	20926000	6.55E-08
chr8	20922001	20927000	2.61E-07
chr8	20923001	20928000	1.17E-06
chr8	20924001	20929000	2.48E-05
chr8	20993001	20998000	2.83E-05
chr8	20994001	20999000	1.64E-05
chr8	21065001	21070000	1.59E-05
chr8	21574001	21579000	1.84E-07

chr8	21575001	21580000	0.001025
chr8	21576001	21581000	0.006259
chr8	21577001	21582000	0.004975
chr8	21945001	21950000	0.009832
chr8	22021001	22026000	0.000415
chr8	22022001	22027000	0.000662
chr8	22024001	22029000	0.000306
chr8	22146001	22151000	0.004795
chr8	22238001	22243000	2.53E-05
chr8	22239001	22244000	0.000112
chr8	22291001	22296000	0.010554
chr8	22456001	22461000	1.84E-10
chr8	22546001	22551000	5.60E-06
chr8	22807001	22812000	1.85E-05
chr8	23603001	23608000	7.09E-05
chr8	23741001	23746000	1.14E-05
chr8	23742001	23747000	0.000721
chr8	24795001	24800000	1.01E-06
chr8	24796001	24801000	2.73E-05
chr8	24797001	24802000	1.81E-05
chr8	24798001	24803000	0.001339
chr8	25037001	25042000	2.82E-09
chr8	25462001	25467000	4.88E-06
chr8	25547001	25552000	0.006929
chr8	25554001	25559000	0.001664
chr8	25567001	25572000	0.000199
chr8	26619001	26624000	6.54E-07
chr8	26622001	26627000	1.57E-08
chr8	26817001	26822000	4.28E-05
chr8	26940001	26945000	0.00022
chr8	27164001	27169000	5.50E-09
chr8	27321001	27326000	1.28E-09
chr8	28486001	28491000	1.70E-06
chr8	28487001	28492000	9.65E-08
chr8	28488001	28493000	6.96E-08
chr8	29260001	29265000	0.000756
chr8	29333001	29338000	1.57E-05
chr8	29945001	29950000	9.37E-07
chr8	29946001	29951000	5.98E-09
chr8	29947001	29952000	2.54E-08
chr8	30066001	30071000	2.28E-05
chr8	30511001	30516000	6.24E-06
chr8	30582001	30587000	1.08E-06
chr8	30584001	30589000	5.40E-05
chr8	30816001	30821000	0.000634
chr8	31365001	31370000	0.004464
chr8	31520001	31525000	2.21E-06
chr8	31521001	31526000	3.24E-05

chr8	31887001	31892000	6.86E-06
chr8	33117001	33122000	8.74E-05
chr8	33429001	33434000	0.001644
chr8	33597001	33602000	0.000422
chr8	33598001	33603000	2.18E-05
chr8	33660001	33665000	1.00E-05
chr8	33676001	33681000	0.001471
chr8	33784001	33789000	0.000183
chr8	33785001	33790000	4.59E-06
chr8	33969001	33974000	1.80E-09
chr8	33970001	33975000	1.22E-09
chr8	34155001	34160000	0.003326
chr8	34156001	34161000	0.000496
chr8	34157001	34162000	0.001563
chr8	34308001	34313000	4.73E-07
chr8	34309001	34314000	4.55E-05
chr8	34465001	34470000	2.22E-07
chr8	34482001	34487000	5.18E-05
chr8	34642001	34647000	3.26E-08
chr8	34643001	34648000	8.38E-07
chr8	34644001	34649000	3.37E-05
chr8	34677001	34682000	1.50E-05
chr8	34688001	34693000	3.26E-10
chr8	34836001	34841000	5.64E-06
chr8	34837001	34842000	2.48E-06
chr8	34838001	34843000	2.69E-05
chr8	34845001	34850000	2.76E-09
chr8	35476001	35481000	1.89E-09
chr8	35644001	35649000	3.95E-07
chr8	35645001	35650000	1.91E-07
chr8	35646001	35651000	3.82E-06
chr8	35647001	35652000	3.43E-06
chr8	35648001	35653000	3.04E-07
chr8	35692001	35697000	1.56E-08
chr8	35699001	35704000	0.013338
chr8	35700001	35705000	4.20E-05
chr8	35836001	35841000	6.84E-08
chr8	35837001	35842000	6.85E-06
chr8	36070001	36075000	1.13E-06
chr8	36098001	36103000	2.95E-07
chr8	36099001	36104000	4.50E-07
chr8	36127001	36132000	3.18E-06
chr8	36141001	36146000	8.54E-05
chr8	36161001	36166000	1.69E-07
chr8	36174001	36179000	2.36E-08
chr8	36176001	36181000	1.30E-07
chr8	36208001	36213000	2.50E-08
chr8	36209001	36214000	2.33E-08

chr8	36210001	36215000	1.57E-06
chr8	36211001	36216000	2.94E-07
chr8	36212001	36217000	8.43E-10
chr8	36213001	36218000	7.97E-09
chr8	36325001	36330000	6.94E-06
chr8	36453001	36458000	7.44E-07
chr8	36462001	36467000	0.000271
chr8	36463001	36468000	0.000271
chr8	36501001	36506000	2.19E-13
chr8	36502001	36507000	2.68E-14
chr8	36608001	36613000	1.26E-11
chr8	36614001	36619000	1.39E-08
chr8	36615001	36620000	1.68E-06
chr8	36616001	36621000	3.17E-06
chr8	37448001	37453000	9.32E-08
chr8	37449001	37454000	5.46E-06
chr8	37648001	37653000	0.000154
chr8	37688001	37693000	1.14E-06
chr8	37690001	37695000	4.31E-05
chr8	37778001	37783000	4.70E-06
chr8	37779001	37784000	8.56E-06
chr8	37780001	37785000	0.000315
chr8	37781001	37786000	2.17E-09
chr8	37782001	37787000	1.49E-06
chr8	37800001	37805000	0.000194
chr8	37866001	37871000	1.83E-13
chr8	37867001	37872000	4.19E-13
chr8	37868001	37873000	4.91E-12
chr8	37885001	37890000	2.02E-05
chr8	37886001	37891000	2.14E-05
chr8	37888001	37893000	0.009153
chr8	37958001	37963000	5.06E-09
chr8	37959001	37964000	1.03E-09
chr8	37960001	37965000	1.06E-08
chr8	37962001	37967000	0.000144
chr8	38324001	38329000	6.99E-06
chr8	38986001	38991000	0.001105
chr8	38987001	38992000	0.002843
chr8	39063001	39068000	1.24E-07
chr8	39907001	39912000	1.28E-06
chr8	39908001	39913000	8.28E-08
chr8	40307001	40312000	0.00027
chr8	40711001	40716000	2.59E-07
chr8	40899001	40904000	0.000108
chr8	40968001	40973000	0.000555
chr8	40969001	40974000	6.13E-06
chr8	41211001	41216000	2.16E-06
chr8	41279001	41284000	1.94E-09

chr8	41280001	41285000	8.61E-11
chr8	41369001	41374000	6.71E-06
chr8	41382001	41387000	2.37E-09
chr8	41434001	41439000	1.35E-06
chr8	41478001	41483000	2.63E-05
chr8	42005001	42010000	4.95E-06
chr8	42006001	42011000	9.62E-07
chr8	42007001	42012000	1.20E-08
chr8	42008001	42013000	2.29E-11
chr8	42035001	42040000	2.08E-08
chr8	42308001	42313000	0.001261
chr8	42554001	42559000	8.18E-06
chr8	42555001	42560000	4.18E-08
chr8	42556001	42561000	1.79E-07
chr8	42906001	42911000	3.60E-05
chr8	42907001	42912000	8.11E-19
chr8	42995001	43000000	1.02E-10
chr8	43155001	43160000	9.26E-05
chr8	43156001	43161000	1.75E-06
chr8	43220001	43225000	2.60E-08
chr8	43221001	43226000	5.34E-14
chr8	43222001	43227000	3.81E-15
chr8	43223001	43228000	1.08E-13
chr8	43224001	43229000	7.11E-13
chr8	43225001	43230000	9.53E-07
chr8	43244001	43249000	1.35E-06
chr8	43252001	43257000	4.93E-05
chr8	43296001	43301000	3.36E-06
chr8	43425001	43430000	4.01E-05
chr8	43531001	43536000	1.92E-07
chr8	43670001	43675000	5.86E-07
chr8	43671001	43676000	2.76E-06
chr8	43742001	43747000	4.12E-06
chr8	43772001	43777000	1.30E-07
chr8	43773001	43778000	8.33E-05
chr8	47243001	47248000	2.37E-05
chr8	47261001	47266000	4.29E-08
chr8	47262001	47267000	4.62E-08
chr8	47284001	47289000	1.49E-06
chr8	47285001	47290000	3.31E-07
chr8	47530001	47535000	8.59E-08
chr8	47539001	47544000	6.80E-07
chr8	47540001	47545000	2.20E-08
chr8	47561001	47566000	2.64E-12
chr8	47562001	47567000	4.63E-18
chr8	47563001	47568000	6.41E-13
chr8	47565001	47570000	2.80E-11
chr8	47578001	47583000	7.73E-07

chr8	47579001	47584000	9.26E-09
chr8	47580001	47585000	1.70E-08
chr8	47581001	47586000	1.28E-08
chr8	47582001	47587000	1.05E-07
chr8	47603001	47608000	3.69E-06
chr8	47604001	47609000	5.53E-08
chr8	47651001	47656000	2.41E-07
chr8	47652001	47657000	2.45E-06
chr8	47699001	47704000	0.00099
chr8	47703001	47708000	1.95E-06
chr8	47828001	47833000	1.78E-07
chr8	48290001	48295000	1.24E-11
chr8	48649001	48654000	7.95E-12
chr8	49103001	49108000	7.52E-06
chr8	49129001	49134000	6.30E-07
chr8	49130001	49135000	1.58E-07
chr8	49131001	49136000	2.06E-06
chr8	49139001	49144000	1.18E-08
chr8	49140001	49145000	7.64E-11
chr8	49141001	49146000	4.92E-12
chr8	49142001	49147000	5.05E-12
chr8	49143001	49148000	5.46E-09
chr8	49144001	49149000	6.17E-10
chr8	49161001	49166000	2.44E-12
chr8	49183001	49188000	2.38E-06
chr8	49185001	49190000	1.04E-08
chr8	49186001	49191000	2.76E-13
chr8	49187001	49192000	1.24E-14
chr8	49188001	49193000	2.88E-10
chr8	49908001	49913000	1.74E-10
chr8	49941001	49946000	1.79E-06
chr8	49944001	49949000	5.66E-06
chr8	49956001	49961000	2.35E-13
chr8	49957001	49962000	8.43E-17
chr8	49958001	49963000	1.41E-17
chr8	49971001	49976000	1.32E-11
chr8	49973001	49978000	4.72E-09
chr8	50021001	50026000	4.12E-10
chr8	50032001	50037000	8.46E-16
chr8	50033001	50038000	8.21E-15
chr8	50034001	50039000	1.69E-16
chr8	50035001	50040000	5.16E-16
chr8	50070001	50075000	9.34E-11
chr8	50072001	50077000	2.51E-09
chr8	50073001	50078000	3.43E-10
chr8	50076001	50081000	2.33E-16
chr8	50092001	50097000	1.93E-11
chr8	50097001	50102000	5.25E-10

chr8	50098001	50103000	3.90E-11
chr8	50099001	50104000	4.01E-09
chr8	50106001	50111000	1.48E-11
chr8	50151001	50156000	3.89E-15
chr8	50193001	50198000	6.58E-10
chr8	50205001	50210000	7.73E-12
chr8	50206001	50211000	4.03E-15
chr8	50207001	50212000	3.34E-16
chr8	50208001	50213000	5.95E-18
chr8	50209001	50214000	8.54E-13
chr8	50223001	50228000	2.89E-09
chr8	50224001	50229000	6.21E-10
chr8	50225001	50230000	9.42E-10
chr8	50226001	50231000	1.52E-10
chr8	50237001	50242000	1.77E-11
chr8	50250001	50255000	1.76E-16
chr8	50251001	50256000	1.02E-19
chr8	50252001	50257000	4.74E-17
chr8	50271001	50276000	5.00E-10
chr8	50285001	50290000	1.31E-06
chr8	50296001	50301000	4.86E-10
chr8	50298001	50303000	0.002495
chr8	50300001	50305000	3.85E-06
chr8	50301001	50306000	1.89E-07
chr8	50302001	50307000	2.88E-15
chr8	50303001	50308000	4.27E-18
chr8	50304001	50309000	1.63E-22
chr8	50305001	50310000	1.03E-18
chr8	50306001	50311000	1.96E-19
chr8	50323001	50328000	9.76E-11
chr8	50324001	50329000	5.38E-10
chr8	50325001	50330000	2.29E-12
chr8	50363001	50368000	1.43E-09
chr8	50364001	50369000	9.06E-10
chr8	50376001	50381000	2.14E-10
chr8	50390001	50395000	5.91E-12
chr8	50391001	50396000	1.22E-13
chr8	50392001	50397000	9.01E-12
chr8	50393001	50398000	3.07E-16
chr8	50394001	50399000	4.95E-14
chr8	50412001	50417000	2.31E-07
chr8	50414001	50419000	1.31E-07
chr8	50415001	50420000	7.21E-06
chr8	50416001	50421000	4.82E-07
chr8	50433001	50438000	9.96E-09
chr8	50436001	50441000	4.28E-10
chr8	50451001	50456000	1.16E-09
chr8	50452001	50457000	1.81E-07

chr8	50453001	50458000	2.68E-09
chr8	50454001	50459000	8.23E-10
chr8	50541001	50546000	6.34E-16
chr8	50549001	50554000	2.58E-07
chr8	50550001	50555000	7.85E-08
chr8	50552001	50557000	5.78E-11
chr8	50553001	50558000	1.08E-10
chr8	50581001	50586000	4.07E-12
chr8	50582001	50587000	7.31E-12
chr8	50620001	50625000	7.38E-11
chr8	50643001	50648000	9.63E-12
chr8	50656001	50661000	6.54E-09
chr8	50657001	50662000	2.48E-06
chr8	50802001	50807000	3.60E-06
chr8	50836001	50841000	5.57E-10
chr8	50850001	50855000	1.02E-11
chr8	50902001	50907000	8.14E-10
chr8	50903001	50908000	7.88E-09
chr8	50934001	50939000	8.20E-13
chr8	50935001	50940000	1.10E-13
chr8	51016001	51021000	1.31E-12
chr8	51029001	51034000	8.09E-12
chr8	51030001	51035000	4.58E-06
chr8	51041001	51046000	9.76E-10
chr8	51042001	51047000	1.28E-08
chr8	51088001	51093000	1.01E-07
chr8	51110001	51115000	6.00E-13
chr8	51111001	51116000	9.08E-12
chr8	51156001	51161000	2.34E-10
chr8	51233001	51238000	1.62E-07
chr8	51234001	51239000	1.77E-05
chr8	51326001	51331000	9.30E-17
chr8	51327001	51332000	4.55E-17
chr8	51407001	51412000	6.33E-11
chr8	51416001	51421000	1.14E-11
chr8	51437001	51442000	6.96E-15
chr8	51438001	51443000	6.08E-14
chr8	51538001	51543000	2.71E-14
chr8	51587001	51592000	8.18E-07
chr8	51588001	51593000	1.90E-06
chr8	51589001	51594000	7.13E-10
chr8	51590001	51595000	1.90E-09
chr8	51622001	51627000	1.81E-14
chr8	51623001	51628000	9.79E-16
chr8	51624001	51629000	5.69E-16
chr8	51625001	51630000	3.18E-15
chr8	51626001	51631000	1.04E-13
chr8	51644001	51649000	1.35E-12

chr8	51679001	51684000	5.58E-13
chr8	51681001	51686000	8.20E-07
chr8	51699001	51704000	3.08E-09
chr8	51737001	51742000	1.15E-11
chr8	51741001	51746000	4.17E-12
chr8	51743001	51748000	1.12E-10
chr8	51744001	51749000	9.63E-10
chr8	51745001	51750000	1.69E-07
chr8	51762001	51767000	6.98E-15
chr8	51763001	51768000	2.94E-16
chr8	51783001	51788000	6.29E-14
chr8	51784001	51789000	3.24E-14
chr8	51785001	51790000	4.75E-12
chr8	51793001	51798000	4.11E-08
chr8	51794001	51799000	1.18E-13
chr8	51797001	51802000	2.21E-13
chr8	51816001	51821000	1.79E-10
chr8	51892001	51897000	1.11E-12
chr8	51893001	51898000	8.72E-15
chr8	51902001	51907000	1.46E-13
chr8	51903001	51908000	5.17E-13
chr8	51925001	51930000	1.39E-11
chr8	51926001	51931000	1.89E-13
chr8	51934001	51939000	9.60E-14
chr8	51964001	51969000	5.04E-16
chr8	52004001	52009000	9.39E-13
chr8	52006001	52011000	7.11E-13
chr8	52007001	52012000	9.80E-13
chr8	52008001	52013000	2.74E-11
chr8	52009001	52014000	8.49E-11
chr8	52010001	52015000	3.98E-11
chr8	52043001	52048000	1.09E-13
chr8	52044001	52049000	2.89E-14
chr8	52050001	52055000	1.89E-07
chr8	52091001	52096000	8.52E-07
chr8	52098001	52103000	2.83E-13
chr8	52099001	52104000	2.70E-12
chr8	52111001	52116000	4.61E-14
chr8	52112001	52117000	8.80E-14
chr8	52113001	52118000	3.47E-14
chr8	52114001	52119000	2.08E-13
chr8	52205001	52210000	7.62E-09
chr8	52246001	52251000	8.14E-09
chr8	52265001	52270000	3.04E-11
chr8	52266001	52271000	1.75E-08
chr8	52267001	52272000	5.22E-07
chr8	52412001	52417000	5.63E-10
chr8	52413001	52418000	3.80E-09

chr8	54071001	54076000	0.000106
chr8	54087001	54092000	8.17E-08
chr8	54935001	54940000	1.52E-12
chr8	55109001	55114000	0.000531
chr8	55111001	55116000	8.87E-05
chr8	55112001	55117000	0.00156
chr8	55425001	55430000	7.28E-10
chr8	55429001	55434000	6.59E-14
chr8	55481001	55486000	7.62E-08
chr8	55482001	55487000	2.09E-08
chr8	55483001	55488000	4.50E-08
chr8	55601001	55606000	5.73E-16
chr8	55602001	55607000	4.10E-08
chr8	55701001	55706000	1.16E-10
chr8	55863001	55868000	5.43E-10
chr8	55891001	55896000	9.08E-11
chr8	55892001	55897000	8.81E-11
chr8	55893001	55898000	7.99E-11
chr8	55894001	55899000	4.03E-08
chr8	55939001	55944000	5.97E-12
chr8	55940001	55945000	1.72E-13
chr8	55941001	55946000	2.05E-11
chr8	55968001	55973000	3.76E-14
chr8	55969001	55974000	1.45E-16
chr8	55970001	55975000	2.68E-17
chr8	55971001	55976000	2.31E-14
chr8	56149001	56154000	5.69E-07
chr8	56186001	56191000	7.46E-11
chr8	56374001	56379000	3.53E-09
chr8	56563001	56568000	1.03E-07
chr8	56583001	56588000	1.13E-05
chr8	57052001	57057000	1.75E-07
chr8	57272001	57277000	8.48E-05
chr8	57273001	57278000	0.000432
chr8	57274001	57279000	0.000795
chr8	57715001	57720000	1.28E-10
chr8	57716001	57721000	1.89E-08
chr8	57846001	57851000	1.77E-09
chr8	57930001	57935000	1.98E-06
chr8	57931001	57936000	4.66E-08
chr8	57932001	57937000	3.72E-08
chr8	57933001	57938000	1.80E-09
chr8	58015001	58020000	3.33E-08
chr8	58016001	58021000	1.15E-09
chr8	58034001	58039000	6.85E-08
chr8	58093001	58098000	2.04E-12
chr8	58163001	58168000	4.09E-08
chr8	58236001	58241000	2.22E-12

chr8	58237001	58242000	2.32E-11
chr8	58238001	58243000	1.38E-10
chr8	58250001	58255000	7.73E-10
chr8	58286001	58291000	1.36E-06
chr8	58288001	58293000	5.18E-08
chr8	58289001	58294000	4.68E-06
chr8	58290001	58295000	4.17E-06
chr8	58305001	58310000	2.11E-07
chr8	58312001	58317000	8.77E-10
chr8	58313001	58318000	1.94E-08
chr8	58538001	58543000	2.68E-14
chr8	58539001	58544000	9.97E-10
chr8	58540001	58545000	2.51E-09
chr8	58567001	58572000	1.85E-11
chr8	58600001	58605000	1.02E-08
chr8	58606001	58611000	1.51E-09
chr8	58607001	58612000	5.13E-12
chr8	58609001	58614000	8.69E-10
chr8	58669001	58674000	1.93E-08
chr8	58670001	58675000	3.16E-07
chr8	58671001	58676000	1.09E-07
chr8	58672001	58677000	3.51E-10
chr8	58750001	58755000	3.19E-09
chr8	58820001	58825000	2.90E-10
chr8	58858001	58863000	1.02E-10
chr8	58869001	58874000	2.10E-06
chr8	58870001	58875000	4.48E-09
chr8	59118001	59123000	5.03E-13
chr8	59119001	59124000	2.36E-13
chr8	59120001	59125000	7.53E-14
chr8	59121001	59126000	1.17E-08
chr8	59129001	59134000	3.50E-17
chr8	59130001	59135000	1.00E-16
chr8	59160001	59165000	1.14E-06
chr8	59161001	59166000	9.38E-10
chr8	59162001	59167000	1.34E-14
chr8	59163001	59168000	3.61E-12
chr8	59164001	59169000	4.25E-13
chr8	59165001	59170000	9.21E-16
chr8	59166001	59171000	1.43E-13
chr8	59168001	59173000	2.60E-12
chr8	59195001	59200000	6.76E-14
chr8	59205001	59210000	4.39E-10
chr8	59206001	59211000	7.24E-15
chr8	59207001	59212000	5.90E-17
chr8	59208001	59213000	4.13E-17
chr8	59209001	59214000	4.17E-18
chr8	59251001	59256000	1.05E-07

chr8	59252001	59257000	2.80E-07
chr8	59271001	59276000	9.77E-07
chr8	59423001	59428000	2.41E-12
chr8	59424001	59429000	1.68E-11
chr8	59425001	59430000	6.78E-11
chr8	59426001	59431000	6.79E-10
chr8	59442001	59447000	9.40E-06
chr8	59831001	59836000	3.94E-10
chr8	61161001	61166000	1.18E-14
chr8	61302001	61307000	2.76E-12
chr8	61425001	61430000	1.65E-06
chr8	62052001	62057000	2.13E-16
chr8	62088001	62093000	8.37E-09
chr8	62090001	62095000	1.79E-12
chr8	62097001	62102000	1.79E-11
chr8	62098001	62103000	7.95E-08
chr8	62126001	62131000	2.09E-07
chr8	62127001	62132000	3.25E-08
chr8	62128001	62133000	2.13E-07
chr8	62134001	62139000	2.32E-10
chr8	62148001	62153000	6.90E-12
chr8	62163001	62168000	1.88E-13
chr8	62164001	62169000	3.05E-12
chr8	62165001	62170000	2.50E-14
chr8	62166001	62171000	3.02E-18
chr8	62177001	62182000	1.00E-10
chr8	62178001	62183000	1.72E-14
chr8	62179001	62184000	2.46E-13
chr8	62189001	62194000	3.54E-09
chr8	62191001	62196000	7.23E-09
chr8	62195001	62200000	2.14E-15
chr8	62230001	62235000	2.00E-07
chr8	62241001	62246000	2.00E-09
chr8	62242001	62247000	8.30E-10
chr8	62259001	62264000	8.56E-12
chr8	62260001	62265000	2.52E-14
chr8	62262001	62267000	1.80E-11
chr8	62263001	62268000	6.35E-17
chr8	62264001	62269000	2.00E-15
chr8	62265001	62270000	2.13E-14
chr8	62266001	62271000	6.59E-16
chr8	62267001	62272000	4.47E-13
chr8	62270001	62275000	1.29E-10
chr8	62288001	62293000	7.03E-13
chr8	62289001	62294000	1.30E-14
chr8	62290001	62295000	1.45E-14
chr8	62291001	62296000	3.00E-13
chr8	62294001	62299000	2.13E-10

chr8	62300001	62305000	7.04E-14
chr8	62301001	62306000	2.56E-12
chr8	62302001	62307000	1.46E-12
chr8	62303001	62308000	3.10E-10
chr8	62304001	62309000	5.89E-10
chr8	62305001	62310000	4.96E-11
chr8	62306001	62311000	7.91E-11
chr8	62307001	62312000	1.37E-13
chr8	62308001	62313000	3.86E-13
chr8	62346001	62351000	1.45E-11
chr8	62381001	62386000	5.40E-09
chr8	62382001	62387000	1.96E-11
chr8	62383001	62388000	4.18E-12
chr8	62384001	62389000	7.36E-08
chr8	62385001	62390000	6.16E-10
chr8	62800001	62805000	1.45E-07
chr8	62801001	62806000	1.46E-08
chr8	62802001	62807000	5.56E-09
chr8	62872001	62877000	1.64E-08
chr8	62873001	62878000	9.68E-10
chr8	62874001	62879000	1.09E-09
chr8	62875001	62880000	1.24E-09
chr8	62900001	62905000	1.49E-15
chr8	62901001	62906000	2.86E-09
chr8	62974001	62979000	2.26E-07
chr8	63026001	63031000	6.85E-16
chr8	63027001	63032000	3.60E-14
chr8	63076001	63081000	7.18E-08
chr8	63077001	63082000	5.13E-08
chr8	63099001	63104000	5.23E-11
chr8	63100001	63105000	8.06E-09
chr8	63105001	63110000	1.17E-09
chr8	63106001	63111000	2.14E-10
chr8	63107001	63112000	1.40E-08
chr8	63311001	63316000	5.06E-11
chr8	63312001	63317000	1.12E-11
chr8	63313001	63318000	5.33E-14
chr8	63314001	63319000	3.84E-19
chr8	63315001	63320000	7.57E-16
chr8	63321001	63326000	5.62E-14
chr8	63322001	63327000	2.53E-20
chr8	63323001	63328000	1.71E-19
chr8	63414001	63419000	2.32E-09
chr8	63424001	63429000	1.69E-07
chr8	63439001	63444000	1.21E-11
chr8	63454001	63459000	2.15E-07
chr8	63455001	63460000	1.18E-07
chr8	63456001	63461000	1.55E-08

chr8	63465001	63470000	6.11E-09
chr8	63467001	63472000	4.06E-08
chr8	63523001	63528000	1.78E-08
chr8	63563001	63568000	7.59E-09
chr8	63564001	63569000	1.60E-12
chr8	63565001	63570000	1.06E-13
chr8	63592001	63597000	2.03E-09
chr8	63593001	63598000	1.23E-09
chr8	63594001	63599000	1.11E-16
chr8	63595001	63600000	3.99E-18
chr8	63596001	63601000	5.56E-16
chr8	63597001	63602000	4.99E-10
chr8	63648001	63653000	6.03E-13
chr8	63649001	63654000	1.21E-10
chr8	63650001	63655000	1.58E-12
chr8	63651001	63656000	1.38E-11
chr8	63652001	63657000	5.57E-07
chr8	63682001	63687000	6.90E-11
chr8	63683001	63688000	1.22E-11
chr8	63691001	63696000	2.63E-06
chr8	63692001	63697000	3.34E-07
chr8	63717001	63722000	3.84E-14
chr8	63718001	63723000	3.75E-18
chr8	63719001	63724000	3.84E-15
chr8	63720001	63725000	1.66E-11
chr8	63721001	63726000	2.80E-11
chr8	63722001	63727000	1.18E-07
chr8	63723001	63728000	1.43E-08
chr8	63757001	63762000	8.73E-12
chr8	63808001	63813000	4.84E-06
chr8	63809001	63814000	1.69E-05
chr8	63965001	63970000	3.96E-09
chr8	64019001	64024000	1.57E-16
chr8	64020001	64025000	1.05E-10
chr8	64021001	64026000	5.60E-13
chr8	64022001	64027000	1.06E-11
chr8	64023001	64028000	1.38E-11
chr8	64052001	64057000	3.15E-09
chr8	64162001	64167000	3.21E-05
chr8	64163001	64168000	2.64E-08
chr8	64213001	64218000	3.82E-09
chr8	64282001	64287000	8.77E-11
chr8	64329001	64334000	1.75E-09
chr8	64367001	64372000	1.94E-10
chr8	64368001	64373000	1.51E-10
chr8	64401001	64406000	1.45E-07
chr8	64402001	64407000	1.21E-08
chr8	64423001	64428000	3.86E-07

chr8	64425001	64430000	2.21E-06
chr8	64426001	64431000	1.05E-06
chr8	64427001	64432000	1.60E-06
chr8	64428001	64433000	8.12E-08
chr8	64429001	64434000	5.71E-09
chr8	64430001	64435000	6.09E-07
chr8	65334001	65339000	3.53E-12
chr8	65335001	65340000	7.93E-13
chr8	65337001	65342000	7.18E-18
chr8	65378001	65383000	1.06E-08
chr8	65379001	65384000	4.50E-07
chr8	66550001	66555000	6.39E-09
chr8	68782001	68787000	2.14E-14
chr8	68783001	68788000	2.82E-16
chr8	68784001	68789000	8.99E-17
chr8	68797001	68802000	2.84E-13
chr8	68798001	68803000	4.58E-17
chr8	68799001	68804000	6.40E-20
chr8	68800001	68805000	2.37E-19
chr8	68801001	68806000	6.85E-12
chr8	68802001	68807000	1.66E-16
chr8	68803001	68808000	1.15E-11
chr8	68849001	68854000	1.55E-11
chr8	68951001	68956000	1.42E-10
chr8	69072001	69077000	7.32E-10
chr8	69073001	69078000	6.93E-13
chr8	69074001	69079000	1.90E-16
chr8	69075001	69080000	6.06E-13
chr8	69076001	69081000	3.78E-16
chr8	69077001	69082000	1.84E-13
chr8	69103001	69108000	3.44E-17
chr8	69209001	69214000	5.05E-07
chr8	69258001	69263000	5.49E-08
chr8	69259001	69264000	1.59E-09
chr8	69260001	69265000	7.99E-12
chr8	69261001	69266000	2.99E-08
chr8	69279001	69284000	1.65E-06
chr8	69313001	69318000	4.25E-12
chr8	69320001	69325000	1.04E-10
chr8	69321001	69326000	2.50E-08
chr8	69322001	69327000	1.25E-06
chr8	69323001	69328000	2.08E-06
chr8	69359001	69364000	4.77E-16
chr8	69360001	69365000	3.23E-18
chr8	69361001	69366000	2.09E-20
chr8	69407001	69412000	3.60E-09
chr8	69408001	69413000	1.66E-10
chr8	69409001	69414000	8.11E-09

chr8	69446001	69451000	3.53E-11
chr8	69469001	69474000	3.26E-13
chr8	69470001	69475000	6.41E-13
chr8	69475001	69480000	1.60E-12
chr8	69482001	69487000	2.19E-17
chr8	69505001	69510000	1.06E-07
chr8	69517001	69522000	4.40E-12
chr8	69518001	69523000	2.06E-13
chr8	69519001	69524000	4.30E-15
chr8	69520001	69525000	4.10E-13
chr8	69521001	69526000	1.68E-15
chr8	69522001	69527000	5.40E-16
chr8	69523001	69528000	2.11E-15
chr8	69534001	69539000	2.59E-10
chr8	69535001	69540000	2.26E-10
chr8	69536001	69541000	1.12E-14
chr8	69537001	69542000	2.76E-11
chr8	69544001	69549000	4.65E-12
chr8	69545001	69550000	5.64E-17
chr8	69546001	69551000	1.37E-17
chr8	69547001	69552000	1.71E-17
chr8	69548001	69553000	3.73E-17
chr8	69568001	69573000	1.50E-08
chr8	69569001	69574000	9.73E-14
chr8	69570001	69575000	3.03E-12
chr8	69571001	69576000	5.87E-14
chr8	69572001	69577000	1.42E-15
chr8	69573001	69578000	5.80E-13
chr8	69574001	69579000	1.25E-09
chr8	69601001	69606000	1.44E-11
chr8	69607001	69612000	7.66E-20
chr8	69608001	69613000	2.96E-18
chr8	69621001	69626000	2.99E-14
chr8	69627001	69632000	1.16E-13
chr8	69628001	69633000	1.84E-15
chr8	69634001	69639000	1.13E-13
chr8	69635001	69640000	3.75E-11
chr8	69636001	69641000	8.83E-11
chr8	69637001	69642000	7.80E-13
chr8	69638001	69643000	9.76E-12
chr8	69684001	69689000	6.92E-12
chr8	69703001	69708000	4.38E-10
chr8	69704001	69709000	2.80E-10
chr8	69715001	69720000	4.82E-16
chr8	69716001	69721000	1.47E-13
chr8	69717001	69722000	1.91E-12
chr8	69718001	69723000	1.81E-14
chr8	69719001	69724000	7.35E-13

chr8	69720001	69725000	1.63E-09
chr8	69737001	69742000	7.68E-10
chr8	69742001	69747000	8.82E-12
chr8	69743001	69748000	3.89E-10
chr8	69758001	69763000	1.61E-13
chr8	69759001	69764000	1.01E-18
chr8	69760001	69765000	8.50E-21
chr8	69761001	69766000	1.28E-15
chr8	69772001	69777000	1.15E-09
chr8	69797001	69802000	1.13E-12
chr8	69812001	69817000	8.99E-15
chr8	69813001	69818000	3.73E-21
chr8	69814001	69819000	2.54E-18
chr8	69815001	69820000	3.23E-17
chr8	69816001	69821000	5.99E-13
chr8	69817001	69822000	1.40E-13
chr8	69818001	69823000	2.06E-06
chr8	69819001	69824000	7.79E-10
chr8	69831001	69836000	3.91E-11
chr8	69834001	69839000	1.69E-13
chr8	69835001	69840000	2.28E-15
chr8	69836001	69841000	1.13E-10
chr8	69837001	69842000	3.91E-16
chr8	69838001	69843000	3.82E-16
chr8	69839001	69844000	7.36E-13
chr8	69850001	69855000	6.93E-10
chr8	69851001	69856000	1.35E-10
chr8	69852001	69857000	8.19E-12
chr8	69853001	69858000	1.37E-12
chr8	69854001	69859000	1.53E-11
chr8	69909001	69914000	1.53E-16
chr8	69911001	69916000	3.11E-13
chr8	69912001	69917000	1.73E-16
chr8	69913001	69918000	3.09E-10
chr8	69956001	69961000	3.30E-10
chr8	69968001	69973000	3.13E-11
chr8	69969001	69974000	2.23E-09
chr8	69982001	69987000	2.39E-17
chr8	69985001	69990000	7.65E-17
chr8	70029001	70034000	6.29E-09
chr8	70031001	70036000	6.44E-09
chr8	70045001	70050000	7.47E-09
chr8	70091001	70096000	1.25E-08
chr8	70145001	70150000	6.52E-15
chr8	70180001	70185000	2.93E-13
chr8	70181001	70186000	8.26E-16
chr8	70182001	70187000	2.91E-13
chr8	70196001	70201000	3.98E-10

chr8	70198001	70203000	1.42E-12
chr8	70199001	70204000	7.64E-08
chr8	70200001	70205000	2.72E-12
chr8	70201001	70206000	2.79E-12
chr8	70204001	70209000	2.24E-09
chr8	70212001	70217000	6.68E-12
chr8	70255001	70260000	3.26E-08
chr8	70257001	70262000	4.75E-15
chr8	70331001	70336000	4.27E-09
chr8	70332001	70337000	1.21E-09
chr8	70333001	70338000	2.56E-11
chr8	70334001	70339000	1.83E-12
chr8	70347001	70352000	7.20E-08
chr8	70348001	70353000	6.07E-10
chr8	70362001	70367000	5.56E-07
chr8	70425001	70430000	6.45E-05
chr8	70524001	70529000	3.81E-10
chr8	70589001	70594000	8.25E-16
chr8	70592001	70597000	1.95E-16
chr8	70670001	70675000	2.14E-10
chr8	70696001	70701000	1.03E-07
chr8	70697001	70702000	4.39E-09
chr8	70698001	70703000	3.15E-10
chr8	70703001	70708000	2.29E-08
chr8	70704001	70709000	1.70E-07
chr8	70720001	70725000	5.11E-14
chr8	70731001	70736000	1.39E-08
chr8	70732001	70737000	4.11E-10
chr8	70733001	70738000	1.33E-07
chr8	70734001	70739000	1.18E-07
chr8	70746001	70751000	2.16E-10
chr8	70784001	70789000	5.32E-09
chr8	70785001	70790000	9.45E-13
chr8	70786001	70791000	1.69E-06
chr8	70788001	70793000	2.40E-05
chr8	70830001	70835000	3.62E-06
chr8	70838001	70843000	1.62E-09
chr8	70873001	70878000	4.77E-10
chr8	70874001	70879000	3.16E-11
chr8	70875001	70880000	2.71E-08
chr8	70876001	70881000	2.81E-11
chr8	70877001	70882000	2.93E-08
chr8	70880001	70885000	0.000249
chr8	70923001	70928000	1.35E-08
chr8	70925001	70930000	8.62E-11
chr8	70941001	70946000	6.37E-07
chr8	70979001	70984000	0.000451
chr8	71011001	71016000	5.59E-07

chr8	71440001	71445000	1.72E-13
chr8	71441001	71446000	4.82E-13
chr8	71448001	71453000	3.14E-11
chr8	71449001	71454000	1.52E-05
chr8	71522001	71527000	7.30E-09
chr8	71523001	71528000	3.86E-09
chr8	71524001	71529000	1.81E-06
chr8	71659001	71664000	2.48E-07
chr8	71660001	71665000	6.75E-08
chr8	71668001	71673000	1.75E-08
chr8	71669001	71674000	2.88E-09
chr8	71685001	71690000	2.90E-06
chr8	71687001	71692000	1.34E-14
chr8	71688001	71693000	7.46E-17
chr8	71702001	71707000	7.24E-06
chr8	71708001	71713000	1.09E-06
chr8	71709001	71714000	3.95E-09
chr8	71710001	71715000	2.02E-11
chr8	71711001	71716000	2.47E-10
chr8	71712001	71717000	2.29E-10
chr8	71713001	71718000	5.20E-08
chr8	71714001	71719000	2.66E-07
chr8	71737001	71742000	6.25E-15
chr8	71740001	71745000	2.13E-15
chr8	71752001	71757000	6.46E-08
chr8	71753001	71758000	3.83E-07
chr8	71754001	71759000	5.77E-07
chr8	71756001	71761000	0.021644
chr8	71783001	71788000	3.69E-11
chr8	71784001	71789000	1.77E-11
chr8	71785001	71790000	7.52E-14
chr8	71786001	71791000	2.22E-14
chr8	71787001	71792000	3.24E-15
chr8	71788001	71793000	1.84E-10
chr8	71789001	71794000	9.55E-13
chr8	71790001	71795000	1.63E-09
chr8	71791001	71796000	5.35E-08
chr8	71816001	71821000	5.15E-14
chr8	71817001	71822000	6.78E-11
chr8	71822001	71827000	1.35E-09
chr8	71823001	71828000	9.45E-10
chr8	71824001	71829000	3.11E-11
chr8	71825001	71830000	1.36E-10
chr8	71826001	71831000	8.99E-11
chr8	71827001	71832000	7.67E-07
chr8	71859001	71864000	1.01E-17
chr8	71860001	71865000	2.91E-16
chr8	71861001	71866000	1.49E-13

chr8	71873001	71878000	2.31E-14
chr8	71874001	71879000	1.64E-12
chr8	71882001	71887000	3.33E-13
chr8	71885001	71890000	2.12E-14
chr8	71886001	71891000	5.12E-15
chr8	71888001	71893000	4.50E-15
chr8	71889001	71894000	5.90E-18
chr8	71890001	71895000	4.80E-15
chr8	71891001	71896000	3.75E-20
chr8	71892001	71897000	1.95E-22
chr8	71893001	71898000	2.91E-18
chr8	71894001	71899000	3.19E-18
chr8	71895001	71900000	1.53E-15
chr8	71904001	71909000	1.78E-07
chr8	71905001	71910000	1.05E-08
chr8	71906001	71911000	7.49E-12
chr8	71907001	71912000	2.46E-10
chr8	71938001	71943000	2.41E-08
chr8	71940001	71945000	3.67E-06
chr8	71941001	71946000	5.09E-06
chr8	71971001	71976000	3.58E-16
chr8	71972001	71977000	1.12E-15
chr8	71973001	71978000	5.55E-15
chr8	71981001	71986000	1.42E-05
chr8	71982001	71987000	3.52E-07
chr8	71983001	71988000	7.11E-10
chr8	71984001	71989000	7.11E-10
chr8	71985001	71990000	6.37E-07
chr8	71986001	71991000	2.16E-11
chr8	71987001	71992000	3.44E-06
chr8	72000001	72005000	7.87E-16
chr8	72001001	72006000	1.00E-13
chr8	72002001	72007000	1.19E-13
chr8	72021001	72026000	5.04E-11
chr8	72022001	72027000	9.39E-16
chr8	72023001	72028000	2.50E-17
chr8	72024001	72029000	5.49E-14
chr8	72025001	72030000	1.67E-15
chr8	72026001	72031000	3.30E-12
chr8	72063001	72068000	9.80E-11
chr8	72069001	72074000	3.77E-09
chr8	72071001	72076000	1.14E-09
chr8	72100001	72105000	8.81E-08
chr8	72112001	72117000	7.98E-11
chr8	72133001	72138000	5.27E-11
chr8	72134001	72139000	1.84E-12
chr8	72390001	72395000	2.14E-15
chr8	72391001	72396000	3.12E-14

chr8	72392001	72397000	9.18E-16
chr8	72393001	72398000	1.38E-12
chr8	72394001	72399000	1.75E-09
chr8	72513001	72518000	1.10E-11
chr8	72538001	72543000	5.48E-16
chr8	72539001	72544000	1.00E-14
chr8	72540001	72545000	3.81E-15
chr8	72541001	72546000	1.01E-12
chr8	72542001	72547000	4.46E-12
chr8	72546001	72551000	1.22E-08
chr8	72548001	72553000	1.41E-11
chr8	72549001	72554000	9.08E-10
chr8	72550001	72555000	1.61E-14
chr8	72551001	72556000	2.69E-16
chr8	72552001	72557000	4.74E-18
chr8	72571001	72576000	5.63E-12
chr8	72572001	72577000	1.49E-08
chr8	72573001	72578000	1.45E-14
chr8	72615001	72620000	7.78E-12
chr8	72617001	72622000	1.35E-13
chr8	72618001	72623000	1.06E-13
chr8	72619001	72624000	5.15E-14
chr8	72643001	72648000	8.66E-09
chr8	72785001	72790000	6.71E-13
chr8	72786001	72791000	5.03E-09
chr8	72787001	72792000	1.80E-08
chr8	72789001	72794000	2.50E-10
chr8	72793001	72798000	8.10E-07
chr8	72802001	72807000	7.80E-06
chr8	72803001	72808000	8.23E-06
chr8	72809001	72814000	7.23E-09
chr8	72810001	72815000	1.71E-11
chr8	72811001	72816000	8.17E-10
chr8	72812001	72817000	3.31E-09
chr8	72828001	72833000	2.23E-09
chr8	72829001	72834000	9.52E-13
chr8	72830001	72835000	6.69E-13
chr8	72832001	72837000	4.50E-14
chr8	73025001	73030000	7.94E-13
chr8	73220001	73225000	0.000101
chr8	74884001	74889000	4.60E-05
chr8	75363001	75368000	0.008095
chr8	76487001	76492000	1.04E-13
chr8	76489001	76494000	1.20E-14
chr8	76548001	76553000	1.98E-05
chr8	76549001	76554000	4.57E-10
chr8	76550001	76555000	3.04E-08
chr8	76649001	76654000	1.69E-07

chr8	76787001	76792000	8.89E-14
chr8	76806001	76811000	6.59E-09
chr8	76807001	76812000	1.27E-09
chr8	77249001	77254000	7.52E-14
chr8	77400001	77405000	6.81E-12
chr8	77529001	77534000	7.60E-14
chr8	77530001	77535000	6.60E-18
chr8	77531001	77536000	2.03E-16
chr8	77532001	77537000	4.99E-14
chr8	77649001	77654000	1.66E-08
chr8	77732001	77737000	4.26E-08
chr8	77744001	77749000	3.41E-10
chr8	77828001	77833000	6.54E-09
chr8	77840001	77845000	1.54E-11
chr8	77841001	77846000	5.13E-14
chr8	77848001	77853000	3.90E-17
chr8	77849001	77854000	1.63E-15
chr8	77850001	77855000	1.08E-14
chr8	77851001	77856000	4.01E-14
chr8	77852001	77857000	1.23E-09
chr8	77868001	77873000	3.36E-08
chr8	77869001	77874000	1.38E-07
chr8	77954001	77959000	7.36E-11
chr8	77955001	77960000	8.55E-10
chr8	78085001	78090000	2.96E-14
chr8	78092001	78097000	5.52E-11
chr8	78102001	78107000	7.76E-09
chr8	78103001	78108000	1.34E-10
chr8	78137001	78142000	5.96E-08
chr8	78198001	78203000	2.50E-11
chr8	78199001	78204000	1.12E-08
chr8	78249001	78254000	6.07E-10
chr8	78346001	78351000	2.44E-16
chr8	78347001	78352000	2.01E-10
chr8	78348001	78353000	4.48E-10
chr8	78349001	78354000	5.98E-09
chr8	78350001	78355000	1.16E-09
chr8	78398001	78403000	3.16E-18
chr8	78399001	78404000	8.78E-18
chr8	78402001	78407000	3.06E-10
chr8	78434001	78439000	1.82E-13
chr8	78608001	78613000	2.64E-11
chr8	78609001	78614000	1.47E-13
chr8	78704001	78709000	2.86E-08
chr8	78738001	78743000	7.19E-11
chr8	78739001	78744000	1.02E-11
chr8	78801001	78806000	1.23E-08
chr8	78825001	78830000	4.07E-09

chr8	78849001	78854000	6.70E-10
chr8	78946001	78951000	3.48E-12
chr8	78947001	78952000	7.11E-13
chr8	78967001	78972000	8.74E-06
chr8	78968001	78973000	3.94E-08
chr8	79174001	79179000	1.18E-09
chr8	79181001	79186000	8.77E-05
chr8	79182001	79187000	2.04E-05
chr8	79190001	79195000	7.77E-15
chr8	79191001	79196000	1.65E-13
chr8	79192001	79197000	5.63E-13
chr8	79194001	79199000	1.74E-16
chr8	79195001	79200000	7.57E-19
chr8	79196001	79201000	9.58E-14
chr8	79198001	79203000	1.86E-16
chr8	79199001	79204000	2.40E-11
chr8	79285001	79290000	5.54E-11
chr8	79286001	79291000	2.33E-15
chr8	79287001	79292000	3.97E-19
chr8	79288001	79293000	5.94E-16
chr8	79294001	79299000	2.12E-10
chr8	79295001	79300000	5.75E-07
chr8	79344001	79349000	3.28E-12
chr8	79365001	79370000	6.23E-11
chr8	79378001	79383000	1.35E-12
chr8	79380001	79385000	2.45E-16
chr8	79844001	79849000	1.99E-10
chr8	79929001	79934000	2.16E-13
chr8	80001001	80006000	0.000116
chr8	80061001	80066000	2.89E-07
chr8	80064001	80069000	4.04E-15
chr8	80068001	80073000	2.45E-14
chr8	80083001	80088000	1.03E-10
chr8	80084001	80089000	8.05E-13
chr8	80085001	80090000	9.95E-14
chr8	80086001	80091000	2.53E-15
chr8	80087001	80092000	9.27E-15
chr8	80101001	80106000	1.75E-11
chr8	80130001	80135000	3.74E-16
chr8	80131001	80136000	1.01E-15
chr8	80132001	80137000	2.99E-16
chr8	80133001	80138000	2.58E-17
chr8	80134001	80139000	1.78E-15
chr8	80135001	80140000	1.15E-10
chr8	80142001	80147000	2.94E-05
chr8	80189001	80194000	5.73E-07
chr8	80190001	80195000	5.14E-10
chr8	80198001	80203000	7.22E-13

chr8	80199001	80204000	1.09E-12
chr8	80213001	80218000	1.30E-14
chr8	80224001	80229000	3.02E-18
chr8	80225001	80230000	5.65E-14
chr8	80293001	80298000	3.79E-09
chr8	80294001	80299000	1.44E-15
chr8	80295001	80300000	2.05E-14
chr8	80335001	80340000	1.01E-15
chr8	80336001	80341000	3.97E-14
chr8	80337001	80342000	2.08E-13
chr8	80338001	80343000	9.95E-19
chr8	80339001	80344000	2.86E-20
chr8	80340001	80345000	2.65E-21
chr8	80341001	80346000	9.47E-20
chr8	80342001	80347000	1.47E-19
chr8	80350001	80355000	6.14E-11
chr8	80361001	80366000	5.38E-13
chr8	80362001	80367000	2.29E-16
chr8	80363001	80368000	1.30E-15
chr8	80379001	80384000	9.46E-11
chr8	80380001	80385000	2.38E-09
chr8	80431001	80436000	1.70E-09
chr8	80469001	80474000	6.47E-09
chr8	80470001	80475000	1.89E-10
chr8	80471001	80476000	5.63E-13
chr8	80472001	80477000	3.40E-15
chr8	80473001	80478000	3.24E-15
chr8	80474001	80479000	1.49E-12
chr8	81080001	81085000	4.97E-20
chr8	81081001	81086000	4.62E-20
chr8	81487001	81492000	5.77E-10
chr8	81490001	81495000	1.27E-10
chr8	81781001	81786000	7.26E-10
chr8	81782001	81787000	1.41E-17
chr8	82218001	82223000	3.19E-12
chr8	82860001	82865000	1.34E-11
chr8	82861001	82866000	6.46E-11
chr8	82862001	82867000	4.57E-10
chr8	82863001	82868000	2.43E-09
chr8	83015001	83020000	3.33E-07
chr8	83016001	83021000	4.12E-09
chr8	83040001	83045000	0.000336
chr8	83076001	83081000	8.38E-09
chr8	83077001	83082000	3.33E-10
chr8	83143001	83148000	6.87E-08
chr8	83184001	83189000	1.52E-08
chr8	83185001	83190000	3.30E-06
chr8	83186001	83191000	0.000544

chr8	83203001	83208000	4.01E-10
chr8	83204001	83209000	1.54E-09
chr8	83385001	83390000	4.88E-09
chr8	83393001	83398000	3.32E-09
chr8	83394001	83399000	3.18E-07
chr8	83398001	83403000	1.31E-13
chr8	83400001	83405000	4.87E-15
chr8	83401001	83406000	2.90E-17
chr8	83402001	83407000	3.26E-16
chr8	83447001	83452000	1.51E-12
chr8	83448001	83453000	1.27E-11
chr8	83452001	83457000	1.42E-15
chr8	83455001	83460000	2.95E-16
chr8	83484001	83489000	1.74E-12
chr8	83485001	83490000	1.06E-10
chr8	83491001	83496000	9.23E-08
chr8	83492001	83497000	4.24E-10
chr8	83523001	83528000	1.21E-11
chr8	83524001	83529000	3.93E-11
chr8	83546001	83551000	9.10E-09
chr8	83549001	83554000	1.86E-09
chr8	83646001	83651000	1.14E-11
chr8	83704001	83709000	9.13E-14
chr8	83705001	83710000	7.80E-15
chr8	83731001	83736000	1.44E-09
chr8	83732001	83737000	5.85E-07
chr8	83849001	83854000	2.03E-06
chr8	83916001	83921000	4.91E-12
chr8	83917001	83922000	4.31E-14
chr8	83919001	83924000	9.61E-14
chr8	83931001	83936000	1.13E-09
chr8	84011001	84016000	1.60E-07
chr8	84116001	84121000	3.17E-07
chr8	84126001	84131000	1.10E-13
chr8	84127001	84132000	2.92E-18
chr8	84128001	84133000	3.30E-16
chr8	84129001	84134000	1.75E-12
chr8	84130001	84135000	5.70E-12
chr8	84135001	84140000	1.47E-09
chr8	84136001	84141000	1.27E-09
chr8	84147001	84152000	7.63E-09
chr8	84156001	84161000	5.54E-15
chr8	84159001	84164000	3.79E-07
chr8	84160001	84165000	2.08E-06
chr8	84161001	84166000	1.60E-08
chr8	84162001	84167000	1.26E-08
chr8	84188001	84193000	2.28E-12
chr8	84522001	84527000	3.07E-10

chr8	84523001	84528000	1.60E-09
chr8	84524001	84529000	3.93E-10
chr8	84611001	84616000	0.000208
chr8	84672001	84677000	1.26E-11
chr8	84719001	84724000	6.49E-20
chr8	84720001	84725000	1.29E-15
chr8	84721001	84726000	1.50E-22
chr8	84722001	84727000	1.08E-18
chr8	84738001	84743000	1.26E-17
chr8	84739001	84744000	1.59E-14
chr8	84741001	84746000	2.84E-13
chr8	84752001	84757000	2.79E-10
chr8	84753001	84758000	8.83E-11
chr8	84764001	84769000	1.79E-10
chr8	84765001	84770000	1.02E-09
chr8	84766001	84771000	1.59E-09
chr8	84767001	84772000	2.16E-15
chr8	84768001	84773000	1.09E-14
chr8	84769001	84774000	1.05E-13
chr8	84770001	84775000	1.38E-11
chr8	84774001	84779000	6.78E-10
chr8	84776001	84781000	7.79E-15
chr8	84781001	84786000	4.63E-05
chr8	84817001	84822000	2.17E-15
chr8	84818001	84823000	2.06E-16
chr8	84820001	84825000	6.97E-13
chr8	84832001	84837000	3.84E-09
chr8	84833001	84838000	1.78E-10
chr8	84834001	84839000	6.61E-11
chr8	84835001	84840000	1.30E-10
chr8	84836001	84841000	1.07E-10
chr8	84839001	84844000	7.88E-12
chr8	84840001	84845000	3.58E-12
chr8	84841001	84846000	1.68E-12
chr8	84930001	84935000	3.24E-17
chr8	84931001	84936000	3.96E-20
chr8	84932001	84937000	1.26E-13
chr8	84971001	84976000	4.77E-09
chr8	84983001	84988000	3.14E-08
chr8	84984001	84989000	8.44E-09
chr8	84985001	84990000	1.70E-13
chr8	84986001	84991000	5.52E-15
chr8	84987001	84992000	3.77E-15
chr8	84988001	84993000	2.45E-17
chr8	84989001	84994000	1.89E-19
chr8	85013001	85018000	1.76E-16
chr8	85014001	85019000	8.58E-19
chr8	85031001	85036000	1.22E-09

chr8	85048001	85053000	2.22E-17
chr8	85049001	85054000	1.64E-18
chr8	85057001	85062000	1.22E-11
chr8	85058001	85063000	5.88E-14
chr8	85059001	85064000	6.32E-20
chr8	85060001	85065000	7.96E-14
chr8	85061001	85066000	1.50E-16
chr8	85062001	85067000	1.54E-13
chr8	85063001	85068000	1.31E-11
chr8	85138001	85143000	4.67E-09
chr8	85139001	85144000	9.66E-16
chr8	85140001	85145000	8.21E-12
chr8	85173001	85178000	3.05E-08
chr8	85174001	85179000	1.11E-10
chr8	85241001	85246000	1.56E-14
chr8	85259001	85264000	2.25E-14
chr8	85260001	85265000	1.88E-12
chr8	85262001	85267000	8.88E-19
chr8	85263001	85268000	5.03E-23
chr8	85272001	85277000	7.29E-09
chr8	85274001	85279000	3.01E-12
chr8	85281001	85286000	3.55E-15
chr8	85316001	85321000	2.40E-06
chr8	85318001	85323000	1.97E-12
chr8	85319001	85324000	5.23E-11
chr8	85320001	85325000	1.62E-12
chr8	85321001	85326000	1.30E-11
chr8	85327001	85332000	2.52E-12
chr8	85330001	85335000	3.38E-11
chr8	85331001	85336000	2.49E-11
chr8	85358001	85363000	8.12E-16
chr8	85359001	85364000	1.95E-17
chr8	85360001	85365000	2.59E-14
chr8	85397001	85402000	1.13E-19
chr8	85398001	85403000	5.50E-12
chr8	85399001	85404000	1.73E-12
chr8	85400001	85405000	6.31E-09
chr8	85401001	85406000	1.70E-10
chr8	85426001	85431000	2.80E-10
chr8	85427001	85432000	2.72E-11
chr8	85429001	85434000	5.65E-13
chr8	85433001	85438000	3.74E-12
chr8	85434001	85439000	1.73E-12
chr8	85435001	85440000	1.12E-21
chr8	85436001	85441000	1.20E-22
chr8	85437001	85442000	1.63E-22
chr8	85438001	85443000	2.71E-11
chr8	85442001	85447000	1.28E-14

chr8	85443001	85448000	6.81E-18
chr8	85457001	85462000	1.20E-15
chr8	85458001	85463000	2.73E-15
chr8	85459001	85464000	1.67E-16
chr8	85460001	85465000	1.40E-13
chr8	85461001	85466000	2.50E-10
chr8	85470001	85475000	8.89E-10
chr8	85481001	85486000	2.54E-09
chr8	85482001	85487000	4.68E-11
chr8	85483001	85488000	8.02E-14
chr8	85484001	85489000	4.88E-14
chr8	85485001	85490000	4.92E-12
chr8	85503001	85508000	1.29E-13
chr8	85504001	85509000	4.12E-10
chr8	85664001	85669000	1.26E-09
chr8	85665001	85670000	1.66E-13
chr8	85666001	85671000	1.99E-13
chr8	85667001	85672000	1.33E-13
chr8	85683001	85688000	4.98E-14
chr8	85684001	85689000	1.55E-12
chr8	85685001	85690000	5.25E-13
chr8	85713001	85718000	5.48E-11
chr8	85721001	85726000	7.46E-11
chr8	85722001	85727000	3.12E-09
chr8	85749001	85754000	1.48E-08
chr8	85788001	85793000	2.75E-10
chr8	85789001	85794000	2.89E-11
chr8	85802001	85807000	2.96E-12
chr8	85803001	85808000	9.73E-21
chr8	85804001	85809000	5.53E-12
chr8	85805001	85810000	2.17E-12
chr8	85817001	85822000	1.52E-09
chr8	85818001	85823000	2.81E-12
chr8	85819001	85824000	3.81E-11
chr8	85821001	85826000	4.90E-08
chr8	85849001	85854000	2.94E-14
chr8	85850001	85855000	6.48E-16
chr8	85851001	85856000	2.43E-10
chr8	85852001	85857000	6.68E-09
chr8	85853001	85858000	3.45E-13
chr8	85854001	85859000	1.35E-12
chr8	85859001	85864000	4.47E-11
chr8	85860001	85865000	6.13E-10
chr8	85865001	85870000	2.51E-12
chr8	85866001	85871000	5.24E-11
chr8	85867001	85872000	9.78E-09
chr8	85868001	85873000	1.00E-08
chr8	85869001	85874000	2.76E-11

chr8	85870001	85875000	2.48E-10
chr8	85871001	85876000	2.78E-11
chr8	85872001	85877000	1.25E-12
chr8	85873001	85878000	6.71E-10
chr8	85894001	85899000	4.80E-10
chr8	85897001	85902000	1.59E-11
chr8	85898001	85903000	1.92E-11
chr8	85946001	85951000	5.08E-11
chr8	85947001	85952000	3.38E-14
chr8	85948001	85953000	3.38E-14
chr8	85949001	85954000	3.52E-14
chr8	85963001	85968000	1.34E-10
chr8	85964001	85969000	1.44E-11
chr8	86178001	86183000	1.67E-10
chr8	86233001	86238000	4.31E-13
chr8	86234001	86239000	8.75E-13
chr8	86305001	86310000	1.27E-06
chr8	86306001	86311000	7.32E-08
chr8	86307001	86312000	2.64E-08
chr8	86308001	86313000	1.37E-06
chr8	86309001	86314000	3.49E-10
chr8	86357001	86362000	2.18E-10
chr8	86391001	86396000	5.43E-13
chr8	86392001	86397000	3.03E-12
chr8	86393001	86398000	2.49E-09
chr8	86394001	86399000	4.93E-10
chr8	86395001	86400000	1.26E-12
chr8	86396001	86401000	8.49E-10
chr8	86406001	86411000	8.42E-12
chr8	86446001	86451000	5.48E-11
chr8	86447001	86452000	6.96E-11
chr8	86483001	86488000	9.39E-09
chr8	86776001	86781000	0.000254
chr8	86887001	86892000	1.38E-07
chr8	86904001	86909000	8.84E-13
chr8	86905001	86910000	3.38E-12
chr8	86906001	86911000	2.00E-12
chr8	86907001	86912000	4.16E-14
chr8	86908001	86913000	4.15E-14
chr8	86946001	86951000	2.14E-10
chr8	86958001	86963000	4.10E-17
chr8	86997001	87002000	2.38E-09
chr8	87004001	87009000	4.12E-11
chr8	87005001	87010000	1.76E-12
chr8	87006001	87011000	7.82E-15
chr8	87007001	87012000	4.18E-13
chr8	87008001	87013000	1.23E-09
chr8	87021001	87026000	7.40E-10

chr8	87023001	87028000	9.51E-11
chr8	87024001	87029000	1.79E-13
chr8	87025001	87030000	2.89E-18
chr8	87032001	87037000	4.98E-07
chr8	87033001	87038000	1.53E-06
chr8	87103001	87108000	6.84E-10
chr8	87143001	87148000	3.28E-07
chr8	87181001	87186000	6.15E-08
chr8	87182001	87187000	2.61E-08
chr8	87183001	87188000	5.71E-08
chr8	87185001	87190000	1.72E-10
chr8	87186001	87191000	1.07E-09
chr8	87195001	87200000	2.11E-10
chr8	87196001	87201000	7.27E-11
chr8	87197001	87202000	9.73E-12
chr8	87198001	87203000	2.28E-11
chr8	87199001	87204000	1.39E-13
chr8	87200001	87205000	1.92E-09
chr8	87201001	87206000	2.21E-10
chr8	87226001	87231000	2.69E-14
chr8	87259001	87264000	1.24E-15
chr8	87260001	87265000	4.25E-15
chr8	87261001	87266000	5.99E-11
chr8	87270001	87275000	2.15E-07
chr8	87272001	87277000	1.76E-05
chr8	87273001	87278000	9.42E-07
chr8	87319001	87324000	1.34E-09
chr8	87320001	87325000	1.64E-08
chr8	87321001	87326000	1.49E-07
chr8	87658001	87663000	7.49E-15
chr8	87668001	87673000	1.36E-09
chr8	87669001	87674000	2.83E-12
chr8	87707001	87712000	5.87E-19
chr8	87708001	87713000	6.05E-18
chr8	87709001	87714000	3.43E-16
chr8	87710001	87715000	4.58E-16
chr8	87725001	87730000	8.43E-08
chr8	87726001	87731000	1.17E-11
chr8	87727001	87732000	1.08E-16
chr8	87728001	87733000	7.41E-16
chr8	87729001	87734000	4.41E-14
chr8	87730001	87735000	3.95E-15
chr8	87731001	87736000	1.02E-08
chr8	87738001	87743000	8.48E-15
chr8	87739001	87744000	1.17E-12
chr8	87740001	87745000	1.58E-16
chr8	87741001	87746000	1.46E-13
chr8	87742001	87747000	1.80E-09

chr8	87743001	87748000	3.18E-09
chr8	87744001	87749000	5.85E-13
chr8	87752001	87757000	1.36E-16
chr8	87783001	87788000	2.08E-11
chr8	87796001	87801000	5.24E-09
chr8	87810001	87815000	2.30E-09
chr8	87837001	87842000	6.89E-10
chr8	87838001	87843000	1.44E-16
chr8	87839001	87844000	1.10E-18
chr8	87840001	87845000	3.77E-23
chr8	87841001	87846000	7.07E-22
chr8	87842001	87847000	1.59E-19
chr8	87843001	87848000	1.87E-12
chr8	87844001	87849000	1.16E-12
chr8	87853001	87858000	2.26E-08
chr8	87863001	87868000	4.51E-08
chr8	87874001	87879000	5.11E-11
chr8	87875001	87880000	5.88E-09
chr8	87883001	87888000	1.66E-11
chr8	87884001	87889000	1.69E-13
chr8	87885001	87890000	1.68E-11
chr8	87886001	87891000	1.06E-09
chr8	87887001	87892000	2.96E-15
chr8	87888001	87893000	2.18E-13
chr8	87889001	87894000	3.28E-12
chr8	87890001	87895000	4.33E-08
chr8	87891001	87896000	1.68E-09
chr8	87899001	87904000	8.92E-11
chr8	87948001	87953000	9.34E-08
chr8	87949001	87954000	1.83E-09
chr8	87981001	87986000	7.67E-09
chr8	87982001	87987000	8.33E-10
chr8	87983001	87988000	8.72E-11
chr8	87984001	87989000	1.13E-11
chr8	87985001	87990000	6.78E-10
chr8	87986001	87991000	2.73E-09
chr8	87987001	87992000	1.65E-09
chr8	87988001	87993000	5.58E-09
chr8	87997001	88002000	4.82E-09
chr8	87999001	88004000	6.29E-09
chr8	88001001	88006000	7.18E-10
chr8	88012001	88017000	7.11E-10
chr8	88013001	88018000	2.09E-09
chr8	88014001	88019000	1.72E-09
chr8	88028001	88033000	1.53E-15
chr8	88044001	88049000	2.22E-18
chr8	88045001	88050000	2.40E-28
chr8	88046001	88051000	7.33E-23

chr8	88047001	88052000	1.02E-19
chr8	88048001	88053000	1.03E-14
chr8	88057001	88062000	2.62E-11
chr8	88058001	88063000	1.93E-12
chr8	88059001	88064000	7.56E-14
chr8	88060001	88065000	7.15E-14
chr8	88061001	88066000	1.32E-07
chr8	88062001	88067000	3.03E-07
chr8	88063001	88068000	5.24E-06
chr8	88090001	88095000	7.94E-18
chr8	88091001	88096000	1.33E-13
chr8	88096001	88101000	9.41E-07
chr8	88098001	88103000	1.22E-09
chr8	88099001	88104000	2.72E-09
chr8	88110001	88115000	2.78E-16
chr8	88111001	88116000	9.45E-21
chr8	88112001	88117000	1.09E-24
chr8	88113001	88118000	5.84E-21
chr8	88114001	88119000	2.28E-20
chr8	88115001	88120000	1.70E-12
chr8	88116001	88121000	1.82E-08
chr8	88117001	88122000	5.01E-11
chr8	88118001	88123000	1.55E-11
chr8	88125001	88130000	1.04E-13
chr8	88126001	88131000	1.77E-16
chr8	88127001	88132000	9.50E-18
chr8	88128001	88133000	1.75E-16
chr8	88129001	88134000	2.21E-13
chr8	88137001	88142000	1.25E-11
chr8	88138001	88143000	1.31E-09
chr8	88139001	88144000	7.77E-10
chr8	88140001	88145000	1.71E-09
chr8	88141001	88146000	4.32E-06
chr8	88158001	88163000	1.20E-13
chr8	88162001	88167000	8.46E-14
chr8	88187001	88192000	1.22E-12
chr8	88202001	88207000	5.30E-16
chr8	88203001	88208000	2.49E-13
chr8	88213001	88218000	2.10E-16
chr8	88223001	88228000	3.69E-10
chr8	88224001	88229000	1.15E-17
chr8	88225001	88230000	9.16E-17
chr8	88226001	88231000	1.61E-17
chr8	88227001	88232000	7.58E-17
chr8	88236001	88241000	9.69E-15
chr8	88237001	88242000	5.69E-13
chr8	88239001	88244000	7.25E-16
chr8	88251001	88256000	9.01E-14

chr8	88252001	88257000	5.51E-17
chr8	88253001	88258000	2.74E-24
chr8	88254001	88259000	1.22E-20
chr8	88255001	88260000	8.44E-19
chr8	88298001	88303000	6.80E-14
chr8	88299001	88304000	2.17E-15
chr8	88319001	88324000	3.47E-11
chr8	88326001	88331000	1.22E-10
chr8	88327001	88332000	1.89E-10
chr8	88328001	88333000	1.35E-11
chr8	88329001	88334000	2.33E-09
chr8	88330001	88335000	5.73E-11
chr8	88331001	88336000	8.97E-10
chr8	88343001	88348000	1.97E-10
chr8	88344001	88349000	9.96E-10
chr8	88345001	88350000	8.11E-08
chr8	88346001	88351000	1.64E-08
chr8	88348001	88353000	4.51E-08
chr8	88362001	88367000	2.28E-17
chr8	88368001	88373000	6.27E-09
chr8	88369001	88374000	5.64E-09
chr8	88370001	88375000	1.82E-10
chr8	88371001	88376000	1.00E-08
chr8	88372001	88377000	3.85E-07
chr8	88411001	88416000	5.42E-10
chr8	88412001	88417000	6.80E-12
chr8	88414001	88419000	1.09E-10
chr8	88415001	88420000	5.24E-12
chr8	88422001	88427000	9.87E-11
chr8	88423001	88428000	5.52E-10
chr8	88424001	88429000	4.13E-09
chr8	88429001	88434000	9.26E-15
chr8	88450001	88455000	1.01E-17
chr8	88451001	88456000	5.00E-17
chr8	88452001	88457000	4.63E-18
chr8	88453001	88458000	1.59E-19
chr8	88454001	88459000	1.08E-13
chr8	88462001	88467000	1.50E-09
chr8	88463001	88468000	1.95E-11
chr8	88464001	88469000	1.72E-10
chr8	88465001	88470000	7.71E-12
chr8	88466001	88471000	2.62E-11
chr8	88467001	88472000	5.40E-13
chr8	88468001	88473000	3.33E-13
chr8	88472001	88477000	8.44E-14
chr8	88480001	88485000	3.13E-10
chr8	88481001	88486000	2.48E-09
chr8	88487001	88492000	8.02E-20

chr8	88488001	88493000	9.38E-18
chr8	88489001	88494000	1.25E-19
chr8	88490001	88495000	2.53E-19
chr8	88491001	88496000	7.01E-17
chr8	88500001	88505000	1.52E-17
chr8	88501001	88506000	2.56E-15
chr8	88518001	88523000	4.30E-11
chr8	88519001	88524000	1.21E-12
chr8	88537001	88542000	3.65E-19
chr8	88538001	88543000	3.76E-18
chr8	88539001	88544000	3.26E-13
chr8	88540001	88545000	4.37E-14
chr8	88541001	88546000	1.99E-11
chr8	88543001	88548000	1.95E-05
chr8	88544001	88549000	9.32E-07
chr8	88545001	88550000	2.50E-07
chr8	88567001	88572000	4.04E-12
chr8	88569001	88574000	1.37E-17
chr8	88575001	88580000	2.75E-11
chr8	88576001	88581000	1.12E-11
chr8	88577001	88582000	1.60E-09
chr8	88578001	88583000	1.57E-11
chr8	88579001	88584000	1.91E-09
chr8	88580001	88585000	1.00E-06
chr8	88610001	88615000	1.38E-14
chr8	88632001	88637000	1.64E-14
chr8	88633001	88638000	6.33E-14
chr8	88634001	88639000	1.25E-17
chr8	88644001	88649000	3.24E-13
chr8	88645001	88650000	1.38E-14
chr8	88646001	88651000	3.21E-14
chr8	88647001	88652000	1.10E-14
chr8	88675001	88680000	9.84E-12
chr8	88676001	88681000	2.59E-15
chr8	88677001	88682000	1.40E-12
chr8	88699001	88704000	1.02E-08
chr8	88712001	88717000	6.24E-08
chr8	88713001	88718000	2.14E-07
chr8	88714001	88719000	4.22E-07
chr8	88725001	88730000	5.57E-08
chr8	88731001	88736000	1.47E-06
chr8	88732001	88737000	8.91E-10
chr8	88733001	88738000	5.76E-11
chr8	88734001	88739000	4.70E-09
chr8	88735001	88740000	6.24E-13
chr8	88736001	88741000	2.02E-11
chr8	88737001	88742000	1.68E-09
chr8	88738001	88743000	3.27E-09

chr8	88746001	88751000	1.46E-11
chr8	88775001	88780000	6.43E-09
chr8	88777001	88782000	5.41E-09
chr8	88779001	88784000	9.25E-11
chr8	88780001	88785000	8.79E-10
chr8	88781001	88786000	1.28E-11
chr8	88791001	88796000	1.67E-11
chr8	88792001	88797000	3.38E-14
chr8	88793001	88798000	1.37E-15
chr8	88794001	88799000	8.85E-14
chr8	88795001	88800000	3.23E-15
chr8	88796001	88801000	2.90E-12
chr8	88797001	88802000	1.55E-10
chr8	88802001	88807000	2.97E-12
chr8	88812001	88817000	1.66E-12
chr8	88813001	88818000	9.50E-15
chr8	88814001	88819000	1.44E-14
chr8	88815001	88820000	5.48E-11
chr8	88816001	88821000	7.81E-15
chr8	88817001	88822000	1.09E-12
chr8	88818001	88823000	1.43E-11
chr8	88819001	88824000	4.49E-12
chr8	88820001	88825000	6.83E-13
chr8	88821001	88826000	1.00E-14
chr8	88828001	88833000	2.84E-14
chr8	88832001	88837000	5.88E-18
chr8	88833001	88838000	2.87E-13
chr8	88834001	88839000	8.52E-14
chr8	88835001	88840000	1.33E-10
chr8	88841001	88846000	7.20E-13
chr8	88847001	88852000	8.13E-09
chr8	88848001	88853000	7.60E-11
chr8	88849001	88854000	2.05E-11
chr8	88850001	88855000	1.26E-10
chr8	88851001	88856000	2.72E-12
chr8	88852001	88857000	4.69E-10
chr8	88853001	88858000	1.74E-06
chr8	88854001	88859000	6.91E-10
chr8	88855001	88860000	2.10E-12
chr8	88856001	88861000	3.38E-10
chr8	88867001	88872000	1.01E-17
chr8	88868001	88873000	1.65E-21
chr8	88869001	88874000	1.72E-20
chr8	88870001	88875000	1.64E-21
chr8	88871001	88876000	3.54E-16
chr8	88872001	88877000	8.15E-15
chr8	88879001	88884000	4.44E-11
chr8	88880001	88885000	5.57E-13

chr8	88888001	88893000	1.14E-16
chr8	88889001	88894000	1.39E-17
chr8	88890001	88895000	9.28E-16
chr8	88891001	88896000	6.16E-12
chr8	88892001	88897000	1.07E-11
chr8	88893001	88898000	2.81E-12
chr8	88912001	88917000	8.24E-08
chr8	88943001	88948000	7.07E-21
chr8	88944001	88949000	2.04E-24
chr8	88945001	88950000	2.24E-23
chr8	88947001	88952000	1.09E-14
chr8	88951001	88956000	4.44E-09
chr8	88953001	88958000	2.76E-12
chr8	88954001	88959000	8.49E-15
chr8	88965001	88970000	3.69E-12
chr8	88966001	88971000	3.51E-15
chr8	88979001	88984000	5.57E-18
chr8	88980001	88985000	6.69E-21
chr8	88999001	89004000	1.16E-19
chr8	89000001	89005000	3.72E-21
chr8	89001001	89006000	1.35E-18
chr8	89002001	89007000	4.22E-18
chr8	89037001	89042000	3.53E-11
chr8	89042001	89047000	4.42E-13
chr8	89043001	89048000	1.44E-18
chr8	89065001	89070000	1.11E-19
chr8	89066001	89071000	1.41E-16
chr8	89111001	89116000	1.73E-13
chr8	89112001	89117000	1.92E-17
chr8	89113001	89118000	1.22E-20
chr8	89114001	89119000	9.38E-18
chr8	89115001	89120000	2.97E-18
chr8	89116001	89121000	1.86E-12
chr8	89117001	89122000	3.76E-12
chr8	89118001	89123000	5.63E-09
chr8	89164001	89169000	2.35E-07
chr8	89165001	89170000	1.80E-10
chr8	89166001	89171000	1.29E-10
chr8	89167001	89172000	2.12E-13
chr8	89169001	89174000	1.38E-18
chr8	89170001	89175000	1.05E-20
chr8	89171001	89176000	7.24E-19
chr8	89182001	89187000	5.94E-16
chr8	89183001	89188000	2.96E-22
chr8	89184001	89189000	4.19E-21
chr8	89211001	89216000	1.72E-12
chr8	89212001	89217000	9.61E-11
chr8	89218001	89223000	4.09E-11

chr8	89219001	89224000	3.12E-09
chr8	89220001	89225000	4.46E-10
chr8	89263001	89268000	6.15E-07
chr8	89272001	89277000	1.39E-09
chr8	89273001	89278000	8.50E-09
chr8	89274001	89279000	1.33E-11
chr8	89275001	89280000	1.00E-09
chr8	89290001	89295000	5.59E-12
chr8	89296001	89301000	1.98E-10
chr8	89316001	89321000	2.14E-09
chr8	89343001	89348000	4.42E-10
chr8	89346001	89351000	1.06E-08
chr8	89371001	89376000	1.68E-07
chr8	89376001	89381000	3.28E-14
chr8	89377001	89382000	3.75E-14
chr8	89378001	89383000	5.19E-16
chr8	89379001	89384000	8.59E-20
chr8	89380001	89385000	3.28E-14
chr8	89381001	89386000	1.64E-12
chr8	89389001	89394000	2.64E-09
chr8	89391001	89396000	2.99E-09
chr8	89392001	89397000	3.10E-09
chr8	89397001	89402000	5.21E-12
chr8	89398001	89403000	4.94E-15
chr8	89414001	89419000	1.88E-13
chr8	89415001	89420000	1.19E-10
chr8	89416001	89421000	9.78E-12
chr8	89417001	89422000	3.68E-08
chr8	89418001	89423000	2.07E-13
chr8	89424001	89429000	3.76E-11
chr8	89425001	89430000	7.88E-10
chr8	89426001	89431000	8.60E-12
chr8	89427001	89432000	2.52E-13
chr8	89428001	89433000	3.63E-14
chr8	89439001	89444000	3.54E-11
chr8	89446001	89451000	2.84E-12
chr8	89447001	89452000	2.79E-15
chr8	89448001	89453000	5.32E-18
chr8	89462001	89467000	2.66E-08
chr8	89478001	89483000	2.28E-14
chr8	89479001	89484000	9.77E-16
chr8	89480001	89485000	7.95E-17
chr8	89481001	89486000	7.43E-18
chr8	89482001	89487000	8.89E-08
chr8	89483001	89488000	4.11E-06
chr8	89494001	89499000	3.44E-09
chr8	89497001	89502000	1.47E-12
chr8	89498001	89503000	2.50E-14

chr8	89534001	89539000	3.86E-11
chr8	89535001	89540000	1.60E-14
chr8	89536001	89541000	2.63E-11
chr8	89537001	89542000	3.22E-14
chr8	89538001	89543000	9.08E-19
chr8	89539001	89544000	2.32E-17
chr8	89540001	89545000	5.44E-14
chr8	89541001	89546000	1.34E-15
chr8	89542001	89547000	8.73E-14
chr8	89543001	89548000	3.67E-12
chr8	89544001	89549000	1.02E-11
chr8	89545001	89550000	2.67E-11
chr8	89547001	89552000	2.13E-11
chr8	89548001	89553000	1.72E-07
chr8	89549001	89554000	5.42E-08
chr8	89561001	89566000	4.39E-12
chr8	89562001	89567000	2.25E-12
chr8	89583001	89588000	3.73E-18
chr8	89584001	89589000	2.62E-19
chr8	89585001	89590000	4.30E-20
chr8	89586001	89591000	4.23E-24
chr8	89587001	89592000	1.54E-19
chr8	89592001	89597000	1.80E-10
chr8	89593001	89598000	1.27E-12
chr8	89594001	89599000	7.70E-11
chr8	89595001	89600000	7.39E-16
chr8	89596001	89601000	8.66E-10
chr8	89597001	89602000	7.68E-12
chr8	89598001	89603000	1.30E-09
chr8	89599001	89604000	6.66E-11
chr8	89600001	89605000	1.23E-12
chr8	89601001	89606000	6.10E-13
chr8	89624001	89629000	1.90E-11
chr8	89625001	89630000	3.84E-11
chr8	89626001	89631000	2.45E-11
chr8	89635001	89640000	1.72E-08
chr8	89667001	89672000	6.04E-11
chr8	89669001	89674000	7.21E-08
chr8	89670001	89675000	1.10E-07
chr8	89693001	89698000	2.24E-14
chr8	89703001	89708000	5.18E-13
chr8	89704001	89709000	4.24E-17
chr8	89705001	89710000	5.23E-19
chr8	89706001	89711000	1.71E-17
chr8	89707001	89712000	9.78E-12
chr8	89708001	89713000	1.46E-12
chr8	89709001	89714000	9.21E-09
chr8	89710001	89715000	5.43E-10

chr8	89712001	89717000	1.77E-10
chr8	89718001	89723000	7.72E-19
chr8	89719001	89724000	2.23E-24
chr8	89720001	89725000	5.17E-29
chr8	89721001	89726000	3.67E-29
chr8	89722001	89727000	5.22E-20
chr8	89785001	89790000	4.83E-16
chr8	89786001	89791000	1.95E-12
chr8	89787001	89792000	8.25E-14
chr8	89788001	89793000	8.24E-16
chr8	89789001	89794000	1.37E-17
chr8	89790001	89795000	2.20E-16
chr8	89791001	89796000	2.28E-18
chr8	89829001	89834000	1.57E-12
chr8	89835001	89840000	5.00E-11
chr8	89844001	89849000	4.38E-07
chr8	89871001	89876000	8.34E-12
chr8	89872001	89877000	5.68E-20
chr8	89873001	89878000	3.91E-11
chr8	89874001	89879000	5.61E-10
chr8	89875001	89880000	5.19E-14
chr8	89876001	89881000	1.30E-11
chr8	89878001	89883000	4.92E-11
chr8	89889001	89894000	5.81E-09
chr8	89906001	89911000	1.95E-08
chr8	89907001	89912000	1.79E-11
chr8	89915001	89920000	4.62E-08
chr8	89957001	89962000	1.21E-14
chr8	89958001	89963000	7.40E-14
chr8	89959001	89964000	7.29E-11
chr8	89960001	89965000	1.21E-10
chr8	89961001	89966000	1.08E-12
chr8	89963001	89968000	6.23E-14
chr8	89964001	89969000	4.29E-15
chr8	89965001	89970000	5.46E-16
chr8	89982001	89987000	3.84E-12
chr8	89993001	89998000	4.80E-10
chr8	89994001	89999000	8.47E-14
chr8	89995001	90000000	4.15E-13
chr8	90006001	90011000	1.53E-16
chr8	90007001	90012000	1.04E-20
chr8	90008001	90013000	4.64E-18
chr8	90009001	90014000	7.17E-16
chr8	90010001	90015000	8.85E-13
chr8	90024001	90029000	1.51E-13
chr8	90026001	90031000	3.95E-10
chr8	90027001	90032000	5.92E-07
chr8	90028001	90033000	1.01E-08

chr8	90029001	90034000	6.55E-10
chr8	90030001	90035000	2.52E-11
chr8	90031001	90036000	4.57E-10
chr8	90032001	90037000	6.68E-12
chr8	90035001	90040000	1.79E-10
chr8	90073001	90078000	3.91E-10
chr8	90074001	90079000	5.24E-11
chr8	90075001	90080000	2.37E-10
chr8	90076001	90081000	1.37E-15
chr8	90077001	90082000	5.05E-16
chr8	90078001	90083000	1.99E-11
chr8	90079001	90084000	9.33E-09
chr8	90080001	90085000	1.23E-06
chr8	90086001	90091000	3.16E-07
chr8	90087001	90092000	1.18E-06
chr8	90088001	90093000	5.65E-09
chr8	90089001	90094000	2.11E-08
chr8	90090001	90095000	1.10E-06
chr8	90092001	90097000	1.78E-08
chr8	90096001	90101000	2.59E-15
chr8	90098001	90103000	3.22E-15
chr8	90109001	90114000	8.81E-15
chr8	90110001	90115000	6.95E-12
chr8	90111001	90116000	8.26E-14
chr8	90176001	90181000	2.89E-10
chr8	90177001	90182000	1.51E-05
chr8	90179001	90184000	3.06E-06
chr8	90204001	90209000	1.16E-16
chr8	90205001	90210000	5.19E-19
chr8	90206001	90211000	3.21E-10
chr8	90257001	90262000	7.60E-09
chr8	90471001	90476000	4.07E-10
chr8	90640001	90645000	1.21E-10
chr8	90681001	90686000	1.35E-10
chr8	90683001	90688000	5.08E-09
chr8	90684001	90689000	1.54E-11
chr8	90685001	90690000	1.47E-08
chr8	90691001	90696000	9.91E-08
chr8	90692001	90697000	4.76E-08
chr8	90693001	90698000	5.14E-11
chr8	90694001	90699000	3.15E-13
chr8	91107001	91112000	8.38E-11
chr8	91465001	91470000	1.11E-07
chr8	91466001	91471000	6.24E-09
chr8	91489001	91494000	1.58E-06
chr8	91730001	91735000	4.19E-13
chr8	91859001	91864000	9.52E-12
chr8	91860001	91865000	1.29E-12

chr8	91861001	91866000	9.73E-12
chr8	91898001	91903000	8.95E-16
chr8	91899001	91904000	3.86E-13
chr8	91963001	91968000	7.00E-09
chr8	92316001	92321000	7.23E-07
chr8	92317001	92322000	2.53E-07
chr8	92507001	92512000	6.24E-11
chr8	92508001	92513000	3.29E-10
chr8	92509001	92514000	1.09E-08
chr8	92510001	92515000	3.66E-12
chr8	92511001	92516000	8.61E-14
chr8	92512001	92517000	8.01E-11
chr8	92513001	92518000	3.44E-10
chr8	92514001	92519000	9.83E-08
chr8	92574001	92579000	1.40E-05
chr8	92575001	92580000	6.43E-06
chr8	92576001	92581000	1.99E-06
chr8	92579001	92584000	3.88E-11
chr8	92580001	92585000	2.66E-14
chr8	92581001	92586000	7.94E-14
chr8	92582001	92587000	2.48E-14
chr8	92624001	92629000	4.67E-09
chr8	92626001	92631000	2.09E-11
chr8	92627001	92632000	5.93E-12
chr8	92641001	92646000	1.91E-19
chr8	92652001	92657000	1.75E-05
chr8	92672001	92677000	2.67E-05
chr8	92673001	92678000	8.39E-08
chr8	92674001	92679000	4.23E-12
chr8	92675001	92680000	6.01E-10
chr8	92676001	92681000	9.22E-11
chr8	92681001	92686000	5.25E-11
chr8	92682001	92687000	1.48E-08
chr8	92693001	92698000	5.35E-09
chr8	92694001	92699000	1.09E-07
chr8	92695001	92700000	7.09E-09
chr8	92696001	92701000	1.57E-07
chr8	92697001	92702000	9.33E-06
chr8	92698001	92703000	5.26E-06
chr8	92710001	92715000	8.80E-05
chr8	92711001	92716000	4.33E-05
chr8	92712001	92717000	0.000344
chr8	92721001	92726000	4.34E-11
chr8	92722001	92727000	3.42E-12
chr8	92729001	92734000	2.85E-09
chr8	92735001	92740000	1.09E-12
chr8	92741001	92746000	2.82E-08
chr8	92753001	92758000	1.09E-08

chr8	92760001	92765000	1.27E-09
chr8	92790001	92795000	2.47E-12
chr8	92791001	92796000	8.24E-12
chr8	92792001	92797000	1.47E-11
chr8	92793001	92798000	1.87E-14
chr8	92828001	92833000	2.46E-09
chr8	92853001	92858000	2.14E-11
chr8	92888001	92893000	2.28E-16
chr8	92889001	92894000	1.13E-14
chr8	92890001	92895000	9.44E-14
chr8	92891001	92896000	3.05E-15
chr8	92892001	92897000	7.66E-12
chr8	92913001	92918000	1.96E-14
chr8	92914001	92919000	7.72E-17
chr8	92915001	92920000	1.19E-19
chr8	92916001	92921000	6.08E-16
chr8	92919001	92924000	1.51E-10
chr8	92928001	92933000	6.62E-10
chr8	92929001	92934000	9.32E-09
chr8	92930001	92935000	4.68E-10
chr8	93217001	93222000	4.49E-07
chr8	93218001	93223000	3.37E-07
chr8	93219001	93224000	7.76E-09
chr8	93220001	93225000	7.67E-09
chr8	93221001	93226000	2.01E-08
chr8	93223001	93228000	1.13E-06
chr8	93227001	93232000	1.51E-09
chr8	93229001	93234000	6.60E-11
chr8	93230001	93235000	1.09E-12
chr8	93231001	93236000	6.02E-09
chr8	93233001	93238000	1.16E-08
chr8	93234001	93239000	5.58E-09
chr8	93235001	93240000	2.25E-07
chr8	93245001	93250000	1.90E-17
chr8	93246001	93251000	1.25E-14
chr8	93247001	93252000	6.95E-14
chr8	93255001	93260000	8.15E-09
chr8	93322001	93327000	3.21E-08
chr8	93331001	93336000	5.91E-15
chr8	93337001	93342000	2.53E-08
chr8	93338001	93343000	2.59E-11
chr8	93339001	93344000	7.13E-12
chr8	93340001	93345000	2.70E-10
chr8	93341001	93346000	2.00E-10
chr8	93346001	93351000	3.99E-05
chr8	93348001	93353000	5.34E-07
chr8	93349001	93354000	3.52E-09
chr8	93350001	93355000	2.57E-10

chr8	93351001	93356000	6.82E-09
chr8	93352001	93357000	4.41E-06
chr8	93353001	93358000	2.09E-06
chr8	93406001	93411000	1.44E-16
chr8	93433001	93438000	2.12E-11
chr8	93440001	93445000	3.77E-15
chr8	93441001	93446000	2.88E-18
chr8	93442001	93447000	2.69E-18
chr8	93469001	93474000	2.67E-10
chr8	93538001	93543000	2.62E-06
chr8	93698001	93703000	9.93E-11
chr8	93777001	93782000	2.70E-10
chr8	94056001	94061000	4.90E-13
chr8	94058001	94063000	4.92E-12
chr8	94418001	94423000	0.002845
chr8	96126001	96131000	4.24E-09
chr8	96127001	96132000	9.57E-13
chr8	96453001	96458000	7.22E-13
chr8	96607001	96612000	1.07E-06
chr8	96750001	96755000	6.19E-10
chr8	96821001	96826000	1.01E-10
chr8	96822001	96827000	6.57E-10
chr8	96844001	96849000	1.78E-09
chr8	96866001	96871000	7.58E-10
chr8	96867001	96872000	2.09E-07
chr8	96868001	96873000	4.42E-08
chr8	97690001	97695000	6.58E-11
chr8	97691001	97696000	3.83E-06
chr8	97692001	97697000	5.33E-06
chr8	97917001	97922000	4.00E-06
chr8	97929001	97934000	4.09E-11
chr8	98464001	98469000	5.43E-06
chr8	98465001	98470000	8.72E-09
chr8	98478001	98483000	1.62E-08
chr8	98501001	98506000	3.27E-07
chr8	98515001	98520000	1.07E-07
chr8	98653001	98658000	4.36E-21
chr8	98767001	98772000	0.000254
chr8	98768001	98773000	0.000185
chr8	98769001	98774000	0.000236
chr8	101514001	101519000	8.75E-17
chr8	102089001	102094000	9.98E-13
chr8	102090001	102095000	3.72E-16
chr8	102447001	102452000	3.91E-15
chr8	102448001	102453000	3.37E-11
chr8	103135001	103140000	9.55E-16
chr8	103741001	103746000	3.20E-10
chr8	103980001	103985000	2.91E-12

chr8	103981001	103986000	2.78E-12
chr8	104597001	104602000	3.00E-07
chr8	104598001	104603000	4.75E-08
chr8	104599001	104604000	2.30E-10
chr8	104600001	104605000	3.72E-07
chr8	105381001	105386000	1.81E-14
chr8	105382001	105387000	1.81E-13
chr8	105614001	105619000	4.53E-20
chr8	105615001	105620000	2.70E-18
chr8	105616001	105621000	2.72E-22
chr8	105617001	105622000	6.60E-19
chr8	105618001	105623000	1.50E-09
chr8	105648001	105653000	1.29E-10
chr8	105649001	105654000	8.79E-10
chr8	105651001	105656000	1.71E-07
chr8	105717001	105722000	1.21E-07
chr8	105718001	105723000	8.97E-11
chr8	105719001	105724000	2.77E-10
chr8	105725001	105730000	1.52E-09
chr8	105728001	105733000	1.05E-12
chr8	105729001	105734000	6.20E-12
chr8	105730001	105735000	3.04E-12
chr8	105768001	105773000	2.30E-12
chr8	105769001	105774000	4.87E-13
chr8	105770001	105775000	2.64E-13
chr8	105771001	105776000	8.37E-17
chr8	105773001	105778000	9.62E-16
chr8	105787001	105792000	8.51E-16
chr8	105788001	105793000	2.99E-17
chr8	105789001	105794000	2.59E-13
chr8	105790001	105795000	2.39E-11
chr8	105791001	105796000	1.81E-13
chr8	105792001	105797000	6.45E-11
chr8	105793001	105798000	1.11E-11
chr8	105794001	105799000	1.71E-14
chr8	105805001	105810000	2.44E-16
chr8	105806001	105811000	1.34E-16
chr8	105807001	105812000	6.43E-18
chr8	105808001	105813000	1.00E-17
chr8	105835001	105840000	2.76E-08
chr8	105836001	105841000	6.95E-09
chr8	105837001	105842000	5.15E-09
chr8	105842001	105847000	7.21E-17
chr8	105843001	105848000	4.57E-15
chr8	105844001	105849000	2.01E-16
chr8	105845001	105850000	3.23E-12
chr8	105846001	105851000	1.60E-14
chr8	105855001	105860000	4.25E-08

chr8	105859001	105864000	1.91E-05
chr8	105860001	105865000	5.79E-08
chr8	105862001	105867000	2.05E-12
chr8	105863001	105868000	1.13E-13
chr8	105864001	105869000	2.10E-12
chr8	105865001	105870000	1.49E-12
chr8	105866001	105871000	4.86E-13
chr8	105867001	105872000	8.52E-10
chr8	105872001	105877000	7.25E-11
chr8	105873001	105878000	1.03E-10
chr8	105879001	105884000	5.66E-10
chr8	105880001	105885000	1.48E-12
chr8	105882001	105887000	5.70E-16
chr8	105883001	105888000	3.27E-17
chr8	105891001	105896000	1.49E-09
chr8	105892001	105897000	2.94E-12
chr8	105893001	105898000	1.83E-13
chr8	105894001	105899000	1.20E-13
chr8	105896001	105901000	4.05E-11
chr8	105897001	105902000	6.62E-12
chr8	105907001	105912000	2.50E-08
chr8	105909001	105914000	7.41E-10
chr8	105911001	105916000	1.02E-12
chr8	105913001	105918000	3.35E-19
chr8	105914001	105919000	3.79E-16
chr8	105915001	105920000	7.20E-16
chr8	105916001	105921000	5.60E-12
chr8	105922001	105927000	1.29E-16
chr8	105923001	105928000	1.48E-16
chr8	105924001	105929000	8.36E-19
chr8	105931001	105936000	1.78E-13
chr8	105932001	105937000	1.32E-14
chr8	105933001	105938000	2.86E-13
chr8	105934001	105939000	7.33E-14
chr8	105935001	105940000	3.98E-11
chr8	105978001	105983000	8.18E-10
chr8	105989001	105994000	1.43E-13
chr8	105990001	105995000	2.66E-20
chr8	105991001	105996000	3.40E-19
chr8	105992001	105997000	6.55E-15
chr8	106023001	106028000	2.04E-13
chr8	106024001	106029000	3.43E-16
chr8	106025001	106030000	2.96E-15
chr8	106026001	106031000	1.45E-15
chr8	106027001	106032000	1.07E-16
chr8	106028001	106033000	1.64E-13
chr8	106029001	106034000	4.56E-11
chr8	106045001	106050000	5.40E-14

chr8	106046001	106051000	4.29E-10
chr8	106055001	106060000	3.47E-08
chr8	106064001	106069000	2.81E-15
chr8	106065001	106070000	5.53E-19
chr8	106066001	106071000	1.38E-18
chr8	106067001	106072000	6.28E-21
chr8	106068001	106073000	6.82E-18
chr8	106069001	106074000	1.30E-10
chr8	106070001	106075000	3.14E-06
chr8	106076001	106081000	4.92E-11
chr8	106077001	106082000	9.89E-12
chr8	106078001	106083000	1.09E-10
chr8	106079001	106084000	2.16E-10
chr8	106080001	106085000	2.16E-10
chr8	106120001	106125000	2.15E-12
chr8	106128001	106133000	5.92E-16
chr8	106129001	106134000	1.29E-15
chr8	106135001	106140000	1.46E-16
chr8	106136001	106141000	8.44E-19
chr8	106142001	106147000	2.08E-12
chr8	106143001	106148000	5.57E-19
chr8	106144001	106149000	1.01E-21
chr8	106145001	106150000	1.09E-23
chr8	106146001	106151000	9.35E-23
chr8	106147001	106152000	1.45E-26
chr8	106148001	106153000	1.16E-26
chr8	106149001	106154000	4.30E-22
chr8	106150001	106155000	2.57E-25
chr8	106151001	106156000	3.22E-22
chr8	106161001	106166000	9.57E-16
chr8	106169001	106174000	1.62E-09
chr8	106192001	106197000	1.58E-11
chr8	106194001	106199000	3.18E-07
chr8	106201001	106206000	6.81E-10
chr8	106202001	106207000	7.89E-14
chr8	106203001	106208000	1.16E-11
chr8	106204001	106209000	4.82E-15
chr8	106205001	106210000	4.44E-15
chr8	106206001	106211000	5.75E-13
chr8	106207001	106212000	5.14E-10
chr8	106208001	106213000	6.95E-09
chr8	106210001	106215000	8.21E-09
chr8	106219001	106224000	2.56E-06
chr8	106235001	106240000	5.29E-09
chr8	106236001	106241000	3.28E-14
chr8	106237001	106242000	2.84E-15
chr8	106238001	106243000	2.52E-16
chr8	106239001	106244000	2.97E-11

chr8	106240001	106245000	6.10E-11
chr8	106241001	106246000	1.02E-08
chr8	106260001	106265000	2.37E-13
chr8	106268001	106273000	3.63E-07
chr8	106269001	106274000	2.98E-07
chr8	106270001	106275000	1.43E-09
chr8	106271001	106276000	3.22E-09
chr8	106272001	106277000	1.90E-08
chr8	106278001	106283000	4.63E-10
chr8	106279001	106284000	1.41E-11
chr8	106280001	106285000	3.12E-14
chr8	106281001	106286000	1.93E-18
chr8	106282001	106287000	9.97E-16
chr8	106283001	106288000	5.45E-13
chr8	106293001	106298000	6.12E-15
chr8	106294001	106299000	2.59E-17
chr8	106295001	106300000	6.66E-21
chr8	106296001	106301000	9.81E-24
chr8	106297001	106302000	7.29E-20
chr8	106298001	106303000	2.22E-14
chr8	106315001	106320000	2.40E-07
chr8	106371001	106376000	1.03E-12
chr8	106718001	106723000	1.08E-15
chr8	106719001	106724000	4.44E-17
chr8	106746001	106751000	3.54E-13
chr8	107133001	107138000	4.32E-14
chr8	107134001	107139000	1.13E-08
chr8	107211001	107216000	7.14E-14
chr8	107212001	107217000	1.87E-10
chr8	107213001	107218000	3.51E-11
chr8	107263001	107268000	3.48E-12
chr8	107264001	107269000	1.05E-12
chr8	107303001	107308000	3.33E-11
chr8	107304001	107309000	3.38E-12
chr8	107305001	107310000	1.87E-17
chr8	107306001	107311000	7.98E-17
chr8	107323001	107328000	1.12E-12
chr8	107378001	107383000	6.11E-14
chr8	107379001	107384000	1.94E-17
chr8	107380001	107385000	9.00E-16
chr8	107381001	107386000	4.96E-18
chr8	107382001	107387000	6.73E-14
chr8	107396001	107401000	1.29E-11
chr8	107582001	107587000	3.40E-08
chr8	107583001	107588000	5.59E-08
chr8	107584001	107589000	1.32E-07
chr8	108659001	108664000	5.43E-05
chr8	108660001	108665000	3.14E-06

chr8	108661001	108666000	1.60E-06
chr8	108662001	108667000	2.94E-07
chr8	108663001	108668000	1.54E-06
chr8	109827001	109832000	1.33E-09
chr8	110771001	110776000	5.72E-16
chr8	110772001	110777000	6.73E-19
chr8	110773001	110778000	3.00E-15
chr8	110774001	110779000	2.62E-14
chr8	110775001	110780000	4.76E-11
chr8	110875001	110880000	4.54E-12
chr8	110883001	110888000	1.41E-06
chr8	110949001	110954000	1.02E-10
chr8	110950001	110955000	2.29E-11
chr8	110951001	110956000	8.78E-14
chr8	111073001	111078000	5.22E-12
chr8	111074001	111079000	2.47E-12
chr8	111231001	111236000	2.02E-11
chr8	111232001	111237000	5.79E-12
chr8	111233001	111238000	6.45E-09
chr8	111326001	111331000	1.57E-09
chr8	111560001	111565000	0.000224
chr8	112144001	112149000	7.99E-12
chr8	112145001	112150000	1.66E-13
chr8	112147001	112152000	3.72E-13
chr8	112148001	112153000	8.89E-11
chr8	112239001	112244000	2.26E-09
chr8	112435001	112440000	1.29E-06
chr8	112436001	112441000	1.78E-06
chr8	112437001	112442000	1.51E-07
chr8	112470001	112475000	6.63E-11
chr8	112599001	112604000	1.04E-13
chr8	112994001	112999000	2.87E-13
chr8	112995001	113000000	1.76E-12
chr8	112996001	113001000	3.94E-12
chr8	113066001	113071000	2.82E-14
chr8	113067001	113072000	8.06E-13
chr8	113068001	113073000	4.07E-09
chr8	113080001	113085000	4.57E-09
chr8	113081001	113086000	1.44E-08
chr8	113085001	113090000	1.50E-07
chr8	113086001	113091000	1.50E-07
chr8	113087001	113092000	2.52E-08
chr8	113088001	113093000	1.24E-08
chr8	113199001	113204000	7.29E-08
chr8	113348001	113353000	7.53E-18
chr8	113435001	113440000	2.06E-10
chr8	113451001	113456000	3.90E-09
chr8	113452001	113457000	7.27E-08

chr8	113467001	113472000	3.36E-07
chr8	113605001	113610000	1.02E-11
chr8	113606001	113611000	3.81E-12
chr8	113666001	113671000	2.45E-09
chr8	113680001	113685000	1.70E-09
chr8	113681001	113686000	4.24E-10
chr8	113741001	113746000	7.94E-17
chr8	113842001	113847000	1.50E-07
chr8	113843001	113848000	3.91E-05
chr8	113856001	113861000	9.54E-07
chr8	113896001	113901000	6.34E-16
chr8	113897001	113902000	1.45E-15
chr8	113932001	113937000	1.07E-27
chr8	114034001	114039000	2.76E-09
chr8	114040001	114045000	2.15E-08
chr8	114041001	114046000	3.22E-08
chr8	114042001	114047000	5.77E-07
chr8	114043001	114048000	2.94E-06
chr8	114074001	114079000	3.28E-12
chr8	114077001	114082000	1.22E-14
chr8	114078001	114083000	8.45E-10
chr8	114106001	114111000	4.22E-13
chr8	114107001	114112000	1.88E-11
chr8	114108001	114113000	3.09E-14
chr8	114109001	114114000	1.08E-15
chr8	114149001	114154000	2.55E-09
chr8	114150001	114155000	2.16E-09
chr8	114157001	114162000	5.91E-12
chr8	114158001	114163000	1.49E-13
chr8	114162001	114167000	6.07E-13
chr8	114195001	114200000	1.77E-17
chr8	114214001	114219000	1.56E-11
chr8	114215001	114220000	6.64E-14
chr8	114216001	114221000	1.27E-16
chr8	114276001	114281000	1.18E-12
chr8	114277001	114282000	3.61E-17
chr8	114298001	114303000	7.50E-12
chr8	114299001	114304000	2.91E-13
chr8	114300001	114305000	8.11E-11
chr8	114301001	114306000	1.35E-11
chr8	114344001	114349000	1.20E-14
chr8	114345001	114350000	7.71E-13
chr8	114346001	114351000	1.22E-12
chr8	114365001	114370000	8.93E-11
chr8	114366001	114371000	4.04E-15
chr8	114367001	114372000	4.13E-13
chr8	114463001	114468000	1.60E-13
chr8	114474001	114479000	7.48E-16

chr8	114475001	114480000	2.06E-16
chr8	114484001	114489000	3.21E-13
chr8	114524001	114529000	3.59E-14
chr8	114525001	114530000	9.56E-14
chr8	114563001	114568000	6.68E-10
chr8	114564001	114569000	5.62E-12
chr8	114565001	114570000	7.58E-10
chr8	114577001	114582000	1.30E-07
chr8	114642001	114647000	4.49E-11
chr8	114643001	114648000	3.30E-12
chr8	114645001	114650000	1.42E-09
chr8	114672001	114677000	6.42E-15
chr8	114673001	114678000	8.52E-14
chr8	114681001	114686000	8.64E-13
chr8	114692001	114697000	4.45E-09
chr8	114699001	114704000	2.78E-17
chr8	114700001	114705000	1.32E-17
chr8	114701001	114706000	3.93E-17
chr8	114703001	114708000	7.77E-14
chr8	114704001	114709000	3.46E-11
chr8	114705001	114710000	9.60E-11
chr8	114706001	114711000	3.02E-12
chr8	114707001	114712000	3.09E-08
chr8	114712001	114717000	5.54E-17
chr8	114713001	114718000	9.37E-13
chr8	114714001	114719000	1.60E-12
chr8	114715001	114720000	1.78E-13
chr8	114716001	114721000	1.30E-12
chr8	114765001	114770000	3.85E-15
chr8	114777001	114782000	2.77E-09
chr8	114778001	114783000	4.03E-09
chr8	114779001	114784000	1.25E-07
chr8	114780001	114785000	7.65E-11
chr8	114781001	114786000	4.91E-09
chr8	114782001	114787000	9.41E-12
chr8	114783001	114788000	1.32E-16
chr8	114784001	114789000	6.13E-19
chr8	114808001	114813000	2.83E-12
chr8	114812001	114817000	3.10E-14
chr8	114813001	114818000	1.13E-13
chr8	114814001	114819000	6.20E-12
chr8	114824001	114829000	3.53E-11
chr8	114827001	114832000	2.68E-09
chr8	114828001	114833000	5.22E-13
chr8	114854001	114859000	1.31E-10
chr8	114855001	114860000	9.54E-12
chr8	114856001	114861000	6.56E-13
chr8	114857001	114862000	8.81E-12

chr8	114904001	114909000	4.87E-13
chr8	114905001	114910000	4.10E-14
chr8	114906001	114911000	3.26E-13
chr8	114907001	114912000	1.36E-11
chr8	114911001	114916000	5.01E-13
chr8	114912001	114917000	9.22E-13
chr8	114913001	114918000	9.30E-12
chr8	114914001	114919000	6.41E-13
chr8	114915001	114920000	2.74E-12
chr8	114916001	114921000	1.49E-08
chr8	114950001	114955000	3.00E-11
chr8	114958001	114963000	1.30E-12
chr8	114960001	114965000	1.67E-14
chr8	114969001	114974000	3.95E-19
chr8	114970001	114975000	2.95E-16
chr8	114992001	114997000	1.11E-11
chr8	114993001	114998000	6.69E-09
chr8	114994001	114999000	3.86E-08
chr8	115089001	115094000	1.22E-14
chr8	115108001	115113000	1.18E-12
chr8	115109001	115114000	2.92E-12
chr8	115135001	115140000	6.31E-16
chr8	115141001	115146000	1.50E-08
chr8	115166001	115171000	5.83E-11
chr8	115167001	115172000	1.54E-11
chr8	115178001	115183000	3.76E-16
chr8	115231001	115236000	3.76E-13
chr8	115241001	115246000	1.84E-16
chr8	115242001	115247000	6.87E-20
chr8	115243001	115248000	1.10E-21
chr8	115244001	115249000	1.39E-21
chr8	115250001	115255000	9.03E-24
chr8	115251001	115256000	1.07E-25
chr8	115252001	115257000	1.02E-25
chr8	115254001	115259000	1.09E-26
chr8	115282001	115287000	5.64E-10
chr8	115381001	115386000	3.47E-14
chr8	115382001	115387000	1.77E-15
chr8	115383001	115388000	1.29E-16
chr8	115384001	115389000	1.89E-17
chr8	115385001	115390000	6.24E-18
chr8	115386001	115391000	7.58E-10
chr8	115387001	115392000	2.38E-09
chr8	115388001	115393000	3.72E-10
chr8	115567001	115572000	1.31E-12
chr8	115568001	115573000	1.12E-17
chr8	115977001	115982000	2.25E-13
chr8	116043001	116048000	1.32E-11

chr8	116072001	116077000	2.77E-11
chr8	116073001	116078000	8.17E-10
chr8	116079001	116084000	1.57E-18
chr8	116080001	116085000	1.10E-19
chr8	116146001	116151000	3.27E-14
chr8	116147001	116152000	8.23E-13
chr8	116148001	116153000	6.24E-10
chr8	116233001	116238000	4.84E-08
chr8	116405001	116410000	7.54E-10
chr8	116406001	116411000	1.96E-09
chr8	116694001	116699000	5.42E-19
chr8	116695001	116700000	1.09E-18
chr8	116773001	116778000	3.75E-14
chr8	116774001	116779000	5.55E-11
chr8	116775001	116780000	1.50E-11
chr8	116776001	116781000	6.98E-10
chr8	116777001	116782000	4.07E-14
chr8	116778001	116783000	1.17E-10
chr8	116779001	116784000	1.70E-09
chr8	116801001	116806000	1.80E-08
chr8	116853001	116858000	6.47E-12
chr8	116855001	116860000	1.83E-12
chr8	116883001	116888000	5.82E-19
chr8	116884001	116889000	5.73E-16
chr8	116885001	116890000	9.16E-16
chr8	116954001	116959000	3.59E-13
chr8	116955001	116960000	1.31E-16
chr8	116956001	116961000	2.49E-17
chr8	116992001	116997000	5.58E-15
chr8	116993001	116998000	8.41E-15
chr8	116994001	116999000	3.29E-10
chr8	117022001	117027000	5.97E-19
chr8	117023001	117028000	1.15E-14
chr8	117025001	117030000	5.20E-14
chr8	117026001	117031000	2.95E-12
chr8	117027001	117032000	2.02E-13
chr8	117029001	117034000	6.04E-15
chr8	117030001	117035000	4.52E-13
chr8	117031001	117036000	6.32E-17
chr8	117032001	117037000	6.43E-15
chr8	117033001	117038000	1.94E-17
chr8	117047001	117052000	7.91E-12
chr8	117061001	117066000	7.82E-25
chr8	117062001	117067000	1.54E-22
chr8	117063001	117068000	6.33E-22
chr8	117065001	117070000	5.11E-18
chr8	117066001	117071000	6.17E-16
chr8	117067001	117072000	6.07E-21

chr8	117068001	117073000	1.85E-13
chr8	117069001	117074000	2.11E-12
chr8	117081001	117086000	1.05E-11
chr8	117141001	117146000	7.87E-13
chr8	117151001	117156000	1.07E-15
chr8	117152001	117157000	2.64E-14
chr8	117153001	117158000	6.23E-17
chr8	117154001	117159000	1.57E-23
chr8	117155001	117160000	5.74E-21
chr8	117156001	117161000	1.06E-19
chr8	117157001	117162000	8.09E-18
chr8	117158001	117163000	2.07E-18
chr8	117207001	117212000	2.43E-15
chr8	117220001	117225000	2.05E-17
chr8	117221001	117226000	1.50E-12
chr8	117222001	117227000	1.45E-13
chr8	117223001	117228000	5.17E-13
chr8	117229001	117234000	4.41E-13
chr8	117230001	117235000	2.62E-15
chr8	117231001	117236000	3.97E-10
chr8	117241001	117246000	2.19E-15
chr8	117242001	117247000	2.41E-14
chr8	117281001	117286000	5.86E-07
chr8	117282001	117287000	7.21E-06
chr8	117295001	117300000	8.90E-12
chr8	117304001	117309000	3.18E-14
chr8	117306001	117311000	1.25E-17
chr8	117380001	117385000	2.97E-13
chr8	117381001	117386000	2.36E-13
chr8	117382001	117387000	2.86E-13
chr8	117389001	117394000	7.08E-09
chr8	117390001	117395000	1.40E-10
chr8	117408001	117413000	2.05E-08
chr8	117990001	117995000	4.49E-08
chr8	117991001	117996000	5.43E-12
chr8	117992001	117997000	9.33E-10
chr8	117993001	117998000	7.22E-09
chr8	118077001	118082000	8.84E-18
chr8	118078001	118083000	5.85E-13
chr8	118079001	118084000	1.51E-12
chr8	118080001	118085000	5.42E-11
chr8	118142001	118147000	1.10E-16
chr8	118219001	118224000	1.31E-09
chr8	118346001	118351000	5.50E-10
chr8	118357001	118362000	1.25E-11
chr8	118358001	118363000	2.65E-09
chr8	118359001	118364000	8.45E-12
chr8	118360001	118365000	1.63E-07

chr8	118366001	118371000	5.05E-10
chr8	118392001	118397000	8.58E-13
chr8	118447001	118452000	2.46E-12
chr8	118454001	118459000	6.09E-11
chr8	118456001	118461000	8.35E-14
chr8	118458001	118463000	1.36E-13
chr8	118467001	118472000	1.36E-08
chr8	118468001	118473000	2.44E-06
chr8	118469001	118474000	8.93E-08
chr8	118470001	118475000	9.73E-07
chr8	118472001	118477000	1.91E-11
chr8	118473001	118478000	2.47E-14
chr8	118499001	118504000	1.09E-07
chr8	118510001	118515000	1.53E-10
chr8	118511001	118516000	3.61E-08
chr8	118523001	118528000	2.16E-11
chr8	118524001	118529000	1.55E-11
chr8	118559001	118564000	5.53E-13
chr8	118560001	118565000	7.18E-16
chr8	118569001	118574000	5.56E-12
chr8	118570001	118575000	6.19E-11
chr8	118571001	118576000	1.09E-09
chr8	118573001	118578000	6.35E-12
chr8	118591001	118596000	2.96E-10
chr8	118592001	118597000	9.73E-15
chr8	118593001	118598000	2.05E-17
chr8	118594001	118599000	5.72E-12
chr8	118595001	118600000	1.05E-11
chr8	118596001	118601000	2.68E-10
chr8	118605001	118610000	4.36E-09
chr8	118606001	118611000	1.05E-06
chr8	118607001	118612000	2.71E-10
chr8	118608001	118613000	1.53E-08
chr8	118609001	118614000	5.50E-08
chr8	118610001	118615000	1.77E-07
chr8	118630001	118635000	1.14E-10
chr8	118631001	118636000	5.36E-11
chr8	118632001	118637000	7.07E-11
chr8	118633001	118638000	2.36E-13
chr8	118634001	118639000	8.44E-19
chr8	118644001	118649000	2.73E-17
chr8	118645001	118650000	2.57E-21
chr8	118646001	118651000	3.26E-17
chr8	118683001	118688000	9.44E-09
chr8	118684001	118689000	1.62E-11
chr8	118685001	118690000	1.84E-12
chr8	118686001	118691000	1.64E-12
chr8	118687001	118692000	5.52E-10

chr8	118689001	118694000	2.63E-09
chr8	118705001	118710000	1.60E-08
chr8	118717001	118722000	7.62E-13
chr8	118719001	118724000	1.30E-11
chr8	118749001	118754000	8.57E-14
chr8	118752001	118757000	1.66E-14
chr8	118753001	118758000	1.65E-16
chr8	119714001	119719000	2.01E-13
chr8	119717001	119722000	4.03E-14
chr8	120177001	120182000	4.17E-10
chr8	120441001	120446000	1.42E-06
chr8	121367001	121372000	5.21E-13
chr8	121368001	121373000	6.91E-12
chr8	121617001	121622000	1.39E-08
chr8	121750001	121755000	7.28E-10
chr8	123577001	123582000	2.11E-10
chr8	123578001	123583000	6.38E-13
chr8	123579001	123584000	6.46E-13
chr8	124054001	124059000	4.86E-12
chr8	124082001	124087000	3.39E-12
chr8	124550001	124555000	4.50E-13
chr8	124552001	124557000	2.98E-09
chr8	124982001	124987000	4.63E-10
chr8	124983001	124988000	2.32E-12
chr8	124984001	124989000	2.70E-12
chr8	125037001	125042000	6.51E-09
chr8	125842001	125847000	2.85E-07
chr8	125843001	125848000	1.25E-07
chr8	126269001	126274000	4.72E-12
chr8	126695001	126700000	9.52E-10
chr8	126696001	126701000	9.39E-10
chr8	126697001	126702000	3.13E-13
chr8	126698001	126703000	7.66E-11
chr8	126719001	126724000	2.51E-09
chr8	126720001	126725000	2.06E-09
chr8	126721001	126726000	4.36E-07
chr8	126722001	126727000	5.82E-08
chr8	126723001	126728000	4.46E-10
chr8	126727001	126732000	5.15E-14
chr8	126728001	126733000	6.00E-13
chr8	126729001	126734000	1.99E-09
chr8	126730001	126735000	4.63E-08
chr8	126745001	126750000	2.73E-10
chr8	126746001	126751000	5.98E-11
chr8	126747001	126752000	8.67E-11
chr8	126748001	126753000	7.15E-10
chr8	126749001	126754000	1.18E-09
chr8	126755001	126760000	1.78E-09

chr8	126756001	126761000	2.39E-09
chr8	126765001	126770000	5.66E-10
chr8	126766001	126771000	2.68E-12
chr8	126815001	126820000	3.42E-09
chr8	126836001	126841000	1.13E-09
chr8	126851001	126856000	2.34E-09
chr8	126905001	126910000	2.01E-07
chr8	126906001	126911000	7.79E-07
chr8	126907001	126912000	1.27E-07
chr8	126908001	126913000	1.25E-07
chr8	126932001	126937000	8.99E-21
chr8	126933001	126938000	1.28E-10
chr8	126935001	126940000	3.33E-11
chr8	126946001	126951000	2.43E-14
chr8	126962001	126967000	1.31E-11
chr8	126963001	126968000	2.85E-15
chr8	126964001	126969000	1.61E-13
chr8	126972001	126977000	2.92E-08
chr8	126973001	126978000	2.65E-08
chr8	126974001	126979000	2.26E-08
chr8	126988001	126993000	3.26E-09
chr8	126990001	126995000	2.20E-08
chr8	126991001	126996000	9.33E-10
chr8	126992001	126997000	7.29E-09
chr8	126993001	126998000	2.37E-07
chr8	126994001	126999000	4.47E-06
chr8	126995001	127000000	7.58E-08
chr8	127025001	127030000	8.78E-18
chr8	127038001	127043000	8.91E-11
chr8	127041001	127046000	5.60E-12
chr8	127042001	127047000	2.65E-16
chr8	127043001	127048000	1.22E-13
chr8	127044001	127049000	1.58E-11
chr8	127045001	127050000	9.49E-16
chr8	127046001	127051000	2.01E-10
chr8	127047001	127052000	1.92E-07
chr8	127048001	127053000	8.36E-08
chr8	127049001	127054000	1.24E-08
chr8	127051001	127056000	1.38E-10
chr8	127068001	127073000	1.80E-16
chr8	127071001	127076000	5.34E-11
chr8	127072001	127077000	1.34E-15
chr8	127094001	127099000	1.88E-13
chr8	127096001	127101000	4.90E-10
chr8	127108001	127113000	5.62E-15
chr8	127109001	127114000	1.33E-13
chr8	127110001	127115000	7.58E-18
chr8	127111001	127116000	2.34E-14

chr8	127129001	127134000	1.26E-13
chr8	127130001	127135000	2.83E-16
chr8	127131001	127136000	1.98E-15
chr8	127132001	127137000	2.30E-16
chr8	127133001	127138000	1.45E-15
chr8	127134001	127139000	7.89E-12
chr8	127135001	127140000	3.89E-08
chr8	127148001	127153000	4.42E-12
chr8	127149001	127154000	1.46E-17
chr8	127150001	127155000	7.64E-17
chr8	127151001	127156000	3.15E-16
chr8	127152001	127157000	5.65E-12
chr8	127153001	127158000	7.30E-15
chr8	127154001	127159000	9.98E-11
chr8	127155001	127160000	1.22E-07
chr8	127156001	127161000	1.09E-10
chr8	127157001	127162000	3.14E-12
chr8	127158001	127163000	4.31E-09
chr8	127159001	127164000	2.39E-08
chr8	127160001	127165000	1.70E-08
chr8	127191001	127196000	1.64E-10
chr8	127192001	127197000	7.13E-11
chr8	127283001	127288000	2.06E-09
chr8	127284001	127289000	7.20E-12
chr8	127285001	127290000	1.87E-14
chr8	127607001	127612000	2.75E-15
chr8	127608001	127613000	1.02E-13
chr8	127629001	127634000	9.45E-12
chr8	127650001	127655000	2.40E-12
chr8	127659001	127664000	1.64E-08
chr8	127660001	127665000	2.50E-07
chr8	127661001	127666000	8.30E-10
chr8	127662001	127667000	1.23E-15
chr8	127663001	127668000	6.37E-13
chr8	127664001	127669000	5.19E-15
chr8	127665001	127670000	1.02E-12
chr8	127687001	127692000	2.41E-06
chr8	127702001	127707000	2.65E-10
chr8	127718001	127723000	3.18E-08
chr8	127719001	127724000	7.01E-11
chr8	127727001	127732000	1.63E-20
chr8	127728001	127733000	8.24E-13
chr8	127735001	127740000	1.57E-10
chr8	127736001	127741000	4.17E-11
chr8	128255001	128260000	1.95E-07
chr8	128276001	128281000	7.94E-14
chr8	128277001	128282000	4.06E-16
chr8	128278001	128283000	6.41E-16

chr8	128303001	128308000	1.32E-15
chr8	128306001	128311000	1.80E-14
chr8	128488001	128493000	1.07E-18
chr8	128489001	128494000	4.66E-27
chr8	128490001	128495000	5.67E-23
chr8	128491001	128496000	1.36E-17
chr8	128804001	128809000	3.20E-11
chr8	128811001	128816000	1.86E-13
chr8	128812001	128817000	2.02E-11
chr8	128813001	128818000	6.48E-09
chr8	129468001	129473000	0.002682
chr8	129809001	129814000	8.68E-14
chr8	129810001	129815000	2.12E-14
chr8	129845001	129850000	2.70E-10
chr8	129846001	129851000	1.29E-11
chr8	129847001	129852000	1.02E-11
chr8	130031001	130036000	6.27E-09
chr8	130032001	130037000	1.57E-09
chr8	130142001	130147000	2.14E-16
chr8	130143001	130148000	4.04E-22
chr8	130144001	130149000	1.38E-19
chr8	130146001	130151000	1.46E-16
chr8	130149001	130154000	1.48E-15
chr8	130150001	130155000	2.04E-15
chr8	130225001	130230000	4.32E-11
chr8	130268001	130273000	1.09E-05
chr8	130311001	130316000	7.85E-09
chr8	130385001	130390000	1.02E-10
chr8	130386001	130391000	1.85E-08
chr8	130395001	130400000	7.03E-13
chr8	130396001	130401000	5.27E-16
chr8	130432001	130437000	1.11E-07
chr8	130433001	130438000	5.57E-10
chr8	130434001	130439000	3.72E-08
chr8	130436001	130441000	1.26E-12
chr8	130437001	130442000	5.28E-13
chr8	130438001	130443000	2.66E-15
chr8	130439001	130444000	6.32E-16
chr8	130440001	130445000	9.98E-10
chr8	130539001	130544000	6.94E-12
chr8	130611001	130616000	8.24E-11
chr8	130613001	130618000	1.11E-08
chr8	130699001	130704000	1.45E-08
chr8	130700001	130705000	3.66E-19
chr8	130816001	130821000	1.33E-10
chr8	130817001	130822000	3.47E-08
chr8	130818001	130823000	2.73E-10
chr8	130819001	130824000	8.84E-08

chr8	130820001	130825000	2.08E-09
chr8	130994001	130999000	0.000527
chr8	130995001	131000000	0.000143
chr8	131509001	131514000	3.35E-07
chr8	131510001	131515000	7.53E-07
chr8	131529001	131534000	2.27E-09
chr8	131530001	131535000	1.23E-07
chr8	131552001	131557000	3.38E-12
chr8	131553001	131558000	3.62E-10
chr8	131599001	131604000	2.57E-10
chr8	131674001	131679000	8.66E-12
chr8	131675001	131680000	1.32E-10
chr8	131676001	131681000	1.88E-09
chr8	131698001	131703000	1.67E-07
chr8	131746001	131751000	1.32E-09
chr8	131791001	131796000	3.26E-11
chr8	131792001	131797000	3.45E-13
chr8	131793001	131798000	7.88E-15
chr8	131823001	131828000	3.02E-16
chr8	131824001	131829000	2.91E-17
chr8	131831001	131836000	3.25E-09
chr8	131840001	131845000	2.47E-12
chr8	131841001	131846000	1.28E-14
chr8	131853001	131858000	1.27E-11
chr8	131854001	131859000	1.39E-09
chr8	131855001	131860000	6.85E-09
chr8	131857001	131862000	2.32E-07
chr8	131861001	131866000	1.42E-09
chr8	131862001	131867000	3.05E-11
chr8	131866001	131871000	2.73E-09
chr8	131896001	131901000	7.95E-10
chr8	131897001	131902000	2.63E-10
chr8	131898001	131903000	7.64E-17
chr8	131899001	131904000	2.09E-13
chr8	131919001	131924000	1.24E-14
chr8	131921001	131926000	1.68E-09
chr8	131935001	131940000	3.76E-12
chr8	131936001	131941000	1.53E-12
chr8	131937001	131942000	1.09E-12
chr8	131941001	131946000	9.13E-09
chr8	131943001	131948000	6.13E-09
chr8	131953001	131958000	1.67E-15
chr8	131954001	131959000	3.44E-14
chr8	131964001	131969000	1.71E-15
chr8	131965001	131970000	3.82E-19
chr8	131966001	131971000	8.47E-24
chr8	131967001	131972000	2.55E-19
chr8	131968001	131973000	9.01E-18

chr8	131969001	131974000	1.43E-13
chr8	131972001	131977000	6.67E-10
chr8	131975001	131980000	5.80E-09
chr8	131982001	131987000	1.53E-09
chr8	131983001	131988000	4.40E-13
chr8	131995001	132000000	5.53E-12
chr8	131996001	132001000	4.74E-09
chr8	131997001	132002000	1.32E-09
chr8	131998001	132003000	6.15E-13
chr8	132016001	132021000	5.07E-10
chr8	132022001	132027000	1.19E-11
chr8	132024001	132029000	7.99E-11
chr8	132025001	132030000	6.34E-16
chr8	132026001	132031000	9.39E-11
chr8	132028001	132033000	1.50E-08
chr8	132065001	132070000	6.54E-11
chr8	132087001	132092000	1.04E-14
chr8	132088001	132093000	1.14E-14
chr8	132089001	132094000	9.19E-12
chr8	132094001	132099000	1.76E-09
chr8	132113001	132118000	7.84E-10
chr8	132114001	132119000	3.55E-12
chr8	132115001	132120000	3.38E-13
chr8	132116001	132121000	1.61E-12
chr8	132125001	132130000	4.54E-16
chr8	132126001	132131000	1.28E-15
chr8	132127001	132132000	2.32E-14
chr8	132128001	132133000	3.72E-16
chr8	132129001	132134000	1.08E-11
chr8	132142001	132147000	2.01E-09
chr8	132188001	132193000	8.86E-15
chr8	132189001	132194000	7.88E-13
chr8	132190001	132195000	3.88E-11
chr8	132191001	132196000	6.11E-09
chr8	132192001	132197000	3.22E-06
chr8	132205001	132210000	4.60E-09
chr8	132214001	132219000	2.62E-08
chr8	132215001	132220000	3.43E-12
chr8	132216001	132221000	8.27E-15
chr8	132217001	132222000	4.84E-16
chr8	132230001	132235000	5.96E-09
chr8	132338001	132343000	3.05E-07
chr8	132339001	132344000	2.37E-08
chr8	132341001	132346000	1.10E-07
chr8	132455001	132460000	1.30E-13
chr8	132456001	132461000	9.62E-12
chr8	132496001	132501000	1.88E-19
chr8	132497001	132502000	1.70E-20

chr8	132498001	132503000	2.26E-19
chr8	132662001	132667000	4.07E-13
chr8	132663001	132668000	2.78E-14
chr8	132664001	132669000	4.55E-15
chr8	132665001	132670000	6.31E-09
chr8	132666001	132671000	3.69E-10
chr8	132668001	132673000	2.49E-11
chr8	132753001	132758000	2.37E-20
chr8	132754001	132759000	5.68E-22
chr8	132755001	132760000	2.83E-25
chr8	132756001	132761000	1.38E-16
chr8	132761001	132766000	6.35E-18
chr8	132762001	132767000	1.24E-20
chr8	132763001	132768000	4.18E-16
chr8	132764001	132769000	2.08E-15
chr8	132765001	132770000	1.31E-09
chr8	132779001	132784000	7.51E-13
chr8	132780001	132785000	2.13E-14
chr8	132781001	132786000	1.33E-12
chr8	132795001	132800000	3.95E-14
chr8	132796001	132801000	8.28E-16
chr8	132837001	132842000	2.12E-09
chr8	132838001	132843000	5.93E-09
chr8	132841001	132846000	2.91E-13
chr8	132847001	132852000	7.30E-15
chr8	132848001	132853000	2.40E-17
chr8	132849001	132854000	5.47E-19
chr8	132862001	132867000	1.12E-14
chr8	132863001	132868000	2.38E-15
chr8	132864001	132869000	3.93E-17
chr8	132891001	132896000	6.67E-15
chr8	132892001	132897000	1.45E-14
chr8	132893001	132898000	7.35E-17
chr8	132894001	132899000	1.26E-11
chr8	132895001	132900000	7.03E-11
chr8	133033001	133038000	4.21E-06
chr8	133034001	133039000	9.71E-07
chr8	133039001	133044000	5.39E-14
chr8	133040001	133045000	7.32E-16
chr8	133041001	133046000	1.25E-18
chr8	133042001	133047000	3.13E-12
chr8	133043001	133048000	2.80E-13
chr8	133045001	133050000	3.73E-11
chr8	133057001	133062000	7.36E-12
chr8	133058001	133063000	3.08E-13
chr8	133059001	133064000	1.91E-13
chr8	133060001	133065000	3.69E-14
chr8	133061001	133066000	4.75E-11

chr8	133068001	133073000	7.89E-17
chr8	133095001	133100000	2.21E-18
chr8	133096001	133101000	2.07E-10
chr8	133099001	133104000	3.29E-11
chr8	133100001	133105000	1.62E-16
chr8	133101001	133106000	9.28E-15
chr8	133162001	133167000	7.07E-13
chr8	133163001	133168000	3.99E-14
chr8	133174001	133179000	3.73E-10
chr8	133175001	133180000	2.29E-14
chr8	133176001	133181000	1.73E-13
chr8	133177001	133182000	5.55E-12
chr8	133188001	133193000	7.96E-10
chr8	133189001	133194000	8.05E-11
chr8	133190001	133195000	1.44E-09
chr8	133191001	133196000	2.06E-10
chr8	133192001	133197000	1.08E-10
chr8	133193001	133198000	1.74E-11
chr8	133194001	133199000	3.41E-17
chr8	133195001	133200000	4.97E-16
chr8	133196001	133201000	2.46E-17
chr8	133197001	133202000	4.02E-13
chr8	133198001	133203000	2.33E-12
chr8	133211001	133216000	1.84E-14
chr8	133212001	133217000	8.32E-17
chr8	133213001	133218000	3.16E-18
chr8	133214001	133219000	2.58E-20
chr8	133215001	133220000	8.23E-19
chr8	133216001	133221000	3.44E-17
chr8	133217001	133222000	4.79E-12
chr8	133218001	133223000	9.03E-10
chr8	133224001	133229000	1.10E-11
chr8	133225001	133230000	6.42E-11
chr8	133241001	133246000	1.25E-07
chr8	133242001	133247000	2.53E-09
chr8	133243001	133248000	1.65E-10
chr8	133259001	133264000	5.10E-09
chr8	133261001	133266000	4.82E-11
chr8	133262001	133267000	6.63E-16
chr8	133263001	133268000	2.22E-14
chr8	133264001	133269000	2.80E-13
chr8	133265001	133270000	1.46E-17
chr8	133266001	133271000	3.12E-12
chr8	133267001	133272000	7.32E-09
chr8	133269001	133274000	1.32E-12
chr8	133280001	133285000	5.47E-13
chr8	133281001	133286000	3.85E-15
chr8	133282001	133287000	1.50E-16

chr8	133283001	133288000	3.01E-14
chr8	133284001	133289000	1.98E-14
chr8	133285001	133290000	1.27E-10
chr8	133292001	133297000	3.80E-08
chr8	133293001	133298000	5.20E-09
chr8	133294001	133299000	1.18E-09
chr8	133295001	133300000	3.63E-10
chr8	133296001	133301000	9.19E-08
chr8	133297001	133302000	1.19E-07
chr8	133298001	133303000	3.18E-05
chr8	133340001	133345000	1.01E-06
chr8	133341001	133346000	2.83E-09
chr8	133342001	133347000	2.80E-11
chr8	133343001	133348000	5.07E-10
chr8	133344001	133349000	1.16E-09
chr8	133345001	133350000	7.26E-12
chr8	133346001	133351000	4.46E-10
chr8	133347001	133352000	4.09E-09
chr8	133348001	133353000	1.33E-14
chr8	133357001	133362000	1.53E-16
chr8	133358001	133363000	7.14E-16
chr8	133359001	133364000	1.23E-17
chr8	133360001	133365000	3.57E-14
chr8	133384001	133389000	8.04E-18
chr8	133387001	133392000	8.09E-22
chr8	133388001	133393000	3.89E-17
chr8	133390001	133395000	6.08E-13
chr8	133411001	133416000	3.62E-10
chr8	133416001	133421000	7.75E-13
chr8	133417001	133422000	9.77E-13
chr8	133418001	133423000	1.22E-13
chr8	133419001	133424000	1.83E-13
chr8	133420001	133425000	4.38E-14
chr8	133423001	133428000	5.39E-14
chr8	133424001	133429000	9.94E-19
chr8	133425001	133430000	3.08E-16
chr8	133426001	133431000	1.43E-26
chr8	133427001	133432000	1.89E-22
chr8	133428001	133433000	1.69E-21
chr8	133429001	133434000	8.98E-24
chr8	133430001	133435000	4.28E-25
chr8	133438001	133443000	7.25E-16
chr8	133439001	133444000	8.57E-16
chr8	133440001	133445000	1.41E-13
chr8	133463001	133468000	4.15E-14
chr8	133470001	133475000	1.78E-14
chr8	133472001	133477000	2.91E-17
chr8	133474001	133479000	1.10E-21

chr8	133476001	133481000	1.24E-16
chr8	133477001	133482000	8.71E-17
chr8	133478001	133483000	1.05E-17
chr8	133479001	133484000	4.08E-20
chr8	133480001	133485000	1.45E-22
chr8	133481001	133486000	2.57E-21
chr8	133484001	133489000	2.68E-06
chr8	133485001	133490000	2.19E-07
chr8	133499001	133504000	6.79E-13
chr8	133501001	133506000	2.98E-15
chr8	133503001	133508000	5.07E-16
chr8	133504001	133509000	2.56E-13
chr8	133513001	133518000	2.10E-11
chr8	133514001	133519000	1.40E-12
chr8	133515001	133520000	7.20E-13
chr8	133516001	133521000	1.17E-10
chr8	133517001	133522000	3.86E-11
chr8	133519001	133524000	4.07E-13
chr8	133521001	133526000	3.82E-12
chr8	133522001	133527000	2.05E-14
chr8	133528001	133533000	1.17E-12
chr8	133529001	133534000	1.80E-12
chr8	133530001	133535000	4.44E-11
chr8	133531001	133536000	2.63E-10
chr8	133537001	133542000	3.43E-16
chr8	133545001	133550000	5.97E-13
chr8	133549001	133554000	3.30E-14
chr8	133551001	133556000	2.90E-15
chr8	133554001	133559000	1.60E-11
chr8	133555001	133560000	2.71E-14
chr8	133556001	133561000	9.33E-13
chr8	133557001	133562000	2.39E-13
chr8	133558001	133563000	5.42E-14
chr8	133559001	133564000	2.98E-13
chr8	133560001	133565000	8.97E-11
chr8	133561001	133566000	5.41E-12
chr8	133565001	133570000	3.80E-17
chr8	133566001	133571000	3.85E-21
chr8	133567001	133572000	3.03E-18
chr8	133573001	133578000	3.01E-09
chr8	133579001	133584000	6.41E-11
chr8	133580001	133585000	4.60E-13
chr8	133581001	133586000	2.56E-14
chr8	133582001	133587000	1.22E-11
chr8	133583001	133588000	5.24E-13
chr8	133584001	133589000	8.42E-14
chr8	133585001	133590000	3.18E-15
chr8	133593001	133598000	1.16E-12

chr8	133594001	133599000	1.62E-13
chr8	133597001	133602000	1.50E-10
chr8	133598001	133603000	1.13E-08
chr8	133600001	133605000	7.83E-08
chr8	133602001	133607000	6.73E-05
chr8	133613001	133618000	6.94E-11
chr8	133614001	133619000	5.48E-14
chr8	133615001	133620000	7.81E-16
chr8	133616001	133621000	2.14E-17
chr8	133617001	133622000	5.45E-18
chr8	133738001	133743000	6.76E-07
chr8	133740001	133745000	1.78E-08
chr8	133770001	133775000	1.48E-13
chr8	133866001	133871000	6.81E-11
chr8	133907001	133912000	1.39E-13
chr8	133955001	133960000	9.44E-13
chr8	133957001	133962000	2.74E-13
chr8	133958001	133963000	8.88E-09
chr8	133986001	133991000	4.66E-09
chr8	133988001	133993000	5.51E-05
chr8	133989001	133994000	2.67E-09
chr8	134147001	134152000	5.80E-10
chr8	134309001	134314000	6.44E-11
chr8	134546001	134551000	1.43E-09
chr8	134603001	134608000	2.14E-09
chr8	134604001	134609000	5.93E-11
chr8	134605001	134610000	1.79E-10
chr8	134607001	134612000	1.38E-13
chr8	134608001	134613000	1.63E-13
chr8	134614001	134619000	1.35E-06
chr8	134615001	134620000	5.27E-06
chr8	134622001	134627000	6.97E-08
chr8	134642001	134647000	1.22E-10
chr8	134643001	134648000	1.40E-11
chr8	134644001	134649000	1.08E-08
chr8	134654001	134659000	1.47E-14
chr8	134656001	134661000	3.06E-13
chr8	134657001	134662000	4.14E-13
chr8	134658001	134663000	2.60E-15
chr8	134659001	134664000	3.55E-14
chr8	134697001	134702000	7.92E-10
chr8	134698001	134703000	9.14E-10
chr8	134699001	134704000	1.34E-11
chr8	134700001	134705000	8.52E-07
chr8	134709001	134714000	2.54E-10
chr8	134715001	134720000	2.69E-09
chr8	134716001	134721000	6.38E-09
chr8	134717001	134722000	4.16E-10

chr8	134724001	134729000	6.03E-10
chr8	134725001	134730000	5.64E-10
chr8	134752001	134757000	2.33E-13
chr8	134753001	134758000	6.16E-12
chr8	134754001	134759000	5.96E-14
chr8	134755001	134760000	2.32E-19
chr8	134756001	134761000	1.33E-19
chr8	134767001	134772000	6.35E-18
chr8	134789001	134794000	3.31E-17
chr8	134790001	134795000	1.11E-20
chr8	134799001	134804000	0.000119
chr8	134800001	134805000	2.09E-05
chr8	134801001	134806000	2.85E-07
chr8	134802001	134807000	6.21E-06
chr8	134803001	134808000	2.76E-06
chr8	134804001	134809000	1.58E-05
chr8	134815001	134820000	1.46E-11
chr8	134857001	134862000	2.23E-12
chr8	134872001	134877000	5.20E-14
chr8	134882001	134887000	7.05E-13
chr8	134883001	134888000	1.41E-17
chr8	134884001	134889000	2.08E-17
chr8	134886001	134891000	8.38E-12
chr8	134891001	134896000	1.01E-08
chr8	134893001	134898000	3.03E-12
chr8	134894001	134899000	4.20E-08
chr8	134916001	134921000	3.73E-13
chr8	134917001	134922000	1.45E-15
chr8	134919001	134924000	2.56E-07
chr8	134920001	134925000	6.63E-09
chr8	134921001	134926000	3.12E-10
chr8	134922001	134927000	2.77E-08
chr8	134923001	134928000	2.66E-15
chr8	134924001	134929000	3.44E-17
chr8	134925001	134930000	4.94E-14
chr8	134926001	134931000	1.21E-11
chr8	134927001	134932000	2.86E-12
chr8	134932001	134937000	6.78E-08
chr8	134934001	134939000	6.36E-09
chr8	134935001	134940000	1.50E-10
chr8	134936001	134941000	3.58E-09
chr8	134945001	134950000	1.31E-10
chr8	134946001	134951000	2.37E-11
chr8	134947001	134952000	1.47E-11
chr8	134948001	134953000	1.53E-08
chr8	134966001	134971000	9.95E-06
chr8	134967001	134972000	4.27E-06
chr8	134980001	134985000	4.76E-08

chr8	134984001	134989000	1.59E-15
chr8	135022001	135027000	1.01E-11
chr8	135023001	135028000	4.49E-18
chr8	135024001	135029000	3.63E-16
chr8	135025001	135030000	1.98E-13
chr8	135026001	135031000	1.83E-14
chr8	135027001	135032000	1.80E-14
chr8	135028001	135033000	4.54E-12
chr8	135029001	135034000	1.16E-12
chr8	135030001	135035000	6.08E-14
chr8	135031001	135036000	6.78E-10
chr8	135032001	135037000	3.65E-11
chr8	135033001	135038000	1.26E-08
chr8	135034001	135039000	9.34E-08
chr8	135035001	135040000	1.21E-07
chr8	135041001	135046000	7.99E-14
chr8	135047001	135052000	2.91E-15
chr8	135048001	135053000	2.27E-13
chr8	135049001	135054000	1.27E-12
chr8	135050001	135055000	1.30E-11
chr8	135051001	135056000	4.63E-10
chr8	135053001	135058000	1.93E-07
chr8	135060001	135065000	2.00E-08
chr8	135061001	135066000	1.13E-09
chr8	135065001	135070000	7.46E-09
chr8	135067001	135072000	9.63E-10
chr8	135079001	135084000	1.81E-14
chr8	135090001	135095000	1.99E-09
chr8	135091001	135096000	1.74E-08
chr8	135092001	135097000	6.21E-08
chr8	135093001	135098000	2.18E-09
chr8	135094001	135099000	1.12E-07
chr8	135095001	135100000	1.40E-06
chr8	135096001	135101000	3.13E-08
chr8	135097001	135102000	1.31E-12
chr8	135098001	135103000	8.84E-11
chr8	135109001	135114000	2.94E-09
chr8	135110001	135115000	2.44E-11
chr8	135111001	135116000	4.82E-13
chr8	135112001	135117000	1.88E-14
chr8	135113001	135118000	2.18E-12
chr8	135114001	135119000	2.31E-17
chr8	135115001	135120000	1.53E-16
chr8	135116001	135121000	1.07E-08
chr8	135117001	135122000	5.52E-09
chr8	135118001	135123000	8.01E-10
chr8	135120001	135125000	1.29E-11
chr8	135121001	135126000	3.08E-13

chr8	135122001	135127000	2.20E-14
chr8	135123001	135128000	7.49E-11
chr8	135130001	135135000	1.88E-14
chr8	135134001	135139000	1.12E-17
chr8	135142001	135147000	7.40E-18
chr8	135143001	135148000	1.52E-15
chr8	135144001	135149000	9.10E-22
chr8	135145001	135150000	7.89E-17
chr8	135146001	135151000	9.26E-19
chr8	135155001	135160000	6.25E-12
chr8	135156001	135161000	9.48E-17
chr8	135157001	135162000	2.22E-17
chr8	135158001	135163000	5.41E-17
chr8	135159001	135164000	7.22E-12
chr8	135172001	135177000	8.25E-12
chr8	135175001	135180000	1.25E-14
chr8	135176001	135181000	3.92E-11
chr8	135179001	135184000	1.83E-09
chr8	135180001	135185000	7.32E-10
chr8	135181001	135186000	1.46E-18
chr8	135182001	135187000	3.34E-18
chr8	135183001	135188000	7.86E-16
chr8	135184001	135189000	4.64E-17
chr8	135185001	135190000	1.36E-15
chr8	135189001	135194000	6.42E-14
chr8	135198001	135203000	8.58E-13
chr8	135205001	135210000	2.29E-11
chr8	135215001	135220000	1.46E-15
chr8	135216001	135221000	6.41E-21
chr8	135217001	135222000	2.09E-14
chr8	135218001	135223000	1.27E-14
chr8	135219001	135224000	8.00E-08
chr8	135234001	135239000	3.42E-09
chr8	135235001	135240000	1.88E-11
chr8	135237001	135242000	1.24E-10
chr8	135238001	135243000	8.03E-09
chr8	135239001	135244000	2.17E-10
chr8	135240001	135245000	1.35E-07
chr8	135242001	135247000	1.37E-06
chr8	135243001	135248000	1.72E-08
chr8	135244001	135249000	2.94E-09
chr8	135245001	135250000	2.64E-08
chr8	135264001	135269000	1.05E-11
chr8	135265001	135270000	3.10E-11
chr8	135266001	135271000	3.87E-14
chr8	135267001	135272000	1.08E-14
chr8	135268001	135273000	2.20E-15
chr8	135269001	135274000	1.28E-12

chr8	135270001	135275000	2.77E-09
chr8	135271001	135276000	8.27E-11
chr8	135272001	135277000	3.20E-11
chr8	135273001	135278000	2.09E-13
chr8	135274001	135279000	9.60E-11
chr8	135277001	135282000	1.53E-14
chr8	135279001	135284000	1.09E-14
chr8	135280001	135285000	5.83E-15
chr8	135282001	135287000	4.50E-11
chr8	135283001	135288000	1.13E-13
chr8	135284001	135289000	2.29E-17
chr8	135285001	135290000	2.02E-16
chr8	135286001	135291000	1.46E-18
chr8	135287001	135292000	1.63E-15
chr8	135302001	135307000	9.62E-13
chr8	135303001	135308000	4.06E-09
chr8	135310001	135315000	6.91E-08
chr8	135312001	135317000	1.08E-08
chr8	135313001	135318000	7.46E-09
chr8	135314001	135319000	6.63E-08
chr8	135316001	135321000	7.41E-09
chr8	135320001	135325000	2.37E-09
chr8	135322001	135327000	1.40E-09
chr8	135326001	135331000	1.80E-13
chr8	135327001	135332000	3.25E-11
chr8	135328001	135333000	4.20E-13
chr8	135329001	135334000	8.04E-14
chr8	135330001	135335000	1.05E-09
chr8	135331001	135336000	6.69E-13
chr8	135332001	135337000	2.58E-15
chr8	135333001	135338000	6.94E-17
chr8	135334001	135339000	6.77E-19
chr8	135335001	135340000	9.39E-24
chr8	135336001	135341000	1.79E-16
chr8	135337001	135342000	6.91E-21
chr8	135338001	135343000	2.28E-17
chr8	135339001	135344000	5.48E-15
chr8	135340001	135345000	1.45E-08
chr8	135341001	135346000	3.00E-12
chr8	135346001	135351000	2.04E-10
chr8	135347001	135352000	1.32E-12
chr8	135348001	135353000	6.32E-13
chr8	135349001	135354000	4.79E-19
chr8	135350001	135355000	8.56E-22
chr8	135351001	135356000	5.80E-15
chr8	135352001	135357000	3.03E-14
chr8	135353001	135358000	1.86E-11
chr8	135376001	135381000	1.80E-12

chr8	135377001	135382000	4.47E-12
chr8	135385001	135390000	6.23E-11
chr8	135387001	135392000	5.91E-12
chr8	135407001	135412000	9.16E-10
chr8	135408001	135413000	1.75E-09
chr8	135409001	135414000	2.13E-08
chr8	135410001	135415000	1.68E-09
chr8	135418001	135423000	5.49E-12
chr8	135419001	135424000	3.23E-10
chr8	135425001	135430000	1.84E-07
chr8	135426001	135431000	1.23E-05
chr8	135430001	135435000	7.93E-09
chr8	135431001	135436000	2.78E-09
chr8	135432001	135437000	8.37E-11
chr8	135433001	135438000	5.16E-14
chr8	135434001	135439000	1.01E-15
chr8	135435001	135440000	7.52E-16
chr8	135436001	135441000	1.14E-17
chr8	135437001	135442000	3.08E-17
chr8	135438001	135443000	5.75E-12
chr8	135444001	135449000	1.27E-08
chr8	135447001	135452000	3.08E-07
chr8	135458001	135463000	5.14E-11
chr8	135459001	135464000	1.06E-09
chr8	135460001	135465000	3.56E-10
chr8	135461001	135466000	5.54E-11
chr8	135475001	135480000	2.54E-13
chr8	135780001	135785000	5.14E-18
chr8	135781001	135786000	1.81E-12
chr8	135862001	135867000	5.99E-05
chr8	135869001	135874000	2.50E-14
chr8	135870001	135875000	5.96E-13
chr8	135920001	135925000	9.13E-08
chr8	135932001	135937000	4.99E-07
chr8	135936001	135941000	4.16E-14
chr8	135938001	135943000	8.29E-13
chr8	135974001	135979000	1.39E-08
chr8	135975001	135980000	1.89E-10
chr8	135976001	135981000	1.48E-09
chr8	135977001	135982000	4.54E-09
chr8	135978001	135983000	1.48E-10
chr8	135979001	135984000	2.99E-11
chr8	136002001	136007000	1.62E-15
chr8	136003001	136008000	8.36E-19
chr8	136004001	136009000	2.08E-21
chr8	136006001	136011000	1.58E-17
chr8	136007001	136012000	6.01E-21
chr8	136009001	136014000	7.64E-17

chr8	136017001	136022000	7.76E-12
chr8	136018001	136023000	4.00E-16
chr8	136019001	136024000	1.55E-14
chr8	136020001	136025000	1.74E-06
chr8	136021001	136026000	2.79E-07
chr8	136023001	136028000	4.99E-09
chr8	136024001	136029000	2.66E-12
chr8	136025001	136030000	6.78E-13
chr8	136026001	136031000	1.56E-11
chr8	136027001	136032000	3.69E-15
chr8	136033001	136038000	9.34E-09
chr8	136034001	136039000	8.38E-11
chr8	136038001	136043000	5.90E-19
chr8	136039001	136044000	1.27E-18
chr8	136040001	136045000	4.60E-25
chr8	136041001	136046000	6.22E-18
chr8	136042001	136047000	3.16E-17
chr8	136047001	136052000	3.46E-13
chr8	136049001	136054000	1.68E-11
chr8	136051001	136056000	2.96E-12
chr8	136053001	136058000	5.27E-11
chr8	136059001	136064000	2.09E-09
chr8	136065001	136070000	1.75E-11
chr8	136066001	136071000	5.13E-22
chr8	136067001	136072000	1.12E-19
chr8	136068001	136073000	9.16E-17
chr8	136069001	136074000	9.99E-19
chr8	136070001	136075000	2.92E-15
chr8	136082001	136087000	1.76E-09
chr8	136083001	136088000	5.61E-10
chr8	136084001	136089000	5.01E-10
chr8	136090001	136095000	1.44E-12
chr8	136091001	136096000	1.36E-12
chr8	136099001	136104000	4.92E-17
chr8	136100001	136105000	1.18E-13
chr8	136107001	136112000	2.54E-10
chr8	136121001	136126000	1.14E-09
chr8	136122001	136127000	5.65E-10
chr8	136124001	136129000	2.40E-05
chr8	136125001	136130000	5.28E-07
chr8	136126001	136131000	1.01E-07
chr8	136127001	136132000	3.06E-10
chr8	136128001	136133000	4.78E-09
chr8	136135001	136140000	1.86E-10
chr8	136145001	136150000	3.63E-08
chr8	136146001	136151000	2.33E-09
chr8	136147001	136152000	3.58E-08
chr8	136148001	136153000	3.88E-09

chr8	136154001	136159000	2.05E-11
chr8	136155001	136160000	1.49E-12
chr8	136156001	136161000	3.47E-11
chr8	136165001	136170000	7.34E-11
chr8	136166001	136171000	1.26E-09
chr8	136167001	136172000	1.20E-12
chr8	136168001	136173000	4.76E-11
chr8	136169001	136174000	1.47E-11
chr8	136175001	136180000	1.21E-06
chr8	136198001	136203000	1.07E-15
chr8	136199001	136204000	1.01E-17
chr8	136200001	136205000	1.38E-15
chr8	136202001	136207000	1.22E-17
chr8	136204001	136209000	3.78E-12
chr8	136205001	136210000	2.66E-11
chr8	136206001	136211000	2.80E-14
chr8	136207001	136212000	6.61E-12
chr8	136208001	136213000	1.17E-11
chr8	136211001	136216000	1.31E-11
chr8	136213001	136218000	1.86E-11
chr8	136214001	136219000	4.39E-11
chr8	136215001	136220000	1.61E-07
chr8	136238001	136243000	2.84E-10
chr8	136242001	136247000	2.46E-11
chr8	136306001	136311000	2.26E-19
chr8	136307001	136312000	3.84E-15
chr8	136365001	136370000	3.21E-13
chr8	136366001	136371000	1.31E-12
chr8	136465001	136470000	5.11E-09
chr8	136469001	136474000	4.02E-22
chr8	136491001	136496000	3.03E-12
chr8	136680001	136685000	5.28E-08
chr8	136688001	136693000	2.02E-12
chr8	136689001	136694000	1.57E-11
chr8	136690001	136695000	4.17E-09
chr8	136760001	136765000	8.20E-10
chr8	136761001	136766000	1.04E-08
chr8	136796001	136801000	3.26E-10
chr8	136798001	136803000	3.21E-07
chr8	136799001	136804000	5.31E-09
chr8	136800001	136805000	9.43E-10
chr8	136814001	136819000	5.63E-11
chr8	136815001	136820000	6.94E-09
chr8	136817001	136822000	8.26E-09
chr8	136818001	136823000	1.53E-07
chr8	136821001	136826000	1.74E-13
chr8	136827001	136832000	5.77E-10
chr8	136828001	136833000	8.51E-12

chr8	136838001	136843000	1.43E-09
chr8	136846001	136851000	7.84E-17
chr8	136847001	136852000	5.85E-16
chr8	136872001	136877000	1.14E-17
chr8	136873001	136878000	1.75E-16
chr8	136874001	136879000	1.56E-12
chr8	136875001	136880000	5.84E-12
chr8	136903001	136908000	1.88E-10
chr8	136912001	136917000	2.83E-17
chr8	136913001	136918000	3.56E-14
chr8	136924001	136929000	1.91E-12
chr8	136925001	136930000	6.74E-10
chr8	136926001	136931000	1.83E-11
chr8	136951001	136956000	5.39E-07
chr8	136952001	136957000	6.05E-22
chr8	136953001	136958000	1.08E-21
chr8	136954001	136959000	3.13E-24
chr8	136955001	136960000	3.59E-19
chr8	136956001	136961000	1.14E-16
chr8	136959001	136964000	9.67E-10
chr8	136965001	136970000	7.33E-08
chr8	136966001	136971000	1.06E-09
chr8	136967001	136972000	5.76E-13
chr8	136968001	136973000	2.26E-14
chr8	136969001	136974000	2.39E-13
chr8	137001001	137006000	1.10E-12
chr8	137002001	137007000	9.12E-08
chr8	137071001	137076000	1.38E-07
chr8	137072001	137077000	3.48E-12
chr8	137073001	137078000	1.10E-13
chr8	137083001	137088000	2.57E-07
chr8	137084001	137089000	6.07E-08
chr8	137085001	137090000	2.87E-09
chr8	137096001	137101000	1.34E-09
chr8	137173001	137178000	2.18E-12
chr8	137220001	137225000	5.36E-13
chr8	137228001	137233000	6.42E-14
chr8	137245001	137250000	1.13E-11
chr8	137261001	137266000	1.01E-13
chr8	137262001	137267000	1.26E-13
chr8	137281001	137286000	2.42E-17
chr8	137282001	137287000	7.15E-17
chr8	137290001	137295000	1.55E-17
chr8	137292001	137297000	5.56E-17
chr8	137293001	137298000	9.37E-16
chr8	137294001	137299000	5.04E-20
chr8	137296001	137301000	3.22E-12
chr8	137297001	137302000	9.86E-12

chr8	137314001	137319000	5.41E-19
chr8	137321001	137326000	2.34E-17
chr8	137322001	137327000	9.73E-19
chr8	137323001	137328000	2.51E-16
chr8	137330001	137335000	5.22E-13
chr8	137331001	137336000	7.91E-16
chr8	137332001	137337000	2.42E-16
chr8	137371001	137376000	1.37E-18
chr8	137411001	137416000	1.30E-09
chr8	137412001	137417000	1.70E-10
chr8	137418001	137423000	7.50E-15
chr8	137419001	137424000	2.42E-15
chr8	137420001	137425000	1.25E-16
chr8	137421001	137426000	1.49E-18
chr8	137422001	137427000	6.18E-14
chr8	137428001	137433000	4.22E-11
chr8	137429001	137434000	3.39E-14
chr8	137437001	137442000	1.12E-06
chr8	137440001	137445000	1.77E-08
chr8	137458001	137463000	5.52E-17
chr8	137459001	137464000	4.63E-15
chr8	137460001	137465000	5.32E-08
chr8	137461001	137466000	1.47E-12
chr8	137493001	137498000	7.06E-13
chr8	137518001	137523000	9.33E-20
chr8	137519001	137524000	3.50E-16
chr8	137539001	137544000	8.05E-10
chr8	137542001	137547000	8.52E-13
chr8	137543001	137548000	2.30E-13
chr8	137549001	137554000	3.57E-16
chr8	137550001	137555000	2.76E-12
chr8	137551001	137556000	1.16E-12
chr8	137557001	137562000	2.57E-14
chr8	137558001	137563000	1.86E-16
chr8	137559001	137564000	2.16E-19
chr8	137560001	137565000	5.37E-23
chr8	137561001	137566000	6.01E-26
chr8	137562001	137567000	3.39E-23
chr8	137568001	137573000	8.52E-14
chr8	137569001	137574000	1.14E-09
chr8	137570001	137575000	4.71E-14
chr8	137571001	137576000	1.58E-12
chr8	137572001	137577000	1.18E-17
chr8	137582001	137587000	7.29E-08
chr8	137583001	137588000	3.88E-08
chr8	137607001	137612000	1.86E-11
chr8	137608001	137613000	1.81E-10
chr8	137614001	137619000	5.95E-08

chr8	137633001	137638000	6.01E-13
chr8	137635001	137640000	4.46E-15
chr8	137648001	137653000	1.99E-16
chr8	137649001	137654000	2.77E-18
chr8	137650001	137655000	3.48E-16
chr8	137651001	137656000	1.42E-18
chr8	137652001	137657000	3.51E-11
chr8	137653001	137658000	1.97E-15
chr8	137654001	137659000	1.87E-13
chr8	137655001	137660000	2.55E-11
chr8	137657001	137662000	6.01E-14
chr8	137667001	137672000	4.68E-09
chr8	137706001	137711000	3.43E-10
chr8	137720001	137725000	2.04E-11
chr8	137721001	137726000	1.85E-14
chr8	137722001	137727000	5.03E-12
chr8	137723001	137728000	6.17E-10
chr8	137740001	137745000	2.52E-16
chr8	137760001	137765000	6.30E-09
chr8	137761001	137766000	1.16E-07
chr8	137762001	137767000	2.21E-10
chr8	137770001	137775000	1.60E-17
chr8	137773001	137778000	3.00E-13
chr8	137822001	137827000	1.05E-14
chr8	137823001	137828000	6.39E-15
chr8	137824001	137829000	9.49E-21
chr8	137825001	137830000	1.34E-26
chr8	137826001	137831000	5.84E-27
chr8	137868001	137873000	1.30E-12
chr8	137869001	137874000	5.79E-21
chr8	137870001	137875000	3.55E-25
chr8	137871001	137876000	7.42E-22
chr8	137872001	137877000	8.55E-20
chr8	137885001	137890000	1.61E-13
chr8	137915001	137920000	7.35E-13
chr8	137916001	137921000	4.78E-11
chr8	137929001	137934000	1.45E-13
chr8	137930001	137935000	5.47E-16
chr8	137932001	137937000	2.58E-14
chr8	137948001	137953000	2.46E-16
chr8	137971001	137976000	4.68E-18
chr8	137972001	137977000	9.10E-20
chr8	137973001	137978000	6.48E-18
chr8	137974001	137979000	1.74E-20
chr8	137975001	137980000	1.43E-18
chr8	137976001	137981000	1.03E-13
chr8	137977001	137982000	2.58E-15
chr8	137984001	137989000	5.13E-09

chr8	137992001	137997000	9.85E-17
chr8	138001001	138006000	2.28E-21
chr8	138002001	138007000	6.21E-18
chr8	138003001	138008000	7.57E-11
chr8	138004001	138009000	6.80E-12
chr8	138009001	138014000	3.84E-15
chr8	138010001	138015000	2.44E-16
chr8	138067001	138072000	1.08E-25
chr8	138068001	138073000	1.35E-24
chr8	138069001	138074000	9.53E-23
chr8	138084001	138089000	1.68E-14
chr8	138085001	138090000	3.13E-13
chr8	138086001	138091000	5.18E-12
chr8	138104001	138109000	2.13E-14
chr8	138105001	138110000	5.87E-14
chr8	138111001	138116000	5.05E-20
chr8	138112001	138117000	2.33E-23
chr8	138113001	138118000	1.71E-24
chr8	138114001	138119000	7.04E-27
chr8	138115001	138120000	7.69E-25
chr8	138116001	138121000	2.68E-21
chr8	138117001	138122000	1.88E-21
chr8	138120001	138125000	1.69E-26
chr8	138130001	138135000	9.56E-10
chr8	138131001	138136000	2.45E-13
chr8	138133001	138138000	3.28E-14
chr8	138137001	138142000	1.23E-10
chr8	138138001	138143000	1.19E-11
chr8	138143001	138148000	6.63E-20
chr8	138146001	138151000	1.12E-14
chr8	138147001	138152000	5.34E-12
chr8	138168001	138173000	5.32E-15
chr8	138169001	138174000	2.13E-15
chr8	138175001	138180000	2.00E-11
chr8	138176001	138181000	1.87E-12
chr8	138184001	138189000	9.93E-15
chr8	138185001	138190000	3.59E-13
chr8	138192001	138197000	4.38E-16
chr8	138205001	138210000	1.25E-13
chr8	138212001	138217000	2.94E-13
chr8	138235001	138240000	1.76E-12
chr8	138236001	138241000	9.04E-10
chr8	138237001	138242000	1.10E-07
chr8	138247001	138252000	1.01E-07
chr8	138257001	138262000	1.70E-10
chr8	138258001	138263000	7.25E-11
chr8	138259001	138264000	4.07E-13
chr8	138260001	138265000	5.52E-15

chr8	138261001	138266000	3.12E-16
chr8	138272001	138277000	3.81E-09
chr8	138281001	138286000	1.51E-11
chr8	138282001	138287000	2.35E-15
chr8	138306001	138311000	1.99E-09
chr8	138315001	138320000	3.82E-13
chr8	138316001	138321000	1.50E-11
chr8	138317001	138322000	6.94E-17
chr8	138318001	138323000	5.85E-18
chr8	138319001	138324000	5.88E-15
chr8	138371001	138376000	3.91E-15
chr8	138407001	138412000	8.07E-12
chr8	138408001	138413000	3.20E-11
chr8	138409001	138414000	1.46E-13
chr8	138410001	138415000	1.98E-08
chr8	138411001	138416000	1.91E-07
chr8	138412001	138417000	1.29E-05
chr8	138413001	138418000	1.29E-08
chr8	138414001	138419000	1.26E-06
chr8	138415001	138420000	6.28E-07
chr8	138416001	138421000	5.97E-07
chr8	138417001	138422000	3.50E-07
chr8	138429001	138434000	6.44E-08
chr8	138439001	138444000	1.03E-11
chr8	138440001	138445000	9.94E-12
chr8	138441001	138446000	8.41E-14
chr8	138444001	138449000	5.57E-14
chr8	138481001	138486000	3.93E-13
chr8	138482001	138487000	1.10E-12
chr8	138550001	138555000	6.70E-16
chr8	138610001	138615000	3.76E-20
chr8	138611001	138616000	1.50E-31
chr8	138612001	138617000	6.91E-32
chr8	138613001	138618000	8.14E-36
chr8	138614001	138619000	3.93E-38
chr8	138615001	138620000	5.20E-30
chr8	138616001	138621000	1.10E-19
chr8	138617001	138622000	1.11E-20
chr8	138628001	138633000	1.31E-16
chr8	138629001	138634000	7.97E-16
chr8	138642001	138647000	6.70E-12
chr8	138643001	138648000	1.07E-11
chr8	138671001	138676000	1.51E-15
chr8	138672001	138677000	5.76E-15
chr8	138693001	138698000	9.21E-13
chr8	138694001	138699000	8.82E-16
chr8	138695001	138700000	2.97E-15
chr8	138696001	138701000	3.85E-14

chr8	138697001	138702000	1.88E-16
chr8	138698001	138703000	4.15E-15
chr8	138699001	138704000	2.09E-13
chr8	138700001	138705000	3.87E-07
chr8	138705001	138710000	3.43E-16
chr8	138706001	138711000	4.21E-14
chr8	138707001	138712000	4.17E-18
chr8	138730001	138735000	3.82E-17
chr8	138731001	138736000	3.26E-16
chr8	138732001	138737000	1.83E-17
chr8	138739001	138744000	6.03E-07
chr8	138748001	138753000	4.90E-11
chr8	138766001	138771000	2.62E-09
chr8	138783001	138788000	4.12E-10
chr8	138818001	138823000	2.32E-09
chr8	138820001	138825000	1.36E-09
chr8	138824001	138829000	2.83E-19
chr8	138825001	138830000	8.24E-17
chr8	138826001	138831000	5.10E-20
chr8	138827001	138832000	2.02E-19
chr8	138828001	138833000	6.86E-25
chr8	138829001	138834000	1.42E-11
chr8	138830001	138835000	4.26E-17
chr8	138840001	138845000	4.62E-14
chr8	138841001	138846000	3.82E-15
chr8	138842001	138847000	1.39E-14
chr8	138867001	138872000	1.12E-08
chr8	138869001	138874000	1.37E-09
chr8	138870001	138875000	1.44E-09
chr8	138904001	138909000	2.41E-11
chr8	138905001	138910000	4.19E-11
chr8	138920001	138925000	7.71E-17
chr8	138923001	138928000	7.68E-12
chr8	138937001	138942000	4.95E-21
chr8	138938001	138943000	7.69E-26
chr8	138939001	138944000	6.08E-29
chr8	138940001	138945000	1.93E-30
chr8	138941001	138946000	2.34E-31
chr8	138942001	138947000	2.09E-30
chr8	138943001	138948000	4.00E-25
chr8	138944001	138949000	2.35E-25
chr8	138945001	138950000	6.92E-18
chr8	138952001	138957000	2.00E-17
chr8	138960001	138965000	2.34E-17
chr8	138961001	138966000	6.44E-18
chr8	138964001	138969000	2.82E-13
chr8	138978001	138983000	5.91E-16
chr8	138979001	138984000	1.80E-14

chr8	138980001	138985000	3.84E-13
chr8	139001001	139006000	3.51E-21
chr8	139002001	139007000	1.48E-24
chr8	139003001	139008000	1.81E-20
chr8	139004001	139009000	5.57E-18
chr8	139005001	139010000	4.25E-11
chr8	139017001	139022000	5.48E-07
chr8	139018001	139023000	2.00E-07
chr8	139019001	139024000	5.19E-05
chr8	139020001	139025000	3.89E-13
chr8	139021001	139026000	2.32E-12
chr8	139039001	139044000	1.17E-15
chr8	139040001	139045000	1.72E-14
chr8	139069001	139074000	2.78E-09
chr8	139070001	139075000	2.56E-10
chr8	139071001	139076000	6.57E-15
chr8	139072001	139077000	6.41E-14
chr8	139073001	139078000	1.74E-15
chr8	139075001	139080000	8.21E-17
chr8	139076001	139081000	1.13E-13
chr8	139077001	139082000	1.74E-14
chr8	139117001	139122000	4.61E-13
chr8	139118001	139123000	1.87E-12
chr8	139125001	139130000	2.50E-12
chr8	139187001	139192000	1.99E-20
chr8	139188001	139193000	3.18E-17
chr8	139189001	139194000	2.32E-15
chr8	139198001	139203000	8.18E-12
chr8	139199001	139204000	8.16E-10
chr8	139200001	139205000	5.41E-11
chr8	139206001	139211000	1.32E-11
chr8	139207001	139212000	1.30E-14
chr8	139208001	139213000	1.29E-16
chr8	139220001	139225000	4.37E-14
chr8	139230001	139235000	2.65E-15
chr8	139231001	139236000	9.73E-16
chr8	139232001	139237000	2.22E-17
chr8	139233001	139238000	4.05E-16
chr8	139234001	139239000	6.52E-17
chr8	139235001	139240000	9.51E-14
chr8	139247001	139252000	7.03E-11
chr8	139248001	139253000	2.03E-13
chr8	139249001	139254000	3.37E-10
chr8	139259001	139264000	6.07E-14
chr8	139260001	139265000	2.83E-12
chr8	139261001	139266000	7.92E-14
chr8	139262001	139267000	3.76E-14
chr8	139263001	139268000	8.26E-12

chr8	139283001	139288000	1.26E-16
chr8	139285001	139290000	1.63E-20
chr8	139286001	139291000	1.21E-20
chr8	139287001	139292000	2.15E-17
chr8	139288001	139293000	3.93E-14
chr8	139289001	139294000	6.00E-15
chr8	139291001	139296000	5.23E-18
chr8	139293001	139298000	3.53E-17
chr8	139294001	139299000	4.26E-16
chr8	139295001	139300000	2.29E-12
chr8	139303001	139308000	9.45E-10
chr8	139318001	139323000	1.98E-06
chr8	139331001	139336000	1.52E-12
chr8	139332001	139337000	2.54E-15
chr8	139333001	139338000	1.23E-14
chr8	139334001	139339000	6.49E-16
chr8	139335001	139340000	3.52E-15
chr8	139338001	139343000	3.29E-08
chr8	139339001	139344000	1.76E-10
chr8	139340001	139345000	1.03E-09
chr8	139341001	139346000	3.25E-11
chr8	139342001	139347000	4.62E-13
chr8	139356001	139361000	1.24E-12
chr8	139357001	139362000	2.89E-12
chr8	139373001	139378000	1.22E-13
chr8	139374001	139379000	8.49E-14
chr8	139375001	139380000	2.06E-13
chr8	139387001	139392000	1.27E-09
chr8	139388001	139393000	1.44E-11
chr8	139395001	139400000	6.62E-11
chr8	139396001	139401000	7.01E-12
chr8	139397001	139402000	4.89E-14
chr8	139398001	139403000	7.76E-20
chr8	139399001	139404000	2.10E-13
chr8	139401001	139406000	5.62E-11
chr8	139414001	139419000	6.52E-09
chr8	139416001	139421000	1.33E-10
chr8	139420001	139425000	1.56E-14
chr8	139426001	139431000	4.39E-07
chr8	139427001	139432000	2.51E-09
chr8	139428001	139433000	1.05E-08
chr8	139429001	139434000	7.54E-09
chr8	139467001	139472000	1.46E-10
chr8	139468001	139473000	3.66E-08
chr8	139469001	139474000	3.88E-12
chr8	139498001	139503000	3.44E-12
chr8	139499001	139504000	3.62E-12
chr8	139500001	139505000	2.26E-12

chr8	139516001	139521000	1.08E-15
chr8	139518001	139523000	8.62E-16
chr8	139519001	139524000	9.41E-13
chr8	139520001	139525000	5.58E-21
chr8	139521001	139526000	2.64E-13
chr8	139522001	139527000	3.29E-17
chr8	139523001	139528000	2.41E-12
chr8	139524001	139529000	3.76E-14
chr8	139525001	139530000	4.62E-07
chr8	139526001	139531000	1.37E-08
chr8	139556001	139561000	9.07E-12
chr8	139567001	139572000	8.13E-08
chr8	139584001	139589000	1.09E-09
chr8	139585001	139590000	1.42E-12
chr8	139586001	139591000	1.23E-11
chr8	139587001	139592000	1.37E-10
chr8	139588001	139593000	8.11E-13
chr8	139615001	139620000	1.95E-21
chr8	139616001	139621000	1.82E-14
chr8	139621001	139626000	2.57E-10
chr8	139622001	139627000	1.78E-09
chr8	139623001	139628000	1.45E-11
chr8	139624001	139629000	2.03E-12
chr8	139625001	139630000	8.37E-17
chr8	139626001	139631000	2.92E-18
chr8	139627001	139632000	1.60E-18
chr8	139628001	139633000	7.63E-16
chr8	139629001	139634000	1.62E-13
chr8	139630001	139635000	4.18E-07
chr8	139642001	139647000	2.20E-08
chr8	139663001	139668000	4.01E-15
chr8	139674001	139679000	1.85E-10
chr8	139678001	139683000	4.27E-07
chr8	139679001	139684000	1.69E-10
chr8	139680001	139685000	1.31E-11
chr8	139686001	139691000	1.61E-12
chr8	139693001	139698000	1.32E-13
chr8	139694001	139699000	2.20E-19
chr8	139695001	139700000	2.14E-16
chr8	139745001	139750000	5.38E-13
chr8	139759001	139764000	1.08E-11
chr8	139760001	139765000	3.13E-20
chr8	139761001	139766000	1.08E-21
chr8	139762001	139767000	8.59E-19
chr8	139778001	139783000	2.37E-10
chr8	139795001	139800000	7.42E-10
chr8	139796001	139801000	2.44E-10
chr8	139809001	139814000	1.75E-14

chr8	139863001	139868000	6.62E-11
chr8	139864001	139869000	3.81E-09
chr8	139865001	139870000	9.24E-12
chr8	139895001	139900000	1.67E-10
chr8	139914001	139919000	2.12E-11
chr8	139915001	139920000	1.06E-10
chr8	139916001	139921000	2.20E-11
chr8	139917001	139922000	4.06E-11
chr8	139969001	139974000	3.13E-08
chr8	140000001	140005000	2.75E-06
chr8	140016001	140021000	3.33E-08
chr8	140085001	140090000	3.11E-11
chr8	140086001	140091000	5.41E-21
chr8	140087001	140092000	1.34E-19
chr8	140088001	140093000	3.69E-23
chr8	140089001	140094000	1.18E-19
chr8	140115001	140120000	5.34E-11
chr8	140116001	140121000	1.99E-14
chr8	140117001	140122000	1.32E-13
chr8	140157001	140162000	1.48E-08
chr8	140158001	140163000	5.28E-11
chr8	140159001	140164000	8.49E-14
chr8	140160001	140165000	6.50E-09
chr8	140165001	140170000	4.40E-09
chr8	140166001	140171000	1.34E-09
chr8	140167001	140172000	1.52E-08
chr8	140191001	140196000	5.09E-09
chr8	140195001	140200000	2.08E-09
chr8	140196001	140201000	1.66E-14
chr8	140197001	140202000	2.12E-15
chr8	140198001	140203000	9.36E-14
chr8	140199001	140204000	5.72E-14
chr8	140206001	140211000	4.26E-12
chr8	140264001	140269000	7.06E-07
chr8	140267001	140272000	1.92E-13
chr8	140271001	140276000	6.85E-17
chr8	140272001	140277000	7.96E-11
chr8	140400001	140405000	1.37E-10
chr8	140481001	140486000	2.08E-10
chr8	140482001	140487000	4.13E-11
chr8	140483001	140488000	1.27E-13
chr8	140492001	140497000	8.77E-09
chr8	140493001	140498000	1.87E-11
chr8	140534001	140539000	6.68E-09
chr8	140535001	140540000	2.40E-09
chr8	140537001	140542000	1.69E-06
chr8	140542001	140547000	5.54E-13
chr8	140555001	140560000	3.00E-15

chr8	140556001	140561000	5.97E-19
chr8	140557001	140562000	8.52E-19
chr8	140558001	140563000	2.20E-17
chr8	140559001	140564000	4.23E-16
chr8	140560001	140565000	7.46E-09
chr8	140576001	140581000	4.14E-07
chr8	140598001	140603000	1.00E-14
chr8	140599001	140604000	5.53E-13
chr8	140606001	140611000	4.73E-05
chr8	140607001	140612000	1.98E-07
chr8	140620001	140625000	1.38E-13
chr8	141464001	141469000	7.95E-13
chr8	141472001	141477000	3.30E-14
chr8	141518001	141523000	4.73E-09
chr8	142117001	142122000	2.78E-08
chr8	142189001	142194000	1.51E-17
chr8	142427001	142432000	7.62E-08
chr8	142473001	142478000	4.88E-12
chr8	142485001	142490000	6.37E-08
chr8	142498001	142503000	1.57E-07
chr8	142514001	142519000	7.45E-09
chr8	142546001	142551000	4.80E-06
chr8	142547001	142552000	7.26E-09
chr8	142555001	142560000	3.48E-08
chr8	142556001	142561000	3.00E-06
chr8	142559001	142564000	1.21E-08
chr8	142560001	142565000	3.80E-08
chr8	142561001	142566000	4.26E-12
chr8	142562001	142567000	4.44E-11
chr8	142563001	142568000	4.47E-11
chr8	142569001	142574000	3.50E-06
chr8	142585001	142590000	1.53E-08
chr8	142604001	142609000	1.89E-08
chr8	142605001	142610000	4.56E-10
chr8	142606001	142611000	2.83E-15
chr8	142607001	142612000	5.28E-19
chr8	142608001	142613000	5.97E-19
chr8	142626001	142631000	3.29E-15
chr8	142627001	142632000	2.61E-26
chr8	142628001	142633000	3.98E-15
chr8	142629001	142634000	2.19E-09
chr8	142630001	142635000	4.45E-08
chr8	142668001	142673000	1.68E-10
chr8	142669001	142674000	1.50E-12
chr8	142670001	142675000	8.85E-11
chr8	142671001	142676000	9.64E-13
chr8	142672001	142677000	4.05E-12
chr8	142673001	142678000	9.28E-09

chr8	142675001	142680000	7.55E-05
chr8	142677001	142682000	1.81E-06
chr8	142678001	142683000	8.58E-07
chr8	142701001	142706000	9.31E-12
chr8	142702001	142707000	9.71E-17
chr8	142703001	142708000	1.22E-19
chr8	142704001	142709000	9.18E-23
chr8	142705001	142710000	1.53E-25
chr8	142706001	142711000	3.67E-19
chr8	142707001	142712000	1.21E-13
chr8	142708001	142713000	1.38E-14
chr8	142709001	142714000	1.28E-10
chr8	142714001	142719000	1.45E-08
chr8	142715001	142720000	1.28E-15
chr8	142716001	142721000	1.87E-15
chr8	142717001	142722000	1.84E-12
chr8	142718001	142723000	5.99E-18
chr8	142719001	142724000	5.37E-18
chr8	142720001	142725000	1.03E-13
chr8	142721001	142726000	3.13E-13
chr8	142722001	142727000	5.75E-15
chr8	142723001	142728000	3.12E-12
chr8	142724001	142729000	0.000193
chr8	142725001	142730000	0.00014
chr8	142726001	142731000	4.54E-06
chr8	142727001	142732000	9.15E-07
chr8	142851001	142856000	1.01E-09
chr8	142864001	142869000	1.49E-09
chr8	142870001	142875000	2.44E-10
chr8	142871001	142876000	1.81E-08
chr8	142887001	142892000	2.97E-14
chr8	142888001	142893000	5.30E-10
chr8	142890001	142895000	7.76E-10
chr8	142894001	142899000	1.55E-17
chr8	142895001	142900000	3.63E-18
chr8	142896001	142901000	4.49E-17
chr8	142897001	142902000	3.36E-20
chr8	142898001	142903000	8.36E-19
chr8	142915001	142920000	3.20E-21
chr8	142935001	142940000	7.29E-15
chr8	142936001	142941000	1.94E-15
chr8	142937001	142942000	4.35E-19
chr8	142939001	142944000	2.27E-16
chr8	142943001	142948000	5.50E-11
chr8	142946001	142951000	1.11E-18
chr8	142947001	142952000	1.44E-17
chr8	142948001	142953000	4.56E-17
chr8	142949001	142954000	1.12E-16

chr8	142950001	142955000	1.16E-18
chr8	142992001	142997000	1.83E-12
chr8	143014001	143019000	6.95E-12
chr8	143055001	143060000	1.95E-13
chr8	143056001	143061000	1.88E-15
chr8	143058001	143063000	1.62E-13
chr8	143059001	143064000	2.07E-12
chr8	143066001	143071000	5.21E-12
chr8	143067001	143072000	3.10E-14
chr8	143068001	143073000	1.26E-08
chr8	143069001	143074000	2.46E-12
chr8	143070001	143075000	3.36E-12
chr8	143087001	143092000	1.53E-09
chr8	143088001	143093000	4.80E-12
chr8	143089001	143094000	2.38E-08
chr8	143094001	143099000	8.19E-11
chr8	143095001	143100000	2.88E-11
chr8	143103001	143108000	1.27E-07
chr8	143104001	143109000	6.99E-10
chr8	143127001	143132000	3.83E-13
chr8	143128001	143133000	3.52E-17
chr8	143129001	143134000	3.13E-12
chr8	143159001	143164000	5.83E-11
chr8	143160001	143165000	1.34E-09
chr8	143170001	143175000	4.99E-11
chr8	143171001	143176000	1.92E-08
chr8	143172001	143177000	1.96E-06
chr8	143181001	143186000	3.63E-09
chr8	143182001	143187000	2.99E-09
chr8	143184001	143189000	5.73E-09
chr8	143231001	143236000	4.09E-07
chr8	143232001	143237000	0.000245
chr8	143277001	143282000	3.50E-09
chr8	143278001	143283000	4.96E-10
chr8	143280001	143285000	2.01E-08
chr8	143281001	143286000	3.46E-08
chr8	143288001	143293000	1.50E-05
chr8	143291001	143296000	3.01E-05
chr8	143295001	143300000	3.36E-06
chr8	143303001	143308000	4.91E-07
chr8	143304001	143309000	2.99E-06
chr8	143305001	143310000	1.02E-07
chr8	143306001	143311000	3.17E-09
chr8	143307001	143312000	1.08E-09
chr8	143308001	143313000	5.80E-06
chr8	143309001	143314000	1.96E-07
chr8	143310001	143315000	1.17E-06
chr8	143329001	143334000	5.73E-10

chr8	143330001	143335000	2.66E-07
chr8	143331001	143336000	8.67E-07
chr8	143332001	143337000	2.35E-05
chr8	143485001	143490000	1.12E-06
chr8	143498001	143503000	0.010447
chr8	143509001	143514000	3.01E-10
chr8	143517001	143522000	4.04E-11
chr8	143518001	143523000	3.50E-11
chr8	143519001	143524000	3.95E-09
chr8	143520001	143525000	1.92E-09
chr8	143521001	143526000	4.30E-06
chr8	143522001	143527000	6.76E-06
chr8	143531001	143536000	5.15E-05
chr8	143563001	143568000	4.15E-14
chr8	143573001	143578000	5.58E-07
chr8	143574001	143579000	5.02E-07
chr8	143575001	143580000	3.78E-08
chr8	143576001	143581000	1.54E-06
chr8	143577001	143582000	1.59E-05
chr8	143583001	143588000	6.57E-06
chr8	143603001	143608000	4.83E-14
chr8	143604001	143609000	6.83E-07
chr8	143616001	143621000	4.47E-07
chr8	143617001	143622000	0.000117
chr8	143618001	143623000	1.68E-06
chr8	143619001	143624000	1.32E-06
chr8	143620001	143625000	1.26E-06
chr8	143621001	143626000	1.44E-14
chr8	143622001	143627000	1.60E-15
chr8	143624001	143629000	3.06E-19
chr8	143625001	143630000	7.39E-15
chr8	143631001	143636000	4.22E-13
chr8	143632001	143637000	2.43E-14
chr8	143633001	143638000	5.76E-15
chr8	143635001	143640000	1.74E-19
chr8	143637001	143642000	5.23E-15
chr8	143638001	143643000	2.34E-18
chr8	143645001	143650000	1.19E-14
chr8	143647001	143652000	2.29E-05
chr8	143648001	143653000	2.17E-06
chr8	143649001	143654000	2.14E-06
chr8	143650001	143655000	5.35E-08
chr8	143660001	143665000	1.22E-09
chr8	143668001	143673000	2.26E-05
chr8	143669001	143674000	3.90E-10
chr8	143670001	143675000	2.96E-10
chr8	143671001	143676000	9.46E-13
chr8	143672001	143677000	8.64E-13

chr8	143673001	143678000	2.71E-12
chr8	143674001	143679000	6.55E-09
chr8	143684001	143689000	1.65E-12
chr8	143685001	143690000	5.28E-14
chr8	143707001	143712000	3.15E-11
chr8	143708001	143713000	1.66E-09
chr8	143803001	143808000	9.50E-08
chr8	143804001	143809000	9.59E-09
chr8	143829001	143834000	8.55E-07
chr8	143830001	143835000	6.86E-07
chr8	143831001	143836000	1.96E-07
chr8	143832001	143837000	1.49E-07
chr8	143833001	143838000	3.14E-09
chr8	143836001	143841000	3.98E-13
chr8	143843001	143848000	5.93E-17
chr8	143844001	143849000	3.07E-16
chr8	143845001	143850000	3.31E-08
chr8	143854001	143859000	0.001004
chr8	143899001	143904000	8.38E-10
chr8	143900001	143905000	4.03E-12
chr8	143901001	143906000	2.87E-11
chr8	143928001	143933000	8.38E-12
chr8	143929001	143934000	1.41E-11
chr8	143930001	143935000	2.87E-10
chr8	143931001	143936000	5.97E-10
chr8	143937001	143942000	6.90E-10
chr8	143943001	143948000	9.34E-08
chr8	143944001	143949000	3.81E-09
chr8	143952001	143957000	6.49E-09
chr8	143953001	143958000	2.95E-08
chr8	143954001	143959000	3.60E-07
chr8	143962001	143967000	5.17E-09
chr8	143963001	143968000	4.21E-10
chr8	143964001	143969000	6.29E-09
chr8	143965001	143970000	8.26E-10
chr8	143966001	143971000	4.09E-10
chr8	143979001	143984000	5.59E-09
chr8	143980001	143985000	4.35E-11
chr8	143981001	143986000	3.79E-16
chr8	143982001	143987000	8.95E-16
chr8	143983001	143988000	2.34E-11
chr8	143989001	143994000	1.09E-08
chr8	143990001	143995000	1.56E-06
chr8	144003001	144008000	1.51E-14
chr8	144004001	144009000	3.62E-17
chr8	144005001	144010000	4.48E-13
chr8	144006001	144011000	1.43E-14
chr8	144014001	144019000	3.50E-10

chr8	144023001	144028000	0.000114
chr8	144024001	144029000	1.79E-10
chr8	144025001	144030000	9.47E-13
chr8	144026001	144031000	2.65E-12
chr8	144027001	144032000	2.94E-12
chr8	144028001	144033000	8.59E-14
chr8	144030001	144035000	2.69E-12
chr8	144031001	144036000	4.48E-16
chr8	144032001	144037000	3.24E-18
chr8	144033001	144038000	2.89E-18
chr8	144034001	144039000	1.16E-19
chr8	144035001	144040000	2.95E-16
chr8	144054001	144059000	4.13E-19
chr8	144055001	144060000	4.94E-15
chr8	144075001	144080000	4.03E-10
chr8	144095001	144100000	1.47E-09
chr8	144096001	144101000	4.95E-07
chr8	144097001	144102000	3.62E-17
chr8	144098001	144103000	2.05E-14
chr8	144099001	144104000	1.05E-08
chr8	144103001	144108000	0.001044
chr8	144104001	144109000	5.11E-05
chr8	144124001	144129000	5.65E-05
chr8	144143001	144148000	3.39E-08
chr8	144144001	144149000	8.56E-11
chr8	144145001	144150000	1.14E-09
chr8	144146001	144151000	1.70E-09
chr8	144151001	144156000	9.14E-09
chr8	144152001	144157000	1.49E-12
chr8	144204001	144209000	4.12E-08
chr8	144205001	144210000	1.72E-08
chr8	144227001	144232000	4.04E-06
chr8	144237001	144242000	4.72E-07
chr8	144238001	144243000	2.90E-05
chr8	144239001	144244000	6.06E-07
chr8	144245001	144250000	1.32E-08
chr8	144246001	144251000	2.37E-11
chr8	144247001	144252000	9.47E-10
chr8	144248001	144253000	2.50E-21
chr8	144249001	144254000	6.72E-11
chr8	144250001	144255000	1.25E-13
chr8	144251001	144256000	3.77E-12
chr8	144252001	144257000	5.13E-11
chr8	144254001	144259000	3.74E-12
chr8	144262001	144267000	3.78E-16
chr8	144263001	144268000	6.70E-22
chr8	144264001	144269000	6.26E-21
chr8	144265001	144270000	9.23E-29

chr8	144266001	144271000	3.56E-22
chr8	144267001	144272000	2.83E-10
chr8	144274001	144279000	1.44E-14
chr8	144275001	144280000	6.52E-19
chr8	144276001	144281000	1.31E-19
chr8	144277001	144282000	3.67E-26
chr8	144278001	144283000	2.04E-23
chr8	144279001	144284000	1.18E-20
chr8	144280001	144285000	1.75E-15
chr8	144281001	144286000	3.78E-19
chr8	144282001	144287000	2.68E-14
chr8	144306001	144311000	3.16E-07
chr8	144308001	144313000	2.10E-07
chr8	144314001	144319000	2.07E-06
chr8	144315001	144320000	6.87E-07
chr8	144352001	144357000	0.000231
chr8	144367001	144372000	0.001988
chr8	144460001	144465000	0.014138
chr8	144467001	144472000	0.000101
chr8	144481001	144486000	1.51E-06
chr8	144482001	144487000	8.35E-07
chr8	144500001	144505000	8.03E-07
chr8	144501001	144506000	4.63E-09
chr8	144513001	144518000	4.93E-05
chr8	144632001	144637000	1.42E-06
chr8	144633001	144638000	3.17E-06
chr8	144648001	144653000	0.004745
chr8	144652001	144657000	2.35E-05
chr8	144676001	144681000	4.99E-07
chr8	144677001	144682000	6.53E-09
chr8	144687001	144692000	4.61E-08
chr8	144688001	144693000	3.86E-10
chr8	144699001	144704000	2.79E-05
chr8	144700001	144705000	9.41E-06
chr8	144702001	144707000	0.000774
chr8	144743001	144748000	4.00E-06
chr8	144744001	144749000	1.25E-07
chr8	144745001	144750000	1.72E-06
chr8	144766001	144771000	1.51E-24
chr8	144767001	144772000	4.43E-17
chr8	144813001	144818000	0.00689
chr8	144817001	144822000	1.92E-06
chr8	144919001	144924000	1.28E-06
chr8	144944001	144949000	6.45E-13
chr8	144958001	144963000	0.000475
chr8	144959001	144964000	2.53E-06
chr8	144960001	144965000	1.14E-07
chr8	144961001	144966000	1.92E-06

chr8	144962001	144967000	1.34E-10
chr8	144963001	144968000	9.25E-10
chr8	144973001	144978000	1.11E-20
chr8	144974001	144979000	9.93E-23
chr8	144975001	144980000	2.80E-24
chr8	144976001	144981000	7.20E-27
chr8	144977001	144982000	3.67E-20
chr8	144978001	144983000	7.46E-19
chr8	145012001	145017000	5.45E-07
chr8	145018001	145023000	1.82E-06
chr8	145020001	145025000	6.54E-05
chr8	145021001	145026000	2.67E-06
chr8	145024001	145029000	0.00104
chr8	145026001	145031000	1.64E-07
chr8	145045001	145050000	0.002191
chr8	145046001	145051000	0.001075
chr8	145085001	145090000	6.13E-07
chr8	145086001	145091000	0.000188
chr8	145094001	145099000	3.62E-09
chr8	145095001	145100000	1.17E-11
chr8	145098001	145103000	6.90E-11
chr8	145129001	145134000	2.43E-06
chr8	145164001	145169000	0.002289
chr8	145165001	145170000	7.81E-07
chr8	145168001	145173000	3.01E-06
chr8	145185001	145190000	0.001037
chr8	145285001	145290000	4.87E-07
chr8	145286001	145291000	1.62E-06
chr8	145287001	145292000	0.000772
chr8	145288001	145293000	0.001135
chr8	145564001	145569000	2.20E-07
chr8	145565001	145570000	5.01E-08
chr8	145566001	145571000	0.006392
chr8	145581001	145586000	2.09E-05
chr8	145582001	145587000	0.00049
chr8	145669001	145674000	2.99E-07
chr8	145670001	145675000	2.57E-05
chr8	145693001	145698000	0.002008
chr8	145724001	145729000	1.68E-05
chr8	145725001	145730000	1.26E-06
chr8	145726001	145731000	5.52E-12
chr8	145730001	145735000	5.57E-13
chr8	145746001	145751000	9.62E-05
chr8	145747001	145752000	6.80E-10
chr8	145748001	145753000	4.51E-08
chr8	145907001	145912000	4.50E-06
chr8	145908001	145913000	2.00E-11
chr8	145909001	145914000	7.22E-12

chr8	145977001	145982000	4.37E-14
chr8	145978001	145983000	6.98E-10
chr8	145979001	145984000	3.09E-11
chr8	145980001	145985000	1.11E-20
chr8	145981001	145986000	6.23E-21
chr8	146046001	146051000	3.21E-08
chr8	146048001	146053000	5.84E-05
chr8	146075001	146080000	0.000437
chr8	146076001	146081000	1.33E-10
chr8	146077001	146082000	1.13E-17
chr8	146078001	146083000	4.29E-14
chr8	146079001	146084000	2.10E-09
chr8	146122001	146127000	2.12E-18
chr8	146123001	146128000	2.86E-17
chr8	146125001	146130000	2.91E-11
chr8	146128001	146133000	3.27E-09
chr8	146135001	146140000	0.000157
chr8	146177001	146182000	1.20E-08
chr8	146178001	146183000	1.78E-13
chr8	146186001	146191000	6.69E-06
chr8	146187001	146192000	1.92E-06
chr8	146188001	146193000	0.000222
chr8	146189001	146194000	0.000114
chr8	146190001	146195000	0.002072
chr8	146193001	146198000	1.79E-06
chr9	24001	29000	0.000701
chr9	688001	693000	0.028111
chr9	774001	779000	0.000547
chr9	838001	843000	0.000374
chr9	844001	849000	3.23E-06
chr9	845001	850000	9.03E-07
chr9	846001	851000	0.000781
chr9	2856001	2861000	1.38E-07
chr9	5292001	5297000	1.44E-05
chr9	6210001	6215000	0.000303
chr9	6370001	6375000	0.001523
chr9	6656001	6661000	0.000594
chr9	6712001	6717000	3.74E-08
chr9	6716001	6721000	0.001911
chr9	6756001	6761000	1.68E-08
chr9	6758001	6763000	4.23E-05
chr9	6759001	6764000	1.01E-06
chr9	7282001	7287000	3.95E-09
chr9	7283001	7288000	4.79E-09
chr9	7284001	7289000	3.68E-08
chr9	7310001	7315000	0.000554
chr9	7311001	7316000	0.000102
chr9	7587001	7592000	1.03E-05

chr9	8092001	8097000	8.00E-08
chr9	8289001	8294000	3.73E-05
chr9	8762001	8767000	7.08E-06
chr9	8962001	8967000	0.00498
chr9	8963001	8968000	0.019491
chr9	10178001	10183000	0.000575
chr9	10550001	10555000	2.35E-06
chr9	10790001	10795000	0.000259
chr9	11299001	11304000	2.09E-05
chr9	11300001	11305000	1.11E-05
chr9	12657001	12662000	5.08E-05
chr9	15023001	15028000	2.53E-05
chr9	15106001	15111000	1.51E-06
chr9	16194001	16199000	8.26E-06
chr9	16737001	16742000	1.13E-07
chr9	16877001	16882000	0.000115
chr9	16941001	16946000	1.21E-08
chr9	17054001	17059000	3.07E-05
chr9	17055001	17060000	0.004433
chr9	18714001	18719000	9.47E-06
chr9	19225001	19230000	7.88E-09
chr9	19226001	19231000	5.73E-09
chr9	21231001	21236000	0.00212
chr9	22377001	22382000	6.94E-09
chr9	22683001	22688000	7.02E-07
chr9	23770001	23775000	8.51E-06
chr9	24675001	24680000	1.82E-08
chr9	25357001	25362000	4.05E-10
chr9	25358001	25363000	6.32E-11
chr9	25958001	25963000	1.00E-04
chr9	25959001	25964000	6.21E-05
chr9	26711001	26716000	5.76E-06
chr9	26736001	26741000	0.000177
chr9	27572001	27577000	1.43E-16
chr9	27573001	27578000	6.17E-23
chr9	27995001	28000000	4.44E-06
chr9	29251001	29256000	6.66E-05
chr9	29252001	29257000	2.17E-05
chr9	29509001	29514000	0.001335
chr9	30050001	30055000	2.23E-06
chr9	30978001	30983000	3.67E-05
chr9	30994001	30999000	4.11E-05
chr9	31108001	31113000	8.06E-06
chr9	31121001	31126000	8.83E-08
chr9	31122001	31127000	5.10E-09
chr9	31123001	31128000	2.33E-11
chr9	31124001	31129000	1.85E-06
chr9	31393001	31398000	0.000276

chr9	32069001	32074000	2.12E-09
chr9	32070001	32075000	2.10E-07
chr9	32071001	32076000	1.52E-07
chr9	32072001	32077000	1.31E-05
chr9	32086001	32091000	1.95E-06
chr9	32372001	32377000	2.47E-05
chr9	33240001	33245000	0.001953
chr9	33412001	33417000	3.68E-05
chr9	33423001	33428000	0.000684
chr9	33424001	33429000	0.00484
chr9	33465001	33470000	0.003155
chr9	33466001	33471000	0.000364
chr9	33597001	33602000	0.004699
chr9	33707001	33712000	1.32E-05
chr9	33814001	33819000	0.000238
chr9	33815001	33820000	3.83E-05
chr9	33999001	34004000	0.016171
chr9	34429001	34434000	0.018662
chr9	34430001	34435000	0.004839
chr9	34431001	34436000	0.001053
chr9	34454001	34459000	1.31E-14
chr9	34455001	34460000	6.32E-12
chr9	34456001	34461000	1.47E-12
chr9	34457001	34462000	2.02E-13
chr9	34458001	34463000	2.74E-12
chr9	34473001	34478000	0.00025
chr9	34474001	34479000	1.73E-05
chr9	34475001	34480000	4.33E-06
chr9	34495001	34500000	1.52E-05
chr9	34545001	34550000	0.000807
chr9	34546001	34551000	9.63E-05
chr9	34547001	34552000	0.001805
chr9	34548001	34553000	0.00033
chr9	34549001	34554000	0.001291
chr9	34582001	34587000	8.97E-05
chr9	34664001	34669000	0.000161
chr9	34671001	34676000	4.56E-07
chr9	34672001	34677000	1.63E-06
chr9	34674001	34679000	5.37E-08
chr9	34675001	34680000	4.88E-07
chr9	34746001	34751000	0.000144
chr9	34856001	34861000	0.0002
chr9	34857001	34862000	0.000174
chr9	34858001	34863000	0.000836
chr9	34984001	34989000	1.10E-05
chr9	35076001	35081000	8.02E-05
chr9	35077001	35082000	1.01E-05
chr9	35134001	35139000	0.001155

chr9	35158001	35163000	1.70E-09
chr9	35159001	35164000	4.26E-08
chr9	35180001	35185000	2.75E-05
chr9	35181001	35186000	0.000161
chr9	35605001	35610000	8.35E-05
chr9	35606001	35611000	8.99E-05
chr9	35686001	35691000	0.002566
chr9	35911001	35916000	0.021474
chr9	36016001	36021000	0.002753
chr9	36017001	36022000	7.23E-05
chr9	36446001	36451000	0.002813
chr9	36507001	36512000	0.004294
chr9	36508001	36513000	0.00079
chr9	36533001	36538000	0.014329
chr9	36534001	36539000	0.043824
chr9	36536001	36541000	0.002872
chr9	36570001	36575000	1.62E-10
chr9	36571001	36576000	2.30E-13
chr9	36572001	36577000	3.90E-17
chr9	36573001	36578000	5.78E-16
chr9	36743001	36748000	0.005616
chr9	36744001	36749000	0.000209
chr9	36745001	36750000	0.000426
chr9	36991001	36996000	0.003286
chr9	37484001	37489000	1.05E-06
chr9	38307001	38312000	6.82E-05
chr9	38803001	38808000	6.51E-05
chr9	38872001	38877000	0.005356
chr9	38873001	38878000	0.001669
chr9	38874001	38879000	0.001669
chr9	38959001	38964000	0.005054
chr9	38991001	38996000	0.010802
chr9	38992001	38997000	0.00215
chr9	38993001	38998000	0.000141
chr9	38994001	38999000	0.000141
chr9	38995001	39000000	0.000875
chr9	39461001	39466000	2.76E-05
chr9	39462001	39467000	0.003522
chr9	39798001	39803000	5.37E-05
chr9	39799001	39804000	0.001243
chr9	39800001	39805000	0.00435
chr9	39801001	39806000	0.003371
chr9	39808001	39813000	0.001639
chr9	39809001	39814000	0.00254
chr9	39810001	39815000	0.001922
chr9	39823001	39828000	0.016929
chr9	40326001	40331000	0.000497
chr9	40530001	40535000	0.00036

chr9	40552001	40557000	0.0004
chr9	40587001	40592000	0.000227
chr9	40642001	40647000	0.00092
chr9	40643001	40648000	0.000917
chr9	40652001	40657000	0.004034
chr9	40653001	40658000	0.003845
chr9	40692001	40697000	0.000394
chr9	40833001	40838000	0.000988
chr9	40872001	40877000	0.005918
chr9	40873001	40878000	0.005918
chr9	40886001	40891000	0.001119
chr9	40887001	40892000	0.001103
chr9	40918001	40923000	0.036571
chr9	40919001	40924000	0.036571
chr9	41258001	41263000	0.027834
chr9	41259001	41264000	0.027834
chr9	41260001	41265000	0.022652
chr9	41771001	41776000	0.026646
chr9	41925001	41930000	0.001447
chr9	41926001	41931000	0.003207
chr9	41927001	41932000	0.000618
chr9	41928001	41933000	0.000217
chr9	41929001	41934000	0.001778
chr9	41968001	41973000	0.000682
chr9	41969001	41974000	1.34E-06
chr9	41978001	41983000	0.006236
chr9	41980001	41985000	0.018879
chr9	41981001	41986000	0.005461
chr9	42093001	42098000	0.020736
chr9	42094001	42099000	0.020736
chr9	42095001	42100000	0.020736
chr9	42096001	42101000	0.010118
chr9	42097001	42102000	0.010118
chr9	42282001	42287000	0.030977
chr9	42283001	42288000	0.024155
chr9	42531001	42536000	6.58E-05
chr9	42941001	42946000	0.001289
chr9	42980001	42985000	0.000758
chr9	42981001	42986000	0.000758
chr9	42982001	42987000	0.000556
chr9	42997001	43002000	3.30E-05
chr9	42998001	43003000	6.48E-06
chr9	42999001	43004000	6.48E-06
chr9	43000001	43005000	1.43E-05
chr9	43381001	43386000	0.001129
chr9	43459001	43464000	0.000142
chr9	43481001	43486000	3.54E-08
chr9	43593001	43598000	0.000101

chr9	43596001	43601000	0.000156
chr9	43597001	43602000	0.001497
chr9	43736001	43741000	1.71E-05
chr9	43737001	43742000	8.73E-06
chr9	43738001	43743000	0.000101
chr9	43773001	43778000	0.000254
chr9	43774001	43779000	0.000337
chr9	43894001	43899000	0.000584
chr9	43895001	43900000	0.001002
chr9	43896001	43901000	0.002247
chr9	43897001	43902000	0.017146
chr9	43903001	43908000	1.05E-06
chr9	43904001	43909000	9.34E-06
chr9	43905001	43910000	0.000561
chr9	43915001	43920000	0.014484
chr9	43916001	43921000	0.016509
chr9	43917001	43922000	0.01892
chr9	43936001	43941000	0.035711
chr9	43937001	43942000	0.035711
chr9	44155001	44160000	0.002339
chr9	44156001	44161000	0.000616
chr9	44424001	44429000	0.000182
chr9	44874001	44879000	1.11E-05
chr9	45427001	45432000	0.010112
chr9	45436001	45441000	0.000731
chr9	45437001	45442000	3.94E-09
chr9	45438001	45443000	5.76E-07
chr9	45439001	45444000	8.97E-07
chr9	45440001	45445000	1.97E-05
chr9	45443001	45448000	0.000305
chr9	45655001	45660000	0.008842
chr9	45731001	45736000	0.006217
chr9	45769001	45774000	0.030132
chr9	45781001	45786000	0.007952
chr9	45922001	45927000	0.015223
chr9	45923001	45928000	0.015223
chr9	45924001	45929000	0.015901
chr9	45925001	45930000	0.043697
chr9	65785001	65790000	0.000221
chr9	65786001	65791000	0.000221
chr9	66038001	66043000	0.032692
chr9	66048001	66053000	0.022818
chr9	66714001	66719000	0.020903
chr9	67065001	67070000	0.002001
chr9	67238001	67243000	0.000507
chr9	67239001	67244000	0.027702
chr9	67240001	67245000	0.007157
chr9	67832001	67837000	0.003889

chr9	67833001	67838000	0.007169
chr9	67918001	67923000	0.000118
chr9	67936001	67941000	0.005971
chr9	67937001	67942000	0.005971
chr9	67938001	67943000	0.005971
chr9	67944001	67949000	0.018941
chr9	67960001	67965000	0.000487
chr9	67961001	67966000	0.000487
chr9	68267001	68272000	0.017805
chr9	68284001	68289000	6.05E-07
chr9	68297001	68302000	0.000255
chr9	68312001	68317000	0.006141
chr9	68513001	68518000	0.021175
chr9	69432001	69437000	0.00858
chr9	69433001	69438000	0.025832
chr9	69443001	69448000	0.004281
chr9	69456001	69461000	1.09E-07
chr9	69457001	69462000	5.02E-06
chr9	69483001	69488000	0.031056
chr9	69545001	69550000	0.019523
chr9	69551001	69556000	0.000336
chr9	69638001	69643000	6.54E-05
chr9	69639001	69644000	3.24E-06
chr9	69857001	69862000	0.000526
chr9	69858001	69863000	0.000156
chr9	69859001	69864000	4.94E-06
chr9	69860001	69865000	0.001728
chr9	69861001	69866000	0.003053
chr9	69862001	69867000	0.003994
chr9	69863001	69868000	0.028437
chr9	69868001	69873000	0.003841
chr9	69880001	69885000	0.009448
chr9	69937001	69942000	0.007453
chr9	70159001	70164000	0.006471
chr9	70160001	70165000	0.021477
chr9	70161001	70166000	0.021477
chr9	70162001	70167000	0.021477
chr9	70554001	70559000	0.000129
chr9	70555001	70560000	4.28E-05
chr9	70556001	70561000	6.19E-05
chr9	70557001	70562000	9.14E-05
chr9	70700001	70705000	3.57E-05
chr9	70702001	70707000	0.001012
chr9	70712001	70717000	4.90E-06
chr9	70713001	70718000	7.88E-07
chr9	73770001	73775000	8.29E-09
chr9	73868001	73873000	1.71E-07
chr9	73869001	73874000	2.21E-08

chr9	73870001	73875000	1.82E-07
chr9	75195001	75200000	0.002063
chr9	76377001	76382000	1.98E-05
chr9	77922001	77927000	4.97E-05
chr9	78302001	78307000	0.000445
chr9	78309001	78314000	0.000157
chr9	78310001	78315000	0.000972
chr9	78388001	78393000	1.73E-05
chr9	78473001	78478000	0.003867
chr9	78945001	78950000	2.19E-07
chr9	78946001	78951000	7.28E-06
chr9	79008001	79013000	2.92E-10
chr9	79371001	79376000	2.48E-06
chr9	80719001	80724000	0.00025
chr9	80851001	80856000	9.50E-11
chr9	81763001	81768000	2.70E-08
chr9	81764001	81769000	3.82E-10
chr9	81835001	81840000	1.19E-06
chr9	81836001	81841000	7.32E-07
chr9	82535001	82540000	3.70E-05
chr9	82643001	82648000	4.07E-06
chr9	82844001	82849000	8.88E-05
chr9	82855001	82860000	2.75E-08
chr9	82856001	82861000	1.34E-07
chr9	82857001	82862000	3.75E-10
chr9	82858001	82863000	9.17E-09
chr9	82859001	82864000	1.05E-08
chr9	84167001	84172000	0.001344
chr9	84479001	84484000	0.004826
chr9	84765001	84770000	4.19E-05
chr9	85089001	85094000	1.35E-07
chr9	85232001	85237000	3.20E-08
chr9	85233001	85238000	7.54E-06
chr9	85252001	85257000	0.002743
chr9	85529001	85534000	0.0013
chr9	86045001	86050000	3.72E-06
chr9	86046001	86051000	1.53E-06
chr9	86155001	86160000	6.04E-05
chr9	86156001	86161000	3.05E-06
chr9	86157001	86162000	0.000483
chr9	86567001	86572000	1.01E-12
chr9	86568001	86573000	8.03E-09
chr9	86569001	86574000	5.19E-10
chr9	86570001	86575000	2.84E-09
chr9	86571001	86576000	3.72E-08
chr9	88480001	88485000	0.002929
chr9	88710001	88715000	5.79E-08
chr9	88711001	88716000	2.26E-09

chr9	88712001	88717000	4.85E-08
chr9	88768001	88773000	0.001257
chr9	88794001	88799000	0.015446
chr9	88795001	88800000	0.006005
chr9	88796001	88801000	0.008155
chr9	88868001	88873000	0.000246
chr9	89545001	89550000	3.79E-10
chr9	89547001	89552000	5.43E-11
chr9	89548001	89553000	3.30E-07
chr9	89910001	89915000	4.10E-05
chr9	89911001	89916000	1.74E-05
chr9	89912001	89917000	1.86E-05
chr9	89957001	89962000	1.29E-09
chr9	89958001	89963000	2.38E-09
chr9	89959001	89964000	3.80E-05
chr9	89960001	89965000	5.73E-05
chr9	90069001	90074000	0.002617
chr9	90071001	90076000	6.72E-06
chr9	90436001	90441000	3.35E-11
chr9	90437001	90442000	2.39E-11
chr9	90438001	90443000	2.38E-06
chr9	90483001	90488000	7.66E-06
chr9	90484001	90489000	2.03E-06
chr9	90497001	90502000	0.003853
chr9	90649001	90654000	4.02E-05
chr9	92047001	92052000	0.000805
chr9	92048001	92053000	0.00025
chr9	92219001	92224000	3.61E-13
chr9	94483001	94488000	5.33E-08
chr9	94526001	94531000	0.004569
chr9	94527001	94532000	0.006067
chr9	94749001	94754000	0.000279
chr9	94902001	94907000	4.12E-05
chr9	94903001	94908000	1.97E-06
chr9	95674001	95679000	2.42E-05
chr9	95731001	95736000	0.001056
chr9	95732001	95737000	0.000336
chr9	95822001	95827000	8.15E-05
chr9	95823001	95828000	9.79E-05
chr9	95942001	95947000	0.022548
chr9	96066001	96071000	0.003768
chr9	96361001	96366000	1.09E-05
chr9	96362001	96367000	7.00E-05
chr9	96488001	96493000	3.02E-05
chr9	96674001	96679000	0.000255
chr9	96676001	96681000	0.000172
chr9	97087001	97092000	0.000875
chr9	97136001	97141000	1.30E-06

chr9	97137001	97142000	0.000935
chr9	97155001	97160000	0.017687
chr9	97465001	97470000	3.99E-05
chr9	97671001	97676000	5.94E-08
chr9	97672001	97677000	1.26E-07
chr9	99073001	99078000	0.003583
chr9	99136001	99141000	0.000591
chr9	99137001	99142000	0.012815
chr9	99756001	99761000	6.78E-06
chr9	100059001	100064000	2.99E-07
chr9	100060001	100065000	4.80E-07
chr9	100314001	100319000	4.91E-06
chr9	100315001	100320000	4.60E-06
chr9	100568001	100573000	0.000254
chr9	100649001	100654000	0.000257
chr9	100954001	100959000	0.000391
chr9	101018001	101023000	8.85E-08
chr9	101019001	101024000	2.30E-08
chr9	101267001	101272000	2.17E-09
chr9	101268001	101273000	3.49E-10
chr9	101556001	101561000	9.57E-10
chr9	101707001	101712000	6.05E-06
chr9	101708001	101713000	2.96E-06
chr9	101730001	101735000	7.04E-11
chr9	101731001	101736000	3.56E-10
chr9	101732001	101737000	1.61E-07
chr9	101733001	101738000	5.81E-11
chr9	101734001	101739000	1.15E-09
chr9	101735001	101740000	5.58E-08
chr9	101737001	101742000	1.22E-10
chr9	101764001	101769000	5.14E-06
chr9	102459001	102464000	3.09E-08
chr9	102614001	102619000	5.38E-05
chr9	102664001	102669000	1.22E-09
chr9	103878001	103883000	0.033877
chr9	104218001	104223000	2.72E-08
chr9	104526001	104531000	1.39E-05
chr9	104528001	104533000	3.81E-05
chr9	104768001	104773000	0.000123
chr9	104769001	104774000	0.000113
chr9	104847001	104852000	6.85E-06
chr9	105102001	105107000	1.56E-05
chr9	105228001	105233000	2.03E-05
chr9	105237001	105242000	2.74E-08
chr9	106294001	106299000	5.08E-05
chr9	107726001	107731000	1.70E-11
chr9	107727001	107732000	1.10E-14
chr9	107728001	107733000	3.58E-11

chr9	107729001	107734000	2.22E-10
chr9	107730001	107735000	3.01E-06
chr9	107950001	107955000	7.07E-10
chr9	107951001	107956000	4.14E-12
chr9	108415001	108420000	1.22E-12
chr9	108416001	108421000	6.80E-09
chr9	108417001	108422000	1.10E-07
chr9	108418001	108423000	2.94E-06
chr9	109024001	109029000	2.14E-08
chr9	110239001	110244000	1.12E-06
chr9	111957001	111962000	1.12E-05
chr9	112090001	112095000	0.005096
chr9	112737001	112742000	0.000135
chr9	114263001	114268000	0.000823
chr9	114420001	114425000	0.001976
chr9	115651001	115656000	0.024379
chr9	115652001	115657000	0.005277
chr9	115708001	115713000	3.64E-07
chr9	115723001	115728000	0.000675
chr9	115909001	115914000	1.29E-09
chr9	115910001	115915000	2.97E-10
chr9	117216001	117221000	0.00013
chr9	117329001	117334000	6.22E-06
chr9	117330001	117335000	2.28E-06
chr9	117331001	117336000	2.62E-08
chr9	117654001	117659000	0.000148
chr9	117655001	117660000	8.66E-05
chr9	117656001	117661000	6.91E-05
chr9	119768001	119773000	0.000609
chr9	119905001	119910000	7.54E-07
chr9	120169001	120174000	7.95E-06
chr9	120170001	120175000	1.31E-05
chr9	120497001	120502000	4.10E-06
chr9	120499001	120504000	8.31E-09
chr9	120558001	120563000	4.16E-10
chr9	120559001	120564000	4.13E-08
chr9	120673001	120678000	4.11E-05
chr9	120729001	120734000	6.49E-10
chr9	120730001	120735000	9.76E-10
chr9	120838001	120843000	5.86E-09
chr9	120943001	120948000	6.32E-09
chr9	120944001	120949000	2.41E-07
chr9	120962001	120967000	1.28E-06
chr9	120963001	120968000	1.22E-05
chr9	120964001	120969000	2.21E-07
chr9	121298001	121303000	2.49E-07
chr9	121486001	121491000	9.26E-07
chr9	121498001	121503000	0.000112

chr9	121538001	121543000	1.60E-06
chr9	121648001	121653000	1.96E-10
chr9	121649001	121654000	4.17E-15
chr9	121731001	121736000	3.67E-05
chr9	122200001	122205000	1.81E-09
chr9	122260001	122265000	5.68E-05
chr9	122261001	122266000	1.56E-05
chr9	122262001	122267000	2.80E-06
chr9	122263001	122268000	4.61E-07
chr9	122442001	122447000	2.34E-07
chr9	122457001	122462000	3.43E-06
chr9	122503001	122508000	4.30E-05
chr9	122504001	122509000	4.58E-05
chr9	122531001	122536000	1.06E-06
chr9	122607001	122612000	1.99E-08
chr9	123002001	123007000	8.30E-08
chr9	123065001	123070000	0.002497
chr9	123482001	123487000	6.84E-06
chr9	123483001	123488000	0.000618
chr9	123484001	123489000	4.33E-05
chr9	123631001	123636000	1.04E-06
chr9	124185001	124190000	0.000913
chr9	124186001	124191000	0.013312
chr9	124218001	124223000	3.99E-06
chr9	124219001	124224000	1.24E-05
chr9	124327001	124332000	0.011535
chr9	124328001	124333000	6.89E-05
chr9	124755001	124760000	1.11E-05
chr9	124883001	124888000	7.54E-08
chr9	124884001	124889000	4.11E-07
chr9	124925001	124930000	1.87E-05
chr9	125097001	125102000	2.28E-06
chr9	125099001	125104000	0.00014
chr9	125130001	125135000	7.64E-07
chr9	125131001	125136000	4.27E-06
chr9	125132001	125137000	1.24E-07
chr9	125133001	125138000	8.99E-09
chr9	125544001	125549000	3.82E-05
chr9	125689001	125694000	1.31E-10
chr9	126094001	126099000	3.71E-08
chr9	126860001	126865000	0.017532
chr9	126861001	126866000	0.007972
chr9	126862001	126867000	0.01249
chr9	126871001	126876000	2.51E-05
chr9	126898001	126903000	0.001459
chr9	126906001	126911000	5.09E-08
chr9	126979001	126984000	2.07E-05
chr9	127019001	127024000	0.001392

chr9	127028001	127033000	4.93E-06
chr9	127029001	127034000	6.70E-05
chr9	127030001	127035000	3.28E-06
chr9	127174001	127179000	0.000298
chr9	127176001	127181000	7.51E-07
chr9	127177001	127182000	4.76E-08
chr9	127250001	127255000	0.000281
chr9	127251001	127256000	0.000602
chr9	127261001	127266000	0.003948
chr9	127262001	127267000	0.003948
chr9	127626001	127631000	2.45E-06
chr9	128149001	128154000	0.000171
chr9	128384001	128389000	1.28E-05
chr9	128826001	128831000	4.50E-06
chr9	128936001	128941000	5.21E-05
chr9	129258001	129263000	5.06E-09
chr9	129294001	129299000	1.28E-05
chr9	129669001	129674000	0.000471
chr9	129670001	129675000	0.000122
chr9	129672001	129677000	9.24E-07
chr9	129673001	129678000	2.43E-10
chr9	129674001	129679000	1.86E-09
chr9	129675001	129680000	6.85E-07
chr9	129677001	129682000	5.67E-06
chr9	129699001	129704000	1.97E-05
chr9	129882001	129887000	1.63E-05
chr9	130159001	130164000	5.07E-07
chr9	130160001	130165000	1.07E-10
chr9	130182001	130187000	1.22E-06
chr9	130186001	130191000	5.33E-08
chr9	130207001	130212000	1.55E-05
chr9	130330001	130335000	0.000782
chr9	130340001	130345000	2.47E-05
chr9	130341001	130346000	0.000303
chr9	130419001	130424000	5.44E-08
chr9	130420001	130425000	3.73E-09
chr9	130421001	130426000	3.72E-10
chr9	130422001	130427000	1.50E-08
chr9	130483001	130488000	0.000108
chr9	130486001	130491000	1.29E-05
chr9	130488001	130493000	6.20E-07
chr9	130532001	130537000	1.04E-07
chr9	130533001	130538000	1.20E-06
chr9	130534001	130539000	9.03E-08
chr9	130622001	130627000	4.31E-06
chr9	130687001	130692000	0.005556
chr9	130704001	130709000	5.59E-05
chr9	130705001	130710000	5.58E-06

chr9	130706001	130711000	2.08E-06
chr9	130721001	130726000	0.001026
chr9	130722001	130727000	0.00349
chr9	130740001	130745000	2.08E-06
chr9	130741001	130746000	5.78E-10
chr9	130742001	130747000	4.00E-05
chr9	130766001	130771000	2.18E-10
chr9	130767001	130772000	1.16E-06
chr9	130768001	130773000	0.003112
chr9	130965001	130970000	2.28E-05
chr9	130966001	130971000	2.86E-08
chr9	130975001	130980000	0.001215
chr9	131418001	131423000	9.02E-05
chr9	131419001	131424000	1.07E-05
chr9	131422001	131427000	0.00598
chr9	131540001	131545000	0.002645
chr9	131541001	131546000	0.003522
chr9	131542001	131547000	0.000642
chr9	131637001	131642000	6.54E-05
chr9	131649001	131654000	0.000249
chr9	131650001	131655000	0.000209
chr9	131651001	131656000	9.78E-05
chr9	131687001	131692000	0.001478
chr9	131688001	131693000	0.005097
chr9	131829001	131834000	0.026033
chr9	131946001	131951000	5.35E-06
chr9	131947001	131952000	1.82E-07
chr9	131948001	131953000	1.59E-06
chr9	132094001	132099000	1.91E-05
chr9	132227001	132232000	0.008682
chr9	132228001	132233000	0.000315
chr9	132229001	132234000	0.047943
chr9	132243001	132248000	0.003016
chr9	132245001	132250000	0.003102
chr9	132246001	132251000	0.003696
chr9	132247001	132252000	0.001223
chr9	132248001	132253000	6.61E-05
chr9	132285001	132290000	0.00148
chr9	132286001	132291000	0.007784
chr9	132309001	132314000	0.024392
chr9	132311001	132316000	0.000137
chr9	132312001	132317000	0.001834
chr9	132326001	132331000	2.31E-05
chr9	132357001	132362000	8.03E-08
chr9	132358001	132363000	4.61E-13
chr9	132359001	132364000	3.04E-14
chr9	132360001	132365000	3.04E-14
chr9	132367001	132372000	0.000401

chr9	132368001	132373000	0.002157
chr9	132369001	132374000	1.73E-07
chr9	132370001	132375000	3.77E-08
chr9	132373001	132378000	5.33E-07
chr9	132434001	132439000	0.019361
chr9	132469001	132474000	5.42E-07
chr9	132485001	132490000	0.003859
chr9	132512001	132517000	1.37E-08
chr9	132513001	132518000	4.23E-11
chr9	132514001	132519000	3.63E-08
chr9	132558001	132563000	0.000194
chr9	132592001	132597000	1.33E-07
chr9	132593001	132598000	1.59E-06
chr9	132595001	132600000	1.00E-05
chr9	132627001	132632000	6.47E-05
chr9	132785001	132790000	3.98E-05
chr9	132930001	132935000	0.003723
chr9	132931001	132936000	0.003723
chr9	132934001	132939000	0.000129
chr9	133018001	133023000	7.58E-05
chr9	133060001	133065000	3.07E-05
chr9	133061001	133066000	0.014661
chr9	133267001	133272000	0.004655
chr9	133304001	133309000	4.34E-07
chr9	133305001	133310000	2.77E-07
chr9	133316001	133321000	9.38E-05
chr9	133415001	133420000	0.004757
chr9	133431001	133436000	4.94E-05
chr9	133432001	133437000	4.72E-06
chr9	133433001	133438000	6.77E-07
chr9	133434001	133439000	1.30E-05
chr9	133435001	133440000	1.32E-06
chr9	133565001	133570000	2.14E-14
chr9	133566001	133571000	9.22E-19
chr9	133567001	133572000	1.54E-12
chr9	133816001	133821000	3.29E-05
chr9	133925001	133930000	0.005307
chr9	133968001	133973000	2.62E-09
chr9	133969001	133974000	6.16E-09
chr9	133970001	133975000	4.60E-08
chr9	134130001	134135000	0.000884
chr9	134131001	134136000	0.000229
chr9	134152001	134157000	9.62E-11
chr9	134153001	134158000	0.000719
chr9	134178001	134183000	0.019014
chr9	134179001	134184000	0.019978
chr9	134182001	134187000	7.78E-07
chr9	134185001	134190000	0.001085

chr9	134235001	134240000	0.007738
chr9	134254001	134259000	3.72E-05
chr9	134255001	134260000	0.000389
chr9	134662001	134667000	8.41E-05
chr9	134663001	134668000	2.46E-09
chr9	134664001	134669000	2.01E-06
chr9	134665001	134670000	5.13E-07
chr9	134666001	134671000	3.30E-06
chr9	135044001	135049000	3.46E-05
chr9	135090001	135095000	0.000569
chr9	135124001	135129000	2.06E-05
chr9	135125001	135130000	1.92E-06
chr9	135126001	135131000	1.20E-09
chr9	135127001	135132000	1.58E-10
chr9	135128001	135133000	9.95E-10
chr9	135359001	135364000	8.58E-09
chr9	135444001	135449000	0.002667
chr9	135445001	135450000	0.000894
chr9	135446001	135451000	0.00082
chr9	135620001	135625000	5.60E-14
chr9	136021001	136026000	8.87E-08
chr9	136078001	136083000	3.44E-09
chr9	136079001	136084000	2.81E-06
chr9	136080001	136085000	9.23E-08
chr9	136081001	136086000	2.72E-10
chr9	136131001	136136000	0.013577
chr9	136132001	136137000	0.002801
chr9	136172001	136177000	0.000421
chr9	136198001	136203000	4.09E-05
chr9	136260001	136265000	2.57E-06
chr9	136261001	136266000	1.54E-05
chr9	136262001	136267000	3.04E-05
chr9	136374001	136379000	1.02E-06
chr9	136499001	136504000	2.11E-05
chr9	136575001	136580000	0.028212
chr9	136584001	136589000	8.92E-08
chr9	136586001	136591000	1.50E-08
chr9	136587001	136592000	1.64E-06
chr9	136588001	136593000	8.65E-09
chr9	136592001	136597000	4.25E-07
chr9	136888001	136893000	5.00E-05
chr9	136889001	136894000	5.71E-05
chr9	136997001	137002000	3.54E-06
chr9	136998001	137003000	1.35E-09
chr9	137133001	137138000	3.98E-07
chr9	137219001	137224000	0.007654
chr9	137334001	137339000	1.29E-06
chr9	137335001	137340000	8.03E-08

chr9	137336001	137341000	3.77E-06
chr9	137337001	137342000	3.40E-06
chr9	137443001	137448000	0.002122
chr9	137446001	137451000	0.000162
chr9	137447001	137452000	0.00019
chr9	137459001	137464000	5.93E-09
chr9	137468001	137473000	2.39E-08
chr9	137469001	137474000	1.56E-06
chr9	137470001	137475000	2.19E-06
chr9	137479001	137484000	0.000139
chr9	137506001	137511000	8.46E-11
chr9	137507001	137512000	1.95E-10
chr9	137508001	137513000	4.48E-05
chr9	137509001	137514000	0.004552
chr9	137510001	137515000	0.000177
chr9	137511001	137516000	0.001093
chr9	137545001	137550000	0.002429
chr9	137589001	137594000	2.29E-08
chr9	137613001	137618000	5.41E-08
chr9	137614001	137619000	6.58E-08
chr9	137616001	137621000	5.95E-10
chr9	137718001	137723000	4.35E-05
chr9	137885001	137890000	1.26E-07
chr9	137957001	137962000	4.85E-05
chr9	137968001	137973000	1.73E-06
chr9	138016001	138021000	1.58E-07
chr9	138017001	138022000	4.11E-08
chr9	138018001	138023000	2.37E-15
chr9	138285001	138290000	0.000404
chr9	138286001	138291000	3.90E-05
chr9	138353001	138358000	0.000146
chr9	138354001	138359000	0.006422
chr9	138355001	138360000	0.004618
chr9	138452001	138457000	2.43E-05
chr9	138454001	138459000	7.82E-08
chr9	138477001	138482000	0.000115
chr9	138481001	138486000	0.000171
chr9	138482001	138487000	0.001197
chr9	138485001	138490000	6.48E-05
chr9	138492001	138497000	4.53E-07
chr9	138493001	138498000	8.78E-09
chr9	138494001	138499000	1.05E-06
chr9	138608001	138613000	0.002061
chr9	138618001	138623000	0.000245
chr9	138620001	138625000	0.002497
chr9	138640001	138645000	3.84E-06
chr9	138641001	138646000	0.000238
chr9	138674001	138679000	0.000491

chr9	138840001	138845000	1.01E-09
chr9	138849001	138854000	2.36E-06
chr9	138850001	138855000	6.24E-06
chr9	138883001	138888000	2.82E-05
chr9	138894001	138899000	0.000405
chr9	139093001	139098000	2.71E-06
chr9	139094001	139099000	2.21E-07
chr9	139272001	139277000	0.027569
chr9	139301001	139306000	9.46E-05
chr9	139437001	139442000	8.95E-12
chr9	139438001	139443000	4.34E-09
chr9	139643001	139648000	1.60E-05
chr9	139645001	139650000	4.11E-07
chr9	139693001	139698000	1.13E-05
chr9	139698001	139703000	1.16E-12
chr9	139733001	139738000	1.32E-07
chr9	139741001	139746000	7.25E-14
chr9	139742001	139747000	2.40E-08
chr9	139743001	139748000	9.31E-09
chr9	139744001	139749000	0.000191
chr9	139776001	139781000	1.82E-05
chr9	139777001	139782000	2.49E-05
chr9	139778001	139783000	1.20E-06
chr9	139779001	139784000	2.80E-15
chr9	139780001	139785000	8.42E-18
chr9	139792001	139797000	0.000117
chr9	139864001	139869000	0.048428
chr9	139893001	139898000	1.61E-05
chr9	139941001	139946000	0.000162
chr9	139942001	139947000	5.63E-06
chr9	139943001	139948000	5.63E-06
chr9	139988001	139993000	1.16E-05
chr9	140006001	140011000	0.020451
chr9	140082001	140087000	7.12E-05
chr9	140083001	140088000	2.30E-05
chr9	140112001	140117000	8.21E-05
chr9	140113001	140118000	8.21E-05
chr9	140130001	140135000	0.004464
chr9	140173001	140178000	0.000599
chr9	140174001	140179000	0.000208
chr9	140209001	140214000	0.033253
chr9	140234001	140239000	0.002674
chr9	140241001	140246000	6.25E-05
chr9	140242001	140247000	0.004071
chr9	140264001	140269000	2.62E-05
chr9	140265001	140270000	2.22E-05
chr9	140276001	140281000	5.22E-05
chr9	140285001	140290000	2.86E-05

chr9	140350001	140355000	0.000384
chr9	140351001	140356000	8.25E-05
chr9	140394001	140399000	0.000329
chr9	140395001	140400000	8.58E-05
chr9	140396001	140401000	7.84E-06
chr9	140397001	140402000	2.85E-05
chr9	140792001	140797000	3.66E-05
chr9	141037001	141042000	0.001527
chr9	141040001	141045000	5.73E-05
chr9	141041001	141046000	8.27E-07
chr9	141042001	141047000	1.73E-05
chr10	84001	89000	0.027739
chr10	830001	835000	0.000123
chr10	997001	1002000	3.88E-06
chr10	998001	1003000	0.000321
chr10	1201001	1206000	7.53E-08
chr10	1216001	1221000	1.23E-12
chr10	1250001	1255000	1.10E-06
chr10	1717001	1722000	2.82E-09
chr10	1741001	1746000	0.000614
chr10	1841001	1846000	6.33E-10
chr10	2274001	2279000	2.64E-05
chr10	2275001	2280000	7.92E-06
chr10	2378001	2383000	7.40E-12
chr10	2562001	2567000	2.14E-11
chr10	2713001	2718000	6.01E-07
chr10	2714001	2719000	1.37E-06
chr10	2726001	2731000	1.27E-10
chr10	2727001	2732000	2.32E-14
chr10	2728001	2733000	1.80E-11
chr10	2745001	2750000	7.14E-09
chr10	2746001	2751000	4.85E-08
chr10	2752001	2757000	1.67E-06
chr10	2760001	2765000	7.42E-11
chr10	3210001	3215000	5.48E-17
chr10	3211001	3216000	4.54E-20
chr10	3212001	3217000	5.69E-13
chr10	3213001	3218000	6.00E-13
chr10	3214001	3219000	2.25E-12
chr10	3307001	3312000	3.59E-08
chr10	5650001	5655000	4.24E-07
chr10	5693001	5698000	5.19E-07
chr10	5694001	5699000	9.51E-10
chr10	5695001	5700000	7.22E-10
chr10	5696001	5701000	2.32E-10
chr10	6085001	6090000	3.98E-09
chr10	6110001	6115000	6.36E-07
chr10	6186001	6191000	3.60E-05

chr10	6236001	6241000	3.13E-07
chr10	6238001	6243000	1.50E-10
chr10	6239001	6244000	4.56E-09
chr10	6240001	6245000	8.57E-07
chr10	6241001	6246000	1.23E-05
chr10	6294001	6299000	1.82E-05
chr10	6295001	6300000	1.57E-05
chr10	6296001	6301000	1.07E-05
chr10	6297001	6302000	3.78E-06
chr10	6298001	6303000	3.27E-05
chr10	6356001	6361000	1.17E-06
chr10	6357001	6362000	1.44E-07
chr10	6447001	6452000	3.34E-09
chr10	6448001	6453000	4.44E-08
chr10	6457001	6462000	0.000152
chr10	6458001	6463000	0.000347
chr10	6481001	6486000	7.95E-07
chr10	6724001	6729000	1.73E-07
chr10	7010001	7015000	4.75E-09
chr10	7011001	7016000	5.59E-08
chr10	7053001	7058000	1.36E-09
chr10	7054001	7059000	1.64E-09
chr10	7055001	7060000	1.30E-10
chr10	7105001	7110000	2.50E-06
chr10	7107001	7112000	3.97E-07
chr10	7108001	7113000	3.52E-06
chr10	7401001	7406000	7.68E-10
chr10	7482001	7487000	7.82E-07
chr10	7483001	7488000	2.28E-08
chr10	7568001	7573000	5.27E-08
chr10	7569001	7574000	2.74E-08
chr10	7570001	7575000	5.47E-12
chr10	7571001	7576000	1.58E-09
chr10	7591001	7596000	0.000642
chr10	7634001	7639000	0.002181
chr10	7642001	7647000	2.73E-07
chr10	7678001	7683000	7.54E-05
chr10	7679001	7684000	0.000199
chr10	7681001	7686000	2.30E-06
chr10	7685001	7690000	4.98E-05
chr10	7687001	7692000	0.00039
chr10	7688001	7693000	0.001122
chr10	7856001	7861000	5.29E-09
chr10	7858001	7863000	6.33E-12
chr10	7859001	7864000	1.67E-15
chr10	7860001	7865000	5.15E-22
chr10	8168001	8173000	0.000139
chr10	8169001	8174000	1.95E-07

chr10	8170001	8175000	1.40E-05
chr10	8209001	8214000	2.79E-08
chr10	8210001	8215000	2.30E-06
chr10	8216001	8221000	1.41E-09
chr10	8217001	8222000	1.82E-05
chr10	8224001	8229000	1.09E-13
chr10	8249001	8254000	1.26E-07
chr10	8280001	8285000	1.29E-08
chr10	8281001	8286000	3.08E-09
chr10	8282001	8287000	1.76E-08
chr10	8288001	8293000	8.38E-07
chr10	8379001	8384000	5.52E-11
chr10	8397001	8402000	2.29E-08
chr10	8399001	8404000	8.96E-09
chr10	8400001	8405000	1.23E-07
chr10	8454001	8459000	1.54E-06
chr10	8499001	8504000	4.23E-08
chr10	8549001	8554000	3.22E-08
chr10	8551001	8556000	3.82E-09
chr10	8567001	8572000	1.72E-14
chr10	8568001	8573000	2.88E-13
chr10	8583001	8588000	4.60E-09
chr10	8585001	8590000	2.77E-09
chr10	8614001	8619000	5.79E-08
chr10	8615001	8620000	2.24E-09
chr10	8621001	8626000	7.42E-12
chr10	8622001	8627000	1.72E-11
chr10	8623001	8628000	3.05E-07
chr10	8663001	8668000	9.70E-09
chr10	8664001	8669000	8.46E-08
chr10	8666001	8671000	1.20E-06
chr10	8856001	8861000	5.15E-10
chr10	8955001	8960000	3.07E-09
chr10	8984001	8989000	1.30E-12
chr10	8985001	8990000	8.57E-13
chr10	8986001	8991000	6.32E-12
chr10	8987001	8992000	1.31E-16
chr10	8988001	8993000	4.55E-15
chr10	9165001	9170000	4.50E-10
chr10	9175001	9180000	2.82E-06
chr10	9722001	9727000	9.22E-07
chr10	10039001	10044000	1.53E-07
chr10	10149001	10154000	5.97E-06
chr10	10494001	10499000	5.69E-12
chr10	11267001	11272000	3.40E-09
chr10	11407001	11412000	1.03E-05
chr10	11417001	11422000	2.13E-06
chr10	11418001	11423000	7.79E-06

chr10	11729001	11734000	9.04E-07
chr10	11915001	11920000	1.90E-05
chr10	12094001	12099000	4.49E-06
chr10	12095001	12100000	2.28E-06
chr10	12169001	12174000	0.000301
chr10	12171001	12176000	4.53E-06
chr10	12914001	12919000	1.65E-07
chr10	13267001	13272000	0.000314
chr10	13480001	13485000	8.48E-12
chr10	13858001	13863000	6.22E-08
chr10	13859001	13864000	6.75E-07
chr10	14055001	14060000	8.75E-07
chr10	14057001	14062000	4.70E-07
chr10	14432001	14437000	2.83E-05
chr10	14726001	14731000	8.30E-08
chr10	14727001	14732000	3.61E-07
chr10	14853001	14858000	6.18E-08
chr10	14997001	15002000	2.06E-14
chr10	15089001	15094000	1.17E-10
chr10	15090001	15095000	1.44E-06
chr10	15091001	15096000	7.68E-07
chr10	15126001	15131000	1.51E-06
chr10	15507001	15512000	8.22E-07
chr10	15608001	15613000	1.35E-07
chr10	15706001	15711000	3.42E-08
chr10	15708001	15713000	2.82E-10
chr10	16122001	16127000	1.26E-11
chr10	16123001	16128000	1.19E-11
chr10	16124001	16129000	1.32E-09
chr10	16248001	16253000	5.52E-09
chr10	16249001	16254000	3.53E-09
chr10	16251001	16256000	6.57E-12
chr10	16252001	16257000	7.06E-11
chr10	16301001	16306000	5.14E-07
chr10	17064001	17069000	4.96E-05
chr10	17286001	17291000	3.45E-05
chr10	17287001	17292000	2.11E-06
chr10	17288001	17293000	3.44E-06
chr10	17289001	17294000	2.84E-07
chr10	17291001	17296000	7.99E-06
chr10	17292001	17297000	2.62E-05
chr10	17658001	17663000	0.000406
chr10	17785001	17790000	0.017193
chr10	17813001	17818000	0.004852
chr10	18022001	18027000	0.002137
chr10	18023001	18028000	0.002137
chr10	18024001	18029000	0.002137
chr10	18025001	18030000	0.002137

chr10	18026001	18031000	0.002137
chr10	18086001	18091000	0.007388
chr10	18087001	18092000	0.007388
chr10	18088001	18093000	0.005486
chr10	18102001	18107000	4.27E-05
chr10	18103001	18108000	0.000707
chr10	18104001	18109000	0.002037
chr10	18105001	18110000	0.011519
chr10	18204001	18209000	0.000725
chr10	18701001	18706000	6.02E-07
chr10	18702001	18707000	9.17E-08
chr10	18916001	18921000	1.13E-05
chr10	18992001	18997000	8.92E-05
chr10	19327001	19332000	7.39E-08
chr10	19328001	19333000	5.42E-10
chr10	19349001	19354000	3.80E-07
chr10	19400001	19405000	3.39E-06
chr10	19649001	19654000	8.55E-07
chr10	19650001	19655000	1.39E-08
chr10	19651001	19656000	8.07E-08
chr10	19821001	19826000	1.97E-11
chr10	19936001	19941000	1.93E-07
chr10	19960001	19965000	3.72E-09
chr10	19961001	19966000	2.57E-06
chr10	20135001	20140000	1.95E-11
chr10	20136001	20141000	4.43E-11
chr10	20137001	20142000	1.03E-11
chr10	20138001	20143000	1.50E-10
chr10	20158001	20163000	2.52E-06
chr10	20224001	20229000	8.43E-08
chr10	20372001	20377000	7.69E-06
chr10	20666001	20671000	3.04E-10
chr10	21566001	21571000	1.66E-05
chr10	21567001	21572000	6.82E-09
chr10	21568001	21573000	0.000199
chr10	21582001	21587000	9.30E-06
chr10	21687001	21692000	0.000165
chr10	22292001	22297000	2.86E-06
chr10	23866001	23871000	3.08E-08
chr10	23906001	23911000	6.15E-09
chr10	23936001	23941000	8.51E-10
chr10	24053001	24058000	7.07E-06
chr10	24199001	24204000	1.50E-05
chr10	24207001	24212000	3.82E-05
chr10	25464001	25469000	3.32E-06
chr10	25465001	25470000	1.69E-05
chr10	25531001	25536000	0.000133
chr10	25902001	25907000	2.45E-05

chr10	26027001	26032000	0.000202
chr10	26159001	26164000	0.000419
chr10	26266001	26271000	5.03E-05
chr10	26514001	26519000	7.14E-05
chr10	26591001	26596000	3.70E-07
chr10	26766001	26771000	4.07E-05
chr10	28778001	28783000	4.83E-07
chr10	28779001	28784000	1.83E-05
chr10	28924001	28929000	0.010233
chr10	29012001	29017000	0.000118
chr10	29013001	29018000	0.001866
chr10	29123001	29128000	4.31E-05
chr10	29673001	29678000	1.96E-07
chr10	30835001	30840000	3.25E-06
chr10	30836001	30841000	4.76E-06
chr10	30874001	30879000	0.000349
chr10	30875001	30880000	3.80E-06
chr10	31031001	31036000	8.36E-06
chr10	31065001	31070000	2.77E-08
chr10	31066001	31071000	3.91E-09
chr10	31067001	31072000	1.77E-06
chr10	31885001	31890000	0.000781
chr10	31992001	31997000	6.66E-05
chr10	32000001	32005000	2.26E-07
chr10	32687001	32692000	0.000207
chr10	32688001	32693000	8.33E-05
chr10	32711001	32716000	2.64E-07
chr10	33549001	33554000	1.51E-08
chr10	35095001	35100000	3.13E-05
chr10	35096001	35101000	1.16E-06
chr10	35097001	35102000	1.05E-06
chr10	35379001	35384000	3.27E-08
chr10	35480001	35485000	6.78E-08
chr10	35555001	35560000	0.003918
chr10	35556001	35561000	0.005485
chr10	35559001	35564000	0.021211
chr10	35903001	35908000	3.56E-05
chr10	35904001	35909000	1.22E-06
chr10	37005001	37010000	9.31E-08
chr10	37030001	37035000	2.52E-06
chr10	37095001	37100000	1.86E-06
chr10	37096001	37101000	4.48E-09
chr10	37098001	37103000	8.15E-08
chr10	37101001	37106000	1.87E-05
chr10	37137001	37142000	4.89E-06
chr10	37138001	37143000	1.78E-07
chr10	37740001	37745000	2.86E-05
chr10	37741001	37746000	1.60E-05

chr10	37742001	37747000	3.74E-05
chr10	37743001	37748000	0.000118
chr10	37970001	37975000	2.55E-06
chr10	38274001	38279000	3.23E-05
chr10	38275001	38280000	5.61E-06
chr10	38278001	38283000	0.000258
chr10	38742001	38747000	0.001854
chr10	42461001	42466000	4.06E-08
chr10	42855001	42860000	1.22E-05
chr10	42885001	42890000	3.14E-07
chr10	42893001	42898000	1.85E-05
chr10	42894001	42899000	1.11E-06
chr10	42947001	42952000	3.59E-06
chr10	42948001	42953000	2.59E-05
chr10	43267001	43272000	7.62E-05
chr10	43268001	43273000	1.10E-05
chr10	43362001	43367000	4.09E-08
chr10	43485001	43490000	0.002091
chr10	43568001	43573000	2.26E-06
chr10	43569001	43574000	2.04E-09
chr10	43570001	43575000	4.91E-07
chr10	43571001	43576000	2.87E-07
chr10	43572001	43577000	3.32E-05
chr10	43723001	43728000	6.49E-05
chr10	43751001	43756000	0.002181
chr10	43829001	43834000	0.004389
chr10	43840001	43845000	1.01E-05
chr10	43841001	43846000	8.56E-05
chr10	43842001	43847000	2.70E-06
chr10	43855001	43860000	4.81E-08
chr10	43856001	43861000	7.18E-07
chr10	43857001	43862000	3.84E-05
chr10	43871001	43876000	0.000211
chr10	43872001	43877000	0.000187
chr10	43902001	43907000	5.30E-20
chr10	43903001	43908000	1.89E-20
chr10	43904001	43909000	2.56E-14
chr10	43905001	43910000	1.23E-15
chr10	43911001	43916000	1.06E-06
chr10	43912001	43917000	7.89E-07
chr10	43914001	43919000	0.000888
chr10	43946001	43951000	2.61E-09
chr10	43947001	43952000	2.29E-08
chr10	43994001	43999000	1.05E-06
chr10	43995001	44000000	0.000184
chr10	44008001	44013000	2.99E-09
chr10	44413001	44418000	1.99E-12
chr10	44414001	44419000	2.31E-09

chr10	44415001	44420000	7.15E-10
chr10	44416001	44421000	1.02E-06
chr10	44417001	44422000	1.57E-06
chr10	44444001	44449000	1.45E-12
chr10	44445001	44450000	2.43E-13
chr10	44446001	44451000	3.08E-09
chr10	44447001	44452000	6.67E-09
chr10	44448001	44453000	8.60E-07
chr10	44449001	44454000	8.02E-07
chr10	44450001	44455000	0.000306
chr10	44481001	44486000	1.09E-08
chr10	44636001	44641000	2.60E-10
chr10	44637001	44642000	2.83E-09
chr10	44652001	44657000	0.0005
chr10	44661001	44666000	9.14E-12
chr10	44696001	44701000	3.76E-05
chr10	44707001	44712000	0.00022
chr10	44708001	44713000	1.17E-06
chr10	44709001	44714000	0.0002
chr10	44742001	44747000	1.04E-07
chr10	44744001	44749000	0.000229
chr10	44972001	44977000	0.000424
chr10	44975001	44980000	2.71E-05
chr10	45275001	45280000	8.46E-06
chr10	45353001	45358000	0.000237
chr10	45758001	45763000	5.96E-05
chr10	45759001	45764000	0.000952
chr10	45936001	45941000	1.67E-06
chr10	46167001	46172000	2.59E-08
chr10	46295001	46300000	0.015378
chr10	46588001	46593000	0.004073
chr10	47159001	47164000	0.001286
chr10	47408001	47413000	0.000913
chr10	47960001	47965000	3.61E-06
chr10	48177001	48182000	0.027507
chr10	48854001	48859000	0.029464
chr10	48855001	48860000	0.029464
chr10	49268001	49273000	0.003806
chr10	49808001	49813000	2.82E-12
chr10	49809001	49814000	1.56E-12
chr10	49810001	49815000	8.66E-12
chr10	49811001	49816000	5.23E-08
chr10	50389001	50394000	8.28E-10
chr10	50390001	50395000	2.65E-09
chr10	50407001	50412000	8.41E-09
chr10	50408001	50413000	3.90E-10
chr10	50425001	50430000	0.000822
chr10	50443001	50448000	0.000127

chr10	50444001	50449000	4.02E-05
chr10	50769001	50774000	7.88E-05
chr10	50799001	50804000	0.001241
chr10	50806001	50811000	0.00067
chr10	50807001	50812000	5.01E-05
chr10	50872001	50877000	9.97E-05
chr10	50972001	50977000	1.71E-05
chr10	50973001	50978000	6.36E-06
chr10	51543001	51548000	2.26E-08
chr10	51773001	51778000	0.000173
chr10	51775001	51780000	0.000786
chr10	51781001	51786000	6.86E-05
chr10	51976001	51981000	0.003923
chr10	51997001	52002000	0.011373
chr10	52855001	52860000	4.09E-07
chr10	52856001	52861000	1.97E-07
chr10	54504001	54509000	6.89E-05
chr10	54928001	54933000	7.62E-09
chr10	54931001	54936000	3.37E-09
chr10	55218001	55223000	1.76E-08
chr10	55324001	55329000	0.001858
chr10	55443001	55448000	6.30E-09
chr10	55444001	55449000	1.32E-09
chr10	55497001	55502000	2.25E-06
chr10	55518001	55523000	1.39E-07
chr10	55521001	55526000	8.36E-06
chr10	55601001	55606000	8.31E-06
chr10	56099001	56104000	1.35E-05
chr10	56100001	56105000	6.05E-07
chr10	56132001	56137000	1.99E-07
chr10	56133001	56138000	2.19E-07
chr10	56193001	56198000	3.91E-06
chr10	56355001	56360000	9.45E-05
chr10	56592001	56597000	3.19E-13
chr10	56610001	56615000	2.87E-09
chr10	56669001	56674000	7.30E-05
chr10	57041001	57046000	1.82E-16
chr10	57042001	57047000	1.08E-15
chr10	57048001	57053000	1.76E-09
chr10	57098001	57103000	2.39E-09
chr10	57129001	57134000	5.74E-07
chr10	57237001	57242000	0.007889
chr10	57239001	57244000	0.006811
chr10	57240001	57245000	0.006803
chr10	57435001	57440000	7.97E-10
chr10	57772001	57777000	0.003109
chr10	58268001	58273000	1.40E-09
chr10	58558001	58563000	5.97E-07

chr10	59144001	59149000	2.76E-09
chr10	59342001	59347000	7.74E-05
chr10	59343001	59348000	5.59E-07
chr10	59797001	59802000	6.56E-05
chr10	59943001	59948000	0.000724
chr10	60090001	60095000	4.55E-09
chr10	60659001	60664000	1.53E-07
chr10	60730001	60735000	0.00104
chr10	61041001	61046000	4.84E-06
chr10	61081001	61086000	2.39E-05
chr10	61662001	61667000	1.68E-10
chr10	61663001	61668000	9.99E-16
chr10	63177001	63182000	1.41E-05
chr10	63178001	63183000	3.18E-05
chr10	64169001	64174000	1.88E-07
chr10	64511001	64516000	4.15E-05
chr10	64875001	64880000	0.001367
chr10	65029001	65034000	5.77E-09
chr10	65563001	65568000	1.63E-06
chr10	65564001	65569000	1.23E-05
chr10	65596001	65601000	0.000485
chr10	65697001	65702000	0.00456
chr10	65975001	65980000	8.88E-05
chr10	67620001	67625000	0.005778
chr10	67770001	67775000	6.80E-06
chr10	68092001	68097000	4.26E-10
chr10	68434001	68439000	5.96E-07
chr10	68472001	68477000	1.29E-09
chr10	69159001	69164000	0.001331
chr10	69330001	69335000	2.13E-07
chr10	69331001	69336000	5.20E-09
chr10	69332001	69337000	2.85E-09
chr10	69333001	69338000	2.11E-05
chr10	69334001	69339000	5.86E-06
chr10	69524001	69529000	1.46E-11
chr10	69608001	69613000	0.002425
chr10	69645001	69650000	4.67E-11
chr10	69832001	69837000	1.50E-08
chr10	69833001	69838000	5.42E-09
chr10	69834001	69839000	7.68E-09
chr10	69941001	69946000	1.73E-05
chr10	70801001	70806000	5.45E-08
chr10	70803001	70808000	1.02E-05
chr10	70804001	70809000	2.60E-05
chr10	70880001	70885000	5.06E-16
chr10	70881001	70886000	6.18E-20
chr10	70882001	70887000	2.06E-19
chr10	70883001	70888000	6.96E-09

chr10	70884001	70889000	9.54E-06
chr10	71046001	71051000	0.013924
chr10	71075001	71080000	6.31E-12
chr10	71076001	71081000	4.01E-07
chr10	71078001	71083000	0.000655
chr10	71079001	71084000	0.001534
chr10	71169001	71174000	8.80E-05
chr10	71170001	71175000	0.00014
chr10	71281001	71286000	0.000698
chr10	71282001	71287000	1.45E-06
chr10	71283001	71288000	1.66E-05
chr10	71284001	71289000	1.66E-05
chr10	71296001	71301000	0.001878
chr10	71298001	71303000	0.000238
chr10	71299001	71304000	2.19E-05
chr10	71300001	71305000	3.86E-07
chr10	71301001	71306000	4.16E-05
chr10	71302001	71307000	1.69E-05
chr10	71320001	71325000	0.000493
chr10	71321001	71326000	4.44E-05
chr10	71395001	71400000	9.14E-08
chr10	71445001	71450000	2.46E-05
chr10	71446001	71451000	0.000127
chr10	71453001	71458000	0.000293
chr10	71455001	71460000	7.95E-05
chr10	71649001	71654000	4.32E-07
chr10	71650001	71655000	3.00E-08
chr10	71675001	71680000	0.000147
chr10	71677001	71682000	0.000249
chr10	71902001	71907000	6.05E-13
chr10	71903001	71908000	9.86E-09
chr10	71904001	71909000	2.66E-07
chr10	72081001	72086000	0.0006
chr10	72151001	72156000	2.83E-05
chr10	72209001	72214000	0.000163
chr10	72279001	72284000	0.000317
chr10	72300001	72305000	0.001667
chr10	72301001	72306000	0.000671
chr10	72675001	72680000	4.14E-05
chr10	72973001	72978000	0.000509
chr10	73008001	73013000	0.000106
chr10	73270001	73275000	2.09E-05
chr10	73271001	73276000	9.02E-06
chr10	73317001	73322000	9.72E-06
chr10	73318001	73323000	1.81E-07
chr10	73319001	73324000	1.77E-08
chr10	73320001	73325000	2.34E-07
chr10	73321001	73326000	5.30E-06

chr10	73411001	73416000	0.005196
chr10	73412001	73417000	0.013082
chr10	73548001	73553000	5.50E-05
chr10	73566001	73571000	9.90E-07
chr10	73628001	73633000	3.84E-06
chr10	73814001	73819000	1.18E-05
chr10	73818001	73823000	0.000106
chr10	73846001	73851000	3.68E-05
chr10	74002001	74007000	0.003455
chr10	74016001	74021000	0.001518
chr10	74385001	74390000	1.54E-11
chr10	74923001	74928000	4.12E-05
chr10	74924001	74929000	5.91E-07
chr10	75040001	75045000	0.000305
chr10	75401001	75406000	0.000715
chr10	75504001	75509000	1.33E-09
chr10	75528001	75533000	5.45E-07
chr10	75529001	75534000	1.22E-07
chr10	75749001	75754000	5.56E-07
chr10	75750001	75755000	1.09E-08
chr10	75751001	75756000	4.84E-06
chr10	76011001	76016000	2.87E-05
chr10	76058001	76063000	0.023089
chr10	76551001	76556000	9.77E-08
chr10	76552001	76557000	4.46E-12
chr10	76553001	76558000	5.90E-10
chr10	76554001	76559000	6.29E-08
chr10	76555001	76560000	3.57E-07
chr10	76582001	76587000	2.22E-05
chr10	76583001	76588000	1.32E-05
chr10	76584001	76589000	1.06E-07
chr10	76585001	76590000	1.89E-07
chr10	76586001	76591000	2.25E-06
chr10	76992001	76997000	0.000374
chr10	78363001	78368000	6.88E-07
chr10	78595001	78600000	1.15E-06
chr10	79665001	79670000	5.43E-07
chr10	79827001	79832000	3.91E-05
chr10	79828001	79833000	3.22E-06
chr10	79841001	79846000	0.000187
chr10	79842001	79847000	0.001196
chr10	79859001	79864000	7.89E-06
chr10	79860001	79865000	0.000568
chr10	79899001	79904000	0.000251
chr10	79934001	79939000	1.28E-08
chr10	79935001	79940000	3.09E-08
chr10	79936001	79941000	1.07E-09
chr10	79937001	79942000	6.37E-09

chr10	79938001	79943000	7.91E-09
chr10	80039001	80044000	6.17E-05
chr10	80093001	80098000	0.000176
chr10	80094001	80099000	0.00024
chr10	80097001	80102000	2.87E-06
chr10	80099001	80104000	1.42E-05
chr10	80108001	80113000	8.46E-07
chr10	80133001	80138000	9.73E-07
chr10	80166001	80171000	7.90E-07
chr10	80174001	80179000	2.76E-07
chr10	80372001	80377000	0.00072
chr10	80373001	80378000	0.001069
chr10	80482001	80487000	1.97E-06
chr10	80483001	80488000	1.08E-06
chr10	80484001	80489000	3.91E-07
chr10	80787001	80792000	4.51E-05
chr10	80788001	80793000	7.06E-05
chr10	81101001	81106000	0.001248
chr10	81102001	81107000	3.91E-05
chr10	81103001	81108000	1.20E-14
chr10	81104001	81109000	5.83E-15
chr10	81105001	81110000	1.75E-08
chr10	81106001	81111000	3.35E-07
chr10	81107001	81112000	1.69E-06
chr10	81201001	81206000	0.017638
chr10	81202001	81207000	6.01E-08
chr10	81291001	81296000	0.000297
chr10	81878001	81883000	0.004118
chr10	81962001	81967000	1.41E-15
chr10	81963001	81968000	1.52E-19
chr10	81964001	81969000	1.37E-17
chr10	81995001	82000000	0.005547
chr10	82009001	82014000	0.00606
chr10	82088001	82093000	0.000431
chr10	82089001	82094000	0.000857
chr10	82090001	82095000	2.51E-06
chr10	82091001	82096000	1.23E-06
chr10	82092001	82097000	6.19E-06
chr10	82093001	82098000	8.32E-06
chr10	82230001	82235000	9.16E-07
chr10	82379001	82384000	5.96E-08
chr10	82434001	82439000	1.67E-12
chr10	82435001	82440000	4.27E-11
chr10	82456001	82461000	2.84E-05
chr10	82917001	82922000	9.34E-06
chr10	82990001	82995000	6.40E-07
chr10	82991001	82996000	2.33E-08
chr10	83023001	83028000	1.16E-10

chr10	83024001	83029000	8.45E-08
chr10	83096001	83101000	1.59E-10
chr10	83114001	83119000	3.69E-06
chr10	83115001	83120000	1.33E-06
chr10	83116001	83121000	1.36E-06
chr10	83209001	83214000	0.001643
chr10	83284001	83289000	7.71E-07
chr10	83285001	83290000	4.09E-08
chr10	83286001	83291000	2.52E-07
chr10	83310001	83315000	1.46E-06
chr10	83311001	83316000	2.16E-06
chr10	83312001	83317000	2.27E-06
chr10	83313001	83318000	1.91E-05
chr10	83398001	83403000	6.90E-07
chr10	83450001	83455000	6.93E-06
chr10	83733001	83738000	6.40E-07
chr10	83770001	83775000	9.15E-08
chr10	83771001	83776000	1.68E-07
chr10	83780001	83785000	7.42E-07
chr10	83818001	83823000	1.81E-07
chr10	83819001	83824000	3.66E-06
chr10	83870001	83875000	1.86E-12
chr10	83871001	83876000	4.27E-12
chr10	83884001	83889000	6.26E-12
chr10	83885001	83890000	3.71E-11
chr10	83886001	83891000	1.04E-08
chr10	83985001	83990000	2.16E-06
chr10	83986001	83991000	2.83E-08
chr10	83987001	83992000	1.29E-08
chr10	84014001	84019000	3.42E-09
chr10	84057001	84062000	5.61E-07
chr10	84058001	84063000	3.05E-08
chr10	84059001	84064000	1.85E-10
chr10	84060001	84065000	2.82E-11
chr10	84061001	84066000	1.78E-10
chr10	84127001	84132000	7.78E-05
chr10	84128001	84133000	3.25E-05
chr10	84194001	84199000	2.88E-07
chr10	84220001	84225000	5.16E-08
chr10	84221001	84226000	8.35E-10
chr10	84222001	84227000	2.07E-05
chr10	84613001	84618000	5.69E-09
chr10	84614001	84619000	1.16E-09
chr10	84620001	84625000	7.00E-11
chr10	84621001	84626000	1.84E-12
chr10	84622001	84627000	8.26E-11
chr10	84653001	84658000	3.41E-10
chr10	84654001	84659000	1.73E-11

chr10	84655001	84660000	2.97E-08
chr10	84656001	84661000	1.66E-12
chr10	84657001	84662000	5.61E-14
chr10	84699001	84704000	1.87E-05
chr10	84730001	84735000	6.38E-06
chr10	84736001	84741000	4.51E-05
chr10	84763001	84768000	1.26E-07
chr10	84764001	84769000	3.20E-08
chr10	84846001	84851000	1.75E-06
chr10	84848001	84853000	8.04E-08
chr10	84849001	84854000	1.52E-06
chr10	84884001	84889000	7.46E-10
chr10	84885001	84890000	1.84E-08
chr10	84886001	84891000	1.84E-08
chr10	84887001	84892000	2.07E-06
chr10	84913001	84918000	1.28E-09
chr10	84914001	84919000	7.40E-08
chr10	84915001	84920000	1.59E-10
chr10	84916001	84921000	7.29E-09
chr10	84926001	84931000	1.73E-06
chr10	84927001	84932000	7.07E-06
chr10	84928001	84933000	3.17E-06
chr10	84929001	84934000	5.75E-05
chr10	84977001	84982000	3.62E-06
chr10	84978001	84983000	4.82E-05
chr10	85005001	85010000	7.14E-06
chr10	85006001	85011000	2.29E-07
chr10	85007001	85012000	8.64E-06
chr10	85008001	85013000	8.66E-05
chr10	85145001	85150000	1.34E-06
chr10	85185001	85190000	7.07E-08
chr10	85194001	85199000	3.35E-07
chr10	85195001	85200000	1.97E-08
chr10	85325001	85330000	3.27E-07
chr10	85326001	85331000	2.43E-08
chr10	85327001	85332000	4.47E-07
chr10	85328001	85333000	2.19E-08
chr10	85329001	85334000	5.19E-07
chr10	85331001	85336000	4.13E-05
chr10	85332001	85337000	0.000403
chr10	85350001	85355000	1.99E-06
chr10	85417001	85422000	4.44E-07
chr10	85505001	85510000	1.42E-07
chr10	85506001	85511000	6.92E-07
chr10	85507001	85512000	9.53E-07
chr10	85508001	85513000	4.19E-07
chr10	85509001	85514000	0.000615
chr10	85521001	85526000	1.71E-06

chr10	85522001	85527000	6.88E-07
chr10	85550001	85555000	1.29E-07
chr10	85551001	85556000	1.68E-07
chr10	85560001	85565000	6.51E-06
chr10	85561001	85566000	1.40E-07
chr10	85562001	85567000	1.11E-08
chr10	85666001	85671000	7.24E-05
chr10	85667001	85672000	1.36E-05
chr10	85668001	85673000	0.000304
chr10	85669001	85674000	0.000478
chr10	85670001	85675000	0.001252
chr10	85678001	85683000	2.00E-07
chr10	85680001	85685000	1.83E-06
chr10	85728001	85733000	4.02E-06
chr10	85759001	85764000	9.59E-07
chr10	85789001	85794000	3.93E-05
chr10	85891001	85896000	8.25E-06
chr10	85892001	85897000	1.42E-08
chr10	85893001	85898000	1.70E-06
chr10	85894001	85899000	3.56E-09
chr10	85929001	85934000	2.66E-10
chr10	85930001	85935000	2.39E-09
chr10	85964001	85969000	0.000263
chr10	85965001	85970000	1.12E-05
chr10	86012001	86017000	3.07E-07
chr10	86013001	86018000	5.29E-11
chr10	86021001	86026000	6.04E-06
chr10	86064001	86069000	0.000771
chr10	86321001	86326000	4.35E-10
chr10	86361001	86366000	0.000152
chr10	86393001	86398000	2.56E-05
chr10	86463001	86468000	9.35E-08
chr10	86505001	86510000	6.49E-06
chr10	86697001	86702000	1.48E-06
chr10	86924001	86929000	6.11E-07
chr10	87143001	87148000	7.49E-05
chr10	87144001	87149000	1.59E-05
chr10	87229001	87234000	0.000153
chr10	87359001	87364000	5.65E-05
chr10	87360001	87365000	7.35E-07
chr10	87372001	87377000	4.35E-06
chr10	87373001	87378000	6.91E-07
chr10	87374001	87379000	1.70E-09
chr10	87429001	87434000	4.85E-06
chr10	87508001	87513000	2.68E-05
chr10	87509001	87514000	6.87E-05
chr10	87510001	87515000	9.94E-05
chr10	87535001	87540000	6.07E-07

chr10	87536001	87541000	1.97E-06
chr10	87537001	87542000	2.21E-05
chr10	87560001	87565000	1.20E-06
chr10	87561001	87566000	6.37E-05
chr10	87648001	87653000	3.65E-07
chr10	87723001	87728000	0.002949
chr10	87738001	87743000	1.10E-05
chr10	87739001	87744000	5.93E-05
chr10	87740001	87745000	0.001195
chr10	87787001	87792000	3.22E-08
chr10	87788001	87793000	1.65E-07
chr10	87789001	87794000	7.92E-08
chr10	87790001	87795000	3.51E-08
chr10	87835001	87840000	1.18E-05
chr10	87836001	87841000	4.37E-07
chr10	87837001	87842000	1.57E-06
chr10	87838001	87843000	5.07E-08
chr10	87839001	87844000	1.96E-08
chr10	87856001	87861000	1.33E-07
chr10	87858001	87863000	4.76E-08
chr10	87881001	87886000	1.24E-07
chr10	87887001	87892000	4.28E-05
chr10	87888001	87893000	9.16E-06
chr10	87889001	87894000	2.32E-05
chr10	87890001	87895000	4.07E-06
chr10	87891001	87896000	8.90E-08
chr10	87917001	87922000	1.16E-09
chr10	87918001	87923000	7.91E-10
chr10	87919001	87924000	3.17E-13
chr10	87920001	87925000	1.94E-05
chr10	87934001	87939000	1.47E-06
chr10	87936001	87941000	9.09E-10
chr10	87937001	87942000	3.67E-10
chr10	87938001	87943000	7.31E-12
chr10	88044001	88049000	0.000253
chr10	88045001	88050000	3.50E-05
chr10	88046001	88051000	4.07E-07
chr10	88047001	88052000	9.58E-08
chr10	88048001	88053000	2.16E-07
chr10	88061001	88066000	0.000696
chr10	88062001	88067000	1.37E-06
chr10	88088001	88093000	1.64E-06
chr10	88089001	88094000	0.000549
chr10	88090001	88095000	8.45E-07
chr10	88091001	88096000	8.20E-07
chr10	88092001	88097000	2.11E-05
chr10	88103001	88108000	0.003713
chr10	88104001	88109000	0.002095

chr10	88105001	88110000	0.018015
chr10	88106001	88111000	0.004727
chr10	88108001	88113000	0.006562
chr10	88109001	88114000	0.002138
chr10	88110001	88115000	0.000279
chr10	88111001	88116000	0.000172
chr10	88112001	88117000	0.003672
chr10	88157001	88162000	1.10E-11
chr10	88158001	88163000	2.29E-06
chr10	88160001	88165000	0.000427
chr10	88161001	88166000	0.000359
chr10	88167001	88172000	4.11E-07
chr10	88425001	88430000	0.005556
chr10	88426001	88431000	0.003562
chr10	88427001	88432000	0.009018
chr10	88788001	88793000	0.003961
chr10	88789001	88794000	0.009267
chr10	88839001	88844000	1.77E-08
chr10	88840001	88845000	0.000397
chr10	88961001	88966000	1.33E-13
chr10	90482001	90487000	2.26E-07
chr10	91749001	91754000	8.88E-07
chr10	91870001	91875000	0.003325
chr10	91871001	91876000	0.001451
chr10	91954001	91959000	0.000593
chr10	91955001	91960000	0.000585
chr10	92045001	92050000	0.003873
chr10	92174001	92179000	4.21E-06
chr10	92246001	92251000	1.93E-09
chr10	92419001	92424000	3.70E-06
chr10	92705001	92710000	2.44E-05
chr10	92708001	92713000	3.08E-05
chr10	92906001	92911000	2.61E-05
chr10	93417001	93422000	6.22E-10
chr10	93418001	93423000	7.68E-10
chr10	93419001	93424000	2.41E-08
chr10	93930001	93935000	0.004588
chr10	94018001	94023000	0.000881
chr10	94020001	94025000	0.00134
chr10	94120001	94125000	3.95E-06
chr10	94121001	94126000	0.000369
chr10	94142001	94147000	0.005169
chr10	94167001	94172000	3.44E-06
chr10	94168001	94173000	6.41E-07
chr10	94169001	94174000	2.00E-06
chr10	95203001	95208000	1.33E-05
chr10	95204001	95209000	1.04E-05
chr10	95205001	95210000	3.40E-06

chr10	95337001	95342000	2.92E-06
chr10	95338001	95343000	2.83E-08
chr10	95339001	95344000	4.50E-07
chr10	95570001	95575000	8.06E-09
chr10	95622001	95627000	1.20E-07
chr10	95623001	95628000	3.99E-07
chr10	95782001	95787000	0.000303
chr10	95783001	95788000	7.13E-05
chr10	96301001	96306000	0.00156
chr10	96302001	96307000	2.50E-05
chr10	96414001	96419000	0.000308
chr10	96597001	96602000	1.17E-09
chr10	97412001	97417000	1.59E-13
chr10	97413001	97418000	8.07E-12
chr10	97414001	97419000	6.38E-12
chr10	97415001	97420000	1.95E-07
chr10	97416001	97421000	2.92E-07
chr10	97480001	97485000	2.50E-06
chr10	97716001	97721000	9.44E-05
chr10	97717001	97722000	1.85E-07
chr10	97718001	97723000	2.78E-07
chr10	97719001	97724000	8.16E-09
chr10	97720001	97725000	3.50E-07
chr10	98928001	98933000	7.07E-07
chr10	98929001	98934000	4.75E-09
chr10	98930001	98935000	2.23E-10
chr10	98943001	98948000	0.000879
chr10	98959001	98964000	1.06E-11
chr10	98960001	98965000	2.19E-10
chr10	98961001	98966000	3.88E-08
chr10	98967001	98972000	6.13E-10
chr10	98968001	98973000	7.11E-12
chr10	99075001	99080000	4.64E-05
chr10	99077001	99082000	1.46E-07
chr10	99201001	99206000	2.04E-10
chr10	99202001	99207000	9.51E-14
chr10	99549001	99554000	0.005001
chr10	99550001	99555000	0.027642
chr10	99610001	99615000	0.000312
chr10	100042001	100047000	9.68E-06
chr10	100251001	100256000	0.007303
chr10	100383001	100388000	2.71E-11
chr10	100460001	100465000	0.00431
chr10	100510001	100515000	0.001354
chr10	100583001	100588000	4.36E-06
chr10	100980001	100985000	1.89E-06
chr10	101230001	101235000	2.45E-08
chr10	101265001	101270000	9.86E-05

chr10	101620001	101625000	3.35E-06
chr10	101628001	101633000	7.34E-05
chr10	101797001	101802000	0.000404
chr10	101799001	101804000	0.000473
chr10	101985001	101990000	5.30E-10
chr10	101986001	101991000	2.21E-13
chr10	102046001	102051000	3.22E-06
chr10	102074001	102079000	5.80E-07
chr10	102275001	102280000	5.01E-07
chr10	102276001	102281000	5.90E-08
chr10	102347001	102352000	0.000146
chr10	102356001	102361000	0.000129
chr10	102418001	102423000	8.80E-06
chr10	102419001	102424000	0.002023
chr10	102420001	102425000	0.000171
chr10	102421001	102426000	0.000679
chr10	102422001	102427000	0.001122
chr10	102538001	102543000	0.00118
chr10	102539001	102544000	0.001087
chr10	102772001	102777000	4.53E-06
chr10	102837001	102842000	0.000595
chr10	102865001	102870000	1.44E-05
chr10	102866001	102871000	0.003004
chr10	103057001	103062000	0.000334
chr10	103086001	103091000	3.80E-06
chr10	103087001	103092000	4.99E-05
chr10	103450001	103455000	3.90E-06
chr10	103451001	103456000	2.90E-10
chr10	103452001	103457000	5.58E-13
chr10	103453001	103458000	2.27E-16
chr10	103454001	103459000	1.16E-10
chr10	103473001	103478000	9.54E-08
chr10	103584001	103589000	2.46E-08
chr10	103730001	103735000	8.29E-06
chr10	103823001	103828000	0.015064
chr10	103834001	103839000	0.001242
chr10	103837001	103842000	0.010641
chr10	103926001	103931000	1.74E-08
chr10	103927001	103932000	1.90E-06
chr10	104188001	104193000	3.98E-09
chr10	104189001	104194000	6.78E-08
chr10	104190001	104195000	1.70E-07
chr10	104191001	104196000	6.02E-06
chr10	104192001	104197000	2.24E-05
chr10	104208001	104213000	0.000101
chr10	104217001	104222000	7.21E-07
chr10	104263001	104268000	5.60E-08
chr10	104474001	104479000	0.000665

chr10	104605001	104610000	3.14E-06
chr10	104749001	104754000	7.05E-09
chr10	104952001	104957000	3.03E-07
chr10	105017001	105022000	0.001315
chr10	105018001	105023000	0.004321
chr10	105033001	105038000	4.05E-05
chr10	105035001	105040000	7.18E-05
chr10	105036001	105041000	1.23E-05
chr10	105037001	105042000	2.67E-05
chr10	105218001	105223000	0.00169
chr10	105250001	105255000	0.003057
chr10	105252001	105257000	0.011235
chr10	105253001	105258000	0.001777
chr10	105313001	105318000	4.46E-07
chr10	105420001	105425000	3.43E-06
chr10	105435001	105440000	5.10E-07
chr10	105437001	105442000	6.97E-06
chr10	106125001	106130000	1.60E-08
chr10	106126001	106131000	1.52E-10
chr10	106153001	106158000	2.51E-06
chr10	106162001	106167000	7.26E-11
chr10	106194001	106199000	3.34E-07
chr10	106245001	106250000	6.62E-05
chr10	106246001	106251000	0.000286
chr10	106267001	106272000	0.000262
chr10	106349001	106354000	1.72E-08
chr10	106352001	106357000	6.70E-08
chr10	106353001	106358000	1.96E-08
chr10	106402001	106407000	6.72E-05
chr10	106486001	106491000	1.12E-05
chr10	106487001	106492000	2.72E-06
chr10	106529001	106534000	1.39E-06
chr10	106530001	106535000	1.24E-06
chr10	106725001	106730000	9.75E-09
chr10	106726001	106731000	9.73E-11
chr10	106727001	106732000	4.25E-10
chr10	106845001	106850000	4.10E-07
chr10	106846001	106851000	7.05E-07
chr10	106847001	106852000	3.36E-08
chr10	106848001	106853000	1.01E-09
chr10	106849001	106854000	1.57E-07
chr10	106851001	106856000	4.59E-07
chr10	106860001	106865000	1.42E-08
chr10	106861001	106866000	4.76E-08
chr10	106935001	106940000	2.73E-12
chr10	106936001	106941000	2.64E-11
chr10	106937001	106942000	1.47E-07
chr10	107066001	107071000	3.22E-11

chr10	107080001	107085000	8.60E-11
chr10	107081001	107086000	4.18E-11
chr10	107082001	107087000	1.51E-07
chr10	107083001	107088000	7.78E-08
chr10	107151001	107156000	1.28E-13
chr10	107200001	107205000	1.19E-07
chr10	107240001	107245000	7.74E-14
chr10	107241001	107246000	3.03E-12
chr10	107242001	107247000	1.38E-12
chr10	107280001	107285000	7.06E-07
chr10	107319001	107324000	5.89E-06
chr10	107320001	107325000	1.19E-05
chr10	107321001	107326000	1.25E-05
chr10	107322001	107327000	2.41E-06
chr10	107323001	107328000	8.46E-07
chr10	107406001	107411000	1.00E-06
chr10	107412001	107417000	4.53E-09
chr10	107425001	107430000	0.000203
chr10	107426001	107431000	1.14E-05
chr10	107428001	107433000	1.60E-05
chr10	107463001	107468000	1.40E-07
chr10	107464001	107469000	4.46E-08
chr10	107522001	107527000	2.91E-08
chr10	107570001	107575000	6.32E-08
chr10	107572001	107577000	7.67E-08
chr10	107573001	107578000	8.31E-07
chr10	107612001	107617000	4.47E-06
chr10	107695001	107700000	1.60E-05
chr10	107963001	107968000	5.99E-09
chr10	107964001	107969000	1.15E-11
chr10	107965001	107970000	6.86E-09
chr10	108027001	108032000	0.012532
chr10	108028001	108033000	0.0049
chr10	108553001	108558000	3.08E-07
chr10	108554001	108559000	7.99E-07
chr10	108556001	108561000	1.85E-06
chr10	108559001	108564000	3.64E-05
chr10	108565001	108570000	0.00022
chr10	108566001	108571000	0.003041
chr10	108624001	108629000	0.000146
chr10	108626001	108631000	2.13E-05
chr10	108627001	108632000	0.000155
chr10	108701001	108706000	7.95E-07
chr10	108778001	108783000	1.71E-06
chr10	108929001	108934000	0.000512
chr10	108930001	108935000	2.13E-08
chr10	108931001	108936000	4.03E-10
chr10	108943001	108948000	1.41E-09

chr10	108967001	108972000	5.53E-06
chr10	108968001	108973000	1.51E-05
chr10	108974001	108979000	7.37E-06
chr10	108975001	108980000	1.53E-06
chr10	109037001	109042000	8.72E-09
chr10	109174001	109179000	8.09E-07
chr10	109280001	109285000	5.62E-08
chr10	109319001	109324000	2.34E-05
chr10	109321001	109326000	0.000229
chr10	109356001	109361000	1.88E-06
chr10	109357001	109362000	2.14E-08
chr10	109358001	109363000	2.95E-08
chr10	109453001	109458000	3.90E-06
chr10	109522001	109527000	2.80E-11
chr10	109523001	109528000	6.24E-11
chr10	109524001	109529000	5.38E-12
chr10	109525001	109530000	1.58E-13
chr10	109526001	109531000	3.45E-13
chr10	109527001	109532000	1.95E-07
chr10	109528001	109533000	7.29E-07
chr10	109732001	109737000	2.93E-05
chr10	109785001	109790000	2.21E-08
chr10	109884001	109889000	2.42E-08
chr10	110073001	110078000	4.58E-05
chr10	110541001	110546000	2.33E-06
chr10	110570001	110575000	4.36E-06
chr10	110677001	110682000	0.00416
chr10	110842001	110847000	0.000844
chr10	110913001	110918000	1.31E-06
chr10	110914001	110919000	3.36E-05
chr10	110927001	110932000	4.57E-07
chr10	110928001	110933000	1.48E-07
chr10	110943001	110948000	2.57E-07
chr10	111408001	111413000	8.13E-12
chr10	111409001	111414000	2.15E-11
chr10	111410001	111415000	1.47E-11
chr10	111411001	111416000	2.17E-08
chr10	111568001	111573000	0.000565
chr10	111569001	111574000	0.009155
chr10	111570001	111575000	0.009155
chr10	111577001	111582000	0.000504
chr10	111763001	111768000	6.09E-10
chr10	111764001	111769000	8.23E-08
chr10	111929001	111934000	4.44E-09
chr10	111931001	111936000	6.63E-11
chr10	112197001	112202000	0.000126
chr10	112199001	112204000	6.74E-05
chr10	112600001	112605000	0.000584

chr10	112817001	112822000	0.011696
chr10	112818001	112823000	0.010656
chr10	112852001	112857000	1.37E-05
chr10	113093001	113098000	1.27E-05
chr10	113094001	113099000	1.82E-06
chr10	113335001	113340000	7.69E-06
chr10	113336001	113341000	4.86E-05
chr10	113362001	113367000	1.66E-05
chr10	113407001	113412000	3.61E-07
chr10	113408001	113413000	5.80E-07
chr10	113847001	113852000	1.49E-07
chr10	114070001	114075000	1.32E-09
chr10	114071001	114076000	1.97E-13
chr10	114072001	114077000	5.16E-12
chr10	114073001	114078000	1.93E-14
chr10	115337001	115342000	0.0024
chr10	115338001	115343000	0.000735
chr10	115718001	115723000	3.65E-09
chr10	117103001	117108000	1.62E-07
chr10	117104001	117109000	1.37E-07
chr10	117556001	117561000	2.67E-07
chr10	117702001	117707000	6.43E-05
chr10	118296001	118301000	3.82E-05
chr10	118299001	118304000	5.70E-07
chr10	118300001	118305000	2.36E-08
chr10	118373001	118378000	1.92E-05
chr10	118786001	118791000	0.008403
chr10	118792001	118797000	6.27E-05
chr10	118793001	118798000	9.63E-05
chr10	118820001	118825000	8.67E-10
chr10	118957001	118962000	9.32E-05
chr10	119221001	119226000	7.57E-05
chr10	119222001	119227000	8.58E-05
chr10	119253001	119258000	1.73E-09
chr10	119255001	119260000	1.78E-09
chr10	119357001	119362000	8.27E-09
chr10	119358001	119363000	2.01E-10
chr10	119454001	119459000	1.22E-06
chr10	119528001	119533000	1.44E-05
chr10	119649001	119654000	3.88E-08
chr10	119650001	119655000	2.17E-07
chr10	119651001	119656000	1.99E-05
chr10	119978001	119983000	2.31E-07
chr10	119979001	119984000	1.23E-06
chr10	120750001	120755000	0.001263
chr10	120751001	120756000	1.41E-07
chr10	121301001	121306000	0.000726
chr10	121415001	121420000	1.01E-05

chr10	121436001	121441000	0.003049
chr10	121437001	121442000	0.002759
chr10	122121001	122126000	2.80E-06
chr10	122193001	122198000	2.13E-05
chr10	122236001	122241000	3.51E-06
chr10	122352001	122357000	1.13E-05
chr10	122514001	122519000	3.20E-07
chr10	122515001	122520000	1.87E-05
chr10	122915001	122920000	5.76E-05
chr10	123702001	123707000	9.98E-05
chr10	124439001	124444000	8.07E-07
chr10	124440001	124445000	8.27E-10
chr10	124441001	124446000	1.77E-06
chr10	124609001	124614000	0.000156
chr10	124710001	124715000	3.70E-10
chr10	124969001	124974000	5.34E-08
chr10	124975001	124980000	3.65E-06
chr10	124976001	124981000	6.51E-05
chr10	124978001	124983000	1.05E-06
chr10	124995001	125000000	8.33E-06
chr10	125048001	125053000	2.63E-05
chr10	125083001	125088000	2.57E-06
chr10	125084001	125089000	1.12E-05
chr10	125233001	125238000	3.35E-09
chr10	125234001	125239000	1.36E-09
chr10	125301001	125306000	5.09E-06
chr10	125330001	125335000	5.61E-07
chr10	125331001	125336000	1.55E-05
chr10	125411001	125416000	0.000664
chr10	125412001	125417000	4.84E-05
chr10	125413001	125418000	1.03E-05
chr10	125414001	125419000	2.50E-06
chr10	125438001	125443000	6.72E-05
chr10	125439001	125444000	2.89E-06
chr10	125677001	125682000	0.00023
chr10	125695001	125700000	0.000294
chr10	125952001	125957000	2.42E-07
chr10	125953001	125958000	1.88E-08
chr10	126122001	126127000	0.020517
chr10	126157001	126162000	2.81E-07
chr10	126158001	126163000	1.40E-08
chr10	126160001	126165000	2.61E-06
chr10	126166001	126171000	0.000491
chr10	126601001	126606000	1.62E-06
chr10	126602001	126607000	1.11E-13
chr10	126604001	126609000	7.15E-11
chr10	126605001	126610000	1.87E-11
chr10	126606001	126611000	3.45E-08

chr10	126837001	126842000	1.21E-06
chr10	126838001	126843000	7.97E-09
chr10	127743001	127748000	1.24E-08
chr10	128615001	128620000	0.001403
chr10	129340001	129345000	4.32E-07
chr10	129401001	129406000	1.04E-05
chr10	129402001	129407000	4.19E-05
chr10	129450001	129455000	8.79E-07
chr10	129507001	129512000	5.73E-06
chr10	129517001	129522000	0.003891
chr10	129640001	129645000	2.36E-06
chr10	129663001	129668000	1.38E-05
chr10	129921001	129926000	2.71E-09
chr10	129924001	129929000	7.51E-10
chr10	129954001	129959000	1.95E-06
chr10	129966001	129971000	1.32E-05
chr10	129967001	129972000	9.96E-05
chr10	130002001	130007000	0.003853
chr10	130064001	130069000	3.39E-07
chr10	130245001	130250000	6.13E-06
chr10	130246001	130251000	4.26E-07
chr10	130289001	130294000	2.39E-05
chr10	130507001	130512000	7.17E-09
chr10	130508001	130513000	8.25E-07
chr10	130509001	130514000	5.67E-10
chr10	130510001	130515000	1.66E-09
chr10	130593001	130598000	1.34E-07
chr10	130595001	130600000	4.08E-08
chr10	130614001	130619000	4.96E-08
chr10	130615001	130620000	3.47E-08
chr10	130647001	130652000	1.03E-10
chr10	130648001	130653000	1.51E-17
chr10	130649001	130654000	7.86E-19
chr10	130650001	130655000	1.73E-19
chr10	130685001	130690000	1.34E-06
chr10	130762001	130767000	2.24E-10
chr10	130775001	130780000	1.38E-05
chr10	130782001	130787000	2.83E-08
chr10	130783001	130788000	3.58E-08
chr10	130807001	130812000	6.72E-08
chr10	130808001	130813000	2.66E-09
chr10	130809001	130814000	1.29E-06
chr10	130898001	130903000	5.02E-08
chr10	130916001	130921000	0.000108
chr10	131112001	131117000	0.000959
chr10	131113001	131118000	0.000873
chr10	131727001	131732000	2.76E-08
chr10	131728001	131733000	6.74E-07

chr10	131799001	131804000	4.45E-05
chr10	131842001	131847000	4.79E-11
chr10	131843001	131848000	7.27E-15
chr10	131844001	131849000	5.79E-07
chr10	131984001	131989000	0.005094
chr10	131989001	131994000	6.37E-05
chr10	131990001	131995000	4.65E-06
chr10	131991001	131996000	8.34E-05
chr10	132027001	132032000	2.76E-06
chr10	132028001	132033000	1.53E-05
chr10	132043001	132048000	4.18E-07
chr10	132250001	132255000	5.09E-06
chr10	132251001	132256000	0.000833
chr10	132429001	132434000	1.04E-09
chr10	132460001	132465000	1.36E-11
chr10	132464001	132469000	4.46E-09
chr10	132493001	132498000	2.30E-07
chr10	132582001	132587000	0.007287
chr10	132605001	132610000	3.99E-05
chr10	132649001	132654000	9.74E-09
chr10	132714001	132719000	1.64E-17
chr10	133183001	133188000	0.000118
chr10	133210001	133215000	2.20E-05
chr10	133315001	133320000	1.13E-09
chr10	133316001	133321000	1.76E-08
chr10	133317001	133322000	1.91E-09
chr10	133318001	133323000	1.54E-09
chr10	133328001	133333000	0.000258
chr10	133337001	133342000	1.14E-08
chr10	133338001	133343000	2.57E-10
chr10	133454001	133459000	0.001406
chr10	133660001	133665000	0.000174
chr10	133661001	133666000	0.000133
chr10	133662001	133667000	9.05E-05
chr10	133834001	133839000	5.89E-05
chr10	133864001	133869000	6.69E-07
chr10	133888001	133893000	0.000808
chr10	133906001	133911000	3.07E-05
chr10	133907001	133912000	0.0011
chr10	133917001	133922000	1.79E-05
chr10	133918001	133923000	2.06E-05
chr10	133934001	133939000	2.55E-10
chr10	133935001	133940000	1.19E-10
chr10	133973001	133978000	2.57E-05
chr10	134034001	134039000	5.04E-05
chr10	134101001	134106000	6.43E-10
chr10	134102001	134107000	6.52E-11
chr10	134215001	134220000	0.024717

chr10	134217001	134222000	4.36E-08
chr10	134221001	134226000	0.000225
chr10	134229001	134234000	4.73E-07
chr10	134230001	134235000	1.51E-09
chr10	134231001	134236000	9.19E-05
chr10	134232001	134237000	8.69E-08
chr10	134233001	134238000	7.87E-06
chr10	134264001	134269000	1.54E-05
chr10	134265001	134270000	5.56E-05
chr10	134266001	134271000	4.84E-05
chr10	134267001	134272000	0.002693
chr10	134277001	134282000	0.000124
chr10	134300001	134305000	7.12E-05
chr10	134301001	134306000	0.000141
chr10	134465001	134470000	0.000503
chr10	134467001	134472000	2.61E-10
chr10	134468001	134473000	1.56E-09
chr10	134555001	134560000	5.91E-06
chr10	134559001	134564000	6.66E-06
chr10	134723001	134728000	0.004897
chr10	134724001	134729000	3.52E-05
chr10	134751001	134756000	3.97E-10
chr10	134752001	134757000	1.64E-08
chr10	134855001	134860000	5.42E-08
chr10	134856001	134861000	1.46E-07
chr10	134857001	134862000	7.27E-06
chr10	134858001	134863000	1.31E-06
chr10	134864001	134869000	1.11E-07
chr10	134865001	134870000	3.36E-09
chr10	134866001	134871000	2.81E-06
chr10	134867001	134872000	9.41E-07
chr10	134871001	134876000	0.00011
chr10	134872001	134877000	6.30E-11
chr10	134873001	134878000	6.83E-11
chr10	134922001	134927000	1.69E-05
chr10	134923001	134928000	1.92E-07
chr10	134957001	134962000	6.23E-15
chr10	135017001	135022000	3.31E-10
chr10	135018001	135023000	1.41E-06
chr10	135032001	135037000	2.46E-08
chr10	135033001	135038000	6.01E-07
chr10	135034001	135039000	3.53E-08
chr10	135075001	135080000	0.001944
chr10	135126001	135131000	4.81E-11
chr10	135127001	135132000	3.23E-08
chr10	135146001	135151000	0.008023
chr10	135271001	135276000	1.55E-05
chr10	135272001	135277000	1.44E-06

chr10	135273001	135278000	5.92E-07
chr10	135274001	135279000	3.19E-09
chr10	135276001	135281000	3.88E-06
chr10	135386001	135391000	0.000761
chr11	168001	173000	0.009076
chr11	169001	174000	0.041173
chr11	170001	175000	0.041173
chr11	208001	213000	6.93E-06
chr11	285001	290000	1.77E-09
chr11	286001	291000	1.82E-15
chr11	287001	292000	4.63E-08
chr11	348001	353000	0.000171
chr11	367001	372000	0.007666
chr11	504001	509000	9.76E-09
chr11	505001	510000	1.24E-11
chr11	506001	511000	4.77E-07
chr11	549001	554000	0.004167
chr11	743001	748000	0.000128
chr11	744001	749000	0.000118
chr11	745001	750000	5.15E-06
chr11	746001	751000	4.04E-05
chr11	776001	781000	0.00839
chr11	793001	798000	4.06E-08
chr11	794001	799000	1.49E-11
chr11	796001	801000	4.51E-13
chr11	797001	802000	1.91E-13
chr11	798001	803000	8.91E-09
chr11	815001	820000	0.027295
chr11	816001	821000	0.00186
chr11	1049001	1054000	0.001329
chr11	1050001	1055000	1.27E-08
chr11	1051001	1056000	2.30E-05
chr11	1113001	1118000	3.34E-06
chr11	1114001	1119000	0.001707
chr11	1255001	1260000	1.90E-06
chr11	1260001	1265000	0.008065
chr11	1261001	1266000	0.009599
chr11	1262001	1267000	0.000737
chr11	1416001	1421000	0.000238
chr11	1475001	1480000	0.000104
chr11	1572001	1577000	0.000188
chr11	1624001	1629000	0.003557
chr11	1625001	1630000	0.003889
chr11	1650001	1655000	3.16E-05
chr11	1651001	1656000	2.64E-05
chr11	1674001	1679000	1.04E-05
chr11	1782001	1787000	7.60E-05
chr11	1783001	1788000	1.18E-05

chr11	1792001	1797000	6.26E-05
chr11	1793001	1798000	9.37E-07
chr11	1795001	1800000	0.009154
chr11	1796001	1801000	0.046049
chr11	1888001	1893000	2.65E-05
chr11	1889001	1894000	4.01E-07
chr11	1890001	1895000	7.80E-08
chr11	1895001	1900000	0.010109
chr11	1908001	1913000	0.000265
chr11	1990001	1995000	5.17E-05
chr11	2050001	2055000	0.005389
chr11	2051001	2056000	0.005228
chr11	2316001	2321000	6.15E-07
chr11	2318001	2323000	0.000147
chr11	2327001	2332000	0.007152
chr11	2452001	2457000	5.33E-07
chr11	2453001	2458000	2.00E-06
chr11	2454001	2459000	5.38E-06
chr11	2684001	2689000	0.001466
chr11	2745001	2750000	2.51E-06
chr11	2813001	2818000	8.78E-09
chr11	2868001	2873000	0.009622
chr11	2879001	2884000	0.021041
chr11	2880001	2885000	0.013071
chr11	3013001	3018000	3.54E-13
chr11	3221001	3226000	4.17E-07
chr11	3222001	3227000	3.29E-10
chr11	3249001	3254000	0.000591
chr11	3250001	3255000	0.000114
chr11	3263001	3268000	3.75E-06
chr11	3264001	3269000	2.96E-07
chr11	3265001	3270000	2.96E-07
chr11	3266001	3271000	0.00122
chr11	3267001	3272000	0.002908
chr11	3338001	3343000	0.012031
chr11	3339001	3344000	0.012031
chr11	3340001	3345000	0.00884
chr11	3356001	3361000	0.000399
chr11	3357001	3362000	5.68E-05
chr11	3400001	3405000	4.25E-07
chr11	3766001	3771000	8.89E-06
chr11	3859001	3864000	5.10E-06
chr11	3860001	3865000	4.74E-06
chr11	3861001	3866000	1.06E-05
chr11	3862001	3867000	1.40E-05
chr11	3877001	3882000	0.040791
chr11	4311001	4316000	5.37E-05
chr11	4312001	4317000	2.36E-05

chr11	4313001	4318000	0.004776
chr11	4315001	4320000	0.035117
chr11	4437001	4442000	2.56E-06
chr11	4652001	4657000	5.53E-06
chr11	4667001	4672000	0.000203
chr11	5173001	5178000	2.86E-08
chr11	5453001	5458000	2.06E-06
chr11	5703001	5708000	6.60E-06
chr11	6001001	6006000	0.000125
chr11	6280001	6285000	1.71E-06
chr11	6398001	6403000	0.001207
chr11	6399001	6404000	0.000134
chr11	6629001	6634000	4.24E-09
chr11	6630001	6635000	3.00E-14
chr11	6631001	6636000	2.05E-14
chr11	6632001	6637000	2.17E-12
chr11	6633001	6638000	7.24E-11
chr11	6648001	6653000	0.000146
chr11	6700001	6705000	2.92E-10
chr11	6701001	6706000	7.13E-14
chr11	6707001	6712000	0.001815
chr11	8375001	8380000	2.72E-05
chr11	9384001	9389000	3.79E-12
chr11	9385001	9390000	6.20E-09
chr11	9403001	9408000	1.57E-08
chr11	9404001	9409000	3.00E-10
chr11	9406001	9411000	1.47E-09
chr11	9622001	9627000	0.002566
chr11	9994001	9999000	7.48E-09
chr11	11046001	11051000	4.28E-06
chr11	11047001	11052000	8.58E-06
chr11	11048001	11053000	1.92E-05
chr11	11222001	11227000	1.06E-12
chr11	12395001	12400000	9.52E-21
chr11	12396001	12401000	1.97E-21
chr11	12743001	12748000	5.98E-11
chr11	13799001	13804000	3.59E-08
chr11	14071001	14076000	0.000164
chr11	14075001	14080000	2.39E-07
chr11	14137001	14142000	9.70E-08
chr11	14409001	14414000	0.000423
chr11	14613001	14618000	6.03E-05
chr11	14669001	14674000	0.000165
chr11	15172001	15177000	2.68E-05
chr11	17293001	17298000	1.64E-08
chr11	17294001	17299000	4.71E-10
chr11	17412001	17417000	9.32E-05
chr11	17442001	17447000	1.19E-07

chr11	17623001	17628000	2.34E-05
chr11	17624001	17629000	9.66E-06
chr11	17753001	17758000	1.19E-07
chr11	18274001	18279000	0.004517
chr11	18749001	18754000	1.84E-05
chr11	18778001	18783000	9.73E-06
chr11	20381001	20386000	4.49E-07
chr11	20382001	20387000	1.46E-10
chr11	20383001	20388000	2.33E-07
chr11	20717001	20722000	1.00E-07
chr11	20718001	20723000	1.03E-06
chr11	20719001	20724000	7.22E-08
chr11	21715001	21720000	9.57E-07
chr11	21716001	21721000	4.15E-06
chr11	21717001	21722000	6.58E-06
chr11	21760001	21765000	0.000464
chr11	21761001	21766000	1.57E-06
chr11	21795001	21800000	5.85E-07
chr11	21796001	21801000	3.07E-06
chr11	21873001	21878000	1.94E-06
chr11	21874001	21879000	1.40E-07
chr11	22060001	22065000	1.34E-09
chr11	22140001	22145000	4.41E-05
chr11	22141001	22146000	7.00E-05
chr11	22142001	22147000	4.89E-05
chr11	24054001	24059000	0.000833
chr11	26834001	26839000	1.86E-08
chr11	26838001	26843000	5.26E-11
chr11	27490001	27495000	5.64E-09
chr11	27597001	27602000	3.87E-07
chr11	27971001	27976000	1.09E-05
chr11	27972001	27977000	1.07E-05
chr11	27973001	27978000	4.11E-06
chr11	27974001	27979000	1.32E-05
chr11	27975001	27980000	1.46E-05
chr11	28801001	28806000	0.001223
chr11	29288001	29293000	3.98E-08
chr11	29289001	29294000	1.42E-08
chr11	29290001	29295000	1.95E-09
chr11	29742001	29747000	7.44E-06
chr11	29923001	29928000	6.36E-05
chr11	30227001	30232000	9.20E-07
chr11	30716001	30721000	4.47E-09
chr11	30717001	30722000	2.00E-10
chr11	30718001	30723000	4.25E-12
chr11	30719001	30724000	3.84E-09
chr11	30997001	31002000	1.05E-05
chr11	31149001	31154000	1.58E-06

chr11	31166001	31171000	1.25E-09
chr11	31851001	31856000	5.88E-08
chr11	32109001	32114000	5.12E-10
chr11	32110001	32115000	2.64E-12
chr11	32111001	32116000	1.89E-10
chr11	32112001	32117000	2.87E-10
chr11	32113001	32118000	2.18E-08
chr11	32282001	32287000	1.45E-06
chr11	32406001	32411000	1.65E-07
chr11	33036001	33041000	4.08E-07
chr11	33056001	33061000	0.000371
chr11	33522001	33527000	5.11E-07
chr11	33523001	33528000	5.68E-08
chr11	34706001	34711000	3.24E-09
chr11	35937001	35942000	2.43E-05
chr11	36425001	36430000	4.50E-05
chr11	36795001	36800000	2.46E-05
chr11	36796001	36801000	3.89E-07
chr11	37327001	37332000	7.97E-06
chr11	37328001	37333000	8.19E-05
chr11	37548001	37553000	1.11E-08
chr11	38485001	38490000	5.33E-07
chr11	38486001	38491000	9.40E-09
chr11	38487001	38492000	1.58E-07
chr11	39034001	39039000	1.49E-07
chr11	39522001	39527000	2.10E-08
chr11	39738001	39743000	2.40E-12
chr11	39741001	39746000	3.57E-06
chr11	39883001	39888000	2.90E-07
chr11	39884001	39889000	2.46E-07
chr11	39890001	39895000	0.000275
chr11	40365001	40370000	4.57E-06
chr11	40672001	40677000	3.90E-05
chr11	40885001	40890000	1.90E-06
chr11	40886001	40891000	5.71E-08
chr11	41003001	41008000	2.16E-05
chr11	41159001	41164000	2.20E-08
chr11	41160001	41165000	8.26E-07
chr11	41776001	41781000	8.25E-06
chr11	41894001	41899000	1.06E-07
chr11	41974001	41979000	0.000239
chr11	41977001	41982000	0.000203
chr11	41978001	41983000	0.000561
chr11	41979001	41984000	0.002128
chr11	42393001	42398000	0.000536
chr11	42399001	42404000	1.79E-09
chr11	42400001	42405000	5.08E-06
chr11	42501001	42506000	0.004409

chr11	42953001	42958000	0.002487
chr11	42990001	42995000	0.000635
chr11	42991001	42996000	0.000125
chr11	42992001	42997000	0.000173
chr11	43537001	43542000	2.99E-06
chr11	43699001	43704000	6.10E-07
chr11	43700001	43705000	1.53E-08
chr11	44035001	44040000	0.001151
chr11	44048001	44053000	1.29E-05
chr11	44376001	44381000	1.46E-06
chr11	44663001	44668000	9.45E-05
chr11	44664001	44669000	0.00023
chr11	44778001	44783000	1.11E-08
chr11	44943001	44948000	6.17E-05
chr11	44944001	44949000	4.79E-06
chr11	44945001	44950000	1.24E-06
chr11	44946001	44951000	3.54E-10
chr11	44947001	44952000	2.71E-10
chr11	44948001	44953000	2.85E-10
chr11	44949001	44954000	1.75E-09
chr11	44955001	44960000	3.93E-05
chr11	44956001	44961000	8.07E-07
chr11	45107001	45112000	1.88E-05
chr11	45122001	45127000	0.00032
chr11	45377001	45382000	4.72E-09
chr11	45495001	45500000	9.15E-07
chr11	45496001	45501000	3.38E-12
chr11	45497001	45502000	3.49E-12
chr11	45498001	45503000	1.29E-10
chr11	45499001	45504000	4.58E-11
chr11	45500001	45505000	8.11E-10
chr11	45501001	45506000	5.63E-06
chr11	45579001	45584000	0.000578
chr11	45595001	45600000	1.31E-05
chr11	45625001	45630000	7.93E-05
chr11	45641001	45646000	0.001766
chr11	45663001	45668000	0.012527
chr11	45687001	45692000	0.001096
chr11	45925001	45930000	0.005409
chr11	45942001	45947000	0.00475
chr11	46354001	46359000	4.74E-05
chr11	46402001	46407000	4.17E-06
chr11	46717001	46722000	2.80E-05
chr11	47733001	47738000	2.28E-05
chr11	47906001	47911000	7.23E-06
chr11	47907001	47912000	3.77E-06
chr11	48099001	48104000	5.15E-06
chr11	48100001	48105000	1.54E-05

chr11	48101001	48106000	1.66E-08
chr11	48102001	48107000	9.90E-07
chr11	48103001	48108000	5.68E-07
chr11	48223001	48228000	0.004652
chr11	48224001	48229000	0.000619
chr11	48236001	48241000	6.69E-07
chr11	48573001	48578000	1.63E-05
chr11	48628001	48633000	1.34E-05
chr11	48635001	48640000	8.01E-09
chr11	48636001	48641000	1.36E-08
chr11	48637001	48642000	2.47E-08
chr11	48973001	48978000	0.000793
chr11	48974001	48979000	0.000235
chr11	48975001	48980000	5.26E-06
chr11	48976001	48981000	5.25E-07
chr11	49129001	49134000	9.89E-07
chr11	49758001	49763000	1.04E-05
chr11	50029001	50034000	0.000277
chr11	50158001	50163000	0.004185
chr11	50183001	50188000	0.005443
chr11	50185001	50190000	0.003111
chr11	50186001	50191000	0.010114
chr11	50187001	50192000	0.000301
chr11	50188001	50193000	0.000108
chr11	50266001	50271000	6.42E-05
chr11	51097001	51102000	0.012994
chr11	51098001	51103000	0.019899
chr11	51128001	51133000	0.018795
chr11	51129001	51134000	0.0147
chr11	51345001	51350000	0.003256
chr11	54776001	54781000	0.002241
chr11	54777001	54782000	0.002241
chr11	54988001	54993000	0.000185
chr11	55375001	55380000	0.000181
chr11	55376001	55381000	0.000113
chr11	55377001	55382000	1.34E-05
chr11	55435001	55440000	0.001872
chr11	55437001	55442000	7.48E-05
chr11	55880001	55885000	2.60E-09
chr11	56023001	56028000	0.002677
chr11	56024001	56029000	0.002751
chr11	56130001	56135000	9.78E-10
chr11	56758001	56763000	0.000124
chr11	56872001	56877000	1.44E-05
chr11	56886001	56891000	6.43E-07
chr11	56887001	56892000	1.10E-06
chr11	57200001	57205000	1.05E-07
chr11	57227001	57232000	7.81E-09

chr11	57228001	57233000	1.04E-11
chr11	57282001	57287000	4.90E-07
chr11	57506001	57511000	8.43E-06
chr11	57509001	57514000	8.44E-05
chr11	57647001	57652000	1.14E-06
chr11	57648001	57653000	3.04E-05
chr11	57649001	57654000	0.000416
chr11	57664001	57669000	0.00026
chr11	57665001	57670000	0.001799
chr11	57666001	57671000	0.000953
chr11	57667001	57672000	7.98E-07
chr11	57668001	57673000	2.44E-09
chr11	57669001	57674000	2.69E-08
chr11	57670001	57675000	9.64E-10
chr11	57671001	57676000	7.34E-07
chr11	57833001	57838000	0.004478
chr11	58142001	58147000	3.94E-05
chr11	58143001	58148000	5.30E-06
chr11	58145001	58150000	6.10E-05
chr11	58195001	58200000	0.000311
chr11	58196001	58201000	0.000459
chr11	58197001	58202000	8.70E-05
chr11	58558001	58563000	9.35E-06
chr11	58592001	58597000	1.46E-05
chr11	59167001	59172000	0.004126
chr11	59267001	59272000	5.20E-06
chr11	59268001	59273000	2.25E-08
chr11	59309001	59314000	8.20E-07
chr11	59310001	59315000	1.48E-05
chr11	59311001	59316000	1.08E-05
chr11	59332001	59337000	4.48E-09
chr11	59880001	59885000	8.21E-06
chr11	60274001	60279000	2.52E-08
chr11	60557001	60562000	0.000246
chr11	60559001	60564000	9.43E-05
chr11	60568001	60573000	7.50E-14
chr11	60569001	60574000	3.15E-12
chr11	60621001	60626000	5.86E-05
chr11	60691001	60696000	1.28E-06
chr11	60692001	60697000	2.71E-11
chr11	60737001	60742000	3.09E-06
chr11	60738001	60743000	3.72E-05
chr11	60783001	60788000	6.98E-06
chr11	60784001	60789000	1.10E-09
chr11	60785001	60790000	3.15E-07
chr11	60955001	60960000	9.76E-10
chr11	61044001	61049000	5.42E-07
chr11	61046001	61051000	8.07E-07

chr11	61099001	61104000	1.53E-08
chr11	61100001	61105000	4.23E-10
chr11	61272001	61277000	0.006569
chr11	61273001	61278000	0.019266
chr11	61274001	61279000	0.022419
chr11	61277001	61282000	3.51E-05
chr11	61278001	61283000	7.09E-05
chr11	61279001	61284000	7.08E-06
chr11	61539001	61544000	0.009377
chr11	61556001	61561000	4.30E-07
chr11	61557001	61562000	6.03E-10
chr11	61578001	61583000	1.07E-05
chr11	61668001	61673000	9.38E-06
chr11	61669001	61674000	9.06E-05
chr11	61670001	61675000	0.000229
chr11	61821001	61826000	0.000755
chr11	61875001	61880000	3.68E-06
chr11	62046001	62051000	0.000341
chr11	62047001	62052000	0.000241
chr11	62179001	62184000	0.006049
chr11	62206001	62211000	0.00069
chr11	62313001	62318000	9.80E-05
chr11	62387001	62392000	1.20E-06
chr11	62388001	62393000	3.07E-05
chr11	62514001	62519000	2.94E-09
chr11	62569001	62574000	3.78E-06
chr11	62570001	62575000	2.82E-06
chr11	62644001	62649000	1.64E-10
chr11	62645001	62650000	1.64E-10
chr11	62666001	62671000	0.043882
chr11	62740001	62745000	0.001902
chr11	62747001	62752000	0.006066
chr11	62748001	62753000	0.001941
chr11	62749001	62754000	0.000347
chr11	62864001	62869000	0.003806
chr11	63532001	63537000	0.000101
chr11	63533001	63538000	4.69E-06
chr11	63562001	63567000	0.002682
chr11	63626001	63631000	1.25E-05
chr11	63685001	63690000	0.006031
chr11	63686001	63691000	0.006031
chr11	63729001	63734000	0.001018
chr11	63763001	63768000	0.000184
chr11	63770001	63775000	1.03E-06
chr11	63771001	63776000	8.37E-09
chr11	63772001	63777000	4.63E-07
chr11	63773001	63778000	1.62E-07
chr11	63774001	63779000	1.96E-08

chr11	63916001	63921000	5.28E-08
chr11	63917001	63922000	2.80E-11
chr11	63918001	63923000	3.73E-13
chr11	63919001	63924000	8.21E-12
chr11	63920001	63925000	3.08E-06
chr11	63975001	63980000	0.028038
chr11	63990001	63995000	0.000213
chr11	63991001	63996000	0.000634
chr11	64009001	64014000	4.21E-06
chr11	64010001	64015000	4.21E-06
chr11	64122001	64127000	9.65E-05
chr11	64123001	64128000	1.38E-05
chr11	64137001	64142000	1.15E-05
chr11	64138001	64143000	1.15E-05
chr11	64139001	64144000	1.15E-05
chr11	64361001	64366000	4.65E-06
chr11	64362001	64367000	3.24E-06
chr11	64363001	64368000	1.30E-05
chr11	64364001	64369000	8.09E-07
chr11	64371001	64376000	0.000192
chr11	64420001	64425000	0.000365
chr11	64491001	64496000	0.003881
chr11	64492001	64497000	9.27E-05
chr11	64493001	64498000	8.20E-10
chr11	64494001	64499000	6.65E-12
chr11	64495001	64500000	2.06E-08
chr11	64496001	64501000	1.25E-06
chr11	64636001	64641000	0.001516
chr11	64644001	64649000	1.09E-05
chr11	64645001	64650000	0.000125
chr11	64679001	64684000	0.000906
chr11	64680001	64685000	0.020138
chr11	64750001	64755000	0.000204
chr11	64793001	64798000	2.10E-13
chr11	64794001	64799000	5.60E-17
chr11	64795001	64800000	4.67E-16
chr11	64978001	64983000	0.020208
chr11	65152001	65157000	0.000103
chr11	65153001	65158000	1.48E-05
chr11	65259001	65264000	1.10E-06
chr11	65260001	65265000	1.88E-09
chr11	65261001	65266000	1.94E-06
chr11	65305001	65310000	3.21E-08
chr11	65306001	65311000	6.17E-05
chr11	65307001	65312000	0.000339
chr11	65312001	65317000	9.63E-05
chr11	65317001	65322000	8.69E-05
chr11	65318001	65323000	0.000361

chr11	65321001	65326000	0.000162
chr11	65323001	65328000	0.001205
chr11	65347001	65352000	0.001202
chr11	65357001	65362000	1.57E-06
chr11	65383001	65388000	6.04E-14
chr11	65478001	65483000	4.41E-11
chr11	65495001	65500000	5.49E-09
chr11	65496001	65501000	3.57E-07
chr11	65554001	65559000	3.09E-09
chr11	65555001	65560000	2.94E-16
chr11	65556001	65561000	8.17E-06
chr11	65557001	65562000	2.72E-05
chr11	65558001	65563000	0.000409
chr11	65604001	65609000	5.31E-06
chr11	65633001	65638000	1.40E-06
chr11	65711001	65716000	0.004787
chr11	65744001	65749000	1.78E-05
chr11	65745001	65750000	0.000694
chr11	65765001	65770000	1.70E-15
chr11	65766001	65771000	1.57E-16
chr11	65767001	65772000	7.34E-18
chr11	65768001	65773000	4.40E-17
chr11	65769001	65774000	7.44E-21
chr11	65785001	65790000	3.51E-05
chr11	65786001	65791000	0.001438
chr11	65795001	65800000	1.94E-05
chr11	65796001	65801000	9.89E-06
chr11	66036001	66041000	1.79E-05
chr11	66046001	66051000	1.41E-15
chr11	66047001	66052000	3.16E-17
chr11	66048001	66053000	2.59E-15
chr11	66049001	66054000	1.86E-08
chr11	66050001	66055000	4.12E-11
chr11	66080001	66085000	0.004126
chr11	66102001	66107000	3.62E-07
chr11	66103001	66108000	0.001234
chr11	66104001	66109000	6.01E-05
chr11	66124001	66129000	5.79E-06
chr11	66125001	66130000	1.06E-05
chr11	66135001	66140000	5.31E-05
chr11	66138001	66143000	1.14E-06
chr11	66174001	66179000	8.82E-05
chr11	66175001	66180000	1.08E-06
chr11	66176001	66181000	1.07E-07
chr11	66177001	66182000	0.000427
chr11	66205001	66210000	7.81E-06
chr11	66206001	66211000	9.68E-05
chr11	66208001	66213000	6.70E-08

chr11	66209001	66214000	3.86E-07
chr11	66210001	66215000	6.93E-06
chr11	66211001	66216000	2.49E-06
chr11	66217001	66222000	0.000742
chr11	66247001	66252000	8.37E-06
chr11	66249001	66254000	0.003647
chr11	66308001	66313000	0.00048
chr11	66309001	66314000	0.003846
chr11	66310001	66315000	9.97E-05
chr11	66325001	66330000	2.99E-08
chr11	66326001	66331000	7.30E-06
chr11	66331001	66336000	0.000492
chr11	66332001	66337000	1.89E-06
chr11	66333001	66338000	5.35E-06
chr11	66334001	66339000	1.10E-05
chr11	66335001	66340000	1.50E-06
chr11	66343001	66348000	1.24E-07
chr11	66486001	66491000	7.52E-06
chr11	66487001	66492000	4.29E-06
chr11	66507001	66512000	8.04E-06
chr11	66508001	66513000	1.39E-05
chr11	66628001	66633000	0.002167
chr11	66630001	66635000	0.003129
chr11	66748001	66753000	0.001191
chr11	66749001	66754000	0.000153
chr11	66805001	66810000	6.87E-07
chr11	66806001	66811000	1.33E-05
chr11	66807001	66812000	3.93E-05
chr11	66846001	66851000	0.023943
chr11	66881001	66886000	3.46E-10
chr11	66882001	66887000	2.95E-11
chr11	66883001	66888000	1.41E-09
chr11	66884001	66889000	2.24E-11
chr11	67054001	67059000	0.000229
chr11	67055001	67060000	2.39E-07
chr11	67081001	67086000	1.22E-11
chr11	67082001	67087000	6.50E-18
chr11	67083001	67088000	1.48E-17
chr11	67084001	67089000	1.64E-08
chr11	67085001	67090000	7.48E-10
chr11	67110001	67115000	3.92E-09
chr11	67111001	67116000	1.57E-06
chr11	67123001	67128000	0.000137
chr11	67142001	67147000	1.07E-05
chr11	67179001	67184000	6.40E-08
chr11	67180001	67185000	1.56E-07
chr11	67181001	67186000	2.84E-06
chr11	67182001	67187000	7.46E-09

chr11	67217001	67222000	0.021607
chr11	67218001	67223000	0.010526
chr11	67231001	67236000	0.000214
chr11	67270001	67275000	7.00E-08
chr11	67271001	67276000	5.01E-07
chr11	67272001	67277000	1.29E-05
chr11	67282001	67287000	6.75E-07
chr11	67283001	67288000	7.81E-10
chr11	67284001	67289000	7.17E-07
chr11	67285001	67290000	5.04E-06
chr11	67298001	67303000	8.18E-05
chr11	67310001	67315000	5.43E-06
chr11	67322001	67327000	1.36E-11
chr11	67324001	67329000	8.05E-09
chr11	67459001	67464000	5.84E-14
chr11	67460001	67465000	9.25E-11
chr11	67461001	67466000	3.99E-10
chr11	67559001	67564000	2.97E-06
chr11	67571001	67576000	4.23E-09
chr11	67572001	67577000	9.37E-11
chr11	67601001	67606000	1.13E-06
chr11	67602001	67607000	2.62E-06
chr11	67758001	67763000	0.001696
chr11	67759001	67764000	0.000259
chr11	67760001	67765000	2.71E-05
chr11	67785001	67790000	1.03E-12
chr11	67786001	67791000	1.61E-13
chr11	67787001	67792000	5.97E-15
chr11	67788001	67793000	7.10E-14
chr11	67793001	67798000	0.001474
chr11	67805001	67810000	3.85E-05
chr11	67863001	67868000	7.35E-05
chr11	67864001	67869000	1.03E-06
chr11	67866001	67871000	0.001182
chr11	68063001	68068000	0.000934
chr11	68150001	68155000	0.017638
chr11	68151001	68156000	0.000118
chr11	68213001	68218000	0.000974
chr11	68214001	68219000	0.000881
chr11	68221001	68226000	0.000298
chr11	68222001	68227000	0.001476
chr11	68431001	68436000	9.13E-11
chr11	68449001	68454000	8.97E-12
chr11	68450001	68455000	6.60E-18
chr11	68451001	68456000	9.15E-26
chr11	68452001	68457000	4.97E-23
chr11	68637001	68642000	1.99E-09
chr11	68644001	68649000	0.000887

chr11	68762001	68767000	4.31E-08
chr11	68763001	68768000	3.19E-08
chr11	68800001	68805000	0.001044
chr11	68804001	68809000	0.000517
chr11	68963001	68968000	1.41E-06
chr11	69188001	69193000	1.94E-12
chr11	69189001	69194000	3.97E-08
chr11	69190001	69195000	2.76E-11
chr11	69192001	69197000	2.29E-13
chr11	69447001	69452000	1.03E-18
chr11	69482001	69487000	1.41E-10
chr11	69486001	69491000	1.16E-12
chr11	69487001	69492000	4.99E-14
chr11	69489001	69494000	1.72E-13
chr11	69557001	69562000	6.07E-05
chr11	69558001	69563000	1.96E-07
chr11	69559001	69564000	9.96E-08
chr11	69560001	69565000	4.19E-09
chr11	69561001	69566000	4.70E-08
chr11	69632001	69637000	6.80E-11
chr11	69633001	69638000	3.69E-07
chr11	69923001	69928000	8.37E-06
chr11	70045001	70050000	1.06E-06
chr11	70115001	70120000	9.59E-13
chr11	70116001	70121000	3.37E-16
chr11	70117001	70122000	1.44E-12
chr11	70416001	70421000	3.33E-08
chr11	70449001	70454000	2.52E-08
chr11	70450001	70455000	1.02E-11
chr11	70451001	70456000	4.39E-11
chr11	70658001	70663000	2.21E-07
chr11	70659001	70664000	8.29E-09
chr11	70994001	70999000	3.55E-09
chr11	71235001	71240000	1.20E-05
chr11	71288001	71293000	1.02E-08
chr11	71357001	71362000	7.52E-05
chr11	71373001	71378000	0.000349
chr11	71488001	71493000	9.24E-10
chr11	71489001	71494000	3.24E-11
chr11	71493001	71498000	2.17E-11
chr11	71494001	71499000	5.53E-11
chr11	71496001	71501000	2.46E-10
chr11	71497001	71502000	6.32E-12
chr11	71788001	71793000	6.71E-08
chr11	71789001	71794000	1.94E-07
chr11	71790001	71795000	1.69E-07
chr11	71876001	71881000	3.53E-07
chr11	72211001	72216000	2.32E-05

chr11	72267001	72272000	0.000415
chr11	72289001	72294000	1.04E-05
chr11	72290001	72295000	1.21E-05
chr11	72305001	72310000	1.60E-06
chr11	72306001	72311000	1.25E-05
chr11	72307001	72312000	3.53E-06
chr11	72311001	72316000	1.89E-05
chr11	72365001	72370000	0.000201
chr11	72485001	72490000	0.001437
chr11	72486001	72491000	0.001876
chr11	72487001	72492000	0.005875
chr11	72488001	72493000	0.014426
chr11	72489001	72494000	0.00081
chr11	72500001	72505000	0.010667
chr11	72881001	72886000	0.000506
chr11	72882001	72887000	6.81E-06
chr11	72972001	72977000	3.20E-07
chr11	72973001	72978000	2.14E-07
chr11	72974001	72979000	0.000124
chr11	72975001	72980000	0.001089
chr11	73061001	73066000	1.09E-12
chr11	73062001	73067000	4.45E-11
chr11	73356001	73361000	2.80E-06
chr11	73357001	73362000	1.33E-06
chr11	73358001	73363000	6.62E-07
chr11	73495001	73500000	1.15E-08
chr11	73583001	73588000	9.97E-11
chr11	74213001	74218000	1.85E-05
chr11	74697001	74702000	2.97E-13
chr11	74698001	74703000	1.26E-09
chr11	74842001	74847000	4.98E-06
chr11	74923001	74928000	0.0013
chr11	74925001	74930000	0.012405
chr11	74940001	74945000	0.000216
chr11	74941001	74946000	1.20E-05
chr11	74942001	74947000	7.18E-06
chr11	74943001	74948000	4.51E-05
chr11	74944001	74949000	1.96E-06
chr11	74945001	74950000	0.000147
chr11	74948001	74953000	1.47E-11
chr11	75050001	75055000	8.58E-05
chr11	75051001	75056000	4.82E-07
chr11	75061001	75066000	0.000349
chr11	75062001	75067000	0.000561
chr11	75079001	75084000	2.77E-06
chr11	75207001	75212000	0.001873
chr11	75226001	75231000	2.58E-08
chr11	75227001	75232000	1.28E-08

chr11	75228001	75233000	7.77E-08
chr11	75229001	75234000	3.46E-07
chr11	75230001	75235000	6.22E-06
chr11	75234001	75239000	8.03E-06
chr11	75235001	75240000	5.83E-09
chr11	75237001	75242000	4.25E-06
chr11	75238001	75243000	1.12E-06
chr11	75380001	75385000	5.25E-15
chr11	75526001	75531000	2.66E-06
chr11	75969001	75974000	7.03E-06
chr11	75970001	75975000	2.21E-06
chr11	75971001	75976000	1.06E-06
chr11	76306001	76311000	1.24E-05
chr11	76380001	76385000	2.42E-05
chr11	76414001	76419000	9.28E-06
chr11	76415001	76420000	2.55E-07
chr11	76421001	76426000	3.93E-05
chr11	76776001	76781000	1.13E-06
chr11	76777001	76782000	2.65E-08
chr11	76866001	76871000	1.09E-06
chr11	76867001	76872000	4.88E-08
chr11	77786001	77791000	1.28E-10
chr11	77896001	77901000	4.96E-10
chr11	81159001	81164000	7.98E-12
chr11	81422001	81427000	2.10E-06
chr11	81423001	81428000	4.67E-05
chr11	81543001	81548000	6.07E-09
chr11	81544001	81549000	8.50E-10
chr11	81570001	81575000	7.21E-13
chr11	81571001	81576000	2.91E-11
chr11	81573001	81578000	7.80E-10
chr11	81588001	81593000	7.06E-12
chr11	81709001	81714000	1.67E-07
chr11	81986001	81991000	1.47E-09
chr11	82210001	82215000	2.30E-06
chr11	82211001	82216000	6.54E-06
chr11	82234001	82239000	1.55E-09
chr11	82235001	82240000	1.88E-16
chr11	82236001	82241000	3.69E-14
chr11	82237001	82242000	7.89E-14
chr11	82238001	82243000	5.38E-16
chr11	82997001	83002000	4.15E-18
chr11	83131001	83136000	6.80E-10
chr11	83132001	83137000	3.15E-08
chr11	83134001	83139000	1.87E-05
chr11	83353001	83358000	7.68E-10
chr11	83854001	83859000	1.21E-10
chr11	84213001	84218000	2.81E-06

chr11	84489001	84494000	1.78E-14
chr11	84710001	84715000	2.59E-09
chr11	84916001	84921000	2.26E-10
chr11	84917001	84922000	1.06E-13
chr11	84918001	84923000	2.42E-05
chr11	84919001	84924000	8.91E-07
chr11	84920001	84925000	3.68E-07
chr11	84952001	84957000	1.03E-08
chr11	85016001	85021000	9.01E-09
chr11	85022001	85027000	2.26E-05
chr11	85130001	85135000	6.71E-08
chr11	85131001	85136000	9.40E-07
chr11	85133001	85138000	0.000129
chr11	85226001	85231000	1.80E-05
chr11	85227001	85232000	2.38E-05
chr11	85228001	85233000	1.04E-07
chr11	85229001	85234000	5.61E-09
chr11	85459001	85464000	9.35E-09
chr11	85497001	85502000	2.43E-05
chr11	85566001	85571000	1.46E-08
chr11	86561001	86566000	9.82E-17
chr11	86562001	86567000	2.12E-17
chr11	86563001	86568000	4.82E-16
chr11	86564001	86569000	1.10E-19
chr11	86744001	86749000	1.27E-17
chr11	86745001	86750000	2.22E-16
chr11	86746001	86751000	9.56E-12
chr11	87110001	87115000	3.58E-08
chr11	87111001	87116000	1.57E-10
chr11	87112001	87117000	1.72E-09
chr11	87211001	87216000	2.13E-06
chr11	87213001	87218000	3.04E-07
chr11	87462001	87467000	2.66E-07
chr11	87463001	87468000	1.38E-05
chr11	87999001	88004000	0.008107
chr11	88308001	88313000	1.23E-06
chr11	88309001	88314000	5.48E-08
chr11	88310001	88315000	9.54E-12
chr11	88486001	88491000	1.41E-06
chr11	88487001	88492000	6.25E-09
chr11	88488001	88493000	1.18E-09
chr11	88489001	88494000	1.11E-09
chr11	88614001	88619000	0.000717
chr11	88615001	88620000	0.000141
chr11	88616001	88621000	0.000319
chr11	88819001	88824000	3.12E-11
chr11	88820001	88825000	6.76E-10
chr11	88821001	88826000	6.67E-10

chr11	88822001	88827000	2.24E-11
chr11	88856001	88861000	0.000215
chr11	88857001	88862000	0.002262
chr11	89363001	89368000	3.00E-10
chr11	89575001	89580000	0.000874
chr11	89576001	89581000	0.000418
chr11	89577001	89582000	0.002215
chr11	89578001	89583000	0.002215
chr11	90250001	90255000	8.33E-09
chr11	90424001	90429000	0.000776
chr11	90559001	90564000	3.49E-08
chr11	91441001	91446000	9.49E-07
chr11	91590001	91595000	0.000963
chr11	91711001	91716000	4.32E-05
chr11	93049001	93054000	5.44E-07
chr11	93085001	93090000	7.85E-08
chr11	93086001	93091000	4.29E-11
chr11	93563001	93568000	0.000769
chr11	94275001	94280000	5.12E-13
chr11	94276001	94281000	6.19E-11
chr11	94346001	94351000	0.000315
chr11	94347001	94352000	5.82E-05
chr11	94813001	94818000	0.000264
chr11	96464001	96469000	8.12E-09
chr11	97267001	97272000	5.12E-09
chr11	97268001	97273000	1.24E-07
chr11	97304001	97309000	7.91E-10
chr11	97442001	97447000	2.82E-10
chr11	98268001	98273000	0.00028
chr11	98421001	98426000	1.27E-05
chr11	98422001	98427000	0.000266
chr11	98468001	98473000	0.000122
chr11	98644001	98649000	4.05E-13
chr11	99759001	99764000	1.41E-08
chr11	99760001	99765000	2.24E-11
chr11	99761001	99766000	3.22E-08
chr11	99762001	99767000	4.26E-09
chr11	99763001	99768000	3.22E-09
chr11	100287001	100292000	0.000216
chr11	100646001	100651000	6.05E-10
chr11	100678001	100683000	6.57E-11
chr11	101166001	101171000	0.000306
chr11	101167001	101172000	5.68E-05
chr11	101168001	101173000	1.60E-05
chr11	101227001	101232000	5.20E-07
chr11	102842001	102847000	2.10E-05
chr11	103162001	103167000	3.49E-06
chr11	103371001	103376000	9.20E-09

chr11	103373001	103378000	1.27E-07
chr11	103644001	103649000	3.89E-05
chr11	103780001	103785000	0.002452
chr11	104265001	104270000	8.72E-06
chr11	104266001	104271000	8.54E-08
chr11	104351001	104356000	2.31E-05
chr11	104383001	104388000	9.06E-07
chr11	104647001	104652000	7.49E-05
chr11	105228001	105233000	0.000125
chr11	105574001	105579000	1.57E-07
chr11	105576001	105581000	5.08E-09
chr11	106317001	106322000	1.51E-08
chr11	106442001	106447000	8.56E-06
chr11	106994001	106999000	5.77E-05
chr11	107436001	107441000	1.59E-17
chr11	107538001	107543000	4.12E-05
chr11	107707001	107712000	1.79E-06
chr11	107708001	107713000	1.29E-09
chr11	107709001	107714000	3.86E-07
chr11	107724001	107729000	2.29E-09
chr11	107780001	107785000	0.000158
chr11	110395001	110400000	4.61E-09
chr11	110396001	110401000	3.65E-06
chr11	111749001	111754000	1.66E-10
chr11	112607001	112612000	5.85E-06
chr11	112608001	112613000	1.63E-05
chr11	112609001	112614000	1.40E-06
chr11	112610001	112615000	2.36E-05
chr11	112673001	112678000	6.03E-06
chr11	112767001	112772000	2.85E-07
chr11	113270001	113275000	0.00536
chr11	113891001	113896000	1.18E-06
chr11	113892001	113897000	1.04E-07
chr11	113931001	113936000	0.00063
chr11	114145001	114150000	4.29E-07
chr11	114192001	114197000	3.57E-07
chr11	114193001	114198000	4.57E-07
chr11	114194001	114199000	9.72E-06
chr11	114195001	114200000	1.01E-06
chr11	114196001	114201000	0.000276
chr11	114198001	114203000	0.000458
chr11	114796001	114801000	1.58E-07
chr11	115006001	115011000	5.65E-06
chr11	115007001	115012000	2.07E-05
chr11	115430001	115435000	0.00066
chr11	116150001	116155000	6.91E-06
chr11	116151001	116156000	3.39E-05
chr11	116284001	116289000	0.004469

chr11	116512001	116517000	0.00013
chr11	116513001	116518000	4.23E-08
chr11	116514001	116519000	1.26E-06
chr11	116515001	116520000	1.80E-05
chr11	116516001	116521000	4.27E-05
chr11	116608001	116613000	3.69E-05
chr11	117014001	117019000	1.01E-06
chr11	117015001	117020000	1.76E-06
chr11	117054001	117059000	1.33E-05
chr11	117055001	117060000	6.57E-05
chr11	117056001	117061000	2.23E-05
chr11	117057001	117062000	1.76E-05
chr11	117313001	117318000	1.72E-05
chr11	117348001	117353000	0.000232
chr11	117378001	117383000	7.63E-07
chr11	117457001	117462000	9.43E-05
chr11	117544001	117549000	1.55E-05
chr11	117786001	117791000	4.54E-05
chr11	117788001	117793000	2.59E-08
chr11	117789001	117794000	4.72E-08
chr11	117790001	117795000	1.27E-08
chr11	118474001	118479000	0.016801
chr11	118794001	118799000	2.78E-12
chr11	118796001	118801000	2.33E-09
chr11	118797001	118802000	1.33E-07
chr11	118827001	118832000	0.000548
chr11	118828001	118833000	9.94E-06
chr11	118858001	118863000	1.15E-07
chr11	118859001	118864000	1.43E-07
chr11	118927001	118932000	5.40E-16
chr11	118928001	118933000	9.87E-12
chr11	118964001	118969000	4.88E-08
chr11	118965001	118970000	3.48E-18
chr11	118966001	118971000	4.05E-17
chr11	118968001	118973000	8.28E-07
chr11	118969001	118974000	1.32E-10
chr11	119054001	119059000	0.000472
chr11	119091001	119096000	6.84E-05
chr11	119092001	119097000	0.000131
chr11	119371001	119376000	8.69E-05
chr11	119372001	119377000	7.50E-07
chr11	119373001	119378000	0.01127
chr11	119457001	119462000	9.02E-07
chr11	119458001	119463000	1.52E-07
chr11	120053001	120058000	0.000219
chr11	120054001	120059000	0.000234
chr11	120055001	120060000	0.000551
chr11	120201001	120206000	5.27E-08

chr11	120406001	120411000	2.10E-09
chr11	121210001	121215000	2.39E-09
chr11	122389001	122394000	0.000301
chr11	122970001	122975000	0.000887
chr11	122998001	123003000	0.00015
chr11	122999001	123004000	0.006043
chr11	123507001	123512000	9.51E-09
chr11	123508001	123513000	2.74E-09
chr11	123509001	123514000	9.99E-13
chr11	123510001	123515000	3.08E-15
chr11	123511001	123516000	1.01E-14
chr11	123512001	123517000	1.15E-05
chr11	123513001	123518000	2.97E-06
chr11	123722001	123727000	4.82E-05
chr11	123804001	123809000	3.24E-05
chr11	124266001	124271000	5.14E-08
chr11	124267001	124272000	3.24E-07
chr11	124268001	124273000	6.25E-10
chr11	124269001	124274000	9.56E-12
chr11	125458001	125463000	8.17E-09
chr11	125591001	125596000	0.000128
chr11	126225001	126230000	0.00206
chr11	126313001	126318000	2.29E-06
chr11	126370001	126375000	2.48E-05
chr11	126371001	126376000	9.22E-07
chr11	126372001	126377000	7.91E-07
chr11	126374001	126379000	3.29E-12
chr11	126375001	126380000	6.06E-09
chr11	126718001	126723000	9.50E-06
chr11	127569001	127574000	1.61E-11
chr11	127593001	127598000	2.52E-13
chr11	127594001	127599000	2.73E-15
chr11	127595001	127600000	6.41E-15
chr11	127628001	127633000	4.17E-09
chr11	127669001	127674000	9.16E-10
chr11	127670001	127675000	1.09E-05
chr11	127697001	127702000	3.44E-07
chr11	127780001	127785000	1.70E-06
chr11	127781001	127786000	1.11E-09
chr11	127862001	127867000	4.99E-10
chr11	127863001	127868000	1.56E-14
chr11	127864001	127869000	2.42E-13
chr11	127929001	127934000	3.16E-07
chr11	127956001	127961000	1.45E-06
chr11	127957001	127962000	5.83E-06
chr11	127958001	127963000	5.01E-06
chr11	127959001	127964000	1.80E-09
chr11	127960001	127965000	1.07E-08

chr11	127968001	127973000	1.50E-08
chr11	127969001	127974000	1.47E-11
chr11	128008001	128013000	5.07E-14
chr11	128012001	128017000	4.80E-11
chr11	128792001	128797000	8.37E-13
chr11	128796001	128801000	2.10E-05
chr11	128797001	128802000	0.000195
chr11	130079001	130084000	3.93E-05
chr11	130086001	130091000	0.000192
chr11	130290001	130295000	1.84E-05
chr11	130292001	130297000	4.57E-07
chr11	130293001	130298000	8.84E-06
chr11	130294001	130299000	0.000381
chr11	130523001	130528000	6.46E-07
chr11	130524001	130529000	9.98E-09
chr11	130525001	130530000	2.07E-08
chr11	131059001	131064000	3.63E-08
chr11	131125001	131130000	1.01E-08
chr11	131293001	131298000	5.29E-07
chr11	131294001	131299000	3.33E-09
chr11	131457001	131462000	9.52E-06
chr11	131479001	131484000	4.26E-16
chr11	131480001	131485000	6.69E-18
chr11	131481001	131486000	1.97E-16
chr11	131746001	131751000	5.34E-11
chr11	131754001	131759000	4.36E-13
chr11	131755001	131760000	2.46E-12
chr11	131955001	131960000	2.17E-09
chr11	131976001	131981000	1.54E-17
chr11	131978001	131983000	1.10E-15
chr11	131979001	131984000	7.86E-19
chr11	131980001	131985000	2.59E-15
chr11	132003001	132008000	5.11E-14
chr11	132004001	132009000	9.22E-14
chr11	132011001	132016000	2.13E-08
chr11	132025001	132030000	4.29E-11
chr11	132027001	132032000	1.35E-08
chr11	132028001	132033000	4.55E-09
chr11	132029001	132034000	3.79E-10
chr11	132044001	132049000	1.26E-10
chr11	132074001	132079000	5.91E-14
chr11	132075001	132080000	7.77E-15
chr11	132076001	132081000	3.48E-13
chr11	132077001	132082000	3.80E-14
chr11	132141001	132146000	1.01E-11
chr11	132142001	132147000	1.98E-12
chr11	132143001	132148000	4.10E-23
chr11	132144001	132149000	5.60E-21

chr11	132161001	132166000	2.32E-14
chr11	132162001	132167000	8.36E-14
chr11	132163001	132168000	2.02E-16
chr11	132164001	132169000	1.86E-22
chr11	132165001	132170000	1.00E-15
chr11	132181001	132186000	1.64E-11
chr11	132182001	132187000	4.49E-14
chr11	132183001	132188000	4.64E-14
chr11	132184001	132189000	2.56E-13
chr11	132275001	132280000	2.65E-07
chr11	132315001	132320000	8.44E-08
chr11	132442001	132447000	1.88E-10
chr11	132443001	132448000	3.87E-11
chr11	132455001	132460000	2.23E-11
chr11	132494001	132499000	1.21E-10
chr11	132500001	132505000	8.23E-11
chr11	132509001	132514000	1.77E-13
chr11	132534001	132539000	2.68E-12
chr11	132543001	132548000	3.25E-10
chr11	132554001	132559000	1.92E-11
chr11	132555001	132560000	3.19E-10
chr11	132556001	132561000	1.29E-12
chr11	132557001	132562000	5.82E-12
chr11	132590001	132595000	9.97E-10
chr11	132591001	132596000	7.91E-11
chr11	132743001	132748000	1.60E-14
chr11	132744001	132749000	1.08E-21
chr11	132745001	132750000	2.76E-18
chr11	132746001	132751000	3.84E-16
chr11	132803001	132808000	1.16E-10
chr11	132848001	132853000	1.81E-17
chr11	132959001	132964000	1.22E-10
chr11	132960001	132965000	4.24E-11
chr11	133035001	133040000	9.94E-13
chr11	133176001	133181000	1.27E-11
chr11	133177001	133182000	2.32E-11
chr11	133581001	133586000	4.25E-10
chr11	133801001	133806000	1.26E-06
chr11	133802001	133807000	6.02E-07
chr11	133803001	133808000	8.85E-07
chr11	133804001	133809000	4.96E-12
chr11	133805001	133810000	5.08E-07
chr11	133806001	133811000	6.69E-07
chr11	133826001	133831000	7.39E-14
chr11	133827001	133832000	1.93E-18
chr11	133840001	133845000	2.30E-13
chr11	133841001	133846000	4.03E-16
chr11	133842001	133847000	6.82E-14

chr11	133843001	133848000	1.96E-09
chr11	133881001	133886000	7.10E-10
chr11	133882001	133887000	1.96E-10
chr11	133883001	133888000	1.28E-13
chr11	133884001	133889000	9.30E-12
chr11	133885001	133890000	2.65E-11
chr11	134094001	134099000	6.02E-13
chr11	134396001	134401000	1.42E-23
chr11	134397001	134402000	2.62E-22
chr11	134399001	134404000	4.48E-13
chr11	134400001	134405000	2.13E-15
chr11	134401001	134406000	4.78E-11
chr11	134694001	134699000	1.08E-09
chr11	134695001	134700000	5.05E-10
chr11	134705001	134710000	8.22E-11
chr11	134706001	134711000	7.09E-11
chr11	134722001	134727000	2.86E-20
chr11	134723001	134728000	1.19E-12
chr11	134724001	134729000	9.74E-14
chr11	134759001	134764000	8.50E-08
chr11	134769001	134774000	3.45E-20
chr11	134845001	134850000	3.70E-15
chr11	134846001	134851000	2.19E-16
chr11	134868001	134873000	1.17E-08
chr11	134911001	134916000	7.26E-06
chr11	134912001	134917000	2.50E-07
chr11	134913001	134918000	8.92E-09
chr12	208001	213000	4.78E-09
chr12	498001	503000	2.24E-09
chr12	856001	861000	3.90E-10
chr12	857001	862000	5.04E-13
chr12	858001	863000	0.000113
chr12	1766001	1771000	7.35E-11
chr12	1767001	1772000	1.37E-12
chr12	1768001	1773000	2.44E-09
chr12	1960001	1965000	4.55E-11
chr12	1963001	1968000	7.29E-10
chr12	1964001	1969000	4.88E-09
chr12	1965001	1970000	5.75E-08
chr12	1966001	1971000	7.55E-09
chr12	1993001	1998000	1.98E-11
chr12	2012001	2017000	7.48E-06
chr12	2170001	2175000	1.55E-18
chr12	2175001	2180000	2.87E-13
chr12	2199001	2204000	3.15E-11
chr12	2240001	2245000	7.98E-05
chr12	2241001	2246000	4.14E-07
chr12	2313001	2318000	8.46E-16

chr12	2314001	2319000	2.75E-16
chr12	2315001	2320000	7.51E-16
chr12	2316001	2321000	1.68E-18
chr12	2372001	2377000	1.65E-12
chr12	2373001	2378000	9.82E-17
chr12	2374001	2379000	2.34E-15
chr12	2385001	2390000	1.72E-10
chr12	2386001	2391000	3.06E-12
chr12	2428001	2433000	1.60E-13
chr12	2455001	2460000	8.44E-11
chr12	2494001	2499000	2.97E-16
chr12	2495001	2500000	1.64E-34
chr12	2496001	2501000	5.75E-24
chr12	2505001	2510000	1.15E-13
chr12	2506001	2511000	1.81E-15
chr12	2507001	2512000	5.40E-18
chr12	2508001	2513000	1.51E-21
chr12	2509001	2514000	5.88E-24
chr12	2510001	2515000	1.89E-24
chr12	2511001	2516000	3.13E-28
chr12	2512001	2517000	1.51E-19
chr12	2513001	2518000	3.65E-17
chr12	2514001	2519000	1.20E-17
chr12	2529001	2534000	4.76E-13
chr12	2530001	2535000	7.13E-14
chr12	2536001	2541000	5.67E-09
chr12	2537001	2542000	4.00E-08
chr12	2538001	2543000	1.43E-11
chr12	2539001	2544000	1.62E-08
chr12	2551001	2556000	1.65E-12
chr12	2552001	2557000	4.82E-11
chr12	2567001	2572000	5.26E-16
chr12	2577001	2582000	7.67E-16
chr12	2578001	2583000	5.86E-16
chr12	2581001	2586000	1.28E-15
chr12	2582001	2587000	1.39E-15
chr12	2583001	2588000	5.79E-16
chr12	2584001	2589000	8.91E-18
chr12	2585001	2590000	1.48E-14
chr12	2593001	2598000	9.71E-15
chr12	2594001	2599000	3.25E-22
chr12	2595001	2600000	1.61E-20
chr12	2604001	2609000	6.79E-17
chr12	2605001	2610000	5.40E-13
chr12	2619001	2624000	2.61E-16
chr12	2628001	2633000	5.39E-11
chr12	2636001	2641000	4.06E-17
chr12	2637001	2642000	1.39E-18

chr12	2638001	2643000	9.43E-19
chr12	2639001	2644000	2.10E-23
chr12	2640001	2645000	1.24E-18
chr12	2641001	2646000	1.88E-17
chr12	2642001	2647000	1.92E-14
chr12	2643001	2648000	1.00E-16
chr12	2644001	2649000	6.98E-13
chr12	2645001	2650000	5.50E-12
chr12	2666001	2671000	1.48E-12
chr12	2678001	2683000	5.76E-09
chr12	2679001	2684000	3.14E-09
chr12	2680001	2685000	2.77E-06
chr12	2688001	2693000	4.17E-12
chr12	2689001	2694000	2.91E-11
chr12	2690001	2695000	8.82E-11
chr12	2691001	2696000	3.79E-09
chr12	2703001	2708000	2.28E-10
chr12	2719001	2724000	2.05E-11
chr12	2720001	2725000	7.88E-13
chr12	2721001	2726000	6.32E-07
chr12	2772001	2777000	3.67E-14
chr12	2773001	2778000	1.08E-13
chr12	2774001	2779000	1.49E-18
chr12	2775001	2780000	1.37E-23
chr12	2776001	2781000	3.70E-16
chr12	2777001	2782000	1.42E-17
chr12	2796001	2801000	4.71E-14
chr12	2843001	2848000	4.32E-09
chr12	2845001	2850000	4.29E-11
chr12	2872001	2877000	3.01E-10
chr12	2873001	2878000	1.57E-06
chr12	2874001	2879000	3.17E-08
chr12	2878001	2883000	1.05E-07
chr12	2879001	2884000	2.69E-11
chr12	2880001	2885000	9.87E-11
chr12	2881001	2886000	7.12E-15
chr12	2889001	2894000	3.47E-11
chr12	2891001	2896000	5.36E-14
chr12	2892001	2897000	3.56E-14
chr12	2893001	2898000	4.53E-12
chr12	4005001	4010000	6.04E-14
chr12	4007001	4012000	1.98E-12
chr12	4009001	4014000	2.79E-15
chr12	4170001	4175000	1.01E-21
chr12	4171001	4176000	4.24E-24
chr12	4172001	4177000	4.09E-22
chr12	4173001	4178000	8.62E-22
chr12	4174001	4179000	4.43E-15

chr12	4175001	4180000	1.10E-13
chr12	4181001	4186000	4.73E-15
chr12	4185001	4190000	9.58E-12
chr12	4475001	4480000	5.93E-09
chr12	4487001	4492000	4.68E-09
chr12	4488001	4493000	3.34E-06
chr12	4489001	4494000	2.68E-05
chr12	4501001	4506000	1.33E-08
chr12	4502001	4507000	2.31E-11
chr12	4503001	4508000	4.34E-11
chr12	4519001	4524000	1.32E-14
chr12	4574001	4579000	3.89E-11
chr12	4575001	4580000	4.06E-12
chr12	4577001	4582000	4.87E-15
chr12	4584001	4589000	3.99E-14
chr12	4585001	4590000	4.62E-18
chr12	4586001	4591000	2.63E-15
chr12	4587001	4592000	1.28E-14
chr12	4588001	4593000	8.20E-17
chr12	4694001	4699000	2.78E-09
chr12	4695001	4700000	1.49E-10
chr12	4696001	4701000	3.31E-10
chr12	4697001	4702000	3.85E-14
chr12	4818001	4823000	1.38E-15
chr12	4850001	4855000	3.25E-14
chr12	4851001	4856000	8.28E-18
chr12	4852001	4857000	8.02E-18
chr12	4853001	4858000	2.25E-14
chr12	4854001	4859000	1.59E-15
chr12	4855001	4860000	4.09E-18
chr12	4856001	4861000	2.35E-16
chr12	4857001	4862000	1.35E-14
chr12	4858001	4863000	1.08E-13
chr12	4859001	4864000	6.16E-13
chr12	4860001	4865000	2.27E-09
chr12	4861001	4866000	5.10E-09
chr12	4862001	4867000	1.24E-08
chr12	4863001	4868000	1.83E-07
chr12	4864001	4869000	1.18E-10
chr12	4865001	4870000	2.30E-09
chr12	4866001	4871000	3.44E-07
chr12	4867001	4872000	1.58E-06
chr12	4870001	4875000	1.82E-07
chr12	4871001	4876000	7.92E-12
chr12	4872001	4877000	8.20E-12
chr12	4886001	4891000	3.67E-10
chr12	4898001	4903000	2.82E-11
chr12	4900001	4905000	2.96E-14

chr12	4906001	4911000	1.26E-07
chr12	4907001	4912000	9.25E-12
chr12	4908001	4913000	5.43E-10
chr12	4909001	4914000	4.17E-11
chr12	4910001	4915000	8.16E-15
chr12	4911001	4916000	4.58E-13
chr12	4940001	4945000	3.46E-12
chr12	4941001	4946000	7.58E-10
chr12	4942001	4947000	3.87E-11
chr12	4984001	4989000	1.42E-12
chr12	4985001	4990000	3.60E-13
chr12	4986001	4991000	5.85E-16
chr12	5044001	5049000	2.06E-19
chr12	5045001	5050000	1.24E-18
chr12	5046001	5051000	2.07E-19
chr12	5047001	5052000	9.36E-19
chr12	5048001	5053000	1.06E-17
chr12	5067001	5072000	1.09E-12
chr12	5096001	5101000	2.90E-11
chr12	5103001	5108000	1.29E-10
chr12	5104001	5109000	2.50E-08
chr12	5113001	5118000	9.31E-09
chr12	5114001	5119000	2.30E-10
chr12	5115001	5120000	1.22E-10
chr12	5116001	5121000	1.42E-08
chr12	5156001	5161000	1.05E-10
chr12	5157001	5162000	3.03E-10
chr12	5167001	5172000	3.51E-10
chr12	5168001	5173000	5.71E-10
chr12	5169001	5174000	3.29E-09
chr12	5170001	5175000	2.19E-15
chr12	5171001	5176000	1.34E-13
chr12	5190001	5195000	1.75E-24
chr12	5220001	5225000	5.36E-07
chr12	5635001	5640000	8.41E-18
chr12	5636001	5641000	1.84E-21
chr12	5637001	5642000	1.06E-18
chr12	5638001	5643000	9.21E-18
chr12	5640001	5645000	1.08E-05
chr12	5641001	5646000	1.33E-06
chr12	5648001	5653000	8.84E-15
chr12	5651001	5656000	4.64E-06
chr12	5760001	5765000	1.89E-16
chr12	5761001	5766000	1.71E-19
chr12	5762001	5767000	2.49E-21
chr12	5763001	5768000	1.01E-17
chr12	5764001	5769000	1.34E-15
chr12	5783001	5788000	2.40E-07

chr12	5790001	5795000	5.08E-09
chr12	5860001	5865000	1.93E-09
chr12	5872001	5877000	2.12E-16
chr12	5873001	5878000	1.79E-11
chr12	5874001	5879000	8.33E-10
chr12	5882001	5887000	1.67E-17
chr12	5883001	5888000	2.78E-16
chr12	5884001	5889000	1.09E-13
chr12	5925001	5930000	5.14E-11
chr12	5926001	5931000	7.44E-09
chr12	5931001	5936000	1.14E-08
chr12	5932001	5937000	6.34E-09
chr12	5933001	5938000	1.35E-09
chr12	5934001	5939000	4.75E-14
chr12	5935001	5940000	3.55E-16
chr12	6053001	6058000	3.47E-12
chr12	6054001	6059000	3.92E-13
chr12	6176001	6181000	1.90E-14
chr12	6177001	6182000	8.36E-14
chr12	6178001	6183000	1.78E-09
chr12	6198001	6203000	1.69E-05
chr12	6199001	6204000	1.85E-06
chr12	6303001	6308000	7.26E-15
chr12	6304001	6309000	1.22E-17
chr12	6305001	6310000	5.59E-27
chr12	6306001	6311000	7.89E-27
chr12	6307001	6312000	5.02E-29
chr12	6308001	6313000	2.08E-14
chr12	6309001	6314000	1.57E-10
chr12	6310001	6315000	1.32E-07
chr12	6478001	6483000	1.71E-11
chr12	6479001	6484000	3.63E-13
chr12	6480001	6485000	3.11E-15
chr12	6481001	6486000	3.78E-13
chr12	6482001	6487000	8.97E-15
chr12	6483001	6488000	5.15E-06
chr12	6674001	6679000	5.12E-09
chr12	6798001	6803000	1.17E-09
chr12	6974001	6979000	4.13E-06
chr12	6981001	6986000	5.29E-10
chr12	6982001	6987000	4.09E-08
chr12	6993001	6998000	5.01E-08
chr12	6994001	6999000	3.07E-07
chr12	7000001	7005000	0.001748
chr12	7019001	7024000	1.77E-09
chr12	7020001	7025000	5.22E-11
chr12	7021001	7026000	2.30E-14
chr12	7022001	7027000	7.62E-12

chr12	7023001	7028000	9.29E-06
chr12	7043001	7048000	3.04E-08
chr12	7054001	7059000	4.01E-08
chr12	7069001	7074000	2.69E-05
chr12	7070001	7075000	1.79E-11
chr12	7071001	7076000	1.94E-07
chr12	7078001	7083000	4.47E-11
chr12	7079001	7084000	6.51E-10
chr12	7122001	7127000	1.85E-15
chr12	7125001	7130000	1.71E-10
chr12	7279001	7284000	1.73E-05
chr12	7280001	7285000	0.00046
chr12	7281001	7286000	7.24E-05
chr12	7282001	7287000	0.0012
chr12	7325001	7330000	1.49E-08
chr12	7326001	7331000	2.15E-09
chr12	7380001	7385000	1.36E-08
chr12	7450001	7455000	4.18E-13
chr12	7451001	7456000	9.05E-16
chr12	7452001	7457000	5.77E-15
chr12	7540001	7545000	4.44E-09
chr12	7571001	7576000	4.21E-06
chr12	7573001	7578000	0.000257
chr12	7596001	7601000	3.07E-07
chr12	7619001	7624000	2.64E-09
chr12	7669001	7674000	1.49E-08
chr12	7675001	7680000	1.10E-07
chr12	7702001	7707000	9.73E-07
chr12	7703001	7708000	9.54E-12
chr12	7704001	7709000	5.33E-10
chr12	7770001	7775000	9.48E-09
chr12	7819001	7824000	2.51E-05
chr12	7883001	7888000	2.13E-06
chr12	7899001	7904000	0.000657
chr12	7935001	7940000	1.85E-07
chr12	7936001	7941000	7.77E-07
chr12	8161001	8166000	5.86E-06
chr12	8162001	8167000	2.48E-05
chr12	8169001	8174000	1.37E-11
chr12	8170001	8175000	3.55E-13
chr12	8171001	8176000	3.87E-08
chr12	8172001	8177000	1.45E-07
chr12	8175001	8180000	1.46E-14
chr12	8176001	8181000	1.89E-13
chr12	8303001	8308000	1.37E-07
chr12	8304001	8309000	1.00E-08
chr12	8415001	8420000	5.44E-08
chr12	8442001	8447000	1.25E-16

chr12	8453001	8458000	2.21E-10
chr12	8464001	8469000	4.10E-08
chr12	8477001	8482000	7.68E-06
chr12	8478001	8483000	1.59E-06
chr12	8480001	8485000	2.21E-06
chr12	8497001	8502000	0.004786
chr12	8498001	8503000	0.018705
chr12	8513001	8518000	1.18E-07
chr12	8514001	8519000	2.17E-07
chr12	8635001	8640000	1.24E-09
chr12	8636001	8641000	6.81E-09
chr12	8637001	8642000	7.44E-11
chr12	8638001	8643000	1.62E-14
chr12	8639001	8644000	1.14E-12
chr12	8645001	8650000	8.87E-15
chr12	8649001	8654000	7.34E-19
chr12	8652001	8657000	9.30E-15
chr12	8655001	8660000	2.95E-14
chr12	8656001	8661000	5.14E-15
chr12	8661001	8666000	5.75E-11
chr12	8662001	8667000	1.64E-13
chr12	8663001	8668000	4.43E-13
chr12	8664001	8669000	1.60E-12
chr12	8740001	8745000	2.75E-11
chr12	8743001	8748000	1.00E-08
chr12	8843001	8848000	5.59E-09
chr12	9130001	9135000	1.35E-11
chr12	9131001	9136000	2.52E-10
chr12	9148001	9153000	4.76E-17
chr12	9149001	9154000	3.29E-22
chr12	9150001	9155000	3.33E-22
chr12	9151001	9156000	2.73E-26
chr12	9152001	9157000	2.79E-26
chr12	9153001	9158000	5.84E-18
chr12	9154001	9159000	1.06E-15
chr12	9155001	9160000	1.20E-14
chr12	9305001	9310000	8.96E-12
chr12	9306001	9311000	3.12E-11
chr12	9307001	9312000	1.23E-06
chr12	9557001	9562000	1.55E-10
chr12	10148001	10153000	1.85E-14
chr12	10431001	10436000	2.56E-14
chr12	10432001	10437000	4.92E-15
chr12	10510001	10515000	5.09E-06
chr12	10511001	10516000	0.000223
chr12	10512001	10517000	2.65E-07
chr12	10620001	10625000	1.31E-10
chr12	10621001	10626000	9.34E-10

chr12	10622001	10627000	8.41E-13
chr12	10623001	10628000	2.58E-14
chr12	10686001	10691000	1.20E-08
chr12	10722001	10727000	3.41E-13
chr12	10946001	10951000	2.61E-11
chr12	11407001	11412000	1.36E-09
chr12	11408001	11413000	2.45E-13
chr12	11409001	11414000	2.03E-14
chr12	11410001	11415000	3.36E-11
chr12	11411001	11416000	6.31E-10
chr12	11450001	11455000	3.59E-06
chr12	11467001	11472000	3.14E-10
chr12	11527001	11532000	1.24E-16
chr12	11552001	11557000	2.30E-11
chr12	11563001	11568000	4.66E-12
chr12	11564001	11569000	7.54E-11
chr12	11565001	11570000	1.03E-09
chr12	11566001	11571000	8.61E-10
chr12	11609001	11614000	3.71E-07
chr12	11610001	11615000	3.57E-07
chr12	11611001	11616000	5.09E-10
chr12	12031001	12036000	4.66E-14
chr12	12488001	12493000	2.17E-10
chr12	12564001	12569000	0.000149
chr12	12565001	12570000	4.19E-06
chr12	12566001	12571000	0.002594
chr12	12957001	12962000	1.48E-09
chr12	13396001	13401000	2.43E-08
chr12	13397001	13402000	7.94E-10
chr12	13434001	13439000	1.54E-10
chr12	13466001	13471000	5.63E-13
chr12	13467001	13472000	7.17E-10
chr12	13468001	13473000	1.26E-10
chr12	13499001	13504000	1.38E-05
chr12	13742001	13747000	2.79E-13
chr12	13743001	13748000	3.26E-10
chr12	13744001	13749000	3.22E-10
chr12	13745001	13750000	3.04E-09
chr12	13774001	13779000	5.29E-20
chr12	13775001	13780000	1.40E-21
chr12	13776001	13781000	2.28E-21
chr12	13777001	13782000	2.61E-18
chr12	13778001	13783000	5.66E-16
chr12	13807001	13812000	1.77E-21
chr12	13808001	13813000	2.08E-23
chr12	13821001	13826000	4.34E-09
chr12	13823001	13828000	6.89E-09
chr12	13824001	13829000	2.46E-10

chr12	13825001	13830000	9.58E-08
chr12	13826001	13831000	1.43E-08
chr12	13827001	13832000	4.30E-10
chr12	13828001	13833000	1.35E-09
chr12	13829001	13834000	1.77E-08
chr12	13870001	13875000	2.10E-14
chr12	13872001	13877000	2.17E-12
chr12	13898001	13903000	1.36E-13
chr12	13904001	13909000	1.73E-15
chr12	13929001	13934000	1.15E-11
chr12	13930001	13935000	5.94E-19
chr12	13931001	13936000	2.76E-16
chr12	13932001	13937000	1.89E-14
chr12	13933001	13938000	3.38E-11
chr12	13934001	13939000	1.91E-11
chr12	13955001	13960000	8.41E-10
chr12	13956001	13961000	2.88E-16
chr12	13957001	13962000	4.63E-14
chr12	13958001	13963000	4.35E-14
chr12	13960001	13965000	1.43E-13
chr12	13982001	13987000	7.40E-12
chr12	14006001	14011000	1.30E-10
chr12	14007001	14012000	2.87E-15
chr12	14008001	14013000	3.16E-18
chr12	14010001	14015000	5.63E-18
chr12	14057001	14062000	5.88E-14
chr12	14058001	14063000	2.83E-20
chr12	14059001	14064000	5.60E-13
chr12	14143001	14148000	4.10E-15
chr12	14144001	14149000	2.75E-17
chr12	14152001	14157000	2.69E-18
chr12	14158001	14163000	7.60E-14
chr12	14159001	14164000	6.32E-15
chr12	14160001	14165000	1.82E-11
chr12	14161001	14166000	2.36E-11
chr12	14162001	14167000	8.65E-13
chr12	14163001	14168000	2.48E-16
chr12	14164001	14169000	3.86E-13
chr12	14165001	14170000	9.41E-12
chr12	14171001	14176000	9.62E-16
chr12	14183001	14188000	6.87E-14
chr12	14184001	14189000	1.62E-15
chr12	14185001	14190000	4.09E-20
chr12	14186001	14191000	2.17E-16
chr12	14204001	14209000	3.11E-10
chr12	14260001	14265000	2.08E-09
chr12	14267001	14272000	5.95E-13
chr12	14268001	14273000	7.60E-09

chr12	14269001	14274000	2.84E-08
chr12	14273001	14278000	1.33E-09
chr12	14296001	14301000	1.18E-11
chr12	14297001	14302000	4.14E-10
chr12	14483001	14488000	9.95E-06
chr12	14484001	14489000	2.04E-10
chr12	14485001	14490000	2.50E-10
chr12	14487001	14492000	1.65E-07
chr12	14737001	14742000	4.94E-06
chr12	14738001	14743000	4.07E-08
chr12	14739001	14744000	9.54E-07
chr12	14740001	14745000	4.64E-06
chr12	14741001	14746000	7.44E-09
chr12	14772001	14777000	4.22E-11
chr12	14835001	14840000	3.65E-10
chr12	14872001	14877000	1.41E-07
chr12	14873001	14878000	5.39E-07
chr12	15170001	15175000	1.92E-13
chr12	15282001	15287000	3.75E-13
chr12	15283001	15288000	4.01E-10
chr12	15462001	15467000	2.23E-10
chr12	15464001	15469000	2.39E-09
chr12	15600001	15605000	0.000149
chr12	15607001	15612000	1.33E-08
chr12	15608001	15613000	7.35E-06
chr12	15651001	15656000	5.21E-14
chr12	15652001	15657000	6.70E-14
chr12	15668001	15673000	2.12E-07
chr12	16243001	16248000	1.32E-15
chr12	16254001	16259000	6.24E-10
chr12	16255001	16260000	3.92E-07
chr12	16256001	16261000	2.32E-05
chr12	16257001	16262000	4.37E-08
chr12	16258001	16263000	1.37E-09
chr12	16384001	16389000	2.60E-10
chr12	16385001	16390000	2.98E-12
chr12	17243001	17248000	2.17E-15
chr12	17244001	17249000	3.49E-15
chr12	17245001	17250000	1.90E-18
chr12	17246001	17251000	7.94E-17
chr12	17343001	17348000	9.09E-10
chr12	17356001	17361000	2.88E-09
chr12	17471001	17476000	6.69E-10
chr12	17680001	17685000	2.40E-13
chr12	17681001	17686000	5.68E-15
chr12	17682001	17687000	3.20E-15
chr12	17683001	17688000	1.41E-17
chr12	17684001	17689000	2.30E-14

chr12	17685001	17690000	5.95E-14
chr12	17686001	17691000	7.00E-13
chr12	17687001	17692000	4.39E-13
chr12	17688001	17693000	4.86E-11
chr12	17697001	17702000	2.56E-07
chr12	17712001	17717000	9.17E-16
chr12	17713001	17718000	9.13E-15
chr12	17842001	17847000	1.42E-11
chr12	17948001	17953000	8.48E-07
chr12	17949001	17954000	1.59E-07
chr12	17950001	17955000	4.56E-07
chr12	17982001	17987000	2.32E-06
chr12	18099001	18104000	7.35E-10
chr12	18100001	18105000	1.20E-09
chr12	18179001	18184000	1.50E-10
chr12	18180001	18185000	1.03E-12
chr12	18324001	18329000	1.10E-07
chr12	18325001	18330000	5.67E-10
chr12	18326001	18331000	2.63E-12
chr12	18327001	18332000	4.79E-19
chr12	18328001	18333000	2.58E-20
chr12	18329001	18334000	1.80E-18
chr12	18330001	18335000	3.18E-16
chr12	18331001	18336000	2.90E-12
chr12	19550001	19555000	6.28E-10
chr12	19551001	19556000	1.09E-09
chr12	19552001	19557000	2.04E-09
chr12	20043001	20048000	2.66E-13
chr12	20045001	20050000	1.22E-16
chr12	20080001	20085000	1.51E-18
chr12	20084001	20089000	1.71E-15
chr12	20091001	20096000	6.99E-19
chr12	20127001	20132000	6.07E-15
chr12	20128001	20133000	1.09E-22
chr12	20129001	20134000	6.82E-24
chr12	20130001	20135000	2.16E-17
chr12	20131001	20136000	2.87E-15
chr12	20156001	20161000	2.16E-11
chr12	20218001	20223000	2.76E-12
chr12	20219001	20224000	7.02E-15
chr12	20220001	20225000	3.78E-14
chr12	20221001	20226000	1.91E-13
chr12	20222001	20227000	3.93E-11
chr12	20223001	20228000	8.45E-10
chr12	20224001	20229000	9.47E-13
chr12	20268001	20273000	9.98E-17
chr12	20292001	20297000	4.74E-14
chr12	20294001	20299000	2.15E-09

chr12	20295001	20300000	2.84E-09
chr12	20299001	20304000	1.38E-14
chr12	20323001	20328000	7.08E-10
chr12	20401001	20406000	1.81E-08
chr12	20403001	20408000	4.73E-09
chr12	20437001	20442000	1.82E-14
chr12	20446001	20451000	5.09E-14
chr12	20447001	20452000	1.09E-07
chr12	20448001	20453000	9.18E-12
chr12	20484001	20489000	9.34E-10
chr12	20485001	20490000	3.68E-08
chr12	20709001	20714000	1.36E-17
chr12	20783001	20788000	2.97E-15
chr12	20784001	20789000	1.45E-16
chr12	20807001	20812000	5.21E-08
chr12	20808001	20813000	3.93E-08
chr12	20809001	20814000	8.10E-09
chr12	20810001	20815000	4.54E-10
chr12	20811001	20816000	2.91E-09
chr12	20812001	20817000	2.24E-08
chr12	20813001	20818000	4.94E-07
chr12	20816001	20821000	9.41E-08
chr12	20839001	20844000	6.28E-12
chr12	20840001	20845000	1.05E-14
chr12	20841001	20846000	8.74E-15
chr12	20842001	20847000	8.24E-10
chr12	21100001	21105000	3.91E-08
chr12	21101001	21106000	1.14E-07
chr12	21113001	21118000	3.23E-07
chr12	21155001	21160000	2.86E-10
chr12	21159001	21164000	6.74E-10
chr12	21162001	21167000	2.48E-07
chr12	21166001	21171000	1.19E-09
chr12	21167001	21172000	5.15E-14
chr12	21186001	21191000	3.43E-11
chr12	21187001	21192000	1.75E-11
chr12	21226001	21231000	2.41E-08
chr12	21227001	21232000	2.47E-08
chr12	21228001	21233000	1.11E-07
chr12	21229001	21234000	8.73E-09
chr12	21245001	21250000	4.39E-14
chr12	21331001	21336000	9.72E-14
chr12	21333001	21338000	9.46E-11
chr12	21334001	21339000	7.05E-10
chr12	21342001	21347000	3.82E-11
chr12	21735001	21740000	3.32E-09
chr12	21891001	21896000	1.73E-08
chr12	22071001	22076000	1.14E-11

chr12	22194001	22199000	4.43E-16
chr12	22195001	22200000	3.26E-17
chr12	22758001	22763000	5.85E-08
chr12	23036001	23041000	8.39E-15
chr12	23302001	23307000	1.62E-08
chr12	25287001	25292000	0.00027
chr12	25330001	25335000	2.61E-14
chr12	25331001	25336000	4.24E-11
chr12	25639001	25644000	7.40E-16
chr12	25739001	25744000	2.55E-09
chr12	25740001	25745000	6.88E-09
chr12	25741001	25746000	1.75E-07
chr12	25757001	25762000	2.82E-12
chr12	25760001	25765000	4.34E-17
chr12	25761001	25766000	7.29E-16
chr12	25774001	25779000	8.13E-16
chr12	25775001	25780000	1.24E-10
chr12	25856001	25861000	1.51E-09
chr12	26035001	26040000	5.62E-14
chr12	26045001	26050000	9.07E-12
chr12	26057001	26062000	2.41E-09
chr12	26058001	26063000	6.21E-10
chr12	26059001	26064000	2.88E-09
chr12	27037001	27042000	4.65E-07
chr12	27174001	27179000	2.30E-08
chr12	28760001	28765000	9.66E-07
chr12	28940001	28945000	5.05E-10
chr12	28942001	28947000	3.24E-09
chr12	28959001	28964000	2.25E-14
chr12	28964001	28969000	4.20E-13
chr12	28965001	28970000	5.10E-14
chr12	28966001	28971000	2.68E-15
chr12	28982001	28987000	5.62E-10
chr12	28983001	28988000	1.23E-10
chr12	28996001	29001000	5.52E-08
chr12	29002001	29007000	4.26E-15
chr12	29005001	29010000	5.05E-17
chr12	29058001	29063000	6.46E-14
chr12	29059001	29064000	2.46E-16
chr12	29060001	29065000	8.58E-16
chr12	29061001	29066000	9.73E-19
chr12	29062001	29067000	2.08E-15
chr12	29128001	29133000	7.68E-07
chr12	29239001	29244000	1.17E-05
chr12	29245001	29250000	8.03E-11
chr12	29246001	29251000	1.31E-09
chr12	29259001	29264000	7.18E-16
chr12	29260001	29265000	5.76E-14

chr12	29261001	29266000	6.35E-17
chr12	29262001	29267000	1.79E-17
chr12	29263001	29268000	6.80E-10
chr12	29279001	29284000	5.72E-14
chr12	29280001	29285000	2.38E-11
chr12	29566001	29571000	2.41E-07
chr12	29579001	29584000	2.99E-08
chr12	29581001	29586000	1.28E-07
chr12	29582001	29587000	4.42E-11
chr12	29806001	29811000	1.15E-11
chr12	29840001	29845000	4.18E-13
chr12	29841001	29846000	1.29E-13
chr12	29842001	29847000	3.93E-12
chr12	29977001	29982000	1.27E-08
chr12	29978001	29983000	1.08E-07
chr12	29979001	29984000	4.64E-07
chr12	29980001	29985000	1.60E-07
chr12	30341001	30346000	4.05E-11
chr12	30462001	30467000	2.85E-06
chr12	30463001	30468000	9.62E-08
chr12	30464001	30469000	4.91E-08
chr12	30476001	30481000	7.85E-05
chr12	30496001	30501000	1.62E-12
chr12	30934001	30939000	1.21E-12
chr12	30967001	30972000	1.98E-07
chr12	30968001	30973000	3.52E-07
chr12	30969001	30974000	2.73E-07
chr12	30970001	30975000	1.49E-07
chr12	30981001	30986000	2.34E-11
chr12	30982001	30987000	2.89E-09
chr12	31107001	31112000	4.78E-08
chr12	31108001	31113000	1.29E-09
chr12	31109001	31114000	8.55E-12
chr12	31111001	31116000	1.36E-12
chr12	31120001	31125000	8.16E-14
chr12	31228001	31233000	1.71E-13
chr12	31229001	31234000	7.73E-14
chr12	31230001	31235000	1.18E-11
chr12	31231001	31236000	2.93E-14
chr12	31382001	31387000	2.70E-06
chr12	31478001	31483000	5.17E-17
chr12	31479001	31484000	3.76E-13
chr12	31743001	31748000	2.85E-13
chr12	31744001	31749000	4.93E-10
chr12	31752001	31757000	1.34E-05
chr12	31753001	31758000	5.68E-05
chr12	32060001	32065000	2.20E-09
chr12	32828001	32833000	1.82E-16

chr12	33189001	33194000	4.56E-09
chr12	33196001	33201000	8.39E-15
chr12	33230001	33235000	1.65E-09
chr12	33233001	33238000	3.71E-11
chr12	33234001	33239000	9.42E-11
chr12	33235001	33240000	2.35E-10
chr12	33266001	33271000	4.92E-10
chr12	33267001	33272000	2.01E-11
chr12	33300001	33305000	1.82E-05
chr12	33301001	33306000	1.34E-05
chr12	33302001	33307000	1.54E-05
chr12	33336001	33341000	2.24E-07
chr12	33345001	33350000	0.000181
chr12	33346001	33351000	1.43E-06
chr12	33461001	33466000	1.44E-12
chr12	33462001	33467000	4.49E-09
chr12	33463001	33468000	1.52E-06
chr12	33505001	33510000	1.80E-16
chr12	33506001	33511000	5.05E-17
chr12	33507001	33512000	1.32E-19
chr12	33508001	33513000	1.24E-20
chr12	33509001	33514000	1.50E-15
chr12	33526001	33531000	3.59E-23
chr12	33528001	33533000	2.44E-24
chr12	33566001	33571000	1.29E-16
chr12	33598001	33603000	3.66E-16
chr12	33599001	33604000	2.72E-16
chr12	33694001	33699000	1.14E-10
chr12	33714001	33719000	2.61E-08
chr12	33724001	33729000	1.59E-07
chr12	33725001	33730000	2.54E-09
chr12	33726001	33731000	1.41E-08
chr12	33807001	33812000	1.36E-13
chr12	33814001	33819000	1.06E-10
chr12	33815001	33820000	3.42E-12
chr12	33816001	33821000	6.36E-12
chr12	33817001	33822000	5.70E-08
chr12	33857001	33862000	1.61E-15
chr12	33858001	33863000	1.50E-16
chr12	33859001	33864000	4.03E-14
chr12	33860001	33865000	2.85E-14
chr12	33868001	33873000	6.72E-15
chr12	33910001	33915000	4.92E-12
chr12	33911001	33916000	2.66E-10
chr12	33912001	33917000	3.52E-09
chr12	33913001	33918000	2.63E-08
chr12	33930001	33935000	4.06E-13
chr12	33931001	33936000	1.22E-14

chr12	33932001	33937000	4.64E-15
chr12	33933001	33938000	1.28E-13
chr12	33934001	33939000	6.05E-07
chr12	33935001	33940000	2.78E-09
chr12	33936001	33941000	1.71E-09
chr12	33937001	33942000	3.04E-09
chr12	33938001	33943000	1.33E-10
chr12	33939001	33944000	6.60E-09
chr12	33953001	33958000	1.76E-12
chr12	33970001	33975000	1.76E-08
chr12	34003001	34008000	6.17E-10
chr12	34004001	34009000	1.89E-14
chr12	34005001	34010000	4.20E-16
chr12	34006001	34011000	2.88E-16
chr12	34007001	34012000	2.59E-20
chr12	34008001	34013000	1.66E-17
chr12	34009001	34014000	8.99E-17
chr12	34010001	34015000	1.25E-11
chr12	34011001	34016000	3.26E-13
chr12	34012001	34017000	4.30E-11
chr12	34013001	34018000	3.47E-07
chr12	34027001	34032000	2.09E-13
chr12	34028001	34033000	2.52E-12
chr12	34029001	34034000	4.39E-09
chr12	34076001	34081000	1.01E-08
chr12	34077001	34082000	8.78E-08
chr12	34078001	34083000	1.59E-07
chr12	34090001	34095000	1.79E-09
chr12	34099001	34104000	1.19E-11
chr12	34100001	34105000	6.91E-12
chr12	34101001	34106000	1.98E-09
chr12	34102001	34107000	2.22E-08
chr12	34109001	34114000	5.92E-16
chr12	34111001	34116000	2.94E-10
chr12	34112001	34117000	1.32E-09
chr12	34130001	34135000	1.65E-11
chr12	34131001	34136000	1.09E-11
chr12	34132001	34137000	9.39E-13
chr12	34225001	34230000	5.55E-10
chr12	34227001	34232000	1.85E-07
chr12	34283001	34288000	2.10E-06
chr12	34312001	34317000	6.56E-08
chr12	34326001	34331000	2.30E-12
chr12	34327001	34332000	2.47E-10
chr12	34328001	34333000	1.02E-09
chr12	34334001	34339000	5.20E-14
chr12	34335001	34340000	4.35E-14
chr12	34336001	34341000	1.78E-14

chr12	34337001	34342000	1.26E-13
chr12	34338001	34343000	6.56E-09
chr12	34439001	34444000	1.05E-07
chr12	34440001	34445000	3.92E-06
chr12	34441001	34446000	1.94E-06
chr12	34442001	34447000	1.03E-10
chr12	34512001	34517000	4.87E-15
chr12	34513001	34518000	1.59E-15
chr12	34514001	34519000	4.68E-13
chr12	34515001	34520000	1.62E-11
chr12	34522001	34527000	7.59E-11
chr12	34523001	34528000	4.93E-07
chr12	34525001	34530000	2.59E-05
chr12	34526001	34531000	1.32E-08
chr12	34533001	34538000	9.90E-10
chr12	34534001	34539000	4.13E-12
chr12	34535001	34540000	2.13E-12
chr12	34536001	34541000	1.66E-13
chr12	34537001	34542000	9.14E-11
chr12	34539001	34544000	9.26E-08
chr12	34541001	34546000	1.27E-06
chr12	34542001	34547000	4.27E-09
chr12	34651001	34656000	5.38E-06
chr12	34779001	34784000	5.32E-12
chr12	37962001	37967000	9.89E-07
chr12	37963001	37968000	2.57E-06
chr12	38139001	38144000	2.57E-09
chr12	38257001	38262000	2.68E-07
chr12	38373001	38378000	2.40E-06
chr12	38380001	38385000	0.000421
chr12	38381001	38386000	7.97E-05
chr12	38431001	38436000	2.14E-05
chr12	38442001	38447000	1.38E-05
chr12	38602001	38607000	6.67E-10
chr12	38614001	38619000	4.57E-12
chr12	38679001	38684000	2.49E-11
chr12	38826001	38831000	9.47E-10
chr12	39010001	39015000	6.78E-09
chr12	39028001	39033000	3.44E-10
chr12	39029001	39034000	1.55E-12
chr12	39030001	39035000	4.84E-12
chr12	39031001	39036000	5.17E-11
chr12	39032001	39037000	1.90E-07
chr12	39086001	39091000	9.00E-13
chr12	39098001	39103000	9.77E-10
chr12	39099001	39104000	1.02E-09
chr12	39148001	39153000	1.06E-09
chr12	39149001	39154000	1.17E-11

chr12	39150001	39155000	4.03E-14
chr12	39151001	39156000	2.87E-13
chr12	39169001	39174000	1.23E-07
chr12	39170001	39175000	2.06E-11
chr12	39171001	39176000	2.10E-09
chr12	39172001	39177000	6.03E-10
chr12	39173001	39178000	2.78E-09
chr12	39174001	39179000	1.76E-09
chr12	39176001	39181000	7.02E-08
chr12	39177001	39182000	7.77E-06
chr12	39178001	39183000	2.61E-06
chr12	39456001	39461000	3.75E-13
chr12	39457001	39462000	5.22E-13
chr12	39507001	39512000	1.65E-09
chr12	39603001	39608000	1.33E-09
chr12	40125001	40130000	6.40E-16
chr12	40126001	40131000	2.28E-16
chr12	40127001	40132000	2.92E-11
chr12	40137001	40142000	1.84E-10
chr12	40138001	40143000	1.08E-08
chr12	40139001	40144000	1.71E-09
chr12	40140001	40145000	3.26E-10
chr12	40208001	40213000	2.11E-12
chr12	40209001	40214000	4.81E-14
chr12	40230001	40235000	6.01E-10
chr12	40239001	40244000	1.53E-05
chr12	40271001	40276000	4.00E-10
chr12	40343001	40348000	1.29E-09
chr12	40469001	40474000	3.34E-15
chr12	40470001	40475000	2.53E-17
chr12	40471001	40476000	1.98E-18
chr12	40472001	40477000	2.77E-14
chr12	40473001	40478000	2.36E-14
chr12	40474001	40479000	1.05E-11
chr12	40475001	40480000	2.16E-15
chr12	40476001	40481000	1.94E-14
chr12	40477001	40482000	2.24E-14
chr12	40478001	40483000	4.10E-12
chr12	40485001	40490000	1.68E-11
chr12	40789001	40794000	2.97E-13
chr12	40802001	40807000	1.99E-08
chr12	40803001	40808000	7.07E-07
chr12	40896001	40901000	1.16E-11
chr12	40897001	40902000	7.31E-09
chr12	40935001	40940000	6.52E-10
chr12	40936001	40941000	2.55E-07
chr12	40942001	40947000	1.63E-09
chr12	40943001	40948000	4.60E-12

chr12	40944001	40949000	1.95E-12
chr12	40945001	40950000	1.34E-11
chr12	40946001	40951000	2.03E-12
chr12	40950001	40955000	2.03E-07
chr12	40966001	40971000	3.66E-09
chr12	42204001	42209000	1.06E-07
chr12	42659001	42664000	2.87E-08
chr12	43007001	43012000	7.15E-11
chr12	43008001	43013000	1.42E-12
chr12	43009001	43014000	7.95E-17
chr12	43010001	43015000	3.42E-15
chr12	43011001	43016000	1.16E-15
chr12	43012001	43017000	6.49E-16
chr12	43013001	43018000	1.14E-17
chr12	43046001	43051000	1.41E-08
chr12	43047001	43052000	2.08E-09
chr12	43049001	43054000	1.46E-10
chr12	43095001	43100000	1.58E-12
chr12	43096001	43101000	2.69E-13
chr12	43097001	43102000	1.05E-06
chr12	43098001	43103000	1.52E-07
chr12	43099001	43104000	1.30E-09
chr12	43113001	43118000	0.000775
chr12	43275001	43280000	2.24E-15
chr12	43276001	43281000	2.12E-14
chr12	43303001	43308000	3.44E-08
chr12	43304001	43309000	6.29E-13
chr12	43415001	43420000	4.96E-08
chr12	43442001	43447000	5.90E-18
chr12	43443001	43448000	5.16E-16
chr12	43446001	43451000	1.22E-16
chr12	43455001	43460000	1.10E-10
chr12	43466001	43471000	2.62E-13
chr12	43467001	43472000	5.86E-12
chr12	43477001	43482000	1.12E-09
chr12	43478001	43483000	9.60E-11
chr12	43479001	43484000	1.64E-08
chr12	43480001	43485000	6.93E-09
chr12	43481001	43486000	8.30E-05
chr12	43519001	43524000	1.04E-10
chr12	43521001	43526000	4.74E-08
chr12	43534001	43539000	1.04E-10
chr12	43536001	43541000	1.30E-08
chr12	43537001	43542000	3.92E-08
chr12	43538001	43543000	1.90E-07
chr12	43580001	43585000	2.95E-12
chr12	43581001	43586000	1.18E-14
chr12	43582001	43587000	2.12E-14

chr12	43583001	43588000	4.54E-20
chr12	43617001	43622000	7.69E-20
chr12	43688001	43693000	6.93E-14
chr12	43689001	43694000	6.88E-14
chr12	43690001	43695000	1.21E-12
chr12	43691001	43696000	6.26E-11
chr12	43729001	43734000	0.002898
chr12	43730001	43735000	3.55E-05
chr12	43737001	43742000	1.80E-08
chr12	43738001	43743000	2.99E-10
chr12	43779001	43784000	2.14E-15
chr12	43780001	43785000	1.54E-13
chr12	43850001	43855000	3.61E-11
chr12	43851001	43856000	5.92E-12
chr12	43852001	43857000	1.67E-14
chr12	43853001	43858000	4.64E-20
chr12	43854001	43859000	4.00E-17
chr12	43855001	43860000	1.46E-14
chr12	43856001	43861000	1.06E-14
chr12	43939001	43944000	9.10E-12
chr12	43976001	43981000	7.67E-12
chr12	43988001	43993000	1.18E-12
chr12	43989001	43994000	1.42E-11
chr12	43990001	43995000	6.04E-16
chr12	44004001	44009000	2.40E-08
chr12	44034001	44039000	2.07E-10
chr12	44051001	44056000	7.02E-13
chr12	44052001	44057000	2.38E-12
chr12	44053001	44058000	3.55E-12
chr12	44054001	44059000	2.93E-09
chr12	44068001	44073000	3.93E-08
chr12	44872001	44877000	6.03E-07
chr12	44874001	44879000	8.19E-09
chr12	44922001	44927000	3.88E-11
chr12	44923001	44928000	1.31E-10
chr12	44925001	44930000	3.34E-14
chr12	44946001	44951000	7.71E-12
chr12	44947001	44952000	6.67E-13
chr12	45008001	45013000	3.88E-14
chr12	45052001	45057000	1.44E-08
chr12	45053001	45058000	3.27E-11
chr12	45054001	45059000	4.14E-12
chr12	45055001	45060000	9.02E-15
chr12	45088001	45093000	4.69E-11
chr12	45109001	45114000	2.89E-12
chr12	45110001	45115000	3.77E-10
chr12	45205001	45210000	1.87E-09
chr12	45257001	45262000	6.58E-05

chr12	45270001	45275000	3.46E-16
chr12	45353001	45358000	4.56E-07
chr12	45478001	45483000	3.10E-12
chr12	45484001	45489000	1.15E-13
chr12	45485001	45490000	2.65E-12
chr12	45524001	45529000	5.72E-08
chr12	45537001	45542000	4.32E-06
chr12	45538001	45543000	2.49E-06
chr12	45539001	45544000	8.16E-07
chr12	46419001	46424000	0.001646
chr12	46420001	46425000	1.63E-05
chr12	46421001	46426000	3.82E-08
chr12	46433001	46438000	4.86E-11
chr12	46662001	46667000	1.00E-15
chr12	46686001	46691000	3.09E-05
chr12	46687001	46692000	4.86E-10
chr12	46688001	46693000	4.33E-10
chr12	46719001	46724000	1.17E-08
chr12	46940001	46945000	1.33E-10
chr12	47277001	47282000	3.57E-15
chr12	47425001	47430000	8.61E-12
chr12	47905001	47910000	0.000588
chr12	47906001	47911000	9.79E-05
chr12	47907001	47912000	9.40E-05
chr12	48134001	48139000	2.00E-05
chr12	48298001	48303000	1.41E-07
chr12	48548001	48553000	2.51E-11
chr12	48551001	48556000	1.54E-05
chr12	48573001	48578000	4.88E-10
chr12	48574001	48579000	7.89E-11
chr12	48575001	48580000	3.23E-06
chr12	48576001	48581000	3.22E-07
chr12	48723001	48728000	2.20E-05
chr12	48724001	48729000	1.87E-09
chr12	48822001	48827000	8.28E-09
chr12	48823001	48828000	6.22E-08
chr12	48978001	48983000	0.000187
chr12	49257001	49262000	3.22E-08
chr12	49258001	49263000	1.38E-08
chr12	49259001	49264000	3.58E-08
chr12	49386001	49391000	0.000127
chr12	49387001	49392000	7.52E-05
chr12	49464001	49469000	0.000769
chr12	49502001	49507000	1.22E-07
chr12	49503001	49508000	3.13E-08
chr12	49656001	49661000	1.23E-08
chr12	49657001	49662000	1.55E-10
chr12	49658001	49663000	2.26E-13

chr12	49659001	49664000	6.60E-14
chr12	49660001	49665000	6.87E-10
chr12	49681001	49686000	8.50E-13
chr12	49682001	49687000	4.55E-10
chr12	49684001	49689000	3.53E-06
chr12	49685001	49690000	3.38E-07
chr12	49717001	49722000	0.003603
chr12	49927001	49932000	1.05E-09
chr12	49928001	49933000	5.86E-05
chr12	49929001	49934000	1.76E-06
chr12	49930001	49935000	6.29E-06
chr12	49931001	49936000	2.40E-05
chr12	49932001	49937000	0.002671
chr12	49957001	49962000	7.58E-08
chr12	50169001	50174000	0.000419
chr12	50170001	50175000	5.57E-05
chr12	50219001	50224000	5.21E-07
chr12	50220001	50225000	7.48E-10
chr12	50221001	50226000	3.53E-10
chr12	50222001	50227000	2.02E-10
chr12	50356001	50361000	1.20E-05
chr12	50357001	50362000	3.85E-06
chr12	50358001	50363000	8.59E-08
chr12	50359001	50364000	1.92E-05
chr12	50361001	50366000	0.000244
chr12	50504001	50509000	0.002238
chr12	50760001	50765000	0.00386
chr12	51155001	51160000	1.21E-08
chr12	51156001	51161000	3.29E-12
chr12	51157001	51162000	6.40E-09
chr12	51340001	51345000	0.001224
chr12	51639001	51644000	0.004703
chr12	51640001	51645000	0.001431
chr12	52211001	52216000	0.014887
chr12	52231001	52236000	0.019162
chr12	52257001	52262000	1.76E-06
chr12	52273001	52278000	3.68E-05
chr12	52275001	52280000	1.13E-05
chr12	52542001	52547000	6.38E-05
chr12	52543001	52548000	0.001822
chr12	52546001	52551000	0.002882
chr12	52698001	52703000	3.35E-08
chr12	52699001	52704000	9.89E-11
chr12	52700001	52705000	2.85E-08
chr12	52701001	52706000	4.44E-10
chr12	52702001	52707000	2.26E-07
chr12	52881001	52886000	3.17E-08
chr12	52882001	52887000	2.18E-10

chr12	52883001	52888000	3.41E-11
chr12	52884001	52889000	6.33E-14
chr12	52885001	52890000	3.10E-13
chr12	52886001	52891000	2.62E-08
chr12	52971001	52976000	4.95E-10
chr12	52972001	52977000	9.50E-06
chr12	53137001	53142000	5.65E-05
chr12	53166001	53171000	1.49E-05
chr12	53213001	53218000	1.62E-06
chr12	53265001	53270000	0.001016
chr12	53296001	53301000	3.78E-07
chr12	53297001	53302000	6.18E-08
chr12	53317001	53322000	8.79E-07
chr12	53319001	53324000	0.002413
chr12	53340001	53345000	0.000104
chr12	53341001	53346000	5.96E-08
chr12	53342001	53347000	9.21E-09
chr12	53343001	53348000	3.43E-06
chr12	53344001	53349000	5.24E-05
chr12	53391001	53396000	0.001509
chr12	53392001	53397000	0.001435
chr12	53445001	53450000	1.27E-10
chr12	53467001	53472000	9.19E-06
chr12	53469001	53474000	3.17E-09
chr12	53570001	53575000	1.81E-08
chr12	53588001	53593000	5.62E-06
chr12	53591001	53596000	5.37E-10
chr12	53604001	53609000	5.18E-05
chr12	53605001	53610000	1.60E-05
chr12	53619001	53624000	0.002589
chr12	53620001	53625000	0.020994
chr12	53622001	53627000	0.008008
chr12	53623001	53628000	0.013874
chr12	53646001	53651000	0.020236
chr12	53659001	53664000	0.004344
chr12	53660001	53665000	0.001103
chr12	53685001	53690000	3.83E-12
chr12	53686001	53691000	8.42E-15
chr12	53687001	53692000	3.42E-10
chr12	53712001	53717000	5.53E-08
chr12	53719001	53724000	2.47E-09
chr12	53732001	53737000	0.008045
chr12	53895001	53900000	2.72E-06
chr12	54150001	54155000	1.13E-07
chr12	54151001	54156000	1.66E-07
chr12	54152001	54157000	2.62E-07
chr12	54520001	54525000	1.72E-06
chr12	54549001	54554000	7.04E-05

chr12	54584001	54589000	0.000176
chr12	54595001	54600000	0.002772
chr12	54597001	54602000	3.28E-05
chr12	54598001	54603000	5.35E-05
chr12	54601001	54606000	2.63E-05
chr12	54913001	54918000	4.51E-06
chr12	54969001	54974000	1.45E-06
chr12	54970001	54975000	5.37E-09
chr12	54971001	54976000	1.00E-08
chr12	54972001	54977000	8.59E-06
chr12	54973001	54978000	1.87E-06
chr12	55015001	55020000	6.02E-06
chr12	55019001	55024000	5.23E-06
chr12	55020001	55025000	8.66E-07
chr12	55037001	55042000	3.24E-10
chr12	55038001	55043000	1.21E-08
chr12	55039001	55044000	1.92E-08
chr12	55210001	55215000	0.000199
chr12	55211001	55216000	0.000204
chr12	55315001	55320000	1.03E-09
chr12	55316001	55321000	1.12E-09
chr12	55661001	55666000	2.31E-08
chr12	55679001	55684000	7.81E-09
chr12	55908001	55913000	5.29E-06
chr12	55909001	55914000	6.92E-08
chr12	55910001	55915000	1.31E-07
chr12	55945001	55950000	1.43E-05
chr12	55976001	55981000	0.000107
chr12	56049001	56054000	8.65E-08
chr12	56087001	56092000	0.000729
chr12	56088001	56093000	0.000547
chr12	56089001	56094000	0.000137
chr12	56090001	56095000	0.001173
chr12	56091001	56096000	0.00227
chr12	56097001	56102000	0.003933
chr12	56133001	56138000	6.65E-06
chr12	56240001	56245000	0.006588
chr12	56331001	56336000	0.000251
chr12	56332001	56337000	0.000219
chr12	56333001	56338000	2.36E-06
chr12	56431001	56436000	5.90E-11
chr12	56468001	56473000	0.029599
chr12	56523001	56528000	6.43E-11
chr12	56658001	56663000	0.000122
chr12	56705001	56710000	4.91E-07
chr12	56706001	56711000	1.28E-11
chr12	56707001	56712000	2.20E-12
chr12	56708001	56713000	1.71E-06

chr12	56709001	56714000	2.02E-09
chr12	56710001	56715000	0.000251
chr12	56723001	56728000	1.48E-15
chr12	56724001	56729000	1.66E-14
chr12	56725001	56730000	2.39E-14
chr12	56726001	56731000	1.03E-07
chr12	57024001	57029000	0.000359
chr12	57083001	57088000	1.46E-05
chr12	57179001	57184000	7.62E-05
chr12	57180001	57185000	0.000509
chr12	57328001	57333000	5.56E-05
chr12	57333001	57338000	0.00168
chr12	57334001	57339000	0.00168
chr12	57335001	57340000	0.000343
chr12	57344001	57349000	0.004884
chr12	57345001	57350000	0.007968
chr12	57346001	57351000	0.028459
chr12	57347001	57352000	5.61E-05
chr12	57348001	57353000	0.000131
chr12	57473001	57478000	0.000644
chr12	57481001	57486000	6.43E-16
chr12	57482001	57487000	5.40E-13
chr12	57483001	57488000	4.88E-06
chr12	57627001	57632000	0.029687
chr12	57943001	57948000	3.32E-07
chr12	57944001	57949000	0.000137
chr12	58006001	58011000	6.91E-06
chr12	58007001	58012000	2.14E-07
chr12	58008001	58013000	3.03E-08
chr12	58034001	58039000	0.001082
chr12	58036001	58041000	0.003795
chr12	58072001	58077000	0.000569
chr12	58073001	58078000	6.31E-06
chr12	58074001	58079000	1.71E-05
chr12	58076001	58081000	2.43E-09
chr12	58077001	58082000	3.17E-05
chr12	58117001	58122000	3.77E-05
chr12	58135001	58140000	7.15E-11
chr12	58136001	58141000	3.03E-11
chr12	58137001	58142000	4.18E-11
chr12	58138001	58143000	7.04E-09
chr12	58159001	58164000	3.32E-13
chr12	58160001	58165000	3.89E-15
chr12	58417001	58422000	1.42E-06
chr12	58572001	58577000	3.29E-06
chr12	59660001	59665000	9.49E-06
chr12	59661001	59666000	8.45E-07
chr12	59766001	59771000	2.30E-10

chr12	59767001	59772000	3.74E-10
chr12	59876001	59881000	1.35E-05
chr12	59968001	59973000	2.02E-05
chr12	59969001	59974000	4.34E-06
chr12	60002001	60007000	1.84E-09
chr12	60003001	60008000	9.18E-10
chr12	60004001	60009000	9.36E-10
chr12	60005001	60010000	1.68E-08
chr12	60136001	60141000	1.34E-09
chr12	60293001	60298000	4.46E-11
chr12	60294001	60299000	1.66E-09
chr12	60639001	60644000	5.94E-06
chr12	60640001	60645000	6.64E-08
chr12	60670001	60675000	2.22E-06
chr12	60671001	60676000	3.35E-06
chr12	60672001	60677000	1.08E-06
chr12	60673001	60678000	2.55E-06
chr12	60767001	60772000	1.32E-07
chr12	60768001	60773000	0.000341
chr12	60769001	60774000	0.000121
chr12	60770001	60775000	9.53E-05
chr12	60905001	60910000	1.21E-09
chr12	60906001	60911000	1.49E-08
chr12	60907001	60912000	4.45E-09
chr12	60998001	61003000	3.78E-06
chr12	61061001	61066000	3.17E-07
chr12	61071001	61076000	2.01E-05
chr12	61081001	61086000	3.22E-07
chr12	61082001	61087000	1.61E-07
chr12	61083001	61088000	2.35E-08
chr12	61084001	61089000	1.27E-08
chr12	61139001	61144000	2.61E-06
chr12	61140001	61145000	7.09E-06
chr12	61141001	61146000	2.44E-05
chr12	61142001	61147000	0.000181
chr12	61164001	61169000	1.03E-12
chr12	61165001	61170000	5.77E-13
chr12	61175001	61180000	6.71E-07
chr12	61176001	61181000	1.45E-08
chr12	61177001	61182000	6.07E-10
chr12	61179001	61184000	2.73E-10
chr12	61204001	61209000	8.23E-13
chr12	61324001	61329000	2.96E-14
chr12	61325001	61330000	2.58E-12
chr12	61326001	61331000	8.35E-13
chr12	61403001	61408000	5.01E-06
chr12	61404001	61409000	0.000387
chr12	61405001	61410000	1.32E-06

chr12	61407001	61412000	6.37E-09
chr12	61416001	61421000	4.61E-05
chr12	61417001	61422000	7.13E-07
chr12	61789001	61794000	1.51E-08
chr12	61790001	61795000	2.23E-07
chr12	61921001	61926000	1.81E-07
chr12	61961001	61966000	4.95E-06
chr12	61994001	61999000	5.58E-09
chr12	62020001	62025000	3.13E-07
chr12	62021001	62026000	6.19E-07
chr12	62022001	62027000	1.64E-08
chr12	62036001	62041000	4.12E-12
chr12	62037001	62042000	1.95E-13
chr12	62060001	62065000	6.27E-07
chr12	62092001	62097000	1.80E-10
chr12	62093001	62098000	1.68E-14
chr12	62094001	62099000	1.27E-16
chr12	62095001	62100000	9.72E-16
chr12	62096001	62101000	7.15E-10
chr12	62117001	62122000	2.93E-07
chr12	62126001	62131000	1.63E-05
chr12	62127001	62132000	2.80E-07
chr12	62128001	62133000	6.13E-07
chr12	62129001	62134000	5.34E-12
chr12	62130001	62135000	2.96E-11
chr12	62131001	62136000	2.90E-08
chr12	62163001	62168000	0.000242
chr12	62181001	62186000	2.59E-08
chr12	62182001	62187000	1.08E-07
chr12	62183001	62188000	1.41E-07
chr12	62184001	62189000	6.60E-10
chr12	62290001	62295000	2.75E-05
chr12	62291001	62296000	1.55E-05
chr12	62292001	62297000	1.47E-06
chr12	62293001	62298000	4.45E-07
chr12	62294001	62299000	4.75E-05
chr12	62295001	62300000	1.62E-05
chr12	62296001	62301000	1.91E-09
chr12	62297001	62302000	9.15E-09
chr12	62405001	62410000	3.35E-10
chr12	63015001	63020000	9.17E-05
chr12	63016001	63021000	3.28E-05
chr12	63017001	63022000	4.80E-05
chr12	63018001	63023000	5.57E-07
chr12	63019001	63024000	2.93E-07
chr12	63384001	63389000	3.93E-10
chr12	63570001	63575000	3.62E-05
chr12	63571001	63576000	5.23E-06

chr12	65152001	65157000	6.23E-12
chr12	68377001	68382000	1.41E-07
chr12	69200001	69205000	1.75E-16
chr12	69201001	69206000	3.41E-21
chr12	73204001	73209000	9.26E-09
chr12	73205001	73210000	3.32E-09
chr12	73206001	73211000	1.28E-06
chr12	74359001	74364000	2.88E-08
chr12	75822001	75827000	6.63E-05
chr12	76478001	76483000	4.55E-12
chr12	76513001	76518000	0.000262
chr12	82087001	82092000	5.53E-07
chr12	82287001	82292000	1.19E-07
chr12	84036001	84041000	3.26E-10
chr12	84037001	84042000	1.72E-11
chr12	84120001	84125000	3.33E-07
chr12	86586001	86591000	1.06E-07
chr12	88532001	88537000	2.39E-11
chr12	88533001	88538000	1.47E-09
chr12	88534001	88539000	3.38E-11
chr12	88535001	88540000	3.24E-15
chr12	93061001	93066000	0.001027
chr12	93062001	93067000	0.000191
chr12	93529001	93534000	5.76E-08
chr12	94267001	94272000	1.57E-06
chr12	94859001	94864000	0.000392
chr12	95201001	95206000	2.33E-09
chr12	95202001	95207000	9.97E-09
chr12	95304001	95309000	1.58E-10
chr12	95332001	95337000	6.05E-10
chr12	95333001	95338000	6.48E-08
chr12	95335001	95340000	3.08E-06
chr12	95493001	95498000	6.65E-08
chr12	95494001	95499000	4.03E-08
chr12	95495001	95500000	7.25E-08
chr12	95792001	95797000	0.000146
chr12	96231001	96236000	0.000123
chr12	96298001	96303000	1.39E-05
chr12	96498001	96503000	2.37E-10
chr12	96499001	96504000	1.05E-08
chr12	96707001	96712000	6.09E-06
chr12	97735001	97740000	6.73E-05
chr12	98877001	98882000	9.47E-05
chr12	98959001	98964000	1.25E-14
chr12	98960001	98965000	7.98E-15
chr12	99131001	99136000	2.44E-09
chr12	99133001	99138000	5.24E-10
chr12	99134001	99139000	1.61E-11

chr12	99565001	99570000	4.28E-08
chr12	100007001	100012000	3.61E-07
chr12	100008001	100013000	1.77E-06
chr12	100072001	100077000	7.40E-07
chr12	100759001	100764000	6.48E-09
chr12	100760001	100765000	1.31E-11
chr12	100761001	100766000	1.06E-09
chr12	100762001	100767000	2.68E-10
chr12	100936001	100941000	3.33E-05
chr12	100945001	100950000	8.58E-08
chr12	100947001	100952000	6.40E-11
chr12	100963001	100968000	2.30E-06
chr12	101236001	101241000	6.35E-09
chr12	102282001	102287000	0.026821
chr12	103389001	103394000	0.000194
chr12	104020001	104025000	8.05E-08
chr12	104764001	104769000	2.33E-06
chr12	104847001	104852000	1.22E-07
chr12	104929001	104934000	1.06E-12
chr12	104930001	104935000	2.91E-11
chr12	105486001	105491000	3.62E-12
chr12	106614001	106619000	2.02E-05
chr12	106615001	106620000	1.51E-07
chr12	106616001	106621000	5.84E-12
chr12	106617001	106622000	6.64E-12
chr12	106680001	106685000	1.80E-08
chr12	107638001	107643000	2.35E-06
chr12	107640001	107645000	2.17E-06
chr12	107641001	107646000	1.91E-07
chr12	107685001	107690000	3.02E-07
chr12	107686001	107691000	2.17E-08
chr12	107874001	107879000	1.76E-05
chr12	107875001	107880000	0.001021
chr12	107876001	107881000	0.000162
chr12	107877001	107882000	3.23E-05
chr12	108179001	108184000	2.24E-06
chr12	108180001	108185000	8.30E-06
chr12	108197001	108202000	1.49E-08
chr12	108198001	108203000	6.98E-11
chr12	108199001	108204000	3.47E-09
chr12	108200001	108205000	3.73E-10
chr12	108213001	108218000	3.51E-12
chr12	108214001	108219000	1.42E-12
chr12	108215001	108220000	3.21E-12
chr12	108319001	108324000	9.74E-10
chr12	108362001	108367000	1.32E-07
chr12	108363001	108368000	2.42E-09
chr12	108364001	108369000	1.82E-09

chr12	108365001	108370000	2.26E-08
chr12	108377001	108382000	3.06E-08
chr12	108385001	108390000	6.72E-09
chr12	108494001	108499000	7.07E-07
chr12	108495001	108500000	1.05E-08
chr12	108496001	108501000	8.69E-08
chr12	108497001	108502000	2.02E-09
chr12	108498001	108503000	1.27E-11
chr12	108505001	108510000	5.50E-08
chr12	108506001	108511000	2.48E-10
chr12	108507001	108512000	6.31E-09
chr12	108508001	108513000	9.43E-08
chr12	108509001	108514000	2.05E-06
chr12	108573001	108578000	2.04E-06
chr12	108609001	108614000	2.76E-08
chr12	108639001	108644000	1.22E-07
chr12	108640001	108645000	6.04E-05
chr12	108641001	108646000	6.05E-06
chr12	108642001	108647000	1.66E-05
chr12	108655001	108660000	3.55E-08
chr12	108656001	108661000	1.17E-08
chr12	108657001	108662000	5.67E-09
chr12	108659001	108664000	1.81E-05
chr12	108670001	108675000	4.44E-07
chr12	108671001	108676000	1.00E-10
chr12	108672001	108677000	1.56E-12
chr12	108673001	108678000	4.84E-09
chr12	108674001	108679000	9.30E-09
chr12	108680001	108685000	2.85E-08
chr12	108681001	108686000	1.06E-07
chr12	108682001	108687000	8.29E-09
chr12	108683001	108688000	3.09E-08
chr12	108684001	108689000	1.01E-10
chr12	108685001	108690000	2.29E-07
chr12	108686001	108691000	2.72E-09
chr12	108700001	108705000	1.71E-07
chr12	108701001	108706000	7.45E-05
chr12	108702001	108707000	0.000798
chr12	108703001	108708000	0.000199
chr12	108704001	108709000	1.42E-06
chr12	108711001	108716000	1.63E-08
chr12	108712001	108717000	4.70E-08
chr12	108713001	108718000	2.82E-09
chr12	108714001	108719000	1.63E-06
chr12	108715001	108720000	2.38E-07
chr12	108769001	108774000	7.44E-06
chr12	108770001	108775000	6.27E-05
chr12	108781001	108786000	5.94E-06

chr12	108798001	108803000	1.11E-06
chr12	108799001	108804000	2.10E-07
chr12	108800001	108805000	3.61E-07
chr12	108801001	108806000	8.50E-10
chr12	108802001	108807000	4.22E-12
chr12	108803001	108808000	9.26E-11
chr12	108804001	108809000	4.51E-07
chr12	108806001	108811000	4.90E-06
chr12	108818001	108823000	1.18E-06
chr12	108819001	108824000	3.36E-07
chr12	108832001	108837000	2.41E-07
chr12	108833001	108838000	8.57E-09
chr12	108834001	108839000	1.17E-10
chr12	108835001	108840000	2.07E-10
chr12	108836001	108841000	5.08E-08
chr12	108842001	108847000	2.90E-10
chr12	108843001	108848000	3.16E-11
chr12	108844001	108849000	1.50E-12
chr12	108956001	108961000	7.14E-08
chr12	108957001	108962000	5.35E-09
chr12	109002001	109007000	2.32E-10
chr12	109003001	109008000	1.03E-09
chr12	109004001	109009000	5.56E-06
chr12	109006001	109011000	2.84E-05
chr12	109009001	109014000	2.89E-06
chr12	109010001	109015000	1.20E-09
chr12	109011001	109016000	2.82E-08
chr12	109012001	109017000	6.68E-08
chr12	109148001	109153000	0.003013
chr12	109367001	109372000	0.000298
chr12	109450001	109455000	1.16E-07
chr12	109472001	109477000	8.15E-05
chr12	109571001	109576000	0.002586
chr12	109572001	109577000	0.000107
chr12	109573001	109578000	0.000896
chr12	109634001	109639000	3.42E-07
chr12	109635001	109640000	3.77E-06
chr12	109636001	109641000	2.02E-08
chr12	109637001	109642000	1.27E-06
chr12	109698001	109703000	1.63E-05
chr12	109699001	109704000	5.44E-06
chr12	109700001	109705000	7.26E-08
chr12	109701001	109706000	3.44E-08
chr12	109702001	109707000	6.07E-08
chr12	109706001	109711000	2.31E-05
chr12	109721001	109726000	0.00736
chr12	109743001	109748000	2.11E-09
chr12	109744001	109749000	1.67E-08

chr12	109745001	109750000	6.26E-09
chr12	109746001	109751000	3.46E-09
chr12	109771001	109776000	4.70E-13
chr12	109772001	109777000	2.62E-08
chr12	109773001	109778000	8.79E-08
chr12	109846001	109851000	6.09E-08
chr12	109847001	109852000	2.85E-07
chr12	109848001	109853000	2.73E-06
chr12	109849001	109854000	1.66E-08
chr12	109863001	109868000	9.00E-05
chr12	109864001	109869000	2.79E-09
chr12	109865001	109870000	2.36E-10
chr12	109866001	109871000	2.64E-06
chr12	110032001	110037000	2.55E-05
chr12	110033001	110038000	5.39E-06
chr12	110034001	110039000	4.27E-06
chr12	110254001	110259000	4.89E-06
chr12	110255001	110260000	4.37E-06
chr12	110263001	110268000	0.000118
chr12	110264001	110269000	1.15E-05
chr12	110265001	110270000	0.000316
chr12	110275001	110280000	1.15E-07
chr12	110276001	110281000	6.83E-10
chr12	110498001	110503000	0.000324
chr12	110714001	110719000	1.99E-06
chr12	110715001	110720000	1.05E-06
chr12	110773001	110778000	2.31E-05
chr12	110841001	110846000	1.75E-12
chr12	111123001	111128000	3.33E-05
chr12	111124001	111129000	1.29E-13
chr12	111125001	111130000	2.14E-09
chr12	111126001	111131000	1.44E-13
chr12	111314001	111319000	2.83E-05
chr12	111415001	111420000	1.52E-05
chr12	111416001	111421000	2.55E-07
chr12	111417001	111422000	2.56E-11
chr12	111430001	111435000	1.28E-08
chr12	111431001	111436000	1.41E-08
chr12	111432001	111437000	3.29E-05
chr12	111476001	111481000	1.69E-11
chr12	111486001	111491000	1.88E-07
chr12	111487001	111492000	2.85E-11
chr12	111488001	111493000	2.52E-11
chr12	111489001	111494000	1.51E-10
chr12	111490001	111495000	3.79E-11
chr12	111504001	111509000	1.83E-05
chr12	111505001	111510000	2.31E-06
chr12	111521001	111526000	6.07E-05

chr12	111565001	111570000	0.000319
chr12	111569001	111574000	4.54E-07
chr12	111570001	111575000	1.50E-06
chr12	111591001	111596000	6.76E-13
chr12	111592001	111597000	3.24E-10
chr12	111593001	111598000	3.03E-07
chr12	111605001	111610000	2.63E-06
chr12	111653001	111658000	1.07E-10
chr12	111654001	111659000	4.79E-10
chr12	111668001	111673000	5.27E-06
chr12	111669001	111674000	0.000118
chr12	111691001	111696000	7.02E-05
chr12	111744001	111749000	0.023624
chr12	111746001	111751000	0.000184
chr12	111747001	111752000	1.65E-06
chr12	111748001	111753000	2.09E-06
chr12	111749001	111754000	3.52E-05
chr12	111750001	111755000	1.06E-05
chr12	111754001	111759000	0.001905
chr12	111775001	111780000	6.31E-05
chr12	111776001	111781000	0.000112
chr12	111777001	111782000	3.16E-05
chr12	111793001	111798000	7.89E-06
chr12	111830001	111835000	1.76E-05
chr12	111832001	111837000	1.88E-07
chr12	111833001	111838000	4.51E-10
chr12	111834001	111839000	5.98E-11
chr12	112036001	112041000	0.000392
chr12	112270001	112275000	2.20E-05
chr12	112446001	112451000	7.11E-08
chr12	112447001	112452000	1.40E-11
chr12	112448001	112453000	1.00E-12
chr12	112449001	112454000	2.41E-14
chr12	112541001	112546000	1.22E-06
chr12	112542001	112547000	1.43E-14
chr12	112543001	112548000	1.00E-16
chr12	112544001	112549000	5.24E-09
chr12	112545001	112550000	5.63E-11
chr12	112546001	112551000	3.29E-07
chr12	112845001	112850000	8.76E-08
chr12	112846001	112851000	5.09E-07
chr12	112847001	112852000	5.41E-07
chr12	112853001	112858000	9.15E-09
chr12	112854001	112859000	2.94E-06
chr12	112982001	112987000	0.000502
chr12	113047001	113052000	3.54E-06
chr12	113048001	113053000	4.44E-06
chr12	113098001	113103000	6.92E-05

chr12	113099001	113104000	5.51E-06
chr12	113139001	113144000	7.92E-05
chr12	113140001	113145000	2.13E-05
chr12	113156001	113161000	3.61E-05
chr12	113295001	113300000	6.62E-09
chr12	113460001	113465000	0.000152
chr12	113481001	113486000	0.000272
chr12	113487001	113492000	1.90E-07
chr12	113488001	113493000	3.50E-06
chr12	113500001	113505000	1.30E-06
chr12	113501001	113506000	7.28E-09
chr12	113502001	113507000	9.11E-09
chr12	113503001	113508000	9.92E-08
chr12	113504001	113509000	0.000148
chr12	113527001	113532000	9.75E-06
chr12	113528001	113533000	1.58E-05
chr12	113530001	113535000	0.000753
chr12	113621001	113626000	2.35E-09
chr12	113623001	113628000	9.66E-07
chr12	113624001	113629000	2.95E-05
chr12	113655001	113660000	5.76E-18
chr12	113656001	113661000	3.35E-19
chr12	113657001	113662000	8.09E-11
chr12	113827001	113832000	3.52E-05
chr12	113864001	113869000	9.13E-11
chr12	113865001	113870000	0.005356
chr12	113872001	113877000	1.31E-07
chr12	113873001	113878000	8.31E-10
chr12	113874001	113879000	1.73E-08
chr12	113886001	113891000	8.67E-06
chr12	114038001	114043000	4.14E-09
chr12	114039001	114044000	7.67E-10
chr12	114040001	114045000	1.95E-10
chr12	114041001	114046000	2.30E-10
chr12	114042001	114047000	9.87E-09
chr12	114062001	114067000	0.002473
chr12	114184001	114189000	0.000332
chr12	114440001	114445000	4.25E-08
chr12	114441001	114446000	1.82E-07
chr12	114744001	114749000	2.43E-07
chr12	114745001	114750000	5.48E-07
chr12	114746001	114751000	9.91E-07
chr12	114765001	114770000	0.000591
chr12	114767001	114772000	9.25E-06
chr12	114768001	114773000	3.58E-05
chr12	114769001	114774000	0.000309
chr12	114785001	114790000	1.77E-06
chr12	114786001	114791000	1.26E-06

chr12	114852001	114857000	2.75E-06
chr12	114962001	114967000	5.52E-10
chr12	114963001	114968000	0.000714
chr12	114980001	114985000	5.75E-08
chr12	114981001	114986000	3.45E-11
chr12	114982001	114987000	2.67E-10
chr12	114997001	115002000	0.000805
chr12	115035001	115040000	0.006022
chr12	115088001	115093000	0.003574
chr12	116754001	116759000	5.35E-16
chr12	116976001	116981000	7.23E-11
chr12	116993001	116998000	1.43E-06
chr12	116994001	116999000	8.19E-09
chr12	116995001	117000000	4.20E-07
chr12	116996001	117001000	1.33E-06
chr12	116997001	117002000	6.34E-06
chr12	117173001	117178000	3.12E-11
chr12	117174001	117179000	2.70E-18
chr12	117175001	117180000	2.27E-15
chr12	117176001	117181000	1.32E-15
chr12	117179001	117184000	0.001162
chr12	117334001	117339000	7.85E-05
chr12	117335001	117340000	1.48E-05
chr12	117336001	117341000	4.03E-05
chr12	117343001	117348000	5.51E-09
chr12	117344001	117349000	5.69E-05
chr12	117532001	117537000	5.81E-08
chr12	117533001	117538000	3.76E-11
chr12	117534001	117539000	2.08E-10
chr12	117535001	117540000	1.46E-07
chr12	117536001	117541000	2.54E-07
chr12	117548001	117553000	0.002759
chr12	117638001	117643000	0.002658
chr12	117639001	117644000	0.002031
chr12	117694001	117699000	7.56E-09
chr12	117695001	117700000	6.10E-08
chr12	117696001	117701000	9.31E-08
chr12	117697001	117702000	2.47E-05
chr12	117742001	117747000	1.61E-06
chr12	117743001	117748000	1.35E-07
chr12	117787001	117792000	1.11E-06
chr12	117788001	117793000	6.76E-08
chr12	117857001	117862000	1.21E-06
chr12	117882001	117887000	2.61E-09
chr12	118387001	118392000	0.00024
chr12	118388001	118393000	5.16E-05
chr12	118440001	118445000	2.97E-05
chr12	118565001	118570000	1.19E-07

chr12	118566001	118571000	1.51E-08
chr12	118567001	118572000	9.92E-08
chr12	118569001	118574000	2.82E-05
chr12	118926001	118931000	2.84E-06
chr12	118927001	118932000	4.15E-09
chr12	118928001	118933000	1.60E-09
chr12	118929001	118934000	5.55E-07
chr12	118930001	118935000	6.95E-06
chr12	118931001	118936000	1.92E-06
chr12	118938001	118943000	5.04E-13
chr12	118939001	118944000	1.91E-10
chr12	118940001	118945000	4.04E-15
chr12	118941001	118946000	1.31E-09
chr12	118942001	118947000	6.21E-10
chr12	118944001	118949000	1.45E-05
chr12	118951001	118956000	5.38E-06
chr12	118952001	118957000	2.96E-06
chr12	118953001	118958000	5.30E-07
chr12	118954001	118959000	4.27E-07
chr12	119006001	119011000	3.24E-07
chr12	119007001	119012000	5.98E-11
chr12	119043001	119048000	1.97E-08
chr12	119063001	119068000	2.94E-06
chr12	119077001	119082000	2.05E-11
chr12	119102001	119107000	3.09E-05
chr12	119116001	119121000	0.000293
chr12	119142001	119147000	1.23E-06
chr12	119181001	119186000	2.03E-08
chr12	119188001	119193000	5.91E-08
chr12	119206001	119211000	2.31E-05
chr12	119272001	119277000	2.39E-10
chr12	119273001	119278000	1.53E-10
chr12	119274001	119279000	2.02E-08
chr12	119289001	119294000	1.82E-06
chr12	119295001	119300000	3.40E-08
chr12	119296001	119301000	1.51E-05
chr12	119532001	119537000	4.28E-06
chr12	119703001	119708000	7.65E-07
chr12	119704001	119709000	1.20E-05
chr12	119707001	119712000	3.23E-07
chr12	119708001	119713000	6.95E-09
chr12	119726001	119731000	3.12E-08
chr12	119787001	119792000	5.21E-05
chr12	119938001	119943000	1.79E-07
chr12	119940001	119945000	9.82E-08
chr12	119941001	119946000	7.43E-10
chr12	119942001	119947000	2.14E-09
chr12	119943001	119948000	3.68E-06

chr12	119944001	119949000	6.18E-05
chr12	119945001	119950000	0.000345
chr12	119946001	119951000	8.97E-06
chr12	119961001	119966000	0.03419
chr12	119992001	119997000	4.47E-06
chr12	120011001	120016000	7.99E-08
chr12	120315001	120320000	4.08E-07
chr12	120535001	120540000	0.000221
chr12	120536001	120541000	0.016756
chr12	120537001	120542000	3.66E-09
chr12	120538001	120543000	1.55E-09
chr12	120539001	120544000	4.82E-10
chr12	120557001	120562000	9.31E-06
chr12	120558001	120563000	1.53E-05
chr12	120717001	120722000	0.001048
chr12	120753001	120758000	5.04E-08
chr12	120754001	120759000	2.15E-07
chr12	120755001	120760000	3.14E-07
chr12	120827001	120832000	0.000386
chr12	120855001	120860000	4.85E-06
chr12	120856001	120861000	2.24E-06
chr12	120857001	120862000	2.49E-08
chr12	120858001	120863000	3.24E-05
chr12	120866001	120871000	4.67E-06
chr12	121021001	121026000	0.000339
chr12	121022001	121027000	2.20E-07
chr12	121046001	121051000	6.99E-09
chr12	121084001	121089000	7.60E-05
chr12	121120001	121125000	1.70E-12
chr12	121382001	121387000	9.26E-06
chr12	121439001	121444000	3.96E-05
chr12	121440001	121445000	2.94E-06
chr12	121441001	121446000	6.46E-07
chr12	121530001	121535000	4.44E-10
chr12	121531001	121536000	8.64E-09
chr12	121532001	121537000	3.72E-07
chr12	121533001	121538000	2.00E-10
chr12	121591001	121596000	5.34E-05
chr12	121592001	121597000	3.07E-06
chr12	121665001	121670000	7.02E-05
chr12	121684001	121689000	1.06E-08
chr12	121685001	121690000	8.79E-06
chr12	121687001	121692000	1.70E-10
chr12	121790001	121795000	4.87E-09
chr12	122103001	122108000	0.000107
chr12	122105001	122110000	2.05E-05
chr12	122215001	122220000	1.45E-10
chr12	122216001	122221000	2.30E-12

chr12	122219001	122224000	0.017144
chr12	122317001	122322000	7.18E-05
chr12	122318001	122323000	0.00475
chr12	122324001	122329000	1.72E-05
chr12	122325001	122330000	1.04E-06
chr12	122326001	122331000	2.63E-08
chr12	122327001	122332000	6.75E-08
chr12	122367001	122372000	0.000176
chr12	122368001	122373000	5.61E-05
chr12	122667001	122672000	1.13E-06
chr12	122679001	122684000	2.11E-05
chr12	122682001	122687000	3.22E-06
chr12	123474001	123479000	8.93E-10
chr12	123475001	123480000	8.97E-08
chr12	123476001	123481000	1.48E-08
chr12	123477001	123482000	1.08E-05
chr12	123478001	123483000	2.62E-06
chr12	123849001	123854000	1.97E-05
chr12	123850001	123855000	3.84E-06
chr12	123931001	123936000	6.42E-06
chr12	123994001	123999000	0.002842
chr12	123995001	124000000	0.0058
chr12	124043001	124048000	0.000517
chr12	124056001	124061000	0.000799
chr12	124114001	124119000	5.18E-10
chr12	124115001	124120000	1.11E-08
chr12	124116001	124121000	3.60E-08
chr12	124117001	124122000	8.72E-05
chr12	124118001	124123000	2.85E-06
chr12	124386001	124391000	8.99E-08
chr12	124392001	124397000	4.62E-08
chr12	124576001	124581000	0.000207
chr12	124615001	124620000	0.004725
chr12	124785001	124790000	5.59E-05
chr12	124786001	124791000	6.84E-05
chr12	124793001	124798000	0.000337
chr12	124794001	124799000	2.39E-06
chr12	124947001	124952000	1.46E-12
chr12	125048001	125053000	2.27E-06
chr12	125323001	125328000	1.21E-09
chr12	125413001	125418000	4.05E-06
chr12	125425001	125430000	2.92E-05
chr12	125546001	125551000	1.59E-07
chr12	125726001	125731000	4.78E-08
chr12	125739001	125744000	5.00E-11
chr12	125742001	125747000	3.54E-15
chr12	125743001	125748000	7.26E-13
chr12	125791001	125796000	3.88E-10

chr12	125792001	125797000	8.31E-09
chr12	125842001	125847000	4.48E-10
chr12	125859001	125864000	1.01E-08
chr12	125860001	125865000	7.77E-10
chr12	125879001	125884000	1.68E-06
chr12	125880001	125885000	1.34E-07
chr12	125881001	125886000	1.37E-05
chr12	125944001	125949000	1.98E-09
chr12	125945001	125950000	1.14E-09
chr12	125946001	125951000	4.85E-08
chr12	125978001	125983000	0.000674
chr12	125979001	125984000	9.60E-05
chr12	126042001	126047000	2.61E-05
chr12	126043001	126048000	2.36E-07
chr12	126097001	126102000	1.35E-05
chr12	126100001	126105000	1.37E-06
chr12	126108001	126113000	6.24E-09
chr12	126109001	126114000	3.55E-12
chr12	126110001	126115000	2.46E-06
chr12	126172001	126177000	1.35E-05
chr12	126173001	126178000	3.52E-06
chr12	126174001	126179000	2.41E-07
chr12	126194001	126199000	5.72E-10
chr12	126195001	126200000	2.77E-07
chr12	126196001	126201000	1.19E-07
chr12	126229001	126234000	7.10E-07
chr12	126276001	126281000	2.47E-09
chr12	126281001	126286000	3.38E-11
chr12	126282001	126287000	1.02E-08
chr12	126284001	126289000	1.55E-12
chr12	126299001	126304000	7.56E-08
chr12	126302001	126307000	5.10E-09
chr12	126303001	126308000	3.18E-07
chr12	126327001	126332000	1.75E-10
chr12	126375001	126380000	4.53E-09
chr12	126376001	126381000	2.99E-11
chr12	126377001	126382000	1.63E-14
chr12	126378001	126383000	8.61E-14
chr12	126405001	126410000	5.16E-08
chr12	126577001	126582000	3.85E-14
chr12	126578001	126583000	5.62E-11
chr12	126579001	126584000	1.53E-12
chr12	126580001	126585000	9.99E-16
chr12	126581001	126586000	5.90E-15
chr12	126583001	126588000	1.74E-07
chr12	126584001	126589000	2.24E-07
chr12	126597001	126602000	4.03E-07
chr12	126598001	126603000	4.97E-07

chr12	126599001	126604000	2.89E-08
chr12	126633001	126638000	2.86E-10
chr12	126634001	126639000	1.74E-08
chr12	126635001	126640000	1.10E-06
chr12	126665001	126670000	1.52E-08
chr12	126666001	126671000	4.92E-09
chr12	126770001	126775000	1.23E-07
chr12	126772001	126777000	1.92E-06
chr12	126782001	126787000	1.16E-07
chr12	126838001	126843000	1.82E-11
chr12	126839001	126844000	5.10E-13
chr12	126870001	126875000	1.63E-07
chr12	126880001	126885000	3.48E-08
chr12	126881001	126886000	4.03E-10
chr12	126893001	126898000	3.40E-08
chr12	126894001	126899000	1.11E-09
chr12	126966001	126971000	1.52E-16
chr12	126967001	126972000	8.48E-13
chr12	127065001	127070000	1.76E-09
chr12	127136001	127141000	1.09E-14
chr12	127163001	127168000	3.60E-08
chr12	127164001	127169000	1.33E-06
chr12	127204001	127209000	7.16E-09
chr12	127206001	127211000	1.43E-07
chr12	127254001	127259000	8.62E-09
chr12	127304001	127309000	1.87E-06
chr12	127315001	127320000	4.33E-06
chr12	127321001	127326000	1.64E-06
chr12	127322001	127327000	5.71E-08
chr12	127324001	127329000	4.03E-14
chr12	127325001	127330000	3.39E-14
chr12	127355001	127360000	5.12E-12
chr12	127356001	127361000	3.74E-13
chr12	127357001	127362000	1.75E-10
chr12	127358001	127363000	4.41E-10
chr12	127371001	127376000	4.32E-08
chr12	127460001	127465000	5.52E-08
chr12	127650001	127655000	2.77E-09
chr12	127707001	127712000	2.16E-05
chr12	127902001	127907000	4.17E-10
chr12	127903001	127908000	1.40E-15
chr12	127904001	127909000	1.46E-19
chr12	127905001	127910000	1.45E-21
chr12	127906001	127911000	4.63E-21
chr12	127907001	127912000	6.70E-18
chr12	127939001	127944000	4.69E-06
chr12	127940001	127945000	5.93E-12
chr12	128008001	128013000	1.99E-07

chr12	128025001	128030000	9.74E-11
chr12	128026001	128031000	1.68E-12
chr12	128053001	128058000	4.62E-16
chr12	128054001	128059000	3.96E-15
chr12	128055001	128060000	4.22E-14
chr12	128056001	128061000	1.16E-12
chr12	128060001	128065000	5.74E-11
chr12	128061001	128066000	5.71E-09
chr12	128062001	128067000	1.59E-08
chr12	128163001	128168000	2.91E-07
chr12	128334001	128339000	3.20E-11
chr12	128421001	128426000	5.70E-13
chr12	128422001	128427000	3.32E-12
chr12	128423001	128428000	2.60E-17
chr12	128424001	128429000	5.13E-08
chr12	128522001	128527000	2.49E-08
chr12	128547001	128552000	1.12E-10
chr12	128558001	128563000	8.16E-08
chr12	128691001	128696000	3.58E-05
chr12	128692001	128697000	1.21E-06
chr12	128693001	128698000	4.75E-06
chr12	128733001	128738000	2.43E-07
chr12	128734001	128739000	2.29E-07
chr12	128735001	128740000	4.40E-08
chr12	128786001	128791000	3.74E-10
chr12	128824001	128829000	5.48E-15
chr12	128832001	128837000	4.12E-06
chr12	128838001	128843000	4.37E-10
chr12	128839001	128844000	4.68E-09
chr12	128852001	128857000	2.20E-09
chr12	128884001	128889000	1.21E-10
chr12	128902001	128907000	1.70E-17
chr12	128903001	128908000	1.84E-13
chr12	128904001	128909000	5.97E-15
chr12	128905001	128910000	4.48E-14
chr12	128916001	128921000	5.56E-07
chr12	128938001	128943000	2.63E-10
chr12	128941001	128946000	6.03E-08
chr12	128942001	128947000	1.13E-07
chr12	128946001	128951000	1.36E-09
chr12	128992001	128997000	1.99E-08
chr12	129008001	129013000	2.24E-08
chr12	129010001	129015000	1.64E-11
chr12	129075001	129080000	3.63E-10
chr12	129079001	129084000	3.37E-09
chr12	129090001	129095000	2.73E-06
chr12	129091001	129096000	9.58E-06
chr12	129111001	129116000	7.93E-13

chr12	129112001	129117000	3.27E-12
chr12	129113001	129118000	4.92E-12
chr12	129122001	129127000	1.38E-05
chr12	129162001	129167000	8.11E-08
chr12	129170001	129175000	2.28E-10
chr12	129171001	129176000	1.64E-12
chr12	129173001	129178000	4.48E-11
chr12	129185001	129190000	1.01E-06
chr12	129193001	129198000	5.34E-12
chr12	129221001	129226000	2.33E-06
chr12	129229001	129234000	8.76E-09
chr12	129231001	129236000	2.14E-09
chr12	129232001	129237000	3.76E-09
chr12	129256001	129261000	2.41E-08
chr12	129258001	129263000	1.70E-07
chr12	129259001	129264000	9.42E-08
chr12	129260001	129265000	1.58E-07
chr12	129366001	129371000	6.88E-10
chr12	129367001	129372000	3.11E-09
chr12	129455001	129460000	0.000276
chr12	129457001	129462000	0.000187
chr12	129458001	129463000	2.76E-08
chr12	129597001	129602000	1.76E-09
chr12	129598001	129603000	2.87E-12
chr12	129599001	129604000	1.70E-12
chr12	129645001	129650000	1.05E-10
chr12	129673001	129678000	0.000781
chr12	129701001	129706000	1.30E-12
chr12	129736001	129741000	1.16E-13
chr12	129737001	129742000	4.45E-17
chr12	129738001	129743000	2.53E-13
chr12	129739001	129744000	4.41E-12
chr12	129751001	129756000	3.84E-07
chr12	129753001	129758000	6.01E-09
chr12	129754001	129759000	2.32E-08
chr12	129831001	129836000	6.77E-11
chr12	129832001	129837000	6.23E-11
chr12	129833001	129838000	1.05E-11
chr12	129834001	129839000	4.63E-09
chr12	129835001	129840000	7.65E-09
chr12	129837001	129842000	2.45E-08
chr12	129838001	129843000	1.23E-09
chr12	129841001	129846000	7.92E-09
chr12	130208001	130213000	8.05E-10
chr12	130269001	130274000	3.27E-14
chr12	130407001	130412000	3.84E-08
chr12	130470001	130475000	1.42E-08
chr12	130471001	130476000	4.62E-07

chr12	130472001	130477000	7.43E-07
chr12	130503001	130508000	9.07E-07
chr12	130504001	130509000	1.88E-09
chr12	130505001	130510000	1.21E-05
chr12	130962001	130967000	2.31E-05
chr12	130963001	130968000	3.84E-06
chr12	131066001	131071000	1.75E-05
chr12	131328001	131333000	0.001361
chr12	131392001	131397000	0.001095
chr12	131434001	131439000	4.57E-12
chr12	131435001	131440000	1.25E-11
chr12	131436001	131441000	5.15E-17
chr12	131437001	131442000	1.15E-11
chr12	131438001	131443000	1.73E-15
chr12	131476001	131481000	4.98E-09
chr12	131477001	131482000	8.67E-10
chr12	131478001	131483000	1.18E-09
chr12	131634001	131639000	9.83E-08
chr12	131732001	131737000	5.53E-05
chr12	131733001	131738000	0.000141
chr12	131756001	131761000	1.74E-05
chr12	131757001	131762000	4.50E-07
chr12	131758001	131763000	4.47E-07
chr12	131808001	131813000	4.69E-09
chr12	131856001	131861000	2.10E-15
chr12	131857001	131862000	6.86E-16
chr12	131963001	131968000	4.55E-05
chr12	131964001	131969000	3.20E-06
chr12	131965001	131970000	8.52E-10
chr12	131966001	131971000	3.45E-07
chr12	132025001	132030000	9.38E-06
chr12	132026001	132031000	8.84E-07
chr12	132129001	132134000	2.19E-07
chr12	132130001	132135000	8.41E-08
chr12	132136001	132141000	5.88E-18
chr12	132137001	132142000	5.07E-19
chr12	132138001	132143000	2.39E-13
chr12	132171001	132176000	3.01E-19
chr12	132185001	132190000	0.000707
chr12	132186001	132191000	0.000654
chr12	132187001	132192000	2.37E-05
chr12	132316001	132321000	9.53E-07
chr12	132317001	132322000	7.32E-07
chr12	132384001	132389000	0.000387
chr12	132641001	132646000	0.001605
chr12	132642001	132647000	0.00057
chr12	132672001	132677000	0.000241
chr12	132673001	132678000	0.008049

chr12	132674001	132679000	4.44E-08
chr12	132675001	132680000	9.13E-08
chr12	132676001	132681000	6.19E-12
chr12	132700001	132705000	4.49E-08
chr12	132701001	132706000	1.55E-11
chr12	132702001	132707000	2.90E-12
chr12	132703001	132708000	3.08E-12
chr12	132704001	132709000	2.87E-06
chr12	132845001	132850000	3.68E-08
chr12	132846001	132851000	1.39E-06
chr12	132886001	132891000	1.95E-06
chr12	132906001	132911000	3.24E-10
chr12	132907001	132912000	9.80E-16
chr12	132908001	132913000	1.92E-13
chr12	132909001	132914000	1.97E-11
chr12	132913001	132918000	6.97E-06
chr12	132922001	132927000	4.42E-07
chr12	132923001	132928000	1.34E-10
chr12	132924001	132929000	1.02E-05
chr12	132974001	132979000	9.38E-08
chr12	133004001	133009000	0.000199
chr12	133029001	133034000	3.22E-08
chr12	133030001	133035000	7.17E-06
chr12	133031001	133036000	2.06E-07
chr12	133032001	133037000	3.73E-08
chr12	133045001	133050000	2.94E-07
chr12	133052001	133057000	3.75E-07
chr12	133056001	133061000	5.05E-07
chr12	133174001	133179000	3.18E-09
chr12	133175001	133180000	6.60E-13
chr12	133176001	133181000	1.21E-12
chr12	133177001	133182000	2.88E-09
chr12	133261001	133266000	5.73E-05
chr12	133262001	133267000	3.75E-06
chr12	133263001	133268000	9.04E-13
chr12	133264001	133269000	1.08E-08
chr12	133790001	133795000	0.000841
chr12	133791001	133796000	0.000149
chr12	133804001	133809000	0.000891
chr13	19144001	19149000	0.000135
chr13	19145001	19150000	6.49E-05
chr13	19325001	19330000	1.26E-07
chr13	19338001	19343000	1.81E-07
chr13	19339001	19344000	3.05E-09
chr13	19340001	19345000	2.42E-06
chr13	19520001	19525000	2.76E-05
chr13	19531001	19536000	0.000103
chr13	19566001	19571000	0.005958

chr13	20027001	20032000	6.77E-07
chr13	20090001	20095000	0.001121
chr13	20091001	20096000	0.000636
chr13	20092001	20097000	0.000285
chr13	20115001	20120000	0.000172
chr13	20116001	20121000	0.000961
chr13	20139001	20144000	1.46E-05
chr13	21769001	21774000	2.15E-06
chr13	22085001	22090000	1.96E-07
chr13	22323001	22328000	7.11E-09
chr13	23388001	23393000	7.01E-05
chr13	24905001	24910000	7.45E-07
chr13	24906001	24911000	3.18E-07
chr13	25754001	25759000	1.78E-07
chr13	27000001	27005000	1.70E-05
chr13	27439001	27444000	4.15E-07
chr13	27440001	27445000	4.00E-06
chr13	27459001	27464000	1.17E-05
chr13	27460001	27465000	3.80E-08
chr13	27461001	27466000	4.09E-09
chr13	28426001	28431000	3.47E-09
chr13	28559001	28564000	9.21E-06
chr13	28610001	28615000	3.93E-05
chr13	28652001	28657000	0.000242
chr13	28708001	28713000	3.25E-09
chr13	29186001	29191000	9.15E-08
chr13	29641001	29646000	3.44E-06
chr13	29642001	29647000	2.26E-08
chr13	29643001	29648000	5.10E-10
chr13	29662001	29667000	0.000381
chr13	30217001	30222000	0.000566
chr13	30424001	30429000	3.66E-13
chr13	30965001	30970000	3.15E-10
chr13	30966001	30971000	1.02E-09
chr13	30967001	30972000	6.72E-08
chr13	33426001	33431000	1.03E-09
chr13	33428001	33433000	1.77E-08
chr13	33429001	33434000	1.21E-07
chr13	33432001	33437000	5.88E-08
chr13	33721001	33726000	1.83E-09
chr13	34707001	34712000	8.68E-12
chr13	35366001	35371000	6.73E-05
chr13	35367001	35372000	8.09E-05
chr13	35495001	35500000	1.36E-05
chr13	35970001	35975000	1.18E-07
chr13	36204001	36209000	9.86E-10
chr13	36249001	36254000	1.01E-09
chr13	36250001	36255000	2.13E-08

chr13	37712001	37717000	8.53E-06
chr13	37850001	37855000	4.45E-06
chr13	38037001	38042000	7.75E-08
chr13	38038001	38043000	2.32E-08
chr13	38077001	38082000	2.04E-05
chr13	38078001	38083000	2.10E-05
chr13	38659001	38664000	0.000298
chr13	38724001	38729000	0.000192
chr13	42530001	42535000	3.33E-12
chr13	42531001	42536000	7.06E-12
chr13	42532001	42537000	8.30E-10
chr13	42610001	42615000	4.48E-08
chr13	42611001	42616000	3.02E-13
chr13	43137001	43142000	1.09E-05
chr13	45150001	45155000	5.46E-14
chr13	45151001	45156000	4.59E-11
chr13	45221001	45226000	9.44E-07
chr13	45222001	45227000	2.81E-06
chr13	45488001	45493000	8.03E-16
chr13	45489001	45494000	9.28E-19
chr13	45490001	45495000	2.74E-23
chr13	45491001	45496000	3.66E-16
chr13	45492001	45497000	2.25E-15
chr13	45886001	45891000	2.24E-07
chr13	45914001	45919000	1.43E-06
chr13	46141001	46146000	2.47E-11
chr13	46890001	46895000	0.000194
chr13	46891001	46896000	4.28E-05
chr13	47038001	47043000	0.000216
chr13	47039001	47044000	0.000278
chr13	47040001	47045000	3.34E-05
chr13	47368001	47373000	5.94E-11
chr13	47498001	47503000	5.60E-05
chr13	48185001	48190000	2.49E-10
chr13	48444001	48449000	0.001637
chr13	48463001	48468000	2.11E-06
chr13	48464001	48469000	4.42E-07
chr13	49178001	49183000	1.26E-09
chr13	49179001	49184000	4.19E-09
chr13	49181001	49186000	2.42E-05
chr13	49182001	49187000	4.67E-05
chr13	49185001	49190000	9.28E-05
chr13	49186001	49191000	0.000119
chr13	49200001	49205000	4.62E-10
chr13	49201001	49206000	1.04E-09
chr13	49202001	49207000	1.42E-07
chr13	50421001	50426000	0.000958
chr13	50930001	50935000	9.62E-09

chr13	50931001	50936000	1.47E-10
chr13	52026001	52031000	2.25E-09
chr13	52027001	52032000	6.07E-11
chr13	52637001	52642000	7.00E-06
chr13	52807001	52812000	0.000468
chr13	52813001	52818000	2.40E-06
chr13	52870001	52875000	1.04E-11
chr13	53059001	53064000	3.69E-08
chr13	53060001	53065000	4.17E-06
chr13	53113001	53118000	9.95E-08
chr13	53114001	53119000	2.79E-07
chr13	53444001	53449000	2.27E-08
chr13	54503001	54508000	4.48E-08
chr13	55487001	55492000	1.57E-06
chr13	55658001	55663000	2.73E-07
chr13	55817001	55822000	3.09E-10
chr13	55818001	55823000	7.23E-09
chr13	55963001	55968000	1.42E-13
chr13	56020001	56025000	1.68E-10
chr13	56021001	56026000	1.79E-11
chr13	56022001	56027000	2.85E-07
chr13	56075001	56080000	1.99E-05
chr13	56234001	56239000	7.33E-09
chr13	56438001	56443000	7.03E-07
chr13	56439001	56444000	6.53E-05
chr13	56738001	56743000	7.23E-05
chr13	57246001	57251000	2.94E-06
chr13	57664001	57669000	9.81E-07
chr13	57683001	57688000	2.24E-06
chr13	58128001	58133000	7.16E-16
chr13	58468001	58473000	6.68E-12
chr13	58640001	58645000	1.43E-10
chr13	58641001	58646000	6.03E-08
chr13	58702001	58707000	4.34E-10
chr13	58771001	58776000	2.11E-10
chr13	58829001	58834000	1.46E-13
chr13	58830001	58835000	7.60E-14
chr13	58831001	58836000	5.32E-14
chr13	58832001	58837000	2.83E-11
chr13	58833001	58838000	1.16E-07
chr13	58882001	58887000	1.60E-09
chr13	59029001	59034000	9.88E-06
chr13	59055001	59060000	1.91E-10
chr13	59174001	59179000	2.63E-13
chr13	59420001	59425000	2.92E-07
chr13	59421001	59426000	6.10E-07
chr13	59660001	59665000	8.60E-11
chr13	59661001	59666000	4.65E-13

chr13	59819001	59824000	3.09E-08
chr13	59820001	59825000	7.88E-07
chr13	59842001	59847000	5.85E-08
chr13	59951001	59956000	2.67E-05
chr13	59959001	59964000	1.45E-08
chr13	60171001	60176000	2.85E-08
chr13	60172001	60177000	3.75E-07
chr13	60173001	60178000	8.21E-07
chr13	60174001	60179000	7.34E-07
chr13	60175001	60180000	4.74E-05
chr13	60966001	60971000	2.00E-21
chr13	60967001	60972000	2.45E-14
chr13	62279001	62284000	0.000276
chr13	62298001	62303000	4.04E-06
chr13	62748001	62753000	3.21E-06
chr13	63390001	63395000	1.36E-09
chr13	63661001	63666000	0.000429
chr13	64072001	64077000	8.35E-08
chr13	64306001	64311000	0.000129
chr13	64334001	64339000	0.010381
chr13	64626001	64631000	4.76E-05
chr13	64627001	64632000	4.91E-05
chr13	64628001	64633000	1.27E-05
chr13	64670001	64675000	3.41E-06
chr13	64775001	64780000	5.75E-08
chr13	64776001	64781000	2.73E-06
chr13	64805001	64810000	0.001408
chr13	64932001	64937000	3.09E-09
chr13	64941001	64946000	2.92E-07
chr13	64983001	64988000	7.28E-05
chr13	65047001	65052000	1.20E-06
chr13	65601001	65606000	2.21E-06
chr13	65602001	65607000	0.000129
chr13	65772001	65777000	2.87E-07
chr13	65773001	65778000	6.66E-07
chr13	65774001	65779000	4.25E-07
chr13	65775001	65780000	2.09E-07
chr13	66392001	66397000	0.0001
chr13	67107001	67112000	3.03E-09
chr13	67108001	67113000	3.60E-09
chr13	67851001	67856000	1.56E-09
chr13	70222001	70227000	0.000299
chr13	70870001	70875000	8.29E-08
chr13	71118001	71123000	7.05E-07
chr13	71291001	71296000	2.81E-11
chr13	71598001	71603000	8.91E-12
chr13	71599001	71604000	1.24E-11
chr13	72483001	72488000	2.47E-13

chr13	72484001	72489000	1.19E-15
chr13	72485001	72490000	4.23E-11
chr13	72487001	72492000	1.19E-09
chr13	72843001	72848000	1.50E-06
chr13	72863001	72868000	4.68E-07
chr13	73038001	73043000	1.32E-10
chr13	73168001	73173000	9.00E-05
chr13	73175001	73180000	1.56E-09
chr13	73199001	73204000	1.36E-08
chr13	74851001	74856000	0.000247
chr13	75134001	75139000	3.82E-11
chr13	76124001	76129000	2.57E-12
chr13	76205001	76210000	1.15E-09
chr13	76449001	76454000	3.35E-09
chr13	76573001	76578000	0.006367
chr13	76752001	76757000	7.09E-06
chr13	78234001	78239000	2.58E-05
chr13	78238001	78243000	2.78E-08
chr13	78239001	78244000	4.55E-09
chr13	78240001	78245000	3.13E-10
chr13	78241001	78246000	2.71E-10
chr13	78429001	78434000	5.04E-06
chr13	78539001	78544000	3.87E-08
chr13	78616001	78621000	3.83E-08
chr13	78640001	78645000	2.49E-08
chr13	78641001	78646000	5.55E-09
chr13	78676001	78681000	1.35E-09
chr13	79510001	79515000	7.96E-07
chr13	79511001	79516000	1.27E-05
chr13	79512001	79517000	1.41E-05
chr13	80787001	80792000	1.13E-09
chr13	81089001	81094000	9.53E-09
chr13	81261001	81266000	0.000258
chr13	82331001	82336000	6.43E-11
chr13	82332001	82337000	1.26E-11
chr13	82333001	82338000	1.38E-10
chr13	82979001	82984000	1.88E-09
chr13	83555001	83560000	1.49E-09
chr13	83556001	83561000	5.22E-09
chr13	83557001	83562000	6.47E-06
chr13	83761001	83766000	1.63E-08
chr13	84255001	84260000	1.84E-07
chr13	85363001	85368000	0.003765
chr13	86906001	86911000	0.001034
chr13	86945001	86950000	5.01E-06
chr13	89346001	89351000	2.12E-07
chr13	89349001	89354000	2.81E-07
chr13	89350001	89355000	3.21E-06

chr13	89817001	89822000	3.52E-07
chr13	93643001	93648000	9.71E-08
chr13	96032001	96037000	0.035518
chr13	96041001	96046000	0.000221
chr13	98512001	98517000	0.000134
chr13	99233001	99238000	0.000112
chr13	99328001	99333000	0.000164
chr13	100258001	100263000	0.00014
chr13	100417001	100422000	0.00018
chr13	100578001	100583000	2.11E-09
chr13	100607001	100612000	4.72E-09
chr13	101830001	101835000	1.19E-08
chr13	102894001	102899000	4.19E-09
chr13	103988001	103993000	2.33E-14
chr13	104134001	104139000	0.00015
chr13	104142001	104147000	4.22E-08
chr13	104143001	104148000	3.69E-09
chr13	104313001	104318000	4.67E-11
chr13	104315001	104320000	3.42E-09
chr13	104417001	104422000	8.23E-14
chr13	104418001	104423000	7.02E-12
chr13	104478001	104483000	2.47E-08
chr13	104479001	104484000	3.52E-08
chr13	104883001	104888000	6.92E-09
chr13	105760001	105765000	2.93E-10
chr13	108218001	108223000	3.36E-15
chr13	108219001	108224000	1.87E-12
chr13	109389001	109394000	4.89E-06
chr13	111213001	111218000	2.94E-08
chr13	111214001	111219000	2.49E-12
chr13	112936001	112941000	0.005534
chr13	112937001	112942000	0.017846
chr13	112957001	112962000	0.029153
chr13	112958001	112963000	0.00267
chr13	112959001	112964000	0.002286
chr13	112960001	112965000	0.008556
chr13	113757001	113762000	2.39E-06
chr13	113758001	113763000	0.001132
chr13	113759001	113764000	0.001132
chr13	113858001	113863000	3.29E-22
chr13	113859001	113864000	9.41E-20
chr13	113947001	113952000	2.69E-11
chr13	113949001	113954000	5.28E-11
chr13	114056001	114061000	4.10E-05
chr13	114057001	114062000	4.10E-05
chr13	114066001	114071000	6.81E-06
chr13	114073001	114078000	4.11E-10
chr13	114074001	114079000	2.03E-10

chr13	114213001	114218000	2.36E-11
chr13	114214001	114219000	7.24E-10
chr13	114215001	114220000	1.15E-07
chr13	114217001	114222000	9.17E-08
chr13	114423001	114428000	0.000217
chr13	114424001	114429000	0.000217
chr13	114493001	114498000	6.23E-11
chr13	114508001	114513000	3.57E-05
chr13	114738001	114743000	1.77E-06
chr13	114739001	114744000	1.31E-06
chr13	114740001	114745000	5.64E-07
chr13	114766001	114771000	3.44E-19
chr13	114767001	114772000	9.97E-18
chr13	114768001	114773000	2.31E-13
chr13	114769001	114774000	2.69E-17
chr13	114770001	114775000	5.13E-25
chr14	19142001	19147000	0.000328
chr14	19210001	19215000	0.000928
chr14	19211001	19216000	0.00042
chr14	19229001	19234000	0.008533
chr14	19230001	19235000	0.010647
chr14	19237001	19242000	0.001531
chr14	19238001	19243000	0.001769
chr14	19284001	19289000	0.002752
chr14	19285001	19290000	0.001226
chr14	19286001	19291000	0.001226
chr14	19292001	19297000	0.004183
chr14	19300001	19305000	2.34E-05
chr14	19301001	19306000	6.00E-06
chr14	19302001	19307000	8.07E-06
chr14	19303001	19308000	7.42E-06
chr14	19307001	19312000	7.26E-05
chr14	19308001	19313000	0.001577
chr14	19309001	19314000	0.000309
chr14	19341001	19346000	0.031237
chr14	19490001	19495000	0.003204
chr14	19491001	19496000	0.000423
chr14	19492001	19497000	0.000423
chr14	19637001	19642000	5.42E-07
chr14	19743001	19748000	6.02E-13
chr14	19744001	19749000	1.70E-09
chr14	19828001	19833000	2.10E-08
chr14	19829001	19834000	1.55E-07
chr14	19830001	19835000	1.81E-07
chr14	20552001	20557000	0.01437
chr14	21246001	21251000	2.35E-05
chr14	21595001	21600000	5.32E-05
chr14	21847001	21852000	3.24E-09

chr14	22163001	22168000	1.15E-06
chr14	22355001	22360000	4.46E-08
chr14	22509001	22514000	2.25E-06
chr14	22510001	22515000	2.84E-06
chr14	22517001	22522000	1.59E-08
chr14	22569001	22574000	2.97E-11
chr14	23104001	23109000	0.001309
chr14	23105001	23110000	0.000699
chr14	23200001	23205000	4.82E-05
chr14	23201001	23206000	7.06E-05
chr14	23202001	23207000	0.000487
chr14	23203001	23208000	2.90E-06
chr14	23319001	23324000	2.05E-06
chr14	23352001	23357000	6.66E-13
chr14	23353001	23358000	1.83E-14
chr14	23354001	23359000	7.26E-10
chr14	23366001	23371000	0.005486
chr14	23367001	23372000	0.000425
chr14	23383001	23388000	7.36E-08
chr14	23384001	23389000	6.91E-06
chr14	23389001	23394000	0.00052
chr14	23515001	23520000	6.68E-05
chr14	23516001	23521000	2.62E-06
chr14	23536001	23541000	1.89E-05
chr14	23537001	23542000	1.49E-08
chr14	23538001	23543000	3.08E-06
chr14	23540001	23545000	3.23E-07
chr14	23562001	23567000	1.62E-24
chr14	23578001	23583000	1.70E-07
chr14	23579001	23584000	2.27E-07
chr14	23580001	23585000	1.01E-07
chr14	23620001	23625000	3.38E-06
chr14	23621001	23626000	4.67E-07
chr14	23768001	23773000	8.01E-07
chr14	23769001	23774000	1.86E-07
chr14	23934001	23939000	1.28E-10
chr14	23981001	23986000	4.35E-05
chr14	23982001	23987000	3.68E-06
chr14	24111001	24116000	8.16E-06
chr14	24112001	24117000	1.73E-07
chr14	24113001	24118000	1.42E-09
chr14	24114001	24119000	9.26E-08
chr14	24115001	24120000	3.71E-06
chr14	24197001	24202000	1.89E-06
chr14	24233001	24238000	0.000463
chr14	24241001	24246000	9.32E-12
chr14	24255001	24260000	5.39E-06
chr14	24265001	24270000	4.48E-06

chr14	24278001	24283000	1.30E-08
chr14	24279001	24284000	9.36E-10
chr14	24280001	24285000	1.32E-06
chr14	24311001	24316000	2.64E-05
chr14	24312001	24317000	1.79E-09
chr14	24313001	24318000	1.11E-07
chr14	24319001	24324000	1.86E-05
chr14	24320001	24325000	1.62E-08
chr14	24527001	24532000	1.24E-05
chr14	24608001	24613000	1.21E-06
chr14	24654001	24659000	9.84E-10
chr14	24655001	24660000	1.96E-10
chr14	24656001	24661000	1.48E-11
chr14	24657001	24662000	6.13E-14
chr14	24736001	24741000	4.57E-07
chr14	24737001	24742000	2.94E-06
chr14	24767001	24772000	2.29E-09
chr14	24768001	24773000	8.65E-11
chr14	24778001	24783000	2.87E-12
chr14	24779001	24784000	1.40E-09
chr14	24780001	24785000	1.38E-12
chr14	24781001	24786000	1.74E-07
chr14	24912001	24917000	1.15E-11
chr14	24946001	24951000	0.000405
chr14	24947001	24952000	0.000877
chr14	24956001	24961000	0.002221
chr14	25035001	25040000	5.38E-05
chr14	25076001	25081000	8.32E-05
chr14	25077001	25082000	7.29E-06
chr14	25078001	25083000	1.05E-05
chr14	25137001	25142000	6.31E-07
chr14	25152001	25157000	4.78E-11
chr14	25154001	25159000	2.49E-10
chr14	25214001	25219000	2.30E-07
chr14	25215001	25220000	2.87E-06
chr14	25236001	25241000	1.53E-10
chr14	25251001	25256000	3.30E-09
chr14	25256001	25261000	5.58E-06
chr14	25257001	25262000	2.68E-08
chr14	25258001	25263000	2.29E-08
chr14	25260001	25265000	1.13E-08
chr14	25298001	25303000	1.61E-08
chr14	25550001	25555000	9.55E-08
chr14	25701001	25706000	1.73E-08
chr14	25702001	25707000	1.57E-08
chr14	25980001	25985000	7.52E-10
chr14	26001001	26006000	0.001176
chr14	26036001	26041000	1.97E-05

chr14	26037001	26042000	1.20E-06
chr14	26038001	26043000	1.45E-11
chr14	26039001	26044000	7.01E-12
chr14	26040001	26045000	1.60E-12
chr14	26132001	26137000	3.26E-12
chr14	26133001	26138000	7.36E-10
chr14	26134001	26139000	4.94E-09
chr14	26135001	26140000	1.72E-08
chr14	26136001	26141000	5.02E-07
chr14	26156001	26161000	0.000142
chr14	26200001	26205000	1.04E-05
chr14	26207001	26212000	0.000213
chr14	26208001	26213000	0.000194
chr14	26331001	26336000	7.37E-11
chr14	26332001	26337000	2.32E-08
chr14	26384001	26389000	4.65E-10
chr14	26409001	26414000	1.05E-13
chr14	26771001	26776000	1.00E-11
chr14	26772001	26777000	2.87E-12
chr14	27208001	27213000	3.56E-07
chr14	30396001	30401000	1.86E-11
chr14	30397001	30402000	8.29E-14
chr14	30413001	30418000	4.44E-08
chr14	30538001	30543000	9.42E-07
chr14	31495001	31500000	1.36E-06
chr14	31496001	31501000	2.28E-05
chr14	31889001	31894000	2.41E-09
chr14	32348001	32353000	4.13E-06
chr14	34786001	34791000	0.000179
chr14	35338001	35343000	1.75E-07
chr14	35339001	35344000	1.01E-06
chr14	35340001	35345000	4.30E-06
chr14	35587001	35592000	2.13E-11
chr14	38583001	38588000	9.67E-06
chr14	38736001	38741000	0.000414
chr14	38914001	38919000	8.19E-09
chr14	38915001	38920000	8.11E-07
chr14	38916001	38921000	3.79E-06
chr14	38940001	38945000	4.84E-08
chr14	39093001	39098000	7.19E-06
chr14	39099001	39104000	0.005236
chr14	39100001	39105000	0.004457
chr14	39111001	39116000	0.000259
chr14	39579001	39584000	8.84E-11
chr14	39580001	39585000	6.16E-13
chr14	40353001	40358000	0.000564
chr14	40374001	40379000	4.66E-08
chr14	40375001	40380000	6.80E-07

chr14	40659001	40664000	0.001026
chr14	41693001	41698000	6.65E-05
chr14	41874001	41879000	4.60E-07
chr14	41875001	41880000	4.66E-07
chr14	42752001	42757000	7.61E-13
chr14	43084001	43089000	6.29E-09
chr14	43395001	43400000	4.77E-07
chr14	44696001	44701000	4.23E-06
chr14	44697001	44702000	1.58E-05
chr14	45048001	45053000	3.37E-06
chr14	45049001	45054000	2.45E-09
chr14	45050001	45055000	7.09E-07
chr14	45058001	45063000	6.63E-07
chr14	45973001	45978000	9.94E-06
chr14	46047001	46052000	0.002183
chr14	46919001	46924000	4.54E-07
chr14	47084001	47089000	0.00034
chr14	47092001	47097000	3.30E-08
chr14	47093001	47098000	2.37E-07
chr14	47096001	47101000	9.74E-10
chr14	47149001	47154000	1.29E-09
chr14	47150001	47155000	2.79E-13
chr14	47414001	47419000	9.03E-08
chr14	47550001	47555000	6.28E-10
chr14	47551001	47556000	2.14E-06
chr14	47570001	47575000	3.82E-09
chr14	47571001	47576000	4.84E-10
chr14	47572001	47577000	3.55E-09
chr14	48031001	48036000	2.44E-06
chr14	48032001	48037000	5.74E-06
chr14	48111001	48116000	9.47E-10
chr14	48410001	48415000	1.88E-08
chr14	48411001	48416000	9.08E-12
chr14	48412001	48417000	3.46E-10
chr14	48413001	48418000	1.77E-08
chr14	48578001	48583000	4.34E-12
chr14	48874001	48879000	4.18E-05
chr14	49219001	49224000	1.24E-10
chr14	49220001	49225000	2.35E-10
chr14	49221001	49226000	4.43E-08
chr14	49287001	49292000	1.78E-08
chr14	49294001	49299000	3.43E-10
chr14	49295001	49300000	9.69E-11
chr14	49296001	49301000	6.60E-09
chr14	49298001	49303000	4.73E-09
chr14	49364001	49369000	1.95E-12
chr14	49365001	49370000	2.50E-11
chr14	49422001	49427000	7.14E-11

chr14	49423001	49428000	1.46E-09
chr14	49488001	49493000	7.28E-09
chr14	49756001	49761000	3.56E-07
chr14	49792001	49797000	1.83E-05
chr14	49816001	49821000	1.42E-07
chr14	49817001	49822000	1.37E-08
chr14	50087001	50092000	4.76E-15
chr14	50088001	50093000	2.39E-10
chr14	50101001	50106000	3.79E-06
chr14	50324001	50329000	1.67E-07
chr14	50333001	50338000	4.57E-07
chr14	50334001	50339000	3.14E-06
chr14	50335001	50340000	1.18E-06
chr14	50336001	50341000	8.25E-05
chr14	50337001	50342000	0.000193
chr14	50426001	50431000	3.13E-10
chr14	51340001	51345000	6.05E-08
chr14	51599001	51604000	3.61E-05
chr14	51600001	51605000	6.60E-07
chr14	51640001	51645000	4.85E-07
chr14	52342001	52347000	1.30E-09
chr14	52343001	52348000	3.80E-09
chr14	52489001	52494000	1.39E-07
chr14	52490001	52495000	2.74E-05
chr14	52504001	52509000	1.54E-11
chr14	52505001	52510000	1.49E-11
chr14	52544001	52549000	2.37E-09
chr14	52545001	52550000	1.55E-11
chr14	52546001	52551000	8.66E-07
chr14	52547001	52552000	5.13E-05
chr14	52553001	52558000	8.81E-13
chr14	52554001	52559000	6.93E-13
chr14	52555001	52560000	5.41E-14
chr14	52578001	52583000	5.87E-05
chr14	52579001	52584000	1.61E-06
chr14	52580001	52585000	7.91E-06
chr14	52640001	52645000	2.33E-07
chr14	52659001	52664000	4.67E-06
chr14	52660001	52665000	0.000143
chr14	52702001	52707000	9.85E-07
chr14	53396001	53401000	7.61E-11
chr14	53397001	53402000	9.14E-10
chr14	53398001	53403000	1.57E-07
chr14	53664001	53669000	6.48E-06
chr14	53665001	53670000	2.98E-05
chr14	54162001	54167000	6.50E-08
chr14	54163001	54168000	2.30E-07
chr14	54164001	54169000	1.15E-07

chr14	54542001	54547000	2.51E-06
chr14	56885001	56890000	4.67E-08
chr14	57852001	57857000	4.08E-09
chr14	57853001	57858000	1.19E-15
chr14	57896001	57901000	3.82E-07
chr14	58597001	58602000	2.56E-08
chr14	58642001	58647000	0.000146
chr14	58928001	58933000	1.14E-08
chr14	59427001	59432000	8.58E-13
chr14	59428001	59433000	3.17E-17
chr14	59429001	59434000	1.27E-12
chr14	59431001	59436000	3.29E-13
chr14	59465001	59470000	0.000376
chr14	59466001	59471000	6.35E-06
chr14	59542001	59547000	1.32E-05
chr14	59996001	60001000	1.22E-05
chr14	60109001	60114000	1.50E-06
chr14	60110001	60115000	1.44E-07
chr14	60111001	60116000	3.43E-07
chr14	60112001	60117000	2.25E-05
chr14	60113001	60118000	8.63E-06
chr14	60119001	60124000	7.26E-08
chr14	60559001	60564000	1.51E-18
chr14	60710001	60715000	3.43E-16
chr14	60711001	60716000	3.93E-11
chr14	60712001	60717000	2.79E-12
chr14	61012001	61017000	0.000754
chr14	62225001	62230000	2.36E-08
chr14	62798001	62803000	0.000584
chr14	63618001	63623000	1.09E-06
chr14	63619001	63624000	1.39E-07
chr14	63620001	63625000	4.44E-07
chr14	64195001	64200000	4.24E-07
chr14	64208001	64213000	4.24E-05
chr14	64209001	64214000	5.75E-05
chr14	64221001	64226000	2.03E-09
chr14	64222001	64227000	5.96E-06
chr14	65220001	65225000	3.28E-07
chr14	65512001	65517000	9.36E-08
chr14	66429001	66434000	2.47E-07
chr14	66673001	66678000	0.00013
chr14	66674001	66679000	5.24E-05
chr14	66683001	66688000	1.91E-07
chr14	66684001	66689000	2.13E-07
chr14	66686001	66691000	2.55E-06
chr14	66687001	66692000	4.11E-05
chr14	66706001	66711000	1.14E-06
chr14	66707001	66712000	2.51E-05

chr14	69148001	69153000	2.64E-07
chr14	69468001	69473000	0.000141
chr14	69469001	69474000	1.39E-05
chr14	69470001	69475000	1.10E-05
chr14	69471001	69476000	5.57E-06
chr14	69472001	69477000	6.72E-05
chr14	69946001	69951000	1.03E-06
chr14	69965001	69970000	2.79E-06
chr14	70008001	70013000	2.06E-06
chr14	70041001	70046000	1.71E-08
chr14	70412001	70417000	7.26E-07
chr14	70413001	70418000	1.66E-06
chr14	70523001	70528000	7.79E-09
chr14	70536001	70541000	5.92E-08
chr14	70644001	70649000	2.80E-09
chr14	70702001	70707000	7.24E-08
chr14	70703001	70708000	1.19E-06
chr14	70704001	70709000	8.04E-08
chr14	70705001	70710000	4.45E-11
chr14	70706001	70711000	6.11E-11
chr14	70903001	70908000	1.74E-08
chr14	70904001	70909000	2.81E-11
chr14	70905001	70910000	1.05E-06
chr14	70983001	70988000	0.000112
chr14	70989001	70994000	9.69E-06
chr14	70991001	70996000	2.03E-05
chr14	70992001	70997000	1.14E-08
chr14	70993001	70998000	2.80E-09
chr14	71013001	71018000	0.002842
chr14	71156001	71161000	1.35E-05
chr14	71694001	71699000	0.000133
chr14	72475001	72480000	3.47E-08
chr14	72476001	72481000	1.08E-08
chr14	72600001	72605000	2.28E-09
chr14	72613001	72618000	1.41E-11
chr14	72617001	72622000	2.04E-12
chr14	72704001	72709000	1.12E-10
chr14	72705001	72710000	3.49E-11
chr14	72707001	72712000	1.62E-10
chr14	72908001	72913000	4.94E-08
chr14	72909001	72914000	5.43E-08
chr14	72911001	72916000	4.09E-07
chr14	73009001	73014000	3.27E-11
chr14	73010001	73015000	1.07E-11
chr14	73011001	73016000	4.40E-09
chr14	73012001	73017000	4.39E-09
chr14	73067001	73072000	8.59E-08
chr14	73078001	73083000	4.73E-08

chr14	73108001	73113000	2.17E-07
chr14	73109001	73114000	1.25E-07
chr14	73190001	73195000	6.65E-06
chr14	73210001	73215000	3.85E-06
chr14	73211001	73216000	3.61E-07
chr14	73212001	73217000	5.62E-07
chr14	73277001	73282000	3.20E-07
chr14	73278001	73283000	1.67E-08
chr14	73285001	73290000	7.62E-05
chr14	73286001	73291000	0.000282
chr14	73293001	73298000	0.000114
chr14	73319001	73324000	3.81E-10
chr14	73335001	73340000	8.40E-07
chr14	73337001	73342000	1.52E-08
chr14	73341001	73346000	1.20E-09
chr14	73701001	73706000	2.93E-10
chr14	74163001	74168000	0.000348
chr14	74409001	74414000	0.000128
chr14	74550001	74555000	1.43E-07
chr14	74551001	74556000	5.59E-09
chr14	74693001	74698000	3.53E-08
chr14	74694001	74699000	7.68E-11
chr14	74830001	74835000	2.98E-06
chr14	74831001	74836000	1.53E-09
chr14	74868001	74873000	1.64E-06
chr14	74869001	74874000	7.70E-06
chr14	74889001	74894000	5.19E-07
chr14	74890001	74895000	2.47E-06
chr14	74891001	74896000	9.72E-06
chr14	75075001	75080000	4.02E-07
chr14	75077001	75082000	4.02E-07
chr14	75412001	75417000	6.18E-08
chr14	75413001	75418000	2.29E-13
chr14	75414001	75419000	1.77E-08
chr14	75415001	75420000	2.20E-08
chr14	75416001	75421000	3.95E-07
chr14	75417001	75422000	0.000123
chr14	75526001	75531000	1.75E-06
chr14	75890001	75895000	7.63E-11
chr14	75891001	75896000	1.63E-11
chr14	75892001	75897000	9.79E-12
chr14	75893001	75898000	1.83E-07
chr14	75954001	75959000	4.81E-08
chr14	75955001	75960000	7.70E-08
chr14	76298001	76303000	7.25E-05
chr14	76614001	76619000	5.85E-12
chr14	76615001	76620000	1.34E-10
chr14	76807001	76812000	2.74E-07

chr14	76859001	76864000	1.76E-09
chr14	77150001	77155000	2.06E-05
chr14	77151001	77156000	2.18E-08
chr14	77153001	77158000	8.59E-10
chr14	77154001	77159000	6.72E-10
chr14	77199001	77204000	5.16E-07
chr14	77314001	77319000	2.20E-06
chr14	77315001	77320000	6.02E-05
chr14	77497001	77502000	2.74E-10
chr14	77601001	77606000	6.01E-06
chr14	77700001	77705000	8.38E-05
chr14	77701001	77706000	1.67E-05
chr14	77702001	77707000	6.47E-06
chr14	77703001	77708000	6.09E-06
chr14	77785001	77790000	1.74E-11
chr14	77786001	77791000	4.58E-11
chr14	77787001	77792000	8.53E-12
chr14	78411001	78416000	0.000156
chr14	78508001	78513000	6.11E-05
chr14	78509001	78514000	9.68E-06
chr14	78517001	78522000	2.00E-08
chr14	78528001	78533000	7.42E-05
chr14	78643001	78648000	1.24E-07
chr14	78892001	78897000	2.91E-05
chr14	78961001	78966000	2.02E-06
chr14	79130001	79135000	6.26E-10
chr14	79190001	79195000	1.70E-07
chr14	79261001	79266000	1.74E-07
chr14	79375001	79380000	5.56E-07
chr14	79378001	79383000	1.91E-09
chr14	79487001	79492000	2.98E-05
chr14	79488001	79493000	3.12E-06
chr14	79489001	79494000	3.98E-05
chr14	82252001	82257000	5.98E-08
chr14	82294001	82299000	2.19E-08
chr14	82295001	82300000	7.80E-09
chr14	82374001	82379000	3.02E-09
chr14	82401001	82406000	2.11E-09
chr14	82402001	82407000	5.40E-08
chr14	82403001	82408000	2.76E-07
chr14	82404001	82409000	4.42E-09
chr14	82405001	82410000	1.33E-07
chr14	82449001	82454000	1.37E-09
chr14	82500001	82505000	4.12E-06
chr14	82501001	82506000	1.17E-06
chr14	82672001	82677000	1.61E-10
chr14	85021001	85026000	4.72E-06
chr14	85029001	85034000	3.24E-05

chr14	85045001	85050000	0.000129
chr14	86555001	86560000	6.22E-10
chr14	86556001	86561000	4.97E-11
chr14	86581001	86586000	1.06E-11
chr14	86582001	86587000	1.80E-10
chr14	86759001	86764000	3.94E-10
chr14	86828001	86833000	7.40E-07
chr14	87204001	87209000	3.81E-10
chr14	87205001	87210000	4.16E-11
chr14	87206001	87211000	2.38E-10
chr14	87297001	87302000	6.45E-05
chr14	87298001	87303000	0.000115
chr14	87513001	87518000	2.11E-14
chr14	87514001	87519000	3.15E-12
chr14	87576001	87581000	3.70E-08
chr14	87577001	87582000	1.69E-08
chr14	87636001	87641000	4.19E-09
chr14	87713001	87718000	1.53E-05
chr14	87714001	87719000	1.50E-06
chr14	87715001	87720000	1.89E-06
chr14	87716001	87721000	3.01E-08
chr14	87717001	87722000	2.14E-08
chr14	87718001	87723000	2.76E-12
chr14	87719001	87724000	2.98E-11
chr14	87720001	87725000	2.45E-12
chr14	87722001	87727000	1.74E-12
chr14	87723001	87728000	1.83E-10
chr14	87725001	87730000	1.26E-11
chr14	87742001	87747000	6.77E-13
chr14	87767001	87772000	3.27E-09
chr14	87930001	87935000	5.10E-13
chr14	87932001	87937000	1.83E-13
chr14	87933001	87938000	1.69E-09
chr14	87934001	87939000	2.94E-10
chr14	87967001	87972000	2.83E-08
chr14	88028001	88033000	3.51E-07
chr14	88051001	88056000	4.26E-09
chr14	88060001	88065000	2.62E-08
chr14	88070001	88075000	3.05E-07
chr14	88097001	88102000	5.79E-10
chr14	88098001	88103000	2.05E-10
chr14	88099001	88104000	1.20E-09
chr14	88101001	88106000	1.05E-11
chr14	88106001	88111000	1.50E-07
chr14	88155001	88160000	7.55E-10
chr14	88335001	88340000	7.75E-08
chr14	88336001	88341000	1.45E-05
chr14	88575001	88580000	3.55E-06

chr14	89883001	89888000	0.000166
chr14	90850001	90855000	4.17E-11
chr14	92338001	92343000	3.18E-07
chr14	92503001	92508000	3.85E-14
chr14	92505001	92510000	3.01E-15
chr14	92506001	92511000	9.37E-16
chr14	92584001	92589000	2.31E-07
chr14	92585001	92590000	1.05E-07
chr14	92586001	92591000	1.02E-09
chr14	93150001	93155000	0.000798
chr14	93797001	93802000	3.63E-13
chr14	93838001	93843000	1.85E-08
chr14	93901001	93906000	5.73E-09
chr14	93902001	93907000	2.86E-08
chr14	93903001	93908000	5.37E-10
chr14	93904001	93909000	1.69E-10
chr14	93978001	93983000	2.01E-06
chr14	93979001	93984000	2.69E-06
chr14	93980001	93985000	2.69E-06
chr14	94032001	94037000	1.60E-09
chr14	94045001	94050000	1.12E-08
chr14	94063001	94068000	1.53E-09
chr14	94064001	94069000	2.44E-10
chr14	94336001	94341000	0.001345
chr14	94368001	94373000	3.98E-07
chr14	94369001	94374000	1.34E-10
chr14	94370001	94375000	2.93E-08
chr14	94501001	94506000	0.000156
chr14	94502001	94507000	3.55E-05
chr14	94590001	94595000	1.06E-05
chr14	94591001	94596000	0.000801
chr14	94932001	94937000	4.26E-07
chr14	94939001	94944000	1.04E-05
chr14	94940001	94945000	1.24E-05
chr14	94941001	94946000	5.28E-05
chr14	94942001	94947000	9.04E-05
chr14	95052001	95057000	3.40E-05
chr14	95053001	95058000	0.00023
chr14	95054001	95059000	0.000162
chr14	95057001	95062000	0.000429
chr14	95193001	95198000	5.30E-06
chr14	95318001	95323000	4.22E-07
chr14	95348001	95353000	5.55E-06
chr14	95349001	95354000	2.76E-07
chr14	95350001	95355000	8.34E-07
chr14	95393001	95398000	2.74E-08
chr14	95461001	95466000	1.03E-06
chr14	95802001	95807000	1.62E-07

chr14	95803001	95808000	3.41E-07
chr14	95804001	95809000	1.03E-08
chr14	95805001	95810000	4.36E-10
chr14	95806001	95811000	1.57E-07
chr14	95885001	95890000	4.53E-05
chr14	95952001	95957000	4.05E-05
chr14	96049001	96054000	9.71E-09
chr14	96050001	96055000	2.06E-09
chr14	96051001	96056000	1.28E-08
chr14	96066001	96071000	2.25E-08
chr14	96142001	96147000	3.73E-06
chr14	96143001	96148000	1.84E-05
chr14	96256001	96261000	1.89E-07
chr14	96257001	96262000	1.02E-09
chr14	96569001	96574000	4.36E-08
chr14	96570001	96575000	4.43E-07
chr14	96669001	96674000	0.004406
chr14	97105001	97110000	2.45E-12
chr14	97106001	97111000	2.34E-09
chr14	97537001	97542000	3.04E-07
chr14	97614001	97619000	0.004252
chr14	97689001	97694000	1.14E-10
chr14	97690001	97695000	5.78E-10
chr14	97691001	97696000	5.90E-10
chr14	97720001	97725000	7.07E-16
chr14	97721001	97726000	7.75E-16
chr14	97722001	97727000	1.33E-14
chr14	97814001	97819000	1.39E-09
chr14	97972001	97977000	2.95E-08
chr14	97973001	97978000	1.44E-07
chr14	98087001	98092000	2.32E-09
chr14	98112001	98117000	1.00E-09
chr14	98113001	98118000	5.59E-11
chr14	98157001	98162000	1.51E-05
chr14	98275001	98280000	8.89E-12
chr14	98322001	98327000	6.44E-08
chr14	98323001	98328000	1.18E-06
chr14	98324001	98329000	1.21E-08
chr14	98380001	98385000	1.31E-05
chr14	98381001	98386000	1.89E-05
chr14	98382001	98387000	1.59E-05
chr14	98933001	98938000	1.24E-06
chr14	99426001	99431000	1.55E-05
chr14	100064001	100069000	4.12E-05
chr14	100109001	100114000	5.90E-07
chr14	100110001	100115000	1.16E-05
chr14	100180001	100185000	1.53E-06
chr14	100201001	100206000	0.005462

chr14	100218001	100223000	7.96E-07
chr14	100219001	100224000	9.53E-17
chr14	100220001	100225000	1.50E-12
chr14	100221001	100226000	5.69E-12
chr14	100222001	100227000	6.58E-10
chr14	100420001	100425000	3.99E-08
chr14	100421001	100426000	1.96E-09
chr14	100422001	100427000	1.05E-10
chr14	100603001	100608000	0.00018
chr14	100613001	100618000	0.002709
chr14	100727001	100732000	9.80E-06
chr14	100770001	100775000	6.48E-05
chr14	101074001	101079000	0.006308
chr14	101075001	101080000	0.000857
chr14	101076001	101081000	2.32E-06
chr14	101077001	101082000	1.77E-07
chr14	101122001	101127000	0.001092
chr14	101152001	101157000	1.38E-05
chr14	101155001	101160000	7.42E-09
chr14	101164001	101169000	6.79E-08
chr14	101165001	101170000	1.23E-08
chr14	101229001	101234000	1.88E-08
chr14	101230001	101235000	4.50E-08
chr14	101232001	101237000	1.49E-08
chr14	101299001	101304000	4.74E-05
chr14	101566001	101571000	3.04E-05
chr14	101574001	101579000	1.84E-08
chr14	101575001	101580000	3.49E-10
chr14	101576001	101581000	3.19E-06
chr14	101577001	101582000	2.51E-09
chr14	101578001	101583000	3.47E-09
chr14	101731001	101736000	1.09E-09
chr14	101732001	101737000	6.53E-11
chr14	101929001	101934000	8.33E-08
chr14	101930001	101935000	2.96E-08
chr14	101931001	101936000	5.02E-08
chr14	101991001	101996000	7.94E-05
chr14	101992001	101997000	7.27E-07
chr14	101993001	101998000	2.94E-07
chr14	102013001	102018000	3.26E-05
chr14	102014001	102019000	1.85E-07
chr14	102184001	102189000	1.32E-06
chr14	102185001	102190000	4.14E-08
chr14	102186001	102191000	9.40E-09
chr14	102187001	102192000	4.48E-05
chr14	102188001	102193000	1.19E-06
chr14	103009001	103014000	0.000168
chr14	103390001	103395000	3.65E-09

chr14	103391001	103396000	3.65E-09
chr14	103392001	103397000	8.33E-09
chr14	103393001	103398000	1.66E-10
chr14	103394001	103399000	1.28E-07
chr14	103562001	103567000	5.95E-07
chr14	103563001	103568000	5.76E-05
chr14	103623001	103628000	3.99E-08
chr14	103778001	103783000	1.45E-05
chr14	103779001	103784000	2.15E-06
chr14	103834001	103839000	1.80E-06
chr14	103843001	103848000	0.001025
chr14	103844001	103849000	0.000494
chr14	104009001	104014000	0.001725
chr14	104010001	104015000	0.004243
chr14	104067001	104072000	8.26E-05
chr14	104068001	104073000	0.00035
chr14	104069001	104074000	6.66E-06
chr14	104070001	104075000	2.44E-05
chr14	104071001	104076000	3.23E-05
chr14	104072001	104077000	7.84E-05
chr14	104073001	104078000	0.000318
chr14	104333001	104338000	2.01E-08
chr14	104334001	104339000	6.62E-11
chr14	104335001	104340000	2.06E-10
chr14	104336001	104341000	7.54E-15
chr14	104337001	104342000	7.72E-16
chr14	104338001	104343000	4.35E-16
chr14	104339001	104344000	8.76E-08
chr14	104383001	104388000	1.43E-13
chr14	104384001	104389000	6.11E-15
chr14	104416001	104421000	3.48E-09
chr14	104447001	104452000	3.53E-07
chr14	104569001	104574000	4.64E-05
chr14	104570001	104575000	2.41E-08
chr14	104571001	104576000	1.26E-09
chr14	104572001	104577000	2.07E-09
chr14	104573001	104578000	4.39E-08
chr14	104596001	104601000	1.57E-09
chr14	104597001	104602000	2.14E-10
chr14	104637001	104642000	7.19E-06
chr14	104638001	104643000	0.000151
chr14	104639001	104644000	1.49E-16
chr14	104640001	104645000	8.07E-17
chr14	104641001	104646000	2.29E-16
chr14	104642001	104647000	1.98E-06
chr14	104648001	104653000	3.50E-09
chr14	104649001	104654000	5.07E-15
chr14	104650001	104655000	3.87E-14

chr14	104651001	104656000	7.63E-12
chr14	104652001	104657000	7.57E-17
chr14	104661001	104666000	1.36E-12
chr14	104663001	104668000	3.21E-10
chr14	104664001	104669000	3.53E-15
chr14	104665001	104670000	1.62E-10
chr14	104680001	104685000	0.001436
chr14	104681001	104686000	0.001349
chr14	104682001	104687000	0.00144
chr14	104695001	104700000	0.000226
chr14	104697001	104702000	0.000199
chr14	104729001	104734000	9.16E-09
chr14	104767001	104772000	6.19E-09
chr14	104768001	104773000	5.65E-09
chr14	104769001	104774000	1.23E-07
chr14	104801001	104806000	7.69E-12
chr14	104802001	104807000	4.94E-12
chr14	104803001	104808000	5.87E-13
chr14	104821001	104826000	2.12E-10
chr14	104822001	104827000	6.87E-13
chr14	104836001	104841000	3.88E-08
chr14	104912001	104917000	4.48E-07
chr14	105146001	105151000	8.26E-06
chr14	105147001	105152000	3.83E-08
chr14	105197001	105202000	0.003419
chr14	105333001	105338000	2.29E-09
chr14	105334001	105339000	1.50E-06
chr14	105335001	105340000	2.40E-06
chr14	105482001	105487000	1.10E-06
chr14	105483001	105488000	2.57E-09
chr14	105538001	105543000	1.86E-07
chr14	105539001	105544000	0.002697
chr14	105541001	105546000	0.000353
chr14	105555001	105560000	9.12E-06
chr14	105557001	105562000	0.00048
chr14	105558001	105563000	1.09E-08
chr14	105560001	105565000	6.89E-08
chr14	105842001	105847000	2.87E-06
chr14	105883001	105888000	7.76E-12
chr14	105884001	105889000	1.26E-05
chr14	106052001	106057000	0.001371
chr14	106053001	106058000	0.001371
chr14	106084001	106089000	5.97E-06
chr14	106085001	106090000	2.83E-06
chr14	106086001	106091000	1.27E-05
chr14	106094001	106099000	3.77E-07
chr14	106144001	106149000	0.002214
chr14	106145001	106150000	0.000205

chr14	106147001	106152000	2.16E-09
chr14	106148001	106153000	2.49E-06
chr14	106154001	106159000	0.006541
chr14	106168001	106173000	0.0003
chr14	106169001	106174000	1.51E-07
chr14	106170001	106175000	2.00E-05
chr14	106176001	106181000	0.000265
chr14	106177001	106182000	0.000166
chr14	106180001	106185000	0.001417
chr14	106238001	106243000	0.000194
chr14	106301001	106306000	1.58E-06
chr14	106303001	106308000	9.01E-07
chr14	106304001	106309000	7.88E-07
chr14	106316001	106321000	2.91E-06
chr14	106317001	106322000	5.07E-07
chr14	106330001	106335000	3.78E-09
chr14	106331001	106336000	2.80E-07
chr14	106332001	106337000	6.33E-08
chr14	106365001	106370000	6.81E-07
chr14	106366001	106371000	1.76E-05
chr14	106428001	106433000	0.000448
chr14	106429001	106434000	6.08E-05
chr14	106437001	106442000	1.80E-11
chr14	106538001	106543000	0.012966
chr14	106539001	106544000	0.012966
chr14	106540001	106545000	0.012966
chr15	20198001	20203000	0.00968
chr15	20232001	20237000	0.013281
chr15	20531001	20536000	0.000463
chr15	21973001	21978000	1.94E-05
chr15	21974001	21979000	5.12E-05
chr15	22804001	22809000	1.57E-05
chr15	22805001	22810000	0.000106
chr15	22829001	22834000	0.000874
chr15	23085001	23090000	8.15E-09
chr15	23086001	23091000	3.26E-07
chr15	23469001	23474000	0.000354
chr15	23577001	23582000	7.66E-05
chr15	23578001	23583000	7.66E-05
chr15	23640001	23645000	2.37E-07
chr15	23778001	23783000	1.20E-09
chr15	23779001	23784000	8.29E-08
chr15	23780001	23785000	4.73E-08
chr15	24271001	24276000	1.66E-07
chr15	24272001	24277000	4.14E-06
chr15	24477001	24482000	5.45E-06
chr15	24478001	24483000	6.21E-09
chr15	24479001	24484000	1.27E-07

chr15	24842001	24847000	1.13E-07
chr15	24843001	24848000	1.32E-07
chr15	24957001	24962000	3.93E-06
chr15	24973001	24978000	2.10E-08
chr15	24974001	24979000	1.05E-09
chr15	25083001	25088000	3.16E-09
chr15	25084001	25089000	1.72E-06
chr15	25085001	25090000	6.82E-09
chr15	25086001	25091000	8.09E-10
chr15	25171001	25176000	0.001365
chr15	25172001	25177000	0.004059
chr15	25173001	25178000	0.001216
chr15	25174001	25179000	0.000912
chr15	25474001	25479000	0.000682
chr15	25999001	26004000	2.83E-07
chr15	26395001	26400000	1.52E-06
chr15	26396001	26401000	1.53E-06
chr15	26522001	26527000	9.73E-11
chr15	26523001	26528000	1.18E-09
chr15	26563001	26568000	2.03E-10
chr15	26626001	26631000	1.81E-17
chr15	26627001	26632000	2.21E-17
chr15	26628001	26633000	4.74E-15
chr15	26700001	26705000	3.66E-11
chr15	26701001	26706000	6.91E-09
chr15	26702001	26707000	1.45E-11
chr15	26703001	26708000	2.67E-10
chr15	26704001	26709000	5.01E-07
chr15	26710001	26715000	1.59E-06
chr15	27147001	27152000	1.76E-06
chr15	27467001	27472000	1.90E-06
chr15	27856001	27861000	4.71E-11
chr15	27857001	27862000	7.22E-11
chr15	28142001	28147000	6.62E-07
chr15	28165001	28170000	4.79E-09
chr15	28185001	28190000	1.16E-08
chr15	28682001	28687000	6.36E-07
chr15	28683001	28688000	3.76E-05
chr15	28684001	28689000	1.42E-05
chr15	28685001	28690000	0.008202
chr15	28743001	28748000	0.009176
chr15	28749001	28754000	0.000163
chr15	28830001	28835000	1.06E-14
chr15	28831001	28836000	2.91E-17
chr15	28952001	28957000	0.023689
chr15	29128001	29133000	3.42E-06
chr15	29205001	29210000	0.008629
chr15	29562001	29567000	1.97E-17

chr15	29759001	29764000	3.23E-12
chr15	29760001	29765000	2.28E-13
chr15	29888001	29893000	1.40E-07
chr15	29890001	29895000	4.62E-09
chr15	29905001	29910000	0.000201
chr15	29906001	29911000	0.000235
chr15	29907001	29912000	6.57E-06
chr15	29908001	29913000	4.22E-06
chr15	30394001	30399000	0.001002
chr15	30471001	30476000	0.000117
chr15	30472001	30477000	6.84E-05
chr15	30473001	30478000	6.84E-05
chr15	30474001	30479000	3.20E-05
chr15	30504001	30509000	8.32E-07
chr15	30505001	30510000	3.33E-06
chr15	30602001	30607000	0.006091
chr15	30656001	30661000	0.010034
chr15	30876001	30881000	0.010383
chr15	31101001	31106000	8.81E-06
chr15	31148001	31153000	7.91E-07
chr15	31149001	31154000	8.88E-07
chr15	31150001	31155000	9.83E-07
chr15	31689001	31694000	8.23E-06
chr15	31995001	32000000	0.000262
chr15	32014001	32019000	3.17E-07
chr15	32429001	32434000	7.18E-06
chr15	32547001	32552000	3.62E-07
chr15	32608001	32613000	0.00925
chr15	32609001	32614000	0.000506
chr15	32610001	32615000	0.002144
chr15	32611001	32616000	0.000385
chr15	32612001	32617000	0.002249
chr15	32613001	32618000	0.023311
chr15	32668001	32673000	0.003176
chr15	32685001	32690000	0.009377
chr15	32686001	32691000	0.009887
chr15	32687001	32692000	0.003336
chr15	32688001	32693000	0.001408
chr15	33563001	33568000	2.57E-07
chr15	34811001	34816000	3.23E-05
chr15	34812001	34817000	5.09E-05
chr15	35070001	35075000	2.85E-10
chr15	35568001	35573000	0.000836
chr15	37838001	37843000	8.43E-08
chr15	40341001	40346000	3.08E-05
chr15	40562001	40567000	5.62E-07
chr15	40572001	40577000	0.000226
chr15	40599001	40604000	0.008179

chr15	40600001	40605000	0.040154
chr15	40601001	40606000	0.004602
chr15	40858001	40863000	1.71E-09
chr15	40861001	40866000	1.85E-05
chr15	41052001	41057000	2.72E-10
chr15	41053001	41058000	9.17E-09
chr15	41054001	41059000	9.79E-16
chr15	41134001	41139000	0.000313
chr15	41162001	41167000	0.000358
chr15	41213001	41218000	1.61E-06
chr15	41214001	41219000	2.45E-06
chr15	41215001	41220000	4.35E-07
chr15	41216001	41221000	4.65E-09
chr15	41474001	41479000	0.000318
chr15	41477001	41482000	0.000766
chr15	41692001	41697000	1.08E-05
chr15	41693001	41698000	1.62E-06
chr15	41694001	41699000	6.09E-12
chr15	41695001	41700000	5.01E-08
chr15	42121001	42126000	1.92E-11
chr15	42122001	42127000	3.12E-08
chr15	42123001	42128000	9.25E-08
chr15	42124001	42129000	1.73E-07
chr15	42135001	42140000	0.000764
chr15	42905001	42910000	2.61E-06
chr15	42945001	42950000	2.48E-09
chr15	44010001	44015000	2.46E-06
chr15	44088001	44093000	0.000365
chr15	44482001	44487000	7.53E-08
chr15	44483001	44488000	2.53E-08
chr15	44484001	44489000	3.09E-05
chr15	44485001	44490000	5.39E-06
chr15	44486001	44491000	4.08E-05
chr15	44527001	44532000	4.65E-09
chr15	45476001	45481000	4.63E-05
chr15	45477001	45482000	0.000458
chr15	45478001	45483000	3.84E-06
chr15	45479001	45484000	1.67E-08
chr15	45480001	45485000	2.11E-08
chr15	45538001	45543000	0.000575
chr15	47132001	47137000	2.69E-10
chr15	47270001	47275000	0.00036
chr15	49850001	49855000	1.10E-05
chr15	49851001	49856000	3.93E-05
chr15	50666001	50671000	0.001159
chr15	51095001	51100000	0.000274
chr15	52468001	52473000	4.86E-06
chr15	52469001	52474000	1.51E-07

chr15	53405001	53410000	2.44E-05
chr15	53406001	53411000	5.26E-06
chr15	53407001	53412000	8.33E-07
chr15	55713001	55718000	0.01613
chr15	56078001	56083000	3.77E-06
chr15	58414001	58419000	2.74E-13
chr15	59041001	59046000	3.66E-05
chr15	59042001	59047000	3.29E-06
chr15	59049001	59054000	1.65E-06
chr15	59883001	59888000	0.000128
chr15	61615001	61620000	2.42E-11
chr15	61616001	61621000	2.41E-10
chr15	61617001	61622000	1.99E-10
chr15	61714001	61719000	4.83E-05
chr15	62039001	62044000	1.79E-08
chr15	63738001	63743000	3.49E-08
chr15	63739001	63744000	7.76E-08
chr15	63740001	63745000	3.66E-07
chr15	63741001	63746000	4.25E-07
chr15	65100001	65105000	1.40E-05
chr15	65101001	65106000	3.45E-07
chr15	65155001	65160000	0.005872
chr15	65156001	65161000	0.016101
chr15	65157001	65162000	0.018795
chr15	65421001	65426000	4.13E-05
chr15	65476001	65481000	4.78E-09
chr15	65477001	65482000	9.82E-09
chr15	66151001	66156000	0.000249
chr15	66152001	66157000	0.000249
chr15	66248001	66253000	8.74E-10
chr15	66305001	66310000	9.06E-05
chr15	66307001	66312000	0.001611
chr15	66308001	66313000	0.000153
chr15	66351001	66356000	0.000424
chr15	66352001	66357000	0.000559
chr15	66648001	66653000	1.91E-06
chr15	66649001	66654000	3.19E-06
chr15	67062001	67067000	1.79E-05
chr15	68499001	68504000	1.32E-05
chr15	68546001	68551000	2.71E-09
chr15	68549001	68554000	1.25E-08
chr15	68746001	68751000	3.31E-05
chr15	68867001	68872000	5.90E-11
chr15	68962001	68967000	6.32E-05
chr15	68963001	68968000	7.08E-07
chr15	69753001	69758000	3.97E-10
chr15	69754001	69759000	2.63E-09
chr15	69755001	69760000	6.17E-06

chr15	70172001	70177000	5.03E-07
chr15	70173001	70178000	1.72E-07
chr15	70267001	70272000	2.99E-05
chr15	72571001	72576000	9.34E-05
chr15	72724001	72729000	3.22E-06
chr15	72937001	72942000	0.002814
chr15	73335001	73340000	1.24E-07
chr15	74129001	74134000	2.42E-06
chr15	74178001	74183000	0.000143
chr15	74215001	74220000	8.02E-06
chr15	74216001	74221000	1.69E-06
chr15	74217001	74222000	3.06E-06
chr15	74218001	74223000	0.000226
chr15	74219001	74224000	0.000158
chr15	74388001	74393000	4.00E-08
chr15	74389001	74394000	9.59E-08
chr15	74390001	74395000	2.25E-08
chr15	74391001	74396000	1.09E-07
chr15	74411001	74416000	7.46E-06
chr15	74466001	74471000	0.000613
chr15	74510001	74515000	0.000104
chr15	74511001	74516000	0.000726
chr15	74707001	74712000	0.000138
chr15	74708001	74713000	5.42E-06
chr15	74709001	74714000	1.88E-06
chr15	74711001	74716000	0.000554
chr15	74803001	74808000	0.000201
chr15	74817001	74822000	0.004507
chr15	74888001	74893000	1.97E-07
chr15	74889001	74894000	1.44E-05
chr15	74890001	74895000	1.52E-08
chr15	74998001	75003000	4.62E-07
chr15	74999001	75004000	4.60E-06
chr15	75042001	75047000	0.003766
chr15	75043001	75048000	1.48E-06
chr15	75044001	75049000	0.000294
chr15	75051001	75056000	0.003326
chr15	75077001	75082000	7.83E-05
chr15	75078001	75083000	0.003256
chr15	75278001	75283000	8.78E-05
chr15	75392001	75397000	0.000261
chr15	75393001	75398000	0.001859
chr15	75624001	75629000	5.79E-11
chr15	75656001	75661000	6.35E-10
chr15	75972001	75977000	2.19E-05
chr15	75973001	75978000	3.11E-08
chr15	75987001	75992000	0.000157
chr15	76424001	76429000	0.000494

chr15	76425001	76430000	0.001617
chr15	76601001	76606000	0.000316
chr15	76681001	76686000	3.96E-05
chr15	77956001	77961000	4.10E-05
chr15	77957001	77962000	0.000532
chr15	77996001	78001000	0.000175
chr15	77997001	78002000	1.53E-05
chr15	78102001	78107000	4.48E-05
chr15	78553001	78558000	8.37E-11
chr15	78554001	78559000	4.24E-13
chr15	78556001	78561000	3.47E-14
chr15	78858001	78863000	8.25E-10
chr15	79148001	79153000	1.35E-12
chr15	79399001	79404000	0.012417
chr15	79569001	79574000	5.15E-06
chr15	79782001	79787000	1.42E-12
chr15	79783001	79788000	7.78E-12
chr15	79793001	79798000	0.001562
chr15	80109001	80114000	1.73E-06
chr15	80110001	80115000	2.86E-05
chr15	80587001	80592000	3.01E-07
chr15	81465001	81470000	0.000298
chr15	82332001	82337000	1.04E-09
chr15	82333001	82338000	1.92E-12
chr15	83210001	83215000	0.022886
chr15	83344001	83349000	0.000274
chr15	83345001	83350000	0.000889
chr15	83346001	83351000	8.12E-06
chr15	83414001	83419000	1.97E-07
chr15	83415001	83420000	1.22E-10
chr15	84162001	84167000	2.69E-05
chr15	84905001	84910000	0.001926
chr15	84906001	84911000	0.001926
chr15	84944001	84949000	0.014399
chr15	84945001	84950000	0.030271
chr15	84946001	84951000	0.02029
chr15	84947001	84952000	4.19E-06
chr15	84948001	84953000	1.01E-05
chr15	84949001	84954000	0.002954
chr15	85142001	85147000	1.02E-17
chr15	85143001	85148000	6.92E-17
chr15	85144001	85149000	4.46E-15
chr15	85519001	85524000	6.42E-09
chr15	86328001	86333000	0.000136
chr15	86354001	86359000	0.001435
chr15	86368001	86373000	1.64E-05
chr15	86397001	86402000	4.15E-06
chr15	86398001	86403000	5.01E-06

chr15	86399001	86404000	0.00025
chr15	86471001	86476000	0.000149
chr15	86930001	86935000	5.73E-08
chr15	87069001	87074000	1.17E-07
chr15	87070001	87075000	2.01E-09
chr15	87071001	87076000	9.08E-10
chr15	87112001	87117000	5.47E-06
chr15	87113001	87118000	3.63E-07
chr15	87114001	87119000	1.76E-05
chr15	87241001	87246000	5.34E-05
chr15	87242001	87247000	6.55E-09
chr15	87243001	87248000	1.15E-08
chr15	87244001	87249000	1.73E-09
chr15	87356001	87361000	1.67E-07
chr15	87597001	87602000	7.64E-17
chr15	87697001	87702000	1.54E-05
chr15	87865001	87870000	1.01E-05
chr15	88240001	88245000	0.000224
chr15	88241001	88246000	7.66E-06
chr15	88242001	88247000	1.24E-08
chr15	88350001	88355000	1.03E-09
chr15	88363001	88368000	2.21E-07
chr15	88431001	88436000	3.97E-06
chr15	88511001	88516000	4.30E-06
chr15	88512001	88517000	3.34E-08
chr15	88513001	88518000	4.69E-08
chr15	88514001	88519000	1.02E-08
chr15	88515001	88520000	1.79E-08
chr15	88516001	88521000	4.38E-07
chr15	88518001	88523000	1.14E-06
chr15	88521001	88526000	9.94E-07
chr15	88522001	88527000	3.02E-06
chr15	88695001	88700000	5.96E-10
chr15	88698001	88703000	5.50E-11
chr15	88701001	88706000	1.02E-08
chr15	88742001	88747000	1.19E-08
chr15	88784001	88789000	1.37E-05
chr15	88785001	88790000	1.07E-05
chr15	88794001	88799000	0.006798
chr15	88795001	88800000	7.82E-05
chr15	88796001	88801000	0.00087
chr15	88800001	88805000	1.88E-09
chr15	88837001	88842000	6.27E-12
chr15	88838001	88843000	5.62E-10
chr15	88839001	88844000	4.53E-12
chr15	88840001	88845000	1.46E-16
chr15	88889001	88894000	1.93E-10
chr15	88890001	88895000	2.11E-13

chr15	88891001	88896000	8.69E-13
chr15	88892001	88897000	3.12E-12
chr15	88893001	88898000	6.03E-09
chr15	89099001	89104000	2.70E-17
chr15	89119001	89124000	3.98E-08
chr15	89121001	89126000	6.77E-06
chr15	89262001	89267000	2.46E-11
chr15	89393001	89398000	0.000149
chr15	89710001	89715000	5.95E-06
chr15	90105001	90110000	6.22E-06
chr15	90106001	90111000	2.49E-05
chr15	90107001	90112000	0.000227
chr15	90114001	90119000	2.24E-14
chr15	90116001	90121000	3.37E-11
chr15	90117001	90122000	3.39E-10
chr15	90701001	90706000	0.000372
chr15	90702001	90707000	4.55E-06
chr15	90703001	90708000	2.83E-05
chr15	90762001	90767000	0.000249
chr15	90763001	90768000	8.54E-06
chr15	90764001	90769000	1.17E-05
chr15	90807001	90812000	2.91E-09
chr15	90808001	90813000	1.67E-09
chr15	90809001	90814000	4.94E-07
chr15	91188001	91193000	3.72E-06
chr15	91256001	91261000	2.13E-07
chr15	91257001	91262000	5.84E-07
chr15	91258001	91263000	3.95E-11
chr15	91259001	91264000	3.93E-14
chr15	91714001	91719000	1.94E-09
chr15	92292001	92297000	0.000108
chr15	93198001	93203000	2.90E-06
chr15	93343001	93348000	3.84E-07
chr15	93345001	93350000	8.42E-06
chr15	93346001	93351000	2.18E-07
chr15	93349001	93354000	1.90E-05
chr15	93352001	93357000	2.75E-14
chr15	95133001	95138000	1.72E-09
chr15	95280001	95285000	3.34E-14
chr15	95281001	95286000	3.07E-14
chr15	95322001	95327000	9.38E-06
chr15	95323001	95328000	1.16E-05
chr15	95370001	95375000	3.17E-08
chr15	95372001	95377000	4.77E-08
chr15	95414001	95419000	6.71E-07
chr15	97368001	97373000	1.43E-07
chr15	97644001	97649000	4.55E-06
chr15	97645001	97650000	1.50E-09

chr15	97647001	97652000	3.93E-10
chr15	97648001	97653000	3.77E-10
chr15	97693001	97698000	1.95E-07
chr15	97706001	97711000	3.06E-12
chr15	97822001	97827000	2.21E-05
chr15	97823001	97828000	4.51E-08
chr15	97824001	97829000	3.03E-07
chr15	97825001	97830000	1.52E-09
chr15	98127001	98132000	7.98E-12
chr15	98156001	98161000	3.92E-10
chr15	98699001	98704000	1.55E-09
chr15	98700001	98705000	9.39E-08
chr15	98801001	98806000	5.25E-08
chr15	98886001	98891000	1.40E-07
chr15	99030001	99035000	4.11E-06
chr15	99052001	99057000	7.20E-08
chr15	99158001	99163000	0.000108
chr15	102035001	102040000	1.47E-06
chr16	281001	286000	7.44E-13
chr16	282001	287000	2.25E-10
chr16	283001	288000	3.69E-12
chr16	284001	289000	1.76E-12
chr16	285001	290000	1.78E-12
chr16	492001	497000	5.34E-06
chr16	573001	578000	1.95E-13
chr16	574001	579000	5.07E-18
chr16	575001	580000	1.01E-14
chr16	601001	606000	2.20E-06
chr16	602001	607000	3.67E-07
chr16	603001	608000	9.26E-07
chr16	604001	609000	2.13E-11
chr16	641001	646000	1.17E-05
chr16	671001	676000	2.07E-05
chr16	684001	689000	7.14E-07
chr16	685001	690000	3.11E-06
chr16	722001	727000	2.90E-08
chr16	736001	741000	6.06E-08
chr16	754001	759000	0.005314
chr16	757001	762000	6.39E-13
chr16	758001	763000	3.32E-09
chr16	759001	764000	4.63E-07
chr16	1031001	1036000	5.46E-06
chr16	1044001	1049000	0.005317
chr16	1045001	1050000	0.000201
chr16	1046001	1051000	0.001015
chr16	1047001	1052000	6.24E-06
chr16	1056001	1061000	1.26E-07
chr16	1062001	1067000	4.59E-10

chr16	1063001	1068000	2.71E-09
chr16	1064001	1069000	2.37E-13
chr16	1065001	1070000	1.13E-12
chr16	1066001	1071000	2.27E-10
chr16	1109001	1114000	0.000146
chr16	1115001	1120000	5.53E-07
chr16	1116001	1121000	1.02E-08
chr16	1117001	1122000	8.55E-07
chr16	1118001	1123000	0.005576
chr16	1173001	1178000	0.027108
chr16	1180001	1185000	0.000823
chr16	1186001	1191000	2.39E-11
chr16	1212001	1217000	1.13E-06
chr16	1213001	1218000	4.65E-08
chr16	1215001	1220000	9.23E-09
chr16	1216001	1221000	2.88E-11
chr16	1217001	1222000	8.54E-08
chr16	1236001	1241000	0.001216
chr16	1254001	1259000	0.002156
chr16	1260001	1265000	1.55E-05
chr16	1261001	1266000	4.83E-05
chr16	1262001	1267000	0.001616
chr16	1303001	1308000	0.004574
chr16	1305001	1310000	3.30E-07
chr16	1306001	1311000	3.31E-07
chr16	1307001	1312000	1.84E-07
chr16	1308001	1313000	2.16E-07
chr16	1355001	1360000	2.34E-10
chr16	1383001	1388000	1.50E-07
chr16	1529001	1534000	2.97E-10
chr16	1530001	1535000	2.84E-10
chr16	1531001	1536000	3.22E-08
chr16	1575001	1580000	8.67E-07
chr16	2008001	2013000	0.000169
chr16	2009001	2014000	3.58E-05
chr16	2022001	2027000	2.63E-06
chr16	2049001	2054000	0.001062
chr16	2050001	2055000	1.33E-07
chr16	2052001	2057000	0.001341
chr16	2053001	2058000	4.15E-06
chr16	2054001	2059000	2.15E-05
chr16	2067001	2072000	0.000545
chr16	2068001	2073000	2.91E-06
chr16	2069001	2074000	9.08E-05
chr16	2074001	2079000	6.67E-05
chr16	2075001	2080000	0.000111
chr16	2103001	2108000	0.000109
chr16	2104001	2109000	5.26E-05

chr16	2105001	2110000	1.02E-07
chr16	2197001	2202000	0.01122
chr16	2228001	2233000	7.24E-06
chr16	2252001	2257000	0.001538
chr16	2271001	2276000	5.45E-08
chr16	2519001	2524000	0.017337
chr16	2522001	2527000	2.79E-08
chr16	2691001	2696000	0.000342
chr16	2692001	2697000	0.002312
chr16	2759001	2764000	3.23E-09
chr16	2760001	2765000	9.12E-14
chr16	2761001	2766000	2.64E-14
chr16	2762001	2767000	1.78E-15
chr16	2763001	2768000	7.69E-07
chr16	2783001	2788000	5.27E-06
chr16	2784001	2789000	0.003557
chr16	2785001	2790000	0.007173
chr16	2786001	2791000	0.000276
chr16	2787001	2792000	0.006317
chr16	2817001	2822000	0.000536
chr16	2908001	2913000	4.10E-05
chr16	3010001	3015000	0.026485
chr16	3011001	3016000	0.000265
chr16	3019001	3024000	0.000242
chr16	3152001	3157000	1.27E-07
chr16	3153001	3158000	1.68E-06
chr16	3154001	3159000	8.85E-07
chr16	3155001	3160000	4.83E-05
chr16	3156001	3161000	7.13E-06
chr16	3301001	3306000	1.47E-05
chr16	3302001	3307000	3.65E-06
chr16	3303001	3308000	2.39E-05
chr16	3661001	3666000	1.93E-12
chr16	4008001	4013000	1.67E-13
chr16	4009001	4014000	1.48E-09
chr16	4029001	4034000	0.000943
chr16	4030001	4035000	1.51E-05
chr16	4031001	4036000	0.000102
chr16	4104001	4109000	5.12E-10
chr16	4163001	4168000	2.22E-09
chr16	4230001	4235000	3.13E-09
chr16	4231001	4236000	0.000304
chr16	4232001	4237000	3.42E-05
chr16	4233001	4238000	1.04E-07
chr16	4246001	4251000	0.002139
chr16	4299001	4304000	0.0004
chr16	4300001	4305000	0.000137
chr16	4301001	4306000	3.37E-05

chr16	4302001	4307000	0.000133
chr16	4303001	4308000	6.25E-05
chr16	4365001	4370000	0.000711
chr16	4366001	4371000	0.011896
chr16	4699001	4704000	0.048238
chr16	4784001	4789000	1.20E-07
chr16	4791001	4796000	5.73E-05
chr16	4792001	4797000	5.06E-05
chr16	4793001	4798000	1.02E-05
chr16	4941001	4946000	0.000328
chr16	4981001	4986000	0.007204
chr16	4982001	4987000	0.002286
chr16	4983001	4988000	0.00293
chr16	4993001	4998000	0.033858
chr16	5084001	5089000	7.78E-05
chr16	5086001	5091000	9.27E-06
chr16	5188001	5193000	8.66E-06
chr16	5349001	5354000	1.35E-06
chr16	5362001	5367000	3.61E-10
chr16	5386001	5391000	8.53E-06
chr16	5474001	5479000	1.03E-08
chr16	5475001	5480000	7.32E-06
chr16	5565001	5570000	1.00E-05
chr16	5580001	5585000	3.71E-05
chr16	6475001	6480000	1.17E-08
chr16	6610001	6615000	3.67E-08
chr16	6611001	6616000	1.53E-10
chr16	6815001	6820000	6.70E-08
chr16	7128001	7133000	1.38E-09
chr16	7129001	7134000	3.91E-11
chr16	7130001	7135000	1.49E-10
chr16	7211001	7216000	1.28E-07
chr16	7212001	7217000	1.49E-06
chr16	7213001	7218000	1.17E-08
chr16	7214001	7219000	1.36E-09
chr16	7215001	7220000	1.40E-08
chr16	7270001	7275000	2.17E-07
chr16	7526001	7531000	2.97E-10
chr16	7757001	7762000	1.52E-10
chr16	8373001	8378000	1.72E-06
chr16	8382001	8387000	4.42E-14
chr16	8383001	8388000	5.22E-10
chr16	8457001	8462000	6.23E-10
chr16	8504001	8509000	3.30E-07
chr16	8505001	8510000	1.65E-07
chr16	8506001	8511000	6.00E-06
chr16	8845001	8850000	0.000542
chr16	9149001	9154000	5.62E-05

chr16	9150001	9155000	3.58E-05
chr16	9151001	9156000	6.34E-05
chr16	9182001	9187000	1.47E-08
chr16	9230001	9235000	3.08E-06
chr16	9253001	9258000	4.40E-05
chr16	9254001	9259000	1.63E-06
chr16	9257001	9262000	3.79E-07
chr16	9258001	9263000	3.48E-05
chr16	9282001	9287000	6.58E-06
chr16	9283001	9288000	9.84E-05
chr16	9334001	9339000	1.94E-08
chr16	9335001	9340000	1.08E-08
chr16	9336001	9341000	1.93E-07
chr16	9337001	9342000	4.25E-07
chr16	9582001	9587000	5.26E-09
chr16	9647001	9652000	1.72E-09
chr16	9648001	9653000	2.18E-08
chr16	10129001	10134000	7.05E-07
chr16	10130001	10135000	7.29E-08
chr16	10449001	10454000	1.61E-07
chr16	10997001	11002000	0.000272
chr16	11346001	11351000	1.44E-05
chr16	11390001	11395000	8.28E-07
chr16	11391001	11396000	1.83E-07
chr16	11392001	11397000	2.03E-07
chr16	11393001	11398000	1.05E-07
chr16	11608001	11613000	0.000907
chr16	11835001	11840000	4.30E-08
chr16	11836001	11841000	2.65E-07
chr16	11881001	11886000	5.41E-08
chr16	11882001	11887000	5.16E-09
chr16	11883001	11888000	4.29E-07
chr16	12008001	12013000	6.87E-13
chr16	12039001	12044000	4.85E-05
chr16	12040001	12045000	6.37E-05
chr16	12893001	12898000	1.36E-07
chr16	13016001	13021000	1.19E-09
chr16	13017001	13022000	9.30E-10
chr16	13027001	13032000	2.62E-07
chr16	13028001	13033000	8.62E-08
chr16	13029001	13034000	1.43E-07
chr16	13102001	13107000	4.32E-07
chr16	13103001	13108000	4.04E-08
chr16	13125001	13130000	7.02E-06
chr16	13176001	13181000	8.02E-06
chr16	13196001	13201000	9.71E-10
chr16	13200001	13205000	9.76E-06
chr16	13201001	13206000	3.01E-05

chr16	13243001	13248000	7.15E-08
chr16	13344001	13349000	3.78E-08
chr16	13345001	13350000	1.06E-09
chr16	13409001	13414000	2.36E-10
chr16	13410001	13415000	2.30E-09
chr16	13492001	13497000	1.48E-05
chr16	13508001	13513000	7.63E-08
chr16	13509001	13514000	1.59E-07
chr16	13510001	13515000	2.02E-09
chr16	13511001	13516000	2.07E-10
chr16	13512001	13517000	4.49E-08
chr16	13788001	13793000	5.68E-09
chr16	13789001	13794000	5.18E-11
chr16	13790001	13795000	1.27E-11
chr16	13853001	13858000	2.24E-08
chr16	13854001	13859000	1.66E-08
chr16	13913001	13918000	1.96E-09
chr16	13914001	13919000	1.88E-09
chr16	13915001	13920000	2.13E-10
chr16	13916001	13921000	3.73E-08
chr16	13918001	13923000	9.07E-10
chr16	13978001	13983000	2.04E-11
chr16	14900001	14905000	0.01551
chr16	14901001	14906000	0.00018
chr16	15409001	15414000	0.006959
chr16	15422001	15427000	0.002263
chr16	15553001	15558000	1.69E-05
chr16	15624001	15629000	1.01E-05
chr16	15848001	15853000	6.00E-05
chr16	15895001	15900000	2.62E-05
chr16	16042001	16047000	0.000177
chr16	16088001	16093000	0.000274
chr16	16089001	16094000	6.73E-06
chr16	16350001	16355000	0.003513
chr16	16500001	16505000	0.011252
chr16	16551001	16556000	0.000193
chr16	16552001	16557000	0.001898
chr16	16553001	16558000	0.010923
chr16	16555001	16560000	0.001095
chr16	16572001	16577000	0.007676
chr16	16574001	16579000	0.022169
chr16	16686001	16691000	0.000268
chr16	16697001	16702000	7.68E-06
chr16	16733001	16738000	1.35E-06
chr16	16919001	16924000	1.76E-05
chr16	16920001	16925000	0.000134
chr16	17588001	17593000	2.64E-06
chr16	17589001	17594000	3.35E-05

chr16	17627001	17632000	2.01E-07
chr16	17849001	17854000	2.26E-05
chr16	17883001	17888000	1.54E-07
chr16	17884001	17889000	1.75E-09
chr16	17890001	17895000	5.73E-05
chr16	17891001	17896000	1.08E-06
chr16	18022001	18027000	8.27E-13
chr16	18023001	18028000	1.09E-12
chr16	18024001	18029000	2.98E-11
chr16	18026001	18031000	3.31E-11
chr16	18063001	18068000	1.69E-06
chr16	18183001	18188000	1.90E-05
chr16	18317001	18322000	0.001389
chr16	18318001	18323000	0.001953
chr16	18319001	18324000	0.00072
chr16	18320001	18325000	0.000662
chr16	18334001	18339000	0.001357
chr16	18335001	18340000	9.08E-06
chr16	18336001	18341000	1.10E-05
chr16	18356001	18361000	0.003999
chr16	18357001	18362000	0.013024
chr16	18358001	18363000	0.013024
chr16	18359001	18364000	0.013024
chr16	18360001	18365000	0.014617
chr16	18364001	18369000	0.044931
chr16	18366001	18371000	0.048303
chr16	18371001	18376000	0.006015
chr16	18375001	18380000	0.005765
chr16	18376001	18381000	0.001034
chr16	18377001	18382000	1.05E-05
chr16	18696001	18701000	0.048793
chr16	18697001	18702000	0.048793
chr16	18759001	18764000	0.001743
chr16	18769001	18774000	0.000277
chr16	18831001	18836000	6.31E-06
chr16	18832001	18837000	0.04765
chr16	19463001	19468000	1.31E-05
chr16	19464001	19469000	4.57E-05
chr16	19465001	19470000	0.000146
chr16	20147001	20152000	6.20E-06
chr16	20370001	20375000	5.83E-08
chr16	20371001	20376000	7.30E-09
chr16	20372001	20377000	6.10E-07
chr16	20388001	20393000	5.06E-08
chr16	20389001	20394000	1.34E-08
chr16	20390001	20395000	6.73E-08
chr16	20391001	20396000	1.94E-10
chr16	20408001	20413000	7.68E-07

chr16	20410001	20415000	1.90E-07
chr16	20447001	20452000	3.55E-06
chr16	20448001	20453000	8.48E-06
chr16	20462001	20467000	0.005547
chr16	20477001	20482000	0.000114
chr16	20478001	20483000	3.09E-05
chr16	20479001	20484000	1.96E-07
chr16	20480001	20485000	1.52E-07
chr16	20482001	20487000	5.37E-07
chr16	20494001	20499000	2.74E-05
chr16	20551001	20556000	8.34E-09
chr16	20736001	20741000	1.88E-05
chr16	20817001	20822000	2.58E-11
chr16	20818001	20823000	1.23E-15
chr16	20909001	20914000	4.24E-09
chr16	20910001	20915000	4.99E-09
chr16	21226001	21231000	1.47E-07
chr16	21227001	21232000	4.46E-08
chr16	21716001	21721000	5.32E-05
chr16	21761001	21766000	0.000778
chr16	21762001	21767000	7.95E-06
chr16	22130001	22135000	0.000453
chr16	22131001	22136000	0.000512
chr16	22928001	22933000	2.75E-06
chr16	22969001	22974000	1.08E-07
chr16	22970001	22975000	1.95E-08
chr16	22972001	22977000	5.49E-05
chr16	23314001	23319000	1.96E-08
chr16	23387001	23392000	0.003568
chr16	23431001	23436000	0.007375
chr16	23461001	23466000	1.38E-08
chr16	23462001	23467000	2.27E-11
chr16	23463001	23468000	1.83E-09
chr16	23464001	23469000	1.25E-10
chr16	23517001	23522000	3.25E-07
chr16	23519001	23524000	4.02E-05
chr16	23543001	23548000	0.000373
chr16	23757001	23762000	0.000281
chr16	23758001	23763000	1.78E-05
chr16	23767001	23772000	3.32E-05
chr16	23878001	23883000	1.04E-07
chr16	23879001	23884000	2.90E-08
chr16	23880001	23885000	2.95E-08
chr16	23883001	23888000	1.33E-07
chr16	23884001	23889000	2.73E-06
chr16	23922001	23927000	6.85E-07
chr16	23934001	23939000	5.80E-08
chr16	23935001	23940000	5.78E-09

chr16	23944001	23949000	2.28E-05
chr16	23945001	23950000	1.37E-08
chr16	23946001	23951000	1.73E-09
chr16	23947001	23952000	2.26E-10
chr16	23996001	24001000	6.07E-13
chr16	23997001	24002000	4.93E-17
chr16	23998001	24003000	6.94E-15
chr16	23999001	24004000	2.04E-10
chr16	24013001	24018000	4.73E-08
chr16	24165001	24170000	2.18E-06
chr16	24166001	24171000	8.85E-09
chr16	24167001	24172000	2.12E-08
chr16	24247001	24252000	0.000466
chr16	24248001	24253000	0.000221
chr16	24249001	24254000	1.85E-06
chr16	24250001	24255000	3.55E-06
chr16	24341001	24346000	2.01E-05
chr16	24351001	24356000	2.67E-11
chr16	24419001	24424000	2.00E-05
chr16	24714001	24719000	8.23E-05
chr16	24876001	24881000	8.66E-05
chr16	24877001	24882000	1.29E-05
chr16	24905001	24910000	0.003959
chr16	24907001	24912000	0.002362
chr16	24920001	24925000	0.005072
chr16	25033001	25038000	0.000148
chr16	25035001	25040000	3.78E-05
chr16	25066001	25071000	0.000899
chr16	25067001	25072000	0.000141
chr16	25074001	25079000	1.95E-07
chr16	25077001	25082000	8.14E-08
chr16	25078001	25083000	1.40E-09
chr16	25210001	25215000	5.08E-06
chr16	25357001	25362000	2.86E-05
chr16	25358001	25363000	0.000142
chr16	25359001	25364000	0.000127
chr16	26169001	26174000	8.40E-09
chr16	26170001	26175000	8.44E-08
chr16	26253001	26258000	1.80E-09
chr16	26254001	26259000	1.07E-12
chr16	26255001	26260000	3.29E-10
chr16	26256001	26261000	2.23E-13
chr16	26317001	26322000	1.32E-10
chr16	26318001	26323000	8.12E-10
chr16	26665001	26670000	2.44E-10
chr16	26679001	26684000	1.80E-08
chr16	26680001	26685000	9.60E-09
chr16	26707001	26712000	3.79E-06

chr16	26771001	26776000	1.41E-06
chr16	26844001	26849000	8.84E-05
chr16	26845001	26850000	1.20E-08
chr16	26846001	26851000	6.46E-08
chr16	26956001	26961000	4.68E-08
chr16	27172001	27177000	0.003603
chr16	27173001	27178000	0.00014
chr16	27233001	27238000	3.18E-08
chr16	27363001	27368000	1.49E-05
chr16	27398001	27403000	1.65E-08
chr16	27399001	27404000	1.30E-06
chr16	27411001	27416000	2.00E-06
chr16	27412001	27417000	1.42E-05
chr16	27556001	27561000	1.34E-07
chr16	27785001	27790000	5.36E-06
chr16	27805001	27810000	0.000191
chr16	27925001	27930000	2.14E-05
chr16	27992001	27997000	0.000912
chr16	27995001	28000000	0.000382
chr16	28071001	28076000	0.000441
chr16	28241001	28246000	0.024645
chr16	28243001	28248000	0.001066
chr16	28244001	28249000	0.006852
chr16	28246001	28251000	0.013073
chr16	28290001	28295000	6.69E-05
chr16	28301001	28306000	1.05E-07
chr16	28302001	28307000	1.10E-09
chr16	28303001	28308000	4.41E-09
chr16	28304001	28309000	1.76E-08
chr16	28325001	28330000	0.006557
chr16	28326001	28331000	0.006557
chr16	28383001	28388000	0.023645
chr16	28511001	28516000	4.96E-06
chr16	28632001	28637000	0.001312
chr16	28810001	28815000	0.004021
chr16	28811001	28816000	0.026587
chr16	28812001	28817000	0.026587
chr16	28813001	28818000	0.00475
chr16	28823001	28828000	7.38E-07
chr16	28888001	28893000	0.001481
chr16	28889001	28894000	0.01431
chr16	28934001	28939000	0.002066
chr16	28935001	28940000	6.22E-05
chr16	28936001	28941000	8.55E-07
chr16	28937001	28942000	3.71E-05
chr16	29105001	29110000	6.72E-05
chr16	29402001	29407000	9.95E-15
chr16	29604001	29609000	4.00E-16

chr16	29605001	29610000	8.71E-14
chr16	29606001	29611000	2.92E-13
chr16	29607001	29612000	3.23E-10
chr16	29613001	29618000	0.001674
chr16	29623001	29628000	0.000377
chr16	29624001	29629000	2.07E-07
chr16	29648001	29653000	0.002148
chr16	29817001	29822000	2.66E-05
chr16	29884001	29889000	0.011896
chr16	29952001	29957000	2.34E-05
chr16	29953001	29958000	2.45E-05
chr16	30013001	30018000	1.42E-05
chr16	30014001	30019000	6.59E-06
chr16	30033001	30038000	0.000164
chr16	30094001	30099000	0.013794
chr16	30201001	30206000	1.50E-05
chr16	30202001	30207000	7.70E-10
chr16	30359001	30364000	0.00018
chr16	30360001	30365000	1.18E-06
chr16	30361001	30366000	2.37E-06
chr16	30362001	30367000	5.70E-20
chr16	30363001	30368000	1.58E-11
chr16	30386001	30391000	7.54E-05
chr16	30387001	30392000	8.28E-05
chr16	30388001	30393000	4.59E-07
chr16	30389001	30394000	4.87E-10
chr16	30390001	30395000	2.89E-06
chr16	30569001	30574000	6.46E-06
chr16	30592001	30597000	2.02E-14
chr16	30593001	30598000	5.73E-09
chr16	30617001	30622000	2.53E-13
chr16	30618001	30623000	7.54E-09
chr16	30619001	30624000	6.15E-10
chr16	30620001	30625000	3.28E-07
chr16	30645001	30650000	1.34E-08
chr16	30749001	30754000	3.98E-05
chr16	30904001	30909000	0.002611
chr16	30905001	30910000	6.34E-06
chr16	30951001	30956000	3.24E-12
chr16	30952001	30957000	3.31E-13
chr16	30953001	30958000	2.05E-05
chr16	30965001	30970000	2.54E-10
chr16	30967001	30972000	7.82E-10
chr16	30968001	30973000	3.95E-08
chr16	30969001	30974000	1.21E-07
chr16	31010001	31015000	0.000245
chr16	31011001	31016000	0.000774
chr16	31084001	31089000	8.77E-05

chr16	31085001	31090000	0.000361
chr16	31150001	31155000	0.00016
chr16	31151001	31156000	4.02E-06
chr16	31158001	31163000	0.013197
chr16	31224001	31229000	3.56E-06
chr16	31418001	31423000	0.007435
chr16	31588001	31593000	1.85E-06
chr16	32005001	32010000	0.002053
chr16	32300001	32305000	0.00437
chr16	32779001	32784000	0.008859
chr16	32780001	32785000	0.008859
chr16	32783001	32788000	0.001352
chr16	32935001	32940000	9.04E-05
chr16	32954001	32959000	0.000328
chr16	32955001	32960000	0.000418
chr16	32962001	32967000	0.000723
chr16	33018001	33023000	8.51E-05
chr16	33655001	33660000	0.000333
chr16	33662001	33667000	6.16E-06
chr16	33784001	33789000	0.015279
chr16	33785001	33790000	0.015279
chr16	33798001	33803000	0.002624
chr16	34208001	34213000	1.47E-08
chr16	34215001	34220000	9.58E-06
chr16	34803001	34808000	1.49E-05
chr16	35188001	35193000	3.49E-12
chr16	35189001	35194000	4.58E-14
chr16	35190001	35195000	4.78E-09
chr16	46659001	46664000	0.00017
chr16	46660001	46665000	6.40E-05
chr16	46661001	46666000	1.97E-06
chr16	46663001	46668000	1.53E-06
chr16	47003001	47008000	1.49E-08
chr16	47064001	47069000	1.17E-05
chr16	47830001	47835000	3.57E-07
chr16	47831001	47836000	2.44E-08
chr16	47832001	47837000	4.31E-09
chr16	48730001	48735000	7.38E-09
chr16	48731001	48736000	1.53E-10
chr16	48739001	48744000	6.92E-06
chr16	48942001	48947000	1.18E-11
chr16	48943001	48948000	3.07E-10
chr16	49261001	49266000	1.06E-11
chr16	49262001	49267000	5.66E-11
chr16	49263001	49268000	4.44E-10
chr16	49264001	49269000	3.15E-07
chr16	49265001	49270000	7.42E-06
chr16	49363001	49368000	4.08E-12

chr16	49364001	49369000	7.26E-09
chr16	49365001	49370000	8.71E-06
chr16	49467001	49472000	1.49E-08
chr16	49468001	49473000	1.11E-08
chr16	49469001	49474000	5.26E-10
chr16	49506001	49511000	4.29E-05
chr16	49507001	49512000	0.00011
chr16	49509001	49514000	3.45E-05
chr16	49510001	49515000	8.62E-05
chr16	49555001	49560000	5.32E-10
chr16	49556001	49561000	1.21E-08
chr16	49814001	49819000	1.05E-07
chr16	50498001	50503000	3.02E-12
chr16	50499001	50504000	1.32E-11
chr16	53084001	53089000	2.95E-06
chr16	53111001	53116000	3.01E-05
chr16	53112001	53117000	4.97E-06
chr16	53113001	53118000	2.71E-07
chr16	53114001	53119000	8.27E-07
chr16	53132001	53137000	4.37E-06
chr16	53133001	53138000	4.28E-08
chr16	55811001	55816000	0.001802
chr16	55820001	55825000	0.000374
chr16	55919001	55924000	1.00E-07
chr16	55920001	55925000	1.70E-06
chr16	55921001	55926000	3.40E-07
chr16	55969001	55974000	5.18E-06
chr16	56123001	56128000	0.000145
chr16	56250001	56255000	1.20E-06
chr16	56251001	56256000	1.21E-05
chr16	56377001	56382000	0.024072
chr16	56378001	56383000	0.023646
chr16	56379001	56384000	0.000844
chr16	56485001	56490000	6.38E-12
chr16	56644001	56649000	0.000267
chr16	56647001	56652000	0.005957
chr16	56760001	56765000	9.00E-08
chr16	56961001	56966000	0.002864
chr16	57116001	57121000	1.31E-09
chr16	57117001	57122000	1.61E-09
chr16	57118001	57123000	1.77E-13
chr16	57329001	57334000	7.54E-11
chr16	57341001	57346000	0.000761
chr16	57413001	57418000	6.24E-06
chr16	57414001	57419000	1.62E-06
chr16	57415001	57420000	7.03E-08
chr16	57416001	57421000	5.52E-07
chr16	57417001	57422000	1.28E-05

chr16	57494001	57499000	1.92E-07
chr16	57560001	57565000	0.000566
chr16	57566001	57571000	0.039377
chr16	57655001	57660000	1.02E-08
chr16	58103001	58108000	3.00E-06
chr16	58104001	58109000	0.001355
chr16	58105001	58110000	0.001329
chr16	58106001	58111000	0.000915
chr16	58126001	58131000	0.001537
chr16	58442001	58447000	1.55E-06
chr16	58443001	58448000	4.72E-06
chr16	58539001	58544000	0.006394
chr16	58657001	58662000	0.015364
chr16	58691001	58696000	0.001033
chr16	58811001	58816000	0.00029
chr16	58812001	58817000	4.02E-05
chr16	58822001	58827000	4.69E-05
chr16	58832001	58837000	3.70E-06
chr16	58834001	58839000	5.38E-05
chr16	58835001	58840000	0.000117
chr16	58836001	58841000	0.00054
chr16	58837001	58842000	0.001982
chr16	58838001	58843000	0.000121
chr16	58849001	58854000	2.25E-05
chr16	58900001	58905000	1.63E-10
chr16	58901001	58906000	5.27E-12
chr16	58902001	58907000	9.50E-10
chr16	58916001	58921000	2.66E-06
chr16	58940001	58945000	2.20E-08
chr16	58941001	58946000	1.53E-09
chr16	58974001	58979000	9.05E-06
chr16	58975001	58980000	1.84E-07
chr16	59017001	59022000	0.001084
chr16	59019001	59024000	0.000177
chr16	59022001	59027000	6.15E-06
chr16	59036001	59041000	1.16E-08
chr16	59079001	59084000	3.47E-07
chr16	59080001	59085000	1.97E-06
chr16	59105001	59110000	3.19E-09
chr16	59182001	59187000	2.12E-10
chr16	59408001	59413000	8.44E-10
chr16	59453001	59458000	1.79E-08
chr16	59454001	59459000	6.55E-08
chr16	59455001	59460000	9.02E-07
chr16	59461001	59466000	3.22E-08
chr16	59673001	59678000	9.33E-11
chr16	59682001	59687000	0.000157
chr16	59683001	59688000	0.000527

chr16	59840001	59845000	1.61E-07
chr16	59841001	59846000	1.89E-06
chr16	59842001	59847000	2.45E-08
chr16	59983001	59988000	4.63E-10
chr16	60026001	60031000	9.55E-06
chr16	60118001	60123000	2.32E-10
chr16	60119001	60124000	2.32E-10
chr16	60120001	60125000	5.96E-11
chr16	60170001	60175000	4.86E-05
chr16	60179001	60184000	1.52E-05
chr16	60343001	60348000	3.07E-07
chr16	60344001	60349000	2.53E-06
chr16	60382001	60387000	2.88E-09
chr16	60595001	60600000	6.00E-06
chr16	60613001	60618000	1.10E-11
chr16	60634001	60639000	2.02E-06
chr16	60880001	60885000	4.37E-08
chr16	60881001	60886000	2.38E-08
chr16	60882001	60887000	4.59E-07
chr16	60910001	60915000	3.38E-05
chr16	61104001	61109000	6.12E-08
chr16	62169001	62174000	2.90E-09
chr16	62170001	62175000	1.74E-10
chr16	62171001	62176000	2.49E-10
chr16	62172001	62177000	2.07E-11
chr16	62173001	62178000	8.88E-10
chr16	62273001	62278000	2.01E-09
chr16	62407001	62412000	4.35E-05
chr16	62408001	62413000	6.30E-05
chr16	62427001	62432000	2.80E-07
chr16	62450001	62455000	4.66E-07
chr16	62497001	62502000	3.14E-08
chr16	62498001	62503000	3.10E-06
chr16	62499001	62504000	0.001531
chr16	62500001	62505000	3.82E-05
chr16	62501001	62506000	0.00014
chr16	62502001	62507000	0.000403
chr16	62516001	62521000	8.42E-09
chr16	62569001	62574000	1.55E-09
chr16	62710001	62715000	2.74E-06
chr16	62711001	62716000	1.57E-10
chr16	62712001	62717000	5.07E-12
chr16	62713001	62718000	2.51E-10
chr16	62877001	62882000	2.65E-08
chr16	62885001	62890000	4.77E-08
chr16	62976001	62981000	1.10E-11
chr16	62995001	63000000	2.19E-10
chr16	62996001	63001000	7.60E-09

chr16	62997001	63002000	1.93E-08
chr16	62998001	63003000	2.17E-08
chr16	63037001	63042000	1.71E-07
chr16	64596001	64601000	2.83E-05
chr16	64597001	64602000	0.001624
chr16	64796001	64801000	1.63E-07
chr16	64870001	64875000	3.41E-06
chr16	64871001	64876000	2.22E-06
chr16	65053001	65058000	0.000183
chr16	66364001	66369000	0.000281
chr16	66640001	66645000	1.14E-05
chr16	66870001	66875000	8.39E-07
chr16	67032001	67037000	0.000159
chr16	67033001	67038000	2.15E-06
chr16	67034001	67039000	1.41E-05
chr16	67035001	67040000	4.59E-07
chr16	67043001	67048000	0.006449
chr16	67200001	67205000	1.10E-05
chr16	67201001	67206000	4.82E-06
chr16	67281001	67286000	1.86E-17
chr16	67282001	67287000	1.41E-07
chr16	67283001	67288000	1.58E-07
chr16	67301001	67306000	0.000401
chr16	67462001	67467000	0.00747
chr16	67495001	67500000	0.002574
chr16	67554001	67559000	0.000543
chr16	67568001	67573000	6.33E-07
chr16	67569001	67574000	9.54E-05
chr16	67571001	67576000	0.004654
chr16	67674001	67679000	1.03E-05
chr16	67675001	67680000	4.57E-12
chr16	67676001	67681000	4.09E-10
chr16	67677001	67682000	5.28E-10
chr16	67679001	67684000	2.68E-10
chr16	67680001	67685000	0.000138
chr16	67681001	67686000	0.000182
chr16	67690001	67695000	1.19E-05
chr16	67693001	67698000	1.39E-10
chr16	67836001	67841000	9.55E-05
chr16	67837001	67842000	1.98E-09
chr16	67839001	67844000	1.21E-10
chr16	67864001	67869000	6.06E-07
chr16	67865001	67870000	2.72E-08
chr16	67866001	67871000	4.90E-10
chr16	67867001	67872000	1.30E-15
chr16	67967001	67972000	2.02E-06
chr16	68269001	68274000	3.03E-15
chr16	68294001	68299000	2.57E-05

chr16	68342001	68347000	3.55E-09
chr16	68436001	68441000	1.84E-13
chr16	68437001	68442000	4.44E-13
chr16	68438001	68443000	1.81E-12
chr16	68439001	68444000	1.38E-11
chr16	68477001	68482000	1.62E-05
chr16	68478001	68483000	0.000106
chr16	68509001	68514000	7.24E-07
chr16	68571001	68576000	0.000167
chr16	68603001	68608000	0.017206
chr16	68604001	68609000	0.038685
chr16	68648001	68653000	1.06E-07
chr16	68738001	68743000	0.000567
chr16	68770001	68775000	5.79E-07
chr16	68788001	68793000	4.55E-07
chr16	69128001	69133000	0.000401
chr16	69216001	69221000	4.92E-06
chr16	69415001	69420000	4.33E-09
chr16	69417001	69422000	4.36E-11
chr16	69755001	69760000	7.60E-06
chr16	69756001	69761000	3.93E-06
chr16	69757001	69762000	1.46E-08
chr16	69990001	69995000	0.003465
chr16	69991001	69996000	0.001841
chr16	70237001	70242000	2.28E-05
chr16	70281001	70286000	2.57E-11
chr16	70363001	70368000	1.44E-06
chr16	70364001	70369000	1.59E-05
chr16	70365001	70370000	1.12E-05
chr16	70459001	70464000	7.03E-06
chr16	70460001	70465000	1.78E-10
chr16	70461001	70466000	1.52E-08
chr16	70552001	70557000	5.02E-06
chr16	70618001	70623000	1.12E-08
chr16	70619001	70624000	3.17E-09
chr16	70620001	70625000	4.84E-09
chr16	70621001	70626000	1.85E-09
chr16	70715001	70720000	0.000111
chr16	70716001	70721000	8.81E-06
chr16	70717001	70722000	3.22E-05
chr16	70718001	70723000	0.001241
chr16	70859001	70864000	0.000309
chr16	70865001	70870000	3.06E-05
chr16	70866001	70871000	0.00078
chr16	70869001	70874000	0.000226
chr16	70904001	70909000	1.92E-05
chr16	70935001	70940000	2.24E-06
chr16	70936001	70941000	7.43E-05

chr16	70937001	70942000	0.000176
chr16	70938001	70943000	9.92E-06
chr16	70944001	70949000	2.39E-05
chr16	70945001	70950000	2.53E-05
chr16	70946001	70951000	0.000275
chr16	70947001	70952000	0.000617
chr16	71116001	71121000	6.92E-07
chr16	71117001	71122000	9.85E-07
chr16	71122001	71127000	7.05E-06
chr16	71135001	71140000	1.48E-05
chr16	71155001	71160000	1.06E-06
chr16	71187001	71192000	5.36E-05
chr16	71188001	71193000	9.41E-06
chr16	71231001	71236000	3.76E-08
chr16	71232001	71237000	3.97E-08
chr16	71233001	71238000	7.85E-08
chr16	71234001	71239000	2.23E-06
chr16	71266001	71271000	0.000108
chr16	71411001	71416000	7.30E-05
chr16	71412001	71417000	5.26E-06
chr16	71444001	71449000	3.67E-09
chr16	71445001	71450000	1.73E-07
chr16	71446001	71451000	1.93E-06
chr16	71619001	71624000	1.05E-06
chr16	71620001	71625000	2.31E-07
chr16	71658001	71663000	5.09E-06
chr16	71753001	71758000	2.68E-08
chr16	71917001	71922000	2.12E-05
chr16	72038001	72043000	5.64E-15
chr16	73202001	73207000	3.76E-08
chr16	73203001	73208000	5.33E-10
chr16	73391001	73396000	5.79E-09
chr16	75144001	75149000	2.93E-08
chr16	75596001	75601000	1.21E-11
chr16	75597001	75602000	1.41E-13
chr16	75598001	75603000	1.59E-11
chr16	75599001	75604000	9.35E-08
chr16	75600001	75605000	6.72E-06
chr16	75888001	75893000	1.77E-05
chr16	76076001	76081000	7.16E-08
chr16	76077001	76082000	8.42E-07
chr16	76132001	76137000	6.44E-06
chr16	76294001	76299000	1.83E-05
chr16	76295001	76300000	1.96E-08
chr16	76364001	76369000	7.06E-13
chr16	76365001	76370000	1.08E-13
chr16	76366001	76371000	3.95E-09
chr16	76538001	76543000	0.000234

chr16	77154001	77159000	1.17E-09
chr16	77155001	77160000	6.86E-13
chr16	77156001	77161000	1.05E-06
chr16	77631001	77636000	3.89E-06
chr16	77854001	77859000	1.20E-08
chr16	77855001	77860000	5.30E-08
chr16	77930001	77935000	2.30E-05
chr16	78078001	78083000	7.55E-09
chr16	78129001	78134000	1.38E-11
chr16	78130001	78135000	5.27E-09
chr16	78131001	78136000	8.88E-15
chr16	78132001	78137000	2.93E-09
chr16	78133001	78138000	1.58E-09
chr16	79272001	79277000	1.29E-05
chr16	79388001	79393000	8.51E-09
chr16	79389001	79394000	8.71E-08
chr16	79581001	79586000	5.95E-07
chr16	79878001	79883000	7.47E-09
chr16	80405001	80410000	1.11E-06
chr16	80406001	80411000	2.77E-08
chr16	80407001	80412000	7.15E-07
chr16	80408001	80413000	1.10E-06
chr16	80473001	80478000	4.17E-10
chr16	80474001	80479000	1.66E-14
chr16	80475001	80480000	7.66E-16
chr16	80476001	80481000	1.26E-13
chr16	81151001	81156000	7.32E-05
chr16	81152001	81157000	1.96E-06
chr16	81613001	81618000	7.06E-09
chr16	81766001	81771000	0.027812
chr16	81767001	81772000	1.50E-08
chr16	81768001	81773000	5.27E-09
chr16	81769001	81774000	6.53E-08
chr16	81770001	81775000	8.25E-09
chr16	81771001	81776000	4.12E-06
chr16	81792001	81797000	9.29E-08
chr16	81809001	81814000	1.04E-10
chr16	81810001	81815000	5.46E-10
chr16	81811001	81816000	5.14E-07
chr16	81812001	81817000	8.85E-09
chr16	81813001	81818000	2.23E-05
chr16	81854001	81859000	0.00104
chr16	81855001	81860000	0.001177
chr16	81861001	81866000	0.000194
chr16	81862001	81867000	0.002955
chr16	83669001	83674000	0.001989
chr16	83672001	83677000	0.01892
chr16	84183001	84188000	0.000706

chr16	84237001	84242000	1.54E-07
chr16	84357001	84362000	2.03E-06
chr16	84651001	84656000	6.81E-09
chr16	84872001	84877000	1.15E-07
chr16	85045001	85050000	9.46E-09
chr16	85332001	85337000	1.16E-07
chr16	85410001	85415000	0.00013
chr16	85450001	85455000	4.09E-06
chr16	85452001	85457000	0.000211
chr16	85453001	85458000	0.000638
chr16	85454001	85459000	0.000228
chr16	85600001	85605000	1.30E-05
chr16	85601001	85606000	1.09E-05
chr16	85602001	85607000	6.33E-07
chr16	85640001	85645000	0.004961
chr16	85684001	85689000	3.65E-09
chr16	85685001	85690000	0.000321
chr16	85749001	85754000	0.001702
chr16	85750001	85755000	2.91E-05
chr16	85751001	85756000	0.000249
chr16	85752001	85757000	1.48E-05
chr16	85753001	85758000	0.000788
chr16	85852001	85857000	7.68E-09
chr16	85853001	85858000	7.71E-10
chr16	85854001	85859000	3.50E-08
chr16	86115001	86120000	2.32E-07
chr16	86172001	86177000	9.40E-11
chr16	86173001	86178000	6.41E-09
chr16	86259001	86264000	1.49E-07
chr16	86265001	86270000	6.74E-07
chr16	86652001	86657000	2.90E-09
chr16	86714001	86719000	1.58E-05
chr16	87020001	87025000	2.18E-07
chr16	87021001	87026000	2.09E-10
chr16	87022001	87027000	5.04E-06
chr16	87182001	87187000	0.000174
chr16	87558001	87563000	3.84E-06
chr16	87559001	87564000	2.59E-06
chr16	87571001	87576000	8.88E-09
chr16	87572001	87577000	1.38E-10
chr16	87622001	87627000	7.54E-06
chr16	87623001	87628000	7.35E-06
chr16	87624001	87629000	1.87E-10
chr16	87625001	87630000	6.17E-11
chr16	87626001	87631000	1.03E-13
chr16	87627001	87632000	3.48E-12
chr16	87628001	87633000	4.33E-09
chr16	87629001	87634000	1.09E-06

chr16	87635001	87640000	2.34E-06
chr16	87650001	87655000	0.000361
chr16	87651001	87656000	0.000172
chr16	87699001	87704000	7.24E-06
chr16	87700001	87705000	9.23E-05
chr16	87701001	87706000	0.000655
chr16	87809001	87814000	6.99E-07
chr16	87914001	87919000	8.69E-05
chr16	87915001	87920000	2.34E-05
chr16	88116001	88121000	7.04E-09
chr16	88117001	88122000	7.59E-10
chr16	88118001	88123000	2.73E-13
chr16	88119001	88124000	3.81E-12
chr16	88136001	88141000	0.000258
chr16	88162001	88167000	2.53E-14
chr16	88232001	88237000	2.35E-06
chr16	88233001	88238000	5.13E-07
chr16	88293001	88298000	2.89E-10
chr16	88505001	88510000	8.86E-06
chr16	88506001	88511000	3.08E-06
chr16	88507001	88512000	1.41E-08
chr16	88508001	88513000	2.26E-06
chr16	88562001	88567000	0.006466
chr16	88598001	88603000	4.88E-05
chr16	88599001	88604000	0.000671
chr16	88600001	88605000	0.000174
chr16	88631001	88636000	9.42E-13
chr16	88632001	88637000	6.70E-32
chr16	88633001	88638000	2.34E-22
chr16	88698001	88703000	4.22E-05
chr16	88699001	88704000	1.97E-08
chr16	88700001	88705000	6.29E-08
chr16	88728001	88733000	2.31E-07
chr16	88729001	88734000	0.0002
chr16	88736001	88741000	6.85E-11
chr16	88737001	88742000	2.34E-09
chr16	88823001	88828000	2.83E-08
chr16	88824001	88829000	4.83E-05
chr16	88849001	88854000	0.00011
chr16	88850001	88855000	5.21E-08
chr16	88851001	88856000	1.27E-12
chr16	88852001	88857000	1.20E-06
chr16	88867001	88872000	3.21E-07
chr16	88920001	88925000	9.18E-06
chr16	88946001	88951000	4.00E-12
chr16	88947001	88952000	1.13E-16
chr16	88948001	88953000	2.26E-13
chr16	88949001	88954000	1.76E-08

chr16	88969001	88974000	3.10E-07
chr16	88970001	88975000	2.54E-05
chr16	89046001	89051000	5.49E-06
chr16	89079001	89084000	2.30E-07
chr16	89102001	89107000	1.17E-08
chr16	89104001	89109000	7.05E-09
chr16	89118001	89123000	1.12E-05
chr16	89119001	89124000	1.99E-07
chr16	89125001	89130000	7.24E-09
chr16	89126001	89131000	2.26E-08
chr16	89127001	89132000	5.93E-07
chr16	89218001	89223000	6.65E-06
chr16	89264001	89269000	5.68E-11
chr16	89277001	89282000	8.08E-05
chr16	89538001	89543000	3.14E-05
chr16	89542001	89547000	5.02E-09
chr16	89556001	89561000	1.55E-05
chr16	89557001	89562000	2.77E-05
chr16	89573001	89578000	9.43E-08
chr16	89625001	89630000	1.82E-05
chr16	89626001	89631000	1.80E-05
chr16	89652001	89657000	0.000786
chr16	89685001	89690000	2.17E-06
chr16	89751001	89756000	2.46E-05
chr16	89752001	89757000	0.04085
chr16	89753001	89758000	0.04085
chr16	89773001	89778000	0.001448
chr16	89774001	89779000	0.001448
chr16	89787001	89792000	4.59E-05
chr16	89877001	89882000	1.85E-09
chr16	89878001	89883000	2.74E-11
chr16	89879001	89884000	7.03E-08
chr16	89923001	89928000	1.58E-06
chr16	89989001	89994000	0.000107
chr16	89990001	89995000	3.39E-06
chr16	89991001	89996000	3.59E-07
chr16	89992001	89997000	7.34E-09
chr16	90110001	90115000	0.000392
chr16	90111001	90116000	6.28E-06
chr16	90112001	90117000	1.44E-07
chr16	90113001	90118000	5.60E-09
chr16	90114001	90119000	1.80E-08
chr17	234001	239000	5.91E-07
chr17	235001	240000	5.91E-07
chr17	411001	416000	2.08E-08
chr17	498001	503000	0.001211
chr17	631001	636000	1.38E-06
chr17	819001	824000	2.74E-08

chr17	820001	825000	2.21E-10
chr17	980001	985000	0.047693
chr17	981001	986000	3.72E-05
chr17	982001	987000	2.97E-06
chr17	1034001	1039000	1.17E-05
chr17	1047001	1052000	6.61E-07
chr17	1048001	1053000	2.22E-07
chr17	1049001	1054000	9.64E-08
chr17	1050001	1055000	1.55E-09
chr17	1051001	1056000	1.11E-09
chr17	1087001	1092000	4.68E-08
chr17	1103001	1108000	3.13E-05
chr17	1104001	1109000	0.000191
chr17	1128001	1133000	0.001048
chr17	1129001	1134000	0.000581
chr17	1176001	1181000	1.87E-08
chr17	1230001	1235000	1.24E-05
chr17	1231001	1236000	2.22E-06
chr17	1300001	1305000	3.01E-08
chr17	1316001	1321000	0.001533
chr17	1365001	1370000	5.21E-07
chr17	1366001	1371000	3.82E-06
chr17	1385001	1390000	1.25E-11
chr17	1492001	1497000	1.47E-05
chr17	1493001	1498000	2.01E-06
chr17	1494001	1499000	2.56E-05
chr17	1551001	1556000	0.000566
chr17	1552001	1557000	0.000653
chr17	1591001	1596000	7.77E-05
chr17	1592001	1597000	0.000868
chr17	1641001	1646000	0.013732
chr17	1642001	1647000	0.018974
chr17	1649001	1654000	2.20E-09
chr17	1650001	1655000	1.32E-08
chr17	1651001	1656000	6.25E-11
chr17	1652001	1657000	8.87E-09
chr17	1670001	1675000	0.000326
chr17	1679001	1684000	3.34E-12
chr17	1875001	1880000	1.36E-06
chr17	1913001	1918000	1.40E-09
chr17	1914001	1919000	6.72E-11
chr17	2039001	2044000	3.49E-06
chr17	2040001	2045000	1.13E-05
chr17	2220001	2225000	1.91E-06
chr17	2236001	2241000	1.69E-05
chr17	2237001	2242000	5.11E-06
chr17	2238001	2243000	1.13E-10
chr17	2239001	2244000	1.32E-06

chr17	2418001	2423000	0.009814
chr17	2635001	2640000	3.79E-08
chr17	2636001	2641000	7.53E-08
chr17	2642001	2647000	2.02E-06
chr17	2643001	2648000	4.55E-09
chr17	2655001	2660000	1.04E-06
chr17	2656001	2661000	1.24E-06
chr17	2657001	2662000	8.26E-07
chr17	2658001	2663000	0.008214
chr17	2659001	2664000	0.000121
chr17	2789001	2794000	2.97E-05
chr17	2790001	2795000	3.55E-05
chr17	2795001	2800000	6.07E-11
chr17	2811001	2816000	6.32E-06
chr17	2812001	2817000	2.30E-05
chr17	2860001	2865000	9.93E-06
chr17	2963001	2968000	6.73E-06
chr17	2964001	2969000	0.000152
chr17	3075001	3080000	5.11E-05
chr17	3076001	3081000	6.17E-05
chr17	3181001	3186000	9.24E-07
chr17	3204001	3209000	2.37E-07
chr17	3205001	3210000	2.32E-07
chr17	3206001	3211000	2.07E-08
chr17	3246001	3251000	1.87E-05
chr17	3335001	3340000	1.53E-07
chr17	3336001	3341000	1.17E-07
chr17	3364001	3369000	3.06E-05
chr17	3688001	3693000	5.78E-05
chr17	3689001	3694000	2.92E-05
chr17	3821001	3826000	1.08E-05
chr17	3822001	3827000	0.000192
chr17	4265001	4270000	0.00076
chr17	4462001	4467000	1.01E-06
chr17	4475001	4480000	3.41E-07
chr17	4476001	4481000	1.80E-08
chr17	4483001	4488000	0.000814
chr17	4484001	4489000	0.000248
chr17	4485001	4490000	0.000173
chr17	4486001	4491000	0.000352
chr17	4541001	4546000	1.09E-06
chr17	4555001	4560000	2.97E-05
chr17	4556001	4561000	2.69E-05
chr17	4557001	4562000	4.38E-06
chr17	4558001	4563000	1.41E-06
chr17	4610001	4615000	4.08E-06
chr17	4611001	4616000	0.003844
chr17	4691001	4696000	0.007033

chr17	4732001	4737000	0.003737
chr17	4735001	4740000	1.38E-06
chr17	4804001	4809000	1.09E-05
chr17	4805001	4810000	3.91E-06
chr17	4806001	4811000	3.78E-05
chr17	4841001	4846000	0.000113
chr17	4842001	4847000	0.000172
chr17	5155001	5160000	5.44E-06
chr17	5572001	5577000	0.000122
chr17	5645001	5650000	8.37E-09
chr17	5646001	5651000	2.24E-08
chr17	5651001	5656000	3.21E-08
chr17	5652001	5657000	2.72E-07
chr17	5653001	5658000	1.24E-07
chr17	5654001	5659000	2.59E-06
chr17	5676001	5681000	1.83E-09
chr17	5677001	5682000	1.25E-10
chr17	5685001	5690000	4.35E-08
chr17	5686001	5691000	9.85E-08
chr17	5696001	5701000	4.16E-11
chr17	5697001	5702000	1.83E-09
chr17	5708001	5713000	2.05E-06
chr17	5709001	5714000	1.02E-06
chr17	5710001	5715000	1.14E-06
chr17	5711001	5716000	4.02E-10
chr17	5735001	5740000	3.10E-09
chr17	5738001	5743000	8.89E-08
chr17	5758001	5763000	4.74E-05
chr17	5759001	5764000	0.000171
chr17	5760001	5765000	0.000238
chr17	5832001	5837000	8.98E-08
chr17	5834001	5839000	2.31E-06
chr17	5835001	5840000	4.03E-10
chr17	5836001	5841000	5.37E-17
chr17	5837001	5842000	1.93E-13
chr17	5838001	5843000	2.30E-16
chr17	5839001	5844000	9.29E-16
chr17	6023001	6028000	4.60E-08
chr17	6024001	6029000	1.35E-07
chr17	6025001	6030000	0.000153
chr17	6029001	6034000	6.91E-08
chr17	6052001	6057000	4.50E-08
chr17	6053001	6058000	7.03E-10
chr17	6108001	6113000	4.68E-10
chr17	6109001	6114000	4.99E-11
chr17	6110001	6115000	1.32E-07
chr17	6135001	6140000	3.55E-08
chr17	6177001	6182000	2.17E-08

chr17	6231001	6236000	3.09E-06
chr17	6232001	6237000	3.77E-05
chr17	6240001	6245000	2.00E-09
chr17	6241001	6246000	1.71E-08
chr17	6242001	6247000	9.47E-09
chr17	6326001	6331000	3.48E-06
chr17	6327001	6332000	1.29E-07
chr17	6328001	6333000	2.52E-06
chr17	6329001	6334000	6.97E-06
chr17	6450001	6455000	1.07E-05
chr17	6451001	6456000	3.39E-05
chr17	6467001	6472000	3.87E-09
chr17	6468001	6473000	3.11E-06
chr17	6576001	6581000	1.73E-09
chr17	6619001	6624000	9.33E-06
chr17	6627001	6632000	2.14E-07
chr17	6725001	6730000	1.08E-06
chr17	6926001	6931000	0.000469
chr17	6936001	6941000	3.38E-07
chr17	6937001	6942000	1.66E-09
chr17	6938001	6943000	1.50E-07
chr17	6939001	6944000	2.98E-06
chr17	6940001	6945000	0.000539
chr17	6996001	7001000	4.31E-06
chr17	6997001	7002000	3.63E-07
chr17	6998001	7003000	5.73E-07
chr17	7055001	7060000	8.07E-05
chr17	7078001	7083000	0.000877
chr17	7079001	7084000	0.000208
chr17	7121001	7126000	0.012643
chr17	7122001	7127000	0.002218
chr17	7206001	7211000	3.01E-13
chr17	7238001	7243000	3.01E-09
chr17	7239001	7244000	3.65E-11
chr17	7240001	7245000	3.42E-10
chr17	7241001	7246000	8.14E-10
chr17	7242001	7247000	1.07E-05
chr17	7255001	7260000	0.001358
chr17	7302001	7307000	7.61E-05
chr17	7308001	7313000	0.000679
chr17	7309001	7314000	0.000112
chr17	7310001	7315000	8.30E-05
chr17	7311001	7316000	7.27E-05
chr17	7312001	7317000	5.37E-05
chr17	7313001	7318000	3.38E-05
chr17	7357001	7362000	0.000107
chr17	7457001	7462000	9.63E-07
chr17	7464001	7469000	1.03E-08

chr17	7515001	7520000	3.61E-09
chr17	7516001	7521000	4.91E-09
chr17	7517001	7522000	1.55E-07
chr17	7518001	7523000	1.18E-06
chr17	7677001	7682000	4.45E-06
chr17	7679001	7684000	2.28E-05
chr17	7747001	7752000	1.98E-07
chr17	7748001	7753000	1.98E-07
chr17	7902001	7907000	5.36E-07
chr17	7904001	7909000	3.14E-05
chr17	8007001	8012000	3.55E-06
chr17	8008001	8013000	1.57E-05
chr17	8009001	8014000	5.89E-05
chr17	8037001	8042000	1.12E-10
chr17	8041001	8046000	3.32E-10
chr17	8147001	8152000	1.67E-07
chr17	8148001	8153000	5.15E-14
chr17	8192001	8197000	0.000104
chr17	8212001	8217000	8.02E-06
chr17	8213001	8218000	3.76E-05
chr17	8231001	8236000	9.70E-07
chr17	8232001	8237000	1.20E-07
chr17	8233001	8238000	6.58E-06
chr17	8283001	8288000	1.77E-09
chr17	8327001	8332000	1.38E-07
chr17	8789001	8794000	7.07E-05
chr17	8987001	8992000	4.54E-10
chr17	8988001	8993000	2.58E-09
chr17	8990001	8995000	1.90E-08
chr17	8991001	8996000	1.22E-11
chr17	9003001	9008000	2.46E-06
chr17	9505001	9510000	2.04E-07
chr17	9719001	9724000	0.00031
chr17	9771001	9776000	2.91E-07
chr17	9783001	9788000	1.67E-06
chr17	9784001	9789000	3.57E-05
chr17	9785001	9790000	1.26E-05
chr17	9822001	9827000	1.52E-06
chr17	9823001	9828000	5.04E-05
chr17	9824001	9829000	1.88E-09
chr17	9825001	9830000	8.46E-08
chr17	9885001	9890000	9.68E-09
chr17	9886001	9891000	1.91E-09
chr17	9887001	9892000	1.23E-08
chr17	9888001	9893000	4.82E-10
chr17	9967001	9972000	1.57E-09
chr17	10080001	10085000	1.75E-05
chr17	10121001	10126000	1.26E-06

chr17	10122001	10127000	3.56E-05
chr17	10123001	10128000	8.39E-05
chr17	10138001	10143000	8.78E-06
chr17	10139001	10144000	1.48E-05
chr17	10655001	10660000	2.63E-06
chr17	10656001	10661000	7.68E-07
chr17	10804001	10809000	1.06E-05
chr17	10909001	10914000	1.42E-06
chr17	10916001	10921000	3.16E-05
chr17	10959001	10964000	1.59E-06
chr17	10985001	10990000	9.88E-07
chr17	11290001	11295000	3.23E-06
chr17	11376001	11381000	0.009008
chr17	11397001	11402000	0.000116
chr17	11435001	11440000	1.80E-07
chr17	11436001	11441000	7.06E-06
chr17	11437001	11442000	1.22E-05
chr17	11659001	11664000	1.34E-11
chr17	11702001	11707000	5.93E-08
chr17	11704001	11709000	1.36E-06
chr17	11800001	11805000	2.57E-09
chr17	11840001	11845000	1.65E-06
chr17	11842001	11847000	1.17E-06
chr17	12344001	12349000	0.009234
chr17	12359001	12364000	2.83E-06
chr17	13175001	13180000	6.29E-06
chr17	13514001	13519000	1.19E-07
chr17	13566001	13571000	8.74E-08
chr17	13567001	13572000	4.76E-07
chr17	13568001	13573000	9.99E-06
chr17	13569001	13574000	3.34E-06
chr17	14148001	14153000	4.69E-13
chr17	14150001	14155000	6.61E-12
chr17	14256001	14261000	2.06E-07
chr17	14282001	14287000	3.92E-10
chr17	14312001	14317000	9.00E-05
chr17	14313001	14318000	7.17E-05
chr17	14359001	14364000	0.000227
chr17	14360001	14365000	5.92E-06
chr17	14436001	14441000	1.50E-08
chr17	14894001	14899000	1.96E-11
chr17	14896001	14901000	1.10E-11
chr17	15031001	15036000	3.38E-12
chr17	15041001	15046000	1.49E-05
chr17	15042001	15047000	8.38E-08
chr17	15043001	15048000	1.14E-09
chr17	15051001	15056000	1.58E-08
chr17	15052001	15057000	2.37E-07

chr17	15076001	15081000	7.28E-10
chr17	15499001	15504000	0.000659
chr17	15545001	15550000	5.40E-08
chr17	15860001	15865000	2.03E-05
chr17	15948001	15953000	8.53E-13
chr17	15949001	15954000	5.32E-18
chr17	16289001	16294000	9.87E-12
chr17	16290001	16295000	3.93E-10
chr17	16345001	16350000	0.001618
chr17	16346001	16351000	0.000448
chr17	16353001	16358000	0.000204
chr17	16390001	16395000	2.10E-07
chr17	16391001	16396000	2.80E-10
chr17	16392001	16397000	0.000133
chr17	16393001	16398000	2.13E-05
chr17	16441001	16446000	0.00014
chr17	16598001	16603000	9.01E-10
chr17	16599001	16604000	8.35E-11
chr17	16600001	16605000	7.08E-10
chr17	16601001	16606000	4.55E-11
chr17	16602001	16607000	1.67E-05
chr17	16721001	16726000	2.79E-06
chr17	16722001	16727000	5.84E-05
chr17	16760001	16765000	1.27E-08
chr17	16761001	16766000	2.42E-08
chr17	16828001	16833000	7.35E-09
chr17	16829001	16834000	4.40E-07
chr17	16830001	16835000	5.96E-06
chr17	16831001	16836000	1.86E-06
chr17	16832001	16837000	7.30E-07
chr17	17044001	17049000	6.34E-09
chr17	17045001	17050000	4.24E-09
chr17	17138001	17143000	2.27E-09
chr17	17139001	17144000	1.21E-12
chr17	17140001	17145000	4.52E-10
chr17	17200001	17205000	2.73E-06
chr17	17201001	17206000	1.08E-05
chr17	17202001	17207000	8.38E-06
chr17	17257001	17262000	1.97E-05
chr17	17398001	17403000	0.000176
chr17	17491001	17496000	1.42E-06
chr17	17492001	17497000	1.76E-06
chr17	17596001	17601000	0.000401
chr17	17597001	17602000	0.000277
chr17	17598001	17603000	0.000138
chr17	17639001	17644000	0.000907
chr17	17640001	17645000	0.000628
chr17	17642001	17647000	3.82E-06

chr17	17719001	17724000	4.52E-06
chr17	18043001	18048000	2.40E-05
chr17	18055001	18060000	5.10E-05
chr17	18056001	18061000	1.07E-05
chr17	18057001	18062000	5.95E-06
chr17	18058001	18063000	0.000636
chr17	18143001	18148000	0.005726
chr17	18144001	18149000	0.000436
chr17	18145001	18150000	1.27E-05
chr17	18146001	18151000	4.02E-09
chr17	18147001	18152000	8.62E-07
chr17	18218001	18223000	8.97E-05
chr17	18281001	18286000	0.005773
chr17	18322001	18327000	0.000389
chr17	18354001	18359000	2.40E-05
chr17	18544001	18549000	0.001693
chr17	18838001	18843000	2.07E-05
chr17	18920001	18925000	0.003935
chr17	19136001	19141000	1.79E-06
chr17	19137001	19142000	1.12E-06
chr17	19138001	19143000	5.72E-07
chr17	19139001	19144000	1.58E-06
chr17	19140001	19145000	2.22E-09
chr17	19262001	19267000	0.000977
chr17	19369001	19374000	4.34E-10
chr17	19370001	19375000	2.79E-09
chr17	19372001	19377000	3.85E-10
chr17	19549001	19554000	9.34E-06
chr17	19619001	19624000	0.005071
chr17	19620001	19625000	0.001222
chr17	19623001	19628000	1.63E-10
chr17	19624001	19629000	7.76E-10
chr17	19626001	19631000	2.71E-08
chr17	19770001	19775000	8.80E-07
chr17	19954001	19959000	5.13E-06
chr17	20462001	20467000	5.58E-08
chr17	20463001	20468000	4.61E-06
chr17	20998001	21003000	6.02E-08
chr17	20999001	21004000	3.67E-07
chr17	21000001	21005000	3.33E-06
chr17	21001001	21006000	9.48E-13
chr17	21002001	21007000	3.08E-12
chr17	21262001	21267000	5.87E-05
chr17	21263001	21268000	1.63E-06
chr17	21671001	21676000	0.000784
chr17	21672001	21677000	0.000134
chr17	21733001	21738000	0.000231
chr17	21840001	21845000	5.18E-06

chr17	21980001	21985000	2.76E-09
chr17	21981001	21986000	4.38E-10
chr17	21982001	21987000	2.69E-10
chr17	22167001	22172000	4.33E-06
chr17	25370001	25375000	8.68E-09
chr17	25565001	25570000	0.002199
chr17	25656001	25661000	4.93E-07
chr17	25657001	25662000	5.88E-14
chr17	25658001	25663000	6.90E-14
chr17	25659001	25664000	1.18E-15
chr17	25748001	25753000	5.00E-05
chr17	25749001	25754000	0.000166
chr17	25750001	25755000	0.000148
chr17	26082001	26087000	5.25E-05
chr17	26353001	26358000	3.17E-09
chr17	26354001	26359000	5.62E-10
chr17	26355001	26360000	6.49E-07
chr17	26364001	26369000	2.34E-11
chr17	26365001	26370000	4.14E-06
chr17	26576001	26581000	7.38E-05
chr17	26644001	26649000	3.83E-08
chr17	26693001	26698000	5.75E-05
chr17	26694001	26699000	0.000254
chr17	26708001	26713000	0.005036
chr17	26709001	26714000	0.000177
chr17	26781001	26786000	1.44E-13
chr17	26782001	26787000	2.82E-12
chr17	26783001	26788000	2.03E-10
chr17	26898001	26903000	9.07E-06
chr17	26926001	26931000	4.02E-09
chr17	27073001	27078000	0.001714
chr17	27074001	27079000	0.003204
chr17	27189001	27194000	0.001693
chr17	27310001	27315000	0.000547
chr17	27311001	27316000	6.14E-05
chr17	27345001	27350000	2.83E-08
chr17	27347001	27352000	2.06E-08
chr17	27348001	27353000	9.02E-15
chr17	27349001	27354000	2.91E-08
chr17	27503001	27508000	0.003905
chr17	27650001	27655000	4.35E-05
chr17	27651001	27656000	0.000277
chr17	27714001	27719000	5.77E-07
chr17	27715001	27720000	0.000132
chr17	27943001	27948000	2.20E-05
chr17	28257001	28262000	8.20E-08
chr17	28258001	28263000	3.30E-07
chr17	28988001	28993000	0.020218

chr17	29018001	29023000	1.32E-06
chr17	29154001	29159000	2.26E-12
chr17	29232001	29237000	1.17E-11
chr17	29250001	29255000	0.000155
chr17	29295001	29300000	5.71E-07
chr17	29715001	29720000	0.000104
chr17	29716001	29721000	5.43E-08
chr17	29811001	29816000	1.06E-09
chr17	29924001	29929000	0.004929
chr17	29927001	29932000	7.23E-08
chr17	29981001	29986000	0.000114
chr17	30248001	30253000	2.36E-09
chr17	30249001	30254000	1.93E-08
chr17	30250001	30255000	2.35E-08
chr17	30385001	30390000	0.014347
chr17	30400001	30405000	0.000754
chr17	30401001	30406000	0.019523
chr17	31152001	31157000	2.32E-07
chr17	31339001	31344000	4.53E-05
chr17	31414001	31419000	3.64E-05
chr17	31485001	31490000	8.44E-07
chr17	31538001	31543000	2.33E-07
chr17	31614001	31619000	3.52E-06
chr17	31678001	31683000	1.05E-05
chr17	31689001	31694000	2.37E-06
chr17	31744001	31749000	3.56E-08
chr17	31745001	31750000	4.82E-07
chr17	31762001	31767000	1.09E-07
chr17	31820001	31825000	4.36E-05
chr17	31848001	31853000	1.00E-08
chr17	31849001	31854000	2.27E-09
chr17	31850001	31855000	1.13E-08
chr17	31880001	31885000	2.92E-07
chr17	31922001	31927000	1.83E-11
chr17	31923001	31928000	3.78E-08
chr17	32064001	32069000	3.79E-07
chr17	32065001	32070000	1.58E-05
chr17	32080001	32085000	9.42E-06
chr17	32089001	32094000	9.77E-05
chr17	32142001	32147000	2.98E-06
chr17	32197001	32202000	6.02E-06
chr17	32198001	32203000	9.15E-07
chr17	32429001	32434000	1.37E-10
chr17	32553001	32558000	0.002981
chr17	32660001	32665000	1.49E-08
chr17	32799001	32804000	4.05E-09
chr17	32800001	32805000	4.77E-07
chr17	32816001	32821000	5.07E-08

chr17	32817001	32822000	3.42E-08
chr17	32819001	32824000	2.17E-06
chr17	32820001	32825000	2.02E-05
chr17	32877001	32882000	9.31E-08
chr17	32972001	32977000	8.46E-08
chr17	32973001	32978000	2.09E-07
chr17	32992001	32997000	7.72E-08
chr17	32994001	32999000	8.02E-10
chr17	33002001	33007000	8.11E-06
chr17	33043001	33048000	2.15E-05
chr17	33045001	33050000	1.57E-05
chr17	33220001	33225000	4.86E-05
chr17	33221001	33226000	0.000236
chr17	33445001	33450000	0.000113
chr17	33879001	33884000	1.88E-07
chr17	33906001	33911000	4.05E-06
chr17	34062001	34067000	2.00E-05
chr17	34064001	34069000	1.19E-05
chr17	34087001	34092000	1.36E-14
chr17	34088001	34093000	3.68E-11
chr17	34089001	34094000	8.50E-12
chr17	34334001	34339000	0.000113
chr17	34337001	34342000	0.000931
chr17	34372001	34377000	0.000541
chr17	34616001	34621000	0.035887
chr17	34617001	34622000	0.007569
chr17	34618001	34623000	0.005861
chr17	34783001	34788000	6.74E-08
chr17	34838001	34843000	3.47E-08
chr17	34886001	34891000	4.04E-05
chr17	35018001	35023000	1.31E-07
chr17	35019001	35024000	4.28E-08
chr17	35020001	35025000	1.77E-06
chr17	35074001	35079000	1.23E-14
chr17	35077001	35082000	1.07E-15
chr17	35078001	35083000	1.82E-15
chr17	35143001	35148000	3.95E-06
chr17	35144001	35149000	4.05E-06
chr17	35214001	35219000	5.05E-07
chr17	35215001	35220000	1.42E-06
chr17	35216001	35221000	5.64E-05
chr17	35217001	35222000	2.12E-05
chr17	35249001	35254000	2.15E-05
chr17	35250001	35255000	2.39E-05
chr17	35251001	35256000	1.05E-08
chr17	35252001	35257000	9.35E-08
chr17	35253001	35258000	2.86E-05
chr17	35655001	35660000	6.37E-07

chr17	35656001	35661000	1.42E-06
chr17	35713001	35718000	1.16E-05
chr17	35715001	35720000	1.51E-08
chr17	35750001	35755000	3.46E-08
chr17	35751001	35756000	6.38E-07
chr17	35763001	35768000	0.00018
chr17	35765001	35770000	9.16E-05
chr17	36223001	36228000	2.70E-05
chr17	36265001	36270000	1.17E-06
chr17	36266001	36271000	8.55E-07
chr17	36267001	36272000	1.07E-06
chr17	36268001	36273000	9.99E-07
chr17	36820001	36825000	0.000177
chr17	36821001	36826000	1.40E-05
chr17	36822001	36827000	0.00036
chr17	36828001	36833000	4.16E-08
chr17	36829001	36834000	9.09E-07
chr17	36861001	36866000	1.19E-05
chr17	36884001	36889000	0.000121
chr17	37028001	37033000	0.000558
chr17	37029001	37034000	0.000862
chr17	37030001	37035000	0.000175
chr17	37179001	37184000	3.09E-12
chr17	37207001	37212000	1.69E-06
chr17	37236001	37241000	0.008449
chr17	37263001	37268000	5.18E-05
chr17	37264001	37269000	0.000332
chr17	37265001	37270000	8.61E-08
chr17	37266001	37271000	4.29E-06
chr17	37267001	37272000	6.71E-06
chr17	37299001	37304000	1.17E-06
chr17	37380001	37385000	0.0051
chr17	37388001	37393000	6.29E-06
chr17	37390001	37395000	4.89E-05
chr17	37558001	37563000	4.40E-07
chr17	37619001	37624000	3.65E-06
chr17	37828001	37833000	0.000948
chr17	37907001	37912000	0.001384
chr17	37908001	37913000	0.009128
chr17	37909001	37914000	0.006127
chr17	38167001	38172000	3.19E-05
chr17	38208001	38213000	3.55E-08
chr17	38210001	38215000	1.70E-06
chr17	38226001	38231000	0.011896
chr17	38345001	38350000	0.000316
chr17	38440001	38445000	6.64E-09
chr17	38441001	38446000	1.77E-10
chr17	38442001	38447000	3.95E-10

chr17	38444001	38449000	2.64E-14
chr17	38605001	38610000	7.84E-05
chr17	38823001	38828000	0.000386
chr17	39779001	39784000	0.000103
chr17	39780001	39785000	8.57E-06
chr17	39809001	39814000	0.002709
chr17	39925001	39930000	3.29E-09
chr17	39926001	39931000	2.85E-09
chr17	39940001	39945000	0.001117
chr17	40199001	40204000	4.50E-07
chr17	40200001	40205000	0.000286
chr17	40201001	40206000	6.33E-05
chr17	40202001	40207000	3.65E-05
chr17	40246001	40251000	0.002967
chr17	40282001	40287000	4.96E-06
chr17	40291001	40296000	2.35E-05
chr17	40327001	40332000	0.001431
chr17	40328001	40333000	4.00E-08
chr17	40329001	40334000	3.25E-06
chr17	40330001	40335000	6.20E-07
chr17	40331001	40336000	0.003559
chr17	40332001	40337000	0.008448
chr17	40438001	40443000	8.24E-08
chr17	40439001	40444000	7.49E-14
chr17	40440001	40445000	5.89E-18
chr17	40573001	40578000	0.019385
chr17	40574001	40579000	0.000302
chr17	40575001	40580000	0.00216
chr17	40601001	40606000	0.000499
chr17	40696001	40701000	4.01E-06
chr17	40697001	40702000	0.000896
chr17	40698001	40703000	0.00026
chr17	40699001	40704000	0.00016
chr17	40700001	40705000	1.70E-09
chr17	40707001	40712000	0.000302
chr17	40710001	40715000	5.78E-08
chr17	40711001	40716000	4.00E-09
chr17	40712001	40717000	4.00E-09
chr17	40715001	40720000	0.002218
chr17	40718001	40723000	8.86E-10
chr17	40719001	40724000	4.34E-12
chr17	40728001	40733000	1.14E-06
chr17	40729001	40734000	4.20E-06
chr17	40730001	40735000	5.08E-05
chr17	40757001	40762000	5.87E-06
chr17	40758001	40763000	3.72E-05
chr17	40788001	40793000	1.18E-05
chr17	40829001	40834000	1.06E-07

chr17	40830001	40835000	3.16E-05
chr17	40886001	40891000	1.13E-06
chr17	40913001	40918000	0.018795
chr17	40921001	40926000	9.70E-08
chr17	40922001	40927000	1.13E-05
chr17	40923001	40928000	3.16E-06
chr17	41036001	41041000	9.80E-07
chr17	41121001	41126000	0.004766
chr17	41129001	41134000	4.64E-10
chr17	41130001	41135000	5.52E-08
chr17	41151001	41156000	1.87E-05
chr17	41152001	41157000	9.59E-06
chr17	41153001	41158000	1.83E-05
chr17	41362001	41367000	1.90E-06
chr17	41363001	41368000	5.95E-11
chr17	41364001	41369000	5.59E-09
chr17	41472001	41477000	2.43E-05
chr17	41473001	41478000	5.94E-11
chr17	41474001	41479000	3.42E-10
chr17	41475001	41480000	2.34E-11
chr17	41476001	41481000	7.23E-05
chr17	41507001	41512000	2.09E-08
chr17	41508001	41513000	7.84E-08
chr17	41509001	41514000	1.31E-05
chr17	41624001	41629000	2.64E-07
chr17	41826001	41831000	0.000114
chr17	41852001	41857000	6.15E-12
chr17	41853001	41858000	4.02E-09
chr17	41854001	41859000	4.75E-08
chr17	41953001	41958000	0.000694
chr17	41954001	41959000	0.000409
chr17	42017001	42022000	0.000133
chr17	42053001	42058000	0.000197
chr17	42054001	42059000	1.91E-06
chr17	42078001	42083000	3.89E-05
chr17	42080001	42085000	0.001984
chr17	42114001	42119000	0.024476
chr17	42259001	42264000	0.000217
chr17	42260001	42265000	5.04E-10
chr17	42262001	42267000	8.38E-11
chr17	42263001	42268000	1.54E-11
chr17	42321001	42326000	0.000312
chr17	42322001	42327000	0.000362
chr17	42354001	42359000	0.005765
chr17	42387001	42392000	0.000184
chr17	42437001	42442000	0.009298
chr17	42775001	42780000	1.97E-05
chr17	42820001	42825000	0.014487

chr17	42821001	42826000	0.011768
chr17	42856001	42861000	0.013804
chr17	42872001	42877000	0.000162
chr17	42873001	42878000	6.51E-05
chr17	42896001	42901000	8.97E-07
chr17	42897001	42902000	9.68E-05
chr17	42898001	42903000	1.35E-07
chr17	42977001	42982000	1.30E-08
chr17	43009001	43014000	0.001396
chr17	43051001	43056000	9.40E-06
chr17	43052001	43057000	0.000141
chr17	43088001	43093000	0.000265
chr17	43724001	43729000	0.002207
chr17	43725001	43730000	0.00012
chr17	43726001	43731000	0.000142
chr17	43744001	43749000	0.006052
chr17	43745001	43750000	0.006052
chr17	43994001	43999000	0.000114
chr17	44103001	44108000	0.009073
chr17	44189001	44194000	0.000285
chr17	44339001	44344000	2.28E-05
chr17	44340001	44345000	7.29E-06
chr17	44466001	44471000	0.003689
chr17	44467001	44472000	0.003689
chr17	44468001	44473000	0.016082
chr17	45052001	45057000	1.85E-07
chr17	45059001	45064000	9.63E-07
chr17	45327001	45332000	0.00051
chr17	45328001	45333000	3.65E-07
chr17	45331001	45336000	2.67E-06
chr17	45604001	45609000	1.99E-07
chr17	45605001	45610000	6.10E-09
chr17	45606001	45611000	1.86E-05
chr17	45607001	45612000	6.64E-05
chr17	45711001	45716000	0.000322
chr17	45838001	45843000	3.81E-06
chr17	45839001	45844000	2.79E-06
chr17	45858001	45863000	0.00012
chr17	45947001	45952000	2.36E-05
chr17	45948001	45953000	9.35E-05
chr17	45949001	45954000	8.63E-05
chr17	45950001	45955000	1.67E-09
chr17	45955001	45960000	7.74E-05
chr17	45956001	45961000	0.00032
chr17	46015001	46020000	2.87E-14
chr17	46016001	46021000	4.62E-12
chr17	46017001	46022000	7.76E-07
chr17	46018001	46023000	1.70E-06

chr17	46019001	46024000	0.000135
chr17	46033001	46038000	0.00059
chr17	46110001	46115000	2.80E-08
chr17	46954001	46959000	0.000344
chr17	46956001	46961000	0.000415
chr17	47019001	47024000	0.001244
chr17	47042001	47047000	0.027257
chr17	47043001	47048000	0.008892
chr17	47045001	47050000	0.000849
chr17	47059001	47064000	9.20E-06
chr17	47060001	47065000	2.20E-06
chr17	47061001	47066000	1.07E-07
chr17	47062001	47067000	1.25E-11
chr17	47063001	47068000	1.78E-08
chr17	47064001	47069000	5.89E-07
chr17	47065001	47070000	1.02E-07
chr17	47084001	47089000	3.62E-05
chr17	47085001	47090000	2.21E-06
chr17	47086001	47091000	1.32E-08
chr17	47087001	47092000	4.81E-08
chr17	47264001	47269000	7.41E-05
chr17	47265001	47270000	1.86E-24
chr17	47266001	47271000	1.24E-20
chr17	47267001	47272000	2.50E-13
chr17	47319001	47324000	5.62E-05
chr17	47320001	47325000	5.94E-06
chr17	47355001	47360000	0.001112
chr17	47452001	47457000	0.009321
chr17	47495001	47500000	5.41E-05
chr17	47496001	47501000	0.000211
chr17	47831001	47836000	0.001267
chr17	47949001	47954000	0.001281
chr17	47996001	48001000	0.004221
chr17	48090001	48095000	6.78E-05
chr17	48091001	48096000	6.32E-06
chr17	48207001	48212000	0.009856
chr17	48208001	48213000	0.009856
chr17	48266001	48271000	0.00036
chr17	48339001	48344000	6.60E-05
chr17	48356001	48361000	6.16E-06
chr17	48384001	48389000	0.003074
chr17	48385001	48390000	3.21E-05
chr17	48386001	48391000	0.00041
chr17	48446001	48451000	1.99E-09
chr17	48447001	48452000	3.76E-09
chr17	48649001	48654000	1.18E-05
chr17	48707001	48712000	0.000255
chr17	48741001	48746000	0.003324

chr17	48742001	48747000	0.000332
chr17	48785001	48790000	4.65E-06
chr17	48918001	48923000	3.12E-07
chr17	48919001	48924000	4.78E-07
chr17	49197001	49202000	2.25E-10
chr17	49198001	49203000	6.76E-07
chr17	49462001	49467000	0.00018
chr17	49463001	49468000	8.67E-06
chr17	49467001	49472000	9.41E-11
chr17	49511001	49516000	7.11E-08
chr17	49546001	49551000	2.81E-06
chr17	49938001	49943000	1.06E-06
chr17	49939001	49944000	7.55E-06
chr17	49940001	49945000	1.39E-05
chr17	49964001	49969000	6.72E-10
chr17	49971001	49976000	3.30E-09
chr17	50101001	50106000	0.001449
chr17	50124001	50129000	8.93E-07
chr17	50209001	50214000	0.000123
chr17	50220001	50225000	3.54E-10
chr17	50221001	50226000	1.95E-11
chr17	50222001	50227000	2.07E-11
chr17	50254001	50259000	8.19E-07
chr17	50255001	50260000	5.42E-07
chr17	50557001	50562000	2.09E-09
chr17	50839001	50844000	1.35E-05
chr17	51082001	51087000	4.77E-08
chr17	51125001	51130000	1.48E-06
chr17	51479001	51484000	4.72E-11
chr17	51480001	51485000	2.19E-12
chr17	51481001	51486000	2.00E-13
chr17	51569001	51574000	0.000365
chr17	51570001	51575000	4.66E-07
chr17	51571001	51576000	1.99E-07
chr17	51943001	51948000	1.51E-08
chr17	52017001	52022000	1.09E-06
chr17	52472001	52477000	2.96E-08
chr17	52605001	52610000	3.44E-06
chr17	52606001	52611000	2.03E-09
chr17	52813001	52818000	9.48E-07
chr17	52932001	52937000	1.35E-05
chr17	54665001	54670000	3.89E-08
chr17	54792001	54797000	3.26E-09
chr17	54793001	54798000	6.19E-10
chr17	54797001	54802000	1.21E-08
chr17	55977001	55982000	1.61E-05
chr17	55978001	55983000	0.000111
chr17	55979001	55984000	0.000303

chr17	55988001	55993000	1.57E-05
chr17	56151001	56156000	8.84E-05
chr17	56344001	56349000	6.64E-05
chr17	56345001	56350000	2.38E-05
chr17	56346001	56351000	8.91E-07
chr17	56347001	56352000	2.85E-05
chr17	56378001	56383000	7.46E-06
chr17	56379001	56384000	5.47E-06
chr17	56380001	56385000	7.88E-07
chr17	56603001	56608000	0.020138
chr17	56604001	56609000	0.020138
chr17	57905001	57910000	3.75E-05
chr17	57921001	57926000	0.000102
chr17	57930001	57935000	1.07E-07
chr17	57931001	57936000	5.22E-06
chr17	57932001	57937000	2.90E-06
chr17	58202001	58207000	0.000872
chr17	58604001	58609000	2.64E-08
chr17	59473001	59478000	2.19E-12
chr17	59511001	59516000	4.23E-05
chr17	60003001	60008000	1.36E-07
chr17	60004001	60009000	2.14E-08
chr17	60343001	60348000	5.14E-06
chr17	60344001	60349000	6.60E-05
chr17	60345001	60350000	9.96E-05
chr17	60346001	60351000	6.79E-06
chr17	61519001	61524000	2.17E-07
chr17	61520001	61525000	3.74E-07
chr17	61521001	61526000	3.91E-11
chr17	61522001	61527000	2.40E-09
chr17	61523001	61528000	5.89E-05
chr17	61613001	61618000	4.62E-07
chr17	61614001	61619000	2.34E-07
chr17	62079001	62084000	0.00477
chr17	62103001	62108000	8.48E-07
chr17	62203001	62208000	4.16E-14
chr17	62204001	62209000	5.63E-10
chr17	62206001	62211000	4.23E-07
chr17	62207001	62212000	6.48E-07
chr17	62969001	62974000	2.03E-10
chr17	62970001	62975000	4.16E-14
chr17	62971001	62976000	1.61E-17
chr17	62972001	62977000	1.01E-13
chr17	66079001	66084000	5.70E-07
chr17	71254001	71259000	6.43E-05
chr17	71285001	71290000	0.002012
chr17	71426001	71431000	4.14E-06
chr17	72256001	72261000	1.22E-09

chr17	72319001	72324000	9.37E-09
chr17	72334001	72339000	0.003331
chr17	72362001	72367000	0.017165
chr17	72423001	72428000	5.94E-06
chr17	72424001	72429000	5.16E-06
chr17	72425001	72430000	4.78E-05
chr17	72516001	72521000	1.09E-05
chr17	72673001	72678000	1.07E-06
chr17	72836001	72841000	0.000136
chr17	72837001	72842000	0.000141
chr17	72882001	72887000	9.78E-06
chr17	72885001	72890000	7.96E-07
chr17	73030001	73035000	3.20E-15
chr17	73071001	73076000	0.002434
chr17	73072001	73077000	3.97E-05
chr17	73073001	73078000	8.79E-07
chr17	73178001	73183000	2.08E-10
chr17	73197001	73202000	0.000123
chr17	73198001	73203000	3.04E-05
chr17	73199001	73204000	3.69E-12
chr17	73200001	73205000	3.24E-14
chr17	73201001	73206000	5.98E-12
chr17	73255001	73260000	2.13E-08
chr17	73256001	73261000	4.92E-07
chr17	73282001	73287000	2.20E-31
chr17	73283001	73288000	2.49E-35
chr17	73284001	73289000	1.12E-15
chr17	73285001	73290000	4.52E-09
chr17	73286001	73291000	1.05E-07
chr17	73401001	73406000	4.46E-06
chr17	73417001	73422000	0.00048
chr17	73418001	73423000	8.48E-05
chr17	73427001	73432000	0.000335
chr17	73428001	73433000	0.000183
chr17	73511001	73516000	1.85E-06
chr17	73512001	73517000	9.16E-11
chr17	73518001	73523000	2.82E-05
chr17	73519001	73524000	0.00398
chr17	73607001	73612000	0.000127
chr17	73608001	73613000	0.000467
chr17	73629001	73634000	0.001564
chr17	73745001	73750000	0.011111
chr17	73891001	73896000	4.00E-05
chr17	73892001	73897000	1.17E-05
chr17	73933001	73938000	1.20E-05
chr17	73935001	73940000	5.79E-05
chr17	73936001	73941000	1.05E-07
chr17	74127001	74132000	4.10E-07

chr17	74128001	74133000	0.000267
chr17	74129001	74134000	0.000181
chr17	74130001	74135000	4.97E-05
chr17	74380001	74385000	0.000367
chr17	74402001	74407000	0.000595
chr17	74403001	74408000	0.002415
chr17	74484001	74489000	1.51E-05
chr17	74549001	74554000	1.21E-20
chr17	74629001	74634000	8.72E-09
chr17	74630001	74635000	2.45E-10
chr17	74815001	74820000	0.000548
chr17	74816001	74821000	0.000293
chr17	74833001	74838000	1.10E-05
chr17	74865001	74870000	0.000817
chr17	74892001	74897000	3.44E-05
chr17	74973001	74978000	1.63E-05
chr17	74981001	74986000	4.48E-07
chr17	74984001	74989000	2.17E-05
chr17	74997001	75002000	8.56E-09
chr17	74998001	75003000	9.41E-10
chr17	75070001	75075000	2.78E-06
chr17	75071001	75076000	1.04E-06
chr17	75072001	75077000	7.82E-08
chr17	75073001	75078000	0.006366
chr17	75239001	75244000	0.003599
chr17	75240001	75245000	9.82E-08
chr17	75241001	75246000	7.83E-07
chr17	75323001	75328000	0.000159
chr17	75364001	75369000	1.25E-07
chr17	75365001	75370000	5.18E-08
chr17	75632001	75637000	0.000366
chr17	75690001	75695000	0.000221
chr17	75874001	75879000	0.00132
chr17	75875001	75880000	1.87E-05
chr17	75876001	75881000	1.77E-08
chr17	75877001	75882000	1.77E-08
chr17	75878001	75883000	4.62E-06
chr17	75879001	75884000	2.61E-07
chr17	75954001	75959000	1.18E-19
chr17	76132001	76137000	2.52E-07
chr17	76354001	76359000	9.07E-05
chr17	76527001	76532000	2.50E-08
chr17	76528001	76533000	2.29E-09
chr17	76529001	76534000	9.07E-12
chr17	76530001	76535000	2.39E-12
chr17	76531001	76536000	2.40E-12
chr17	77048001	77053000	3.07E-05
chr17	77055001	77060000	4.29E-05

chr17	77056001	77061000	7.62E-05
chr17	77100001	77105000	8.80E-09
chr17	77101001	77106000	3.51E-08
chr17	77102001	77107000	4.13E-12
chr17	77103001	77108000	1.88E-13
chr17	77104001	77109000	3.99E-09
chr17	77119001	77124000	2.94E-09
chr17	77139001	77144000	0.000737
chr17	77141001	77146000	0.002596
chr17	77145001	77150000	0.009345
chr17	77156001	77161000	0.000361
chr17	77179001	77184000	6.97E-06
chr17	77199001	77204000	9.19E-05
chr17	77200001	77205000	5.85E-08
chr17	77201001	77206000	5.11E-13
chr17	77202001	77207000	1.21E-19
chr17	77203001	77208000	2.19E-15
chr17	77264001	77269000	1.20E-07
chr17	77265001	77270000	1.86E-05
chr17	77266001	77271000	8.96E-08
chr17	77267001	77272000	1.57E-08
chr17	77268001	77273000	7.94E-08
chr17	77366001	77371000	7.56E-08
chr17	77367001	77372000	3.78E-05
chr17	77447001	77452000	7.10E-09
chr17	77539001	77544000	7.03E-08
chr17	77540001	77545000	1.12E-06
chr17	77541001	77546000	1.18E-06
chr17	77759001	77764000	1.57E-07
chr17	77760001	77765000	7.35E-10
chr17	77902001	77907000	8.25E-06
chr17	77903001	77908000	0.000107
chr17	77944001	77949000	5.41E-05
chr17	78075001	78080000	1.36E-08
chr17	78164001	78169000	3.85E-05
chr17	78165001	78170000	5.01E-06
chr17	78166001	78171000	2.51E-08
chr17	78228001	78233000	2.10E-06
chr17	78483001	78488000	1.55E-07
chr17	78972001	78977000	1.82E-09
chr17	78973001	78978000	6.87E-11
chr17	78974001	78979000	4.26E-08
chr17	79037001	79042000	6.26E-06
chr17	79038001	79043000	0.001352
chr17	79218001	79223000	1.05E-05
chr17	79220001	79225000	6.60E-08
chr17	79222001	79227000	3.93E-09
chr17	79282001	79287000	0.000116

chr17	79384001	79389000	0.001296
chr17	79387001	79392000	3.89E-06
chr17	79388001	79393000	1.14E-07
chr17	79431001	79436000	1.35E-11
chr17	79464001	79469000	0.00026
chr17	79465001	79470000	5.42E-05
chr17	79466001	79471000	1.16E-06
chr17	79467001	79472000	2.96E-05
chr17	79468001	79473000	0.000425
chr17	79479001	79484000	6.70E-11
chr17	79480001	79485000	6.70E-11
chr17	79481001	79486000	5.96E-12
chr17	79482001	79487000	7.06E-10
chr17	79483001	79488000	1.99E-07
chr17	79493001	79498000	0.000331
chr17	79532001	79537000	5.77E-06
chr17	79533001	79538000	4.41E-06
chr17	79609001	79614000	0.000186
chr17	79610001	79615000	5.21E-05
chr17	79776001	79781000	4.57E-07
chr17	79844001	79849000	1.03E-06
chr17	79847001	79852000	3.39E-06
chr17	79871001	79876000	8.22E-06
chr17	79921001	79926000	0.002431
chr17	79922001	79927000	0.000114
chr17	79923001	79928000	1.75E-06
chr17	79924001	79929000	0.000307
chr17	80006001	80011000	1.06E-09
chr17	80007001	80012000	4.45E-06
chr17	80009001	80014000	0.019134
chr17	80052001	80057000	0.009189
chr17	80087001	80092000	0.000197
chr17	80088001	80093000	1.23E-05
chr17	80602001	80607000	3.15E-20
chr17	80603001	80608000	1.04E-19
chr17	80604001	80609000	4.85E-09
chr17	80605001	80610000	0.000589
chr17	80651001	80656000	4.00E-07
chr17	80653001	80658000	3.32E-07
chr17	80668001	80673000	0.00023
chr17	80674001	80679000	3.14E-06
chr17	80708001	80713000	0.000198
chr17	80709001	80714000	0.000648
chr17	80931001	80936000	2.71E-07
chr17	81029001	81034000	5.14E-10
chr17	81030001	81035000	1.79E-13
chr17	81031001	81036000	2.67E-14
chr17	81032001	81037000	9.00E-14

chr17	81033001	81038000	1.46E-19
chr17	81034001	81039000	6.77E-07
chr17	81036001	81041000	8.09E-12
chr17	81037001	81042000	8.78E-10
chr17	81040001	81045000	4.41E-06
chr17	81058001	81063000	2.40E-08
chr17	81064001	81069000	2.62E-14
chr18	79001	84000	0.000592
chr18	501001	506000	0.000557
chr18	937001	942000	2.73E-09
chr18	1941001	1946000	2.76E-05
chr18	1942001	1947000	0.00025
chr18	2865001	2870000	5.44E-07
chr18	4742001	4747000	6.32E-06
chr18	4934001	4939000	3.04E-07
chr18	4935001	4940000	1.92E-07
chr18	4936001	4941000	8.59E-08
chr18	4937001	4942000	4.04E-08
chr18	4943001	4948000	0.000892
chr18	4951001	4956000	9.83E-05
chr18	4952001	4957000	6.09E-06
chr18	4953001	4958000	0.000736
chr18	5214001	5219000	1.98E-06
chr18	5224001	5229000	1.28E-05
chr18	5225001	5230000	2.52E-06
chr18	5562001	5567000	5.94E-11
chr18	5873001	5878000	1.16E-06
chr18	6454001	6459000	1.26E-06
chr18	6925001	6930000	1.16E-08
chr18	6926001	6931000	1.42E-11
chr18	6927001	6932000	3.06E-14
chr18	7098001	7103000	7.58E-07
chr18	7302001	7307000	1.68E-05
chr18	7480001	7485000	9.93E-07
chr18	7532001	7537000	4.09E-06
chr18	7533001	7538000	1.84E-06
chr18	7534001	7539000	6.75E-06
chr18	10316001	10321000	4.31E-05
chr18	11248001	11253000	1.35E-08
chr18	11345001	11350000	8.86E-06
chr18	11346001	11351000	0.000132
chr18	11347001	11352000	3.05E-05
chr18	11508001	11513000	7.06E-07
chr18	11907001	11912000	5.74E-07
chr18	11908001	11913000	3.15E-13
chr18	11993001	11998000	8.40E-07
chr18	11994001	11999000	5.57E-09
chr18	11995001	12000000	6.62E-08

chr18	12038001	12043000	0.000731
chr18	12219001	12224000	2.38E-06
chr18	12250001	12255000	6.71E-05
chr18	12392001	12397000	0.002234
chr18	12416001	12421000	0.000951
chr18	12417001	12422000	4.09E-06
chr18	12418001	12423000	0.000647
chr18	12419001	12424000	7.70E-05
chr18	12676001	12681000	1.64E-08
chr18	12677001	12682000	3.79E-07
chr18	12679001	12684000	1.30E-07
chr18	13323001	13328000	1.40E-07
chr18	13324001	13329000	1.26E-07
chr18	13622001	13627000	9.65E-06
chr18	13820001	13825000	9.80E-11
chr18	13821001	13826000	6.43E-08
chr18	13883001	13888000	0.000701
chr18	14046001	14051000	1.65E-07
chr18	14047001	14052000	1.45E-07
chr18	14049001	14054000	3.03E-08
chr18	14223001	14228000	2.04E-05
chr18	14856001	14861000	0.000984
chr18	15213001	15218000	1.93E-06
chr18	15214001	15219000	1.44E-07
chr18	15320001	15325000	2.89E-08
chr18	18692001	18697000	2.01E-13
chr18	18715001	18720000	0.003256
chr18	18716001	18721000	2.77E-06
chr18	18793001	18798000	6.41E-06
chr18	18869001	18874000	2.65E-08
chr18	18870001	18875000	9.37E-10
chr18	19215001	19220000	6.72E-06
chr18	19216001	19221000	2.21E-05
chr18	19808001	19813000	1.60E-06
chr18	19809001	19814000	6.32E-09
chr18	19810001	19815000	7.63E-08
chr18	20712001	20717000	4.15E-10
chr18	21975001	21980000	4.92E-10
chr18	22581001	22586000	0.000671
chr18	22700001	22705000	5.24E-05
chr18	23075001	23080000	8.79E-05
chr18	23274001	23279000	2.87E-06
chr18	24563001	24568000	1.68E-05
chr18	27460001	27465000	0.000218
chr18	27536001	27541000	3.31E-08
chr18	27573001	27578000	2.23E-05
chr18	27574001	27579000	0.002404
chr18	28363001	28368000	0.000349

chr18	28364001	28369000	0.002597
chr18	29518001	29523000	6.08E-11
chr18	29520001	29525000	5.83E-13
chr18	29521001	29526000	9.60E-20
chr18	29532001	29537000	3.23E-05
chr18	29960001	29965000	4.63E-06
chr18	30849001	30854000	6.01E-05
chr18	31154001	31159000	3.09E-14
chr18	31393001	31398000	3.73E-06
chr18	31394001	31399000	7.50E-06
chr18	31699001	31704000	1.59E-05
chr18	33106001	33111000	5.62E-07
chr18	33363001	33368000	7.00E-05
chr18	33466001	33471000	0.000425
chr18	33535001	33540000	0.012626
chr18	33763001	33768000	0.001418
chr18	33764001	33769000	0.000994
chr18	33877001	33882000	1.58E-10
chr18	34865001	34870000	0.00013
chr18	34885001	34890000	0.0031
chr18	34958001	34963000	0.000379
chr18	34959001	34964000	0.013836
chr18	34961001	34966000	0.008299
chr18	34962001	34967000	9.07E-07
chr18	35065001	35070000	0.001286
chr18	35071001	35076000	4.70E-06
chr18	35084001	35089000	0.001033
chr18	35086001	35091000	0.00095
chr18	35087001	35092000	2.76E-06
chr18	35467001	35472000	2.78E-06
chr18	35468001	35473000	3.11E-05
chr18	35469001	35474000	4.12E-06
chr18	35530001	35535000	6.19E-05
chr18	35706001	35711000	7.36E-05
chr18	36052001	36057000	1.15E-06
chr18	36395001	36400000	1.22E-05
chr18	39144001	39149000	0.001122
chr18	39996001	40001000	4.40E-07
chr18	39997001	40002000	2.80E-05
chr18	39998001	40003000	4.57E-05
chr18	40333001	40338000	4.43E-07
chr18	40793001	40798000	6.67E-08
chr18	40794001	40799000	1.19E-06
chr18	40915001	40920000	2.09E-05
chr18	41917001	41922000	0.000118
chr18	41918001	41923000	0.000341
chr18	41986001	41991000	0.000121
chr18	41987001	41992000	0.000138

chr18	41988001	41993000	0.000608
chr18	42059001	42064000	0.002324
chr18	42502001	42507000	0.002639
chr18	42743001	42748000	4.69E-05
chr18	43034001	43039000	4.94E-05
chr18	43101001	43106000	0.001467
chr18	43112001	43117000	1.27E-09
chr18	43113001	43118000	1.85E-08
chr18	43114001	43119000	2.97E-07
chr18	43115001	43120000	3.46E-07
chr18	43265001	43270000	8.93E-05
chr18	43266001	43271000	1.98E-06
chr18	43407001	43412000	0.000704
chr18	43902001	43907000	2.49E-05
chr18	43909001	43914000	3.15E-14
chr18	43910001	43915000	1.38E-12
chr18	43911001	43916000	2.13E-08
chr18	44157001	44162000	0.005579
chr18	44202001	44207000	2.70E-05
chr18	44203001	44208000	4.44E-07
chr18	44204001	44209000	6.98E-07
chr18	44258001	44263000	9.33E-05
chr18	44698001	44703000	9.65E-07
chr18	44751001	44756000	8.77E-07
chr18	44870001	44875000	0.000751
chr18	44871001	44876000	4.09E-06
chr18	44872001	44877000	2.52E-06
chr18	45043001	45048000	0.000118
chr18	45044001	45049000	0.000172
chr18	45091001	45096000	4.44E-07
chr18	45285001	45290000	3.57E-07
chr18	45315001	45320000	0.000267
chr18	45661001	45666000	6.03E-11
chr18	46160001	46165000	0.000106
chr18	46358001	46363000	2.65E-05
chr18	46359001	46364000	0.00028
chr18	46360001	46365000	0.00046
chr18	46416001	46421000	6.99E-05
chr18	47742001	47747000	0.000224
chr18	47743001	47748000	1.80E-08
chr18	48289001	48294000	0.001331
chr18	48290001	48295000	0.000385
chr18	48291001	48296000	0.000627
chr18	48659001	48664000	7.84E-08
chr18	48661001	48666000	1.66E-07
chr18	48778001	48783000	0.040157
chr18	49066001	49071000	0.005246
chr18	49164001	49169000	0.00052

chr18	49328001	49333000	4.04E-06
chr18	49481001	49486000	0.000149
chr18	49561001	49566000	1.55E-05
chr18	49668001	49673000	0.000906
chr18	49669001	49674000	4.62E-05
chr18	49670001	49675000	0.001307
chr18	50372001	50377000	2.80E-09
chr18	50870001	50875000	5.34E-05
chr18	50871001	50876000	0.000117
chr18	50898001	50903000	9.74E-12
chr18	50899001	50904000	4.66E-12
chr18	51068001	51073000	3.48E-05
chr18	51356001	51361000	1.46E-05
chr18	51357001	51362000	5.75E-08
chr18	51358001	51363000	1.43E-07
chr18	51359001	51364000	3.74E-05
chr18	51426001	51431000	0.00029
chr18	51427001	51432000	6.07E-05
chr18	51440001	51445000	7.58E-10
chr18	51441001	51446000	2.37E-10
chr18	51594001	51599000	6.02E-05
chr18	51595001	51600000	3.96E-05
chr18	51880001	51885000	6.18E-13
chr18	52492001	52497000	9.83E-07
chr18	52617001	52622000	0.001458
chr18	54798001	54803000	3.61E-06
chr18	54838001	54843000	2.27E-07
chr18	54839001	54844000	1.50E-09
chr18	54862001	54867000	0.000114
chr18	54979001	54984000	0.000286
chr18	54992001	54997000	1.37E-05
chr18	54993001	54998000	1.43E-05
chr18	55016001	55021000	4.48E-09
chr18	55070001	55075000	0.000351
chr18	55076001	55081000	0.000161
chr18	55077001	55082000	0.000854
chr18	55299001	55304000	0.00196
chr18	55669001	55674000	0.000585
chr18	56335001	56340000	2.55E-07
chr18	56435001	56440000	0.000334
chr18	56666001	56671000	0.001159
chr18	56667001	56672000	0.002057
chr18	56668001	56673000	0.002942
chr18	59618001	59623000	6.11E-06
chr18	60131001	60136000	0.000106
chr18	60132001	60137000	1.06E-05
chr18	60261001	60266000	1.67E-08
chr18	60262001	60267000	8.73E-12

chr18	60263001	60268000	5.22E-11
chr18	60264001	60269000	5.90E-13
chr18	60343001	60348000	0.000475
chr18	62103001	62108000	0.003156
chr18	62104001	62109000	0.000733
chr18	62559001	62564000	5.16E-06
chr18	62560001	62565000	6.39E-06
chr18	63068001	63073000	3.62E-08
chr18	63069001	63074000	5.82E-07
chr18	63200001	63205000	0.000133
chr18	67369001	67374000	9.66E-07
chr18	67370001	67375000	2.10E-07
chr18	68132001	68137000	0.000144
chr18	68425001	68430000	7.04E-13
chr18	68426001	68431000	3.14E-11
chr18	68448001	68453000	1.89E-09
chr18	68545001	68550000	5.07E-08
chr18	68637001	68642000	3.43E-07
chr18	69327001	69332000	1.11E-06
chr18	69328001	69333000	4.03E-07
chr18	69593001	69598000	3.52E-07
chr18	69853001	69858000	2.94E-06
chr18	69854001	69859000	3.38E-06
chr18	70262001	70267000	7.73E-08
chr18	73101001	73106000	3.14E-12
chr18	73587001	73592000	3.06E-05
chr18	73799001	73804000	3.05E-07
chr18	74529001	74534000	2.15E-07
chr18	74532001	74537000	1.66E-09
chr18	76127001	76132000	0.000628
chr18	76128001	76133000	8.96E-05
chr18	76224001	76229000	6.06E-06
chr18	76225001	76230000	4.59E-05
chr18	76319001	76324000	1.24E-09
chr18	76320001	76325000	2.31E-10
chr18	76321001	76326000	8.14E-09
chr18	76322001	76327000	1.78E-07
chr18	76411001	76416000	2.31E-07
chr18	76412001	76417000	2.15E-07
chr18	77153001	77158000	6.94E-09
chr18	77155001	77160000	2.88E-08
chr18	77548001	77553000	1.44E-09
chr18	77549001	77554000	1.08E-09
chr18	77709001	77714000	0.00046
chr18	77710001	77715000	0.000152
chr18	77718001	77723000	1.93E-10
chr18	77747001	77752000	4.62E-06
chr18	77848001	77853000	0.000197

chr19	228001	233000	0.027905
chr19	403001	408000	0.000172
chr19	407001	412000	0.007298
chr19	408001	413000	0.009622
chr19	422001	427000	1.40E-05
chr19	423001	428000	2.18E-05
chr19	424001	429000	5.93E-09
chr19	435001	440000	0.006714
chr19	436001	441000	0.004326
chr19	437001	442000	8.77E-07
chr19	438001	443000	8.08E-06
chr19	439001	444000	4.55E-06
chr19	440001	445000	1.83E-06
chr19	461001	466000	0.044199
chr19	483001	488000	0.005531
chr19	491001	496000	0.006083
chr19	492001	497000	0.003748
chr19	493001	498000	0.003748
chr19	509001	514000	0.002251
chr19	510001	515000	2.09E-05
chr19	511001	516000	8.08E-08
chr19	522001	527000	0.016181
chr19	552001	557000	0.000376
chr19	553001	558000	0.002708
chr19	574001	579000	0.003111
chr19	584001	589000	9.78E-09
chr19	585001	590000	3.29E-08
chr19	586001	591000	4.20E-06
chr19	692001	697000	0.003171
chr19	693001	698000	2.80E-06
chr19	694001	699000	4.19E-06
chr19	695001	700000	2.68E-05
chr19	700001	705000	1.26E-05
chr19	725001	730000	0.002407
chr19	726001	731000	0.002278
chr19	825001	830000	0.004828
chr19	842001	847000	0.002122
chr19	846001	851000	0.000833
chr19	912001	917000	0.004991
chr19	913001	918000	0.004991
chr19	914001	919000	0.004991
chr19	916001	921000	6.09E-10
chr19	920001	925000	4.39E-09
chr19	921001	926000	1.94E-07
chr19	922001	927000	1.94E-07
chr19	923001	928000	2.35E-07
chr19	924001	929000	6.40E-10
chr19	1002001	1007000	1.21E-05

chr19	1003001	1008000	1.31E-05
chr19	1023001	1028000	1.25E-05
chr19	1024001	1029000	0.000618
chr19	1059001	1064000	9.82E-05
chr19	1061001	1066000	5.19E-08
chr19	1086001	1091000	1.63E-05
chr19	1087001	1092000	7.43E-05
chr19	1103001	1108000	0.00012
chr19	1104001	1109000	0.001511
chr19	1105001	1110000	0.001511
chr19	1111001	1116000	6.11E-07
chr19	1112001	1117000	6.11E-07
chr19	1113001	1118000	6.11E-07
chr19	1256001	1261000	1.04E-06
chr19	1260001	1265000	1.90E-08
chr19	1301001	1306000	2.31E-05
chr19	1302001	1307000	0.000753
chr19	1310001	1315000	0.00039
chr19	1313001	1318000	1.21E-09
chr19	1314001	1319000	2.50E-08
chr19	1354001	1359000	2.39E-07
chr19	1394001	1399000	0.000102
chr19	1470001	1475000	4.73E-08
chr19	1572001	1577000	0.004249
chr19	1573001	1578000	0.008639
chr19	1591001	1596000	1.69E-08
chr19	1592001	1597000	7.43E-05
chr19	1593001	1598000	7.98E-05
chr19	1647001	1652000	1.61E-08
chr19	1648001	1653000	1.62E-07
chr19	1649001	1654000	6.09E-17
chr19	1650001	1655000	2.03E-14
chr19	1651001	1656000	1.99E-14
chr19	1652001	1657000	1.82E-07
chr19	1653001	1658000	2.21E-07
chr19	1676001	1681000	0.005671
chr19	1692001	1697000	0.003683
chr19	1693001	1698000	0.003752
chr19	1715001	1720000	0.034937
chr19	1766001	1771000	1.52E-05
chr19	1798001	1803000	5.92E-05
chr19	1799001	1804000	7.44E-05
chr19	1809001	1814000	3.12E-05
chr19	1810001	1815000	7.73E-08
chr19	1811001	1816000	0.000102
chr19	1812001	1817000	7.77E-06
chr19	1813001	1818000	7.77E-06
chr19	1884001	1889000	0.000286

chr19	1885001	1890000	0.001209
chr19	1886001	1891000	0.001488
chr19	1928001	1933000	5.32E-08
chr19	1929001	1934000	8.49E-06
chr19	1938001	1943000	1.39E-06
chr19	1939001	1944000	5.01E-08
chr19	1942001	1947000	2.49E-12
chr19	1943001	1948000	2.19E-06
chr19	2092001	2097000	1.69E-07
chr19	2241001	2246000	5.78E-05
chr19	2294001	2299000	6.30E-05
chr19	2298001	2303000	2.13E-20
chr19	2299001	2304000	8.34E-20
chr19	2300001	2305000	1.29E-22
chr19	2301001	2306000	5.87E-17
chr19	2355001	2360000	3.82E-05
chr19	2356001	2361000	0.000119
chr19	2377001	2382000	0.045066
chr19	2378001	2383000	0.00793
chr19	2409001	2414000	0.000182
chr19	2410001	2415000	0.000182
chr19	2411001	2416000	1.66E-06
chr19	2412001	2417000	0.000185
chr19	2415001	2420000	0.000421
chr19	2416001	2421000	0.003321
chr19	2417001	2422000	0.011432
chr19	2418001	2423000	0.007758
chr19	2419001	2424000	0.001922
chr19	2462001	2467000	5.16E-05
chr19	2478001	2483000	5.29E-08
chr19	2490001	2495000	0.00034
chr19	2498001	2503000	0.000115
chr19	2505001	2510000	2.31E-06
chr19	2506001	2511000	5.13E-09
chr19	2507001	2512000	4.85E-09
chr19	2508001	2513000	1.60E-06
chr19	2509001	2514000	0.000146
chr19	2580001	2585000	0.013238
chr19	2581001	2586000	2.67E-06
chr19	2593001	2598000	0.001226
chr19	2594001	2599000	0.004002
chr19	2608001	2613000	0.022033
chr19	2662001	2667000	0.000437
chr19	2663001	2668000	0.000103
chr19	2664001	2669000	3.24E-07
chr19	2665001	2670000	4.62E-07
chr19	2666001	2671000	6.65E-05
chr19	2669001	2674000	0.002849

chr19	2677001	2682000	0.000279
chr19	2678001	2683000	0.000594
chr19	2684001	2689000	2.98E-05
chr19	2772001	2777000	5.35E-06
chr19	2773001	2778000	3.33E-06
chr19	2958001	2963000	9.92E-08
chr19	2960001	2965000	5.08E-08
chr19	2961001	2966000	0.00512
chr19	2962001	2967000	0.007588
chr19	3021001	3026000	1.34E-05
chr19	3022001	3027000	9.48E-07
chr19	3024001	3029000	7.39E-06
chr19	3025001	3030000	0.002138
chr19	3026001	3031000	0.005907
chr19	3058001	3063000	0.000542
chr19	3091001	3096000	6.43E-05
chr19	3092001	3097000	0.000143
chr19	3093001	3098000	0.007247
chr19	3101001	3106000	9.62E-09
chr19	3102001	3107000	1.08E-07
chr19	3103001	3108000	1.08E-07
chr19	3104001	3109000	1.48E-05
chr19	3116001	3121000	0.000867
chr19	3129001	3134000	0.000587
chr19	3130001	3135000	0.000897
chr19	3131001	3136000	0.000105
chr19	3218001	3223000	0.005611
chr19	3220001	3225000	7.09E-10
chr19	3221001	3226000	7.09E-10
chr19	3236001	3241000	0.012417
chr19	3263001	3268000	1.48E-05
chr19	3274001	3279000	0.000822
chr19	3320001	3325000	0.014458
chr19	3339001	3344000	0.003023
chr19	3343001	3348000	0.000305
chr19	3344001	3349000	0.005097
chr19	3345001	3350000	0.000304
chr19	3346001	3351000	0.000162
chr19	3365001	3370000	0.009329
chr19	3366001	3371000	0.000274
chr19	3433001	3438000	7.46E-07
chr19	3434001	3439000	3.03E-07
chr19	3435001	3440000	8.91E-07
chr19	3436001	3441000	5.83E-06
chr19	3507001	3512000	0.000264
chr19	3517001	3522000	3.81E-05
chr19	3572001	3577000	0.000569
chr19	3573001	3578000	0.001064

chr19	3574001	3579000	0.008532
chr19	3666001	3671000	0.001033
chr19	3711001	3716000	3.29E-08
chr19	3712001	3717000	7.44E-09
chr19	3713001	3718000	7.44E-09
chr19	3715001	3720000	1.33E-07
chr19	3719001	3724000	7.46E-09
chr19	3720001	3725000	2.11E-06
chr19	3762001	3767000	8.83E-05
chr19	3770001	3775000	0.000165
chr19	3773001	3778000	0.032747
chr19	3807001	3812000	0.002515
chr19	3808001	3813000	1.32E-10
chr19	3809001	3814000	4.41E-10
chr19	3810001	3815000	2.72E-05
chr19	3811001	3816000	0.000202
chr19	3812001	3817000	0.001675
chr19	3859001	3864000	0.000332
chr19	3860001	3865000	3.51E-05
chr19	3862001	3867000	1.01E-06
chr19	3863001	3868000	6.27E-06
chr19	3864001	3869000	6.27E-06
chr19	3865001	3870000	0.000155
chr19	3874001	3879000	0.000631
chr19	3875001	3880000	3.80E-05
chr19	3884001	3889000	9.44E-06
chr19	3914001	3919000	0.001117
chr19	3916001	3921000	0.001018
chr19	4075001	4080000	0.001935
chr19	4076001	4081000	0.012074
chr19	4077001	4082000	0.012074
chr19	4078001	4083000	0.008199
chr19	4079001	4084000	0.020208
chr19	4202001	4207000	0.000687
chr19	4203001	4208000	0.0004
chr19	4204001	4209000	0.000197
chr19	4218001	4223000	0.031921
chr19	4226001	4231000	0.000773
chr19	4228001	4233000	0.001292
chr19	4229001	4234000	9.98E-05
chr19	4230001	4235000	0.000391
chr19	4231001	4236000	9.68E-09
chr19	4232001	4237000	4.34E-07
chr19	4233001	4238000	8.55E-08
chr19	4234001	4239000	7.57E-10
chr19	4235001	4240000	0.009096
chr19	4236001	4241000	0.018864
chr19	4294001	4299000	6.52E-05

chr19	4295001	4300000	0.00054
chr19	4304001	4309000	7.85E-05
chr19	4305001	4310000	5.98E-05
chr19	4456001	4461000	0.000282
chr19	4539001	4544000	0.000185
chr19	4540001	4545000	2.71E-05
chr19	4557001	4562000	3.82E-06
chr19	4558001	4563000	4.73E-09
chr19	4559001	4564000	0.000306
chr19	4582001	4587000	0.012278
chr19	4605001	4610000	0.000687
chr19	4606001	4611000	0.000139
chr19	4770001	4775000	5.26E-05
chr19	4771001	4776000	0.000685
chr19	4864001	4869000	1.45E-06
chr19	4879001	4884000	0.025542
chr19	4880001	4885000	0.002362
chr19	4881001	4886000	0.001382
chr19	4882001	4887000	0.011544
chr19	5209001	5214000	0.000345
chr19	5210001	5215000	0.000452
chr19	5397001	5402000	0.002452
chr19	5423001	5428000	4.11E-05
chr19	5424001	5429000	2.02E-08
chr19	5425001	5430000	1.46E-05
chr19	5481001	5486000	0.002129
chr19	5514001	5519000	9.98E-06
chr19	5516001	5521000	0.000109
chr19	5517001	5522000	0.000143
chr19	5524001	5529000	0.004439
chr19	5566001	5571000	0.023985
chr19	5675001	5680000	0.013351
chr19	5733001	5738000	0.000984
chr19	5734001	5739000	0.000636
chr19	5742001	5747000	5.62E-07
chr19	5743001	5748000	5.62E-07
chr19	5744001	5749000	2.26E-06
chr19	5745001	5750000	8.98E-07
chr19	5751001	5756000	0.000449
chr19	5789001	5794000	1.07E-12
chr19	5790001	5795000	4.24E-13
chr19	5818001	5823000	0.002387
chr19	6172001	6177000	0.000472
chr19	6345001	6350000	0.012709
chr19	6346001	6351000	0.00197
chr19	6347001	6352000	0.000985
chr19	6348001	6353000	0.000985
chr19	6350001	6355000	0.002912

chr19	6351001	6356000	0.020535
chr19	6464001	6469000	6.10E-05
chr19	6613001	6618000	0.00273
chr19	6640001	6645000	0.003937
chr19	6641001	6646000	0.003351
chr19	6642001	6647000	0.00333
chr19	6643001	6648000	0.010863
chr19	6706001	6711000	0.000682
chr19	6707001	6712000	0.00239
chr19	6708001	6713000	0.002254
chr19	6709001	6714000	0.001122
chr19	6801001	6806000	2.02E-07
chr19	6858001	6863000	0.000113
chr19	6859001	6864000	2.16E-08
chr19	6860001	6865000	4.13E-07
chr19	6862001	6867000	1.97E-08
chr19	6863001	6868000	1.60E-07
chr19	7061001	7066000	2.70E-05
chr19	7062001	7067000	0.000134
chr19	7063001	7068000	0.000182
chr19	7137001	7142000	7.48E-05
chr19	7199001	7204000	8.61E-07
chr19	7200001	7205000	3.98E-10
chr19	7201001	7206000	1.15E-09
chr19	7202001	7207000	1.98E-09
chr19	7250001	7255000	0.000315
chr19	7251001	7256000	6.18E-05
chr19	7252001	7257000	1.74E-05
chr19	7253001	7258000	2.61E-05
chr19	7393001	7398000	0.040071
chr19	7432001	7437000	1.15E-05
chr19	7468001	7473000	6.01E-05
chr19	7487001	7492000	3.74E-05
chr19	7550001	7555000	3.79E-05
chr19	7551001	7556000	2.96E-07
chr19	7552001	7557000	6.90E-08
chr19	7553001	7558000	3.22E-06
chr19	7554001	7559000	2.16E-08
chr19	7615001	7620000	1.26E-06
chr19	7616001	7621000	2.01E-08
chr19	7617001	7622000	7.97E-08
chr19	7618001	7623000	9.98E-16
chr19	7619001	7624000	9.76E-09
chr19	7695001	7700000	6.60E-07
chr19	7697001	7702000	4.33E-06
chr19	7706001	7711000	0.000488
chr19	7707001	7712000	9.04E-06
chr19	7708001	7713000	9.63E-08

chr19	7721001	7726000	3.09E-05
chr19	7722001	7727000	4.71E-06
chr19	7723001	7728000	2.05E-05
chr19	7725001	7730000	0.000484
chr19	7779001	7784000	0.000117
chr19	7780001	7785000	6.64E-06
chr19	7781001	7786000	1.69E-05
chr19	7782001	7787000	2.03E-05
chr19	7798001	7803000	0.00025
chr19	7800001	7805000	6.52E-05
chr19	7802001	7807000	5.47E-06
chr19	7807001	7812000	0.001623
chr19	7809001	7814000	0.000338
chr19	7817001	7822000	2.81E-07
chr19	7820001	7825000	3.76E-06
chr19	7821001	7826000	4.89E-06
chr19	7822001	7827000	0.000112
chr19	7881001	7886000	0.004262
chr19	7882001	7887000	4.18E-05
chr19	8066001	8071000	5.64E-05
chr19	8109001	8114000	0.004586
chr19	8160001	8165000	2.81E-06
chr19	8248001	8253000	0.009234
chr19	8249001	8254000	0.000878
chr19	8250001	8255000	0.000282
chr19	8251001	8256000	0.000372
chr19	8253001	8258000	0.030968
chr19	8285001	8290000	5.24E-07
chr19	8286001	8291000	1.05E-08
chr19	8287001	8292000	3.86E-07
chr19	8288001	8293000	3.37E-09
chr19	8289001	8294000	3.08E-11
chr19	8382001	8387000	1.37E-05
chr19	8394001	8399000	6.78E-05
chr19	8395001	8400000	6.78E-05
chr19	8497001	8502000	0.00435
chr19	8499001	8504000	9.04E-06
chr19	8610001	8615000	7.41E-05
chr19	8611001	8616000	7.41E-05
chr19	8612001	8617000	2.43E-05
chr19	8613001	8618000	0.000452
chr19	8623001	8628000	0.00042
chr19	8624001	8629000	0.000757
chr19	8633001	8638000	0.013619
chr19	8735001	8740000	3.80E-05
chr19	8736001	8741000	2.44E-05
chr19	8737001	8742000	1.19E-05
chr19	8738001	8743000	3.46E-06

chr19	8739001	8744000	2.03E-05
chr19	8756001	8761000	3.18E-05
chr19	8757001	8762000	4.75E-05
chr19	8758001	8763000	2.32E-05
chr19	8764001	8769000	0.005378
chr19	8765001	8770000	0.037483
chr19	8766001	8771000	0.002544
chr19	8767001	8772000	0.012231
chr19	8853001	8858000	0.045328
chr19	8854001	8859000	3.90E-06
chr19	8855001	8860000	1.47E-08
chr19	8937001	8942000	4.23E-05
chr19	8938001	8943000	8.10E-05
chr19	8939001	8944000	0.000355
chr19	8940001	8945000	0.001728
chr19	9161001	9166000	4.70E-05
chr19	9162001	9167000	2.42E-08
chr19	9164001	9169000	1.45E-07
chr19	9332001	9337000	3.69E-06
chr19	9334001	9339000	0.000113
chr19	9633001	9638000	0.00082
chr19	9785001	9790000	0.011304
chr19	9797001	9802000	0.000667
chr19	9798001	9803000	5.29E-05
chr19	9863001	9868000	6.85E-05
chr19	9991001	9996000	0.000255
chr19	9992001	9997000	0.000913
chr19	10019001	10024000	0.002317
chr19	10022001	10027000	0.000558
chr19	10045001	10050000	0.003153
chr19	10181001	10186000	1.43E-05
chr19	10182001	10187000	1.43E-05
chr19	10183001	10188000	0.029606
chr19	10184001	10189000	0.009215
chr19	10195001	10200000	0.000213
chr19	10196001	10201000	5.44E-09
chr19	10197001	10202000	5.44E-09
chr19	10213001	10218000	6.01E-05
chr19	10214001	10219000	3.39E-06
chr19	10215001	10220000	4.08E-06
chr19	10216001	10221000	2.38E-07
chr19	10301001	10306000	4.94E-05
chr19	10302001	10307000	8.39E-05
chr19	10303001	10308000	0.004141
chr19	10319001	10324000	0.000129
chr19	10320001	10325000	0.003002
chr19	10382001	10387000	0.000586
chr19	10447001	10452000	0.018303

chr19	10448001	10453000	0.001219
chr19	10490001	10495000	9.82E-09
chr19	10520001	10525000	0.000183
chr19	10612001	10617000	1.77E-06
chr19	10613001	10618000	4.46E-08
chr19	10614001	10619000	1.80E-07
chr19	10615001	10620000	2.69E-07
chr19	10616001	10621000	0.000116
chr19	10681001	10686000	5.44E-05
chr19	10682001	10687000	3.84E-06
chr19	10817001	10822000	0.000311
chr19	10948001	10953000	0.000124
chr19	10949001	10954000	1.12E-06
chr19	11035001	11040000	9.94E-11
chr19	11036001	11041000	3.21E-12
chr19	11040001	11045000	4.11E-05
chr19	11043001	11048000	0.044504
chr19	11352001	11357000	5.08E-08
chr19	11353001	11358000	7.07E-05
chr19	11375001	11380000	0.000286
chr19	11462001	11467000	5.08E-05
chr19	11463001	11468000	8.36E-05
chr19	11465001	11470000	9.16E-16
chr19	11466001	11471000	1.52E-14
chr19	11467001	11472000	0.000161
chr19	11468001	11473000	0.000268
chr19	11469001	11474000	0.000389
chr19	11470001	11475000	0.004473
chr19	11473001	11478000	0.001676
chr19	11492001	11497000	8.35E-10
chr19	11493001	11498000	4.23E-09
chr19	11537001	11542000	7.20E-05
chr19	11538001	11543000	6.02E-06
chr19	11542001	11547000	1.10E-07
chr19	11543001	11548000	1.75E-05
chr19	11585001	11590000	0.000165
chr19	11588001	11593000	0.003207
chr19	11593001	11598000	0.006075
chr19	11594001	11599000	0.000205
chr19	11595001	11600000	2.29E-06
chr19	11612001	11617000	1.21E-10
chr19	11613001	11618000	9.34E-09
chr19	11614001	11619000	3.17E-06
chr19	11615001	11620000	5.61E-10
chr19	11616001	11621000	1.94E-09
chr19	11649001	11654000	0.011277
chr19	11747001	11752000	0.001774
chr19	11813001	11818000	4.52E-08

chr19	11814001	11819000	3.43E-07
chr19	11845001	11850000	2.43E-05
chr19	11857001	11862000	0.013134
chr19	11872001	11877000	2.07E-06
chr19	11873001	11878000	0.000347
chr19	12033001	12038000	1.87E-05
chr19	12034001	12039000	0.003234
chr19	12120001	12125000	2.27E-11
chr19	12163001	12168000	0.024315
chr19	12178001	12183000	8.21E-06
chr19	12179001	12184000	4.58E-05
chr19	12419001	12424000	0.000415
chr19	12420001	12425000	3.36E-05
chr19	12421001	12426000	1.71E-12
chr19	12422001	12427000	1.90E-07
chr19	12423001	12428000	2.58E-07
chr19	12424001	12429000	6.30E-06
chr19	12425001	12430000	3.29E-07
chr19	12444001	12449000	0.000575
chr19	12595001	12600000	9.19E-10
chr19	12602001	12607000	0.001409
chr19	12603001	12608000	0.001424
chr19	12758001	12763000	0.003266
chr19	12759001	12764000	0.000165
chr19	12848001	12853000	8.56E-07
chr19	12859001	12864000	2.23E-06
chr19	12860001	12865000	2.01E-06
chr19	12861001	12866000	7.48E-05
chr19	12866001	12871000	2.94E-06
chr19	12867001	12872000	0.000762
chr19	12882001	12887000	1.29E-13
chr19	12883001	12888000	1.16E-06
chr19	12884001	12889000	8.00E-07
chr19	12885001	12890000	1.57E-08
chr19	12886001	12891000	5.04E-10
chr19	12935001	12940000	1.59E-05
chr19	12938001	12943000	1.85E-05
chr19	12974001	12979000	3.17E-12
chr19	12975001	12980000	2.76E-09
chr19	12976001	12981000	3.16E-06
chr19	12977001	12982000	3.53E-05
chr19	13002001	13007000	0.0001
chr19	13055001	13060000	2.87E-13
chr19	13056001	13061000	5.15E-11
chr19	13057001	13062000	6.30E-11
chr19	13301001	13306000	0.009887
chr19	13315001	13320000	2.63E-05
chr19	13323001	13328000	0.047302

chr19	13324001	13329000	0.011005
chr19	13336001	13341000	0.000442
chr19	13337001	13342000	0.000746
chr19	13424001	13429000	0.001069
chr19	13425001	13430000	0.000262
chr19	13428001	13433000	0.000569
chr19	13483001	13488000	0.002689
chr19	13484001	13489000	0.007978
chr19	13500001	13505000	0.0044
chr19	13532001	13537000	0.009983
chr19	13533001	13538000	0.002362
chr19	13534001	13539000	0.001393
chr19	13555001	13560000	0.000132
chr19	13573001	13578000	9.45E-05
chr19	13574001	13579000	0.000283
chr19	13604001	13609000	1.50E-05
chr19	13635001	13640000	0.000127
chr19	13636001	13641000	2.35E-05
chr19	13698001	13703000	0.000603
chr19	13699001	13704000	4.07E-06
chr19	13769001	13774000	2.78E-06
chr19	13770001	13775000	6.96E-06
chr19	13779001	13784000	0.001617
chr19	13814001	13819000	0.0032
chr19	13815001	13820000	7.72E-05
chr19	13831001	13836000	0.000464
chr19	13832001	13837000	0.001852
chr19	13833001	13838000	0.005332
chr19	13834001	13839000	0.001082
chr19	13836001	13841000	2.23E-05
chr19	13837001	13842000	1.60E-06
chr19	13838001	13843000	1.52E-06
chr19	13839001	13844000	5.62E-05
chr19	13840001	13845000	0.000648
chr19	13871001	13876000	4.95E-06
chr19	13935001	13940000	0.002375
chr19	13952001	13957000	0.001599
chr19	13975001	13980000	0.00054
chr19	13990001	13995000	1.33E-05
chr19	13991001	13996000	2.59E-06
chr19	13992001	13997000	0.002061
chr19	14124001	14129000	0.001147
chr19	14126001	14131000	0.001483
chr19	14127001	14132000	0.011004
chr19	14274001	14279000	0.002459
chr19	14373001	14378000	0.000989
chr19	14382001	14387000	0.005677
chr19	14448001	14453000	0.001319

chr19	14449001	14454000	0.001319
chr19	14532001	14537000	0.000728
chr19	14533001	14538000	0.001539
chr19	14637001	14642000	1.69E-08
chr19	14688001	14693000	0.000859
chr19	14694001	14699000	9.48E-06
chr19	14696001	14701000	0.001075
chr19	14697001	14702000	2.85E-05
chr19	14703001	14708000	0.003193
chr19	14704001	14709000	0.000206
chr19	14759001	14764000	0.002674
chr19	14762001	14767000	0.000207
chr19	14782001	14787000	0.000104
chr19	14783001	14788000	3.06E-05
chr19	14784001	14789000	8.43E-13
chr19	14785001	14790000	4.29E-10
chr19	14945001	14950000	1.68E-08
chr19	15006001	15011000	5.73E-06
chr19	15043001	15048000	0.001544
chr19	15044001	15049000	0.019451
chr19	15108001	15113000	0.003959
chr19	15109001	15114000	0.001382
chr19	15110001	15115000	0.003217
chr19	15130001	15135000	0.000135
chr19	15131001	15136000	0.007513
chr19	15133001	15138000	0.001181
chr19	15134001	15139000	0.0022
chr19	15164001	15169000	0.000654
chr19	15193001	15198000	0.000111
chr19	15331001	15336000	0.000146
chr19	15332001	15337000	1.16E-05
chr19	15333001	15338000	4.95E-07
chr19	15334001	15339000	5.80E-06
chr19	15340001	15345000	2.85E-05
chr19	15560001	15565000	9.02E-05
chr19	15561001	15566000	4.35E-06
chr19	15562001	15567000	1.24E-05
chr19	15563001	15568000	1.74E-05
chr19	15564001	15569000	0.004717
chr19	15633001	15638000	0.001438
chr19	15642001	15647000	0.000487
chr19	15689001	15694000	7.64E-07
chr19	15690001	15695000	2.10E-07
chr19	15724001	15729000	0.007313
chr19	15736001	15741000	5.04E-09
chr19	15944001	15949000	2.77E-10
chr19	15945001	15950000	1.35E-08
chr19	15946001	15951000	6.67E-08

chr19	15969001	15974000	2.84E-06
chr19	15970001	15975000	2.48E-06
chr19	15971001	15976000	2.46E-06
chr19	16000001	16005000	0.000281
chr19	16034001	16039000	0.000509
chr19	16187001	16192000	1.76E-07
chr19	16188001	16193000	1.02E-08
chr19	16218001	16223000	0.000327
chr19	16286001	16291000	3.55E-08
chr19	16287001	16292000	1.08E-08
chr19	16288001	16293000	6.60E-08
chr19	16290001	16295000	3.94E-07
chr19	16564001	16569000	0.001243
chr19	16649001	16654000	1.84E-15
chr19	16650001	16655000	4.86E-09
chr19	16651001	16656000	4.86E-10
chr19	16652001	16657000	4.17E-06
chr19	16691001	16696000	4.86E-05
chr19	16692001	16697000	8.63E-06
chr19	16693001	16698000	1.03E-05
chr19	16737001	16742000	4.10E-09
chr19	16738001	16743000	5.35E-15
chr19	16739001	16744000	9.65E-08
chr19	16769001	16774000	9.81E-06
chr19	16770001	16775000	1.69E-07
chr19	16771001	16776000	5.17E-05
chr19	16806001	16811000	0.007914
chr19	16819001	16824000	9.97E-05
chr19	16845001	16850000	0.022872
chr19	16847001	16852000	0.01232
chr19	16848001	16853000	0.001971
chr19	17002001	17007000	0.002519
chr19	17003001	17008000	0.001422
chr19	17004001	17009000	0.00147
chr19	17085001	17090000	0.000318
chr19	17123001	17128000	0.004138
chr19	17125001	17130000	0.003309
chr19	17135001	17140000	0.014182
chr19	17136001	17141000	0.013789
chr19	17319001	17324000	0.020574
chr19	17321001	17326000	0.002779
chr19	17324001	17329000	0.000649
chr19	17325001	17330000	1.59E-05
chr19	17326001	17331000	0.003069
chr19	17327001	17332000	0.003069
chr19	17343001	17348000	0.000525
chr19	17414001	17419000	9.65E-06
chr19	17447001	17452000	0.000359

chr19	17448001	17453000	1.09E-05
chr19	17450001	17455000	9.16E-06
chr19	17530001	17535000	1.32E-05
chr19	17531001	17536000	4.63E-07
chr19	17618001	17623000	0.00077
chr19	17626001	17631000	0.004719
chr19	17627001	17632000	0.006586
chr19	17628001	17633000	0.011485
chr19	17791001	17796000	0.006987
chr19	17819001	17824000	0.001673
chr19	17871001	17876000	0.009793
chr19	17873001	17878000	0.006354
chr19	17884001	17889000	0.000616
chr19	17885001	17890000	0.005474
chr19	17886001	17891000	0.028735
chr19	17887001	17892000	0.005271
chr19	17954001	17959000	1.20E-05
chr19	17956001	17961000	5.30E-05
chr19	17984001	17989000	0.000659
chr19	18118001	18123000	0.00143
chr19	18119001	18124000	0.003105
chr19	18224001	18229000	6.72E-05
chr19	18281001	18286000	4.29E-05
chr19	18282001	18287000	4.38E-05
chr19	18283001	18288000	9.00E-07
chr19	18284001	18289000	5.77E-07
chr19	18287001	18292000	2.35E-06
chr19	18328001	18333000	0.000362
chr19	18351001	18356000	0.000126
chr19	18362001	18367000	4.50E-06
chr19	18363001	18368000	5.78E-08
chr19	18364001	18369000	6.97E-09
chr19	18365001	18370000	3.98E-05
chr19	18366001	18371000	4.46E-08
chr19	18453001	18458000	0.006091
chr19	18454001	18459000	0.006091
chr19	18475001	18480000	0.000191
chr19	18476001	18481000	0.000392
chr19	18477001	18482000	0.000384
chr19	18536001	18541000	1.40E-05
chr19	18537001	18542000	8.63E-06
chr19	18538001	18543000	8.37E-07
chr19	18593001	18598000	8.42E-05
chr19	18594001	18599000	3.06E-07
chr19	18595001	18600000	1.35E-06
chr19	18627001	18632000	2.05E-06
chr19	18628001	18633000	6.45E-06
chr19	18629001	18634000	2.21E-06

chr19	18722001	18727000	1.68E-05
chr19	18869001	18874000	0.014818
chr19	18899001	18904000	0.002399
chr19	18900001	18905000	0.045328
chr19	18901001	18906000	0.024279
chr19	18908001	18913000	0.012705
chr19	19004001	19009000	4.85E-06
chr19	19005001	19010000	6.61E-07
chr19	19006001	19011000	0.001412
chr19	19069001	19074000	1.09E-05
chr19	19070001	19075000	2.84E-06
chr19	19169001	19174000	0.000389
chr19	19228001	19233000	4.43E-05
chr19	19250001	19255000	4.22E-07
chr19	19300001	19305000	3.78E-08
chr19	19301001	19306000	1.12E-06
chr19	19318001	19323000	0.00076
chr19	19319001	19324000	0.000339
chr19	19320001	19325000	0.000309
chr19	19350001	19355000	0.010863
chr19	19365001	19370000	8.15E-05
chr19	19379001	19384000	0.000334
chr19	19380001	19385000	4.94E-05
chr19	19383001	19388000	3.56E-13
chr19	19384001	19389000	9.56E-10
chr19	19619001	19624000	3.77E-05
chr19	19644001	19649000	8.38E-07
chr19	19709001	19714000	0.003401
chr19	19710001	19715000	0.009255
chr19	19711001	19716000	0.0017
chr19	19712001	19717000	0.006625
chr19	19736001	19741000	2.85E-05
chr19	19798001	19803000	0.000258
chr19	19811001	19816000	5.92E-06
chr19	19812001	19817000	1.37E-05
chr19	19948001	19953000	2.75E-05
chr19	20019001	20024000	2.32E-08
chr19	20048001	20053000	1.81E-05
chr19	20172001	20177000	0.000143
chr19	20406001	20411000	0.000601
chr19	20407001	20412000	0.001686
chr19	20408001	20413000	0.001432
chr19	20409001	20414000	0.00107
chr19	20474001	20479000	0.000151
chr19	20666001	20671000	0.000189
chr19	20667001	20672000	0.001459
chr19	20668001	20673000	0.000717
chr19	20669001	20674000	0.016355

chr19	20728001	20733000	0.004469
chr19	20737001	20742000	0.000257
chr19	20744001	20749000	1.15E-06
chr19	21149001	21154000	0.000284
chr19	21204001	21209000	6.76E-06
chr19	21448001	21453000	0.00022
chr19	21460001	21465000	2.76E-06
chr19	21461001	21466000	2.54E-08
chr19	21462001	21467000	2.09E-09
chr19	21509001	21514000	9.43E-09
chr19	21510001	21515000	1.71E-07
chr19	21511001	21516000	3.63E-08
chr19	21512001	21517000	0.000298
chr19	21520001	21525000	7.86E-06
chr19	21543001	21548000	2.19E-05
chr19	21544001	21549000	3.62E-05
chr19	21735001	21740000	0.001172
chr19	21766001	21771000	4.31E-07
chr19	21767001	21772000	1.53E-13
chr19	21768001	21773000	7.42E-17
chr19	21769001	21774000	2.03E-17
chr19	21770001	21775000	0.000396
chr19	21771001	21776000	0.000209
chr19	21916001	21921000	0.002027
chr19	22221001	22226000	1.15E-05
chr19	22223001	22228000	6.23E-05
chr19	22224001	22229000	1.15E-05
chr19	22382001	22387000	0.000241
chr19	22383001	22388000	0.00024
chr19	22386001	22391000	0.000389
chr19	22423001	22428000	0.000282
chr19	22424001	22429000	2.61E-06
chr19	22425001	22430000	1.11E-05
chr19	22426001	22431000	5.10E-06
chr19	22427001	22432000	1.21E-06
chr19	22434001	22439000	0.000904
chr19	22435001	22440000	8.94E-05
chr19	22436001	22441000	0.04024
chr19	22437001	22442000	0.044504
chr19	22449001	22454000	0.000118
chr19	22450001	22455000	0.000238
chr19	22451001	22456000	0.000836
chr19	22530001	22535000	0.000338
chr19	22531001	22536000	4.04E-06
chr19	22532001	22537000	5.65E-08
chr19	22533001	22538000	2.40E-07
chr19	22534001	22539000	1.99E-05
chr19	22543001	22548000	3.02E-11

chr19	22544001	22549000	3.69E-11
chr19	22545001	22550000	4.62E-12
chr19	22547001	22552000	8.45E-11
chr19	22883001	22888000	9.32E-05
chr19	22993001	22998000	0.00032
chr19	22994001	22999000	0.000121
chr19	22995001	23000000	3.50E-05
chr19	23052001	23057000	7.59E-07
chr19	23053001	23058000	1.11E-08
chr19	23054001	23059000	1.97E-09
chr19	23056001	23061000	0.001362
chr19	23106001	23111000	0.000116
chr19	23231001	23236000	1.05E-05
chr19	23232001	23237000	1.50E-06
chr19	23315001	23320000	0.00011
chr19	23322001	23327000	8.18E-08
chr19	23325001	23330000	1.72E-06
chr19	23456001	23461000	4.80E-07
chr19	23457001	23462000	9.83E-07
chr19	23503001	23508000	3.52E-06
chr19	23504001	23509000	2.57E-09
chr19	23505001	23510000	2.39E-09
chr19	23554001	23559000	1.25E-07
chr19	23555001	23560000	8.43E-07
chr19	23556001	23561000	8.95E-07
chr19	23648001	23653000	0.005674
chr19	23649001	23654000	0.030585
chr19	23650001	23655000	0.001092
chr19	23710001	23715000	1.04E-06
chr19	23741001	23746000	0.000224
chr19	23742001	23747000	8.84E-05
chr19	23743001	23748000	9.71E-05
chr19	23744001	23749000	0.000315
chr19	23786001	23791000	0.017982
chr19	23841001	23846000	0.029289
chr19	23865001	23870000	2.48E-08
chr19	23866001	23871000	3.67E-06
chr19	23867001	23872000	1.03E-05
chr19	23868001	23873000	7.34E-06
chr19	23869001	23874000	4.89E-05
chr19	23912001	23917000	0.000253
chr19	23984001	23989000	0.000329
chr19	23985001	23990000	0.000221
chr19	24032001	24037000	6.34E-08
chr19	27926001	27931000	0.000954
chr19	28381001	28386000	0.000144
chr19	28382001	28387000	5.76E-06
chr19	28383001	28388000	7.39E-07

chr19	28384001	28389000	4.67E-05
chr19	28385001	28390000	2.07E-07
chr19	28386001	28391000	1.78E-07
chr19	28387001	28392000	2.29E-07
chr19	28490001	28495000	1.55E-09
chr19	28491001	28496000	1.75E-07
chr19	28492001	28497000	1.53E-07
chr19	28493001	28498000	8.57E-10
chr19	28623001	28628000	3.58E-07
chr19	28624001	28629000	4.05E-10
chr19	28625001	28630000	6.64E-13
chr19	28626001	28631000	3.23E-11
chr19	28663001	28668000	1.06E-06
chr19	28687001	28692000	5.48E-10
chr19	28688001	28693000	1.68E-10
chr19	28689001	28694000	2.61E-09
chr19	28828001	28833000	5.11E-06
chr19	28829001	28834000	1.04E-06
chr19	28883001	28888000	5.33E-05
chr19	28889001	28894000	1.91E-08
chr19	28890001	28895000	5.76E-08
chr19	28891001	28896000	3.73E-09
chr19	28954001	28959000	0.002174
chr19	28960001	28965000	1.63E-05
chr19	28961001	28966000	0.000158
chr19	29026001	29031000	2.63E-08
chr19	29143001	29148000	3.42E-05
chr19	29145001	29150000	9.46E-06
chr19	29146001	29151000	2.93E-05
chr19	29319001	29324000	0.00027
chr19	29320001	29325000	0.001391
chr19	29398001	29403000	3.08E-08
chr19	29472001	29477000	7.43E-05
chr19	29506001	29511000	0.002395
chr19	29512001	29517000	1.90E-06
chr19	29574001	29579000	2.39E-05
chr19	29627001	29632000	2.24E-06
chr19	29638001	29643000	2.27E-07
chr19	29656001	29661000	0.000164
chr19	29657001	29662000	0.000344
chr19	29658001	29663000	0.000145
chr19	29708001	29713000	3.42E-06
chr19	29727001	29732000	7.19E-06
chr19	29744001	29749000	6.75E-07
chr19	29745001	29750000	8.32E-07
chr19	29828001	29833000	0.000206
chr19	29891001	29896000	4.44E-06
chr19	29930001	29935000	8.72E-08

chr19	29931001	29936000	1.20E-08
chr19	29932001	29937000	5.41E-10
chr19	29934001	29939000	3.03E-08
chr19	29966001	29971000	0.000145
chr19	29967001	29972000	1.11E-06
chr19	29968001	29973000	0.000325
chr19	29969001	29974000	2.46E-05
chr19	29999001	30004000	0.000497
chr19	30038001	30043000	1.86E-05
chr19	30039001	30044000	7.80E-06
chr19	30040001	30045000	0.000118
chr19	30041001	30046000	0.000178
chr19	30119001	30124000	1.33E-06
chr19	30157001	30162000	3.62E-16
chr19	30327001	30332000	0.005303
chr19	30328001	30333000	0.000304
chr19	30343001	30348000	0.000793
chr19	30350001	30355000	3.56E-07
chr19	30351001	30356000	2.80E-09
chr19	30352001	30357000	6.40E-08
chr19	30353001	30358000	8.95E-10
chr19	30354001	30359000	8.86E-12
chr19	30355001	30360000	9.37E-10
chr19	30356001	30361000	3.93E-06
chr19	30378001	30383000	0.00098
chr19	30389001	30394000	0.000304
chr19	30404001	30409000	0.000689
chr19	30429001	30434000	7.46E-11
chr19	30430001	30435000	9.65E-11
chr19	30956001	30961000	5.31E-07
chr19	30957001	30962000	2.93E-06
chr19	31089001	31094000	2.17E-07
chr19	31090001	31095000	2.28E-08
chr19	31091001	31096000	1.22E-07
chr19	31212001	31217000	6.75E-09
chr19	31213001	31218000	9.08E-10
chr19	31214001	31219000	3.23E-10
chr19	31241001	31246000	0.000391
chr19	31242001	31247000	3.65E-06
chr19	31243001	31248000	5.31E-08
chr19	31272001	31277000	2.57E-07
chr19	31273001	31278000	3.45E-08
chr19	31274001	31279000	4.60E-08
chr19	31275001	31280000	1.48E-09
chr19	31347001	31352000	5.91E-06
chr19	31416001	31421000	2.94E-08
chr19	31449001	31454000	1.06E-05
chr19	31482001	31487000	4.74E-11

chr19	31538001	31543000	1.80E-07
chr19	31539001	31544000	1.37E-09
chr19	31540001	31545000	1.48E-08
chr19	31541001	31546000	1.13E-07
chr19	31542001	31547000	1.42E-05
chr19	31543001	31548000	3.97E-05
chr19	31620001	31625000	1.35E-07
chr19	31708001	31713000	1.69E-07
chr19	31821001	31826000	2.56E-07
chr19	31860001	31865000	9.93E-07
chr19	31872001	31877000	0.000145
chr19	31967001	31972000	2.00E-07
chr19	31968001	31973000	1.19E-06
chr19	31970001	31975000	3.00E-05
chr19	32025001	32030000	4.86E-08
chr19	32038001	32043000	6.50E-07
chr19	32095001	32100000	1.16E-08
chr19	32096001	32101000	2.57E-11
chr19	32097001	32102000	1.06E-11
chr19	32107001	32112000	1.16E-10
chr19	32108001	32113000	5.54E-10
chr19	32109001	32114000	1.08E-07
chr19	32110001	32115000	6.97E-06
chr19	32130001	32135000	4.12E-05
chr19	32217001	32222000	5.16E-08
chr19	32218001	32223000	1.03E-07
chr19	32219001	32224000	7.15E-10
chr19	32293001	32298000	3.17E-06
chr19	32413001	32418000	1.05E-06
chr19	32474001	32479000	5.75E-07
chr19	32475001	32480000	2.77E-11
chr19	32476001	32481000	5.20E-09
chr19	32477001	32482000	1.15E-07
chr19	32495001	32500000	1.25E-08
chr19	32551001	32556000	5.95E-05
chr19	32552001	32557000	5.33E-06
chr19	32553001	32558000	9.04E-07
chr19	32564001	32569000	1.19E-05
chr19	32565001	32570000	1.95E-05
chr19	32572001	32577000	4.55E-07
chr19	32576001	32581000	5.46E-08
chr19	32578001	32583000	1.54E-06
chr19	32579001	32584000	3.23E-05
chr19	32649001	32654000	3.38E-09
chr19	32650001	32655000	4.86E-08
chr19	32716001	32721000	0.000177
chr19	32813001	32818000	5.00E-08
chr19	32893001	32898000	2.44E-10

chr19	32896001	32901000	3.19E-14
chr19	33528001	33533000	1.76E-06
chr19	33529001	33534000	7.86E-06
chr19	33530001	33535000	9.26E-08
chr19	33570001	33575000	2.93E-13
chr19	33571001	33576000	1.22E-13
chr19	33572001	33577000	3.39E-12
chr19	33646001	33651000	1.61E-08
chr19	33647001	33652000	4.97E-08
chr19	33648001	33653000	9.18E-08
chr19	33649001	33654000	3.19E-05
chr19	33859001	33864000	3.08E-10
chr19	33882001	33887000	0.000312
chr19	33958001	33963000	1.58E-06
chr19	33990001	33995000	4.10E-09
chr19	34009001	34014000	2.78E-06
chr19	34011001	34016000	7.82E-05
chr19	34023001	34028000	6.66E-05
chr19	34024001	34029000	3.42E-07
chr19	34025001	34030000	2.77E-06
chr19	34026001	34031000	1.86E-07
chr19	34089001	34094000	0.001557
chr19	34091001	34096000	0.000687
chr19	34104001	34109000	1.45E-06
chr19	34106001	34111000	7.94E-07
chr19	34107001	34112000	4.86E-06
chr19	34108001	34113000	7.30E-08
chr19	34243001	34248000	1.64E-05
chr19	34244001	34249000	1.25E-06
chr19	34250001	34255000	0.00041
chr19	34352001	34357000	0.000248
chr19	34353001	34358000	5.08E-07
chr19	34388001	34393000	0.00048
chr19	34432001	34437000	8.86E-08
chr19	34451001	34456000	0.000263
chr19	34531001	34536000	2.25E-07
chr19	34532001	34537000	1.77E-05
chr19	34606001	34611000	5.70E-05
chr19	34629001	34634000	1.23E-05
chr19	34631001	34636000	2.10E-06
chr19	34633001	34638000	2.39E-06
chr19	34659001	34664000	1.69E-07
chr19	35211001	35216000	0.000316
chr19	35212001	35217000	2.62E-06
chr19	35390001	35395000	8.74E-06
chr19	35391001	35396000	4.78E-06
chr19	35392001	35397000	2.46E-06
chr19	35393001	35398000	3.46E-06

chr19	35394001	35399000	1.56E-05
chr19	35527001	35532000	8.28E-07
chr19	35588001	35593000	3.49E-07
chr19	35632001	35637000	2.17E-10
chr19	35633001	35638000	4.23E-13
chr19	35634001	35639000	7.24E-10
chr19	35645001	35650000	0.00039
chr19	35648001	35653000	1.19E-06
chr19	35662001	35667000	3.33E-06
chr19	35672001	35677000	9.59E-09
chr19	35673001	35678000	9.67E-09
chr19	35693001	35698000	3.64E-07
chr19	35694001	35699000	2.94E-07
chr19	35695001	35700000	3.12E-11
chr19	35696001	35701000	1.82E-07
chr19	35705001	35710000	2.51E-05
chr19	35741001	35746000	3.71E-05
chr19	35814001	35819000	7.43E-08
chr19	35821001	35826000	6.83E-05
chr19	35841001	35846000	4.18E-05
chr19	35941001	35946000	1.86E-06
chr19	35942001	35947000	4.27E-06
chr19	36009001	36014000	4.78E-05
chr19	36010001	36015000	4.62E-06
chr19	36039001	36044000	0.010162
chr19	36051001	36056000	2.57E-05
chr19	36052001	36057000	7.36E-06
chr19	36071001	36076000	3.60E-05
chr19	36132001	36137000	0.006311
chr19	36154001	36159000	1.68E-07
chr19	36155001	36160000	0.001872
chr19	36157001	36162000	0.001835
chr19	36158001	36163000	3.24E-06
chr19	36159001	36164000	0.001805
chr19	36203001	36208000	0.000916
chr19	36239001	36244000	0.00031
chr19	36263001	36268000	0.006597
chr19	36316001	36321000	1.79E-05
chr19	36317001	36322000	8.48E-06
chr19	36318001	36323000	7.03E-07
chr19	36319001	36324000	2.03E-05
chr19	36355001	36360000	1.15E-05
chr19	36356001	36361000	3.14E-06
chr19	36385001	36390000	0.000166
chr19	36403001	36408000	0.004089
chr19	36404001	36409000	0.003528
chr19	36419001	36424000	0.000144
chr19	36420001	36425000	0.000223

chr19	36422001	36427000	0.000185
chr19	36423001	36428000	2.75E-06
chr19	36424001	36429000	8.64E-05
chr19	36425001	36430000	8.05E-06
chr19	36426001	36431000	2.58E-15
chr19	36427001	36432000	4.87E-10
chr19	36449001	36454000	0.003384
chr19	36450001	36455000	1.06E-05
chr19	36451001	36456000	7.80E-09
chr19	36452001	36457000	1.26E-07
chr19	36453001	36458000	2.04E-07
chr19	36454001	36459000	4.03E-07
chr19	36464001	36469000	0.011626
chr19	36465001	36470000	6.75E-05
chr19	36466001	36471000	0.000385
chr19	36467001	36472000	0.001168
chr19	36478001	36483000	0.016723
chr19	36479001	36484000	0.016723
chr19	36601001	36606000	0.000164
chr19	36602001	36607000	0.000125
chr19	36615001	36620000	7.48E-13
chr19	36616001	36621000	0.000316
chr19	36617001	36622000	1.81E-05
chr19	36618001	36623000	0.002811
chr19	36742001	36747000	1.47E-05
chr19	36802001	36807000	1.10E-05
chr19	36803001	36808000	1.74E-07
chr19	36804001	36809000	5.69E-06
chr19	36806001	36811000	2.29E-06
chr19	37120001	37125000	2.97E-09
chr19	37121001	37126000	4.02E-09
chr19	37123001	37128000	5.78E-05
chr19	37154001	37159000	2.58E-05
chr19	37155001	37160000	0.000175
chr19	37288001	37293000	6.91E-07
chr19	37342001	37347000	4.65E-08
chr19	37419001	37424000	6.08E-09
chr19	37420001	37425000	2.07E-08
chr19	37421001	37426000	1.19E-11
chr19	37422001	37427000	2.72E-10
chr19	37481001	37486000	1.52E-06
chr19	37482001	37487000	2.42E-07
chr19	37507001	37512000	1.03E-07
chr19	37535001	37540000	0.000122
chr19	37611001	37616000	2.40E-06
chr19	37699001	37704000	2.58E-07
chr19	37700001	37705000	1.02E-06
chr19	37701001	37706000	4.99E-08

chr19	37743001	37748000	3.60E-11
chr19	37744001	37749000	4.06E-15
chr19	37745001	37750000	3.43E-09
chr19	37795001	37800000	8.67E-05
chr19	37796001	37801000	1.83E-05
chr19	37855001	37860000	3.67E-06
chr19	37888001	37893000	8.96E-12
chr19	37889001	37894000	1.45E-11
chr19	37989001	37994000	9.34E-07
chr19	37990001	37995000	1.04E-08
chr19	37991001	37996000	2.89E-11
chr19	37992001	37997000	5.90E-05
chr19	37998001	38003000	3.73E-08
chr19	38007001	38012000	1.22E-08
chr19	38099001	38104000	2.11E-13
chr19	38100001	38105000	4.24E-12
chr19	38101001	38106000	2.55E-14
chr19	38102001	38107000	1.08E-12
chr19	38233001	38238000	0.000592
chr19	38308001	38313000	4.62E-08
chr19	38347001	38352000	1.95E-08
chr19	38353001	38358000	0.000463
chr19	38367001	38372000	1.05E-05
chr19	38368001	38373000	3.87E-06
chr19	38534001	38539000	2.16E-05
chr19	38701001	38706000	3.25E-06
chr19	38702001	38707000	4.12E-05
chr19	38726001	38731000	0.00029
chr19	38727001	38732000	5.97E-06
chr19	38742001	38747000	2.20E-05
chr19	38743001	38748000	1.06E-08
chr19	38744001	38749000	1.34E-10
chr19	38745001	38750000	3.41E-11
chr19	38746001	38751000	2.81E-09
chr19	38752001	38757000	0.00027
chr19	38753001	38758000	0.000301
chr19	38754001	38759000	9.34E-06
chr19	38755001	38760000	0.000273
chr19	38818001	38823000	6.41E-05
chr19	38819001	38824000	4.19E-07
chr19	38821001	38826000	9.14E-06
chr19	38831001	38836000	0.001049
chr19	38851001	38856000	0.000597
chr19	38854001	38859000	0.000158
chr19	38878001	38883000	0.000382
chr19	38886001	38891000	0.000761
chr19	38887001	38892000	0.000236
chr19	38900001	38905000	2.86E-06

chr19	38901001	38906000	9.60E-05
chr19	38928001	38933000	5.64E-05
chr19	38929001	38934000	1.42E-05
chr19	38930001	38935000	5.45E-05
chr19	38931001	38936000	0.000211
chr19	38943001	38948000	1.16E-10
chr19	38944001	38949000	0.000423
chr19	38946001	38951000	2.42E-08
chr19	38947001	38952000	5.10E-09
chr19	38948001	38953000	7.39E-09
chr19	38949001	38954000	3.95E-07
chr19	38954001	38959000	0.014072
chr19	38955001	38960000	0.006559
chr19	38956001	38961000	0.006974
chr19	38957001	38962000	0.005447
chr19	38958001	38963000	0.045033
chr19	39011001	39016000	0.001046
chr19	39049001	39054000	1.43E-08
chr19	39050001	39055000	9.17E-09
chr19	39051001	39056000	4.54E-22
chr19	39052001	39057000	1.63E-16
chr19	39053001	39058000	5.20E-07
chr19	39054001	39059000	0.007847
chr19	39091001	39096000	0.000856
chr19	39092001	39097000	0.00022
chr19	39285001	39290000	4.15E-07
chr19	39290001	39295000	1.72E-05
chr19	39326001	39331000	0.00197
chr19	39327001	39332000	0.00013
chr19	39352001	39357000	0.001363
chr19	39353001	39358000	2.81E-08
chr19	39354001	39359000	1.37E-05
chr19	39420001	39425000	3.22E-08
chr19	39442001	39447000	0.003296
chr19	39448001	39453000	4.36E-05
chr19	39461001	39466000	6.80E-11
chr19	39462001	39467000	2.54E-11
chr19	39463001	39468000	2.45E-11
chr19	39484001	39489000	0.00584
chr19	39485001	39490000	1.06E-05
chr19	39493001	39498000	0.000898
chr19	39494001	39499000	0.000534
chr19	39525001	39530000	5.41E-06
chr19	39526001	39531000	2.67E-06
chr19	39527001	39532000	3.14E-07
chr19	39528001	39533000	2.41E-08
chr19	39529001	39534000	7.92E-07
chr19	39530001	39535000	3.72E-06

chr19	39548001	39553000	4.18E-05
chr19	39581001	39586000	0.003128
chr19	39582001	39587000	4.75E-05
chr19	39583001	39588000	0.000315
chr19	39584001	39589000	0.000437
chr19	39585001	39590000	0.002858
chr19	39586001	39591000	0.002858
chr19	39612001	39617000	5.02E-10
chr19	39615001	39620000	6.11E-08
chr19	39616001	39621000	5.34E-06
chr19	39690001	39695000	0.014662
chr19	39731001	39736000	0.002157
chr19	39732001	39737000	5.96E-05
chr19	39733001	39738000	0.000535
chr19	39734001	39739000	0.003133
chr19	39773001	39778000	4.38E-07
chr19	39808001	39813000	1.55E-05
chr19	39810001	39815000	0.0061
chr19	39892001	39897000	1.31E-09
chr19	40001001	40006000	4.18E-11
chr19	40002001	40007000	1.24E-16
chr19	40003001	40008000	1.05E-08
chr19	40004001	40009000	4.44E-06
chr19	40005001	40010000	0.0004
chr19	40037001	40042000	8.38E-07
chr19	40038001	40043000	2.03E-05
chr19	40045001	40050000	1.09E-07
chr19	40046001	40051000	1.40E-07
chr19	40101001	40106000	1.40E-05
chr19	40112001	40117000	2.90E-05
chr19	40113001	40118000	0.000239
chr19	40298001	40303000	2.55E-09
chr19	40323001	40328000	1.79E-11
chr19	40324001	40329000	2.46E-13
chr19	40347001	40352000	1.68E-06
chr19	40348001	40353000	3.02E-05
chr19	40413001	40418000	0.00602
chr19	40414001	40419000	0.004655
chr19	40415001	40420000	6.55E-05
chr19	40420001	40425000	0.001497
chr19	40455001	40460000	1.22E-05
chr19	40456001	40461000	3.84E-06
chr19	40457001	40462000	5.21E-06
chr19	40601001	40606000	4.10E-06
chr19	40608001	40613000	0.000139
chr19	40609001	40614000	4.04E-05
chr19	40610001	40615000	0.009561
chr19	40621001	40626000	0.000368

chr19	40622001	40627000	0.000531
chr19	40623001	40628000	0.001639
chr19	40670001	40675000	6.30E-05
chr19	40675001	40680000	0.003206
chr19	40716001	40721000	7.63E-07
chr19	40717001	40722000	2.62E-07
chr19	40718001	40723000	6.08E-07
chr19	40719001	40724000	6.03E-06
chr19	40728001	40733000	0.008936
chr19	40729001	40734000	0.000169
chr19	40911001	40916000	0.001028
chr19	40940001	40945000	0.001371
chr19	40967001	40972000	2.05E-06
chr19	40968001	40973000	0.003216
chr19	40971001	40976000	2.15E-07
chr19	40986001	40991000	1.81E-05
chr19	40987001	40992000	2.12E-05
chr19	40988001	40993000	1.07E-05
chr19	40989001	40994000	0.000112
chr19	40990001	40995000	0.000244
chr19	41069001	41074000	0.020903
chr19	41072001	41077000	1.40E-07
chr19	41075001	41080000	1.60E-07
chr19	41078001	41083000	0.014944
chr19	41079001	41084000	0.014944
chr19	41080001	41085000	0.000194
chr19	41081001	41086000	4.15E-05
chr19	41082001	41087000	1.63E-05
chr19	41096001	41101000	3.92E-13
chr19	41097001	41102000	2.99E-09
chr19	41098001	41103000	4.27E-08
chr19	41108001	41113000	4.44E-06
chr19	41109001	41114000	1.04E-06
chr19	41110001	41115000	1.04E-06
chr19	41111001	41116000	0.000553
chr19	41112001	41117000	1.42E-06
chr19	41113001	41118000	0.001085
chr19	41117001	41122000	2.48E-06
chr19	41118001	41123000	1.41E-08
chr19	41119001	41124000	8.10E-09
chr19	41120001	41125000	1.08E-08
chr19	41141001	41146000	0.003324
chr19	41165001	41170000	5.82E-09
chr19	41166001	41171000	4.04E-13
chr19	41167001	41172000	2.85E-07
chr19	41169001	41174000	0.000134
chr19	41217001	41222000	2.41E-11
chr19	41218001	41223000	7.22E-19

chr19	41219001	41224000	7.29E-20
chr19	41220001	41225000	5.12E-10
chr19	41278001	41283000	0.002016
chr19	41345001	41350000	1.35E-06
chr19	41346001	41351000	2.35E-06
chr19	41347001	41352000	2.89E-07
chr19	41348001	41353000	7.17E-06
chr19	41354001	41359000	0.002754
chr19	41355001	41360000	0.000731
chr19	41468001	41473000	6.58E-06
chr19	41469001	41474000	6.38E-06
chr19	41482001	41487000	0.011446
chr19	41517001	41522000	0.000298
chr19	41520001	41525000	2.74E-07
chr19	41521001	41526000	7.80E-07
chr19	41522001	41527000	2.09E-08
chr19	41523001	41528000	5.93E-14
chr19	41524001	41529000	6.19E-12
chr19	41525001	41530000	8.55E-05
chr19	41526001	41531000	4.28E-05
chr19	41567001	41572000	1.25E-05
chr19	41568001	41573000	0.000219
chr19	41569001	41574000	2.56E-05
chr19	41571001	41576000	1.81E-06
chr19	41616001	41621000	4.07E-06
chr19	41617001	41622000	7.31E-07
chr19	41764001	41769000	2.99E-09
chr19	41853001	41858000	0.000747
chr19	41878001	41883000	0.00987
chr19	41881001	41886000	3.28E-05
chr19	41882001	41887000	0.000159
chr19	41946001	41951000	8.24E-10
chr19	42042001	42047000	9.34E-09
chr19	42078001	42083000	1.38E-05
chr19	42079001	42084000	5.55E-05
chr19	42080001	42085000	9.69E-05
chr19	42081001	42086000	8.83E-05
chr19	42090001	42095000	0.000639
chr19	42091001	42096000	0.000345
chr19	42092001	42097000	0.000181
chr19	42155001	42160000	0.005241
chr19	42199001	42204000	0.002355
chr19	42200001	42205000	0.002349
chr19	42238001	42243000	2.59E-06
chr19	42311001	42316000	0.018015
chr19	42324001	42329000	2.81E-05
chr19	42357001	42362000	6.86E-06
chr19	42358001	42363000	1.74E-05

chr19	42360001	42365000	2.06E-06
chr19	42418001	42423000	3.72E-07
chr19	42419001	42424000	1.26E-10
chr19	42420001	42425000	1.21E-09
chr19	42421001	42426000	5.10E-11
chr19	42452001	42457000	9.32E-06
chr19	42453001	42458000	2.31E-10
chr19	42466001	42471000	9.04E-08
chr19	42467001	42472000	8.56E-09
chr19	42468001	42473000	7.56E-07
chr19	42469001	42474000	3.77E-06
chr19	42478001	42483000	1.13E-05
chr19	42479001	42484000	2.47E-06
chr19	42503001	42508000	3.06E-07
chr19	42504001	42509000	1.05E-05
chr19	42505001	42510000	1.03E-05
chr19	42506001	42511000	2.78E-06
chr19	42507001	42512000	4.10E-05
chr19	42522001	42527000	4.67E-05
chr19	42537001	42542000	2.83E-14
chr19	42538001	42543000	1.48E-10
chr19	42539001	42544000	6.99E-06
chr19	42590001	42595000	0.000204
chr19	42591001	42596000	0.000556
chr19	42592001	42597000	0.002276
chr19	42677001	42682000	3.11E-05
chr19	42693001	42698000	0.002814
chr19	42698001	42703000	0.000176
chr19	42699001	42704000	4.52E-05
chr19	42700001	42705000	6.47E-05
chr19	42825001	42830000	1.62E-07
chr19	42897001	42902000	9.34E-09
chr19	42898001	42903000	4.46E-07
chr19	42900001	42905000	9.66E-06
chr19	43098001	43103000	0.000124
chr19	43128001	43133000	0.000908
chr19	43129001	43134000	0.000108
chr19	43130001	43135000	0.004737
chr19	43134001	43139000	1.25E-05
chr19	43135001	43140000	0.000214
chr19	43145001	43150000	3.60E-05
chr19	43159001	43164000	1.98E-05
chr19	43160001	43165000	6.05E-07
chr19	43185001	43190000	9.98E-05
chr19	43186001	43191000	9.04E-05
chr19	43210001	43215000	5.14E-06
chr19	43211001	43216000	2.05E-06
chr19	43291001	43296000	0.001638

chr19	43292001	43297000	0.001969
chr19	43293001	43298000	0.000979
chr19	43343001	43348000	0.000796
chr19	43355001	43360000	4.55E-05
chr19	43356001	43361000	0.001865
chr19	43392001	43397000	3.62E-10
chr19	43393001	43398000	1.56E-09
chr19	43469001	43474000	4.18E-06
chr19	43491001	43496000	1.04E-11
chr19	43492001	43497000	3.10E-10
chr19	43493001	43498000	1.68E-09
chr19	43522001	43527000	3.07E-05
chr19	43554001	43559000	1.12E-05
chr19	43601001	43606000	3.07E-06
chr19	43704001	43709000	0.00038
chr19	43705001	43710000	0.000152
chr19	43723001	43728000	5.61E-06
chr19	43726001	43731000	4.44E-05
chr19	43729001	43734000	1.17E-06
chr19	43740001	43745000	5.01E-06
chr19	43741001	43746000	9.47E-06
chr19	43742001	43747000	6.75E-05
chr19	43743001	43748000	0.002244
chr19	43744001	43749000	0.002361
chr19	43752001	43757000	1.54E-06
chr19	43799001	43804000	1.09E-07
chr19	43800001	43805000	7.01E-07
chr19	43808001	43813000	0.000237
chr19	43874001	43879000	8.66E-06
chr19	43875001	43880000	2.32E-05
chr19	43964001	43969000	5.78E-06
chr19	43965001	43970000	1.02E-09
chr19	43966001	43971000	3.12E-05
chr19	43967001	43972000	6.83E-05
chr19	43974001	43979000	0.000724
chr19	43975001	43980000	0.002494
chr19	43977001	43982000	0.002935
chr19	44003001	44008000	1.10E-05
chr19	44025001	44030000	3.00E-07
chr19	44031001	44036000	0.001518
chr19	44078001	44083000	0.000424
chr19	44079001	44084000	2.55E-09
chr19	44095001	44100000	0.001453
chr19	44129001	44134000	4.73E-06
chr19	44191001	44196000	0.011432
chr19	44281001	44286000	0.000185
chr19	44284001	44289000	0.000315
chr19	44285001	44290000	0.000369

chr19	44363001	44368000	7.95E-07
chr19	44519001	44524000	8.97E-09
chr19	44520001	44525000	2.62E-05
chr19	44521001	44526000	7.76E-05
chr19	44948001	44953000	7.11E-14
chr19	44949001	44954000	4.29E-15
chr19	45028001	45033000	1.80E-05
chr19	45029001	45034000	6.28E-07
chr19	45030001	45035000	1.07E-07
chr19	45031001	45036000	3.28E-06
chr19	45032001	45037000	4.01E-05
chr19	45034001	45039000	0.004668
chr19	45036001	45041000	0.00481
chr19	45045001	45050000	5.23E-05
chr19	45104001	45109000	3.17E-07
chr19	45250001	45255000	0.00047
chr19	45251001	45256000	0.000328
chr19	45283001	45288000	6.02E-08
chr19	45287001	45292000	3.36E-07
chr19	45403001	45408000	2.96E-07
chr19	45404001	45409000	1.28E-10
chr19	45405001	45410000	6.26E-10
chr19	45406001	45411000	3.10E-09
chr19	45422001	45427000	0.00017
chr19	45446001	45451000	0.000193
chr19	45452001	45457000	0.030239
chr19	45502001	45507000	3.82E-05
chr19	45516001	45521000	0.000835
chr19	45517001	45522000	0.001089
chr19	45518001	45523000	0.000214
chr19	45519001	45524000	0.000641
chr19	45590001	45595000	2.63E-07
chr19	45679001	45684000	0.000238
chr19	45680001	45685000	0.000932
chr19	45681001	45686000	2.11E-09
chr19	45682001	45687000	4.94E-09
chr19	45683001	45688000	1.07E-05
chr19	45734001	45739000	1.14E-05
chr19	45735001	45740000	4.74E-05
chr19	45806001	45811000	3.45E-06
chr19	45824001	45829000	0.005301
chr19	45851001	45856000	0.001205
chr19	45904001	45909000	1.62E-07
chr19	45956001	45961000	0.000668
chr19	46132001	46137000	4.57E-06
chr19	46147001	46152000	2.75E-09
chr19	46148001	46153000	2.04E-11
chr19	46158001	46163000	3.55E-05

chr19	46178001	46183000	0.000522
chr19	46181001	46186000	0.001716
chr19	46215001	46220000	0.000162
chr19	46230001	46235000	0.001862
chr19	46231001	46236000	0.001863
chr19	46232001	46237000	0.00013
chr19	46233001	46238000	9.24E-07
chr19	46244001	46249000	0.000868
chr19	46245001	46250000	0.001202
chr19	46249001	46254000	0.002537
chr19	46257001	46262000	0.000115
chr19	46278001	46283000	0.003044
chr19	46279001	46284000	2.05E-05
chr19	46383001	46388000	0.004121
chr19	46385001	46390000	9.95E-07
chr19	46386001	46391000	9.76E-08
chr19	46387001	46392000	6.39E-10
chr19	46388001	46393000	3.00E-07
chr19	46389001	46394000	6.79E-08
chr19	46403001	46408000	0.000337
chr19	46404001	46409000	0.002659
chr19	46405001	46410000	0.000561
chr19	46448001	46453000	7.80E-07
chr19	46568001	46573000	1.46E-06
chr19	46719001	46724000	2.36E-06
chr19	46727001	46732000	0.032798
chr19	46728001	46733000	1.09E-05
chr19	46729001	46734000	5.88E-07
chr19	46730001	46735000	1.05E-05
chr19	46762001	46767000	2.32E-05
chr19	46785001	46790000	7.65E-06
chr19	46806001	46811000	0.011641
chr19	46807001	46812000	0.003407
chr19	46822001	46827000	1.85E-05
chr19	46846001	46851000	7.86E-05
chr19	46931001	46936000	0.005856
chr19	46932001	46937000	0.002162
chr19	46933001	46938000	3.55E-05
chr19	46941001	46946000	0.000129
chr19	46943001	46948000	0.000856
chr19	46950001	46955000	0.001145
chr19	46985001	46990000	0.000246
chr19	46987001	46992000	0.000239
chr19	46998001	47003000	8.86E-05
chr19	46999001	47004000	0.000134
chr19	47002001	47007000	8.49E-06
chr19	47040001	47045000	2.21E-05
chr19	47043001	47048000	5.07E-05

chr19	47044001	47049000	0.00052
chr19	47050001	47055000	0.001692
chr19	47197001	47202000	4.32E-09
chr19	47198001	47203000	0.004958
chr19	47199001	47204000	0.010415
chr19	47229001	47234000	0.002061
chr19	47247001	47252000	2.93E-05
chr19	47248001	47253000	2.86E-05
chr19	47491001	47496000	0.000418
chr19	47617001	47622000	2.08E-08
chr19	47621001	47626000	2.33E-06
chr19	47629001	47634000	9.13E-06
chr19	47710001	47715000	5.16E-06
chr19	47779001	47784000	0.007258
chr19	47780001	47785000	0.000467
chr19	47781001	47786000	0.00024
chr19	47782001	47787000	0.001397
chr19	47809001	47814000	0.000546
chr19	47810001	47815000	0.002693
chr19	47811001	47816000	0.000426
chr19	47818001	47823000	0.000118
chr19	47853001	47858000	1.05E-07
chr19	47954001	47959000	0.000303
chr19	47956001	47961000	0.00396
chr19	47957001	47962000	0.002985
chr19	47958001	47963000	0.000118
chr19	47961001	47966000	1.49E-05
chr19	47970001	47975000	3.16E-05
chr19	47971001	47976000	4.86E-05
chr19	47972001	47977000	1.44E-07
chr19	47973001	47978000	0.000453
chr19	48029001	48034000	1.20E-06
chr19	48030001	48035000	0.000894
chr19	48083001	48088000	0.00049
chr19	48100001	48105000	3.50E-05
chr19	48101001	48106000	0.000348
chr19	48102001	48107000	2.05E-08
chr19	48103001	48108000	2.36E-10
chr19	48104001	48109000	2.09E-09
chr19	48249001	48254000	2.58E-05
chr19	48257001	48262000	0.000972
chr19	48258001	48263000	0.000703
chr19	48268001	48273000	1.95E-05
chr19	48277001	48282000	3.11E-08
chr19	48278001	48283000	5.54E-06
chr19	48319001	48324000	0.006372
chr19	48329001	48334000	3.43E-09
chr19	48330001	48335000	6.84E-07

chr19	48331001	48336000	4.57E-05
chr19	48332001	48337000	1.66E-06
chr19	48333001	48338000	0.000495
chr19	48357001	48362000	9.08E-07
chr19	48367001	48372000	0.000103
chr19	48385001	48390000	2.81E-06
chr19	48387001	48392000	4.84E-06
chr19	48471001	48476000	0.000228
chr19	48472001	48477000	4.29E-09
chr19	48473001	48478000	1.85E-11
chr19	48474001	48479000	8.96E-10
chr19	48506001	48511000	2.54E-06
chr19	48513001	48518000	2.43E-07
chr19	48514001	48519000	2.57E-07
chr19	48554001	48559000	6.07E-05
chr19	48555001	48560000	1.85E-05
chr19	48688001	48693000	0.000974
chr19	48770001	48775000	1.31E-10
chr19	48773001	48778000	5.94E-06
chr19	48774001	48779000	9.88E-08
chr19	48862001	48867000	0.000222
chr19	48865001	48870000	1.77E-08
chr19	48866001	48871000	7.08E-14
chr19	48894001	48899000	0.001178
chr19	48947001	48952000	2.56E-08
chr19	48948001	48953000	2.34E-19
chr19	48949001	48954000	2.12E-11
chr19	48985001	48990000	3.41E-06
chr19	48986001	48991000	8.67E-11
chr19	48987001	48992000	7.41E-10
chr19	49001001	49006000	0.035711
chr19	49047001	49052000	0.026871
chr19	49074001	49079000	0.011093
chr19	49127001	49132000	5.80E-05
chr19	49157001	49162000	0.000736
chr19	49158001	49163000	4.30E-05
chr19	49179001	49184000	2.00E-05
chr19	49180001	49185000	0.000127
chr19	49195001	49200000	3.54E-06
chr19	49237001	49242000	3.21E-08
chr19	49238001	49243000	2.34E-08
chr19	49255001	49260000	1.66E-08
chr19	49256001	49261000	2.28E-08
chr19	49257001	49262000	2.52E-07
chr19	49384001	49389000	8.09E-08
chr19	49422001	49427000	0.008117
chr19	49425001	49430000	0.030717
chr19	49444001	49449000	0.003595

chr19	49445001	49450000	0.003595
chr19	49466001	49471000	1.94E-06
chr19	49467001	49472000	4.43E-06
chr19	49468001	49473000	0.000145
chr19	49469001	49474000	0.002277
chr19	49536001	49541000	1.00E-05
chr19	49542001	49547000	6.26E-07
chr19	49543001	49548000	2.47E-08
chr19	49544001	49549000	1.61E-08
chr19	49545001	49550000	0.000401
chr19	49546001	49551000	0.000273
chr19	49547001	49552000	0.010296
chr19	49584001	49589000	3.79E-06
chr19	49585001	49590000	2.07E-08
chr19	49586001	49591000	1.87E-08
chr19	49587001	49592000	2.91E-07
chr19	49620001	49625000	1.11E-11
chr19	49621001	49626000	3.50E-14
chr19	49622001	49627000	3.90E-06
chr19	49635001	49640000	0.013121
chr19	49636001	49641000	0.001077
chr19	49647001	49652000	1.25E-06
chr19	49648001	49653000	4.82E-06
chr19	49649001	49654000	3.33E-08
chr19	49707001	49712000	1.38E-06
chr19	49709001	49714000	3.44E-06
chr19	49710001	49715000	2.54E-05
chr19	49711001	49716000	1.18E-08
chr19	49801001	49806000	1.50E-07
chr19	49802001	49807000	8.15E-09
chr19	49803001	49808000	3.81E-09
chr19	49804001	49809000	4.20E-08
chr19	49860001	49865000	0.0162
chr19	49878001	49883000	9.61E-06
chr19	49892001	49897000	0.005002
chr19	49968001	49973000	1.27E-10
chr19	49969001	49974000	1.27E-10
chr19	49973001	49978000	0.008091
chr19	49976001	49981000	0.00094
chr19	50053001	50058000	2.15E-10
chr19	50054001	50059000	4.71E-07
chr19	50055001	50060000	8.80E-08
chr19	50056001	50061000	5.68E-08
chr19	50057001	50062000	1.28E-09
chr19	50169001	50174000	0.000116
chr19	50250001	50255000	0.003933
chr19	50251001	50256000	0.0049
chr19	50252001	50257000	0.008753

chr19	50350001	50355000	9.09E-06
chr19	50351001	50356000	2.00E-06
chr19	50363001	50368000	1.17E-08
chr19	50372001	50377000	0.000716
chr19	50403001	50408000	7.07E-07
chr19	50404001	50409000	2.79E-07
chr19	50405001	50410000	5.03E-07
chr19	50431001	50436000	1.44E-09
chr19	50432001	50437000	7.12E-12
chr19	50433001	50438000	4.40E-07
chr19	50472001	50477000	4.27E-05
chr19	50572001	50577000	4.91E-06
chr19	50580001	50585000	0.006318
chr19	50641001	50646000	0.001288
chr19	50651001	50656000	3.74E-08
chr19	50686001	50691000	0.000106
chr19	50687001	50692000	0.000103
chr19	50693001	50698000	3.04E-07
chr19	50700001	50705000	3.34E-06
chr19	50701001	50706000	0.000112
chr19	50702001	50707000	3.66E-05
chr19	50736001	50741000	0.031672
chr19	50759001	50764000	0.012017
chr19	50931001	50936000	2.80E-06
chr19	50932001	50937000	0.000277
chr19	50934001	50939000	4.70E-07
chr19	51020001	51025000	1.43E-12
chr19	51021001	51026000	2.92E-12
chr19	51022001	51027000	5.88E-12
chr19	51053001	51058000	0.000153
chr19	51055001	51060000	1.53E-05
chr19	51056001	51061000	2.52E-07
chr19	51059001	51064000	1.15E-05
chr19	51070001	51075000	0.007453
chr19	51118001	51123000	1.70E-09
chr19	51119001	51124000	6.51E-11
chr19	51120001	51125000	1.44E-09
chr19	51121001	51126000	2.06E-11
chr19	51122001	51127000	1.37E-07
chr19	51123001	51128000	0.001618
chr19	51159001	51164000	1.74E-07
chr19	51165001	51170000	0.000373
chr19	51166001	51171000	9.03E-06
chr19	51188001	51193000	5.29E-05
chr19	51203001	51208000	1.58E-05
chr19	51211001	51216000	8.38E-07
chr19	51254001	51259000	2.35E-05
chr19	51255001	51260000	2.38E-06

chr19	51256001	51261000	3.03E-07
chr19	51257001	51262000	0.000172
chr19	51269001	51274000	1.82E-06
chr19	51277001	51282000	5.82E-07
chr19	51278001	51283000	2.05E-07
chr19	51279001	51284000	5.17E-07
chr19	51280001	51285000	9.35E-07
chr19	51281001	51286000	1.63E-05
chr19	51282001	51287000	0.001369
chr19	51390001	51395000	7.41E-06
chr19	51432001	51437000	3.36E-09
chr19	51670001	51675000	2.32E-08
chr19	51671001	51676000	3.60E-09
chr19	51672001	51677000	9.94E-08
chr19	51732001	51737000	0.000232
chr19	51760001	51765000	1.67E-05
chr19	51761001	51766000	4.74E-08
chr19	51775001	51780000	2.56E-07
chr19	51801001	51806000	1.35E-09
chr19	51819001	51824000	0.000639
chr19	51862001	51867000	0.000592
chr19	51865001	51870000	1.34E-05
chr19	51866001	51871000	9.67E-10
chr19	51907001	51912000	1.08E-05
chr19	51936001	51941000	2.50E-07
chr19	51971001	51976000	7.70E-06
chr19	52000001	52005000	5.94E-05
chr19	52001001	52006000	8.32E-07
chr19	52007001	52012000	7.43E-07
chr19	52008001	52013000	3.80E-07
chr19	52009001	52014000	0.000172
chr19	52043001	52048000	1.51E-05
chr19	52052001	52057000	1.05E-06
chr19	52053001	52058000	1.74E-11
chr19	52055001	52060000	1.42E-08
chr19	52097001	52102000	2.41E-07
chr19	52112001	52117000	9.42E-07
chr19	52128001	52133000	2.58E-08
chr19	52129001	52134000	5.09E-06
chr19	52254001	52259000	1.96E-08
chr19	52255001	52260000	3.25E-07
chr19	52490001	52495000	4.58E-12
chr19	52650001	52655000	4.73E-05
chr19	52651001	52656000	2.34E-05
chr19	52652001	52657000	3.75E-06
chr19	52747001	52752000	4.70E-09
chr19	52756001	52761000	2.42E-07
chr19	52757001	52762000	9.36E-11

chr19	52758001	52763000	2.46E-11
chr19	52845001	52850000	1.42E-06
chr19	52847001	52852000	1.42E-12
chr19	52848001	52853000	8.95E-12
chr19	52849001	52854000	2.76E-08
chr19	52874001	52879000	7.20E-13
chr19	52875001	52880000	1.01E-12
chr19	52876001	52881000	1.29E-09
chr19	52877001	52882000	8.26E-13
chr19	52878001	52883000	7.73E-05
chr19	52881001	52886000	3.18E-06
chr19	52897001	52902000	7.32E-07
chr19	52898001	52903000	8.15E-07
chr19	52905001	52910000	1.52E-06
chr19	52907001	52912000	1.12E-07
chr19	52964001	52969000	9.84E-12
chr19	52997001	53002000	1.20E-05
chr19	52998001	53003000	0.000386
chr19	53010001	53015000	1.16E-08
chr19	53198001	53203000	0.000715
chr19	53199001	53204000	0.000531
chr19	53320001	53325000	9.64E-12
chr19	53321001	53326000	4.23E-08
chr19	53402001	53407000	1.97E-07
chr19	53403001	53408000	6.91E-07
chr19	53417001	53422000	0.000863
chr19	53425001	53430000	8.31E-07
chr19	53426001	53431000	1.60E-08
chr19	53427001	53432000	2.36E-05
chr19	53428001	53433000	1.90E-06
chr19	53492001	53497000	3.23E-07
chr19	53493001	53498000	0.004847
chr19	53494001	53499000	1.03E-07
chr19	53495001	53500000	4.63E-12
chr19	53496001	53501000	5.63E-14
chr19	53497001	53502000	6.25E-07
chr19	53498001	53503000	5.67E-09
chr19	53686001	53691000	2.38E-05
chr19	53687001	53692000	3.45E-06
chr19	53688001	53693000	0.000144
chr19	53690001	53695000	1.94E-06
chr19	53691001	53696000	2.79E-06
chr19	53692001	53697000	8.82E-10
chr19	53693001	53698000	8.31E-10
chr19	53694001	53699000	8.15E-15
chr19	53741001	53746000	2.61E-10
chr19	53742001	53747000	2.90E-09
chr19	53743001	53748000	6.90E-07

chr19	53749001	53754000	1.66E-07
chr19	53750001	53755000	7.03E-08
chr19	53751001	53756000	2.54E-08
chr19	54022001	54027000	9.66E-10
chr19	54023001	54028000	8.06E-11
chr19	54096001	54101000	7.93E-05
chr19	54097001	54102000	1.65E-05
chr19	54098001	54103000	2.26E-05
chr19	54129001	54134000	0.03755
chr19	54168001	54173000	0.001099
chr19	54169001	54174000	8.38E-05
chr19	54170001	54175000	1.51E-06
chr19	54196001	54201000	4.98E-08
chr19	54197001	54202000	3.34E-07
chr19	54240001	54245000	1.96E-10
chr19	54241001	54246000	2.17E-09
chr19	54242001	54247000	1.17E-07
chr19	54249001	54254000	2.49E-05
chr19	54292001	54297000	1.09E-05
chr19	54293001	54298000	9.93E-06
chr19	54294001	54299000	4.58E-05
chr19	54296001	54301000	0.001417
chr19	54298001	54303000	9.14E-05
chr19	54299001	54304000	8.98E-05
chr19	54300001	54305000	2.39E-06
chr19	54362001	54367000	4.56E-06
chr19	54363001	54368000	1.63E-06
chr19	54364001	54369000	3.04E-05
chr19	54381001	54386000	5.13E-07
chr19	54391001	54396000	6.91E-08
chr19	54392001	54397000	6.00E-07
chr19	54397001	54402000	1.51E-06
chr19	54398001	54403000	9.26E-10
chr19	54399001	54404000	1.16E-06
chr19	54400001	54405000	0.002718
chr19	54411001	54416000	4.03E-08
chr19	54412001	54417000	1.29E-08
chr19	54413001	54418000	1.68E-05
chr19	54414001	54419000	4.85E-10
chr19	54415001	54420000	0.000215
chr19	54416001	54421000	8.71E-05
chr19	54417001	54422000	4.98E-08
chr19	54418001	54423000	7.63E-08
chr19	54419001	54424000	3.05E-07
chr19	54420001	54425000	7.27E-07
chr19	54421001	54426000	1.64E-05
chr19	54425001	54430000	0.04765
chr19	54428001	54433000	0.011878

chr19	54443001	54448000	2.66E-08
chr19	54444001	54449000	6.98E-08
chr19	54445001	54450000	1.14E-06
chr19	54446001	54451000	1.51E-06
chr19	54448001	54453000	6.58E-10
chr19	54449001	54454000	1.11E-06
chr19	54450001	54455000	2.22E-07
chr19	54451001	54456000	1.06E-07
chr19	54452001	54457000	1.43E-05
chr19	54453001	54458000	9.53E-05
chr19	54454001	54459000	1.93E-07
chr19	54457001	54462000	3.14E-13
chr19	54463001	54468000	1.39E-05
chr19	54464001	54469000	2.20E-06
chr19	54465001	54470000	3.80E-11
chr19	54466001	54471000	6.24E-09
chr19	54467001	54472000	1.55E-10
chr19	54468001	54473000	1.20E-08
chr19	54479001	54484000	5.61E-07
chr19	54484001	54489000	0.00014
chr19	54500001	54505000	2.75E-06
chr19	54513001	54518000	9.47E-07
chr19	54550001	54555000	4.98E-08
chr19	54584001	54589000	1.53E-08
chr19	54603001	54608000	2.92E-10
chr19	54604001	54609000	4.39E-05
chr19	54606001	54611000	2.34E-07
chr19	54675001	54680000	0.000175
chr19	54676001	54681000	4.05E-05
chr19	54691001	54696000	0.008117
chr19	54755001	54760000	0.000106
chr19	54758001	54763000	4.77E-05
chr19	54759001	54764000	5.61E-06
chr19	54760001	54765000	5.21E-06
chr19	54761001	54766000	5.55E-09
chr19	54818001	54823000	2.75E-05
chr19	54819001	54824000	1.52E-05
chr19	54823001	54828000	9.40E-07
chr19	54824001	54829000	3.34E-09
chr19	54825001	54830000	4.62E-08
chr19	54852001	54857000	3.37E-06
chr19	54853001	54858000	6.61E-05
chr19	54854001	54859000	3.27E-06
chr19	54910001	54915000	6.30E-05
chr19	54912001	54917000	0.000274
chr19	54914001	54919000	0.016232
chr19	54956001	54961000	2.37E-14
chr19	54957001	54962000	2.12E-12

chr19	54960001	54965000	4.43E-11
chr19	54969001	54974000	2.23E-06
chr19	54976001	54981000	7.70E-09
chr19	54982001	54987000	0.000134
chr19	54983001	54988000	1.25E-10
chr19	54984001	54989000	4.91E-09
chr19	54997001	55002000	1.59E-07
chr19	55035001	55040000	3.76E-16
chr19	55036001	55041000	4.51E-15
chr19	55037001	55042000	5.92E-16
chr19	55055001	55060000	1.39E-10
chr19	55086001	55091000	0.000441
chr19	55173001	55178000	0.000347
chr19	55174001	55179000	4.06E-05
chr19	55176001	55181000	0.002434
chr19	55216001	55221000	8.82E-05
chr19	55269001	55274000	3.62E-07
chr19	55286001	55291000	3.85E-07
chr19	55330001	55335000	0.001141
chr19	55398001	55403000	4.51E-05
chr19	55409001	55414000	1.14E-05
chr19	55421001	55426000	9.54E-06
chr19	55422001	55427000	5.74E-05
chr19	55424001	55429000	6.27E-07
chr19	55425001	55430000	3.74E-05
chr19	55445001	55450000	0.00205
chr19	55528001	55533000	0.006146
chr19	55580001	55585000	6.69E-05
chr19	55582001	55587000	0.00186
chr19	55714001	55719000	3.37E-13
chr19	55715001	55720000	1.54E-10
chr19	55716001	55721000	3.41E-07
chr19	55717001	55722000	9.02E-08
chr19	55721001	55726000	4.24E-09
chr19	55762001	55767000	0.002772
chr19	55769001	55774000	1.84E-08
chr19	55770001	55775000	4.49E-10
chr19	55813001	55818000	0.000322
chr19	55817001	55822000	0.002362
chr19	55846001	55851000	7.03E-08
chr19	55897001	55902000	4.29E-06
chr19	55943001	55948000	0.003942
chr19	55944001	55949000	0.000128
chr19	55945001	55950000	0.000135
chr19	55946001	55951000	0.027313
chr19	55947001	55952000	0.016733
chr19	55956001	55961000	5.37E-08
chr19	55957001	55962000	1.59E-08

chr19	55982001	55987000	1.79E-09
chr19	55983001	55988000	1.28E-09
chr19	55984001	55989000	2.31E-10
chr19	56001001	56006000	5.26E-05
chr19	56002001	56007000	6.29E-06
chr19	56003001	56008000	0.000114
chr19	56058001	56063000	4.47E-07
chr19	56061001	56066000	8.95E-09
chr19	56076001	56081000	6.39E-05
chr19	56113001	56118000	3.03E-06
chr19	56132001	56137000	1.43E-10
chr19	56133001	56138000	7.67E-12
chr19	56134001	56139000	2.40E-11
chr19	56135001	56140000	9.26E-12
chr19	56136001	56141000	1.57E-10
chr19	56139001	56144000	9.13E-07
chr19	56182001	56187000	3.99E-10
chr19	56269001	56274000	2.58E-17
chr19	56299001	56304000	0.000109
chr19	56362001	56367000	1.00E-05
chr19	56363001	56368000	8.86E-07
chr19	56415001	56420000	6.21E-08
chr19	56416001	56421000	2.54E-10
chr19	56417001	56422000	8.32E-06
chr19	56418001	56423000	9.53E-07
chr19	56514001	56519000	1.27E-07
chr19	56515001	56520000	9.07E-06
chr19	56516001	56521000	0.000215
chr19	56536001	56541000	1.63E-05
chr19	56613001	56618000	1.86E-10
chr19	56614001	56619000	5.04E-13
chr19	56615001	56620000	5.97E-10
chr19	56642001	56647000	0.000113
chr19	56674001	56679000	7.28E-06
chr19	56675001	56680000	7.79E-08
chr19	56841001	56846000	3.87E-09
chr19	56868001	56873000	5.14E-09
chr19	56899001	56904000	1.90E-09
chr19	56935001	56940000	1.24E-07
chr19	56955001	56960000	1.72E-06
chr19	56956001	56961000	3.53E-05
chr19	56969001	56974000	2.22E-06
chr19	56974001	56979000	2.05E-05
chr19	56975001	56980000	1.76E-06
chr19	56976001	56981000	1.68E-06
chr19	57000001	57005000	1.64E-08
chr19	57003001	57008000	7.70E-07
chr19	57004001	57009000	1.05E-08

chr19	57005001	57010000	1.61E-08
chr19	57025001	57030000	6.90E-12
chr19	57135001	57140000	7.68E-06
chr19	57136001	57141000	6.59E-05
chr19	57173001	57178000	0.00532
chr19	57174001	57179000	0.00013
chr19	57175001	57180000	7.15E-06
chr19	57176001	57181000	7.15E-06
chr19	57177001	57182000	0.000497
chr19	57178001	57183000	0.000369
chr19	57179001	57184000	0.001341
chr19	57182001	57187000	0.001477
chr19	57183001	57188000	8.13E-10
chr19	57184001	57189000	2.40E-08
chr19	57185001	57190000	1.72E-12
chr19	57186001	57191000	2.96E-13
chr19	57187001	57192000	1.55E-10
chr19	57188001	57193000	3.42E-10
chr19	57189001	57194000	2.26E-07
chr19	57209001	57214000	7.14E-06
chr19	57398001	57403000	1.05E-06
chr19	57399001	57404000	2.41E-08
chr19	57400001	57405000	3.25E-09
chr19	57557001	57562000	1.42E-06
chr19	57569001	57574000	9.69E-07
chr19	57570001	57575000	0.00011
chr19	57634001	57639000	8.51E-11
chr19	57636001	57641000	2.85E-08
chr19	57668001	57673000	2.26E-05
chr19	57696001	57701000	0.000339
chr19	58151001	58156000	5.81E-09
chr19	58152001	58157000	2.14E-10
chr19	58153001	58158000	1.23E-06
chr19	58154001	58159000	1.40E-06
chr19	58155001	58160000	8.73E-08
chr19	58173001	58178000	8.40E-10
chr19	58174001	58179000	1.34E-10
chr19	58175001	58180000	7.88E-10
chr19	58230001	58235000	0.000304
chr19	58231001	58236000	0.000107
chr19	58281001	58286000	2.53E-05
chr19	58434001	58439000	2.43E-11
chr19	58458001	58463000	0.001569
chr19	58459001	58464000	0.00025
chr19	58544001	58549000	1.43E-06
chr19	58545001	58550000	2.63E-07
chr19	58546001	58551000	5.21E-09
chr19	58573001	58578000	1.09E-07

chr19	58574001	58579000	1.36E-08
chr19	58575001	58580000	8.98E-10
chr19	58576001	58581000	5.05E-12
chr19	58577001	58582000	1.56E-10
chr19	58579001	58584000	7.62E-06
chr19	58581001	58586000	0.000198
chr19	58583001	58588000	0.000169
chr19	58584001	58589000	0.001047
chr19	58585001	58590000	0.001169
chr19	58671001	58676000	4.21E-05
chr19	58685001	58690000	4.41E-09
chr19	58826001	58831000	1.38E-07
chr19	58827001	58832000	1.07E-06
chr19	58879001	58884000	5.33E-08
chr19	58880001	58885000	2.90E-07
chr19	58889001	58894000	1.17E-08
chr19	58890001	58895000	0.000422
chr19	58891001	58896000	0.025049
chr19	58937001	58942000	5.05E-05
chr19	58938001	58943000	0.000341
chr19	58947001	58952000	0.000364
chr19	58948001	58953000	0.000129
chr19	58949001	58954000	0.001315
chr19	58950001	58955000	3.58E-05
chr19	58972001	58977000	4.03E-05
chr19	59021001	59026000	0.039169
chr19	59026001	59031000	5.98E-06
chr19	59027001	59032000	3.14E-08
chr19	59029001	59034000	2.91E-06
chr19	59030001	59035000	1.61E-05
chr19	59046001	59051000	0.003194
chr19	59047001	59052000	0.003168
chr19	59056001	59061000	9.61E-08
chr19	59084001	59089000	8.75E-12
chr19	59085001	59090000	1.11E-09
chr19	59086001	59091000	6.51E-11
chr19	59087001	59092000	2.26E-10
chr20	79001	84000	4.35E-05
chr20	90001	95000	5.48E-07
chr20	361001	366000	0.002214
chr20	585001	590000	1.19E-07
chr20	586001	591000	3.17E-08
chr20	587001	592000	4.40E-09
chr20	596001	601000	2.84E-09
chr20	597001	602000	1.59E-07
chr20	598001	603000	9.53E-07
chr20	712001	717000	5.00E-05
chr20	723001	728000	2.78E-05

chr20	733001	738000	1.93E-05
chr20	734001	739000	0.000126
chr20	780001	785000	6.99E-09
chr20	781001	786000	5.94E-07
chr20	897001	902000	1.00E-06
chr20	898001	903000	9.90E-07
chr20	899001	904000	6.05E-07
chr20	900001	905000	1.04E-06
chr20	916001	921000	0.000149
chr20	937001	942000	1.70E-06
chr20	938001	943000	1.01E-07
chr20	939001	944000	1.45E-06
chr20	940001	945000	4.44E-09
chr20	941001	946000	4.35E-09
chr20	962001	967000	2.33E-06
chr20	963001	968000	0.000994
chr20	964001	969000	0.000892
chr20	993001	998000	8.80E-05
chr20	994001	999000	4.05E-06
chr20	1048001	1053000	5.11E-08
chr20	1049001	1054000	1.13E-06
chr20	1506001	1511000	5.18E-10
chr20	1535001	1540000	1.72E-07
chr20	1536001	1541000	1.39E-06
chr20	1591001	1596000	0.044908
chr20	1626001	1631000	1.31E-06
chr20	1628001	1633000	1.13E-06
chr20	1629001	1634000	4.74E-06
chr20	1630001	1635000	2.43E-05
chr20	1631001	1636000	3.45E-06
chr20	1639001	1644000	8.38E-10
chr20	1672001	1677000	5.29E-07
chr20	1673001	1678000	8.02E-07
chr20	1674001	1679000	1.11E-06
chr20	1675001	1680000	1.01E-06
chr20	1676001	1681000	4.19E-07
chr20	1719001	1724000	0.000398
chr20	1720001	1725000	4.96E-05
chr20	1722001	1727000	0.000701
chr20	1723001	1728000	1.19E-07
chr20	1815001	1820000	4.30E-06
chr20	1826001	1831000	4.04E-06
chr20	1957001	1962000	3.09E-06
chr20	2003001	2008000	3.13E-09
chr20	2048001	2053000	1.64E-09
chr20	2142001	2147000	9.40E-09
chr20	2147001	2152000	2.54E-06
chr20	2196001	2201000	3.90E-06

chr20	2197001	2202000	1.77E-05
chr20	2218001	2223000	1.98E-07
chr20	2278001	2283000	1.34E-09
chr20	2279001	2284000	3.25E-11
chr20	2280001	2285000	6.09E-08
chr20	2281001	2286000	0.000336
chr20	2282001	2287000	6.56E-06
chr20	2283001	2288000	0.00013
chr20	2313001	2318000	2.97E-06
chr20	2360001	2365000	2.97E-07
chr20	2361001	2366000	1.82E-05
chr20	2375001	2380000	2.77E-05
chr20	2376001	2381000	1.40E-07
chr20	2377001	2382000	9.22E-08
chr20	2384001	2389000	3.62E-06
chr20	2385001	2390000	1.23E-09
chr20	2386001	2391000	3.82E-05
chr20	2411001	2416000	2.16E-08
chr20	2412001	2417000	4.28E-07
chr20	2413001	2418000	6.04E-06
chr20	2414001	2419000	8.13E-07
chr20	2415001	2420000	4.29E-05
chr20	2504001	2509000	7.15E-06
chr20	2505001	2510000	6.62E-06
chr20	2525001	2530000	3.18E-05
chr20	2526001	2531000	7.04E-07
chr20	2527001	2532000	4.01E-09
chr20	2535001	2540000	5.61E-08
chr20	2539001	2544000	9.07E-06
chr20	2545001	2550000	2.52E-06
chr20	2547001	2552000	3.38E-09
chr20	2548001	2553000	6.11E-07
chr20	2614001	2619000	9.71E-05
chr20	2634001	2639000	3.59E-09
chr20	2672001	2677000	1.20E-09
chr20	2673001	2678000	9.74E-07
chr20	2674001	2679000	1.17E-07
chr20	2675001	2680000	5.78E-07
chr20	2733001	2738000	1.73E-05
chr20	2735001	2740000	4.27E-07
chr20	2736001	2741000	1.94E-08
chr20	2799001	2804000	1.10E-07
chr20	2800001	2805000	9.32E-07
chr20	2808001	2813000	2.07E-06
chr20	2809001	2814000	6.36E-07
chr20	3070001	3075000	0.000344
chr20	3071001	3076000	9.01E-05
chr20	3072001	3077000	0.000431

chr20	3103001	3108000	0.026212
chr20	3146001	3151000	0.015378
chr20	3149001	3154000	0.010022
chr20	3180001	3185000	0.000939
chr20	3195001	3200000	2.43E-11
chr20	3196001	3201000	2.50E-06
chr20	3667001	3672000	7.58E-05
chr20	3668001	3673000	5.15E-05
chr20	3749001	3754000	6.51E-12
chr20	3855001	3860000	0.004881
chr20	3857001	3862000	5.29E-07
chr20	3858001	3863000	1.39E-05
chr20	3994001	3999000	1.72E-07
chr20	4148001	4153000	2.78E-06
chr20	4149001	4154000	2.22E-05
chr20	4195001	4200000	0.000291
chr20	4235001	4240000	2.24E-07
chr20	4236001	4241000	1.43E-08
chr20	4314001	4319000	4.93E-09
chr20	4315001	4320000	2.32E-11
chr20	4316001	4321000	2.69E-14
chr20	4317001	4322000	2.14E-15
chr20	4318001	4323000	9.57E-16
chr20	4324001	4329000	2.31E-08
chr20	4326001	4331000	7.06E-06
chr20	4380001	4385000	2.05E-07
chr20	4382001	4387000	9.56E-06
chr20	4479001	4484000	5.54E-11
chr20	4480001	4485000	1.79E-12
chr20	4481001	4486000	2.52E-13
chr20	4482001	4487000	5.88E-18
chr20	4519001	4524000	1.36E-05
chr20	4590001	4595000	5.72E-05
chr20	4592001	4597000	1.79E-06
chr20	4593001	4598000	2.14E-06
chr20	4818001	4823000	0.000247
chr20	5224001	5229000	2.21E-09
chr20	5225001	5230000	2.78E-11
chr20	5226001	5231000	2.29E-10
chr20	5325001	5330000	1.10E-09
chr20	5406001	5411000	0.000169
chr20	5407001	5412000	5.81E-06
chr20	5408001	5413000	5.24E-08
chr20	5409001	5414000	2.53E-07
chr20	5411001	5416000	1.69E-08
chr20	5476001	5481000	0.000336
chr20	5778001	5783000	4.91E-05
chr20	6290001	6295000	0.001269

chr20	6781001	6786000	4.70E-08
chr20	7576001	7581000	1.18E-06
chr20	7577001	7582000	8.08E-08
chr20	7578001	7583000	4.43E-05
chr20	7764001	7769000	2.61E-12
chr20	9838001	9843000	6.19E-08
chr20	11708001	11713000	2.19E-05
chr20	11760001	11765000	4.79E-12
chr20	11761001	11766000	6.91E-07
chr20	11762001	11767000	5.50E-08
chr20	11983001	11988000	1.03E-08
chr20	12012001	12017000	7.08E-07
chr20	12066001	12071000	1.81E-14
chr20	12236001	12241000	6.35E-11
chr20	12237001	12242000	6.53E-14
chr20	12238001	12243000	1.18E-15
chr20	12239001	12244000	1.63E-16
chr20	12240001	12245000	1.49E-14
chr20	12253001	12258000	1.95E-05
chr20	12288001	12293000	7.35E-12
chr20	12405001	12410000	0.000224
chr20	12704001	12709000	1.71E-07
chr20	12800001	12805000	9.74E-10
chr20	12947001	12952000	2.90E-08
chr20	12948001	12953000	1.30E-10
chr20	12949001	12954000	7.62E-14
chr20	13834001	13839000	1.72E-07
chr20	13851001	13856000	4.39E-12
chr20	13852001	13857000	1.68E-10
chr20	13869001	13874000	1.39E-06
chr20	13896001	13901000	9.41E-05
chr20	13912001	13917000	7.44E-10
chr20	13933001	13938000	5.84E-06
chr20	14000001	14005000	3.79E-08
chr20	14061001	14066000	0.00111
chr20	14086001	14091000	1.61E-08
chr20	14087001	14092000	1.40E-07
chr20	14145001	14150000	1.20E-05
chr20	14146001	14151000	8.74E-07
chr20	14147001	14152000	1.46E-07
chr20	14265001	14270000	5.88E-07
chr20	14266001	14271000	8.90E-08
chr20	14730001	14735000	2.13E-07
chr20	15043001	15048000	7.25E-10
chr20	15045001	15050000	1.03E-07
chr20	15046001	15051000	1.25E-07
chr20	15509001	15514000	5.80E-09
chr20	15589001	15594000	3.50E-11

chr20	15634001	15639000	5.98E-12
chr20	15636001	15641000	2.14E-09
chr20	15657001	15662000	8.66E-05
chr20	15666001	15671000	3.73E-06
chr20	15843001	15848000	1.07E-09
chr20	15844001	15849000	2.78E-09
chr20	15845001	15850000	3.67E-10
chr20	16126001	16131000	2.30E-05
chr20	16127001	16132000	4.24E-06
chr20	16237001	16242000	3.58E-05
chr20	16238001	16243000	1.45E-08
chr20	16648001	16653000	2.16E-07
chr20	16699001	16704000	0.001216
chr20	16707001	16712000	3.70E-10
chr20	16708001	16713000	1.13E-11
chr20	16709001	16714000	1.20E-08
chr20	16735001	16740000	1.30E-08
chr20	17055001	17060000	4.72E-07
chr20	17878001	17883000	3.59E-06
chr20	17910001	17915000	1.72E-06
chr20	17972001	17977000	9.51E-05
chr20	18037001	18042000	1.78E-07
chr20	18038001	18043000	4.13E-07
chr20	18206001	18211000	5.41E-07
chr20	18310001	18315000	3.50E-05
chr20	18333001	18338000	0.000107
chr20	18706001	18711000	0.001008
chr20	18857001	18862000	3.13E-05
chr20	18963001	18968000	5.80E-06
chr20	19619001	19624000	9.72E-09
chr20	19995001	20000000	2.09E-28
chr20	19996001	20001000	1.69E-32
chr20	19997001	20002000	4.09E-37
chr20	19998001	20003000	2.09E-28
chr20	20099001	20104000	3.94E-12
chr20	20291001	20296000	1.33E-06
chr20	20876001	20881000	1.50E-05
chr20	20964001	20969000	2.74E-08
chr20	21009001	21014000	2.13E-06
chr20	21023001	21028000	1.42E-06
chr20	21036001	21041000	9.75E-14
chr20	21587001	21592000	0.000141
chr20	21708001	21713000	7.94E-17
chr20	21709001	21714000	4.38E-13
chr20	21715001	21720000	3.34E-10
chr20	21859001	21864000	5.90E-09
chr20	21860001	21865000	4.00E-06
chr20	21861001	21866000	4.11E-05

chr20	21941001	21946000	1.52E-05
chr20	21942001	21947000	1.52E-05
chr20	21947001	21952000	4.40E-08
chr20	21948001	21953000	1.62E-07
chr20	21962001	21967000	1.00E-06
chr20	21963001	21968000	2.11E-06
chr20	21971001	21976000	9.93E-08
chr20	21972001	21977000	2.03E-06
chr20	21973001	21978000	6.58E-06
chr20	21974001	21979000	1.29E-06
chr20	21992001	21997000	2.40E-08
chr20	22015001	22020000	8.50E-13
chr20	22016001	22021000	2.56E-14
chr20	22017001	22022000	2.37E-14
chr20	22018001	22023000	6.92E-14
chr20	22070001	22075000	3.40E-06
chr20	22100001	22105000	4.98E-06
chr20	22168001	22173000	4.82E-06
chr20	22170001	22175000	7.13E-07
chr20	22271001	22276000	1.31E-07
chr20	22273001	22278000	1.37E-10
chr20	22469001	22474000	1.93E-07
chr20	22470001	22475000	1.10E-07
chr20	22647001	22652000	0.000782
chr20	22648001	22653000	0.000539
chr20	22763001	22768000	3.61E-10
chr20	22764001	22769000	1.11E-09
chr20	22765001	22770000	1.03E-10
chr20	22766001	22771000	7.31E-09
chr20	23012001	23017000	0.001603
chr20	23274001	23279000	6.80E-07
chr20	23275001	23280000	4.98E-08
chr20	23276001	23281000	4.81E-08
chr20	23673001	23678000	7.02E-11
chr20	23694001	23699000	2.88E-08
chr20	23714001	23719000	0.000127
chr20	23715001	23720000	2.52E-06
chr20	23726001	23731000	5.39E-06
chr20	23727001	23732000	7.29E-06
chr20	23728001	23733000	3.78E-07
chr20	23729001	23734000	1.20E-06
chr20	23732001	23737000	3.42E-07
chr20	23740001	23745000	1.62E-06
chr20	23741001	23746000	3.82E-06
chr20	23742001	23747000	2.95E-05
chr20	23743001	23748000	0.003119
chr20	23805001	23810000	8.72E-06
chr20	23840001	23845000	2.40E-09

chr20	23841001	23846000	1.00E-07
chr20	24239001	24244000	1.88E-07
chr20	24240001	24245000	2.24E-07
chr20	24241001	24246000	1.61E-07
chr20	24393001	24398000	9.70E-10
chr20	24477001	24482000	6.43E-10
chr20	24478001	24483000	8.22E-10
chr20	24479001	24484000	8.28E-14
chr20	24480001	24485000	4.51E-07
chr20	24589001	24594000	3.82E-06
chr20	24919001	24924000	0.000113
chr20	24920001	24925000	5.89E-05
chr20	25601001	25606000	2.09E-14
chr20	26319001	26324000	0.009155
chr20	29849001	29854000	6.43E-11
chr20	29934001	29939000	0.001142
chr20	29936001	29941000	0.000143
chr20	29938001	29943000	6.70E-05
chr20	29939001	29944000	2.65E-07
chr20	30025001	30030000	0.006298
chr20	30063001	30068000	4.35E-07
chr20	30064001	30069000	1.63E-06
chr20	30153001	30158000	0.021535
chr20	30163001	30168000	0.00189
chr20	30249001	30254000	0.002031
chr20	30409001	30414000	2.17E-06
chr20	30471001	30476000	1.64E-05
chr20	30472001	30477000	1.09E-06
chr20	30473001	30478000	2.31E-05
chr20	30605001	30610000	4.86E-08
chr20	30606001	30611000	1.50E-07
chr20	30613001	30618000	0.000122
chr20	30614001	30619000	8.22E-06
chr20	30647001	30652000	0.004287
chr20	30648001	30653000	0.000731
chr20	30677001	30682000	1.08E-06
chr20	31106001	31111000	1.87E-05
chr20	31107001	31112000	2.35E-05
chr20	31108001	31113000	4.89E-08
chr20	31110001	31115000	0.000144
chr20	31111001	31116000	6.07E-06
chr20	31112001	31117000	2.60E-06
chr20	31184001	31189000	6.76E-05
chr20	31185001	31190000	2.05E-05
chr20	31202001	31207000	0.000811
chr20	31229001	31234000	7.97E-06
chr20	31230001	31235000	2.13E-07
chr20	31231001	31236000	0.000331

chr20	31232001	31237000	8.23E-06
chr20	31364001	31369000	0.014261
chr20	31519001	31524000	1.99E-06
chr20	31551001	31556000	5.83E-06
chr20	31607001	31612000	5.79E-06
chr20	31608001	31613000	9.90E-08
chr20	31609001	31614000	1.86E-06
chr20	31616001	31621000	7.64E-05
chr20	31618001	31623000	8.31E-07
chr20	31642001	31647000	4.73E-07
chr20	31643001	31648000	3.75E-06
chr20	31674001	31679000	2.22E-07
chr20	31717001	31722000	2.98E-12
chr20	31718001	31723000	7.53E-11
chr20	31761001	31766000	1.21E-07
chr20	31779001	31784000	3.66E-10
chr20	31780001	31785000	2.93E-10
chr20	31781001	31786000	5.96E-10
chr20	31841001	31846000	1.21E-07
chr20	31933001	31938000	0.000172
chr20	31984001	31989000	1.64E-06
chr20	32028001	32033000	0.001193
chr20	32031001	32036000	2.57E-06
chr20	32032001	32037000	1.64E-05
chr20	32065001	32070000	1.96E-05
chr20	32286001	32291000	1.18E-08
chr20	32287001	32292000	1.09E-07
chr20	32288001	32293000	5.92E-09
chr20	32289001	32294000	0.001978
chr20	32448001	32453000	2.79E-06
chr20	32449001	32454000	4.35E-07
chr20	32450001	32455000	6.32E-08
chr20	32823001	32828000	3.09E-05
chr20	33100001	33105000	2.86E-15
chr20	33130001	33135000	1.53E-07
chr20	33131001	33136000	6.06E-06
chr20	33132001	33137000	1.93E-08
chr20	33133001	33138000	3.78E-08
chr20	33262001	33267000	3.79E-08
chr20	33263001	33268000	1.49E-09
chr20	33264001	33269000	1.40E-10
chr20	33265001	33270000	1.47E-08
chr20	33297001	33302000	0.000253
chr20	33445001	33450000	0.047238
chr20	33570001	33575000	1.98E-11
chr20	33571001	33576000	1.79E-05
chr20	33782001	33787000	0.000192
chr20	34129001	34134000	1.03E-05

chr20	34162001	34167000	0.001676
chr20	34379001	34384000	0.00055
chr20	34671001	34676000	8.51E-07
chr20	34852001	34857000	0.000335
chr20	34854001	34859000	4.36E-05
chr20	34863001	34868000	3.42E-07
chr20	34864001	34869000	3.92E-08
chr20	34865001	34870000	3.86E-10
chr20	34866001	34871000	5.59E-13
chr20	34867001	34872000	3.74E-12
chr20	34893001	34898000	0.000315
chr20	34894001	34899000	0.000357
chr20	34895001	34900000	0.000357
chr20	34973001	34978000	2.08E-08
chr20	34974001	34979000	2.34E-08
chr20	34975001	34980000	5.74E-06
chr20	34978001	34983000	0.009708
chr20	35014001	35019000	1.51E-07
chr20	35016001	35021000	3.67E-05
chr20	35043001	35048000	8.49E-06
chr20	35044001	35049000	3.48E-05
chr20	35045001	35050000	7.92E-07
chr20	35046001	35051000	5.99E-06
chr20	35047001	35052000	2.75E-09
chr20	35048001	35053000	3.17E-07
chr20	35049001	35054000	2.51E-07
chr20	35061001	35066000	3.19E-11
chr20	35062001	35067000	7.37E-09
chr20	35063001	35068000	4.53E-05
chr20	35069001	35074000	8.57E-07
chr20	35070001	35075000	3.44E-06
chr20	35071001	35076000	2.53E-07
chr20	35072001	35077000	1.53E-07
chr20	35085001	35090000	0.006832
chr20	35086001	35091000	0.002416
chr20	35169001	35174000	1.19E-07
chr20	35170001	35175000	3.69E-08
chr20	35303001	35308000	3.21E-08
chr20	35341001	35346000	1.55E-06
chr20	35487001	35492000	7.67E-06
chr20	35734001	35739000	3.93E-08
chr20	35735001	35740000	9.64E-09
chr20	35736001	35741000	4.99E-08
chr20	36011001	36016000	0.004507
chr20	36078001	36083000	8.58E-05
chr20	36152001	36157000	1.56E-07
chr20	36160001	36165000	4.09E-09
chr20	36161001	36166000	6.80E-08

chr20	36162001	36167000	3.09E-06
chr20	36163001	36168000	2.62E-05
chr20	36164001	36169000	5.95E-09
chr20	36230001	36235000	4.18E-09
chr20	36231001	36236000	3.97E-09
chr20	36232001	36237000	1.68E-11
chr20	36233001	36238000	1.65E-09
chr20	36234001	36239000	9.06E-09
chr20	36304001	36309000	0.000747
chr20	36530001	36535000	6.76E-05
chr20	37265001	37270000	2.19E-08
chr20	37330001	37335000	1.67E-07
chr20	37331001	37336000	3.92E-06
chr20	37332001	37337000	3.23E-07
chr20	37338001	37343000	7.63E-06
chr20	37339001	37344000	3.45E-06
chr20	37435001	37440000	1.03E-05
chr20	37436001	37441000	7.34E-05
chr20	37501001	37506000	6.12E-05
chr20	37502001	37507000	6.39E-07
chr20	37503001	37508000	4.44E-08
chr20	37504001	37509000	3.64E-09
chr20	37505001	37510000	2.14E-10
chr20	37507001	37512000	4.82E-08
chr20	37517001	37522000	6.49E-05
chr20	37520001	37525000	0.001129
chr20	37521001	37526000	0.000109
chr20	37691001	37696000	0.000101
chr20	37709001	37714000	6.36E-07
chr20	37710001	37715000	3.02E-07
chr20	37711001	37716000	5.45E-06
chr20	37800001	37805000	1.05E-07
chr20	37801001	37806000	3.83E-06
chr20	37812001	37817000	0.000564
chr20	37857001	37862000	7.56E-08
chr20	37870001	37875000	1.05E-05
chr20	37872001	37877000	3.36E-05
chr20	37931001	37936000	7.17E-09
chr20	37932001	37937000	7.07E-08
chr20	37933001	37938000	2.08E-10
chr20	37934001	37939000	7.99E-13
chr20	37935001	37940000	2.43E-09
chr20	37986001	37991000	1.40E-08
chr20	37987001	37992000	4.33E-09
chr20	37988001	37993000	1.09E-07
chr20	38003001	38008000	4.26E-07
chr20	38009001	38014000	8.81E-07
chr20	38010001	38015000	1.69E-07

chr20	38028001	38033000	5.36E-06
chr20	38040001	38045000	2.45E-06
chr20	38041001	38046000	5.98E-08
chr20	38042001	38047000	3.76E-11
chr20	38043001	38048000	9.81E-11
chr20	38044001	38049000	8.24E-10
chr20	38045001	38050000	2.65E-08
chr20	38104001	38109000	1.01E-06
chr20	38106001	38111000	2.50E-07
chr20	38128001	38133000	2.58E-07
chr20	38129001	38134000	1.50E-07
chr20	38130001	38135000	6.32E-08
chr20	38189001	38194000	1.24E-08
chr20	38190001	38195000	7.90E-10
chr20	38199001	38204000	1.93E-08
chr20	38302001	38307000	1.65E-08
chr20	38333001	38338000	1.30E-09
chr20	38334001	38339000	5.32E-07
chr20	38336001	38341000	1.91E-10
chr20	38340001	38345000	1.03E-12
chr20	38355001	38360000	1.01E-09
chr20	38392001	38397000	2.22E-07
chr20	38422001	38427000	1.50E-06
chr20	38592001	38597000	4.90E-07
chr20	38593001	38598000	5.22E-06
chr20	38603001	38608000	1.79E-07
chr20	38604001	38609000	6.98E-09
chr20	38605001	38610000	1.41E-07
chr20	38606001	38611000	1.59E-07
chr20	38683001	38688000	3.06E-08
chr20	38686001	38691000	8.76E-05
chr20	38687001	38692000	2.87E-05
chr20	38709001	38714000	3.64E-08
chr20	38772001	38777000	7.68E-09
chr20	38773001	38778000	4.28E-10
chr20	38849001	38854000	4.60E-07
chr20	38999001	39004000	8.15E-10
chr20	39022001	39027000	1.87E-09
chr20	39023001	39028000	3.69E-06
chr20	39024001	39029000	1.98E-06
chr20	39025001	39030000	4.90E-07
chr20	39026001	39031000	2.43E-07
chr20	39028001	39033000	1.20E-08
chr20	39050001	39055000	0.001501
chr20	39052001	39057000	1.03E-06
chr20	39054001	39059000	3.20E-06
chr20	39145001	39150000	3.41E-07
chr20	39146001	39151000	1.13E-08

chr20	39147001	39152000	6.85E-06
chr20	39158001	39163000	5.53E-05
chr20	39159001	39164000	2.30E-06
chr20	39181001	39186000	2.87E-05
chr20	39182001	39187000	1.76E-07
chr20	39183001	39188000	1.13E-12
chr20	39184001	39189000	1.03E-09
chr20	39185001	39190000	2.01E-08
chr20	39190001	39195000	1.28E-06
chr20	39298001	39303000	2.33E-05
chr20	39375001	39380000	1.73E-09
chr20	39376001	39381000	4.46E-08
chr20	39377001	39382000	1.32E-08
chr20	39378001	39383000	4.95E-07
chr20	39384001	39389000	1.26E-07
chr20	39388001	39393000	1.02E-10
chr20	39429001	39434000	2.82E-06
chr20	39435001	39440000	3.92E-07
chr20	39444001	39449000	1.15E-05
chr20	39445001	39450000	5.30E-07
chr20	39463001	39468000	1.70E-05
chr20	39464001	39469000	7.96E-06
chr20	39465001	39470000	0.000486
chr20	39466001	39471000	0.001368
chr20	39467001	39472000	0.000492
chr20	39468001	39473000	0.000663
chr20	39469001	39474000	6.62E-06
chr20	39470001	39475000	4.57E-09
chr20	39471001	39476000	3.47E-13
chr20	39472001	39477000	4.51E-14
chr20	39473001	39478000	3.38E-13
chr20	39491001	39496000	2.84E-06
chr20	39513001	39518000	1.99E-06
chr20	39514001	39519000	3.39E-06
chr20	39527001	39532000	3.31E-06
chr20	39529001	39534000	2.29E-05
chr20	39536001	39541000	1.32E-09
chr20	39538001	39543000	3.74E-11
chr20	39539001	39544000	5.78E-13
chr20	39946001	39951000	6.48E-08
chr20	39964001	39969000	2.76E-06
chr20	40352001	40357000	7.42E-07
chr20	40379001	40384000	6.34E-11
chr20	40380001	40385000	9.16E-11
chr20	40381001	40386000	4.62E-11
chr20	40425001	40430000	4.26E-07
chr20	40545001	40550000	1.77E-07
chr20	40586001	40591000	2.31E-05

chr20	40644001	40649000	5.87E-05
chr20	40645001	40650000	1.31E-05
chr20	40646001	40651000	0.000164
chr20	40727001	40732000	3.00E-06
chr20	40768001	40773000	0.001147
chr20	40826001	40831000	4.45E-05
chr20	40864001	40869000	1.80E-07
chr20	40865001	40870000	1.18E-07
chr20	40866001	40871000	9.91E-07
chr20	40913001	40918000	5.17E-07
chr20	41364001	41369000	1.69E-05
chr20	41648001	41653000	2.29E-05
chr20	41649001	41654000	8.10E-07
chr20	41709001	41714000	1.10E-07
chr20	41724001	41729000	0.00013
chr20	41901001	41906000	4.06E-06
chr20	41903001	41908000	2.87E-07
chr20	41904001	41909000	2.32E-06
chr20	41910001	41915000	2.04E-05
chr20	41923001	41928000	2.22E-14
chr20	41924001	41929000	6.42E-14
chr20	41925001	41930000	1.99E-08
chr20	41926001	41931000	3.66E-06
chr20	42004001	42009000	4.84E-08
chr20	42006001	42011000	3.54E-08
chr20	42022001	42027000	0.00016
chr20	42023001	42028000	0.000162
chr20	42039001	42044000	8.67E-07
chr20	42040001	42045000	4.42E-05
chr20	42272001	42277000	0.014618
chr20	42344001	42349000	0.002353
chr20	42346001	42351000	0.000108
chr20	42347001	42352000	7.94E-08
chr20	42348001	42353000	5.19E-07
chr20	42401001	42406000	4.80E-08
chr20	42402001	42407000	2.14E-12
chr20	42403001	42408000	1.18E-11
chr20	42404001	42409000	6.83E-08
chr20	42405001	42410000	2.40E-07
chr20	42434001	42439000	1.21E-06
chr20	42435001	42440000	4.35E-06
chr20	42436001	42441000	0.000402
chr20	42495001	42500000	4.34E-09
chr20	42496001	42501000	8.79E-08
chr20	42497001	42502000	1.06E-08
chr20	42526001	42531000	1.45E-06
chr20	42527001	42532000	1.73E-07
chr20	42528001	42533000	1.30E-05

chr20	42529001	42534000	6.02E-12
chr20	42530001	42535000	1.10E-07
chr20	42678001	42683000	7.53E-11
chr20	42679001	42684000	1.71E-11
chr20	42680001	42685000	2.45E-06
chr20	42734001	42739000	3.50E-05
chr20	42743001	42748000	0.000225
chr20	42744001	42749000	0.001946
chr20	42745001	42750000	0.000312
chr20	42855001	42860000	2.40E-13
chr20	42856001	42861000	4.06E-14
chr20	42857001	42862000	3.02E-16
chr20	42858001	42863000	5.33E-12
chr20	42859001	42864000	4.49E-07
chr20	42868001	42873000	5.76E-08
chr20	42869001	42874000	3.08E-10
chr20	42870001	42875000	2.67E-08
chr20	42903001	42908000	7.01E-08
chr20	43033001	43038000	1.47E-05
chr20	43034001	43039000	3.73E-07
chr20	43035001	43040000	2.23E-05
chr20	43066001	43071000	1.18E-06
chr20	43067001	43072000	1.79E-05
chr20	43156001	43161000	5.55E-10
chr20	43157001	43162000	5.03E-10
chr20	43343001	43348000	3.71E-05
chr20	43471001	43476000	8.43E-10
chr20	43481001	43486000	3.51E-06
chr20	43483001	43488000	4.46E-06
chr20	43736001	43741000	1.11E-07
chr20	43910001	43915000	2.09E-08
chr20	43911001	43916000	9.69E-08
chr20	43914001	43919000	1.54E-06
chr20	44163001	44168000	1.70E-07
chr20	44228001	44233000	9.78E-10
chr20	44255001	44260000	6.09E-07
chr20	44296001	44301000	0.000268
chr20	44297001	44302000	9.97E-05
chr20	44298001	44303000	4.83E-07
chr20	44299001	44304000	1.50E-08
chr20	44300001	44305000	1.41E-10
chr20	44301001	44306000	1.28E-09
chr20	44302001	44307000	4.04E-10
chr20	44329001	44334000	0.000424
chr20	44359001	44364000	0.014661
chr20	44360001	44365000	0.005172
chr20	44372001	44377000	7.11E-09
chr20	44373001	44378000	3.13E-08

chr20	44374001	44379000	2.96E-09
chr20	44375001	44380000	2.77E-07
chr20	44376001	44381000	1.43E-08
chr20	44377001	44382000	0.000377
chr20	44534001	44539000	5.02E-07
chr20	44535001	44540000	1.41E-09
chr20	44536001	44541000	1.33E-07
chr20	44537001	44542000	3.24E-09
chr20	44558001	44563000	1.74E-05
chr20	44559001	44564000	6.03E-05
chr20	44613001	44618000	5.32E-08
chr20	44614001	44619000	0.000151
chr20	44616001	44621000	0.000116
chr20	44887001	44892000	3.79E-06
chr20	44888001	44893000	9.01E-08
chr20	44898001	44903000	7.36E-05
chr20	44899001	44904000	1.28E-08
chr20	45075001	45080000	3.27E-08
chr20	45076001	45081000	1.05E-12
chr20	45077001	45082000	1.61E-09
chr20	45078001	45083000	2.60E-09
chr20	45079001	45084000	2.37E-08
chr20	45157001	45162000	1.17E-07
chr20	45158001	45163000	1.15E-07
chr20	45159001	45164000	2.66E-07
chr20	45160001	45165000	9.97E-09
chr20	45161001	45166000	5.98E-07
chr20	45231001	45236000	2.89E-06
chr20	45232001	45237000	9.75E-08
chr20	45265001	45270000	2.08E-06
chr20	45266001	45271000	5.08E-07
chr20	45338001	45343000	0.005485
chr20	45945001	45950000	1.61E-08
chr20	45946001	45951000	6.95E-09
chr20	45947001	45952000	7.33E-09
chr20	45948001	45953000	1.91E-07
chr20	45986001	45991000	0.000151
chr20	45989001	45994000	1.27E-05
chr20	46008001	46013000	0.000105
chr20	46009001	46014000	0.001292
chr20	46010001	46015000	1.13E-06
chr20	46011001	46016000	4.66E-09
chr20	46337001	46342000	6.30E-08
chr20	46338001	46343000	9.41E-10
chr20	46339001	46344000	4.72E-08
chr20	46409001	46414000	1.31E-09
chr20	46410001	46415000	4.04E-06
chr20	46466001	46471000	0.001756

chr20	46669001	46674000	4.54E-14
chr20	46670001	46675000	3.85E-10
chr20	46676001	46681000	5.59E-07
chr20	46677001	46682000	0.000129
chr20	46748001	46753000	7.15E-09
chr20	46850001	46855000	9.22E-07
chr20	46851001	46856000	1.02E-06
chr20	46853001	46858000	1.13E-06
chr20	46854001	46859000	1.14E-06
chr20	46900001	46905000	2.96E-05
chr20	46901001	46906000	6.14E-05
chr20	46943001	46948000	3.91E-09
chr20	46966001	46971000	2.61E-08
chr20	46980001	46985000	1.79E-05
chr20	46981001	46986000	0.000166
chr20	47034001	47039000	2.71E-08
chr20	47035001	47040000	5.81E-09
chr20	47081001	47086000	1.24E-07
chr20	47103001	47108000	4.35E-10
chr20	47105001	47110000	1.94E-09
chr20	47134001	47139000	3.44E-05
chr20	47135001	47140000	0.000269
chr20	47165001	47170000	5.17E-16
chr20	47166001	47171000	9.25E-12
chr20	47225001	47230000	5.46E-12
chr20	47226001	47231000	1.55E-11
chr20	47227001	47232000	9.47E-12
chr20	47269001	47274000	3.10E-06
chr20	47270001	47275000	5.71E-08
chr20	47326001	47331000	5.52E-09
chr20	47349001	47354000	0.000395
chr20	47482001	47487000	8.84E-06
chr20	47483001	47488000	6.05E-07
chr20	47484001	47489000	5.74E-07
chr20	47485001	47490000	1.05E-07
chr20	47514001	47519000	6.56E-06
chr20	47515001	47520000	4.53E-05
chr20	47516001	47521000	2.23E-05
chr20	47521001	47526000	0.001075
chr20	47522001	47527000	4.79E-06
chr20	47523001	47528000	8.44E-08
chr20	47524001	47529000	4.60E-06
chr20	47525001	47530000	7.19E-05
chr20	47835001	47840000	2.00E-10
chr20	47893001	47898000	3.26E-07
chr20	47927001	47932000	2.42E-07
chr20	47928001	47933000	1.48E-06
chr20	47929001	47934000	7.55E-10

chr20	47930001	47935000	1.69E-09
chr20	47931001	47936000	8.57E-11
chr20	47933001	47938000	4.77E-09
chr20	47947001	47952000	0.000177
chr20	47953001	47958000	1.63E-05
chr20	47954001	47959000	6.62E-09
chr20	47955001	47960000	6.51E-10
chr20	48024001	48029000	6.05E-07
chr20	48025001	48030000	2.30E-08
chr20	48069001	48074000	1.45E-09
chr20	48070001	48075000	3.02E-11
chr20	48071001	48076000	1.59E-11
chr20	48072001	48077000	1.47E-11
chr20	48073001	48078000	1.68E-13
chr20	48074001	48079000	2.49E-13
chr20	48113001	48118000	2.08E-06
chr20	48114001	48119000	5.88E-06
chr20	48126001	48131000	6.46E-10
chr20	48133001	48138000	5.78E-06
chr20	48227001	48232000	5.27E-05
chr20	48233001	48238000	0.004026
chr20	48389001	48394000	0.000276
chr20	48684001	48689000	7.07E-07
chr20	48685001	48690000	6.22E-06
chr20	48686001	48691000	8.59E-06
chr20	48804001	48809000	9.46E-06
chr20	48805001	48810000	0.001168
chr20	48808001	48813000	0.000566
chr20	48883001	48888000	0.000284
chr20	48884001	48889000	0.000798
chr20	49079001	49084000	5.53E-07
chr20	49238001	49243000	1.18E-05
chr20	49239001	49244000	1.76E-05
chr20	49591001	49596000	4.42E-05
chr20	49592001	49597000	5.36E-10
chr20	49593001	49598000	7.75E-10
chr20	49600001	49605000	0.000101
chr20	49619001	49624000	0.000514
chr20	49621001	49626000	2.71E-07
chr20	49688001	49693000	1.01E-05
chr20	49725001	49730000	1.69E-08
chr20	49726001	49731000	1.52E-09
chr20	49727001	49732000	2.12E-11
chr20	49728001	49733000	5.64E-08
chr20	49868001	49873000	0.000107
chr20	49870001	49875000	6.85E-06
chr20	49871001	49876000	1.96E-05
chr20	49872001	49877000	2.92E-06

chr20	49873001	49878000	1.65E-05
chr20	49974001	49979000	0.00028
chr20	49997001	50002000	6.84E-07
chr20	50112001	50117000	3.62E-10
chr20	50155001	50160000	5.17E-10
chr20	50171001	50176000	1.42E-13
chr20	50172001	50177000	5.02E-10
chr20	50173001	50178000	1.50E-12
chr20	50174001	50179000	3.41E-10
chr20	50297001	50302000	5.66E-09
chr20	50379001	50384000	2.68E-06
chr20	50380001	50385000	1.39E-06
chr20	50381001	50386000	1.72E-09
chr20	50382001	50387000	3.69E-07
chr20	50440001	50445000	0.005737
chr20	50544001	50549000	1.29E-06
chr20	50579001	50584000	6.36E-12
chr20	50602001	50607000	0.000553
chr20	50604001	50609000	0.000557
chr20	50673001	50678000	6.83E-07
chr20	50675001	50680000	6.00E-10
chr20	50693001	50698000	2.60E-06
chr20	50815001	50820000	1.87E-08
chr20	50816001	50821000	6.83E-10
chr20	50817001	50822000	1.91E-07
chr20	50819001	50824000	0.006571
chr20	51110001	51115000	1.05E-14
chr20	51111001	51116000	4.21E-15
chr20	52137001	52142000	1.03E-05
chr20	52356001	52361000	3.46E-09
chr20	53088001	53093000	1.27E-16
chr20	53089001	53094000	9.09E-14
chr20	53470001	53475000	1.49E-09
chr20	53521001	53526000	2.59E-09
chr20	53522001	53527000	1.16E-07
chr20	53529001	53534000	1.80E-13
chr20	53550001	53555000	1.52E-10
chr20	53586001	53591000	3.53E-09
chr20	53596001	53601000	1.49E-10
chr20	53626001	53631000	1.49E-09
chr20	53627001	53632000	8.52E-11
chr20	53699001	53704000	0.000144
chr20	53800001	53805000	1.29E-08
chr20	53801001	53806000	4.71E-10
chr20	53818001	53823000	2.26E-08
chr20	53819001	53824000	2.39E-05
chr20	54045001	54050000	1.77E-08
chr20	54102001	54107000	1.30E-05

chr20	54245001	54250000	3.60E-09
chr20	54247001	54252000	2.41E-11
chr20	54260001	54265000	1.02E-06
chr20	54286001	54291000	9.46E-09
chr20	54287001	54292000	2.29E-09
chr20	54288001	54293000	4.45E-10
chr20	54595001	54600000	4.90E-08
chr20	54599001	54604000	7.82E-10
chr20	54642001	54647000	0.000113
chr20	54661001	54666000	5.95E-05
chr20	54668001	54673000	1.51E-05
chr20	54669001	54674000	2.48E-06
chr20	54755001	54760000	2.03E-08
chr20	54772001	54777000	8.17E-05
chr20	54842001	54847000	0.000152
chr20	54906001	54911000	1.67E-08
chr20	54907001	54912000	3.95E-09
chr20	54908001	54913000	8.69E-08
chr20	54929001	54934000	3.91E-06
chr20	55118001	55123000	3.08E-07
chr20	55149001	55154000	2.01E-05
chr20	55150001	55155000	1.71E-05
chr20	55151001	55156000	6.60E-07
chr20	55152001	55157000	0.000118
chr20	55283001	55288000	0.000896
chr20	55338001	55343000	6.42E-06
chr20	55822001	55827000	2.39E-05
chr20	55989001	55994000	8.34E-06
chr20	56280001	56285000	4.87E-09
chr20	56281001	56286000	2.27E-09
chr20	56282001	56287000	1.60E-12
chr20	56283001	56288000	2.11E-09
chr20	56284001	56289000	0.000171
chr20	56469001	56474000	3.03E-05
chr20	56538001	56543000	3.36E-07
chr20	56539001	56544000	2.85E-06
chr20	56540001	56545000	4.43E-07
chr20	56752001	56757000	2.91E-07
chr20	56753001	56758000	4.34E-08
chr20	56754001	56759000	1.36E-05
chr20	57093001	57098000	2.91E-06
chr20	57141001	57146000	2.85E-08
chr20	57153001	57158000	1.93E-12
chr20	57185001	57190000	1.32E-09
chr20	57186001	57191000	2.79E-09
chr20	57210001	57215000	1.71E-05
chr20	57211001	57216000	1.78E-06
chr20	57316001	57321000	3.42E-05

chr20	57317001	57322000	1.70E-08
chr20	57536001	57541000	8.05E-08
chr20	57550001	57555000	1.86E-05
chr20	57643001	57648000	7.84E-08
chr20	57644001	57649000	1.41E-08
chr20	57650001	57655000	5.70E-11
chr20	57651001	57656000	2.98E-08
chr20	57677001	57682000	3.17E-07
chr20	57678001	57683000	2.60E-08
chr20	57679001	57684000	1.53E-08
chr20	57680001	57685000	2.48E-09
chr20	57681001	57686000	1.63E-06
chr20	57695001	57700000	7.97E-09
chr20	57701001	57706000	7.26E-10
chr20	57702001	57707000	8.01E-12
chr20	57703001	57708000	2.18E-12
chr20	57704001	57709000	3.29E-11
chr20	57739001	57744000	2.31E-14
chr20	57758001	57763000	2.36E-06
chr20	57796001	57801000	6.33E-19
chr20	57797001	57802000	2.15E-22
chr20	57798001	57803000	8.54E-13
chr20	57850001	57855000	1.72E-14
chr20	57851001	57856000	2.99E-19
chr20	57852001	57857000	5.27E-10
chr20	57853001	57858000	2.53E-14
chr20	57854001	57859000	5.06E-14
chr20	57857001	57862000	9.84E-09
chr20	57918001	57923000	5.09E-07
chr20	57922001	57927000	8.57E-11
chr20	57937001	57942000	7.62E-09
chr20	57938001	57943000	8.22E-09
chr20	57958001	57963000	2.32E-05
chr20	58094001	58099000	1.70E-05
chr20	58134001	58139000	1.98E-11
chr20	58135001	58140000	3.04E-11
chr20	58136001	58141000	3.75E-08
chr20	58137001	58142000	5.28E-10
chr20	58148001	58153000	1.73E-07
chr20	58160001	58165000	3.41E-11
chr20	58172001	58177000	1.37E-05
chr20	58175001	58180000	4.21E-08
chr20	58178001	58183000	0.002153
chr20	58213001	58218000	0.001245
chr20	58296001	58301000	1.43E-07
chr20	58313001	58318000	6.51E-08
chr20	58315001	58320000	9.24E-08
chr20	58316001	58321000	4.28E-08

chr20	58329001	58334000	4.16E-07
chr20	58828001	58833000	4.11E-07
chr20	59686001	59691000	5.74E-12
chr20	59687001	59692000	2.25E-12
chr20	59688001	59693000	1.31E-12
chr20	59689001	59694000	1.51E-10
chr20	59721001	59726000	1.51E-08
chr20	59723001	59728000	2.27E-06
chr20	59746001	59751000	4.80E-05
chr20	59748001	59753000	4.16E-05
chr20	59793001	59798000	1.05E-10
chr20	59975001	59980000	3.05E-10
chr20	60032001	60037000	1.96E-06
chr20	60068001	60073000	3.05E-07
chr20	60104001	60109000	5.73E-11
chr20	60105001	60110000	9.21E-12
chr20	60106001	60111000	7.33E-12
chr20	60107001	60112000	1.15E-11
chr20	60108001	60113000	2.85E-09
chr20	60114001	60119000	2.15E-13
chr20	60115001	60120000	1.99E-14
chr20	60122001	60127000	1.36E-11
chr20	60123001	60128000	5.38E-14
chr20	60151001	60156000	3.49E-11
chr20	60153001	60158000	4.01E-09
chr20	60154001	60159000	6.12E-08
chr20	60179001	60184000	7.72E-08
chr20	60180001	60185000	3.41E-07
chr20	60181001	60186000	1.46E-08
chr20	60182001	60187000	1.38E-09
chr20	60368001	60373000	0.006391
chr20	60378001	60383000	7.18E-10
chr20	60435001	60440000	8.69E-07
chr20	60436001	60441000	9.79E-10
chr20	60455001	60460000	2.29E-07
chr20	60466001	60471000	2.84E-07
chr20	60467001	60472000	2.05E-05
chr20	60488001	60493000	0.000354
chr20	60503001	60508000	1.78E-06
chr20	60504001	60509000	1.33E-10
chr20	60505001	60510000	2.28E-11
chr20	60506001	60511000	3.86E-10
chr20	60507001	60512000	1.17E-09
chr20	60638001	60643000	2.04E-14
chr20	60639001	60644000	1.36E-07
chr20	60640001	60645000	2.61E-12
chr20	60641001	60646000	1.29E-10
chr20	60679001	60684000	2.77E-05

chr20	60683001	60688000	0.001199
chr20	60722001	60727000	8.79E-07
chr20	60729001	60734000	0.001295
chr20	60876001	60881000	1.02E-05
chr20	60892001	60897000	0.001859
chr20	60924001	60929000	0.007895
chr20	60980001	60985000	6.13E-05
chr20	61021001	61026000	1.73E-07
chr20	61046001	61051000	0.026169
chr20	61047001	61052000	0.004639
chr20	61066001	61071000	4.11E-10
chr20	61067001	61072000	1.66E-10
chr20	61068001	61073000	1.71E-10
chr20	61069001	61074000	7.92E-08
chr20	61070001	61075000	0.000266
chr20	61079001	61084000	0.000124
chr20	61080001	61085000	4.04E-05
chr20	61083001	61088000	2.03E-05
chr20	61090001	61095000	2.25E-05
chr20	61139001	61144000	5.71E-05
chr20	61140001	61145000	8.81E-07
chr20	61141001	61146000	2.13E-05
chr20	61142001	61147000	3.21E-06
chr20	61161001	61166000	3.75E-05
chr20	61175001	61180000	1.39E-07
chr20	61176001	61181000	7.69E-07
chr20	61177001	61182000	4.52E-06
chr20	61178001	61183000	4.06E-05
chr20	61187001	61192000	1.76E-08
chr20	61205001	61210000	5.18E-06
chr20	61290001	61295000	3.20E-05
chr20	61321001	61326000	3.56E-10
chr20	61322001	61327000	5.58E-09
chr20	61355001	61360000	5.50E-05
chr20	61356001	61361000	1.68E-07
chr20	61357001	61362000	1.40E-10
chr20	61380001	61385000	7.32E-11
chr20	61381001	61386000	3.71E-13
chr20	61382001	61387000	2.21E-09
chr20	61383001	61388000	6.55E-08
chr20	61401001	61406000	2.01E-05
chr20	61402001	61407000	1.83E-06
chr20	61403001	61408000	1.93E-09
chr20	61404001	61409000	1.81E-09
chr20	61405001	61410000	1.08E-12
chr20	61406001	61411000	4.06E-06
chr20	61408001	61413000	0.000133
chr20	61409001	61414000	1.53E-06

chr20	61599001	61604000	1.08E-07
chr20	61600001	61605000	2.13E-07
chr20	61697001	61702000	0.000312
chr20	61699001	61704000	0.001322
chr20	61700001	61705000	0.002077
chr20	61719001	61724000	4.46E-07
chr20	61742001	61747000	3.39E-07
chr20	61743001	61748000	3.05E-07
chr20	61752001	61757000	2.32E-09
chr20	61779001	61784000	1.39E-08
chr20	61853001	61858000	8.23E-06
chr20	61854001	61859000	0.000104
chr20	61883001	61888000	3.48E-05
chr20	61884001	61889000	0.000303
chr20	61885001	61890000	9.75E-08
chr20	61886001	61891000	4.12E-09
chr20	61887001	61892000	5.49E-10
chr20	61921001	61926000	0.001404
chr20	61937001	61942000	0.000285
chr20	61939001	61944000	6.43E-07
chr20	61940001	61945000	2.75E-05
chr20	61941001	61946000	0.001151
chr20	61947001	61952000	3.54E-05
chr20	61948001	61953000	9.39E-05
chr20	61949001	61954000	0.000193
chr20	62034001	62039000	5.87E-05
chr20	62035001	62040000	1.73E-07
chr20	62036001	62041000	0.001104
chr20	62046001	62051000	3.25E-05
chr20	62082001	62087000	0.003103
chr20	62084001	62089000	0.008184
chr20	62125001	62130000	4.55E-10
chr20	62126001	62131000	6.37E-12
chr20	62128001	62133000	3.40E-10
chr20	62129001	62134000	3.37E-11
chr20	62130001	62135000	7.47E-08
chr20	62166001	62171000	2.35E-05
chr20	62167001	62172000	4.93E-05
chr20	62168001	62173000	0.000538
chr20	62181001	62186000	0.000257
chr20	62182001	62187000	1.18E-08
chr20	62185001	62190000	4.86E-09
chr20	62200001	62205000	1.89E-07
chr20	62254001	62259000	3.44E-18
chr20	62255001	62260000	1.17E-14
chr20	62256001	62261000	3.31E-09
chr20	62258001	62263000	7.52E-12
chr20	62259001	62264000	1.42E-05

chr20	62260001	62265000	1.02E-05
chr20	62261001	62266000	2.48E-05
chr20	62262001	62267000	1.09E-06
chr20	62268001	62273000	0.002316
chr20	62269001	62274000	0.006399
chr20	62270001	62275000	0.006399
chr20	62312001	62317000	2.68E-06
chr20	62313001	62318000	2.68E-06
chr20	62386001	62391000	2.19E-09
chr20	62398001	62403000	0.004856
chr20	62412001	62417000	6.87E-09
chr20	62413001	62418000	1.79E-06
chr20	62451001	62456000	3.96E-10
chr20	62470001	62475000	0.000114
chr20	62481001	62486000	1.06E-09
chr20	62583001	62588000	8.28E-09
chr20	62586001	62591000	2.49E-06
chr20	62743001	62748000	2.19E-07
chr20	62744001	62749000	7.66E-07
chr20	62745001	62750000	2.73E-06
chr20	62746001	62751000	4.34E-06
chr20	62803001	62808000	0.037786
chr20	62853001	62858000	1.27E-08
chr20	62917001	62922000	0.013001
chr20	62918001	62923000	0.004797
chr20	62919001	62924000	0.000212
chr20	62922001	62927000	0.003674
chr21	9771001	9776000	0.023694
chr21	9874001	9879000	0.000655
chr21	9875001	9880000	7.75E-06
chr21	9952001	9957000	0.015508
chr21	9954001	9959000	0.03945
chr21	9960001	9965000	0.023624
chr21	9967001	9972000	0.003799
chr21	9968001	9973000	0.00083
chr21	9969001	9974000	1.35E-06
chr21	9970001	9975000	1.00E-04
chr21	10104001	10109000	0.015061
chr21	14469001	14474000	0.000227
chr21	15584001	15589000	5.88E-07
chr21	15600001	15605000	0.000356
chr21	16497001	16502000	5.75E-07
chr21	18984001	18989000	6.09E-08
chr21	19191001	19196000	7.37E-10
chr21	20082001	20087000	2.23E-05
chr21	20083001	20088000	4.35E-05
chr21	20219001	20224000	1.06E-05
chr21	20220001	20225000	4.66E-05

chr21	20221001	20226000	0.001934
chr21	22940001	22945000	1.21E-08
chr21	22941001	22946000	4.53E-08
chr21	22942001	22947000	2.39E-08
chr21	22943001	22948000	2.93E-08
chr21	23397001	23402000	1.53E-06
chr21	23463001	23468000	7.84E-09
chr21	23464001	23469000	3.33E-08
chr21	23465001	23470000	1.73E-07
chr21	23466001	23471000	3.00E-07
chr21	24750001	24755000	0.000102
chr21	25197001	25202000	2.29E-06
chr21	25220001	25225000	1.09E-05
chr21	25384001	25389000	0.00107
chr21	25385001	25390000	0.000798
chr21	25961001	25966000	7.26E-10
chr21	25962001	25967000	1.27E-09
chr21	26114001	26119000	1.39E-06
chr21	27019001	27024000	3.46E-06
chr21	27174001	27179000	9.38E-07
chr21	27201001	27206000	0.002593
chr21	27202001	27207000	1.40E-06
chr21	27747001	27752000	1.12E-07
chr21	27749001	27754000	1.35E-06
chr21	30493001	30498000	2.34E-05
chr21	31237001	31242000	2.17E-08
chr21	31238001	31243000	9.31E-10
chr21	31522001	31527000	0.000156
chr21	31523001	31528000	3.18E-07
chr21	32206001	32211000	9.79E-07
chr21	32253001	32258000	9.40E-07
chr21	32428001	32433000	4.36E-05
chr21	32456001	32461000	5.38E-10
chr21	32984001	32989000	0.000603
chr21	34497001	34502000	1.67E-09
chr21	34498001	34503000	3.03E-11
chr21	34525001	34530000	0.000255
chr21	34863001	34868000	4.39E-05
chr21	34864001	34869000	0.000243
chr21	35666001	35671000	1.08E-06
chr21	35730001	35735000	7.64E-09
chr21	35731001	35736000	1.34E-06
chr21	35732001	35737000	3.11E-07
chr21	35733001	35738000	2.03E-05
chr21	37251001	37256000	2.06E-05
chr21	37252001	37257000	2.83E-05
chr21	37289001	37294000	0.000104
chr21	37956001	37961000	0.004421

chr21	37957001	37962000	4.98E-06
chr21	38345001	38350000	6.24E-05
chr21	38719001	38724000	3.76E-05
chr21	38720001	38725000	0.00059
chr21	39223001	39228000	0.000318
chr21	39224001	39229000	0.000914
chr21	39313001	39318000	6.47E-09
chr21	39314001	39319000	4.26E-09
chr21	40275001	40280000	3.37E-07
chr21	40755001	40760000	1.46E-09
chr21	40756001	40761000	1.58E-08
chr21	41279001	41284000	1.53E-07
chr21	41280001	41285000	5.60E-08
chr21	41281001	41286000	1.67E-07
chr21	41551001	41556000	0.000901
chr21	42717001	42722000	0.032708
chr21	42832001	42837000	8.42E-05
chr21	42833001	42838000	0.000888
chr21	43294001	43299000	6.03E-09
chr21	43480001	43485000	3.01E-05
chr21	43481001	43486000	8.13E-06
chr21	43635001	43640000	0.000549
chr21	44073001	44078000	3.87E-16
chr21	44074001	44079000	3.12E-16
chr21	44277001	44282000	0.001816
chr21	44462001	44467000	1.98E-06
chr21	44581001	44586000	2.12E-08
chr21	44789001	44794000	0.001026
chr21	44790001	44795000	5.24E-05
chr21	44791001	44796000	0.000186
chr21	44792001	44797000	0.000468
chr21	44799001	44804000	7.46E-05
chr21	44817001	44822000	0.001098
chr21	45122001	45127000	0.002861
chr21	45230001	45235000	4.26E-08
chr21	45231001	45236000	1.22E-07
chr21	45232001	45237000	4.54E-09
chr21	45233001	45238000	0.017749
chr21	45418001	45423000	0.002826
chr21	45553001	45558000	3.93E-10
chr21	45656001	45661000	6.56E-06
chr21	45660001	45665000	1.12E-07
chr21	45661001	45666000	1.06E-06
chr21	45675001	45680000	0.003185
chr21	45676001	45681000	0.002005
chr21	45677001	45682000	0.004261
chr21	45678001	45683000	0.007148
chr21	46054001	46059000	0.000156

chr21	46055001	46060000	0.000128
chr21	46303001	46308000	0.000868
chr21	46304001	46309000	0.000538
chr21	46305001	46310000	0.003062
chr21	46306001	46311000	0.012921
chr21	46336001	46341000	1.09E-06
chr21	46609001	46614000	0.002231
chr21	46661001	46666000	0.000243
chr21	46825001	46830000	3.17E-05
chr21	46826001	46831000	2.23E-05
chr21	47178001	47183000	2.40E-12
chr21	47179001	47184000	3.07E-08
chr21	47180001	47185000	1.01E-08
chr21	47181001	47186000	1.37E-07
chr21	47318001	47323000	2.98E-06
chr21	47333001	47338000	0.002175
chr21	47550001	47555000	0.002604
chr21	47569001	47574000	0.000342
chr21	47738001	47743000	0.000145
chr21	47739001	47744000	8.83E-05
chr21	47874001	47879000	1.49E-07
chr21	47875001	47880000	1.32E-06
chr21	47877001	47882000	2.47E-05
chr21	47878001	47883000	7.43E-05
chr22	16046001	16051000	4.19E-05
chr22	16047001	16052000	5.24E-08
chr22	16048001	16053000	2.34E-08
chr22	16153001	16158000	1.84E-05
chr22	16154001	16159000	8.25E-06
chr22	16492001	16497000	0.003804
chr22	16509001	16514000	7.76E-05
chr22	16510001	16515000	0.001586
chr22	16511001	16516000	0.000466
chr22	16522001	16527000	0.029892
chr22	16529001	16534000	0.000596
chr22	16530001	16535000	3.09E-05
chr22	16546001	16551000	7.62E-07
chr22	16555001	16560000	6.30E-05
chr22	16591001	16596000	8.16E-06
chr22	16592001	16597000	2.34E-07
chr22	16593001	16598000	6.39E-08
chr22	16601001	16606000	0.000305
chr22	16611001	16616000	1.26E-05
chr22	16612001	16617000	3.38E-06
chr22	16613001	16618000	2.16E-06
chr22	16614001	16619000	1.33E-09
chr22	16616001	16621000	2.69E-08
chr22	16617001	16622000	1.19E-09

chr22	16618001	16623000	1.69E-09
chr22	16619001	16624000	1.44E-05
chr22	16640001	16645000	1.63E-07
chr22	16669001	16674000	0.003655
chr22	16689001	16694000	5.83E-07
chr22	16690001	16695000	2.03E-06
chr22	16691001	16696000	1.40E-05
chr22	16962001	16967000	0.000759
chr22	17002001	17007000	6.98E-07
chr22	17005001	17010000	2.26E-07
chr22	17006001	17011000	7.22E-06
chr22	17040001	17045000	0.000389
chr22	17041001	17046000	0.003131
chr22	17046001	17051000	9.16E-06
chr22	17074001	17079000	2.01E-07
chr22	17075001	17080000	3.28E-06
chr22	17076001	17081000	0.002543
chr22	17313001	17318000	2.61E-06
chr22	17320001	17325000	2.42E-09
chr22	17321001	17326000	1.76E-05
chr22	17342001	17347000	3.18E-07
chr22	17343001	17348000	2.19E-07
chr22	17415001	17420000	6.48E-07
chr22	17436001	17441000	3.95E-07
chr22	17437001	17442000	3.50E-06
chr22	17438001	17443000	1.41E-05
chr22	17439001	17444000	3.41E-10
chr22	17440001	17445000	9.33E-13
chr22	17441001	17446000	1.05E-06
chr22	17451001	17456000	8.95E-11
chr22	17452001	17457000	1.53E-10
chr22	17453001	17458000	1.02E-07
chr22	17454001	17459000	2.60E-07
chr22	17455001	17460000	1.59E-10
chr22	17470001	17475000	0.000473
chr22	17486001	17491000	1.06E-07
chr22	17489001	17494000	2.32E-06
chr22	17492001	17497000	6.33E-06
chr22	17525001	17530000	3.43E-07
chr22	17526001	17531000	6.23E-06
chr22	17534001	17539000	3.61E-07
chr22	17604001	17609000	1.26E-06
chr22	17605001	17610000	9.46E-07
chr22	17606001	17611000	6.42E-06
chr22	17679001	17684000	1.52E-06
chr22	17681001	17686000	1.35E-06
chr22	17682001	17687000	2.44E-08
chr22	17683001	17688000	1.71E-07

chr22	17685001	17690000	6.13E-07
chr22	17686001	17691000	5.57E-06
chr22	17780001	17785000	2.65E-07
chr22	17782001	17787000	0.000148
chr22	17835001	17840000	4.20E-09
chr22	17934001	17939000	8.02E-10
chr22	18044001	18049000	3.73E-09
chr22	18045001	18050000	5.88E-09
chr22	18054001	18059000	1.22E-05
chr22	18055001	18060000	0.000217
chr22	18056001	18061000	0.041679
chr22	18060001	18065000	0.002759
chr22	18061001	18066000	0.002759
chr22	18062001	18067000	6.34E-07
chr22	18063001	18068000	5.44E-07
chr22	18108001	18113000	0.000121
chr22	18109001	18114000	8.66E-05
chr22	18110001	18115000	4.03E-05
chr22	18117001	18122000	0.034314
chr22	18118001	18123000	2.14E-07
chr22	18254001	18259000	4.65E-09
chr22	18255001	18260000	4.65E-11
chr22	18256001	18261000	9.64E-12
chr22	18556001	18561000	3.75E-18
chr22	18557001	18562000	4.11E-22
chr22	18558001	18563000	1.23E-11
chr22	18559001	18564000	1.04E-15
chr22	18560001	18565000	8.62E-17
chr22	18633001	18638000	9.72E-14
chr22	18705001	18710000	0.000839
chr22	18706001	18711000	0.000839
chr22	18763001	18768000	2.47E-08
chr22	18764001	18769000	9.31E-08
chr22	18765001	18770000	1.21E-08
chr22	18766001	18771000	6.27E-09
chr22	18767001	18772000	2.83E-10
chr22	18768001	18773000	9.21E-10
chr22	18769001	18774000	1.53E-13
chr22	18805001	18810000	0.001139
chr22	18832001	18837000	6.44E-06
chr22	18839001	18844000	2.04E-06
chr22	18840001	18845000	2.06E-07
chr22	18841001	18846000	7.39E-09
chr22	18909001	18914000	0.00107
chr22	18941001	18946000	0.013462
chr22	19003001	19008000	3.36E-05
chr22	19004001	19009000	1.60E-05
chr22	19005001	19010000	2.11E-05

chr22	19017001	19022000	0.003564
chr22	19018001	19023000	0.000733
chr22	19139001	19144000	0.000178
chr22	19140001	19145000	5.74E-06
chr22	19141001	19146000	1.76E-06
chr22	19153001	19158000	1.07E-07
chr22	19166001	19171000	1.49E-06
chr22	19281001	19286000	4.42E-05
chr22	19282001	19287000	3.07E-05
chr22	19717001	19722000	0.003358
chr22	19738001	19743000	2.58E-06
chr22	19838001	19843000	3.96E-09
chr22	19975001	19980000	4.43E-05
chr22	20008001	20013000	4.15E-06
chr22	20103001	20108000	2.18E-12
chr22	20104001	20109000	1.27E-12
chr22	20168001	20173000	1.77E-08
chr22	20181001	20186000	3.68E-06
chr22	20182001	20187000	2.26E-05
chr22	20311001	20316000	3.34E-05
chr22	20333001	20338000	0.000128
chr22	20334001	20339000	0.000363
chr22	20335001	20340000	0.000709
chr22	20336001	20341000	0.00674
chr22	20343001	20348000	4.91E-08
chr22	20344001	20349000	3.43E-11
chr22	20345001	20350000	8.54E-10
chr22	20346001	20351000	1.11E-07
chr22	20347001	20352000	6.15E-07
chr22	20348001	20353000	3.30E-08
chr22	20349001	20354000	2.95E-05
chr22	20455001	20460000	6.46E-05
chr22	20456001	20461000	6.46E-05
chr22	20457001	20462000	0.000405
chr22	20659001	20664000	0.020736
chr22	20768001	20773000	5.15E-10
chr22	20769001	20774000	1.27E-08
chr22	20770001	20775000	2.15E-07
chr22	20771001	20776000	1.79E-07
chr22	20772001	20777000	2.33E-08
chr22	20779001	20784000	1.01E-12
chr22	20780001	20785000	1.26E-12
chr22	20781001	20786000	9.73E-09
chr22	20787001	20792000	0.000231
chr22	20850001	20855000	0.000658
chr22	20960001	20965000	1.12E-05
chr22	21307001	21312000	4.40E-09
chr22	21308001	21313000	1.28E-13

chr22	21309001	21314000	6.39E-10
chr22	21310001	21315000	3.80E-07
chr22	21311001	21316000	0.005731
chr22	21330001	21335000	0.004175
chr22	21411001	21416000	4.48E-05
chr22	21449001	21454000	7.68E-07
chr22	21451001	21456000	3.07E-05
chr22	21463001	21468000	8.48E-05
chr22	21464001	21469000	0.00264
chr22	21522001	21527000	0.005639
chr22	21523001	21528000	0.007089
chr22	21524001	21529000	6.78E-08
chr22	21525001	21530000	1.44E-08
chr22	21526001	21531000	7.98E-08
chr22	21550001	21555000	6.17E-08
chr22	21551001	21556000	3.98E-05
chr22	21596001	21601000	0.000188
chr22	21630001	21635000	3.02E-05
chr22	21799001	21804000	6.55E-09
chr22	21983001	21988000	2.72E-09
chr22	21984001	21989000	2.62E-09
chr22	21985001	21990000	2.12E-12
chr22	21986001	21991000	4.84E-11
chr22	22002001	22007000	5.83E-10
chr22	22010001	22015000	6.59E-06
chr22	22011001	22016000	1.56E-10
chr22	22012001	22017000	6.72E-09
chr22	22019001	22024000	0.022419
chr22	22054001	22059000	2.82E-10
chr22	22055001	22060000	7.92E-11
chr22	22070001	22075000	0.000328
chr22	22071001	22076000	1.94E-07
chr22	22072001	22077000	1.47E-05
chr22	22086001	22091000	2.21E-05
chr22	22087001	22092000	8.48E-06
chr22	22088001	22093000	4.32E-14
chr22	22089001	22094000	1.70E-20
chr22	22090001	22095000	7.62E-15
chr22	22279001	22284000	7.73E-07
chr22	22280001	22285000	1.66E-07
chr22	22353001	22358000	5.22E-08
chr22	22533001	22538000	0.000177
chr22	22684001	22689000	9.75E-10
chr22	22685001	22690000	6.12E-10
chr22	22692001	22697000	1.88E-07
chr22	22784001	22789000	3.63E-09
chr22	22811001	22816000	5.85E-08
chr22	22838001	22843000	2.09E-05

chr22	22840001	22845000	1.10E-08
chr22	22886001	22891000	4.88E-06
chr22	22889001	22894000	5.09E-05
chr22	22897001	22902000	2.43E-08
chr22	22920001	22925000	1.12E-12
chr22	22928001	22933000	1.03E-06
chr22	22929001	22934000	2.41E-06
chr22	22930001	22935000	2.07E-07
chr22	22932001	22937000	2.05E-08
chr22	22946001	22951000	3.76E-06
chr22	22947001	22952000	9.34E-06
chr22	22987001	22992000	0.000509
chr22	23003001	23008000	2.19E-07
chr22	23004001	23009000	5.65E-07
chr22	23005001	23010000	1.07E-06
chr22	23006001	23011000	4.92E-06
chr22	23007001	23012000	4.14E-06
chr22	23043001	23048000	2.39E-06
chr22	23085001	23090000	1.10E-07
chr22	23087001	23092000	3.10E-05
chr22	23234001	23239000	3.09E-06
chr22	23235001	23240000	1.15E-07
chr22	23236001	23241000	3.50E-07
chr22	23237001	23242000	4.47E-06
chr22	23336001	23341000	0.003213
chr22	23455001	23460000	0.000782
chr22	23456001	23461000	0.000117
chr22	23654001	23659000	0.00278
chr22	23655001	23660000	0.010254
chr22	23717001	23722000	0.000833
chr22	23728001	23733000	2.60E-05
chr22	23729001	23734000	0.000646
chr22	23838001	23843000	1.89E-05
chr22	23844001	23849000	0.000408
chr22	23893001	23898000	1.41E-05
chr22	23900001	23905000	7.14E-06
chr22	23901001	23906000	2.24E-07
chr22	23902001	23907000	1.45E-08
chr22	23903001	23908000	1.60E-07
chr22	23913001	23918000	0.001006
chr22	23921001	23926000	1.53E-05
chr22	23933001	23938000	6.28E-06
chr22	23934001	23939000	1.92E-06
chr22	23935001	23940000	9.50E-05
chr22	23943001	23948000	3.28E-06
chr22	23944001	23949000	5.50E-08
chr22	23945001	23950000	6.87E-09
chr22	23946001	23951000	4.12E-06

chr22	23947001	23952000	8.32E-07
chr22	23948001	23953000	1.15E-05
chr22	24117001	24122000	0.010631
chr22	24119001	24124000	8.55E-05
chr22	24120001	24125000	0.00013
chr22	24122001	24127000	7.55E-05
chr22	24123001	24128000	3.37E-05
chr22	24125001	24130000	0.000292
chr22	24177001	24182000	0.000875
chr22	24179001	24184000	9.58E-08
chr22	24180001	24185000	3.20E-09
chr22	24181001	24186000	7.35E-09
chr22	24365001	24370000	0.013482
chr22	24575001	24580000	0.006399
chr22	24576001	24581000	0.010018
chr22	24591001	24596000	0.000231
chr22	24814001	24819000	3.30E-06
chr22	24918001	24923000	8.51E-08
chr22	24919001	24924000	1.32E-06
chr22	24977001	24982000	0.000681
chr22	24978001	24983000	2.30E-05
chr22	24979001	24984000	3.80E-06
chr22	24988001	24993000	7.87E-06
chr22	24989001	24994000	0.001637
chr22	25019001	25024000	0.000611
chr22	25330001	25335000	0.000168
chr22	25331001	25336000	0.000154
chr22	25332001	25337000	0.001424
chr22	25333001	25338000	0.002894
chr22	25334001	25339000	7.84E-06
chr22	25800001	25805000	0.003369
chr22	25801001	25806000	4.43E-06
chr22	26262001	26267000	2.75E-07
chr22	26456001	26461000	1.68E-06
chr22	27038001	27043000	1.30E-06
chr22	27039001	27044000	1.94E-07
chr22	27040001	27045000	4.68E-05
chr22	27199001	27204000	4.90E-10
chr22	27225001	27230000	1.68E-07
chr22	27340001	27345000	3.02E-10
chr22	27368001	27373000	7.64E-07
chr22	27369001	27374000	5.58E-09
chr22	27370001	27375000	1.09E-09
chr22	27403001	27408000	6.35E-10
chr22	27417001	27422000	2.02E-09
chr22	27418001	27423000	7.37E-11
chr22	27419001	27424000	2.10E-13
chr22	27420001	27425000	1.67E-12

chr22	27439001	27444000	4.07E-07
chr22	27440001	27445000	4.56E-06
chr22	27512001	27517000	1.20E-09
chr22	27554001	27559000	2.17E-05
chr22	27615001	27620000	0.000297
chr22	27647001	27652000	5.38E-06
chr22	27709001	27714000	5.57E-12
chr22	27779001	27784000	1.25E-07
chr22	27780001	27785000	2.94E-06
chr22	27899001	27904000	0.000111
chr22	27901001	27906000	0.000153
chr22	27914001	27919000	4.35E-07
chr22	28031001	28036000	2.13E-08
chr22	28032001	28037000	1.00E-07
chr22	28033001	28038000	2.99E-06
chr22	28052001	28057000	3.01E-06
chr22	28060001	28065000	5.13E-05
chr22	28061001	28066000	3.13E-07
chr22	28062001	28067000	6.98E-05
chr22	28063001	28068000	8.45E-05
chr22	28066001	28071000	0.001999
chr22	28080001	28085000	1.42E-06
chr22	28152001	28157000	8.44E-07
chr22	28158001	28163000	4.12E-11
chr22	28159001	28164000	9.47E-07
chr22	28164001	28169000	4.91E-07
chr22	28325001	28330000	6.12E-13
chr22	28326001	28331000	1.59E-12
chr22	29463001	29468000	2.94E-08
chr22	29464001	29469000	1.76E-13
chr22	29465001	29470000	1.15E-11
chr22	29466001	29471000	3.72E-13
chr22	29467001	29472000	9.58E-12
chr22	29474001	29479000	7.40E-06
chr22	29811001	29816000	0.000368
chr22	29849001	29854000	0.000311
chr22	29850001	29855000	0.000281
chr22	29863001	29868000	1.09E-11
chr22	29864001	29869000	4.71E-10
chr22	29865001	29870000	1.36E-08
chr22	29866001	29871000	8.93E-11
chr22	29973001	29978000	4.02E-06
chr22	29974001	29979000	1.26E-06
chr22	30275001	30280000	2.18E-08
chr22	30276001	30281000	3.33E-11
chr22	30639001	30644000	0.009737
chr22	30646001	30651000	0.000136
chr22	30647001	30652000	4.43E-06

chr22	30648001	30653000	3.47E-06
chr22	30921001	30926000	1.30E-05
chr22	30934001	30939000	1.20E-15
chr22	30935001	30940000	1.83E-12
chr22	30936001	30941000	3.06E-14
chr22	30937001	30942000	4.60E-10
chr22	30938001	30943000	1.72E-05
chr22	30945001	30950000	1.98E-06
chr22	31021001	31026000	0.026855
chr22	31028001	31033000	2.12E-10
chr22	31029001	31034000	2.17E-07
chr22	31030001	31035000	0.000474
chr22	31031001	31036000	2.22E-06
chr22	31032001	31037000	1.85E-06
chr22	31159001	31164000	3.38E-06
chr22	31480001	31485000	1.17E-05
chr22	31643001	31648000	0.001872
chr22	31644001	31649000	0.000893
chr22	31736001	31741000	4.06E-05
chr22	32007001	32012000	0.000466
chr22	32063001	32068000	1.61E-05
chr22	32435001	32440000	1.85E-06
chr22	32551001	32556000	5.08E-10
chr22	32651001	32656000	1.82E-06
chr22	32652001	32657000	5.37E-07
chr22	32693001	32698000	3.76E-07
chr22	32709001	32714000	3.93E-05
chr22	32715001	32720000	1.29E-05
chr22	32728001	32733000	7.04E-10
chr22	33050001	33055000	0.032747
chr22	33113001	33118000	4.37E-06
chr22	33166001	33171000	2.52E-08
chr22	33190001	33195000	4.72E-11
chr22	33253001	33258000	1.87E-08
chr22	33379001	33384000	6.33E-06
chr22	33394001	33399000	2.15E-10
chr22	33395001	33400000	5.84E-11
chr22	33396001	33401000	7.38E-09
chr22	34443001	34448000	3.59E-08
chr22	34444001	34449000	2.49E-13
chr22	34445001	34450000	8.27E-13
chr22	34446001	34451000	1.06E-11
chr22	34465001	34470000	2.06E-08
chr22	34466001	34471000	7.00E-09
chr22	34609001	34614000	1.12E-06
chr22	34610001	34615000	2.61E-05
chr22	34662001	34667000	0.000293
chr22	34663001	34668000	3.10E-06

chr22	34664001	34669000	3.31E-07
chr22	34675001	34680000	3.42E-07
chr22	34688001	34693000	5.07E-09
chr22	34760001	34765000	1.04E-06
chr22	34927001	34932000	4.49E-12
chr22	34928001	34933000	2.01E-08
chr22	34948001	34953000	3.60E-11
chr22	35023001	35028000	7.30E-07
chr22	35025001	35030000	1.73E-07
chr22	35026001	35031000	5.27E-10
chr22	35027001	35032000	3.51E-09
chr22	35211001	35216000	1.40E-08
chr22	35239001	35244000	0.000182
chr22	35240001	35245000	4.82E-05
chr22	35243001	35248000	1.25E-09
chr22	35247001	35252000	7.16E-10
chr22	35249001	35254000	1.66E-09
chr22	35250001	35255000	1.23E-08
chr22	35251001	35256000	1.31E-08
chr22	35373001	35378000	2.88E-05
chr22	35391001	35396000	1.15E-09
chr22	35417001	35422000	0.00015
chr22	35419001	35424000	1.19E-06
chr22	35420001	35425000	2.26E-08
chr22	35476001	35481000	2.12E-07
chr22	35477001	35482000	1.17E-06
chr22	35483001	35488000	1.46E-09
chr22	35484001	35489000	1.57E-08
chr22	35587001	35592000	1.65E-16
chr22	35588001	35593000	7.94E-17
chr22	35589001	35594000	8.88E-15
chr22	35590001	35595000	1.39E-14
chr22	35607001	35612000	2.63E-07
chr22	35611001	35616000	1.03E-07
chr22	35612001	35617000	4.61E-07
chr22	35613001	35618000	1.25E-07
chr22	35614001	35619000	1.70E-08
chr22	35615001	35620000	2.40E-08
chr22	35764001	35769000	0.00518
chr22	35765001	35770000	0.006783
chr22	35773001	35778000	1.53E-06
chr22	35791001	35796000	3.34E-06
chr22	35851001	35856000	1.81E-07
chr22	36458001	36463000	4.92E-06
chr22	36491001	36496000	1.04E-10
chr22	36826001	36831000	7.19E-06
chr22	36923001	36928000	5.94E-08
chr22	36924001	36929000	1.24E-10

chr22	36969001	36974000	1.59E-12
chr22	36970001	36975000	3.31E-14
chr22	36971001	36976000	1.92E-12
chr22	36972001	36977000	2.99E-11
chr22	36973001	36978000	1.80E-10
chr22	36974001	36979000	3.24E-07
chr22	36991001	36996000	1.11E-07
chr22	36992001	36997000	1.30E-09
chr22	37082001	37087000	5.54E-08
chr22	37083001	37088000	3.92E-09
chr22	37084001	37089000	3.98E-11
chr22	37085001	37090000	2.62E-10
chr22	37086001	37091000	1.75E-09
chr22	37104001	37109000	0.000675
chr22	37105001	37110000	2.17E-06
chr22	37106001	37111000	3.63E-08
chr22	37107001	37112000	2.14E-07
chr22	37108001	37113000	4.19E-08
chr22	37109001	37114000	1.41E-08
chr22	37122001	37127000	1.61E-07
chr22	37123001	37128000	1.16E-06
chr22	37144001	37149000	0.000148
chr22	37145001	37150000	9.13E-06
chr22	37146001	37151000	7.89E-08
chr22	37168001	37173000	5.61E-09
chr22	37170001	37175000	1.37E-08
chr22	37172001	37177000	2.18E-09
chr22	37188001	37193000	8.10E-11
chr22	37189001	37194000	7.39E-10
chr22	37190001	37195000	7.44E-09
chr22	37191001	37196000	3.52E-08
chr22	37214001	37219000	1.44E-05
chr22	37268001	37273000	2.58E-07
chr22	37269001	37274000	3.69E-09
chr22	37270001	37275000	2.84E-08
chr22	37271001	37276000	5.93E-08
chr22	37272001	37277000	1.27E-07
chr22	37293001	37298000	0.000213
chr22	37347001	37352000	1.37E-06
chr22	37440001	37445000	5.89E-06
chr22	37441001	37446000	1.68E-07
chr22	37442001	37447000	7.26E-09
chr22	37464001	37469000	1.89E-05
chr22	37465001	37470000	1.16E-06
chr22	37466001	37471000	1.83E-12
chr22	37467001	37472000	3.47E-10
chr22	37468001	37473000	3.21E-06
chr22	37479001	37484000	3.55E-07

chr22	37480001	37485000	3.61E-06
chr22	37481001	37486000	9.82E-06
chr22	37482001	37487000	1.19E-05
chr22	37575001	37580000	2.03E-08
chr22	37576001	37581000	8.87E-13
chr22	37577001	37582000	6.00E-13
chr22	37578001	37583000	1.99E-09
chr22	37661001	37666000	2.73E-09
chr22	37679001	37684000	5.01E-05
chr22	37688001	37693000	2.81E-06
chr22	37689001	37694000	0.00014
chr22	37709001	37714000	6.05E-08
chr22	37710001	37715000	8.14E-08
chr22	37711001	37716000	7.22E-08
chr22	37712001	37717000	3.06E-06
chr22	37762001	37767000	0.000337
chr22	37763001	37768000	2.03E-07
chr22	37764001	37769000	5.00E-08
chr22	37765001	37770000	5.87E-10
chr22	37766001	37771000	2.24E-11
chr22	37767001	37772000	4.50E-12
chr22	37768001	37773000	6.80E-06
chr22	37769001	37774000	1.57E-05
chr22	37792001	37797000	5.43E-09
chr22	37795001	37800000	5.12E-08
chr22	37806001	37811000	0.000504
chr22	37807001	37812000	8.84E-06
chr22	37810001	37815000	3.78E-07
chr22	37904001	37909000	6.66E-06
chr22	37905001	37910000	2.57E-06
chr22	37906001	37911000	2.24E-06
chr22	37907001	37912000	1.37E-07
chr22	37937001	37942000	7.92E-08
chr22	37938001	37943000	0.000104
chr22	37972001	37977000	0.021132
chr22	37995001	38000000	3.87E-06
chr22	38032001	38037000	0.000103
chr22	38196001	38201000	4.15E-14
chr22	38198001	38203000	1.58E-17
chr22	38199001	38204000	6.63E-19
chr22	38200001	38205000	3.53E-20
chr22	38201001	38206000	2.96E-09
chr22	38202001	38207000	1.13E-08
chr22	38203001	38208000	9.57E-07
chr22	38204001	38209000	5.97E-06
chr22	38218001	38223000	0.00015
chr22	38289001	38294000	1.93E-07
chr22	38290001	38295000	6.91E-11

chr22	38291001	38296000	3.29E-13
chr22	38292001	38297000	7.55E-12
chr22	38350001	38355000	4.32E-05
chr22	38401001	38406000	5.78E-07
chr22	38413001	38418000	7.75E-06
chr22	38432001	38437000	0.00012
chr22	38433001	38438000	4.39E-06
chr22	38434001	38439000	1.91E-06
chr22	38435001	38440000	0.000183
chr22	38436001	38441000	0.000191
chr22	38504001	38509000	0.007029
chr22	38576001	38581000	4.67E-06
chr22	38577001	38582000	6.48E-08
chr22	38664001	38669000	0.00013
chr22	38665001	38670000	5.55E-07
chr22	38666001	38671000	8.32E-07
chr22	38667001	38672000	3.35E-07
chr22	38668001	38673000	5.70E-05
chr22	38828001	38833000	0.000256
chr22	38829001	38834000	8.05E-07
chr22	38830001	38835000	4.68E-07
chr22	38851001	38856000	0.000732
chr22	39076001	39081000	9.53E-09
chr22	39077001	39082000	1.22E-06
chr22	39225001	39230000	0.001367
chr22	39244001	39249000	4.41E-05
chr22	39294001	39299000	0.000115
chr22	39330001	39335000	3.61E-08
chr22	39331001	39336000	3.10E-07
chr22	39381001	39386000	0.02119
chr22	39463001	39468000	3.43E-08
chr22	39464001	39469000	2.78E-08
chr22	39568001	39573000	3.07E-05
chr22	39610001	39615000	5.53E-07
chr22	39613001	39618000	5.07E-05
chr22	39634001	39639000	1.88E-07
chr22	39649001	39654000	0.000113
chr22	39650001	39655000	7.71E-07
chr22	39657001	39662000	1.12E-05
chr22	39658001	39663000	0.000102
chr22	39659001	39664000	5.53E-05
chr22	39837001	39842000	1.23E-06
chr22	39865001	39870000	3.48E-05
chr22	39879001	39884000	6.51E-07
chr22	39880001	39885000	8.69E-06
chr22	39938001	39943000	2.27E-08
chr22	39939001	39944000	3.83E-10
chr22	39940001	39945000	2.70E-06

chr22	39941001	39946000	7.97E-07
chr22	39942001	39947000	4.69E-06
chr22	39943001	39948000	2.29E-05
chr22	39945001	39950000	4.86E-05
chr22	39971001	39976000	1.10E-08
chr22	39972001	39977000	1.31E-06
chr22	39973001	39978000	1.91E-05
chr22	39994001	39999000	0.000376
chr22	39995001	40000000	7.09E-05
chr22	40047001	40052000	1.73E-08
chr22	40048001	40053000	7.68E-06
chr22	41038001	41043000	0.000491
chr22	41386001	41391000	0.03973
chr22	41387001	41392000	0.007078
chr22	41388001	41393000	0.018198
chr22	41694001	41699000	8.40E-08
chr22	41695001	41700000	6.73E-07
chr22	41697001	41702000	4.57E-06
chr22	41767001	41772000	3.13E-06
chr22	41836001	41841000	7.94E-07
chr22	41837001	41842000	1.51E-08
chr22	41864001	41869000	8.36E-14
chr22	41957001	41962000	0.000427
chr22	41992001	41997000	1.46E-07
chr22	42013001	42018000	9.47E-17
chr22	42014001	42019000	9.13E-19
chr22	42015001	42020000	2.50E-12
chr22	42016001	42021000	5.12E-22
chr22	42017001	42022000	1.30E-17
chr22	42148001	42153000	6.46E-05
chr22	42149001	42154000	0.000163
chr22	42192001	42197000	2.75E-10
chr22	42193001	42198000	7.72E-09
chr22	42307001	42312000	0.008193
chr22	42332001	42337000	0.000971
chr22	42752001	42757000	1.97E-07
chr22	42782001	42787000	0.001752
chr22	42845001	42850000	0.01099
chr22	42932001	42937000	3.90E-05
chr22	42945001	42950000	0.000378
chr22	42973001	42978000	0.001452
chr22	43056001	43061000	0.000141
chr22	43057001	43062000	0.000225
chr22	43116001	43121000	0.008105
chr22	43133001	43138000	6.35E-08
chr22	43159001	43164000	1.92E-06
chr22	43160001	43165000	4.28E-06
chr22	43161001	43166000	4.11E-05

chr22	43162001	43167000	0.008587
chr22	43164001	43169000	0.007052
chr22	43480001	43485000	5.31E-06
chr22	43484001	43489000	0.000159
chr22	43539001	43544000	1.25E-09
chr22	43540001	43545000	9.70E-05
chr22	43541001	43546000	0.000302
chr22	43548001	43553000	0.000297
chr22	43549001	43554000	5.66E-05
chr22	43550001	43555000	5.54E-08
chr22	43551001	43556000	2.91E-07
chr22	43552001	43557000	9.04E-08
chr22	43553001	43558000	7.38E-05
chr22	43578001	43583000	7.47E-17
chr22	43579001	43584000	1.46E-12
chr22	43580001	43585000	5.20E-18
chr22	43581001	43586000	1.04E-18
chr22	43582001	43587000	6.03E-19
chr22	43583001	43588000	0.000399
chr22	43604001	43609000	3.88E-05
chr22	43605001	43610000	1.98E-06
chr22	43671001	43676000	1.29E-05
chr22	43672001	43677000	2.00E-06
chr22	43673001	43678000	6.06E-08
chr22	43674001	43679000	5.81E-08
chr22	43675001	43680000	3.36E-06
chr22	43789001	43794000	1.28E-05
chr22	43908001	43913000	0.001746
chr22	43909001	43914000	0.0006
chr22	44048001	44053000	1.33E-06
chr22	44206001	44211000	2.73E-08
chr22	44208001	44213000	5.59E-13
chr22	44250001	44255000	0.000299
chr22	44252001	44257000	9.14E-05
chr22	44261001	44266000	4.91E-07
chr22	44280001	44285000	0.000673
chr22	44281001	44286000	7.73E-05
chr22	44282001	44287000	1.32E-08
chr22	44291001	44296000	8.56E-05
chr22	44302001	44307000	0.000246
chr22	44303001	44308000	0.001191
chr22	44731001	44736000	4.60E-06
chr22	44732001	44737000	7.08E-08
chr22	44733001	44738000	1.28E-11
chr22	44734001	44739000	4.29E-05
chr22	44796001	44801000	3.27E-06
chr22	44968001	44973000	3.63E-05
chr22	45031001	45036000	4.61E-05

chr22	45114001	45119000	0.000577
chr22	45273001	45278000	6.89E-07
chr22	45274001	45279000	2.04E-08
chr22	45275001	45280000	7.05E-06
chr22	45366001	45371000	1.24E-07
chr22	45367001	45372000	1.08E-07
chr22	45374001	45379000	1.87E-05
chr22	45376001	45381000	5.34E-06
chr22	45445001	45450000	0.000686
chr22	45466001	45471000	5.55E-05
chr22	45475001	45480000	3.71E-05
chr22	45476001	45481000	2.76E-05
chr22	45589001	45594000	8.88E-11
chr22	45590001	45595000	1.20E-11
chr22	45591001	45596000	5.97E-13
chr22	45592001	45597000	3.32E-13
chr22	45684001	45689000	1.30E-05
chr22	45685001	45690000	6.44E-05
chr22	45686001	45691000	0.002477
chr22	46374001	46379000	3.19E-06
chr22	46395001	46400000	0.001619
chr22	46419001	46424000	1.51E-05
chr22	46420001	46425000	0.00012
chr22	46526001	46531000	0.019144
chr22	46527001	46532000	0.001523
chr22	46659001	46664000	1.26E-13
chr22	46660001	46665000	2.14E-10
chr22	46661001	46666000	3.01E-08
chr22	46663001	46668000	5.27E-08
chr22	46919001	46924000	1.66E-05
chr22	46921001	46926000	3.26E-06
chr22	46922001	46927000	3.30E-08
chr22	46930001	46935000	2.40E-09
chr22	46940001	46945000	2.86E-11
chr22	46941001	46946000	1.41E-11
chr22	47055001	47060000	0.000141
chr22	47057001	47062000	1.86E-05
chr22	47157001	47162000	1.61E-12
chr22	47627001	47632000	1.32E-07
chr22	47651001	47656000	7.39E-06
chr22	47652001	47657000	1.05E-05
chr22	47653001	47658000	2.30E-05
chr22	47782001	47787000	4.36E-06
chr22	47969001	47974000	1.02E-09
chr22	48021001	48026000	0.003596
chr22	48280001	48285000	2.10E-06
chr22	48302001	48307000	2.47E-05
chr22	48490001	48495000	9.91E-07

chr22	48491001	48496000	2.26E-05
chr22	48619001	48624000	2.13E-08
chr22	48635001	48640000	8.48E-06
chr22	48636001	48641000	6.34E-05
chr22	48637001	48642000	1.87E-07
chr22	48638001	48643000	6.98E-07
chr22	48669001	48674000	1.54E-05
chr22	48845001	48850000	3.49E-07
chr22	48846001	48851000	1.72E-07
chr22	48892001	48897000	5.02E-07
chr22	49037001	49042000	1.58E-08
chr22	49038001	49043000	3.58E-08
chr22	49039001	49044000	9.97E-05
chr22	49068001	49073000	1.00E-07
chr22	49069001	49074000	5.76E-06
chr22	49070001	49075000	2.57E-08
chr22	49071001	49076000	1.64E-07
chr22	49072001	49077000	3.18E-09
chr22	49133001	49138000	1.34E-05
chr22	49134001	49139000	1.81E-05
chr22	49148001	49153000	0.003683
chr22	49149001	49154000	0.004215
chr22	49150001	49155000	0.005209
chr22	49198001	49203000	2.85E-07
chr22	49224001	49229000	7.23E-09
chr22	49225001	49230000	4.34E-09
chr22	49237001	49242000	2.04E-06
chr22	49383001	49388000	8.48E-06
chr22	49384001	49389000	1.01E-05
chr22	49546001	49551000	0.00428
chr22	49804001	49809000	2.53E-09
chr22	49888001	49893000	1.23E-15
chr22	49889001	49894000	2.27E-14
chr22	49890001	49895000	2.87E-11
chr22	49891001	49896000	3.34E-12
chr22	49959001	49964000	1.71E-06
chr22	50010001	50015000	0.000474
chr22	50032001	50037000	4.67E-05
chr22	50055001	50060000	2.96E-07
chr22	50056001	50061000	4.84E-07
chr22	50057001	50062000	6.93E-05
chr22	50080001	50085000	3.52E-08
chr22	50081001	50086000	1.21E-08
chr22	50082001	50087000	9.60E-10
chr22	50083001	50088000	1.12E-15
chr22	50084001	50089000	3.67E-14
chr22	50085001	50090000	9.93E-12
chr22	50086001	50091000	7.85E-08

chr22	50087001	50092000	2.07E-07
chr22	50097001	50102000	4.25E-06
chr22	50098001	50103000	1.21E-06
chr22	50218001	50223000	0.000629
chr22	50238001	50243000	0.000145
chr22	50320001	50325000	2.94E-05
chr22	50321001	50326000	0.004395
chr22	50325001	50330000	6.09E-05
chr22	50326001	50331000	4.37E-07
chr22	50327001	50332000	0.000144
chr22	50328001	50333000	0.004483
chr22	50329001	50334000	0.018854
chr22	50336001	50341000	2.07E-08
chr22	50337001	50342000	2.90E-09
chr22	50338001	50343000	1.04E-07
chr22	50339001	50344000	3.30E-09
chr22	50519001	50524000	2.05E-09
chr22	50656001	50661000	0.002401
chr22	50658001	50663000	0.012316
chr22	50748001	50753000	2.34E-05
chr22	50749001	50754000	5.96E-05
chr22	50752001	50757000	4.80E-06
chr22	50753001	50758000	3.40E-06
chr22	50755001	50760000	3.07E-07
chr22	50959001	50964000	0.000102
chr22	50960001	50965000	0.000102
chr22	50961001	50966000	1.13E-06
chr22	50962001	50967000	5.34E-05
chr22	50983001	50988000	0.000234
chr22	50986001	50991000	2.79E-08
chr22	51176001	51181000	2.45E-05
chrX	2737001	2742000	0.000714
chrX	2738001	2743000	7.15E-06
chrX	2739001	2744000	1.55E-05
chrX	2898001	2903000	0.000285
chrX	2924001	2929000	3.01E-05
chrX	3862001	3867000	9.66E-08
chrX	3863001	3868000	4.80E-08
chrX	3865001	3870000	1.79E-05
chrX	3928001	3933000	6.85E-05
chrX	3929001	3934000	7.14E-06
chrX	3930001	3935000	1.13E-06
chrX	3944001	3949000	0.000209
chrX	4047001	4052000	3.25E-07
chrX	4301001	4306000	1.57E-05
chrX	4302001	4307000	2.74E-07
chrX	5039001	5044000	4.94E-05
chrX	5040001	5045000	0.000366

chrX	5041001	5046000	0.000103
chrX	5242001	5247000	0.000175
chrX	5243001	5248000	0.000268
chrX	5705001	5710000	4.20E-05
chrX	5999001	6004000	8.56E-05
chrX	6243001	6248000	0.000223
chrX	6244001	6249000	0.001049
chrX	6425001	6430000	1.13E-08
chrX	6426001	6431000	4.10E-09
chrX	9409001	9414000	8.82E-05
chrX	9433001	9438000	4.76E-09
chrX	9673001	9678000	1.91E-05
chrX	11834001	11839000	0.000441
chrX	12107001	12112000	0.011472
chrX	12774001	12779000	4.88E-05
chrX	14382001	14387000	0.000719
chrX	14467001	14472000	0.010573
chrX	14575001	14580000	5.12E-06
chrX	14911001	14916000	0.002916
chrX	15159001	15164000	2.41E-06
chrX	15245001	15250000	9.29E-08
chrX	15930001	15935000	0.001427
chrX	16028001	16033000	0.000419
chrX	17298001	17303000	0.001141
chrX	17875001	17880000	1.96E-08
chrX	18400001	18405000	0.005158
chrX	19166001	19171000	0.000153
chrX	19390001	19395000	0.002049
chrX	19425001	19430000	7.80E-06
chrX	20047001	20052000	6.93E-05
chrX	21945001	21950000	2.40E-05
chrX	22986001	22991000	5.64E-05
chrX	23658001	23663000	0.000669
chrX	23768001	23773000	0.0025
chrX	23814001	23819000	0.002758
chrX	24164001	24169000	1.20E-09
chrX	24179001	24184000	1.03E-08
chrX	24180001	24185000	1.28E-07
chrX	24181001	24186000	3.84E-06
chrX	24352001	24357000	0.001323
chrX	24479001	24484000	8.34E-06
chrX	25437001	25442000	8.17E-06
chrX	25617001	25622000	0.000254
chrX	25696001	25701000	7.40E-06
chrX	25697001	25702000	2.68E-05
chrX	25771001	25776000	0.000803
chrX	25772001	25777000	2.14E-05
chrX	26486001	26491000	6.19E-08

chrX	26487001	26492000	2.82E-09
chrX	26488001	26493000	8.21E-09
chrX	26592001	26597000	0.00104
chrX	26898001	26903000	0.000389
chrX	27057001	27062000	5.97E-05
chrX	27196001	27201000	0.00022
chrX	27396001	27401000	0.00759
chrX	28055001	28060000	5.00E-06
chrX	28056001	28061000	2.33E-07
chrX	28408001	28413000	1.12E-08
chrX	28409001	28414000	2.98E-08
chrX	28410001	28415000	2.78E-06
chrX	28567001	28572000	1.26E-05
chrX	28568001	28573000	1.05E-06
chrX	29399001	29404000	0.002136
chrX	29414001	29419000	0.000503
chrX	30232001	30237000	0.000981
chrX	30579001	30584000	0.003891
chrX	30809001	30814000	0.01983
chrX	30810001	30815000	0.002831
chrX	30811001	30816000	0.006102
chrX	33559001	33564000	2.45E-05
chrX	33682001	33687000	1.05E-05
chrX	33874001	33879000	1.82E-05
chrX	33937001	33942000	6.45E-05
chrX	34090001	34095000	6.39E-05
chrX	34350001	34355000	3.48E-05
chrX	34464001	34469000	5.17E-05
chrX	34465001	34470000	5.17E-05
chrX	34563001	34568000	6.81E-06
chrX	34564001	34569000	1.42E-05
chrX	35777001	35782000	7.99E-06
chrX	36743001	36748000	2.14E-05
chrX	36744001	36749000	2.72E-06
chrX	36745001	36750000	2.72E-05
chrX	36805001	36810000	0.000324
chrX	36806001	36811000	0.000324
chrX	36807001	36812000	0.000295
chrX	37097001	37102000	0.003599
chrX	37144001	37149000	0.029512
chrX	37398001	37403000	1.33E-06
chrX	37399001	37404000	5.11E-07
chrX	37400001	37405000	9.03E-09
chrX	38190001	38195000	5.59E-05
chrX	38288001	38293000	4.12E-06
chrX	38969001	38974000	9.27E-05
chrX	39159001	39164000	0.002542
chrX	39249001	39254000	0.000332

chrX	39586001	39591000	2.55E-05
chrX	39587001	39592000	9.08E-07
chrX	39588001	39593000	0.002668
chrX	39589001	39594000	0.00073
chrX	39590001	39595000	0.000216
chrX	39990001	39995000	0.001681
chrX	40022001	40027000	1.33E-05
chrX	40148001	40153000	0.019619
chrX	40155001	40160000	3.23E-05
chrX	40628001	40633000	8.38E-09
chrX	40767001	40772000	0.00872
chrX	41109001	41114000	0.000104
chrX	41110001	41115000	3.23E-05
chrX	41148001	41153000	0.000412
chrX	41149001	41154000	0.000774
chrX	41156001	41161000	0.009587
chrX	42570001	42575000	0.000211
chrX	44571001	44576000	0.000328
chrX	44572001	44577000	0.006931
chrX	44652001	44657000	4.55E-06
chrX	44655001	44660000	0.00206
chrX	45741001	45746000	9.96E-05
chrX	45786001	45791000	6.09E-06
chrX	45794001	45799000	1.76E-12
chrX	45795001	45800000	5.31E-09
chrX	45796001	45801000	1.44E-09
chrX	45797001	45802000	1.31E-08
chrX	45822001	45827000	6.07E-07
chrX	45823001	45828000	9.48E-09
chrX	45831001	45836000	4.09E-08
chrX	45832001	45837000	1.07E-05
chrX	45833001	45838000	8.39E-06
chrX	45834001	45839000	2.29E-09
chrX	45850001	45855000	4.29E-05
chrX	45851001	45856000	1.49E-05
chrX	45852001	45857000	1.23E-08
chrX	45853001	45858000	8.10E-07
chrX	45854001	45859000	1.07E-05
chrX	45876001	45881000	1.51E-06
chrX	45877001	45882000	1.96E-08
chrX	45878001	45883000	1.33E-08
chrX	45879001	45884000	6.46E-08
chrX	45880001	45885000	1.89E-06
chrX	45910001	45915000	2.38E-08
chrX	45913001	45918000	3.70E-07
chrX	45914001	45919000	2.21E-05
chrX	45915001	45920000	5.85E-08
chrX	45916001	45921000	2.01E-06

chrX	45917001	45922000	7.35E-07
chrX	45937001	45942000	6.59E-09
chrX	45938001	45943000	1.15E-08
chrX	45939001	45944000	5.27E-08
chrX	45958001	45963000	1.82E-06
chrX	45959001	45964000	1.70E-05
chrX	45960001	45965000	0.002555
chrX	46642001	46647000	0.047657
chrX	47137001	47142000	6.39E-05
chrX	47417001	47422000	4.16E-07
chrX	47418001	47423000	1.83E-09
chrX	47419001	47424000	1.14E-06
chrX	47420001	47425000	1.29E-07
chrX	47482001	47487000	0.000139
chrX	47483001	47488000	0.004551
chrX	47509001	47514000	0.013147
chrX	47631001	47636000	0.002759
chrX	47632001	47637000	0.002389
chrX	47633001	47638000	0.001144
chrX	47635001	47640000	5.42E-05
chrX	47926001	47931000	1.22E-07
chrX	47928001	47933000	0.000182
chrX	47929001	47934000	0.000154
chrX	48227001	48232000	7.21E-09
chrX	48266001	48271000	0.034994
chrX	48267001	48272000	0.034994
chrX	48268001	48273000	0.001556
chrX	48269001	48274000	0.004023
chrX	48270001	48275000	0.004288
chrX	48288001	48293000	0.00504
chrX	48444001	48449000	0.005434
chrX	48454001	48459000	0.005972
chrX	48699001	48704000	2.89E-05
chrX	48700001	48705000	0.00074
chrX	48745001	48750000	2.44E-06
chrX	48746001	48751000	5.63E-07
chrX	48811001	48816000	7.75E-09
chrX	48812001	48817000	1.36E-06
chrX	48813001	48818000	3.88E-08
chrX	48814001	48819000	3.07E-05
chrX	48815001	48820000	0.0021
chrX	48816001	48821000	6.72E-06
chrX	48869001	48874000	1.16E-07
chrX	48870001	48875000	1.49E-06
chrX	48871001	48876000	3.12E-06
chrX	48872001	48877000	4.60E-08
chrX	48878001	48883000	1.48E-07
chrX	48879001	48884000	7.23E-05

chrX	48880001	48885000	0.002118
chrX	48881001	48886000	0.000197
chrX	48882001	48887000	0.000168
chrX	48889001	48894000	0.008461
chrX	48890001	48895000	0.016351
chrX	48908001	48913000	0.012686
chrX	48930001	48935000	3.20E-14
chrX	48931001	48936000	1.65E-10
chrX	48977001	48982000	5.79E-06
chrX	48980001	48985000	6.41E-05
chrX	48991001	48996000	0.006942
chrX	49005001	49010000	0.036619
chrX	49020001	49025000	0.002302
chrX	49021001	49026000	0.002302
chrX	49022001	49027000	0.000742
chrX	49024001	49029000	5.15E-05
chrX	49047001	49052000	0.000283
chrX	49064001	49069000	0.014972
chrX	49066001	49071000	0.003308
chrX	49137001	49142000	5.59E-05
chrX	49138001	49143000	2.92E-05
chrX	49443001	49448000	0.000307
chrX	49490001	49495000	6.43E-05
chrX	49491001	49496000	1.91E-05
chrX	49492001	49497000	5.34E-08
chrX	49493001	49498000	1.10E-05
chrX	49494001	49499000	3.81E-06
chrX	49628001	49633000	4.99E-05
chrX	49773001	49778000	0.000981
chrX	50630001	50635000	0.005581
chrX	50708001	50713000	1.24E-05
chrX	50709001	50714000	0.00021
chrX	50899001	50904000	0.027522
chrX	50900001	50905000	0.001149
chrX	51188001	51193000	0.028185
chrX	51720001	51725000	0.000166
chrX	51722001	51727000	1.64E-07
chrX	51723001	51728000	9.54E-07
chrX	51724001	51729000	2.19E-08
chrX	51838001	51843000	0.000106
chrX	51839001	51844000	0.000196
chrX	52030001	52035000	0.000153
chrX	53249001	53254000	0.030968
chrX	53250001	53255000	7.88E-15
chrX	53251001	53256000	4.13E-11
chrX	53252001	53257000	5.22E-11
chrX	53253001	53258000	8.25E-11
chrX	53254001	53259000	2.92E-12

chrX	53291001	53296000	0.00047
chrX	53292001	53297000	7.29E-05
chrX	53293001	53298000	0.00068
chrX	53379001	53384000	3.53E-06
chrX	53500001	53505000	0.002068
chrX	53501001	53506000	0.003208
chrX	53537001	53542000	0.008009
chrX	53893001	53898000	3.16E-08
chrX	53894001	53899000	5.96E-08
chrX	54453001	54458000	0.001812
chrX	54533001	54538000	1.13E-06
chrX	54947001	54952000	0.000917
chrX	55406001	55411000	1.63E-08
chrX	55724001	55729000	0.000937
chrX	55826001	55831000	0.001234
chrX	56148001	56153000	0.001067
chrX	56511001	56516000	0.000786
chrX	56722001	56727000	0.031237
chrX	56723001	56728000	0.018201
chrX	56916001	56921000	0.001795
chrX	56917001	56922000	0.008106
chrX	57046001	57051000	0.00396
chrX	57539001	57544000	1.02E-05
chrX	57540001	57545000	4.36E-06
chrX	57732001	57737000	4.33E-06
chrX	57734001	57739000	9.97E-09
chrX	57735001	57740000	3.32E-06
chrX	57824001	57829000	0.00027
chrX	57896001	57901000	0.000274
chrX	61842001	61847000	4.99E-05
chrX	62949001	62954000	0.000288
chrX	63084001	63089000	0.000335
chrX	63174001	63179000	0.000102
chrX	63175001	63180000	0.000174
chrX	63176001	63181000	0.00079
chrX	63182001	63187000	0.020586
chrX	63183001	63188000	0.008295
chrX	63225001	63230000	0.00037
chrX	63226001	63231000	7.44E-06
chrX	63227001	63232000	3.03E-05
chrX	63265001	63270000	0.000917
chrX	63596001	63601000	0.001466
chrX	63684001	63689000	0.002361
chrX	63806001	63811000	1.15E-05
chrX	63815001	63820000	0.000679
chrX	63860001	63865000	0.000153
chrX	63898001	63903000	0.000643
chrX	64391001	64396000	0.000288

chrX	64438001	64443000	0.004246
chrX	64439001	64444000	0.001822
chrX	64603001	64608000	0.000819
chrX	64604001	64609000	1.78E-06
chrX	64605001	64610000	1.05E-06
chrX	64606001	64611000	7.88E-07
chrX	64607001	64612000	9.95E-05
chrX	64642001	64647000	0.000126
chrX	64643001	64648000	1.14E-06
chrX	64644001	64649000	0.037483
chrX	64645001	64650000	0.006809
chrX	64674001	64679000	0.003044
chrX	64675001	64680000	0.002093
chrX	64708001	64713000	2.51E-08
chrX	64756001	64761000	0.000244
chrX	65235001	65240000	0.007741
chrX	65348001	65353000	6.02E-05
chrX	65349001	65354000	0.001634
chrX	65434001	65439000	3.29E-05
chrX	65435001	65440000	2.05E-06
chrX	65436001	65441000	3.49E-05
chrX	65437001	65442000	6.57E-05
chrX	65438001	65443000	4.54E-05
chrX	65449001	65454000	0.000983
chrX	65475001	65480000	0.003711
chrX	65612001	65617000	7.80E-05
chrX	65613001	65618000	7.80E-05
chrX	65720001	65725000	2.78E-05
chrX	65721001	65726000	0.000388
chrX	65754001	65759000	9.40E-05
chrX	65853001	65858000	2.10E-10
chrX	65854001	65859000	7.68E-10
chrX	65855001	65860000	2.39E-10
chrX	65856001	65861000	1.81E-09
chrX	65857001	65862000	4.05E-09
chrX	66476001	66481000	2.80E-05
chrX	66525001	66530000	0.015342
chrX	66627001	66632000	0.000191
chrX	66634001	66639000	1.10E-05
chrX	66692001	66697000	1.78E-05
chrX	66906001	66911000	1.50E-06
chrX	67072001	67077000	0.000104
chrX	67078001	67083000	0.002693
chrX	67080001	67085000	0.000226
chrX	67126001	67131000	0.014588
chrX	67215001	67220000	1.81E-05
chrX	67218001	67223000	6.24E-06
chrX	67358001	67363000	2.68E-07

chrX	67591001	67596000	5.41E-05
chrX	67680001	67685000	0.000157
chrX	67708001	67713000	5.00E-07
chrX	67770001	67775000	3.97E-06
chrX	67771001	67776000	0.000238
chrX	68534001	68539000	0.006346
chrX	68543001	68548000	0.00468
chrX	68577001	68582000	0.020243
chrX	68687001	68692000	0.002255
chrX	68961001	68966000	8.86E-05
chrX	68962001	68967000	2.05E-05
chrX	69335001	69340000	0.000514
chrX	69671001	69676000	0.002077
chrX	69841001	69846000	7.80E-05
chrX	69844001	69849000	9.23E-06
chrX	69845001	69850000	1.83E-07
chrX	70078001	70083000	7.12E-05
chrX	70095001	70100000	0.000108
chrX	70096001	70101000	8.45E-05
chrX	70103001	70108000	0.010533
chrX	70364001	70369000	7.26E-07
chrX	70366001	70371000	0.005779
chrX	70572001	70577000	0.000729
chrX	70842001	70847000	0.002891
chrX	70922001	70927000	0.002247
chrX	71507001	71512000	0.001028
chrX	71931001	71936000	2.95E-06
chrX	72121001	72126000	0.008055
chrX	72178001	72183000	0.001178
chrX	72179001	72184000	0.001178
chrX	72180001	72185000	0.001178
chrX	72181001	72186000	0.001911
chrX	72182001	72187000	0.001911
chrX	72222001	72227000	0.004041
chrX	72418001	72423000	0.022872
chrX	72583001	72588000	4.38E-06
chrX	72584001	72589000	3.16E-06
chrX	72705001	72710000	0.000512
chrX	72706001	72711000	0.002869
chrX	72964001	72969000	4.25E-05
chrX	72975001	72980000	0.00043
chrX	73013001	73018000	0.000875
chrX	73512001	73517000	7.47E-05
chrX	73623001	73628000	0.001588
chrX	73628001	73633000	0.000309
chrX	73699001	73704000	0.000121
chrX	73700001	73705000	0.000333
chrX	73713001	73718000	0.000501

chrX	73714001	73719000	0.000179
chrX	74116001	74121000	0.000169
chrX	74117001	74122000	0.000592
chrX	74118001	74123000	0.000198
chrX	74119001	74124000	0.000336
chrX	74120001	74125000	1.43E-05
chrX	74239001	74244000	0.013749
chrX	74447001	74452000	0.004741
chrX	74596001	74601000	9.30E-05
chrX	74606001	74611000	6.09E-05
chrX	74607001	74612000	0.000137
chrX	74608001	74613000	0.00048
chrX	74978001	74983000	0.000285
chrX	75018001	75023000	0.000753
chrX	75164001	75169000	4.78E-05
chrX	75166001	75171000	1.22E-05
chrX	75167001	75172000	7.58E-05
chrX	75168001	75173000	2.09E-05
chrX	75243001	75248000	0.000303
chrX	75244001	75249000	1.94E-05
chrX	75357001	75362000	0.000295
chrX	75358001	75363000	0.000346
chrX	75359001	75364000	0.001585
chrX	75360001	75365000	0.003072
chrX	75361001	75366000	9.25E-06
chrX	75368001	75373000	0.030367
chrX	75568001	75573000	0.000215
chrX	75569001	75574000	0.000112
chrX	75570001	75575000	0.001354
chrX	75583001	75588000	7.42E-07
chrX	75603001	75608000	0.000852
chrX	75611001	75616000	3.76E-06
chrX	75907001	75912000	0.007093
chrX	75908001	75913000	0.003528
chrX	75923001	75928000	7.46E-05
chrX	76055001	76060000	0.000325
chrX	76069001	76074000	0.001284
chrX	76122001	76127000	0.010601
chrX	76123001	76128000	0.000423
chrX	76124001	76129000	0.00027
chrX	76140001	76145000	0.000549
chrX	76320001	76325000	0.001027
chrX	76426001	76431000	2.42E-05
chrX	76427001	76432000	1.09E-05
chrX	76428001	76433000	2.25E-05
chrX	76429001	76434000	9.91E-05
chrX	76480001	76485000	0.000773
chrX	76481001	76486000	0.001805

chrX	76482001	76487000	0.008951
chrX	76496001	76501000	0.002781
chrX	76497001	76502000	0.000765
chrX	76523001	76528000	0.000461
chrX	76630001	76635000	0.000249
chrX	76631001	76636000	9.33E-05
chrX	76632001	76637000	0.00118
chrX	76633001	76638000	0.000309
chrX	76634001	76639000	0.001871
chrX	77022001	77027000	0.002147
chrX	77023001	77028000	0.002147
chrX	77186001	77191000	0.000596
chrX	77187001	77192000	8.43E-05
chrX	77188001	77193000	0.000398
chrX	77314001	77319000	0.000171
chrX	77503001	77508000	0.000817
chrX	77504001	77509000	0.001651
chrX	77995001	78000000	1.44E-05
chrX	77996001	78001000	0.000275
chrX	77997001	78002000	0.000169
chrX	78143001	78148000	2.62E-05
chrX	78217001	78222000	1.62E-06
chrX	78354001	78359000	3.69E-05
chrX	78355001	78360000	1.49E-05
chrX	78843001	78848000	0.001923
chrX	78971001	78976000	0.001455
chrX	79068001	79073000	0.000714
chrX	79321001	79326000	0.00397
chrX	79351001	79356000	0.000538
chrX	79508001	79513000	3.62E-08
chrX	79660001	79665000	3.95E-11
chrX	79661001	79666000	9.21E-08
chrX	80075001	80080000	4.45E-05
chrX	80086001	80091000	0.012672
chrX	81072001	81077000	0.004153
chrX	81251001	81256000	9.59E-05
chrX	81253001	81258000	0.002725
chrX	81331001	81336000	0.00011
chrX	81332001	81337000	1.08E-06
chrX	81534001	81539000	8.09E-07
chrX	81535001	81540000	5.29E-06
chrX	81615001	81620000	1.81E-07
chrX	82092001	82097000	0.001699
chrX	82095001	82100000	0.001093
chrX	82184001	82189000	0.004266
chrX	82686001	82691000	6.19E-08
chrX	82687001	82692000	5.78E-07
chrX	82909001	82914000	0.000484

chrX	82910001	82915000	0.00104
chrX	82911001	82916000	0.000431
chrX	83001001	83006000	1.19E-05
chrX	83700001	83705000	0.008355
chrX	83857001	83862000	0.004384
chrX	84457001	84462000	0.000574
chrX	84458001	84463000	0.000103
chrX	84980001	84985000	3.71E-05
chrX	85060001	85065000	0.02296
chrX	85452001	85457000	0.00032
chrX	85453001	85458000	0.000118
chrX	85455001	85460000	3.40E-06
chrX	85456001	85461000	2.28E-06
chrX	85499001	85504000	0.002148
chrX	85500001	85505000	0.000214
chrX	85501001	85506000	0.000122
chrX	85657001	85662000	5.27E-05
chrX	85816001	85821000	2.86E-05
chrX	85894001	85899000	0.000364
chrX	86109001	86114000	1.26E-05
chrX	86218001	86223000	0.005113
chrX	86219001	86224000	0.002855
chrX	86220001	86225000	0.000171
chrX	86277001	86282000	0.001993
chrX	86278001	86283000	0.011126
chrX	86279001	86284000	0.008709
chrX	86685001	86690000	4.55E-10
chrX	86686001	86691000	2.86E-08
chrX	86714001	86719000	0.000211
chrX	86728001	86733000	2.35E-06
chrX	86729001	86734000	6.82E-07
chrX	86730001	86735000	1.53E-07
chrX	86798001	86803000	0.001135
chrX	86839001	86844000	0.007887
chrX	87015001	87020000	0.008269
chrX	87227001	87232000	0.000141
chrX	87767001	87772000	8.56E-06
chrX	88012001	88017000	6.83E-05
chrX	88013001	88018000	0.00024
chrX	88160001	88165000	0.003169
chrX	88223001	88228000	0.005378
chrX	88537001	88542000	0.013132
chrX	88653001	88658000	0.000704
chrX	88662001	88667000	0.000841
chrX	88667001	88672000	2.25E-06
chrX	88760001	88765000	0.001187
chrX	88761001	88766000	0.00042
chrX	88932001	88937000	0.000204

chrX	88933001	88938000	9.02E-05
chrX	89127001	89132000	0.000232
chrX	89128001	89133000	0.000134
chrX	89129001	89134000	3.57E-05
chrX	89130001	89135000	0.000509
chrX	89131001	89136000	0.002598
chrX	89157001	89162000	0.001276
chrX	89350001	89355000	8.21E-06
chrX	89455001	89460000	0.000226
chrX	90344001	90349000	0.001089
chrX	90401001	90406000	2.64E-05
chrX	90402001	90407000	1.07E-05
chrX	90417001	90422000	4.67E-07
chrX	90449001	90454000	0.000724
chrX	90566001	90571000	2.13E-06
chrX	90567001	90572000	8.28E-07
chrX	90674001	90679000	1.54E-05
chrX	90898001	90903000	0.000212
chrX	90899001	90904000	0.00013
chrX	91209001	91214000	1.89E-06
chrX	91210001	91215000	4.47E-06
chrX	91240001	91245000	0.000498
chrX	91241001	91246000	0.004831
chrX	91242001	91247000	0.000951
chrX	91243001	91248000	0.001115
chrX	91244001	91249000	0.0003
chrX	91245001	91250000	7.82E-05
chrX	91260001	91265000	0.000748
chrX	91271001	91276000	0.008764
chrX	91471001	91476000	0.000764
chrX	91472001	91477000	0.000204
chrX	91473001	91478000	1.55E-05
chrX	91591001	91596000	5.61E-07
chrX	91812001	91817000	0.004844
chrX	91813001	91818000	0.00399
chrX	91905001	91910000	8.03E-07
chrX	91906001	91911000	1.66E-06
chrX	92025001	92030000	9.20E-05
chrX	92026001	92031000	0.00011
chrX	92027001	92032000	0.000762
chrX	92040001	92045000	0.000643
chrX	92274001	92279000	0.000123
chrX	92276001	92281000	2.62E-07
chrX	92891001	92896000	2.14E-06
chrX	92892001	92897000	9.75E-06
chrX	93204001	93209000	0.018792
chrX	93538001	93543000	2.06E-06
chrX	94147001	94152000	2.47E-05

chrX	94566001	94571000	0.000994
chrX	95036001	95041000	2.51E-06
chrX	96584001	96589000	1.95E-05
chrX	97693001	97698000	0.000451
chrX	97715001	97720000	0.000799
chrX	97785001	97790000	0.000826
chrX	97789001	97794000	0.000726
chrX	97814001	97819000	5.48E-06
chrX	97837001	97842000	1.90E-05
chrX	97968001	97973000	3.70E-05
chrX	97969001	97974000	6.80E-07
chrX	97993001	97998000	2.91E-08
chrX	97994001	97999000	1.12E-07
chrX	97995001	98000000	5.53E-08
chrX	98112001	98117000	9.54E-05
chrX	98506001	98511000	0.003047
chrX	98508001	98513000	0.006509
chrX	99322001	99327000	1.02E-05
chrX	99323001	99328000	4.17E-06
chrX	100302001	100307000	8.38E-05
chrX	100303001	100308000	0.000861
chrX	100304001	100309000	2.66E-14
chrX	100305001	100310000	1.44E-11
chrX	100306001	100311000	2.21E-13
chrX	100326001	100331000	0.001279
chrX	100516001	100521000	0.006789
chrX	101081001	101086000	8.29E-05
chrX	101135001	101140000	1.96E-05
chrX	101224001	101229000	2.71E-05
chrX	101225001	101230000	4.02E-06
chrX	101273001	101278000	6.70E-05
chrX	101484001	101489000	0.0049
chrX	101485001	101490000	0.0049
chrX	101486001	101491000	0.000202
chrX	101487001	101492000	0.000202
chrX	101488001	101493000	7.37E-05
chrX	101650001	101655000	0.008943
chrX	101651001	101656000	0.008943
chrX	101736001	101741000	0.000811
chrX	102224001	102229000	0.015668
chrX	102315001	102320000	9.71E-05
chrX	102362001	102367000	0.033816
chrX	102403001	102408000	0.001499
chrX	102404001	102409000	0.001969
chrX	102859001	102864000	4.48E-07
chrX	102860001	102865000	7.63E-08
chrX	102861001	102866000	5.44E-08
chrX	102862001	102867000	3.57E-09

chrX	102863001	102868000	3.10E-07
chrX	103169001	103174000	2.88E-07
chrX	103227001	103232000	0.038984
chrX	103228001	103233000	0.038984
chrX	103229001	103234000	0.038984
chrX	103230001	103235000	0.038984
chrX	103231001	103236000	0.038984
chrX	103327001	103332000	9.04E-05
chrX	103328001	103333000	0.00066
chrX	103329001	103334000	0.005949
chrX	103796001	103801000	9.58E-06
chrX	104599001	104604000	0.000439
chrX	104617001	104622000	0.005516
chrX	104630001	104635000	2.02E-06
chrX	104645001	104650000	0.0007
chrX	105539001	105544000	0.014053
chrX	105762001	105767000	2.04E-05
chrX	105763001	105768000	2.45E-06
chrX	106287001	106292000	1.87E-05
chrX	106629001	106634000	0.000152
chrX	106640001	106645000	1.55E-05
chrX	106657001	106662000	0.001521
chrX	106674001	106679000	0.000183
chrX	106675001	106680000	0.009168
chrX	106802001	106807000	8.46E-05
chrX	106867001	106872000	2.89E-06
chrX	106917001	106922000	1.87E-06
chrX	107019001	107024000	9.01E-14
chrX	107020001	107025000	3.93E-14
chrX	107242001	107247000	1.07E-05
chrX	108123001	108128000	0.002466
chrX	108124001	108129000	0.003458
chrX	108163001	108168000	0.000207
chrX	108212001	108217000	0.000331
chrX	108243001	108248000	2.07E-05
chrX	108249001	108254000	0.00923
chrX	108395001	108400000	0.001751
chrX	108396001	108401000	0.006131
chrX	108831001	108836000	4.06E-06
chrX	108832001	108837000	3.60E-06
chrX	108996001	109001000	0.000509
chrX	109032001	109037000	0.000155
chrX	109033001	109038000	0.000361
chrX	109116001	109121000	6.98E-07
chrX	109117001	109122000	5.75E-08
chrX	110097001	110102000	0.002964
chrX	110118001	110123000	0.002738
chrX	110856001	110861000	3.61E-05

chrX	110882001	110887000	6.51E-07
chrX	111806001	111811000	0.00796
chrX	111965001	111970000	0.000419
chrX	112081001	112086000	2.63E-07
chrX	112082001	112087000	6.65E-07
chrX	112083001	112088000	5.68E-09
chrX	112255001	112260000	0.000478
chrX	112256001	112261000	7.75E-05
chrX	112326001	112331000	2.41E-05
chrX	112533001	112538000	1.14E-05
chrX	112534001	112539000	2.99E-06
chrX	112535001	112540000	2.23E-05
chrX	112564001	112569000	0.000793
chrX	112584001	112589000	0.00032
chrX	112585001	112590000	0.000616
chrX	112586001	112591000	0.006041
chrX	112587001	112592000	0.00216
chrX	112606001	112611000	0.000452
chrX	112940001	112945000	0.000314
chrX	114165001	114170000	0.002907
chrX	114196001	114201000	0.000297
chrX	114341001	114346000	1.37E-05
chrX	114357001	114362000	8.32E-11
chrX	114923001	114928000	0.016687
chrX	114926001	114931000	1.80E-06
chrX	114929001	114934000	2.42E-05
chrX	114930001	114935000	0.000519
chrX	114932001	114937000	0.000117
chrX	114933001	114938000	9.87E-06
chrX	114934001	114939000	3.80E-05
chrX	115133001	115138000	4.21E-05
chrX	115134001	115139000	1.06E-05
chrX	115135001	115140000	0.001498
chrX	115136001	115141000	3.55E-05
chrX	116067001	116072000	0.001792
chrX	116068001	116073000	0.000119
chrX	116173001	116178000	0.000265
chrX	116174001	116179000	0.000318
chrX	116175001	116180000	0.002222
chrX	116490001	116495000	0.000943
chrX	116491001	116496000	0.000943
chrX	116492001	116497000	0.000623
chrX	116584001	116589000	0.000181
chrX	116585001	116590000	0.00055
chrX	116715001	116720000	0.000402
chrX	116808001	116813000	0.005193
chrX	116809001	116814000	0.000542
chrX	116810001	116815000	0.000133

chrX	117687001	117692000	0.001905
chrX	118544001	118549000	9.37E-06
chrX	118545001	118550000	2.83E-05
chrX	118546001	118551000	9.44E-05
chrX	118695001	118700000	0.004435
chrX	118825001	118830000	1.45E-11
chrX	118826001	118831000	8.68E-14
chrX	118839001	118844000	0.000387
chrX	118840001	118845000	0.000462
chrX	118841001	118846000	0.000644
chrX	118863001	118868000	0.001929
chrX	118874001	118879000	0.026892
chrX	118885001	118890000	0.02052
chrX	118892001	118897000	0.013849
chrX	118924001	118929000	1.53E-09
chrX	118999001	119004000	0.010195
chrX	119000001	119005000	4.04E-05
chrX	119001001	119006000	0.000438
chrX	119002001	119007000	0.000283
chrX	119420001	119425000	0.000664
chrX	119471001	119476000	0.001795
chrX	120390001	120395000	0.001832
chrX	121451001	121456000	2.13E-05
chrX	121581001	121586000	1.10E-08
chrX	121582001	121587000	4.88E-10
chrX	121834001	121839000	3.10E-05
chrX	121948001	121953000	0.000294
chrX	122029001	122034000	0.00321
chrX	122030001	122035000	0.000347
chrX	122031001	122036000	0.000447
chrX	122032001	122037000	0.000471
chrX	122033001	122038000	0.004399
chrX	122187001	122192000	0.000455
chrX	122702001	122707000	0.020102
chrX	122704001	122709000	0.004399
chrX	122724001	122729000	0.000384
chrX	122885001	122890000	0.009552
chrX	122886001	122891000	0.002425
chrX	122887001	122892000	0.002425
chrX	122900001	122905000	0.000105
chrX	122901001	122906000	2.97E-05
chrX	122918001	122923000	4.60E-06
chrX	123068001	123073000	0.000546
chrX	123069001	123074000	0.000877
chrX	123075001	123080000	0.001704
chrX	123091001	123096000	7.04E-06
chrX	123094001	123099000	0.001025
chrX	123313001	123318000	0.000697

chrX	123848001	123853000	2.76E-05
chrX	123849001	123854000	2.17E-07
chrX	124320001	124325000	1.45E-08
chrX	124321001	124326000	6.42E-08
chrX	124322001	124327000	1.01E-06
chrX	124692001	124697000	0.003409
chrX	125158001	125163000	1.22E-05
chrX	125166001	125171000	0.005253
chrX	125802001	125807000	0.017537
chrX	126377001	126382000	0.016101
chrX	126665001	126670000	2.12E-05
chrX	127247001	127252000	1.69E-06
chrX	127248001	127253000	1.39E-05
chrX	127249001	127254000	4.17E-06
chrX	127387001	127392000	0.003364
chrX	127444001	127449000	2.64E-06
chrX	127545001	127550000	9.81E-05
chrX	127622001	127627000	1.04E-05
chrX	128089001	128094000	0.00128
chrX	128103001	128108000	0.000367
chrX	128104001	128109000	0.000297
chrX	128561001	128566000	0.003604
chrX	130180001	130185000	2.50E-07
chrX	130181001	130186000	9.87E-09
chrX	131703001	131708000	7.16E-05
chrX	131887001	131892000	0.000471
chrX	132575001	132580000	0.023879
chrX	132576001	132581000	0.001399
chrX	132577001	132582000	0.000421
chrX	132578001	132583000	0.002178
chrX	132627001	132632000	0.000937
chrX	133200001	133205000	5.27E-05
chrX	133224001	133229000	0.005206
chrX	134048001	134053000	0.002288
chrX	134166001	134171000	2.93E-07
chrX	135346001	135351000	0.002652
chrX	135347001	135352000	0.004991
chrX	135773001	135778000	0.000272
chrX	135774001	135779000	2.99E-07
chrX	135857001	135862000	0.003393
chrX	136687001	136692000	0.001146
chrX	136688001	136693000	8.05E-06
chrX	136689001	136694000	8.74E-06
chrX	136690001	136695000	1.03E-05
chrX	136777001	136782000	0.000349
chrX	136778001	136783000	0.000135
chrX	136785001	136790000	0.000112
chrX	136986001	136991000	5.16E-05

chrX	136988001	136993000	9.39E-05
chrX	137020001	137025000	0.001414
chrX	137228001	137233000	0.004849
chrX	137287001	137292000	9.56E-07
chrX	137288001	137293000	1.71E-07
chrX	137290001	137295000	0.00031
chrX	137323001	137328000	5.67E-05
chrX	138011001	138016000	0.001286
chrX	138157001	138162000	7.65E-08
chrX	138287001	138292000	7.27E-08
chrX	138495001	138500000	0.002501
chrX	138496001	138501000	0.001574
chrX	138497001	138502000	0.029062
chrX	138498001	138503000	0.010253
chrX	139022001	139027000	2.30E-06
chrX	139023001	139028000	5.10E-06
chrX	139059001	139064000	0.013836
chrX	139081001	139086000	7.09E-06
chrX	139082001	139087000	2.23E-06
chrX	139488001	139493000	1.05E-06
chrX	139490001	139495000	1.69E-06
chrX	139491001	139496000	1.56E-05
chrX	139520001	139525000	1.32E-07
chrX	139521001	139526000	2.01E-08
chrX	139622001	139627000	0.00013
chrX	140127001	140132000	0.000203
chrX	140134001	140139000	0.035638
chrX	140420001	140425000	0.000638
chrX	140526001	140531000	0.000404
chrX	140527001	140532000	2.74E-05
chrX	140528001	140533000	1.34E-05
chrX	140529001	140534000	6.90E-07
chrX	140635001	140640000	3.16E-05
chrX	140679001	140684000	1.45E-05
chrX	140680001	140685000	1.87E-05
chrX	143170001	143175000	0.00157
chrX	143171001	143176000	0.011871
chrX	143234001	143239000	2.29E-05
chrX	143235001	143240000	7.51E-05
chrX	143236001	143241000	2.96E-06
chrX	143237001	143242000	3.61E-06
chrX	143267001	143272000	0.004709
chrX	143268001	143273000	0.004709
chrX	144337001	144342000	1.15E-05
chrX	144338001	144343000	0.000192
chrX	144355001	144360000	0.000105
chrX	144356001	144361000	3.36E-05
chrX	144369001	144374000	0.00664

chrX	144385001	144390000	0.000173
chrX	144386001	144391000	0.001301
chrX	144387001	144392000	0.000371
chrX	144388001	144393000	4.27E-06
chrX	144389001	144394000	1.17E-05
chrX	144421001	144426000	0.002694
chrX	144525001	144530000	9.62E-07
chrX	144526001	144531000	3.18E-07
chrX	144527001	144532000	4.22E-07
chrX	144581001	144586000	0.018771
chrX	144625001	144630000	6.43E-06
chrX	145380001	145385000	5.10E-09
chrX	145381001	145386000	1.01E-07
chrX	145415001	145420000	1.17E-05
chrX	145416001	145421000	7.16E-06
chrX	145544001	145549000	1.71E-09
chrX	145568001	145573000	0.000484
chrX	145570001	145575000	7.08E-05
chrX	145632001	145637000	2.53E-06
chrX	145633001	145638000	2.03E-06
chrX	146093001	146098000	0.000259
chrX	146096001	146101000	5.98E-08
chrX	146097001	146102000	1.06E-07
chrX	146107001	146112000	7.24E-06
chrX	146343001	146348000	3.12E-05
chrX	146344001	146349000	1.15E-05
chrX	146345001	146350000	8.94E-06
chrX	146737001	146742000	0.000112
chrX	147341001	147346000	0.005461
chrX	147342001	147347000	0.000304
chrX	147343001	147348000	0.000226
chrX	147391001	147396000	2.68E-05
chrX	147430001	147435000	6.96E-07
chrX	147504001	147509000	2.94E-07
chrX	148239001	148244000	0.000452
chrX	148351001	148356000	0.000819
chrX	148736001	148741000	0.016786
chrX	149322001	149327000	0.000453
chrX	149348001	149353000	0.01629
chrX	149449001	149454000	9.34E-07
chrX	149487001	149492000	0.001916
chrX	149488001	149493000	0.000144
chrX	149489001	149494000	4.51E-05
chrX	149490001	149495000	4.50E-05
chrX	149491001	149496000	0.004183
chrX	149676001	149681000	0.005907
chrX	149683001	149688000	0.0002
chrX	150063001	150068000	0.000312

chrX	150250001	150255000	0.000276
chrX	150256001	150261000	0.000276
chrX	150328001	150333000	0.002584
chrX	150329001	150334000	0.000271
chrX	150504001	150509000	0.000182
chrX	150505001	150510000	3.65E-06
chrX	150737001	150742000	3.64E-07
chrX	150738001	150743000	1.71E-05
chrX	150767001	150772000	3.43E-05
chrX	150768001	150773000	0.000323
chrX	150769001	150774000	1.91E-06
chrX	150770001	150775000	5.53E-06
chrX	150771001	150776000	1.59E-06
chrX	150861001	150866000	5.67E-05
chrX	150889001	150894000	0.000432
chrX	151062001	151067000	2.35E-05
chrX	151087001	151092000	0.001294
chrX	151148001	151153000	0.000131
chrX	151529001	151534000	7.90E-05
chrX	151530001	151535000	1.76E-05
chrX	151531001	151536000	1.65E-07
chrX	151567001	151572000	9.40E-06
chrX	151568001	151573000	2.92E-06
chrX	151621001	151626000	1.41E-05
chrX	151661001	151666000	6.26E-07
chrX	151662001	151667000	2.19E-07
chrX	151698001	151703000	1.55E-06
chrX	151889001	151894000	0.001815
chrX	151890001	151895000	0.000473
chrX	151891001	151896000	0.000336
chrX	151963001	151968000	0.000126
chrX	151964001	151969000	0.000144
chrX	152080001	152085000	0.021678
chrX	152170001	152175000	0.003049
chrX	152192001	152197000	0.000217
chrX	152193001	152198000	0.000503
chrX	152743001	152748000	0.047234
chrX	152861001	152866000	1.40E-06
chrX	152863001	152868000	1.05E-08
chrX	152864001	152869000	2.48E-05
chrX	152886001	152891000	6.45E-09
chrX	152887001	152892000	5.54E-07
chrX	152930001	152935000	0.00329
chrX	153136001	153141000	5.52E-05
chrX	153175001	153180000	1.19E-05
chrX	153176001	153181000	7.45E-07
chrX	153208001	153213000	0.000685
chrX	153232001	153237000	0.000135

chrX	153661001	153666000	0.003509
chrX	153683001	153688000	0.000289
chrX	153711001	153716000	0.00135
chrX	153715001	153720000	2.33E-05
chrX	153716001	153721000	5.08E-06
chrX	153717001	153722000	1.10E-05
chrX	153719001	153724000	1.18E-06
chrX	153987001	153992000	0.00023
chrX	153988001	153993000	2.16E-05
chrX	154732001	154737000	0.011223
chrX	154770001	154775000	0.004809
chrY	2660001	2665000	0.007866
chrY	2803001	2808000	0.015246
chrY	2990001	2995000	0.014995
chrY	2991001	2996000	0.014082
chrY	2992001	2997000	0.004445
chrY	2993001	2998000	0.000448
chrY	3024001	3029000	0.002076
chrY	3025001	3030000	0.000678
chrY	3026001	3031000	5.20E-05
chrY	3034001	3039000	0.007961
chrY	3035001	3040000	0.007961
chrY	3043001	3048000	0.011942
chrY	3055001	3060000	0.030717
chrY	3056001	3061000	0.037483
chrY	3057001	3062000	0.013267
chrY	3058001	3063000	0.006269
chrY	3059001	3064000	0.008199
chrY	3060001	3065000	0.026871
chrY	3076001	3081000	0.007367
chrY	3111001	3116000	0.002806
chrY	3117001	3122000	0.01416
chrY	3138001	3143000	0.001453
chrY	3139001	3144000	0.000664
chrY	3140001	3145000	0.002427
chrY	3141001	3146000	0.003002
chrY	3142001	3147000	0.032445
chrY	3144001	3149000	0.023645
chrY	3167001	3172000	0.010717
chrY	3168001	3173000	0.000804
chrY	3169001	3174000	0.005258
chrY	3177001	3182000	0.008108
chrY	3178001	3183000	0.000283
chrY	3180001	3185000	0.000382
chrY	3181001	3186000	0.000164
chrY	3186001	3191000	0.014101
chrY	3194001	3199000	0.005
chrY	3195001	3200000	0.007199

chrY	3196001	3201000	0.000211
chrY	3197001	3202000	0.00049
chrY	3223001	3228000	0.026419
chrY	3224001	3229000	0.028458
chrY	3248001	3253000	0.001985
chrY	3271001	3276000	0.048532
chrY	3273001	3278000	9.42E-05
chrY	3274001	3279000	0.000214
chrY	3281001	3286000	0.000525
chrY	3316001	3321000	0.002376
chrY	3317001	3322000	1.97E-06
chrY	3335001	3340000	0.020234
chrY	3336001	3341000	0.001549
chrY	3337001	3342000	0.001549
chrY	3350001	3355000	0.008179
chrY	3351001	3356000	0.023068
chrY	3385001	3390000	5.37E-05
chrY	3386001	3391000	0.00063
chrY	3387001	3392000	1.47E-06
chrY	3396001	3401000	0.009791
chrY	3422001	3427000	0.022993
chrY	3423001	3428000	0.000198
chrY	3424001	3429000	0.000198
chrY	3426001	3431000	3.14E-06
chrY	3430001	3435000	0.007258
chrY	3431001	3436000	0.009825
chrY	3432001	3437000	0.006177
chrY	3433001	3438000	0.021033
chrY	3434001	3439000	0.001445
chrY	3458001	3463000	0.003708
chrY	3462001	3467000	0.012971
chrY	3464001	3469000	0.021074
chrY	3465001	3470000	0.021074
chrY	3466001	3471000	0.031154
chrY	3473001	3478000	0.002399
chrY	3475001	3480000	0.001562
chrY	3481001	3486000	0.00094
chrY	3492001	3497000	0.002673
chrY	3512001	3517000	0.00489
chrY	3519001	3524000	0.000944
chrY	3521001	3526000	0.000247
chrY	3522001	3527000	0.003479
chrY	3523001	3528000	0.007918
chrY	3524001	3529000	0.010554
chrY	3527001	3532000	0.000889
chrY	3528001	3533000	0.002268
chrY	3529001	3534000	0.0008
chrY	3530001	3535000	0.007249

chrY	3531001	3536000	0.003053
chrY	3541001	3546000	0.000979
chrY	3542001	3547000	0.005726
chrY	3543001	3548000	0.000925
chrY	3544001	3549000	0.005678
chrY	3545001	3550000	0.001919
chrY	3546001	3551000	0.000186
chrY	3547001	3552000	9.46E-08
chrY	3548001	3553000	0.00064
chrY	3574001	3579000	0.012283
chrY	3575001	3580000	0.003908
chrY	3576001	3581000	0.019523
chrY	3577001	3582000	0.047069
chrY	3578001	3583000	0.047069
chrY	3579001	3584000	0.030664
chrY	3580001	3585000	0.019363
chrY	3581001	3586000	0.038242
chrY	3591001	3596000	0.009839
chrY	3592001	3597000	0.000902
chrY	3593001	3598000	4.20E-05
chrY	3594001	3599000	0.000124
chrY	3595001	3600000	2.96E-05
chrY	3603001	3608000	0.017846
chrY	3604001	3609000	0.017846
chrY	3605001	3610000	0.021535
chrY	3615001	3620000	0.000455
chrY	3616001	3621000	2.58E-05
chrY	3617001	3622000	3.39E-06
chrY	3638001	3643000	0.019178
chrY	3639001	3644000	0.019363
chrY	3640001	3645000	0.034976
chrY	3641001	3646000	0.006512
chrY	3642001	3647000	0.006512
chrY	3645001	3650000	0.013865
chrY	3651001	3656000	0.009086
chrY	3656001	3661000	0.030585
chrY	3657001	3662000	0.006346
chrY	3673001	3678000	0.001542
chrY	3674001	3679000	0.004548
chrY	3675001	3680000	0.004548
chrY	3676001	3681000	0.004548
chrY	3687001	3692000	0.015717
chrY	3702001	3707000	0.003414
chrY	3708001	3713000	0.000273
chrY	3727001	3732000	0.018538
chrY	3728001	3733000	0.008432
chrY	3729001	3734000	0.017812
chrY	3730001	3735000	0.001245

chrY	3731001	3736000	0.000598
chrY	3735001	3740000	0.013481
chrY	3747001	3752000	0.002553
chrY	3757001	3762000	0.000856
chrY	3758001	3763000	0.007657
chrY	3759001	3764000	0.007657
chrY	3761001	3766000	0.02041
chrY	3763001	3768000	0.035738
chrY	3764001	3769000	0.035738
chrY	3783001	3788000	0.021535
chrY	3785001	3790000	0.011232
chrY	3786001	3791000	0.002427
chrY	3787001	3792000	0.002427
chrY	3795001	3800000	0.011223
chrY	3796001	3801000	0.003343
chrY	3797001	3802000	0.001664
chrY	3798001	3803000	0.000948
chrY	3884001	3889000	0.005952
chrY	3885001	3890000	0.000946
chrY	3886001	3891000	0.016574
chrY	3887001	3892000	0.003183
chrY	3888001	3893000	0.003183
chrY	3895001	3900000	0.000272
chrY	3911001	3916000	0.007062
chrY	3913001	3918000	8.91E-05
chrY	3914001	3919000	0.000277
chrY	3917001	3922000	0.000981
chrY	3981001	3986000	6.12E-06
chrY	3982001	3987000	1.16E-05
chrY	3992001	3997000	0.023985
chrY	3993001	3998000	0.013032
chrY	3995001	4000000	0.031445
chrY	4007001	4012000	0.004054
chrY	4008001	4013000	0.004054
chrY	4009001	4014000	0.020717
chrY	4018001	4023000	0.010381
chrY	4020001	4025000	0.041406
chrY	4021001	4026000	0.041406
chrY	4022001	4027000	0.024214
chrY	4173001	4178000	0.022537
chrY	4174001	4179000	0.024789
chrY	4200001	4205000	0.022746
chrY	4213001	4218000	0.002762
chrY	4245001	4250000	0.001596
chrY	4246001	4251000	0.00529
chrY	4247001	4252000	0.00437
chrY	4248001	4253000	0.013916
chrY	4260001	4265000	0.012027

chrY	4261001	4266000	0.012027
chrY	4262001	4267000	0.012499
chrY	4278001	4283000	0.001041
chrY	4280001	4285000	0.007982
chrY	4317001	4322000	0.011232
chrY	4321001	4326000	0.033302
chrY	4322001	4327000	0.010436
chrY	4323001	4328000	0.006403
chrY	4324001	4329000	0.002523
chrY	4326001	4331000	0.005636
chrY	4374001	4379000	0.008439
chrY	4407001	4412000	0.000598
chrY	4408001	4413000	0.000598
chrY	4409001	4414000	0.011319
chrY	4410001	4415000	0.000569
chrY	4411001	4416000	0.000125
chrY	4416001	4421000	0.005753
chrY	4417001	4422000	0.002699
chrY	4418001	4423000	0.010802
chrY	4430001	4435000	0.022155
chrY	4431001	4436000	4.59E-06
chrY	4432001	4437000	2.96E-05
chrY	4433001	4438000	0.0006
chrY	4434001	4439000	1.80E-05
chrY	4435001	4440000	1.80E-05
chrY	4436001	4441000	0.033974
chrY	4442001	4447000	0.000879
chrY	4465001	4470000	0.023092
chrY	4473001	4478000	0.002969
chrY	4474001	4479000	0.000382
chrY	4475001	4480000	0.005279
chrY	4476001	4481000	0.017573
chrY	4477001	4482000	0.035859
chrY	4478001	4483000	0.026122
chrY	4480001	4485000	0.001285
chrY	4481001	4486000	0.001285
chrY	4540001	4545000	0.015853
chrY	4585001	4590000	0.000157
chrY	4586001	4591000	0.000631
chrY	4587001	4592000	0.005008
chrY	4614001	4619000	0.00104
chrY	4615001	4620000	0.002583
chrY	4635001	4640000	0.000113
chrY	4636001	4641000	0.000585
chrY	4658001	4663000	0.007739
chrY	4659001	4664000	0.001487
chrY	4660001	4665000	0.001245
chrY	4661001	4666000	0.003179

chrY	4664001	4669000	0.028185
chrY	4687001	4692000	3.77E-05
chrY	4688001	4693000	6.86E-05
chrY	4689001	4694000	0.001509
chrY	4691001	4696000	0.034595
chrY	4701001	4706000	0.00487
chrY	4702001	4707000	0.00093
chrY	4703001	4708000	0.001402
chrY	4704001	4709000	0.003063
chrY	4705001	4710000	0.008623
chrY	4708001	4713000	0.007676
chrY	4709001	4714000	0.002494
chrY	4710001	4715000	0.002494
chrY	4711001	4716000	0.006211
chrY	4712001	4717000	0.016752
chrY	4724001	4729000	0.00841
chrY	4725001	4730000	0.00841
chrY	4726001	4731000	0.000913
chrY	4727001	4732000	0.000913
chrY	4743001	4748000	0.026918
chrY	4763001	4768000	0.002673
chrY	4764001	4769000	0.000511
chrY	4765001	4770000	3.45E-05
chrY	4766001	4771000	3.45E-05
chrY	4767001	4772000	0.000345
chrY	4793001	4798000	0.007198
chrY	4794001	4799000	0.004664
chrY	4795001	4800000	0.039112
chrY	4806001	4811000	0.027994
chrY	4807001	4812000	0.027994
chrY	4808001	4813000	0.021027
chrY	4809001	4814000	0.039213
chrY	4810001	4815000	0.009587
chrY	4825001	4830000	0.013061
chrY	4826001	4831000	0.007854
chrY	4837001	4842000	0.005438
chrY	4844001	4849000	0.003616
chrY	4872001	4877000	0.009913
chrY	4873001	4878000	0.011619
chrY	4874001	4879000	0.011619
chrY	4888001	4893000	0.043824
chrY	4895001	4900000	0.000808
chrY	4896001	4901000	0.000157
chrY	4897001	4902000	0.000865
chrY	4898001	4903000	0.016744
chrY	4899001	4904000	0.001417
chrY	4913001	4918000	2.34E-08
chrY	4914001	4919000	4.31E-05

chrY	4915001	4920000	0.000186
chrY	4916001	4921000	0.000161
chrY	4921001	4926000	0.001803
chrY	4922001	4927000	0.000603
chrY	4923001	4928000	0.013326
chrY	4941001	4946000	0.009661
chrY	4942001	4947000	0.000594
chrY	4943001	4948000	0.000213
chrY	4944001	4949000	0.001058
chrY	4945001	4950000	0.002429
chrY	4946001	4951000	0.002221
chrY	4982001	4987000	0.00021
chrY	4987001	4992000	0.001348
chrY	4988001	4993000	0.00041
chrY	4989001	4994000	0.001609
chrY	4990001	4995000	0.000967
chrY	4991001	4996000	0.001053
chrY	5002001	5007000	0.005899
chrY	5003001	5008000	0.000162
chrY	5004001	5009000	0.000114
chrY	5005001	5010000	9.23E-06
chrY	5009001	5014000	0.006063
chrY	5010001	5015000	0.016268
chrY	5011001	5016000	0.003642
chrY	5012001	5017000	0.005121
chrY	5013001	5018000	0.005121
chrY	5014001	5019000	0.005952
chrY	5017001	5022000	0.008865
chrY	5018001	5023000	0.008865
chrY	5021001	5026000	0.014941
chrY	5024001	5029000	2.96E-05
chrY	5025001	5030000	1.70E-07
chrY	5034001	5039000	0.003727
chrY	5042001	5047000	0.001276
chrY	5043001	5048000	0.002579
chrY	5044001	5049000	0.000725
chrY	5045001	5050000	2.18E-05
chrY	5046001	5051000	0.005864
chrY	5047001	5052000	0.00598
chrY	5048001	5053000	0.000949
chrY	5049001	5054000	0.000283
chrY	5059001	5064000	0.000944
chrY	5060001	5065000	0.002836
chrY	5061001	5066000	0.012792
chrY	5066001	5071000	0.001103
chrY	5067001	5072000	0.008979
chrY	5068001	5073000	0.004626
chrY	5069001	5074000	0.00793

chrY	5090001	5095000	0.000661
chrY	5091001	5096000	7.69E-05
chrY	5092001	5097000	7.13E-06
chrY	5100001	5105000	0.010554
chrY	5101001	5106000	0.00437
chrY	5102001	5107000	0.024261
chrY	5157001	5162000	0.000275
chrY	5158001	5163000	0.000825
chrY	5159001	5164000	0.001346
chrY	5160001	5165000	0.012887
chrY	5162001	5167000	0.013061
chrY	5163001	5168000	0.017378
chrY	5166001	5171000	9.81E-05
chrY	5167001	5172000	0.012499
chrY	5173001	5178000	0.003801
chrY	5174001	5179000	0.000711
chrY	5175001	5180000	0.000729
chrY	5176001	5181000	0.000277
chrY	5198001	5203000	3.70E-05
chrY	5199001	5204000	0.001822
chrY	5205001	5210000	0.009775
chrY	5239001	5244000	0.001824
chrY	5240001	5245000	0.000736
chrY	5242001	5247000	0.023406
chrY	5243001	5248000	0.023406
chrY	5258001	5263000	0.009033
chrY	5259001	5264000	0.001555
chrY	5260001	5265000	0.000761
chrY	5261001	5266000	0.001065
chrY	5272001	5277000	0.006495
chrY	5273001	5278000	0.007555
chrY	5274001	5279000	0.005017
chrY	5275001	5280000	0.008545
chrY	5276001	5281000	0.003131
chrY	5278001	5283000	0.018438
chrY	5282001	5287000	0.000104
chrY	5285001	5290000	6.27E-07
chrY	5286001	5291000	6.74E-09
chrY	5287001	5292000	0.009689
chrY	5290001	5295000	0.000766
chrY	5291001	5296000	0.004324
chrY	5292001	5297000	0.000196
chrY	5332001	5337000	0.004224
chrY	5333001	5338000	0.008009
chrY	5342001	5347000	0.002399
chrY	5407001	5412000	0.045887
chrY	5417001	5422000	0.02515
chrY	5418001	5423000	0.02515

chrY	5521001	5526000	0.014394
chrY	5745001	5750000	0.00137
chrY	5746001	5751000	0.009818
chrY	5747001	5752000	0.000949
chrY	5748001	5753000	0.00888
chrY	5771001	5776000	0.014697
chrY	5772001	5777000	0.014697
chrY	5773001	5778000	0.024345
chrY	5775001	5780000	0.017193
chrY	5896001	5901000	0.007392
chrY	5930001	5935000	0.003512
chrY	6032001	6037000	0.000439
chrY	6033001	6038000	4.40E-05
chrY	6034001	6039000	9.14E-05
chrY	6104001	6109000	0.007249
chrY	6115001	6120000	6.88E-07
chrY	6116001	6121000	4.37E-05
chrY	6117001	6122000	1.68E-05
chrY	6118001	6123000	0.000334
chrY	6119001	6124000	0.000881
chrY	6412001	6417000	0.002889
chrY	6413001	6418000	0.002889
chrY	7274001	7279000	0.024261
chrY	7275001	7280000	0.013577
chrY	7276001	7281000	0.008062
chrY	7279001	7284000	0.030239
chrY	7306001	7311000	0.000496
chrY	7307001	7312000	0.015625
chrY	7330001	7335000	1.10E-05
chrY	7331001	7336000	8.65E-07
chrY	7332001	7337000	6.40E-07
chrY	7333001	7338000	0.002233
chrY	7347001	7352000	0.003303
chrY	7348001	7353000	0.000291
chrY	7349001	7354000	0.003687
chrY	7368001	7373000	0.009332
chrY	7369001	7374000	0.009332
chrY	7370001	7375000	0.005767
chrY	7371001	7376000	0.007213
chrY	7372001	7377000	0.010047
chrY	7393001	7398000	0.041515
chrY	7394001	7399000	0.021769
chrY	7423001	7428000	0.023624
chrY	7424001	7429000	0.023624
chrY	7425001	7430000	0.008662
chrY	7426001	7431000	0.000364
chrY	7427001	7432000	8.92E-05
chrY	7456001	7461000	9.43E-06

chrY	7507001	7512000	0.000274
chrY	7508001	7513000	0.010924
chrY	7509001	7514000	0.014563
chrY	7511001	7516000	0.004936
chrY	7514001	7519000	0.000432
chrY	7523001	7528000	0.001117
chrY	7540001	7545000	2.24E-06
chrY	7551001	7556000	0.012106
chrY	7552001	7557000	0.021051
chrY	7553001	7558000	0.003904
chrY	7554001	7559000	0.018602
chrY	7555001	7560000	0.018602
chrY	7572001	7577000	0.031772
chrY	7588001	7593000	0.040071
chrY	7603001	7608000	0.013296
chrY	7606001	7611000	0.009689
chrY	7607001	7612000	0.002681
chrY	7611001	7616000	1.79E-05
chrY	7620001	7625000	0.002186
chrY	7621001	7626000	0.020983
chrY	7622001	7627000	0.025961
chrY	7624001	7629000	0.003549
chrY	7626001	7631000	0.000751
chrY	7627001	7632000	0.00145
chrY	7638001	7643000	0.000693
chrY	7639001	7644000	0.006751
chrY	7640001	7645000	0.004423
chrY	7641001	7646000	0.001304
chrY	7642001	7647000	0.000532
chrY	7643001	7648000	0.010178
chrY	7675001	7680000	0.001623
chrY	7676001	7681000	0.005847
chrY	7677001	7682000	0.0019
chrY	7733001	7738000	0.007696
chrY	7734001	7739000	0.00358
chrY	7735001	7740000	0.009714
chrY	7736001	7741000	0.000904
chrY	7737001	7742000	3.03E-05
chrY	7738001	7743000	0.004176
chrY	7739001	7744000	0.004176
chrY	7751001	7756000	0.00565
chrY	7753001	7758000	0.026044
chrY	7853001	7858000	0.001291
chrY	7854001	7859000	0.001664
chrY	7857001	7862000	0.00157
chrY	7858001	7863000	0.007066
chrY	7859001	7864000	0.0032
chrY	7860001	7865000	0.006248

chrY	7861001	7866000	0.014419
chrY	7875001	7880000	0.003917
chrY	7928001	7933000	0.00084
chrY	7931001	7936000	2.29E-06
chrY	7932001	7937000	5.58E-06
chrY	7933001	7938000	0.004663
chrY	7941001	7946000	0.006249
chrY	7943001	7948000	0.012499
chrY	7944001	7949000	0.008295
chrY	7945001	7950000	0.000693
chrY	7947001	7952000	0.002329
chrY	7961001	7966000	0.001297
chrY	7962001	7967000	0.001297
chrY	7963001	7968000	0.001886
chrY	8010001	8015000	0.012716
chrY	8056001	8061000	0.014307
chrY	8057001	8062000	0.000847
chrY	8058001	8063000	6.08E-05
chrY	8059001	8064000	0.000146
chrY	8060001	8065000	0.000985
chrY	8126001	8131000	0.007982
chrY	8127001	8132000	0.024345
chrY	8160001	8165000	0.005087
chrY	8161001	8166000	0.014338
chrY	8194001	8199000	0.000626
chrY	8195001	8200000	0.003595
chrY	8196001	8201000	0.008541
chrY	8227001	8232000	0.001497
chrY	8228001	8233000	0.00348
chrY	8229001	8234000	0.00348
chrY	8248001	8253000	0.006024
chrY	8256001	8261000	0.000154
chrY	8274001	8279000	0.000133
chrY	8275001	8280000	0.00116
chrY	8277001	8282000	0.003666
chrY	8278001	8283000	0.000118
chrY	8285001	8290000	0.000263
chrY	8286001	8291000	0.002229
chrY	8287001	8292000	0.000282
chrY	8340001	8345000	0.032423
chrY	8359001	8364000	0.000271
chrY	8361001	8366000	0.000767
chrY	8362001	8367000	0.002506
chrY	8372001	8377000	0.008395
chrY	8373001	8378000	0.011079
chrY	8392001	8397000	0.001558
chrY	8481001	8486000	0.011828
chrY	8482001	8487000	0.006748

chrY	8498001	8503000	0.004663
chrY	8499001	8504000	0.007676
chrY	8500001	8505000	0.007127
chrY	8502001	8507000	0.002234
chrY	8563001	8568000	0.001408
chrY	8565001	8570000	0.025491
chrY	8566001	8571000	0.031237
chrY	8677001	8682000	0.002441
chrY	8678001	8683000	5.29E-05
chrY	8679001	8684000	8.53E-06
chrY	8680001	8685000	3.26E-05
chrY	8681001	8686000	1.27E-05
chrY	8729001	8734000	0.026752
chrY	8751001	8756000	0.001709
chrY	8752001	8757000	3.49E-05
chrY	8753001	8758000	1.58E-05
chrY	8754001	8759000	0.000856
chrY	8755001	8760000	0.002504
chrY	8756001	8761000	0.010988
chrY	8779001	8784000	0.029309
chrY	8782001	8787000	0.000616
chrY	8783001	8788000	0.004888
chrY	8834001	8839000	0.009647
chrY	8841001	8846000	0.001371
chrY	8842001	8847000	0.005844
chrY	8978001	8983000	0.033395
chrY	9038001	9043000	0.038225
chrY	9039001	9044000	0.001643
chrY	9040001	9045000	0.001595
chrY	9041001	9046000	0.001595
chrY	9042001	9047000	0.01434
chrY	9078001	9083000	0.035425
chrY	9083001	9088000	0.002321
chrY	9084001	9089000	0.000588
chrY	9085001	9090000	0.000126
chrY	9086001	9091000	0.0012
chrY	9087001	9092000	0.00239
chrY	9122001	9127000	0.018201
chrY	9123001	9128000	0.016574
chrY	9145001	9150000	0.000347
chrY	9146001	9151000	8.77E-05
chrY	9147001	9152000	1.79E-08
chrY	9148001	9153000	3.01E-07
chrY	9149001	9154000	6.22E-07
chrY	9150001	9155000	7.52E-05
chrY	9151001	9156000	6.51E-05
chrY	9158001	9163000	0.020717
chrY	9160001	9165000	0.000147

chrY	9161001	9166000	0.000303
chrY	9162001	9167000	0.000213
chrY	9185001	9190000	1.56E-07
chrY	9186001	9191000	1.56E-07
chrY	9189001	9194000	6.03E-08
chrY	9190001	9195000	6.03E-08
chrY	9191001	9196000	6.03E-08
chrY	9194001	9199000	6.89E-05
chrY	9195001	9200000	6.89E-05
chrY	9196001	9201000	3.04E-05
chrY	9198001	9203000	5.79E-06
chrY	9293001	9298000	0.000156
chrY	9294001	9299000	0.000156
chrY	9295001	9300000	0.000156
chrY	9302001	9307000	1.98E-06
chrY	9303001	9308000	4.88E-08
chrY	9304001	9309000	4.88E-08
chrY	9310001	9315000	9.64E-07
chrY	9311001	9316000	9.64E-07
chrY	9361001	9366000	0.000175
chrY	9374001	9379000	0.00036
chrY	9376001	9381000	0.000531
chrY	9377001	9382000	0.000108
chrY	9380001	9385000	0.016723
chrY	9387001	9392000	0.012195
chrY	9388001	9393000	0.001445
chrY	9389001	9394000	9.98E-05
chrY	9390001	9395000	0.000323
chrY	9391001	9396000	0.000502
chrY	9404001	9409000	0.042634
chrY	9410001	9415000	0.006534
chrY	9437001	9442000	0.005302
chrY	9438001	9443000	0.005293
chrY	9439001	9444000	0.005293
chrY	9440001	9445000	0.005293
chrY	9451001	9456000	0.00046
chrY	9452001	9457000	0.000443
chrY	9454001	9459000	0.000539
chrY	9477001	9482000	0.040071
chrY	9486001	9491000	0.001946
chrY	9755001	9760000	0.002527
chrY	9756001	9761000	0.001448
chrY	9757001	9762000	6.60E-05
chrY	9758001	9763000	0.000103
chrY	9759001	9764000	8.54E-08
chrY	9760001	9765000	1.49E-06
chrY	9761001	9766000	3.84E-05
chrY	9762001	9767000	0.000481

chrY	9763001	9768000	0.000367
chrY	9793001	9798000	4.23E-05
chrY	9794001	9799000	0.000118
chrY	9795001	9800000	9.57E-05
chrY	9796001	9801000	4.84E-06
chrY	9797001	9802000	7.94E-06
chrY	9812001	9817000	0.003719
chrY	9813001	9818000	0.000124
chrY	9814001	9819000	0.000512
chrY	9827001	9832000	0.004384
chrY	9843001	9848000	0.003385
chrY	9844001	9849000	0.004332
chrY	9845001	9850000	0.017651
chrY	9865001	9870000	0.000887
chrY	9866001	9871000	0.00026
chrY	9867001	9872000	1.77E-06
chrY	9868001	9873000	3.50E-07
chrY	9869001	9874000	1.09E-06
chrY	9870001	9875000	0.000261
chrY	9875001	9880000	0.001763
chrY	9876001	9881000	0.000227
chrY	9971001	9976000	6.80E-06
chrY	10002001	10007000	1.86E-07
chrY	10003001	10008000	5.75E-07
chrY	10004001	10009000	1.54E-07
chrY	10005001	10010000	3.56E-08
chrY	10082001	10087000	0.008705
chrY	10083001	10088000	0.008705
chrY	13302001	13307000	0.004751
chrY	13386001	13391000	0.000643
chrY	13387001	13392000	5.84E-05
chrY	13388001	13393000	6.40E-05
chrY	13390001	13395000	0.000387
chrY	13547001	13552000	0.007216
chrY	13548001	13553000	0.00058
chrY	13641001	13646000	4.87E-07
chrY	13830001	13835000	0.000823
chrY	13831001	13836000	0.001522
chrY	13852001	13857000	0.001915
chrY	14957001	14962000	0.011586
chrY	15095001	15100000	0.005034
chrY	15096001	15101000	0.000274
chrY	15097001	15102000	0.000274
chrY	15098001	15103000	0.000274
chrY	15099001	15104000	0.000563
chrY	17033001	17038000	0.018656
chrY	17034001	17039000	0.018656
chrY	17035001	17040000	0.007607

chrY	17036001	17041000	0.006189
