# Supplementary material for: DNA methylome profiling of circulating tumor cells in lung cancer at single base-pair resolution
Source: Oncogene. 2021 Feb 9;40(10):1884–95. doi: 10.1038/s41388-021-01657-0 (PMC7946637; doi:10.1038/s41388-021-01657-0)
Supplement: Supplementary file 7 — Supplementary TableS2 [file 41388_2021_1657_MOESM7_ESM.pdf]

Supplemental Table S2. List of identified DMRs between CTCs and tumor tissues

chr	start	end	qvalue	direction (CTCs compared to tumor)
chr1	1100001	1105000	3.80E-07	hyper
chr1	1102001	1107000	3.78E-07	
chr1	1103001	1108000	2.38E-07	
chr1	1408001	1413000	2.42E-19	
chr1	1545001	1550000	3.88E-09	
chr1	1593001	1598000	3.94E-14	
chr1	1594001	1599000	1.13E-13	
chr1	1659001	1664000	2.34E-06	
chr1	1660001	1665000	3.50E-07	
chr1	1830001	1835000	1.56E-06	
chr1	2118001	2123000	7.23E-29	
chr1	2119001	2124000	4.67E-33	
chr1	2120001	2125000	6.84E-24	
chr1	2121001	2126000	3.48E-23	
chr1	2130001	2135000	1.67E-06	
chr1	2162001	2167000	9.79E-15	
chr1	2281001	2286000	5.23E-10	
chr1	2423001	2428000	6.55E-24	
chr1	2424001	2429000	4.18E-14	
chr1	2425001	2430000	1.33E-14	
chr1	2426001	2431000	1.40E-13	
chr1	2427001	2432000	3.27E-11	
chr1	2463001	2468000	7.74E-16	
chr1	2466001	2471000	5.98E-13	
chr1	3153001	3158000	1.48E-06	
chr1	3154001	3159000	1.23E-08	
chr1	3155001	3160000	1.36E-07	
chr1	3373001	3378000	6.67E-15	
chr1	3374001	3379000	1.87E-16	
chr1	3375001	3380000	1.62E-19	
chr1	3634001	3639000	1.85E-19	
chr1	4218001	4223000	1.15E-08	
chr1	4219001	4224000	4.60E-08	
chr1	4603001	4608000	1.46E-08	
chr1	4914001	4919000	0.001312821	
chr1	4915001	4920000	0.001806456	
chr1	4942001	4947000	0.002133645	
chr1	4943001	4948000	0.004437546	
chr1	5051001	5056000	2.14E-06	
chr1	5090001	5095000	0.000146076	
chr1	5091001	5096000	9.64E-06	
chr1	5599001	5604000	0.000939892	
chr1	5600001	5605000	2.50E-05	
chr1	5603001	5608000	0.008386639	
chr1	6053001	6058000	2.31E-09	

chr1	6109001	6114000	2.04E-15
chr1	6111001	6116000	6.25E-24
chr1	6125001	6130000	1.26E-10
chr1	6152001	6157000	6.84E-11
chr1	6153001	6158000	6.25E-11
chr1	6183001	6188000	1.64E-06
chr1	6184001	6189000	2.37E-08
chr1	6185001	6190000	6.01E-05
chr1	6226001	6231000	9.99E-13
chr1	6552001	6557000	3.18E-05
chr1	6789001	6794000	2.25E-09
chr1	6790001	6795000	1.02E-10
chr1	7131001	7136000	9.85E-13
chr1	7132001	7137000	1.08E-10
chr1	7330001	7335000	4.88E-05
chr1	7331001	7336000	2.57E-05
chr1	7332001	7337000	1.93E-05
chr1	7333001	7338000	2.89E-05
chr1	7640001	7645000	2.70E-06
chr1	7658001	7663000	1.12E-07
chr1	7659001	7664000	4.20E-12
chr1	7954001	7959000	5.09E-08
chr1	7955001	7960000	0.000462185
chr1	8073001	8078000	3.44E-09
chr1	8128001	8133000	1.91E-10
chr1	8129001	8134000	3.33E-10
chr1	8130001	8135000	5.75E-10
chr1	8134001	8139000	2.58E-06
chr1	8156001	8161000	7.45E-07
chr1	8157001	8162000	1.54E-08
chr1	8978001	8983000	1.11E-09
chr1	9127001	9132000	1.46E-06
chr1	9128001	9133000	5.73E-06
chr1	9190001	9195000	1.45E-09
chr1	9347001	9352000	3.42E-09
chr1	9774001	9779000	8.32E-08
chr1	9775001	9780000	0.000137813
chr1	9950001	9955000	9.27E-12
chr1	9951001	9956000	3.22E-13
chr1	10511001	10516000	2.47E-06
chr1	10512001	10517000	2.56E-09
chr1	10908001	10913000	2.39E-09
chr1	11091001	11096000	4.78E-09
chr1	11092001	11097000	5.47E-09
chr1	11093001	11098000	2.64E-17
chr1	11110001	11115000	9.51E-09
chr1	11111001	11116000	4.41E-11
chr1	11112001	11117000	4.18E-11

chr1	11571001	11576000	0.000144679
chr1	11617001	11622000	4.62E-09
chr1	11939001	11944000	4.42E-05
chr1	12050001	12055000	1.40E-08
chr1	12128001	12133000	0.000143648
chr1	14362001	14367000	8.00E-07
chr1	15781001	15786000	7.15E-05
chr1	15782001	15787000	1.59E-05
chr1	15783001	15788000	0.00023715
chr1	15825001	15830000	8.65E-06
chr1	16447001	16452000	2.85E-10
chr1	17419001	17424000	0.009039462
chr1	17463001	17468000	9.87E-06
chr1	17483001	17488000	0.000133872
chr1	17547001	17552000	5.33E-08
chr1	17586001	17591000	0.000592655
chr1	17767001	17772000	2.67E-17
chr1	17768001	17773000	1.12E-13
chr1	19026001	19031000	1.05E-15
chr1	19357001	19362000	3.66E-14
chr1	19979001	19984000	0.000278261
chr1	20001001	20006000	1.02E-10
chr1	20312001	20317000	5.49E-11
chr1	20856001	20861000	1.29E-10
chr1	20919001	20924000	4.56E-06
chr1	21034001	21039000	1.71E-10
chr1	21048001	21053000	0.000254598
chr1	21049001	21054000	0.000879856
chr1	21050001	21055000	0.001152812
chr1	21348001	21353000	5.50E-14
chr1	21349001	21354000	7.54E-11
chr1	21508001	21513000	2.55E-10
chr1	21840001	21845000	6.24E-09
chr1	21843001	21848000	7.18E-14
chr1	21900001	21905000	5.02E-06
chr1	21961001	21966000	6.01E-05
chr1	22313001	22318000	1.58E-09
chr1	22314001	22319000	8.26E-09
chr1	22315001	22320000	0.000153858
chr1	22397001	22402000	1.08E-05
chr1	22398001	22403000	6.35E-14
chr1	22399001	22404000	1.31E-08
chr1	22400001	22405000	4.39E-11
chr1	22401001	22406000	1.72E-06
chr1	22402001	22407000	4.89E-07
chr1	22403001	22408000	2.87E-05
chr1	22404001	22409000	8.15E-06
chr1	22630001	22635000	5.18E-06

chr1	22631001	22636000	1.19E-06
chr1	22737001	22742000	8.10E-11
chr1	22738001	22743000	4.00E-09
chr1	22760001	22765000	3.94E-06
chr1	23244001	23249000	6.43E-11
chr1	23245001	23250000	1.75E-08
chr1	23246001	23251000	5.97E-08
chr1	23247001	23252000	7.77E-08
chr1	23248001	23253000	1.22E-07
chr1	23249001	23254000	4.65E-13
chr1	23250001	23255000	7.75E-13
chr1	23251001	23256000	8.23E-10
chr1	23293001	23298000	4.84E-05
chr1	23886001	23891000	3.31E-09
chr1	24000001	24005000	9.15E-06
chr1	24212001	24217000	0.00011282
chr1	24396001	24401000	5.74E-13
chr1	24397001	24402000	2.07E-19
chr1	24467001	24472000	9.53E-07
chr1	24917001	24922000	4.28E-06
chr1	25093001	25098000	2.64E-06
chr1	25094001	25099000	1.08E-05
chr1	25095001	25100000	3.54E-07
chr1	25911001	25916000	2.26E-10
chr1	25912001	25917000	2.29E-06
chr1	25918001	25923000	3.52E-16
chr1	25919001	25924000	3.21E-14
chr1	26354001	26359000	4.68E-05
chr1	26355001	26360000	5.06E-06
chr1	26512001	26517000	7.52E-05
chr1	26540001	26545000	0.001811708
chr1	26709001	26714000	3.25E-07
chr1	26716001	26721000	1.88E-05
chr1	26717001	26722000	2.52E-05
chr1	26731001	26736000	7.36E-12
chr1	26732001	26737000	4.35E-17
chr1	26920001	26925000	2.55E-10
chr1	26921001	26926000	2.64E-07
chr1	26922001	26927000	9.14E-07
chr1	27029001	27034000	1.44E-06
chr1	27356001	27361000	5.09E-10
chr1	27407001	27412000	4.30E-08
chr1	27409001	27414000	5.68E-08
chr1	27410001	27415000	2.75E-09
chr1	27670001	27675000	1.08E-05
chr1	27761001	27766000	7.21E-06
chr1	27762001	27767000	0.009246511
chr1	28765001	28770000	7.02E-05

chr1	28834001	28839000	3.78E-06
chr1	29078001	29083000	9.86E-05
chr1	29149001	29154000	5.78E-06
chr1	29252001	29257000	6.55E-09
chr1	29253001	29258000	1.01E-10
chr1	29501001	29506000	3.31E-06
chr1	29502001	29507000	3.12E-14
chr1	29595001	29600000	0.000514033
chr1	29774001	29779000	1.56E-05
chr1	30183001	30188000	0.000125812
chr1	30224001	30229000	5.98E-11
chr1	30225001	30230000	3.96E-10
chr1	30226001	30231000	2.26E-06
chr1	30227001	30232000	1.74E-06
chr1	30228001	30233000	2.35E-05
chr1	30402001	30407000	4.28E-08
chr1	30527001	30532000	1.65E-10
chr1	30528001	30533000	4.03E-12
chr1	30529001	30534000	4.80E-12
chr1	30548001	30553000	4.38E-07
chr1	30904001	30909000	0.000980037
chr1	31584001	31589000	7.14E-07
chr1	31585001	31590000	6.04E-05
chr1	31586001	31591000	0.000301748
chr1	31644001	31649000	4.65E-24
chr1	31645001	31650000	1.69E-27
chr1	31898001	31903000	3.04E-06
chr1	31899001	31904000	1.62E-09
chr1	32089001	32094000	1.67E-11
chr1	32090001	32095000	5.22E-09
chr1	32450001	32455000	5.92E-10
chr1	32677001	32682000	0.000149415
chr1	32726001	32731000	3.88E-06
chr1	32730001	32735000	1.89E-08
chr1	32731001	32736000	7.24E-06
chr1	32910001	32915000	6.93E-17
chr1	32912001	32917000	2.03E-18
chr1	32913001	32918000	7.25E-24
chr1	33143001	33148000	1.16E-06
chr1	33144001	33149000	4.52E-06
chr1	33182001	33187000	0.000377829
chr1	33224001	33229000	0.000162083
chr1	33234001	33239000	9.82E-09
chr1	33353001	33358000	0.000630333
chr1	33439001	33444000	9.58E-18
chr1	33440001	33445000	4.85E-12
chr1	33771001	33776000	1.41E-27
chr1	33772001	33777000	1.01E-39

chr1	34095001	34100000	3.77E-19
chr1	35057001	35062000	2.53E-05
chr1	35514001	35519000	0.000139019
chr1	36108001	36113000	1.02E-11
chr1	36555001	36560000	4.69E-15
chr1	37035001	37040000	2.37E-05
chr1	37358001	37363000	0.008659637
chr1	38418001	38423000	0.000933047
chr1	38419001	38424000	0.00014269
chr1	39659001	39664000	9.03E-06
chr1	39660001	39665000	2.50E-09
chr1	40072001	40077000	0.000223225
chr1	40618001	40623000	0.000111128
chr1	41175001	41180000	1.12E-06
chr1	41176001	41181000	2.41E-07
chr1	42235001	42240000	5.79E-07
chr1	43364001	43369000	9.19E-11
chr1	43365001	43370000	4.46E-11
chr1	43366001	43371000	1.25E-06
chr1	43378001	43383000	1.56E-10
chr1	44014001	44019000	1.06E-12
chr1	44015001	44020000	2.12E-14
chr1	44016001	44021000	2.83E-14
chr1	44227001	44232000	6.04E-10
chr1	44304001	44309000	5.25E-09
chr1	44788001	44793000	1.60E-09
chr1	44988001	44993000	7.85E-16
chr1	44989001	44994000	6.60E-15
chr1	45182001	45187000	2.92E-07
chr1	45183001	45188000	2.17E-12
chr1	45186001	45191000	2.06E-10
chr1	45187001	45192000	2.11E-08
chr1	45634001	45639000	7.64E-06
chr1	45635001	45640000	2.57E-06
chr1	46330001	46335000	0.002903351
chr1	47554001	47559000	0.000360841
chr1	47555001	47560000	0.001094085
chr1	48244001	48249000	9.00E-08
chr1	48996001	49001000	0.000540493
chr1	48997001	49002000	7.74E-05
chr1	49492001	49497000	1.16E-05
chr1	49509001	49514000	0.010825259
chr1	49648001	49653000	0.00015732
chr1	49649001	49654000	5.03E-07
chr1	49650001	49655000	4.69E-05
chr1	49651001	49656000	4.91E-05
chr1	49652001	49657000	0.000378201
chr1	50259001	50264000	9.20E-11

chr1	50260001	50265000	1.37E-09
chr1	50499001	50504000	9.46E-06
chr1	50500001	50505000	4.85E-06
chr1	50774001	50779000	6.74E-05
chr1	51068001	51073000	1.42E-07
chr1	51069001	51074000	1.54E-07
chr1	51494001	51499000	3.49E-08
chr1	51495001	51500000	1.14E-09
chr1	51496001	51501000	9.11E-09
chr1	51602001	51607000	0.000153948
chr1	51646001	51651000	4.34E-05
chr1	53407001	53412000	2.12E-08
chr1	53477001	53482000	8.22E-12
chr1	53478001	53483000	5.04E-10
chr1	53479001	53484000	2.09E-08
chr1	53480001	53485000	0.000816868
chr1	53885001	53890000	0.007682481
chr1	54464001	54469000	1.94E-06
chr1	55031001	55036000	4.70E-09
chr1	55032001	55037000	1.02E-07
chr1	55057001	55062000	0.002976716
chr1	55058001	55063000	0.003304412
chr1	55131001	55136000	0.006170262
chr1	55321001	55326000	5.94E-07
chr1	55322001	55327000	1.57E-08
chr1	55354001	55359000	0.000484612
chr1	55423001	55428000	0.000724243
chr1	55900001	55905000	0.000206523
chr1	56042001	56047000	1.46E-07
chr1	56202001	56207000	3.85E-05
chr1	56302001	56307000	5.11E-05
chr1	57089001	57094000	0.000429629
chr1	57090001	57095000	0.000230568
chr1	57091001	57096000	0.000448212
chr1	58057001	58062000	0.000420716
chr1	58413001	58418000	0.004881222
chr1	58449001	58454000	7.63E-10
chr1	58450001	58455000	7.53E-09
chr1	58451001	58456000	1.09E-06
chr1	58598001	58603000	0.000130256
chr1	58675001	58680000	8.55E-06
chr1	58678001	58683000	0.000883602
chr1	59049001	59054000	8.25E-06
chr1	59051001	59056000	5.26E-08
chr1	59271001	59276000	4.26E-14
chr1	59272001	59277000	5.19E-10
chr1	59274001	59279000	1.70E-09
chr1	59588001	59593000	2.59E-05

chr1	59644001	59649000	3.34E-05
chr1	59645001	59650000	7.69E-05
chr1	60529001	60534000	0.024794565
chr1	60716001	60721000	1.30E-06
chr1	61606001	61611000	3.17E-06
chr1	62835001	62840000	2.20E-08
chr1	63208001	63213000	7.20E-05
chr1	63209001	63214000	0.000293702
chr1	63210001	63215000	0.002663077
chr1	63211001	63216000	0.000501721
chr1	63400001	63405000	0.00212167
chr1	63412001	63417000	1.42E-08
chr1	63413001	63418000	0.000583042
chr1	63631001	63636000	0.000186631
chr1	64080001	64085000	1.80E-11
chr1	64261001	64266000	2.22E-09
chr1	64262001	64267000	2.08E-09
chr1	64263001	64268000	6.34E-09
chr1	64264001	64269000	4.64E-08
chr1	64588001	64593000	8.37E-06
chr1	64633001	64638000	4.98E-08
chr1	64634001	64639000	3.76E-10
chr1	64635001	64640000	5.03E-10
chr1	64636001	64641000	5.61E-06
chr1	65290001	65295000	2.42E-05
chr1	65555001	65560000	4.35E-05
chr1	65573001	65578000	3.02E-06
chr1	65698001	65703000	6.32E-06
chr1	65887001	65892000	6.15E-14
chr1	66040001	66045000	0.000694584
chr1	66041001	66046000	6.04E-05
chr1	66042001	66047000	0.000394127
chr1	66361001	66366000	0.000200666
chr1	66420001	66425000	0.000873808
chr1	66534001	66539000	7.11E-05
chr1	66535001	66540000	1.53E-05
chr1	66543001	66548000	1.01E-05
chr1	66544001	66549000	0.000116218
chr1	66546001	66551000	0.005297635
chr1	67055001	67060000	2.01E-05
chr1	67149001	67154000	1.87E-05
chr1	67150001	67155000	0.006763871
chr1	67444001	67449000	2.42E-05
chr1	67445001	67450000	6.18E-06
chr1	67573001	67578000	0.000246148
chr1	68023001	68028000	6.11E-12
chr1	68024001	68029000	1.23E-14
chr1	68025001	68030000	1.26E-14

chr1	68026001	68031000	1.78E-10
chr1	68027001	68032000	2.64E-09
chr1	68080001	68085000	5.23E-07
chr1	68186001	68191000	0.00031719
chr1	68355001	68360000	0.000204602
chr1	68476001	68481000	0.00045323
chr1	68632001	68637000	5.12E-06
chr1	68633001	68638000	0.000104841
chr1	68634001	68639000	7.71E-06
chr1	68635001	68640000	7.92E-05
chr1	68670001	68675000	0.000303883
chr1	68793001	68798000	0.000328468
chr1	68794001	68799000	0.001926134
chr1	68969001	68974000	2.24E-08
chr1	68970001	68975000	2.59E-08
chr1	69069001	69074000	0.001226885
chr1	69216001	69221000	7.84E-05
chr1	69307001	69312000	0.001879302
chr1	69321001	69326000	0.0001471
chr1	69322001	69327000	1.28E-05
chr1	69323001	69328000	1.08E-06
chr1	69324001	69329000	4.19E-06
chr1	69330001	69335000	5.58E-05
chr1	69440001	69445000	3.46E-05
chr1	69441001	69446000	1.47E-05
chr1	69442001	69447000	9.71E-06
chr1	69443001	69448000	0.000238125
chr1	69444001	69449000	0.002201945
chr1	69517001	69522000	1.23E-06
chr1	69539001	69544000	0.000137422
chr1	69540001	69545000	1.79E-09
chr1	69541001	69546000	8.38E-11
chr1	69542001	69547000	9.09E-09
chr1	69543001	69548000	2.91E-08
chr1	69544001	69549000	6.48E-08
chr1	69572001	69577000	0.000856987
chr1	69595001	69600000	7.28E-08
chr1	69680001	69685000	0.000226739
chr1	69739001	69744000	0.001002523
chr1	69740001	69745000	0.002610105
chr1	69798001	69803000	0.001307932
chr1	69799001	69804000	0.000480778
chr1	69850001	69855000	0.007270839
chr1	69899001	69904000	0.003519629
chr1	69901001	69906000	0.00960655
chr1	69915001	69920000	0.000438178
chr1	69916001	69921000	0.000114163
chr1	69917001	69922000	0.000741399

chr1	69939001	69944000	3.48E-05
chr1	69940001	69945000	3.50E-05
chr1	69947001	69952000	0.001776304
chr1	69960001	69965000	0.001547264
chr1	70084001	70089000	2.13E-05
chr1	70154001	70159000	0.000585835
chr1	70191001	70196000	8.06E-06
chr1	70192001	70197000	4.66E-05
chr1	70328001	70333000	2.28E-09
chr1	70329001	70334000	3.98E-15
chr1	70330001	70335000	4.90E-13
chr1	70927001	70932000	1.48E-05
chr1	70928001	70933000	1.25E-05
chr1	71132001	71137000	7.39E-06
chr1	71173001	71178000	0.000201281
chr1	71265001	71270000	0.014768481
chr1	71353001	71358000	1.54E-07
chr1	71388001	71393000	0.004784377
chr1	71389001	71394000	0.001320669
chr1	71405001	71410000	9.98E-06
chr1	71467001	71472000	1.99E-05
chr1	71468001	71473000	5.87E-06
chr1	71469001	71474000	8.31E-06
chr1	71470001	71475000	1.90E-05
chr1	71471001	71476000	4.20E-05
chr1	71808001	71813000	0.000441972
chr1	71840001	71845000	0.001083215
chr1	72062001	72067000	7.60E-07
chr1	72063001	72068000	2.49E-06
chr1	72156001	72161000	1.24E-05
chr1	72348001	72353000	0.000303775
chr1	72906001	72911000	1.64E-05
chr1	73050001	73055000	0.000816407
chr1	73051001	73056000	0.000897053
chr1	73052001	73057000	0.000781496
chr1	73053001	73058000	0.000650678
chr1	73105001	73110000	3.14E-05
chr1	73193001	73198000	6.52E-06
chr1	73194001	73199000	1.30E-05
chr1	73461001	73466000	0.000247687
chr1	73462001	73467000	0.00043024
chr1	73748001	73753000	0.000283753
chr1	73749001	73754000	0.000283753
chr1	74301001	74306000	1.94E-10
chr1	74319001	74324000	0.021474838
chr1	74320001	74325000	0.000124124
chr1	74321001	74326000	0.000259585
chr1	74979001	74984000	0.000734538

chr1	75129001	75134000	0.002073476
chr1	75130001	75135000	0.000786563
chr1	75131001	75136000	0.006255689
chr1	75132001	75137000	0.002698764
chr1	75133001	75138000	0.009771182
chr1	75134001	75139000	0.002526268
chr1	75489001	75494000	0.000893575
chr1	75490001	75495000	2.95E-05
chr1	75491001	75496000	8.21E-05
chr1	75492001	75497000	0.000160463
chr1	75493001	75498000	2.93E-05
chr1	75628001	75633000	0.000995786
chr1	75630001	75635000	0.000477333
chr1	75712001	75717000	4.32E-05
chr1	75713001	75718000	9.77E-06
chr1	75714001	75719000	5.96E-05
chr1	75715001	75720000	4.74E-05
chr1	75902001	75907000	0.007347303
chr1	76334001	76339000	0.018595245
chr1	76581001	76586000	2.05E-09
chr1	76582001	76587000	2.09E-10
chr1	76703001	76708000	0.001200054
chr1	76720001	76725000	0.000343477
chr1	76721001	76726000	0.002010599
chr1	76823001	76828000	3.59E-05
chr1	76912001	76917000	0.000182736
chr1	76913001	76918000	9.56E-05
chr1	77106001	77111000	0.000163988
chr1	77107001	77112000	0.000306725
chr1	77140001	77145000	7.72E-05
chr1	77250001	77255000	6.70E-06
chr1	77251001	77256000	7.71E-05
chr1	77410001	77415000	0.00012726
chr1	77411001	77416000	2.53E-05
chr1	77412001	77417000	2.01E-05
chr1	77413001	77418000	2.15E-06
chr1	77427001	77432000	1.62E-05
chr1	77702001	77707000	9.64E-06
chr1	77703001	77708000	3.54E-06
chr1	77704001	77709000	2.74E-05
chr1	77705001	77710000	5.88E-05
chr1	77749001	77754000	0.000277631
chr1	77750001	77755000	0.000595133
chr1	77771001	77776000	5.92E-09
chr1	77772001	77777000	2.01E-07
chr1	77854001	77859000	1.09E-06
chr1	77884001	77889000	0.001106805
chr1	77937001	77942000	0.000456229

chr1	77939001	77944000	0.001396871
chr1	78469001	78474000	2.09E-06
chr1	78470001	78475000	6.16E-06
chr1	78814001	78819000	1.73E-05
chr1	79140001	79145000	1.16E-09
chr1	79141001	79146000	9.37E-06
chr1	79142001	79147000	5.13E-06
chr1	79144001	79149000	0.001741124
chr1	79204001	79209000	0.000566006
chr1	79205001	79210000	0.001188062
chr1	79224001	79229000	2.04E-05
chr1	79225001	79230000	8.57E-05
chr1	79226001	79231000	9.46E-05
chr1	79227001	79232000	0.000137963
chr1	79350001	79355000	0.008144686
chr1	79506001	79511000	0.000956734
chr1	79573001	79578000	3.14E-07
chr1	79664001	79669000	8.33E-08
chr1	79693001	79698000	1.06E-05
chr1	79694001	79699000	2.83E-05
chr1	79868001	79873000	0.002658279
chr1	79897001	79902000	5.87E-09
chr1	80096001	80101000	0.005371778
chr1	80097001	80102000	8.80E-05
chr1	80098001	80103000	1.68E-05
chr1	80099001	80104000	1.25E-05
chr1	80731001	80736000	0.000166081
chr1	80732001	80737000	3.86E-05
chr1	80733001	80738000	5.85E-05
chr1	80735001	80740000	0.000659294
chr1	80856001	80861000	0.005411731
chr1	80891001	80896000	0.000609982
chr1	80981001	80986000	0.026922348
chr1	81077001	81082000	4.07E-05
chr1	81147001	81152000	0.008822146
chr1	81148001	81153000	0.011029462
chr1	81182001	81187000	0.000129565
chr1	81354001	81359000	5.23E-05
chr1	81355001	81360000	1.92E-05
chr1	81356001	81361000	1.33E-06
chr1	82144001	82149000	1.96E-06
chr1	84335001	84340000	0.000985718
chr1	84336001	84341000	0.000835576
chr1	84429001	84434000	0.001431142
chr1	84484001	84489000	0.006898028
chr1	84497001	84502000	0.003391716
chr1	85063001	85068000	6.33E-06
chr1	85598001	85603000	0.000320357

chr1	85871001	85876000	0.00023747
chr1	85872001	85877000	6.41E-07
chr1	85873001	85878000	1.09E-06
chr1	86119001	86124000	0.000276432
chr1	86260001	86265000	9.57E-05
chr1	86301001	86306000	4.05E-07
chr1	86302001	86307000	7.46E-06
chr1	86363001	86368000	0.016018291
chr1	86553001	86558000	8.68E-07
chr1	86554001	86559000	1.25E-05
chr1	86650001	86655000	1.39E-06
chr1	86651001	86656000	3.19E-08
chr1	86652001	86657000	1.83E-07
chr1	86653001	86658000	1.83E-07
chr1	86654001	86659000	2.62E-05
chr1	86702001	86707000	2.09E-07
chr1	86746001	86751000	0.001607001
chr1	86925001	86930000	0.001143021
chr1	87216001	87221000	5.86E-05
chr1	87568001	87573000	0.002327563
chr1	87569001	87574000	0.003426209
chr1	87570001	87575000	0.000232927
chr1	88065001	88070000	4.80E-08
chr1	89590001	89595000	3.95E-18
chr1	89591001	89596000	4.97E-16
chr1	90017001	90022000	0.001680717
chr1	90724001	90729000	7.34E-06
chr1	90725001	90730000	4.26E-05
chr1	90726001	90731000	3.42E-06
chr1	91002001	91007000	8.34E-07
chr1	91705001	91710000	0.007657857
chr1	92320001	92325000	0.006580464
chr1	92410001	92415000	3.51E-12
chr1	92411001	92416000	7.35E-12
chr1	92487001	92492000	1.97E-09
chr1	92500001	92505000	7.36E-06
chr1	93322001	93327000	2.83E-07
chr1	93769001	93774000	3.33E-05
chr1	95510001	95515000	0.000138373
chr1	95739001	95744000	0.000265064
chr1	95865001	95870000	4.78E-07
chr1	95941001	95946000	0.000941732
chr1	95957001	95962000	1.09E-05
chr1	95987001	95992000	0.000335697
chr1	95988001	95993000	0.000203488
chr1	95989001	95994000	0.000324978
chr1	95991001	95996000	5.60E-05
chr1	96311001	96316000	0.004931326

chr1	96433001	96438000	0.000477521
chr1	96434001	96439000	0.000478152
chr1	96495001	96500000	5.25E-06
chr1	96589001	96594000	0.005119219
chr1	96627001	96632000	9.19E-07
chr1	96923001	96928000	0.000697299
chr1	96924001	96929000	0.000333469
chr1	96960001	96965000	0.000108042
chr1	96961001	96966000	0.001297803
chr1	97397001	97402000	0.002044294
chr1	97398001	97403000	0.001139184
chr1	97399001	97404000	0.001139184
chr1	97400001	97405000	1.94E-05
chr1	97717001	97722000	0.000134455
chr1	98487001	98492000	0.002215174
chr1	98551001	98556000	0.001808471
chr1	98577001	98582000	4.52E-05
chr1	98657001	98662000	0.000478297
chr1	98670001	98675000	0.000292705
chr1	98671001	98676000	0.000377382
chr1	98672001	98677000	0.00055301
chr1	98848001	98853000	0.000172493
chr1	98849001	98854000	0.000154056
chr1	98956001	98961000	0.000142184
chr1	98957001	98962000	0.000139606
chr1	98990001	98995000	0.001327517
chr1	98991001	98996000	0.001914119
chr1	98992001	98997000	0.002738978
chr1	99029001	99034000	0.000134923
chr1	99030001	99035000	0.000318757
chr1	99031001	99036000	0.002872437
chr1	99032001	99037000	0.005334987
chr1	99276001	99281000	0.006092333
chr1	99278001	99283000	0.000133552
chr1	99279001	99284000	0.000157991
chr1	99280001	99285000	0.000239395
chr1	99393001	99398000	3.19E-09
chr1	99445001	99450000	0.00333955
chr1	99831001	99836000	0.000141758
chr1	99891001	99896000	0.005019951
chr1	99933001	99938000	0.000102037
chr1	99988001	99993000	0.00020271
chr1	100232001	100237000	5.26E-08
chr1	100790001	100795000	0.00040974
chr1	101793001	101798000	2.97E-08
chr1	101794001	101799000	1.35E-07
chr1	102032001	102037000	0.000230243
chr1	102034001	102039000	1.60E-05

chr1	102035001	102040000	2.99E-05
chr1	102159001	102164000	2.53E-05
chr1	102386001	102391000	0.001093905
chr1	102439001	102444000	0.022901097
chr1	102591001	102596000	1.03E-06
chr1	102628001	102633000	0.001887572
chr1	102631001	102636000	0.005843842
chr1	102632001	102637000	0.003121812
chr1	102668001	102673000	0.002971222
chr1	102706001	102711000	0.000112622
chr1	102753001	102758000	0.001998301
chr1	102754001	102759000	0.002647699
chr1	102859001	102864000	0.000223241
chr1	102959001	102964000	0.018037637
chr1	102979001	102984000	0.00818882
chr1	102999001	103004000	3.77E-05
chr1	103000001	103005000	4.33E-06
chr1	103142001	103147000	0.006924682
chr1	103276001	103281000	1.33E-05
chr1	103277001	103282000	4.57E-05
chr1	103655001	103660000	4.12E-09
chr1	104695001	104700000	1.97E-06
chr1	104811001	104816000	0.001047985
chr1	104812001	104817000	0.000440676
chr1	104832001	104837000	0.001414105
chr1	104834001	104839000	2.26E-05
chr1	104933001	104938000	1.34E-05
chr1	104934001	104939000	4.82E-05
chr1	104935001	104940000	0.000129488
chr1	105317001	105322000	5.63E-06
chr1	105318001	105323000	1.53E-05
chr1	105431001	105436000	1.11E-09
chr1	105444001	105449000	0.000155568
chr1	105445001	105450000	1.24E-07
chr1	105446001	105451000	2.75E-09
chr1	105455001	105460000	3.67E-07
chr1	105521001	105526000	0.000130007
chr1	105524001	105529000	0.000395403
chr1	105586001	105591000	0.000355001
chr1	105587001	105592000	0.000303365
chr1	105599001	105604000	1.46E-06
chr1	105600001	105605000	4.49E-06
chr1	105633001	105638000	0.007279145
chr1	105635001	105640000	1.16E-05
chr1	105684001	105689000	1.42E-05
chr1	105767001	105772000	0.002918356
chr1	105768001	105773000	0.004543049
chr1	105798001	105803000	8.14E-12

chr1	105863001	105868000	7.97E-10
chr1	105864001	105869000	1.40E-09
chr1	105865001	105870000	2.22E-07
chr1	105867001	105872000	9.01E-08
chr1	105976001	105981000	3.21E-06
chr1	105978001	105983000	5.06E-06
chr1	105979001	105984000	1.70E-06
chr1	106097001	106102000	0.005225105
chr1	106180001	106185000	3.98E-06
chr1	106181001	106186000	1.51E-06
chr1	106319001	106324000	6.23E-05
chr1	106320001	106325000	0.000271661
chr1	106321001	106326000	0.000686211
chr1	106427001	106432000	4.37E-05
chr1	106517001	106522000	2.51E-06
chr1	106518001	106523000	6.10E-05
chr1	106519001	106524000	0.00017086
chr1	106549001	106554000	5.73E-06
chr1	106550001	106555000	1.83E-05
chr1	106551001	106556000	2.83E-07
chr1	106552001	106557000	1.42E-06
chr1	106575001	106580000	0.001839059
chr1	106576001	106581000	0.019568444
chr1	106579001	106584000	0.023400454
chr1	106605001	106610000	0.023281814
chr1	106608001	106613000	0.006097305
chr1	106731001	106736000	7.21E-06
chr1	106732001	106737000	1.92E-05
chr1	106750001	106755000	0.015183314
chr1	106752001	106757000	2.41E-05
chr1	106847001	106852000	0.002725803
chr1	106848001	106853000	0.004801828
chr1	106904001	106909000	0.001867574
chr1	106941001	106946000	0.000273084
chr1	107101001	107106000	0.013199752
chr1	107197001	107202000	0.00256962
chr1	107330001	107335000	7.36E-12
chr1	107333001	107338000	1.33E-11
chr1	107334001	107339000	9.79E-10
chr1	107355001	107360000	0.000774448
chr1	107356001	107361000	0.000363355
chr1	107357001	107362000	4.30E-05
chr1	107358001	107363000	0.000175529
chr1	107359001	107364000	0.000512628
chr1	107404001	107409000	5.36E-07
chr1	107405001	107410000	2.08E-06
chr1	107443001	107448000	1.17E-05
chr1	107445001	107450000	2.12E-05

chr1	107446001	107451000	4.62E-05
chr1	107447001	107452000	0.00170257
chr1	107522001	107527000	0.008897117
chr1	107652001	107657000	0.001646993
chr1	107722001	107727000	0.001184776
chr1	107970001	107975000	2.15E-05
chr1	107971001	107976000	2.42E-05
chr1	108218001	108223000	0.004746339
chr1	108419001	108424000	0.002230315
chr1	108420001	108425000	1.95E-05
chr1	108864001	108869000	1.15E-07
chr1	109364001	109369000	5.41E-05
chr1	109365001	109370000	2.40E-05
chr1	109366001	109371000	2.05E-06
chr1	109368001	109373000	0.000141031
chr1	109592001	109597000	0.000285481
chr1	109627001	109632000	0.000689802
chr1	109871001	109876000	2.39E-06
chr1	110037001	110042000	1.40E-09
chr1	110113001	110118000	1.44E-05
chr1	110932001	110937000	0.00082919
chr1	110933001	110938000	0.000181566
chr1	111032001	111037000	2.90E-05
chr1	111187001	111192000	0.000534847
chr1	111370001	111375000	0.001982566
chr1	111371001	111376000	0.000801887
chr1	111372001	111377000	0.00153402
chr1	111447001	111452000	0.004042601
chr1	111507001	111512000	1.03E-05
chr1	111508001	111513000	5.13E-05
chr1	112928001	112933000	0.000468676
chr1	113002001	113007000	0.000119273
chr1	113941001	113946000	0.001026455
chr1	114580001	114585000	8.10E-07
chr1	114872001	114877000	5.07E-06
chr1	114919001	114924000	0.000406331
chr1	115046001	115051000	6.60E-12
chr1	115047001	115052000	1.64E-12
chr1	115498001	115503000	0.000137378
chr1	115855001	115860000	2.50E-05
chr1	115856001	115861000	8.25E-07
chr1	116419001	116424000	3.19E-11
chr1	116461001	116466000	4.54E-05
chr1	116462001	116467000	8.48E-06
chr1	116910001	116915000	2.50E-20
chr1	116918001	116923000	9.52E-11
chr1	116919001	116924000	3.71E-07
chr1	117260001	117265000	0.00018915

chr1	118313001	118318000	0.000359486
chr1	118557001	118562000	0.004697615
chr1	118631001	118636000	0.002445553
chr1	118632001	118637000	0.000508593
chr1	118693001	118698000	1.48E-07
chr1	118870001	118875000	0.000886643
chr1	119133001	119138000	0.00251701
chr1	119441001	119446000	3.49E-05
chr1	119552001	119557000	0.001460314
chr1	119818001	119823000	0.001854912
chr1	119819001	119824000	1.17E-07
chr1	119820001	119825000	5.06E-07
chr1	119821001	119826000	1.74E-05
chr1	119822001	119827000	3.51E-05
chr1	120007001	120012000	1.64E-06
chr1	120389001	120394000	9.63E-07
chr1	120760001	120765000	7.24E-06
chr1	121235001	121240000	1.43E-10
chr1	121236001	121241000	7.43E-10
chr1	121237001	121242000	1.24E-10
chr1	121333001	121338000	8.93E-05
chr1	144578001	144583000	6.02E-07
chr1	145383001	145388000	1.70E-29
chr1	145694001	145699000	2.25E-07
chr1	146516001	146521000	0.000325824
chr1	146607001	146612000	3.22E-07
chr1	146608001	146613000	2.09E-07
chr1	146609001	146614000	3.73E-07
chr1	146707001	146712000	1.95E-08
chr1	147205001	147210000	8.26E-06
chr1	147292001	147297000	1.73E-08
chr1	148187001	148192000	0.00068571
chr1	149678001	149683000	1.52E-11
chr1	149907001	149912000	3.44E-20
chr1	149931001	149936000	4.38E-11
chr1	150545001	150550000	3.10E-14
chr1	150894001	150899000	6.08E-38
chr1	151678001	151683000	3.28E-24
chr1	151839001	151844000	1.03E-15
chr1	151840001	151845000	1.40E-20
chr1	152581001	152586000	6.33E-05
chr1	152809001	152814000	8.85E-06
chr1	153586001	153591000	1.58E-08
chr1	153701001	153706000	5.29E-14
chr1	154902001	154907000	6.52E-06
chr1	154988001	154993000	1.15E-16
chr1	155072001	155077000	3.68E-15
chr1	155073001	155078000	1.08E-14

chr1	155157001	155162000	1.22E-10
chr1	155565001	155570000	2.01E-19
chr1	155566001	155571000	1.40E-24
chr1	155567001	155572000	5.40E-28
chr1	155568001	155573000	1.81E-18
chr1	155580001	155585000	1.23E-06
chr1	155942001	155947000	1.38E-07
chr1	156148001	156153000	1.34E-14
chr1	156149001	156154000	3.43E-14
chr1	156262001	156267000	8.38E-06
chr1	156836001	156841000	8.12E-12
chr1	157998001	158003000	5.20E-10
chr1	158512001	158517000	0.000164858
chr1	158649001	158654000	9.47E-06
chr1	158688001	158693000	0.000877934
chr1	158884001	158889000	0.012650985
chr1	159198001	159203000	1.65E-14
chr1	159199001	159204000	7.72E-16
chr1	159451001	159456000	1.10E-09
chr1	159452001	159457000	1.89E-13
chr1	159453001	159458000	9.06E-13
chr1	159454001	159459000	1.51E-14
chr1	159455001	159460000	1.31E-13
chr1	159487001	159492000	2.36E-10
chr1	159488001	159493000	7.65E-09
chr1	159898001	159903000	5.56E-17
chr1	162678001	162683000	1.29E-06
chr1	163357001	163362000	6.27E-06
chr1	163358001	163363000	4.19E-06
chr1	163420001	163425000	1.65E-06
chr1	163421001	163426000	3.64E-06
chr1	163566001	163571000	2.45E-05
chr1	163567001	163572000	0.00019857
chr1	163568001	163573000	0.000154775
chr1	163869001	163874000	0.001657722
chr1	164568001	164573000	1.81E-11
chr1	166165001	166170000	3.22E-06
chr1	166166001	166171000	9.35E-08
chr1	166167001	166172000	1.43E-06
chr1	167114001	167119000	0.001783306
chr1	168375001	168380000	6.80E-06
chr1	168802001	168807000	4.69E-24
chr1	168803001	168808000	5.14E-37
chr1	168804001	168809000	4.44E-43
chr1	168805001	168810000	4.10E-37
chr1	168873001	168878000	3.64E-05
chr1	169581001	169586000	0.000406814
chr1	169894001	169899000	3.65E-05

chr1	170116001	170121000	3.85E-08
chr1	170117001	170122000	1.48E-06
chr1	170119001	170124000	8.39E-05
chr1	171814001	171819000	0.000721912
chr1	171879001	171884000	2.09E-13
chr1	172983001	172988000	1.76E-07
chr1	173238001	173243000	8.97E-05
chr1	174840001	174845000	8.11E-21
chr1	174841001	174846000	6.62E-19
chr1	174842001	174847000	7.97E-20
chr1	174844001	174849000	0.000104279
chr1	174930001	174935000	1.82E-08
chr1	174931001	174936000	3.46E-07
chr1	174934001	174939000	2.30E-07
chr1	174957001	174962000	2.28E-06
chr1	175252001	175257000	0.00016884
chr1	176369001	176374000	0.00168314
chr1	176370001	176375000	0.002158439
chr1	176575001	176580000	1.73E-05
chr1	176576001	176581000	0.001248728
chr1	177166001	177171000	2.79E-07
chr1	177282001	177287000	1.89E-05
chr1	177283001	177288000	1.03E-05
chr1	177284001	177289000	2.55E-05
chr1	178308001	178313000	4.68E-07
chr1	179686001	179691000	0.005212722
chr1	179898001	179903000	3.17E-07
chr1	179906001	179911000	0.010799406
chr1	181442001	181447000	1.88E-15
chr1	181443001	181448000	2.14E-13
chr1	181458001	181463000	4.29E-08
chr1	181460001	181465000	2.38E-07
chr1	182966001	182971000	8.62E-08
chr1	182967001	182972000	2.35E-05
chr1	184215001	184220000	3.54E-05
chr1	184275001	184280000	5.10E-09
chr1	185003001	185008000	0.00010787
chr1	185535001	185540000	1.43E-07
chr1	185653001	185658000	6.67E-07
chr1	185654001	185659000	0.000286044
chr1	185905001	185910000	0.025196288
chr1	186117001	186122000	2.43E-06
chr1	186118001	186123000	7.98E-08
chr1	186119001	186124000	7.88E-05
chr1	186120001	186125000	0.000170568
chr1	186121001	186126000	0.000151508
chr1	186447001	186452000	5.67E-05
chr1	186448001	186453000	4.86E-05

chr1	186449001	186454000	0.00011365
chr1	186761001	186766000	0.000163216
chr1	187002001	187007000	0.014242359
chr1	187003001	187008000	0.004741945
chr1	187047001	187052000	4.77E-06
chr1	187128001	187133000	8.54E-20
chr1	187222001	187227000	0.002521717
chr1	187223001	187228000	3.01E-06
chr1	187224001	187229000	2.64E-06
chr1	187291001	187296000	0.000496
chr1	187378001	187383000	4.74E-08
chr1	187379001	187384000	0.00025548
chr1	187380001	187385000	0.003325213
chr1	187386001	187391000	0.008443385
chr1	187387001	187392000	0.010790777
chr1	187582001	187587000	2.83E-12
chr1	187616001	187621000	3.32E-13
chr1	187617001	187622000	0.001375077
chr1	187618001	187623000	0.000114331
chr1	187619001	187624000	0.00017504
chr1	187620001	187625000	4.32E-05
chr1	187951001	187956000	0.001369133
chr1	187984001	187989000	1.08E-07
chr1	187985001	187990000	1.77E-06
chr1	187986001	187991000	6.48E-07
chr1	188008001	188013000	0.000476421
chr1	188009001	188014000	0.003024519
chr1	188010001	188015000	0.004810295
chr1	188011001	188016000	0.000757702
chr1	188012001	188017000	0.002040283
chr1	188126001	188131000	6.69E-07
chr1	188212001	188217000	0.000130776
chr1	188233001	188238000	1.10E-06
chr1	188398001	188403000	0.003759445
chr1	188401001	188406000	1.81E-13
chr1	188402001	188407000	9.69E-13
chr1	188403001	188408000	8.41E-11
chr1	188612001	188617000	0.000198961
chr1	188670001	188675000	0.000634943
chr1	188671001	188676000	0.000194651
chr1	188692001	188697000	0.000100798
chr1	188819001	188824000	0.013140681
chr1	188909001	188914000	0.010862284
chr1	189015001	189020000	8.07E-10
chr1	189210001	189215000	1.52E-05
chr1	189375001	189380000	6.49E-10
chr1	189376001	189381000	2.51E-12
chr1	189377001	189382000	1.41E-11

chr1	189420001	189425000	0.000112993
chr1	189421001	189426000	0.00025596
chr1	189422001	189427000	0.00015975
chr1	189561001	189566000	0.002426552
chr1	189562001	189567000	0.000694673
chr1	189563001	189568000	0.000627596
chr1	189564001	189569000	0.018615712
chr1	189615001	189620000	0.000582414
chr1	189617001	189622000	0.00040339
chr1	189991001	189996000	1.74E-09
chr1	190079001	190084000	0.000437351
chr1	190080001	190085000	0.000565228
chr1	190086001	190091000	8.74E-06
chr1	190118001	190123000	3.42E-09
chr1	190321001	190326000	0.000404895
chr1	190563001	190568000	0.000183988
chr1	190564001	190569000	0.000198432
chr1	190565001	190570000	2.25E-05
chr1	190566001	190571000	5.59E-05
chr1	190646001	190651000	5.38E-05
chr1	190722001	190727000	0.002883753
chr1	190723001	190728000	0.002574825
chr1	190724001	190729000	0.003195913
chr1	190725001	190730000	0.007076768
chr1	190726001	190731000	0.004981448
chr1	190889001	190894000	0.00088588
chr1	190905001	190910000	3.72E-05
chr1	190906001	190911000	3.05E-05
chr1	190913001	190918000	0.00026458
chr1	190941001	190946000	0.00784398
chr1	190942001	190947000	0.01432735
chr1	190995001	191000000	9.70E-13
chr1	191033001	191038000	1.51E-06
chr1	191034001	191039000	1.04E-05
chr1	191111001	191116000	0.001087875
chr1	191178001	191183000	0.000597691
chr1	191179001	191184000	2.91E-05
chr1	191451001	191456000	0.000414257
chr1	191452001	191457000	0.001206785
chr1	191476001	191481000	7.44E-08
chr1	191477001	191482000	8.39E-05
chr1	191536001	191541000	0.000713793
chr1	191537001	191542000	0.001215633
chr1	191538001	191543000	0.001311073
chr1	191539001	191544000	0.000973788
chr1	191540001	191545000	0.000743437
chr1	191544001	191549000	1.48E-05
chr1	191545001	191550000	3.67E-07

chr1	191580001	191585000	1.87E-09
chr1	191668001	191673000	2.09E-06
chr1	191750001	191755000	0.000491045
chr1	191751001	191756000	7.42E-05
chr1	191792001	191797000	4.93E-06
chr1	191807001	191812000	5.36E-05
chr1	191813001	191818000	3.55E-08
chr1	191814001	191819000	4.29E-08
chr1	191815001	191820000	4.01E-08
chr1	191837001	191842000	1.53E-05
chr1	191838001	191843000	1.83E-05
chr1	191863001	191868000	0.007028847
chr1	191967001	191972000	5.02E-10
chr1	191969001	191974000	1.01E-09
chr1	191989001	191994000	0.000488012
chr1	191990001	191995000	0.00021821
chr1	192012001	192017000	5.22E-07
chr1	192032001	192037000	0.000350547
chr1	192172001	192177000	2.67E-06
chr1	192173001	192178000	9.95E-07
chr1	192256001	192261000	0.002795964
chr1	192257001	192262000	0.003247082
chr1	192302001	192307000	3.53E-05
chr1	192303001	192308000	0.000165511
chr1	192304001	192309000	0.000156512
chr1	192310001	192315000	0.000929283
chr1	192320001	192325000	4.43E-05
chr1	192321001	192326000	5.58E-08
chr1	192322001	192327000	9.32E-05
chr1	192417001	192422000	0.000136707
chr1	192727001	192732000	0.00792045
chr1	192728001	192733000	0.000562541
chr1	193694001	193699000	0.000659238
chr1	193808001	193813000	0.00190115
chr1	193809001	193814000	0.001853476
chr1	194220001	194225000	0.00225029
chr1	194355001	194360000	0.000273855
chr1	194378001	194383000	0.001264233
chr1	194621001	194626000	0.000242367
chr1	194782001	194787000	0.000129236
chr1	194783001	194788000	5.19E-05
chr1	194830001	194835000	8.19E-05
chr1	195071001	195076000	0.000157305
chr1	195072001	195077000	7.96E-05
chr1	195085001	195090000	0.00319561
chr1	195086001	195091000	0.004181747
chr1	195087001	195092000	0.005365917
chr1	195088001	195093000	0.000424878

chr1	195089001	195094000	0.001821109
chr1	195624001	195629000	8.66E-05
chr1	195744001	195749000	0.000216518
chr1	195998001	196003000	3.19E-05
chr1	196175001	196180000	1.90E-05
chr1	196410001	196415000	0.007532342
chr1	196412001	196417000	0.006835029
chr1	196729001	196734000	0.000250699
chr1	196813001	196818000	2.27E-08
chr1	196814001	196819000	2.66E-08
chr1	196815001	196820000	2.34E-08
chr1	196841001	196846000	1.36E-09
chr1	196884001	196889000	0.000726084
chr1	196886001	196891000	6.89E-05
chr1	196887001	196892000	6.77E-05
chr1	197116001	197121000	8.17E-18
chr1	197287001	197292000	0.00115581
chr1	197288001	197293000	0.002167204
chr1	197359001	197364000	0.008584974
chr1	197404001	197409000	0.012603856
chr1	198053001	198058000	0.000205421
chr1	198054001	198059000	1.79E-05
chr1	198055001	198060000	1.57E-05
chr1	198056001	198061000	0.000378468
chr1	198085001	198090000	1.31E-05
chr1	198593001	198598000	3.93E-09
chr1	198734001	198739000	0.002058216
chr1	198764001	198769000	1.98E-06
chr1	198765001	198770000	6.20E-07
chr1	198766001	198771000	0.000106825
chr1	198767001	198772000	0.005780622
chr1	198823001	198828000	1.52E-06
chr1	198824001	198829000	1.02E-09
chr1	198825001	198830000	1.13E-08
chr1	198826001	198831000	5.28E-09
chr1	198828001	198833000	2.24E-07
chr1	198939001	198944000	2.80E-05
chr1	198974001	198979000	2.03E-08
chr1	199077001	199082000	3.22E-07
chr1	199096001	199101000	7.31E-05
chr1	199104001	199109000	6.64E-06
chr1	199105001	199110000	1.04E-06
chr1	199191001	199196000	5.08E-08
chr1	199204001	199209000	5.11E-05
chr1	199205001	199210000	7.30E-05
chr1	199206001	199211000	0.000562541
chr1	199278001	199283000	1.13E-06
chr1	199279001	199284000	1.09E-06

chr1	199280001	199285000	6.87E-06
chr1	199641001	199646000	5.73E-08
chr1	199642001	199647000	4.59E-06
chr1	199652001	199657000	0.006450756
chr1	199687001	199692000	9.06E-07
chr1	199688001	199693000	2.83E-09
chr1	199689001	199694000	7.03E-08
chr1	199733001	199738000	0.000208589
chr1	199734001	199739000	0.000460015
chr1	199735001	199740000	0.000104616
chr1	199736001	199741000	0.000369854
chr1	199799001	199804000	0.000685501
chr1	199864001	199869000	1.91E-08
chr1	199884001	199889000	4.21E-10
chr1	199885001	199890000	3.28E-07
chr1	199886001	199891000	6.24E-07
chr1	200026001	200031000	4.06E-18
chr1	200027001	200032000	1.64E-17
chr1	200028001	200033000	1.57E-13
chr1	200029001	200034000	1.18E-14
chr1	200030001	200035000	4.82E-11
chr1	200153001	200158000	3.84E-16
chr1	200432001	200437000	7.55E-07
chr1	200433001	200438000	1.97E-07
chr1	200476001	200481000	3.22E-17
chr1	200477001	200482000	2.69E-21
chr1	201935001	201940000	1.29E-09
chr1	202545001	202550000	3.29E-06
chr1	202546001	202551000	4.90E-08
chr1	203289001	203294000	3.47E-07
chr1	204612001	204617000	3.34E-11
chr1	204613001	204618000	2.81E-05
chr1	205240001	205245000	1.11E-10
chr1	205339001	205344000	1.16E-11
chr1	205340001	205345000	1.65E-13
chr1	205548001	205553000	1.06E-21
chr1	205549001	205554000	4.11E-18
chr1	207047001	207052000	9.58E-08
chr1	207048001	207053000	4.15E-07
chr1	208871001	208876000	0.0009931
chr1	209088001	209093000	2.38E-10
chr1	209195001	209200000	0.001199958
chr1	209342001	209347000	8.12E-07
chr1	209426001	209431000	2.69E-08
chr1	210029001	210034000	3.40E-32
chr1	210030001	210035000	3.63E-31
chr1	210031001	210036000	3.07E-34
chr1	210032001	210037000	1.28E-29

chr1	210159001	210164000	2.39E-05
chr1	210822001	210827000	1.10E-06
chr1	211505001	211510000	1.15E-20
chr1	212681001	212686000	1.20E-11
chr1	212894001	212899000	1.98E-07
chr1	213814001	213819000	9.68E-05
chr1	214443001	214448000	0.000232827
chr1	214855001	214860000	1.83E-06
chr1	214856001	214861000	3.67E-07
chr1	214960001	214965000	0.000177722
chr1	215136001	215141000	1.14E-05
chr1	215138001	215143000	1.31E-06
chr1	215139001	215144000	2.14E-07
chr1	215140001	215145000	8.55E-09
chr1	215229001	215234000	7.85E-12
chr1	215230001	215235000	3.73E-10
chr1	215231001	215236000	1.59E-10
chr1	215232001	215237000	2.21E-09
chr1	215458001	215463000	0.000147191
chr1	215479001	215484000	8.48E-05
chr1	215517001	215522000	0.009991335
chr1	215573001	215578000	0.000436967
chr1	215574001	215579000	0.000276316
chr1	215579001	215584000	1.02E-09
chr1	215711001	215716000	8.26E-06
chr1	215712001	215717000	3.76E-06
chr1	215877001	215882000	4.39E-06
chr1	215932001	215937000	0.000262156
chr1	215934001	215939000	9.55E-05
chr1	215935001	215940000	6.38E-06
chr1	216068001	216073000	4.04E-09
chr1	216217001	216222000	0.019889663
chr1	216218001	216223000	0.012267176
chr1	216389001	216394000	4.46E-05
chr1	216504001	216509000	0.001208657
chr1	217042001	217047000	1.36E-07
chr1	217134001	217139000	8.30E-09
chr1	217148001	217153000	5.10E-05
chr1	217149001	217154000	0.000141758
chr1	217515001	217520000	0.00020869
chr1	217517001	217522000	1.32E-05
chr1	218107001	218112000	0.000723944
chr1	218218001	218223000	0.000924581
chr1	218219001	218224000	0.000273204
chr1	218341001	218346000	0.000891759
chr1	218342001	218347000	0.001817229
chr1	218547001	218552000	1.60E-17
chr1	219006001	219011000	0.000319143

chr1	219766001	219771000	6.87E-05
chr1	220734001	220739000	8.03E-05
chr1	220735001	220740000	6.96E-05
chr1	221074001	221079000	3.09E-07
chr1	221185001	221190000	1.05E-10
chr1	221202001	221207000	0.000416192
chr1	221423001	221428000	0.000821808
chr1	221424001	221429000	0.00143035
chr1	221425001	221430000	0.000287825
chr1	221629001	221634000	0.000475742
chr1	221659001	221664000	8.26E-09
chr1	221660001	221665000	1.66E-06
chr1	221689001	221694000	0.000492736
chr1	221800001	221805000	0.007621013
chr1	222353001	222358000	2.91E-13
chr1	222354001	222359000	3.96E-14
chr1	222355001	222360000	1.71E-11
chr1	222455001	222460000	0.000377829
chr1	222456001	222461000	1.15E-06
chr1	222571001	222576000	3.17E-07
chr1	222572001	222577000	1.48E-06
chr1	222573001	222578000	6.51E-09
chr1	222939001	222944000	5.02E-05
chr1	222950001	222955000	0.000816795
chr1	222951001	222956000	1.89E-05
chr1	223097001	223102000	0.000169874
chr1	224061001	224066000	9.62E-09
chr1	224062001	224067000	3.52E-08
chr1	224221001	224226000	0.000212409
chr1	224222001	224227000	0.001101858
chr1	226810001	226815000	1.17E-22
chr1	226811001	226816000	1.07E-18
chr1	227429001	227434000	1.06E-09
chr1	227430001	227435000	6.72E-10
chr1	227457001	227462000	1.52E-05
chr1	227458001	227463000	2.43E-05
chr1	229367001	229372000	1.42E-12
chr1	230940001	230945000	5.47E-10
chr1	231586001	231591000	9.43E-08
chr1	233439001	233444000	1.17E-06
chr1	234282001	234287000	1.10E-11
chr1	234283001	234288000	1.80E-09
chr1	234754001	234759000	4.55E-13
chr1	234755001	234760000	8.06E-13
chr1	234862001	234867000	1.01E-20
chr1	236250001	236255000	5.87E-12
chr1	237547001	237552000	7.03E-09
chr1	237971001	237976000	6.18E-09

chr1	238069001	238074000	0.000416208
chr1	238070001	238075000	0.000416291
chr1	238212001	238217000	0.000157069
chr1	238506001	238511000	9.26E-07
chr1	238998001	239003000	0.000250793
chr1	238999001	239004000	0.000627867
chr1	240345001	240350000	1.49E-11
chr1	240401001	240406000	4.42E-17
chr1	240402001	240407000	1.23E-10
chr1	240403001	240408000	2.58E-10
chr1	240539001	240544000	0.00078137
chr1	240540001	240545000	0.000280335
chr1	240595001	240600000	2.26E-08
chr1	240607001	240612000	2.75E-07
chr1	240627001	240632000	2.28E-05
chr1	240695001	240700000	1.37E-05
chr1	240696001	240701000	1.33E-05
chr1	240733001	240738000	1.59E-05
chr1	240735001	240740000	2.96E-09
chr1	240867001	240872000	1.75E-10
chr1	240905001	240910000	0.000271774
chr1	241347001	241352000	8.37E-07
chr1	241348001	241353000	1.04E-06
chr1	241349001	241354000	8.58E-12
chr1	241532001	241537000	0.002931231
chr1	241533001	241538000	0.003960062
chr1	241899001	241904000	0.00576248
chr1	242180001	242185000	2.90E-14
chr1	242323001	242328000	2.39E-09
chr1	242324001	242329000	1.16E-09
chr1	242547001	242552000	1.73E-07
chr1	242721001	242726000	1.04E-07
chr1	242722001	242727000	1.68E-10
chr1	243167001	243172000	1.41E-05
chr1	243168001	243173000	1.68E-06
chr1	243195001	243200000	0.002017378
chr1	245848001	245853000	7.16E-15
chr1	247922001	247927000	5.32E-09
chr1	247923001	247928000	1.31E-09
chr1	247924001	247929000	3.82E-11
chr1	247925001	247930000	8.11E-11
chr1	247958001	247963000	7.31E-06
chr1	247959001	247964000	7.52E-06
chr1	247962001	247967000	4.50E-06
chr1	248361001	248366000	0.004868055
chr2	168001	173000	2.40E-08
chr2	169001	174000	3.29E-07
chr2	614001	619000	1.13E-08

chr2	615001	620000	8.56E-10
chr2	616001	621000	6.55E-07
chr2	617001	622000	1.52E-11
chr2	618001	623000	9.07E-08
chr2	894001	899000	1.55E-07
chr2	895001	900000	5.76E-10
chr2	1116001	1121000	1.15E-10
chr2	1118001	1123000	1.20E-08
chr2	1572001	1577000	2.11E-11
chr2	1890001	1895000	2.17E-12
chr2	1977001	1982000	3.21E-08
chr2	1985001	1990000	1.24E-08
chr2	2082001	2087000	6.69E-05
chr2	2432001	2437000	7.90E-07
chr2	2433001	2438000	1.97E-07
chr2	3579001	3584000	2.50E-19
chr2	3580001	3585000	7.75E-17
chr2	4032001	4037000	0.000401258
chr2	4418001	4423000	0.000127453
chr2	4484001	4489000	2.55E-08
chr2	4485001	4490000	4.76E-06
chr2	4486001	4491000	6.72E-05
chr2	4487001	4492000	4.88E-05
chr2	4675001	4680000	2.25E-06
chr2	4676001	4681000	0.000249244
chr2	4743001	4748000	1.63E-06
chr2	4937001	4942000	0.000197545
chr2	4938001	4943000	1.17E-06
chr2	4939001	4944000	1.92E-06
chr2	5200001	5205000	4.31E-09
chr2	5201001	5206000	2.04E-08
chr2	5202001	5207000	4.87E-07
chr2	5223001	5228000	6.14E-06
chr2	5554001	5559000	8.97E-07
chr2	5573001	5578000	5.30E-05
chr2	6140001	6145000	2.15E-07
chr2	6142001	6147000	3.56E-08
chr2	6375001	6380000	1.94E-11
chr2	6376001	6381000	1.54E-16
chr2	6433001	6438000	5.87E-05
chr2	6817001	6822000	5.54E-06
chr2	6829001	6834000	0.000214914
chr2	7290001	7295000	1.56E-05
chr2	7297001	7302000	0.000526147
chr2	7298001	7303000	0.00071181
chr2	7727001	7732000	0.000934357
chr2	7757001	7762000	2.17E-05
chr2	7793001	7798000	0.00515584

chr2	7844001	7849000	1.35E-07
chr2	8251001	8256000	6.92E-07
chr2	8976001	8981000	2.59E-15
chr2	9526001	9531000	4.65E-23
chr2	10151001	10156000	4.19E-46
chr2	10152001	10157000	1.16E-47
chr2	10153001	10158000	3.93E-37
chr2	10154001	10159000	1.69E-22
chr2	10389001	10394000	7.96E-07
chr2	11036001	11041000	3.16E-09
chr2	11038001	11043000	2.51E-10
chr2	11039001	11044000	2.25E-08
chr2	12207001	12212000	1.10E-12
chr2	12208001	12213000	2.15E-11
chr2	12568001	12573000	3.83E-06
chr2	12596001	12601000	4.91E-10
chr2	12597001	12602000	2.01E-05
chr2	12924001	12929000	1.97E-09
chr2	12925001	12930000	9.09E-12
chr2	13297001	13302000	0.000445797
chr2	13641001	13646000	0.000181502
chr2	13659001	13664000	0.001126611
chr2	13735001	13740000	0.000269071
chr2	13897001	13902000	0.00030818
chr2	16649001	16654000	1.31E-06
chr2	16917001	16922000	1.95E-09
chr2	16997001	17002000	0.001700798
chr2	16998001	17003000	0.001568418
chr2	17238001	17243000	0.000715113
chr2	17272001	17277000	0.000448421
chr2	17292001	17297000	0.005945583
chr2	17293001	17298000	0.006314578
chr2	17598001	17603000	0.00488822
chr2	17600001	17605000	0.005519412
chr2	17601001	17606000	0.005550234
chr2	18186001	18191000	5.89E-09
chr2	18205001	18210000	0.000182188
chr2	18250001	18255000	0.000822831
chr2	18291001	18296000	0.00085763
chr2	18360001	18365000	0.00687123
chr2	18361001	18366000	0.00927776
chr2	18390001	18395000	2.76E-05
chr2	19150001	19155000	1.88E-05
chr2	19151001	19156000	6.30E-06
chr2	20634001	20639000	1.12E-08
chr2	20635001	20640000	1.91E-08
chr2	21440001	21445000	0.003548126
chr2	21541001	21546000	0.000612886

chr2	21581001	21586000	6.12E-08
chr2	21639001	21644000	0.000146369
chr2	21725001	21730000	1.88E-05
chr2	21964001	21969000	4.55E-05
chr2	22058001	22063000	0.000175212
chr2	22190001	22195000	3.28E-05
chr2	22191001	22196000	2.64E-05
chr2	22215001	22220000	0.00015248
chr2	22216001	22221000	0.000176411
chr2	22234001	22239000	5.31E-05
chr2	22416001	22421000	2.72E-07
chr2	22417001	22422000	2.11E-06
chr2	22418001	22423000	1.01E-05
chr2	22511001	22516000	0.013890264
chr2	23040001	23045000	0.000193929
chr2	23042001	23047000	0.000144985
chr2	23314001	23319000	0.000797452
chr2	23379001	23384000	7.25E-05
chr2	23506001	23511000	2.43E-05
chr2	23507001	23512000	1.08E-05
chr2	23825001	23830000	4.12E-07
chr2	24369001	24374000	0.000417546
chr2	26366001	26371000	9.91E-11
chr2	26367001	26372000	8.10E-13
chr2	26835001	26840000	2.61E-10
chr2	26836001	26841000	3.50E-11
chr2	26906001	26911000	2.28E-14
chr2	27412001	27417000	1.51E-11
chr2	27501001	27506000	7.68E-16
chr2	27502001	27507000	2.58E-17
chr2	27503001	27508000	1.57E-10
chr2	28079001	28084000	5.29E-05
chr2	28639001	28644000	5.33E-10
chr2	28640001	28645000	1.11E-07
chr2	28642001	28647000	1.12E-10
chr2	28643001	28648000	8.54E-13
chr2	28734001	28739000	6.65E-06
chr2	28938001	28943000	7.90E-11
chr2	29099001	29104000	2.52E-06
chr2	30106001	30111000	0.000584395
chr2	30107001	30112000	8.16E-05
chr2	30618001	30623000	2.21E-09
chr2	30619001	30624000	4.07E-07
chr2	30922001	30927000	5.13E-05
chr2	31734001	31739000	0.000170093
chr2	31864001	31869000	0.007065124
chr2	31986001	31991000	0.001068539
chr2	32182001	32187000	9.05E-07

chr2	33085001	33090000	1.87E-09
chr2	33442001	33447000	5.39E-11
chr2	33443001	33448000	5.83E-08
chr2	33452001	33457000	3.04E-07
chr2	33667001	33672000	1.51E-09
chr2	33765001	33770000	1.58E-07
chr2	33766001	33771000	1.18E-06
chr2	34082001	34087000	3.32E-09
chr2	34219001	34224000	0.000548776
chr2	34318001	34323000	0.000681213
chr2	34572001	34577000	0.000792041
chr2	34677001	34682000	0.001179845
chr2	34788001	34793000	8.77E-07
chr2	34789001	34794000	5.55E-06
chr2	35043001	35048000	0.000279055
chr2	35045001	35050000	0.00094011
chr2	35046001	35051000	0.000150007
chr2	35047001	35052000	1.50E-06
chr2	35426001	35431000	0.011785392
chr2	35603001	35608000	0.002274601
chr2	35632001	35637000	0.000667984
chr2	35633001	35638000	5.42E-05
chr2	35678001	35683000	0.00013786
chr2	36408001	36413000	0.000513857
chr2	37602001	37607000	1.44E-16
chr2	37603001	37608000	8.84E-18
chr2	37814001	37819000	8.47E-07
chr2	40019001	40024000	0.002220317
chr2	40134001	40139000	0.000634654
chr2	40151001	40156000	5.92E-07
chr2	40152001	40157000	9.99E-07
chr2	40153001	40158000	1.04E-08
chr2	40154001	40159000	2.47E-06
chr2	40203001	40208000	2.17E-05
chr2	40267001	40272000	1.78E-10
chr2	40268001	40273000	1.38E-10
chr2	40289001	40294000	3.81E-06
chr2	40290001	40295000	5.09E-06
chr2	40291001	40296000	9.58E-06
chr2	40323001	40328000	0.000644672
chr2	40325001	40330000	0.000154725
chr2	40326001	40331000	6.39E-06
chr2	40328001	40333000	3.27E-05
chr2	40329001	40334000	7.84E-08
chr2	40350001	40355000	0.001422236
chr2	40367001	40372000	9.83E-08
chr2	40368001	40373000	1.03E-05
chr2	40424001	40429000	0.001015033

chr2	40475001	40480000	1.55E-06
chr2	40485001	40490000	3.29E-07
chr2	40504001	40509000	1.67E-05
chr2	40507001	40512000	0.000372445
chr2	40508001	40513000	0.000373304
chr2	40517001	40522000	0.000107002
chr2	40622001	40627000	0.004671408
chr2	40669001	40674000	5.09E-08
chr2	40670001	40675000	7.51E-07
chr2	40750001	40755000	1.16E-05
chr2	40901001	40906000	4.50E-08
chr2	40902001	40907000	3.13E-07
chr2	40903001	40908000	1.89E-07
chr2	40904001	40909000	9.36E-08
chr2	40905001	40910000	1.44E-12
chr2	41046001	41051000	0.001040779
chr2	41047001	41052000	0.000718391
chr2	41048001	41053000	0.001092241
chr2	41049001	41054000	0.000695836
chr2	41050001	41055000	0.000722117
chr2	41160001	41165000	2.14E-05
chr2	41161001	41166000	1.04E-05
chr2	41162001	41167000	4.83E-06
chr2	41164001	41169000	0.000103205
chr2	41187001	41192000	0.000339815
chr2	41188001	41193000	0.000235054
chr2	41239001	41244000	5.20E-07
chr2	41262001	41267000	0.007518402
chr2	41413001	41418000	0.00016862
chr2	41416001	41421000	0.000145331
chr2	41417001	41422000	4.08E-06
chr2	41553001	41558000	0.002827097
chr2	41627001	41632000	7.51E-05
chr2	41628001	41633000	0.000523162
chr2	41629001	41634000	0.000955414
chr2	41643001	41648000	0.001699185
chr2	41644001	41649000	0.001309465
chr2	41645001	41650000	0.000914135
chr2	41697001	41702000	5.71E-09
chr2	41698001	41703000	4.87E-09
chr2	41699001	41704000	3.49E-08
chr2	41700001	41705000	0.019777101
chr2	41701001	41706000	0.021382916
chr2	41728001	41733000	0.000978033
chr2	41832001	41837000	0.001100866
chr2	41833001	41838000	0.000419271
chr2	41834001	41839000	1.75E-07
chr2	41835001	41840000	3.76E-09

chr2	41836001	41841000	7.47E-06
chr2	41896001	41901000	8.77E-08
chr2	41928001	41933000	0.000102329
chr2	41970001	41975000	1.75E-09
chr2	41976001	41981000	0.00075837
chr2	42476001	42481000	0.000902602
chr2	42673001	42678000	1.97E-11
chr2	42862001	42867000	5.50E-11
chr2	42863001	42868000	2.50E-11
chr2	43298001	43303000	8.61E-15
chr2	43299001	43304000	4.78E-14
chr2	43300001	43305000	6.57E-12
chr2	44288001	44293000	1.37E-19
chr2	44289001	44294000	1.80E-17
chr2	44290001	44295000	6.00E-09
chr2	44292001	44297000	5.66E-06
chr2	44467001	44472000	1.05E-16
chr2	44468001	44473000	1.32E-19
chr2	44471001	44476000	7.01E-08
chr2	46340001	46345000	1.40E-07
chr2	47571001	47576000	7.37E-21
chr2	47572001	47577000	3.21E-10
chr2	47803001	47808000	3.54E-05
chr2	48984001	48989000	0.001431718
chr2	49246001	49251000	7.89E-06
chr2	49258001	49263000	9.44E-05
chr2	49610001	49615000	9.13E-10
chr2	49611001	49616000	1.16E-09
chr2	49629001	49634000	1.05E-09
chr2	49631001	49636000	2.02E-10
chr2	49788001	49793000	0.000589938
chr2	49789001	49794000	0.000456624
chr2	49790001	49795000	0.000423231
chr2	49895001	49900000	5.10E-10
chr2	49896001	49901000	3.75E-09
chr2	49897001	49902000	4.82E-10
chr2	49899001	49904000	3.84E-05
chr2	50015001	50020000	3.59E-06
chr2	50017001	50022000	0.001646102
chr2	50084001	50089000	0.001060646
chr2	50085001	50090000	0.001507144
chr2	50086001	50091000	0.000234645
chr2	50121001	50126000	0.005965944
chr2	50145001	50150000	0.005026623
chr2	50146001	50151000	0.003566668
chr2	50180001	50185000	1.93E-06
chr2	50357001	50362000	6.76E-07
chr2	50358001	50363000	4.14E-06

chr2	50359001	50364000	2.26E-05
chr2	50541001	50546000	0.002454917
chr2	50562001	50567000	8.95E-08
chr2	50564001	50569000	1.64E-05
chr2	50634001	50639000	7.16E-13
chr2	50636001	50641000	3.85E-16
chr2	50643001	50648000	8.72E-05
chr2	50644001	50649000	3.32E-05
chr2	50645001	50650000	0.000459042
chr2	50646001	50651000	0.000732388
chr2	50657001	50662000	5.96E-11
chr2	50658001	50663000	1.29E-10
chr2	50746001	50751000	0.000277022
chr2	50759001	50764000	3.60E-05
chr2	50800001	50805000	2.00E-05
chr2	50801001	50806000	2.95E-05
chr2	51017001	51022000	0.000932172
chr2	51316001	51321000	4.77E-07
chr2	51342001	51347000	9.59E-08
chr2	51380001	51385000	1.06E-07
chr2	51398001	51403000	0.000497495
chr2	51526001	51531000	2.32E-05
chr2	51527001	51532000	0.000402557
chr2	51528001	51533000	0.00830056
chr2	51572001	51577000	4.67E-13
chr2	51573001	51578000	7.46E-17
chr2	51574001	51579000	8.21E-15
chr2	51575001	51580000	2.16E-15
chr2	51603001	51608000	0.00063025
chr2	51604001	51609000	0.001334489
chr2	51653001	51658000	5.81E-05
chr2	51659001	51664000	1.55E-06
chr2	51715001	51720000	0.001894003
chr2	52040001	52045000	0.014158049
chr2	52041001	52046000	0.00743613
chr2	52042001	52047000	0.008678603
chr2	52044001	52049000	1.74E-05
chr2	52050001	52055000	0.001653826
chr2	52051001	52056000	4.85E-06
chr2	52104001	52109000	0.018529556
chr2	52107001	52112000	8.18E-05
chr2	52167001	52172000	0.002441704
chr2	52168001	52173000	0.004215512
chr2	52256001	52261000	0.006689764
chr2	52257001	52262000	0.010303673
chr2	52272001	52277000	3.69E-08
chr2	52315001	52320000	0.0009122
chr2	52503001	52508000	1.71E-05

chr2	52504001	52509000	7.58E-06
chr2	52505001	52510000	8.03E-05
chr2	52506001	52511000	5.66E-05
chr2	52555001	52560000	0.001481482
chr2	52600001	52605000	1.73E-06
chr2	52640001	52645000	0.000362245
chr2	52641001	52646000	0.000683798
chr2	52666001	52671000	4.23E-08
chr2	52667001	52672000	1.73E-05
chr2	52723001	52728000	2.26E-05
chr2	52724001	52729000	8.18E-05
chr2	52725001	52730000	0.002477624
chr2	52789001	52794000	4.63E-05
chr2	52790001	52795000	6.47E-05
chr2	52972001	52977000	0.001042125
chr2	52973001	52978000	0.001452085
chr2	52974001	52979000	0.000762787
chr2	53061001	53066000	0.002814223
chr2	53067001	53072000	0.000428847
chr2	53193001	53198000	1.23E-08
chr2	53194001	53199000	1.62E-08
chr2	53455001	53460000	4.01E-25
chr2	53456001	53461000	6.08E-25
chr2	53457001	53462000	7.82E-25
chr2	53530001	53535000	3.08E-15
chr2	53531001	53536000	3.16E-17
chr2	53532001	53537000	1.09E-08
chr2	53548001	53553000	4.52E-12
chr2	53564001	53569000	1.68E-33
chr2	53565001	53570000	1.06E-39
chr2	53566001	53571000	2.05E-34
chr2	53567001	53572000	1.45E-33
chr2	53568001	53573000	2.98E-26
chr2	53569001	53574000	3.58E-19
chr2	53585001	53590000	1.41E-20
chr2	53586001	53591000	4.59E-18
chr2	53587001	53592000	3.20E-12
chr2	53594001	53599000	2.98E-19
chr2	54277001	54282000	2.59E-06
chr2	54278001	54283000	1.80E-06
chr2	54430001	54435000	1.04E-07
chr2	54431001	54436000	1.30E-09
chr2	54591001	54596000	0.000742937
chr2	54631001	54636000	9.55E-12
chr2	54632001	54637000	4.53E-11
chr2	54633001	54638000	5.75E-18
chr2	54634001	54639000	1.02E-15
chr2	54688001	54693000	1.10E-10

chr2	54689001	54694000	8.85E-07
chr2	54690001	54695000	6.86E-15
chr2	54691001	54696000	9.69E-08
chr2	54899001	54904000	1.75E-23
chr2	54900001	54905000	1.82E-22
chr2	55846001	55851000	1.24E-17
chr2	55997001	56002000	3.26E-12
chr2	55998001	56003000	1.45E-11
chr2	56128001	56133000	0.00157278
chr2	56298001	56303000	0.000230729
chr2	56469001	56474000	0.000476653
chr2	56515001	56520000	3.28E-06
chr2	56913001	56918000	0.000645162
chr2	56942001	56947000	2.97E-09
chr2	56977001	56982000	0.000164818
chr2	56978001	56983000	0.002174394
chr2	56979001	56984000	0.003045793
chr2	57010001	57015000	0.000869925
chr2	57011001	57016000	0.000291689
chr2	57025001	57030000	3.06E-06
chr2	57026001	57031000	1.28E-06
chr2	57027001	57032000	2.65E-06
chr2	57028001	57033000	9.15E-06
chr2	57029001	57034000	1.67E-05
chr2	57030001	57035000	0.00016614
chr2	57031001	57036000	0.004945405
chr2	57385001	57390000	0.002482259
chr2	57407001	57412000	0.003942339
chr2	57416001	57421000	1.14E-06
chr2	57451001	57456000	1.48E-06
chr2	57480001	57485000	0.000140347
chr2	57504001	57509000	0.013504662
chr2	57541001	57546000	0.000572638
chr2	57553001	57558000	0.007830335
chr2	57673001	57678000	1.05E-07
chr2	57718001	57723000	2.37E-07
chr2	57734001	57739000	4.99E-06
chr2	57739001	57744000	0.001605822
chr2	57740001	57745000	1.45E-05
chr2	57801001	57806000	0.001020604
chr2	57802001	57807000	2.85E-05
chr2	57803001	57808000	0.000434944
chr2	58024001	58029000	0.005159725
chr2	58515001	58520000	0.002079403
chr2	58516001	58521000	0.000810062
chr2	58517001	58522000	0.003819905
chr2	58518001	58523000	0.00106249
chr2	58533001	58538000	6.60E-05

chr2	58657001	58662000	1.64E-05
chr2	58658001	58663000	2.04E-05
chr2	58659001	58664000	0.003200003
chr2	58689001	58694000	5.87E-10
chr2	58888001	58893000	0.003600051
chr2	58925001	58930000	0.000389865
chr2	58926001	58931000	0.000914204
chr2	58974001	58979000	0.000912302
chr2	58999001	59004000	2.95E-05
chr2	59000001	59005000	0.000109784
chr2	59001001	59006000	0.002006611
chr2	59002001	59007000	0.001258574
chr2	59056001	59061000	0.003490147
chr2	59073001	59078000	1.82E-05
chr2	59134001	59139000	7.32E-05
chr2	59216001	59221000	3.03E-06
chr2	59229001	59234000	0.000287438
chr2	59230001	59235000	0.000146809
chr2	59320001	59325000	3.81E-07
chr2	59460001	59465000	1.64E-06
chr2	59462001	59467000	1.77E-06
chr2	59463001	59468000	1.13E-07
chr2	59617001	59622000	0.017671493
chr2	59820001	59825000	0.00050879
chr2	59878001	59883000	0.00302923
chr2	61873001	61878000	7.70E-05
chr2	61986001	61991000	5.54E-17
chr2	62793001	62798000	1.11E-07
chr2	63967001	63972000	0.000627732
chr2	63968001	63973000	0.00236206
chr2	64339001	64344000	6.16E-05
chr2	64439001	64444000	1.67E-07
chr2	64440001	64445000	1.32E-08
chr2	65876001	65881000	0.000995646
chr2	66225001	66230000	0.005797017
chr2	66286001	66291000	0.000588066
chr2	67178001	67183000	0.004798427
chr2	67347001	67352000	1.75E-05
chr2	67371001	67376000	0.000186439
chr2	67372001	67377000	0.000250461
chr2	67405001	67410000	0.000120032
chr2	67406001	67411000	0.000107285
chr2	67843001	67848000	0.006343347
chr2	67867001	67872000	5.98E-05
chr2	68473001	68478000	3.44E-24
chr2	68495001	68500000	1.41E-10
chr2	68830001	68835000	1.60E-05
chr2	68857001	68862000	0.001138916

chr2	68894001	68899000	0.000480183
chr2	68977001	68982000	9.80E-20
chr2	70026001	70031000	1.42E-07
chr2	70348001	70353000	4.29E-19
chr2	70349001	70354000	1.86E-12
chr2	70351001	70356000	6.27E-12
chr2	70352001	70357000	4.23E-28
chr2	71033001	71038000	2.02E-14
chr2	72047001	72052000	0.000720682
chr2	72306001	72311000	1.90E-11
chr2	72348001	72353000	4.22E-07
chr2	73163001	73168000	7.07E-16
chr2	73164001	73169000	3.12E-12
chr2	74913001	74918000	7.37E-09
chr2	74961001	74966000	1.19E-06
chr2	74962001	74967000	0.000111183
chr2	75123001	75128000	4.74E-08
chr2	75124001	75129000	6.31E-08
chr2	75304001	75309000	2.69E-08
chr2	75540001	75545000	0.000143256
chr2	75973001	75978000	0.001316305
chr2	75974001	75979000	0.00606499
chr2	75975001	75980000	0.003873174
chr2	75976001	75981000	0.000898043
chr2	75977001	75982000	0.000220155
chr2	76120001	76125000	7.37E-05
chr2	76121001	76126000	8.22E-07
chr2	76122001	76127000	9.57E-07
chr2	76123001	76128000	2.48E-05
chr2	76124001	76129000	0.00017398
chr2	76148001	76153000	0.0075407
chr2	76439001	76444000	1.66E-06
chr2	76440001	76445000	1.17E-05
chr2	76491001	76496000	6.32E-06
chr2	76492001	76497000	3.10E-06
chr2	76516001	76521000	5.48E-05
chr2	76539001	76544000	6.00E-08
chr2	76638001	76643000	1.44E-09
chr2	76639001	76644000	7.10E-08
chr2	76640001	76645000	8.43E-08
chr2	76689001	76694000	0.00889017
chr2	76701001	76706000	2.38E-06
chr2	76702001	76707000	6.11E-06
chr2	77013001	77018000	7.25E-05
chr2	77014001	77019000	0.000388108
chr2	77015001	77020000	0.000214182
chr2	77016001	77021000	3.36E-07
chr2	77434001	77439000	6.02E-05

chr2	77436001	77441000	0.000165961
chr2	77437001	77442000	8.69E-05
chr2	77577001	77582000	0.008165837
chr2	77578001	77583000	0.001548046
chr2	77589001	77594000	0.001002109
chr2	77741001	77746000	0.0029399
chr2	78027001	78032000	0.000275058
chr2	78028001	78033000	0.000211542
chr2	78086001	78091000	4.59E-13
chr2	78551001	78556000	0.00030584
chr2	78552001	78557000	0.000169373
chr2	78553001	78558000	8.68E-05
chr2	78554001	78559000	0.001366396
chr2	78584001	78589000	6.56E-09
chr2	78585001	78590000	8.95E-11
chr2	78586001	78591000	1.04E-10
chr2	78746001	78751000	9.12E-06
chr2	78916001	78921000	0.014064074
chr2	79109001	79114000	0.000176717
chr2	79110001	79115000	7.10E-05
chr2	79161001	79166000	2.74E-06
chr2	79162001	79167000	9.28E-07
chr2	79426001	79431000	0.000231316
chr2	79427001	79432000	0.005306045
chr2	79445001	79450000	6.91E-12
chr2	79446001	79451000	8.78E-07
chr2	79587001	79592000	0.012062125
chr2	79788001	79793000	0.000647817
chr2	79789001	79794000	0.000133345
chr2	79790001	79795000	4.93E-05
chr2	79873001	79878000	6.17E-08
chr2	79874001	79879000	0.000108955
chr2	79935001	79940000	9.13E-05
chr2	79936001	79941000	2.45E-05
chr2	79956001	79961000	0.000153587
chr2	79960001	79965000	6.96E-05
chr2	80029001	80034000	4.63E-07
chr2	80030001	80035000	1.10E-06
chr2	80031001	80036000	1.43E-10
chr2	80040001	80045000	1.48E-06
chr2	80096001	80101000	5.94E-06
chr2	80132001	80137000	2.54E-15
chr2	80208001	80213000	1.14E-07
chr2	80256001	80261000	2.65E-06
chr2	80257001	80262000	4.03E-05
chr2	80399001	80404000	8.89E-06
chr2	80433001	80438000	4.40E-07
chr2	80672001	80677000	1.34E-05

chr2	80673001	80678000	0.000412755
chr2	80705001	80710000	0.003572116
chr2	80722001	80727000	5.12E-05
chr2	80723001	80728000	0.000117663
chr2	80726001	80731000	0.001027319
chr2	80836001	80841000	0.01688675
chr2	80905001	80910000	2.84E-08
chr2	80906001	80911000	4.84E-09
chr2	80907001	80912000	1.25E-07
chr2	80908001	80913000	7.75E-08
chr2	80973001	80978000	0.001685839
chr2	80975001	80980000	0.003071053
chr2	81096001	81101000	0.000139436
chr2	81217001	81222000	1.16E-07
chr2	81390001	81395000	9.03E-05
chr2	81697001	81702000	0.000573742
chr2	81698001	81703000	0.000546763
chr2	81699001	81704000	0.00030935
chr2	82060001	82065000	1.42E-06
chr2	82522001	82527000	0.000185255
chr2	82845001	82850000	0.008617467
chr2	82979001	82984000	8.78E-06
chr2	82980001	82985000	4.98E-06
chr2	82981001	82986000	9.98E-06
chr2	83033001	83038000	0.000458727
chr2	83058001	83063000	1.91E-06
chr2	83084001	83089000	0.001186144
chr2	83116001	83121000	0.000171309
chr2	83117001	83122000	0.000174691
chr2	83154001	83159000	2.53E-05
chr2	83184001	83189000	4.75E-11
chr2	83246001	83251000	7.12E-05
chr2	83247001	83252000	0.000447318
chr2	83268001	83273000	4.44E-06
chr2	83269001	83274000	8.08E-07
chr2	83306001	83311000	2.19E-06
chr2	83307001	83312000	2.07E-12
chr2	83310001	83315000	1.56E-09
chr2	83465001	83470000	1.23E-05
chr2	83466001	83471000	1.41E-05
chr2	83554001	83559000	0.000753738
chr2	83555001	83560000	0.000179123
chr2	83611001	83616000	0.002401507
chr2	83612001	83617000	0.001696395
chr2	83837001	83842000	0.000124139
chr2	83866001	83871000	0.000300542
chr2	84040001	84045000	0.018083384
chr2	84345001	84350000	0.000828601

chr2	84364001	84369000	6.75E-05
chr2	84374001	84379000	5.39E-05
chr2	84375001	84380000	3.86E-06
chr2	84376001	84381000	9.45E-05
chr2	84377001	84382000	0.000572967
chr2	84389001	84394000	6.33E-07
chr2	84391001	84396000	0.000589983
chr2	84549001	84554000	7.62E-06
chr2	85200001	85205000	1.14E-15
chr2	86011001	86016000	1.93E-15
chr2	86170001	86175000	0.004260928
chr2	86707001	86712000	2.38E-15
chr2	86916001	86921000	4.62E-05
chr2	87377001	87382000	0.004587023
chr2	87395001	87400000	0.00011468
chr2	87461001	87466000	0.008657916
chr2	87745001	87750000	0.000151157
chr2	87746001	87751000	6.87E-06
chr2	87747001	87752000	5.21E-07
chr2	87955001	87960000	3.78E-12
chr2	87956001	87961000	3.03E-13
chr2	88190001	88195000	0.009067912
chr2	88206001	88211000	0.000484177
chr2	88207001	88212000	0.00145914
chr2	89132001	89137000	0.000320596
chr2	89135001	89140000	5.79E-05
chr2	89136001	89141000	0.000662081
chr2	89154001	89159000	6.90E-14
chr2	89217001	89222000	7.11E-20
chr2	89272001	89277000	2.32E-07
chr2	89274001	89279000	5.90E-06
chr2	89381001	89386000	0.000116784
chr2	89383001	89388000	5.92E-06
chr2	89449001	89454000	0.000173598
chr2	89450001	89455000	7.63E-05
chr2	89496001	89501000	2.07E-05
chr2	89497001	89502000	5.11E-07
chr2	89912001	89917000	0.000266558
chr2	89913001	89918000	4.81E-05
chr2	89980001	89985000	0.017516186
chr2	90041001	90046000	0.001007403
chr2	90042001	90047000	6.96E-05
chr2	90043001	90048000	1.01E-05
chr2	90144001	90149000	4.08E-07
chr2	95341001	95346000	8.17E-05
chr2	95970001	95975000	8.83E-17
chr2	96252001	96257000	0.002544723
chr2	96438001	96443000	6.65E-07

chr2	96721001	96726000	0.002938713
chr2	96867001	96872000	6.70E-12
chr2	97241001	97246000	0.000583498
chr2	97591001	97596000	8.33E-11
chr2	97592001	97597000	9.99E-11
chr2	97593001	97598000	9.70E-11
chr2	97972001	97977000	0.001954205
chr2	98676001	98681000	0.001722272
chr2	98677001	98682000	0.001073128
chr2	99089001	99094000	1.38E-11
chr2	99090001	99095000	1.49E-16
chr2	99798001	99803000	5.43E-06
chr2	99859001	99864000	1.89E-06
chr2	99860001	99865000	2.30E-08
chr2	100285001	100290000	5.28E-05
chr2	100286001	100291000	1.52E-08
chr2	101198001	101203000	0.00787564
chr2	102190001	102195000	4.53E-06
chr2	103402001	103407000	4.77E-09
chr2	103706001	103711000	1.73E-05
chr2	103712001	103717000	0.002412063
chr2	104056001	104061000	2.54E-06
chr2	104058001	104063000	1.27E-07
chr2	104059001	104064000	4.77E-08
chr2	104060001	104065000	1.68E-11
chr2	104465001	104470000	3.27E-05
chr2	104466001	104471000	0.000125646
chr2	104467001	104472000	2.43E-05
chr2	104772001	104777000	0.016202572
chr2	104773001	104778000	0.015485399
chr2	105084001	105089000	3.95E-07
chr2	105105001	105110000	0.000155204
chr2	105133001	105138000	6.28E-09
chr2	105134001	105139000	1.46E-07
chr2	105537001	105542000	0.002059851
chr2	106747001	106752000	6.63E-08
chr2	107222001	107227000	0.000535759
chr2	107295001	107300000	0.019292039
chr2	107296001	107301000	0.026685767
chr2	107420001	107425000	7.88E-09
chr2	107456001	107461000	8.52E-12
chr2	107661001	107666000	0.002336184
chr2	107750001	107755000	1.66E-06
chr2	107751001	107756000	1.63E-07
chr2	107752001	107757000	6.44E-06
chr2	107894001	107899000	0.008615306
chr2	108073001	108078000	5.58E-10
chr2	108074001	108079000	2.46E-10

chr2	108170001	108175000	0.006618823
chr2	108231001	108236000	0.000309501
chr2	108232001	108237000	0.002627714
chr2	108292001	108297000	0.000747153
chr2	108293001	108298000	0.001010333
chr2	108356001	108361000	0.002253549
chr2	108357001	108362000	0.003882316
chr2	108358001	108363000	0.006911655
chr2	108977001	108982000	0.000499695
chr2	109208001	109213000	3.59E-09
chr2	109249001	109254000	1.98E-07
chr2	109250001	109255000	2.59E-09
chr2	109717001	109722000	1.23E-06
chr2	110849001	110854000	8.87E-07
chr2	110850001	110855000	2.56E-05
chr2	111105001	111110000	0.018186358
chr2	111769001	111774000	0.000185981
chr2	112315001	112320000	5.14E-06
chr2	112316001	112321000	6.38E-06
chr2	113921001	113926000	5.24E-05
chr2	113922001	113927000	2.02E-05
chr2	114447001	114452000	0.000368211
chr2	114633001	114638000	5.72E-13
chr2	114634001	114639000	3.12E-11
chr2	114635001	114640000	6.53E-16
chr2	114750001	114755000	0.000931088
chr2	119463001	119468000	4.53E-06
chr2	119845001	119850000	0.000351892
chr2	119953001	119958000	4.97E-14
chr2	119954001	119959000	2.08E-13
chr2	119955001	119960000	3.58E-15
chr2	120242001	120247000	1.87E-12
chr2	120243001	120248000	2.35E-16
chr2	120294001	120299000	4.45E-06
chr2	120990001	120995000	3.33E-07
chr2	121325001	121330000	4.12E-05
chr2	122058001	122063000	5.01E-07
chr2	122060001	122065000	1.59E-05
chr2	122090001	122095000	3.58E-09
chr2	123367001	123372000	0.003309602
chr2	123368001	123373000	0.003067135
chr2	123554001	123559000	4.57E-06
chr2	123714001	123719000	2.11E-07
chr2	124347001	124352000	0.000406861
chr2	124427001	124432000	0.000121326
chr2	124965001	124970000	0.000672401
chr2	125098001	125103000	0.00134155
chr2	125099001	125104000	0.000530638

chr2	125106001	125111000	4.75E-06
chr2	125107001	125112000	6.95E-06
chr2	125287001	125292000	6.83E-10
chr2	125288001	125293000	4.76E-11
chr2	125289001	125294000	1.83E-10
chr2	125398001	125403000	2.43E-06
chr2	125649001	125654000	0.00011002
chr2	125703001	125708000	2.01E-07
chr2	125704001	125709000	6.03E-06
chr2	126211001	126216000	0.037993719
chr2	126212001	126217000	0.00257353
chr2	126252001	126257000	0.006954493
chr2	126545001	126550000	0.001033815
chr2	126570001	126575000	2.11E-13
chr2	126674001	126679000	5.15E-07
chr2	127246001	127251000	7.88E-17
chr2	127247001	127252000	1.94E-18
chr2	127248001	127253000	1.15E-15
chr2	127951001	127956000	1.07E-18
chr2	127952001	127957000	1.08E-11
chr2	128407001	128412000	5.67E-12
chr2	128416001	128421000	5.11E-05
chr2	128491001	128496000	0.005650668
chr2	128712001	128717000	7.62E-05
chr2	129056001	129061000	0.000187873
chr2	129640001	129645000	0.000339355
chr2	129941001	129946000	1.68E-05
chr2	130155001	130160000	4.30E-05
chr2	130156001	130161000	0.000386032
chr2	130296001	130301000	0.000134223
chr2	130297001	130302000	0.000319514
chr2	130414001	130419000	2.26E-05
chr2	130415001	130420000	0.000116358
chr2	130516001	130521000	7.18E-11
chr2	131164001	131169000	0.000102639
chr2	131165001	131170000	0.00048448
chr2	131166001	131171000	0.000758397
chr2	131167001	131172000	0.001040757
chr2	131168001	131173000	0.000481376
chr2	131430001	131435000	0.011095021
chr2	131527001	131532000	1.02E-06
chr2	131793001	131798000	1.31E-25
chr2	131794001	131799000	3.56E-12
chr2	131795001	131800000	1.51E-12
chr2	131796001	131801000	3.22E-10
chr2	131797001	131802000	3.05E-10
chr2	131988001	131993000	0.000431317
chr2	132076001	132081000	5.38E-05

chr2	132077001	132082000	4.18E-05
chr2	132078001	132083000	3.18E-06
chr2	132079001	132084000	7.21E-05
chr2	132523001	132528000	3.94E-10
chr2	132544001	132549000	2.70E-06
chr2	132545001	132550000	2.88E-05
chr2	132546001	132551000	6.86E-05
chr2	132714001	132719000	0.000132083
chr2	135692001	135697000	0.004976897
chr2	135693001	135698000	1.35E-09
chr2	136553001	136558000	0.000361303
chr2	137269001	137274000	0.024858525
chr2	137270001	137275000	0.00053837
chr2	137338001	137343000	2.81E-06
chr2	137339001	137344000	3.52E-13
chr2	137340001	137345000	2.02E-15
chr2	137341001	137346000	7.07E-16
chr2	137342001	137347000	3.20E-13
chr2	137343001	137348000	4.57E-12
chr2	137754001	137759000	0.000145553
chr2	137755001	137760000	0.000266635
chr2	137929001	137934000	1.80E-06
chr2	137930001	137935000	9.35E-06
chr2	137931001	137936000	0.001588231
chr2	137932001	137937000	0.002065502
chr2	138159001	138164000	0.001558013
chr2	139033001	139038000	4.51E-05
chr2	139504001	139509000	1.24E-07
chr2	139519001	139524000	0.000652378
chr2	139628001	139633000	0.000218306
chr2	139629001	139634000	8.35E-06
chr2	139735001	139740000	1.07E-06
chr2	139736001	139741000	6.55E-07
chr2	139774001	139779000	2.42E-08
chr2	139775001	139780000	1.69E-07
chr2	139776001	139781000	0.001692643
chr2	139786001	139791000	2.25E-05
chr2	139787001	139792000	3.13E-06
chr2	139788001	139793000	0.000227854
chr2	139869001	139874000	3.27E-05
chr2	139870001	139875000	9.66E-06
chr2	139871001	139876000	2.86E-05
chr2	139872001	139877000	4.79E-06
chr2	139873001	139878000	1.82E-08
chr2	140090001	140095000	0.002036559
chr2	140235001	140240000	7.52E-06
chr2	140243001	140248000	0.000778613
chr2	140283001	140288000	0.002289136

chr2	140409001	140414000	0.000183757
chr2	140411001	140416000	5.37E-05
chr2	140412001	140417000	3.59E-05
chr2	140413001	140418000	0.000101659
chr2	140507001	140512000	7.30E-09
chr2	140508001	140513000	5.80E-08
chr2	140571001	140576000	6.08E-14
chr2	140642001	140647000	1.05E-07
chr2	140678001	140683000	6.57E-28
chr2	140680001	140685000	1.81E-20
chr2	140681001	140686000	5.38E-26
chr2	140682001	140687000	5.32E-22
chr2	140691001	140696000	1.98E-17
chr2	141282001	141287000	0.004826139
chr2	141418001	141423000	0.003243907
chr2	141419001	141424000	0.001670887
chr2	141871001	141876000	8.75E-06
chr2	141881001	141886000	0.00321132
chr2	141884001	141889000	0.001135179
chr2	142298001	142303000	0.001430629
chr2	142465001	142470000	0.001593722
chr2	142466001	142471000	0.000168372
chr2	142737001	142742000	0.000146523
chr2	142738001	142743000	3.25E-05
chr2	142831001	142836000	0.002198484
chr2	143145001	143150000	1.98E-06
chr2	143146001	143151000	9.41E-07
chr2	143147001	143152000	9.31E-06
chr2	143201001	143206000	5.45E-06
chr2	143202001	143207000	2.41E-06
chr2	143232001	143237000	1.14E-05
chr2	143241001	143246000	0.002380474
chr2	143270001	143275000	3.59E-08
chr2	143271001	143276000	2.41E-05
chr2	143272001	143277000	4.64E-05
chr2	143628001	143633000	2.31E-09
chr2	143630001	143635000	9.86E-06
chr2	143631001	143636000	1.24E-08
chr2	143632001	143637000	2.29E-09
chr2	143940001	143945000	2.49E-09
chr2	144089001	144094000	4.22E-05
chr2	144090001	144095000	1.12E-06
chr2	144236001	144241000	0.001357955
chr2	144237001	144242000	9.16E-05
chr2	144238001	144243000	0.00010179
chr2	144306001	144311000	0.000386674
chr2	144307001	144312000	0.000268672
chr2	144309001	144314000	0.000685258

chr2	144512001	144517000	2.96E-05
chr2	145156001	145161000	1.55E-06
chr2	145363001	145368000	2.17E-06
chr2	145364001	145369000	1.36E-06
chr2	145457001	145462000	0.000472189
chr2	145458001	145463000	0.001145383
chr2	145459001	145464000	0.0009662
chr2	145510001	145515000	0.001046012
chr2	145511001	145516000	0.001582407
chr2	145512001	145517000	0.002031671
chr2	145536001	145541000	5.15E-05
chr2	145821001	145826000	0.001563657
chr2	145822001	145827000	0.001529933
chr2	145863001	145868000	7.45E-08
chr2	145864001	145869000	1.78E-08
chr2	145865001	145870000	1.74E-08
chr2	145866001	145871000	5.00E-08
chr2	145938001	145943000	0.000642536
chr2	145939001	145944000	0.003314461
chr2	146319001	146324000	0.014678812
chr2	146821001	146826000	0.000334293
chr2	146822001	146827000	3.51E-07
chr2	146920001	146925000	0.000655443
chr2	146921001	146926000	3.84E-05
chr2	146946001	146951000	0.000600523
chr2	146949001	146954000	6.40E-05
chr2	147186001	147191000	5.59E-06
chr2	147187001	147192000	0.000128485
chr2	147188001	147193000	0.000154687
chr2	147206001	147211000	0.011125678
chr2	147412001	147417000	0.00026606
chr2	147739001	147744000	8.53E-05
chr2	148185001	148190000	0.000650869
chr2	148187001	148192000	2.45E-05
chr2	148188001	148193000	2.40E-05
chr2	148392001	148397000	0.00240073
chr2	149358001	149363000	1.60E-05
chr2	149552001	149557000	7.93E-07
chr2	149650001	149655000	0.000483897
chr2	149845001	149850000	1.29E-10
chr2	149846001	149851000	3.98E-12
chr2	150273001	150278000	7.42E-10
chr2	150530001	150535000	4.44E-05
chr2	150621001	150626000	0.001246631
chr2	150640001	150645000	5.07E-08
chr2	150641001	150646000	8.36E-11
chr2	150717001	150722000	0.000367015
chr2	151135001	151140000	1.26E-05

chr2	151256001	151261000	0.004118865
chr2	151701001	151706000	0.000222577
chr2	151708001	151713000	0.000499679
chr2	151709001	151714000	0.005191154
chr2	151764001	151769000	0.002783748
chr2	151907001	151912000	7.43E-09
chr2	151908001	151913000	4.59E-06
chr2	151929001	151934000	4.63E-06
chr2	151930001	151935000	3.40E-06
chr2	152210001	152215000	0.001261368
chr2	152211001	152216000	0.00201661
chr2	152913001	152918000	1.53E-05
chr2	152914001	152919000	0.007964453
chr2	153198001	153203000	0.031501254
chr2	153709001	153714000	2.58E-05
chr2	153745001	153750000	0.001880897
chr2	153771001	153776000	0.01085103
chr2	153772001	153777000	0.001160256
chr2	153804001	153809000	9.12E-07
chr2	153805001	153810000	4.65E-05
chr2	153919001	153924000	1.82E-05
chr2	154271001	154276000	0.000303448
chr2	154272001	154277000	0.000781
chr2	154696001	154701000	0.002544395
chr2	154697001	154702000	0.000956718
chr2	154734001	154739000	0.001630011
chr2	154826001	154831000	0.000128408
chr2	154827001	154832000	0.001057617
chr2	155044001	155049000	6.36E-10
chr2	155045001	155050000	1.01E-07
chr2	155046001	155051000	4.96E-07
chr2	155047001	155052000	1.85E-08
chr2	155048001	155053000	9.38E-08
chr2	155230001	155235000	3.32E-08
chr2	156040001	156045000	4.40E-05
chr2	156254001	156259000	0.002155773
chr2	156255001	156260000	0.00186822
chr2	157769001	157774000	4.28E-07
chr2	157770001	157775000	0.000142249
chr2	158537001	158542000	1.61E-06
chr2	158538001	158543000	6.38E-07
chr2	158539001	158544000	1.06E-07
chr2	158878001	158883000	1.14E-07
chr2	158913001	158918000	1.79E-05
chr2	159049001	159054000	0.007972114
chr2	159936001	159941000	8.11E-10
chr2	159937001	159942000	3.01E-09
chr2	159938001	159943000	2.74E-07

chr2	159948001	159953000	1.62E-14
chr2	160649001	160654000	1.23E-10
chr2	160866001	160871000	4.11E-06
chr2	160868001	160873000	1.59E-05
chr2	160869001	160874000	3.02E-05
chr2	161054001	161059000	0.005384153
chr2	161055001	161060000	0.000153214
chr2	161290001	161295000	4.88E-05
chr2	161436001	161441000	0.000324569
chr2	161437001	161442000	0.000108967
chr2	161666001	161671000	0.00017057
chr2	161667001	161672000	0.000322527
chr2	161849001	161854000	1.35E-07
chr2	162326001	162331000	7.36E-06
chr2	162327001	162332000	6.46E-05
chr2	162413001	162418000	0.001861341
chr2	162839001	162844000	8.77E-05
chr2	163248001	163253000	0.017922332
chr2	163460001	163465000	0.000320801
chr2	163515001	163520000	0.000122813
chr2	163516001	163521000	0.000125116
chr2	163517001	163522000	7.05E-05
chr2	163737001	163742000	6.42E-05
chr2	163799001	163804000	0.005863314
chr2	163982001	163987000	0.000881274
chr2	164113001	164118000	4.12E-05
chr2	164115001	164120000	4.47E-05
chr2	164129001	164134000	0.000149288
chr2	164240001	164245000	6.57E-05
chr2	164261001	164266000	5.86E-11
chr2	164366001	164371000	5.69E-05
chr2	164393001	164398000	0.000438978
chr2	164485001	164490000	4.46E-06
chr2	164486001	164491000	2.19E-06
chr2	164487001	164492000	9.59E-06
chr2	164507001	164512000	1.12E-09
chr2	164508001	164513000	6.10E-07
chr2	164509001	164514000	0.005748893
chr2	164510001	164515000	0.003056692
chr2	164511001	164516000	2.92E-05
chr2	164512001	164517000	0.000862505
chr2	164753001	164758000	0.001381248
chr2	164754001	164759000	0.001747276
chr2	164813001	164818000	4.05E-10
chr2	164917001	164922000	0.0003385
chr2	164918001	164923000	0.006658273
chr2	164919001	164924000	0.00244339
chr2	166098001	166103000	0.000795354

chr2	166179001	166184000	6.14E-13
chr2	166550001	166555000	0.000753534
chr2	166551001	166556000	0.00102678
chr2	166679001	166684000	0.000468737
chr2	166680001	166685000	0.000297617
chr2	167344001	167349000	3.18E-07
chr2	167373001	167378000	8.60E-07
chr2	167807001	167812000	1.23E-07
chr2	168152001	168157000	0.000842726
chr2	168989001	168994000	2.80E-10
chr2	168990001	168995000	1.77E-07
chr2	169133001	169138000	0.001318754
chr2	169748001	169753000	0.001874783
chr2	170011001	170016000	0.000412635
chr2	170227001	170232000	9.45E-08
chr2	170228001	170233000	2.11E-05
chr2	170515001	170520000	0.003479804
chr2	170642001	170647000	7.31E-05
chr2	170643001	170648000	1.80E-07
chr2	171102001	171107000	0.000273242
chr2	171278001	171283000	8.33E-07
chr2	171280001	171285000	8.36E-11
chr2	171633001	171638000	0.000199964
chr2	172383001	172388000	7.13E-08
chr2	172443001	172448000	0.000124154
chr2	172444001	172449000	1.11E-08
chr2	172445001	172450000	0.001403727
chr2	172454001	172459000	5.53E-06
chr2	172455001	172460000	4.45E-06
chr2	172456001	172461000	4.64E-05
chr2	172753001	172758000	6.69E-10
chr2	172754001	172759000	4.71E-10
chr2	173004001	173009000	0.003186726
chr2	173792001	173797000	2.10E-07
chr2	173793001	173798000	0.000644895
chr2	173794001	173799000	8.95E-05
chr2	173795001	173800000	5.44E-10
chr2	173796001	173801000	5.52E-07
chr2	174322001	174327000	2.34E-11
chr2	175210001	175215000	0.000126089
chr2	176229001	176234000	0.002548606
chr2	176233001	176238000	0.000223846
chr2	176767001	176772000	0.000203367
chr2	177352001	177357000	4.39E-12
chr2	177353001	177358000	5.41E-15
chr2	177354001	177359000	4.79E-15
chr2	177445001	177450000	4.90E-05
chr2	177448001	177453000	0.004720573

chr2	177516001	177521000	0.000409125
chr2	177721001	177726000	1.52E-05
chr2	177920001	177925000	1.82E-10
chr2	178178001	178183000	0.000131605
chr2	178214001	178219000	1.68E-07
chr2	178604001	178609000	0.000333283
chr2	178703001	178708000	4.78E-06
chr2	178733001	178738000	0.000446308
chr2	178734001	178739000	0.000754751
chr2	178744001	178749000	0.002212904
chr2	178745001	178750000	0.000558794
chr2	178887001	178892000	0.000287231
chr2	178890001	178895000	1.03E-05
chr2	179184001	179189000	1.52E-05
chr2	179651001	179656000	0.004858807
chr2	179652001	179657000	0.004848891
chr2	180174001	180179000	4.20E-05
chr2	180195001	180200000	0.011277744
chr2	180197001	180202000	0.000284125
chr2	180198001	180203000	3.59E-09
chr2	180274001	180279000	0.007305856
chr2	180361001	180366000	0.000442019
chr2	180362001	180367000	0.016362913
chr2	180363001	180368000	0.004917399
chr2	180442001	180447000	5.72E-05
chr2	180501001	180506000	0.004080058
chr2	180899001	180904000	0.003074972
chr2	180957001	180962000	0.001098506
chr2	180959001	180964000	0.001306062
chr2	180960001	180965000	0.001267284
chr2	180968001	180973000	0.003523891
chr2	181291001	181296000	0.000141458
chr2	181292001	181297000	0.000283442
chr2	181358001	181363000	2.00E-05
chr2	181462001	181467000	6.67E-05
chr2	181464001	181469000	6.51E-06
chr2	181551001	181556000	0.00020258
chr2	181552001	181557000	1.23E-07
chr2	181709001	181714000	0.000279687
chr2	181738001	181743000	0.000155763
chr2	181739001	181744000	0.018549846
chr2	181778001	181783000	5.28E-05
chr2	181816001	181821000	0.001004844
chr2	181817001	181822000	0.001083751
chr2	182039001	182044000	2.07E-05
chr2	182137001	182142000	0.000282425
chr2	182534001	182539000	0.006396074
chr2	182535001	182540000	0.004794547

chr2	182605001	182610000	0.000115071
chr2	182671001	182676000	6.07E-05
chr2	182720001	182725000	0.001157427
chr2	183115001	183120000	0.000310253
chr2	183363001	183368000	3.45E-06
chr2	183393001	183398000	3.49E-05
chr2	183394001	183399000	0.000779752
chr2	183467001	183472000	0.000567477
chr2	183478001	183483000	0.002535692
chr2	183479001	183484000	0.006120076
chr2	183480001	183485000	0.008586616
chr2	183534001	183539000	2.49E-11
chr2	183535001	183540000	9.74E-11
chr2	184136001	184141000	0.003358654
chr2	184383001	184388000	0.001169373
chr2	184559001	184564000	0.000132609
chr2	184560001	184565000	7.75E-05
chr2	184561001	184566000	0.003044847
chr2	184944001	184949000	1.52E-05
chr2	184978001	184983000	3.09E-11
chr2	184979001	184984000	3.02E-09
chr2	184980001	184985000	8.37E-09
chr2	184989001	184994000	0.012332084
chr2	185293001	185298000	0.000671791
chr2	185384001	185389000	8.41E-06
chr2	185385001	185390000	1.80E-07
chr2	185472001	185477000	0.001009491
chr2	185784001	185789000	2.38E-05
chr2	186050001	186055000	0.000175773
chr2	186127001	186132000	0.000763914
chr2	186220001	186225000	6.29E-10
chr2	186317001	186322000	0.0068465
chr2	186426001	186431000	0.000933736
chr2	186427001	186432000	0.002450185
chr2	186817001	186822000	0.000576882
chr2	186967001	186972000	2.41E-05
chr2	186968001	186973000	7.38E-07
chr2	187352001	187357000	0.002154889
chr2	187571001	187576000	0.000616163
chr2	187636001	187641000	1.38E-07
chr2	187637001	187642000	1.13E-09
chr2	187723001	187728000	2.92E-06
chr2	187986001	187991000	5.57E-05
chr2	188323001	188328000	1.03E-07
chr2	188353001	188358000	9.09E-05
chr2	188374001	188379000	3.84E-13
chr2	188416001	188421000	7.11E-19
chr2	188417001	188422000	1.46E-36

chr2	188418001	188423000	1.88E-27
chr2	188719001	188724000	0.001472219
chr2	188720001	188725000	0.002954861
chr2	188809001	188814000	6.33E-08
chr2	189095001	189100000	1.08E-05
chr2	189106001	189111000	0.005037384
chr2	189187001	189192000	0.005830845
chr2	189201001	189206000	3.40E-05
chr2	189247001	189252000	1.08E-05
chr2	189248001	189253000	1.48E-05
chr2	189572001	189577000	1.45E-05
chr2	189573001	189578000	5.68E-06
chr2	189661001	189666000	1.10E-05
chr2	189662001	189667000	2.02E-05
chr2	189663001	189668000	4.91E-07
chr2	189664001	189669000	3.64E-05
chr2	189724001	189729000	9.52E-06
chr2	189836001	189841000	0.005845689
chr2	189837001	189842000	0.003802585
chr2	190252001	190257000	0.003957248
chr2	190254001	190259000	0.004442327
chr2	190817001	190822000	0.000240001
chr2	190818001	190823000	3.50E-06
chr2	190819001	190824000	6.63E-06
chr2	190850001	190855000	0.017313144
chr2	190945001	190950000	1.60E-05
chr2	191406001	191411000	0.004398239
chr2	191891001	191896000	2.21E-05
chr2	192342001	192347000	8.16E-11
chr2	192343001	192348000	3.83E-09
chr2	192344001	192349000	7.32E-10
chr2	192345001	192350000	2.00E-05
chr2	192346001	192351000	5.29E-05
chr2	192378001	192383000	8.56E-09
chr2	192379001	192384000	6.87E-07
chr2	192743001	192748000	2.71E-08
chr2	192744001	192749000	1.21E-07
chr2	192791001	192796000	0.000147587
chr2	192859001	192864000	0.000138803
chr2	192933001	192938000	8.26E-05
chr2	193112001	193117000	3.94E-05
chr2	193454001	193459000	2.34E-05
chr2	193455001	193460000	5.11E-06
chr2	193456001	193461000	3.60E-05
chr2	193501001	193506000	0.000916421
chr2	193633001	193638000	1.36E-05
chr2	193867001	193872000	0.00030315
chr2	194211001	194216000	0.000287108

chr2	194212001	194217000	0.000195555
chr2	194324001	194329000	3.45E-05
chr2	194326001	194331000	1.65E-07
chr2	194327001	194332000	1.56E-06
chr2	194328001	194333000	3.38E-05
chr2	194339001	194344000	4.88E-05
chr2	194340001	194345000	0.001945139
chr2	194341001	194346000	0.002542529
chr2	194342001	194347000	0.005320447
chr2	194343001	194348000	0.00303995
chr2	194388001	194393000	0.000195228
chr2	194389001	194394000	6.78E-06
chr2	194390001	194395000	4.58E-06
chr2	194584001	194589000	0.026509086
chr2	194795001	194800000	3.08E-05
chr2	195025001	195030000	3.10E-05
chr2	195136001	195141000	2.51E-05
chr2	195581001	195586000	0.000199669
chr2	195652001	195657000	1.81E-05
chr2	195839001	195844000	2.94E-05
chr2	196049001	196054000	0.000199111
chr2	196050001	196055000	0.00130026
chr2	196069001	196074000	0.026629074
chr2	196137001	196142000	0.000464155
chr2	196224001	196229000	0.007572752
chr2	196225001	196230000	0.011171025
chr2	196306001	196311000	0.005329166
chr2	196307001	196312000	0.005312684
chr2	196333001	196338000	1.39E-05
chr2	196939001	196944000	0.00309109
chr2	196940001	196945000	0.000641014
chr2	196943001	196948000	0.004167216
chr2	196965001	196970000	0.000636742
chr2	196966001	196971000	0.001194295
chr2	197029001	197034000	1.30E-05
chr2	197096001	197101000	1.02E-05
chr2	197372001	197377000	0.000568625
chr2	197373001	197378000	6.04E-06
chr2	197411001	197416000	0.000814522
chr2	197449001	197454000	0.002617559
chr2	197450001	197455000	0.00045394
chr2	198753001	198758000	0.003981675
chr2	198754001	198759000	0.007261613
chr2	199037001	199042000	5.27E-07
chr2	199038001	199043000	0.000195428
chr2	199078001	199083000	3.47E-06
chr2	199081001	199086000	1.28E-06
chr2	199082001	199087000	4.88E-06

chr2	199138001	199143000	3.61E-05
chr2	199139001	199144000	1.86E-05
chr2	199140001	199145000	2.86E-05
chr2	199429001	199434000	3.15E-05
chr2	199431001	199436000	0.005067009
chr2	199432001	199437000	0.006567577
chr2	199520001	199525000	0.016375204
chr2	199521001	199526000	0.023988838
chr2	199949001	199954000	6.10E-05
chr2	199950001	199955000	8.71E-05
chr2	199951001	199956000	0.000214995
chr2	200024001	200029000	2.56E-05
chr2	200025001	200030000	6.32E-06
chr2	200027001	200032000	1.12E-05
chr2	200029001	200034000	5.31E-05
chr2	200070001	200075000	3.77E-08
chr2	200207001	200212000	0.00012475
chr2	200209001	200214000	6.40E-08
chr2	200210001	200215000	1.78E-08
chr2	200213001	200218000	4.33E-05
chr2	200790001	200795000	0.001671982
chr2	200868001	200873000	0.001774595
chr2	200886001	200891000	0.000394631
chr2	202279001	202284000	1.73E-08
chr2	202394001	202399000	7.21E-08
chr2	202395001	202400000	3.14E-07
chr2	203132001	203137000	9.86E-13
chr2	203142001	203147000	3.00E-08
chr2	203243001	203248000	1.25E-06
chr2	204722001	204727000	1.59E-05
chr2	205090001	205095000	1.39E-06
chr2	205091001	205096000	1.27E-09
chr2	205885001	205890000	1.56E-08
chr2	207074001	207079000	9.50E-05
chr2	207075001	207080000	2.26E-08
chr2	207076001	207081000	1.12E-07
chr2	207585001	207590000	0.000432352
chr2	208311001	208316000	0.000861609
chr2	208520001	208525000	1.92E-09
chr2	208586001	208591000	7.80E-10
chr2	208675001	208680000	1.63E-07
chr2	209027001	209032000	3.03E-06
chr2	209222001	209227000	5.80E-12
chr2	209223001	209228000	2.48E-13
chr2	209224001	209229000	1.51E-13
chr2	209447001	209452000	0.001160396
chr2	209448001	209453000	0.001532947
chr2	209449001	209454000	2.63E-05

chr2	209595001	209600000	4.93E-07
chr2	209720001	209725000	0.000529002
chr2	209721001	209726000	0.033941813
chr2	209734001	209739000	0.005350236
chr2	209830001	209835000	1.89E-06
chr2	210069001	210074000	1.12E-13
chr2	210070001	210075000	2.65E-25
chr2	210071001	210076000	2.27E-22
chr2	210072001	210077000	3.70E-18
chr2	210073001	210078000	6.39E-16
chr2	210074001	210079000	3.15E-09
chr2	210200001	210205000	7.57E-06
chr2	211093001	211098000	4.21E-08
chr2	211094001	211099000	1.82E-06
chr2	212071001	212076000	1.87E-06
chr2	212096001	212101000	0.000622821
chr2	212182001	212187000	0.004559048
chr2	212183001	212188000	0.003820627
chr2	212184001	212189000	0.00304894
chr2	212219001	212224000	0.007974493
chr2	212721001	212726000	5.07E-08
chr2	212736001	212741000	0.002019967
chr2	212854001	212859000	4.06E-05
chr2	212855001	212860000	3.58E-05
chr2	212975001	212980000	0.002625354
chr2	213147001	213152000	3.53E-17
chr2	213148001	213153000	4.04E-10
chr2	213332001	213337000	0.001202716
chr2	213473001	213478000	0.000331147
chr2	213476001	213481000	0.000189842
chr2	213574001	213579000	0.000462498
chr2	213685001	213690000	0.024262858
chr2	213908001	213913000	0.000289541
chr2	214576001	214581000	4.55E-07
chr2	214577001	214582000	4.07E-06
chr2	214606001	214611000	8.74E-14
chr2	214618001	214623000	0.000744615
chr2	214621001	214626000	0.000363275
chr2	214676001	214681000	9.73E-05
chr2	214757001	214762000	0.005889216
chr2	214783001	214788000	0.00019575
chr2	214860001	214865000	0.00013526
chr2	214905001	214910000	0.001701693
chr2	214906001	214911000	0.000810917
chr2	214907001	214912000	0.005078172
chr2	214914001	214919000	4.99E-08
chr2	214915001	214920000	2.99E-09
chr2	215114001	215119000	5.61E-08

chr2	215115001	215120000	4.09E-07
chr2	215116001	215121000	3.92E-06
chr2	215328001	215333000	3.79E-05
chr2	215368001	215373000	5.20E-11
chr2	215369001	215374000	5.67E-11
chr2	215400001	215405000	0.000129862
chr2	215745001	215750000	0.001238782
chr2	215746001	215751000	0.000197733
chr2	215772001	215777000	0.000984449
chr2	216000001	216005000	7.95E-08
chr2	216001001	216006000	1.47E-07
chr2	216303001	216308000	8.16E-06
chr2	216469001	216474000	0.000757119
chr2	216487001	216492000	1.23E-05
chr2	216590001	216595000	0.020969501
chr2	216592001	216597000	6.41E-06
chr2	216593001	216598000	8.23E-06
chr2	216594001	216599000	0.00020964
chr2	216595001	216600000	9.14E-05
chr2	216758001	216763000	5.91E-16
chr2	216759001	216764000	6.87E-13
chr2	216783001	216788000	3.38E-09
chr2	217135001	217140000	1.56E-08
chr2	217813001	217818000	2.45E-06
chr2	218040001	218045000	0.002359072
chr2	218041001	218046000	0.00056362
chr2	218155001	218160000	2.11E-05
chr2	218244001	218249000	6.17E-09
chr2	218245001	218250000	1.14E-06
chr2	218530001	218535000	5.60E-05
chr2	218532001	218537000	5.42E-06
chr2	219094001	219099000	1.64E-06
chr2	219237001	219242000	7.23E-07
chr2	219294001	219299000	0.000695117
chr2	219461001	219466000	2.46E-05
chr2	219642001	219647000	2.69E-15
chr2	220463001	220468000	7.12E-11
chr2	220523001	220528000	1.46E-07
chr2	221082001	221087000	2.12E-08
chr2	221084001	221089000	7.77E-05
chr2	221881001	221886000	4.57E-07
chr2	225302001	225307000	5.49E-16
chr2	225567001	225572000	6.76E-08
chr2	225568001	225573000	5.99E-07
chr2	225808001	225813000	2.76E-08
chr2	225810001	225815000	0.000836999
chr2	225816001	225821000	1.44E-05
chr2	225844001	225849000	3.85E-12

chr2	225946001	225951000	1.53E-05
chr2	226274001	226279000	8.46E-05
chr2	226417001	226422000	1.73E-06
chr2	226522001	226527000	0.00112409
chr2	226533001	226538000	9.41E-05
chr2	226601001	226606000	1.45E-07
chr2	226604001	226609000	3.71E-14
chr2	227155001	227160000	8.78E-06
chr2	227156001	227161000	2.64E-05
chr2	227157001	227162000	1.17E-06
chr2	227209001	227214000	0.000326364
chr2	227216001	227221000	0.001178452
chr2	227217001	227222000	9.83E-05
chr2	227684001	227689000	6.21E-06
chr2	227685001	227690000	4.52E-06
chr2	227686001	227691000	2.10E-05
chr2	228056001	228061000	0.001274995
chr2	228140001	228145000	7.37E-06
chr2	228267001	228272000	1.65E-08
chr2	228439001	228444000	0.000359774
chr2	228440001	228445000	0.000749982
chr2	228441001	228446000	6.87E-05
chr2	228442001	228447000	0.000127093
chr2	229078001	229083000	0.011642255
chr2	229103001	229108000	0.000175127
chr2	230585001	230590000	7.51E-05
chr2	231425001	231430000	0.002580621
chr2	232262001	232267000	2.27E-09
chr2	232452001	232457000	3.44E-07
chr2	232494001	232499000	1.05E-06
chr2	232828001	232833000	8.33E-08
chr2	233249001	233254000	3.54E-30
chr2	233250001	233255000	5.59E-20
chr2	234952001	234957000	0.001499382
chr2	235037001	235042000	2.08E-06
chr2	235597001	235602000	1.67E-07
chr2	236498001	236503000	1.04E-08
chr2	237804001	237809000	1.30E-05
chr2	237805001	237810000	1.12E-08
chr2	238195001	238200000	4.65E-16
chr2	238715001	238720000	0.006643051
chr2	239776001	239781000	2.05E-10
chr2	239966001	239971000	1.03E-18
chr2	240583001	240588000	2.94E-12
chr2	240585001	240590000	1.92E-10
chr2	240586001	240591000	1.66E-11
chr2	240587001	240592000	6.85E-06
chr2	241377001	241382000	2.12E-09

chr2	242158001	242163000	3.60E-15
chr2	242330001	242335000	2.23E-11
chr2	242331001	242336000	5.70E-09
chr2	242707001	242712000	3.89E-11
chr2	242708001	242713000	2.44E-11
chr2	242709001	242714000	5.70E-16
chr2	242710001	242715000	5.83E-14
chr2	242711001	242716000	1.64E-12
chr2	242841001	242846000	1.56E-19
chr2	242842001	242847000	5.56E-20
chr3	405001	410000	4.43E-05
chr3	752001	757000	1.47E-05
chr3	753001	758000	1.19E-05
chr3	1076001	1081000	8.25E-06
chr3	1240001	1245000	5.24E-05
chr3	2359001	2364000	0.001716772
chr3	2360001	2365000	1.33E-05
chr3	2772001	2777000	7.39E-08
chr3	3740001	3745000	0.00711743
chr3	4191001	4196000	4.90E-05
chr3	4192001	4197000	0.000119695
chr3	4332001	4337000	7.54E-07
chr3	4392001	4397000	1.47E-08
chr3	4623001	4628000	4.76E-06
chr3	4935001	4940000	4.33E-06
chr3	4936001	4941000	6.57E-06
chr3	5052001	5057000	0.000288234
chr3	5316001	5321000	9.99E-05
chr3	5317001	5322000	2.66E-05
chr3	5318001	5323000	8.80E-05
chr3	5409001	5414000	0.000607856
chr3	5540001	5545000	4.29E-05
chr3	5600001	5605000	5.10E-05
chr3	5601001	5606000	1.33E-07
chr3	5777001	5782000	2.27E-05
chr3	5833001	5838000	1.74E-07
chr3	6183001	6188000	0.000162999
chr3	6976001	6981000	4.98E-08
chr3	6977001	6982000	1.27E-08
chr3	7399001	7404000	0.000119133
chr3	7400001	7405000	0.002048369
chr3	7699001	7704000	4.21E-08
chr3	8068001	8073000	5.88E-05
chr3	9010001	9015000	3.63E-05
chr3	9552001	9557000	6.93E-05
chr3	9553001	9558000	8.90E-05
chr3	9567001	9572000	0.000792555
chr3	9925001	9930000	1.61E-05

chr3	9927001	9932000	3.69E-06
chr3	10552001	10557000	0.000351754
chr3	11134001	11139000	0.000156117
chr3	11265001	11270000	3.05E-09
chr3	11266001	11271000	5.83E-08
chr3	11460001	11465000	7.82E-05
chr3	12114001	12119000	4.84E-05
chr3	12115001	12120000	4.86E-05
chr3	12157001	12162000	0.000470261
chr3	12211001	12216000	0.000104006
chr3	12272001	12277000	2.24E-05
chr3	12273001	12278000	3.34E-06
chr3	12274001	12279000	3.19E-08
chr3	12487001	12492000	1.11E-06
chr3	12688001	12693000	3.08E-05
chr3	12689001	12694000	3.02E-05
chr3	12757001	12762000	9.62E-10
chr3	12758001	12763000	4.30E-12
chr3	12759001	12764000	1.67E-15
chr3	12760001	12765000	9.40E-13
chr3	12761001	12766000	2.77E-12
chr3	13237001	13242000	4.55E-08
chr3	13238001	13243000	8.57E-07
chr3	13300001	13305000	1.09E-08
chr3	13554001	13559000	1.14E-07
chr3	13566001	13571000	7.28E-08
chr3	13568001	13573000	6.46E-07
chr3	13569001	13574000	4.35E-09
chr3	13854001	13859000	0.013813377
chr3	14264001	14269000	1.10E-07
chr3	14330001	14335000	1.42E-06
chr3	14374001	14379000	1.58E-11
chr3	14375001	14380000	5.13E-12
chr3	14401001	14406000	3.56E-05
chr3	14645001	14650000	0.002651522
chr3	15355001	15360000	1.09E-08
chr3	15664001	15669000	4.63E-05
chr3	15665001	15670000	4.33E-07
chr3	15667001	15672000	3.99E-10
chr3	15668001	15673000	2.13E-09
chr3	15669001	15674000	5.47E-14
chr3	16096001	16101000	3.74E-09
chr3	16097001	16102000	2.08E-12
chr3	16799001	16804000	0.00062622
chr3	18149001	18154000	0.000265565
chr3	18150001	18155000	7.10E-05
chr3	18217001	18222000	6.33E-05
chr3	18235001	18240000	0.002013727

chr3	18631001	18636000	0.002212305
chr3	18632001	18637000	0.026538766
chr3	19085001	19090000	0.000283491
chr3	19345001	19350000	0.000631558
chr3	19739001	19744000	1.07E-07
chr3	20148001	20153000	0.011551529
chr3	20326001	20331000	0.000285669
chr3	20438001	20443000	8.51E-06
chr3	20447001	20452000	8.02E-06
chr3	20682001	20687000	1.47E-05
chr3	20833001	20838000	0.027767966
chr3	20834001	20839000	0.009714682
chr3	20879001	20884000	0.001230877
chr3	20880001	20885000	0.00139649
chr3	20881001	20886000	0.004408967
chr3	21747001	21752000	2.49E-06
chr3	21995001	22000000	0.003345833
chr3	22041001	22046000	0.000185269
chr3	22227001	22232000	2.06E-06
chr3	22935001	22940000	0.00415762
chr3	22946001	22951000	0.00289568
chr3	22947001	22952000	9.38E-08
chr3	22948001	22953000	3.07E-05
chr3	22949001	22954000	0.000304261
chr3	23000001	23005000	8.26E-05
chr3	23868001	23873000	0.001243655
chr3	23962001	23967000	0.00015185
chr3	24083001	24088000	0.000997703
chr3	24179001	24184000	0.000205774
chr3	24180001	24185000	1.02E-05
chr3	24329001	24334000	0.004140934
chr3	24467001	24472000	0.000130811
chr3	24468001	24473000	0.000362744
chr3	24583001	24588000	0.006041766
chr3	24585001	24590000	0.005348396
chr3	24586001	24591000	0.00049074
chr3	24587001	24592000	0.001399467
chr3	24724001	24729000	0.001971679
chr3	25006001	25011000	1.24E-05
chr3	25862001	25867000	4.70E-05
chr3	25863001	25868000	8.64E-05
chr3	26066001	26071000	1.32E-06
chr3	26067001	26072000	1.68E-06
chr3	26491001	26496000	4.76E-05
chr3	26641001	26646000	0.00089727
chr3	26642001	26647000	0.000626927
chr3	27574001	27579000	4.84E-10
chr3	27575001	27580000	7.74E-11

chr3	29102001	29107000	0.000893038
chr3	29410001	29415000	9.77E-06
chr3	29564001	29569000	6.22E-05
chr3	30050001	30055000	2.82E-09
chr3	30051001	30056000	8.56E-11
chr3	30052001	30057000	5.30E-10
chr3	30053001	30058000	1.64E-09
chr3	30440001	30445000	5.25E-05
chr3	30682001	30687000	0.001508892
chr3	30694001	30699000	1.39E-06
chr3	30695001	30700000	1.21E-07
chr3	32266001	32271000	9.49E-05
chr3	33086001	33091000	6.37E-05
chr3	33210001	33215000	0.00398775
chr3	34033001	34038000	0.004691967
chr3	34035001	34040000	0.002439252
chr3	34101001	34106000	0.00028215
chr3	34221001	34226000	0.000110921
chr3	34240001	34245000	0.005099579
chr3	34445001	34450000	1.88E-05
chr3	34446001	34451000	4.69E-07
chr3	34447001	34452000	2.09E-07
chr3	34603001	34608000	3.96E-05
chr3	34604001	34609000	4.79E-05
chr3	34634001	34639000	0.020356535
chr3	34849001	34854000	0.003461319
chr3	34850001	34855000	0.012860155
chr3	34851001	34856000	0.013897503
chr3	34852001	34857000	0.018958939
chr3	35666001	35671000	0.000838429
chr3	35767001	35772000	0.000691413
chr3	36171001	36176000	0.022012084
chr3	36326001	36331000	5.62E-07
chr3	36414001	36419000	0.000273814
chr3	36794001	36799000	3.78E-05
chr3	37186001	37191000	0.000194887
chr3	37961001	37966000	4.08E-05
chr3	37962001	37967000	2.07E-06
chr3	38788001	38793000	0.001953174
chr3	39957001	39962000	6.81E-06
chr3	39958001	39963000	1.93E-07
chr3	39959001	39964000	3.73E-06
chr3	39960001	39965000	0.000187838
chr3	40433001	40438000	0.001889233
chr3	40720001	40725000	0.000247
chr3	41472001	41477000	1.37E-06
chr3	41709001	41714000	0.002550307
chr3	41835001	41840000	0.000981702

chr3	41954001	41959000	1.89E-05
chr3	42487001	42492000	0.001698726
chr3	43475001	43480000	0.013678603
chr3	43856001	43861000	8.84E-05
chr3	43897001	43902000	3.11E-08
chr3	43898001	43903000	3.86E-08
chr3	43899001	43904000	1.87E-12
chr3	43900001	43905000	7.57E-09
chr3	44081001	44086000	0.000520103
chr3	44263001	44268000	7.47E-05
chr3	44334001	44339000	0.004049433
chr3	45594001	45599000	4.51E-07
chr3	45595001	45600000	1.57E-05
chr3	45636001	45641000	3.37E-05
chr3	45906001	45911000	1.16E-07
chr3	46301001	46306000	8.92E-06
chr3	46692001	46697000	0.00251576
chr3	47268001	47273000	1.36E-06
chr3	47651001	47656000	0.000781903
chr3	47652001	47657000	0.001382317
chr3	47831001	47836000	4.84E-09
chr3	48181001	48186000	9.04E-09
chr3	49307001	49312000	1.30E-05
chr3	49308001	49313000	4.70E-06
chr3	49309001	49314000	0.000153436
chr3	49389001	49394000	0.002254975
chr3	49598001	49603000	0.003898316
chr3	49978001	49983000	2.63E-08
chr3	50406001	50411000	1.37E-06
chr3	50529001	50534000	0.000392955
chr3	50530001	50535000	0.000453529
chr3	50634001	50639000	7.76E-07
chr3	50635001	50640000	8.26E-08
chr3	50637001	50642000	2.33E-06
chr3	50638001	50643000	8.25E-06
chr3	50812001	50817000	0.002763059
chr3	50823001	50828000	0.003157571
chr3	51111001	51116000	0.000850533
chr3	51172001	51177000	6.80E-11
chr3	51173001	51178000	1.12E-11
chr3	51212001	51217000	0.002064936
chr3	51242001	51247000	0.000737439
chr3	51799001	51804000	7.35E-05
chr3	51800001	51805000	0.000393043
chr3	51897001	51902000	0.004339585
chr3	52021001	52026000	2.20E-07
chr3	52022001	52027000	2.40E-08
chr3	52222001	52227000	0.000334938

chr3	52521001	52526000	2.52E-15
chr3	52523001	52528000	3.13E-13
chr3	52549001	52554000	4.16E-11
chr3	52550001	52555000	7.53E-16
chr3	52551001	52556000	6.03E-13
chr3	52552001	52557000	3.27E-13
chr3	52639001	52644000	0.003060945
chr3	52822001	52827000	0.001211491
chr3	53004001	53009000	3.76E-05
chr3	53089001	53094000	0.002060749
chr3	53241001	53246000	1.23E-06
chr3	53252001	53257000	3.09E-05
chr3	53253001	53258000	2.03E-05
chr3	53308001	53313000	3.47E-06
chr3	53661001	53666000	3.31E-09
chr3	53837001	53842000	0.009010627
chr3	53935001	53940000	0.008021571
chr3	53936001	53941000	0.005639638
chr3	56371001	56376000	0.002761468
chr3	56787001	56792000	4.97E-09
chr3	56788001	56793000	3.48E-05
chr3	56790001	56795000	0.001098763
chr3	56829001	56834000	8.29E-06
chr3	57528001	57533000	9.81E-05
chr3	57680001	57685000	4.14E-08
chr3	57681001	57686000	2.59E-05
chr3	57788001	57793000	2.57E-05
chr3	57789001	57794000	5.95E-11
chr3	58464001	58469000	1.22E-05
chr3	58465001	58470000	0.000198729
chr3	58466001	58471000	0.001847645
chr3	58514001	58519000	1.11E-05
chr3	58515001	58520000	3.53E-05
chr3	58550001	58555000	9.57E-10
chr3	58551001	58556000	9.09E-15
chr3	58591001	58596000	7.06E-05
chr3	58592001	58597000	7.84E-05
chr3	58594001	58599000	5.13E-06
chr3	58610001	58615000	9.02E-11
chr3	58895001	58900000	0.002476618
chr3	59052001	59057000	0.001058762
chr3	59850001	59855000	0.004842801
chr3	60191001	60196000	0.010194475
chr3	60192001	60197000	0.006228133
chr3	60259001	60264000	2.24E-11
chr3	60260001	60265000	1.50E-13
chr3	61227001	61232000	4.35E-06
chr3	61494001	61499000	0.003097933

chr3	61756001	61761000	2.05E-08
chr3	61777001	61782000	1.05E-05
chr3	61826001	61831000	0.001969779
chr3	62347001	62352000	2.64E-07
chr3	62348001	62353000	9.11E-08
chr3	62471001	62476000	0.000204734
chr3	62484001	62489000	5.38E-07
chr3	62485001	62490000	1.41E-07
chr3	62486001	62491000	6.11E-07
chr3	62925001	62930000	7.07E-05
chr3	63071001	63076000	0.000263433
chr3	64301001	64306000	2.16E-05
chr3	64302001	64307000	1.94E-06
chr3	64303001	64308000	5.59E-06
chr3	64304001	64309000	1.11E-05
chr3	64305001	64310000	1.43E-05
chr3	64380001	64385000	0.003107399
chr3	64384001	64389000	3.23E-05
chr3	64385001	64390000	0.000137882
chr3	64413001	64418000	0.001230928
chr3	64649001	64654000	2.96E-05
chr3	64650001	64655000	2.68E-06
chr3	64651001	64656000	3.86E-08
chr3	64652001	64657000	0.000967411
chr3	65001001	65006000	6.14E-08
chr3	65725001	65730000	0.00244695
chr3	67780001	67785000	0.001541908
chr3	67782001	67787000	0.020583277
chr3	68037001	68042000	0.000261561
chr3	68038001	68043000	0.000352873
chr3	68287001	68292000	0.008493337
chr3	68658001	68663000	0.010595286
chr3	68662001	68667000	0.000127977
chr3	69135001	69140000	4.61E-05
chr3	69167001	69172000	3.95E-14
chr3	69210001	69215000	5.25E-05
chr3	69211001	69216000	2.21E-05
chr3	69448001	69453000	0.004377446
chr3	69449001	69454000	0.001036319
chr3	69450001	69455000	3.97E-06
chr3	69729001	69734000	0.000220971
chr3	69730001	69735000	7.15E-05
chr3	69731001	69736000	3.73E-05
chr3	69882001	69887000	3.97E-06
chr3	69883001	69888000	1.78E-08
chr3	69889001	69894000	5.47E-06
chr3	69902001	69907000	0.000350143
chr3	70203001	70208000	0.002082539

chr3	70825001	70830000	1.76E-05
chr3	70909001	70914000	0.000307229
chr3	71108001	71113000	0.00363724
chr3	71175001	71180000	1.62E-10
chr3	71240001	71245000	9.12E-08
chr3	71241001	71246000	4.71E-08
chr3	71363001	71368000	5.08E-08
chr3	71364001	71369000	1.23E-07
chr3	71749001	71754000	0.000734287
chr3	72541001	72546000	0.000194911
chr3	72793001	72798000	1.72E-12
chr3	73368001	73373000	3.91E-05
chr3	73369001	73374000	5.09E-05
chr3	73377001	73382000	0.000348249
chr3	73501001	73506000	9.32E-06
chr3	73585001	73590000	0.000205146
chr3	73822001	73827000	0.006440693
chr3	73854001	73859000	4.13E-09
chr3	74388001	74393000	0.000153205
chr3	74389001	74394000	0.003744345
chr3	75759001	75764000	0.000612616
chr3	75876001	75881000	0.000134137
chr3	75959001	75964000	2.85E-08
chr3	75960001	75965000	1.01E-08
chr3	75989001	75994000	8.72E-05
chr3	75991001	75996000	1.57E-05
chr3	77424001	77429000	0.006221254
chr3	77918001	77923000	0.000152103
chr3	77945001	77950000	0.002442665
chr3	78579001	78584000	0.000253351
chr3	78580001	78585000	0.001354428
chr3	78581001	78586000	0.002118424
chr3	78839001	78844000	0.001800968
chr3	78840001	78845000	0.000450882
chr3	78895001	78900000	0.00119722
chr3	80019001	80024000	0.005410054
chr3	80213001	80218000	0.000286044
chr3	80471001	80476000	0.017369391
chr3	80906001	80911000	1.65E-05
chr3	80908001	80913000	0.001946087
chr3	81490001	81495000	1.32E-07
chr3	81705001	81710000	3.36E-06
chr3	81934001	81939000	1.65E-05
chr3	81936001	81941000	0.000145714
chr3	81938001	81943000	0.000179864
chr3	82504001	82509000	4.47E-05
chr3	82530001	82535000	0.001783731
chr3	83923001	83928000	3.92E-06

chr3	83938001	83943000	0.001656508
chr3	83939001	83944000	0.000221801
chr3	83940001	83945000	0.000276016
chr3	83941001	83946000	5.78E-05
chr3	83980001	83985000	3.08E-07
chr3	83981001	83986000	2.18E-09
chr3	83982001	83987000	1.00E-08
chr3	83983001	83988000	1.29E-06
chr3	84284001	84289000	1.78E-05
chr3	84285001	84290000	1.73E-06
chr3	84706001	84711000	0.024221195
chr3	84822001	84827000	0.000141231
chr3	84823001	84828000	0.000425572
chr3	84824001	84829000	0.000950479
chr3	85189001	85194000	1.06E-06
chr3	85190001	85195000	8.75E-06
chr3	85707001	85712000	0.005318124
chr3	85708001	85713000	0.003075305
chr3	85828001	85833000	0.003670363
chr3	85935001	85940000	4.65E-05
chr3	85936001	85941000	3.20E-06
chr3	85948001	85953000	0.002530652
chr3	86363001	86368000	0.009100252
chr3	86806001	86811000	4.31E-05
chr3	87189001	87194000	0.009733
chr3	87236001	87241000	0.002176039
chr3	87238001	87243000	0.00289054
chr3	87344001	87349000	3.41E-05
chr3	87436001	87441000	0.001080176
chr3	87732001	87737000	4.07E-05
chr3	88267001	88272000	6.32E-08
chr3	88428001	88433000	9.37E-07
chr3	88429001	88434000	7.56E-07
chr3	88430001	88435000	2.09E-06
chr3	88574001	88579000	0.002883043
chr3	88787001	88792000	1.78E-06
chr3	88788001	88793000	9.34E-05
chr3	88961001	88966000	0.000768843
chr3	89011001	89016000	5.21E-05
chr3	89188001	89193000	0.001961877
chr3	89189001	89194000	0.004409321
chr3	89190001	89195000	0.008756052
chr3	89353001	89358000	0.001588669
chr3	89387001	89392000	3.45E-06
chr3	89490001	89495000	8.75E-05
chr3	89491001	89496000	6.10E-05
chr3	89521001	89526000	1.98E-14
chr3	89522001	89527000	7.31E-12

chr3	89523001	89528000	2.07E-14
chr3	89610001	89615000	0.008432262
chr3	89611001	89616000	0.012530559
chr3	89723001	89728000	0.008876516
chr3	89724001	89729000	0.014028727
chr3	89773001	89778000	0.006148323
chr3	89803001	89808000	1.36E-07
chr3	89804001	89809000	1.02E-06
chr3	89805001	89810000	9.10E-07
chr3	89869001	89874000	0.000453679
chr3	89870001	89875000	0.00097201
chr3	89980001	89985000	0.001642566
chr3	90071001	90076000	2.20E-07
chr3	90334001	90339000	0.001309306
chr3	90441001	90446000	2.92E-05
chr3	90442001	90447000	6.79E-05
chr3	93909001	93914000	0.000349639
chr3	93936001	93941000	2.40E-05
chr3	93943001	93948000	7.00E-05
chr3	93944001	93949000	1.07E-05
chr3	93969001	93974000	0.000225702
chr3	93970001	93975000	0.000609982
chr3	94054001	94059000	0.000384753
chr3	94619001	94624000	0.002983529
chr3	94867001	94872000	0.001182384
chr3	95178001	95183000	2.83E-05
chr3	95208001	95213000	0.003402028
chr3	95439001	95444000	8.18E-05
chr3	95978001	95983000	7.78E-05
chr3	96039001	96044000	3.21E-05
chr3	96041001	96046000	5.71E-06
chr3	96042001	96047000	5.35E-09
chr3	96084001	96089000	8.86E-05
chr3	96085001	96090000	4.38E-05
chr3	96174001	96179000	0.001924796
chr3	96363001	96368000	9.57E-10
chr3	96365001	96370000	3.71E-10
chr3	96621001	96626000	0.009162521
chr3	96758001	96763000	4.92E-07
chr3	96965001	96970000	0.001171369
chr3	96996001	97001000	8.10E-05
chr3	97149001	97154000	0.000335906
chr3	97338001	97343000	0.001541939
chr3	97422001	97427000	7.29E-07
chr3	97438001	97443000	0.007543131
chr3	97716001	97721000	0.000237443
chr3	97717001	97722000	0.00020092
chr3	97718001	97723000	0.000177221

chr3	97719001	97724000	0.00021393
chr3	97728001	97733000	5.15E-10
chr3	97729001	97734000	6.58E-09
chr3	97730001	97735000	0.000507838
chr3	97825001	97830000	0.001247315
chr3	97826001	97831000	4.83E-06
chr3	97827001	97832000	1.05E-05
chr3	98107001	98112000	0.001646255
chr3	98108001	98113000	0.005642025
chr3	98380001	98385000	2.35E-08
chr3	98381001	98386000	8.70E-10
chr3	98382001	98387000	4.48E-08
chr3	98825001	98830000	7.23E-06
chr3	98826001	98831000	4.46E-06
chr3	98827001	98832000	2.16E-07
chr3	98828001	98833000	7.51E-05
chr3	98829001	98834000	1.62E-11
chr3	98842001	98847000	0.000253341
chr3	98920001	98925000	5.25E-08
chr3	98921001	98926000	1.39E-06
chr3	99215001	99220000	5.09E-05
chr3	99216001	99221000	1.05E-05
chr3	99217001	99222000	8.53E-06
chr3	99252001	99257000	5.74E-05
chr3	99409001	99414000	3.09E-06
chr3	99473001	99478000	0.001039665
chr3	99474001	99479000	0.000858193
chr3	99507001	99512000	0.000225726
chr3	99508001	99513000	6.52E-07
chr3	100296001	100301000	0.002596107
chr3	100413001	100418000	2.84E-09
chr3	100414001	100419000	6.85E-09
chr3	100500001	100505000	0.001864016
chr3	100513001	100518000	0.002362812
chr3	100711001	100716000	1.67E-12
chr3	100712001	100717000	1.98E-09
chr3	100713001	100718000	2.04E-11
chr3	100745001	100750000	3.61E-05
chr3	100818001	100823000	0.000541413
chr3	100819001	100824000	0.000385509
chr3	100866001	100871000	8.23E-05
chr3	100867001	100872000	0.000225941
chr3	100878001	100883000	1.11E-05
chr3	100879001	100884000	1.58E-06
chr3	100880001	100885000	3.10E-09
chr3	100881001	100886000	3.17E-05
chr3	100896001	100901000	8.52E-06
chr3	100921001	100926000	3.84E-06

chr3	100987001	100992000	1.25E-08
chr3	100988001	100993000	1.13E-06
chr3	101153001	101158000	1.18E-08
chr3	101154001	101159000	8.12E-09
chr3	101331001	101336000	1.37E-08
chr3	101406001	101411000	3.31E-20
chr3	101547001	101552000	6.08E-06
chr3	101895001	101900000	4.42E-12
chr3	101944001	101949000	0.001182232
chr3	101945001	101950000	3.97E-06
chr3	101946001	101951000	7.16E-05
chr3	102015001	102020000	0.010622078
chr3	102080001	102085000	2.08E-06
chr3	102193001	102198000	0.000479586
chr3	102194001	102199000	0.000331791
chr3	102297001	102302000	0.001548046
chr3	102298001	102303000	0.000776998
chr3	102314001	102319000	0.000103998
chr3	102319001	102324000	0.010303673
chr3	102671001	102676000	8.14E-05
chr3	102722001	102727000	0.000312789
chr3	102845001	102850000	3.18E-08
chr3	102907001	102912000	7.14E-05
chr3	103002001	103007000	0.000343881
chr3	103003001	103008000	0.000391584
chr3	103230001	103235000	4.70E-05
chr3	103231001	103236000	8.50E-06
chr3	103339001	103344000	0.003056329
chr3	103484001	103489000	3.94E-05
chr3	103585001	103590000	2.27E-07
chr3	103586001	103591000	4.57E-08
chr3	103587001	103592000	1.32E-07
chr3	103588001	103593000	2.22E-09
chr3	103707001	103712000	4.28E-05
chr3	103708001	103713000	2.73E-06
chr3	103711001	103716000	2.94E-10
chr3	103822001	103827000	1.06E-06
chr3	103823001	103828000	4.07E-06
chr3	103866001	103871000	0.00191108
chr3	103867001	103872000	0.002456832
chr3	103868001	103873000	0.001334432
chr3	103906001	103911000	9.41E-06
chr3	104030001	104035000	9.63E-06
chr3	104077001	104082000	2.10E-11
chr3	104237001	104242000	1.34E-07
chr3	104238001	104243000	1.63E-10
chr3	104255001	104260000	0.00103216
chr3	104446001	104451000	0.001040359

chr3	104612001	104617000	0.003978538
chr3	104654001	104659000	5.93E-07
chr3	104764001	104769000	9.29E-08
chr3	104765001	104770000	3.60E-05
chr3	104862001	104867000	5.14E-06
chr3	104896001	104901000	1.79E-06
chr3	104898001	104903000	5.82E-10
chr3	104951001	104956000	0.004746339
chr3	104964001	104969000	0.000298705
chr3	105057001	105062000	7.26E-10
chr3	105612001	105617000	0.000269701
chr3	106055001	106060000	8.19E-05
chr3	106090001	106095000	3.55E-06
chr3	106543001	106548000	0.001030086
chr3	106607001	106612000	0.00035357
chr3	106608001	106613000	1.17E-06
chr3	106688001	106693000	0.000186909
chr3	106690001	106695000	4.93E-07
chr3	106691001	106696000	9.88E-11
chr3	106692001	106697000	2.25E-06
chr3	106905001	106910000	5.62E-05
chr3	106906001	106911000	8.12E-05
chr3	106907001	106912000	0.00083203
chr3	106940001	106945000	0.00141325
chr3	107218001	107223000	1.62E-05
chr3	107219001	107224000	5.34E-06
chr3	107220001	107225000	2.41E-05
chr3	108702001	108707000	5.58E-06
chr3	108703001	108708000	1.26E-05
chr3	108704001	108709000	5.50E-07
chr3	108887001	108892000	7.93E-06
chr3	108890001	108895000	7.84E-06
chr3	109056001	109061000	1.11E-09
chr3	109070001	109075000	0.0001111
chr3	109088001	109093000	0.000169185
chr3	109090001	109095000	0.000148608
chr3	109314001	109319000	5.77E-06
chr3	109315001	109320000	4.40E-05
chr3	109324001	109329000	8.37E-05
chr3	109325001	109330000	6.89E-05
chr3	109340001	109345000	1.44E-07
chr3	109341001	109346000	5.78E-14
chr3	109342001	109347000	6.11E-15
chr3	109343001	109348000	1.55E-13
chr3	109565001	109570000	0.000346668
chr3	109567001	109572000	0.000817262
chr3	109569001	109574000	0.00508097
chr3	109573001	109578000	0.015805505

chr3	109574001	109579000	0.013778839
chr3	109721001	109726000	1.09E-08
chr3	109785001	109790000	6.76E-09
chr3	109786001	109791000	7.83E-09
chr3	109787001	109792000	1.96E-05
chr3	109814001	109819000	0.001537956
chr3	109821001	109826000	9.09E-05
chr3	109822001	109827000	2.49E-05
chr3	110095001	110100000	6.17E-05
chr3	110096001	110101000	0.000152184
chr3	110164001	110169000	0.000416148
chr3	110232001	110237000	0.010399395
chr3	110233001	110238000	0.006702267
chr3	110273001	110278000	0.021330198
chr3	110451001	110456000	0.001279004
chr3	110452001	110457000	3.54E-10
chr3	110453001	110458000	2.12E-10
chr3	110454001	110459000	3.73E-09
chr3	110455001	110460000	9.47E-09
chr3	110722001	110727000	0.000670878
chr3	110725001	110730000	2.70E-06
chr3	110726001	110731000	0.000783296
chr3	110752001	110757000	2.76E-08
chr3	110809001	110814000	9.06E-10
chr3	110902001	110907000	0.000278855
chr3	110914001	110919000	4.43E-06
chr3	111057001	111062000	0.000144679
chr3	111058001	111063000	4.57E-05
chr3	111059001	111064000	4.36E-05
chr3	111183001	111188000	0.009932365
chr3	111184001	111189000	0.001333085
chr3	111185001	111190000	0.001440563
chr3	111483001	111488000	4.99E-06
chr3	111519001	111524000	1.95E-05
chr3	112355001	112360000	2.43E-13
chr3	112356001	112361000	5.40E-17
chr3	112357001	112362000	1.66E-14
chr3	112358001	112363000	2.71E-15
chr3	112795001	112800000	1.06E-06
chr3	112796001	112801000	1.87E-06
chr3	112798001	112803000	2.31E-10
chr3	112799001	112804000	8.28E-08
chr3	112833001	112838000	1.81E-06
chr3	112834001	112839000	5.24E-12
chr3	112835001	112840000	7.21E-10
chr3	112860001	112865000	2.62E-08
chr3	113261001	113266000	4.54E-07
chr3	113262001	113267000	2.99E-05

chr3	113562001	113567000	2.57E-06
chr3	113563001	113568000	2.81E-05
chr3	114135001	114140000	0.000342965
chr3	115062001	115067000	2.17E-06
chr3	115221001	115226000	0.0007834
chr3	115222001	115227000	0.000511299
chr3	116487001	116492000	9.56E-06
chr3	116488001	116493000	6.55E-06
chr3	116489001	116494000	5.61E-05
chr3	116584001	116589000	0.010161812
chr3	116709001	116714000	2.80E-06
chr3	116710001	116715000	6.31E-06
chr3	116711001	116716000	4.86E-06
chr3	116712001	116717000	1.13E-05
chr3	116713001	116718000	1.30E-06
chr3	116732001	116737000	2.83E-05
chr3	116820001	116825000	1.42E-06
chr3	116821001	116826000	1.47E-06
chr3	116851001	116856000	7.96E-05
chr3	117059001	117064000	0.000557018
chr3	117122001	117127000	0.025440423
chr3	117396001	117401000	0.001273592
chr3	117543001	117548000	5.95E-05
chr3	117643001	117648000	0.001349946
chr3	117822001	117827000	2.76E-09
chr3	117823001	117828000	3.15E-11
chr3	117825001	117830000	2.26E-20
chr3	117826001	117831000	3.03E-17
chr3	117964001	117969000	0.001238789
chr3	117966001	117971000	0.000169021
chr3	117967001	117972000	0.005894526
chr3	118219001	118224000	6.15E-06
chr3	118447001	118452000	0.001070404
chr3	118454001	118459000	1.25E-05
chr3	118576001	118581000	0.001015414
chr3	118599001	118604000	5.57E-08
chr3	119487001	119492000	1.58E-08
chr3	119915001	119920000	0.000352005
chr3	119916001	119921000	0.002076599
chr3	119917001	119922000	0.000169373
chr3	120203001	120208000	0.000690791
chr3	120603001	120608000	2.52E-06
chr3	120639001	120644000	4.70E-06
chr3	120649001	120654000	0.000255952
chr3	120773001	120778000	0.014259967
chr3	121720001	121725000	5.92E-15
chr3	123403001	123408000	1.31E-06
chr3	123404001	123409000	7.73E-06

chr3	123436001	123441000	3.75E-10
chr3	123437001	123442000	5.90E-12
chr3	123438001	123443000	4.02E-13
chr3	123439001	123444000	1.30E-13
chr3	123440001	123445000	6.08E-09
chr3	123580001	123585000	2.14E-06
chr3	123686001	123691000	3.23E-05
chr3	123974001	123979000	8.69E-05
chr3	124120001	124125000	9.72E-05
chr3	124121001	124126000	1.39E-06
chr3	124246001	124251000	4.75E-05
chr3	124292001	124297000	3.08E-05
chr3	124293001	124298000	7.53E-06
chr3	124310001	124315000	3.45E-11
chr3	124476001	124481000	1.47E-23
chr3	124478001	124483000	5.54E-25
chr3	124479001	124484000	7.77E-22
chr3	124480001	124485000	1.29E-23
chr3	124622001	124627000	7.22E-07
chr3	124718001	124723000	1.14E-08
chr3	126675001	126680000	4.70E-11
chr3	126791001	126796000	2.90E-12
chr3	126792001	126797000	3.23E-12
chr3	126954001	126959000	7.33E-05
chr3	127096001	127101000	3.85E-06
chr3	127097001	127102000	1.67E-06
chr3	127254001	127259000	4.70E-08
chr3	127341001	127346000	5.36E-15
chr3	127342001	127347000	8.04E-13
chr3	127429001	127434000	8.56E-08
chr3	127430001	127435000	2.23E-08
chr3	127452001	127457000	6.65E-09
chr3	127453001	127458000	1.87E-09
chr3	127529001	127534000	1.09E-09
chr3	127670001	127675000	0.00110431
chr3	127672001	127677000	1.24E-07
chr3	127673001	127678000	1.18E-09
chr3	127674001	127679000	3.29E-09
chr3	127682001	127687000	3.37E-07
chr3	127755001	127760000	5.05E-08
chr3	127756001	127761000	5.32E-06
chr3	128131001	128136000	1.30E-07
chr3	128252001	128257000	1.21E-05
chr3	128254001	128259000	1.14E-07
chr3	128653001	128658000	5.39E-10
chr3	129165001	129170000	2.41E-08
chr3	129946001	129951000	0.000355677
chr3	131027001	131032000	0.000284694

chr3	131277001	131282000	1.77E-05
chr3	131278001	131283000	8.27E-06
chr3	131440001	131445000	4.99E-05
chr3	131597001	131602000	1.46E-10
chr3	131598001	131603000	7.19E-09
chr3	131599001	131604000	4.73E-12
chr3	131600001	131605000	0.000354789
chr3	131633001	131638000	0.000334521
chr3	131634001	131639000	0.000277836
chr3	131635001	131640000	0.00022625
chr3	131636001	131641000	0.000170622
chr3	131637001	131642000	1.70E-05
chr3	131722001	131727000	0.000713123
chr3	131758001	131763000	0.001237604
chr3	131796001	131801000	1.04E-09
chr3	131797001	131802000	2.81E-09
chr3	131812001	131817000	4.33E-05
chr3	131813001	131818000	2.65E-05
chr3	131814001	131819000	6.80E-05
chr3	131834001	131839000	0.001319064
chr3	131870001	131875000	3.17E-06
chr3	131871001	131876000	2.14E-06
chr3	131872001	131877000	3.76E-07
chr3	132035001	132040000	6.74E-06
chr3	132799001	132804000	0.000192462
chr3	132819001	132824000	0.000709074
chr3	132926001	132931000	3.70E-05
chr3	132985001	132990000	2.42E-05
chr3	133467001	133472000	1.31E-09
chr3	133479001	133484000	0.000166931
chr3	133552001	133557000	1.37E-07
chr3	134112001	134117000	4.49E-13
chr3	134114001	134119000	3.77E-17
chr3	134115001	134120000	1.53E-17
chr3	134116001	134121000	1.40E-14
chr3	134118001	134123000	6.11E-07
chr3	134517001	134522000	2.01E-05
chr3	134518001	134523000	0.000906884
chr3	134753001	134758000	9.05E-13
chr3	135020001	135025000	0.001163147
chr3	135070001	135075000	0.015355925
chr3	135091001	135096000	0.005147822
chr3	135145001	135150000	9.00E-06
chr3	136589001	136594000	6.67E-09
chr3	137161001	137166000	0.000967982
chr3	137162001	137167000	0.000310943
chr3	137163001	137168000	0.000418579
chr3	137164001	137169000	0.00049148

chr3	137203001	137208000	0.000111419
chr3	137204001	137209000	0.000407294
chr3	137205001	137210000	0.000563048
chr3	137504001	137509000	7.86E-05
chr3	137561001	137566000	0.001228481
chr3	137725001	137730000	1.11E-11
chr3	137726001	137731000	2.59E-11
chr3	138038001	138043000	0.00016015
chr3	138039001	138044000	4.56E-05
chr3	138040001	138045000	4.92E-06
chr3	138189001	138194000	2.03E-11
chr3	138554001	138559000	1.81E-09
chr3	138578001	138583000	4.18E-05
chr3	138579001	138584000	7.75E-07
chr3	138580001	138585000	1.17E-06
chr3	138581001	138586000	3.54E-10
chr3	138715001	138720000	1.08E-15
chr3	138716001	138721000	7.17E-14
chr3	138717001	138722000	8.18E-16
chr3	138732001	138737000	4.94E-14
chr3	138733001	138738000	1.12E-14
chr3	138755001	138760000	0.000567839
chr3	138851001	138856000	0.00175705
chr3	138852001	138857000	0.001362341
chr3	139803001	139808000	5.55E-06
chr3	139804001	139809000	4.18E-06
chr3	139819001	139824000	3.47E-05
chr3	139827001	139832000	1.43E-06
chr3	139828001	139833000	1.90E-06
chr3	139829001	139834000	4.39E-08
chr3	139830001	139835000	3.70E-09
chr3	140066001	140071000	0.000267408
chr3	140098001	140103000	0.000156173
chr3	140114001	140119000	8.46E-05
chr3	140261001	140266000	4.86E-05
chr3	140262001	140267000	6.69E-05
chr3	140460001	140465000	0.000321121
chr3	140942001	140947000	0.001791417
chr3	141099001	141104000	8.04E-14
chr3	142161001	142166000	6.82E-23
chr3	142565001	142570000	9.49E-09
chr3	142566001	142571000	7.54E-16
chr3	142567001	142572000	1.24E-14
chr3	142676001	142681000	0.001954464
chr3	142841001	142846000	1.28E-13
chr3	143900001	143905000	0.001456507
chr3	144038001	144043000	0.000430514
chr3	144535001	144540000	0.001565583

chr3	144536001	144541000	0.000965313
chr3	144685001	144690000	2.61E-08
chr3	144686001	144691000	1.23E-10
chr3	144687001	144692000	1.28E-09
chr3	144688001	144693000	2.99E-12
chr3	144689001	144694000	1.25E-10
chr3	144692001	144697000	1.63E-05
chr3	144693001	144698000	0.001349967
chr3	144694001	144699000	0.001101604
chr3	144695001	144700000	0.00072047
chr3	144744001	144749000	0.004083222
chr3	144935001	144940000	0.001077223
chr3	145046001	145051000	0.0002383
chr3	145189001	145194000	4.36E-07
chr3	145190001	145195000	6.65E-07
chr3	145617001	145622000	6.23E-08
chr3	145740001	145745000	0.000121459
chr3	145742001	145747000	1.04E-06
chr3	145743001	145748000	1.41E-05
chr3	146141001	146146000	0.002019139
chr3	146142001	146147000	0.003497193
chr3	146152001	146157000	0.000452118
chr3	146323001	146328000	6.45E-05
chr3	146324001	146329000	2.00E-05
chr3	146356001	146361000	5.58E-07
chr3	146404001	146409000	0.001386297
chr3	146424001	146429000	0.001640703
chr3	146428001	146433000	0.001436707
chr3	146430001	146435000	0.002100554
chr3	146435001	146440000	0.002791853
chr3	146600001	146605000	0.0009206
chr3	146601001	146606000	0.001295335
chr3	146629001	146634000	3.09E-05
chr3	146630001	146635000	3.57E-08
chr3	146631001	146636000	4.88E-08
chr3	146632001	146637000	5.17E-08
chr3	146633001	146638000	2.63E-08
chr3	146651001	146656000	0.000193733
chr3	146653001	146658000	6.16E-06
chr3	146710001	146715000	0.000905839
chr3	146711001	146716000	0.000236277
chr3	146712001	146717000	0.000362887
chr3	146753001	146758000	0.007353426
chr3	146763001	146768000	5.87E-06
chr3	146764001	146769000	3.08E-07
chr3	146765001	146770000	1.20E-06
chr3	146808001	146813000	0.006836118
chr3	146823001	146828000	0.000528895

chr3	146853001	146858000	7.06E-06
chr3	146889001	146894000	0.000773856
chr3	146958001	146963000	3.70E-09
chr3	147017001	147022000	0.000640806
chr3	147018001	147023000	1.10E-06
chr3	147019001	147024000	9.72E-07
chr3	147020001	147025000	9.71E-06
chr3	147026001	147031000	0.006427407
chr3	147027001	147032000	0.003715237
chr3	147193001	147198000	7.77E-11
chr3	147194001	147199000	3.89E-10
chr3	147236001	147241000	0.010685479
chr3	147342001	147347000	0.009304304
chr3	147352001	147357000	3.42E-06
chr3	147353001	147358000	0.008357981
chr3	147520001	147525000	0.001593527
chr3	147544001	147549000	1.28E-06
chr3	147547001	147552000	1.11E-07
chr3	147548001	147553000	8.84E-07
chr3	147554001	147559000	4.36E-06
chr3	147570001	147575000	0.027241407
chr3	147610001	147615000	0.003457727
chr3	147640001	147645000	0.000125161
chr3	147641001	147646000	2.41E-06
chr3	147658001	147663000	3.02E-05
chr3	147695001	147700000	1.13E-05
chr3	147698001	147703000	1.09E-07
chr3	147736001	147741000	0.000900027
chr3	147793001	147798000	0.000283499
chr3	147885001	147890000	9.36E-05
chr3	147894001	147899000	8.98E-16
chr3	147917001	147922000	6.49E-05
chr3	147918001	147923000	2.13E-05
chr3	147952001	147957000	0.000140912
chr3	147953001	147958000	0.0003075
chr3	147961001	147966000	3.42E-05
chr3	147962001	147967000	4.12E-06
chr3	147965001	147970000	7.61E-06
chr3	148049001	148054000	0.002581075
chr3	148140001	148145000	1.01E-07
chr3	148174001	148179000	2.02E-08
chr3	148250001	148255000	0.000387001
chr3	148251001	148256000	0.000761088
chr3	148252001	148257000	0.001406235
chr3	148262001	148267000	6.24E-05
chr3	148344001	148349000	4.70E-10
chr3	148346001	148351000	1.90E-08
chr3	148362001	148367000	0.008901798

chr3	148389001	148394000	0.000606943
chr3	148401001	148406000	9.79E-05
chr3	148425001	148430000	0.002302611
chr3	148449001	148454000	1.36E-09
chr3	148450001	148455000	3.30E-11
chr3	148526001	148531000	1.16E-09
chr3	148527001	148532000	3.17E-10
chr3	148528001	148533000	1.22E-10
chr3	148529001	148534000	4.45E-12
chr3	148530001	148535000	8.84E-07
chr3	148567001	148572000	5.69E-13
chr3	148568001	148573000	4.13E-13
chr3	148575001	148580000	0.000305898
chr3	148576001	148581000	0.000339663
chr3	148586001	148591000	0.000814751
chr3	148604001	148609000	9.51E-07
chr3	148630001	148635000	2.61E-06
chr3	148631001	148636000	4.25E-07
chr3	148632001	148637000	0.000131503
chr3	148633001	148638000	0.000535758
chr3	148634001	148639000	0.000443404
chr3	148636001	148641000	6.88E-06
chr3	148637001	148642000	4.50E-07
chr3	148646001	148651000	9.72E-05
chr3	148647001	148652000	0.000128611
chr3	148648001	148653000	5.49E-05
chr3	148669001	148674000	0.000103031
chr3	148961001	148966000	6.06E-05
chr3	149083001	149088000	1.92E-14
chr3	149084001	149089000	9.06E-08
chr3	149086001	149091000	2.07E-14
chr3	149103001	149108000	1.11E-06
chr3	149104001	149109000	1.53E-11
chr3	149477001	149482000	0.000984816
chr3	149478001	149483000	0.00091017
chr3	149706001	149711000	6.62E-13
chr3	149713001	149718000	3.02E-05
chr3	149714001	149719000	0.003081634
chr3	149748001	149753000	2.84E-07
chr3	149749001	149754000	9.57E-08
chr3	149763001	149768000	1.32E-05
chr3	149798001	149803000	7.25E-21
chr3	149805001	149810000	6.27E-08
chr3	149826001	149831000	9.67E-09
chr3	149846001	149851000	5.60E-10
chr3	149847001	149852000	1.20E-12
chr3	149848001	149853000	6.01E-10
chr3	149849001	149854000	1.28E-05

chr3	149850001	149855000	1.63E-05
chr3	149992001	149997000	9.44E-07
chr3	149993001	149998000	8.02E-06
chr3	149994001	149999000	2.17E-05
chr3	149996001	150001000	9.58E-05
chr3	150088001	150093000	0.002465051
chr3	150090001	150095000	0.000413939
chr3	150091001	150096000	0.001044041
chr3	150213001	150218000	1.76E-07
chr3	150357001	150362000	0.001996943
chr3	150466001	150471000	2.52E-21
chr3	150467001	150472000	6.52E-21
chr3	150468001	150473000	1.77E-23
chr3	150536001	150541000	9.86E-20
chr3	150658001	150663000	8.18E-07
chr3	150659001	150664000	1.10E-07
chr3	150682001	150687000	2.79E-09
chr3	150685001	150690000	2.21E-05
chr3	150703001	150708000	0.000675489
chr3	150704001	150709000	0.001443572
chr3	151660001	151665000	8.79E-06
chr3	151675001	151680000	1.23E-05
chr3	151676001	151681000	1.57E-06
chr3	151677001	151682000	1.30E-09
chr3	151679001	151684000	0.000148471
chr3	151741001	151746000	9.14E-07
chr3	151742001	151747000	2.90E-06
chr3	151743001	151748000	1.33E-06
chr3	151771001	151776000	0.000852468
chr3	151772001	151777000	0.001449671
chr3	151773001	151778000	0.000148344
chr3	151774001	151779000	1.49E-05
chr3	151775001	151780000	4.50E-05
chr3	151777001	151782000	0.002024595
chr3	151854001	151859000	6.95E-05
chr3	152248001	152253000	3.44E-05
chr3	152249001	152254000	0.00094576
chr3	153525001	153530000	0.000402213
chr3	153570001	153575000	0.010083035
chr3	153571001	153576000	0.006826509
chr3	153620001	153625000	8.11E-10
chr3	153621001	153626000	0.001231557
chr3	153642001	153647000	1.21E-08
chr3	153675001	153680000	5.82E-06
chr3	153676001	153681000	1.09E-06
chr3	153730001	153735000	0.000228143
chr3	153731001	153736000	0.000228143
chr3	153771001	153776000	6.78E-05

chr3	153853001	153858000	3.76E-05
chr3	153874001	153879000	7.24E-05
chr3	153875001	153880000	0.000111014
chr3	153902001	153907000	6.35E-09
chr3	153903001	153908000	9.54E-11
chr3	153947001	153952000	0.017897151
chr3	154237001	154242000	0.003751167
chr3	154275001	154280000	0.000104695
chr3	154276001	154281000	0.007940933
chr3	154380001	154385000	7.51E-05
chr3	154428001	154433000	4.28E-07
chr3	154429001	154434000	1.12E-06
chr3	154472001	154477000	3.86E-09
chr3	154473001	154478000	2.20E-10
chr3	154474001	154479000	1.39E-11
chr3	154475001	154480000	8.55E-14
chr3	154476001	154481000	3.05E-07
chr3	154495001	154500000	0.000900025
chr3	154502001	154507000	8.69E-06
chr3	154545001	154550000	0.000138757
chr3	154546001	154551000	0.000152324
chr3	154547001	154552000	0.000410409
chr3	154607001	154612000	3.26E-28
chr3	154614001	154619000	0.003960883
chr3	154732001	154737000	4.75E-06
chr3	154733001	154738000	5.18E-05
chr3	154734001	154739000	2.44E-05
chr3	154764001	154769000	8.36E-07
chr3	154781001	154786000	0.000487873
chr3	154782001	154787000	0.000223084
chr3	154784001	154789000	0.001704101
chr3	154823001	154828000	5.68E-05
chr3	154869001	154874000	0.000331261
chr3	154870001	154875000	3.87E-05
chr3	154871001	154876000	0.000140636
chr3	154872001	154877000	1.01E-05
chr3	154873001	154878000	0.000808017
chr3	154881001	154886000	0.000113426
chr3	154882001	154887000	0.001516581
chr3	154890001	154895000	0.006170812
chr3	154891001	154896000	1.29E-05
chr3	154892001	154897000	5.69E-08
chr3	154893001	154898000	2.08E-06
chr3	154894001	154899000	9.55E-10
chr3	154946001	154951000	2.25E-05
chr3	154972001	154977000	2.60E-05
chr3	155014001	155019000	0.000844446
chr3	155015001	155020000	0.001715633

chr3	155064001	155069000	0.003820587
chr3	155065001	155070000	0.00114198
chr3	155066001	155071000	0.001647807
chr3	155072001	155077000	2.57E-09
chr3	155416001	155421000	1.95E-07
chr3	155417001	155422000	5.37E-06
chr3	155429001	155434000	2.30E-05
chr3	155802001	155807000	3.01E-08
chr3	155810001	155815000	3.84E-07
chr3	155811001	155816000	4.02E-06
chr3	155812001	155817000	0.000153082
chr3	155846001	155851000	3.55E-10
chr3	155847001	155852000	4.13E-10
chr3	155848001	155853000	1.25E-05
chr3	155849001	155854000	0.000366725
chr3	155870001	155875000	3.48E-06
chr3	155871001	155876000	7.23E-07
chr3	155872001	155877000	8.47E-10
chr3	155873001	155878000	0.000381021
chr3	155927001	155932000	2.05E-06
chr3	156114001	156119000	3.69E-13
chr3	156115001	156120000	1.14E-13
chr3	156116001	156121000	1.12E-12
chr3	156154001	156159000	0.000205319
chr3	156162001	156167000	2.31E-08
chr3	156163001	156168000	8.43E-07
chr3	156164001	156169000	0.000187275
chr3	156195001	156200000	1.92E-05
chr3	156490001	156495000	4.23E-07
chr3	156493001	156498000	3.36E-06
chr3	156584001	156589000	0.01154418
chr3	156599001	156604000	1.55E-08
chr3	156600001	156605000	5.07E-12
chr3	156601001	156606000	3.37E-11
chr3	156602001	156607000	1.50E-11
chr3	156610001	156615000	4.35E-07
chr3	156611001	156616000	3.04E-07
chr3	156626001	156631000	3.96E-05
chr3	156627001	156632000	1.34E-06
chr3	156655001	156660000	1.41E-08
chr3	156656001	156661000	1.82E-09
chr3	156657001	156662000	2.93E-09
chr3	156658001	156663000	4.21E-08
chr3	156659001	156664000	4.73E-10
chr3	156670001	156675000	4.36E-06
chr3	156671001	156676000	1.15E-05
chr3	156672001	156677000	0.00028168
chr3	156690001	156695000	0.000177663

chr3	156691001	156696000	4.08E-07
chr3	156692001	156697000	1.28E-09
chr3	156693001	156698000	1.45E-05
chr3	156701001	156706000	3.37E-05
chr3	156710001	156715000	1.59E-08
chr3	156711001	156716000	6.08E-10
chr3	156713001	156718000	4.19E-10
chr3	156736001	156741000	1.05E-06
chr3	156931001	156936000	2.58E-11
chr3	156935001	156940000	1.43E-07
chr3	156998001	157003000	6.00E-06
chr3	157006001	157011000	1.96E-12
chr3	157010001	157015000	2.80E-08
chr3	157037001	157042000	8.10E-05
chr3	157038001	157043000	1.39E-05
chr3	157039001	157044000	2.52E-05
chr3	157040001	157045000	6.83E-09
chr3	157041001	157046000	1.05E-10
chr3	157042001	157047000	4.23E-07
chr3	157043001	157048000	1.76E-06
chr3	157050001	157055000	8.47E-05
chr3	157051001	157056000	0.000151172
chr3	157075001	157080000	7.18E-12
chr3	157076001	157081000	2.83E-13
chr3	157077001	157082000	1.12E-13
chr3	157078001	157083000	2.81E-12
chr3	157079001	157084000	5.14E-10
chr3	157085001	157090000	3.44E-08
chr3	157086001	157091000	2.81E-09
chr3	157087001	157092000	3.65E-09
chr3	157089001	157094000	1.20E-11
chr3	157090001	157095000	9.39E-10
chr3	157091001	157096000	3.62E-10
chr3	157092001	157097000	3.63E-07
chr3	157104001	157109000	8.74E-11
chr3	157116001	157121000	1.23E-07
chr3	157123001	157128000	4.61E-05
chr3	157124001	157129000	0.003090749
chr3	157169001	157174000	6.39E-06
chr3	157170001	157175000	1.28E-05
chr3	157171001	157176000	9.34E-07
chr3	157172001	157177000	6.18E-07
chr3	157189001	157194000	9.65E-08
chr3	157190001	157195000	1.03E-08
chr3	157191001	157196000	5.00E-08
chr3	157205001	157210000	0.014158642
chr3	157212001	157217000	2.42E-07
chr3	157218001	157223000	1.29E-05

chr3	157219001	157224000	0.000166755
chr3	157236001	157241000	3.57E-09
chr3	157237001	157242000	4.24E-09
chr3	157238001	157243000	1.73E-09
chr3	157239001	157244000	8.70E-10
chr3	157255001	157260000	6.73E-10
chr3	157308001	157313000	7.62E-05
chr3	157309001	157314000	3.12E-05
chr3	157384001	157389000	5.70E-06
chr3	157385001	157390000	9.61E-05
chr3	157426001	157431000	1.02E-22
chr3	157428001	157433000	2.95E-14
chr3	157429001	157434000	6.42E-09
chr3	157457001	157462000	0.002715577
chr3	157458001	157463000	0.000520973
chr3	157459001	157464000	0.001264907
chr3	157480001	157485000	3.90E-05
chr3	157481001	157486000	0.000484002
chr3	157482001	157487000	7.68E-06
chr3	157483001	157488000	0.000711296
chr3	157484001	157489000	0.000160634
chr3	157485001	157490000	2.66E-05
chr3	157487001	157492000	1.80E-05
chr3	157488001	157493000	2.60E-06
chr3	157505001	157510000	4.95E-07
chr3	157506001	157511000	1.17E-05
chr3	157538001	157543000	0.000508401
chr3	157539001	157544000	0.000122588
chr3	157544001	157549000	4.18E-06
chr3	157545001	157550000	9.86E-07
chr3	157546001	157551000	1.37E-07
chr3	157547001	157552000	1.15E-06
chr3	157569001	157574000	4.49E-06
chr3	157571001	157576000	0.002593765
chr3	157572001	157577000	0.000748159
chr3	157658001	157663000	0.009880334
chr3	157659001	157664000	0.019080577
chr3	157660001	157665000	0.008087335
chr3	157661001	157666000	0.015332517
chr3	157669001	157674000	3.50E-07
chr3	157670001	157675000	3.05E-14
chr3	157671001	157676000	3.60E-10
chr3	157672001	157677000	1.30E-12
chr3	157673001	157678000	2.96E-13
chr3	157765001	157770000	0.000807391
chr3	157766001	157771000	2.68E-05
chr3	157767001	157772000	2.82E-05
chr3	157768001	157773000	0.000107328

chr3	157794001	157799000	6.30E-07
chr3	157795001	157800000	6.79E-10
chr3	157796001	157801000	5.23E-08
chr3	157797001	157802000	0.000329393
chr3	157798001	157803000	0.000549544
chr3	158044001	158049000	1.11E-16
chr3	158045001	158050000	5.28E-17
chr3	158046001	158051000	5.03E-19
chr3	158047001	158052000	7.91E-25
chr3	158589001	158594000	2.93E-07
chr3	158590001	158595000	2.00E-07
chr3	158597001	158602000	1.64E-05
chr3	158685001	158690000	6.75E-07
chr3	158740001	158745000	3.19E-07
chr3	158741001	158746000	6.30E-09
chr3	158742001	158747000	7.92E-08
chr3	158743001	158748000	1.81E-07
chr3	158744001	158749000	1.20E-07
chr3	158745001	158750000	9.35E-05
chr3	158746001	158751000	0.000201792
chr3	158837001	158842000	0.000139893
chr3	158838001	158843000	2.10E-05
chr3	158839001	158844000	9.47E-08
chr3	158840001	158845000	1.44E-08
chr3	158841001	158846000	6.04E-07
chr3	158842001	158847000	0.000168275
chr3	158901001	158906000	1.52E-12
chr3	158902001	158907000	5.42E-12
chr3	158903001	158908000	3.05E-13
chr3	159015001	159020000	1.76E-06
chr3	159016001	159021000	2.06E-06
chr3	159029001	159034000	0.01511558
chr3	159030001	159035000	3.26E-08
chr3	159031001	159036000	4.44E-07
chr3	159042001	159047000	5.30E-06
chr3	159043001	159048000	6.72E-07
chr3	159044001	159049000	4.44E-08
chr3	159045001	159050000	3.38E-08
chr3	159046001	159051000	3.61E-06
chr3	159047001	159052000	5.71E-05
chr3	159048001	159053000	7.77E-05
chr3	159163001	159168000	3.89E-07
chr3	159250001	159255000	0.001130109
chr3	159251001	159256000	5.23E-05
chr3	159252001	159257000	0.000481622
chr3	159254001	159259000	0.00262369
chr3	159255001	159260000	0.001454749
chr3	159261001	159266000	0.00899501

chr3	159262001	159267000	0.004843722
chr3	159263001	159268000	0.001273478
chr3	159264001	159269000	0.003090882
chr3	159282001	159287000	6.44E-05
chr3	159283001	159288000	0.000171177
chr3	159399001	159404000	3.55E-05
chr3	159652001	159657000	1.15E-06
chr3	159663001	159668000	2.21E-06
chr3	159664001	159669000	2.22E-05
chr3	160327001	160332000	6.16E-09
chr3	160328001	160333000	2.25E-09
chr3	160329001	160334000	1.53E-05
chr3	160335001	160340000	1.50E-06
chr3	160336001	160341000	1.15E-06
chr3	160341001	160346000	1.27E-05
chr3	160342001	160347000	0.000160586
chr3	160384001	160389000	1.05E-15
chr3	160385001	160390000	2.03E-18
chr3	160386001	160391000	2.54E-17
chr3	160387001	160392000	2.44E-08
chr3	160446001	160451000	7.69E-05
chr3	160447001	160452000	2.02E-05
chr3	160448001	160453000	1.63E-05
chr3	160479001	160484000	0.000567205
chr3	160481001	160486000	6.01E-09
chr3	160506001	160511000	0.00088588
chr3	160507001	160512000	0.00086863
chr3	160611001	160616000	1.61E-05
chr3	160612001	160617000	1.94E-06
chr3	160613001	160618000	3.03E-06
chr3	160614001	160619000	4.02E-06
chr3	160615001	160620000	1.35E-05
chr3	160684001	160689000	1.21E-07
chr3	160687001	160692000	1.41E-09
chr3	160708001	160713000	0.001699138
chr3	160709001	160714000	9.71E-05
chr3	160710001	160715000	3.45E-06
chr3	160711001	160716000	6.27E-06
chr3	160712001	160717000	1.42E-05
chr3	160713001	160718000	4.14E-05
chr3	160714001	160719000	5.27E-05
chr3	160775001	160780000	1.61E-05
chr3	160776001	160781000	3.43E-05
chr3	160789001	160794000	6.62E-05
chr3	160841001	160846000	0.000868748
chr3	160845001	160850000	0.010647136
chr3	160846001	160851000	0.014739229
chr3	160854001	160859000	7.54E-09

chr3	160855001	160860000	2.64E-09
chr3	161040001	161045000	4.05E-06
chr3	161064001	161069000	9.27E-11
chr3	161065001	161070000	1.08E-11
chr3	161183001	161188000	0.002356015
chr3	161184001	161189000	0.003036445
chr3	161206001	161211000	0.001156936
chr3	161207001	161212000	0.000100209
chr3	161210001	161215000	0.000575269
chr3	161211001	161216000	5.95E-05
chr3	161212001	161217000	4.86E-07
chr3	161213001	161218000	1.71E-08
chr3	161214001	161219000	7.37E-10
chr3	161215001	161220000	3.28E-11
chr3	161216001	161221000	6.77E-06
chr3	161226001	161231000	0.000563142
chr3	161227001	161232000	0.001361047
chr3	161228001	161233000	6.49E-06
chr3	161284001	161289000	0.004350546
chr3	161285001	161290000	0.000107432
chr3	161286001	161291000	9.37E-05
chr3	161287001	161292000	3.73E-07
chr3	161301001	161306000	8.40E-06
chr3	161309001	161314000	0.000954512
chr3	161310001	161315000	0.001084032
chr3	161331001	161336000	0.000520138
chr3	161342001	161347000	0.010421305
chr3	161343001	161348000	0.001327109
chr3	161344001	161349000	1.64E-05
chr3	161345001	161350000	2.11E-05
chr3	161346001	161351000	0.000126736
chr3	161460001	161465000	7.48E-05
chr3	161461001	161466000	0.000907349
chr3	161519001	161524000	0.004026079
chr3	161586001	161591000	0.000983522
chr3	161587001	161592000	0.000187816
chr3	161588001	161593000	0.000180416
chr3	161612001	161617000	0.000247267
chr3	161613001	161618000	0.000192577
chr3	161676001	161681000	0.000213785
chr3	161688001	161693000	1.34E-06
chr3	161689001	161694000	1.33E-06
chr3	161690001	161695000	2.22E-06
chr3	161812001	161817000	0.000268615
chr3	161813001	161818000	6.66E-06
chr3	161814001	161819000	1.25E-05
chr3	161828001	161833000	0.0002658
chr3	161840001	161845000	2.64E-05

chr3	161842001	161847000	0.004564279
chr3	161843001	161848000	0.002183564
chr3	162039001	162044000	2.37E-05
chr3	162046001	162051000	0.008118465
chr3	162050001	162055000	4.19E-05
chr3	162135001	162140000	1.69E-05
chr3	162148001	162153000	0.011586808
chr3	162163001	162168000	0.00079242
chr3	162164001	162169000	3.26E-07
chr3	162165001	162170000	4.03E-08
chr3	162196001	162201000	0.000230568
chr3	162199001	162204000	1.20E-05
chr3	162237001	162242000	1.93E-06
chr3	162238001	162243000	9.86E-08
chr3	162257001	162262000	0.00937372
chr3	162263001	162268000	0.000145376
chr3	162264001	162269000	4.76E-05
chr3	162313001	162318000	3.63E-22
chr3	162319001	162324000	4.04E-13
chr3	162320001	162325000	3.60E-12
chr3	162352001	162357000	6.70E-05
chr3	162489001	162494000	0.00089567
chr3	162744001	162749000	0.017306769
chr3	162758001	162763000	3.14E-10
chr3	162761001	162766000	2.47E-08
chr3	162762001	162767000	3.98E-05
chr3	162917001	162922000	0.000119255
chr3	162918001	162923000	0.000103274
chr3	162919001	162924000	0.000110126
chr3	162920001	162925000	0.000430921
chr3	162933001	162938000	0.002843176
chr3	162994001	162999000	3.59E-05
chr3	162995001	163000000	0.005329166
chr3	163022001	163027000	9.84E-06
chr3	163176001	163181000	1.05E-06
chr3	163493001	163498000	1.74E-05
chr3	163666001	163671000	1.28E-10
chr3	163709001	163714000	3.83E-06
chr3	163710001	163715000	6.49E-07
chr3	163711001	163716000	3.59E-07
chr3	163712001	163717000	2.89E-10
chr3	163713001	163718000	7.70E-12
chr3	163768001	163773000	0.000130773
chr3	163925001	163930000	5.02E-05
chr3	163926001	163931000	0.004204913
chr3	164096001	164101000	4.37E-06
chr3	164097001	164102000	7.57E-07
chr3	164098001	164103000	2.03E-05

chr3	164177001	164182000	3.82E-05
chr3	164240001	164245000	3.44E-05
chr3	164302001	164307000	4.26E-06
chr3	164447001	164452000	0.000134588
chr3	164448001	164453000	5.78E-05
chr3	164497001	164502000	3.72E-26
chr3	164553001	164558000	0.001045325
chr3	164560001	164565000	0.000146793
chr3	164617001	164622000	1.90E-05
chr3	164618001	164623000	2.47E-05
chr3	164888001	164893000	3.10E-05
chr3	164926001	164931000	0.000299106
chr3	165056001	165061000	2.18E-11
chr3	165057001	165062000	2.02E-09
chr3	165059001	165064000	1.59E-09
chr3	165153001	165158000	0.000130677
chr3	165156001	165161000	0.00035748
chr3	165316001	165321000	0.00380455
chr3	165317001	165322000	0.003024519
chr3	165318001	165323000	0.001488433
chr3	165319001	165324000	0.001187613
chr3	165442001	165447000	1.48E-06
chr3	165560001	165565000	1.55E-06
chr3	165561001	165566000	2.76E-07
chr3	165568001	165573000	0.002609477
chr3	165569001	165574000	0.001538292
chr3	165666001	165671000	0.000148935
chr3	165667001	165672000	0.00016549
chr3	165701001	165706000	2.05E-07
chr3	165803001	165808000	0.000989169
chr3	165848001	165853000	2.29E-05
chr3	165940001	165945000	0.001178842
chr3	165966001	165971000	5.03E-06
chr3	165967001	165972000	5.16E-07
chr3	165968001	165973000	0.000514365
chr3	165992001	165997000	8.52E-06
chr3	165993001	165998000	1.08E-06
chr3	165994001	165999000	6.92E-08
chr3	166042001	166047000	1.45E-05
chr3	166047001	166052000	0.001524023
chr3	166048001	166053000	0.000932736
chr3	166065001	166070000	3.88E-05
chr3	166102001	166107000	2.07E-06
chr3	166272001	166277000	0.003078893
chr3	166273001	166278000	0.002397905
chr3	166296001	166301000	0.003626652
chr3	166317001	166322000	2.47E-08
chr3	166318001	166323000	1.22E-09

chr3	166319001	166324000	4.75E-08
chr3	166326001	166331000	0.003178729
chr3	166327001	166332000	0.000366897
chr3	166369001	166374000	0.001580021
chr3	166422001	166427000	0.004664389
chr3	166494001	166499000	1.51E-06
chr3	166495001	166500000	4.04E-07
chr3	166496001	166501000	1.41E-11
chr3	166497001	166502000	3.58E-10
chr3	166636001	166641000	0.000509126
chr3	166689001	166694000	2.13E-20
chr3	166709001	166714000	8.46E-06
chr3	166710001	166715000	1.06E-10
chr3	166711001	166716000	1.12E-12
chr3	166717001	166722000	4.67E-10
chr3	166718001	166723000	3.88E-11
chr3	166723001	166728000	7.88E-05
chr3	166724001	166729000	4.91E-07
chr3	166773001	166778000	4.34E-05
chr3	166801001	166806000	0.000128916
chr3	166810001	166815000	0.0003988
chr3	166871001	166876000	0.0002048
chr3	166879001	166884000	0.000349924
chr3	166906001	166911000	1.01E-07
chr3	166907001	166912000	9.43E-07
chr3	166909001	166914000	0.00386766
chr3	166957001	166962000	0.001506484
chr3	167037001	167042000	0.000903106
chr3	167068001	167073000	0.000607849
chr3	167069001	167074000	0.000241698
chr3	167183001	167188000	0.000748896
chr3	167184001	167189000	0.000505654
chr3	167185001	167190000	0.000609101
chr3	167253001	167258000	0.015980453
chr3	167480001	167485000	1.60E-11
chr3	167481001	167486000	1.92E-09
chr3	167483001	167488000	6.36E-08
chr3	167506001	167511000	4.27E-05
chr3	167523001	167528000	0.000135107
chr3	167539001	167544000	1.12E-16
chr3	167678001	167683000	0.00024052
chr3	167679001	167684000	0.000275416
chr3	167700001	167705000	2.31E-06
chr3	167701001	167706000	5.11E-07
chr3	167702001	167707000	3.56E-06
chr3	167703001	167708000	2.00E-05
chr3	167709001	167714000	6.34E-06
chr3	167713001	167718000	1.13E-05

chr3	167835001	167840000	5.10E-09
chr3	167837001	167842000	1.35E-14
chr3	167838001	167843000	6.66E-15
chr3	167839001	167844000	1.11E-06
chr3	167840001	167845000	7.94E-07
chr3	167841001	167846000	5.73E-08
chr3	167853001	167858000	7.96E-05
chr3	167855001	167860000	0.001149575
chr3	167856001	167861000	0.002165367
chr3	167920001	167925000	7.31E-07
chr3	167939001	167944000	2.19E-08
chr3	168009001	168014000	4.11E-07
chr3	168013001	168018000	0.002137728
chr3	168189001	168194000	8.68E-07
chr3	168191001	168196000	2.89E-05
chr3	168200001	168205000	4.79E-05
chr3	168470001	168475000	0.001599555
chr3	168565001	168570000	0.003432115
chr3	168602001	168607000	0.000498948
chr3	168683001	168688000	2.25E-05
chr3	168684001	168689000	1.43E-05
chr3	168685001	168690000	5.34E-05
chr3	169658001	169663000	1.12E-07
chr3	170265001	170270000	3.59E-07
chr3	170266001	170271000	3.85E-08
chr3	170267001	170272000	8.53E-12
chr3	170406001	170411000	3.57E-21
chr3	171594001	171599000	6.80E-08
chr3	171813001	171818000	4.33E-08
chr3	171814001	171819000	3.05E-08
chr3	171858001	171863000	1.02E-12
chr3	171934001	171939000	0.000253951
chr3	172233001	172238000	2.93E-07
chr3	172234001	172239000	1.76E-07
chr3	172236001	172241000	7.97E-06
chr3	172279001	172284000	6.72E-08
chr3	172463001	172468000	4.78E-27
chr3	172558001	172563000	1.10E-11
chr3	172615001	172620000	1.43E-06
chr3	172658001	172663000	1.32E-07
chr3	172684001	172689000	1.12E-07
chr3	172685001	172690000	6.58E-07
chr3	172717001	172722000	2.21E-06
chr3	172727001	172732000	3.98E-25
chr3	172729001	172734000	4.30E-05
chr3	172747001	172752000	4.84E-07
chr3	172748001	172753000	3.47E-07
chr3	172749001	172754000	3.17E-07

chr3	172765001	172770000	6.80E-11
chr3	172813001	172818000	3.49E-05
chr3	172814001	172819000	3.13E-05
chr3	172815001	172820000	6.51E-08
chr3	172816001	172821000	1.64E-09
chr3	172832001	172837000	0.000146749
chr3	172839001	172844000	4.47E-08
chr3	172840001	172845000	5.67E-09
chr3	172850001	172855000	0.000648565
chr3	172851001	172856000	4.27E-06
chr3	172852001	172857000	0.000181625
chr3	172862001	172867000	1.07E-09
chr3	172909001	172914000	1.03E-08
chr3	172910001	172915000	3.33E-07
chr3	172911001	172916000	2.96E-07
chr3	172912001	172917000	3.00E-09
chr3	172913001	172918000	2.17E-09
chr3	172916001	172921000	0.000166107
chr3	172938001	172943000	4.18E-05
chr3	172939001	172944000	2.20E-07
chr3	172958001	172963000	0.002933259
chr3	172959001	172964000	0.004080927
chr3	172960001	172965000	1.05E-05
chr3	173007001	173012000	4.92E-08
chr3	173008001	173013000	3.00E-06
chr3	173031001	173036000	1.16E-06
chr3	173047001	173052000	5.86E-05
chr3	173048001	173053000	0.000143351
chr3	173049001	173054000	1.47E-09
chr3	173050001	173055000	1.44E-10
chr3	173053001	173058000	2.06E-11
chr3	173093001	173098000	0.003549894
chr3	173094001	173099000	0.004350962
chr3	173131001	173136000	4.43E-09
chr3	173153001	173158000	3.09E-05
chr3	173200001	173205000	3.92E-06
chr3	173201001	173206000	3.53E-06
chr3	173202001	173207000	4.43E-05
chr3	173223001	173228000	6.07E-05
chr3	173253001	173258000	8.25E-05
chr3	173275001	173280000	8.96E-13
chr3	173276001	173281000	1.44E-14
chr3	173331001	173336000	0.001205393
chr3	173362001	173367000	9.65E-09
chr3	173363001	173368000	0.001004754
chr3	173372001	173377000	0.007029578
chr3	173373001	173378000	0.000642535
chr3	173374001	173379000	0.000425789

chr3	173375001	173380000	0.000613187
chr3	173376001	173381000	0.000321635
chr3	173382001	173387000	4.62E-10
chr3	173383001	173388000	5.78E-10
chr3	173385001	173390000	1.31E-13
chr3	173386001	173391000	1.96E-12
chr3	173392001	173397000	7.14E-05
chr3	173394001	173399000	1.72E-05
chr3	173441001	173446000	1.35E-11
chr3	173442001	173447000	1.29E-12
chr3	173458001	173463000	7.66E-06
chr3	173459001	173464000	2.50E-07
chr3	173460001	173465000	1.47E-05
chr3	173479001	173484000	0.000776876
chr3	173500001	173505000	7.33E-05
chr3	173501001	173506000	4.63E-05
chr3	173502001	173507000	5.94E-06
chr3	173507001	173512000	2.50E-05
chr3	173508001	173513000	3.85E-05
chr3	173509001	173514000	4.28E-05
chr3	173510001	173515000	0.000432331
chr3	173511001	173516000	0.000515432
chr3	173522001	173527000	1.11E-05
chr3	173523001	173528000	9.76E-06
chr3	173524001	173529000	3.95E-05
chr3	173525001	173530000	1.45E-05
chr3	173526001	173531000	2.71E-05
chr3	173527001	173532000	0.000334473
chr3	173539001	173544000	8.13E-05
chr3	173595001	173600000	4.01E-08
chr3	173596001	173601000	5.79E-09
chr3	173597001	173602000	5.18E-13
chr3	173598001	173603000	1.77E-13
chr3	173599001	173604000	9.50E-17
chr3	173600001	173605000	2.55E-10
chr3	173604001	173609000	1.58E-06
chr3	173656001	173661000	4.31E-05
chr3	173657001	173662000	1.49E-05
chr3	173658001	173663000	8.72E-07
chr3	173659001	173664000	2.64E-05
chr3	173660001	173665000	1.89E-10
chr3	173661001	173666000	1.13E-07
chr3	173679001	173684000	6.96E-11
chr3	173680001	173685000	2.47E-15
chr3	173681001	173686000	6.19E-16
chr3	173682001	173687000	8.19E-11
chr3	173683001	173688000	6.72E-10
chr3	173709001	173714000	4.91E-08

chr3	173710001	173715000	3.58E-09
chr3	173711001	173716000	4.61E-12
chr3	173735001	173740000	1.82E-06
chr3	173736001	173741000	7.60E-05
chr3	173762001	173767000	8.93E-06
chr3	173765001	173770000	9.93E-05
chr3	173824001	173829000	5.76E-05
chr3	173825001	173830000	4.56E-06
chr3	173826001	173831000	2.90E-05
chr3	173827001	173832000	0.000796119
chr3	173828001	173833000	3.35E-05
chr3	173896001	173901000	7.99E-05
chr3	173938001	173943000	1.90E-10
chr3	173939001	173944000	3.55E-09
chr3	173940001	173945000	7.12E-09
chr3	174048001	174053000	0.000134784
chr3	174049001	174054000	0.00056164
chr3	174050001	174055000	0.000220084
chr3	174292001	174297000	4.80E-07
chr3	174293001	174298000	2.83E-07
chr3	174341001	174346000	1.27E-08
chr3	174342001	174347000	7.19E-09
chr3	174380001	174385000	3.68E-05
chr3	174381001	174386000	5.22E-05
chr3	174424001	174429000	6.77E-06
chr3	174445001	174450000	0.002987775
chr3	174446001	174451000	0.002632147
chr3	175366001	175371000	3.40E-06
chr3	175699001	175704000	6.04E-07
chr3	175700001	175705000	2.05E-10
chr3	175701001	175706000	4.04E-08
chr3	175703001	175708000	5.79E-11
chr3	175704001	175709000	4.13E-09
chr3	175705001	175710000	9.84E-07
chr3	175706001	175711000	3.03E-07
chr3	175707001	175712000	2.07E-07
chr3	175773001	175778000	0.00122542
chr3	175818001	175823000	0.000355425
chr3	175821001	175826000	1.97E-06
chr3	175822001	175827000	9.20E-07
chr3	175902001	175907000	6.67E-10
chr3	175903001	175908000	8.82E-15
chr3	175904001	175909000	1.39E-14
chr3	175905001	175910000	0.000619769
chr3	175932001	175937000	0.00306978
chr3	175938001	175943000	1.50E-11
chr3	175955001	175960000	0.001947559
chr3	175993001	175998000	7.35E-06

chr3	176021001	176026000	6.85E-06
chr3	176022001	176027000	3.62E-08
chr3	176023001	176028000	1.49E-06
chr3	176024001	176029000	1.90E-07
chr3	176025001	176030000	1.06E-19
chr3	176026001	176031000	5.44E-18
chr3	176027001	176032000	8.22E-16
chr3	176028001	176033000	1.87E-13
chr3	176045001	176050000	5.42E-05
chr3	176093001	176098000	0.000159911
chr3	176094001	176099000	3.23E-07
chr3	176095001	176100000	7.79E-07
chr3	176096001	176101000	1.10E-05
chr3	176097001	176102000	6.02E-05
chr3	176108001	176113000	0.000850962
chr3	176109001	176114000	0.004717877
chr3	176110001	176115000	0.001251038
chr3	176111001	176116000	0.000237133
chr3	176112001	176117000	7.95E-08
chr3	176113001	176118000	8.26E-08
chr3	176122001	176127000	0.000306164
chr3	176185001	176190000	4.03E-05
chr3	176197001	176202000	1.97E-08
chr3	176200001	176205000	1.63E-10
chr3	176201001	176206000	1.49E-12
chr3	176207001	176212000	0.000135772
chr3	176208001	176213000	0.00011618
chr3	176209001	176214000	0.009794795
chr3	176243001	176248000	1.02E-06
chr3	176244001	176249000	4.28E-10
chr3	176245001	176250000	8.72E-10
chr3	176290001	176295000	0.000257184
chr3	176294001	176299000	0.00935588
chr3	176351001	176356000	8.99E-06
chr3	176355001	176360000	0.007819433
chr3	176367001	176372000	0.000318768
chr3	176416001	176421000	6.67E-07
chr3	176509001	176514000	4.84E-07
chr3	176510001	176515000	3.90E-07
chr3	176524001	176529000	9.30E-06
chr3	176525001	176530000	1.30E-05
chr3	176559001	176564000	6.14E-08
chr3	176645001	176650000	4.29E-09
chr3	176646001	176651000	3.53E-09
chr3	176647001	176652000	9.33E-12
chr3	176650001	176655000	6.00E-19
chr3	176652001	176657000	1.84E-18
chr3	176653001	176658000	1.29E-28

chr3	176654001	176659000	1.09E-28
chr3	176656001	176661000	1.10E-09
chr3	176825001	176830000	4.38E-09
chr3	176826001	176831000	1.19E-08
chr3	176827001	176832000	7.99E-07
chr3	177095001	177100000	8.28E-14
chr3	177163001	177168000	9.45E-06
chr3	177164001	177169000	3.00E-10
chr3	177165001	177170000	3.57E-17
chr3	177166001	177171000	1.29E-16
chr3	177237001	177242000	2.09E-27
chr3	177826001	177831000	5.39E-05
chr3	178106001	178111000	0.000489871
chr3	178107001	178112000	0.000185522
chr3	178133001	178138000	8.30E-07
chr3	178134001	178139000	2.98E-06
chr3	178135001	178140000	9.31E-07
chr3	178136001	178141000	0.000316406
chr3	178196001	178201000	5.35E-12
chr3	178197001	178202000	2.66E-11
chr3	178198001	178203000	5.42E-14
chr3	178199001	178204000	2.30E-10
chr3	178204001	178209000	3.63E-05
chr3	178217001	178222000	3.86E-13
chr3	178218001	178223000	3.02E-15
chr3	178219001	178224000	4.82E-14
chr3	178220001	178225000	5.37E-10
chr3	178221001	178226000	1.07E-05
chr3	178480001	178485000	6.76E-05
chr3	178482001	178487000	1.46E-07
chr3	178592001	178597000	5.25E-06
chr3	178605001	178610000	1.15E-09
chr3	178606001	178611000	2.07E-08
chr3	178607001	178612000	1.23E-06
chr3	178645001	178650000	1.04E-06
chr3	178646001	178651000	4.84E-08
chr3	178670001	178675000	8.08E-05
chr3	178683001	178688000	4.30E-08
chr3	179161001	179166000	6.88E-08
chr3	179171001	179176000	9.92E-15
chr3	179194001	179199000	5.31E-09
chr3	179195001	179200000	6.56E-12
chr3	179196001	179201000	4.98E-11
chr3	179197001	179202000	3.19E-10
chr3	179275001	179280000	2.76E-13
chr3	179526001	179531000	5.62E-09
chr3	179527001	179532000	3.39E-09
chr3	179528001	179533000	2.94E-10

chr3	179529001	179534000	3.18E-08
chr3	179570001	179575000	0.00029649
chr3	179571001	179576000	0.000746975
chr3	179580001	179585000	5.81E-12
chr3	179619001	179624000	5.40E-09
chr3	179620001	179625000	1.92E-08
chr3	179621001	179626000	6.58E-07
chr3	179709001	179714000	0.001183678
chr3	179711001	179716000	2.87E-06
chr3	179712001	179717000	6.09E-12
chr3	179713001	179718000	3.69E-17
chr3	179714001	179719000	1.54E-14
chr3	179715001	179720000	9.53E-14
chr3	179721001	179726000	2.51E-10
chr3	179722001	179727000	1.25E-10
chr3	179723001	179728000	1.04E-12
chr3	179724001	179729000	2.88E-11
chr3	179725001	179730000	3.07E-13
chr3	179726001	179731000	5.92E-07
chr3	179758001	179763000	7.03E-06
chr3	179759001	179764000	2.46E-06
chr3	179762001	179767000	8.84E-05
chr3	179763001	179768000	9.69E-07
chr3	179774001	179779000	0.000772747
chr3	179775001	179780000	0.002012814
chr3	179797001	179802000	1.11E-15
chr3	179798001	179803000	1.73E-15
chr3	179799001	179804000	6.58E-17
chr3	179800001	179805000	9.56E-18
chr3	179801001	179806000	2.45E-19
chr3	179889001	179894000	1.12E-10
chr3	179890001	179895000	2.19E-11
chr3	179891001	179896000	9.08E-11
chr3	179895001	179900000	0.013484517
chr3	179963001	179968000	0.00033024
chr3	179964001	179969000	0.000292485
chr3	179965001	179970000	2.74E-06
chr3	179966001	179971000	2.88E-08
chr3	179967001	179972000	0.000683882
chr3	179990001	179995000	1.24E-05
chr3	179993001	179998000	4.10E-06
chr3	180004001	180009000	5.50E-08
chr3	180005001	180010000	4.67E-09
chr3	180052001	180057000	0.000354548
chr3	180162001	180167000	7.66E-10
chr3	180225001	180230000	0.015116907
chr3	180345001	180350000	0.001102753
chr3	180346001	180351000	0.002842229

chr3	180401001	180406000	2.12E-10
chr3	180402001	180407000	9.89E-11
chr3	180403001	180408000	2.00E-07
chr3	180404001	180409000	1.21E-06
chr3	180414001	180419000	8.84E-05
chr3	180415001	180420000	1.21E-09
chr3	180416001	180421000	1.22E-09
chr3	180417001	180422000	6.30E-08
chr3	180418001	180423000	3.57E-08
chr3	180419001	180424000	1.76E-08
chr3	180466001	180471000	0.000154635
chr3	180467001	180472000	1.04E-05
chr3	180483001	180488000	5.67E-07
chr3	180507001	180512000	0.000481132
chr3	180779001	180784000	2.84E-06
chr3	180780001	180785000	1.08E-05
chr3	181018001	181023000	0.000102443
chr3	181019001	181024000	1.35E-05
chr3	181217001	181222000	2.70E-05
chr3	181218001	181223000	3.66E-05
chr3	181378001	181383000	2.20E-05
chr3	181379001	181384000	0.000301318
chr3	181554001	181559000	3.46E-05
chr3	181555001	181560000	1.44E-06
chr3	181556001	181561000	1.45E-15
chr3	181631001	181636000	5.74E-11
chr3	181671001	181676000	4.50E-10
chr3	181896001	181901000	6.35E-14
chr3	181897001	181902000	6.28E-20
chr3	182486001	182491000	8.87E-06
chr3	183151001	183156000	1.27E-07
chr3	183417001	183422000	1.11E-23
chr3	183692001	183697000	1.94E-22
chr3	183693001	183698000	2.90E-19
chr3	183707001	183712000	8.37E-25
chr3	185644001	185649000	3.60E-14
chr3	186086001	186091000	1.93E-07
chr3	186526001	186531000	1.17E-20
chr3	186546001	186551000	1.04E-11
chr3	186547001	186552000	6.28E-13
chr3	186978001	186983000	1.81E-08
chr3	187221001	187226000	1.06E-08
chr3	187222001	187227000	7.82E-09
chr3	187223001	187228000	2.87E-08
chr3	187627001	187632000	1.26E-07
chr3	187629001	187634000	3.90E-08
chr3	189028001	189033000	0.000112229
chr3	189347001	189352000	7.52E-08

chr3	189348001	189353000	1.23E-07
chr3	189534001	189539000	2.12E-05
chr3	189916001	189921000	2.11E-06
chr3	189917001	189922000	8.74E-07
chr3	189982001	189987000	2.61E-05
chr3	189983001	189988000	2.07E-07
chr3	190555001	190560000	0.000419453
chr3	190607001	190612000	0.000362195
chr3	190608001	190613000	0.000953781
chr3	190624001	190629000	6.92E-05
chr3	190738001	190743000	0.001595182
chr3	190998001	191003000	9.07E-05
chr3	191139001	191144000	0.001050533
chr3	191171001	191176000	3.67E-07
chr3	191172001	191177000	2.50E-09
chr3	191173001	191178000	7.67E-11
chr3	191174001	191179000	6.10E-09
chr3	191254001	191259000	0.002024242
chr3	191480001	191485000	0.000591909
chr3	191483001	191488000	0.001728329
chr3	191484001	191489000	0.001013853
chr3	191539001	191544000	4.81E-08
chr3	191542001	191547000	0.000344122
chr3	191564001	191569000	7.96E-08
chr3	191565001	191570000	6.46E-08
chr3	191566001	191571000	2.40E-09
chr3	191567001	191572000	1.16E-08
chr3	191568001	191573000	3.37E-13
chr3	191574001	191579000	2.64E-06
chr3	191575001	191580000	2.93E-08
chr3	191628001	191633000	0.003532308
chr3	191629001	191634000	0.001892785
chr3	191681001	191686000	2.53E-07
chr3	191686001	191691000	8.23E-12
chr3	191723001	191728000	1.49E-05
chr3	191724001	191729000	6.01E-06
chr3	191725001	191730000	7.91E-08
chr3	191726001	191731000	1.75E-07
chr3	191746001	191751000	8.48E-09
chr3	191747001	191752000	3.98E-08
chr3	191748001	191753000	3.16E-06
chr3	191749001	191754000	8.61E-06
chr3	191757001	191762000	6.60E-13
chr3	191758001	191763000	3.21E-11
chr3	191759001	191764000	3.75E-13
chr3	191760001	191765000	7.04E-12
chr3	191886001	191891000	0.002026629
chr3	191896001	191901000	4.13E-06

chr3	191915001	191920000	5.58E-07
chr3	191916001	191921000	0.000630608
chr3	191917001	191922000	0.000934393
chr3	191919001	191924000	4.92E-05
chr3	191920001	191925000	2.20E-05
chr3	191921001	191926000	2.02E-05
chr3	191922001	191927000	2.25E-05
chr3	191923001	191928000	1.37E-05
chr3	191928001	191933000	4.55E-10
chr3	191930001	191935000	3.23E-10
chr3	191931001	191936000	1.72E-14
chr3	191932001	191937000	1.36E-09
chr3	191934001	191939000	5.93E-09
chr3	191940001	191945000	0.000427134
chr3	191965001	191970000	3.30E-05
chr3	191966001	191971000	2.96E-07
chr3	191967001	191972000	6.57E-05
chr3	191975001	191980000	5.35E-06
chr3	191976001	191981000	1.61E-06
chr3	191996001	192001000	8.86E-05
chr3	192006001	192011000	0.001036366
chr3	192026001	192031000	4.65E-09
chr3	192028001	192033000	0.000119298
chr3	192082001	192087000	1.23E-06
chr3	192090001	192095000	4.13E-06
chr3	192091001	192096000	1.65E-12
chr3	192108001	192113000	0.000846259
chr3	192141001	192146000	9.07E-08
chr3	192142001	192147000	4.12E-07
chr3	192143001	192148000	1.09E-06
chr3	192247001	192252000	1.64E-05
chr3	192453001	192458000	0.001513321
chr3	192502001	192507000	5.31E-19
chr3	192787001	192792000	5.29E-05
chr3	192788001	192793000	8.07E-06
chr3	192825001	192830000	0.00030474
chr3	192883001	192888000	0.000269031
chr3	192932001	192937000	8.65E-06
chr3	193465001	193470000	0.000124168
chr3	193466001	193471000	1.39E-06
chr3	193467001	193472000	2.67E-09
chr3	193557001	193562000	1.38E-21
chr3	193558001	193563000	4.65E-33
chr3	194440001	194445000	7.43E-16
chr3	195172001	195177000	3.02E-22
chr3	195600001	195605000	4.65E-11
chr3	196431001	196436000	6.44E-12
chr3	196432001	196437000	1.78E-15

chr3	196433001	196438000	9.90E-15
chr3	197243001	197248000	4.99E-20
chr3	197244001	197249000	1.13E-19
chr3	197327001	197332000	1.48E-49
chr3	197635001	197640000	7.05E-23
chr3	197636001	197641000	4.10E-29
chr3	197696001	197701000	3.09E-09
chr4	114001	119000	1.97E-05
chr4	115001	120000	4.15E-06
chr4	670001	675000	2.52E-06
chr4	677001	682000	0.000417047
chr4	679001	684000	4.53E-10
chr4	807001	812000	6.17E-16
chr4	808001	813000	3.11E-14
chr4	809001	814000	1.32E-13
chr4	1031001	1036000	1.71E-12
chr4	1038001	1043000	5.55E-20
chr4	1063001	1068000	2.28E-17
chr4	1184001	1189000	2.21E-27
chr4	1186001	1191000	5.90E-22
chr4	1618001	1623000	1.05E-14
chr4	1802001	1807000	7.43E-15
chr4	2028001	2033000	2.24E-09
chr4	2245001	2250000	6.81E-21
chr4	2246001	2251000	4.80E-13
chr4	2247001	2252000	7.78E-17
chr4	2248001	2253000	3.14E-15
chr4	2319001	2324000	2.43E-11
chr4	2370001	2375000	1.04E-09
chr4	3308001	3313000	2.76E-05
chr4	3309001	3314000	6.20E-07
chr4	3479001	3484000	2.76E-21
chr4	3480001	3485000	7.86E-17
chr4	3481001	3486000	3.72E-20
chr4	3482001	3487000	4.16E-14
chr4	4337001	4342000	4.24E-18
chr4	4338001	4343000	1.11E-14
chr4	4413001	4418000	1.16E-09
chr4	4414001	4419000	2.78E-10
chr4	4917001	4922000	0.00075827
chr4	6241001	6246000	8.66E-07
chr4	6242001	6247000	3.83E-08
chr4	6325001	6330000	7.05E-10
chr4	6327001	6332000	1.06E-11
chr4	6372001	6377000	4.44E-09
chr4	6401001	6406000	5.13E-12
chr4	6457001	6462000	4.07E-10
chr4	7119001	7124000	1.89E-07

chr4	8239001	8244000	7.52E-05
chr4	8435001	8440000	1.70E-17
chr4	8436001	8441000	8.52E-16
chr4	10044001	10049000	2.38E-07
chr4	10502001	10507000	9.35E-17
chr4	10538001	10543000	3.10E-06
chr4	10539001	10544000	1.64E-06
chr4	10760001	10765000	0.00021823
chr4	11503001	11508000	0.003373944
chr4	11685001	11690000	0.000122161
chr4	11686001	11691000	4.71E-05
chr4	11710001	11715000	0.000368209
chr4	11711001	11716000	0.00089769
chr4	11837001	11842000	0.000361116
chr4	11847001	11852000	4.95E-05
chr4	12097001	12102000	0.000735973
chr4	12098001	12103000	0.000750304
chr4	12228001	12233000	1.14E-07
chr4	12266001	12271000	0.000148486
chr4	12535001	12540000	3.70E-07
chr4	12536001	12541000	1.44E-05
chr4	12577001	12582000	8.15E-05
chr4	12847001	12852000	4.68E-06
chr4	12848001	12853000	5.75E-06
chr4	12849001	12854000	1.19E-05
chr4	12850001	12855000	5.39E-06
chr4	13115001	13120000	5.80E-05
chr4	13116001	13121000	0.000107399
chr4	13117001	13122000	0.000144681
chr4	13140001	13145000	8.25E-06
chr4	13141001	13146000	3.51E-07
chr4	13248001	13253000	6.49E-08
chr4	13512001	13517000	0.000810499
chr4	13727001	13732000	2.31E-05
chr4	13856001	13861000	4.21E-09
chr4	13992001	13997000	4.78E-05
chr4	13993001	13998000	9.85E-05
chr4	13994001	13999000	2.47E-06
chr4	13996001	14001000	3.61E-06
chr4	14068001	14073000	6.05E-06
chr4	14069001	14074000	2.98E-06
chr4	14070001	14075000	6.61E-05
chr4	14071001	14076000	2.26E-05
chr4	14130001	14135000	9.24E-05
chr4	14131001	14136000	0.000355022
chr4	14227001	14232000	3.90E-05
chr4	14233001	14238000	0.003672646
chr4	14280001	14285000	0.000352549

chr4	14307001	14312000	1.52E-11
chr4	14308001	14313000	1.32E-18
chr4	14309001	14314000	4.91E-07
chr4	14316001	14321000	0.003368437
chr4	14341001	14346000	0.014852117
chr4	14342001	14347000	0.000622552
chr4	14343001	14348000	0.001759534
chr4	14344001	14349000	0.000452716
chr4	14362001	14367000	4.87E-09
chr4	14459001	14464000	2.03E-05
chr4	14464001	14469000	1.07E-05
chr4	14466001	14471000	7.27E-06
chr4	14467001	14472000	7.27E-06
chr4	14553001	14558000	5.13E-06
chr4	14554001	14559000	6.00E-06
chr4	14555001	14560000	0.000302995
chr4	14556001	14561000	0.000612217
chr4	14575001	14580000	2.94E-08
chr4	14631001	14636000	0.000320948
chr4	14632001	14637000	0.000603283
chr4	14633001	14638000	0.001792731
chr4	14634001	14639000	5.98E-06
chr4	14635001	14640000	0.000342876
chr4	14682001	14687000	1.41E-06
chr4	14695001	14700000	0.022808746
chr4	14747001	14752000	0.00812591
chr4	14835001	14840000	0.006319998
chr4	15312001	15317000	0.001358564
chr4	15345001	15350000	0.000268415
chr4	15362001	15367000	0.00039132
chr4	15372001	15377000	1.96E-10
chr4	15373001	15378000	9.02E-11
chr4	15374001	15379000	3.94E-10
chr4	15375001	15380000	6.05E-10
chr4	15727001	15732000	7.95E-08
chr4	15728001	15733000	3.54E-11
chr4	15788001	15793000	7.37E-06
chr4	15789001	15794000	9.35E-06
chr4	16270001	16275000	0.000361738
chr4	16357001	16362000	3.93E-05
chr4	16719001	16724000	0.003626882
chr4	16984001	16989000	0.001447776
chr4	16985001	16990000	0.00223574
chr4	16986001	16991000	0.00300457
chr4	16987001	16992000	0.005361826
chr4	16989001	16994000	0.001401124
chr4	17146001	17151000	1.27E-08
chr4	17170001	17175000	0.009134872

chr4	17203001	17208000	0.000135085
chr4	17371001	17376000	0.000164849
chr4	17450001	17455000	9.44E-07
chr4	17649001	17654000	1.22E-06
chr4	17650001	17655000	0.000132388
chr4	18053001	18058000	0.00153155
chr4	18148001	18153000	7.19E-06
chr4	18250001	18255000	0.004589015
chr4	18328001	18333000	0.019146627
chr4	18329001	18334000	0.017630522
chr4	18523001	18528000	0.000144353
chr4	18594001	18599000	0.000998326
chr4	18763001	18768000	0.00409149
chr4	18764001	18769000	0.024794565
chr4	18831001	18836000	0.001716736
chr4	18832001	18837000	0.00596115
chr4	18868001	18873000	0.004539814
chr4	18869001	18874000	0.001160236
chr4	18870001	18875000	0.001107023
chr4	18895001	18900000	3.92E-05
chr4	18896001	18901000	0.000630053
chr4	18976001	18981000	0.000187464
chr4	19149001	19154000	0.000111518
chr4	19285001	19290000	0.000324231
chr4	19342001	19347000	0.000158395
chr4	19367001	19372000	0.00151922
chr4	19369001	19374000	0.000362795
chr4	19434001	19439000	0.003800241
chr4	19510001	19515000	0.004531005
chr4	19511001	19516000	1.01E-05
chr4	19512001	19517000	1.65E-05
chr4	19546001	19551000	0.001220146
chr4	19800001	19805000	0.004694648
chr4	19801001	19806000	0.001489937
chr4	19802001	19807000	0.005211312
chr4	19855001	19860000	0.001569978
chr4	19870001	19875000	3.20E-07
chr4	20007001	20012000	0.000880664
chr4	20064001	20069000	0.011968751
chr4	20065001	20070000	0.000789637
chr4	20154001	20159000	0.009733
chr4	20289001	20294000	0.000358388
chr4	20610001	20615000	3.96E-07
chr4	20890001	20895000	9.14E-05
chr4	20893001	20898000	3.13E-07
chr4	20894001	20899000	1.02E-05
chr4	21059001	21064000	0.002231872
chr4	21092001	21097000	0.00166435

chr4	21094001	21099000	0.001944261
chr4	21518001	21523000	1.80E-06
chr4	21519001	21524000	2.64E-06
chr4	21591001	21596000	0.001484602
chr4	21592001	21597000	0.008132384
chr4	21609001	21614000	0.008348652
chr4	21770001	21775000	9.07E-08
chr4	21809001	21814000	0.000617321
chr4	21887001	21892000	0.000793603
chr4	21910001	21915000	0.002285918
chr4	21965001	21970000	7.29E-05
chr4	21966001	21971000	0.000120764
chr4	21967001	21972000	0.000392345
chr4	21968001	21973000	0.001186599
chr4	22043001	22048000	0.002297698
chr4	22102001	22107000	0.013457321
chr4	22103001	22108000	0.000718905
chr4	22112001	22117000	0.001267052
chr4	22182001	22187000	7.80E-05
chr4	22183001	22188000	4.74E-10
chr4	22184001	22189000	3.96E-10
chr4	22185001	22190000	3.17E-09
chr4	22186001	22191000	2.04E-09
chr4	22213001	22218000	0.000130677
chr4	22254001	22259000	0.001157729
chr4	22277001	22282000	0.000792555
chr4	22711001	22716000	0.000636381
chr4	22765001	22770000	3.58E-07
chr4	22771001	22776000	4.57E-05
chr4	22841001	22846000	0.000266728
chr4	23133001	23138000	1.70E-08
chr4	23211001	23216000	0.001300368
chr4	23257001	23262000	0.000934995
chr4	23402001	23407000	0.005019756
chr4	23570001	23575000	0.000690215
chr4	23571001	23576000	0.000130907
chr4	23942001	23947000	3.13E-05
chr4	24157001	24162000	2.64E-05
chr4	24158001	24163000	4.94E-05
chr4	24268001	24273000	6.61E-05
chr4	24721001	24726000	1.26E-06
chr4	25251001	25256000	3.37E-13
chr4	25556001	25561000	0.000958234
chr4	25987001	25992000	4.74E-16
chr4	26154001	26159000	7.74E-06
chr4	26324001	26329000	0.000119775
chr4	26328001	26333000	2.12E-10
chr4	26540001	26545000	8.37E-08

chr4	26541001	26546000	1.70E-09
chr4	26542001	26547000	1.92E-07
chr4	27046001	27051000	3.71E-07
chr4	27047001	27052000	1.80E-13
chr4	27048001	27053000	2.23E-14
chr4	27049001	27054000	6.52E-29
chr4	27050001	27055000	1.60E-26
chr4	27121001	27126000	4.77E-05
chr4	27146001	27151000	4.87E-08
chr4	27179001	27184000	2.31E-08
chr4	27180001	27185000	1.26E-06
chr4	27355001	27360000	0.010686427
chr4	27460001	27465000	0.001600276
chr4	27461001	27466000	0.000473104
chr4	27478001	27483000	0.000209217
chr4	27482001	27487000	1.53E-16
chr4	27483001	27488000	3.65E-14
chr4	27570001	27575000	0.001074653
chr4	27610001	27615000	0.003137059
chr4	27624001	27629000	0.003832856
chr4	27710001	27715000	2.62E-05
chr4	27751001	27756000	4.66E-07
chr4	27762001	27767000	0.001062619
chr4	27763001	27768000	0.002061956
chr4	27766001	27771000	0.000626664
chr4	27838001	27843000	0.001250887
chr4	27918001	27923000	0.014046121
chr4	27926001	27931000	0.015010503
chr4	27927001	27932000	0.02299824
chr4	27928001	27933000	0.024599568
chr4	27953001	27958000	4.65E-07
chr4	27954001	27959000	2.38E-07
chr4	27955001	27960000	3.04E-09
chr4	27957001	27962000	0.000235563
chr4	28004001	28009000	0.002708101
chr4	28005001	28010000	0.000280878
chr4	28006001	28011000	7.70E-05
chr4	28033001	28038000	0.000701393
chr4	28146001	28151000	0.001286782
chr4	28148001	28153000	2.33E-05
chr4	28150001	28155000	1.08E-05
chr4	28151001	28156000	5.54E-06
chr4	28216001	28221000	0.006009061
chr4	28217001	28222000	0.012675762
chr4	28222001	28227000	0.000466418
chr4	28395001	28400000	1.95E-05
chr4	28396001	28401000	0.000496321
chr4	28423001	28428000	4.73E-06

chr4	28424001	28429000	4.06E-05
chr4	28425001	28430000	1.78E-05
chr4	28554001	28559000	0.00600758
chr4	28565001	28570000	0.000627589
chr4	28566001	28571000	0.000160005
chr4	28654001	28659000	0.000645659
chr4	28659001	28664000	5.54E-05
chr4	28660001	28665000	1.70E-05
chr4	28661001	28666000	9.12E-07
chr4	28662001	28667000	0.000115136
chr4	28698001	28703000	8.21E-07
chr4	28699001	28704000	9.28E-07
chr4	28825001	28830000	6.03E-13
chr4	28826001	28831000	4.59E-18
chr4	28827001	28832000	2.29E-15
chr4	28828001	28833000	1.39E-15
chr4	28829001	28834000	2.12E-14
chr4	28900001	28905000	6.45E-09
chr4	28901001	28906000	1.18E-08
chr4	28902001	28907000	0.002439951
chr4	28903001	28908000	0.001749894
chr4	29003001	29008000	4.93E-06
chr4	29043001	29048000	0.00019193
chr4	29163001	29168000	2.76E-05
chr4	29215001	29220000	6.18E-05
chr4	29272001	29277000	0.002657982
chr4	29273001	29278000	0.002562063
chr4	29340001	29345000	0.002333925
chr4	29423001	29428000	0.000267373
chr4	29605001	29610000	2.40E-05
chr4	29606001	29611000	1.22E-05
chr4	29607001	29612000	1.01E-06
chr4	29608001	29613000	2.30E-05
chr4	29690001	29695000	1.11E-05
chr4	29744001	29749000	0.002779394
chr4	29752001	29757000	0.010974032
chr4	29753001	29758000	0.004940149
chr4	29992001	29997000	0.000736044
chr4	30285001	30290000	0.000222816
chr4	30421001	30426000	0.000789262
chr4	30500001	30505000	0.011576503
chr4	30694001	30699000	6.03E-05
chr4	31186001	31191000	4.01E-12
chr4	31261001	31266000	1.91E-10
chr4	31262001	31267000	1.27E-07
chr4	31273001	31278000	3.40E-06
chr4	31274001	31279000	3.30E-05
chr4	31324001	31329000	0.002889736

chr4	31325001	31330000	0.002207942
chr4	31329001	31334000	2.15E-06
chr4	31331001	31336000	4.79E-06
chr4	31332001	31337000	2.55E-06
chr4	31333001	31338000	1.91E-07
chr4	31358001	31363000	0.017074184
chr4	31664001	31669000	2.63E-08
chr4	31665001	31670000	6.27E-08
chr4	31984001	31989000	0.000112088
chr4	31985001	31990000	0.000302016
chr4	32035001	32040000	0.001412079
chr4	32118001	32123000	2.78E-08
chr4	32171001	32176000	0.000663013
chr4	32172001	32177000	0.003605801
chr4	32580001	32585000	7.11E-06
chr4	32624001	32629000	0.001641173
chr4	32752001	32757000	0.005821696
chr4	32753001	32758000	0.004289628
chr4	32754001	32759000	0.012583113
chr4	33069001	33074000	6.35E-05
chr4	33070001	33075000	9.68E-07
chr4	33071001	33076000	0.00025137
chr4	33248001	33253000	1.72E-10
chr4	33249001	33254000	3.38E-14
chr4	33250001	33255000	1.19E-13
chr4	33284001	33289000	0.000779271
chr4	33285001	33290000	0.000272683
chr4	33305001	33310000	0.000245194
chr4	33306001	33311000	0.000263606
chr4	33307001	33312000	4.38E-07
chr4	33308001	33313000	2.29E-06
chr4	33309001	33314000	1.17E-05
chr4	33310001	33315000	0.000274378
chr4	33311001	33316000	0.000123041
chr4	33313001	33318000	3.30E-05
chr4	33314001	33319000	5.02E-05
chr4	33315001	33320000	6.76E-06
chr4	33316001	33321000	5.63E-06
chr4	33317001	33322000	1.92E-06
chr4	33318001	33323000	0.00033155
chr4	33499001	33504000	0.006098659
chr4	33500001	33505000	3.58E-05
chr4	33501001	33506000	0.000349896
chr4	33502001	33507000	0.000408451
chr4	33503001	33508000	0.009130318
chr4	33505001	33510000	1.50E-05
chr4	33506001	33511000	0.000710844
chr4	33669001	33674000	0.001011838

chr4	33798001	33803000	0.005444057
chr4	33904001	33909000	3.89E-07
chr4	33905001	33910000	8.20E-08
chr4	34006001	34011000	8.67E-08
chr4	34007001	34012000	1.01E-11
chr4	34008001	34013000	2.43E-15
chr4	34009001	34014000	6.63E-12
chr4	34010001	34015000	1.23E-10
chr4	34011001	34016000	6.24E-09
chr4	34012001	34017000	1.41E-10
chr4	34013001	34018000	1.66E-07
chr4	34014001	34019000	3.09E-09
chr4	34015001	34020000	2.74E-06
chr4	34059001	34064000	0.001333005
chr4	34119001	34124000	1.46E-09
chr4	34120001	34125000	1.33E-09
chr4	34121001	34126000	1.44E-08
chr4	34122001	34127000	4.99E-11
chr4	34123001	34128000	3.82E-11
chr4	34157001	34162000	0.000976844
chr4	34158001	34163000	0.000296934
chr4	34159001	34164000	0.000104075
chr4	34160001	34165000	0.00016841
chr4	34194001	34199000	1.18E-05
chr4	34195001	34200000	1.99E-07
chr4	34196001	34201000	3.36E-07
chr4	34197001	34202000	0.000164215
chr4	34252001	34257000	0.005224592
chr4	34253001	34258000	0.008165837
chr4	34279001	34284000	0.002539922
chr4	34280001	34285000	6.61E-05
chr4	34298001	34303000	0.014580127
chr4	34299001	34304000	0.020320409
chr4	34324001	34329000	7.82E-07
chr4	34437001	34442000	0.000677621
chr4	34438001	34443000	0.001326599
chr4	34440001	34445000	0.000446876
chr4	34441001	34446000	0.003289057
chr4	34474001	34479000	0.000316256
chr4	34546001	34551000	0.002068855
chr4	34547001	34552000	0.003938829
chr4	34556001	34561000	1.52E-05
chr4	34882001	34887000	9.08E-15
chr4	34885001	34890000	3.20E-14
chr4	34996001	35001000	0.007747934
chr4	34997001	35002000	0.006787206
chr4	35035001	35040000	1.09E-13
chr4	35184001	35189000	0.000117665

chr4	35964001	35969000	3.79E-05
chr4	36322001	36327000	1.52E-05
chr4	36323001	36328000	3.94E-06
chr4	36522001	36527000	3.40E-05
chr4	36668001	36673000	0.000663099
chr4	36669001	36674000	0.002089902
chr4	36689001	36694000	0.000145896
chr4	36795001	36800000	0.000232259
chr4	37762001	37767000	6.35E-06
chr4	38806001	38811000	3.91E-14
chr4	39353001	39358000	8.25E-06
chr4	39354001	39359000	4.59E-07
chr4	39443001	39448000	6.54E-07
chr4	39701001	39706000	1.13E-10
chr4	39813001	39818000	5.65E-13
chr4	39814001	39819000	7.12E-14
chr4	40457001	40462000	4.49E-07
chr4	40458001	40463000	1.67E-09
chr4	40460001	40465000	4.16E-10
chr4	40547001	40552000	1.98E-06
chr4	40548001	40553000	2.46E-06
chr4	40549001	40554000	9.05E-06
chr4	40585001	40590000	4.96E-12
chr4	40975001	40980000	1.28E-06
chr4	41210001	41215000	4.88E-11
chr4	41219001	41224000	4.33E-05
chr4	41333001	41338000	3.88E-05
chr4	41705001	41710000	0.000351025
chr4	41706001	41711000	0.000258383
chr4	41709001	41714000	0.004406074
chr4	41711001	41716000	0.000950301
chr4	42869001	42874000	2.32E-05
chr4	42871001	42876000	0.026639583
chr4	42926001	42931000	6.34E-05
chr4	42927001	42932000	0.00819389
chr4	43009001	43014000	0.001427355
chr4	43200001	43205000	0.000503727
chr4	43223001	43228000	0.000325306
chr4	43467001	43472000	0.003369432
chr4	43476001	43481000	0.001807458
chr4	43477001	43482000	0.000557023
chr4	43553001	43558000	2.00E-07
chr4	43712001	43717000	0.002100827
chr4	43713001	43718000	0.00049954
chr4	43963001	43968000	8.13E-06
chr4	44037001	44042000	0.011239163
chr4	44170001	44175000	1.77E-08
chr4	44314001	44319000	1.78E-06

chr4	44315001	44320000	6.00E-06
chr4	44409001	44414000	9.68E-06
chr4	44469001	44474000	3.14E-06
chr4	44470001	44475000	1.33E-05
chr4	44516001	44521000	2.82E-09
chr4	44531001	44536000	0.00258258
chr4	44533001	44538000	0.000116592
chr4	44534001	44539000	0.000107022
chr4	44535001	44540000	0.000107338
chr4	44612001	44617000	5.80E-08
chr4	44613001	44618000	1.11E-08
chr4	44614001	44619000	0.000101564
chr4	44615001	44620000	0.000359304
chr4	44661001	44666000	1.04E-10
chr4	44821001	44826000	1.49E-06
chr4	44822001	44827000	5.25E-10
chr4	44823001	44828000	1.64E-09
chr4	44824001	44829000	1.92E-11
chr4	44849001	44854000	1.12E-06
chr4	44850001	44855000	4.09E-05
chr4	44851001	44856000	1.28E-07
chr4	44852001	44857000	6.13E-06
chr4	44860001	44865000	0.02066097
chr4	44952001	44957000	0.021157163
chr4	44964001	44969000	0.002943503
chr4	44985001	44990000	3.66E-06
chr4	44986001	44991000	4.89E-06
chr4	44987001	44992000	2.05E-07
chr4	44988001	44993000	2.91E-05
chr4	45040001	45045000	0.00011548
chr4	45119001	45124000	0.004713809
chr4	45120001	45125000	0.006411186
chr4	45160001	45165000	3.64E-05
chr4	45161001	45166000	0.000347035
chr4	45162001	45167000	1.45E-05
chr4	45185001	45190000	0.001952604
chr4	45313001	45318000	0.000334198
chr4	45436001	45441000	0.008247208
chr4	45437001	45442000	0.017909494
chr4	45438001	45443000	0.005893454
chr4	45641001	45646000	1.51E-05
chr4	45642001	45647000	5.95E-05
chr4	45759001	45764000	0.001394192
chr4	45866001	45871000	2.55E-05
chr4	45867001	45872000	3.93E-05
chr4	45868001	45873000	6.76E-05
chr4	45902001	45907000	0.00391907
chr4	45969001	45974000	9.26E-05

chr4	46111001	46116000	0.00492756
chr4	46280001	46285000	0.001822892
chr4	46374001	46379000	9.45E-08
chr4	46409001	46414000	1.30E-05
chr4	46427001	46432000	0.014903637
chr4	46457001	46462000	0.001184243
chr4	46528001	46533000	8.25E-06
chr4	46529001	46534000	0.000491149
chr4	46624001	46629000	1.12E-07
chr4	46625001	46630000	0.00174056
chr4	46631001	46636000	5.58E-05
chr4	46632001	46637000	0.001479249
chr4	46633001	46638000	0.000511102
chr4	46643001	46648000	0.000885969
chr4	46657001	46662000	6.75E-05
chr4	46658001	46663000	1.11E-05
chr4	46982001	46987000	0.000603955
chr4	46983001	46988000	0.000591865
chr4	47086001	47091000	0.001195112
chr4	47087001	47092000	0.000845276
chr4	47088001	47093000	0.000230816
chr4	47211001	47216000	2.31E-06
chr4	47212001	47217000	2.89E-07
chr4	47213001	47218000	3.36E-07
chr4	47214001	47219000	1.77E-06
chr4	47258001	47263000	0.002373547
chr4	47336001	47341000	0.000390831
chr4	47337001	47342000	0.000384707
chr4	47343001	47348000	0.000203619
chr4	47363001	47368000	0.001241236
chr4	47364001	47369000	0.000675488
chr4	47407001	47412000	5.85E-07
chr4	47408001	47413000	1.64E-07
chr4	47409001	47414000	3.06E-07
chr4	47816001	47821000	5.48E-06
chr4	47817001	47822000	2.46E-05
chr4	47818001	47823000	0.000168407
chr4	47933001	47938000	1.45E-05
chr4	48259001	48264000	0.002725803
chr4	48290001	48295000	7.06E-10
chr4	48291001	48296000	5.92E-09
chr4	48292001	48297000	7.18E-07
chr4	48701001	48706000	0.002394985
chr4	48756001	48761000	0.000574722
chr4	52967001	52972000	0.001400552
chr4	52976001	52981000	0.001532696
chr4	53010001	53015000	0.003753858
chr4	53011001	53016000	0.002221541

chr4	53145001	53150000	0.000375529
chr4	53169001	53174000	0.000125577
chr4	53349001	53354000	0.000233474
chr4	53645001	53650000	2.18E-08
chr4	53849001	53854000	2.55E-07
chr4	53930001	53935000	0.003006932
chr4	54470001	54475000	3.11E-05
chr4	54774001	54779000	3.73E-08
chr4	54793001	54798000	7.26E-08
chr4	55033001	55038000	1.65E-07
chr4	55034001	55039000	4.42E-06
chr4	55035001	55040000	1.41E-05
chr4	55119001	55124000	6.56E-06
chr4	55389001	55394000	1.23E-08
chr4	55446001	55451000	1.11E-08
chr4	55510001	55515000	0.000162125
chr4	55535001	55540000	3.93E-10
chr4	55536001	55541000	2.75E-09
chr4	55792001	55797000	7.05E-05
chr4	55793001	55798000	4.44E-05
chr4	56149001	56154000	0.000909065
chr4	56729001	56734000	4.69E-10
chr4	57028001	57033000	1.20E-07
chr4	57029001	57034000	1.33E-08
chr4	57030001	57035000	1.20E-06
chr4	57031001	57036000	1.14E-06
chr4	57050001	57055000	1.27E-16
chr4	57051001	57056000	6.37E-14
chr4	57053001	57058000	5.96E-07
chr4	57548001	57553000	2.00E-08
chr4	57549001	57554000	1.80E-07
chr4	58012001	58017000	2.27E-05
chr4	58013001	58018000	1.41E-06
chr4	58014001	58019000	6.39E-08
chr4	58015001	58020000	3.11E-07
chr4	58146001	58151000	1.41E-05
chr4	58157001	58162000	0.001613815
chr4	58199001	58204000	1.15E-05
chr4	58200001	58205000	2.52E-06
chr4	58290001	58295000	7.38E-05
chr4	58406001	58411000	0.000522688
chr4	58466001	58471000	2.33E-06
chr4	58609001	58614000	8.84E-05
chr4	58611001	58616000	0.000345513
chr4	58658001	58663000	6.92E-07
chr4	58913001	58918000	2.13E-05
chr4	58914001	58919000	0.000331418
chr4	58916001	58921000	0.000819205

chr4	58924001	58929000	0.001661224
chr4	59110001	59115000	4.24E-05
chr4	59214001	59219000	0.000160835
chr4	59825001	59830000	0.005098029
chr4	59927001	59932000	0.000553299
chr4	60198001	60203000	4.71E-06
chr4	60199001	60204000	5.04E-06
chr4	60200001	60205000	1.87E-06
chr4	60201001	60206000	1.21E-06
chr4	60238001	60243000	2.42E-07
chr4	60404001	60409000	4.40E-05
chr4	60405001	60410000	2.99E-07
chr4	60406001	60411000	3.57E-09
chr4	60407001	60412000	1.89E-10
chr4	60408001	60413000	3.76E-10
chr4	60409001	60414000	2.02E-06
chr4	60410001	60415000	0.000346288
chr4	60620001	60625000	0.000224803
chr4	60660001	60665000	1.46E-05
chr4	60661001	60666000	1.87E-05
chr4	60662001	60667000	3.65E-06
chr4	60663001	60668000	3.03E-06
chr4	60664001	60669000	0.000227573
chr4	60726001	60731000	0.004836168
chr4	60748001	60753000	3.43E-11
chr4	60921001	60926000	4.21E-09
chr4	60923001	60928000	1.84E-07
chr4	60944001	60949000	5.45E-05
chr4	61125001	61130000	2.65E-08
chr4	61126001	61131000	5.31E-08
chr4	61127001	61132000	4.05E-07
chr4	61220001	61225000	0.00165341
chr4	61221001	61226000	2.88E-05
chr4	61223001	61228000	1.49E-05
chr4	61224001	61229000	1.34E-05
chr4	61491001	61496000	2.17E-07
chr4	61492001	61497000	1.86E-07
chr4	61493001	61498000	6.12E-07
chr4	61557001	61562000	0.000726822
chr4	61567001	61572000	7.34E-10
chr4	61568001	61573000	3.35E-11
chr4	61569001	61574000	2.63E-12
chr4	61750001	61755000	3.68E-05
chr4	61808001	61813000	0.002133994
chr4	61809001	61814000	0.000938967
chr4	62003001	62008000	0.000107186
chr4	62056001	62061000	6.81E-05
chr4	62201001	62206000	6.76E-05

chr4	62202001	62207000	7.09E-05
chr4	62203001	62208000	0.000199856
chr4	62430001	62435000	0.000690338
chr4	62468001	62473000	0.0001071
chr4	62469001	62474000	3.09E-05
chr4	62526001	62531000	1.16E-12
chr4	62693001	62698000	0.000268031
chr4	62774001	62779000	9.27E-06
chr4	62775001	62780000	9.93E-07
chr4	63132001	63137000	2.77E-08
chr4	63133001	63138000	5.67E-07
chr4	63134001	63139000	1.13E-06
chr4	63181001	63186000	0.007307974
chr4	63182001	63187000	0.002964335
chr4	63226001	63231000	1.88E-06
chr4	63227001	63232000	1.36E-06
chr4	63286001	63291000	0.001144192
chr4	63287001	63292000	0.005553043
chr4	63296001	63301000	1.85E-07
chr4	63297001	63302000	3.08E-05
chr4	63343001	63348000	0.002937543
chr4	63396001	63401000	7.20E-07
chr4	63397001	63402000	3.63E-08
chr4	63404001	63409000	0.017155584
chr4	63405001	63410000	0.000660984
chr4	63408001	63413000	1.55E-07
chr4	63409001	63414000	6.08E-07
chr4	63520001	63525000	1.90E-06
chr4	63521001	63526000	3.04E-06
chr4	63522001	63527000	8.04E-06
chr4	63579001	63584000	0.00057435
chr4	63580001	63585000	0.001075036
chr4	63581001	63586000	0.000501173
chr4	63583001	63588000	2.52E-14
chr4	63587001	63592000	1.15E-10
chr4	63894001	63899000	0.001301046
chr4	63905001	63910000	0.002189108
chr4	63906001	63911000	0.000311575
chr4	63974001	63979000	9.48E-05
chr4	64117001	64122000	3.24E-05
chr4	64488001	64493000	3.64E-09
chr4	64489001	64494000	1.34E-09
chr4	64490001	64495000	9.93E-08
chr4	64739001	64744000	0.000138669
chr4	64740001	64745000	0.000357373
chr4	64741001	64746000	0.000549425
chr4	64751001	64756000	0.002328031
chr4	65050001	65055000	0.000429617

chr4	65586001	65591000	6.47E-09
chr4	65587001	65592000	4.47E-10
chr4	65588001	65593000	2.76E-09
chr4	65589001	65594000	5.05E-08
chr4	65590001	65595000	1.28E-07
chr4	65597001	65602000	1.71E-05
chr4	65642001	65647000	0.004225676
chr4	65718001	65723000	0.001778239
chr4	65757001	65762000	0.002275458
chr4	65758001	65763000	0.011181716
chr4	65799001	65804000	3.85E-07
chr4	65822001	65827000	1.19E-09
chr4	65873001	65878000	0.000217585
chr4	66035001	66040000	1.61E-07
chr4	66036001	66041000	1.42E-07
chr4	66037001	66042000	1.11E-07
chr4	66102001	66107000	1.27E-05
chr4	66199001	66204000	5.46E-05
chr4	66201001	66206000	6.71E-05
chr4	66214001	66219000	0.00051688
chr4	66270001	66275000	0.001018478
chr4	66271001	66276000	0.000603283
chr4	66281001	66286000	0.000479897
chr4	66350001	66355000	8.72E-07
chr4	66351001	66356000	4.04E-10
chr4	66352001	66357000	1.59E-10
chr4	66353001	66358000	3.95E-11
chr4	66354001	66359000	1.21E-08
chr4	66583001	66588000	0.000722204
chr4	66716001	66721000	1.87E-05
chr4	66955001	66960000	3.30E-08
chr4	67064001	67069000	0.001469726
chr4	67276001	67281000	0.000569424
chr4	67277001	67282000	0.00083701
chr4	67278001	67283000	4.78E-06
chr4	67286001	67291000	0.000530778
chr4	67289001	67294000	0.007656064
chr4	67338001	67343000	2.27E-05
chr4	67339001	67344000	0.000106872
chr4	67577001	67582000	5.75E-12
chr4	67699001	67704000	0.000895623
chr4	67701001	67706000	0.003717932
chr4	67702001	67707000	0.003293587
chr4	67727001	67732000	0.001816806
chr4	67728001	67733000	0.000169668
chr4	67856001	67861000	0.000264509
chr4	67857001	67862000	1.87E-11
chr4	67858001	67863000	4.13E-10

chr4	67859001	67864000	3.32E-09
chr4	67860001	67865000	6.97E-08
chr4	67883001	67888000	0.00149302
chr4	67887001	67892000	0.011490626
chr4	67895001	67900000	0.001487435
chr4	67896001	67901000	0.000527878
chr4	68013001	68018000	3.22E-12
chr4	68015001	68020000	1.53E-09
chr4	68016001	68021000	1.71E-10
chr4	68017001	68022000	1.82E-10
chr4	68092001	68097000	2.03E-09
chr4	68093001	68098000	2.01E-09
chr4	68094001	68099000	2.51E-11
chr4	68095001	68100000	6.07E-14
chr4	68096001	68101000	1.90E-13
chr4	68214001	68219000	1.90E-05
chr4	68215001	68220000	0.000483846
chr4	68251001	68256000	6.17E-08
chr4	68294001	68299000	3.09E-05
chr4	68295001	68300000	3.88E-05
chr4	68880001	68885000	0.025095312
chr4	69990001	69995000	7.47E-05
chr4	69991001	69996000	0.000126758
chr4	70216001	70221000	0.001908502
chr4	70217001	70222000	0.000777384
chr4	70218001	70223000	0.000509481
chr4	70219001	70224000	0.000200684
chr4	70584001	70589000	0.000894022
chr4	70596001	70601000	2.88E-11
chr4	70597001	70602000	3.46E-13
chr4	70598001	70603000	4.61E-11
chr4	70613001	70618000	1.13E-08
chr4	70626001	70631000	8.15E-15
chr4	70636001	70641000	0.000283473
chr4	71186001	71191000	0.028548906
chr4	71596001	71601000	1.87E-08
chr4	71757001	71762000	4.91E-09
chr4	71759001	71764000	4.26E-11
chr4	71989001	71994000	4.23E-05
chr4	72013001	72018000	1.16E-05
chr4	72019001	72024000	5.18E-05
chr4	72030001	72035000	1.91E-07
chr4	72047001	72052000	0.000494933
chr4	72107001	72112000	1.30E-10
chr4	72235001	72240000	6.33E-07
chr4	72236001	72241000	6.82E-10
chr4	72253001	72258000	0.003006026
chr4	72254001	72259000	0.000377243

chr4	72558001	72563000	0.001703816
chr4	72559001	72564000	3.94E-05
chr4	72560001	72565000	0.00012668
chr4	72628001	72633000	0.000646381
chr4	72764001	72769000	0.000148198
chr4	72765001	72770000	0.000148198
chr4	72808001	72813000	0.000357041
chr4	72853001	72858000	0.000735646
chr4	72854001	72859000	2.65E-06
chr4	72855001	72860000	5.36E-07
chr4	72856001	72861000	1.05E-08
chr4	72973001	72978000	0.000393425
chr4	72974001	72979000	0.000463065
chr4	72975001	72980000	0.000484769
chr4	73311001	73316000	7.79E-05
chr4	73312001	73317000	3.67E-05
chr4	73313001	73318000	6.29E-05
chr4	73314001	73319000	8.72E-05
chr4	73387001	73392000	0.002364653
chr4	73389001	73394000	0.001637255
chr4	73390001	73395000	0.000839813
chr4	73415001	73420000	0.000582783
chr4	73426001	73431000	0.0005867
chr4	73525001	73530000	0.004551444
chr4	74294001	74299000	9.66E-05
chr4	74295001	74300000	1.30E-05
chr4	74368001	74373000	0.011204262
chr4	74516001	74521000	0.01007564
chr4	74517001	74522000	0.002986281
chr4	74546001	74551000	0.000359731
chr4	74547001	74552000	0.000729
chr4	74548001	74553000	0.00047878
chr4	74557001	74562000	0.00119619
chr4	74642001	74647000	7.85E-06
chr4	74710001	74715000	0.00212167
chr4	74812001	74817000	7.90E-05
chr4	74813001	74818000	7.55E-08
chr4	75223001	75228000	3.75E-05
chr4	75529001	75534000	6.02E-05
chr4	75581001	75586000	7.99E-07
chr4	75582001	75587000	1.54E-05
chr4	75600001	75605000	5.95E-05
chr4	75601001	75606000	0.000299412
chr4	75602001	75607000	0.000552574
chr4	75609001	75614000	5.60E-06
chr4	75610001	75615000	1.10E-07
chr4	75611001	75616000	5.04E-05
chr4	76248001	76253000	0.003490958

chr4	78365001	78370000	0.001126993
chr4	78366001	78371000	0.001039083
chr4	79670001	79675000	3.31E-05
chr4	79931001	79936000	0.000207498
chr4	79949001	79954000	0.000178251
chr4	79950001	79955000	3.45E-07
chr4	79951001	79956000	4.40E-08
chr4	79952001	79957000	3.22E-07
chr4	79965001	79970000	2.00E-06
chr4	80049001	80054000	0.001123595
chr4	80210001	80215000	0.000434112
chr4	80211001	80216000	0.000171496
chr4	80212001	80217000	9.38E-05
chr4	80227001	80232000	9.86E-08
chr4	80263001	80268000	8.97E-07
chr4	80424001	80429000	0.001470722
chr4	80425001	80430000	0.001806019
chr4	80631001	80636000	0.000496826
chr4	80632001	80637000	0.001087515
chr4	80729001	80734000	0.003709206
chr4	80773001	80778000	0.001417744
chr4	80774001	80779000	0.000813952
chr4	80799001	80804000	0.000297132
chr4	81269001	81274000	4.45E-05
chr4	81671001	81676000	0.001690142
chr4	81674001	81679000	1.38E-06
chr4	81675001	81680000	1.65E-09
chr4	81815001	81820000	0.000771198
chr4	81816001	81821000	0.000785357
chr4	81817001	81822000	0.000518129
chr4	81818001	81823000	0.001824752
chr4	81819001	81824000	0.000469395
chr4	81841001	81846000	0.00488039
chr4	81842001	81847000	0.002723664
chr4	81929001	81934000	0.010980553
chr4	82299001	82304000	4.66E-06
chr4	82642001	82647000	4.03E-06
chr4	82955001	82960000	0.002057588
chr4	82984001	82989000	0.003575014
chr4	83706001	83711000	2.20E-07
chr4	84277001	84282000	2.41E-06
chr4	84562001	84567000	1.27E-07
chr4	84692001	84697000	0.000144284
chr4	84693001	84698000	1.54E-06
chr4	84719001	84724000	0.000150936
chr4	84897001	84902000	0.000193189
chr4	85374001	85379000	0.000615736
chr4	85600001	85605000	2.24E-08

chr4	85601001	85606000	7.68E-11
chr4	85602001	85607000	2.09E-10
chr4	85985001	85990000	0.007551381
chr4	86067001	86072000	3.54E-09
chr4	86068001	86073000	2.76E-09
chr4	86235001	86240000	0.000396397
chr4	87099001	87104000	0.000131847
chr4	87450001	87455000	9.86E-06
chr4	87866001	87871000	1.63E-06
chr4	87931001	87936000	2.20E-06
chr4	87932001	87937000	9.40E-05
chr4	88166001	88171000	0.000236402
chr4	88181001	88186000	5.60E-06
chr4	88184001	88189000	9.13E-07
chr4	88225001	88230000	1.73E-07
chr4	88442001	88447000	5.25E-07
chr4	88548001	88553000	4.13E-05
chr4	88549001	88554000	0.000168049
chr4	88550001	88555000	0.00252424
chr4	88849001	88854000	7.40E-05
chr4	89064001	89069000	7.26E-06
chr4	89065001	89070000	4.85E-06
chr4	89066001	89071000	3.61E-10
chr4	89067001	89072000	1.05E-08
chr4	89068001	89073000	1.92E-05
chr4	89941001	89946000	3.27E-06
chr4	89976001	89981000	0.003672739
chr4	90212001	90217000	0.000356121
chr4	90213001	90218000	0.001782421
chr4	90230001	90235000	3.23E-07
chr4	90317001	90322000	0.000940612
chr4	90363001	90368000	2.68E-06
chr4	90364001	90369000	2.23E-06
chr4	90456001	90461000	0.005137149
chr4	90505001	90510000	0.004745348
chr4	90573001	90578000	0.001488938
chr4	90574001	90579000	0.001109688
chr4	90974001	90979000	1.02E-05
chr4	90975001	90980000	1.18E-05
chr4	91010001	91015000	1.83E-05
chr4	91011001	91016000	0.004150677
chr4	91038001	91043000	0.003755644
chr4	91190001	91195000	7.92E-05
chr4	91191001	91196000	2.71E-05
chr4	91192001	91197000	0.004531005
chr4	91874001	91879000	1.32E-06
chr4	91875001	91880000	1.74E-06
chr4	91876001	91881000	3.56E-07

chr4	91878001	91883000	0.000103885
chr4	91965001	91970000	8.37E-05
chr4	92367001	92372000	2.30E-07
chr4	92370001	92375000	2.60E-09
chr4	92371001	92376000	4.52E-06
chr4	92756001	92761000	0.000242827
chr4	92759001	92764000	5.40E-06
chr4	92760001	92765000	3.08E-05
chr4	92762001	92767000	7.47E-05
chr4	92763001	92768000	6.02E-05
chr4	92797001	92802000	0.000680526
chr4	92875001	92880000	0.002249737
chr4	93469001	93474000	0.00010301
chr4	93686001	93691000	0.000630266
chr4	94012001	94017000	5.23E-05
chr4	94061001	94066000	5.57E-05
chr4	94262001	94267000	0.000378781
chr4	94324001	94329000	6.94E-06
chr4	94375001	94380000	4.23E-05
chr4	94503001	94508000	1.91E-05
chr4	94504001	94509000	2.39E-05
chr4	94681001	94686000	3.82E-07
chr4	94682001	94687000	9.56E-10
chr4	94683001	94688000	2.48E-09
chr4	94684001	94689000	0.000738989
chr4	94685001	94690000	1.02E-05
chr4	94686001	94691000	0.001392288
chr4	94765001	94770000	0.001152207
chr4	94767001	94772000	0.000580466
chr4	94768001	94773000	0.000375311
chr4	94939001	94944000	0.002948176
chr4	94940001	94945000	0.003339155
chr4	95046001	95051000	0.006766526
chr4	95642001	95647000	9.68E-07
chr4	95722001	95727000	0.00518443
chr4	95730001	95735000	3.06E-05
chr4	95767001	95772000	0.002094563
chr4	95768001	95773000	0.005333738
chr4	95769001	95774000	0.003347789
chr4	95770001	95775000	2.88E-05
chr4	95852001	95857000	1.42E-07
chr4	96271001	96276000	0.001577819
chr4	96335001	96340000	0.003744851
chr4	96336001	96341000	0.003416936
chr4	96375001	96380000	2.14E-06
chr4	96376001	96381000	4.53E-06
chr4	96449001	96454000	0.000577498
chr4	96450001	96455000	0.000274378

chr4	96674001	96679000	0.005642567
chr4	96742001	96747000	0.001004919
chr4	96743001	96748000	0.000135795
chr4	97106001	97111000	0.001907909
chr4	97226001	97231000	8.24E-07
chr4	97227001	97232000	6.40E-07
chr4	97228001	97233000	7.13E-06
chr4	97307001	97312000	0.017969248
chr4	97377001	97382000	0.000106997
chr4	97378001	97383000	8.44E-05
chr4	97627001	97632000	0.001709225
chr4	97628001	97633000	0.001698073
chr4	97629001	97634000	0.000846958
chr4	97630001	97635000	0.000787429
chr4	97631001	97636000	5.91E-05
chr4	97672001	97677000	0.001944204
chr4	97673001	97678000	0.001415954
chr4	97674001	97679000	0.000689171
chr4	97852001	97857000	4.43E-05
chr4	97944001	97949000	1.94E-06
chr4	98121001	98126000	5.43E-09
chr4	98640001	98645000	2.70E-05
chr4	98641001	98646000	1.88E-05
chr4	98642001	98647000	2.63E-07
chr4	98643001	98648000	1.41E-05
chr4	98644001	98649000	9.97E-06
chr4	98804001	98809000	0.001656987
chr4	99636001	99641000	0.000141623
chr4	99828001	99833000	7.68E-08
chr4	99829001	99834000	4.16E-09
chr4	100683001	100688000	0.000205963
chr4	100899001	100904000	0.001031144
chr4	100975001	100980000	0.000857442
chr4	101201001	101206000	0.000956171
chr4	101202001	101207000	0.00110063
chr4	101203001	101208000	0.000604922
chr4	101204001	101209000	0.003900723
chr4	101205001	101210000	0.001151258
chr4	101341001	101346000	3.38E-05
chr4	101342001	101347000	5.68E-05
chr4	101548001	101553000	3.24E-06
chr4	103058001	103063000	0.000622397
chr4	103103001	103108000	0.000950093
chr4	103391001	103396000	0.011851374
chr4	103930001	103935000	2.88E-06
chr4	104121001	104126000	0.003887343
chr4	104202001	104207000	0.000335441
chr4	104231001	104236000	0.001816945

chr4	104497001	104502000	0.00038209
chr4	104498001	104503000	0.000650126
chr4	104640001	104645000	1.78E-12
chr4	104641001	104646000	9.91E-08
chr4	104662001	104667000	2.34E-06
chr4	104663001	104668000	3.31E-06
chr4	104670001	104675000	9.41E-05
chr4	105137001	105142000	0.001731548
chr4	105656001	105661000	0.000455306
chr4	105657001	105662000	0.000927892
chr4	105672001	105677000	0.009602302
chr4	105781001	105786000	0.002078438
chr4	105982001	105987000	0.000182367
chr4	106069001	106074000	0.000484088
chr4	106264001	106269000	2.96E-06
chr4	106474001	106479000	0.00115094
chr4	106515001	106520000	0.000183674
chr4	106521001	106526000	0.005310072
chr4	106522001	106527000	0.005249269
chr4	107291001	107296000	4.64E-05
chr4	107293001	107298000	0.003589645
chr4	107294001	107299000	0.00241517
chr4	107345001	107350000	0.002066134
chr4	107358001	107363000	4.20E-05
chr4	107359001	107364000	7.98E-05
chr4	107360001	107365000	0.000204057
chr4	107426001	107431000	0.002279493
chr4	107427001	107432000	0.000616396
chr4	107506001	107511000	0.01119597
chr4	107704001	107709000	0.000597429
chr4	107839001	107844000	0.00604466
chr4	108387001	108392000	0.022421251
chr4	108807001	108812000	2.48E-06
chr4	108808001	108813000	2.48E-06
chr4	108809001	108814000	1.00E-06
chr4	109148001	109153000	4.27E-07
chr4	109177001	109182000	1.60E-06
chr4	109178001	109183000	4.81E-06
chr4	110722001	110727000	2.67E-08
chr4	111045001	111050000	0.001685488
chr4	111128001	111133000	2.82E-06
chr4	111140001	111145000	0.000471209
chr4	111141001	111146000	0.000540624
chr4	111268001	111273000	0.005310072
chr4	111276001	111281000	0.001550249
chr4	111278001	111283000	0.000332827
chr4	111279001	111284000	0.00373443
chr4	111280001	111285000	0.002672377

chr4	112187001	112192000	0.003121293
chr4	112226001	112231000	6.60E-06
chr4	112228001	112233000	4.46E-06
chr4	112229001	112234000	7.39E-07
chr4	112230001	112235000	7.29E-11
chr4	112231001	112236000	2.35E-07
chr4	112272001	112277000	0.000691632
chr4	112273001	112278000	0.002192378
chr4	112274001	112279000	0.005487109
chr4	112275001	112280000	0.024599664
chr4	112294001	112299000	0.002487809
chr4	112572001	112577000	2.65E-05
chr4	112993001	112998000	1.50E-06
chr4	113218001	113223000	0.00078008
chr4	113425001	113430000	2.41E-11
chr4	113450001	113455000	3.59E-06
chr4	113451001	113456000	1.46E-11
chr4	113452001	113457000	5.08E-05
chr4	113453001	113458000	0.000171533
chr4	113641001	113646000	3.75E-06
chr4	113672001	113677000	0.000355676
chr4	113713001	113718000	0.000214208
chr4	113808001	113813000	0.001066706
chr4	113828001	113833000	9.86E-16
chr4	113829001	113834000	3.08E-14
chr4	113830001	113835000	3.05E-12
chr4	113831001	113836000	2.10E-11
chr4	113932001	113937000	7.19E-08
chr4	114065001	114070000	2.75E-06
chr4	114066001	114071000	7.17E-07
chr4	114067001	114072000	0.00084343
chr4	114366001	114371000	3.51E-07
chr4	115687001	115692000	0.007994013
chr4	115688001	115693000	0.003441094
chr4	115903001	115908000	0.000699659
chr4	117045001	117050000	0.002453236
chr4	117140001	117145000	0.000729379
chr4	118623001	118628000	0.000722386
chr4	118897001	118902000	1.18E-05
chr4	119445001	119450000	1.71E-06
chr4	119446001	119451000	8.36E-06
chr4	119946001	119951000	3.35E-10
chr4	120202001	120207000	0.000446568
chr4	120205001	120210000	3.08E-06
chr4	120843001	120848000	6.72E-12
chr4	120844001	120849000	1.67E-11
chr4	120863001	120868000	2.60E-06
chr4	121145001	121150000	0.00273017

chr4	121351001	121356000	0.000211439
chr4	121644001	121649000	2.44E-05
chr4	121713001	121718000	0.003284253
chr4	121714001	121719000	0.000690899
chr4	121787001	121792000	0.005785157
chr4	122064001	122069000	0.000572039
chr4	122160001	122165000	0.00014377
chr4	122340001	122345000	2.78E-06
chr4	122517001	122522000	0.018344516
chr4	122518001	122523000	0.00622702
chr4	122519001	122524000	0.001984511
chr4	122571001	122576000	0.000201687
chr4	122947001	122952000	0.000775404
chr4	122973001	122978000	0.003034168
chr4	123296001	123301000	4.20E-07
chr4	123297001	123302000	1.32E-09
chr4	123851001	123856000	4.69E-05
chr4	123911001	123916000	3.64E-08
chr4	124307001	124312000	1.74E-07
chr4	124330001	124335000	1.99E-06
chr4	124331001	124336000	8.80E-07
chr4	124337001	124342000	4.97E-06
chr4	124338001	124343000	6.06E-06
chr4	124339001	124344000	3.72E-07
chr4	125087001	125092000	0.000308341
chr4	125181001	125186000	0.000496626
chr4	125182001	125187000	0.000346953
chr4	125211001	125216000	4.64E-05
chr4	125212001	125217000	4.42E-05
chr4	125292001	125297000	1.62E-06
chr4	125294001	125299000	2.31E-06
chr4	125295001	125300000	8.71E-05
chr4	125344001	125349000	0.01182388
chr4	126490001	126495000	0.00034531
chr4	126491001	126496000	1.18E-05
chr4	126492001	126497000	3.84E-05
chr4	126535001	126540000	8.64E-07
chr4	126637001	126642000	0.002102581
chr4	127200001	127205000	0.00125779
chr4	127201001	127206000	0.001945855
chr4	127202001	127207000	0.00196824
chr4	127357001	127362000	9.11E-05
chr4	127359001	127364000	4.89E-07
chr4	127360001	127365000	1.02E-05
chr4	127608001	127613000	0.015417598
chr4	127609001	127614000	0.019399886
chr4	128032001	128037000	0.003213058
chr4	128033001	128038000	0.002904239

chr4	128034001	128039000	0.002646965
chr4	128065001	128070000	0.004389317
chr4	128066001	128071000	0.005072658
chr4	128068001	128073000	0.000859794
chr4	128191001	128196000	0.000626573
chr4	128331001	128336000	7.31E-06
chr4	128439001	128444000	0.011935771
chr4	128651001	128656000	9.04E-17
chr4	129168001	129173000	0.004205072
chr4	129359001	129364000	7.92E-05
chr4	129367001	129372000	3.01E-06
chr4	129564001	129569000	9.58E-13
chr4	129565001	129570000	2.02E-06
chr4	129566001	129571000	7.59E-07
chr4	129596001	129601000	1.97E-05
chr4	129597001	129602000	8.20E-06
chr4	129673001	129678000	4.45E-08
chr4	129674001	129679000	4.76E-11
chr4	129698001	129703000	1.01E-05
chr4	130077001	130082000	0.006036239
chr4	130110001	130115000	0.000319513
chr4	130123001	130128000	0.000375029
chr4	130185001	130190000	0.000107167
chr4	130335001	130340000	1.04E-07
chr4	130387001	130392000	0.003770869
chr4	130706001	130711000	0.001335079
chr4	130708001	130713000	0.001459725
chr4	130812001	130817000	0.000143441
chr4	131057001	131062000	0.000686959
chr4	131228001	131233000	0.001469233
chr4	131494001	131499000	0.002207871
chr4	131495001	131500000	0.002699845
chr4	131631001	131636000	0.001614916
chr4	132241001	132246000	1.89E-05
chr4	132242001	132247000	5.77E-05
chr4	132726001	132731000	8.62E-15
chr4	132727001	132732000	1.41E-15
chr4	132728001	132733000	1.06E-08
chr4	132729001	132734000	4.06E-05
chr4	132730001	132735000	1.89E-09
chr4	133030001	133035000	0.008635087
chr4	133031001	133036000	0.003948237
chr4	133631001	133636000	2.62E-05
chr4	133632001	133637000	8.75E-06
chr4	133633001	133638000	6.66E-06
chr4	133634001	133639000	7.08E-05
chr4	133829001	133834000	1.77E-05
chr4	133830001	133835000	7.52E-06

chr4	133831001	133836000	1.22E-05
chr4	133832001	133837000	1.58E-05
chr4	133843001	133848000	0.0004313
chr4	133844001	133849000	0.000733441
chr4	133860001	133865000	0.000466202
chr4	134806001	134811000	1.46E-05
chr4	134807001	134812000	2.13E-07
chr4	134808001	134813000	1.69E-07
chr4	134809001	134814000	1.67E-07
chr4	135086001	135091000	1.90E-06
chr4	135087001	135092000	0.000409282
chr4	135687001	135692000	0.004056664
chr4	135688001	135693000	0.001232026
chr4	135689001	135694000	0.001984511
chr4	136071001	136076000	9.53E-05
chr4	136112001	136117000	0.001009084
chr4	136113001	136118000	0.000273112
chr4	136114001	136119000	0.000569996
chr4	136448001	136453000	0.00366195
chr4	136558001	136563000	0.000107216
chr4	136872001	136877000	0.003814362
chr4	137264001	137269000	0.002256023
chr4	137325001	137330000	0.000584703
chr4	137651001	137656000	3.01E-06
chr4	137652001	137657000	4.61E-09
chr4	139007001	139012000	2.50E-21
chr4	139008001	139013000	3.05E-27
chr4	139009001	139014000	1.07E-19
chr4	139010001	139015000	2.49E-21
chr4	139270001	139275000	5.71E-05
chr4	139457001	139462000	0.000792638
chr4	140117001	140122000	2.56E-05
chr4	140586001	140591000	8.01E-14
chr4	140587001	140592000	3.36E-13
chr4	141279001	141284000	1.21E-09
chr4	141280001	141285000	2.91E-09
chr4	141281001	141286000	2.33E-09
chr4	141887001	141892000	2.32E-06
chr4	141888001	141893000	1.38E-05
chr4	141889001	141894000	0.001524655
chr4	142077001	142082000	0.000202539
chr4	142355001	142360000	0.002168058
chr4	142356001	142361000	0.002131485
chr4	142387001	142392000	0.000126063
chr4	142388001	142393000	2.77E-07
chr4	142768001	142773000	0.000591726
chr4	142770001	142775000	8.93E-08
chr4	142817001	142822000	3.27E-07

chr4	142913001	142918000	1.23E-08
chr4	142914001	142919000	0.000965654
chr4	142915001	142920000	0.000307712
chr4	142916001	142921000	0.000633006
chr4	143045001	143050000	9.16E-06
chr4	143047001	143052000	6.35E-05
chr4	143048001	143053000	0.000293922
chr4	143401001	143406000	0.00468631
chr4	143402001	143407000	0.006510181
chr4	143529001	143534000	0.000301354
chr4	143578001	143583000	3.27E-07
chr4	143579001	143584000	2.05E-08
chr4	143581001	143586000	9.13E-09
chr4	143630001	143635000	0.002111968
chr4	143631001	143636000	0.001452007
chr4	143649001	143654000	0.000127171
chr4	143650001	143655000	5.75E-05
chr4	143814001	143819000	7.17E-06
chr4	144004001	144009000	0.000544821
chr4	144263001	144268000	3.44E-07
chr4	144301001	144306000	1.58E-07
chr4	144302001	144307000	7.18E-06
chr4	144449001	144454000	2.04E-08
chr4	144696001	144701000	0.006768536
chr4	144697001	144702000	0.005707275
chr4	144698001	144703000	0.006787628
chr4	145092001	145097000	1.57E-06
chr4	145093001	145098000	1.28E-06
chr4	145284001	145289000	0.004138331
chr4	145517001	145522000	0.000219111
chr4	145518001	145523000	4.12E-05
chr4	145519001	145524000	0.000249583
chr4	145549001	145554000	1.04E-05
chr4	145579001	145584000	2.48E-08
chr4	146875001	146880000	1.15E-05
chr4	146876001	146881000	1.55E-06
chr4	147162001	147167000	1.13E-17
chr4	147163001	147168000	8.64E-16
chr4	147986001	147991000	0.003213986
chr4	147987001	147992000	0.004338137
chr4	148366001	148371000	0.005223155
chr4	148435001	148440000	5.00E-09
chr4	149310001	149315000	5.50E-06
chr4	149311001	149316000	5.08E-06
chr4	149525001	149530000	0.000249092
chr4	149751001	149756000	1.93E-06
chr4	150065001	150070000	0.000722798
chr4	150330001	150335000	0.000223067

chr4	150727001	150732000	1.78E-08
chr4	150728001	150733000	6.29E-06
chr4	150729001	150734000	3.53E-06
chr4	150809001	150814000	0.00085221
chr4	151055001	151060000	6.50E-05
chr4	151064001	151069000	0.00126367
chr4	151082001	151087000	0.00011488
chr4	151083001	151088000	1.03E-06
chr4	151084001	151089000	4.60E-06
chr4	151099001	151104000	3.99E-05
chr4	151100001	151105000	3.91E-06
chr4	151101001	151106000	6.69E-05
chr4	151114001	151119000	1.58E-05
chr4	151154001	151159000	7.61E-11
chr4	151161001	151166000	9.62E-06
chr4	152113001	152118000	0.000323701
chr4	152114001	152119000	4.96E-05
chr4	152723001	152728000	2.20E-06
chr4	152969001	152974000	1.31E-07
chr4	152970001	152975000	7.43E-06
chr4	152971001	152976000	1.46E-05
chr4	153431001	153436000	1.47E-08
chr4	154993001	154998000	9.56E-05
chr4	155390001	155395000	4.98E-05
chr4	155477001	155482000	0.000561567
chr4	155493001	155498000	0.007248997
chr4	155555001	155560000	1.33E-05
chr4	155556001	155561000	2.72E-08
chr4	155557001	155562000	1.13E-07
chr4	155558001	155563000	5.54E-09
chr4	155559001	155564000	1.60E-07
chr4	155560001	155565000	1.90E-06
chr4	155561001	155566000	0.000298079
chr4	155805001	155810000	0.025825642
chr4	155824001	155829000	3.70E-05
chr4	155877001	155882000	0.017677976
chr4	155878001	155883000	0.018697015
chr4	155915001	155920000	0.001268483
chr4	156321001	156326000	5.67E-06
chr4	156411001	156416000	8.44E-06
chr4	156412001	156417000	4.11E-08
chr4	156907001	156912000	1.89E-10
chr4	156908001	156913000	1.18E-10
chr4	156909001	156914000	7.96E-10
chr4	157303001	157308000	5.54E-05
chr4	157420001	157425000	0.000570673
chr4	157439001	157444000	0.002835571
chr4	157490001	157495000	9.90E-08

chr4	157491001	157496000	7.78E-07
chr4	157564001	157569000	1.93E-06
chr4	157911001	157916000	3.06E-05
chr4	157912001	157917000	0.00018865
chr4	158348001	158353000	0.000106443
chr4	158365001	158370000	6.61E-06
chr4	158378001	158383000	1.35E-06
chr4	158379001	158384000	1.90E-06
chr4	158380001	158385000	8.88E-07
chr4	158556001	158561000	2.36E-07
chr4	158557001	158562000	3.78E-09
chr4	158558001	158563000	1.32E-08
chr4	158559001	158564000	4.39E-06
chr4	158570001	158575000	0.002315677
chr4	158571001	158576000	0.003210551
chr4	159200001	159205000	0.00582232
chr4	159475001	159480000	0.000770416
chr4	159476001	159481000	0.007216433
chr4	159691001	159696000	0.001571862
chr4	159738001	159743000	4.95E-05
chr4	159922001	159927000	0.000825757
chr4	160369001	160374000	8.20E-07
chr4	160538001	160543000	0.000267429
chr4	160942001	160947000	6.28E-05
chr4	161255001	161260000	0.003365033
chr4	161256001	161261000	9.88E-08
chr4	161297001	161302000	1.93E-08
chr4	161330001	161335000	5.03E-05
chr4	161380001	161385000	1.08E-07
chr4	161381001	161386000	1.56E-06
chr4	161382001	161387000	3.83E-05
chr4	161593001	161598000	2.12E-05
chr4	161619001	161624000	2.03E-05
chr4	161620001	161625000	5.87E-05
chr4	161637001	161642000	9.86E-10
chr4	161638001	161643000	9.78E-11
chr4	161639001	161644000	4.33E-08
chr4	161640001	161645000	8.71E-08
chr4	161690001	161695000	0.000212474
chr4	161715001	161720000	0.001440628
chr4	161716001	161721000	4.54E-05
chr4	161717001	161722000	1.02E-05
chr4	162164001	162169000	1.21E-07
chr4	162165001	162170000	2.11E-06
chr4	162293001	162298000	9.39E-05
chr4	162554001	162559000	1.69E-05
chr4	162661001	162666000	0.000585856
chr4	162790001	162795000	1.11E-05

chr4	162911001	162916000	0.001518819
chr4	162912001	162917000	0.000157991
chr4	163484001	163489000	0.006140214
chr4	163555001	163560000	1.33E-05
chr4	163595001	163600000	0.001627819
chr4	163809001	163814000	0.002895008
chr4	163813001	163818000	0.000988492
chr4	163988001	163993000	3.16E-05
chr4	164222001	164227000	1.92E-08
chr4	164223001	164228000	1.41E-08
chr4	164296001	164301000	0.00379602
chr4	164586001	164591000	0.004771385
chr4	165252001	165257000	0.000997145
chr4	165253001	165258000	0.000823358
chr4	165666001	165671000	0.004769064
chr4	165690001	165695000	1.47E-06
chr4	165691001	165696000	6.03E-05
chr4	166040001	166045000	0.002761945
chr4	166351001	166356000	9.40E-06
chr4	166720001	166725000	8.41E-11
chr4	166826001	166831000	0.000166682
chr4	166883001	166888000	0.000252968
chr4	166980001	166985000	0.000112408
chr4	167095001	167100000	0.003658108
chr4	167179001	167184000	0.013161768
chr4	167301001	167306000	0.000830819
chr4	167341001	167346000	0.000177196
chr4	167370001	167375000	5.49E-05
chr4	167478001	167483000	1.56E-05
chr4	167480001	167485000	5.31E-06
chr4	167606001	167611000	0.002480749
chr4	167648001	167653000	1.20E-05
chr4	167649001	167654000	7.59E-06
chr4	167844001	167849000	0.015470275
chr4	167888001	167893000	0.004491784
chr4	167889001	167894000	0.007067596
chr4	167915001	167920000	0.000849985
chr4	167916001	167921000	0.000881074
chr4	167917001	167922000	0.00100419
chr4	168158001	168163000	0.00160615
chr4	168219001	168224000	1.90E-05
chr4	168410001	168415000	0.001826266
chr4	168411001	168416000	1.89E-05
chr4	168542001	168547000	0.003544444
chr4	168555001	168560000	0.008598256
chr4	168557001	168562000	9.61E-08
chr4	168558001	168563000	1.11E-08
chr4	168559001	168564000	0.000132115

chr4	168753001	168758000	0.000129332
chr4	168754001	168759000	0.000169306
chr4	168761001	168766000	0.000367894
chr4	168881001	168886000	0.003903024
chr4	168882001	168887000	0.000858099
chr4	168884001	168889000	0.007633444
chr4	168935001	168940000	0.000219767
chr4	168936001	168941000	0.000204357
chr4	168938001	168943000	5.53E-05
chr4	168939001	168944000	4.79E-05
chr4	168943001	168948000	1.53E-06
chr4	169044001	169049000	0.001869014
chr4	169071001	169076000	3.52E-09
chr4	169072001	169077000	6.08E-10
chr4	169073001	169078000	2.90E-12
chr4	169074001	169079000	4.88E-13
chr4	169403001	169408000	0.002210956
chr4	169434001	169439000	2.28E-05
chr4	169470001	169475000	1.16E-07
chr4	169491001	169496000	1.63E-07
chr4	169524001	169529000	2.70E-09
chr4	170052001	170057000	1.90E-07
chr4	170053001	170058000	1.78E-06
chr4	170100001	170105000	6.33E-10
chr4	170101001	170106000	3.01E-10
chr4	170170001	170175000	1.74E-07
chr4	170272001	170277000	7.25E-05
chr4	170534001	170539000	9.24E-05
chr4	171251001	171256000	8.74E-09
chr4	171315001	171320000	0.002254934
chr4	171316001	171321000	0.000915092
chr4	171318001	171323000	0.006541948
chr4	171765001	171770000	0.00026133
chr4	171767001	171772000	4.96E-05
chr4	171770001	171775000	0.014096817
chr4	171771001	171776000	0.011175962
chr4	171804001	171809000	3.15E-06
chr4	171821001	171826000	5.26E-06
chr4	171822001	171827000	4.87E-07
chr4	171823001	171828000	1.60E-07
chr4	171824001	171829000	1.44E-09
chr4	171825001	171830000	2.19E-05
chr4	171826001	171831000	0.000572992
chr4	172135001	172140000	0.00214598
chr4	172136001	172141000	0.002060533
chr4	172137001	172142000	0.000746728
chr4	172138001	172143000	0.00079914
chr4	172358001	172363000	0.004000599

chr4	172359001	172364000	0.001078593
chr4	172360001	172365000	1.86E-12
chr4	172361001	172366000	1.38E-12
chr4	172363001	172368000	1.50E-09
chr4	172364001	172369000	6.77E-07
chr4	172385001	172390000	0.000224259
chr4	172552001	172557000	0.000247987
chr4	172989001	172994000	0.001514835
chr4	172992001	172997000	4.69E-06
chr4	173092001	173097000	1.11E-08
chr4	173161001	173166000	0.007828482
chr4	173188001	173193000	0.00408116
chr4	173189001	173194000	0.004281279
chr4	173190001	173195000	0.029413203
chr4	173261001	173266000	0.008188387
chr4	173262001	173267000	0.004289628
chr4	173391001	173396000	0.000264748
chr4	173483001	173488000	4.33E-07
chr4	173598001	173603000	0.005980036
chr4	173599001	173604000	0.007974867
chr4	173695001	173700000	0.000582181
chr4	174569001	174574000	2.98E-09
chr4	174570001	174575000	1.21E-07
chr4	174571001	174576000	1.12E-08
chr4	174602001	174607000	1.36E-05
chr4	174603001	174608000	2.69E-07
chr4	174604001	174609000	1.69E-06
chr4	174650001	174655000	0.000171263
chr4	174651001	174656000	0.000227076
chr4	174665001	174670000	0.029050264
chr4	174764001	174769000	0.000295289
chr4	174819001	174824000	1.82E-06
chr4	174820001	174825000	3.12E-06
chr4	174821001	174826000	2.64E-06
chr4	174822001	174827000	1.89E-06
chr4	174894001	174899000	5.45E-16
chr4	174895001	174900000	1.68E-19
chr4	174899001	174904000	0.000102034
chr4	175096001	175101000	0.000153658
chr4	175121001	175126000	0.00023243
chr4	175147001	175152000	0.001303242
chr4	175306001	175311000	0.004142631
chr4	175406001	175411000	0.000136391
chr4	175570001	175575000	0.003732618
chr4	175606001	175611000	0.000952846
chr4	175607001	175612000	0.000856599
chr4	175634001	175639000	0.000549481
chr4	175635001	175640000	0.000431637

chr4	175680001	175685000	0.000286745
chr4	175681001	175686000	2.26E-05
chr4	175752001	175757000	0.001150472
chr4	175859001	175864000	0.000686815
chr4	175860001	175865000	0.001418982
chr4	175880001	175885000	1.78E-08
chr4	175917001	175922000	0.002897378
chr4	175955001	175960000	0.019851166
chr4	176106001	176111000	0.003984278
chr4	176107001	176112000	0.001004822
chr4	176313001	176318000	0.000345164
chr4	176391001	176396000	2.97E-11
chr4	176392001	176397000	2.62E-10
chr4	176393001	176398000	1.89E-08
chr4	176502001	176507000	4.89E-05
chr4	176503001	176508000	7.68E-05
chr4	176532001	176537000	8.02E-08
chr4	176533001	176538000	1.05E-05
chr4	176578001	176583000	0.000356293
chr4	176579001	176584000	0.000256411
chr4	176580001	176585000	4.68E-05
chr4	176888001	176893000	4.63E-05
chr4	176889001	176894000	4.25E-05
chr4	176914001	176919000	8.68E-05
chr4	177111001	177116000	3.13E-06
chr4	177261001	177266000	7.04E-07
chr4	177293001	177298000	0.00237032
chr4	177310001	177315000	9.86E-05
chr4	177417001	177422000	5.65E-06
chr4	177448001	177453000	0.000133691
chr4	177449001	177454000	0.000459284
chr4	177450001	177455000	0.001808904
chr4	177451001	177456000	0.000418234
chr4	177452001	177457000	0.000131568
chr4	177454001	177459000	0.004764979
chr4	177496001	177501000	0.00029041
chr4	177497001	177502000	0.000199107
chr4	177553001	177558000	1.46E-09
chr4	177554001	177559000	6.43E-11
chr4	177555001	177560000	4.61E-10
chr4	177556001	177561000	2.91E-09
chr4	177557001	177562000	2.26E-07
chr4	177558001	177563000	4.16E-06
chr4	177721001	177726000	2.17E-07
chr4	177723001	177728000	0.002073124
chr4	177724001	177729000	0.002733772
chr4	177769001	177774000	0.003025238
chr4	177790001	177795000	9.69E-05

chr4	177791001	177796000	0.005720301
chr4	177877001	177882000	0.000376037
chr4	177912001	177917000	4.25E-05
chr4	177913001	177918000	6.12E-06
chr4	177914001	177919000	1.18E-05
chr4	177915001	177920000	1.07E-05
chr4	177930001	177935000	0.000494988
chr4	177933001	177938000	1.49E-05
chr4	177945001	177950000	5.43E-09
chr4	177946001	177951000	4.67E-10
chr4	178004001	178009000	0.000248714
chr4	178005001	178010000	0.001091138
chr4	178006001	178011000	0.000384978
chr4	178027001	178032000	0.002184476
chr4	178072001	178077000	0.001064315
chr4	178185001	178190000	0.000803492
chr4	178344001	178349000	2.96E-05
chr4	178371001	178376000	3.89E-07
chr4	178716001	178721000	6.80E-08
chr4	178784001	178789000	5.11E-05
chr4	178800001	178805000	0.001173124
chr4	178801001	178806000	6.20E-05
chr4	179039001	179044000	0.001033162
chr4	179041001	179046000	0.004522885
chr4	179042001	179047000	0.004417961
chr4	179053001	179058000	8.59E-07
chr4	179054001	179059000	4.69E-13
chr4	179078001	179083000	6.95E-05
chr4	179217001	179222000	0.000138842
chr4	179218001	179223000	9.09E-06
chr4	179382001	179387000	0.000293258
chr4	179523001	179528000	0.007142425
chr4	179896001	179901000	6.23E-08
chr4	179898001	179903000	2.74E-08
chr4	179899001	179904000	7.02E-08
chr4	179900001	179905000	6.77E-12
chr4	179918001	179923000	0.000292195
chr4	180004001	180009000	8.64E-09
chr4	180007001	180012000	3.63E-05
chr4	180077001	180082000	5.39E-09
chr4	180078001	180083000	7.06E-08
chr4	180124001	180129000	9.66E-06
chr4	180194001	180199000	0.003217113
chr4	180366001	180371000	0.000852872
chr4	180367001	180372000	0.000340568
chr4	180470001	180475000	1.32E-07
chr4	180513001	180518000	5.18E-06
chr4	180519001	180524000	9.41E-07

chr4	180520001	180525000	1.93E-06
chr4	180521001	180526000	6.66E-06
chr4	180522001	180527000	0.014546382
chr4	180660001	180665000	0.000627355
chr4	180810001	180815000	0.000815898
chr4	181147001	181152000	0.001321918
chr4	181148001	181153000	0.002174837
chr4	181267001	181272000	0.000635468
chr4	181268001	181273000	0.00036699
chr4	181269001	181274000	0.000127803
chr4	181270001	181275000	3.71E-05
chr4	181283001	181288000	3.66E-05
chr4	181348001	181353000	5.93E-09
chr4	181560001	181565000	1.96E-06
chr4	181562001	181567000	1.17E-05
chr4	181585001	181590000	2.14E-24
chr4	181586001	181591000	9.21E-18
chr4	181587001	181592000	1.70E-14
chr4	181656001	181661000	0.000786861
chr4	181657001	181662000	0.001173218
chr4	181658001	181663000	0.00321782
chr4	181666001	181671000	0.000782334
chr4	181728001	181733000	1.76E-06
chr4	181729001	181734000	7.58E-07
chr4	181730001	181735000	2.07E-07
chr4	181740001	181745000	0.001155943
chr4	181749001	181754000	0.00062292
chr4	181827001	181832000	0.001080504
chr4	182448001	182453000	0.002176598
chr4	182587001	182592000	0.000195788
chr4	182588001	182593000	0.000401008
chr4	182589001	182594000	0.000168275
chr4	182590001	182595000	0.000875294
chr4	182632001	182637000	1.52E-05
chr4	182730001	182735000	2.74E-06
chr4	182804001	182809000	3.23E-12
chr4	183268001	183273000	2.91E-11
chr4	183269001	183274000	5.69E-09
chr4	183270001	183275000	4.57E-08
chr4	183560001	183565000	9.99E-05
chr4	186438001	186443000	6.08E-05
chr4	186439001	186444000	0.000147089
chr4	186440001	186445000	0.00626838
chr4	187837001	187842000	1.08E-08
chr4	188548001	188553000	0.000911402
chr4	189121001	189126000	1.15E-07
chr4	189524001	189529000	0.000315505
chr4	190045001	190050000	0.000708243

chr4	190481001	190486000	3.48E-09
chr4	190483001	190488000	9.56E-13
chr4	190484001	190489000	7.88E-08
chr5	391001	396000	9.60E-27
chr5	392001	397000	4.22E-30
chr5	8397001	8402000	5.91E-06
chr5	8651001	8656000	0.001237359
chr5	8852001	8857000	0.000194363
chr5	8914001	8919000	2.01E-05
chr5	8916001	8921000	4.31E-05
chr5	11986001	11991000	2.76E-05
chr5	11987001	11992000	2.34E-05
chr5	11988001	11993000	6.45E-05
chr5	11989001	11994000	3.50E-05
chr5	12043001	12048000	1.66E-09
chr5	12044001	12049000	7.08E-08
chr5	12068001	12073000	4.78E-09
chr5	12069001	12074000	4.26E-05
chr5	12147001	12152000	0.000123596
chr5	12148001	12153000	1.72E-05
chr5	12948001	12953000	6.01E-07
chr5	12949001	12954000	4.03E-06
chr5	12950001	12955000	1.35E-06
chr5	12980001	12985000	7.38E-08
chr5	12982001	12987000	2.84E-06
chr5	12983001	12988000	1.41E-06
chr5	12984001	12989000	7.18E-07
chr5	13095001	13100000	0.005743906
chr5	13143001	13148000	1.64E-05
chr5	13169001	13174000	6.46E-05
chr5	13512001	13517000	0.00043192
chr5	13513001	13518000	5.01E-05
chr5	13526001	13531000	6.33E-06
chr5	13550001	13555000	5.04E-06
chr5	13551001	13556000	2.12E-06
chr5	13565001	13570000	3.08E-05
chr5	13798001	13803000	8.54E-05
chr5	14625001	14630000	5.88E-07
chr5	15319001	15324000	0.020197105
chr5	15940001	15945000	5.55E-05
chr5	17278001	17283000	1.57E-09
chr5	17279001	17284000	5.89E-10
chr5	17281001	17286000	4.22E-08
chr5	17760001	17765000	0.000313084
chr5	17761001	17766000	4.59E-05
chr5	17830001	17835000	0.002090479
chr5	17831001	17836000	1.53E-05
chr5	18003001	18008000	1.34E-06

chr5	18178001	18183000	0.001154908
chr5	18200001	18205000	4.06E-10
chr5	18411001	18416000	0.001254115
chr5	18482001	18487000	0.002146555
chr5	18499001	18504000	0.004364052
chr5	18778001	18783000	0.000152817
chr5	18808001	18813000	0.000363254
chr5	18872001	18877000	4.47E-07
chr5	18874001	18879000	8.63E-08
chr5	18932001	18937000	6.97E-06
chr5	18950001	18955000	4.94E-05
chr5	18951001	18956000	8.21E-05
chr5	19084001	19089000	0.000298923
chr5	19131001	19136000	9.42E-06
chr5	19167001	19172000	0.004690708
chr5	19380001	19385000	9.55E-06
chr5	19520001	19525000	7.67E-11
chr5	19550001	19555000	0.000382068
chr5	19602001	19607000	0.013065511
chr5	19740001	19745000	7.84E-05
chr5	19845001	19850000	0.000894288
chr5	19997001	20002000	2.80E-06
chr5	19998001	20003000	1.24E-05
chr5	20185001	20190000	1.47E-05
chr5	20500001	20505000	8.86E-08
chr5	20501001	20506000	2.60E-07
chr5	20551001	20556000	9.05E-05
chr5	20552001	20557000	2.60E-05
chr5	20564001	20569000	0.000732699
chr5	20604001	20609000	0.00142328
chr5	20606001	20611000	0.00036534
chr5	20664001	20669000	0.014856995
chr5	20758001	20763000	2.61E-08
chr5	20771001	20776000	0.001107214
chr5	21016001	21021000	1.95E-06
chr5	21371001	21376000	1.17E-05
chr5	21745001	21750000	2.68E-14
chr5	22047001	22052000	0.000397842
chr5	22048001	22053000	0.000282587
chr5	22054001	22059000	6.28E-06
chr5	22055001	22060000	1.90E-05
chr5	22056001	22061000	2.89E-06
chr5	22269001	22274000	0.000192369
chr5	22270001	22275000	0.000317812
chr5	22271001	22276000	0.000215402
chr5	22272001	22277000	2.83E-05
chr5	22317001	22322000	0.000145493
chr5	22332001	22337000	6.02E-08

chr5	22339001	22344000	0.000619175
chr5	22390001	22395000	3.83E-07
chr5	22480001	22485000	0.003719325
chr5	22481001	22486000	0.003103615
chr5	22482001	22487000	8.69E-06
chr5	22592001	22597000	5.07E-06
chr5	23327001	23332000	6.61E-05
chr5	23344001	23349000	0.00092485
chr5	23358001	23363000	1.42E-06
chr5	23396001	23401000	1.45E-07
chr5	23405001	23410000	0.000355442
chr5	23406001	23411000	0.000116383
chr5	23717001	23722000	6.90E-05
chr5	23718001	23723000	2.72E-05
chr5	23838001	23843000	0.001136638
chr5	23839001	23844000	0.001275021
chr5	23842001	23847000	0.011770506
chr5	23843001	23848000	1.60E-09
chr5	23844001	23849000	5.56E-09
chr5	23845001	23850000	1.30E-08
chr5	23846001	23851000	4.11E-08
chr5	23920001	23925000	6.00E-06
chr5	24056001	24061000	2.80E-05
chr5	24057001	24062000	3.61E-06
chr5	24063001	24068000	0.002673505
chr5	24131001	24136000	0.000222544
chr5	24132001	24137000	0.00016855
chr5	24133001	24138000	2.58E-05
chr5	24134001	24139000	1.34E-05
chr5	24295001	24300000	2.84E-08
chr5	24296001	24301000	1.17E-08
chr5	24297001	24302000	5.85E-06
chr5	24298001	24303000	2.00E-06
chr5	24328001	24333000	5.21E-05
chr5	24381001	24386000	0.001548806
chr5	24466001	24471000	3.06E-08
chr5	24481001	24486000	0.000427144
chr5	24544001	24549000	0.005285217
chr5	24545001	24550000	1.54E-06
chr5	24546001	24551000	3.86E-05
chr5	24552001	24557000	0.000289422
chr5	24557001	24562000	0.016084024
chr5	24558001	24563000	0.017438122
chr5	24559001	24564000	0.029949748
chr5	24573001	24578000	3.98E-11
chr5	24594001	24599000	0.00257095
chr5	24645001	24650000	6.78E-15
chr5	24724001	24729000	1.25E-07

chr5	24725001	24730000	8.68E-11
chr5	24742001	24747000	9.34E-08
chr5	24915001	24920000	0.001806382
chr5	24970001	24975000	6.12E-06
chr5	24971001	24976000	1.97E-06
chr5	24972001	24977000	8.26E-06
chr5	24973001	24978000	1.85E-05
chr5	25036001	25041000	8.00E-09
chr5	25037001	25042000	5.56E-09
chr5	25147001	25152000	0.000278465
chr5	25148001	25153000	0.003709972
chr5	25294001	25299000	0.000212923
chr5	25295001	25300000	2.09E-07
chr5	25339001	25344000	0.000105395
chr5	25340001	25345000	5.59E-06
chr5	25431001	25436000	4.43E-07
chr5	25432001	25437000	2.56E-07
chr5	25441001	25446000	6.51E-06
chr5	25442001	25447000	1.55E-05
chr5	25588001	25593000	2.80E-07
chr5	25589001	25594000	4.18E-06
chr5	25685001	25690000	0.014839999
chr5	25747001	25752000	0.000268407
chr5	25761001	25766000	1.06E-05
chr5	25762001	25767000	0.000500818
chr5	25948001	25953000	0.004815545
chr5	25949001	25954000	0.006894159
chr5	26019001	26024000	0.004122522
chr5	26077001	26082000	1.58E-06
chr5	26278001	26283000	1.07E-08
chr5	26280001	26285000	1.02E-05
chr5	26351001	26356000	5.58E-06
chr5	26352001	26357000	7.91E-06
chr5	26353001	26358000	8.04E-05
chr5	26420001	26425000	0.009956939
chr5	26557001	26562000	0.011266333
chr5	26596001	26601000	4.20E-05
chr5	26597001	26602000	0.000198131
chr5	26616001	26621000	2.55E-06
chr5	26617001	26622000	1.68E-06
chr5	26619001	26624000	0.007251056
chr5	26729001	26734000	8.28E-05
chr5	26940001	26945000	0.004939836
chr5	26994001	26999000	0.001123152
chr5	26996001	27001000	0.000474874
chr5	26997001	27002000	1.99E-05
chr5	26998001	27003000	5.31E-06
chr5	27001001	27006000	0.000195542

chr5	27002001	27007000	0.000337405
chr5	27003001	27008000	0.001105758
chr5	27004001	27009000	0.000994159
chr5	27005001	27010000	0.000412949
chr5	27179001	27184000	0.004062695
chr5	27180001	27185000	0.000248396
chr5	27181001	27186000	0.001327
chr5	27182001	27187000	0.003152519
chr5	27183001	27188000	0.006958226
chr5	27244001	27249000	2.38E-08
chr5	27245001	27250000	0.000223225
chr5	27278001	27283000	3.23E-11
chr5	27279001	27284000	6.33E-12
chr5	27280001	27285000	3.26E-11
chr5	27281001	27286000	2.96E-10
chr5	27282001	27287000	4.08E-09
chr5	27336001	27341000	0.02501258
chr5	27337001	27342000	0.01862061
chr5	27338001	27343000	0.012967121
chr5	27484001	27489000	7.28E-05
chr5	27485001	27490000	0.000114027
chr5	27486001	27491000	0.000178772
chr5	27504001	27509000	1.02E-07
chr5	27557001	27562000	0.001804202
chr5	27718001	27723000	0.000438749
chr5	27759001	27764000	7.93E-09
chr5	27903001	27908000	2.71E-05
chr5	28010001	28015000	7.27E-08
chr5	28011001	28016000	2.40E-08
chr5	28034001	28039000	0.00822693
chr5	28137001	28142000	1.02E-05
chr5	28234001	28239000	0.003287053
chr5	28235001	28240000	0.002115738
chr5	28824001	28829000	0.038975135
chr5	28970001	28975000	1.55E-05
chr5	28971001	28976000	1.02E-07
chr5	29151001	29156000	0.026629074
chr5	29301001	29306000	1.91E-12
chr5	29302001	29307000	9.49E-10
chr5	29303001	29308000	1.74E-10
chr5	29304001	29309000	3.79E-12
chr5	29305001	29310000	6.46E-11
chr5	29639001	29644000	2.05E-05
chr5	29655001	29660000	0.00028005
chr5	29692001	29697000	1.42E-05
chr5	29742001	29747000	0.004649797
chr5	29978001	29983000	1.23E-06
chr5	29979001	29984000	2.28E-06

chr5	29980001	29985000	0.000128096
chr5	29981001	29986000	3.03E-06
chr5	30045001	30050000	8.10E-06
chr5	30081001	30086000	5.33E-05
chr5	30083001	30088000	2.57E-05
chr5	30289001	30294000	0.005754812
chr5	30326001	30331000	0.013401886
chr5	30327001	30332000	0.028529539
chr5	30460001	30465000	0.017943392
chr5	30550001	30555000	0.003440173
chr5	30551001	30556000	0.003403863
chr5	30590001	30595000	2.92E-05
chr5	30600001	30605000	0.00105875
chr5	30601001	30606000	0.000287849
chr5	30725001	30730000	0.000785779
chr5	30726001	30731000	0.000295085
chr5	30727001	30732000	0.000363864
chr5	30728001	30733000	0.001280201
chr5	30730001	30735000	0.000793914
chr5	30789001	30794000	1.14E-05
chr5	30790001	30795000	0.000221062
chr5	30791001	30796000	5.44E-07
chr5	30792001	30797000	1.01E-05
chr5	31268001	31273000	9.79E-13
chr5	32175001	32180000	1.56E-13
chr5	32529001	32534000	3.73E-09
chr5	33061001	33066000	8.10E-09
chr5	33062001	33067000	2.50E-09
chr5	33063001	33068000	5.89E-08
chr5	33064001	33069000	3.10E-07
chr5	33132001	33137000	8.31E-05
chr5	33181001	33186000	0.000815493
chr5	33182001	33187000	9.05E-07
chr5	33183001	33188000	1.14E-05
chr5	33184001	33189000	2.80E-05
chr5	33221001	33226000	1.21E-06
chr5	33273001	33278000	0.003792778
chr5	33280001	33285000	6.25E-08
chr5	33287001	33292000	0.001852294
chr5	33288001	33293000	0.002747156
chr5	33296001	33301000	7.82E-12
chr5	33336001	33341000	2.44E-06
chr5	33397001	33402000	0.003823476
chr5	33398001	33403000	0.005346054
chr5	38659001	38664000	1.91E-17
chr5	39437001	39442000	2.49E-05
chr5	39547001	39552000	3.76E-06
chr5	39577001	39582000	7.46E-05

chr5	41345001	41350000	3.93E-06
chr5	41542001	41547000	0.001305177
chr5	42043001	42048000	3.12E-06
chr5	42069001	42074000	4.92E-05
chr5	42071001	42076000	3.15E-05
chr5	43270001	43275000	1.27E-10
chr5	43271001	43276000	8.62E-11
chr5	43511001	43516000	2.41E-28
chr5	44048001	44053000	0.009434229
chr5	44098001	44103000	0.00073014
chr5	44472001	44477000	0.00113183
chr5	44794001	44799000	0.02654662
chr5	44864001	44869000	1.88E-07
chr5	44866001	44871000	5.21E-08
chr5	44867001	44872000	4.64E-08
chr5	44868001	44873000	2.11E-08
chr5	44943001	44948000	0.007018923
chr5	45301001	45306000	0.006820593
chr5	45302001	45307000	0.006060608
chr5	45527001	45532000	0.001198902
chr5	45966001	45971000	0.000237854
chr5	49447001	49452000	0.000606532
chr5	49639001	49644000	0.002866642
chr5	50408001	50413000	6.28E-11
chr5	50409001	50414000	1.62E-10
chr5	50568001	50573000	7.00E-05
chr5	50569001	50574000	0.000304089
chr5	50609001	50614000	0.000103396
chr5	50704001	50709000	6.39E-06
chr5	50713001	50718000	0.001508372
chr5	50737001	50742000	2.36E-06
chr5	50797001	50802000	0.000790245
chr5	50798001	50803000	0.00031072
chr5	50845001	50850000	0.00064236
chr5	50846001	50851000	0.001766115
chr5	50985001	50990000	0.000463137
chr5	50987001	50992000	0.000900811
chr5	50988001	50993000	0.000774636
chr5	51039001	51044000	1.41E-05
chr5	51040001	51045000	3.71E-06
chr5	51041001	51046000	1.58E-06
chr5	51042001	51047000	5.15E-09
chr5	51340001	51345000	0.00665378
chr5	51357001	51362000	0.005371035
chr5	51358001	51363000	0.0020958
chr5	51359001	51364000	0.014028727
chr5	51430001	51435000	7.17E-07
chr5	52821001	52826000	0.011170506

chr5	53646001	53651000	0.003993803
chr5	53647001	53652000	2.98E-06
chr5	53648001	53653000	6.76E-07
chr5	54490001	54495000	0.000655706
chr5	54492001	54497000	0.000201619
chr5	54872001	54877000	4.48E-09
chr5	54893001	54898000	2.73E-10
chr5	54894001	54899000	1.30E-08
chr5	56471001	56476000	2.12E-25
chr5	57152001	57157000	6.03E-11
chr5	57153001	57158000	6.52E-11
chr5	57196001	57201000	1.03E-05
chr5	57313001	57318000	0.002126102
chr5	57373001	57378000	3.86E-09
chr5	57450001	57455000	2.26E-05
chr5	57451001	57456000	5.96E-05
chr5	57478001	57483000	1.08E-08
chr5	57479001	57484000	1.55E-06
chr5	57480001	57485000	0.008710521
chr5	57631001	57636000	0.00041641
chr5	57632001	57637000	0.000212255
chr5	57704001	57709000	6.32E-07
chr5	57705001	57710000	1.90E-08
chr5	57706001	57711000	3.43E-08
chr5	57735001	57740000	0.000103264
chr5	57860001	57865000	1.57E-19
chr5	57911001	57916000	6.50E-07
chr5	57912001	57917000	4.31E-07
chr5	58100001	58105000	6.15E-08
chr5	58101001	58106000	3.60E-09
chr5	58302001	58307000	3.46E-05
chr5	58731001	58736000	7.39E-06
chr5	59513001	59518000	0.007864378
chr5	60504001	60509000	1.99E-05
chr5	60505001	60510000	4.03E-05
chr5	60574001	60579000	2.64E-07
chr5	63927001	63932000	2.48E-06
chr5	64550001	64555000	0.000437849
chr5	64551001	64556000	0.000337074
chr5	64777001	64782000	1.04E-21
chr5	64778001	64783000	1.13E-13
chr5	65224001	65229000	4.04E-15
chr5	66175001	66180000	1.53E-05
chr5	68344001	68349000	2.32E-05
chr5	70081001	70086000	0.000547276
chr5	70623001	70628000	4.78E-05
chr5	71539001	71544000	0.013393765
chr5	72009001	72014000	6.67E-05

chr5	72525001	72530000	4.83E-19
chr5	72727001	72732000	2.61E-09
chr5	72728001	72733000	1.01E-18
chr5	72837001	72842000	1.93E-06
chr5	72957001	72962000	0.000660929
chr5	72985001	72990000	2.62E-07
chr5	73652001	73657000	1.28E-06
chr5	73653001	73658000	8.58E-06
chr5	76278001	76283000	2.27E-06
chr5	76519001	76524000	0.006743605
chr5	81395001	81400000	0.000207299
chr5	81646001	81651000	2.08E-06
chr5	82666001	82671000	0.001895176
chr5	82667001	82672000	0.005223907
chr5	82856001	82861000	3.59E-13
chr5	82857001	82862000	2.20E-07
chr5	82858001	82863000	1.18E-08
chr5	82859001	82864000	1.47E-09
chr5	82860001	82865000	6.98E-07
chr5	82958001	82963000	0.000934995
chr5	83010001	83015000	1.88E-06
chr5	83806001	83811000	0.000120403
chr5	83816001	83821000	0.00033303
chr5	83974001	83979000	0.000132694
chr5	83976001	83981000	0.002236857
chr5	84040001	84045000	0.006114926
chr5	84041001	84046000	0.0230251
chr5	84042001	84047000	0.00960655
chr5	84052001	84057000	6.02E-05
chr5	84384001	84389000	0.000653902
chr5	84385001	84390000	0.0003852
chr5	84390001	84395000	9.36E-05
chr5	84391001	84396000	5.28E-05
chr5	84392001	84397000	1.36E-05
chr5	84699001	84704000	0.003854813
chr5	84700001	84705000	0.004618913
chr5	84702001	84707000	0.001073223
chr5	84706001	84711000	2.42E-07
chr5	84886001	84891000	0.000198757
chr5	84887001	84892000	0.000389341
chr5	85479001	85484000	4.82E-05
chr5	85480001	85485000	5.91E-05
chr5	85598001	85603000	0.007030157
chr5	85774001	85779000	0.004634286
chr5	85775001	85780000	0.003067438
chr5	85779001	85784000	0.004161713
chr5	85823001	85828000	0.005196251
chr5	86205001	86210000	9.34E-06

chr5	86260001	86265000	0.000872695
chr5	86261001	86266000	0.000238434
chr5	86304001	86309000	2.87E-08
chr5	86305001	86310000	1.03E-08
chr5	86308001	86313000	4.70E-07
chr5	86309001	86314000	0.000341406
chr5	86843001	86848000	1.62E-08
chr5	86844001	86849000	2.57E-07
chr5	86885001	86890000	7.37E-05
chr5	87047001	87052000	0.000473051
chr5	87048001	87053000	8.31E-08
chr5	87814001	87819000	0.002650657
chr5	87992001	87997000	0.005191598
chr5	87993001	87998000	0.001488938
chr5	88080001	88085000	4.38E-12
chr5	88081001	88086000	4.25E-12
chr5	88990001	88995000	0.002005808
chr5	89564001	89569000	0.00320328
chr5	89842001	89847000	8.87E-08
chr5	89843001	89848000	1.95E-09
chr5	89906001	89911000	0.00017595
chr5	90168001	90173000	1.26E-07
chr5	90171001	90176000	0.026661836
chr5	90236001	90241000	0.002239769
chr5	90936001	90941000	0.00017387
chr5	91087001	91092000	0.000269416
chr5	91371001	91376000	6.98E-06
chr5	91587001	91592000	0.000160151
chr5	91588001	91593000	5.84E-05
chr5	91924001	91929000	5.33E-07
chr5	91925001	91930000	3.96E-07
chr5	91927001	91932000	3.20E-07
chr5	92690001	92695000	1.09E-05
chr5	92786001	92791000	0.004582242
chr5	93710001	93715000	1.84E-14
chr5	93711001	93716000	4.74E-14
chr5	93712001	93717000	3.98E-07
chr5	93755001	93760000	0.00108458
chr5	94070001	94075000	6.56E-06
chr5	94266001	94271000	0.021515694
chr5	95061001	95066000	0.002185496
chr5	96230001	96235000	0.00550108
chr5	96232001	96237000	0.001094437
chr5	96727001	96732000	0.002987322
chr5	96728001	96733000	0.000969184
chr5	97509001	97514000	0.002380474
chr5	97755001	97760000	0.001046616
chr5	97836001	97841000	2.29E-07

chr5	97837001	97842000	3.04E-08
chr5	97840001	97845000	9.10E-06
chr5	98031001	98036000	0.013456244
chr5	98212001	98217000	0.005726933
chr5	99314001	99319000	2.91E-08
chr5	100840001	100845000	0.000135913
chr5	101022001	101027000	0.000452596
chr5	101340001	101345000	0.00044908
chr5	101341001	101346000	0.000301963
chr5	101342001	101347000	0.000548013
chr5	101638001	101643000	3.73E-05
chr5	101639001	101644000	0.000128751
chr5	101935001	101940000	5.80E-05
chr5	102556001	102561000	1.08E-10
chr5	102557001	102562000	1.19E-11
chr5	102635001	102640000	5.43E-07
chr5	102636001	102641000	2.16E-07
chr5	102785001	102790000	8.38E-06
chr5	103313001	103318000	5.72E-07
chr5	103314001	103319000	2.98E-06
chr5	103315001	103320000	1.50E-05
chr5	103808001	103813000	5.06E-05
chr5	103809001	103814000	0.000128666
chr5	103811001	103816000	0.000545116
chr5	104201001	104206000	0.000573592
chr5	104202001	104207000	0.001592621
chr5	104417001	104422000	0.000655855
chr5	104418001	104423000	3.80E-05
chr5	104419001	104424000	3.54E-06
chr5	104485001	104490000	0.000147205
chr5	104595001	104600000	0.00712255
chr5	104596001	104601000	9.07E-06
chr5	104597001	104602000	1.30E-05
chr5	104656001	104661000	6.71E-06
chr5	105130001	105135000	5.17E-06
chr5	105131001	105136000	1.85E-05
chr5	105252001	105257000	5.63E-06
chr5	105253001	105258000	3.64E-08
chr5	105448001	105453000	0.000200785
chr5	105689001	105694000	3.18E-05
chr5	105690001	105695000	1.69E-06
chr5	105711001	105716000	0.000255194
chr5	105712001	105717000	0.000652549
chr5	105837001	105842000	0.001706662
chr5	105854001	105859000	9.94E-05
chr5	105855001	105860000	6.35E-05
chr5	106006001	106011000	0.001545141
chr5	106058001	106063000	1.52E-05

chr5	106059001	106064000	6.34E-06
chr5	106148001	106153000	0.005506012
chr5	106400001	106405000	4.67E-05
chr5	106516001	106521000	9.88E-05
chr5	106808001	106813000	5.27E-05
chr5	106809001	106814000	5.59E-06
chr5	107181001	107186000	2.06E-05
chr5	108539001	108544000	8.13E-05
chr5	108850001	108855000	6.53E-05
chr5	109241001	109246000	0.000154616
chr5	109602001	109607000	3.24E-08
chr5	110193001	110198000	0.001045341
chr5	111029001	111034000	0.001804718
chr5	111030001	111035000	0.000204827
chr5	111154001	111159000	0.000405162
chr5	111155001	111160000	2.00E-05
chr5	112289001	112294000	1.50E-05
chr5	112290001	112295000	3.01E-10
chr5	113119001	113124000	7.25E-09
chr5	113120001	113125000	2.02E-07
chr5	114796001	114801000	1.55E-05
chr5	115050001	115055000	0.002379349
chr5	115051001	115056000	5.96E-05
chr5	115470001	115475000	4.54E-08
chr5	117115001	117120000	0.000184123
chr5	117169001	117174000	8.87E-06
chr5	117710001	117715000	0.0001271
chr5	117745001	117750000	4.24E-06
chr5	118357001	118362000	0.000139663
chr5	118358001	118363000	0.000241896
chr5	119228001	119233000	3.67E-05
chr5	119229001	119234000	3.66E-05
chr5	119230001	119235000	5.79E-05
chr5	119424001	119429000	5.17E-05
chr5	119549001	119554000	6.91E-05
chr5	119550001	119555000	0.000305589
chr5	119551001	119556000	0.000650448
chr5	119627001	119632000	9.14E-05
chr5	119628001	119633000	1.05E-05
chr5	119706001	119711000	0.005834634
chr5	119726001	119731000	0.000981938
chr5	119751001	119756000	2.62E-05
chr5	119973001	119978000	0.001226238
chr5	119974001	119979000	0.000257739
chr5	120045001	120050000	0.001182472
chr5	120138001	120143000	0.016262338
chr5	120598001	120603000	1.95E-11
chr5	120599001	120604000	1.87E-10

chr5	120600001	120605000	4.79E-09
chr5	120601001	120606000	0.000866633
chr5	120666001	120671000	1.18E-05
chr5	120667001	120672000	3.86E-05
chr5	120668001	120673000	0.000229815
chr5	120669001	120674000	0.000465433
chr5	120738001	120743000	2.21E-07
chr5	120739001	120744000	0.000297592
chr5	120904001	120909000	0.001202803
chr5	121003001	121008000	4.48E-06
chr5	121078001	121083000	5.94E-08
chr5	121203001	121208000	0.001253222
chr5	121475001	121480000	0.000163713
chr5	121651001	121656000	6.15E-07
chr5	121652001	121657000	7.36E-07
chr5	122043001	122048000	0.000132377
chr5	122453001	122458000	4.24E-07
chr5	123676001	123681000	0.000813664
chr5	124431001	124436000	0.000210674
chr5	126596001	126601000	0.000431073
chr5	126597001	126602000	0.001125822
chr5	126895001	126900000	0.000206747
chr5	127421001	127426000	8.64E-07
chr5	128732001	128737000	0.000169254
chr5	130445001	130450000	8.66E-07
chr5	130613001	130618000	3.40E-06
chr5	131090001	131095000	5.05E-05
chr5	131091001	131096000	5.13E-05
chr5	131092001	131097000	0.012560852
chr5	131399001	131404000	4.34E-05
chr5	131527001	131532000	0.000334835
chr5	133430001	133435000	0.000819539
chr5	133555001	133560000	1.47E-09
chr5	133773001	133778000	0.001669219
chr5	134095001	134100000	4.40E-20
chr5	134394001	134399000	2.64E-05
chr5	134396001	134401000	0.002383077
chr5	134563001	134568000	0.002911788
chr5	135610001	135615000	4.29E-07
chr5	136202001	136207000	0.001056852
chr5	136203001	136208000	0.002108417
chr5	137442001	137447000	8.90E-07
chr5	137443001	137448000	1.63E-08
chr5	137444001	137449000	1.78E-07
chr5	137447001	137452000	9.20E-09
chr5	137599001	137604000	1.07E-05
chr5	137600001	137605000	5.92E-05
chr5	137601001	137606000	1.91E-05

chr5	138482001	138487000	6.47E-07
chr5	138632001	138637000	1.19E-07
chr5	138633001	138638000	3.58E-11
chr5	138634001	138639000	1.08E-09
chr5	138851001	138856000	5.52E-11
chr5	138852001	138857000	4.78E-11
chr5	138853001	138858000	1.55E-13
chr5	138854001	138859000	2.19E-11
chr5	138855001	138860000	2.37E-08
chr5	138943001	138948000	5.51E-23
chr5	139043001	139048000	2.06E-16
chr5	139556001	139561000	6.83E-06
chr5	140231001	140236000	1.34E-05
chr5	140257001	140262000	2.84E-13
chr5	140361001	140366000	3.28E-09
chr5	140362001	140367000	1.87E-11
chr5	140363001	140368000	1.46E-12
chr5	140364001	140369000	7.95E-10
chr5	140365001	140370000	3.13E-09
chr5	141100001	141105000	0.001333439
chr5	141217001	141222000	9.97E-05
chr5	141218001	141223000	2.20E-06
chr5	141219001	141224000	0.00035453
chr5	141696001	141701000	2.35E-07
chr5	141697001	141702000	8.15E-11
chr5	142078001	142083000	0.000352999
chr5	143047001	143052000	0.000132425
chr5	143048001	143053000	0.000676125
chr5	143328001	143333000	0.018632489
chr5	143349001	143354000	2.51E-08
chr5	143414001	143419000	0.000467941
chr5	143454001	143459000	8.57E-06
chr5	143455001	143460000	1.43E-05
chr5	143524001	143529000	0.00212764
chr5	143641001	143646000	0.004965002
chr5	143807001	143812000	0.010385873
chr5	143828001	143833000	0.000600505
chr5	143829001	143834000	0.001767282
chr5	143917001	143922000	0.000119498
chr5	143918001	143923000	1.41E-06
chr5	143919001	143924000	1.14E-07
chr5	143920001	143925000	2.40E-08
chr5	143922001	143927000	4.33E-10
chr5	143957001	143962000	1.60E-07
chr5	143979001	143984000	2.12E-08
chr5	143981001	143986000	0.001011927
chr5	144494001	144499000	0.000284535
chr5	144701001	144706000	0.00061896

chr5	145156001	145161000	1.76E-06
chr5	145578001	145583000	4.36E-13
chr5	145593001	145598000	1.24E-12
chr5	145922001	145927000	8.87E-07
chr5	145923001	145928000	3.60E-07
chr5	145924001	145929000	2.54E-06
chr5	145925001	145930000	8.83E-05
chr5	145926001	145931000	2.33E-06
chr5	146150001	146155000	0.001742117
chr5	147236001	147241000	0.009578394
chr5	147470001	147475000	0.000599973
chr5	147522001	147527000	2.19E-05
chr5	147523001	147528000	3.37E-06
chr5	147524001	147529000	9.09E-07
chr5	147525001	147530000	1.36E-05
chr5	147526001	147531000	1.57E-06
chr5	147838001	147843000	6.78E-05
chr5	148111001	148116000	3.22E-09
chr5	148112001	148117000	3.98E-10
chr5	148113001	148118000	2.49E-10
chr5	148869001	148874000	7.42E-10
chr5	148925001	148930000	3.80E-18
chr5	149497001	149502000	1.73E-08
chr5	149498001	149503000	6.13E-09
chr5	149842001	149847000	8.05E-05
chr5	150081001	150086000	4.76E-16
chr5	151671001	151676000	0.025055084
chr5	152229001	152234000	0.006677255
chr5	152231001	152236000	0.000226909
chr5	152234001	152239000	8.31E-05
chr5	152600001	152605000	2.36E-06
chr5	152601001	152606000	3.80E-07
chr5	152602001	152607000	2.85E-06
chr5	152603001	152608000	4.58E-08
chr5	152769001	152774000	6.73E-06
chr5	152770001	152775000	5.89E-06
chr5	152771001	152776000	1.08E-07
chr5	152772001	152777000	2.83E-06
chr5	153072001	153077000	5.93E-07
chr5	154041001	154046000	0.000622293
chr5	154376001	154381000	3.43E-05
chr5	155064001	155069000	0.000445079
chr5	155443001	155448000	0.000106343
chr5	155476001	155481000	3.17E-05
chr5	155735001	155740000	0.000489618
chr5	155796001	155801000	0.001978678
chr5	155808001	155813000	1.92E-05
chr5	155809001	155814000	1.64E-05

chr5	155810001	155815000	1.18E-07
chr5	155811001	155816000	1.82E-05
chr5	155843001	155848000	0.000234352
chr5	156065001	156070000	2.53E-05
chr5	156066001	156071000	3.90E-07
chr5	156067001	156072000	1.55E-07
chr5	156068001	156073000	6.50E-07
chr5	156076001	156081000	0.000402328
chr5	156104001	156109000	1.70E-07
chr5	156105001	156110000	1.54E-09
chr5	156106001	156111000	1.66E-10
chr5	156107001	156112000	3.86E-13
chr5	156108001	156113000	1.08E-10
chr5	156117001	156122000	7.34E-05
chr5	156118001	156123000	0.001190753
chr5	156119001	156124000	0.000529913
chr5	156120001	156125000	6.85E-06
chr5	156121001	156126000	0.000435996
chr5	156123001	156128000	0.000272059
chr5	156165001	156170000	2.89E-09
chr5	156166001	156171000	2.89E-09
chr5	157341001	157346000	7.21E-08
chr5	158521001	158526000	4.30E-10
chr5	158762001	158767000	7.11E-05
chr5	159572001	159577000	1.56E-05
chr5	159611001	159616000	3.80E-06
chr5	161419001	161424000	0.000164089
chr5	161520001	161525000	8.24E-08
chr5	161521001	161526000	7.68E-06
chr5	161522001	161527000	5.09E-05
chr5	161523001	161528000	4.40E-05
chr5	162108001	162113000	2.42E-09
chr5	163062001	163067000	0.00013632
chr5	163193001	163198000	8.96E-09
chr5	163477001	163482000	0.000128506
chr5	163518001	163523000	0.000578747
chr5	163562001	163567000	5.33E-06
chr5	163580001	163585000	0.00120422
chr5	163627001	163632000	0.000209788
chr5	163734001	163739000	1.37E-05
chr5	163736001	163741000	0.000302698
chr5	164038001	164043000	0.000140624
chr5	164039001	164044000	5.18E-05
chr5	164040001	164045000	1.25E-05
chr5	164041001	164046000	4.96E-05
chr5	164063001	164068000	1.36E-05
chr5	164064001	164069000	6.43E-05
chr5	164127001	164132000	0.000236411

chr5	164167001	164172000	0.01535821
chr5	164200001	164205000	1.52E-07
chr5	164201001	164206000	6.00E-10
chr5	164202001	164207000	1.32E-15
chr5	164203001	164208000	1.64E-14
chr5	164204001	164209000	4.98E-18
chr5	164205001	164210000	2.69E-08
chr5	164206001	164211000	4.96E-08
chr5	164303001	164308000	3.83E-05
chr5	164388001	164393000	0.000350442
chr5	164445001	164450000	1.89E-05
chr5	164446001	164451000	5.04E-05
chr5	164485001	164490000	4.12E-06
chr5	164486001	164491000	2.28E-06
chr5	164487001	164492000	4.96E-05
chr5	165337001	165342000	8.17E-05
chr5	165601001	165606000	0.00068244
chr5	165602001	165607000	0.000706663
chr5	165603001	165608000	0.001305958
chr5	165604001	165609000	0.002078273
chr5	165611001	165616000	6.62E-05
chr5	165615001	165620000	4.63E-07
chr5	165688001	165693000	2.41E-07
chr5	165689001	165694000	1.97E-09
chr5	165690001	165695000	5.26E-07
chr5	165953001	165958000	1.73E-06
chr5	166171001	166176000	7.05E-06
chr5	166866001	166871000	5.55E-06
chr5	167112001	167117000	3.60E-10
chr5	167113001	167118000	8.51E-09
chr5	167114001	167119000	3.63E-08
chr5	167125001	167130000	1.37E-06
chr5	167328001	167333000	4.42E-09
chr5	169123001	169128000	4.55E-10
chr5	169158001	169163000	5.13E-11
chr5	169159001	169164000	7.70E-11
chr5	169184001	169189000	8.73E-06
chr5	169185001	169190000	1.92E-06
chr5	169868001	169873000	2.94E-05
chr5	171661001	171666000	0.000136083
chr5	173745001	173750000	2.15E-08
chr5	173884001	173889000	1.28E-09
chr5	173885001	173890000	3.82E-08
chr5	173925001	173930000	2.48E-07
chr5	174238001	174243000	3.25E-06
chr5	174239001	174244000	6.55E-06
chr5	174240001	174245000	5.08E-05
chr5	175029001	175034000	8.09E-08

chr5	175632001	175637000	0.006139572
chr5	175861001	175866000	4.40E-12
chr5	175862001	175867000	5.20E-08
chr5	176523001	176528000	1.53E-09
chr5	176933001	176938000	8.12E-09
chr5	178941001	178946000	6.42E-08
chr5	179186001	179191000	1.90E-07
chr5	179187001	179192000	1.56E-07
chr5	179235001	179240000	3.54E-10
chr5	179597001	179602000	7.07E-06
chr6	3185001	3190000	1.66E-06
chr6	3913001	3918000	7.65E-13
chr6	5033001	5038000	1.62E-08
chr6	5034001	5039000	9.74E-06
chr6	7612001	7617000	8.15E-10
chr6	8288001	8293000	0.000300679
chr6	8500001	8505000	0.00293658
chr6	8501001	8506000	0.005726933
chr6	8554001	8559000	0.008582324
chr6	8555001	8560000	2.67E-05
chr6	8556001	8561000	6.93E-05
chr6	8939001	8944000	0.001240825
chr6	8940001	8945000	0.000989662
chr6	8941001	8946000	5.13E-05
chr6	8979001	8984000	1.37E-07
chr6	8980001	8985000	1.61E-07
chr6	8981001	8986000	1.76E-08
chr6	8982001	8987000	2.83E-08
chr6	8983001	8988000	2.86E-08
chr6	9083001	9088000	1.65E-06
chr6	9300001	9305000	0.000123749
chr6	9312001	9317000	7.32E-05
chr6	9313001	9318000	4.58E-05
chr6	9375001	9380000	3.50E-07
chr6	9376001	9381000	2.65E-08
chr6	9502001	9507000	0.000241865
chr6	9530001	9535000	0.002320977
chr6	9780001	9785000	0.00010038
chr6	9843001	9848000	0.000748717
chr6	9978001	9983000	1.77E-06
chr6	10005001	10010000	0.001562393
chr6	10394001	10399000	1.44E-11
chr6	10424001	10429000	1.38E-13
chr6	11095001	11100000	1.24E-09
chr6	11140001	11145000	1.50E-13
chr6	11803001	11808000	3.25E-12
chr6	12211001	12216000	1.78E-06
chr6	13648001	13653000	2.08E-05

chr6	13759001	13764000	7.57E-05
chr6	13761001	13766000	3.83E-07
chr6	13865001	13870000	1.93E-07
chr6	13866001	13871000	2.57E-08
chr6	13904001	13909000	0.000239959
chr6	13960001	13965000	4.07E-08
chr6	14072001	14077000	1.64E-07
chr6	14073001	14078000	1.22E-05
chr6	14074001	14079000	3.36E-06
chr6	14075001	14080000	3.91E-10
chr6	14840001	14845000	1.67E-11
chr6	16132001	16137000	0.000674533
chr6	16328001	16333000	3.24E-16
chr6	18322001	18327000	2.38E-07
chr6	18388001	18393000	4.14E-12
chr6	18494001	18499000	5.95E-08
chr6	18613001	18618000	1.54E-08
chr6	18614001	18619000	1.13E-07
chr6	18615001	18620000	3.75E-05
chr6	18620001	18625000	0.000508066
chr6	18621001	18626000	0.00014599
chr6	18622001	18627000	0.000254919
chr6	18770001	18775000	5.40E-07
chr6	18790001	18795000	5.16E-07
chr6	18791001	18796000	1.33E-07
chr6	18948001	18953000	0.005479825
chr6	18949001	18954000	0.002270261
chr6	18950001	18955000	0.002918347
chr6	18951001	18956000	0.000264114
chr6	19379001	19384000	8.10E-08
chr6	19449001	19454000	9.84E-06
chr6	19450001	19455000	3.98E-05
chr6	19788001	19793000	0.000400626
chr6	19923001	19928000	0.000324678
chr6	19924001	19929000	0.00018419
chr6	19925001	19930000	0.000499695
chr6	19936001	19941000	6.31E-07
chr6	19937001	19942000	4.30E-07
chr6	19962001	19967000	2.59E-07
chr6	20235001	20240000	1.76E-25
chr6	20236001	20241000	4.71E-28
chr6	20237001	20242000	1.30E-23
chr6	20238001	20243000	8.70E-20
chr6	20535001	20540000	2.22E-07
chr6	20580001	20585000	2.25E-06
chr6	20581001	20586000	5.36E-10
chr6	20825001	20830000	0.00568838
chr6	21808001	21813000	7.58E-10

chr6	21844001	21849000	9.76E-07
chr6	21911001	21916000	3.73E-06
chr6	21912001	21917000	2.39E-11
chr6	21913001	21918000	3.47E-08
chr6	22848001	22853000	0.000356551
chr6	23238001	23243000	3.88E-05
chr6	23348001	23353000	9.35E-09
chr6	23349001	23354000	3.70E-09
chr6	23383001	23388000	0.000406485
chr6	23411001	23416000	1.11E-06
chr6	23412001	23417000	1.97E-10
chr6	23413001	23418000	5.89E-07
chr6	23414001	23419000	4.22E-07
chr6	23443001	23448000	1.94E-08
chr6	23444001	23449000	5.91E-09
chr6	23508001	23513000	0.002574182
chr6	23965001	23970000	1.06E-08
chr6	23966001	23971000	1.95E-05
chr6	24001001	24006000	5.90E-06
chr6	24054001	24059000	2.12E-05
chr6	24274001	24279000	0.001607746
chr6	24659001	24664000	1.11E-09
chr6	25020001	25025000	6.58E-06
chr6	25136001	25141000	6.10E-29
chr6	25137001	25142000	4.69E-22
chr6	25138001	25143000	2.23E-17
chr6	25311001	25316000	0.000102011
chr6	25410001	25415000	4.00E-06
chr6	25411001	25416000	2.43E-05
chr6	25995001	26000000	1.54E-11
chr6	26470001	26475000	1.25E-06
chr6	26471001	26476000	3.12E-07
chr6	26472001	26477000	4.81E-07
chr6	26531001	26536000	1.34E-11
chr6	26532001	26537000	1.28E-11
chr6	27716001	27721000	1.07E-21
chr6	27717001	27722000	1.83E-36
chr6	27718001	27723000	6.25E-36
chr6	27719001	27724000	1.75E-27
chr6	27721001	27726000	1.32E-12
chr6	27828001	27833000	3.96E-24
chr6	27829001	27834000	7.08E-20
chr6	33459001	33464000	1.38E-09
chr6	33542001	33547000	4.73E-11
chr6	33653001	33658000	0.000169445
chr6	34424001	34429000	1.92E-05
chr6	34425001	34430000	0.000280143
chr6	34466001	34471000	1.00E-12

chr6	34490001	34495000	9.87E-12
chr6	34491001	34496000	4.33E-19
chr6	34492001	34497000	1.90E-21
chr6	34493001	34498000	5.25E-21
chr6	34648001	34653000	1.09E-09
chr6	34649001	34654000	1.32E-06
chr6	35997001	36002000	1.84E-14
chr6	36400001	36405000	8.81E-05
chr6	37174001	37179000	8.95E-08
chr6	37175001	37180000	2.98E-07
chr6	37176001	37181000	2.34E-09
chr6	37177001	37182000	1.06E-08
chr6	37395001	37400000	1.34E-09
chr6	37560001	37565000	6.96E-10
chr6	40449001	40454000	1.41E-07
chr6	40450001	40455000	1.09E-09
chr6	41089001	41094000	9.65E-05
chr6	41372001	41377000	1.20E-06
chr6	41880001	41885000	5.71E-07
chr6	41959001	41964000	3.20E-07
chr6	42139001	42144000	6.07E-10
chr6	42141001	42146000	1.55E-08
chr6	42744001	42749000	2.55E-21
chr6	42745001	42750000	1.88E-09
chr6	43190001	43195000	1.01E-09
chr6	43576001	43581000	3.54E-09
chr6	43664001	43669000	6.16E-08
chr6	43907001	43912000	1.06E-06
chr6	44421001	44426000	5.31E-11
chr6	44422001	44427000	7.44E-11
chr6	46241001	46246000	5.18E-05
chr6	46811001	46816000	0.000349494
chr6	46891001	46896000	2.26E-11
chr6	47009001	47014000	3.32E-06
chr6	47378001	47383000	0.000645703
chr6	47379001	47384000	3.08E-06
chr6	47380001	47385000	0.000383334
chr6	47414001	47419000	0.000449399
chr6	47415001	47420000	1.61E-06
chr6	47417001	47422000	0.000552767
chr6	47793001	47798000	0.000508066
chr6	47794001	47799000	0.001063193
chr6	47795001	47800000	0.00712524
chr6	47800001	47805000	0.000125555
chr6	47857001	47862000	5.13E-06
chr6	47858001	47863000	1.77E-07
chr6	47894001	47899000	2.38E-06
chr6	47900001	47905000	0.008410485

chr6	47959001	47964000	1.30E-05
chr6	47960001	47965000	1.53E-05
chr6	48048001	48053000	3.97E-07
chr6	48050001	48055000	8.64E-06
chr6	48051001	48056000	1.81E-06
chr6	48052001	48057000	3.87E-07
chr6	48053001	48058000	0.000170399
chr6	48054001	48059000	4.17E-05
chr6	48055001	48060000	1.08E-06
chr6	48057001	48062000	2.90E-05
chr6	48104001	48109000	6.24E-13
chr6	48105001	48110000	1.75E-12
chr6	48106001	48111000	3.68E-07
chr6	48107001	48112000	5.25E-08
chr6	48108001	48113000	4.39E-09
chr6	48145001	48150000	3.37E-06
chr6	48146001	48151000	7.70E-06
chr6	48148001	48153000	0.000244417
chr6	48269001	48274000	0.007671502
chr6	48317001	48322000	0.000363417
chr6	48319001	48324000	0.000142351
chr6	48385001	48390000	3.91E-07
chr6	48386001	48391000	3.31E-09
chr6	48394001	48399000	0.001138351
chr6	48395001	48400000	0.000132377
chr6	48454001	48459000	0.000504526
chr6	48530001	48535000	1.05E-05
chr6	48545001	48550000	0.000101738
chr6	48561001	48566000	0.001042671
chr6	48685001	48690000	0.000361326
chr6	48686001	48691000	4.03E-05
chr6	48687001	48692000	2.32E-05
chr6	48688001	48693000	8.95E-05
chr6	48710001	48715000	0.017811141
chr6	48711001	48716000	0.020810294
chr6	48728001	48733000	0.006656287
chr6	48742001	48747000	0.000121753
chr6	48743001	48748000	0.000436734
chr6	48765001	48770000	2.17E-10
chr6	48803001	48808000	0.000182838
chr6	48818001	48823000	0.005565195
chr6	48826001	48831000	1.48E-05
chr6	48862001	48867000	0.018877102
chr6	48901001	48906000	0.000202775
chr6	48902001	48907000	0.000113721
chr6	48960001	48965000	0.000249727
chr6	48987001	48992000	0.002461161
chr6	49199001	49204000	0.000231306

chr6	49223001	49228000	9.26E-08
chr6	49224001	49229000	5.80E-07
chr6	49278001	49283000	0.000707125
chr6	49321001	49326000	5.64E-05
chr6	49499001	49504000	5.46E-06
chr6	49574001	49579000	0.00051608
chr6	49654001	49659000	2.45E-05
chr6	49655001	49660000	2.14E-05
chr6	49834001	49839000	0.000178334
chr6	50301001	50306000	4.48E-07
chr6	50619001	50624000	6.50E-05
chr6	50621001	50626000	3.11E-06
chr6	50622001	50627000	0.000199722
chr6	50713001	50718000	0.000324707
chr6	50714001	50719000	0.004821647
chr6	50779001	50784000	8.23E-10
chr6	50962001	50967000	0.000162973
chr6	51007001	51012000	0.000467632
chr6	51112001	51117000	4.86E-10
chr6	51146001	51151000	9.08E-06
chr6	51318001	51323000	2.88E-06
chr6	51666001	51671000	0.003863363
chr6	51667001	51672000	0.001915406
chr6	51842001	51847000	1.08E-08
chr6	51843001	51848000	4.22E-09
chr6	52484001	52489000	0.000138714
chr6	53288001	53293000	4.03E-11
chr6	53510001	53515000	0.00011365
chr6	53791001	53796000	2.56E-07
chr6	54047001	54052000	0.001532906
chr6	54048001	54053000	0.000785581
chr6	54072001	54077000	0.00028966
chr6	54073001	54078000	0.000190685
chr6	54074001	54079000	0.000153152
chr6	54279001	54284000	0.000958906
chr6	54280001	54285000	0.003587084
chr6	54282001	54287000	0.002040081
chr6	54283001	54288000	0.001213958
chr6	54289001	54294000	0.001381613
chr6	54332001	54337000	0.000204815
chr6	54334001	54339000	0.000184123
chr6	54380001	54385000	1.68E-07
chr6	54561001	54566000	0.000382639
chr6	54562001	54567000	0.001369951
chr6	54591001	54596000	0.001305188
chr6	54592001	54597000	5.99E-05
chr6	54593001	54598000	7.98E-05
chr6	54594001	54599000	0.000942996

chr6	54925001	54930000	2.61E-08
chr6	54997001	55002000	0.004538402
chr6	55162001	55167000	0.000793674
chr6	55163001	55168000	0.000942594
chr6	55181001	55186000	0.002714114
chr6	55210001	55215000	1.49E-08
chr6	55234001	55239000	2.48E-09
chr6	55272001	55277000	4.10E-07
chr6	55302001	55307000	0.001033692
chr6	55380001	55385000	0.021755363
chr6	55386001	55391000	0.007133353
chr6	55484001	55489000	0.002348695
chr6	55492001	55497000	0.00071502
chr6	55493001	55498000	0.000605678
chr6	55530001	55535000	0.004303122
chr6	55531001	55536000	0.00588544
chr6	55532001	55537000	0.011244406
chr6	55534001	55539000	0.000876578
chr6	55543001	55548000	7.01E-06
chr6	55544001	55549000	8.57E-07
chr6	55610001	55615000	0.000274517
chr6	55735001	55740000	1.28E-07
chr6	55736001	55741000	3.62E-10
chr6	55737001	55742000	4.77E-10
chr6	55738001	55743000	2.89E-08
chr6	55739001	55744000	8.04E-06
chr6	55775001	55780000	5.60E-18
chr6	55820001	55825000	2.48E-05
chr6	55843001	55848000	0.000239061
chr6	55860001	55865000	2.31E-07
chr6	55912001	55917000	0.003999641
chr6	55985001	55990000	0.001219201
chr6	56233001	56238000	1.68E-06
chr6	56500001	56505000	8.09E-13
chr6	56501001	56506000	2.35E-18
chr6	56502001	56507000	1.47E-05
chr6	56823001	56828000	0.000345729
chr6	58341001	58346000	3.03E-05
chr6	62613001	62618000	1.38E-05
chr6	63161001	63166000	0.004320144
chr6	63164001	63169000	0.001962484
chr6	63869001	63874000	1.88E-08
chr6	64320001	64325000	8.14E-09
chr6	64322001	64327000	2.86E-07
chr6	64450001	64455000	0.000150776
chr6	64472001	64477000	0.000460102
chr6	64574001	64579000	8.09E-05
chr6	64632001	64637000	4.37E-19

chr6	64633001	64638000	1.44E-19
chr6	64704001	64709000	0.00023994
chr6	64781001	64786000	0.005884558
chr6	64782001	64787000	0.01162666
chr6	64830001	64835000	0.001360975
chr6	64873001	64878000	4.84E-09
chr6	64888001	64893000	2.47E-05
chr6	64889001	64894000	0.00018694
chr6	64890001	64895000	0.000228433
chr6	64927001	64932000	2.29E-06
chr6	64928001	64933000	3.61E-06
chr6	64929001	64934000	0.001323043
chr6	64953001	64958000	9.04E-07
chr6	64954001	64959000	2.34E-06
chr6	64990001	64995000	0.000125755
chr6	65110001	65115000	0.000778176
chr6	65111001	65116000	8.85E-06
chr6	65310001	65315000	0.002497216
chr6	65381001	65386000	0.000171779
chr6	65415001	65420000	1.52E-07
chr6	65416001	65421000	2.92E-07
chr6	65566001	65571000	2.78E-06
chr6	65642001	65647000	0.002325717
chr6	65674001	65679000	6.74E-05
chr6	65675001	65680000	3.03E-05
chr6	65726001	65731000	5.78E-13
chr6	65727001	65732000	1.41E-08
chr6	65728001	65733000	1.82E-06
chr6	65729001	65734000	3.12E-08
chr6	65730001	65735000	4.93E-08
chr6	65908001	65913000	5.38E-09
chr6	65909001	65914000	4.44E-08
chr6	65929001	65934000	4.22E-06
chr6	65930001	65935000	5.46E-07
chr6	65965001	65970000	0.000977504
chr6	66015001	66020000	3.09E-07
chr6	66030001	66035000	4.95E-09
chr6	66031001	66036000	8.84E-11
chr6	66032001	66037000	1.04E-11
chr6	66139001	66144000	1.96E-05
chr6	66140001	66145000	1.51E-05
chr6	66276001	66281000	1.62E-07
chr6	66341001	66346000	8.27E-05
chr6	66342001	66347000	0.003168949
chr6	66483001	66488000	2.11E-10
chr6	66485001	66490000	2.57E-07
chr6	66770001	66775000	2.33E-06
chr6	66771001	66776000	2.01E-05

chr6	66837001	66842000	7.63E-06
chr6	66850001	66855000	0.00027791
chr6	66915001	66920000	0.002159415
chr6	66916001	66921000	3.32E-07
chr6	66917001	66922000	5.59E-06
chr6	66968001	66973000	4.02E-10
chr6	66969001	66974000	9.10E-10
chr6	66975001	66980000	0.001839311
chr6	66976001	66981000	0.002278404
chr6	66998001	67003000	0.000208774
chr6	66999001	67004000	0.000227481
chr6	67050001	67055000	0.002005569
chr6	67271001	67276000	0.023162238
chr6	67274001	67279000	0.013054031
chr6	67304001	67309000	0.004165745
chr6	67450001	67455000	0.016076629
chr6	67547001	67552000	0.005575943
chr6	67607001	67612000	0.000979192
chr6	67629001	67634000	0.004834391
chr6	67632001	67637000	0.000107385
chr6	67633001	67638000	5.35E-05
chr6	67635001	67640000	1.32E-06
chr6	67636001	67641000	3.35E-06
chr6	67639001	67644000	0.00036775
chr6	67734001	67739000	0.002050135
chr6	67735001	67740000	0.001356081
chr6	67773001	67778000	1.62E-15
chr6	67774001	67779000	1.17E-14
chr6	67851001	67856000	9.47E-05
chr6	67852001	67857000	0.002451295
chr6	67964001	67969000	0.000190896
chr6	68367001	68372000	0.000188813
chr6	68821001	68826000	0.000316406
chr6	68822001	68827000	0.000142776
chr6	68858001	68863000	0.00827564
chr6	69387001	69392000	2.69E-05
chr6	69502001	69507000	0.000472176
chr6	69503001	69508000	0.000111256
chr6	69504001	69509000	2.64E-11
chr6	69505001	69510000	3.62E-09
chr6	69506001	69511000	1.82E-07
chr6	69556001	69561000	0.002843124
chr6	69704001	69709000	0.000233705
chr6	69713001	69718000	2.12E-06
chr6	69714001	69719000	5.05E-09
chr6	69715001	69720000	2.38E-08
chr6	69716001	69721000	2.09E-08
chr6	69717001	69722000	9.43E-08

chr6	69718001	69723000	2.00E-05
chr6	69750001	69755000	5.38E-08
chr6	69870001	69875000	0.007112574
chr6	69955001	69960000	4.74E-05
chr6	69973001	69978000	0.008303844
chr6	70027001	70032000	1.12E-05
chr6	70196001	70201000	0.000282655
chr6	70197001	70202000	0.002094563
chr6	70198001	70203000	0.000553219
chr6	71392001	71397000	4.65E-13
chr6	71393001	71398000	2.47E-05
chr6	71701001	71706000	0.001446194
chr6	72117001	72122000	6.44E-05
chr6	72118001	72123000	2.58E-05
chr6	72119001	72124000	3.72E-05
chr6	72250001	72255000	1.51E-05
chr6	72251001	72256000	9.42E-06
chr6	72283001	72288000	0.008675465
chr6	74224001	74229000	1.92E-11
chr6	74286001	74291000	6.30E-10
chr6	74288001	74293000	1.16E-10
chr6	74289001	74294000	1.05E-11
chr6	74290001	74295000	8.18E-12
chr6	75940001	75945000	3.43E-05
chr6	76202001	76207000	6.75E-08
chr6	76674001	76679000	2.36E-07
chr6	76675001	76680000	1.42E-06
chr6	76767001	76772000	0.001013242
chr6	76768001	76773000	7.56E-06
chr6	76769001	76774000	0.000137433
chr6	76977001	76982000	0.000698564
chr6	76978001	76983000	0.000783687
chr6	76979001	76984000	0.0006923
chr6	77402001	77407000	6.31E-05
chr6	77403001	77408000	2.56E-05
chr6	77404001	77409000	0.000149423
chr6	77430001	77435000	0.00053941
chr6	77942001	77947000	6.69E-06
chr6	77943001	77948000	1.10E-08
chr6	77944001	77949000	7.64E-08
chr6	77945001	77950000	1.17E-07
chr6	77946001	77951000	1.13E-06
chr6	78292001	78297000	0.002000182
chr6	78307001	78312000	0.000195043
chr6	78335001	78340000	5.33E-06
chr6	78552001	78557000	0.003586103
chr6	78783001	78788000	0.00525073
chr6	78784001	78789000	0.002537131

chr6	78785001	78790000	0.003379279
chr6	78926001	78931000	7.17E-05
chr6	78946001	78951000	9.03E-05
chr6	78947001	78952000	4.81E-06
chr6	78948001	78953000	0.000278018
chr6	78955001	78960000	4.20E-06
chr6	78977001	78982000	0.000121115
chr6	78978001	78983000	9.14E-05
chr6	79003001	79008000	0.000137233
chr6	79004001	79009000	7.27E-05
chr6	79020001	79025000	0.002496187
chr6	79328001	79333000	0.003980762
chr6	79813001	79818000	0.001253958
chr6	80294001	80299000	3.21E-06
chr6	80342001	80347000	0.000194788
chr6	80457001	80462000	0.000173386
chr6	80458001	80463000	0.000520217
chr6	80521001	80526000	1.85E-08
chr6	80620001	80625000	0.00033596
chr6	80621001	80626000	0.002003119
chr6	81042001	81047000	0.008473074
chr6	81167001	81172000	0.003650541
chr6	81233001	81238000	1.52E-07
chr6	81448001	81453000	8.63E-05
chr6	81449001	81454000	9.48E-05
chr6	81474001	81479000	0.000689171
chr6	81475001	81480000	0.001370299
chr6	81578001	81583000	0.000924988
chr6	81579001	81584000	0.000349896
chr6	81580001	81585000	1.71E-05
chr6	82164001	82169000	4.38E-07
chr6	82393001	82398000	0.005276893
chr6	82394001	82399000	0.005855137
chr6	82497001	82502000	0.000461278
chr6	83245001	83250000	1.09E-05
chr6	83497001	83502000	0.002435829
chr6	84224001	84229000	4.71E-13
chr6	84225001	84230000	2.98E-11
chr6	84226001	84231000	1.01E-07
chr6	84306001	84311000	0.010399395
chr6	84373001	84378000	0.00052903
chr6	84471001	84476000	0.005648353
chr6	84514001	84519000	0.012509028
chr6	84955001	84960000	2.27E-09
chr6	85055001	85060000	0.01046024
chr6	85126001	85131000	1.34E-05
chr6	85177001	85182000	0.0011223
chr6	85442001	85447000	2.18E-11

chr6	85501001	85506000	0.003946164
chr6	85617001	85622000	2.72E-05
chr6	85618001	85623000	4.00E-08
chr6	85773001	85778000	4.41E-07
chr6	85774001	85779000	2.24E-08
chr6	85775001	85780000	8.38E-06
chr6	85776001	85781000	1.81E-12
chr6	85777001	85782000	8.12E-07
chr6	85860001	85865000	0.004911439
chr6	86381001	86386000	0.000165742
chr6	86848001	86853000	5.65E-05
chr6	87302001	87307000	0.001416198
chr6	87303001	87308000	0.000517305
chr6	87418001	87423000	1.50E-06
chr6	87419001	87424000	2.04E-07
chr6	87420001	87425000	2.06E-06
chr6	87772001	87777000	1.10E-05
chr6	87774001	87779000	6.77E-07
chr6	88080001	88085000	1.96E-05
chr6	88486001	88491000	4.88E-06
chr6	88487001	88492000	2.17E-06
chr6	88522001	88527000	8.32E-09
chr6	88524001	88529000	7.01E-07
chr6	88538001	88543000	6.29E-13
chr6	88539001	88544000	7.08E-15
chr6	88540001	88545000	3.34E-15
chr6	88541001	88546000	7.72E-22
chr6	88542001	88547000	2.85E-07
chr6	88844001	88849000	1.05E-07
chr6	88886001	88891000	2.95E-05
chr6	89079001	89084000	5.80E-06
chr6	89213001	89218000	1.41E-05
chr6	89214001	89219000	8.88E-06
chr6	89215001	89220000	6.88E-06
chr6	89216001	89221000	2.45E-05
chr6	89694001	89699000	0.00177496
chr6	90007001	90012000	3.53E-10
chr6	90534001	90539000	9.58E-07
chr6	92057001	92062000	0.000286623
chr6	92058001	92063000	0.000191534
chr6	92059001	92064000	0.000231501
chr6	92060001	92065000	0.000158204
chr6	92061001	92066000	0.000257507
chr6	92147001	92152000	0.000139885
chr6	92257001	92262000	0.001722154
chr6	92340001	92345000	5.23E-06
chr6	92342001	92347000	1.45E-05
chr6	92753001	92758000	0.000235053

chr6	92821001	92826000	5.84E-05
chr6	92822001	92827000	5.13E-05
chr6	93012001	93017000	0.007724263
chr6	93505001	93510000	0.005825344
chr6	94316001	94321000	0.000169284
chr6	94384001	94389000	0.000530691
chr6	96650001	96655000	3.05E-05
chr6	98028001	98033000	0.014363706
chr6	98031001	98036000	0.000272105
chr6	98105001	98110000	0.002689264
chr6	98514001	98519000	0.001955652
chr6	98620001	98625000	1.59E-05
chr6	99067001	99072000	3.53E-06
chr6	99068001	99073000	2.11E-06
chr6	99104001	99109000	0.001429183
chr6	99105001	99110000	0.002444318
chr6	99106001	99111000	0.005683553
chr6	99107001	99112000	0.00420344
chr6	100267001	100272000	1.10E-08
chr6	100356001	100361000	1.48E-05
chr6	100708001	100713000	0.008751349
chr6	100709001	100714000	0.003261919
chr6	100710001	100715000	0.001067532
chr6	101531001	101536000	2.98E-08
chr6	101606001	101611000	9.84E-07
chr6	101852001	101857000	9.30E-07
chr6	101854001	101859000	0.000496833
chr6	102044001	102049000	0.00255263
chr6	102571001	102576000	0.00314543
chr6	102988001	102993000	0.000370652
chr6	103396001	103401000	0.007636477
chr6	103397001	103402000	0.008819122
chr6	103575001	103580000	8.96E-06
chr6	104373001	104378000	0.000147289
chr6	104374001	104379000	0.001392126
chr6	104631001	104636000	7.22E-07
chr6	104632001	104637000	1.15E-05
chr6	104889001	104894000	0.000302372
chr6	104890001	104895000	9.81E-06
chr6	105498001	105503000	4.97E-05
chr6	105749001	105754000	2.74E-12
chr6	105879001	105884000	0.000120297
chr6	106246001	106251000	1.46E-07
chr6	106460001	106465000	3.29E-05
chr6	106541001	106546000	3.57E-12
chr6	106545001	106550000	1.41E-14
chr6	106972001	106977000	8.98E-09
chr6	107054001	107059000	0.000201344

chr6	107072001	107077000	1.25E-13
chr6	109611001	109616000	0.000301407
chr6	109778001	109783000	3.39E-07
chr6	110670001	110675000	1.26E-08
chr6	110671001	110676000	3.03E-07
chr6	110710001	110715000	8.96E-07
chr6	110828001	110833000	7.51E-07
chr6	110835001	110840000	1.02E-07
chr6	111395001	111400000	1.06E-05
chr6	111798001	111803000	1.26E-05
chr6	111806001	111811000	1.66E-06
chr6	112364001	112369000	1.03E-43
chr6	112758001	112763000	0.002571478
chr6	113091001	113096000	0.000378781
chr6	113423001	113428000	0.000723511
chr6	113424001	113429000	0.000714059
chr6	113425001	113430000	0.00101491
chr6	114081001	114086000	0.00058439
chr6	114093001	114098000	3.60E-08
chr6	114094001	114099000	2.48E-06
chr6	115259001	115264000	0.000275282
chr6	115260001	115265000	0.000437419
chr6	115781001	115786000	0.001273694
chr6	115782001	115787000	0.00042289
chr6	115783001	115788000	8.77E-05
chr6	116166001	116171000	0.000100164
chr6	116167001	116172000	5.19E-07
chr6	116189001	116194000	0.000769329
chr6	116222001	116227000	1.47E-05
chr6	116244001	116249000	7.84E-09
chr6	116245001	116250000	2.89E-11
chr6	116653001	116658000	0.000161375
chr6	116851001	116856000	2.56E-09
chr6	117233001	117238000	0.001001901
chr6	117458001	117463000	0.001502575
chr6	118252001	118257000	0.006541688
chr6	118621001	118626000	3.91E-07
chr6	118622001	118627000	8.50E-07
chr6	118623001	118628000	4.38E-07
chr6	118624001	118629000	0.001227716
chr6	118770001	118775000	1.34E-07
chr6	118808001	118813000	3.83E-06
chr6	119012001	119017000	4.18E-07
chr6	119013001	119018000	0.0007415
chr6	119927001	119932000	9.07E-07
chr6	119929001	119934000	0.000170129
chr6	119930001	119935000	4.50E-06
chr6	119931001	119936000	1.83E-06

chr6	120348001	120353000	0.013521323
chr6	120420001	120425000	1.60E-06
chr6	120421001	120426000	4.49E-07
chr6	120423001	120428000	2.74E-06
chr6	120448001	120453000	0.004638116
chr6	120458001	120463000	0.001138916
chr6	120495001	120500000	0.000957414
chr6	120496001	120501000	0.000164152
chr6	120836001	120841000	0.001948578
chr6	120852001	120857000	0.001806907
chr6	120940001	120945000	0.00023892
chr6	121231001	121236000	0.002440129
chr6	121232001	121237000	0.000253236
chr6	121434001	121439000	0.002859118
chr6	122433001	122438000	2.72E-07
chr6	122434001	122439000	6.96E-08
chr6	122676001	122681000	0.0025646
chr6	122677001	122682000	0.002655087
chr6	122809001	122814000	1.74E-08
chr6	122810001	122815000	1.63E-08
chr6	122811001	122816000	4.31E-10
chr6	122812001	122817000	5.80E-11
chr6	122813001	122818000	2.86E-07
chr6	122951001	122956000	0.000141033
chr6	122952001	122957000	8.45E-05
chr6	122953001	122958000	4.19E-05
chr6	123017001	123022000	9.87E-05
chr6	123018001	123023000	0.000511538
chr6	123221001	123226000	0.003686791
chr6	123396001	123401000	0.00046468
chr6	123397001	123402000	6.62E-05
chr6	123410001	123415000	0.029959429
chr6	123617001	123622000	0.006317711
chr6	124056001	124061000	0.019539162
chr6	124057001	124062000	0.010697952
chr6	124224001	124229000	1.01E-08
chr6	124227001	124232000	2.06E-06
chr6	124228001	124233000	6.37E-05
chr6	124292001	124297000	0.000190843
chr6	124293001	124298000	7.21E-05
chr6	124587001	124592000	2.87E-07
chr6	124660001	124665000	2.29E-05
chr6	124675001	124680000	1.20E-06
chr6	125468001	125473000	1.51E-06
chr6	125626001	125631000	1.65E-08
chr6	127098001	127103000	0.000618607
chr6	127099001	127104000	0.000269701
chr6	127100001	127105000	0.000121877

chr6	127106001	127111000	0.000423299
chr6	127107001	127112000	0.000423299
chr6	127108001	127113000	0.002527853
chr6	127704001	127709000	5.59E-11
chr6	127705001	127710000	3.85E-08
chr6	127706001	127711000	1.22E-08
chr6	127754001	127759000	1.59E-09
chr6	127755001	127760000	1.67E-06
chr6	127756001	127761000	1.17E-06
chr6	127777001	127782000	7.41E-09
chr6	127778001	127783000	4.00E-09
chr6	127779001	127784000	9.53E-07
chr6	127781001	127786000	0.000122318
chr6	127783001	127788000	3.17E-05
chr6	127784001	127789000	2.51E-07
chr6	127791001	127796000	3.19E-09
chr6	127841001	127846000	0.000447781
chr6	128152001	128157000	5.38E-05
chr6	128256001	128261000	0.000362815
chr6	128257001	128262000	0.000932679
chr6	129290001	129295000	8.07E-05
chr6	129341001	129346000	0.00029977
chr6	130254001	130259000	0.002130337
chr6	130257001	130262000	2.74E-08
chr6	130636001	130641000	0.001312344
chr6	130644001	130649000	2.85E-06
chr6	130757001	130762000	9.06E-09
chr6	130976001	130981000	0.002178534
chr6	131441001	131446000	0.000296563
chr6	131852001	131857000	1.44E-10
chr6	131853001	131858000	2.06E-11
chr6	131854001	131859000	5.01E-08
chr6	131855001	131860000	1.60E-06
chr6	132158001	132163000	2.78E-09
chr6	132451001	132456000	0.001700021
chr6	132651001	132656000	5.55E-09
chr6	132682001	132687000	0.001109474
chr6	132683001	132688000	0.000271514
chr6	132684001	132689000	0.006161167
chr6	132685001	132690000	0.000103103
chr6	132858001	132863000	3.77E-08
chr6	132859001	132864000	2.48E-08
chr6	132860001	132865000	0.000134957
chr6	133610001	133615000	0.000505313
chr6	133627001	133632000	0.000148859
chr6	133961001	133966000	3.04E-06
chr6	133962001	133967000	1.50E-07
chr6	133963001	133968000	1.95E-07

chr6	134123001	134128000	5.78E-05
chr6	134124001	134129000	1.39E-05
chr6	134150001	134155000	2.40E-05
chr6	134487001	134492000	9.41E-25
chr6	134488001	134493000	3.08E-26
chr6	135092001	135097000	7.42E-09
chr6	135093001	135098000	2.99E-08
chr6	136222001	136227000	2.05E-07
chr6	136547001	136552000	1.63E-12
chr6	136796001	136801000	0.018729575
chr6	137312001	137317000	2.33E-10
chr6	137313001	137318000	7.76E-10
chr6	138192001	138197000	1.03E-08
chr6	138193001	138198000	3.78E-07
chr6	138194001	138199000	1.18E-07
chr6	139327001	139332000	1.30E-06
chr6	140529001	140534000	1.72E-05
chr6	140712001	140717000	0.00717405
chr6	140754001	140759000	1.96E-09
chr6	140755001	140760000	2.58E-10
chr6	140802001	140807000	5.49E-05
chr6	140805001	140810000	1.26E-09
chr6	140806001	140811000	1.72E-08
chr6	140966001	140971000	0.004726891
chr6	141076001	141081000	0.002803419
chr6	141091001	141096000	0.00587751
chr6	141506001	141511000	0.000792504
chr6	141507001	141512000	4.40E-05
chr6	141508001	141513000	2.19E-05
chr6	141510001	141515000	2.01E-05
chr6	141511001	141516000	0.000271055
chr6	141770001	141775000	0.015375386
chr6	141783001	141788000	0.000683799
chr6	142179001	142184000	3.62E-07
chr6	142695001	142700000	0.015192174
chr6	142795001	142800000	7.27E-07
chr6	142931001	142936000	0.002304827
chr6	143138001	143143000	2.22E-05
chr6	143748001	143753000	4.90E-12
chr6	144065001	144070000	2.15E-16
chr6	144280001	144285000	1.13E-13
chr6	145349001	145354000	0.001104059
chr6	145406001	145411000	7.60E-06
chr6	145409001	145414000	0.000991221
chr6	145456001	145461000	1.28E-07
chr6	145478001	145483000	0.000373732
chr6	145479001	145484000	0.000945261
chr6	145480001	145485000	0.009139564

chr6	145542001	145547000	0.000819726
chr6	145554001	145559000	6.56E-07
chr6	145581001	145586000	0.004387136
chr6	145667001	145672000	0.000145032
chr6	145668001	145673000	6.06E-05
chr6	145669001	145674000	0.000397766
chr6	145765001	145770000	0.000357468
chr6	146025001	146030000	0.000592038
chr6	146913001	146918000	0.001171035
chr6	147054001	147059000	0.000455306
chr6	147055001	147060000	2.01E-05
chr6	147332001	147337000	0.000606071
chr6	147395001	147400000	2.98E-05
chr6	147406001	147411000	4.21E-07
chr6	147433001	147438000	0.002665754
chr6	147434001	147439000	2.44E-05
chr6	147435001	147440000	0.000138978
chr6	147467001	147472000	5.87E-05
chr6	147480001	147485000	6.86E-10
chr6	147481001	147486000	9.15E-09
chr6	147603001	147608000	2.20E-05
chr6	148102001	148107000	0.000376781
chr6	148168001	148173000	1.02E-05
chr6	148346001	148351000	4.37E-06
chr6	148353001	148358000	3.22E-07
chr6	151768001	151773000	1.18E-22
chr6	152892001	152897000	0.004242616
chr6	153176001	153181000	0.002006724
chr6	153177001	153182000	7.60E-06
chr6	153271001	153276000	2.64E-07
chr6	153494001	153499000	7.82E-07
chr6	153495001	153500000	1.40E-06
chr6	153496001	153501000	7.22E-05
chr6	153497001	153502000	1.46E-05
chr6	153498001	153503000	0.000187275
chr6	153545001	153550000	0.000228009
chr6	153550001	153555000	7.51E-05
chr6	153658001	153663000	0.003785275
chr6	153672001	153677000	0.000171426
chr6	153705001	153710000	1.71E-06
chr6	153799001	153804000	9.52E-05
chr6	153801001	153806000	0.000798062
chr6	153891001	153896000	9.60E-07
chr6	153893001	153898000	3.62E-08
chr6	153976001	153981000	5.35E-05
chr6	154265001	154270000	0.001649701
chr6	154266001	154271000	0.002240633
chr6	154267001	154272000	0.004670746

chr6	154294001	154299000	9.27E-08
chr6	154295001	154300000	2.55E-05
chr6	154439001	154444000	0.003906992
chr6	154440001	154445000	0.000452772
chr6	155506001	155511000	3.43E-10
chr6	155507001	155512000	7.79E-12
chr6	155775001	155780000	2.35E-05
chr6	155856001	155861000	3.63E-05
chr6	156054001	156059000	3.67E-06
chr6	156055001	156060000	0.000180369
chr6	156086001	156091000	8.83E-08
chr6	156115001	156120000	2.23E-05
chr6	156160001	156165000	0.006480717
chr6	156968001	156973000	3.98E-17
chr6	157956001	157961000	4.57E-11
chr6	158371001	158376000	3.96E-11
chr6	158938001	158943000	5.15E-14
chr6	158939001	158944000	2.81E-09
chr6	158940001	158945000	4.92E-07
chr6	159233001	159238000	9.30E-14
chr6	161204001	161209000	0.000445181
chr6	161206001	161211000	2.76E-05
chr6	161370001	161375000	1.82E-10
chr6	162355001	162360000	2.03E-06
chr6	162595001	162600000	4.19E-10
chr6	162892001	162897000	0.000105794
chr6	163025001	163030000	7.06E-05
chr6	163112001	163117000	0.004389697
chr6	163216001	163221000	1.42E-05
chr6	163218001	163223000	1.46E-08
chr6	163219001	163224000	9.82E-09
chr6	163728001	163733000	7.13E-18
chr6	163729001	163734000	2.66E-18
chr6	163730001	163735000	3.95E-17
chr6	164533001	164538000	9.58E-06
chr6	164551001	164556000	5.33E-05
chr6	164624001	164629000	0.003866973
chr6	164643001	164648000	1.73E-05
chr6	164644001	164649000	3.82E-06
chr6	165036001	165041000	5.69E-05
chr6	165049001	165054000	0.001901636
chr6	165065001	165070000	0.013863638
chr6	165066001	165071000	0.015779236
chr6	166261001	166266000	4.33E-10
chr6	168230001	168235000	6.71E-06
chr6	168332001	168337000	4.29E-07
chr6	169294001	169299000	1.22E-10
chr6	170460001	170465000	2.40E-12

chr6	170461001	170466000	8.36E-09
chr7	1577001	1582000	7.84E-26
chr7	1785001	1790000	1.47E-34
chr7	1786001	1791000	5.13E-29
chr7	2307001	2312000	3.31E-06
chr7	2738001	2743000	1.76E-13
chr7	5646001	5651000	8.49E-21
chr7	5647001	5652000	3.64E-18
chr7	6423001	6428000	1.24E-10
chr7	6693001	6698000	7.60E-11
chr7	6694001	6699000	2.18E-11
chr7	6695001	6700000	7.41E-12
chr7	7717001	7722000	2.96E-07
chr7	7797001	7802000	1.03E-06
chr7	7958001	7963000	0.001437919
chr7	7959001	7964000	0.002368015
chr7	7960001	7965000	9.28E-05
chr7	8015001	8020000	6.88E-08
chr7	8016001	8021000	1.60E-07
chr7	8330001	8335000	3.43E-08
chr7	8331001	8336000	5.47E-10
chr7	8363001	8368000	8.15E-11
chr7	8364001	8369000	5.40E-14
chr7	8365001	8370000	1.17E-13
chr7	8366001	8371000	5.60E-06
chr7	8430001	8435000	2.73E-06
chr7	8467001	8472000	2.05E-06
chr7	8498001	8503000	2.29E-06
chr7	8499001	8504000	7.37E-07
chr7	8500001	8505000	1.10E-05
chr7	8604001	8609000	0.00040904
chr7	8605001	8610000	0.002601205
chr7	8733001	8738000	3.58E-05
chr7	8734001	8739000	5.85E-05
chr7	8735001	8740000	1.68E-05
chr7	8824001	8829000	0.000767693
chr7	8925001	8930000	2.91E-14
chr7	8926001	8931000	5.25E-17
chr7	9556001	9561000	0.000174526
chr7	9557001	9562000	0.00014381
chr7	9648001	9653000	7.67E-05
chr7	9649001	9654000	5.78E-05
chr7	9863001	9868000	9.71E-10
chr7	9866001	9871000	8.16E-07
chr7	10198001	10203000	7.45E-07
chr7	10199001	10204000	1.70E-05
chr7	10503001	10508000	8.50E-25
chr7	10504001	10509000	1.20E-23

chr7	10656001	10661000	2.92E-09
chr7	10657001	10662000	6.64E-09
chr7	10658001	10663000	3.47E-07
chr7	10659001	10664000	8.88E-05
chr7	10798001	10803000	0.002827463
chr7	11557001	11562000	0.002361024
chr7	11948001	11953000	2.53E-05
chr7	11949001	11954000	4.04E-07
chr7	12009001	12014000	0.000499332
chr7	12031001	12036000	0.001374995
chr7	12429001	12434000	5.13E-06
chr7	12519001	12524000	0.001697432
chr7	12520001	12525000	0.000395403
chr7	12523001	12528000	0.000357606
chr7	12527001	12532000	0.000813765
chr7	12634001	12639000	3.61E-11
chr7	12839001	12844000	0.000105539
chr7	12840001	12845000	2.17E-05
chr7	13293001	13298000	1.17E-07
chr7	13294001	13299000	2.66E-06
chr7	13295001	13300000	2.15E-06
chr7	13456001	13461000	2.65E-11
chr7	13457001	13462000	5.25E-14
chr7	13458001	13463000	4.10E-10
chr7	13561001	13566000	2.91E-05
chr7	14831001	14836000	0.004957903
chr7	14832001	14837000	0.008981315
chr7	15244001	15249000	0.001059283
chr7	15260001	15265000	0.007691999
chr7	15484001	15489000	2.04E-08
chr7	15485001	15490000	2.09E-09
chr7	15486001	15491000	1.07E-10
chr7	15487001	15492000	5.62E-07
chr7	15537001	15542000	0.001877537
chr7	15538001	15543000	0.001859565
chr7	15581001	15586000	0.002565329
chr7	16985001	16990000	9.74E-06
chr7	18265001	18270000	4.48E-06
chr7	18290001	18295000	0.001992759
chr7	18438001	18443000	0.00085831
chr7	18539001	18544000	0.000116087
chr7	18589001	18594000	6.68E-06
chr7	18590001	18595000	2.32E-09
chr7	18591001	18596000	6.07E-09
chr7	18592001	18597000	8.57E-08
chr7	19133001	19138000	2.09E-05
chr7	19287001	19292000	8.96E-06
chr7	19288001	19293000	4.75E-06

chr7	19299001	19304000	4.29E-05
chr7	19337001	19342000	3.09E-06
chr7	19341001	19346000	1.28E-05
chr7	19429001	19434000	6.53E-05
chr7	19434001	19439000	3.06E-07
chr7	19435001	19440000	9.27E-08
chr7	19436001	19441000	2.08E-08
chr7	19437001	19442000	7.95E-10
chr7	19463001	19468000	1.65E-06
chr7	19464001	19469000	2.83E-07
chr7	19681001	19686000	0.002719735
chr7	19714001	19719000	5.71E-05
chr7	19794001	19799000	1.65E-08
chr7	19856001	19861000	3.85E-05
chr7	20534001	20539000	0.001656508
chr7	20550001	20555000	0.014017406
chr7	20570001	20575000	0.000236874
chr7	20913001	20918000	1.79E-07
chr7	20914001	20919000	2.25E-09
chr7	20915001	20920000	8.68E-09
chr7	21406001	21411000	3.41E-07
chr7	21456001	21461000	0.000153539
chr7	22881001	22886000	2.90E-08
chr7	25903001	25908000	7.19E-09
chr7	25904001	25909000	8.96E-08
chr7	25905001	25910000	5.89E-08
chr7	26179001	26184000	2.97E-09
chr7	26234001	26239000	6.71E-17
chr7	27052001	27057000	7.01E-06
chr7	27144001	27149000	1.11E-39
chr7	27145001	27150000	1.29E-39
chr7	27146001	27151000	4.43E-28
chr7	27147001	27152000	3.16E-10
chr7	27156001	27161000	4.34E-22
chr7	27157001	27162000	1.07E-11
chr7	27159001	27164000	1.74E-14
chr7	27165001	27170000	2.56E-08
chr7	27233001	27238000	3.05E-18
chr7	28132001	28137000	3.24E-10
chr7	28133001	28138000	1.42E-12
chr7	28249001	28254000	0.004597357
chr7	28877001	28882000	2.14E-16
chr7	28878001	28883000	6.38E-17
chr7	29219001	29224000	9.00E-09
chr7	29294001	29299000	2.38E-11
chr7	29295001	29300000	2.05E-12
chr7	30511001	30516000	2.74E-12
chr7	30766001	30771000	0.000930367

chr7	31596001	31601000	0.011214876
chr7	31684001	31689000	4.38E-05
chr7	31687001	31692000	6.65E-07
chr7	32426001	32431000	4.34E-06
chr7	33946001	33951000	0.001642663
chr7	36477001	36482000	5.37E-11
chr7	36478001	36483000	1.22E-11
chr7	36479001	36484000	2.86E-11
chr7	36536001	36541000	9.83E-10
chr7	37449001	37454000	3.88E-06
chr7	38487001	38492000	1.71E-06
chr7	38971001	38976000	2.03E-11
chr7	40473001	40478000	1.62E-05
chr7	40504001	40509000	0.000122997
chr7	43625001	43630000	1.34E-06
chr7	43626001	43631000	8.37E-05
chr7	43736001	43741000	0.001404573
chr7	43739001	43744000	8.75E-08
chr7	44535001	44540000	5.45E-07
chr7	44536001	44541000	6.17E-07
chr7	44585001	44590000	0.000144611
chr7	44774001	44779000	4.59E-05
chr7	45200001	45205000	0.000153422
chr7	45201001	45206000	3.65E-05
chr7	47677001	47682000	5.17E-16
chr7	48887001	48892000	0.000652633
chr7	48905001	48910000	0.001010605
chr7	48976001	48981000	0.000242724
chr7	49613001	49618000	0.002726917
chr7	49614001	49619000	2.13E-05
chr7	50161001	50166000	0.000722279
chr7	50162001	50167000	0.007023695
chr7	53721001	53726000	0.002631698
chr7	53846001	53851000	6.07E-05
chr7	53899001	53904000	0.007682481
chr7	54224001	54229000	4.99E-05
chr7	54422001	54427000	0.001410314
chr7	54423001	54428000	0.00010933
chr7	54424001	54429000	2.15E-06
chr7	54453001	54458000	0.017961008
chr7	54600001	54605000	0.000662467
chr7	54602001	54607000	0.000131986
chr7	54711001	54716000	0.002276508
chr7	54712001	54717000	0.004591931
chr7	54713001	54718000	0.006558185
chr7	55174001	55179000	1.18E-13
chr7	55175001	55180000	3.60E-23
chr7	56114001	56119000	4.10E-29

chr7	56215001	56220000	5.74E-13
chr7	56216001	56221000	3.96E-10
chr7	56621001	56626000	0.000411994
chr7	56742001	56747000	5.33E-06
chr7	56743001	56748000	3.31E-07
chr7	56744001	56749000	9.74E-07
chr7	58006001	58011000	3.74E-05
chr7	58007001	58012000	8.20E-06
chr7	62325001	62330000	0.007431643
chr7	62713001	62718000	0.00021626
chr7	62715001	62720000	7.12E-05
chr7	63880001	63885000	3.59E-05
chr7	64906001	64911000	2.06E-06
chr7	64907001	64912000	9.09E-05
chr7	64919001	64924000	0.001494352
chr7	65149001	65154000	0.000114515
chr7	65279001	65284000	3.02E-08
chr7	66432001	66437000	1.75E-07
chr7	68183001	68188000	3.31E-05
chr7	70270001	70275000	1.68E-11
chr7	70271001	70276000	8.53E-09
chr7	71493001	71498000	0.00023224
chr7	71494001	71499000	5.33E-05
chr7	71534001	71539000	5.79E-05
chr7	71947001	71952000	8.00E-10
chr7	72703001	72708000	0.000229046
chr7	72866001	72871000	7.01E-11
chr7	73020001	73025000	5.01E-12
chr7	73023001	73028000	1.28E-06
chr7	73239001	73244000	1.63E-12
chr7	73240001	73245000	3.43E-16
chr7	73253001	73258000	5.09E-11
chr7	73263001	73268000	5.63E-07
chr7	73444001	73449000	8.72E-07
chr7	73470001	73475000	2.30E-07
chr7	73624001	73629000	0.000230349
chr7	73761001	73766000	1.32E-10
chr7	73940001	73945000	1.36E-09
chr7	73956001	73961000	1.13E-05
chr7	73957001	73962000	1.32E-07
chr7	74002001	74007000	2.20E-05
chr7	74157001	74162000	0.00053371
chr7	74982001	74987000	0.01181428
chr7	74983001	74988000	0.01181428
chr7	75923001	75928000	0.000910831
chr7	75925001	75930000	5.75E-07
chr7	76071001	76076000	1.00E-06
chr7	76072001	76077000	9.91E-09

chr7	76073001	76078000	0.000268016
chr7	76074001	76079000	0.000227688
chr7	76554001	76559000	0.00046469
chr7	76555001	76560000	1.46E-08
chr7	76556001	76561000	8.97E-06
chr7	76586001	76591000	1.13E-07
chr7	76587001	76592000	1.31E-05
chr7	77699001	77704000	9.80E-08
chr7	77863001	77868000	1.09E-06
chr7	78926001	78931000	0.001082814
chr7	78927001	78932000	0.001398864
chr7	78928001	78933000	0.004775802
chr7	79406001	79411000	3.84E-06
chr7	79407001	79412000	3.23E-07
chr7	79408001	79413000	1.29E-06
chr7	79409001	79414000	1.72E-06
chr7	79411001	79416000	0.000939135
chr7	79636001	79641000	0.000434231
chr7	79717001	79722000	4.76E-05
chr7	79718001	79723000	2.29E-08
chr7	79846001	79851000	0.008022683
chr7	79905001	79910000	3.53E-05
chr7	80293001	80298000	5.12E-05
chr7	80294001	80299000	4.63E-07
chr7	80762001	80767000	1.33E-06
chr7	80763001	80768000	2.52E-07
chr7	80764001	80769000	3.22E-07
chr7	80818001	80823000	3.46E-06
chr7	80860001	80865000	0.00182925
chr7	81166001	81171000	2.31E-05
chr7	82138001	82143000	0.00031855
chr7	82221001	82226000	0.000193248
chr7	82365001	82370000	4.00E-06
chr7	82366001	82371000	1.50E-05
chr7	82737001	82742000	4.22E-05
chr7	83242001	83247000	0.00139649
chr7	83243001	83248000	0.001559969
chr7	83244001	83249000	0.003995736
chr7	83475001	83480000	0.000823531
chr7	83502001	83507000	8.44E-05
chr7	83687001	83692000	1.32E-13
chr7	83875001	83880000	1.14E-06
chr7	83928001	83933000	4.25E-06
chr7	83966001	83971000	0.000572322
chr7	83967001	83972000	0.000527653
chr7	84103001	84108000	5.88E-06
chr7	84104001	84109000	0.000222248
chr7	84105001	84110000	1.80E-05

chr7	84142001	84147000	0.000186194
chr7	84175001	84180000	4.40E-09
chr7	84176001	84181000	0.008027328
chr7	84227001	84232000	0.000253779
chr7	84228001	84233000	8.14E-06
chr7	84229001	84234000	2.57E-06
chr7	84249001	84254000	0.006763871
chr7	84317001	84322000	0.001383882
chr7	84318001	84323000	0.005029384
chr7	84319001	84324000	0.004405796
chr7	84342001	84347000	0.00285695
chr7	84343001	84348000	0.002769684
chr7	84344001	84349000	0.002264501
chr7	84529001	84534000	0.00013478
chr7	85414001	85419000	0.000115734
chr7	85415001	85420000	0.002883013
chr7	85445001	85450000	0.003148015
chr7	85446001	85451000	4.37E-07
chr7	85447001	85452000	1.01E-07
chr7	85990001	85995000	2.21E-05
chr7	86853001	86858000	0.000324341
chr7	87366001	87371000	1.40E-05
chr7	87367001	87372000	3.39E-05
chr7	87717001	87722000	0.004570814
chr7	88081001	88086000	8.93E-06
chr7	88082001	88087000	1.11E-07
chr7	88083001	88088000	0.000171439
chr7	88197001	88202000	0.007985797
chr7	88198001	88203000	0.014795402
chr7	88229001	88234000	0.000650162
chr7	89617001	89622000	0.006110884
chr7	89618001	89623000	0.003269182
chr7	89619001	89624000	0.000436697
chr7	90082001	90087000	0.000195464
chr7	90111001	90116000	0.002478332
chr7	90594001	90599000	1.31E-05
chr7	90727001	90732000	0.00204119
chr7	90728001	90733000	2.18E-08
chr7	90808001	90813000	2.38E-16
chr7	90809001	90814000	2.48E-06
chr7	90810001	90815000	0.000137461
chr7	92233001	92238000	1.52E-08
chr7	92275001	92280000	3.01E-05
chr7	92838001	92843000	0.004071703
chr7	93539001	93544000	0.000389151
chr7	93540001	93545000	0.000165963
chr7	93679001	93684000	8.66E-08
chr7	93824001	93829000	6.95E-06

chr7	94059001	94064000	0.000174693
chr7	94061001	94066000	1.13E-06
chr7	94062001	94067000	1.43E-07
chr7	94299001	94304000	0.000751807
chr7	94522001	94527000	5.34E-06
chr7	94783001	94788000	0.000151421
chr7	95345001	95350000	1.12E-09
chr7	95415001	95420000	6.21E-05
chr7	96002001	96007000	6.82E-06
chr7	96502001	96507000	4.56E-05
chr7	96503001	96508000	3.27E-07
chr7	96707001	96712000	9.66E-08
chr7	96713001	96718000	5.76E-06
chr7	97143001	97148000	5.14E-06
chr7	97144001	97149000	5.09E-07
chr7	97145001	97150000	4.60E-06
chr7	97146001	97151000	5.95E-06
chr7	97147001	97152000	6.61E-06
chr7	97196001	97201000	0.000591519
chr7	97199001	97204000	0.000405825
chr7	97220001	97225000	1.33E-05
chr7	99406001	99411000	8.34E-22
chr7	99747001	99752000	8.88E-12
chr7	99748001	99753000	9.04E-15
chr7	99838001	99843000	1.48E-08
chr7	99840001	99845000	1.27E-11
chr7	100728001	100733000	1.65E-22
chr7	100729001	100734000	4.84E-12
chr7	100992001	100997000	6.33E-07
chr7	101372001	101377000	7.05E-10
chr7	101373001	101378000	1.28E-08
chr7	101510001	101515000	3.17E-10
chr7	101511001	101516000	8.32E-11
chr7	102189001	102194000	0.00528632
chr7	102190001	102195000	0.001566503
chr7	102191001	102196000	0.000589117
chr7	102630001	102635000	1.40E-05
chr7	102989001	102994000	2.08E-13
chr7	103214001	103219000	1.21E-13
chr7	103251001	103256000	6.14E-07
chr7	103252001	103257000	1.91E-08
chr7	103253001	103258000	2.38E-06
chr7	103254001	103259000	0.000685726
chr7	103255001	103260000	2.50E-05
chr7	103267001	103272000	0.005492014
chr7	103360001	103365000	1.34E-05
chr7	103361001	103366000	6.44E-05
chr7	103362001	103367000	4.05E-05

chr7	103363001	103368000	0.000101966
chr7	103369001	103374000	0.000129569
chr7	103442001	103447000	0.00155677
chr7	103491001	103496000	0.000316454
chr7	103541001	103546000	3.37E-06
chr7	103542001	103547000	2.38E-06
chr7	103561001	103566000	1.49E-05
chr7	103562001	103567000	0.000120068
chr7	103672001	103677000	0.000246973
chr7	103673001	103678000	0.00055992
chr7	104086001	104091000	0.000738785
chr7	104591001	104596000	1.28E-08
chr7	104593001	104598000	2.31E-09
chr7	104647001	104652000	1.26E-18
chr7	104984001	104989000	3.58E-09
chr7	105448001	105453000	4.90E-20
chr7	106188001	106193000	0.001127185
chr7	106689001	106694000	1.19E-06
chr7	106771001	106776000	0.000185746
chr7	106772001	106777000	0.000897121
chr7	106800001	106805000	7.84E-06
chr7	107360001	107365000	1.50E-08
chr7	107361001	107366000	1.51E-13
chr7	107362001	107367000	1.22E-09
chr7	107682001	107687000	9.41E-06
chr7	107691001	107696000	5.19E-11
chr7	107783001	107788000	9.87E-11
chr7	107892001	107897000	9.28E-27
chr7	107893001	107898000	1.85E-22
chr7	107894001	107899000	4.92E-27
chr7	108229001	108234000	4.02E-07
chr7	108230001	108235000	1.76E-08
chr7	108251001	108256000	3.84E-10
chr7	108252001	108257000	0.000366285
chr7	108610001	108615000	0.000962968
chr7	108611001	108616000	0.000222975
chr7	108969001	108974000	0.002158607
chr7	109179001	109184000	2.92E-06
chr7	109180001	109185000	2.83E-08
chr7	109181001	109186000	2.17E-07
chr7	109182001	109187000	0.018344583
chr7	109308001	109313000	3.12E-07
chr7	109309001	109314000	2.28E-07
chr7	109310001	109315000	2.60E-07
chr7	109505001	109510000	0.000180048
chr7	109544001	109549000	5.22E-08
chr7	109634001	109639000	5.67E-05
chr7	109732001	109737000	0.018620318

chr7	109770001	109775000	1.40E-06
chr7	109772001	109777000	5.35E-06
chr7	109779001	109784000	0.001531178
chr7	109780001	109785000	0.002890876
chr7	109781001	109786000	0.000853684
chr7	109790001	109795000	2.51E-05
chr7	109791001	109796000	5.57E-05
chr7	109792001	109797000	0.000531622
chr7	109793001	109798000	0.000238326
chr7	109794001	109799000	0.000280736
chr7	109856001	109861000	0.001026001
chr7	109857001	109862000	8.90E-06
chr7	109858001	109863000	1.59E-05
chr7	109859001	109864000	0.000698269
chr7	109900001	109905000	1.16E-09
chr7	109901001	109906000	4.38E-10
chr7	109903001	109908000	1.84E-09
chr7	109904001	109909000	0.000282788
chr7	109973001	109978000	7.53E-15
chr7	109974001	109979000	1.04E-14
chr7	109975001	109980000	0.000109487
chr7	109979001	109984000	3.36E-09
chr7	109996001	110001000	1.21E-06
chr7	110054001	110059000	7.50E-10
chr7	110055001	110060000	1.52E-08
chr7	110221001	110226000	4.15E-12
chr7	110244001	110249000	2.39E-06
chr7	110649001	110654000	1.73E-06
chr7	111316001	111321000	6.95E-06
chr7	111752001	111757000	1.39E-08
chr7	111753001	111758000	5.90E-06
chr7	112000001	112005000	4.35E-13
chr7	112845001	112850000	0.00018034
chr7	113027001	113032000	0.00084736
chr7	113028001	113033000	5.89E-05
chr7	113420001	113425000	6.70E-07
chr7	113439001	113444000	0.0006585
chr7	113658001	113663000	0.000309309
chr7	113666001	113671000	0.002592559
chr7	113667001	113672000	0.003643444
chr7	114166001	114171000	0.000954498
chr7	114194001	114199000	2.43E-08
chr7	114195001	114200000	2.07E-08
chr7	114350001	114355000	5.12E-07
chr7	114351001	114356000	0.00015135
chr7	114403001	114408000	1.79E-11
chr7	114405001	114410000	3.30E-10
chr7	114440001	114445000	0.000247514

chr7	115321001	115326000	0.016932928
chr7	115498001	115503000	0.001455922
chr7	115658001	115663000	3.28E-06
chr7	115659001	115664000	4.58E-06
chr7	115678001	115683000	3.76E-06
chr7	115679001	115684000	3.76E-06
chr7	115898001	115903000	0.000182474
chr7	116299001	116304000	2.85E-05
chr7	116446001	116451000	1.20E-05
chr7	116447001	116452000	1.50E-05
chr7	116757001	116762000	1.94E-06
chr7	116758001	116763000	1.94E-06
chr7	116759001	116764000	1.27E-07
chr7	116951001	116956000	0.000179687
chr7	116952001	116957000	0.000159215
chr7	117077001	117082000	8.01E-06
chr7	117078001	117083000	8.06E-05
chr7	117079001	117084000	3.25E-07
chr7	117896001	117901000	0.001815642
chr7	117897001	117902000	1.22E-05
chr7	117936001	117941000	0.003879285
chr7	117947001	117952000	2.82E-05
chr7	117948001	117953000	1.06E-05
chr7	117962001	117967000	0.00416834
chr7	118098001	118103000	0.000573124
chr7	118099001	118104000	0.003298653
chr7	118141001	118146000	0.000733364
chr7	118225001	118230000	0.000117969
chr7	118226001	118231000	9.00E-05
chr7	118227001	118232000	5.59E-06
chr7	118228001	118233000	2.50E-05
chr7	118475001	118480000	0.001100866
chr7	118622001	118627000	4.44E-06
chr7	119091001	119096000	0.000126202
chr7	119117001	119122000	9.65E-05
chr7	119460001	119465000	5.39E-06
chr7	120359001	120364000	1.87E-10
chr7	120360001	120365000	2.06E-08
chr7	120361001	120366000	6.99E-11
chr7	120554001	120559000	3.57E-06
chr7	120555001	120560000	4.95E-07
chr7	121937001	121942000	3.98E-16
chr7	122070001	122075000	0.00240073
chr7	122699001	122704000	0.005821696
chr7	122962001	122967000	0.00011737
chr7	122963001	122968000	2.66E-05
chr7	122964001	122969000	8.78E-05
chr7	123053001	123058000	0.008079968

chr7	123424001	123429000	0.001139401
chr7	123606001	123611000	4.05E-05
chr7	123607001	123612000	0.000323394
chr7	123709001	123714000	1.64E-06
chr7	123710001	123715000	8.29E-06
chr7	123711001	123716000	9.88E-07
chr7	123712001	123717000	8.25E-06
chr7	123713001	123718000	8.06E-05
chr7	123871001	123876000	9.96E-06
chr7	123872001	123877000	3.52E-05
chr7	123873001	123878000	1.90E-06
chr7	123889001	123894000	2.56E-05
chr7	124130001	124135000	7.05E-05
chr7	124144001	124149000	9.68E-07
chr7	124850001	124855000	0.001728608
chr7	124893001	124898000	7.17E-05
chr7	124894001	124899000	2.64E-05
chr7	124964001	124969000	8.88E-08
chr7	124965001	124970000	2.12E-07
chr7	125066001	125071000	6.50E-06
chr7	125361001	125366000	5.66E-05
chr7	125362001	125367000	2.34E-05
chr7	125363001	125368000	2.31E-06
chr7	125364001	125369000	3.36E-06
chr7	125625001	125630000	1.32E-06
chr7	125940001	125945000	0.000397661
chr7	126024001	126029000	0.00035625
chr7	126068001	126073000	0.000154946
chr7	126090001	126095000	3.17E-05
chr7	126091001	126096000	7.70E-05
chr7	126092001	126097000	3.64E-06
chr7	126093001	126098000	0.000156205
chr7	126922001	126927000	3.44E-06
chr7	127273001	127278000	5.88E-05
chr7	127904001	127909000	1.89E-06
chr7	128162001	128167000	5.18E-10
chr7	128163001	128168000	2.46E-10
chr7	128309001	128314000	4.17E-11
chr7	128496001	128501000	4.44E-05
chr7	128979001	128984000	5.98E-09
chr7	129609001	129614000	0.002245813
chr7	130574001	130579000	1.47E-09
chr7	130583001	130588000	6.92E-18
chr7	130584001	130589000	4.37E-07
chr7	130585001	130590000	2.85E-06
chr7	130591001	130596000	1.12E-15
chr7	130592001	130597000	3.80E-15
chr7	130593001	130598000	6.66E-23

chr7	130678001	130683000	4.86E-11
chr7	130739001	130744000	3.12E-10
chr7	130741001	130746000	4.08E-13
chr7	130742001	130747000	6.58E-10
chr7	131432001	131437000	0.000460277
chr7	131433001	131438000	0.000260101
chr7	134313001	134318000	0.004372401
chr7	134693001	134698000	1.50E-05
chr7	135559001	135564000	1.03E-05
chr7	135560001	135565000	2.24E-05
chr7	136229001	136234000	0.000444444
chr7	137795001	137800000	0.000296455
chr7	138408001	138413000	3.82E-10
chr7	138759001	138764000	7.38E-09
chr7	140173001	140178000	1.40E-16
chr7	140289001	140294000	6.59E-10
chr7	140574001	140579000	5.03E-10
chr7	141783001	141788000	2.33E-06
chr7	141860001	141865000	2.28E-09
chr7	141885001	141890000	4.31E-08
chr7	141886001	141891000	5.28E-08
chr7	141887001	141892000	4.39E-10
chr7	141888001	141893000	1.53E-09
chr7	141889001	141894000	4.85E-06
chr7	141899001	141904000	0.000533109
chr7	142594001	142599000	5.74E-08
chr7	143177001	143182000	0.000198816
chr7	143649001	143654000	0.000764638
chr7	144521001	144526000	4.95E-08
chr7	144524001	144529000	1.66E-12
chr7	144605001	144610000	3.48E-05
chr7	144757001	144762000	5.61E-06
chr7	144758001	144763000	8.61E-05
chr7	144759001	144764000	0.000231774
chr7	144760001	144765000	0.000231774
chr7	144793001	144798000	0.015401951
chr7	144794001	144799000	0.011366434
chr7	144795001	144800000	0.002575227
chr7	144796001	144801000	0.000139193
chr7	144889001	144894000	0.002985591
chr7	144961001	144966000	0.000870833
chr7	145122001	145127000	5.23E-07
chr7	145143001	145148000	1.11E-05
chr7	145144001	145149000	5.13E-05
chr7	145185001	145190000	0.000232825
chr7	145285001	145290000	0.000200898
chr7	145572001	145577000	0.00034784
chr7	145862001	145867000	0.000694252

chr7	145935001	145940000	2.94E-06
chr7	146016001	146021000	0.001514019
chr7	146017001	146022000	0.001487435
chr7	146018001	146023000	0.001617219
chr7	146160001	146165000	0.000390216
chr7	146161001	146166000	0.000194532
chr7	146174001	146179000	5.57E-10
chr7	146175001	146180000	1.90E-09
chr7	146194001	146199000	0.018255622
chr7	146487001	146492000	5.67E-09
chr7	146488001	146493000	3.75E-08
chr7	146531001	146536000	0.007066738
chr7	146552001	146557000	6.05E-06
chr7	146553001	146558000	3.67E-07
chr7	146586001	146591000	0.00022149
chr7	146587001	146592000	0.000309206
chr7	146638001	146643000	3.90E-07
chr7	146639001	146644000	3.75E-07
chr7	146640001	146645000	4.72E-07
chr7	146799001	146804000	2.03E-07
chr7	146992001	146997000	0.000127218
chr7	146993001	146998000	7.09E-05
chr7	147122001	147127000	0.000885083
chr7	147123001	147128000	0.000807396
chr7	147124001	147129000	0.004019419
chr7	147260001	147265000	1.51E-05
chr7	147543001	147548000	4.44E-06
chr7	148720001	148725000	7.32E-18
chr7	149421001	149426000	0.001240111
chr7	150049001	150054000	2.88E-20
chr7	150050001	150055000	3.69E-18
chr7	150051001	150056000	3.34E-11
chr7	150077001	150082000	2.02E-15
chr7	150479001	150484000	1.81E-05
chr7	150480001	150485000	2.41E-05
chr7	150821001	150826000	0.001136188
chr7	150937001	150942000	1.09E-09
chr7	150965001	150970000	9.26E-09
chr7	151124001	151129000	8.16E-07
chr7	152281001	152286000	1.97E-05
chr7	154266001	154271000	0.000582026
chr7	154267001	154272000	0.000252952
chr7	154268001	154273000	4.98E-05
chr7	155292001	155297000	5.43E-07
chr7	155293001	155298000	1.77E-05
chr7	155294001	155299000	9.13E-05
chr7	155605001	155610000	4.70E-05
chr7	158510001	158515000	5.44E-05

chr7	158511001	158516000	0.000686501
chr8	177001	182000	4.83E-12
chr8	1952001	1957000	1.38E-16
chr8	1953001	1958000	3.17E-10
chr8	3079001	3084000	1.33E-07
chr8	3080001	3085000	2.89E-09
chr8	3082001	3087000	1.75E-13
chr8	4109001	4114000	6.99E-07
chr8	5864001	5869000	0.000480813
chr8	6092001	6097000	8.71E-06
chr8	7684001	7689000	0.00955559
chr8	7685001	7690000	0.015258169
chr8	7686001	7691000	0.005067341
chr8	7704001	7709000	0.008172142
chr8	7768001	7773000	2.30E-06
chr8	7770001	7775000	6.10E-07
chr8	8515001	8520000	2.84E-06
chr8	8586001	8591000	8.83E-07
chr8	8932001	8937000	3.83E-09
chr8	9010001	9015000	3.99E-26
chr8	9011001	9016000	1.68E-26
chr8	9012001	9017000	2.57E-21
chr8	9013001	9018000	4.91E-10
chr8	9122001	9127000	0.003721727
chr8	9247001	9252000	3.89E-06
chr8	10456001	10461000	5.99E-06
chr8	10457001	10462000	4.99E-08
chr8	10458001	10463000	2.43E-06
chr8	10459001	10464000	1.48E-06
chr8	10595001	10600000	0.000490217
chr8	10596001	10601000	7.94E-07
chr8	11299001	11304000	2.17E-08
chr8	12649001	12654000	1.30E-12
chr8	12762001	12767000	0.000168858
chr8	12765001	12770000	1.12E-07
chr8	12766001	12771000	1.36E-10
chr8	12794001	12799000	0.000239061
chr8	14714001	14719000	0.004053854
chr8	14715001	14720000	0.000759757
chr8	15133001	15138000	3.64E-09
chr8	15134001	15139000	1.52E-06
chr8	15248001	15253000	0.003560837
chr8	16118001	16123000	0.000702841
chr8	16587001	16592000	0.008799835
chr8	16588001	16593000	0.003993803
chr8	16589001	16594000	0.000273712
chr8	16898001	16903000	8.61E-06
chr8	17346001	17351000	9.37E-07

chr8	17347001	17352000	8.90E-09
chr8	18022001	18027000	2.01E-05
chr8	18899001	18904000	0.000536205
chr8	18949001	18954000	1.25E-06
chr8	18950001	18955000	3.01E-09
chr8	18951001	18956000	1.11E-08
chr8	18952001	18957000	7.40E-05
chr8	18997001	19002000	7.29E-08
chr8	18998001	19003000	8.78E-11
chr8	19124001	19129000	7.72E-05
chr8	20488001	20493000	0.000237365
chr8	20489001	20494000	2.29E-05
chr8	20491001	20496000	4.18E-08
chr8	20534001	20539000	0.000495069
chr8	21133001	21138000	2.28E-05
chr8	21135001	21140000	2.87E-05
chr8	22024001	22029000	2.27E-05
chr8	22807001	22812000	4.46E-07
chr8	23706001	23711000	4.72E-14
chr8	23713001	23718000	1.13E-05
chr8	23714001	23719000	5.78E-06
chr8	23715001	23720000	4.32E-07
chr8	23740001	23745000	8.41E-06
chr8	23741001	23746000	0.000243943
chr8	23742001	23747000	0.003259748
chr8	23771001	23776000	1.01E-05
chr8	23773001	23778000	3.94E-07
chr8	23774001	23779000	5.40E-07
chr8	23831001	23836000	3.18E-09
chr8	25951001	25956000	0.001617256
chr8	25957001	25962000	0.037006064
chr8	26007001	26012000	9.69E-05
chr8	27700001	27705000	1.03E-06
chr8	27753001	27758000	7.27E-06
chr8	27951001	27956000	3.13E-21
chr8	27972001	27977000	1.23E-10
chr8	28322001	28327000	8.22E-10
chr8	28323001	28328000	1.02E-08
chr8	28324001	28329000	1.73E-07
chr8	28485001	28490000	3.62E-10
chr8	28486001	28491000	1.77E-09
chr8	28488001	28493000	3.31E-11
chr8	29753001	29758000	1.72E-10
chr8	29754001	29759000	9.64E-10
chr8	29755001	29760000	1.17E-07
chr8	29814001	29819000	0.003439922
chr8	29905001	29910000	3.71E-07
chr8	29906001	29911000	1.16E-05

chr8	29932001	29937000	1.49E-05
chr8	29945001	29950000	4.28E-08
chr8	29947001	29952000	1.25E-08
chr8	29978001	29983000	6.53E-05
chr8	30389001	30394000	2.05E-05
chr8	30520001	30525000	4.57E-10
chr8	30521001	30526000	3.41E-07
chr8	30756001	30761000	2.55E-05
chr8	30816001	30821000	0.008690968
chr8	30817001	30822000	0.018951848
chr8	31038001	31043000	3.42E-10
chr8	31435001	31440000	2.65E-07
chr8	31500001	31505000	0.000993559
chr8	31518001	31523000	0.000100569
chr8	31520001	31525000	0.000105206
chr8	31622001	31627000	1.16E-05
chr8	31624001	31629000	0.000299563
chr8	31635001	31640000	8.83E-05
chr8	31650001	31655000	5.88E-08
chr8	31651001	31656000	2.19E-07
chr8	31652001	31657000	2.26E-07
chr8	31756001	31761000	2.82E-09
chr8	31757001	31762000	6.49E-09
chr8	31819001	31824000	4.47E-06
chr8	31821001	31826000	0.000102932
chr8	31822001	31827000	3.84E-07
chr8	31837001	31842000	0.001956047
chr8	31838001	31843000	0.002032388
chr8	31845001	31850000	0.000283286
chr8	31858001	31863000	0.000121559
chr8	31911001	31916000	5.71E-05
chr8	31912001	31917000	4.49E-07
chr8	31913001	31918000	0.007306804
chr8	31914001	31919000	9.69E-05
chr8	32408001	32413000	0.000691445
chr8	32726001	32731000	0.000147105
chr8	32727001	32732000	6.22E-05
chr8	32962001	32967000	0.000297015
chr8	33091001	33096000	2.89E-07
chr8	33321001	33326000	0.000445735
chr8	33427001	33432000	3.60E-08
chr8	33785001	33790000	0.002097283
chr8	34481001	34486000	9.04E-05
chr8	34623001	34628000	4.87E-07
chr8	34785001	34790000	0.005638605
chr8	34799001	34804000	0.001190483
chr8	34815001	34820000	0.0014574
chr8	34835001	34840000	2.75E-05

chr8	34844001	34849000	8.32E-08
chr8	34845001	34850000	2.89E-09
chr8	34862001	34867000	0.00213527
chr8	34863001	34868000	0.000652479
chr8	34889001	34894000	0.001214832
chr8	34891001	34896000	0.003138739
chr8	34925001	34930000	0.002642863
chr8	34967001	34972000	0.007703453
chr8	35000001	35005000	2.73E-05
chr8	35001001	35006000	5.18E-05
chr8	35002001	35007000	1.30E-05
chr8	35003001	35008000	3.05E-05
chr8	35004001	35009000	3.38E-06
chr8	35252001	35257000	0.000216582
chr8	35647001	35652000	0.000590013
chr8	36351001	36356000	0.000964692
chr8	36352001	36357000	0.000701355
chr8	37638001	37643000	1.01E-10
chr8	37751001	37756000	4.98E-15
chr8	37780001	37785000	9.80E-11
chr8	37781001	37786000	2.08E-16
chr8	37782001	37787000	4.87E-15
chr8	38035001	38040000	1.80E-20
chr8	38388001	38393000	8.50E-07
chr8	38603001	38608000	8.33E-06
chr8	38632001	38637000	1.39E-10
chr8	38806001	38811000	1.22E-11
chr8	38807001	38812000	3.00E-08
chr8	39927001	39932000	7.38E-07
chr8	39928001	39933000	2.47E-06
chr8	39962001	39967000	0.008100767
chr8	40011001	40016000	2.13E-26
chr8	40012001	40017000	1.52E-28
chr8	40013001	40018000	5.77E-15
chr8	40165001	40170000	2.42E-05
chr8	40166001	40171000	3.70E-06
chr8	40557001	40562000	6.64E-08
chr8	40558001	40563000	2.38E-09
chr8	40709001	40714000	8.19E-09
chr8	41369001	41374000	6.12E-07
chr8	41478001	41483000	2.18E-21
chr8	41479001	41484000	4.60E-18
chr8	41480001	41485000	1.72E-18
chr8	43091001	43096000	4.08E-08
chr8	43591001	43596000	2.21E-06
chr8	43592001	43597000	4.52E-06
chr8	43593001	43598000	0.000176183
chr8	43594001	43599000	0.000207149

chr8	43595001	43600000	0.000395093
chr8	43631001	43636000	9.89E-06
chr8	43632001	43637000	5.04E-05
chr8	43633001	43638000	1.92E-05
chr8	43634001	43639000	0.001644977
chr8	43637001	43642000	0.002352866
chr8	43640001	43645000	8.02E-05
chr8	43641001	43646000	7.85E-05
chr8	43671001	43676000	1.11E-05
chr8	43672001	43677000	0.001302005
chr8	47484001	47489000	3.10E-08
chr8	48288001	48293000	7.33E-10
chr8	48289001	48294000	1.12E-13
chr8	48290001	48295000	2.84E-17
chr8	48291001	48296000	2.31E-17
chr8	48292001	48297000	7.74E-15
chr8	49131001	49136000	2.63E-06
chr8	49846001	49851000	0.000585479
chr8	49847001	49852000	0.001297116
chr8	49888001	49893000	9.06E-41
chr8	49889001	49894000	2.57E-52
chr8	49890001	49895000	5.46E-68
chr8	49891001	49896000	5.27E-20
chr8	49892001	49897000	5.94E-12
chr8	49893001	49898000	1.19E-08
chr8	49899001	49904000	0.000127638
chr8	49921001	49926000	6.76E-07
chr8	50033001	50038000	0.000172807
chr8	50034001	50039000	0.000116271
chr8	50035001	50040000	0.000276991
chr8	50043001	50048000	1.06E-07
chr8	50096001	50101000	6.19E-05
chr8	50097001	50102000	0.000144993
chr8	50098001	50103000	0.000188615
chr8	50099001	50104000	2.90E-07
chr8	50155001	50160000	0.000437493
chr8	50156001	50161000	0.000314457
chr8	50157001	50162000	0.001178884
chr8	50158001	50163000	7.57E-05
chr8	50204001	50209000	2.79E-16
chr8	50205001	50210000	4.87E-15
chr8	50206001	50211000	1.46E-15
chr8	50207001	50212000	4.08E-15
chr8	50208001	50213000	1.65E-13
chr8	50233001	50238000	2.88E-08
chr8	50237001	50242000	0.003716548
chr8	50238001	50243000	2.03E-06
chr8	50239001	50244000	2.61E-06

chr8	50240001	50245000	2.52E-07
chr8	50241001	50246000	1.26E-08
chr8	50254001	50259000	9.09E-10
chr8	50351001	50356000	0.000925515
chr8	50353001	50358000	0.000102415
chr8	50365001	50370000	0.00019788
chr8	50412001	50417000	1.06E-05
chr8	50413001	50418000	1.89E-07
chr8	50448001	50453000	6.90E-10
chr8	50450001	50455000	1.53E-08
chr8	50514001	50519000	0.000346552
chr8	50515001	50520000	0.000361028
chr8	50516001	50521000	1.25E-05
chr8	50517001	50522000	4.26E-05
chr8	50592001	50597000	1.25E-06
chr8	50593001	50598000	0.002112411
chr8	50604001	50609000	5.83E-06
chr8	50679001	50684000	0.021825014
chr8	50719001	50724000	0.000110797
chr8	50720001	50725000	7.47E-06
chr8	50836001	50841000	1.59E-06
chr8	50837001	50842000	7.13E-06
chr8	50838001	50843000	7.28E-05
chr8	50867001	50872000	7.90E-09
chr8	50868001	50873000	8.11E-07
chr8	50878001	50883000	0.001145876
chr8	50887001	50892000	1.10E-11
chr8	50888001	50893000	3.55E-18
chr8	50889001	50894000	3.40E-20
chr8	50890001	50895000	3.54E-18
chr8	50891001	50896000	2.31E-15
chr8	50892001	50897000	3.76E-13
chr8	50893001	50898000	1.28E-08
chr8	50894001	50899000	3.48E-08
chr8	50895001	50900000	9.78E-09
chr8	50908001	50913000	6.37E-06
chr8	50909001	50914000	2.41E-09
chr8	50910001	50915000	3.80E-11
chr8	50911001	50916000	6.61E-14
chr8	50912001	50917000	1.55E-13
chr8	50934001	50939000	0.000914863
chr8	50935001	50940000	6.06E-05
chr8	50936001	50941000	0.000732518
chr8	50963001	50968000	0.000306839
chr8	50964001	50969000	0.000151536
chr8	50965001	50970000	0.004223461
chr8	50966001	50971000	0.001984321
chr8	50967001	50972000	0.001850975

chr8	50973001	50978000	0.001067328
chr8	50982001	50987000	8.11E-08
chr8	50983001	50988000	1.38E-16
chr8	51003001	51008000	2.81E-06
chr8	51004001	51009000	9.68E-08
chr8	51005001	51010000	3.77E-06
chr8	51006001	51011000	3.92E-05
chr8	51018001	51023000	1.41E-06
chr8	51019001	51024000	9.76E-10
chr8	51020001	51025000	4.70E-11
chr8	51021001	51026000	5.02E-07
chr8	51022001	51027000	2.59E-05
chr8	51030001	51035000	0.004259469
chr8	51037001	51042000	0.000186549
chr8	51047001	51052000	1.11E-08
chr8	51050001	51055000	1.12E-09
chr8	51053001	51058000	4.56E-06
chr8	51087001	51092000	1.06E-07
chr8	51088001	51093000	8.76E-13
chr8	51089001	51094000	9.52E-06
chr8	51145001	51150000	0.00036916
chr8	51146001	51151000	7.41E-07
chr8	51147001	51152000	7.14E-05
chr8	51148001	51153000	7.33E-05
chr8	51149001	51154000	8.60E-07
chr8	51152001	51157000	5.82E-07
chr8	51185001	51190000	2.59E-07
chr8	51186001	51191000	3.39E-09
chr8	51187001	51192000	1.82E-09
chr8	51189001	51194000	3.40E-08
chr8	51190001	51195000	0.000158152
chr8	51214001	51219000	0.000372431
chr8	51254001	51259000	0.003287441
chr8	51255001	51260000	1.19E-08
chr8	51256001	51261000	1.35E-06
chr8	51257001	51262000	1.76E-07
chr8	51258001	51263000	1.12E-27
chr8	51261001	51266000	5.94E-20
chr8	51262001	51267000	1.54E-21
chr8	51264001	51269000	2.70E-07
chr8	51272001	51277000	0.001131186
chr8	51273001	51278000	0.000111304
chr8	51274001	51279000	0.001622345
chr8	51283001	51288000	0.000875494
chr8	51284001	51289000	0.00020718
chr8	51285001	51290000	1.02E-05
chr8	51304001	51309000	9.48E-11
chr8	51305001	51310000	2.94E-14

chr8	51319001	51324000	3.47E-08
chr8	51320001	51325000	1.34E-08
chr8	51322001	51327000	7.27E-10
chr8	51323001	51328000	3.37E-11
chr8	51324001	51329000	1.68E-07
chr8	51356001	51361000	1.15E-05
chr8	51358001	51363000	4.59E-07
chr8	51362001	51367000	4.46E-08
chr8	51363001	51368000	6.44E-10
chr8	51364001	51369000	2.08E-07
chr8	51378001	51383000	5.75E-06
chr8	51405001	51410000	0.019846768
chr8	51414001	51419000	8.78E-09
chr8	51450001	51455000	2.85E-07
chr8	51468001	51473000	2.74E-06
chr8	51474001	51479000	4.42E-06
chr8	51475001	51480000	2.56E-07
chr8	51476001	51481000	2.87E-07
chr8	51477001	51482000	2.04E-08
chr8	51492001	51497000	1.76E-06
chr8	51547001	51552000	1.66E-05
chr8	51548001	51553000	0.000182896
chr8	51568001	51573000	6.61E-06
chr8	51569001	51574000	1.32E-07
chr8	51571001	51576000	4.66E-06
chr8	51572001	51577000	1.50E-08
chr8	51573001	51578000	3.11E-05
chr8	51574001	51579000	0.000122148
chr8	51589001	51594000	2.50E-06
chr8	51590001	51595000	2.34E-05
chr8	51596001	51601000	0.00032319
chr8	51605001	51610000	6.85E-14
chr8	51611001	51616000	1.65E-06
chr8	51622001	51627000	1.95E-07
chr8	51623001	51628000	6.52E-09
chr8	51624001	51629000	6.45E-07
chr8	51657001	51662000	4.64E-05
chr8	51665001	51670000	1.36E-05
chr8	51666001	51671000	9.32E-08
chr8	51667001	51672000	4.44E-08
chr8	51668001	51673000	1.35E-08
chr8	51674001	51679000	2.46E-08
chr8	51675001	51680000	4.56E-10
chr8	51699001	51704000	4.49E-09
chr8	51700001	51705000	1.80E-07
chr8	51701001	51706000	2.85E-07
chr8	51702001	51707000	0.000402122
chr8	51727001	51732000	0.001684836

chr8	51728001	51733000	0.002722675
chr8	51729001	51734000	0.000117307
chr8	51751001	51756000	1.15E-05
chr8	51752001	51757000	0.000460428
chr8	51753001	51758000	9.41E-06
chr8	51754001	51759000	0.000159127
chr8	51794001	51799000	4.76E-05
chr8	51813001	51818000	2.19E-11
chr8	51814001	51819000	6.35E-11
chr8	51815001	51820000	6.55E-09
chr8	51961001	51966000	4.79E-06
chr8	51987001	51992000	6.91E-07
chr8	52047001	52052000	2.58E-13
chr8	52048001	52053000	4.24E-14
chr8	52049001	52054000	3.26E-13
chr8	52050001	52055000	1.92E-13
chr8	52051001	52056000	2.98E-12
chr8	52057001	52062000	1.44E-08
chr8	52058001	52063000	3.94E-11
chr8	52059001	52064000	1.51E-06
chr8	52071001	52076000	4.45E-16
chr8	52101001	52106000	0.000128073
chr8	52102001	52107000	4.99E-05
chr8	52229001	52234000	6.15E-08
chr8	52263001	52268000	5.56E-09
chr8	52276001	52281000	2.23E-08
chr8	52640001	52645000	0.000423844
chr8	53172001	53177000	3.34E-07
chr8	53182001	53187000	1.22E-20
chr8	53183001	53188000	7.21E-20
chr8	53232001	53237000	1.35E-05
chr8	53234001	53239000	2.56E-05
chr8	53739001	53744000	2.44E-08
chr8	53740001	53745000	1.15E-06
chr8	53741001	53746000	3.19E-06
chr8	55527001	55532000	8.23E-07
chr8	55652001	55657000	3.51E-06
chr8	55735001	55740000	7.89E-09
chr8	55736001	55741000	1.81E-16
chr8	55737001	55742000	5.99E-18
chr8	55738001	55743000	1.60E-11
chr8	55933001	55938000	5.47E-10
chr8	56150001	56155000	0.000188324
chr8	57235001	57240000	2.34E-05
chr8	57236001	57241000	0.000241949
chr8	57575001	57580000	2.84E-09
chr8	57576001	57581000	3.27E-07
chr8	57577001	57582000	2.22E-07

chr8	57608001	57613000	3.10E-07
chr8	57609001	57614000	6.25E-06
chr8	57610001	57615000	4.50E-05
chr8	57611001	57616000	2.91E-05
chr8	57612001	57617000	2.46E-06
chr8	57716001	57721000	4.54E-05
chr8	57735001	57740000	1.83E-05
chr8	57806001	57811000	8.48E-06
chr8	57910001	57915000	9.57E-07
chr8	57933001	57938000	1.84E-12
chr8	57934001	57939000	7.75E-13
chr8	57935001	57940000	7.50E-07
chr8	57950001	57955000	2.37E-06
chr8	58038001	58043000	3.27E-05
chr8	58197001	58202000	1.54E-06
chr8	58198001	58203000	2.51E-08
chr8	58199001	58204000	4.87E-06
chr8	58350001	58355000	0.000478279
chr8	58412001	58417000	9.92E-06
chr8	58432001	58437000	5.12E-05
chr8	58538001	58543000	6.70E-05
chr8	58585001	58590000	0.000412764
chr8	58631001	58636000	5.91E-09
chr8	58632001	58637000	3.38E-07
chr8	58642001	58647000	0.000798099
chr8	58654001	58659000	1.89E-06
chr8	58769001	58774000	0.000181107
chr8	58770001	58775000	1.06E-07
chr8	58771001	58776000	2.44E-07
chr8	58772001	58777000	7.78E-06
chr8	58880001	58885000	0.001112679
chr8	58900001	58905000	5.10E-10
chr8	59133001	59138000	4.50E-11
chr8	59134001	59139000	9.32E-09
chr8	59650001	59655000	3.28E-08
chr8	59662001	59667000	3.14E-07
chr8	59737001	59742000	1.69E-07
chr8	59738001	59743000	5.57E-06
chr8	59831001	59836000	4.44E-13
chr8	60045001	60050000	4.00E-06
chr8	60046001	60051000	1.49E-05
chr8	60047001	60052000	1.25E-08
chr8	60048001	60053000	0.00074125
chr8	60131001	60136000	2.18E-06
chr8	60133001	60138000	1.96E-10
chr8	60156001	60161000	1.05E-07
chr8	60175001	60180000	9.15E-10
chr8	60189001	60194000	5.93E-07

chr8	60209001	60214000	7.61E-07
chr8	60239001	60244000	1.09E-07
chr8	60240001	60245000	7.21E-08
chr8	60319001	60324000	0.000553477
chr8	60346001	60351000	2.77E-10
chr8	60455001	60460000	1.22E-13
chr8	60508001	60513000	4.08E-05
chr8	60547001	60552000	0.000337074
chr8	60548001	60553000	4.29E-05
chr8	60549001	60554000	0.000606508
chr8	60550001	60555000	0.000720225
chr8	60604001	60609000	7.04E-05
chr8	60605001	60610000	6.84E-05
chr8	60606001	60611000	0.000201793
chr8	60607001	60612000	0.000119826
chr8	60752001	60757000	3.57E-06
chr8	60825001	60830000	3.07E-06
chr8	60955001	60960000	2.06E-13
chr8	60975001	60980000	0.001429525
chr8	60976001	60981000	0.000628594
chr8	62007001	62012000	3.51E-17
chr8	62008001	62013000	7.92E-19
chr8	62140001	62145000	8.76E-13
chr8	62141001	62146000	1.98E-10
chr8	62150001	62155000	9.68E-08
chr8	62151001	62156000	8.14E-09
chr8	62152001	62157000	1.66E-06
chr8	62161001	62166000	6.99E-06
chr8	62167001	62172000	1.61E-13
chr8	62168001	62173000	1.46E-13
chr8	62230001	62235000	0.010866967
chr8	62234001	62239000	0.00027965
chr8	62235001	62240000	5.87E-07
chr8	62236001	62241000	1.02E-07
chr8	62238001	62243000	2.93E-08
chr8	62242001	62247000	4.58E-11
chr8	62286001	62291000	5.55E-14
chr8	62287001	62292000	1.75E-18
chr8	62288001	62293000	4.18E-22
chr8	62289001	62294000	1.50E-14
chr8	62300001	62305000	9.19E-11
chr8	62301001	62306000	6.93E-13
chr8	62302001	62307000	6.84E-12
chr8	62335001	62340000	3.07E-08
chr8	62336001	62341000	4.32E-10
chr8	62337001	62342000	1.53E-10
chr8	62338001	62343000	1.87E-10
chr8	62340001	62345000	1.33E-08

chr8	62341001	62346000	1.65E-08
chr8	62345001	62350000	0.000151972
chr8	62346001	62351000	1.06E-05
chr8	62347001	62352000	9.81E-05
chr8	62356001	62361000	6.24E-05
chr8	62371001	62376000	1.55E-05
chr8	62372001	62377000	2.06E-05
chr8	62382001	62387000	2.97E-14
chr8	62383001	62388000	3.56E-13
chr8	62406001	62411000	0.000243274
chr8	62407001	62412000	2.35E-05
chr8	62689001	62694000	2.00E-10
chr8	62690001	62695000	3.07E-11
chr8	62702001	62707000	6.16E-13
chr8	62723001	62728000	6.93E-15
chr8	62724001	62729000	6.70E-18
chr8	62725001	62730000	6.24E-16
chr8	62733001	62738000	2.52E-12
chr8	62734001	62739000	3.76E-11
chr8	62736001	62741000	2.62E-06
chr8	62752001	62757000	5.49E-10
chr8	62753001	62758000	2.27E-09
chr8	62764001	62769000	4.99E-07
chr8	62765001	62770000	1.47E-06
chr8	62766001	62771000	2.85E-06
chr8	62767001	62772000	3.30E-05
chr8	62774001	62779000	6.22E-06
chr8	62775001	62780000	2.25E-07
chr8	62776001	62781000	4.24E-08
chr8	62777001	62782000	4.51E-07
chr8	62778001	62783000	2.72E-05
chr8	62799001	62804000	3.05E-08
chr8	62800001	62805000	1.76E-10
chr8	62801001	62806000	9.77E-07
chr8	62812001	62817000	1.25E-18
chr8	62813001	62818000	1.41E-20
chr8	62814001	62819000	5.64E-19
chr8	62815001	62820000	1.09E-20
chr8	62830001	62835000	1.84E-08
chr8	62831001	62836000	2.59E-07
chr8	62832001	62837000	5.74E-08
chr8	62837001	62842000	5.87E-11
chr8	62838001	62843000	2.02E-11
chr8	62839001	62844000	4.67E-10
chr8	62883001	62888000	7.47E-05
chr8	62885001	62890000	5.12E-07
chr8	62899001	62904000	3.85E-05
chr8	62916001	62921000	4.42E-07

chr8	62917001	62922000	1.25E-10
chr8	62918001	62923000	2.37E-12
chr8	62928001	62933000	0.000288528
chr8	62956001	62961000	1.77E-13
chr8	62957001	62962000	4.22E-11
chr8	62958001	62963000	2.83E-09
chr8	63024001	63029000	0.000340913
chr8	63025001	63030000	0.000653591
chr8	63031001	63036000	0.000778562
chr8	63034001	63039000	3.76E-05
chr8	63035001	63040000	1.11E-05
chr8	63036001	63041000	3.97E-07
chr8	63037001	63042000	1.46E-09
chr8	63038001	63043000	7.80E-09
chr8	63039001	63044000	5.20E-05
chr8	63040001	63045000	9.46E-05
chr8	63042001	63047000	2.87E-19
chr8	63043001	63048000	5.70E-19
chr8	63044001	63049000	7.70E-16
chr8	63045001	63050000	2.05E-16
chr8	63046001	63051000	1.15E-10
chr8	63047001	63052000	0.000440023
chr8	63061001	63066000	2.95E-05
chr8	63062001	63067000	1.89E-05
chr8	63064001	63069000	6.45E-06
chr8	63065001	63070000	0.000140413
chr8	63082001	63087000	0.006426131
chr8	63083001	63088000	0.00893426
chr8	63085001	63090000	0.000239009
chr8	63088001	63093000	2.66E-24
chr8	63089001	63094000	2.52E-20
chr8	63090001	63095000	2.27E-21
chr8	63091001	63096000	1.19E-20
chr8	63092001	63097000	5.98E-16
chr8	63106001	63111000	3.95E-07
chr8	63107001	63112000	3.58E-10
chr8	63108001	63113000	8.49E-09
chr8	63109001	63114000	4.34E-08
chr8	63113001	63118000	4.26E-08
chr8	63114001	63119000	9.17E-07
chr8	63127001	63132000	7.43E-06
chr8	63130001	63135000	0.00026343
chr8	63146001	63151000	0.000109399
chr8	63152001	63157000	6.56E-13
chr8	63181001	63186000	2.30E-05
chr8	63182001	63187000	1.27E-05
chr8	63224001	63229000	3.44E-10
chr8	63244001	63249000	1.53E-06

chr8	63262001	63267000	2.69E-05
chr8	63264001	63269000	8.03E-10
chr8	63306001	63311000	1.50E-21
chr8	63307001	63312000	5.33E-25
chr8	63312001	63317000	3.63E-13
chr8	63313001	63318000	4.61E-16
chr8	63314001	63319000	1.62E-16
chr8	63315001	63320000	3.23E-16
chr8	63322001	63327000	6.96E-09
chr8	63323001	63328000	6.20E-11
chr8	63324001	63329000	1.15E-11
chr8	63327001	63332000	4.46E-09
chr8	63328001	63333000	1.37E-07
chr8	63329001	63334000	2.01E-05
chr8	63330001	63335000	2.17E-05
chr8	63331001	63336000	6.18E-07
chr8	63347001	63352000	1.70E-06
chr8	63354001	63359000	3.97E-05
chr8	63414001	63419000	3.50E-12
chr8	63415001	63420000	1.23E-10
chr8	63423001	63428000	4.54E-07
chr8	63424001	63429000	0.008978469
chr8	63428001	63433000	4.93E-05
chr8	63438001	63443000	2.44E-07
chr8	63463001	63468000	8.56E-05
chr8	63464001	63469000	0.000265406
chr8	63465001	63470000	0.000321444
chr8	63485001	63490000	1.29E-05
chr8	63487001	63492000	4.68E-05
chr8	63496001	63501000	3.94E-06
chr8	63497001	63502000	2.61E-06
chr8	63498001	63503000	0.000414887
chr8	63522001	63527000	9.57E-06
chr8	63523001	63528000	4.95E-08
chr8	63531001	63536000	9.79E-10
chr8	63532001	63537000	1.86E-07
chr8	63533001	63538000	1.44E-05
chr8	63534001	63539000	3.52E-06
chr8	63535001	63540000	8.73E-11
chr8	63550001	63555000	3.64E-07
chr8	63551001	63556000	2.15E-05
chr8	63568001	63573000	1.09E-06
chr8	63589001	63594000	4.75E-11
chr8	63596001	63601000	1.42E-05
chr8	63632001	63637000	0.000174252
chr8	63633001	63638000	0.000126106
chr8	63666001	63671000	0.001046582
chr8	63667001	63672000	0.001046582

chr8	63668001	63673000	0.001513169
chr8	63669001	63674000	0.000224342
chr8	63683001	63688000	2.13E-05
chr8	63735001	63740000	0.000104654
chr8	63736001	63741000	0.000148842
chr8	63737001	63742000	0.000305281
chr8	63778001	63783000	1.39E-26
chr8	63782001	63787000	6.14E-18
chr8	63795001	63800000	3.33E-05
chr8	63806001	63811000	7.35E-07
chr8	63807001	63812000	4.01E-10
chr8	63809001	63814000	5.68E-08
chr8	64409001	64414000	5.54E-09
chr8	64410001	64415000	4.63E-10
chr8	64423001	64428000	1.71E-10
chr8	64425001	64430000	5.20E-09
chr8	64427001	64432000	0.012471553
chr8	64544001	64549000	6.47E-08
chr8	64595001	64600000	5.19E-06
chr8	64597001	64602000	0.000194761
chr8	64599001	64604000	0.000244565
chr8	64750001	64755000	0.002258467
chr8	64751001	64756000	0.002375595
chr8	64752001	64757000	0.002786339
chr8	64760001	64765000	0.00029321
chr8	64761001	64766000	0.000371905
chr8	64762001	64767000	0.000542725
chr8	64769001	64774000	1.96E-06
chr8	64803001	64808000	2.14E-10
chr8	64804001	64809000	2.85E-13
chr8	64805001	64810000	4.87E-10
chr8	64806001	64811000	1.39E-10
chr8	64807001	64812000	1.03E-09
chr8	64829001	64834000	0.007747256
chr8	64868001	64873000	0.000374796
chr8	65037001	65042000	9.79E-05
chr8	65038001	65043000	0.000360787
chr8	65103001	65108000	3.94E-09
chr8	65104001	65109000	7.89E-09
chr8	65378001	65383000	9.88E-08
chr8	65379001	65384000	7.10E-06
chr8	65437001	65442000	0.00058297
chr8	65438001	65443000	7.14E-05
chr8	65439001	65444000	0.000248123
chr8	65455001	65460000	0.00181319
chr8	65480001	65485000	1.04E-05
chr8	65481001	65486000	1.89E-05
chr8	65939001	65944000	0.000769348

chr8	65940001	65945000	5.05E-05
chr8	65941001	65946000	3.63E-08
chr8	65942001	65947000	2.31E-08
chr8	66020001	66025000	0.000207
chr8	66021001	66026000	0.000392735
chr8	66221001	66226000	0.004374539
chr8	66549001	66554000	3.89E-16
chr8	67092001	67097000	0.001275061
chr8	69013001	69018000	0.007398136
chr8	69069001	69074000	0.001408232
chr8	69074001	69079000	7.64E-10
chr8	69077001	69082000	1.36E-12
chr8	69261001	69266000	0.00262399
chr8	69262001	69267000	4.70E-07
chr8	69263001	69268000	2.23E-05
chr8	69413001	69418000	6.24E-11
chr8	69414001	69419000	1.12E-07
chr8	69415001	69420000	1.82E-07
chr8	69422001	69427000	1.89E-05
chr8	69489001	69494000	0.001322939
chr8	69492001	69497000	0.000266896
chr8	69530001	69535000	1.33E-08
chr8	69531001	69536000	3.83E-11
chr8	69547001	69552000	1.20E-06
chr8	69548001	69553000	6.78E-07
chr8	69549001	69554000	2.63E-06
chr8	69550001	69555000	8.88E-06
chr8	69551001	69556000	1.57E-06
chr8	69568001	69573000	9.66E-05
chr8	69569001	69574000	3.46E-07
chr8	69580001	69585000	9.81E-05
chr8	69585001	69590000	1.87E-09
chr8	69586001	69591000	3.79E-07
chr8	69614001	69619000	2.83E-08
chr8	69615001	69620000	1.07E-07
chr8	69616001	69621000	2.70E-08
chr8	69617001	69622000	6.34E-10
chr8	69618001	69623000	5.81E-15
chr8	69619001	69624000	7.35E-13
chr8	69620001	69625000	1.85E-10
chr8	69621001	69626000	5.20E-12
chr8	69642001	69647000	2.17E-05
chr8	69643001	69648000	4.11E-05
chr8	69644001	69649000	2.15E-05
chr8	69645001	69650000	0.000322935
chr8	69663001	69668000	4.15E-08
chr8	69681001	69686000	0.001072136
chr8	69691001	69696000	2.47E-08

chr8	69692001	69697000	7.17E-06
chr8	69693001	69698000	2.08E-07
chr8	69694001	69699000	1.44E-09
chr8	69695001	69700000	1.13E-10
chr8	69696001	69701000	6.12E-05
chr8	69697001	69702000	2.33E-07
chr8	69698001	69703000	8.13E-07
chr8	69699001	69704000	1.22E-05
chr8	69710001	69715000	4.20E-05
chr8	69711001	69716000	5.15E-05
chr8	69712001	69717000	1.96E-05
chr8	69713001	69718000	1.05E-05
chr8	69714001	69719000	7.15E-06
chr8	69718001	69723000	9.94E-15
chr8	69720001	69725000	7.41E-19
chr8	69727001	69732000	3.99E-07
chr8	69737001	69742000	2.20E-11
chr8	69738001	69743000	2.68E-12
chr8	69739001	69744000	1.45E-08
chr8	69740001	69745000	1.68E-08
chr8	69741001	69746000	3.25E-09
chr8	69742001	69747000	5.95E-05
chr8	69744001	69749000	0.000353586
chr8	69745001	69750000	9.20E-05
chr8	69746001	69751000	0.001299754
chr8	69752001	69757000	1.87E-09
chr8	69753001	69758000	1.36E-10
chr8	69761001	69766000	1.15E-09
chr8	69762001	69767000	8.26E-06
chr8	69763001	69768000	0.00056488
chr8	69775001	69780000	3.99E-07
chr8	69777001	69782000	1.15E-06
chr8	69779001	69784000	8.06E-10
chr8	69781001	69786000	2.10E-09
chr8	69782001	69787000	2.14E-08
chr8	69783001	69788000	3.38E-08
chr8	69819001	69824000	4.05E-08
chr8	69820001	69825000	3.55E-08
chr8	69821001	69826000	1.61E-07
chr8	69822001	69827000	2.67E-06
chr8	69823001	69828000	3.61E-06
chr8	69831001	69836000	5.79E-10
chr8	69832001	69837000	9.64E-09
chr8	69833001	69838000	1.68E-10
chr8	69834001	69839000	1.84E-12
chr8	69835001	69840000	1.44E-12
chr8	69836001	69841000	3.31E-07
chr8	69837001	69842000	5.43E-31

chr8	69838001	69843000	1.86E-32
chr8	69839001	69844000	2.62E-29
chr8	69840001	69845000	1.08E-26
chr8	69841001	69846000	7.52E-24
chr8	69843001	69848000	0.002301374
chr8	69845001	69850000	5.20E-05
chr8	69846001	69851000	0.000178735
chr8	69847001	69852000	0.000114137
chr8	69848001	69853000	2.99E-08
chr8	69849001	69854000	1.58E-07
chr8	69850001	69855000	1.65E-08
chr8	69851001	69856000	7.67E-11
chr8	69852001	69857000	1.11E-11
chr8	69853001	69858000	1.57E-06
chr8	69854001	69859000	3.90E-07
chr8	69855001	69860000	3.60E-05
chr8	69889001	69894000	1.84E-24
chr8	69890001	69895000	2.14E-22
chr8	69898001	69903000	4.00E-08
chr8	69909001	69914000	2.97E-18
chr8	69912001	69917000	2.06E-19
chr8	69921001	69926000	1.06E-05
chr8	69922001	69927000	9.44E-08
chr8	69923001	69928000	1.53E-12
chr8	69924001	69929000	3.74E-10
chr8	69925001	69930000	3.61E-11
chr8	69926001	69931000	5.35E-08
chr8	69937001	69942000	1.65E-18
chr8	69938001	69943000	1.95E-22
chr8	69939001	69944000	1.93E-27
chr8	69940001	69945000	6.96E-28
chr8	69941001	69946000	1.04E-27
chr8	69950001	69955000	4.91E-05
chr8	69951001	69956000	0.000199282
chr8	69952001	69957000	0.000466968
chr8	69980001	69985000	1.29E-05
chr8	69981001	69986000	9.65E-22
chr8	69982001	69987000	7.12E-25
chr8	69983001	69988000	7.54E-34
chr8	69984001	69989000	2.12E-28
chr8	69985001	69990000	9.37E-27
chr8	69997001	70002000	1.66E-14
chr8	69998001	70003000	1.73E-12
chr8	69999001	70004000	4.15E-11
chr8	70000001	70005000	8.32E-09
chr8	70001001	70006000	3.14E-08
chr8	70016001	70021000	1.46E-06
chr8	70017001	70022000	2.15E-09

chr8	70018001	70023000	3.73E-09
chr8	70019001	70024000	2.54E-08
chr8	70020001	70025000	2.90E-08
chr8	70021001	70026000	5.92E-05
chr8	70054001	70059000	7.00E-08
chr8	70055001	70060000	2.22E-10
chr8	70056001	70061000	9.47E-09
chr8	70057001	70062000	2.86E-18
chr8	70058001	70063000	4.75E-14
chr8	70059001	70064000	7.11E-14
chr8	70060001	70065000	5.88E-13
chr8	70061001	70066000	1.72E-12
chr8	70089001	70094000	7.44E-06
chr8	70090001	70095000	2.91E-08
chr8	70091001	70096000	1.38E-09
chr8	70092001	70097000	2.25E-09
chr8	70101001	70106000	0.000195259
chr8	70105001	70110000	9.96E-10
chr8	70106001	70111000	2.09E-11
chr8	70107001	70112000	2.10E-08
chr8	70108001	70113000	2.01E-08
chr8	70109001	70114000	8.59E-11
chr8	70110001	70115000	9.89E-08
chr8	70111001	70116000	4.17E-08
chr8	70112001	70117000	1.04E-10
chr8	70113001	70118000	1.65E-11
chr8	70114001	70119000	7.13E-10
chr8	70117001	70122000	5.33E-06
chr8	70118001	70123000	3.25E-08
chr8	70121001	70126000	3.93E-07
chr8	70131001	70136000	0.000142178
chr8	70132001	70137000	5.72E-06
chr8	70141001	70146000	0.014572343
chr8	70142001	70147000	0.002060133
chr8	70143001	70148000	6.55E-06
chr8	70144001	70149000	3.91E-06
chr8	70145001	70150000	7.10E-07
chr8	70172001	70177000	3.99E-07
chr8	70173001	70178000	8.31E-08
chr8	70174001	70179000	9.90E-09
chr8	70175001	70180000	3.65E-08
chr8	70258001	70263000	1.96E-08
chr8	70283001	70288000	9.24E-06
chr8	70284001	70289000	1.45E-07
chr8	70285001	70290000	9.08E-14
chr8	70286001	70291000	8.44E-12
chr8	70287001	70292000	1.25E-12
chr8	70347001	70352000	1.20E-07

chr8	70349001	70354000	3.66E-11
chr8	70350001	70355000	4.43E-08
chr8	70391001	70396000	1.28E-06
chr8	70406001	70411000	9.52E-06
chr8	70419001	70424000	4.74E-08
chr8	70431001	70436000	2.46E-12
chr8	70432001	70437000	7.01E-12
chr8	70433001	70438000	2.92E-16
chr8	70434001	70439000	2.84E-14
chr8	70464001	70469000	1.78E-09
chr8	70495001	70500000	6.42E-08
chr8	70496001	70501000	1.70E-06
chr8	70498001	70503000	2.13E-11
chr8	70502001	70507000	1.26E-16
chr8	70503001	70508000	9.42E-13
chr8	70504001	70509000	2.27E-12
chr8	70517001	70522000	4.96E-17
chr8	70518001	70523000	2.37E-16
chr8	70519001	70524000	2.18E-14
chr8	70520001	70525000	3.27E-13
chr8	70521001	70526000	1.89E-11
chr8	70588001	70593000	1.16E-12
chr8	70589001	70594000	2.95E-23
chr8	70590001	70595000	3.75E-23
chr8	70591001	70596000	9.11E-20
chr8	70592001	70597000	2.58E-20
chr8	70593001	70598000	8.79E-16
chr8	70608001	70613000	6.62E-14
chr8	70609001	70614000	1.09E-09
chr8	70667001	70672000	4.57E-16
chr8	70669001	70674000	8.40E-16
chr8	70670001	70675000	1.20E-18
chr8	70671001	70676000	1.72E-11
chr8	70698001	70703000	8.33E-08
chr8	70699001	70704000	4.11E-11
chr8	70703001	70708000	3.29E-07
chr8	70760001	70765000	2.84E-16
chr8	70761001	70766000	8.86E-16
chr8	70762001	70767000	3.23E-16
chr8	70763001	70768000	1.64E-20
chr8	70764001	70769000	1.42E-18
chr8	70765001	70770000	3.78E-15
chr8	70783001	70788000	5.64E-22
chr8	70784001	70789000	5.82E-36
chr8	70785001	70790000	7.46E-39
chr8	70787001	70792000	0.000403212
chr8	70788001	70793000	4.66E-06
chr8	70789001	70794000	4.50E-08

chr8	70811001	70816000	4.72E-12
chr8	70837001	70842000	8.34E-18
chr8	70838001	70843000	2.12E-14
chr8	70849001	70854000	3.56E-09
chr8	70850001	70855000	3.07E-10
chr8	70908001	70913000	1.73E-26
chr8	70909001	70914000	7.89E-24
chr8	70910001	70915000	2.10E-19
chr8	71328001	71333000	1.26E-07
chr8	71385001	71390000	2.19E-05
chr8	71429001	71434000	7.74E-05
chr8	71522001	71527000	8.20E-12
chr8	71523001	71528000	2.49E-12
chr8	71524001	71529000	2.11E-12
chr8	71598001	71603000	4.75E-12
chr8	71613001	71618000	1.25E-11
chr8	71685001	71690000	5.44E-05
chr8	71703001	71708000	1.03E-06
chr8	71704001	71709000	6.69E-05
chr8	71741001	71746000	7.28E-08
chr8	71771001	71776000	1.52E-13
chr8	71772001	71777000	1.04E-14
chr8	71773001	71778000	1.70E-11
chr8	71774001	71779000	5.27E-07
chr8	71775001	71780000	1.68E-10
chr8	71781001	71786000	0.000334621
chr8	71784001	71789000	1.81E-07
chr8	71816001	71821000	7.20E-15
chr8	71817001	71822000	1.45E-11
chr8	71826001	71831000	3.03E-06
chr8	71827001	71832000	1.33E-11
chr8	71828001	71833000	3.21E-10
chr8	71829001	71834000	8.59E-10
chr8	71830001	71835000	7.33E-08
chr8	71861001	71866000	4.90E-16
chr8	71862001	71867000	5.53E-11
chr8	71863001	71868000	8.65E-13
chr8	71892001	71897000	2.42E-24
chr8	71893001	71898000	1.46E-19
chr8	71906001	71911000	1.14E-06
chr8	71922001	71927000	2.35E-14
chr8	71946001	71951000	0.000129081
chr8	71947001	71952000	2.26E-07
chr8	71948001	71953000	8.76E-06
chr8	71950001	71955000	6.17E-08
chr8	71951001	71956000	5.51E-08
chr8	71971001	71976000	5.65E-13
chr8	71972001	71977000	1.66E-11

chr8	71973001	71978000	3.97E-11
chr8	71974001	71979000	1.86E-12
chr8	71975001	71980000	1.28E-09
chr8	71981001	71986000	9.34E-06
chr8	71982001	71987000	0.000840755
chr8	71983001	71988000	0.002334413
chr8	71984001	71989000	0.002334413
chr8	72009001	72014000	1.66E-07
chr8	72010001	72015000	1.41E-08
chr8	72016001	72021000	0.001096646
chr8	72017001	72022000	0.000780115
chr8	72021001	72026000	8.70E-09
chr8	72022001	72027000	2.01E-10
chr8	72029001	72034000	2.69E-08
chr8	72030001	72035000	1.11E-07
chr8	72031001	72036000	1.51E-11
chr8	72032001	72037000	2.82E-09
chr8	72033001	72038000	3.28E-06
chr8	72034001	72039000	2.12E-13
chr8	72035001	72040000	1.98E-11
chr8	72079001	72084000	1.66E-07
chr8	72094001	72099000	7.15E-05
chr8	72112001	72117000	3.89E-13
chr8	72113001	72118000	3.81E-12
chr8	72114001	72119000	1.32E-08
chr8	72115001	72120000	3.20E-07
chr8	72123001	72128000	1.49E-06
chr8	72181001	72186000	2.46E-09
chr8	72200001	72205000	1.53E-06
chr8	72227001	72232000	1.47E-13
chr8	72228001	72233000	4.80E-15
chr8	72229001	72234000	2.91E-19
chr8	72230001	72235000	5.73E-22
chr8	72231001	72236000	1.52E-22
chr8	72232001	72237000	3.63E-12
chr8	72233001	72238000	1.21E-12
chr8	72234001	72239000	1.63E-07
chr8	72287001	72292000	1.78E-11
chr8	72288001	72293000	8.33E-12
chr8	72297001	72302000	9.23E-10
chr8	72298001	72303000	1.65E-08
chr8	72299001	72304000	8.42E-10
chr8	72313001	72318000	8.09E-05
chr8	72334001	72339000	2.22E-10
chr8	72335001	72340000	2.29E-10
chr8	72336001	72341000	3.21E-12
chr8	72337001	72342000	2.73E-12
chr8	72357001	72362000	0.000935751

chr8	72358001	72363000	6.05E-05
chr8	72359001	72364000	0.000419894
chr8	72387001	72392000	0.000955603
chr8	72411001	72416000	6.08E-09
chr8	72412001	72417000	6.73E-10
chr8	72413001	72418000	5.41E-09
chr8	72414001	72419000	5.25E-07
chr8	72415001	72420000	9.92E-09
chr8	72416001	72421000	1.64E-05
chr8	72440001	72445000	1.44E-05
chr8	72441001	72446000	5.65E-06
chr8	72444001	72449000	0.000131805
chr8	72446001	72451000	0.000839076
chr8	72453001	72458000	6.14E-12
chr8	72454001	72459000	3.48E-20
chr8	72469001	72474000	5.10E-40
chr8	72470001	72475000	1.34E-52
chr8	72471001	72476000	3.09E-07
chr8	72472001	72477000	5.41E-06
chr8	72473001	72478000	0.000172341
chr8	72487001	72492000	1.36E-06
chr8	72488001	72493000	1.35E-07
chr8	72489001	72494000	1.73E-13
chr8	72499001	72504000	0.003837269
chr8	72505001	72510000	3.28E-05
chr8	72513001	72518000	2.46E-19
chr8	72514001	72519000	2.16E-21
chr8	72515001	72520000	1.37E-16
chr8	72539001	72544000	0.000167734
chr8	72540001	72545000	8.43E-07
chr8	72543001	72548000	9.61E-07
chr8	72544001	72549000	5.06E-06
chr8	72550001	72555000	7.87E-12
chr8	72551001	72556000	8.62E-13
chr8	72552001	72557000	7.43E-09
chr8	72594001	72599000	0.000719727
chr8	72610001	72615000	0.016050663
chr8	72611001	72616000	1.28E-08
chr8	72612001	72617000	3.93E-10
chr8	72614001	72619000	4.58E-16
chr8	72615001	72620000	1.57E-12
chr8	72633001	72638000	0.000304823
chr8	72634001	72639000	4.50E-12
chr8	72642001	72647000	5.52E-06
chr8	72643001	72648000	6.11E-07
chr8	72644001	72649000	3.40E-05
chr8	72645001	72650000	1.96E-06
chr8	72648001	72653000	3.44E-05

chr8	72649001	72654000	6.81E-09
chr8	72683001	72688000	0.000256638
chr8	72726001	72731000	1.94E-09
chr8	72727001	72732000	4.10E-09
chr8	72835001	72840000	0.000907373
chr8	73244001	73249000	1.14E-05
chr8	73282001	73287000	0.001636
chr8	73375001	73380000	6.18E-25
chr8	75103001	75108000	0.000167594
chr8	75335001	75340000	1.22E-10
chr8	75336001	75341000	1.53E-05
chr8	75337001	75342000	8.66E-06
chr8	75338001	75343000	1.47E-05
chr8	75363001	75368000	0.012513927
chr8	75364001	75369000	0.000173288
chr8	75824001	75829000	8.43E-05
chr8	76111001	76116000	5.20E-06
chr8	76130001	76135000	6.00E-05
chr8	76279001	76284000	5.42E-06
chr8	76539001	76544000	1.50E-09
chr8	76588001	76593000	0.000437719
chr8	76704001	76709000	3.86E-05
chr8	76729001	76734000	1.61E-09
chr8	76730001	76735000	4.50E-09
chr8	76731001	76736000	1.07E-08
chr8	76755001	76760000	9.81E-06
chr8	76756001	76761000	1.61E-08
chr8	76805001	76810000	0.005057174
chr8	76806001	76811000	0.001699708
chr8	76897001	76902000	0.000477376
chr8	76898001	76903000	0.008921845
chr8	77086001	77091000	9.71E-06
chr8	77141001	77146000	1.53E-05
chr8	77142001	77147000	1.80E-05
chr8	77143001	77148000	1.09E-05
chr8	77145001	77150000	0.000921159
chr8	77237001	77242000	0.000310981
chr8	77242001	77247000	9.99E-07
chr8	77243001	77248000	6.18E-08
chr8	77246001	77251000	1.11E-06
chr8	77260001	77265000	9.74E-08
chr8	77267001	77272000	0.000525414
chr8	77282001	77287000	1.01E-05
chr8	77283001	77288000	3.47E-06
chr8	77284001	77289000	2.84E-06
chr8	77352001	77357000	1.59E-19
chr8	77353001	77358000	3.16E-18
chr8	77354001	77359000	3.01E-13

chr8	77373001	77378000	0.02849212
chr8	77374001	77379000	0.026722195
chr8	77414001	77419000	2.04E-05
chr8	77415001	77420000	3.38E-05
chr8	77453001	77458000	0.001433344
chr8	77461001	77466000	2.93E-06
chr8	77470001	77475000	0.000144307
chr8	77488001	77493000	1.75E-05
chr8	77501001	77506000	0.000440874
chr8	77502001	77507000	0.000692751
chr8	77503001	77508000	0.000286952
chr8	77504001	77509000	0.000291357
chr8	77511001	77516000	0.000240696
chr8	77517001	77522000	4.28E-09
chr8	77518001	77523000	1.01E-07
chr8	77530001	77535000	7.41E-05
chr8	77532001	77537000	7.90E-09
chr8	77542001	77547000	4.30E-07
chr8	77578001	77583000	0.013718295
chr8	77623001	77628000	2.05E-08
chr8	77624001	77629000	2.20E-08
chr8	77625001	77630000	8.90E-09
chr8	77650001	77655000	7.86E-06
chr8	77651001	77656000	9.33E-07
chr8	77652001	77657000	3.17E-13
chr8	77653001	77658000	9.06E-14
chr8	77654001	77659000	7.74E-15
chr8	77672001	77677000	1.19E-08
chr8	77675001	77680000	1.60E-10
chr8	77676001	77681000	5.68E-05
chr8	77677001	77682000	0.000167456
chr8	77681001	77686000	8.52E-12
chr8	77682001	77687000	8.62E-13
chr8	77683001	77688000	1.18E-13
chr8	77684001	77689000	6.76E-12
chr8	77685001	77690000	9.56E-08
chr8	77699001	77704000	0.007458123
chr8	77703001	77708000	1.33E-08
chr8	77704001	77709000	5.23E-08
chr8	77705001	77710000	1.46E-06
chr8	77735001	77740000	1.01E-06
chr8	77736001	77741000	9.19E-08
chr8	77737001	77742000	6.94E-09
chr8	77740001	77745000	6.36E-10
chr8	77741001	77746000	5.68E-11
chr8	77742001	77747000	8.75E-09
chr8	77743001	77748000	1.21E-07
chr8	77744001	77749000	1.10E-08

chr8	77745001	77750000	8.66E-09
chr8	77746001	77751000	8.12E-09
chr8	77747001	77752000	3.30E-08
chr8	77789001	77794000	1.13E-05
chr8	77790001	77795000	1.07E-05
chr8	77797001	77802000	3.49E-06
chr8	77798001	77803000	9.64E-08
chr8	77818001	77823000	8.57E-06
chr8	77820001	77825000	1.62E-06
chr8	77821001	77826000	1.04E-06
chr8	77822001	77827000	1.15E-05
chr8	77836001	77841000	5.07E-10
chr8	77837001	77842000	4.98E-09
chr8	77839001	77844000	8.34E-10
chr8	77840001	77845000	5.36E-06
chr8	77841001	77846000	0.002438712
chr8	77851001	77856000	3.75E-11
chr8	77852001	77857000	0.000181662
chr8	77868001	77873000	0.002421149
chr8	77869001	77874000	3.75E-09
chr8	77872001	77877000	2.10E-05
chr8	77907001	77912000	1.67E-08
chr8	77946001	77951000	2.10E-06
chr8	77947001	77952000	1.35E-06
chr8	77948001	77953000	5.48E-07
chr8	77961001	77966000	4.87E-06
chr8	77991001	77996000	1.48E-18
chr8	77992001	77997000	2.96E-17
chr8	78020001	78025000	4.87E-07
chr8	78021001	78026000	3.70E-07
chr8	78030001	78035000	1.70E-07
chr8	78060001	78065000	0.00055609
chr8	78073001	78078000	3.62E-05
chr8	78074001	78079000	0.001859061
chr8	78083001	78088000	0.000225086
chr8	78084001	78089000	9.83E-06
chr8	78085001	78090000	1.13E-06
chr8	78086001	78091000	1.26E-07
chr8	78087001	78092000	2.23E-07
chr8	78088001	78093000	4.40E-16
chr8	78089001	78094000	2.87E-15
chr8	78090001	78095000	8.48E-17
chr8	78091001	78096000	8.68E-17
chr8	78092001	78097000	2.06E-14
chr8	78111001	78116000	6.15E-05
chr8	78128001	78133000	0.002280795
chr8	78129001	78134000	2.95E-06
chr8	78140001	78145000	5.81E-06

chr8	78141001	78146000	7.92E-05
chr8	78142001	78147000	0.000247503
chr8	78143001	78148000	5.30E-05
chr8	78183001	78188000	0.002881431
chr8	78201001	78206000	4.14E-07
chr8	78202001	78207000	2.35E-09
chr8	78203001	78208000	5.03E-11
chr8	78204001	78209000	2.36E-10
chr8	78205001	78210000	1.46E-10
chr8	78206001	78211000	1.23E-06
chr8	78207001	78212000	5.17E-06
chr8	78208001	78213000	2.01E-08
chr8	78209001	78214000	5.72E-08
chr8	78210001	78215000	1.00E-06
chr8	78211001	78216000	3.55E-05
chr8	78212001	78217000	3.91E-06
chr8	78285001	78290000	0.000626999
chr8	78301001	78306000	3.13E-06
chr8	78302001	78307000	9.81E-07
chr8	78385001	78390000	0.000449915
chr8	78386001	78391000	4.89E-05
chr8	78393001	78398000	0.020155478
chr8	78396001	78401000	0.002240239
chr8	78402001	78407000	0.000319379
chr8	78410001	78415000	0.000532382
chr8	78411001	78416000	0.000467669
chr8	78414001	78419000	0.00034154
chr8	78438001	78443000	0.007652738
chr8	78469001	78474000	0.000662327
chr8	78482001	78487000	2.69E-10
chr8	78498001	78503000	2.76E-08
chr8	78500001	78505000	2.39E-06
chr8	78514001	78519000	3.54E-07
chr8	78548001	78553000	5.38E-08
chr8	78584001	78589000	5.19E-06
chr8	78585001	78590000	1.84E-06
chr8	78586001	78591000	1.77E-07
chr8	78587001	78592000	1.15E-05
chr8	78663001	78668000	4.52E-06
chr8	78683001	78688000	2.90E-06
chr8	78694001	78699000	8.34E-06
chr8	78695001	78700000	9.18E-09
chr8	78702001	78707000	2.38E-08
chr8	78715001	78720000	3.74E-05
chr8	78747001	78752000	1.69E-05
chr8	78748001	78753000	0.000348448
chr8	78770001	78775000	0.000837078
chr8	78771001	78776000	0.001339065

chr8	78774001	78779000	4.80E-05
chr8	78801001	78806000	0.000116817
chr8	78808001	78813000	5.67E-12
chr8	78809001	78814000	6.54E-10
chr8	78812001	78817000	4.38E-10
chr8	78815001	78820000	0.000857317
chr8	78834001	78839000	3.03E-05
chr8	78835001	78840000	8.15E-05
chr8	78866001	78871000	7.51E-25
chr8	78867001	78872000	3.91E-18
chr8	78868001	78873000	1.02E-18
chr8	78869001	78874000	1.25E-22
chr8	78870001	78875000	0.000164978
chr8	78871001	78876000	5.33E-05
chr8	78872001	78877000	7.15E-10
chr8	78873001	78878000	3.22E-09
chr8	78874001	78879000	1.77E-08
chr8	78875001	78880000	1.72E-08
chr8	78876001	78881000	5.18E-07
chr8	78877001	78882000	3.93E-05
chr8	78929001	78934000	9.50E-06
chr8	78930001	78935000	3.63E-06
chr8	78931001	78936000	1.47E-06
chr8	78932001	78937000	6.62E-07
chr8	78933001	78938000	7.10E-05
chr8	78942001	78947000	2.62E-05
chr8	78962001	78967000	3.58E-09
chr8	78963001	78968000	6.33E-10
chr8	78964001	78969000	4.28E-08
chr8	78965001	78970000	1.41E-06
chr8	78966001	78971000	1.23E-07
chr8	78974001	78979000	0.000589306
chr8	78975001	78980000	8.08E-11
chr8	78976001	78981000	5.59E-11
chr8	78977001	78982000	3.92E-10
chr8	78978001	78983000	1.24E-08
chr8	78979001	78984000	3.14E-06
chr8	78984001	78989000	9.77E-05
chr8	78985001	78990000	2.52E-05
chr8	78992001	78997000	7.00E-13
chr8	78993001	78998000	8.38E-13
chr8	78994001	78999000	2.69E-12
chr8	78995001	79000000	2.52E-12
chr8	79041001	79046000	0.000188331
chr8	79042001	79047000	4.83E-07
chr8	79043001	79048000	5.30E-10
chr8	79044001	79049000	6.81E-11
chr8	79045001	79050000	8.40E-13

chr8	79046001	79051000	1.70E-08
chr8	79047001	79052000	2.36E-06
chr8	79048001	79053000	0.001194113
chr8	79052001	79057000	0.000190474
chr8	79054001	79059000	0.000127035
chr8	79055001	79060000	0.000587692
chr8	79063001	79068000	1.87E-06
chr8	79064001	79069000	7.11E-05
chr8	79065001	79070000	6.50E-07
chr8	79086001	79091000	0.000712871
chr8	79087001	79092000	4.15E-07
chr8	79088001	79093000	4.00E-06
chr8	79089001	79094000	2.44E-06
chr8	79144001	79149000	1.53E-05
chr8	79174001	79179000	8.12E-06
chr8	79175001	79180000	1.22E-05
chr8	79176001	79181000	5.72E-05
chr8	79181001	79186000	8.97E-05
chr8	79182001	79187000	0.000113628
chr8	79194001	79199000	1.14E-07
chr8	79195001	79200000	1.24E-07
chr8	79211001	79216000	0.001169842
chr8	79290001	79295000	2.40E-08
chr8	79291001	79296000	1.43E-07
chr8	79298001	79303000	7.14E-06
chr8	79299001	79304000	0.000113991
chr8	79346001	79351000	1.66E-06
chr8	79365001	79370000	1.81E-06
chr8	79372001	79377000	0.000524682
chr8	79391001	79396000	1.68E-07
chr8	79392001	79397000	6.84E-10
chr8	79468001	79473000	6.96E-14
chr8	79469001	79474000	7.54E-14
chr8	79470001	79475000	2.63E-15
chr8	79471001	79476000	1.07E-15
chr8	79652001	79657000	6.39E-10
chr8	79770001	79775000	1.20E-17
chr8	79827001	79832000	2.81E-05
chr8	79866001	79871000	9.63E-10
chr8	79921001	79926000	0.000520606
chr8	79922001	79927000	0.000488749
chr8	79937001	79942000	0.000237384
chr8	79938001	79943000	0.000258768
chr8	79952001	79957000	9.06E-05
chr8	79954001	79959000	0.001434632
chr8	79958001	79963000	0.004766932
chr8	79962001	79967000	5.62E-05
chr8	79975001	79980000	7.06E-07

chr8	80065001	80070000	0.000330436
chr8	80160001	80165000	1.48E-06
chr8	80161001	80166000	4.76E-07
chr8	80162001	80167000	9.01E-06
chr8	80163001	80168000	0.000116848
chr8	80164001	80169000	6.97E-05
chr8	80165001	80170000	0.000122404
chr8	80177001	80182000	0.000103377
chr8	80178001	80183000	0.000145904
chr8	80189001	80194000	1.44E-07
chr8	80190001	80195000	7.26E-06
chr8	80191001	80196000	8.77E-05
chr8	80192001	80197000	1.24E-05
chr8	80211001	80216000	8.91E-36
chr8	80223001	80228000	8.75E-05
chr8	80268001	80273000	3.72E-08
chr8	80269001	80274000	1.02E-09
chr8	80270001	80275000	2.13E-09
chr8	80325001	80330000	6.06E-09
chr8	80365001	80370000	0.001581058
chr8	80381001	80386000	0.000141866
chr8	80430001	80435000	4.22E-10
chr8	80431001	80436000	1.91E-11
chr8	80432001	80437000	3.75E-09
chr8	80433001	80438000	7.84E-08
chr8	80507001	80512000	1.36E-06
chr8	80508001	80513000	9.04E-07
chr8	80509001	80514000	7.58E-08
chr8	80510001	80515000	5.61E-07
chr8	80548001	80553000	2.40E-05
chr8	81954001	81959000	5.75E-08
chr8	82042001	82047000	3.47E-15
chr8	82158001	82163000	4.53E-05
chr8	82186001	82191000	0.000187503
chr8	82362001	82367000	7.61E-05
chr8	82363001	82368000	1.43E-05
chr8	82364001	82369000	7.13E-05
chr8	82859001	82864000	0.002444006
chr8	82860001	82865000	0.001810044
chr8	82867001	82872000	1.31E-05
chr8	82911001	82916000	1.50E-06
chr8	82912001	82917000	1.32E-07
chr8	82913001	82918000	1.88E-05
chr8	82914001	82919000	7.55E-08
chr8	82917001	82922000	5.52E-08
chr8	82918001	82923000	5.93E-08
chr8	82944001	82949000	3.54E-05
chr8	82945001	82950000	0.00050045

chr8	82946001	82951000	0.002091911
chr8	82947001	82952000	0.000663094
chr8	82949001	82954000	1.78E-11
chr8	82950001	82955000	2.83E-12
chr8	82951001	82956000	8.24E-12
chr8	82952001	82957000	4.93E-11
chr8	82953001	82958000	1.34E-10
chr8	82956001	82961000	1.17E-09
chr8	82962001	82967000	2.08E-06
chr8	82972001	82977000	9.29E-11
chr8	83028001	83033000	0.002611749
chr8	83100001	83105000	2.50E-42
chr8	83128001	83133000	1.36E-09
chr8	83129001	83134000	7.02E-10
chr8	83130001	83135000	0.000135225
chr8	83152001	83157000	3.38E-07
chr8	83153001	83158000	2.49E-09
chr8	83154001	83159000	1.00E-12
chr8	83155001	83160000	3.89E-11
chr8	83156001	83161000	5.78E-10
chr8	83189001	83194000	0.000415868
chr8	83190001	83195000	5.37E-07
chr8	83203001	83208000	2.26E-08
chr8	83218001	83223000	0.001686441
chr8	83248001	83253000	7.11E-11
chr8	83249001	83254000	2.67E-10
chr8	83319001	83324000	5.98E-10
chr8	83340001	83345000	2.89E-05
chr8	83364001	83369000	6.08E-18
chr8	83387001	83392000	5.94E-07
chr8	83388001	83393000	4.01E-07
chr8	83389001	83394000	6.77E-08
chr8	83390001	83395000	4.81E-07
chr8	83398001	83403000	4.71E-09
chr8	83399001	83404000	2.92E-09
chr8	83400001	83405000	3.07E-10
chr8	83401001	83406000	8.05E-12
chr8	83402001	83407000	2.09E-09
chr8	83423001	83428000	0.000546693
chr8	83431001	83436000	7.69E-05
chr8	83432001	83437000	0.002149124
chr8	83447001	83452000	7.94E-24
chr8	83448001	83453000	6.51E-22
chr8	83449001	83454000	6.51E-12
chr8	83455001	83460000	4.06E-06
chr8	83456001	83461000	0.003516463
chr8	83457001	83462000	0.002369188
chr8	83466001	83471000	8.44E-05

chr8	83467001	83472000	1.09E-05
chr8	83468001	83473000	1.46E-06
chr8	83485001	83490000	0.000211917
chr8	83486001	83491000	0.000101452
chr8	83489001	83494000	2.41E-10
chr8	83490001	83495000	1.76E-12
chr8	83509001	83514000	2.77E-13
chr8	83510001	83515000	7.78E-14
chr8	83511001	83516000	1.67E-16
chr8	83512001	83517000	1.27E-14
chr8	83519001	83524000	3.28E-11
chr8	83520001	83525000	5.47E-15
chr8	83521001	83526000	1.65E-13
chr8	83522001	83527000	1.26E-15
chr8	83523001	83528000	1.72E-11
chr8	83524001	83529000	1.26E-10
chr8	83525001	83530000	5.20E-08
chr8	83526001	83531000	6.27E-09
chr8	83547001	83552000	2.55E-15
chr8	83548001	83553000	1.22E-16
chr8	83549001	83554000	2.84E-16
chr8	83550001	83555000	2.76E-14
chr8	83551001	83556000	3.74E-13
chr8	83571001	83576000	1.22E-14
chr8	83572001	83577000	4.08E-16
chr8	83573001	83578000	3.53E-14
chr8	83643001	83648000	0.000187415
chr8	83644001	83649000	3.01E-09
chr8	83645001	83650000	1.13E-10
chr8	83646001	83651000	5.77E-09
chr8	83647001	83652000	3.65E-07
chr8	83657001	83662000	0.000452393
chr8	83684001	83689000	2.81E-12
chr8	83687001	83692000	0.002211213
chr8	83688001	83693000	0.010793194
chr8	83700001	83705000	3.29E-05
chr8	83738001	83743000	2.72E-07
chr8	83787001	83792000	1.61E-05
chr8	83788001	83793000	8.52E-13
chr8	83789001	83794000	1.23E-22
chr8	83810001	83815000	3.78E-05
chr8	83854001	83859000	1.87E-11
chr8	83855001	83860000	1.78E-18
chr8	83878001	83883000	1.76E-06
chr8	83879001	83884000	3.42E-08
chr8	83880001	83885000	2.24E-06
chr8	83887001	83892000	5.85E-05
chr8	83888001	83893000	0.000804325

chr8	83889001	83894000	0.000939412
chr8	83912001	83917000	0.02463006
chr8	83913001	83918000	0.012738203
chr8	83914001	83919000	0.021284185
chr8	83916001	83921000	0.004228711
chr8	83929001	83934000	1.54E-06
chr8	83930001	83935000	5.90E-09
chr8	83931001	83936000	8.39E-08
chr8	83942001	83947000	4.46E-06
chr8	83943001	83948000	0.000358763
chr8	83944001	83949000	0.000253123
chr8	83945001	83950000	0.000861064
chr8	83946001	83951000	0.000375211
chr8	83963001	83968000	1.06E-22
chr8	83964001	83969000	1.89E-23
chr8	83965001	83970000	5.11E-18
chr8	83966001	83971000	4.67E-15
chr8	84016001	84021000	4.68E-08
chr8	84017001	84022000	4.18E-10
chr8	84018001	84023000	1.38E-09
chr8	84056001	84061000	3.55E-07
chr8	84057001	84062000	4.32E-07
chr8	84074001	84079000	1.53E-05
chr8	84075001	84080000	7.50E-06
chr8	84128001	84133000	5.14E-08
chr8	84129001	84134000	1.96E-12
chr8	84130001	84135000	5.30E-07
chr8	84131001	84136000	2.07E-05
chr8	84148001	84153000	5.63E-05
chr8	84150001	84155000	4.07E-11
chr8	84151001	84156000	1.56E-09
chr8	84152001	84157000	6.84E-10
chr8	84153001	84158000	3.32E-09
chr8	84154001	84159000	9.93E-12
chr8	84155001	84160000	0.002487954
chr8	84188001	84193000	1.16E-07
chr8	84189001	84194000	6.36E-06
chr8	84190001	84195000	6.54E-05
chr8	84191001	84196000	4.23E-05
chr8	84192001	84197000	7.26E-05
chr8	84220001	84225000	1.57E-09
chr8	84242001	84247000	2.47E-07
chr8	84379001	84384000	5.27E-06
chr8	84392001	84397000	6.88E-07
chr8	84452001	84457000	4.35E-11
chr8	84497001	84502000	0.019523258
chr8	84500001	84505000	0.002724209
chr8	84501001	84506000	0.001636455

chr8	84506001	84511000	1.23E-06
chr8	84507001	84512000	8.80E-07
chr8	84508001	84513000	6.47E-06
chr8	84510001	84515000	4.68E-15
chr8	84511001	84516000	9.77E-15
chr8	84523001	84528000	3.76E-15
chr8	84532001	84537000	1.43E-10
chr8	84533001	84538000	1.64E-17
chr8	84534001	84539000	1.83E-15
chr8	84535001	84540000	5.38E-14
chr8	84536001	84541000	3.91E-07
chr8	84566001	84571000	3.66E-09
chr8	84567001	84572000	4.58E-10
chr8	84568001	84573000	3.41E-09
chr8	84582001	84587000	5.33E-05
chr8	84583001	84588000	5.25E-05
chr8	84584001	84589000	0.000171538
chr8	84585001	84590000	0.000228744
chr8	84586001	84591000	5.21E-05
chr8	84587001	84592000	0.002721356
chr8	84600001	84605000	0.000322197
chr8	84601001	84606000	2.72E-09
chr8	84631001	84636000	1.81E-15
chr8	84638001	84643000	3.05E-07
chr8	84648001	84653000	0.000637601
chr8	84649001	84654000	4.51E-05
chr8	84650001	84655000	0.000151089
chr8	84683001	84688000	4.35E-12
chr8	84684001	84689000	1.09E-12
chr8	84685001	84690000	7.94E-14
chr8	84687001	84692000	2.65E-12
chr8	84691001	84696000	0.004589196
chr8	84709001	84714000	3.88E-05
chr8	84710001	84715000	2.12E-13
chr8	84711001	84716000	8.91E-12
chr8	84712001	84717000	1.86E-13
chr8	84713001	84718000	1.13E-11
chr8	84714001	84719000	5.54E-11
chr8	84738001	84743000	9.55E-06
chr8	84745001	84750000	1.26E-08
chr8	84746001	84751000	7.49E-09
chr8	84747001	84752000	9.61E-09
chr8	84748001	84753000	1.74E-08
chr8	84749001	84754000	1.24E-05
chr8	84750001	84755000	0.000510033
chr8	84751001	84756000	2.53E-05
chr8	84752001	84757000	4.99E-06
chr8	84775001	84780000	0.00074427

chr8	84829001	84834000	0.000195495
chr8	84831001	84836000	1.46E-05
chr8	84832001	84837000	1.49E-07
chr8	84833001	84838000	2.36E-08
chr8	84839001	84844000	1.73E-12
chr8	84840001	84845000	2.65E-12
chr8	84841001	84846000	4.17E-11
chr8	84842001	84847000	4.78E-10
chr8	84843001	84848000	1.43E-09
chr8	84878001	84883000	2.96E-11
chr8	84887001	84892000	2.06E-05
chr8	84888001	84893000	6.29E-06
chr8	84890001	84895000	7.93E-09
chr8	84923001	84928000	0.000227712
chr8	84944001	84949000	2.48E-06
chr8	84950001	84955000	2.93E-05
chr8	84971001	84976000	1.20E-10
chr8	84972001	84977000	2.77E-11
chr8	84973001	84978000	6.70E-09
chr8	85003001	85008000	0.003313095
chr8	85004001	85009000	0.003224504
chr8	85005001	85010000	0.001113171
chr8	85012001	85017000	0.000785037
chr8	85025001	85030000	3.56E-06
chr8	85026001	85031000	8.77E-06
chr8	85027001	85032000	6.67E-07
chr8	85028001	85033000	1.22E-08
chr8	85029001	85034000	1.02E-08
chr8	85030001	85035000	1.98E-09
chr8	85031001	85036000	1.92E-12
chr8	85032001	85037000	3.91E-12
chr8	85049001	85054000	0.001116286
chr8	85077001	85082000	3.93E-09
chr8	85084001	85089000	0.003362541
chr8	85085001	85090000	0.000103636
chr8	85099001	85104000	0.006931181
chr8	85100001	85105000	0.001871782
chr8	85101001	85106000	0.002483414
chr8	85118001	85123000	4.80E-05
chr8	85119001	85124000	9.83E-06
chr8	85120001	85125000	3.28E-08
chr8	85121001	85126000	1.21E-08
chr8	85122001	85127000	1.61E-07
chr8	85147001	85152000	6.62E-12
chr8	85148001	85153000	2.07E-13
chr8	85149001	85154000	1.56E-21
chr8	85150001	85155000	6.01E-21
chr8	85151001	85156000	4.44E-18

chr8	85152001	85157000	1.03E-10
chr8	85153001	85158000	1.59E-11
chr8	85154001	85159000	2.37E-05
chr8	85155001	85160000	3.61E-05
chr8	85156001	85161000	1.71E-05
chr8	85157001	85162000	1.79E-05
chr8	85198001	85203000	1.79E-11
chr8	85199001	85204000	5.95E-08
chr8	85201001	85206000	1.12E-06
chr8	85202001	85207000	5.64E-06
chr8	85248001	85253000	0.004483048
chr8	85257001	85262000	0.001368497
chr8	85258001	85263000	0.000361729
chr8	85260001	85265000	3.42E-05
chr8	85261001	85266000	2.77E-05
chr8	85270001	85275000	1.06E-05
chr8	85271001	85276000	1.45E-06
chr8	85272001	85277000	1.11E-05
chr8	85281001	85286000	0.000236157
chr8	85284001	85289000	5.63E-07
chr8	85285001	85290000	0.000126786
chr8	85295001	85300000	0.000359149
chr8	85309001	85314000	9.50E-10
chr8	85310001	85315000	1.49E-09
chr8	85343001	85348000	0.00046293
chr8	85344001	85349000	5.26E-05
chr8	85347001	85352000	3.88E-05
chr8	85348001	85353000	2.16E-05
chr8	85351001	85356000	2.14E-05
chr8	85354001	85359000	0.000137162
chr8	85355001	85360000	1.82E-11
chr8	85356001	85361000	2.35E-15
chr8	85357001	85362000	7.24E-12
chr8	85358001	85363000	1.08E-10
chr8	85359001	85364000	5.77E-08
chr8	85393001	85398000	3.37E-08
chr8	85394001	85399000	5.61E-08
chr8	85395001	85400000	5.26E-08
chr8	85396001	85401000	1.87E-09
chr8	85397001	85402000	3.95E-11
chr8	85461001	85466000	4.09E-05
chr8	85462001	85467000	0.000276755
chr8	85463001	85468000	0.001516146
chr8	85464001	85469000	0.001657477
chr8	85465001	85470000	0.001730033
chr8	85497001	85502000	5.35E-08
chr8	85498001	85503000	1.05E-09
chr8	85505001	85510000	0.005266564

chr8	85517001	85522000	1.12E-11
chr8	85519001	85524000	1.22E-11
chr8	85534001	85539000	0.000139526
chr8	85713001	85718000	9.56E-05
chr8	85714001	85719000	2.14E-05
chr8	85730001	85735000	6.69E-06
chr8	85765001	85770000	4.99E-12
chr8	85766001	85771000	3.31E-10
chr8	85789001	85794000	0.006162318
chr8	85790001	85795000	0.005159042
chr8	85794001	85799000	0.001092515
chr8	85795001	85800000	5.83E-05
chr8	85796001	85801000	0.000538833
chr8	85797001	85802000	0.00354693
chr8	85803001	85808000	5.68E-10
chr8	85804001	85809000	2.41E-09
chr8	85805001	85810000	5.01E-07
chr8	85806001	85811000	3.40E-07
chr8	85819001	85824000	0.000376313
chr8	85854001	85859000	1.49E-09
chr8	85855001	85860000	2.29E-08
chr8	85869001	85874000	3.82E-06
chr8	85880001	85885000	2.07E-05
chr8	85898001	85903000	0.002994162
chr8	85900001	85905000	3.42E-09
chr8	85901001	85906000	1.20E-11
chr8	85902001	85907000	2.70E-10
chr8	85903001	85908000	5.48E-12
chr8	85904001	85909000	9.28E-17
chr8	86011001	86016000	1.75E-09
chr8	86176001	86181000	7.72E-08
chr8	86177001	86182000	3.02E-07
chr8	86339001	86344000	6.73E-08
chr8	86340001	86345000	1.15E-08
chr8	86341001	86346000	1.24E-07
chr8	86414001	86419000	3.97E-06
chr8	86501001	86506000	5.56E-05
chr8	86994001	86999000	3.44E-05
chr8	87066001	87071000	3.27E-08
chr8	87067001	87072000	1.92E-08
chr8	87099001	87104000	1.19E-05
chr8	87100001	87105000	6.04E-10
chr8	87101001	87106000	1.12E-11
chr8	87126001	87131000	4.00E-11
chr8	87192001	87197000	1.15E-05
chr8	87194001	87199000	2.06E-07
chr8	87226001	87231000	1.00E-07
chr8	87227001	87232000	2.47E-07

chr8	87228001	87233000	9.78E-08
chr8	87229001	87234000	3.48E-07
chr8	87230001	87235000	1.36E-08
chr8	87231001	87236000	4.01E-08
chr8	87232001	87237000	1.33E-06
chr8	87259001	87264000	1.09E-05
chr8	87260001	87265000	5.61E-08
chr8	87316001	87321000	1.66E-07
chr8	87317001	87322000	4.89E-08
chr8	87318001	87323000	9.25E-11
chr8	87594001	87599000	0.000136902
chr8	87628001	87633000	1.75E-08
chr8	87629001	87634000	1.16E-08
chr8	87630001	87635000	1.14E-12
chr8	87631001	87636000	6.58E-12
chr8	87663001	87668000	1.10E-06
chr8	87664001	87669000	1.70E-07
chr8	87683001	87688000	6.01E-09
chr8	87705001	87710000	1.17E-21
chr8	87767001	87772000	0.0005662
chr8	87788001	87793000	3.96E-07
chr8	87837001	87842000	9.89E-10
chr8	87838001	87843000	1.65E-10
chr8	87839001	87844000	5.08E-12
chr8	87840001	87845000	1.17E-08
chr8	87841001	87846000	1.68E-08
chr8	87844001	87849000	0.000684864
chr8	87848001	87853000	1.07E-05
chr8	87863001	87868000	9.87E-08
chr8	87864001	87869000	3.77E-08
chr8	87865001	87870000	2.54E-09
chr8	87866001	87871000	7.41E-08
chr8	87867001	87872000	5.07E-10
chr8	87883001	87888000	2.02E-09
chr8	87884001	87889000	2.29E-09
chr8	87885001	87890000	8.43E-09
chr8	87888001	87893000	3.82E-10
chr8	87889001	87894000	5.64E-11
chr8	87890001	87895000	1.78E-09
chr8	87891001	87896000	1.88E-09
chr8	87917001	87922000	0.000799017
chr8	87927001	87932000	1.09E-07
chr8	87928001	87933000	2.78E-09
chr8	87980001	87985000	4.18E-06
chr8	87988001	87993000	7.78E-11
chr8	87989001	87994000	4.63E-09
chr8	87990001	87995000	8.56E-10
chr8	87991001	87996000	0.02654662

chr8	88018001	88023000	0.000439138
chr8	88019001	88024000	2.70E-05
chr8	88020001	88025000	2.81E-06
chr8	88021001	88026000	6.05E-05
chr8	88022001	88027000	1.78E-05
chr8	88024001	88029000	0.000498189
chr8	88039001	88044000	1.35E-05
chr8	88041001	88046000	1.32E-07
chr8	88042001	88047000	5.12E-06
chr8	88043001	88048000	7.22E-06
chr8	88044001	88049000	5.11E-14
chr8	88045001	88050000	6.09E-15
chr8	88046001	88051000	4.71E-11
chr8	88047001	88052000	9.47E-11
chr8	88048001	88053000	9.81E-12
chr8	88049001	88054000	2.43E-06
chr8	88050001	88055000	6.41E-06
chr8	88051001	88056000	1.04E-05
chr8	88110001	88115000	1.37E-13
chr8	88111001	88116000	8.32E-25
chr8	88112001	88117000	5.40E-27
chr8	88113001	88118000	2.01E-25
chr8	88114001	88119000	5.49E-24
chr8	88115001	88120000	9.25E-18
chr8	88116001	88121000	3.78E-05
chr8	88117001	88122000	2.97E-06
chr8	88118001	88123000	4.42E-07
chr8	88119001	88124000	1.36E-06
chr8	88158001	88163000	5.78E-11
chr8	88159001	88164000	4.49E-09
chr8	88160001	88165000	2.79E-09
chr8	88161001	88166000	2.28E-12
chr8	88162001	88167000	2.97E-16
chr8	88164001	88169000	8.87E-08
chr8	88165001	88170000	1.64E-07
chr8	88166001	88171000	1.76E-06
chr8	88168001	88173000	0.000307066
chr8	88187001	88192000	4.91E-09
chr8	88188001	88193000	2.82E-11
chr8	88189001	88194000	2.51E-11
chr8	88190001	88195000	2.39E-10
chr8	88191001	88196000	7.11E-10
chr8	88241001	88246000	4.02E-26
chr8	88256001	88261000	0.000472978
chr8	88288001	88293000	0.006954493
chr8	88405001	88410000	0.001672981
chr8	88406001	88411000	0.005490847
chr8	88407001	88412000	0.000457717

chr8	88408001	88413000	0.000142333
chr8	88414001	88419000	0.000110636
chr8	88417001	88422000	1.54E-13
chr8	88418001	88423000	7.95E-15
chr8	88419001	88424000	6.01E-15
chr8	88420001	88425000	4.44E-15
chr8	88421001	88426000	1.46E-05
chr8	88456001	88461000	0.00014528
chr8	88457001	88462000	8.03E-06
chr8	88458001	88463000	1.17E-05
chr8	88459001	88464000	3.62E-05
chr8	88460001	88465000	0.000198822
chr8	88467001	88472000	2.10E-13
chr8	88468001	88473000	3.33E-13
chr8	88490001	88495000	0.001158374
chr8	88519001	88524000	0.000825583
chr8	88537001	88542000	0.000141744
chr8	88538001	88543000	0.000201455
chr8	88539001	88544000	0.000505891
chr8	88540001	88545000	0.003248571
chr8	88545001	88550000	0.000248947
chr8	88579001	88584000	0.000280654
chr8	88585001	88590000	0.0017115
chr8	88607001	88612000	0.000126538
chr8	88608001	88613000	9.24E-05
chr8	88609001	88614000	4.38E-05
chr8	88610001	88615000	4.82E-06
chr8	88713001	88718000	4.45E-06
chr8	88714001	88719000	8.55E-10
chr8	88728001	88733000	0.00398847
chr8	88732001	88737000	7.91E-12
chr8	88733001	88738000	1.26E-11
chr8	88734001	88739000	1.70E-12
chr8	88735001	88740000	7.29E-15
chr8	88736001	88741000	3.47E-10
chr8	88737001	88742000	2.01E-10
chr8	88738001	88743000	1.04E-10
chr8	88739001	88744000	4.24E-11
chr8	88740001	88745000	7.92E-10
chr8	88741001	88746000	2.20E-09
chr8	88788001	88793000	2.70E-10
chr8	88789001	88794000	4.13E-09
chr8	88790001	88795000	3.53E-08
chr8	88791001	88796000	1.60E-07
chr8	88812001	88817000	0.000510757
chr8	88819001	88824000	1.42E-08
chr8	88820001	88825000	1.21E-12
chr8	88823001	88828000	3.20E-10

chr8	88824001	88829000	0.00019077
chr8	88826001	88831000	2.36E-05
chr8	88827001	88832000	8.48E-06
chr8	88828001	88833000	1.27E-05
chr8	88834001	88839000	7.74E-11
chr8	88849001	88854000	5.61E-11
chr8	88850001	88855000	8.76E-12
chr8	88868001	88873000	1.18E-13
chr8	88869001	88874000	3.46E-15
chr8	88870001	88875000	1.60E-17
chr8	88871001	88876000	5.57E-07
chr8	88872001	88877000	5.81E-09
chr8	88873001	88878000	3.59E-09
chr8	88874001	88879000	1.00E-07
chr8	88897001	88902000	0.000532596
chr8	88903001	88908000	6.55E-18
chr8	88904001	88909000	4.01E-17
chr8	88910001	88915000	3.21E-12
chr8	88925001	88930000	1.49E-05
chr8	88926001	88931000	0.000928318
chr8	88929001	88934000	0.00083251
chr8	88930001	88935000	0.000820066
chr8	88931001	88936000	0.000423061
chr8	88951001	88956000	6.56E-07
chr8	88955001	88960000	4.89E-11
chr8	88958001	88963000	0.001075713
chr8	88959001	88964000	0.001850502
chr8	88979001	88984000	1.90E-41
chr8	88996001	89001000	0.00072964
chr8	88997001	89002000	1.83E-10
chr8	88998001	89003000	1.60E-10
chr8	88999001	89004000	2.09E-13
chr8	89000001	89005000	2.86E-11
chr8	89001001	89006000	2.90E-11
chr8	89009001	89014000	2.58E-10
chr8	89030001	89035000	8.37E-08
chr8	89035001	89040000	5.07E-05
chr8	89037001	89042000	1.10E-07
chr8	89039001	89044000	8.10E-05
chr8	89041001	89046000	0.000231983
chr8	89064001	89069000	0.006586559
chr8	89068001	89073000	0.000531023
chr8	89069001	89074000	0.000312177
chr8	89080001	89085000	0.000751495
chr8	89088001	89093000	1.93E-09
chr8	89089001	89094000	1.29E-09
chr8	89096001	89101000	1.81E-09
chr8	89097001	89102000	5.05E-10

chr8	89098001	89103000	8.25E-09
chr8	89099001	89104000	2.87E-08
chr8	89106001	89111000	0.008918558
chr8	89107001	89112000	0.008884152
chr8	89126001	89131000	3.34E-09
chr8	89127001	89132000	1.32E-10
chr8	89128001	89133000	2.89E-08
chr8	89129001	89134000	5.87E-05
chr8	89183001	89188000	1.75E-21
chr8	89184001	89189000	1.96E-20
chr8	89185001	89190000	2.65E-19
chr8	89186001	89191000	6.67E-16
chr8	89187001	89192000	5.42E-15
chr8	89211001	89216000	4.73E-11
chr8	89212001	89217000	5.03E-12
chr8	89213001	89218000	8.58E-12
chr8	89214001	89219000	2.18E-12
chr8	89215001	89220000	7.94E-10
chr8	89218001	89223000	1.78E-07
chr8	89219001	89224000	2.90E-09
chr8	89220001	89225000	1.07E-08
chr8	89221001	89226000	6.70E-07
chr8	89226001	89231000	3.49E-08
chr8	89227001	89232000	5.83E-06
chr8	89258001	89263000	0.001190133
chr8	89264001	89269000	0.00020721
chr8	89265001	89270000	0.000132377
chr8	89266001	89271000	5.88E-06
chr8	89267001	89272000	6.12E-06
chr8	89276001	89281000	0.000170412
chr8	89293001	89298000	6.45E-08
chr8	89294001	89299000	1.26E-12
chr8	89295001	89300000	1.87E-14
chr8	89296001	89301000	9.08E-14
chr8	89297001	89302000	1.26E-11
chr8	89325001	89330000	0.000122516
chr8	89364001	89369000	5.62E-07
chr8	89368001	89373000	0.000733568
chr8	89371001	89376000	0.000585856
chr8	89391001	89396000	3.63E-11
chr8	89392001	89397000	1.01E-11
chr8	89393001	89398000	1.02E-17
chr8	89394001	89399000	2.11E-20
chr8	89395001	89400000	4.05E-32
chr8	89396001	89401000	8.75E-21
chr8	89397001	89402000	3.75E-20
chr8	89398001	89403000	6.71E-21
chr8	89399001	89404000	2.22E-23

chr8	89420001	89425000	0.00612216
chr8	89426001	89431000	8.54E-07
chr8	89428001	89433000	6.34E-08
chr8	89430001	89435000	3.78E-09
chr8	89446001	89451000	5.75E-08
chr8	89465001	89470000	2.25E-12
chr8	89466001	89471000	3.93E-11
chr8	89467001	89472000	7.50E-09
chr8	89468001	89473000	6.81E-14
chr8	89469001	89474000	3.50E-13
chr8	89477001	89482000	1.81E-12
chr8	89478001	89483000	4.26E-13
chr8	89495001	89500000	0.003609386
chr8	89498001	89503000	0.012374026
chr8	89510001	89515000	0.000176715
chr8	89511001	89516000	2.17E-05
chr8	89531001	89536000	6.81E-06
chr8	89532001	89537000	5.79E-07
chr8	89595001	89600000	1.95E-13
chr8	89596001	89601000	2.89E-12
chr8	89597001	89602000	3.61E-12
chr8	89607001	89612000	0.002348816
chr8	89624001	89629000	1.69E-09
chr8	89625001	89630000	0.003319584
chr8	89626001	89631000	0.00499849
chr8	89636001	89641000	0.000480409
chr8	89637001	89642000	0.000336939
chr8	89638001	89643000	0.000318248
chr8	89652001	89657000	5.38E-07
chr8	89653001	89658000	4.33E-07
chr8	89684001	89689000	1.43E-17
chr8	89685001	89690000	2.03E-21
chr8	89686001	89691000	3.77E-20
chr8	89687001	89692000	2.56E-18
chr8	89688001	89693000	1.57E-19
chr8	89691001	89696000	9.15E-13
chr8	89692001	89697000	1.86E-15
chr8	89693001	89698000	1.45E-11
chr8	89703001	89708000	1.04E-08
chr8	89704001	89709000	2.68E-11
chr8	89705001	89710000	3.36E-14
chr8	89706001	89711000	5.87E-18
chr8	89707001	89712000	5.83E-07
chr8	89718001	89723000	5.71E-07
chr8	89719001	89724000	2.14E-09
chr8	89720001	89725000	1.34E-14
chr8	89721001	89726000	7.23E-17
chr8	89722001	89727000	1.50E-17

chr8	89723001	89728000	4.46E-13
chr8	89724001	89729000	1.88E-09
chr8	89736001	89741000	2.23E-09
chr8	89737001	89742000	3.13E-09
chr8	89744001	89749000	4.21E-13
chr8	89745001	89750000	3.21E-14
chr8	89746001	89751000	2.58E-13
chr8	89747001	89752000	3.83E-14
chr8	89773001	89778000	7.19E-06
chr8	89774001	89779000	0.000147432
chr8	89793001	89798000	6.68E-07
chr8	89794001	89799000	1.49E-06
chr8	89795001	89800000	0.000635548
chr8	89802001	89807000	0.001581073
chr8	89803001	89808000	0.004743976
chr8	89804001	89809000	0.008021853
chr8	89810001	89815000	0.002244278
chr8	89811001	89816000	0.001700502
chr8	89826001	89831000	2.10E-10
chr8	89827001	89832000	2.25E-12
chr8	89828001	89833000	9.01E-14
chr8	89829001	89834000	2.35E-17
chr8	89830001	89835000	1.98E-16
chr8	89851001	89856000	0.000164773
chr8	89871001	89876000	3.14E-25
chr8	89872001	89877000	1.19E-32
chr8	89873001	89878000	1.69E-17
chr8	89874001	89879000	1.38E-16
chr8	89875001	89880000	5.14E-18
chr8	89876001	89881000	0.005667463
chr8	89885001	89890000	0.000118669
chr8	89893001	89898000	3.11E-07
chr8	89899001	89904000	0.00014794
chr8	89900001	89905000	5.06E-05
chr8	89907001	89912000	4.80E-12
chr8	89926001	89931000	0.000122627
chr8	89945001	89950000	1.79E-15
chr8	89947001	89952000	2.03E-14
chr8	89954001	89959000	0.000592565
chr8	89955001	89960000	0.000322441
chr8	89957001	89962000	0.000157629
chr8	89958001	89963000	7.61E-05
chr8	89961001	89966000	5.23E-08
chr8	89962001	89967000	1.43E-06
chr8	89963001	89968000	2.52E-06
chr8	89964001	89969000	6.51E-07
chr8	89965001	89970000	2.31E-05
chr8	89974001	89979000	1.54E-05

chr8	89975001	89980000	2.64E-06
chr8	89976001	89981000	7.95E-07
chr8	89977001	89982000	6.15E-07
chr8	89978001	89983000	6.10E-06
chr8	89979001	89984000	0.000796726
chr8	89980001	89985000	0.004817941
chr8	89983001	89988000	6.33E-07
chr8	89984001	89989000	3.06E-10
chr8	89985001	89990000	1.30E-09
chr8	89986001	89991000	4.79E-09
chr8	89987001	89992000	1.03E-08
chr8	89991001	89996000	0.000524558
chr8	89992001	89997000	0.000720164
chr8	90003001	90008000	9.76E-05
chr8	90004001	90009000	6.28E-05
chr8	90022001	90027000	4.33E-09
chr8	90023001	90028000	1.32E-08
chr8	90024001	90029000	7.16E-07
chr8	90030001	90035000	6.32E-06
chr8	90031001	90036000	7.52E-08
chr8	90032001	90037000	1.88E-09
chr8	90033001	90038000	1.76E-10
chr8	90034001	90039000	1.31E-12
chr8	90035001	90040000	4.09E-13
chr8	90036001	90041000	2.45E-09
chr8	90037001	90042000	8.60E-07
chr8	90038001	90043000	2.55E-06
chr8	90056001	90061000	4.38E-05
chr8	90062001	90067000	9.67E-06
chr8	90076001	90081000	0.001545656
chr8	90117001	90122000	5.85E-07
chr8	90118001	90123000	0.000494057
chr8	90119001	90124000	0.000546366
chr8	90131001	90136000	0.000311078
chr8	90138001	90143000	0.007705019
chr8	90187001	90192000	1.83E-06
chr8	90188001	90193000	2.18E-08
chr8	90189001	90194000	2.15E-08
chr8	90190001	90195000	6.61E-07
chr8	90191001	90196000	4.54E-06
chr8	90204001	90209000	0.000221404
chr8	90277001	90282000	4.36E-07
chr8	90278001	90283000	3.75E-08
chr8	90290001	90295000	1.55E-05
chr8	90303001	90308000	0.000186172
chr8	90304001	90309000	5.27E-05
chr8	90321001	90326000	2.28E-05
chr8	90322001	90327000	1.10E-05

chr8	90323001	90328000	0.000816372
chr8	90324001	90329000	0.000201385
chr8	90325001	90330000	0.000137201
chr8	90328001	90333000	0.000578168
chr8	90330001	90335000	0.000260355
chr8	90331001	90336000	0.000312236
chr8	90332001	90337000	0.002426017
chr8	90333001	90338000	0.001347774
chr8	90336001	90341000	2.49E-07
chr8	90362001	90367000	0.000570031
chr8	90363001	90368000	0.000149856
chr8	90375001	90380000	4.19E-10
chr8	90376001	90381000	7.88E-10
chr8	90377001	90382000	3.01E-10
chr8	90378001	90383000	8.58E-10
chr8	90379001	90384000	2.16E-07
chr8	90408001	90413000	5.00E-07
chr8	90413001	90418000	2.71E-05
chr8	90414001	90419000	3.60E-05
chr8	90428001	90433000	0.000648891
chr8	90457001	90462000	3.52E-08
chr8	90458001	90463000	1.54E-09
chr8	90459001	90464000	2.77E-10
chr8	90460001	90465000	1.64E-09
chr8	90461001	90466000	1.80E-07
chr8	90480001	90485000	7.32E-09
chr8	90488001	90493000	8.30E-08
chr8	90489001	90494000	7.07E-09
chr8	90490001	90495000	1.06E-08
chr8	90491001	90496000	6.10E-10
chr8	90492001	90497000	0.000471385
chr8	90515001	90520000	0.000304911
chr8	90533001	90538000	6.88E-07
chr8	90534001	90539000	3.82E-07
chr8	90535001	90540000	4.83E-07
chr8	90590001	90595000	6.62E-16
chr8	90591001	90596000	7.81E-15
chr8	90614001	90619000	0.001570733
chr8	90615001	90620000	0.002775937
chr8	90627001	90632000	3.48E-07
chr8	90645001	90650000	9.88E-05
chr8	90646001	90651000	1.69E-12
chr8	90647001	90652000	8.49E-12
chr8	90648001	90653000	4.23E-13
chr8	90649001	90654000	2.07E-12
chr8	90650001	90655000	9.22E-16
chr8	90651001	90656000	1.30E-10
chr8	90652001	90657000	4.99E-12

chr8	90856001	90861000	2.59E-13
chr8	90857001	90862000	1.37E-07
chr8	90999001	91004000	1.27E-16
chr8	91106001	91111000	1.45E-31
chr8	91107001	91112000	9.70E-18
chr8	91114001	91119000	1.41E-05
chr8	91128001	91133000	1.21E-06
chr8	91130001	91135000	0.000363748
chr8	91131001	91136000	0.000170728
chr8	91306001	91311000	4.03E-06
chr8	91319001	91324000	0.000403969
chr8	91320001	91325000	0.00015861
chr8	91381001	91386000	1.01E-05
chr8	91382001	91387000	4.35E-05
chr8	91393001	91398000	0.000141266
chr8	91416001	91421000	0.00013593
chr8	91417001	91422000	3.03E-08
chr8	91457001	91462000	6.43E-05
chr8	91458001	91463000	3.56E-05
chr8	91460001	91465000	4.03E-07
chr8	91464001	91469000	1.64E-06
chr8	91543001	91548000	1.48E-05
chr8	91775001	91780000	4.76E-06
chr8	91818001	91823000	2.57E-12
chr8	91819001	91824000	8.35E-12
chr8	91831001	91836000	0.001813227
chr8	91898001	91903000	6.71E-07
chr8	91899001	91904000	4.30E-07
chr8	91939001	91944000	2.02E-05
chr8	91945001	91950000	1.44E-06
chr8	92243001	92248000	3.55E-08
chr8	92244001	92249000	7.91E-08
chr8	92245001	92250000	5.93E-09
chr8	92270001	92275000	1.49E-07
chr8	92271001	92276000	9.91E-08
chr8	92272001	92277000	1.43E-05
chr8	92313001	92318000	0.001302068
chr8	92314001	92319000	0.001512228
chr8	92329001	92334000	1.41E-07
chr8	92330001	92335000	2.04E-07
chr8	92331001	92336000	2.44E-07
chr8	92338001	92343000	1.78E-05
chr8	92339001	92344000	0.000232278
chr8	92401001	92406000	1.91E-10
chr8	92423001	92428000	0.002181778
chr8	92424001	92429000	0.000356354
chr8	92425001	92430000	9.25E-05
chr8	92480001	92485000	5.51E-06

chr8	92482001	92487000	3.84E-05
chr8	92483001	92488000	9.66E-07
chr8	92487001	92492000	1.43E-05
chr8	92488001	92493000	5.66E-05
chr8	92489001	92494000	2.94E-05
chr8	92490001	92495000	3.64E-05
chr8	92525001	92530000	0.000683882
chr8	92527001	92532000	0.002278835
chr8	92528001	92533000	0.0004065
chr8	92625001	92630000	0.000323597
chr8	92626001	92631000	1.56E-05
chr8	92627001	92632000	0.000873087
chr8	92635001	92640000	0.010299141
chr8	92649001	92654000	2.48E-08
chr8	92650001	92655000	4.55E-10
chr8	92651001	92656000	8.89E-11
chr8	92652001	92657000	2.47E-10
chr8	92664001	92669000	5.45E-05
chr8	92686001	92691000	0.002182669
chr8	92687001	92692000	4.79E-07
chr8	92688001	92693000	2.43E-09
chr8	92689001	92694000	1.42E-11
chr8	92690001	92695000	1.36E-12
chr8	92691001	92696000	5.42E-08
chr8	92759001	92764000	9.63E-06
chr8	92760001	92765000	1.15E-05
chr8	92841001	92846000	3.12E-06
chr8	92896001	92901000	0.013470908
chr8	92910001	92915000	5.22E-10
chr8	92936001	92941000	5.20E-12
chr8	92937001	92942000	6.31E-10
chr8	92966001	92971000	6.43E-09
chr8	93087001	93092000	8.56E-05
chr8	93218001	93223000	8.94E-13
chr8	93219001	93224000	1.23E-14
chr8	93220001	93225000	9.13E-19
chr8	93221001	93226000	3.41E-23
chr8	93222001	93227000	5.80E-23
chr8	93224001	93229000	5.23E-05
chr8	93459001	93464000	0.000114584
chr8	93511001	93516000	6.63E-05
chr8	93512001	93517000	0.000137444
chr8	93539001	93544000	0.024411221
chr8	93749001	93754000	1.64E-06
chr8	93750001	93755000	1.80E-06
chr8	94085001	94090000	4.59E-05
chr8	94086001	94091000	3.95E-05
chr8	94125001	94130000	5.05E-09

chr8	94153001	94158000	0.000401585
chr8	94154001	94159000	0.000598735
chr8	94156001	94161000	0.002278404
chr8	94157001	94162000	0.001256667
chr8	94182001	94187000	1.14E-05
chr8	94257001	94262000	1.47E-08
chr8	94258001	94263000	0.000108642
chr8	94484001	94489000	4.95E-11
chr8	94588001	94593000	6.73E-09
chr8	94589001	94594000	7.96E-10
chr8	95347001	95352000	0.000170605
chr8	96065001	96070000	6.29E-15
chr8	96149001	96154000	2.90E-08
chr8	96803001	96808000	0.002501036
chr8	97621001	97626000	6.92E-10
chr8	97622001	97627000	1.26E-10
chr8	97623001	97628000	7.60E-11
chr8	97682001	97687000	1.13E-08
chr8	97740001	97745000	1.56E-06
chr8	97747001	97752000	0.002890689
chr8	97868001	97873000	9.42E-08
chr8	97891001	97896000	5.05E-07
chr8	97892001	97897000	7.55E-07
chr8	97955001	97960000	0.005458608
chr8	98018001	98023000	1.97E-13
chr8	98019001	98024000	5.09E-12
chr8	98395001	98400000	3.94E-06
chr8	98397001	98402000	1.53E-06
chr8	98477001	98482000	1.47E-05
chr8	98582001	98587000	0.007258785
chr8	98745001	98750000	5.72E-15
chr8	98746001	98751000	2.45E-16
chr8	99086001	99091000	8.10E-08
chr8	99348001	99353000	7.09E-09
chr8	100010001	100015000	0.000862435
chr8	101675001	101680000	3.18E-23
chr8	101957001	101962000	1.29E-15
chr8	102585001	102590000	9.25E-17
chr8	104533001	104538000	0.000437878
chr8	104598001	104603000	0.00149289
chr8	104856001	104861000	4.13E-05
chr8	104866001	104871000	0.000207335
chr8	104867001	104872000	0.000251763
chr8	104878001	104883000	2.42E-08
chr8	105054001	105059000	6.00E-07
chr8	105118001	105123000	0.000486977
chr8	105119001	105124000	0.000589349
chr8	105174001	105179000	0.003195026

chr8	105202001	105207000	0.000913164
chr8	105220001	105225000	1.43E-13
chr8	105459001	105464000	4.62E-05
chr8	105604001	105609000	0.016551677
chr8	105605001	105610000	0.000618033
chr8	105606001	105611000	0.001945025
chr8	105648001	105653000	9.54E-20
chr8	105649001	105654000	7.65E-18
chr8	105672001	105677000	1.53E-07
chr8	105704001	105709000	0.000101863
chr8	105707001	105712000	0.000808993
chr8	105708001	105713000	3.05E-08
chr8	105716001	105721000	7.03E-11
chr8	105717001	105722000	2.19E-11
chr8	105718001	105723000	1.89E-12
chr8	105722001	105727000	1.63E-05
chr8	105723001	105728000	3.00E-07
chr8	105724001	105729000	2.39E-07
chr8	105725001	105730000	1.87E-08
chr8	105726001	105731000	0.000655432
chr8	105727001	105732000	0.0001114
chr8	105735001	105740000	5.20E-08
chr8	105736001	105741000	3.94E-10
chr8	105737001	105742000	4.98E-14
chr8	105738001	105743000	9.36E-13
chr8	105772001	105777000	5.20E-13
chr8	105773001	105778000	2.11E-16
chr8	105785001	105790000	2.86E-14
chr8	105786001	105791000	7.31E-17
chr8	105787001	105792000	3.88E-19
chr8	105788001	105793000	5.66E-20
chr8	105802001	105807000	1.02E-09
chr8	105806001	105811000	6.06E-09
chr8	105829001	105834000	7.13E-08
chr8	105830001	105835000	2.88E-09
chr8	105833001	105838000	9.16E-07
chr8	105837001	105842000	0.000209228
chr8	105838001	105843000	1.17E-05
chr8	105864001	105869000	1.29E-06
chr8	105865001	105870000	3.01E-06
chr8	105879001	105884000	0.001767676
chr8	105897001	105902000	7.46E-07
chr8	105900001	105905000	3.50E-19
chr8	105903001	105908000	9.72E-12
chr8	105904001	105909000	6.96E-13
chr8	105957001	105962000	0.002539264
chr8	105993001	105998000	0.000216193
chr8	106028001	106033000	3.76E-06

chr8	106029001	106034000	4.97E-06
chr8	106039001	106044000	0.001547399
chr8	106040001	106045000	2.64E-14
chr8	106041001	106046000	2.55E-13
chr8	106044001	106049000	2.49E-09
chr8	106080001	106085000	3.54E-06
chr8	106081001	106086000	3.89E-05
chr8	106082001	106087000	0.001171377
chr8	106083001	106088000	9.09E-05
chr8	106084001	106089000	8.66E-05
chr8	106139001	106144000	0.009515536
chr8	106140001	106145000	0.014982667
chr8	106167001	106172000	5.93E-08
chr8	106202001	106207000	7.04E-06
chr8	106203001	106208000	8.33E-08
chr8	106229001	106234000	0.000564634
chr8	106297001	106302000	1.35E-16
chr8	106314001	106319000	0.000913206
chr8	106315001	106320000	6.64E-07
chr8	106385001	106390000	0.017954611
chr8	106386001	106391000	0.003145352
chr8	106387001	106392000	0.004834997
chr8	106403001	106408000	2.38E-10
chr8	106510001	106515000	5.05E-10
chr8	106651001	106656000	1.72E-05
chr8	106690001	106695000	0.000430335
chr8	106697001	106702000	0.000145742
chr8	106717001	106722000	1.64E-07
chr8	106718001	106723000	4.57E-09
chr8	106719001	106724000	3.88E-09
chr8	106720001	106725000	8.59E-08
chr8	106766001	106771000	2.77E-06
chr8	106767001	106772000	1.37E-06
chr8	106787001	106792000	0.003141562
chr8	106807001	106812000	1.11E-19
chr8	106841001	106846000	0.000371086
chr8	106842001	106847000	0.001402705
chr8	106867001	106872000	0.000446884
chr8	106885001	106890000	0.000124629
chr8	106886001	106891000	0.000159551
chr8	107160001	107165000	1.80E-06
chr8	107177001	107182000	0.001096755
chr8	107211001	107216000	8.38E-07
chr8	107241001	107246000	6.21E-05
chr8	107242001	107247000	1.04E-05
chr8	107243001	107248000	1.63E-07
chr8	107244001	107249000	2.97E-06
chr8	107276001	107281000	0.000253487

chr8	107298001	107303000	5.10E-06
chr8	107299001	107304000	9.85E-06
chr8	107300001	107305000	5.93E-05
chr8	107324001	107329000	6.86E-06
chr8	107520001	107525000	0.000628224
chr8	108133001	108138000	1.98E-22
chr8	108134001	108139000	2.37E-20
chr8	108262001	108267000	8.53E-06
chr8	108263001	108268000	2.32E-06
chr8	108264001	108269000	0.000618392
chr8	108361001	108366000	0.001728608
chr8	108570001	108575000	2.39E-07
chr8	108582001	108587000	5.71E-07
chr8	108790001	108795000	4.19E-07
chr8	108948001	108953000	4.07E-09
chr8	109199001	109204000	6.70E-06
chr8	109807001	109812000	0.000155044
chr8	109808001	109813000	9.51E-05
chr8	109809001	109814000	9.51E-05
chr8	109810001	109815000	0.000819378
chr8	109856001	109861000	0.000266533
chr8	109858001	109863000	3.93E-06
chr8	109923001	109928000	0.000225014
chr8	110530001	110535000	3.17E-08
chr8	110531001	110536000	1.02E-09
chr8	110532001	110537000	6.52E-08
chr8	110728001	110733000	7.12E-05
chr8	110731001	110736000	0.000238411
chr8	110732001	110737000	0.006183563
chr8	110772001	110777000	0.000123017
chr8	110881001	110886000	3.38E-08
chr8	110882001	110887000	1.85E-07
chr8	111149001	111154000	0.020023618
chr8	111251001	111256000	1.72E-05
chr8	111252001	111257000	1.53E-06
chr8	111315001	111320000	0.001180518
chr8	111321001	111326000	0.000191949
chr8	111493001	111498000	0.001404575
chr8	111567001	111572000	0.002003872
chr8	111602001	111607000	0.018135235
chr8	111603001	111608000	0.016981278
chr8	111604001	111609000	0.025394149
chr8	111605001	111610000	0.016788496
chr8	111669001	111674000	0.000251966
chr8	111670001	111675000	0.002479012
chr8	111734001	111739000	9.01E-06
chr8	111748001	111753000	7.20E-06
chr8	111749001	111754000	0.000202475

chr8	111754001	111759000	8.07E-09
chr8	111756001	111761000	1.64E-08
chr8	111814001	111819000	0.002651317
chr8	111815001	111820000	0.000292893
chr8	111816001	111821000	0.000289772
chr8	111860001	111865000	3.69E-14
chr8	111861001	111866000	2.66E-12
chr8	111862001	111867000	9.37E-13
chr8	111863001	111868000	1.34E-12
chr8	111864001	111869000	5.70E-07
chr8	111866001	111871000	5.65E-06
chr8	111884001	111889000	8.13E-06
chr8	111910001	111915000	0.000686353
chr8	111911001	111916000	0.00124259
chr8	111912001	111917000	0.000859605
chr8	112127001	112132000	6.76E-08
chr8	112128001	112133000	3.34E-09
chr8	112129001	112134000	4.31E-09
chr8	112200001	112205000	0.000389647
chr8	112201001	112206000	9.18E-05
chr8	112268001	112273000	6.03E-06
chr8	112271001	112276000	2.46E-06
chr8	112287001	112292000	0.000870217
chr8	112314001	112319000	1.06E-08
chr8	112315001	112320000	2.66E-07
chr8	112316001	112321000	1.53E-07
chr8	112317001	112322000	7.64E-06
chr8	112318001	112323000	1.00E-06
chr8	112390001	112395000	0.000221888
chr8	112456001	112461000	0.000462178
chr8	112533001	112538000	0.002820489
chr8	112534001	112539000	3.13E-08
chr8	112535001	112540000	9.42E-09
chr8	112536001	112541000	2.00E-09
chr8	112537001	112542000	6.34E-07
chr8	112538001	112543000	6.05E-06
chr8	112554001	112559000	2.58E-06
chr8	112555001	112560000	1.44E-05
chr8	112556001	112561000	9.97E-08
chr8	112557001	112562000	5.62E-11
chr8	112559001	112564000	3.62E-15
chr8	112561001	112566000	2.35E-15
chr8	112562001	112567000	3.84E-14
chr8	112588001	112593000	0.005288453
chr8	112604001	112609000	1.31E-14
chr8	112605001	112610000	2.59E-17
chr8	112625001	112630000	0.001920417
chr8	112626001	112631000	0.001439307

chr8	112656001	112661000	1.21E-07
chr8	112673001	112678000	7.05E-08
chr8	112674001	112679000	7.02E-09
chr8	112699001	112704000	5.87E-06
chr8	112749001	112754000	0.005314522
chr8	112750001	112755000	0.00730128
chr8	112751001	112756000	0.010059296
chr8	112760001	112765000	7.65E-15
chr8	112761001	112766000	1.91E-13
chr8	112762001	112767000	1.76E-15
chr8	112790001	112795000	2.60E-05
chr8	112791001	112796000	1.53E-06
chr8	112792001	112797000	1.32E-05
chr8	112793001	112798000	4.13E-06
chr8	112794001	112799000	0.000192975
chr8	112852001	112857000	0.002371269
chr8	112853001	112858000	0.00205145
chr8	112854001	112859000	1.74E-06
chr8	112939001	112944000	3.18E-16
chr8	112940001	112945000	6.56E-19
chr8	112941001	112946000	6.29E-24
chr8	112942001	112947000	4.57E-19
chr8	112943001	112948000	2.64E-17
chr8	113014001	113019000	1.96E-05
chr8	113116001	113121000	3.64E-05
chr8	113118001	113123000	1.16E-09
chr8	113140001	113145000	1.47E-12
chr8	113162001	113167000	8.09E-08
chr8	113171001	113176000	2.54E-05
chr8	113173001	113178000	3.80E-05
chr8	113179001	113184000	8.57E-06
chr8	113184001	113189000	1.17E-05
chr8	113236001	113241000	9.12E-10
chr8	113237001	113242000	1.90E-11
chr8	113278001	113283000	0.000367646
chr8	113280001	113285000	0.011065643
chr8	113326001	113331000	8.45E-05
chr8	113328001	113333000	0.000558057
chr8	113329001	113334000	0.000496883
chr8	113330001	113335000	0.000720916
chr8	113349001	113354000	0.000953244
chr8	113350001	113355000	0.0022022
chr8	113351001	113356000	8.92E-05
chr8	113380001	113385000	4.22E-10
chr8	113384001	113389000	4.06E-07
chr8	113422001	113427000	2.78E-06
chr8	113441001	113446000	0.000588978
chr8	113442001	113447000	0.001274307

chr8	113443001	113448000	0.000223511
chr8	113477001	113482000	1.86E-05
chr8	113504001	113509000	8.04E-21
chr8	113505001	113510000	4.57E-20
chr8	113506001	113511000	9.61E-21
chr8	113507001	113512000	9.24E-20
chr8	113508001	113513000	4.12E-25
chr8	113509001	113514000	1.21E-07
chr8	113552001	113557000	0.005453422
chr8	113553001	113558000	3.35E-06
chr8	113653001	113658000	0.002607193
chr8	113656001	113661000	5.71E-08
chr8	113680001	113685000	6.29E-07
chr8	113681001	113686000	6.04E-11
chr8	113682001	113687000	4.18E-11
chr8	113683001	113688000	7.75E-12
chr8	113684001	113689000	5.96E-09
chr8	113685001	113690000	1.00E-05
chr8	113687001	113692000	0.000717389
chr8	113708001	113713000	1.81E-09
chr8	113709001	113714000	1.29E-10
chr8	113710001	113715000	1.94E-10
chr8	113711001	113716000	1.52E-09
chr8	113717001	113722000	0.014792998
chr8	113721001	113726000	3.07E-06
chr8	113722001	113727000	6.14E-07
chr8	113723001	113728000	9.06E-09
chr8	113758001	113763000	1.25E-05
chr8	113759001	113764000	1.90E-05
chr8	113768001	113773000	4.69E-11
chr8	113769001	113774000	5.26E-13
chr8	113839001	113844000	3.36E-10
chr8	113840001	113845000	1.28E-09
chr8	113841001	113846000	3.42E-08
chr8	113842001	113847000	3.00E-08
chr8	113843001	113848000	8.75E-08
chr8	113903001	113908000	8.91E-06
chr8	113931001	113936000	2.70E-07
chr8	113932001	113937000	5.89E-06
chr8	113933001	113938000	2.88E-07
chr8	113934001	113939000	2.36E-09
chr8	113935001	113940000	5.29E-06
chr8	113942001	113947000	0.001546311
chr8	113945001	113950000	1.59E-05
chr8	113946001	113951000	0.000116563
chr8	113947001	113952000	0.000727782
chr8	113959001	113964000	0.000111573
chr8	113960001	113965000	5.30E-05

chr8	113996001	114001000	1.60E-06
chr8	113997001	114002000	2.26E-05
chr8	114000001	114005000	0.000883533
chr8	114023001	114028000	0.001485689
chr8	114050001	114055000	1.17E-05
chr8	114051001	114056000	2.52E-06
chr8	114052001	114057000	2.89E-06
chr8	114053001	114058000	7.83E-07
chr8	114054001	114059000	2.29E-15
chr8	114055001	114060000	2.43E-23
chr8	114105001	114110000	0.001715127
chr8	114106001	114111000	0.000158326
chr8	114107001	114112000	0.000339059
chr8	114193001	114198000	0.00169098
chr8	114199001	114204000	3.62E-05
chr8	114215001	114220000	0.000443206
chr8	114216001	114221000	0.000247791
chr8	114272001	114277000	0.001489542
chr8	114273001	114278000	0.001488947
chr8	114344001	114349000	0.020795209
chr8	114345001	114350000	0.026457348
chr8	114346001	114351000	0.022804984
chr8	114366001	114371000	0.020378702
chr8	114367001	114372000	0.004271903
chr8	114395001	114400000	0.001478658
chr8	114433001	114438000	5.06E-05
chr8	114453001	114458000	4.38E-10
chr8	114454001	114459000	2.80E-10
chr8	114455001	114460000	2.62E-05
chr8	114456001	114461000	1.79E-05
chr8	114457001	114462000	0.000339582
chr8	114461001	114466000	5.84E-07
chr8	114462001	114467000	3.39E-08
chr8	114463001	114468000	5.74E-09
chr8	114478001	114483000	0.000128216
chr8	114479001	114484000	2.81E-06
chr8	114480001	114485000	3.50E-07
chr8	114481001	114486000	7.77E-09
chr8	114482001	114487000	1.12E-06
chr8	114500001	114505000	2.27E-05
chr8	114518001	114523000	4.58E-07
chr8	114519001	114524000	5.15E-09
chr8	114520001	114525000	1.17E-13
chr8	114554001	114559000	1.13E-05
chr8	114582001	114587000	2.25E-09
chr8	114586001	114591000	1.21E-11
chr8	114603001	114608000	3.23E-07
chr8	114604001	114609000	7.07E-05

chr8	114605001	114610000	0.000135541
chr8	114606001	114611000	1.55E-05
chr8	114607001	114612000	0.00153414
chr8	114630001	114635000	1.67E-05
chr8	114631001	114636000	0.002079742
chr8	114633001	114638000	0.001658799
chr8	114634001	114639000	0.000155292
chr8	114647001	114652000	3.60E-06
chr8	114648001	114653000	0.000408573
chr8	114664001	114669000	9.16E-07
chr8	114665001	114670000	2.15E-09
chr8	114666001	114671000	2.90E-08
chr8	114667001	114672000	2.70E-12
chr8	114668001	114673000	4.73E-13
chr8	114669001	114674000	6.83E-14
chr8	114670001	114675000	5.49E-12
chr8	114671001	114676000	6.94E-10
chr8	114694001	114699000	6.03E-11
chr8	114695001	114700000	5.98E-17
chr8	114696001	114701000	3.92E-16
chr8	114697001	114702000	1.96E-19
chr8	114698001	114703000	5.28E-11
chr8	114699001	114704000	3.93E-10
chr8	114706001	114711000	1.07E-14
chr8	114707001	114712000	2.76E-10
chr8	114715001	114720000	2.03E-06
chr8	114765001	114770000	0.000744053
chr8	114767001	114772000	0.001851415
chr8	114778001	114783000	4.30E-07
chr8	114779001	114784000	1.15E-06
chr8	114780001	114785000	2.34E-08
chr8	114781001	114786000	3.89E-06
chr8	114782001	114787000	5.53E-06
chr8	114784001	114789000	0.001389533
chr8	114785001	114790000	0.007042795
chr8	114787001	114792000	0.001480028
chr8	114806001	114811000	0.001061346
chr8	114807001	114812000	0.000563528
chr8	114808001	114813000	6.60E-06
chr8	114809001	114814000	0.000584571
chr8	114810001	114815000	0.000515328
chr8	114814001	114819000	0.000246944
chr8	114815001	114820000	0.000539814
chr8	114828001	114833000	3.45E-06
chr8	114853001	114858000	0.00120255
chr8	114854001	114859000	0.000242781
chr8	114855001	114860000	5.68E-07
chr8	114856001	114861000	1.02E-09

chr8	114857001	114862000	2.06E-09
chr8	114860001	114865000	1.65E-07
chr8	114861001	114866000	3.24E-05
chr8	114863001	114868000	8.08E-07
chr8	114864001	114869000	0.000145062
chr8	114865001	114870000	0.007618558
chr8	114866001	114871000	0.007296272
chr8	114867001	114872000	7.74E-11
chr8	114875001	114880000	0.001150007
chr8	114886001	114891000	2.05E-20
chr8	114887001	114892000	9.94E-19
chr8	114888001	114893000	3.64E-19
chr8	114889001	114894000	2.37E-07
chr8	114895001	114900000	1.49E-14
chr8	114896001	114901000	2.82E-17
chr8	114943001	114948000	0.002195897
chr8	114965001	114970000	4.87E-05
chr8	114966001	114971000	0.000387109
chr8	114969001	114974000	2.18E-17
chr8	114970001	114975000	3.39E-18
chr8	114971001	114976000	1.27E-19
chr8	114972001	114977000	1.88E-18
chr8	114973001	114978000	2.12E-16
chr8	114979001	114984000	9.60E-06
chr8	114980001	114985000	7.02E-06
chr8	114981001	114986000	8.46E-06
chr8	115044001	115049000	2.60E-06
chr8	115045001	115050000	5.16E-07
chr8	115046001	115051000	1.45E-06
chr8	115047001	115052000	1.34E-06
chr8	115048001	115053000	0.000178268
chr8	115049001	115054000	0.006699518
chr8	115050001	115055000	0.007324351
chr8	115069001	115074000	5.90E-09
chr8	115070001	115075000	1.82E-10
chr8	115071001	115076000	3.03E-11
chr8	115072001	115077000	4.62E-12
chr8	115073001	115078000	2.51E-10
chr8	115123001	115128000	1.17E-09
chr8	115124001	115129000	2.20E-09
chr8	115134001	115139000	1.94E-07
chr8	115135001	115140000	1.54E-10
chr8	115136001	115141000	3.78E-08
chr8	115137001	115142000	1.75E-08
chr8	115138001	115143000	0.001884732
chr8	115205001	115210000	0.00978546
chr8	115217001	115222000	2.55E-14
chr8	115242001	115247000	3.95E-08

chr8	115282001	115287000	0.006819565
chr8	115285001	115290000	0.003467353
chr8	115335001	115340000	0.000658831
chr8	115336001	115341000	3.98E-05
chr8	115337001	115342000	8.56E-06
chr8	115349001	115354000	9.55E-09
chr8	115369001	115374000	3.55E-08
chr8	115397001	115402000	3.41E-21
chr8	115467001	115472000	1.27E-15
chr8	115468001	115473000	3.81E-14
chr8	115522001	115527000	2.00E-07
chr8	115523001	115528000	1.81E-08
chr8	115530001	115535000	6.88E-13
chr8	115532001	115537000	7.73E-07
chr8	115534001	115539000	8.90E-05
chr8	115545001	115550000	4.19E-11
chr8	115546001	115551000	9.12E-08
chr8	115547001	115552000	5.03E-09
chr8	115548001	115553000	1.36E-09
chr8	115550001	115555000	2.35E-08
chr8	115551001	115556000	7.97E-09
chr8	115552001	115557000	1.32E-08
chr8	115553001	115558000	5.91E-09
chr8	115554001	115559000	6.91E-08
chr8	115568001	115573000	0.000147586
chr8	115669001	115674000	0.000616241
chr8	115748001	115753000	6.53E-06
chr8	115777001	115782000	0.000404599
chr8	115802001	115807000	0.003345875
chr8	115803001	115808000	0.00298262
chr8	115804001	115809000	0.001585155
chr8	115888001	115893000	5.75E-08
chr8	115923001	115928000	5.25E-07
chr8	115955001	115960000	8.54E-05
chr8	115969001	115974000	6.88E-05
chr8	115975001	115980000	5.72E-10
chr8	115976001	115981000	4.74E-11
chr8	115977001	115982000	3.00E-09
chr8	115982001	115987000	0.008672355
chr8	115983001	115988000	0.001073936
chr8	116009001	116014000	4.40E-12
chr8	116010001	116015000	1.23E-10
chr8	116011001	116016000	4.74E-08
chr8	116012001	116017000	1.35E-08
chr8	116022001	116027000	7.14E-09
chr8	116025001	116030000	5.12E-06
chr8	116026001	116031000	1.19E-05
chr8	116029001	116034000	0.00033178

chr8	116030001	116035000	0.00029605
chr8	116036001	116041000	5.46E-06
chr8	116060001	116065000	0.000189676
chr8	116071001	116076000	1.28E-13
chr8	116072001	116077000	4.67E-07
chr8	116073001	116078000	7.79E-07
chr8	116074001	116079000	1.29E-06
chr8	116076001	116081000	0.009735557
chr8	116077001	116082000	0.008206162
chr8	116078001	116083000	0.000825286
chr8	116081001	116086000	2.60E-10
chr8	116137001	116142000	0.00094406
chr8	116138001	116143000	0.002004537
chr8	116146001	116151000	3.33E-17
chr8	116147001	116152000	7.47E-20
chr8	116190001	116195000	0.007095544
chr8	116191001	116196000	0.00013448
chr8	116315001	116320000	4.33E-05
chr8	116335001	116340000	0.000104321
chr8	116336001	116341000	1.23E-05
chr8	116337001	116342000	5.26E-05
chr8	116406001	116411000	1.96E-07
chr8	116639001	116644000	1.66E-06
chr8	116640001	116645000	1.14E-05
chr8	116700001	116705000	0.00083146
chr8	116734001	116739000	4.36E-10
chr8	116735001	116740000	1.49E-08
chr8	116752001	116757000	1.98E-06
chr8	116753001	116758000	1.94E-05
chr8	116754001	116759000	3.04E-05
chr8	116760001	116765000	1.23E-08
chr8	116771001	116776000	2.09E-09
chr8	116772001	116777000	1.05E-17
chr8	116773001	116778000	4.58E-17
chr8	116774001	116779000	5.26E-18
chr8	116775001	116780000	1.33E-15
chr8	116776001	116781000	4.51E-12
chr8	116795001	116800000	5.86E-07
chr8	116796001	116801000	1.53E-11
chr8	116797001	116802000	1.17E-08
chr8	116819001	116824000	0.000111221
chr8	116820001	116825000	0.000156869
chr8	116883001	116888000	0.003019145
chr8	116884001	116889000	0.00029022
chr8	116886001	116891000	1.50E-05
chr8	116916001	116921000	2.22E-08
chr8	116917001	116922000	1.06E-08
chr8	116918001	116923000	1.99E-10

chr8	116919001	116924000	1.21E-07
chr8	116924001	116929000	5.01E-06
chr8	116972001	116977000	1.01E-05
chr8	116973001	116978000	1.53E-06
chr8	116974001	116979000	3.11E-06
chr8	116975001	116980000	6.93E-05
chr8	116976001	116981000	6.23E-05
chr8	116992001	116997000	0.000605175
chr8	116993001	116998000	0.000118352
chr8	116994001	116999000	2.31E-05
chr8	116996001	117001000	1.51E-08
chr8	116997001	117002000	5.43E-08
chr8	116998001	117003000	1.82E-07
chr8	117000001	117005000	9.30E-08
chr8	117001001	117006000	0.000578665
chr8	117002001	117007000	1.97E-05
chr8	117003001	117008000	2.17E-06
chr8	117004001	117009000	2.85E-06
chr8	117021001	117026000	7.13E-05
chr8	117029001	117034000	2.41E-09
chr8	117030001	117035000	3.19E-05
chr8	117031001	117036000	0.003617688
chr8	117033001	117038000	5.82E-06
chr8	117034001	117039000	7.27E-06
chr8	117035001	117040000	8.89E-07
chr8	117059001	117064000	1.95E-09
chr8	117060001	117065000	4.51E-14
chr8	117061001	117066000	1.19E-14
chr8	117062001	117067000	1.26E-12
chr8	117063001	117068000	2.68E-08
chr8	117064001	117069000	6.85E-06
chr8	117065001	117070000	0.00037409
chr8	117067001	117072000	3.59E-06
chr8	117068001	117073000	1.06E-05
chr8	117069001	117074000	0.000142142
chr8	117073001	117078000	3.32E-08
chr8	117074001	117079000	3.70E-09
chr8	117075001	117080000	1.13E-07
chr8	117115001	117120000	2.40E-13
chr8	117116001	117121000	2.18E-09
chr8	117130001	117135000	1.63E-10
chr8	117131001	117136000	1.58E-16
chr8	117132001	117137000	3.61E-17
chr8	117133001	117138000	4.55E-15
chr8	117134001	117139000	5.53E-11
chr8	117135001	117140000	2.19E-06
chr8	117141001	117146000	0.001257691
chr8	117143001	117148000	6.16E-11

chr8	117171001	117176000	2.70E-14
chr8	117172001	117177000	3.96E-11
chr8	117173001	117178000	2.19E-10
chr8	117174001	117179000	2.19E-05
chr8	117176001	117181000	0.002486888
chr8	117177001	117182000	2.77E-07
chr8	117191001	117196000	7.58E-07
chr8	117192001	117197000	2.22E-08
chr8	117193001	117198000	8.32E-12
chr8	117206001	117211000	1.05E-28
chr8	117207001	117212000	8.97E-32
chr8	117217001	117222000	9.23E-08
chr8	117218001	117223000	4.30E-09
chr8	117219001	117224000	2.41E-11
chr8	117220001	117225000	2.23E-09
chr8	117230001	117235000	6.15E-19
chr8	117231001	117236000	5.14E-16
chr8	117232001	117237000	4.64E-15
chr8	117234001	117239000	4.22E-09
chr8	117249001	117254000	2.17E-06
chr8	117250001	117255000	4.94E-05
chr8	117288001	117293000	0.007208568
chr8	117289001	117294000	0.001097609
chr8	117295001	117300000	1.76E-13
chr8	117304001	117309000	1.54E-06
chr8	117305001	117310000	2.10E-45
chr8	117306001	117311000	7.20E-43
chr8	117307001	117312000	2.84E-44
chr8	117308001	117313000	2.84E-39
chr8	117315001	117320000	3.84E-08
chr8	117316001	117321000	5.75E-08
chr8	117408001	117413000	0.002397576
chr8	117498001	117503000	1.30E-10
chr8	117942001	117947000	1.40E-08
chr8	117943001	117948000	7.12E-09
chr8	117944001	117949000	7.77E-10
chr8	117990001	117995000	3.01E-07
chr8	117991001	117996000	8.26E-09
chr8	118012001	118017000	1.47E-09
chr8	118013001	118018000	7.68E-10
chr8	118014001	118019000	3.90E-08
chr8	118015001	118020000	6.49E-10
chr8	118040001	118045000	1.10E-06
chr8	118049001	118054000	4.20E-10
chr8	118064001	118069000	2.48E-15
chr8	118065001	118070000	6.23E-14
chr8	118066001	118071000	0.000260811
chr8	118077001	118082000	4.28E-10

chr8	118078001	118083000	7.00E-11
chr8	118079001	118084000	1.12E-06
chr8	118108001	118113000	2.90E-08
chr8	118112001	118117000	0.002810399
chr8	118129001	118134000	7.62E-09
chr8	118130001	118135000	1.68E-07
chr8	118131001	118136000	8.78E-08
chr8	118169001	118174000	2.20E-09
chr8	118170001	118175000	3.41E-08
chr8	118171001	118176000	4.70E-07
chr8	118219001	118224000	0.000460812
chr8	118220001	118225000	8.41E-06
chr8	118233001	118238000	0.000390406
chr8	118234001	118239000	1.22E-05
chr8	118235001	118240000	4.05E-11
chr8	118253001	118258000	5.21E-09
chr8	118283001	118288000	1.28E-05
chr8	118284001	118289000	1.10E-06
chr8	118285001	118290000	6.59E-07
chr8	118293001	118298000	1.40E-09
chr8	118298001	118303000	0.000256672
chr8	118299001	118304000	2.99E-05
chr8	118300001	118305000	1.59E-05
chr8	118323001	118328000	7.30E-12
chr8	118324001	118329000	2.01E-12
chr8	118325001	118330000	1.83E-14
chr8	118326001	118331000	2.63E-13
chr8	118401001	118406000	2.89E-08
chr8	118402001	118407000	1.13E-05
chr8	118474001	118479000	0.008211081
chr8	118493001	118498000	2.47E-06
chr8	118559001	118564000	2.47E-05
chr8	118612001	118617000	1.38E-07
chr8	118669001	118674000	0.000241328
chr8	118686001	118691000	1.00E-08
chr8	118687001	118692000	0.000174502
chr8	118689001	118694000	4.17E-07
chr8	118690001	118695000	4.17E-07
chr8	118691001	118696000	0.000519332
chr8	118692001	118697000	8.96E-05
chr8	119775001	119780000	0.003972817
chr8	119776001	119781000	0.003129306
chr8	119777001	119782000	0.00016956
chr8	119851001	119856000	8.02E-14
chr8	119900001	119905000	0.000136114
chr8	119901001	119906000	4.46E-05
chr8	119902001	119907000	0.000860071
chr8	119949001	119954000	0.001963257

chr8	120064001	120069000	0.002012052
chr8	120345001	120350000	4.83E-11
chr8	120731001	120736000	7.31E-05
chr8	120732001	120737000	5.81E-05
chr8	121750001	121755000	2.00E-08
chr8	121828001	121833000	0.000205172
chr8	121989001	121994000	0.000927307
chr8	121990001	121995000	0.000110865
chr8	122010001	122015000	0.002588651
chr8	122011001	122016000	0.001162992
chr8	122048001	122053000	1.16E-05
chr8	122185001	122190000	1.42E-07
chr8	122188001	122193000	0.004425427
chr8	122189001	122194000	0.001130817
chr8	122801001	122806000	1.35E-19
chr8	122901001	122906000	3.12E-05
chr8	124747001	124752000	2.67E-07
chr8	125036001	125041000	0.001332055
chr8	126645001	126650000	3.32E-24
chr8	126646001	126651000	1.75E-31
chr8	126647001	126652000	1.26E-37
chr8	126723001	126728000	1.36E-10
chr8	126992001	126997000	0.000640289
chr8	126993001	126998000	0.000160544
chr8	126994001	126999000	0.000535343
chr8	127148001	127153000	8.36E-08
chr8	127149001	127154000	4.86E-08
chr8	127224001	127229000	5.78E-06
chr8	127225001	127230000	2.59E-06
chr8	127226001	127231000	1.43E-05
chr8	127246001	127251000	6.07E-06
chr8	127247001	127252000	4.58E-07
chr8	127256001	127261000	0.000394991
chr8	127264001	127269000	0.000843493
chr8	127265001	127270000	0.000661804
chr8	127287001	127292000	4.00E-20
chr8	127315001	127320000	4.85E-07
chr8	127316001	127321000	3.19E-05
chr8	127373001	127378000	5.45E-06
chr8	127376001	127381000	1.58E-11
chr8	127377001	127382000	3.67E-11
chr8	127540001	127545000	6.62E-10
chr8	127653001	127658000	1.58E-05
chr8	128187001	128192000	0.000113298
chr8	128224001	128229000	1.73E-05
chr8	128225001	128230000	1.41E-10
chr8	128226001	128231000	3.86E-08
chr8	128255001	128260000	4.55E-10

chr8	128280001	128285000	0.002187845
chr8	128281001	128286000	0.000564007
chr8	128282001	128287000	0.006276427
chr8	128356001	128361000	0.001660364
chr8	128431001	128436000	1.67E-08
chr8	128464001	128469000	4.23E-10
chr8	128465001	128470000	3.00E-12
chr8	128489001	128494000	9.41E-25
chr8	128490001	128495000	2.66E-20
chr8	128491001	128496000	9.29E-14
chr8	128492001	128497000	6.56E-17
chr8	128523001	128528000	5.53E-12
chr8	128524001	128529000	9.10E-13
chr8	128525001	128530000	6.67E-06
chr8	128526001	128531000	2.26E-06
chr8	128527001	128532000	0.005778706
chr8	129552001	129557000	2.35E-11
chr8	129557001	129562000	2.00E-06
chr8	129601001	129606000	2.80E-08
chr8	129641001	129646000	5.89E-05
chr8	129642001	129647000	2.74E-07
chr8	129643001	129648000	1.09E-08
chr8	129644001	129649000	8.84E-11
chr8	129645001	129650000	2.53E-10
chr8	129741001	129746000	0.00082162
chr8	129789001	129794000	2.41E-06
chr8	129793001	129798000	0.004182249
chr8	129941001	129946000	0.000166186
chr8	129945001	129950000	1.88E-07
chr8	129991001	129996000	0.00295765
chr8	130102001	130107000	2.78E-08
chr8	130103001	130108000	1.19E-11
chr8	130219001	130224000	0.000670451
chr8	130258001	130263000	3.21E-14
chr8	130269001	130274000	1.64E-06
chr8	130270001	130275000	3.47E-08
chr8	130271001	130276000	2.19E-09
chr8	130272001	130277000	1.28E-09
chr8	130273001	130278000	9.24E-10
chr8	130296001	130301000	0.000982559
chr8	130402001	130407000	2.11E-05
chr8	130592001	130597000	6.55E-14
chr8	131337001	131342000	2.31E-14
chr8	131338001	131343000	3.25E-15
chr8	131562001	131567000	3.01E-16
chr8	131611001	131616000	3.67E-21
chr8	131612001	131617000	2.77E-17
chr8	131613001	131618000	1.91E-18

chr8	131671001	131676000	5.13E-15
chr8	131672001	131677000	4.27E-13
chr8	131673001	131678000	2.26E-12
chr8	131674001	131679000	1.21E-15
chr8	131675001	131680000	8.67E-12
chr8	131911001	131916000	5.83E-17
chr8	132016001	132021000	2.08E-08
chr8	132042001	132047000	8.81E-06
chr8	132100001	132105000	0.000918744
chr8	132101001	132106000	5.16E-06
chr8	132113001	132118000	1.08E-07
chr8	132114001	132119000	3.26E-08
chr8	132115001	132120000	1.87E-05
chr8	132141001	132146000	0.000543719
chr8	132142001	132147000	0.00242928
chr8	132180001	132185000	1.72E-13
chr8	132181001	132186000	2.41E-09
chr8	132185001	132190000	3.29E-05
chr8	132187001	132192000	3.77E-05
chr8	132188001	132193000	2.43E-07
chr8	132194001	132199000	5.24E-11
chr8	132195001	132200000	1.00E-11
chr8	132196001	132201000	1.09E-15
chr8	132197001	132202000	7.78E-18
chr8	132198001	132203000	8.09E-15
chr8	132241001	132246000	0.006073224
chr8	132255001	132260000	7.09E-05
chr8	132257001	132262000	0.000631795
chr8	132380001	132385000	0.001115508
chr8	132382001	132387000	2.01E-07
chr8	132387001	132392000	0.002807322
chr8	132388001	132393000	0.002344163
chr8	132397001	132402000	0.001155712
chr8	132413001	132418000	1.06E-06
chr8	132496001	132501000	6.13E-27
chr8	132497001	132502000	4.23E-31
chr8	132498001	132503000	1.91E-34
chr8	132499001	132504000	8.71E-25
chr8	132522001	132527000	1.69E-15
chr8	132530001	132535000	0.010077194
chr8	132597001	132602000	0.000171019
chr8	132598001	132603000	0.000149519
chr8	132683001	132688000	0.000849426
chr8	132684001	132689000	0.001498121
chr8	132694001	132699000	1.51E-05
chr8	132696001	132701000	6.59E-05
chr8	132706001	132711000	0.004102965
chr8	132805001	132810000	1.06E-13

chr8	132816001	132821000	6.48E-17
chr8	132836001	132841000	4.61E-17
chr8	132841001	132846000	0.000410532
chr8	132883001	132888000	1.50E-07
chr8	132884001	132889000	5.41E-09
chr8	132885001	132890000	7.25E-11
chr8	133097001	133102000	2.13E-20
chr8	133195001	133200000	3.23E-12
chr8	133210001	133215000	8.71E-15
chr8	133211001	133216000	1.00E-11
chr8	133212001	133217000	1.58E-11
chr8	133241001	133246000	5.81E-10
chr8	133242001	133247000	3.87E-16
chr8	133258001	133263000	4.63E-28
chr8	133259001	133264000	1.74E-33
chr8	133260001	133265000	3.17E-26
chr8	133261001	133266000	1.41E-30
chr8	133262001	133267000	8.10E-31
chr8	133267001	133272000	7.16E-13
chr8	133268001	133273000	1.88E-15
chr8	133280001	133285000	7.09E-13
chr8	133281001	133286000	9.55E-14
chr8	133282001	133287000	1.08E-12
chr8	133283001	133288000	2.05E-13
chr8	133292001	133297000	2.33E-16
chr8	133307001	133312000	3.21E-07
chr8	133481001	133486000	3.84E-11
chr8	133482001	133487000	1.98E-06
chr8	135010001	135015000	5.09E-09
chr8	135011001	135016000	1.03E-09
chr8	135031001	135036000	1.34E-08
chr8	135032001	135037000	3.17E-08
chr8	135072001	135077000	8.55E-06
chr8	135073001	135078000	1.25E-07
chr8	135074001	135079000	0.000823531
chr8	135075001	135080000	6.29E-06
chr8	135131001	135136000	0.00012407
chr8	135142001	135147000	0.001083898
chr8	135221001	135226000	1.86E-07
chr8	135222001	135227000	1.03E-08
chr8	135223001	135228000	2.39E-07
chr8	135224001	135229000	6.18E-09
chr8	135240001	135245000	4.80E-08
chr8	135254001	135259000	5.02E-05
chr8	135311001	135316000	3.00E-06
chr8	135349001	135354000	3.94E-11
chr8	135410001	135415000	3.59E-07
chr8	136491001	136496000	4.45E-08

chr8	136544001	136549000	9.16E-14
chr8	136932001	136937000	2.36E-09
chr8	136933001	136938000	9.67E-08
chr8	137165001	137170000	8.26E-05
chr8	137379001	137384000	8.85E-08
chr8	137505001	137510000	0.000869435
chr8	137506001	137511000	0.000419742
chr8	137535001	137540000	7.07E-06
chr8	137536001	137541000	0.001515504
chr8	137538001	137543000	3.16E-17
chr8	137736001	137741000	0.000191498
chr8	137894001	137899000	0.000221799
chr8	137895001	137900000	0.027138404
chr8	137925001	137930000	1.33E-07
chr8	137926001	137931000	9.97E-08
chr8	138067001	138072000	0.001618003
chr8	138071001	138076000	0.010096208
chr8	138084001	138089000	0.001369975
chr8	138085001	138090000	0.001296977
chr8	138128001	138133000	3.06E-21
chr8	138129001	138134000	2.27E-10
chr8	138130001	138135000	8.11E-11
chr8	138131001	138136000	1.19E-11
chr8	138246001	138251000	5.53E-14
chr8	138273001	138278000	6.71E-05
chr8	138274001	138279000	0.000136391
chr8	138275001	138280000	9.54E-06
chr8	138276001	138281000	0.000201639
chr8	138283001	138288000	1.78E-16
chr8	138340001	138345000	1.77E-09
chr8	138359001	138364000	7.04E-09
chr8	138388001	138393000	3.66E-05
chr8	138389001	138394000	0.000211739
chr8	138390001	138395000	6.61E-05
chr8	138520001	138525000	7.24E-09
chr8	138521001	138526000	2.17E-09
chr8	138565001	138570000	4.87E-11
chr8	138588001	138593000	1.44E-08
chr8	138724001	138729000	5.67E-06
chr8	138740001	138745000	3.17E-09
chr8	138942001	138947000	5.68E-17
chr8	139089001	139094000	2.64E-08
chr8	139172001	139177000	3.48E-13
chr8	139449001	139454000	5.46E-10
chr8	139450001	139455000	2.37E-06
chr8	139630001	139635000	8.67E-17
chr8	139642001	139647000	0.000317948
chr8	139865001	139870000	9.99E-17

chr8	139990001	139995000	1.40E-09
chr8	140071001	140076000	4.84E-08
chr8	142066001	142071000	5.17E-26
chr8	142288001	142293000	3.98E-47
chr8	142289001	142294000	4.27E-47
chr8	142605001	142610000	1.41E-28
chr8	142606001	142611000	2.77E-33
chr8	142607001	142612000	9.12E-35
chr8	143103001	143108000	3.00E-17
chr8	143104001	143109000	3.18E-23
chr8	143171001	143176000	5.37E-11
chr8	143310001	143315000	5.19E-20
chr8	143311001	143316000	5.80E-20
chr8	143312001	143317000	2.72E-21
chr8	143332001	143337000	2.24E-09
chr8	143333001	143338000	1.39E-07
chr8	143573001	143578000	1.88E-13
chr8	143576001	143581000	3.70E-19
chr8	143577001	143582000	1.31E-19
chr8	143613001	143618000	2.19E-13
chr8	143687001	143692000	2.16E-20
chr8	143688001	143693000	1.05E-16
chr8	143852001	143857000	3.70E-22
chr8	143853001	143858000	2.28E-24
chr8	143868001	143873000	1.68E-08
chr8	143990001	143995000	5.33E-10
chr8	144075001	144080000	3.17E-08
chr8	144104001	144109000	4.19E-19
chr8	144125001	144130000	2.24E-25
chr8	144136001	144141000	2.17E-44
chr8	144137001	144142000	1.99E-39
chr8	144455001	144460000	1.08E-32
chr8	144456001	144461000	8.24E-39
chr8	144459001	144464000	1.11E-33
chr8	144460001	144465000	4.27E-28
chr8	144461001	144466000	5.34E-16
chr8	144462001	144467000	1.05E-19
chr8	144474001	144479000	4.85E-10
chr8	144497001	144502000	6.38E-17
chr8	144498001	144503000	1.34E-16
chr8	144499001	144504000	3.72E-10
chr8	144504001	144509000	2.82E-11
chr8	144505001	144510000	1.91E-37
chr8	144944001	144949000	1.25E-40
chr8	144946001	144951000	4.97E-31
chr8	144947001	144952000	7.95E-30
chr8	144961001	144966000	3.04E-21
chr8	144962001	144967000	1.35E-28

chr8	145020001	145025000	3.07E-10
chr8	145140001	145145000	5.73E-21
chr8	145166001	145171000	1.13E-28
chr8	145167001	145172000	7.89E-28
chr8	145287001	145292000	2.51E-26
chr8	145288001	145293000	1.36E-20
chr8	145540001	145545000	1.43E-27
chr8	145724001	145729000	7.47E-10
chr8	145915001	145920000	3.54E-27
chr8	145916001	145921000	5.80E-27
chr8	146270001	146275000	9.26E-12
chr8	146271001	146276000	6.56E-20
chr9	487001	492000	6.26E-07
chr9	688001	693000	0.016882667
chr9	1606001	1611000	0.0010148
chr9	2936001	2941000	0.000143375
chr9	2950001	2955000	0.000189141
chr9	3014001	3019000	0.00233988
chr9	3016001	3021000	1.06E-12
chr9	3017001	3022000	7.57E-11
chr9	3018001	3023000	2.76E-10
chr9	3019001	3024000	6.83E-08
chr9	3079001	3084000	0.000697005
chr9	3080001	3085000	0.001722093
chr9	3717001	3722000	0.000169769
chr9	3729001	3734000	1.89E-10
chr9	6656001	6661000	0.004236184
chr9	7284001	7289000	8.35E-08
chr9	7508001	7513000	0.000310481
chr9	7509001	7514000	6.08E-05
chr9	7511001	7516000	0.00040974
chr9	7585001	7590000	7.82E-05
chr9	7728001	7733000	4.57E-06
chr9	7772001	7777000	0.00066522
chr9	7773001	7778000	2.80E-05
chr9	7841001	7846000	0.000180569
chr9	7886001	7891000	0.00061273
chr9	8022001	8027000	0.000710911
chr9	8023001	8028000	2.17E-06
chr9	8024001	8029000	2.23E-07
chr9	8210001	8215000	6.25E-08
chr9	8265001	8270000	0.00182107
chr9	8266001	8271000	0.000688387
chr9	8442001	8447000	0.000942094
chr9	8873001	8878000	0.000209464
chr9	8945001	8950000	3.73E-07
chr9	8946001	8951000	1.41E-06
chr9	9577001	9582000	0.000277874

chr9	9874001	9879000	0.004309992
chr9	9875001	9880000	0.003190751
chr9	9876001	9881000	0.000121826
chr9	10432001	10437000	1.98E-05
chr9	10433001	10438000	1.04E-05
chr9	10434001	10439000	4.44E-05
chr9	10695001	10700000	0.013758037
chr9	11230001	11235000	0.000179124
chr9	11424001	11429000	0.000135943
chr9	11425001	11430000	0.00013432
chr9	12298001	12303000	0.003227386
chr9	12300001	12305000	3.83E-06
chr9	12362001	12367000	0.000181283
chr9	12657001	12662000	6.27E-06
chr9	12777001	12782000	2.31E-07
chr9	13295001	13300000	8.42E-06
chr9	13320001	13325000	3.75E-11
chr9	13604001	13609000	7.63E-05
chr9	14091001	14096000	0.005289777
chr9	14307001	14312000	3.28E-14
chr9	15722001	15727000	0.00259231
chr9	15723001	15728000	0.001464556
chr9	15724001	15729000	0.001524362
chr9	15886001	15891000	0.002011619
chr9	16877001	16882000	1.74E-07
chr9	16973001	16978000	2.45E-10
chr9	17001001	17006000	1.28E-06
chr9	17051001	17056000	8.30E-06
chr9	17052001	17057000	3.33E-06
chr9	17053001	17058000	2.58E-05
chr9	17054001	17059000	1.69E-08
chr9	17055001	17060000	0.00150309
chr9	17517001	17522000	4.11E-07
chr9	17518001	17523000	6.85E-07
chr9	17654001	17659000	8.78E-05
chr9	17760001	17765000	3.12E-05
chr9	17811001	17816000	1.34E-05
chr9	17812001	17817000	2.88E-05
chr9	17829001	17834000	0.000156056
chr9	17950001	17955000	0.000254738
chr9	18251001	18256000	6.11E-18
chr9	18252001	18257000	1.87E-15
chr9	18253001	18258000	1.50E-15
chr9	18254001	18259000	1.72E-20
chr9	18255001	18260000	2.41E-11
chr9	18352001	18357000	3.36E-06
chr9	18353001	18358000	5.29E-05
chr9	18363001	18368000	3.05E-09

chr9	18366001	18371000	3.90E-12
chr9	18700001	18705000	4.35E-07
chr9	18868001	18873000	8.79E-05
chr9	18879001	18884000	0.000237649
chr9	18880001	18885000	0.000136294
chr9	18881001	18886000	1.73E-06
chr9	18882001	18887000	2.38E-06
chr9	19154001	19159000	1.54E-05
chr9	19205001	19210000	1.25E-10
chr9	19206001	19211000	3.86E-11
chr9	19207001	19212000	2.24E-05
chr9	19646001	19651000	2.29E-05
chr9	19729001	19734000	0.001223189
chr9	19790001	19795000	0.003336532
chr9	20628001	20633000	0.001074696
chr9	21349001	21354000	0.001509213
chr9	21919001	21924000	0.000120484
chr9	22239001	22244000	8.35E-05
chr9	22240001	22245000	2.51E-05
chr9	22241001	22246000	6.61E-05
chr9	22257001	22262000	0.003254004
chr9	22269001	22274000	0.000419559
chr9	22271001	22276000	2.06E-06
chr9	22272001	22277000	9.67E-07
chr9	22273001	22278000	4.96E-07
chr9	22298001	22303000	3.35E-06
chr9	22365001	22370000	1.52E-05
chr9	23280001	23285000	0.000120921
chr9	23281001	23286000	4.22E-05
chr9	23282001	23287000	0.000180569
chr9	23337001	23342000	0.005345875
chr9	23559001	23564000	1.12E-07
chr9	24044001	24049000	2.36E-07
chr9	24045001	24050000	1.86E-11
chr9	24047001	24052000	6.55E-06
chr9	24062001	24067000	0.000164138
chr9	24212001	24217000	0.00084947
chr9	24213001	24218000	0.00044165
chr9	24423001	24428000	8.84E-10
chr9	24424001	24429000	1.65E-08
chr9	24481001	24486000	0.006137127
chr9	24562001	24567000	0.000423545
chr9	24563001	24568000	0.011642255
chr9	24625001	24630000	0.000621694
chr9	24626001	24631000	0.000520167
chr9	24627001	24632000	0.001462978
chr9	24674001	24679000	1.54E-05
chr9	24675001	24680000	8.21E-10

chr9	24676001	24681000	1.52E-06
chr9	24797001	24802000	0.000280383
chr9	24798001	24803000	0.000208341
chr9	24799001	24804000	0.008061236
chr9	24819001	24824000	0.000117248
chr9	24899001	24904000	0.001307103
chr9	24908001	24913000	0.00312654
chr9	24910001	24915000	0.004457277
chr9	25174001	25179000	1.18E-05
chr9	25175001	25180000	1.18E-05
chr9	25178001	25183000	2.48E-05
chr9	25193001	25198000	0.000185215
chr9	25277001	25282000	1.56E-06
chr9	25278001	25283000	2.11E-06
chr9	25279001	25284000	2.75E-06
chr9	25280001	25285000	1.11E-06
chr9	25355001	25360000	3.18E-05
chr9	25358001	25363000	5.27E-06
chr9	25497001	25502000	0.000230054
chr9	25498001	25503000	0.000588429
chr9	25499001	25504000	0.00019466
chr9	25785001	25790000	0.000862177
chr9	25902001	25907000	0.000588718
chr9	25960001	25965000	0.000186985
chr9	26259001	26264000	0.000116217
chr9	26267001	26272000	1.97E-05
chr9	26268001	26273000	2.23E-08
chr9	26269001	26274000	1.76E-07
chr9	26270001	26275000	0.000315598
chr9	26501001	26506000	2.60E-06
chr9	26502001	26507000	3.22E-07
chr9	26503001	26508000	2.68E-07
chr9	27277001	27282000	2.32E-06
chr9	27958001	27963000	1.45E-05
chr9	27959001	27964000	3.01E-05
chr9	27960001	27965000	4.93E-05
chr9	28072001	28077000	1.40E-05
chr9	28073001	28078000	0.000371595
chr9	28117001	28122000	4.41E-07
chr9	28119001	28124000	1.11E-05
chr9	28120001	28125000	1.06E-05
chr9	28121001	28126000	8.52E-06
chr9	28140001	28145000	0.000977256
chr9	28141001	28146000	5.27E-06
chr9	28164001	28169000	0.000885233
chr9	28165001	28170000	7.76E-05
chr9	28176001	28181000	0.001517378
chr9	28600001	28605000	1.89E-05

chr9	28601001	28606000	0.000121352
chr9	28602001	28607000	0.000893725
chr9	28603001	28608000	0.000786161
chr9	28641001	28646000	1.28E-07
chr9	28643001	28648000	0.000265587
chr9	28777001	28782000	0.008069373
chr9	28778001	28783000	0.006639338
chr9	28798001	28803000	2.00E-05
chr9	28799001	28804000	1.97E-05
chr9	28800001	28805000	8.58E-06
chr9	28818001	28823000	1.05E-05
chr9	28819001	28824000	8.06E-06
chr9	28820001	28825000	2.06E-06
chr9	28842001	28847000	0.000257797
chr9	28866001	28871000	8.21E-06
chr9	28867001	28872000	7.70E-08
chr9	28868001	28873000	1.22E-05
chr9	28869001	28874000	2.56E-05
chr9	28969001	28974000	2.36E-08
chr9	28970001	28975000	2.30E-07
chr9	28971001	28976000	2.40E-05
chr9	28980001	28985000	0.000357606
chr9	29296001	29301000	6.37E-05
chr9	29297001	29302000	0.000741611
chr9	29389001	29394000	0.000266811
chr9	29390001	29395000	0.000145285
chr9	29440001	29445000	9.04E-05
chr9	29441001	29446000	7.52E-06
chr9	29442001	29447000	1.62E-05
chr9	29443001	29448000	9.38E-08
chr9	29444001	29449000	2.93E-06
chr9	29506001	29511000	0.00111928
chr9	29509001	29514000	0.000865108
chr9	29668001	29673000	4.98E-11
chr9	29862001	29867000	8.27E-05
chr9	29863001	29868000	2.73E-05
chr9	29895001	29900000	1.14E-05
chr9	29896001	29901000	3.92E-06
chr9	29897001	29902000	1.90E-05
chr9	29961001	29966000	0.00035357
chr9	29962001	29967000	8.11E-05
chr9	29963001	29968000	0.000231636
chr9	29965001	29970000	5.03E-08
chr9	30050001	30055000	0.005827125
chr9	30084001	30089000	0.000668062
chr9	30435001	30440000	6.33E-06
chr9	30726001	30731000	0.003564728
chr9	30727001	30732000	0.000438145

chr9	30851001	30856000	4.88E-11
chr9	30900001	30905000	8.22E-06
chr9	30901001	30906000	2.28E-07
chr9	30902001	30907000	1.98E-06
chr9	30903001	30908000	4.93E-06
chr9	30976001	30981000	0.000292538
chr9	31017001	31022000	2.18E-05
chr9	31066001	31071000	0.010871337
chr9	31082001	31087000	4.45E-05
chr9	31099001	31104000	8.05E-07
chr9	31100001	31105000	9.93E-07
chr9	31110001	31115000	0.002287613
chr9	31111001	31116000	0.000150216
chr9	31120001	31125000	6.44E-07
chr9	31121001	31126000	8.62E-07
chr9	31122001	31127000	7.78E-11
chr9	31123001	31128000	1.48E-11
chr9	31124001	31129000	6.42E-05
chr9	31302001	31307000	0.000364451
chr9	31304001	31309000	0.00010443
chr9	31649001	31654000	1.96E-05
chr9	31650001	31655000	1.51E-05
chr9	31769001	31774000	4.47E-05
chr9	31770001	31775000	1.83E-09
chr9	31771001	31776000	6.41E-09
chr9	31798001	31803000	0.000415403
chr9	31829001	31834000	0.000535399
chr9	31911001	31916000	7.88E-10
chr9	31912001	31917000	3.84E-10
chr9	31913001	31918000	2.18E-10
chr9	31928001	31933000	0.001414074
chr9	32303001	32308000	0.000138793
chr9	32788001	32793000	0.001273397
chr9	32790001	32795000	1.73E-06
chr9	33442001	33447000	7.46E-09
chr9	33871001	33876000	8.21E-14
chr9	33999001	34004000	0.00014469
chr9	34120001	34125000	3.90E-17
chr9	34546001	34551000	5.14E-05
chr9	36004001	36009000	0.000413204
chr9	36743001	36748000	0.001139022
chr9	36744001	36749000	1.93E-05
chr9	36990001	36995000	2.68E-06
chr9	36991001	36996000	6.76E-06
chr9	36992001	36997000	6.15E-08
chr9	36993001	36998000	7.10E-08
chr9	37019001	37024000	1.61E-05
chr9	37020001	37025000	2.63E-06

chr9	38178001	38183000	1.78E-07
chr9	38294001	38299000	8.10E-06
chr9	38307001	38312000	5.57E-07
chr9	38373001	38378000	1.46E-11
chr9	39070001	39075000	0.007140374
chr9	39071001	39076000	8.21E-05
chr9	39072001	39077000	0.001879507
chr9	40380001	40385000	0.001324737
chr9	40529001	40534000	0.001958703
chr9	40530001	40535000	0.000568947
chr9	40581001	40586000	0.000775626
chr9	40582001	40587000	0.000943543
chr9	40583001	40588000	0.000894565
chr9	40584001	40589000	0.006625872
chr9	40585001	40590000	0.002251277
chr9	40811001	40816000	1.54E-09
chr9	40812001	40817000	6.74E-08
chr9	42182001	42187000	0.000791407
chr9	42183001	42188000	0.000791407
chr9	42266001	42271000	0.00024273
chr9	42835001	42840000	0.031952045
chr9	42949001	42954000	4.97E-06
chr9	42950001	42955000	8.41E-07
chr9	42951001	42956000	2.55E-07
chr9	42952001	42957000	1.34E-08
chr9	42993001	42998000	0.000656707
chr9	42994001	42999000	0.000656707
chr9	42997001	43002000	2.06E-07
chr9	43593001	43598000	3.12E-05
chr9	45437001	45442000	1.11E-07
chr9	45439001	45444000	2.76E-08
chr9	46763001	46768000	0.000819535
chr9	46764001	46769000	0.000819535
chr9	65625001	65630000	3.16E-05
chr9	66763001	66768000	0.000365843
chr9	67069001	67074000	0.005875854
chr9	67935001	67940000	4.46E-06
chr9	68297001	68302000	8.46E-05
chr9	68673001	68678000	9.28E-06
chr9	68823001	68828000	0.002022982
chr9	68824001	68829000	1.36E-05
chr9	68833001	68838000	7.29E-06
chr9	69351001	69356000	3.84E-08
chr9	69399001	69404000	0.001482035
chr9	69426001	69431000	1.97E-06
chr9	69427001	69432000	5.77E-06
chr9	69428001	69433000	1.72E-05
chr9	69432001	69437000	0.004668379

chr9	69456001	69461000	1.07E-05
chr9	69457001	69462000	0.000248768
chr9	69500001	69505000	0.000720003
chr9	69654001	69659000	0.001195365
chr9	69655001	69660000	0.001195365
chr9	69656001	69661000	0.001195365
chr9	69869001	69874000	0.005272853
chr9	69870001	69875000	0.005272853
chr9	69871001	69876000	0.022287421
chr9	70003001	70008000	2.05E-05
chr9	70999001	71004000	1.19E-06
chr9	72398001	72403000	0.002130833
chr9	73758001	73763000	0.000552341
chr9	73869001	73874000	0.001357087
chr9	74371001	74376000	1.09E-06
chr9	74372001	74377000	6.01E-07
chr9	74595001	74600000	3.91E-06
chr9	75195001	75200000	3.50E-05
chr9	75791001	75796000	2.28E-05
chr9	75792001	75797000	0.002306008
chr9	75793001	75798000	0.004944374
chr9	75988001	75993000	0.000737555
chr9	76256001	76261000	4.23E-06
chr9	76257001	76262000	3.03E-07
chr9	76488001	76493000	1.98E-11
chr9	76489001	76494000	2.37E-10
chr9	76490001	76495000	7.36E-11
chr9	76491001	76496000	4.10E-10
chr9	76503001	76508000	0.015338896
chr9	76544001	76549000	0.008562764
chr9	77182001	77187000	3.82E-10
chr9	77704001	77709000	1.15E-08
chr9	78073001	78078000	0.000208943
chr9	78074001	78079000	3.49E-05
chr9	78075001	78080000	0.000513784
chr9	78309001	78314000	0.001150472
chr9	78310001	78315000	0.001490217
chr9	78945001	78950000	4.69E-12
chr9	78946001	78951000	7.04E-12
chr9	78947001	78952000	4.78E-14
chr9	78948001	78953000	1.62E-07
chr9	79295001	79300000	6.80E-05
chr9	79442001	79447000	7.78E-06
chr9	79443001	79448000	4.26E-05
chr9	79487001	79492000	2.35E-08
chr9	79490001	79495000	6.44E-08
chr9	79778001	79783000	5.57E-09
chr9	80453001	80458000	3.19E-08

chr9	80719001	80724000	7.98E-05
chr9	81140001	81145000	1.46E-06
chr9	81209001	81214000	3.61E-08
chr9	81210001	81215000	5.80E-08
chr9	81247001	81252000	1.39E-07
chr9	81248001	81253000	1.37E-07
chr9	81249001	81254000	4.87E-08
chr9	81326001	81331000	2.73E-05
chr9	81332001	81337000	1.67E-07
chr9	81382001	81387000	3.16E-14
chr9	81383001	81388000	8.83E-12
chr9	81402001	81407000	0.000325666
chr9	81403001	81408000	4.71E-05
chr9	81449001	81454000	0.00035436
chr9	81450001	81455000	0.000370652
chr9	81451001	81456000	0.000205949
chr9	81452001	81457000	0.000343648
chr9	81475001	81480000	0.000858851
chr9	81481001	81486000	6.35E-05
chr9	81482001	81487000	4.87E-05
chr9	81493001	81498000	1.21E-07
chr9	81535001	81540000	0.000507557
chr9	81537001	81542000	3.92E-07
chr9	81538001	81543000	8.61E-07
chr9	81539001	81544000	1.64E-07
chr9	81554001	81559000	1.22E-05
chr9	81555001	81560000	1.03E-05
chr9	81556001	81561000	0.001145593
chr9	81580001	81585000	0.000239897
chr9	81635001	81640000	6.12E-05
chr9	81636001	81641000	0.000238017
chr9	81653001	81658000	3.12E-06
chr9	81654001	81659000	0.000152056
chr9	81725001	81730000	4.40E-10
chr9	81726001	81731000	7.50E-12
chr9	81744001	81749000	4.32E-08
chr9	81745001	81750000	8.64E-06
chr9	81746001	81751000	1.79E-06
chr9	81783001	81788000	0.002487813
chr9	81835001	81840000	9.23E-06
chr9	81839001	81844000	1.77E-06
chr9	81917001	81922000	5.31E-08
chr9	81918001	81923000	4.05E-09
chr9	81989001	81994000	0.002740048
chr9	81991001	81996000	0.001717153
chr9	82001001	82006000	0.000257589
chr9	82002001	82007000	3.20E-12
chr9	82297001	82302000	2.32E-07

chr9	82423001	82428000	0.000711354
chr9	82562001	82567000	0.001197512
chr9	82772001	82777000	0.003399683
chr9	82785001	82790000	0.000169051
chr9	83159001	83164000	0.009456209
chr9	83558001	83563000	0.000431864
chr9	83656001	83661000	0.000479117
chr9	83738001	83743000	0.00296966
chr9	83952001	83957000	0.000261528
chr9	84168001	84173000	7.00E-08
chr9	84169001	84174000	5.16E-06
chr9	84428001	84433000	0.001314476
chr9	84641001	84646000	0.017369391
chr9	84642001	84647000	0.000839098
chr9	84643001	84648000	1.92E-09
chr9	84644001	84649000	1.09E-08
chr9	84645001	84650000	9.97E-07
chr9	84646001	84651000	8.67E-07
chr9	84781001	84786000	0.000222523
chr9	84826001	84831000	7.01E-08
chr9	84867001	84872000	0.000610338
chr9	84970001	84975000	0.000490606
chr9	84971001	84976000	9.37E-05
chr9	84972001	84977000	0.000140261
chr9	84973001	84978000	0.000524605
chr9	85006001	85011000	0.000605334
chr9	85009001	85014000	0.009130318
chr9	85010001	85015000	0.009519139
chr9	85029001	85034000	0.000141282
chr9	85124001	85129000	1.93E-05
chr9	85126001	85131000	1.41E-05
chr9	85158001	85163000	8.61E-07
chr9	85159001	85164000	2.15E-08
chr9	85232001	85237000	4.46E-05
chr9	85233001	85238000	0.000207407
chr9	85241001	85246000	0.000889805
chr9	85242001	85247000	0.000128396
chr9	85243001	85248000	0.000142669
chr9	85244001	85249000	0.000462664
chr9	85285001	85290000	1.03E-06
chr9	85456001	85461000	6.34E-06
chr9	88867001	88872000	1.69E-05
chr9	88868001	88873000	8.81E-08
chr9	89548001	89553000	5.14E-05
chr9	90054001	90059000	7.42E-05
chr9	90055001	90060000	7.99E-06
chr9	90069001	90074000	0.001568309
chr9	90071001	90076000	1.54E-05

chr9	90224001	90229000	4.55E-09
chr9	90569001	90574000	1.65E-08
chr9	90570001	90575000	3.17E-08
chr9	92092001	92097000	6.44E-08
chr9	92927001	92932000	1.29E-06
chr9	92928001	92933000	1.33E-07
chr9	93355001	93360000	0.000438217
chr9	94482001	94487000	1.06E-11
chr9	94483001	94488000	4.45E-13
chr9	95104001	95109000	4.89E-05
chr9	95673001	95678000	4.36E-17
chr9	95674001	95679000	1.35E-11
chr9	95715001	95720000	1.03E-12
chr9	95716001	95721000	4.97E-14
chr9	95731001	95736000	6.33E-11
chr9	95864001	95869000	7.50E-05
chr9	96361001	96366000	4.91E-05
chr9	96466001	96471000	4.01E-08
chr9	96467001	96472000	7.29E-08
chr9	96930001	96935000	2.12E-07
chr9	96931001	96936000	5.00E-09
chr9	97465001	97470000	4.16E-09
chr9	97466001	97471000	5.13E-08
chr9	97812001	97817000	0.000390558
chr9	99631001	99636000	7.68E-06
chr9	99640001	99645000	2.67E-10
chr9	100315001	100320000	1.81E-09
chr9	100316001	100321000	4.07E-14
chr9	100317001	100322000	3.18E-12
chr9	100650001	100655000	1.99E-06
chr9	100834001	100839000	4.30E-09
chr9	100981001	100986000	0.00037381
chr9	102287001	102292000	0.002792728
chr9	102321001	102326000	1.91E-05
chr9	102627001	102632000	0.000238525
chr9	103774001	103779000	0.009130318
chr9	103890001	103895000	6.77E-06
chr9	104425001	104430000	0.002641673
chr9	104477001	104482000	1.63E-07
chr9	104479001	104484000	8.88E-07
chr9	104526001	104531000	0.003537221
chr9	104574001	104579000	0.003154311
chr9	104583001	104588000	4.95E-06
chr9	104768001	104773000	0.002143726
chr9	104769001	104774000	0.002670592
chr9	104845001	104850000	4.10E-11
chr9	104846001	104851000	1.34E-11
chr9	104847001	104852000	6.62E-12

chr9	104848001	104853000	2.87E-08
chr9	104849001	104854000	0.006166972
chr9	105236001	105241000	6.63E-07
chr9	105237001	105242000	1.06E-06
chr9	105238001	105243000	4.00E-06
chr9	105298001	105303000	2.88E-05
chr9	105299001	105304000	9.71E-06
chr9	105760001	105765000	0.000137358
chr9	106181001	106186000	4.61E-06
chr9	106182001	106187000	1.62E-05
chr9	106701001	106706000	0.000992931
chr9	106707001	106712000	0.002318887
chr9	106708001	106713000	0.00264199
chr9	106709001	106714000	0.001070465
chr9	106757001	106762000	4.52E-21
chr9	106849001	106854000	6.45E-08
chr9	107002001	107007000	9.40E-06
chr9	107004001	107009000	2.18E-05
chr9	107005001	107010000	0.001030488
chr9	107090001	107095000	1.56E-12
chr9	107091001	107096000	1.08E-11
chr9	107950001	107955000	2.80E-15
chr9	107951001	107956000	1.07E-13
chr9	109158001	109163000	0.000237941
chr9	109212001	109217000	1.20E-06
chr9	109213001	109218000	7.81E-06
chr9	109394001	109399000	0.001932377
chr9	109677001	109682000	3.68E-09
chr9	109846001	109851000	0.000499332
chr9	110047001	110052000	6.03E-11
chr9	111202001	111207000	0.00509168
chr9	111904001	111909000	7.92E-09
chr9	112566001	112571000	9.31E-05
chr9	112567001	112572000	3.46E-07
chr9	112568001	112573000	7.55E-05
chr9	113382001	113387000	0.000532772
chr9	114111001	114116000	0.000751119
chr9	114675001	114680000	2.55E-10
chr9	114676001	114681000	9.46E-11
chr9	114677001	114682000	1.85E-09
chr9	115121001	115126000	7.20E-09
chr9	115301001	115306000	8.99E-07
chr9	115302001	115307000	8.77E-07
chr9	115303001	115308000	9.89E-07
chr9	115482001	115487000	0.004186062
chr9	116143001	116148000	7.74E-07
chr9	117646001	117651000	0.000255579
chr9	118009001	118014000	0.003487991

chr9	118010001	118015000	0.00367058
chr9	118738001	118743000	0.000722843
chr9	118739001	118744000	0.000175746
chr9	118850001	118855000	0.002833171
chr9	118851001	118856000	0.00248329
chr9	119634001	119639000	2.94E-07
chr9	119635001	119640000	0.001263713
chr9	119734001	119739000	6.38E-07
chr9	119735001	119740000	8.18E-07
chr9	119736001	119741000	5.53E-06
chr9	119737001	119742000	1.54E-05
chr9	120167001	120172000	2.64E-09
chr9	120168001	120173000	1.79E-11
chr9	120169001	120174000	3.39E-07
chr9	120624001	120629000	0.000763986
chr9	120671001	120676000	1.18E-05
chr9	120673001	120678000	0.000483195
chr9	120703001	120708000	0.003045962
chr9	120858001	120863000	3.45E-08
chr9	120922001	120927000	0.000816526
chr9	120923001	120928000	0.002447683
chr9	120948001	120953000	0.008236391
chr9	121072001	121077000	0.002357272
chr9	121073001	121078000	0.000871542
chr9	121138001	121143000	2.35E-05
chr9	121139001	121144000	3.23E-07
chr9	121140001	121145000	0.000133053
chr9	121192001	121197000	0.000971504
chr9	121210001	121215000	3.86E-06
chr9	121212001	121217000	2.60E-05
chr9	121296001	121301000	1.20E-06
chr9	121297001	121302000	1.70E-06
chr9	121298001	121303000	5.16E-06
chr9	121299001	121304000	0.000355865
chr9	121302001	121307000	5.00E-05
chr9	121351001	121356000	3.76E-05
chr9	121352001	121357000	5.86E-09
chr9	121353001	121358000	8.14E-09
chr9	121394001	121399000	0.000191434
chr9	121395001	121400000	6.15E-05
chr9	121407001	121412000	3.02E-05
chr9	121418001	121423000	3.90E-05
chr9	121424001	121429000	6.80E-06
chr9	121430001	121435000	8.34E-09
chr9	121444001	121449000	5.91E-07
chr9	121456001	121461000	0.000319387
chr9	121459001	121464000	0.003524029
chr9	121472001	121477000	0.000809316

chr9	121485001	121490000	1.05E-05
chr9	121497001	121502000	0.028040812
chr9	121498001	121503000	5.23E-05
chr9	121499001	121504000	0.00059096
chr9	121548001	121553000	4.47E-07
chr9	121549001	121554000	4.23E-07
chr9	121818001	121823000	0.000729509
chr9	121982001	121987000	2.14E-05
chr9	122027001	122032000	5.01E-08
chr9	122030001	122035000	1.06E-11
chr9	122031001	122036000	3.15E-06
chr9	122032001	122037000	1.12E-06
chr9	122185001	122190000	0.004217452
chr9	122210001	122215000	1.08E-06
chr9	122211001	122216000	1.23E-06
chr9	122212001	122217000	0.000131874
chr9	122213001	122218000	0.000606711
chr9	122260001	122265000	0.000902879
chr9	122287001	122292000	0.000249174
chr9	122417001	122422000	4.95E-09
chr9	122418001	122423000	2.81E-11
chr9	122419001	122424000	2.29E-09
chr9	123382001	123387000	1.29E-09
chr9	123482001	123487000	7.09E-06
chr9	123484001	123489000	3.16E-05
chr9	123508001	123513000	4.17E-09
chr9	123509001	123514000	2.08E-08
chr9	123957001	123962000	0.000111836
chr9	123958001	123963000	0.00032739
chr9	128188001	128193000	4.03E-16
chr9	128189001	128194000	2.04E-16
chr9	128384001	128389000	5.52E-07
chr9	128386001	128391000	0.000114983
chr9	128704001	128709000	0.000699587
chr9	128707001	128712000	0.003167181
chr9	128727001	128732000	1.85E-17
chr9	128729001	128734000	3.84E-11
chr9	129720001	129725000	2.27E-10
chr9	130409001	130414000	7.81E-12
chr9	130410001	130415000	2.90E-11
chr9	130420001	130425000	2.68E-14
chr9	130421001	130426000	2.76E-18
chr9	130422001	130427000	1.72E-18
chr9	130423001	130428000	6.14E-20
chr9	130622001	130627000	4.70E-16
chr9	130664001	130669000	1.49E-11
chr9	130665001	130670000	8.44E-11
chr9	130666001	130671000	1.79E-10

chr9	130667001	130672000	1.27E-10
chr9	130837001	130842000	6.07E-05
chr9	130838001	130843000	0.000119532
chr9	130839001	130844000	2.72E-06
chr9	130840001	130845000	1.16E-06
chr9	131648001	131653000	4.84E-11
chr9	131649001	131654000	1.07E-12
chr9	131650001	131655000	1.84E-11
chr9	131946001	131951000	2.36E-11
chr9	131947001	131952000	6.69E-14
chr9	131948001	131953000	1.33E-12
chr9	132080001	132085000	1.18E-09
chr9	132111001	132116000	8.85E-08
chr9	132469001	132474000	6.14E-05
chr9	132627001	132632000	9.61E-08
chr9	133431001	133436000	6.01E-06
chr9	133432001	133437000	1.16E-06
chr9	133433001	133438000	1.04E-07
chr9	133821001	133826000	1.26E-12
chr9	133822001	133827000	6.64E-12
chr9	134130001	134135000	1.02E-07
chr9	134273001	134278000	4.66E-10
chr9	134550001	134555000	6.24E-14
chr9	136195001	136200000	6.10E-12
chr9	136198001	136203000	5.77E-06
chr9	136261001	136266000	5.21E-06
chr9	136357001	136362000	9.34E-06
chr9	137092001	137097000	4.56E-09
chr9	137479001	137484000	0.000173011
chr9	137545001	137550000	7.43E-06
chr9	137546001	137551000	6.17E-07
chr9	137547001	137552000	1.58E-06
chr9	138018001	138023000	2.80E-12
chr9	138353001	138358000	2.52E-07
chr9	138793001	138798000	2.00E-14
chr9	138894001	138899000	0.000110696
chr9	139091001	139096000	5.17E-13
chr9	139092001	139097000	2.77E-09
chr9	139093001	139098000	9.57E-11
chr9	139094001	139099000	1.10E-11
chr9	139272001	139277000	4.04E-05
chr9	139273001	139278000	2.03E-05
chr9	139792001	139797000	3.03E-08
chr9	139988001	139993000	6.21E-11
chr9	140198001	140203000	6.68E-15
chr9	140283001	140288000	2.94E-15
chr9	140284001	140289000	3.42E-14
chr9	140505001	140510000	4.13E-11

chr10	825001	830000	3.28E-10
chr10	1016001	1021000	1.39E-18
chr10	1939001	1944000	2.22E-10
chr10	2021001	2026000	1.08E-06
chr10	2126001	2131000	1.31E-07
chr10	2179001	2184000	6.04E-07
chr10	2236001	2241000	8.12E-05
chr10	2243001	2248000	0.000868735
chr10	2244001	2249000	0.009301877
chr10	2273001	2278000	0.000306806
chr10	2274001	2279000	9.80E-06
chr10	2275001	2280000	5.54E-05
chr10	2319001	2324000	1.46E-07
chr10	2320001	2325000	3.00E-09
chr10	2378001	2383000	2.87E-10
chr10	2379001	2384000	2.03E-10
chr10	2390001	2395000	4.91E-07
chr10	2391001	2396000	2.86E-06
chr10	2702001	2707000	0.000627734
chr10	2724001	2729000	1.21E-09
chr10	2726001	2731000	1.19E-08
chr10	2727001	2732000	2.90E-12
chr10	2746001	2751000	0.00078507
chr10	2849001	2854000	1.59E-09
chr10	3238001	3243000	2.49E-12
chr10	3239001	3244000	4.89E-13
chr10	3327001	3332000	4.52E-10
chr10	3510001	3515000	5.36E-14
chr10	3541001	3546000	3.70E-08
chr10	3542001	3547000	4.75E-07
chr10	3914001	3919000	3.93E-10
chr10	3975001	3980000	2.01E-11
chr10	3976001	3981000	4.21E-13
chr10	3977001	3982000	3.43E-12
chr10	4189001	4194000	9.31E-18
chr10	4985001	4990000	1.23E-05
chr10	4986001	4991000	0.001530916
chr10	4987001	4992000	1.59E-05
chr10	5117001	5122000	8.30E-05
chr10	5310001	5315000	0.000439092
chr10	5656001	5661000	2.41E-08
chr10	5657001	5662000	5.82E-10
chr10	5658001	5663000	2.78E-05
chr10	5659001	5664000	3.35E-06
chr10	5694001	5699000	3.41E-15
chr10	5695001	5700000	7.37E-15
chr10	5905001	5910000	1.12E-08
chr10	6136001	6141000	1.33E-06

chr10	6236001	6241000	2.40E-05
chr10	6296001	6301000	5.48E-06
chr10	6297001	6302000	3.74E-06
chr10	6357001	6362000	8.36E-06
chr10	6457001	6462000	0.000549854
chr10	6458001	6463000	0.000231205
chr10	6660001	6665000	2.52E-07
chr10	6661001	6666000	9.46E-08
chr10	6662001	6667000	1.88E-07
chr10	6663001	6668000	4.09E-07
chr10	6664001	6669000	2.07E-08
chr10	6665001	6670000	3.45E-10
chr10	6666001	6671000	4.28E-09
chr10	6667001	6672000	2.16E-09
chr10	6668001	6673000	1.17E-09
chr10	6711001	6716000	0.000227222
chr10	6724001	6729000	0.00019985
chr10	6749001	6754000	1.26E-08
chr10	6750001	6755000	2.98E-10
chr10	6751001	6756000	9.70E-12
chr10	6752001	6757000	2.99E-08
chr10	6794001	6799000	2.22E-08
chr10	6795001	6800000	2.06E-08
chr10	6796001	6801000	8.28E-11
chr10	6797001	6802000	2.47E-07
chr10	6798001	6803000	2.00E-06
chr10	6807001	6812000	1.54E-11
chr10	6808001	6813000	4.19E-11
chr10	6811001	6816000	3.38E-07
chr10	6854001	6859000	1.62E-06
chr10	6855001	6860000	5.87E-07
chr10	6897001	6902000	5.05E-06
chr10	6964001	6969000	1.72E-07
chr10	7011001	7016000	2.96E-06
chr10	7052001	7057000	0.002153269
chr10	7053001	7058000	0.000171263
chr10	7074001	7079000	0.000134446
chr10	7679001	7684000	3.81E-07
chr10	7685001	7690000	4.10E-05
chr10	7686001	7691000	0.000777706
chr10	7687001	7692000	0.000664193
chr10	7688001	7693000	0.00018529
chr10	7782001	7787000	7.49E-08
chr10	8068001	8073000	1.25E-07
chr10	8168001	8173000	9.17E-06
chr10	8169001	8174000	2.95E-08
chr10	8170001	8175000	1.05E-08
chr10	8171001	8176000	6.15E-11

chr10	8210001	8215000	7.09E-06
chr10	8284001	8289000	5.75E-09
chr10	8285001	8290000	6.53E-08
chr10	8286001	8291000	4.49E-08
chr10	8288001	8293000	4.84E-06
chr10	8289001	8294000	1.33E-06
chr10	8290001	8295000	1.43E-05
chr10	8291001	8296000	9.88E-06
chr10	8313001	8318000	3.46E-05
chr10	8314001	8319000	5.89E-06
chr10	8315001	8320000	1.73E-05
chr10	8332001	8337000	0.002151943
chr10	8388001	8393000	1.59E-10
chr10	8389001	8394000	1.51E-11
chr10	8390001	8395000	1.27E-11
chr10	8391001	8396000	1.22E-09
chr10	8392001	8397000	2.24E-08
chr10	8397001	8402000	5.72E-08
chr10	8398001	8403000	2.86E-07
chr10	8399001	8404000	5.55E-08
chr10	8400001	8405000	1.02E-06
chr10	8432001	8437000	0.000484612
chr10	8433001	8438000	0.000350663
chr10	8435001	8440000	0.003903414
chr10	8446001	8451000	0.011402937
chr10	8456001	8461000	3.59E-05
chr10	8457001	8462000	1.23E-05
chr10	8458001	8463000	1.64E-07
chr10	8473001	8478000	7.91E-05
chr10	8492001	8497000	1.07E-08
chr10	8493001	8498000	1.86E-08
chr10	8500001	8505000	1.71E-05
chr10	8517001	8522000	6.17E-05
chr10	8549001	8554000	1.21E-11
chr10	8550001	8555000	2.66E-10
chr10	8551001	8556000	6.67E-23
chr10	8564001	8569000	1.69E-06
chr10	8585001	8590000	0.001514818
chr10	8613001	8618000	4.72E-06
chr10	8614001	8619000	2.88E-05
chr10	8615001	8620000	1.70E-06
chr10	8621001	8626000	1.66E-05
chr10	8654001	8659000	0.002859118
chr10	8690001	8695000	0.000199333
chr10	8691001	8696000	0.000799446
chr10	8707001	8712000	0.000179314
chr10	8742001	8747000	2.42E-08
chr10	8745001	8750000	0.00084175

chr10	8751001	8756000	0.000301504
chr10	8777001	8782000	6.24E-06
chr10	8779001	8784000	5.44E-05
chr10	8790001	8795000	4.75E-07
chr10	8805001	8810000	0.000141771
chr10	8879001	8884000	2.99E-06
chr10	8885001	8890000	0.007593099
chr10	8886001	8891000	0.000318724
chr10	8887001	8892000	0.0021904
chr10	8890001	8895000	0.004241264
chr10	8898001	8903000	0.000251029
chr10	8914001	8919000	0.002590141
chr10	8915001	8920000	0.00157994
chr10	8916001	8921000	7.18E-05
chr10	8917001	8922000	0.000812007
chr10	8918001	8923000	0.025132385
chr10	8943001	8948000	0.000698858
chr10	9000001	9005000	0.000154549
chr10	9005001	9010000	0.003806131
chr10	9047001	9052000	5.18E-07
chr10	9054001	9059000	0.000404499
chr10	9057001	9062000	3.35E-05
chr10	9058001	9063000	9.18E-06
chr10	9059001	9064000	2.07E-05
chr10	9061001	9066000	0.002222663
chr10	9106001	9111000	1.35E-07
chr10	9133001	9138000	1.08E-07
chr10	9147001	9152000	0.000289276
chr10	9162001	9167000	4.76E-06
chr10	9163001	9168000	2.45E-06
chr10	9171001	9176000	2.04E-08
chr10	9219001	9224000	6.33E-06
chr10	9220001	9225000	1.39E-08
chr10	9261001	9266000	3.19E-05
chr10	9342001	9347000	0.000109059
chr10	9373001	9378000	5.72E-11
chr10	9770001	9775000	2.38E-05
chr10	9773001	9778000	1.10E-07
chr10	9774001	9779000	5.84E-07
chr10	10230001	10235000	0.002605297
chr10	10231001	10236000	0.002427354
chr10	10232001	10237000	0.004232976
chr10	10864001	10869000	4.14E-12
chr10	10865001	10870000	6.83E-12
chr10	10923001	10928000	0.005454329
chr10	10942001	10947000	2.26E-05
chr10	11034001	11039000	0.001648933
chr10	11417001	11422000	2.60E-05

chr10	11418001	11423000	7.13E-06
chr10	11572001	11577000	0.000189062
chr10	11592001	11597000	0.000699029
chr10	11594001	11599000	1.29E-09
chr10	13021001	13026000	2.35E-08
chr10	13134001	13139000	1.57E-07
chr10	13850001	13855000	1.60E-12
chr10	14051001	14056000	2.97E-06
chr10	14055001	14060000	4.61E-09
chr10	14650001	14655000	5.37E-07
chr10	14700001	14705000	1.29E-08
chr10	14853001	14858000	1.68E-07
chr10	14864001	14869000	1.68E-10
chr10	14865001	14870000	4.58E-10
chr10	15241001	15246000	6.92E-09
chr10	15629001	15634000	0.000348499
chr10	15974001	15979000	0.000158148
chr10	16241001	16246000	0.011889648
chr10	16296001	16301000	7.00E-11
chr10	16379001	16384000	9.95E-07
chr10	16380001	16385000	1.94E-06
chr10	16405001	16410000	9.19E-07
chr10	16406001	16411000	3.66E-06
chr10	16413001	16418000	0.004859673
chr10	16940001	16945000	9.27E-05
chr10	17064001	17069000	0.00024897
chr10	17570001	17575000	3.54E-05
chr10	17571001	17576000	7.50E-08
chr10	17572001	17577000	2.57E-07
chr10	17777001	17782000	2.74E-05
chr10	18511001	18516000	1.67E-10
chr10	18598001	18603000	8.87E-12
chr10	18662001	18667000	0.000252264
chr10	18663001	18668000	0.00089284
chr10	18675001	18680000	7.90E-06
chr10	18703001	18708000	4.28E-07
chr10	18704001	18709000	5.16E-08
chr10	18705001	18710000	9.88E-08
chr10	18706001	18711000	2.17E-05
chr10	18935001	18940000	0.000302766
chr10	18949001	18954000	2.73E-11
chr10	18977001	18982000	2.49E-09
chr10	18978001	18983000	9.91E-09
chr10	18992001	18997000	1.13E-08
chr10	19025001	19030000	2.09E-06
chr10	19318001	19323000	0.002509078
chr10	19349001	19354000	4.03E-08
chr10	19452001	19457000	7.71E-05

chr10	19453001	19458000	2.51E-07
chr10	19454001	19459000	9.57E-07
chr10	19927001	19932000	0.000997437
chr10	19928001	19933000	0.000520502
chr10	20010001	20015000	0.001834766
chr10	20011001	20016000	0.001530989
chr10	20137001	20142000	0.001713499
chr10	20509001	20514000	0.000584061
chr10	20511001	20516000	2.21E-05
chr10	20647001	20652000	0.000180088
chr10	20772001	20777000	0.005089451
chr10	20773001	20778000	0.000149459
chr10	20774001	20779000	0.000405132
chr10	20775001	20780000	0.000208731
chr10	20776001	20781000	0.003343613
chr10	20887001	20892000	0.002276949
chr10	20888001	20893000	0.002158638
chr10	21050001	21055000	0.001858454
chr10	21506001	21511000	7.25E-06
chr10	21578001	21583000	9.51E-06
chr10	21579001	21584000	3.39E-10
chr10	21580001	21585000	2.71E-18
chr10	21581001	21586000	2.00E-22
chr10	21582001	21587000	1.58E-16
chr10	21687001	21692000	6.02E-09
chr10	21688001	21693000	6.87E-10
chr10	22336001	22341000	0.000146702
chr10	22715001	22720000	1.07E-08
chr10	22716001	22721000	2.57E-06
chr10	23580001	23585000	0.003411647
chr10	23603001	23608000	1.87E-06
chr10	24190001	24195000	0.001403641
chr10	24191001	24196000	0.001300245
chr10	24910001	24915000	5.45E-05
chr10	25138001	25143000	1.82E-09
chr10	25564001	25569000	0.000480506
chr10	25580001	25585000	4.37E-07
chr10	25581001	25586000	2.80E-07
chr10	25582001	25587000	8.97E-08
chr10	25583001	25588000	1.12E-08
chr10	25619001	25624000	3.26E-05
chr10	25707001	25712000	2.48E-05
chr10	25708001	25713000	6.20E-05
chr10	25744001	25749000	0.001328439
chr10	25810001	25815000	1.04E-07
chr10	25870001	25875000	1.32E-09
chr10	26159001	26164000	6.64E-05
chr10	26160001	26165000	8.96E-12

chr10	26161001	26166000	1.88E-17
chr10	26162001	26167000	9.72E-12
chr10	26163001	26168000	5.11E-09
chr10	26164001	26169000	3.76E-09
chr10	26182001	26187000	1.22E-05
chr10	26185001	26190000	3.59E-07
chr10	26186001	26191000	3.55E-06
chr10	26357001	26362000	0.001019767
chr10	26976001	26981000	0.000302526
chr10	26977001	26982000	0.005596594
chr10	26979001	26984000	0.001073128
chr10	27144001	27149000	4.53E-13
chr10	27688001	27693000	0.002364653
chr10	27691001	27696000	0.000157955
chr10	27769001	27774000	0.000172054
chr10	28089001	28094000	0.000791559
chr10	28969001	28974000	1.05E-10
chr10	29047001	29052000	6.33E-06
chr10	29115001	29120000	2.59E-08
chr10	29123001	29128000	4.03E-06
chr10	29124001	29129000	3.37E-06
chr10	29471001	29476000	9.52E-08
chr10	29472001	29477000	1.89E-08
chr10	29544001	29549000	1.66E-11
chr10	29545001	29550000	1.16E-12
chr10	29592001	29597000	2.98E-07
chr10	29638001	29643000	2.41E-06
chr10	29639001	29644000	1.10E-05
chr10	29921001	29926000	6.02E-09
chr10	30114001	30119000	2.94E-11
chr10	30825001	30830000	5.29E-06
chr10	31295001	31300000	0.005102416
chr10	31909001	31914000	1.21E-11
chr10	32170001	32175000	0.001684781
chr10	33464001	33469000	1.59E-07
chr10	33490001	33495000	3.72E-07
chr10	33602001	33607000	6.34E-09
chr10	34927001	34932000	8.88E-07
chr10	35512001	35517000	1.91E-10
chr10	35513001	35518000	8.26E-10
chr10	35952001	35957000	3.86E-05
chr10	36339001	36344000	0.013252652
chr10	36474001	36479000	3.19E-05
chr10	36475001	36480000	3.76E-05
chr10	36476001	36481000	0.000403271
chr10	36984001	36989000	9.14E-10
chr10	37005001	37010000	5.28E-09
chr10	37006001	37011000	3.59E-09

chr10	37030001	37035000	4.07E-07
chr10	37369001	37374000	2.05E-11
chr10	37370001	37375000	1.99E-10
chr10	37371001	37376000	7.15E-10
chr10	37383001	37388000	7.11E-05
chr10	37738001	37743000	0.000212832
chr10	37740001	37745000	3.91E-05
chr10	37741001	37746000	5.41E-05
chr10	37770001	37775000	4.21E-07
chr10	37771001	37776000	2.64E-06
chr10	38374001	38379000	0.000482523
chr10	38378001	38383000	4.46E-10
chr10	38711001	38716000	6.48E-05
chr10	38889001	38894000	5.99E-14
chr10	38890001	38895000	7.86E-18
chr10	42592001	42597000	0
chr10	42603001	42608000	5.63E-20
chr10	42852001	42857000	9.26E-10
chr10	43196001	43201000	9.16E-09
chr10	43197001	43202000	4.69E-08
chr10	43198001	43203000	0.00031299
chr10	43228001	43233000	0.004984681
chr10	43738001	43743000	4.50E-18
chr10	44444001	44449000	0.006050163
chr10	44682001	44687000	0.000287942
chr10	45547001	45552000	0.000103061
chr10	45548001	45553000	3.73E-05
chr10	45549001	45554000	7.47E-05
chr10	48525001	48530000	5.59E-06
chr10	50389001	50394000	3.77E-10
chr10	53139001	53144000	3.58E-05
chr10	53140001	53145000	1.10E-05
chr10	53141001	53146000	4.11E-06
chr10	53427001	53432000	0.001183547
chr10	53428001	53433000	0.001783673
chr10	53583001	53588000	0.000357749
chr10	54020001	54025000	0.000225298
chr10	54116001	54121000	0.00026878
chr10	54137001	54142000	1.54E-08
chr10	54265001	54270000	6.39E-05
chr10	54276001	54281000	1.32E-06
chr10	54277001	54282000	5.24E-06
chr10	54403001	54408000	0.009755471
chr10	54467001	54472000	0.003575388
chr10	54559001	54564000	2.16E-05
chr10	54570001	54575000	0.000155775
chr10	54571001	54576000	6.93E-06
chr10	54572001	54577000	7.10E-05

chr10	54573001	54578000	1.36E-05
chr10	54652001	54657000	0.000215203
chr10	54689001	54694000	7.79E-05
chr10	54690001	54695000	3.33E-05
chr10	54726001	54731000	0.000294856
chr10	54727001	54732000	0.00251701
chr10	54729001	54734000	0.000528669
chr10	54783001	54788000	0.000506258
chr10	54825001	54830000	2.28E-07
chr10	54826001	54831000	5.23E-07
chr10	55291001	55296000	1.47E-05
chr10	55292001	55297000	1.23E-07
chr10	55789001	55794000	0.004431128
chr10	55790001	55795000	0.005130975
chr10	55861001	55866000	0.000102652
chr10	55891001	55896000	0.000382617
chr10	56209001	56214000	0.006542585
chr10	56210001	56215000	0.001096438
chr10	56297001	56302000	9.44E-07
chr10	56326001	56331000	3.65E-05
chr10	56330001	56335000	1.17E-10
chr10	56477001	56482000	0.000215891
chr10	56478001	56483000	0.000286015
chr10	56493001	56498000	0.000417121
chr10	56547001	56552000	2.82E-05
chr10	56548001	56553000	2.00E-05
chr10	56549001	56554000	1.84E-05
chr10	56550001	56555000	1.30E-05
chr10	56551001	56556000	0.000671871
chr10	56663001	56668000	0.000122534
chr10	56952001	56957000	4.18E-06
chr10	56953001	56958000	2.22E-06
chr10	56954001	56959000	3.99E-11
chr10	56955001	56960000	1.64E-10
chr10	57031001	57036000	1.35E-06
chr10	57032001	57037000	5.28E-07
chr10	57075001	57080000	0.000228662
chr10	57076001	57081000	0.000118917
chr10	57077001	57082000	3.19E-05
chr10	57078001	57083000	0.000519314
chr10	57423001	57428000	3.96E-11
chr10	57424001	57429000	8.49E-14
chr10	57425001	57430000	1.17E-14
chr10	57426001	57431000	1.14E-14
chr10	57427001	57432000	5.42E-14
chr10	57428001	57433000	9.86E-07
chr10	58054001	58059000	0.000294077
chr10	58056001	58061000	3.32E-05

chr10	58057001	58062000	1.16E-05
chr10	58058001	58063000	1.21E-05
chr10	58193001	58198000	0.002953974
chr10	58194001	58199000	0.003283566
chr10	58241001	58246000	0.002365972
chr10	58255001	58260000	7.36E-05
chr10	58256001	58261000	0.000263745
chr10	58558001	58563000	0.000351424
chr10	58745001	58750000	0.000838761
chr10	59019001	59024000	2.63E-05
chr10	59043001	59048000	0.015477108
chr10	59216001	59221000	0.00047584
chr10	59217001	59222000	0.000406685
chr10	59247001	59252000	0.000236795
chr10	59248001	59253000	3.13E-05
chr10	59249001	59254000	0.005094883
chr10	59362001	59367000	0.000125249
chr10	59363001	59368000	7.27E-05
chr10	59364001	59369000	0.000178371
chr10	59365001	59370000	0.000412585
chr10	59416001	59421000	0.00118814
chr10	59617001	59622000	6.16E-05
chr10	59624001	59629000	0.007681247
chr10	59638001	59643000	0.000103766
chr10	59719001	59724000	0.002058153
chr10	59725001	59730000	5.56E-05
chr10	59726001	59731000	0.000154649
chr10	59727001	59732000	2.12E-05
chr10	59730001	59735000	0.000258594
chr10	59755001	59760000	0.006333031
chr10	59849001	59854000	0.000124496
chr10	59862001	59867000	1.13E-05
chr10	59863001	59868000	3.43E-07
chr10	59935001	59940000	0.00798651
chr10	59941001	59946000	0.000188156
chr10	59942001	59947000	0.000320393
chr10	59943001	59948000	0.020628942
chr10	60194001	60199000	0.001374149
chr10	60195001	60200000	0.00212626
chr10	60196001	60201000	0.0024098
chr10	60197001	60202000	0.000149871
chr10	60213001	60218000	0.004570814
chr10	60231001	60236000	1.82E-05
chr10	60274001	60279000	1.77E-10
chr10	60275001	60280000	7.66E-07
chr10	60276001	60281000	8.90E-08
chr10	60277001	60282000	2.27E-08
chr10	60303001	60308000	0.00755991

chr10	60304001	60309000	0.003978538
chr10	60305001	60310000	0.000456038
chr10	60314001	60319000	1.11E-06
chr10	60315001	60320000	4.58E-06
chr10	60316001	60321000	1.06E-05
chr10	60318001	60323000	7.98E-07
chr10	60330001	60335000	2.32E-07
chr10	60331001	60336000	0.000179037
chr10	60379001	60384000	1.68E-05
chr10	60388001	60393000	5.95E-08
chr10	60395001	60400000	0.000630266
chr10	60396001	60401000	0.001325129
chr10	60397001	60402000	1.49E-05
chr10	60426001	60431000	0.000196641
chr10	60427001	60432000	0.000453709
chr10	60428001	60433000	7.72E-05
chr10	60429001	60434000	1.19E-05
chr10	60438001	60443000	6.62E-07
chr10	60460001	60465000	1.24E-06
chr10	60475001	60480000	6.48E-05
chr10	60500001	60505000	5.20E-06
chr10	60521001	60526000	0.000860648
chr10	60539001	60544000	6.14E-05
chr10	60540001	60545000	0.002553929
chr10	60541001	60546000	0.002036166
chr10	60593001	60598000	4.30E-11
chr10	60594001	60599000	9.79E-11
chr10	60652001	60657000	0.000208639
chr10	60653001	60658000	7.19E-05
chr10	60702001	60707000	0.000182887
chr10	60729001	60734000	0.00018936
chr10	60731001	60736000	0.002783817
chr10	60742001	60747000	0.000179441
chr10	60881001	60886000	0.000214208
chr10	60884001	60889000	0.000189978
chr10	60893001	60898000	6.29E-13
chr10	61011001	61016000	0.000913046
chr10	61012001	61017000	0.001262012
chr10	61140001	61145000	7.90E-05
chr10	61141001	61146000	0.000695668
chr10	61194001	61199000	0.002514784
chr10	61195001	61200000	0.0050125
chr10	61240001	61245000	0.000221755
chr10	61241001	61246000	8.12E-05
chr10	61242001	61247000	7.71E-05
chr10	61313001	61318000	9.08E-05
chr10	61314001	61319000	0.000424965
chr10	61320001	61325000	1.10E-05

chr10	61923001	61928000	2.21E-05
chr10	61940001	61945000	1.28E-10
chr10	61941001	61946000	3.65E-07
chr10	62194001	62199000	3.58E-06
chr10	62586001	62591000	2.21E-05
chr10	62723001	62728000	0.000114274
chr10	63367001	63372000	2.91E-09
chr10	63368001	63373000	3.01E-07
chr10	63379001	63384000	0.000306105
chr10	63458001	63463000	1.56E-05
chr10	63551001	63556000	8.37E-06
chr10	63594001	63599000	2.52E-09
chr10	63595001	63600000	3.65E-12
chr10	63664001	63669000	0.000215768
chr10	63699001	63704000	8.08E-05
chr10	63749001	63754000	4.59E-06
chr10	63750001	63755000	2.54E-08
chr10	63794001	63799000	3.99E-28
chr10	63795001	63800000	8.52E-30
chr10	64290001	64295000	0.000747546
chr10	64678001	64683000	5.13E-08
chr10	64917001	64922000	0.000216123
chr10	65026001	65031000	3.00E-23
chr10	65027001	65032000	4.72E-16
chr10	65028001	65033000	9.28E-12
chr10	65029001	65034000	1.09E-10
chr10	65562001	65567000	0.000236874
chr10	65578001	65583000	5.31E-06
chr10	65579001	65584000	4.60E-06
chr10	65813001	65818000	0.00018187
chr10	67548001	67553000	1.74E-07
chr10	67685001	67690000	0.00044544
chr10	67948001	67953000	0.014999379
chr10	67955001	67960000	1.92E-06
chr10	68283001	68288000	0.003161864
chr10	68284001	68289000	0.001300602
chr10	68425001	68430000	9.09E-05
chr10	68426001	68431000	0.000203072
chr10	68427001	68432000	7.58E-05
chr10	68613001	68618000	0.001331616
chr10	68708001	68713000	0.002637797
chr10	68716001	68721000	0.001374916
chr10	68798001	68803000	0.000262706
chr10	68799001	68804000	0.001233144
chr10	69283001	69288000	0.001159492
chr10	69359001	69364000	0.000797132
chr10	69387001	69392000	0.014768481
chr10	69538001	69543000	0.002308194

chr10	69593001	69598000	0.0021049
chr10	69954001	69959000	5.97E-05
chr10	69955001	69960000	2.14E-07
chr10	70627001	70632000	7.59E-10
chr10	70801001	70806000	5.81E-09
chr10	70844001	70849000	2.33E-12
chr10	70845001	70850000	2.57E-11
chr10	70847001	70852000	8.02E-08
chr10	70863001	70868000	1.31E-09
chr10	70981001	70986000	2.31E-14
chr10	70982001	70987000	3.24E-13
chr10	71169001	71174000	5.70E-07
chr10	71170001	71175000	3.53E-06
chr10	71282001	71287000	0.000309377
chr10	72028001	72033000	4.73E-11
chr10	72029001	72034000	1.89E-11
chr10	72032001	72037000	1.67E-10
chr10	72474001	72479000	5.93E-10
chr10	73005001	73010000	5.55E-08
chr10	73008001	73013000	1.14E-05
chr10	73011001	73016000	2.09E-11
chr10	73205001	73210000	5.86E-11
chr10	73273001	73278000	2.51E-06
chr10	73495001	73500000	6.39E-06
chr10	73548001	73553000	8.84E-10
chr10	73627001	73632000	6.33E-08
chr10	73628001	73633000	3.07E-10
chr10	73629001	73634000	4.65E-08
chr10	73814001	73819000	1.32E-08
chr10	73817001	73822000	2.31E-08
chr10	73818001	73823000	5.98E-10
chr10	74383001	74388000	9.90E-21
chr10	74384001	74389000	4.73E-28
chr10	74385001	74390000	6.62E-29
chr10	75333001	75338000	0.000723511
chr10	75334001	75339000	0.000174647
chr10	75515001	75520000	3.22E-07
chr10	75749001	75754000	8.75E-08
chr10	75750001	75755000	2.52E-10
chr10	76010001	76015000	5.89E-05
chr10	76011001	76016000	2.72E-08
chr10	76012001	76017000	1.89E-05
chr10	76484001	76489000	6.24E-05
chr10	76485001	76490000	0.00011986
chr10	76551001	76556000	8.78E-06
chr10	76552001	76557000	1.32E-21
chr10	76553001	76558000	1.42E-17
chr10	76554001	76559000	4.54E-13

chr10	77529001	77534000	5.08E-09
chr10	77530001	77535000	2.98E-12
chr10	77531001	77536000	9.88E-15
chr10	77532001	77537000	1.90E-06
chr10	78310001	78315000	8.07E-07
chr10	78363001	78368000	6.39E-06
chr10	78521001	78526000	5.91E-05
chr10	78569001	78574000	5.62E-06
chr10	79507001	79512000	0.00081859
chr10	79795001	79800000	1.07E-08
chr10	80644001	80649000	2.16E-06
chr10	81101001	81106000	0.002214908
chr10	81387001	81392000	1.95E-06
chr10	81735001	81740000	0.036664948
chr10	81736001	81741000	2.98E-05
chr10	81826001	81831000	0.002864847
chr10	81827001	81832000	0.000965684
chr10	81879001	81884000	0.000399639
chr10	82091001	82096000	0.000409381
chr10	82678001	82683000	2.39E-07
chr10	82920001	82925000	6.51E-06
chr10	82968001	82973000	0.000579716
chr10	82969001	82974000	0.000551495
chr10	82970001	82975000	0.00171182
chr10	83002001	83007000	0.001040749
chr10	83037001	83042000	0.000308907
chr10	83038001	83043000	0.000771382
chr10	83077001	83082000	1.10E-06
chr10	83091001	83096000	0.006527831
chr10	83092001	83097000	0.005034105
chr10	83114001	83119000	8.01E-06
chr10	83115001	83120000	3.67E-06
chr10	83116001	83121000	1.46E-07
chr10	83638001	83643000	5.17E-05
chr10	83674001	83679000	0.004900835
chr10	84091001	84096000	2.76E-06
chr10	84092001	84097000	3.63E-05
chr10	84267001	84272000	0.003763541
chr10	84268001	84273000	0.000538854
chr10	84296001	84301000	1.05E-05
chr10	84297001	84302000	0.000141983
chr10	84353001	84358000	5.06E-05
chr10	84383001	84388000	0.009614681
chr10	84384001	84389000	0.00261984
chr10	84443001	84448000	0.000263651
chr10	84444001	84449000	0.000205407
chr10	84445001	84450000	6.97E-05
chr10	84446001	84451000	4.11E-06

chr10	84447001	84452000	1.71E-06
chr10	84448001	84453000	6.28E-06
chr10	84452001	84457000	3.01E-06
chr10	84622001	84627000	0.000387874
chr10	84623001	84628000	0.000763896
chr10	84844001	84849000	0.002200184
chr10	84906001	84911000	0.002766703
chr10	84968001	84973000	0.000211467
chr10	84969001	84974000	0.000611217
chr10	84992001	84997000	0.005512465
chr10	84993001	84998000	0.005029071
chr10	85187001	85192000	6.17E-06
chr10	85428001	85433000	0.001113742
chr10	85450001	85455000	8.95E-05
chr10	85521001	85526000	0.003757953
chr10	85551001	85556000	5.60E-05
chr10	85564001	85569000	0.001817106
chr10	85565001	85570000	0.005440033
chr10	85566001	85571000	0.010383858
chr10	85577001	85582000	0.020934629
chr10	85578001	85583000	0.002997079
chr10	85650001	85655000	0.017630915
chr10	85708001	85713000	0.002788575
chr10	85774001	85779000	0.007279741
chr10	85840001	85845000	0.001564792
chr10	85928001	85933000	2.00E-09
chr10	85929001	85934000	4.26E-12
chr10	85930001	85935000	2.52E-09
chr10	86362001	86367000	5.50E-07
chr10	86363001	86368000	8.22E-09
chr10	86364001	86369000	1.16E-09
chr10	86365001	86370000	2.05E-09
chr10	86722001	86727000	4.28E-05
chr10	86993001	86998000	1.96E-10
chr10	86994001	86999000	5.73E-11
chr10	86995001	87000000	5.65E-10
chr10	87195001	87200000	0.001665184
chr10	87283001	87288000	0.001724929
chr10	87291001	87296000	7.95E-07
chr10	87292001	87297000	1.03E-05
chr10	87408001	87413000	7.87E-08
chr10	87510001	87515000	0.001610715
chr10	87517001	87522000	8.03E-05
chr10	87536001	87541000	2.65E-05
chr10	87587001	87592000	4.34E-05
chr10	87602001	87607000	0.002093043
chr10	87603001	87608000	0.006603036
chr10	87666001	87671000	0.000403872

chr10	87786001	87791000	8.51E-06
chr10	87891001	87896000	3.66E-06
chr10	87919001	87924000	2.77E-09
chr10	87934001	87939000	3.71E-15
chr10	87935001	87940000	2.57E-17
chr10	88089001	88094000	0.00016091
chr10	88090001	88095000	5.24E-07
chr10	88091001	88096000	4.73E-06
chr10	88103001	88108000	0.000751239
chr10	88106001	88111000	0.000366774
chr10	88393001	88398000	9.55E-08
chr10	88837001	88842000	5.08E-10
chr10	88838001	88843000	6.87E-13
chr10	88839001	88844000	8.89E-19
chr10	88840001	88845000	7.62E-08
chr10	89352001	89357000	3.11E-06
chr10	89353001	89358000	6.44E-08
chr10	89354001	89359000	3.80E-06
chr10	90144001	90149000	2.89E-06
chr10	90145001	90150000	2.83E-07
chr10	90146001	90151000	0.000408002
chr10	90404001	90409000	9.24E-06
chr10	90405001	90410000	2.07E-05
chr10	90406001	90411000	3.34E-06
chr10	90409001	90414000	0.001941341
chr10	90475001	90480000	0.002167657
chr10	90476001	90481000	0.001710144
chr10	90483001	90488000	1.61E-05
chr10	90484001	90489000	1.01E-13
chr10	90485001	90490000	7.36E-11
chr10	90521001	90526000	0.000410609
chr10	90522001	90527000	0.000801525
chr10	90707001	90712000	4.01E-05
chr10	91191001	91196000	7.61E-07
chr10	91192001	91197000	2.77E-05
chr10	91193001	91198000	3.60E-05
chr10	91372001	91377000	1.64E-06
chr10	91388001	91393000	1.15E-09
chr10	91427001	91432000	0.001835451
chr10	91818001	91823000	4.28E-05
chr10	91819001	91824000	1.85E-05
chr10	92110001	92115000	2.05E-06
chr10	92153001	92158000	0.005362246
chr10	92154001	92159000	0.008602418
chr10	92349001	92354000	0.002140195
chr10	92737001	92742000	2.50E-05
chr10	92739001	92744000	3.23E-06
chr10	92904001	92909000	5.17E-05

chr10	92905001	92910000	6.53E-07
chr10	92906001	92911000	7.65E-06
chr10	93171001	93176000	0.000224505
chr10	93415001	93420000	0.0006213
chr10	93416001	93421000	0.000226241
chr10	93417001	93422000	1.65E-06
chr10	93418001	93423000	1.65E-06
chr10	93419001	93424000	1.66E-07
chr10	93420001	93425000	2.56E-06
chr10	93421001	93426000	1.69E-05
chr10	93464001	93469000	0.00084969
chr10	93684001	93689000	8.39E-09
chr10	93688001	93693000	7.26E-07
chr10	93931001	93936000	0.000554114
chr10	94140001	94145000	1.97E-05
chr10	94509001	94514000	3.63E-08
chr10	94510001	94515000	4.14E-10
chr10	95337001	95342000	1.76E-08
chr10	95338001	95343000	2.01E-12
chr10	95339001	95344000	2.91E-13
chr10	95587001	95592000	0.00012562
chr10	95588001	95593000	5.09E-05
chr10	95839001	95844000	0.003595137
chr10	96025001	96030000	4.17E-07
chr10	96507001	96512000	5.39E-05
chr10	96706001	96711000	3.40E-06
chr10	96707001	96712000	6.50E-07
chr10	96976001	96981000	0.004722826
chr10	97501001	97506000	0.009951543
chr10	97502001	97507000	0.00142181
chr10	97575001	97580000	1.31E-07
chr10	97576001	97581000	4.72E-06
chr10	97868001	97873000	0.003677374
chr10	98573001	98578000	0.000447673
chr10	98618001	98623000	2.23E-07
chr10	98822001	98827000	3.76E-13
chr10	98823001	98828000	6.31E-13
chr10	98959001	98964000	4.15E-10
chr10	98960001	98965000	9.08E-12
chr10	98961001	98966000	2.34E-07
chr10	98967001	98972000	3.58E-20
chr10	98968001	98973000	5.98E-17
chr10	99541001	99546000	1.08E-20
chr10	99542001	99547000	9.27E-18
chr10	99543001	99548000	9.14E-16
chr10	100251001	100256000	0.007032773
chr10	100252001	100257000	0.020185837
chr10	100529001	100534000	0.000624182

chr10	100674001	100679000	0.000535146
chr10	100847001	100852000	0.005064675
chr10	100978001	100983000	0.01144957
chr10	100980001	100985000	0.001340671
chr10	101033001	101038000	1.23E-08
chr10	101034001	101039000	1.16E-08
chr10	101035001	101040000	4.10E-08
chr10	101036001	101041000	6.11E-05
chr10	101281001	101286000	7.70E-10
chr10	101620001	101625000	3.31E-06
chr10	102074001	102079000	4.21E-05
chr10	102449001	102454000	5.15E-09
chr10	102538001	102543000	0.001865969
chr10	102539001	102544000	0.000427624
chr10	102837001	102842000	0.000114073
chr10	102978001	102983000	6.80E-16
chr10	102979001	102984000	2.45E-10
chr10	103584001	103589000	3.30E-21
chr10	103585001	103590000	5.22E-32
chr10	103928001	103933000	3.66E-11
chr10	104407001	104412000	6.36E-07
chr10	104529001	104534000	6.72E-06
chr10	104535001	104540000	4.74E-14
chr10	104746001	104751000	3.07E-11
chr10	104747001	104752000	2.28E-11
chr10	104748001	104753000	4.49E-10
chr10	105014001	105019000	4.55E-09
chr10	105435001	105440000	2.08E-09
chr10	105965001	105970000	4.45E-05
chr10	106093001	106098000	1.68E-09
chr10	106126001	106131000	1.78E-07
chr10	106197001	106202000	8.81E-11
chr10	106295001	106300000	0.000572638
chr10	106296001	106301000	5.67E-05
chr10	106555001	106560000	7.99E-11
chr10	106556001	106561000	1.07E-08
chr10	106557001	106562000	2.73E-07
chr10	106558001	106563000	1.68E-06
chr10	106559001	106564000	6.65E-07
chr10	106570001	106575000	3.17E-06
chr10	106821001	106826000	5.95E-11
chr10	106869001	106874000	6.15E-05
chr10	106870001	106875000	7.03E-05
chr10	106871001	106876000	1.22E-05
chr10	106872001	106877000	0.000144302
chr10	106979001	106984000	0.00442059
chr10	107001001	107006000	9.47E-07
chr10	107007001	107012000	4.10E-05

chr10	107008001	107013000	0.000395054
chr10	107046001	107051000	0.000729027
chr10	107066001	107071000	2.01E-05
chr10	107271001	107276000	0.000190187
chr10	107298001	107303000	0.001022795
chr10	107345001	107350000	0.014618204
chr10	107346001	107351000	0.014364849
chr10	107383001	107388000	0.007621268
chr10	107406001	107411000	0.000284004
chr10	107412001	107417000	0.004599643
chr10	107415001	107420000	0.005917976
chr10	107463001	107468000	9.02E-05
chr10	107465001	107470000	0.002913093
chr10	107466001	107471000	0.001380173
chr10	107506001	107511000	0.000111305
chr10	107519001	107524000	2.25E-05
chr10	107522001	107527000	2.13E-06
chr10	107523001	107528000	2.77E-05
chr10	107577001	107582000	1.30E-07
chr10	107578001	107583000	1.30E-07
chr10	107579001	107584000	5.42E-09
chr10	107593001	107598000	0.000654294
chr10	107594001	107599000	1.95E-05
chr10	107595001	107600000	0.000106549
chr10	107612001	107617000	0.000802512
chr10	107639001	107644000	0.000854668
chr10	107663001	107668000	6.32E-06
chr10	107664001	107669000	0.000304749
chr10	107665001	107670000	0.000194304
chr10	107699001	107704000	5.04E-05
chr10	107945001	107950000	0.000225568
chr10	107968001	107973000	1.01E-05
chr10	108047001	108052000	7.14E-08
chr10	108048001	108053000	6.67E-08
chr10	108058001	108063000	0.000826843
chr10	108059001	108064000	3.14E-05
chr10	108060001	108065000	3.49E-05
chr10	108061001	108066000	0.000312675
chr10	108062001	108067000	0.000414176
chr10	108090001	108095000	0.00112409
chr10	108168001	108173000	0.002320471
chr10	108778001	108783000	1.07E-05
chr10	109034001	109039000	0.00070421
chr10	109036001	109041000	0.005363559
chr10	109160001	109165000	4.88E-06
chr10	109161001	109166000	1.02E-06
chr10	109162001	109167000	4.40E-07
chr10	109163001	109168000	5.75E-06

chr10	109164001	109169000	4.49E-06
chr10	109264001	109269000	0.022691925
chr10	109265001	109270000	0.014505838
chr10	109266001	109271000	0.007751226
chr10	109267001	109272000	0.000995372
chr10	109655001	109660000	0.00030098
chr10	109795001	109800000	0.004103168
chr10	109829001	109834000	0.011985041
chr10	109830001	109835000	0.00098371
chr10	109831001	109836000	0.001845874
chr10	109832001	109837000	0.00148486
chr10	109907001	109912000	0.000968002
chr10	109908001	109913000	0.001526054
chr10	109952001	109957000	4.70E-05
chr10	109953001	109958000	3.14E-05
chr10	109955001	109960000	5.01E-05
chr10	109991001	109996000	0.000337453
chr10	110062001	110067000	0.001145479
chr10	110135001	110140000	4.44E-06
chr10	110136001	110141000	3.88E-06
chr10	110239001	110244000	0.002137111
chr10	110537001	110542000	0.009794588
chr10	110584001	110589000	0.011566767
chr10	110585001	110590000	0.00072622
chr10	110586001	110591000	0.001851022
chr10	110809001	110814000	0.000816588
chr10	110810001	110815000	0.000215418
chr10	110811001	110816000	0.001863779
chr10	110812001	110817000	0.004637583
chr10	110822001	110827000	7.69E-07
chr10	110823001	110828000	1.49E-07
chr10	110929001	110934000	2.38E-07
chr10	110930001	110935000	0.000136989
chr10	110972001	110977000	0.000729581
chr10	111118001	111123000	2.61E-05
chr10	111119001	111124000	0.00047911
chr10	111331001	111336000	0.002392309
chr10	111516001	111521000	4.82E-09
chr10	111760001	111765000	5.51E-11
chr10	111761001	111766000	6.77E-12
chr10	112304001	112309000	1.06E-09
chr10	113265001	113270000	7.77E-05
chr10	114501001	114506000	0.00017362
chr10	114502001	114507000	7.72E-08
chr10	114503001	114508000	2.75E-08
chr10	114968001	114973000	0.010314684
chr10	115300001	115305000	1.09E-08
chr10	116228001	116233000	3.10E-09

chr10	116313001	116318000	0.000138714
chr10	116381001	116386000	0.000107307
chr10	116685001	116690000	0.005313976
chr10	117056001	117061000	0.000142262
chr10	117556001	117561000	2.55E-08
chr10	117557001	117562000	8.50E-07
chr10	117559001	117564000	3.72E-05
chr10	118277001	118282000	0.000578058
chr10	119008001	119013000	2.13E-08
chr10	119186001	119191000	9.61E-07
chr10	119409001	119414000	1.06E-05
chr10	119411001	119416000	0.000596838
chr10	119540001	119545000	1.92E-09
chr10	119818001	119823000	0.00188378
chr10	119858001	119863000	0.000108196
chr10	119859001	119864000	3.27E-05
chr10	119861001	119866000	1.56E-05
chr10	120049001	120054000	2.92E-07
chr10	120050001	120055000	6.70E-10
chr10	120051001	120056000	3.33E-08
chr10	120217001	120222000	1.76E-05
chr10	120278001	120283000	1.73E-06
chr10	120327001	120332000	4.57E-08
chr10	120533001	120538000	0.000119151
chr10	120750001	120755000	2.58E-06
chr10	120751001	120756000	5.19E-09
chr10	120834001	120839000	1.05E-08
chr10	120887001	120892000	1.44E-12
chr10	120888001	120893000	3.62E-21
chr10	120889001	120894000	1.74E-20
chr10	120890001	120895000	5.69E-23
chr10	120891001	120896000	7.84E-23
chr10	121350001	121355000	6.16E-18
chr10	121412001	121417000	1.44E-14
chr10	121413001	121418000	1.05E-12
chr10	121436001	121441000	5.10E-05
chr10	121889001	121894000	0.000679201
chr10	121892001	121897000	8.13E-09
chr10	122083001	122088000	2.05E-10
chr10	122085001	122090000	2.85E-06
chr10	122086001	122091000	4.66E-06
chr10	122093001	122098000	1.94E-05
chr10	122164001	122169000	6.04E-05
chr10	122165001	122170000	1.91E-07
chr10	122272001	122277000	2.47E-07
chr10	122291001	122296000	0.000170518
chr10	122351001	122356000	6.02E-08
chr10	122352001	122357000	1.52E-07

chr10	122353001	122358000	5.70E-06
chr10	122393001	122398000	5.77E-07
chr10	123498001	123503000	0.005513907
chr10	123702001	123707000	1.51E-08
chr10	123778001	123783000	5.73E-16
chr10	123779001	123784000	2.65E-18
chr10	123780001	123785000	2.10E-20
chr10	123781001	123786000	5.52E-18
chr10	123801001	123806000	2.09E-09
chr10	123963001	123968000	3.93E-05
chr10	124063001	124068000	1.66E-08
chr10	124064001	124069000	4.48E-07
chr10	124065001	124070000	8.71E-13
chr10	124066001	124071000	1.60E-12
chr10	124439001	124444000	4.02E-05
chr10	124440001	124445000	1.05E-05
chr10	124441001	124446000	3.75E-08
chr10	124442001	124447000	2.05E-08
chr10	124650001	124655000	3.17E-05
chr10	124995001	125000000	1.16E-06
chr10	125029001	125034000	3.38E-09
chr10	125413001	125418000	1.99E-05
chr10	125414001	125419000	3.01E-06
chr10	126140001	126145000	7.65E-06
chr10	126299001	126304000	0.000402911
chr10	126300001	126305000	0.000149961
chr10	126685001	126690000	4.29E-09
chr10	126688001	126693000	9.21E-05
chr10	126837001	126842000	2.30E-12
chr10	126838001	126843000	2.77E-17
chr10	126839001	126844000	1.96E-21
chr10	127394001	127399000	1.32E-07
chr10	127397001	127402000	2.50E-08
chr10	127463001	127468000	4.90E-07
chr10	127743001	127748000	2.45E-09
chr10	127745001	127750000	1.04E-08
chr10	128615001	128620000	0.000121587
chr10	130685001	130690000	5.07E-06
chr10	130782001	130787000	6.34E-05
chr10	130825001	130830000	7.78E-22
chr10	131990001	131995000	1.66E-09
chr10	131991001	131996000	3.16E-10
chr10	132582001	132587000	0.007583859
chr10	133210001	133215000	1.27E-05
chr10	133808001	133813000	2.54E-08
chr10	133809001	133814000	3.62E-09
chr10	133906001	133911000	1.08E-05
chr10	133934001	133939000	6.94E-19

chr10	133935001	133940000	3.26E-17
chr10	133936001	133941000	1.62E-17
chr10	133938001	133943000	5.58E-14
chr10	133988001	133993000	0.003609573
chr10	134037001	134042000	2.84E-12
chr10	134055001	134060000	6.50E-06
chr10	134082001	134087000	9.52E-14
chr10	134214001	134219000	0.00592535
chr10	134279001	134284000	2.58E-11
chr10	134280001	134285000	1.11E-08
chr10	134554001	134559000	3.92E-15
chr10	134555001	134560000	3.65E-15
chr10	134556001	134561000	5.09E-14
chr10	134558001	134563000	5.58E-09
chr10	135126001	135131000	8.80E-24
chr10	135127001	135132000	2.38E-21
chr10	135386001	135391000	8.98E-06
chr11	1113001	1118000	2.98E-07
chr11	1259001	1264000	2.27E-09
chr11	1474001	1479000	2.89E-06
chr11	1475001	1480000	1.20E-07
chr11	1478001	1483000	1.92E-07
chr11	1624001	1629000	0.001500443
chr11	1775001	1780000	0.000237673
chr11	1791001	1796000	1.64E-06
chr11	1969001	1974000	4.06E-11
chr11	3007001	3012000	5.79E-19
chr11	3238001	3243000	2.02E-15
chr11	3741001	3746000	4.56E-06
chr11	3742001	3747000	1.26E-08
chr11	4624001	4629000	1.29E-07
chr11	4730001	4735000	1.35E-05
chr11	4779001	4784000	1.66E-05
chr11	5173001	5178000	1.02E-06
chr11	5210001	5215000	0.000154905
chr11	5820001	5825000	0.002422235
chr11	6398001	6403000	2.49E-05
chr11	6399001	6404000	0.000178664
chr11	6634001	6639000	1.61E-09
chr11	7205001	7210000	0.001344054
chr11	7206001	7211000	0.000371439
chr11	7221001	7226000	1.11E-05
chr11	7275001	7280000	8.21E-06
chr11	8737001	8742000	3.28E-07
chr11	8934001	8939000	1.13E-08
chr11	9349001	9354000	0.000259642
chr11	9720001	9725000	4.90E-11
chr11	12585001	12590000	5.43E-05

chr11	12816001	12821000	1.08E-07
chr11	13112001	13117000	2.64E-07
chr11	13113001	13118000	3.17E-08
chr11	13114001	13119000	3.64E-08
chr11	13359001	13364000	8.01E-15
chr11	15301001	15306000	0.000395953
chr11	15323001	15328000	0.005022115
chr11	15525001	15530000	0.00746591
chr11	15526001	15531000	5.27E-07
chr11	16414001	16419000	0.000198889
chr11	16892001	16897000	5.24E-05
chr11	20762001	20767000	3.93E-05
chr11	20763001	20768000	0.000102449
chr11	21108001	21113000	1.35E-10
chr11	21362001	21367000	0.000115704
chr11	21659001	21664000	0.002012013
chr11	21660001	21665000	4.82E-06
chr11	21661001	21666000	0.001922176
chr11	21803001	21808000	0.000385247
chr11	21862001	21867000	0.002889777
chr11	21906001	21911000	0.004445689
chr11	21938001	21943000	3.98E-05
chr11	21939001	21944000	0.001028257
chr11	21994001	21999000	5.07E-06
chr11	21997001	22002000	1.28E-06
chr11	22135001	22140000	1.93E-05
chr11	22136001	22141000	1.55E-05
chr11	22137001	22142000	1.24E-05
chr11	22140001	22145000	0.007058571
chr11	22141001	22146000	0.003605573
chr11	22142001	22147000	0.001823362
chr11	22438001	22443000	0.004644924
chr11	22498001	22503000	0.006476331
chr11	22560001	22565000	6.57E-05
chr11	22561001	22566000	2.40E-05
chr11	22562001	22567000	1.95E-05
chr11	25391001	25396000	0.001104714
chr11	25998001	26003000	4.25E-05
chr11	26280001	26285000	3.69E-07
chr11	26282001	26287000	8.63E-05
chr11	26369001	26374000	1.17E-11
chr11	27588001	27593000	3.98E-05
chr11	27755001	27760000	8.58E-06
chr11	27814001	27819000	0.001377632
chr11	27815001	27820000	9.97E-06
chr11	27822001	27827000	2.13E-06
chr11	27861001	27866000	0.003161965
chr11	27968001	27973000	2.13E-06

chr11	28577001	28582000	0.001172872
chr11	28578001	28583000	0.001583819
chr11	28715001	28720000	4.88E-06
chr11	28883001	28888000	9.26E-05
chr11	28974001	28979000	0.000246486
chr11	28975001	28980000	0.000141405
chr11	28976001	28981000	0.000195257
chr11	28980001	28985000	3.29E-05
chr11	29030001	29035000	0.002515555
chr11	29031001	29036000	0.005650668
chr11	29078001	29083000	0.001381294
chr11	29190001	29195000	0.012562931
chr11	30223001	30228000	0.0003277
chr11	30398001	30403000	8.39E-06
chr11	30399001	30404000	6.23E-05
chr11	30546001	30551000	2.37E-08
chr11	30547001	30552000	4.38E-09
chr11	30548001	30553000	1.34E-09
chr11	30682001	30687000	5.52E-06
chr11	30683001	30688000	6.39E-07
chr11	30684001	30689000	3.73E-07
chr11	30685001	30690000	2.61E-05
chr11	30686001	30691000	1.82E-05
chr11	30971001	30976000	0.00048043
chr11	31022001	31027000	7.33E-06
chr11	31023001	31028000	6.71E-06
chr11	31244001	31249000	0.002033792
chr11	31245001	31250000	0.001425881
chr11	32010001	32015000	4.11E-05
chr11	32132001	32137000	0.000233266
chr11	32159001	32164000	9.30E-08
chr11	32217001	32222000	5.34E-05
chr11	33444001	33449000	0.000198404
chr11	33518001	33523000	5.38E-07
chr11	33519001	33524000	4.99E-06
chr11	33520001	33525000	1.56E-06
chr11	33521001	33526000	1.08E-08
chr11	33522001	33527000	5.80E-07
chr11	34747001	34752000	0.001148529
chr11	34770001	34775000	1.28E-06
chr11	35024001	35029000	1.05E-09
chr11	35025001	35030000	4.22E-10
chr11	35062001	35067000	8.37E-07
chr11	35259001	35264000	3.00E-05
chr11	35260001	35265000	1.38E-05
chr11	35507001	35512000	4.38E-12
chr11	35508001	35513000	3.12E-08
chr11	35633001	35638000	3.83E-09

chr11	36498001	36503000	4.59E-07
chr11	36499001	36504000	5.51E-08
chr11	37004001	37009000	0.000283878
chr11	37005001	37010000	0.000412873
chr11	37058001	37063000	0.001463037
chr11	37061001	37066000	2.34E-06
chr11	37433001	37438000	8.13E-05
chr11	37434001	37439000	0.000122161
chr11	37463001	37468000	3.09E-07
chr11	37466001	37471000	2.63E-09
chr11	37532001	37537000	0.001767453
chr11	37609001	37614000	1.83E-08
chr11	37610001	37615000	3.36E-08
chr11	37629001	37634000	4.16E-06
chr11	37636001	37641000	0.002382348
chr11	37773001	37778000	0.000137211
chr11	37774001	37779000	0.000561135
chr11	37790001	37795000	5.55E-07
chr11	37990001	37995000	6.25E-06
chr11	38025001	38030000	0.000120435
chr11	38159001	38164000	0.011375933
chr11	38345001	38350000	0.003177897
chr11	38346001	38351000	2.94E-05
chr11	38441001	38446000	0.000710886
chr11	38442001	38447000	2.53E-05
chr11	38443001	38448000	0.000146922
chr11	38444001	38449000	4.53E-05
chr11	38445001	38450000	0.005412298
chr11	38481001	38486000	1.17E-08
chr11	38556001	38561000	0.000282795
chr11	38677001	38682000	0.000448537
chr11	38678001	38683000	0.000598779
chr11	38764001	38769000	0.001193629
chr11	38781001	38786000	6.60E-05
chr11	38782001	38787000	6.52E-05
chr11	38783001	38788000	0.000885632
chr11	38784001	38789000	1.80E-05
chr11	38785001	38790000	0.019020009
chr11	39270001	39275000	0.001197485
chr11	39792001	39797000	3.54E-06
chr11	39793001	39798000	1.01E-06
chr11	40050001	40055000	3.33E-05
chr11	40238001	40243000	6.86E-05
chr11	40409001	40414000	1.44E-05
chr11	40410001	40415000	1.35E-05
chr11	41014001	41019000	0.001067671
chr11	41066001	41071000	7.75E-05
chr11	41226001	41231000	0.005532503

chr11	41289001	41294000	0.012888979
chr11	41395001	41400000	0.000581189
chr11	41397001	41402000	0.000894854
chr11	41895001	41900000	0.013854343
chr11	42640001	42645000	2.83E-05
chr11	42641001	42646000	7.22E-05
chr11	42719001	42724000	0.000226909
chr11	42721001	42726000	0.000464155
chr11	42983001	42988000	0.000189046
chr11	42984001	42989000	0.000155116
chr11	42985001	42990000	0.000624326
chr11	43174001	43179000	4.63E-08
chr11	43175001	43180000	6.77E-11
chr11	44433001	44438000	0.000109796
chr11	44434001	44439000	0.000840797
chr11	44943001	44948000	1.11E-05
chr11	45663001	45668000	0.009309511
chr11	45762001	45767000	4.05E-11
chr11	45763001	45768000	6.74E-11
chr11	45764001	45769000	5.65E-12
chr11	46405001	46410000	8.11E-08
chr11	47922001	47927000	0.000341705
chr11	48100001	48105000	3.21E-14
chr11	48101001	48106000	1.09E-15
chr11	48102001	48107000	5.77E-10
chr11	48113001	48118000	9.49E-11
chr11	48693001	48698000	1.86E-05
chr11	49741001	49746000	0.006029156
chr11	51546001	51551000	0.002207207
chr11	54922001	54927000	0.000996834
chr11	55472001	55477000	0.00150722
chr11	55893001	55898000	0.006311897
chr11	55894001	55899000	0.005021367
chr11	55895001	55900000	0.007472036
chr11	56521001	56526000	6.26E-06
chr11	57960001	57965000	0.004915816
chr11	58592001	58597000	7.71E-05
chr11	58593001	58598000	0.00036949
chr11	59090001	59095000	0.010256944
chr11	59475001	59480000	0.000843215
chr11	59937001	59942000	6.54E-06
chr11	59938001	59943000	3.93E-07
chr11	60700001	60705000	9.83E-11
chr11	60701001	60706000	1.41E-12
chr11	61749001	61754000	3.90E-11
chr11	62049001	62054000	4.00E-06
chr11	62317001	62322000	5.99E-08
chr11	63433001	63438000	1.45E-06

chr11	63502001	63507000	1.17E-06
chr11	63687001	63692000	2.53E-05
chr11	63688001	63693000	1.01E-06
chr11	63918001	63923000	3.99E-10
chr11	63919001	63924000	6.15E-10
chr11	63920001	63925000	1.02E-07
chr11	64146001	64151000	0.001056078
chr11	64724001	64729000	1.27E-07
chr11	64725001	64730000	8.63E-08
chr11	64727001	64732000	6.41E-08
chr11	64793001	64798000	1.23E-18
chr11	64794001	64799000	1.23E-22
chr11	64795001	64800000	3.45E-32
chr11	65145001	65150000	5.92E-06
chr11	65146001	65151000	4.33E-08
chr11	65344001	65349000	5.27E-08
chr11	65345001	65350000	2.80E-06
chr11	65357001	65362000	1.41E-15
chr11	65359001	65364000	1.27E-13
chr11	65383001	65388000	1.18E-17
chr11	65631001	65636000	1.22E-24
chr11	65632001	65637000	2.75E-20
chr11	65633001	65638000	6.13E-22
chr11	65634001	65639000	4.86E-20
chr11	65635001	65640000	3.38E-18
chr11	65711001	65716000	6.51E-09
chr11	65744001	65749000	4.33E-06
chr11	65745001	65750000	2.51E-05
chr11	66047001	66052000	5.60E-23
chr11	66048001	66053000	2.93E-27
chr11	66049001	66054000	3.00E-23
chr11	66050001	66055000	8.02E-29
chr11	66067001	66072000	7.18E-10
chr11	66150001	66155000	1.27E-13
chr11	66151001	66156000	6.23E-14
chr11	66218001	66223000	6.19E-05
chr11	66219001	66224000	0.000120307
chr11	66308001	66313000	1.91E-17
chr11	66408001	66413000	8.73E-19
chr11	66483001	66488000	6.34E-16
chr11	66485001	66490000	1.06E-13
chr11	66486001	66491000	2.37E-10
chr11	66632001	66637000	4.77E-24
chr11	66633001	66638000	6.07E-22
chr11	66634001	66639000	8.71E-22
chr11	66635001	66640000	9.90E-22
chr11	66636001	66641000	7.61E-21
chr11	67142001	67147000	8.71E-08

chr11	67261001	67266000	2.44E-13
chr11	67263001	67268000	6.80E-11
chr11	67282001	67287000	2.47E-07
chr11	67310001	67315000	1.59E-09
chr11	67324001	67329000	3.47E-19
chr11	67344001	67349000	5.15E-13
chr11	67345001	67350000	3.65E-24
chr11	67458001	67463000	3.48E-10
chr11	67459001	67464000	7.07E-17
chr11	67461001	67466000	6.40E-15
chr11	67865001	67870000	7.69E-14
chr11	67866001	67871000	4.21E-13
chr11	68221001	68226000	1.02E-05
chr11	68804001	68809000	2.98E-06
chr11	68853001	68858000	2.76E-13
chr11	69703001	69708000	1.07E-76
chr11	70578001	70583000	1.78E-15
chr11	70659001	70664000	5.27E-15
chr11	70677001	70682000	2.41E-19
chr11	71876001	71881000	2.43E-08
chr11	72289001	72294000	4.18E-06
chr11	72305001	72310000	7.02E-08
chr11	73025001	73030000	1.36E-10
chr11	73035001	73040000	1.78E-12
chr11	73036001	73041000	1.10E-11
chr11	73037001	73042000	1.39E-10
chr11	73038001	73043000	1.49E-11
chr11	73039001	73044000	1.89E-06
chr11	73061001	73066000	3.78E-23
chr11	73427001	73432000	4.43E-06
chr11	74081001	74086000	0.007091704
chr11	74192001	74197000	7.21E-06
chr11	75380001	75385000	3.12E-17
chr11	76021001	76026000	6.19E-06
chr11	76744001	76749000	2.04E-11
chr11	76745001	76750000	1.46E-14
chr11	77289001	77294000	7.32E-08
chr11	77988001	77993000	3.01E-15
chr11	78268001	78273000	3.73E-11
chr11	79440001	79445000	3.94E-05
chr11	79441001	79446000	1.38E-05
chr11	79442001	79447000	1.37E-08
chr11	79583001	79588000	0.000486531
chr11	79786001	79791000	6.39E-08
chr11	80469001	80474000	0.01075608
chr11	80800001	80805000	2.54E-06
chr11	81003001	81008000	1.42E-06
chr11	81422001	81427000	6.14E-05

chr11	81423001	81428000	0.000536202
chr11	81585001	81590000	4.17E-05
chr11	81586001	81591000	0.000216755
chr11	81587001	81592000	0.003614991
chr11	81618001	81623000	8.99E-09
chr11	81619001	81624000	4.89E-10
chr11	81680001	81685000	0.000300597
chr11	81888001	81893000	1.69E-23
chr11	82013001	82018000	1.00E-11
chr11	82014001	82019000	8.99E-09
chr11	82015001	82020000	4.81E-07
chr11	82132001	82137000	1.85E-05
chr11	82133001	82138000	1.15E-05
chr11	83331001	83336000	0.002695637
chr11	83368001	83373000	0.000366897
chr11	83571001	83576000	0.000407977
chr11	83733001	83738000	2.69E-07
chr11	83734001	83739000	6.18E-05
chr11	83735001	83740000	0.000480761
chr11	83922001	83927000	0.000153366
chr11	84084001	84089000	0.000895847
chr11	84828001	84833000	0.000530345
chr11	85065001	85070000	3.86E-05
chr11	85770001	85775000	1.70E-06
chr11	87110001	87115000	1.88E-07
chr11	87244001	87249000	0.002599205
chr11	87415001	87420000	0.000299126
chr11	87532001	87537000	0.00020081
chr11	87577001	87582000	1.69E-08
chr11	87607001	87612000	0.000234614
chr11	87608001	87613000	0.000463866
chr11	87621001	87626000	0.000806784
chr11	87622001	87627000	7.00E-05
chr11	87646001	87651000	3.37E-05
chr11	87822001	87827000	5.97E-06
chr11	88017001	88022000	4.67E-07
chr11	88295001	88300000	0.000747153
chr11	88323001	88328000	5.89E-06
chr11	88489001	88494000	2.93E-07
chr11	88614001	88619000	0.003521563
chr11	88616001	88621000	0.000298054
chr11	88639001	88644000	0.00125323
chr11	88640001	88645000	0.001311664
chr11	88641001	88646000	0.001770284
chr11	88787001	88792000	0.000142031
chr11	88833001	88838000	3.77E-05
chr11	88872001	88877000	0.003436882
chr11	88873001	88878000	0.010033229

chr11	88874001	88879000	0.015220916
chr11	88875001	88880000	0.018693077
chr11	88974001	88979000	0.000306839
chr11	89468001	89473000	0.000815434
chr11	89470001	89475000	3.35E-09
chr11	90089001	90094000	0.001324366
chr11	90250001	90255000	8.06E-07
chr11	90251001	90256000	4.58E-07
chr11	90326001	90331000	0.000802835
chr11	90327001	90332000	0.000665107
chr11	90661001	90666000	9.55E-05
chr11	90742001	90747000	1.33E-05
chr11	90808001	90813000	0.002207045
chr11	90973001	90978000	5.40E-06
chr11	91124001	91129000	8.14E-05
chr11	91156001	91161000	0.001283549
chr11	91203001	91208000	0.00027865
chr11	91447001	91452000	0.003356018
chr11	91778001	91783000	0.000394707
chr11	91779001	91784000	1.58E-05
chr11	91780001	91785000	0.000272162
chr11	91790001	91795000	0.005840383
chr11	91874001	91879000	0.000133357
chr11	92002001	92007000	2.06E-06
chr11	92052001	92057000	0.000236676
chr11	92144001	92149000	9.37E-10
chr11	92145001	92150000	2.69E-09
chr11	92146001	92151000	6.14E-08
chr11	92147001	92152000	7.18E-07
chr11	92748001	92753000	1.81E-07
chr11	92749001	92754000	4.10E-07
chr11	92956001	92961000	1.13E-07
chr11	92957001	92962000	8.82E-07
chr11	93534001	93539000	5.18E-05
chr11	93712001	93717000	0.000188209
chr11	94054001	94059000	8.66E-06
chr11	94736001	94741000	0.00682589
chr11	95068001	95073000	4.65E-09
chr11	95069001	95074000	5.70E-08
chr11	95070001	95075000	8.24E-13
chr11	95071001	95076000	5.36E-08
chr11	95662001	95667000	2.24E-06
chr11	95692001	95697000	0.000266245
chr11	95983001	95988000	9.32E-12
chr11	95984001	95989000	9.22E-15
chr11	96463001	96468000	1.73E-07
chr11	96464001	96469000	2.43E-15
chr11	96465001	96470000	6.50E-08

chr11	96466001	96471000	1.96E-05
chr11	96762001	96767000	1.30E-06
chr11	96763001	96768000	5.63E-08
chr11	96774001	96779000	0.006756522
chr11	96838001	96843000	0.006630718
chr11	96839001	96844000	0.003574792
chr11	97059001	97064000	6.46E-05
chr11	97060001	97065000	4.13E-06
chr11	97378001	97383000	3.06E-06
chr11	97843001	97848000	4.71E-09
chr11	97844001	97849000	1.17E-14
chr11	97845001	97850000	9.53E-10
chr11	97846001	97851000	3.42E-11
chr11	97859001	97864000	0.00385214
chr11	97860001	97865000	0.000806023
chr11	97861001	97866000	0.000161245
chr11	97862001	97867000	0.00014582
chr11	97895001	97900000	0.010272463
chr11	97896001	97901000	0.003993803
chr11	97897001	97902000	0.001160236
chr11	97995001	98000000	0.001385548
chr11	97996001	98001000	0.001369983
chr11	98037001	98042000	3.09E-09
chr11	98038001	98043000	8.95E-07
chr11	98039001	98044000	5.21E-06
chr11	98266001	98271000	8.67E-06
chr11	98267001	98272000	0.000561452
chr11	98309001	98314000	0.005806263
chr11	98328001	98333000	0.000113984
chr11	98342001	98347000	0.004487111
chr11	98642001	98647000	0.000101239
chr11	98643001	98648000	5.22E-06
chr11	98644001	98649000	1.65E-05
chr11	98659001	98664000	0.00010944
chr11	98724001	98729000	2.74E-06
chr11	98725001	98730000	5.20E-06
chr11	98933001	98938000	0.011613942
chr11	99043001	99048000	1.52E-05
chr11	99220001	99225000	2.26E-06
chr11	99309001	99314000	0.001843523
chr11	99510001	99515000	0.000297753
chr11	99544001	99549000	0.001156023
chr11	99545001	99550000	0.007859794
chr11	99708001	99713000	0.000785736
chr11	99709001	99714000	6.47E-05
chr11	99747001	99752000	8.87E-10
chr11	99748001	99753000	4.16E-11
chr11	99749001	99754000	2.08E-11

chr11	99947001	99952000	1.35E-07
chr11	99948001	99953000	2.14E-07
chr11	100047001	100052000	0.00039056
chr11	100062001	100067000	0.002334323
chr11	100063001	100068000	0.000638135
chr11	100354001	100359000	6.68E-09
chr11	100381001	100386000	0.000400893
chr11	100446001	100451000	1.42E-05
chr11	100447001	100452000	2.77E-06
chr11	100928001	100933000	0.017264165
chr11	101894001	101899000	4.87E-07
chr11	102183001	102188000	7.25E-11
chr11	102903001	102908000	3.77E-06
chr11	103084001	103089000	0.000115572
chr11	103085001	103090000	0.00078137
chr11	103311001	103316000	0.001207237
chr11	103335001	103340000	1.57E-07
chr11	103392001	103397000	8.47E-07
chr11	103416001	103421000	3.77E-09
chr11	103417001	103422000	2.99E-09
chr11	103457001	103462000	0.000108935
chr11	103474001	103479000	0.000281379
chr11	103558001	103563000	0.001793787
chr11	103634001	103639000	0.003506991
chr11	103644001	103649000	5.40E-12
chr11	103647001	103652000	3.56E-07
chr11	103692001	103697000	4.86E-08
chr11	103693001	103698000	2.05E-07
chr11	103694001	103699000	1.05E-05
chr11	103765001	103770000	2.28E-11
chr11	103766001	103771000	1.16E-09
chr11	103871001	103876000	2.01E-07
chr11	103906001	103911000	2.80E-08
chr11	103907001	103912000	9.02E-10
chr11	103908001	103913000	2.70E-09
chr11	103943001	103948000	0.003506631
chr11	103944001	103949000	0.000435832
chr11	104143001	104148000	3.78E-07
chr11	104145001	104150000	0.001922322
chr11	104146001	104151000	0.001103778
chr11	104147001	104152000	0.000837915
chr11	104785001	104790000	0.015812323
chr11	104786001	104791000	3.08E-05
chr11	104787001	104792000	9.52E-06
chr11	104837001	104842000	3.44E-14
chr11	105078001	105083000	5.60E-05
chr11	105079001	105084000	0.000306765
chr11	105566001	105571000	0.000249363

chr11	105567001	105572000	1.05E-05
chr11	105568001	105573000	0.000365228
chr11	105569001	105574000	4.80E-05
chr11	105573001	105578000	0.00013526
chr11	105659001	105664000	0.014417247
chr11	105788001	105793000	0.000304185
chr11	105789001	105794000	0.000186391
chr11	105790001	105795000	0.000125934
chr11	105800001	105805000	0.003851796
chr11	105954001	105959000	1.54E-08
chr11	105955001	105960000	1.68E-08
chr11	106073001	106078000	1.84E-05
chr11	106074001	106079000	1.29E-07
chr11	106297001	106302000	0.004360586
chr11	106317001	106322000	3.78E-14
chr11	106380001	106385000	0.009169822
chr11	106560001	106565000	1.03E-10
chr11	106583001	106588000	4.12E-06
chr11	106584001	106589000	5.51E-06
chr11	106585001	106590000	3.96E-06
chr11	106600001	106605000	0.001069546
chr11	106601001	106606000	0.001474359
chr11	106602001	106607000	0.001815399
chr11	107707001	107712000	2.04E-07
chr11	108003001	108008000	5.36E-08
chr11	109454001	109459000	0.00070078
chr11	109806001	109811000	0.000563439
chr11	110204001	110209000	0.000121629
chr11	110205001	110210000	8.99E-08
chr11	110207001	110212000	8.54E-08
chr11	110624001	110629000	0.000816159
chr11	110855001	110860000	0.000118203
chr11	110856001	110861000	0.001175273
chr11	111242001	111247000	0.00010622
chr11	112764001	112769000	0.000312069
chr11	113114001	113119000	1.10E-07
chr11	114159001	114164000	3.21E-05
chr11	114196001	114201000	4.67E-05
chr11	116163001	116168000	2.22E-06
chr11	117056001	117061000	0.000420673
chr11	117786001	117791000	5.27E-10
chr11	117787001	117792000	2.27E-09
chr11	117788001	117793000	3.81E-10
chr11	117789001	117794000	1.67E-08
chr11	117790001	117795000	7.06E-07
chr11	117917001	117922000	1.26E-07
chr11	118199001	118204000	1.76E-05
chr11	118858001	118863000	6.24E-10

chr11	118859001	118864000	3.26E-09
chr11	120201001	120206000	1.66E-13
chr11	122183001	122188000	7.92E-06
chr11	122184001	122189000	5.47E-07
chr11	122937001	122942000	6.86E-11
chr11	122969001	122974000	3.89E-08
chr11	122970001	122975000	1.10E-09
chr11	122971001	122976000	2.01E-10
chr11	122972001	122977000	4.56E-07
chr11	124222001	124227000	0.000194932
chr11	124223001	124228000	1.30E-05
chr11	124828001	124833000	0.000107585
chr11	127577001	127582000	5.09E-07
chr11	127578001	127583000	5.60E-06
chr11	127594001	127599000	1.17E-05
chr11	127632001	127637000	2.44E-08
chr11	127633001	127638000	5.55E-08
chr11	127672001	127677000	0.0005201
chr11	127695001	127700000	1.60E-12
chr11	127696001	127701000	6.62E-07
chr11	127697001	127702000	0.000213212
chr11	127791001	127796000	0.000146952
chr11	127969001	127974000	0.000491565
chr11	128139001	128144000	0.002132562
chr11	128343001	128348000	2.00E-09
chr11	128472001	128477000	9.16E-06
chr11	129088001	129093000	6.99E-06
chr11	129089001	129094000	9.89E-06
chr11	129696001	129701000	1.77E-09
chr11	130085001	130090000	5.74E-07
chr11	130086001	130091000	9.35E-08
chr11	131597001	131602000	3.59E-12
chr11	131900001	131905000	4.61E-12
chr11	131901001	131906000	5.27E-11
chr11	131912001	131917000	4.51E-11
chr11	131914001	131919000	9.65E-12
chr11	132016001	132021000	1.87E-11
chr11	132025001	132030000	4.65E-12
chr11	132034001	132039000	1.39E-05
chr11	132079001	132084000	1.36E-14
chr11	132144001	132149000	3.10E-09
chr11	132145001	132150000	3.72E-07
chr12	177001	182000	3.30E-09
chr12	207001	212000	1.82E-29
chr12	208001	213000	5.92E-28
chr12	646001	651000	9.58E-44
chr12	647001	652000	1.13E-30
chr12	1198001	1203000	2.55E-12

chr12	1933001	1938000	2.18E-29
chr12	2272001	2277000	5.84E-22
chr12	2273001	2278000	7.64E-12
chr12	2274001	2279000	2.51E-10
chr12	2488001	2493000	1.52E-22
chr12	2507001	2512000	3.25E-18
chr12	2677001	2682000	5.95E-10
chr12	2678001	2683000	9.34E-09
chr12	2773001	2778000	1.32E-20
chr12	3679001	3684000	1.61E-13
chr12	4678001	4683000	1.14E-17
chr12	4808001	4813000	2.65E-17
chr12	4810001	4815000	1.60E-15
chr12	4998001	5003000	0.000133551
chr12	5036001	5041000	3.25E-07
chr12	5073001	5078000	4.63E-13
chr12	5074001	5079000	2.61E-11
chr12	5075001	5080000	6.10E-16
chr12	5076001	5081000	8.01E-12
chr12	5157001	5162000	1.11E-14
chr12	5167001	5172000	1.22E-12
chr12	5494001	5499000	0.000337898
chr12	5760001	5765000	5.64E-06
chr12	5871001	5876000	0.000147462
chr12	6177001	6182000	2.22E-31
chr12	6178001	6183000	2.47E-31
chr12	6334001	6339000	5.94E-45
chr12	6335001	6340000	9.38E-46
chr12	6336001	6341000	1.29E-46
chr12	6479001	6484000	1.23E-32
chr12	6993001	6998000	1.09E-22
chr12	6994001	6999000	3.78E-17
chr12	6995001	7000000	3.71E-27
chr12	7322001	7327000	0.00529207
chr12	7382001	7387000	0.00013637
chr12	7579001	7584000	6.14E-06
chr12	7580001	7585000	1.04E-06
chr12	7581001	7586000	1.06E-08
chr12	7739001	7744000	6.64E-09
chr12	8308001	8313000	8.70E-05
chr12	8309001	8314000	4.65E-07
chr12	8310001	8315000	7.61E-08
chr12	8844001	8849000	7.55E-14
chr12	9556001	9561000	2.11E-24
chr12	10868001	10873000	2.86E-09
chr12	10869001	10874000	6.28E-09
chr12	10924001	10929000	3.36E-08
chr12	10933001	10938000	3.75E-17

chr12	10934001	10939000	2.29E-14
chr12	10935001	10940000	5.36E-16
chr12	10936001	10941000	1.89E-15
chr12	10958001	10963000	0.021648852
chr12	10965001	10970000	0.007069313
chr12	11357001	11362000	0.003345199
chr12	11383001	11388000	1.15E-09
chr12	11384001	11389000	1.32E-10
chr12	11385001	11390000	9.04E-11
chr12	11386001	11391000	1.88E-25
chr12	11387001	11392000	3.67E-18
chr12	11456001	11461000	5.03E-05
chr12	11479001	11484000	0.000780753
chr12	11480001	11485000	0.00045722
chr12	11481001	11486000	1.74E-05
chr12	11482001	11487000	0.000296306
chr12	11536001	11541000	0.000538808
chr12	11561001	11566000	3.96E-09
chr12	11609001	11614000	0.001469598
chr12	11642001	11647000	5.78E-07
chr12	11643001	11648000	5.96E-07
chr12	11644001	11649000	8.43E-09
chr12	11645001	11650000	1.88E-07
chr12	11664001	11669000	5.78E-10
chr12	11665001	11670000	2.74E-09
chr12	11666001	11671000	3.58E-07
chr12	12031001	12036000	1.92E-15
chr12	12032001	12037000	1.86E-11
chr12	12434001	12439000	1.16E-11
chr12	13345001	13350000	4.56E-15
chr12	13457001	13462000	8.52E-07
chr12	13458001	13463000	4.87E-07
chr12	13505001	13510000	1.57E-07
chr12	13506001	13511000	4.45E-07
chr12	13507001	13512000	0.001386325
chr12	13741001	13746000	4.74E-06
chr12	13806001	13811000	1.88E-07
chr12	13817001	13822000	0.000497833
chr12	13819001	13824000	0.000218778
chr12	13843001	13848000	0.000556971
chr12	13888001	13893000	2.05E-09
chr12	13902001	13907000	1.93E-16
chr12	13903001	13908000	6.66E-14
chr12	13919001	13924000	9.67E-09
chr12	13932001	13937000	4.03E-11
chr12	13941001	13946000	9.13E-17
chr12	13942001	13947000	2.18E-16
chr12	13943001	13948000	2.29E-13

chr12	13961001	13966000	7.62E-05
chr12	13969001	13974000	0.005360425
chr12	13970001	13975000	9.34E-06
chr12	13992001	13997000	7.71E-08
chr12	14001001	14006000	9.73E-15
chr12	14017001	14022000	1.67E-23
chr12	14046001	14051000	9.70E-09
chr12	14047001	14052000	3.72E-11
chr12	14048001	14053000	4.84E-10
chr12	14049001	14054000	4.15E-14
chr12	14050001	14055000	1.14E-11
chr12	14051001	14056000	1.79E-05
chr12	14056001	14061000	0.001623901
chr12	14116001	14121000	2.62E-11
chr12	14117001	14122000	0.000704053
chr12	14119001	14124000	0.000470798
chr12	14150001	14155000	2.14E-12
chr12	14151001	14156000	5.20E-16
chr12	14152001	14157000	9.63E-16
chr12	14171001	14176000	5.91E-11
chr12	14172001	14177000	1.02E-09
chr12	14173001	14178000	3.16E-08
chr12	14309001	14314000	8.93E-07
chr12	14667001	14672000	1.09E-16
chr12	14758001	14763000	3.61E-06
chr12	14759001	14764000	5.95E-07
chr12	14760001	14765000	2.20E-06
chr12	14761001	14766000	0.004809913
chr12	14762001	14767000	0.001258261
chr12	14804001	14809000	2.50E-08
chr12	14815001	14820000	4.70E-23
chr12	14816001	14821000	1.54E-26
chr12	14817001	14822000	1.26E-29
chr12	14818001	14823000	6.29E-28
chr12	14819001	14824000	1.15E-15
chr12	14827001	14832000	0.000646429
chr12	14845001	14850000	1.17E-07
chr12	15236001	15241000	0.003265105
chr12	15237001	15242000	0.003206832
chr12	15282001	15287000	7.25E-06
chr12	15319001	15324000	7.50E-07
chr12	15320001	15325000	2.04E-07
chr12	15321001	15326000	8.07E-08
chr12	15322001	15327000	3.73E-06
chr12	15323001	15328000	4.57E-05
chr12	15324001	15329000	3.84E-05
chr12	15325001	15330000	9.02E-05
chr12	15331001	15336000	4.04E-05

chr12	15346001	15351000	0.000842776
chr12	15440001	15445000	0.000641758
chr12	15449001	15454000	0.000699861
chr12	15464001	15469000	8.23E-07
chr12	15465001	15470000	2.58E-10
chr12	15466001	15471000	9.38E-09
chr12	15467001	15472000	2.01E-08
chr12	15468001	15473000	1.68E-10
chr12	15469001	15474000	2.27E-08
chr12	15539001	15544000	2.92E-05
chr12	15577001	15582000	1.08E-11
chr12	15578001	15583000	1.81E-09
chr12	15579001	15584000	5.39E-07
chr12	15580001	15585000	7.62E-07
chr12	15599001	15604000	0.000659752
chr12	15600001	15605000	0.002960085
chr12	15602001	15607000	0.001910718
chr12	15603001	15608000	0.001394046
chr12	15634001	15639000	0.002207072
chr12	15635001	15640000	0.002706475
chr12	15636001	15641000	1.93E-06
chr12	15637001	15642000	2.82E-05
chr12	15651001	15656000	4.56E-08
chr12	15653001	15658000	0.002419329
chr12	15673001	15678000	8.05E-07
chr12	15682001	15687000	6.51E-05
chr12	15683001	15688000	0.000117867
chr12	15684001	15689000	1.57E-05
chr12	15685001	15690000	1.90E-08
chr12	15686001	15691000	9.67E-07
chr12	15739001	15744000	3.71E-15
chr12	16216001	16221000	2.31E-05
chr12	16269001	16274000	0.000243843
chr12	16286001	16291000	1.98E-06
chr12	16434001	16439000	2.08E-06
chr12	16441001	16446000	2.68E-10
chr12	16442001	16447000	1.03E-13
chr12	16888001	16893000	4.75E-07
chr12	16981001	16986000	0.000392602
chr12	16982001	16987000	0.000148208
chr12	17083001	17088000	0.000870364
chr12	17138001	17143000	4.96E-10
chr12	17139001	17144000	2.25E-09
chr12	17140001	17145000	1.84E-07
chr12	17184001	17189000	1.78E-08
chr12	17239001	17244000	0.003578645
chr12	17240001	17245000	0.002774467
chr12	17241001	17246000	0.000942828

chr12	17242001	17247000	0.00184534
chr12	17243001	17248000	0.001568032
chr12	17246001	17251000	2.14E-11
chr12	17358001	17363000	1.38E-12
chr12	17359001	17364000	1.29E-12
chr12	17360001	17365000	1.38E-10
chr12	17592001	17597000	0.000757119
chr12	17594001	17599000	0.000487074
chr12	17600001	17605000	4.74E-07
chr12	17658001	17663000	0.003392879
chr12	17680001	17685000	4.50E-08
chr12	17681001	17686000	7.60E-08
chr12	17702001	17707000	0.007830616
chr12	17703001	17708000	0.009638763
chr12	17704001	17709000	0.000321233
chr12	17705001	17710000	0.002026791
chr12	17759001	17764000	2.14E-09
chr12	17796001	17801000	0.004692117
chr12	17829001	17834000	9.36E-07
chr12	17830001	17835000	3.83E-07
chr12	17831001	17836000	3.45E-07
chr12	17832001	17837000	1.73E-07
chr12	17834001	17839000	0.001088484
chr12	17835001	17840000	0.001779629
chr12	17836001	17841000	0.002269038
chr12	17860001	17865000	5.71E-05
chr12	18069001	18074000	6.49E-07
chr12	18081001	18086000	0.007724177
chr12	18082001	18087000	0.001660536
chr12	18083001	18088000	0.002660396
chr12	18084001	18089000	0.001186392
chr12	18085001	18090000	0.000785915
chr12	18099001	18104000	1.63E-08
chr12	18140001	18145000	0.001118778
chr12	18216001	18221000	9.46E-10
chr12	18287001	18292000	1.93E-06
chr12	18364001	18369000	0.001069232
chr12	18365001	18370000	0.005082119
chr12	18366001	18371000	0.000382108
chr12	18367001	18372000	0.000990246
chr12	18368001	18373000	0.006728558
chr12	18375001	18380000	8.93E-05
chr12	18598001	18603000	0.000791266
chr12	18625001	18630000	1.28E-06
chr12	18762001	18767000	0.001138853
chr12	18763001	18768000	0.003338533
chr12	18825001	18830000	0.000325226
chr12	19174001	19179000	0.000315442

chr12	19175001	19180000	0.000440658
chr12	19176001	19181000	1.20E-08
chr12	19178001	19183000	1.29E-06
chr12	19594001	19599000	1.57E-16
chr12	19899001	19904000	9.10E-06
chr12	19902001	19907000	0.000128693
chr12	19910001	19915000	0.000492269
chr12	19953001	19958000	2.82E-07
chr12	19956001	19961000	2.14E-05
chr12	19957001	19962000	3.37E-06
chr12	19958001	19963000	1.06E-06
chr12	19961001	19966000	0.00052646
chr12	19970001	19975000	6.57E-05
chr12	19993001	19998000	1.19E-07
chr12	20001001	20006000	2.33E-13
chr12	20002001	20007000	3.55E-12
chr12	20003001	20008000	3.94E-09
chr12	20004001	20009000	2.27E-07
chr12	20011001	20016000	3.77E-13
chr12	20012001	20017000	8.27E-14
chr12	20013001	20018000	5.05E-14
chr12	20015001	20020000	1.04E-16
chr12	20024001	20029000	6.22E-10
chr12	20025001	20030000	1.23E-07
chr12	20028001	20033000	7.38E-08
chr12	20037001	20042000	3.69E-11
chr12	20041001	20046000	5.25E-06
chr12	20059001	20064000	0.001485987
chr12	20060001	20065000	4.98E-05
chr12	20061001	20066000	6.84E-05
chr12	20083001	20088000	0.007255044
chr12	20084001	20089000	0.004703734
chr12	20085001	20090000	0.008956214
chr12	20114001	20119000	0.00301693
chr12	20115001	20120000	0.002259014
chr12	20128001	20133000	0.004826114
chr12	20132001	20137000	0.008166602
chr12	20153001	20158000	5.54E-05
chr12	20155001	20160000	3.58E-05
chr12	20156001	20161000	8.94E-06
chr12	20178001	20183000	0.00012805
chr12	20179001	20184000	6.11E-05
chr12	20180001	20185000	2.33E-05
chr12	20182001	20187000	5.38E-05
chr12	20223001	20228000	7.37E-07
chr12	20224001	20229000	2.68E-06
chr12	20225001	20230000	7.34E-05
chr12	20226001	20231000	0.000267244

chr12	20268001	20273000	2.88E-35
chr12	20290001	20295000	1.22E-06
chr12	20291001	20296000	1.10E-06
chr12	20292001	20297000	2.51E-09
chr12	20293001	20298000	1.19E-10
chr12	20294001	20299000	1.10E-10
chr12	20295001	20300000	1.73E-05
chr12	20307001	20312000	2.95E-07
chr12	20329001	20334000	2.15E-06
chr12	20335001	20340000	2.91E-06
chr12	20336001	20341000	8.22E-08
chr12	20360001	20365000	4.44E-08
chr12	20361001	20366000	3.37E-07
chr12	20362001	20367000	1.05E-07
chr12	20363001	20368000	1.43E-05
chr12	20364001	20369000	2.05E-05
chr12	20375001	20380000	8.43E-06
chr12	20376001	20381000	3.06E-10
chr12	20377001	20382000	1.38E-13
chr12	20378001	20383000	1.48E-17
chr12	20379001	20384000	5.37E-14
chr12	20380001	20385000	1.93E-13
chr12	20382001	20387000	6.18E-15
chr12	20383001	20388000	9.29E-13
chr12	20384001	20389000	6.54E-06
chr12	20385001	20390000	0.000632018
chr12	20410001	20415000	9.16E-07
chr12	20423001	20428000	0.000620888
chr12	20424001	20429000	0.001182808
chr12	20425001	20430000	0.00124444
chr12	20438001	20443000	0.000376031
chr12	20466001	20471000	0.006281668
chr12	20467001	20472000	0.006389504
chr12	20468001	20473000	3.68E-06
chr12	20534001	20539000	7.63E-07
chr12	20535001	20540000	1.20E-06
chr12	20536001	20541000	1.93E-06
chr12	20549001	20554000	3.07E-07
chr12	20550001	20555000	1.22E-08
chr12	20551001	20556000	2.69E-10
chr12	20552001	20557000	3.00E-11
chr12	20553001	20558000	5.16E-11
chr12	20583001	20588000	0.000216547
chr12	20585001	20590000	0.000122808
chr12	20591001	20596000	1.88E-06
chr12	20612001	20617000	0.000842167
chr12	20613001	20618000	0.0013405
chr12	20684001	20689000	1.60E-20

chr12	20685001	20690000	1.11E-18
chr12	20686001	20691000	3.36E-21
chr12	20700001	20705000	2.29E-30
chr12	20706001	20711000	2.80E-09
chr12	20709001	20714000	4.14E-08
chr12	20710001	20715000	5.15E-06
chr12	20712001	20717000	8.38E-06
chr12	20728001	20733000	3.36E-05
chr12	20729001	20734000	4.57E-05
chr12	20752001	20757000	6.21E-07
chr12	20753001	20758000	2.27E-06
chr12	20754001	20759000	5.01E-06
chr12	20755001	20760000	1.94E-05
chr12	20793001	20798000	7.00E-06
chr12	20794001	20799000	1.45E-06
chr12	20811001	20816000	0.000108323
chr12	20838001	20843000	0.000825163
chr12	20881001	20886000	0.005459384
chr12	20883001	20888000	2.77E-05
chr12	20887001	20892000	0.001544949
chr12	20938001	20943000	3.25E-05
chr12	20963001	20968000	0.000372254
chr12	20964001	20969000	0.0003978
chr12	21100001	21105000	3.94E-05
chr12	21101001	21106000	6.91E-07
chr12	21112001	21117000	0.000202743
chr12	21226001	21231000	1.99E-13
chr12	21227001	21232000	2.78E-10
chr12	21228001	21233000	2.53E-08
chr12	21229001	21234000	1.69E-09
chr12	21230001	21235000	0.00021653
chr12	21245001	21250000	0.003599122
chr12	21275001	21280000	0.003446038
chr12	21311001	21316000	0.011091404
chr12	21699001	21704000	2.56E-05
chr12	21700001	21705000	2.90E-06
chr12	21701001	21706000	1.86E-05
chr12	21702001	21707000	1.35E-05
chr12	21894001	21899000	0.000224717
chr12	21905001	21910000	6.81E-07
chr12	21992001	21997000	1.18E-08
chr12	21993001	21998000	4.47E-08
chr12	21994001	21999000	4.94E-08
chr12	22058001	22063000	0.018026632
chr12	22564001	22569000	6.58E-12
chr12	22740001	22745000	1.75E-14
chr12	23060001	23065000	0.000531112
chr12	23061001	23066000	0.001688509

chr12	23062001	23067000	4.30E-05
chr12	23192001	23197000	0.001520658
chr12	23286001	23291000	0.000153651
chr12	23472001	23477000	1.11E-07
chr12	23473001	23478000	8.00E-10
chr12	23979001	23984000	4.05E-06
chr12	25548001	25553000	5.26E-06
chr12	25549001	25554000	2.49E-08
chr12	25763001	25768000	3.37E-09
chr12	25820001	25825000	7.35E-09
chr12	25896001	25901000	0.000317251
chr12	25914001	25919000	0.000524902
chr12	25915001	25920000	4.53E-05
chr12	25916001	25921000	5.87E-05
chr12	25917001	25922000	0.000138611
chr12	25918001	25923000	0.000128212
chr12	25929001	25934000	3.45E-05
chr12	25966001	25971000	2.51E-08
chr12	25967001	25972000	6.38E-08
chr12	25968001	25973000	1.04E-08
chr12	25980001	25985000	2.65E-08
chr12	25983001	25988000	3.44E-06
chr12	26035001	26040000	1.37E-39
chr12	26054001	26059000	0.000117881
chr12	26246001	26251000	3.65E-07
chr12	26739001	26744000	1.26E-05
chr12	26934001	26939000	1.06E-11
chr12	26951001	26956000	3.85E-09
chr12	27299001	27304000	2.79E-18
chr12	27300001	27305000	2.18E-16
chr12	27329001	27334000	6.41E-05
chr12	27330001	27335000	7.79E-06
chr12	27612001	27617000	2.93E-30
chr12	27613001	27618000	1.25E-31
chr12	27964001	27969000	2.29E-06
chr12	27983001	27988000	8.52E-12
chr12	27984001	27989000	2.43E-11
chr12	27985001	27990000	2.03E-12
chr12	28051001	28056000	2.10E-07
chr12	28720001	28725000	2.76E-06
chr12	28729001	28734000	1.02E-09
chr12	28744001	28749000	9.14E-05
chr12	28784001	28789000	1.84E-09
chr12	28851001	28856000	3.51E-07
chr12	28942001	28947000	0.000486652
chr12	29029001	29034000	0.000213108
chr12	29046001	29051000	0.000126266
chr12	29086001	29091000	2.92E-06

chr12	29099001	29104000	3.28E-06
chr12	29262001	29267000	2.81E-08
chr12	29263001	29268000	7.42E-07
chr12	29834001	29839000	1.98E-05
chr12	29836001	29841000	2.90E-08
chr12	30240001	30245000	0.000131792
chr12	30241001	30246000	0.000145191
chr12	30242001	30247000	0.000481812
chr12	31520001	31525000	5.44E-49
chr12	31521001	31526000	5.73E-69
chr12	31522001	31527000	2.97E-66
chr12	31523001	31528000	4.09E-53
chr12	31752001	31757000	6.98E-23
chr12	31753001	31758000	1.70E-24
chr12	31754001	31759000	6.03E-22
chr12	31755001	31760000	7.67E-15
chr12	32571001	32576000	1.10E-25
chr12	32807001	32812000	1.10E-27
chr12	32808001	32813000	3.35E-25
chr12	32809001	32814000	3.46E-19
chr12	33141001	33146000	0.001043709
chr12	33142001	33147000	0.002780628
chr12	33234001	33239000	0.000445909
chr12	33235001	33240000	0.004045405
chr12	33306001	33311000	2.46E-06
chr12	33452001	33457000	1.18E-07
chr12	33455001	33460000	7.11E-06
chr12	33456001	33461000	1.20E-05
chr12	33526001	33531000	0.002296545
chr12	33527001	33532000	0.003776704
chr12	33542001	33547000	1.10E-06
chr12	33567001	33572000	1.86E-07
chr12	33568001	33573000	1.64E-07
chr12	33569001	33574000	1.06E-06
chr12	33570001	33575000	6.24E-06
chr12	33604001	33609000	2.12E-08
chr12	33605001	33610000	2.26E-09
chr12	33606001	33611000	1.42E-08
chr12	33607001	33612000	5.53E-09
chr12	33650001	33655000	1.11E-05
chr12	33707001	33712000	3.22E-05
chr12	33708001	33713000	4.55E-06
chr12	33709001	33714000	0.000618033
chr12	33725001	33730000	0.000117027
chr12	33731001	33736000	0.000433452
chr12	33734001	33739000	8.74E-09
chr12	33735001	33740000	6.54E-08
chr12	33736001	33741000	6.30E-07

chr12	33745001	33750000	0.000341927
chr12	33764001	33769000	0.006176837
chr12	33765001	33770000	0.006710715
chr12	33770001	33775000	4.33E-05
chr12	33771001	33776000	0.000292035
chr12	33772001	33777000	0.007146738
chr12	33800001	33805000	2.39E-05
chr12	33801001	33806000	0.000129209
chr12	33802001	33807000	0.000464951
chr12	33918001	33923000	0.000137225
chr12	33928001	33933000	1.26E-07
chr12	33929001	33934000	2.80E-08
chr12	33930001	33935000	3.67E-07
chr12	33977001	33982000	0.000628226
chr12	33978001	33983000	0.001585651
chr12	33996001	34001000	0.00147143
chr12	34075001	34080000	2.16E-06
chr12	34076001	34081000	1.94E-06
chr12	34088001	34093000	4.42E-07
chr12	34089001	34094000	5.03E-06
chr12	34130001	34135000	3.54E-05
chr12	34131001	34136000	5.18E-05
chr12	34132001	34137000	0.000223227
chr12	34139001	34144000	0.003454409
chr12	34148001	34153000	8.55E-05
chr12	34149001	34154000	4.81E-05
chr12	34341001	34346000	8.72E-08
chr12	34342001	34347000	3.40E-08
chr12	34343001	34348000	1.55E-08
chr12	34344001	34349000	1.07E-09
chr12	34379001	34384000	4.59E-06
chr12	34711001	34716000	0.000567943
chr12	34845001	34850000	2.42E-08
chr12	34846001	34851000	3.50E-10
chr12	34847001	34852000	1.13E-07
chr12	37976001	37981000	8.75E-09
chr12	37977001	37982000	9.36E-09
chr12	38122001	38127000	4.79E-05
chr12	38185001	38190000	0.001352238
chr12	38366001	38371000	7.32E-10
chr12	38367001	38372000	5.24E-08
chr12	38368001	38373000	3.53E-07
chr12	38369001	38374000	3.11E-08
chr12	38520001	38525000	3.79E-09
chr12	38521001	38526000	8.39E-11
chr12	38522001	38527000	9.16E-09
chr12	38523001	38528000	3.83E-07
chr12	38525001	38530000	1.66E-09

chr12	38526001	38531000	1.40E-14
chr12	38570001	38575000	0.00121692
chr12	38571001	38576000	0.000875166
chr12	38606001	38611000	0.002754197
chr12	38607001	38612000	6.99E-07
chr12	38608001	38613000	1.22E-05
chr12	38609001	38614000	4.45E-05
chr12	38610001	38615000	1.17E-05
chr12	38665001	38670000	2.48E-06
chr12	38858001	38863000	0.00128487
chr12	38875001	38880000	0.000253311
chr12	38940001	38945000	9.78E-11
chr12	38941001	38946000	9.35E-12
chr12	38996001	39001000	2.63E-05
chr12	39099001	39104000	0.000195726
chr12	39100001	39105000	0.000301156
chr12	39111001	39116000	8.15E-12
chr12	39115001	39120000	1.27E-10
chr12	39153001	39158000	7.72E-09
chr12	39154001	39159000	1.38E-08
chr12	39155001	39160000	1.68E-07
chr12	39171001	39176000	0.004617328
chr12	39173001	39178000	0.005996336
chr12	39174001	39179000	7.11E-07
chr12	39175001	39180000	3.50E-05
chr12	39176001	39181000	7.31E-06
chr12	39177001	39182000	6.37E-06
chr12	39178001	39183000	7.18E-08
chr12	39230001	39235000	0.001042217
chr12	39231001	39236000	0.000608483
chr12	39232001	39237000	0.000386774
chr12	39233001	39238000	6.10E-06
chr12	39234001	39239000	2.93E-05
chr12	39260001	39265000	0.000177675
chr12	39261001	39266000	8.94E-05
chr12	39278001	39283000	0.001059283
chr12	39301001	39306000	1.10E-05
chr12	39347001	39352000	1.03E-09
chr12	39348001	39353000	1.33E-08
chr12	39398001	39403000	0.004216833
chr12	39400001	39405000	1.13E-05
chr12	39408001	39413000	2.53E-05
chr12	39409001	39414000	1.15E-05
chr12	39454001	39459000	8.70E-16
chr12	39499001	39504000	0.002224735
chr12	39501001	39506000	0.001843899
chr12	39509001	39514000	2.84E-05
chr12	39675001	39680000	5.15E-05

chr12	39676001	39681000	8.53E-09
chr12	39894001	39899000	6.86E-07
chr12	39895001	39900000	3.28E-06
chr12	39934001	39939000	0.003714602
chr12	39935001	39940000	2.37E-05
chr12	39936001	39941000	4.57E-06
chr12	39937001	39942000	5.65E-06
chr12	39938001	39943000	4.03E-05
chr12	40078001	40083000	5.76E-05
chr12	40079001	40084000	0.00018902
chr12	40081001	40086000	0.001282766
chr12	40108001	40113000	0.00031124
chr12	40109001	40114000	0.000364761
chr12	40156001	40161000	3.34E-07
chr12	40165001	40170000	4.10E-08
chr12	40167001	40172000	9.45E-08
chr12	40171001	40176000	1.98E-07
chr12	40204001	40209000	3.48E-07
chr12	40205001	40210000	1.44E-08
chr12	40302001	40307000	7.39E-21
chr12	40303001	40308000	3.25E-18
chr12	40304001	40309000	2.33E-13
chr12	40305001	40310000	9.07E-16
chr12	40306001	40311000	1.31E-13
chr12	40342001	40347000	0.000762334
chr12	40383001	40388000	4.37E-05
chr12	40477001	40482000	2.23E-08
chr12	40478001	40483000	1.14E-07
chr12	40479001	40484000	3.70E-07
chr12	40536001	40541000	4.93E-07
chr12	40537001	40542000	3.22E-11
chr12	40538001	40543000	3.47E-10
chr12	40539001	40544000	2.89E-12
chr12	40540001	40545000	7.72E-13
chr12	40541001	40546000	1.05E-08
chr12	40562001	40567000	5.37E-07
chr12	40564001	40569000	7.70E-10
chr12	40565001	40570000	2.63E-11
chr12	40575001	40580000	0.000154011
chr12	40576001	40581000	8.57E-05
chr12	40577001	40582000	0.000405199
chr12	40578001	40583000	0.000112444
chr12	40593001	40598000	4.30E-11
chr12	40594001	40599000	3.83E-11
chr12	40772001	40777000	2.60E-05
chr12	40820001	40825000	3.48E-10
chr12	40821001	40826000	8.82E-10
chr12	40822001	40827000	2.77E-10

chr12	40823001	40828000	1.64E-10
chr12	40824001	40829000	1.95E-09
chr12	40834001	40839000	0.000150114
chr12	40835001	40840000	0.000133659
chr12	40864001	40869000	0.000211959
chr12	40868001	40873000	1.48E-05
chr12	40924001	40929000	4.27E-07
chr12	40945001	40950000	0.000252422
chr12	40946001	40951000	0.000342944
chr12	40947001	40952000	0.003001295
chr12	41047001	41052000	0.000772955
chr12	41285001	41290000	0.00257198
chr12	41532001	41537000	0.001081957
chr12	41644001	41649000	0.000857563
chr12	41784001	41789000	0.002101856
chr12	41785001	41790000	0.000409619
chr12	41807001	41812000	0.000394508
chr12	41853001	41858000	7.03E-05
chr12	41854001	41859000	1.21E-06
chr12	41855001	41860000	1.35E-06
chr12	41856001	41861000	7.16E-08
chr12	41857001	41862000	1.24E-07
chr12	42919001	42924000	8.75E-17
chr12	42920001	42925000	6.10E-14
chr12	42988001	42993000	8.27E-07
chr12	42989001	42994000	4.77E-09
chr12	42990001	42995000	8.55E-10
chr12	42991001	42996000	8.82E-09
chr12	42992001	42997000	1.37E-10
chr12	42994001	42999000	1.62E-11
chr12	43082001	43087000	0.000633396
chr12	43095001	43100000	1.04E-09
chr12	43096001	43101000	5.73E-09
chr12	43112001	43117000	3.46E-05
chr12	43171001	43176000	3.35E-11
chr12	43211001	43216000	2.62E-07
chr12	43214001	43219000	5.46E-06
chr12	43215001	43220000	0.000231352
chr12	43228001	43233000	1.21E-10
chr12	43229001	43234000	1.29E-12
chr12	43230001	43235000	1.07E-13
chr12	43231001	43236000	1.66E-15
chr12	43234001	43239000	4.33E-05
chr12	43271001	43276000	8.16E-05
chr12	43272001	43277000	0.002315366
chr12	43273001	43278000	0.002389841
chr12	43279001	43284000	0.00049812
chr12	43293001	43298000	1.13E-07

chr12	43305001	43310000	9.14E-07
chr12	43306001	43311000	1.34E-06
chr12	43308001	43313000	5.31E-06
chr12	43323001	43328000	1.28E-09
chr12	43324001	43329000	2.71E-08
chr12	43325001	43330000	1.47E-10
chr12	43326001	43331000	4.24E-13
chr12	43327001	43332000	2.44E-10
chr12	43374001	43379000	4.09E-05
chr12	43389001	43394000	6.77E-14
chr12	43397001	43402000	1.08E-12
chr12	43398001	43403000	7.76E-13
chr12	43399001	43404000	4.53E-11
chr12	43405001	43410000	2.77E-05
chr12	43406001	43411000	1.17E-06
chr12	43407001	43412000	2.26E-06
chr12	43408001	43413000	3.07E-06
chr12	43409001	43414000	2.72E-05
chr12	43416001	43421000	9.67E-07
chr12	43417001	43422000	9.06E-08
chr12	43418001	43423000	1.18E-05
chr12	43424001	43429000	6.61E-06
chr12	43425001	43430000	4.61E-07
chr12	43426001	43431000	6.22E-07
chr12	43466001	43471000	8.85E-07
chr12	43467001	43472000	0.000140872
chr12	43494001	43499000	7.14E-05
chr12	43502001	43507000	5.00E-06
chr12	43503001	43508000	3.35E-05
chr12	43504001	43509000	0.001996272
chr12	43536001	43541000	4.16E-06
chr12	43537001	43542000	7.28E-05
chr12	43538001	43543000	0.000391529
chr12	43539001	43544000	1.23E-05
chr12	43540001	43545000	0.000324554
chr12	43541001	43546000	0.004995886
chr12	43553001	43558000	1.28E-07
chr12	43554001	43559000	8.31E-08
chr12	43555001	43560000	1.09E-07
chr12	43568001	43573000	1.10E-12
chr12	43569001	43574000	1.52E-15
chr12	43570001	43575000	7.29E-15
chr12	43571001	43576000	1.87E-11
chr12	43572001	43577000	1.08E-10
chr12	43589001	43594000	1.53E-06
chr12	43591001	43596000	1.47E-08
chr12	43598001	43603000	6.64E-08
chr12	43599001	43604000	4.85E-07

chr12	43600001	43605000	7.38E-07
chr12	43601001	43606000	3.59E-10
chr12	43602001	43607000	4.72E-08
chr12	43639001	43644000	3.93E-08
chr12	43640001	43645000	5.13E-06
chr12	43641001	43646000	1.21E-07
chr12	43656001	43661000	0.000553215
chr12	43657001	43662000	5.30E-05
chr12	43658001	43663000	0.000315203
chr12	43659001	43664000	6.17E-08
chr12	43660001	43665000	1.44E-07
chr12	43661001	43666000	4.93E-08
chr12	43662001	43667000	5.62E-06
chr12	43663001	43668000	8.54E-06
chr12	43676001	43681000	2.35E-08
chr12	43677001	43682000	1.26E-09
chr12	43678001	43683000	2.29E-10
chr12	43679001	43684000	6.70E-12
chr12	43681001	43686000	8.79E-10
chr12	43682001	43687000	9.70E-06
chr12	43688001	43693000	6.87E-13
chr12	43707001	43712000	0.000425988
chr12	43736001	43741000	0.000479455
chr12	43737001	43742000	3.82E-06
chr12	43738001	43743000	1.58E-07
chr12	43739001	43744000	0.000624757
chr12	43740001	43745000	0.000607661
chr12	43741001	43746000	7.99E-06
chr12	43742001	43747000	5.54E-09
chr12	43743001	43748000	2.27E-06
chr12	43753001	43758000	8.43E-07
chr12	43754001	43759000	9.16E-08
chr12	43792001	43797000	0.001947142
chr12	43847001	43852000	8.36E-09
chr12	43882001	43887000	1.65E-14
chr12	43883001	43888000	5.84E-14
chr12	43884001	43889000	1.13E-12
chr12	43885001	43890000	8.20E-13
chr12	43940001	43945000	8.92E-21
chr12	43976001	43981000	0.00058137
chr12	43992001	43997000	5.93E-05
chr12	44049001	44054000	0.000200075
chr12	44080001	44085000	1.47E-05
chr12	44082001	44087000	2.94E-12
chr12	44083001	44088000	3.81E-12
chr12	44094001	44099000	2.03E-10
chr12	44108001	44113000	5.83E-07
chr12	44109001	44114000	1.20E-05

chr12	44110001	44115000	3.53E-05
chr12	44805001	44810000	0.000735064
chr12	44857001	44862000	8.92E-05
chr12	44875001	44880000	0.000709499
chr12	44876001	44881000	0.00431758
chr12	44883001	44888000	1.58E-11
chr12	44884001	44889000	8.63E-12
chr12	44885001	44890000	1.56E-13
chr12	44886001	44891000	6.53E-13
chr12	44896001	44901000	2.41E-16
chr12	44982001	44987000	3.14E-08
chr12	45031001	45036000	5.34E-11
chr12	45108001	45113000	0.000678753
chr12	45109001	45114000	0.000748688
chr12	45110001	45115000	0.001957726
chr12	45187001	45192000	8.88E-05
chr12	45201001	45206000	0.000222054
chr12	45202001	45207000	0.000253463
chr12	45203001	45208000	9.17E-05
chr12	45204001	45209000	2.02E-05
chr12	45205001	45210000	3.63E-07
chr12	45246001	45251000	5.23E-07
chr12	45372001	45377000	1.14E-05
chr12	45373001	45378000	4.16E-05
chr12	45374001	45379000	7.43E-05
chr12	45375001	45380000	2.48E-05
chr12	45505001	45510000	0.000714605
chr12	46005001	46010000	3.93E-14
chr12	46009001	46014000	2.24E-12
chr12	46497001	46502000	3.17E-07
chr12	46599001	46604000	0.002605589
chr12	46724001	46729000	0.000507264
chr12	46794001	46799000	0.000451067
chr12	46836001	46841000	1.50E-10
chr12	46946001	46951000	0.002121475
chr12	46947001	46952000	0.00032847
chr12	46951001	46956000	1.70E-09
chr12	46952001	46957000	2.41E-09
chr12	46953001	46958000	2.31E-07
chr12	48723001	48728000	1.87E-08
chr12	48724001	48729000	2.47E-18
chr12	49677001	49682000	1.56E-13
chr12	49680001	49685000	1.24E-20
chr12	49681001	49686000	1.34E-24
chr12	49682001	49687000	9.15E-17
chr12	49684001	49689000	1.92E-06
chr12	49721001	49726000	5.41E-07
chr12	49904001	49909000	0.007477291

chr12	50037001	50042000	9.07E-05
chr12	50079001	50084000	1.26E-06
chr12	50080001	50085000	1.40E-09
chr12	50081001	50086000	1.77E-07
chr12	50362001	50367000	2.13E-11
chr12	50363001	50368000	2.08E-09
chr12	50364001	50369000	1.38E-14
chr12	50365001	50370000	5.03E-13
chr12	50651001	50656000	3.84E-08
chr12	50796001	50801000	1.08E-22
chr12	52771001	52776000	1.36E-09
chr12	52884001	52889000	1.37E-19
chr12	52885001	52890000	3.41E-20
chr12	52886001	52891000	1.62E-15
chr12	52971001	52976000	9.45E-11
chr12	53275001	53280000	1.20E-07
chr12	53276001	53281000	2.27E-10
chr12	53277001	53282000	4.31E-08
chr12	53392001	53397000	2.02E-06
chr12	53587001	53592000	3.40E-28
chr12	53588001	53593000	7.29E-40
chr12	53589001	53594000	1.23E-41
chr12	53590001	53595000	4.59E-40
chr12	53591001	53596000	7.56E-39
chr12	53616001	53621000	0.000385297
chr12	54035001	54040000	1.07E-08
chr12	54520001	54525000	1.73E-14
chr12	54521001	54526000	4.22E-06
chr12	54523001	54528000	1.88E-12
chr12	54601001	54606000	1.37E-07
chr12	54677001	54682000	4.38E-06
chr12	54742001	54747000	7.80E-07
chr12	54743001	54748000	5.79E-06
chr12	55315001	55320000	8.30E-07
chr12	55316001	55321000	1.50E-06
chr12	55444001	55449000	1.56E-11
chr12	55445001	55450000	4.01E-14
chr12	55446001	55451000	2.85E-09
chr12	55447001	55452000	1.04E-08
chr12	56361001	56366000	1.30E-09
chr12	56637001	56642000	4.12E-05
chr12	56852001	56857000	1.61E-10
chr12	57067001	57072000	0.000161448
chr12	57076001	57081000	7.90E-15
chr12	57179001	57184000	7.22E-12
chr12	57180001	57185000	9.16E-15
chr12	57182001	57187000	5.27E-15
chr12	57249001	57254000	0.000281242

chr12	57271001	57276000	5.48E-06
chr12	57999001	58004000	9.47E-15
chr12	58008001	58013000	5.80E-08
chr12	58587001	58592000	9.33E-08
chr12	58806001	58811000	0.001152801
chr12	58807001	58812000	0.000920293
chr12	58923001	58928000	0.000625187
chr12	58993001	58998000	0.000508132
chr12	59769001	59774000	1.36E-05
chr12	59773001	59778000	0.000184566
chr12	59803001	59808000	0.013730786
chr12	59804001	59809000	0.007783622
chr12	59827001	59832000	4.42E-05
chr12	59904001	59909000	0.000227043
chr12	59905001	59910000	0.00011717
chr12	59915001	59920000	0.004159573
chr12	59982001	59987000	5.96E-07
chr12	59984001	59989000	1.78E-06
chr12	60257001	60262000	2.40E-11
chr12	60258001	60263000	2.64E-05
chr12	60259001	60264000	4.62E-05
chr12	60260001	60265000	6.12E-05
chr12	60465001	60470000	0.000253613
chr12	60466001	60471000	1.26E-05
chr12	60557001	60562000	0.004074893
chr12	60609001	60614000	0.001681313
chr12	60621001	60626000	1.10E-09
chr12	60622001	60627000	9.64E-09
chr12	60623001	60628000	4.77E-08
chr12	60677001	60682000	7.10E-05
chr12	60695001	60700000	5.28E-05
chr12	60889001	60894000	1.11E-05
chr12	60890001	60895000	5.48E-06
chr12	60891001	60896000	8.14E-06
chr12	61032001	61037000	8.24E-07
chr12	61033001	61038000	5.84E-08
chr12	61034001	61039000	2.27E-07
chr12	61035001	61040000	3.01E-06
chr12	61036001	61041000	1.92E-05
chr12	61090001	61095000	3.46E-07
chr12	61091001	61096000	1.03E-06
chr12	61094001	61099000	3.92E-08
chr12	61360001	61365000	0.003060889
chr12	61379001	61384000	0.00216077
chr12	61532001	61537000	0.000442956
chr12	61533001	61538000	0.000677721
chr12	61542001	61547000	3.97E-05
chr12	61610001	61615000	0.000443384

chr12	61767001	61772000	0.010579463
chr12	61923001	61928000	0.002685102
chr12	61995001	62000000	0.003396889
chr12	62016001	62021000	3.61E-07
chr12	62017001	62022000	1.97E-06
chr12	62018001	62023000	9.77E-07
chr12	62019001	62024000	4.87E-06
chr12	62209001	62214000	2.86E-05
chr12	62308001	62313000	2.16E-06
chr12	62309001	62314000	4.61E-05
chr12	62310001	62315000	0.002795937
chr12	63014001	63019000	2.94E-12
chr12	63016001	63021000	1.08E-10
chr12	63018001	63023000	4.44E-08
chr12	63312001	63317000	0.002741945
chr12	63470001	63475000	1.49E-06
chr12	63526001	63531000	0.006751238
chr12	63527001	63532000	0.005810368
chr12	63562001	63567000	0.000148053
chr12	63683001	63688000	6.37E-11
chr12	63684001	63689000	1.77E-12
chr12	63685001	63690000	3.09E-14
chr12	63686001	63691000	1.40E-09
chr12	63691001	63696000	0.004621273
chr12	63702001	63707000	5.60E-07
chr12	63704001	63709000	5.04E-05
chr12	63899001	63904000	7.17E-05
chr12	64006001	64011000	0.00016934
chr12	64007001	64012000	0.00016934
chr12	64105001	64110000	0.013186402
chr12	64322001	64327000	6.86E-06
chr12	64323001	64328000	5.77E-05
chr12	64741001	64746000	0.001008075
chr12	65992001	65997000	0.000113206
chr12	66348001	66353000	0.003470216
chr12	66646001	66651000	0.000377506
chr12	66647001	66652000	0.005271048
chr12	67483001	67488000	0.002609922
chr12	67608001	67613000	7.17E-08
chr12	67748001	67753000	4.36E-11
chr12	68004001	68009000	9.59E-06
chr12	68114001	68119000	7.70E-05
chr12	68349001	68354000	0.000144448
chr12	68362001	68367000	0.00039132
chr12	68527001	68532000	0.000219526
chr12	68596001	68601000	9.69E-11
chr12	68597001	68602000	1.26E-12
chr12	68598001	68603000	1.76E-10

chr12	68621001	68626000	0.000312921
chr12	69455001	69460000	2.91E-07
chr12	69458001	69463000	1.01E-08
chr12	69674001	69679000	0.003952456
chr12	69675001	69680000	0.001878252
chr12	69865001	69870000	7.78E-18
chr12	69866001	69871000	1.89E-17
chr12	70381001	70386000	6.65E-06
chr12	70661001	70666000	2.09E-08
chr12	71549001	71554000	1.59E-06
chr12	71554001	71559000	1.37E-05
chr12	71876001	71881000	9.24E-05
chr12	71966001	71971000	4.98E-06
chr12	72334001	72339000	9.23E-05
chr12	72582001	72587000	0.000266269
chr12	72583001	72588000	0.00037913
chr12	72584001	72589000	7.81E-09
chr12	72585001	72590000	1.93E-07
chr12	72605001	72610000	0.001058008
chr12	72606001	72611000	0.001887439
chr12	72607001	72612000	0.002457346
chr12	72608001	72613000	0.002310736
chr12	72626001	72631000	6.15E-05
chr12	72627001	72632000	0.003740988
chr12	72697001	72702000	6.49E-05
chr12	72698001	72703000	6.49E-06
chr12	73375001	73380000	3.99E-12
chr12	73378001	73383000	2.47E-11
chr12	73711001	73716000	4.20E-05
chr12	74026001	74031000	0.000668294
chr12	74160001	74165000	0.003073949
chr12	74161001	74166000	0.005138387
chr12	74162001	74167000	0.007147714
chr12	74204001	74209000	0.001272778
chr12	74360001	74365000	3.50E-05
chr12	74582001	74587000	7.82E-06
chr12	74980001	74985000	5.45E-19
chr12	75253001	75258000	0.00023059
chr12	75588001	75593000	4.49E-05
chr12	75589001	75594000	2.34E-05
chr12	75875001	75880000	7.94E-13
chr12	75876001	75881000	7.83E-11
chr12	76112001	76117000	3.24E-08
chr12	76588001	76593000	7.99E-06
chr12	76589001	76594000	2.04E-05
chr12	76591001	76596000	0.000223577
chr12	76650001	76655000	1.72E-09
chr12	76651001	76656000	1.10E-06

chr12	77400001	77405000	1.03E-15
chr12	77401001	77406000	2.01E-14
chr12	77402001	77407000	1.54E-18
chr12	77403001	77408000	3.15E-16
chr12	77593001	77598000	0.002571189
chr12	77613001	77618000	8.71E-07
chr12	77870001	77875000	0.00014414
chr12	77871001	77876000	4.99E-06
chr12	77872001	77877000	2.30E-06
chr12	77902001	77907000	0.000580534
chr12	77903001	77908000	0.001598843
chr12	78127001	78132000	0.002724526
chr12	78391001	78396000	7.65E-10
chr12	78392001	78397000	6.89E-11
chr12	78393001	78398000	1.33E-10
chr12	78394001	78399000	8.50E-10
chr12	79068001	79073000	0.002511083
chr12	79183001	79188000	1.19E-22
chr12	79802001	79807000	0.000170605
chr12	79803001	79808000	0.000411215
chr12	79804001	79809000	4.92E-06
chr12	79805001	79810000	0.000747979
chr12	79806001	79811000	0.001214681
chr12	79807001	79812000	0.000346501
chr12	80667001	80672000	0.004176452
chr12	80747001	80752000	3.11E-05
chr12	80748001	80753000	4.17E-05
chr12	80749001	80754000	0.000722433
chr12	80794001	80799000	0.000687158
chr12	80918001	80923000	1.21E-05
chr12	81171001	81176000	0.000232155
chr12	81229001	81234000	0.001434735
chr12	81818001	81823000	0.002166988
chr12	81901001	81906000	0.00480248
chr12	81902001	81907000	9.68E-05
chr12	81925001	81930000	5.90E-06
chr12	82632001	82637000	0.000576022
chr12	82633001	82638000	7.49E-05
chr12	82662001	82667000	0.000154351
chr12	82664001	82669000	9.49E-07
chr12	82665001	82670000	4.27E-06
chr12	83272001	83277000	1.51E-07
chr12	83757001	83762000	0.005451725
chr12	83805001	83810000	1.80E-07
chr12	83806001	83811000	3.31E-07
chr12	83816001	83821000	0.001687969
chr12	84008001	84013000	0.000115117
chr12	84119001	84124000	2.63E-06

chr12	84236001	84241000	0.003627791
chr12	84238001	84243000	0.010336871
chr12	84273001	84278000	0.000199111
chr12	84993001	84998000	4.33E-07
chr12	85067001	85072000	0.002671809
chr12	85174001	85179000	1.86E-10
chr12	85175001	85180000	2.72E-09
chr12	85177001	85182000	3.07E-09
chr12	85842001	85847000	8.95E-06
chr12	86068001	86073000	0.006161449
chr12	86221001	86226000	0.008082924
chr12	86375001	86380000	0.004088838
chr12	86377001	86382000	0.004207545
chr12	86442001	86447000	0.000724681
chr12	86560001	86565000	3.63E-05
chr12	86775001	86780000	7.15E-10
chr12	86776001	86781000	3.69E-10
chr12	86851001	86856000	1.31E-10
chr12	86907001	86912000	6.20E-08
chr12	86908001	86913000	9.86E-08
chr12	86909001	86914000	7.99E-08
chr12	86911001	86916000	1.21E-05
chr12	86997001	87002000	0.002888095
chr12	86998001	87003000	0.003145446
chr12	86999001	87004000	0.000709083
chr12	87000001	87005000	0.000680326
chr12	87001001	87006000	1.21E-05
chr12	87002001	87007000	1.75E-05
chr12	87003001	87008000	1.01E-05
chr12	87004001	87009000	8.10E-05
chr12	87142001	87147000	0.018344755
chr12	87181001	87186000	0.008402255
chr12	87339001	87344000	0.003518592
chr12	87340001	87345000	0.005470182
chr12	87357001	87362000	1.09E-09
chr12	87358001	87363000	3.98E-10
chr12	87359001	87364000	3.23E-09
chr12	87402001	87407000	6.34E-11
chr12	87404001	87409000	2.33E-11
chr12	87405001	87410000	1.80E-14
chr12	87406001	87411000	3.50E-13
chr12	87593001	87598000	0.000147481
chr12	87594001	87599000	0.000204029
chr12	87596001	87601000	0.000118993
chr12	87652001	87657000	0.003243907
chr12	87653001	87658000	0.000185635
chr12	87654001	87659000	0.000157371
chr12	87743001	87748000	0.000122829

chr12	87799001	87804000	8.86E-05
chr12	87801001	87806000	4.09E-06
chr12	87893001	87898000	0.00015018
chr12	88038001	88043000	0.00165341
chr12	88102001	88107000	0.001290981
chr12	88151001	88156000	0.012112501
chr12	88152001	88157000	0.000104885
chr12	88153001	88158000	8.44E-05
chr12	88154001	88159000	0.001828274
chr12	88191001	88196000	0.001970728
chr12	88345001	88350000	1.05E-08
chr12	88346001	88351000	9.36E-11
chr12	88347001	88352000	2.93E-12
chr12	88945001	88950000	9.66E-06
chr12	88968001	88973000	1.24E-08
chr12	89194001	89199000	0.006385616
chr12	89899001	89904000	3.50E-07
chr12	89900001	89905000	2.77E-08
chr12	90916001	90921000	1.05E-06
chr12	90933001	90938000	0.003312181
chr12	91501001	91506000	5.17E-05
chr12	91502001	91507000	7.97E-05
chr12	91660001	91665000	0.007538102
chr12	91680001	91685000	0.000334094
chr12	91707001	91712000	0.000427037
chr12	91805001	91810000	0.001112605
chr12	92530001	92535000	5.72E-08
chr12	93061001	93066000	6.86E-07
chr12	93062001	93067000	2.68E-05
chr12	93410001	93415000	1.88E-07
chr12	94165001	94170000	5.63E-07
chr12	95333001	95338000	1.39E-13
chr12	95605001	95610000	1.02E-08
chr12	95773001	95778000	1.33E-08
chr12	96231001	96236000	2.02E-05
chr12	96298001	96303000	6.11E-16
chr12	97157001	97162000	4.50E-05
chr12	97405001	97410000	0.003186947
chr12	97407001	97412000	0.008285655
chr12	97408001	97413000	0.011625697
chr12	97678001	97683000	1.91E-05
chr12	97688001	97693000	0.000388143
chr12	97689001	97694000	0.000355316
chr12	97690001	97695000	8.95E-07
chr12	97691001	97696000	5.36E-07
chr12	97692001	97697000	3.45E-06
chr12	97706001	97711000	2.48E-05
chr12	97815001	97820000	2.08E-06

chr12	97816001	97821000	3.86E-08
chr12	97817001	97822000	2.58E-08
chr12	97818001	97823000	3.33E-09
chr12	97819001	97824000	7.04E-10
chr12	98040001	98045000	0.000250835
chr12	98147001	98152000	0.000184266
chr12	98148001	98153000	0.000185145
chr12	98149001	98154000	0.006762899
chr12	98168001	98173000	2.47E-09
chr12	98169001	98174000	3.07E-09
chr12	98628001	98633000	3.03E-05
chr12	98629001	98634000	9.98E-06
chr12	98630001	98635000	0.001308217
chr12	98662001	98667000	9.78E-06
chr12	98879001	98884000	0.000853549
chr12	98880001	98885000	1.99E-19
chr12	98881001	98886000	1.19E-19
chr12	98882001	98887000	1.57E-17
chr12	98883001	98888000	6.86E-13
chr12	98956001	98961000	4.75E-17
chr12	98957001	98962000	1.26E-19
chr12	98958001	98963000	1.42E-22
chr12	98959001	98964000	9.51E-28
chr12	98960001	98965000	1.18E-28
chr12	99040001	99045000	1.33E-08
chr12	99234001	99239000	0.000104545
chr12	99375001	99380000	2.79E-06
chr12	99466001	99471000	1.52E-05
chr12	99467001	99472000	2.73E-05
chr12	99468001	99473000	2.13E-05
chr12	99475001	99480000	2.11E-06
chr12	99481001	99486000	3.70E-05
chr12	99615001	99620000	5.34E-05
chr12	99616001	99621000	6.71E-05
chr12	99691001	99696000	8.44E-06
chr12	99835001	99840000	0.000421976
chr12	99836001	99841000	0.002184686
chr12	99913001	99918000	1.82E-05
chr12	99928001	99933000	0.000170183
chr12	100237001	100242000	0.00024224
chr12	100238001	100243000	4.35E-05
chr12	100239001	100244000	3.10E-05
chr12	100241001	100246000	0.000283701
chr12	100265001	100270000	0.00052956
chr12	100327001	100332000	0.000561222
chr12	100328001	100333000	0.000262994
chr12	100362001	100367000	8.25E-05
chr12	100759001	100764000	2.00E-05

chr12	100760001	100765000	1.31E-06
chr12	100761001	100766000	1.19E-11
chr12	101216001	101221000	3.02E-06
chr12	101234001	101239000	0.000641124
chr12	101293001	101298000	2.02E-08
chr12	101333001	101338000	7.24E-08
chr12	101334001	101339000	3.74E-08
chr12	101335001	101340000	1.14E-07
chr12	101336001	101341000	8.84E-08
chr12	101344001	101349000	3.07E-08
chr12	101345001	101350000	3.70E-07
chr12	101578001	101583000	4.64E-08
chr12	101666001	101671000	1.38E-07
chr12	101667001	101672000	4.84E-08
chr12	102829001	102834000	5.47E-05
chr12	104668001	104673000	3.58E-14
chr12	104669001	104674000	7.69E-12
chr12	104670001	104675000	2.67E-08
chr12	104761001	104766000	1.18E-08
chr12	104762001	104767000	7.65E-10
chr12	104763001	104768000	1.67E-09
chr12	104764001	104769000	1.13E-09
chr12	104765001	104770000	7.19E-08
chr12	104794001	104799000	0.004326446
chr12	105189001	105194000	4.71E-07
chr12	106681001	106686000	2.40E-10
chr12	107132001	107137000	3.41E-11
chr12	107162001	107167000	2.16E-08
chr12	107504001	107509000	2.24E-05
chr12	107524001	107529000	6.82E-07
chr12	107525001	107530000	2.38E-05
chr12	107696001	107701000	6.65E-05
chr12	107697001	107702000	0.000112001
chr12	107716001	107721000	0.000192286
chr12	107717001	107722000	0.00027402
chr12	107871001	107876000	4.34E-07
chr12	107874001	107879000	6.52E-07
chr12	107877001	107882000	3.48E-11
chr12	107878001	107883000	3.04E-10
chr12	107879001	107884000	0.000109033
chr12	107880001	107885000	9.04E-06
chr12	108044001	108049000	2.27E-17
chr12	108046001	108051000	5.51E-09
chr12	108047001	108052000	5.90E-09
chr12	108232001	108237000	1.99E-05
chr12	108370001	108375000	0.000950301
chr12	108371001	108376000	0.00020184
chr12	109002001	109007000	3.10E-10

chr12	109269001	109274000	1.62E-05
chr12	109420001	109425000	0.008112839
chr12	109421001	109426000	0.000453277
chr12	109636001	109641000	4.71E-12
chr12	110161001	110166000	6.55E-06
chr12	110773001	110778000	1.20E-09
chr12	111015001	111020000	6.42E-15
chr12	111299001	111304000	0.000130698
chr12	111417001	111422000	1.50E-11
chr12	111474001	111479000	2.75E-18
chr12	111475001	111480000	2.98E-22
chr12	111476001	111481000	1.63E-21
chr12	111512001	111517000	9.45E-15
chr12	111513001	111518000	5.40E-17
chr12	111593001	111598000	1.22E-07
chr12	111611001	111616000	4.75E-11
chr12	111746001	111751000	7.88E-07
chr12	111747001	111752000	1.29E-06
chr12	112565001	112570000	6.26E-18
chr12	112566001	112571000	8.20E-13
chr12	112567001	112572000	3.61E-12
chr12	113131001	113136000	0.000134402
chr12	113157001	113162000	0.000156188
chr12	113211001	113216000	1.31E-08
chr12	113214001	113219000	3.55E-10
chr12	113215001	113220000	5.42E-11
chr12	113269001	113274000	8.56E-05
chr12	113526001	113531000	6.11E-10
chr12	113528001	113533000	7.91E-10
chr12	113624001	113629000	1.13E-06
chr12	113864001	113869000	1.95E-12
chr12	114979001	114984000	9.84E-08
chr12	114980001	114985000	4.92E-08
chr12	116994001	116999000	1.81E-09
chr12	117526001	117531000	1.30E-06
chr12	117586001	117591000	1.97E-16
chr12	117587001	117592000	8.28E-20
chr12	119063001	119068000	0.000866694
chr12	119184001	119189000	0.000782224
chr12	119185001	119190000	7.46E-05
chr12	119295001	119300000	2.42E-06
chr12	119467001	119472000	5.11E-05
chr12	120445001	120450000	2.68E-12
chr12	120446001	120451000	5.73E-10
chr12	120558001	120563000	2.48E-06
chr12	120708001	120713000	3.73E-09
chr12	120709001	120714000	7.04E-14
chr12	121102001	121107000	1.47E-09

chr12	121363001	121368000	5.90E-05
chr12	121398001	121403000	1.04E-09
chr12	121684001	121689000	3.44E-35
chr12	121685001	121690000	1.14E-31
chr12	121686001	121691000	3.41E-31
chr12	121687001	121692000	3.78E-30
chr12	122026001	122031000	1.05E-22
chr12	122027001	122032000	2.73E-20
chr12	122102001	122107000	2.56E-07
chr12	122103001	122108000	1.13E-08
chr12	122137001	122142000	1.01E-14
chr12	122219001	122224000	6.25E-19
chr12	122317001	122322000	0.000295435
chr12	122430001	122435000	7.53E-18
chr12	123467001	123472000	1.06E-07
chr12	123624001	123629000	1.93E-08
chr12	123850001	123855000	9.42E-16
chr12	124551001	124556000	2.57E-10
chr12	124794001	124799000	9.49E-14
chr12	124795001	124800000	8.88E-15
chr12	126377001	126382000	1.31E-16
chr12	126378001	126383000	3.55E-15
chr12	127014001	127019000	2.59E-05
chr12	127022001	127027000	0.000132502
chr12	127023001	127028000	7.79E-05
chr12	127061001	127066000	0.000741756
chr12	127707001	127712000	0.020507049
chr12	127708001	127713000	0.009597516
chr12	128058001	128063000	4.71E-09
chr12	128059001	128064000	4.30E-08
chr12	128060001	128065000	3.71E-12
chr12	128123001	128128000	0.000238522
chr12	128904001	128909000	1.27E-09
chr12	128941001	128946000	1.16E-06
chr12	129717001	129722000	4.08E-05
chr12	130504001	130509000	1.27E-07
chr12	131436001	131441000	1.09E-22
chr12	132360001	132365000	1.07E-08
chr12	132669001	132674000	9.98E-11
chr12	132815001	132820000	4.05E-10
chr12	133116001	133121000	4.91E-16
chr12	133161001	133166000	2.85E-11
chr12	133162001	133167000	2.04E-07
chr13	19139001	19144000	5.66E-09
chr13	19140001	19145000	1.20E-07
chr13	19141001	19146000	1.04E-05
chr13	19142001	19147000	1.54E-10
chr13	19145001	19150000	0.004459469

chr13	20092001	20097000	3.90E-07
chr13	23334001	23339000	3.41E-13
chr13	23335001	23340000	2.59E-12
chr13	23394001	23399000	0.000462224
chr13	23575001	23580000	2.14E-05
chr13	23576001	23581000	6.61E-05
chr13	23598001	23603000	0.005383615
chr13	23610001	23615000	0.000188241
chr13	24482001	24487000	0.001055807
chr13	24483001	24488000	0.000411215
chr13	24484001	24489000	9.39E-06
chr13	24905001	24910000	0.003052857
chr13	25601001	25606000	3.92E-05
chr13	25683001	25688000	0.000458658
chr13	25992001	25997000	0.00047573
chr13	25993001	25998000	1.91E-05
chr13	27238001	27243000	9.42E-06
chr13	27239001	27244000	5.40E-05
chr13	28090001	28095000	4.97E-08
chr13	28432001	28437000	3.22E-08
chr13	28434001	28439000	0.000251749
chr13	28483001	28488000	1.66E-06
chr13	28484001	28489000	1.60E-06
chr13	29090001	29095000	0.000731388
chr13	30382001	30387000	0.000552029
chr13	30965001	30970000	9.51E-12
chr13	30966001	30971000	3.64E-15
chr13	31049001	31054000	3.65E-05
chr13	31186001	31191000	0.001765751
chr13	32056001	32061000	0.003484222
chr13	32267001	32272000	0.001711928
chr13	32287001	32292000	5.77E-06
chr13	32288001	32293000	9.35E-06
chr13	32335001	32340000	0.000262934
chr13	32336001	32341000	0.001985187
chr13	32501001	32506000	6.86E-06
chr13	33425001	33430000	8.23E-06
chr13	33426001	33431000	0.003150595
chr13	33626001	33631000	1.44E-06
chr13	33852001	33857000	0.006433785
chr13	33982001	33987000	0.000222938
chr13	34565001	34570000	7.84E-05
chr13	34566001	34571000	8.85E-07
chr13	34567001	34572000	1.03E-07
chr13	34573001	34578000	7.65E-10
chr13	34650001	34655000	3.20E-08
chr13	34682001	34687000	1.31E-06
chr13	34683001	34688000	2.88E-08

chr13	34684001	34689000	5.60E-08
chr13	34685001	34690000	6.55E-08
chr13	34723001	34728000	2.69E-06
chr13	34775001	34780000	7.93E-07
chr13	34776001	34781000	5.12E-07
chr13	34777001	34782000	6.48E-09
chr13	34788001	34793000	1.50E-07
chr13	34789001	34794000	2.85E-07
chr13	34790001	34795000	1.16E-07
chr13	34791001	34796000	0.001572154
chr13	34795001	34800000	4.21E-05
chr13	34796001	34801000	2.94E-05
chr13	34936001	34941000	1.27E-08
chr13	34990001	34995000	8.22E-10
chr13	34991001	34996000	6.04E-12
chr13	34992001	34997000	3.55E-09
chr13	34993001	34998000	5.60E-08
chr13	35355001	35360000	1.30E-05
chr13	35356001	35361000	2.40E-06
chr13	35368001	35373000	0.005326488
chr13	35425001	35430000	0.000846959
chr13	35426001	35431000	0.009331577
chr13	35451001	35456000	5.11E-06
chr13	35484001	35489000	1.21E-07
chr13	35485001	35490000	4.11E-06
chr13	35486001	35491000	2.23E-06
chr13	35524001	35529000	2.26E-07
chr13	35585001	35590000	0.000170084
chr13	35586001	35591000	0.000235748
chr13	35587001	35592000	0.0003303
chr13	35613001	35618000	0.000239324
chr13	35614001	35619000	0.000279348
chr13	35716001	35721000	0.001552183
chr13	35796001	35801000	0.000262117
chr13	35797001	35802000	0.000178297
chr13	35798001	35803000	0.006810682
chr13	35924001	35929000	3.42E-06
chr13	35925001	35930000	0.000107307
chr13	35931001	35936000	0.000997803
chr13	35932001	35937000	0.000566908
chr13	35933001	35938000	4.30E-05
chr13	35934001	35939000	7.69E-05
chr13	35970001	35975000	0.001510031
chr13	35982001	35987000	0.000298866
chr13	35983001	35988000	0.000119624
chr13	36019001	36024000	0.000409927
chr13	36152001	36157000	6.85E-08
chr13	36155001	36160000	1.35E-08

chr13	36203001	36208000	7.10E-11
chr13	36204001	36209000	1.34E-12
chr13	36205001	36210000	8.00E-12
chr13	36206001	36211000	2.10E-10
chr13	36338001	36343000	1.66E-10
chr13	36365001	36370000	5.63E-09
chr13	36366001	36371000	1.08E-09
chr13	36367001	36372000	4.35E-10
chr13	36368001	36373000	6.34E-14
chr13	36369001	36374000	9.23E-10
chr13	36458001	36463000	1.59E-06
chr13	36459001	36464000	2.64E-06
chr13	36460001	36465000	1.43E-06
chr13	36500001	36505000	0.000181254
chr13	36501001	36506000	1.71E-05
chr13	36503001	36508000	6.00E-07
chr13	36507001	36512000	1.00E-10
chr13	36520001	36525000	1.12E-07
chr13	36550001	36555000	5.56E-12
chr13	36551001	36556000	2.98E-09
chr13	36595001	36600000	8.39E-05
chr13	36596001	36601000	4.25E-06
chr13	36597001	36602000	7.83E-07
chr13	36625001	36630000	7.55E-05
chr13	36626001	36631000	0.000187596
chr13	36784001	36789000	3.26E-12
chr13	36802001	36807000	0.00112324
chr13	36806001	36811000	0.001793397
chr13	36933001	36938000	3.15E-05
chr13	36948001	36953000	0.000306712
chr13	37205001	37210000	0.001921421
chr13	37505001	37510000	3.87E-08
chr13	37709001	37714000	0.001065576
chr13	37710001	37715000	0.000152406
chr13	37712001	37717000	0.000178208
chr13	37713001	37718000	0.000187747
chr13	37714001	37719000	0.00206226
chr13	37765001	37770000	1.78E-05
chr13	37850001	37855000	3.40E-08
chr13	37871001	37876000	1.92E-07
chr13	37872001	37877000	5.75E-09
chr13	37937001	37942000	1.02E-05
chr13	38012001	38017000	5.19E-07
chr13	38013001	38018000	4.35E-07
chr13	38014001	38019000	3.43E-07
chr13	38020001	38025000	8.16E-06
chr13	38021001	38026000	9.75E-05
chr13	38052001	38057000	2.43E-06

chr13	38053001	38058000	8.96E-07
chr13	38077001	38082000	9.28E-09
chr13	38078001	38083000	3.12E-11
chr13	38079001	38084000	9.09E-06
chr13	38105001	38110000	1.95E-06
chr13	38108001	38113000	3.29E-07
chr13	38175001	38180000	2.63E-05
chr13	38203001	38208000	0.000140889
chr13	38485001	38490000	0.013999529
chr13	38490001	38495000	8.85E-05
chr13	38491001	38496000	0.000105544
chr13	38492001	38497000	8.25E-06
chr13	38493001	38498000	6.41E-06
chr13	38494001	38499000	2.96E-05
chr13	38500001	38505000	0.002641272
chr13	38501001	38506000	0.002025787
chr13	38502001	38507000	0.000736071
chr13	38503001	38508000	0.000981689
chr13	38514001	38519000	8.96E-05
chr13	38537001	38542000	0.005513907
chr13	38574001	38579000	1.56E-05
chr13	38575001	38580000	5.83E-06
chr13	38576001	38581000	1.16E-05
chr13	38753001	38758000	0.002616383
chr13	38944001	38949000	0.000331921
chr13	38945001	38950000	3.97E-06
chr13	38946001	38951000	3.10E-07
chr13	38947001	38952000	9.08E-07
chr13	38948001	38953000	3.01E-05
chr13	38949001	38954000	1.93E-05
chr13	38964001	38969000	8.96E-07
chr13	38965001	38970000	6.66E-07
chr13	38966001	38971000	0.000117184
chr13	39208001	39213000	1.23E-07
chr13	39209001	39214000	1.34E-05
chr13	39210001	39215000	0.001715633
chr13	39274001	39279000	0.003867455
chr13	39358001	39363000	4.94E-05
chr13	39482001	39487000	2.08E-06
chr13	39483001	39488000	4.24E-07
chr13	39488001	39493000	4.85E-13
chr13	39499001	39504000	1.38E-05
chr13	39834001	39839000	9.87E-10
chr13	40111001	40116000	1.67E-09
chr13	40112001	40117000	1.69E-07
chr13	40113001	40118000	1.22E-06
chr13	40114001	40119000	9.61E-06
chr13	40154001	40159000	3.45E-08

chr13	40402001	40407000	6.87E-05
chr13	41557001	41562000	4.30E-13
chr13	41565001	41570000	4.98E-06
chr13	41567001	41572000	3.86E-10
chr13	41568001	41573000	1.07E-09
chr13	41575001	41580000	0.001182514
chr13	41714001	41719000	7.86E-07
chr13	42226001	42231000	0.00021491
chr13	43227001	43232000	1.66E-05
chr13	43228001	43233000	2.00E-09
chr13	43294001	43299000	0.003033958
chr13	43804001	43809000	1.04E-05
chr13	43848001	43853000	2.05E-06
chr13	43938001	43943000	3.61E-10
chr13	43939001	43944000	6.64E-06
chr13	43985001	43990000	2.13E-14
chr13	44042001	44047000	0.003066541
chr13	44044001	44049000	0.000108133
chr13	44045001	44050000	0.000967694
chr13	44046001	44051000	0.00289682
chr13	44369001	44374000	0.000130513
chr13	44370001	44375000	0.000249467
chr13	44371001	44376000	0.002362697
chr13	44477001	44482000	0.000136418
chr13	44478001	44483000	4.72E-05
chr13	44580001	44585000	0.002493806
chr13	44598001	44603000	9.45E-07
chr13	45221001	45226000	6.19E-05
chr13	45627001	45632000	4.33E-12
chr13	45629001	45634000	7.56E-11
chr13	45630001	45635000	4.06E-10
chr13	45695001	45700000	1.45E-14
chr13	46437001	46442000	9.40E-08
chr13	46644001	46649000	8.77E-05
chr13	46646001	46651000	0.000256224
chr13	46802001	46807000	3.44E-06
chr13	46804001	46809000	3.85E-05
chr13	47095001	47100000	0.000267314
chr13	47402001	47407000	1.26E-07
chr13	47787001	47792000	0.000233662
chr13	47788001	47793000	0.000248303
chr13	47789001	47794000	0.000407649
chr13	48010001	48015000	2.35E-05
chr13	48017001	48022000	0.000416529
chr13	48018001	48023000	3.15E-05
chr13	48227001	48232000	0.000963445
chr13	48236001	48241000	0.001105915
chr13	48252001	48257000	7.38E-05

chr13	48281001	48286000	1.63E-07
chr13	48282001	48287000	7.10E-05
chr13	48385001	48390000	0.000498091
chr13	48463001	48468000	5.56E-09
chr13	48464001	48469000	7.95E-06
chr13	48814001	48819000	7.30E-05
chr13	49178001	49183000	0.000130799
chr13	49179001	49184000	0.000228605
chr13	49501001	49506000	2.91E-15
chr13	49516001	49521000	5.05E-05
chr13	49874001	49879000	0.000173353
chr13	50657001	50662000	5.79E-11
chr13	50928001	50933000	6.93E-06
chr13	50929001	50934000	4.36E-07
chr13	50930001	50935000	5.33E-13
chr13	50931001	50936000	3.93E-15
chr13	51809001	51814000	1.23E-06
chr13	53060001	53065000	9.54E-09
chr13	53113001	53118000	6.86E-07
chr13	53114001	53119000	1.68E-06
chr13	53333001	53338000	1.26E-07
chr13	53366001	53371000	4.61E-07
chr13	53408001	53413000	2.84E-06
chr13	53569001	53574000	4.34E-07
chr13	53683001	53688000	2.32E-06
chr13	53699001	53704000	9.51E-07
chr13	53700001	53705000	1.15E-07
chr13	53701001	53706000	1.27E-05
chr13	53720001	53725000	7.26E-08
chr13	53740001	53745000	4.53E-05
chr13	53741001	53746000	0.007703285
chr13	53766001	53771000	0.000203683
chr13	53779001	53784000	0.004892116
chr13	53785001	53790000	0.000558926
chr13	53786001	53791000	0.000657254
chr13	53787001	53792000	0.001351713
chr13	53805001	53810000	3.52E-08
chr13	53821001	53826000	3.46E-07
chr13	53822001	53827000	4.72E-07
chr13	53823001	53828000	2.69E-06
chr13	53824001	53829000	1.96E-06
chr13	53925001	53930000	2.50E-06
chr13	53960001	53965000	0.000750237
chr13	54003001	54008000	1.42E-05
chr13	54195001	54200000	9.75E-17
chr13	54196001	54201000	2.07E-18
chr13	54197001	54202000	1.99E-18
chr13	54198001	54203000	7.61E-25

chr13	54199001	54204000	2.76E-19
chr13	54269001	54274000	0.000594112
chr13	54314001	54319000	0.000242817
chr13	54315001	54320000	0.000270045
chr13	54316001	54321000	0.000188835
chr13	54485001	54490000	4.79E-05
chr13	54512001	54517000	0.004010574
chr13	54513001	54518000	0.001433814
chr13	54515001	54520000	0.000514565
chr13	54516001	54521000	0.000245243
chr13	54517001	54522000	0.000247612
chr13	55088001	55093000	0.000873061
chr13	55089001	55094000	0.000127227
chr13	55306001	55311000	1.59E-18
chr13	55505001	55510000	8.01E-07
chr13	55524001	55529000	0.002964356
chr13	55556001	55561000	0.002929488
chr13	55557001	55562000	0.003105005
chr13	55564001	55569000	2.64E-05
chr13	55565001	55570000	7.92E-07
chr13	55820001	55825000	0.000680496
chr13	55892001	55897000	0.001277823
chr13	56020001	56025000	4.34E-08
chr13	56021001	56026000	1.98E-06
chr13	56109001	56114000	0.002741958
chr13	56110001	56115000	0.00024161
chr13	56111001	56116000	0.000671742
chr13	56268001	56273000	0.000963445
chr13	56450001	56455000	8.41E-08
chr13	56451001	56456000	1.03E-06
chr13	56461001	56466000	5.99E-05
chr13	56579001	56584000	0.001196341
chr13	56598001	56603000	0.002842611
chr13	56717001	56722000	7.41E-07
chr13	56718001	56723000	1.49E-07
chr13	56871001	56876000	0.007619693
chr13	56872001	56877000	0.000816545
chr13	56873001	56878000	0.001017458
chr13	56899001	56904000	0.000582181
chr13	56900001	56905000	0.000602804
chr13	56901001	56906000	3.29E-05
chr13	57152001	57157000	0.001187819
chr13	57153001	57158000	0.000657671
chr13	57160001	57165000	2.86E-06
chr13	57664001	57669000	0.000103547
chr13	57756001	57761000	0.000185049
chr13	58594001	58599000	3.19E-08
chr13	58595001	58600000	3.80E-09

chr13	58596001	58601000	9.49E-09
chr13	58829001	58834000	2.76E-16
chr13	58830001	58835000	3.04E-17
chr13	58831001	58836000	6.12E-16
chr13	58832001	58837000	4.75E-12
chr13	58833001	58838000	2.67E-10
chr13	58869001	58874000	2.72E-05
chr13	58870001	58875000	1.23E-05
chr13	58871001	58876000	0.00019154
chr13	58887001	58892000	0.000546241
chr13	58907001	58912000	6.78E-06
chr13	59010001	59015000	2.31E-05
chr13	59011001	59016000	2.96E-06
chr13	59012001	59017000	1.22E-06
chr13	59018001	59023000	0.000261155
chr13	59043001	59048000	0.001430233
chr13	59301001	59306000	6.97E-05
chr13	59656001	59661000	6.32E-13
chr13	59757001	59762000	0.000149028
chr13	59986001	59991000	3.43E-06
chr13	59987001	59992000	5.56E-05
chr13	60763001	60768000	0.000388287
chr13	60765001	60770000	0.001571862
chr13	60809001	60814000	3.06E-07
chr13	60810001	60815000	1.66E-06
chr13	60909001	60914000	0.000823252
chr13	61326001	61331000	1.00E-09
chr13	61328001	61333000	2.68E-11
chr13	61462001	61467000	0.006843416
chr13	61463001	61468000	0.001251042
chr13	61464001	61469000	4.18E-05
chr13	61465001	61470000	7.86E-05
chr13	61538001	61543000	2.18E-05
chr13	61540001	61545000	0.000849849
chr13	61541001	61546000	0.000136235
chr13	61576001	61581000	0.003218616
chr13	61593001	61598000	0.002621187
chr13	61594001	61599000	0.002173153
chr13	61595001	61600000	6.76E-08
chr13	61596001	61601000	3.99E-10
chr13	61597001	61602000	8.95E-10
chr13	61605001	61610000	0.001598618
chr13	61717001	61722000	0.000125859
chr13	61720001	61725000	1.86E-05
chr13	61736001	61741000	0.000576031
chr13	61737001	61742000	0.000293547
chr13	61751001	61756000	9.76E-07
chr13	61752001	61757000	3.06E-07

chr13	61753001	61758000	6.35E-07
chr13	61915001	61920000	0.000187308
chr13	61916001	61921000	0.002433978
chr13	62233001	62238000	0.001330767
chr13	62525001	62530000	0.000240732
chr13	62607001	62612000	1.72E-08
chr13	62608001	62613000	0.000159849
chr13	62626001	62631000	2.31E-05
chr13	62764001	62769000	4.20E-08
chr13	62774001	62779000	2.53E-09
chr13	62879001	62884000	0.001647978
chr13	62888001	62893000	3.73E-05
chr13	62925001	62930000	4.98E-13
chr13	62926001	62931000	2.23E-12
chr13	63013001	63018000	3.90E-12
chr13	63014001	63019000	3.99E-11
chr13	63015001	63020000	3.40E-08
chr13	63059001	63064000	0.000418507
chr13	63095001	63100000	0.014023115
chr13	63275001	63280000	2.96E-05
chr13	63276001	63281000	5.69E-05
chr13	63277001	63282000	0.000126341
chr13	63278001	63283000	0.000300529
chr13	63288001	63293000	0.014901771
chr13	63289001	63294000	0.015697907
chr13	63296001	63301000	0.015449874
chr13	63313001	63318000	3.91E-05
chr13	63314001	63319000	4.17E-05
chr13	63360001	63365000	1.89E-07
chr13	63361001	63366000	6.98E-05
chr13	63407001	63412000	6.31E-08
chr13	63437001	63442000	4.03E-07
chr13	63438001	63443000	3.90E-11
chr13	63439001	63444000	1.11E-10
chr13	63624001	63629000	0.000183915
chr13	63849001	63854000	8.83E-05
chr13	63851001	63856000	0.000185271
chr13	63852001	63857000	0.011283887
chr13	64011001	64016000	6.89E-05
chr13	64014001	64019000	0.00052531
chr13	64306001	64311000	0.000716788
chr13	64441001	64446000	3.65E-05
chr13	64442001	64447000	0.000275024
chr13	64628001	64633000	0.006007915
chr13	64629001	64634000	0.000261448
chr13	64932001	64937000	0.000466263
chr13	64933001	64938000	0.000370224
chr13	65046001	65051000	0.001643431

chr13	65047001	65052000	0.001524765
chr13	65242001	65247000	9.19E-06
chr13	65243001	65248000	3.97E-07
chr13	65255001	65260000	1.84E-05
chr13	65256001	65261000	6.94E-05
chr13	65257001	65262000	7.33E-06
chr13	65258001	65263000	3.06E-06
chr13	65259001	65264000	8.15E-05
chr13	65300001	65305000	7.62E-09
chr13	65356001	65361000	0.027241407
chr13	65357001	65362000	0.00016905
chr13	65378001	65383000	1.68E-06
chr13	65814001	65819000	7.37E-05
chr13	65815001	65820000	8.33E-05
chr13	65844001	65849000	4.37E-06
chr13	65847001	65852000	3.40E-06
chr13	65944001	65949000	1.30E-07
chr13	65945001	65950000	7.57E-06
chr13	65946001	65951000	1.31E-09
chr13	66174001	66179000	0.000432261
chr13	66305001	66310000	0.00083683
chr13	66306001	66311000	0.000178897
chr13	66344001	66349000	0.022874457
chr13	66345001	66350000	0.006364425
chr13	66392001	66397000	2.48E-05
chr13	66456001	66461000	0.007053813
chr13	66457001	66462000	4.49E-07
chr13	66458001	66463000	4.11E-07
chr13	66459001	66464000	3.90E-07
chr13	66472001	66477000	0.004273059
chr13	66483001	66488000	2.04E-08
chr13	66484001	66489000	9.13E-07
chr13	66529001	66534000	6.64E-05
chr13	66542001	66547000	4.46E-10
chr13	66543001	66548000	1.72E-09
chr13	66545001	66550000	1.26E-09
chr13	66546001	66551000	2.55E-10
chr13	66556001	66561000	1.09E-05
chr13	66557001	66562000	2.72E-06
chr13	66558001	66563000	0.000110111
chr13	66559001	66564000	4.33E-05
chr13	66574001	66579000	0.000514773
chr13	66643001	66648000	0.000982307
chr13	66644001	66649000	3.00E-05
chr13	66645001	66650000	2.05E-05
chr13	66775001	66780000	3.08E-05
chr13	66838001	66843000	2.64E-06
chr13	66855001	66860000	1.40E-06

chr13	66892001	66897000	2.75E-06
chr13	66975001	66980000	0.000272871
chr13	66986001	66991000	2.21E-05
chr13	66987001	66992000	2.75E-05
chr13	67037001	67042000	0.000762032
chr13	67051001	67056000	0.000542925
chr13	67257001	67262000	0.011328535
chr13	67324001	67329000	0.022356667
chr13	67325001	67330000	0.025095312
chr13	67326001	67331000	0.028583784
chr13	67382001	67387000	4.27E-05
chr13	67424001	67429000	7.44E-06
chr13	67448001	67453000	9.25E-08
chr13	67449001	67454000	8.39E-08
chr13	67451001	67456000	2.38E-07
chr13	67500001	67505000	0.002010255
chr13	67593001	67598000	6.82E-10
chr13	67594001	67599000	2.04E-07
chr13	67614001	67619000	8.53E-09
chr13	67615001	67620000	1.74E-08
chr13	67616001	67621000	6.85E-08
chr13	67819001	67824000	0.001477385
chr13	67820001	67825000	0.00077235
chr13	67821001	67826000	0.000513758
chr13	68241001	68246000	7.59E-10
chr13	68242001	68247000	3.29E-11
chr13	68243001	68248000	3.92E-10
chr13	68325001	68330000	2.80E-06
chr13	68428001	68433000	2.14E-09
chr13	68429001	68434000	1.65E-08
chr13	68490001	68495000	2.56E-05
chr13	68729001	68734000	0.000436734
chr13	68761001	68766000	0.001135406
chr13	69384001	69389000	0.021602795
chr13	69517001	69522000	2.68E-05
chr13	69518001	69523000	8.04E-07
chr13	69590001	69595000	0.001799412
chr13	69591001	69596000	0.004093
chr13	69619001	69624000	2.24E-05
chr13	70118001	70123000	7.03E-05
chr13	70119001	70124000	0.00030548
chr13	70127001	70132000	3.12E-05
chr13	70214001	70219000	0.004057182
chr13	70367001	70372000	0.002692352
chr13	70368001	70373000	0.00286476
chr13	70393001	70398000	0.000759992
chr13	70394001	70399000	0.000203807
chr13	70565001	70570000	0.000148901

chr13	70612001	70617000	0.000105452
chr13	70613001	70618000	6.73E-05
chr13	70687001	70692000	0.000754417
chr13	70764001	70769000	0.000476348
chr13	70792001	70797000	0.005313327
chr13	70799001	70804000	3.13E-05
chr13	70907001	70912000	1.04E-06
chr13	70922001	70927000	0.00350983
chr13	70923001	70928000	0.004026192
chr13	70924001	70929000	0.005536784
chr13	70925001	70930000	0.003656311
chr13	71012001	71017000	4.85E-07
chr13	71013001	71018000	5.64E-08
chr13	71025001	71030000	1.05E-07
chr13	71026001	71031000	4.62E-09
chr13	71027001	71032000	9.56E-09
chr13	71122001	71127000	3.74E-12
chr13	71123001	71128000	8.62E-12
chr13	71266001	71271000	2.53E-05
chr13	71287001	71292000	0.001473706
chr13	71306001	71311000	6.21E-05
chr13	71307001	71312000	0.000222637
chr13	71324001	71329000	0.000361078
chr13	71325001	71330000	7.30E-06
chr13	71390001	71395000	1.48E-06
chr13	71391001	71396000	8.84E-07
chr13	71443001	71448000	2.93E-10
chr13	71483001	71488000	6.03E-06
chr13	71487001	71492000	9.43E-06
chr13	71585001	71590000	7.79E-10
chr13	71586001	71591000	1.05E-06
chr13	71645001	71650000	0.000997733
chr13	71657001	71662000	0.000141436
chr13	71716001	71721000	0.000653886
chr13	71783001	71788000	0.000629541
chr13	71791001	71796000	0.000529795
chr13	71865001	71870000	1.04E-08
chr13	71867001	71872000	3.86E-05
chr13	71877001	71882000	0.000781279
chr13	71903001	71908000	0.008601247
chr13	71967001	71972000	0.00111857
chr13	72021001	72026000	4.51E-05
chr13	72022001	72027000	1.17E-05
chr13	72214001	72219000	8.51E-05
chr13	72232001	72237000	0.000111083
chr13	72499001	72504000	2.28E-06
chr13	72500001	72505000	3.69E-06
chr13	72598001	72603000	0.003020465

chr13	72845001	72850000	3.76E-06
chr13	72864001	72869000	0.000888038
chr13	72865001	72870000	0.00016785
chr13	72866001	72871000	8.24E-05
chr13	72933001	72938000	0.000114396
chr13	72967001	72972000	0.001786182
chr13	72968001	72973000	0.000402271
chr13	73037001	73042000	0.002999453
chr13	73168001	73173000	0.000202129
chr13	73232001	73237000	0.009653643
chr13	73261001	73266000	0.000271942
chr13	73708001	73713000	2.50E-07
chr13	73709001	73714000	1.38E-11
chr13	73710001	73715000	1.10E-13
chr13	73711001	73716000	4.15E-14
chr13	73712001	73717000	5.36E-15
chr13	73973001	73978000	3.86E-07
chr13	74179001	74184000	6.55E-06
chr13	74595001	74600000	2.59E-06
chr13	74766001	74771000	0.003238139
chr13	74798001	74803000	0.005058713
chr13	74815001	74820000	5.48E-10
chr13	74849001	74854000	0.003706139
chr13	74850001	74855000	0.000973602
chr13	74851001	74856000	0.000375804
chr13	74898001	74903000	1.09E-09
chr13	74899001	74904000	3.01E-12
chr13	75020001	75025000	0.005947543
chr13	75021001	75026000	0.004276642
chr13	75022001	75027000	0.008370288
chr13	75023001	75028000	0.004826816
chr13	75133001	75138000	8.75E-05
chr13	75134001	75139000	0.000141232
chr13	75135001	75140000	0.000250938
chr13	75136001	75141000	0.000217263
chr13	75417001	75422000	0.000221801
chr13	75418001	75423000	0.014123229
chr13	75461001	75466000	4.37E-06
chr13	75463001	75468000	8.43E-06
chr13	75621001	75626000	8.58E-05
chr13	75872001	75877000	7.71E-08
chr13	75873001	75878000	4.49E-08
chr13	75946001	75951000	5.29E-11
chr13	75992001	75997000	0.002189121
chr13	76245001	76250000	1.95E-05
chr13	76246001	76251000	0.000121377
chr13	76247001	76252000	0.001727127
chr13	76296001	76301000	0.004093911

chr13	76437001	76442000	1.09E-18
chr13	76438001	76443000	7.25E-18
chr13	76439001	76444000	4.69E-19
chr13	76440001	76445000	3.63E-17
chr13	76878001	76883000	0.000315113
chr13	76879001	76884000	0.000238177
chr13	76882001	76887000	0.001564781
chr13	77080001	77085000	1.29E-05
chr13	77089001	77094000	0.000532284
chr13	77194001	77199000	0.000235754
chr13	77197001	77202000	0.00245178
chr13	77198001	77203000	0.000297407
chr13	77374001	77379000	1.92E-08
chr13	77380001	77385000	5.22E-05
chr13	77382001	77387000	4.36E-05
chr13	77411001	77416000	0.003887885
chr13	77476001	77481000	3.74E-05
chr13	77477001	77482000	9.82E-09
chr13	77535001	77540000	6.62E-07
chr13	77932001	77937000	1.35E-06
chr13	78049001	78054000	7.53E-18
chr13	78050001	78055000	9.67E-13
chr13	78052001	78057000	1.73E-09
chr13	78098001	78103000	0.000171884
chr13	78137001	78142000	0.010768376
chr13	78153001	78158000	0.000303533
chr13	78541001	78546000	2.36E-05
chr13	78561001	78566000	3.25E-05
chr13	78676001	78681000	8.65E-05
chr13	78742001	78747000	0.000244864
chr13	78911001	78916000	3.35E-05
chr13	79639001	79644000	0.013767463
chr13	79763001	79768000	7.07E-06
chr13	79764001	79769000	9.29E-05
chr13	79765001	79770000	0.000400278
chr13	79973001	79978000	0.00267783
chr13	80787001	80792000	1.96E-09
chr13	80809001	80814000	1.86E-06
chr13	80810001	80815000	1.00E-07
chr13	81411001	81416000	0.000110107
chr13	81551001	81556000	3.59E-06
chr13	81802001	81807000	0.000638205
chr13	82088001	82093000	6.30E-11
chr13	82089001	82094000	2.47E-07
chr13	82090001	82095000	1.39E-08
chr13	82091001	82096000	0.007808578
chr13	82118001	82123000	0.000735973
chr13	82121001	82126000	0.009291194

chr13	82122001	82127000	0.005721096
chr13	82239001	82244000	0.000337715
chr13	82459001	82464000	0.000148161
chr13	82489001	82494000	6.14E-08
chr13	82540001	82545000	0.000257326
chr13	82541001	82546000	0.000158716
chr13	82542001	82547000	0.000110214
chr13	82861001	82866000	0.000206549
chr13	82914001	82919000	0.000195608
chr13	82988001	82993000	2.72E-05
chr13	83212001	83217000	0.000157128
chr13	83213001	83218000	0.00025725
chr13	83214001	83219000	0.000571941
chr13	83327001	83332000	0.002680615
chr13	83328001	83333000	0.001265258
chr13	83407001	83412000	0.006084009
chr13	83408001	83413000	0.004872263
chr13	83542001	83547000	4.67E-06
chr13	83544001	83549000	2.36E-06
chr13	83546001	83551000	1.29E-06
chr13	83699001	83704000	4.08E-05
chr13	83700001	83705000	1.93E-05
chr13	83701001	83706000	0.000104809
chr13	83702001	83707000	0.000104809
chr13	84098001	84103000	0.000435601
chr13	84109001	84114000	7.23E-07
chr13	84110001	84115000	0.0013833
chr13	84111001	84116000	0.001009408
chr13	84181001	84186000	0.004821716
chr13	84274001	84279000	2.16E-05
chr13	84353001	84358000	2.25E-06
chr13	84354001	84359000	1.16E-07
chr13	84499001	84504000	1.04E-07
chr13	84500001	84505000	6.05E-08
chr13	84501001	84506000	9.56E-08
chr13	84502001	84507000	7.56E-10
chr13	84503001	84508000	5.61E-07
chr13	84633001	84638000	0.00269553
chr13	84765001	84770000	0.000736099
chr13	84945001	84950000	0.000104986
chr13	84946001	84951000	9.54E-05
chr13	84990001	84995000	1.80E-05
chr13	85000001	85005000	3.60E-06
chr13	85001001	85006000	6.58E-06
chr13	85079001	85084000	6.66E-06
chr13	85080001	85085000	3.57E-07
chr13	85081001	85086000	3.27E-08
chr13	85410001	85415000	3.25E-06

chr13	85411001	85416000	2.15E-07
chr13	85605001	85610000	0.000620683
chr13	85606001	85611000	0.002022104
chr13	85607001	85612000	0.002022104
chr13	85896001	85901000	3.76E-09
chr13	86396001	86401000	0.000468918
chr13	86467001	86472000	3.96E-05
chr13	86746001	86751000	0.000108207
chr13	87509001	87514000	1.35E-06
chr13	87510001	87515000	0.000943608
chr13	87511001	87516000	4.46E-12
chr13	87703001	87708000	4.81E-07
chr13	87937001	87942000	8.73E-06
chr13	87938001	87943000	0.000158125
chr13	87939001	87944000	0.00021927
chr13	88043001	88048000	0.003682982
chr13	88044001	88049000	0.004182249
chr13	88045001	88050000	0.000187629
chr13	88046001	88051000	0.000106856
chr13	88047001	88052000	0.000642675
chr13	88261001	88266000	6.40E-08
chr13	88263001	88268000	1.12E-07
chr13	88264001	88269000	8.19E-07
chr13	88312001	88317000	0.002918043
chr13	88713001	88718000	6.65E-05
chr13	88741001	88746000	1.15E-05
chr13	88742001	88747000	0.000216001
chr13	88743001	88748000	8.77E-06
chr13	88939001	88944000	5.26E-07
chr13	88940001	88945000	5.79E-15
chr13	89020001	89025000	0.00013567
chr13	89021001	89026000	0.000793757
chr13	89300001	89305000	0.001424871
chr13	89411001	89416000	1.60E-06
chr13	89412001	89417000	6.69E-06
chr13	89413001	89418000	3.99E-07
chr13	89414001	89419000	5.55E-05
chr13	89467001	89472000	0.000141821
chr13	89472001	89477000	7.70E-05
chr13	89786001	89791000	0.000757597
chr13	89787001	89792000	0.001812816
chr13	89788001	89793000	0.002293019
chr13	89961001	89966000	0.004691773
chr13	90212001	90217000	2.19E-05
chr13	90277001	90282000	0.000180279
chr13	90278001	90283000	0.00013295
chr13	90285001	90290000	0.00276496
chr13	90286001	90291000	0.00220474

chr13	90354001	90359000	1.47E-06
chr13	90640001	90645000	1.67E-08
chr13	90691001	90696000	5.53E-06
chr13	90692001	90697000	0.00013121
chr13	91055001	91060000	5.24E-09
chr13	91057001	91062000	0.00060627
chr13	91126001	91131000	6.79E-05
chr13	91188001	91193000	0.003346625
chr13	91189001	91194000	0.003588247
chr13	91330001	91335000	1.67E-05
chr13	91331001	91336000	2.50E-05
chr13	91408001	91413000	3.62E-06
chr13	91409001	91414000	1.34E-05
chr13	91411001	91416000	0.000455111
chr13	91456001	91461000	1.55E-06
chr13	91457001	91462000	7.37E-06
chr13	91461001	91466000	0.001353155
chr13	91675001	91680000	0.00049677
chr13	91676001	91681000	0.002197343
chr13	91677001	91682000	9.48E-05
chr13	91705001	91710000	1.96E-06
chr13	91763001	91768000	0.00024425
chr13	91791001	91796000	1.49E-07
chr13	91852001	91857000	0.000664211
chr13	91853001	91858000	0.00197193
chr13	91862001	91867000	0.001091212
chr13	91863001	91868000	0.001932302
chr13	92213001	92218000	3.32E-05
chr13	92227001	92232000	1.58E-07
chr13	92370001	92375000	5.00E-08
chr13	92455001	92460000	5.17E-07
chr13	92457001	92462000	1.41E-05
chr13	92822001	92827000	2.08E-06
chr13	92954001	92959000	0.00241334
chr13	92963001	92968000	2.08E-06
chr13	92983001	92988000	7.11E-05
chr13	92984001	92989000	4.49E-05
chr13	93040001	93045000	0.001670102
chr13	93051001	93056000	1.27E-06
chr13	93053001	93058000	0.001311467
chr13	93222001	93227000	1.42E-05
chr13	93223001	93228000	2.29E-05
chr13	93224001	93229000	1.65E-05
chr13	93251001	93256000	0.000575923
chr13	93254001	93259000	0.005533094
chr13	93276001	93281000	8.54E-08
chr13	93285001	93290000	0.000724089
chr13	93326001	93331000	0.001586034

chr13	93429001	93434000	1.77E-09
chr13	93474001	93479000	0.000949085
chr13	93553001	93558000	0.000794022
chr13	93642001	93647000	0.000446055
chr13	93643001	93648000	0.017630915
chr13	93644001	93649000	0.000501189
chr13	93645001	93650000	0.001280032
chr13	93769001	93774000	4.98E-10
chr13	93770001	93775000	2.92E-10
chr13	93771001	93776000	1.07E-08
chr13	93772001	93777000	8.19E-08
chr13	93773001	93778000	2.24E-12
chr13	93802001	93807000	1.70E-06
chr13	93803001	93808000	0.000120437
chr13	93817001	93822000	3.38E-07
chr13	93874001	93879000	2.93E-14
chr13	93943001	93948000	5.29E-11
chr13	93981001	93986000	2.28E-07
chr13	94011001	94016000	2.57E-05
chr13	94012001	94017000	0.000161574
chr13	94013001	94018000	7.00E-05
chr13	94019001	94024000	1.63E-05
chr13	94020001	94025000	0.000102361
chr13	94021001	94026000	2.50E-07
chr13	94022001	94027000	4.98E-06
chr13	94043001	94048000	0.005086714
chr13	94101001	94106000	1.03E-07
chr13	94156001	94161000	2.14E-06
chr13	94274001	94279000	3.17E-05
chr13	94282001	94287000	2.30E-08
chr13	94283001	94288000	1.39E-06
chr13	94284001	94289000	5.10E-11
chr13	94285001	94290000	1.00E-09
chr13	94295001	94300000	0.000182118
chr13	94372001	94377000	0.000177428
chr13	94407001	94412000	0.001530814
chr13	94408001	94413000	0.001001522
chr13	94424001	94429000	0.0001415
chr13	94443001	94448000	2.48E-08
chr13	94444001	94449000	1.63E-10
chr13	94451001	94456000	0.000267204
chr13	94522001	94527000	0.00059407
chr13	94586001	94591000	0.000451328
chr13	94629001	94634000	0.003184072
chr13	94630001	94635000	0.002525201
chr13	94740001	94745000	0.00746591
chr13	94876001	94881000	2.05E-05
chr13	94877001	94882000	1.69E-05

chr13	94878001	94883000	2.26E-05
chr13	94919001	94924000	2.77E-09
chr13	94920001	94925000	3.75E-10
chr13	95100001	95105000	0.000290767
chr13	95101001	95106000	0.000503219
chr13	96940001	96945000	4.11E-05
chr13	96941001	96946000	2.84E-05
chr13	97737001	97742000	1.63E-07
chr13	97738001	97743000	4.93E-10
chr13	97877001	97882000	3.27E-09
chr13	97878001	97883000	2.28E-07
chr13	98590001	98595000	2.03E-10
chr13	100393001	100398000	2.91E-10
chr13	100394001	100399000	1.78E-10
chr13	100417001	100422000	3.78E-05
chr13	100691001	100696000	7.64E-06
chr13	101163001	101168000	7.46E-16
chr13	101165001	101170000	1.30E-19
chr13	101881001	101886000	0.000128357
chr13	101891001	101896000	2.85E-05
chr13	101895001	101900000	0.000316844
chr13	101902001	101907000	1.06E-07
chr13	101903001	101908000	1.13E-06
chr13	101912001	101917000	4.27E-05
chr13	102023001	102028000	0.000294426
chr13	102313001	102318000	0.002837787
chr13	102477001	102482000	7.38E-06
chr13	102499001	102504000	1.44E-06
chr13	102549001	102554000	0.002041659
chr13	102591001	102596000	0.000244797
chr13	102623001	102628000	2.36E-05
chr13	102653001	102658000	0.00054049
chr13	102655001	102660000	0.000238434
chr13	102656001	102661000	1.54E-05
chr13	102789001	102794000	5.57E-08
chr13	102790001	102795000	1.68E-09
chr13	102791001	102796000	5.66E-10
chr13	102792001	102797000	7.28E-08
chr13	102793001	102798000	4.67E-10
chr13	102794001	102799000	3.27E-06
chr13	102795001	102800000	6.11E-06
chr13	102801001	102806000	9.79E-08
chr13	102829001	102834000	0.000181531
chr13	102830001	102835000	6.92E-06
chr13	102834001	102839000	2.99E-05
chr13	102875001	102880000	0.000402338
chr13	103157001	103162000	0.004525035
chr13	103158001	103163000	0.011511705

chr13	103379001	103384000	2.03E-08
chr13	103627001	103632000	1.50E-10
chr13	103731001	103736000	0.000726907
chr13	103870001	103875000	2.31E-13
chr13	103871001	103876000	1.22E-14
chr13	103872001	103877000	2.24E-17
chr13	103873001	103878000	5.08E-19
chr13	103874001	103879000	1.97E-09
chr13	103894001	103899000	5.90E-05
chr13	103908001	103913000	0.00072189
chr13	103938001	103943000	6.85E-09
chr13	104022001	104027000	0.000431825
chr13	104028001	104033000	0.000911875
chr13	104029001	104034000	0.000326953
chr13	104082001	104087000	6.10E-05
chr13	104083001	104088000	0.005412298
chr13	104104001	104109000	0.000301717
chr13	104201001	104206000	9.37E-05
chr13	104202001	104207000	4.07E-05
chr13	104224001	104229000	0.000223285
chr13	104302001	104307000	0.010081315
chr13	104308001	104313000	2.64E-07
chr13	104315001	104320000	6.88E-07
chr13	104316001	104321000	9.36E-09
chr13	104326001	104331000	0.000125666
chr13	104405001	104410000	0.000360679
chr13	104409001	104414000	8.11E-06
chr13	104799001	104804000	0.000246486
chr13	105190001	105195000	4.63E-05
chr13	105191001	105196000	1.85E-05
chr13	105360001	105365000	0.000864466
chr13	105947001	105952000	1.91E-06
chr13	105989001	105994000	0.000145135
chr13	106593001	106598000	1.06E-11
chr13	106594001	106599000	4.30E-09
chr13	106795001	106800000	0.000485704
chr13	107990001	107995000	0.005522662
chr13	108022001	108027000	7.28E-05
chr13	108023001	108028000	0.002319228
chr13	108043001	108048000	5.65E-08
chr13	108049001	108054000	2.21E-06
chr13	108149001	108154000	0.002069695
chr13	108151001	108156000	0.00137061
chr13	108215001	108220000	5.91E-10
chr13	108219001	108224000	2.08E-07
chr13	108656001	108661000	3.29E-06
chr13	108675001	108680000	0.000381778
chr13	108676001	108681000	6.36E-05

chr13	108677001	108682000	2.26E-06
chr13	108708001	108713000	0.002703995
chr13	109354001	109359000	0.007056539
chr13	111466001	111471000	5.70E-28
chr13	112814001	112819000	6.02E-21
chr13	113757001	113762000	9.41E-13
chr13	113758001	113763000	2.59E-10
chr13	113759001	113764000	1.07E-08
chr13	114066001	114071000	3.70E-11
chr13	114213001	114218000	3.54E-16
chr13	114766001	114771000	1.42E-20
chr13	114767001	114772000	2.76E-19
chr13	115097001	115102000	1.45E-18
chr14	19405001	19410000	0.002126077
chr14	19659001	19664000	0.00061228
chr14	19709001	19714000	1.26E-11
chr14	19710001	19715000	3.61E-11
chr14	19851001	19856000	2.94E-05
chr14	21732001	21737000	6.18E-19
chr14	22158001	22163000	7.06E-09
chr14	22591001	22596000	3.81E-05
chr14	23038001	23043000	5.54E-11
chr14	23039001	23044000	5.84E-09
chr14	23724001	23729000	0.000388863
chr14	24113001	24118000	1.93E-07
chr14	24114001	24119000	1.52E-06
chr14	24235001	24240000	0.01553793
chr14	24236001	24241000	8.38E-07
chr14	24278001	24283000	3.65E-07
chr14	24279001	24284000	1.06E-07
chr14	24782001	24787000	1.50E-15
chr14	25315001	25320000	0.000501274
chr14	25477001	25482000	2.79E-07
chr14	25528001	25533000	0.000348258
chr14	25529001	25534000	0.000164344
chr14	25530001	25535000	0.000519713
chr14	25779001	25784000	1.88E-06
chr14	25780001	25785000	4.50E-06
chr14	25908001	25913000	5.32E-05
chr14	26037001	26042000	0.000170146
chr14	26038001	26043000	0.000238864
chr14	26127001	26132000	0.00778809
chr14	26327001	26332000	0.000195446
chr14	26329001	26334000	1.28E-05
chr14	26330001	26335000	5.95E-05
chr14	26331001	26336000	5.93E-05
chr14	26332001	26337000	0.00011187
chr14	26333001	26338000	0.000614991

chr14	26372001	26377000	0.007515642
chr14	26373001	26378000	0.000241505
chr14	26989001	26994000	3.30E-06
chr14	26990001	26995000	7.95E-06
chr14	27234001	27239000	2.32E-07
chr14	27355001	27360000	5.64E-07
chr14	27356001	27361000	1.50E-06
chr14	27487001	27492000	6.66E-06
chr14	27488001	27493000	1.63E-06
chr14	27489001	27494000	2.03E-06
chr14	27574001	27579000	4.38E-05
chr14	27575001	27580000	6.97E-06
chr14	27576001	27581000	7.30E-06
chr14	27577001	27582000	7.70E-05
chr14	27655001	27660000	0.016339082
chr14	27800001	27805000	0.002522826
chr14	27938001	27943000	0.003300198
chr14	28280001	28285000	0.001645488
chr14	28281001	28286000	0.001295174
chr14	28282001	28287000	0.001987541
chr14	28336001	28341000	1.99E-08
chr14	28805001	28810000	1.74E-09
chr14	28806001	28811000	1.83E-10
chr14	28814001	28819000	3.06E-09
chr14	28963001	28968000	0.000556898
chr14	28964001	28969000	4.68E-05
chr14	28967001	28972000	2.43E-07
chr14	29079001	29084000	2.08E-08
chr14	29080001	29085000	4.03E-09
chr14	29081001	29086000	5.48E-09
chr14	29082001	29087000	6.59E-08
chr14	29083001	29088000	6.79E-07
chr14	29207001	29212000	5.30E-08
chr14	29208001	29213000	6.43E-08
chr14	29628001	29633000	0.000546599
chr14	29728001	29733000	0.005735356
chr14	29778001	29783000	0.010383858
chr14	29779001	29784000	0.010383858
chr14	29793001	29798000	8.94E-08
chr14	29794001	29799000	9.75E-08
chr14	29795001	29800000	1.21E-05
chr14	29889001	29894000	0.000606996
chr14	29934001	29939000	0.000281845
chr14	29935001	29940000	0.00084959
chr14	29938001	29943000	0.025410548
chr14	29959001	29964000	4.17E-10
chr14	29960001	29965000	3.84E-09
chr14	29961001	29966000	5.84E-09

chr14	30235001	30240000	1.21E-06
chr14	30470001	30475000	2.72E-06
chr14	30471001	30476000	4.14E-07
chr14	30472001	30477000	8.46E-05
chr14	30473001	30478000	1.15E-06
chr14	30990001	30995000	2.07E-11
chr14	30991001	30996000	1.68E-08
chr14	31017001	31022000	3.81E-16
chr14	31460001	31465000	9.95E-05
chr14	32348001	32353000	1.03E-06
chr14	32460001	32465000	5.77E-10
chr14	34786001	34791000	0.003323951
chr14	34815001	34820000	0.001442336
chr14	35881001	35886000	4.02E-08
chr14	36725001	36730000	0.001211865
chr14	36906001	36911000	5.90E-19
chr14	36907001	36912000	8.77E-20
chr14	36908001	36913000	7.12E-22
chr14	36909001	36914000	3.33E-20
chr14	37411001	37416000	7.75E-10
chr14	37729001	37734000	0.007761723
chr14	38285001	38290000	0.003358401
chr14	38286001	38291000	0.012258302
chr14	38287001	38292000	6.63E-07
chr14	38288001	38293000	4.70E-08
chr14	38499001	38504000	0.000426368
chr14	38506001	38511000	5.24E-05
chr14	38988001	38993000	0.000283223
chr14	40049001	40054000	1.34E-05
chr14	40212001	40217000	0.001280266
chr14	40213001	40218000	0.001026263
chr14	40488001	40493000	0.002011715
chr14	40489001	40494000	0.002278113
chr14	40490001	40495000	0.000168964
chr14	40492001	40497000	2.02E-06
chr14	40495001	40500000	0.000477878
chr14	40666001	40671000	0.000243641
chr14	41108001	41113000	5.72E-10
chr14	41109001	41114000	6.70E-10
chr14	41211001	41216000	5.93E-06
chr14	41319001	41324000	1.45E-11
chr14	41320001	41325000	3.84E-08
chr14	41443001	41448000	5.01E-07
chr14	41444001	41449000	5.84E-07
chr14	41454001	41459000	2.42E-06
chr14	41707001	41712000	0.024065311
chr14	41737001	41742000	0.00077051
chr14	41738001	41743000	0.002385744

chr14	41788001	41793000	7.76E-05
chr14	41789001	41794000	0.000712273
chr14	41790001	41795000	0.001662974
chr14	42294001	42299000	9.10E-10
chr14	42537001	42542000	5.96E-06
chr14	42542001	42547000	8.28E-05
chr14	42543001	42548000	2.37E-05
chr14	42544001	42549000	3.56E-05
chr14	42545001	42550000	3.56E-05
chr14	42626001	42631000	0.003236987
chr14	42628001	42633000	0.000759004
chr14	42629001	42634000	0.006775993
chr14	42927001	42932000	0.000526633
chr14	42928001	42933000	0.002897923
chr14	43082001	43087000	3.41E-07
chr14	43269001	43274000	0.00020652
chr14	43270001	43275000	2.78E-07
chr14	43271001	43276000	8.65E-08
chr14	43272001	43277000	1.94E-06
chr14	43395001	43400000	4.01E-10
chr14	43505001	43510000	0.002024521
chr14	43506001	43511000	0.000130637
chr14	43595001	43600000	0.000255953
chr14	43596001	43601000	0.000179072
chr14	43606001	43611000	8.45E-05
chr14	44049001	44054000	3.59E-05
chr14	44050001	44055000	2.16E-05
chr14	44104001	44109000	3.47E-05
chr14	44506001	44511000	0.002762996
chr14	44557001	44562000	0.000128749
chr14	44558001	44563000	0.000222731
chr14	44727001	44732000	1.60E-07
chr14	44728001	44733000	4.73E-08
chr14	44729001	44734000	2.84E-09
chr14	45195001	45200000	7.55E-05
chr14	45298001	45303000	3.08E-05
chr14	45777001	45782000	0.000234471
chr14	45809001	45814000	0.002296277
chr14	45810001	45815000	0.000199333
chr14	46030001	46035000	0.000905082
chr14	46251001	46256000	0.000233017
chr14	46859001	46864000	6.77E-08
chr14	47005001	47010000	0.000113968
chr14	47006001	47011000	0.00114792
chr14	47187001	47192000	0.000281859
chr14	47360001	47365000	5.47E-05
chr14	47601001	47606000	1.47E-05
chr14	47602001	47607000	7.46E-07

chr14	47603001	47608000	9.88E-07
chr14	47724001	47729000	0.000500646
chr14	47725001	47730000	9.86E-05
chr14	47762001	47767000	0.001570733
chr14	47872001	47877000	9.31E-05
chr14	47873001	47878000	6.29E-09
chr14	47874001	47879000	2.16E-09
chr14	47875001	47880000	1.11E-10
chr14	47901001	47906000	0.000423705
chr14	47903001	47908000	8.87E-05
chr14	47904001	47909000	0.000687311
chr14	47925001	47930000	0.002816567
chr14	47927001	47932000	3.49E-05
chr14	47956001	47961000	6.13E-06
chr14	47957001	47962000	1.66E-06
chr14	47958001	47963000	0.000233321
chr14	47979001	47984000	8.80E-10
chr14	47989001	47994000	0.000492009
chr14	47990001	47995000	0.000136372
chr14	47992001	47997000	0.001107519
chr14	48003001	48008000	4.04E-05
chr14	48102001	48107000	2.02E-05
chr14	48130001	48135000	3.73E-17
chr14	48131001	48136000	4.03E-09
chr14	48238001	48243000	0.000221936
chr14	48239001	48244000	0.000103416
chr14	48256001	48261000	0.001936732
chr14	48301001	48306000	4.81E-07
chr14	48399001	48404000	0.000155234
chr14	48475001	48480000	5.56E-06
chr14	48649001	48654000	8.14E-06
chr14	48650001	48655000	2.77E-07
chr14	48890001	48895000	0.005071678
chr14	48922001	48927000	0.001472677
chr14	48977001	48982000	0.000597144
chr14	49018001	49023000	0.003895559
chr14	49026001	49031000	3.59E-06
chr14	49030001	49035000	5.90E-08
chr14	49031001	49036000	3.46E-11
chr14	49146001	49151000	0.000264632
chr14	49147001	49152000	0.000283217
chr14	49148001	49153000	0.000327192
chr14	49149001	49154000	2.31E-06
chr14	49189001	49194000	5.22E-05
chr14	49190001	49195000	0.001652441
chr14	49191001	49196000	6.69E-07
chr14	49192001	49197000	1.54E-05
chr14	49193001	49198000	1.04E-07

chr14	49194001	49199000	1.73E-07
chr14	49195001	49200000	7.99E-09
chr14	49217001	49222000	6.15E-08
chr14	49218001	49223000	7.73E-08
chr14	49219001	49224000	8.05E-05
chr14	49220001	49225000	0.000176877
chr14	49226001	49231000	3.98E-06
chr14	49227001	49232000	4.06E-06
chr14	49243001	49248000	0.000304378
chr14	49244001	49249000	6.67E-05
chr14	49254001	49259000	4.73E-06
chr14	49255001	49260000	5.49E-06
chr14	49256001	49261000	7.84E-07
chr14	49422001	49427000	4.89E-08
chr14	49433001	49438000	2.04E-06
chr14	49434001	49439000	2.22E-05
chr14	49477001	49482000	0.002806364
chr14	49478001	49483000	0.003389439
chr14	49479001	49484000	0.000614773
chr14	49480001	49485000	0.000310089
chr14	49545001	49550000	1.94E-05
chr14	49570001	49575000	1.15E-09
chr14	49623001	49628000	1.19E-12
chr14	50332001	50337000	4.06E-20
chr14	50333001	50338000	1.54E-18
chr14	50334001	50339000	6.10E-16
chr14	50336001	50341000	4.21E-07
chr14	50426001	50431000	4.55E-13
chr14	50436001	50441000	2.14E-18
chr14	50437001	50442000	3.52E-15
chr14	50438001	50443000	4.44E-15
chr14	50439001	50444000	1.08E-18
chr14	51328001	51333000	5.21E-13
chr14	51582001	51587000	6.56E-05
chr14	51583001	51588000	2.21E-07
chr14	51584001	51589000	5.93E-05
chr14	51585001	51590000	0.003982997
chr14	51586001	51591000	0.00451017
chr14	51640001	51645000	0.00016181
chr14	51651001	51656000	0.000125335
chr14	51652001	51657000	2.64E-05
chr14	51831001	51836000	5.29E-19
chr14	51832001	51837000	1.48E-14
chr14	52340001	52345000	4.19E-07
chr14	52341001	52346000	2.28E-07
chr14	52342001	52347000	4.56E-07
chr14	52344001	52349000	0.000126012
chr14	52489001	52494000	5.95E-06

chr14	52490001	52495000	7.33E-06
chr14	52546001	52551000	3.70E-08
chr14	52547001	52552000	3.73E-07
chr14	52548001	52553000	1.90E-05
chr14	52549001	52554000	7.72E-07
chr14	52550001	52555000	0.000904348
chr14	52876001	52881000	1.60E-11
chr14	52877001	52882000	5.52E-12
chr14	53290001	53295000	4.01E-09
chr14	53846001	53851000	0.00184826
chr14	54143001	54148000	0.000106359
chr14	54344001	54349000	7.95E-10
chr14	54608001	54613000	4.98E-05
chr14	54731001	54736000	9.67E-08
chr14	55344001	55349000	6.76E-10
chr14	55598001	55603000	1.81E-10
chr14	55890001	55895000	9.97E-06
chr14	56067001	56072000	4.19E-09
chr14	56076001	56081000	1.41E-12
chr14	57251001	57256000	0.000885602
chr14	58704001	58709000	9.57E-14
chr14	58705001	58710000	8.21E-20
chr14	58706001	58711000	3.49E-20
chr14	58927001	58932000	0.000196079
chr14	59173001	59178000	0.000167411
chr14	59261001	59266000	3.57E-11
chr14	59271001	59276000	0.001136716
chr14	59274001	59279000	0.000461376
chr14	59345001	59350000	2.01E-05
chr14	59440001	59445000	4.28E-06
chr14	59441001	59446000	3.61E-08
chr14	59466001	59471000	3.06E-05
chr14	59513001	59518000	0.00110431
chr14	60173001	60178000	0.000241341
chr14	60174001	60179000	0.000134324
chr14	60222001	60227000	0.000263433
chr14	60462001	60467000	3.51E-06
chr14	60649001	60654000	4.46E-19
chr14	60650001	60655000	9.30E-14
chr14	60651001	60656000	5.47E-13
chr14	60967001	60972000	0.000151492
chr14	61644001	61649000	1.12E-13
chr14	61945001	61950000	8.94E-10
chr14	61987001	61992000	6.44E-05
chr14	61991001	61996000	8.76E-17
chr14	62334001	62339000	0.005368078
chr14	62488001	62493000	6.64E-07
chr14	62489001	62494000	4.35E-05

chr14	62794001	62799000	0.000372867
chr14	62926001	62931000	0.002324373
chr14	63113001	63118000	4.40E-08
chr14	63114001	63119000	7.22E-09
chr14	63115001	63120000	8.23E-11
chr14	63375001	63380000	3.75E-11
chr14	63618001	63623000	0.001325991
chr14	63619001	63624000	2.58E-05
chr14	63680001	63685000	5.35E-05
chr14	63681001	63686000	0.000631027
chr14	64209001	64214000	2.82E-16
chr14	64210001	64215000	2.08E-18
chr14	64221001	64226000	3.06E-08
chr14	64223001	64228000	2.29E-11
chr14	65510001	65515000	2.42E-09
chr14	65512001	65517000	2.37E-06
chr14	66822001	66827000	3.20E-05
chr14	66823001	66828000	2.95E-06
chr14	66895001	66900000	0.000928013
chr14	68320001	68325000	2.55E-14
chr14	68426001	68431000	3.69E-10
chr14	68625001	68630000	5.62E-09
chr14	68627001	68632000	1.51E-09
chr14	68628001	68633000	1.13E-08
chr14	68629001	68634000	2.32E-09
chr14	68639001	68644000	0.00033942
chr14	68714001	68719000	0.000307817
chr14	69832001	69837000	2.79E-10
chr14	69843001	69848000	3.04E-07
chr14	70037001	70042000	1.58E-20
chr14	70295001	70300000	2.27E-17
chr14	70297001	70302000	1.01E-07
chr14	70383001	70388000	2.80E-15
chr14	70384001	70389000	7.27E-21
chr14	70385001	70390000	3.12E-16
chr14	70404001	70409000	2.60E-12
chr14	70477001	70482000	2.03E-15
chr14	70478001	70483000	4.50E-13
chr14	70479001	70484000	2.13E-13
chr14	70536001	70541000	2.24E-10
chr14	70543001	70548000	4.02E-11
chr14	70544001	70549000	2.10E-08
chr14	70910001	70915000	0.000103653
chr14	70911001	70916000	6.94E-05
chr14	71013001	71018000	0.000912609
chr14	71076001	71081000	1.05E-06
chr14	72651001	72656000	6.27E-05
chr14	74206001	74211000	2.51E-06

chr14	74292001	74297000	4.73E-10
chr14	74293001	74298000	3.19E-09
chr14	74341001	74346000	1.38E-14
chr14	74409001	74414000	2.89E-06
chr14	75236001	75241000	1.92E-08
chr14	75413001	75418000	6.68E-14
chr14	76259001	76264000	1.74E-05
chr14	76287001	76292000	3.01E-11
chr14	76288001	76293000	2.71E-13
chr14	76298001	76303000	2.58E-07
chr14	77292001	77297000	9.85E-06
chr14	77665001	77670000	2.89E-11
chr14	77666001	77671000	1.11E-10
chr14	77667001	77672000	3.11E-09
chr14	77700001	77705000	8.12E-07
chr14	77720001	77725000	6.40E-12
chr14	77721001	77726000	9.02E-11
chr14	77768001	77773000	2.96E-14
chr14	77769001	77774000	6.21E-12
chr14	77770001	77775000	4.06E-12
chr14	78417001	78422000	2.12E-07
chr14	78453001	78458000	1.11E-08
chr14	78454001	78459000	1.52E-09
chr14	78469001	78474000	9.19E-05
chr14	78470001	78475000	3.64E-06
chr14	78471001	78476000	1.47E-05
chr14	78509001	78514000	2.37E-05
chr14	78510001	78515000	5.06E-05
chr14	78511001	78516000	3.66E-06
chr14	78771001	78776000	9.59E-09
chr14	78781001	78786000	1.66E-08
chr14	78926001	78931000	7.55E-06
chr14	78993001	78998000	3.67E-07
chr14	78994001	78999000	8.57E-05
chr14	78995001	79000000	8.67E-11
chr14	79046001	79051000	1.23E-07
chr14	79365001	79370000	1.09E-07
chr14	79485001	79490000	8.35E-06
chr14	79489001	79494000	0.000231542
chr14	79793001	79798000	1.14E-09
chr14	80289001	80294000	0.000229726
chr14	80384001	80389000	1.27E-06
chr14	80474001	80479000	0.000127432
chr14	80475001	80480000	0.000115674
chr14	80597001	80602000	0.003154311
chr14	80649001	80654000	1.84E-10
chr14	80650001	80655000	1.12E-09
chr14	80651001	80656000	2.14E-09

chr14	80735001	80740000	0.005801658
chr14	80746001	80751000	8.32E-05
chr14	80838001	80843000	8.26E-06
chr14	80855001	80860000	0.000404998
chr14	80856001	80861000	1.31E-06
chr14	80857001	80862000	0.000257739
chr14	80997001	81002000	4.16E-06
chr14	80998001	81003000	7.07E-07
chr14	81239001	81244000	0.000102037
chr14	81426001	81431000	2.90E-05
chr14	81924001	81929000	3.37E-13
chr14	81927001	81932000	9.33E-26
chr14	81928001	81933000	1.70E-21
chr14	82082001	82087000	9.20E-08
chr14	82083001	82088000	4.25E-09
chr14	82187001	82192000	0.000376497
chr14	82188001	82193000	0.003436157
chr14	82267001	82272000	5.01E-07
chr14	82304001	82309000	9.84E-08
chr14	82305001	82310000	3.99E-10
chr14	82385001	82390000	2.38E-15
chr14	82387001	82392000	1.25E-14
chr14	82388001	82393000	1.16E-13
chr14	82389001	82394000	9.18E-15
chr14	82404001	82409000	2.29E-06
chr14	82441001	82446000	0.004332852
chr14	82444001	82449000	5.10E-05
chr14	82445001	82450000	0.000461547
chr14	82446001	82451000	0.000154609
chr14	82447001	82452000	4.17E-06
chr14	82623001	82628000	7.29E-05
chr14	82624001	82629000	1.52E-05
chr14	82627001	82632000	6.62E-05
chr14	82758001	82763000	2.90E-06
chr14	82762001	82767000	1.42E-05
chr14	83078001	83083000	0.003707152
chr14	83162001	83167000	0.000821846
chr14	83227001	83232000	0.00037456
chr14	83376001	83381000	3.42E-05
chr14	83377001	83382000	4.01E-06
chr14	83378001	83383000	8.54E-07
chr14	83379001	83384000	9.63E-08
chr14	83380001	83385000	9.76E-09
chr14	83381001	83386000	4.45E-05
chr14	83383001	83388000	0.000973602
chr14	83419001	83424000	0.002975583
chr14	83420001	83425000	0.000903946
chr14	83421001	83426000	0.000839813

chr14	83422001	83427000	0.001987541
chr14	83423001	83428000	0.001257563
chr14	83444001	83449000	0.000144293
chr14	83451001	83456000	1.48E-06
chr14	84294001	84299000	0.004975997
chr14	84339001	84344000	9.02E-10
chr14	84437001	84442000	1.03E-07
chr14	84539001	84544000	1.44E-06
chr14	84699001	84704000	1.06E-05
chr14	84700001	84705000	4.14E-06
chr14	84906001	84911000	1.28E-06
chr14	84907001	84912000	6.47E-06
chr14	84937001	84942000	7.82E-07
chr14	85013001	85018000	0.002251977
chr14	85065001	85070000	9.09E-05
chr14	85213001	85218000	0.002922522
chr14	85214001	85219000	0.000422005
chr14	85215001	85220000	4.96E-06
chr14	85216001	85221000	1.27E-05
chr14	85217001	85222000	5.06E-07
chr14	85218001	85223000	2.71E-05
chr14	85221001	85226000	0.001134478
chr14	85365001	85370000	0.000112251
chr14	85513001	85518000	6.00E-07
chr14	85533001	85538000	4.97E-08
chr14	85534001	85539000	3.42E-08
chr14	85535001	85540000	1.27E-06
chr14	85559001	85564000	0.002216796
chr14	85560001	85565000	0.001179312
chr14	85561001	85566000	0.000453609
chr14	85562001	85567000	0.000789474
chr14	85676001	85681000	0.000103927
chr14	85765001	85770000	5.04E-08
chr14	85766001	85771000	3.00E-07
chr14	85905001	85910000	2.45E-08
chr14	85906001	85911000	2.60E-07
chr14	86255001	86260000	4.92E-06
chr14	86256001	86261000	2.45E-05
chr14	86257001	86262000	3.21E-06
chr14	86258001	86263000	6.12E-06
chr14	86470001	86475000	0.000197532
chr14	86482001	86487000	4.17E-05
chr14	86483001	86488000	1.53E-06
chr14	86484001	86489000	4.88E-05
chr14	86485001	86490000	7.53E-05
chr14	86525001	86530000	8.09E-05
chr14	86535001	86540000	3.52E-10
chr14	86536001	86541000	1.58E-09

chr14	86548001	86553000	0.011804298
chr14	86554001	86559000	0.001621656
chr14	86555001	86560000	5.98E-05
chr14	86556001	86561000	6.14E-05
chr14	86557001	86562000	0.000423701
chr14	86558001	86563000	0.001192814
chr14	86603001	86608000	0.000422005
chr14	86604001	86609000	0.000206889
chr14	86607001	86612000	1.32E-05
chr14	86608001	86613000	1.43E-06
chr14	86609001	86614000	1.67E-05
chr14	86610001	86615000	2.23E-07
chr14	86640001	86645000	1.23E-06
chr14	86641001	86646000	0.000155644
chr14	86678001	86683000	0.001919718
chr14	86696001	86701000	0.00773554
chr14	86726001	86731000	5.65E-05
chr14	86743001	86748000	0.000205155
chr14	86744001	86749000	2.43E-05
chr14	86745001	86750000	1.42E-05
chr14	86746001	86751000	7.38E-06
chr14	86751001	86756000	0.001011135
chr14	86752001	86757000	9.04E-06
chr14	86753001	86758000	7.54E-06
chr14	86811001	86816000	0.005714154
chr14	86894001	86899000	1.21E-07
chr14	86895001	86900000	6.57E-08
chr14	86948001	86953000	4.18E-06
chr14	86985001	86990000	2.35E-05
chr14	87120001	87125000	0.005678246
chr14	87121001	87126000	0.005379997
chr14	87175001	87180000	0.000488957
chr14	87182001	87187000	4.44E-05
chr14	87189001	87194000	0.00036347
chr14	87190001	87195000	0.000540738
chr14	87191001	87196000	7.78E-05
chr14	87204001	87209000	0.000189982
chr14	87205001	87210000	2.35E-05
chr14	87206001	87211000	2.92E-05
chr14	87207001	87212000	0.000823452
chr14	87230001	87235000	0.000199985
chr14	87242001	87247000	0.000108577
chr14	87251001	87256000	0.000162993
chr14	87303001	87308000	2.48E-05
chr14	87304001	87309000	0.000455256
chr14	87305001	87310000	0.000707704
chr14	87306001	87311000	0.000271255
chr14	87313001	87318000	0.003735463

chr14	87458001	87463000	4.05E-08
chr14	87492001	87497000	1.98E-09
chr14	87493001	87498000	5.09E-10
chr14	87504001	87509000	8.02E-06
chr14	87505001	87510000	4.07E-05
chr14	87506001	87511000	3.81E-05
chr14	87565001	87570000	0.000743437
chr14	87566001	87571000	0.000243572
chr14	87567001	87572000	0.00065242
chr14	87568001	87573000	0.000468697
chr14	87569001	87574000	0.012201981
chr14	87586001	87591000	0.000101611
chr14	87608001	87613000	1.01E-07
chr14	87609001	87614000	5.78E-05
chr14	87620001	87625000	2.81E-06
chr14	87621001	87626000	1.80E-07
chr14	87641001	87646000	2.29E-05
chr14	87643001	87648000	6.93E-05
chr14	87644001	87649000	9.36E-06
chr14	87645001	87650000	8.17E-05
chr14	87649001	87654000	2.34E-06
chr14	87656001	87661000	0.000233156
chr14	87658001	87663000	0.003813192
chr14	87661001	87666000	2.36E-06
chr14	87662001	87667000	2.56E-05
chr14	87681001	87686000	1.40E-05
chr14	87682001	87687000	1.61E-06
chr14	87683001	87688000	6.34E-09
chr14	87684001	87689000	2.49E-08
chr14	87695001	87700000	0.000646134
chr14	87696001	87701000	0.000680426
chr14	87697001	87702000	4.10E-12
chr14	87698001	87703000	7.18E-12
chr14	87699001	87704000	2.35E-10
chr14	87713001	87718000	5.20E-08
chr14	87714001	87719000	4.64E-10
chr14	87715001	87720000	4.15E-10
chr14	87716001	87721000	5.04E-14
chr14	87717001	87722000	3.54E-10
chr14	87725001	87730000	3.63E-06
chr14	87726001	87731000	0.001053871
chr14	87739001	87744000	0.001948696
chr14	87740001	87745000	0.00158306
chr14	87749001	87754000	6.10E-07
chr14	87750001	87755000	7.65E-06
chr14	87751001	87756000	0.00029215
chr14	87752001	87757000	0.000976166
chr14	87753001	87758000	0.00033539

chr14	87754001	87759000	0.001696507
chr14	87774001	87779000	0.00121132
chr14	87775001	87780000	0.000944452
chr14	87784001	87789000	3.61E-05
chr14	87785001	87790000	2.26E-05
chr14	87786001	87791000	0.000154559
chr14	87787001	87792000	0.000149242
chr14	87788001	87793000	0.000524194
chr14	87789001	87794000	0.00041441
chr14	87795001	87800000	4.19E-06
chr14	87799001	87804000	4.53E-06
chr14	87827001	87832000	7.96E-06
chr14	87857001	87862000	0.002529021
chr14	87871001	87876000	0.006940389
chr14	87913001	87918000	1.43E-06
chr14	87914001	87919000	3.01E-08
chr14	87915001	87920000	3.57E-08
chr14	87926001	87931000	2.31E-07
chr14	87927001	87932000	1.41E-06
chr14	87928001	87933000	1.28E-08
chr14	87991001	87996000	1.70E-06
chr14	87994001	87999000	8.61E-10
chr14	87995001	88000000	1.58E-10
chr14	87998001	88003000	0.000889387
chr14	88049001	88054000	1.95E-05
chr14	88052001	88057000	0.000142893
chr14	90548001	90553000	0.000130143
chr14	90549001	90554000	3.84E-06
chr14	90550001	90555000	2.33E-09
chr14	90551001	90556000	8.13E-08
chr14	91683001	91688000	1.66E-06
chr14	93809001	93814000	1.24E-19
chr14	93849001	93854000	4.22E-05
chr14	93910001	93915000	5.70E-07
chr14	93998001	94003000	6.85E-10
chr14	94198001	94203000	2.62E-10
chr14	94590001	94595000	1.01E-06
chr14	94592001	94597000	1.59E-09
chr14	94649001	94654000	1.06E-05
chr14	95986001	95991000	4.30E-10
chr14	96279001	96284000	1.98E-08
chr14	96280001	96285000	1.17E-11
chr14	96281001	96286000	1.59E-10
chr14	96282001	96287000	2.47E-08
chr14	97316001	97321000	0.000985718
chr14	97537001	97542000	5.47E-11
chr14	97538001	97543000	4.61E-11
chr14	97887001	97892000	0.00016031

chr14	98972001	98977000	0.000292409
chr14	99133001	99138000	0.006230935
chr14	100061001	100066000	7.73E-11
chr14	100062001	100067000	1.57E-06
chr14	100064001	100069000	2.21E-06
chr14	100539001	100544000	7.03E-18
chr14	101025001	101030000	8.88E-09
chr14	101026001	101031000	1.03E-16
chr14	101027001	101032000	2.04E-15
chr14	101074001	101079000	5.07E-13
chr14	101076001	101081000	4.37E-12
chr14	101077001	101082000	1.48E-11
chr14	101229001	101234000	3.58E-11
chr14	101299001	101304000	2.96E-05
chr14	101381001	101386000	9.96E-07
chr14	101382001	101387000	2.25E-06
chr14	101425001	101430000	2.90E-06
chr14	101575001	101580000	1.74E-14
chr14	101577001	101582000	4.02E-13
chr14	101578001	101583000	9.75E-17
chr14	102184001	102189000	8.36E-11
chr14	102651001	102656000	1.77E-10
chr14	104333001	104338000	5.18E-15
chr14	104334001	104339000	1.34E-18
chr14	104335001	104340000	2.79E-24
chr14	104336001	104341000	2.57E-15
chr14	104337001	104342000	1.69E-14
chr14	104338001	104343000	1.34E-14
chr14	104339001	104344000	2.80E-12
chr14	104568001	104573000	2.51E-15
chr14	104569001	104574000	3.12E-13
chr14	104570001	104575000	1.30E-20
chr14	104571001	104576000	5.50E-26
chr14	104572001	104577000	4.43E-26
chr14	104573001	104578000	7.41E-26
chr14	104574001	104579000	1.18E-22
chr14	104575001	104580000	4.66E-15
chr14	104596001	104601000	3.12E-13
chr14	104639001	104644000	4.62E-28
chr14	104640001	104645000	4.61E-26
chr14	104641001	104646000	2.81E-26
chr14	104642001	104647000	3.03E-21
chr14	104643001	104648000	3.11E-28
chr14	104682001	104687000	0.000632537
chr14	104703001	104708000	1.33E-15
chr14	104767001	104772000	4.02E-14
chr14	104768001	104773000	6.90E-11
chr14	105539001	105544000	1.35E-07

chr14	105542001	105547000	1.88E-11
chr14	105842001	105847000	3.46E-07
chr14	106332001	106337000	5.90E-10
chr14	106333001	106338000	3.84E-15
chr14	106488001	106493000	7.54E-11
chr14	106592001	106597000	0.000277429
chr14	106693001	106698000	0.000191099
chr14	106735001	106740000	1.65E-05
chr15	20786001	20791000	0.000175503
chr15	23815001	23820000	3.23E-10
chr15	24376001	24381000	2.24E-06
chr15	24377001	24382000	1.11E-07
chr15	24379001	24384000	0.000246798
chr15	26528001	26533000	1.26E-06
chr15	26530001	26535000	0.000332622
chr15	26702001	26707000	4.21E-07
chr15	26769001	26774000	3.68E-05
chr15	29037001	29042000	0.001221384
chr15	30472001	30477000	0.001350276
chr15	30473001	30478000	9.05E-05
chr15	31076001	31081000	0.011521479
chr15	31079001	31084000	3.54E-08
chr15	31080001	31085000	4.27E-08
chr15	31101001	31106000	1.42E-08
chr15	31186001	31191000	4.47E-06
chr15	31187001	31192000	9.72E-08
chr15	31995001	32000000	0.001466601
chr15	32570001	32575000	0.000414874
chr15	32861001	32866000	0.002059829
chr15	33003001	33008000	1.40E-13
chr15	33004001	33009000	1.25E-16
chr15	33133001	33138000	2.00E-07
chr15	33491001	33496000	6.03E-21
chr15	33492001	33497000	3.77E-17
chr15	33493001	33498000	8.48E-17
chr15	34907001	34912000	0.001105126
chr15	35143001	35148000	5.94E-07
chr15	36365001	36370000	0.006345461
chr15	36366001	36371000	0.004849706
chr15	36367001	36372000	0.003762786
chr15	36647001	36652000	0.000331866
chr15	37194001	37199000	5.08E-05
chr15	37443001	37448000	0.000114941
chr15	37741001	37746000	0.003872754
chr15	37779001	37784000	1.60E-11
chr15	37780001	37785000	3.29E-10
chr15	37781001	37786000	5.09E-10
chr15	38032001	38037000	1.76E-05

chr15	38105001	38110000	0.001240095
chr15	38136001	38141000	0.004420774
chr15	38164001	38169000	9.34E-07
chr15	38175001	38180000	4.92E-06
chr15	38682001	38687000	8.77E-05
chr15	38977001	38982000	0.000218509
chr15	39334001	39339000	0.000231657
chr15	39336001	39341000	0.00019008
chr15	39458001	39463000	0.001632445
chr15	40127001	40132000	0.00229416
chr15	40420001	40425000	9.12E-09
chr15	40562001	40567000	7.94E-12
chr15	40563001	40568000	4.64E-12
chr15	40793001	40798000	1.09E-05
chr15	40794001	40799000	3.05E-14
chr15	41691001	41696000	1.42E-23
chr15	41694001	41699000	1.74E-24
chr15	42121001	42126000	4.58E-20
chr15	42123001	42128000	8.60E-10
chr15	44008001	44013000	0.003561791
chr15	44009001	44014000	0.000364381
chr15	44010001	44015000	4.74E-06
chr15	44313001	44318000	0.003243198
chr15	44314001	44319000	0.001422158
chr15	44315001	44320000	1.04E-08
chr15	44343001	44348000	0.000154351
chr15	44357001	44362000	0.005423637
chr15	44358001	44363000	0.006576066
chr15	44383001	44388000	2.76E-07
chr15	44421001	44426000	3.46E-11
chr15	44422001	44427000	9.34E-16
chr15	44423001	44428000	1.99E-14
chr15	44449001	44454000	4.81E-05
chr15	44450001	44455000	2.61E-10
chr15	44460001	44465000	5.93E-12
chr15	44461001	44466000	2.12E-11
chr15	44462001	44467000	3.66E-09
chr15	44527001	44532000	7.28E-16
chr15	44528001	44533000	4.49E-13
chr15	45486001	45491000	7.83E-19
chr15	45577001	45582000	1.60E-05
chr15	45733001	45738000	4.00E-10
chr15	45771001	45776000	0.000641493
chr15	46021001	46026000	1.99E-09
chr15	46134001	46139000	2.51E-05
chr15	46135001	46140000	3.25E-07
chr15	46186001	46191000	5.44E-05
chr15	46281001	46286000	9.70E-06

chr15	46404001	46409000	0.000775806
chr15	46405001	46410000	0.001515562
chr15	46444001	46449000	0.003096116
chr15	46476001	46481000	9.95E-08
chr15	46477001	46482000	2.11E-09
chr15	46478001	46483000	2.11E-09
chr15	46537001	46542000	1.34E-08
chr15	46538001	46543000	6.38E-06
chr15	46686001	46691000	1.02E-05
chr15	46688001	46693000	0.00014422
chr15	46856001	46861000	0.000108069
chr15	47023001	47028000	0.000734538
chr15	47119001	47124000	0.000161754
chr15	47396001	47401000	0.000907459
chr15	47634001	47639000	0.003211588
chr15	47637001	47642000	5.00E-05
chr15	47639001	47644000	0.000130128
chr15	47640001	47645000	1.38E-06
chr15	47661001	47666000	2.25E-10
chr15	48024001	48029000	0.001942535
chr15	48025001	48030000	0.000398636
chr15	48117001	48122000	6.35E-05
chr15	48174001	48179000	1.45E-06
chr15	48175001	48180000	2.54E-06
chr15	48176001	48181000	1.76E-06
chr15	48416001	48421000	3.31E-05
chr15	49717001	49722000	0.000735536
chr15	49718001	49723000	0.000851357
chr15	49719001	49724000	0.001678032
chr15	49720001	49725000	0.000413736
chr15	50461001	50466000	1.27E-09
chr15	50462001	50467000	2.41E-11
chr15	53069001	53074000	2.33E-05
chr15	53320001	53325000	0.021288951
chr15	53947001	53952000	2.57E-07
chr15	53948001	53953000	1.18E-05
chr15	54007001	54012000	2.10E-05
chr15	54009001	54014000	5.75E-06
chr15	54118001	54123000	2.00E-06
chr15	54121001	54126000	1.51E-05
chr15	54122001	54127000	0.000217553
chr15	54124001	54129000	8.89E-06
chr15	54187001	54192000	6.94E-14
chr15	54455001	54460000	4.79E-05
chr15	54459001	54464000	0.000237781
chr15	55712001	55717000	0.007798007
chr15	56078001	56083000	1.10E-07
chr15	57503001	57508000	8.13E-06

chr15	57504001	57509000	1.34E-06
chr15	57505001	57510000	0.000430672
chr15	57673001	57678000	3.56E-14
chr15	59147001	59152000	3.00E-06
chr15	60712001	60717000	3.25E-06
chr15	63544001	63549000	9.55E-08
chr15	65593001	65598000	2.86E-28
chr15	66305001	66310000	1.35E-08
chr15	66306001	66311000	1.07E-06
chr15	66307001	66312000	0.000158175
chr15	66308001	66313000	4.74E-06
chr15	67158001	67163000	6.07E-05
chr15	67693001	67698000	3.67E-10
chr15	68544001	68549000	9.72E-09
chr15	68553001	68558000	3.15E-05
chr15	68554001	68559000	0.000103946
chr15	68557001	68562000	4.88E-05
chr15	68595001	68600000	1.35E-12
chr15	68596001	68601000	1.39E-12
chr15	71031001	71036000	8.95E-08
chr15	72070001	72075000	2.60E-13
chr15	74164001	74169000	3.46E-07
chr15	74374001	74379000	0.016692442
chr15	74568001	74573000	1.88E-10
chr15	74710001	74715000	2.78E-18
chr15	74711001	74716000	5.32E-13
chr15	75042001	75047000	3.19E-10
chr15	75043001	75048000	1.25E-11
chr15	75044001	75049000	5.30E-07
chr15	75269001	75274000	2.34E-08
chr15	75306001	75311000	7.75E-06
chr15	75942001	75947000	4.67E-15
chr15	76681001	76686000	3.45E-06
chr15	77317001	77322000	0.00345913
chr15	78102001	78107000	5.06E-07
chr15	80360001	80365000	1.82E-14
chr15	80361001	80366000	6.83E-11
chr15	81691001	81696000	7.84E-06
chr15	84947001	84952000	0.000319077
chr15	85726001	85731000	2.80E-06
chr15	85983001	85988000	1.18E-09
chr15	85984001	85989000	1.18E-09
chr15	85985001	85990000	4.73E-10
chr15	87356001	87361000	2.97E-07
chr15	87521001	87526000	2.41E-05
chr15	87699001	87704000	0.002984882
chr15	87949001	87954000	5.75E-05
chr15	89881001	89886000	5.94E-11

chr15	89944001	89949000	6.66E-14
chr15	90100001	90105000	3.31E-07
chr15	90105001	90110000	8.72E-07
chr15	90107001	90112000	5.04E-06
chr15	90108001	90113000	3.52E-05
chr15	90188001	90193000	2.51E-09
chr15	90299001	90304000	1.57E-09
chr15	91187001	91192000	7.34E-09
chr15	91425001	91430000	4.22E-06
chr15	91586001	91591000	4.95E-06
chr15	92611001	92616000	6.73E-15
chr15	92612001	92617000	1.61E-12
chr15	93345001	93350000	3.24E-13
chr15	93346001	93351000	2.00E-15
chr15	95211001	95216000	1.12E-10
chr15	95212001	95217000	1.63E-10
chr15	95681001	95686000	0.001528558
chr15	95790001	95795000	6.42E-05
chr15	97295001	97300000	7.49E-07
chr15	97536001	97541000	0.001004747
chr15	97683001	97688000	4.44E-07
chr15	97684001	97689000	2.23E-07
chr15	97824001	97829000	0.001420462
chr15	97825001	97830000	0.000844906
chr15	97868001	97873000	0.001835205
chr15	97869001	97874000	0.003281886
chr15	97870001	97875000	0.004737104
chr15	97885001	97890000	3.45E-06
chr15	97886001	97891000	6.96E-08
chr15	97887001	97892000	1.89E-08
chr15	97942001	97947000	8.25E-06
chr15	97997001	98002000	0.013052921
chr15	98010001	98015000	1.34E-05
chr15	98129001	98134000	0.005000412
chr15	98242001	98247000	0.001047728
chr15	98357001	98362000	1.70E-06
chr15	98358001	98363000	1.05E-05
chr15	98359001	98364000	5.10E-06
chr15	102348001	102353000	0.000164725
chr15	102363001	102368000	0.002686384
chr15	102429001	102434000	0.000377823
chr15	102439001	102444000	3.65E-05
chr16	492001	497000	7.63E-15
chr16	602001	607000	4.73E-16
chr16	603001	608000	3.22E-15
chr16	605001	610000	1.83E-19
chr16	620001	625000	4.21E-26
chr16	641001	646000	4.00E-09

chr16	722001	727000	2.14E-18
chr16	823001	828000	3.52E-12
chr16	1152001	1157000	1.36E-07
chr16	1153001	1158000	1.62E-10
chr16	1154001	1159000	7.77E-11
chr16	1196001	1201000	3.34E-25
chr16	1209001	1214000	3.64E-19
chr16	1210001	1215000	1.49E-19
chr16	1211001	1216000	2.08E-16
chr16	1213001	1218000	9.52E-28
chr16	1214001	1219000	1.06E-24
chr16	1215001	1220000	4.86E-26
chr16	1216001	1221000	1.70E-27
chr16	1217001	1222000	9.18E-26
chr16	1254001	1259000	0.003808254
chr16	1573001	1578000	8.67E-12
chr16	1574001	1579000	4.66E-10
chr16	1575001	1580000	3.57E-12
chr16	2053001	2058000	1.45E-19
chr16	2054001	2059000	7.86E-19
chr16	2691001	2696000	0.000226652
chr16	2785001	2790000	2.65E-09
chr16	2786001	2791000	1.87E-05
chr16	2976001	2981000	1.70E-14
chr16	2977001	2982000	2.35E-15
chr16	4136001	4141000	1.14E-20
chr16	4137001	4142000	6.08E-25
chr16	4699001	4704000	6.72E-07
chr16	4700001	4705000	4.87E-10
chr16	4792001	4797000	1.31E-07
chr16	6632001	6637000	2.52E-08
chr16	6633001	6638000	6.09E-10
chr16	6776001	6781000	0.000648279
chr16	7092001	7097000	2.23E-05
chr16	7093001	7098000	2.19E-05
chr16	7296001	7301000	1.48E-06
chr16	9239001	9244000	3.61E-13
chr16	9241001	9246000	1.92E-12
chr16	11392001	11397000	4.02E-11
chr16	11393001	11398000	4.30E-09
chr16	11394001	11399000	1.65E-08
chr16	11551001	11556000	1.02E-15
chr16	11875001	11880000	1.19E-10
chr16	11877001	11882000	2.22E-14
chr16	11880001	11885000	2.24E-15
chr16	11881001	11886000	1.87E-13
chr16	13103001	13108000	7.05E-10
chr16	13105001	13110000	2.15E-11

chr16	15409001	15414000	0.003372832
chr16	15738001	15743000	1.81E-15
chr16	16097001	16102000	5.26E-06
chr16	16572001	16577000	0.000443975
chr16	16573001	16578000	9.51E-05
chr16	17921001	17926000	1.80E-06
chr16	18553001	18558000	4.03E-08
chr16	18554001	18559000	3.68E-07
chr16	18555001	18560000	3.03E-07
chr16	21232001	21237000	1.01E-07
chr16	21233001	21238000	8.79E-07
chr16	23387001	23392000	0.002420155
chr16	23388001	23393000	0.00082651
chr16	23543001	23548000	3.35E-09
chr16	23544001	23549000	3.83E-08
chr16	23877001	23882000	1.34E-07
chr16	23996001	24001000	4.36E-06
chr16	23997001	24002000	4.30E-15
chr16	23998001	24003000	1.59E-14
chr16	24877001	24882000	2.77E-05
chr16	25054001	25059000	3.57E-09
chr16	25055001	25060000	1.68E-09
chr16	25066001	25071000	5.68E-08
chr16	26374001	26379000	0.000694379
chr16	26457001	26462000	2.64E-06
chr16	26559001	26564000	0.000561872
chr16	26560001	26565000	0.00084162
chr16	30012001	30017000	3.83E-19
chr16	30013001	30018000	2.06E-24
chr16	30014001	30019000	3.71E-24
chr16	30015001	30020000	1.00E-22
chr16	30049001	30054000	1.72E-16
chr16	30050001	30055000	1.09E-13
chr16	30360001	30365000	3.95E-06
chr16	30361001	30366000	3.30E-05
chr16	30504001	30509000	5.69E-11
chr16	30749001	30754000	4.56E-16
chr16	30750001	30755000	7.39E-12
chr16	31137001	31142000	1.88E-13
chr16	31138001	31143000	1.08E-13
chr16	31139001	31144000	1.52E-16
chr16	31140001	31145000	4.10E-09
chr16	31141001	31146000	3.22E-10
chr16	31886001	31891000	1.36E-10
chr16	32999001	33004000	0.004654391
chr16	33299001	33304000	1.69E-07
chr16	33683001	33688000	0.000697314
chr16	33774001	33779000	4.65E-08

chr16	34304001	34309000	0.009888667
chr16	34329001	34334000	1.34E-06
chr16	34563001	34568000	6.79E-05
chr16	34564001	34569000	9.43E-05
chr16	34565001	34570000	0.000643239
chr16	34567001	34572000	0.001015544
chr16	47777001	47782000	0.000141974
chr16	48178001	48183000	9.03E-12
chr16	48179001	48184000	5.86E-11
chr16	49266001	49271000	0.000125056
chr16	49975001	49980000	0.000295695
chr16	51530001	51535000	5.10E-08
chr16	51531001	51536000	2.96E-07
chr16	51903001	51908000	0.005426
chr16	52543001	52548000	0.000415406
chr16	52545001	52550000	7.47E-05
chr16	52586001	52591000	7.91E-05
chr16	53113001	53118000	6.70E-06
chr16	55585001	55590000	0.009801807
chr16	55599001	55604000	1.41E-05
chr16	55665001	55670000	3.82E-05
chr16	55924001	55929000	2.87E-05
chr16	56379001	56384000	3.97E-17
chr16	56380001	56385000	7.97E-18
chr16	56968001	56973000	1.52E-14
chr16	56969001	56974000	7.06E-08
chr16	57118001	57123000	1.08E-09
chr16	57212001	57217000	0.000149724
chr16	57341001	57346000	4.76E-05
chr16	57414001	57419000	1.30E-07
chr16	57616001	57621000	3.40E-09
chr16	57635001	57640000	4.12E-19
chr16	58102001	58107000	1.32E-10
chr16	58538001	58543000	7.47E-07
chr16	58539001	58544000	4.99E-05
chr16	58657001	58662000	1.41E-09
chr16	58966001	58971000	6.85E-08
chr16	59079001	59084000	1.31E-07
chr16	59080001	59085000	2.16E-08
chr16	59147001	59152000	3.51E-06
chr16	59204001	59209000	8.68E-09
chr16	59356001	59361000	1.01E-07
chr16	59410001	59415000	0.012865564
chr16	59464001	59469000	4.95E-05
chr16	59466001	59471000	0.000441755
chr16	59535001	59540000	1.79E-06
chr16	59566001	59571000	3.54E-07
chr16	59570001	59575000	0.000179569

chr16	59571001	59576000	0.000300538
chr16	59774001	59779000	0.014138328
chr16	59956001	59961000	0.000536588
chr16	59982001	59987000	4.46E-07
chr16	59983001	59988000	6.41E-11
chr16	60000001	60005000	0.001728354
chr16	60001001	60006000	1.77E-05
chr16	60002001	60007000	0.000185363
chr16	60118001	60123000	2.55E-10
chr16	60119001	60124000	3.86E-06
chr16	60479001	60484000	5.18E-06
chr16	60812001	60817000	0.000224305
chr16	60894001	60899000	0.00038313
chr16	60912001	60917000	0.001080314
chr16	61008001	61013000	0.002147854
chr16	61156001	61161000	0.003576702
chr16	61202001	61207000	8.06E-05
chr16	61342001	61347000	0.016357961
chr16	61343001	61348000	0.003740174
chr16	61344001	61349000	0.006573679
chr16	61401001	61406000	2.87E-08
chr16	61402001	61407000	3.40E-07
chr16	61409001	61414000	0.001493243
chr16	61419001	61424000	2.07E-09
chr16	61610001	61615000	4.92E-06
chr16	61646001	61651000	0.000324243
chr16	61647001	61652000	0.00200027
chr16	61691001	61696000	1.81E-05
chr16	61692001	61697000	0.000139224
chr16	61757001	61762000	0.00401976
chr16	61915001	61920000	5.95E-06
chr16	61916001	61921000	4.23E-06
chr16	61986001	61991000	9.10E-05
chr16	62030001	62035000	0.0029995
chr16	62063001	62068000	0.000983669
chr16	62171001	62176000	5.04E-07
chr16	62172001	62177000	4.59E-06
chr16	62173001	62178000	6.72E-06
chr16	62174001	62179000	7.33E-08
chr16	62268001	62273000	0.000127499
chr16	62345001	62350000	0.000704842
chr16	62346001	62351000	0.014928499
chr16	62502001	62507000	0.005223414
chr16	62514001	62519000	1.33E-05
chr16	62549001	62554000	0.000182775
chr16	62579001	62584000	0.004237094
chr16	62580001	62585000	0.003339155
chr16	62581001	62586000	0.005256408

chr16	62666001	62671000	3.16E-06
chr16	62667001	62672000	3.08E-06
chr16	62668001	62673000	3.57E-07
chr16	62669001	62674000	5.34E-07
chr16	62705001	62710000	0.001769621
chr16	62722001	62727000	0.000119677
chr16	62723001	62728000	0.00036652
chr16	62966001	62971000	8.03E-06
chr16	62995001	63000000	1.14E-05
chr16	62996001	63001000	0.000232966
chr16	63269001	63274000	0.000123435
chr16	63271001	63276000	1.47E-05
chr16	63272001	63277000	2.78E-06
chr16	63455001	63460000	0.000144177
chr16	63456001	63461000	1.40E-05
chr16	63861001	63866000	0.000317247
chr16	63862001	63867000	0.000977278
chr16	64332001	64337000	4.07E-13
chr16	64333001	64338000	5.50E-17
chr16	64334001	64339000	4.70E-11
chr16	64336001	64341000	1.06E-08
chr16	64368001	64373000	2.40E-05
chr16	64369001	64374000	2.44E-05
chr16	64370001	64375000	0.005537822
chr16	64596001	64601000	0.000544821
chr16	64597001	64602000	0.004112008
chr16	64739001	64744000	7.64E-06
chr16	64898001	64903000	7.14E-07
chr16	64899001	64904000	1.95E-06
chr16	64914001	64919000	0.000142817
chr16	65137001	65142000	0.006577229
chr16	65173001	65178000	2.37E-06
chr16	65175001	65180000	1.19E-06
chr16	65363001	65368000	0.004934731
chr16	65367001	65372000	0.000669882
chr16	65368001	65373000	0.000784676
chr16	65369001	65374000	0.000281335
chr16	65596001	65601000	0.003933447
chr16	65597001	65602000	0.002872551
chr16	65995001	66000000	4.51E-07
chr16	65996001	66001000	2.97E-09
chr16	65997001	66002000	1.01E-06
chr16	65998001	66003000	3.89E-07
chr16	66364001	66369000	1.51E-06
chr16	66389001	66394000	9.68E-11
chr16	66775001	66780000	5.24E-09
chr16	67136001	67141000	7.03E-09
chr16	67544001	67549000	2.53E-05

chr16	67545001	67550000	3.06E-05
chr16	67676001	67681000	2.33E-19
chr16	67678001	67683000	8.70E-30
chr16	67679001	67684000	2.22E-29
chr16	68788001	68793000	1.79E-05
chr16	69426001	69431000	3.74E-11
chr16	69797001	69802000	3.24E-07
chr16	70363001	70368000	2.26E-11
chr16	70365001	70370000	1.80E-14
chr16	70460001	70465000	5.63E-07
chr16	70461001	70466000	7.64E-07
chr16	70462001	70467000	1.47E-08
chr16	70463001	70468000	5.85E-07
chr16	70552001	70557000	1.17E-13
chr16	70553001	70558000	1.97E-24
chr16	70619001	70624000	6.40E-13
chr16	70620001	70625000	1.96E-15
chr16	70859001	70864000	7.98E-08
chr16	71232001	71237000	0.000113649
chr16	71870001	71875000	1.16E-06
chr16	72192001	72197000	4.48E-06
chr16	72193001	72198000	2.37E-07
chr16	73202001	73207000	2.70E-14
chr16	73203001	73208000	3.02E-23
chr16	73204001	73209000	1.04E-23
chr16	73704001	73709000	0.000205929
chr16	74463001	74468000	1.31E-12
chr16	74690001	74695000	5.56E-15
chr16	75584001	75589000	6.85E-13
chr16	76363001	76368000	1.54E-05
chr16	76492001	76497000	1.21E-05
chr16	76742001	76747000	0.001038107
chr16	76766001	76771000	1.10E-05
chr16	76860001	76865000	9.40E-07
chr16	77058001	77063000	0.002578989
chr16	77437001	77442000	0.003589645
chr16	77630001	77635000	1.44E-05
chr16	81613001	81618000	1.16E-13
chr16	81767001	81772000	4.80E-08
chr16	84183001	84188000	0.000104551
chr16	84841001	84846000	6.64E-17
chr16	84873001	84878000	2.29E-14
chr16	84874001	84879000	2.06E-16
chr16	85682001	85687000	2.84E-16
chr16	85685001	85690000	4.36E-10
chr16	87914001	87919000	1.27E-05
chr16	87915001	87920000	9.47E-09
chr16	88118001	88123000	8.86E-30

chr16	88119001	88124000	3.44E-25
chr16	88120001	88125000	2.82E-22
chr16	88162001	88167000	3.50E-18
chr16	88232001	88237000	1.09E-06
chr16	88293001	88298000	2.03E-20
chr16	88823001	88828000	9.18E-11
chr16	88824001	88829000	5.76E-11
chr16	88946001	88951000	1.85E-11
chr16	88947001	88952000	2.22E-16
chr16	89218001	89223000	8.43E-17
chr16	89327001	89332000	5.74E-17
chr16	89328001	89333000	2.95E-17
chr16	89877001	89882000	2.16E-09
chr17	820001	825000	1.39E-10
chr17	833001	838000	2.41E-08
chr17	982001	987000	1.30E-09
chr17	1276001	1281000	4.06E-15
chr17	1494001	1499000	1.06E-08
chr17	1592001	1597000	0.001109433
chr17	1650001	1655000	1.92E-06
chr17	1651001	1656000	2.76E-08
chr17	1679001	1684000	2.64E-31
chr17	1680001	1685000	4.14E-23
chr17	1682001	1687000	2.15E-20
chr17	2417001	2422000	0.000516366
chr17	2589001	2594000	1.29E-18
chr17	2792001	2797000	3.67E-16
chr17	2831001	2836000	1.02E-08
chr17	2832001	2837000	8.11E-09
chr17	2875001	2880000	4.64E-10
chr17	3144001	3149000	0.011693529
chr17	3363001	3368000	5.87E-16
chr17	3364001	3369000	1.09E-08
chr17	3365001	3370000	1.69E-12
chr17	3495001	3500000	0.002985866
chr17	4476001	4481000	6.30E-10
chr17	5708001	5713000	1.24E-08
chr17	5709001	5714000	6.24E-09
chr17	5710001	5715000	5.75E-07
chr17	5711001	5716000	2.94E-08
chr17	5734001	5739000	8.64E-13
chr17	5735001	5740000	1.69E-15
chr17	5736001	5741000	2.51E-09
chr17	5836001	5841000	1.22E-10
chr17	6452001	6457000	5.39E-10
chr17	6468001	6473000	9.68E-13
chr17	6997001	7002000	5.01E-12
chr17	7248001	7253000	3.00E-07

chr17	7310001	7315000	5.00E-08
chr17	7311001	7316000	3.55E-12
chr17	7312001	7317000	6.10E-12
chr17	7313001	7318000	4.51E-13
chr17	7325001	7330000	8.72E-11
chr17	7457001	7462000	2.06E-21
chr17	7458001	7463000	2.69E-14
chr17	7652001	7657000	2.42E-10
chr17	7783001	7788000	2.68E-05
chr17	8676001	8681000	1.84E-07
chr17	8678001	8683000	1.02E-09
chr17	8679001	8684000	0.001949279
chr17	8789001	8794000	2.45E-05
chr17	9751001	9756000	3.83E-09
chr17	9752001	9757000	8.11E-16
chr17	9753001	9758000	5.52E-14
chr17	9824001	9829000	2.07E-10
chr17	10259001	10264000	3.34E-08
chr17	10374001	10379000	0.004681984
chr17	10375001	10380000	0.000441193
chr17	10376001	10381000	0.000715659
chr17	10377001	10382000	0.000418751
chr17	10656001	10661000	2.53E-07
chr17	10834001	10839000	4.74E-09
chr17	10835001	10840000	3.28E-09
chr17	11435001	11440000	1.84E-07
chr17	11839001	11844000	5.72E-07
chr17	13073001	13078000	2.72E-05
chr17	14281001	14286000	7.30E-09
chr17	14282001	14287000	3.51E-07
chr17	14344001	14349000	1.34E-08
chr17	14345001	14350000	4.60E-08
chr17	14602001	14607000	1.85E-09
chr17	14711001	14716000	0.000264015
chr17	14763001	14768000	0.000233315
chr17	14901001	14906000	2.07E-07
chr17	14902001	14907000	4.33E-08
chr17	15139001	15144000	7.79E-06
chr17	15948001	15953000	5.95E-09
chr17	15949001	15954000	4.93E-08
chr17	16343001	16348000	1.79E-18
chr17	16346001	16351000	7.38E-08
chr17	16388001	16393000	4.00E-07
chr17	16440001	16445000	9.30E-10
chr17	16634001	16639000	3.46E-05
chr17	16642001	16647000	0.015434684
chr17	16643001	16648000	0.001377595
chr17	16790001	16795000	1.71E-10

chr17	16791001	16796000	2.46E-08
chr17	16828001	16833000	1.38E-08
chr17	17074001	17079000	2.25E-14
chr17	17198001	17203000	1.49E-07
chr17	17282001	17287000	7.95E-16
chr17	17283001	17288000	5.95E-18
chr17	17644001	17649000	1.56E-15
chr17	17719001	17724000	5.16E-08
chr17	18041001	18046000	2.73E-11
chr17	18042001	18047000	2.93E-11
chr17	18043001	18048000	4.33E-12
chr17	18057001	18062000	6.30E-09
chr17	18838001	18843000	9.60E-08
chr17	18839001	18844000	8.62E-12
chr17	19142001	19147000	3.15E-23
chr17	19143001	19148000	2.48E-18
chr17	19144001	19149000	4.22E-11
chr17	19413001	19418000	3.28E-10
chr17	19954001	19959000	1.98E-05
chr17	20388001	20393000	3.27E-13
chr17	20687001	20692000	1.67E-06
chr17	22221001	22226000	0.00215818
chr17	25534001	25539000	1.05E-06
chr17	25622001	25627000	5.29E-13
chr17	25623001	25628000	2.02E-09
chr17	25832001	25837000	1.29E-16
chr17	25833001	25838000	8.03E-14
chr17	26379001	26384000	0.003524549
chr17	26781001	26786000	9.39E-40
chr17	26782001	26787000	2.83E-40
chr17	26783001	26788000	3.77E-24
chr17	27073001	27078000	2.27E-11
chr17	27074001	27079000	1.63E-09
chr17	27650001	27655000	6.18E-07
chr17	30231001	30236000	1.99E-09
chr17	30249001	30254000	8.46E-10
chr17	30250001	30255000	4.32E-10
chr17	30385001	30390000	0.000330288
chr17	30386001	30391000	0.000703537
chr17	30400001	30405000	7.14E-05
chr17	32217001	32222000	6.77E-06
chr17	32966001	32971000	9.45E-09
chr17	32972001	32977000	1.38E-09
chr17	34062001	34067000	1.27E-06
chr17	34760001	34765000	1.34E-05
chr17	34902001	34907000	3.84E-14
chr17	35195001	35200000	1.03E-12
chr17	35250001	35255000	3.66E-10

chr17	35750001	35755000	2.67E-07
chr17	36814001	36819000	8.89E-09
chr17	36815001	36820000	1.82E-08
chr17	36820001	36825000	4.92E-10
chr17	36943001	36948000	0.001598637
chr17	37688001	37693000	0.000616997
chr17	37829001	37834000	1.46E-06
chr17	38206001	38211000	5.69E-22
chr17	38258001	38263000	0.002153086
chr17	38259001	38264000	0.000401604
chr17	38512001	38517000	5.59E-12
chr17	38605001	38610000	1.12E-06
chr17	38617001	38622000	7.29E-07
chr17	38707001	38712000	3.32E-17
chr17	39807001	39812000	7.16E-06
chr17	39956001	39961000	3.89E-07
chr17	40111001	40116000	0.000125165
chr17	40581001	40586000	0.000282612
chr17	40736001	40741000	5.32E-11
chr17	40737001	40742000	2.36E-09
chr17	40738001	40743000	4.86E-08
chr17	41002001	41007000	1.75E-13
chr17	41068001	41073000	3.32E-05
chr17	41119001	41124000	2.42E-05
chr17	41121001	41126000	5.65E-09
chr17	41467001	41472000	7.12E-16
chr17	41506001	41511000	4.24E-19
chr17	41507001	41512000	1.02E-17
chr17	41508001	41513000	3.78E-14
chr17	41509001	41514000	1.37E-09
chr17	41526001	41531000	1.24E-06
chr17	42265001	42270000	2.95E-15
chr17	42387001	42392000	4.22E-09
chr17	42642001	42647000	0.000348049
chr17	42669001	42674000	5.65E-10
chr17	42670001	42675000	1.21E-07
chr17	42821001	42826000	0.006718493
chr17	42871001	42876000	2.46E-10
chr17	42872001	42877000	1.45E-09
chr17	42873001	42878000	1.39E-13
chr17	42896001	42901000	5.54E-07
chr17	42898001	42903000	9.93E-09
chr17	42914001	42919000	1.75E-12
chr17	42977001	42982000	2.34E-08
chr17	43055001	43060000	2.10E-13
chr17	43139001	43144000	3.26E-16
chr17	43724001	43729000	7.53E-08
chr17	43725001	43730000	1.79E-08

chr17	43792001	43797000	4.89E-05
chr17	43808001	43813000	0.000131877
chr17	43809001	43814000	4.71E-05
chr17	43920001	43925000	4.56E-05
chr17	43921001	43926000	4.00E-05
chr17	44040001	44045000	0.005232191
chr17	44041001	44046000	0.003015142
chr17	44111001	44116000	0.002185131
chr17	44226001	44231000	9.05E-09
chr17	44347001	44352000	0.000386918
chr17	44368001	44373000	0.001843424
chr17	45188001	45193000	5.12E-09
chr17	45798001	45803000	5.75E-16
chr17	46057001	46062000	4.15E-07
chr17	46079001	46084000	0.000236077
chr17	46205001	46210000	1.76E-09
chr17	46627001	46632000	2.01E-32
chr17	46633001	46638000	4.28E-11
chr17	46634001	46639000	3.46E-10
chr17	46649001	46654000	3.40E-15
chr17	48049001	48054000	7.16E-05
chr17	48541001	48546000	3.00E-05
chr17	48757001	48762000	2.74E-05
chr17	48918001	48923000	7.35E-11
chr17	48919001	48924000	2.83E-10
chr17	48930001	48935000	5.42E-15
chr17	49192001	49197000	3.57E-11
chr17	50219001	50224000	0.000238353
chr17	50472001	50477000	0.00404432
chr17	50473001	50478000	0.001314127
chr17	50568001	50573000	0.000344435
chr17	50579001	50584000	6.09E-05
chr17	50580001	50585000	6.68E-06
chr17	50581001	50586000	3.99E-05
chr17	50632001	50637000	0.006049088
chr17	50792001	50797000	0.001551584
chr17	50852001	50857000	0.000745166
chr17	50956001	50961000	1.72E-10
chr17	51125001	51130000	0.000411182
chr17	51320001	51325000	1.75E-09
chr17	51552001	51557000	5.80E-05
chr17	51553001	51558000	4.26E-05
chr17	51554001	51559000	0.000652195
chr17	51672001	51677000	6.67E-05
chr17	51686001	51691000	0.002185789
chr17	51687001	51692000	8.07E-05
chr17	51688001	51693000	1.46E-05
chr17	51689001	51694000	1.82E-05

chr17	51690001	51695000	0.001321191
chr17	51691001	51696000	0.001157324
chr17	51728001	51733000	0.000502983
chr17	51937001	51942000	0.001951891
chr17	52379001	52384000	0.024827721
chr17	52472001	52477000	0.000573319
chr17	52697001	52702000	3.78E-10
chr17	52698001	52703000	7.71E-08
chr17	52699001	52704000	7.53E-05
chr17	53506001	53511000	9.76E-06
chr17	53690001	53695000	2.37E-06
chr17	55988001	55993000	4.69E-07
chr17	56151001	56156000	2.76E-05
chr17	56430001	56435000	1.37E-15
chr17	56733001	56738000	4.67E-17
chr17	57716001	57721000	4.53E-14
chr17	57717001	57722000	2.94E-13
chr17	57861001	57866000	1.76E-17
chr17	57862001	57867000	3.19E-26
chr17	57863001	57868000	6.01E-26
chr17	57864001	57869000	3.00E-12
chr17	57902001	57907000	3.39E-10
chr17	57903001	57908000	5.29E-07
chr17	57904001	57909000	1.77E-08
chr17	57922001	57927000	6.71E-09
chr17	58189001	58194000	1.92E-13
chr17	58220001	58225000	1.03E-09
chr17	58221001	58226000	3.43E-07
chr17	58222001	58227000	1.85E-12
chr17	59511001	59516000	4.09E-05
chr17	60343001	60348000	1.66E-06
chr17	60344001	60349000	5.95E-07
chr17	60345001	60350000	1.54E-08
chr17	60346001	60351000	5.12E-08
chr17	67628001	67633000	0.002057177
chr17	68137001	68142000	0.000234622
chr17	68544001	68549000	1.50E-08
chr17	68545001	68550000	5.34E-08
chr17	69530001	69535000	0.001069786
chr17	70055001	70060000	1.07E-09
chr17	72334001	72339000	0.000109233
chr17	73286001	73291000	1.33E-52
chr17	73607001	73612000	8.68E-22
chr17	73608001	73613000	9.73E-23
chr17	73769001	73774000	1.74E-16
chr17	74074001	74079000	7.71E-11
chr17	74129001	74134000	8.54E-11
chr17	74130001	74135000	1.16E-15

chr17	74131001	74136000	1.59E-16
chr17	74402001	74407000	2.43E-13
chr17	74727001	74732000	2.28E-26
chr17	74892001	74897000	5.57E-15
chr17	74893001	74898000	1.53E-15
chr17	74933001	74938000	4.37E-21
chr17	75070001	75075000	1.63E-17
chr17	75071001	75076000	9.34E-20
chr17	75072001	75077000	7.44E-17
chr17	75143001	75148000	5.58E-19
chr17	75259001	75264000	3.26E-08
chr17	75317001	75322000	1.10E-25
chr17	75319001	75324000	1.33E-24
chr17	75320001	75325000	1.84E-43
chr17	75321001	75326000	4.75E-36
chr17	75324001	75329000	3.62E-16
chr17	75876001	75881000	1.46E-07
chr17	75877001	75882000	5.14E-07
chr17	76121001	76126000	7.81E-17
chr17	76378001	76383000	5.07E-17
chr17	76379001	76384000	2.24E-17
chr17	76380001	76385000	2.28E-16
chr17	76849001	76854000	3.22E-13
chr17	76934001	76939000	1.53E-06
chr17	76935001	76940000	6.51E-07
chr17	76936001	76941000	2.08E-07
chr17	76967001	76972000	2.73E-09
chr17	76969001	76974000	3.10E-06
chr17	76970001	76975000	2.04E-09
chr17	77099001	77104000	1.81E-11
chr17	77100001	77105000	4.14E-13
chr17	77101001	77106000	7.51E-11
chr17	77103001	77108000	4.72E-16
chr17	77142001	77147000	3.48E-08
chr17	77143001	77148000	2.98E-07
chr17	77144001	77149000	9.48E-12
chr17	77808001	77813000	5.74E-28
chr17	77886001	77891000	4.12E-17
chr17	78162001	78167000	9.52E-27
chr17	78163001	78168000	9.31E-26
chr17	78164001	78169000	1.67E-24
chr17	78165001	78170000	9.32E-25
chr17	78228001	78233000	7.02E-11
chr17	78972001	78977000	1.97E-15
chr17	78973001	78978000	2.29E-21
chr17	79038001	79043000	4.98E-12
chr17	79393001	79398000	3.78E-10
chr17	79398001	79403000	2.17E-09

chr17	79503001	79508000	4.59E-13
chr17	79504001	79509000	5.70E-08
chr17	79530001	79535000	1.77E-12
chr17	79531001	79536000	2.19E-11
chr17	79532001	79537000	6.53E-13
chr17	79533001	79538000	6.69E-14
chr17	79687001	79692000	1.25E-10
chr17	80088001	80093000	3.17E-10
chr17	80192001	80197000	5.76E-09
chr17	80193001	80198000	3.93E-10
chr17	80569001	80574000	2.98E-32
chr17	80570001	80575000	1.07E-31
chr17	80571001	80576000	3.65E-31
chr17	80699001	80704000	1.91E-21
chr17	80702001	80707000	1.56E-21
chr17	80795001	80800000	2.28E-19
chr17	80834001	80839000	6.22E-53
chr17	80916001	80921000	2.12E-06
chr17	80917001	80922000	9.66E-06
chr18	962001	967000	8.04E-08
chr18	1232001	1237000	7.89E-07
chr18	1234001	1239000	2.05E-05
chr18	1235001	1240000	6.69E-05
chr18	1264001	1269000	0.002072457
chr18	1265001	1270000	1.44E-07
chr18	1266001	1271000	8.46E-08
chr18	1279001	1284000	4.49E-05
chr18	1280001	1285000	4.96E-05
chr18	1281001	1286000	1.17E-07
chr18	1282001	1287000	3.12E-09
chr18	1283001	1288000	3.99E-09
chr18	1491001	1496000	3.42E-08
chr18	1582001	1587000	0.001413215
chr18	1706001	1711000	0.000121483
chr18	3277001	3282000	2.37E-06
chr18	3453001	3458000	4.81E-16
chr18	3603001	3608000	1.13E-19
chr18	3604001	3609000	2.22E-23
chr18	3605001	3610000	8.47E-22
chr18	4714001	4719000	3.86E-05
chr18	4760001	4765000	8.92E-06
chr18	4832001	4837000	1.06E-13
chr18	4833001	4838000	3.90E-14
chr18	4834001	4839000	1.31E-13
chr18	4925001	4930000	3.71E-08
chr18	4926001	4931000	1.03E-07
chr18	4927001	4932000	1.14E-07
chr18	4988001	4993000	0.011242179

chr18	6717001	6722000	9.46E-05
chr18	6749001	6754000	0.001364397
chr18	6769001	6774000	5.78E-10
chr18	6770001	6775000	1.12E-12
chr18	8071001	8076000	4.60E-05
chr18	9113001	9118000	1.94E-09
chr18	9114001	9119000	6.15E-09
chr18	9115001	9120000	4.09E-09
chr18	9181001	9186000	4.37E-06
chr18	9260001	9265000	1.11E-10
chr18	9326001	9331000	1.97E-06
chr18	9327001	9332000	2.13E-06
chr18	9958001	9963000	7.07E-15
chr18	9959001	9964000	5.84E-15
chr18	9960001	9965000	4.37E-14
chr18	10232001	10237000	2.34E-13
chr18	14576001	14581000	7.47E-06
chr18	14577001	14582000	1.62E-09
chr18	15267001	15272000	0.001144906
chr18	15316001	15321000	3.56E-08
chr18	15320001	15325000	2.64E-05
chr18	18693001	18698000	9.45E-09
chr18	18747001	18752000	2.76E-06
chr18	18773001	18778000	1.95E-07
chr18	18793001	18798000	2.54E-09
chr18	18794001	18799000	6.37E-09
chr18	18795001	18800000	8.97E-07
chr18	18796001	18801000	2.23E-06
chr18	18836001	18841000	0.000458708
chr18	18837001	18842000	0.000212992
chr18	18859001	18864000	1.46E-05
chr18	18860001	18865000	4.04E-07
chr18	18861001	18866000	9.78E-08
chr18	18862001	18867000	2.09E-06
chr18	18868001	18873000	4.20E-08
chr18	18869001	18874000	1.80E-07
chr18	18870001	18875000	3.70E-07
chr18	18886001	18891000	1.06E-07
chr18	18911001	18916000	2.04E-08
chr18	18920001	18925000	1.14E-05
chr18	18921001	18926000	1.11E-05
chr18	18922001	18927000	9.69E-08
chr18	18931001	18936000	5.82E-11
chr18	18938001	18943000	1.32E-10
chr18	18978001	18983000	3.83E-09
chr18	18998001	19003000	2.59E-08
chr18	18999001	19004000	4.93E-08
chr18	19000001	19005000	1.16E-07

chr18	19013001	19018000	2.65E-08
chr18	20811001	20816000	1.03E-21
chr18	22457001	22462000	5.10E-07
chr18	22459001	22464000	7.71E-10
chr18	22581001	22586000	0.000180942
chr18	22700001	22705000	2.11E-05
chr18	22725001	22730000	1.50E-06
chr18	22726001	22731000	2.29E-05
chr18	22727001	22732000	5.81E-06
chr18	24547001	24552000	5.48E-08
chr18	24584001	24589000	8.22E-06
chr18	24660001	24665000	0.000935531
chr18	24664001	24669000	0.000191674
chr18	24745001	24750000	1.06E-07
chr18	24774001	24779000	0.009735053
chr18	26748001	26753000	9.41E-09
chr18	26821001	26826000	5.69E-05
chr18	27171001	27176000	0.002024114
chr18	27172001	27177000	0.001636851
chr18	28853001	28858000	3.08E-07
chr18	28854001	28859000	7.61E-07
chr18	29959001	29964000	5.13E-08
chr18	29960001	29965000	2.40E-07
chr18	30341001	30346000	0.000726017
chr18	30651001	30656000	9.45E-05
chr18	30762001	30767000	3.27E-06
chr18	30821001	30826000	1.52E-05
chr18	30915001	30920000	1.88E-05
chr18	31393001	31398000	9.91E-05
chr18	31443001	31448000	0.002050397
chr18	31444001	31449000	0.000241131
chr18	31479001	31484000	1.29E-05
chr18	31528001	31533000	2.18E-05
chr18	31529001	31534000	4.01E-06
chr18	31695001	31700000	5.25E-10
chr18	31755001	31760000	0.000164922
chr18	31936001	31941000	0.000431652
chr18	31937001	31942000	3.04E-05
chr18	31938001	31943000	3.27E-07
chr18	31939001	31944000	1.74E-06
chr18	31995001	32000000	4.93E-10
chr18	32087001	32092000	1.37E-05
chr18	32223001	32228000	7.79E-10
chr18	32224001	32229000	3.89E-14
chr18	32225001	32230000	3.44E-14
chr18	32226001	32231000	1.70E-16
chr18	32365001	32370000	8.87E-07
chr18	32440001	32445000	3.49E-14

chr18	32650001	32655000	6.20E-09
chr18	33974001	33979000	8.74E-05
chr18	34043001	34048000	1.35E-07
chr18	34051001	34056000	8.03E-09
chr18	34270001	34275000	1.14E-15
chr18	34271001	34276000	4.43E-14
chr18	34272001	34277000	9.30E-15
chr18	34884001	34889000	0.001008475
chr18	34918001	34923000	1.22E-05
chr18	34958001	34963000	0.010489788
chr18	34959001	34964000	0.000208507
chr18	34960001	34965000	0.000306563
chr18	34961001	34966000	3.84E-05
chr18	34962001	34967000	2.69E-11
chr18	34963001	34968000	1.76E-06
chr18	35071001	35076000	7.01E-07
chr18	35072001	35077000	3.02E-08
chr18	35136001	35141000	8.03E-08
chr18	35137001	35142000	4.20E-11
chr18	35327001	35332000	5.68E-11
chr18	35328001	35333000	8.44E-11
chr18	35463001	35468000	2.61E-06
chr18	35464001	35469000	1.00E-07
chr18	35465001	35470000	1.45E-12
chr18	35466001	35471000	3.76E-10
chr18	35467001	35472000	6.55E-12
chr18	35468001	35473000	7.75E-07
chr18	35530001	35535000	0.000221452
chr18	35544001	35549000	2.88E-06
chr18	35590001	35595000	3.30E-07
chr18	35706001	35711000	0.002503973
chr18	35715001	35720000	4.31E-08
chr18	35718001	35723000	8.09E-07
chr18	35976001	35981000	0.005175927
chr18	36162001	36167000	0.001585872
chr18	36355001	36360000	0.002384464
chr18	36356001	36361000	0.000883584
chr18	36387001	36392000	0.001924602
chr18	36536001	36541000	0.001810042
chr18	36788001	36793000	2.49E-05
chr18	37164001	37169000	7.49E-06
chr18	37165001	37170000	5.88E-07
chr18	37166001	37171000	1.10E-06
chr18	37351001	37356000	0.000141811
chr18	37443001	37448000	4.69E-05
chr18	37708001	37713000	0.00259231
chr18	37958001	37963000	0.000493534
chr18	38035001	38040000	0.006568509

chr18	38244001	38249000	5.01E-05
chr18	38264001	38269000	0.000734381
chr18	38335001	38340000	0.001706404
chr18	38524001	38529000	2.35E-07
chr18	38558001	38563000	0.00044882
chr18	38629001	38634000	9.01E-06
chr18	38630001	38635000	1.33E-06
chr18	38631001	38636000	2.65E-05
chr18	38632001	38637000	8.35E-05
chr18	38778001	38783000	4.82E-06
chr18	38779001	38784000	5.71E-05
chr18	38830001	38835000	7.72E-05
chr18	38831001	38836000	7.25E-05
chr18	38917001	38922000	0.000792555
chr18	39021001	39026000	0.001375893
chr18	39085001	39090000	1.33E-07
chr18	39134001	39139000	1.98E-05
chr18	39135001	39140000	1.42E-05
chr18	39136001	39141000	4.44E-06
chr18	39238001	39243000	4.82E-09
chr18	39350001	39355000	1.52E-10
chr18	39381001	39386000	0.000599865
chr18	39430001	39435000	0.000277439
chr18	39523001	39528000	8.41E-06
chr18	39673001	39678000	0.008248544
chr18	39675001	39680000	0.000143094
chr18	39781001	39786000	3.81E-05
chr18	39808001	39813000	1.13E-07
chr18	39949001	39954000	2.84E-05
chr18	39993001	39998000	1.05E-14
chr18	39994001	39999000	2.25E-13
chr18	39995001	40000000	3.24E-13
chr18	39996001	40001000	6.26E-13
chr18	39997001	40002000	1.57E-10
chr18	39998001	40003000	8.75E-06
chr18	40037001	40042000	0.000102532
chr18	40070001	40075000	3.73E-05
chr18	40430001	40435000	0.000126466
chr18	40431001	40436000	0.000126871
chr18	40529001	40534000	3.87E-05
chr18	40530001	40535000	1.39E-05
chr18	40618001	40623000	1.47E-05
chr18	40704001	40709000	3.55E-06
chr18	40725001	40730000	1.13E-08
chr18	40726001	40731000	1.20E-07
chr18	40733001	40738000	8.34E-06
chr18	40793001	40798000	1.66E-09
chr18	40794001	40799000	6.21E-10

chr18	40806001	40811000	9.56E-07
chr18	40910001	40915000	1.18E-08
chr18	40911001	40916000	2.36E-07
chr18	40912001	40917000	8.78E-06
chr18	40919001	40924000	8.04E-06
chr18	40921001	40926000	1.00E-09
chr18	40936001	40941000	0.005871369
chr18	40981001	40986000	0.000130449
chr18	40982001	40987000	6.67E-05
chr18	40983001	40988000	5.13E-06
chr18	40998001	41003000	3.26E-05
chr18	41037001	41042000	0.002984557
chr18	41144001	41149000	0.000645986
chr18	41145001	41150000	4.49E-05
chr18	41290001	41295000	0.001064418
chr18	41291001	41296000	0.001277127
chr18	41292001	41297000	0.000117052
chr18	41551001	41556000	0.000670268
chr18	41588001	41593000	0.000172323
chr18	41589001	41594000	0.000359774
chr18	41590001	41595000	0.000665178
chr18	41611001	41616000	9.61E-08
chr18	42545001	42550000	2.10E-07
chr18	42710001	42715000	2.21E-06
chr18	42776001	42781000	0.000195552
chr18	42781001	42786000	8.15E-07
chr18	42782001	42787000	1.34E-07
chr18	42960001	42965000	4.82E-05
chr18	43034001	43039000	1.51E-06
chr18	43306001	43311000	1.04E-08
chr18	43307001	43312000	1.12E-12
chr18	44255001	44260000	5.80E-09
chr18	44256001	44261000	1.60E-10
chr18	44257001	44262000	6.15E-05
chr18	44258001	44263000	9.80E-08
chr18	44259001	44264000	1.40E-09
chr18	44870001	44875000	7.99E-06
chr18	45089001	45094000	4.72E-05
chr18	45090001	45095000	3.20E-06
chr18	45091001	45096000	6.49E-06
chr18	45509001	45514000	0.001786178
chr18	46158001	46163000	8.28E-06
chr18	46160001	46165000	1.44E-05
chr18	46469001	46474000	1.69E-10
chr18	46470001	46475000	9.73E-11
chr18	48147001	48152000	1.17E-09
chr18	49161001	49166000	0.002456832
chr18	49162001	49167000	0.000102812

chr18	49164001	49169000	0.001173368
chr18	49430001	49435000	3.47E-05
chr18	49431001	49436000	4.53E-05
chr18	49432001	49437000	9.09E-05
chr18	49433001	49438000	5.34E-05
chr18	49481001	49486000	0.000144703
chr18	49497001	49502000	0.002114652
chr18	49536001	49541000	6.07E-05
chr18	49537001	49542000	0.000439422
chr18	49602001	49607000	0.000292645
chr18	49609001	49614000	2.61E-06
chr18	49610001	49615000	0.000212513
chr18	49611001	49616000	0.001364083
chr18	49612001	49617000	4.26E-05
chr18	49626001	49631000	5.46E-05
chr18	49660001	49665000	0.000125021
chr18	49668001	49673000	0.000132166
chr18	49670001	49675000	0.000385621
chr18	49748001	49753000	2.87E-05
chr18	49749001	49754000	0.000159005
chr18	49813001	49818000	0.005503268
chr18	49871001	49876000	0.000139147
chr18	49872001	49877000	0.001440103
chr18	49873001	49878000	0.000711635
chr18	49874001	49879000	0.000170978
chr18	49950001	49955000	7.95E-10
chr18	49951001	49956000	1.42E-08
chr18	49952001	49957000	2.04E-06
chr18	49953001	49958000	3.91E-07
chr18	50068001	50073000	0.000358781
chr18	50172001	50177000	1.56E-07
chr18	50173001	50178000	8.78E-07
chr18	50174001	50179000	5.97E-06
chr18	50175001	50180000	0.000134128
chr18	50176001	50181000	0.000419455
chr18	50371001	50376000	0.000955657
chr18	50372001	50377000	2.35E-05
chr18	50404001	50409000	0.000164035
chr18	50549001	50554000	2.62E-07
chr18	50550001	50555000	3.76E-09
chr18	50553001	50558000	7.30E-06
chr18	50729001	50734000	0.000648014
chr18	50750001	50755000	2.51E-05
chr18	50751001	50756000	5.14E-05
chr18	50764001	50769000	0.000245732
chr18	50765001	50770000	2.80E-06
chr18	50766001	50771000	2.30E-06
chr18	50767001	50772000	2.15E-07

chr18	50797001	50802000	4.89E-06
chr18	50798001	50803000	1.45E-05
chr18	50848001	50853000	0.000132596
chr18	50849001	50854000	0.000342486
chr18	50898001	50903000	2.20E-11
chr18	50899001	50904000	9.52E-14
chr18	50900001	50905000	1.89E-12
chr18	50901001	50906000	3.88E-11
chr18	51030001	51035000	6.25E-14
chr18	51031001	51036000	4.08E-11
chr18	51032001	51037000	6.43E-11
chr18	51033001	51038000	2.45E-18
chr18	51034001	51039000	1.36E-09
chr18	51058001	51063000	1.67E-08
chr18	51068001	51073000	0.000254245
chr18	51218001	51223000	9.82E-09
chr18	51273001	51278000	0.001852223
chr18	51439001	51444000	6.47E-14
chr18	51440001	51445000	5.82E-14
chr18	51441001	51446000	3.06E-11
chr18	53574001	53579000	0.000139544
chr18	53575001	53580000	0.000912484
chr18	53710001	53715000	4.44E-05
chr18	54238001	54243000	9.79E-12
chr18	54239001	54244000	9.61E-40
chr18	54240001	54245000	7.14E-22
chr18	54241001	54246000	1.17E-06
chr18	54772001	54777000	2.01E-06
chr18	54773001	54778000	3.41E-05
chr18	54832001	54837000	3.11E-06
chr18	54833001	54838000	9.88E-06
chr18	54839001	54844000	4.56E-10
chr18	54856001	54861000	8.89E-05
chr18	54858001	54863000	0.00011852
chr18	54859001	54864000	8.11E-07
chr18	54860001	54865000	6.42E-07
chr18	54861001	54866000	7.46E-07
chr18	54862001	54867000	8.33E-07
chr18	55298001	55303000	5.65E-07
chr18	55837001	55842000	0.000445434
chr18	56159001	56164000	1.42E-09
chr18	56666001	56671000	0.002527728
chr18	56667001	56672000	0.001228608
chr18	56918001	56923000	8.21E-10
chr18	56919001	56924000	5.58E-10
chr18	57098001	57103000	1.32E-10
chr18	57150001	57155000	5.84E-07
chr18	57152001	57157000	6.85E-09

chr18	57380001	57385000	2.53E-06
chr18	57381001	57386000	1.54E-07
chr18	57651001	57656000	5.69E-19
chr18	57652001	57657000	9.13E-16
chr18	57653001	57658000	1.14E-11
chr18	57655001	57660000	0.000210391
chr18	57826001	57831000	4.90E-06
chr18	58289001	58294000	4.11E-08
chr18	58290001	58295000	5.78E-07
chr18	58291001	58296000	0.001145873
chr18	58492001	58497000	4.34E-06
chr18	58493001	58498000	1.92E-06
chr18	58727001	58732000	4.26E-05
chr18	58728001	58733000	0.000227166
chr18	58729001	58734000	5.65E-07
chr18	58730001	58735000	0.000206856
chr18	58766001	58771000	6.69E-07
chr18	58770001	58775000	0.000225509
chr18	58842001	58847000	9.36E-05
chr18	58843001	58848000	1.91E-06
chr18	58844001	58849000	1.78E-08
chr18	59027001	59032000	8.20E-07
chr18	59122001	59127000	0.000272242
chr18	59123001	59128000	0.00038821
chr18	59238001	59243000	6.05E-06
chr18	59617001	59622000	2.08E-10
chr18	59618001	59623000	5.06E-10
chr18	59712001	59717000	4.28E-06
chr18	59713001	59718000	0.000139009
chr18	60343001	60348000	2.25E-05
chr18	61204001	61209000	0.000716708
chr18	61400001	61405000	0.001144425
chr18	61508001	61513000	2.41E-10
chr18	61509001	61514000	2.71E-10
chr18	61510001	61515000	2.22E-11
chr18	61577001	61582000	1.88E-06
chr18	61580001	61585000	0.000194563
chr18	61703001	61708000	1.42E-05
chr18	61704001	61709000	5.31E-05
chr18	61705001	61710000	1.40E-06
chr18	61810001	61815000	1.71E-05
chr18	61811001	61816000	1.03E-06
chr18	61812001	61817000	1.03E-06
chr18	61852001	61857000	0.000213375
chr18	61854001	61859000	9.17E-07
chr18	61923001	61928000	4.83E-06
chr18	61989001	61994000	1.06E-06
chr18	61990001	61995000	0.000147827

chr18	61991001	61996000	0.000195322
chr18	62040001	62045000	2.65E-08
chr18	62041001	62046000	1.11E-06
chr18	62043001	62048000	6.12E-05
chr18	62058001	62063000	8.38E-05
chr18	62059001	62064000	0.000207052
chr18	62103001	62108000	8.47E-05
chr18	62104001	62109000	0.001026116
chr18	62114001	62119000	3.19E-09
chr18	62130001	62135000	6.82E-06
chr18	62200001	62205000	7.03E-05
chr18	62201001	62206000	2.80E-07
chr18	62202001	62207000	1.02E-05
chr18	62203001	62208000	1.41E-05
chr18	62289001	62294000	2.26E-06
chr18	62290001	62295000	1.17E-06
chr18	62316001	62321000	5.66E-12
chr18	62317001	62322000	1.46E-14
chr18	62588001	62593000	4.41E-05
chr18	62589001	62594000	9.93E-07
chr18	62590001	62595000	1.31E-07
chr18	62591001	62596000	1.97E-12
chr18	62593001	62598000	1.00E-11
chr18	62594001	62599000	1.67E-10
chr18	62595001	62600000	1.57E-08
chr18	62610001	62615000	1.93E-07
chr18	62672001	62677000	0.000222938
chr18	62673001	62678000	1.34E-05
chr18	62674001	62679000	6.26E-06
chr18	62675001	62680000	0.000148859
chr18	62707001	62712000	1.27E-14
chr18	62708001	62713000	3.48E-14
chr18	62709001	62714000	9.23E-12
chr18	62710001	62715000	1.47E-10
chr18	62723001	62728000	0.002954861
chr18	62724001	62729000	3.06E-06
chr18	62851001	62856000	1.04E-09
chr18	62852001	62857000	1.02E-11
chr18	62861001	62866000	0.000365374
chr18	62862001	62867000	0.000380368
chr18	62968001	62973000	0.000365458
chr18	62969001	62974000	0.000365458
chr18	62975001	62980000	7.02E-05
chr18	63092001	63097000	0.000184215
chr18	63351001	63356000	1.13E-06
chr18	63892001	63897000	0.000107674
chr18	63915001	63920000	0.001523077
chr18	63916001	63921000	0.003940513

chr18	63939001	63944000	2.94E-06
chr18	63940001	63945000	5.14E-05
chr18	64528001	64533000	0.001033345
chr18	64529001	64534000	1.52E-15
chr18	64530001	64535000	2.48E-13
chr18	64531001	64536000	2.94E-16
chr18	64532001	64537000	2.75E-18
chr18	64896001	64901000	2.44E-06
chr18	64897001	64902000	1.11E-05
chr18	66147001	66152000	2.08E-05
chr18	66148001	66153000	5.11E-05
chr18	66315001	66320000	4.81E-06
chr18	66619001	66624000	1.15E-05
chr18	66620001	66625000	4.49E-05
chr18	66621001	66626000	4.47E-05
chr18	67369001	67374000	1.83E-06
chr18	67385001	67390000	1.29E-05
chr18	68127001	68132000	3.25E-11
chr18	68130001	68135000	4.03E-07
chr18	68132001	68137000	0.000104942
chr18	68158001	68163000	1.33E-07
chr18	68159001	68164000	5.70E-08
chr18	68160001	68165000	4.62E-10
chr18	68162001	68167000	8.32E-18
chr18	68164001	68169000	8.06E-24
chr18	68165001	68170000	2.11E-19
chr18	68166001	68171000	9.69E-20
chr18	68168001	68173000	5.85E-08
chr18	68169001	68174000	1.10E-05
chr18	68196001	68201000	4.57E-06
chr18	68260001	68265000	1.64E-06
chr18	68280001	68285000	5.02E-05
chr18	68326001	68331000	4.46E-08
chr18	68448001	68453000	2.03E-09
chr18	68449001	68454000	5.99E-08
chr18	68516001	68521000	6.79E-06
chr18	68526001	68531000	1.02E-05
chr18	68527001	68532000	0.000188798
chr18	68700001	68705000	1.00E-06
chr18	68701001	68706000	3.60E-07
chr18	68900001	68905000	1.96E-10
chr18	68901001	68906000	7.98E-10
chr18	69057001	69062000	1.10E-05
chr18	69168001	69173000	0.000132975
chr18	69297001	69302000	9.78E-08
chr18	69335001	69340000	7.45E-05
chr18	69355001	69360000	7.44E-05
chr18	69491001	69496000	0.000871542

chr18	69492001	69497000	0.003095848
chr18	69662001	69667000	2.41E-05
chr18	70020001	70025000	5.17E-06
chr18	70192001	70197000	5.17E-06
chr18	70193001	70198000	4.74E-07
chr18	70194001	70199000	3.41E-05
chr18	70798001	70803000	1.45E-07
chr18	70862001	70867000	8.83E-06
chr18	70972001	70977000	1.33E-12
chr18	70974001	70979000	7.19E-15
chr18	70975001	70980000	5.35E-15
chr18	71063001	71068000	1.18E-05
chr18	71346001	71351000	4.70E-07
chr18	71359001	71364000	3.47E-07
chr18	71661001	71666000	0.000702953
chr18	71662001	71667000	0.004624539
chr18	73113001	73118000	1.28E-11
chr18	73946001	73951000	1.02E-07
chr18	73949001	73954000	3.43E-06
chr18	74419001	74424000	1.04E-07
chr18	74708001	74713000	7.40E-18
chr18	74709001	74714000	1.49E-13
chr18	76323001	76328000	1.42E-07
chr18	76397001	76402000	2.79E-14
chr18	76398001	76403000	5.69E-15
chr18	76547001	76552000	1.38E-06
chr18	77548001	77553000	3.70E-13
chr18	77549001	77554000	2.41E-11
chr18	77716001	77721000	5.28E-16
chr18	77719001	77724000	5.74E-22
chr18	77720001	77725000	2.27E-15
chr18	77721001	77726000	3.32E-11
chr19	403001	408000	6.83E-11
chr19	404001	409000	1.41E-08
chr19	455001	460000	1.60E-11
chr19	510001	515000	5.87E-06
chr19	511001	516000	6.65E-09
chr19	512001	517000	3.58E-06
chr19	513001	518000	6.12E-07
chr19	553001	558000	6.68E-09
chr19	736001	741000	4.30E-08
chr19	1062001	1067000	6.50E-13
chr19	1256001	1261000	7.40E-09
chr19	1647001	1652000	1.81E-12
chr19	1813001	1818000	1.24E-11
chr19	2409001	2414000	5.31E-06
chr19	2410001	2415000	8.13E-06
chr19	2411001	2416000	1.49E-08

chr19	2417001	2422000	1.48E-06
chr19	2418001	2423000	2.00E-07
chr19	2419001	2424000	1.57E-07
chr19	2498001	2503000	1.85E-06
chr19	2506001	2511000	2.06E-14
chr19	2507001	2512000	1.85E-14
chr19	2508001	2513000	2.05E-08
chr19	2757001	2762000	2.13E-10
chr19	2758001	2763000	3.19E-11
chr19	2960001	2965000	4.00E-10
chr19	2961001	2966000	2.96E-08
chr19	2962001	2967000	2.13E-08
chr19	3039001	3044000	5.91E-06
chr19	3040001	3045000	1.40E-07
chr19	3041001	3046000	2.32E-07
chr19	3100001	3105000	2.27E-14
chr19	3101001	3106000	7.02E-16
chr19	3102001	3107000	1.03E-16
chr19	3103001	3108000	3.09E-16
chr19	3436001	3441000	1.98E-24
chr19	3808001	3813000	6.00E-07
chr19	4231001	4236000	1.94E-08
chr19	4452001	4457000	8.93E-15
chr19	4453001	4458000	5.60E-09
chr19	4880001	4885000	0.000658461
chr19	4917001	4922000	0.000237279
chr19	5752001	5757000	1.50E-08
chr19	5821001	5826000	7.75E-06
chr19	5822001	5827000	2.64E-07
chr19	5863001	5868000	0.000610822
chr19	5864001	5869000	0.002082736
chr19	6061001	6066000	2.81E-13
chr19	6062001	6067000	1.07E-15
chr19	6063001	6068000	4.09E-19
chr19	6267001	6272000	0.000123451
chr19	6349001	6354000	9.82E-06
chr19	6425001	6430000	1.83E-18
chr19	6975001	6980000	0.00017941
chr19	7062001	7067000	0.000168384
chr19	7063001	7068000	3.60E-05
chr19	7310001	7315000	3.40E-10
chr19	7311001	7316000	2.28E-07
chr19	7312001	7317000	1.18E-07
chr19	7313001	7318000	1.07E-10
chr19	7314001	7319000	1.81E-13
chr19	7468001	7473000	8.21E-07
chr19	7604001	7609000	3.50E-14
chr19	7615001	7620000	1.18E-27

chr19	7616001	7621000	1.26E-47
chr19	7617001	7622000	1.81E-64
chr19	7618001	7623000	1.45E-58
chr19	7619001	7624000	2.75E-47
chr19	7620001	7625000	1.85E-20
chr19	7696001	7701000	0.002793997
chr19	7720001	7725000	2.85E-10
chr19	7721001	7726000	1.33E-11
chr19	7722001	7727000	2.96E-12
chr19	7723001	7728000	2.56E-08
chr19	8249001	8254000	3.61E-05
chr19	8250001	8255000	3.94E-05
chr19	8251001	8256000	6.21E-05
chr19	8287001	8292000	6.64E-14
chr19	8288001	8293000	8.78E-14
chr19	8497001	8502000	1.92E-05
chr19	8524001	8529000	4.02E-15
chr19	9633001	9638000	0.00151974
chr19	9798001	9803000	0.002477624
chr19	10393001	10398000	6.69E-14
chr19	10394001	10399000	3.99E-18
chr19	10508001	10513000	1.53E-17
chr19	10949001	10954000	5.02E-05
chr19	11055001	11060000	2.71E-10
chr19	11056001	11061000	5.22E-11
chr19	11057001	11062000	3.13E-09
chr19	11066001	11071000	4.78E-05
chr19	11073001	11078000	5.11E-12
chr19	11183001	11188000	1.09E-13
chr19	11350001	11355000	8.98E-12
chr19	11352001	11357000	2.16E-12
chr19	11466001	11471000	3.95E-08
chr19	11516001	11521000	1.15E-07
chr19	11537001	11542000	5.76E-08
chr19	11593001	11598000	0.001667518
chr19	11594001	11599000	1.63E-09
chr19	11595001	11600000	3.90E-08
chr19	12421001	12426000	5.84E-09
chr19	12660001	12665000	3.93E-15
chr19	12973001	12978000	4.20E-07
chr19	12974001	12979000	1.66E-22
chr19	12975001	12980000	2.16E-21
chr19	13057001	13062000	3.98E-14
chr19	13126001	13131000	6.28E-08
chr19	13129001	13134000	2.95E-11
chr19	13172001	13177000	1.03E-09
chr19	13320001	13325000	0.007386722
chr19	13321001	13326000	0.006959773

chr19	13322001	13327000	0.001621042
chr19	13323001	13328000	0.002099093
chr19	13324001	13329000	0.005043453
chr19	13338001	13343000	1.91E-08
chr19	13371001	13376000	4.54E-08
chr19	13407001	13412000	1.31E-20
chr19	13408001	13413000	8.23E-22
chr19	13409001	13414000	8.70E-25
chr19	13483001	13488000	0.000237345
chr19	13484001	13489000	0.002509951
chr19	13505001	13510000	1.39E-07
chr19	13573001	13578000	1.45E-07
chr19	13574001	13579000	5.26E-06
chr19	13777001	13782000	2.13E-07
chr19	13778001	13783000	1.03E-08
chr19	13779001	13784000	7.44E-08
chr19	13814001	13819000	6.03E-06
chr19	13815001	13820000	7.17E-06
chr19	13910001	13915000	0.000161574
chr19	13991001	13996000	3.11E-06
chr19	13992001	13997000	0.000320483
chr19	14124001	14129000	5.51E-08
chr19	14128001	14133000	2.59E-05
chr19	14129001	14134000	0.001858245
chr19	14192001	14197000	2.87E-09
chr19	14272001	14277000	2.20E-08
chr19	14783001	14788000	4.08E-09
chr19	16188001	16193000	3.79E-14
chr19	16390001	16395000	4.64E-15
chr19	16692001	16697000	5.45E-07
chr19	16693001	16698000	4.43E-08
chr19	16694001	16699000	1.04E-07
chr19	17321001	17326000	0.0004727
chr19	17433001	17438000	3.26E-08
chr19	17434001	17439000	2.94E-07
chr19	17435001	17440000	2.18E-12
chr19	17898001	17903000	8.37E-08
chr19	17899001	17904000	3.14E-08
chr19	18305001	18310000	3.35E-10
chr19	18351001	18356000	3.66E-08
chr19	18364001	18369000	3.85E-19
chr19	18365001	18370000	1.19E-14
chr19	18453001	18458000	0.000371965
chr19	18454001	18459000	0.000191067
chr19	18609001	18614000	2.15E-10
chr19	18611001	18616000	1.47E-11
chr19	18869001	18874000	0.001684576
chr19	18947001	18952000	2.72E-13

chr19	19304001	19309000	3.21E-11
chr19	19305001	19310000	2.64E-11
chr19	19383001	19388000	4.10E-15
chr19	19384001	19389000	1.17E-09
chr19	19619001	19624000	2.32E-15
chr19	19620001	19625000	1.77E-17
chr19	19711001	19716000	4.93E-05
chr19	19712001	19717000	6.53E-05
chr19	19947001	19952000	0.000769713
chr19	20691001	20696000	7.57E-06
chr19	20855001	20860000	0.025437117
chr19	21406001	21411000	0.000318403
chr19	21513001	21518000	0.00026017
chr19	21683001	21688000	1.60E-10
chr19	21916001	21921000	0.013314632
chr19	21918001	21923000	2.40E-07
chr19	22883001	22888000	2.99E-05
chr19	23106001	23111000	3.35E-07
chr19	23107001	23112000	1.53E-07
chr19	23556001	23561000	6.06E-05
chr19	23864001	23869000	8.94E-06
chr19	23897001	23902000	0.002027958
chr19	24311001	24316000	0.000179879
chr19	24471001	24476000	0.000292951
chr19	27749001	27754000	0.000442382
chr19	28847001	28852000	0.000318767
chr19	28848001	28853000	8.60E-05
chr19	29447001	29452000	0.001021617
chr19	30157001	30162000	3.68E-21
chr19	30386001	30391000	3.03E-06
chr19	30387001	30392000	1.83E-06
chr19	30388001	30393000	4.08E-06
chr19	33557001	33562000	0.001220234
chr19	33646001	33651000	8.29E-05
chr19	33648001	33653000	7.98E-05
chr19	33859001	33864000	8.75E-23
chr19	34732001	34737000	1.36E-07
chr19	34757001	34762000	1.64E-14
chr19	35588001	35593000	9.22E-08
chr19	35695001	35700000	6.59E-09
chr19	35741001	35746000	3.76E-11
chr19	37421001	37426000	3.41E-07
chr19	37904001	37909000	1.18E-05
chr19	37905001	37910000	7.04E-08
chr19	38390001	38395000	4.62E-08
chr19	38700001	38705000	3.24E-08
chr19	38701001	38706000	1.23E-10
chr19	38702001	38707000	1.24E-10

chr19	38703001	38708000	1.04E-08
chr19	38704001	38709000	1.66E-09
chr19	38803001	38808000	1.08E-08
chr19	39286001	39291000	1.95E-18
chr19	39287001	39292000	3.22E-17
chr19	39288001	39293000	4.09E-17
chr19	39289001	39294000	8.80E-19
chr19	39290001	39295000	7.17E-15
chr19	39352001	39357000	4.70E-17
chr19	39353001	39358000	2.27E-19
chr19	39354001	39359000	2.26E-14
chr19	39442001	39447000	0.00318192
chr19	39484001	39489000	0.004960705
chr19	39485001	39490000	5.21E-06
chr19	40031001	40036000	1.27E-11
chr19	40347001	40352000	3.21E-07
chr19	40348001	40353000	6.04E-08
chr19	40609001	40614000	2.47E-05
chr19	40610001	40615000	1.40E-06
chr19	40716001	40721000	5.32E-09
chr19	40717001	40722000	2.08E-11
chr19	41117001	41122000	5.38E-21
chr19	41289001	41294000	0.000305657
chr19	41306001	41311000	0.009432975
chr19	41308001	41313000	0.000104406
chr19	41325001	41330000	1.05E-05
chr19	41468001	41473000	8.33E-08
chr19	41469001	41474000	3.98E-07
chr19	41904001	41909000	3.17E-08
chr19	42375001	42380000	7.70E-13
chr19	42465001	42470000	0.00015007
chr19	42466001	42471000	3.95E-07
chr19	42467001	42472000	2.17E-11
chr19	42468001	42473000	3.65E-12
chr19	42477001	42482000	6.76E-16
chr19	42478001	42483000	7.83E-22
chr19	42480001	42485000	3.39E-10
chr19	42507001	42512000	3.42E-09
chr19	42537001	42542000	8.56E-12
chr19	42538001	42543000	7.85E-10
chr19	42566001	42571000	3.25E-11
chr19	42590001	42595000	0.001392192
chr19	42591001	42596000	0.000221127
chr19	42693001	42698000	0.00015569
chr19	42696001	42701000	3.61E-06
chr19	43343001	43348000	9.36E-05
chr19	43356001	43361000	1.99E-05
chr19	43613001	43618000	3.67E-07

chr19	43704001	43709000	0.000431239
chr19	43730001	43735000	7.16E-05
chr19	43731001	43736000	0.000141705
chr19	43732001	43737000	0.002453707
chr19	43752001	43757000	1.20E-13
chr19	43765001	43770000	4.71E-14
chr19	43767001	43772000	2.28E-11
chr19	43768001	43773000	9.76E-13
chr19	43769001	43774000	1.33E-12
chr19	43791001	43796000	3.70E-06
chr19	43792001	43797000	1.75E-06
chr19	43799001	43804000	0.009033187
chr19	43808001	43813000	5.52E-07
chr19	43846001	43851000	1.10E-11
chr19	43847001	43852000	2.63E-11
chr19	43874001	43879000	9.15E-08
chr19	43875001	43880000	8.57E-08
chr19	44519001	44524000	0.000101405
chr19	44520001	44525000	0.000739011
chr19	44612001	44617000	1.87E-15
chr19	45104001	45109000	2.73E-09
chr19	45105001	45110000	5.23E-14
chr19	45106001	45111000	1.82E-09
chr19	45107001	45112000	5.33E-09
chr19	45257001	45262000	1.47E-09
chr19	45258001	45263000	9.60E-07
chr19	45515001	45520000	1.74E-15
chr19	45516001	45521000	1.91E-12
chr19	45517001	45522000	5.96E-12
chr19	45518001	45523000	1.96E-10
chr19	45519001	45524000	1.27E-09
chr19	45765001	45770000	0.00041953
chr19	45797001	45802000	0.000194506
chr19	45798001	45803000	4.69E-05
chr19	45806001	45811000	1.85E-08
chr19	45830001	45835000	8.06E-05
chr19	46036001	46041000	5.65E-06
chr19	46148001	46153000	8.83E-11
chr19	46215001	46220000	4.00E-16
chr19	46276001	46281000	1.27E-07
chr19	46299001	46304000	1.98E-05
chr19	46300001	46305000	1.03E-06
chr19	46319001	46324000	9.69E-10
chr19	46532001	46537000	0.00120778
chr19	46533001	46538000	0.000264258
chr19	46534001	46539000	2.47E-06
chr19	46933001	46938000	5.88E-07
chr19	46965001	46970000	1.13E-09

chr19	46966001	46971000	2.06E-19
chr19	46967001	46972000	9.51E-21
chr19	46968001	46973000	2.49E-15
chr19	46969001	46974000	1.97E-09
chr19	47367001	47372000	8.02E-08
chr19	47368001	47373000	2.07E-09
chr19	47538001	47543000	1.02E-21
chr19	47539001	47544000	2.61E-20
chr19	47540001	47545000	0.000116488
chr19	47621001	47626000	1.96E-14
chr19	47655001	47660000	4.46E-15
chr19	47852001	47857000	6.49E-12
chr19	47853001	47858000	6.16E-23
chr19	47883001	47888000	1.20E-09
chr19	48249001	48254000	1.36E-09
chr19	48257001	48262000	3.91E-09
chr19	48258001	48263000	7.62E-11
chr19	48674001	48679000	1.14E-10
chr19	49117001	49122000	4.12E-26
chr19	49179001	49184000	1.06E-09
chr19	49237001	49242000	2.31E-25
chr19	49309001	49314000	7.53E-08
chr19	49369001	49374000	6.63E-15
chr19	49370001	49375000	1.42E-13
chr19	49381001	49386000	1.64E-27
chr19	49382001	49387000	7.50E-24
chr19	49383001	49388000	3.41E-23
chr19	49384001	49389000	1.11E-19
chr19	49426001	49431000	7.50E-07
chr19	49546001	49551000	1.56E-13
chr19	49710001	49715000	1.31E-12
chr19	49711001	49716000	4.81E-14
chr19	49968001	49973000	1.22E-32
chr19	49969001	49974000	6.55E-28
chr19	49970001	49975000	1.01E-16
chr19	49971001	49976000	1.95E-14
chr19	50000001	50005000	7.82E-10
chr19	50053001	50058000	1.63E-21
chr19	50054001	50059000	6.35E-23
chr19	50055001	50060000	1.53E-37
chr19	50365001	50370000	1.52E-26
chr19	50403001	50408000	3.43E-09
chr19	50404001	50409000	1.25E-10
chr19	50405001	50410000	1.24E-08
chr19	50990001	50995000	2.32E-07
chr19	51020001	51025000	1.05E-17
chr19	51527001	51532000	2.62E-11
chr19	51528001	51533000	1.85E-12

chr19	52739001	52744000	3.18E-09
chr19	52740001	52745000	5.87E-09
chr19	52758001	52763000	2.13E-13
chr19	55651001	55656000	2.11E-12
chr19	56003001	56008000	4.51E-07
chr19	56045001	56050000	0.000292282
chr19	56167001	56172000	2.19E-23
chr19	56613001	56618000	3.61E-16
chr19	56614001	56619000	1.66E-16
chr19	56615001	56620000	4.62E-20
chr19	56673001	56678000	9.36E-05
chr19	56674001	56679000	1.19E-05
chr19	56841001	56846000	1.50E-05
chr19	56933001	56938000	0.000577214
chr19	57126001	57131000	1.60E-12
chr19	57127001	57132000	3.10E-11
chr19	57129001	57134000	7.50E-18
chr19	57130001	57135000	2.21E-17
chr19	57133001	57138000	4.61E-12
chr19	57134001	57139000	2.52E-13
chr19	57135001	57140000	2.74E-11
chr19	57136001	57141000	2.36E-09
chr19	57844001	57849000	7.57E-19
chr19	57845001	57850000	3.89E-16
chr19	58151001	58156000	1.58E-08
chr19	58152001	58157000	8.20E-09
chr19	58153001	58158000	6.66E-05
chr19	58154001	58159000	1.24E-06
chr19	58155001	58160000	1.32E-09
chr19	58879001	58884000	1.67E-22
chr19	58880001	58885000	1.59E-16
chr19	58972001	58977000	2.55E-11
chr19	58973001	58978000	2.66E-12
chr20	796001	801000	6.72E-05
chr20	1778001	1783000	0.000210131
chr20	2196001	2201000	0.004372524
chr20	2276001	2281000	8.87E-16
chr20	2277001	2282000	6.17E-13
chr20	2278001	2283000	1.64E-14
chr20	2280001	2285000	1.15E-06
chr20	2313001	2318000	2.92E-10
chr20	3057001	3062000	3.08E-06
chr20	3668001	3673000	5.03E-08
chr20	3855001	3860000	3.64E-08
chr20	3857001	3862000	2.06E-09
chr20	3858001	3863000	1.49E-08
chr20	4439001	4444000	3.19E-05
chr20	4440001	4445000	6.15E-07

chr20	4441001	4446000	2.15E-06
chr20	4442001	4447000	0.000160611
chr20	4480001	4485000	2.49E-09
chr20	4481001	4486000	2.47E-11
chr20	4482001	4487000	2.71E-20
chr20	4592001	4597000	1.35E-13
chr20	4593001	4598000	3.16E-12
chr20	4703001	4708000	2.58E-18
chr20	4704001	4709000	7.39E-17
chr20	5623001	5628000	6.55E-16
chr20	5625001	5630000	1.97E-15
chr20	5626001	5631000	4.70E-23
chr20	5679001	5684000	3.33E-11
chr20	5680001	5685000	9.47E-21
chr20	5681001	5686000	1.80E-19
chr20	5682001	5687000	1.14E-20
chr20	5778001	5783000	9.20E-05
chr20	5877001	5882000	0.000437198
chr20	6290001	6295000	0.000887481
chr20	6376001	6381000	0.006995603
chr20	6377001	6382000	0.00477352
chr20	6614001	6619000	9.11E-05
chr20	6615001	6620000	9.24E-09
chr20	6616001	6621000	1.25E-07
chr20	6781001	6786000	3.70E-10
chr20	6900001	6905000	4.88E-13
chr20	7045001	7050000	1.13E-05
chr20	7046001	7051000	6.22E-07
chr20	7047001	7052000	1.11E-06
chr20	7176001	7181000	1.98E-06
chr20	7178001	7183000	0.000513486
chr20	7179001	7184000	0.000953085
chr20	7187001	7192000	9.74E-05
chr20	7349001	7354000	0.002174768
chr20	7370001	7375000	0.00020608
chr20	7373001	7378000	2.67E-06
chr20	7374001	7379000	1.15E-05
chr20	7688001	7693000	7.20E-07
chr20	7769001	7774000	7.76E-12
chr20	7770001	7775000	3.32E-11
chr20	7798001	7803000	0.00096273
chr20	7800001	7805000	0.000154623
chr20	8469001	8474000	0.001547965
chr20	8837001	8842000	3.16E-07
chr20	9042001	9047000	1.60E-07
chr20	9710001	9715000	0.001831087
chr20	9739001	9744000	9.67E-06
chr20	9740001	9745000	1.65E-07

chr20	9827001	9832000	1.94E-05
chr20	9982001	9987000	1.15E-05
chr20	9983001	9988000	1.22E-06
chr20	10048001	10053000	0.000163603
chr20	10050001	10055000	0.000298503
chr20	10079001	10084000	6.80E-05
chr20	10080001	10085000	5.02E-05
chr20	10154001	10159000	1.55E-08
chr20	10155001	10160000	1.91E-11
chr20	10156001	10161000	9.79E-09
chr20	10204001	10209000	5.42E-06
chr20	10205001	10210000	1.46E-06
chr20	10206001	10211000	2.94E-05
chr20	10207001	10212000	7.71E-06
chr20	10278001	10283000	1.51E-10
chr20	10283001	10288000	5.08E-05
chr20	10284001	10289000	7.16E-08
chr20	10285001	10290000	1.25E-06
chr20	10316001	10321000	0.000208199
chr20	11591001	11596000	5.19E-06
chr20	11592001	11597000	9.91E-06
chr20	11669001	11674000	4.87E-06
chr20	11670001	11675000	2.59E-07
chr20	11671001	11676000	6.60E-08
chr20	11682001	11687000	8.11E-06
chr20	11761001	11766000	6.00E-06
chr20	11773001	11778000	7.91E-10
chr20	11774001	11779000	3.37E-07
chr20	11775001	11780000	4.91E-06
chr20	11813001	11818000	2.64E-09
chr20	11959001	11964000	4.59E-06
chr20	12012001	12017000	1.96E-09
chr20	12013001	12018000	7.83E-20
chr20	12014001	12019000	1.24E-15
chr20	12059001	12064000	0.000144048
chr20	12060001	12065000	0.000220543
chr20	12072001	12077000	9.50E-08
chr20	12080001	12085000	4.96E-05
chr20	12194001	12199000	0.000227042
chr20	12235001	12240000	0.000500351
chr20	12236001	12241000	1.43E-05
chr20	12237001	12242000	2.58E-05
chr20	12252001	12257000	0.000110769
chr20	12326001	12331000	3.98E-07
chr20	12377001	12382000	0.005639309
chr20	12380001	12385000	0.002807658
chr20	12382001	12387000	9.76E-06
chr20	12383001	12388000	2.28E-05

chr20	12384001	12389000	0.000164621
chr20	12440001	12445000	2.32E-05
chr20	12460001	12465000	2.65E-06
chr20	12461001	12466000	7.03E-05
chr20	12598001	12603000	1.97E-10
chr20	12599001	12604000	2.06E-09
chr20	12600001	12605000	1.07E-09
chr20	12601001	12606000	2.05E-07
chr20	12602001	12607000	0.000630149
chr20	12603001	12608000	0.000748784
chr20	12606001	12611000	0.000335019
chr20	12607001	12612000	0.000108205
chr20	12608001	12613000	1.87E-11
chr20	12609001	12614000	1.59E-08
chr20	12610001	12615000	1.56E-08
chr20	12702001	12707000	0.000194231
chr20	12703001	12708000	1.89E-05
chr20	12706001	12711000	5.51E-05
chr20	12707001	12712000	0.00022619
chr20	12894001	12899000	3.48E-08
chr20	12970001	12975000	5.18E-08
chr20	12974001	12979000	3.13E-05
chr20	12975001	12980000	3.13E-05
chr20	13178001	13183000	8.25E-08
chr20	13179001	13184000	6.82E-11
chr20	13180001	13185000	1.24E-10
chr20	13862001	13867000	1.53E-13
chr20	13863001	13868000	4.70E-15
chr20	13899001	13904000	2.39E-06
chr20	13982001	13987000	7.06E-11
chr20	13983001	13988000	2.58E-06
chr20	13994001	13999000	4.60E-12
chr20	13997001	14002000	6.89E-13
chr20	13998001	14003000	3.85E-12
chr20	13999001	14004000	2.44E-12
chr20	14000001	14005000	1.53E-12
chr20	14049001	14054000	8.06E-13
chr20	14128001	14133000	3.42E-05
chr20	14129001	14134000	4.83E-05
chr20	14131001	14136000	1.20E-06
chr20	14143001	14148000	7.59E-06
chr20	14146001	14151000	1.40E-08
chr20	14147001	14152000	1.11E-09
chr20	14183001	14188000	2.28E-06
chr20	14263001	14268000	2.94E-08
chr20	14272001	14277000	3.67E-07
chr20	14273001	14278000	9.38E-08
chr20	14274001	14279000	0.00059678

chr20	14275001	14280000	0.001440651
chr20	14277001	14282000	0.001360632
chr20	14278001	14283000	9.62E-05
chr20	14319001	14324000	5.66E-08
chr20	14320001	14325000	2.28E-08
chr20	14321001	14326000	5.14E-09
chr20	14348001	14353000	0.000501598
chr20	14349001	14354000	0.000434989
chr20	14350001	14355000	0.003043556
chr20	14351001	14356000	6.74E-05
chr20	14569001	14574000	1.50E-06
chr20	14570001	14575000	0.000129969
chr20	14587001	14592000	7.98E-06
chr20	14605001	14610000	2.43E-09
chr20	14606001	14611000	2.77E-08
chr20	14607001	14612000	3.69E-06
chr20	14658001	14663000	0.019587236
chr20	14685001	14690000	1.04E-07
chr20	14689001	14694000	0.000143926
chr20	14690001	14695000	0.001304336
chr20	14691001	14696000	0.000174459
chr20	14692001	14697000	5.70E-05
chr20	14912001	14917000	3.66E-06
chr20	14914001	14919000	5.34E-06
chr20	15010001	15015000	6.13E-26
chr20	15011001	15016000	1.42E-32
chr20	15012001	15017000	1.04E-32
chr20	15013001	15018000	1.56E-23
chr20	15042001	15047000	4.97E-11
chr20	15043001	15048000	1.74E-22
chr20	15044001	15049000	3.97E-20
chr20	15045001	15050000	7.42E-21
chr20	15046001	15051000	2.90E-16
chr20	15047001	15052000	5.13E-16
chr20	15050001	15055000	9.54E-06
chr20	15087001	15092000	0.000172954
chr20	15100001	15105000	4.44E-07
chr20	15124001	15129000	2.52E-10
chr20	15198001	15203000	8.14E-11
chr20	15199001	15204000	1.87E-07
chr20	15211001	15216000	2.11E-09
chr20	15250001	15255000	1.97E-06
chr20	15251001	15256000	4.03E-06
chr20	15296001	15301000	3.19E-05
chr20	15302001	15307000	0.001579368
chr20	15450001	15455000	2.92E-07
chr20	15657001	15662000	0.000142472
chr20	15666001	15671000	8.74E-08

chr20	15667001	15672000	3.76E-06
chr20	15712001	15717000	2.16E-05
chr20	15713001	15718000	2.16E-05
chr20	15714001	15719000	1.15E-05
chr20	15735001	15740000	3.50E-08
chr20	15769001	15774000	0.000847262
chr20	15844001	15849000	1.41E-05
chr20	15894001	15899000	0.000260579
chr20	15945001	15950000	0.000109862
chr20	15967001	15972000	0.008191122
chr20	15969001	15974000	2.54E-05
chr20	15970001	15975000	0.000105795
chr20	15971001	15976000	3.82E-05
chr20	16035001	16040000	1.82E-08
chr20	16069001	16074000	1.42E-07
chr20	16099001	16104000	0.000419248
chr20	16205001	16210000	2.46E-07
chr20	16239001	16244000	3.15E-06
chr20	17972001	17977000	6.45E-09
chr20	18042001	18047000	1.30E-08
chr20	18206001	18211000	6.73E-09
chr20	18706001	18711000	6.18E-05
chr20	19374001	19379000	8.77E-05
chr20	21494001	21499000	1.01E-23
chr20	21495001	21500000	2.06E-38
chr20	21496001	21501000	3.08E-44
chr20	21601001	21606000	0.001207981
chr20	21949001	21954000	0.00076601
chr20	21950001	21955000	0.000293922
chr20	21951001	21956000	0.000531027
chr20	22319001	22324000	9.37E-18
chr20	22647001	22652000	1.17E-06
chr20	22648001	22653000	5.08E-06
chr20	22649001	22654000	9.02E-05
chr20	24165001	24170000	1.67E-05
chr20	24166001	24171000	0.004269733
chr20	24406001	24411000	7.22E-06
chr20	25013001	25018000	1.28E-17
chr20	25167001	25172000	5.61E-07
chr20	26285001	26290000	0.001965629
chr20	26286001	26291000	0.004462457
chr20	29449001	29454000	9.95E-10
chr20	29450001	29455000	3.52E-09
chr20	29451001	29456000	6.32E-10
chr20	29452001	29457000	3.57E-08
chr20	29453001	29458000	1.74E-08
chr20	30010001	30015000	5.37E-12
chr20	30011001	30016000	1.61E-10

chr20	30296001	30301000	3.53E-17
chr20	30297001	30302000	1.28E-16
chr20	30613001	30618000	2.10E-09
chr20	30614001	30619000	1.13E-09
chr20	30788001	30793000	2.86E-18
chr20	31230001	31235000	3.36E-11
chr20	31231001	31236000	2.81E-10
chr20	31483001	31488000	4.93E-10
chr20	31866001	31871000	5.89E-11
chr20	32286001	32291000	7.62E-10
chr20	32287001	32292000	2.80E-07
chr20	32288001	32293000	2.84E-09
chr20	32400001	32405000	3.35E-21
chr20	32449001	32454000	1.71E-11
chr20	32628001	32633000	3.84E-20
chr20	32717001	32722000	1.34E-21
chr20	32908001	32913000	7.56E-10
chr20	33132001	33137000	9.90E-13
chr20	33133001	33138000	2.48E-28
chr20	33134001	33139000	3.23E-26
chr20	33135001	33140000	7.22E-24
chr20	33136001	33141000	5.12E-20
chr20	33570001	33575000	3.39E-12
chr20	33571001	33576000	8.99E-13
chr20	34318001	34323000	4.42E-05
chr20	34570001	34575000	1.49E-14
chr20	34571001	34576000	8.57E-16
chr20	34572001	34577000	2.46E-18
chr20	34573001	34578000	1.08E-11
chr20	34577001	34582000	3.00E-08
chr20	34578001	34583000	2.68E-09
chr20	35061001	35066000	6.05E-09
chr20	35071001	35076000	7.24E-09
chr20	35072001	35077000	5.11E-09
chr20	35170001	35175000	1.06E-08
chr20	35249001	35254000	6.68E-08
chr20	35250001	35255000	4.17E-07
chr20	35303001	35308000	7.53E-18
chr20	35820001	35825000	4.75E-09
chr20	36164001	36169000	2.57E-08
chr20	36179001	36184000	1.37E-17
chr20	37087001	37092000	9.98E-14
chr20	37088001	37093000	3.22E-17
chr20	37089001	37094000	1.61E-20
chr20	37812001	37817000	0.015476894
chr20	38188001	38193000	0.000448212
chr20	38654001	38659000	0.000206878
chr20	39026001	39031000	7.45E-06

chr20	39050001	39055000	2.17E-08
chr20	39051001	39056000	1.59E-09
chr20	39052001	39057000	2.28E-10
chr20	39053001	39058000	2.78E-08
chr20	39054001	39059000	4.04E-11
chr20	39183001	39188000	2.18E-06
chr20	39184001	39189000	5.59E-06
chr20	39630001	39635000	6.92E-09
chr20	39631001	39636000	4.10E-11
chr20	39632001	39637000	9.30E-06
chr20	39677001	39682000	1.43E-05
chr20	39678001	39683000	4.55E-08
chr20	39720001	39725000	1.44E-14
chr20	39760001	39765000	5.08E-11
chr20	40545001	40550000	6.19E-06
chr20	40546001	40551000	1.97E-07
chr20	40547001	40552000	1.74E-06
chr20	40548001	40553000	7.48E-07
chr20	42344001	42349000	8.05E-08
chr20	42346001	42351000	5.56E-11
chr20	42347001	42352000	7.87E-10
chr20	42348001	42353000	5.83E-09
chr20	42495001	42500000	3.57E-06
chr20	42844001	42849000	1.47E-12
chr20	43190001	43195000	8.05E-07
chr20	43192001	43197000	2.36E-08
chr20	43456001	43461000	0.014642967
chr20	43481001	43486000	1.38E-07
chr20	43869001	43874000	0.00611221
chr20	44230001	44235000	0.000138811
chr20	44376001	44381000	3.24E-06
chr20	44534001	44539000	1.24E-08
chr20	44546001	44551000	4.44E-11
chr20	44719001	44724000	5.16E-22
chr20	45948001	45953000	8.09E-13
chr20	46011001	46016000	1.60E-11
chr20	46119001	46124000	0.003170568
chr20	46396001	46401000	2.96E-08
chr20	46402001	46407000	7.21E-16
chr20	46527001	46532000	4.80E-06
chr20	46528001	46533000	3.32E-06
chr20	47455001	47460000	3.43E-07
chr20	47515001	47520000	8.67E-06
chr20	47516001	47521000	1.12E-05
chr20	47947001	47952000	1.11E-15
chr20	47948001	47953000	1.74E-19
chr20	47949001	47954000	3.38E-16
chr20	47950001	47955000	6.52E-18

chr20	47951001	47956000	4.79E-21
chr20	47952001	47957000	1.94E-18
chr20	47953001	47958000	3.68E-15
chr20	47974001	47979000	1.96E-11
chr20	47975001	47980000	2.28E-09
chr20	48133001	48138000	5.72E-09
chr20	48506001	48511000	1.82E-17
chr20	49130001	49135000	3.53E-09
chr20	49131001	49136000	7.84E-14
chr20	49590001	49595000	2.01E-08
chr20	49591001	49596000	2.43E-08
chr20	49592001	49597000	1.18E-20
chr20	49593001	49598000	2.94E-19
chr20	49594001	49599000	7.17E-12
chr20	49616001	49621000	8.74E-07
chr20	49871001	49876000	1.35E-05
chr20	49872001	49877000	1.27E-05
chr20	49995001	50000000	1.22E-11
chr20	49996001	50001000	1.22E-12
chr20	49997001	50002000	4.25E-11
chr20	50170001	50175000	7.94E-16
chr20	50171001	50176000	3.03E-29
chr20	50172001	50177000	1.39E-26
chr20	50173001	50178000	1.38E-13
chr20	50297001	50302000	7.18E-11
chr20	50543001	50548000	5.57E-16
chr20	50544001	50549000	3.54E-10
chr20	50545001	50550000	1.06E-11
chr20	50568001	50573000	2.08E-09
chr20	50569001	50574000	4.39E-08
chr20	50602001	50607000	2.97E-06
chr20	50664001	50669000	1.71E-14
chr20	50693001	50698000	9.04E-09
chr20	50697001	50702000	2.43E-22
chr20	50698001	50703000	5.28E-19
chr20	51109001	51114000	2.96E-23
chr20	51110001	51115000	7.81E-16
chr20	51111001	51116000	2.00E-14
chr20	52195001	52200000	2.30E-31
chr20	52196001	52201000	1.65E-24
chr20	52226001	52231000	4.79E-05
chr20	52238001	52243000	2.03E-09
chr20	52239001	52244000	7.79E-10
chr20	52483001	52488000	2.02E-06
chr20	52637001	52642000	5.37E-05
chr20	52638001	52643000	1.71E-05
chr20	53358001	53363000	2.97E-05
chr20	53474001	53479000	0.003849648

chr20	53476001	53481000	2.82E-05
chr20	53550001	53555000	5.26E-06
chr20	53551001	53556000	3.29E-06
chr20	53639001	53644000	1.50E-06
chr20	53854001	53859000	4.03E-05
chr20	54043001	54048000	2.17E-08
chr20	54044001	54049000	0.000183382
chr20	54145001	54150000	1.19E-10
chr20	54148001	54153000	7.25E-05
chr20	54443001	54448000	0.000898806
chr20	54444001	54449000	0.000114228
chr20	54649001	54654000	3.92E-06
chr20	54650001	54655000	4.31E-06
chr20	54656001	54661000	0.002890876
chr20	54709001	54714000	0.001727924
chr20	54755001	54760000	3.39E-06
chr20	55151001	55156000	7.80E-08
chr20	55989001	55994000	1.67E-08
chr20	56289001	56294000	2.36E-24
chr20	56420001	56425000	1.30E-06
chr20	56430001	56435000	2.49E-05
chr20	56537001	56542000	7.44E-10
chr20	56538001	56543000	9.02E-09
chr20	56539001	56544000	6.88E-08
chr20	56788001	56793000	4.84E-08
chr20	57644001	57649000	8.85E-12
chr20	57645001	57650000	6.59E-12
chr20	57646001	57651000	5.75E-08
chr20	57647001	57652000	5.82E-10
chr20	57659001	57664000	9.86E-12
chr20	57660001	57665000	1.77E-10
chr20	57661001	57666000	1.94E-08
chr20	57662001	57667000	1.79E-07
chr20	57793001	57798000	4.85E-23
chr20	57814001	57819000	2.10E-14
chr20	58287001	58292000	3.25E-05
chr20	60506001	60511000	5.20E-10
chr20	60507001	60512000	1.49E-10
chr20	61066001	61071000	4.02E-21
chr20	61067001	61072000	2.19E-15
chr20	61068001	61073000	5.11E-13
chr20	61186001	61191000	2.98E-09
chr20	61187001	61192000	5.47E-08
chr20	61205001	61210000	3.92E-11
chr20	61290001	61295000	1.98E-09
chr20	61292001	61297000	1.76E-09
chr20	61383001	61388000	1.06E-16
chr20	61403001	61408000	1.14E-15

chr20	61404001	61409000	5.60E-13
chr20	61405001	61410000	3.53E-26
chr20	61407001	61412000	2.39E-15
chr20	61408001	61413000	7.78E-16
chr20	61409001	61414000	2.11E-16
chr20	61921001	61926000	6.74E-09
chr20	61938001	61943000	2.93E-06
chr20	61940001	61945000	1.93E-05
chr20	61941001	61946000	6.58E-06
chr20	62035001	62040000	7.99E-09
chr20	62106001	62111000	8.65E-06
chr20	62107001	62112000	2.87E-08
chr20	62125001	62130000	1.00E-18
chr20	62126001	62131000	2.72E-15
chr20	62260001	62265000	3.94E-11
chr20	62262001	62267000	2.40E-19
chr20	62386001	62391000	4.28E-13
chr20	62470001	62475000	1.07E-14
chr20	62481001	62486000	1.37E-15
chr20	62539001	62544000	1.54E-08
chr20	62818001	62823000	1.67E-05
chr20	62915001	62920000	4.53E-05
chr20	62916001	62921000	3.93E-05
chr21	9969001	9974000	0.000358464
chr21	9970001	9975000	0.003523927
chr21	9973001	9978000	0.013829075
chr21	14542001	14547000	0.000249299
chr21	15321001	15326000	0.001243655
chr21	15600001	15605000	0.000392252
chr21	16079001	16084000	6.28E-05
chr21	16165001	16170000	0.019167578
chr21	16208001	16213000	0.000272173
chr21	16495001	16500000	0.000170661
chr21	16496001	16501000	7.24E-05
chr21	16497001	16502000	2.01E-08
chr21	16589001	16594000	2.71E-09
chr21	16975001	16980000	0.000144765
chr21	16976001	16981000	0.000364768
chr21	17055001	17060000	0.000338272
chr21	17789001	17794000	5.16E-05
chr21	17790001	17795000	1.26E-05
chr21	17791001	17796000	1.11E-05
chr21	18621001	18626000	0.001240825
chr21	18622001	18627000	0.004982104
chr21	18770001	18775000	0.000270755
chr21	19438001	19443000	0.003090788
chr21	19439001	19444000	5.37E-05
chr21	19440001	19445000	5.76E-05

chr21	19441001	19446000	0.000154816
chr21	19589001	19594000	9.11E-05
chr21	19662001	19667000	1.91E-07
chr21	19753001	19758000	0.000819242
chr21	19754001	19759000	0.000746128
chr21	19828001	19833000	0.001529417
chr21	19829001	19834000	0.001360531
chr21	19885001	19890000	0.000893643
chr21	20040001	20045000	6.42E-05
chr21	20138001	20143000	0.001461504
chr21	20164001	20169000	3.56E-07
chr21	20287001	20292000	7.93E-10
chr21	20432001	20437000	0.001890544
chr21	20459001	20464000	0.001815081
chr21	20460001	20465000	0.00802739
chr21	20461001	20466000	0.005422964
chr21	20575001	20580000	5.33E-07
chr21	20576001	20581000	1.08E-07
chr21	20577001	20582000	8.13E-09
chr21	20579001	20584000	0.000220631
chr21	20580001	20585000	0.001383262
chr21	20600001	20605000	0.000682919
chr21	20755001	20760000	0.000181433
chr21	20783001	20788000	6.00E-09
chr21	20986001	20991000	1.11E-07
chr21	21511001	21516000	1.12E-05
chr21	21582001	21587000	8.57E-07
chr21	21911001	21916000	9.22E-10
chr21	22023001	22028000	3.38E-11
chr21	22051001	22056000	3.94E-05
chr21	22229001	22234000	0.004710112
chr21	22253001	22258000	0.000950664
chr21	22254001	22259000	0.003033333
chr21	22408001	22413000	0.000222145
chr21	22409001	22414000	3.44E-05
chr21	22410001	22415000	0.005583522
chr21	22502001	22507000	4.93E-05
chr21	22541001	22546000	0.002285918
chr21	22607001	22612000	6.55E-07
chr21	22608001	22613000	2.89E-08
chr21	22609001	22614000	9.11E-07
chr21	22661001	22666000	0.002752953
chr21	22662001	22667000	0.00084969
chr21	22806001	22811000	0.007519749
chr21	22808001	22813000	0.000174854
chr21	22848001	22853000	1.65E-06
chr21	22850001	22855000	2.36E-07
chr21	22851001	22856000	1.28E-07

chr21	22877001	22882000	5.60E-07
chr21	22971001	22976000	2.25E-08
chr21	22972001	22977000	4.33E-09
chr21	22973001	22978000	2.33E-07
chr21	23314001	23319000	2.95E-05
chr21	23315001	23320000	1.66E-05
chr21	23465001	23470000	3.30E-06
chr21	23466001	23471000	2.25E-07
chr21	23467001	23472000	1.78E-07
chr21	23517001	23522000	2.03E-06
chr21	23518001	23523000	1.90E-07
chr21	23772001	23777000	0.001823027
chr21	23783001	23788000	1.19E-05
chr21	23784001	23789000	1.50E-05
chr21	23977001	23982000	2.16E-05
chr21	24026001	24031000	0.000306712
chr21	24082001	24087000	1.15E-06
chr21	24207001	24212000	0.001830596
chr21	24372001	24377000	0.001382342
chr21	24558001	24563000	3.07E-05
chr21	24593001	24598000	0.000111927
chr21	24680001	24685000	0.003919002
chr21	24728001	24733000	0.000173997
chr21	24729001	24734000	0.000420924
chr21	24730001	24735000	3.27E-05
chr21	24731001	24736000	0.000106177
chr21	24764001	24769000	3.04E-05
chr21	24765001	24770000	0.000272639
chr21	24805001	24810000	8.99E-06
chr21	24817001	24822000	0.003574792
chr21	24818001	24823000	0.000959254
chr21	24906001	24911000	0.000732729
chr21	24909001	24914000	1.72E-06
chr21	24981001	24986000	4.29E-07
chr21	24982001	24987000	7.34E-08
chr21	24983001	24988000	3.63E-05
chr21	25080001	25085000	2.02E-06
chr21	25081001	25086000	4.58E-10
chr21	25082001	25087000	6.04E-06
chr21	25083001	25088000	4.52E-06
chr21	25084001	25089000	2.55E-06
chr21	25085001	25090000	1.36E-05
chr21	25320001	25325000	1.63E-08
chr21	25384001	25389000	0.003286767
chr21	25388001	25393000	2.01E-09
chr21	25389001	25394000	7.27E-09
chr21	25390001	25395000	3.71E-07
chr21	25391001	25396000	1.56E-08

chr21	25392001	25397000	7.22E-05
chr21	25692001	25697000	0.002226788
chr21	25693001	25698000	2.58E-06
chr21	25694001	25699000	8.03E-06
chr21	25695001	25700000	1.65E-05
chr21	25857001	25862000	3.39E-05
chr21	25858001	25863000	0.000298824
chr21	25961001	25966000	1.29E-07
chr21	25962001	25967000	1.77E-08
chr21	26058001	26063000	9.16E-07
chr21	26215001	26220000	0.000141983
chr21	26299001	26304000	0.000336042
chr21	26420001	26425000	9.80E-05
chr21	26421001	26426000	1.55E-05
chr21	26623001	26628000	0.007236987
chr21	27621001	27626000	1.80E-05
chr21	27622001	27627000	9.24E-10
chr21	27623001	27628000	2.88E-09
chr21	27625001	27630000	8.17E-10
chr21	27723001	27728000	0.000606411
chr21	27724001	27729000	0.000729027
chr21	27769001	27774000	1.39E-05
chr21	28060001	28065000	7.98E-11
chr21	28061001	28066000	8.68E-10
chr21	28062001	28067000	8.02E-09
chr21	29050001	29055000	0.006279571
chr21	29051001	29056000	0.000959701
chr21	29052001	29057000	0.007326836
chr21	29053001	29058000	0.006221254
chr21	29054001	29059000	0.007471842
chr21	29787001	29792000	0.000114286
chr21	31082001	31087000	0.000808432
chr21	31823001	31828000	6.68E-05
chr21	31965001	31970000	0.00010537
chr21	32013001	32018000	4.04E-05
chr21	32014001	32019000	8.41E-05
chr21	32456001	32461000	1.07E-17
chr21	32457001	32462000	1.75E-16
chr21	33033001	33038000	3.89E-10
chr21	33079001	33084000	6.56E-06
chr21	33080001	33085000	2.72E-06
chr21	34655001	34660000	1.77E-06
chr21	34750001	34755000	9.50E-10
chr21	34916001	34921000	1.02E-27
chr21	34917001	34922000	2.28E-15
chr21	35143001	35148000	3.67E-12
chr21	35894001	35899000	1.86E-17
chr21	35895001	35900000	4.50E-18

chr21	35896001	35901000	4.86E-12
chr21	35897001	35902000	1.93E-10
chr21	37612001	37617000	0.000800215
chr21	37630001	37635000	5.83E-12
chr21	37631001	37636000	1.67E-12
chr21	37632001	37637000	1.21E-08
chr21	37957001	37962000	0.000247373
chr21	38406001	38411000	2.42E-12
chr21	39313001	39318000	2.02E-10
chr21	39314001	39319000	1.82E-10
chr21	39315001	39320000	5.30E-09
chr21	39400001	39405000	1.72E-06
chr21	39527001	39532000	1.88E-06
chr21	39528001	39533000	1.89E-06
chr21	39683001	39688000	1.84E-05
chr21	40275001	40280000	2.09E-14
chr21	41279001	41284000	3.74E-10
chr21	41280001	41285000	1.24E-12
chr21	41281001	41286000	1.37E-16
chr21	41282001	41287000	4.81E-15
chr21	41283001	41288000	2.13E-10
chr21	41615001	41620000	6.66E-06
chr21	41661001	41666000	1.90E-08
chr21	41662001	41667000	1.36E-07
chr21	41663001	41668000	3.15E-11
chr21	41810001	41815000	0.000103005
chr21	42168001	42173000	1.15E-05
chr21	43480001	43485000	1.04E-06
chr21	44073001	44078000	7.69E-14
chr21	46264001	46269000	6.81E-12
chr21	46336001	46341000	1.04E-17
chr21	47333001	47338000	0.00209637
chr21	47608001	47613000	6.42E-11
chr22	16097001	16102000	6.57E-05
chr22	16156001	16161000	2.43E-06
chr22	16181001	16186000	1.18E-05
chr22	16219001	16224000	0.000380562
chr22	16220001	16225000	2.81E-09
chr22	16221001	16226000	3.70E-10
chr22	16222001	16227000	3.35E-09
chr22	16641001	16646000	0.001373204
chr22	17199001	17204000	1.16E-11
chr22	17201001	17206000	1.48E-05
chr22	17202001	17207000	3.85E-05
chr22	17529001	17534000	0.005530629
chr22	17540001	17545000	5.51E-19
chr22	17541001	17546000	9.33E-18
chr22	17542001	17547000	2.01E-17

chr22	17543001	17548000	2.46E-13
chr22	17544001	17549000	1.36E-12
chr22	17545001	17550000	4.96E-08
chr22	17604001	17609000	2.05E-07
chr22	17606001	17611000	1.74E-06
chr22	17757001	17762000	1.05E-08
chr22	17954001	17959000	1.52E-11
chr22	17978001	17983000	4.87E-10
chr22	18040001	18045000	3.48E-19
chr22	18744001	18749000	9.39E-11
chr22	18745001	18750000	7.43E-11
chr22	18746001	18751000	7.47E-13
chr22	18804001	18809000	6.34E-06
chr22	18856001	18861000	6.63E-08
chr22	18857001	18862000	2.49E-06
chr22	18973001	18978000	4.77E-22
chr22	18976001	18981000	4.87E-22
chr22	18977001	18982000	4.30E-33
chr22	18986001	18991000	2.19E-12
chr22	19002001	19007000	0.000127668
chr22	19003001	19008000	4.06E-12
chr22	19004001	19009000	6.33E-14
chr22	19005001	19010000	3.17E-14
chr22	19006001	19011000	1.10E-19
chr22	19007001	19012000	2.72E-12
chr22	20125001	20130000	7.58E-14
chr22	20313001	20318000	4.50E-05
chr22	20657001	20662000	1.11E-05
chr22	20941001	20946000	8.33E-08
chr22	20942001	20947000	5.18E-13
chr22	20943001	20948000	1.35E-15
chr22	20944001	20949000	2.42E-15
chr22	21048001	21053000	1.23E-10
chr22	21273001	21278000	1.36E-23
chr22	21274001	21279000	4.82E-15
chr22	21408001	21413000	4.40E-09
chr22	21411001	21416000	5.91E-08
chr22	21449001	21454000	5.99E-09
chr22	21450001	21455000	3.23E-07
chr22	21451001	21456000	8.26E-10
chr22	21630001	21635000	1.10E-09
chr22	21799001	21804000	6.28E-17
chr22	22054001	22059000	3.29E-31
chr22	22055001	22060000	1.92E-30
chr22	22064001	22069000	1.22E-23
chr22	22280001	22285000	1.34E-11
chr22	22491001	22496000	0.00646178
chr22	23106001	23111000	1.99E-09

chr22	23667001	23672000	7.00E-08
chr22	23717001	23722000	3.16E-08
chr22	23728001	23733000	6.51E-07
chr22	23729001	23734000	3.45E-07
chr22	23893001	23898000	8.67E-08
chr22	23899001	23904000	6.14E-15
chr22	23900001	23905000	5.26E-12
chr22	23901001	23906000	9.70E-13
chr22	23902001	23907000	4.39E-16
chr22	23934001	23939000	1.55E-11
chr22	23945001	23950000	4.93E-12
chr22	23946001	23951000	2.48E-12
chr22	23947001	23952000	4.51E-13
chr22	23948001	23953000	2.81E-12
chr22	24122001	24127000	2.68E-16
chr22	24123001	24128000	2.55E-16
chr22	24368001	24373000	1.71E-12
chr22	24643001	24648000	0.001051868
chr22	24952001	24957000	6.76E-17
chr22	24978001	24983000	4.32E-15
chr22	24979001	24984000	6.83E-16
chr22	25019001	25024000	2.67E-05
chr22	25338001	25343000	8.80E-15
chr22	25509001	25514000	1.67E-11
chr22	26058001	26063000	2.92E-15
chr22	26059001	26064000	4.46E-13
chr22	26060001	26065000	3.39E-12
chr22	26651001	26656000	5.73E-12
chr22	26652001	26657000	2.16E-07
chr22	26944001	26949000	0.000419576
chr22	27553001	27558000	1.40E-13
chr22	27554001	27559000	7.58E-12
chr22	27555001	27560000	7.07E-08
chr22	27586001	27591000	2.32E-09
chr22	28324001	28329000	2.10E-07
chr22	28325001	28330000	1.89E-10
chr22	28326001	28331000	4.28E-06
chr22	29211001	29216000	8.47E-07
chr22	29369001	29374000	8.54E-22
chr22	29370001	29375000	5.70E-34
chr22	29371001	29376000	4.85E-32
chr22	29706001	29711000	4.69E-10
chr22	30098001	30103000	4.95E-15
chr22	30121001	30126000	3.63E-18
chr22	30122001	30127000	3.21E-13
chr22	30255001	30260000	1.10E-14
chr22	30766001	30771000	5.38E-05
chr22	31643001	31648000	5.82E-05

chr22	31644001	31649000	2.82E-05
chr22	31645001	31650000	1.79E-06
chr22	32021001	32026000	1.70E-10
chr22	32458001	32463000	5.94E-08
chr22	32475001	32480000	7.20E-12
chr22	32643001	32648000	5.93E-08
chr22	32644001	32649000	5.50E-08
chr22	32645001	32650000	5.21E-09
chr22	32651001	32656000	5.12E-07
chr22	34513001	34518000	2.20E-05
chr22	34514001	34519000	0.000453319
chr22	34814001	34819000	1.64E-05
chr22	35247001	35252000	5.04E-06
chr22	35309001	35314000	4.72E-13
chr22	35387001	35392000	1.22E-07
chr22	35428001	35433000	1.60E-15
chr22	35429001	35434000	8.15E-15
chr22	35480001	35485000	1.71E-08
chr22	35481001	35486000	1.47E-08
chr22	35482001	35487000	1.73E-08
chr22	35607001	35612000	1.03E-09
chr22	35611001	35616000	3.32E-07
chr22	35988001	35993000	6.72E-08
chr22	36728001	36733000	2.48E-15
chr22	36768001	36773000	5.77E-11
chr22	36953001	36958000	3.45E-09
chr22	36954001	36959000	1.45E-13
chr22	36955001	36960000	3.67E-16
chr22	37105001	37110000	9.37E-07
chr22	37417001	37422000	1.46E-16
chr22	37418001	37423000	6.49E-15
chr22	37441001	37446000	1.26E-07
chr22	37577001	37582000	2.58E-07
chr22	37578001	37583000	8.02E-07
chr22	37759001	37764000	6.05E-11
chr22	37763001	37768000	1.01E-08
chr22	37764001	37769000	3.74E-10
chr22	37765001	37770000	4.30E-11
chr22	37766001	37771000	2.61E-27
chr22	37767001	37772000	6.89E-35
chr22	37768001	37773000	4.20E-34
chr22	37769001	37774000	1.80E-31
chr22	37770001	37775000	3.17E-29
chr22	37771001	37776000	3.95E-10
chr22	37798001	37803000	6.09E-14
chr22	37799001	37804000	5.75E-16
chr22	37800001	37805000	3.54E-14
chr22	37801001	37806000	1.38E-11

chr22	37802001	37807000	1.49E-11
chr22	37855001	37860000	2.77E-12
chr22	37959001	37964000	2.21E-08
chr22	37960001	37965000	3.19E-08
chr22	38134001	38139000	1.74E-10
chr22	38135001	38140000	5.44E-11
chr22	38179001	38184000	3.25E-14
chr22	38288001	38293000	1.37E-12
chr22	38289001	38294000	2.58E-11
chr22	38290001	38295000	3.45E-12
chr22	38433001	38438000	9.18E-15
chr22	38434001	38439000	2.56E-12
chr22	38796001	38801000	1.14E-32
chr22	38830001	38835000	2.32E-11
chr22	39256001	39261000	2.19E-10
chr22	39290001	39295000	1.62E-05
chr22	39293001	39298000	3.18E-11
chr22	39294001	39299000	9.92E-09
chr22	39295001	39300000	2.26E-07
chr22	39296001	39301000	1.45E-07
chr22	39316001	39321000	7.38E-18
chr22	39318001	39323000	3.96E-18
chr22	39360001	39365000	3.32E-14
chr22	39381001	39386000	0.000166123
chr22	39528001	39533000	5.96E-16
chr22	39529001	39534000	1.64E-16
chr22	39710001	39715000	7.18E-09
chr22	39882001	39887000	3.32E-14
chr22	39883001	39888000	9.83E-16
chr22	39942001	39947000	1.98E-11
chr22	39943001	39948000	1.97E-09
chr22	39944001	39949000	6.03E-07
chr22	39945001	39950000	1.83E-08
chr22	39946001	39951000	1.83E-07
chr22	39954001	39959000	3.83E-07
chr22	39967001	39972000	5.62E-12
chr22	39977001	39982000	1.42E-06
chr22	39980001	39985000	2.74E-13
chr22	39983001	39988000	2.52E-25
chr22	39995001	40000000	2.42E-10
chr22	40047001	40052000	4.41E-08
chr22	40570001	40575000	2.80E-12
chr22	41767001	41772000	8.67E-08
chr22	42332001	42337000	1.61E-13
chr22	42333001	42338000	8.20E-10
chr22	42858001	42863000	2.32E-13
chr22	43053001	43058000	6.56E-09
chr22	43144001	43149000	5.56E-09

chr22	43145001	43150000	5.15E-09
chr22	43480001	43485000	1.96E-17
chr22	43550001	43555000	3.44E-16
chr22	43551001	43556000	1.08E-11
chr22	43552001	43557000	8.12E-14
chr22	43553001	43558000	1.89E-10
chr22	43605001	43610000	2.99E-06
chr22	43749001	43754000	5.19E-09
chr22	43844001	43849000	4.29E-08
chr22	43905001	43910000	9.55E-05
chr22	43906001	43911000	2.35E-05
chr22	43907001	43912000	4.74E-05
chr22	43955001	43960000	3.57E-10
chr22	44291001	44296000	7.09E-10
chr22	44587001	44592000	1.53E-08
chr22	44588001	44593000	4.21E-09
chr22	44718001	44723000	1.10E-20
chr22	44720001	44725000	1.43E-20
chr22	44733001	44738000	6.15E-10
chr22	45142001	45147000	9.94E-17
chr22	45144001	45149000	3.68E-12
chr22	45273001	45278000	3.52E-15
chr22	45274001	45279000	3.76E-15
chr22	45445001	45450000	0.000139696
chr22	45591001	45596000	1.12E-13
chr22	45592001	45597000	8.79E-14
chr22	45593001	45598000	1.02E-19
chr22	45640001	45645000	4.68E-08
chr22	45641001	45646000	6.96E-14
chr22	45863001	45868000	1.19E-08
chr22	45927001	45932000	3.37E-07
chr22	45928001	45933000	2.28E-08
chr22	45943001	45948000	1.59E-12
chr22	45945001	45950000	8.37E-14
chr22	45996001	46001000	2.06E-11
chr22	45997001	46002000	5.66E-14
chr22	45998001	46003000	7.59E-15
chr22	46512001	46517000	6.44E-07
chr22	46925001	46930000	5.28E-17
chr22	47055001	47060000	1.62E-07
chr22	47056001	47061000	1.25E-08
chr22	47057001	47062000	6.51E-07
chr22	48122001	48127000	0.00447563
chr22	48923001	48928000	1.31E-06
chr22	49072001	49077000	3.66E-09
chr22	49389001	49394000	1.03E-09
chr22	49390001	49395000	2.42E-11
chr22	50336001	50341000	6.64E-12

chr22	50519001	50524000	8.97E-14
chr22	50947001	50952000	1.07E-16
chr22	50976001	50981000	9.02E-26
chr22	50977001	50982000	4.45E-30
chrX	3854001	3859000	7.19E-10
chrX	3855001	3860000	1.09E-08
chrX	3856001	3861000	4.87E-08
chrX	4012001	4017000	0.004420774
chrX	4013001	4018000	0.004214802
chrX	4014001	4019000	0.000787704
chrX	4041001	4046000	0.000428194
chrX	4043001	4048000	0.000211424
chrX	4044001	4049000	3.33E-05
chrX	4137001	4142000	1.99E-07
chrX	4138001	4143000	1.73E-06
chrX	4199001	4204000	0.000482456
chrX	4200001	4205000	0.000154866
chrX	4201001	4206000	0.000204638
chrX	4202001	4207000	6.96E-07
chrX	4203001	4208000	1.67E-06
chrX	4204001	4209000	4.84E-06
chrX	4205001	4210000	6.77E-05
chrX	4206001	4211000	0.000292186
chrX	4298001	4303000	0.000557023
chrX	4299001	4304000	0.001827603
chrX	4505001	4510000	8.26E-05
chrX	4560001	4565000	0.000239959
chrX	4573001	4578000	0.000791951
chrX	4637001	4642000	6.28E-05
chrX	4719001	4724000	0.000359758
chrX	4721001	4726000	0.002011109
chrX	4780001	4785000	4.88E-05
chrX	4903001	4908000	5.33E-06
chrX	4977001	4982000	4.15E-06
chrX	5156001	5161000	0.001308295
chrX	5157001	5162000	0.000295212
chrX	5159001	5164000	0.000659427
chrX	5160001	5165000	0.000336991
chrX	5357001	5362000	0.000361326
chrX	6000001	6005000	1.61E-06
chrX	6001001	6006000	0.000144253
chrX	7295001	7300000	0.003438665
chrX	7297001	7302000	8.96E-05
chrX	7298001	7303000	0.000162914
chrX	7324001	7329000	1.79E-05
chrX	7325001	7330000	0.000106208
chrX	7379001	7384000	4.03E-07
chrX	7454001	7459000	0.003416857

chrX	7567001	7572000	0.001377942
chrX	8038001	8043000	0.000620102
chrX	8394001	8399000	1.76E-05
chrX	8396001	8401000	0.000467633
chrX	8557001	8562000	0.000331694
chrX	8558001	8563000	0.000318612
chrX	8559001	8564000	6.77E-05
chrX	8800001	8805000	3.08E-05
chrX	8851001	8856000	0.004440817
chrX	8852001	8857000	0.001315461
chrX	8853001	8858000	0.001315461
chrX	9053001	9058000	7.38E-05
chrX	9054001	9059000	4.99E-07
chrX	9963001	9968000	4.55E-14
chrX	9964001	9969000	5.97E-12
chrX	10336001	10341000	0.000953947
chrX	10580001	10585000	0.001618504
chrX	10581001	10586000	0.000475767
chrX	10870001	10875000	2.30E-05
chrX	10871001	10876000	1.04E-05
chrX	11667001	11672000	0.000513698
chrX	11888001	11893000	6.02E-06
chrX	11912001	11917000	1.73E-07
chrX	11913001	11918000	6.24E-07
chrX	11914001	11919000	5.73E-07
chrX	11915001	11920000	0.00031955
chrX	11916001	11921000	0.00017408
chrX	11917001	11922000	0.000140724
chrX	11965001	11970000	0.000807243
chrX	11966001	11971000	0.000992578
chrX	12147001	12152000	7.08E-07
chrX	12236001	12241000	7.91E-05
chrX	12246001	12251000	1.08E-05
chrX	12247001	12252000	1.66E-06
chrX	12248001	12253000	4.53E-07
chrX	12249001	12254000	0.001537468
chrX	12250001	12255000	0.000432917
chrX	12251001	12256000	0.001294999
chrX	12270001	12275000	0.000222588
chrX	12271001	12276000	0.000353569
chrX	12341001	12346000	0.000477521
chrX	12342001	12347000	0.001854397
chrX	12343001	12348000	0.001330438
chrX	12345001	12350000	0.004897742
chrX	12415001	12420000	0.00087838
chrX	12416001	12421000	0.000287965
chrX	12519001	12524000	5.29E-05
chrX	12520001	12525000	0.00038364

chrX	12521001	12526000	0.002116449
chrX	12522001	12527000	0.002200184
chrX	12661001	12666000	0.001123385
chrX	12995001	13000000	9.69E-11
chrX	12996001	13001000	7.58E-08
chrX	13140001	13145000	4.53E-14
chrX	13141001	13146000	1.36E-12
chrX	13142001	13147000	6.87E-12
chrX	13721001	13726000	0.000734839
chrX	14128001	14133000	3.21E-10
chrX	14440001	14445000	7.26E-09
chrX	14471001	14476000	0.008286783
chrX	14766001	14771000	0.000539449
chrX	15140001	15145000	0.015982201
chrX	15141001	15146000	0.02308026
chrX	15142001	15147000	0.023039408
chrX	15143001	15148000	0.026523328
chrX	15158001	15163000	3.37E-05
chrX	15159001	15164000	2.00E-05
chrX	15160001	15165000	8.34E-07
chrX	15161001	15166000	5.02E-05
chrX	15260001	15265000	2.55E-06
chrX	15261001	15266000	4.26E-07
chrX	15431001	15436000	9.41E-05
chrX	16134001	16139000	0.001091493
chrX	16789001	16794000	1.41E-11
chrX	17054001	17059000	5.82E-07
chrX	17055001	17060000	1.16E-07
chrX	17056001	17061000	9.22E-07
chrX	17278001	17283000	0.000218824
chrX	17279001	17284000	0.001302072
chrX	17280001	17285000	0.000431986
chrX	17302001	17307000	1.54E-06
chrX	17308001	17313000	0.000234795
chrX	17309001	17314000	1.76E-05
chrX	17510001	17515000	3.00E-05
chrX	17511001	17516000	6.73E-06
chrX	17574001	17579000	8.71E-06
chrX	17575001	17580000	1.75E-09
chrX	17749001	17754000	8.25E-08
chrX	17750001	17755000	1.25E-09
chrX	17847001	17852000	0.00665312
chrX	17848001	17853000	2.63E-06
chrX	17849001	17854000	8.70E-07
chrX	17962001	17967000	2.54E-05
chrX	18408001	18413000	4.34E-07
chrX	18409001	18414000	7.32E-09
chrX	20509001	20514000	0.001101187

chrX	20735001	20740000	4.98E-05
chrX	21046001	21051000	0.001436439
chrX	21047001	21052000	0.000301778
chrX	21048001	21053000	0.001308308
chrX	21049001	21054000	0.005983587
chrX	21943001	21948000	1.25E-10
chrX	21944001	21949000	2.77E-13
chrX	21945001	21950000	9.54E-12
chrX	22111001	22116000	4.67E-06
chrX	22113001	22118000	6.99E-05
chrX	22154001	22159000	1.28E-10
chrX	22344001	22349000	4.47E-07
chrX	22345001	22350000	4.89E-07
chrX	22346001	22351000	2.69E-07
chrX	22347001	22352000	0.000106398
chrX	22366001	22371000	1.61E-05
chrX	22367001	22372000	1.01E-05
chrX	22431001	22436000	0.000179352
chrX	22499001	22504000	0.001717993
chrX	22532001	22537000	3.31E-05
chrX	22924001	22929000	1.90E-05
chrX	23747001	23752000	0.006520401
chrX	23771001	23776000	0.000234506
chrX	23813001	23818000	1.48E-06
chrX	23814001	23819000	1.49E-05
chrX	24178001	24183000	1.72E-06
chrX	24179001	24184000	7.96E-08
chrX	24180001	24185000	1.44E-07
chrX	24181001	24186000	2.83E-07
chrX	24591001	24596000	0.000244702
chrX	24651001	24656000	9.45E-08
chrX	25047001	25052000	1.74E-10
chrX	25254001	25259000	1.01E-10
chrX	25271001	25276000	0.005889342
chrX	25397001	25402000	0.005293598
chrX	25463001	25468000	2.86E-05
chrX	25521001	25526000	0.003908459
chrX	25576001	25581000	4.08E-05
chrX	25755001	25760000	7.95E-05
chrX	25756001	25761000	4.10E-05
chrX	25824001	25829000	2.28E-07
chrX	25826001	25831000	0.001722568
chrX	26388001	26393000	5.46E-07
chrX	26571001	26576000	3.64E-20
chrX	26592001	26597000	7.71E-08
chrX	27110001	27115000	3.51E-05
chrX	27111001	27116000	2.86E-08
chrX	27112001	27117000	0.000673546

chrX	27206001	27211000	0.000612974
chrX	27409001	27414000	4.56E-07
chrX	27411001	27416000	5.75E-07
chrX	27412001	27417000	5.54E-07
chrX	27464001	27469000	0.001039679
chrX	27466001	27471000	0.003976719
chrX	27467001	27472000	1.37E-05
chrX	27468001	27473000	9.10E-06
chrX	27470001	27475000	2.00E-05
chrX	27477001	27482000	1.08E-09
chrX	27573001	27578000	0.002460455
chrX	27588001	27593000	2.32E-05
chrX	27590001	27595000	1.22E-08
chrX	27591001	27596000	8.97E-10
chrX	27592001	27597000	2.03E-08
chrX	27593001	27598000	2.70E-06
chrX	27634001	27639000	0.014140185
chrX	27649001	27654000	0.002986815
chrX	27654001	27659000	0.000522402
chrX	27705001	27710000	1.81E-05
chrX	27843001	27848000	8.81E-08
chrX	28275001	28280000	2.60E-05
chrX	28870001	28875000	0.001125004
chrX	29248001	29253000	8.60E-06
chrX	29249001	29254000	3.44E-06
chrX	29319001	29324000	0.000109621
chrX	29364001	29369000	0.012088463
chrX	29419001	29424000	3.32E-08
chrX	29420001	29425000	2.22E-08
chrX	29439001	29444000	0.012452952
chrX	29576001	29581000	0.026765961
chrX	29577001	29582000	0.001746301
chrX	29578001	29583000	0.002264293
chrX	29709001	29714000	0.002169176
chrX	29846001	29851000	0.003045449
chrX	29955001	29960000	0.001835651
chrX	30055001	30060000	0.00027097
chrX	30073001	30078000	3.78E-05
chrX	30074001	30079000	0.000172146
chrX	30388001	30393000	0.000890815
chrX	30753001	30758000	1.14E-07
chrX	30754001	30759000	4.14E-06
chrX	30987001	30992000	7.15E-06
chrX	31294001	31299000	1.17E-05
chrX	31329001	31334000	2.56E-05
chrX	31453001	31458000	3.00E-09
chrX	31465001	31470000	0.000194404
chrX	31615001	31620000	1.15E-05

chrX	31617001	31622000	1.01E-06
chrX	32285001	32290000	9.20E-07
chrX	32286001	32291000	1.66E-07
chrX	32287001	32292000	4.72E-08
chrX	33087001	33092000	2.11E-06
chrX	33458001	33463000	0.00707991
chrX	33962001	33967000	3.00E-05
chrX	33963001	33968000	0.000947433
chrX	33964001	33969000	0.003357704
chrX	33970001	33975000	0.000117053
chrX	33971001	33976000	0.001095059
chrX	34628001	34633000	0.000585258
chrX	34645001	34650000	0.00016074
chrX	34646001	34651000	0.000472176
chrX	34888001	34893000	0.000182065
chrX	34889001	34894000	0.000199597
chrX	34985001	34990000	0.000812007
chrX	34988001	34993000	0.001649419
chrX	34989001	34994000	0.001341191
chrX	35343001	35348000	0.008952752
chrX	36893001	36898000	0.001375374
chrX	37188001	37193000	3.01E-05
chrX	37189001	37194000	2.90E-05
chrX	37190001	37195000	2.57E-06
chrX	37191001	37196000	2.78E-05
chrX	37596001	37601000	2.69E-05
chrX	37837001	37842000	0.001197205
chrX	37838001	37843000	0.00075539
chrX	37839001	37844000	0.000293903
chrX	37991001	37996000	3.31E-06
chrX	38285001	38290000	0.000661902
chrX	39047001	39052000	0.002671534
chrX	39048001	39053000	0.000538325
chrX	39433001	39438000	5.83E-11
chrX	39434001	39439000	1.44E-09
chrX	39940001	39945000	9.17E-19
chrX	39941001	39946000	1.01E-16
chrX	40148001	40153000	0.000262355
chrX	40737001	40742000	7.22E-08
chrX	40779001	40784000	0.000858386
chrX	40780001	40785000	0.00145914
chrX	40789001	40794000	3.77E-10
chrX	41149001	41154000	2.74E-06
chrX	41150001	41155000	5.13E-05
chrX	41151001	41156000	3.39E-10
chrX	41195001	41200000	6.71E-07
chrX	41680001	41685000	0.000713168
chrX	41681001	41686000	0.000689126

chrX	41682001	41687000	0.001104714
chrX	41683001	41688000	1.92E-05
chrX	41829001	41834000	0.002484272
chrX	41851001	41856000	0.002719066
chrX	41852001	41857000	0.003978538
chrX	42503001	42508000	0.001144663
chrX	42788001	42793000	0.001067532
chrX	43077001	43082000	0.005350819
chrX	43592001	43597000	1.99E-05
chrX	44070001	44075000	1.35E-07
chrX	44302001	44307000	0.000134677
chrX	45036001	45041000	0.002151371
chrX	45053001	45058000	2.20E-05
chrX	45154001	45159000	0.001322786
chrX	45458001	45463000	7.37E-05
chrX	45731001	45736000	7.93E-14
chrX	45732001	45737000	4.33E-11
chrX	45733001	45738000	5.11E-14
chrX	45776001	45781000	0.002387214
chrX	45785001	45790000	1.03E-06
chrX	45800001	45805000	1.19E-05
chrX	45802001	45807000	3.47E-06
chrX	45805001	45810000	5.43E-05
chrX	45806001	45811000	2.28E-05
chrX	45807001	45812000	0.000912589
chrX	45808001	45813000	0.000754138
chrX	45809001	45814000	0.000268862
chrX	45821001	45826000	1.39E-08
chrX	45822001	45827000	2.09E-07
chrX	45823001	45828000	3.52E-10
chrX	45824001	45829000	9.10E-07
chrX	45828001	45833000	5.67E-10
chrX	45829001	45834000	1.81E-13
chrX	45830001	45835000	3.93E-14
chrX	45831001	45836000	6.15E-14
chrX	45832001	45837000	2.67E-13
chrX	45833001	45838000	1.88E-05
chrX	45837001	45842000	0.000588605
chrX	45841001	45846000	1.36E-15
chrX	45843001	45848000	4.26E-19
chrX	45873001	45878000	0.000221763
chrX	45874001	45879000	1.83E-10
chrX	45875001	45880000	4.27E-10
chrX	45876001	45881000	7.87E-11
chrX	45877001	45882000	4.66E-08
chrX	45878001	45883000	3.97E-07
chrX	45885001	45890000	0.000501005
chrX	45888001	45893000	4.37E-07

chrX	45889001	45894000	2.64E-06
chrX	45897001	45902000	0.000149187
chrX	45898001	45903000	5.29E-05
chrX	45899001	45904000	5.29E-05
chrX	45900001	45905000	0.001065382
chrX	45908001	45913000	6.40E-05
chrX	45909001	45914000	7.17E-07
chrX	45910001	45915000	1.09E-10
chrX	45911001	45916000	6.56E-09
chrX	45912001	45917000	3.13E-11
chrX	45913001	45918000	2.70E-15
chrX	45914001	45919000	8.96E-16
chrX	45915001	45920000	2.30E-11
chrX	45935001	45940000	1.18E-17
chrX	45936001	45941000	5.44E-18
chrX	45937001	45942000	6.15E-17
chrX	45938001	45943000	2.85E-14
chrX	45939001	45944000	4.44E-13
chrX	45953001	45958000	1.72E-05
chrX	45954001	45959000	1.23E-06
chrX	45955001	45960000	2.77E-07
chrX	45956001	45961000	2.52E-08
chrX	45957001	45962000	5.83E-08
chrX	46199001	46204000	2.69E-05
chrX	46210001	46215000	0.000108324
chrX	46572001	46577000	0.000910832
chrX	46648001	46653000	3.11E-07
chrX	46649001	46654000	2.40E-08
chrX	47240001	47245000	2.96E-05
chrX	47786001	47791000	5.25E-06
chrX	48326001	48331000	0.000328274
chrX	48436001	48441000	4.37E-05
chrX	48873001	48878000	1.53E-13
chrX	48883001	48888000	2.12E-16
chrX	48895001	48900000	6.26E-10
chrX	48928001	48933000	3.09E-11
chrX	48929001	48934000	9.02E-13
chrX	49049001	49054000	6.79E-09
chrX	49085001	49090000	6.30E-14
chrX	49632001	49637000	0.00059096
chrX	49647001	49652000	7.48E-05
chrX	51466001	51471000	1.52E-05
chrX	51782001	51787000	0.007728751
chrX	53048001	53053000	2.50E-06
chrX	53474001	53479000	0.001879845
chrX	53475001	53480000	0.000114682
chrX	53481001	53486000	6.97E-08
chrX	53489001	53494000	3.58E-05

chrX	53656001	53661000	0.001625019
chrX	53684001	53689000	7.26E-09
chrX	53935001	53940000	3.52E-05
chrX	53936001	53941000	0.00055353
chrX	55587001	55592000	0.001720804
chrX	58068001	58073000	0.002836667
chrX	61881001	61886000	0.018957727
chrX	61882001	61887000	0.018192439
chrX	62346001	62351000	0.035894832
chrX	62687001	62692000	0.004382848
chrX	62691001	62696000	0.001081595
chrX	62717001	62722000	0.000336957
chrX	63179001	63184000	0.002809917
chrX	64706001	64711000	1.84E-11
chrX	64707001	64712000	6.09E-13
chrX	64708001	64713000	2.86E-07
chrX	65437001	65442000	1.56E-06
chrX	65482001	65487000	0.008167602
chrX	65838001	65843000	1.32E-07
chrX	65839001	65844000	2.85E-08
chrX	65850001	65855000	6.33E-07
chrX	65851001	65856000	5.90E-07
chrX	65852001	65857000	1.55E-05
chrX	65996001	66001000	0.034517906
chrX	66755001	66760000	0.001312496
chrX	66756001	66761000	0.001661726
chrX	67011001	67016000	0.013788129
chrX	67078001	67083000	0.000602232
chrX	67497001	67502000	3.53E-06
chrX	67669001	67674000	2.33E-06
chrX	67670001	67675000	1.45E-08
chrX	67671001	67676000	6.19E-08
chrX	67672001	67677000	7.17E-08
chrX	67673001	67678000	2.80E-07
chrX	67680001	67685000	1.15E-05
chrX	67708001	67713000	5.37E-07
chrX	69441001	69446000	5.50E-06
chrX	69844001	69849000	0.001062205
chrX	70051001	70056000	0.004271112
chrX	70230001	70235000	3.22E-06
chrX	70402001	70407000	6.89E-05
chrX	72295001	72300000	3.49E-09
chrX	72296001	72301000	4.78E-09
chrX	72297001	72302000	1.68E-07
chrX	72627001	72632000	0.000868632
chrX	72927001	72932000	0.005235702
chrX	72928001	72933000	0.003226156
chrX	73518001	73523000	9.02E-05

chrX	73519001	73524000	3.30E-05
chrX	73586001	73591000	0.013291195
chrX	73627001	73632000	3.73E-07
chrX	73709001	73714000	8.46E-05
chrX	73710001	73715000	8.64E-09
chrX	73774001	73779000	8.80E-05
chrX	74083001	74088000	0.000137262
chrX	74084001	74089000	0.000181984
chrX	74085001	74090000	0.000126334
chrX	74542001	74547000	2.32E-05
chrX	74586001	74591000	0.000634036
chrX	75618001	75623000	4.57E-05
chrX	75619001	75624000	9.52E-05
chrX	76496001	76501000	0.000494965
chrX	76497001	76502000	0.000115126
chrX	77171001	77176000	3.44E-08
chrX	77172001	77177000	6.19E-08
chrX	77187001	77192000	2.04E-10
chrX	77188001	77193000	5.00E-15
chrX	78142001	78147000	0.018958455
chrX	78664001	78669000	1.24E-05
chrX	78665001	78670000	1.86E-05
chrX	78666001	78671000	1.23E-05
chrX	79149001	79154000	5.56E-05
chrX	79284001	79289000	3.48E-05
chrX	79285001	79290000	6.73E-05
chrX	79337001	79342000	0.001743368
chrX	79338001	79343000	0.003242295
chrX	79733001	79738000	6.50E-06
chrX	79746001	79751000	4.33E-06
chrX	80811001	80816000	2.60E-06
chrX	80812001	80817000	1.34E-06
chrX	81534001	81539000	0.002025229
chrX	82008001	82013000	8.92E-05
chrX	82009001	82014000	1.84E-05
chrX	82010001	82015000	8.00E-13
chrX	82011001	82016000	1.27E-09
chrX	82012001	82017000	2.35E-05
chrX	82282001	82287000	0.001301586
chrX	82967001	82972000	3.01E-05
chrX	82988001	82993000	0.000594606
chrX	82989001	82994000	0.00048676
chrX	82990001	82995000	0.000560792
chrX	83539001	83544000	0.000477405
chrX	83540001	83545000	0.001171743
chrX	83541001	83546000	0.000880705
chrX	83855001	83860000	3.07E-05
chrX	84084001	84089000	0.017910321

chrX	84135001	84140000	0.000454337
chrX	84136001	84141000	0.000883663
chrX	84709001	84714000	0.007393526
chrX	84710001	84715000	0.004674706
chrX	85387001	85392000	5.98E-05
chrX	85394001	85399000	0.005470754
chrX	85927001	85932000	0.000115111
chrX	86038001	86043000	2.52E-05
chrX	86039001	86044000	0.000109475
chrX	86655001	86660000	1.38E-05
chrX	86717001	86722000	0.000142468
chrX	86718001	86723000	9.15E-05
chrX	87766001	87771000	9.11E-16
chrX	87768001	87773000	4.62E-15
chrX	87769001	87774000	6.38E-13
chrX	87770001	87775000	3.02E-11
chrX	88284001	88289000	0.006366801
chrX	88285001	88290000	0.006471127
chrX	91399001	91404000	3.42E-05
chrX	91460001	91465000	0.004230562
chrX	91461001	91466000	0.0005679
chrX	91462001	91467000	0.000719756
chrX	92414001	92419000	0.001132564
chrX	92794001	92799000	0.004463895
chrX	93910001	93915000	0.000813783
chrX	94147001	94152000	6.35E-09
chrX	94148001	94153000	1.60E-08
chrX	94149001	94154000	7.95E-07
chrX	94190001	94195000	0.00023418
chrX	94311001	94316000	0.000192597
chrX	94312001	94317000	0.00014656
chrX	94315001	94320000	2.59E-05
chrX	95304001	95309000	1.71E-06
chrX	95747001	95752000	0.001372113
chrX	96029001	96034000	0.000808451
chrX	96059001	96064000	3.10E-08
chrX	96060001	96065000	4.15E-08
chrX	96061001	96066000	6.07E-09
chrX	96390001	96395000	0.003474904
chrX	96514001	96519000	0.000204897
chrX	96621001	96626000	0.000185268
chrX	98589001	98594000	0.000453739
chrX	99776001	99781000	5.36E-09
chrX	100462001	100467000	1.99E-05
chrX	100922001	100927000	3.76E-05
chrX	101430001	101435000	7.53E-07
chrX	102223001	102228000	0.016761654
chrX	102394001	102399000	1.44E-06

chrX	102395001	102400000	3.01E-09
chrX	102396001	102401000	1.48E-08
chrX	102409001	102414000	0.000331908
chrX	102893001	102898000	0.000869302
chrX	103071001	103076000	3.76E-07
chrX	103072001	103077000	4.24E-13
chrX	103073001	103078000	2.37E-10
chrX	103074001	103079000	1.72E-09
chrX	103195001	103200000	3.09E-08
chrX	103196001	103201000	8.16E-05
chrX	103814001	103819000	0.002160139
chrX	103906001	103911000	0.004807528
chrX	103907001	103912000	0.003909216
chrX	103985001	103990000	0.000502625
chrX	103986001	103991000	0.000133053
chrX	104538001	104543000	0.001313256
chrX	104539001	104544000	0.000985792
chrX	104616001	104621000	0.000845139
chrX	104617001	104622000	0.000211802
chrX	104618001	104623000	4.71E-06
chrX	105105001	105110000	0.017256666
chrX	105106001	105111000	0.021231006
chrX	105479001	105484000	9.22E-05
chrX	106018001	106023000	1.08E-06
chrX	106128001	106133000	0.012234009
chrX	106223001	106228000	0.000172153
chrX	106290001	106295000	0.004112008
chrX	106303001	106308000	1.12E-06
chrX	106336001	106341000	0.004180859
chrX	106453001	106458000	3.56E-10
chrX	106468001	106473000	3.09E-06
chrX	106631001	106636000	0.014866256
chrX	106674001	106679000	1.87E-05
chrX	106675001	106680000	0.013483359
chrX	107212001	107217000	0.015258169
chrX	107371001	107376000	6.40E-08
chrX	107372001	107377000	1.85E-07
chrX	107541001	107546000	1.45E-06
chrX	107543001	107548000	0.004836019
chrX	107705001	107710000	1.46E-05
chrX	107706001	107711000	6.20E-06
chrX	107940001	107945000	6.31E-07
chrX	107941001	107946000	4.48E-08
chrX	107942001	107947000	1.66E-08
chrX	107943001	107948000	3.05E-08
chrX	107944001	107949000	0.005940017
chrX	108462001	108467000	3.95E-07
chrX	108463001	108468000	8.85E-07

chrX	108464001	108469000	9.62E-05
chrX	108624001	108629000	0.003596423
chrX	108625001	108630000	0.008035608
chrX	108831001	108836000	4.95E-05
chrX	108930001	108935000	0.00186411
chrX	108932001	108937000	0.000380185
chrX	108995001	109000000	6.38E-06
chrX	108996001	109001000	3.02E-05
chrX	109094001	109099000	0.0013557
chrX	109095001	109100000	0.004609298
chrX	109096001	109101000	6.43E-07
chrX	109116001	109121000	1.23E-19
chrX	109117001	109122000	5.52E-21
chrX	109564001	109569000	0.000289097
chrX	109648001	109653000	4.77E-08
chrX	109749001	109754000	4.64E-07
chrX	109752001	109757000	0.000993371
chrX	109967001	109972000	0.000539962
chrX	110082001	110087000	0.000164926
chrX	110225001	110230000	0.001512346
chrX	110243001	110248000	0.01108006
chrX	110244001	110249000	0.001405623
chrX	110245001	110250000	0.003732393
chrX	110376001	110381000	0.0007986
chrX	110536001	110541000	4.38E-06
chrX	110609001	110614000	0.013407351
chrX	110684001	110689000	0.011181969
chrX	110685001	110690000	0.007765837
chrX	110709001	110714000	0.001393699
chrX	111162001	111167000	0.000222303
chrX	111490001	111495000	6.76E-06
chrX	111491001	111496000	1.57E-05
chrX	111492001	111497000	2.11E-06
chrX	112225001	112230000	6.89E-07
chrX	112584001	112589000	0.002856733
chrX	112585001	112590000	0.002904856
chrX	112799001	112804000	6.75E-05
chrX	113043001	113048000	0.00079495
chrX	113044001	113049000	0.000154559
chrX	113152001	113157000	4.28E-10
chrX	113271001	113276000	0.000444228
chrX	113272001	113277000	0.000249676
chrX	113314001	113319000	2.30E-05
chrX	114390001	114395000	0.00012668
chrX	114537001	114542000	0.000405487
chrX	114578001	114583000	6.25E-10
chrX	114579001	114584000	4.82E-09
chrX	114580001	114585000	1.15E-09

chrX	114581001	114586000	3.05E-09
chrX	114582001	114587000	4.84E-10
chrX	114583001	114588000	5.55E-06
chrX	114585001	114590000	2.54E-06
chrX	114643001	114648000	1.56E-05
chrX	114715001	114720000	0.017190652
chrX	114716001	114721000	0.018037637
chrX	114733001	114738000	1.52E-06
chrX	114826001	114831000	3.10E-06
chrX	114827001	114832000	9.80E-07
chrX	114828001	114833000	2.16E-06
chrX	114830001	114835000	2.79E-09
chrX	114832001	114837000	1.17E-08
chrX	114833001	114838000	7.89E-09
chrX	114834001	114839000	9.44E-08
chrX	114890001	114895000	9.26E-11
chrX	114891001	114896000	1.40E-13
chrX	114892001	114897000	1.20E-13
chrX	114893001	114898000	4.01E-11
chrX	114894001	114899000	1.23E-08
chrX	114919001	114924000	7.58E-05
chrX	114920001	114925000	1.16E-05
chrX	114929001	114934000	9.98E-07
chrX	114930001	114935000	1.77E-08
chrX	115104001	115109000	1.31E-07
chrX	115342001	115347000	0.006114239
chrX	115547001	115552000	1.01E-07
chrX	115624001	115629000	0.026444853
chrX	116679001	116684000	0.000549891
chrX	116897001	116902000	8.88E-07
chrX	117000001	117005000	1.37E-05
chrX	117385001	117390000	2.24E-05
chrX	117386001	117391000	0.000587331
chrX	117589001	117594000	7.45E-05
chrX	117591001	117596000	0.000842384
chrX	117662001	117667000	1.50E-07
chrX	117678001	117683000	0.007715872
chrX	117758001	117763000	1.50E-05
chrX	117759001	117764000	1.75E-05
chrX	117979001	117984000	5.61E-05
chrX	118141001	118146000	2.00E-08
chrX	118142001	118147000	5.14E-07
chrX	118227001	118232000	0.002367251
chrX	118228001	118233000	0.000298635
chrX	118519001	118524000	3.86E-07
chrX	118695001	118700000	2.15E-05
chrX	118815001	118820000	6.04E-08
chrX	118860001	118865000	1.18E-06

chrX	118861001	118866000	2.75E-08
chrX	118862001	118867000	5.05E-10
chrX	118863001	118868000	1.22E-11
chrX	118864001	118869000	1.56E-10
chrX	119465001	119470000	2.74E-05
chrX	119815001	119820000	0.002001926
chrX	119870001	119875000	0.00795708
chrX	119910001	119915000	0.000208701
chrX	119911001	119916000	9.05E-07
chrX	119912001	119917000	1.78E-05
chrX	119913001	119918000	0.000164564
chrX	119914001	119919000	5.41E-05
chrX	119915001	119920000	2.10E-05
chrX	119985001	119990000	0.000749052
chrX	119994001	119999000	4.58E-05
chrX	120359001	120364000	0.000258504
chrX	120603001	120608000	0.000571596
chrX	120914001	120919000	0.00830364
chrX	120980001	120985000	3.43E-08
chrX	120981001	120986000	2.87E-06
chrX	120982001	120987000	1.01E-05
chrX	120983001	120988000	4.20E-06
chrX	120984001	120989000	2.88E-08
chrX	121158001	121163000	0.001577882
chrX	121185001	121190000	0.003631979
chrX	121186001	121191000	0.001024524
chrX	121252001	121257000	0.000319182
chrX	121710001	121715000	0.001397428
chrX	121711001	121716000	0.001035107
chrX	121712001	121717000	0.000205832
chrX	121740001	121745000	0.009439746
chrX	121928001	121933000	2.56E-06
chrX	122422001	122427000	0.010381711
chrX	122541001	122546000	0.003284341
chrX	122548001	122553000	0.002430242
chrX	122646001	122651000	1.92E-12
chrX	122900001	122905000	8.12E-07
chrX	122901001	122906000	1.42E-05
chrX	122937001	122942000	4.79E-05
chrX	123075001	123080000	0.002689724
chrX	123696001	123701000	0.000470957
chrX	124355001	124360000	0.000582177
chrX	124356001	124361000	0.001977518
chrX	124357001	124362000	0.001500805
chrX	124359001	124364000	0.000822068
chrX	124509001	124514000	0.000370379
chrX	124510001	124515000	0.000288049
chrX	124511001	124516000	0.000905079

chrX	124693001	124698000	0.005951491
chrX	124696001	124701000	0.002672377
chrX	124703001	124708000	0.013333674
chrX	125105001	125110000	0.001200909
chrX	125457001	125462000	0.027288393
chrX	126977001	126982000	1.65E-05
chrX	127198001	127203000	0.002079852
chrX	127338001	127343000	0.000309401
chrX	127339001	127344000	0.00300965
chrX	127458001	127463000	2.76E-05
chrX	127459001	127464000	4.08E-05
chrX	127495001	127500000	0.000323712
chrX	127496001	127501000	0.000134923
chrX	127598001	127603000	5.97E-05
chrX	127698001	127703000	0.000124678
chrX	127699001	127704000	0.000521926
chrX	127787001	127792000	2.95E-05
chrX	128086001	128091000	0.012195737
chrX	128087001	128092000	0.020143445
chrX	128088001	128093000	0.021326207
chrX	128158001	128163000	0.000254919
chrX	129373001	129378000	1.86E-06
chrX	129374001	129379000	4.29E-07
chrX	129980001	129985000	5.95E-06
chrX	130106001	130111000	0.000869961
chrX	130110001	130115000	1.06E-07
chrX	130154001	130159000	0.000676572
chrX	130585001	130590000	0.004526294
chrX	130753001	130758000	0.000451966
chrX	130754001	130759000	0.00021783
chrX	130755001	130760000	0.00010559
chrX	130756001	130761000	1.14E-05
chrX	130769001	130774000	0.003385375
chrX	130770001	130775000	0.000655995
chrX	130979001	130984000	1.91E-06
chrX	130980001	130985000	7.47E-07
chrX	131887001	131892000	0.000584049
chrX	131949001	131954000	0.000446704
chrX	132010001	132015000	0.001778254
chrX	132819001	132824000	3.44E-09
chrX	132820001	132825000	1.32E-08
chrX	133200001	133205000	0.010852014
chrX	135192001	135197000	0.000126238
chrX	135346001	135351000	0.000377859
chrX	135347001	135352000	1.32E-05
chrX	135348001	135353000	0.004903422
chrX	135462001	135467000	0.001242654
chrX	135463001	135468000	0.000437419

chrX	135464001	135469000	0.000261783
chrX	135465001	135470000	0.001447804
chrX	135466001	135471000	0.001491107
chrX	135872001	135877000	6.56E-06
chrX	136221001	136226000	0.003511885
chrX	136726001	136731000	2.81E-07
chrX	136727001	136732000	3.97E-08
chrX	136729001	136734000	7.91E-07
chrX	137112001	137117000	9.65E-05
chrX	137113001	137118000	2.90E-10
chrX	137321001	137326000	0.007391428
chrX	137453001	137458000	0.00083965
chrX	137854001	137859000	3.99E-05
chrX	137875001	137880000	2.16E-07
chrX	137876001	137881000	1.33E-10
chrX	137911001	137916000	3.14E-05
chrX	137921001	137926000	0.001592436
chrX	137922001	137927000	0.001386078
chrX	137928001	137933000	0.000589071
chrX	138000001	138005000	0.021911981
chrX	138029001	138034000	0.000309142
chrX	138030001	138035000	0.002252216
chrX	138031001	138036000	0.002545943
chrX	138210001	138215000	0.003219837
chrX	138280001	138285000	2.43E-05
chrX	138670001	138675000	0.002400188
chrX	139022001	139027000	3.41E-06
chrX	139023001	139028000	0.000374435
chrX	139470001	139475000	0.00056351
chrX	139726001	139731000	0.008070849
chrX	139993001	139998000	3.25E-08
chrX	140311001	140316000	0.002172207
chrX	140481001	140486000	6.28E-05
chrX	140527001	140532000	0.001354143
chrX	140528001	140533000	8.47E-05
chrX	140529001	140534000	3.97E-05
chrX	140553001	140558000	1.86E-05
chrX	140554001	140559000	6.14E-09
chrX	140635001	140640000	4.12E-09
chrX	140636001	140641000	6.58E-05
chrX	140637001	140642000	0.000100498
chrX	140638001	140643000	0.000100498
chrX	140694001	140699000	6.96E-05
chrX	140695001	140700000	0.000465263
chrX	140829001	140834000	6.56E-05
chrX	140836001	140841000	0.004108
chrX	141821001	141826000	0.001040676
chrX	141822001	141827000	2.73E-05

chrX	142177001	142182000	0.000124571
chrX	142306001	142311000	0.001558152
chrX	142814001	142819000	1.31E-07
chrX	143122001	143127000	0.000764813
chrX	143123001	143128000	0.000157954
chrX	143124001	143129000	1.18E-06
chrX	143260001	143265000	0.000417
chrX	143494001	143499000	1.16E-05
chrX	143495001	143500000	1.95E-09
chrX	143496001	143501000	2.49E-09
chrX	143497001	143502000	9.26E-08
chrX	143756001	143761000	0.007132975
chrX	143880001	143885000	0.000801244
chrX	143881001	143886000	0.002482234
chrX	144884001	144889000	4.04E-06
chrX	144885001	144890000	9.08E-07
chrX	144886001	144891000	1.39E-06
chrX	145097001	145102000	4.03E-09
chrX	145098001	145103000	0.000293742
chrX	145378001	145383000	0.01064533
chrX	145431001	145436000	0.001318212
chrX	145432001	145437000	4.61E-06
chrX	145433001	145438000	1.95E-05
chrX	145464001	145469000	0.000549425
chrX	145466001	145471000	0.000274972
chrX	145833001	145838000	0.00046081
chrX	145835001	145840000	6.06E-09
chrX	145943001	145948000	2.84E-06
chrX	146627001	146632000	5.75E-05
chrX	146628001	146633000	8.27E-05
chrX	146629001	146634000	8.60E-05
chrX	146705001	146710000	0.000297777
chrX	146708001	146713000	5.54E-05
chrX	147129001	147134000	1.10E-12
chrX	147130001	147135000	2.66E-12
chrX	148204001	148209000	2.14E-05
chrX	148205001	148210000	1.59E-07
chrX	148206001	148211000	1.62E-07
chrX	148789001	148794000	2.48E-08
chrX	149452001	149457000	0.004442225
chrX	150583001	150588000	0.005725637
chrX	150584001	150589000	0.001788227
chrX	150717001	150722000	1.97E-12
chrX	150718001	150723000	2.54E-15
chrX	151499001	151504000	3.22E-06
chrX	151500001	151505000	3.97E-06
chrX	151501001	151506000	2.63E-06
chrX	151510001	151515000	0.001661878

chrX	151529001	151534000	2.27E-05	
chrX	151530001	151535000	0.000225418	
chrX	151705001	151710000	4.25E-05	
chrX	151706001	151711000	0.000110383	
chrX	151708001	151713000	0.000372605	
chrX	151964001	151969000	0.001057924	
chrX	152168001	152173000	5.58E-07	
chrX	153110001	153115000	1.30E-08	
chrX	153208001	153213000	6.01E-17	
chrX	153982001	153987000	3.70E-06	
chrX	154041001	154046000	8.93E-08	
chrX	154187001	154192000	0.000184975	
chrX	154188001	154193000	0.000309298	
chrX	154825001	154830000	2.19E-05	
chrY	14961001	14966000	0.006119051	
chr1	52001	57000	2.28E-06	hypo
chr1	93001	98000	0.000780422	
chr1	758001	763000	6.97E-25	
chr1	759001	764000	9.02E-69	
chr1	760001	765000	1.80E-87	
chr1	761001	766000	1.15E-74	
chr1	762001	767000	2.10E-45	
chr1	763001	768000	5.37E-14	
chr1	898001	903000	1.36E-14	
chr1	899001	904000	3.55E-12	
chr1	900001	905000	3.69E-17	
chr1	953001	958000	0.011668602	
chr1	1209001	1214000	1.43E-14	
chr1	1335001	1340000	1.96E-17	
chr1	1338001	1343000	3.03E-43	
chr1	1339001	1344000	1.42E-45	
chr1	1370001	1375000	0.000189849	
chr1	1586001	1591000	2.89E-53	
chr1	1587001	1592000	1.67E-46	
chr1	1588001	1593000	3.31E-44	
chr1	1590001	1595000	9.32E-19	
chr1	1621001	1626000	4.84E-36	
chr1	1651001	1656000	8.47E-08	
chr1	1652001	1657000	4.88E-11	
chr1	1705001	1710000	1.06E-50	
chr1	1706001	1711000	7.43E-21	
chr1	2154001	2159000	2.03E-33	
chr1	2155001	2160000	2.44E-58	
chr1	2156001	2161000	8.45E-55	
chr1	2157001	2162000	1.40E-48	
chr1	2319001	2324000	3.91E-19	
chr1	2982001	2987000	6.13E-05	
chr1	3130001	3135000	2.36E-23	

chr1	3163001	3168000	5.16E-12
chr1	3164001	3169000	8.93E-18
chr1	3213001	3218000	3.94E-10
chr1	3537001	3542000	2.55E-12
chr1	3538001	3543000	8.56E-28
chr1	3539001	3544000	1.22E-28
chr1	3540001	3545000	2.65E-29
chr1	3541001	3546000	5.41E-34
chr1	3567001	3572000	9.51E-30
chr1	3568001	3573000	1.66E-25
chr1	3812001	3817000	2.04E-07
chr1	3813001	3818000	1.00E-14
chr1	3815001	3820000	3.32E-13
chr1	4301001	4306000	0.000127987
chr1	5274001	5279000	0.000219647
chr1	5290001	5295000	0.008531568
chr1	5362001	5367000	1.00E-05
chr1	5413001	5418000	2.84E-05
chr1	5699001	5704000	2.56E-07
chr1	6259001	6264000	1.47E-25
chr1	6261001	6266000	4.28E-20
chr1	6262001	6267000	1.15E-23
chr1	6263001	6268000	1.57E-23
chr1	6308001	6313000	0.004529629
chr1	6309001	6314000	0.000271055
chr1	6416001	6421000	0.000529848
chr1	6505001	6510000	7.90E-06
chr1	6506001	6511000	9.55E-06
chr1	6673001	6678000	1.38E-29
chr1	6674001	6679000	2.54E-29
chr1	6675001	6680000	1.72E-16
chr1	6682001	6687000	2.23E-19
chr1	6683001	6688000	6.13E-22
chr1	6684001	6689000	2.54E-13
chr1	6685001	6690000	2.70E-07
chr1	6761001	6766000	1.85E-25
chr1	6840001	6845000	2.07E-18
chr1	6842001	6847000	7.45E-20
chr1	6843001	6848000	2.63E-38
chr1	6844001	6849000	3.03E-33
chr1	7827001	7832000	6.82E-79
chr1	7828001	7833000	1.03E-55
chr1	7829001	7834000	8.24E-79
chr1	7830001	7835000	1.58E-79
chr1	7831001	7836000	9.61E-57
chr1	8019001	8024000	6.74E-15
chr1	8020001	8025000	1.99E-21
chr1	8021001	8026000	3.67E-22

chr1	8028001	8033000	6.84E-32
chr1	8029001	8034000	1.66E-37
chr1	8030001	8035000	3.92E-35
chr1	8031001	8036000	1.75E-34
chr1	8032001	8037000	6.60E-18
chr1	8081001	8086000	5.18E-32
chr1	8082001	8087000	1.88E-59
chr1	8086001	8091000	7.13E-28
chr1	8374001	8379000	3.68E-12
chr1	8375001	8380000	3.04E-18
chr1	8376001	8381000	2.17E-23
chr1	8377001	8382000	3.95E-30
chr1	8378001	8383000	1.45E-29
chr1	8873001	8878000	2.47E-26
chr1	8938001	8943000	4.62E-37
chr1	8939001	8944000	3.72E-45
chr1	9184001	9189000	2.96E-15
chr1	9185001	9190000	6.05E-21
chr1	9242001	9247000	3.14E-09
chr1	9292001	9297000	8.80E-28
chr1	9293001	9298000	2.04E-37
chr1	9294001	9299000	3.45E-49
chr1	9295001	9300000	2.82E-18
chr1	9352001	9357000	2.53E-10
chr1	9353001	9358000	5.67E-12
chr1	9485001	9490000	7.08E-09
chr1	9486001	9491000	1.31E-09
chr1	9487001	9492000	5.49E-11
chr1	9488001	9493000	2.45E-09
chr1	9489001	9494000	1.52E-11
chr1	9644001	9649000	1.92E-30
chr1	9645001	9650000	1.05E-13
chr1	9966001	9971000	3.69E-38
chr1	10089001	10094000	9.36E-18
chr1	10090001	10095000	5.64E-26
chr1	10266001	10271000	2.46E-49
chr1	10459001	10464000	3.20E-19
chr1	10856001	10861000	6.51E-35
chr1	11776001	11781000	2.50E-14
chr1	11778001	11783000	3.01E-07
chr1	11779001	11784000	4.56E-07
chr1	11864001	11869000	2.64E-17
chr1	11865001	11870000	2.01E-33
chr1	11866001	11871000	3.56E-20
chr1	12075001	12080000	8.61E-08
chr1	12076001	12081000	9.39E-09
chr1	12120001	12125000	1.05E-05
chr1	12121001	12126000	0.000796316

chr1	12223001	12228000	1.48E-13
chr1	12227001	12232000	4.52E-14
chr1	12288001	12293000	2.68E-14
chr1	12290001	12295000	2.14E-31
chr1	13204001	13209000	0.000370239
chr1	13947001	13952000	7.09E-13
chr1	14022001	14027000	1.44E-17
chr1	14071001	14076000	5.50E-35
chr1	14072001	14077000	8.86E-64
chr1	14073001	14078000	2.61E-69
chr1	14074001	14079000	4.42E-67
chr1	14075001	14080000	5.20E-32
chr1	14076001	14081000	2.41E-38
chr1	14175001	14180000	0.003961297
chr1	14245001	14250000	9.40E-06
chr1	14246001	14251000	2.43E-06
chr1	14247001	14252000	2.47E-13
chr1	14248001	14253000	5.91E-09
chr1	14383001	14388000	0.000493202
chr1	14485001	14490000	0.003438415
chr1	14486001	14491000	4.07E-05
chr1	14514001	14519000	1.96E-06
chr1	14515001	14520000	2.42E-06
chr1	14516001	14521000	2.68E-07
chr1	14629001	14634000	6.92E-05
chr1	14632001	14637000	0.001070323
chr1	14638001	14643000	0.001066145
chr1	14680001	14685000	2.34E-06
chr1	14921001	14926000	6.69E-15
chr1	14923001	14928000	2.03E-20
chr1	14925001	14930000	9.32E-23
chr1	15246001	15251000	1.17E-08
chr1	15247001	15252000	0.000865116
chr1	15248001	15253000	1.23E-09
chr1	15249001	15254000	1.63E-09
chr1	15250001	15255000	3.83E-18
chr1	15251001	15256000	1.57E-08
chr1	15476001	15481000	1.24E-34
chr1	15480001	15485000	2.05E-11
chr1	15569001	15574000	4.40E-08
chr1	15573001	15578000	8.00E-11
chr1	15732001	15737000	3.33E-05
chr1	15733001	15738000	8.59E-08
chr1	15853001	15858000	2.41E-28
chr1	16008001	16013000	5.64E-09
chr1	16009001	16014000	6.24E-08
chr1	16082001	16087000	1.01E-09
chr1	16083001	16088000	2.52E-12

chr1	16084001	16089000	2.18E-12
chr1	16085001	16090000	4.61E-15
chr1	16299001	16304000	1.66E-18
chr1	16300001	16305000	1.06E-14
chr1	16302001	16307000	6.53E-17
chr1	16337001	16342000	5.47E-06
chr1	16467001	16472000	4.26E-06
chr1	16482001	16487000	1.55E-12
chr1	16529001	16534000	5.62E-09
chr1	16674001	16679000	3.92E-28
chr1	16675001	16680000	5.13E-23
chr1	16676001	16681000	3.00E-26
chr1	16678001	16683000	1.20E-20
chr1	16679001	16684000	5.37E-15
chr1	16763001	16768000	1.16E-10
chr1	16764001	16769000	1.55E-13
chr1	16765001	16770000	1.55E-19
chr1	16766001	16771000	1.77E-15
chr1	16767001	16772000	5.24E-17
chr1	16825001	16830000	1.32E-21
chr1	16940001	16945000	2.22E-156
chr1	17067001	17072000	3.34E-05
chr1	17068001	17073000	3.34E-05
chr1	17069001	17074000	3.34E-05
chr1	17236001	17241000	4.16E-36
chr1	17237001	17242000	2.59E-32
chr1	17238001	17243000	5.19E-34
chr1	17336001	17341000	2.27E-05
chr1	17376001	17381000	4.41E-14
chr1	17624001	17629000	3.83E-10
chr1	17625001	17630000	8.50E-12
chr1	17862001	17867000	3.10E-08
chr1	17864001	17869000	2.29E-13
chr1	17865001	17870000	2.30E-18
chr1	17866001	17871000	6.12E-22
chr1	19278001	19283000	5.07E-40
chr1	19279001	19284000	1.04E-44
chr1	19281001	19286000	5.47E-46
chr1	19282001	19287000	2.38E-53
chr1	19283001	19288000	6.95E-51
chr1	19574001	19579000	8.81E-22
chr1	19575001	19580000	3.43E-26
chr1	19576001	19581000	1.11E-20
chr1	19634001	19639000	2.13E-19
chr1	19635001	19640000	1.98E-20
chr1	19636001	19641000	7.95E-22
chr1	19637001	19642000	1.73E-15
chr1	19638001	19643000	1.52E-19

chr1	19639001	19644000	1.30E-08
chr1	19971001	19976000	2.52E-05
chr1	20122001	20127000	2.56E-35
chr1	20123001	20128000	1.43E-17
chr1	20124001	20129000	1.75E-09
chr1	20185001	20190000	1.19E-11
chr1	20186001	20191000	4.81E-12
chr1	20187001	20192000	4.97E-12
chr1	20188001	20193000	1.55E-11
chr1	20189001	20194000	7.51E-11
chr1	20209001	20214000	2.20E-50
chr1	20746001	20751000	0.000154774
chr1	20830001	20835000	1.31E-25
chr1	20831001	20836000	1.74E-23
chr1	20832001	20837000	5.00E-39
chr1	20833001	20838000	5.23E-31
chr1	20834001	20839000	9.30E-23
chr1	21065001	21070000	0.010535738
chr1	21113001	21118000	3.27E-11
chr1	21503001	21508000	1.53E-20
chr1	21612001	21617000	2.11E-18
chr1	21613001	21618000	6.73E-20
chr1	21614001	21619000	9.73E-14
chr1	21615001	21620000	3.73E-07
chr1	21616001	21621000	6.75E-07
chr1	21672001	21677000	5.19E-11
chr1	21766001	21771000	1.83E-33
chr1	23346001	23351000	5.12E-47
chr1	23425001	23430000	0.000225373
chr1	23500001	23505000	1.34E-10
chr1	23501001	23506000	9.96E-19
chr1	23502001	23507000	1.18E-22
chr1	23503001	23508000	2.10E-17
chr1	23539001	23544000	8.34E-13
chr1	23670001	23675000	6.12E-26
chr1	23810001	23815000	1.04E-09
chr1	23853001	23858000	1.27E-09
chr1	23854001	23859000	2.99E-10
chr1	23855001	23860000	2.81E-15
chr1	23856001	23861000	1.02E-12
chr1	23857001	23862000	5.61E-12
chr1	24065001	24070000	1.10E-06
chr1	24066001	24071000	1.86E-12
chr1	24126001	24131000	1.06E-18
chr1	24127001	24132000	7.82E-14
chr1	24152001	24157000	5.24E-05
chr1	24190001	24195000	4.55E-23
chr1	24229001	24234000	4.90E-08

chr1	24510001	24515000	6.33E-09
chr1	24513001	24518000	2.90E-13
chr1	24969001	24974000	8.09E-47
chr1	24970001	24975000	4.12E-46
chr1	25170001	25175000	8.34E-18
chr1	25171001	25176000	1.94E-18
chr1	25173001	25178000	1.33E-20
chr1	25174001	25179000	2.35E-28
chr1	25175001	25180000	3.49E-22
chr1	25203001	25208000	5.84E-09
chr1	25574001	25579000	8.64E-21
chr1	25752001	25757000	1.19E-24
chr1	25753001	25758000	1.00E-35
chr1	25755001	25760000	2.58E-59
chr1	25756001	25761000	1.15E-72
chr1	25757001	25762000	1.79E-64
chr1	25758001	25763000	3.69E-27
chr1	25866001	25871000	7.76E-23
chr1	25867001	25872000	6.84E-15
chr1	25868001	25873000	1.36E-25
chr1	25869001	25874000	2.05E-30
chr1	25870001	25875000	3.72E-07
chr1	25939001	25944000	1.11E-17
chr1	26122001	26127000	3.49E-31
chr1	26124001	26129000	1.01E-05
chr1	26125001	26130000	1.47E-09
chr1	26126001	26131000	2.35E-06
chr1	26181001	26186000	1.40E-21
chr1	26183001	26188000	1.06E-14
chr1	26184001	26189000	8.93E-27
chr1	26200001	26205000	9.15E-08
chr1	26201001	26206000	1.62E-07
chr1	26484001	26489000	8.30E-14
chr1	26485001	26490000	1.40E-06
chr1	26489001	26494000	7.11E-17
chr1	26492001	26497000	3.55E-14
chr1	26493001	26498000	5.09E-11
chr1	26495001	26500000	3.95E-06
chr1	26496001	26501000	1.24E-08
chr1	26556001	26561000	1.21E-08
chr1	26825001	26830000	5.04E-10
chr1	26826001	26831000	1.10E-29
chr1	26827001	26832000	2.34E-45
chr1	26852001	26857000	3.21E-08
chr1	26853001	26858000	3.48E-08
chr1	27068001	27073000	1.75E-06
chr1	27149001	27154000	6.55E-27
chr1	27150001	27155000	1.36E-17

chr1	27151001	27156000	6.98E-17
chr1	27152001	27157000	5.13E-15
chr1	27187001	27192000	5.05E-25
chr1	27222001	27227000	2.11E-20
chr1	27248001	27253000	8.52E-29
chr1	27557001	27562000	8.84E-21
chr1	27558001	27563000	1.49E-08
chr1	27559001	27564000	2.36E-10
chr1	27560001	27565000	2.16E-12
chr1	27561001	27566000	9.39E-10
chr1	27648001	27653000	4.24E-26
chr1	27812001	27817000	2.02E-38
chr1	27854001	27859000	8.90E-15
chr1	27930001	27935000	2.22E-11
chr1	27956001	27961000	5.02E-12
chr1	28052001	28057000	2.34E-40
chr1	28053001	28058000	1.26E-14
chr1	28153001	28158000	2.35E-09
chr1	28154001	28159000	2.95E-18
chr1	28156001	28161000	1.61E-26
chr1	28157001	28162000	1.07E-22
chr1	28241001	28246000	2.63E-18
chr1	28558001	28563000	6.72E-32
chr1	28559001	28564000	2.99E-46
chr1	28560001	28565000	2.15E-47
chr1	28582001	28587000	1.30E-06
chr1	28693001	28698000	4.10E-12
chr1	28694001	28699000	1.06E-12
chr1	28695001	28700000	5.59E-12
chr1	28696001	28701000	2.04E-09
chr1	28844001	28849000	5.73E-11
chr1	28845001	28850000	2.95E-09
chr1	28875001	28880000	1.43E-21
chr1	28876001	28881000	2.59E-18
chr1	28877001	28882000	4.39E-15
chr1	28878001	28883000	1.07E-19
chr1	28915001	28920000	6.16E-16
chr1	28966001	28971000	3.12E-16
chr1	28967001	28972000	2.10E-16
chr1	28968001	28973000	1.08E-14
chr1	28969001	28974000	2.97E-14
chr1	29508001	29513000	8.16E-24
chr1	29564001	29569000	1.43E-17
chr1	29586001	29591000	3.19E-12
chr1	30619001	30624000	9.08E-10
chr1	31063001	31068000	5.84E-06
chr1	31064001	31069000	9.80E-05
chr1	31065001	31070000	3.53E-05

chr1	31190001	31195000	1.02E-19
chr1	31276001	31281000	1.74E-11
chr1	31277001	31282000	1.49E-10
chr1	31627001	31632000	2.50E-12
chr1	31712001	31717000	3.19E-21
chr1	31769001	31774000	2.27E-15
chr1	31770001	31775000	7.88E-12
chr1	31863001	31868000	5.32E-33
chr1	31864001	31869000	2.21E-17
chr1	31885001	31890000	1.81E-19
chr1	31886001	31891000	6.10E-26
chr1	32224001	32229000	2.51E-20
chr1	32225001	32230000	1.89E-06
chr1	32252001	32257000	8.54E-08
chr1	32253001	32258000	2.16E-05
chr1	32475001	32480000	2.99E-07
chr1	32479001	32484000	1.72E-26
chr1	32537001	32542000	1.44E-14
chr1	32671001	32676000	9.78E-07
chr1	32706001	32711000	4.33E-26
chr1	32797001	32802000	6.32E-09
chr1	32798001	32803000	9.48E-07
chr1	32799001	32804000	2.37E-08
chr1	32814001	32819000	3.75E-17
chr1	32815001	32820000	3.63E-13
chr1	32816001	32821000	5.43E-15
chr1	32817001	32822000	7.84E-13
chr1	32855001	32860000	4.35E-12
chr1	32966001	32971000	1.57E-16
chr1	33001001	33006000	2.80E-25
chr1	33002001	33007000	5.76E-30
chr1	33003001	33008000	7.10E-29
chr1	33004001	33009000	1.61E-29
chr1	33005001	33010000	4.72E-08
chr1	33117001	33122000	4.21E-11
chr1	33164001	33169000	1.71E-32
chr1	33165001	33170000	9.29E-18
chr1	33215001	33220000	1.29E-18
chr1	33279001	33284000	2.65E-19
chr1	33281001	33286000	6.14E-17
chr1	33282001	33287000	1.78E-52
chr1	33283001	33288000	1.68E-26
chr1	33336001	33341000	4.88E-07
chr1	33434001	33439000	3.87E-19
chr1	33435001	33440000	1.61E-09
chr1	33498001	33503000	6.91E-15
chr1	33499001	33504000	1.16E-14
chr1	33500001	33505000	7.16E-19

chr1	33501001	33506000	5.87E-22
chr1	33502001	33507000	1.60E-22
chr1	33642001	33647000	1.14E-14
chr1	33643001	33648000	4.53E-17
chr1	33644001	33649000	7.30E-17
chr1	33645001	33650000	6.78E-26
chr1	33717001	33722000	8.21E-10
chr1	33718001	33723000	1.98E-15
chr1	33719001	33724000	5.14E-08
chr1	33814001	33819000	2.40E-37
chr1	33815001	33820000	2.29E-37
chr1	33892001	33897000	3.59E-62
chr1	33893001	33898000	7.34E-72
chr1	33894001	33899000	2.49E-61
chr1	33895001	33900000	7.34E-64
chr1	33896001	33901000	4.12E-43
chr1	33938001	33943000	1.61E-12
chr1	34352001	34357000	3.71E-07
chr1	34353001	34358000	4.58E-07
chr1	34354001	34359000	1.77E-06
chr1	34407001	34412000	6.83E-05
chr1	34580001	34585000	0.00396648
chr1	34581001	34586000	0.005346575
chr1	34597001	34602000	7.66E-05
chr1	34598001	34603000	9.32E-07
chr1	34599001	34604000	1.20E-05
chr1	34740001	34745000	2.19E-05
chr1	34773001	34778000	1.05E-08
chr1	34774001	34779000	4.46E-09
chr1	34775001	34780000	5.67E-06
chr1	34844001	34849000	1.38E-09
chr1	34861001	34866000	3.35E-05
chr1	34862001	34867000	0.00138674
chr1	34880001	34885000	5.42E-07
chr1	34892001	34897000	2.35E-07
chr1	34950001	34955000	0.00256551
chr1	34986001	34991000	3.62E-06
chr1	35007001	35012000	5.90E-05
chr1	35141001	35146000	6.78E-07
chr1	35144001	35149000	1.19E-05
chr1	35145001	35150000	1.47E-08
chr1	35246001	35251000	9.31E-27
chr1	35247001	35252000	1.91E-18
chr1	35321001	35326000	2.70E-15
chr1	35322001	35327000	9.53E-27
chr1	35323001	35328000	1.63E-23
chr1	35324001	35329000	2.46E-27
chr1	35325001	35330000	8.17E-24

chr1	35451001	35456000	2.07E-11
chr1	35543001	35548000	1.61E-09
chr1	35545001	35550000	5.31E-12
chr1	36034001	36039000	3.38E-07
chr1	36182001	36187000	1.79E-14
chr1	36183001	36188000	1.65E-13
chr1	36184001	36189000	2.28E-19
chr1	36235001	36240000	1.02E-15
chr1	36344001	36349000	5.70E-15
chr1	36345001	36350000	1.28E-10
chr1	36617001	36622000	4.70E-15
chr1	36768001	36773000	1.05E-15
chr1	36769001	36774000	7.46E-21
chr1	36770001	36775000	4.02E-18
chr1	36771001	36776000	1.58E-15
chr1	36772001	36777000	4.01E-19
chr1	36847001	36852000	3.26E-12
chr1	36912001	36917000	4.71E-10
chr1	36913001	36918000	9.56E-08
chr1	36914001	36919000	2.80E-08
chr1	36915001	36920000	6.13E-19
chr1	36916001	36921000	1.76E-17
chr1	36925001	36930000	1.17E-17
chr1	36926001	36931000	2.85E-13
chr1	36927001	36932000	3.09E-11
chr1	37935001	37940000	3.53E-13
chr1	37936001	37941000	3.31E-14
chr1	38015001	38020000	2.42E-36
chr1	38016001	38021000	1.21E-23
chr1	38017001	38022000	1.18E-21
chr1	38151001	38156000	6.14E-15
chr1	38152001	38157000	3.76E-19
chr1	38153001	38158000	5.42E-32
chr1	38214001	38219000	1.91E-11
chr1	38215001	38220000	5.82E-09
chr1	38216001	38221000	6.43E-06
chr1	38217001	38222000	2.90E-07
chr1	38255001	38260000	3.76E-26
chr1	38256001	38261000	1.17E-27
chr1	38257001	38262000	1.07E-22
chr1	38258001	38263000	7.75E-23
chr1	38259001	38264000	8.20E-09
chr1	38269001	38274000	6.55E-19
chr1	38270001	38275000	1.47E-16
chr1	38271001	38276000	6.47E-29
chr1	38272001	38277000	1.86E-20
chr1	38273001	38278000	1.83E-26
chr1	38274001	38279000	1.11E-27

chr1	38321001	38326000	1.05E-24
chr1	38322001	38327000	1.70E-16
chr1	38323001	38328000	2.88E-12
chr1	38453001	38458000	2.29E-17
chr1	38454001	38459000	3.06E-19
chr1	38455001	38460000	1.98E-25
chr1	38457001	38462000	6.75E-16
chr1	38468001	38473000	4.57E-07
chr1	38469001	38474000	2.70E-09
chr1	38470001	38475000	5.40E-29
chr1	38471001	38476000	2.58E-17
chr1	38780001	38785000	5.55E-06
chr1	38781001	38786000	2.66E-08
chr1	38782001	38787000	8.57E-08
chr1	38783001	38788000	2.44E-08
chr1	38784001	38789000	1.24E-08
chr1	39040001	39045000	3.99E-22
chr1	39095001	39100000	5.54E-07
chr1	39265001	39270000	3.29E-10
chr1	39320001	39325000	5.56E-08
chr1	39321001	39326000	1.85E-15
chr1	39334001	39339000	6.91E-12
chr1	39337001	39342000	1.18E-38
chr1	39338001	39343000	2.34E-44
chr1	39339001	39344000	4.18E-31
chr1	39543001	39548000	1.17E-16
chr1	39571001	39576000	3.29E-13
chr1	39870001	39875000	1.32E-36
chr1	39871001	39876000	2.89E-47
chr1	39872001	39877000	7.62E-37
chr1	39873001	39878000	1.16E-33
chr1	39952001	39957000	2.51E-24
chr1	40039001	40044000	4.30E-24
chr1	40040001	40045000	1.14E-37
chr1	40041001	40046000	2.58E-59
chr1	40042001	40047000	1.74E-45
chr1	40103001	40108000	3.14E-07
chr1	40104001	40109000	1.58E-10
chr1	40105001	40110000	4.21E-09
chr1	40155001	40160000	3.65E-06
chr1	40156001	40161000	3.40E-07
chr1	40157001	40162000	7.96E-12
chr1	40254001	40259000	3.63E-13
chr1	40308001	40313000	1.61E-05
chr1	40309001	40314000	2.71E-12
chr1	40363001	40368000	8.89E-12
chr1	40365001	40370000	4.65E-18
chr1	40367001	40372000	2.69E-32

chr1	40368001	40373000	3.00E-14
chr1	40416001	40421000	4.67E-09
chr1	40558001	40563000	1.09E-13
chr1	40559001	40564000	3.15E-14
chr1	40560001	40565000	1.99E-14
chr1	40561001	40566000	7.87E-21
chr1	40562001	40567000	1.42E-18
chr1	40563001	40568000	1.53E-10
chr1	40782001	40787000	8.92E-11
chr1	40838001	40843000	1.77E-36
chr1	40840001	40845000	2.67E-26
chr1	40938001	40943000	1.05E-41
chr1	40939001	40944000	9.93E-41
chr1	40940001	40945000	2.13E-30
chr1	40941001	40946000	2.78E-33
chr1	40942001	40947000	3.93E-32
chr1	40943001	40948000	3.52E-15
chr1	40970001	40975000	1.78E-29
chr1	40971001	40976000	3.15E-37
chr1	40972001	40977000	3.70E-39
chr1	40973001	40978000	1.21E-38
chr1	40974001	40979000	2.91E-41
chr1	40993001	40998000	1.40E-16
chr1	40994001	40999000	1.16E-16
chr1	40995001	41000000	6.28E-22
chr1	40996001	41001000	3.66E-19
chr1	40997001	41002000	1.31E-08
chr1	41249001	41254000	3.12E-06
chr1	41327001	41332000	2.62E-11
chr1	41441001	41446000	3.81E-13
chr1	41445001	41450000	6.32E-20
chr1	41702001	41707000	8.49E-14
chr1	41703001	41708000	3.78E-28
chr1	41708001	41713000	1.20E-12
chr1	41799001	41804000	0.004531125
chr1	41829001	41834000	0.00058475
chr1	41830001	41835000	1.06E-09
chr1	41831001	41836000	3.18E-09
chr1	41844001	41849000	3.26E-08
chr1	41980001	41985000	2.20E-11
chr1	42123001	42128000	9.08E-54
chr1	42124001	42129000	9.42E-49
chr1	42125001	42130000	5.52E-37
chr1	42126001	42131000	3.74E-31
chr1	42127001	42132000	1.27E-17
chr1	42128001	42133000	1.27E-14
chr1	42385001	42390000	3.75E-08
chr1	42499001	42504000	6.13E-26

chr1	42500001	42505000	1.69E-30
chr1	42501001	42506000	1.84E-36
chr1	42796001	42801000	9.27E-39
chr1	42797001	42802000	1.66E-34
chr1	42842001	42847000	2.91E-20
chr1	42843001	42848000	3.35E-33
chr1	42844001	42849000	1.82E-59
chr1	42845001	42850000	1.66E-63
chr1	42846001	42851000	2.16E-88
chr1	43232001	43237000	1.04E-18
chr1	43233001	43238000	2.92E-11
chr1	43820001	43825000	7.41E-40
chr1	43821001	43826000	5.61E-42
chr1	43822001	43827000	5.57E-39
chr1	43996001	44001000	2.06E-13
chr1	43997001	44002000	5.46E-11
chr1	44169001	44174000	4.00E-29
chr1	44170001	44175000	6.85E-48
chr1	44171001	44176000	6.15E-55
chr1	44172001	44177000	1.02E-72
chr1	44173001	44178000	2.30E-76
chr1	44402001	44407000	1.02E-08
chr1	44410001	44415000	2.22E-12
chr1	44412001	44417000	3.52E-09
chr1	44439001	44444000	2.22E-11
chr1	44440001	44445000	5.58E-15
chr1	44441001	44446000	2.88E-13
chr1	44442001	44447000	1.18E-09
chr1	44674001	44679000	6.63E-10
chr1	44816001	44821000	4.69E-24
chr1	44817001	44822000	1.03E-14
chr1	44818001	44823000	2.02E-17
chr1	44819001	44824000	1.88E-16
chr1	44820001	44825000	2.53E-15
chr1	44867001	44872000	8.65E-59
chr1	44868001	44873000	1.29E-51
chr1	44881001	44886000	1.03E-16
chr1	44882001	44887000	1.19E-20
chr1	44883001	44888000	1.75E-20
chr1	45093001	45098000	1.14E-12
chr1	45201001	45206000	2.36E-08
chr1	45205001	45210000	8.34E-32
chr1	45261001	45266000	7.93E-07
chr1	45262001	45267000	1.05E-10
chr1	45476001	45481000	2.72E-12
chr1	45477001	45482000	3.25E-08
chr1	45667001	45672000	1.73E-68
chr1	45668001	45673000	3.04E-61

chr1	45669001	45674000	3.01E-65
chr1	45765001	45770000	7.92E-10
chr1	45769001	45774000	4.65E-17
chr1	45801001	45806000	4.28E-25
chr1	45961001	45966000	9.78E-07
chr1	45962001	45967000	1.03E-10
chr1	45964001	45969000	3.23E-05
chr1	45965001	45970000	6.24E-07
chr1	45986001	45991000	9.57E-28
chr1	45987001	45992000	8.61E-29
chr1	46016001	46021000	1.37E-05
chr1	46049001	46054000	2.38E-11
chr1	46268001	46273000	1.82E-46
chr1	46269001	46274000	5.21E-53
chr1	46599001	46604000	1.52E-06
chr1	46661001	46666000	3.77E-05
chr1	46663001	46668000	3.65E-08
chr1	46664001	46669000	6.09E-07
chr1	46709001	46714000	4.06E-08
chr1	46710001	46715000	2.23E-11
chr1	46711001	46716000	3.59E-09
chr1	46712001	46717000	1.66E-15
chr1	46713001	46718000	8.75E-13
chr1	46928001	46933000	3.27E-25
chr1	46929001	46934000	4.76E-06
chr1	46947001	46952000	1.27E-08
chr1	46996001	47001000	4.26E-10
chr1	46997001	47002000	1.09E-10
chr1	46998001	47003000	2.00E-21
chr1	46999001	47004000	5.21E-19
chr1	47006001	47011000	1.71E-05
chr1	47065001	47070000	2.15E-28
chr1	47066001	47071000	9.79E-25
chr1	47067001	47072000	9.24E-21
chr1	47068001	47073000	8.69E-25
chr1	47069001	47074000	3.63E-27
chr1	47079001	47084000	0.000289304
chr1	47080001	47085000	1.12E-08
chr1	47081001	47086000	1.85E-05
chr1	47082001	47087000	5.58E-08
chr1	47129001	47134000	1.86E-12
chr1	47132001	47137000	1.01E-10
chr1	47133001	47138000	1.81E-13
chr1	47134001	47139000	4.33E-08
chr1	47183001	47188000	5.29E-18
chr1	47457001	47462000	1.68E-11
chr1	47504001	47509000	0.004757007
chr1	47775001	47780000	7.25E-16

chr1	47795001	47800000	2.11E-17
chr1	47799001	47804000	5.14E-18
chr1	47800001	47805000	1.49E-07
chr1	47880001	47885000	7.55E-16
chr1	47902001	47907000	5.71E-26
chr1	47903001	47908000	3.33E-28
chr1	47904001	47909000	3.26E-08
chr1	47970001	47975000	1.58E-12
chr1	47971001	47976000	1.77E-18
chr1	47972001	47977000	2.60E-19
chr1	47973001	47978000	2.05E-11
chr1	48462001	48467000	6.27E-23
chr1	48463001	48468000	8.68E-20
chr1	48609001	48614000	1.45E-07
chr1	48610001	48615000	3.98E-07
chr1	48611001	48616000	7.72E-08
chr1	48612001	48617000	4.22E-06
chr1	48645001	48650000	8.77E-05
chr1	48646001	48651000	4.71E-05
chr1	48647001	48652000	0.000206982
chr1	48747001	48752000	1.23E-05
chr1	48748001	48753000	0.001928963
chr1	48797001	48802000	1.29E-08
chr1	49238001	49243000	9.40E-10
chr1	49242001	49247000	8.46E-13
chr1	49265001	49270000	0.001409723
chr1	49266001	49271000	0.00026564
chr1	49421001	49426000	0.006188665
chr1	49424001	49429000	1.48E-05
chr1	49425001	49430000	5.40E-05
chr1	49426001	49431000	0.000305885
chr1	49497001	49502000	0.010856472
chr1	49591001	49596000	0.00079768
chr1	49596001	49601000	2.98E-06
chr1	49668001	49673000	6.53E-05
chr1	49777001	49782000	0.000374818
chr1	49778001	49783000	0.000491785
chr1	49779001	49784000	0.00035145
chr1	49931001	49936000	0.000712168
chr1	49997001	50002000	3.23E-06
chr1	50014001	50019000	3.81E-12
chr1	50015001	50020000	9.39E-10
chr1	50095001	50100000	0.001553942
chr1	50096001	50101000	9.71E-05
chr1	50097001	50102000	0.001940631
chr1	50144001	50149000	8.73E-05
chr1	50145001	50150000	0.00042982
chr1	50146001	50151000	0.000404564

chr1	50297001	50302000	1.67E-09
chr1	50370001	50375000	0.004881916
chr1	50371001	50376000	0.002940071
chr1	50429001	50434000	2.82E-05
chr1	50510001	50515000	3.35E-19
chr1	50511001	50516000	7.52E-21
chr1	50512001	50517000	3.64E-27
chr1	50513001	50518000	5.62E-30
chr1	50548001	50553000	0.016009494
chr1	50848001	50853000	0.000290625
chr1	50849001	50854000	0.00227142
chr1	50871001	50876000	0.01517217
chr1	51421001	51426000	1.01E-58
chr1	51439001	51444000	1.09E-50
chr1	51473001	51478000	2.68E-05
chr1	51792001	51797000	1.21E-09
chr1	51796001	51801000	1.80E-07
chr1	51878001	51883000	1.11E-08
chr1	51981001	51986000	2.73E-26
chr1	51982001	51987000	1.25E-14
chr1	51983001	51988000	4.84E-20
chr1	51984001	51989000	1.79E-25
chr1	52082001	52087000	4.90E-13
chr1	52339001	52344000	1.99E-21
chr1	52452001	52457000	6.60E-19
chr1	52453001	52458000	6.90E-08
chr1	52454001	52459000	8.91E-06
chr1	52455001	52460000	6.94E-06
chr1	52456001	52461000	9.21E-07
chr1	52494001	52499000	9.77E-20
chr1	52495001	52500000	6.80E-25
chr1	52496001	52501000	2.78E-23
chr1	52497001	52502000	1.56E-28
chr1	52498001	52503000	1.94E-36
chr1	52499001	52504000	1.89E-25
chr1	52603001	52608000	3.75E-16
chr1	52832001	52837000	1.28E-26
chr1	52833001	52838000	3.60E-28
chr1	52834001	52839000	1.28E-24
chr1	52870001	52875000	1.88E-15
chr1	52885001	52890000	4.81E-10
chr1	52887001	52892000	4.07E-05
chr1	52888001	52893000	9.54E-05
chr1	53161001	53166000	3.07E-19
chr1	53192001	53197000	2.04E-13
chr1	53304001	53309000	2.87E-22
chr1	53305001	53310000	1.15E-23
chr1	53306001	53311000	2.78E-33

chr1	53307001	53312000	4.91E-22
chr1	53308001	53313000	4.43E-23
chr1	53383001	53388000	2.28E-18
chr1	53384001	53389000	9.72E-14
chr1	53389001	53394000	7.05E-22
chr1	53527001	53532000	0.000224427
chr1	53658001	53663000	2.76E-19
chr1	53686001	53691000	2.03E-19
chr1	53700001	53705000	2.07E-09
chr1	53703001	53708000	9.18E-12
chr1	53704001	53709000	1.62E-15
chr1	53788001	53793000	1.27E-30
chr1	53789001	53794000	2.93E-29
chr1	53790001	53795000	1.29E-25
chr1	53793001	53798000	9.54E-08
chr1	54202001	54207000	1.38E-13
chr1	54203001	54208000	1.28E-17
chr1	54204001	54209000	1.19E-25
chr1	54205001	54210000	9.57E-17
chr1	54299001	54304000	1.22E-36
chr1	54300001	54305000	8.85E-34
chr1	54407001	54412000	3.34E-33
chr1	54666001	54671000	1.94E-21
chr1	55003001	55008000	3.89E-11
chr1	55924001	55929000	7.38E-05
chr1	56061001	56066000	0.001096191
chr1	56192001	56197000	0.00484883
chr1	56852001	56857000	0.003419956
chr1	57043001	57048000	1.18E-32
chr1	57044001	57049000	2.58E-54
chr1	57045001	57050000	5.00E-17
chr1	57111001	57116000	9.92E-35
chr1	57119001	57124000	0.011085846
chr1	57246001	57251000	8.56E-05
chr1	57253001	57258000	2.48E-07
chr1	57337001	57342000	8.25E-06
chr1	57358001	57363000	8.33E-05
chr1	57366001	57371000	0.001816402
chr1	57398001	57403000	2.95E-07
chr1	57401001	57406000	0.00167218
chr1	57511001	57516000	1.21E-05
chr1	57579001	57584000	6.93E-06
chr1	57605001	57610000	0.002187956
chr1	57606001	57611000	2.09E-05
chr1	57708001	57713000	0.001539948
chr1	57710001	57715000	0.00030044
chr1	57711001	57716000	0.000664528
chr1	57727001	57732000	1.04E-09

chr1	57728001	57733000	8.23E-12
chr1	57743001	57748000	1.47E-06
chr1	57744001	57749000	0.000322271
chr1	57745001	57750000	0.000112885
chr1	57746001	57751000	0.000129973
chr1	57747001	57752000	6.35E-06
chr1	57754001	57759000	1.31E-05
chr1	57755001	57760000	7.29E-08
chr1	57843001	57848000	3.09E-05
chr1	57844001	57849000	5.24E-05
chr1	57845001	57850000	3.33E-05
chr1	57846001	57851000	0.000458234
chr1	57847001	57852000	0.001944261
chr1	57854001	57859000	6.66E-06
chr1	57857001	57862000	1.95E-08
chr1	57884001	57889000	1.73E-18
chr1	57968001	57973000	0.000276624
chr1	58012001	58017000	2.04E-07
chr1	58013001	58018000	5.48E-07
chr1	58016001	58021000	3.50E-07
chr1	58019001	58024000	0.000409704
chr1	58146001	58151000	1.03E-07
chr1	58147001	58152000	1.24E-07
chr1	58148001	58153000	1.58E-09
chr1	58149001	58154000	3.02E-11
chr1	58150001	58155000	1.44E-10
chr1	58151001	58156000	2.39E-05
chr1	58275001	58280000	0.000628739
chr1	58276001	58281000	1.28E-05
chr1	58715001	58720000	6.40E-23
chr1	58824001	58829000	0.003525521
chr1	58834001	58839000	8.02E-07
chr1	58958001	58963000	7.41E-05
chr1	59041001	59046000	2.43E-25
chr1	59042001	59047000	2.58E-36
chr1	59043001	59048000	6.98E-12
chr1	59078001	59083000	9.80E-05
chr1	59079001	59084000	0.000426121
chr1	59161001	59166000	1.15E-13
chr1	59162001	59167000	8.57E-21
chr1	59163001	59168000	1.40E-26
chr1	59164001	59169000	2.73E-36
chr1	59165001	59170000	4.10E-36
chr1	59249001	59254000	2.92E-15
chr1	59250001	59255000	6.29E-16
chr1	59282001	59287000	8.43E-33
chr1	59365001	59370000	8.58E-22
chr1	59612001	59617000	5.87E-22

chr1	59613001	59618000	5.01E-10
chr1	59684001	59689000	8.89E-05
chr1	59685001	59690000	0.000100339
chr1	59719001	59724000	2.02E-09
chr1	59720001	59725000	6.99E-09
chr1	59850001	59855000	2.80E-10
chr1	60144001	60149000	0.005482185
chr1	60276001	60281000	3.65E-34
chr1	60277001	60282000	1.36E-27
chr1	60278001	60283000	3.29E-29
chr1	60279001	60284000	2.50E-28
chr1	60280001	60285000	3.48E-30
chr1	60388001	60393000	8.17E-12
chr1	60500001	60505000	0.000704818
chr1	60514001	60519000	0.000189047
chr1	60519001	60524000	0.000267225
chr1	60520001	60525000	0.000171339
chr1	60835001	60840000	0.004966784
chr1	60836001	60841000	0.007980796
chr1	60912001	60917000	0.007424485
chr1	60913001	60918000	0.003393545
chr1	61021001	61026000	7.27E-05
chr1	61022001	61027000	0.000394684
chr1	61023001	61028000	0.00043558
chr1	61060001	61065000	2.52E-18
chr1	61061001	61066000	1.27E-14
chr1	61062001	61067000	1.05E-16
chr1	61063001	61068000	4.20E-12
chr1	61064001	61069000	3.13E-09
chr1	61231001	61236000	0.000659787
chr1	61298001	61303000	1.51E-06
chr1	61504001	61509000	1.78E-38
chr1	61505001	61510000	2.67E-24
chr1	61506001	61511000	4.34E-17
chr1	61507001	61512000	1.12E-16
chr1	61508001	61513000	3.47E-13
chr1	61521001	61526000	6.53E-20
chr1	61522001	61527000	1.05E-13
chr1	61523001	61528000	2.91E-13
chr1	61548001	61553000	8.69E-62
chr1	61549001	61554000	1.40E-40
chr1	62204001	62209000	2.31E-22
chr1	62586001	62591000	2.86E-07
chr1	62780001	62785000	3.78E-12
chr1	62781001	62786000	2.25E-18
chr1	62782001	62787000	3.28E-14
chr1	62783001	62788000	1.13E-15
chr1	62784001	62789000	9.25E-24

chr1	62898001	62903000	1.61E-29
chr1	63104001	63109000	0.000396956
chr1	63105001	63110000	0.00046872
chr1	63106001	63111000	0.001881151
chr1	63149001	63154000	2.13E-25
chr1	63150001	63155000	1.75E-44
chr1	63151001	63156000	1.64E-32
chr1	63152001	63157000	2.51E-25
chr1	63154001	63159000	1.23E-12
chr1	63238001	63243000	1.05E-08
chr1	63239001	63244000	6.61E-05
chr1	63245001	63250000	4.01E-26
chr1	63246001	63251000	3.89E-22
chr1	63247001	63252000	4.36E-26
chr1	63248001	63253000	4.63E-24
chr1	63249001	63254000	9.49E-28
chr1	63250001	63255000	3.16E-07
chr1	63557001	63562000	7.06E-05
chr1	63558001	63563000	4.06E-05
chr1	63559001	63564000	0.001938856
chr1	63829001	63834000	1.51E-09
chr1	63830001	63835000	3.45E-10
chr1	63831001	63836000	3.34E-26
chr1	63832001	63837000	8.62E-42
chr1	63833001	63838000	1.60E-58
chr1	63986001	63991000	4.83E-43
chr1	63987001	63992000	1.27E-56
chr1	63988001	63993000	1.14E-47
chr1	63989001	63994000	1.53E-19
chr1	64054001	64059000	3.01E-15
chr1	64055001	64060000	3.13E-89
chr1	64056001	64061000	2.09E-93
chr1	64057001	64062000	3.90E-91
chr1	64058001	64063000	3.02E-90
chr1	64059001	64064000	3.33E-89
chr1	64060001	64065000	3.30E-20
chr1	64235001	64240000	2.56E-12
chr1	64239001	64244000	4.08E-29
chr1	64240001	64245000	1.17E-33
chr1	64505001	64510000	0.0050125
chr1	64764001	64769000	0.003300254
chr1	64779001	64784000	3.50E-05
chr1	64805001	64810000	0.000364933
chr1	64843001	64848000	0.006660085
chr1	64931001	64936000	1.97E-09
chr1	64982001	64987000	0.005657283
chr1	64983001	64988000	0.000301693
chr1	65174001	65179000	0.000298293

chr1	65206001	65211000	6.17E-87
chr1	65207001	65212000	1.54E-69
chr1	65208001	65213000	9.45E-57
chr1	65209001	65214000	3.32E-52
chr1	65329001	65334000	5.49E-14
chr1	65427001	65432000	4.84E-23
chr1	65432001	65437000	3.31E-48
chr1	65435001	65440000	2.92E-08
chr1	65436001	65441000	6.23E-08
chr1	65464001	65469000	2.45E-21
chr1	65468001	65473000	1.32E-19
chr1	65609001	65614000	1.10E-34
chr1	65610001	65615000	2.75E-43
chr1	65611001	65616000	6.65E-37
chr1	65612001	65617000	3.10E-16
chr1	65720001	65725000	1.06E-21
chr1	65771001	65776000	7.57E-36
chr1	65774001	65779000	6.02E-30
chr1	65775001	65780000	1.92E-24
chr1	65881001	65886000	1.62E-18
chr1	66024001	66029000	4.73E-05
chr1	66130001	66135000	9.81E-06
chr1	66131001	66136000	1.56E-08
chr1	66132001	66137000	8.12E-10
chr1	66348001	66353000	0.014668185
chr1	66437001	66442000	0.012131436
chr1	66484001	66489000	2.92E-07
chr1	66522001	66527000	0.022874457
chr1	66523001	66528000	0.012527992
chr1	66551001	66556000	0.002428456
chr1	66999001	67004000	2.26E-09
chr1	67270001	67275000	3.19E-07
chr1	67271001	67276000	1.13E-07
chr1	67311001	67316000	0.00301713
chr1	67312001	67317000	0.001400318
chr1	67313001	67318000	0.000655864
chr1	67378001	67383000	1.27E-07
chr1	67514001	67519000	8.29E-16
chr1	67515001	67520000	6.34E-57
chr1	67520001	67525000	1.35E-07
chr1	67692001	67697000	0.00016897
chr1	68147001	68152000	1.52E-49
chr1	68150001	68155000	3.18E-49
chr1	68151001	68156000	1.65E-54
chr1	68152001	68157000	1.36E-44
chr1	68298001	68303000	8.17E-50
chr1	68299001	68304000	2.43E-32
chr1	68698001	68703000	1.94E-10

chr1	68958001	68963000	1.05E-27
chr1	68962001	68967000	3.47E-21
chr1	68963001	68968000	2.91E-08
chr1	68966001	68971000	2.94E-05
chr1	69252001	69257000	4.98E-05
chr1	69453001	69458000	0.000915166
chr1	69460001	69465000	1.97E-09
chr1	69461001	69466000	3.95E-06
chr1	69701001	69706000	0.002757366
chr1	70028001	70033000	5.83E-16
chr1	70029001	70034000	1.23E-36
chr1	70030001	70035000	5.51E-36
chr1	70035001	70040000	2.74E-10
chr1	70312001	70317000	0.001382078
chr1	70815001	70820000	2.18E-23
chr1	70816001	70821000	1.62E-59
chr1	70817001	70822000	6.29E-50
chr1	70818001	70823000	5.64E-45
chr1	70819001	70824000	1.82E-40
chr1	70820001	70825000	3.10E-49
chr1	70872001	70877000	2.58E-26
chr1	71341001	71346000	8.27E-05
chr1	71369001	71374000	0.001714459
chr1	71370001	71375000	0.001307892
chr1	71542001	71547000	3.58E-16
chr1	71543001	71548000	6.09E-15
chr1	71614001	71619000	0.001110237
chr1	71709001	71714000	0.000552745
chr1	71753001	71758000	0.00033685
chr1	72562001	72567000	0.003029266
chr1	73880001	73885000	0.001085031
chr1	73895001	73900000	0.00026631
chr1	74109001	74114000	0.010266674
chr1	74111001	74116000	0.010502869
chr1	74373001	74378000	0.003105951
chr1	74374001	74379000	0.001439757
chr1	74490001	74495000	1.95E-07
chr1	74491001	74496000	0.000204575
chr1	74659001	74664000	6.30E-20
chr1	74660001	74665000	7.04E-19
chr1	74661001	74666000	1.53E-23
chr1	74662001	74667000	5.44E-26
chr1	74663001	74668000	2.83E-21
chr1	74664001	74669000	4.97E-06
chr1	74707001	74712000	0.000421642
chr1	74737001	74742000	8.19E-06
chr1	74748001	74753000	1.11E-07
chr1	74750001	74755000	5.81E-06

chr1	74751001	74756000	8.24E-05
chr1	74752001	74757000	1.50E-06
chr1	74840001	74845000	0.026031511
chr1	74841001	74846000	5.24E-06
chr1	74850001	74855000	8.77E-07
chr1	74851001	74856000	3.41E-06
chr1	74852001	74857000	5.02E-05
chr1	74853001	74858000	3.84E-06
chr1	75120001	75125000	0.004491636
chr1	75137001	75142000	7.15E-17
chr1	75138001	75143000	2.46E-13
chr1	75139001	75144000	1.69E-12
chr1	75145001	75150000	0.002940372
chr1	75151001	75156000	0.000373993
chr1	75152001	75157000	0.000131429
chr1	75153001	75158000	4.87E-05
chr1	75194001	75199000	2.93E-14
chr1	75297001	75302000	0.001617989
chr1	75359001	75364000	4.92E-05
chr1	76097001	76102000	5.64E-06
chr1	76098001	76103000	1.44E-05
chr1	76099001	76104000	0.000183435
chr1	76100001	76105000	9.81E-05
chr1	76185001	76190000	9.12E-08
chr1	76186001	76191000	4.50E-61
chr1	76187001	76192000	6.12E-50
chr1	76188001	76193000	2.51E-43
chr1	76189001	76194000	1.31E-26
chr1	76190001	76195000	5.30E-20
chr1	76427001	76432000	6.34E-05
chr1	76429001	76434000	0.001135708
chr1	76682001	76687000	0.002503774
chr1	76683001	76688000	4.85E-07
chr1	76962001	76967000	0.003255601
chr1	76963001	76968000	1.66E-06
chr1	76964001	76969000	1.76E-07
chr1	76965001	76970000	6.12E-08
chr1	76966001	76971000	7.23E-06
chr1	76967001	76972000	7.30E-07
chr1	77036001	77041000	0.023394385
chr1	77114001	77119000	0.000161118
chr1	77115001	77120000	0.000541345
chr1	77197001	77202000	0.006527859
chr1	77198001	77203000	8.68E-08
chr1	77199001	77204000	2.44E-05
chr1	77310001	77315000	0.000843852
chr1	77311001	77316000	0.000960568
chr1	77329001	77334000	3.05E-49

chr1	77331001	77336000	2.09E-41
chr1	77468001	77473000	2.08E-06
chr1	77680001	77685000	1.08E-22
chr1	77681001	77686000	5.32E-21
chr1	77718001	77723000	0.000182694
chr1	77719001	77724000	8.42E-05
chr1	77744001	77749000	9.60E-32
chr1	77843001	77848000	1.08E-05
chr1	77844001	77849000	8.52E-07
chr1	77845001	77850000	0.000995982
chr1	78221001	78226000	2.20E-42
chr1	78222001	78227000	4.63E-35
chr1	78223001	78228000	5.40E-36
chr1	78224001	78229000	2.12E-28
chr1	78225001	78230000	3.95E-08
chr1	78241001	78246000	1.16E-15
chr1	78242001	78247000	1.91E-19
chr1	78243001	78248000	3.37E-24
chr1	78244001	78249000	3.06E-23
chr1	78245001	78250000	2.99E-28
chr1	78350001	78355000	9.00E-10
chr1	78512001	78517000	4.94E-15
chr1	78824001	78829000	1.24E-08
chr1	78952001	78957000	9.05E-27
chr1	79106001	79111000	0.001656508
chr1	79107001	79112000	0.000206716
chr1	79688001	79693000	0.000221168
chr1	79689001	79694000	0.000574576
chr1	80062001	80067000	0.005979099
chr1	80064001	80069000	0.006874398
chr1	80404001	80409000	2.05E-09
chr1	80462001	80467000	9.32E-07
chr1	80463001	80468000	1.66E-06
chr1	80464001	80469000	9.57E-07
chr1	80478001	80483000	0.000134826
chr1	80479001	80484000	0.000305965
chr1	81065001	81070000	0.002750209
chr1	81251001	81256000	5.94E-06
chr1	81252001	81257000	8.85E-08
chr1	81253001	81258000	1.00E-07
chr1	81442001	81447000	0.000704757
chr1	81831001	81836000	0.003278381
chr1	81832001	81837000	0.003278381
chr1	81833001	81838000	0.002394114
chr1	81897001	81902000	0.000434678
chr1	81925001	81930000	0.005390305
chr1	81926001	81931000	0.003001609
chr1	82014001	82019000	0.000924917

chr1	82056001	82061000	0.001919239
chr1	82262001	82267000	2.24E-28
chr1	82526001	82531000	0.001731707
chr1	82858001	82863000	0.002178358
chr1	82933001	82938000	0.006407362
chr1	83316001	83321000	6.34E-06
chr1	83386001	83391000	0.00211086
chr1	83389001	83394000	0.002226113
chr1	83801001	83806000	0.000299341
chr1	83885001	83890000	3.32E-05
chr1	84019001	84024000	1.29E-06
chr1	84020001	84025000	3.44E-07
chr1	84185001	84190000	0.001501754
chr1	84186001	84191000	0.000778675
chr1	84187001	84192000	4.64E-05
chr1	84257001	84262000	0.001669986
chr1	84307001	84312000	0.016040357
chr1	84322001	84327000	6.68E-27
chr1	84323001	84328000	1.10E-32
chr1	84324001	84329000	1.19E-30
chr1	84325001	84330000	5.57E-28
chr1	84326001	84331000	3.60E-32
chr1	84460001	84465000	4.90E-37
chr1	84462001	84467000	1.18E-46
chr1	84463001	84468000	1.28E-35
chr1	84539001	84544000	1.31E-70
chr1	84540001	84545000	4.52E-57
chr1	84541001	84546000	1.06E-65
chr1	84542001	84547000	2.85E-65
chr1	84543001	84548000	7.80E-77
chr1	84544001	84549000	3.16E-46
chr1	84768001	84773000	0.007440627
chr1	84929001	84934000	0.000640854
chr1	84942001	84947000	4.29E-21
chr1	84943001	84948000	9.22E-24
chr1	84944001	84949000	1.06E-21
chr1	84945001	84950000	3.81E-21
chr1	85004001	85009000	0.000976774
chr1	85082001	85087000	6.89E-35
chr1	85083001	85088000	8.60E-22
chr1	85084001	85089000	2.13E-17
chr1	85151001	85156000	1.09E-78
chr1	85152001	85157000	6.13E-103
chr1	85153001	85158000	1.52E-62
chr1	85154001	85159000	2.16E-73
chr1	85156001	85161000	2.02E-62
chr1	85159001	85164000	9.77E-06
chr1	85268001	85273000	1.63E-11

chr1	85354001	85359000	1.85E-17
chr1	85355001	85360000	3.10E-20
chr1	85356001	85361000	4.68E-23
chr1	85416001	85421000	1.02E-06
chr1	85417001	85422000	9.66E-07
chr1	85458001	85463000	1.23E-37
chr1	85459001	85464000	1.54E-17
chr1	85466001	85471000	0.001145615
chr1	85478001	85483000	1.96E-05
chr1	85509001	85514000	2.51E-26
chr1	85510001	85515000	1.54E-49
chr1	85511001	85516000	3.53E-53
chr1	85512001	85517000	3.42E-41
chr1	85513001	85518000	3.21E-24
chr1	85514001	85519000	1.61E-34
chr1	85526001	85531000	2.16E-10
chr1	85528001	85533000	1.81E-06
chr1	85570001	85575000	0.000303858
chr1	85571001	85576000	6.50E-05
chr1	85574001	85579000	0.000337552
chr1	85575001	85580000	0.00042176
chr1	85666001	85671000	7.46E-69
chr1	85667001	85672000	7.91E-51
chr1	85721001	85726000	1.91E-39
chr1	85722001	85727000	4.99E-39
chr1	85723001	85728000	6.82E-39
chr1	85724001	85729000	1.61E-32
chr1	85725001	85730000	3.78E-48
chr1	85742001	85747000	8.11E-29
chr1	85772001	85777000	9.80E-12
chr1	85773001	85778000	2.33E-11
chr1	85925001	85930000	1.15E-09
chr1	85929001	85934000	9.25E-53
chr1	85930001	85935000	2.35E-38
chr1	86038001	86043000	1.96E-22
chr1	86045001	86050000	1.41E-27
chr1	86269001	86274000	0.011379736
chr1	86270001	86275000	0.012180786
chr1	86457001	86462000	0.00699587
chr1	86738001	86743000	0.000136126
chr1	86739001	86744000	8.56E-06
chr1	86740001	86745000	7.41E-08
chr1	86741001	86746000	9.81E-08
chr1	86742001	86747000	9.98E-07
chr1	86761001	86766000	1.92E-05
chr1	86778001	86783000	0.000466369
chr1	86857001	86862000	3.21E-67
chr1	86858001	86863000	1.18E-51

chr1	86859001	86864000	2.01E-57
chr1	86860001	86865000	2.30E-58
chr1	86861001	86866000	3.97E-32
chr1	87039001	87044000	0.020598508
chr1	87040001	87045000	0.01410638
chr1	87041001	87046000	0.01475703
chr1	87050001	87055000	3.46E-06
chr1	87051001	87056000	3.93E-05
chr1	87054001	87059000	3.45E-06
chr1	87133001	87138000	0.017483426
chr1	87134001	87139000	0.002058117
chr1	87166001	87171000	3.75E-25
chr1	87170001	87175000	7.67E-51
chr1	87291001	87296000	0.004610002
chr1	87380001	87385000	1.53E-59
chr1	87381001	87386000	8.94E-42
chr1	88001001	88006000	0.000514269
chr1	88134001	88139000	2.66E-05
chr1	88419001	88424000	1.00E-06
chr1	88487001	88492000	0.00018248
chr1	88668001	88673000	0.000155433
chr1	88723001	88728000	2.66E-13
chr1	88724001	88729000	6.60E-15
chr1	88725001	88730000	1.42E-14
chr1	88726001	88731000	5.84E-16
chr1	88748001	88753000	0.009495525
chr1	88749001	88754000	0.002086555
chr1	88838001	88843000	0.000851054
chr1	88839001	88844000	1.37E-06
chr1	88840001	88845000	0.000190098
chr1	88851001	88856000	0.002383606
chr1	88852001	88857000	0.000257491
chr1	88855001	88860000	1.13E-06
chr1	88895001	88900000	9.06E-05
chr1	88896001	88901000	0.002097283
chr1	88897001	88902000	0.000359927
chr1	88927001	88932000	1.93E-30
chr1	88988001	88993000	0.009385755
chr1	88989001	88994000	0.007896566
chr1	89025001	89030000	0.00465834
chr1	89150001	89155000	6.16E-48
chr1	89434001	89439000	0.0066399
chr1	89551001	89556000	3.99E-08
chr1	89552001	89557000	9.00E-08
chr1	89601001	89606000	0.000962635
chr1	89664001	89669000	1.45E-13
chr1	89723001	89728000	0.000574235
chr1	89724001	89729000	0.000706513

chr1	89725001	89730000	6.96E-05
chr1	89726001	89731000	2.83E-06
chr1	89796001	89801000	0.002076567
chr1	89797001	89802000	6.24E-05
chr1	89973001	89978000	0.003357017
chr1	89974001	89979000	0.010296641
chr1	89986001	89991000	6.86E-21
chr1	89990001	89995000	4.83E-29
chr1	90032001	90037000	2.31E-05
chr1	90094001	90099000	4.65E-32
chr1	90287001	90292000	1.44E-20
chr1	90307001	90312000	1.34E-23
chr1	90308001	90313000	3.12E-31
chr1	90309001	90314000	2.78E-44
chr1	90456001	90461000	8.25E-15
chr1	90461001	90466000	3.08E-24
chr1	90837001	90842000	4.70E-06
chr1	90928001	90933000	0.00095961
chr1	90930001	90935000	0.001360371
chr1	91058001	91063000	0.000926599
chr1	91059001	91064000	6.99E-05
chr1	91060001	91065000	1.08E-08
chr1	91076001	91081000	5.07E-05
chr1	91077001	91082000	0.000214785
chr1	91078001	91083000	1.94E-05
chr1	91248001	91253000	0.004830374
chr1	91571001	91576000	3.86E-10
chr1	91637001	91642000	9.34E-08
chr1	91765001	91770000	2.39E-05
chr1	91777001	91782000	0.002712337
chr1	91822001	91827000	0.000227893
chr1	91865001	91870000	5.42E-27
chr1	91866001	91871000	5.22E-47
chr1	91867001	91872000	3.54E-48
chr1	91868001	91873000	3.62E-40
chr1	91869001	91874000	1.59E-31
chr1	91870001	91875000	9.26E-25
chr1	91886001	91891000	1.57E-05
chr1	91962001	91967000	3.16E-34
chr1	91963001	91968000	2.00E-24
chr1	91964001	91969000	1.63E-36
chr1	91965001	91970000	1.03E-30
chr1	91966001	91971000	2.31E-33
chr1	92169001	92174000	0.000485716
chr1	92541001	92546000	2.45E-06
chr1	92545001	92550000	1.48E-22
chr1	92760001	92765000	1.88E-35
chr1	92762001	92767000	1.64E-44

chr1	92763001	92768000	1.29E-48
chr1	92826001	92831000	3.31E-06
chr1	92895001	92900000	2.26E-10
chr1	92896001	92901000	1.02E-07
chr1	92952001	92957000	9.76E-11
chr1	93153001	93158000	3.70E-05
chr1	93374001	93379000	2.24E-06
chr1	93375001	93380000	3.20E-05
chr1	93412001	93417000	0.00050949
chr1	93426001	93431000	1.87E-22
chr1	93427001	93432000	4.18E-22
chr1	93645001	93650000	3.57E-44
chr1	93646001	93651000	1.55E-41
chr1	93650001	93655000	0.013639347
chr1	93656001	93661000	0.000599046
chr1	93807001	93812000	1.15E-34
chr1	93913001	93918000	3.32E-50
chr1	93914001	93919000	3.36E-32
chr1	94145001	94150000	2.04E-30
chr1	94146001	94151000	9.68E-41
chr1	94147001	94152000	1.10E-50
chr1	94269001	94274000	0.010472125
chr1	94270001	94275000	0.023847908
chr1	94311001	94316000	1.94E-38
chr1	94312001	94317000	7.56E-53
chr1	94313001	94318000	1.48E-31
chr1	94370001	94375000	1.08E-40
chr1	94703001	94708000	1.21E-38
chr1	94708001	94713000	5.94E-40
chr1	94709001	94714000	1.79E-49
chr1	94710001	94715000	3.55E-58
chr1	94711001	94716000	1.86E-56
chr1	94712001	94717000	3.25E-39
chr1	94713001	94718000	5.04E-58
chr1	94879001	94884000	9.65E-53
chr1	94884001	94889000	3.96E-50
chr1	95007001	95012000	1.70E-32
chr1	95281001	95286000	9.35E-18
chr1	95391001	95396000	3.22E-23
chr1	95392001	95397000	1.78E-39
chr1	95579001	95584000	3.61E-48
chr1	95583001	95588000	5.46E-44
chr1	95695001	95700000	2.74E-42
chr1	95696001	95701000	8.20E-45
chr1	95697001	95702000	6.75E-58
chr1	95698001	95703000	1.42E-41
chr1	95699001	95704000	1.79E-35
chr1	95700001	95705000	4.05E-24

chr1	95827001	95832000	0.001244485
chr1	95923001	95928000	9.82E-07
chr1	95924001	95929000	2.13E-07
chr1	96209001	96214000	0.002087849
chr1	96345001	96350000	0.00158387
chr1	96414001	96419000	1.30E-06
chr1	96415001	96420000	9.68E-09
chr1	96416001	96421000	2.79E-10
chr1	96427001	96432000	0.002051103
chr1	96428001	96433000	0.002610933
chr1	96429001	96434000	0.000679159
chr1	97075001	97080000	0.00208836
chr1	97183001	97188000	2.32E-37
chr1	97184001	97189000	2.43E-32
chr1	97185001	97190000	5.93E-35
chr1	97186001	97191000	6.92E-33
chr1	97187001	97192000	4.66E-27
chr1	97275001	97280000	3.07E-07
chr1	97359001	97364000	0.000623335
chr1	97360001	97365000	1.41E-05
chr1	97361001	97366000	7.90E-06
chr1	97362001	97367000	1.58E-05
chr1	97363001	97368000	9.98E-05
chr1	97470001	97475000	3.30E-06
chr1	97471001	97476000	8.71E-06
chr1	97472001	97477000	3.93E-07
chr1	98080001	98085000	8.47E-07
chr1	98081001	98086000	6.97E-07
chr1	98082001	98087000	1.72E-07
chr1	98083001	98088000	1.87E-06
chr1	98371001	98376000	0.003314667
chr1	98372001	98377000	0.00057069
chr1	98386001	98391000	3.70E-42
chr1	98518001	98523000	1.17E-26
chr1	98519001	98524000	4.54E-35
chr1	99123001	99128000	4.69E-28
chr1	99126001	99131000	5.93E-39
chr1	99127001	99132000	1.03E-42
chr1	99252001	99257000	6.46E-06
chr1	99253001	99258000	0.000133721
chr1	99254001	99259000	0.000633054
chr1	99255001	99260000	0.005286862
chr1	99332001	99337000	0.005951589
chr1	99333001	99338000	8.70E-05
chr1	99334001	99339000	1.95E-05
chr1	99335001	99340000	3.74E-05
chr1	99342001	99347000	5.60E-11
chr1	99465001	99470000	1.69E-15

chr1	99466001	99471000	8.52E-40
chr1	99467001	99472000	1.58E-37
chr1	99468001	99473000	6.89E-31
chr1	99631001	99636000	0.005405782
chr1	99673001	99678000	1.18E-05
chr1	99675001	99680000	0.005931491
chr1	99763001	99768000	0.003197303
chr1	99968001	99973000	7.98E-06
chr1	100311001	100316000	7.34E-24
chr1	100431001	100436000	1.75E-21
chr1	100504001	100509000	1.20E-40
chr1	100594001	100599000	1.05E-32
chr1	100599001	100604000	8.54E-16
chr1	100727001	100732000	6.39E-73
chr1	100728001	100733000	2.01E-73
chr1	100729001	100734000	9.24E-62
chr1	100730001	100735000	2.01E-40
chr1	100859001	100864000	2.95E-10
chr1	101001001	101006000	1.25E-18
chr1	101002001	101007000	2.91E-25
chr1	101003001	101008000	1.50E-28
chr1	101004001	101009000	1.35E-26
chr1	101005001	101010000	5.13E-43
chr1	101006001	101011000	0.000734053
chr1	101044001	101049000	0.000534299
chr1	101045001	101050000	0.009664274
chr1	101062001	101067000	1.88E-05
chr1	101063001	101068000	6.74E-06
chr1	101361001	101366000	4.25E-36
chr1	101362001	101367000	2.85E-19
chr1	101487001	101492000	5.75E-41
chr1	101488001	101493000	5.90E-32
chr1	101489001	101494000	1.52E-26
chr1	101490001	101495000	4.43E-23
chr1	101491001	101496000	1.63E-25
chr1	101581001	101586000	0.009027447
chr1	101582001	101587000	0.007725249
chr1	101725001	101730000	0.001262168
chr1	101726001	101731000	0.000341449
chr1	101853001	101858000	0.005045412
chr1	102001001	102006000	0.000553222
chr1	102002001	102007000	0.003812091
chr1	102003001	102008000	0.000181621
chr1	102138001	102143000	0.000901528
chr1	102139001	102144000	0.009434126
chr1	102162001	102167000	0.003021669
chr1	102163001	102168000	0.00252761
chr1	102164001	102169000	0.002463302

chr1	102185001	102190000	0.000249304
chr1	102190001	102195000	2.95E-05
chr1	103073001	103078000	0.017348096
chr1	103078001	103083000	0.027247393
chr1	103079001	103084000	0.022951026
chr1	103240001	103245000	1.53E-05
chr1	103241001	103246000	0.001518684
chr1	103330001	103335000	0.000226339
chr1	103508001	103513000	0.002540516
chr1	104391001	104396000	0.000257626
chr1	104392001	104397000	0.002312994
chr1	104498001	104503000	5.88E-05
chr1	104640001	104645000	4.53E-06
chr1	104641001	104646000	1.28E-05
chr1	104798001	104803000	0.002745233
chr1	106118001	106123000	0.000138691
chr1	106438001	106443000	0.000668591
chr1	106439001	106444000	0.000616163
chr1	106440001	106445000	0.000169149
chr1	106791001	106796000	0.009909452
chr1	107545001	107550000	0.000511339
chr1	107546001	107551000	0.000410895
chr1	107595001	107600000	1.01E-126
chr1	107596001	107601000	7.33E-89
chr1	107597001	107602000	2.62E-79
chr1	107598001	107603000	1.54E-81
chr1	107599001	107604000	3.60E-57
chr1	107600001	107605000	2.52E-32
chr1	107716001	107721000	0.000129606
chr1	107717001	107722000	0.000122196
chr1	107718001	107723000	0.000296832
chr1	107719001	107724000	0.000209867
chr1	107720001	107725000	0.00017374
chr1	107862001	107867000	0.000232033
chr1	108131001	108136000	0.015215309
chr1	108246001	108251000	0.004084028
chr1	108453001	108458000	0.00026614
chr1	108490001	108495000	0.003047298
chr1	108697001	108702000	1.41E-06
chr1	108743001	108748000	8.42E-21
chr1	109199001	109204000	1.02E-29
chr1	109200001	109205000	5.78E-37
chr1	109201001	109206000	8.30E-44
chr1	109202001	109207000	1.92E-34
chr1	109203001	109208000	3.66E-26
chr1	109204001	109209000	5.24E-16
chr1	109230001	109235000	2.47E-23
chr1	109285001	109290000	2.74E-35

chr1	109286001	109291000	3.69E-37
chr1	109287001	109292000	4.91E-32
chr1	109288001	109293000	6.40E-46
chr1	109289001	109294000	4.20E-44
chr1	109415001	109420000	6.35E-38
chr1	109416001	109421000	3.10E-27
chr1	109417001	109422000	1.34E-40
chr1	109418001	109423000	2.88E-34
chr1	109501001	109506000	1.56E-36
chr1	109502001	109507000	2.52E-40
chr1	109580001	109585000	1.72E-20
chr1	109581001	109586000	1.56E-36
chr1	109582001	109587000	5.18E-36
chr1	109583001	109588000	2.65E-43
chr1	109584001	109589000	7.12E-40
chr1	109640001	109645000	3.83E-14
chr1	109641001	109646000	2.98E-17
chr1	109642001	109647000	2.48E-16
chr1	109752001	109757000	9.54E-20
chr1	109753001	109758000	6.48E-27
chr1	109754001	109759000	4.19E-30
chr1	109821001	109826000	6.26E-09
chr1	109940001	109945000	1.36E-24
chr1	109969001	109974000	5.48E-18
chr1	110051001	110056000	2.28E-11
chr1	110052001	110057000	4.19E-30
chr1	110158001	110163000	3.13E-20
chr1	110159001	110164000	6.87E-22
chr1	110523001	110528000	4.54E-21
chr1	110524001	110529000	1.05E-17
chr1	110525001	110530000	1.16E-31
chr1	110526001	110531000	2.11E-24
chr1	110527001	110532000	1.61E-32
chr1	110575001	110580000	3.32E-34
chr1	110576001	110581000	3.56E-32
chr1	110577001	110582000	1.24E-27
chr1	110612001	110617000	1.42E-10
chr1	110613001	110618000	7.03E-11
chr1	110749001	110754000	9.89E-14
chr1	110876001	110881000	4.30E-24
chr1	111221001	111226000	3.97E-05
chr1	111222001	111227000	8.31E-08
chr1	111223001	111228000	5.58E-07
chr1	111360001	111365000	0.0157872
chr1	111501001	111506000	2.80E-15
chr1	111502001	111507000	4.20E-52
chr1	111503001	111508000	3.61E-43
chr1	111504001	111509000	1.09E-24

chr1	111519001	111524000	0.00053314
chr1	111520001	111525000	0.000606007
chr1	111683001	111688000	8.66E-26
chr1	111745001	111750000	3.38E-25
chr1	111746001	111751000	9.57E-40
chr1	111747001	111752000	5.23E-30
chr1	111856001	111861000	9.59E-08
chr1	111857001	111862000	4.86E-07
chr1	111880001	111885000	2.37E-09
chr1	111881001	111886000	8.15E-11
chr1	111882001	111887000	1.11E-16
chr1	111883001	111888000	1.08E-16
chr1	111884001	111889000	2.17E-33
chr1	111885001	111890000	5.45E-69
chr1	111886001	111891000	1.80E-53
chr1	111887001	111892000	4.73E-39
chr1	111888001	111893000	1.89E-25
chr1	111889001	111894000	3.20E-27
chr1	111930001	111935000	0.000576336
chr1	111931001	111936000	0.003668751
chr1	112046001	112051000	8.08E-13
chr1	112158001	112163000	3.34E-30
chr1	112159001	112164000	5.94E-22
chr1	112160001	112165000	1.10E-25
chr1	112161001	112166000	8.79E-38
chr1	112162001	112167000	4.85E-56
chr1	112277001	112282000	7.71E-15
chr1	112294001	112299000	5.94E-18
chr1	112532001	112537000	3.10E-13
chr1	112647001	112652000	0.014976487
chr1	112758001	112763000	2.50E-06
chr1	113014001	113019000	3.50E-09
chr1	113015001	113020000	1.27E-08
chr1	113040001	113045000	1.04E-36
chr1	113041001	113046000	8.64E-32
chr1	113042001	113047000	1.88E-24
chr1	113049001	113054000	4.75E-07
chr1	113213001	113218000	3.19E-32
chr1	113215001	113220000	8.27E-41
chr1	113216001	113221000	1.17E-51
chr1	113217001	113222000	1.87E-64
chr1	113218001	113223000	5.29E-13
chr1	113249001	113254000	8.42E-15
chr1	113957001	113962000	1.25E-09
chr1	114301001	114306000	1.13E-29
chr1	114302001	114307000	2.18E-38
chr1	114350001	114355000	3.61E-56
chr1	114354001	114359000	3.47E-42

chr1	114355001	114360000	2.32E-12
chr1	114443001	114448000	4.60E-33
chr1	114444001	114449000	8.16E-35
chr1	114445001	114450000	8.58E-13
chr1	114446001	114451000	6.65E-25
chr1	114447001	114452000	3.17E-27
chr1	114843001	114848000	1.37E-15
chr1	114844001	114849000	9.57E-18
chr1	114845001	114850000	2.35E-18
chr1	115208001	115213000	1.29E-40
chr1	115209001	115214000	2.10E-31
chr1	115211001	115216000	3.33E-42
chr1	115212001	115217000	1.60E-71
chr1	115213001	115218000	5.51E-34
chr1	115215001	115220000	0.001478999
chr1	115255001	115260000	1.80E-20
chr1	115318001	115323000	1.12E-23
chr1	115319001	115324000	1.25E-64
chr1	115320001	115325000	6.92E-49
chr1	115321001	115326000	1.40E-53
chr1	115322001	115327000	3.73E-37
chr1	115323001	115328000	9.61E-31
chr1	115377001	115382000	1.61E-06
chr1	115378001	115383000	0.000461607
chr1	115479001	115484000	8.82E-08
chr1	115573001	115578000	0.009174637
chr1	115628001	115633000	4.28E-09
chr1	115629001	115634000	5.44E-15
chr1	115630001	115635000	6.34E-23
chr1	115631001	115636000	2.88E-18
chr1	115632001	115637000	9.93E-19
chr1	115651001	115656000	3.29E-13
chr1	115849001	115854000	0.019627651
chr1	116377001	116382000	1.55E-30
chr1	116514001	116519000	1.51E-21
chr1	116515001	116520000	5.65E-33
chr1	116516001	116521000	2.68E-41
chr1	116517001	116522000	8.17E-39
chr1	116518001	116523000	2.04E-44
chr1	116519001	116524000	2.92E-32
chr1	116622001	116627000	9.61E-05
chr1	116623001	116628000	9.86E-06
chr1	116690001	116695000	3.13E-18
chr1	116691001	116696000	1.18E-14
chr1	116692001	116697000	3.53E-14
chr1	116693001	116698000	3.48E-15
chr1	116694001	116699000	4.75E-18
chr1	116755001	116760000	0.000336483

chr1	117108001	117113000	5.98E-14
chr1	117109001	117114000	2.44E-21
chr1	117448001	117453000	5.69E-30
chr1	117449001	117454000	8.90E-29
chr1	117451001	117456000	8.51E-38
chr1	117453001	117458000	2.09E-22
chr1	117662001	117667000	1.53E-33
chr1	117664001	117669000	9.77E-42
chr1	117665001	117670000	8.92E-14
chr1	118415001	118420000	0.000744771
chr1	118467001	118472000	2.37E-17
chr1	118472001	118477000	2.14E-39
chr1	118564001	118569000	0.000928684
chr1	118565001	118570000	0.000764888
chr1	118566001	118571000	0.002370978
chr1	118567001	118572000	0.000958805
chr1	118568001	118573000	0.001367976
chr1	118576001	118581000	0.005329783
chr1	118723001	118728000	1.18E-11
chr1	118724001	118729000	1.19E-13
chr1	118725001	118730000	3.58E-14
chr1	118727001	118732000	1.46E-11
chr1	119083001	119088000	2.25E-05
chr1	119194001	119199000	0.000305095
chr1	119195001	119200000	7.57E-08
chr1	119196001	119201000	1.06E-07
chr1	119214001	119219000	5.88E-05
chr1	119215001	119220000	0.000121115
chr1	119281001	119286000	0.000168893
chr1	119282001	119287000	0.008808197
chr1	119292001	119297000	2.54E-09
chr1	119293001	119298000	0.000275342
chr1	119434001	119439000	0.004423318
chr1	119474001	119479000	0.011883299
chr1	119550001	119555000	1.80E-10
chr1	119601001	119606000	3.76E-07
chr1	119738001	119743000	0.000185748
chr1	119796001	119801000	0.001274466
chr1	119952001	119957000	0.026849312
chr1	120186001	120191000	1.01E-33
chr1	120188001	120193000	1.28E-38
chr1	120189001	120194000	1.65E-46
chr1	120190001	120195000	5.31E-39
chr1	120191001	120196000	7.75E-26
chr1	120612001	120617000	1.61E-50
chr1	121203001	121208000	0.000213924
chr1	121223001	121228000	0.000703964
chr1	121224001	121229000	0.010856472

chr1	121257001	121262000	5.69E-26
chr1	121258001	121263000	5.11E-20
chr1	121259001	121264000	2.29E-20
chr1	142857001	142862000	0.004414142
chr1	142858001	142863000	8.81E-06
chr1	142859001	142864000	3.75E-05
chr1	142919001	142924000	1.19E-06
chr1	143721001	143726000	2.70E-05
chr1	143722001	143727000	4.29E-05
chr1	143723001	143728000	0.000285204
chr1	143744001	143749000	7.91E-92
chr1	143745001	143750000	7.30E-11
chr1	144515001	144520000	1.50E-13
chr1	144516001	144521000	5.28E-81
chr1	144517001	144522000	8.55E-88
chr1	144520001	144525000	2.45E-40
chr1	144521001	144526000	1.59E-24
chr1	144593001	144598000	1.35E-51
chr1	145204001	145209000	2.54E-112
chr1	145205001	145210000	2.63E-205
chr1	145477001	145482000	3.27E-24
chr1	145571001	145576000	1.65E-41
chr1	145572001	145577000	2.26E-57
chr1	145573001	145578000	2.83E-28
chr1	145574001	145579000	3.11E-10
chr1	145575001	145580000	3.02E-20
chr1	145588001	145593000	2.70E-26
chr1	145589001	145594000	2.41E-38
chr1	145822001	145827000	5.06E-63
chr1	145824001	145829000	6.81E-49
chr1	145825001	145830000	1.14E-43
chr1	145826001	145831000	7.99E-41
chr1	146952001	146957000	7.84E-06
chr1	147071001	147076000	2.42E-34
chr1	147138001	147143000	4.32E-63
chr1	147358001	147363000	1.56E-11
chr1	147400001	147405000	2.41E-89
chr1	147772001	147777000	2.88E-11
chr1	148174001	148179000	5.01E-31
chr1	148175001	148180000	2.65E-55
chr1	148176001	148181000	1.77E-64
chr1	148556001	148561000	2.70E-86
chr1	148563001	148568000	1.01E-06
chr1	148564001	148569000	1.01E-06
chr1	148565001	148570000	1.21E-12
chr1	148594001	148599000	4.71E-21
chr1	148595001	148600000	2.02E-19
chr1	148596001	148601000	3.06E-23

chr1	148597001	148602000	1.89E-43
chr1	148598001	148603000	7.56E-28
chr1	148599001	148604000	6.20E-14
chr1	148669001	148674000	4.24E-05
chr1	148670001	148675000	0.000212724
chr1	148790001	148795000	0.008255222
chr1	148791001	148796000	0.000525968
chr1	148792001	148797000	0.000525968
chr1	148865001	148870000	6.19E-12
chr1	149375001	149380000	1.16E-24
chr1	149379001	149384000	1.48E-16
chr1	149380001	149385000	3.35E-08
chr1	149386001	149391000	2.84E-10
chr1	149387001	149392000	2.52E-10
chr1	149389001	149394000	1.57E-12
chr1	149392001	149397000	3.49E-10
chr1	149393001	149398000	2.10E-08
chr1	149708001	149713000	5.60E-40
chr1	149709001	149714000	1.64E-36
chr1	149710001	149715000	1.89E-35
chr1	149711001	149716000	1.36E-31
chr1	149712001	149717000	1.18E-18
chr1	149760001	149765000	2.26E-35
chr1	149763001	149768000	8.96E-28
chr1	149764001	149769000	1.35E-52
chr1	149785001	149790000	3.19E-25
chr1	149895001	149900000	1.92E-28
chr1	149896001	149901000	1.06E-33
chr1	150118001	150123000	8.48E-47
chr1	150517001	150522000	4.91E-49
chr1	150670001	150675000	1.15E-06
chr1	150979001	150984000	4.22E-28
chr1	151020001	151025000	2.27E-41
chr1	151032001	151037000	7.08E-23
chr1	151255001	151260000	2.73E-15
chr1	151431001	151436000	1.72E-27
chr1	151582001	151587000	1.57E-61
chr1	151583001	151588000	3.83E-61
chr1	151584001	151589000	5.02E-74
chr1	151585001	151590000	1.15E-30
chr1	152028001	152033000	1.94E-11
chr1	152106001	152111000	0.000425103
chr1	152107001	152112000	1.39E-06
chr1	152108001	152113000	8.85E-06
chr1	152109001	152114000	3.00E-05
chr1	152251001	152256000	1.33E-17
chr1	152253001	152258000	5.61E-09
chr1	152254001	152259000	2.60E-08

chr1	152255001	152260000	3.56E-07
chr1	152256001	152261000	2.32E-08
chr1	152257001	152262000	1.84E-12
chr1	152258001	152263000	9.29E-11
chr1	152259001	152264000	2.57E-11
chr1	152260001	152265000	2.91E-08
chr1	152295001	152300000	0.00037012
chr1	152296001	152301000	0.000238762
chr1	152297001	152302000	6.43E-05
chr1	152598001	152603000	5.50E-09
chr1	152760001	152765000	1.18E-05
chr1	152783001	152788000	6.00E-15
chr1	152784001	152789000	5.40E-12
chr1	152817001	152822000	3.46E-06
chr1	152818001	152823000	2.03E-08
chr1	152819001	152824000	4.32E-09
chr1	152845001	152850000	3.04E-09
chr1	152846001	152851000	3.62E-09
chr1	152847001	152852000	4.29E-18
chr1	152927001	152932000	6.19E-16
chr1	152928001	152933000	1.41E-19
chr1	152929001	152934000	1.11E-20
chr1	152930001	152935000	5.61E-17
chr1	153008001	153013000	8.84E-10
chr1	153010001	153015000	5.19E-09
chr1	153011001	153016000	3.00E-10
chr1	153012001	153017000	3.63E-10
chr1	153156001	153161000	6.93E-11
chr1	153157001	153162000	3.10E-17
chr1	153158001	153163000	2.56E-13
chr1	153159001	153164000	8.22E-14
chr1	153240001	153245000	7.95E-08
chr1	153292001	153297000	1.30E-11
chr1	153647001	153652000	2.34E-15
chr1	153648001	153653000	2.88E-13
chr1	153649001	153654000	8.31E-09
chr1	154301001	154306000	5.35E-14
chr1	154374001	154379000	1.59E-19
chr1	154375001	154380000	1.01E-14
chr1	154633001	154638000	3.70E-07
chr1	154733001	154738000	1.85E-27
chr1	155019001	155024000	4.47E-19
chr1	155047001	155052000	2.46E-13
chr1	155048001	155053000	7.43E-22
chr1	155049001	155054000	2.05E-20
chr1	155162001	155167000	9.23E-21
chr1	155163001	155168000	1.50E-17
chr1	155228001	155233000	5.07E-13

chr1	155229001	155234000	8.29E-17
chr1	155230001	155235000	2.28E-17
chr1	155231001	155236000	9.75E-29
chr1	155232001	155237000	6.15E-14
chr1	155294001	155299000	2.01E-16
chr1	155601001	155606000	0.004110058
chr1	155657001	155662000	4.59E-48
chr1	155658001	155663000	3.71E-69
chr1	155823001	155828000	1.22E-27
chr1	155824001	155829000	6.78E-26
chr1	155901001	155906000	3.61E-14
chr1	156021001	156026000	1.63E-18
chr1	156024001	156029000	1.69E-19
chr1	156080001	156085000	1.83E-15
chr1	156159001	156164000	3.35E-26
chr1	156258001	156263000	2.89E-05
chr1	156308001	156313000	1.61E-24
chr1	156629001	156634000	1.58E-09
chr1	156675001	156680000	6.18E-07
chr1	156720001	156725000	3.08E-42
chr1	156721001	156726000	3.78E-37
chr1	157011001	157016000	7.29E-54
chr1	157014001	157019000	2.48E-38
chr1	157015001	157020000	6.45E-46
chr1	157103001	157108000	1.12E-26
chr1	157107001	157112000	2.72E-73
chr1	157108001	157113000	7.32E-72
chr1	157187001	157192000	4.32E-12
chr1	157188001	157193000	1.53E-17
chr1	157189001	157194000	1.47E-16
chr1	157200001	157205000	5.43E-10
chr1	157201001	157206000	1.17E-08
chr1	157210001	157215000	7.53E-09
chr1	157211001	157216000	1.34E-08
chr1	157212001	157217000	5.63E-11
chr1	157230001	157235000	0.000259015
chr1	157231001	157236000	1.04E-06
chr1	157232001	157237000	3.61E-06
chr1	157233001	157238000	1.86E-08
chr1	157234001	157239000	3.85E-07
chr1	157254001	157259000	5.85E-13
chr1	157255001	157260000	3.75E-15
chr1	157267001	157272000	2.93E-09
chr1	157285001	157290000	7.18E-10
chr1	157301001	157306000	4.32E-07
chr1	157302001	157307000	2.54E-09
chr1	157303001	157308000	2.01E-13
chr1	157304001	157309000	3.51E-12

chr1	157360001	157365000	1.15E-06
chr1	157363001	157368000	4.99E-05
chr1	157364001	157369000	8.97E-07
chr1	157365001	157370000	5.50E-09
chr1	157533001	157538000	1.33E-09
chr1	157551001	157556000	5.29E-10
chr1	157560001	157565000	3.76E-05
chr1	157561001	157566000	3.10E-07
chr1	157562001	157567000	8.12E-08
chr1	157573001	157578000	3.68E-06
chr1	157574001	157579000	0.000917187
chr1	157630001	157635000	5.48E-07
chr1	157631001	157636000	9.88E-07
chr1	157633001	157638000	2.68E-08
chr1	157634001	157639000	2.05E-06
chr1	157635001	157640000	0.013242623
chr1	157636001	157641000	0.010010392
chr1	157643001	157648000	0.001551354
chr1	157873001	157878000	4.97E-13
chr1	157874001	157879000	1.34E-10
chr1	157875001	157880000	2.92E-07
chr1	157876001	157881000	9.30E-07
chr1	157897001	157902000	1.35E-08
chr1	157909001	157914000	1.64E-10
chr1	157910001	157915000	2.19E-10
chr1	157962001	157967000	3.28E-19
chr1	157963001	157968000	1.40E-13
chr1	158139001	158144000	0.00391495
chr1	158182001	158187000	2.53E-09
chr1	158183001	158188000	3.89E-14
chr1	158184001	158189000	9.99E-17
chr1	158190001	158195000	1.51E-12
chr1	158352001	158357000	0.000169306
chr1	158362001	158367000	5.50E-05
chr1	158363001	158368000	3.16E-05
chr1	158364001	158369000	8.89E-06
chr1	158365001	158370000	1.97E-06
chr1	158366001	158371000	1.26E-06
chr1	158486001	158491000	0.000147071
chr1	158487001	158492000	2.24E-06
chr1	158488001	158493000	0.00018579
chr1	158533001	158538000	0.000215768
chr1	158570001	158575000	0.003591573
chr1	158744001	158749000	1.18E-06
chr1	158745001	158750000	7.25E-08
chr1	158746001	158751000	5.70E-08
chr1	158747001	158752000	4.69E-08
chr1	158748001	158753000	5.26E-05

chr1	158871001	158876000	0.000531677
chr1	158872001	158877000	5.38E-06
chr1	158873001	158878000	5.38E-06
chr1	158874001	158879000	1.02E-05
chr1	158890001	158895000	2.66E-06
chr1	158891001	158896000	3.64E-05
chr1	158892001	158897000	7.19E-06
chr1	158893001	158898000	0.000813311
chr1	158894001	158899000	0.000685467
chr1	159051001	159056000	4.84E-12
chr1	159052001	159057000	1.59E-08
chr1	159098001	159103000	0.002698705
chr1	159132001	159137000	3.06E-06
chr1	159357001	159362000	1.71E-10
chr1	159358001	159363000	1.14E-10
chr1	159359001	159364000	5.29E-11
chr1	159360001	159365000	4.31E-11
chr1	159849001	159854000	3.66E-08
chr1	159912001	159917000	1.52E-14
chr1	159913001	159918000	2.93E-24
chr1	159914001	159919000	2.48E-31
chr1	159915001	159920000	6.76E-30
chr1	160171001	160176000	7.26E-26
chr1	160172001	160177000	4.95E-27
chr1	160173001	160178000	1.23E-31
chr1	160174001	160179000	2.67E-15
chr1	160232001	160237000	2.27E-20
chr1	160366001	160371000	1.67E-06
chr1	160409001	160414000	8.89E-07
chr1	160423001	160428000	1.22E-06
chr1	160425001	160430000	3.02E-06
chr1	160506001	160511000	2.62E-07
chr1	160507001	160512000	2.63E-07
chr1	160561001	160566000	1.17E-06
chr1	160617001	160622000	1.84E-08
chr1	160631001	160636000	1.79E-10
chr1	160632001	160637000	1.01E-08
chr1	160633001	160638000	1.82E-12
chr1	160634001	160639000	2.37E-13
chr1	160635001	160640000	1.73E-13
chr1	161008001	161013000	2.54E-10
chr1	161064001	161069000	3.45E-13
chr1	161065001	161070000	1.64E-10
chr1	161066001	161071000	7.35E-21
chr1	161146001	161151000	2.44E-22
chr1	161147001	161152000	1.40E-23
chr1	161405001	161410000	5.69E-13
chr1	161406001	161411000	2.02E-19

chr1	161407001	161412000	1.01E-14
chr1	161490001	161495000	1.88E-29
chr1	161494001	161499000	4.76E-16
chr1	161697001	161702000	1.09E-19
chr1	162035001	162040000	4.51E-37
chr1	162036001	162041000	1.82E-34
chr1	162037001	162042000	1.38E-42
chr1	162038001	162043000	1.28E-31
chr1	162039001	162044000	5.11E-35
chr1	162463001	162468000	1.81E-22
chr1	162464001	162469000	9.38E-20
chr1	162465001	162470000	4.06E-46
chr1	162466001	162471000	2.16E-53
chr1	162467001	162472000	5.29E-50
chr1	162527001	162532000	6.68E-86
chr1	162528001	162533000	1.71E-93
chr1	162529001	162534000	6.44E-121
chr1	162530001	162535000	4.54E-101
chr1	162531001	162536000	1.88E-104
chr1	163620001	163625000	4.92E-08
chr1	163710001	163715000	0.000632016
chr1	163888001	163893000	0.000314819
chr1	163929001	163934000	3.26E-05
chr1	163930001	163935000	0.000122499
chr1	163931001	163936000	0.000152056
chr1	163932001	163937000	0.000142128
chr1	164104001	164109000	1.28E-06
chr1	164105001	164110000	2.40E-07
chr1	164237001	164242000	5.24E-15
chr1	164238001	164243000	1.04E-12
chr1	164353001	164358000	4.88E-06
chr1	164402001	164407000	0.000132378
chr1	164681001	164686000	1.40E-17
chr1	164682001	164687000	5.01E-14
chr1	164847001	164852000	0.003240848
chr1	164848001	164853000	0.002450156
chr1	164930001	164935000	9.48E-05
chr1	164955001	164960000	8.16E-05
chr1	164963001	164968000	2.32E-05
chr1	165007001	165012000	0.00262591
chr1	165021001	165026000	1.33E-05
chr1	165211001	165216000	6.53E-06
chr1	165212001	165217000	4.42E-05
chr1	165213001	165218000	0.002895412
chr1	165416001	165421000	2.56E-08
chr1	165417001	165422000	3.61E-08
chr1	165418001	165423000	1.18E-08
chr1	165596001	165601000	5.00E-39

chr1	165598001	165603000	1.02E-69
chr1	165599001	165604000	1.49E-58
chr1	165600001	165605000	3.73E-57
chr1	165663001	165668000	5.26E-42
chr1	165667001	165672000	8.33E-18
chr1	165733001	165738000	1.82E-25
chr1	165734001	165739000	5.83E-33
chr1	165735001	165740000	4.95E-58
chr1	165736001	165741000	1.83E-56
chr1	165737001	165742000	1.11E-67
chr1	165738001	165743000	3.40E-50
chr1	165792001	165797000	3.81E-32
chr1	165793001	165798000	3.32E-56
chr1	165797001	165802000	4.05E-32
chr1	165934001	165939000	1.73E-09
chr1	165973001	165978000	2.07E-14
chr1	165974001	165979000	1.94E-17
chr1	166180001	166185000	8.44E-10
chr1	166181001	166186000	1.76E-09
chr1	166183001	166188000	2.09E-08
chr1	166186001	166191000	7.85E-07
chr1	166187001	166192000	9.45E-07
chr1	166210001	166215000	0.000140193
chr1	166212001	166217000	0.000328408
chr1	166213001	166218000	1.88E-05
chr1	166229001	166234000	0.00010109
chr1	166286001	166291000	6.65E-11
chr1	166287001	166292000	2.28E-13
chr1	166288001	166293000	6.14E-13
chr1	166289001	166294000	1.00E-07
chr1	166369001	166374000	6.32E-09
chr1	166370001	166375000	3.82E-09
chr1	166521001	166526000	2.48E-08
chr1	166522001	166527000	5.13E-06
chr1	166523001	166528000	9.78E-08
chr1	166524001	166529000	1.15E-05
chr1	166561001	166566000	9.34E-05
chr1	166562001	166567000	0.000792191
chr1	166616001	166621000	0.004728043
chr1	166670001	166675000	0.000255987
chr1	166679001	166684000	0.000116571
chr1	166680001	166685000	6.45E-06
chr1	166681001	166686000	1.32E-07
chr1	166703001	166708000	0.000140887
chr1	166724001	166729000	0.000120628
chr1	166725001	166730000	0.001132564
chr1	166726001	166731000	3.14E-05
chr1	166760001	166765000	0.000189476

chr1	166771001	166776000	5.01E-07
chr1	166799001	166804000	4.79E-08
chr1	166801001	166806000	7.89E-07
chr1	166804001	166809000	2.03E-72
chr1	166805001	166810000	1.84E-69
chr1	166806001	166811000	1.90E-58
chr1	166807001	166812000	8.31E-37
chr1	166920001	166925000	8.99E-06
chr1	166921001	166926000	0.000763259
chr1	166981001	166986000	2.61E-09
chr1	166982001	166987000	2.97E-12
chr1	166983001	166988000	6.51E-05
chr1	167185001	167190000	9.20E-53
chr1	167186001	167191000	6.82E-53
chr1	167190001	167195000	5.34E-71
chr1	167191001	167196000	1.17E-48
chr1	167298001	167303000	0.01157403
chr1	167420001	167425000	3.84E-30
chr1	167421001	167426000	4.71E-27
chr1	167422001	167427000	2.85E-30
chr1	167423001	167428000	2.43E-36
chr1	167424001	167429000	5.68E-27
chr1	167518001	167523000	2.10E-38
chr1	167519001	167524000	6.71E-32
chr1	167520001	167525000	2.62E-54
chr1	167521001	167526000	3.16E-41
chr1	167522001	167527000	2.07E-33
chr1	167803001	167808000	2.40E-09
chr1	167953001	167958000	0.000236183
chr1	167977001	167982000	2.08E-05
chr1	167978001	167983000	8.23E-08
chr1	168106001	168111000	1.80E-47
chr1	168191001	168196000	6.92E-18
chr1	168313001	168318000	3.50E-07
chr1	168525001	168530000	0.000574598
chr1	168785001	168790000	5.38E-06
chr1	168786001	168791000	1.64E-06
chr1	168788001	168793000	1.16E-05
chr1	169163001	169168000	0.00984817
chr1	169348001	169353000	0.017391791
chr1	169400001	169405000	0.001181681
chr1	169401001	169406000	0.002637639
chr1	169450001	169455000	1.87E-59
chr1	169571001	169576000	1.01E-07
chr1	169572001	169577000	1.75E-09
chr1	169573001	169578000	6.18E-06
chr1	169596001	169601000	1.14E-06
chr1	169597001	169602000	1.76E-05

chr1	169683001	169688000	2.68E-08
chr1	170039001	170044000	2.23E-50
chr1	170040001	170045000	4.92E-34
chr1	170041001	170046000	1.89E-40
chr1	170042001	170047000	1.95E-40
chr1	170086001	170091000	0.000489174
chr1	170087001	170092000	0.000342635
chr1	170099001	170104000	0.015632245
chr1	170146001	170151000	2.73E-05
chr1	170180001	170185000	1.47E-09
chr1	170356001	170361000	1.76E-06
chr1	170371001	170376000	1.01E-06
chr1	170372001	170377000	1.04E-05
chr1	170373001	170378000	1.26E-05
chr1	170374001	170379000	3.56E-05
chr1	170375001	170380000	6.61E-05
chr1	170389001	170394000	5.34E-07
chr1	170390001	170395000	0.001457057
chr1	170391001	170396000	2.79E-09
chr1	170408001	170413000	0.030994668
chr1	170449001	170454000	4.22E-07
chr1	170450001	170455000	8.30E-08
chr1	170453001	170458000	3.14E-08
chr1	170454001	170459000	1.61E-06
chr1	170455001	170460000	1.41E-06
chr1	170456001	170461000	2.61E-08
chr1	170457001	170462000	5.86E-08
chr1	170496001	170501000	3.83E-16
chr1	170607001	170612000	0.000581902
chr1	170608001	170613000	0.000171359
chr1	170640001	170645000	1.66E-22
chr1	170727001	170732000	4.17E-06
chr1	170729001	170734000	3.70E-08
chr1	170730001	170735000	1.67E-10
chr1	170731001	170736000	2.48E-07
chr1	170733001	170738000	7.53E-06
chr1	170734001	170739000	0.001015033
chr1	170783001	170788000	1.39E-10
chr1	170784001	170789000	1.62E-08
chr1	170831001	170836000	0.000109769
chr1	170901001	170906000	3.03E-05
chr1	170908001	170913000	2.86E-05
chr1	170916001	170921000	0.001297619
chr1	170938001	170943000	8.00E-06
chr1	170939001	170944000	7.80E-06
chr1	170965001	170970000	8.48E-05
chr1	171016001	171021000	2.23E-06
chr1	171017001	171022000	7.68E-09

chr1	171018001	171023000	1.16E-08
chr1	171026001	171031000	2.55E-05
chr1	171027001	171032000	0.008703193
chr1	171044001	171049000	7.83E-08
chr1	171045001	171050000	6.41E-08
chr1	171046001	171051000	1.13E-05
chr1	171063001	171068000	8.21E-05
chr1	171082001	171087000	0.00044525
chr1	171101001	171106000	5.65E-05
chr1	171117001	171122000	3.98E-06
chr1	171120001	171125000	2.64E-06
chr1	171121001	171126000	1.17E-06
chr1	171122001	171127000	2.42E-05
chr1	171123001	171128000	2.33E-06
chr1	171129001	171134000	0.000739703
chr1	171130001	171135000	0.002684234
chr1	171135001	171140000	0.000170091
chr1	171136001	171141000	0.000644541
chr1	171228001	171233000	6.59E-08
chr1	171245001	171250000	1.61E-06
chr1	171246001	171251000	4.39E-07
chr1	171247001	171252000	5.06E-05
chr1	171249001	171254000	5.09E-06
chr1	171251001	171256000	0.002040773
chr1	171253001	171258000	2.14E-05
chr1	171254001	171259000	2.83E-06
chr1	171255001	171260000	2.94E-06
chr1	171298001	171303000	1.75E-10
chr1	171299001	171304000	6.93E-12
chr1	171302001	171307000	0.002540475
chr1	171315001	171320000	1.84E-05
chr1	171316001	171321000	3.07E-06
chr1	171806001	171811000	1.16E-64
chr1	171807001	171812000	6.84E-60
chr1	171808001	171813000	2.12E-55
chr1	171809001	171814000	4.35E-78
chr1	171810001	171815000	1.36E-81
chr1	171811001	171816000	4.11E-29
chr1	172072001	172077000	2.09E-09
chr1	172073001	172078000	9.65E-12
chr1	172076001	172081000	8.23E-15
chr1	172497001	172502000	1.78E-25
chr1	172707001	172712000	0.007301868
chr1	172793001	172798000	0.000419588
chr1	172794001	172799000	1.15E-06
chr1	172795001	172800000	0.000246723
chr1	172834001	172839000	0.007540196
chr1	172877001	172882000	3.89E-10

chr1	172978001	172983000	1.15E-06
chr1	172979001	172984000	1.34E-05
chr1	172980001	172985000	0.004795679
chr1	173220001	173225000	0.000132442
chr1	173269001	173274000	0.001504837
chr1	173498001	173503000	0.002454586
chr1	173499001	173504000	0.001937253
chr1	173500001	173505000	0.006624648
chr1	173680001	173685000	3.00E-24
chr1	173789001	173794000	5.01E-32
chr1	174124001	174129000	8.54E-40
chr1	174125001	174130000	4.38E-39
chr1	174471001	174476000	0.008954942
chr1	174816001	174821000	2.45E-08
chr1	175176001	175181000	8.32E-05
chr1	175177001	175182000	6.67E-05
chr1	175178001	175183000	4.20E-07
chr1	175179001	175184000	9.05E-05
chr1	175453001	175458000	3.40E-08
chr1	175592001	175597000	6.42E-12
chr1	175593001	175598000	1.23E-12
chr1	175594001	175599000	9.97E-12
chr1	175595001	175600000	5.05E-08
chr1	175780001	175785000	1.31E-05
chr1	175782001	175787000	8.19E-07
chr1	175795001	175800000	0.000181045
chr1	176555001	176560000	6.64E-06
chr1	176556001	176561000	0.001775486
chr1	176605001	176610000	9.33E-06
chr1	176611001	176616000	2.39E-05
chr1	176612001	176617000	1.59E-06
chr1	176705001	176710000	0.000669199
chr1	176706001	176711000	2.61E-05
chr1	176771001	176776000	9.09E-07
chr1	176780001	176785000	7.97E-05
chr1	176781001	176786000	7.51E-05
chr1	176877001	176882000	2.57E-07
chr1	176885001	176890000	5.12E-11
chr1	176887001	176892000	5.93E-07
chr1	176888001	176893000	2.80E-06
chr1	177059001	177064000	5.14E-06
chr1	177077001	177082000	2.03E-13
chr1	177081001	177086000	2.92E-10
chr1	177132001	177137000	3.68E-24
chr1	177133001	177138000	3.27E-25
chr1	177146001	177151000	1.24E-27
chr1	177149001	177154000	4.32E-28
chr1	177259001	177264000	0.000462378

chr1	177355001	177360000	0.001409076
chr1	177398001	177403000	0.000781
chr1	177399001	177404000	5.95E-07
chr1	177400001	177405000	2.60E-10
chr1	177401001	177406000	5.76E-08
chr1	177403001	177408000	0.000297332
chr1	177498001	177503000	0.002845652
chr1	177500001	177505000	2.81E-05
chr1	177511001	177516000	0.000509662
chr1	177512001	177517000	0.000312221
chr1	177535001	177540000	4.72E-10
chr1	177562001	177567000	0.003063491
chr1	178063001	178068000	4.68E-55
chr1	178339001	178344000	3.65E-07
chr1	178690001	178695000	1.12E-95
chr1	178691001	178696000	1.24E-78
chr1	178693001	178698000	3.46E-72
chr1	179047001	179052000	2.81E-56
chr1	179049001	179054000	1.31E-35
chr1	179050001	179055000	3.20E-16
chr1	179051001	179056000	6.90E-37
chr1	179194001	179199000	6.37E-29
chr1	179195001	179200000	5.86E-15
chr1	179196001	179201000	1.66E-33
chr1	179197001	179202000	3.23E-48
chr1	179198001	179203000	5.20E-45
chr1	179489001	179494000	1.07E-08
chr1	179490001	179495000	0.001819391
chr1	179493001	179498000	8.46E-07
chr1	179494001	179499000	6.38E-06
chr1	179497001	179502000	2.49E-08
chr1	179645001	179650000	1.70E-11
chr1	179646001	179651000	3.58E-10
chr1	179649001	179654000	6.74E-06
chr1	179650001	179655000	1.08E-16
chr1	179651001	179656000	1.86E-16
chr1	179652001	179657000	3.25E-18
chr1	179653001	179658000	2.05E-17
chr1	179654001	179659000	3.69E-24
chr1	179655001	179660000	6.74E-16
chr1	179656001	179661000	3.89E-19
chr1	179657001	179662000	7.74E-19
chr1	179658001	179663000	1.05E-17
chr1	179660001	179665000	4.07E-11
chr1	179697001	179702000	3.09E-09
chr1	179698001	179703000	6.35E-12
chr1	179699001	179704000	4.10E-11
chr1	179852001	179857000	3.91E-45

chr1	179920001	179925000	3.52E-39
chr1	179924001	179929000	8.17E-47
chr1	180119001	180124000	2.51E-23
chr1	180277001	180282000	4.03E-05
chr1	180597001	180602000	6.84E-36
chr1	180598001	180603000	1.18E-34
chr1	180600001	180605000	2.50E-47
chr1	180601001	180606000	1.13E-60
chr1	180879001	180884000	2.78E-19
chr1	180880001	180885000	1.83E-33
chr1	180881001	180886000	1.77E-25
chr1	180991001	180996000	1.55E-11
chr1	181072001	181077000	3.67E-23
chr1	181074001	181079000	8.07E-15
chr1	181662001	181667000	0.003733127
chr1	181832001	181837000	0.000625521
chr1	181833001	181838000	5.75E-07
chr1	181834001	181839000	1.04E-06
chr1	181835001	181840000	1.06E-05
chr1	181994001	181999000	7.28E-37
chr1	181995001	182000000	1.59E-57
chr1	181996001	182001000	5.51E-52
chr1	181997001	182002000	5.64E-37
chr1	181999001	182004000	4.07E-24
chr1	182154001	182159000	5.67E-06
chr1	182155001	182160000	4.14E-08
chr1	182156001	182161000	5.18E-05
chr1	182360001	182365000	4.71E-61
chr1	182361001	182366000	1.32E-34
chr1	182428001	182433000	0.000488929
chr1	182429001	182434000	8.95E-05
chr1	182430001	182435000	0.000212896
chr1	182486001	182491000	0.004906964
chr1	182756001	182761000	1.03E-36
chr1	182757001	182762000	1.18E-41
chr1	182758001	182763000	9.40E-52
chr1	183151001	183156000	1.55E-23
chr1	184006001	184011000	6.28E-29
chr1	184021001	184026000	2.95E-20
chr1	184069001	184074000	1.42E-09
chr1	184070001	184075000	1.91E-06
chr1	184351001	184356000	1.88E-27
chr1	184352001	184357000	1.81E-44
chr1	184719001	184724000	8.20E-96
chr1	184724001	184729000	1.90E-32
chr1	184759001	184764000	2.14E-13
chr1	184966001	184971000	3.62E-22
chr1	185379001	185384000	2.45E-08

chr1	185380001	185385000	2.03E-11
chr1	185642001	185647000	4.55E-05
chr1	185786001	185791000	0.003897677
chr1	185965001	185970000	0.000159911
chr1	186547001	186552000	0.000840041
chr1	186645001	186650000	9.01E-48
chr1	187017001	187022000	0.002727205
chr1	187771001	187776000	3.20E-06
chr1	188362001	188367000	1.31E-06
chr1	188945001	188950000	3.57E-10
chr1	188993001	188998000	1.40E-06
chr1	188994001	188999000	1.56E-06
chr1	188995001	189000000	3.77E-07
chr1	189033001	189038000	0.001437335
chr1	189034001	189039000	0.002893795
chr1	189180001	189185000	2.95E-06
chr1	189585001	189590000	0.001307681
chr1	190877001	190882000	0.001500481
chr1	192579001	192584000	0.000115794
chr1	192580001	192585000	8.37E-07
chr1	192581001	192586000	1.03E-05
chr1	192582001	192587000	4.95E-06
chr1	193074001	193079000	7.81E-95
chr1	193086001	193091000	3.36E-39
chr1	193087001	193092000	2.72E-84
chr1	193604001	193609000	1.81E-06
chr1	193941001	193946000	1.62E-05
chr1	195812001	195817000	2.23E-06
chr1	196192001	196197000	1.43E-08
chr1	196299001	196304000	3.69E-05
chr1	196300001	196305000	5.42E-05
chr1	196301001	196306000	9.58E-10
chr1	196302001	196307000	4.72E-07
chr1	196705001	196710000	0.000589889
chr1	197744001	197749000	8.34E-93
chr1	197867001	197872000	8.66E-45
chr1	197868001	197873000	2.73E-69
chr1	197869001	197874000	3.64E-71
chr1	197870001	197875000	3.29E-63
chr1	197871001	197876000	3.15E-52
chr1	197872001	197877000	1.10E-48
chr1	198275001	198280000	8.51E-14
chr1	199294001	199299000	0.000170469
chr1	200271001	200276000	3.85E-27
chr1	200374001	200379000	2.22E-45
chr1	200375001	200380000	1.98E-60
chr1	200376001	200381000	8.73E-39
chr1	200377001	200382000	4.48E-23

chr1	200704001	200709000	8.09E-39
chr1	200856001	200861000	8.25E-19
chr1	200857001	200862000	4.63E-28
chr1	200858001	200863000	3.32E-33
chr1	200859001	200864000	3.62E-22
chr1	201081001	201086000	3.83E-24
chr1	201082001	201087000	3.06E-39
chr1	201083001	201088000	3.46E-45
chr1	201119001	201124000	6.77E-23
chr1	201120001	201125000	9.31E-22
chr1	201476001	201481000	8.19E-24
chr1	201947001	201952000	1.08E-47
chr1	201948001	201953000	1.34E-55
chr1	201949001	201954000	3.13E-58
chr1	201950001	201955000	2.11E-55
chr1	201951001	201956000	5.26E-37
chr1	201952001	201957000	1.33E-20
chr1	202109001	202114000	2.73E-20
chr1	202313001	202318000	3.25E-45
chr1	202679001	202684000	5.92E-22
chr1	202772001	202777000	3.04E-26
chr1	202773001	202778000	8.52E-34
chr1	202774001	202779000	1.25E-37
chr1	202853001	202858000	2.72E-19
chr1	202854001	202859000	1.41E-41
chr1	202855001	202860000	1.03E-55
chr1	202856001	202861000	7.55E-64
chr1	202892001	202897000	8.67E-49
chr1	202893001	202898000	4.33E-24
chr1	202894001	202899000	1.65E-30
chr1	202923001	202928000	2.37E-41
chr1	202976001	202981000	2.00E-56
chr1	203201001	203206000	7.92E-10
chr1	203765001	203770000	1.02E-21
chr1	204380001	204385000	9.60E-36
chr1	204650001	204655000	4.56E-48
chr1	204654001	204659000	5.48E-19
chr1	205176001	205181000	1.25E-22
chr1	205177001	205182000	3.14E-33
chr1	205178001	205183000	3.78E-35
chr1	205179001	205184000	1.53E-32
chr1	205180001	205185000	1.20E-48
chr1	205197001	205202000	3.00E-29
chr1	205538001	205543000	2.68E-08
chr1	205557001	205562000	9.72E-14
chr1	205780001	205785000	2.53E-71
chr1	205781001	205786000	2.45E-66
chr1	205782001	205787000	1.11E-69

chr1	206321001	206326000	0.002099578
chr1	206798001	206803000	8.38E-06
chr1	206808001	206813000	4.43E-52
chr1	206809001	206814000	9.41E-43
chr1	207079001	207084000	2.76E-36
chr1	207283001	207288000	0.00052581
chr1	207299001	207304000	7.36E-05
chr1	207354001	207359000	0.002808487
chr1	207355001	207360000	0.001202507
chr1	207356001	207361000	1.73E-07
chr1	207357001	207362000	9.78E-05
chr1	207495001	207500000	1.21E-70
chr1	207505001	207510000	0.002338953
chr1	207508001	207513000	6.53E-06
chr1	207577001	207582000	0.002897378
chr1	207584001	207589000	2.34E-06
chr1	207623001	207628000	6.44E-25
chr1	207624001	207629000	4.41E-36
chr1	207625001	207630000	7.31E-37
chr1	207626001	207631000	3.85E-36
chr1	207627001	207632000	6.51E-35
chr1	207628001	207633000	1.12E-28
chr1	207665001	207670000	8.63E-40
chr1	207666001	207671000	1.26E-39
chr1	207667001	207672000	2.79E-43
chr1	207668001	207673000	1.46E-42
chr1	207838001	207843000	2.46E-36
chr1	207839001	207844000	1.24E-33
chr1	208084001	208089000	3.97E-30
chr1	208099001	208104000	0.000123136
chr1	208128001	208133000	1.41E-16
chr1	208129001	208134000	3.17E-16
chr1	208130001	208135000	9.67E-19
chr1	208131001	208136000	8.65E-20
chr1	208510001	208515000	0.000461236
chr1	208997001	209002000	0.000162552
chr1	208998001	209003000	2.37E-06
chr1	208999001	209004000	2.22E-05
chr1	209771001	209776000	2.43E-07
chr1	209845001	209850000	1.59E-29
chr1	209848001	209853000	4.89E-18
chr1	209849001	209854000	4.22E-21
chr1	210001001	210006000	6.40E-29
chr1	210111001	210116000	7.11E-39
chr1	210205001	210210000	9.37E-06
chr1	210206001	210211000	2.49E-05
chr1	210207001	210212000	1.90E-07
chr1	210208001	210213000	1.37E-08

chr1	210209001	210214000	7.99E-08
chr1	210213001	210218000	0.000885691
chr1	210237001	210242000	1.84E-09
chr1	210240001	210245000	0.004191936
chr1	210401001	210406000	6.27E-13
chr1	210402001	210407000	7.29E-81
chr1	210403001	210408000	1.75E-63
chr1	210404001	210409000	1.75E-73
chr1	210405001	210410000	5.66E-73
chr1	210406001	210411000	2.17E-65
chr1	210407001	210412000	2.68E-23
chr1	210461001	210466000	5.87E-24
chr1	210526001	210531000	2.26E-05
chr1	211336001	211341000	0.000405281
chr1	211337001	211342000	0.000936196
chr1	211341001	211346000	2.60E-11
chr1	211342001	211347000	2.29E-15
chr1	211344001	211349000	1.77E-07
chr1	211428001	211433000	1.24E-31
chr1	211429001	211434000	2.55E-38
chr1	211431001	211436000	3.15E-38
chr1	211432001	211437000	5.38E-35
chr1	211433001	211438000	3.27E-26
chr1	211496001	211501000	5.38E-34
chr1	211585001	211590000	6.07E-30
chr1	211746001	211751000	3.61E-06
chr1	211747001	211752000	1.13E-70
chr1	211752001	211757000	2.02E-22
chr1	211844001	211849000	2.10E-45
chr1	211845001	211850000	3.21E-46
chr1	211846001	211851000	9.61E-44
chr1	211999001	212004000	2.43E-37
chr1	212000001	212005000	1.06E-50
chr1	212001001	212006000	5.06E-53
chr1	212002001	212007000	1.03E-46
chr1	212003001	212008000	8.73E-40
chr1	212004001	212009000	7.53E-23
chr1	212588001	212593000	8.27E-25
chr1	212602001	212607000	8.62E-38
chr1	212606001	212611000	2.11E-19
chr1	212727001	212732000	1.67E-20
chr1	212728001	212733000	7.37E-57
chr1	212729001	212734000	1.49E-47
chr1	212730001	212735000	2.85E-36
chr1	212731001	212736000	4.92E-30
chr1	212732001	212737000	1.01E-30
chr1	212782001	212787000	6.39E-39
chr1	213027001	213032000	7.14E-17

chr1	213028001	213033000	8.52E-15
chr1	213029001	213034000	6.15E-15
chr1	213030001	213035000	2.87E-16
chr1	213031001	213036000	1.26E-32
chr1	213032001	213037000	1.06E-19
chr1	213120001	213125000	2.13E-30
chr1	213121001	213126000	8.48E-29
chr1	213124001	213129000	9.37E-32
chr1	213184001	213189000	4.07E-51
chr1	213220001	213225000	2.21E-30
chr1	213221001	213226000	1.15E-18
chr1	213222001	213227000	4.88E-16
chr1	213223001	213228000	1.91E-16
chr1	213224001	213229000	1.14E-21
chr1	213732001	213737000	9.85E-05
chr1	213733001	213738000	1.60E-05
chr1	214163001	214168000	1.60E-07
chr1	214164001	214169000	2.04E-05
chr1	214303001	214308000	0.002725307
chr1	214304001	214309000	3.03E-05
chr1	214450001	214455000	1.79E-63
chr1	214451001	214456000	1.60E-62
chr1	214452001	214457000	2.43E-58
chr1	214453001	214458000	8.71E-43
chr1	214720001	214725000	1.32E-49
chr1	214721001	214726000	1.84E-36
chr1	214724001	214729000	9.78E-32
chr1	215210001	215215000	0.000879492
chr1	215211001	215216000	0.001910069
chr1	215212001	215217000	0.001429183
chr1	215213001	215218000	0.000402977
chr1	215252001	215257000	1.85E-17
chr1	215286001	215291000	0.001963843
chr1	215741001	215746000	2.99E-28
chr1	216755001	216760000	4.56E-08
chr1	216756001	216761000	8.41E-08
chr1	216757001	216762000	4.34E-06
chr1	216771001	216776000	1.64E-24
chr1	216772001	216777000	6.18E-22
chr1	216773001	216778000	3.44E-28
chr1	216774001	216779000	5.03E-35
chr1	216854001	216859000	0.000392301
chr1	216855001	216860000	8.15E-05
chr1	216959001	216964000	0.001069271
chr1	216960001	216965000	0.001189331
chr1	216973001	216978000	0.003104983
chr1	217032001	217037000	3.32E-05
chr1	217243001	217248000	8.41E-05

chr1	217410001	217415000	7.28E-06
chr1	217468001	217473000	0.016330413
chr1	218050001	218055000	0.000550617
chr1	218096001	218101000	1.77E-22
chr1	218097001	218102000	3.59E-21
chr1	218098001	218103000	8.21E-30
chr1	218147001	218152000	0.001081957
chr1	218264001	218269000	0.000102858
chr1	218286001	218291000	0.002217555
chr1	218514001	218519000	6.22E-11
chr1	218515001	218520000	3.99E-52
chr1	219223001	219228000	1.32E-05
chr1	219315001	219320000	0.000374475
chr1	219337001	219342000	0.003488858
chr1	219590001	219595000	0.00380539
chr1	219591001	219596000	0.001596327
chr1	219796001	219801000	0.000262505
chr1	219797001	219802000	0.000518018
chr1	219892001	219897000	0.003798058
chr1	220265001	220270000	8.34E-45
chr1	220490001	220495000	7.87E-08
chr1	220520001	220525000	5.86E-14
chr1	220521001	220526000	1.55E-13
chr1	220816001	220821000	1.50E-14
chr1	220920001	220925000	4.61E-40
chr1	220922001	220927000	1.06E-18
chr1	220956001	220961000	2.91E-62
chr1	221916001	221921000	6.32E-66
chr1	222306001	222311000	0.001688566
chr1	222307001	222312000	0.001090843
chr1	222308001	222313000	0.004835681
chr1	222758001	222763000	2.68E-15
chr1	222787001	222792000	1.07E-41
chr1	222789001	222794000	2.11E-50
chr1	222790001	222795000	3.43E-55
chr1	222791001	222796000	4.90E-59
chr1	222813001	222818000	6.47E-35
chr1	222816001	222821000	1.24E-41
chr1	222817001	222822000	6.02E-67
chr1	223304001	223309000	1.66E-14
chr1	223305001	223310000	1.77E-21
chr1	223306001	223311000	4.74E-15
chr1	223307001	223312000	2.69E-16
chr1	223896001	223901000	6.13E-41
chr1	223897001	223902000	5.56E-35
chr1	223898001	223903000	1.12E-25
chr1	223899001	223904000	1.64E-25
chr1	224541001	224546000	2.32E-31

chr1	224622001	224627000	1.00E-39
chr1	224691001	224696000	3.61E-15
chr1	224799001	224804000	2.93E-53
chr1	225017001	225022000	2.93E-06
chr1	225113001	225118000	1.01E-62
chr1	225114001	225119000	7.21E-83
chr1	225115001	225120000	1.91E-93
chr1	225116001	225121000	2.71E-92
chr1	225117001	225122000	2.93E-69
chr1	225255001	225260000	8.47E-05
chr1	225336001	225341000	2.52E-06
chr1	225393001	225398000	0.000842592
chr1	225531001	225536000	5.51E-05
chr1	225532001	225537000	0.000282582
chr1	225961001	225966000	1.70E-36
chr1	225993001	225998000	8.99E-58
chr1	225994001	225999000	1.03E-51
chr1	226068001	226073000	4.04E-38
chr1	226069001	226074000	1.25E-37
chr1	226070001	226075000	1.90E-29
chr1	226110001	226115000	2.44E-47
chr1	226111001	226116000	4.70E-40
chr1	226492001	226497000	3.61E-41
chr1	226493001	226498000	9.27E-44
chr1	226494001	226499000	1.06E-19
chr1	226495001	226500000	1.67E-21
chr1	226496001	226501000	8.06E-28
chr1	226497001	226502000	3.52E-27
chr1	226595001	226600000	4.84E-36
chr1	227054001	227059000	5.38E-25
chr1	227056001	227061000	6.70E-24
chr1	227057001	227062000	8.35E-25
chr1	227058001	227063000	3.55E-23
chr1	227730001	227735000	1.74E-12
chr1	228130001	228135000	4.11E-24
chr1	228131001	228136000	1.28E-50
chr1	228132001	228137000	1.03E-10
chr1	228242001	228247000	1.05E-23
chr1	228243001	228248000	3.03E-33
chr1	228266001	228271000	6.46E-37
chr1	228296001	228301000	2.43E-32
chr1	228297001	228302000	3.97E-24
chr1	228353001	228358000	4.02E-35
chr1	228590001	228595000	2.66E-24
chr1	228675001	228680000	8.07E-16
chr1	228689001	228694000	2.50E-17
chr1	228690001	228695000	1.87E-10
chr1	228691001	228696000	1.02E-09

chr1	228833001	228838000	9.95E-05
chr1	228867001	228872000	1.40E-63
chr1	228868001	228873000	1.53E-32
chr1	228870001	228875000	1.46E-30
chr1	228871001	228876000	2.54E-19
chr1	229402001	229407000	1.59E-37
chr1	229403001	229408000	1.97E-50
chr1	229404001	229409000	7.58E-57
chr1	229405001	229410000	5.55E-47
chr1	229406001	229411000	1.25E-65
chr1	229407001	229412000	9.51E-50
chr1	229474001	229479000	2.64E-43
chr1	229639001	229644000	1.29E-30
chr1	229689001	229694000	9.81E-33
chr1	229690001	229695000	2.59E-63
chr1	229691001	229696000	9.85E-63
chr1	229692001	229697000	2.37E-49
chr1	229693001	229698000	6.36E-46
chr1	229694001	229699000	1.44E-39
chr1	229757001	229762000	1.06E-67
chr1	229944001	229949000	7.42E-05
chr1	229945001	229950000	0.000125735
chr1	230198001	230203000	5.29E-31
chr1	230236001	230241000	2.13E-13
chr1	230237001	230242000	1.29E-14
chr1	230686001	230691000	0.008300727
chr1	230712001	230717000	2.26E-08
chr1	231002001	231007000	6.12E-29
chr1	231003001	231008000	2.58E-28
chr1	231004001	231009000	8.14E-53
chr1	231171001	231176000	1.08E-79
chr1	231172001	231177000	6.42E-53
chr1	231173001	231178000	3.60E-40
chr1	231174001	231179000	1.20E-28
chr1	231175001	231180000	2.00E-29
chr1	231372001	231377000	2.04E-40
chr1	231546001	231551000	0.000334322
chr1	231557001	231562000	8.47E-90
chr1	231558001	231563000	2.38E-39
chr1	231758001	231763000	4.25E-26
chr1	232137001	232142000	0.003992772
chr1	232761001	232766000	3.01E-44
chr1	232766001	232771000	6.65E-52
chr1	232937001	232942000	9.55E-25
chr1	232938001	232943000	4.24E-37
chr1	232939001	232944000	1.81E-34
chr1	232940001	232945000	4.23E-29
chr1	232941001	232946000	1.43E-30

chr1	233428001	233433000	2.96E-27
chr1	233429001	233434000	4.60E-19
chr1	233430001	233435000	7.15E-30
chr1	233431001	233436000	1.73E-21
chr1	233459001	233464000	1.39E-74
chr1	233460001	233465000	6.17E-87
chr1	233461001	233466000	1.64E-50
chr1	233463001	233468000	4.18E-83
chr1	233464001	233469000	2.12E-65
chr1	233720001	233725000	9.54E-09
chr1	233721001	233726000	2.17E-12
chr1	233722001	233727000	1.25E-11
chr1	233723001	233728000	7.88E-11
chr1	233724001	233729000	4.18E-08
chr1	233750001	233755000	1.05E-34
chr1	234008001	234013000	7.30E-06
chr1	234036001	234041000	7.83E-56
chr1	234350001	234355000	2.74E-15
chr1	234614001	234619000	2.53E-43
chr1	235667001	235672000	2.09E-71
chr1	235668001	235673000	1.80E-85
chr1	236030001	236035000	6.63E-36
chr1	236349001	236354000	3.14E-17
chr1	236440001	236445000	3.38E-20
chr1	236685001	236690000	1.14E-48
chr1	236686001	236691000	9.91E-60
chr1	236687001	236692000	5.71E-72
chr1	236877001	236882000	1.02E-06
chr1	237130001	237135000	6.72E-13
chr1	237230001	237235000	2.00E-14
chr1	237423001	237428000	5.89E-06
chr1	237424001	237429000	7.37E-09
chr1	237425001	237430000	9.53E-08
chr1	237446001	237451000	8.64E-06
chr1	237447001	237452000	1.33E-09
chr1	237558001	237563000	0.000488609
chr1	237559001	237564000	0.000892524
chr1	237560001	237565000	0.000603828
chr1	237574001	237579000	1.54E-06
chr1	237577001	237582000	1.08E-05
chr1	237578001	237583000	3.33E-05
chr1	237579001	237584000	1.98E-05
chr1	237580001	237585000	3.74E-05
chr1	237635001	237640000	4.28E-06
chr1	237705001	237710000	1.31E-06
chr1	237706001	237711000	8.03E-09
chr1	237763001	237768000	3.90E-12
chr1	238151001	238156000	0.000837055

chr1	238152001	238157000	2.72E-07
chr1	238219001	238224000	0.000275457
chr1	238256001	238261000	0.002998285
chr1	238323001	238328000	8.56E-08
chr1	238378001	238383000	3.95E-06
chr1	238379001	238384000	0.001001901
chr1	238793001	238798000	0.001720864
chr1	238794001	238799000	0.000341204
chr1	239043001	239048000	0.002305316
chr1	239183001	239188000	0.002351586
chr1	239507001	239512000	0.000139955
chr1	239533001	239538000	3.56E-05
chr1	239545001	239550000	1.50E-40
chr1	239611001	239616000	0.007940933
chr1	239633001	239638000	6.78E-06
chr1	239634001	239639000	0.004960082
chr1	239667001	239672000	0.0005278
chr1	239688001	239693000	0.000293311
chr1	239798001	239803000	1.77E-05
chr1	239801001	239806000	9.93E-12
chr1	240161001	240166000	1.15E-50
chr1	240252001	240257000	9.03E-28
chr1	240253001	240258000	8.84E-51
chr1	240254001	240259000	2.51E-52
chr1	240255001	240260000	3.69E-58
chr1	240256001	240261000	3.55E-39
chr1	240431001	240436000	0.002550595
chr1	240775001	240780000	8.68E-20
chr1	241053001	241058000	1.67E-06
chr1	241103001	241108000	5.18E-05
chr1	241120001	241125000	1.93E-07
chr1	241153001	241158000	2.57E-15
chr1	241154001	241159000	6.58E-17
chr1	241155001	241160000	1.38E-12
chr1	241292001	241297000	1.26E-06
chr1	241293001	241298000	0.001416823
chr1	241321001	241326000	3.31E-13
chr1	241507001	241512000	0.007558567
chr1	241508001	241513000	0.00571257
chr1	241520001	241525000	2.98E-24
chr1	241583001	241588000	1.03E-33
chr1	241584001	241589000	4.09E-23
chr1	241711001	241716000	7.38E-13
chr1	241712001	241717000	5.69E-14
chr1	241713001	241718000	5.84E-12
chr1	241714001	241719000	1.28E-10
chr1	241773001	241778000	1.01E-05
chr1	242007001	242012000	1.26E-61

chr1	242516001	242521000	0.015949928
chr1	242889001	242894000	0.009593732
chr1	243379001	243384000	0.002000109
chr1	243583001	243588000	1.02E-14
chr1	243584001	243589000	9.64E-14
chr1	243585001	243590000	1.14E-17
chr1	244008001	244013000	3.93E-35
chr1	244009001	244014000	3.59E-35
chr1	244010001	244015000	1.68E-44
chr1	244011001	244016000	9.05E-68
chr1	244012001	244017000	1.06E-50
chr1	244013001	244018000	2.71E-29
chr1	244492001	244497000	2.72E-08
chr1	244493001	244498000	4.41E-09
chr1	244494001	244499000	4.98E-09
chr1	244569001	244574000	0.00285699
chr1	244610001	244615000	5.94E-62
chr1	244612001	244617000	2.68E-88
chr1	244615001	244620000	1.42E-37
chr1	244620001	244625000	5.32E-42
chr1	244621001	244626000	4.91E-39
chr1	244622001	244627000	3.51E-36
chr1	244623001	244628000	1.93E-38
chr1	244624001	244629000	1.70E-53
chr1	244812001	244817000	5.17E-39
chr1	244890001	244895000	2.40E-18
chr1	244891001	244896000	1.60E-28
chr1	244892001	244897000	2.69E-27
chr1	244893001	244898000	5.08E-34
chr1	244894001	244899000	3.73E-21
chr1	245082001	245087000	5.08E-31
chr1	245129001	245134000	1.33E-88
chr1	245130001	245135000	2.07E-67
chr1	245133001	245138000	3.26E-50
chr1	245790001	245795000	2.57E-11
chr1	246884001	246889000	3.93E-40
chr1	246885001	246890000	3.24E-57
chr1	246886001	246891000	1.11E-75
chr1	246887001	246892000	9.73E-64
chr1	247012001	247017000	3.37E-08
chr1	247091001	247096000	6.46E-45
chr1	247095001	247100000	4.11E-19
chr1	247237001	247242000	1.52E-51
chr1	247238001	247243000	2.12E-36
chr1	247239001	247244000	1.92E-47
chr1	247240001	247245000	8.23E-69
chr1	247241001	247246000	2.37E-86
chr1	247242001	247247000	3.42E-53

chr1	247263001	247268000	2.99E-51
chr1	247264001	247269000	9.01E-61
chr1	247265001	247270000	2.07E-38
chr1	247266001	247271000	2.23E-45
chr1	247267001	247272000	2.45E-42
chr1	247270001	247275000	2.99E-27
chr1	247271001	247276000	1.06E-37
chr1	247272001	247277000	1.21E-34
chr1	247273001	247278000	4.52E-22
chr1	247369001	247374000	4.61E-41
chr1	247370001	247375000	7.34E-36
chr1	247371001	247376000	3.01E-30
chr1	247374001	247379000	5.37E-16
chr1	247414001	247419000	8.64E-07
chr1	247415001	247420000	1.03E-09
chr1	247416001	247421000	1.49E-10
chr1	247417001	247422000	4.75E-09
chr1	247418001	247423000	3.74E-05
chr1	247549001	247554000	2.06E-10
chr1	247723001	247728000	6.37E-10
chr1	247765001	247770000	7.86E-10
chr1	247766001	247771000	1.57E-07
chr1	247767001	247772000	2.27E-07
chr1	247914001	247919000	1.77E-17
chr1	248186001	248191000	0.00020969
chr1	248254001	248259000	0.00163685
chr1	248369001	248374000	1.42E-08
chr1	248370001	248375000	1.72E-07
chr1	248371001	248376000	6.99E-08
chr1	248372001	248377000	7.01E-07
chr1	248373001	248378000	0.000136634
chr1	248374001	248379000	0.000680597
chr1	248375001	248380000	7.76E-05
chr1	248481001	248486000	1.61E-09
chr1	248482001	248487000	1.48E-09
chr1	248537001	248542000	0.00035678
chr1	248546001	248551000	1.03E-06
chr1	248615001	248620000	3.81E-06
chr1	248616001	248621000	3.03E-11
chr1	248617001	248622000	8.47E-06
chr1	248772001	248777000	0.000145237
chr1	248774001	248779000	1.45E-08
chr1	248775001	248780000	1.18E-06
chr1	248776001	248781000	3.13E-06
chr1	248830001	248835000	2.59E-10
chr1	248831001	248836000	2.05E-12
chr1	248907001	248912000	0.008548199
chr1	249155001	249160000	4.86E-19

chr1	249156001	249161000	5.01E-21
chr1	249157001	249162000	3.18E-16
chr1	249196001	249201000	9.31E-70
chr1	249197001	249202000	1.68E-59
chr1	249198001	249203000	7.93E-45
chr1	249199001	249204000	3.21E-37
chr1	249200001	249205000	1.09E-26
chr2	24001	29000	0.00011326
chr2	204001	209000	0.001550451
chr2	264001	269000	5.59E-82
chr2	265001	270000	1.19E-21
chr2	528001	533000	0.002500157
chr2	1039001	1044000	4.62E-07
chr2	1040001	1045000	3.09E-07
chr2	1042001	1047000	2.51E-08
chr2	1360001	1365000	0.00123948
chr2	1393001	1398000	1.74E-11
chr2	1394001	1399000	1.12E-10
chr2	1395001	1400000	5.39E-12
chr2	2413001	2418000	2.16E-08
chr2	2414001	2419000	3.13E-08
chr2	2613001	2618000	3.72E-21
chr2	3019001	3024000	0.000241122
chr2	3090001	3095000	2.91E-05
chr2	3379001	3384000	4.52E-46
chr2	3382001	3387000	4.68E-35
chr2	3383001	3388000	2.03E-39
chr2	3518001	3523000	6.58E-35
chr2	3601001	3606000	1.15E-95
chr2	3602001	3607000	2.86E-80
chr2	3603001	3608000	1.07E-70
chr2	3605001	3610000	4.10E-55
chr2	3606001	3611000	1.65E-30
chr2	3619001	3624000	5.84E-50
chr2	3620001	3625000	2.98E-48
chr2	3621001	3626000	1.16E-41
chr2	3623001	3628000	1.12E-53
chr2	3895001	3900000	9.38E-06
chr2	3896001	3901000	4.16E-07
chr2	4322001	4327000	6.31E-05
chr2	4834001	4839000	1.70E-05
chr2	4863001	4868000	2.72E-05
chr2	5384001	5389000	8.33E-06
chr2	5527001	5532000	0.002043105
chr2	5528001	5533000	0.002764783
chr2	5715001	5720000	2.04E-05
chr2	5716001	5721000	8.74E-05
chr2	5828001	5833000	2.88E-48

chr2	5829001	5834000	6.16E-39
chr2	5830001	5835000	3.72E-20
chr2	5832001	5837000	1.20E-32
chr2	6704001	6709000	9.87E-06
chr2	6705001	6710000	2.46E-05
chr2	6706001	6711000	6.61E-06
chr2	6713001	6718000	1.89E-06
chr2	6975001	6980000	1.96E-05
chr2	6977001	6982000	1.47E-05
chr2	6978001	6983000	0.000141366
chr2	7001001	7006000	3.32E-45
chr2	7005001	7010000	1.64E-22
chr2	7013001	7018000	7.47E-05
chr2	7014001	7019000	3.73E-06
chr2	7015001	7020000	2.12E-05
chr2	7016001	7021000	7.20E-06
chr2	7053001	7058000	1.74E-24
chr2	7054001	7059000	6.20E-29
chr2	7055001	7060000	2.50E-34
chr2	7056001	7061000	7.69E-29
chr2	7283001	7288000	2.04E-06
chr2	7557001	7562000	0.000125532
chr2	7558001	7563000	9.50E-05
chr2	7624001	7629000	1.15E-14
chr2	7625001	7630000	7.67E-13
chr2	7626001	7631000	6.91E-15
chr2	7627001	7632000	5.97E-15
chr2	7628001	7633000	3.04E-13
chr2	7629001	7634000	1.74E-05
chr2	7630001	7635000	4.21E-06
chr2	8317001	8322000	0.00087838
chr2	9139001	9144000	1.28E-72
chr2	9144001	9149000	3.06E-49
chr2	9342001	9347000	1.14E-32
chr2	9343001	9348000	2.69E-68
chr2	9344001	9349000	7.26E-64
chr2	9345001	9350000	2.09E-73
chr2	9347001	9352000	2.27E-55
chr2	9559001	9564000	7.15E-55
chr2	9563001	9568000	8.04E-43
chr2	9602001	9607000	2.03E-46
chr2	9603001	9608000	2.99E-43
chr2	9604001	9609000	1.08E-59
chr2	9605001	9610000	4.17E-62
chr2	9606001	9611000	7.74E-56
chr2	9979001	9984000	9.30E-36
chr2	10087001	10092000	1.49E-28
chr2	10088001	10093000	6.51E-36

chr2	10089001	10094000	1.62E-33
chr2	10090001	10095000	2.97E-27
chr2	10091001	10096000	5.98E-56
chr2	10182001	10187000	3.01E-15
chr2	10183001	10188000	3.69E-21
chr2	10220001	10225000	3.23E-38
chr2	10583001	10588000	1.07E-42
chr2	10584001	10589000	6.69E-53
chr2	10585001	10590000	3.30E-31
chr2	10586001	10591000	4.10E-50
chr2	10587001	10592000	3.77E-44
chr2	10588001	10593000	1.50E-27
chr2	10857001	10862000	6.21E-22
chr2	10858001	10863000	4.69E-22
chr2	10859001	10864000	1.36E-19
chr2	11484001	11489000	2.30E-57
chr2	11485001	11490000	2.43E-45
chr2	11601001	11606000	1.62E-19
chr2	11602001	11607000	2.27E-41
chr2	11623001	11628000	7.64E-43
chr2	12108001	12113000	4.27E-05
chr2	12194001	12199000	1.86E-11
chr2	12359001	12364000	8.79E-07
chr2	12360001	12365000	1.14E-12
chr2	12361001	12366000	1.38E-13
chr2	12362001	12367000	3.81E-11
chr2	12363001	12368000	6.72E-08
chr2	12547001	12552000	0.006340518
chr2	12603001	12608000	1.88E-08
chr2	12636001	12641000	2.72E-11
chr2	12637001	12642000	3.61E-08
chr2	12638001	12643000	1.27E-07
chr2	12750001	12755000	0.001802552
chr2	12852001	12857000	7.87E-35
chr2	12959001	12964000	1.88E-07
chr2	12960001	12965000	1.06E-09
chr2	13412001	13417000	6.49E-05
chr2	13413001	13418000	0.000691283
chr2	14428001	14433000	0.00012133
chr2	14742001	14747000	0.000122438
chr2	14759001	14764000	8.11E-06
chr2	14760001	14765000	1.08E-05
chr2	14798001	14803000	8.30E-06
chr2	14805001	14810000	0.002540838
chr2	14806001	14811000	0.008620195
chr2	15070001	15075000	0.009316867
chr2	15071001	15076000	0.00022976
chr2	15417001	15422000	0.001155138

chr2	15574001	15579000	1.42E-07
chr2	15732001	15737000	7.19E-27
chr2	16038001	16043000	0.000126964
chr2	16039001	16044000	0.000162552
chr2	16040001	16045000	0.000165413
chr2	16041001	16046000	0.000144132
chr2	16480001	16485000	1.56E-07
chr2	16522001	16527000	9.16E-05
chr2	16523001	16528000	7.60E-06
chr2	16589001	16594000	8.02E-07
chr2	16590001	16595000	8.29E-08
chr2	16601001	16606000	0.006538495
chr2	16689001	16694000	5.90E-21
chr2	16734001	16739000	0.000809466
chr2	17614001	17619000	0.000257881
chr2	17615001	17620000	0.00018143
chr2	17616001	17621000	0.000384587
chr2	17619001	17624000	1.39E-06
chr2	17620001	17625000	1.14E-06
chr2	17621001	17626000	9.40E-07
chr2	17622001	17627000	2.41E-12
chr2	17623001	17628000	3.50E-10
chr2	17624001	17629000	1.95E-08
chr2	17631001	17636000	3.25E-08
chr2	17632001	17637000	6.09E-06
chr2	17633001	17638000	1.80E-06
chr2	17634001	17639000	1.60E-08
chr2	17635001	17640000	7.00E-08
chr2	17636001	17641000	1.29E-06
chr2	17654001	17659000	6.40E-07
chr2	17655001	17660000	1.04E-07
chr2	17656001	17661000	1.10E-09
chr2	17695001	17700000	2.21E-12
chr2	17696001	17701000	1.52E-13
chr2	17697001	17702000	2.85E-16
chr2	17698001	17703000	4.72E-16
chr2	17699001	17704000	2.56E-15
chr2	17735001	17740000	0.000108469
chr2	17747001	17752000	1.59E-05
chr2	17748001	17753000	9.51E-05
chr2	17749001	17754000	0.000208622
chr2	17750001	17755000	0.00054797
chr2	17844001	17849000	0.002164711
chr2	17845001	17850000	0.003419345
chr2	17898001	17903000	0.003448736
chr2	17930001	17935000	8.23E-41
chr2	17934001	17939000	1.35E-76
chr2	17935001	17940000	6.13E-66

chr2	17936001	17941000	5.21E-05
chr2	18012001	18017000	1.34E-16
chr2	18059001	18064000	1.03E-23
chr2	18060001	18065000	6.28E-24
chr2	18537001	18542000	0.000816408
chr2	18588001	18593000	4.05E-05
chr2	18589001	18594000	0.000100571
chr2	18590001	18595000	0.000114426
chr2	18591001	18596000	0.000371098
chr2	18592001	18597000	0.000823156
chr2	18603001	18608000	0.000186599
chr2	18604001	18609000	4.25E-06
chr2	18638001	18643000	2.21E-06
chr2	18639001	18644000	8.79E-06
chr2	18736001	18741000	0.003108991
chr2	18737001	18742000	4.01E-84
chr2	18738001	18743000	6.43E-55
chr2	18739001	18744000	2.00E-43
chr2	18740001	18745000	2.16E-40
chr2	18741001	18746000	3.83E-46
chr2	18742001	18747000	1.07E-15
chr2	18804001	18809000	0.007308974
chr2	18887001	18892000	0.002425063
chr2	18918001	18923000	3.42E-05
chr2	18924001	18929000	8.90E-06
chr2	18953001	18958000	4.11E-07
chr2	19179001	19184000	1.86E-11
chr2	19180001	19185000	1.23E-13
chr2	19255001	19260000	0.000130161
chr2	19256001	19261000	0.029381244
chr2	19350001	19355000	0.000100772
chr2	19583001	19588000	1.06E-20
chr2	19652001	19657000	0.005683841
chr2	19713001	19718000	9.34E-06
chr2	19714001	19719000	1.69E-05
chr2	19907001	19912000	0.006068554
chr2	19908001	19913000	0.000141054
chr2	20186001	20191000	1.04E-36
chr2	20187001	20192000	5.62E-27
chr2	20188001	20193000	4.31E-25
chr2	20419001	20424000	3.43E-08
chr2	20420001	20425000	5.65E-10
chr2	20424001	20429000	5.67E-24
chr2	21021001	21026000	2.11E-28
chr2	21050001	21055000	1.37E-05
chr2	21052001	21057000	6.00E-06
chr2	21408001	21413000	1.83E-06
chr2	21445001	21450000	9.04E-05

chr2	21670001	21675000	0.001100288
chr2	21700001	21705000	0.002197343
chr2	21744001	21749000	2.33E-12
chr2	21912001	21917000	4.11E-05
chr2	21913001	21918000	2.57E-05
chr2	22074001	22079000	0.000410777
chr2	22086001	22091000	3.74E-06
chr2	22087001	22092000	2.99E-07
chr2	22088001	22093000	5.08E-05
chr2	22141001	22146000	2.34E-06
chr2	22208001	22213000	0.001077453
chr2	22210001	22215000	9.73E-06
chr2	22211001	22216000	4.59E-06
chr2	22212001	22217000	1.24E-05
chr2	22313001	22318000	3.63E-05
chr2	22314001	22319000	1.45E-05
chr2	22315001	22320000	1.46E-05
chr2	22316001	22321000	0.00139652
chr2	22364001	22369000	0.001965115
chr2	22365001	22370000	0.001032271
chr2	22366001	22371000	0.000455938
chr2	22367001	22372000	0.000912422
chr2	22368001	22373000	0.002547999
chr2	22455001	22460000	4.51E-07
chr2	22456001	22461000	6.80E-07
chr2	22457001	22462000	7.42E-06
chr2	22543001	22548000	2.16E-08
chr2	22544001	22549000	7.21E-05
chr2	22545001	22550000	4.40E-05
chr2	22546001	22551000	2.36E-05
chr2	22547001	22552000	8.98E-05
chr2	22613001	22618000	0.009181745
chr2	22614001	22619000	0.005785406
chr2	22615001	22620000	0.002476813
chr2	22661001	22666000	0.000530427
chr2	22662001	22667000	0.00135671
chr2	22664001	22669000	0.000868954
chr2	22756001	22761000	0.000290939
chr2	22757001	22762000	0.000243322
chr2	22770001	22775000	0.000947502
chr2	22787001	22792000	0.000653975
chr2	22876001	22881000	7.17E-05
chr2	22879001	22884000	5.20E-05
chr2	22981001	22986000	0.000372445
chr2	23131001	23136000	0.003276883
chr2	23156001	23161000	1.15E-05
chr2	23157001	23162000	0.003971159
chr2	23251001	23256000	4.80E-06

chr2	23445001	23450000	9.12E-07
chr2	23604001	23609000	5.21E-36
chr2	23605001	23610000	1.25E-50
chr2	23606001	23611000	2.09E-39
chr2	23607001	23612000	2.23E-25
chr2	23608001	23613000	1.82E-33
chr2	23609001	23614000	1.34E-24
chr2	24037001	24042000	2.25E-05
chr2	24038001	24043000	1.65E-15
chr2	24150001	24155000	4.29E-18
chr2	24295001	24300000	5.92E-24
chr2	24296001	24301000	3.91E-23
chr2	24299001	24304000	4.18E-20
chr2	24300001	24305000	1.80E-11
chr2	24303001	24308000	1.79E-14
chr2	24304001	24309000	2.54E-08
chr2	24306001	24311000	1.11E-10
chr2	24307001	24312000	1.56E-19
chr2	24342001	24347000	2.27E-32
chr2	24343001	24348000	1.01E-34
chr2	24344001	24349000	1.06E-92
chr2	24345001	24350000	9.70E-94
chr2	24346001	24351000	8.28E-103
chr2	24709001	24714000	3.52E-26
chr2	24710001	24715000	8.66E-43
chr2	24752001	24757000	5.51E-10
chr2	24796001	24801000	0.003112443
chr2	24797001	24802000	0.003324687
chr2	24854001	24859000	0.00344077
chr2	24855001	24860000	0.000952683
chr2	24856001	24861000	0.000250193
chr2	25190001	25195000	7.18E-38
chr2	25265001	25270000	8.87E-20
chr2	25891001	25896000	1.90E-19
chr2	25892001	25897000	6.05E-22
chr2	25893001	25898000	3.91E-26
chr2	25894001	25899000	1.12E-31
chr2	25895001	25900000	5.45E-38
chr2	25896001	25901000	3.20E-37
chr2	26046001	26051000	0.004221543
chr2	26101001	26106000	6.06E-31
chr2	26205001	26210000	1.95E-19
chr2	26403001	26408000	1.44E-09
chr2	26404001	26409000	4.71E-10
chr2	26405001	26410000	7.57E-17
chr2	26468001	26473000	1.05E-15
chr2	26724001	26729000	3.66E-16
chr2	26725001	26730000	1.10E-20

chr2	26987001	26992000	1.15E-08
chr2	27009001	27014000	4.95E-23
chr2	27070001	27075000	3.99E-18
chr2	27071001	27076000	1.03E-17
chr2	27072001	27077000	4.45E-23
chr2	27256001	27261000	1.11E-12
chr2	27290001	27295000	1.57E-34
chr2	27291001	27296000	1.69E-35
chr2	27292001	27297000	1.94E-22
chr2	27337001	27342000	2.31E-09
chr2	27353001	27358000	5.06E-28
chr2	27354001	27359000	1.59E-28
chr2	27355001	27360000	9.16E-32
chr2	27356001	27361000	3.13E-20
chr2	27357001	27362000	3.55E-20
chr2	27436001	27441000	5.64E-33
chr2	27437001	27442000	8.03E-20
chr2	27438001	27443000	4.13E-24
chr2	27439001	27444000	1.74E-29
chr2	27440001	27445000	1.48E-30
chr2	27882001	27887000	5.20E-36
chr2	27887001	27892000	2.04E-10
chr2	27889001	27894000	2.27E-20
chr2	27890001	27895000	1.71E-19
chr2	27937001	27942000	7.02E-13
chr2	27938001	27943000	9.90E-23
chr2	27992001	27997000	5.77E-30
chr2	27993001	27998000	2.31E-42
chr2	27994001	27999000	1.84E-34
chr2	29093001	29098000	2.51E-22
chr2	29113001	29118000	4.88E-33
chr2	29533001	29538000	1.74E-14
chr2	29534001	29539000	1.63E-13
chr2	29535001	29540000	5.37E-15
chr2	29536001	29541000	2.90E-13
chr2	29729001	29734000	6.22E-08
chr2	29730001	29735000	1.44E-10
chr2	29731001	29736000	4.64E-12
chr2	29751001	29756000	2.11E-13
chr2	29762001	29767000	4.76E-05
chr2	29777001	29782000	0.000539512
chr2	29779001	29784000	0.012160244
chr2	29865001	29870000	1.99E-10
chr2	29880001	29885000	3.73E-05
chr2	30045001	30050000	0.001661363
chr2	30240001	30245000	3.12E-06
chr2	30255001	30260000	3.18E-08
chr2	30669001	30674000	7.91E-40

chr2	30670001	30675000	5.10E-46
chr2	30704001	30709000	4.11E-08
chr2	31015001	31020000	0.002312677
chr2	31356001	31361000	8.91E-29
chr2	31358001	31363000	2.22E-35
chr2	31359001	31364000	8.95E-37
chr2	31360001	31365000	4.07E-46
chr2	31361001	31366000	5.50E-37
chr2	31452001	31457000	1.42E-52
chr2	31453001	31458000	3.54E-29
chr2	31454001	31459000	2.61E-25
chr2	31456001	31461000	1.56E-13
chr2	31674001	31679000	0.001349946
chr2	31949001	31954000	6.38E-08
chr2	31951001	31956000	0.000382255
chr2	32230001	32235000	2.71E-21
chr2	32231001	32236000	6.80E-47
chr2	32235001	32240000	2.04E-25
chr2	32577001	32582000	1.54E-08
chr2	32578001	32583000	1.04E-25
chr2	32579001	32584000	7.41E-10
chr2	32580001	32585000	3.86E-24
chr2	32581001	32586000	1.16E-20
chr2	32582001	32587000	9.94E-37
chr2	33172001	33177000	1.79E-46
chr2	33820001	33825000	1.21E-35
chr2	33824001	33829000	1.13E-44
chr2	33911001	33916000	9.26E-09
chr2	33912001	33917000	2.54E-11
chr2	34251001	34256000	0.007897731
chr2	34252001	34257000	0.001957736
chr2	34253001	34258000	0.010620841
chr2	34416001	34421000	6.67E-05
chr2	34479001	34484000	9.30E-05
chr2	34534001	34539000	1.57E-08
chr2	34535001	34540000	8.90E-06
chr2	34820001	34825000	0.001479915
chr2	35005001	35010000	0.002942369
chr2	35006001	35011000	0.005817341
chr2	35735001	35740000	3.18E-05
chr2	35736001	35741000	1.20E-06
chr2	36032001	36037000	2.50E-05
chr2	36378001	36383000	0.000253865
chr2	36458001	36463000	0.000926302
chr2	36459001	36464000	0.001055649
chr2	36460001	36465000	0.000914878
chr2	36463001	36468000	9.73E-15
chr2	36464001	36469000	2.49E-15

chr2	36465001	36470000	2.11E-14
chr2	36466001	36471000	2.97E-13
chr2	36508001	36513000	5.29E-05
chr2	36599001	36604000	2.99E-18
chr2	36600001	36605000	1.13E-12
chr2	36601001	36606000	2.38E-13
chr2	36602001	36607000	6.42E-15
chr2	36937001	36942000	9.97E-13
chr2	36938001	36943000	3.23E-12
chr2	36939001	36944000	2.62E-10
chr2	37189001	37194000	1.28E-45
chr2	37307001	37312000	6.63E-34
chr2	37382001	37387000	6.96E-13
chr2	37383001	37388000	2.79E-15
chr2	37419001	37424000	1.93E-23
chr2	37552001	37557000	5.11E-25
chr2	37568001	37573000	9.31E-28
chr2	37569001	37574000	1.03E-35
chr2	37570001	37575000	3.32E-32
chr2	37571001	37576000	3.17E-33
chr2	37572001	37577000	4.76E-37
chr2	37669001	37674000	0.003552458
chr2	37898001	37903000	3.00E-27
chr2	37899001	37904000	3.87E-31
chr2	38148001	38153000	1.54E-30
chr2	38152001	38157000	7.89E-38
chr2	38375001	38380000	8.16E-09
chr2	38604001	38609000	1.80E-32
chr2	39098001	39103000	2.92E-18
chr2	39893001	39898000	1.27E-43
chr2	40022001	40027000	0.022303066
chr2	40023001	40028000	0.007806191
chr2	40024001	40029000	0.013757079
chr2	41009001	41014000	1.11E-14
chr2	41389001	41394000	0.00152301
chr2	42068001	42073000	1.05E-30
chr2	42270001	42275000	1.24E-11
chr2	42392001	42397000	2.19E-37
chr2	42393001	42398000	9.09E-33
chr2	42394001	42399000	1.86E-27
chr2	42721001	42726000	3.61E-31
chr2	43453001	43458000	4.83E-51
chr2	43454001	43459000	1.95E-44
chr2	43862001	43867000	1.56E-28
chr2	43863001	43868000	7.30E-49
chr2	43864001	43869000	7.93E-76
chr2	43899001	43904000	8.39E-28
chr2	43900001	43905000	4.61E-18

chr2	44391001	44396000	1.20E-48
chr2	44392001	44397000	1.04E-58
chr2	44589001	44594000	2.41E-34
chr2	44678001	44683000	0.012820205
chr2	44679001	44684000	0.013612913
chr2	44680001	44685000	3.97E-08
chr2	44899001	44904000	0.001636787
chr2	45392001	45397000	1.26E-77
chr2	45873001	45878000	6.20E-36
chr2	46439001	46444000	0.010684911
chr2	46499001	46504000	2.07E-10
chr2	46500001	46505000	3.32E-08
chr2	46501001	46506000	6.15E-11
chr2	46503001	46508000	1.87E-16
chr2	46520001	46525000	1.07E-13
chr2	46770001	46775000	2.99E-16
chr2	46839001	46844000	2.14E-42
chr2	46840001	46845000	1.08E-42
chr2	46841001	46846000	4.75E-58
chr2	46842001	46847000	1.22E-54
chr2	46843001	46848000	8.31E-33
chr2	46922001	46927000	1.72E-33
chr2	46925001	46930000	5.23E-61
chr2	46926001	46931000	6.36E-81
chr2	46927001	46932000	1.27E-11
chr2	47138001	47143000	3.09E-18
chr2	47163001	47168000	3.46E-14
chr2	47592001	47597000	8.92E-16
chr2	47593001	47598000	7.48E-11
chr2	47594001	47599000	1.76E-16
chr2	47595001	47600000	2.05E-19
chr2	48010001	48015000	7.34E-29
chr2	48011001	48016000	2.64E-15
chr2	48212001	48217000	4.48E-06
chr2	48213001	48218000	2.31E-06
chr2	48220001	48225000	0.00425651
chr2	48221001	48226000	0.001840247
chr2	48537001	48542000	7.06E-34
chr2	48541001	48546000	1.14E-60
chr2	48542001	48547000	1.98E-54
chr2	48665001	48670000	6.15E-48
chr2	48666001	48671000	1.48E-36
chr2	48668001	48673000	7.79E-17
chr2	48817001	48822000	2.83E-06
chr2	48863001	48868000	1.14E-05
chr2	48865001	48870000	3.39E-05
chr2	49394001	49399000	1.46E-06
chr2	49542001	49547000	1.22E-05

chr2	49592001	49597000	0.000302135
chr2	49593001	49598000	1.12E-05
chr2	49740001	49745000	0.003268569
chr2	49741001	49746000	0.002872854
chr2	49806001	49811000	2.73E-08
chr2	49807001	49812000	4.12E-07
chr2	49808001	49813000	3.41E-06
chr2	49866001	49871000	0.002834637
chr2	49867001	49872000	0.003257043
chr2	49954001	49959000	0.000430056
chr2	49955001	49960000	1.42E-07
chr2	49956001	49961000	9.35E-07
chr2	50237001	50242000	0.001027601
chr2	50262001	50267000	0.00625528
chr2	50312001	50317000	0.000683799
chr2	50313001	50318000	0.000771876
chr2	50703001	50708000	0.008708143
chr2	50704001	50709000	0.001464727
chr2	51423001	51428000	0.000173758
chr2	51766001	51771000	0.003690296
chr2	51932001	51937000	6.56E-06
chr2	52008001	52013000	0.001337376
chr2	52652001	52657000	0.000259294
chr2	52748001	52753000	0.001277199
chr2	52890001	52895000	0.003485352
chr2	53557001	53562000	1.67E-26
chr2	54014001	54019000	7.68E-31
chr2	54193001	54198000	3.19E-22
chr2	54338001	54343000	8.64E-26
chr2	54339001	54344000	9.33E-27
chr2	54342001	54347000	5.62E-23
chr2	54641001	54646000	5.12E-08
chr2	54642001	54647000	9.54E-09
chr2	54679001	54684000	3.15E-43
chr2	54680001	54685000	5.86E-47
chr2	54781001	54786000	3.81E-35
chr2	54782001	54787000	3.54E-20
chr2	54952001	54957000	4.02E-34
chr2	55210001	55215000	0.00149727
chr2	55446001	55451000	3.76E-16
chr2	55742001	55747000	6.83E-33
chr2	55745001	55750000	7.93E-48
chr2	55916001	55921000	1.39E-51
chr2	56145001	56150000	8.40E-18
chr2	56151001	56156000	1.11E-10
chr2	56407001	56412000	3.93E-25
chr2	56751001	56756000	0.000357713
chr2	56752001	56757000	3.05E-05

chr2	56753001	56758000	2.54E-05
chr2	56754001	56759000	0.00279849
chr2	57464001	57469000	0.000277164
chr2	57770001	57775000	0.000346216
chr2	57946001	57951000	4.38E-10
chr2	57948001	57953000	4.80E-11
chr2	58166001	58171000	0.006639338
chr2	58269001	58274000	1.47E-40
chr2	58270001	58275000	7.77E-52
chr2	58274001	58279000	1.38E-34
chr2	58407001	58412000	0.012295044
chr2	58460001	58465000	0.000114664
chr2	58461001	58466000	0.000114247
chr2	58463001	58468000	1.13E-07
chr2	58466001	58471000	1.15E-48
chr2	58467001	58472000	9.40E-49
chr2	58468001	58473000	5.80E-55
chr2	58918001	58923000	4.82E-06
chr2	59128001	59133000	7.75E-05
chr2	59129001	59134000	0.00321086
chr2	59365001	59370000	0.000356817
chr2	59366001	59371000	0.000169824
chr2	59445001	59450000	0.007092979
chr2	59446001	59451000	0.004495561
chr2	59497001	59502000	0.00126846
chr2	59498001	59503000	0.006713208
chr2	59499001	59504000	8.59E-05
chr2	59500001	59505000	9.18E-06
chr2	59501001	59506000	0.00019899
chr2	59543001	59548000	0.000981863
chr2	59544001	59549000	0.000866117
chr2	59545001	59550000	0.00079027
chr2	59546001	59551000	0.0006684
chr2	60022001	60027000	7.19E-05
chr2	60023001	60028000	2.71E-05
chr2	60143001	60148000	0.000193041
chr2	60145001	60150000	2.10E-05
chr2	60211001	60216000	3.40E-05
chr2	60250001	60255000	1.11E-10
chr2	60325001	60330000	9.72E-12
chr2	60326001	60331000	1.71E-09
chr2	60327001	60332000	4.74E-10
chr2	60382001	60387000	0.000709529
chr2	60410001	60415000	1.95E-05
chr2	60428001	60433000	8.87E-12
chr2	60429001	60434000	8.39E-17
chr2	60430001	60435000	1.79E-11
chr2	60431001	60436000	1.26E-06

chr2	60432001	60437000	4.30E-07
chr2	60640001	60645000	4.24E-11
chr2	61226001	61231000	0.000955427
chr2	61227001	61232000	8.09E-06
chr2	61228001	61233000	1.65E-11
chr2	61229001	61234000	1.43E-10
chr2	61293001	61298000	1.87E-70
chr2	61693001	61698000	9.78E-49
chr2	61694001	61699000	2.54E-61
chr2	61695001	61700000	2.56E-54
chr2	61765001	61770000	1.45E-17
chr2	61917001	61922000	4.53E-14
chr2	62128001	62133000	8.65E-31
chr2	62129001	62134000	5.75E-28
chr2	62197001	62202000	2.74E-10
chr2	62198001	62203000	1.16E-12
chr2	62277001	62282000	0.002320133
chr2	62278001	62283000	0.003776809
chr2	62326001	62331000	2.87E-09
chr2	62419001	62424000	1.17E-17
chr2	62728001	62733000	2.58E-49
chr2	62729001	62734000	1.22E-104
chr2	62730001	62735000	1.56E-98
chr2	62731001	62736000	1.87E-67
chr2	62732001	62737000	9.21E-70
chr2	62733001	62738000	3.70E-58
chr2	62929001	62934000	9.60E-31
chr2	62933001	62938000	5.17E-33
chr2	63050001	63055000	0.006762852
chr2	63271001	63276000	7.33E-17
chr2	63287001	63292000	1.66E-18
chr2	63356001	63361000	0.001736826
chr2	63534001	63539000	3.54E-05
chr2	63664001	63669000	0.014965975
chr2	63811001	63816000	9.05E-51
chr2	64063001	64068000	2.54E-32
chr2	64064001	64069000	9.34E-40
chr2	64246001	64251000	5.36E-44
chr2	64677001	64682000	3.81E-45
chr2	64681001	64686000	6.97E-63
chr2	64871001	64876000	1.03E-21
chr2	64872001	64877000	7.09E-13
chr2	64876001	64881000	1.11E-50
chr2	64880001	64885000	7.60E-78
chr2	64881001	64886000	3.00E-64
chr2	64899001	64904000	3.96E-06
chr2	64995001	65000000	1.40E-41
chr2	65088001	65093000	1.20E-07

chr2	65279001	65284000	1.49E-21
chr2	65653001	65658000	2.21E-29
chr2	65654001	65659000	3.24E-54
chr2	65655001	65660000	4.94E-47
chr2	65676001	65681000	3.78E-07
chr2	65677001	65682000	5.50E-07
chr2	65678001	65683000	1.78E-06
chr2	66030001	66035000	0.01385398
chr2	66629001	66634000	0.000175678
chr2	66631001	66636000	0.000231523
chr2	66632001	66637000	4.10E-06
chr2	67068001	67073000	2.66E-05
chr2	67163001	67168000	0.009581333
chr2	67595001	67600000	7.29E-08
chr2	67620001	67625000	1.13E-42
chr2	68148001	68153000	0.000380059
chr2	68385001	68390000	1.98E-43
chr2	68615001	68620000	2.79E-07
chr2	68616001	68621000	4.51E-07
chr2	68623001	68628000	2.50E-05
chr2	68624001	68629000	5.98E-11
chr2	68625001	68630000	1.68E-07
chr2	68715001	68720000	3.43E-07
chr2	68731001	68736000	6.11E-07
chr2	68732001	68737000	5.42E-06
chr2	68748001	68753000	0.001748576
chr2	68782001	68787000	7.76E-06
chr2	68866001	68871000	3.50E-47
chr2	68867001	68872000	1.71E-42
chr2	68868001	68873000	3.09E-35
chr2	68869001	68874000	3.65E-25
chr2	68917001	68922000	1.72E-05
chr2	69166001	69171000	6.08E-21
chr2	69226001	69231000	5.70E-05
chr2	69272001	69277000	8.88E-05
chr2	69614001	69619000	3.96E-41
chr2	69664001	69669000	4.24E-23
chr2	70052001	70057000	1.99E-35
chr2	70062001	70067000	0.00058475
chr2	70137001	70142000	1.55E-55
chr2	70524001	70529000	1.23E-52
chr2	70525001	70530000	9.34E-50
chr2	70526001	70531000	3.12E-47
chr2	70527001	70532000	2.28E-33
chr2	70776001	70781000	7.20E-47
chr2	70777001	70782000	3.28E-47
chr2	70778001	70783000	6.67E-36
chr2	70990001	70995000	2.14E-92

chr2	70991001	70996000	3.72E-103
chr2	70992001	70997000	1.66E-67
chr2	70993001	70998000	1.04E-24
chr2	70994001	70999000	1.24E-18
chr2	71094001	71099000	1.79E-21
chr2	71134001	71139000	9.00E-19
chr2	71693001	71698000	9.12E-34
chr2	71694001	71699000	7.01E-21
chr2	72131001	72136000	0.000116673
chr2	72132001	72137000	0.000383093
chr2	72572001	72577000	0.004310788
chr2	73089001	73094000	1.66E-11
chr2	73110001	73115000	3.23E-19
chr2	73294001	73299000	1.30E-46
chr2	73339001	73344000	8.72E-31
chr2	73340001	73345000	7.19E-23
chr2	73379001	73384000	7.96E-07
chr2	73380001	73385000	7.30E-07
chr2	73381001	73386000	2.32E-06
chr2	73507001	73512000	1.87E-08
chr2	73508001	73513000	8.38E-11
chr2	73509001	73514000	5.33E-12
chr2	73510001	73515000	2.40E-16
chr2	73511001	73516000	2.49E-21
chr2	73613001	73618000	4.23E-29
chr2	74004001	74009000	7.22E-31
chr2	74005001	74010000	4.08E-33
chr2	74343001	74348000	3.07E-40
chr2	74344001	74349000	5.59E-50
chr2	74345001	74350000	8.76E-50
chr2	74405001	74410000	7.87E-16
chr2	74421001	74426000	2.23E-33
chr2	74422001	74427000	2.83E-29
chr2	74423001	74428000	2.18E-27
chr2	74425001	74430000	2.07E-20
chr2	74688001	74693000	6.08E-16
chr2	74705001	74710000	1.68E-32
chr2	74755001	74760000	2.40E-25
chr2	74756001	74761000	1.06E-20
chr2	74772001	74777000	6.84E-20
chr2	74773001	74778000	9.91E-17
chr2	74774001	74779000	6.03E-23
chr2	74775001	74780000	2.88E-18
chr2	74776001	74781000	7.66E-14
chr2	74778001	74783000	3.21E-09
chr2	74877001	74882000	1.44E-35
chr2	74881001	74886000	7.73E-23
chr2	75057001	75062000	1.74E-20

chr2	75058001	75063000	1.32E-08
chr2	75061001	75066000	8.90E-51
chr2	75062001	75067000	3.83E-24
chr2	75181001	75186000	4.29E-41
chr2	75182001	75187000	8.42E-32
chr2	75183001	75188000	2.81E-60
chr2	75184001	75189000	3.09E-63
chr2	75185001	75190000	8.98E-67
chr2	75423001	75428000	3.93E-37
chr2	75427001	75432000	1.27E-57
chr2	75475001	75480000	9.11E-05
chr2	75476001	75481000	2.05E-05
chr2	75477001	75482000	0.004678065
chr2	75783001	75788000	1.46E-32
chr2	75784001	75789000	1.48E-28
chr2	75785001	75790000	3.03E-28
chr2	75786001	75791000	8.19E-29
chr2	75787001	75792000	2.14E-36
chr2	76369001	76374000	0.001340203
chr2	76370001	76375000	0.013274077
chr2	76511001	76516000	0.000680966
chr2	76512001	76517000	0.006227974
chr2	77480001	77485000	0.002196395
chr2	77481001	77486000	0.002545868
chr2	77482001	77487000	0.001958993
chr2	77776001	77781000	0.014149092
chr2	77869001	77874000	4.37E-06
chr2	77870001	77875000	3.14E-07
chr2	77871001	77876000	1.79E-08
chr2	77872001	77877000	2.75E-07
chr2	78020001	78025000	0.014991855
chr2	78065001	78070000	0.017378726
chr2	78638001	78643000	0.006203114
chr2	78639001	78644000	0.000673437
chr2	78894001	78899000	0.002417637
chr2	78924001	78929000	3.63E-08
chr2	79456001	79461000	3.08E-06
chr2	79507001	79512000	1.12E-06
chr2	80273001	80278000	1.17E-05
chr2	80274001	80279000	6.81E-08
chr2	80275001	80280000	2.93E-12
chr2	80276001	80281000	7.84E-12
chr2	80287001	80292000	0.001943837
chr2	80640001	80645000	4.82E-05
chr2	80897001	80902000	0.001441497
chr2	81030001	81035000	2.31E-05
chr2	81031001	81036000	0.000127869
chr2	81165001	81170000	0.000539617

chr2	81316001	81321000	0.000218168
chr2	81317001	81322000	0.002012525
chr2	81318001	81323000	0.001249908
chr2	81513001	81518000	3.78E-06
chr2	81598001	81603000	0.005003998
chr2	81830001	81835000	4.18E-05
chr2	81831001	81836000	0.00047038
chr2	81832001	81837000	0.00125779
chr2	81972001	81977000	1.75E-05
chr2	82271001	82276000	1.46E-05
chr2	82272001	82277000	0.000304148
chr2	82273001	82278000	3.06E-05
chr2	82362001	82367000	0.000539652
chr2	82859001	82864000	0.001307844
chr2	82860001	82865000	0.004011876
chr2	82862001	82867000	0.000635088
chr2	82863001	82868000	0.005217593
chr2	83410001	83415000	0.000220813
chr2	83411001	83416000	7.67E-05
chr2	83656001	83661000	0.000235086
chr2	83843001	83848000	3.35E-08
chr2	84155001	84160000	0.000589349
chr2	84156001	84161000	0.000170971
chr2	84158001	84163000	0.000509102
chr2	84159001	84164000	0.0009656
chr2	84478001	84483000	2.60E-05
chr2	84681001	84686000	1.81E-15
chr2	84729001	84734000	6.69E-09
chr2	84730001	84735000	4.48E-10
chr2	84745001	84750000	4.74E-06
chr2	84746001	84751000	9.29E-12
chr2	84747001	84752000	1.33E-11
chr2	84748001	84753000	1.40E-12
chr2	84749001	84754000	3.75E-13
chr2	84751001	84756000	0.002353178
chr2	84752001	84757000	0.000137646
chr2	84753001	84758000	0.000443975
chr2	84784001	84789000	1.23E-06
chr2	84788001	84793000	1.05E-07
chr2	84791001	84796000	5.59E-06
chr2	84792001	84797000	9.29E-09
chr2	84811001	84816000	3.21E-08
chr2	84812001	84817000	5.16E-07
chr2	84813001	84818000	9.16E-09
chr2	84868001	84873000	5.92E-05
chr2	84869001	84874000	0.003860939
chr2	84876001	84881000	0.000219873
chr2	84877001	84882000	2.33E-05

chr2	84948001	84953000	6.95E-07
chr2	84950001	84955000	7.53E-09
chr2	84982001	84987000	0.000100973
chr2	84983001	84988000	1.60E-06
chr2	84997001	85002000	0.00072337
chr2	84998001	85003000	7.00E-05
chr2	84999001	85004000	0.001846393
chr2	85837001	85842000	6.57E-18
chr2	85838001	85843000	7.10E-22
chr2	85839001	85844000	1.31E-30
chr2	86115001	86120000	6.37E-35
chr2	86635001	86640000	7.97E-10
chr2	86636001	86641000	5.61E-08
chr2	86637001	86642000	1.17E-12
chr2	86638001	86643000	1.33E-12
chr2	86639001	86644000	1.54E-17
chr2	86640001	86645000	2.44E-08
chr2	86641001	86646000	1.75E-06
chr2	86663001	86668000	8.78E-29
chr2	86664001	86669000	1.18E-35
chr2	86669001	86674000	6.22E-31
chr2	86790001	86795000	5.39E-38
chr2	86850001	86855000	4.29E-38
chr2	86882001	86887000	0.000152199
chr2	86883001	86888000	1.66E-05
chr2	86884001	86889000	3.95E-08
chr2	86885001	86890000	1.28E-10
chr2	86942001	86947000	1.86E-09
chr2	86943001	86948000	4.40E-60
chr2	86944001	86949000	1.44E-41
chr2	86945001	86950000	3.64E-36
chr2	86946001	86951000	6.02E-20
chr2	87043001	87048000	0.005558591
chr2	87086001	87091000	2.95E-17
chr2	87087001	87092000	8.27E-28
chr2	87088001	87093000	1.32E-20
chr2	87298001	87303000	3.31E-33
chr2	87303001	87308000	2.35E-54
chr2	87330001	87335000	3.42E-05
chr2	87333001	87338000	0.000907202
chr2	87637001	87642000	9.21E-12
chr2	87880001	87885000	1.58E-27
chr2	87881001	87886000	6.05E-28
chr2	87882001	87887000	4.65E-34
chr2	87883001	87888000	3.76E-34
chr2	88350001	88355000	3.33E-28
chr2	88351001	88356000	3.22E-54
chr2	88352001	88357000	2.23E-50

chr2	88353001	88358000	9.35E-58
chr2	88354001	88359000	3.00E-70
chr2	88355001	88360000	2.21E-77
chr2	88498001	88503000	1.11E-14
chr2	88499001	88504000	4.29E-14
chr2	88500001	88505000	2.59E-19
chr2	88631001	88636000	3.83E-11
chr2	88633001	88638000	8.47E-06
chr2	88922001	88927000	6.47E-29
chr2	88926001	88931000	1.42E-47
chr2	88927001	88932000	1.98E-34
chr2	89091001	89096000	0.000375409
chr2	89443001	89448000	2.62E-08
chr2	89530001	89535000	2.28E-05
chr2	89827001	89832000	4.40E-18
chr2	89828001	89833000	3.89E-34
chr2	89829001	89834000	9.70E-40
chr2	89830001	89835000	8.73E-40
chr2	89831001	89836000	4.30E-34
chr2	89837001	89842000	1.12E-42
chr2	89838001	89843000	4.00E-77
chr2	89839001	89844000	2.97E-85
chr2	89840001	89845000	2.62E-66
chr2	89841001	89846000	3.69E-65
chr2	89850001	89855000	2.69E-19
chr2	89851001	89856000	1.44E-20
chr2	89950001	89955000	0.002612631
chr2	90050001	90055000	0.000167465
chr2	90051001	90056000	0.000307712
chr2	90122001	90127000	0.001569425
chr2	90377001	90382000	1.94E-40
chr2	90378001	90383000	2.55E-40
chr2	90379001	90384000	2.55E-40
chr2	90380001	90385000	2.55E-40
chr2	90381001	90386000	2.55E-40
chr2	90444001	90449000	8.80E-20
chr2	91705001	91710000	7.19E-07
chr2	91706001	91711000	1.99E-11
chr2	91707001	91712000	2.04E-11
chr2	91737001	91742000	1.07E-06
chr2	91993001	91998000	2.50E-26
chr2	91994001	91999000	2.50E-26
chr2	91995001	92000000	6.39E-27
chr2	92240001	92245000	2.67E-08
chr2	92270001	92275000	5.25E-18
chr2	92271001	92276000	3.78E-16
chr2	95576001	95581000	0.002408518
chr2	95785001	95790000	1.18E-28

chr2	95786001	95791000	5.40E-42
chr2	95787001	95792000	8.82E-52
chr2	95820001	95825000	6.46E-08
chr2	95821001	95826000	1.62E-16
chr2	95822001	95827000	1.47E-15
chr2	95828001	95833000	9.02E-13
chr2	95829001	95834000	4.52E-10
chr2	95830001	95835000	8.69E-25
chr2	95831001	95836000	1.99E-22
chr2	95871001	95876000	6.62E-18
chr2	95872001	95877000	6.44E-32
chr2	95873001	95878000	3.08E-34
chr2	96064001	96069000	7.58E-48
chr2	96065001	96070000	3.58E-40
chr2	96088001	96093000	0.000422807
chr2	96089001	96094000	2.91E-06
chr2	96090001	96095000	1.46E-06
chr2	96093001	96098000	2.23E-08
chr2	96190001	96195000	9.18E-26
chr2	96191001	96196000	5.09E-19
chr2	96192001	96197000	1.36E-22
chr2	96484001	96489000	1.07E-07
chr2	96653001	96658000	1.25E-45
chr2	96654001	96659000	3.81E-43
chr2	96655001	96660000	1.76E-28
chr2	96656001	96661000	2.74E-28
chr2	96657001	96662000	2.91E-26
chr2	96927001	96932000	5.11E-16
chr2	96928001	96933000	5.62E-20
chr2	96931001	96936000	5.91E-17
chr2	96985001	96990000	4.04E-18
chr2	96986001	96991000	1.26E-20
chr2	96987001	96992000	3.74E-09
chr2	96999001	97004000	1.25E-30
chr2	97001001	97006000	1.33E-22
chr2	97478001	97483000	7.67E-29
chr2	97519001	97524000	7.78E-17
chr2	97520001	97525000	1.25E-21
chr2	97521001	97526000	6.88E-25
chr2	97534001	97539000	2.17E-09
chr2	97535001	97540000	1.17E-09
chr2	97774001	97779000	2.80E-26
chr2	97775001	97780000	4.36E-30
chr2	97776001	97781000	1.46E-52
chr2	97777001	97782000	8.49E-57
chr2	97778001	97783000	1.68E-52
chr2	97779001	97784000	3.29E-72
chr2	97836001	97841000	1.90E-07

chr2	98202001	98207000	3.52E-31
chr2	98204001	98209000	6.36E-36
chr2	98205001	98210000	7.29E-35
chr2	98207001	98212000	1.09E-20
chr2	98260001	98265000	3.93E-16
chr2	98261001	98266000	2.62E-19
chr2	98262001	98267000	6.46E-24
chr2	98280001	98285000	1.15E-09
chr2	98548001	98553000	0.014262767
chr2	98612001	98617000	9.65E-37
chr2	98818001	98823000	0.007809224
chr2	99057001	99062000	2.88E-43
chr2	99058001	99063000	2.41E-42
chr2	99059001	99064000	2.41E-35
chr2	99061001	99066000	2.73E-49
chr2	99224001	99229000	2.11E-56
chr2	99225001	99230000	8.76E-36
chr2	99548001	99553000	1.46E-28
chr2	99549001	99554000	6.53E-21
chr2	99550001	99555000	5.85E-21
chr2	99552001	99557000	2.44E-08
chr2	99793001	99798000	1.34E-14
chr2	99948001	99953000	4.55E-43
chr2	99954001	99959000	2.05E-12
chr2	100534001	100539000	5.34E-09
chr2	100757001	100762000	1.36E-16
chr2	100933001	100938000	2.40E-24
chr2	100934001	100939000	2.85E-56
chr2	100937001	100942000	3.68E-37
chr2	100938001	100943000	6.98E-54
chr2	101431001	101436000	3.88E-34
chr2	101432001	101437000	2.59E-52
chr2	101433001	101438000	8.18E-53
chr2	101434001	101439000	2.67E-50
chr2	101435001	101440000	2.07E-61
chr2	101436001	101441000	5.79E-63
chr2	101437001	101442000	1.28E-47
chr2	101520001	101525000	8.40E-11
chr2	101763001	101768000	3.66E-32
chr2	101764001	101769000	1.16E-23
chr2	101765001	101770000	1.05E-24
chr2	101865001	101870000	8.90E-52
chr2	101866001	101871000	4.98E-41
chr2	101920001	101925000	2.27E-19
chr2	101999001	102004000	9.61E-31
chr2	102000001	102005000	3.04E-22
chr2	102001001	102006000	1.24E-39
chr2	102002001	102007000	1.84E-45

chr2	102003001	102008000	6.05E-50
chr2	102004001	102009000	1.40E-46
chr2	102086001	102091000	2.30E-21
chr2	102947001	102952000	4.89E-05
chr2	102968001	102973000	3.75E-37
chr2	102969001	102974000	7.22E-20
chr2	102970001	102975000	1.58E-22
chr2	102971001	102976000	9.32E-19
chr2	102972001	102977000	2.82E-25
chr2	102992001	102997000	2.48E-09
chr2	103016001	103021000	4.34E-07
chr2	103042001	103047000	0.000670882
chr2	103043001	103048000	4.40E-05
chr2	103044001	103049000	4.57E-06
chr2	103045001	103050000	1.30E-05
chr2	103085001	103090000	0.000114022
chr2	103086001	103091000	5.28E-06
chr2	103087001	103092000	0.000138146
chr2	103124001	103129000	1.05E-05
chr2	103130001	103135000	1.91E-05
chr2	103212001	103217000	4.16E-05
chr2	103213001	103218000	1.31E-05
chr2	103231001	103236000	5.84E-32
chr2	103232001	103237000	1.58E-20
chr2	103233001	103238000	4.48E-25
chr2	103236001	103241000	4.78E-19
chr2	103262001	103267000	6.48E-07
chr2	103353001	103358000	2.82E-16
chr2	103499001	103504000	8.29E-07
chr2	103500001	103505000	3.66E-08
chr2	103503001	103508000	2.69E-08
chr2	103534001	103539000	1.99E-07
chr2	103535001	103540000	4.00E-09
chr2	103567001	103572000	0.002638894
chr2	103670001	103675000	0.008497957
chr2	103676001	103681000	9.34E-06
chr2	103677001	103682000	3.51E-05
chr2	103785001	103790000	2.59E-07
chr2	103786001	103791000	0.000766653
chr2	103822001	103827000	0.000419455
chr2	103850001	103855000	0.002130871
chr2	103925001	103930000	6.12E-09
chr2	103927001	103932000	1.15E-13
chr2	103940001	103945000	0.006550861
chr2	103941001	103946000	0.008043091
chr2	104005001	104010000	1.83E-05
chr2	104106001	104111000	2.57E-06
chr2	104149001	104154000	0.002962568

chr2	104227001	104232000	0.009434384
chr2	104233001	104238000	1.39E-05
chr2	104234001	104239000	1.45E-05
chr2	104235001	104240000	4.73E-09
chr2	104236001	104241000	1.55E-08
chr2	104237001	104242000	7.92E-06
chr2	104312001	104317000	8.81E-08
chr2	104318001	104323000	0.004769064
chr2	104319001	104324000	0.004360043
chr2	104325001	104330000	0.00090718
chr2	104341001	104346000	5.18E-07
chr2	104342001	104347000	3.40E-06
chr2	104350001	104355000	0.000134163
chr2	104415001	104420000	3.04E-10
chr2	104416001	104421000	1.30E-08
chr2	104448001	104453000	4.41E-06
chr2	104529001	104534000	5.20E-05
chr2	104556001	104561000	2.56E-05
chr2	104562001	104567000	0.009928642
chr2	104695001	104700000	7.04E-06
chr2	104801001	104806000	0.002778521
chr2	104802001	104807000	0.000730694
chr2	104803001	104808000	0.004830043
chr2	104867001	104872000	2.63E-10
chr2	104868001	104873000	6.71E-07
chr2	104869001	104874000	8.02E-05
chr2	104870001	104875000	0.000684619
chr2	105192001	105197000	9.16E-13
chr2	105193001	105198000	3.86E-06
chr2	105194001	105199000	0.000595406
chr2	105340001	105345000	4.96E-05
chr2	105455001	105460000	1.63E-20
chr2	105518001	105523000	4.44E-08
chr2	105601001	105606000	0.000360339
chr2	105607001	105612000	0.000283106
chr2	105655001	105660000	1.72E-12
chr2	105760001	105765000	1.22E-13
chr2	105942001	105947000	1.56E-15
chr2	105943001	105948000	1.33E-14
chr2	105944001	105949000	2.89E-11
chr2	105945001	105950000	3.11E-15
chr2	105946001	105951000	9.81E-29
chr2	105954001	105959000	3.94E-14
chr2	106017001	106022000	9.63E-10
chr2	106018001	106023000	2.28E-08
chr2	106318001	106323000	0.00021491
chr2	106319001	106324000	0.00040532
chr2	106320001	106325000	0.000194911

chr2	106337001	106342000	0.000617768
chr2	106361001	106366000	3.83E-32
chr2	106458001	106463000	6.19E-08
chr2	106459001	106464000	9.92E-05
chr2	106514001	106519000	3.63E-07
chr2	106810001	106815000	1.66E-16
chr2	106841001	106846000	9.49E-05
chr2	106968001	106973000	0.000972756
chr2	106969001	106974000	0.000714293
chr2	107069001	107074000	0.000720309
chr2	107090001	107095000	4.88E-08
chr2	107099001	107104000	9.49E-13
chr2	107100001	107105000	9.12E-16
chr2	107101001	107106000	2.79E-12
chr2	107102001	107107000	3.11E-12
chr2	107103001	107108000	6.20E-05
chr2	107241001	107246000	0.000358615
chr2	107242001	107247000	1.33E-07
chr2	107243001	107248000	0.000243943
chr2	107280001	107285000	0.000198788
chr2	107332001	107337000	0.00017694
chr2	107333001	107338000	0.000229157
chr2	107363001	107368000	1.84E-06
chr2	107364001	107369000	4.48E-08
chr2	107365001	107370000	0.00101998
chr2	107399001	107404000	0.010187889
chr2	107441001	107446000	2.43E-08
chr2	107442001	107447000	1.30E-07
chr2	107699001	107704000	0.002389044
chr2	107700001	107705000	0.001866917
chr2	107701001	107706000	0.002732134
chr2	107714001	107719000	0.0003277
chr2	107856001	107861000	3.13E-05
chr2	107857001	107862000	6.86E-05
chr2	107858001	107863000	0.000719109
chr2	107859001	107864000	2.14E-05
chr2	107933001	107938000	0.003895559
chr2	107941001	107946000	2.72E-07
chr2	107942001	107947000	2.24E-07
chr2	107943001	107948000	4.92E-07
chr2	107944001	107949000	2.54E-07
chr2	108286001	108291000	7.28E-05
chr2	108287001	108292000	0.000189725
chr2	108288001	108293000	0.000136463
chr2	108396001	108401000	0.000424399
chr2	108397001	108402000	0.000532746
chr2	108450001	108455000	0.001507045
chr2	108581001	108586000	0.000144026

chr2	108582001	108587000	1.14E-07
chr2	108583001	108588000	4.08E-08
chr2	108584001	108589000	9.94E-09
chr2	108585001	108590000	2.29E-09
chr2	108586001	108591000	1.04E-07
chr2	108594001	108599000	0.000568849
chr2	108595001	108600000	0.001075346
chr2	108603001	108608000	2.53E-22
chr2	108650001	108655000	1.11E-11
chr2	108701001	108706000	0.00709654
chr2	108702001	108707000	0.001335588
chr2	109146001	109151000	1.10E-35
chr2	109147001	109152000	1.05E-35
chr2	109148001	109153000	1.57E-42
chr2	109149001	109154000	7.49E-50
chr2	109150001	109155000	1.66E-45
chr2	109151001	109156000	3.28E-28
chr2	109399001	109404000	1.13E-19
chr2	109654001	109659000	1.77E-07
chr2	109744001	109749000	2.45E-26
chr2	110368001	110373000	3.69E-33
chr2	110370001	110375000	4.11E-29
chr2	110371001	110376000	1.05E-30
chr2	110372001	110377000	5.55E-45
chr2	110373001	110378000	3.39E-18
chr2	110494001	110499000	2.28E-07
chr2	110515001	110520000	7.88E-08
chr2	110516001	110521000	1.55E-16
chr2	110517001	110522000	4.89E-17
chr2	110518001	110523000	3.69E-24
chr2	110934001	110939000	0.003789185
chr2	110935001	110940000	0.002209307
chr2	110959001	110964000	4.83E-26
chr2	111486001	111491000	8.89E-30
chr2	111487001	111492000	7.53E-25
chr2	111880001	111885000	4.83E-53
chr2	111889001	111894000	1.09E-11
chr2	112101001	112106000	0.004054023
chr2	112103001	112108000	0.001021743
chr2	112119001	112124000	3.63E-15
chr2	112651001	112656000	3.90E-14
chr2	112652001	112657000	7.25E-26
chr2	112653001	112658000	5.78E-14
chr2	112935001	112940000	8.91E-28
chr2	112936001	112941000	1.96E-15
chr2	113008001	113013000	7.22E-26
chr2	113009001	113014000	7.22E-34
chr2	113010001	113015000	3.02E-32

chr2	113011001	113016000	5.93E-41
chr2	113012001	113017000	2.23E-50
chr2	113033001	113038000	4.59E-23
chr2	113180001	113185000	0.002588948
chr2	113187001	113192000	3.54E-15
chr2	113208001	113213000	0.00050055
chr2	113221001	113226000	4.14E-05
chr2	113235001	113240000	7.19E-37
chr2	113337001	113342000	4.01E-29
chr2	113338001	113343000	7.49E-32
chr2	113521001	113526000	1.77E-30
chr2	113522001	113527000	1.60E-34
chr2	113612001	113617000	0.000994736
chr2	113613001	113618000	0.000374113
chr2	113617001	113622000	0.000248065
chr2	113758001	113763000	3.46E-05
chr2	113915001	113920000	1.19E-13
chr2	113952001	113957000	1.52E-17
chr2	113953001	113958000	3.27E-10
chr2	113954001	113959000	7.26E-17
chr2	113955001	113960000	1.75E-10
chr2	113956001	113961000	2.63E-18
chr2	114296001	114301000	5.00E-07
chr2	114297001	114302000	5.97E-13
chr2	114298001	114303000	8.53E-10
chr2	114299001	114304000	4.68E-12
chr2	114378001	114383000	2.95E-05
chr2	114380001	114385000	2.23E-12
chr2	114381001	114386000	7.15E-07
chr2	114382001	114387000	4.34E-06
chr2	114383001	114388000	8.32E-09
chr2	114384001	114389000	4.86E-14
chr2	114409001	114414000	2.96E-18
chr2	114514001	114519000	7.65E-26
chr2	114688001	114693000	0.001612866
chr2	114831001	114836000	1.60E-05
chr2	114996001	115001000	0.001231183
chr2	115002001	115007000	4.05E-06
chr2	115003001	115008000	4.11E-06
chr2	115004001	115009000	9.30E-06
chr2	115011001	115016000	0.000354526
chr2	115012001	115017000	3.21E-05
chr2	115013001	115018000	9.65E-05
chr2	115014001	115019000	0.0003012
chr2	115068001	115073000	0.02220117
chr2	115091001	115096000	0.00132337
chr2	115133001	115138000	0.000302259
chr2	115161001	115166000	0.001958538

chr2	115162001	115167000	0.00010601
chr2	115163001	115168000	3.03E-11
chr2	115164001	115169000	5.75E-11
chr2	115165001	115170000	4.88E-11
chr2	115166001	115171000	1.10E-13
chr2	115187001	115192000	0.015728026
chr2	115200001	115205000	0.001515809
chr2	115201001	115206000	2.37E-05
chr2	115202001	115207000	3.30E-06
chr2	115203001	115208000	3.65E-05
chr2	115331001	115336000	0.001119334
chr2	115370001	115375000	7.79E-06
chr2	115371001	115376000	7.41E-05
chr2	115447001	115452000	0.000400542
chr2	115448001	115453000	0.000182438
chr2	115498001	115503000	3.13E-06
chr2	115499001	115504000	1.09E-05
chr2	115519001	115524000	0.000290938
chr2	115521001	115526000	4.87E-06
chr2	115522001	115527000	2.10E-05
chr2	115560001	115565000	0.003124975
chr2	115561001	115566000	0.000394586
chr2	115568001	115573000	5.09E-08
chr2	115569001	115574000	6.52E-08
chr2	115570001	115575000	2.71E-05
chr2	115571001	115576000	0.000111873
chr2	115590001	115595000	0.000137776
chr2	115591001	115596000	1.54E-08
chr2	115592001	115597000	7.96E-14
chr2	115593001	115598000	4.21E-09
chr2	115594001	115599000	1.55E-08
chr2	115595001	115600000	4.98E-05
chr2	115624001	115629000	1.21E-06
chr2	115650001	115655000	1.40E-16
chr2	115651001	115656000	2.40E-18
chr2	115652001	115657000	8.61E-20
chr2	115653001	115658000	5.91E-23
chr2	115654001	115659000	3.35E-13
chr2	115655001	115660000	2.50E-06
chr2	115656001	115661000	0.00031712
chr2	115657001	115662000	0.000444074
chr2	115658001	115663000	0.005855548
chr2	115673001	115678000	9.12E-05
chr2	115687001	115692000	2.30E-06
chr2	115688001	115693000	0.00112409
chr2	115708001	115713000	3.79E-07
chr2	115785001	115790000	0.000206091
chr2	115792001	115797000	3.10E-05

chr2	115798001	115803000	0.000725892
chr2	115837001	115842000	2.92E-05
chr2	115838001	115843000	0.000258368
chr2	115839001	115844000	3.63E-05
chr2	115876001	115881000	0.006370538
chr2	115879001	115884000	0.000176692
chr2	115881001	115886000	0.000219408
chr2	115884001	115889000	0.000697539
chr2	115885001	115890000	0.000121292
chr2	115886001	115891000	0.007505463
chr2	115887001	115892000	5.45E-05
chr2	115888001	115893000	1.87E-05
chr2	115914001	115919000	3.11E-27
chr2	115915001	115920000	8.72E-39
chr2	115968001	115973000	6.00E-06
chr2	115969001	115974000	1.79E-07
chr2	115970001	115975000	1.33E-06
chr2	115971001	115976000	5.72E-07
chr2	115972001	115977000	5.71E-05
chr2	116046001	116051000	6.98E-05
chr2	116055001	116060000	2.05E-05
chr2	116059001	116064000	1.38E-06
chr2	116060001	116065000	0.002996797
chr2	116131001	116136000	8.30E-07
chr2	116132001	116137000	3.97E-07
chr2	116148001	116153000	3.76E-06
chr2	116149001	116154000	8.35E-08
chr2	116155001	116160000	0.001013592
chr2	116156001	116161000	0.001673916
chr2	116157001	116162000	0.008247765
chr2	116169001	116174000	0.000931544
chr2	116181001	116186000	5.78E-08
chr2	116185001	116190000	3.62E-05
chr2	116289001	116294000	7.32E-05
chr2	116290001	116295000	5.23E-05
chr2	116291001	116296000	0.00046543
chr2	116300001	116305000	0.000834776
chr2	116301001	116306000	0.000580261
chr2	116302001	116307000	0.000263463
chr2	116333001	116338000	9.78E-05
chr2	116334001	116339000	0.00061873
chr2	116387001	116392000	0.001935481
chr2	116469001	116474000	7.36E-08
chr2	116470001	116475000	3.64E-07
chr2	116471001	116476000	5.66E-08
chr2	116503001	116508000	9.34E-08
chr2	116504001	116509000	4.12E-07
chr2	116598001	116603000	0.000119304

chr2	116599001	116604000	4.15E-05
chr2	116600001	116605000	1.51E-05
chr2	116645001	116650000	4.23E-05
chr2	116646001	116651000	1.59E-06
chr2	116694001	116699000	0.003170824
chr2	116702001	116707000	0.000168131
chr2	116703001	116708000	5.15E-07
chr2	116704001	116709000	5.42E-05
chr2	116705001	116710000	7.74E-05
chr2	116706001	116711000	0.002896953
chr2	116715001	116720000	4.50E-05
chr2	116716001	116721000	1.88E-06
chr2	116735001	116740000	5.72E-05
chr2	116736001	116741000	2.75E-07
chr2	116737001	116742000	7.55E-07
chr2	116769001	116774000	0.000203448
chr2	116783001	116788000	2.80E-05
chr2	116801001	116806000	1.08E-05
chr2	116802001	116807000	2.03E-07
chr2	116850001	116855000	7.27E-05
chr2	116874001	116879000	5.61E-09
chr2	116875001	116880000	1.75E-10
chr2	116970001	116975000	4.61E-05
chr2	116996001	117001000	0.00049634
chr2	117092001	117097000	9.39E-14
chr2	117093001	117098000	4.12E-10
chr2	117113001	117118000	0.000198203
chr2	117209001	117214000	1.73E-05
chr2	117210001	117215000	5.20E-09
chr2	117211001	117216000	7.54E-08
chr2	117212001	117217000	4.63E-08
chr2	117216001	117221000	0.000285852
chr2	117217001	117222000	0.000690792
chr2	117255001	117260000	4.81E-06
chr2	117372001	117377000	0.000245814
chr2	117472001	117477000	1.16E-05
chr2	117473001	117478000	5.09E-06
chr2	117474001	117479000	3.18E-06
chr2	117475001	117480000	4.51E-05
chr2	117611001	117616000	9.95E-07
chr2	117612001	117617000	4.58E-08
chr2	117613001	117618000	1.83E-06
chr2	117614001	117619000	4.84E-06
chr2	117698001	117703000	0.00122152
chr2	117699001	117704000	0.00202943
chr2	117700001	117705000	0.000225232
chr2	117701001	117706000	0.003706876
chr2	117708001	117713000	0.000600126

chr2	117720001	117725000	0.000176081
chr2	117740001	117745000	1.90E-07
chr2	117763001	117768000	1.53E-06
chr2	117764001	117769000	0.001542536
chr2	117823001	117828000	0.000409886
chr2	117841001	117846000	0.000124287
chr2	117905001	117910000	2.58E-05
chr2	117963001	117968000	8.82E-06
chr2	117964001	117969000	1.98E-06
chr2	117965001	117970000	4.73E-07
chr2	117966001	117971000	6.39E-06
chr2	117967001	117972000	0.001353466
chr2	117979001	117984000	0.000390946
chr2	118114001	118119000	6.75E-07
chr2	118144001	118149000	0.000594967
chr2	118173001	118178000	0.003017323
chr2	118175001	118180000	0.013016265
chr2	118232001	118237000	5.46E-06
chr2	118238001	118243000	0.000356096
chr2	118279001	118284000	6.21E-10
chr2	118280001	118285000	5.06E-10
chr2	118281001	118286000	2.60E-10
chr2	118282001	118287000	9.11E-15
chr2	118283001	118288000	3.94E-12
chr2	118284001	118289000	1.59E-08
chr2	118285001	118290000	4.39E-07
chr2	118287001	118292000	0.000271587
chr2	118288001	118293000	0.000359197
chr2	118339001	118344000	2.19E-05
chr2	118340001	118345000	8.17E-06
chr2	118341001	118346000	0.002451458
chr2	118363001	118368000	6.49E-09
chr2	118376001	118381000	1.76E-13
chr2	118389001	118394000	0.000998651
chr2	118390001	118395000	0.000245094
chr2	118469001	118474000	2.52E-08
chr2	118470001	118475000	1.65E-08
chr2	118471001	118476000	3.33E-08
chr2	118557001	118562000	1.90E-13
chr2	118559001	118564000	3.42E-10
chr2	118560001	118565000	1.00E-11
chr2	118651001	118656000	7.84E-06
chr2	118652001	118657000	2.01E-10
chr2	118653001	118658000	1.32E-08
chr2	118654001	118659000	1.58E-08
chr2	118766001	118771000	6.23E-10
chr2	118900001	118905000	8.55E-20
chr2	118939001	118944000	5.72E-18

chr2	118982001	118987000	3.77E-12
chr2	119156001	119161000	0.000148158
chr2	119229001	119234000	3.48E-06
chr2	119401001	119406000	1.63E-07
chr2	119402001	119407000	2.12E-07
chr2	119403001	119408000	4.67E-05
chr2	119451001	119456000	4.23E-10
chr2	119452001	119457000	2.72E-14
chr2	119453001	119458000	8.63E-19
chr2	119454001	119459000	7.35E-12
chr2	119654001	119659000	1.87E-06
chr2	119795001	119800000	1.99E-05
chr2	119977001	119982000	1.25E-12
chr2	119978001	119983000	2.01E-14
chr2	120365001	120370000	0.000248768
chr2	120366001	120371000	6.21E-10
chr2	120367001	120372000	1.54E-11
chr2	120513001	120518000	8.31E-36
chr2	120514001	120519000	5.40E-29
chr2	120517001	120522000	5.61E-35
chr2	120622001	120627000	0.006470924
chr2	120766001	120771000	7.57E-24
chr2	120767001	120772000	9.78E-44
chr2	120768001	120773000	3.16E-42
chr2	120769001	120774000	1.40E-26
chr2	120770001	120775000	1.98E-52
chr2	120771001	120776000	1.58E-21
chr2	120799001	120804000	0.000317611
chr2	120800001	120805000	0.000167897
chr2	120977001	120982000	1.28E-20
chr2	121011001	121016000	3.11E-36
chr2	121492001	121497000	1.04E-21
chr2	121934001	121939000	5.81E-07
chr2	122038001	122043000	2.93E-21
chr2	122065001	122070000	1.19E-05
chr2	122511001	122516000	7.90E-29
chr2	122512001	122517000	2.16E-33
chr2	122513001	122518000	5.42E-38
chr2	122615001	122620000	7.11E-08
chr2	122616001	122621000	2.66E-07
chr2	122685001	122690000	2.56E-09
chr2	122686001	122691000	1.79E-13
chr2	122687001	122692000	1.40E-11
chr2	122752001	122757000	1.45E-08
chr2	122753001	122758000	1.42E-11
chr2	122871001	122876000	2.18E-05
chr2	122872001	122877000	2.13E-05
chr2	122873001	122878000	0.000151709

chr2	122876001	122881000	2.22E-06
chr2	122899001	122904000	8.43E-09
chr2	123144001	123149000	7.91E-08
chr2	123145001	123150000	7.53E-06
chr2	123164001	123169000	0.000649275
chr2	123165001	123170000	0.00023419
chr2	123218001	123223000	0.001008624
chr2	123230001	123235000	0.003682831
chr2	123231001	123236000	2.36E-05
chr2	123232001	123237000	6.50E-05
chr2	123338001	123343000	1.05E-06
chr2	123340001	123345000	4.20E-07
chr2	123355001	123360000	0.000301168
chr2	123359001	123364000	8.39E-06
chr2	123362001	123367000	0.000114377
chr2	123382001	123387000	0.001123474
chr2	123435001	123440000	0.003852261
chr2	123748001	123753000	4.07E-07
chr2	123815001	123820000	2.23E-05
chr2	123852001	123857000	4.19E-06
chr2	123853001	123858000	1.52E-10
chr2	124004001	124009000	2.58E-06
chr2	124005001	124010000	1.00E-05
chr2	124006001	124011000	6.88E-05
chr2	124007001	124012000	9.39E-07
chr2	124067001	124072000	0.000902744
chr2	124068001	124073000	0.000175119
chr2	124206001	124211000	5.52E-05
chr2	124214001	124219000	3.78E-06
chr2	124268001	124273000	0.00059156
chr2	124289001	124294000	0.000133925
chr2	124294001	124299000	2.47E-06
chr2	124480001	124485000	1.25E-07
chr2	124551001	124556000	0.002384131
chr2	124587001	124592000	0.000283593
chr2	124668001	124673000	1.87E-09
chr2	124680001	124685000	0.00010961
chr2	124681001	124686000	0.001700798
chr2	124735001	124740000	5.15E-05
chr2	124769001	124774000	0.000276896
chr2	124804001	124809000	9.56E-05
chr2	124811001	124816000	3.47E-07
chr2	124832001	124837000	0.020114159
chr2	124833001	124838000	0.014680384
chr2	124840001	124845000	0.000176638
chr2	124934001	124939000	3.23E-07
chr2	124935001	124940000	4.57E-06
chr2	125120001	125125000	3.81E-06

chr2	125121001	125126000	6.25E-08
chr2	125122001	125127000	3.47E-09
chr2	125123001	125128000	2.87E-07
chr2	125243001	125248000	3.22E-07
chr2	125260001	125265000	2.58E-05
chr2	125263001	125268000	0.001207902
chr2	125264001	125269000	0.000366342
chr2	125339001	125344000	1.69E-05
chr2	125367001	125372000	4.29E-10
chr2	125408001	125413000	5.02E-06
chr2	125409001	125414000	2.04E-06
chr2	125411001	125416000	0.001351263
chr2	125412001	125417000	0.001116636
chr2	125426001	125431000	3.80E-05
chr2	125427001	125432000	6.41E-06
chr2	125512001	125517000	0.000186599
chr2	125644001	125649000	0.000533612
chr2	125768001	125773000	3.42E-06
chr2	125769001	125774000	1.19E-05
chr2	125770001	125775000	1.01E-05
chr2	125772001	125777000	0.000122976
chr2	125782001	125787000	6.04E-07
chr2	125783001	125788000	1.74E-05
chr2	125798001	125803000	0.001094085
chr2	125799001	125804000	7.85E-08
chr2	125838001	125843000	0.00010972
chr2	126087001	126092000	0.008966429
chr2	126304001	126309000	7.89E-05
chr2	126305001	126310000	4.64E-05
chr2	126374001	126379000	2.44E-06
chr2	126375001	126380000	1.75E-06
chr2	126577001	126582000	1.19E-06
chr2	126578001	126583000	2.24E-06
chr2	126588001	126593000	2.17E-05
chr2	126590001	126595000	3.62E-06
chr2	126591001	126596000	5.88E-07
chr2	126592001	126597000	2.80E-05
chr2	126594001	126599000	1.51E-06
chr2	126751001	126756000	0.000362868
chr2	126795001	126800000	7.35E-05
chr2	126824001	126829000	0.000432518
chr2	126825001	126830000	0.001383308
chr2	126826001	126831000	0.000283612
chr2	126827001	126832000	0.001293506
chr2	126834001	126839000	3.28E-05
chr2	126840001	126845000	9.26E-09
chr2	126841001	126846000	4.35E-13
chr2	126842001	126847000	1.83E-11

chr2	126843001	126848000	2.24E-14
chr2	126844001	126849000	1.26E-10
chr2	126845001	126850000	1.16E-07
chr2	126903001	126908000	4.50E-05
chr2	126915001	126920000	3.47E-08
chr2	126916001	126921000	3.15E-08
chr2	126953001	126958000	3.92E-12
chr2	126954001	126959000	1.05E-06
chr2	127032001	127037000	0.001063736
chr2	127097001	127102000	0.001037268
chr2	127098001	127103000	0.000289471
chr2	127099001	127104000	0.000449851
chr2	127129001	127134000	7.11E-11
chr2	127278001	127283000	1.43E-05
chr2	127294001	127299000	0.001086576
chr2	127295001	127300000	0.000104167
chr2	127296001	127301000	6.07E-05
chr2	127311001	127316000	6.21E-13
chr2	127312001	127317000	3.04E-13
chr2	127313001	127318000	1.86E-08
chr2	127314001	127319000	3.70E-06
chr2	127315001	127320000	3.50E-08
chr2	127355001	127360000	0.000784155
chr2	127596001	127601000	1.08E-12
chr2	127597001	127602000	3.34E-09
chr2	127680001	127685000	0.000161974
chr2	127682001	127687000	0.000364363
chr2	127683001	127688000	3.45E-06
chr2	127684001	127689000	1.11E-08
chr2	127685001	127690000	3.68E-11
chr2	127708001	127713000	7.12E-05
chr2	127709001	127714000	1.90E-06
chr2	127860001	127865000	2.67E-08
chr2	127972001	127977000	1.17E-18
chr2	127973001	127978000	1.04E-31
chr2	128047001	128052000	1.87E-26
chr2	128279001	128284000	8.22E-52
chr2	128304001	128309000	1.07E-05
chr2	128457001	128462000	5.64E-31
chr2	128459001	128464000	2.07E-43
chr2	128639001	128644000	2.33E-12
chr2	128640001	128645000	3.49E-15
chr2	128641001	128646000	1.28E-22
chr2	128644001	128649000	7.74E-22
chr2	128785001	128790000	1.82E-27
chr2	128786001	128791000	1.88E-20
chr2	128986001	128991000	2.71E-14
chr2	128987001	128992000	2.24E-26

chr2	128988001	128993000	5.17E-15
chr2	128989001	128994000	8.10E-11
chr2	128990001	128995000	3.39E-13
chr2	128991001	128996000	1.10E-10
chr2	129108001	129113000	2.63E-07
chr2	129109001	129114000	2.19E-06
chr2	129110001	129115000	6.99E-05
chr2	129236001	129241000	0.001001244
chr2	129612001	129617000	6.54E-06
chr2	129648001	129653000	4.03E-06
chr2	129649001	129654000	3.18E-07
chr2	129650001	129655000	2.55E-10
chr2	129651001	129656000	1.48E-06
chr2	129921001	129926000	0.000113507
chr2	129983001	129988000	0.001247256
chr2	129992001	129997000	6.91E-06
chr2	130231001	130236000	8.94E-06
chr2	130352001	130357000	1.97E-11
chr2	130354001	130359000	5.69E-09
chr2	130363001	130368000	3.73E-08
chr2	130364001	130369000	1.65E-08
chr2	130379001	130384000	1.45E-09
chr2	130672001	130677000	2.27E-07
chr2	131109001	131114000	2.23E-08
chr2	131112001	131117000	1.25E-18
chr2	131113001	131118000	2.84E-23
chr2	131125001	131130000	5.58E-15
chr2	131128001	131133000	5.98E-07
chr2	131129001	131134000	8.71E-22
chr2	131130001	131135000	1.43E-15
chr2	131181001	131186000	1.07E-18
chr2	131207001	131212000	1.90E-07
chr2	131513001	131518000	2.97E-12
chr2	131552001	131557000	1.59E-14
chr2	131594001	131599000	1.22E-09
chr2	131717001	131722000	3.00E-14
chr2	131718001	131723000	2.95E-13
chr2	131719001	131724000	2.37E-14
chr2	132181001	132186000	5.58E-10
chr2	132183001	132188000	9.48E-08
chr2	132194001	132199000	3.58E-05
chr2	132215001	132220000	1.54E-05
chr2	132216001	132221000	0.000389944
chr2	132217001	132222000	4.03E-06
chr2	132218001	132223000	0.000189653
chr2	132245001	132250000	3.53E-14
chr2	132430001	132435000	1.37E-25
chr2	132431001	132436000	6.78E-33

chr2	132439001	132444000	4.05E-34
chr2	132440001	132445000	2.01E-33
chr2	132661001	132666000	0.000534107
chr2	132667001	132672000	8.98E-06
chr2	132668001	132673000	4.04E-06
chr2	132669001	132674000	2.75E-06
chr2	132845001	132850000	0.018737103
chr2	132884001	132889000	4.64E-05
chr2	132885001	132890000	0.000876823
chr2	132899001	132904000	7.40E-07
chr2	133169001	133174000	5.25E-14
chr2	133170001	133175000	2.81E-13
chr2	133239001	133244000	4.50E-06
chr2	133389001	133394000	1.82E-07
chr2	133390001	133395000	1.67E-06
chr2	133422001	133427000	1.11E-11
chr2	133423001	133428000	7.47E-23
chr2	133428001	133433000	1.96E-34
chr2	133480001	133485000	2.86E-07
chr2	133481001	133486000	4.02E-10
chr2	133482001	133487000	1.48E-07
chr2	133483001	133488000	1.76E-07
chr2	133689001	133694000	8.42E-05
chr2	133794001	133799000	0.004208676
chr2	133812001	133817000	5.75E-07
chr2	133813001	133818000	8.33E-07
chr2	133814001	133819000	2.54E-06
chr2	133817001	133822000	0.000462026
chr2	133818001	133823000	0.000657689
chr2	133924001	133929000	3.65E-05
chr2	133938001	133943000	0.000488827
chr2	133963001	133968000	3.87E-06
chr2	133973001	133978000	0.00193653
chr2	133986001	133991000	0.002489832
chr2	134022001	134027000	7.33E-17
chr2	134023001	134028000	6.61E-34
chr2	134024001	134029000	1.18E-32
chr2	134067001	134072000	1.15E-05
chr2	134132001	134137000	0.0196344
chr2	134157001	134162000	9.29E-05
chr2	134158001	134163000	4.45E-05
chr2	134159001	134164000	3.55E-05
chr2	134204001	134209000	3.58E-07
chr2	134205001	134210000	2.41E-05
chr2	134222001	134227000	1.55E-07
chr2	134273001	134278000	7.55E-09
chr2	134274001	134279000	5.73E-09
chr2	134400001	134405000	3.72E-05

chr2	134401001	134406000	6.60E-17
chr2	134406001	134411000	0.000157159
chr2	134407001	134412000	2.02E-06
chr2	134408001	134413000	5.42E-07
chr2	134409001	134414000	3.11E-07
chr2	134422001	134427000	0.000605538
chr2	134423001	134428000	0.000413402
chr2	134424001	134429000	0.000302611
chr2	134425001	134430000	0.000582455
chr2	134494001	134499000	0.000603622
chr2	134542001	134547000	0.000827916
chr2	134659001	134664000	2.63E-05
chr2	134660001	134665000	0.001229416
chr2	134661001	134666000	0.000476314
chr2	134662001	134667000	6.93E-05
chr2	134707001	134712000	0.00016042
chr2	134708001	134713000	1.06E-07
chr2	134781001	134786000	3.80E-23
chr2	134782001	134787000	2.17E-24
chr2	134783001	134788000	1.61E-22
chr2	134784001	134789000	3.36E-21
chr2	134794001	134799000	1.38E-07
chr2	134795001	134800000	3.81E-08
chr2	134873001	134878000	1.11E-11
chr2	135090001	135095000	2.90E-05
chr2	135455001	135460000	5.00E-07
chr2	135475001	135480000	5.12E-35
chr2	135476001	135481000	1.04E-40
chr2	135486001	135491000	4.87E-07
chr2	135487001	135492000	7.74E-09
chr2	135501001	135506000	7.17E-08
chr2	135502001	135507000	1.98E-11
chr2	135503001	135508000	5.09E-14
chr2	135504001	135509000	2.07E-16
chr2	135505001	135510000	2.04E-10
chr2	135510001	135515000	9.74E-06
chr2	135672001	135677000	8.30E-51
chr2	135785001	135790000	8.39E-06
chr2	136285001	136290000	6.97E-21
chr2	136286001	136291000	1.15E-16
chr2	136287001	136292000	5.06E-19
chr2	136288001	136293000	2.23E-20
chr2	136289001	136294000	5.83E-21
chr2	136495001	136500000	3.14E-39
chr2	136629001	136634000	1.71E-28
chr2	136742001	136747000	1.58E-20
chr2	136743001	136748000	7.74E-29
chr2	137069001	137074000	1.58E-08

chr2	137070001	137075000	6.66E-06
chr2	137381001	137386000	2.18E-05
chr2	137382001	137387000	0.00104715
chr2	137420001	137425000	4.26E-06
chr2	137421001	137426000	5.80E-05
chr2	137433001	137438000	0.011928577
chr2	137542001	137547000	0.000312406
chr2	137799001	137804000	2.36E-05
chr2	137877001	137882000	0.004024675
chr2	137878001	137883000	0.007784953
chr2	137880001	137885000	0.003196607
chr2	138186001	138191000	0.002638228
chr2	138197001	138202000	0.00018609
chr2	138308001	138313000	5.97E-07
chr2	138323001	138328000	6.03E-10
chr2	138324001	138329000	1.90E-12
chr2	138325001	138330000	2.09E-13
chr2	138326001	138331000	1.29E-13
chr2	138376001	138381000	5.23E-10
chr2	138378001	138383000	2.45E-12
chr2	138405001	138410000	0.003016124
chr2	138406001	138411000	0.001183733
chr2	138407001	138412000	5.90E-07
chr2	138498001	138503000	2.29E-05
chr2	138499001	138504000	9.42E-06
chr2	138500001	138505000	0.000669157
chr2	138536001	138541000	0.02915925
chr2	138545001	138550000	0.000611467
chr2	138829001	138834000	6.07E-05
chr2	139096001	139101000	0.029396232
chr2	139532001	139537000	1.14E-06
chr2	139810001	139815000	0.003822558
chr2	139811001	139816000	0.001411322
chr2	139812001	139817000	0.000675827
chr2	139985001	139990000	0.000449485
chr2	140280001	140285000	1.28E-06
chr2	140281001	140286000	2.29E-05
chr2	140730001	140735000	7.85E-12
chr2	140947001	140952000	0.000567114
chr2	141536001	141541000	2.36E-06
chr2	141537001	141542000	2.46E-06
chr2	141613001	141618000	0.002559167
chr2	141781001	141786000	0.034732835
chr2	142127001	142132000	0.011043041
chr2	142128001	142133000	0.006308801
chr2	142143001	142148000	0.001826242
chr2	142144001	142149000	0.001501248
chr2	142237001	142242000	0.003538811

chr2	142238001	142243000	1.83E-06
chr2	142239001	142244000	4.43E-08
chr2	142240001	142245000	1.56E-05
chr2	142241001	142246000	9.38E-07
chr2	142242001	142247000	0.002787335
chr2	142291001	142296000	0.016980391
chr2	142489001	142494000	0.001229223
chr2	142781001	142786000	0.009600279
chr2	142782001	142787000	0.007735527
chr2	143351001	143356000	4.82E-10
chr2	143352001	143357000	1.26E-08
chr2	143353001	143358000	2.15E-09
chr2	143354001	143359000	1.19E-08
chr2	143355001	143360000	3.06E-07
chr2	143524001	143529000	0.011065761
chr2	143780001	143785000	0.00026688
chr2	143797001	143802000	5.90E-08
chr2	143798001	143803000	8.78E-11
chr2	143799001	143804000	5.98E-10
chr2	143800001	143805000	4.45E-08
chr2	143801001	143806000	8.20E-07
chr2	143923001	143928000	0.001085391
chr2	143924001	143929000	2.06E-06
chr2	143963001	143968000	0.001221987
chr2	144168001	144173000	1.61E-05
chr2	144174001	144179000	0.001353771
chr2	144220001	144225000	0.000198076
chr2	144279001	144284000	7.86E-06
chr2	144280001	144285000	2.34E-07
chr2	144281001	144286000	5.91E-06
chr2	144282001	144287000	5.70E-08
chr2	144283001	144288000	1.62E-08
chr2	144334001	144339000	1.24E-05
chr2	144422001	144427000	0.001505537
chr2	144641001	144646000	1.67E-05
chr2	144690001	144695000	2.05E-56
chr2	144691001	144696000	4.04E-42
chr2	144692001	144697000	3.00E-47
chr2	144693001	144698000	1.77E-35
chr2	144694001	144699000	1.24E-23
chr2	144743001	144748000	0.000805419
chr2	144744001	144749000	0.000854537
chr2	144890001	144895000	4.51E-05
chr2	144893001	144898000	6.61E-06
chr2	144937001	144942000	1.58E-05
chr2	144939001	144944000	1.40E-05
chr2	145014001	145019000	0.005066554
chr2	145087001	145092000	7.34E-61

chr2	145088001	145093000	3.06E-62
chr2	145090001	145095000	2.07E-13
chr2	145223001	145228000	0.000262116
chr2	145224001	145229000	0.000966793
chr2	145225001	145230000	0.000488732
chr2	145280001	145285000	3.66E-23
chr2	145281001	145286000	1.85E-21
chr2	145282001	145287000	3.42E-07
chr2	145418001	145423000	0.003264787
chr2	145479001	145484000	0.000419583
chr2	145480001	145485000	0.001695888
chr2	145701001	145706000	4.54E-06
chr2	145702001	145707000	3.54E-08
chr2	145703001	145708000	4.71E-13
chr2	146259001	146264000	0.000119298
chr2	146260001	146265000	2.33E-05
chr2	146261001	146266000	4.74E-05
chr2	146262001	146267000	1.44E-06
chr2	146668001	146673000	0.001343991
chr2	146747001	146752000	0.001847585
chr2	146847001	146852000	0.000735716
chr2	146894001	146899000	0.000788109
chr2	147001001	147006000	0.0009498
chr2	147002001	147007000	8.41E-05
chr2	147003001	147008000	5.96E-05
chr2	147004001	147009000	0.000516165
chr2	147388001	147393000	1.45E-10
chr2	147389001	147394000	1.38E-07
chr2	147390001	147395000	7.15E-08
chr2	147391001	147396000	7.73E-06
chr2	147542001	147547000	2.43E-06
chr2	147543001	147548000	1.53E-07
chr2	147671001	147676000	0.00018609
chr2	147672001	147677000	0.000221763
chr2	147673001	147678000	0.007972654
chr2	147703001	147708000	0.000290041
chr2	147978001	147983000	0.002608461
chr2	148039001	148044000	4.50E-05
chr2	148118001	148123000	0.000847067
chr2	148215001	148220000	0.000206892
chr2	148516001	148521000	1.65E-05
chr2	148517001	148522000	1.37E-06
chr2	148518001	148523000	1.46E-07
chr2	148529001	148534000	0.002495469
chr2	148685001	148690000	4.51E-07
chr2	148778001	148783000	6.72E-50
chr2	149266001	149271000	3.29E-21
chr2	149267001	149272000	4.78E-27

chr2	149397001	149402000	1.68E-20
chr2	149398001	149403000	5.35E-35
chr2	149628001	149633000	3.47E-42
chr2	149629001	149634000	1.75E-41
chr2	149630001	149635000	1.32E-34
chr2	149798001	149803000	2.86E-07
chr2	149891001	149896000	8.91E-27
chr2	149892001	149897000	2.89E-28
chr2	149924001	149929000	8.85E-05
chr2	150184001	150189000	6.78E-48
chr2	150185001	150190000	2.15E-54
chr2	150186001	150191000	1.74E-50
chr2	150187001	150192000	2.15E-49
chr2	150192001	150197000	0.000225026
chr2	150193001	150198000	5.97E-05
chr2	150194001	150199000	0.001181823
chr2	150439001	150444000	8.13E-27
chr2	150444001	150449000	1.55E-27
chr2	150769001	150774000	0.003775758
chr2	150770001	150775000	0.003003781
chr2	150978001	150983000	8.28E-06
chr2	151337001	151342000	3.16E-14
chr2	151501001	151506000	4.60E-05
chr2	151502001	151507000	1.57E-05
chr2	151520001	151525000	0.002711039
chr2	151680001	151685000	0.000388117
chr2	151681001	151686000	0.000839832
chr2	151682001	151687000	0.000839832
chr2	151773001	151778000	2.02E-23
chr2	151774001	151779000	4.51E-23
chr2	151919001	151924000	0.005703531
chr2	151967001	151972000	4.98E-07
chr2	151968001	151973000	8.93E-06
chr2	151969001	151974000	2.88E-06
chr2	152039001	152044000	6.24E-11
chr2	152040001	152045000	1.27E-06
chr2	152118001	152123000	1.51E-26
chr2	152141001	152146000	1.21E-23
chr2	152262001	152267000	4.34E-08
chr2	152266001	152271000	2.75E-38
chr2	152680001	152685000	2.29E-51
chr2	152681001	152686000	6.72E-41
chr2	153032001	153037000	1.46E-40
chr2	153192001	153197000	5.40E-17
chr2	153265001	153270000	0.006237572
chr2	153267001	153272000	2.39E-05
chr2	153322001	153327000	4.20E-05
chr2	153614001	153619000	0.002971571

chr2	153820001	153825000	0.000240468
chr2	153821001	153826000	0.001028573
chr2	153825001	153830000	0.000971408
chr2	153856001	153861000	0.005979099
chr2	153978001	153983000	0.004318142
chr2	154027001	154032000	0.002088795
chr2	154028001	154033000	0.001488938
chr2	154044001	154049000	0.000383471
chr2	154045001	154050000	0.000239851
chr2	154046001	154051000	0.000152529
chr2	154115001	154120000	5.74E-05
chr2	154116001	154121000	0.000202721
chr2	154154001	154159000	0.024682538
chr2	154288001	154293000	9.78E-06
chr2	154289001	154294000	1.55E-09
chr2	154335001	154340000	1.21E-43
chr2	154384001	154389000	0.000696714
chr2	154385001	154390000	7.51E-06
chr2	154392001	154397000	0.000995349
chr2	154393001	154398000	0.002154889
chr2	154427001	154432000	0.001092863
chr2	154518001	154523000	0.000583042
chr2	154519001	154524000	1.32E-05
chr2	154520001	154525000	5.66E-06
chr2	154523001	154528000	1.95E-06
chr2	154567001	154572000	0.00124473
chr2	154718001	154723000	0.000887346
chr2	154719001	154724000	7.09E-05
chr2	154723001	154728000	3.50E-11
chr2	154724001	154729000	8.35E-20
chr2	154974001	154979000	0.015836
chr2	154975001	154980000	0.015836
chr2	155473001	155478000	9.88E-21
chr2	155474001	155479000	4.93E-20
chr2	155475001	155480000	8.10E-17
chr2	155476001	155481000	2.80E-15
chr2	155770001	155775000	1.43E-05
chr2	156155001	156160000	0.01802726
chr2	156436001	156441000	0.000108566
chr2	156475001	156480000	0.00269119
chr2	156476001	156481000	0.004656439
chr2	156818001	156823000	5.60E-13
chr2	157091001	157096000	0.008986863
chr2	157192001	157197000	4.29E-06
chr2	157194001	157199000	1.41E-23
chr2	157197001	157202000	2.62E-32
chr2	157198001	157203000	1.92E-55
chr2	157199001	157204000	8.64E-34

chr2	157288001	157293000	2.05E-25
chr2	157292001	157297000	2.71E-37
chr2	157293001	157298000	1.06E-18
chr2	157342001	157347000	4.41E-09
chr2	157343001	157348000	3.50E-11
chr2	157613001	157618000	0.000588076
chr2	157968001	157973000	0.001553404
chr2	157994001	157999000	0.000487084
chr2	158362001	158367000	0.00536327
chr2	158363001	158368000	0.00536327
chr2	158364001	158369000	0.008974851
chr2	158365001	158370000	7.35E-06
chr2	158406001	158411000	0.001267175
chr2	158480001	158485000	8.71E-15
chr2	158481001	158486000	1.92E-30
chr2	158482001	158487000	1.57E-46
chr2	158483001	158488000	1.45E-44
chr2	158484001	158489000	3.27E-44
chr2	158485001	158490000	2.34E-44
chr2	158732001	158737000	5.57E-21
chr2	158802001	158807000	0.000666566
chr2	159068001	159073000	0.001707423
chr2	159182001	159187000	2.51E-07
chr2	159309001	159314000	2.58E-19
chr2	159310001	159315000	1.44E-24
chr2	159779001	159784000	9.58E-05
chr2	159780001	159785000	8.62E-05
chr2	159821001	159826000	6.29E-39
chr2	160138001	160143000	7.41E-32
chr2	160139001	160144000	8.43E-49
chr2	160140001	160145000	1.81E-51
chr2	160141001	160146000	4.28E-58
chr2	160142001	160147000	7.56E-30
chr2	160143001	160148000	4.83E-29
chr2	160366001	160371000	0.00074777
chr2	160367001	160372000	0.002544098
chr2	160368001	160373000	0.018487001
chr2	160564001	160569000	6.80E-29
chr2	160636001	160641000	1.84E-06
chr2	160637001	160642000	1.94E-06
chr2	160654001	160659000	1.45E-48
chr2	160693001	160698000	0.000263916
chr2	160919001	160924000	7.77E-11
chr2	160978001	160983000	8.03E-05
chr2	160994001	160999000	7.52E-08
chr2	161161001	161166000	5.58E-09
chr2	161211001	161216000	0.001831405
chr2	161264001	161269000	1.81E-50

chr2	161345001	161350000	1.20E-81
chr2	161346001	161351000	2.55E-62
chr2	161348001	161353000	1.02E-38
chr2	161349001	161354000	6.50E-54
chr2	161350001	161355000	3.98E-18
chr2	161564001	161569000	0.000454141
chr2	161793001	161798000	0.016102755
chr2	161908001	161913000	0.001060896
chr2	162017001	162022000	5.49E-33
chr2	162090001	162095000	9.94E-29
chr2	162160001	162165000	3.44E-38
chr2	162266001	162271000	3.42E-10
chr2	162267001	162272000	1.52E-29
chr2	162351001	162356000	0.002059092
chr2	162352001	162357000	0.001625532
chr2	162394001	162399000	1.53E-06
chr2	162395001	162400000	1.79E-07
chr2	162396001	162401000	9.16E-05
chr2	162476001	162481000	2.45E-06
chr2	162477001	162482000	3.64E-11
chr2	162571001	162576000	0.000886145
chr2	162599001	162604000	8.06E-07
chr2	162603001	162608000	1.36E-09
chr2	162741001	162746000	3.38E-06
chr2	162917001	162922000	3.69E-05
chr2	162918001	162923000	8.64E-06
chr2	162919001	162924000	0.00450096
chr2	163074001	163079000	6.60E-07
chr2	163075001	163080000	2.85E-07
chr2	163076001	163081000	2.00E-07
chr2	163077001	163082000	7.80E-11
chr2	163141001	163146000	3.07E-07
chr2	163142001	163147000	9.46E-05
chr2	163170001	163175000	1.36E-17
chr2	163171001	163176000	6.05E-38
chr2	163175001	163180000	2.36E-32
chr2	163196001	163201000	1.47E-36
chr2	163200001	163205000	7.20E-35
chr2	163201001	163206000	1.27E-12
chr2	163716001	163721000	4.87E-05
chr2	163775001	163780000	0.022779107
chr2	164281001	164286000	0.000192784
chr2	164285001	164290000	0.001022281
chr2	164286001	164291000	0.00034984
chr2	164287001	164292000	7.56E-05
chr2	164593001	164598000	1.36E-25
chr2	165033001	165038000	0.001268891
chr2	165141001	165146000	0.00078929

chr2	165431001	165436000	7.57E-07
chr2	165432001	165437000	1.87E-06
chr2	165435001	165440000	3.75E-06
chr2	165473001	165478000	4.17E-12
chr2	165475001	165480000	1.29E-19
chr2	165478001	165483000	9.19E-14
chr2	165693001	165698000	6.13E-47
chr2	165694001	165699000	2.35E-50
chr2	165698001	165703000	2.88E-07
chr2	165807001	165812000	4.12E-14
chr2	165808001	165813000	1.38E-16
chr2	165809001	165814000	6.65E-26
chr2	165810001	165815000	3.57E-26
chr2	165811001	165816000	6.78E-21
chr2	166104001	166109000	0.00164158
chr2	166322001	166327000	4.85E-05
chr2	166323001	166328000	6.58E-07
chr2	166324001	166329000	2.70E-05
chr2	166325001	166330000	1.52E-05
chr2	166326001	166331000	4.03E-06
chr2	166519001	166524000	2.02E-06
chr2	166520001	166525000	2.57E-07
chr2	166521001	166526000	4.25E-08
chr2	166522001	166527000	3.64E-09
chr2	166523001	166528000	1.96E-08
chr2	166626001	166631000	0.00652286
chr2	166634001	166639000	0.018845459
chr2	166648001	166653000	2.71E-38
chr2	166649001	166654000	8.40E-43
chr2	166650001	166655000	3.09E-53
chr2	166651001	166656000	1.04E-22
chr2	166659001	166664000	0.000153328
chr2	167058001	167063000	0.009650093
chr2	167059001	167064000	0.025362899
chr2	167060001	167065000	0.00409697
chr2	167061001	167066000	0.002371049
chr2	167219001	167224000	0.000256787
chr2	167232001	167237000	1.13E-29
chr2	167362001	167367000	6.42E-09
chr2	167450001	167455000	0.00348944
chr2	167451001	167456000	0.00350267
chr2	167479001	167484000	5.38E-07
chr2	167543001	167548000	9.07E-05
chr2	167544001	167549000	0.0009122
chr2	167660001	167665000	0.001518978
chr2	167661001	167666000	0.000276571
chr2	167662001	167667000	0.003018677
chr2	167742001	167747000	0.000199019

chr2	167947001	167952000	1.21E-05
chr2	168183001	168188000	2.74E-07
chr2	168282001	168287000	3.28E-09
chr2	168581001	168586000	0.000126474
chr2	169101001	169106000	5.76E-38
chr2	169102001	169107000	1.29E-76
chr2	169103001	169108000	7.96E-53
chr2	169104001	169109000	7.11E-23
chr2	169269001	169274000	1.46E-11
chr2	169270001	169275000	2.65E-08
chr2	169271001	169276000	1.71E-07
chr2	169308001	169313000	5.96E-20
chr2	169309001	169314000	8.16E-09
chr2	169311001	169316000	4.93E-26
chr2	169312001	169317000	4.95E-47
chr2	169313001	169318000	4.84E-29
chr2	169477001	169482000	0.00078999
chr2	170163001	170168000	3.28E-10
chr2	170214001	170219000	8.74E-25
chr2	170336001	170341000	1.01E-22
chr2	170437001	170442000	1.23E-20
chr2	170546001	170551000	4.13E-09
chr2	170590001	170595000	1.34E-22
chr2	170677001	170682000	7.92E-24
chr2	170682001	170687000	3.25E-43
chr2	170683001	170688000	1.95E-58
chr2	170684001	170689000	3.93E-29
chr2	171572001	171577000	3.39E-25
chr2	171573001	171578000	1.09E-33
chr2	171781001	171786000	4.24E-36
chr2	171782001	171787000	2.63E-32
chr2	171783001	171788000	1.93E-32
chr2	171784001	171789000	1.80E-38
chr2	171786001	171791000	1.33E-29
chr2	172016001	172021000	3.91E-58
chr2	172017001	172022000	2.09E-57
chr2	172269001	172274000	0.000465797
chr2	172289001	172294000	9.40E-57
chr2	172290001	172295000	2.80E-88
chr2	172291001	172296000	6.60E-61
chr2	172377001	172382000	1.05E-42
chr2	172378001	172383000	7.79E-35
chr2	172578001	172583000	1.13E-06
chr2	172579001	172584000	2.62E-06
chr2	172580001	172585000	2.08E-06
chr2	172748001	172753000	1.54E-29
chr2	172749001	172754000	1.29E-30
chr2	172860001	172865000	1.45E-12

chr2	173034001	173039000	2.47E-47
chr2	173035001	173040000	3.71E-32
chr2	173036001	173041000	2.18E-36
chr2	173288001	173293000	4.78E-26
chr2	173290001	173295000	1.07E-26
chr2	173291001	173296000	1.79E-49
chr2	173292001	173297000	1.08E-53
chr2	173421001	173426000	1.07E-14
chr2	173596001	173601000	1.66E-21
chr2	173597001	173602000	6.97E-21
chr2	173598001	173603000	1.10E-22
chr2	173599001	173604000	1.86E-24
chr2	173600001	173605000	9.88E-31
chr2	173936001	173941000	3.24E-43
chr2	173937001	173942000	2.40E-35
chr2	173941001	173946000	1.82E-28
chr2	173989001	173994000	6.81E-05
chr2	173990001	173995000	0.000143482
chr2	173991001	173996000	0.000266818
chr2	174038001	174043000	0.000153996
chr2	174197001	174202000	0.000841614
chr2	174198001	174203000	0.00021893
chr2	174332001	174337000	0.001227451
chr2	174985001	174990000	0.005777856
chr2	174986001	174991000	0.009878916
chr2	175109001	175114000	7.33E-27
chr2	175110001	175115000	1.96E-49
chr2	175111001	175116000	3.63E-53
chr2	175112001	175117000	8.00E-51
chr2	175113001	175118000	7.53E-47
chr2	175347001	175352000	5.83E-24
chr2	175348001	175353000	9.92E-33
chr2	175351001	175356000	1.75E-43
chr2	175352001	175357000	2.91E-19
chr2	175477001	175482000	0.001266497
chr2	175478001	175483000	0.007680558
chr2	175543001	175548000	1.45E-30
chr2	175547001	175552000	5.44E-26
chr2	175593001	175598000	7.91E-16
chr2	175865001	175870000	1.65E-48
chr2	175866001	175871000	5.92E-66
chr2	175867001	175872000	3.22E-68
chr2	175868001	175873000	1.96E-82
chr2	175869001	175874000	5.32E-64
chr2	175870001	175875000	8.87E-59
chr2	176028001	176033000	1.97E-62
chr2	176029001	176034000	4.51E-70
chr2	176030001	176035000	2.22E-78

chr2	176031001	176036000	1.22E-69
chr2	176032001	176037000	4.68E-71
chr2	176033001	176038000	3.61E-74
chr2	176041001	176046000	7.79E-11
chr2	176042001	176047000	3.62E-40
chr2	176043001	176048000	5.16E-36
chr2	176044001	176049000	1.56E-22
chr2	176045001	176050000	5.06E-26
chr2	176046001	176051000	3.34E-27
chr2	176072001	176077000	0.000274456
chr2	176080001	176085000	8.90E-05
chr2	176081001	176086000	0.000626797
chr2	176178001	176183000	0.008205223
chr2	176253001	176258000	6.14E-06
chr2	176310001	176315000	5.01E-05
chr2	176311001	176316000	1.61E-05
chr2	176312001	176317000	0.000108215
chr2	176313001	176318000	8.51E-05
chr2	176314001	176319000	7.30E-05
chr2	176771001	176776000	0.008205223
chr2	176772001	176777000	0.002358465
chr2	176773001	176778000	4.89E-14
chr2	176867001	176872000	1.18E-18
chr2	177004001	177009000	1.21E-30
chr2	177005001	177010000	7.84E-26
chr2	177041001	177046000	1.17E-05
chr2	177042001	177047000	1.29E-06
chr2	177130001	177135000	4.35E-35
chr2	177131001	177136000	8.56E-39
chr2	177132001	177137000	4.70E-45
chr2	177133001	177138000	6.66E-88
chr2	177134001	177139000	1.17E-84
chr2	177271001	177276000	0.003679296
chr2	177272001	177277000	0.004159841
chr2	177499001	177504000	1.02E-34
chr2	177500001	177505000	5.00E-33
chr2	177501001	177506000	6.14E-32
chr2	177502001	177507000	9.77E-32
chr2	177549001	177554000	0.004276337
chr2	177664001	177669000	1.61E-06
chr2	177665001	177670000	9.66E-06
chr2	177702001	177707000	0.001589435
chr2	177705001	177710000	0.010036385
chr2	177743001	177748000	0.001912642
chr2	177925001	177930000	0.000525554
chr2	178164001	178169000	0.012456313
chr2	178198001	178203000	7.42E-05
chr2	178253001	178258000	2.50E-64

chr2	178254001	178259000	4.84E-67
chr2	178255001	178260000	7.58E-74
chr2	178256001	178261000	2.58E-62
chr2	178257001	178262000	1.71E-44
chr2	178441001	178446000	0.001921499
chr2	178822001	178827000	0.000195824
chr2	178823001	178828000	7.65E-06
chr2	178824001	178829000	3.76E-06
chr2	178825001	178830000	0.000450681
chr2	178973001	178978000	1.65E-42
chr2	178974001	178979000	2.89E-42
chr2	178975001	178980000	4.79E-45
chr2	178976001	178981000	3.48E-33
chr2	178977001	178982000	3.15E-46
chr2	179054001	179059000	1.34E-24
chr2	179055001	179060000	8.12E-49
chr2	179056001	179061000	6.79E-53
chr2	179057001	179062000	5.13E-37
chr2	179059001	179064000	3.29E-57
chr2	179060001	179065000	6.15E-15
chr2	179111001	179116000	2.57E-05
chr2	179274001	179279000	1.83E-14
chr2	179344001	179349000	1.37E-19
chr2	179345001	179350000	1.12E-23
chr2	179346001	179351000	9.29E-08
chr2	179387001	179392000	3.22E-20
chr2	179388001	179393000	3.24E-18
chr2	179587001	179592000	0.001191641
chr2	179588001	179593000	3.66E-05
chr2	179600001	179605000	1.69E-05
chr2	179632001	179637000	0.002571824
chr2	179633001	179638000	0.002906665
chr2	180127001	180132000	1.77E-40
chr2	180128001	180133000	2.56E-47
chr2	180129001	180134000	7.50E-46
chr2	180158001	180163000	0.002036782
chr2	180282001	180287000	5.65E-06
chr2	180283001	180288000	8.63E-08
chr2	180284001	180289000	1.26E-07
chr2	180285001	180290000	8.38E-08
chr2	180721001	180726000	8.15E-28
chr2	180722001	180727000	6.04E-36
chr2	180723001	180728000	4.31E-33
chr2	180724001	180729000	1.37E-29
chr2	180755001	180760000	0.00143454
chr2	180756001	180761000	0.000668805
chr2	181722001	181727000	0.00024193
chr2	181799001	181804000	0.000568936

chr2	181800001	181805000	6.81E-06
chr2	181801001	181806000	5.97E-07
chr2	181802001	181807000	1.20E-06
chr2	181803001	181808000	5.04E-05
chr2	181823001	181828000	0.000374618
chr2	181824001	181829000	0.001635254
chr2	181825001	181830000	0.001217769
chr2	181840001	181845000	6.30E-32
chr2	181841001	181846000	3.44E-81
chr2	182303001	182308000	0.00410977
chr2	182468001	182473000	0.003204003
chr2	182517001	182522000	7.27E-77
chr2	182518001	182523000	4.40E-61
chr2	182519001	182524000	3.13E-41
chr2	182520001	182525000	3.67E-34
chr2	182521001	182526000	1.37E-22
chr2	182654001	182659000	2.09E-05
chr2	182655001	182660000	4.04E-07
chr2	182752001	182757000	2.71E-57
chr2	182932001	182937000	0.000233147
chr2	183576001	183581000	6.32E-49
chr2	183577001	183582000	8.10E-49
chr2	183581001	183586000	3.23E-52
chr2	184442001	184447000	0.00071711
chr2	184883001	184888000	0.002482869
chr2	185001001	185006000	8.67E-06
chr2	185002001	185007000	7.94E-05
chr2	185003001	185008000	1.18E-05
chr2	185463001	185468000	4.39E-54
chr2	185626001	185631000	4.38E-06
chr2	185627001	185632000	2.42E-05
chr2	185628001	185633000	0.000126041
chr2	185630001	185635000	1.84E-05
chr2	185956001	185961000	0.00081851
chr2	185957001	185962000	0.000155244
chr2	185958001	185963000	0.000304294
chr2	186006001	186011000	1.33E-08
chr2	186007001	186012000	7.19E-09
chr2	186009001	186014000	8.83E-11
chr2	186311001	186316000	9.69E-05
chr2	186312001	186317000	2.63E-06
chr2	186313001	186318000	2.20E-05
chr2	186344001	186349000	1.18E-06
chr2	186345001	186350000	7.86E-07
chr2	186346001	186351000	4.82E-07
chr2	186348001	186353000	1.50E-06
chr2	186389001	186394000	0.003682271
chr2	186678001	186683000	0.009264563

chr2	186679001	186684000	0.002215208
chr2	186758001	186763000	4.74E-05
chr2	186759001	186764000	5.71E-11
chr2	186760001	186765000	2.24E-11
chr2	186761001	186766000	1.78E-10
chr2	186792001	186797000	0.002646572
chr2	186793001	186798000	0.00064495
chr2	187554001	187559000	4.78E-24
chr2	187555001	187560000	4.66E-19
chr2	187556001	187561000	1.06E-22
chr2	187557001	187562000	3.87E-29
chr2	187558001	187563000	8.98E-27
chr2	187559001	187564000	3.43E-05
chr2	187888001	187893000	0.000739707
chr2	188594001	188599000	0.010535912
chr2	188595001	188600000	0.001332704
chr2	188818001	188823000	6.72E-06
chr2	189152001	189157000	1.10E-33
chr2	189153001	189158000	3.37E-59
chr2	189154001	189159000	7.57E-49
chr2	189156001	189161000	1.12E-57
chr2	189157001	189162000	2.34E-53
chr2	189356001	189361000	9.21E-05
chr2	189855001	189860000	0.006153352
chr2	190212001	190217000	0.020290786
chr2	190301001	190306000	2.43E-13
chr2	190306001	190311000	2.92E-21
chr2	190381001	190386000	0.00010237
chr2	190443001	190448000	1.56E-37
chr2	190444001	190449000	3.21E-46
chr2	190445001	190450000	2.11E-43
chr2	190447001	190452000	0.00278298
chr2	190478001	190483000	0.000344914
chr2	190529001	190534000	0.003744301
chr2	190535001	190540000	9.88E-28
chr2	190536001	190541000	4.18E-24
chr2	190537001	190542000	9.08E-27
chr2	190538001	190543000	8.07E-33
chr2	190539001	190544000	1.58E-25
chr2	191041001	191046000	1.37E-37
chr2	191042001	191047000	3.57E-24
chr2	191044001	191049000	5.45E-55
chr2	191045001	191050000	4.09E-63
chr2	191183001	191188000	3.04E-26
chr2	191184001	191189000	2.69E-38
chr2	191204001	191209000	1.72E-27
chr2	191206001	191211000	3.28E-21
chr2	191268001	191273000	3.42E-24

chr2	191269001	191274000	4.00E-32
chr2	191270001	191275000	1.12E-37
chr2	191271001	191276000	1.45E-51
chr2	191272001	191277000	1.14E-36
chr2	191273001	191278000	2.06E-32
chr2	191275001	191280000	2.32E-06
chr2	191277001	191282000	5.04E-06
chr2	191399001	191404000	2.95E-69
chr2	191427001	191432000	0.003373911
chr2	191509001	191514000	6.04E-39
chr2	191512001	191517000	4.26E-46
chr2	191513001	191518000	3.52E-73
chr2	191514001	191519000	7.19E-61
chr2	191843001	191848000	1.65E-13
chr2	191844001	191849000	9.49E-15
chr2	191878001	191883000	3.62E-41
chr2	191881001	191886000	1.06E-34
chr2	191882001	191887000	8.35E-29
chr2	191885001	191890000	4.40E-21
chr2	192105001	192110000	1.37E-32
chr2	192106001	192111000	1.50E-48
chr2	192107001	192112000	2.18E-30
chr2	192108001	192113000	6.38E-17
chr2	192109001	192114000	5.94E-33
chr2	192110001	192115000	5.33E-31
chr2	192184001	192189000	0.003494693
chr2	192494001	192499000	2.84E-05
chr2	192499001	192504000	2.75E-05
chr2	192500001	192505000	8.18E-07
chr2	192501001	192506000	2.10E-06
chr2	192502001	192507000	1.49E-05
chr2	192503001	192508000	0.000107477
chr2	192514001	192519000	0.001882313
chr2	192538001	192543000	5.15E-45
chr2	192539001	192544000	1.32E-26
chr2	192627001	192632000	3.18E-06
chr2	192629001	192634000	4.90E-12
chr2	192702001	192707000	3.24E-06
chr2	193223001	193228000	3.31E-09
chr2	194346001	194351000	0.000262151
chr2	194347001	194352000	0.000235896
chr2	194348001	194353000	0.000408018
chr2	194349001	194354000	0.000548776
chr2	194395001	194400000	0.015389503
chr2	194552001	194557000	8.80E-05
chr2	194553001	194558000	0.018374144
chr2	194897001	194902000	0.001524391
chr2	195063001	195068000	0.006626968

chr2	195065001	195070000	0.016919339
chr2	195303001	195308000	0.002776204
chr2	195304001	195309000	0.00350155
chr2	195557001	195562000	0.006708563
chr2	195563001	195568000	1.07E-05
chr2	195636001	195641000	0.003497445
chr2	196522001	196527000	3.25E-46
chr2	196826001	196831000	0.001971394
chr2	196827001	196832000	0.001430199
chr2	196931001	196936000	4.41E-34
chr2	196932001	196937000	3.47E-32
chr2	196933001	196938000	5.83E-32
chr2	197157001	197162000	1.77E-07
chr2	197453001	197458000	3.48E-48
chr2	197454001	197459000	7.19E-40
chr2	197455001	197460000	7.65E-42
chr2	197500001	197505000	1.02E-52
chr2	197501001	197506000	4.20E-55
chr2	197502001	197507000	3.49E-62
chr2	197503001	197508000	6.28E-57
chr2	197504001	197509000	2.05E-30
chr2	197660001	197665000	3.72E-16
chr2	197661001	197666000	2.25E-15
chr2	197662001	197667000	4.62E-14
chr2	197772001	197777000	0.004621147
chr2	197773001	197778000	0.016011995
chr2	197786001	197791000	3.21E-33
chr2	197787001	197792000	1.65E-34
chr2	197868001	197873000	0.025175604
chr2	197950001	197955000	0.000600992
chr2	198175001	198180000	2.44E-37
chr2	198299001	198304000	9.17E-19
chr2	198376001	198381000	7.42E-15
chr2	198380001	198385000	1.95E-24
chr2	198470001	198475000	2.93E-08
chr2	198471001	198476000	1.42E-05
chr2	198566001	198571000	7.30E-64
chr2	198567001	198572000	1.02E-45
chr2	198669001	198674000	3.50E-59
chr2	198670001	198675000	1.31E-21
chr2	198672001	198677000	4.58E-05
chr2	198746001	198751000	0.000658828
chr2	198903001	198908000	0.000493726
chr2	198904001	198909000	0.000142459
chr2	198905001	198910000	5.20E-06
chr2	198906001	198911000	5.16E-07
chr2	198907001	198912000	9.02E-09
chr2	198952001	198957000	0.000196422

chr2	198953001	198958000	0.000148441
chr2	199200001	199205000	0.010041654
chr2	199241001	199246000	4.25E-05
chr2	199286001	199291000	0.000360987
chr2	199495001	199500000	0.000126548
chr2	199633001	199638000	0.001461451
chr2	199714001	199719000	0.000179844
chr2	199715001	199720000	0.000179844
chr2	199766001	199771000	0.000758084
chr2	199767001	199772000	6.46E-05
chr2	199768001	199773000	3.58E-06
chr2	199769001	199774000	1.31E-06
chr2	199770001	199775000	1.92E-05
chr2	200101001	200106000	1.87E-05
chr2	200250001	200255000	0.000947929
chr2	200316001	200321000	2.91E-21
chr2	200317001	200322000	3.45E-32
chr2	200379001	200384000	0.000905168
chr2	200460001	200465000	0.000134013
chr2	200461001	200466000	0.001295262
chr2	200711001	200716000	4.37E-43
chr2	200771001	200776000	1.94E-21
chr2	200772001	200777000	1.69E-40
chr2	200773001	200778000	5.94E-64
chr2	200774001	200779000	4.89E-56
chr2	200776001	200781000	2.05E-51
chr2	200820001	200825000	2.55E-66
chr2	200821001	200826000	3.89E-17
chr2	201004001	201009000	0.001230807
chr2	201114001	201119000	2.15E-06
chr2	201167001	201172000	1.84E-52
chr2	201316001	201321000	5.29E-06
chr2	201370001	201375000	9.34E-53
chr2	201371001	201376000	2.88E-43
chr2	201372001	201377000	6.25E-24
chr2	201373001	201378000	2.92E-20
chr2	201672001	201677000	8.91E-24
chr2	201827001	201832000	8.43E-38
chr2	201828001	201833000	1.63E-60
chr2	202503001	202508000	8.96E-34
chr2	202505001	202510000	2.33E-43
chr2	202506001	202511000	2.81E-58
chr2	202507001	202512000	4.44E-59
chr2	202898001	202903000	4.49E-08
chr2	202899001	202904000	1.37E-07
chr2	203036001	203041000	1.25E-06
chr2	203101001	203106000	8.86E-10
chr2	203102001	203107000	4.86E-09

chr2	203103001	203108000	8.29E-13
chr2	203449001	203454000	2.67E-09
chr2	203451001	203456000	5.13E-09
chr2	203452001	203457000	2.29E-09
chr2	203453001	203458000	3.85E-09
chr2	203495001	203500000	1.41E-13
chr2	203496001	203501000	1.12E-31
chr2	203498001	203503000	3.63E-28
chr2	203499001	203504000	3.51E-40
chr2	203500001	203505000	1.45E-47
chr2	203902001	203907000	6.72E-11
chr2	203903001	203908000	8.65E-10
chr2	203904001	203909000	6.61E-11
chr2	204099001	204104000	5.70E-32
chr2	204100001	204105000	1.18E-27
chr2	204101001	204106000	6.45E-46
chr2	204102001	204107000	2.95E-38
chr2	204193001	204198000	1.17E-21
chr2	204266001	204271000	0.003632198
chr2	204399001	204404000	2.01E-34
chr2	204400001	204405000	3.37E-40
chr2	204925001	204930000	0.009545035
chr2	204926001	204931000	2.72E-06
chr2	204927001	204932000	2.71E-05
chr2	204938001	204943000	5.64E-06
chr2	204939001	204944000	6.19E-06
chr2	205135001	205140000	0.009459395
chr2	205405001	205410000	2.35E-21
chr2	205406001	205411000	2.04E-73
chr2	205407001	205412000	8.87E-46
chr2	205408001	205413000	1.00E-54
chr2	205409001	205414000	1.35E-55
chr2	205410001	205415000	1.14E-58
chr2	206073001	206078000	0.000246567
chr2	206197001	206202000	0.008583784
chr2	206198001	206203000	0.000133367
chr2	206199001	206204000	8.39E-05
chr2	206200001	206205000	0.000472094
chr2	206375001	206380000	0.001335989
chr2	206550001	206555000	9.71E-21
chr2	206790001	206795000	2.11E-17
chr2	206950001	206955000	2.26E-35
chr2	207139001	207144000	3.19E-46
chr2	207140001	207145000	3.16E-12
chr2	207290001	207295000	1.42E-07
chr2	207304001	207309000	2.66E-24
chr2	207306001	207311000	1.81E-17
chr2	207307001	207312000	2.04E-25

chr2	207308001	207313000	3.43E-47
chr2	207382001	207387000	0.002973072
chr2	207593001	207598000	3.24E-05
chr2	207627001	207632000	1.73E-25
chr2	207628001	207633000	8.71E-24
chr2	207629001	207634000	2.26E-22
chr2	207630001	207635000	1.27E-39
chr2	207774001	207779000	0.003843219
chr2	207775001	207780000	0.004931482
chr2	208030001	208035000	2.97E-53
chr2	208031001	208036000	5.43E-58
chr2	208390001	208395000	3.63E-57
chr2	208391001	208396000	3.51E-45
chr2	208486001	208491000	3.60E-20
chr2	208489001	208494000	2.54E-12
chr2	208490001	208495000	6.07E-11
chr2	208542001	208547000	5.20E-19
chr2	208543001	208548000	1.97E-33
chr2	208544001	208549000	4.09E-37
chr2	208545001	208550000	2.51E-35
chr2	208546001	208551000	4.28E-32
chr2	208576001	208581000	5.49E-24
chr2	208633001	208638000	5.83E-36
chr2	208634001	208639000	3.55E-32
chr2	208731001	208736000	0.002513264
chr2	208886001	208891000	3.63E-38
chr2	208887001	208892000	4.01E-29
chr2	208888001	208893000	1.24E-28
chr2	208889001	208894000	2.90E-22
chr2	208890001	208895000	2.06E-23
chr2	208958001	208963000	0.002808014
chr2	208959001	208964000	0.002808014
chr2	209250001	209255000	0.001349728
chr2	209258001	209263000	1.50E-13
chr2	209259001	209264000	6.82E-12
chr2	209260001	209265000	2.33E-15
chr2	209268001	209273000	5.98E-10
chr2	209269001	209274000	4.51E-16
chr2	209270001	209275000	9.02E-14
chr2	209271001	209276000	1.66E-15
chr2	209525001	209530000	4.66E-06
chr2	209552001	209557000	9.52E-05
chr2	209553001	209558000	0.000643708
chr2	209554001	209559000	0.000304477
chr2	209555001	209560000	0.017481658
chr2	209672001	209677000	0.0001504
chr2	209821001	209826000	2.24E-06
chr2	209882001	209887000	3.38E-06

chr2	209899001	209904000	0.004843687
chr2	210079001	210084000	1.37E-05
chr2	210284001	210289000	1.97E-23
chr2	210285001	210290000	7.18E-28
chr2	210289001	210294000	3.95E-16
chr2	210748001	210753000	0.015851487
chr2	210867001	210872000	1.29E-36
chr2	210929001	210934000	0.000109326
chr2	210930001	210935000	3.45E-05
chr2	211085001	211090000	2.13E-30
chr2	211088001	211093000	1.29E-34
chr2	211104001	211109000	4.27E-05
chr2	211206001	211211000	0.002335754
chr2	211529001	211534000	0.001890601
chr2	211595001	211600000	0.001849782
chr2	211831001	211836000	0.002681971
chr2	211890001	211895000	8.31E-05
chr2	211934001	211939000	0.000476299
chr2	211935001	211940000	0.001238605
chr2	211936001	211941000	0.001229028
chr2	211937001	211942000	0.001398306
chr2	212359001	212364000	0.002692173
chr2	212575001	212580000	0.002005569
chr2	212681001	212686000	0.001359872
chr2	212863001	212868000	0.000839192
chr2	212864001	212869000	0.008868951
chr2	213396001	213401000	9.85E-06
chr2	213402001	213407000	5.39E-23
chr2	213403001	213408000	1.24E-25
chr2	213481001	213486000	1.62E-07
chr2	213643001	213648000	0.000215433
chr2	213756001	213761000	0.001014886
chr2	214029001	214034000	0.000424515
chr2	214030001	214035000	0.001083996
chr2	214144001	214149000	0.003456961
chr2	214145001	214150000	5.28E-37
chr2	214146001	214151000	9.92E-62
chr2	214147001	214152000	6.66E-58
chr2	214148001	214153000	1.93E-53
chr2	214149001	214154000	7.56E-42
chr2	214217001	214222000	4.10E-07
chr2	214587001	214592000	0.002391074
chr2	215393001	215398000	0.001652457
chr2	215486001	215491000	0.000392928
chr2	215487001	215492000	0.000163341
chr2	215540001	215545000	0.00013028
chr2	215679001	215684000	0.003257518
chr2	215680001	215685000	2.32E-05

chr2	215681001	215686000	5.29E-05
chr2	215682001	215687000	4.71E-05
chr2	215739001	215744000	1.18E-08
chr2	215740001	215745000	6.62E-08
chr2	215799001	215804000	0.000745867
chr2	215800001	215805000	0.000186306
chr2	215801001	215806000	1.35E-06
chr2	215802001	215807000	0.000142685
chr2	215805001	215810000	0.000143168
chr2	215828001	215833000	2.02E-11
chr2	215829001	215834000	3.13E-14
chr2	215917001	215922000	0.007560373
chr2	216172001	216177000	1.03E-32
chr2	216173001	216178000	8.24E-29
chr2	216174001	216179000	3.74E-31
chr2	216876001	216881000	2.29E-26
chr2	216877001	216882000	9.09E-25
chr2	216878001	216883000	3.03E-20
chr2	216942001	216947000	1.16E-29
chr2	216943001	216948000	1.06E-22
chr2	216970001	216975000	8.42E-18
chr2	216971001	216976000	4.83E-21
chr2	216972001	216977000	4.31E-19
chr2	216974001	216979000	4.94E-18
chr2	217494001	217499000	1.14E-23
chr2	217495001	217500000	1.93E-17
chr2	217563001	217568000	1.88E-06
chr2	217808001	217813000	3.56E-06
chr2	217809001	217814000	3.98E-05
chr2	217962001	217967000	8.48E-05
chr2	217966001	217971000	1.22E-07
chr2	218023001	218028000	0.000181381
chr2	218405001	218410000	0.000797396
chr2	218406001	218411000	0.000965654
chr2	218538001	218543000	2.07E-10
chr2	218539001	218544000	5.70E-12
chr2	218540001	218545000	4.32E-10
chr2	218541001	218546000	9.74E-08
chr2	218542001	218547000	0.000145754
chr2	218543001	218548000	0.00719628
chr2	218634001	218639000	0.000429337
chr2	218839001	218844000	0.00017433
chr2	218871001	218876000	3.28E-11
chr2	218926001	218931000	1.02E-08
chr2	218951001	218956000	1.85E-06
chr2	218976001	218981000	0.001011717
chr2	219077001	219082000	2.55E-27
chr2	219078001	219083000	4.94E-25

chr2	219079001	219084000	1.23E-29
chr2	219080001	219085000	1.03E-17
chr2	219270001	219275000	1.65E-08
chr2	219271001	219276000	1.76E-13
chr2	219428001	219433000	3.23E-16
chr2	219429001	219434000	3.02E-45
chr2	219532001	219537000	4.43E-16
chr2	219536001	219541000	1.77E-11
chr2	219571001	219576000	9.90E-40
chr2	219572001	219577000	3.03E-35
chr2	219573001	219578000	3.88E-34
chr2	219575001	219580000	1.31E-27
chr2	219744001	219749000	1.03E-06
chr2	219753001	219758000	1.28E-30
chr2	219754001	219759000	7.18E-14
chr2	219755001	219760000	1.55E-12
chr2	219756001	219761000	2.00E-10
chr2	219925001	219930000	5.12E-21
chr2	220021001	220026000	1.37E-25
chr2	220022001	220027000	8.41E-21
chr2	220023001	220028000	6.26E-25
chr2	220024001	220029000	8.13E-17
chr2	220025001	220030000	9.33E-11
chr2	220037001	220042000	3.79E-10
chr2	220067001	220072000	1.54E-24
chr2	220116001	220121000	4.71E-17
chr2	220117001	220122000	1.54E-17
chr2	220118001	220123000	4.67E-14
chr2	220169001	220174000	1.98E-15
chr2	220170001	220175000	2.83E-20
chr2	220171001	220176000	5.09E-14
chr2	220252001	220257000	2.46E-37
chr2	220280001	220285000	0.001376815
chr2	220303001	220308000	4.42E-06
chr2	220407001	220412000	9.72E-53
chr2	220433001	220438000	4.56E-26
chr2	220434001	220439000	9.95E-42
chr2	220435001	220440000	1.46E-32
chr2	220490001	220495000	1.90E-06
chr2	220813001	220818000	0.007812864
chr2	220840001	220845000	0.000139663
chr2	220842001	220847000	4.91E-05
chr2	221210001	221215000	0.000153406
chr2	221211001	221216000	0.000138948
chr2	221212001	221217000	0.001198645
chr2	221213001	221218000	3.26E-05
chr2	221265001	221270000	8.90E-07
chr2	221266001	221271000	1.64E-05

chr2	221267001	221272000	8.88E-06
chr2	221268001	221273000	3.76E-05
chr2	221925001	221930000	0.00048179
chr2	221926001	221931000	0.001292161
chr2	221994001	221999000	0.003787145
chr2	221995001	222000000	0.000198015
chr2	222326001	222331000	9.15E-09
chr2	222327001	222332000	1.04E-07
chr2	222329001	222334000	8.78E-09
chr2	222330001	222335000	7.37E-08
chr2	222432001	222437000	1.98E-16
chr2	222433001	222438000	2.23E-23
chr2	222508001	222513000	0.005851676
chr2	222740001	222745000	8.78E-05
chr2	222741001	222746000	0.00010733
chr2	222938001	222943000	1.33E-06
chr2	222939001	222944000	1.81E-05
chr2	222988001	222993000	1.61E-10
chr2	222989001	222994000	1.44E-06
chr2	222990001	222995000	9.09E-08
chr2	222991001	222996000	2.53E-07
chr2	223012001	223017000	0.000903308
chr2	223016001	223021000	5.51E-06
chr2	223047001	223052000	2.47E-07
chr2	223048001	223053000	1.01E-06
chr2	223049001	223054000	1.03E-08
chr2	223050001	223055000	2.94E-07
chr2	223078001	223083000	0.005265739
chr2	223079001	223084000	0.008424149
chr2	223099001	223104000	2.14E-06
chr2	223100001	223105000	4.24E-13
chr2	223140001	223145000	6.83E-12
chr2	223141001	223146000	5.44E-12
chr2	223184001	223189000	4.57E-24
chr2	223220001	223225000	4.47E-06
chr2	223221001	223226000	7.07E-06
chr2	223222001	223227000	1.71E-06
chr2	223284001	223289000	1.90E-08
chr2	223285001	223290000	1.76E-21
chr2	223286001	223291000	1.18E-20
chr2	223287001	223292000	1.59E-16
chr2	223721001	223726000	5.91E-55
chr2	223952001	223957000	0.001603035
chr2	224060001	224065000	0.00584471
chr2	224216001	224221000	0.00012187
chr2	224351001	224356000	2.37E-06
chr2	224473001	224478000	8.11E-12
chr2	224474001	224479000	5.86E-28

chr2	224475001	224480000	1.82E-20
chr2	224476001	224481000	2.15E-22
chr2	224477001	224482000	3.10E-20
chr2	224478001	224483000	5.16E-19
chr2	224501001	224506000	0.01876607
chr2	224818001	224823000	1.51E-36
chr2	224819001	224824000	6.20E-40
chr2	224899001	224904000	2.42E-25
chr2	224900001	224905000	7.99E-22
chr2	224903001	224908000	3.35E-30
chr2	225307001	225312000	1.15E-22
chr2	225400001	225405000	0.004935692
chr2	225445001	225450000	3.98E-61
chr2	225446001	225451000	6.29E-69
chr2	225447001	225452000	1.85E-50
chr2	225449001	225454000	3.88E-42
chr2	225543001	225548000	2.21E-06
chr2	225904001	225909000	6.50E-26
chr2	225905001	225910000	1.69E-40
chr2	225906001	225911000	1.15E-55
chr2	225907001	225912000	3.65E-37
chr2	225926001	225931000	4.37E-06
chr2	225927001	225932000	1.71E-05
chr2	226241001	226246000	0.003046007
chr2	226455001	226460000	0.000537829
chr2	226481001	226486000	4.52E-90
chr2	226482001	226487000	1.13E-90
chr2	226483001	226488000	7.73E-86
chr2	226484001	226489000	1.38E-78
chr2	226485001	226490000	1.51E-67
chr2	226892001	226897000	3.46E-11
chr2	226893001	226898000	2.51E-13
chr2	226894001	226899000	4.30E-15
chr2	227390001	227395000	0.001530412
chr2	227391001	227396000	0.002598743
chr2	227696001	227701000	8.83E-52
chr2	227700001	227705000	2.22E-52
chr2	227813001	227818000	0.000317296
chr2	227825001	227830000	6.04E-05
chr2	227826001	227831000	4.42E-05
chr2	227827001	227832000	1.31E-08
chr2	228024001	228029000	4.31E-49
chr2	228025001	228030000	3.96E-53
chr2	228026001	228031000	2.83E-46
chr2	228027001	228032000	5.39E-52
chr2	228029001	228034000	3.21E-28
chr2	228187001	228192000	2.20E-38
chr2	228188001	228193000	2.11E-52

chr2	228189001	228194000	1.67E-44
chr2	228190001	228195000	1.45E-55
chr2	228191001	228196000	8.76E-08
chr2	228192001	228197000	1.05E-07
chr2	228193001	228198000	1.31E-05
chr2	228332001	228337000	1.14E-19
chr2	228333001	228338000	3.05E-36
chr2	228471001	228476000	4.70E-08
chr2	228472001	228477000	6.65E-07
chr2	228552001	228557000	0.000301734
chr2	228553001	228558000	0.000488672
chr2	228679001	228684000	4.39E-12
chr2	228680001	228685000	4.16E-13
chr2	228681001	228686000	2.14E-11
chr2	228732001	228737000	3.85E-31
chr2	228733001	228738000	3.23E-22
chr2	228734001	228739000	5.67E-25
chr2	228735001	228740000	1.32E-24
chr2	228736001	228741000	1.31E-17
chr2	228843001	228848000	0.000237324
chr2	228916001	228921000	2.15E-06
chr2	228987001	228992000	0.00081849
chr2	229115001	229120000	8.64E-09
chr2	229117001	229122000	7.10E-07
chr2	229215001	229220000	2.98E-09
chr2	229216001	229221000	1.54E-08
chr2	229217001	229222000	4.33E-08
chr2	229272001	229277000	0.002541772
chr2	229273001	229278000	0.002120322
chr2	229302001	229307000	1.44E-07
chr2	229303001	229308000	4.14E-08
chr2	229304001	229309000	0.000644964
chr2	229305001	229310000	0.000323364
chr2	229306001	229311000	0.000460288
chr2	229644001	229649000	8.81E-06
chr2	229645001	229650000	6.06E-05
chr2	229658001	229663000	1.44E-06
chr2	229865001	229870000	0.017256666
chr2	229867001	229872000	0.016532973
chr2	229868001	229873000	0.015526214
chr2	229943001	229948000	5.27E-05
chr2	230030001	230035000	2.18E-06
chr2	230031001	230036000	8.34E-05
chr2	230032001	230037000	2.68E-07
chr2	230084001	230089000	8.20E-08
chr2	230085001	230090000	1.56E-10
chr2	230086001	230091000	6.68E-07
chr2	230103001	230108000	0.000181077

chr2	230104001	230109000	1.71E-05
chr2	230105001	230110000	0.000570208
chr2	230146001	230151000	7.80E-05
chr2	230392001	230397000	0.000232084
chr2	230786001	230791000	6.25E-12
chr2	230787001	230792000	2.30E-18
chr2	230927001	230932000	5.84E-18
chr2	230928001	230933000	7.46E-39
chr2	231016001	231021000	1.86E-08
chr2	231187001	231192000	1.57E-05
chr2	231189001	231194000	1.34E-19
chr2	231190001	231195000	1.53E-21
chr2	231191001	231196000	1.00E-38
chr2	231192001	231197000	0.000111879
chr2	231217001	231222000	1.72E-06
chr2	231272001	231277000	5.78E-26
chr2	231273001	231278000	1.67E-18
chr2	231274001	231279000	8.18E-20
chr2	231275001	231280000	4.63E-20
chr2	231276001	231281000	1.21E-13
chr2	231281001	231286000	1.18E-05
chr2	231345001	231350000	1.59E-12
chr2	231573001	231578000	3.34E-29
chr2	231578001	231583000	1.12E-55
chr2	231709001	231714000	3.55E-10
chr2	231918001	231923000	2.32E-27
chr2	231919001	231924000	2.35E-50
chr2	231920001	231925000	3.40E-68
chr2	231921001	231926000	6.72E-69
chr2	231922001	231927000	5.82E-16
chr2	232059001	232064000	5.01E-34
chr2	232060001	232065000	4.62E-36
chr2	232061001	232066000	9.08E-27
chr2	232324001	232329000	7.91E-30
chr2	232325001	232330000	1.12E-47
chr2	232328001	232333000	3.90E-15
chr2	232329001	232334000	6.07E-28
chr2	232375001	232380000	2.46E-18
chr2	232376001	232381000	5.61E-17
chr2	232377001	232382000	6.04E-19
chr2	232378001	232383000	1.32E-30
chr2	232379001	232384000	1.59E-11
chr2	232523001	232528000	1.08E-26
chr2	232640001	232645000	8.61E-09
chr2	232641001	232646000	2.21E-54
chr2	232642001	232647000	7.12E-47
chr2	232643001	232648000	1.26E-55
chr2	232644001	232649000	3.16E-61

chr2	232645001	232650000	9.20E-59
chr2	232646001	232651000	2.94E-26
chr2	232647001	232652000	1.20E-80
chr2	232648001	232653000	9.45E-72
chr2	232649001	232654000	6.05E-68
chr2	232650001	232655000	1.42E-51
chr2	232651001	232656000	7.59E-41
chr2	232761001	232766000	2.55E-13
chr2	232762001	232767000	9.08E-13
chr2	232786001	232791000	8.24E-15
chr2	232787001	232792000	1.22E-15
chr2	232790001	232795000	6.11E-11
chr2	232822001	232827000	1.48E-17
chr2	232823001	232828000	5.09E-11
chr2	233346001	233351000	4.01E-10
chr2	233347001	233352000	5.48E-11
chr2	233348001	233353000	3.19E-10
chr2	233349001	233354000	4.65E-14
chr2	233411001	233416000	3.66E-24
chr2	233412001	233417000	1.55E-25
chr2	233413001	233418000	2.44E-32
chr2	233414001	233419000	1.44E-20
chr2	233415001	233420000	2.72E-19
chr2	233494001	233499000	3.36E-22
chr2	233495001	233500000	3.02E-12
chr2	233562001	233567000	1.06E-22
chr2	233788001	233793000	7.11E-08
chr2	234156001	234161000	8.37E-36
chr2	234157001	234162000	1.14E-40
chr2	234158001	234163000	3.78E-24
chr2	234159001	234164000	7.35E-37
chr2	234160001	234165000	3.02E-66
chr2	234260001	234265000	2.33E-15
chr2	234261001	234266000	4.21E-10
chr2	234262001	234267000	2.79E-11
chr2	234263001	234268000	6.14E-39
chr2	234471001	234476000	4.81E-34
chr2	234472001	234477000	2.47E-31
chr2	235404001	235409000	2.08E-28
chr2	235405001	235410000	3.69E-27
chr2	235814001	235819000	7.83E-06
chr2	235856001	235861000	6.10E-20
chr2	236307001	236312000	0.001230342
chr2	236398001	236403000	7.05E-24
chr2	236399001	236404000	5.25E-34
chr2	236574001	236579000	6.86E-27
chr2	236575001	236580000	1.16E-32
chr2	236576001	236581000	1.38E-24

chr2	236577001	236582000	4.85E-42
chr2	236578001	236583000	7.69E-40
chr2	236579001	236584000	7.50E-26
chr2	237187001	237192000	0.004426796
chr2	237188001	237193000	9.24E-05
chr2	237189001	237194000	2.89E-05
chr2	237235001	237240000	2.15E-09
chr2	237236001	237241000	1.74E-11
chr2	237318001	237323000	8.90E-07
chr2	237334001	237339000	6.01E-08
chr2	237335001	237340000	1.84E-08
chr2	237336001	237341000	2.58E-06
chr2	237337001	237342000	5.73E-05
chr2	237358001	237363000	3.54E-09
chr2	237366001	237371000	1.83E-10
chr2	237399001	237404000	4.79E-05
chr2	237982001	237987000	2.73E-09
chr2	237990001	237995000	1.17E-36
chr2	237991001	237996000	8.10E-35
chr2	237992001	237997000	1.09E-54
chr2	237993001	237998000	4.63E-66
chr2	237994001	237999000	2.30E-43
chr2	238383001	238388000	3.06E-23
chr2	238384001	238389000	2.13E-27
chr2	238391001	238396000	8.89E-12
chr2	238392001	238397000	1.77E-11
chr2	238393001	238398000	1.24E-10
chr2	238461001	238466000	0.000222013
chr2	238534001	238539000	1.94E-11
chr2	238535001	238540000	6.07E-12
chr2	238536001	238541000	2.85E-12
chr2	238596001	238601000	1.46E-08
chr2	238597001	238602000	2.43E-16
chr2	238598001	238603000	2.76E-15
chr2	238599001	238604000	2.44E-21
chr2	238871001	238876000	7.55E-10
chr2	238872001	238877000	2.98E-13
chr2	238873001	238878000	9.08E-22
chr2	238875001	238880000	5.90E-38
chr2	238876001	238881000	1.91E-25
chr2	238965001	238970000	1.62E-29
chr2	238967001	238972000	2.71E-79
chr2	238968001	238973000	2.18E-53
chr2	238969001	238974000	5.83E-54
chr2	238970001	238975000	6.01E-27
chr2	239107001	239112000	7.75E-43
chr2	239144001	239149000	3.08E-09
chr2	239146001	239151000	4.06E-06

chr2	239148001	239153000	2.58E-09
chr2	239197001	239202000	4.88E-23
chr2	239225001	239230000	9.51E-36
chr2	239226001	239231000	1.76E-15
chr2	239675001	239680000	1.88E-09
chr2	239756001	239761000	2.44E-19
chr2	239757001	239762000	1.01E-26
chr2	239758001	239763000	2.70E-20
chr2	240320001	240325000	1.23E-18
chr2	240322001	240327000	3.33E-43
chr2	240323001	240328000	2.07E-30
chr2	240999001	241004000	5.49E-05
chr2	241008001	241013000	0.001293962
chr2	241015001	241020000	0.010151391
chr2	241027001	241032000	1.17E-15
chr2	241028001	241033000	7.89E-14
chr2	241029001	241034000	4.12E-09
chr2	241030001	241035000	4.43E-17
chr2	241031001	241036000	2.72E-26
chr2	241035001	241040000	2.81E-11
chr2	241052001	241057000	4.17E-05
chr2	241178001	241183000	0.000415221
chr2	241503001	241508000	5.38E-25
chr2	241758001	241763000	3.87E-11
chr2	242037001	242042000	4.82E-08
chr2	242084001	242089000	5.17E-26
chr2	242085001	242090000	3.18E-38
chr2	242087001	242092000	4.83E-27
chr2	242211001	242216000	1.86E-32
chr2	242212001	242217000	4.03E-24
chr2	242250001	242255000	8.96E-46
chr2	242251001	242256000	1.34E-55
chr2	242252001	242257000	1.01E-34
chr2	242253001	242258000	1.25E-15
chr2	242255001	242260000	1.60E-14
chr2	242295001	242300000	2.67E-24
chr2	242296001	242301000	1.51E-13
chr2	242443001	242448000	3.34E-06
chr2	242444001	242449000	2.55E-08
chr2	242445001	242450000	6.24E-34
chr2	242446001	242451000	3.86E-29
chr2	242447001	242452000	1.48E-39
chr2	242448001	242453000	4.26E-22
chr2	242572001	242577000	1.41E-16
chr2	242573001	242578000	6.15E-37
chr2	242574001	242579000	1.54E-40
chr2	242575001	242580000	4.18E-20
chr2	242576001	242581000	2.26E-22

chr2	242577001	242582000	8.41E-14
chr2	242622001	242627000	2.19E-20
chr2	242623001	242628000	1.09E-24
chr2	242624001	242629000	1.32E-17
chr2	242637001	242642000	9.39E-15
chr2	242638001	242643000	2.36E-10
chr2	242639001	242644000	2.53E-07
chr2	243029001	243034000	2.23E-40
chr2	243031001	243036000	3.01E-41
chr2	243156001	243161000	0.002733344
chr2	243157001	243162000	0.000975905
chr3	74001	79000	8.97E-06
chr3	234001	239000	4.92E-12
chr3	235001	240000	4.01E-25
chr3	236001	241000	4.69E-35
chr3	237001	242000	8.58E-44
chr3	239001	244000	6.67E-39
chr3	496001	501000	0.002144244
chr3	526001	531000	0.031696785
chr3	574001	579000	1.53E-12
chr3	577001	582000	1.21E-13
chr3	747001	752000	3.25E-05
chr3	748001	753000	0.000404849
chr3	801001	806000	9.28E-05
chr3	803001	808000	4.54E-06
chr3	831001	836000	4.29E-05
chr3	832001	837000	0.000198138
chr3	862001	867000	6.68E-07
chr3	998001	1003000	3.67E-08
chr3	999001	1004000	1.44E-10
chr3	1000001	1005000	6.99E-12
chr3	1001001	1006000	3.79E-10
chr3	1046001	1051000	3.44E-05
chr3	1062001	1067000	0.003337425
chr3	1063001	1068000	0.000833768
chr3	1064001	1069000	1.30E-05
chr3	1065001	1070000	1.03E-05
chr3	1099001	1104000	4.99E-06
chr3	1100001	1105000	2.05E-08
chr3	1101001	1106000	1.59E-09
chr3	1102001	1107000	5.45E-10
chr3	1103001	1108000	6.03E-10
chr3	1104001	1109000	4.05E-10
chr3	1105001	1110000	1.84E-05
chr3	1192001	1197000	5.71E-06
chr3	1193001	1198000	9.95E-07
chr3	1194001	1199000	9.16E-06
chr3	1195001	1200000	2.41E-05

chr3	1196001	1201000	1.25E-06
chr3	1215001	1220000	1.57E-07
chr3	1375001	1380000	0.00228061
chr3	1407001	1412000	1.43E-05
chr3	1408001	1413000	8.69E-06
chr3	1409001	1414000	5.43E-05
chr3	1413001	1418000	0.001069008
chr3	1446001	1451000	0.003524062
chr3	1539001	1544000	0.005198779
chr3	1540001	1545000	0.000127822
chr3	1541001	1546000	0.000148025
chr3	1542001	1547000	0.000145063
chr3	1543001	1548000	3.45E-05
chr3	1598001	1603000	3.49E-05
chr3	1599001	1604000	8.95E-06
chr3	1700001	1705000	1.83E-07
chr3	1701001	1706000	1.60E-09
chr3	1702001	1707000	2.82E-09
chr3	1801001	1806000	0.009444092
chr3	1802001	1807000	0.009444092
chr3	1827001	1832000	0.035806468
chr3	1831001	1836000	0.002110224
chr3	1870001	1875000	0.004588725
chr3	1892001	1897000	5.20E-08
chr3	1893001	1898000	1.94E-08
chr3	1894001	1899000	1.04E-06
chr3	1931001	1936000	0.000978568
chr3	1932001	1937000	4.92E-06
chr3	1933001	1938000	0.000126397
chr3	1959001	1964000	3.01E-06
chr3	1960001	1965000	0.00287544
chr3	1966001	1971000	2.83E-07
chr3	1967001	1972000	1.56E-11
chr3	1968001	1973000	7.09E-09
chr3	1984001	1989000	1.75E-06
chr3	1996001	2001000	1.63E-07
chr3	1997001	2002000	1.35E-09
chr3	1998001	2003000	6.62E-09
chr3	1999001	2004000	1.69E-09
chr3	2385001	2390000	0.000299039
chr3	2407001	2412000	2.60E-12
chr3	2503001	2508000	4.06E-20
chr3	2504001	2509000	7.55E-19
chr3	2505001	2510000	2.80E-16
chr3	2506001	2511000	1.27E-13
chr3	2518001	2523000	2.35E-07
chr3	2519001	2524000	3.29E-06
chr3	2520001	2525000	2.44E-05

chr3	2521001	2526000	2.60E-06
chr3	2531001	2536000	2.73E-06
chr3	2532001	2537000	4.11E-06
chr3	2533001	2538000	1.34E-09
chr3	2534001	2539000	1.59E-09
chr3	2536001	2541000	9.38E-06
chr3	2537001	2542000	8.58E-07
chr3	2539001	2544000	0.000482718
chr3	2540001	2545000	0.000417939
chr3	2541001	2546000	7.11E-07
chr3	2542001	2547000	7.29E-06
chr3	2553001	2558000	9.34E-08
chr3	2565001	2570000	1.08E-05
chr3	2566001	2571000	1.22E-05
chr3	2567001	2572000	0.000466469
chr3	2646001	2651000	0.012259136
chr3	2647001	2652000	0.011480047
chr3	2650001	2655000	2.98E-05
chr3	2705001	2710000	0.0002539
chr3	2706001	2711000	0.000246486
chr3	2716001	2721000	8.07E-06
chr3	2717001	2722000	6.52E-07
chr3	2731001	2736000	2.09E-05
chr3	2733001	2738000	6.08E-06
chr3	2734001	2739000	5.42E-06
chr3	2750001	2755000	0.000495233
chr3	2782001	2787000	0.000337031
chr3	2783001	2788000	1.39E-07
chr3	2800001	2805000	2.14E-13
chr3	2801001	2806000	2.74E-09
chr3	2828001	2833000	3.97E-06
chr3	2831001	2836000	5.54E-05
chr3	2932001	2937000	7.34E-05
chr3	3034001	3039000	1.36E-05
chr3	3141001	3146000	0.028873299
chr3	3142001	3147000	8.93E-06
chr3	3217001	3222000	8.95E-20
chr3	3218001	3223000	9.62E-29
chr3	3219001	3224000	1.48E-30
chr3	3220001	3225000	1.45E-27
chr3	3221001	3226000	8.19E-33
chr3	3226001	3231000	0.000264114
chr3	3228001	3233000	1.66E-05
chr3	3229001	3234000	8.22E-05
chr3	3329001	3334000	0.000296029
chr3	3332001	3337000	3.37E-06
chr3	3336001	3341000	0.001333456
chr3	3353001	3358000	0.008188983

chr3	3354001	3359000	0.001211865
chr3	3355001	3360000	4.29E-07
chr3	3356001	3361000	6.59E-10
chr3	3357001	3362000	1.32E-08
chr3	3381001	3386000	2.18E-07
chr3	3382001	3387000	3.44E-06
chr3	3383001	3388000	2.26E-05
chr3	3384001	3389000	0.000125386
chr3	3403001	3408000	4.58E-05
chr3	3404001	3409000	3.54E-06
chr3	3436001	3441000	3.11E-05
chr3	3518001	3523000	0.000489469
chr3	3520001	3525000	8.52E-05
chr3	3521001	3526000	9.50E-06
chr3	3522001	3527000	1.85E-05
chr3	3558001	3563000	0.001979331
chr3	3559001	3564000	0.001176205
chr3	3570001	3575000	0.00092857
chr3	3571001	3576000	0.000851016
chr3	3632001	3637000	7.10E-06
chr3	3734001	3739000	4.52E-06
chr3	3765001	3770000	8.05E-06
chr3	3810001	3815000	8.88E-09
chr3	3836001	3841000	2.27E-41
chr3	3837001	3842000	3.70E-49
chr3	3873001	3878000	8.46E-05
chr3	3924001	3929000	6.82E-06
chr3	3925001	3930000	0.00015292
chr3	3926001	3931000	0.000178847
chr3	3927001	3932000	2.66E-07
chr3	3984001	3989000	0.000709352
chr3	3985001	3990000	0.00020012
chr3	3986001	3991000	0.000137457
chr3	3988001	3993000	0.006067841
chr3	4028001	4033000	0.000429394
chr3	4044001	4049000	2.35E-05
chr3	4045001	4050000	0.000288685
chr3	4102001	4107000	0.003368441
chr3	4158001	4163000	0.00161567
chr3	4202001	4207000	1.82E-07
chr3	4204001	4209000	3.67E-09
chr3	4205001	4210000	8.46E-08
chr3	4206001	4211000	3.63E-09
chr3	4208001	4213000	4.02E-08
chr3	4213001	4218000	1.03E-07
chr3	4214001	4219000	6.13E-10
chr3	4215001	4220000	3.24E-09
chr3	4216001	4221000	1.57E-11

chr3	4217001	4222000	4.65E-12
chr3	4218001	4223000	2.42E-06
chr3	4219001	4224000	1.62E-07
chr3	4245001	4250000	0.009390848
chr3	4255001	4260000	4.43E-08
chr3	4263001	4268000	0.000544451
chr3	4277001	4282000	0.007820777
chr3	4279001	4284000	0.000484787
chr3	4280001	4285000	1.57E-05
chr3	4289001	4294000	4.26E-06
chr3	4296001	4301000	2.83E-06
chr3	4300001	4305000	1.95E-06
chr3	4306001	4311000	3.08E-07
chr3	4398001	4403000	5.04E-09
chr3	4502001	4507000	1.56E-07
chr3	4503001	4508000	3.96E-10
chr3	4504001	4509000	4.07E-25
chr3	4505001	4510000	4.25E-22
chr3	4506001	4511000	1.16E-16
chr3	4507001	4512000	3.61E-10
chr3	4508001	4513000	7.54E-10
chr3	4556001	4561000	2.19E-08
chr3	4557001	4562000	2.06E-06
chr3	4581001	4586000	1.85E-06
chr3	4893001	4898000	2.88E-08
chr3	4910001	4915000	4.24E-09
chr3	5225001	5230000	2.17E-49
chr3	5226001	5231000	2.18E-43
chr3	5229001	5234000	5.71E-31
chr3	5330001	5335000	0.010343475
chr3	5367001	5372000	0.000319744
chr3	5497001	5502000	0.000198755
chr3	5499001	5504000	0.002053601
chr3	5500001	5505000	0.004516089
chr3	5512001	5517000	0.000179747
chr3	5513001	5518000	0.000186747
chr3	5535001	5540000	0.004868949
chr3	5563001	5568000	0.003591815
chr3	5565001	5570000	0.0072209
chr3	5628001	5633000	3.44E-05
chr3	5950001	5955000	9.79E-07
chr3	6063001	6068000	0.000502922
chr3	6188001	6193000	0.001460707
chr3	6299001	6304000	1.49E-05
chr3	6300001	6305000	3.11E-05
chr3	6326001	6331000	0.000136855
chr3	6328001	6333000	3.84E-05
chr3	6329001	6334000	1.75E-05

chr3	6330001	6335000	4.47E-05
chr3	6341001	6346000	8.26E-06
chr3	6369001	6374000	2.90E-08
chr3	6370001	6375000	6.17E-07
chr3	6371001	6376000	5.13E-06
chr3	6372001	6377000	1.89E-05
chr3	6449001	6454000	0.00578417
chr3	6520001	6525000	0.000470317
chr3	6521001	6526000	0.000154728
chr3	6522001	6527000	7.45E-05
chr3	6560001	6565000	0.001052574
chr3	6569001	6574000	0.00053537
chr3	6627001	6632000	0.000687994
chr3	6642001	6647000	0.000462161
chr3	6778001	6783000	0.003455445
chr3	6779001	6784000	0.001127159
chr3	6840001	6845000	0.000221247
chr3	6841001	6846000	8.64E-05
chr3	6865001	6870000	3.51E-06
chr3	6866001	6871000	0.000293517
chr3	6868001	6873000	9.41E-06
chr3	6869001	6874000	2.48E-06
chr3	6870001	6875000	0.000230729
chr3	6871001	6876000	9.14E-05
chr3	6872001	6877000	6.32E-05
chr3	6915001	6920000	6.67E-05
chr3	6918001	6923000	0.000129819
chr3	6987001	6992000	6.73E-10
chr3	7062001	7067000	5.16E-06
chr3	7069001	7074000	0.000356039
chr3	7072001	7077000	0.001137242
chr3	7212001	7217000	0.000202324
chr3	7261001	7266000	0.000178603
chr3	7299001	7304000	0.000462649
chr3	7373001	7378000	3.77E-05
chr3	7447001	7452000	5.30E-06
chr3	7448001	7453000	5.14E-05
chr3	7449001	7454000	8.66E-06
chr3	7456001	7461000	3.28E-05
chr3	7478001	7483000	0.00014337
chr3	7479001	7484000	0.00014337
chr3	7590001	7595000	2.12E-05
chr3	7612001	7617000	1.23E-06
chr3	7613001	7618000	8.28E-07
chr3	7614001	7619000	2.09E-07
chr3	7615001	7620000	2.13E-05
chr3	7633001	7638000	8.97E-05
chr3	7634001	7639000	2.53E-06

chr3	7636001	7641000	3.57E-05
chr3	7637001	7642000	5.65E-06
chr3	7669001	7674000	0.001913472
chr3	7670001	7675000	0.002159097
chr3	7671001	7676000	0.001918273
chr3	7732001	7737000	3.46E-06
chr3	7733001	7738000	1.71E-05
chr3	7736001	7741000	0.001886748
chr3	7902001	7907000	4.47E-06
chr3	7914001	7919000	4.53E-05
chr3	7928001	7933000	0.000247837
chr3	7934001	7939000	0.00015643
chr3	7941001	7946000	2.96E-05
chr3	7942001	7947000	1.42E-06
chr3	7943001	7948000	1.53E-05
chr3	7944001	7949000	2.95E-07
chr3	7945001	7950000	3.21E-06
chr3	7946001	7951000	1.39E-08
chr3	7947001	7952000	3.10E-08
chr3	7948001	7953000	2.75E-07
chr3	7977001	7982000	6.74E-10
chr3	7978001	7983000	1.54E-10
chr3	7979001	7984000	1.80E-10
chr3	7987001	7992000	0.000142261
chr3	7988001	7993000	4.95E-06
chr3	7989001	7994000	0.000122283
chr3	7990001	7995000	7.04E-05
chr3	8021001	8026000	1.82E-05
chr3	8022001	8027000	2.00E-06
chr3	8028001	8033000	1.83E-05
chr3	8029001	8034000	0.000446687
chr3	8039001	8044000	6.02E-07
chr3	8040001	8045000	1.26E-05
chr3	8085001	8090000	4.59E-05
chr3	8088001	8093000	0.00145521
chr3	8089001	8094000	0.000936099
chr3	8090001	8095000	0.000333491
chr3	8091001	8096000	0.000595991
chr3	8136001	8141000	0.000271607
chr3	8230001	8235000	0.009660504
chr3	8231001	8236000	0.009597516
chr3	8232001	8237000	0.002168541
chr3	8250001	8255000	0.000140636
chr3	8251001	8256000	6.02E-05
chr3	8253001	8258000	0.000192008
chr3	8354001	8359000	0.003263771
chr3	8355001	8360000	0.000581123
chr3	8371001	8376000	1.03E-05

chr3	8372001	8377000	1.03E-07
chr3	8401001	8406000	0.012953982
chr3	8402001	8407000	0.005320493
chr3	8403001	8408000	0.000730857
chr3	8404001	8409000	4.62E-05
chr3	8405001	8410000	1.08E-08
chr3	8406001	8411000	4.62E-08
chr3	8407001	8412000	2.01E-07
chr3	8443001	8448000	0.000164089
chr3	8867001	8872000	0.000783898
chr3	9000001	9005000	1.87E-08
chr3	9057001	9062000	4.25E-07
chr3	9400001	9405000	6.56E-08
chr3	9744001	9749000	2.15E-05
chr3	9881001	9886000	3.79E-33
chr3	9882001	9887000	6.13E-35
chr3	9883001	9888000	5.86E-34
chr3	9956001	9961000	0.000225334
chr3	9957001	9962000	1.23E-12
chr3	9970001	9975000	9.68E-17
chr3	9971001	9976000	5.14E-15
chr3	9985001	9990000	7.82E-05
chr3	9986001	9991000	2.08E-06
chr3	9987001	9992000	2.69E-06
chr3	9993001	9998000	3.46E-05
chr3	10025001	10030000	3.53E-16
chr3	10026001	10031000	2.30E-21
chr3	10027001	10032000	7.08E-12
chr3	10052001	10057000	3.70E-13
chr3	10063001	10068000	3.42E-08
chr3	10153001	10158000	2.63E-20
chr3	10154001	10159000	6.43E-24
chr3	10157001	10162000	2.58E-09
chr3	10203001	10208000	0.000766451
chr3	10286001	10291000	1.18E-14
chr3	10593001	10598000	4.15E-13
chr3	10745001	10750000	2.23E-19
chr3	10853001	10858000	2.33E-22
chr3	10854001	10859000	4.56E-22
chr3	10856001	10861000	4.20E-06
chr3	11178001	11183000	1.51E-16
chr3	11179001	11184000	1.53E-15
chr3	11195001	11200000	1.69E-19
chr3	11196001	11201000	3.27E-19
chr3	11760001	11765000	1.50E-24
chr3	11761001	11766000	1.49E-16
chr3	11886001	11891000	2.17E-16
chr3	11887001	11892000	8.15E-16

chr3	11888001	11893000	3.70E-17
chr3	11924001	11929000	3.54E-05
chr3	11925001	11930000	0.001502536
chr3	11981001	11986000	1.82E-08
chr3	12021001	12026000	0.00024122
chr3	12034001	12039000	2.75E-05
chr3	12041001	12046000	2.01E-14
chr3	12042001	12047000	1.59E-12
chr3	12043001	12048000	9.95E-12
chr3	12101001	12106000	7.70E-05
chr3	12124001	12129000	0.000286364
chr3	12125001	12130000	0.000988989
chr3	12325001	12330000	1.47E-40
chr3	12326001	12331000	1.21E-47
chr3	12327001	12332000	1.78E-60
chr3	12328001	12333000	4.11E-92
chr3	12329001	12334000	9.18E-108
chr3	12330001	12335000	7.16E-71
chr3	12356001	12361000	1.12E-05
chr3	12357001	12362000	4.77E-06
chr3	12358001	12363000	0.001695018
chr3	12526001	12531000	4.16E-21
chr3	13009001	13014000	1.16E-14
chr3	13032001	13037000	5.47E-08
chr3	13036001	13041000	8.33E-40
chr3	13457001	13462000	1.70E-06
chr3	13458001	13463000	1.25E-10
chr3	13459001	13464000	2.09E-08
chr3	13460001	13465000	9.89E-11
chr3	13461001	13466000	1.16E-19
chr3	13462001	13467000	8.38E-05
chr3	13587001	13592000	0.001174148
chr3	13928001	13933000	0.000105729
chr3	13983001	13988000	2.92E-06
chr3	13989001	13994000	4.33E-06
chr3	13990001	13995000	0.00082976
chr3	13997001	14002000	3.01E-06
chr3	13998001	14003000	2.41E-06
chr3	13999001	14004000	6.24E-07
chr3	14162001	14167000	3.77E-15
chr3	14215001	14220000	1.62E-18
chr3	14216001	14221000	1.97E-34
chr3	14217001	14222000	5.47E-33
chr3	14218001	14223000	2.31E-32
chr3	14219001	14224000	8.23E-34
chr3	14220001	14225000	1.75E-23
chr3	14442001	14447000	1.16E-06
chr3	14689001	14694000	5.49E-23

chr3	14690001	14695000	1.13E-24
chr3	14691001	14696000	4.17E-25
chr3	14692001	14697000	5.39E-25
chr3	14693001	14698000	4.70E-29
chr3	14785001	14790000	5.07E-05
chr3	14786001	14791000	2.22E-05
chr3	14988001	14993000	8.39E-24
chr3	14989001	14994000	2.02E-14
chr3	15104001	15109000	7.10E-19
chr3	15105001	15110000	6.50E-25
chr3	15106001	15111000	2.48E-36
chr3	15136001	15141000	1.38E-32
chr3	15137001	15142000	6.30E-34
chr3	15138001	15143000	2.12E-40
chr3	15139001	15144000	3.02E-19
chr3	15140001	15145000	1.04E-17
chr3	15241001	15246000	3.74E-08
chr3	15242001	15247000	3.04E-09
chr3	15243001	15248000	2.77E-40
chr3	15244001	15249000	5.80E-41
chr3	15245001	15250000	6.63E-32
chr3	15246001	15251000	6.62E-25
chr3	15248001	15253000	9.83E-12
chr3	15310001	15315000	4.75E-05
chr3	15311001	15316000	1.83E-05
chr3	15373001	15378000	1.31E-05
chr3	15464001	15469000	1.34E-25
chr3	15465001	15470000	7.13E-20
chr3	15466001	15471000	2.18E-19
chr3	15467001	15472000	7.04E-21
chr3	15468001	15473000	3.65E-18
chr3	15469001	15474000	5.82E-17
chr3	15559001	15564000	1.10E-08
chr3	15638001	15643000	9.01E-18
chr3	15821001	15826000	0.000995208
chr3	15896001	15901000	7.74E-18
chr3	15897001	15902000	4.06E-57
chr3	15898001	15903000	5.90E-30
chr3	15899001	15904000	2.50E-36
chr3	15900001	15905000	4.93E-33
chr3	15901001	15906000	5.88E-44
chr3	15902001	15907000	3.03E-21
chr3	16026001	16031000	0.001008031
chr3	16027001	16032000	0.000247505
chr3	16087001	16092000	0.006978448
chr3	16638001	16643000	2.09E-05
chr3	16921001	16926000	1.11E-18
chr3	16922001	16927000	4.70E-30

chr3	16930001	16935000	0.000197665
chr3	16976001	16981000	0.000205414
chr3	17876001	17881000	0.000570685
chr3	17896001	17901000	0.002483368
chr3	17954001	17959000	0.01186134
chr3	18245001	18250000	4.32E-06
chr3	18407001	18412000	1.52E-06
chr3	18408001	18413000	9.97E-07
chr3	18409001	18414000	2.48E-09
chr3	18410001	18415000	4.00E-07
chr3	18411001	18416000	1.88E-06
chr3	18461001	18466000	8.48E-13
chr3	18467001	18472000	4.41E-12
chr3	18481001	18486000	1.19E-50
chr3	18482001	18487000	7.93E-52
chr3	18483001	18488000	5.62E-35
chr3	18484001	18489000	4.22E-24
chr3	18486001	18491000	1.23E-09
chr3	18582001	18587000	0.000143496
chr3	18583001	18588000	0.000877317
chr3	18606001	18611000	0.021386942
chr3	18650001	18655000	0.000386662
chr3	18653001	18658000	0.000359054
chr3	18741001	18746000	0.010920066
chr3	18742001	18747000	0.022000849
chr3	18743001	18748000	0.024307694
chr3	18744001	18749000	0.001318259
chr3	18745001	18750000	0.001124103
chr3	18746001	18751000	0.001833942
chr3	18815001	18820000	0.00281663
chr3	18838001	18843000	0.000190025
chr3	18839001	18844000	4.84E-05
chr3	18877001	18882000	0.001939868
chr3	18878001	18883000	2.00E-06
chr3	18901001	18906000	4.30E-05
chr3	18930001	18935000	7.04E-07
chr3	18931001	18936000	5.87E-07
chr3	18932001	18937000	2.11E-06
chr3	18933001	18938000	8.07E-05
chr3	18949001	18954000	0.001263166
chr3	18955001	18960000	0.003443651
chr3	18997001	19002000	2.36E-07
chr3	18998001	19003000	3.31E-07
chr3	18999001	19004000	4.11E-09
chr3	19000001	19005000	3.80E-09
chr3	19184001	19189000	9.21E-10
chr3	19445001	19450000	0.000790304
chr3	19446001	19451000	0.002927162

chr3	19447001	19452000	0.001823188
chr3	19585001	19590000	0.021764514
chr3	19586001	19591000	0.004437223
chr3	19622001	19627000	1.66E-06
chr3	19623001	19628000	8.69E-07
chr3	19624001	19629000	0.000126185
chr3	19691001	19696000	4.09E-06
chr3	19696001	19701000	0.002195607
chr3	19820001	19825000	8.24E-06
chr3	19821001	19826000	0.000320153
chr3	19822001	19827000	0.000171052
chr3	19882001	19887000	0.000372404
chr3	19888001	19893000	0.004020899
chr3	19889001	19894000	0.000244224
chr3	19891001	19896000	0.005865707
chr3	19984001	19989000	9.73E-20
chr3	19986001	19991000	6.56E-29
chr3	19987001	19992000	1.40E-29
chr3	19988001	19993000	8.02E-34
chr3	19989001	19994000	1.37E-28
chr3	20082001	20087000	1.65E-08
chr3	20282001	20287000	9.44E-05
chr3	20283001	20288000	4.43E-05
chr3	20284001	20289000	3.80E-05
chr3	20285001	20290000	0.000113993
chr3	20286001	20291000	1.93E-05
chr3	20453001	20458000	0.001638265
chr3	20454001	20459000	0.000216936
chr3	20455001	20460000	0.000222731
chr3	20456001	20461000	0.00012224
chr3	20457001	20462000	0.005087604
chr3	20465001	20470000	0.001852063
chr3	20466001	20471000	1.08E-08
chr3	20467001	20472000	6.93E-10
chr3	20470001	20475000	1.10E-14
chr3	20490001	20495000	0.023229813
chr3	20491001	20496000	0.025205648
chr3	20492001	20497000	0.014475107
chr3	20499001	20504000	0.000253355
chr3	20551001	20556000	6.32E-06
chr3	20552001	20557000	1.10E-05
chr3	20553001	20558000	1.79E-05
chr3	20554001	20559000	0.001936644
chr3	20561001	20566000	2.63E-09
chr3	20562001	20567000	2.25E-10
chr3	20563001	20568000	4.33E-08
chr3	20656001	20661000	3.85E-05
chr3	20665001	20670000	0.000181718

chr3	20717001	20722000	0.000568998
chr3	20812001	20817000	1.68E-05
chr3	20813001	20818000	0.000135943
chr3	20814001	20819000	6.33E-05
chr3	20815001	20820000	0.004821716
chr3	20858001	20863000	5.38E-15
chr3	20859001	20864000	6.27E-14
chr3	20884001	20889000	0.017345865
chr3	20930001	20935000	0.005240099
chr3	20931001	20936000	0.007819147
chr3	20955001	20960000	1.12E-09
chr3	20956001	20961000	7.43E-09
chr3	21050001	21055000	5.78E-05
chr3	21051001	21056000	0.010256944
chr3	21059001	21064000	0.000895199
chr3	21140001	21145000	0.004283934
chr3	21141001	21146000	0.00235107
chr3	21153001	21158000	0.001908227
chr3	21154001	21159000	1.10E-05
chr3	21155001	21160000	3.20E-05
chr3	21156001	21161000	0.000135775
chr3	21157001	21162000	2.91E-05
chr3	21210001	21215000	0.027286118
chr3	21222001	21227000	3.44E-05
chr3	21223001	21228000	2.56E-05
chr3	21224001	21229000	0.000726349
chr3	21268001	21273000	0.000204635
chr3	21269001	21274000	0.030949176
chr3	21270001	21275000	0.027055606
chr3	21347001	21352000	2.01E-06
chr3	21348001	21353000	3.48E-07
chr3	21442001	21447000	0.000566006
chr3	21535001	21540000	3.83E-06
chr3	21551001	21556000	1.43E-05
chr3	21552001	21557000	1.23E-09
chr3	21553001	21558000	7.47E-05
chr3	21554001	21559000	0.000119848
chr3	21558001	21563000	8.37E-06
chr3	21575001	21580000	9.07E-07
chr3	21576001	21581000	1.05E-09
chr3	21577001	21582000	1.00E-11
chr3	21578001	21583000	2.64E-10
chr3	21597001	21602000	4.00E-10
chr3	21653001	21658000	5.04E-05
chr3	21656001	21661000	0.000146149
chr3	21766001	21771000	3.51E-08
chr3	21802001	21807000	1.65E-06
chr3	21829001	21834000	8.42E-05

chr3	21895001	21900000	0.000228344
chr3	22195001	22200000	0.000396653
chr3	22205001	22210000	0.002233013
chr3	22206001	22211000	0.003227725
chr3	22247001	22252000	0.002956374
chr3	22248001	22253000	0.003934586
chr3	22409001	22414000	8.65E-15
chr3	22411001	22416000	3.33E-31
chr3	22412001	22417000	9.32E-28
chr3	22413001	22418000	1.52E-28
chr3	22414001	22419000	1.34E-28
chr3	22423001	22428000	0.000301208
chr3	22437001	22442000	8.40E-06
chr3	22498001	22503000	0.000964466
chr3	22499001	22504000	0.00661161
chr3	22500001	22505000	0.02271435
chr3	22582001	22587000	0.001363361
chr3	22637001	22642000	2.28E-05
chr3	22638001	22643000	3.23E-05
chr3	22656001	22661000	1.33E-08
chr3	22657001	22662000	4.34E-08
chr3	22658001	22663000	2.00E-09
chr3	22687001	22692000	0.002961638
chr3	22688001	22693000	0.005091446
chr3	22784001	22789000	2.20E-06
chr3	22869001	22874000	2.22E-08
chr3	23240001	23245000	1.00E-28
chr3	23245001	23250000	6.63E-08
chr3	23391001	23396000	1.58E-12
chr3	23392001	23397000	3.47E-10
chr3	23393001	23398000	3.03E-09
chr3	23843001	23848000	1.91E-09
chr3	23844001	23849000	1.48E-12
chr3	23845001	23850000	1.48E-28
chr3	23846001	23851000	7.06E-27
chr3	23849001	23854000	3.13E-22
chr3	23850001	23855000	2.25E-20
chr3	23852001	23857000	7.67E-16
chr3	23954001	23959000	1.47E-28
chr3	23955001	23960000	1.01E-33
chr3	23983001	23988000	3.31E-11
chr3	23986001	23991000	1.45E-42
chr3	23987001	23992000	6.46E-46
chr3	24398001	24403000	0.005356506
chr3	24531001	24536000	2.69E-37
chr3	24532001	24537000	3.52E-34
chr3	24533001	24538000	3.54E-20
chr3	24534001	24539000	1.28E-22

chr3	24535001	24540000	3.69E-17
chr3	24536001	24541000	4.55E-20
chr3	24537001	24542000	1.61E-13
chr3	24866001	24871000	8.36E-12
chr3	24867001	24872000	1.39E-16
chr3	24868001	24873000	3.60E-22
chr3	24869001	24874000	2.00E-20
chr3	24870001	24875000	2.39E-25
chr3	24871001	24876000	1.52E-31
chr3	25094001	25099000	0.00127795
chr3	25123001	25128000	0.005221025
chr3	25263001	25268000	0.002653979
chr3	25264001	25269000	0.000771703
chr3	25265001	25270000	0.000435954
chr3	25266001	25271000	4.56E-05
chr3	25289001	25294000	5.29E-06
chr3	25328001	25333000	0.001339887
chr3	25407001	25412000	0.014217824
chr3	25704001	25709000	2.11E-32
chr3	25705001	25710000	1.25E-35
chr3	25706001	25711000	7.09E-39
chr3	25932001	25937000	4.10E-06
chr3	25933001	25938000	0.000149546
chr3	25956001	25961000	3.24E-08
chr3	25957001	25962000	2.82E-08
chr3	25958001	25963000	1.15E-07
chr3	25959001	25964000	1.13E-08
chr3	25967001	25972000	8.47E-06
chr3	25968001	25973000	0.000108167
chr3	26024001	26029000	0.008648337
chr3	26038001	26043000	4.53E-10
chr3	26040001	26045000	1.87E-05
chr3	26100001	26105000	0.002111307
chr3	26101001	26106000	0.00014284
chr3	26146001	26151000	0.011108837
chr3	26148001	26153000	0.001064425
chr3	26149001	26154000	0.003200909
chr3	26161001	26166000	0.000509058
chr3	26168001	26173000	1.32E-12
chr3	26176001	26181000	0.000156137
chr3	26177001	26182000	2.73E-05
chr3	26178001	26183000	4.14E-05
chr3	26241001	26246000	0.024973524
chr3	26258001	26263000	1.90E-05
chr3	26259001	26264000	5.29E-18
chr3	26260001	26265000	2.95E-17
chr3	26261001	26266000	2.32E-06
chr3	26475001	26480000	3.28E-06

chr3	26500001	26505000	0.000306898
chr3	26501001	26506000	5.01E-06
chr3	26518001	26523000	0.0002181
chr3	26519001	26524000	0.000386032
chr3	26526001	26531000	0.000846539
chr3	26527001	26532000	0.001491418
chr3	26530001	26535000	0.00175862
chr3	26570001	26575000	1.27E-07
chr3	26611001	26616000	0.000427811
chr3	26612001	26617000	0.000197594
chr3	26614001	26619000	0.000236795
chr3	26628001	26633000	6.02E-05
chr3	26629001	26634000	9.74E-07
chr3	26720001	26725000	5.05E-06
chr3	26721001	26726000	8.04E-09
chr3	26722001	26727000	6.62E-07
chr3	26723001	26728000	1.93E-07
chr3	26753001	26758000	0.010829597
chr3	26755001	26760000	0.006162217
chr3	26756001	26761000	0.000251191
chr3	26757001	26762000	0.000728453
chr3	26758001	26763000	0.000668449
chr3	26759001	26764000	0.000228052
chr3	26822001	26827000	2.57E-07
chr3	26823001	26828000	5.16E-10
chr3	26824001	26829000	2.32E-12
chr3	26825001	26830000	2.78E-11
chr3	26826001	26831000	0.0001591
chr3	26831001	26836000	5.30E-06
chr3	26832001	26837000	0.000150716
chr3	26835001	26840000	0.000174869
chr3	26893001	26898000	0.002011134
chr3	26894001	26899000	0.000201992
chr3	26918001	26923000	1.08E-05
chr3	27001001	27006000	2.55E-05
chr3	27010001	27015000	0.000127346
chr3	27011001	27016000	0.000489907
chr3	27012001	27017000	0.000572625
chr3	27033001	27038000	2.25E-12
chr3	27034001	27039000	3.23E-09
chr3	27048001	27053000	3.65E-07
chr3	27049001	27054000	1.95E-08
chr3	27050001	27055000	2.89E-05
chr3	27081001	27086000	0.000143684
chr3	27223001	27228000	0.019630629
chr3	27269001	27274000	2.86E-05
chr3	27406001	27411000	8.84E-18
chr3	27407001	27412000	5.07E-25

chr3	27408001	27413000	3.79E-31
chr3	27409001	27414000	2.02E-24
chr3	27410001	27415000	1.52E-11
chr3	27521001	27526000	1.06E-25
chr3	27522001	27527000	2.96E-22
chr3	27523001	27528000	6.39E-16
chr3	27670001	27675000	1.02E-28
chr3	27671001	27676000	6.85E-18
chr3	27672001	27677000	1.21E-19
chr3	27673001	27678000	1.43E-15
chr3	27674001	27679000	7.20E-18
chr3	27749001	27754000	2.74E-09
chr3	27759001	27764000	2.62E-09
chr3	27899001	27904000	0.000556379
chr3	27900001	27905000	1.08E-05
chr3	27901001	27906000	3.62E-06
chr3	27902001	27907000	1.74E-08
chr3	27903001	27908000	4.40E-06
chr3	27904001	27909000	4.10E-05
chr3	27908001	27913000	0.002045785
chr3	27974001	27979000	0.000845574
chr3	28001001	28006000	0.002358363
chr3	28028001	28033000	0.012073457
chr3	28033001	28038000	0.003368296
chr3	28051001	28056000	0.000753368
chr3	28070001	28075000	0.000441659
chr3	28074001	28079000	0.006404273
chr3	28128001	28133000	0.010022285
chr3	28147001	28152000	0.000940108
chr3	28278001	28283000	1.29E-13
chr3	28279001	28284000	9.56E-28
chr3	28280001	28285000	2.21E-26
chr3	28281001	28286000	2.53E-24
chr3	28283001	28288000	1.22E-18
chr3	28385001	28390000	0.000599102
chr3	28389001	28394000	2.70E-39
chr3	28390001	28395000	3.94E-41
chr3	28480001	28485000	0.000107875
chr3	28481001	28486000	8.92E-05
chr3	28574001	28579000	0.000108438
chr3	28575001	28580000	8.59E-05
chr3	28605001	28610000	0.000464572
chr3	28606001	28611000	2.03E-06
chr3	28680001	28685000	0.000488446
chr3	28681001	28686000	0.00034581
chr3	28700001	28705000	0.034070449
chr3	28732001	28737000	0.000193367
chr3	28733001	28738000	0.000174647

chr3	28741001	28746000	0.000255598
chr3	28742001	28747000	0.000109365
chr3	28769001	28774000	0.011490626
chr3	28869001	28874000	0.027192494
chr3	28870001	28875000	0.001095241
chr3	28914001	28919000	0.000761329
chr3	28973001	28978000	8.35E-05
chr3	29009001	29014000	0.003822002
chr3	29078001	29083000	1.87E-05
chr3	29207001	29212000	8.84E-06
chr3	29326001	29331000	7.73E-05
chr3	29327001	29332000	9.84E-05
chr3	29673001	29678000	4.47E-05
chr3	29674001	29679000	0.000498697
chr3	29675001	29680000	0.000778319
chr3	29676001	29681000	0.000128621
chr3	29677001	29682000	1.06E-08
chr3	29828001	29833000	0.000939456
chr3	29902001	29907000	5.12E-05
chr3	30406001	30411000	0.000125744
chr3	30598001	30603000	0.001359598
chr3	30654001	30659000	0.004889803
chr3	30856001	30861000	0.000125425
chr3	30857001	30862000	1.51E-05
chr3	30908001	30913000	0.000449737
chr3	30932001	30937000	1.99E-30
chr3	30933001	30938000	2.53E-31
chr3	30934001	30939000	1.02E-30
chr3	30935001	30940000	2.31E-29
chr3	30936001	30941000	2.98E-24
chr3	31131001	31136000	2.29E-06
chr3	31569001	31574000	2.04E-22
chr3	32017001	32022000	9.01E-05
chr3	32018001	32023000	6.96E-23
chr3	32019001	32024000	1.80E-11
chr3	32020001	32025000	1.20E-10
chr3	32021001	32026000	5.90E-10
chr3	32022001	32027000	2.72E-23
chr3	32023001	32028000	1.79E-15
chr3	32275001	32280000	2.32E-10
chr3	32276001	32281000	8.00E-12
chr3	32277001	32282000	1.49E-10
chr3	32279001	32284000	8.60E-16
chr3	32280001	32285000	1.25E-08
chr3	32439001	32444000	7.47E-14
chr3	32440001	32445000	4.40E-05
chr3	32459001	32464000	4.43E-10
chr3	32515001	32520000	0.000504368

chr3	32540001	32545000	8.94E-24
chr3	32541001	32546000	1.76E-12
chr3	32544001	32549000	3.58E-14
chr3	32612001	32617000	4.00E-15
chr3	32722001	32727000	4.81E-14
chr3	32725001	32730000	5.44E-26
chr3	32819001	32824000	9.62E-08
chr3	33151001	33156000	2.03E-47
chr3	33256001	33261000	1.02E-42
chr3	33257001	33262000	2.56E-47
chr3	33258001	33263000	2.83E-29
chr3	33260001	33265000	1.13E-12
chr3	33314001	33319000	1.33E-16
chr3	33315001	33320000	7.00E-18
chr3	33316001	33321000	1.25E-14
chr3	33341001	33346000	0.004757157
chr3	33464001	33469000	1.22E-10
chr3	33803001	33808000	1.47E-05
chr3	33836001	33841000	1.22E-20
chr3	33837001	33842000	3.26E-21
chr3	33838001	33843000	2.51E-23
chr3	33839001	33844000	6.72E-22
chr3	33840001	33845000	1.31E-28
chr3	34179001	34184000	0.000653501
chr3	34211001	34216000	1.25E-06
chr3	34212001	34217000	1.89E-06
chr3	34213001	34218000	1.13E-06
chr3	34214001	34219000	0.000541954
chr3	34306001	34311000	0.013459157
chr3	34372001	34377000	1.52E-06
chr3	34385001	34390000	2.21E-05
chr3	34386001	34391000	1.93E-05
chr3	34412001	34417000	0.006601889
chr3	34468001	34473000	3.78E-12
chr3	34469001	34474000	4.86E-12
chr3	34470001	34475000	1.31E-11
chr3	34492001	34497000	3.21E-05
chr3	34493001	34498000	1.82E-08
chr3	34494001	34499000	1.26E-05
chr3	34495001	34500000	9.61E-08
chr3	34496001	34501000	1.50E-07
chr3	34499001	34504000	0.000663697
chr3	34505001	34510000	4.78E-08
chr3	34506001	34511000	1.26E-06
chr3	34508001	34513000	1.86E-09
chr3	34509001	34514000	4.75E-07
chr3	34540001	34545000	0.000268481
chr3	34544001	34549000	0.000473117

chr3	34545001	34550000	0.001258061
chr3	34627001	34632000	0.000505461
chr3	34628001	34633000	0.000176874
chr3	34629001	34634000	9.90E-07
chr3	34630001	34635000	0.00093231
chr3	34731001	34736000	1.21E-10
chr3	34740001	34745000	0.001366546
chr3	34741001	34746000	0.001366546
chr3	34750001	34755000	1.57E-07
chr3	34788001	34793000	0.010740014
chr3	34789001	34794000	0.002008744
chr3	34790001	34795000	0.000300551
chr3	34791001	34796000	0.00105999
chr3	34792001	34797000	0.000155335
chr3	34808001	34813000	6.04E-05
chr3	34809001	34814000	5.95E-05
chr3	34844001	34849000	0.009311486
chr3	34870001	34875000	0.003176668
chr3	34871001	34876000	0.002038882
chr3	34872001	34877000	9.17E-06
chr3	34893001	34898000	0.001246959
chr3	34914001	34919000	0.014844369
chr3	35003001	35008000	0.014417247
chr3	35004001	35009000	0.027433551
chr3	35083001	35088000	0.008056839
chr3	35084001	35089000	0.008056839
chr3	35085001	35090000	0.005389673
chr3	35334001	35339000	0.000256841
chr3	35365001	35370000	0.019445838
chr3	35428001	35433000	0.000321466
chr3	35436001	35441000	0.012759249
chr3	35480001	35485000	1.52E-09
chr3	35499001	35504000	2.14E-05
chr3	35500001	35505000	0.000574727
chr3	35537001	35542000	0.006155757
chr3	35559001	35564000	0.021009212
chr3	35614001	35619000	0.000451082
chr3	35615001	35620000	0.00015883
chr3	35616001	35621000	0.000948184
chr3	35617001	35622000	2.50E-05
chr3	35625001	35630000	0.000235568
chr3	35626001	35631000	0.000308121
chr3	35627001	35632000	0.015612742
chr3	35628001	35633000	0.018469734
chr3	35896001	35901000	2.54E-13
chr3	35897001	35902000	2.36E-09
chr3	36138001	36143000	0.003036656
chr3	36139001	36144000	0.016180425

chr3	36194001	36199000	0.000679233
chr3	36195001	36200000	0.000938019
chr3	36196001	36201000	0.000747079
chr3	36207001	36212000	3.19E-05
chr3	36208001	36213000	1.37E-06
chr3	36209001	36214000	0.023748726
chr3	36264001	36269000	9.24E-05
chr3	36417001	36422000	1.79E-10
chr3	36418001	36423000	7.38E-32
chr3	36419001	36424000	9.62E-26
chr3	36420001	36425000	1.96E-21
chr3	36421001	36426000	7.62E-19
chr3	36422001	36427000	7.95E-17
chr3	36436001	36441000	0.019788225
chr3	36601001	36606000	0.001530269
chr3	36625001	36630000	0.014696885
chr3	36626001	36631000	0.018385359
chr3	36837001	36842000	1.46E-07
chr3	36838001	36843000	8.77E-09
chr3	36839001	36844000	3.02E-11
chr3	36982001	36987000	1.70E-14
chr3	36983001	36988000	3.43E-24
chr3	36984001	36989000	3.27E-25
chr3	36985001	36990000	6.41E-27
chr3	36986001	36991000	9.25E-33
chr3	37035001	37040000	1.47E-12
chr3	37217001	37222000	2.55E-18
chr3	37280001	37285000	1.55E-44
chr3	37281001	37286000	1.99E-33
chr3	37282001	37287000	2.13E-27
chr3	37284001	37289000	8.28E-16
chr3	37285001	37290000	2.47E-10
chr3	37626001	37631000	2.62E-06
chr3	37627001	37632000	1.03E-07
chr3	37628001	37633000	5.00E-08
chr3	37652001	37657000	3.33E-11
chr3	37653001	37658000	7.91E-14
chr3	37654001	37659000	3.45E-13
chr3	37693001	37698000	1.95E-06
chr3	37720001	37725000	6.69E-11
chr3	37721001	37726000	2.69E-07
chr3	37722001	37727000	1.16E-10
chr3	37723001	37728000	5.23E-11
chr3	37724001	37729000	6.78E-13
chr3	37725001	37730000	3.13E-09
chr3	37726001	37731000	3.62E-08
chr3	37727001	37732000	9.75E-06
chr3	37728001	37733000	3.85E-05

chr3	37792001	37797000	9.62E-05
chr3	37793001	37798000	2.89E-05
chr3	37794001	37799000	0.000101524
chr3	37898001	37903000	4.39E-13
chr3	37899001	37904000	4.12E-10
chr3	38062001	38067000	5.14E-09
chr3	38066001	38071000	5.95E-06
chr3	38067001	38072000	4.33E-05
chr3	38068001	38073000	4.34E-07
chr3	38076001	38081000	3.04E-09
chr3	38174001	38179000	4.15E-27
chr3	38202001	38207000	2.93E-42
chr3	38207001	38212000	9.01E-29
chr3	38385001	38390000	1.25E-18
chr3	38386001	38391000	8.67E-24
chr3	38387001	38392000	1.22E-32
chr3	38388001	38393000	1.96E-39
chr3	38491001	38496000	2.47E-09
chr3	38492001	38497000	1.50E-10
chr3	38496001	38501000	2.95E-07
chr3	38533001	38538000	1.81E-22
chr3	38699001	38704000	0.004006299
chr3	38709001	38714000	8.57E-05
chr3	38710001	38715000	6.17E-06
chr3	38711001	38716000	8.74E-06
chr3	38712001	38717000	5.18E-06
chr3	38713001	38718000	2.68E-08
chr3	38714001	38719000	1.37E-08
chr3	38715001	38720000	9.93E-07
chr3	38716001	38721000	3.38E-08
chr3	38717001	38722000	6.75E-07
chr3	38893001	38898000	3.28E-07
chr3	38899001	38904000	1.17E-05
chr3	38900001	38905000	0.000183988
chr3	38907001	38912000	0.000146079
chr3	38924001	38929000	5.18E-06
chr3	38925001	38930000	2.76E-06
chr3	39089001	39094000	5.13E-15
chr3	39144001	39149000	1.12E-19
chr3	39145001	39150000	2.15E-26
chr3	39146001	39151000	8.01E-34
chr3	39147001	39152000	2.02E-37
chr3	39148001	39153000	1.30E-14
chr3	39149001	39154000	3.78E-08
chr3	39195001	39200000	1.26E-08
chr3	39218001	39223000	2.74E-23
chr3	39219001	39224000	1.10E-28
chr3	39220001	39225000	5.47E-21

chr3	39221001	39226000	2.09E-13
chr3	39222001	39227000	1.35E-08
chr3	39448001	39453000	3.56E-08
chr3	39507001	39512000	5.25E-06
chr3	39529001	39534000	6.17E-05
chr3	39560001	39565000	0.000953409
chr3	39604001	39609000	0.001474817
chr3	39605001	39610000	2.15E-05
chr3	39630001	39635000	0.001300245
chr3	39716001	39721000	0.00032739
chr3	39717001	39722000	4.39E-06
chr3	39718001	39723000	3.48E-06
chr3	39756001	39761000	0.001342148
chr3	39782001	39787000	7.89E-06
chr3	39790001	39795000	0.002055344
chr3	39903001	39908000	0.004449455
chr3	39904001	39909000	0.007259129
chr3	39905001	39910000	0.001193089
chr3	39985001	39990000	1.82E-05
chr3	39986001	39991000	0.000947624
chr3	39987001	39992000	0.000947624
chr3	39988001	39993000	5.75E-05
chr3	39989001	39994000	4.54E-05
chr3	40042001	40047000	0.009611025
chr3	40075001	40080000	0.000402193
chr3	40209001	40214000	1.36E-05
chr3	40210001	40215000	0.000108392
chr3	40311001	40316000	1.30E-06
chr3	40346001	40351000	1.32E-20
chr3	40347001	40352000	9.69E-26
chr3	40348001	40353000	1.24E-28
chr3	40349001	40354000	7.28E-28
chr3	40350001	40355000	2.32E-27
chr3	40351001	40356000	1.53E-22
chr3	40403001	40408000	0.001756239
chr3	40424001	40429000	5.36E-05
chr3	40426001	40431000	0.001599684
chr3	40490001	40495000	1.12E-32
chr3	40491001	40496000	6.29E-18
chr3	40492001	40497000	3.81E-19
chr3	40493001	40498000	8.36E-19
chr3	40494001	40499000	3.83E-17
chr3	40495001	40500000	1.80E-11
chr3	40496001	40501000	7.74E-19
chr3	40497001	40502000	8.28E-18
chr3	40498001	40503000	1.00E-13
chr3	40499001	40504000	8.07E-09
chr3	40516001	40521000	2.31E-12

chr3	40517001	40522000	1.89E-11
chr3	40518001	40523000	3.32E-14
chr3	40544001	40549000	1.61E-13
chr3	40545001	40550000	5.64E-20
chr3	40546001	40551000	3.84E-16
chr3	40547001	40552000	1.44E-17
chr3	40562001	40567000	8.68E-19
chr3	40563001	40568000	8.47E-26
chr3	40564001	40569000	8.56E-18
chr3	40565001	40570000	4.60E-22
chr3	40566001	40571000	1.35E-18
chr3	40636001	40641000	1.62E-05
chr3	40897001	40902000	1.31E-06
chr3	40898001	40903000	1.19E-05
chr3	41225001	41230000	0.00380809
chr3	41236001	41241000	1.16E-51
chr3	41237001	41242000	3.05E-48
chr3	41259001	41264000	0.00440977
chr3	41260001	41265000	0.002175233
chr3	41261001	41266000	0.006104105
chr3	41578001	41583000	0.000164998
chr3	41808001	41813000	0.000354983
chr3	41999001	42004000	5.37E-19
chr3	42000001	42005000	4.81E-16
chr3	42001001	42006000	3.78E-16
chr3	42003001	42008000	3.60E-08
chr3	42050001	42055000	4.74E-08
chr3	42051001	42056000	1.55E-10
chr3	42053001	42058000	5.07E-10
chr3	42054001	42059000	4.00E-19
chr3	42055001	42060000	5.96E-17
chr3	42503001	42508000	0.001393799
chr3	42619001	42624000	1.46E-32
chr3	42620001	42625000	3.03E-23
chr3	42621001	42626000	4.55E-13
chr3	42622001	42627000	3.51E-22
chr3	42623001	42628000	7.74E-23
chr3	42630001	42635000	3.77E-19
chr3	42631001	42636000	2.01E-26
chr3	42632001	42637000	8.07E-29
chr3	42637001	42642000	2.72E-15
chr3	42638001	42643000	3.49E-37
chr3	42639001	42644000	3.42E-41
chr3	42640001	42645000	6.91E-43
chr3	42642001	42647000	8.28E-23
chr3	42810001	42815000	1.14E-13
chr3	42813001	42818000	6.44E-12
chr3	42814001	42819000	7.29E-15

chr3	42841001	42846000	3.20E-15
chr3	42842001	42847000	3.72E-18
chr3	42843001	42848000	2.31E-29
chr3	42844001	42849000	6.16E-39
chr3	42845001	42850000	1.22E-39
chr3	42846001	42851000	1.72E-25
chr3	42895001	42900000	6.43E-08
chr3	42896001	42901000	3.89E-09
chr3	42918001	42923000	2.36E-13
chr3	42919001	42924000	1.21E-11
chr3	42920001	42925000	1.10E-11
chr3	42922001	42927000	1.90E-12
chr3	42942001	42947000	6.38E-11
chr3	42943001	42948000	2.62E-22
chr3	42944001	42949000	8.04E-16
chr3	42945001	42950000	2.99E-13
chr3	42946001	42951000	8.80E-17
chr3	43189001	43194000	1.92E-05
chr3	43558001	43563000	4.05E-05
chr3	43712001	43717000	0.001331123
chr3	43728001	43733000	3.73E-26
chr3	43875001	43880000	0.004686202
chr3	44032001	44037000	7.15E-23
chr3	44033001	44038000	5.28E-22
chr3	44034001	44039000	1.30E-19
chr3	44191001	44196000	6.33E-06
chr3	44316001	44321000	0.005380755
chr3	44359001	44364000	0.00026105
chr3	44360001	44365000	2.03E-05
chr3	44624001	44629000	1.02E-09
chr3	44662001	44667000	2.29E-12
chr3	44663001	44668000	1.94E-11
chr3	44665001	44670000	5.64E-16
chr3	44666001	44671000	4.97E-10
chr3	44686001	44691000	4.57E-24
chr3	44687001	44692000	7.02E-25
chr3	44688001	44693000	4.88E-25
chr3	44689001	44694000	1.17E-18
chr3	44690001	44695000	1.34E-20
chr3	44732001	44737000	3.15E-07
chr3	44733001	44738000	9.71E-10
chr3	44734001	44739000	8.63E-10
chr3	44750001	44755000	6.62E-12
chr3	44899001	44904000	8.05E-14
chr3	44900001	44905000	1.64E-12
chr3	44901001	44906000	4.81E-17
chr3	44902001	44907000	4.40E-16
chr3	44903001	44908000	1.14E-09

chr3	45013001	45018000	2.96E-26
chr3	45014001	45019000	1.44E-33
chr3	45017001	45022000	4.85E-25
chr3	45018001	45023000	4.35E-14
chr3	45262001	45267000	1.88E-21
chr3	45263001	45268000	6.88E-41
chr3	45264001	45269000	8.79E-23
chr3	45265001	45270000	4.35E-27
chr3	45266001	45271000	1.53E-16
chr3	45267001	45272000	2.03E-18
chr3	45425001	45430000	1.17E-21
chr3	45426001	45431000	4.05E-29
chr3	45427001	45432000	4.99E-19
chr3	45428001	45433000	9.59E-12
chr3	45631001	45636000	5.53E-06
chr3	45731001	45736000	3.10E-17
chr3	45834001	45839000	1.40E-10
chr3	45835001	45840000	5.29E-12
chr3	45836001	45841000	2.59E-13
chr3	45837001	45842000	8.70E-15
chr3	45879001	45884000	1.06E-19
chr3	45880001	45885000	3.69E-21
chr3	45881001	45886000	2.65E-26
chr3	45882001	45887000	7.05E-22
chr3	45883001	45888000	1.84E-19
chr3	46032001	46037000	1.54E-06
chr3	46037001	46042000	1.90E-10
chr3	46140001	46145000	0.002388761
chr3	46225001	46230000	0.000180679
chr3	46264001	46269000	1.96E-05
chr3	46267001	46272000	0.001160051
chr3	46276001	46281000	3.54E-06
chr3	46294001	46299000	0.013698003
chr3	46295001	46300000	0.000652574
chr3	46296001	46301000	0.002802984
chr3	46327001	46332000	0.000979015
chr3	46328001	46333000	5.44E-06
chr3	46330001	46335000	1.55E-07
chr3	46375001	46380000	0.000281459
chr3	46606001	46611000	1.66E-08
chr3	46607001	46612000	3.95E-08
chr3	46730001	46735000	1.35E-09
chr3	46731001	46736000	5.43E-10
chr3	46732001	46737000	9.52E-11
chr3	46733001	46738000	0.000680366
chr3	46742001	46747000	0.003283706
chr3	46883001	46888000	5.31E-08
chr3	46920001	46925000	2.23E-05

chr3	47133001	47138000	5.14E-05
chr3	47200001	47205000	2.81E-09
chr3	47201001	47206000	6.68E-22
chr3	47248001	47253000	8.97E-06
chr3	47249001	47254000	1.85E-05
chr3	47251001	47256000	0.000147884
chr3	47252001	47257000	0.000691168
chr3	47319001	47324000	6.21E-23
chr3	47320001	47325000	3.24E-37
chr3	47321001	47326000	4.15E-31
chr3	47322001	47327000	9.35E-26
chr3	47323001	47328000	8.03E-25
chr3	47324001	47329000	5.48E-25
chr3	47420001	47425000	5.43E-11
chr3	47421001	47426000	1.25E-09
chr3	47422001	47427000	4.94E-09
chr3	47512001	47517000	1.16E-05
chr3	47513001	47518000	1.87E-07
chr3	47514001	47519000	3.10E-07
chr3	47515001	47520000	4.95E-20
chr3	47516001	47521000	8.40E-18
chr3	47517001	47522000	5.96E-20
chr3	47550001	47555000	6.12E-06
chr3	47551001	47556000	1.11E-08
chr3	47616001	47621000	1.06E-11
chr3	47617001	47622000	1.38E-12
chr3	47618001	47623000	1.84E-17
chr3	47620001	47625000	1.13E-14
chr3	47844001	47849000	2.17E-08
chr3	47862001	47867000	7.67E-09
chr3	47863001	47868000	2.69E-05
chr3	48126001	48131000	1.07E-06
chr3	48129001	48134000	0.000627803
chr3	48130001	48135000	0.000376714
chr3	48226001	48231000	1.84E-15
chr3	48228001	48233000	6.01E-09
chr3	48229001	48234000	9.04E-08
chr3	48281001	48286000	2.81E-11
chr3	48282001	48287000	6.73E-14
chr3	48384001	48389000	5.05E-06
chr3	48385001	48390000	1.57E-05
chr3	48386001	48391000	0.001570368
chr3	48477001	48482000	8.96E-14
chr3	48483001	48488000	5.12E-12
chr3	48484001	48489000	3.53E-32
chr3	48485001	48490000	6.32E-25
chr3	48486001	48491000	9.29E-23
chr3	48487001	48492000	7.92E-24

chr3	48488001	48493000	2.50E-09
chr3	48590001	48595000	0.001138505
chr3	48645001	48650000	7.03E-20
chr3	48671001	48676000	1.97E-13
chr3	48672001	48677000	1.20E-08
chr3	48701001	48706000	1.08E-13
chr3	48722001	48727000	6.31E-10
chr3	48723001	48728000	2.99E-14
chr3	48932001	48937000	1.39E-16
chr3	48933001	48938000	3.79E-07
chr3	49023001	49028000	4.23E-08
chr3	49024001	49029000	1.48E-09
chr3	49025001	49030000	9.59E-10
chr3	49027001	49032000	2.60E-05
chr3	49043001	49048000	5.39E-12
chr3	49044001	49049000	1.70E-12
chr3	49067001	49072000	6.74E-05
chr3	49138001	49143000	1.28E-07
chr3	49139001	49144000	0.00201339
chr3	49140001	49145000	0.000307683
chr3	49393001	49398000	3.19E-19
chr3	49394001	49399000	8.57E-24
chr3	49395001	49400000	1.42E-24
chr3	49396001	49401000	8.61E-08
chr3	49573001	49578000	1.15E-09
chr3	49574001	49579000	1.90E-09
chr3	49941001	49946000	0.000629668
chr3	50123001	50128000	1.02E-05
chr3	50124001	50129000	1.04E-06
chr3	50126001	50131000	8.92E-25
chr3	50127001	50132000	1.83E-09
chr3	50188001	50193000	2.18E-26
chr3	50189001	50194000	1.33E-22
chr3	50190001	50195000	9.39E-19
chr3	50327001	50332000	4.48E-05
chr3	50328001	50333000	8.75E-07
chr3	50329001	50334000	6.13E-07
chr3	50361001	50366000	9.19E-06
chr3	50364001	50369000	4.01E-10
chr3	50365001	50370000	7.21E-06
chr3	50395001	50400000	4.29E-09
chr3	50396001	50401000	9.14E-17
chr3	50601001	50606000	2.14E-09
chr3	50602001	50607000	1.40E-15
chr3	50711001	50716000	2.24E-15
chr3	50712001	50717000	2.54E-22
chr3	50803001	50808000	1.41E-06
chr3	50877001	50882000	0.01017596

chr3	51014001	51019000	6.68E-19
chr3	51184001	51189000	5.64E-06
chr3	51218001	51223000	0.01145541
chr3	51418001	51423000	0.004645673
chr3	51422001	51427000	1.44E-06
chr3	51424001	51429000	6.81E-07
chr3	51573001	51578000	4.59E-07
chr3	51815001	51820000	0.003494144
chr3	51998001	52003000	0.000269002
chr3	51999001	52004000	4.48E-07
chr3	52000001	52005000	1.57E-09
chr3	52001001	52006000	1.30E-05
chr3	52025001	52030000	2.58E-10
chr3	52027001	52032000	2.66E-23
chr3	52028001	52033000	2.28E-16
chr3	52029001	52034000	7.06E-16
chr3	52184001	52189000	1.72E-14
chr3	52185001	52190000	8.97E-15
chr3	52188001	52193000	6.11E-19
chr3	52231001	52236000	4.39E-11
chr3	52232001	52237000	1.85E-15
chr3	52273001	52278000	1.76E-05
chr3	52318001	52323000	5.42E-10
chr3	52319001	52324000	6.76E-12
chr3	52443001	52448000	4.74E-25
chr3	52444001	52449000	3.26E-27
chr3	52488001	52493000	4.13E-13
chr3	52489001	52494000	1.22E-11
chr3	52570001	52575000	3.18E-12
chr3	52720001	52725000	1.15E-09
chr3	52929001	52934000	9.67E-10
chr3	52930001	52935000	1.16E-12
chr3	52931001	52936000	3.29E-11
chr3	53074001	53079000	1.05E-06
chr3	53075001	53080000	6.40E-31
chr3	53076001	53081000	2.00E-24
chr3	53077001	53082000	1.77E-22
chr3	53078001	53083000	9.22E-14
chr3	53079001	53084000	4.69E-15
chr3	53080001	53085000	1.63E-06
chr3	53377001	53382000	2.36E-49
chr3	53378001	53383000	1.86E-30
chr3	53379001	53384000	3.81E-38
chr3	53380001	53385000	1.22E-35
chr3	53381001	53386000	1.54E-43
chr3	53488001	53493000	0.00033303
chr3	53489001	53494000	0.000309876
chr3	53665001	53670000	1.44E-08

chr3	53666001	53671000	7.00E-10
chr3	53667001	53672000	4.29E-08
chr3	53668001	53673000	1.24E-08
chr3	53877001	53882000	3.86E-08
chr3	53878001	53883000	2.90E-13
chr3	53879001	53884000	2.28E-17
chr3	53880001	53885000	7.30E-17
chr3	53911001	53916000	5.08E-18
chr3	53912001	53917000	1.30E-47
chr3	53913001	53918000	8.07E-37
chr3	53914001	53919000	5.77E-47
chr3	53915001	53920000	2.20E-49
chr3	53916001	53921000	2.23E-49
chr3	54005001	54010000	2.21E-05
chr3	54268001	54273000	5.33E-05
chr3	54269001	54274000	4.35E-05
chr3	54373001	54378000	2.19E-08
chr3	54374001	54379000	9.91E-07
chr3	54414001	54419000	1.39E-10
chr3	54420001	54425000	0.000168506
chr3	54467001	54472000	0.000138239
chr3	54468001	54473000	0.00015049
chr3	54539001	54544000	0.000107299
chr3	54581001	54586000	8.42E-05
chr3	54582001	54587000	2.16E-06
chr3	54599001	54604000	7.89E-05
chr3	54622001	54627000	0.000205229
chr3	54625001	54630000	1.14E-06
chr3	54705001	54710000	1.11E-05
chr3	54752001	54757000	1.68E-07
chr3	54794001	54799000	1.84E-06
chr3	54795001	54800000	6.62E-06
chr3	54796001	54801000	8.39E-07
chr3	54798001	54803000	8.10E-05
chr3	54799001	54804000	6.43E-05
chr3	54800001	54805000	8.29E-07
chr3	54801001	54806000	4.59E-07
chr3	54802001	54807000	2.05E-06
chr3	54820001	54825000	0.000112654
chr3	54821001	54826000	0.001324737
chr3	54844001	54849000	1.20E-06
chr3	54847001	54852000	7.97E-07
chr3	55130001	55135000	0.001261869
chr3	55131001	55136000	0.001616214
chr3	55245001	55250000	0.000710984
chr3	55247001	55252000	0.01361875
chr3	55248001	55253000	0.017119495
chr3	55456001	55461000	0.018121752

chr3	55523001	55528000	1.09E-15
chr3	55524001	55529000	1.55E-14
chr3	55791001	55796000	0.001790783
chr3	56119001	56124000	0.007746396
chr3	56120001	56125000	0.002113876
chr3	56121001	56126000	0.000731691
chr3	56122001	56127000	0.000164383
chr3	56123001	56128000	0.001511166
chr3	56189001	56194000	0.005880792
chr3	56190001	56195000	0.000482997
chr3	56243001	56248000	4.44E-07
chr3	56261001	56266000	0.001363361
chr3	56347001	56352000	0.005833587
chr3	56411001	56416000	9.74E-05
chr3	56412001	56417000	1.03E-05
chr3	56462001	56467000	2.66E-06
chr3	56463001	56468000	7.38E-07
chr3	56464001	56469000	1.18E-06
chr3	56502001	56507000	4.97E-08
chr3	56507001	56512000	0.001509917
chr3	56509001	56514000	0.000614694
chr3	56557001	56562000	0.000961354
chr3	56558001	56563000	0.000106843
chr3	56559001	56564000	0.000879602
chr3	56590001	56595000	6.16E-24
chr3	56591001	56596000	9.98E-21
chr3	56610001	56615000	7.46E-06
chr3	56712001	56717000	1.97E-19
chr3	56713001	56718000	1.38E-05
chr3	56717001	56722000	1.45E-09
chr3	56870001	56875000	0.000463175
chr3	56905001	56910000	1.55E-05
chr3	56906001	56911000	9.66E-06
chr3	56908001	56913000	1.25E-05
chr3	56909001	56914000	3.81E-05
chr3	56924001	56929000	3.25E-07
chr3	57108001	57113000	2.41E-19
chr3	57109001	57114000	2.34E-13
chr3	57110001	57115000	1.22E-14
chr3	57112001	57117000	9.13E-13
chr3	57257001	57262000	1.37E-25
chr3	57258001	57263000	6.76E-29
chr3	57259001	57264000	7.92E-16
chr3	57261001	57266000	0.000104924
chr3	57378001	57383000	0.005688628
chr3	57537001	57542000	9.36E-19
chr3	57538001	57543000	3.38E-22
chr3	57539001	57544000	9.83E-18

chr3	57542001	57547000	2.37E-14
chr3	57674001	57679000	1.48E-09
chr3	57737001	57742000	5.56E-11
chr3	57739001	57744000	1.57E-25
chr3	57740001	57745000	4.55E-33
chr3	57741001	57746000	1.70E-24
chr3	57742001	57747000	6.18E-30
chr3	57832001	57837000	2.08E-08
chr3	57991001	57996000	2.08E-06
chr3	57994001	57999000	0.000102452
chr3	57995001	58000000	0.013701744
chr3	58219001	58224000	1.62E-22
chr3	58220001	58225000	9.37E-30
chr3	58221001	58226000	3.29E-31
chr3	58222001	58227000	4.40E-23
chr3	58223001	58228000	7.51E-24
chr3	58315001	58320000	0.000121463
chr3	58415001	58420000	2.78E-20
chr3	58416001	58421000	1.27E-09
chr3	58418001	58423000	2.73E-14
chr3	58419001	58424000	1.32E-19
chr3	58478001	58483000	1.07E-17
chr3	58890001	58895000	7.57E-05
chr3	59031001	59036000	6.14E-37
chr3	59212001	59217000	0.000568088
chr3	59213001	59218000	0.00251513
chr3	59332001	59337000	3.11E-08
chr3	59395001	59400000	0.000232335
chr3	59397001	59402000	3.50E-06
chr3	59529001	59534000	8.33E-05
chr3	59602001	59607000	6.54E-12
chr3	59603001	59608000	7.97E-14
chr3	59713001	59718000	2.30E-07
chr3	59869001	59874000	0.003711281
chr3	59870001	59875000	0.0032713
chr3	60123001	60128000	0.000241152
chr3	60198001	60203000	1.84E-05
chr3	60199001	60204000	1.19E-05
chr3	60200001	60205000	7.06E-06
chr3	60289001	60294000	0.010433091
chr3	60400001	60405000	0.005217352
chr3	60448001	60453000	7.96E-05
chr3	60598001	60603000	0.000453278
chr3	60599001	60604000	3.53E-05
chr3	60644001	60649000	0.000555489
chr3	60645001	60650000	0.001622846
chr3	60646001	60651000	4.18E-05
chr3	60729001	60734000	0.000101943

chr3	60870001	60875000	0.003771551
chr3	61030001	61035000	2.07E-08
chr3	61257001	61262000	0.001902224
chr3	61305001	61310000	0.002474824
chr3	61306001	61311000	0.008785227
chr3	61307001	61312000	0.004391287
chr3	61398001	61403000	0.00107421
chr3	61447001	61452000	0.000251311
chr3	62300001	62305000	8.82E-17
chr3	62301001	62306000	2.59E-20
chr3	62302001	62307000	9.62E-13
chr3	62303001	62308000	1.86E-15
chr3	62304001	62309000	7.03E-15
chr3	62305001	62310000	1.05E-14
chr3	62502001	62507000	0.002153539
chr3	62503001	62508000	1.14E-06
chr3	62504001	62509000	1.96E-11
chr3	62805001	62810000	0.000260566
chr3	62832001	62837000	1.70E-05
chr3	62834001	62839000	4.77E-07
chr3	62889001	62894000	0.000338138
chr3	62896001	62901000	0.00060492
chr3	62898001	62903000	1.12E-05
chr3	62899001	62904000	0.000161653
chr3	62900001	62905000	7.55E-06
chr3	62901001	62906000	0.000574518
chr3	62909001	62914000	0.000697669
chr3	62910001	62915000	0.000422149
chr3	62911001	62916000	7.00E-06
chr3	62987001	62992000	6.16E-05
chr3	63027001	63032000	2.20E-06
chr3	63028001	63033000	1.94E-06
chr3	63216001	63221000	0.000722727
chr3	63217001	63222000	0.000721166
chr3	63218001	63223000	0.000544326
chr3	63219001	63224000	1.90E-05
chr3	63238001	63243000	4.82E-09
chr3	63239001	63244000	2.70E-11
chr3	63264001	63269000	9.48E-15
chr3	63295001	63300000	0.005073423
chr3	63362001	63367000	0.012066926
chr3	63363001	63368000	0.003123897
chr3	63364001	63369000	0.000349659
chr3	63365001	63370000	0.000489714
chr3	63366001	63371000	0.012369821
chr3	63368001	63373000	0.000115203
chr3	63389001	63394000	0.000159293
chr3	63390001	63395000	0.000317281

chr3	63431001	63436000	7.32E-06
chr3	63480001	63485000	3.85E-06
chr3	63481001	63486000	6.27E-06
chr3	63482001	63487000	2.86E-06
chr3	63483001	63488000	0.000246596
chr3	63620001	63625000	0.003321781
chr3	63621001	63626000	0.002405486
chr3	63622001	63627000	0.000279252
chr3	63674001	63679000	0.00309039
chr3	63734001	63739000	1.49E-07
chr3	63735001	63740000	6.45E-05
chr3	63803001	63808000	0.016934147
chr3	63804001	63809000	0.014546382
chr3	63844001	63849000	2.11E-19
chr3	63845001	63850000	1.65E-39
chr3	63846001	63851000	1.81E-44
chr3	63847001	63852000	4.20E-24
chr3	63848001	63853000	6.52E-21
chr3	63849001	63854000	3.09E-30
chr3	63850001	63855000	5.17E-20
chr3	63893001	63898000	3.99E-38
chr3	63894001	63899000	3.80E-27
chr3	63895001	63900000	1.53E-25
chr3	63993001	63998000	0.001175974
chr3	64004001	64009000	3.40E-37
chr3	64005001	64010000	7.58E-34
chr3	64006001	64011000	6.32E-33
chr3	64007001	64012000	7.31E-33
chr3	64008001	64013000	2.93E-34
chr3	64009001	64014000	1.41E-29
chr3	64012001	64017000	9.39E-10
chr3	64013001	64018000	2.22E-10
chr3	64428001	64433000	2.93E-22
chr3	64429001	64434000	6.49E-23
chr3	64430001	64435000	2.23E-20
chr3	64538001	64543000	2.80E-06
chr3	64539001	64544000	2.63E-05
chr3	64563001	64568000	2.17E-05
chr3	64564001	64569000	6.81E-05
chr3	64659001	64664000	0.000505165
chr3	64660001	64665000	0.000434938
chr3	64661001	64666000	0.004147642
chr3	64673001	64678000	8.62E-45
chr3	64734001	64739000	0.000114219
chr3	64841001	64846000	0.002610296
chr3	64860001	64865000	5.45E-05
chr3	64861001	64866000	0.000151517
chr3	64862001	64867000	0.016599503

chr3	64863001	64868000	0.000199258
chr3	64865001	64870000	0.001750479
chr3	64885001	64890000	0.002878717
chr3	64918001	64923000	1.37E-05
chr3	64931001	64936000	8.22E-05
chr3	64932001	64937000	0.000190366
chr3	64933001	64938000	0.000324764
chr3	64955001	64960000	1.63E-06
chr3	65040001	65045000	1.24E-09
chr3	65041001	65046000	6.29E-12
chr3	65062001	65067000	1.52E-09
chr3	65063001	65068000	8.39E-09
chr3	65064001	65069000	5.85E-10
chr3	65065001	65070000	4.60E-06
chr3	65074001	65079000	1.45E-07
chr3	65076001	65081000	1.66E-06
chr3	65077001	65082000	9.39E-07
chr3	65197001	65202000	0.000682977
chr3	65210001	65215000	0.004691973
chr3	65224001	65229000	1.07E-05
chr3	65232001	65237000	0.001171623
chr3	65233001	65238000	2.11E-05
chr3	65298001	65303000	0.000111821
chr3	65486001	65491000	0.004538685
chr3	65531001	65536000	3.96E-16
chr3	65532001	65537000	4.12E-15
chr3	65533001	65538000	0.000117775
chr3	65534001	65539000	2.64E-07
chr3	65535001	65540000	1.00E-21
chr3	65579001	65584000	5.34E-16
chr3	65580001	65585000	6.72E-16
chr3	65581001	65586000	9.74E-14
chr3	65582001	65587000	2.70E-18
chr3	65583001	65588000	2.74E-18
chr3	65868001	65873000	0.002632724
chr3	66024001	66029000	2.79E-16
chr3	66025001	66030000	4.61E-16
chr3	66149001	66154000	0.009281922
chr3	66150001	66155000	9.15E-05
chr3	66151001	66156000	3.69E-05
chr3	66157001	66162000	2.38E-05
chr3	66158001	66163000	9.43E-05
chr3	66351001	66356000	0.000592798
chr3	66551001	66556000	7.47E-34
chr3	66655001	66660000	0.003438724
chr3	66694001	66699000	0.000217052
chr3	66723001	66728000	0.000373018
chr3	66724001	66729000	0.000426139

chr3	66728001	66733000	2.86E-05
chr3	66730001	66735000	6.39E-05
chr3	66731001	66736000	0.000123308
chr3	66732001	66737000	0.000235941
chr3	66733001	66738000	0.00390184
chr3	66734001	66739000	0.001110248
chr3	66735001	66740000	0.000884461
chr3	66783001	66788000	0.000254486
chr3	66815001	66820000	6.02E-06
chr3	66823001	66828000	1.72E-05
chr3	66844001	66849000	9.50E-13
chr3	66845001	66850000	2.75E-14
chr3	66846001	66851000	1.02E-13
chr3	66847001	66852000	8.46E-15
chr3	66853001	66858000	7.58E-08
chr3	67044001	67049000	5.11E-16
chr3	67045001	67050000	1.11E-23
chr3	67046001	67051000	1.80E-30
chr3	67047001	67052000	7.94E-38
chr3	67048001	67053000	1.54E-54
chr3	67085001	67090000	0.000276412
chr3	67105001	67110000	0.020637987
chr3	67242001	67247000	6.65E-05
chr3	67257001	67262000	0.000311736
chr3	67343001	67348000	0.006431965
chr3	67370001	67375000	0.000379864
chr3	67371001	67376000	0.000419455
chr3	67431001	67436000	2.28E-05
chr3	67700001	67705000	2.14E-13
chr3	67701001	67706000	9.52E-12
chr3	67702001	67707000	4.89E-17
chr3	67703001	67708000	1.29E-25
chr3	67704001	67709000	4.77E-29
chr3	67792001	67797000	0.001049845
chr3	67877001	67882000	0.002046494
chr3	67887001	67892000	0.001198902
chr3	67893001	67898000	1.13E-06
chr3	67894001	67899000	1.49E-06
chr3	67906001	67911000	9.54E-05
chr3	68018001	68023000	0.00663619
chr3	68046001	68051000	0.013231326
chr3	68053001	68058000	1.89E-13
chr3	68054001	68059000	2.59E-16
chr3	68055001	68060000	2.42E-15
chr3	68056001	68061000	3.55E-21
chr3	68057001	68062000	1.01E-23
chr3	68080001	68085000	0.000368183
chr3	68178001	68183000	0.001714244

chr3	68231001	68236000	2.04E-05
chr3	68232001	68237000	3.54E-05
chr3	68234001	68239000	0.006533551
chr3	68282001	68287000	0.002752353
chr3	68403001	68408000	0.0020267
chr3	68460001	68465000	5.53E-05
chr3	68461001	68466000	0.000696395
chr3	68523001	68528000	0.003317908
chr3	68527001	68532000	0.006187775
chr3	68615001	68620000	4.60E-06
chr3	68648001	68653000	2.25E-07
chr3	68649001	68654000	5.08E-09
chr3	68650001	68655000	3.04E-08
chr3	68672001	68677000	1.42E-07
chr3	68673001	68678000	5.47E-06
chr3	68674001	68679000	0.000498148
chr3	68675001	68680000	0.000119593
chr3	68681001	68686000	4.08E-07
chr3	68682001	68687000	4.20E-07
chr3	68683001	68688000	1.74E-06
chr3	68687001	68692000	0.001731691
chr3	68688001	68693000	0.000560324
chr3	68702001	68707000	0.002365159
chr3	68730001	68735000	0.001684291
chr3	68731001	68736000	1.60E-06
chr3	68732001	68737000	1.11E-05
chr3	68733001	68738000	2.36E-05
chr3	68734001	68739000	5.38E-07
chr3	68767001	68772000	8.53E-09
chr3	68768001	68773000	1.62E-05
chr3	68769001	68774000	2.39E-08
chr3	68821001	68826000	2.56E-08
chr3	68822001	68827000	6.73E-08
chr3	68876001	68881000	1.98E-12
chr3	68885001	68890000	0.00025852
chr3	69006001	69011000	1.93E-05
chr3	69007001	69012000	0.000664063
chr3	69059001	69064000	1.16E-22
chr3	69060001	69065000	3.05E-25
chr3	69061001	69066000	2.75E-38
chr3	69062001	69067000	3.67E-53
chr3	69063001	69068000	2.73E-22
chr3	69096001	69101000	5.85E-43
chr3	69097001	69102000	7.12E-50
chr3	69098001	69103000	2.30E-26
chr3	69099001	69104000	3.41E-24
chr3	69100001	69105000	1.35E-35
chr3	69101001	69106000	2.79E-33

chr3	69125001	69130000	7.26E-23
chr3	69126001	69131000	1.17E-24
chr3	69127001	69132000	1.47E-15
chr3	69190001	69195000	0.002776852
chr3	69191001	69196000	0.004365681
chr3	69192001	69197000	0.001961882
chr3	69223001	69228000	0.00034132
chr3	69224001	69229000	2.05E-06
chr3	69225001	69230000	1.10E-05
chr3	69396001	69401000	8.38E-06
chr3	69398001	69403000	2.26E-05
chr3	69399001	69404000	2.66E-05
chr3	69400001	69405000	0.00067254
chr3	69401001	69406000	0.000691445
chr3	69431001	69436000	7.11E-10
chr3	69432001	69437000	1.05E-23
chr3	69433001	69438000	1.98E-19
chr3	69434001	69439000	2.22E-12
chr3	69435001	69440000	2.42E-12
chr3	69586001	69591000	1.24E-11
chr3	69587001	69592000	5.18E-49
chr3	69588001	69593000	3.51E-51
chr3	69589001	69594000	3.44E-46
chr3	69590001	69595000	7.89E-24
chr3	69591001	69596000	1.08E-45
chr3	69592001	69597000	1.70E-07
chr3	69595001	69600000	0.001851034
chr3	69596001	69601000	5.86E-05
chr3	69597001	69602000	0.001120053
chr3	69620001	69625000	0.009126506
chr3	69622001	69627000	1.04E-06
chr3	69623001	69628000	5.68E-06
chr3	69667001	69672000	0.000164185
chr3	69668001	69673000	0.00030314
chr3	69754001	69759000	0.004995151
chr3	70075001	70080000	0.000383993
chr3	70076001	70081000	0.000591519
chr3	70077001	70082000	0.000213456
chr3	70078001	70083000	9.66E-06
chr3	70079001	70084000	1.80E-08
chr3	70080001	70085000	1.25E-07
chr3	70081001	70086000	7.95E-07
chr3	70082001	70087000	2.08E-06
chr3	70083001	70088000	0.001581963
chr3	70092001	70097000	0.000255608
chr3	70094001	70099000	0.000101726
chr3	70106001	70111000	3.32E-06
chr3	70107001	70112000	3.02E-05

chr3	70181001	70186000	3.34E-05
chr3	70256001	70261000	0.004067658
chr3	70274001	70279000	0.002526574
chr3	70275001	70280000	0.002073124
chr3	70448001	70453000	0.000725783
chr3	70449001	70454000	0.001301108
chr3	70506001	70511000	7.60E-05
chr3	70507001	70512000	2.33E-05
chr3	70508001	70513000	2.77E-07
chr3	70509001	70514000	2.98E-07
chr3	70510001	70515000	2.04E-08
chr3	70718001	70723000	0.004940234
chr3	70719001	70724000	4.43E-06
chr3	70720001	70725000	1.92E-06
chr3	70721001	70726000	1.62E-05
chr3	70733001	70738000	3.62E-07
chr3	70734001	70739000	3.45E-11
chr3	70735001	70740000	2.29E-09
chr3	70736001	70741000	1.24E-07
chr3	70737001	70742000	2.88E-13
chr3	70784001	70789000	0.002558449
chr3	70786001	70791000	0.000155991
chr3	70787001	70792000	5.01E-05
chr3	70989001	70994000	8.47E-06
chr3	70990001	70995000	0.000138947
chr3	71544001	71549000	5.36E-06
chr3	71545001	71550000	2.64E-06
chr3	71626001	71631000	1.07E-21
chr3	71627001	71632000	2.81E-27
chr3	71628001	71633000	1.57E-18
chr3	71769001	71774000	1.94E-09
chr3	71770001	71775000	4.07E-24
chr3	71771001	71776000	2.51E-29
chr3	71772001	71777000	1.20E-46
chr3	71773001	71778000	1.16E-42
chr3	71774001	71779000	1.17E-41
chr3	71798001	71803000	2.82E-17
chr3	71799001	71804000	2.78E-28
chr3	71800001	71805000	3.76E-24
chr3	71801001	71806000	6.68E-44
chr3	71802001	71807000	8.69E-30
chr3	71803001	71808000	5.68E-40
chr3	71823001	71828000	4.20E-07
chr3	71824001	71829000	1.31E-07
chr3	71825001	71830000	3.28E-07
chr3	71826001	71831000	7.52E-05
chr3	71830001	71835000	1.27E-19
chr3	71831001	71836000	1.60E-12

chr3	71832001	71837000	1.99E-13
chr3	71833001	71838000	1.48E-20
chr3	71834001	71839000	1.56E-21
chr3	72438001	72443000	0.011791476
chr3	72495001	72500000	1.75E-10
chr3	72496001	72501000	1.53E-14
chr3	73170001	73175000	0.002209753
chr3	73263001	73268000	5.42E-08
chr3	73264001	73269000	2.13E-07
chr3	73313001	73318000	1.68E-06
chr3	73357001	73362000	3.54E-05
chr3	73644001	73649000	0.002317829
chr3	73734001	73739000	4.10E-06
chr3	73758001	73763000	7.79E-05
chr3	73803001	73808000	0.003399732
chr3	73804001	73809000	0.000357759
chr3	73907001	73912000	4.45E-05
chr3	73908001	73913000	2.58E-05
chr3	73935001	73940000	0.000214905
chr3	73937001	73942000	0.002800196
chr3	74032001	74037000	0.005657283
chr3	74033001	74038000	0.004408549
chr3	74139001	74144000	0.003007419
chr3	74156001	74161000	1.17E-05
chr3	74178001	74183000	1.87E-06
chr3	74180001	74185000	2.73E-09
chr3	74206001	74211000	0.000242021
chr3	74207001	74212000	1.15E-07
chr3	74279001	74284000	2.32E-06
chr3	74280001	74285000	2.57E-06
chr3	74281001	74286000	2.15E-05
chr3	74282001	74287000	1.88E-05
chr3	74283001	74288000	1.08E-06
chr3	74309001	74314000	0.011921064
chr3	74310001	74315000	0.016758852
chr3	74318001	74323000	0.006697278
chr3	74342001	74347000	0.000332816
chr3	74362001	74367000	0.000483405
chr3	74434001	74439000	0.000133398
chr3	74435001	74440000	8.66E-06
chr3	74443001	74448000	0.000293107
chr3	74445001	74450000	5.33E-06
chr3	74481001	74486000	0.000194596
chr3	74482001	74487000	4.67E-07
chr3	74483001	74488000	1.24E-05
chr3	74503001	74508000	9.98E-07
chr3	74504001	74509000	1.15E-05
chr3	74510001	74515000	0.001115555

chr3	74511001	74516000	0.000311183
chr3	74512001	74517000	0.000275332
chr3	74514001	74519000	0.000764856
chr3	74619001	74624000	0.000263644
chr3	74663001	74668000	5.73E-17
chr3	74694001	74699000	1.26E-05
chr3	74695001	74700000	2.90E-05
chr3	74696001	74701000	8.55E-06
chr3	74713001	74718000	2.43E-05
chr3	74756001	74761000	9.04E-05
chr3	74877001	74882000	1.12E-09
chr3	75052001	75057000	1.74E-06
chr3	75053001	75058000	8.70E-08
chr3	75061001	75066000	0.001481607
chr3	75179001	75184000	0.002399674
chr3	75234001	75239000	8.52E-05
chr3	75303001	75308000	0.002894153
chr3	75346001	75351000	4.85E-07
chr3	75347001	75352000	8.23E-05
chr3	75348001	75353000	9.03E-06
chr3	75349001	75354000	0.000420716
chr3	75364001	75369000	0.000425178
chr3	75365001	75370000	3.13E-05
chr3	75427001	75432000	2.15E-08
chr3	75428001	75433000	1.58E-11
chr3	75429001	75434000	1.05E-10
chr3	75430001	75435000	1.77E-10
chr3	75431001	75436000	2.60E-07
chr3	75480001	75485000	6.77E-11
chr3	75481001	75486000	9.78E-18
chr3	75482001	75487000	2.72E-35
chr3	75483001	75488000	2.83E-46
chr3	75484001	75489000	9.27E-37
chr3	75582001	75587000	0.018290907
chr3	75603001	75608000	0.002882513
chr3	75608001	75613000	0.000236817
chr3	75951001	75956000	3.24E-20
chr3	75952001	75957000	1.66E-12
chr3	75953001	75958000	1.57E-11
chr3	75954001	75959000	4.52E-10
chr3	76036001	76041000	0.000480776
chr3	76219001	76224000	5.22E-06
chr3	76457001	76462000	3.69E-05
chr3	76491001	76496000	5.00E-05
chr3	76492001	76497000	2.03E-07
chr3	76493001	76498000	5.63E-05
chr3	76494001	76499000	6.32E-06
chr3	76495001	76500000	0.000220915

chr3	76558001	76563000	1.33E-09
chr3	76769001	76774000	1.30E-06
chr3	76771001	76776000	5.64E-08
chr3	76772001	76777000	2.41E-07
chr3	76773001	76778000	1.81E-06
chr3	76795001	76800000	0.000400587
chr3	76796001	76801000	7.43E-06
chr3	76797001	76802000	9.42E-05
chr3	76798001	76803000	0.000283331
chr3	76827001	76832000	0.002080266
chr3	76828001	76833000	0.002841067
chr3	76834001	76839000	0.000621908
chr3	76964001	76969000	0.000291398
chr3	77045001	77050000	0.000936282
chr3	77083001	77088000	4.21E-38
chr3	77084001	77089000	1.23E-64
chr3	77085001	77090000	4.57E-68
chr3	77087001	77092000	1.70E-62
chr3	77089001	77094000	8.92E-27
chr3	77188001	77193000	0.004339216
chr3	77198001	77203000	2.90E-05
chr3	77352001	77357000	3.33E-06
chr3	77353001	77358000	1.40E-05
chr3	77396001	77401000	0.001390983
chr3	77397001	77402000	0.000824453
chr3	77398001	77403000	0.004692309
chr3	77399001	77404000	0.003357268
chr3	77538001	77543000	0.001442851
chr3	77787001	77792000	0.000662327
chr3	77789001	77794000	0.00137205
chr3	77970001	77975000	0.001192732
chr3	78081001	78086000	3.71E-08
chr3	78082001	78087000	2.76E-05
chr3	78083001	78088000	1.24E-05
chr3	78084001	78089000	0.000324482
chr3	78427001	78432000	0.000386192
chr3	78770001	78775000	0.000343034
chr3	79062001	79067000	1.11E-05
chr3	79063001	79068000	4.73E-24
chr3	79064001	79069000	8.41E-33
chr3	79065001	79070000	2.79E-25
chr3	79066001	79071000	2.52E-22
chr3	79067001	79072000	5.55E-29
chr3	79068001	79073000	4.31E-27
chr3	79170001	79175000	2.57E-09
chr3	79171001	79176000	6.00E-12
chr3	79172001	79177000	6.91E-10
chr3	79173001	79178000	1.68E-12

chr3	79174001	79179000	1.71E-12
chr3	79175001	79180000	0.002642034
chr3	79243001	79248000	1.78E-05
chr3	79296001	79301000	0.005749931
chr3	79297001	79302000	0.001143028
chr3	79343001	79348000	0.002950518
chr3	79693001	79698000	0.00294235
chr3	79694001	79699000	0.005383587
chr3	80122001	80127000	2.04E-05
chr3	80646001	80651000	1.99E-06
chr3	80654001	80659000	1.66E-06
chr3	80655001	80660000	1.83E-05
chr3	80656001	80661000	5.76E-05
chr3	80657001	80662000	0.000105029
chr3	80718001	80723000	0.000157963
chr3	80755001	80760000	4.42E-09
chr3	80756001	80761000	7.44E-11
chr3	80853001	80858000	1.48E-07
chr3	80854001	80859000	1.00E-06
chr3	80957001	80962000	5.32E-07
chr3	80958001	80963000	2.73E-09
chr3	80959001	80964000	6.41E-08
chr3	80998001	81003000	1.13E-05
chr3	80999001	81004000	6.33E-05
chr3	81000001	81005000	1.06E-05
chr3	81001001	81006000	2.83E-08
chr3	81002001	81007000	1.06E-06
chr3	81166001	81171000	0.000342543
chr3	81217001	81222000	0.000143527
chr3	81286001	81291000	5.95E-06
chr3	81349001	81354000	0.004920368
chr3	81534001	81539000	1.51E-05
chr3	81715001	81720000	0.010449268
chr3	81716001	81721000	0.013034235
chr3	81717001	81722000	0.00202544
chr3	81806001	81811000	2.37E-09
chr3	81810001	81815000	4.71E-14
chr3	82275001	82280000	1.54E-05
chr3	82276001	82281000	4.44E-05
chr3	82277001	82282000	6.70E-05
chr3	82278001	82283000	0.000158512
chr3	82429001	82434000	0.000542936
chr3	82482001	82487000	0.000209704
chr3	82483001	82488000	0.000995254
chr3	82576001	82581000	6.64E-05
chr3	82577001	82582000	0.000164467
chr3	82649001	82654000	0.000799935
chr3	82685001	82690000	0.000105134

chr3	82787001	82792000	0.000108396
chr3	82852001	82857000	0.000163446
chr3	82887001	82892000	0.000138541
chr3	82953001	82958000	0.011377414
chr3	82954001	82959000	0.014356118
chr3	83174001	83179000	0.003266358
chr3	83175001	83180000	0.00269952
chr3	83176001	83181000	0.013251786
chr3	83289001	83294000	0.005217617
chr3	83331001	83336000	0.006772618
chr3	83480001	83485000	3.69E-05
chr3	83481001	83486000	3.50E-05
chr3	83482001	83487000	1.02E-05
chr3	83483001	83488000	9.85E-05
chr3	84366001	84371000	2.75E-05
chr3	84420001	84425000	0.001237551
chr3	84449001	84454000	7.22E-06
chr3	84592001	84597000	1.03E-06
chr3	84665001	84670000	0.000239357
chr3	84682001	84687000	0.000133183
chr3	84841001	84846000	0.014611332
chr3	84859001	84864000	3.31E-08
chr3	84860001	84865000	2.92E-11
chr3	84861001	84866000	1.11E-13
chr3	84862001	84867000	1.16E-15
chr3	84863001	84868000	5.46E-13
chr3	84915001	84920000	0.000396495
chr3	84938001	84943000	2.99E-05
chr3	84981001	84986000	0.000489751
chr3	84982001	84987000	0.000960662
chr3	84983001	84988000	0.000476852
chr3	84985001	84990000	5.47E-08
chr3	85003001	85008000	3.32E-13
chr3	85004001	85009000	5.66E-31
chr3	85145001	85150000	0.002366837
chr3	85270001	85275000	3.50E-07
chr3	85369001	85374000	3.16E-10
chr3	85370001	85375000	0.000144968
chr3	85445001	85450000	4.35E-06
chr3	85643001	85648000	0.013342307
chr3	85644001	85649000	0.008712046
chr3	85645001	85650000	0.009697262
chr3	85888001	85893000	0.000838354
chr3	85889001	85894000	0.004982277
chr3	85890001	85895000	0.008331504
chr3	85994001	85999000	1.51E-06
chr3	86099001	86104000	0.00212626
chr3	86147001	86152000	9.36E-06

chr3	86209001	86214000	0.000753257
chr3	86409001	86414000	0.007155809
chr3	86411001	86416000	0.005270972
chr3	86412001	86417000	0.00386368
chr3	86413001	86418000	0.002469211
chr3	86530001	86535000	0.005064517
chr3	87035001	87040000	1.25E-29
chr3	87037001	87042000	1.42E-27
chr3	87038001	87043000	9.90E-27
chr3	87039001	87044000	4.11E-30
chr3	87040001	87045000	1.32E-20
chr3	87134001	87139000	2.55E-14
chr3	87135001	87140000	3.05E-11
chr3	87136001	87141000	4.00E-09
chr3	87271001	87276000	8.47E-27
chr3	87272001	87277000	4.90E-62
chr3	87273001	87278000	1.65E-48
chr3	87274001	87279000	2.48E-53
chr3	87275001	87280000	1.34E-37
chr3	87276001	87281000	3.92E-40
chr3	87277001	87282000	3.05E-08
chr3	87317001	87322000	0.014074638
chr3	87494001	87499000	0.000135009
chr3	87495001	87500000	6.90E-05
chr3	87507001	87512000	0.00026334
chr3	87524001	87529000	0.002988654
chr3	87525001	87530000	0.002766665
chr3	87528001	87533000	0.008968994
chr3	87542001	87547000	4.26E-09
chr3	87543001	87548000	4.97E-10
chr3	87751001	87756000	0.002227794
chr3	87752001	87757000	0.001316211
chr3	87772001	87777000	1.94E-06
chr3	87773001	87778000	7.22E-05
chr3	87774001	87779000	0.000253406
chr3	87775001	87780000	0.000599406
chr3	87819001	87824000	0.00028846
chr3	88020001	88025000	0.000160371
chr3	88021001	88026000	1.87E-06
chr3	88022001	88027000	0.000867429
chr3	88103001	88108000	1.23E-53
chr3	88194001	88199000	1.52E-33
chr3	88195001	88200000	3.63E-40
chr3	88233001	88238000	2.49E-06
chr3	88291001	88296000	0.023062989
chr3	88334001	88339000	0.00082463
chr3	88340001	88345000	0.000305275
chr3	88554001	88559000	1.67E-06

chr3	89152001	89157000	5.11E-19
chr3	89159001	89164000	2.22E-28
chr3	89160001	89165000	5.16E-35
chr3	89161001	89166000	8.01E-31
chr3	89164001	89169000	5.88E-08
chr3	89560001	89565000	0.000253647
chr3	89561001	89566000	0.000225161
chr3	93688001	93693000	4.47E-22
chr3	93689001	93694000	1.02E-18
chr3	93692001	93697000	1.05E-30
chr3	93699001	93704000	8.94E-30
chr3	93916001	93921000	0.021970675
chr3	94130001	94135000	0.000140816
chr3	94193001	94198000	0.003053981
chr3	94194001	94199000	0.013437579
chr3	94195001	94200000	0.007002179
chr3	94316001	94321000	0.000197299
chr3	94831001	94836000	0.000913225
chr3	95383001	95388000	6.38E-06
chr3	95384001	95389000	1.90E-05
chr3	95519001	95524000	6.36E-05
chr3	95520001	95525000	6.61E-06
chr3	95523001	95528000	3.61E-06
chr3	95607001	95612000	1.96E-05
chr3	95793001	95798000	1.90E-05
chr3	95794001	95799000	5.30E-05
chr3	95795001	95800000	0.000382257
chr3	95928001	95933000	1.65E-05
chr3	95929001	95934000	0.000413825
chr3	95973001	95978000	6.86E-06
chr3	96009001	96014000	0.004195974
chr3	96078001	96083000	0.021266641
chr3	96273001	96278000	0.000112486
chr3	96274001	96279000	0.002230702
chr3	96275001	96280000	0.004047312
chr3	96276001	96281000	0.003454335
chr3	96320001	96325000	4.22E-05
chr3	96533001	96538000	3.94E-28
chr3	97479001	97484000	2.21E-52
chr3	97480001	97485000	8.14E-53
chr3	97481001	97486000	5.13E-49
chr3	97482001	97487000	9.54E-48
chr3	97483001	97488000	6.75E-31
chr3	97484001	97489000	5.08E-07
chr3	97536001	97541000	9.76E-72
chr3	97539001	97544000	2.52E-59
chr3	97541001	97546000	3.65E-51
chr3	97547001	97552000	0.000411989

chr3	97599001	97604000	3.73E-05
chr3	97600001	97605000	5.74E-05
chr3	97601001	97606000	0.000271856
chr3	97691001	97696000	9.34E-39
chr3	97844001	97849000	2.79E-05
chr3	97845001	97850000	2.38E-05
chr3	97851001	97856000	0.013349712
chr3	97916001	97921000	2.16E-05
chr3	98074001	98079000	0.002384549
chr3	98075001	98080000	1.63E-05
chr3	98077001	98082000	1.52E-05
chr3	98078001	98083000	8.92E-06
chr3	98098001	98103000	0.000978033
chr3	98099001	98104000	0.001604624
chr3	98101001	98106000	0.0013411
chr3	98148001	98153000	0.00149857
chr3	98237001	98242000	6.89E-69
chr3	98238001	98243000	7.41E-51
chr3	98307001	98312000	1.26E-42
chr3	98308001	98313000	7.41E-113
chr3	98309001	98314000	1.23E-122
chr3	98310001	98315000	2.65E-44
chr3	98311001	98316000	4.67E-41
chr3	98312001	98317000	1.07E-52
chr3	98341001	98346000	0.0068547
chr3	98344001	98349000	5.15E-05
chr3	98521001	98526000	9.54E-20
chr3	98565001	98570000	0.018817559
chr3	98912001	98917000	5.02E-09
chr3	98913001	98918000	4.51E-07
chr3	98989001	98994000	0.04567179
chr3	99006001	99011000	0.013599159
chr3	99075001	99080000	0.002064898
chr3	99128001	99133000	0.00168505
chr3	99134001	99139000	0.001018227
chr3	99585001	99590000	0.010534398
chr3	99595001	99600000	2.81E-37
chr3	99689001	99694000	6.17E-08
chr3	99690001	99695000	2.70E-10
chr3	99849001	99854000	8.24E-07
chr3	100115001	100120000	1.73E-23
chr3	100116001	100121000	1.00E-24
chr3	100117001	100122000	1.87E-23
chr3	100119001	100124000	1.28E-24
chr3	100120001	100125000	1.87E-16
chr3	100424001	100429000	2.11E-25
chr3	100428001	100433000	1.48E-32
chr3	100446001	100451000	0.002248718

chr3	100447001	100452000	0.000625095
chr3	101279001	101284000	4.76E-16
chr3	101280001	101285000	2.86E-13
chr3	101391001	101396000	5.97E-59
chr3	101498001	101503000	1.21E-45
chr3	101564001	101569000	2.83E-96
chr3	102218001	102223000	0.022651622
chr3	103758001	103763000	0.011560806
chr3	103759001	103764000	0.010421191
chr3	103761001	103766000	0.021995422
chr3	104114001	104119000	0.000449913
chr3	105080001	105085000	4.50E-14
chr3	105588001	105593000	8.36E-17
chr3	105918001	105923000	0.001090779
chr3	106254001	106259000	0.000387356
chr3	106576001	106581000	0.002515274
chr3	106957001	106962000	1.80E-27
chr3	106958001	106963000	1.19E-24
chr3	106959001	106964000	2.32E-22
chr3	106960001	106965000	3.01E-05
chr3	107144001	107149000	1.53E-29
chr3	107145001	107150000	1.15E-30
chr3	107242001	107247000	9.13E-25
chr3	107243001	107248000	1.11E-35
chr3	107244001	107249000	7.64E-40
chr3	107809001	107814000	4.04E-33
chr3	107936001	107941000	9.75E-14
chr3	107937001	107942000	6.07E-33
chr3	108027001	108032000	2.58E-05
chr3	108123001	108128000	0.000383397
chr3	108124001	108129000	0.000536799
chr3	108157001	108162000	0.010059532
chr3	108161001	108166000	6.74E-05
chr3	108162001	108167000	0.000641493
chr3	108256001	108261000	0.003583623
chr3	108257001	108262000	0.001593293
chr3	108303001	108308000	4.42E-14
chr3	108304001	108309000	1.53E-58
chr3	108422001	108427000	0.00612723
chr3	108423001	108428000	5.25E-05
chr3	108424001	108429000	1.13E-06
chr3	108425001	108430000	2.96E-06
chr3	108426001	108431000	4.79E-05
chr3	108462001	108467000	8.73E-06
chr3	108463001	108468000	4.68E-08
chr3	108500001	108505000	0.003181228
chr3	108521001	108526000	0.000705911
chr3	108774001	108779000	3.95E-07

chr3	108775001	108780000	2.49E-07
chr3	108791001	108796000	1.97E-12
chr3	108792001	108797000	0.000774686
chr3	108793001	108798000	0.000533689
chr3	108794001	108799000	0.000478689
chr3	108795001	108800000	0.000695917
chr3	108796001	108801000	0.002138296
chr3	108797001	108802000	5.31E-05
chr3	108798001	108803000	2.23E-06
chr3	108799001	108804000	5.59E-07
chr3	108800001	108805000	5.81E-07
chr3	108801001	108806000	1.61E-06
chr3	108804001	108809000	6.12E-05
chr3	108805001	108810000	3.23E-05
chr3	108956001	108961000	5.48E-07
chr3	108958001	108963000	1.07E-05
chr3	110129001	110134000	0.006895186
chr3	110790001	110795000	6.64E-39
chr3	110791001	110796000	1.69E-35
chr3	110982001	110987000	0.000274008
chr3	111016001	111021000	5.66E-05
chr3	111337001	111342000	0.003374067
chr3	111693001	111698000	2.14E-45
chr3	111696001	111701000	3.28E-48
chr3	111698001	111703000	3.62E-24
chr3	111805001	111810000	1.20E-35
chr3	111935001	111940000	0.002474656
chr3	112053001	112058000	0.001145297
chr3	112188001	112193000	4.55E-06
chr3	112189001	112194000	2.08E-06
chr3	112190001	112195000	2.64E-06
chr3	112191001	112196000	5.83E-05
chr3	112658001	112663000	0.00070763
chr3	112705001	112710000	2.74E-21
chr3	112707001	112712000	5.90E-49
chr3	112708001	112713000	1.56E-45
chr3	112709001	112714000	2.97E-41
chr3	112710001	112715000	3.20E-26
chr3	112738001	112743000	2.41E-28
chr3	113234001	113239000	1.94E-33
chr3	113640001	113645000	7.23E-06
chr3	113869001	113874000	4.34E-08
chr3	113870001	113875000	6.91E-08
chr3	113871001	113876000	5.11E-10
chr3	113945001	113950000	0.001453542
chr3	114029001	114034000	0.000217537
chr3	114339001	114344000	1.53E-11
chr3	114340001	114345000	1.72E-09

chr3	114572001	114577000	0.002695252
chr3	114573001	114578000	0.000122084
chr3	114574001	114579000	2.23E-07
chr3	114861001	114866000	1.34E-21
chr3	114862001	114867000	6.33E-41
chr3	115179001	115184000	0.004871521
chr3	115373001	115378000	4.50E-63
chr3	115374001	115379000	1.73E-58
chr3	115375001	115380000	8.58E-49
chr3	115439001	115444000	0.005933176
chr3	115498001	115503000	3.83E-15
chr3	115499001	115504000	1.99E-59
chr3	115500001	115505000	2.08E-43
chr3	115667001	115672000	0.005383545
chr3	115718001	115723000	0.001384228
chr3	116037001	116042000	7.90E-06
chr3	116059001	116064000	3.85E-05
chr3	116060001	116065000	7.13E-06
chr3	116087001	116092000	5.15E-05
chr3	116088001	116093000	0.001843261
chr3	116113001	116118000	0.000145122
chr3	116114001	116119000	3.11E-05
chr3	116143001	116148000	0.013016265
chr3	116159001	116164000	2.75E-18
chr3	116261001	116266000	0.000943857
chr3	116314001	116319000	0.001095638
chr3	116339001	116344000	0.001466706
chr3	116340001	116345000	0.001156675
chr3	116341001	116346000	0.000805967
chr3	116342001	116347000	0.000798338
chr3	116343001	116348000	6.17E-05
chr3	116423001	116428000	9.98E-06
chr3	116424001	116429000	1.46E-05
chr3	116511001	116516000	0.000834086
chr3	116512001	116517000	0.000823571
chr3	116513001	116518000	0.002389839
chr3	116623001	116628000	7.35E-05
chr3	116625001	116630000	0.000597759
chr3	116779001	116784000	4.24E-09
chr3	116780001	116785000	6.72E-09
chr3	116878001	116883000	2.73E-06
chr3	116879001	116884000	0.00080383
chr3	117409001	117414000	0.000103436
chr3	117480001	117485000	0.001137113
chr3	117481001	117486000	0.000236405
chr3	117954001	117959000	0.001825912
chr3	117955001	117960000	0.001059221
chr3	118027001	118032000	0.002649224

chr3	118134001	118139000	0.00026114
chr3	118135001	118140000	0.001082814
chr3	118137001	118142000	0.000142525
chr3	118138001	118143000	6.21E-05
chr3	118139001	118144000	0.001015536
chr3	118407001	118412000	1.33E-10
chr3	118408001	118413000	1.92E-11
chr3	118409001	118414000	6.67E-11
chr3	118410001	118415000	1.19E-11
chr3	118749001	118754000	1.40E-41
chr3	118750001	118755000	1.05E-44
chr3	118751001	118756000	1.63E-44
chr3	118955001	118960000	6.32E-24
chr3	119009001	119014000	1.02E-25
chr3	119011001	119016000	1.01E-27
chr3	119013001	119018000	2.09E-26
chr3	119037001	119042000	9.19E-27
chr3	119038001	119043000	2.39E-37
chr3	119039001	119044000	1.20E-28
chr3	119042001	119047000	5.34E-31
chr3	119177001	119182000	0.000657014
chr3	119178001	119183000	7.51E-57
chr3	119179001	119184000	4.15E-45
chr3	119180001	119185000	3.16E-29
chr3	119181001	119186000	2.24E-34
chr3	119182001	119187000	3.49E-47
chr3	119183001	119188000	6.83E-40
chr3	119184001	119189000	1.16E-51
chr3	119185001	119190000	3.13E-39
chr3	119186001	119191000	3.93E-29
chr3	119187001	119192000	2.50E-15
chr3	119394001	119399000	4.22E-50
chr3	119395001	119400000	2.26E-55
chr3	119396001	119401000	6.94E-60
chr3	119417001	119422000	8.96E-14
chr3	119418001	119423000	1.88E-19
chr3	119419001	119424000	4.44E-23
chr3	119420001	119425000	1.76E-25
chr3	119421001	119426000	1.76E-20
chr3	119814001	119819000	4.88E-35
chr3	120000001	120005000	1.94E-26
chr3	120001001	120006000	1.28E-16
chr3	120063001	120068000	9.11E-46
chr3	120064001	120069000	2.98E-64
chr3	120068001	120073000	1.54E-28
chr3	120088001	120093000	3.95E-05
chr3	120089001	120094000	1.66E-05
chr3	120090001	120095000	3.25E-07

chr3	120315001	120320000	2.07E-20
chr3	120457001	120462000	3.34E-29
chr3	120627001	120632000	6.86E-24
chr3	120667001	120672000	9.63E-13
chr3	120668001	120673000	8.62E-12
chr3	120807001	120812000	2.10E-06
chr3	120914001	120919000	1.26E-08
chr3	120963001	120968000	0.000192453
chr3	120986001	120991000	0.016543308
chr3	121052001	121057000	7.16E-07
chr3	121062001	121067000	8.97E-07
chr3	121063001	121068000	5.54E-09
chr3	121102001	121107000	0.000193684
chr3	121260001	121265000	2.18E-17
chr3	121463001	121468000	1.51E-09
chr3	121554001	121559000	1.94E-37
chr3	121662001	121667000	0.001150909
chr3	121663001	121668000	3.35E-06
chr3	121664001	121669000	5.18E-05
chr3	121741001	121746000	4.40E-15
chr3	121748001	121753000	0.000996659
chr3	121750001	121755000	0.000401957
chr3	121751001	121756000	2.24E-05
chr3	121752001	121757000	2.59E-05
chr3	121754001	121759000	5.04E-05
chr3	121755001	121760000	4.66E-05
chr3	121756001	121761000	0.006261412
chr3	121784001	121789000	0.000269286
chr3	121898001	121903000	7.02E-09
chr3	122074001	122079000	1.07E-05
chr3	122103001	122108000	1.03E-23
chr3	122113001	122118000	2.57E-05
chr3	122130001	122135000	8.88E-17
chr3	122228001	122233000	6.74E-13
chr3	122279001	122284000	7.21E-34
chr3	122280001	122285000	7.44E-15
chr3	122281001	122286000	4.88E-14
chr3	122282001	122287000	8.49E-15
chr3	122283001	122288000	1.53E-11
chr3	122514001	122519000	1.65E-44
chr3	122742001	122747000	4.54E-10
chr3	122743001	122748000	2.73E-08
chr3	122746001	122751000	5.01E-13
chr3	122781001	122786000	8.18E-19
chr3	122782001	122787000	1.14E-31
chr3	122916001	122921000	1.20E-28
chr3	122917001	122922000	7.95E-30
chr3	122918001	122923000	2.05E-39

chr3	122919001	122924000	2.92E-37
chr3	122920001	122925000	2.59E-42
chr3	122921001	122926000	1.70E-26
chr3	123601001	123606000	2.13E-16
chr3	123602001	123607000	1.09E-16
chr3	123603001	123608000	1.62E-24
chr3	123694001	123699000	1.89E-08
chr3	123747001	123752000	3.04E-16
chr3	123748001	123753000	6.18E-12
chr3	123749001	123754000	1.84E-07
chr3	123931001	123936000	3.27E-05
chr3	123932001	123937000	9.74E-05
chr3	123933001	123938000	6.03E-05
chr3	124110001	124115000	3.30E-06
chr3	124595001	124600000	2.98E-05
chr3	124601001	124606000	5.14E-24
chr3	124602001	124607000	1.75E-23
chr3	124603001	124608000	1.50E-18
chr3	124604001	124609000	5.23E-37
chr3	124605001	124610000	9.27E-24
chr3	124771001	124776000	8.29E-14
chr3	124772001	124777000	4.89E-16
chr3	124773001	124778000	7.72E-20
chr3	124774001	124779000	9.80E-26
chr3	124860001	124865000	4.22E-07
chr3	125071001	125076000	2.46E-12
chr3	125072001	125077000	1.79E-14
chr3	125073001	125078000	4.34E-07
chr3	125091001	125096000	3.08E-32
chr3	125092001	125097000	1.38E-36
chr3	125094001	125099000	7.86E-25
chr3	125236001	125241000	6.67E-19
chr3	125237001	125242000	5.08E-17
chr3	125238001	125243000	5.47E-19
chr3	125239001	125244000	1.26E-12
chr3	125314001	125319000	5.20E-32
chr3	125385001	125390000	0.016917854
chr3	125523001	125528000	1.10E-07
chr3	125524001	125529000	2.23E-08
chr3	125631001	125636000	1.91E-14
chr3	125802001	125807000	2.58E-17
chr3	125883001	125888000	0.003535971
chr3	125899001	125904000	1.98E-11
chr3	125912001	125917000	0.013932481
chr3	125957001	125962000	0.009065781
chr3	125958001	125963000	0.012398688
chr3	126002001	126007000	2.56E-14
chr3	126028001	126033000	6.11E-13

chr3	126369001	126374000	5.43E-70
chr3	126418001	126423000	8.73E-16
chr3	126419001	126424000	2.99E-24
chr3	126420001	126425000	9.39E-27
chr3	126421001	126426000	4.53E-22
chr3	126697001	126702000	3.96E-18
chr3	126698001	126703000	1.57E-28
chr3	126699001	126704000	3.11E-19
chr3	127219001	127224000	0.003625544
chr3	127313001	127318000	9.13E-18
chr3	127314001	127319000	1.46E-16
chr3	127315001	127320000	2.80E-20
chr3	127316001	127321000	3.11E-24
chr3	127317001	127322000	1.66E-18
chr3	127541001	127546000	2.99E-22
chr3	127766001	127771000	2.59E-40
chr3	127767001	127772000	2.29E-45
chr3	127768001	127773000	3.97E-30
chr3	127769001	127774000	8.16E-31
chr3	127771001	127776000	3.94E-35
chr3	127868001	127873000	5.24E-26
chr3	127869001	127874000	1.54E-18
chr3	127870001	127875000	6.89E-28
chr3	127871001	127876000	7.49E-37
chr3	127872001	127877000	9.94E-43
chr3	128395001	128400000	2.19E-21
chr3	128396001	128401000	7.03E-28
chr3	128397001	128402000	6.74E-18
chr3	128398001	128403000	1.29E-17
chr3	128399001	128404000	3.52E-13
chr3	128440001	128445000	1.13E-11
chr3	128875001	128880000	8.38E-10
chr3	128993001	128998000	2.82E-29
chr3	128994001	128999000	1.32E-49
chr3	128995001	129000000	8.70E-35
chr3	128996001	129001000	1.01E-33
chr3	128997001	129002000	3.29E-36
chr3	128998001	129003000	1.40E-28
chr3	129145001	129150000	6.68E-17
chr3	129146001	129151000	2.48E-37
chr3	129147001	129152000	9.61E-37
chr3	129154001	129159000	4.37E-29
chr3	129155001	129160000	1.26E-29
chr3	129345001	129350000	1.44E-27
chr3	129346001	129351000	1.22E-27
chr3	129368001	129373000	1.89E-08
chr3	129509001	129514000	4.50E-14
chr3	129607001	129612000	1.82E-30

chr3	129608001	129613000	8.19E-35
chr3	129609001	129614000	3.34E-42
chr3	129610001	129615000	2.39E-34
chr3	129611001	129616000	4.04E-20
chr3	129612001	129617000	5.78E-16
chr3	129908001	129913000	6.18E-08
chr3	130035001	130040000	0.006517691
chr3	130036001	130041000	0.006209696
chr3	130037001	130042000	0.007742936
chr3	130056001	130061000	0.010321181
chr3	130190001	130195000	0.010959626
chr3	130196001	130201000	6.33E-06
chr3	130211001	130216000	1.18E-06
chr3	130231001	130236000	7.55E-20
chr3	130233001	130238000	7.95E-30
chr3	130234001	130239000	3.53E-43
chr3	130235001	130240000	4.97E-56
chr3	130236001	130241000	6.31E-44
chr3	130250001	130255000	4.58E-05
chr3	130267001	130272000	4.96E-06
chr3	130274001	130279000	5.97E-05
chr3	130325001	130330000	0.0051618
chr3	130326001	130331000	9.23E-05
chr3	130332001	130337000	5.79E-05
chr3	130333001	130338000	8.92E-05
chr3	130442001	130447000	5.29E-07
chr3	130593001	130598000	1.01E-08
chr3	130594001	130599000	1.38E-06
chr3	130613001	130618000	6.63E-18
chr3	130743001	130748000	6.49E-29
chr3	130744001	130749000	2.52E-32
chr3	130745001	130750000	3.86E-35
chr3	130797001	130802000	0.002913393
chr3	130799001	130804000	5.69E-05
chr3	130800001	130805000	0.000179548
chr3	131076001	131081000	1.77E-18
chr3	131077001	131082000	1.01E-18
chr3	131078001	131083000	3.04E-11
chr3	131079001	131084000	9.31E-17
chr3	131080001	131085000	5.02E-19
chr3	131081001	131086000	1.67E-13
chr3	131096001	131101000	6.87E-41
chr3	131097001	131102000	7.88E-36
chr3	131098001	131103000	6.71E-28
chr3	131099001	131104000	2.25E-16
chr3	131100001	131105000	3.91E-12
chr3	131135001	131140000	0.016403494
chr3	131138001	131143000	0.000638077

chr3	131420001	131425000	0.017521854
chr3	131703001	131708000	0.007182795
chr3	131704001	131709000	0.002870809
chr3	131705001	131710000	0.002833909
chr3	132131001	132136000	5.12E-16
chr3	132132001	132137000	2.73E-51
chr3	132374001	132379000	3.58E-22
chr3	132432001	132437000	2.39E-07
chr3	132433001	132438000	5.23E-06
chr3	132434001	132439000	3.03E-06
chr3	132436001	132441000	2.52E-19
chr3	132437001	132442000	1.23E-42
chr3	132438001	132443000	4.27E-47
chr3	132439001	132444000	8.73E-46
chr3	132440001	132445000	9.45E-45
chr3	132441001	132446000	4.99E-38
chr3	132644001	132649000	0.005060542
chr3	132645001	132650000	0.000320147
chr3	132646001	132651000	0.000581994
chr3	132648001	132653000	0.000291228
chr3	132731001	132736000	5.66E-05
chr3	132753001	132758000	6.24E-41
chr3	132789001	132794000	0.012057958
chr3	132790001	132795000	0.000312675
chr3	132810001	132815000	1.22E-05
chr3	132811001	132816000	1.98E-05
chr3	132859001	132864000	0.011912438
chr3	132860001	132865000	0.001697639
chr3	133520001	133525000	1.21E-24
chr3	133521001	133526000	8.26E-30
chr3	133522001	133527000	6.92E-39
chr3	133523001	133528000	1.34E-31
chr3	133744001	133749000	1.66E-40
chr3	133745001	133750000	1.02E-39
chr3	133746001	133751000	1.22E-35
chr3	133747001	133752000	3.85E-30
chr3	133748001	133753000	1.25E-37
chr3	134028001	134033000	6.64E-06
chr3	134487001	134492000	0.001047363
chr3	134500001	134505000	0.000356771
chr3	134908001	134913000	1.77E-06
chr3	134910001	134915000	2.18E-06
chr3	134970001	134975000	2.52E-10
chr3	135134001	135139000	9.50E-05
chr3	135135001	135140000	5.89E-06
chr3	135136001	135141000	0.000672072
chr3	135153001	135158000	1.78E-05
chr3	135312001	135317000	0.000244702

chr3	135313001	135318000	0.003770429
chr3	135314001	135319000	0.000497495
chr3	135359001	135364000	4.56E-05
chr3	135405001	135410000	3.41E-05
chr3	135406001	135411000	2.36E-08
chr3	135556001	135561000	0.000208794
chr3	135680001	135685000	6.25E-18
chr3	135681001	135686000	4.40E-26
chr3	135682001	135687000	6.76E-47
chr3	135683001	135688000	4.93E-48
chr3	135684001	135689000	2.15E-41
chr3	135685001	135690000	3.11E-24
chr3	135700001	135705000	9.23E-09
chr3	135701001	135706000	7.30E-09
chr3	135718001	135723000	4.98E-05
chr3	135838001	135843000	0.000679212
chr3	135965001	135970000	2.12E-13
chr3	136237001	136242000	3.67E-06
chr3	136238001	136243000	4.37E-06
chr3	136538001	136543000	8.75E-40
chr3	136539001	136544000	2.74E-17
chr3	136576001	136581000	1.45E-10
chr3	136577001	136582000	2.32E-10
chr3	136580001	136585000	1.54E-18
chr3	136581001	136586000	2.89E-14
chr3	136859001	136864000	3.68E-08
chr3	137024001	137029000	0.000183602
chr3	137025001	137030000	5.04E-05
chr3	137026001	137031000	4.14E-06
chr3	137032001	137037000	2.90E-08
chr3	137040001	137045000	6.33E-07
chr3	137041001	137046000	2.67E-07
chr3	137042001	137047000	1.42E-07
chr3	137082001	137087000	1.49E-05
chr3	137083001	137088000	8.94E-07
chr3	137088001	137093000	1.29E-08
chr3	137099001	137104000	6.59E-07
chr3	137100001	137105000	6.51E-06
chr3	137132001	137137000	3.99E-13
chr3	137150001	137155000	2.72E-08
chr3	137151001	137156000	4.45E-09
chr3	137152001	137157000	1.02E-08
chr3	137153001	137158000	1.54E-05
chr3	137154001	137159000	9.15E-06
chr3	137155001	137160000	2.69E-05
chr3	137156001	137161000	1.37E-05
chr3	137192001	137197000	0.000231181
chr3	137193001	137198000	3.24E-06

chr3	137194001	137199000	1.13E-06
chr3	137214001	137219000	0.001600383
chr3	137215001	137220000	0.000189673
chr3	137216001	137221000	0.002904873
chr3	137230001	137235000	0.003124977
chr3	137231001	137236000	0.003482232
chr3	137258001	137263000	9.27E-05
chr3	137266001	137271000	0.000104055
chr3	137267001	137272000	0.001708497
chr3	137276001	137281000	9.98E-09
chr3	137434001	137439000	4.43E-05
chr3	137516001	137521000	0.00011739
chr3	137517001	137522000	1.35E-06
chr3	137518001	137523000	2.46E-06
chr3	137528001	137533000	0.007764408
chr3	137541001	137546000	0.000118352
chr3	137554001	137559000	1.48E-10
chr3	137555001	137560000	6.26E-07
chr3	137651001	137656000	0.000801839
chr3	137654001	137659000	0.001072007
chr3	137655001	137660000	9.22E-09
chr3	137656001	137661000	5.11E-09
chr3	137687001	137692000	3.74E-08
chr3	137688001	137693000	1.44E-14
chr3	137689001	137694000	9.64E-13
chr3	137690001	137695000	9.78E-14
chr3	137829001	137834000	3.21E-13
chr3	137906001	137911000	4.59E-40
chr3	138029001	138034000	0.000613743
chr3	138067001	138072000	7.05E-13
chr3	138309001	138314000	7.40E-33
chr3	138313001	138318000	4.24E-34
chr3	138323001	138328000	1.77E-05
chr3	138327001	138332000	1.75E-21
chr3	138422001	138427000	7.77E-09
chr3	138423001	138428000	1.22E-08
chr3	138424001	138429000	2.24E-08
chr3	138746001	138751000	5.95E-09
chr3	138747001	138752000	9.80E-08
chr3	138790001	138795000	0.000236179
chr3	138791001	138796000	0.005800748
chr3	138960001	138965000	0.023816203
chr3	139063001	139068000	8.06E-22
chr3	139109001	139114000	1.09E-10
chr3	139214001	139219000	6.11E-06
chr3	139215001	139220000	7.69E-05
chr3	139392001	139397000	6.41E-21
chr3	139393001	139398000	1.15E-31

chr3	139394001	139399000	1.26E-30
chr3	139395001	139400000	7.55E-29
chr3	139396001	139401000	3.19E-38
chr3	139430001	139435000	0.000250498
chr3	139431001	139436000	0.000669938
chr3	139439001	139444000	0.000404212
chr3	139559001	139564000	3.70E-05
chr3	139646001	139651000	4.48E-06
chr3	139647001	139652000	2.06E-08
chr3	139649001	139654000	1.04E-27
chr3	139650001	139655000	4.55E-38
chr3	139712001	139717000	4.49E-11
chr3	139824001	139829000	0.000502647
chr3	140051001	140056000	6.82E-06
chr3	140373001	140378000	6.41E-06
chr3	140482001	140487000	2.93E-06
chr3	140483001	140488000	3.39E-09
chr3	140484001	140489000	4.49E-09
chr3	140485001	140490000	4.05E-09
chr3	140486001	140491000	1.41E-09
chr3	140500001	140505000	0.000521027
chr3	140501001	140506000	0.000423038
chr3	140502001	140507000	0.000291682
chr3	140503001	140508000	0.003936392
chr3	140519001	140524000	2.70E-10
chr3	140544001	140549000	0.00088671
chr3	140545001	140550000	0.000214592
chr3	140654001	140659000	5.40E-07
chr3	140655001	140660000	3.24E-08
chr3	140656001	140661000	1.58E-53
chr3	140657001	140662000	2.59E-63
chr3	140658001	140663000	8.69E-59
chr3	140659001	140664000	8.46E-76
chr3	140660001	140665000	1.27E-57
chr3	140661001	140666000	2.95E-31
chr3	140718001	140723000	0.000450416
chr3	140950001	140955000	2.04E-12
chr3	140951001	140956000	8.98E-18
chr3	141028001	141033000	6.01E-24
chr3	141029001	141034000	2.45E-25
chr3	141030001	141035000	3.50E-45
chr3	141081001	141086000	8.81E-07
chr3	141121001	141126000	6.70E-46
chr3	141201001	141206000	7.88E-24
chr3	141202001	141207000	8.58E-38
chr3	141206001	141211000	4.17E-29
chr3	141377001	141382000	2.47E-16
chr3	141379001	141384000	4.50E-16

chr3	141452001	141457000	7.38E-13
chr3	141453001	141458000	6.97E-48
chr3	141866001	141871000	3.35E-26
chr3	141868001	141873000	8.74E-30
chr3	142032001	142037000	7.51E-05
chr3	142439001	142444000	3.66E-23
chr3	142440001	142445000	1.97E-28
chr3	142441001	142446000	8.02E-41
chr3	142442001	142447000	7.93E-44
chr3	142603001	142608000	1.22E-59
chr3	142604001	142609000	5.31E-74
chr3	142605001	142610000	4.95E-82
chr3	142606001	142611000	1.84E-68
chr3	142607001	142612000	2.37E-58
chr3	142608001	142613000	4.34E-18
chr3	142744001	142749000	0.00093591
chr3	142745001	142750000	0.000376501
chr3	143011001	143016000	5.85E-05
chr3	143263001	143268000	4.08E-07
chr3	143273001	143278000	6.19E-05
chr3	143274001	143279000	0.000103812
chr3	143275001	143280000	9.16E-05
chr3	143305001	143310000	0.00355524
chr3	143335001	143340000	3.20E-10
chr3	143382001	143387000	2.80E-14
chr3	143383001	143388000	2.36E-09
chr3	143384001	143389000	1.05E-10
chr3	143406001	143411000	0.000521881
chr3	143407001	143412000	0.000234879
chr3	143466001	143471000	5.63E-10
chr3	143467001	143472000	1.50E-09
chr3	143576001	143581000	3.53E-05
chr3	143577001	143582000	2.70E-05
chr3	143685001	143690000	9.65E-24
chr3	144017001	144022000	0.000260456
chr3	144094001	144099000	0.005254544
chr3	144394001	144399000	0.002735167
chr3	144573001	144578000	8.48E-05
chr3	144574001	144579000	0.000718924
chr3	144577001	144582000	0.00020389
chr3	145084001	145089000	0.023389005
chr3	145489001	145494000	0.004061537
chr3	145879001	145884000	6.22E-19
chr3	145964001	145969000	1.79E-47
chr3	145968001	145973000	1.53E-16
chr3	146187001	146192000	3.65E-28
chr3	146262001	146267000	1.55E-83
chr3	146386001	146391000	0.038951356

chr3	146387001	146392000	0.001759729
chr3	146388001	146393000	0.002249263
chr3	148705001	148710000	5.89E-78
chr3	148709001	148714000	1.32E-91
chr3	148893001	148898000	0.000237649
chr3	148894001	148899000	1.44E-08
chr3	149682001	149687000	0.000459154
chr3	150317001	150322000	6.37E-75
chr3	150803001	150808000	1.51E-69
chr3	150804001	150809000	1.27E-53
chr3	151015001	151020000	0.000682947
chr3	151046001	151051000	0.003171112
chr3	151048001	151053000	0.001160936
chr3	151174001	151179000	1.11E-49
chr3	151175001	151180000	1.69E-36
chr3	151176001	151181000	1.46E-33
chr3	151177001	151182000	1.49E-43
chr3	151178001	151183000	2.60E-44
chr3	151258001	151263000	0.00051203
chr3	151336001	151341000	0.005717112
chr3	152044001	152049000	6.28E-06
chr3	152045001	152050000	4.66E-05
chr3	152091001	152096000	1.51E-07
chr3	152350001	152355000	0.000159729
chr3	152422001	152427000	4.21E-05
chr3	152649001	152654000	3.83E-08
chr3	152650001	152655000	1.55E-11
chr3	152651001	152656000	1.35E-10
chr3	152652001	152657000	1.01E-06
chr3	153758001	153763000	0.001789882
chr3	155519001	155524000	3.88E-26
chr3	156273001	156278000	4.47E-10
chr3	156320001	156325000	7.30E-23
chr3	156321001	156326000	5.13E-25
chr3	156322001	156327000	1.99E-25
chr3	156323001	156328000	1.44E-22
chr3	156359001	156364000	4.01E-17
chr3	156360001	156365000	6.89E-16
chr3	156361001	156366000	6.34E-13
chr3	156387001	156392000	4.36E-23
chr3	157155001	157160000	2.03E-81
chr3	157881001	157886000	3.51E-05
chr3	157882001	157887000	1.04E-05
chr3	157883001	157888000	1.90E-05
chr3	157884001	157889000	0.003102019
chr3	157885001	157890000	0.002846391
chr3	158113001	158118000	4.23E-18
chr3	158284001	158289000	2.43E-50

chr3	158285001	158290000	7.71E-56
chr3	158289001	158294000	2.18E-36
chr3	158832001	158837000	1.27E-05
chr3	159130001	159135000	8.30E-05
chr3	159702001	159707000	4.35E-28
chr3	159706001	159711000	2.08E-26
chr3	160818001	160823000	1.21E-68
chr3	160819001	160824000	2.57E-72
chr3	160820001	160825000	2.58E-76
chr3	160821001	160826000	4.43E-75
chr3	160822001	160827000	4.22E-56
chr3	160823001	160828000	1.03E-45
chr3	161085001	161090000	5.84E-46
chr3	163157001	163162000	0.009317711
chr3	163727001	163732000	0.000466218
chr3	164773001	164778000	0.023791677
chr3	165010001	165015000	0.000108611
chr3	165194001	165199000	1.14E-16
chr3	166192001	166197000	0.018317776
chr3	167097001	167102000	1.27E-27
chr3	167098001	167103000	3.50E-27
chr3	167762001	167767000	0.002117666
chr3	168867001	168872000	1.21E-40
chr3	168873001	168878000	3.81E-08
chr3	168874001	168879000	1.92E-08
chr3	169061001	169066000	1.07E-07
chr3	169062001	169067000	4.54E-07
chr3	169106001	169111000	0.010516672
chr3	169165001	169170000	0.010553359
chr3	169290001	169295000	7.21E-10
chr3	169291001	169296000	4.22E-12
chr3	169292001	169297000	2.18E-12
chr3	169293001	169298000	3.05E-12
chr3	169478001	169483000	9.19E-58
chr3	169479001	169484000	1.26E-73
chr3	169480001	169485000	2.80E-85
chr3	169481001	169486000	3.96E-60
chr3	169482001	169487000	9.74E-62
chr3	169483001	169488000	3.02E-45
chr3	169484001	169489000	6.31E-34
chr3	169526001	169531000	6.96E-56
chr3	169756001	169761000	1.06E-31
chr3	169825001	169830000	3.87E-26
chr3	169826001	169831000	5.30E-21
chr3	169827001	169832000	1.14E-17
chr3	169828001	169833000	8.45E-18
chr3	169829001	169834000	3.25E-16
chr3	169938001	169943000	8.08E-50

chr3	169939001	169944000	1.69E-70
chr3	169940001	169945000	3.06E-108
chr3	170070001	170075000	5.53E-44
chr3	170071001	170076000	5.53E-77
chr3	170512001	170517000	8.69E-05
chr3	170588001	170593000	5.61E-31
chr3	170621001	170626000	3.09E-30
chr3	170657001	170662000	1.22E-05
chr3	170791001	170796000	0.006740213
chr3	170792001	170797000	0.007090148
chr3	170895001	170900000	6.42E-11
chr3	170896001	170901000	1.23E-15
chr3	170897001	170902000	2.30E-15
chr3	170898001	170903000	2.32E-09
chr3	170904001	170909000	0.000344261
chr3	170933001	170938000	0.000699739
chr3	170968001	170973000	4.34E-11
chr3	170969001	170974000	1.46E-09
chr3	170980001	170985000	2.54E-06
chr3	170981001	170986000	3.03E-07
chr3	170982001	170987000	2.57E-05
chr3	171014001	171019000	0.00058313
chr3	171100001	171105000	1.57E-07
chr3	171101001	171106000	3.93E-08
chr3	171102001	171107000	2.39E-10
chr3	171103001	171108000	3.32E-06
chr3	171125001	171130000	3.00E-12
chr3	171126001	171131000	4.45E-12
chr3	171321001	171326000	3.00E-05
chr3	171322001	171327000	0.000147755
chr3	171323001	171328000	2.26E-05
chr3	171528001	171533000	7.57E-43
chr3	172951001	172956000	0.001152896
chr3	173116001	173121000	1.07E-10
chr3	174159001	174164000	2.73E-46
chr3	174996001	175001000	0.020542964
chr3	177455001	177460000	0.000354588
chr3	177933001	177938000	1.42E-07
chr3	177934001	177939000	2.17E-07
chr3	177935001	177940000	2.07E-08
chr3	177936001	177941000	9.04E-11
chr3	178338001	178343000	0.004166322
chr3	178438001	178443000	0.00270367
chr3	178493001	178498000	4.87E-07
chr3	178785001	178790000	5.74E-67
chr3	178893001	178898000	1.90E-05
chr3	178894001	178899000	6.70E-08
chr3	178895001	178900000	8.26E-07

chr3	178896001	178901000	8.07E-06
chr3	178975001	178980000	3.24E-31
chr3	179025001	179030000	0.003456351
chr3	179207001	179212000	4.44E-05
chr3	179318001	179323000	1.41E-36
chr3	179972001	179977000	0.001565073
chr3	180320001	180325000	2.95E-44
chr3	181181001	181186000	0.000323258
chr3	181238001	181243000	0.000370379
chr3	181295001	181300000	0.009000872
chr3	181313001	181318000	1.04E-05
chr3	181314001	181319000	1.96E-06
chr3	181439001	181444000	1.54E-48
chr3	181440001	181445000	9.87E-86
chr3	182512001	182517000	1.75E-15
chr3	182698001	182703000	6.44E-34
chr3	182813001	182818000	4.32E-73
chr3	182972001	182977000	3.85E-85
chr3	183142001	183147000	5.09E-57
chr3	183143001	183148000	5.66E-41
chr3	183144001	183149000	3.82E-55
chr3	183411001	183416000	3.47E-25
chr3	183539001	183544000	3.90E-40
chr3	183542001	183547000	1.28E-33
chr3	184028001	184033000	2.55E-31
chr3	184425001	184430000	6.67E-29
chr3	184525001	184530000	1.09E-10
chr3	184526001	184531000	5.80E-28
chr3	184527001	184532000	2.67E-34
chr3	184528001	184533000	5.83E-37
chr3	184529001	184534000	2.98E-31
chr3	184530001	184535000	5.97E-38
chr3	184866001	184871000	3.45E-42
chr3	185531001	185536000	5.93E-09
chr3	185543001	185548000	8.71E-79
chr3	186280001	186285000	5.09E-22
chr3	186365001	186370000	0.001692172
chr3	186420001	186425000	9.18E-07
chr3	186644001	186649000	9.47E-52
chr3	186857001	186862000	2.26E-41
chr3	186883001	186888000	1.17E-09
chr3	187090001	187095000	2.86E-08
chr3	187103001	187108000	5.21E-05
chr3	187104001	187109000	3.39E-06
chr3	187105001	187110000	3.13E-07
chr3	187106001	187111000	1.22E-07
chr3	187168001	187173000	3.05E-06
chr3	187196001	187201000	3.84E-06

chr3	187468001	187473000	7.61E-08
chr3	187967001	187972000	3.87E-06
chr3	188109001	188114000	0.000100429
chr3	188139001	188144000	4.68E-06
chr3	188227001	188232000	0.000135959
chr3	188295001	188300000	4.19E-07
chr3	188296001	188301000	3.78E-10
chr3	188297001	188302000	3.61E-10
chr3	188329001	188334000	3.06E-05
chr3	188330001	188335000	1.57E-06
chr3	188331001	188336000	5.26E-07
chr3	188332001	188337000	2.69E-09
chr3	188333001	188338000	1.48E-11
chr3	188343001	188348000	6.70E-06
chr3	188404001	188409000	7.16E-12
chr3	188405001	188410000	8.37E-10
chr3	188453001	188458000	0.000565484
chr3	188464001	188469000	9.45E-08
chr3	188465001	188470000	1.06E-09
chr3	188466001	188471000	3.86E-10
chr3	188467001	188472000	3.45E-07
chr3	188528001	188533000	8.67E-06
chr3	188529001	188534000	7.30E-07
chr3	188535001	188540000	2.82E-09
chr3	188536001	188541000	1.54E-09
chr3	188537001	188542000	6.81E-07
chr3	188579001	188584000	0.000128704
chr3	188580001	188585000	4.34E-07
chr3	188581001	188586000	4.26E-07
chr3	188582001	188587000	4.19E-06
chr3	188583001	188588000	1.45E-06
chr3	188727001	188732000	3.23E-05
chr3	188805001	188810000	0.000751247
chr3	188986001	188991000	3.68E-16
chr3	188989001	188994000	1.61E-11
chr3	188990001	188995000	3.79E-08
chr3	189124001	189129000	1.94E-10
chr3	189125001	189130000	2.63E-10
chr3	189126001	189131000	1.81E-11
chr3	189127001	189132000	3.42E-13
chr3	189128001	189133000	1.28E-10
chr3	189796001	189801000	2.74E-10
chr3	189838001	189843000	1.21E-38
chr3	189868001	189873000	3.82E-07
chr3	189931001	189936000	0.001012458
chr3	189942001	189947000	3.69E-05
chr3	189944001	189949000	3.06E-07
chr3	189945001	189950000	6.79E-09

chr3	189946001	189951000	0.00011652
chr3	190010001	190015000	3.11E-05
chr3	190138001	190143000	5.78E-11
chr3	190139001	190144000	5.59E-09
chr3	190140001	190145000	1.33E-07
chr3	190141001	190146000	3.63E-07
chr3	190142001	190147000	1.14E-06
chr3	190170001	190175000	1.74E-06
chr3	190227001	190232000	1.46E-79
chr3	190242001	190247000	6.40E-06
chr3	190294001	190299000	1.76E-09
chr3	190343001	190348000	1.95E-06
chr3	191047001	191052000	3.79E-37
chr3	191310001	191315000	0.005219174
chr3	191874001	191879000	8.75E-14
chr3	191875001	191880000	4.22E-13
chr3	191876001	191881000	6.18E-13
chr3	192363001	192368000	0.005601109
chr3	192440001	192445000	1.95E-15
chr3	192888001	192893000	5.26E-14
chr3	192941001	192946000	2.65E-06
chr3	192954001	192959000	2.87E-59
chr3	192955001	192960000	1.27E-93
chr3	192956001	192961000	1.26E-75
chr3	192957001	192962000	8.78E-66
chr3	192959001	192964000	5.10E-36
chr3	193051001	193056000	5.12E-08
chr3	193103001	193108000	8.73E-10
chr3	193126001	193131000	1.44E-06
chr3	193157001	193162000	0.000177612
chr3	193169001	193174000	1.87E-10
chr3	193190001	193195000	2.39E-09
chr3	193191001	193196000	3.31E-12
chr3	193193001	193198000	7.72E-12
chr3	193194001	193199000	3.21E-10
chr3	193210001	193215000	6.19E-06
chr3	193221001	193226000	0.000367575
chr3	193222001	193227000	0.000101068
chr3	193917001	193922000	1.92E-25
chr3	194204001	194209000	2.99E-87
chr3	194207001	194212000	1.09E-75
chr3	194824001	194829000	6.61E-26
chr3	194825001	194830000	5.07E-27
chr3	194826001	194831000	6.95E-26
chr3	194992001	194997000	1.49E-51
chr3	195163001	195168000	1.86E-60
chr3	195715001	195720000	2.73E-130
chr3	196040001	196045000	5.48E-44

chr3	196230001	196235000	3.05E-31
chr3	196291001	196296000	1.04E-68
chr3	196355001	196360000	3.10E-37
chr3	196590001	196595000	3.21E-40
chr3	196692001	196697000	1.59E-34
chr3	197020001	197025000	6.95E-47
chr3	197138001	197143000	3.28E-11
chr4	21001	26000	4.73E-07
chr4	22001	27000	5.24E-08
chr4	89001	94000	0.000204808
chr4	232001	237000	1.73E-16
chr4	233001	238000	1.97E-19
chr4	234001	239000	3.71E-20
chr4	326001	331000	2.41E-25
chr4	327001	332000	4.24E-24
chr4	330001	335000	1.51E-21
chr4	331001	336000	1.64E-27
chr4	332001	337000	7.65E-21
chr4	463001	468000	4.68E-29
chr4	488001	493000	7.79E-35
chr4	489001	494000	5.47E-31
chr4	663001	668000	9.32E-16
chr4	664001	669000	2.72E-45
chr4	665001	670000	7.88E-34
chr4	666001	671000	3.11E-30
chr4	770001	775000	6.00E-22
chr4	771001	776000	1.68E-41
chr4	923001	928000	1.66E-22
chr4	924001	929000	1.06E-39
chr4	925001	930000	5.09E-40
chr4	926001	931000	2.75E-27
chr4	964001	969000	1.86E-13
chr4	965001	970000	1.50E-10
chr4	966001	971000	5.29E-18
chr4	1003001	1008000	3.53E-07
chr4	1103001	1108000	2.11E-10
chr4	1152001	1157000	0.000140177
chr4	1241001	1246000	9.51E-08
chr4	1242001	1247000	3.63E-22
chr4	1682001	1687000	2.51E-20
chr4	1709001	1714000	1.53E-19
chr4	1718001	1723000	1.54E-07
chr4	1792001	1797000	9.03E-07
chr4	1853001	1858000	2.16E-29
chr4	1854001	1859000	6.97E-28
chr4	1855001	1860000	2.08E-33
chr4	1856001	1861000	1.04E-27
chr4	1857001	1862000	7.99E-18

chr4	1868001	1873000	1.15E-19
chr4	1869001	1874000	1.78E-31
chr4	1871001	1876000	3.52E-26
chr4	2006001	2011000	1.41E-37
chr4	2007001	2012000	1.31E-43
chr4	2010001	2015000	9.09E-41
chr4	2011001	2016000	4.22E-20
chr4	2039001	2044000	1.01E-43
chr4	2040001	2045000	7.74E-31
chr4	2057001	2062000	1.22E-22
chr4	2058001	2063000	1.27E-09
chr4	2416001	2421000	1.43E-17
chr4	2466001	2471000	2.89E-18
chr4	2467001	2472000	5.11E-38
chr4	2468001	2473000	7.12E-43
chr4	2469001	2474000	3.92E-47
chr4	2470001	2475000	4.51E-30
chr4	2471001	2476000	6.13E-22
chr4	2533001	2538000	2.16E-09
chr4	2536001	2541000	4.40E-20
chr4	2537001	2542000	1.00E-18
chr4	2538001	2543000	4.07E-11
chr4	2757001	2762000	7.64E-11
chr4	2792001	2797000	2.19E-10
chr4	2793001	2798000	1.51E-09
chr4	2794001	2799000	6.65E-08
chr4	2795001	2800000	1.54E-06
chr4	2817001	2822000	6.26E-05
chr4	2820001	2825000	1.53E-06
chr4	2841001	2846000	3.43E-21
chr4	3295001	3300000	8.46E-10
chr4	4069001	4074000	4.91E-06
chr4	4070001	4075000	6.05E-09
chr4	4071001	4076000	4.10E-10
chr4	4082001	4087000	1.62E-10
chr4	4083001	4088000	4.46E-08
chr4	4084001	4089000	1.04E-06
chr4	4105001	4110000	2.90E-12
chr4	4106001	4111000	3.50E-08
chr4	4384001	4389000	1.48E-09
chr4	4500001	4505000	8.12E-06
chr4	4539001	4544000	5.20E-61
chr4	4540001	4545000	3.74E-57
chr4	4541001	4546000	6.09E-40
chr4	4542001	4547000	1.34E-58
chr4	4543001	4548000	1.19E-54
chr4	4573001	4578000	2.41E-07
chr4	4574001	4579000	3.55E-10

chr4	4575001	4580000	8.24E-11
chr4	4739001	4744000	6.60E-07
chr4	5111001	5116000	0.0032713
chr4	5112001	5117000	7.72E-07
chr4	5113001	5118000	1.86E-08
chr4	5114001	5119000	6.85E-08
chr4	5155001	5160000	1.15E-12
chr4	5171001	5176000	3.71E-09
chr4	5172001	5177000	1.48E-13
chr4	5181001	5186000	1.85E-08
chr4	5253001	5258000	5.11E-07
chr4	5292001	5297000	6.08E-07
chr4	5293001	5298000	8.65E-09
chr4	5316001	5321000	3.81E-06
chr4	5317001	5322000	1.26E-08
chr4	5318001	5323000	1.92E-08
chr4	5417001	5422000	1.11E-08
chr4	5432001	5437000	1.90E-05
chr4	5489001	5494000	2.24E-06
chr4	5644001	5649000	2.08E-08
chr4	6201001	6206000	2.31E-12
chr4	6267001	6272000	7.51E-21
chr4	6468001	6473000	2.82E-21
chr4	6469001	6474000	9.21E-12
chr4	6564001	6569000	1.03E-10
chr4	6565001	6570000	3.48E-11
chr4	6643001	6648000	1.72E-31
chr4	6783001	6788000	8.21E-42
chr4	6784001	6789000	1.19E-52
chr4	6785001	6790000	3.44E-21
chr4	6911001	6916000	4.73E-22
chr4	6984001	6989000	1.75E-38
chr4	7040001	7045000	3.67E-16
chr4	7041001	7046000	6.15E-25
chr4	7042001	7047000	1.45E-33
chr4	7043001	7048000	1.38E-21
chr4	7044001	7049000	3.24E-16
chr4	7065001	7070000	9.83E-56
chr4	7066001	7071000	3.80E-48
chr4	7067001	7072000	1.16E-23
chr4	7101001	7106000	6.84E-06
chr4	7102001	7107000	1.40E-06
chr4	7103001	7108000	8.16E-09
chr4	7190001	7195000	4.04E-09
chr4	8155001	8160000	4.32E-12
chr4	8156001	8161000	4.79E-25
chr4	8426001	8431000	3.26E-24
chr4	8427001	8432000	7.80E-36

chr4	8428001	8433000	2.28E-36
chr4	8429001	8434000	2.21E-26
chr4	8430001	8435000	7.41E-25
chr4	8709001	8714000	2.65E-09
chr4	8710001	8715000	4.00E-09
chr4	8842001	8847000	2.86E-08
chr4	8923001	8928000	0.00018541
chr4	8929001	8934000	0.001012198
chr4	8961001	8966000	1.40E-06
chr4	9048001	9053000	1.53E-08
chr4	9150001	9155000	6.48E-07
chr4	9236001	9241000	1.91E-20
chr4	9237001	9242000	3.00E-37
chr4	9238001	9243000	1.99E-43
chr4	9239001	9244000	1.99E-43
chr4	9240001	9245000	1.14E-45
chr4	9366001	9371000	1.45E-21
chr4	9373001	9378000	7.35E-07
chr4	9374001	9379000	3.34E-06
chr4	9413001	9418000	0.000466798
chr4	9414001	9419000	0.001111324
chr4	9415001	9420000	0.001416198
chr4	9438001	9443000	0.0009931
chr4	9458001	9463000	3.71E-08
chr4	9460001	9465000	9.95E-11
chr4	9472001	9477000	1.05E-05
chr4	9523001	9528000	3.08E-08
chr4	9524001	9529000	2.03E-11
chr4	9525001	9530000	4.98E-10
chr4	9531001	9536000	0.000250161
chr4	9532001	9537000	1.35E-05
chr4	9580001	9585000	9.98E-08
chr4	9581001	9586000	1.12E-08
chr4	9582001	9587000	1.30E-10
chr4	9596001	9601000	4.21E-05
chr4	9634001	9639000	1.71E-06
chr4	9636001	9641000	7.37E-06
chr4	9637001	9642000	4.95E-11
chr4	9638001	9643000	9.39E-12
chr4	9683001	9688000	4.21E-11
chr4	10209001	10214000	8.95E-05
chr4	10211001	10216000	1.29E-05
chr4	10220001	10225000	4.70E-11
chr4	10222001	10227000	7.50E-07
chr4	10223001	10228000	5.64E-06
chr4	10251001	10256000	2.19E-16
chr4	10330001	10335000	4.53E-07
chr4	10454001	10459000	1.31E-66

chr4	10455001	10460000	2.80E-39
chr4	10456001	10461000	5.14E-28
chr4	10682001	10687000	2.59E-06
chr4	10730001	10735000	5.05E-05
chr4	10731001	10736000	6.72E-06
chr4	10894001	10899000	0.000533408
chr4	11018001	11023000	0.000386033
chr4	11156001	11161000	0.00053872
chr4	11157001	11162000	0.000234305
chr4	11190001	11195000	6.21E-06
chr4	11425001	11430000	1.56E-11
chr4	11426001	11431000	2.22E-18
chr4	11430001	11435000	8.76E-38
chr4	11798001	11803000	4.12E-05
chr4	12064001	12069000	8.16E-05
chr4	12222001	12227000	0.000115206
chr4	12223001	12228000	9.58E-05
chr4	12464001	12469000	4.67E-07
chr4	12491001	12496000	0.000822228
chr4	12492001	12497000	0.002380474
chr4	13015001	13020000	0.000581123
chr4	13099001	13104000	1.27E-05
chr4	13405001	13410000	1.10E-13
chr4	13481001	13486000	4.39E-32
chr4	13484001	13489000	2.34E-38
chr4	13485001	13490000	7.44E-56
chr4	13486001	13491000	1.25E-19
chr4	13624001	13629000	2.92E-17
chr4	13625001	13630000	2.47E-41
chr4	13626001	13631000	2.94E-43
chr4	13629001	13634000	1.65E-34
chr4	14391001	14396000	0.008241183
chr4	14469001	14474000	0.001812816
chr4	15005001	15010000	6.52E-71
chr4	15006001	15011000	1.55E-44
chr4	15166001	15171000	0.005294406
chr4	15214001	15219000	0.003975501
chr4	15215001	15220000	0.001986859
chr4	15216001	15221000	0.003484212
chr4	15217001	15222000	0.004225863
chr4	15451001	15456000	0.000860703
chr4	15798001	15803000	4.86E-05
chr4	15835001	15840000	7.39E-06
chr4	15836001	15841000	1.30E-05
chr4	15837001	15842000	3.81E-08
chr4	15846001	15851000	1.44E-09
chr4	15847001	15852000	5.39E-07
chr4	15848001	15853000	1.49E-07

chr4	16029001	16034000	0.002010061
chr4	16229001	16234000	7.87E-33
chr4	16230001	16235000	4.28E-11
chr4	16328001	16333000	0.001319064
chr4	16329001	16334000	0.00299263
chr4	16330001	16335000	0.00283939
chr4	16613001	16618000	1.07E-06
chr4	16674001	16679000	1.04E-06
chr4	16675001	16680000	6.03E-07
chr4	16816001	16821000	1.92E-07
chr4	16817001	16822000	5.30E-08
chr4	16818001	16823000	1.53E-06
chr4	16877001	16882000	0.000483064
chr4	16948001	16953000	1.10E-06
chr4	16949001	16954000	7.92E-07
chr4	16952001	16957000	1.45E-05
chr4	17080001	17085000	1.55E-06
chr4	17390001	17395000	8.76E-06
chr4	17778001	17783000	1.45E-28
chr4	17806001	17811000	3.67E-13
chr4	18022001	18027000	1.80E-55
chr4	18023001	18028000	1.62E-56
chr4	18024001	18029000	3.40E-26
chr4	18029001	18034000	0.004207849
chr4	18030001	18035000	0.003427924
chr4	18315001	18320000	0.00035758
chr4	18674001	18679000	1.87E-05
chr4	18727001	18732000	0.008286783
chr4	19059001	19064000	5.03E-22
chr4	19060001	19065000	1.94E-13
chr4	19143001	19148000	0.003623181
chr4	19155001	19160000	0.002187509
chr4	19156001	19161000	0.00178814
chr4	19157001	19162000	0.003234005
chr4	19551001	19556000	0.005119179
chr4	20448001	20453000	0.001646093
chr4	20502001	20507000	0.029416646
chr4	20699001	20704000	1.76E-33
chr4	20700001	20705000	3.03E-30
chr4	20701001	20706000	2.45E-33
chr4	20702001	20707000	3.26E-39
chr4	20739001	20744000	1.16E-25
chr4	20740001	20745000	8.33E-20
chr4	20741001	20746000	1.11E-21
chr4	20742001	20747000	4.33E-14
chr4	20743001	20748000	1.24E-16
chr4	20848001	20853000	0.003340973
chr4	20849001	20854000	0.004649981

chr4	20872001	20877000	4.87E-05
chr4	20873001	20878000	2.87E-05
chr4	20876001	20881000	2.72E-05
chr4	20983001	20988000	1.78E-12
chr4	21099001	21104000	0.002103033
chr4	21100001	21105000	0.00178667
chr4	21160001	21165000	0.005309306
chr4	21223001	21228000	4.79E-07
chr4	21224001	21229000	8.20E-09
chr4	21225001	21230000	1.02E-11
chr4	21226001	21231000	3.31E-08
chr4	21227001	21232000	3.58E-09
chr4	21235001	21240000	2.96E-05
chr4	21236001	21241000	8.70E-05
chr4	21241001	21246000	7.65E-05
chr4	21301001	21306000	1.30E-08
chr4	21302001	21307000	1.79E-10
chr4	21303001	21308000	8.85E-13
chr4	21304001	21309000	3.46E-15
chr4	21305001	21310000	5.55E-14
chr4	21306001	21311000	8.34E-15
chr4	21333001	21338000	0.000615596
chr4	21368001	21373000	0.000518434
chr4	21379001	21384000	0.000206225
chr4	21380001	21385000	0.000277252
chr4	21381001	21386000	0.00192141
chr4	21523001	21528000	0.011797495
chr4	21695001	21700000	2.43E-09
chr4	21696001	21701000	4.98E-13
chr4	21697001	21702000	1.24E-11
chr4	21698001	21703000	3.67E-11
chr4	21699001	21704000	2.36E-09
chr4	22134001	22139000	0.003981675
chr4	22263001	22268000	0.000337527
chr4	22517001	22522000	1.02E-30
chr4	22985001	22990000	0.004209737
chr4	23227001	23232000	0.003461449
chr4	23228001	23233000	0.000441347
chr4	23277001	23282000	0.001670688
chr4	23278001	23283000	0.002575943
chr4	23618001	23623000	0.004271112
chr4	24039001	24044000	0.007497421
chr4	24129001	24134000	6.92E-06
chr4	24130001	24135000	8.02E-06
chr4	24910001	24915000	7.37E-24
chr4	24911001	24916000	2.15E-28
chr4	24912001	24917000	6.69E-24
chr4	24913001	24918000	7.38E-34

chr4	24914001	24919000	9.97E-44
chr4	25028001	25033000	6.27E-16
chr4	25029001	25034000	7.56E-13
chr4	25031001	25036000	1.35E-13
chr4	25032001	25037000	5.23E-13
chr4	25157001	25162000	9.63E-42
chr4	25158001	25163000	4.18E-28
chr4	25231001	25236000	6.46E-36
chr4	25232001	25237000	1.23E-44
chr4	25233001	25238000	4.31E-33
chr4	25234001	25239000	7.72E-39
chr4	25235001	25240000	2.46E-31
chr4	25264001	25269000	1.80E-06
chr4	25265001	25270000	9.42E-07
chr4	25266001	25271000	1.86E-06
chr4	25313001	25318000	7.99E-21
chr4	25314001	25319000	1.94E-23
chr4	25864001	25869000	3.53E-14
chr4	26210001	26215000	2.83E-07
chr4	26382001	26387000	5.07E-10
chr4	26383001	26388000	2.64E-09
chr4	26384001	26389000	1.94E-09
chr4	26863001	26868000	1.33E-31
chr4	27221001	27226000	0.000724035
chr4	27338001	27343000	0.000256368
chr4	27589001	27594000	0.000559319
chr4	27792001	27797000	0.001052949
chr4	28528001	28533000	0.000999718
chr4	29934001	29939000	0.000149196
chr4	29969001	29974000	0.018915928
chr4	29970001	29975000	0.013964587
chr4	29971001	29976000	0.004898335
chr4	29972001	29977000	0.005793535
chr4	30011001	30016000	0.00064643
chr4	30669001	30674000	0.001410031
chr4	30723001	30728000	1.39E-26
chr4	30839001	30844000	1.30E-05
chr4	31316001	31321000	4.06E-05
chr4	31747001	31752000	1.85E-08
chr4	31748001	31753000	1.85E-08
chr4	31870001	31875000	2.14E-11
chr4	34964001	34969000	0.004445063
chr4	35741001	35746000	5.46E-05
chr4	35742001	35747000	3.49E-06
chr4	35974001	35979000	0.000338017
chr4	36246001	36251000	7.94E-30
chr4	36405001	36410000	0.007898772
chr4	36406001	36411000	0.003181071

chr4	36407001	36412000	0.003962051
chr4	36408001	36413000	0.007745971
chr4	36652001	36657000	0.001027365
chr4	36841001	36846000	0.028603909
chr4	36842001	36847000	0.026759457
chr4	37129001	37134000	0.000112783
chr4	37130001	37135000	6.45E-05
chr4	37131001	37136000	0.000254871
chr4	37233001	37238000	2.94E-05
chr4	37300001	37305000	0.001171412
chr4	37329001	37334000	8.88E-05
chr4	37453001	37458000	4.11E-39
chr4	37454001	37459000	6.36E-52
chr4	37455001	37460000	4.51E-58
chr4	37683001	37688000	1.52E-23
chr4	37978001	37983000	8.84E-35
chr4	37979001	37984000	1.74E-33
chr4	38294001	38299000	0.003581427
chr4	38661001	38666000	4.89E-37
chr4	38662001	38667000	1.87E-35
chr4	38663001	38668000	6.01E-31
chr4	38664001	38669000	2.51E-23
chr4	39030001	39035000	0.000187415
chr4	39042001	39047000	2.10E-14
chr4	39043001	39048000	1.60E-21
chr4	39045001	39050000	7.67E-35
chr4	39046001	39051000	7.03E-34
chr4	39047001	39052000	6.15E-18
chr4	39179001	39184000	2.14E-15
chr4	39333001	39338000	0.000338398
chr4	39380001	39385000	0.00055036
chr4	39455001	39460000	1.92E-08
chr4	39456001	39461000	1.98E-52
chr4	39458001	39463000	3.22E-49
chr4	39459001	39464000	3.83E-45
chr4	39460001	39465000	1.79E-52
chr4	39695001	39700000	1.90E-15
chr4	39974001	39979000	2.25E-12
chr4	39975001	39980000	2.09E-20
chr4	39976001	39981000	2.26E-29
chr4	39977001	39982000	6.83E-40
chr4	39978001	39983000	3.21E-38
chr4	39979001	39984000	4.03E-24
chr4	40057001	40062000	3.56E-37
chr4	40058001	40063000	3.90E-37
chr4	40747001	40752000	1.03E-19
chr4	40748001	40753000	1.64E-43
chr4	40749001	40754000	8.82E-39

chr4	40750001	40755000	1.16E-41
chr4	40751001	40756000	1.40E-49
chr4	40752001	40757000	1.10E-47
chr4	41259001	41264000	1.16E-31
chr4	41356001	41361000	4.90E-06
chr4	41358001	41363000	2.36E-19
chr4	41359001	41364000	5.44E-18
chr4	41582001	41587000	6.74E-06
chr4	41583001	41588000	1.30E-06
chr4	41642001	41647000	8.86E-12
chr4	41643001	41648000	7.65E-11
chr4	41733001	41738000	0.000237985
chr4	41734001	41739000	0.001919717
chr4	41792001	41797000	3.49E-06
chr4	41869001	41874000	1.08E-13
chr4	41937001	41942000	1.59E-34
chr4	42206001	42211000	0.000510708
chr4	42395001	42400000	3.86E-17
chr4	42549001	42554000	1.89E-06
chr4	42550001	42555000	1.99E-07
chr4	42654001	42659000	7.79E-31
chr4	42657001	42662000	1.68E-61
chr4	42658001	42663000	8.55E-56
chr4	42659001	42664000	1.24E-41
chr4	42707001	42712000	2.58E-07
chr4	42708001	42713000	0.000208933
chr4	42709001	42714000	0.000260775
chr4	42710001	42715000	0.00133528
chr4	42734001	42739000	0.00422733
chr4	42748001	42753000	0.000304809
chr4	42834001	42839000	1.30E-06
chr4	42835001	42840000	1.88E-08
chr4	42836001	42841000	4.15E-06
chr4	42837001	42842000	4.53E-05
chr4	42848001	42853000	9.18E-07
chr4	42849001	42854000	1.94E-06
chr4	42850001	42855000	1.97E-07
chr4	42851001	42856000	1.38E-06
chr4	42852001	42857000	9.47E-07
chr4	43416001	43421000	2.69E-06
chr4	43417001	43422000	5.80E-09
chr4	43418001	43423000	1.01E-07
chr4	43611001	43616000	0.001689226
chr4	43819001	43824000	0.000694671
chr4	43820001	43825000	0.00111589
chr4	43822001	43827000	0.000488149
chr4	43891001	43896000	1.39E-07
chr4	43892001	43897000	8.30E-08

chr4	43900001	43905000	0.004575149
chr4	44141001	44146000	0.004817617
chr4	44449001	44454000	2.82E-41
chr4	44450001	44455000	4.39E-73
chr4	44451001	44456000	1.90E-15
chr4	44726001	44731000	1.76E-25
chr4	44727001	44732000	5.47E-26
chr4	44728001	44733000	1.31E-27
chr4	45273001	45278000	0.010472707
chr4	45391001	45396000	0.001729084
chr4	45392001	45397000	0.001337105
chr4	45450001	45455000	1.50E-08
chr4	45451001	45456000	1.33E-08
chr4	45452001	45457000	9.89E-08
chr4	46139001	46144000	1.41E-08
chr4	46185001	46190000	0.003390744
chr4	46220001	46225000	0.000130151
chr4	46221001	46226000	0.000133801
chr4	46289001	46294000	3.02E-05
chr4	46355001	46360000	0.001018478
chr4	46359001	46364000	0.00056909
chr4	46611001	46616000	2.09E-09
chr4	46613001	46618000	4.13E-12
chr4	46996001	47001000	1.75E-14
chr4	47034001	47039000	1.66E-30
chr4	47097001	47102000	0.000661305
chr4	47157001	47162000	0.005990232
chr4	47487001	47492000	1.35E-38
chr4	47605001	47610000	0.001712976
chr4	47660001	47665000	3.88E-06
chr4	47661001	47666000	6.48E-06
chr4	47802001	47807000	4.53E-09
chr4	47831001	47836000	4.70E-05
chr4	47945001	47950000	0.000103285
chr4	48014001	48019000	3.05E-35
chr4	48015001	48020000	9.77E-43
chr4	48016001	48021000	4.89E-38
chr4	48017001	48022000	4.84E-30
chr4	48019001	48024000	1.33E-14
chr4	48210001	48215000	6.34E-06
chr4	48211001	48216000	1.21E-07
chr4	48212001	48217000	4.45E-08
chr4	48267001	48272000	8.27E-28
chr4	48268001	48273000	1.58E-22
chr4	48270001	48275000	1.18E-20
chr4	48324001	48329000	0.012113048
chr4	48344001	48349000	7.78E-25
chr4	48416001	48421000	2.23E-06

chr4	48481001	48486000	9.85E-08
chr4	48482001	48487000	6.09E-10
chr4	48483001	48488000	1.99E-11
chr4	48484001	48489000	1.51E-19
chr4	48831001	48836000	4.86E-31
chr4	48833001	48838000	1.03E-19
chr4	48945001	48950000	3.60E-05
chr4	49126001	49131000	2.92E-16
chr4	49127001	49132000	2.45E-16
chr4	49133001	49138000	1.17E-07
chr4	49134001	49139000	4.04E-08
chr4	49143001	49148000	1.34E-64
chr4	49144001	49149000	1.23E-47
chr4	52704001	52709000	1.29E-23
chr4	52705001	52710000	2.59E-65
chr4	52706001	52711000	1.94E-67
chr4	52707001	52712000	6.95E-69
chr4	52709001	52714000	3.63E-57
chr4	52710001	52715000	1.34E-37
chr4	52711001	52716000	5.06E-25
chr4	52900001	52905000	4.28E-19
chr4	52901001	52906000	7.52E-24
chr4	52902001	52907000	4.37E-20
chr4	52903001	52908000	8.55E-23
chr4	52904001	52909000	3.12E-23
chr4	52913001	52918000	6.96E-47
chr4	53151001	53156000	0.016262338
chr4	53521001	53526000	5.70E-28
chr4	53574001	53579000	6.83E-21
chr4	53576001	53581000	2.27E-48
chr4	53577001	53582000	1.75E-45
chr4	53578001	53583000	7.75E-59
chr4	53579001	53584000	2.71E-37
chr4	53614001	53619000	5.00E-23
chr4	53615001	53620000	3.39E-27
chr4	53616001	53621000	6.42E-33
chr4	53617001	53622000	6.02E-47
chr4	53680001	53685000	1.22E-10
chr4	53681001	53686000	8.45E-10
chr4	53724001	53729000	1.67E-56
chr4	53725001	53730000	1.66E-64
chr4	53726001	53731000	9.13E-60
chr4	53727001	53732000	3.61E-30
chr4	53728001	53733000	6.90E-30
chr4	54227001	54232000	2.16E-31
chr4	54239001	54244000	8.00E-23
chr4	54240001	54245000	1.44E-48
chr4	54241001	54246000	4.67E-47

chr4	54242001	54247000	1.16E-40
chr4	54243001	54248000	9.74E-57
chr4	54244001	54249000	8.02E-50
chr4	54401001	54406000	7.54E-06
chr4	54568001	54573000	3.26E-10
chr4	54640001	54645000	0.000450044
chr4	54916001	54921000	5.80E-05
chr4	54917001	54922000	2.91E-06
chr4	54918001	54923000	1.96E-05
chr4	54925001	54930000	1.24E-06
chr4	54926001	54931000	1.66E-46
chr4	54928001	54933000	6.51E-33
chr4	54929001	54934000	4.30E-31
chr4	54930001	54935000	4.01E-40
chr4	54969001	54974000	2.13E-07
chr4	55328001	55333000	1.14E-06
chr4	55614001	55619000	3.96E-08
chr4	55910001	55915000	2.77E-08
chr4	55987001	55992000	6.54E-29
chr4	56258001	56263000	1.64E-65
chr4	56655001	56660000	6.71E-19
chr4	56656001	56661000	1.94E-14
chr4	56657001	56662000	2.44E-13
chr4	56658001	56663000	1.13E-12
chr4	56659001	56664000	5.77E-13
chr4	56715001	56720000	3.32E-18
chr4	56717001	56722000	1.46E-49
chr4	56718001	56723000	2.59E-49
chr4	56719001	56724000	1.05E-49
chr4	56720001	56725000	1.88E-21
chr4	56814001	56819000	7.32E-52
chr4	56815001	56820000	2.04E-63
chr4	56916001	56921000	1.13E-18
chr4	57303001	57308000	5.94E-11
chr4	57334001	57339000	1.77E-22
chr4	57378001	57383000	2.88E-07
chr4	57379001	57384000	1.37E-06
chr4	57392001	57397000	2.44E-09
chr4	57393001	57398000	9.69E-15
chr4	57394001	57399000	1.19E-19
chr4	57395001	57400000	9.00E-21
chr4	57516001	57521000	8.95E-05
chr4	57684001	57689000	7.51E-14
chr4	57685001	57690000	4.15E-18
chr4	57686001	57691000	1.83E-13
chr4	57838001	57843000	1.09E-14
chr4	57845001	57850000	8.81E-27
chr4	58385001	58390000	0.003157571

chr4	58422001	58427000	1.26E-09
chr4	58423001	58428000	9.69E-07
chr4	58424001	58429000	1.74E-08
chr4	58554001	58559000	0.003536842
chr4	58696001	58701000	0.00921846
chr4	58697001	58702000	0.009894835
chr4	58698001	58703000	0.005412179
chr4	58699001	58704000	0.006309656
chr4	58789001	58794000	3.44E-05
chr4	58822001	58827000	7.59E-06
chr4	58823001	58828000	1.29E-06
chr4	58824001	58829000	7.46E-06
chr4	58886001	58891000	6.99E-05
chr4	58887001	58892000	4.87E-05
chr4	59353001	59358000	0.001233784
chr4	59365001	59370000	0.002956091
chr4	60008001	60013000	1.48E-06
chr4	60777001	60782000	0.000140023
chr4	60778001	60783000	0.000194459
chr4	60779001	60784000	1.42E-05
chr4	60780001	60785000	8.83E-05
chr4	61174001	61179000	0.000219598
chr4	61175001	61180000	6.87E-05
chr4	61973001	61978000	9.94E-07
chr4	61974001	61979000	2.22E-07
chr4	62061001	62066000	6.06E-14
chr4	62062001	62067000	3.10E-58
chr4	62125001	62130000	0.000129177
chr4	62699001	62704000	0.003261249
chr4	63687001	63692000	1.11E-08
chr4	63689001	63694000	1.99E-09
chr4	64197001	64202000	6.34E-09
chr4	64368001	64373000	6.46E-05
chr4	64590001	64595000	0.000674523
chr4	64591001	64596000	0.000114229
chr4	64604001	64609000	7.08E-05
chr4	64711001	64716000	0.002658407
chr4	64712001	64717000	0.002873349
chr4	64763001	64768000	5.56E-06
chr4	64764001	64769000	5.81E-08
chr4	64765001	64770000	1.47E-06
chr4	65488001	65493000	0.000830568
chr4	65489001	65494000	7.22E-06
chr4	66011001	66016000	4.81E-06
chr4	66133001	66138000	4.86E-05
chr4	66134001	66139000	9.91E-08
chr4	66135001	66140000	0.000634092
chr4	68109001	68114000	0.012795403

chr4	68319001	68324000	9.45E-05
chr4	68562001	68567000	4.59E-22
chr4	68564001	68569000	5.31E-27
chr4	68566001	68571000	2.18E-26
chr4	68590001	68595000	0.000424333
chr4	68591001	68596000	0.000148109
chr4	68691001	68696000	0.004922662
chr4	68692001	68697000	0.009573245
chr4	68865001	68870000	0.004950745
chr4	69068001	69073000	0.004587023
chr4	69069001	69074000	0.001685938
chr4	69070001	69075000	0.001217419
chr4	69131001	69136000	8.65E-06
chr4	69613001	69618000	0.001690916
chr4	69614001	69619000	0.002301157
chr4	70044001	70049000	6.09E-05
chr4	70163001	70168000	0.000937213
chr4	70685001	70690000	0.001042624
chr4	70874001	70879000	7.28E-08
chr4	70999001	71004000	3.30E-07
chr4	71242001	71247000	4.60E-06
chr4	71331001	71336000	7.42E-06
chr4	71554001	71559000	3.52E-25
chr4	71566001	71571000	1.91E-65
chr4	71646001	71651000	3.87E-23
chr4	71647001	71652000	4.71E-26
chr4	71648001	71653000	1.54E-26
chr4	71649001	71654000	2.47E-19
chr4	71650001	71655000	7.02E-12
chr4	71764001	71769000	5.00E-18
chr4	71765001	71770000	1.33E-14
chr4	71766001	71771000	1.08E-15
chr4	71767001	71772000	5.88E-23
chr4	71768001	71773000	3.11E-22
chr4	71855001	71860000	2.99E-65
chr4	71856001	71861000	6.10E-52
chr4	71857001	71862000	9.27E-36
chr4	71858001	71863000	3.33E-39
chr4	71859001	71864000	1.39E-44
chr4	72053001	72058000	9.15E-38
chr4	72738001	72743000	7.11E-05
chr4	72739001	72744000	8.94E-06
chr4	72740001	72745000	6.46E-05
chr4	72741001	72746000	3.62E-05
chr4	72933001	72938000	0.000384193
chr4	72934001	72939000	0.001253896
chr4	72935001	72940000	0.000635927
chr4	73434001	73439000	2.23E-46

chr4	73435001	73440000	9.90E-44
chr4	73698001	73703000	0.001018525
chr4	73699001	73704000	4.19E-05
chr4	74085001	74090000	0.001626952
chr4	74086001	74091000	2.42E-06
chr4	74125001	74130000	1.83E-27
chr4	74337001	74342000	0.000155511
chr4	74402001	74407000	1.70E-07
chr4	74604001	74609000	0.000165482
chr4	74612001	74617000	0.001026839
chr4	74698001	74703000	2.88E-18
chr4	74699001	74704000	1.28E-19
chr4	74700001	74705000	5.15E-26
chr4	74701001	74706000	4.80E-23
chr4	74702001	74707000	1.19E-25
chr4	74718001	74723000	1.32E-08
chr4	74719001	74724000	8.77E-07
chr4	74731001	74736000	9.13E-61
chr4	74734001	74739000	1.47E-45
chr4	74735001	74740000	1.42E-47
chr4	74805001	74810000	1.85E-06
chr4	74807001	74812000	6.98E-11
chr4	74843001	74848000	2.39E-09
chr4	74844001	74849000	1.25E-10
chr4	74845001	74850000	7.64E-11
chr4	74852001	74857000	2.99E-05
chr4	74853001	74858000	4.91E-05
chr4	74855001	74860000	0.000260688
chr4	74860001	74865000	1.19E-43
chr4	74861001	74866000	2.42E-45
chr4	74862001	74867000	3.34E-57
chr4	74863001	74868000	1.63E-44
chr4	74864001	74869000	4.12E-41
chr4	74899001	74904000	1.77E-09
chr4	74900001	74905000	8.55E-70
chr4	74904001	74909000	3.33E-53
chr4	74947001	74952000	0.002561411
chr4	75006001	75011000	0.01368923
chr4	75019001	75024000	4.84E-70
chr4	75022001	75027000	2.06E-75
chr4	75024001	75029000	2.46E-38
chr4	75093001	75098000	1.39E-06
chr4	75094001	75099000	7.91E-06
chr4	75095001	75100000	0.000812861
chr4	75096001	75101000	0.003234442
chr4	75097001	75102000	0.001633921
chr4	75116001	75121000	6.28E-06
chr4	75117001	75122000	1.23E-06

chr4	75118001	75123000	3.78E-05
chr4	75119001	75124000	2.34E-07
chr4	75236001	75241000	0.0003162
chr4	75259001	75264000	1.93E-05
chr4	75558001	75563000	0.000169275
chr4	75715001	75720000	2.52E-67
chr4	75716001	75721000	1.08E-55
chr4	75717001	75722000	7.46E-37
chr4	76126001	76131000	0.00248315
chr4	76246001	76251000	1.43E-05
chr4	76408001	76413000	0.000287641
chr4	76409001	76414000	0.001937705
chr4	76435001	76440000	1.01E-28
chr4	76439001	76444000	1.54E-42
chr4	76443001	76448000	1.78E-05
chr4	76551001	76556000	1.87E-19
chr4	76552001	76557000	6.27E-33
chr4	76553001	76558000	3.02E-23
chr4	76554001	76559000	8.43E-19
chr4	76555001	76560000	2.10E-24
chr4	76556001	76561000	1.74E-22
chr4	76862001	76867000	1.70E-21
chr4	76907001	76912000	1.26E-13
chr4	76909001	76914000	1.48E-26
chr4	77070001	77075000	0.000229958
chr4	77130001	77135000	5.37E-46
chr4	77131001	77136000	5.50E-48
chr4	77132001	77137000	4.81E-64
chr4	77133001	77138000	1.37E-40
chr4	77134001	77139000	3.73E-45
chr4	77135001	77140000	4.11E-41
chr4	77168001	77173000	7.14E-31
chr4	77169001	77174000	4.30E-24
chr4	77170001	77175000	3.59E-34
chr4	77171001	77176000	1.76E-26
chr4	77172001	77177000	9.93E-25
chr4	77179001	77184000	0.008179963
chr4	77228001	77233000	3.00E-15
chr4	77241001	77246000	0.000132103
chr4	77339001	77344000	7.57E-28
chr4	77340001	77345000	6.74E-17
chr4	77341001	77346000	6.96E-12
chr4	77606001	77611000	5.64E-49
chr4	77814001	77819000	1.45E-13
chr4	77818001	77823000	1.84E-20
chr4	77819001	77824000	1.35E-37
chr4	77866001	77871000	1.70E-39
chr4	77991001	77996000	2.80E-06

chr4	78078001	78083000	2.71E-58
chr4	78079001	78084000	4.52E-20
chr4	78299001	78304000	8.27E-07
chr4	78300001	78305000	1.44E-09
chr4	78420001	78425000	0.002223053
chr4	78464001	78469000	8.25E-05
chr4	78466001	78471000	9.24E-06
chr4	78467001	78472000	1.46E-08
chr4	78468001	78473000	2.19E-06
chr4	78469001	78474000	2.59E-08
chr4	78470001	78475000	1.18E-10
chr4	78471001	78476000	3.89E-07
chr4	78473001	78478000	7.29E-06
chr4	78702001	78707000	0.007861536
chr4	78740001	78745000	5.50E-39
chr4	78741001	78746000	1.60E-25
chr4	78980001	78985000	3.30E-35
chr4	79692001	79697000	1.69E-20
chr4	79693001	79698000	1.87E-64
chr4	79694001	79699000	2.50E-50
chr4	79697001	79702000	4.61E-43
chr4	80756001	80761000	0.001287195
chr4	80757001	80762000	0.00114566
chr4	80758001	80763000	0.000347664
chr4	80759001	80764000	7.57E-05
chr4	80890001	80895000	0.016030085
chr4	80924001	80929000	0.004438018
chr4	80925001	80930000	0.003026541
chr4	80994001	80999000	5.60E-35
chr4	81123001	81128000	2.17E-26
chr4	81257001	81262000	1.25E-33
chr4	81471001	81476000	0.000108982
chr4	81478001	81483000	4.74E-09
chr4	81479001	81484000	4.04E-07
chr4	81480001	81485000	1.20E-05
chr4	81637001	81642000	0.000312642
chr4	81670001	81675000	0.005960962
chr4	81947001	81952000	6.70E-16
chr4	81948001	81953000	7.47E-24
chr4	81952001	81957000	9.86E-11
chr4	82021001	82026000	2.67E-17
chr4	82131001	82136000	1.26E-24
chr4	82132001	82137000	4.73E-16
chr4	82133001	82138000	1.82E-25
chr4	82134001	82139000	3.84E-30
chr4	82135001	82140000	2.58E-33
chr4	82136001	82141000	2.37E-27
chr4	82536001	82541000	0.020563982

chr4	82571001	82576000	0.000759907
chr4	82671001	82676000	1.67E-07
chr4	82672001	82677000	1.10E-07
chr4	82673001	82678000	0.00032644
chr4	82674001	82679000	1.52E-07
chr4	82675001	82680000	1.60E-05
chr4	82677001	82682000	0.000328943
chr4	82727001	82732000	1.29E-05
chr4	82731001	82736000	0.000251578
chr4	82804001	82809000	0.000411193
chr4	82921001	82926000	0.000167085
chr4	82934001	82939000	0.001060743
chr4	82935001	82940000	8.75E-05
chr4	82961001	82966000	7.65E-18
chr4	82964001	82969000	5.00E-24
chr4	82965001	82970000	1.30E-39
chr4	83014001	83019000	0.000191099
chr4	83034001	83039000	1.25E-05
chr4	83079001	83084000	6.23E-10
chr4	83080001	83085000	3.74E-12
chr4	83081001	83086000	1.14E-08
chr4	83136001	83141000	0.011098997
chr4	83158001	83163000	4.25E-07
chr4	83159001	83164000	2.42E-08
chr4	83160001	83165000	8.26E-10
chr4	83161001	83166000	7.13E-10
chr4	83179001	83184000	0.009216604
chr4	83206001	83211000	6.47E-14
chr4	83479001	83484000	3.41E-16
chr4	83480001	83485000	8.99E-32
chr4	83481001	83486000	1.00E-38
chr4	83482001	83487000	1.29E-25
chr4	83483001	83488000	2.19E-23
chr4	83715001	83720000	1.63E-29
chr4	83716001	83721000	1.76E-24
chr4	83718001	83723000	8.82E-25
chr4	83719001	83724000	3.69E-41
chr4	83720001	83725000	2.36E-16
chr4	83780001	83785000	0.004207484
chr4	83781001	83786000	0.000455614
chr4	83812001	83817000	1.32E-35
chr4	83822001	83827000	5.97E-25
chr4	84201001	84206000	3.58E-42
chr4	84202001	84207000	8.87E-37
chr4	84256001	84261000	1.72E-12
chr4	84402001	84407000	2.67E-16
chr4	84403001	84408000	2.12E-16
chr4	84404001	84409000	1.76E-12

chr4	84405001	84410000	4.03E-14
chr4	84446001	84451000	3.81E-17
chr4	84447001	84452000	2.73E-12
chr4	84448001	84453000	2.19E-09
chr4	84457001	84462000	1.26E-31
chr4	84774001	84779000	0.005458296
chr4	84816001	84821000	0.016168977
chr4	85170001	85175000	0.001043443
chr4	85172001	85177000	0.001385835
chr4	85173001	85178000	0.00075513
chr4	85174001	85179000	0.000277836
chr4	85209001	85214000	0.005865674
chr4	85239001	85244000	0.000251905
chr4	85240001	85245000	0.000150854
chr4	85499001	85504000	1.06E-33
chr4	85500001	85505000	5.41E-40
chr4	85883001	85888000	1.48E-38
chr4	85884001	85889000	9.19E-20
chr4	85885001	85890000	4.78E-32
chr4	85886001	85891000	2.41E-36
chr4	85887001	85892000	5.50E-27
chr4	86246001	86251000	2.41E-06
chr4	86359001	86364000	0.012493386
chr4	86660001	86665000	0.000129431
chr4	86661001	86666000	7.21E-05
chr4	87492001	87497000	0.024752805
chr4	87511001	87516000	1.32E-59
chr4	87821001	87826000	1.60E-17
chr4	87822001	87827000	1.28E-10
chr4	87823001	87828000	2.01E-13
chr4	87824001	87829000	6.01E-12
chr4	88141001	88146000	7.14E-43
chr4	88339001	88344000	2.23E-29
chr4	88340001	88345000	8.48E-15
chr4	88591001	88596000	0.000383666
chr4	88593001	88598000	5.17E-05
chr4	88672001	88677000	0.000131241
chr4	88693001	88698000	4.48E-08
chr4	88694001	88699000	4.57E-07
chr4	88695001	88700000	2.38E-08
chr4	88696001	88701000	5.72E-06
chr4	88728001	88733000	0.000115393
chr4	88833001	88838000	0.002158638
chr4	88924001	88929000	1.20E-47
chr4	88925001	88930000	7.14E-50
chr4	88926001	88931000	3.32E-48
chr4	88927001	88932000	1.29E-49
chr4	88928001	88933000	2.17E-53

chr4	88929001	88934000	1.19E-33
chr4	89075001	89080000	4.93E-25
chr4	89076001	89081000	5.44E-29
chr4	89077001	89082000	8.11E-27
chr4	89078001	89083000	7.02E-16
chr4	89079001	89084000	2.78E-17
chr4	89080001	89085000	1.28E-13
chr4	89201001	89206000	9.08E-23
chr4	89296001	89301000	3.47E-25
chr4	89297001	89302000	4.17E-51
chr4	89298001	89303000	3.77E-47
chr4	89299001	89304000	3.82E-45
chr4	89300001	89305000	7.10E-55
chr4	89381001	89386000	0.008072041
chr4	89440001	89445000	1.94E-49
chr4	89441001	89446000	1.23E-39
chr4	89442001	89447000	4.34E-34
chr4	89443001	89448000	1.69E-35
chr4	89444001	89449000	5.92E-24
chr4	89513001	89518000	6.85E-41
chr4	89514001	89519000	9.00E-32
chr4	89534001	89539000	0.009916794
chr4	89535001	89540000	0.006613896
chr4	89987001	89992000	3.44E-05
chr4	89988001	89993000	0.000420832
chr4	90016001	90021000	6.49E-07
chr4	90030001	90035000	1.91E-35
chr4	90031001	90036000	7.12E-56
chr4	90032001	90037000	7.20E-68
chr4	90224001	90229000	6.38E-30
chr4	90463001	90468000	0.004899964
chr4	90496001	90501000	0.019316346
chr4	91044001	91049000	4.33E-25
chr4	91045001	91050000	1.78E-50
chr4	91046001	91051000	3.46E-49
chr4	91047001	91052000	2.05E-73
chr4	91048001	91053000	3.38E-47
chr4	91049001	91054000	7.88E-47
chr4	91186001	91191000	0.000769371
chr4	91835001	91840000	1.20E-05
chr4	91836001	91841000	5.05E-07
chr4	91837001	91842000	9.76E-13
chr4	91981001	91986000	5.05E-06
chr4	91982001	91987000	1.14E-05
chr4	91983001	91988000	9.35E-07
chr4	92055001	92060000	8.16E-07
chr4	92057001	92062000	3.75E-06
chr4	92058001	92063000	2.15E-05

chr4	92059001	92064000	0.000179922
chr4	92273001	92278000	6.51E-07
chr4	92274001	92279000	1.85E-06
chr4	92338001	92343000	0.020406476
chr4	92691001	92696000	0.016703212
chr4	92807001	92812000	0.002236213
chr4	92846001	92851000	0.002028857
chr4	93221001	93226000	1.22E-26
chr4	93650001	93655000	0.00071141
chr4	94110001	94115000	0.000433483
chr4	94111001	94116000	3.16E-06
chr4	94112001	94117000	3.14E-06
chr4	94113001	94118000	1.50E-06
chr4	94438001	94443000	0.00145025
chr4	94691001	94696000	0.004834391
chr4	94812001	94817000	0.003635403
chr4	94842001	94847000	0.003525521
chr4	94843001	94848000	0.004761404
chr4	94887001	94892000	7.11E-05
chr4	94998001	95003000	0.003654008
chr4	95329001	95334000	6.97E-07
chr4	95330001	95335000	1.00E-05
chr4	95373001	95378000	1.45E-30
chr4	95574001	95579000	0.000238618
chr4	95676001	95681000	1.01E-28
chr4	95677001	95682000	3.95E-39
chr4	95678001	95683000	4.19E-33
chr4	95679001	95684000	3.17E-50
chr4	96153001	96158000	0.003097808
chr4	96562001	96567000	0.014067936
chr4	96639001	96644000	0.002984518
chr4	96640001	96645000	0.002543346
chr4	96716001	96721000	8.10E-08
chr4	97480001	97485000	0.007336639
chr4	98173001	98178000	0.00019985
chr4	98202001	98207000	0.00041327
chr4	98203001	98208000	2.18E-05
chr4	98204001	98209000	9.24E-06
chr4	98352001	98357000	0.000246189
chr4	98891001	98896000	9.96E-06
chr4	98892001	98897000	9.82E-06
chr4	98894001	98899000	8.70E-05
chr4	99079001	99084000	0.001124103
chr4	99182001	99187000	4.72E-39
chr4	99244001	99249000	3.22E-08
chr4	99245001	99250000	9.69E-08
chr4	99279001	99284000	1.62E-05
chr4	99282001	99287000	2.33E-05

chr4	99574001	99579000	3.36E-22
chr4	99648001	99653000	3.31E-06
chr4	99649001	99654000	2.05E-09
chr4	99650001	99655000	0.002373674
chr4	99651001	99656000	0.002625814
chr4	99681001	99686000	0.000182852
chr4	99845001	99850000	1.73E-51
chr4	99846001	99851000	1.11E-40
chr4	99916001	99921000	1.02E-24
chr4	99917001	99922000	1.55E-14
chr4	100049001	100054000	0.024162633
chr4	100052001	100057000	0.007161759
chr4	100104001	100109000	1.76E-07
chr4	100105001	100110000	1.76E-09
chr4	100138001	100143000	1.99E-05
chr4	100139001	100144000	6.81E-05
chr4	100166001	100171000	1.69E-05
chr4	100483001	100488000	2.19E-20
chr4	100484001	100489000	2.80E-32
chr4	100811001	100816000	9.55E-68
chr4	100812001	100817000	5.28E-59
chr4	100813001	100818000	3.49E-49
chr4	100814001	100819000	2.70E-37
chr4	100815001	100820000	9.31E-21
chr4	100961001	100966000	9.40E-05
chr4	101042001	101047000	0.003042896
chr4	101052001	101057000	0.00716933
chr4	101053001	101058000	0.016437075
chr4	101193001	101198000	0.000440836
chr4	101273001	101278000	0.001385543
chr4	101275001	101280000	0.004390596
chr4	101702001	101707000	0.002488102
chr4	101703001	101708000	1.98E-05
chr4	101704001	101709000	0.008437336
chr4	101781001	101786000	7.55E-07
chr4	101782001	101787000	3.66E-05
chr4	101785001	101790000	1.17E-07
chr4	102020001	102025000	0.000348521
chr4	102021001	102026000	1.08E-05
chr4	102022001	102027000	0.000135701
chr4	102262001	102267000	3.30E-15
chr4	102263001	102268000	7.40E-43
chr4	102264001	102269000	7.63E-45
chr4	102265001	102270000	1.93E-29
chr4	102266001	102271000	2.85E-12
chr4	102267001	102272000	3.16E-34
chr4	102268001	102273000	2.10E-52
chr4	102269001	102274000	5.07E-45

chr4	102707001	102712000	1.14E-14
chr4	102708001	102713000	4.90E-44
chr4	102709001	102714000	8.60E-42
chr4	102712001	102717000	2.58E-18
chr4	103024001	103029000	0.000959284
chr4	103025001	103030000	0.000571926
chr4	103026001	103031000	6.91E-06
chr4	103377001	103382000	8.84E-10
chr4	103677001	103682000	2.26E-15
chr4	103790001	103795000	2.33E-25
chr4	103791001	103796000	1.38E-11
chr4	103963001	103968000	3.73E-06
chr4	103993001	103998000	6.58E-50
chr4	103994001	103999000	4.92E-53
chr4	103995001	104000000	1.97E-79
chr4	103996001	104001000	1.15E-77
chr4	103997001	104002000	2.97E-77
chr4	104255001	104260000	0.006615785
chr4	104256001	104261000	0.001202212
chr4	104322001	104327000	0.000349357
chr4	104323001	104328000	0.000506998
chr4	104324001	104329000	0.01317465
chr4	104325001	104330000	1.56E-05
chr4	104378001	104383000	0.014771264
chr4	104410001	104415000	0.00676685
chr4	104525001	104530000	4.90E-06
chr4	104526001	104531000	3.89E-05
chr4	104619001	104624000	6.23E-05
chr4	104620001	104625000	3.43E-05
chr4	104621001	104626000	1.26E-05
chr4	104622001	104627000	4.45E-05
chr4	104730001	104735000	9.51E-09
chr4	104731001	104736000	1.50E-07
chr4	104816001	104821000	6.88E-06
chr4	104898001	104903000	0.007682481
chr4	104899001	104904000	0.002929128
chr4	104900001	104905000	0.003317071
chr4	104990001	104995000	0.00048771
chr4	105058001	105063000	8.57E-05
chr4	105088001	105093000	0.003062562
chr4	105299001	105304000	6.84E-06
chr4	105300001	105305000	3.80E-05
chr4	105407001	105412000	3.09E-10
chr4	105408001	105413000	1.65E-33
chr4	105410001	105415000	9.10E-23
chr4	105663001	105668000	0.009767339
chr4	105664001	105669000	0.018687698
chr4	106004001	106009000	0.007436213

chr4	106063001	106068000	7.19E-76
chr4	106064001	106069000	6.44E-55
chr4	106390001	106395000	9.54E-25
chr4	106570001	106575000	0.011251739
chr4	106625001	106630000	1.21E-12
chr4	106629001	106634000	3.60E-13
chr4	106630001	106635000	1.79E-08
chr4	106805001	106810000	3.89E-06
chr4	106806001	106811000	0.000362056
chr4	107224001	107229000	0.006438111
chr4	107232001	107237000	2.29E-05
chr4	107233001	107238000	5.81E-34
chr4	107486001	107491000	0.001097595
chr4	107629001	107634000	5.39E-05
chr4	107630001	107635000	0.000112927
chr4	107631001	107636000	7.48E-06
chr4	107632001	107637000	0.000232326
chr4	107633001	107638000	0.00083701
chr4	107635001	107640000	4.21E-06
chr4	107636001	107641000	2.46E-06
chr4	107637001	107642000	3.13E-06
chr4	107638001	107643000	1.92E-06
chr4	107794001	107799000	0.001567155
chr4	107877001	107882000	0.000338954
chr4	107878001	107883000	0.005052353
chr4	107879001	107884000	0.003412779
chr4	107891001	107896000	3.59E-05
chr4	107892001	107897000	0.000401287
chr4	107951001	107956000	2.38E-13
chr4	107952001	107957000	6.80E-24
chr4	107980001	107985000	9.92E-05
chr4	107981001	107986000	4.82E-05
chr4	107982001	107987000	0.000635348
chr4	108119001	108124000	0.000150791
chr4	108382001	108387000	0.002661859
chr4	108413001	108418000	0.000952225
chr4	108507001	108512000	5.69E-06
chr4	108509001	108514000	5.17E-07
chr4	108510001	108515000	1.66E-05
chr4	108511001	108516000	2.69E-05
chr4	108538001	108543000	0.002896613
chr4	108741001	108746000	2.52E-09
chr4	108742001	108747000	3.52E-34
chr4	108743001	108748000	5.33E-35
chr4	108744001	108749000	1.33E-26
chr4	108745001	108750000	1.05E-29
chr4	108746001	108751000	2.08E-34
chr4	108848001	108853000	3.85E-43

chr4	108849001	108854000	2.25E-21
chr4	108850001	108855000	2.35E-26
chr4	108853001	108858000	2.60E-11
chr4	108906001	108911000	4.39E-33
chr4	109082001	109087000	0.000718079
chr4	109092001	109097000	1.30E-35
chr4	109093001	109098000	7.31E-55
chr4	109094001	109099000	1.02E-59
chr4	109438001	109443000	6.93E-05
chr4	109567001	109572000	1.42E-26
chr4	109621001	109626000	0.000723745
chr4	109622001	109627000	0.00327201
chr4	109681001	109686000	8.73E-14
chr4	109682001	109687000	2.14E-14
chr4	109685001	109690000	3.69E-06
chr4	109718001	109723000	7.56E-07
chr4	109719001	109724000	4.47E-09
chr4	109720001	109725000	1.09E-07
chr4	109823001	109828000	3.39E-06
chr4	109867001	109872000	8.34E-07
chr4	109868001	109873000	7.11E-06
chr4	109869001	109874000	7.77E-05
chr4	109944001	109949000	2.76E-05
chr4	109945001	109950000	7.70E-05
chr4	109946001	109951000	8.18E-07
chr4	109947001	109952000	4.09E-07
chr4	109948001	109953000	2.55E-10
chr4	109949001	109954000	1.18E-06
chr4	110012001	110017000	0.000391028
chr4	110013001	110018000	2.14E-05
chr4	110014001	110019000	6.55E-05
chr4	110015001	110020000	0.000104404
chr4	110016001	110021000	0.001077262
chr4	110022001	110027000	0.01046831
chr4	110123001	110128000	5.27E-05
chr4	110219001	110224000	1.13E-15
chr4	110224001	110229000	4.62E-11
chr4	110256001	110261000	0.000251083
chr4	110262001	110267000	4.85E-14
chr4	110350001	110355000	1.48E-46
chr4	110351001	110356000	1.53E-29
chr4	110353001	110358000	3.83E-16
chr4	110477001	110482000	7.86E-21
chr4	110478001	110483000	1.77E-20
chr4	110479001	110484000	1.81E-70
chr4	110480001	110485000	5.31E-87
chr4	110481001	110486000	1.79E-92
chr4	110482001	110487000	2.70E-32

chr4	110618001	110623000	2.12E-08
chr4	110619001	110624000	1.33E-07
chr4	110620001	110625000	1.22E-12
chr4	110624001	110629000	7.46E-30
chr4	110646001	110651000	2.64E-12
chr4	110737001	110742000	4.68E-20
chr4	110828001	110833000	0.00064356
chr4	110922001	110927000	0.000123039
chr4	111113001	111118000	1.68E-14
chr4	111413001	111418000	0.001429662
chr4	111414001	111419000	0.001252203
chr4	111626001	111631000	0.020688126
chr4	111627001	111632000	0.021818043
chr4	112172001	112177000	0.000718292
chr4	112889001	112894000	3.40E-05
chr4	113062001	113067000	4.77E-21
chr4	113065001	113070000	2.61E-57
chr4	113066001	113071000	9.68E-61
chr4	113067001	113072000	6.41E-41
chr4	113148001	113153000	6.76E-38
chr4	113149001	113154000	8.43E-51
chr4	113150001	113155000	7.59E-47
chr4	113151001	113156000	2.47E-52
chr4	113152001	113157000	4.03E-44
chr4	113153001	113158000	2.53E-62
chr4	113368001	113373000	0.0002484
chr4	113414001	113419000	2.36E-05
chr4	113445001	113450000	7.19E-18
chr4	113591001	113596000	0.006602281
chr4	113623001	113628000	4.30E-11
chr4	113624001	113629000	2.19E-13
chr4	113625001	113630000	3.02E-10
chr4	113626001	113631000	4.89E-22
chr4	113627001	113632000	4.41E-25
chr4	114185001	114190000	4.99E-06
chr4	114682001	114687000	8.98E-29
chr4	114900001	114905000	1.70E-19
chr4	115040001	115045000	0.001035542
chr4	115098001	115103000	0.00046962
chr4	115195001	115200000	0.002292846
chr4	115196001	115201000	0.000406934
chr4	115197001	115202000	0.001639697
chr4	115198001	115203000	0.002158439
chr4	115199001	115204000	0.002042084
chr4	115378001	115383000	4.97E-07
chr4	115379001	115384000	2.91E-09
chr4	115548001	115553000	0.000966541
chr4	115559001	115564000	5.96E-09

chr4	115631001	115636000	0.003179171
chr4	115632001	115637000	0.000134123
chr4	115657001	115662000	0.001073906
chr4	115872001	115877000	0.000398487
chr4	115873001	115878000	0.000557197
chr4	115874001	115879000	0.00051289
chr4	115985001	115990000	0.003172228
chr4	115987001	115992000	0.010820468
chr4	116228001	116233000	0.001163747
chr4	116307001	116312000	0.000416525
chr4	116315001	116320000	0.0006558
chr4	116316001	116321000	0.000181529
chr4	116317001	116322000	0.001773546
chr4	116378001	116383000	1.59E-05
chr4	116440001	116445000	0.0019107
chr4	116506001	116511000	0.006331833
chr4	116507001	116512000	0.005472718
chr4	116571001	116576000	1.51E-07
chr4	116779001	116784000	0.000235497
chr4	116883001	116888000	0.005086871
chr4	116998001	117003000	5.01E-07
chr4	117000001	117005000	2.85E-07
chr4	117001001	117006000	1.65E-07
chr4	117050001	117055000	0.001174726
chr4	117092001	117097000	0.000407175
chr4	117093001	117098000	0.000412246
chr4	117301001	117306000	4.69E-05
chr4	117730001	117735000	7.90E-16
chr4	117777001	117782000	8.66E-05
chr4	117778001	117783000	0.000120561
chr4	117847001	117852000	1.58E-19
chr4	117890001	117895000	0.025825642
chr4	118002001	118007000	1.58E-41
chr4	118003001	118008000	8.74E-50
chr4	118004001	118009000	1.19E-54
chr4	118005001	118010000	6.59E-54
chr4	118006001	118011000	1.75E-38
chr4	118050001	118055000	0.000171134
chr4	118051001	118056000	0.003048914
chr4	118119001	118124000	0.001938119
chr4	118120001	118125000	6.68E-05
chr4	118543001	118548000	0.0015744
chr4	118544001	118549000	0.006064261
chr4	118608001	118613000	0.00030269
chr4	118659001	118664000	0.000186669
chr4	118663001	118668000	0.003270596
chr4	118925001	118930000	4.63E-05
chr4	118926001	118931000	7.78E-06

chr4	118931001	118936000	6.06E-06
chr4	118932001	118937000	0.000265337
chr4	118933001	118938000	3.83E-05
chr4	118993001	118998000	3.63E-05
chr4	118994001	118999000	9.00E-07
chr4	119269001	119274000	5.60E-12
chr4	119271001	119276000	1.28E-14
chr4	119272001	119277000	4.32E-11
chr4	119273001	119278000	1.22E-16
chr4	119274001	119279000	4.13E-18
chr4	119345001	119350000	1.46E-06
chr4	119756001	119761000	3.94E-19
chr4	119757001	119762000	2.40E-19
chr4	119805001	119810000	7.26E-08
chr4	119806001	119811000	3.02E-12
chr4	119807001	119812000	1.04E-08
chr4	119809001	119814000	2.21E-12
chr4	119810001	119815000	2.75E-10
chr4	120134001	120139000	9.86E-36
chr4	120151001	120156000	0.000278253
chr4	120216001	120221000	8.65E-07
chr4	120264001	120269000	7.55E-05
chr4	120371001	120376000	7.62E-29
chr4	120436001	120441000	0.001047985
chr4	120544001	120549000	1.02E-28
chr4	120550001	120555000	2.32E-21
chr4	121363001	121368000	0.003337425
chr4	121448001	121453000	1.18E-05
chr4	121473001	121478000	5.75E-06
chr4	121502001	121507000	0.000600259
chr4	121503001	121508000	0.000925015
chr4	121504001	121509000	0.000119973
chr4	121505001	121510000	7.49E-05
chr4	121509001	121514000	1.14E-05
chr4	121862001	121867000	3.18E-06
chr4	121874001	121879000	0.001394111
chr4	122298001	122303000	4.75E-22
chr4	122299001	122304000	1.52E-23
chr4	122300001	122305000	1.09E-30
chr4	122301001	122306000	7.52E-30
chr4	122302001	122307000	4.34E-28
chr4	122456001	122461000	0.001286739
chr4	122463001	122468000	6.89E-05
chr4	122464001	122469000	4.79E-05
chr4	122613001	122618000	7.92E-24
chr4	122628001	122633000	1.99E-10
chr4	122631001	122636000	9.69E-21
chr4	122632001	122637000	3.26E-23

chr4	122633001	122638000	6.01E-10
chr4	122718001	122723000	1.41E-23
chr4	122720001	122725000	9.44E-36
chr4	122721001	122726000	4.73E-29
chr4	122722001	122727000	4.80E-31
chr4	122745001	122750000	3.93E-35
chr4	122787001	122792000	8.51E-22
chr4	122788001	122793000	1.08E-16
chr4	122789001	122794000	2.02E-12
chr4	122790001	122795000	6.98E-14
chr4	122791001	122796000	3.27E-14
chr4	122872001	122877000	7.80E-20
chr4	122873001	122878000	1.11E-07
chr4	122917001	122922000	0.000423915
chr4	122918001	122923000	0.000803878
chr4	122999001	123004000	0.000622734
chr4	123027001	123032000	4.79E-10
chr4	123028001	123033000	1.19E-12
chr4	123029001	123034000	7.37E-16
chr4	123030001	123035000	1.29E-17
chr4	123174001	123179000	5.36E-05
chr4	123472001	123477000	0.012254925
chr4	123542001	123547000	4.61E-05
chr4	123543001	123548000	7.79E-05
chr4	123651001	123656000	1.51E-54
chr4	123652001	123657000	5.28E-61
chr4	123653001	123658000	4.28E-63
chr4	123654001	123659000	5.01E-34
chr4	123744001	123749000	2.26E-39
chr4	123745001	123750000	2.64E-31
chr4	123746001	123751000	2.39E-38
chr4	123747001	123752000	1.24E-32
chr4	123748001	123753000	4.30E-30
chr4	123864001	123869000	1.05E-13
chr4	123865001	123870000	2.94E-13
chr4	123866001	123871000	1.04E-11
chr4	124153001	124158000	0.003419393
chr4	124320001	124325000	4.10E-19
chr4	124636001	124641000	2.71E-07
chr4	124741001	124746000	0.001049589
chr4	124887001	124892000	0.004762341
chr4	125598001	125603000	0.000382813
chr4	125627001	125632000	0.006309363
chr4	125633001	125638000	7.02E-53
chr4	125634001	125639000	1.84E-21
chr4	125859001	125864000	0.001701032
chr4	126413001	126418000	0.010580977
chr4	126833001	126838000	0.000694346

chr4	126834001	126839000	8.86E-05
chr4	126835001	126840000	7.62E-05
chr4	126836001	126841000	0.000236199
chr4	126837001	126842000	0.000206302
chr4	126937001	126942000	0.002292686
chr4	127037001	127042000	5.27E-09
chr4	127038001	127043000	1.43E-08
chr4	127039001	127044000	7.13E-08
chr4	127040001	127045000	7.72E-08
chr4	127177001	127182000	0.011231699
chr4	127217001	127222000	0.001614651
chr4	127238001	127243000	5.73E-05
chr4	127239001	127244000	0.001290241
chr4	127416001	127421000	0.000153232
chr4	127446001	127451000	0.000983389
chr4	127447001	127452000	0.002893344
chr4	127630001	127635000	0.000308529
chr4	127636001	127641000	0.00403995
chr4	127917001	127922000	0.00625531
chr4	127973001	127978000	8.97E-06
chr4	127976001	127981000	1.54E-05
chr4	128089001	128094000	0.002423188
chr4	128090001	128095000	0.009567929
chr4	128186001	128191000	0.000143575
chr4	128397001	128402000	5.44E-09
chr4	128398001	128403000	7.27E-09
chr4	128422001	128427000	0.000325001
chr4	128539001	128544000	6.98E-16
chr4	128540001	128545000	3.52E-22
chr4	128541001	128546000	2.95E-22
chr4	128542001	128547000	2.14E-16
chr4	128543001	128548000	3.53E-12
chr4	128699001	128704000	5.84E-44
chr4	128703001	128708000	9.42E-51
chr4	128704001	128709000	1.89E-11
chr4	128881001	128886000	1.72E-12
chr4	128982001	128987000	1.94E-30
chr4	128983001	128988000	2.47E-12
chr4	129080001	129085000	0.008151348
chr4	129174001	129179000	0.00089818
chr4	129208001	129213000	3.04E-44
chr4	129209001	129214000	8.29E-61
chr4	129210001	129215000	6.15E-09
chr4	129470001	129475000	5.83E-14
chr4	129473001	129478000	3.72E-13
chr4	129727001	129732000	1.34E-39
chr4	129728001	129733000	5.47E-61
chr4	129729001	129734000	7.18E-68

chr4	129732001	129737000	1.47E-20
chr4	130010001	130015000	1.73E-45
chr4	130011001	130016000	5.42E-56
chr4	130012001	130017000	1.36E-71
chr4	130014001	130019000	7.99E-75
chr4	130495001	130500000	4.68E-05
chr4	130562001	130567000	0.000981733
chr4	130603001	130608000	0.000205928
chr4	130668001	130673000	0.007998825
chr4	130671001	130676000	0.000292406
chr4	130672001	130677000	0.000495142
chr4	130687001	130692000	0.004181072
chr4	130798001	130803000	2.69E-05
chr4	130799001	130804000	8.50E-06
chr4	130800001	130805000	0.000246327
chr4	130801001	130806000	0.001180973
chr4	130826001	130831000	0.000109828
chr4	130915001	130920000	2.97E-05
chr4	130916001	130921000	3.77E-07
chr4	130917001	130922000	1.94E-05
chr4	130918001	130923000	3.15E-05
chr4	130919001	130924000	0.000349619
chr4	131575001	131580000	0.000913343
chr4	131659001	131664000	0.000635677
chr4	131834001	131839000	2.20E-06
chr4	131865001	131870000	2.41E-06
chr4	132060001	132065000	8.69E-05
chr4	132061001	132066000	6.28E-06
chr4	132062001	132067000	1.24E-06
chr4	132063001	132068000	0.0008922
chr4	132209001	132214000	0.002380474
chr4	132277001	132282000	1.43E-06
chr4	132346001	132351000	2.91E-05
chr4	132620001	132625000	0.003284275
chr4	132719001	132724000	9.38E-05
chr4	133050001	133055000	3.12E-18
chr4	133325001	133330000	0.002023535
chr4	133387001	133392000	0.000230406
chr4	133500001	133505000	0.009821011
chr4	133686001	133691000	4.39E-05
chr4	133793001	133798000	0.000411285
chr4	134076001	134081000	0.002542809
chr4	134077001	134082000	0.000484536
chr4	134090001	134095000	0.002865536
chr4	134091001	134096000	0.001837493
chr4	134120001	134125000	4.14E-06
chr4	134121001	134126000	3.79E-07
chr4	134690001	134695000	0.005603093

chr4	134691001	134696000	0.000904076
chr4	134692001	134697000	0.000161179
chr4	134849001	134854000	0.001068057
chr4	134850001	134855000	0.000856732
chr4	135118001	135123000	5.01E-25
chr4	135123001	135128000	0.000218589
chr4	135216001	135221000	0.000329852
chr4	135349001	135354000	5.87E-07
chr4	135350001	135355000	8.09E-07
chr4	135389001	135394000	7.45E-16
chr4	135390001	135395000	1.88E-15
chr4	135391001	135396000	1.54E-12
chr4	135491001	135496000	2.86E-05
chr4	135717001	135722000	5.65E-06
chr4	135718001	135723000	0.000113033
chr4	135841001	135846000	9.97E-05
chr4	135975001	135980000	4.08E-05
chr4	135994001	135999000	8.69E-08
chr4	135995001	136000000	2.22E-08
chr4	135996001	136001000	2.54E-06
chr4	135997001	136002000	1.14E-05
chr4	136730001	136735000	0.004943205
chr4	136769001	136774000	0.000162037
chr4	137447001	137452000	1.95E-05
chr4	137984001	137989000	0.003474656
chr4	138158001	138163000	5.24E-06
chr4	138159001	138164000	4.95E-06
chr4	138160001	138165000	4.46E-07
chr4	138409001	138414000	0.002265473
chr4	138496001	138501000	2.20E-08
chr4	138497001	138502000	1.24E-09
chr4	138498001	138503000	1.01E-08
chr4	138499001	138504000	3.87E-07
chr4	138627001	138632000	0.001731237
chr4	138628001	138633000	8.80E-06
chr4	139435001	139440000	0.026245541
chr4	139452001	139457000	0.000867971
chr4	139468001	139473000	0.043316281
chr4	139727001	139732000	0.000469214
chr4	140000001	140005000	6.14E-34
chr4	140094001	140099000	1.67E-40
chr4	140166001	140171000	4.78E-06
chr4	140167001	140172000	0.000173091
chr4	140196001	140201000	9.16E-14
chr4	140197001	140202000	1.51E-27
chr4	140198001	140203000	3.82E-24
chr4	140199001	140204000	5.03E-22
chr4	140201001	140206000	1.59E-13

chr4	140223001	140228000	1.26E-37
chr4	140375001	140380000	2.69E-37
chr4	140477001	140482000	1.20E-11
chr4	140478001	140483000	5.93E-22
chr4	141075001	141080000	1.15E-10
chr4	141170001	141175000	3.13E-18
chr4	141171001	141176000	7.37E-16
chr4	141295001	141300000	6.97E-10
chr4	141344001	141349000	1.25E-40
chr4	141345001	141350000	1.62E-26
chr4	141346001	141351000	7.26E-32
chr4	141347001	141352000	1.52E-45
chr4	141348001	141353000	1.51E-27
chr4	141431001	141436000	7.04E-09
chr4	141444001	141449000	4.95E-40
chr4	141445001	141450000	1.60E-59
chr4	141547001	141552000	0.008143776
chr4	141672001	141677000	2.99E-09
chr4	141673001	141678000	7.45E-32
chr4	141674001	141679000	9.52E-44
chr4	141675001	141680000	6.89E-52
chr4	141676001	141681000	1.11E-69
chr4	141677001	141682000	1.90E-60
chr4	141701001	141706000	1.51E-17
chr4	141919001	141924000	0.019524265
chr4	142138001	142143000	9.54E-18
chr4	142139001	142144000	1.02E-30
chr4	142140001	142145000	2.05E-23
chr4	142141001	142146000	1.24E-41
chr4	142142001	142147000	2.25E-51
chr4	142466001	142471000	4.82E-07
chr4	142553001	142558000	1.06E-25
chr4	142555001	142560000	3.34E-43
chr4	142556001	142561000	1.44E-47
chr4	142557001	142562000	7.78E-48
chr4	142558001	142563000	1.51E-35
chr4	143080001	143085000	0.00132205
chr4	143214001	143219000	0.000153867
chr4	143324001	143329000	7.62E-06
chr4	143441001	143446000	9.43E-06
chr4	143442001	143447000	0.000215067
chr4	143443001	143448000	1.38E-05
chr4	143444001	143449000	9.57E-06
chr4	143445001	143450000	3.21E-05
chr4	143497001	143502000	0.010807027
chr4	143498001	143503000	0.007616556
chr4	143499001	143504000	0.00261984
chr4	143762001	143767000	2.78E-05

chr4	143768001	143773000	5.38E-27
chr4	143887001	143892000	0.002813461
chr4	144254001	144259000	1.93E-15
chr4	144430001	144435000	3.32E-85
chr4	144431001	144436000	1.13E-96
chr4	144432001	144437000	5.07E-64
chr4	144434001	144439000	7.59E-38
chr4	144435001	144440000	7.18E-49
chr4	144555001	144560000	0.002488515
chr4	144565001	144570000	0.000420832
chr4	144566001	144571000	0.000622978
chr4	144568001	144573000	0.00073387
chr4	144569001	144574000	0.003699631
chr4	144617001	144622000	1.43E-16
chr4	144618001	144623000	2.97E-13
chr4	144620001	144625000	1.93E-15
chr4	144621001	144626000	1.77E-15
chr4	144659001	144664000	1.70E-06
chr4	144671001	144676000	0.000122352
chr4	144672001	144677000	6.52E-06
chr4	144737001	144742000	0.001229546
chr4	144810001	144815000	2.01E-05
chr4	144816001	144821000	0.000187023
chr4	144817001	144822000	1.06E-05
chr4	144838001	144843000	2.99E-05
chr4	144842001	144847000	0.002408436
chr4	144846001	144851000	6.29E-05
chr4	144847001	144852000	1.01E-05
chr4	144848001	144853000	1.41E-06
chr4	144849001	144854000	0.001395034
chr4	144860001	144865000	0.018708005
chr4	144936001	144941000	0.007113468
chr4	144937001	144942000	0.009524981
chr4	145015001	145020000	0.001040676
chr4	145210001	145215000	0.017349008
chr4	145211001	145216000	0.021006817
chr4	145212001	145217000	0.009258396
chr4	145263001	145268000	7.74E-08
chr4	145930001	145935000	4.65E-07
chr4	146002001	146007000	3.07E-05
chr4	146101001	146106000	5.21E-21
chr4	146236001	146241000	5.65E-06
chr4	146237001	146242000	7.53E-05
chr4	146260001	146265000	0.002206975
chr4	146398001	146403000	1.06E-14
chr4	146399001	146404000	1.60E-60
chr4	146400001	146405000	1.38E-55
chr4	146401001	146406000	3.89E-61

chr4	146403001	146408000	6.04E-41
chr4	146479001	146484000	1.47E-06
chr4	146502001	146507000	0.031036192
chr4	146650001	146655000	1.30E-17
chr4	146651001	146656000	5.71E-17
chr4	146653001	146658000	7.25E-15
chr4	146978001	146983000	0.000558583
chr4	147019001	147024000	0.001109035
chr4	147092001	147097000	4.49E-29
chr4	147094001	147099000	2.45E-41
chr4	147095001	147100000	4.02E-61
chr4	147096001	147101000	1.50E-54
chr4	147097001	147102000	4.38E-45
chr4	147515001	147520000	2.39E-06
chr4	147516001	147521000	4.74E-05
chr4	147517001	147522000	5.22E-06
chr4	147518001	147523000	5.72E-06
chr4	147519001	147524000	0.001418982
chr4	147535001	147540000	1.70E-05
chr4	147542001	147547000	0.000162855
chr4	147575001	147580000	1.30E-17
chr4	147576001	147581000	6.48E-21
chr4	147666001	147671000	1.10E-05
chr4	147667001	147672000	1.74E-06
chr4	147668001	147673000	1.52E-12
chr4	147669001	147674000	1.45E-06
chr4	147727001	147732000	2.48E-13
chr4	147728001	147733000	5.29E-09
chr4	147756001	147761000	0.003153175
chr4	147757001	147762000	0.00152218
chr4	147768001	147773000	0.001783981
chr4	147814001	147819000	1.37E-05
chr4	147867001	147872000	9.97E-23
chr4	148015001	148020000	0.004041295
chr4	148016001	148021000	0.004802341
chr4	148037001	148042000	2.17E-05
chr4	148038001	148043000	0.000142214
chr4	148039001	148044000	0.000135424
chr4	148075001	148080000	3.86E-16
chr4	148076001	148081000	3.82E-11
chr4	148077001	148082000	4.71E-14
chr4	148079001	148084000	1.05E-13
chr4	148111001	148116000	3.96E-07
chr4	148112001	148117000	0.000415772
chr4	148398001	148403000	7.51E-19
chr4	148534001	148539000	1.62E-21
chr4	148535001	148540000	6.61E-27
chr4	148653001	148658000	1.36E-53

chr4	148869001	148874000	1.62E-05
chr4	149211001	149216000	0.000458215
chr4	149242001	149247000	6.74E-08
chr4	149366001	149371000	2.56E-27
chr4	149367001	149372000	1.20E-10
chr4	149551001	149556000	0.00050932
chr4	149568001	149573000	0.002362979
chr4	149664001	149669000	0.000433159
chr4	149665001	149670000	0.001285732
chr4	149739001	149744000	5.53E-05
chr4	149740001	149745000	0.000143122
chr4	149741001	149746000	0.000595972
chr4	149830001	149835000	0.002003627
chr4	150232001	150237000	8.95E-05
chr4	150233001	150238000	4.70E-05
chr4	150234001	150239000	3.11E-06
chr4	150255001	150260000	4.44E-05
chr4	150256001	150261000	7.89E-06
chr4	150338001	150343000	0.001427164
chr4	150339001	150344000	0.000515543
chr4	150340001	150345000	0.002015713
chr4	150430001	150435000	0.006511671
chr4	150431001	150436000	0.000659391
chr4	150511001	150516000	6.20E-07
chr4	150576001	150581000	9.35E-05
chr4	150618001	150623000	0.00651535
chr4	150619001	150624000	0.010142263
chr4	150620001	150625000	0.005202992
chr4	150874001	150879000	0.004966784
chr4	150876001	150881000	0.018775312
chr4	150995001	151000000	2.39E-41
chr4	150996001	151001000	1.27E-51
chr4	150997001	151002000	2.12E-49
chr4	150998001	151003000	1.98E-58
chr4	150999001	151004000	5.71E-51
chr4	151000001	151005000	9.15E-43
chr4	151210001	151215000	0.002147225
chr4	151320001	151325000	0.003099043
chr4	151513001	151518000	1.06E-05
chr4	151600001	151605000	0.003084213
chr4	151601001	151606000	0.006486215
chr4	151618001	151623000	6.67E-05
chr4	151745001	151750000	0.000228733
chr4	151865001	151870000	0.001003655
chr4	151867001	151872000	0.000340039
chr4	151936001	151941000	4.75E-27
chr4	151937001	151942000	3.00E-22
chr4	152016001	152021000	1.47E-18

chr4	152246001	152251000	5.10E-28
chr4	152288001	152293000	1.32E-08
chr4	152289001	152294000	1.04E-06
chr4	152290001	152295000	1.13E-06
chr4	152291001	152296000	2.98E-06
chr4	152325001	152330000	5.86E-38
chr4	152326001	152331000	2.53E-44
chr4	152327001	152332000	1.60E-30
chr4	152328001	152333000	1.27E-19
chr4	152329001	152334000	1.18E-09
chr4	152330001	152335000	5.28E-24
chr4	152436001	152441000	1.34E-07
chr4	152437001	152442000	3.65E-07
chr4	152469001	152474000	0.000499068
chr4	152470001	152475000	0.000224103
chr4	152665001	152670000	3.42E-06
chr4	152754001	152759000	6.33E-05
chr4	152755001	152760000	1.63E-05
chr4	152757001	152762000	0.000248668
chr4	152889001	152894000	0.002866801
chr4	152900001	152905000	0.000166389
chr4	153043001	153048000	0.003016124
chr4	153452001	153457000	3.35E-15
chr4	153453001	153458000	2.96E-21
chr4	153455001	153460000	1.11E-21
chr4	153456001	153461000	8.24E-30
chr4	153457001	153462000	2.54E-11
chr4	153597001	153602000	8.73E-12
chr4	153598001	153603000	5.55E-13
chr4	153599001	153604000	8.18E-07
chr4	153701001	153706000	1.19E-50
chr4	153852001	153857000	2.26E-12
chr4	153853001	153858000	3.56E-40
chr4	153854001	153859000	1.79E-38
chr4	153856001	153861000	2.91E-24
chr4	153857001	153862000	2.88E-26
chr4	154070001	154075000	1.40E-26
chr4	154139001	154144000	2.40E-16
chr4	154261001	154266000	8.58E-05
chr4	154383001	154388000	7.60E-14
chr4	154601001	154606000	1.32E-32
chr4	154602001	154607000	5.52E-34
chr4	154603001	154608000	6.23E-24
chr4	154604001	154609000	1.35E-26
chr4	154605001	154610000	8.36E-30
chr4	154679001	154684000	3.92E-13
chr4	154747001	154752000	6.72E-06
chr4	154912001	154917000	0.001933549

chr4	154913001	154918000	1.00E-04
chr4	154914001	154919000	9.61E-06
chr4	154974001	154979000	2.68E-07
chr4	154975001	154980000	7.53E-10
chr4	154976001	154981000	1.53E-09
chr4	155087001	155092000	5.49E-05
chr4	155088001	155093000	3.95E-06
chr4	155089001	155094000	5.18E-08
chr4	155090001	155095000	4.12E-07
chr4	155105001	155110000	0.001544959
chr4	155109001	155114000	2.39E-08
chr4	155111001	155116000	1.30E-05
chr4	155112001	155117000	6.00E-05
chr4	155183001	155188000	1.55E-06
chr4	155226001	155231000	0.006963752
chr4	155299001	155304000	0.000219884
chr4	155338001	155343000	8.94E-24
chr4	155359001	155364000	0.002521717
chr4	155467001	155472000	1.24E-27
chr4	155468001	155473000	2.20E-29
chr4	155469001	155474000	6.09E-24
chr4	155470001	155475000	5.00E-17
chr4	155471001	155476000	8.65E-14
chr4	155544001	155549000	4.50E-19
chr4	155545001	155550000	2.35E-20
chr4	155546001	155551000	1.58E-18
chr4	155547001	155552000	2.53E-21
chr4	155548001	155553000	6.14E-29
chr4	155570001	155575000	0.000424894
chr4	155718001	155723000	6.05E-05
chr4	155719001	155724000	1.07E-05
chr4	155870001	155875000	0.000231319
chr4	155974001	155979000	0.006843416
chr4	156015001	156020000	1.15E-07
chr4	156016001	156021000	3.42E-09
chr4	156017001	156022000	1.09E-05
chr4	156018001	156023000	1.16E-08
chr4	156019001	156024000	1.15E-05
chr4	156020001	156025000	1.55E-06
chr4	156050001	156055000	0.000150998
chr4	156051001	156056000	0.000553697
chr4	156052001	156057000	0.001685027
chr4	156130001	156135000	7.30E-17
chr4	156149001	156154000	0.004520856
chr4	156150001	156155000	0.008480437
chr4	156293001	156298000	4.62E-54
chr4	156294001	156299000	6.53E-60
chr4	156295001	156300000	8.23E-56

chr4	156296001	156301000	1.08E-58
chr4	156297001	156302000	4.51E-45
chr4	156298001	156303000	6.60E-29
chr4	156584001	156589000	3.73E-34
chr4	156676001	156681000	2.20E-35
chr4	156678001	156683000	2.23E-39
chr4	156679001	156684000	3.70E-41
chr4	156680001	156685000	7.39E-46
chr4	156681001	156686000	4.02E-26
chr4	156682001	156687000	0.016786918
chr4	156683001	156688000	0.027145025
chr4	156789001	156794000	6.27E-05
chr4	156873001	156878000	3.63E-31
chr4	156874001	156879000	5.63E-34
chr4	156875001	156880000	1.08E-22
chr4	156968001	156973000	0.002129505
chr4	157993001	157998000	4.18E-52
chr4	157994001	157999000	2.26E-63
chr4	157995001	158000000	4.34E-65
chr4	157996001	158001000	3.09E-45
chr4	157997001	158002000	2.43E-54
chr4	158260001	158265000	0.011875287
chr4	158261001	158266000	0.003641669
chr4	158262001	158267000	0.004271232
chr4	158263001	158268000	0.005903071
chr4	158459001	158464000	3.28E-05
chr4	158460001	158465000	9.62E-05
chr4	158461001	158466000	0.000173495
chr4	158523001	158528000	1.78E-05
chr4	158524001	158529000	8.43E-06
chr4	158525001	158530000	1.41E-05
chr4	159344001	159349000	2.42E-10
chr4	159345001	159350000	1.33E-13
chr4	159422001	159427000	0.000366714
chr4	159511001	159516000	3.46E-12
chr4	159588001	159593000	1.86E-24
chr4	159589001	159594000	3.58E-37
chr4	159590001	159595000	8.55E-56
chr4	159591001	159596000	4.37E-60
chr4	159592001	159597000	4.05E-56
chr4	159593001	159598000	1.59E-77
chr4	159644001	159649000	3.92E-10
chr4	159686001	159691000	1.81E-32
chr4	159687001	159692000	2.22E-44
chr4	159688001	159693000	9.71E-34
chr4	159689001	159694000	5.77E-36
chr4	159690001	159695000	5.88E-39
chr4	160019001	160024000	2.23E-12

chr4	160020001	160025000	2.56E-22
chr4	160021001	160026000	8.07E-23
chr4	160025001	160030000	1.26E-21
chr4	160897001	160902000	2.03E-06
chr4	160898001	160903000	1.29E-05
chr4	161947001	161952000	3.64E-07
chr4	161956001	161961000	0.001079261
chr4	162128001	162133000	0.001003508
chr4	162129001	162134000	0.001118208
chr4	162130001	162135000	0.001071829
chr4	162763001	162768000	0.004142245
chr4	163080001	163085000	0.001401889
chr4	163457001	163462000	0.00079442
chr4	163458001	163463000	0.001857274
chr4	163467001	163472000	3.39E-06
chr4	163468001	163473000	1.93E-06
chr4	163469001	163474000	2.80E-07
chr4	163470001	163475000	1.81E-09
chr4	163587001	163592000	0.003659945
chr4	163884001	163889000	0.000101674
chr4	163885001	163890000	5.06E-05
chr4	163950001	163955000	0.000971911
chr4	164088001	164093000	1.01E-21
chr4	164161001	164166000	0.001343391
chr4	164229001	164234000	0.000302168
chr4	164243001	164248000	0.00037312
chr4	164244001	164249000	0.000297097
chr4	164245001	164250000	0.000222573
chr4	164246001	164251000	0.001622414
chr4	164248001	164253000	2.95E-09
chr4	164342001	164347000	5.35E-05
chr4	164411001	164416000	1.46E-57
chr4	164412001	164417000	2.10E-52
chr4	164413001	164418000	1.40E-24
chr4	164414001	164419000	1.29E-25
chr4	164812001	164817000	0.000427534
chr4	164813001	164818000	0.000756842
chr4	164815001	164820000	0.000334542
chr4	165083001	165088000	0.018026553
chr4	165168001	165173000	0.018192439
chr4	165364001	165369000	0.001863052
chr4	165365001	165370000	0.001533281
chr4	165555001	165560000	6.14E-05
chr4	165556001	165561000	7.45E-06
chr4	165573001	165578000	7.67E-11
chr4	165592001	165597000	5.29E-15
chr4	165874001	165879000	5.06E-25
chr4	165875001	165880000	1.43E-23

chr4	165876001	165881000	6.33E-27
chr4	165877001	165882000	1.90E-18
chr4	165878001	165883000	3.40E-17
chr4	166124001	166129000	2.24E-30
chr4	166128001	166133000	6.15E-38
chr4	166129001	166134000	7.23E-24
chr4	166159001	166164000	0.000363091
chr4	166210001	166215000	3.45E-06
chr4	166300001	166305000	7.42E-42
chr4	166313001	166318000	0.001192946
chr4	166642001	166647000	0.000332743
chr4	166643001	166648000	0.000735702
chr4	166790001	166795000	4.25E-20
chr4	166791001	166796000	2.98E-22
chr4	166792001	166797000	4.27E-38
chr4	166793001	166798000	3.02E-29
chr4	166794001	166799000	1.59E-31
chr4	166795001	166800000	3.80E-22
chr4	166796001	166801000	3.04E-07
chr4	167667001	167672000	9.24E-05
chr4	168151001	168156000	9.53E-34
chr4	168155001	168160000	3.80E-24
chr4	168548001	168553000	4.97E-21
chr4	168549001	168554000	1.18E-12
chr4	168653001	168658000	0.001161416
chr4	168654001	168659000	0.000379996
chr4	168655001	168660000	0.001028551
chr4	169235001	169240000	1.77E-25
chr4	169236001	169241000	3.52E-26
chr4	169237001	169242000	2.63E-24
chr4	169238001	169243000	5.77E-21
chr4	169239001	169244000	2.48E-16
chr4	169449001	169454000	0.003158638
chr4	169587001	169592000	0.000833035
chr4	169737001	169742000	4.58E-08
chr4	169738001	169743000	2.22E-10
chr4	169749001	169754000	3.07E-39
chr4	169750001	169755000	5.04E-39
chr4	169751001	169756000	4.77E-21
chr4	169752001	169757000	2.76E-38
chr4	169753001	169758000	4.91E-59
chr4	169754001	169759000	1.59E-34
chr4	169795001	169800000	2.55E-11
chr4	169796001	169801000	2.24E-09
chr4	169798001	169803000	5.16E-08
chr4	170191001	170196000	1.36E-21
chr4	170192001	170197000	1.41E-19
chr4	170374001	170379000	2.47E-12

chr4	170375001	170380000	5.11E-13
chr4	170438001	170443000	1.45E-06
chr4	170529001	170534000	4.79E-22
chr4	170675001	170680000	5.14E-31
chr4	170943001	170948000	5.72E-71
chr4	170945001	170950000	7.37E-63
chr4	170948001	170953000	7.97E-17
chr4	171006001	171011000	2.84E-33
chr4	171007001	171012000	8.41E-41
chr4	171011001	171016000	3.50E-16
chr4	171042001	171047000	8.07E-05
chr4	171073001	171078000	0.002234287
chr4	171076001	171081000	0.001396895
chr4	171180001	171185000	1.23E-08
chr4	171181001	171186000	0.000249133
chr4	171204001	171209000	0.000259129
chr4	171205001	171210000	0.004378125
chr4	171206001	171211000	0.000151536
chr4	171239001	171244000	3.56E-07
chr4	171240001	171245000	2.24E-06
chr4	171241001	171246000	2.56E-06
chr4	171242001	171247000	8.59E-05
chr4	171564001	171569000	2.73E-05
chr4	171832001	171837000	1.87E-05
chr4	171833001	171838000	6.53E-07
chr4	172148001	172153000	0.002980385
chr4	172149001	172154000	0.002719718
chr4	172178001	172183000	0.016867357
chr4	172179001	172184000	0.02385215
chr4	172248001	172253000	9.80E-05
chr4	172249001	172254000	7.57E-05
chr4	172405001	172410000	2.31E-06
chr4	172406001	172411000	3.04E-06
chr4	172408001	172413000	0.00262031
chr4	172734001	172739000	1.67E-39
chr4	172997001	173002000	0.004457718
chr4	172998001	173003000	0.000985729
chr4	173494001	173499000	3.87E-05
chr4	173495001	173500000	3.59E-06
chr4	173701001	173706000	0.00189928
chr4	174085001	174090000	1.75E-16
chr4	174086001	174091000	5.12E-71
chr4	174087001	174092000	2.99E-65
chr4	174088001	174093000	2.92E-29
chr4	174250001	174255000	5.81E-36
chr4	174251001	174256000	7.15E-50
chr4	174254001	174259000	3.77E-39
chr4	174255001	174260000	3.71E-42

chr4	174286001	174291000	2.74E-11
chr4	174293001	174298000	1.18E-19
chr4	174609001	174614000	0.002740287
chr4	174642001	174647000	1.03E-07
chr4	174643001	174648000	1.68E-07
chr4	174658001	174663000	0.035133298
chr4	175106001	175111000	6.73E-05
chr4	175107001	175112000	5.27E-05
chr4	175205001	175210000	2.51E-26
chr4	175443001	175448000	1.12E-15
chr4	175463001	175468000	0.013070672
chr4	175747001	175752000	5.14E-20
chr4	175748001	175753000	1.29E-21
chr4	175749001	175754000	6.55E-21
chr4	175750001	175755000	6.01E-26
chr4	176167001	176172000	0.003795281
chr4	176168001	176173000	0.005921609
chr4	176514001	176519000	0.013985747
chr4	176919001	176924000	2.74E-17
chr4	176921001	176926000	7.46E-06
chr4	176922001	176927000	5.83E-21
chr4	176923001	176928000	1.09E-46
chr4	176982001	176987000	1.16E-13
chr4	176983001	176988000	1.38E-36
chr4	176984001	176989000	1.16E-36
chr4	176985001	176990000	1.06E-33
chr4	176986001	176991000	1.79E-43
chr4	176987001	176992000	1.51E-43
chr4	177114001	177119000	8.23E-25
chr4	177115001	177120000	1.81E-21
chr4	177116001	177121000	6.31E-23
chr4	177709001	177714000	1.67E-47
chr4	178171001	178176000	3.39E-09
chr4	178172001	178177000	0.006606235
chr4	178358001	178363000	8.53E-07
chr4	178359001	178364000	1.59E-32
chr4	178361001	178366000	2.86E-25
chr4	178363001	178368000	6.42E-19
chr4	178739001	178744000	1.92E-05
chr4	178872001	178877000	0.002728728
chr4	179088001	179093000	1.49E-07
chr4	179090001	179095000	3.42E-07
chr4	179091001	179096000	5.21E-07
chr4	179092001	179097000	1.94E-07
chr4	179351001	179356000	0.001963089
chr4	180345001	180350000	0.002385794
chr4	180346001	180351000	0.003800485
chr4	180667001	180672000	5.70E-06

chr4	180668001	180673000	8.91E-06
chr4	180669001	180674000	0.000213102
chr4	180670001	180675000	0.000298457
chr4	181016001	181021000	0.000154909
chr4	181224001	181229000	0.002059584
chr4	181229001	181234000	0.00432787
chr4	181230001	181235000	0.001442573
chr4	181231001	181236000	7.86E-05
chr4	181375001	181380000	2.95E-10
chr4	181377001	181382000	6.09E-09
chr4	181653001	181658000	0.001751664
chr4	181961001	181966000	0.0013427
chr4	182217001	182222000	0.001473686
chr4	182218001	182223000	0.000669755
chr4	182253001	182258000	0.000179424
chr4	182254001	182259000	2.07E-05
chr4	182255001	182260000	3.40E-07
chr4	182256001	182261000	8.82E-07
chr4	182416001	182421000	0.00010448
chr4	182417001	182422000	0.002340377
chr4	182435001	182440000	3.23E-11
chr4	182436001	182441000	8.03E-09
chr4	182490001	182495000	1.52E-05
chr4	182491001	182496000	2.44E-05
chr4	182492001	182497000	4.73E-05
chr4	182519001	182524000	7.07E-05
chr4	182520001	182525000	0.010234549
chr4	182621001	182626000	4.45E-10
chr4	182698001	182703000	2.09E-08
chr4	182852001	182857000	2.55E-06
chr4	183052001	183057000	6.95E-06
chr4	183053001	183058000	2.04E-07
chr4	183054001	183059000	1.78E-06
chr4	183055001	183060000	6.54E-06
chr4	183066001	183071000	1.46E-38
chr4	183365001	183370000	2.80E-36
chr4	183366001	183371000	8.44E-42
chr4	183367001	183372000	3.50E-27
chr4	183370001	183375000	7.84E-26
chr4	183455001	183460000	1.23E-06
chr4	183727001	183732000	1.44E-10
chr4	183750001	183755000	3.84E-08
chr4	184016001	184021000	2.36E-32
chr4	184017001	184022000	5.36E-26
chr4	184020001	184025000	6.60E-39
chr4	184366001	184371000	2.26E-52
chr4	184421001	184426000	9.19E-41
chr4	184422001	184427000	2.99E-67

chr4	184576001	184581000	7.90E-37
chr4	184577001	184582000	2.96E-40
chr4	184579001	184584000	7.15E-35
chr4	184580001	184585000	2.61E-40
chr4	184714001	184719000	3.15E-53
chr4	184715001	184720000	6.35E-60
chr4	184716001	184721000	1.03E-72
chr4	184717001	184722000	1.79E-81
chr4	184718001	184723000	7.49E-61
chr4	184719001	184724000	3.35E-43
chr4	184827001	184832000	1.83E-17
chr4	184847001	184852000	2.69E-18
chr4	184947001	184952000	1.65E-09
chr4	184957001	184962000	2.40E-33
chr4	184958001	184963000	3.42E-27
chr4	184959001	184964000	9.56E-23
chr4	184960001	184965000	7.69E-18
chr4	184961001	184966000	2.28E-21
chr4	185137001	185142000	8.84E-10
chr4	185138001	185143000	1.17E-10
chr4	185139001	185144000	2.66E-11
chr4	185140001	185145000	8.20E-08
chr4	185381001	185386000	0.000127798
chr4	185390001	185395000	1.59E-24
chr4	185440001	185445000	0.001074419
chr4	185565001	185570000	2.72E-28
chr4	185568001	185573000	7.38E-45
chr4	185569001	185574000	3.06E-30
chr4	185596001	185601000	5.86E-09
chr4	185742001	185747000	5.72E-58
chr4	185743001	185748000	1.04E-45
chr4	185744001	185749000	1.30E-38
chr4	185745001	185750000	1.20E-32
chr4	185746001	185751000	8.35E-40
chr4	185747001	185752000	4.73E-28
chr4	186045001	186050000	7.79E-27
chr4	186046001	186051000	6.56E-22
chr4	186047001	186052000	7.50E-20
chr4	186049001	186054000	3.55E-18
chr4	186060001	186065000	4.93E-23
chr4	186061001	186066000	3.14E-13
chr4	186063001	186068000	6.58E-19
chr4	186064001	186069000	5.41E-25
chr4	186120001	186125000	3.34E-22
chr4	186121001	186126000	1.20E-41
chr4	186122001	186127000	2.24E-18
chr4	186123001	186128000	5.54E-19
chr4	186124001	186129000	5.15E-19

chr4	186126001	186131000	1.82E-12
chr4	186127001	186132000	2.43E-19
chr4	186128001	186133000	2.18E-17
chr4	186135001	186140000	6.63E-11
chr4	186136001	186141000	1.05E-10
chr4	186316001	186321000	4.59E-66
chr4	186317001	186322000	9.02E-78
chr4	186318001	186323000	8.22E-40
chr4	186392001	186397000	1.14E-17
chr4	186452001	186457000	4.56E-31
chr4	186453001	186458000	1.86E-29
chr4	186454001	186459000	4.11E-38
chr4	186455001	186460000	2.24E-49
chr4	186456001	186461000	1.13E-36
chr4	186504001	186509000	2.89E-15
chr4	186627001	186632000	0.003513147
chr4	186628001	186633000	1.31E-09
chr4	186707001	186712000	4.29E-06
chr4	186708001	186713000	2.00E-05
chr4	186709001	186714000	1.52E-06
chr4	187004001	187009000	9.28E-07
chr4	187005001	187010000	0.000455231
chr4	187006001	187011000	0.000929251
chr4	187007001	187012000	0.000108492
chr4	187008001	187013000	8.24E-06
chr4	187061001	187066000	2.31E-13
chr4	187062001	187067000	4.84E-30
chr4	187063001	187068000	5.67E-23
chr4	187064001	187069000	5.96E-31
chr4	187065001	187070000	1.77E-29
chr4	187066001	187071000	5.33E-30
chr4	187100001	187105000	4.52E-10
chr4	187101001	187106000	1.68E-14
chr4	187110001	187115000	1.24E-43
chr4	187111001	187116000	1.65E-63
chr4	187112001	187117000	8.33E-75
chr4	187113001	187118000	2.30E-49
chr4	187139001	187144000	0.00093527
chr4	187140001	187145000	3.59E-06
chr4	187174001	187179000	0.000129103
chr4	187308001	187313000	0.000244948
chr4	187640001	187645000	3.47E-25
chr4	187648001	187653000	6.56E-23
chr4	188497001	188502000	0.000249679
chr4	188522001	188527000	0.005735399
chr4	188574001	188579000	0.000136021
chr4	188764001	188769000	4.89E-05
chr4	189369001	189374000	9.12E-07

chr4	189370001	189375000	1.90E-06
chr4	189531001	189536000	0.005624364
chr4	190054001	190059000	1.31E-21
chr4	190079001	190084000	1.96E-07
chr4	190080001	190085000	2.08E-06
chr4	190081001	190086000	1.29E-06
chr4	190097001	190102000	7.66E-06
chr4	190147001	190152000	0.017498583
chr4	190161001	190166000	5.95E-07
chr4	190209001	190214000	0.000640587
chr4	190286001	190291000	8.27E-05
chr4	190301001	190306000	5.32E-12
chr4	190325001	190330000	0.011214876
chr4	190356001	190361000	9.22E-09
chr4	190390001	190395000	4.36E-05
chr4	190391001	190396000	1.63E-05
chr4	190438001	190443000	0.001657722
chr4	190729001	190734000	3.56E-08
chr4	190730001	190735000	6.80E-10
chr4	190731001	190736000	3.94E-21
chr4	190732001	190737000	5.16E-22
chr4	190769001	190774000	3.10E-06
chr5	120001	125000	1.45E-10
chr5	121001	126000	3.00E-08
chr5	214001	219000	6.42E-28
chr5	340001	345000	9.50E-31
chr5	341001	346000	2.68E-17
chr5	612001	617000	2.89E-37
chr5	888001	893000	3.54E-68
chr5	889001	894000	1.67E-100
chr5	890001	895000	1.38E-38
chr5	892001	897000	1.71E-69
chr5	893001	898000	7.47E-48
chr5	1798001	1803000	3.61E-60
chr5	1929001	1934000	7.01E-16
chr5	1930001	1935000	6.51E-20
chr5	2034001	2039000	3.15E-35
chr5	2232001	2237000	6.82E-09
chr5	2233001	2238000	4.01E-09
chr5	2926001	2931000	1.58E-14
chr5	3166001	3171000	4.45E-06
chr5	3195001	3200000	1.55E-23
chr5	3253001	3258000	7.41E-14
chr5	3296001	3301000	1.54E-08
chr5	3297001	3302000	3.66E-08
chr5	3357001	3362000	2.56E-12
chr5	3358001	3363000	4.49E-07
chr5	3365001	3370000	3.28E-07

chr5	3440001	3445000	7.04E-15
chr5	3475001	3480000	3.34E-09
chr5	3476001	3481000	7.61E-12
chr5	3477001	3482000	1.12E-13
chr5	3498001	3503000	4.71E-10
chr5	3555001	3560000	0.002272155
chr5	3557001	3562000	0.002429379
chr5	3558001	3563000	0.002158336
chr5	3559001	3564000	8.76E-06
chr5	3560001	3565000	4.39E-05
chr5	3562001	3567000	5.63E-16
chr5	3725001	3730000	4.09E-14
chr5	3727001	3732000	1.05E-12
chr5	3756001	3761000	9.52E-12
chr5	3801001	3806000	3.93E-09
chr5	3832001	3837000	1.29E-06
chr5	3833001	3838000	1.30E-05
chr5	3915001	3920000	3.30E-08
chr5	3916001	3921000	5.03E-07
chr5	3951001	3956000	1.39E-10
chr5	3952001	3957000	4.31E-06
chr5	4045001	4050000	5.19E-10
chr5	4046001	4051000	3.94E-09
chr5	4053001	4058000	1.79E-05
chr5	4054001	4059000	0.000359012
chr5	4082001	4087000	5.70E-15
chr5	4112001	4117000	2.55E-21
chr5	4113001	4118000	4.85E-24
chr5	4114001	4119000	1.32E-17
chr5	4150001	4155000	0.000226628
chr5	4421001	4426000	1.05E-05
chr5	4426001	4431000	9.55E-09
chr5	4467001	4472000	0.00080307
chr5	4468001	4473000	0.000130486
chr5	4469001	4474000	6.20E-05
chr5	4470001	4475000	1.30E-05
chr5	4482001	4487000	1.78E-06
chr5	4484001	4489000	0.000533006
chr5	4510001	4515000	2.39E-20
chr5	4512001	4517000	1.11E-29
chr5	4513001	4518000	3.21E-08
chr5	4524001	4529000	4.08E-10
chr5	4549001	4554000	1.54E-10
chr5	4587001	4592000	1.27E-07
chr5	4588001	4593000	5.58E-07
chr5	4589001	4594000	3.31E-10
chr5	4590001	4595000	8.93E-08
chr5	4598001	4603000	3.28E-05

chr5	4599001	4604000	7.66E-05
chr5	4601001	4606000	0.000777957
chr5	4602001	4607000	2.48E-07
chr5	4603001	4608000	4.11E-07
chr5	4606001	4611000	4.72E-18
chr5	4607001	4612000	2.08E-17
chr5	4608001	4613000	1.46E-16
chr5	4609001	4614000	1.05E-19
chr5	4610001	4615000	3.91E-16
chr5	4619001	4624000	6.93E-11
chr5	4622001	4627000	0.000233263
chr5	4653001	4658000	7.19E-08
chr5	4654001	4659000	1.87E-08
chr5	4662001	4667000	1.48E-11
chr5	4663001	4668000	3.59E-16
chr5	4664001	4669000	3.04E-13
chr5	4665001	4670000	6.63E-10
chr5	4666001	4671000	2.13E-09
chr5	4667001	4672000	7.56E-08
chr5	4669001	4674000	2.27E-13
chr5	4681001	4686000	3.39E-05
chr5	4683001	4688000	3.57E-06
chr5	4684001	4689000	2.15E-08
chr5	4685001	4690000	5.69E-07
chr5	4686001	4691000	5.08E-05
chr5	4687001	4692000	0.001932302
chr5	4701001	4706000	2.79E-07
chr5	4702001	4707000	1.34E-08
chr5	4718001	4723000	4.22E-13
chr5	4719001	4724000	5.49E-13
chr5	4746001	4751000	1.18E-14
chr5	4747001	4752000	1.11E-17
chr5	4748001	4753000	2.01E-16
chr5	4749001	4754000	8.21E-11
chr5	4759001	4764000	2.46E-06
chr5	4760001	4765000	2.64E-06
chr5	4761001	4766000	7.32E-09
chr5	4839001	4844000	4.46E-25
chr5	4840001	4845000	2.38E-27
chr5	4841001	4846000	4.30E-31
chr5	4842001	4847000	9.99E-33
chr5	4969001	4974000	1.85E-08
chr5	4970001	4975000	2.11E-05
chr5	4971001	4976000	4.20E-11
chr5	4972001	4977000	1.86E-07
chr5	4995001	5000000	9.20E-07
chr5	4996001	5001000	3.59E-06
chr5	4997001	5002000	1.16E-05

chr5	4999001	5004000	7.69E-05
chr5	5006001	5011000	1.63E-06
chr5	5031001	5036000	1.08E-15
chr5	5098001	5103000	0.000123344
chr5	5423001	5428000	7.51E-52
chr5	5525001	5530000	7.19E-07
chr5	5526001	5531000	5.11E-07
chr5	5527001	5532000	7.24E-08
chr5	5729001	5734000	6.92E-15
chr5	5736001	5741000	1.38E-15
chr5	5737001	5742000	1.23E-15
chr5	5738001	5743000	4.11E-16
chr5	5739001	5744000	1.70E-07
chr5	5740001	5745000	7.91E-07
chr5	5791001	5796000	1.14E-11
chr5	5841001	5846000	9.02E-14
chr5	5851001	5856000	2.24E-12
chr5	5852001	5857000	5.23E-13
chr5	5853001	5858000	1.22E-15
chr5	5854001	5859000	4.00E-10
chr5	5875001	5880000	4.07E-08
chr5	5931001	5936000	2.46E-10
chr5	5955001	5960000	1.83E-06
chr5	5957001	5962000	2.67E-09
chr5	5983001	5988000	0.000752291
chr5	5991001	5996000	9.51E-11
chr5	5992001	5997000	2.74E-10
chr5	5994001	5999000	5.82E-13
chr5	5995001	6000000	3.79E-13
chr5	5996001	6001000	4.83E-10
chr5	6050001	6055000	0.000537761
chr5	6052001	6057000	0.000271199
chr5	6060001	6065000	9.58E-11
chr5	6297001	6302000	8.04E-18
chr5	6298001	6303000	3.01E-20
chr5	6299001	6304000	3.09E-14
chr5	6300001	6305000	1.69E-15
chr5	6374001	6379000	2.13E-68
chr5	6375001	6380000	4.36E-34
chr5	6376001	6381000	3.53E-30
chr5	6377001	6382000	1.30E-33
chr5	6378001	6383000	7.79E-53
chr5	6709001	6714000	5.57E-45
chr5	6710001	6715000	8.95E-52
chr5	6967001	6972000	0.000155134
chr5	6970001	6975000	3.57E-07
chr5	6977001	6982000	7.11E-10
chr5	6984001	6989000	4.16E-09

chr5	6988001	6993000	2.99E-09
chr5	6990001	6995000	2.85E-06
chr5	6991001	6996000	0.000148053
chr5	6993001	6998000	6.95E-07
chr5	7047001	7052000	7.00E-05
chr5	7048001	7053000	8.24E-07
chr5	7049001	7054000	2.89E-06
chr5	7082001	7087000	8.14E-13
chr5	7083001	7088000	4.83E-11
chr5	7118001	7123000	5.08E-06
chr5	7119001	7124000	2.60E-09
chr5	7120001	7125000	2.10E-09
chr5	7121001	7126000	1.34E-11
chr5	7122001	7127000	3.84E-11
chr5	7239001	7244000	1.65E-08
chr5	7241001	7246000	3.69E-08
chr5	7270001	7275000	2.00E-20
chr5	7271001	7276000	2.15E-16
chr5	7311001	7316000	6.70E-10
chr5	7334001	7339000	2.76E-07
chr5	7335001	7340000	1.11E-07
chr5	7336001	7341000	5.94E-05
chr5	7337001	7342000	0.00011234
chr5	7341001	7346000	1.55E-10
chr5	7342001	7347000	7.97E-19
chr5	7343001	7348000	3.29E-20
chr5	7350001	7355000	8.73E-11
chr5	7351001	7356000	3.71E-12
chr5	7352001	7357000	8.63E-13
chr5	7392001	7397000	4.70E-46
chr5	7420001	7425000	1.60E-12
chr5	7421001	7426000	3.20E-14
chr5	7422001	7427000	3.68E-21
chr5	7423001	7428000	1.50E-18
chr5	7424001	7429000	6.78E-15
chr5	7425001	7430000	2.51E-10
chr5	7448001	7453000	1.82E-11
chr5	7450001	7455000	1.39E-07
chr5	7453001	7458000	1.86E-11
chr5	7454001	7459000	1.40E-13
chr5	7455001	7460000	4.84E-13
chr5	7464001	7469000	2.23E-06
chr5	7465001	7470000	1.10E-07
chr5	7466001	7471000	2.49E-07
chr5	7467001	7472000	5.32E-08
chr5	7493001	7498000	5.69E-10
chr5	7508001	7513000	1.39E-12
chr5	7509001	7514000	7.23E-12

chr5	7510001	7515000	1.08E-07
chr5	7511001	7516000	6.37E-11
chr5	7521001	7526000	3.98E-16
chr5	7522001	7527000	1.94E-26
chr5	7523001	7528000	4.04E-22
chr5	7524001	7529000	1.16E-21
chr5	7525001	7530000	3.21E-20
chr5	7526001	7531000	4.67E-15
chr5	7533001	7538000	5.41E-12
chr5	7558001	7563000	1.00E-20
chr5	7559001	7564000	1.26E-20
chr5	7569001	7574000	7.33E-06
chr5	7570001	7575000	0.000284128
chr5	7571001	7576000	0.000167684
chr5	7572001	7577000	1.00E-06
chr5	7573001	7578000	6.58E-05
chr5	7604001	7609000	4.10E-05
chr5	7605001	7610000	7.31E-06
chr5	7606001	7611000	1.13E-09
chr5	7607001	7612000	2.25E-10
chr5	7653001	7658000	1.49E-23
chr5	7654001	7659000	2.98E-20
chr5	7691001	7696000	9.85E-27
chr5	7692001	7697000	2.01E-21
chr5	7704001	7709000	2.31E-18
chr5	7732001	7737000	0.000480183
chr5	7733001	7738000	0.0001535
chr5	7739001	7744000	4.42E-11
chr5	7740001	7745000	1.72E-11
chr5	7741001	7746000	4.20E-13
chr5	7748001	7753000	4.18E-08
chr5	7749001	7754000	1.64E-06
chr5	7750001	7755000	4.33E-06
chr5	7805001	7810000	1.43E-15
chr5	7806001	7811000	3.61E-13
chr5	7807001	7812000	1.15E-22
chr5	7808001	7813000	6.64E-21
chr5	7809001	7814000	7.18E-16
chr5	7846001	7851000	3.41E-20
chr5	7864001	7869000	4.50E-43
chr5	7865001	7870000	2.94E-63
chr5	7918001	7923000	2.08E-05
chr5	7919001	7924000	0.000135534
chr5	7939001	7944000	2.98E-14
chr5	7940001	7945000	2.86E-13
chr5	7950001	7955000	1.02E-11
chr5	8001001	8006000	1.73E-05
chr5	8002001	8007000	7.79E-07

chr5	8016001	8021000	0.009283545
chr5	8037001	8042000	3.46E-05
chr5	8038001	8043000	1.32E-06
chr5	8093001	8098000	2.83E-09
chr5	8228001	8233000	7.34E-10
chr5	8229001	8234000	8.28E-16
chr5	8231001	8236000	8.54E-08
chr5	8237001	8242000	5.33E-08
chr5	8256001	8261000	9.49E-11
chr5	8257001	8262000	3.24E-13
chr5	8258001	8263000	4.92E-10
chr5	8259001	8264000	1.33E-11
chr5	8260001	8265000	4.06E-10
chr5	8453001	8458000	2.36E-25
chr5	8457001	8462000	3.12E-45
chr5	8481001	8486000	7.21E-06
chr5	8482001	8487000	0.001529562
chr5	8483001	8488000	0.000173627
chr5	8558001	8563000	7.83E-06
chr5	8778001	8783000	3.09E-06
chr5	8967001	8972000	0.000456344
chr5	9347001	9352000	0.006662693
chr5	9492001	9497000	0.006311933
chr5	9541001	9546000	3.13E-79
chr5	9805001	9810000	1.65E-06
chr5	9986001	9991000	6.08E-08
chr5	10437001	10442000	5.43E-58
chr5	10564001	10569000	2.27E-17
chr5	10597001	10602000	5.50E-08
chr5	10695001	10700000	1.69E-19
chr5	10761001	10766000	3.82E-27
chr5	11442001	11447000	5.34E-13
chr5	11638001	11643000	3.69E-08
chr5	11639001	11644000	1.16E-06
chr5	11640001	11645000	4.02E-08
chr5	11641001	11646000	4.35E-09
chr5	11642001	11647000	8.43E-06
chr5	11683001	11688000	0.000245987
chr5	11807001	11812000	1.40E-05
chr5	11844001	11849000	6.38E-06
chr5	11845001	11850000	3.51E-07
chr5	11892001	11897000	9.32E-05
chr5	12040001	12045000	7.93E-07
chr5	12219001	12224000	0.000671119
chr5	12220001	12225000	0.001611376
chr5	12291001	12296000	2.52E-05
chr5	12292001	12297000	1.32E-05
chr5	12293001	12298000	5.42E-07

chr5	12294001	12299000	2.68E-06
chr5	12505001	12510000	2.07E-05
chr5	12618001	12623000	0.000439817
chr5	12620001	12625000	0.000378596
chr5	12755001	12760000	6.20E-09
chr5	12756001	12761000	5.43E-08
chr5	13029001	13034000	1.84E-14
chr5	13030001	13035000	0.000322988
chr5	14144001	14149000	9.13E-48
chr5	14660001	14665000	2.02E-52
chr5	14661001	14666000	4.23E-49
chr5	14662001	14667000	1.21E-56
chr5	14663001	14668000	7.71E-57
chr5	14870001	14875000	1.74E-53
chr5	14871001	14876000	2.02E-62
chr5	14872001	14877000	4.02E-57
chr5	15125001	15130000	4.29E-06
chr5	15176001	15181000	1.28E-06
chr5	15177001	15182000	5.87E-05
chr5	15256001	15261000	0.006120117
chr5	15305001	15310000	1.08E-12
chr5	15306001	15311000	9.81E-18
chr5	15336001	15341000	7.92E-06
chr5	15337001	15342000	1.81E-09
chr5	15349001	15354000	0.000399367
chr5	15362001	15367000	3.76E-05
chr5	15373001	15378000	4.78E-06
chr5	15374001	15379000	3.52E-14
chr5	15375001	15380000	2.01E-05
chr5	15376001	15381000	1.56E-08
chr5	15430001	15435000	2.97E-05
chr5	15452001	15457000	7.16E-07
chr5	15454001	15459000	2.58E-07
chr5	15455001	15460000	1.18E-06
chr5	15456001	15461000	4.88E-08
chr5	15457001	15462000	2.47E-07
chr5	15458001	15463000	3.38E-07
chr5	15462001	15467000	5.08E-05
chr5	15463001	15468000	2.78E-06
chr5	15464001	15469000	9.31E-06
chr5	15496001	15501000	3.02E-40
chr5	15500001	15505000	2.57E-53
chr5	15558001	15563000	6.55E-06
chr5	15559001	15564000	8.33E-06
chr5	15567001	15572000	3.10E-13
chr5	15568001	15573000	8.86E-12
chr5	15571001	15576000	5.49E-07
chr5	15623001	15628000	6.38E-09

chr5	15624001	15629000	2.92E-09
chr5	15625001	15630000	1.78E-06
chr5	15632001	15637000	4.21E-09
chr5	15633001	15638000	1.14E-07
chr5	15656001	15661000	6.73E-11
chr5	15657001	15662000	3.90E-10
chr5	15707001	15712000	1.24E-07
chr5	15708001	15713000	1.33E-09
chr5	15764001	15769000	4.35E-10
chr5	15766001	15771000	2.67E-09
chr5	15845001	15850000	0.000115979
chr5	15876001	15881000	5.51E-10
chr5	15877001	15882000	6.36E-09
chr5	16157001	16162000	9.34E-05
chr5	16171001	16176000	2.12E-09
chr5	16172001	16177000	1.55E-09
chr5	16466001	16471000	3.80E-34
chr5	16612001	16617000	1.20E-38
chr5	16613001	16618000	1.11E-42
chr5	16614001	16619000	3.78E-34
chr5	16617001	16622000	5.44E-17
chr5	16934001	16939000	5.04E-54
chr5	16935001	16940000	1.17E-73
chr5	16936001	16941000	1.49E-41
chr5	18447001	18452000	0.001054951
chr5	18448001	18453000	0.000276373
chr5	18585001	18590000	3.69E-07
chr5	18586001	18591000	1.70E-07
chr5	18587001	18592000	1.91E-08
chr5	18588001	18593000	2.65E-09
chr5	19310001	19315000	7.90E-08
chr5	19944001	19949000	0.006879367
chr5	20113001	20118000	0.001791611
chr5	20270001	20275000	0.001048755
chr5	20389001	20394000	0.000857305
chr5	20405001	20410000	1.76E-05
chr5	20406001	20411000	5.33E-05
chr5	20540001	20545000	0.000567724
chr5	20766001	20771000	3.76E-06
chr5	20767001	20772000	9.88E-07
chr5	20894001	20899000	1.13E-07
chr5	21339001	21344000	6.40E-30
chr5	21340001	21345000	7.39E-27
chr5	21341001	21346000	2.48E-25
chr5	21455001	21460000	8.56E-39
chr5	21456001	21461000	6.62E-39
chr5	21457001	21462000	1.73E-22
chr5	21459001	21464000	1.23E-21

chr5	22353001	22358000	0.001010837
chr5	22683001	22688000	1.08E-07
chr5	22684001	22689000	2.34E-08
chr5	22685001	22690000	2.62E-08
chr5	23833001	23838000	1.05E-06
chr5	24860001	24865000	0.000359331
chr5	25268001	25273000	0.00022512
chr5	26336001	26341000	4.03E-05
chr5	26846001	26851000	2.11E-08
chr5	27749001	27754000	1.09E-06
chr5	27972001	27977000	4.49E-05
chr5	28158001	28163000	0.028544926
chr5	28557001	28562000	6.20E-06
chr5	28563001	28568000	0.000473507
chr5	28614001	28619000	0.002699498
chr5	28657001	28662000	2.06E-15
chr5	28658001	28663000	1.07E-16
chr5	28659001	28664000	5.29E-15
chr5	28660001	28665000	2.09E-18
chr5	28661001	28666000	8.13E-16
chr5	28662001	28667000	1.16E-05
chr5	28663001	28668000	0.00375301
chr5	28669001	28674000	4.54E-05
chr5	28735001	28740000	1.93E-07
chr5	28736001	28741000	9.65E-08
chr5	29073001	29078000	0.021561731
chr5	29704001	29709000	0.001319285
chr5	29794001	29799000	0.000439984
chr5	29908001	29913000	0.005623734
chr5	31042001	31047000	1.77E-07
chr5	31189001	31194000	3.25E-07
chr5	31231001	31236000	1.66E-05
chr5	32440001	32445000	9.63E-20
chr5	32441001	32446000	1.10E-09
chr5	32442001	32447000	9.25E-15
chr5	32443001	32448000	2.88E-24
chr5	32444001	32449000	8.04E-18
chr5	32585001	32590000	1.72E-53
chr5	32821001	32826000	0.000573334
chr5	33415001	33420000	0.001922299
chr5	33712001	33717000	0.002161114
chr5	34003001	34008000	1.99E-30
chr5	34004001	34009000	3.00E-42
chr5	34005001	34010000	1.57E-36
chr5	34244001	34249000	5.60E-20
chr5	34245001	34250000	5.76E-19
chr5	34835001	34840000	2.49E-47
chr5	34836001	34841000	2.61E-55

chr5	34837001	34842000	5.12E-60
chr5	34838001	34843000	3.17E-70
chr5	34839001	34844000	6.12E-72
chr5	34911001	34916000	1.62E-61
chr5	34925001	34930000	6.76E-96
chr5	34926001	34931000	2.22E-54
chr5	34927001	34932000	8.72E-56
chr5	35054001	35059000	0.001179021
chr5	35055001	35060000	9.27E-05
chr5	35056001	35061000	0.006154957
chr5	35203001	35208000	0.000687794
chr5	35271001	35276000	6.25E-05
chr5	35272001	35277000	1.61E-05
chr5	35273001	35278000	2.75E-05
chr5	35274001	35279000	2.05E-08
chr5	35344001	35349000	9.08E-07
chr5	35411001	35416000	1.89E-10
chr5	35446001	35451000	2.11E-14
chr5	35447001	35452000	4.59E-11
chr5	35448001	35453000	7.25E-12
chr5	35449001	35454000	1.23E-12
chr5	35450001	35455000	3.31E-12
chr5	35451001	35456000	5.05E-05
chr5	35459001	35464000	0.00252999
chr5	35506001	35511000	4.74E-08
chr5	35507001	35512000	6.37E-10
chr5	35509001	35514000	6.18E-07
chr5	35510001	35515000	2.17E-06
chr5	35564001	35569000	0.000232064
chr5	35613001	35618000	6.46E-12
chr5	35614001	35619000	1.01E-11
chr5	35615001	35620000	5.93E-26
chr5	35616001	35621000	5.00E-32
chr5	35617001	35622000	4.59E-29
chr5	35618001	35623000	9.67E-37
chr5	35694001	35699000	0.008322625
chr5	35948001	35953000	0.000614867
chr5	35973001	35978000	3.17E-09
chr5	35974001	35979000	6.78E-09
chr5	35975001	35980000	1.40E-09
chr5	35976001	35981000	3.73E-06
chr5	35993001	35998000	3.00E-11
chr5	35994001	35999000	8.82E-15
chr5	35995001	36000000	1.13E-11
chr5	36000001	36005000	4.04E-14
chr5	36010001	36015000	0.000223093
chr5	36049001	36054000	5.32E-07
chr5	36237001	36242000	1.13E-40

chr5	36261001	36266000	1.88E-12
chr5	36262001	36267000	6.82E-12
chr5	36302001	36307000	1.17E-05
chr5	36433001	36438000	0.003567707
chr5	36673001	36678000	2.92E-05
chr5	36687001	36692000	5.89E-24
chr5	36688001	36693000	2.58E-30
chr5	36690001	36695000	1.09E-27
chr5	36745001	36750000	1.04E-26
chr5	36841001	36846000	9.86E-09
chr5	36842001	36847000	1.37E-05
chr5	36858001	36863000	0.007883854
chr5	37245001	37250000	7.54E-18
chr5	37246001	37251000	1.50E-23
chr5	37247001	37252000	1.56E-34
chr5	37248001	37253000	1.02E-43
chr5	37249001	37254000	7.53E-47
chr5	37493001	37498000	2.68E-36
chr5	37494001	37499000	2.92E-32
chr5	37495001	37500000	2.85E-36
chr5	37496001	37501000	1.06E-30
chr5	37497001	37502000	2.17E-19
chr5	37956001	37961000	0.000664769
chr5	37957001	37962000	0.000295728
chr5	37958001	37963000	0.00060081
chr5	38271001	38276000	9.99E-05
chr5	38272001	38277000	0.000108781
chr5	38273001	38278000	8.41E-07
chr5	38275001	38280000	0.00054601
chr5	38276001	38281000	1.25E-06
chr5	38277001	38282000	2.00E-06
chr5	38278001	38283000	0.000922866
chr5	38279001	38284000	9.72E-05
chr5	38363001	38368000	3.24E-07
chr5	38588001	38593000	0.002238009
chr5	38816001	38821000	3.22E-05
chr5	39074001	39079000	5.12E-86
chr5	39222001	39227000	1.75E-05
chr5	39223001	39228000	3.84E-06
chr5	39224001	39229000	2.78E-06
chr5	39709001	39714000	0.002661756
chr5	39738001	39743000	3.96E-12
chr5	39789001	39794000	0.002789527
chr5	39790001	39795000	0.002318151
chr5	39882001	39887000	1.94E-06
chr5	40009001	40014000	0.000103858
chr5	40132001	40137000	8.56E-05
chr5	40133001	40138000	0.000830995

chr5	40153001	40158000	0.000192367
chr5	40178001	40183000	1.99E-05
chr5	40270001	40275000	0.000149454
chr5	40514001	40519000	1.82E-05
chr5	40524001	40529000	2.18E-05
chr5	40617001	40622000	0.004635825
chr5	40751001	40756000	7.62E-15
chr5	41208001	41213000	1.68E-09
chr5	41299001	41304000	0.00035234
chr5	41685001	41690000	1.43E-05
chr5	41865001	41870000	7.08E-20
chr5	41866001	41871000	5.15E-37
chr5	41867001	41872000	3.21E-27
chr5	41868001	41873000	2.37E-58
chr5	41869001	41874000	1.03E-54
chr5	41870001	41875000	7.39E-57
chr5	41900001	41905000	8.24E-57
chr5	41901001	41906000	1.42E-54
chr5	41902001	41907000	1.02E-55
chr5	41903001	41908000	3.41E-53
chr5	41904001	41909000	1.66E-49
chr5	41921001	41926000	1.23E-35
chr5	42185001	42190000	0.000638205
chr5	42253001	42258000	7.41E-10
chr5	42270001	42275000	0.000640766
chr5	42272001	42277000	7.70E-05
chr5	42339001	42344000	1.20E-06
chr5	42419001	42424000	6.48E-21
chr5	42426001	42431000	0.000161909
chr5	42430001	42435000	0.011855302
chr5	42442001	42447000	4.23E-05
chr5	42721001	42726000	4.83E-06
chr5	42820001	42825000	1.44E-05
chr5	42864001	42869000	1.34E-12
chr5	42865001	42870000	1.35E-09
chr5	42967001	42972000	1.05E-12
chr5	42996001	43001000	8.34E-15
chr5	42997001	43002000	5.26E-21
chr5	42998001	43003000	2.18E-23
chr5	42999001	43004000	3.99E-31
chr5	43000001	43005000	1.02E-30
chr5	43013001	43018000	2.81E-09
chr5	43014001	43019000	2.39E-20
chr5	43119001	43124000	3.10E-40
chr5	43120001	43125000	9.60E-35
chr5	43188001	43193000	7.18E-41
chr5	43309001	43314000	9.23E-23
chr5	43552001	43557000	1.73E-44

chr5	43598001	43603000	4.21E-23
chr5	43599001	43604000	2.46E-31
chr5	43738001	43743000	2.27E-07
chr5	43750001	43755000	3.75E-10
chr5	43753001	43758000	1.22E-07
chr5	43793001	43798000	1.07E-15
chr5	43806001	43811000	3.61E-11
chr5	43807001	43812000	1.44E-10
chr5	43808001	43813000	2.14E-12
chr5	43830001	43835000	0.00020622
chr5	43831001	43836000	0.000275815
chr5	43832001	43837000	0.000869792
chr5	43833001	43838000	0.000429042
chr5	43875001	43880000	0.001588738
chr5	43884001	43889000	0.000425389
chr5	43885001	43890000	0.000127793
chr5	43886001	43891000	9.20E-05
chr5	43887001	43892000	4.65E-08
chr5	43888001	43893000	3.07E-08
chr5	43897001	43902000	0.000384081
chr5	43898001	43903000	0.000470653
chr5	43899001	43904000	0.000343808
chr5	43918001	43923000	2.07E-06
chr5	43921001	43926000	1.98E-15
chr5	43922001	43927000	3.67E-16
chr5	43923001	43928000	8.33E-12
chr5	43924001	43929000	1.89E-08
chr5	43928001	43933000	5.60E-05
chr5	43929001	43934000	3.74E-06
chr5	43930001	43935000	6.81E-06
chr5	43937001	43942000	1.58E-05
chr5	43965001	43970000	3.53E-06
chr5	43966001	43971000	2.34E-05
chr5	43967001	43972000	8.32E-10
chr5	43968001	43973000	1.92E-09
chr5	43969001	43974000	1.47E-07
chr5	43970001	43975000	1.89E-05
chr5	43971001	43976000	8.19E-06
chr5	43976001	43981000	3.78E-06
chr5	43979001	43984000	4.69E-07
chr5	43980001	43985000	2.00E-06
chr5	43982001	43987000	1.50E-05
chr5	44036001	44041000	0.000103897
chr5	44037001	44042000	3.05E-14
chr5	44043001	44048000	7.69E-17
chr5	44044001	44049000	1.70E-18
chr5	44045001	44050000	6.19E-16
chr5	44046001	44051000	1.03E-10

chr5	44057001	44062000	8.22E-05
chr5	44058001	44063000	0.00299191
chr5	44075001	44080000	3.50E-07
chr5	44076001	44081000	0.004231891
chr5	44086001	44091000	0.000126589
chr5	44088001	44093000	0.000162686
chr5	44089001	44094000	8.91E-05
chr5	44090001	44095000	2.26E-05
chr5	44103001	44108000	3.48E-09
chr5	44135001	44140000	1.55E-09
chr5	44146001	44151000	0.000221568
chr5	44207001	44212000	2.17E-05
chr5	44208001	44213000	9.46E-06
chr5	44209001	44214000	7.03E-08
chr5	44210001	44215000	1.43E-08
chr5	44212001	44217000	2.09E-06
chr5	44213001	44218000	8.99E-07
chr5	44295001	44300000	0.000269433
chr5	44312001	44317000	2.16E-05
chr5	44313001	44318000	1.58E-05
chr5	44314001	44319000	3.80E-05
chr5	44315001	44320000	0.000623469
chr5	44316001	44321000	1.16E-05
chr5	44342001	44347000	0.000494998
chr5	44343001	44348000	0.000142382
chr5	44344001	44349000	0.000238193
chr5	44345001	44350000	0.000638571
chr5	44390001	44395000	3.75E-07
chr5	44408001	44413000	4.10E-05
chr5	44409001	44414000	2.95E-06
chr5	44410001	44415000	1.70E-05
chr5	44411001	44416000	3.53E-07
chr5	44412001	44417000	9.76E-05
chr5	44427001	44432000	0.006780241
chr5	44428001	44433000	0.005212043
chr5	44429001	44434000	0.008584974
chr5	44432001	44437000	0.010956391
chr5	44433001	44438000	0.018320452
chr5	44498001	44503000	0.000505572
chr5	44499001	44504000	0.000588779
chr5	44544001	44549000	2.41E-05
chr5	44545001	44550000	5.96E-09
chr5	44546001	44551000	2.41E-10
chr5	44547001	44552000	2.18E-08
chr5	44564001	44569000	0.000397485
chr5	44573001	44578000	3.41E-09
chr5	44574001	44579000	1.52E-11
chr5	44596001	44601000	1.30E-05

chr5	44597001	44602000	1.05E-05
chr5	44598001	44603000	4.24E-05
chr5	44680001	44685000	8.19E-08
chr5	44681001	44686000	4.11E-08
chr5	44685001	44690000	1.28E-06
chr5	44695001	44700000	0.000223208
chr5	44805001	44810000	1.06E-78
chr5	44806001	44811000	4.16E-68
chr5	44807001	44812000	2.88E-98
chr5	44808001	44813000	5.09E-92
chr5	44809001	44814000	1.34E-97
chr5	44931001	44936000	3.23E-07
chr5	44954001	44959000	2.05E-05
chr5	44957001	44962000	3.85E-07
chr5	44963001	44968000	0.001249909
chr5	44981001	44986000	0.003814362
chr5	45018001	45023000	5.04E-10
chr5	45020001	45025000	2.05E-08
chr5	45024001	45029000	7.96E-07
chr5	45025001	45030000	5.13E-07
chr5	45065001	45070000	2.26E-06
chr5	45066001	45071000	1.66E-07
chr5	45067001	45072000	2.20E-13
chr5	45068001	45073000	8.86E-11
chr5	45160001	45165000	7.89E-09
chr5	45164001	45169000	4.85E-07
chr5	45174001	45179000	1.39E-06
chr5	45197001	45202000	1.01E-06
chr5	45198001	45203000	3.81E-08
chr5	45199001	45204000	1.95E-05
chr5	45267001	45272000	1.80E-06
chr5	45399001	45404000	0.001600849
chr5	45421001	45426000	4.34E-10
chr5	45500001	45505000	0.000955449
chr5	45504001	45509000	0.001210569
chr5	45505001	45510000	0.00032808
chr5	45560001	45565000	6.45E-06
chr5	45574001	45579000	0.000224453
chr5	45581001	45586000	5.07E-15
chr5	45583001	45588000	5.50E-18
chr5	45696001	45701000	1.15E-34
chr5	45715001	45720000	9.14E-06
chr5	45716001	45721000	3.84E-08
chr5	45757001	45762000	1.99E-05
chr5	45800001	45805000	1.04E-08
chr5	45801001	45806000	2.26E-05
chr5	45839001	45844000	2.05E-07
chr5	45840001	45845000	1.27E-07

chr5	45861001	45866000	1.23E-08
chr5	45862001	45867000	3.25E-10
chr5	45863001	45868000	9.41E-07
chr5	45864001	45869000	2.59E-06
chr5	45865001	45870000	9.79E-05
chr5	45892001	45897000	8.57E-06
chr5	45955001	45960000	0.00014451
chr5	45956001	45961000	5.55E-05
chr5	45975001	45980000	6.73E-06
chr5	46002001	46007000	4.45E-05
chr5	46098001	46103000	0.002304169
chr5	46182001	46187000	2.07E-05
chr5	46183001	46188000	4.93E-07
chr5	49552001	49557000	3.38E-08
chr5	49566001	49571000	0.000484898
chr5	49716001	49721000	5.36E-05
chr5	49737001	49742000	6.23E-38
chr5	49787001	49792000	0.000596838
chr5	49810001	49815000	0.002699498
chr5	49812001	49817000	6.29E-10
chr5	49813001	49818000	3.81E-11
chr5	49814001	49819000	1.43E-15
chr5	49815001	49820000	7.15E-15
chr5	49816001	49821000	6.08E-16
chr5	49817001	49822000	0.00116898
chr5	49824001	49829000	1.28E-07
chr5	49845001	49850000	9.47E-07
chr5	49846001	49851000	2.51E-06
chr5	49874001	49879000	0.003455708
chr5	49937001	49942000	0.001939469
chr5	49938001	49943000	0.002254913
chr5	49963001	49968000	6.93E-34
chr5	50046001	50051000	2.10E-05
chr5	50047001	50052000	2.82E-06
chr5	50048001	50053000	2.05E-06
chr5	50049001	50054000	2.32E-06
chr5	50050001	50055000	4.23E-06
chr5	50051001	50056000	0.000628694
chr5	50052001	50057000	0.004618125
chr5	50053001	50058000	0.010945631
chr5	50065001	50070000	5.39E-06
chr5	50107001	50112000	1.44E-08
chr5	50108001	50113000	1.37E-07
chr5	50109001	50114000	4.90E-08
chr5	50280001	50285000	0.000127678
chr5	50350001	50355000	0.002710408
chr5	50720001	50725000	9.54E-06
chr5	50721001	50726000	1.71E-05

chr5	50722001	50727000	2.40E-05
chr5	50969001	50974000	0.000939511
chr5	51611001	51616000	0.000648961
chr5	51657001	51662000	5.02E-05
chr5	51658001	51663000	4.46E-05
chr5	51659001	51664000	0.00012133
chr5	51683001	51688000	0.000894015
chr5	51797001	51802000	0.002370474
chr5	52079001	52084000	5.94E-28
chr5	52080001	52085000	3.36E-33
chr5	52081001	52086000	1.62E-26
chr5	52084001	52089000	8.80E-24
chr5	52097001	52102000	9.41E-08
chr5	52166001	52171000	3.29E-05
chr5	52210001	52215000	0.002955896
chr5	52282001	52287000	3.06E-16
chr5	52283001	52288000	1.58E-23
chr5	52284001	52289000	5.40E-29
chr5	52285001	52290000	6.38E-27
chr5	52296001	52301000	0.000426738
chr5	52297001	52302000	4.96E-05
chr5	52347001	52352000	0.000202025
chr5	52349001	52354000	0.00039459
chr5	52401001	52406000	2.09E-50
chr5	52402001	52407000	2.93E-63
chr5	52403001	52408000	1.42E-67
chr5	52404001	52409000	1.25E-64
chr5	52405001	52410000	1.87E-47
chr5	52538001	52543000	0.000294411
chr5	52770001	52775000	6.81E-13
chr5	52804001	52809000	0.003255446
chr5	53016001	53021000	2.97E-05
chr5	53410001	53415000	0.004770536
chr5	53480001	53485000	0.000528101
chr5	53601001	53606000	2.71E-36
chr5	53602001	53607000	7.07E-86
chr5	53603001	53608000	7.88E-60
chr5	54023001	54028000	0.00038135
chr5	54024001	54029000	1.35E-05
chr5	54470001	54475000	1.48E-56
chr5	54471001	54476000	5.35E-65
chr5	54472001	54477000	6.61E-59
chr5	54521001	54526000	3.63E-34
chr5	54522001	54527000	8.76E-36
chr5	54599001	54604000	1.01E-44
chr5	54603001	54608000	2.51E-49
chr5	55051001	55056000	0.001171959
chr5	55097001	55102000	1.60E-10

chr5	55098001	55103000	1.10E-09
chr5	55143001	55148000	2.00E-05
chr5	55244001	55249000	1.74E-05
chr5	55245001	55250000	0.000388079
chr5	55246001	55251000	7.19E-05
chr5	55291001	55296000	3.66E-30
chr5	55493001	55498000	6.84E-20
chr5	55494001	55499000	9.07E-13
chr5	55495001	55500000	2.53E-10
chr5	55496001	55501000	9.73E-06
chr5	55650001	55655000	3.67E-06
chr5	56106001	56111000	1.24E-25
chr5	56107001	56112000	1.96E-62
chr5	56204001	56209000	9.92E-61
chr5	56205001	56210000	6.02E-98
chr5	56206001	56211000	6.91E-42
chr5	56231001	56236000	0.001590783
chr5	56247001	56252000	9.84E-65
chr5	56248001	56253000	1.88E-60
chr5	56877001	56882000	0.000542359
chr5	57682001	57687000	0.016946686
chr5	57903001	57908000	2.45E-08
chr5	58313001	58318000	9.65E-05
chr5	58327001	58332000	5.76E-05
chr5	58328001	58333000	0.000515662
chr5	58423001	58428000	1.34E-09
chr5	58572001	58577000	0.002416735
chr5	58883001	58888000	5.25E-05
chr5	59064001	59069000	1.91E-13
chr5	59160001	59165000	0.005472156
chr5	59221001	59226000	1.37E-06
chr5	59222001	59227000	9.55E-08
chr5	59223001	59228000	3.91E-06
chr5	59224001	59229000	2.01E-05
chr5	59274001	59279000	0.00384281
chr5	59275001	59280000	0.001818369
chr5	59276001	59281000	0.000530384
chr5	59392001	59397000	0.007269964
chr5	59393001	59398000	0.008244269
chr5	59405001	59410000	2.39E-05
chr5	59468001	59473000	6.64E-05
chr5	59469001	59474000	4.58E-08
chr5	59470001	59475000	2.04E-07
chr5	59471001	59476000	2.26E-06
chr5	59472001	59477000	3.52E-06
chr5	59532001	59537000	0.000339776
chr5	59534001	59539000	0.000280797
chr5	59590001	59595000	0.000111239

chr5	59591001	59596000	0.000177486
chr5	59611001	59616000	2.54E-18
chr5	59617001	59622000	4.14E-05
chr5	59618001	59623000	2.28E-05
chr5	59619001	59624000	0.000213554
chr5	59636001	59641000	7.05E-06
chr5	59638001	59643000	2.39E-05
chr5	59639001	59644000	6.57E-05
chr5	59647001	59652000	0.00047799
chr5	59650001	59655000	0.000337158
chr5	59670001	59675000	1.64E-11
chr5	59720001	59725000	2.17E-08
chr5	59728001	59733000	2.06E-05
chr5	59761001	59766000	2.21E-05
chr5	59762001	59767000	4.51E-06
chr5	59774001	59779000	0.000347092
chr5	59798001	59803000	4.98E-07
chr5	59833001	59838000	1.61E-05
chr5	59834001	59839000	1.92E-06
chr5	60241001	60246000	1.97E-18
chr5	60622001	60627000	4.06E-31
chr5	60623001	60628000	1.43E-21
chr5	60626001	60631000	6.08E-23
chr5	60627001	60632000	9.42E-60
chr5	60628001	60633000	8.58E-98
chr5	60629001	60634000	1.58E-31
chr5	60665001	60670000	2.42E-05
chr5	60917001	60922000	3.48E-09
chr5	60921001	60926000	0.001541106
chr5	60974001	60979000	0.003515373
chr5	61139001	61144000	1.70E-07
chr5	61140001	61145000	3.93E-10
chr5	61141001	61146000	3.54E-07
chr5	61154001	61159000	0.000676082
chr5	61155001	61160000	2.91E-05
chr5	61248001	61253000	1.61E-05
chr5	61349001	61354000	0.004763715
chr5	61416001	61421000	4.24E-08
chr5	61417001	61422000	1.68E-06
chr5	61597001	61602000	1.29E-13
chr5	61598001	61603000	6.49E-17
chr5	61601001	61606000	8.77E-27
chr5	61602001	61607000	5.81E-23
chr5	61679001	61684000	1.57E-05
chr5	61995001	62000000	1.89E-05
chr5	61996001	62001000	1.23E-05
chr5	62021001	62026000	0.001112144
chr5	62024001	62029000	7.78E-05

chr5	62025001	62030000	0.000553646
chr5	62026001	62031000	0.001136586
chr5	62028001	62033000	0.006038164
chr5	62050001	62055000	0.000386214
chr5	62091001	62096000	3.36E-05
chr5	62092001	62097000	4.52E-07
chr5	62094001	62099000	1.05E-06
chr5	62123001	62128000	0.00012742
chr5	62131001	62136000	1.65E-07
chr5	62132001	62137000	4.14E-10
chr5	62133001	62138000	2.51E-09
chr5	62134001	62139000	1.27E-10
chr5	62135001	62140000	6.22E-06
chr5	62139001	62144000	0.010208029
chr5	62140001	62145000	0.000525563
chr5	62144001	62149000	0.002872169
chr5	62152001	62157000	0.000863006
chr5	62154001	62159000	0.000378215
chr5	62162001	62167000	2.55E-09
chr5	62163001	62168000	2.74E-08
chr5	62186001	62191000	2.65E-05
chr5	62187001	62192000	3.78E-08
chr5	62188001	62193000	1.62E-05
chr5	62189001	62194000	8.94E-06
chr5	62260001	62265000	0.0003818
chr5	62263001	62268000	2.41E-06
chr5	62269001	62274000	0.003104548
chr5	62280001	62285000	0.000525938
chr5	62310001	62315000	0.028336593
chr5	62334001	62339000	6.38E-06
chr5	62335001	62340000	4.65E-05
chr5	62336001	62341000	1.22E-06
chr5	62337001	62342000	0.000162709
chr5	62338001	62343000	0.000365366
chr5	62340001	62345000	4.39E-05
chr5	62387001	62392000	1.67E-06
chr5	62410001	62415000	0.000626783
chr5	62411001	62416000	0.000668517
chr5	62420001	62425000	0.011540355
chr5	62421001	62426000	0.003293529
chr5	62423001	62428000	0.002956407
chr5	62424001	62429000	1.15E-06
chr5	62425001	62430000	2.67E-06
chr5	62426001	62431000	0.001648716
chr5	62427001	62432000	0.002423981
chr5	62428001	62433000	0.003425774
chr5	62446001	62451000	5.87E-05
chr5	62450001	62455000	4.08E-07

chr5	62451001	62456000	1.14E-06
chr5	62452001	62457000	1.69E-08
chr5	62487001	62492000	0.002534205
chr5	62488001	62493000	0.000522015
chr5	62489001	62494000	4.05E-08
chr5	62490001	62495000	1.66E-08
chr5	62491001	62496000	1.60E-06
chr5	62492001	62497000	2.06E-05
chr5	62493001	62498000	2.02E-05
chr5	62523001	62528000	0.000148077
chr5	62546001	62551000	1.50E-05
chr5	62547001	62552000	1.39E-05
chr5	62548001	62553000	2.68E-06
chr5	62578001	62583000	0.001846453
chr5	62621001	62626000	0.000112146
chr5	62622001	62627000	0.000294278
chr5	62642001	62647000	0.001291818
chr5	62643001	62648000	0.00047378
chr5	62647001	62652000	0.000186329
chr5	62648001	62653000	0.000236123
chr5	62649001	62654000	0.001275456
chr5	62650001	62655000	0.000201941
chr5	62651001	62656000	7.78E-05
chr5	62652001	62657000	3.03E-07
chr5	62653001	62658000	4.34E-06
chr5	62654001	62659000	1.26E-05
chr5	62655001	62660000	2.60E-05
chr5	62672001	62677000	1.65E-06
chr5	62706001	62711000	0.002577894
chr5	62709001	62714000	0.001207607
chr5	62719001	62724000	1.16E-05
chr5	62720001	62725000	8.10E-05
chr5	62793001	62798000	0.000575852
chr5	62794001	62799000	0.000591778
chr5	62795001	62800000	0.000480174
chr5	62796001	62801000	0.000988492
chr5	62924001	62929000	2.58E-09
chr5	62977001	62982000	7.06E-08
chr5	62996001	63001000	0.001367976
chr5	62997001	63002000	0.001434611
chr5	63045001	63050000	0.013782975
chr5	63048001	63053000	0.002726139
chr5	63115001	63120000	6.30E-05
chr5	63140001	63145000	0.007993346
chr5	63141001	63146000	0.000348763
chr5	63221001	63226000	7.98E-09
chr5	63223001	63228000	2.13E-07
chr5	63224001	63229000	3.69E-07

chr5	63234001	63239000	0.001517398
chr5	63250001	63255000	0.000628035
chr5	63251001	63256000	6.44E-22
chr5	63266001	63271000	1.18E-05
chr5	63276001	63281000	2.40E-08
chr5	63277001	63282000	8.51E-09
chr5	63278001	63283000	2.89E-08
chr5	63279001	63284000	7.56E-11
chr5	63280001	63285000	2.93E-08
chr5	63281001	63286000	1.94E-08
chr5	63333001	63338000	0.000189662
chr5	63352001	63357000	7.74E-05
chr5	63353001	63358000	0.00181591
chr5	63354001	63359000	0.000134942
chr5	63358001	63363000	0.000705076
chr5	63359001	63364000	0.002414888
chr5	63398001	63403000	1.47E-10
chr5	63399001	63404000	3.25E-09
chr5	63400001	63405000	3.97E-05
chr5	63411001	63416000	4.25E-05
chr5	63413001	63418000	0.000313761
chr5	63457001	63462000	1.04E-38
chr5	63459001	63464000	1.44E-37
chr5	63460001	63465000	1.07E-42
chr5	63461001	63466000	1.12E-65
chr5	63568001	63573000	0.009245395
chr5	63571001	63576000	0.017774159
chr5	63699001	63704000	3.33E-05
chr5	63722001	63727000	1.52E-06
chr5	63723001	63728000	5.77E-08
chr5	63724001	63729000	2.71E-10
chr5	63725001	63730000	0.00010118
chr5	63726001	63731000	0.001451517
chr5	63727001	63732000	0.003559349
chr5	63751001	63756000	0.000114264
chr5	63752001	63757000	0.001555608
chr5	63758001	63763000	0.000531228
chr5	63759001	63764000	0.000109802
chr5	63760001	63765000	2.00E-05
chr5	63775001	63780000	4.34E-07
chr5	63797001	63802000	7.85E-08
chr5	63798001	63803000	1.86E-16
chr5	63799001	63804000	6.88E-17
chr5	63800001	63805000	1.47E-27
chr5	63801001	63806000	4.62E-28
chr5	63802001	63807000	5.09E-27
chr5	63915001	63920000	0.001293962
chr5	63916001	63921000	0.008814957

chr5	63982001	63987000	5.41E-30
chr5	63983001	63988000	1.52E-25
chr5	63984001	63989000	1.32E-14
chr5	63985001	63990000	3.00E-15
chr5	63986001	63991000	4.49E-22
chr5	64394001	64399000	2.66E-27
chr5	64395001	64400000	2.57E-30
chr5	64396001	64401000	3.34E-29
chr5	64398001	64403000	2.47E-29
chr5	64399001	64404000	4.54E-14
chr5	64431001	64436000	3.95E-05
chr5	64849001	64854000	0.00304815
chr5	64850001	64855000	0.00289264
chr5	64854001	64859000	1.49E-20
chr5	65016001	65021000	9.10E-62
chr5	65017001	65022000	1.92E-44
chr5	65018001	65023000	6.73E-49
chr5	65112001	65117000	0.00069142
chr5	65216001	65221000	2.52E-16
chr5	65217001	65222000	5.48E-43
chr5	65435001	65440000	6.88E-17
chr5	65436001	65441000	4.39E-60
chr5	65559001	65564000	0.010126164
chr5	65888001	65893000	7.18E-34
chr5	65889001	65894000	5.12E-20
chr5	65890001	65895000	3.99E-26
chr5	65891001	65896000	1.84E-23
chr5	65892001	65897000	2.93E-39
chr5	65893001	65898000	9.94E-25
chr5	66685001	66690000	0.001301072
chr5	66813001	66818000	0.000255201
chr5	66814001	66819000	3.42E-05
chr5	66815001	66820000	0.000130512
chr5	66825001	66830000	0.002811799
chr5	67512001	67517000	1.80E-24
chr5	67698001	67703000	8.25E-07
chr5	67893001	67898000	1.01E-05
chr5	67932001	67937000	5.47E-05
chr5	67992001	67997000	0.00015717
chr5	67993001	67998000	0.000740191
chr5	67994001	67999000	4.87E-06
chr5	68104001	68109000	8.65E-08
chr5	68105001	68110000	1.09E-07
chr5	68137001	68142000	0.00300101
chr5	68387001	68392000	4.96E-23
chr5	68388001	68393000	1.71E-25
chr5	68389001	68394000	7.31E-48
chr5	68390001	68395000	8.46E-40

chr5	68463001	68468000	2.94E-26
chr5	68526001	68531000	7.82E-12
chr5	68706001	68711000	1.16E-18
chr5	68784001	68789000	4.28E-24
chr5	68785001	68790000	8.15E-55
chr5	68786001	68791000	4.10E-38
chr5	68788001	68793000	2.30E-30
chr5	68789001	68794000	4.71E-14
chr5	68851001	68856000	2.08E-23
chr5	70706001	70711000	1.13E-05
chr5	70707001	70712000	1.02E-06
chr5	70708001	70713000	1.10E-06
chr5	70709001	70714000	2.97E-05
chr5	70747001	70752000	2.31E-34
chr5	70749001	70754000	1.06E-34
chr5	70750001	70755000	3.37E-34
chr5	70751001	70756000	6.49E-31
chr5	71216001	71221000	0.0057472
chr5	71231001	71236000	4.86E-15
chr5	71398001	71403000	6.34E-13
chr5	71401001	71406000	2.02E-57
chr5	71402001	71407000	4.75E-64
chr5	71403001	71408000	7.91E-75
chr5	71404001	71409000	9.13E-61
chr5	71574001	71579000	0.000289909
chr5	71604001	71609000	8.23E-50
chr5	72030001	72035000	1.47E-05
chr5	72031001	72036000	0.000107772
chr5	72108001	72113000	1.53E-33
chr5	72112001	72117000	2.13E-68
chr5	72113001	72118000	1.35E-15
chr5	72144001	72149000	3.61E-57
chr5	72145001	72150000	1.06E-07
chr5	72252001	72257000	5.49E-31
chr5	72364001	72369000	0.002143134
chr5	72365001	72370000	0.001480897
chr5	72511001	72516000	1.55E-09
chr5	72512001	72517000	5.31E-33
chr5	72794001	72799000	3.98E-16
chr5	72922001	72927000	6.95E-16
chr5	73706001	73711000	1.75E-07
chr5	73727001	73732000	2.04E-07
chr5	73977001	73982000	1.30E-18
chr5	73978001	73983000	6.51E-06
chr5	73979001	73984000	2.61E-13
chr5	73981001	73986000	1.42E-31
chr5	74062001	74067000	8.87E-96
chr5	74063001	74068000	6.51E-88

chr5	74143001	74148000	0.000325502
chr5	74157001	74162000	1.51E-43
chr5	74158001	74163000	4.47E-52
chr5	74159001	74164000	2.95E-80
chr5	74160001	74165000	4.80E-66
chr5	74161001	74166000	2.95E-55
chr5	74162001	74167000	1.43E-47
chr5	74271001	74276000	1.23E-09
chr5	74272001	74277000	4.21E-09
chr5	74273001	74278000	1.29E-08
chr5	74274001	74279000	1.39E-10
chr5	74275001	74280000	2.18E-10
chr5	74276001	74281000	3.23E-08
chr5	74291001	74296000	1.01E-07
chr5	74293001	74298000	9.08E-09
chr5	74454001	74459000	0.00016442
chr5	74532001	74537000	1.96E-28
chr5	74533001	74538000	4.51E-07
chr5	74804001	74809000	1.01E-46
chr5	74805001	74810000	1.38E-60
chr5	74806001	74811000	5.33E-70
chr5	74807001	74812000	7.83E-69
chr5	75076001	75081000	2.86E-06
chr5	75077001	75082000	0.000117257
chr5	75090001	75095000	2.04E-16
chr5	75091001	75096000	2.77E-15
chr5	75092001	75097000	1.76E-18
chr5	75093001	75098000	4.97E-16
chr5	75134001	75139000	1.73E-07
chr5	75173001	75178000	0.002845929
chr5	75174001	75179000	0.002676131
chr5	75187001	75192000	0.000159404
chr5	75203001	75208000	9.65E-07
chr5	75204001	75209000	2.26E-10
chr5	75205001	75210000	4.71E-06
chr5	75217001	75222000	1.16E-07
chr5	75218001	75223000	0.00017509
chr5	75219001	75224000	0.000354853
chr5	75230001	75235000	6.54E-06
chr5	75241001	75246000	1.81E-06
chr5	75242001	75247000	9.62E-10
chr5	75243001	75248000	2.03E-09
chr5	75244001	75249000	2.37E-08
chr5	75245001	75250000	4.27E-07
chr5	75267001	75272000	2.28E-12
chr5	75268001	75273000	5.83E-09
chr5	75306001	75311000	4.17E-09
chr5	75307001	75312000	2.19E-09

chr5	75308001	75313000	3.20E-05
chr5	75339001	75344000	6.55E-06
chr5	75340001	75345000	7.66E-06
chr5	75341001	75346000	0.000221095
chr5	75348001	75353000	2.45E-05
chr5	75351001	75356000	5.63E-07
chr5	75352001	75357000	3.75E-11
chr5	75353001	75358000	3.43E-05
chr5	75419001	75424000	5.15E-11
chr5	75420001	75425000	8.16E-12
chr5	75421001	75426000	2.25E-12
chr5	75422001	75427000	8.25E-12
chr5	75423001	75428000	4.53E-10
chr5	75425001	75430000	0.010390604
chr5	75426001	75431000	0.001666134
chr5	75427001	75432000	3.11E-05
chr5	75440001	75445000	0.010611694
chr5	75452001	75457000	0.021337487
chr5	75453001	75458000	0.020314839
chr5	75454001	75459000	6.76E-05
chr5	75513001	75518000	2.03E-07
chr5	75514001	75519000	1.91E-05
chr5	75588001	75593000	1.37E-06
chr5	75633001	75638000	5.01E-08
chr5	75634001	75639000	1.27E-09
chr5	75635001	75640000	2.00E-08
chr5	75695001	75700000	8.30E-20
chr5	75696001	75701000	1.19E-14
chr5	75697001	75702000	3.38E-10
chr5	75698001	75703000	3.65E-08
chr5	75699001	75704000	1.24E-09
chr5	75723001	75728000	3.93E-08
chr5	75724001	75729000	6.59E-08
chr5	75731001	75736000	6.24E-07
chr5	75811001	75816000	2.49E-07
chr5	75812001	75817000	6.91E-08
chr5	75813001	75818000	4.00E-08
chr5	75819001	75824000	1.06E-05
chr5	75821001	75826000	3.71E-05
chr5	75822001	75827000	6.43E-05
chr5	75824001	75829000	2.42E-07
chr5	75836001	75841000	4.40E-10
chr5	75837001	75842000	2.51E-11
chr5	75838001	75843000	2.10E-10
chr5	75860001	75865000	1.35E-09
chr5	75861001	75866000	8.04E-09
chr5	75871001	75876000	4.18E-05
chr5	75882001	75887000	6.98E-08

chr5	75883001	75888000	8.88E-10
chr5	75884001	75889000	1.82E-05
chr5	75885001	75890000	1.07E-05
chr5	75918001	75923000	1.78E-12
chr5	75919001	75924000	5.48E-16
chr5	75920001	75925000	4.67E-19
chr5	75921001	75926000	2.30E-13
chr5	75922001	75927000	4.16E-08
chr5	75946001	75951000	0.001686287
chr5	75948001	75953000	7.22E-07
chr5	75949001	75954000	2.41E-08
chr5	75985001	75990000	3.04E-06
chr5	76008001	76013000	1.45E-08
chr5	76009001	76014000	3.01E-23
chr5	76010001	76015000	2.10E-20
chr5	76011001	76016000	4.11E-51
chr5	76012001	76017000	1.02E-32
chr5	76196001	76201000	3.87E-05
chr5	76326001	76331000	1.19E-55
chr5	76369001	76374000	9.34E-30
chr5	76370001	76375000	7.46E-34
chr5	76371001	76376000	2.30E-38
chr5	76372001	76377000	2.02E-21
chr5	76373001	76378000	1.12E-24
chr5	76378001	76383000	8.70E-19
chr5	76379001	76384000	6.71E-23
chr5	76383001	76388000	2.09E-12
chr5	76472001	76477000	3.72E-11
chr5	76502001	76507000	1.24E-11
chr5	76570001	76575000	1.39E-06
chr5	76799001	76804000	7.10E-17
chr5	76844001	76849000	1.47E-05
chr5	76948001	76953000	1.78E-06
chr5	77136001	77141000	2.83E-16
chr5	77585001	77590000	1.36E-16
chr5	77586001	77591000	5.98E-65
chr5	77587001	77592000	2.41E-78
chr5	77588001	77593000	3.56E-64
chr5	77589001	77594000	3.38E-39
chr5	77590001	77595000	2.46E-29
chr5	77591001	77596000	2.65E-17
chr5	77595001	77600000	2.94E-05
chr5	77596001	77601000	1.24E-05
chr5	77597001	77602000	1.97E-05
chr5	77651001	77656000	4.76E-21
chr5	77652001	77657000	5.78E-35
chr5	77653001	77658000	3.39E-24
chr5	77739001	77744000	1.15E-05

chr5	77944001	77949000	3.96E-49
chr5	78235001	78240000	1.40E-09
chr5	78281001	78286000	1.23E-38
chr5	78309001	78314000	0.000997904
chr5	78310001	78315000	0.001622486
chr5	78337001	78342000	0.002955005
chr5	78527001	78532000	1.62E-07
chr5	78528001	78533000	7.11E-30
chr5	78800001	78805000	0.001649883
chr5	78801001	78806000	0.004182542
chr5	78809001	78814000	1.35E-58
chr5	78810001	78815000	6.46E-58
chr5	78901001	78906000	7.56E-07
chr5	78902001	78907000	6.47E-06
chr5	78903001	78908000	1.10E-07
chr5	78904001	78909000	1.20E-16
chr5	79211001	79216000	3.79E-07
chr5	79240001	79245000	1.94E-05
chr5	79262001	79267000	2.50E-06
chr5	79282001	79287000	3.22E-52
chr5	79283001	79288000	2.24E-37
chr5	79284001	79289000	1.95E-41
chr5	79285001	79290000	2.01E-38
chr5	79286001	79291000	4.26E-40
chr5	79307001	79312000	3.88E-09
chr5	79699001	79704000	3.43E-18
chr5	79700001	79705000	2.52E-23
chr5	79701001	79706000	1.05E-19
chr5	79702001	79707000	8.70E-14
chr5	79703001	79708000	1.19E-11
chr5	79779001	79784000	4.29E-32
chr5	79780001	79785000	2.69E-29
chr5	79781001	79786000	5.83E-39
chr5	79782001	79787000	9.65E-27
chr5	79783001	79788000	7.84E-23
chr5	79863001	79868000	1.19E-08
chr5	79864001	79869000	1.23E-16
chr5	79865001	79870000	6.10E-18
chr5	79950001	79955000	2.52E-24
chr5	80344001	80349000	1.59E-05
chr5	80394001	80399000	1.27E-10
chr5	80395001	80400000	1.17E-11
chr5	80397001	80402000	2.35E-06
chr5	80429001	80434000	0.000201987
chr5	81041001	81046000	4.86E-37
chr5	81042001	81047000	1.25E-59
chr5	81043001	81048000	3.29E-37
chr5	81044001	81049000	2.35E-36

chr5	81045001	81050000	1.05E-42
chr5	81046001	81051000	8.61E-48
chr5	81047001	81052000	1.56E-36
chr5	81263001	81268000	1.25E-26
chr5	81264001	81269000	2.51E-40
chr5	81265001	81270000	1.97E-54
chr5	81266001	81271000	6.15E-54
chr5	81267001	81272000	1.92E-44
chr5	81268001	81273000	3.85E-46
chr5	81323001	81328000	0.001429579
chr5	81324001	81329000	0.000800533
chr5	81325001	81330000	0.00022406
chr5	81574001	81579000	2.84E-16
chr5	81832001	81837000	3.31E-06
chr5	81833001	81838000	4.69E-07
chr5	81834001	81839000	0.000239048
chr5	81855001	81860000	2.89E-08
chr5	81887001	81892000	0.000137792
chr5	81945001	81950000	0.000145662
chr5	81946001	81951000	0.000206814
chr5	82181001	82186000	3.35E-05
chr5	82369001	82374000	3.30E-28
chr5	82370001	82375000	1.91E-30
chr5	82371001	82376000	5.33E-25
chr5	83015001	83020000	3.11E-18
chr5	83016001	83021000	3.24E-20
chr5	83017001	83022000	4.90E-26
chr5	83018001	83023000	1.63E-35
chr5	83680001	83685000	5.56E-93
chr5	84190001	84195000	0.001791342
chr5	84714001	84719000	4.40E-08
chr5	84715001	84720000	3.10E-08
chr5	84716001	84721000	1.29E-06
chr5	85252001	85257000	4.89E-05
chr5	85591001	85596000	6.12E-05
chr5	85910001	85915000	1.18E-21
chr5	85911001	85916000	4.44E-19
chr5	85912001	85917000	7.23E-27
chr5	85913001	85918000	3.92E-29
chr5	85927001	85932000	1.55E-05
chr5	85928001	85933000	0.000541935
chr5	85945001	85950000	0.011080349
chr5	86143001	86148000	1.41E-05
chr5	86229001	86234000	8.53E-05
chr5	86357001	86362000	0.002435946
chr5	86430001	86435000	0.002549494
chr5	86431001	86436000	0.003040433
chr5	86432001	86437000	0.001017083

chr5	86433001	86438000	0.000516299
chr5	86465001	86470000	1.08E-06
chr5	86467001	86472000	1.83E-06
chr5	86559001	86564000	8.11E-36
chr5	86560001	86565000	1.86E-42
chr5	86561001	86566000	1.46E-57
chr5	86562001	86567000	7.09E-53
chr5	86563001	86568000	5.32E-28
chr5	86564001	86569000	3.27E-24
chr5	86685001	86690000	0.000825549
chr5	87085001	87090000	0.008771064
chr5	87538001	87543000	4.86E-08
chr5	87539001	87544000	3.06E-07
chr5	87560001	87565000	8.12E-26
chr5	87561001	87566000	3.36E-33
chr5	87563001	87568000	8.23E-43
chr5	87564001	87569000	9.22E-43
chr5	87650001	87655000	0.000454998
chr5	87651001	87656000	0.0052455
chr5	87678001	87683000	0.009972787
chr5	88180001	88185000	3.52E-23
chr5	88228001	88233000	0.013882571
chr5	88244001	88249000	0.000249987
chr5	88276001	88281000	0.00018712
chr5	88278001	88283000	0.000568661
chr5	88280001	88285000	4.46E-07
chr5	88321001	88326000	1.70E-06
chr5	88323001	88328000	1.43E-07
chr5	88324001	88329000	4.90E-05
chr5	88325001	88330000	6.38E-05
chr5	88326001	88331000	0.001069067
chr5	88327001	88332000	0.001807653
chr5	88328001	88333000	0.003335514
chr5	88329001	88334000	0.000746757
chr5	88364001	88369000	0.000240059
chr5	88383001	88388000	2.49E-06
chr5	88519001	88524000	0.000293052
chr5	88605001	88610000	0.005895837
chr5	88685001	88690000	0.021807476
chr5	88921001	88926000	1.41E-06
chr5	88922001	88927000	1.30E-05
chr5	89001001	89006000	0.002134474
chr5	89081001	89086000	0.003402042
chr5	89082001	89087000	0.000131019
chr5	89083001	89088000	7.23E-05
chr5	89084001	89089000	1.15E-05
chr5	89402001	89407000	0.016789285
chr5	89403001	89408000	0.032937261

chr5	89444001	89449000	2.42E-08
chr5	89458001	89463000	7.16E-05
chr5	89502001	89507000	9.02E-09
chr5	89503001	89508000	5.77E-11
chr5	89504001	89509000	7.55E-10
chr5	89505001	89510000	7.21E-08
chr5	89506001	89511000	1.34E-08
chr5	89507001	89512000	2.30E-06
chr5	89508001	89513000	0.000320559
chr5	89509001	89514000	0.000420777
chr5	89678001	89683000	9.18E-05
chr5	89705001	89710000	6.34E-12
chr5	89765001	89770000	5.19E-36
chr5	89766001	89771000	4.81E-42
chr5	89770001	89775000	8.67E-24
chr5	90493001	90498000	0.000517424
chr5	90494001	90499000	0.000972778
chr5	90574001	90579000	1.34E-28
chr5	90671001	90676000	1.62E-28
chr5	90672001	90677000	9.53E-42
chr5	91075001	91080000	3.90E-06
chr5	91076001	91081000	0.000264887
chr5	91439001	91444000	0.000171502
chr5	91440001	91445000	0.000392111
chr5	91441001	91446000	0.000276725
chr5	91442001	91447000	0.000229971
chr5	91801001	91806000	0.000146527
chr5	91802001	91807000	0.000115809
chr5	91803001	91808000	0.000652714
chr5	92411001	92416000	9.12E-07
chr5	92430001	92435000	4.22E-05
chr5	92952001	92957000	1.90E-64
chr5	92953001	92958000	3.03E-72
chr5	92954001	92959000	1.19E-53
chr5	92955001	92960000	1.45E-46
chr5	92957001	92962000	1.22E-29
chr5	93013001	93018000	6.85E-08
chr5	93230001	93235000	0.003676839
chr5	93255001	93260000	1.06E-10
chr5	93256001	93261000	9.46E-07
chr5	93687001	93692000	4.67E-05
chr5	93954001	93959000	6.35E-29
chr5	94615001	94620000	9.34E-53
chr5	94650001	94655000	4.79E-06
chr5	94675001	94680000	6.36E-07
chr5	94885001	94890000	7.85E-18
chr5	94886001	94891000	9.76E-72
chr5	94887001	94892000	2.49E-60

chr5	94888001	94893000	1.12E-42
chr5	94889001	94894000	6.45E-29
chr5	94956001	94961000	2.77E-18
chr5	94957001	94962000	1.12E-14
chr5	95062001	95067000	6.22E-40
chr5	95063001	95068000	3.39E-46
chr5	95064001	95069000	1.96E-82
chr5	95065001	95070000	8.66E-100
chr5	95066001	95071000	2.82E-77
chr5	95067001	95072000	1.69E-76
chr5	95166001	95171000	1.47E-11
chr5	95296001	95301000	2.30E-56
chr5	95297001	95302000	4.99E-57
chr5	95468001	95473000	0.0036176
chr5	95469001	95474000	0.002948032
chr5	95470001	95475000	0.003338046
chr5	95770001	95775000	2.49E-14
chr5	95987001	95992000	0.004221385
chr5	96036001	96041000	7.09E-45
chr5	96037001	96042000	8.55E-65
chr5	96038001	96043000	1.15E-44
chr5	96058001	96063000	0.000179778
chr5	96139001	96144000	2.22E-29
chr5	96140001	96145000	2.25E-20
chr5	96141001	96146000	1.36E-22
chr5	96142001	96147000	1.11E-44
chr5	96143001	96148000	5.30E-47
chr5	96144001	96149000	1.20E-11
chr5	96156001	96161000	7.06E-05
chr5	96266001	96271000	7.06E-41
chr5	96272001	96277000	2.49E-22
chr5	96514001	96519000	1.05E-14
chr5	97006001	97011000	0.000843649
chr5	97007001	97012000	0.000203155
chr5	97008001	97013000	0.002037053
chr5	97009001	97014000	1.39E-05
chr5	97159001	97164000	1.49E-10
chr5	97169001	97174000	0.016946686
chr5	97223001	97228000	0.00202832
chr5	97224001	97229000	0.001231341
chr5	97598001	97603000	0.004492848
chr5	97865001	97870000	0.006974835
chr5	97866001	97871000	0.009295809
chr5	98087001	98092000	0.000203875
chr5	98100001	98105000	2.13E-27
chr5	98259001	98264000	5.16E-12
chr5	98264001	98269000	8.02E-86
chr5	98265001	98270000	1.59E-61

chr5	98523001	98528000	1.55E-06
chr5	98585001	98590000	4.10E-05
chr5	98611001	98616000	3.44E-05
chr5	98612001	98617000	0.004563132
chr5	98613001	98618000	0.005086565
chr5	98834001	98839000	0.002763746
chr5	98929001	98934000	0.004948838
chr5	99116001	99121000	0.001728329
chr5	99117001	99122000	0.005263009
chr5	99164001	99169000	0.000156244
chr5	99228001	99233000	0.000220087
chr5	99307001	99312000	0.004233045
chr5	99576001	99581000	0.000453915
chr5	99603001	99608000	0.0001089
chr5	99604001	99609000	0.000641432
chr5	99615001	99620000	7.07E-06
chr5	99616001	99621000	7.07E-06
chr5	99867001	99872000	9.56E-41
chr5	99868001	99873000	2.28E-43
chr5	99869001	99874000	3.43E-55
chr5	99870001	99875000	3.20E-58
chr5	99871001	99876000	5.60E-84
chr5	100031001	100036000	0.008383545
chr5	100232001	100237000	8.73E-46
chr5	100255001	100260000	5.93E-05
chr5	100257001	100262000	0.000104655
chr5	100258001	100263000	3.61E-05
chr5	100361001	100366000	1.13E-05
chr5	100597001	100602000	3.29E-06
chr5	100683001	100688000	9.07E-07
chr5	100922001	100927000	5.57E-07
chr5	100923001	100928000	1.26E-06
chr5	100924001	100929000	0.000349083
chr5	101233001	101238000	0.002932628
chr5	101374001	101379000	2.33E-06
chr5	101384001	101389000	0.006798515
chr5	101400001	101405000	0.005414532
chr5	101525001	101530000	5.46E-07
chr5	101537001	101542000	1.98E-05
chr5	101586001	101591000	1.23E-06
chr5	101587001	101592000	2.40E-05
chr5	101601001	101606000	1.29E-06
chr5	101602001	101607000	2.24E-06
chr5	101603001	101608000	0.000845808
chr5	101604001	101609000	0.001609813
chr5	101619001	101624000	0.002028799
chr5	101627001	101632000	6.09E-19
chr5	101648001	101653000	7.09E-05

chr5	101649001	101654000	0.000266279
chr5	101674001	101679000	3.92E-08
chr5	101718001	101723000	0.000153203
chr5	101741001	101746000	6.71E-05
chr5	101742001	101747000	0.000518647
chr5	101745001	101750000	0.000766497
chr5	101860001	101865000	3.71E-06
chr5	101861001	101866000	0.003401598
chr5	101881001	101886000	0.000460795
chr5	101894001	101899000	4.31E-09
chr5	101895001	101900000	1.96E-08
chr5	101896001	101901000	9.57E-10
chr5	101897001	101902000	5.92E-07
chr5	101898001	101903000	4.64E-10
chr5	102086001	102091000	3.92E-10
chr5	102087001	102092000	6.62E-08
chr5	102088001	102093000	1.76E-20
chr5	102089001	102094000	2.09E-21
chr5	102090001	102095000	9.90E-29
chr5	102091001	102096000	1.34E-24
chr5	102197001	102202000	1.04E-16
chr5	102199001	102204000	8.41E-27
chr5	102200001	102205000	9.75E-29
chr5	102201001	102206000	6.22E-26
chr5	102330001	102335000	1.55E-05
chr5	102405001	102410000	9.30E-06
chr5	102406001	102411000	1.05E-05
chr5	102546001	102551000	1.98E-05
chr5	102590001	102595000	2.10E-37
chr5	102591001	102596000	7.92E-64
chr5	102592001	102597000	7.27E-78
chr5	102593001	102598000	7.65E-82
chr5	102594001	102599000	7.14E-36
chr5	102595001	102600000	8.85E-38
chr5	102720001	102725000	2.22E-08
chr5	102836001	102841000	0.000290366
chr5	102881001	102886000	0.005111951
chr5	102933001	102938000	0.004363076
chr5	103192001	103197000	0.000599063
chr5	103307001	103312000	0.000164747
chr5	103431001	103436000	1.78E-05
chr5	103831001	103836000	7.10E-06
chr5	103832001	103837000	4.00E-05
chr5	103833001	103838000	9.33E-07
chr5	103834001	103839000	4.91E-06
chr5	104242001	104247000	0.017029208
chr5	104243001	104248000	7.10E-08
chr5	104244001	104249000	1.45E-09

chr5	104245001	104250000	8.06E-11
chr5	104246001	104251000	1.42E-09
chr5	104247001	104252000	9.97E-07
chr5	104289001	104294000	3.09E-05
chr5	104291001	104296000	0.000751074
chr5	104292001	104297000	0.000902588
chr5	104649001	104654000	4.35E-09
chr5	104664001	104669000	3.59E-06
chr5	104666001	104671000	0.004621926
chr5	104772001	104777000	0.006945476
chr5	104844001	104849000	0.005435723
chr5	104937001	104942000	1.30E-07
chr5	105243001	105248000	0.002421246
chr5	105410001	105415000	0.003933118
chr5	106143001	106148000	3.29E-06
chr5	106144001	106149000	3.72E-05
chr5	106903001	106908000	2.00E-13
chr5	107000001	107005000	2.71E-28
chr5	107001001	107006000	7.80E-47
chr5	107002001	107007000	5.63E-47
chr5	107107001	107112000	1.07E-07
chr5	107108001	107113000	9.81E-07
chr5	107712001	107717000	5.90E-37
chr5	107713001	107718000	3.66E-55
chr5	107896001	107901000	0.007253128
chr5	108059001	108064000	4.36E-46
chr5	108075001	108080000	0.000256503
chr5	108264001	108269000	1.93E-10
chr5	108265001	108270000	2.07E-07
chr5	108266001	108271000	1.51E-09
chr5	108741001	108746000	3.49E-35
chr5	108997001	109002000	1.39E-06
chr5	108998001	109003000	7.23E-07
chr5	109009001	109014000	0.012975408
chr5	109020001	109025000	6.97E-15
chr5	109021001	109026000	2.47E-51
chr5	109380001	109385000	0.001017737
chr5	109381001	109386000	0.002710408
chr5	109382001	109387000	0.000129987
chr5	109454001	109459000	1.56E-05
chr5	109455001	109460000	1.30E-05
chr5	109457001	109462000	0.000244948
chr5	109458001	109463000	1.95E-05
chr5	109519001	109524000	0.000113128
chr5	109544001	109549000	0.00095672
chr5	109625001	109630000	0.000288421
chr5	109733001	109738000	0.019894396
chr5	109763001	109768000	0.001352032

chr5	109764001	109769000	0.000373623
chr5	109766001	109771000	0.001076
chr5	109767001	109772000	0.000236604
chr5	109768001	109773000	0.000242814
chr5	109772001	109777000	8.53E-06
chr5	109773001	109778000	2.16E-05
chr5	109903001	109908000	0.00233852
chr5	109912001	109917000	3.24E-05
chr5	109975001	109980000	0.006649729
chr5	110070001	110075000	8.59E-33
chr5	110074001	110079000	2.01E-25
chr5	110075001	110080000	4.51E-15
chr5	110145001	110150000	0.001797482
chr5	110146001	110151000	0.000521421
chr5	110147001	110152000	0.000609656
chr5	110148001	110153000	0.00579228
chr5	110257001	110262000	3.77E-06
chr5	110258001	110263000	2.91E-06
chr5	110259001	110264000	1.43E-06
chr5	110260001	110265000	0.000288466
chr5	110407001	110412000	3.44E-17
chr5	110454001	110459000	0.006816503
chr5	110500001	110505000	0.000755622
chr5	110555001	110560000	6.13E-45
chr5	110556001	110561000	1.08E-64
chr5	110557001	110562000	2.89E-49
chr5	110558001	110563000	1.78E-61
chr5	110559001	110564000	4.49E-46
chr5	110560001	110565000	4.85E-43
chr5	110618001	110623000	0.002205981
chr5	110779001	110784000	2.88E-05
chr5	110843001	110848000	9.72E-29
chr5	110844001	110849000	2.67E-45
chr5	110845001	110850000	9.40E-47
chr5	110848001	110853000	1.47E-43
chr5	110906001	110911000	0.00499504
chr5	111043001	111048000	2.01E-05
chr5	111044001	111049000	6.87E-06
chr5	111093001	111098000	1.02E-35
chr5	111230001	111235000	0.000846731
chr5	111231001	111236000	0.004051581
chr5	111233001	111238000	0.000312218
chr5	111329001	111334000	0.000451875
chr5	111447001	111452000	0.002134793
chr5	111448001	111453000	0.00262315
chr5	111449001	111454000	0.000539064
chr5	111755001	111760000	8.55E-45
chr5	111756001	111761000	3.50E-24

chr5	111850001	111855000	0.000212124
chr5	112039001	112044000	3.20E-44
chr5	112040001	112045000	1.72E-41
chr5	112253001	112258000	4.30E-20
chr5	112254001	112259000	2.18E-15
chr5	112255001	112260000	7.91E-12
chr5	112256001	112261000	4.10E-29
chr5	112257001	112262000	1.14E-22
chr5	112308001	112313000	1.06E-21
chr5	112312001	112317000	2.41E-50
chr5	112313001	112318000	2.37E-26
chr5	112626001	112631000	2.81E-40
chr5	112630001	112635000	4.92E-14
chr5	112665001	112670000	7.03E-06
chr5	112811001	112816000	3.55E-05
chr5	112812001	112817000	0.000341744
chr5	112813001	112818000	5.61E-05
chr5	112993001	112998000	1.14E-05
chr5	112995001	113000000	1.04E-06
chr5	112996001	113001000	9.37E-08
chr5	112997001	113002000	1.16E-07
chr5	113030001	113035000	0.005655901
chr5	113036001	113041000	0.003160243
chr5	113039001	113044000	0.000145644
chr5	113040001	113045000	3.02E-05
chr5	113041001	113046000	7.04E-05
chr5	113061001	113066000	1.51E-05
chr5	113062001	113067000	2.24E-09
chr5	113129001	113134000	0.028440153
chr5	113152001	113157000	2.61E-06
chr5	113153001	113158000	2.06E-06
chr5	113154001	113159000	9.74E-06
chr5	113170001	113175000	5.03E-08
chr5	113171001	113176000	6.89E-10
chr5	113172001	113177000	4.44E-09
chr5	113173001	113178000	5.25E-10
chr5	113272001	113277000	0.001227125
chr5	113333001	113338000	0.001381809
chr5	113359001	113364000	7.50E-06
chr5	113360001	113365000	6.85E-08
chr5	113415001	113420000	0.004541385
chr5	113416001	113421000	0.002963281
chr5	113504001	113509000	2.38E-07
chr5	113505001	113510000	1.16E-05
chr5	113622001	113627000	0.003178729
chr5	113811001	113816000	4.85E-07
chr5	113812001	113817000	1.59E-06
chr5	113815001	113820000	2.37E-07

chr5	113848001	113853000	3.15E-05
chr5	113904001	113909000	0.000154561
chr5	113905001	113910000	1.70E-13
chr5	113906001	113911000	6.67E-07
chr5	113907001	113912000	1.91E-05
chr5	113908001	113913000	3.00E-06
chr5	113909001	113914000	1.77E-07
chr5	113935001	113940000	2.06E-09
chr5	113936001	113941000	1.08E-08
chr5	113937001	113942000	5.28E-10
chr5	113948001	113953000	0.000192453
chr5	113949001	113954000	8.62E-05
chr5	113950001	113955000	0.000192453
chr5	113957001	113962000	1.53E-07
chr5	113958001	113963000	4.72E-07
chr5	113959001	113964000	8.05E-06
chr5	113981001	113986000	6.49E-07
chr5	113982001	113987000	2.43E-07
chr5	113983001	113988000	5.26E-10
chr5	113996001	114001000	1.48E-08
chr5	113999001	114004000	3.24E-06
chr5	114010001	114015000	7.32E-05
chr5	114012001	114017000	0.000187848
chr5	114014001	114019000	0.000886065
chr5	114016001	114021000	5.72E-05
chr5	114071001	114076000	3.89E-08
chr5	114072001	114077000	1.48E-14
chr5	114073001	114078000	5.05E-12
chr5	114074001	114079000	2.12E-11
chr5	114075001	114080000	4.51E-15
chr5	114076001	114081000	2.40E-12
chr5	114082001	114087000	0.001770542
chr5	114083001	114088000	0.00076222
chr5	114087001	114092000	0.000267745
chr5	114094001	114099000	5.14E-06
chr5	114095001	114100000	1.53E-07
chr5	114103001	114108000	0.000208311
chr5	114106001	114111000	3.18E-05
chr5	114147001	114152000	3.55E-06
chr5	114152001	114157000	4.18E-06
chr5	114156001	114161000	0.000628224
chr5	114157001	114162000	0.001140621
chr5	114163001	114168000	0.000312882
chr5	114166001	114171000	5.45E-11
chr5	114167001	114172000	2.93E-09
chr5	114168001	114173000	7.83E-08
chr5	114169001	114174000	4.56E-07
chr5	114196001	114201000	1.31E-09

chr5	114197001	114202000	2.16E-11
chr5	114198001	114203000	3.76E-09
chr5	114199001	114204000	2.20E-09
chr5	114200001	114205000	9.64E-07
chr5	114205001	114210000	0.00071181
chr5	114206001	114211000	0.000134127
chr5	114207001	114212000	0.001277127
chr5	114218001	114223000	5.87E-05
chr5	114219001	114224000	1.89E-05
chr5	114243001	114248000	6.44E-05
chr5	114244001	114249000	2.79E-05
chr5	114245001	114250000	7.81E-06
chr5	114266001	114271000	5.90E-06
chr5	114267001	114272000	5.77E-06
chr5	114268001	114273000	3.48E-06
chr5	114271001	114276000	0.00032724
chr5	114272001	114277000	0.000244064
chr5	114350001	114355000	4.19E-05
chr5	114375001	114380000	1.62E-06
chr5	114376001	114381000	4.15E-07
chr5	114378001	114383000	5.04E-07
chr5	114407001	114412000	8.33E-08
chr5	114408001	114413000	4.27E-10
chr5	114505001	114510000	1.74E-29
chr5	114506001	114511000	8.80E-21
chr5	114511001	114516000	3.03E-18
chr5	114513001	114518000	7.99E-18
chr5	114514001	114519000	3.11E-13
chr5	114515001	114520000	4.45E-23
chr5	114516001	114521000	1.25E-26
chr5	114601001	114606000	7.00E-09
chr5	114632001	114637000	4.85E-23
chr5	114833001	114838000	0.000308135
chr5	114877001	114882000	8.62E-39
chr5	114878001	114883000	8.61E-33
chr5	114879001	114884000	1.99E-31
chr5	114880001	114885000	7.63E-52
chr5	114933001	114938000	9.52E-50
chr5	114934001	114939000	1.88E-34
chr5	114935001	114940000	6.02E-19
chr5	114936001	114941000	5.35E-23
chr5	114937001	114942000	7.04E-21
chr5	114938001	114943000	1.16E-26
chr5	114957001	114962000	1.12E-63
chr5	114958001	114963000	1.99E-41
chr5	114960001	114965000	1.42E-47
chr5	114961001	114966000	1.50E-46
chr5	114962001	114967000	6.17E-12

chr5	115020001	115025000	0.001213296
chr5	115416001	115421000	4.56E-26
chr5	115417001	115422000	6.67E-44
chr5	115418001	115423000	5.27E-28
chr5	115489001	115494000	0.000800238
chr5	115490001	115495000	1.78E-07
chr5	115491001	115496000	3.82E-06
chr5	115492001	115497000	1.30E-08
chr5	115493001	115498000	2.71E-13
chr5	115989001	115994000	5.10E-05
chr5	116299001	116304000	0.000471805
chr5	116344001	116349000	1.98E-07
chr5	116345001	116350000	3.99E-09
chr5	116346001	116351000	2.34E-07
chr5	116347001	116352000	6.05E-08
chr5	116348001	116353000	1.50E-06
chr5	116526001	116531000	0.005560325
chr5	116552001	116557000	3.96E-05
chr5	116679001	116684000	5.72E-10
chr5	116771001	116776000	2.28E-06
chr5	116773001	116778000	1.95E-06
chr5	116774001	116779000	6.32E-08
chr5	116775001	116780000	0.00012439
chr5	116818001	116823000	0.000693261
chr5	116873001	116878000	2.32E-05
chr5	116877001	116882000	0.003544722
chr5	117005001	117010000	6.38E-05
chr5	117070001	117075000	0.000892995
chr5	117208001	117213000	0.003309035
chr5	117253001	117258000	6.03E-06
chr5	117323001	117328000	0.000208161
chr5	117324001	117329000	7.87E-05
chr5	117547001	117552000	0.00104993
chr5	117548001	117553000	0.000553852
chr5	117549001	117554000	0.000670376
chr5	117645001	117650000	9.88E-07
chr5	117646001	117651000	1.05E-05
chr5	117790001	117795000	0.001190752
chr5	117852001	117857000	4.00E-05
chr5	118053001	118058000	2.94E-06
chr5	118074001	118079000	5.24E-05
chr5	118075001	118080000	2.97E-07
chr5	118077001	118082000	9.16E-06
chr5	118078001	118083000	2.48E-06
chr5	118150001	118155000	0.003200559
chr5	118320001	118325000	7.73E-54
chr5	118321001	118326000	3.68E-57
chr5	118322001	118327000	8.09E-42

chr5	118323001	118328000	1.49E-23
chr5	118324001	118329000	5.42E-23
chr5	118403001	118408000	5.87E-41
chr5	118407001	118412000	7.12E-20
chr5	118784001	118789000	9.40E-36
chr5	118785001	118790000	5.58E-24
chr5	118786001	118791000	1.91E-40
chr5	118787001	118792000	4.71E-43
chr5	118788001	118793000	1.25E-28
chr5	118891001	118896000	0.001350644
chr5	118952001	118957000	5.65E-07
chr5	118958001	118963000	3.36E-06
chr5	118959001	118964000	1.06E-05
chr5	118989001	118994000	0.00586494
chr5	119365001	119370000	9.18E-06
chr5	119384001	119389000	2.08E-06
chr5	119385001	119390000	7.72E-08
chr5	119795001	119800000	1.30E-28
chr5	119801001	119806000	8.97E-19
chr5	120101001	120106000	0.031067566
chr5	120186001	120191000	0.000589213
chr5	120377001	120382000	4.68E-06
chr5	120413001	120418000	1.15E-07
chr5	121039001	121044000	0.000572967
chr5	121624001	121629000	7.61E-07
chr5	121643001	121648000	4.22E-31
chr5	121885001	121890000	0.016917854
chr5	121887001	121892000	0.028363567
chr5	121898001	121903000	6.28E-07
chr5	121899001	121904000	1.51E-08
chr5	121900001	121905000	2.09E-08
chr5	121901001	121906000	2.81E-07
chr5	122017001	122022000	5.45E-08
chr5	122018001	122023000	6.74E-07
chr5	122106001	122111000	2.45E-27
chr5	122107001	122112000	1.44E-29
chr5	122108001	122113000	7.44E-30
chr5	122109001	122114000	1.10E-34
chr5	122110001	122115000	1.02E-45
chr5	122111001	122116000	1.01E-30
chr5	122391001	122396000	0.004504064
chr5	122435001	122440000	1.37E-28
chr5	122465001	122470000	0.00055303
chr5	122664001	122669000	0.000642798
chr5	122800001	122805000	0.001456758
chr5	122843001	122848000	2.26E-33
chr5	122844001	122849000	6.25E-54
chr5	122845001	122850000	2.24E-38

chr5	122847001	122852000	1.28E-23
chr5	122848001	122853000	1.21E-49
chr5	123343001	123348000	0.000233885
chr5	123514001	123519000	0.004437348
chr5	123567001	123572000	0.000195841
chr5	123568001	123573000	4.62E-05
chr5	123698001	123703000	1.63E-07
chr5	123699001	123704000	1.87E-06
chr5	124138001	124143000	5.23E-10
chr5	124522001	124527000	4.74E-08
chr5	124523001	124528000	1.70E-05
chr5	124526001	124531000	1.86E-05
chr5	124618001	124623000	6.81E-07
chr5	124619001	124624000	2.93E-06
chr5	124620001	124625000	2.71E-07
chr5	124819001	124824000	2.21E-06
chr5	124820001	124825000	5.48E-07
chr5	124821001	124826000	6.22E-05
chr5	125755001	125760000	1.23E-09
chr5	125758001	125763000	1.63E-31
chr5	125759001	125764000	1.61E-39
chr5	126361001	126366000	3.01E-47
chr5	126362001	126367000	6.44E-49
chr5	126365001	126370000	1.10E-16
chr5	126366001	126371000	1.16E-16
chr5	126488001	126493000	2.98E-08
chr5	126530001	126535000	2.79E-05
chr5	126557001	126562000	1.94E-05
chr5	126681001	126686000	1.49E-06
chr5	126682001	126687000	0.000439835
chr5	126812001	126817000	0.008922903
chr5	126849001	126854000	3.28E-31
chr5	126850001	126855000	5.13E-28
chr5	126851001	126856000	2.80E-38
chr5	126852001	126857000	1.41E-45
chr5	126853001	126858000	2.11E-77
chr5	126854001	126859000	6.30E-17
chr5	127052001	127057000	0.002024568
chr5	127053001	127058000	0.000372466
chr5	127054001	127059000	4.35E-06
chr5	127415001	127420000	7.52E-46
chr5	127419001	127424000	2.18E-55
chr5	127537001	127542000	3.28E-23
chr5	127873001	127878000	1.46E-22
chr5	127874001	127879000	1.30E-18
chr5	128039001	128044000	0.011851151
chr5	128058001	128063000	0.001939224
chr5	128085001	128090000	9.74E-07

chr5	128086001	128091000	0.001014501
chr5	128158001	128163000	1.23E-05
chr5	128173001	128178000	0.000485443
chr5	128232001	128237000	1.18E-08
chr5	128233001	128238000	1.58E-05
chr5	128234001	128239000	0.000141937
chr5	128263001	128268000	0.006432004
chr5	128264001	128269000	7.18E-05
chr5	128293001	128298000	2.69E-12
chr5	128294001	128299000	5.26E-17
chr5	128295001	128300000	2.10E-14
chr5	128296001	128301000	6.03E-31
chr5	128413001	128418000	3.44E-05
chr5	128414001	128419000	7.43E-05
chr5	128415001	128420000	2.80E-05
chr5	128426001	128431000	1.46E-36
chr5	128427001	128432000	3.18E-41
chr5	128428001	128433000	1.02E-57
chr5	128429001	128434000	1.08E-58
chr5	128430001	128435000	7.35E-48
chr5	128495001	128500000	5.24E-09
chr5	128496001	128501000	3.49E-07
chr5	128499001	128504000	3.00E-06
chr5	128545001	128550000	4.70E-06
chr5	128555001	128560000	0.002169596
chr5	128612001	128617000	0.00010966
chr5	128650001	128655000	2.05E-05
chr5	128653001	128658000	1.67E-08
chr5	128654001	128659000	8.67E-09
chr5	128655001	128660000	2.30E-07
chr5	128656001	128661000	2.72E-08
chr5	128662001	128667000	8.34E-05
chr5	128663001	128668000	3.56E-05
chr5	128664001	128669000	0.000167094
chr5	128669001	128674000	0.003018677
chr5	128676001	128681000	0.006918624
chr5	128677001	128682000	0.000212708
chr5	128678001	128683000	3.60E-10
chr5	128679001	128684000	1.81E-09
chr5	128680001	128685000	5.82E-09
chr5	128681001	128686000	9.04E-07
chr5	128709001	128714000	4.32E-07
chr5	128710001	128715000	1.41E-07
chr5	128711001	128716000	8.66E-08
chr5	128712001	128717000	5.07E-07
chr5	128713001	128718000	2.18E-06
chr5	128755001	128760000	0.002289136
chr5	128756001	128761000	0.002789053

chr5	128837001	128842000	0.001786998
chr5	128844001	128849000	0.000995401
chr5	128845001	128850000	1.11E-05
chr5	128846001	128851000	0.003108139
chr5	128865001	128870000	6.81E-06
chr5	128943001	128948000	1.55E-07
chr5	128944001	128949000	9.46E-17
chr5	128945001	128950000	1.04E-18
chr5	128946001	128951000	5.21E-22
chr5	128947001	128952000	2.47E-18
chr5	128948001	128953000	2.82E-16
chr5	128949001	128954000	4.42E-08
chr5	129021001	129026000	1.03E-05
chr5	129066001	129071000	0.001545819
chr5	129067001	129072000	0.00127902
chr5	129068001	129073000	0.000162551
chr5	129118001	129123000	0.000156203
chr5	129148001	129153000	1.38E-07
chr5	129157001	129162000	0.000358159
chr5	129200001	129205000	1.53E-05
chr5	129207001	129212000	8.22E-06
chr5	129208001	129213000	8.15E-06
chr5	129209001	129214000	1.30E-06
chr5	129210001	129215000	4.52E-08
chr5	129211001	129216000	2.16E-05
chr5	129235001	129240000	4.99E-23
chr5	129236001	129241000	8.18E-57
chr5	129261001	129266000	4.66E-07
chr5	129262001	129267000	4.16E-13
chr5	129263001	129268000	1.42E-12
chr5	129317001	129322000	0.000561935
chr5	129318001	129323000	0.00034808
chr5	129319001	129324000	0.00373736
chr5	129320001	129325000	0.00373736
chr5	129327001	129332000	1.14E-06
chr5	129328001	129333000	1.43E-06
chr5	129329001	129334000	1.96E-10
chr5	129330001	129335000	5.49E-05
chr5	129332001	129337000	0.000569096
chr5	129333001	129338000	2.30E-07
chr5	129372001	129377000	0.000654909
chr5	129404001	129409000	0.005769185
chr5	129417001	129422000	2.58E-12
chr5	129418001	129423000	1.08E-09
chr5	129419001	129424000	3.61E-10
chr5	129420001	129425000	8.53E-13
chr5	129421001	129426000	1.26E-10
chr5	129423001	129428000	6.90E-08

chr5	129424001	129429000	2.42E-05
chr5	129427001	129432000	0.001546612
chr5	129476001	129481000	0.006857531
chr5	129477001	129482000	0.00271383
chr5	129512001	129517000	0.000103585
chr5	129513001	129518000	0.000197949
chr5	129588001	129593000	0.003607997
chr5	129682001	129687000	0.004832397
chr5	129683001	129688000	0.010424853
chr5	129831001	129836000	0.000409365
chr5	129832001	129837000	5.05E-05
chr5	129849001	129854000	0.003614426
chr5	129873001	129878000	2.28E-06
chr5	129874001	129879000	9.31E-08
chr5	129876001	129881000	3.49E-06
chr5	129924001	129929000	7.54E-06
chr5	129925001	129930000	3.58E-06
chr5	129926001	129931000	1.95E-05
chr5	129927001	129932000	1.73E-07
chr5	129928001	129933000	5.77E-12
chr5	129940001	129945000	0.002514387
chr5	129941001	129946000	2.62E-05
chr5	129965001	129970000	2.26E-09
chr5	130000001	130005000	6.32E-09
chr5	130002001	130007000	1.97E-05
chr5	130024001	130029000	6.58E-05
chr5	130035001	130040000	0.000278657
chr5	130069001	130074000	5.89E-08
chr5	130070001	130075000	5.53E-07
chr5	130071001	130076000	0.000108771
chr5	130072001	130077000	5.38E-05
chr5	130073001	130078000	0.001335079
chr5	130074001	130079000	0.00142907
chr5	130155001	130160000	3.64E-08
chr5	130184001	130189000	0.001593118
chr5	130188001	130193000	0.003904084
chr5	130199001	130204000	0.015376709
chr5	130229001	130234000	1.76E-12
chr5	130264001	130269000	7.94E-09
chr5	130265001	130270000	1.37E-09
chr5	130266001	130271000	2.20E-09
chr5	130267001	130272000	3.33E-10
chr5	130268001	130273000	6.66E-06
chr5	130269001	130274000	0.00014134
chr5	130277001	130282000	2.24E-14
chr5	130278001	130283000	1.39E-07
chr5	130299001	130304000	0.001699286
chr5	130313001	130318000	0.000288608

chr5	130315001	130320000	0.00034531
chr5	130316001	130321000	0.000519618
chr5	130377001	130382000	0.005340973
chr5	130387001	130392000	2.56E-07
chr5	130595001	130600000	6.45E-11
chr5	131128001	131133000	3.85E-29
chr5	131132001	131137000	1.79E-37
chr5	131133001	131138000	5.28E-19
chr5	131180001	131185000	0.002161952
chr5	131625001	131630000	8.56E-35
chr5	131701001	131706000	7.66E-14
chr5	131702001	131707000	1.39E-10
chr5	131703001	131708000	3.37E-10
chr5	131704001	131709000	1.01E-11
chr5	131705001	131710000	8.00E-11
chr5	131742001	131747000	2.15E-34
chr5	131743001	131748000	9.33E-21
chr5	131825001	131830000	2.56E-23
chr5	131826001	131831000	1.05E-32
chr5	131828001	131833000	9.17E-30
chr5	131829001	131834000	2.72E-15
chr5	131830001	131835000	3.48E-11
chr5	131888001	131893000	4.80E-19
chr5	131988001	131993000	9.18E-28
chr5	131989001	131994000	2.53E-23
chr5	131990001	131995000	4.19E-20
chr5	131991001	131996000	7.00E-12
chr5	132070001	132075000	8.64E-18
chr5	132071001	132076000	1.33E-13
chr5	132072001	132077000	1.08E-12
chr5	132073001	132078000	3.21E-22
chr5	132111001	132116000	1.60E-23
chr5	132112001	132117000	2.63E-25
chr5	132113001	132118000	1.61E-17
chr5	132145001	132150000	1.30E-20
chr5	132146001	132151000	1.36E-10
chr5	132163001	132168000	1.30E-08
chr5	132164001	132169000	3.34E-17
chr5	132165001	132170000	8.18E-18
chr5	132166001	132171000	1.87E-20
chr5	132198001	132203000	2.07E-26
chr5	132199001	132204000	5.31E-26
chr5	132200001	132205000	1.65E-29
chr5	132201001	132206000	2.03E-17
chr5	132202001	132207000	8.88E-16
chr5	132298001	132303000	2.30E-08
chr5	132299001	132304000	2.48E-12
chr5	132386001	132391000	4.58E-22

chr5	132387001	132392000	1.71E-22
chr5	132388001	132393000	3.82E-22
chr5	133301001	133306000	1.38E-15
chr5	133302001	133307000	2.32E-17
chr5	133304001	133309000	4.94E-33
chr5	133402001	133407000	1.19E-09
chr5	133449001	133454000	7.18E-05
chr5	133450001	133455000	4.77E-08
chr5	133512001	133517000	1.34E-08
chr5	133561001	133566000	7.86E-50
chr5	133562001	133567000	4.80E-30
chr5	133626001	133631000	0.001081959
chr5	133700001	133705000	1.69E-17
chr5	133701001	133706000	7.11E-26
chr5	133702001	133707000	5.05E-26
chr5	133703001	133708000	7.70E-25
chr5	133704001	133709000	7.72E-21
chr5	133747001	133752000	8.46E-14
chr5	133861001	133866000	1.02E-15
chr5	133862001	133867000	1.94E-20
chr5	133964001	133969000	3.88E-12
chr5	133965001	133970000	4.25E-17
chr5	133966001	133971000	1.88E-15
chr5	133967001	133972000	2.62E-16
chr5	133968001	133973000	6.86E-15
chr5	134070001	134075000	3.73E-11
chr5	134073001	134078000	1.02E-16
chr5	134074001	134079000	1.03E-19
chr5	134177001	134182000	9.13E-14
chr5	134236001	134241000	2.30E-20
chr5	134523001	134528000	3.79E-50
chr5	134524001	134529000	2.88E-30
chr5	134525001	134530000	7.88E-29
chr5	134526001	134531000	8.35E-27
chr5	134527001	134532000	2.49E-14
chr5	134730001	134735000	6.93E-29
chr5	134731001	134736000	3.24E-41
chr5	134732001	134737000	3.76E-44
chr5	134733001	134738000	3.55E-58
chr5	134734001	134739000	1.08E-84
chr5	134735001	134740000	2.41E-52
chr5	135199001	135204000	8.38E-06
chr5	135201001	135206000	9.66E-08
chr5	135202001	135207000	1.21E-07
chr5	135360001	135365000	8.75E-15
chr5	135361001	135366000	4.19E-16
chr5	135362001	135367000	1.09E-16
chr5	135363001	135368000	1.05E-18

chr5	135364001	135369000	4.06E-17
chr5	135417001	135422000	1.41E-05
chr5	135528001	135533000	4.40E-11
chr5	135775001	135780000	6.81E-07
chr5	135809001	135814000	3.33E-06
chr5	135810001	135815000	2.11E-06
chr5	135811001	135816000	3.06E-09
chr5	135812001	135817000	1.20E-05
chr5	135813001	135818000	1.41E-05
chr5	135814001	135819000	0.000114043
chr5	135815001	135820000	0.000236897
chr5	135835001	135840000	5.78E-11
chr5	135836001	135841000	5.25E-14
chr5	135983001	135988000	0.001304445
chr5	135984001	135989000	0.000877994
chr5	135985001	135990000	4.06E-05
chr5	136010001	136015000	8.24E-06
chr5	136086001	136091000	6.52E-07
chr5	136193001	136198000	0.000238465
chr5	136194001	136199000	0.00041318
chr5	136195001	136200000	1.08E-05
chr5	136210001	136215000	0.00020027
chr5	136275001	136280000	4.16E-07
chr5	136387001	136392000	5.66E-05
chr5	136555001	136560000	0.000123994
chr5	136830001	136835000	1.67E-05
chr5	136831001	136836000	3.62E-12
chr5	136832001	136837000	1.68E-08
chr5	136834001	136839000	8.12E-26
chr5	137090001	137095000	1.90E-10
chr5	137364001	137369000	4.75E-18
chr5	137366001	137371000	4.52E-19
chr5	137367001	137372000	1.05E-18
chr5	137576001	137581000	2.72E-09
chr5	137577001	137582000	7.14E-08
chr5	137663001	137668000	1.39E-15
chr5	137664001	137669000	2.53E-19
chr5	137665001	137670000	4.79E-15
chr5	137673001	137678000	2.04E-11
chr5	137796001	137801000	6.04E-08
chr5	137800001	137805000	5.61E-35
chr5	137801001	137806000	8.03E-24
chr5	138530001	138535000	1.41E-20
chr5	138531001	138536000	3.21E-27
chr5	138532001	138537000	9.77E-15
chr5	138533001	138538000	6.67E-13
chr5	138534001	138539000	3.37E-22
chr5	138772001	138777000	6.96E-21

chr5	138773001	138778000	4.63E-22
chr5	138774001	138779000	7.60E-28
chr5	138775001	138780000	5.46E-24
chr5	138936001	138941000	3.97E-07
chr5	138937001	138942000	5.41E-13
chr5	139024001	139029000	2.01E-18
chr5	139025001	139030000	5.64E-20
chr5	139125001	139130000	1.39E-10
chr5	139126001	139131000	1.26E-12
chr5	139127001	139132000	4.98E-14
chr5	139163001	139168000	2.08E-14
chr5	139164001	139169000	1.35E-07
chr5	139166001	139171000	1.98E-07
chr5	139279001	139284000	1.75E-05
chr5	139281001	139286000	5.69E-05
chr5	139282001	139287000	1.22E-09
chr5	139283001	139288000	4.22E-12
chr5	139323001	139328000	1.95E-05
chr5	139447001	139452000	0.000434101
chr5	139470001	139475000	1.24E-05
chr5	139471001	139476000	2.67E-06
chr5	139492001	139497000	3.50E-36
chr5	139493001	139498000	5.19E-68
chr5	139494001	139499000	1.60E-82
chr5	139550001	139555000	2.40E-09
chr5	139553001	139558000	2.08E-07
chr5	139726001	139731000	5.28E-11
chr5	139735001	139740000	3.18E-10
chr5	139736001	139741000	7.20E-11
chr5	139737001	139742000	3.67E-11
chr5	139738001	139743000	5.29E-09
chr5	139739001	139744000	1.16E-06
chr5	139742001	139747000	3.07E-07
chr5	139781001	139786000	1.26E-15
chr5	139836001	139841000	0.002735167
chr5	139923001	139928000	1.36E-14
chr5	139924001	139929000	7.70E-08
chr5	139927001	139932000	0.004064383
chr5	139933001	139938000	1.20E-12
chr5	140015001	140020000	3.18E-21
chr5	140016001	140021000	1.52E-55
chr5	140017001	140022000	2.43E-30
chr5	140018001	140023000	1.69E-25
chr5	140019001	140024000	1.39E-19
chr5	140066001	140071000	2.68E-08
chr5	140076001	140081000	1.44E-10
chr5	140077001	140082000	1.97E-10
chr5	140079001	140084000	0.000244949

chr5	140080001	140085000	0.000103178
chr5	140127001	140132000	2.28E-05
chr5	140301001	140306000	5.18E-11
chr5	140449001	140454000	0.000122608
chr5	140851001	140856000	4.31E-13
chr5	140852001	140857000	2.23E-09
chr5	140994001	140999000	1.51E-37
chr5	140998001	141003000	4.50E-34
chr5	141027001	141032000	3.22E-11
chr5	141028001	141033000	7.76E-12
chr5	141029001	141034000	8.01E-17
chr5	141030001	141035000	1.28E-19
chr5	141347001	141352000	1.36E-11
chr5	141348001	141353000	6.60E-24
chr5	141349001	141354000	1.03E-08
chr5	141484001	141489000	1.07E-51
chr5	141485001	141490000	1.67E-42
chr5	141486001	141491000	7.41E-32
chr5	141489001	141494000	7.03E-11
chr5	141732001	141737000	1.14E-16
chr5	142150001	142155000	3.01E-41
chr5	142151001	142156000	2.71E-07
chr5	142433001	142438000	2.55E-05
chr5	142782001	142787000	8.32E-59
chr5	142783001	142788000	5.43E-59
chr5	142784001	142789000	6.53E-60
chr5	142785001	142790000	2.21E-48
chr5	143054001	143059000	0.009408536
chr5	143545001	143550000	4.12E-09
chr5	143546001	143551000	1.12E-34
chr5	143547001	143552000	6.28E-35
chr5	143548001	143553000	2.62E-37
chr5	143549001	143554000	1.05E-30
chr5	143550001	143555000	4.70E-42
chr5	143584001	143589000	1.33E-20
chr5	143613001	143618000	0.003107946
chr5	143629001	143634000	0.022091561
chr5	144013001	144018000	0.025959438
chr5	144184001	144189000	0.0001611
chr5	144188001	144193000	0.000857339
chr5	144251001	144256000	0.002146687
chr5	144255001	144260000	0.008835797
chr5	144321001	144326000	0.005037796
chr5	144322001	144327000	0.00020915
chr5	144325001	144330000	0.000472855
chr5	144463001	144468000	0.004260928
chr5	144464001	144469000	0.00127006
chr5	144465001	144470000	9.60E-05

chr5	144472001	144477000	0.01490735
chr5	144509001	144514000	0.011759635
chr5	144510001	144515000	0.004669982
chr5	144590001	144595000	0.000625407
chr5	144592001	144597000	1.89E-06
chr5	144617001	144622000	0.001216864
chr5	144784001	144789000	0.000240428
chr5	144785001	144790000	5.40E-05
chr5	144786001	144791000	1.56E-06
chr5	145822001	145827000	9.50E-27
chr5	145931001	145936000	0.000733057
chr5	146253001	146258000	1.12E-18
chr5	146257001	146262000	7.67E-19
chr5	146279001	146284000	0.007352004
chr5	146391001	146396000	2.88E-05
chr5	146467001	146472000	5.79E-06
chr5	146468001	146473000	9.18E-06
chr5	146495001	146500000	0.000281862
chr5	146501001	146506000	0.001571216
chr5	146503001	146508000	0.00034375
chr5	146610001	146615000	5.73E-37
chr5	146611001	146616000	2.88E-29
chr5	146612001	146617000	6.68E-26
chr5	146613001	146618000	2.82E-14
chr5	146614001	146619000	1.19E-18
chr5	146872001	146877000	9.13E-08
chr5	146873001	146878000	1.34E-07
chr5	146874001	146879000	9.19E-07
chr5	147064001	147069000	3.99E-07
chr5	147065001	147070000	4.10E-06
chr5	147130001	147135000	0.000147829
chr5	147131001	147136000	1.83E-06
chr5	147132001	147137000	0.000542592
chr5	147157001	147162000	1.19E-05
chr5	147158001	147163000	1.07E-13
chr5	147622001	147627000	0.00214777
chr5	147700001	147705000	1.48E-13
chr5	147912001	147917000	1.10E-05
chr5	147952001	147957000	2.07E-05
chr5	147953001	147958000	2.38E-06
chr5	147954001	147959000	2.78E-08
chr5	147955001	147960000	3.82E-08
chr5	147959001	147964000	6.88E-05
chr5	148048001	148053000	4.83E-13
chr5	148201001	148206000	1.12E-34
chr5	148517001	148522000	1.20E-08
chr5	148733001	148738000	2.33E-26
chr5	148734001	148739000	1.06E-23

chr5	148735001	148740000	1.68E-23
chr5	148736001	148741000	6.91E-17
chr5	148737001	148742000	1.05E-09
chr5	148931001	148936000	1.25E-10
chr5	148961001	148966000	5.35E-13
chr5	149031001	149036000	0.001991609
chr5	149105001	149110000	4.24E-27
chr5	149106001	149111000	3.29E-18
chr5	149108001	149113000	2.92E-22
chr5	149109001	149114000	1.41E-25
chr5	149110001	149115000	4.28E-25
chr5	149336001	149341000	2.16E-27
chr5	149337001	149342000	7.81E-30
chr5	149338001	149343000	3.57E-19
chr5	149376001	149381000	5.73E-36
chr5	149377001	149382000	4.14E-28
chr5	149380001	149385000	7.97E-16
chr5	149733001	149738000	8.82E-17
chr5	149824001	149829000	8.87E-07
chr5	149826001	149831000	1.06E-20
chr5	149865001	149870000	0.00148859
chr5	150138001	150143000	5.68E-09
chr5	150185001	150190000	2.62E-08
chr5	150186001	150191000	8.58E-05
chr5	150187001	150192000	4.45E-09
chr5	150188001	150193000	3.48E-05
chr5	150189001	150194000	7.49E-08
chr5	150190001	150195000	3.81E-06
chr5	150191001	150196000	2.37E-10
chr5	150286001	150291000	1.97E-08
chr5	150287001	150292000	2.66E-06
chr5	150288001	150293000	1.40E-06
chr5	150289001	150294000	5.47E-10
chr5	150290001	150295000	1.53E-06
chr5	150291001	150296000	2.35E-08
chr5	150350001	150355000	1.52E-05
chr5	150397001	150402000	3.71E-19
chr5	150398001	150403000	4.37E-12
chr5	150399001	150404000	9.54E-12
chr5	150599001	150604000	3.97E-13
chr5	150600001	150605000	2.49E-08
chr5	150602001	150607000	2.57E-08
chr5	150603001	150608000	2.39E-13
chr5	150628001	150633000	2.76E-13
chr5	150629001	150634000	5.13E-15
chr5	150630001	150635000	8.75E-18
chr5	150631001	150636000	3.47E-15
chr5	150632001	150637000	6.46E-10

chr5	150771001	150776000	1.33E-14
chr5	150772001	150777000	1.38E-10
chr5	150773001	150778000	1.78E-11
chr5	150774001	150779000	2.29E-11
chr5	150775001	150780000	2.34E-07
chr5	150822001	150827000	5.23E-05
chr5	150823001	150828000	6.80E-46
chr5	150824001	150829000	1.11E-32
chr5	150825001	150830000	4.85E-22
chr5	150826001	150831000	1.90E-21
chr5	150827001	150832000	5.56E-22
chr5	151133001	151138000	1.31E-07
chr5	151134001	151139000	9.22E-16
chr5	151135001	151140000	2.91E-21
chr5	151136001	151141000	5.01E-14
chr5	151137001	151142000	6.70E-12
chr5	151138001	151143000	2.43E-11
chr5	151297001	151302000	1.63E-06
chr5	151298001	151303000	0.000382131
chr5	151354001	151359000	3.17E-09
chr5	151386001	151391000	6.31E-11
chr5	151387001	151392000	6.01E-12
chr5	151422001	151427000	1.13E-06
chr5	151429001	151434000	1.40E-07
chr5	151430001	151435000	1.50E-08
chr5	151464001	151469000	0.000133385
chr5	151554001	151559000	0.000120853
chr5	151555001	151560000	0.000194918
chr5	151556001	151561000	5.25E-05
chr5	151557001	151562000	0.000210115
chr5	151560001	151565000	0.000167682
chr5	151561001	151566000	1.08E-07
chr5	151562001	151567000	1.10E-08
chr5	151563001	151568000	3.54E-09
chr5	151564001	151569000	3.56E-09
chr5	151565001	151570000	6.01E-10
chr5	151566001	151571000	2.86E-06
chr5	151567001	151572000	2.03E-06
chr5	151568001	151573000	2.28E-06
chr5	151583001	151588000	1.55E-10
chr5	151584001	151589000	3.66E-11
chr5	151585001	151590000	3.23E-09
chr5	151586001	151591000	6.32E-10
chr5	151587001	151592000	6.17E-11
chr5	151642001	151647000	1.12E-05
chr5	151657001	151662000	0.000721946
chr5	151658001	151663000	0.000933504
chr5	151717001	151722000	0.00465462

chr5	151732001	151737000	0.000184637
chr5	151733001	151738000	5.31E-05
chr5	151776001	151781000	0.005329984
chr5	151786001	151791000	8.61E-10
chr5	151787001	151792000	2.16E-08
chr5	151793001	151798000	0.000784676
chr5	151794001	151799000	2.64E-06
chr5	151795001	151800000	5.94E-05
chr5	151802001	151807000	5.80E-06
chr5	151808001	151813000	0.000263802
chr5	151863001	151868000	0.000236411
chr5	151866001	151871000	0.007060509
chr5	152041001	152046000	0.005378973
chr5	152083001	152088000	0.005323297
chr5	152084001	152089000	0.001227842
chr5	152250001	152255000	0.006490459
chr5	152322001	152327000	0.000750953
chr5	152411001	152416000	0.000429367
chr5	152537001	152542000	0.00068892
chr5	152565001	152570000	0.0001129
chr5	152566001	152571000	7.69E-05
chr5	152577001	152582000	2.61E-05
chr5	152578001	152583000	0.001628332
chr5	152749001	152754000	0.00087929
chr5	152752001	152757000	0.018197992
chr5	152802001	152807000	0.000226461
chr5	152804001	152809000	0.009281368
chr5	152846001	152851000	0.000222176
chr5	152882001	152887000	6.41E-05
chr5	152927001	152932000	0.002685855
chr5	153110001	153115000	0.001914151
chr5	153224001	153229000	0.004911373
chr5	153416001	153421000	4.07E-51
chr5	153565001	153570000	1.25E-22
chr5	153566001	153571000	4.25E-32
chr5	153567001	153572000	5.26E-29
chr5	153570001	153575000	4.40E-10
chr5	153716001	153721000	4.35E-07
chr5	153820001	153825000	2.24E-12
chr5	153821001	153826000	2.87E-27
chr5	153826001	153831000	4.64E-30
chr5	154130001	154135000	8.52E-22
chr5	154131001	154136000	1.58E-24
chr5	154132001	154137000	3.25E-23
chr5	154313001	154318000	6.37E-79
chr5	154314001	154319000	5.89E-67
chr5	154315001	154320000	2.76E-44
chr5	154607001	154612000	5.76E-05

chr5	154664001	154669000	3.65E-07
chr5	154706001	154711000	7.42E-08
chr5	154841001	154846000	0.000430125
chr5	154880001	154885000	4.62E-06
chr5	154882001	154887000	0.00028039
chr5	154883001	154888000	0.000469159
chr5	154897001	154902000	0.002777802
chr5	154927001	154932000	9.57E-07
chr5	154928001	154933000	1.55E-06
chr5	155276001	155281000	2.45E-05
chr5	155277001	155282000	6.67E-06
chr5	155309001	155314000	0.000154604
chr5	155686001	155691000	0.001179845
chr5	155725001	155730000	0.000231419
chr5	155987001	155992000	0.000146964
chr5	155988001	155993000	0.000255877
chr5	155991001	155996000	1.82E-06
chr5	155992001	155997000	5.54E-05
chr5	155995001	156000000	3.40E-05
chr5	156223001	156228000	0.010259863
chr5	156320001	156325000	4.55E-05
chr5	156321001	156326000	7.06E-06
chr5	156322001	156327000	0.000100487
chr5	156689001	156694000	7.59E-29
chr5	156690001	156695000	1.28E-34
chr5	156691001	156696000	1.01E-54
chr5	156692001	156697000	7.69E-40
chr5	156693001	156698000	1.21E-50
chr5	156883001	156888000	1.20E-47
chr5	156884001	156889000	3.01E-37
chr5	156885001	156890000	1.31E-16
chr5	156886001	156891000	1.90E-15
chr5	157002001	157007000	3.34E-37
chr5	157095001	157100000	8.21E-11
chr5	157096001	157101000	9.48E-23
chr5	157097001	157102000	4.76E-25
chr5	157098001	157103000	1.03E-26
chr5	157154001	157159000	5.43E-20
chr5	157155001	157160000	9.29E-13
chr5	157166001	157171000	4.27E-21
chr5	157167001	157172000	7.96E-27
chr5	157168001	157173000	5.73E-18
chr5	157169001	157174000	5.19E-24
chr5	157170001	157175000	3.09E-30
chr5	157171001	157176000	2.19E-18
chr5	157286001	157291000	6.62E-09
chr5	157555001	157560000	0.000124128
chr5	157637001	157642000	0.000113469

chr5	157638001	157643000	0.00044322
chr5	157640001	157645000	9.56E-06
chr5	157642001	157647000	0.000135077
chr5	157645001	157650000	1.16E-05
chr5	157646001	157651000	3.62E-05
chr5	157647001	157652000	1.33E-06
chr5	157648001	157653000	2.99E-07
chr5	157697001	157702000	4.16E-10
chr5	157698001	157703000	2.94E-09
chr5	157758001	157763000	3.16E-06
chr5	157779001	157784000	0.000161461
chr5	157785001	157790000	4.09E-05
chr5	157837001	157842000	0.000123855
chr5	157854001	157859000	1.35E-05
chr5	157919001	157924000	0.004624539
chr5	157936001	157941000	1.12E-07
chr5	157937001	157942000	0.000190825
chr5	157986001	157991000	3.39E-09
chr5	157987001	157992000	1.86E-10
chr5	157999001	158004000	1.17E-06
chr5	158002001	158007000	1.18E-05
chr5	158030001	158035000	2.19E-05
chr5	158031001	158036000	1.14E-05
chr5	158032001	158037000	2.21E-07
chr5	158033001	158038000	3.15E-08
chr5	158034001	158039000	6.11E-05
chr5	158036001	158041000	2.91E-05
chr5	158038001	158043000	0.000693108
chr5	158039001	158044000	3.94E-05
chr5	158078001	158083000	0.000272228
chr5	158079001	158084000	0.000475694
chr5	158107001	158112000	4.08E-11
chr5	158108001	158113000	2.45E-09
chr5	158109001	158114000	3.25E-08
chr5	158122001	158127000	2.67E-10
chr5	158156001	158161000	0.000228389
chr5	158158001	158163000	7.67E-06
chr5	158191001	158196000	4.11E-05
chr5	158194001	158199000	1.17E-05
chr5	158195001	158200000	9.40E-07
chr5	158214001	158219000	0.000119279
chr5	158230001	158235000	0.000135835
chr5	158231001	158236000	1.44E-10
chr5	158232001	158237000	5.38E-11
chr5	158233001	158238000	3.88E-21
chr5	158234001	158239000	8.73E-20
chr5	158235001	158240000	9.62E-14
chr5	158236001	158241000	3.82E-08

chr5	158237001	158242000	4.18E-08
chr5	158253001	158258000	1.51E-06
chr5	158254001	158259000	1.00E-07
chr5	158255001	158260000	3.68E-06
chr5	158408001	158413000	0.00013943
chr5	158410001	158415000	2.81E-05
chr5	158411001	158416000	8.15E-08
chr5	158419001	158424000	4.04E-07
chr5	158494001	158499000	2.24E-05
chr5	158495001	158500000	0.000111225
chr5	158496001	158501000	1.12E-06
chr5	158497001	158502000	2.27E-07
chr5	158500001	158505000	3.74E-11
chr5	158501001	158506000	2.87E-11
chr5	158511001	158516000	0.001301026
chr5	158636001	158641000	2.04E-25
chr5	158685001	158690000	1.06E-08
chr5	158686001	158691000	3.49E-48
chr5	158687001	158692000	1.40E-35
chr5	158690001	158695000	1.27E-12
chr5	158754001	158759000	1.28E-23
chr5	158755001	158760000	1.68E-21
chr5	158756001	158761000	1.09E-16
chr5	158757001	158762000	8.64E-16
chr5	158788001	158793000	2.04E-07
chr5	158802001	158807000	6.63E-05
chr5	158803001	158808000	3.00E-05
chr5	158804001	158809000	7.64E-05
chr5	159196001	159201000	1.57E-23
chr5	159197001	159202000	9.00E-29
chr5	159198001	159203000	1.49E-30
chr5	159199001	159204000	2.57E-30
chr5	159200001	159205000	6.77E-40
chr5	159542001	159547000	1.26E-14
chr5	159544001	159549000	9.87E-20
chr5	159546001	159551000	1.38E-13
chr5	159735001	159740000	2.67E-11
chr5	159736001	159741000	1.48E-19
chr5	159737001	159742000	1.71E-32
chr5	159738001	159743000	8.00E-30
chr5	159739001	159744000	3.57E-36
chr5	159955001	159960000	1.77E-05
chr5	159968001	159973000	0.000870781
chr5	159993001	159998000	1.09E-06
chr5	160001001	160006000	1.60E-05
chr5	160002001	160007000	6.34E-05
chr5	160022001	160027000	3.60E-05
chr5	160122001	160127000	0.001531137

chr5	160139001	160144000	0.000156827
chr5	160140001	160145000	3.32E-05
chr5	160543001	160548000	9.45E-09
chr5	160604001	160609000	0.007188471
chr5	160629001	160634000	0.012729259
chr5	160693001	160698000	0.001526605
chr5	160695001	160700000	0.001691713
chr5	160696001	160701000	0.004443525
chr5	160741001	160746000	7.54E-07
chr5	160742001	160747000	2.89E-07
chr5	160743001	160748000	2.85E-06
chr5	160786001	160791000	1.72E-06
chr5	160787001	160792000	4.72E-07
chr5	160788001	160793000	2.00E-06
chr5	160789001	160794000	7.16E-08
chr5	160804001	160809000	7.93E-08
chr5	160841001	160846000	0.000135467
chr5	160842001	160847000	1.68E-06
chr5	160843001	160848000	1.12E-07
chr5	160844001	160849000	1.96E-07
chr5	160845001	160850000	1.09E-06
chr5	160849001	160854000	0.000490606
chr5	160850001	160855000	0.000365575
chr5	160872001	160877000	0.000115725
chr5	160897001	160902000	1.25E-05
chr5	160898001	160903000	1.77E-05
chr5	160899001	160904000	0.001689208
chr5	160907001	160912000	7.82E-05
chr5	160908001	160913000	1.18E-05
chr5	160909001	160914000	4.64E-07
chr5	160910001	160915000	5.23E-07
chr5	160911001	160916000	1.87E-06
chr5	160937001	160942000	0.000828194
chr5	160953001	160958000	1.15E-05
chr5	160954001	160959000	5.02E-05
chr5	160960001	160965000	0.000385195
chr5	160966001	160971000	0.005639248
chr5	160967001	160972000	3.50E-09
chr5	160988001	160993000	0.035388721
chr5	160989001	160994000	0.003564728
chr5	161019001	161024000	3.64E-05
chr5	161020001	161025000	0.000199769
chr5	161021001	161026000	0.000329191
chr5	161022001	161027000	0.00021997
chr5	161027001	161032000	9.43E-09
chr5	161028001	161033000	1.26E-07
chr5	161072001	161077000	0.000119601
chr5	161078001	161083000	0.000164456

chr5	161098001	161103000	7.43E-05
chr5	161104001	161109000	1.74E-06
chr5	161105001	161110000	1.16E-08
chr5	161125001	161130000	2.44E-07
chr5	161126001	161131000	8.44E-07
chr5	161127001	161132000	1.37E-06
chr5	161128001	161133000	2.86E-05
chr5	161140001	161145000	5.12E-06
chr5	161144001	161149000	0.002085682
chr5	161146001	161151000	0.001231994
chr5	161152001	161157000	0.00045858
chr5	161182001	161187000	3.29E-05
chr5	161194001	161199000	3.42E-06
chr5	161195001	161200000	2.83E-07
chr5	161209001	161214000	0.00096367
chr5	161210001	161215000	0.000374205
chr5	161211001	161216000	0.000150525
chr5	161228001	161233000	0.005503268
chr5	161264001	161269000	3.29E-05
chr5	161265001	161270000	0.000248519
chr5	161266001	161271000	0.000140043
chr5	161349001	161354000	1.49E-13
chr5	161350001	161355000	1.90E-14
chr5	161432001	161437000	0.000207736
chr5	161444001	161449000	8.42E-11
chr5	161445001	161450000	8.48E-07
chr5	161503001	161508000	0.002042915
chr5	161581001	161586000	0.004239133
chr5	161586001	161591000	4.20E-05
chr5	161595001	161600000	1.68E-05
chr5	161596001	161601000	5.54E-07
chr5	161598001	161603000	2.74E-07
chr5	161599001	161604000	7.15E-07
chr5	161600001	161605000	2.83E-06
chr5	161601001	161606000	1.34E-07
chr5	161602001	161607000	6.87E-08
chr5	161622001	161627000	0.000257739
chr5	161624001	161629000	1.52E-06
chr5	161625001	161630000	1.13E-06
chr5	161641001	161646000	0.002786626
chr5	161693001	161698000	2.67E-08
chr5	161694001	161699000	1.57E-07
chr5	161695001	161700000	1.25E-06
chr5	161696001	161701000	1.82E-08
chr5	161697001	161702000	0.001298021
chr5	161722001	161727000	0.000120535
chr5	161725001	161730000	1.39E-05
chr5	161741001	161746000	9.11E-10

chr5	161742001	161747000	1.76E-07
chr5	161743001	161748000	3.78E-08
chr5	161744001	161749000	6.93E-08
chr5	161745001	161750000	0.000300436
chr5	161751001	161756000	3.73E-08
chr5	161752001	161757000	4.97E-07
chr5	161753001	161758000	3.87E-06
chr5	161754001	161759000	2.47E-05
chr5	161763001	161768000	0.000221751
chr5	161764001	161769000	0.000182658
chr5	161772001	161777000	1.28E-09
chr5	161788001	161793000	0.00154569
chr5	161789001	161794000	0.003256148
chr5	161799001	161804000	0.001346765
chr5	161800001	161805000	0.000520773
chr5	161801001	161806000	0.0035681
chr5	161802001	161807000	0.005010518
chr5	161807001	161812000	0.001431579
chr5	161808001	161813000	4.66E-08
chr5	161809001	161814000	1.03E-06
chr5	161810001	161815000	5.52E-06
chr5	161811001	161816000	8.77E-06
chr5	161812001	161817000	2.80E-05
chr5	161816001	161821000	7.75E-05
chr5	161817001	161822000	0.000242468
chr5	161825001	161830000	2.56E-05
chr5	161826001	161831000	7.55E-07
chr5	161827001	161832000	6.24E-07
chr5	161828001	161833000	1.97E-05
chr5	161829001	161834000	3.97E-06
chr5	161830001	161835000	0.000477147
chr5	161831001	161836000	0.000215523
chr5	161832001	161837000	6.70E-06
chr5	161833001	161838000	1.28E-09
chr5	161834001	161839000	6.73E-10
chr5	161835001	161840000	1.88E-10
chr5	161836001	161841000	4.52E-09
chr5	161916001	161921000	0.006324743
chr5	161918001	161923000	0.002712831
chr5	161948001	161953000	4.49E-06
chr5	161969001	161974000	0.000830739
chr5	161975001	161980000	0.001203885
chr5	161976001	161981000	8.12E-05
chr5	161977001	161982000	4.14E-06
chr5	161985001	161990000	1.11E-05
chr5	161990001	161995000	0.000335773
chr5	162005001	162010000	2.43E-10
chr5	162007001	162012000	1.96E-10

chr5	162008001	162013000	1.24E-11
chr5	162009001	162014000	7.87E-12
chr5	162023001	162028000	2.42E-08
chr5	162024001	162029000	4.61E-09
chr5	162025001	162030000	5.36E-07
chr5	162034001	162039000	2.40E-06
chr5	162035001	162040000	5.73E-05
chr5	162036001	162041000	4.48E-06
chr5	162037001	162042000	1.46E-06
chr5	162038001	162043000	9.24E-05
chr5	162143001	162148000	5.35E-06
chr5	162144001	162149000	2.24E-07
chr5	162145001	162150000	4.53E-06
chr5	162149001	162154000	7.13E-05
chr5	162150001	162155000	0.000402541
chr5	162151001	162156000	1.44E-05
chr5	162152001	162157000	1.01E-05
chr5	162164001	162169000	1.88E-06
chr5	162165001	162170000	8.10E-06
chr5	162167001	162172000	7.85E-06
chr5	162173001	162178000	2.07E-05
chr5	162174001	162179000	3.93E-06
chr5	162175001	162180000	1.23E-05
chr5	162177001	162182000	1.80E-06
chr5	162179001	162184000	1.19E-05
chr5	162180001	162185000	1.51E-05
chr5	162181001	162186000	6.41E-05
chr5	162188001	162193000	5.04E-05
chr5	162189001	162194000	5.25E-05
chr5	162190001	162195000	8.08E-05
chr5	162191001	162196000	3.96E-06
chr5	162233001	162238000	5.82E-07
chr5	162234001	162239000	2.35E-09
chr5	162235001	162240000	2.09E-06
chr5	162261001	162266000	1.36E-06
chr5	162262001	162267000	2.44E-06
chr5	162268001	162273000	0.000920404
chr5	162276001	162281000	4.77E-06
chr5	162345001	162350000	3.04E-05
chr5	162346001	162351000	2.28E-05
chr5	162348001	162353000	5.30E-06
chr5	162349001	162354000	3.24E-05
chr5	162360001	162365000	9.89E-06
chr5	162393001	162398000	0.002188667
chr5	162394001	162399000	0.005373522
chr5	162401001	162406000	0.000443689
chr5	162403001	162408000	0.000769757
chr5	162428001	162433000	0.007440676

chr5	162432001	162437000	3.75E-06
chr5	162433001	162438000	6.28E-06
chr5	162434001	162439000	1.56E-05
chr5	162489001	162494000	3.85E-07
chr5	162490001	162495000	3.55E-07
chr5	162509001	162514000	1.27E-08
chr5	162530001	162535000	7.11E-05
chr5	162531001	162536000	3.36E-05
chr5	162543001	162548000	1.19E-09
chr5	162544001	162549000	3.93E-15
chr5	162566001	162571000	7.60E-06
chr5	162567001	162572000	4.95E-05
chr5	162575001	162580000	3.50E-10
chr5	162643001	162648000	3.84E-05
chr5	162644001	162649000	2.94E-08
chr5	162645001	162650000	4.10E-07
chr5	162685001	162690000	5.79E-09
chr5	162686001	162691000	4.54E-10
chr5	162687001	162692000	1.01E-06
chr5	162694001	162699000	0.000293282
chr5	162702001	162707000	0.000114257
chr5	162703001	162708000	8.57E-05
chr5	162704001	162709000	3.83E-06
chr5	162722001	162727000	7.80E-05
chr5	162756001	162761000	5.24E-05
chr5	162860001	162865000	7.26E-22
chr5	162882001	162887000	5.44E-36
chr5	162883001	162888000	1.12E-48
chr5	163050001	163055000	0.000203987
chr5	163250001	163255000	0.008772378
chr5	164076001	164081000	1.18E-05
chr5	164079001	164084000	1.30E-05
chr5	164191001	164196000	0.000133705
chr5	164192001	164197000	3.29E-06
chr5	164535001	164540000	0.001047939
chr5	164592001	164597000	0.009886632
chr5	164615001	164620000	0.000652638
chr5	164616001	164621000	0.000604117
chr5	164617001	164622000	0.000357996
chr5	164714001	164719000	0.002654882
chr5	164862001	164867000	1.45E-05
chr5	164863001	164868000	1.23E-05
chr5	164864001	164869000	9.82E-06
chr5	164865001	164870000	2.71E-05
chr5	164867001	164872000	3.28E-05
chr5	165154001	165159000	6.93E-10
chr5	165230001	165235000	5.24E-07
chr5	165231001	165236000	5.23E-07

chr5	165232001	165237000	4.74E-06
chr5	165240001	165245000	0.012538013
chr5	165353001	165358000	2.85E-08
chr5	165354001	165359000	3.00E-09
chr5	165355001	165360000	1.51E-08
chr5	165356001	165361000	6.94E-09
chr5	165357001	165362000	6.33E-06
chr5	165358001	165363000	0.015669953
chr5	165371001	165376000	8.62E-05
chr5	165777001	165782000	3.84E-07
chr5	165849001	165854000	5.95E-06
chr5	165851001	165856000	0.015415208
chr5	166079001	166084000	1.76E-06
chr5	166112001	166117000	0.000145132
chr5	166373001	166378000	0.000448032
chr5	166927001	166932000	0.002646048
chr5	167474001	167479000	1.29E-08
chr5	167714001	167719000	4.83E-26
chr5	167715001	167720000	1.73E-28
chr5	167716001	167721000	1.19E-35
chr5	167717001	167722000	1.99E-27
chr5	167909001	167914000	7.16E-18
chr5	167910001	167915000	3.45E-23
chr5	167911001	167916000	6.26E-35
chr5	167912001	167917000	5.22E-37
chr5	167913001	167918000	2.23E-43
chr5	167914001	167919000	6.54E-19
chr5	168005001	168010000	2.83E-30
chr5	168006001	168011000	3.24E-48
chr5	168049001	168054000	0.000194177
chr5	168491001	168496000	1.37E-09
chr5	168587001	168592000	1.21E-11
chr5	168588001	168593000	1.21E-13
chr5	168621001	168626000	5.94E-10
chr5	168622001	168627000	2.13E-10
chr5	168624001	168629000	3.92E-08
chr5	168784001	168789000	5.16E-06
chr5	168785001	168790000	2.46E-05
chr5	168818001	168823000	3.95E-06
chr5	168900001	168905000	0.002117765
chr5	168980001	168985000	0.000855875
chr5	168981001	168986000	0.009915208
chr5	168982001	168987000	0.004456526
chr5	169662001	169667000	0.020442335
chr5	170153001	170158000	0.00065408
chr5	170284001	170289000	3.49E-10
chr5	170285001	170290000	9.95E-25
chr5	170286001	170291000	8.69E-25

chr5	170287001	170292000	1.03E-14
chr5	170288001	170293000	2.35E-22
chr5	170289001	170294000	2.32E-23
chr5	170847001	170852000	1.10E-11
chr5	170997001	171002000	1.87E-05
chr5	171153001	171158000	2.34E-06
chr5	171154001	171159000	2.06E-05
chr5	171611001	171616000	4.03E-16
chr5	171706001	171711000	4.52E-21
chr5	171877001	171882000	5.72E-26
chr5	171880001	171885000	6.92E-20
chr5	172064001	172069000	1.69E-22
chr5	172065001	172070000	8.56E-30
chr5	172066001	172071000	2.96E-23
chr5	172067001	172072000	1.29E-30
chr5	172068001	172073000	2.03E-21
chr5	172257001	172262000	9.93E-09
chr5	172382001	172387000	2.58E-29
chr5	172384001	172389000	3.03E-26
chr5	172385001	172390000	2.63E-20
chr5	172386001	172391000	5.54E-23
chr5	172407001	172412000	3.66E-06
chr5	172571001	172576000	8.35E-11
chr5	173039001	173044000	2.77E-30
chr5	173437001	173442000	0.001423134
chr5	173468001	173473000	1.43E-18
chr5	173469001	173474000	5.98E-15
chr5	173470001	173475000	1.94E-07
chr5	174285001	174290000	2.62E-12
chr5	174286001	174291000	7.69E-09
chr5	174329001	174334000	1.16E-08
chr5	174334001	174339000	2.46E-12
chr5	174335001	174340000	3.33E-12
chr5	174392001	174397000	2.04E-05
chr5	174484001	174489000	1.60E-10
chr5	174485001	174490000	3.65E-10
chr5	174486001	174491000	1.30E-11
chr5	174487001	174492000	2.91E-11
chr5	174568001	174573000	0.002817364
chr5	174586001	174591000	1.32E-08
chr5	174693001	174698000	6.42E-06
chr5	174694001	174699000	1.15E-06
chr5	174741001	174746000	0.000105653
chr5	174826001	174831000	7.33E-06
chr5	174829001	174834000	3.35E-05
chr5	174830001	174835000	4.07E-05
chr5	174869001	174874000	1.23E-18
chr5	174901001	174906000	6.91E-22

chr5	174902001	174907000	3.58E-39
chr5	174903001	174908000	1.04E-38
chr5	174904001	174909000	3.63E-46
chr5	174905001	174910000	3.96E-48
chr5	174957001	174962000	2.08E-35
chr5	174958001	174963000	1.54E-28
chr5	174959001	174964000	9.72E-31
chr5	174960001	174965000	2.36E-29
chr5	174961001	174966000	5.41E-29
chr5	175152001	175157000	3.47E-06
chr5	175153001	175158000	5.66E-06
chr5	175617001	175622000	3.80E-06
chr5	175619001	175624000	1.23E-07
chr5	175625001	175630000	1.30E-24
chr5	175626001	175631000	7.55E-40
chr5	175661001	175666000	1.05E-25
chr5	175662001	175667000	5.91E-24
chr5	175663001	175668000	3.73E-27
chr5	175664001	175669000	3.29E-24
chr5	175665001	175670000	3.21E-28
chr5	175681001	175686000	0.00133155
chr5	175682001	175687000	0.000289507
chr5	175784001	175789000	1.15E-26
chr5	175785001	175790000	3.25E-21
chr5	175786001	175791000	3.37E-26
chr5	175787001	175792000	1.81E-15
chr5	175788001	175793000	6.55E-29
chr5	175789001	175794000	1.11E-16
chr5	175790001	175795000	6.78E-13
chr5	175791001	175796000	1.20E-05
chr5	175792001	175797000	2.50E-07
chr5	175842001	175847000	2.38E-10
chr5	175843001	175848000	3.09E-13
chr5	176033001	176038000	1.29E-08
chr5	176035001	176040000	7.47E-08
chr5	176429001	176434000	1.13E-60
chr5	176430001	176435000	2.14E-41
chr5	176432001	176437000	5.04E-24
chr5	176433001	176438000	3.45E-27
chr5	176560001	176565000	1.95E-18
chr5	176561001	176566000	1.79E-20
chr5	176871001	176876000	8.03E-07
chr5	176872001	176877000	8.52E-15
chr5	176873001	176878000	7.07E-17
chr5	176877001	176882000	1.20E-07
chr5	176878001	176883000	5.63E-08
chr5	176976001	176981000	5.06E-07
chr5	176977001	176982000	2.26E-14

chr5	176978001	176983000	2.43E-08
chr5	176979001	176984000	7.16E-14
chr5	176980001	176985000	3.24E-09
chr5	176981001	176986000	1.44E-17
chr5	177094001	177099000	8.90E-29
chr5	177095001	177100000	9.20E-76
chr5	177096001	177101000	3.68E-38
chr5	177097001	177102000	2.82E-24
chr5	177098001	177103000	3.58E-05
chr5	177099001	177104000	3.20E-20
chr5	177144001	177149000	0.001970414
chr5	177168001	177173000	0.000118775
chr5	177169001	177174000	0.001496231
chr5	177171001	177176000	0.000850211
chr5	177172001	177177000	0.023291183
chr5	177452001	177457000	1.27E-11
chr5	177453001	177458000	8.13E-09
chr5	177454001	177459000	1.10E-08
chr5	177455001	177460000	1.31E-10
chr5	177470001	177475000	4.34E-05
chr5	177558001	177563000	4.83E-16
chr5	177627001	177632000	6.44E-19
chr5	177656001	177661000	6.22E-13
chr5	177657001	177662000	1.92E-15
chr5	177658001	177663000	9.01E-15
chr5	177659001	177664000	2.66E-21
chr5	178017001	178022000	5.45E-15
chr5	178153001	178158000	7.22E-09
chr5	178157001	178162000	1.10E-19
chr5	178283001	178288000	3.02E-40
chr5	178284001	178289000	1.16E-36
chr5	178285001	178290000	4.38E-16
chr5	178286001	178291000	1.28E-12
chr5	178287001	178292000	1.37E-32
chr5	178318001	178323000	9.73E-31
chr5	178319001	178324000	1.12E-54
chr5	178320001	178325000	1.12E-48
chr5	178321001	178326000	7.97E-46
chr5	178322001	178327000	4.89E-34
chr5	178323001	178328000	1.65E-22
chr5	178348001	178353000	4.31E-08
chr5	178449001	178454000	4.62E-24
chr5	178510001	178515000	1.90E-06
chr5	178511001	178516000	7.03E-06
chr5	178512001	178517000	5.19E-07
chr5	178768001	178773000	2.40E-08
chr5	178802001	178807000	1.55E-09
chr5	179051001	179056000	6.32E-13

chr5	179121001	179126000	6.40E-12
chr5	179159001	179164000	7.19E-36
chr5	179160001	179165000	2.79E-38
chr5	179229001	179234000	4.47E-13
chr5	179330001	179335000	3.35E-18
chr5	179331001	179336000	3.58E-14
chr5	179332001	179337000	3.15E-18
chr5	179497001	179502000	5.73E-43
chr5	179498001	179503000	3.82E-39
chr5	179499001	179504000	7.67E-22
chr5	179714001	179719000	1.17E-32
chr5	179715001	179720000	7.00E-48
chr5	179776001	179781000	3.99E-06
chr5	179777001	179782000	1.34E-08
chr5	179778001	179783000	6.00E-05
chr5	180076001	180081000	2.24E-15
chr5	180140001	180145000	0.000438107
chr5	180225001	180230000	4.14E-16
chr5	180227001	180232000	1.49E-15
chr5	180228001	180233000	5.61E-17
chr5	180229001	180234000	2.35E-21
chr5	180237001	180242000	2.45E-18
chr5	180254001	180259000	1.43E-17
chr5	180255001	180260000	8.33E-17
chr5	180258001	180263000	6.17E-21
chr5	180284001	180289000	1.78E-43
chr5	180285001	180290000	2.65E-60
chr5	180286001	180291000	5.20E-64
chr5	180287001	180292000	1.01E-52
chr5	180288001	180293000	2.52E-58
chr5	180306001	180311000	1.19E-12
chr5	180307001	180312000	1.93E-09
chr5	180318001	180323000	1.80E-11
chr5	180319001	180324000	3.21E-10
chr5	180329001	180334000	7.37E-13
chr5	180330001	180335000	5.25E-17
chr5	180346001	180351000	0.001037692
chr5	180347001	180352000	3.08E-12
chr5	180348001	180353000	8.61E-14
chr5	180349001	180354000	5.96E-12
chr5	180350001	180355000	3.65E-12
chr5	180351001	180356000	7.95E-14
chr5	180379001	180384000	6.33E-08
chr5	180387001	180392000	4.76E-08
chr5	180388001	180393000	2.18E-10
chr5	180389001	180394000	0.000142133
chr5	180399001	180404000	1.20E-08
chr5	180400001	180405000	1.48E-07

chr5	180436001	180441000	2.02E-19
chr5	180437001	180442000	2.16E-12
chr5	180438001	180443000	1.25E-11
chr5	180446001	180451000	2.96E-07
chr6	688001	693000	4.86E-46
chr6	689001	694000	8.09E-37
chr6	708001	713000	4.57E-22
chr6	709001	714000	1.30E-28
chr6	710001	715000	7.57E-31
chr6	711001	716000	6.38E-29
chr6	849001	854000	1.89E-07
chr6	931001	936000	5.64E-10
chr6	932001	937000	5.51E-08
chr6	1157001	1162000	5.67E-10
chr6	1269001	1274000	4.16E-08
chr6	1553001	1558000	5.06E-16
chr6	1555001	1560000	1.55E-13
chr6	1953001	1958000	1.70E-11
chr6	2018001	2023000	8.38E-07
chr6	2019001	2024000	4.61E-06
chr6	2022001	2027000	2.49E-07
chr6	2241001	2246000	4.67E-28
chr6	2631001	2636000	1.03E-15
chr6	2632001	2637000	9.20E-23
chr6	2761001	2766000	9.75E-34
chr6	2762001	2767000	4.52E-54
chr6	2763001	2768000	3.46E-37
chr6	2764001	2769000	3.54E-33
chr6	2765001	2770000	2.91E-69
chr6	2766001	2771000	8.14E-52
chr6	2814001	2819000	4.62E-05
chr6	2840001	2845000	1.90E-15
chr6	2841001	2846000	1.44E-23
chr6	2842001	2847000	7.41E-17
chr6	2949001	2954000	7.10E-20
chr6	2950001	2955000	4.43E-16
chr6	2951001	2956000	1.16E-28
chr6	2967001	2972000	2.14E-30
chr6	2968001	2973000	8.86E-31
chr6	2969001	2974000	2.55E-27
chr6	2970001	2975000	3.13E-43
chr6	2971001	2976000	2.60E-40
chr6	2972001	2977000	5.95E-27
chr6	2993001	2998000	0.000527963
chr6	2994001	2999000	2.92E-20
chr6	2995001	3000000	3.74E-30
chr6	3050001	3055000	2.85E-22
chr6	3060001	3065000	4.32E-27

chr6	3061001	3066000	6.33E-06
chr6	3063001	3068000	1.91E-21
chr6	3064001	3069000	5.38E-23
chr6	3066001	3071000	3.43E-39
chr6	3067001	3072000	3.71E-51
chr6	3068001	3073000	3.83E-68
chr6	3069001	3074000	1.28E-37
chr6	3114001	3119000	1.56E-18
chr6	3115001	3120000	1.59E-43
chr6	3116001	3121000	2.67E-102
chr6	3117001	3122000	3.19E-83
chr6	3118001	3123000	1.61E-60
chr6	3119001	3124000	1.02E-44
chr6	3157001	3162000	1.12E-11
chr6	3158001	3163000	6.95E-07
chr6	3223001	3228000	5.13E-23
chr6	3224001	3229000	1.59E-25
chr6	3456001	3461000	6.30E-45
chr6	3457001	3462000	1.97E-32
chr6	3458001	3463000	7.28E-13
chr6	3747001	3752000	1.27E-11
chr6	3749001	3754000	7.25E-13
chr6	3750001	3755000	8.51E-39
chr6	3751001	3756000	1.80E-47
chr6	4020001	4025000	2.28E-25
chr6	4021001	4026000	1.43E-41
chr6	4131001	4136000	2.00E-38
chr6	4132001	4137000	6.11E-44
chr6	4648001	4653000	1.27E-08
chr6	4776001	4781000	3.42E-55
chr6	4777001	4782000	1.91E-21
chr6	4806001	4811000	2.72E-09
chr6	4999001	5004000	1.51E-19
chr6	5000001	5005000	3.24E-24
chr6	5001001	5006000	4.10E-23
chr6	5002001	5007000	2.88E-19
chr6	5003001	5008000	2.21E-11
chr6	5080001	5085000	1.58E-31
chr6	5081001	5086000	4.21E-25
chr6	5918001	5923000	6.48E-07
chr6	5943001	5948000	0.000625172
chr6	5944001	5949000	3.15E-05
chr6	5945001	5950000	5.81E-05
chr6	6062001	6067000	0.001131497
chr6	6077001	6082000	1.97E-07
chr6	6110001	6115000	2.54E-06
chr6	6112001	6117000	2.45E-09
chr6	6113001	6118000	1.88E-13

chr6	6114001	6119000	9.01E-12
chr6	6115001	6120000	9.55E-11
chr6	6116001	6121000	9.86E-11
chr6	6117001	6122000	5.68E-09
chr6	6118001	6123000	3.24E-06
chr6	6119001	6124000	3.40E-09
chr6	6120001	6125000	1.44E-07
chr6	6121001	6126000	7.88E-05
chr6	6149001	6154000	4.80E-11
chr6	6234001	6239000	0.000389414
chr6	6235001	6240000	0.000321142
chr6	6236001	6241000	6.96E-06
chr6	6237001	6242000	3.80E-06
chr6	6238001	6243000	0.001597311
chr6	6292001	6297000	0.000580922
chr6	6441001	6446000	1.73E-06
chr6	6442001	6447000	3.25E-07
chr6	6483001	6488000	7.33E-07
chr6	6499001	6504000	1.20E-08
chr6	7047001	7052000	9.87E-17
chr6	7048001	7053000	2.01E-21
chr6	7050001	7055000	4.48E-39
chr6	7051001	7056000	3.93E-31
chr6	7103001	7108000	7.26E-15
chr6	7104001	7109000	1.04E-24
chr6	7107001	7112000	2.71E-24
chr6	7108001	7113000	1.78E-39
chr6	7109001	7114000	2.37E-38
chr6	7110001	7115000	1.60E-12
chr6	7313001	7318000	9.15E-18
chr6	7537001	7542000	3.26E-26
chr6	7538001	7543000	3.59E-36
chr6	7539001	7544000	2.53E-37
chr6	7540001	7545000	2.46E-40
chr6	7541001	7546000	7.66E-36
chr6	7542001	7547000	5.71E-45
chr6	7543001	7548000	3.37E-21
chr6	7906001	7911000	1.39E-12
chr6	7907001	7912000	6.36E-26
chr6	7908001	7913000	1.36E-25
chr6	7909001	7914000	1.13E-34
chr6	7910001	7915000	1.32E-28
chr6	8060001	8065000	2.11E-56
chr6	8061001	8066000	2.62E-37
chr6	8084001	8089000	1.72E-10
chr6	8086001	8091000	7.58E-14
chr6	8102001	8107000	1.27E-16
chr6	8248001	8253000	0.000269239

chr6	8431001	8436000	1.05E-64
chr6	8432001	8437000	1.91E-53
chr6	8433001	8438000	1.64E-64
chr6	8434001	8439000	2.53E-51
chr6	8435001	8440000	1.52E-54
chr6	8436001	8441000	3.46E-18
chr6	8729001	8734000	0.004054843
chr6	8767001	8772000	8.19E-12
chr6	9247001	9252000	7.59E-11
chr6	9248001	9253000	8.78E-11
chr6	9603001	9608000	0.001439915
chr6	9605001	9610000	0.000146977
chr6	9635001	9640000	0.018115609
chr6	10402001	10407000	5.41E-54
chr6	10405001	10410000	7.74E-30
chr6	10690001	10695000	4.49E-26
chr6	10691001	10696000	2.97E-20
chr6	10693001	10698000	1.94E-26
chr6	10744001	10749000	1.81E-10
chr6	10746001	10751000	2.93E-12
chr6	10838001	10843000	2.11E-20
chr6	11044001	11049000	5.55E-17
chr6	11089001	11094000	6.88E-47
chr6	11090001	11095000	2.69E-75
chr6	11091001	11096000	1.12E-63
chr6	11092001	11097000	1.13E-64
chr6	11420001	11425000	8.42E-06
chr6	11421001	11426000	1.25E-06
chr6	11505001	11510000	0.00609419
chr6	11533001	11538000	1.07E-31
chr6	11534001	11539000	3.86E-44
chr6	11535001	11540000	3.87E-47
chr6	11536001	11541000	1.67E-47
chr6	11537001	11542000	6.01E-58
chr6	11538001	11543000	8.26E-66
chr6	11870001	11875000	4.02E-08
chr6	11906001	11911000	0.003961297
chr6	11907001	11912000	0.00046404
chr6	12005001	12010000	6.49E-32
chr6	12405001	12410000	1.64E-10
chr6	12406001	12411000	1.28E-09
chr6	12407001	12412000	2.45E-09
chr6	12417001	12422000	0.00042441
chr6	12480001	12485000	0.000666364
chr6	12481001	12486000	0.000176818
chr6	12482001	12487000	9.46E-05
chr6	12489001	12494000	5.35E-06
chr6	12539001	12544000	1.57E-13

chr6	12574001	12579000	2.91E-11
chr6	12575001	12580000	5.81E-12
chr6	12595001	12600000	5.17E-05
chr6	12633001	12638000	9.16E-05
chr6	12635001	12640000	5.27E-10
chr6	12636001	12641000	5.06E-07
chr6	12702001	12707000	0.001771897
chr6	12703001	12708000	0.001492027
chr6	12704001	12709000	0.003656737
chr6	12705001	12710000	0.000584085
chr6	12769001	12774000	2.63E-08
chr6	12828001	12833000	0.000245198
chr6	12829001	12834000	1.26E-06
chr6	12830001	12835000	5.65E-06
chr6	12834001	12839000	6.68E-05
chr6	12854001	12859000	6.40E-05
chr6	12856001	12861000	1.63E-05
chr6	12857001	12862000	2.24E-05
chr6	13003001	13008000	0.001196275
chr6	13017001	13022000	0.005638101
chr6	13018001	13023000	0.001693777
chr6	13057001	13062000	1.55E-08
chr6	13058001	13063000	6.71E-13
chr6	13059001	13064000	2.84E-10
chr6	13060001	13065000	1.79E-07
chr6	13061001	13066000	8.27E-07
chr6	13062001	13067000	1.83E-05
chr6	13108001	13113000	3.68E-05
chr6	13109001	13114000	0.000172432
chr6	13110001	13115000	7.27E-05
chr6	13111001	13116000	7.51E-08
chr6	13112001	13117000	7.34E-07
chr6	13127001	13132000	5.34E-05
chr6	13174001	13179000	2.85E-11
chr6	13175001	13180000	4.70E-09
chr6	13177001	13182000	1.97E-09
chr6	13178001	13183000	6.79E-13
chr6	13231001	13236000	1.91E-12
chr6	13232001	13237000	0.000422807
chr6	13261001	13266000	2.92E-08
chr6	13482001	13487000	2.18E-13
chr6	13483001	13488000	2.43E-30
chr6	13484001	13489000	3.63E-31
chr6	13485001	13490000	2.78E-30
chr6	13486001	13491000	2.47E-40
chr6	13487001	13492000	3.24E-39
chr6	13488001	13493000	4.22E-17
chr6	13572001	13577000	7.67E-20

chr6	13573001	13578000	2.77E-21
chr6	13574001	13579000	4.88E-39
chr6	13575001	13580000	2.36E-25
chr6	13810001	13815000	2.72E-36
chr6	13811001	13816000	2.05E-32
chr6	13812001	13817000	3.67E-74
chr6	13813001	13818000	6.19E-48
chr6	13814001	13819000	4.05E-43
chr6	13873001	13878000	6.63E-23
chr6	14113001	14118000	3.58E-20
chr6	14114001	14119000	3.02E-23
chr6	14115001	14120000	2.16E-24
chr6	14116001	14121000	1.79E-58
chr6	14117001	14122000	6.47E-79
chr6	14118001	14123000	6.91E-32
chr6	14287001	14292000	2.76E-06
chr6	15241001	15246000	1.05E-21
chr6	15242001	15247000	3.29E-23
chr6	15397001	15402000	3.78E-10
chr6	15658001	15663000	4.07E-30
chr6	15659001	15664000	2.00E-31
chr6	15660001	15665000	2.65E-30
chr6	15661001	15666000	7.60E-31
chr6	15662001	15667000	3.37E-27
chr6	15663001	15668000	4.86E-32
chr6	15714001	15719000	1.04E-09
chr6	15715001	15720000	4.33E-08
chr6	15716001	15721000	3.70E-05
chr6	15717001	15722000	1.80E-06
chr6	15849001	15854000	3.01E-08
chr6	15850001	15855000	3.52E-10
chr6	15899001	15904000	2.43E-06
chr6	16125001	16130000	8.15E-39
chr6	16126001	16131000	3.82E-39
chr6	16756001	16761000	2.48E-82
chr6	17060001	17065000	0.000180942
chr6	17065001	17070000	0.001706826
chr6	17067001	17072000	5.84E-08
chr6	17068001	17073000	6.11E-07
chr6	17099001	17104000	7.73E-31
chr6	17100001	17105000	7.16E-34
chr6	17101001	17106000	1.09E-23
chr6	17102001	17107000	6.75E-18
chr6	17277001	17282000	2.18E-34
chr6	17280001	17285000	3.71E-25
chr6	17281001	17286000	5.81E-31
chr6	17282001	17287000	4.31E-21
chr6	17389001	17394000	2.58E-14

chr6	17390001	17395000	2.61E-24
chr6	17391001	17396000	2.21E-40
chr6	17392001	17397000	1.35E-41
chr6	17394001	17399000	3.90E-22
chr6	17576001	17581000	1.80E-30
chr6	17577001	17582000	1.08E-24
chr6	17578001	17583000	1.92E-49
chr6	17579001	17584000	1.11E-54
chr6	17596001	17601000	5.93E-48
chr6	17597001	17602000	4.42E-30
chr6	17702001	17707000	1.21E-30
chr6	17774001	17779000	4.38E-06
chr6	17944001	17949000	5.68E-08
chr6	18151001	18156000	3.14E-22
chr6	18155001	18160000	2.41E-31
chr6	18156001	18161000	1.11E-23
chr6	18274001	18279000	1.48E-20
chr6	18277001	18282000	7.23E-26
chr6	19080001	19085000	0.000193628
chr6	19089001	19094000	1.91E-05
chr6	19833001	19838000	7.03E-16
chr6	19834001	19839000	4.59E-38
chr6	20212001	20217000	6.45E-22
chr6	20398001	20403000	3.36E-21
chr6	20399001	20404000	4.31E-15
chr6	20402001	20407000	5.14E-14
chr6	20403001	20408000	2.75E-16
chr6	21583001	21588000	2.26E-18
chr6	21589001	21594000	1.43E-19
chr6	21590001	21595000	3.34E-35
chr6	21591001	21596000	5.84E-35
chr6	21661001	21666000	3.97E-26
chr6	21662001	21667000	1.31E-17
chr6	21664001	21669000	3.47E-11
chr6	21665001	21670000	1.66E-11
chr6	21666001	21671000	1.89E-08
chr6	22237001	22242000	0.00011704
chr6	23272001	23277000	0.000181017
chr6	23682001	23687000	0.000259074
chr6	23693001	23698000	0.001037425
chr6	23905001	23910000	6.59E-06
chr6	23911001	23916000	0.000144871
chr6	23912001	23917000	0.0031854
chr6	23915001	23920000	8.81E-05
chr6	23971001	23976000	2.58E-09
chr6	23993001	23998000	2.64E-06
chr6	23994001	23999000	1.68E-06
chr6	24401001	24406000	8.45E-38

chr6	24402001	24407000	1.14E-38
chr6	24403001	24408000	2.59E-54
chr6	24491001	24496000	2.09E-50
chr6	24492001	24497000	1.28E-45
chr6	24493001	24498000	2.37E-49
chr6	24494001	24499000	4.62E-46
chr6	24495001	24500000	1.17E-35
chr6	24642001	24647000	2.77E-30
chr6	24643001	24648000	8.06E-76
chr6	24644001	24649000	7.06E-66
chr6	24645001	24650000	3.04E-61
chr6	24646001	24651000	1.74E-71
chr6	24720001	24725000	2.85E-54
chr6	24721001	24726000	1.06E-44
chr6	24771001	24776000	1.24E-41
chr6	24776001	24781000	1.83E-16
chr6	25076001	25081000	2.48E-06
chr6	25077001	25082000	3.26E-06
chr6	25078001	25083000	3.82E-11
chr6	25079001	25084000	7.08E-08
chr6	25108001	25113000	3.03E-05
chr6	25275001	25280000	2.98E-10
chr6	25277001	25282000	5.07E-15
chr6	25278001	25283000	2.03E-10
chr6	25279001	25284000	7.06E-13
chr6	25988001	25993000	1.11E-63
chr6	25989001	25994000	1.28E-34
chr6	25990001	25995000	1.35E-27
chr6	25991001	25996000	5.22E-28
chr6	25992001	25997000	2.11E-17
chr6	26056001	26061000	1.13E-25
chr6	26076001	26081000	2.04E-08
chr6	26077001	26082000	5.74E-10
chr6	26168001	26173000	5.99E-42
chr6	26236001	26241000	4.59E-13
chr6	26267001	26272000	1.23E-12
chr6	26363001	26368000	2.64E-09
chr6	26522001	26527000	2.44E-49
chr6	26549001	26554000	6.38E-16
chr6	26655001	26660000	1.49E-30
chr6	26656001	26661000	5.66E-34
chr6	26796001	26801000	0.030939884
chr6	26798001	26803000	0.029827847
chr6	26925001	26930000	1.13E-05
chr6	27042001	27047000	9.44E-08
chr6	27101001	27106000	2.32E-36
chr6	27217001	27222000	1.28E-17
chr6	27218001	27223000	2.90E-14

chr6	27219001	27224000	9.69E-18
chr6	27229001	27234000	4.12E-23
chr6	27230001	27235000	8.61E-14
chr6	27338001	27343000	6.38E-21
chr6	27367001	27372000	9.82E-07
chr6	27399001	27404000	0.000333333
chr6	27894001	27899000	0.001103074
chr6	27895001	27900000	0.00162565
chr6	28048001	28053000	3.22E-30
chr6	28068001	28073000	6.55E-23
chr6	28069001	28074000	7.16E-22
chr6	28070001	28075000	7.31E-29
chr6	28071001	28076000	7.37E-28
chr6	28125001	28130000	3.12E-16
chr6	28126001	28131000	3.28E-18
chr6	28127001	28132000	4.81E-21
chr6	28128001	28133000	1.05E-14
chr6	28129001	28134000	1.53E-14
chr6	28160001	28165000	5.06E-07
chr6	28161001	28166000	0.000769531
chr6	28163001	28168000	0.002790664
chr6	28193001	28198000	1.85E-11
chr6	28204001	28209000	0.021198477
chr6	28215001	28220000	1.37E-21
chr6	28216001	28221000	5.03E-22
chr6	28217001	28222000	3.49E-15
chr6	28218001	28223000	8.18E-10
chr6	28220001	28225000	3.63E-07
chr6	28299001	28304000	8.35E-27
chr6	28315001	28320000	1.50E-22
chr6	28347001	28352000	1.19E-20
chr6	28348001	28353000	1.14E-26
chr6	28349001	28354000	4.72E-30
chr6	28350001	28355000	1.01E-30
chr6	28351001	28356000	2.30E-30
chr6	28461001	28466000	0.000386627
chr6	28462001	28467000	0.000220697
chr6	28463001	28468000	9.70E-06
chr6	28475001	28480000	0.003125783
chr6	33587001	33592000	9.23E-10
chr6	33588001	33593000	3.12E-14
chr6	33752001	33757000	7.03E-11
chr6	33753001	33758000	6.76E-22
chr6	33754001	33759000	1.08E-32
chr6	33755001	33760000	2.57E-42
chr6	33756001	33761000	3.68E-52
chr6	34160001	34165000	4.31E-15
chr6	34162001	34167000	1.26E-17

chr6	34163001	34168000	4.89E-14
chr6	34164001	34169000	1.29E-15
chr6	34187001	34192000	9.30E-09
chr6	34188001	34193000	2.94E-11
chr6	34189001	34194000	8.03E-18
chr6	34190001	34195000	1.76E-16
chr6	34191001	34196000	1.52E-12
chr6	34215001	34220000	1.82E-08
chr6	34216001	34221000	1.17E-12
chr6	34356001	34361000	2.76E-44
chr6	34664001	34669000	1.18E-12
chr6	34755001	34760000	1.83E-39
chr6	34756001	34761000	7.76E-37
chr6	34857001	34862000	6.78E-35
chr6	34993001	34998000	0.000110224
chr6	35150001	35155000	1.19E-07
chr6	35227001	35232000	3.73E-17
chr6	35281001	35286000	4.01E-09
chr6	35282001	35287000	3.09E-24
chr6	35283001	35288000	6.23E-17
chr6	35284001	35289000	1.44E-13
chr6	35418001	35423000	5.02E-10
chr6	35419001	35424000	1.60E-06
chr6	35420001	35425000	3.88E-07
chr6	35432001	35437000	2.43E-27
chr6	35433001	35438000	2.69E-35
chr6	35434001	35439000	1.50E-25
chr6	35540001	35545000	1.29E-05
chr6	35541001	35546000	1.09E-08
chr6	35651001	35656000	8.39E-11
chr6	35740001	35745000	3.82E-09
chr6	35742001	35747000	2.32E-08
chr6	35743001	35748000	1.35E-08
chr6	35744001	35749000	6.44E-08
chr6	35773001	35778000	2.11E-07
chr6	35888001	35893000	9.90E-27
chr6	35988001	35993000	2.69E-18
chr6	35989001	35994000	5.60E-17
chr6	35990001	35995000	1.29E-17
chr6	35991001	35996000	8.31E-34
chr6	36160001	36165000	9.72E-47
chr6	36387001	36392000	2.04E-08
chr6	36841001	36846000	1.34E-22
chr6	36842001	36847000	1.31E-35
chr6	37133001	37138000	1.10E-14
chr6	37134001	37139000	4.26E-43
chr6	37135001	37140000	1.62E-19
chr6	37138001	37143000	1.77E-16

chr6	37465001	37470000	9.24E-15
chr6	37782001	37787000	5.40E-22
chr6	37783001	37788000	2.85E-35
chr6	37784001	37789000	2.84E-34
chr6	37785001	37790000	8.79E-38
chr6	37787001	37792000	4.71E-43
chr6	38352001	38357000	2.30E-09
chr6	38353001	38358000	1.28E-09
chr6	38497001	38502000	2.51E-07
chr6	38518001	38523000	1.20E-06
chr6	38521001	38526000	1.00E-06
chr6	38538001	38543000	4.68E-07
chr6	38603001	38608000	5.60E-61
chr6	38604001	38609000	1.18E-44
chr6	38605001	38610000	3.23E-40
chr6	38607001	38612000	3.27E-56
chr6	38608001	38613000	9.83E-21
chr6	38625001	38630000	3.04E-06
chr6	38666001	38671000	2.02E-32
chr6	38667001	38672000	7.12E-27
chr6	38668001	38673000	5.16E-22
chr6	38669001	38674000	2.60E-25
chr6	38670001	38675000	6.64E-24
chr6	38684001	38689000	6.29E-05
chr6	38685001	38690000	0.000148236
chr6	38724001	38729000	2.11E-10
chr6	38725001	38730000	4.10E-07
chr6	38727001	38732000	7.44E-08
chr6	38728001	38733000	4.79E-07
chr6	38729001	38734000	3.33E-06
chr6	38766001	38771000	2.65E-07
chr6	38767001	38772000	2.85E-05
chr6	38768001	38773000	1.86E-05
chr6	38769001	38774000	0.000439452
chr6	38808001	38813000	2.77E-07
chr6	38821001	38826000	9.79E-06
chr6	38822001	38827000	0.000342911
chr6	38823001	38828000	8.29E-07
chr6	38838001	38843000	4.56E-19
chr6	38839001	38844000	2.67E-22
chr6	38840001	38845000	2.75E-27
chr6	38841001	38846000	5.00E-19
chr6	38854001	38859000	1.72E-10
chr6	38869001	38874000	3.53E-09
chr6	38870001	38875000	2.66E-13
chr6	38871001	38876000	1.40E-11
chr6	38872001	38877000	1.31E-07
chr6	38878001	38883000	2.21E-05

chr6	38879001	38884000	3.66E-08
chr6	38880001	38885000	1.37E-05
chr6	38881001	38886000	4.53E-06
chr6	38882001	38887000	6.32E-06
chr6	38883001	38888000	0.000610249
chr6	38896001	38901000	2.03E-08
chr6	38937001	38942000	1.05E-07
chr6	39012001	39017000	3.79E-06
chr6	39013001	39018000	8.88E-06
chr6	39078001	39083000	1.98E-73
chr6	39079001	39084000	1.58E-60
chr6	39083001	39088000	7.40E-12
chr6	39085001	39090000	2.82E-11
chr6	39086001	39091000	4.30E-16
chr6	39087001	39092000	1.48E-13
chr6	39088001	39093000	2.56E-13
chr6	39089001	39094000	2.16E-12
chr6	39281001	39286000	8.30E-07
chr6	39282001	39287000	0.000514881
chr6	39300001	39305000	1.54E-05
chr6	39306001	39311000	4.53E-11
chr6	39319001	39324000	0.005722707
chr6	39440001	39445000	8.84E-05
chr6	39469001	39474000	8.10E-07
chr6	39470001	39475000	4.19E-07
chr6	39471001	39476000	1.07E-06
chr6	39472001	39477000	7.77E-06
chr6	39517001	39522000	1.96E-09
chr6	39532001	39537000	7.90E-11
chr6	39533001	39538000	2.38E-17
chr6	39534001	39539000	7.87E-15
chr6	39535001	39540000	8.68E-16
chr6	39536001	39541000	3.40E-12
chr6	39554001	39559000	5.60E-15
chr6	39555001	39560000	5.41E-15
chr6	39556001	39561000	1.74E-13
chr6	39587001	39592000	2.22E-08
chr6	39588001	39593000	6.23E-08
chr6	39589001	39594000	8.67E-08
chr6	39593001	39598000	0.001247373
chr6	39608001	39613000	6.58E-10
chr6	39609001	39614000	3.71E-08
chr6	39610001	39615000	1.82E-06
chr6	39618001	39623000	1.69E-06
chr6	39648001	39653000	2.74E-05
chr6	39649001	39654000	0.00011461
chr6	39650001	39655000	0.000170917
chr6	39651001	39656000	7.19E-07

chr6	39654001	39659000	9.57E-06
chr6	39660001	39665000	0.000119904
chr6	39661001	39666000	0.000308027
chr6	39670001	39675000	4.71E-05
chr6	39681001	39686000	0.000815829
chr6	39682001	39687000	2.46E-05
chr6	39683001	39688000	1.58E-07
chr6	39684001	39689000	0.000270951
chr6	39685001	39690000	0.000129616
chr6	39694001	39699000	0.002146687
chr6	39706001	39711000	1.77E-10
chr6	39707001	39712000	1.42E-10
chr6	39708001	39713000	7.03E-10
chr6	39713001	39718000	2.73E-06
chr6	39714001	39719000	2.97E-06
chr6	39715001	39720000	1.68E-08
chr6	39726001	39731000	1.31E-06
chr6	39727001	39732000	1.76E-08
chr6	39728001	39733000	6.29E-08
chr6	39763001	39768000	8.93E-07
chr6	39804001	39809000	1.44E-06
chr6	40000001	40005000	9.09E-09
chr6	40143001	40148000	4.89E-05
chr6	40144001	40149000	9.14E-07
chr6	40145001	40150000	6.68E-05
chr6	40551001	40556000	1.85E-07
chr6	41041001	41046000	5.41E-49
chr6	41263001	41268000	1.70E-07
chr6	41510001	41515000	1.14E-36
chr6	41511001	41516000	2.86E-12
chr6	41524001	41529000	8.43E-13
chr6	41525001	41530000	7.45E-16
chr6	41697001	41702000	5.86E-09
chr6	41698001	41703000	9.11E-10
chr6	41909001	41914000	3.67E-09
chr6	42527001	42532000	2.77E-15
chr6	42629001	42634000	4.48E-20
chr6	42713001	42718000	3.80E-12
chr6	42714001	42719000	8.53E-10
chr6	42942001	42947000	6.17E-08
chr6	42945001	42950000	3.46E-25
chr6	42952001	42957000	2.20E-08
chr6	43134001	43139000	1.34E-08
chr6	43135001	43140000	4.74E-21
chr6	43240001	43245000	0.000180613
chr6	43395001	43400000	1.93E-23
chr6	43539001	43544000	5.14E-37
chr6	44037001	44042000	3.88E-20

chr6	44038001	44043000	1.59E-16
chr6	44039001	44044000	2.93E-09
chr6	44265001	44270000	0.000238521
chr6	44281001	44286000	1.54E-14
chr6	44354001	44359000	1.32E-22
chr6	44355001	44360000	1.06E-21
chr6	44888001	44893000	6.88E-05
chr6	44901001	44906000	0.000652008
chr6	45286001	45291000	0.004606088
chr6	45287001	45292000	0.008674188
chr6	45288001	45293000	0.006160735
chr6	45290001	45295000	0.003396871
chr6	45341001	45346000	3.89E-56
chr6	45383001	45388000	7.73E-32
chr6	45390001	45395000	2.23E-63
chr6	45391001	45396000	2.32E-48
chr6	45392001	45397000	2.27E-20
chr6	45848001	45853000	0.000100738
chr6	45858001	45863000	0.00025856
chr6	45859001	45864000	0.000111049
chr6	45864001	45869000	6.76E-10
chr6	45961001	45966000	0.001718861
chr6	45962001	45967000	0.000568257
chr6	45979001	45984000	2.85E-38
chr6	45980001	45985000	7.51E-56
chr6	45981001	45986000	4.52E-52
chr6	45983001	45988000	2.00E-49
chr6	46093001	46098000	9.61E-23
chr6	46097001	46102000	3.26E-27
chr6	46098001	46103000	1.94E-15
chr6	46258001	46263000	0.000513983
chr6	46259001	46264000	0.000131717
chr6	46422001	46427000	0.000692299
chr6	46448001	46453000	1.01E-13
chr6	46449001	46454000	3.87E-12
chr6	46450001	46455000	8.60E-10
chr6	46451001	46456000	7.83E-08
chr6	46452001	46457000	1.47E-05
chr6	46454001	46459000	6.12E-22
chr6	46455001	46460000	6.01E-37
chr6	46458001	46463000	1.46E-37
chr6	46459001	46464000	1.73E-43
chr6	46483001	46488000	0.002879031
chr6	46510001	46515000	2.97E-05
chr6	46511001	46516000	3.20E-07
chr6	46512001	46517000	2.62E-08
chr6	46513001	46518000	1.48E-06
chr6	46560001	46565000	0.000176405

chr6	46561001	46566000	6.73E-06
chr6	46569001	46574000	0.000233744
chr6	46611001	46616000	6.75E-05
chr6	46612001	46617000	3.26E-07
chr6	46621001	46626000	2.47E-29
chr6	46671001	46676000	3.48E-05
chr6	46672001	46677000	8.75E-05
chr6	46673001	46678000	0.000293946
chr6	46696001	46701000	6.86E-07
chr6	46698001	46703000	4.77E-33
chr6	46699001	46704000	9.72E-47
chr6	46700001	46705000	4.56E-33
chr6	46701001	46706000	1.08E-26
chr6	46702001	46707000	8.82E-32
chr6	46703001	46708000	3.86E-39
chr6	47030001	47035000	6.38E-06
chr6	47272001	47277000	1.01E-34
chr6	47277001	47282000	7.56E-44
chr6	47736001	47741000	0.00196749
chr6	47980001	47985000	0.000585449
chr6	48032001	48037000	1.15E-16
chr6	48034001	48039000	1.87E-24
chr6	48035001	48040000	3.57E-25
chr6	48036001	48041000	7.33E-23
chr6	48417001	48422000	0.000238694
chr6	48673001	48678000	7.62E-18
chr6	48676001	48681000	0.022874457
chr6	48677001	48682000	0.029130049
chr6	49430001	49435000	6.78E-25
chr6	49431001	49436000	3.53E-26
chr6	49677001	49682000	1.99E-10
chr6	49678001	49683000	1.10E-09
chr6	49679001	49684000	1.89E-08
chr6	49690001	49695000	0.001680412
chr6	49691001	49696000	0.003606973
chr6	50010001	50015000	3.45E-05
chr6	50014001	50019000	0.000274514
chr6	50198001	50203000	4.68E-06
chr6	50199001	50204000	1.40E-05
chr6	50393001	50398000	0.000172244
chr6	50394001	50399000	3.23E-05
chr6	50395001	50400000	3.71E-07
chr6	50396001	50401000	5.10E-11
chr6	51040001	51045000	9.09E-06
chr6	51360001	51365000	0.000129058
chr6	51414001	51419000	7.26E-11
chr6	51415001	51420000	1.21E-10
chr6	51416001	51421000	2.14E-11

chr6	51417001	51422000	1.87E-11
chr6	51418001	51423000	1.33E-09
chr6	51630001	51635000	0.001980187
chr6	51778001	51783000	0.000259015
chr6	51779001	51784000	3.17E-05
chr6	51814001	51819000	0.002391565
chr6	51836001	51841000	0.000892185
chr6	52145001	52150000	1.84E-30
chr6	52222001	52227000	2.53E-22
chr6	52349001	52354000	0.006955126
chr6	52441001	52446000	1.18E-46
chr6	52442001	52447000	5.67E-42
chr6	52531001	52536000	5.30E-55
chr6	52532001	52537000	1.20E-63
chr6	52533001	52538000	9.21E-53
chr6	52534001	52539000	3.02E-45
chr6	52535001	52540000	5.12E-33
chr6	52842001	52847000	0.014607276
chr6	53208001	53213000	1.93E-19
chr6	53209001	53214000	1.35E-59
chr6	53213001	53218000	4.17E-49
chr6	53300001	53305000	3.14E-06
chr6	53301001	53306000	1.16E-05
chr6	53405001	53410000	7.08E-58
chr6	53406001	53411000	4.59E-84
chr6	53407001	53412000	1.13E-68
chr6	53408001	53413000	2.61E-58
chr6	53409001	53414000	3.27E-57
chr6	53512001	53517000	4.75E-71
chr6	53515001	53520000	1.90E-65
chr6	53516001	53521000	3.45E-58
chr6	53517001	53522000	1.11E-30
chr6	54174001	54179000	0.003787846
chr6	54175001	54180000	0.003080813
chr6	54445001	54450000	0.002994586
chr6	54661001	54666000	0.001360861
chr6	55682001	55687000	0.002976627
chr6	56406001	56411000	6.60E-43
chr6	56407001	56412000	5.24E-49
chr6	56760001	56765000	2.76E-08
chr6	56950001	56955000	2.80E-55
chr6	56951001	56956000	2.88E-61
chr6	56952001	56957000	1.32E-60
chr6	56953001	56958000	3.57E-86
chr6	56954001	56959000	7.91E-76
chr6	56955001	56960000	8.23E-48
chr6	57032001	57037000	1.70E-09
chr6	57033001	57038000	7.34E-147

chr6	57034001	57039000	1.69E-121
chr6	57035001	57040000	9.28E-112
chr6	57036001	57041000	5.62E-81
chr6	57082001	57087000	2.21E-55
chr6	57577001	57582000	0.002672668
chr6	57578001	57583000	2.64E-07
chr6	57579001	57584000	4.05E-08
chr6	57580001	57585000	5.46E-06
chr6	57581001	57586000	5.35E-12
chr6	57582001	57587000	5.53E-09
chr6	57583001	57588000	8.94E-08
chr6	57584001	57589000	1.84E-06
chr6	57597001	57602000	2.40E-11
chr6	57598001	57603000	1.05E-15
chr6	57652001	57657000	0.00098754
chr6	57684001	57689000	2.60E-07
chr6	57685001	57690000	3.69E-07
chr6	57686001	57691000	6.55E-07
chr6	57703001	57708000	0.000107235
chr6	57729001	57734000	0.000157158
chr6	57730001	57735000	5.21E-05
chr6	57731001	57736000	2.33E-06
chr6	57733001	57738000	0.001123152
chr6	57818001	57823000	9.33E-07
chr6	57913001	57918000	0.002639808
chr6	57948001	57953000	0.00089818
chr6	58011001	58016000	9.57E-06
chr6	58207001	58212000	5.88E-06
chr6	58210001	58215000	7.19E-08
chr6	58211001	58216000	8.77E-08
chr6	58212001	58217000	0.000156527
chr6	58312001	58317000	0.000449643
chr6	58313001	58318000	0.000498216
chr6	58358001	58363000	1.17E-05
chr6	58361001	58366000	1.20E-08
chr6	58362001	58367000	1.40E-10
chr6	58363001	58368000	2.87E-09
chr6	58371001	58376000	3.10E-05
chr6	58372001	58377000	2.23E-05
chr6	58699001	58704000	2.75E-06
chr6	58700001	58705000	1.23E-09
chr6	58701001	58706000	2.44E-09
chr6	58747001	58752000	0.006933923
chr6	58748001	58753000	0.007326483
chr6	58749001	58754000	0.00394716
chr6	58750001	58755000	0.008573672
chr6	58768001	58773000	0.002733344
chr6	61882001	61887000	3.68E-06

chr6	61883001	61888000	1.64E-05
chr6	62186001	62191000	2.65E-08
chr6	62187001	62192000	4.06E-06
chr6	62188001	62193000	0.000258797
chr6	62196001	62201000	0.002525628
chr6	62280001	62285000	8.97E-10
chr6	62281001	62286000	6.55E-10
chr6	62282001	62287000	1.58E-12
chr6	62283001	62288000	8.96E-12
chr6	62463001	62468000	2.67E-05
chr6	62529001	62534000	0.01165996
chr6	62654001	62659000	0.003501553
chr6	62847001	62852000	1.25E-08
chr6	62848001	62853000	0.005888248
chr6	62849001	62854000	0.000206436
chr6	62850001	62855000	0.001676533
chr6	62912001	62917000	0.00019028
chr6	62913001	62918000	0.000188993
chr6	62965001	62970000	0.000198313
chr6	62966001	62971000	0.000268959
chr6	62977001	62982000	0.002413709
chr6	63249001	63254000	3.06E-06
chr6	63315001	63320000	0.00016435
chr6	63383001	63388000	0.000895042
chr6	63384001	63389000	0.001988986
chr6	63400001	63405000	1.73E-08
chr6	63420001	63425000	0.001240891
chr6	63493001	63498000	0.003462203
chr6	63522001	63527000	1.89E-05
chr6	63523001	63528000	1.52E-08
chr6	63530001	63535000	0.00057075
chr6	63613001	63618000	1.45E-05
chr6	63694001	63699000	0.002115764
chr6	63695001	63700000	0.000127389
chr6	63818001	63823000	2.90E-05
chr6	63819001	63824000	1.44E-06
chr6	63820001	63825000	1.56E-06
chr6	63833001	63838000	6.48E-05
chr6	63834001	63839000	6.29E-06
chr6	63835001	63840000	8.84E-06
chr6	63841001	63846000	2.81E-08
chr6	63842001	63847000	2.73E-07
chr6	63844001	63849000	1.47E-06
chr6	63845001	63850000	1.16E-06
chr6	63855001	63860000	0.000115508
chr6	63857001	63862000	1.60E-05
chr6	63904001	63909000	6.49E-05
chr6	63905001	63910000	0.000776083

chr6	63976001	63981000	0.002147298
chr6	64283001	64288000	5.52E-56
chr6	64341001	64346000	1.32E-33
chr6	64618001	64623000	0.001223812
chr6	64883001	64888000	2.50E-06
chr6	64972001	64977000	0.000729532
chr6	65419001	65424000	0.003888159
chr6	65420001	65425000	0.002538166
chr6	66933001	66938000	0.008577389
chr6	66934001	66939000	0.011101152
chr6	67232001	67237000	0.009448342
chr6	67586001	67591000	0.000630053
chr6	67587001	67592000	8.96E-05
chr6	67588001	67593000	0.000140453
chr6	67692001	67697000	1.74E-06
chr6	68434001	68439000	8.33E-05
chr6	68436001	68441000	0.000170831
chr6	69141001	69146000	0.005559147
chr6	69144001	69149000	0.0025646
chr6	69264001	69269000	0.010293513
chr6	69567001	69572000	0.003539895
chr6	70329001	70334000	0.000582158
chr6	70339001	70344000	3.51E-06
chr6	70352001	70357000	0.000394191
chr6	70502001	70507000	1.02E-33
chr6	70503001	70508000	1.88E-32
chr6	70504001	70509000	4.34E-36
chr6	70505001	70510000	4.11E-29
chr6	70506001	70511000	6.12E-25
chr6	70553001	70558000	0.001685929
chr6	70554001	70559000	0.000484585
chr6	70589001	70594000	0.001485116
chr6	70630001	70635000	7.75E-06
chr6	70631001	70636000	4.51E-05
chr6	70663001	70668000	0.001755274
chr6	70689001	70694000	7.56E-12
chr6	70714001	70719000	5.74E-07
chr6	70715001	70720000	2.70E-06
chr6	70730001	70735000	0.000487371
chr6	70775001	70780000	6.81E-06
chr6	70841001	70846000	1.94E-08
chr6	70842001	70847000	2.19E-11
chr6	70844001	70849000	1.34E-05
chr6	70845001	70850000	2.43E-05
chr6	70907001	70912000	9.11E-07
chr6	70962001	70967000	0.006766526
chr6	70971001	70976000	0.002771978
chr6	70972001	70977000	0.001216945

chr6	70980001	70985000	3.75E-07
chr6	70981001	70986000	0.000904348
chr6	70982001	70987000	0.002180573
chr6	70983001	70988000	0.001179194
chr6	71065001	71070000	1.45E-05
chr6	71272001	71277000	2.28E-26
chr6	71373001	71378000	1.16E-32
chr6	71374001	71379000	1.41E-34
chr6	71375001	71380000	4.09E-47
chr6	71376001	71381000	1.76E-46
chr6	71377001	71382000	4.90E-50
chr6	71624001	71629000	5.80E-09
chr6	71661001	71666000	3.29E-35
chr6	71662001	71667000	9.86E-36
chr6	71663001	71668000	4.51E-33
chr6	71664001	71669000	1.07E-28
chr6	71665001	71670000	2.79E-28
chr6	71666001	71671000	4.55E-35
chr6	71994001	71999000	5.20E-48
chr6	71995001	72000000	1.40E-36
chr6	71996001	72001000	1.05E-26
chr6	71998001	72003000	9.16E-46
chr6	72128001	72133000	6.54E-56
chr6	72129001	72134000	5.01E-85
chr6	72130001	72135000	5.44E-120
chr6	72131001	72136000	2.05E-08
chr6	72132001	72137000	1.45E-06
chr6	72164001	72169000	0.000847976
chr6	72165001	72170000	0.001092851
chr6	72376001	72381000	9.84E-05
chr6	72378001	72383000	7.66E-07
chr6	72505001	72510000	0.001790446
chr6	72506001	72511000	1.22E-06
chr6	72507001	72512000	2.94E-06
chr6	72508001	72513000	1.46E-07
chr6	72550001	72555000	0.000880664
chr6	72551001	72556000	0.001651419
chr6	72553001	72558000	0.004281279
chr6	72584001	72589000	0.01042079
chr6	72586001	72591000	0.01046831
chr6	72592001	72597000	2.57E-36
chr6	72593001	72598000	5.14E-25
chr6	72729001	72734000	0.011616451
chr6	72730001	72735000	0.011046142
chr6	72824001	72829000	1.20E-05
chr6	73161001	73166000	0.000101892
chr6	73197001	73202000	0.002230702
chr6	73198001	73203000	1.43E-07

chr6	73199001	73204000	1.44E-07
chr6	73200001	73205000	2.36E-07
chr6	73201001	73206000	1.26E-07
chr6	73203001	73208000	0.004053729
chr6	73291001	73296000	9.77E-14
chr6	73293001	73298000	1.99E-10
chr6	73299001	73304000	0.000159448
chr6	73308001	73313000	1.90E-13
chr6	73309001	73314000	9.13E-13
chr6	73310001	73315000	3.36E-11
chr6	73311001	73316000	6.59E-14
chr6	73312001	73317000	1.72E-10
chr6	73331001	73336000	2.53E-24
chr6	73332001	73337000	3.99E-24
chr6	73365001	73370000	2.28E-08
chr6	73428001	73433000	2.26E-06
chr6	73538001	73543000	2.70E-08
chr6	73540001	73545000	8.22E-10
chr6	73541001	73546000	2.07E-07
chr6	73598001	73603000	0.000210414
chr6	73599001	73604000	0.00035836
chr6	73628001	73633000	2.59E-06
chr6	73629001	73634000	2.00E-06
chr6	73651001	73656000	0.000109101
chr6	73734001	73739000	3.60E-05
chr6	73746001	73751000	8.10E-06
chr6	73781001	73786000	5.05E-07
chr6	73782001	73787000	2.23E-06
chr6	73783001	73788000	1.35E-07
chr6	73784001	73789000	8.87E-07
chr6	73785001	73790000	6.10E-06
chr6	73807001	73812000	0.000437001
chr6	73810001	73815000	9.94E-05
chr6	73814001	73819000	0.010152696
chr6	73815001	73820000	0.008196181
chr6	73826001	73831000	0.000291244
chr6	73827001	73832000	1.63E-09
chr6	73828001	73833000	1.45E-08
chr6	73829001	73834000	1.03E-05
chr6	73830001	73835000	9.30E-06
chr6	73834001	73839000	1.28E-05
chr6	73854001	73859000	0.002061455
chr6	73879001	73884000	0.008322625
chr6	74157001	74162000	1.74E-16
chr6	74159001	74164000	1.10E-11
chr6	74160001	74165000	1.26E-12
chr6	74233001	74238000	4.02E-16
chr6	74363001	74368000	9.57E-20

chr6	74401001	74406000	2.83E-24
chr6	74402001	74407000	1.19E-50
chr6	74403001	74408000	2.20E-51
chr6	74404001	74409000	7.28E-59
chr6	74405001	74410000	7.05E-54
chr6	74406001	74411000	9.06E-27
chr6	74432001	74437000	1.78E-06
chr6	74501001	74506000	8.92E-05
chr6	74629001	74634000	6.21E-06
chr6	74630001	74635000	0.000127367
chr6	74676001	74681000	2.12E-05
chr6	74677001	74682000	1.75E-05
chr6	74683001	74688000	4.68E-07
chr6	74684001	74689000	1.30E-05
chr6	74685001	74690000	0.0001545
chr6	74694001	74699000	0.000798029
chr6	74701001	74706000	0.00022177
chr6	74741001	74746000	1.75E-17
chr6	74743001	74748000	4.30E-13
chr6	74744001	74749000	9.42E-10
chr6	74747001	74752000	6.58E-08
chr6	74748001	74753000	5.29E-08
chr6	74749001	74754000	2.89E-07
chr6	74750001	74755000	9.08E-07
chr6	74870001	74875000	0.001180706
chr6	74933001	74938000	3.10E-07
chr6	74934001	74939000	3.35E-11
chr6	74935001	74940000	1.39E-07
chr6	74936001	74941000	2.27E-06
chr6	74943001	74948000	0.001998867
chr6	74944001	74949000	0.000889858
chr6	74945001	74950000	4.30E-05
chr6	74946001	74951000	0.000276238
chr6	74947001	74952000	0.000409988
chr6	74948001	74953000	7.90E-06
chr6	74971001	74976000	0.009381647
chr6	74973001	74978000	0.000231123
chr6	75057001	75062000	1.91E-06
chr6	75058001	75063000	4.17E-05
chr6	75059001	75064000	1.10E-06
chr6	75091001	75096000	0.000409713
chr6	75110001	75115000	3.24E-05
chr6	75111001	75116000	4.02E-05
chr6	75123001	75128000	0.001613497
chr6	75124001	75129000	0.001357117
chr6	75125001	75130000	0.002725119
chr6	75126001	75131000	0.0036262
chr6	75134001	75139000	0.00062195

chr6	75135001	75140000	7.77E-05
chr6	75136001	75141000	6.64E-05
chr6	75137001	75142000	0.00046843
chr6	75138001	75143000	0.002686435
chr6	75146001	75151000	6.30E-05
chr6	75169001	75174000	0.001345489
chr6	75190001	75195000	1.55E-12
chr6	75245001	75250000	3.18E-06
chr6	75301001	75306000	0.004097303
chr6	75379001	75384000	2.04E-05
chr6	75436001	75441000	3.04E-06
chr6	75455001	75460000	0.000108604
chr6	75585001	75590000	6.24E-05
chr6	75586001	75591000	0.001595911
chr6	75659001	75664000	0.003325261
chr6	75660001	75665000	0.0002558
chr6	75661001	75666000	4.35E-05
chr6	75662001	75667000	3.96E-05
chr6	75778001	75783000	0.001659595
chr6	75782001	75787000	0.018197992
chr6	75783001	75788000	5.71E-07
chr6	75784001	75789000	1.60E-07
chr6	75785001	75790000	2.53E-07
chr6	75786001	75791000	6.34E-06
chr6	75787001	75792000	6.34E-06
chr6	75863001	75868000	0.001430498
chr6	75864001	75869000	0.001155127
chr6	75953001	75958000	1.54E-24
chr6	76163001	76168000	0.003300956
chr6	76165001	76170000	0.000200267
chr6	76166001	76171000	0.000783917
chr6	76167001	76172000	0.002560721
chr6	76184001	76189000	6.46E-05
chr6	76209001	76214000	8.81E-06
chr6	76250001	76255000	5.60E-07
chr6	76269001	76274000	0.016841655
chr6	76307001	76312000	9.18E-13
chr6	76311001	76316000	5.43E-46
chr6	76459001	76464000	2.04E-21
chr6	76912001	76917000	0.003138739
chr6	77023001	77028000	0.002542504
chr6	77758001	77763000	4.66E-07
chr6	78014001	78019000	0.020974331
chr6	78015001	78020000	0.025286226
chr6	78016001	78021000	0.029423211
chr6	78200001	78205000	5.61E-05
chr6	78287001	78292000	0.012627084
chr6	78327001	78332000	0.007102839

chr6	78328001	78333000	0.002460503
chr6	78329001	78334000	0.003140182
chr6	78478001	78483000	3.22E-05
chr6	78480001	78485000	0.000151347
chr6	78494001	78499000	0.000527333
chr6	78581001	78586000	0.000136867
chr6	78582001	78587000	8.43E-07
chr6	78583001	78588000	2.55E-05
chr6	78634001	78639000	0.000408412
chr6	78661001	78666000	5.38E-05
chr6	78672001	78677000	0.010015862
chr6	78699001	78704000	5.79E-11
chr6	78700001	78705000	2.62E-10
chr6	78701001	78706000	1.12E-14
chr6	78763001	78768000	0.000733834
chr6	78810001	78815000	0.000333333
chr6	78850001	78855000	0.000533436
chr6	78851001	78856000	0.002576433
chr6	78852001	78857000	0.001022078
chr6	78854001	78859000	3.99E-05
chr6	78855001	78860000	0.00028002
chr6	78897001	78902000	0.000361572
chr6	78960001	78965000	0.007367383
chr6	78997001	79002000	0.000887191
chr6	78998001	79003000	0.000500172
chr6	78999001	79004000	0.000277499
chr6	79320001	79325000	0.004994441
chr6	79382001	79387000	2.93E-05
chr6	79413001	79418000	7.93E-07
chr6	79414001	79419000	1.44E-07
chr6	79415001	79420000	3.67E-06
chr6	79427001	79432000	0.0055681
chr6	79428001	79433000	0.002082998
chr6	79521001	79526000	2.45E-06
chr6	79782001	79787000	3.96E-21
chr6	79783001	79788000	7.87E-72
chr6	79787001	79792000	1.59E-52
chr6	79788001	79793000	1.21E-48
chr6	79939001	79944000	1.77E-29
chr6	79940001	79945000	6.72E-50
chr6	79941001	79946000	6.63E-52
chr6	79942001	79947000	1.51E-48
chr6	79943001	79948000	1.58E-43
chr6	79944001	79949000	8.51E-40
chr6	80242001	80247000	4.11E-38
chr6	80336001	80341000	5.75E-23
chr6	80337001	80342000	2.80E-44
chr6	80577001	80582000	3.30E-11

chr6	80579001	80584000	9.96E-12
chr6	80652001	80657000	2.04E-39
chr6	80709001	80714000	1.90E-09
chr6	80710001	80715000	3.29E-34
chr6	80714001	80719000	1.78E-32
chr6	80816001	80821000	1.09E-29
chr6	80854001	80859000	0.001448916
chr6	81073001	81078000	0.000868748
chr6	81075001	81080000	0.002825955
chr6	81146001	81151000	0.002841124
chr6	81147001	81152000	0.001763645
chr6	81149001	81154000	0.003831114
chr6	81150001	81155000	0.002483284
chr6	81330001	81335000	0.024838358
chr6	81366001	81371000	0.002603119
chr6	81502001	81507000	0.000276034
chr6	81503001	81508000	7.65E-06
chr6	81505001	81510000	8.15E-05
chr6	81506001	81511000	0.000237456
chr6	81509001	81514000	5.30E-05
chr6	81510001	81515000	8.82E-05
chr6	81511001	81516000	9.09E-05
chr6	81550001	81555000	1.27E-05
chr6	81551001	81556000	1.20E-05
chr6	81552001	81557000	0.000164684
chr6	81673001	81678000	0.011101152
chr6	81674001	81679000	2.50E-05
chr6	81675001	81680000	4.45E-05
chr6	81818001	81823000	1.08E-06
chr6	81819001	81824000	3.05E-07
chr6	81841001	81846000	0.010198474
chr6	81927001	81932000	0.00250263
chr6	82001001	82006000	0.009598352
chr6	82029001	82034000	0.007705363
chr6	82030001	82035000	0.000660209
chr6	82031001	82036000	0.001007403
chr6	82032001	82037000	0.002413017
chr6	82463001	82468000	1.45E-55
chr6	82530001	82535000	0.001307777
chr6	82551001	82556000	2.35E-05
chr6	82651001	82656000	0.001815102
chr6	83069001	83074000	2.95E-39
chr6	83070001	83075000	3.79E-45
chr6	83072001	83077000	1.04E-58
chr6	83073001	83078000	1.89E-48
chr6	83074001	83079000	2.21E-51
chr6	83075001	83080000	1.42E-42
chr6	83777001	83782000	1.62E-59

chr6	83899001	83904000	2.85E-24
chr6	83902001	83907000	2.73E-21
chr6	83903001	83908000	2.58E-21
chr6	84039001	84044000	2.33E-08
chr6	84040001	84045000	2.83E-10
chr6	84041001	84046000	1.19E-09
chr6	84042001	84047000	3.87E-10
chr6	84043001	84048000	2.06E-10
chr6	84136001	84141000	2.98E-26
chr6	84217001	84222000	2.63E-11
chr6	84218001	84223000	2.25E-11
chr6	84219001	84224000	5.10E-19
chr6	84220001	84225000	5.99E-16
chr6	84266001	84271000	0.017578117
chr6	84739001	84744000	2.37E-25
chr6	84740001	84745000	2.66E-23
chr6	84741001	84746000	7.53E-27
chr6	84742001	84747000	1.72E-35
chr6	84743001	84748000	4.55E-23
chr6	84807001	84812000	2.46E-05
chr6	85173001	85178000	0.000296147
chr6	85272001	85277000	3.87E-05
chr6	85404001	85409000	0.000120039
chr6	85850001	85855000	0.00346273
chr6	85851001	85856000	0.008371805
chr6	85953001	85958000	4.44E-06
chr6	85954001	85959000	0.001122267
chr6	86010001	86015000	3.52E-11
chr6	86011001	86016000	6.80E-09
chr6	86155001	86160000	5.48E-67
chr6	86299001	86304000	1.21E-37
chr6	86352001	86357000	2.23E-35
chr6	86353001	86358000	1.48E-29
chr6	86388001	86393000	3.55E-26
chr6	86480001	86485000	0.002388761
chr6	86481001	86486000	0.001052369
chr6	86487001	86492000	3.56E-05
chr6	86488001	86493000	2.07E-07
chr6	86489001	86494000	0.000850094
chr6	86516001	86521000	4.74E-06
chr6	86517001	86522000	0.000167727
chr6	86518001	86523000	0.000438024
chr6	86582001	86587000	2.65E-05
chr6	86595001	86600000	5.49E-06
chr6	86596001	86601000	3.40E-07
chr6	86597001	86602000	3.95E-10
chr6	86598001	86603000	6.43E-15
chr6	86599001	86604000	5.34E-16

chr6	86600001	86605000	4.02E-07
chr6	86601001	86606000	7.47E-06
chr6	86602001	86607000	2.27E-08
chr6	86608001	86613000	0.001198862
chr6	86609001	86614000	4.20E-05
chr6	86610001	86615000	2.46E-05
chr6	86611001	86616000	4.60E-08
chr6	86612001	86617000	5.28E-08
chr6	86630001	86635000	1.14E-05
chr6	86646001	86651000	7.02E-10
chr6	86647001	86652000	0.001847753
chr6	86648001	86653000	0.000449559
chr6	86659001	86664000	7.58E-05
chr6	86666001	86671000	3.68E-06
chr6	86667001	86672000	8.99E-07
chr6	86699001	86704000	5.73E-05
chr6	86701001	86706000	7.13E-05
chr6	86702001	86707000	0.000114518
chr6	86703001	86708000	9.35E-06
chr6	86715001	86720000	5.77E-18
chr6	86716001	86721000	4.23E-18
chr6	86717001	86722000	2.58E-08
chr6	86739001	86744000	3.62E-09
chr6	86740001	86745000	9.79E-09
chr6	86767001	86772000	6.24E-10
chr6	86773001	86778000	4.94E-12
chr6	86774001	86779000	2.09E-10
chr6	86775001	86780000	8.86E-10
chr6	86798001	86803000	2.95E-06
chr6	86799001	86804000	1.55E-06
chr6	86800001	86805000	1.30E-09
chr6	86801001	86806000	6.04E-09
chr6	86956001	86961000	0.001105768
chr6	86957001	86962000	8.53E-08
chr6	86958001	86963000	1.60E-07
chr6	86959001	86964000	5.03E-08
chr6	86960001	86965000	2.05E-08
chr6	86961001	86966000	9.04E-07
chr6	87043001	87048000	0.001945587
chr6	87044001	87049000	0.0018215
chr6	87045001	87050000	0.001792242
chr6	87046001	87051000	5.07E-05
chr6	87052001	87057000	0.000541961
chr6	87053001	87058000	0.000107553
chr6	87054001	87059000	0.000449297
chr6	87074001	87079000	0.00093527
chr6	87075001	87080000	7.99E-08
chr6	87076001	87081000	0.000592008

chr6	87130001	87135000	0.013638647
chr6	87131001	87136000	0.004780399
chr6	87228001	87233000	0.000110013
chr6	87229001	87234000	0.000172742
chr6	87230001	87235000	0.002227341
chr6	87231001	87236000	7.92E-05
chr6	87234001	87239000	0.001921191
chr6	87258001	87263000	1.32E-06
chr6	87284001	87289000	0.000189555
chr6	87398001	87403000	0.001020698
chr6	87518001	87523000	8.58E-13
chr6	87519001	87524000	1.58E-17
chr6	87520001	87525000	1.06E-14
chr6	87521001	87526000	3.57E-16
chr6	87522001	87527000	1.33E-06
chr6	87554001	87559000	1.74E-06
chr6	87586001	87591000	1.75E-07
chr6	87587001	87592000	5.91E-05
chr6	87616001	87621000	7.26E-08
chr6	87857001	87862000	2.79E-31
chr6	87858001	87863000	3.26E-63
chr6	87859001	87864000	7.63E-68
chr6	88028001	88033000	6.35E-42
chr6	88029001	88034000	3.66E-40
chr6	88030001	88035000	9.91E-41
chr6	88031001	88036000	8.44E-49
chr6	88032001	88037000	1.19E-36
chr6	88113001	88118000	1.72E-38
chr6	88114001	88119000	3.94E-47
chr6	88115001	88120000	3.22E-67
chr6	88116001	88121000	1.06E-61
chr6	88117001	88122000	1.34E-54
chr6	88118001	88123000	1.95E-16
chr6	88295001	88300000	1.46E-32
chr6	88297001	88302000	9.43E-29
chr6	88298001	88303000	3.55E-28
chr6	88299001	88304000	2.54E-41
chr6	89335001	89340000	4.85E-05
chr6	89336001	89341000	9.65E-06
chr6	89668001	89673000	1.39E-09
chr6	89669001	89674000	2.93E-23
chr6	89670001	89675000	5.98E-20
chr6	89671001	89676000	4.70E-16
chr6	89672001	89677000	5.87E-10
chr6	89673001	89678000	1.06E-11
chr6	89823001	89828000	7.65E-24
chr6	89827001	89832000	1.86E-15
chr6	89856001	89861000	1.95E-32

chr6	90117001	90122000	2.34E-39
chr6	90118001	90123000	2.64E-20
chr6	90120001	90125000	3.60E-23
chr6	90121001	90126000	1.99E-21
chr6	90122001	90127000	7.66E-17
chr6	90139001	90144000	6.98E-23
chr6	90140001	90145000	2.13E-24
chr6	90141001	90146000	3.11E-40
chr6	90142001	90147000	1.53E-44
chr6	90143001	90148000	8.97E-33
chr6	90345001	90350000	9.53E-51
chr6	90785001	90790000	5.68E-08
chr6	90802001	90807000	9.16E-12
chr6	90803001	90808000	3.66E-10
chr6	90804001	90809000	7.04E-15
chr6	90805001	90810000	6.23E-14
chr6	90869001	90874000	1.22E-06
chr6	90921001	90926000	4.46E-07
chr6	91001001	91006000	1.28E-53
chr6	91002001	91007000	1.83E-55
chr6	91003001	91008000	2.36E-54
chr6	91004001	91009000	4.42E-46
chr6	91005001	91010000	2.21E-62
chr6	91006001	91011000	1.14E-40
chr6	91033001	91038000	2.41E-08
chr6	91034001	91039000	1.97E-09
chr6	91035001	91040000	1.22E-10
chr6	91036001	91041000	1.80E-11
chr6	91072001	91077000	1.26E-09
chr6	91073001	91078000	6.04E-09
chr6	91075001	91080000	7.13E-05
chr6	91083001	91088000	7.45E-08
chr6	91088001	91093000	0.000908179
chr6	91089001	91094000	0.000291743
chr6	91292001	91297000	9.92E-27
chr6	91307001	91312000	2.15E-06
chr6	91321001	91326000	7.50E-23
chr6	91322001	91327000	1.21E-05
chr6	91324001	91329000	0.000138307
chr6	91329001	91334000	3.34E-09
chr6	91409001	91414000	0.000191128
chr6	91410001	91415000	0.000224923
chr6	91412001	91417000	4.91E-06
chr6	91413001	91418000	2.75E-06
chr6	91453001	91458000	7.87E-08
chr6	91454001	91459000	1.50E-06
chr6	91501001	91506000	3.87E-05
chr6	91502001	91507000	9.26E-08

chr6	91503001	91508000	6.26E-08
chr6	91522001	91527000	5.33E-07
chr6	91523001	91528000	8.94E-07
chr6	91524001	91529000	4.45E-07
chr6	91534001	91539000	5.14E-07
chr6	91535001	91540000	1.79E-07
chr6	91536001	91541000	3.48E-07
chr6	91537001	91542000	4.79E-08
chr6	91538001	91543000	1.94E-07
chr6	91551001	91556000	4.47E-06
chr6	91552001	91557000	4.72E-07
chr6	91589001	91594000	9.54E-07
chr6	91590001	91595000	4.07E-08
chr6	91597001	91602000	1.10E-09
chr6	91633001	91638000	0.002059678
chr6	91634001	91639000	0.006873742
chr6	91635001	91640000	0.002184686
chr6	91636001	91641000	0.002316491
chr6	91677001	91682000	0.004328289
chr6	91678001	91683000	0.001718522
chr6	91679001	91684000	0.000136981
chr6	91844001	91849000	3.28E-05
chr6	91845001	91850000	1.40E-05
chr6	91887001	91892000	0.001306088
chr6	91888001	91893000	0.002000033
chr6	91889001	91894000	0.010820188
chr6	91929001	91934000	9.42E-05
chr6	91930001	91935000	0.002165601
chr6	91931001	91936000	0.003553618
chr6	91954001	91959000	0.002934462
chr6	92037001	92042000	9.68E-06
chr6	92067001	92072000	0.000458186
chr6	92104001	92109000	0.004035951
chr6	92108001	92113000	9.02E-06
chr6	92152001	92157000	0.001952209
chr6	92352001	92357000	0.000205224
chr6	92685001	92690000	1.33E-05
chr6	92736001	92741000	0.00139964
chr6	92737001	92742000	8.98E-07
chr6	92853001	92858000	0.030012111
chr6	92895001	92900000	0.002911428
chr6	93033001	93038000	0.006265181
chr6	93034001	93039000	0.007259129
chr6	93516001	93521000	0.003309994
chr6	93517001	93522000	0.002722128
chr6	93518001	93523000	0.00509168
chr6	93526001	93531000	0.012451999
chr6	94122001	94127000	7.50E-48

chr6	94123001	94128000	1.20E-38
chr6	94186001	94191000	4.37E-05
chr6	94199001	94204000	0.000349569
chr6	94200001	94205000	0.000190641
chr6	94201001	94206000	0.000229284
chr6	94202001	94207000	0.000321131
chr6	94203001	94208000	0.000207274
chr6	94566001	94571000	0.000377445
chr6	94736001	94741000	4.86E-07
chr6	94737001	94742000	2.63E-08
chr6	94738001	94743000	1.14E-05
chr6	94739001	94744000	0.001260743
chr6	95060001	95065000	0.002162362
chr6	95443001	95448000	5.57E-06
chr6	95494001	95499000	5.59E-07
chr6	95594001	95599000	0.01474071
chr6	95848001	95853000	0.004209019
chr6	95865001	95870000	1.87E-05
chr6	95866001	95871000	0.000597508
chr6	95867001	95872000	0.000308974
chr6	95868001	95873000	0.002124381
chr6	95902001	95907000	3.62E-08
chr6	95910001	95915000	0.00260737
chr6	95911001	95916000	3.33E-05
chr6	95949001	95954000	0.018999519
chr6	96000001	96005000	1.13E-05
chr6	96001001	96006000	1.65E-07
chr6	96002001	96007000	4.44E-08
chr6	96003001	96008000	4.55E-07
chr6	96025001	96030000	1.44E-22
chr6	96026001	96031000	9.23E-12
chr6	96068001	96073000	0.006972411
chr6	96157001	96162000	5.39E-05
chr6	96225001	96230000	0.000199858
chr6	96234001	96239000	5.91E-07
chr6	96235001	96240000	1.28E-05
chr6	96317001	96322000	0.027090221
chr6	96460001	96465000	1.08E-24
chr6	96461001	96466000	1.45E-23
chr6	96463001	96468000	2.89E-33
chr6	96464001	96469000	1.29E-21
chr6	96657001	96662000	0.003059754
chr6	96753001	96758000	0.000179302
chr6	96754001	96759000	0.000352694
chr6	96808001	96813000	0.000890445
chr6	96809001	96814000	0.005982827
chr6	96845001	96850000	0.02012928
chr6	96965001	96970000	3.23E-44

chr6	96966001	96971000	1.38E-45
chr6	96968001	96973000	1.56E-53
chr6	96969001	96974000	5.62E-68
chr6	96970001	96975000	9.23E-45
chr6	97138001	97143000	1.48E-07
chr6	97139001	97144000	1.14E-09
chr6	97140001	97145000	2.36E-09
chr6	97141001	97146000	4.35E-08
chr6	97197001	97202000	0.001178452
chr6	97280001	97285000	1.35E-31
chr6	97281001	97286000	3.19E-47
chr6	97283001	97288000	1.64E-37
chr6	97285001	97290000	1.76E-31
chr6	97341001	97346000	2.00E-49
chr6	97368001	97373000	7.32E-23
chr6	97369001	97374000	4.23E-32
chr6	97370001	97375000	4.39E-30
chr6	97371001	97376000	9.71E-24
chr6	97372001	97377000	5.05E-26
chr6	97434001	97439000	4.93E-07
chr6	97618001	97623000	0.001657145
chr6	97728001	97733000	1.76E-24
chr6	97729001	97734000	5.15E-38
chr6	97730001	97735000	2.26E-28
chr6	97731001	97736000	8.75E-14
chr6	97751001	97756000	1.85E-07
chr6	97753001	97758000	2.33E-09
chr6	97897001	97902000	4.84E-05
chr6	97899001	97904000	0.000196446
chr6	98348001	98353000	0.001233673
chr6	98633001	98638000	0.001673887
chr6	98647001	98652000	0.000432694
chr6	98648001	98653000	0.000829353
chr6	98649001	98654000	0.000169167
chr6	98650001	98655000	0.00254932
chr6	98751001	98756000	0.000431377
chr6	98752001	98757000	0.003814472
chr6	99044001	99049000	0.002111626
chr6	99053001	99058000	2.53E-11
chr6	99054001	99059000	6.00E-08
chr6	99058001	99063000	4.26E-06
chr6	99059001	99064000	1.68E-07
chr6	99060001	99065000	2.72E-08
chr6	99061001	99066000	0.000119028
chr6	99062001	99067000	0.000195405
chr6	99132001	99137000	0.00532033
chr6	99134001	99139000	0.002584025
chr6	99135001	99140000	0.002843288

chr6	99159001	99164000	0.000671769
chr6	99160001	99165000	3.41E-09
chr6	99161001	99166000	1.20E-06
chr6	99207001	99212000	0.000236535
chr6	99208001	99213000	6.78E-05
chr6	99209001	99214000	9.22E-06
chr6	99210001	99215000	1.98E-05
chr6	99211001	99216000	0.004685047
chr6	99391001	99396000	9.39E-19
chr6	99395001	99400000	1.60E-13
chr6	99462001	99467000	0.001502203
chr6	99480001	99485000	0.000356119
chr6	99554001	99559000	0.000166656
chr6	99555001	99560000	0.000483086
chr6	99842001	99847000	9.04E-13
chr6	99958001	99963000	4.52E-14
chr6	99959001	99964000	8.11E-27
chr6	99960001	99965000	1.43E-41
chr6	99961001	99966000	1.52E-31
chr6	99963001	99968000	6.26E-28
chr6	100011001	100016000	2.50E-11
chr6	100020001	100025000	0.000109808
chr6	100021001	100026000	0.00029546
chr6	100022001	100027000	5.85E-05
chr6	100023001	100028000	0.000215418
chr6	100024001	100029000	5.69E-05
chr6	100032001	100037000	3.23E-20
chr6	100033001	100038000	7.92E-26
chr6	100166001	100171000	0.000157817
chr6	100167001	100172000	0.000314506
chr6	100168001	100173000	0.000419492
chr6	100220001	100225000	0.004864469
chr6	100409001	100414000	0.000681146
chr6	100410001	100415000	0.002426537
chr6	100413001	100418000	0.000819584
chr6	100677001	100682000	2.87E-13
chr6	101326001	101331000	7.59E-34
chr6	101329001	101334000	4.59E-37
chr6	102232001	102237000	0.002554738
chr6	102462001	102467000	2.37E-06
chr6	102463001	102468000	1.32E-06
chr6	103216001	103221000	0.002519487
chr6	103217001	103222000	4.11E-06
chr6	103603001	103608000	2.19E-07
chr6	103618001	103623000	0.021450908
chr6	103821001	103826000	9.92E-07
chr6	104751001	104756000	0.000209073
chr6	104763001	104768000	0.002641919

chr6	104781001	104786000	8.77E-08
chr6	105040001	105045000	0.000101076
chr6	105303001	105308000	2.42E-37
chr6	105304001	105309000	7.69E-34
chr6	105305001	105310000	2.30E-30
chr6	105306001	105311000	1.62E-39
chr6	105307001	105312000	1.41E-19
chr6	105580001	105585000	2.47E-25
chr6	105581001	105586000	5.94E-36
chr6	105582001	105587000	1.53E-40
chr6	105583001	105588000	2.01E-35
chr6	105584001	105589000	1.51E-38
chr6	105585001	105590000	1.45E-31
chr6	105623001	105628000	5.62E-74
chr6	105624001	105629000	2.93E-79
chr6	105625001	105630000	8.37E-42
chr6	105626001	105631000	1.37E-30
chr6	105627001	105632000	1.71E-37
chr6	105628001	105633000	2.04E-12
chr6	105851001	105856000	4.97E-31
chr6	106022001	106027000	0.027460374
chr6	106023001	106028000	0.00202943
chr6	106024001	106029000	0.001507289
chr6	106159001	106164000	0.000129481
chr6	106764001	106769000	0.000810177
chr6	106769001	106774000	2.44E-11
chr6	106770001	106775000	1.05E-15
chr6	106771001	106776000	1.44E-15
chr6	106772001	106777000	9.27E-21
chr6	106773001	106778000	1.03E-22
chr6	106804001	106809000	9.62E-33
chr6	106805001	106810000	3.00E-26
chr6	106806001	106811000	9.17E-42
chr6	106807001	106812000	5.77E-46
chr6	106808001	106813000	9.64E-49
chr6	107077001	107082000	9.68E-47
chr6	107079001	107084000	1.42E-11
chr6	107432001	107437000	2.96E-16
chr6	107776001	107781000	4.07E-24
chr6	107777001	107782000	2.06E-37
chr6	107778001	107783000	4.50E-37
chr6	107779001	107784000	2.25E-38
chr6	107780001	107785000	1.20E-37
chr6	107781001	107786000	7.01E-31
chr6	107782001	107787000	0.001971842
chr6	107783001	107788000	0.007732179
chr6	107815001	107820000	1.29E-05
chr6	108190001	108195000	1.82E-07

chr6	108391001	108396000	4.04E-38
chr6	108392001	108397000	9.38E-20
chr6	108393001	108398000	1.13E-28
chr6	108394001	108399000	7.34E-23
chr6	108430001	108435000	3.96E-08
chr6	108578001	108583000	4.25E-18
chr6	108580001	108585000	2.41E-17
chr6	108614001	108619000	2.09E-09
chr6	108615001	108620000	1.84E-11
chr6	108616001	108621000	2.05E-16
chr6	109411001	109416000	2.61E-33
chr6	109502001	109507000	3.60E-05
chr6	109503001	109508000	1.42E-06
chr6	109757001	109762000	8.87E-43
chr6	109758001	109763000	2.53E-42
chr6	109759001	109764000	1.10E-32
chr6	109762001	109767000	9.45E-09
chr6	109800001	109805000	5.34E-24
chr6	109801001	109806000	9.73E-15
chr6	109802001	109807000	1.58E-13
chr6	109803001	109808000	5.31E-17
chr6	109804001	109809000	1.07E-44
chr6	109915001	109920000	3.03E-09
chr6	109976001	109981000	0.002488958
chr6	109977001	109982000	0.001495685
chr6	110005001	110010000	4.33E-07
chr6	110012001	110017000	8.80E-39
chr6	110273001	110278000	0.002467949
chr6	110496001	110501000	2.23E-67
chr6	110497001	110502000	3.77E-53
chr6	110500001	110505000	1.22E-35
chr6	110501001	110506000	2.24E-21
chr6	110614001	110619000	0.000238
chr6	110615001	110620000	0.000629459
chr6	110679001	110684000	1.55E-27
chr6	111132001	111137000	2.78E-18
chr6	111275001	111280000	5.21E-10
chr6	111276001	111281000	2.14E-06
chr6	111277001	111282000	2.48E-05
chr6	111278001	111283000	1.38E-05
chr6	111366001	111371000	2.07E-10
chr6	111367001	111372000	1.12E-10
chr6	111404001	111409000	4.30E-29
chr6	111405001	111410000	2.99E-30
chr6	111406001	111411000	1.22E-46
chr6	111407001	111412000	6.81E-35
chr6	111408001	111413000	9.58E-30
chr6	111409001	111414000	4.08E-26

chr6	111563001	111568000	4.38E-06
chr6	111858001	111863000	2.98E-11
chr6	112090001	112095000	1.79E-13
chr6	112091001	112096000	4.46E-08
chr6	112194001	112199000	4.40E-85
chr6	112383001	112388000	0.000595907
chr6	112385001	112390000	0.000295013
chr6	112386001	112391000	0.000401483
chr6	112404001	112409000	3.68E-50
chr6	112405001	112410000	3.97E-71
chr6	112406001	112411000	1.07E-64
chr6	112407001	112412000	3.97E-56
chr6	112692001	112697000	0.00044322
chr6	112693001	112698000	0.000142376
chr6	112852001	112857000	4.94E-08
chr6	112853001	112858000	1.46E-10
chr6	112854001	112859000	1.38E-09
chr6	112855001	112860000	1.17E-08
chr6	112856001	112861000	3.84E-09
chr6	112871001	112876000	2.74E-06
chr6	112872001	112877000	4.04E-07
chr6	112873001	112878000	2.01E-08
chr6	112874001	112879000	1.43E-05
chr6	113026001	113031000	0.001817514
chr6	113027001	113032000	0.000407131
chr6	113029001	113034000	0.005073423
chr6	113059001	113064000	0.020117895
chr6	113068001	113073000	0.002912053
chr6	114179001	114184000	3.53E-07
chr6	114180001	114185000	1.34E-09
chr6	114260001	114265000	0.013595148
chr6	114287001	114292000	5.61E-21
chr6	114288001	114293000	1.31E-47
chr6	114289001	114294000	1.32E-34
chr6	114290001	114295000	6.42E-36
chr6	114372001	114377000	0.000217661
chr6	114493001	114498000	0.001188172
chr6	114523001	114528000	2.31E-05
chr6	114524001	114529000	0.00011103
chr6	114525001	114530000	0.000193104
chr6	114526001	114531000	0.002798569
chr6	114561001	114566000	7.06E-07
chr6	114562001	114567000	2.34E-07
chr6	114563001	114568000	1.60E-05
chr6	114564001	114569000	0.000249052
chr6	114625001	114630000	0.00057085
chr6	114659001	114664000	1.05E-14
chr6	114667001	114672000	8.26E-05

chr6	114668001	114673000	6.46E-05
chr6	114669001	114674000	0.006495211
chr6	114670001	114675000	0.007229405
chr6	114671001	114676000	0.006269509
chr6	114790001	114795000	0.000717364
chr6	114791001	114796000	0.000502177
chr6	114805001	114810000	3.43E-06
chr6	114806001	114811000	2.57E-05
chr6	114820001	114825000	0.000107873
chr6	114829001	114834000	3.26E-05
chr6	114830001	114835000	1.62E-06
chr6	114831001	114836000	3.07E-06
chr6	114848001	114853000	2.84E-10
chr6	114849001	114854000	1.97E-09
chr6	114850001	114855000	4.59E-11
chr6	114851001	114856000	2.88E-11
chr6	114865001	114870000	2.59E-06
chr6	114910001	114915000	7.94E-07
chr6	114911001	114916000	1.28E-09
chr6	114912001	114917000	2.85E-07
chr6	114926001	114931000	0.003441094
chr6	114946001	114951000	0.00049833
chr6	114949001	114954000	3.52E-06
chr6	115007001	115012000	0.003243485
chr6	115008001	115013000	0.0018262
chr6	115124001	115129000	8.58E-08
chr6	115125001	115130000	7.78E-08
chr6	115126001	115131000	1.76E-07
chr6	115279001	115284000	0.012833038
chr6	115280001	115285000	0.00727415
chr6	115413001	115418000	0.003049081
chr6	115528001	115533000	0.005440756
chr6	115529001	115534000	0.015527466
chr6	115591001	115596000	0.000358572
chr6	115592001	115597000	0.000531515
chr6	115809001	115814000	0.00017103
chr6	115822001	115827000	0.008812056
chr6	115927001	115932000	0.002077196
chr6	116422001	116427000	6.87E-41
chr6	116522001	116527000	0.005781903
chr6	116570001	116575000	1.58E-21
chr6	116573001	116578000	1.03E-42
chr6	116574001	116579000	1.71E-54
chr6	116575001	116580000	1.04E-23
chr6	116687001	116692000	2.05E-16
chr6	116779001	116784000	1.69E-19
chr6	116780001	116785000	2.27E-25
chr6	116781001	116786000	2.11E-23

chr6	116782001	116787000	7.54E-21
chr6	116783001	116788000	5.81E-25
chr6	116938001	116943000	2.25E-13
chr6	116949001	116954000	9.92E-05
chr6	117326001	117331000	0.006682426
chr6	117465001	117470000	0.00126061
chr6	117466001	117471000	0.004477188
chr6	117799001	117804000	8.80E-47
chr6	117800001	117805000	1.21E-45
chr6	117801001	117806000	3.79E-46
chr6	117802001	117807000	1.78E-58
chr6	117803001	117808000	1.77E-41
chr6	117804001	117809000	4.59E-38
chr6	117811001	117816000	0.004615539
chr6	117920001	117925000	1.82E-51
chr6	117921001	117926000	2.90E-43
chr6	117924001	117929000	8.71E-13
chr6	117996001	118001000	3.66E-28
chr6	117997001	118002000	1.53E-29
chr6	118092001	118097000	2.12E-07
chr6	118093001	118098000	8.33E-09
chr6	118122001	118127000	2.45E-07
chr6	118160001	118165000	1.55E-07
chr6	118204001	118209000	0.011270776
chr6	118234001	118239000	1.38E-05
chr6	118257001	118262000	0.00893419
chr6	118440001	118445000	0.000832401
chr6	118441001	118446000	0.001479934
chr6	118575001	118580000	7.91E-05
chr6	118967001	118972000	3.05E-34
chr6	118968001	118973000	8.27E-86
chr6	118969001	118974000	2.41E-62
chr6	119211001	119216000	7.10E-52
chr6	119251001	119256000	7.63E-15
chr6	119252001	119257000	2.17E-38
chr6	119253001	119258000	1.09E-25
chr6	119254001	119259000	3.88E-29
chr6	119255001	119260000	2.96E-35
chr6	119256001	119261000	5.53E-34
chr6	119395001	119400000	5.85E-34
chr6	119584001	119589000	0.027903344
chr6	119817001	119822000	0.001542127
chr6	119818001	119823000	0.002625399
chr6	120247001	120252000	1.77E-05
chr6	120248001	120253000	0.00073074
chr6	120249001	120254000	0.001300999
chr6	120764001	120769000	0.005789145
chr6	120765001	120770000	0.003811924

chr6	120766001	120771000	0.002847839
chr6	120767001	120772000	0.000342594
chr6	121019001	121024000	1.61E-22
chr6	121020001	121025000	1.14E-23
chr6	121021001	121026000	2.09E-21
chr6	121176001	121181000	0.000260566
chr6	121177001	121182000	4.22E-05
chr6	121196001	121201000	0.000695657
chr6	121197001	121202000	0.001156585
chr6	121258001	121263000	0.001315047
chr6	121299001	121304000	0.01958502
chr6	121392001	121397000	4.31E-05
chr6	121393001	121398000	0.000216484
chr6	121394001	121399000	0.000428
chr6	121651001	121656000	1.04E-23
chr6	121652001	121657000	8.71E-25
chr6	121653001	121658000	3.57E-31
chr6	121654001	121659000	1.97E-39
chr6	121655001	121660000	6.27E-33
chr6	121918001	121923000	0.009537212
chr6	121919001	121924000	0.007521321
chr6	121920001	121925000	0.009637547
chr6	122212001	122217000	0.020067908
chr6	122488001	122493000	0.000115735
chr6	122489001	122494000	5.63E-05
chr6	122490001	122495000	1.23E-05
chr6	122491001	122496000	8.14E-05
chr6	122492001	122497000	0.000396115
chr6	122626001	122631000	0.00047144
chr6	122716001	122721000	1.54E-16
chr6	122717001	122722000	1.00E-53
chr6	122718001	122723000	1.04E-53
chr6	122719001	122724000	6.31E-41
chr6	122720001	122725000	1.91E-45
chr6	122721001	122726000	9.53E-42
chr6	122847001	122852000	7.10E-07
chr6	122848001	122853000	2.11E-05
chr6	122849001	122854000	9.27E-05
chr6	123106001	123111000	6.78E-39
chr6	123448001	123453000	0.000242496
chr6	123518001	123523000	0.000123875
chr6	125073001	125078000	2.04E-05
chr6	125416001	125421000	2.59E-37
chr6	125475001	125480000	2.96E-20
chr6	125515001	125520000	4.28E-07
chr6	125565001	125570000	8.01E-07
chr6	125618001	125623000	2.70E-57
chr6	125619001	125624000	2.72E-46

chr6	125620001	125625000	3.12E-34
chr6	125621001	125626000	2.78E-45
chr6	125622001	125627000	6.88E-32
chr6	125669001	125674000	0.000139876
chr6	125670001	125675000	0.005137448
chr6	125680001	125685000	5.96E-43
chr6	125681001	125686000	1.89E-37
chr6	126107001	126112000	6.04E-33
chr6	126112001	126117000	8.19E-29
chr6	126462001	126467000	1.10E-11
chr6	126860001	126865000	7.06E-05
chr6	126861001	126866000	8.01E-05
chr6	127031001	127036000	0.000891999
chr6	127032001	127037000	0.001997085
chr6	127033001	127038000	0.005980631
chr6	127111001	127116000	0.000385876
chr6	127112001	127117000	5.05E-05
chr6	127113001	127118000	4.70E-05
chr6	127114001	127119000	0.000129698
chr6	127138001	127143000	0.002305172
chr6	127189001	127194000	0.000136268
chr6	127369001	127374000	2.70E-05
chr6	127370001	127375000	2.78E-05
chr6	127435001	127440000	2.50E-33
chr6	127436001	127441000	8.52E-44
chr6	127437001	127442000	1.54E-42
chr6	127441001	127446000	1.40E-28
chr6	127482001	127487000	0.009749928
chr6	127483001	127488000	0.013138345
chr6	127484001	127489000	0.012894646
chr6	127515001	127520000	0.006952856
chr6	127580001	127585000	0.000168154
chr6	127583001	127588000	2.74E-28
chr6	127660001	127665000	2.17E-25
chr6	127986001	127991000	6.18E-05
chr6	128460001	128465000	0.013855094
chr6	128842001	128847000	6.36E-14
chr6	128920001	128925000	1.41E-05
chr6	128995001	129000000	0.000355067
chr6	129130001	129135000	0.000331279
chr6	129131001	129136000	0.000580018
chr6	129143001	129148000	0.000362306
chr6	129324001	129329000	4.95E-05
chr6	129325001	129330000	7.91E-06
chr6	129326001	129331000	1.29E-06
chr6	129327001	129332000	3.76E-07
chr6	129328001	129333000	3.27E-05
chr6	129360001	129365000	0.004299411

chr6	129581001	129586000	0.000197759
chr6	129582001	129587000	0.000361789
chr6	129671001	129676000	2.01E-06
chr6	129672001	129677000	1.03E-06
chr6	129703001	129708000	0.000334376
chr6	129704001	129709000	0.000417647
chr6	129778001	129783000	7.68E-05
chr6	129779001	129784000	0.000390843
chr6	129787001	129792000	0.000228248
chr6	130084001	130089000	4.32E-30
chr6	130085001	130090000	9.98E-35
chr6	130086001	130091000	3.96E-36
chr6	130087001	130092000	7.54E-35
chr6	130088001	130093000	6.39E-33
chr6	130158001	130163000	2.18E-11
chr6	130204001	130209000	3.95E-05
chr6	130341001	130346000	4.13E-43
chr6	130533001	130538000	0.000461924
chr6	130534001	130539000	0.00013014
chr6	130713001	130718000	3.26E-05
chr6	130714001	130719000	1.76E-05
chr6	130779001	130784000	3.54E-05
chr6	130885001	130890000	6.60E-05
chr6	131114001	131119000	1.36E-05
chr6	131243001	131248000	7.52E-10
chr6	131244001	131249000	5.16E-09
chr6	131298001	131303000	8.04E-05
chr6	131299001	131304000	0.003140901
chr6	131308001	131313000	1.31E-09
chr6	131309001	131314000	7.81E-11
chr6	131311001	131316000	1.36E-09
chr6	131339001	131344000	2.58E-05
chr6	131345001	131350000	1.14E-10
chr6	131346001	131351000	1.12E-08
chr6	131347001	131352000	3.53E-05
chr6	131384001	131389000	4.81E-09
chr6	131452001	131457000	9.13E-59
chr6	131453001	131458000	1.46E-34
chr6	131454001	131459000	1.42E-52
chr6	131455001	131460000	1.27E-56
chr6	131456001	131461000	4.35E-41
chr6	132107001	132112000	1.21E-05
chr6	132125001	132130000	1.99E-38
chr6	132126001	132131000	1.01E-41
chr6	132129001	132134000	2.92E-38
chr6	132407001	132412000	0.002187879
chr6	132408001	132413000	0.003257792
chr6	132409001	132414000	0.004300176

chr6	132718001	132723000	2.13E-42
chr6	132719001	132724000	4.65E-33
chr6	132720001	132725000	2.28E-39
chr6	132721001	132726000	1.41E-35
chr6	132722001	132727000	5.64E-20
chr6	132834001	132839000	8.50E-19
chr6	132865001	132870000	7.91E-09
chr6	132866001	132871000	4.28E-09
chr6	132867001	132872000	1.72E-06
chr6	133245001	133250000	0.002941252
chr6	133410001	133415000	6.21E-05
chr6	133431001	133436000	4.83E-08
chr6	133478001	133483000	0.00036987
chr6	133480001	133485000	0.001162862
chr6	133483001	133488000	2.01E-07
chr6	133484001	133489000	1.28E-10
chr6	133485001	133490000	1.32E-06
chr6	133620001	133625000	1.83E-06
chr6	134155001	134160000	1.36E-28
chr6	134156001	134161000	6.12E-34
chr6	134157001	134162000	3.44E-30
chr6	134158001	134163000	4.77E-33
chr6	134159001	134164000	5.75E-29
chr6	134303001	134308000	0.000785879
chr6	134304001	134309000	0.001817852
chr6	134372001	134377000	8.06E-18
chr6	134373001	134378000	8.44E-32
chr6	134639001	134644000	1.85E-07
chr6	135084001	135089000	0.003120506
chr6	135498001	135503000	8.31E-29
chr6	135503001	135508000	1.07E-25
chr6	135504001	135509000	1.50E-24
chr6	135814001	135819000	1.15E-43
chr6	135819001	135824000	4.60E-30
chr6	135873001	135878000	0.000265487
chr6	135874001	135879000	0.000946972
chr6	136567001	136572000	1.71E-39
chr6	136866001	136871000	3.54E-20
chr6	136867001	136872000	1.80E-82
chr6	136868001	136873000	1.25E-46
chr6	136869001	136874000	1.23E-75
chr6	136870001	136875000	2.10E-75
chr6	136871001	136876000	8.61E-64
chr6	137112001	137117000	3.62E-36
chr6	137113001	137118000	2.58E-73
chr6	137114001	137119000	2.28E-25
chr6	137139001	137144000	5.37E-38
chr6	137141001	137146000	2.50E-19

chr6	137142001	137147000	3.25E-24
chr6	137144001	137149000	1.02E-16
chr6	137212001	137217000	2.13E-12
chr6	137214001	137219000	3.03E-15
chr6	137215001	137220000	1.90E-14
chr6	137238001	137243000	4.72E-24
chr6	137242001	137247000	1.48E-08
chr6	137361001	137366000	9.74E-33
chr6	137427001	137432000	0.003101923
chr6	137442001	137447000	4.55E-06
chr6	137443001	137448000	2.71E-06
chr6	137448001	137453000	1.66E-06
chr6	137728001	137733000	0.002867376
chr6	138184001	138189000	2.83E-20
chr6	138255001	138260000	0.001983272
chr6	138478001	138483000	8.05E-33
chr6	138479001	138484000	8.69E-31
chr6	138480001	138485000	5.53E-47
chr6	138481001	138486000	8.58E-54
chr6	138482001	138487000	5.79E-56
chr6	138483001	138488000	7.21E-68
chr6	138720001	138725000	8.70E-29
chr6	138721001	138726000	5.46E-46
chr6	138722001	138727000	3.72E-30
chr6	138723001	138728000	1.10E-19
chr6	138725001	138730000	1.20E-49
chr6	139009001	139014000	4.04E-41
chr6	139306001	139311000	2.91E-48
chr6	139309001	139314000	2.19E-18
chr6	139345001	139350000	1.93E-22
chr6	139346001	139351000	2.67E-69
chr6	139452001	139457000	1.52E-27
chr6	139455001	139460000	6.76E-31
chr6	139456001	139461000	1.90E-79
chr6	139457001	139462000	4.94E-32
chr6	139695001	139700000	9.89E-33
chr6	139696001	139701000	5.06E-26
chr6	140208001	140213000	0.0007171
chr6	141405001	141410000	0.000548669
chr6	142093001	142098000	6.77E-05
chr6	142125001	142130000	5.28E-05
chr6	142126001	142131000	0.000564215
chr6	142127001	142132000	0.001386758
chr6	142128001	142133000	0.016599808
chr6	142215001	142220000	2.44E-06
chr6	142216001	142221000	2.00E-05
chr6	142217001	142222000	0.000473663
chr6	142447001	142452000	2.27E-07

chr6	142618001	142623000	9.01E-54
chr6	142619001	142624000	5.52E-49
chr6	142620001	142625000	4.27E-69
chr6	142621001	142626000	1.12E-102
chr6	142622001	142627000	8.00E-104
chr6	142623001	142628000	3.66E-78
chr6	143019001	143024000	2.11E-09
chr6	143036001	143041000	6.19E-05
chr6	143110001	143115000	0.007515788
chr6	143118001	143123000	7.80E-06
chr6	143119001	143124000	7.80E-06
chr6	143145001	143150000	2.64E-15
chr6	143146001	143151000	2.03E-15
chr6	143147001	143152000	9.57E-13
chr6	143148001	143153000	1.01E-09
chr6	143162001	143167000	1.26E-08
chr6	143170001	143175000	0.000163513
chr6	143243001	143248000	9.70E-25
chr6	143244001	143249000	3.56E-51
chr6	143245001	143250000	3.35E-48
chr6	143261001	143266000	1.65E-26
chr6	143262001	143267000	1.06E-53
chr6	143267001	143272000	3.25E-22
chr6	143268001	143273000	8.44E-20
chr6	143284001	143289000	1.92E-05
chr6	143285001	143290000	0.000309765
chr6	143287001	143292000	7.53E-09
chr6	143288001	143293000	6.76E-10
chr6	143289001	143294000	3.78E-07
chr6	143290001	143295000	2.11E-07
chr6	143291001	143296000	6.89E-05
chr6	143330001	143335000	3.51E-07
chr6	143381001	143386000	1.85E-26
chr6	143382001	143387000	1.16E-20
chr6	143468001	143473000	1.57E-07
chr6	143501001	143506000	0.000568661
chr6	143537001	143542000	1.50E-17
chr6	143538001	143543000	1.17E-18
chr6	143539001	143544000	4.62E-16
chr6	143540001	143545000	1.58E-09
chr6	143767001	143772000	3.48E-36
chr6	143828001	143833000	9.84E-32
chr6	143829001	143834000	1.96E-49
chr6	143830001	143835000	1.10E-41
chr6	143831001	143836000	5.56E-33
chr6	143832001	143837000	7.35E-34
chr6	143857001	143862000	5.31E-39
chr6	143858001	143863000	7.36E-47

chr6	143927001	143932000	6.61E-07
chr6	144162001	144167000	3.15E-40
chr6	144163001	144168000	3.83E-42
chr6	144164001	144169000	3.99E-54
chr6	144378001	144383000	4.03E-06
chr6	144397001	144402000	2.63E-07
chr6	144414001	144419000	9.75E-42
chr6	144415001	144420000	7.34E-40
chr6	144416001	144421000	2.21E-49
chr6	144417001	144422000	8.36E-40
chr6	144449001	144454000	1.25E-05
chr6	144511001	144516000	3.29E-06
chr6	144533001	144538000	5.16E-22
chr6	144653001	144658000	3.93E-14
chr6	144654001	144659000	5.51E-16
chr6	144679001	144684000	7.29E-06
chr6	144680001	144685000	4.08E-05
chr6	144707001	144712000	4.65E-15
chr6	144708001	144713000	6.62E-14
chr6	144725001	144730000	5.72E-09
chr6	144743001	144748000	2.25E-08
chr6	144744001	144749000	8.41E-09
chr6	144764001	144769000	3.96E-08
chr6	144765001	144770000	1.03E-08
chr6	144767001	144772000	1.15E-08
chr6	144768001	144773000	4.11E-08
chr6	144821001	144826000	0.000104055
chr6	144903001	144908000	6.90E-07
chr6	144904001	144909000	2.89E-06
chr6	145559001	145564000	1.70E-05
chr6	146053001	146058000	2.48E-61
chr6	146054001	146059000	5.11E-73
chr6	146055001	146060000	6.41E-79
chr6	146056001	146061000	1.95E-65
chr6	146131001	146136000	2.42E-83
chr6	146132001	146137000	3.86E-75
chr6	146133001	146138000	4.08E-69
chr6	146136001	146141000	1.71E-26
chr6	146305001	146310000	0.000189219
chr6	146359001	146364000	1.11E-06
chr6	146360001	146365000	2.64E-06
chr6	146362001	146367000	1.74E-06
chr6	146391001	146396000	0.00089237
chr6	146530001	146535000	0.004817617
chr6	146531001	146536000	0.003050952
chr6	146532001	146537000	0.003346625
chr6	146558001	146563000	7.66E-09
chr6	146559001	146564000	1.05E-08

chr6	146584001	146589000	1.50E-10
chr6	146585001	146590000	4.85E-10
chr6	146586001	146591000	6.14E-10
chr6	146587001	146592000	3.67E-10
chr6	146691001	146696000	0.001242446
chr6	146692001	146697000	3.97E-05
chr6	146751001	146756000	3.51E-13
chr6	146752001	146757000	2.58E-11
chr6	146753001	146758000	6.29E-20
chr6	146754001	146759000	3.55E-25
chr6	146755001	146760000	7.23E-21
chr6	146843001	146848000	3.78E-06
chr6	146844001	146849000	0.000346552
chr6	146860001	146865000	2.81E-34
chr6	146861001	146866000	6.38E-29
chr6	146862001	146867000	1.26E-22
chr6	146863001	146868000	2.33E-20
chr6	147103001	147108000	3.21E-05
chr6	147104001	147109000	5.04E-05
chr6	147530001	147535000	1.20E-07
chr6	147797001	147802000	4.13E-13
chr6	147979001	147984000	4.01E-06
chr6	147980001	147985000	0.000131792
chr6	148377001	148382000	2.29E-06
chr6	148407001	148412000	4.50E-05
chr6	148545001	148550000	1.29E-06
chr6	148903001	148908000	5.25E-08
chr6	149034001	149039000	4.36E-08
chr6	149041001	149046000	0.000112885
chr6	149042001	149047000	6.28E-06
chr6	149063001	149068000	1.66E-16
chr6	149064001	149069000	2.94E-44
chr6	149065001	149070000	7.31E-38
chr6	149067001	149072000	5.10E-19
chr6	149068001	149073000	2.52E-20
chr6	149069001	149074000	8.44E-18
chr6	149106001	149111000	1.09E-05
chr6	149121001	149126000	1.10E-10
chr6	149122001	149127000	3.90E-07
chr6	149293001	149298000	3.99E-18
chr6	149387001	149392000	8.75E-09
chr6	149389001	149394000	4.92E-10
chr6	149461001	149466000	3.49E-05
chr6	149547001	149552000	4.47E-09
chr6	149594001	149599000	4.42E-06
chr6	149634001	149639000	4.36E-78
chr6	149635001	149640000	4.35E-72
chr6	150070001	150075000	1.11E-20

chr6	150176001	150181000	6.98E-05
chr6	150180001	150185000	3.92E-21
chr6	150181001	150186000	1.07E-23
chr6	150256001	150261000	1.01E-21
chr6	150355001	150360000	7.17E-15
chr6	150356001	150361000	1.75E-13
chr6	150357001	150362000	1.53E-10
chr6	150464001	150469000	4.10E-41
chr6	150761001	150766000	0.000144659
chr6	150762001	150767000	2.09E-05
chr6	150763001	150768000	9.40E-05
chr6	150765001	150770000	0.001117717
chr6	150792001	150797000	3.14E-10
chr6	150795001	150800000	3.32E-09
chr6	150813001	150818000	1.75E-12
chr6	150814001	150819000	7.81E-14
chr6	150891001	150896000	2.58E-10
chr6	150917001	150922000	1.84E-68
chr6	150918001	150923000	3.46E-67
chr6	150919001	150924000	7.43E-81
chr6	150920001	150925000	1.40E-68
chr6	150921001	150926000	4.10E-62
chr6	150922001	150927000	5.50E-22
chr6	150962001	150967000	0.009634878
chr6	150964001	150969000	1.70E-05
chr6	150965001	150970000	4.60E-05
chr6	151182001	151187000	2.73E-28
chr6	151183001	151188000	3.18E-68
chr6	151557001	151562000	9.08E-25
chr6	151558001	151563000	1.58E-20
chr6	151559001	151564000	8.49E-34
chr6	151560001	151565000	9.02E-39
chr6	151561001	151566000	8.36E-47
chr6	151562001	151567000	7.55E-31
chr6	151645001	151650000	3.37E-15
chr6	151707001	151712000	3.94E-48
chr6	151708001	151713000	8.35E-44
chr6	151859001	151864000	0.003973937
chr6	151860001	151865000	0.004953995
chr6	151882001	151887000	9.60E-05
chr6	151888001	151893000	0.000219806
chr6	152133001	152138000	0.001138645
chr6	152134001	152139000	0.000605589
chr6	152320001	152325000	0.003337317
chr6	152321001	152326000	0.000477408
chr6	152322001	152327000	0.012934796
chr6	152677001	152682000	2.65E-08
chr6	152932001	152937000	0.001945587

chr6	152997001	153002000	0.002808683
chr6	153117001	153122000	0.001059884
chr6	153299001	153304000	4.13E-23
chr6	153319001	153324000	2.39E-35
chr6	153324001	153329000	1.80E-25
chr6	153447001	153452000	8.82E-60
chr6	153618001	153623000	0.000134443
chr6	154028001	154033000	0.000552749
chr6	154227001	154232000	0.000533113
chr6	154497001	154502000	4.48E-09
chr6	154498001	154503000	5.71E-10
chr6	154542001	154547000	4.32E-06
chr6	154775001	154780000	6.27E-07
chr6	154831001	154836000	6.62E-75
chr6	155050001	155055000	7.47E-35
chr6	155051001	155056000	9.35E-28
chr6	155052001	155057000	1.03E-71
chr6	155053001	155058000	5.70E-78
chr6	155054001	155059000	1.29E-66
chr6	155055001	155060000	2.31E-17
chr6	155316001	155321000	6.98E-14
chr6	155317001	155322000	4.05E-25
chr6	155844001	155849000	1.83E-06
chr6	155945001	155950000	8.47E-06
chr6	155946001	155951000	2.04E-06
chr6	155947001	155952000	3.76E-06
chr6	155948001	155953000	4.54E-08
chr6	155949001	155954000	0.000133525
chr6	156283001	156288000	0.003355898
chr6	156325001	156330000	0.000383508
chr6	156557001	156562000	7.57E-06
chr6	156558001	156563000	3.81E-06
chr6	157343001	157348000	3.74E-28
chr6	157691001	157696000	7.11E-22
chr6	157692001	157697000	1.62E-23
chr6	157693001	157698000	3.60E-12
chr6	157694001	157699000	1.38E-18
chr6	157695001	157700000	1.01E-33
chr6	157740001	157745000	8.15E-76
chr6	157741001	157746000	2.14E-77
chr6	157742001	157747000	3.95E-60
chr6	157743001	157748000	3.74E-28
chr6	157797001	157802000	5.31E-74
chr6	157798001	157803000	6.25E-80
chr6	157800001	157805000	1.53E-59
chr6	157801001	157806000	3.10E-97
chr6	157802001	157807000	2.38E-71
chr6	158240001	158245000	3.94E-86

chr6	158241001	158246000	5.53E-80
chr6	158242001	158247000	6.50E-45
chr6	158244001	158249000	1.18E-45
chr6	158584001	158589000	7.11E-14
chr6	158585001	158590000	6.37E-49
chr6	158586001	158591000	9.20E-37
chr6	158587001	158592000	1.59E-46
chr6	158588001	158593000	8.75E-74
chr6	158589001	158594000	1.31E-84
chr6	158649001	158654000	5.74E-45
chr6	158650001	158655000	3.91E-41
chr6	158651001	158656000	4.30E-42
chr6	158653001	158658000	6.96E-32
chr6	158977001	158982000	4.52E-46
chr6	158978001	158983000	1.07E-36
chr6	158979001	158984000	3.28E-44
chr6	158980001	158985000	3.80E-41
chr6	158981001	158986000	2.38E-54
chr6	159239001	159244000	4.90E-22
chr6	159240001	159245000	1.61E-24
chr6	159290001	159295000	3.16E-24
chr6	159291001	159296000	3.57E-29
chr6	159358001	159363000	9.51E-09
chr6	159359001	159364000	6.11E-09
chr6	159360001	159365000	2.97E-08
chr6	159507001	159512000	1.66E-06
chr6	159703001	159708000	3.06E-09
chr6	159704001	159709000	6.76E-08
chr6	159705001	159710000	3.55E-08
chr6	159706001	159711000	2.50E-09
chr6	159707001	159712000	2.00E-07
chr6	159757001	159762000	1.09E-06
chr6	159758001	159763000	1.33E-06
chr6	159764001	159769000	3.17E-06
chr6	159810001	159815000	1.70E-08
chr6	159848001	159853000	1.15E-05
chr6	159896001	159901000	5.31E-06
chr6	159937001	159942000	3.57E-05
chr6	159938001	159943000	8.37E-06
chr6	159940001	159945000	1.72E-08
chr6	159941001	159946000	5.64E-09
chr6	160380001	160385000	7.34E-08
chr6	160381001	160386000	1.32E-06
chr6	160382001	160387000	2.04E-07
chr6	160602001	160607000	1.31E-08
chr6	160635001	160640000	1.48E-09
chr6	160647001	160652000	0.000172978
chr6	160691001	160696000	1.49E-07

chr6	160738001	160743000	0.000613871
chr6	160761001	160766000	2.72E-07
chr6	160765001	160770000	2.12E-14
chr6	160803001	160808000	1.52E-07
chr6	160804001	160809000	1.25E-11
chr6	160805001	160810000	4.65E-07
chr6	160897001	160902000	6.62E-06
chr6	160937001	160942000	3.69E-24
chr6	160938001	160943000	4.19E-38
chr6	160939001	160944000	1.72E-32
chr6	160940001	160945000	1.29E-21
chr6	160941001	160946000	5.84E-16
chr6	160946001	160951000	1.14E-07
chr6	160947001	160952000	1.02E-06
chr6	160971001	160976000	2.76E-13
chr6	160972001	160977000	4.66E-05
chr6	160995001	161000000	4.67E-08
chr6	160996001	161001000	4.10E-05
chr6	160997001	161002000	9.57E-05
chr6	160998001	161003000	6.84E-06
chr6	161022001	161027000	8.86E-12
chr6	161023001	161028000	2.09E-13
chr6	161024001	161029000	5.37E-13
chr6	161025001	161030000	9.36E-17
chr6	161058001	161063000	2.17E-06
chr6	161074001	161079000	4.48E-07
chr6	161075001	161080000	2.80E-07
chr6	161090001	161095000	1.24E-06
chr6	161162001	161167000	3.53E-06
chr6	161163001	161168000	1.98E-09
chr6	161164001	161169000	3.02E-07
chr6	161165001	161170000	1.30E-10
chr6	161166001	161171000	3.68E-07
chr6	161167001	161172000	4.33E-09
chr6	161184001	161189000	3.73E-30
chr6	161408001	161413000	7.78E-59
chr6	161409001	161414000	1.54E-55
chr6	161410001	161415000	1.01E-81
chr6	161411001	161416000	1.86E-101
chr6	161412001	161417000	1.75E-72
chr6	161413001	161418000	6.97E-39
chr6	162579001	162584000	2.20E-08
chr6	162858001	162863000	8.07E-06
chr6	163071001	163076000	2.80E-07
chr6	163072001	163077000	0.003955841
chr6	163125001	163130000	8.17E-07
chr6	163144001	163149000	5.41E-34
chr6	163830001	163835000	3.57E-53

chr6	164249001	164254000	2.65E-11
chr6	164250001	164255000	8.61E-10
chr6	164721001	164726000	1.31E-06
chr6	164770001	164775000	0.000126591
chr6	164771001	164776000	0.000974887
chr6	164772001	164777000	0.000307526
chr6	165081001	165086000	1.30E-07
chr6	165082001	165087000	1.92E-09
chr6	165083001	165088000	3.06E-10
chr6	165084001	165089000	5.26E-10
chr6	165085001	165090000	1.34E-08
chr6	165202001	165207000	3.08E-06
chr6	165297001	165302000	0.000261845
chr6	165298001	165303000	0.00097424
chr6	165371001	165376000	9.58E-05
chr6	165506001	165511000	3.30E-05
chr6	165649001	165654000	6.92E-05
chr6	165650001	165655000	4.89E-06
chr6	165695001	165700000	0.000657556
chr6	165698001	165703000	0.000321296
chr6	165722001	165727000	7.52E-19
chr6	165844001	165849000	3.24E-05
chr6	165845001	165850000	1.47E-06
chr6	165846001	165851000	5.50E-05
chr6	165942001	165947000	1.92E-05
chr6	165955001	165960000	0.000908332
chr6	165979001	165984000	0.002827717
chr6	166070001	166075000	2.16E-32
chr6	166071001	166076000	2.36E-34
chr6	166072001	166077000	1.27E-15
chr6	166091001	166096000	2.42E-07
chr6	166395001	166400000	2.68E-11
chr6	166465001	166470000	5.53E-06
chr6	166466001	166471000	0.000275497
chr6	166521001	166526000	3.56E-06
chr6	166627001	166632000	2.03E-08
chr6	166699001	166704000	1.70E-07
chr6	166791001	166796000	1.64E-35
chr6	166947001	166952000	1.01E-20
chr6	166970001	166975000	1.45E-15
chr6	167407001	167412000	1.95E-30
chr6	167408001	167413000	3.11E-50
chr6	167412001	167417000	1.65E-80
chr6	167413001	167418000	1.08E-46
chr6	167801001	167806000	1.28E-11
chr6	168076001	168081000	2.44E-25
chr6	168077001	168082000	1.51E-20
chr6	168078001	168083000	2.42E-17

chr6	168148001	168153000	1.87E-07
chr6	168149001	168154000	9.84E-08
chr6	168223001	168228000	1.93E-60
chr6	168540001	168545000	0.010935268
chr6	168720001	168725000	7.41E-29
chr6	168728001	168733000	2.00E-17
chr6	168729001	168734000	3.13E-20
chr6	168842001	168847000	2.70E-30
chr6	168869001	168874000	2.07E-14
chr6	168870001	168875000	3.00E-14
chr6	168876001	168881000	1.36E-18
chr6	168877001	168882000	6.81E-14
chr6	168879001	168884000	1.85E-10
chr6	168882001	168887000	1.75E-05
chr6	168884001	168889000	8.29E-06
chr6	169147001	169152000	0.004684231
chr6	169171001	169176000	1.22E-06
chr6	169174001	169179000	3.83E-08
chr6	169207001	169212000	9.04E-10
chr6	169219001	169224000	6.61E-12
chr6	169280001	169285000	2.64E-31
chr6	169463001	169468000	3.61E-06
chr6	169609001	169614000	4.86E-16
chr6	169676001	169681000	0.000265939
chr6	169699001	169704000	6.40E-07
chr6	169700001	169705000	1.66E-07
chr6	169701001	169706000	7.02E-08
chr6	169797001	169802000	4.33E-13
chr6	169798001	169803000	1.41E-14
chr6	169813001	169818000	0.00050021
chr6	169833001	169838000	9.64E-07
chr6	169834001	169839000	5.58E-09
chr6	169835001	169840000	0.000329552
chr6	169859001	169864000	1.32E-07
chr6	169860001	169865000	0.000112673
chr6	170101001	170106000	4.44E-77
chr6	170102001	170107000	9.64E-88
chr6	170103001	170108000	2.21E-11
chr6	170125001	170130000	3.66E-26
chr6	170147001	170152000	3.67E-65
chr6	170148001	170153000	5.74E-67
chr6	170149001	170154000	1.04E-72
chr6	170150001	170155000	1.42E-102
chr6	170151001	170156000	1.17E-97
chr6	170152001	170157000	1.39E-58
chr6	170186001	170191000	3.40E-16
chr6	170611001	170616000	1.58E-60
chr6	170612001	170617000	8.32E-50

chr6	170613001	170618000	1.55E-37
chr6	170614001	170619000	4.96E-34
chr6	170615001	170620000	5.13E-26
chr6	170616001	170621000	4.60E-17
chr6	170867001	170872000	1.54E-34
chr6	170868001	170873000	1.48E-46
chr6	170869001	170874000	4.16E-50
chr6	170870001	170875000	9.93E-51
chr6	170871001	170876000	6.50E-49
chr6	170903001	170908000	0.000196932
chr6	170904001	170909000	0.000264592
chr6	170910001	170915000	6.27E-05
chr6	170932001	170937000	0.000498521
chr6	170933001	170938000	0.000155223
chr6	170950001	170955000	0.000519885
chr7	145001	150000	1.19E-52
chr7	762001	767000	3.71E-10
chr7	763001	768000	1.09E-10
chr7	764001	769000	8.62E-08
chr7	766001	771000	1.86E-08
chr7	856001	861000	1.98E-44
chr7	912001	917000	4.40E-47
chr7	913001	918000	3.42E-43
chr7	914001	919000	1.58E-18
chr7	915001	920000	4.47E-31
chr7	916001	921000	7.60E-19
chr7	1408001	1413000	6.87E-07
chr7	1609001	1614000	4.44E-21
chr7	2277001	2282000	3.04E-19
chr7	2490001	2495000	1.84E-06
chr7	2491001	2496000	4.99E-06
chr7	2594001	2599000	2.75E-09
chr7	2596001	2601000	1.02E-11
chr7	2597001	2602000	2.70E-48
chr7	2598001	2603000	2.17E-69
chr7	2599001	2604000	3.62E-31
chr7	2879001	2884000	7.08E-37
chr7	2880001	2885000	1.68E-19
chr7	2881001	2886000	3.29E-17
chr7	2882001	2887000	2.65E-12
chr7	2883001	2888000	1.49E-15
chr7	3336001	3341000	2.10E-29
chr7	3337001	3342000	6.72E-41
chr7	3340001	3345000	7.74E-45
chr7	3341001	3346000	6.38E-46
chr7	3364001	3369000	0.000381734
chr7	3422001	3427000	2.58E-05
chr7	3431001	3436000	0.005550997

chr7	3432001	3437000	0.005582147
chr7	3451001	3456000	3.48E-10
chr7	3484001	3489000	4.16E-06
chr7	3596001	3601000	7.67E-06
chr7	3647001	3652000	3.99E-13
chr7	3648001	3653000	1.33E-15
chr7	3649001	3654000	1.31E-14
chr7	3650001	3655000	8.00E-11
chr7	3651001	3656000	2.11E-17
chr7	3652001	3657000	3.47E-13
chr7	3653001	3658000	4.36E-10
chr7	3682001	3687000	3.95E-06
chr7	3712001	3717000	5.91E-12
chr7	3728001	3733000	6.14E-06
chr7	3729001	3734000	3.80E-09
chr7	3730001	3735000	2.41E-10
chr7	3731001	3736000	3.09E-10
chr7	3733001	3738000	1.75E-12
chr7	3803001	3808000	0.000396056
chr7	3804001	3809000	0.000470672
chr7	3893001	3898000	8.64E-09
chr7	4158001	4163000	7.31E-10
chr7	5013001	5018000	1.66E-13
chr7	5081001	5086000	8.20E-46
chr7	5082001	5087000	4.09E-45
chr7	5083001	5088000	3.61E-32
chr7	5084001	5089000	5.56E-39
chr7	5085001	5090000	3.06E-27
chr7	5086001	5091000	6.88E-15
chr7	5226001	5231000	3.29E-25
chr7	5227001	5232000	6.89E-33
chr7	5228001	5233000	1.74E-36
chr7	5230001	5235000	8.99E-19
chr7	5816001	5821000	3.06E-24
chr7	5817001	5822000	3.30E-29
chr7	5818001	5823000	1.15E-34
chr7	5819001	5824000	6.13E-24
chr7	5820001	5825000	1.26E-34
chr7	6311001	6316000	1.10E-17
chr7	6384001	6389000	1.98E-08
chr7	6385001	6390000	1.16E-07
chr7	6386001	6391000	0.0001982
chr7	6387001	6392000	2.43E-11
chr7	6483001	6488000	2.22E-11
chr7	6523001	6528000	2.60E-30
chr7	6625001	6630000	2.04E-11
chr7	6626001	6631000	5.67E-07
chr7	6627001	6632000	5.53E-13

chr7	6628001	6633000	9.13E-19
chr7	6629001	6634000	1.69E-17
chr7	6675001	6680000	3.09E-08
chr7	6765001	6770000	2.28E-30
chr7	6766001	6771000	8.86E-30
chr7	6767001	6772000	1.06E-13
chr7	6768001	6773000	6.07E-36
chr7	6769001	6774000	9.24E-44
chr7	8004001	8009000	2.19E-47
chr7	8301001	8306000	5.75E-73
chr7	8302001	8307000	3.06E-54
chr7	8855001	8860000	6.47E-05
chr7	8856001	8861000	0.001432402
chr7	8868001	8873000	4.23E-11
chr7	8869001	8874000	1.07E-06
chr7	9086001	9091000	0.000255959
chr7	9119001	9124000	0.002020914
chr7	9852001	9857000	0.006766526
chr7	9996001	10001000	0.004516516
chr7	10070001	10075000	2.23E-05
chr7	10243001	10248000	1.54E-08
chr7	10374001	10379000	0.000268337
chr7	10375001	10380000	1.04E-05
chr7	10376001	10381000	0.00067147
chr7	10561001	10566000	0.001305018
chr7	10813001	10818000	0.000207787
chr7	10914001	10919000	4.80E-05
chr7	11012001	11017000	1.99E-40
chr7	11013001	11018000	1.43E-45
chr7	11014001	11019000	2.45E-23
chr7	11083001	11088000	1.07E-05
chr7	11262001	11267000	6.02E-06
chr7	11481001	11486000	4.88E-05
chr7	11646001	11651000	0.012505994
chr7	11664001	11669000	0.000515594
chr7	11867001	11872000	1.79E-40
chr7	11872001	11877000	3.83E-08
chr7	12014001	12019000	0.006457331
chr7	12251001	12256000	3.45E-33
chr7	12443001	12448000	8.04E-44
chr7	12606001	12611000	6.27E-22
chr7	12607001	12612000	8.37E-22
chr7	12608001	12613000	8.37E-22
chr7	12610001	12615000	5.59E-39
chr7	13243001	13248000	0.001142771
chr7	13244001	13249000	0.000267408
chr7	13262001	13267000	0.001358702
chr7	13362001	13367000	0.004475851

chr7	13462001	13467000	7.01E-05
chr7	13702001	13707000	0.00925332
chr7	14030001	14035000	9.38E-41
chr7	14031001	14036000	9.60E-24
chr7	14131001	14136000	0.002886201
chr7	14203001	14208000	2.77E-05
chr7	14600001	14605000	0.00297832
chr7	14950001	14955000	5.78E-07
chr7	14951001	14956000	6.68E-07
chr7	14952001	14957000	5.40E-07
chr7	15027001	15032000	9.62E-07
chr7	15046001	15051000	0.005172702
chr7	15313001	15318000	4.54E-07
chr7	15709001	15714000	5.02E-05
chr7	15772001	15777000	0.009338384
chr7	15793001	15798000	0.000122511
chr7	15794001	15799000	2.56E-07
chr7	15815001	15820000	0.000249977
chr7	15841001	15846000	0.002136122
chr7	15932001	15937000	0.034299766
chr7	16108001	16113000	0.000489618
chr7	16270001	16275000	0.000444202
chr7	16314001	16319000	0.00058767
chr7	16315001	16320000	8.16E-05
chr7	16316001	16321000	1.55E-06
chr7	16358001	16363000	2.57E-08
chr7	16359001	16364000	5.92E-11
chr7	16360001	16365000	2.61E-06
chr7	16361001	16366000	1.57E-06
chr7	16362001	16367000	1.15E-05
chr7	16378001	16383000	0.002284981
chr7	16387001	16392000	7.51E-07
chr7	16407001	16412000	0.000228756
chr7	16408001	16413000	3.44E-05
chr7	16409001	16414000	8.94E-06
chr7	16410001	16415000	1.42E-08
chr7	16411001	16416000	2.06E-07
chr7	16412001	16417000	3.63E-06
chr7	16416001	16421000	1.00E-09
chr7	16417001	16422000	7.28E-12
chr7	16456001	16461000	3.43E-35
chr7	16458001	16463000	2.29E-40
chr7	16459001	16464000	1.42E-47
chr7	16461001	16466000	8.47E-37
chr7	16497001	16502000	0.002206636
chr7	16514001	16519000	2.86E-13
chr7	16515001	16520000	4.16E-10
chr7	16516001	16521000	8.22E-10

chr7	16680001	16685000	2.86E-30
chr7	16681001	16686000	1.95E-38
chr7	16686001	16691000	7.08E-36
chr7	16789001	16794000	1.02E-38
chr7	16790001	16795000	1.52E-34
chr7	16791001	16796000	5.75E-34
chr7	16792001	16797000	3.89E-38
chr7	17081001	17086000	0.00093387
chr7	17082001	17087000	0.000186681
chr7	17142001	17147000	0.001618462
chr7	17143001	17148000	0.001310061
chr7	17334001	17339000	8.49E-48
chr7	17425001	17430000	7.01E-05
chr7	17426001	17431000	8.87E-07
chr7	17825001	17830000	4.39E-10
chr7	17979001	17984000	3.63E-48
chr7	17980001	17985000	1.73E-57
chr7	18121001	18126000	8.64E-17
chr7	18122001	18127000	6.07E-51
chr7	18123001	18128000	6.51E-33
chr7	18124001	18129000	4.54E-36
chr7	18125001	18130000	1.49E-41
chr7	18126001	18131000	7.00E-23
chr7	18329001	18334000	0.000124304
chr7	18493001	18498000	1.04E-07
chr7	18494001	18499000	6.51E-08
chr7	18495001	18500000	6.09E-07
chr7	18531001	18536000	1.70E-12
chr7	18532001	18537000	1.40E-10
chr7	18533001	18538000	1.43E-10
chr7	19744001	19749000	2.48E-37
chr7	20147001	20152000	0.001917836
chr7	20370001	20375000	2.36E-36
chr7	20371001	20376000	2.07E-33
chr7	20410001	20415000	0.001613313
chr7	20687001	20692000	1.36E-05
chr7	20688001	20693000	5.48E-06
chr7	20837001	20842000	3.11E-40
chr7	21065001	21070000	4.05E-10
chr7	21066001	21071000	1.05E-11
chr7	21420001	21425000	0.00014468
chr7	21462001	21467000	1.12E-22
chr7	21463001	21468000	3.60E-24
chr7	21578001	21583000	4.26E-65
chr7	21579001	21584000	5.89E-78
chr7	21580001	21585000	2.52E-58
chr7	21581001	21586000	1.22E-52
chr7	21582001	21587000	6.26E-50

chr7	21734001	21739000	2.35E-05
chr7	21735001	21740000	0.000111709
chr7	21736001	21741000	0.000112372
chr7	21857001	21862000	0.000831351
chr7	21858001	21863000	0.002087211
chr7	21859001	21864000	0.003479827
chr7	21985001	21990000	8.33E-32
chr7	22396001	22401000	2.49E-26
chr7	22571001	22576000	1.92E-08
chr7	23048001	23053000	1.36E-17
chr7	23049001	23054000	1.15E-22
chr7	23050001	23055000	1.34E-28
chr7	23051001	23056000	1.77E-32
chr7	23052001	23057000	1.47E-25
chr7	23053001	23058000	1.36E-19
chr7	23144001	23149000	1.06E-48
chr7	23145001	23150000	2.13E-66
chr7	23146001	23151000	1.47E-15
chr7	23567001	23572000	2.10E-35
chr7	23571001	23576000	1.81E-21
chr7	23609001	23614000	1.09E-10
chr7	23715001	23720000	9.78E-39
chr7	23716001	23721000	6.72E-42
chr7	23717001	23722000	5.02E-25
chr7	23718001	23723000	4.60E-32
chr7	23719001	23724000	1.75E-33
chr7	23778001	23783000	1.68E-07
chr7	23779001	23784000	4.80E-07
chr7	23800001	23805000	0.000497319
chr7	23848001	23853000	0.000341491
chr7	23869001	23874000	1.61E-22
chr7	23885001	23890000	2.82E-12
chr7	23886001	23891000	1.70E-15
chr7	23887001	23892000	5.59E-10
chr7	23888001	23893000	4.30E-11
chr7	23889001	23894000	8.76E-13
chr7	23974001	23979000	1.40E-06
chr7	23975001	23980000	1.97E-07
chr7	24047001	24052000	0.002729733
chr7	24048001	24053000	0.000184269
chr7	24070001	24075000	0.01404037
chr7	24121001	24126000	8.00E-10
chr7	24122001	24127000	9.09E-08
chr7	24123001	24128000	1.80E-10
chr7	24124001	24129000	1.14E-14
chr7	24125001	24130000	1.43E-13
chr7	24126001	24131000	4.37E-11
chr7	24127001	24132000	4.79E-14

chr7	24128001	24133000	4.81E-10
chr7	24129001	24134000	1.21E-05
chr7	24133001	24138000	2.16E-08
chr7	24134001	24139000	5.95E-08
chr7	24135001	24140000	2.53E-08
chr7	24136001	24141000	1.50E-06
chr7	24144001	24149000	1.69E-05
chr7	24145001	24150000	3.89E-08
chr7	24146001	24151000	1.50E-08
chr7	24147001	24152000	1.11E-07
chr7	24196001	24201000	1.48E-06
chr7	24213001	24218000	9.78E-05
chr7	24214001	24219000	1.30E-05
chr7	24249001	24254000	4.68E-05
chr7	24250001	24255000	1.13E-07
chr7	24260001	24265000	9.34E-07
chr7	24266001	24271000	1.23E-07
chr7	24267001	24272000	1.13E-07
chr7	24268001	24273000	5.99E-07
chr7	24269001	24274000	3.08E-09
chr7	24270001	24275000	4.07E-06
chr7	24282001	24287000	5.63E-11
chr7	24283001	24288000	2.11E-05
chr7	24284001	24289000	4.14E-06
chr7	24310001	24315000	2.04E-08
chr7	24312001	24317000	7.61E-08
chr7	24313001	24318000	8.82E-12
chr7	24326001	24331000	2.15E-08
chr7	24388001	24393000	5.74E-08
chr7	24389001	24394000	3.09E-10
chr7	24390001	24395000	1.90E-09
chr7	24391001	24396000	9.77E-08
chr7	24392001	24397000	1.33E-08
chr7	24405001	24410000	4.74E-07
chr7	24406001	24411000	1.12E-07
chr7	24407001	24412000	4.53E-06
chr7	24563001	24568000	0.000386382
chr7	24564001	24569000	0.000350768
chr7	24661001	24666000	0.027453331
chr7	24792001	24797000	8.54E-13
chr7	24793001	24798000	1.14E-25
chr7	24794001	24799000	1.53E-38
chr7	24795001	24800000	5.33E-50
chr7	24796001	24801000	1.83E-58
chr7	24797001	24802000	6.62E-57
chr7	24911001	24916000	3.38E-08
chr7	24912001	24917000	2.01E-08
chr7	24913001	24918000	5.91E-06

chr7	24914001	24919000	1.94E-05
chr7	24973001	24978000	1.24E-07
chr7	24974001	24979000	1.18E-07
chr7	24975001	24980000	4.92E-06
chr7	25015001	25020000	4.87E-56
chr7	25016001	25021000	1.27E-38
chr7	25215001	25220000	1.97E-62
chr7	25216001	25221000	6.48E-56
chr7	25217001	25222000	5.89E-54
chr7	25218001	25223000	2.95E-52
chr7	25219001	25224000	1.78E-39
chr7	25220001	25225000	3.66E-23
chr7	25339001	25344000	0.000110745
chr7	25358001	25363000	1.38E-07
chr7	25359001	25364000	1.47E-07
chr7	25361001	25366000	1.20E-07
chr7	25376001	25381000	8.86E-11
chr7	25377001	25382000	1.46E-09
chr7	25401001	25406000	9.31E-08
chr7	25402001	25407000	5.15E-05
chr7	25403001	25408000	1.07E-06
chr7	25404001	25409000	1.46E-06
chr7	25405001	25410000	1.75E-07
chr7	25413001	25418000	0.002321179
chr7	25416001	25421000	2.28E-05
chr7	25469001	25474000	1.03E-08
chr7	25470001	25475000	1.07E-11
chr7	25471001	25476000	2.30E-11
chr7	25472001	25477000	5.99E-11
chr7	25474001	25479000	0.000325994
chr7	25482001	25487000	1.15E-05
chr7	25484001	25489000	7.46E-05
chr7	25638001	25643000	1.05E-09
chr7	25676001	25681000	6.53E-08
chr7	25693001	25698000	0.000490462
chr7	25935001	25940000	3.14E-17
chr7	25990001	25995000	2.69E-19
chr7	25991001	25996000	5.95E-14
chr7	26189001	26194000	2.39E-17
chr7	26327001	26332000	2.14E-22
chr7	26328001	26333000	1.27E-16
chr7	26329001	26334000	4.60E-15
chr7	26330001	26335000	3.38E-16
chr7	26331001	26336000	1.82E-12
chr7	26411001	26416000	3.67E-56
chr7	26412001	26417000	6.58E-54
chr7	26413001	26418000	4.44E-80
chr7	26414001	26419000	8.23E-66

chr7	26415001	26420000	3.83E-52
chr7	26416001	26421000	3.87E-48
chr7	26438001	26443000	5.07E-48
chr7	26726001	26731000	0.015615924
chr7	26815001	26820000	0.000285739
chr7	26897001	26902000	2.52E-10
chr7	26899001	26904000	8.40E-13
chr7	27190001	27195000	6.76E-10
chr7	27339001	27344000	1.34E-06
chr7	27647001	27652000	0.000552626
chr7	28098001	28103000	3.95E-07
chr7	28099001	28104000	1.29E-06
chr7	28215001	28220000	9.48E-63
chr7	28216001	28221000	1.39E-34
chr7	28217001	28222000	3.33E-44
chr7	28218001	28223000	7.84E-36
chr7	28219001	28224000	9.61E-46
chr7	28444001	28449000	2.39E-12
chr7	28450001	28455000	2.06E-29
chr7	28596001	28601000	1.21E-30
chr7	28597001	28602000	9.94E-22
chr7	28598001	28603000	1.93E-32
chr7	28599001	28604000	4.29E-39
chr7	28600001	28605000	2.87E-44
chr7	28643001	28648000	5.90E-11
chr7	28644001	28649000	1.14E-11
chr7	28858001	28863000	8.46E-12
chr7	28859001	28864000	2.77E-09
chr7	28860001	28865000	2.59E-07
chr7	28889001	28894000	4.22E-27
chr7	28983001	28988000	0.00422733
chr7	29233001	29238000	5.64E-36
chr7	29234001	29239000	2.77E-61
chr7	29235001	29240000	1.15E-17
chr7	29725001	29730000	2.60E-20
chr7	29758001	29763000	2.60E-08
chr7	30024001	30029000	3.23E-23
chr7	30025001	30030000	3.82E-24
chr7	30026001	30031000	2.40E-27
chr7	30027001	30032000	3.87E-47
chr7	30028001	30033000	1.28E-54
chr7	30029001	30034000	1.63E-25
chr7	30068001	30073000	6.60E-53
chr7	30170001	30175000	7.33E-28
chr7	30171001	30176000	2.79E-34
chr7	30172001	30177000	2.58E-26
chr7	30174001	30179000	4.39E-30
chr7	30175001	30180000	6.11E-21

chr7	30320001	30325000	1.23E-33
chr7	30516001	30521000	2.30E-14
chr7	30517001	30522000	1.31E-35
chr7	30518001	30523000	2.53E-48
chr7	30540001	30545000	2.98E-14
chr7	30541001	30546000	8.40E-16
chr7	30542001	30547000	4.82E-08
chr7	30543001	30548000	2.84E-09
chr7	30544001	30549000	5.87E-17
chr7	30811001	30816000	1.52E-19
chr7	30986001	30991000	2.61E-05
chr7	30987001	30992000	2.22E-06
chr7	30994001	30999000	3.06E-06
chr7	30995001	31000000	1.26E-06
chr7	30996001	31001000	5.14E-12
chr7	30998001	31003000	6.47E-12
chr7	31218001	31223000	6.95E-07
chr7	31219001	31224000	1.62E-06
chr7	31220001	31225000	4.58E-06
chr7	31224001	31229000	2.45E-05
chr7	31226001	31231000	1.90E-05
chr7	31234001	31239000	3.38E-05
chr7	31235001	31240000	1.95E-06
chr7	31263001	31268000	2.63E-06
chr7	31270001	31275000	1.30E-07
chr7	31282001	31287000	8.73E-05
chr7	31285001	31290000	1.48E-10
chr7	31286001	31291000	2.18E-10
chr7	31325001	31330000	4.48E-06
chr7	31333001	31338000	1.07E-05
chr7	31334001	31339000	0.00023014
chr7	31335001	31340000	9.11E-06
chr7	31337001	31342000	7.75E-07
chr7	31353001	31358000	2.73E-07
chr7	31354001	31359000	1.14E-05
chr7	31366001	31371000	4.21E-07
chr7	31398001	31403000	1.37E-06
chr7	31399001	31404000	3.12E-10
chr7	31401001	31406000	3.40E-07
chr7	31622001	31627000	6.13E-05
chr7	31655001	31660000	1.52E-06
chr7	31920001	31925000	1.79E-06
chr7	31979001	31984000	0.000357474
chr7	32278001	32283000	0.000326712
chr7	32279001	32284000	1.36E-05
chr7	32314001	32319000	7.04E-05
chr7	32315001	32320000	1.31E-06
chr7	32316001	32321000	3.01E-09

chr7	32338001	32343000	5.44E-23
chr7	32430001	32435000	0.001531585
chr7	32431001	32436000	0.000166411
chr7	32531001	32536000	2.45E-34
chr7	32533001	32538000	2.98E-29
chr7	32535001	32540000	1.09E-29
chr7	32763001	32768000	1.10E-76
chr7	32764001	32769000	7.66E-88
chr7	32765001	32770000	1.05E-71
chr7	32766001	32771000	5.78E-83
chr7	32767001	32772000	2.43E-76
chr7	32768001	32773000	3.48E-83
chr7	32981001	32986000	1.05E-16
chr7	32982001	32987000	9.96E-17
chr7	32995001	33000000	4.76E-14
chr7	32996001	33001000	8.59E-26
chr7	32997001	33002000	5.91E-19
chr7	33102001	33107000	8.99E-43
chr7	33169001	33174000	4.53E-26
chr7	33213001	33218000	1.17E-09
chr7	33277001	33282000	0.001221542
chr7	33278001	33283000	0.001265191
chr7	33281001	33286000	0.004060641
chr7	33337001	33342000	0.001278673
chr7	33338001	33343000	4.16E-05
chr7	33368001	33373000	2.81E-13
chr7	33391001	33396000	2.77E-08
chr7	33392001	33397000	5.61E-08
chr7	33401001	33406000	1.67E-08
chr7	33439001	33444000	2.59E-06
chr7	33440001	33445000	3.15E-08
chr7	33489001	33494000	1.31E-08
chr7	33490001	33495000	5.89E-06
chr7	33513001	33518000	1.32E-05
chr7	33514001	33519000	5.67E-06
chr7	33515001	33520000	3.72E-06
chr7	33557001	33562000	6.04E-07
chr7	33558001	33563000	7.17E-06
chr7	33560001	33565000	1.03E-09
chr7	33574001	33579000	3.11E-07
chr7	33576001	33581000	3.30E-06
chr7	33857001	33862000	0.000193779
chr7	33935001	33940000	7.01E-13
chr7	33941001	33946000	1.82E-16
chr7	33942001	33947000	2.34E-41
chr7	33943001	33948000	1.22E-72
chr7	33944001	33949000	7.60E-75
chr7	33945001	33950000	1.01E-34

chr7	34456001	34461000	1.13E-07
chr7	34457001	34462000	3.80E-07
chr7	34593001	34598000	3.48E-11
chr7	34594001	34599000	1.40E-11
chr7	34596001	34601000	4.10E-12
chr7	34597001	34602000	8.74E-08
chr7	34671001	34676000	6.05E-08
chr7	34672001	34677000	1.69E-07
chr7	34673001	34678000	6.89E-06
chr7	34674001	34679000	5.14E-07
chr7	34675001	34680000	3.31E-06
chr7	34676001	34681000	6.71E-06
chr7	34677001	34682000	5.38E-07
chr7	34746001	34751000	0.000416181
chr7	34747001	34752000	5.76E-05
chr7	34748001	34753000	0.00025725
chr7	34780001	34785000	0.000270339
chr7	34829001	34834000	0.003833435
chr7	34831001	34836000	0.013407351
chr7	34832001	34837000	0.007002156
chr7	34833001	34838000	0.006842053
chr7	35072001	35077000	1.76E-48
chr7	35073001	35078000	8.50E-43
chr7	35075001	35080000	4.16E-53
chr7	35076001	35081000	1.76E-51
chr7	35077001	35082000	1.97E-54
chr7	35226001	35231000	8.51E-29
chr7	35284001	35289000	4.72E-11
chr7	35485001	35490000	2.31E-09
chr7	35730001	35735000	1.24E-42
chr7	35778001	35783000	2.46E-18
chr7	35836001	35841000	1.63E-44
chr7	35855001	35860000	0.000348361
chr7	35856001	35861000	0.000120234
chr7	36188001	36193000	2.65E-22
chr7	36189001	36194000	5.93E-24
chr7	36190001	36195000	2.65E-07
chr7	36462001	36467000	0.000466516
chr7	36618001	36623000	0.000164084
chr7	36619001	36624000	2.99E-05
chr7	36620001	36625000	7.71E-06
chr7	37275001	37280000	6.57E-10
chr7	37303001	37308000	5.24E-09
chr7	37390001	37395000	1.19E-21
chr7	37393001	37398000	1.72E-14
chr7	37397001	37402000	3.58E-07
chr7	37398001	37403000	6.14E-08
chr7	37399001	37404000	8.74E-10

chr7	37464001	37469000	1.32E-06
chr7	37473001	37478000	5.89E-07
chr7	37474001	37479000	5.26E-09
chr7	37475001	37480000	1.04E-09
chr7	37483001	37488000	3.00E-12
chr7	37484001	37489000	2.28E-19
chr7	37485001	37490000	1.32E-19
chr7	37486001	37491000	3.23E-17
chr7	37487001	37492000	1.21E-14
chr7	37488001	37493000	8.19E-27
chr7	37515001	37520000	2.90E-05
chr7	37557001	37562000	0.000122017
chr7	37558001	37563000	4.80E-06
chr7	37559001	37564000	4.83E-06
chr7	37560001	37565000	2.03E-06
chr7	37561001	37566000	9.99E-06
chr7	37565001	37570000	1.58E-06
chr7	37566001	37571000	1.06E-11
chr7	37567001	37572000	1.86E-18
chr7	37568001	37573000	2.42E-13
chr7	37569001	37574000	3.97E-10
chr7	37570001	37575000	3.35E-10
chr7	37622001	37627000	1.04E-09
chr7	37624001	37629000	4.15E-10
chr7	37625001	37630000	1.19E-11
chr7	37626001	37631000	4.95E-07
chr7	37628001	37633000	1.53E-07
chr7	37634001	37639000	9.85E-05
chr7	37637001	37642000	1.23E-05
chr7	37650001	37655000	0.000628317
chr7	37651001	37656000	1.94E-10
chr7	37652001	37657000	7.93E-11
chr7	37731001	37736000	2.98E-09
chr7	37732001	37737000	3.00E-10
chr7	37745001	37750000	1.78E-09
chr7	37792001	37797000	1.92E-06
chr7	37795001	37800000	7.20E-07
chr7	37810001	37815000	7.37E-10
chr7	37847001	37852000	4.87E-10
chr7	37848001	37853000	6.91E-08
chr7	37849001	37854000	7.82E-10
chr7	37850001	37855000	1.69E-12
chr7	37890001	37895000	0.000396465
chr7	37902001	37907000	1.74E-05
chr7	37903001	37908000	1.57E-05
chr7	37904001	37909000	5.34E-07
chr7	37905001	37910000	5.41E-08
chr7	37906001	37911000	4.42E-08

chr7	37907001	37912000	1.54E-06
chr7	37917001	37922000	9.14E-05
chr7	37918001	37923000	0.000145887
chr7	37925001	37930000	1.18E-07
chr7	37926001	37931000	6.94E-12
chr7	37951001	37956000	7.69E-24
chr7	38008001	38013000	0.000116544
chr7	38045001	38050000	0.000145132
chr7	38068001	38073000	0.005929993
chr7	38152001	38157000	8.93E-05
chr7	38154001	38159000	2.97E-09
chr7	38158001	38163000	3.49E-06
chr7	38218001	38223000	5.68E-43
chr7	38467001	38472000	9.50E-09
chr7	38468001	38473000	2.98E-11
chr7	38469001	38474000	4.94E-09
chr7	38470001	38475000	8.80E-10
chr7	38471001	38476000	3.97E-07
chr7	38472001	38477000	0.000177511
chr7	38666001	38671000	2.37E-29
chr7	38667001	38672000	6.53E-27
chr7	38668001	38673000	1.14E-21
chr7	38669001	38674000	9.81E-29
chr7	38670001	38675000	3.63E-31
chr7	39014001	39019000	6.88E-13
chr7	39016001	39021000	0.000259921
chr7	39047001	39052000	3.05E-12
chr7	39060001	39065000	1.63E-06
chr7	39061001	39066000	6.36E-06
chr7	39080001	39085000	0.004583323
chr7	39159001	39164000	1.99E-07
chr7	39163001	39168000	2.17E-06
chr7	39373001	39378000	7.67E-09
chr7	39397001	39402000	0.005425117
chr7	39449001	39454000	8.00E-27
chr7	39521001	39526000	1.24E-10
chr7	39522001	39527000	1.53E-10
chr7	39523001	39528000	7.24E-07
chr7	39601001	39606000	3.66E-22
chr7	39603001	39608000	2.80E-56
chr7	39604001	39609000	1.09E-54
chr7	39605001	39610000	7.47E-53
chr7	39606001	39611000	9.12E-51
chr7	39658001	39663000	5.77E-57
chr7	39659001	39664000	6.09E-71
chr7	39660001	39665000	3.53E-60
chr7	39661001	39666000	1.77E-36
chr7	39662001	39667000	7.20E-41

chr7	39663001	39668000	5.82E-28
chr7	39768001	39773000	1.51E-09
chr7	39890001	39895000	3.35E-11
chr7	39891001	39896000	6.13E-13
chr7	39892001	39897000	2.60E-08
chr7	39985001	39990000	2.88E-28
chr7	39986001	39991000	1.73E-39
chr7	39987001	39992000	4.19E-55
chr7	39991001	39996000	3.03E-37
chr7	40193001	40198000	1.28E-09
chr7	40194001	40199000	2.67E-11
chr7	40352001	40357000	9.44E-05
chr7	40427001	40432000	4.44E-08
chr7	40428001	40433000	1.54E-09
chr7	40440001	40445000	2.82E-05
chr7	40467001	40472000	8.76E-06
chr7	40468001	40473000	6.53E-06
chr7	40485001	40490000	7.63E-05
chr7	40492001	40497000	6.19E-06
chr7	40493001	40498000	9.13E-06
chr7	40494001	40499000	2.59E-06
chr7	40495001	40500000	1.25E-07
chr7	40519001	40524000	2.83E-05
chr7	40520001	40525000	1.48E-05
chr7	40533001	40538000	0.000195468
chr7	40534001	40539000	0.000297025
chr7	40538001	40543000	0.013099881
chr7	40633001	40638000	2.02E-06
chr7	40635001	40640000	7.19E-10
chr7	40636001	40641000	2.03E-09
chr7	40637001	40642000	8.01E-10
chr7	40638001	40643000	2.06E-08
chr7	40639001	40644000	3.13E-05
chr7	40828001	40833000	0.002804699
chr7	41681001	41686000	0.000192916
chr7	41842001	41847000	0.000550722
chr7	41931001	41936000	9.61E-07
chr7	41933001	41938000	0.002575943
chr7	41934001	41939000	2.32E-05
chr7	41988001	41993000	1.31E-14
chr7	41989001	41994000	3.23E-11
chr7	41991001	41996000	2.64E-08
chr7	42082001	42087000	2.52E-08
chr7	42098001	42103000	6.29E-06
chr7	42099001	42104000	1.21E-07
chr7	42100001	42105000	2.45E-07
chr7	42101001	42106000	2.45E-05
chr7	42233001	42238000	2.56E-16

chr7	42234001	42239000	1.94E-16
chr7	42235001	42240000	1.68E-16
chr7	42263001	42268000	1.07E-24
chr7	42272001	42277000	2.72E-54
chr7	42287001	42292000	3.37E-11
chr7	42288001	42293000	1.18E-09
chr7	42354001	42359000	9.77E-05
chr7	42355001	42360000	9.45E-05
chr7	42356001	42361000	8.56E-07
chr7	42357001	42362000	4.20E-07
chr7	42366001	42371000	8.05E-10
chr7	42367001	42372000	1.14E-07
chr7	42388001	42393000	1.65E-09
chr7	42389001	42394000	2.23E-11
chr7	42390001	42395000	5.07E-05
chr7	42533001	42538000	7.83E-17
chr7	42592001	42597000	0.00037236
chr7	42593001	42598000	2.36E-05
chr7	42635001	42640000	0.003084976
chr7	42659001	42664000	4.71E-08
chr7	42660001	42665000	2.70E-07
chr7	42689001	42694000	0.000167086
chr7	42690001	42695000	3.71E-08
chr7	42691001	42696000	6.76E-07
chr7	42692001	42697000	1.57E-05
chr7	42693001	42698000	0.000238174
chr7	42746001	42751000	2.30E-11
chr7	42747001	42752000	9.18E-11
chr7	42748001	42753000	2.06E-08
chr7	42749001	42754000	9.78E-08
chr7	42750001	42755000	1.45E-05
chr7	42813001	42818000	4.26E-09
chr7	42814001	42819000	2.83E-08
chr7	42815001	42820000	3.12E-06
chr7	42828001	42833000	8.11E-05
chr7	42831001	42836000	0.004039649
chr7	42888001	42893000	3.18E-05
chr7	42889001	42894000	1.86E-08
chr7	42928001	42933000	4.63E-13
chr7	42947001	42952000	3.50E-54
chr7	42948001	42953000	4.03E-54
chr7	42949001	42954000	1.86E-30
chr7	42950001	42955000	2.99E-23
chr7	42951001	42956000	3.93E-25
chr7	42972001	42977000	2.89E-14
chr7	42992001	42997000	0.00135761
chr7	42993001	42998000	7.63E-05
chr7	43037001	43042000	3.40E-05

chr7	43038001	43043000	0.001424615
chr7	43039001	43044000	0.000778841
chr7	43071001	43076000	0.001054181
chr7	43077001	43082000	0.000143853
chr7	43112001	43117000	7.33E-12
chr7	43127001	43132000	6.87E-05
chr7	43128001	43133000	0.00039863
chr7	43129001	43134000	0.001110635
chr7	43130001	43135000	3.14E-05
chr7	43131001	43136000	3.91E-05
chr7	43149001	43154000	2.93E-11
chr7	43152001	43157000	6.54E-17
chr7	43153001	43158000	1.69E-50
chr7	43154001	43159000	1.00E-07
chr7	43155001	43160000	1.27E-12
chr7	43156001	43161000	5.57E-12
chr7	43157001	43162000	1.79E-09
chr7	43167001	43172000	3.21E-09
chr7	43284001	43289000	2.09E-08
chr7	43616001	43621000	0.000528741
chr7	43618001	43623000	4.26E-50
chr7	43768001	43773000	2.49E-29
chr7	43769001	43774000	1.74E-24
chr7	43941001	43946000	2.59E-11
chr7	43942001	43947000	1.42E-21
chr7	43943001	43948000	6.13E-21
chr7	43944001	43949000	2.46E-32
chr7	43946001	43951000	1.69E-18
chr7	43966001	43971000	2.82E-17
chr7	44077001	44082000	4.27E-11
chr7	44078001	44083000	1.69E-10
chr7	44084001	44089000	5.78E-10
chr7	44236001	44241000	5.87E-33
chr7	44237001	44242000	2.11E-32
chr7	44238001	44243000	4.94E-41
chr7	44239001	44244000	1.94E-48
chr7	44240001	44245000	2.22E-51
chr7	44525001	44530000	4.46E-18
chr7	44528001	44533000	1.74E-36
chr7	44529001	44534000	1.03E-30
chr7	44530001	44535000	4.85E-28
chr7	44565001	44570000	2.63E-05
chr7	44618001	44623000	1.55E-30
chr7	44619001	44624000	1.13E-33
chr7	44620001	44625000	9.91E-33
chr7	44621001	44626000	1.26E-33
chr7	44622001	44627000	2.55E-26
chr7	44784001	44789000	1.67E-19

chr7	44785001	44790000	1.33E-21
chr7	44786001	44791000	1.07E-15
chr7	44788001	44793000	1.02E-13
chr7	44832001	44837000	3.42E-08
chr7	44920001	44925000	3.24E-29
chr7	44922001	44927000	2.60E-23
chr7	44923001	44928000	2.27E-06
chr7	44924001	44929000	3.52E-11
chr7	44925001	44930000	3.72E-16
chr7	45151001	45156000	1.28E-22
chr7	45614001	45619000	2.46E-13
chr7	45804001	45809000	8.53E-44
chr7	45805001	45810000	3.38E-29
chr7	45806001	45811000	2.59E-24
chr7	45807001	45812000	8.65E-31
chr7	45808001	45813000	3.04E-36
chr7	45924001	45929000	1.61E-18
chr7	45925001	45930000	1.29E-24
chr7	45994001	45999000	5.00E-05
chr7	45995001	46000000	0.000316066
chr7	46033001	46038000	0.002873394
chr7	46058001	46063000	1.38E-07
chr7	46059001	46064000	2.21E-05
chr7	46060001	46065000	0.000145843
chr7	46062001	46067000	6.77E-06
chr7	46063001	46068000	1.30E-12
chr7	46064001	46069000	2.50E-10
chr7	46065001	46070000	3.11E-10
chr7	46066001	46071000	1.70E-09
chr7	46067001	46072000	5.05E-08
chr7	46282001	46287000	0.004689609
chr7	46283001	46288000	9.20E-05
chr7	46284001	46289000	4.17E-06
chr7	46285001	46290000	2.09E-06
chr7	46286001	46291000	0.00023728
chr7	46329001	46334000	1.29E-06
chr7	46330001	46335000	7.22E-13
chr7	46331001	46336000	9.02E-14
chr7	46332001	46337000	1.72E-14
chr7	46333001	46338000	2.80E-17
chr7	46334001	46339000	6.62E-10
chr7	46341001	46346000	2.60E-07
chr7	46342001	46347000	3.80E-07
chr7	46343001	46348000	5.21E-07
chr7	46422001	46427000	9.98E-05
chr7	46423001	46428000	0.000280588
chr7	46447001	46452000	0.000250605
chr7	46462001	46467000	0.000134052

chr7	46473001	46478000	1.91E-05
chr7	46474001	46479000	0.000308859
chr7	46490001	46495000	0.000804141
chr7	46548001	46553000	0.001119089
chr7	46729001	46734000	0.000107784
chr7	46730001	46735000	1.98E-05
chr7	46731001	46736000	1.99E-05
chr7	46794001	46799000	0.000340765
chr7	46841001	46846000	9.16E-05
chr7	46854001	46859000	3.30E-06
chr7	46966001	46971000	8.84E-08
chr7	47010001	47015000	0.00173507
chr7	47290001	47295000	3.11E-16
chr7	47291001	47296000	2.55E-11
chr7	47292001	47297000	4.56E-09
chr7	47293001	47298000	7.81E-19
chr7	47294001	47299000	1.98E-20
chr7	47617001	47622000	6.62E-10
chr7	47620001	47625000	3.91E-07
chr7	47621001	47626000	3.12E-17
chr7	48015001	48020000	1.05E-26
chr7	48016001	48021000	1.16E-27
chr7	48019001	48024000	4.58E-32
chr7	48060001	48065000	0.000119363
chr7	48069001	48074000	1.00E-07
chr7	48070001	48075000	1.36E-19
chr7	48071001	48076000	1.03E-34
chr7	48072001	48077000	1.33E-31
chr7	48073001	48078000	1.91E-51
chr7	48074001	48079000	8.82E-46
chr7	48075001	48080000	4.49E-34
chr7	48093001	48098000	2.19E-06
chr7	48125001	48130000	2.87E-22
chr7	48126001	48131000	2.50E-21
chr7	48127001	48132000	1.43E-20
chr7	48128001	48133000	4.08E-13
chr7	48129001	48134000	5.08E-18
chr7	48165001	48170000	4.00E-06
chr7	48248001	48253000	0.001391828
chr7	48275001	48280000	2.78E-07
chr7	48317001	48322000	8.41E-05
chr7	48371001	48376000	1.04E-08
chr7	48485001	48490000	0.003698728
chr7	48521001	48526000	0.001051272
chr7	48522001	48527000	5.78E-08
chr7	48523001	48528000	2.74E-07
chr7	48560001	48565000	0.00013298
chr7	48561001	48566000	0.000105773

chr7	48562001	48567000	3.28E-05
chr7	48568001	48573000	1.98E-06
chr7	48569001	48574000	0.000340672
chr7	48570001	48575000	5.71E-06
chr7	48639001	48644000	1.17E-08
chr7	48641001	48646000	8.92E-11
chr7	48879001	48884000	6.62E-06
chr7	48880001	48885000	7.99E-07
chr7	48980001	48985000	0.016730177
chr7	48981001	48986000	0.000251769
chr7	49022001	49027000	0.000267469
chr7	49036001	49041000	0.000164442
chr7	49037001	49042000	0.000177969
chr7	49038001	49043000	4.54E-05
chr7	49069001	49074000	2.15E-08
chr7	49099001	49104000	8.61E-09
chr7	49100001	49105000	6.21E-06
chr7	49130001	49135000	8.79E-06
chr7	49137001	49142000	4.50E-10
chr7	49151001	49156000	1.23E-07
chr7	49162001	49167000	1.11E-05
chr7	49163001	49168000	1.44E-06
chr7	49166001	49171000	0.007293818
chr7	49169001	49174000	8.76E-05
chr7	49179001	49184000	5.88E-11
chr7	49180001	49185000	3.55E-08
chr7	49181001	49186000	8.49E-08
chr7	49188001	49193000	0.001507826
chr7	49204001	49209000	1.58E-07
chr7	49205001	49210000	1.73E-11
chr7	49217001	49222000	0.000754625
chr7	49256001	49261000	2.95E-05
chr7	49258001	49263000	0.003804275
chr7	49293001	49298000	0.002098532
chr7	49323001	49328000	7.78E-07
chr7	49325001	49330000	1.03E-09
chr7	49345001	49350000	9.75E-10
chr7	49346001	49351000	3.82E-10
chr7	49347001	49352000	2.57E-15
chr7	49348001	49353000	4.31E-10
chr7	49349001	49354000	3.82E-09
chr7	49360001	49365000	0.000287908
chr7	49389001	49394000	0.00863734
chr7	49392001	49397000	1.81E-06
chr7	49393001	49398000	3.64E-07
chr7	49395001	49400000	0.000383805
chr7	49396001	49401000	0.001146871
chr7	49434001	49439000	1.13E-06

chr7	49435001	49440000	1.48E-06
chr7	49441001	49446000	0.001185777
chr7	49442001	49447000	7.15E-06
chr7	49475001	49480000	0.000345182
chr7	49476001	49481000	0.000558074
chr7	49531001	49536000	2.33E-09
chr7	49542001	49547000	9.49E-06
chr7	49570001	49575000	9.10E-08
chr7	49571001	49576000	9.93E-09
chr7	49654001	49659000	0.000121111
chr7	49655001	49660000	2.88E-05
chr7	49703001	49708000	2.17E-13
chr7	49704001	49709000	7.59E-12
chr7	49724001	49729000	6.48E-05
chr7	49725001	49730000	7.44E-05
chr7	49726001	49731000	7.76E-05
chr7	49780001	49785000	3.90E-06
chr7	49781001	49786000	1.72E-05
chr7	49782001	49787000	0.00258258
chr7	49799001	49804000	0.000355406
chr7	49800001	49805000	0.000310709
chr7	49818001	49823000	2.25E-05
chr7	49819001	49824000	9.79E-09
chr7	49820001	49825000	1.78E-05
chr7	49822001	49827000	8.10E-05
chr7	49835001	49840000	3.96E-08
chr7	49865001	49870000	7.65E-08
chr7	49866001	49871000	1.04E-05
chr7	49979001	49984000	0.002651607
chr7	49981001	49986000	0.000185397
chr7	49987001	49992000	0.000119305
chr7	49991001	49996000	8.95E-05
chr7	50003001	50008000	0.000478195
chr7	50004001	50009000	0.000123032
chr7	50005001	50010000	0.000155676
chr7	50006001	50011000	0.000147211
chr7	50011001	50016000	0.000678344
chr7	50025001	50030000	0.000169434
chr7	50036001	50041000	1.43E-06
chr7	50055001	50060000	0.001210962
chr7	50062001	50067000	1.11E-07
chr7	50069001	50074000	2.24E-08
chr7	50250001	50255000	1.26E-08
chr7	50253001	50258000	7.83E-10
chr7	50254001	50259000	3.92E-12
chr7	50255001	50260000	1.18E-08
chr7	50269001	50274000	9.56E-08
chr7	50270001	50275000	1.04E-07

chr7	50363001	50368000	1.04E-17
chr7	50410001	50415000	1.39E-07
chr7	50513001	50518000	3.99E-37
chr7	51379001	51384000	1.93E-46
chr7	51380001	51385000	2.15E-44
chr7	51381001	51386000	1.63E-23
chr7	51382001	51387000	1.37E-28
chr7	51383001	51388000	1.01E-19
chr7	51384001	51389000	6.67E-20
chr7	51594001	51599000	0.000244627
chr7	51595001	51600000	0.000123772
chr7	51824001	51829000	0.000135374
chr7	51845001	51850000	7.62E-07
chr7	51846001	51851000	4.03E-13
chr7	51847001	51852000	4.15E-09
chr7	51848001	51853000	1.49E-06
chr7	51982001	51987000	9.47E-07
chr7	51983001	51988000	8.04E-09
chr7	52002001	52007000	5.58E-05
chr7	52003001	52008000	2.33E-05
chr7	52011001	52016000	2.38E-13
chr7	52012001	52017000	2.31E-09
chr7	52013001	52018000	3.17E-06
chr7	52023001	52028000	2.15E-07
chr7	52024001	52029000	3.43E-08
chr7	52025001	52030000	1.60E-11
chr7	52056001	52061000	4.71E-05
chr7	52057001	52062000	5.61E-09
chr7	52060001	52065000	1.76E-07
chr7	52073001	52078000	1.85E-09
chr7	52074001	52079000	9.72E-10
chr7	52098001	52103000	1.33E-07
chr7	52119001	52124000	0.001846203
chr7	52120001	52125000	0.001183186
chr7	52121001	52126000	1.89E-07
chr7	52142001	52147000	2.41E-07
chr7	52143001	52148000	1.50E-07
chr7	52157001	52162000	2.30E-05
chr7	52193001	52198000	3.81E-11
chr7	52194001	52199000	6.03E-19
chr7	52195001	52200000	1.51E-14
chr7	52196001	52201000	4.07E-15
chr7	52206001	52211000	8.58E-07
chr7	52207001	52212000	1.60E-07
chr7	52208001	52213000	1.60E-07
chr7	52211001	52216000	2.45E-05
chr7	52220001	52225000	6.69E-05
chr7	52281001	52286000	0.000112502

chr7	52282001	52287000	0.000352481
chr7	52283001	52288000	5.85E-05
chr7	52312001	52317000	0.000262071
chr7	52323001	52328000	1.10E-11
chr7	52346001	52351000	3.19E-05
chr7	52364001	52369000	5.42E-08
chr7	52383001	52388000	0.002629544
chr7	52387001	52392000	1.71E-09
chr7	52388001	52393000	2.37E-10
chr7	52389001	52394000	2.55E-10
chr7	52411001	52416000	3.67E-05
chr7	52412001	52417000	1.28E-05
chr7	52474001	52479000	0.00259937
chr7	52475001	52480000	0.000516418
chr7	52476001	52481000	0.005094035
chr7	52559001	52564000	0.001658263
chr7	52560001	52565000	0.002839612
chr7	52581001	52586000	8.08E-07
chr7	52620001	52625000	1.20E-09
chr7	52631001	52636000	1.25E-10
chr7	52632001	52637000	2.58E-10
chr7	52634001	52639000	5.75E-09
chr7	52642001	52647000	1.47E-05
chr7	52643001	52648000	8.53E-06
chr7	52666001	52671000	9.90E-07
chr7	52692001	52697000	6.79E-10
chr7	52776001	52781000	0.000166656
chr7	52777001	52782000	0.001325037
chr7	52822001	52827000	0.000532939
chr7	52850001	52855000	2.37E-05
chr7	52851001	52856000	2.94E-06
chr7	52868001	52873000	3.26E-16
chr7	52869001	52874000	4.96E-07
chr7	52892001	52897000	0.00013024
chr7	52893001	52898000	2.10E-06
chr7	52894001	52899000	9.27E-05
chr7	52926001	52931000	1.06E-07
chr7	52939001	52944000	6.34E-05
chr7	52940001	52945000	2.77E-05
chr7	52968001	52973000	1.55E-05
chr7	52969001	52974000	2.08E-07
chr7	52970001	52975000	3.95E-07
chr7	52971001	52976000	1.90E-06
chr7	52972001	52977000	0.000190453
chr7	53010001	53015000	2.77E-06
chr7	53011001	53016000	0.004391287
chr7	53046001	53051000	5.28E-05
chr7	53047001	53052000	5.02E-05

chr7	53048001	53053000	2.83E-06
chr7	53156001	53161000	9.66E-05
chr7	53157001	53162000	0.000204516
chr7	53160001	53165000	0.00139649
chr7	53244001	53249000	3.66E-07
chr7	53247001	53252000	0.000171626
chr7	53248001	53253000	2.05E-08
chr7	53249001	53254000	8.45E-08
chr7	53270001	53275000	1.27E-05
chr7	53271001	53276000	2.88E-05
chr7	53307001	53312000	5.68E-09
chr7	53308001	53313000	1.02E-11
chr7	53309001	53314000	8.67E-11
chr7	53310001	53315000	1.63E-11
chr7	53395001	53400000	4.61E-06
chr7	53398001	53403000	1.33E-05
chr7	53456001	53461000	2.42E-05
chr7	53458001	53463000	1.13E-06
chr7	53481001	53486000	1.56E-09
chr7	53482001	53487000	9.20E-09
chr7	53483001	53488000	1.32E-11
chr7	53491001	53496000	8.55E-08
chr7	53645001	53650000	7.97E-05
chr7	53800001	53805000	0.000224411
chr7	53801001	53806000	0.000415153
chr7	54077001	54082000	0.000190653
chr7	54105001	54110000	0.000550869
chr7	54322001	54327000	2.72E-05
chr7	54613001	54618000	1.72E-24
chr7	54614001	54619000	5.79E-27
chr7	54643001	54648000	3.11E-06
chr7	54644001	54649000	1.78E-05
chr7	54822001	54827000	1.61E-48
chr7	54823001	54828000	1.29E-62
chr7	54824001	54829000	1.30E-56
chr7	54825001	54830000	1.27E-59
chr7	54905001	54910000	1.17E-11
chr7	54906001	54911000	2.39E-12
chr7	54907001	54912000	3.41E-09
chr7	54952001	54957000	1.23E-19
chr7	54953001	54958000	9.15E-26
chr7	54954001	54959000	1.30E-17
chr7	54955001	54960000	8.22E-15
chr7	54956001	54961000	1.00E-14
chr7	54999001	55004000	8.66E-18
chr7	55000001	55005000	1.58E-29
chr7	55001001	55006000	1.50E-30
chr7	55031001	55036000	2.93E-06

chr7	55050001	55055000	2.52E-09
chr7	55051001	55056000	2.01E-07
chr7	55052001	55057000	4.62E-08
chr7	55053001	55058000	3.68E-07
chr7	55082001	55087000	5.74E-33
chr7	55640001	55645000	3.06E-58
chr7	56333001	56338000	1.03E-18
chr7	56364001	56369000	1.77E-17
chr7	56365001	56370000	4.09E-19
chr7	56366001	56371000	7.77E-14
chr7	56367001	56372000	1.14E-05
chr7	56626001	56631000	1.22E-10
chr7	56628001	56633000	1.04E-09
chr7	56704001	56709000	6.13E-13
chr7	56730001	56735000	2.95E-08
chr7	56731001	56736000	1.95E-07
chr7	56896001	56901000	6.00E-07
chr7	56897001	56902000	0.000117999
chr7	56906001	56911000	9.81E-10
chr7	56927001	56932000	0.000320638
chr7	56928001	56933000	9.29E-05
chr7	56970001	56975000	1.12E-07
chr7	56973001	56978000	1.94E-05
chr7	57058001	57063000	3.63E-05
chr7	57122001	57127000	7.83E-09
chr7	57501001	57506000	7.26E-07
chr7	57529001	57534000	0.003848581
chr7	57668001	57673000	0.006614496
chr7	62469001	62474000	2.79E-11
chr7	62507001	62512000	1.66E-09
chr7	62657001	62662000	2.24E-11
chr7	62668001	62673000	2.81E-13
chr7	62824001	62829000	6.20E-06
chr7	62916001	62921000	0.002395987
chr7	62939001	62944000	2.00E-07
chr7	62940001	62945000	1.70E-07
chr7	63071001	63076000	0.00017366
chr7	63111001	63116000	0.001572383
chr7	63184001	63189000	6.66E-05
chr7	63185001	63190000	1.07E-05
chr7	63186001	63191000	0.000143937
chr7	63243001	63248000	0.000129519
chr7	63259001	63264000	0.000192453
chr7	63261001	63266000	1.80E-18
chr7	63262001	63267000	2.02E-16
chr7	63265001	63270000	1.54E-16
chr7	63272001	63277000	0.000257926
chr7	63273001	63278000	3.29E-06

chr7	63274001	63279000	1.39E-07
chr7	63276001	63281000	6.50E-06
chr7	63285001	63290000	5.48E-10
chr7	63298001	63303000	8.11E-09
chr7	63320001	63325000	1.01E-08
chr7	63378001	63383000	1.02E-06
chr7	63405001	63410000	2.79E-08
chr7	63406001	63411000	5.69E-08
chr7	63424001	63429000	1.46E-10
chr7	63440001	63445000	0.002664225
chr7	63441001	63446000	0.00012542
chr7	63449001	63454000	6.13E-11
chr7	63450001	63455000	3.07E-11
chr7	63451001	63456000	1.12E-05
chr7	63453001	63458000	6.92E-09
chr7	63504001	63509000	5.27E-18
chr7	63505001	63510000	2.81E-21
chr7	63506001	63511000	1.24E-20
chr7	63588001	63593000	5.91E-11
chr7	63605001	63610000	1.82E-07
chr7	63624001	63629000	3.03E-08
chr7	63673001	63678000	0.001867358
chr7	63730001	63735000	2.95E-05
chr7	63731001	63736000	1.10E-08
chr7	63732001	63737000	9.13E-09
chr7	63733001	63738000	4.56E-07
chr7	63734001	63739000	2.52E-07
chr7	63773001	63778000	2.62E-13
chr7	63774001	63779000	3.59E-25
chr7	63775001	63780000	2.64E-07
chr7	63952001	63957000	8.01E-20
chr7	63953001	63958000	1.19E-27
chr7	63954001	63959000	1.35E-42
chr7	63955001	63960000	1.17E-41
chr7	63999001	64004000	7.53E-08
chr7	64063001	64068000	1.79E-10
chr7	64064001	64069000	9.91E-12
chr7	64065001	64070000	2.98E-13
chr7	64066001	64071000	2.03E-11
chr7	64123001	64128000	3.16E-11
chr7	64124001	64129000	3.95E-09
chr7	64254001	64259000	2.46E-17
chr7	64255001	64260000	1.07E-15
chr7	64441001	64446000	0.001408597
chr7	64462001	64467000	9.18E-16
chr7	64463001	64468000	4.52E-14
chr7	64464001	64469000	7.92E-13
chr7	64465001	64470000	3.57E-08

chr7	64537001	64542000	5.69E-31
chr7	64538001	64543000	9.94E-34
chr7	64539001	64544000	1.58E-28
chr7	64708001	64713000	1.88E-09
chr7	64709001	64714000	4.77E-13
chr7	64710001	64715000	1.04E-10
chr7	64711001	64716000	1.26E-22
chr7	64712001	64717000	3.36E-53
chr7	64750001	64755000	0.006622326
chr7	64752001	64757000	0.001541507
chr7	64834001	64839000	6.66E-27
chr7	64835001	64840000	1.57E-26
chr7	64836001	64841000	8.08E-32
chr7	64837001	64842000	6.03E-34
chr7	64838001	64843000	1.74E-27
chr7	64974001	64979000	2.47E-33
chr7	64976001	64981000	2.75E-05
chr7	65211001	65216000	1.34E-12
chr7	65212001	65217000	8.70E-21
chr7	65213001	65218000	3.20E-28
chr7	65214001	65219000	7.38E-38
chr7	65215001	65220000	1.50E-40
chr7	65216001	65221000	2.26E-33
chr7	65231001	65236000	5.91E-35
chr7	65232001	65237000	1.10E-28
chr7	65233001	65238000	1.18E-24
chr7	65334001	65339000	1.78E-59
chr7	65335001	65340000	4.02E-63
chr7	65336001	65341000	5.13E-63
chr7	65337001	65342000	4.40E-54
chr7	65338001	65343000	2.72E-35
chr7	65447001	65452000	4.35E-18
chr7	65536001	65541000	7.14E-08
chr7	65576001	65581000	1.58E-24
chr7	65577001	65582000	2.49E-23
chr7	65578001	65583000	2.34E-21
chr7	65579001	65584000	2.19E-19
chr7	65580001	65585000	8.53E-15
chr7	65666001	65671000	6.67E-31
chr7	65875001	65880000	1.48E-14
chr7	65876001	65881000	6.16E-13
chr7	66115001	66120000	6.70E-50
chr7	66116001	66121000	1.09E-47
chr7	66117001	66122000	1.45E-50
chr7	66119001	66124000	2.48E-37
chr7	66382001	66387000	1.62E-32
chr7	66899001	66904000	3.67E-06
chr7	66929001	66934000	3.29E-05

chr7	67100001	67105000	1.59E-12
chr7	67101001	67106000	3.39E-13
chr7	67103001	67108000	1.12E-07
chr7	67166001	67171000	0.000448561
chr7	67220001	67225000	5.46E-13
chr7	67221001	67226000	7.44E-12
chr7	67222001	67227000	6.56E-13
chr7	67223001	67228000	8.82E-10
chr7	67224001	67229000	7.01E-10
chr7	67288001	67293000	3.90E-10
chr7	67339001	67344000	2.02E-09
chr7	67358001	67363000	1.39E-07
chr7	67359001	67364000	1.40E-07
chr7	67360001	67365000	2.83E-06
chr7	67501001	67506000	0.00013299
chr7	68037001	68042000	1.54E-06
chr7	68038001	68043000	3.38E-09
chr7	68368001	68373000	4.23E-06
chr7	68369001	68374000	1.22E-06
chr7	68370001	68375000	1.82E-06
chr7	68470001	68475000	0.001749779
chr7	68477001	68482000	0.001992103
chr7	68478001	68483000	0.002537729
chr7	68479001	68484000	0.000292073
chr7	68873001	68878000	1.14E-10
chr7	69058001	69063000	5.44E-35
chr7	69059001	69064000	8.94E-41
chr7	69069001	69074000	0.008344979
chr7	69072001	69077000	9.14E-06
chr7	69141001	69146000	3.47E-07
chr7	69142001	69147000	2.20E-10
chr7	69158001	69163000	0.000613413
chr7	69159001	69164000	3.63E-05
chr7	69182001	69187000	1.18E-06
chr7	69240001	69245000	1.74E-07
chr7	69241001	69246000	0.000364869
chr7	69329001	69334000	1.90E-06
chr7	69385001	69390000	2.75E-05
chr7	69417001	69422000	4.41E-06
chr7	69418001	69423000	3.51E-06
chr7	69602001	69607000	0.000181138
chr7	69603001	69608000	0.000103072
chr7	69604001	69609000	0.000129074
chr7	69711001	69716000	1.03E-05
chr7	69793001	69798000	0.000299139
chr7	69794001	69799000	1.47E-06
chr7	69795001	69800000	8.97E-07
chr7	69796001	69801000	9.49E-09

chr7	69806001	69811000	0.000711749
chr7	70154001	70159000	0.000154597
chr7	70155001	70160000	2.47E-13
chr7	70323001	70328000	0.000183858
chr7	70351001	70356000	1.07E-05
chr7	70352001	70357000	0.000172337
chr7	70381001	70386000	6.27E-10
chr7	70382001	70387000	1.51E-05
chr7	70449001	70454000	6.69E-05
chr7	70452001	70457000	7.66E-06
chr7	70453001	70458000	1.05E-08
chr7	70455001	70460000	1.16E-05
chr7	70456001	70461000	1.79E-05
chr7	70503001	70508000	9.86E-10
chr7	70504001	70509000	2.69E-06
chr7	70553001	70558000	1.19E-05
chr7	70555001	70560000	5.04E-09
chr7	70587001	70592000	6.55E-08
chr7	70588001	70593000	7.04E-08
chr7	70589001	70594000	3.11E-08
chr7	70644001	70649000	2.40E-09
chr7	70657001	70662000	3.30E-11
chr7	70673001	70678000	4.10E-08
chr7	70674001	70679000	1.28E-11
chr7	70675001	70680000	6.05E-15
chr7	70676001	70681000	3.73E-12
chr7	70677001	70682000	3.54E-09
chr7	70687001	70692000	8.12E-12
chr7	70688001	70693000	4.20E-13
chr7	70689001	70694000	1.01E-08
chr7	70786001	70791000	2.82E-08
chr7	70787001	70792000	8.20E-11
chr7	70788001	70793000	7.34E-09
chr7	70789001	70794000	1.59E-08
chr7	70790001	70795000	3.85E-05
chr7	70797001	70802000	1.47E-12
chr7	70798001	70803000	8.89E-26
chr7	70799001	70804000	3.23E-21
chr7	70874001	70879000	2.87E-09
chr7	70910001	70915000	2.17E-06
chr7	70911001	70916000	9.57E-07
chr7	70912001	70917000	3.08E-07
chr7	70913001	70918000	2.08E-06
chr7	70914001	70919000	8.22E-08
chr7	70923001	70928000	5.01E-11
chr7	70925001	70930000	6.14E-08
chr7	71018001	71023000	1.43E-06
chr7	71042001	71047000	1.67E-07

chr7	71204001	71209000	1.39E-05
chr7	72294001	72299000	8.97E-15
chr7	72345001	72350000	1.80E-13
chr7	72346001	72351000	1.01E-25
chr7	72347001	72352000	1.17E-25
chr7	72348001	72353000	8.14E-21
chr7	72349001	72354000	1.89E-25
chr7	72350001	72355000	1.02E-47
chr7	72393001	72398000	3.89E-24
chr7	72394001	72399000	2.68E-42
chr7	72395001	72400000	3.48E-70
chr7	72421001	72426000	2.19E-07
chr7	72721001	72726000	3.83E-14
chr7	72967001	72972000	5.62E-27
chr7	73061001	73066000	0.001349465
chr7	73078001	73083000	1.88E-07
chr7	73131001	73136000	3.56E-12
chr7	73132001	73137000	2.28E-15
chr7	73133001	73138000	5.97E-15
chr7	73498001	73503000	1.18E-09
chr7	73700001	73705000	8.18E-17
chr7	73701001	73706000	7.90E-07
chr7	73702001	73707000	6.99E-05
chr7	74305001	74310000	5.92E-10
chr7	74306001	74311000	2.02E-17
chr7	74485001	74490000	1.47E-09
chr7	74486001	74491000	2.75E-07
chr7	74487001	74492000	8.21E-19
chr7	74488001	74493000	3.20E-23
chr7	74489001	74494000	3.12E-15
chr7	74504001	74509000	1.10E-07
chr7	74505001	74510000	5.36E-08
chr7	75067001	75072000	4.01E-13
chr7	75068001	75073000	5.94E-13
chr7	75069001	75074000	1.34E-09
chr7	75070001	75075000	9.06E-10
chr7	75111001	75116000	2.81E-28
chr7	75112001	75117000	2.23E-19
chr7	75152001	75157000	1.79E-13
chr7	75264001	75269000	4.48E-12
chr7	75265001	75270000	4.66E-13
chr7	75266001	75271000	9.95E-15
chr7	75267001	75272000	1.68E-08
chr7	75268001	75273000	1.83E-08
chr7	75364001	75369000	1.27E-06
chr7	75492001	75497000	1.96E-06
chr7	75493001	75498000	3.32E-07
chr7	75494001	75499000	9.73E-09

chr7	75988001	75993000	9.68E-10
chr7	76018001	76023000	2.67E-14
chr7	76019001	76024000	2.29E-15
chr7	76020001	76025000	1.02E-26
chr7	76021001	76026000	7.73E-20
chr7	76022001	76027000	8.75E-10
chr7	76174001	76179000	3.05E-35
chr7	76175001	76180000	7.29E-47
chr7	76176001	76181000	1.09E-20
chr7	76177001	76182000	3.79E-16
chr7	76178001	76183000	5.96E-16
chr7	76179001	76184000	3.75E-18
chr7	76231001	76236000	1.71E-06
chr7	76747001	76752000	4.53E-38
chr7	76748001	76753000	4.89E-35
chr7	76749001	76754000	1.25E-44
chr7	76750001	76755000	1.46E-33
chr7	76751001	76756000	1.05E-35
chr7	77045001	77050000	1.28E-38
chr7	77428001	77433000	6.64E-46
chr7	77649001	77654000	8.51E-31
chr7	77911001	77916000	0.000100369
chr7	77965001	77970000	0.02141183
chr7	78032001	78037000	1.60E-07
chr7	78033001	78038000	1.09E-06
chr7	78188001	78193000	1.34E-06
chr7	78224001	78229000	3.64E-05
chr7	78372001	78377000	0.011333376
chr7	78491001	78496000	0.001171877
chr7	78657001	78662000	0.000131241
chr7	78658001	78663000	0.000528933
chr7	78659001	78664000	0.000736007
chr7	78665001	78670000	0.002760086
chr7	78673001	78678000	0.000288727
chr7	78674001	78679000	8.77E-05
chr7	78714001	78719000	0.00287544
chr7	78739001	78744000	4.29E-08
chr7	78748001	78753000	5.64E-05
chr7	78749001	78754000	5.37E-06
chr7	78750001	78755000	1.34E-07
chr7	79481001	79486000	0.001444275
chr7	80548001	80553000	1.25E-42
chr7	80550001	80555000	0.012716769
chr7	80777001	80782000	0.036664948
chr7	82068001	82073000	2.96E-63
chr7	82069001	82074000	5.61E-54
chr7	82070001	82075000	7.25E-73
chr7	82071001	82076000	7.79E-63

chr7	82072001	82077000	4.33E-57
chr7	82073001	82078000	3.85E-46
chr7	82787001	82792000	1.85E-27
chr7	82788001	82793000	4.53E-41
chr7	82789001	82794000	5.09E-80
chr7	82790001	82795000	1.67E-83
chr7	82791001	82796000	3.27E-74
chr7	82792001	82797000	1.75E-68
chr7	82951001	82956000	0.003206532
chr7	83819001	83824000	0.000212834
chr7	83822001	83827000	4.77E-06
chr7	84608001	84613000	3.47E-07
chr7	84743001	84748000	0.00112037
chr7	84780001	84785000	0.003398757
chr7	84810001	84815000	1.67E-33
chr7	84811001	84816000	8.76E-28
chr7	84832001	84837000	0.000306976
chr7	84833001	84838000	0.001945881
chr7	84834001	84839000	0.002017449
chr7	84842001	84847000	0.018968511
chr7	84843001	84848000	0.008543595
chr7	84880001	84885000	3.61E-08
chr7	84881001	84886000	7.18E-11
chr7	84882001	84887000	1.28E-08
chr7	84892001	84897000	0.001043412
chr7	84893001	84898000	0.000164564
chr7	84894001	84899000	0.000128396
chr7	84976001	84981000	0.000662424
chr7	84980001	84985000	9.16E-05
chr7	84981001	84986000	0.000694813
chr7	84982001	84987000	0.000169126
chr7	84983001	84988000	0.000228156
chr7	84984001	84989000	0.000349896
chr7	84997001	85002000	6.36E-05
chr7	84998001	85003000	0.000404162
chr7	85031001	85036000	0.001959405
chr7	85032001	85037000	0.010219852
chr7	85054001	85059000	4.65E-05
chr7	85055001	85060000	8.04E-06
chr7	85435001	85440000	0.013125894
chr7	85436001	85441000	0.012826252
chr7	85513001	85518000	0.001298135
chr7	85514001	85519000	0.000296096
chr7	85515001	85520000	0.00028069
chr7	85538001	85543000	0.000147463
chr7	85539001	85544000	0.000147463
chr7	85540001	85545000	3.18E-05
chr7	85585001	85590000	0.000329917

chr7	85621001	85626000	5.38E-06
chr7	85731001	85736000	0.000184467
chr7	85732001	85737000	5.19E-06
chr7	85733001	85738000	1.89E-08
chr7	85734001	85739000	9.47E-06
chr7	85760001	85765000	1.21E-13
chr7	85769001	85774000	0.000297164
chr7	85770001	85775000	0.000765795
chr7	85783001	85788000	2.58E-06
chr7	85784001	85789000	8.62E-12
chr7	85785001	85790000	1.94E-12
chr7	85786001	85791000	1.56E-13
chr7	85787001	85792000	4.04E-09
chr7	85822001	85827000	9.66E-06
chr7	85883001	85888000	9.39E-08
chr7	85884001	85889000	3.21E-06
chr7	85965001	85970000	7.07E-22
chr7	85966001	85971000	9.63E-24
chr7	85967001	85972000	1.24E-21
chr7	85968001	85973000	4.51E-19
chr7	85969001	85974000	1.13E-13
chr7	86060001	86065000	0.002901517
chr7	86084001	86089000	1.98E-05
chr7	86099001	86104000	8.38E-05
chr7	86101001	86106000	0.002061956
chr7	86102001	86107000	0.000596095
chr7	86103001	86108000	0.000889322
chr7	86141001	86146000	0.000242469
chr7	86143001	86148000	3.41E-06
chr7	86205001	86210000	0.004505308
chr7	86206001	86211000	0.000674533
chr7	86207001	86212000	0.000600423
chr7	86208001	86213000	0.000526138
chr7	86209001	86214000	9.72E-06
chr7	86213001	86218000	3.39E-11
chr7	86259001	86264000	8.96E-05
chr7	86260001	86265000	0.000620227
chr7	86262001	86267000	0.002209511
chr7	86303001	86308000	0.001261303
chr7	86375001	86380000	0.000239074
chr7	86376001	86381000	1.23E-05
chr7	86377001	86382000	0.00032993
chr7	86378001	86383000	0.000283424
chr7	86542001	86547000	0.001014743
chr7	86543001	86548000	0.003869494
chr7	86544001	86549000	0.002366426
chr7	86545001	86550000	0.000902146
chr7	86665001	86670000	2.46E-12

chr7	86666001	86671000	9.80E-18
chr7	86684001	86689000	2.84E-105
chr7	86685001	86690000	1.41E-95
chr7	86686001	86691000	2.42E-80
chr7	86687001	86692000	9.78E-70
chr7	86721001	86726000	9.77E-10
chr7	86768001	86773000	0.000665964
chr7	86849001	86854000	7.94E-45
chr7	86891001	86896000	1.83E-11
chr7	86898001	86903000	0.00032739
chr7	86899001	86904000	0.000428084
chr7	86900001	86905000	0.000164916
chr7	86944001	86949000	4.11E-18
chr7	86945001	86950000	6.98E-12
chr7	86946001	86951000	2.22E-16
chr7	86947001	86952000	1.55E-17
chr7	86948001	86953000	4.15E-15
chr7	86970001	86975000	2.44E-24
chr7	86971001	86976000	1.64E-35
chr7	86972001	86977000	3.23E-37
chr7	86973001	86978000	5.02E-48
chr7	86974001	86979000	4.84E-55
chr7	86975001	86980000	1.40E-27
chr7	87062001	87067000	0.002303178
chr7	87063001	87068000	0.003980874
chr7	87117001	87122000	0.001489177
chr7	87118001	87123000	0.000205189
chr7	87119001	87124000	6.71E-05
chr7	87120001	87125000	8.15E-05
chr7	87121001	87126000	0.002433636
chr7	87261001	87266000	0.006098659
chr7	87377001	87382000	1.63E-07
chr7	87559001	87564000	4.20E-30
chr7	87560001	87565000	1.01E-19
chr7	87561001	87566000	2.49E-16
chr7	87562001	87567000	7.68E-19
chr7	87563001	87568000	4.14E-18
chr7	87564001	87569000	7.14E-17
chr7	87629001	87634000	0.013518174
chr7	87630001	87635000	0.018472826
chr7	87792001	87797000	1.88E-06
chr7	87793001	87798000	2.14E-05
chr7	87794001	87799000	2.60E-05
chr7	87844001	87849000	9.46E-13
chr7	87934001	87939000	6.88E-16
chr7	87935001	87940000	1.49E-17
chr7	87936001	87941000	1.24E-16
chr7	87954001	87959000	0.003924895

chr7	88059001	88064000	1.07E-06
chr7	88060001	88065000	2.16E-06
chr7	88061001	88066000	7.65E-06
chr7	88160001	88165000	1.18E-05
chr7	88215001	88220000	0.001600276
chr7	88235001	88240000	6.85E-10
chr7	88237001	88242000	2.35E-07
chr7	88250001	88255000	0.003103496
chr7	88407001	88412000	0.003001909
chr7	88441001	88446000	0.000285472
chr7	88442001	88447000	0.003120697
chr7	88471001	88476000	2.79E-06
chr7	88472001	88477000	1.69E-05
chr7	88473001	88478000	4.05E-05
chr7	88474001	88479000	2.13E-05
chr7	88485001	88490000	1.60E-06
chr7	88561001	88566000	0.000525822
chr7	88562001	88567000	6.83E-06
chr7	88570001	88575000	0.002104031
chr7	88690001	88695000	7.42E-07
chr7	88691001	88696000	1.09E-09
chr7	88873001	88878000	1.98E-05
chr7	88874001	88879000	8.14E-06
chr7	88875001	88880000	4.15E-08
chr7	88876001	88881000	2.80E-06
chr7	88915001	88920000	1.93E-05
chr7	88919001	88924000	2.19E-06
chr7	88940001	88945000	3.47E-05
chr7	89003001	89008000	2.34E-05
chr7	89006001	89011000	0.002876906
chr7	89012001	89017000	0.000365376
chr7	89013001	89018000	1.85E-05
chr7	89055001	89060000	4.53E-05
chr7	89111001	89116000	1.37E-07
chr7	89112001	89117000	2.30E-09
chr7	89113001	89118000	6.32E-09
chr7	89114001	89119000	3.07E-07
chr7	89122001	89127000	0.000318757
chr7	89123001	89128000	0.00028117
chr7	89213001	89218000	0.00011902
chr7	89235001	89240000	1.23E-21
chr7	89236001	89241000	8.02E-21
chr7	89237001	89242000	2.31E-20
chr7	89238001	89243000	4.77E-18
chr7	89239001	89244000	7.73E-16
chr7	89334001	89339000	0.004871716
chr7	89341001	89346000	3.96E-05
chr7	89342001	89347000	0.000124225

chr7	89367001	89372000	0.001017973
chr7	89368001	89373000	0.00045748
chr7	89369001	89374000	2.14E-05
chr7	89370001	89375000	3.33E-05
chr7	89405001	89410000	8.16E-05
chr7	89413001	89418000	0.006518372
chr7	89425001	89430000	2.94E-13
chr7	89426001	89431000	7.82E-17
chr7	89427001	89432000	2.16E-16
chr7	89449001	89454000	0.008785727
chr7	89524001	89529000	6.40E-06
chr7	89544001	89549000	0.002741617
chr7	89564001	89569000	0.009313336
chr7	89585001	89590000	1.40E-08
chr7	89586001	89591000	1.20E-06
chr7	89612001	89617000	0.001279513
chr7	89613001	89618000	0.000741555
chr7	89614001	89619000	0.004609298
chr7	89646001	89651000	0.000549785
chr7	89647001	89652000	0.000571447
chr7	89663001	89668000	1.38E-05
chr7	89664001	89669000	1.01E-07
chr7	89665001	89670000	1.10E-07
chr7	89700001	89705000	2.65E-12
chr7	89701001	89706000	2.25E-11
chr7	89702001	89707000	2.66E-10
chr7	89782001	89787000	6.34E-36
chr7	89783001	89788000	3.74E-47
chr7	89808001	89813000	0.00393189
chr7	89837001	89842000	3.82E-24
chr7	89853001	89858000	0.000110516
chr7	89946001	89951000	3.14E-35
chr7	89947001	89952000	1.06E-25
chr7	90009001	90014000	3.18E-08
chr7	90075001	90080000	2.90E-08
chr7	90076001	90081000	4.99E-05
chr7	90152001	90157000	1.73E-10
chr7	90153001	90158000	2.20E-10
chr7	90225001	90230000	5.31E-29
chr7	90845001	90850000	4.41E-07
chr7	90943001	90948000	0.006407365
chr7	91028001	91033000	0.000170084
chr7	91030001	91035000	7.78E-05
chr7	91214001	91219000	3.67E-05
chr7	91215001	91220000	0.000303728
chr7	91292001	91297000	0.002196003
chr7	91387001	91392000	8.50E-06
chr7	91439001	91444000	0.002593765

chr7	91440001	91445000	7.95E-06
chr7	91441001	91446000	1.93E-05
chr7	91759001	91764000	1.78E-43
chr7	91870001	91875000	1.64E-34
chr7	91871001	91876000	5.37E-65
chr7	92072001	92077000	1.61E-52
chr7	92073001	92078000	1.41E-35
chr7	92153001	92158000	6.53E-53
chr7	92259001	92264000	0.001315133
chr7	92260001	92265000	8.43E-05
chr7	92343001	92348000	4.96E-09
chr7	92344001	92349000	6.72E-12
chr7	92345001	92350000	6.72E-12
chr7	92346001	92351000	2.82E-09
chr7	92347001	92352000	1.99E-09
chr7	92373001	92378000	1.52E-05
chr7	92458001	92463000	1.11E-77
chr7	92464001	92469000	8.34E-13
chr7	92465001	92470000	4.80E-23
chr7	92515001	92520000	1.14E-07
chr7	92560001	92565000	1.62E-06
chr7	92707001	92712000	0.00256686
chr7	92708001	92713000	0.001518208
chr7	93058001	93063000	1.16E-06
chr7	93059001	93064000	2.09E-13
chr7	93060001	93065000	3.07E-08
chr7	93062001	93067000	1.87E-07
chr7	93063001	93068000	3.11E-08
chr7	93123001	93128000	0.011804298
chr7	93199001	93204000	3.47E-30
chr7	93200001	93205000	2.21E-79
chr7	93201001	93206000	4.39E-78
chr7	93202001	93207000	4.62E-52
chr7	93218001	93223000	2.47E-14
chr7	93219001	93224000	2.27E-11
chr7	93467001	93472000	0.000721149
chr7	93514001	93519000	7.04E-15
chr7	93548001	93553000	1.06E-18
chr7	93549001	93554000	3.38E-17
chr7	93551001	93556000	2.01E-12
chr7	93784001	93789000	6.06E-13
chr7	93785001	93790000	1.73E-11
chr7	93786001	93791000	1.06E-07
chr7	93787001	93792000	4.72E-07
chr7	93885001	93890000	4.98E-07
chr7	94135001	94140000	4.07E-13
chr7	94389001	94394000	0.001627346
chr7	94390001	94395000	7.34E-05

chr7	94391001	94396000	0.000517109
chr7	94532001	94537000	2.26E-13
chr7	94533001	94538000	1.18E-65
chr7	94534001	94539000	4.86E-64
chr7	94535001	94540000	2.19E-64
chr7	94536001	94541000	2.15E-35
chr7	94537001	94542000	4.87E-38
chr7	94721001	94726000	0.000179002
chr7	95021001	95026000	5.29E-10
chr7	95022001	95027000	2.55E-11
chr7	95060001	95065000	2.09E-17
chr7	95061001	95066000	9.64E-17
chr7	95062001	95067000	5.29E-12
chr7	95064001	95069000	1.34E-28
chr7	95221001	95226000	1.80E-36
chr7	95222001	95227000	6.15E-43
chr7	95223001	95228000	2.28E-34
chr7	95224001	95229000	1.31E-35
chr7	95225001	95230000	5.78E-33
chr7	95226001	95231000	2.85E-21
chr7	95402001	95407000	5.35E-22
chr7	95473001	95478000	0.0002191
chr7	95553001	95558000	3.76E-06
chr7	95558001	95563000	1.55E-05
chr7	95700001	95705000	3.25E-12
chr7	95946001	95951000	4.15E-20
chr7	95947001	95952000	8.28E-57
chr7	95951001	95956000	5.18E-42
chr7	96334001	96339000	4.95E-23
chr7	96335001	96340000	5.82E-37
chr7	96336001	96341000	7.88E-56
chr7	96337001	96342000	1.29E-43
chr7	96624001	96629000	1.26E-46
chr7	96625001	96630000	6.64E-46
chr7	96626001	96631000	7.77E-41
chr7	96628001	96633000	6.13E-25
chr7	96637001	96642000	1.19E-28
chr7	96643001	96648000	9.44E-28
chr7	96644001	96649000	1.07E-36
chr7	96645001	96650000	1.36E-50
chr7	96646001	96651000	1.84E-42
chr7	97061001	97066000	5.75E-05
chr7	97656001	97661000	2.68E-09
chr7	98472001	98477000	2.52E-36
chr7	98741001	98746000	5.74E-49
chr7	99032001	99037000	1.14E-25
chr7	99093001	99098000	7.92E-25
chr7	99094001	99099000	1.52E-27

chr7	99151001	99156000	1.55E-14
chr7	99329001	99334000	4.77E-07
chr7	99361001	99366000	0.000360006
chr7	99512001	99517000	4.60E-35
chr7	99515001	99520000	7.32E-34
chr7	99516001	99521000	6.07E-37
chr7	99517001	99522000	7.91E-30
chr7	99929001	99934000	1.04E-46
chr7	99930001	99935000	1.07E-42
chr7	99931001	99936000	1.75E-32
chr7	99932001	99937000	1.71E-27
chr7	99933001	99938000	3.03E-35
chr7	100132001	100137000	2.25E-46
chr7	100133001	100138000	5.99E-61
chr7	100134001	100139000	2.25E-45
chr7	100135001	100140000	1.30E-30
chr7	100136001	100141000	5.83E-28
chr7	100137001	100142000	7.20E-20
chr7	100468001	100473000	2.81E-13
chr7	100494001	100499000	4.85E-24
chr7	100802001	100807000	2.02E-12
chr7	101006001	101011000	1.87E-26
chr7	101007001	101012000	4.65E-38
chr7	101459001	101464000	1.80E-78
chr7	101460001	101465000	2.12E-83
chr7	102003001	102008000	1.10E-35
chr7	102004001	102009000	7.23E-35
chr7	102453001	102458000	0.001364565
chr7	102454001	102459000	7.92E-06
chr7	102455001	102460000	3.09E-07
chr7	102511001	102516000	7.36E-09
chr7	102624001	102629000	3.42E-06
chr7	102636001	102641000	1.98E-07
chr7	102715001	102720000	5.51E-54
chr7	102916001	102921000	1.03E-46
chr7	102917001	102922000	1.13E-22
chr7	102918001	102923000	8.87E-60
chr7	102919001	102924000	2.36E-51
chr7	102920001	102925000	3.59E-58
chr7	102921001	102926000	7.47E-37
chr7	102933001	102938000	5.56E-29
chr7	102934001	102939000	3.91E-30
chr7	102936001	102941000	1.24E-31
chr7	102937001	102942000	1.37E-28
chr7	103567001	103572000	0.000364239
chr7	103570001	103575000	8.95E-05
chr7	103571001	103576000	6.35E-06
chr7	103703001	103708000	0.000379781

chr7	103870001	103875000	2.04E-12
chr7	103871001	103876000	3.65E-12
chr7	103872001	103877000	5.84E-11
chr7	103873001	103878000	2.38E-06
chr7	103874001	103879000	2.73E-07
chr7	103875001	103880000	1.82E-06
chr7	103887001	103892000	3.57E-07
chr7	103934001	103939000	1.24E-06
chr7	103935001	103940000	8.52E-07
chr7	103939001	103944000	4.38E-07
chr7	103940001	103945000	2.61E-07
chr7	103941001	103946000	6.88E-12
chr7	103942001	103947000	1.60E-08
chr7	103950001	103955000	4.05E-08
chr7	103961001	103966000	0.000264748
chr7	103962001	103967000	6.82E-05
chr7	103963001	103968000	4.31E-05
chr7	104035001	104040000	1.77E-08
chr7	104037001	104042000	1.03E-05
chr7	104039001	104044000	4.15E-06
chr7	104110001	104115000	3.24E-11
chr7	104111001	104116000	2.25E-08
chr7	104227001	104232000	2.96E-14
chr7	104241001	104246000	1.70E-12
chr7	104242001	104247000	8.62E-13
chr7	104281001	104286000	5.40E-08
chr7	104296001	104301000	3.13E-06
chr7	104297001	104302000	8.66E-06
chr7	104299001	104304000	1.87E-08
chr7	104300001	104305000	1.63E-07
chr7	104316001	104321000	1.00E-11
chr7	104318001	104323000	7.00E-18
chr7	104319001	104324000	1.65E-11
chr7	104370001	104375000	4.08E-11
chr7	104423001	104428000	2.00E-12
chr7	104424001	104429000	3.70E-08
chr7	104618001	104623000	0.012662022
chr7	105024001	105029000	4.08E-19
chr7	105025001	105030000	2.80E-33
chr7	105026001	105031000	9.95E-46
chr7	105027001	105032000	2.10E-42
chr7	105028001	105033000	7.00E-35
chr7	105029001	105034000	3.34E-37
chr7	105517001	105522000	3.54E-19
chr7	105650001	105655000	2.38E-28
chr7	105752001	105757000	1.19E-43
chr7	105753001	105758000	2.74E-24
chr7	105925001	105930000	1.08E-80

chr7	106030001	106035000	0.000974272
chr7	106032001	106037000	5.04E-06
chr7	106296001	106301000	3.64E-63
chr7	106297001	106302000	5.35E-81
chr7	106298001	106303000	1.01E-74
chr7	106299001	106304000	1.20E-75
chr7	106300001	106305000	1.56E-81
chr7	106301001	106306000	2.54E-95
chr7	106377001	106382000	6.33E-05
chr7	107216001	107221000	3.15E-82
chr7	107217001	107222000	2.50E-88
chr7	107218001	107223000	1.69E-82
chr7	107219001	107224000	8.81E-74
chr7	107379001	107384000	1.60E-42
chr7	107638001	107643000	7.60E-38
chr7	108091001	108096000	5.65E-23
chr7	108162001	108167000	3.14E-36
chr7	108166001	108171000	1.24E-45
chr7	108211001	108216000	2.04E-07
chr7	108269001	108274000	4.18E-05
chr7	108278001	108283000	0.000147746
chr7	108326001	108331000	0.000275497
chr7	108408001	108413000	3.05E-06
chr7	108409001	108414000	5.96E-05
chr7	108505001	108510000	2.69E-05
chr7	108553001	108558000	6.62E-07
chr7	108554001	108559000	1.62E-07
chr7	108569001	108574000	3.78E-06
chr7	108605001	108610000	0.002508887
chr7	108606001	108611000	0.008688152
chr7	108650001	108655000	6.15E-10
chr7	108651001	108656000	1.28E-08
chr7	108652001	108657000	3.71E-10
chr7	108683001	108688000	0.000228002
chr7	108780001	108785000	3.73E-05
chr7	108781001	108786000	0.000192489
chr7	108947001	108952000	2.27E-07
chr7	108948001	108953000	3.96E-09
chr7	108949001	108954000	4.32E-10
chr7	108950001	108955000	1.47E-09
chr7	109026001	109031000	4.51E-07
chr7	109027001	109032000	2.31E-08
chr7	109140001	109145000	0.001614785
chr7	109141001	109146000	0.003887498
chr7	109158001	109163000	0.004648833
chr7	109159001	109164000	0.001366954
chr7	109160001	109165000	8.63E-05
chr7	109162001	109167000	0.001402358

chr7	109461001	109466000	0.008945553
chr7	109918001	109923000	1.27E-05
chr7	110843001	110848000	0.014189675
chr7	110844001	110849000	0.021900654
chr7	110893001	110898000	0.012916626
chr7	110964001	110969000	0.005060551
chr7	110965001	110970000	2.53E-05
chr7	111198001	111203000	7.94E-37
chr7	111199001	111204000	3.39E-40
chr7	111200001	111205000	4.10E-37
chr7	111202001	111207000	2.17E-24
chr7	111215001	111220000	0.000551521
chr7	111216001	111221000	0.001478813
chr7	112027001	112032000	7.56E-78
chr7	112028001	112033000	1.16E-80
chr7	112029001	112034000	4.12E-71
chr7	112030001	112035000	7.52E-54
chr7	112031001	112036000	9.28E-36
chr7	112032001	112037000	4.77E-25
chr7	112086001	112091000	5.96E-89
chr7	112087001	112092000	3.46E-54
chr7	112289001	112294000	5.15E-05
chr7	112323001	112328000	4.54E-07
chr7	112426001	112431000	2.39E-40
chr7	112427001	112432000	2.00E-35
chr7	112428001	112433000	4.59E-32
chr7	112429001	112434000	2.67E-52
chr7	112430001	112435000	1.79E-64
chr7	112575001	112580000	1.40E-43
chr7	112580001	112585000	5.83E-19
chr7	112608001	112613000	0.000697409
chr7	112697001	112702000	0.000231192
chr7	112698001	112703000	0.001457489
chr7	112745001	112750000	0.001979961
chr7	113312001	113317000	6.02E-05
chr7	113313001	113318000	0.000306105
chr7	113332001	113337000	1.20E-05
chr7	113334001	113339000	6.30E-06
chr7	113389001	113394000	0.001376344
chr7	113726001	113731000	6.50E-43
chr7	113727001	113732000	8.63E-33
chr7	114051001	114056000	0.000147387
chr7	114266001	114271000	6.85E-30
chr7	114267001	114272000	9.17E-26
chr7	114268001	114273000	1.18E-33
chr7	114269001	114274000	1.50E-36
chr7	114270001	114275000	1.97E-38
chr7	115112001	115117000	0.000150599

chr7	115113001	115118000	3.69E-37
chr7	115114001	115119000	5.12E-31
chr7	115210001	115215000	0.004243572
chr7	115307001	115312000	0.005925813
chr7	115450001	115455000	5.65E-05
chr7	115451001	115456000	3.25E-06
chr7	115452001	115457000	1.94E-05
chr7	115807001	115812000	4.58E-06
chr7	115808001	115813000	0.00022877
chr7	115934001	115939000	0.003374756
chr7	115935001	115940000	0.002476999
chr7	115936001	115941000	0.002290246
chr7	115977001	115982000	0.005743401
chr7	115978001	115983000	0.003167144
chr7	115979001	115984000	0.003869193
chr7	115980001	115985000	0.000605029
chr7	115981001	115986000	8.33E-05
chr7	116032001	116037000	0.000158523
chr7	116074001	116079000	6.05E-08
chr7	116076001	116081000	2.70E-07
chr7	116109001	116114000	1.48E-08
chr7	116110001	116115000	2.96E-09
chr7	116135001	116140000	1.00E-49
chr7	116136001	116141000	3.03E-33
chr7	116147001	116152000	4.78E-05
chr7	116148001	116153000	4.65E-12
chr7	116161001	116166000	3.73E-36
chr7	116205001	116210000	0.00965309
chr7	116209001	116214000	0.000738299
chr7	116312001	116317000	5.84E-50
chr7	116498001	116503000	2.47E-34
chr7	116499001	116504000	9.26E-21
chr7	116502001	116507000	3.75E-39
chr7	116594001	116599000	6.94E-31
chr7	116660001	116665000	7.48E-05
chr7	116761001	116766000	0.003494261
chr7	116762001	116767000	0.007448749
chr7	116764001	116769000	0.000106114
chr7	116981001	116986000	0.000178409
chr7	117147001	117152000	0.008876121
chr7	117291001	117296000	3.29E-06
chr7	117292001	117297000	1.12E-05
chr7	117293001	117298000	5.91E-05
chr7	117374001	117379000	1.97E-12
chr7	117375001	117380000	3.05E-13
chr7	117376001	117381000	7.57E-14
chr7	117377001	117382000	2.69E-11
chr7	117479001	117484000	3.73E-05

chr7	117480001	117485000	2.39E-07
chr7	117508001	117513000	9.23E-52
chr7	117509001	117514000	2.78E-78
chr7	117510001	117515000	1.45E-83
chr7	117513001	117518000	6.15E-52
chr7	117523001	117528000	4.79E-07
chr7	117524001	117529000	1.06E-05
chr7	117525001	117530000	0.00024299
chr7	117555001	117560000	2.99E-05
chr7	117630001	117635000	2.90E-05
chr7	117663001	117668000	9.72E-06
chr7	117664001	117669000	5.25E-07
chr7	117665001	117670000	1.33E-07
chr7	117667001	117672000	5.70E-05
chr7	117685001	117690000	2.52E-07
chr7	117686001	117691000	8.97E-06
chr7	117687001	117692000	1.84E-05
chr7	117688001	117693000	0.000256499
chr7	117824001	117829000	7.56E-55
chr7	117850001	117855000	4.17E-21
chr7	118604001	118609000	0.000105286
chr7	118815001	118820000	3.18E-06
chr7	118816001	118821000	3.73E-07
chr7	118959001	118964000	0.00019845
chr7	119267001	119272000	0.003581427
chr7	119985001	119990000	0.011985502
chr7	120258001	120263000	0.00679783
chr7	120492001	120497000	3.01E-11
chr7	120493001	120498000	2.94E-33
chr7	120846001	120851000	0.003998548
chr7	120847001	120852000	0.004166464
chr7	120848001	120853000	0.002059047
chr7	120969001	120974000	6.91E-35
chr7	120970001	120975000	1.37E-51
chr7	121031001	121036000	2.05E-56
chr7	121032001	121037000	3.62E-110
chr7	121033001	121038000	3.32E-81
chr7	121034001	121039000	2.72E-59
chr7	121035001	121040000	1.16E-51
chr7	121036001	121041000	1.20E-55
chr7	121056001	121061000	1.90E-07
chr7	121599001	121604000	1.06E-05
chr7	121736001	121741000	0.001441655
chr7	121737001	121742000	0.00137061
chr7	121738001	121743000	0.000191947
chr7	121739001	121744000	0.000368784
chr7	121740001	121745000	2.55E-06
chr7	121780001	121785000	2.44E-28

chr7	121784001	121789000	1.13E-19
chr7	121790001	121795000	0.000465797
chr7	121981001	121986000	9.14E-05
chr7	122107001	122112000	0.000226835
chr7	122110001	122115000	0.001137181
chr7	122255001	122260000	0.010663355
chr7	122339001	122344000	6.92E-10
chr7	122340001	122345000	2.81E-09
chr7	122342001	122347000	9.56E-08
chr7	122427001	122432000	0.000794869
chr7	122462001	122467000	3.45E-05
chr7	122522001	122527000	1.08E-17
chr7	122648001	122653000	0.002385511
chr7	122659001	122664000	0.000679152
chr7	122660001	122665000	0.001310866
chr7	122983001	122988000	2.26E-08
chr7	122984001	122989000	2.75E-06
chr7	123026001	123031000	4.71E-05
chr7	123027001	123032000	4.15E-05
chr7	123047001	123052000	0.016729072
chr7	123048001	123053000	0.013951328
chr7	123111001	123116000	0.003581838
chr7	123112001	123117000	9.80E-05
chr7	123146001	123151000	8.57E-05
chr7	123193001	123198000	2.45E-42
chr7	123383001	123388000	7.68E-15
chr7	123389001	123394000	8.19E-53
chr7	123672001	123677000	4.66E-22
chr7	123673001	123678000	3.34E-22
chr7	123970001	123975000	0.01724609
chr7	123971001	123976000	0.006156171
chr7	124400001	124405000	2.61E-24
chr7	124401001	124406000	8.29E-24
chr7	124402001	124407000	4.90E-23
chr7	124403001	124408000	6.83E-35
chr7	124405001	124410000	5.76E-31
chr7	124406001	124411000	1.46E-13
chr7	124565001	124570000	8.00E-43
chr7	124566001	124571000	8.36E-41
chr7	124567001	124572000	2.41E-22
chr7	124568001	124573000	2.42E-20
chr7	124744001	124749000	0.001526676
chr7	124956001	124961000	0.000728789
chr7	124957001	124962000	0.001093354
chr7	124958001	124963000	7.39E-06
chr7	124959001	124964000	0.000257909
chr7	124960001	124965000	0.006091092
chr7	125205001	125210000	3.82E-09

chr7	125237001	125242000	0.000494974
chr7	125239001	125244000	0.005050839
chr7	125456001	125461000	2.19E-05
chr7	125458001	125463000	2.42E-05
chr7	125459001	125464000	7.52E-06
chr7	125460001	125465000	1.56E-06
chr7	125991001	125996000	0.008787046
chr7	126057001	126062000	0.003338924
chr7	126058001	126063000	1.92E-07
chr7	126059001	126064000	3.37E-06
chr7	126060001	126065000	0.00023837
chr7	126122001	126127000	0.003557607
chr7	126123001	126128000	0.003785275
chr7	126126001	126131000	0.009015716
chr7	126129001	126134000	0.007607968
chr7	126239001	126244000	2.34E-06
chr7	126547001	126552000	3.57E-05
chr7	126556001	126561000	0.011314258
chr7	126557001	126562000	0.005163027
chr7	126567001	126572000	0.000154084
chr7	126569001	126574000	0.00135844
chr7	126600001	126605000	2.62E-06
chr7	126601001	126606000	8.65E-06
chr7	126602001	126607000	2.28E-05
chr7	126603001	126608000	4.35E-05
chr7	126604001	126609000	0.000178017
chr7	126629001	126634000	7.12E-05
chr7	126671001	126676000	0.004719081
chr7	126686001	126691000	0.000334667
chr7	126687001	126692000	2.50E-05
chr7	126688001	126693000	7.41E-05
chr7	126689001	126694000	0.000321261
chr7	126706001	126711000	0.000131498
chr7	126707001	126712000	0.000192695
chr7	126708001	126713000	3.76E-06
chr7	126718001	126723000	2.36E-07
chr7	126719001	126724000	1.23E-05
chr7	126720001	126725000	7.12E-09
chr7	126721001	126726000	1.16E-07
chr7	126728001	126733000	1.66E-06
chr7	126730001	126735000	8.35E-08
chr7	126731001	126736000	1.20E-07
chr7	126732001	126737000	8.15E-10
chr7	126733001	126738000	2.62E-05
chr7	126734001	126739000	7.27E-07
chr7	126735001	126740000	0.00084849
chr7	126757001	126762000	0.000555762
chr7	126758001	126763000	5.73E-05

chr7	126769001	126774000	9.73E-08
chr7	126770001	126775000	2.89E-07
chr7	126771001	126776000	6.98E-07
chr7	126809001	126814000	0.001839058
chr7	126810001	126815000	3.98E-05
chr7	126811001	126816000	7.72E-05
chr7	126820001	126825000	8.16E-06
chr7	126859001	126864000	8.68E-07
chr7	126860001	126865000	5.59E-08
chr7	126861001	126866000	3.78E-09
chr7	126864001	126869000	5.82E-06
chr7	126871001	126876000	0.000614804
chr7	126872001	126877000	6.37E-06
chr7	126904001	126909000	0.002511325
chr7	126907001	126912000	3.27E-06
chr7	126908001	126913000	3.26E-05
chr7	126914001	126919000	9.58E-06
chr7	126930001	126935000	0.000105609
chr7	126931001	126936000	8.10E-08
chr7	126932001	126937000	1.86E-05
chr7	126934001	126939000	5.68E-06
chr7	126935001	126940000	0.000103817
chr7	126936001	126941000	1.23E-06
chr7	126937001	126942000	2.82E-07
chr7	126959001	126964000	7.85E-07
chr7	126960001	126965000	2.59E-07
chr7	126961001	126966000	5.44E-10
chr7	126962001	126967000	3.14E-08
chr7	126984001	126989000	1.70E-20
chr7	126985001	126990000	2.17E-21
chr7	126986001	126991000	1.40E-47
chr7	126987001	126992000	2.94E-51
chr7	126988001	126993000	1.03E-56
chr7	127027001	127032000	1.71E-73
chr7	127028001	127033000	1.03E-90
chr7	127029001	127034000	5.72E-69
chr7	127030001	127035000	4.28E-66
chr7	127031001	127036000	1.25E-58
chr7	127032001	127037000	2.44E-50
chr7	127033001	127038000	4.13E-29
chr7	127035001	127040000	2.84E-06
chr7	127075001	127080000	0.000205461
chr7	127076001	127081000	8.42E-05
chr7	127084001	127089000	2.71E-06
chr7	127107001	127112000	0.001921421
chr7	127114001	127119000	2.27E-05
chr7	127123001	127128000	0.000128948
chr7	127124001	127129000	0.000178421

chr7	127148001	127153000	0.00021687
chr7	127223001	127228000	1.58E-29
chr7	127224001	127229000	3.27E-40
chr7	127225001	127230000	1.44E-44
chr7	127226001	127231000	4.57E-41
chr7	127227001	127232000	5.34E-18
chr7	127288001	127293000	5.43E-21
chr7	127289001	127294000	5.40E-29
chr7	127290001	127295000	9.07E-28
chr7	127291001	127296000	3.99E-27
chr7	127292001	127297000	2.38E-23
chr7	127393001	127398000	1.01E-06
chr7	127395001	127400000	2.58E-08
chr7	128041001	128046000	3.94E-11
chr7	128043001	128048000	1.56E-08
chr7	128375001	128380000	4.98E-14
chr7	128378001	128383000	8.06E-50
chr7	128379001	128384000	1.95E-40
chr7	128427001	128432000	9.75E-08
chr7	128428001	128433000	3.56E-08
chr7	128508001	128513000	8.43E-14
chr7	128509001	128514000	4.20E-10
chr7	128574001	128579000	4.90E-15
chr7	128575001	128580000	4.36E-14
chr7	128824001	128829000	1.93E-22
chr7	128825001	128830000	1.90E-30
chr7	128826001	128831000	1.24E-32
chr7	128827001	128832000	2.48E-33
chr7	128828001	128833000	2.49E-33
chr7	128829001	128834000	2.39E-20
chr7	129070001	129075000	7.74E-20
chr7	129071001	129076000	2.90E-15
chr7	129072001	129077000	7.27E-14
chr7	129073001	129078000	5.63E-28
chr7	129074001	129079000	3.20E-62
chr7	129138001	129143000	1.49E-22
chr7	129139001	129144000	7.28E-21
chr7	129140001	129145000	5.44E-20
chr7	129142001	129147000	6.37E-17
chr7	129247001	129252000	2.75E-14
chr7	129592001	129597000	5.60E-08
chr7	129841001	129846000	3.50E-38
chr7	129842001	129847000	8.38E-21
chr7	129843001	129848000	1.61E-25
chr7	129844001	129849000	7.13E-36
chr7	129845001	129850000	2.14E-34
chr7	130348001	130353000	9.54E-20
chr7	130349001	130354000	8.77E-72

chr7	130350001	130355000	3.30E-58
chr7	130410001	130415000	0.000127007
chr7	130413001	130418000	0.000257231
chr7	130419001	130424000	9.01E-10
chr7	130450001	130455000	1.48E-06
chr7	130786001	130791000	5.28E-35
chr7	131010001	131015000	1.00E-18
chr7	131012001	131017000	1.69E-32
chr7	131013001	131018000	1.74E-17
chr7	131386001	131391000	0.000324197
chr7	131423001	131428000	0.000145337
chr7	131585001	131590000	1.72E-06
chr7	131626001	131631000	1.05E-16
chr7	131627001	131632000	1.20E-13
chr7	131628001	131633000	1.10E-09
chr7	131661001	131666000	6.09E-06
chr7	131662001	131667000	6.12E-06
chr7	131955001	131960000	0.000809909
chr7	131956001	131961000	6.06E-05
chr7	132246001	132251000	4.86E-05
chr7	132247001	132252000	2.46E-05
chr7	132275001	132280000	5.29E-15
chr7	132762001	132767000	3.64E-86
chr7	132763001	132768000	7.84E-64
chr7	132764001	132769000	2.75E-63
chr7	132765001	132770000	5.44E-47
chr7	132766001	132771000	4.71E-25
chr7	132933001	132938000	5.32E-07
chr7	132993001	132998000	2.64E-05
chr7	132994001	132999000	4.08E-06
chr7	132995001	133000000	1.06E-05
chr7	133208001	133213000	0.000258302
chr7	133595001	133600000	7.94E-05
chr7	133630001	133635000	4.67E-07
chr7	133802001	133807000	0.000381983
chr7	133812001	133817000	3.34E-16
chr7	133997001	134002000	1.83E-57
chr7	133998001	134003000	3.48E-46
chr7	133999001	134004000	6.83E-43
chr7	134000001	134005000	1.92E-35
chr7	134001001	134006000	2.06E-23
chr7	134139001	134144000	5.97E-27
chr7	134140001	134145000	6.11E-27
chr7	134141001	134146000	2.43E-37
chr7	134142001	134147000	1.64E-35
chr7	134143001	134148000	2.97E-46
chr7	134156001	134161000	4.17E-06
chr7	134273001	134278000	2.89E-09

chr7	134274001	134279000	3.40E-08
chr7	134291001	134296000	1.57E-14
chr7	134351001	134356000	9.23E-06
chr7	134439001	134444000	0.000542472
chr7	134518001	134523000	1.48E-09
chr7	134519001	134524000	3.87E-10
chr7	134520001	134525000	6.20E-10
chr7	134669001	134674000	6.37E-33
chr7	134670001	134675000	5.89E-44
chr7	134671001	134676000	1.52E-48
chr7	134709001	134714000	0.002614013
chr7	134710001	134715000	6.69E-06
chr7	134712001	134717000	0.000422653
chr7	134756001	134761000	2.59E-08
chr7	134770001	134775000	1.18E-05
chr7	134771001	134776000	1.46E-09
chr7	134772001	134777000	2.48E-05
chr7	134778001	134783000	0.004512014
chr7	134790001	134795000	1.76E-07
chr7	134791001	134796000	4.96E-08
chr7	134792001	134797000	2.08E-09
chr7	134793001	134798000	1.24E-06
chr7	134794001	134799000	1.12E-07
chr7	134795001	134800000	2.05E-06
chr7	134949001	134954000	6.90E-10
chr7	135344001	135349000	7.35E-08
chr7	135345001	135350000	5.76E-10
chr7	135346001	135351000	6.32E-12
chr7	135650001	135655000	0.003068535
chr7	135651001	135656000	0.000720235
chr7	135657001	135662000	2.34E-32
chr7	135706001	135711000	0.000302277
chr7	135737001	135742000	5.82E-07
chr7	135739001	135744000	3.49E-06
chr7	135752001	135757000	0.00030172
chr7	135970001	135975000	0.004992515
chr7	136037001	136042000	0.002774467
chr7	136038001	136043000	0.000977976
chr7	136179001	136184000	0.000376722
chr7	136285001	136290000	0.000147423
chr7	136286001	136291000	9.59E-05
chr7	136307001	136312000	1.41E-05
chr7	136308001	136313000	1.92E-05
chr7	136332001	136337000	0.000804763
chr7	136433001	136438000	0.00011234
chr7	136754001	136759000	0.010937952
chr7	136784001	136789000	5.33E-07
chr7	136883001	136888000	0.015245392

chr7	136973001	136978000	0.000375452
chr7	136978001	136983000	0.001327851
chr7	136981001	136986000	0.007460045
chr7	136983001	136988000	0.005016968
chr7	137003001	137008000	4.97E-09
chr7	137004001	137009000	1.09E-07
chr7	137023001	137028000	6.54E-06
chr7	137024001	137029000	6.30E-13
chr7	137025001	137030000	1.14E-16
chr7	137055001	137060000	0.000263314
chr7	137056001	137061000	8.20E-06
chr7	137163001	137168000	0.006941488
chr7	137165001	137170000	0.008530329
chr7	137172001	137177000	0.000204318
chr7	137179001	137184000	1.73E-05
chr7	137209001	137214000	1.09E-07
chr7	137372001	137377000	0.000654883
chr7	137373001	137378000	0.000352575
chr7	137442001	137447000	9.56E-11
chr7	137443001	137448000	4.06E-12
chr7	137527001	137532000	1.59E-23
chr7	137579001	137584000	4.68E-10
chr7	137580001	137585000	7.04E-17
chr7	137581001	137586000	1.65E-20
chr7	137582001	137587000	7.77E-13
chr7	137583001	137588000	2.36E-07
chr7	137684001	137689000	7.29E-18
chr7	137685001	137690000	4.36E-34
chr7	137686001	137691000	1.30E-40
chr7	137861001	137866000	2.07E-05
chr7	137862001	137867000	2.69E-08
chr7	137881001	137886000	5.59E-07
chr7	137882001	137887000	2.92E-08
chr7	137894001	137899000	0.00033055
chr7	138013001	138018000	3.43E-10
chr7	138014001	138019000	8.80E-12
chr7	138015001	138020000	3.51E-11
chr7	138141001	138146000	3.41E-26
chr7	138354001	138359000	5.55E-06
chr7	138661001	138666000	2.03E-08
chr7	138717001	138722000	5.61E-26
chr7	138719001	138724000	2.02E-27
chr7	138720001	138725000	3.79E-32
chr7	138814001	138819000	5.30E-23
chr7	138815001	138820000	1.81E-23
chr7	138816001	138821000	1.16E-24
chr7	138817001	138822000	6.73E-27
chr7	138818001	138823000	7.35E-29

chr7	138911001	138916000	1.43E-18
chr7	138912001	138917000	1.59E-53
chr7	138914001	138919000	4.55E-49
chr7	138915001	138920000	2.39E-40
chr7	138916001	138921000	1.09E-40
chr7	139045001	139050000	5.49E-30
chr7	139163001	139168000	2.73E-11
chr7	139164001	139169000	2.31E-13
chr7	139165001	139170000	7.83E-23
chr7	139166001	139171000	9.62E-23
chr7	139167001	139172000	1.11E-20
chr7	139168001	139173000	4.75E-14
chr7	139331001	139336000	1.28E-05
chr7	139477001	139482000	1.37E-28
chr7	139762001	139767000	3.23E-21
chr7	139871001	139876000	1.66E-19
chr7	139872001	139877000	1.09E-49
chr7	139929001	139934000	1.28E-09
chr7	140132001	140137000	0.006535423
chr7	140178001	140183000	2.42E-12
chr7	140339001	140344000	8.40E-12
chr7	140619001	140624000	7.93E-09
chr7	140773001	140778000	6.78E-40
chr7	140774001	140779000	3.67E-45
chr7	141251001	141256000	3.63E-33
chr7	141401001	141406000	1.03E-21
chr7	141435001	141440000	2.00E-16
chr7	141436001	141441000	1.99E-31
chr7	141437001	141442000	4.55E-26
chr7	141438001	141443000	1.44E-16
chr7	141519001	141524000	0.001960056
chr7	141644001	141649000	0.000920516
chr7	141645001	141650000	4.67E-05
chr7	142013001	142018000	2.98E-13
chr7	142016001	142021000	3.40E-05
chr7	142187001	142192000	1.36E-06
chr7	142219001	142224000	7.50E-07
chr7	142323001	142328000	0.027511645
chr7	142353001	142358000	4.60E-07
chr7	142365001	142370000	2.61E-05
chr7	142448001	142453000	5.69E-05
chr7	142528001	142533000	3.61E-08
chr7	142529001	142534000	2.23E-08
chr7	142530001	142535000	2.11E-09
chr7	142531001	142536000	6.11E-09
chr7	142541001	142546000	5.00E-06
chr7	142542001	142547000	4.59E-05
chr7	142552001	142557000	6.98E-14

chr7	142614001	142619000	3.39E-12
chr7	142615001	142620000	5.53E-10
chr7	142616001	142621000	6.69E-08
chr7	142617001	142622000	2.05E-09
chr7	142672001	142677000	3.29E-06
chr7	142678001	142683000	0.000472206
chr7	142679001	142684000	0.000106728
chr7	142680001	142685000	1.00E-06
chr7	142681001	142686000	4.96E-06
chr7	142682001	142687000	0.001317642
chr7	142683001	142688000	0.001407971
chr7	142739001	142744000	1.53E-08
chr7	142740001	142745000	5.15E-07
chr7	142772001	142777000	0.019304167
chr7	142773001	142778000	0.011804571
chr7	142775001	142780000	1.11E-05
chr7	142776001	142781000	1.41E-05
chr7	142843001	142848000	0.000424149
chr7	142956001	142961000	1.73E-09
chr7	142957001	142962000	4.17E-10
chr7	142958001	142963000	9.09E-07
chr7	143057001	143062000	1.64E-06
chr7	143058001	143063000	4.98E-15
chr7	143059001	143064000	2.18E-13
chr7	143073001	143078000	9.71E-31
chr7	143074001	143079000	2.80E-41
chr7	143075001	143080000	1.58E-23
chr7	143076001	143081000	2.75E-11
chr7	143445001	143450000	1.25E-08
chr7	143449001	143454000	3.47E-05
chr7	143594001	143599000	4.92E-43
chr7	143595001	143600000	2.25E-26
chr7	143596001	143601000	2.76E-27
chr7	143843001	143848000	3.67E-08
chr7	143844001	143849000	2.40E-07
chr7	143873001	143878000	3.30E-05
chr7	143889001	143894000	0.000271998
chr7	143891001	143896000	1.05E-09
chr7	143892001	143897000	1.09E-06
chr7	143893001	143898000	1.26E-05
chr7	143931001	143936000	0.003508981
chr7	143957001	143962000	7.23E-07
chr7	143958001	143963000	2.15E-07
chr7	143959001	143964000	5.58E-05
chr7	143981001	143986000	1.46E-07
chr7	143982001	143987000	7.70E-11
chr7	143983001	143988000	6.72E-11
chr7	143984001	143989000	8.20E-05

chr7	144052001	144057000	5.19E-07
chr7	144072001	144077000	1.07E-07
chr7	144073001	144078000	6.42E-08
chr7	144212001	144217000	9.64E-06
chr7	144213001	144218000	8.31E-05
chr7	144245001	144250000	1.58E-06
chr7	144246001	144251000	4.48E-08
chr7	144247001	144252000	1.92E-08
chr7	144386001	144391000	0.000349002
chr7	144387001	144392000	1.82E-05
chr7	144388001	144393000	0.000144247
chr7	144533001	144538000	3.66E-29
chr7	144546001	144551000	1.86E-12
chr7	144580001	144585000	0.000830024
chr7	144581001	144586000	0.002164651
chr7	144623001	144628000	5.33E-05
chr7	144624001	144629000	0.00076367
chr7	144838001	144843000	2.53E-05
chr7	144839001	144844000	7.46E-06
chr7	144952001	144957000	8.67E-05
chr7	144953001	144958000	0.000445827
chr7	145155001	145160000	0.002365159
chr7	145304001	145309000	0.00062152
chr7	145590001	145595000	3.50E-05
chr7	145591001	145596000	1.10E-06
chr7	145592001	145597000	3.87E-06
chr7	145593001	145598000	1.96E-06
chr7	145594001	145599000	4.76E-05
chr7	145653001	145658000	0.007788105
chr7	145951001	145956000	0.008652493
chr7	145978001	145983000	3.99E-20
chr7	145979001	145984000	4.77E-14
chr7	145980001	145985000	1.41E-19
chr7	145981001	145986000	4.32E-16
chr7	145982001	145987000	5.93E-18
chr7	146012001	146017000	4.32E-05
chr7	146263001	146268000	1.18E-06
chr7	146292001	146297000	0.001786782
chr7	146293001	146298000	8.79E-05
chr7	146294001	146299000	0.000856379
chr7	146316001	146321000	5.50E-06
chr7	146317001	146322000	4.71E-05
chr7	146318001	146323000	4.99E-06
chr7	146336001	146341000	8.35E-07
chr7	146337001	146342000	1.22E-10
chr7	146338001	146343000	3.98E-10
chr7	146341001	146346000	1.21E-07
chr7	146366001	146371000	0.000295329

chr7	146803001	146808000	3.50E-05
chr7	146804001	146809000	8.84E-05
chr7	147288001	147293000	0.001937845
chr7	147475001	147480000	0.000181461
chr7	148032001	148037000	2.05E-09
chr7	148033001	148038000	5.17E-19
chr7	148034001	148039000	3.14E-18
chr7	148035001	148040000	2.54E-18
chr7	148036001	148041000	1.45E-21
chr7	148391001	148396000	3.24E-76
chr7	148392001	148397000	4.95E-82
chr7	148579001	148584000	2.34E-65
chr7	148580001	148585000	2.26E-75
chr7	148581001	148586000	4.72E-85
chr7	148582001	148587000	6.81E-41
chr7	148725001	148730000	1.18E-13
chr7	148762001	148767000	1.13E-17
chr7	148785001	148790000	6.17E-21
chr7	148786001	148791000	2.41E-25
chr7	148787001	148792000	1.22E-27
chr7	148788001	148793000	4.49E-23
chr7	148819001	148824000	3.57E-15
chr7	148821001	148826000	2.21E-05
chr7	148822001	148827000	3.71E-19
chr7	148823001	148828000	1.11E-20
chr7	148824001	148829000	5.77E-08
chr7	148840001	148845000	9.25E-19
chr7	148843001	148848000	2.66E-24
chr7	148844001	148849000	1.86E-25
chr7	148845001	148850000	2.10E-38
chr7	148955001	148960000	3.81E-31
chr7	148956001	148961000	1.83E-31
chr7	148957001	148962000	1.46E-13
chr7	148958001	148963000	1.24E-16
chr7	149024001	149029000	2.88E-05
chr7	149036001	149041000	1.36E-05
chr7	149037001	149042000	1.62E-06
chr7	149115001	149120000	3.02E-11
chr7	149152001	149157000	2.60E-19
chr7	149153001	149158000	3.04E-59
chr7	149154001	149159000	3.37E-28
chr7	149155001	149160000	2.61E-22
chr7	149156001	149161000	1.09E-41
chr7	149157001	149162000	4.24E-62
chr7	149190001	149195000	7.46E-36
chr7	149191001	149196000	1.74E-43
chr7	149192001	149197000	7.00E-60
chr7	149193001	149198000	1.15E-38

chr7	149194001	149199000	1.10E-43
chr7	149466001	149471000	8.21E-06
chr7	149535001	149540000	4.68E-33
chr7	149569001	149574000	2.83E-29
chr7	149570001	149575000	9.35E-31
chr7	149571001	149576000	8.64E-22
chr7	149608001	149613000	0.000419332
chr7	149609001	149614000	0.000489803
chr7	149647001	149652000	2.75E-06
chr7	149682001	149687000	5.74E-05
chr7	149683001	149688000	1.35E-10
chr7	149684001	149689000	2.83E-09
chr7	149685001	149690000	1.93E-16
chr7	149686001	149691000	4.02E-13
chr7	149687001	149692000	2.02E-15
chr7	149717001	149722000	0.000122399
chr7	149718001	149723000	0.000571814
chr7	149719001	149724000	7.37E-05
chr7	149720001	149725000	8.18E-09
chr7	149721001	149726000	5.12E-13
chr7	149747001	149752000	2.79E-05
chr7	149748001	149753000	7.56E-08
chr7	149749001	149754000	2.56E-07
chr7	149750001	149755000	1.43E-07
chr7	149771001	149776000	0.01307064
chr7	149773001	149778000	1.40E-06
chr7	149774001	149779000	6.15E-06
chr7	149817001	149822000	4.71E-05
chr7	149819001	149824000	3.22E-13
chr7	149820001	149825000	7.00E-14
chr7	149821001	149826000	6.66E-15
chr7	149822001	149827000	8.38E-14
chr7	149823001	149828000	1.84E-07
chr7	149884001	149889000	0.001292703
chr7	149897001	149902000	1.24E-15
chr7	149898001	149903000	8.03E-15
chr7	149899001	149904000	1.05E-12
chr7	149900001	149905000	2.77E-13
chr7	149950001	149955000	3.21E-07
chr7	149951001	149956000	2.61E-08
chr7	149953001	149958000	2.18E-05
chr7	150016001	150021000	5.30E-16
chr7	150017001	150022000	2.27E-17
chr7	150020001	150025000	4.77E-11
chr7	150061001	150066000	3.98E-25
chr7	150062001	150067000	1.29E-28
chr7	150063001	150068000	7.80E-29
chr7	150188001	150193000	4.96E-05

chr7	150191001	150196000	8.38E-09
chr7	150195001	150200000	6.41E-09
chr7	150226001	150231000	8.71E-06
chr7	150228001	150233000	3.64E-08
chr7	150229001	150234000	1.76E-13
chr7	150230001	150235000	9.04E-13
chr7	150231001	150236000	1.46E-08
chr7	150232001	150237000	1.76E-08
chr7	150247001	150252000	4.30E-05
chr7	150262001	150267000	8.15E-08
chr7	150263001	150268000	4.41E-10
chr7	150282001	150287000	3.81E-05
chr7	150283001	150288000	1.50E-07
chr7	150284001	150289000	8.81E-07
chr7	150285001	150290000	1.52E-05
chr7	150286001	150291000	2.02E-09
chr7	150296001	150301000	1.00E-09
chr7	150297001	150302000	1.21E-07
chr7	150298001	150303000	1.83E-08
chr7	150299001	150304000	1.60E-06
chr7	150317001	150322000	0.000120921
chr7	150326001	150331000	0.002053538
chr7	150328001	150333000	0.002099244
chr7	150351001	150356000	1.87E-05
chr7	150357001	150362000	2.15E-07
chr7	150364001	150369000	0.00283939
chr7	150390001	150395000	2.77E-05
chr7	150420001	150425000	3.42E-08
chr7	150422001	150427000	1.31E-05
chr7	150423001	150428000	2.85E-05
chr7	150424001	150429000	0.000153938
chr7	150425001	150430000	0.000164453
chr7	150493001	150498000	2.44E-13
chr7	150494001	150499000	2.99E-15
chr7	150704001	150709000	0.000300531
chr7	150711001	150716000	0.000111019
chr7	150712001	150717000	0.000133669
chr7	150715001	150720000	2.60E-15
chr7	150860001	150865000	1.39E-22
chr7	150861001	150866000	1.10E-05
chr7	150863001	150868000	1.61E-05
chr7	150864001	150869000	2.38E-07
chr7	150891001	150896000	2.04E-05
chr7	150945001	150950000	1.55E-06
chr7	151034001	151039000	2.82E-37
chr7	151035001	151040000	5.29E-27
chr7	151038001	151043000	9.59E-14
chr7	151103001	151108000	3.13E-19

chr7	151104001	151109000	7.87E-15
chr7	151216001	151221000	1.16E-21
chr7	151217001	151222000	1.03E-19
chr7	151327001	151332000	2.19E-19
chr7	151328001	151333000	9.11E-26
chr7	151329001	151334000	8.90E-37
chr7	151569001	151574000	2.01E-22
chr7	151570001	151575000	1.28E-30
chr7	151573001	151578000	1.01E-25
chr7	151574001	151579000	5.58E-35
chr7	151718001	151723000	8.44E-45
chr7	151719001	151724000	1.10E-44
chr7	151720001	151725000	4.25E-31
chr7	151721001	151726000	2.36E-27
chr7	151722001	151727000	8.48E-23
chr7	152157001	152162000	7.56E-19
chr7	152158001	152163000	8.27E-27
chr7	152159001	152164000	5.62E-28
chr7	152160001	152165000	7.10E-24
chr7	152161001	152166000	7.63E-46
chr7	152369001	152374000	6.77E-22
chr7	152626001	152631000	1.46E-08
chr7	152815001	152820000	8.11E-16
chr7	152818001	152823000	4.00E-11
chr7	152939001	152944000	3.35E-08
chr7	152940001	152945000	2.65E-09
chr7	152941001	152946000	8.80E-11
chr7	152942001	152947000	1.43E-11
chr7	152943001	152948000	6.90E-09
chr7	152970001	152975000	0.001027319
chr7	152979001	152984000	2.22E-10
chr7	152999001	153004000	9.09E-12
chr7	153000001	153005000	6.53E-11
chr7	153001001	153006000	1.97E-07
chr7	153072001	153077000	3.11E-06
chr7	153193001	153198000	2.93E-07
chr7	153194001	153199000	1.53E-06
chr7	153255001	153260000	8.37E-08
chr7	153265001	153270000	5.71E-09
chr7	153266001	153271000	2.19E-09
chr7	153267001	153272000	3.73E-05
chr7	153268001	153273000	0.000266947
chr7	153269001	153274000	6.37E-05
chr7	153336001	153341000	4.17E-11
chr7	153337001	153342000	5.15E-07
chr7	153338001	153343000	1.55E-05
chr7	153352001	153357000	7.33E-06
chr7	153419001	153424000	0.000426873

chr7	153515001	153520000	0.001110149
chr7	153556001	153561000	8.45E-05
chr7	153561001	153566000	1.56E-08
chr7	153562001	153567000	8.07E-09
chr7	153571001	153576000	0.001048664
chr7	153610001	153615000	8.77E-08
chr7	153656001	153661000	2.14E-10
chr7	153657001	153662000	1.13E-11
chr7	153759001	153764000	0.000358061
chr7	153760001	153765000	3.48E-06
chr7	153761001	153766000	8.58E-09
chr7	153762001	153767000	1.37E-10
chr7	153763001	153768000	2.05E-07
chr7	153783001	153788000	1.91E-07
chr7	153784001	153789000	1.25E-07
chr7	153797001	153802000	1.82E-08
chr7	153807001	153812000	0.003942923
chr7	153829001	153834000	3.11E-06
chr7	153831001	153836000	0.002222951
chr7	153832001	153837000	0.003073095
chr7	153847001	153852000	7.81E-14
chr7	153848001	153853000	8.83E-15
chr7	153875001	153880000	0.000198816
chr7	153881001	153886000	1.54E-14
chr7	153895001	153900000	1.91E-12
chr7	153902001	153907000	8.71E-08
chr7	153923001	153928000	0.001005624
chr7	153932001	153937000	0.000120703
chr7	153933001	153938000	0.000146362
chr7	153962001	153967000	5.87E-05
chr7	153963001	153968000	6.26E-06
chr7	153964001	153969000	0.000141266
chr7	153965001	153970000	0.000159975
chr7	153966001	153971000	8.42E-05
chr7	153967001	153972000	4.83E-06
chr7	153979001	153984000	1.20E-09
chr7	154022001	154027000	6.35E-07
chr7	154072001	154077000	1.65E-07
chr7	154074001	154079000	7.17E-08
chr7	154120001	154125000	2.00E-08
chr7	154121001	154126000	1.21E-08
chr7	154142001	154147000	7.30E-06
chr7	154143001	154148000	1.60E-05
chr7	154144001	154149000	6.69E-09
chr7	154151001	154156000	4.48E-07
chr7	154152001	154157000	1.17E-05
chr7	154158001	154163000	9.18E-06
chr7	154159001	154164000	3.06E-06

chr7	154160001	154165000	2.15E-06
chr7	154161001	154166000	2.60E-09
chr7	154162001	154167000	2.98E-05
chr7	154176001	154181000	4.55E-13
chr7	154177001	154182000	1.19E-13
chr7	154222001	154227000	0.000168102
chr7	154261001	154266000	1.67E-07
chr7	154262001	154267000	2.45E-09
chr7	154263001	154268000	1.81E-05
chr7	154385001	154390000	7.52E-13
chr7	154386001	154391000	3.78E-11
chr7	154637001	154642000	5.78E-05
chr7	154790001	154795000	2.40E-37
chr7	154791001	154796000	5.01E-32
chr7	154792001	154797000	3.41E-25
chr7	154793001	154798000	1.06E-41
chr7	154794001	154799000	5.38E-50
chr7	154795001	154800000	6.13E-48
chr7	155133001	155138000	2.25E-10
chr7	155437001	155442000	4.62E-50
chr7	155585001	155590000	9.17E-11
chr7	155925001	155930000	4.52E-10
chr7	155974001	155979000	2.78E-06
chr7	156055001	156060000	3.94E-13
chr7	156063001	156068000	1.85E-06
chr7	156065001	156070000	1.67E-06
chr7	156074001	156079000	4.14E-05
chr7	156075001	156080000	0.000289869
chr7	156077001	156082000	0.000113986
chr7	156078001	156083000	1.43E-06
chr7	156079001	156084000	6.04E-08
chr7	156080001	156085000	1.33E-06
chr7	156081001	156086000	1.16E-07
chr7	156082001	156087000	1.10E-10
chr7	156083001	156088000	1.04E-09
chr7	156084001	156089000	6.33E-08
chr7	156085001	156090000	2.48E-07
chr7	156086001	156091000	1.63E-05
chr7	156116001	156121000	4.78E-06
chr7	156429001	156434000	1.47E-53
chr7	156430001	156435000	1.13E-52
chr7	156431001	156436000	1.10E-45
chr7	156432001	156437000	3.08E-40
chr7	156433001	156438000	1.16E-126
chr7	156685001	156690000	3.98E-09
chr7	156738001	156743000	1.45E-46
chr7	156788001	156793000	1.89E-09
chr7	156798001	156803000	4.99E-13

chr7	156927001	156932000	7.57E-21
chr7	157125001	157130000	2.85E-06
chr7	157241001	157246000	3.52E-14
chr7	157764001	157769000	1.04E-08
chr7	158364001	158369000	9.97E-12
chr7	158493001	158498000	9.83E-39
chr7	158494001	158499000	3.19E-35
chr7	158495001	158500000	1.70E-14
chr7	158609001	158614000	2.14E-13
chr7	158610001	158615000	1.23E-19
chr7	158611001	158616000	7.60E-20
chr7	158648001	158653000	7.54E-19
chr7	158649001	158654000	4.05E-19
chr7	158653001	158658000	9.87E-09
chr7	158846001	158851000	2.16E-12
chr7	158866001	158871000	3.44E-08
chr7	158869001	158874000	2.99E-09
chr7	158935001	158940000	2.69E-60
chr7	158937001	158942000	4.57E-25
chr7	158965001	158970000	3.86E-11
chr7	158966001	158971000	8.54E-14
chr7	158967001	158972000	5.40E-14
chr7	158975001	158980000	0.000268998
chr7	158976001	158981000	6.64E-06
chr7	158977001	158982000	6.64E-06
chr7	158978001	158983000	2.09E-08
chr7	159059001	159064000	1.30E-08
chr7	159066001	159071000	1.04E-06
chr7	159067001	159072000	1.02E-06
chr7	159068001	159073000	1.54E-08
chr7	159078001	159083000	1.80E-05
chr8	180001	185000	3.14E-24
chr8	181001	186000	1.29E-47
chr8	182001	187000	3.73E-60
chr8	214001	219000	3.07E-09
chr8	352001	357000	2.39E-15
chr8	353001	358000	3.30E-25
chr8	354001	359000	1.06E-38
chr8	355001	360000	6.94E-50
chr8	356001	361000	3.11E-60
chr8	357001	362000	2.94E-74
chr8	472001	477000	9.90E-11
chr8	473001	478000	5.13E-09
chr8	474001	479000	1.28E-08
chr8	490001	495000	4.78E-15
chr8	491001	496000	1.17E-21
chr8	493001	498000	1.09E-22
chr8	494001	499000	1.05E-55

chr8	495001	500000	3.19E-55
chr8	527001	532000	1.93E-16
chr8	528001	533000	1.31E-24
chr8	529001	534000	9.06E-22
chr8	530001	535000	5.52E-22
chr8	531001	536000	3.97E-12
chr8	532001	537000	1.31E-09
chr8	633001	638000	6.02E-17
chr8	634001	639000	1.60E-14
chr8	744001	749000	2.94E-10
chr8	745001	750000	2.10E-10
chr8	746001	751000	2.68E-12
chr8	747001	752000	2.51E-14
chr8	777001	782000	1.65E-06
chr8	823001	828000	0.001400999
chr8	824001	829000	7.48E-06
chr8	907001	912000	6.92E-10
chr8	999001	1004000	4.93E-16
chr8	1058001	1063000	2.47E-13
chr8	1371001	1376000	7.71E-11
chr8	1372001	1377000	1.46E-10
chr8	1373001	1378000	4.98E-09
chr8	1533001	1538000	2.93E-15
chr8	1558001	1563000	1.93E-12
chr8	1560001	1565000	4.24E-14
chr8	1566001	1571000	5.15E-21
chr8	1567001	1572000	8.18E-21
chr8	1568001	1573000	7.12E-21
chr8	1712001	1717000	8.70E-08
chr8	1768001	1773000	5.09E-28
chr8	1920001	1925000	1.17E-20
chr8	1921001	1926000	6.27E-32
chr8	1945001	1950000	7.34E-29
chr8	1946001	1951000	5.30E-19
chr8	1947001	1952000	2.62E-13
chr8	2128001	2133000	0.000270486
chr8	2323001	2328000	0.000102919
chr8	2324001	2329000	4.87E-05
chr8	2415001	2420000	1.24E-14
chr8	2416001	2421000	9.95E-19
chr8	2581001	2586000	2.36E-12
chr8	2897001	2902000	4.07E-05
chr8	3340001	3345000	0.000721529
chr8	3417001	3422000	4.62E-10
chr8	3418001	3423000	1.30E-09
chr8	3430001	3435000	6.21E-07
chr8	3692001	3697000	4.76E-06
chr8	3694001	3699000	1.46E-06

chr8	3695001	3700000	4.99E-08
chr8	3743001	3748000	5.04E-05
chr8	3800001	3805000	7.88E-06
chr8	3806001	3811000	2.96E-07
chr8	4037001	4042000	9.41E-06
chr8	4233001	4238000	9.61E-07
chr8	4331001	4336000	6.76E-07
chr8	4332001	4337000	3.18E-08
chr8	4333001	4338000	2.28E-06
chr8	4400001	4405000	0.010466536
chr8	4424001	4429000	1.62E-09
chr8	4425001	4430000	9.66E-17
chr8	4426001	4431000	3.10E-18
chr8	4427001	4432000	3.85E-24
chr8	4428001	4433000	7.98E-23
chr8	4429001	4434000	2.57E-21
chr8	4430001	4435000	1.26E-14
chr8	4431001	4436000	1.07E-11
chr8	4477001	4482000	7.32E-06
chr8	4488001	4493000	5.12E-11
chr8	4496001	4501000	3.57E-08
chr8	4521001	4526000	3.13E-07
chr8	4522001	4527000	3.21E-05
chr8	4524001	4529000	0.003765604
chr8	4566001	4571000	3.29E-05
chr8	4567001	4572000	4.09E-13
chr8	4568001	4573000	7.04E-13
chr8	4569001	4574000	1.37E-13
chr8	4594001	4599000	2.24E-10
chr8	4633001	4638000	1.91E-07
chr8	4673001	4678000	1.08E-06
chr8	4698001	4703000	7.07E-07
chr8	4699001	4704000	1.35E-05
chr8	4740001	4745000	0.010737889
chr8	4753001	4758000	8.68E-10
chr8	4754001	4759000	1.46E-18
chr8	4755001	4760000	8.56E-15
chr8	4756001	4761000	7.56E-11
chr8	4757001	4762000	1.87E-09
chr8	4758001	4763000	1.51E-08
chr8	4762001	4767000	2.77E-09
chr8	4763001	4768000	6.64E-07
chr8	4792001	4797000	0.004201515
chr8	4880001	4885000	0.000529863
chr8	4889001	4894000	1.33E-05
chr8	4939001	4944000	3.85E-13
chr8	4940001	4945000	3.54E-15
chr8	4941001	4946000	1.93E-12

chr8	4942001	4947000	1.28E-08
chr8	4943001	4948000	5.29E-08
chr8	4955001	4960000	1.07E-05
chr8	4956001	4961000	1.36E-05
chr8	4957001	4962000	5.44E-05
chr8	4971001	4976000	5.95E-10
chr8	4972001	4977000	9.94E-06
chr8	5018001	5023000	0.00072213
chr8	5024001	5029000	8.62E-14
chr8	5078001	5083000	1.61E-07
chr8	5079001	5084000	7.72E-05
chr8	5103001	5108000	0.007035519
chr8	5122001	5127000	7.34E-05
chr8	5186001	5191000	0.00012079
chr8	5222001	5227000	1.21E-06
chr8	5223001	5228000	2.42E-06
chr8	5262001	5267000	0.008645908
chr8	5263001	5268000	0.006186546
chr8	5277001	5282000	1.13E-09
chr8	5476001	5481000	4.16E-06
chr8	5477001	5482000	5.97E-05
chr8	5494001	5499000	0.000109466
chr8	5526001	5531000	6.56E-05
chr8	5584001	5589000	2.98E-07
chr8	5593001	5598000	7.97E-07
chr8	5646001	5651000	1.37E-06
chr8	5658001	5663000	0.000416525
chr8	5700001	5705000	0.000956317
chr8	5701001	5706000	4.25E-05
chr8	5765001	5770000	1.96E-12
chr8	5766001	5771000	9.06E-11
chr8	5767001	5772000	2.58E-11
chr8	5784001	5789000	1.83E-12
chr8	5787001	5792000	1.80E-08
chr8	5788001	5793000	2.96E-05
chr8	5846001	5851000	0.000113481
chr8	5847001	5852000	5.10E-07
chr8	5848001	5853000	6.26E-06
chr8	5849001	5854000	8.89E-06
chr8	5850001	5855000	6.72E-06
chr8	5961001	5966000	7.79E-05
chr8	5969001	5974000	0.004292419
chr8	5970001	5975000	0.010475688
chr8	6050001	6055000	3.49E-06
chr8	6051001	6056000	6.77E-07
chr8	6259001	6264000	4.23E-21
chr8	6260001	6265000	6.64E-38
chr8	6261001	6266000	7.40E-43

chr8	6262001	6267000	3.45E-38
chr8	6263001	6268000	7.78E-17
chr8	6264001	6269000	5.43E-15
chr8	6561001	6566000	4.37E-36
chr8	6563001	6568000	1.35E-24
chr8	6564001	6569000	1.59E-38
chr8	6565001	6570000	3.15E-77
chr8	6566001	6571000	2.31E-81
chr8	6949001	6954000	1.63E-11
chr8	6972001	6977000	1.08E-06
chr8	6973001	6978000	3.37E-06
chr8	7017001	7022000	0.016258168
chr8	7062001	7067000	0.008405359
chr8	7063001	7068000	0.005485018
chr8	7158001	7163000	0.001085135
chr8	7175001	7180000	5.39E-06
chr8	7176001	7181000	5.95E-07
chr8	7195001	7200000	6.43E-05
chr8	7198001	7203000	8.03E-07
chr8	7199001	7204000	4.36E-05
chr8	7210001	7215000	1.17E-19
chr8	7211001	7216000	7.01E-35
chr8	7212001	7217000	4.01E-38
chr8	7213001	7218000	1.55E-08
chr8	7214001	7219000	4.56E-08
chr8	7215001	7220000	0.000903221
chr8	7277001	7282000	2.47E-09
chr8	7278001	7283000	3.93E-09
chr8	7311001	7316000	0.000282395
chr8	7312001	7317000	1.57E-09
chr8	7313001	7318000	1.23E-07
chr8	7314001	7319000	4.48E-09
chr8	7315001	7320000	4.74E-07
chr8	7324001	7329000	0.000699723
chr8	7325001	7330000	8.19E-08
chr8	7326001	7331000	8.19E-08
chr8	7328001	7333000	9.31E-07
chr8	7349001	7354000	6.38E-06
chr8	7433001	7438000	4.22E-10
chr8	7446001	7451000	3.08E-06
chr8	7462001	7467000	3.31E-09
chr8	7463001	7468000	4.41E-07
chr8	7668001	7673000	0.012668086
chr8	7695001	7700000	4.82E-06
chr8	7696001	7701000	3.18E-08
chr8	7729001	7734000	0.000149633
chr8	7730001	7735000	1.55E-05
chr8	7780001	7785000	0.00171372

chr8	7879001	7884000	3.37E-53
chr8	7987001	7992000	8.49E-07
chr8	7988001	7993000	1.58E-10
chr8	7989001	7994000	1.47E-09
chr8	7990001	7995000	3.24E-10
chr8	7991001	7996000	3.12E-05
chr8	7997001	8002000	6.27E-09
chr8	7998001	8003000	5.77E-10
chr8	8006001	8011000	1.14E-06
chr8	8012001	8017000	9.16E-08
chr8	8013001	8018000	1.72E-08
chr8	8014001	8019000	3.87E-09
chr8	8021001	8026000	4.85E-05
chr8	8085001	8090000	5.40E-06
chr8	8244001	8249000	3.27E-09
chr8	8749001	8754000	9.70E-08
chr8	8750001	8755000	4.38E-34
chr8	8751001	8756000	2.73E-20
chr8	8816001	8821000	7.04E-17
chr8	9412001	9417000	1.48E-39
chr8	9413001	9418000	3.26E-66
chr8	9414001	9419000	6.99E-08
chr8	9472001	9477000	0.001161412
chr8	9624001	9629000	1.02E-10
chr8	9625001	9630000	5.62E-13
chr8	9626001	9631000	9.55E-17
chr8	9627001	9632000	2.81E-24
chr8	9628001	9633000	1.51E-36
chr8	9629001	9634000	2.13E-17
chr8	9731001	9736000	1.41E-11
chr8	9732001	9737000	1.44E-11
chr8	9733001	9738000	3.69E-12
chr8	9734001	9739000	7.99E-12
chr8	9756001	9761000	2.70E-14
chr8	9757001	9762000	7.11E-24
chr8	9791001	9796000	2.18E-06
chr8	9907001	9912000	4.73E-22
chr8	9908001	9913000	4.52E-33
chr8	9909001	9914000	8.08E-39
chr8	9910001	9915000	7.55E-42
chr8	9911001	9916000	5.59E-59
chr8	9912001	9917000	3.94E-64
chr8	10188001	10193000	1.68E-11
chr8	10192001	10197000	7.06E-07
chr8	10347001	10352000	1.52E-09
chr8	10442001	10447000	3.91E-08
chr8	10692001	10697000	4.56E-19
chr8	10693001	10698000	6.35E-78

chr8	10694001	10699000	2.72E-70
chr8	10695001	10700000	1.30E-38
chr8	10696001	10701000	6.31E-41
chr8	10697001	10702000	4.71E-40
chr8	10736001	10741000	0.000165689
chr8	10786001	10791000	2.23E-13
chr8	10980001	10985000	1.40E-10
chr8	10981001	10986000	3.26E-18
chr8	10982001	10987000	5.65E-15
chr8	11002001	11007000	6.14E-08
chr8	11007001	11012000	2.43E-13
chr8	11008001	11013000	1.29E-13
chr8	11009001	11014000	1.19E-08
chr8	11010001	11015000	7.27E-10
chr8	11055001	11060000	1.28E-18
chr8	11057001	11062000	2.67E-20
chr8	11058001	11063000	8.37E-26
chr8	11059001	11064000	4.49E-18
chr8	11137001	11142000	3.02E-15
chr8	11138001	11143000	2.56E-33
chr8	11139001	11144000	3.92E-36
chr8	11140001	11145000	2.60E-44
chr8	11141001	11146000	2.19E-31
chr8	11142001	11147000	2.18E-35
chr8	11155001	11160000	0.000118159
chr8	11200001	11205000	1.19E-11
chr8	11201001	11206000	1.53E-15
chr8	11322001	11327000	1.06E-16
chr8	11323001	11328000	2.61E-26
chr8	11324001	11329000	6.00E-17
chr8	11452001	11457000	1.01E-09
chr8	11663001	11668000	9.98E-14
chr8	11664001	11669000	1.25E-16
chr8	11665001	11670000	4.74E-14
chr8	11725001	11730000	6.94E-21
chr8	11756001	11761000	2.35E-07
chr8	11757001	11762000	9.50E-07
chr8	11840001	11845000	1.92E-26
chr8	11841001	11846000	5.06E-18
chr8	11842001	11847000	1.81E-18
chr8	11843001	11848000	1.82E-17
chr8	11862001	11867000	0.000575635
chr8	11896001	11901000	1.07E-07
chr8	11897001	11902000	1.07E-07
chr8	11981001	11986000	1.41E-05
chr8	12175001	12180000	0.000173501
chr8	12176001	12181000	1.86E-05
chr8	12177001	12182000	7.68E-05

chr8	12205001	12210000	0.042825625
chr8	12206001	12211000	0.042825625
chr8	12207001	12212000	0.042825625
chr8	12369001	12374000	1.72E-06
chr8	12411001	12416000	7.96E-12
chr8	12459001	12464000	8.94E-08
chr8	12471001	12476000	9.82E-06
chr8	12484001	12489000	6.59E-12
chr8	12487001	12492000	1.51E-06
chr8	12512001	12517000	1.66E-05
chr8	12513001	12518000	2.45E-05
chr8	12514001	12519000	1.65E-06
chr8	12515001	12520000	3.51E-06
chr8	12516001	12521000	1.21E-10
chr8	12517001	12522000	1.14E-09
chr8	12607001	12612000	3.11E-43
chr8	12608001	12613000	2.05E-42
chr8	12804001	12809000	5.37E-19
chr8	12805001	12810000	2.20E-90
chr8	12806001	12811000	1.31E-66
chr8	12807001	12812000	2.23E-50
chr8	12808001	12813000	3.84E-38
chr8	12809001	12814000	4.40E-35
chr8	12989001	12994000	6.07E-09
chr8	12990001	12995000	1.54E-33
chr8	13134001	13139000	2.03E-35
chr8	13173001	13178000	0.000190453
chr8	13308001	13313000	0.017619945
chr8	13309001	13314000	0.001000978
chr8	13327001	13332000	4.44E-05
chr8	13330001	13335000	7.20E-09
chr8	13331001	13336000	1.22E-07
chr8	13332001	13337000	3.32E-07
chr8	13333001	13338000	2.21E-07
chr8	13414001	13419000	1.23E-05
chr8	13503001	13508000	2.54E-05
chr8	13530001	13535000	6.27E-07
chr8	13590001	13595000	2.04E-05
chr8	13591001	13596000	0.000111508
chr8	13592001	13597000	8.31E-06
chr8	13593001	13598000	9.57E-06
chr8	13594001	13599000	6.64E-05
chr8	13603001	13608000	2.20E-07
chr8	13604001	13609000	2.43E-07
chr8	13613001	13618000	0.000421206
chr8	13614001	13619000	6.44E-05
chr8	13705001	13710000	4.33E-08
chr8	13706001	13711000	8.56E-10

chr8	13707001	13712000	1.07E-09
chr8	13708001	13713000	5.57E-07
chr8	13709001	13714000	3.24E-07
chr8	13748001	13753000	0.00051653
chr8	13792001	13797000	0.004367019
chr8	13815001	13820000	0.001264448
chr8	13819001	13824000	0.001471854
chr8	13825001	13830000	0.000136082
chr8	13826001	13831000	3.47E-06
chr8	13857001	13862000	0.002711879
chr8	13953001	13958000	3.39E-07
chr8	13954001	13959000	3.46E-06
chr8	14011001	14016000	8.25E-07
chr8	14013001	14018000	5.68E-06
chr8	14018001	14023000	6.37E-05
chr8	14153001	14158000	6.24E-05
chr8	14157001	14162000	1.80E-05
chr8	14159001	14164000	7.99E-06
chr8	14160001	14165000	1.32E-05
chr8	14191001	14196000	0.00124268
chr8	14192001	14197000	0.001370299
chr8	14297001	14302000	7.47E-05
chr8	14477001	14482000	3.87E-11
chr8	14533001	14538000	0.003030248
chr8	14547001	14552000	0.00787151
chr8	14558001	14563000	1.45E-07
chr8	14575001	14580000	0.001701412
chr8	14642001	14647000	4.31E-06
chr8	14644001	14649000	3.47E-06
chr8	14646001	14651000	2.17E-05
chr8	14647001	14652000	0.000167194
chr8	14648001	14653000	6.28E-05
chr8	14673001	14678000	0.00078203
chr8	14675001	14680000	0.000403826
chr8	14676001	14681000	0.002452324
chr8	14682001	14687000	0.000406568
chr8	14721001	14726000	5.99E-05
chr8	14774001	14779000	0.001320415
chr8	14775001	14780000	0.000367628
chr8	14776001	14781000	0.000250733
chr8	14821001	14826000	2.70E-06
chr8	14822001	14827000	7.20E-06
chr8	14828001	14833000	1.75E-09
chr8	14829001	14834000	3.03E-09
chr8	14830001	14835000	3.28E-10
chr8	14831001	14836000	7.56E-08
chr8	14838001	14843000	2.04E-07
chr8	14839001	14844000	4.56E-08

chr8	14868001	14873000	9.69E-05
chr8	14869001	14874000	2.37E-06
chr8	15016001	15021000	0.000384001
chr8	15054001	15059000	3.91E-05
chr8	15057001	15062000	3.08E-05
chr8	15102001	15107000	7.04E-07
chr8	15115001	15120000	0.00030172
chr8	15116001	15121000	0.000980047
chr8	15124001	15129000	2.35E-06
chr8	15126001	15131000	4.34E-07
chr8	15127001	15132000	2.04E-06
chr8	15128001	15133000	9.85E-06
chr8	15129001	15134000	0.000386447
chr8	15149001	15154000	0.000526266
chr8	15168001	15173000	3.92E-11
chr8	15287001	15292000	0.000391662
chr8	15393001	15398000	8.94E-17
chr8	15395001	15400000	2.33E-16
chr8	15396001	15401000	2.63E-24
chr8	15397001	15402000	5.67E-26
chr8	15398001	15403000	1.36E-11
chr8	15415001	15420000	9.09E-13
chr8	15661001	15666000	1.19E-07
chr8	15662001	15667000	2.48E-08
chr8	15663001	15668000	1.98E-06
chr8	15671001	15676000	4.72E-05
chr8	15679001	15684000	0.005241147
chr8	15693001	15698000	1.42E-05
chr8	15694001	15699000	1.90E-05
chr8	15695001	15700000	8.24E-06
chr8	15819001	15824000	4.35E-05
chr8	15820001	15825000	1.28E-06
chr8	15822001	15827000	3.43E-08
chr8	15824001	15829000	6.03E-10
chr8	15883001	15888000	0.00630841
chr8	15884001	15889000	0.000189913
chr8	15969001	15974000	0.002911207
chr8	16024001	16029000	2.16E-08
chr8	16090001	16095000	4.21E-06
chr8	16091001	16096000	5.74E-09
chr8	16092001	16097000	1.23E-08
chr8	16093001	16098000	0.000184279
chr8	16094001	16099000	8.64E-05
chr8	16156001	16161000	6.38E-06
chr8	16177001	16182000	0.000313768
chr8	16179001	16184000	0.000187002
chr8	16180001	16185000	0.000359486
chr8	16183001	16188000	0.000408815

chr8	16196001	16201000	0.003070424
chr8	16203001	16208000	0.003084072
chr8	16212001	16217000	0.000312026
chr8	16242001	16247000	0.000153061
chr8	16243001	16248000	7.95E-05
chr8	16244001	16249000	0.000228039
chr8	16412001	16417000	1.00E-06
chr8	16492001	16497000	8.86E-06
chr8	16493001	16498000	7.89E-08
chr8	16494001	16499000	5.99E-07
chr8	16524001	16529000	1.11E-15
chr8	16571001	16576000	2.43E-08
chr8	16681001	16686000	7.47E-06
chr8	16682001	16687000	1.68E-05
chr8	16745001	16750000	0.000102919
chr8	16746001	16751000	3.99E-05
chr8	16747001	16752000	1.23E-06
chr8	16748001	16753000	3.33E-06
chr8	16788001	16793000	0.010097493
chr8	16885001	16890000	6.25E-37
chr8	16904001	16909000	0.031050667
chr8	17014001	17019000	1.81E-42
chr8	17099001	17104000	1.17E-30
chr8	17100001	17105000	2.57E-54
chr8	17101001	17106000	1.17E-38
chr8	17102001	17107000	1.11E-56
chr8	17103001	17108000	1.44E-72
chr8	17104001	17109000	4.14E-80
chr8	17105001	17110000	2.44E-10
chr8	17267001	17272000	6.02E-10
chr8	17268001	17273000	3.23E-14
chr8	17269001	17274000	1.13E-14
chr8	17270001	17275000	4.49E-12
chr8	17271001	17276000	3.15E-09
chr8	17350001	17355000	9.66E-36
chr8	17352001	17357000	1.21E-42
chr8	17354001	17359000	1.16E-28
chr8	17659001	17664000	2.30E-26
chr8	17776001	17781000	2.58E-118
chr8	17777001	17782000	4.77E-114
chr8	17778001	17783000	2.64E-119
chr8	17779001	17784000	6.18E-74
chr8	17937001	17942000	4.99E-26
chr8	17941001	17946000	4.56E-45
chr8	18135001	18140000	6.82E-05
chr8	18432001	18437000	0.001734237
chr8	18655001	18660000	7.15E-13
chr8	18656001	18661000	1.44E-12

chr8	18657001	18662000	1.83E-12
chr8	18658001	18663000	2.08E-16
chr8	18659001	18664000	8.75E-12
chr8	18871001	18876000	2.41E-14
chr8	19239001	19244000	2.98E-07
chr8	19436001	19441000	9.91E-07
chr8	19456001	19461000	9.11E-10
chr8	19513001	19518000	6.39E-05
chr8	19514001	19519000	0.000162966
chr8	19515001	19520000	1.03E-05
chr8	19636001	19641000	0.000101033
chr8	19637001	19642000	0.00258695
chr8	19674001	19679000	6.16E-53
chr8	19675001	19680000	2.40E-45
chr8	19754001	19759000	7.65E-06
chr8	19901001	19906000	0.000768966
chr8	19902001	19907000	3.80E-05
chr8	19918001	19923000	0.000142376
chr8	19919001	19924000	3.67E-06
chr8	19920001	19925000	5.77E-05
chr8	19940001	19945000	1.17E-09
chr8	19941001	19946000	2.48E-09
chr8	19942001	19947000	8.38E-09
chr8	19995001	20000000	7.04E-16
chr8	19997001	20002000	9.39E-05
chr8	20050001	20055000	8.18E-19
chr8	20051001	20056000	4.63E-26
chr8	20053001	20058000	4.80E-26
chr8	20160001	20165000	1.85E-07
chr8	20397001	20402000	3.64E-07
chr8	20398001	20403000	2.07E-05
chr8	20898001	20903000	3.22E-05
chr8	21265001	21270000	5.43E-05
chr8	21266001	21271000	6.60E-07
chr8	21290001	21295000	5.72E-08
chr8	21291001	21296000	1.43E-08
chr8	21371001	21376000	1.08E-05
chr8	21372001	21377000	8.10E-08
chr8	21374001	21379000	8.66E-08
chr8	21677001	21682000	3.12E-07
chr8	21678001	21683000	5.33E-07
chr8	21773001	21778000	2.50E-12
chr8	21776001	21781000	6.05E-25
chr8	21777001	21782000	3.12E-33
chr8	21916001	21921000	0.007212808
chr8	21920001	21925000	2.93E-14
chr8	21985001	21990000	9.12E-08
chr8	21987001	21992000	3.36E-06

chr8	22013001	22018000	2.11E-09
chr8	22014001	22019000	6.63E-11
chr8	22220001	22225000	0.000234171
chr8	22418001	22423000	1.76E-06
chr8	22419001	22424000	5.73E-13
chr8	22420001	22425000	7.69E-10
chr8	22421001	22426000	4.54E-10
chr8	22422001	22427000	7.49E-08
chr8	22423001	22428000	5.70E-11
chr8	22522001	22527000	1.06E-09
chr8	22543001	22548000	7.72E-13
chr8	22718001	22723000	4.09E-13
chr8	22719001	22724000	3.66E-13
chr8	22853001	22858000	5.57E-09
chr8	22923001	22928000	1.76E-05
chr8	22957001	22962000	1.85E-05
chr8	22958001	22963000	1.81E-06
chr8	22959001	22964000	3.57E-07
chr8	22960001	22965000	4.12E-07
chr8	23078001	23083000	3.75E-16
chr8	23079001	23084000	2.55E-13
chr8	23082001	23087000	5.71E-10
chr8	23100001	23105000	9.83E-42
chr8	23101001	23106000	2.71E-18
chr8	23141001	23146000	3.15E-28
chr8	23142001	23147000	4.18E-19
chr8	23143001	23148000	1.52E-06
chr8	23145001	23150000	8.61E-07
chr8	23256001	23261000	1.50E-08
chr8	23257001	23262000	5.13E-18
chr8	23259001	23264000	9.16E-18
chr8	23260001	23265000	2.74E-12
chr8	23310001	23315000	3.23E-52
chr8	23311001	23316000	3.59E-68
chr8	23312001	23317000	7.15E-46
chr8	23313001	23318000	6.32E-40
chr8	23314001	23319000	3.19E-16
chr8	23315001	23320000	1.32E-20
chr8	23387001	23392000	1.71E-11
chr8	23500001	23505000	4.67E-06
chr8	23523001	23528000	1.24E-05
chr8	23524001	23529000	9.62E-05
chr8	23535001	23540000	2.30E-09
chr8	23536001	23541000	9.53E-15
chr8	23537001	23542000	1.89E-36
chr8	23538001	23543000	1.80E-29
chr8	23539001	23544000	1.58E-47
chr8	23540001	23545000	3.18E-48

chr8	24117001	24122000	0.00035266
chr8	24118001	24123000	0.000285834
chr8	24119001	24124000	2.02E-06
chr8	24135001	24140000	0.023678455
chr8	24137001	24142000	7.22E-06
chr8	24138001	24143000	6.01E-06
chr8	24139001	24144000	7.35E-09
chr8	24140001	24145000	1.82E-09
chr8	24141001	24146000	2.72E-09
chr8	24164001	24169000	9.54E-05
chr8	24215001	24220000	1.37E-05
chr8	24216001	24221000	2.62E-06
chr8	24233001	24238000	0.004092517
chr8	24250001	24255000	4.64E-05
chr8	24260001	24265000	1.15E-08
chr8	24261001	24266000	4.41E-08
chr8	24262001	24267000	5.87E-10
chr8	24263001	24268000	2.94E-10
chr8	24264001	24269000	3.40E-09
chr8	24304001	24309000	1.45E-10
chr8	24312001	24317000	6.25E-10
chr8	24329001	24334000	0.001738945
chr8	24330001	24335000	0.000187306
chr8	24331001	24336000	0.000462907
chr8	24337001	24342000	2.58E-05
chr8	24338001	24343000	8.55E-09
chr8	24341001	24346000	1.53E-07
chr8	24342001	24347000	1.80E-06
chr8	24354001	24359000	5.93E-05
chr8	24372001	24377000	5.59E-06
chr8	24375001	24380000	0.000441829
chr8	24376001	24381000	1.53E-06
chr8	24418001	24423000	8.15E-10
chr8	24420001	24425000	1.97E-07
chr8	24426001	24431000	2.08E-07
chr8	24427001	24432000	6.19E-09
chr8	24428001	24433000	2.95E-08
chr8	24429001	24434000	1.02E-08
chr8	24430001	24435000	9.49E-07
chr8	24432001	24437000	3.13E-07
chr8	24433001	24438000	6.28E-08
chr8	24434001	24439000	3.09E-06
chr8	24435001	24440000	2.38E-08
chr8	24446001	24451000	4.77E-06
chr8	24448001	24453000	0.000392943
chr8	24474001	24479000	0.000229114
chr8	24475001	24480000	6.76E-09
chr8	24476001	24481000	2.88E-10

chr8	24477001	24482000	1.04E-08
chr8	24478001	24483000	1.01E-08
chr8	24530001	24535000	2.16E-05
chr8	24531001	24536000	1.74E-08
chr8	24533001	24538000	4.30E-07
chr8	24534001	24539000	4.28E-06
chr8	24537001	24542000	0.001978678
chr8	24549001	24554000	0.00063637
chr8	24550001	24555000	1.11E-05
chr8	24585001	24590000	2.19E-05
chr8	24604001	24609000	2.33E-06
chr8	24605001	24610000	1.20E-10
chr8	24606001	24611000	3.67E-09
chr8	24607001	24612000	3.70E-07
chr8	24608001	24613000	2.46E-06
chr8	24615001	24620000	0.00142907
chr8	24628001	24633000	9.26E-06
chr8	24629001	24634000	6.26E-09
chr8	24645001	24650000	0.0001142
chr8	24650001	24655000	0.000213729
chr8	24656001	24661000	0.003638153
chr8	24657001	24662000	0.002544529
chr8	24658001	24663000	0.000813164
chr8	24681001	24686000	0.000839261
chr8	24683001	24688000	0.000221305
chr8	24727001	24732000	0.000331908
chr8	24739001	24744000	2.16E-09
chr8	24741001	24746000	2.75E-07
chr8	24841001	24846000	6.70E-07
chr8	24884001	24889000	1.87E-05
chr8	24885001	24890000	6.33E-05
chr8	24886001	24891000	0.000509189
chr8	24892001	24897000	0.000174248
chr8	24893001	24898000	3.26E-07
chr8	25383001	25388000	3.11E-06
chr8	25570001	25575000	0.000221301
chr8	25571001	25576000	1.27E-07
chr8	25572001	25577000	3.56E-07
chr8	25573001	25578000	2.78E-06
chr8	26236001	26241000	9.30E-41
chr8	26237001	26242000	1.23E-45
chr8	26238001	26243000	2.67E-23
chr8	26239001	26244000	1.01E-23
chr8	26304001	26309000	5.79E-22
chr8	26317001	26322000	1.08E-05
chr8	26412001	26417000	0.000744105
chr8	26644001	26649000	5.22E-05
chr8	26645001	26650000	8.73E-07

chr8	26646001	26651000	1.67E-05
chr8	26647001	26652000	0.000563045
chr8	26658001	26663000	6.75E-09
chr8	26669001	26674000	0.000396999
chr8	26688001	26693000	3.53E-07
chr8	26689001	26694000	2.31E-10
chr8	26690001	26695000	7.21E-07
chr8	26691001	26696000	2.18E-06
chr8	26692001	26697000	7.47E-08
chr8	26693001	26698000	9.95E-05
chr8	26789001	26794000	4.53E-06
chr8	26790001	26795000	7.64E-07
chr8	26791001	26796000	2.29E-06
chr8	26815001	26820000	2.45E-05
chr8	26829001	26834000	1.06E-06
chr8	26991001	26996000	4.77E-05
chr8	26992001	26997000	9.54E-08
chr8	26993001	26998000	4.49E-08
chr8	27003001	27008000	0.000132126
chr8	27010001	27015000	0.000200669
chr8	27011001	27016000	0.000740677
chr8	27024001	27029000	5.26E-06
chr8	27030001	27035000	0.000443979
chr8	27073001	27078000	0.003467877
chr8	27074001	27079000	0.003999881
chr8	27075001	27080000	0.005241602
chr8	27084001	27089000	3.18E-07
chr8	27085001	27090000	3.80E-10
chr8	27179001	27184000	1.35E-14
chr8	27446001	27451000	4.49E-10
chr8	27472001	27477000	2.86E-12
chr8	27487001	27492000	6.75E-17
chr8	27488001	27493000	4.35E-10
chr8	27626001	27631000	3.95E-27
chr8	27627001	27632000	2.15E-28
chr8	27628001	27633000	1.26E-31
chr8	27630001	27635000	7.74E-55
chr8	27631001	27636000	3.84E-52
chr8	27632001	27637000	1.26E-28
chr8	28091001	28096000	3.69E-05
chr8	28139001	28144000	6.94E-09
chr8	28140001	28145000	8.28E-08
chr8	28243001	28248000	6.43E-23
chr8	28257001	28262000	6.17E-11
chr8	28259001	28264000	2.74E-08
chr8	28265001	28270000	4.59E-21
chr8	28266001	28271000	8.77E-13
chr8	28267001	28272000	9.14E-09

chr8	28268001	28273000	3.01E-08
chr8	28351001	28356000	1.45E-35
chr8	28352001	28357000	9.16E-33
chr8	28370001	28375000	1.53E-06
chr8	28554001	28559000	4.69E-21
chr8	28555001	28560000	2.81E-24
chr8	28556001	28561000	1.94E-34
chr8	28557001	28562000	4.15E-45
chr8	28558001	28563000	3.23E-63
chr8	28559001	28564000	1.84E-47
chr8	28743001	28748000	3.75E-23
chr8	28744001	28749000	2.97E-34
chr8	28747001	28752000	5.60E-44
chr8	28748001	28753000	2.63E-42
chr8	28749001	28754000	8.81E-21
chr8	29116001	29121000	3.84E-38
chr8	29117001	29122000	5.59E-30
chr8	29118001	29123000	1.31E-27
chr8	29119001	29124000	1.43E-17
chr8	29120001	29125000	3.23E-17
chr8	29201001	29206000	2.34E-08
chr8	29202001	29207000	7.02E-10
chr8	29203001	29208000	1.97E-14
chr8	29543001	29548000	0.000588859
chr8	29544001	29549000	5.79E-05
chr8	29545001	29550000	0.000383085
chr8	29951001	29956000	3.87E-22
chr8	29952001	29957000	4.21E-21
chr8	29953001	29958000	2.12E-27
chr8	30237001	30242000	5.40E-11
chr8	30585001	30590000	2.21E-06
chr8	30726001	30731000	5.55E-05
chr8	30765001	30770000	8.03E-14
chr8	30767001	30772000	1.43E-13
chr8	30768001	30773000	1.46E-14
chr8	30769001	30774000	1.78E-12
chr8	30886001	30891000	4.99E-33
chr8	30887001	30892000	8.28E-44
chr8	31010001	31015000	0.000594392
chr8	31169001	31174000	0.000935145
chr8	31181001	31186000	3.22E-10
chr8	31185001	31190000	0.000526488
chr8	31187001	31192000	6.65E-09
chr8	31188001	31193000	2.51E-07
chr8	31189001	31194000	1.67E-05
chr8	31194001	31199000	1.07E-06
chr8	31195001	31200000	1.18E-08
chr8	31196001	31201000	1.57E-07

chr8	31258001	31263000	3.73E-06
chr8	31259001	31264000	0.000153445
chr8	31260001	31265000	0.00035101
chr8	31265001	31270000	1.50E-05
chr8	31266001	31271000	1.81E-06
chr8	31268001	31273000	6.65E-05
chr8	31275001	31280000	2.61E-06
chr8	31368001	31373000	8.87E-07
chr8	31421001	31426000	2.66E-09
chr8	32005001	32010000	0.000198316
chr8	32067001	32072000	0.002379348
chr8	32068001	32073000	0.002788575
chr8	32069001	32074000	0.002999276
chr8	32117001	32122000	3.94E-06
chr8	32125001	32130000	0.00387528
chr8	32126001	32131000	0.006644191
chr8	32141001	32146000	5.88E-05
chr8	32234001	32239000	2.63E-06
chr8	32235001	32240000	3.27E-07
chr8	32236001	32241000	1.03E-05
chr8	32268001	32273000	4.01E-07
chr8	32269001	32274000	1.59E-09
chr8	32310001	32315000	6.90E-05
chr8	32404001	32409000	2.17E-29
chr8	32405001	32410000	3.44E-25
chr8	32406001	32411000	1.59E-24
chr8	32432001	32437000	0.004472127
chr8	32433001	32438000	0.002170196
chr8	32434001	32439000	0.002105586
chr8	32435001	32440000	0.00241541
chr8	32776001	32781000	0.000342452
chr8	32777001	32782000	0.002126457
chr8	32789001	32794000	0.016191046
chr8	32818001	32823000	0.00030019
chr8	32909001	32914000	2.38E-07
chr8	33002001	33007000	2.91E-06
chr8	33003001	33008000	6.63E-06
chr8	33004001	33009000	5.78E-05
chr8	33005001	33010000	5.74E-05
chr8	33014001	33019000	0.002930629
chr8	33338001	33343000	1.79E-09
chr8	33339001	33344000	2.33E-09
chr8	33340001	33345000	5.40E-13
chr8	33342001	33347000	7.20E-35
chr8	33343001	33348000	2.18E-07
chr8	33366001	33371000	1.77E-10
chr8	33372001	33377000	1.23E-07
chr8	33421001	33426000	2.70E-05

chr8	33455001	33460000	3.66E-09
chr8	33456001	33461000	6.11E-08
chr8	33457001	33462000	7.73E-14
chr8	33671001	33676000	0.000765296
chr8	33803001	33808000	0.000709456
chr8	34045001	34050000	6.59E-05
chr8	34046001	34051000	1.03E-05
chr8	34133001	34138000	0.006617045
chr8	34222001	34227000	0.003234206
chr8	34223001	34228000	8.35E-05
chr8	34224001	34229000	8.60E-05
chr8	34225001	34230000	0.000191319
chr8	34269001	34274000	0.003301476
chr8	34270001	34275000	5.52E-05
chr8	34271001	34276000	4.27E-05
chr8	34272001	34277000	1.00E-05
chr8	34273001	34278000	0.000976437
chr8	34472001	34477000	3.91E-07
chr8	34473001	34478000	6.33E-07
chr8	34474001	34479000	4.70E-09
chr8	34475001	34480000	1.71E-12
chr8	34476001	34481000	2.96E-12
chr8	34611001	34616000	0.001166646
chr8	35088001	35093000	2.67E-16
chr8	35089001	35094000	1.85E-29
chr8	35091001	35096000	2.49E-18
chr8	35092001	35097000	1.92E-09
chr8	35201001	35206000	0.017498583
chr8	35202001	35207000	0.026608908
chr8	35203001	35208000	0.018376273
chr8	35216001	35221000	0.016776641
chr8	35287001	35292000	6.32E-05
chr8	35312001	35317000	0.00073825
chr8	35421001	35426000	6.99E-08
chr8	35422001	35427000	1.50E-07
chr8	35423001	35428000	6.04E-05
chr8	35442001	35447000	2.18E-05
chr8	35501001	35506000	0.001065864
chr8	35504001	35509000	9.26E-06
chr8	35505001	35510000	6.05E-06
chr8	35534001	35539000	8.41E-05
chr8	35563001	35568000	0.000196087
chr8	35569001	35574000	1.08E-05
chr8	35570001	35575000	8.68E-08
chr8	35571001	35576000	2.34E-07
chr8	35696001	35701000	3.15E-06
chr8	35759001	35764000	0.000472701
chr8	35779001	35784000	5.62E-07

chr8	35815001	35820000	7.88E-06
chr8	35890001	35895000	5.65E-05
chr8	35891001	35896000	0.000562473
chr8	36022001	36027000	9.08E-06
chr8	36023001	36028000	9.26E-09
chr8	36024001	36029000	5.74E-08
chr8	36045001	36050000	1.65E-08
chr8	36110001	36115000	0.008374253
chr8	36111001	36116000	0.002374923
chr8	36112001	36117000	0.003963981
chr8	36195001	36200000	3.44E-05
chr8	36276001	36281000	3.00E-06
chr8	36360001	36365000	3.08E-12
chr8	36641001	36646000	1.29E-08
chr8	36676001	36681000	6.34E-05
chr8	36693001	36698000	0.000101184
chr8	36803001	36808000	0.002832793
chr8	37594001	37599000	1.08E-10
chr8	37616001	37621000	7.86E-48
chr8	37617001	37622000	8.91E-36
chr8	37618001	37623000	2.26E-31
chr8	37619001	37624000	2.79E-32
chr8	37620001	37625000	2.06E-09
chr8	37650001	37655000	1.23E-09
chr8	37651001	37656000	4.38E-08
chr8	37883001	37888000	2.14E-09
chr8	37963001	37968000	5.27E-25
chr8	38029001	38034000	1.08E-32
chr8	38030001	38035000	7.77E-51
chr8	38084001	38089000	6.98E-31
chr8	38085001	38090000	2.34E-36
chr8	38089001	38094000	2.19E-25
chr8	38122001	38127000	3.07E-20
chr8	38123001	38128000	4.63E-21
chr8	38124001	38129000	3.57E-31
chr8	38125001	38130000	1.04E-27
chr8	38126001	38131000	1.79E-45
chr8	38127001	38132000	8.42E-13
chr8	38244001	38249000	1.50E-12
chr8	38326001	38331000	3.57E-25
chr8	38612001	38617000	7.89E-21
chr8	38755001	38760000	3.22E-10
chr8	38849001	38854000	6.02E-28
chr8	38850001	38855000	3.75E-72
chr8	39155001	39160000	0.022571767
chr8	39156001	39161000	0.002112749
chr8	39167001	39172000	0.000162182
chr8	39198001	39203000	7.27E-06

chr8	39199001	39204000	7.52E-06
chr8	39200001	39205000	5.53E-05
chr8	39201001	39206000	0.000119255
chr8	39224001	39229000	0.00283703
chr8	39286001	39291000	3.56E-08
chr8	39290001	39295000	5.54E-09
chr8	39291001	39296000	1.10E-09
chr8	39292001	39297000	1.83E-07
chr8	39293001	39298000	7.72E-06
chr8	39294001	39299000	9.22E-05
chr8	39321001	39326000	0.001001146
chr8	39322001	39327000	4.74E-05
chr8	39323001	39328000	2.71E-05
chr8	39373001	39378000	5.29E-06
chr8	39374001	39379000	4.10E-06
chr8	39396001	39401000	4.89E-10
chr8	39397001	39402000	1.21E-05
chr8	39403001	39408000	2.97E-07
chr8	39404001	39409000	1.75E-05
chr8	39432001	39437000	3.37E-06
chr8	39526001	39531000	0.000416947
chr8	39527001	39532000	0.000285511
chr8	39561001	39566000	2.98E-06
chr8	39622001	39627000	1.22E-06
chr8	39653001	39658000	4.82E-08
chr8	39654001	39659000	1.15E-07
chr8	39663001	39668000	5.40E-08
chr8	39669001	39674000	3.65E-09
chr8	39670001	39675000	1.64E-07
chr8	39671001	39676000	9.72E-07
chr8	39743001	39748000	0.00065559
chr8	39829001	39834000	3.36E-07
chr8	39830001	39835000	1.33E-05
chr8	39831001	39836000	1.07E-12
chr8	40887001	40892000	3.70E-05
chr8	40894001	40899000	1.21E-07
chr8	40895001	40900000	3.81E-09
chr8	40896001	40901000	7.20E-09
chr8	40930001	40935000	5.08E-05
chr8	41205001	41210000	6.21E-06
chr8	41206001	41211000	1.39E-06
chr8	41207001	41212000	3.21E-06
chr8	41344001	41349000	6.01E-07
chr8	41345001	41350000	1.94E-06
chr8	41346001	41351000	1.47E-15
chr8	41347001	41352000	1.17E-31
chr8	41348001	41353000	1.19E-35
chr8	41431001	41436000	8.52E-25

chr8	41432001	41437000	7.52E-20
chr8	41433001	41438000	5.04E-21
chr8	41620001	41625000	8.36E-07
chr8	41622001	41627000	1.61E-06
chr8	41623001	41628000	3.43E-11
chr8	41909001	41914000	1.16E-44
chr8	42010001	42015000	2.15E-41
chr8	42249001	42254000	6.81E-45
chr8	42397001	42402000	2.51E-14
chr8	42693001	42698000	6.53E-20
chr8	42694001	42699000	2.78E-30
chr8	42695001	42700000	4.60E-22
chr8	42696001	42701000	4.19E-21
chr8	42697001	42702000	3.20E-18
chr8	42698001	42703000	1.28E-18
chr8	42944001	42949000	2.96E-24
chr8	43209001	43214000	8.86E-07
chr8	43210001	43215000	2.69E-06
chr8	43264001	43269000	1.51E-05
chr8	43390001	43395000	0.000124542
chr8	43391001	43396000	0.000116662
chr8	43717001	43722000	0.005845883
chr8	47080001	47085000	0.000112909
chr8	47306001	47311000	0.000256774
chr8	47307001	47312000	3.60E-06
chr8	47586001	47591000	4.94E-07
chr8	47786001	47791000	2.54E-06
chr8	47787001	47792000	1.45E-08
chr8	47944001	47949000	0.000600461
chr8	47945001	47950000	0.001216019
chr8	47962001	47967000	1.37E-06
chr8	47963001	47968000	0.001120668
chr8	47970001	47975000	9.95E-08
chr8	47971001	47976000	1.49E-06
chr8	47972001	47977000	1.47E-08
chr8	48096001	48101000	5.49E-58
chr8	48097001	48102000	5.96E-58
chr8	48098001	48103000	9.06E-56
chr8	48099001	48104000	8.31E-46
chr8	48100001	48105000	1.19E-44
chr8	48650001	48655000	2.78E-26
chr8	48651001	48656000	4.70E-24
chr8	48916001	48921000	4.40E-60
chr8	48917001	48922000	2.10E-82
chr8	48918001	48923000	6.30E-86
chr8	48919001	48924000	1.10E-77
chr8	48920001	48925000	9.48E-53
chr8	48921001	48926000	1.34E-35

chr8	49424001	49429000	8.68E-29
chr8	49425001	49430000	1.07E-30
chr8	49427001	49432000	9.51E-36
chr8	52806001	52811000	1.68E-31
chr8	52807001	52812000	1.78E-78
chr8	53400001	53405000	0.000390541
chr8	53401001	53406000	0.000258169
chr8	53622001	53627000	9.61E-70
chr8	53732001	53737000	4.36E-05
chr8	53824001	53829000	5.53E-08
chr8	53967001	53972000	1.65E-05
chr8	54019001	54024000	1.92E-10
chr8	54033001	54038000	1.01E-09
chr8	54159001	54164000	6.80E-45
chr8	54160001	54165000	1.74E-41
chr8	54161001	54166000	1.34E-43
chr8	55294001	55299000	4.78E-24
chr8	55466001	55471000	8.68E-15
chr8	55560001	55565000	3.70E-05
chr8	55682001	55687000	5.22E-07
chr8	55683001	55688000	4.61E-06
chr8	55698001	55703000	1.06E-09
chr8	55798001	55803000	0.028314368
chr8	55845001	55850000	1.83E-06
chr8	55846001	55851000	1.55E-06
chr8	55847001	55852000	6.43E-05
chr8	56037001	56042000	1.41E-05
chr8	56038001	56043000	1.68E-09
chr8	56788001	56793000	6.33E-38
chr8	56793001	56798000	2.40E-16
chr8	57686001	57691000	0.001856086
chr8	58067001	58072000	6.99E-06
chr8	58069001	58074000	5.81E-06
chr8	59323001	59328000	1.11E-75
chr8	59324001	59329000	2.78E-94
chr8	59567001	59572000	3.20E-52
chr8	59568001	59573000	1.33E-94
chr8	59569001	59574000	6.55E-93
chr8	59570001	59575000	4.60E-66
chr8	59571001	59576000	7.31E-65
chr8	59572001	59577000	3.40E-84
chr8	61430001	61435000	7.13E-31
chr8	61560001	61565000	3.03E-41
chr8	61591001	61596000	3.75E-121
chr8	61592001	61597000	5.59E-91
chr8	61936001	61941000	2.41E-23
chr8	63996001	64001000	3.53E-50
chr8	63998001	64003000	4.25E-37

chr8	64081001	64086000	9.60E-121
chr8	64188001	64193000	0.000153865
chr8	64818001	64823000	1.36E-07
chr8	64938001	64943000	5.70E-06
chr8	64939001	64944000	5.82E-07
chr8	64940001	64945000	8.35E-07
chr8	64999001	65004000	4.92E-10
chr8	65067001	65072000	0.000108639
chr8	65159001	65164000	0.011590455
chr8	65160001	65165000	0.000676347
chr8	65161001	65166000	4.88E-05
chr8	65310001	65315000	8.27E-08
chr8	66754001	66759000	5.76E-91
chr8	67034001	67039000	6.79E-18
chr8	67620001	67625000	1.83E-23
chr8	67621001	67626000	9.30E-29
chr8	67625001	67630000	2.36E-34
chr8	67687001	67692000	2.30E-65
chr8	67977001	67982000	1.47E-20
chr8	68251001	68256000	7.32E-63
chr8	68256001	68261000	1.03E-40
chr8	68413001	68418000	2.77E-05
chr8	68535001	68540000	1.14E-05
chr8	68838001	68843000	0.000693261
chr8	69190001	69195000	1.83E-12
chr8	69239001	69244000	1.99E-36
chr8	69400001	69405000	0.003005252
chr8	71316001	71321000	2.70E-54
chr8	71517001	71522000	1.66E-63
chr8	71518001	71523000	2.21E-62
chr8	71519001	71524000	1.11E-67
chr8	71520001	71525000	1.75E-56
chr8	71576001	71581000	5.30E-07
chr8	71577001	71582000	2.65E-35
chr8	73656001	73661000	1.03E-07
chr8	73657001	73662000	2.95E-08
chr8	73658001	73663000	4.88E-07
chr8	73693001	73698000	8.36E-05
chr8	73695001	73700000	2.40E-05
chr8	73769001	73774000	2.00E-06
chr8	73770001	73775000	2.75E-07
chr8	74654001	74659000	1.29E-27
chr8	74786001	74791000	5.12E-59
chr8	74885001	74890000	4.63E-99
chr8	74886001	74891000	1.99E-62
chr8	74887001	74892000	6.14E-88
chr8	74888001	74893000	4.00E-92
chr8	75228001	75233000	1.40E-48

chr8	75262001	75267000	4.52E-39
chr8	75330001	75335000	4.94E-26
chr8	76320001	76325000	5.68E-47
chr8	76367001	76372000	0.004367835
chr8	76721001	76726000	1.43E-14
chr8	76722001	76727000	3.00E-14
chr8	77210001	77215000	0.001525705
chr8	77211001	77216000	0.001599982
chr8	77912001	77917000	2.21E-102
chr8	78614001	78619000	0.004661787
chr8	79712001	79717000	1.19E-49
chr8	79858001	79863000	0.024361876
chr8	80121001	80126000	0.005649967
chr8	80122001	80127000	0.003651762
chr8	80123001	80128000	0.000606906
chr8	80871001	80876000	0.000315228
chr8	80873001	80878000	1.92E-07
chr8	80874001	80879000	1.23E-08
chr8	80890001	80895000	1.52E-24
chr8	81399001	81404000	4.15E-119
chr8	81596001	81601000	3.93E-31
chr8	81597001	81602000	3.68E-28
chr8	81598001	81603000	1.11E-27
chr8	81793001	81798000	0.001789615
chr8	81959001	81964000	2.89E-07
chr8	82019001	82024000	8.14E-52
chr8	82021001	82026000	9.18E-76
chr8	82022001	82027000	3.19E-74
chr8	82632001	82637000	2.68E-59
chr8	82633001	82638000	7.17E-74
chr8	82644001	82649000	7.20E-54
chr8	86085001	86090000	1.15E-89
chr8	86086001	86091000	2.44E-108
chr8	86087001	86092000	7.05E-82
chr8	86088001	86093000	1.45E-51
chr8	86280001	86285000	0.004097652
chr8	86376001	86381000	1.39E-50
chr8	86509001	86514000	2.38E-11
chr8	86511001	86516000	2.18E-09
chr8	86512001	86517000	1.87E-08
chr8	86777001	86782000	1.34E-11
chr8	86778001	86783000	6.21E-17
chr8	86837001	86842000	2.75E-14
chr8	86840001	86845000	4.30E-16
chr8	86980001	86985000	0.000657726
chr8	87350001	87355000	2.23E-31
chr8	87351001	87356000	3.89E-43
chr8	87522001	87527000	2.70E-37

chr8	87523001	87528000	8.59E-35
chr8	87524001	87529000	1.35E-57
chr8	87525001	87530000	1.86E-69
chr8	87526001	87531000	3.45E-66
chr8	87527001	87532000	9.97E-45
chr8	90765001	90770000	1.80E-27
chr8	90792001	90797000	4.50E-10
chr8	90910001	90915000	2.96E-54
chr8	90912001	90917000	4.90E-83
chr8	90913001	90918000	2.50E-90
chr8	90914001	90919000	1.44E-127
chr8	90915001	90920000	9.24E-38
chr8	90992001	90997000	4.57E-101
chr8	91657001	91662000	3.59E-73
chr8	91658001	91663000	2.86E-66
chr8	92048001	92053000	2.10E-25
chr8	93103001	93108000	3.22E-29
chr8	93973001	93978000	1.81E-25
chr8	93974001	93979000	2.49E-73
chr8	93975001	93980000	1.02E-75
chr8	93976001	93981000	7.84E-56
chr8	93977001	93982000	2.41E-34
chr8	93978001	93983000	1.34E-37
chr8	94766001	94771000	7.61E-33
chr8	94767001	94772000	2.39E-25
chr8	94929001	94934000	3.56E-91
chr8	95655001	95660000	4.88E-08
chr8	95732001	95737000	1.58E-60
chr8	95834001	95839000	5.90E-51
chr8	95835001	95840000	6.53E-69
chr8	95957001	95962000	1.76E-74
chr8	95958001	95963000	1.45E-53
chr8	95961001	95966000	1.02E-35
chr8	96276001	96281000	7.85E-30
chr8	96542001	96547000	6.97E-05
chr8	96641001	96646000	5.36E-07
chr8	96655001	96660000	6.07E-07
chr8	96695001	96700000	2.44E-06
chr8	96727001	96732000	1.27E-12
chr8	96919001	96924000	8.89E-08
chr8	96920001	96925000	5.89E-07
chr8	96987001	96992000	1.48E-06
chr8	96988001	96993000	3.52E-10
chr8	96989001	96994000	3.99E-07
chr8	97031001	97036000	4.94E-15
chr8	97077001	97082000	4.97E-05
chr8	97142001	97147000	1.67E-24
chr8	97143001	97148000	4.62E-23

chr8	97144001	97149000	5.92E-20
chr8	97145001	97150000	3.79E-21
chr8	97148001	97153000	1.66E-05
chr8	97211001	97216000	4.17E-06
chr8	98286001	98291000	1.73E-35
chr8	98287001	98292000	7.41E-36
chr8	98288001	98293000	9.04E-50
chr8	98289001	98294000	4.52E-61
chr8	98290001	98295000	2.05E-73
chr8	98349001	98354000	0.00143634
chr8	98350001	98355000	0.001159078
chr8	98784001	98789000	3.92E-27
chr8	98877001	98882000	1.01E-31
chr8	98881001	98886000	6.61E-34
chr8	99077001	99082000	4.82E-46
chr8	99276001	99281000	2.75E-08
chr8	99306001	99311000	5.70E-39
chr8	99646001	99651000	8.27E-06
chr8	99833001	99838000	2.96E-52
chr8	99834001	99839000	1.25E-42
chr8	101114001	101119000	1.70E-12
chr8	101166001	101171000	2.20E-31
chr8	101167001	101172000	7.84E-31
chr8	101168001	101173000	2.29E-43
chr8	101169001	101174000	1.25E-45
chr8	101170001	101175000	1.30E-62
chr8	101224001	101229000	2.78E-79
chr8	101225001	101230000	9.11E-68
chr8	101322001	101327000	7.78E-59
chr8	103246001	103251000	1.59E-34
chr8	103247001	103252000	7.73E-55
chr8	103423001	103428000	8.99E-33
chr8	103424001	103429000	2.53E-37
chr8	103613001	103618000	1.06E-22
chr8	103662001	103667000	2.17E-56
chr8	104033001	104038000	3.99E-46
chr8	104299001	104304000	2.06E-15
chr8	104300001	104305000	1.60E-19
chr8	104301001	104306000	2.49E-24
chr8	104302001	104307000	1.13E-21
chr8	104311001	104316000	9.03E-50
chr8	104379001	104384000	2.07E-39
chr8	104512001	104517000	3.86E-40
chr8	104526001	104531000	4.87E-05
chr8	105601001	105606000	2.38E-60
chr8	106338001	106343000	0.000415844
chr8	106612001	106617000	5.67E-06
chr8	106613001	106618000	3.26E-06

chr8	107278001	107283000	4.83E-74
chr8	107595001	107600000	8.34E-06
chr8	107665001	107670000	2.08E-36
chr8	107666001	107671000	5.70E-55
chr8	107668001	107673000	8.83E-71
chr8	107669001	107674000	1.76E-90
chr8	107670001	107675000	2.61E-95
chr8	108326001	108331000	0.013049461
chr8	108462001	108467000	0.004909535
chr8	108463001	108468000	0.005406614
chr8	108465001	108470000	7.35E-11
chr8	108466001	108471000	2.85E-10
chr8	108467001	108472000	2.17E-10
chr8	108468001	108473000	1.12E-10
chr8	108469001	108474000	4.28E-06
chr8	108903001	108908000	2.00E-20
chr8	108904001	108909000	5.49E-21
chr8	108905001	108910000	1.25E-17
chr8	108907001	108912000	3.38E-20
chr8	108928001	108933000	0.002875357
chr8	108964001	108969000	0.000202469
chr8	109181001	109186000	4.94E-15
chr8	109182001	109187000	1.06E-08
chr8	109183001	109188000	2.17E-07
chr8	109188001	109193000	0.001704645
chr8	109189001	109194000	0.000128079
chr8	109374001	109379000	0.000288866
chr8	109451001	109456000	1.80E-44
chr8	109456001	109461000	1.22E-45
chr8	109662001	109667000	0.001107834
chr8	109663001	109668000	0.000119186
chr8	109689001	109694000	2.04E-06
chr8	109690001	109695000	6.83E-06
chr8	109692001	109697000	0.001220692
chr8	109699001	109704000	5.10E-09
chr8	109710001	109715000	5.73E-05
chr8	109799001	109804000	1.24E-33
chr8	110007001	110012000	0.013542786
chr8	110274001	110279000	4.85E-09
chr8	110346001	110351000	1.39E-61
chr8	110496001	110501000	0.002132562
chr8	110547001	110552000	1.13E-33
chr8	110988001	110993000	5.56E-49
chr8	111081001	111086000	0.00032101
chr8	111082001	111087000	0.000564105
chr8	111117001	111122000	7.91E-05
chr8	111118001	111123000	7.08E-05
chr8	111119001	111124000	0.000196796

chr8	111230001	111235000	0.002332765
chr8	111739001	111744000	3.80E-05
chr8	111740001	111745000	3.97E-06
chr8	111997001	112002000	0.004656616
chr8	113190001	113195000	0.009182584
chr8	116675001	116680000	1.60E-70
chr8	116676001	116681000	8.07E-72
chr8	118375001	118380000	5.61E-05
chr8	118376001	118381000	7.70E-05
chr8	118533001	118538000	1.20E-59
chr8	119124001	119129000	3.79E-54
chr8	119248001	119253000	3.91E-05
chr8	119249001	119254000	1.19E-05
chr8	119251001	119256000	3.11E-06
chr8	119275001	119280000	0.000140216
chr8	119388001	119393000	4.66E-05
chr8	119555001	119560000	9.59E-13
chr8	119612001	119617000	0.000716253
chr8	119613001	119618000	1.08E-07
chr8	119614001	119619000	2.85E-08
chr8	119615001	119620000	2.58E-14
chr8	119616001	119621000	3.48E-11
chr8	120127001	120132000	6.59E-06
chr8	120150001	120155000	0.005304875
chr8	120315001	120320000	1.39E-08
chr8	120424001	120429000	3.37E-50
chr8	120425001	120430000	4.54E-39
chr8	120426001	120431000	7.40E-43
chr8	120427001	120432000	2.95E-56
chr8	120428001	120433000	1.93E-67
chr8	120429001	120434000	1.97E-33
chr8	120505001	120510000	4.93E-08
chr8	120506001	120511000	7.14E-10
chr8	120507001	120512000	3.01E-07
chr8	120579001	120584000	5.24E-06
chr8	120586001	120591000	1.45E-06
chr8	120842001	120847000	5.07E-52
chr8	120843001	120848000	3.96E-62
chr8	120844001	120849000	1.85E-53
chr8	120845001	120850000	3.03E-26
chr8	120863001	120868000	2.48E-29
chr8	121677001	121682000	1.26E-06
chr8	121866001	121871000	2.24E-06
chr8	122269001	122274000	0.000212171
chr8	122270001	122275000	1.11E-05
chr8	122271001	122276000	4.10E-12
chr8	122272001	122277000	9.23E-11
chr8	122273001	122278000	1.79E-11

chr8	122274001	122279000	1.32E-10
chr8	122275001	122280000	1.83E-09
chr8	122751001	122756000	4.97E-10
chr8	122836001	122841000	0.000116358
chr8	122853001	122858000	4.57E-08
chr8	123034001	123039000	6.11E-28
chr8	123035001	123040000	4.38E-19
chr8	123037001	123042000	2.92E-22
chr8	123038001	123043000	1.16E-21
chr8	124124001	124129000	0.004124245
chr8	124282001	124287000	2.99E-93
chr8	124408001	124413000	9.96E-44
chr8	124553001	124558000	3.58E-34
chr8	124779001	124784000	3.95E-68
chr8	124780001	124785000	3.27E-79
chr8	125380001	125385000	1.30E-89
chr8	125547001	125552000	1.20E-43
chr8	125549001	125554000	2.18E-74
chr8	125551001	125556000	1.72E-53
chr8	126006001	126011000	4.41E-83
chr8	126913001	126918000	0.000205972
chr8	127564001	127569000	2.52E-61
chr8	128089001	128094000	0.001699908
chr8	128139001	128144000	0.005445888
chr8	130253001	130258000	5.05E-07
chr8	131370001	131375000	5.70E-57
chr8	133783001	133788000	1.52E-99
chr8	133784001	133789000	3.66E-84
chr8	133785001	133790000	8.01E-97
chr8	133786001	133791000	3.35E-116
chr8	133787001	133792000	1.69E-109
chr8	134178001	134183000	1.46E-07
chr8	138395001	138400000	1.26E-06
chr8	138815001	138820000	4.56E-08
chr8	141467001	141472000	6.91E-62
chr8	142010001	142015000	2.16E-58
chr8	142011001	142016000	1.47E-44
chr8	143749001	143754000	8.81E-39
chr8	143750001	143755000	3.33E-43
chr8	143808001	143813000	4.13E-83
chr8	144715001	144720000	3.88E-29
chr8	144716001	144721000	1.12E-29
chr8	144717001	144722000	3.56E-42
chr8	144718001	144723000	2.89E-75
chr8	145135001	145140000	2.84E-21
chr8	145136001	145141000	5.74E-13
chr8	145137001	145142000	6.15E-08
chr8	145515001	145520000	9.51E-30

chr8	146023001	146028000	1.87E-38
chr8	146052001	146057000	5.61E-30
chr8	146277001	146282000	5.06E-36
chr9	179001	184000	5.54E-07
chr9	180001	185000	0.009035805
chr9	210001	215000	2.59E-50
chr9	211001	216000	2.18E-59
chr9	212001	217000	9.41E-62
chr9	213001	218000	9.66E-66
chr9	214001	219000	5.85E-59
chr9	215001	220000	1.76E-21
chr9	236001	241000	2.56E-07
chr9	363001	368000	5.57E-07
chr9	440001	445000	1.20E-08
chr9	441001	446000	1.25E-07
chr9	451001	456000	1.19E-09
chr9	452001	457000	7.64E-11
chr9	453001	458000	5.31E-15
chr9	454001	459000	5.39E-09
chr9	455001	460000	4.45E-07
chr9	477001	482000	0.000140649
chr9	500001	505000	1.54E-25
chr9	501001	506000	1.06E-24
chr9	503001	508000	6.87E-14
chr9	504001	509000	7.54E-14
chr9	505001	510000	9.42E-30
chr9	556001	561000	6.60E-10
chr9	557001	562000	6.09E-08
chr9	700001	705000	4.28E-12
chr9	701001	706000	2.88E-13
chr9	705001	710000	2.68E-19
chr9	727001	732000	8.90E-11
chr9	728001	733000	2.82E-11
chr9	853001	858000	4.07E-06
chr9	905001	910000	1.09E-09
chr9	906001	911000	3.94E-11
chr9	958001	963000	2.97E-09
chr9	1014001	1019000	2.62E-11
chr9	1105001	1110000	0.000132683
chr9	1106001	1111000	2.88E-05
chr9	1108001	1113000	0.00216924
chr9	1125001	1130000	7.96E-05
chr9	1126001	1131000	3.82E-05
chr9	1127001	1132000	3.10E-06
chr9	1193001	1198000	6.66E-11
chr9	1195001	1200000	1.28E-12
chr9	1196001	1201000	5.75E-06
chr9	1201001	1206000	7.55E-07

chr9	1223001	1228000	2.96E-06
chr9	1271001	1276000	0.000330675
chr9	1285001	1290000	0.001848107
chr9	1289001	1294000	4.73E-05
chr9	1324001	1329000	0.000118313
chr9	1325001	1330000	0.004183577
chr9	1327001	1332000	0.000588972
chr9	1337001	1342000	0.001493127
chr9	1365001	1370000	2.31E-09
chr9	1366001	1371000	8.31E-08
chr9	1367001	1372000	2.01E-07
chr9	1405001	1410000	1.27E-10
chr9	1409001	1414000	3.93E-07
chr9	1452001	1457000	9.79E-05
chr9	1455001	1460000	0.000320352
chr9	1466001	1471000	0.003301756
chr9	1467001	1472000	0.004337562
chr9	1468001	1473000	0.00174983
chr9	1489001	1494000	9.39E-08
chr9	1490001	1495000	1.54E-07
chr9	1491001	1496000	1.81E-09
chr9	1492001	1497000	5.88E-10
chr9	1493001	1498000	6.67E-10
chr9	1494001	1499000	8.45E-05
chr9	1495001	1500000	0.001148677
chr9	1496001	1501000	0.000653334
chr9	1505001	1510000	1.54E-05
chr9	1515001	1520000	2.75E-06
chr9	1524001	1529000	2.51E-08
chr9	1525001	1530000	8.32E-08
chr9	1528001	1533000	2.33E-05
chr9	1529001	1534000	3.00E-06
chr9	1538001	1543000	3.32E-05
chr9	1546001	1551000	0.000365387
chr9	1554001	1559000	2.28E-05
chr9	1580001	1585000	7.36E-05
chr9	1581001	1586000	1.96E-07
chr9	1582001	1587000	1.47E-12
chr9	1583001	1588000	1.30E-12
chr9	1584001	1589000	5.74E-12
chr9	1585001	1590000	7.39E-15
chr9	1586001	1591000	2.31E-13
chr9	1587001	1592000	6.34E-07
chr9	1588001	1593000	9.08E-07
chr9	1589001	1594000	1.93E-08
chr9	1590001	1595000	9.64E-07
chr9	1591001	1596000	9.91E-05
chr9	1592001	1597000	0.000715681

chr9	1593001	1598000	5.20E-05
chr9	1594001	1599000	0.000250415
chr9	1595001	1600000	7.49E-05
chr9	1596001	1601000	9.08E-09
chr9	1597001	1602000	7.53E-08
chr9	1598001	1603000	5.30E-06
chr9	1634001	1639000	1.43E-06
chr9	1635001	1640000	4.31E-08
chr9	1655001	1660000	4.03E-09
chr9	1657001	1662000	1.68E-09
chr9	1689001	1694000	6.98E-14
chr9	1690001	1695000	1.86E-09
chr9	1691001	1696000	5.50E-07
chr9	1692001	1697000	1.59E-06
chr9	1709001	1714000	1.71E-05
chr9	1744001	1749000	9.41E-05
chr9	1766001	1771000	4.32E-08
chr9	1778001	1783000	1.14E-08
chr9	1780001	1785000	8.18E-10
chr9	1784001	1789000	6.01E-06
chr9	1785001	1790000	1.34E-08
chr9	1786001	1791000	1.01E-07
chr9	1800001	1805000	0.000333168
chr9	1813001	1818000	0.00254932
chr9	1814001	1819000	0.001429525
chr9	1818001	1823000	6.12E-09
chr9	1822001	1827000	1.11E-09
chr9	1824001	1829000	2.76E-06
chr9	1825001	1830000	2.45E-06
chr9	1829001	1834000	2.50E-05
chr9	1859001	1864000	2.31E-13
chr9	1881001	1886000	0.000967411
chr9	1888001	1893000	9.22E-07
chr9	1890001	1895000	7.68E-06
chr9	1892001	1897000	1.40E-06
chr9	1901001	1906000	7.62E-05
chr9	1954001	1959000	4.87E-06
chr9	1955001	1960000	3.09E-09
chr9	1974001	1979000	1.32E-05
chr9	2010001	2015000	1.85E-16
chr9	2011001	2016000	3.24E-49
chr9	2012001	2017000	1.11E-53
chr9	2017001	2022000	7.07E-36
chr9	2158001	2163000	3.77E-12
chr9	2376001	2381000	9.96E-05
chr9	2390001	2395000	0.000814808
chr9	2392001	2397000	0.009816498
chr9	2393001	2398000	0.02583298

chr9	2617001	2622000	1.21E-17
chr9	2839001	2844000	9.42E-23
chr9	2840001	2845000	2.83E-23
chr9	3504001	3509000	0.015565007
chr9	3505001	3510000	0.017549712
chr9	3520001	3525000	0.000415439
chr9	3526001	3531000	2.46E-38
chr9	3915001	3920000	0.00031249
chr9	4293001	4298000	5.23E-22
chr9	4294001	4299000	3.87E-21
chr9	4412001	4417000	0.000589275
chr9	4413001	4418000	2.02E-10
chr9	4414001	4419000	5.09E-07
chr9	4452001	4457000	5.78E-05
chr9	4490001	4495000	2.45E-17
chr9	4491001	4496000	1.27E-06
chr9	4658001	4663000	6.48E-13
chr9	4659001	4664000	5.21E-11
chr9	4660001	4665000	3.46E-18
chr9	4664001	4669000	1.83E-15
chr9	4665001	4670000	3.02E-07
chr9	4666001	4671000	4.63E-06
chr9	4736001	4741000	1.23E-15
chr9	4737001	4742000	1.61E-31
chr9	4738001	4743000	3.66E-28
chr9	4739001	4744000	5.94E-15
chr9	4793001	4798000	1.13E-24
chr9	5139001	5144000	0.000252841
chr9	5141001	5146000	4.60E-07
chr9	5156001	5161000	1.66E-05
chr9	5157001	5162000	8.47E-06
chr9	5158001	5163000	5.02E-06
chr9	5159001	5164000	3.58E-06
chr9	5227001	5232000	2.83E-09
chr9	5230001	5235000	1.36E-09
chr9	5264001	5269000	0.003147108
chr9	5297001	5302000	2.18E-05
chr9	5298001	5303000	0.000366364
chr9	5300001	5305000	6.98E-14
chr9	5301001	5306000	5.35E-13
chr9	5302001	5307000	2.61E-07
chr9	5303001	5308000	7.39E-09
chr9	5304001	5309000	2.57E-23
chr9	5305001	5310000	9.76E-08
chr9	5307001	5312000	6.48E-08
chr9	5336001	5341000	4.34E-11
chr9	5337001	5342000	2.23E-11
chr9	5375001	5380000	0.001366963

chr9	5435001	5440000	7.75E-35
chr9	5436001	5441000	4.11E-36
chr9	5437001	5442000	4.86E-37
chr9	5445001	5450000	1.16E-09
chr9	5446001	5451000	2.47E-10
chr9	5447001	5452000	1.10E-11
chr9	5493001	5498000	3.14E-06
chr9	5575001	5580000	0.000201855
chr9	5624001	5629000	1.31E-09
chr9	5833001	5838000	2.39E-09
chr9	6003001	6008000	9.11E-52
chr9	6004001	6009000	4.93E-46
chr9	6005001	6010000	1.53E-54
chr9	6006001	6011000	9.47E-70
chr9	6007001	6012000	8.16E-59
chr9	6008001	6013000	1.15E-35
chr9	6158001	6163000	0.00112409
chr9	6159001	6164000	0.000795682
chr9	6160001	6165000	0.001004844
chr9	6161001	6166000	0.00105889
chr9	6168001	6173000	0.000201824
chr9	6176001	6181000	6.75E-10
chr9	6179001	6184000	1.82E-11
chr9	6409001	6414000	4.11E-33
chr9	6676001	6681000	6.51E-11
chr9	6677001	6682000	2.28E-31
chr9	6678001	6683000	3.24E-22
chr9	6679001	6684000	4.80E-25
chr9	6680001	6685000	2.13E-40
chr9	6681001	6686000	4.86E-28
chr9	6699001	6704000	3.80E-07
chr9	6700001	6705000	6.99E-16
chr9	6701001	6706000	7.30E-16
chr9	6702001	6707000	1.65E-11
chr9	6703001	6708000	1.17E-09
chr9	7164001	7169000	6.16E-06
chr9	7534001	7539000	1.85E-05
chr9	7649001	7654000	2.28E-05
chr9	7795001	7800000	3.22E-58
chr9	7796001	7801000	9.64E-51
chr9	7797001	7802000	3.67E-47
chr9	7798001	7803000	1.10E-34
chr9	7799001	7804000	4.55E-26
chr9	7848001	7853000	0.001641329
chr9	8239001	8244000	6.24E-05
chr9	8248001	8253000	1.83E-05
chr9	8286001	8291000	4.85E-06
chr9	8287001	8292000	4.63E-05

chr9	8430001	8435000	0.0003508
chr9	8486001	8491000	0.000120018
chr9	8826001	8831000	0.021379307
chr9	8997001	9002000	0.000802807
chr9	9059001	9064000	0.012967221
chr9	9060001	9065000	0.000615624
chr9	9097001	9102000	3.18E-05
chr9	9124001	9129000	0.002606396
chr9	9138001	9143000	0.000857263
chr9	9139001	9144000	8.32E-07
chr9	9140001	9145000	0.000658526
chr9	9169001	9174000	0.00134806
chr9	9170001	9175000	0.002762193
chr9	9172001	9177000	0.000186169
chr9	9188001	9193000	0.002087151
chr9	9189001	9194000	0.004180142
chr9	9190001	9195000	0.001355539
chr9	9191001	9196000	0.001398124
chr9	9192001	9197000	0.031317311
chr9	9204001	9209000	1.18E-05
chr9	9211001	9216000	0.013608215
chr9	9347001	9352000	0.002840691
chr9	9348001	9353000	0.006257778
chr9	9349001	9354000	0.003063289
chr9	9618001	9623000	1.35E-06
chr9	9822001	9827000	0.001488051
chr9	9823001	9828000	5.34E-07
chr9	9929001	9934000	1.86E-05
chr9	10268001	10273000	4.34E-05
chr9	10572001	10577000	0.001178453
chr9	10608001	10613000	8.79E-16
chr9	10613001	10618000	6.14E-22
chr9	10627001	10632000	0.000257922
chr9	10668001	10673000	0.000646926
chr9	10716001	10721000	0.001196123
chr9	10717001	10722000	0.002128151
chr9	10718001	10723000	1.27E-05
chr9	10719001	10724000	0.000100757
chr9	10720001	10725000	0.000512186
chr9	10816001	10821000	0.00027742
chr9	10817001	10822000	0.000237428
chr9	10950001	10955000	2.54E-07
chr9	10951001	10956000	4.71E-10
chr9	10975001	10980000	3.24E-05
chr9	11189001	11194000	0.001259697
chr9	11282001	11287000	2.66E-05
chr9	11419001	11424000	0.001429328
chr9	11608001	11613000	0.010116761

chr9	11679001	11684000	0.001152875
chr9	11691001	11696000	0.006809074
chr9	11769001	11774000	1.96E-05
chr9	11770001	11775000	6.25E-05
chr9	11771001	11776000	2.60E-05
chr9	11796001	11801000	0.000702841
chr9	11807001	11812000	0.0002867
chr9	12163001	12168000	8.93E-08
chr9	12213001	12218000	3.50E-06
chr9	12214001	12219000	3.92E-05
chr9	12318001	12323000	0.000375636
chr9	12423001	12428000	0.003663292
chr9	12488001	12493000	0.000324923
chr9	12530001	12535000	8.86E-05
chr9	12531001	12536000	0.001376761
chr9	12532001	12537000	0.000171635
chr9	12771001	12776000	2.08E-29
chr9	12772001	12777000	2.54E-28
chr9	12812001	12817000	0.011958965
chr9	12813001	12818000	0.00176168
chr9	13181001	13186000	0.011403062
chr9	13274001	13279000	2.43E-17
chr9	13275001	13280000	1.08E-23
chr9	13276001	13281000	1.18E-26
chr9	13277001	13282000	9.19E-16
chr9	13278001	13283000	6.38E-19
chr9	13279001	13284000	2.16E-22
chr9	13781001	13786000	0.013937538
chr9	14270001	14275000	2.66E-08
chr9	14315001	14320000	2.45E-19
chr9	14689001	14694000	3.10E-44
chr9	14690001	14695000	1.92E-35
chr9	14691001	14696000	2.07E-42
chr9	14693001	14698000	3.94E-23
chr9	14816001	14821000	5.72E-05
chr9	14818001	14823000	1.86E-06
chr9	14843001	14848000	1.22E-12
chr9	14844001	14849000	3.65E-09
chr9	14893001	14898000	3.79E-05
chr9	14904001	14909000	1.55E-05
chr9	14905001	14910000	1.71E-05
chr9	14906001	14911000	4.32E-07
chr9	14962001	14967000	8.80E-09
chr9	15000001	15005000	5.92E-05
chr9	15001001	15006000	3.87E-06
chr9	15002001	15007000	7.18E-05
chr9	15008001	15013000	0.001744862
chr9	15009001	15014000	3.68E-06

chr9	15012001	15017000	6.59E-06
chr9	15015001	15020000	2.86E-05
chr9	15028001	15033000	9.77E-09
chr9	15029001	15034000	7.50E-06
chr9	15030001	15035000	3.78E-06
chr9	15031001	15036000	2.89E-08
chr9	15032001	15037000	2.07E-13
chr9	15033001	15038000	5.39E-09
chr9	15034001	15039000	6.81E-10
chr9	15069001	15074000	3.90E-05
chr9	15070001	15075000	0.001981666
chr9	15302001	15307000	3.19E-23
chr9	15303001	15308000	6.21E-47
chr9	15304001	15309000	1.26E-39
chr9	15305001	15310000	1.75E-41
chr9	15306001	15311000	2.53E-42
chr9	15307001	15312000	1.65E-34
chr9	15506001	15511000	8.16E-105
chr9	15507001	15512000	6.97E-76
chr9	15508001	15513000	8.73E-111
chr9	15509001	15514000	1.95E-75
chr9	15510001	15515000	3.16E-61
chr9	15511001	15516000	6.97E-26
chr9	15765001	15770000	2.55E-05
chr9	16226001	16231000	1.20E-09
chr9	16535001	16540000	1.50E-10
chr9	16536001	16541000	9.20E-15
chr9	16537001	16542000	1.81E-12
chr9	16538001	16543000	5.10E-09
chr9	16539001	16544000	6.21E-07
chr9	16722001	16727000	9.33E-20
chr9	16866001	16871000	1.80E-19
chr9	16867001	16872000	2.45E-16
chr9	16869001	16874000	4.28E-15
chr9	16870001	16875000	5.80E-09
chr9	16946001	16951000	0.000222505
chr9	17133001	17138000	1.90E-24
chr9	17134001	17139000	1.50E-26
chr9	17135001	17140000	6.54E-46
chr9	17267001	17272000	2.91E-07
chr9	17268001	17273000	3.69E-06
chr9	17357001	17362000	0.000115342
chr9	17358001	17363000	0.001178575
chr9	17359001	17364000	0.000565502
chr9	17360001	17365000	0.001355076
chr9	17574001	17579000	3.23E-17
chr9	17575001	17580000	1.05E-27
chr9	17576001	17581000	2.38E-49

chr9	17577001	17582000	5.91E-70
chr9	17578001	17583000	1.28E-69
chr9	17579001	17584000	8.50E-54
chr9	17940001	17945000	0.000352227
chr9	18087001	18092000	5.43E-05
chr9	18088001	18093000	3.51E-05
chr9	18089001	18094000	0.000336582
chr9	18223001	18228000	0.000731627
chr9	18471001	18476000	3.51E-11
chr9	18472001	18477000	1.99E-11
chr9	18473001	18478000	6.84E-15
chr9	18474001	18479000	3.61E-08
chr9	19125001	19130000	2.87E-09
chr9	19126001	19131000	2.29E-08
chr9	19127001	19132000	9.24E-07
chr9	19380001	19385000	3.49E-16
chr9	19870001	19875000	6.27E-07
chr9	19871001	19876000	3.12E-07
chr9	19912001	19917000	0.000231283
chr9	19930001	19935000	1.12E-18
chr9	19931001	19936000	7.34E-16
chr9	19932001	19937000	1.68E-18
chr9	19933001	19938000	8.26E-16
chr9	19954001	19959000	5.19E-08
chr9	19955001	19960000	1.15E-11
chr9	19956001	19961000	1.35E-11
chr9	19957001	19962000	6.86E-20
chr9	19958001	19963000	1.06E-18
chr9	19959001	19964000	7.61E-10
chr9	19989001	19994000	0.000380849
chr9	20020001	20025000	8.88E-07
chr9	20022001	20027000	1.20E-05
chr9	20026001	20031000	2.08E-06
chr9	20033001	20038000	1.89E-05
chr9	20034001	20039000	5.94E-07
chr9	20035001	20040000	1.63E-10
chr9	20036001	20041000	9.33E-09
chr9	20037001	20042000	4.75E-10
chr9	20038001	20043000	5.12E-05
chr9	20079001	20084000	9.34E-05
chr9	20080001	20085000	3.84E-08
chr9	20081001	20086000	1.78E-08
chr9	20082001	20087000	3.67E-07
chr9	20151001	20156000	0.000320008
chr9	20152001	20157000	4.74E-05
chr9	20159001	20164000	1.25E-08
chr9	20160001	20165000	3.78E-11
chr9	20161001	20166000	6.53E-08

chr9	20162001	20167000	2.43E-06
chr9	20163001	20168000	0.00021717
chr9	20164001	20169000	0.007983487
chr9	20174001	20179000	0.000560693
chr9	20223001	20228000	0.004039448
chr9	20226001	20231000	3.75E-05
chr9	20253001	20258000	0.00027717
chr9	20293001	20298000	1.49E-08
chr9	20300001	20305000	0.001781596
chr9	20378001	20383000	0.000152936
chr9	20407001	20412000	0.002692549
chr9	20616001	20621000	1.37E-26
chr9	20617001	20622000	1.17E-39
chr9	20618001	20623000	1.69E-42
chr9	20647001	20652000	0.004540832
chr9	20654001	20659000	2.07E-29
chr9	20655001	20660000	2.01E-12
chr9	20656001	20661000	3.95E-12
chr9	20657001	20662000	1.12E-09
chr9	20658001	20663000	1.33E-10
chr9	20684001	20689000	9.38E-20
chr9	21134001	21139000	0.000553588
chr9	21146001	21151000	5.52E-06
chr9	21181001	21186000	1.20E-07
chr9	21182001	21187000	1.22E-06
chr9	21183001	21188000	1.19E-05
chr9	21184001	21189000	0.001488427
chr9	21185001	21190000	0.001547823
chr9	21186001	21191000	0.000391146
chr9	21187001	21192000	3.16E-10
chr9	21204001	21209000	0.000103886
chr9	21238001	21243000	6.23E-05
chr9	21239001	21244000	0.000894206
chr9	21240001	21245000	0.000894206
chr9	21282001	21287000	0.000998673
chr9	21359001	21364000	2.87E-06
chr9	21396001	21401000	5.06E-18
chr9	21441001	21446000	2.63E-08
chr9	21459001	21464000	0.000242055
chr9	21500001	21505000	0.000701771
chr9	21501001	21506000	0.000268964
chr9	21529001	21534000	0.000217524
chr9	21531001	21536000	0.000934416
chr9	21548001	21553000	7.85E-06
chr9	21549001	21554000	3.78E-07
chr9	21555001	21560000	7.01E-73
chr9	21556001	21561000	1.49E-67
chr9	21557001	21562000	2.62E-73

chr9	21558001	21563000	4.80E-66
chr9	21559001	21564000	7.94E-97
chr9	21560001	21565000	1.70E-34
chr9	21574001	21579000	0.011088955
chr9	21581001	21586000	2.37E-07
chr9	21597001	21602000	1.11E-06
chr9	21618001	21623000	7.60E-05
chr9	21704001	21709000	3.77E-06
chr9	21706001	21711000	8.28E-06
chr9	21707001	21712000	6.96E-07
chr9	21710001	21715000	1.18E-05
chr9	21757001	21762000	6.34E-05
chr9	21765001	21770000	0.000397436
chr9	21766001	21771000	2.90E-05
chr9	21767001	21772000	0.000911977
chr9	21768001	21773000	1.07E-05
chr9	21769001	21774000	0.000188706
chr9	21770001	21775000	5.94E-06
chr9	21781001	21786000	0.001031363
chr9	21783001	21788000	0.000938059
chr9	21801001	21806000	1.90E-29
chr9	21990001	21995000	6.42E-68
chr9	21991001	21996000	4.13E-71
chr9	21992001	21997000	1.13E-86
chr9	21993001	21998000	2.80E-56
chr9	21994001	21999000	6.12E-28
chr9	21995001	22000000	5.12E-26
chr9	21996001	22001000	2.08E-16
chr9	22001001	22006000	0.000131992
chr9	22007001	22012000	3.54E-41
chr9	22008001	22013000	8.26E-37
chr9	22009001	22014000	5.89E-33
chr9	22442001	22447000	8.98E-18
chr9	22443001	22448000	3.79E-56
chr9	22444001	22449000	5.62E-62
chr9	22445001	22450000	3.23E-62
chr9	22447001	22452000	1.33E-55
chr9	22755001	22760000	1.57E-23
chr9	22756001	22761000	1.39E-16
chr9	22757001	22762000	6.75E-16
chr9	22758001	22763000	5.73E-21
chr9	22759001	22764000	1.19E-15
chr9	23455001	23460000	0.000990958
chr9	23457001	23462000	0.00116928
chr9	23483001	23488000	0.012967221
chr9	23850001	23855000	5.69E-19
chr9	23942001	23947000	0.015017214
chr9	24557001	24562000	0.00927776

chr9	25118001	25123000	0.001000297
chr9	25121001	25126000	4.84E-05
chr9	25327001	25332000	0.001460702
chr9	25433001	25438000	0.000180787
chr9	25673001	25678000	4.42E-30
chr9	25674001	25679000	6.09E-38
chr9	25675001	25680000	2.87E-55
chr9	25676001	25681000	1.75E-55
chr9	25677001	25682000	8.85E-37
chr9	25678001	25683000	5.95E-55
chr9	25832001	25837000	0.002098515
chr9	25844001	25849000	2.83E-05
chr9	25845001	25850000	5.17E-05
chr9	25846001	25851000	4.03E-05
chr9	26235001	26240000	0.001367263
chr9	26367001	26372000	0.001089432
chr9	26369001	26374000	0.000160005
chr9	26430001	26435000	0.003361644
chr9	26434001	26439000	0.00041338
chr9	26707001	26712000	5.91E-05
chr9	26708001	26713000	0.001238246
chr9	26888001	26893000	9.28E-33
chr9	26889001	26894000	3.52E-26
chr9	26892001	26897000	3.60E-08
chr9	27214001	27219000	0.000115026
chr9	27215001	27220000	5.59E-05
chr9	27216001	27221000	0.000104167
chr9	27217001	27222000	4.93E-07
chr9	27218001	27223000	4.66E-05
chr9	27510001	27515000	0.004935787
chr9	27529001	27534000	1.70E-44
chr9	27825001	27830000	0.000242014
chr9	28124001	28129000	0.004227346
chr9	28172001	28177000	6.59E-05
chr9	28173001	28178000	3.56E-05
chr9	29208001	29213000	9.30E-33
chr9	29288001	29293000	0.015316858
chr9	29399001	29404000	0.00660531
chr9	29400001	29405000	0.00718887
chr9	29761001	29766000	0.006545248
chr9	29795001	29800000	3.48E-06
chr9	30182001	30187000	5.38E-05
chr9	30471001	30476000	0.016599808
chr9	31020001	31025000	0.007433209
chr9	31950001	31955000	0.003609373
chr9	32384001	32389000	1.29E-20
chr9	32526001	32531000	2.92E-11
chr9	32546001	32551000	9.32E-29

chr9	32596001	32601000	1.87E-05
chr9	32597001	32602000	0.000270893
chr9	32648001	32653000	6.54E-06
chr9	32649001	32654000	1.45E-05
chr9	32678001	32683000	3.41E-05
chr9	32679001	32684000	3.09E-05
chr9	32776001	32781000	1.93E-07
chr9	32841001	32846000	8.00E-07
chr9	32932001	32937000	4.99E-06
chr9	32997001	33002000	3.32E-23
chr9	32998001	33003000	6.21E-23
chr9	32999001	33004000	1.21E-16
chr9	33000001	33005000	8.02E-23
chr9	33001001	33006000	1.52E-14
chr9	33021001	33026000	3.13E-20
chr9	33040001	33045000	1.19E-20
chr9	33044001	33049000	6.16E-19
chr9	33286001	33291000	2.17E-22
chr9	33290001	33295000	6.03E-25
chr9	33510001	33515000	1.36E-22
chr9	33511001	33516000	2.90E-15
chr9	33672001	33677000	5.24E-15
chr9	33673001	33678000	5.21E-39
chr9	33674001	33679000	3.98E-44
chr9	33675001	33680000	2.93E-36
chr9	33676001	33681000	4.98E-39
chr9	33812001	33817000	4.41E-17
chr9	33813001	33818000	4.66E-21
chr9	33815001	33820000	1.38E-11
chr9	33816001	33821000	1.39E-32
chr9	33817001	33822000	5.35E-18
chr9	34126001	34131000	1.27E-07
chr9	34174001	34179000	2.78E-08
chr9	34175001	34180000	9.41E-12
chr9	34176001	34181000	3.30E-07
chr9	34178001	34183000	1.20E-20
chr9	34179001	34184000	2.10E-13
chr9	34374001	34379000	1.85E-13
chr9	34521001	34526000	0.000121994
chr9	34522001	34527000	6.11E-09
chr9	34523001	34528000	6.23E-14
chr9	34578001	34583000	1.95E-06
chr9	34637001	34642000	6.23E-12
chr9	34697001	34702000	6.13E-19
chr9	34701001	34706000	1.17E-20
chr9	34780001	34785000	1.61E-08
chr9	34861001	34866000	0.003964341
chr9	34923001	34928000	4.92E-06

chr9	34954001	34959000	1.58E-12
chr9	34956001	34961000	1.58E-13
chr9	34957001	34962000	5.29E-11
chr9	34958001	34963000	5.39E-10
chr9	35070001	35075000	1.79E-23
chr9	35071001	35076000	1.56E-33
chr9	35072001	35077000	3.16E-35
chr9	35075001	35080000	3.64E-15
chr9	35094001	35099000	9.98E-06
chr9	35095001	35100000	3.07E-07
chr9	35096001	35101000	3.07E-08
chr9	35098001	35103000	2.38E-05
chr9	35109001	35114000	1.49E-07
chr9	35116001	35121000	7.70E-12
chr9	35643001	35648000	1.76E-07
chr9	35654001	35659000	7.24E-10
chr9	35661001	35666000	1.57E-07
chr9	35662001	35667000	3.49E-13
chr9	35663001	35668000	1.00E-10
chr9	35664001	35669000	3.45E-12
chr9	35665001	35670000	4.50E-08
chr9	35744001	35749000	1.90E-10
chr9	35745001	35750000	5.58E-21
chr9	35746001	35751000	8.63E-16
chr9	35748001	35753000	3.27E-12
chr9	35749001	35754000	2.17E-22
chr9	35753001	35758000	2.16E-13
chr9	35755001	35760000	1.49E-08
chr9	35756001	35761000	2.02E-15
chr9	35757001	35762000	8.04E-19
chr9	35825001	35830000	9.27E-07
chr9	35826001	35831000	4.89E-08
chr9	35827001	35832000	5.37E-08
chr9	35879001	35884000	5.99E-05
chr9	35880001	35885000	1.08E-07
chr9	35881001	35886000	4.32E-05
chr9	36032001	36037000	9.45E-21
chr9	36033001	36038000	6.98E-36
chr9	36034001	36039000	2.49E-25
chr9	36035001	36040000	4.24E-26
chr9	36036001	36041000	2.33E-17
chr9	36037001	36042000	4.91E-18
chr9	36073001	36078000	0.00949066
chr9	36132001	36137000	2.95E-24
chr9	36133001	36138000	2.96E-15
chr9	36134001	36139000	2.75E-14
chr9	36135001	36140000	3.76E-19
chr9	36136001	36141000	2.31E-20

chr9	36254001	36259000	1.04E-17
chr9	36255001	36260000	1.19E-17
chr9	36396001	36401000	2.02E-33
chr9	36484001	36489000	6.36E-26
chr9	36487001	36492000	1.28E-29
chr9	36568001	36573000	1.02E-13
chr9	36982001	36987000	9.24E-07
chr9	37080001	37085000	1.46E-37
chr9	37418001	37423000	3.00E-07
chr9	37419001	37424000	7.32E-05
chr9	37420001	37425000	2.02E-06
chr9	37572001	37577000	1.36E-14
chr9	37573001	37578000	2.07E-30
chr9	37574001	37579000	1.42E-37
chr9	37575001	37580000	1.00E-41
chr9	37576001	37581000	4.88E-42
chr9	37588001	37593000	1.78E-41
chr9	37589001	37594000	3.04E-34
chr9	37648001	37653000	2.68E-14
chr9	37649001	37654000	3.21E-14
chr9	37650001	37655000	1.52E-16
chr9	37780001	37785000	5.56E-12
chr9	37801001	37806000	1.28E-17
chr9	38421001	38426000	5.46E-23
chr9	38422001	38427000	6.33E-27
chr9	38423001	38428000	9.56E-33
chr9	38424001	38429000	1.19E-33
chr9	38615001	38620000	7.77E-15
chr9	38616001	38621000	9.76E-84
chr9	38617001	38622000	1.06E-82
chr9	38618001	38623000	8.65E-63
chr9	38619001	38624000	1.00E-80
chr9	38621001	38626000	1.78E-41
chr9	38622001	38627000	1.03E-21
chr9	38775001	38780000	0.008799835
chr9	38792001	38797000	1.86E-07
chr9	38793001	38798000	8.82E-11
chr9	38794001	38799000	8.46E-10
chr9	38795001	38800000	7.04E-05
chr9	38948001	38953000	0.000106944
chr9	38949001	38954000	0.003108785
chr9	39024001	39029000	1.30E-05
chr9	39025001	39030000	4.25E-05
chr9	39741001	39746000	0.000543342
chr9	40626001	40631000	0.000311193
chr9	40787001	40792000	7.97E-39
chr9	40788001	40793000	3.26E-32
chr9	40789001	40794000	2.71E-32

chr9	41952001	41957000	1.90E-47
chr9	41953001	41958000	5.10E-60
chr9	41954001	41959000	2.73E-66
chr9	42341001	42346000	1.39E-07
chr9	42367001	42372000	2.79E-12
chr9	43129001	43134000	1.07E-36
chr9	43130001	43135000	5.77E-16
chr9	43131001	43136000	1.55E-11
chr9	43132001	43137000	3.16E-13
chr9	43438001	43443000	0.00363266
chr9	43473001	43478000	2.92E-06
chr9	43474001	43479000	4.34E-07
chr9	43475001	43480000	3.30E-07
chr9	43476001	43481000	0.001218349
chr9	43560001	43565000	0.000997988
chr9	43561001	43566000	0.003249626
chr9	43562001	43567000	0.00647671
chr9	43577001	43582000	0.000699723
chr9	43578001	43583000	4.02E-09
chr9	43579001	43584000	7.27E-09
chr9	43580001	43585000	9.23E-09
chr9	43581001	43586000	4.59E-08
chr9	43582001	43587000	7.36E-08
chr9	43635001	43640000	3.32E-05
chr9	43636001	43641000	4.33E-06
chr9	43637001	43642000	5.96E-05
chr9	43638001	43643000	0.000108569
chr9	43762001	43767000	0.000169284
chr9	43765001	43770000	1.29E-05
chr9	43766001	43771000	1.29E-05
chr9	43768001	43773000	0.003231199
chr9	43769001	43774000	0.00048327
chr9	43884001	43889000	0.00088381
chr9	44071001	44076000	4.63E-06
chr9	44072001	44077000	5.66E-08
chr9	44084001	44089000	0.003430727
chr9	44168001	44173000	2.93E-06
chr9	44169001	44174000	1.00E-05
chr9	44203001	44208000	3.91E-06
chr9	44204001	44209000	5.07E-07
chr9	44205001	44210000	3.96E-06
chr9	44207001	44212000	2.82E-06
chr9	44226001	44231000	7.13E-10
chr9	44234001	44239000	0.000136032
chr9	44399001	44404000	2.07E-10
chr9	44400001	44405000	1.74E-12
chr9	44401001	44406000	1.74E-12
chr9	44415001	44420000	6.99E-16

chr9	44420001	44425000	3.42E-12
chr9	44888001	44893000	2.19E-07
chr9	44889001	44894000	4.31E-10
chr9	44890001	44895000	1.94E-14
chr9	44891001	44896000	2.14E-10
chr9	44893001	44898000	0.000498722
chr9	44894001	44899000	0.009099385
chr9	44902001	44907000	1.56E-05
chr9	45645001	45650000	0.001123219
chr9	45646001	45651000	0.00484883
chr9	45722001	45727000	3.78E-12
chr9	46019001	46024000	0.003545841
chr9	46101001	46106000	0.002587504
chr9	46102001	46107000	0.004271112
chr9	46122001	46127000	0.000619984
chr9	46301001	46306000	0.03560161
chr9	65558001	65563000	0.003805916
chr9	66670001	66675000	0.007888633
chr9	66677001	66682000	1.53E-06
chr9	66678001	66683000	2.56E-05
chr9	66679001	66684000	2.54E-05
chr9	66702001	66707000	0.003581041
chr9	66703001	66708000	0.002920641
chr9	66979001	66984000	0.00062622
chr9	66980001	66985000	0.00062622
chr9	66981001	66986000	0.000951925
chr9	67923001	67928000	4.09E-05
chr9	68225001	68230000	2.23E-08
chr9	68226001	68231000	5.60E-10
chr9	68306001	68311000	0.000182483
chr9	68308001	68313000	4.17E-06
chr9	68324001	68329000	3.36E-07
chr9	68661001	68666000	0.008220025
chr9	68790001	68795000	6.51E-06
chr9	68796001	68801000	0.000380288
chr9	68805001	68810000	4.02E-09
chr9	68806001	68811000	5.33E-10
chr9	68807001	68812000	8.83E-13
chr9	69122001	69127000	0.009159645
chr9	69148001	69153000	1.42E-07
chr9	69377001	69382000	6.34E-15
chr9	69378001	69383000	6.13E-17
chr9	69379001	69384000	2.23E-19
chr9	69380001	69385000	8.12E-14
chr9	69381001	69386000	0.000126681
chr9	69505001	69510000	0.000463777
chr9	69506001	69511000	9.20E-05
chr9	69648001	69653000	6.02E-05

chr9	69652001	69657000	3.52E-05
chr9	70685001	70690000	3.73E-05
chr9	70686001	70691000	2.14E-05
chr9	70687001	70692000	2.14E-05
chr9	70967001	70972000	2.06E-23
chr9	70968001	70973000	5.99E-12
chr9	70969001	70974000	8.47E-11
chr9	70970001	70975000	2.10E-07
chr9	70972001	70977000	0.000136547
chr9	71071001	71076000	0.003030779
chr9	71072001	71077000	0.008136225
chr9	71120001	71125000	6.25E-05
chr9	71316001	71321000	2.55E-22
chr9	71320001	71325000	1.86E-29
chr9	71321001	71326000	1.42E-18
chr9	71322001	71327000	3.22E-06
chr9	71390001	71395000	6.21E-42
chr9	71391001	71396000	1.11E-48
chr9	71392001	71397000	7.77E-40
chr9	71393001	71398000	4.05E-39
chr9	71394001	71399000	1.92E-44
chr9	71395001	71400000	6.59E-44
chr9	71461001	71466000	0.000174135
chr9	71522001	71527000	0.000288715
chr9	71646001	71651000	4.65E-12
chr9	71648001	71653000	1.30E-13
chr9	71649001	71654000	5.31E-12
chr9	71650001	71655000	1.11E-18
chr9	71785001	71790000	2.77E-27
chr9	71786001	71791000	5.43E-23
chr9	71787001	71792000	3.44E-14
chr9	71788001	71793000	3.56E-13
chr9	71935001	71940000	4.89E-25
chr9	72239001	72244000	0.000996903
chr9	72283001	72288000	8.71E-45
chr9	72284001	72289000	1.55E-42
chr9	72287001	72292000	2.29E-32
chr9	72445001	72450000	5.50E-05
chr9	72499001	72504000	5.25E-05
chr9	72518001	72523000	0.002083944
chr9	73030001	73035000	1.17E-30
chr9	73156001	73161000	8.64E-08
chr9	73159001	73164000	6.63E-05
chr9	73258001	73263000	4.64E-07
chr9	73259001	73264000	8.64E-06
chr9	73277001	73282000	6.65E-07
chr9	73285001	73290000	2.50E-05
chr9	73287001	73292000	5.18E-05

chr9	73288001	73293000	4.55E-07
chr9	73290001	73295000	8.31E-07
chr9	73457001	73462000	0.000572403
chr9	73458001	73463000	0.000120187
chr9	73467001	73472000	2.18E-05
chr9	73468001	73473000	3.52E-07
chr9	73469001	73474000	7.41E-08
chr9	73470001	73475000	8.92E-07
chr9	73471001	73476000	7.02E-06
chr9	73514001	73519000	1.76E-05
chr9	73515001	73520000	1.65E-06
chr9	73516001	73521000	3.62E-06
chr9	73517001	73522000	8.63E-09
chr9	73537001	73542000	2.05E-05
chr9	73538001	73543000	2.87E-05
chr9	73541001	73546000	0.000252748
chr9	73557001	73562000	0.021269933
chr9	73562001	73567000	0.003238382
chr9	73642001	73647000	0.000895287
chr9	73653001	73658000	0.000389946
chr9	73654001	73659000	0.000235649
chr9	73671001	73676000	1.65E-05
chr9	73672001	73677000	5.84E-07
chr9	73764001	73769000	1.68E-06
chr9	73789001	73794000	1.38E-05
chr9	73800001	73805000	0.006333031
chr9	73838001	73843000	2.48E-06
chr9	73839001	73844000	6.05E-05
chr9	73937001	73942000	1.55E-09
chr9	73938001	73943000	2.12E-08
chr9	73949001	73954000	4.21E-06
chr9	73950001	73955000	1.42E-05
chr9	73951001	73956000	2.05E-06
chr9	73990001	73995000	4.02E-05
chr9	74008001	74013000	4.09E-05
chr9	74009001	74014000	7.54E-05
chr9	74010001	74015000	2.89E-06
chr9	74013001	74018000	1.29E-07
chr9	74068001	74073000	0.001091306
chr9	74193001	74198000	6.23E-05
chr9	74250001	74255000	0.001927594
chr9	74379001	74384000	9.46E-09
chr9	74381001	74386000	1.78E-13
chr9	74382001	74387000	5.46E-14
chr9	74383001	74388000	4.65E-15
chr9	74384001	74389000	1.25E-12
chr9	74760001	74765000	5.63E-08
chr9	74762001	74767000	1.20E-13

chr9	74763001	74768000	1.43E-15
chr9	74764001	74769000	2.49E-16
chr9	74926001	74931000	1.50E-09
chr9	74927001	74932000	1.97E-06
chr9	74928001	74933000	0.000192782
chr9	74979001	74984000	1.75E-06
chr9	74980001	74985000	5.75E-12
chr9	75263001	75268000	0.000296666
chr9	75541001	75546000	0.000155521
chr9	75543001	75548000	0.000503106
chr9	75611001	75616000	2.21E-06
chr9	75612001	75617000	1.79E-07
chr9	75852001	75857000	0.000680935
chr9	75992001	75997000	1.67E-06
chr9	75993001	75998000	2.23E-06
chr9	76002001	76007000	2.04E-08
chr9	76003001	76008000	8.78E-06
chr9	76072001	76077000	7.34E-09
chr9	76101001	76106000	0.00028169
chr9	76102001	76107000	0.000248694
chr9	76140001	76145000	0.00074418
chr9	76337001	76342000	1.67E-05
chr9	76359001	76364000	6.87E-05
chr9	76360001	76365000	0.000338002
chr9	76370001	76375000	2.10E-05
chr9	76393001	76398000	3.27E-07
chr9	76410001	76415000	0.001411152
chr9	76411001	76416000	0.003247667
chr9	76412001	76417000	0.00131055
chr9	76413001	76418000	0.000197103
chr9	76414001	76419000	0.001926039
chr9	76447001	76452000	9.63E-07
chr9	76448001	76453000	6.90E-07
chr9	76449001	76454000	3.50E-06
chr9	76515001	76520000	0.000942481
chr9	76520001	76525000	0.000120387
chr9	76521001	76526000	1.19E-05
chr9	76522001	76527000	5.44E-05
chr9	76681001	76686000	2.48E-06
chr9	76820001	76825000	2.80E-07
chr9	76821001	76826000	2.18E-08
chr9	76822001	76827000	1.63E-08
chr9	76823001	76828000	4.31E-08
chr9	76830001	76835000	0.00012068
chr9	76831001	76836000	6.83E-05
chr9	76835001	76840000	7.00E-06
chr9	76837001	76842000	5.28E-06
chr9	76860001	76865000	0.02136852

chr9	76861001	76866000	0.013269864
chr9	76862001	76867000	0.022702129
chr9	76863001	76868000	0.024411221
chr9	76871001	76876000	0.00041706
chr9	76872001	76877000	0.000230564
chr9	76873001	76878000	0.002265047
chr9	76875001	76880000	6.14E-09
chr9	76876001	76881000	1.21E-08
chr9	76976001	76981000	0.000949452
chr9	77061001	77066000	5.00E-06
chr9	77092001	77097000	0.000807295
chr9	77093001	77098000	2.73E-06
chr9	77094001	77099000	7.42E-05
chr9	77095001	77100000	0.000231507
chr9	77096001	77101000	0.000369152
chr9	77097001	77102000	5.06E-05
chr9	77104001	77109000	0.000494939
chr9	77105001	77110000	0.000585827
chr9	77106001	77111000	0.000295668
chr9	77107001	77112000	7.24E-21
chr9	77108001	77113000	1.96E-34
chr9	77113001	77118000	6.57E-30
chr9	77114001	77119000	2.33E-19
chr9	77116001	77121000	0.001100215
chr9	77117001	77122000	0.005705497
chr9	77165001	77170000	4.94E-06
chr9	77166001	77171000	2.22E-07
chr9	77203001	77208000	4.67E-05
chr9	77244001	77249000	2.21E-05
chr9	77245001	77250000	2.33E-05
chr9	77329001	77334000	3.33E-06
chr9	77330001	77335000	1.82E-05
chr9	77331001	77336000	7.39E-07
chr9	77332001	77337000	0.000283775
chr9	77362001	77367000	4.54E-08
chr9	77498001	77503000	1.75E-25
chr9	77499001	77504000	1.35E-36
chr9	77500001	77505000	1.15E-33
chr9	77501001	77506000	1.48E-37
chr9	77502001	77507000	1.41E-27
chr9	77563001	77568000	3.46E-41
chr9	77564001	77569000	1.76E-49
chr9	77565001	77570000	6.39E-57
chr9	77567001	77572000	7.98E-57
chr9	77638001	77643000	6.92E-61
chr9	77639001	77644000	3.68E-62
chr9	77643001	77648000	3.77E-15
chr9	77832001	77837000	9.13E-08

chr9	77833001	77838000	7.71E-12
chr9	77834001	77839000	1.29E-09
chr9	77835001	77840000	0.000740929
chr9	77845001	77850000	1.08E-10
chr9	77862001	77867000	4.70E-06
chr9	77863001	77868000	9.61E-09
chr9	77864001	77869000	3.71E-12
chr9	77865001	77870000	2.74E-14
chr9	77866001	77871000	2.79E-09
chr9	77897001	77902000	0.001966496
chr9	77919001	77924000	1.35E-05
chr9	77920001	77925000	3.77E-06
chr9	77921001	77926000	0.000107643
chr9	77929001	77934000	0.000501765
chr9	77941001	77946000	0.000120807
chr9	77992001	77997000	0.014868207
chr9	77993001	77998000	0.002728821
chr9	78010001	78015000	0.006230968
chr9	78011001	78016000	1.70E-06
chr9	78019001	78024000	0.000434178
chr9	78052001	78057000	3.15E-10
chr9	78053001	78058000	8.06E-15
chr9	78082001	78087000	2.52E-08
chr9	78104001	78109000	0.005122685
chr9	78122001	78127000	4.42E-06
chr9	78123001	78128000	1.00E-05
chr9	78134001	78139000	5.15E-10
chr9	78137001	78142000	2.42E-08
chr9	78155001	78160000	5.39E-05
chr9	78156001	78161000	9.22E-05
chr9	78230001	78235000	0.000127341
chr9	78334001	78339000	0.000827572
chr9	78348001	78353000	0.000156298
chr9	78349001	78354000	2.06E-05
chr9	78350001	78355000	5.87E-05
chr9	78351001	78356000	0.000291321
chr9	78356001	78361000	1.16E-08
chr9	78357001	78362000	1.91E-08
chr9	78358001	78363000	3.68E-07
chr9	78453001	78458000	9.96E-05
chr9	78507001	78512000	4.57E-30
chr9	78551001	78556000	1.40E-06
chr9	78604001	78609000	4.51E-10
chr9	78687001	78692000	0.000111482
chr9	78688001	78693000	0.000147673
chr9	79074001	79079000	4.30E-28
chr9	79788001	79793000	7.75E-31
chr9	79789001	79794000	2.55E-26

chr9	79792001	79797000	9.41E-39
chr9	79793001	79798000	2.22E-19
chr9	80258001	80263000	9.57E-21
chr9	80259001	80264000	3.77E-14
chr9	80308001	80313000	0.003446746
chr9	80767001	80772000	5.75E-07
chr9	80768001	80773000	3.90E-06
chr9	80769001	80774000	4.38E-07
chr9	80784001	80789000	0.00061936
chr9	80785001	80790000	2.46E-05
chr9	80812001	80817000	6.24E-06
chr9	80907001	80912000	8.87E-20
chr9	80908001	80913000	5.52E-30
chr9	81972001	81977000	4.54E-05
chr9	81973001	81978000	1.08E-07
chr9	81974001	81979000	2.47E-06
chr9	82187001	82192000	8.90E-43
chr9	82188001	82193000	5.66E-38
chr9	82498001	82503000	1.22E-05
chr9	82733001	82738000	1.77E-10
chr9	82734001	82739000	2.52E-08
chr9	82919001	82924000	0.006284002
chr9	82920001	82925000	0.001398365
chr9	83043001	83048000	0.002210706
chr9	83058001	83063000	0.001985336
chr9	83059001	83064000	0.002296164
chr9	83068001	83073000	0.000605538
chr9	83198001	83203000	0.012025967
chr9	83200001	83205000	0.001485793
chr9	83304001	83309000	0.000896618
chr9	83306001	83311000	0.002249599
chr9	83526001	83531000	0.000298077
chr9	83527001	83532000	2.80E-05
chr9	83550001	83555000	0.000341809
chr9	83551001	83556000	0.000544109
chr9	83552001	83557000	0.003034837
chr9	84059001	84064000	1.72E-05
chr9	84060001	84065000	1.30E-06
chr9	84061001	84066000	2.15E-06
chr9	84062001	84067000	4.66E-06
chr9	85722001	85727000	0.014829798
chr9	85902001	85907000	0.00016134
chr9	86238001	86243000	2.15E-08
chr9	86428001	86433000	3.47E-13
chr9	86675001	86680000	0.000665023
chr9	87073001	87078000	6.72E-07
chr9	87188001	87193000	1.51E-06
chr9	87207001	87212000	1.50E-05

chr9	87210001	87215000	9.85E-08
chr9	87247001	87252000	1.23E-10
chr9	87248001	87253000	8.28E-10
chr9	87249001	87254000	1.04E-13
chr9	87250001	87255000	6.30E-12
chr9	87251001	87256000	6.31E-14
chr9	87252001	87257000	7.51E-05
chr9	87645001	87650000	0.006963639
chr9	87646001	87651000	0.010329606
chr9	87723001	87728000	1.16E-06
chr9	87782001	87787000	0.000377829
chr9	87783001	87788000	3.99E-06
chr9	87818001	87823000	5.60E-08
chr9	87819001	87824000	1.26E-07
chr9	87820001	87825000	1.79E-07
chr9	87821001	87826000	1.85E-05
chr9	87885001	87890000	1.80E-06
chr9	87886001	87891000	5.53E-06
chr9	87937001	87942000	8.11E-06
chr9	87940001	87945000	5.43E-05
chr9	87973001	87978000	1.31E-05
chr9	87974001	87979000	5.86E-05
chr9	87992001	87997000	5.32E-05
chr9	88352001	88357000	4.08E-39
chr9	88353001	88358000	2.49E-27
chr9	88551001	88556000	4.75E-16
chr9	88552001	88557000	5.13E-43
chr9	88553001	88558000	6.73E-51
chr9	88714001	88719000	1.39E-31
chr9	88893001	88898000	6.71E-31
chr9	88968001	88973000	1.71E-34
chr9	88969001	88974000	2.45E-41
chr9	88988001	88993000	9.26E-05
chr9	88989001	88994000	5.73E-05
chr9	89087001	89092000	3.95E-08
chr9	89132001	89137000	8.00E-16
chr9	89133001	89138000	2.46E-17
chr9	89134001	89139000	1.70E-18
chr9	89135001	89140000	1.78E-06
chr9	89142001	89147000	3.91E-08
chr9	89143001	89148000	1.51E-08
chr9	89144001	89149000	1.55E-10
chr9	89177001	89182000	1.27E-07
chr9	89180001	89185000	1.38E-05
chr9	89196001	89201000	1.12E-12
chr9	89197001	89202000	1.11E-12
chr9	89211001	89216000	3.75E-08
chr9	89213001	89218000	4.71E-06

chr9	89214001	89219000	2.96E-05
chr9	89219001	89224000	1.52E-06
chr9	89232001	89237000	2.60E-06
chr9	89240001	89245000	2.97E-10
chr9	89241001	89246000	5.58E-10
chr9	89259001	89264000	2.58E-15
chr9	89260001	89265000	1.34E-17
chr9	89261001	89266000	2.96E-12
chr9	89262001	89267000	6.14E-10
chr9	89263001	89268000	7.57E-10
chr9	89312001	89317000	9.14E-07
chr9	89348001	89353000	1.31E-09
chr9	89349001	89354000	2.15E-11
chr9	89350001	89355000	1.16E-12
chr9	89351001	89356000	1.85E-11
chr9	89352001	89357000	6.99E-14
chr9	89353001	89358000	6.88E-10
chr9	89373001	89378000	1.59E-07
chr9	89418001	89423000	4.21E-09
chr9	89419001	89424000	3.12E-13
chr9	89459001	89464000	3.46E-10
chr9	89460001	89465000	2.76E-08
chr9	89461001	89466000	4.68E-10
chr9	89462001	89467000	3.29E-09
chr9	89502001	89507000	0.000681418
chr9	89540001	89545000	1.32E-09
chr9	89541001	89546000	7.90E-07
chr9	89590001	89595000	3.24E-06
chr9	89592001	89597000	1.09E-07
chr9	89593001	89598000	1.96E-09
chr9	89604001	89609000	0.006404694
chr9	89702001	89707000	2.34E-07
chr9	89703001	89708000	1.04E-09
chr9	89704001	89709000	2.69E-07
chr9	89705001	89710000	5.22E-08
chr9	89725001	89730000	0.000559279
chr9	89726001	89731000	7.23E-09
chr9	89727001	89732000	8.49E-08
chr9	89755001	89760000	8.49E-25
chr9	89756001	89761000	3.17E-15
chr9	89757001	89762000	3.44E-27
chr9	89765001	89770000	3.80E-15
chr9	89766001	89771000	1.63E-09
chr9	89767001	89772000	9.73E-15
chr9	89768001	89773000	1.22E-05
chr9	89775001	89780000	1.83E-07
chr9	89776001	89781000	0.002016483
chr9	89810001	89815000	8.28E-11

chr9	89811001	89816000	1.97E-15
chr9	89812001	89817000	6.50E-12
chr9	89818001	89823000	2.88E-05
chr9	89834001	89839000	2.42E-09
chr9	89835001	89840000	8.62E-09
chr9	89867001	89872000	1.49E-06
chr9	89902001	89907000	1.49E-06
chr9	89903001	89908000	6.03E-08
chr9	89904001	89909000	7.40E-08
chr9	89905001	89910000	1.71E-05
chr9	89914001	89919000	2.65E-05
chr9	89976001	89981000	9.48E-06
chr9	89979001	89984000	2.49E-06
chr9	90108001	90113000	7.69E-35
chr9	90109001	90114000	8.85E-69
chr9	90110001	90115000	2.12E-55
chr9	90111001	90116000	1.32E-53
chr9	90337001	90342000	8.62E-23
chr9	90338001	90343000	2.72E-25
chr9	90339001	90344000	1.29E-32
chr9	90340001	90345000	3.49E-26
chr9	90341001	90346000	7.27E-32
chr9	90354001	90359000	0.000206216
chr9	90542001	90547000	1.12E-05
chr9	90589001	90594000	1.54E-12
chr9	90687001	90692000	3.14E-10
chr9	90690001	90695000	1.49E-08
chr9	90691001	90696000	1.24E-09
chr9	90737001	90742000	0.0065829
chr9	90811001	90816000	6.41E-06
chr9	90812001	90817000	1.80E-05
chr9	91144001	91149000	1.21E-11
chr9	91145001	91150000	5.74E-14
chr9	91146001	91151000	2.35E-14
chr9	91147001	91152000	2.82E-08
chr9	91322001	91327000	5.88E-06
chr9	91452001	91457000	0.000322081
chr9	91453001	91458000	0.000163307
chr9	91454001	91459000	0.000325666
chr9	91593001	91598000	3.15E-05
chr9	91600001	91605000	2.94E-09
chr9	91644001	91649000	3.18E-07
chr9	91695001	91700000	3.44E-06
chr9	91704001	91709000	4.11E-06
chr9	91738001	91743000	8.97E-05
chr9	91739001	91744000	2.08E-13
chr9	91761001	91766000	9.03E-09
chr9	91762001	91767000	2.72E-10

chr9	91764001	91769000	9.35E-12
chr9	91806001	91811000	0.009495258
chr9	91880001	91885000	9.68E-07
chr9	91905001	91910000	0.000928863
chr9	91921001	91926000	5.45E-30
chr9	92112001	92117000	4.55E-08
chr9	92914001	92919000	0.008583784
chr9	93168001	93173000	0.001932649
chr9	93405001	93410000	6.59E-32
chr9	93984001	93989000	1.41E-18
chr9	93985001	93990000	1.10E-18
chr9	93986001	93991000	2.76E-14
chr9	94119001	94124000	2.60E-18
chr9	94120001	94125000	3.60E-30
chr9	94123001	94128000	2.00E-11
chr9	94124001	94129000	3.23E-36
chr9	94186001	94191000	2.11E-40
chr9	94303001	94308000	0.000170734
chr9	94397001	94402000	0.001022078
chr9	94398001	94403000	6.33E-05
chr9	94399001	94404000	5.80E-05
chr9	94463001	94468000	1.68E-07
chr9	94711001	94716000	1.33E-11
chr9	94712001	94717000	2.17E-13
chr9	94873001	94878000	6.04E-25
chr9	95083001	95088000	2.41E-43
chr9	95084001	95089000	3.73E-41
chr9	95085001	95090000	8.60E-43
chr9	95432001	95437000	3.74E-13
chr9	96148001	96153000	1.22E-06
chr9	96210001	96215000	3.40E-63
chr9	96329001	96334000	5.07E-18
chr9	96338001	96343000	5.81E-08
chr9	96339001	96344000	1.01E-06
chr9	96788001	96793000	1.85E-14
chr9	96789001	96794000	1.81E-39
chr9	96790001	96795000	7.71E-40
chr9	96791001	96796000	2.67E-34
chr9	96792001	96797000	2.68E-38
chr9	96793001	96798000	3.95E-34
chr9	96924001	96929000	2.98E-32
chr9	96925001	96930000	1.39E-21
chr9	97133001	97138000	3.63E-16
chr9	97763001	97768000	2.86E-91
chr9	97764001	97769000	1.57E-72
chr9	98071001	98076000	1.64E-17
chr9	98072001	98077000	1.26E-20
chr9	98073001	98078000	6.38E-23

chr9	98074001	98079000	6.86E-25
chr9	98075001	98080000	7.66E-49
chr9	98076001	98081000	2.03E-46
chr9	98077001	98082000	2.39E-46
chr9	98078001	98083000	2.48E-39
chr9	98079001	98084000	4.72E-37
chr9	98278001	98283000	3.32E-11
chr9	98279001	98284000	1.76E-08
chr9	98511001	98516000	1.00E-17
chr9	98633001	98638000	4.09E-49
chr9	99180001	99185000	7.02E-43
chr9	99181001	99186000	1.04E-15
chr9	99208001	99213000	1.54E-32
chr9	99324001	99329000	4.06E-19
chr9	99325001	99330000	3.57E-40
chr9	99326001	99331000	2.47E-41
chr9	99327001	99332000	5.08E-42
chr9	99328001	99333000	5.15E-44
chr9	99329001	99334000	4.59E-36
chr9	99377001	99382000	3.93E-30
chr9	99378001	99383000	5.31E-20
chr9	99380001	99385000	8.99E-17
chr9	99381001	99386000	3.74E-32
chr9	99412001	99417000	1.13E-12
chr9	99413001	99418000	2.71E-75
chr9	99414001	99419000	6.11E-49
chr9	99415001	99420000	3.72E-53
chr9	99416001	99421000	2.68E-41
chr9	99417001	99422000	2.83E-49
chr9	99773001	99778000	1.42E-23
chr9	99774001	99779000	9.53E-19
chr9	99775001	99780000	3.22E-24
chr9	99797001	99802000	4.26E-42
chr9	99798001	99803000	3.88E-37
chr9	99799001	99804000	4.21E-38
chr9	99800001	99805000	5.26E-23
chr9	99801001	99806000	3.93E-28
chr9	99941001	99946000	0.003552455
chr9	100170001	100175000	8.57E-42
chr9	100171001	100176000	2.10E-42
chr9	100172001	100177000	8.96E-41
chr9	100173001	100178000	3.47E-52
chr9	100174001	100179000	1.49E-76
chr9	100264001	100269000	2.77E-09
chr9	100390001	100395000	9.87E-15
chr9	100391001	100396000	8.38E-56
chr9	100392001	100397000	1.36E-61
chr9	100455001	100460000	4.01E-41

chr9	100456001	100461000	5.79E-40
chr9	100562001	100567000	1.69E-13
chr9	100564001	100569000	1.36E-12
chr9	100698001	100703000	0.000276594
chr9	100740001	100745000	2.45E-12
chr9	100741001	100746000	8.32E-34
chr9	100742001	100747000	2.89E-37
chr9	100745001	100750000	2.77E-36
chr9	100746001	100751000	4.60E-31
chr9	100814001	100819000	3.53E-26
chr9	100815001	100820000	1.26E-34
chr9	100816001	100821000	8.84E-25
chr9	100817001	100822000	1.73E-25
chr9	100818001	100823000	1.03E-15
chr9	100877001	100882000	6.35E-14
chr9	100878001	100883000	9.01E-11
chr9	101469001	101474000	5.74E-12
chr9	101470001	101475000	1.22E-16
chr9	101471001	101476000	2.86E-12
chr9	101566001	101571000	2.56E-32
chr9	101567001	101572000	2.39E-34
chr9	101568001	101573000	3.24E-24
chr9	101569001	101574000	1.57E-25
chr9	101868001	101873000	7.85E-26
chr9	102312001	102317000	0.000840969
chr9	102533001	102538000	0.000780058
chr9	102577001	102582000	7.78E-26
chr9	102578001	102583000	1.30E-48
chr9	102669001	102674000	5.06E-32
chr9	102787001	102792000	1.25E-05
chr9	102788001	102793000	6.25E-05
chr9	103110001	103115000	1.47E-67
chr9	103111001	103116000	3.42E-77
chr9	103112001	103117000	2.53E-59
chr9	103113001	103118000	2.54E-63
chr9	103114001	103119000	2.52E-74
chr9	103115001	103120000	1.41E-69
chr9	103231001	103236000	2.79E-33
chr9	103232001	103237000	4.51E-24
chr9	103233001	103238000	2.91E-51
chr9	103234001	103239000	3.69E-43
chr9	103235001	103240000	7.23E-46
chr9	103236001	103241000	2.08E-24
chr9	103361001	103366000	1.17E-17
chr9	103585001	103590000	4.90E-10
chr9	103586001	103591000	5.26E-13
chr9	103594001	103599000	0.000260671
chr9	103595001	103600000	1.73E-05

chr9	103596001	103601000	1.84E-05
chr9	103634001	103639000	4.36E-07
chr9	103635001	103640000	3.33E-05
chr9	103657001	103662000	0.000149983
chr9	103670001	103675000	2.45E-05
chr9	103699001	103704000	1.81E-08
chr9	103819001	103824000	0.006510536
chr9	103837001	103842000	0.000731244
chr9	103987001	103992000	1.67E-05
chr9	104102001	104107000	0.00084676
chr9	104157001	104162000	4.27E-35
chr9	104158001	104163000	1.70E-35
chr9	104159001	104164000	3.29E-33
chr9	104160001	104165000	7.92E-21
chr9	104161001	104166000	2.08E-27
chr9	104207001	104212000	1.06E-19
chr9	104208001	104213000	1.32E-21
chr9	104209001	104214000	8.34E-18
chr9	104210001	104215000	6.19E-17
chr9	104211001	104216000	2.47E-13
chr9	104249001	104254000	2.74E-33
chr9	104466001	104471000	6.16E-26
chr9	104792001	104797000	1.50E-06
chr9	104818001	104823000	1.27E-05
chr9	105022001	105027000	3.30E-05
chr9	105023001	105028000	0.00014567
chr9	105056001	105061000	0.027474765
chr9	105057001	105062000	0.025839962
chr9	105564001	105569000	6.87E-05
chr9	105771001	105776000	0.001049509
chr9	106045001	106050000	2.38E-07
chr9	106046001	106051000	1.54E-08
chr9	106047001	106052000	9.32E-09
chr9	106048001	106053000	2.49E-06
chr9	106086001	106091000	0.013229451
chr9	106123001	106128000	0.004445259
chr9	106557001	106562000	0.008510393
chr9	106984001	106989000	2.55E-05
chr9	106985001	106990000	6.61E-05
chr9	107277001	107282000	0.000599614
chr9	107381001	107386000	0.006605453
chr9	107439001	107444000	2.55E-07
chr9	107440001	107445000	5.39E-08
chr9	107441001	107446000	1.27E-06
chr9	107442001	107447000	1.70E-05
chr9	107481001	107486000	0.000120665
chr9	107509001	107514000	8.99E-51
chr9	107510001	107515000	4.79E-46

chr9	107526001	107531000	5.23E-37
chr9	108002001	108007000	1.54E-27
chr9	108007001	108012000	1.18E-26
chr9	108206001	108211000	6.34E-09
chr9	108208001	108213000	8.99E-26
chr9	108209001	108214000	1.25E-34
chr9	108210001	108215000	1.77E-44
chr9	108675001	108680000	0.000107823
chr9	108676001	108681000	0.000230406
chr9	108746001	108751000	7.67E-05
chr9	108781001	108786000	5.85E-06
chr9	108782001	108787000	1.80E-08
chr9	108783001	108788000	3.65E-07
chr9	108784001	108789000	7.86E-07
chr9	108859001	108864000	0.0017982
chr9	108860001	108865000	0.00036768
chr9	108861001	108866000	0.000122702
chr9	108862001	108867000	0.000466297
chr9	108929001	108934000	3.98E-06
chr9	108930001	108935000	0.006337138
chr9	108988001	108993000	3.99E-05
chr9	108998001	109003000	1.85E-06
chr9	109016001	109021000	3.29E-08
chr9	109017001	109022000	9.70E-10
chr9	109558001	109563000	3.41E-07
chr9	109626001	109631000	1.99E-10
chr9	110041001	110046000	9.12E-44
chr9	111460001	111465000	4.64E-05
chr9	111697001	111702000	4.77E-08
chr9	111775001	111780000	5.72E-39
chr9	111925001	111930000	1.68E-09
chr9	111926001	111931000	8.52E-08
chr9	111927001	111932000	1.34E-12
chr9	111928001	111933000	8.17E-11
chr9	111929001	111934000	4.10E-16
chr9	112080001	112085000	3.30E-23
chr9	112081001	112086000	2.89E-22
chr9	112082001	112087000	9.87E-45
chr9	112083001	112088000	3.63E-32
chr9	112256001	112261000	4.34E-16
chr9	112538001	112543000	8.05E-19
chr9	112539001	112544000	6.16E-19
chr9	112540001	112545000	5.30E-20
chr9	112542001	112547000	5.26E-18
chr9	113090001	113095000	2.01E-06
chr9	113140001	113145000	6.91E-08
chr9	113141001	113146000	1.79E-07
chr9	113147001	113152000	3.99E-05

chr9	113156001	113161000	0.00080405
chr9	113157001	113162000	0.000333725
chr9	113218001	113223000	0.004830043
chr9	113233001	113238000	9.08E-07
chr9	113234001	113239000	1.57E-06
chr9	113287001	113292000	0.000213019
chr9	113343001	113348000	3.66E-06
chr9	113344001	113349000	1.33E-07
chr9	113369001	113374000	9.74E-05
chr9	113370001	113375000	0.000355745
chr9	113371001	113376000	1.03E-06
chr9	113372001	113377000	7.20E-09
chr9	113373001	113378000	2.02E-08
chr9	113374001	113379000	4.57E-06
chr9	113375001	113380000	0.000540319
chr9	113390001	113395000	0.000806023
chr9	113404001	113409000	4.89E-05
chr9	113420001	113425000	0.000506828
chr9	113421001	113426000	0.000388731
chr9	113425001	113430000	2.65E-05
chr9	113426001	113431000	1.61E-05
chr9	113448001	113453000	0.000395865
chr9	113544001	113549000	0.000355065
chr9	113605001	113610000	5.44E-07
chr9	113631001	113636000	1.12E-06
chr9	113632001	113637000	2.62E-05
chr9	113742001	113747000	0.000247529
chr9	113799001	113804000	1.41E-39
chr9	113800001	113805000	1.78E-46
chr9	113801001	113806000	3.84E-16
chr9	113912001	113917000	0.0035765
chr9	114393001	114398000	1.31E-22
chr9	114419001	114424000	1.11E-10
chr9	114424001	114429000	9.09E-18
chr9	114654001	114659000	8.15E-40
chr9	114655001	114660000	1.13E-74
chr9	114687001	114692000	0.00115621
chr9	114688001	114693000	2.74E-05
chr9	114689001	114694000	2.24E-06
chr9	114933001	114938000	8.98E-22
chr9	114934001	114939000	8.88E-19
chr9	114935001	114940000	2.31E-15
chr9	114936001	114941000	4.76E-18
chr9	114937001	114942000	2.40E-08
chr9	115141001	115146000	2.18E-19
chr9	115142001	115147000	4.67E-21
chr9	115244001	115249000	3.56E-30
chr9	115245001	115250000	2.98E-49

chr9	115246001	115251000	1.49E-51
chr9	115247001	115252000	9.81E-59
chr9	115248001	115253000	3.75E-59
chr9	115249001	115254000	1.07E-87
chr9	115250001	115255000	2.55E-08
chr9	115480001	115485000	7.21E-10
chr9	115509001	115514000	9.37E-32
chr9	115510001	115515000	5.43E-18
chr9	115511001	115516000	4.18E-20
chr9	115513001	115518000	2.63E-10
chr9	115655001	115660000	0.005019082
chr9	115685001	115690000	6.42E-09
chr9	115686001	115691000	1.30E-10
chr9	115687001	115692000	2.32E-11
chr9	115688001	115693000	5.30E-12
chr9	115689001	115694000	2.99E-07
chr9	115698001	115703000	2.24E-05
chr9	115734001	115739000	2.04E-06
chr9	115737001	115742000	1.15E-10
chr9	115738001	115743000	2.43E-09
chr9	115739001	115744000	2.92E-08
chr9	115789001	115794000	2.00E-06
chr9	115979001	115984000	8.78E-27
chr9	115980001	115985000	1.10E-25
chr9	115981001	115986000	1.62E-15
chr9	115982001	115987000	5.80E-25
chr9	115983001	115988000	1.17E-31
chr9	115984001	115989000	1.58E-07
chr9	116037001	116042000	8.26E-19
chr9	116107001	116112000	1.02E-10
chr9	116139001	116144000	0.000643643
chr9	116163001	116168000	3.55E-20
chr9	116226001	116231000	7.93E-32
chr9	116472001	116477000	1.59E-09
chr9	116491001	116496000	4.32E-06
chr9	116607001	116612000	5.72E-05
chr9	116637001	116642000	8.55E-15
chr9	116638001	116643000	8.00E-19
chr9	116787001	116792000	4.60E-11
chr9	116950001	116955000	0.00014504
chr9	117054001	117059000	5.04E-06
chr9	117160001	117165000	5.73E-12
chr9	117262001	117267000	9.81E-12
chr9	118291001	118296000	0.000216585
chr9	118350001	118355000	8.67E-07
chr9	118351001	118356000	2.79E-09
chr9	118352001	118357000	4.35E-09
chr9	118353001	118358000	1.07E-08

chr9	118582001	118587000	0.001057168
chr9	118583001	118588000	5.45E-06
chr9	118584001	118589000	5.24E-05
chr9	118642001	118647000	6.67E-07
chr9	118912001	118917000	6.18E-32
chr9	119223001	119228000	7.19E-09
chr9	119224001	119229000	1.78E-10
chr9	119271001	119276000	8.75E-10
chr9	119278001	119283000	2.43E-05
chr9	119280001	119285000	5.35E-07
chr9	119284001	119289000	4.16E-06
chr9	119286001	119291000	1.40E-05
chr9	119287001	119292000	7.12E-06
chr9	119288001	119293000	3.08E-05
chr9	119516001	119521000	5.04E-05
chr9	119529001	119534000	0.000167319
chr9	119918001	119923000	6.65E-06
chr9	119936001	119941000	0.005725637
chr9	120363001	120368000	0.000237443
chr9	121222001	121227000	0.002214295
chr9	121746001	121751000	8.11E-05
chr9	123338001	123343000	3.39E-25
chr9	123339001	123344000	3.20E-21
chr9	123340001	123345000	1.83E-23
chr9	123476001	123481000	3.99E-16
chr9	123477001	123482000	5.35E-18
chr9	123551001	123556000	5.91E-59
chr9	123552001	123557000	8.08E-34
chr9	123601001	123606000	4.05E-18
chr9	123689001	123694000	3.31E-20
chr9	123963001	123968000	4.05E-13
chr9	124117001	124122000	6.55E-07
chr9	124118001	124123000	1.80E-08
chr9	124156001	124161000	7.19E-06
chr9	124169001	124174000	9.52E-05
chr9	124262001	124267000	2.59E-11
chr9	124349001	124354000	2.59E-06
chr9	124357001	124362000	5.02E-26
chr9	124358001	124363000	1.66E-30
chr9	124359001	124364000	9.15E-06
chr9	124361001	124366000	8.16E-31
chr9	124362001	124367000	2.51E-14
chr9	124409001	124414000	1.62E-10
chr9	124410001	124415000	4.44E-11
chr9	124411001	124416000	2.62E-06
chr9	124414001	124419000	7.00E-11
chr9	124457001	124462000	2.42E-13
chr9	124459001	124464000	8.84E-10

chr9	124460001	124465000	1.96E-10
chr9	124461001	124466000	1.37E-11
chr9	124851001	124856000	4.85E-20
chr9	124852001	124857000	1.13E-15
chr9	124917001	124922000	1.54E-27
chr9	124972001	124977000	5.16E-17
chr9	124990001	124995000	6.32E-26
chr9	125179001	125184000	0.009298943
chr9	125191001	125196000	8.89E-05
chr9	125258001	125263000	5.30E-06
chr9	125309001	125314000	1.52E-06
chr9	125310001	125315000	3.01E-05
chr9	125311001	125316000	1.85E-06
chr9	125334001	125339000	4.97E-07
chr9	125368001	125373000	3.22E-06
chr9	125406001	125411000	3.26E-07
chr9	125407001	125412000	1.06E-08
chr9	125409001	125414000	2.60E-10
chr9	125410001	125415000	3.59E-10
chr9	125514001	125519000	0.000116835
chr9	125515001	125520000	0.001485083
chr9	125517001	125522000	1.05E-05
chr9	125667001	125672000	2.06E-32
chr9	125670001	125675000	0.000239676
chr9	125671001	125676000	6.76E-50
chr9	125672001	125677000	7.24E-30
chr9	125694001	125699000	2.51E-09
chr9	126026001	126031000	2.73E-26
chr9	126027001	126032000	2.54E-28
chr9	126028001	126033000	2.33E-25
chr9	126030001	126035000	1.50E-24
chr9	126031001	126036000	6.71E-07
chr9	126807001	126812000	3.50E-11
chr9	127016001	127021000	7.14E-19
chr9	127527001	127532000	6.56E-12
chr9	127528001	127533000	8.08E-31
chr9	127529001	127534000	6.40E-23
chr9	127535001	127540000	6.20E-13
chr9	127699001	127704000	6.72E-13
chr9	127703001	127708000	2.43E-45
chr9	127901001	127906000	9.28E-15
chr9	127903001	127908000	2.06E-35
chr9	127947001	127952000	1.41E-25
chr9	127948001	127953000	1.02E-25
chr9	127962001	127967000	1.22E-12
chr9	128300001	128305000	2.68E-06
chr9	128465001	128470000	1.34E-37
chr9	128466001	128471000	1.14E-55

chr9	128467001	128472000	2.75E-41
chr9	128468001	128473000	2.00E-35
chr9	128469001	128474000	8.38E-24
chr9	128510001	128515000	3.55E-30
chr9	128648001	128653000	1.69E-29
chr9	128649001	128654000	5.05E-36
chr9	128650001	128655000	7.24E-37
chr9	128651001	128656000	4.57E-32
chr9	128652001	128657000	6.10E-44
chr9	128670001	128675000	1.76E-07
chr9	128673001	128678000	5.22E-06
chr9	128896001	128901000	0.002628506
chr9	128897001	128902000	0.000808787
chr9	129084001	129089000	8.33E-21
chr9	129085001	129090000	7.24E-31
chr9	129086001	129091000	1.88E-41
chr9	129087001	129092000	4.28E-43
chr9	129088001	129093000	1.25E-23
chr9	129089001	129094000	3.86E-56
chr9	129483001	129488000	2.45E-09
chr9	129484001	129489000	1.06E-10
chr9	129563001	129568000	9.64E-26
chr9	129564001	129569000	1.64E-21
chr9	129565001	129570000	5.24E-23
chr9	129566001	129571000	2.32E-25
chr9	129622001	129627000	7.71E-33
chr9	129623001	129628000	1.10E-27
chr9	129982001	129987000	6.58E-58
chr9	129983001	129988000	5.44E-93
chr9	129984001	129989000	5.60E-65
chr9	129985001	129990000	2.03E-44
chr9	129986001	129991000	2.27E-20
chr9	129987001	129992000	9.90E-35
chr9	130214001	130219000	4.91E-11
chr9	130367001	130372000	2.84E-16
chr9	130370001	130375000	1.13E-31
chr9	130371001	130376000	8.47E-28
chr9	130372001	130377000	2.27E-28
chr9	130373001	130378000	4.62E-18
chr9	130374001	130379000	2.06E-15
chr9	130375001	130380000	1.92E-06
chr9	130495001	130500000	2.75E-07
chr9	130561001	130566000	1.40E-37
chr9	130562001	130567000	3.11E-13
chr9	130563001	130568000	2.12E-11
chr9	130564001	130569000	4.71E-15
chr9	130743001	130748000	8.01E-06
chr9	130918001	130923000	3.44E-17

chr9	130919001	130924000	2.28E-18
chr9	130920001	130925000	5.13E-12
chr9	130949001	130954000	1.32E-07
chr9	130961001	130966000	3.67E-08
chr9	131101001	131106000	8.02E-11
chr9	131102001	131107000	1.65E-16
chr9	131123001	131128000	7.44E-09
chr9	131214001	131219000	8.11E-12
chr9	131217001	131222000	8.49E-10
chr9	131218001	131223000	3.73E-25
chr9	131219001	131224000	3.92E-10
chr9	131263001	131268000	2.55E-08
chr9	131264001	131269000	8.00E-11
chr9	131266001	131271000	8.78E-11
chr9	131267001	131272000	8.10E-15
chr9	131310001	131315000	2.16E-22
chr9	131314001	131319000	8.46E-23
chr9	131315001	131320000	1.01E-19
chr9	131461001	131466000	1.59E-08
chr9	131462001	131467000	2.66E-09
chr9	131545001	131550000	4.67E-17
chr9	131546001	131551000	9.86E-16
chr9	131547001	131552000	1.55E-25
chr9	131548001	131553000	7.08E-19
chr9	131549001	131554000	1.55E-09
chr9	131577001	131582000	1.70E-33
chr9	131644001	131649000	6.90E-11
chr9	131709001	131714000	4.66E-13
chr9	131710001	131715000	1.69E-11
chr9	131932001	131937000	2.12E-10
chr9	132142001	132147000	4.34E-09
chr9	132172001	132177000	7.67E-09
chr9	132173001	132178000	4.64E-05
chr9	132175001	132180000	3.03E-06
chr9	132176001	132181000	1.99E-08
chr9	132241001	132246000	2.01E-05
chr9	132457001	132462000	5.11E-21
chr9	132458001	132463000	1.30E-18
chr9	132459001	132464000	7.85E-21
chr9	132565001	132570000	8.69E-12
chr9	132582001	132587000	2.71E-06
chr9	132583001	132588000	2.94E-09
chr9	132584001	132589000	1.25E-11
chr9	133452001	133457000	1.13E-11
chr9	133453001	133458000	1.07E-22
chr9	133454001	133459000	6.57E-18
chr9	133455001	133460000	1.69E-17
chr9	133552001	133557000	5.22E-06

chr9	133584001	133589000	2.70E-16
chr9	133585001	133590000	2.44E-12
chr9	133588001	133593000	4.69E-23
chr9	133706001	133711000	1.25E-36
chr9	133707001	133712000	5.24E-40
chr9	133708001	133713000	1.68E-25
chr9	133709001	133714000	1.05E-11
chr9	133710001	133715000	4.40E-22
chr9	133711001	133716000	3.55E-08
chr9	134158001	134163000	1.54E-14
chr9	134221001	134226000	3.17E-08
chr9	134266001	134271000	2.91E-15
chr9	134267001	134272000	4.05E-25
chr9	134416001	134421000	5.93E-21
chr9	134417001	134422000	2.30E-28
chr9	134418001	134423000	3.95E-44
chr9	134419001	134424000	6.53E-60
chr9	134420001	134425000	7.47E-69
chr9	134421001	134426000	1.53E-48
chr9	134612001	134617000	5.96E-07
chr9	134613001	134618000	4.81E-06
chr9	135541001	135546000	4.22E-32
chr9	135572001	135577000	8.84E-05
chr9	135573001	135578000	7.63E-05
chr9	135752001	135757000	1.12E-22
chr9	135815001	135820000	3.31E-31
chr9	135816001	135821000	3.16E-21
chr9	135848001	135853000	4.44E-08
chr9	135901001	135906000	5.68E-10
chr9	135904001	135909000	4.20E-28
chr9	135905001	135910000	4.81E-29
chr9	135996001	136001000	7.23E-09
chr9	136608001	136613000	1.83E-05
chr9	136852001	136857000	1.62E-08
chr9	136853001	136858000	1.79E-14
chr9	136929001	136934000	0.000871393
chr9	136932001	136937000	1.40E-07
chr9	136933001	136938000	0.000240064
chr9	138798001	138803000	1.21E-11
chr9	138799001	138804000	1.20E-15
chr9	138984001	138989000	2.21E-10
chr9	138985001	138990000	7.79E-15
chr9	138986001	138991000	1.26E-09
chr9	138987001	138992000	1.58E-05
chr9	139010001	139015000	3.59E-09
chr9	139290001	139295000	5.71E-13
chr9	139291001	139296000	5.34E-12
chr9	139292001	139297000	9.62E-13

chr9	139293001	139298000	4.11E-13
chr9	139374001	139379000	7.34E-35
chr9	139964001	139969000	1.41E-10
chr9	139971001	139976000	2.33E-10
chr9	140020001	140025000	1.65E-25
chr9	140021001	140026000	1.57E-25
chr9	140022001	140027000	9.54E-21
chr9	140023001	140028000	2.32E-32
chr9	140024001	140029000	4.95E-23
chr9	140091001	140096000	2.16E-10
chr9	140093001	140098000	0.002255806
chr9	140094001	140099000	6.46E-15
chr9	140095001	140100000	1.20E-12
chr9	140147001	140152000	1.76E-10
chr9	140148001	140153000	2.50E-13
chr9	140149001	140154000	9.64E-21
chr9	140313001	140318000	4.24E-24
chr9	140314001	140319000	4.09E-33
chr9	140315001	140320000	4.10E-29
chr9	140316001	140321000	3.90E-25
chr9	140317001	140322000	3.82E-25
chr9	140445001	140450000	1.41E-09
chr9	140480001	140485000	6.88E-15
chr9	140485001	140490000	6.92E-06
chr9	140509001	140514000	0.000132779
chr9	140913001	140918000	4.70E-08
chr9	140914001	140919000	1.07E-08
chr9	140916001	140921000	2.51E-07
chr10	78001	83000	1.96E-05
chr10	262001	267000	5.56E-05
chr10	263001	268000	0.000208992
chr10	732001	737000	3.87E-09
chr10	733001	738000	1.35E-14
chr10	1032001	1037000	2.15E-22
chr10	1033001	1038000	3.81E-21
chr10	1034001	1039000	4.82E-18
chr10	1086001	1091000	7.15E-12
chr10	1087001	1092000	4.49E-06
chr10	1088001	1093000	5.00E-05
chr10	1095001	1100000	2.37E-36
chr10	1097001	1102000	1.15E-19
chr10	1098001	1103000	1.03E-38
chr10	1099001	1104000	1.44E-36
chr10	1100001	1105000	7.29E-50
chr10	1101001	1106000	1.09E-33
chr10	1102001	1107000	1.66E-39
chr10	1775001	1780000	2.54E-39
chr10	1776001	1781000	1.22E-35

chr10	1787001	1792000	3.39E-12
chr10	1792001	1797000	1.76E-05
chr10	1918001	1923000	9.73E-09
chr10	1919001	1924000	4.11E-11
chr10	2031001	2036000	3.09E-07
chr10	2302001	2307000	0.00195246
chr10	2678001	2683000	0.001047728
chr10	2785001	2790000	0.000376387
chr10	2829001	2834000	2.53E-06
chr10	2832001	2837000	4.28E-05
chr10	2916001	2921000	5.72E-10
chr10	2917001	2922000	3.88E-09
chr10	3105001	3110000	4.71E-18
chr10	3111001	3116000	1.95E-23
chr10	3827001	3832000	2.46E-39
chr10	4013001	4018000	0.000625952
chr10	4488001	4493000	1.24E-05
chr10	4824001	4829000	3.28E-06
chr10	4825001	4830000	1.31E-07
chr10	4826001	4831000	9.87E-08
chr10	4827001	4832000	3.89E-08
chr10	4866001	4871000	1.03E-25
chr10	4867001	4872000	1.06E-22
chr10	4868001	4873000	1.27E-55
chr10	5123001	5128000	1.14E-05
chr10	5133001	5138000	4.71E-07
chr10	5134001	5139000	9.35E-06
chr10	5454001	5459000	1.97E-12
chr10	5730001	5735000	1.18E-35
chr10	5731001	5736000	5.62E-39
chr10	5732001	5737000	5.04E-40
chr10	5927001	5932000	1.05E-32
chr10	6015001	6020000	4.03E-22
chr10	6017001	6022000	2.09E-17
chr10	6018001	6023000	1.72E-15
chr10	6019001	6024000	3.68E-24
chr10	6130001	6135000	4.64E-27
chr10	6131001	6136000	1.39E-32
chr10	6201001	6206000	1.09E-35
chr10	6202001	6207000	1.33E-41
chr10	6203001	6208000	9.78E-24
chr10	6204001	6209000	5.64E-12
chr10	6581001	6586000	9.78E-10
chr10	6622001	6627000	1.25E-18
chr10	7007001	7012000	0.001050128
chr10	7182001	7187000	0.000735747
chr10	7183001	7188000	0.001463037
chr10	7528001	7533000	1.74E-05

chr10	7825001	7830000	2.06E-40
chr10	7826001	7831000	7.43E-42
chr10	7831001	7836000	2.30E-07
chr10	9793001	9798000	3.99E-06
chr10	9794001	9799000	9.78E-06
chr10	9851001	9856000	0.009231329
chr10	10140001	10145000	0.001854818
chr10	10141001	10146000	0.001166888
chr10	10142001	10147000	0.001973782
chr10	10143001	10148000	0.002711039
chr10	10189001	10194000	0.009338491
chr10	10395001	10400000	6.84E-05
chr10	10438001	10443000	0.000674383
chr10	10451001	10456000	0.000179352
chr10	10810001	10815000	2.85E-10
chr10	11055001	11060000	3.83E-29
chr10	11060001	11065000	7.06E-33
chr10	11207001	11212000	1.41E-12
chr10	11525001	11530000	2.37E-05
chr10	11555001	11560000	2.96E-07
chr10	11556001	11561000	2.52E-07
chr10	11557001	11562000	1.03E-05
chr10	11649001	11654000	3.84E-18
chr10	11652001	11657000	7.32E-29
chr10	11653001	11658000	3.02E-31
chr10	11861001	11866000	6.93E-59
chr10	11862001	11867000	8.07E-51
chr10	11863001	11868000	2.78E-35
chr10	11864001	11869000	1.36E-52
chr10	11865001	11870000	2.80E-46
chr10	12080001	12085000	4.27E-41
chr10	12081001	12086000	3.94E-35
chr10	12167001	12172000	1.36E-33
chr10	12168001	12173000	8.11E-20
chr10	12387001	12392000	1.52E-12
chr10	12661001	12666000	1.30E-19
chr10	12662001	12667000	2.98E-25
chr10	12663001	12668000	1.06E-17
chr10	12664001	12669000	6.40E-18
chr10	12665001	12670000	1.19E-23
chr10	13039001	13044000	3.18E-26
chr10	13040001	13045000	1.10E-14
chr10	13142001	13147000	7.51E-20
chr10	13337001	13342000	7.53E-28
chr10	13482001	13487000	8.44E-18
chr10	13566001	13571000	5.33E-32
chr10	13567001	13572000	2.12E-31
chr10	13568001	13573000	2.88E-42

chr10	13569001	13574000	3.36E-44
chr10	13570001	13575000	6.84E-64
chr10	13571001	13576000	8.15E-58
chr10	13820001	13825000	7.83E-05
chr10	14211001	14216000	1.03E-17
chr10	14212001	14217000	3.20E-28
chr10	14213001	14218000	2.90E-15
chr10	14435001	14440000	0.000114584
chr10	14642001	14647000	9.35E-22
chr10	14645001	14650000	9.46E-28
chr10	14646001	14651000	9.70E-24
chr10	14875001	14880000	5.23E-38
chr10	14876001	14881000	2.51E-68
chr10	14877001	14882000	2.72E-59
chr10	14878001	14883000	4.44E-79
chr10	14879001	14884000	5.45E-56
chr10	14880001	14885000	5.45E-31
chr10	14916001	14921000	4.52E-52
chr10	14917001	14922000	6.30E-51
chr10	14918001	14923000	1.76E-65
chr10	14919001	14924000	9.06E-63
chr10	14920001	14925000	9.99E-76
chr10	14921001	14926000	9.25E-46
chr10	15206001	15211000	1.30E-26
chr10	15207001	15212000	5.69E-33
chr10	15208001	15213000	7.05E-30
chr10	15210001	15215000	1.59E-20
chr10	15408001	15413000	3.02E-19
chr10	15409001	15414000	2.94E-35
chr10	15410001	15415000	1.40E-37
chr10	15411001	15416000	3.01E-17
chr10	15583001	15588000	0.00169463
chr10	15618001	15623000	7.02E-08
chr10	15619001	15624000	2.35E-09
chr10	15716001	15721000	1.72E-05
chr10	15718001	15723000	1.00E-07
chr10	15719001	15724000	7.42E-06
chr10	15761001	15766000	1.59E-08
chr10	15897001	15902000	3.02E-13
chr10	15898001	15903000	1.01E-30
chr10	15899001	15904000	1.87E-25
chr10	15900001	15905000	7.16E-33
chr10	15901001	15906000	1.41E-32
chr10	15902001	15907000	5.67E-31
chr10	16559001	16564000	7.94E-26
chr10	16563001	16568000	2.41E-23
chr10	17104001	17109000	1.28E-05
chr10	17105001	17110000	1.03E-06

chr10	17199001	17204000	6.22E-06
chr10	17200001	17205000	2.16E-08
chr10	17201001	17206000	1.73E-12
chr10	17202001	17207000	1.77E-13
chr10	17243001	17248000	3.95E-33
chr10	17250001	17255000	0.01810975
chr10	17251001	17256000	0.007864122
chr10	17360001	17365000	0.001115885
chr10	17370001	17375000	2.21E-10
chr10	17371001	17376000	6.20E-09
chr10	17476001	17481000	0.000243552
chr10	17491001	17496000	9.61E-17
chr10	17659001	17664000	6.22E-09
chr10	17730001	17735000	0.000762533
chr10	18338001	18343000	4.38E-05
chr10	18351001	18356000	3.07E-13
chr10	18352001	18357000	3.32E-08
chr10	18425001	18430000	2.45E-14
chr10	18476001	18481000	0.006857531
chr10	18550001	18555000	0.000267102
chr10	18940001	18945000	2.78E-10
chr10	18944001	18949000	6.53E-40
chr10	19067001	19072000	3.55E-06
chr10	19089001	19094000	0.012025563
chr10	19158001	19163000	0.002003302
chr10	19309001	19314000	0.007715651
chr10	19561001	19566000	2.48E-06
chr10	19657001	19662000	0.006818534
chr10	20381001	20386000	0.000179749
chr10	20382001	20387000	0.000189101
chr10	20383001	20388000	0.005453422
chr10	20384001	20389000	0.003728553
chr10	20385001	20390000	0.000489589
chr10	20481001	20486000	1.60E-05
chr10	20482001	20487000	0.000633006
chr10	20483001	20488000	0.000394512
chr10	20484001	20489000	0.000427883
chr10	20557001	20562000	0.014761719
chr10	20746001	20751000	3.67E-09
chr10	20893001	20898000	0.000145755
chr10	20905001	20910000	3.59E-12
chr10	20906001	20911000	1.07E-08
chr10	21005001	21010000	9.09E-07
chr10	21006001	21011000	4.43E-07
chr10	21007001	21012000	1.01E-05
chr10	21463001	21468000	3.17E-19
chr10	21779001	21784000	2.40E-21
chr10	21801001	21806000	3.24E-33

chr10	21802001	21807000	2.42E-26
chr10	21803001	21808000	2.23E-30
chr10	22359001	22364000	3.03E-07
chr10	22361001	22366000	3.05E-06
chr10	22604001	22609000	9.97E-67
chr10	22605001	22610000	2.39E-108
chr10	22606001	22611000	2.05E-113
chr10	22607001	22612000	2.88E-129
chr10	22608001	22613000	8.24E-93
chr10	22609001	22614000	6.41E-68
chr10	22613001	22618000	1.10E-40
chr10	22614001	22619000	2.89E-41
chr10	22615001	22620000	1.13E-13
chr10	22633001	22638000	3.23E-21
chr10	22634001	22639000	6.54E-26
chr10	22726001	22731000	3.69E-35
chr10	22806001	22811000	5.05E-05
chr10	22935001	22940000	2.89E-13
chr10	22998001	23003000	7.75E-19
chr10	22999001	23004000	4.44E-12
chr10	23000001	23005000	1.78E-12
chr10	23001001	23006000	1.55E-21
chr10	23002001	23007000	1.38E-26
chr10	23003001	23008000	8.11E-23
chr10	23039001	23044000	8.44E-05
chr10	23040001	23045000	9.51E-06
chr10	23041001	23046000	8.27E-06
chr10	23042001	23047000	4.96E-07
chr10	23043001	23048000	1.52E-08
chr10	23046001	23051000	1.23E-06
chr10	23074001	23079000	4.47E-05
chr10	23101001	23106000	3.07E-06
chr10	23176001	23181000	6.09E-08
chr10	23177001	23182000	3.90E-06
chr10	23212001	23217000	4.91E-26
chr10	23213001	23218000	4.12E-25
chr10	23214001	23219000	1.22E-31
chr10	23215001	23220000	2.76E-30
chr10	23216001	23221000	1.51E-26
chr10	23217001	23222000	2.64E-10
chr10	23229001	23234000	0.001481907
chr10	23284001	23289000	1.81E-05
chr10	23285001	23290000	5.86E-05
chr10	23299001	23304000	0.000407119
chr10	23309001	23314000	1.22E-08
chr10	23310001	23315000	5.74E-08
chr10	23380001	23385000	6.77E-22
chr10	23495001	23500000	2.64E-05

chr10	23574001	23579000	0.000651734
chr10	23616001	23621000	6.82E-08
chr10	23617001	23622000	1.32E-08
chr10	23618001	23623000	8.60E-14
chr10	23619001	23624000	1.16E-16
chr10	23620001	23625000	1.07E-26
chr10	23623001	23628000	1.00E-07
chr10	23624001	23629000	1.71E-07
chr10	23625001	23630000	1.26E-06
chr10	23633001	23638000	8.09E-08
chr10	23636001	23641000	0.001616371
chr10	23673001	23678000	7.78E-05
chr10	23674001	23679000	0.000132697
chr10	23723001	23728000	5.64E-41
chr10	23724001	23729000	6.61E-73
chr10	23741001	23746000	0.000175092
chr10	23877001	23882000	5.61E-07
chr10	23899001	23904000	7.34E-06
chr10	23965001	23970000	6.84E-06
chr10	23966001	23971000	2.22E-05
chr10	23990001	23995000	8.36E-08
chr10	24007001	24012000	6.94E-05
chr10	24008001	24013000	6.14E-05
chr10	24087001	24092000	0.000151938
chr10	24301001	24306000	0.002339359
chr10	24302001	24307000	0.002642359
chr10	24334001	24339000	0.000319605
chr10	24335001	24340000	0.000102653
chr10	24336001	24341000	2.76E-05
chr10	24337001	24342000	6.78E-07
chr10	24338001	24343000	8.37E-08
chr10	24339001	24344000	0.000408421
chr10	24349001	24354000	2.21E-09
chr10	24350001	24355000	4.93E-09
chr10	24351001	24356000	3.51E-09
chr10	24431001	24436000	0.003780647
chr10	24467001	24472000	0.002531814
chr10	24468001	24473000	0.00459166
chr10	24493001	24498000	1.16E-15
chr10	25011001	25016000	1.60E-17
chr10	25012001	25017000	2.03E-18
chr10	25237001	25242000	2.37E-25
chr10	25240001	25245000	8.31E-23
chr10	25241001	25246000	6.09E-23
chr10	25302001	25307000	2.31E-38
chr10	25303001	25308000	5.43E-39
chr10	25305001	25310000	6.38E-35
chr10	25306001	25311000	1.66E-35

chr10	25573001	25578000	0.000189845
chr10	26309001	26314000	0.000118304
chr10	26310001	26315000	0.00181745
chr10	26334001	26339000	1.17E-05
chr10	26404001	26409000	0.006093439
chr10	26431001	26436000	7.72E-07
chr10	26432001	26437000	7.98E-08
chr10	26434001	26439000	1.16E-05
chr10	26435001	26440000	1.02E-05
chr10	26493001	26498000	0.000401287
chr10	26579001	26584000	7.93E-08
chr10	26580001	26585000	3.32E-07
chr10	26807001	26812000	0.001749475
chr10	26808001	26813000	1.47E-06
chr10	26852001	26857000	1.35E-10
chr10	26853001	26858000	4.55E-10
chr10	26854001	26859000	6.34E-15
chr10	26855001	26860000	1.05E-19
chr10	26856001	26861000	1.13E-21
chr10	26911001	26916000	1.34E-20
chr10	26982001	26987000	2.06E-53
chr10	26984001	26989000	1.95E-61
chr10	27250001	27255000	2.00E-05
chr10	27385001	27390000	4.31E-41
chr10	27386001	27391000	1.33E-38
chr10	27387001	27392000	2.31E-18
chr10	27388001	27393000	7.52E-13
chr10	27444001	27449000	1.27E-39
chr10	27519001	27524000	5.75E-07
chr10	27520001	27525000	1.54E-05
chr10	27521001	27526000	4.09E-05
chr10	27530001	27535000	9.80E-16
chr10	27547001	27552000	2.83E-27
chr10	27550001	27555000	3.12E-05
chr10	27577001	27582000	0.000265127
chr10	27578001	27583000	0.000241644
chr10	27621001	27626000	0.016388913
chr10	27789001	27794000	5.07E-20
[truncated: 378,717 more chars]
